# Integrating bulk and single-cell transcriptome profiling to uncover diagnostic biomarkers and regulatory mechanisms of oxidative stress in spinal cord injury

**DOI:** 10.4103/NRR.NRR-D-24-00693

**Published:** 2025-01-13

**Authors:** Jianfeng Li, Kuileung Tong, Jiaxiang Zhou, Shiming Li, Zhongyuan He, Fuan Wang, Hongkun Chen, Haizhen Li, Gang Cheng, Junhong Li, Zhiyu Zhou, Manman Gao

**Affiliations:** 1 Innovation Platform of Regeneration and Repair of Spinal Cord and Nerve Injury, Department of Orthopedics, The Seventh Affiliated Hospital, Sun Yat-sen University, Shenzhen, Guangdong Province, China; 2 Guangdong Provincial Key Laboratory of Orthopedics and Traumatology, Orthopedic Research Institute/Department of Spinal Surgery, The First Affiliated Hospital, Sun Yat-sen University, Guangzhou, Guangdong Province, China; 3 Department of Orthopedics, The Third Affiliated Hospital, Sun Yat-sen University, Guangzhou, Guangdong Province, China; 4 Department of Orthopedic Surgery, The Affiliated Suzhou Hospital of Nanjing Medical University, Gusu School, Nanjing Medical University, Suzhou, Jiangsu Province, China; 5 Department of Orthopedics, The Second Affiliated Hospital, Chongqing Medical University, Chongqing, China; 6 Department of Orthopedics and Trauma, The Affiliated Hospital of Yunnan University, Yunnan University, Kunming, Yunnan Province, China; 7 Orthopedic Research Institute, Fuzhou Second General Hospital, Fuzhou, Fujian Province, China; 8 Department of Pediatric Orthopedic, Fuzhou Second General Hospital, Fuzhou, Fujian Province, China; 9 Fujian Provincial Clinical Medical Research Center for First Aid and Rehabilitation in Orthopaedic Trauma (2020Y2014), Fuzhou Second General Hospital, Fuzhou, Fujian Province, China

**Keywords:** bioinformatics analysis, diagnostic biomarker, drug intervention, expression characteristics, immune change, oxidative stress, regulation mechanism, severity of the illness, spinal cord injury, spinal cord repair

## Abstract

Oxidative stress significantly contributes to secondary damage after spinal cord injury. Despite its importance, research on oxidative stress in spinal cord injury remains limited. Investigating the expression and regulation of oxidative stress–related genes could enhance the diagnosis and treatment of spinal cord injury. In this study, we analyzed the sequencing data of human blood samples and injured mouse spinal cord tissue that were sourced from GEO databases and identified diagnostic biomarkers associated with the severity of spinal cord injury. We also explored the expression patterns of oxidative stress–related genes, potential regulatory mechanisms, and therapeutic drugs. To validate our findings, we performed immunofluorescence and quantitative polymerase chain reaction to assess gene expression in the injured spinal cord. Our results revealed biomarkers associated with oxidative stress and immune responses across different levels of spinal cord injury in humans. We identified differentially expressed oxidative stress–related genes and key hub genes in injured mouse spinal cord tissue and revealed their temporal expression patterns at both the tissue and single-cell levels. We also clarified the signaling pathways associated with oxidative stress and identified ligand-receptor pairs among various cell types at different time points after injury. Furthermore, we discovered microRNAs, long non-coding RNAs, and transcription factors that regulate these hub genes and revealed their roles in modulating gene expression at various stages after spinal cord injury. We also identified drugs targeting these hub genes. The findings from this study not only aid in identifying diagnostic biomarkers that reflect the severity of spinal cord injury, but also provide insights into the expression dynamics of oxidative stress-related genes. In addition, the study reveals potential regulatory mechanisms and identifies potential drugs to treat patients with spinal cord injury.

## Graphical abstract

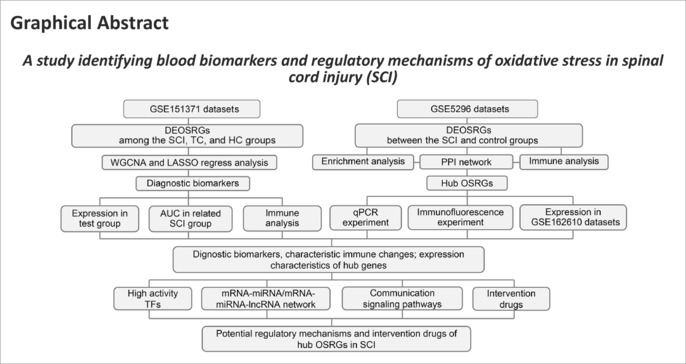

## Introduction

Spinal cord injury (SCI) represents a severe trauma to the central nervous system and frequently leads to motor and sensory dysfunction below the injury site (Ahuja et al., 2017; Quadri et al., 2020; Barbiellini Amidei et al., 2022). SCI is categorized into primary and secondary phases (Ahuja et al., 2017; Anjum et al., 2020). Primary SCI results from direct mechanical damage to tissue and cells. In contrast, secondary SCI emerges from a series of reactions triggered by oxidative stress, immune inflammation, tissue hemorrhage, hypoxia, and cellular death (Ahuja et al., 2017; Anjum et al., 2020; Ding et al., 2025). Oxidative stress arises from an imbalance between reactive oxygen species and the body’s antioxidant defense and is pivotal in exacerbating secondary SCI by generating excessive free radicals and cytotoxic responses (Jia et al., 2012; Sies and Jones, 2020).

The American Spinal Injury Association Impairment Scale (AIS) grades SCI severity from A to E, with A being the most severe and E representing normal sensory and motor functions (Zrzavy et al., 2021). In the clinical setting, SCI diagnosis and evaluation primarily depend on magnetic resonance imaging and specialized physical examinations. However, magnetic resonance imaging is not feasible in patients with metal implants or those too ill for transport. In addition, specialized physical examinations might not accurately assess SCI severity because of factors such as spinal shock, concussion, and possible biases in symptom reporting (Marquis et al., 2015; Li et al., 2023). Research indicates that monitoring changes in blood biomarkers can provide insights into SCI severity (Heller et al., 2021).

Oxidative stress reactions occur rapidly in both the injured tissue and blood during the acute phase of spinal cord injury (SCI) (Marquis et al., 2015; Takahashi et al., 2019; Zrzavy et al., 2021; Li et al., 2024b). Therefore, the detection of blood biomarkers associated with oxidative stress could offer profound clinical insights into SCI severity (Marquis et al., 2015; Zhang et al., 2021; Li et al., 2024c). While prior research has suggested that oxidative changes in red blood cells after SCI may serve as potential biomarkers (Zhang et al., 2021), the role of oxidative stress changes in bloodborne immune cells remains less clear. However, studies have confirmed that alterations do occur in these cells after SCI (Heller et al., 2021; Li et al., 2023). Consequently, we propose that oxidative stress modifications in immune cells may provide valuable diagnostic biomarkers for assessing SCI severity.

Research has demonstrated that excessive production of reactive oxygen species and lipid peroxidation are pivotal mechanisms underlying oxidative stress–induced injury in SCI (Lam et al., 2013; Catalá and Díaz, 2016). Attenuating the oxidative stress response may mitigate secondary injury and enhance functional recovery (Lv et al., 2019; Zhang et al., 2023). Although blood and spinal cord tissues differ, numerous genes are concurrently activated in both under disease or injury conditions (David and Kroner, 2011; Gillespie et al., 2024). Furthermore, the pathophysiological mechanisms of SCI are similar across various species (Ahuja et al., 2017; Rodrigues et al., 2018). However, the regulatory mechanisms governing oxidative stress responses in SCI have not yet been completely elucidated. Given the absence of sequencing data from injured human spinal cord tissue, and the conservation of gene expression patterns between humans and mice, a comparative cross-species analysis is warranted (Brawand et al., 2011; GTEx Consortium, 2020). Thus, a comprehensive analysis of sequencing data from a mouse SCI model could provide insights into the regulatory mechanisms of oxidative stress after SCI. This study aimed to identify oxidative stress-related diagnostic biomarkers for SCI and elucidate the expression characteristics and regulatory mechanisms of oxidative stress–related genes (OSRGs) in SCI. We also identified and further examined drugs that might modulate these genes. Our efforts will provide some new insights into SCI diagnosis and treatment.

## Methods

### Data source and general information

We accessed datasets GSE5296, GSE162610, and GSE151371 from the Gene Expression Omnibus (GEO) database (https://www.ncbi.nlm.nih.gov/gds). Dataset GSE5296 used Allen’s method to establish a moderate SCI model at the T8 segment in female C57BL/6 mice. It included a control group (*n* = 2) and six post-injury time points (0.5, 4, 24, 72 hours and 7 and 28 days), with three mice at each time point. GSE162610 similarly employed Allen’s method to induce moderate SCI at the T8 segment in female C57BL/6 mice. It also included a control group (*n* = 5) and three post-injury time points (1, 3, and 7 days) with respective mouse counts of five, three, and three (Milich et al., 2021). GSE151371 contained blood samples from three groups: 10 healthy uninjured controls (HC), 10 trauma controls without central nervous system injury (TC), and 38 SCI patients. SCI patients were stratified into four grades according to the AIS: AIS A (*n* = 12), AIS B (*n* = 4), AIS C (*n* = 6), and AIS D (*n* = 11). OSRGs were sourced from the Gene Set Enrichment Analysis (GSEA; https://www.gsea-msigdb.org/gsea/index.jsp) (Subramanian et al., 2005).

### Identification of differentially expressed genes and hub genes

The “Bioconductor limma” R package was employed to identify differentially expressed genes and differentially expressed OSRGs (DEOSRGs) between the injury and control groups (Yu et al., 2012). An adjusted *P*-value threshold of < 0.05 was used for gene selection. Visualization of these results was performed using the “limma” and “pheatmap” packages. We further analyzed DEOSRGs by constructing time-point specific protein-protein interaction (PPI) networks using the STRING database (https://cn.string-db.org/) (Szklarczyk et al., 2023), setting a medium confidence threshold of 0.4 and excluding unconnected nodes. Proteins present in at least four time-point specific PPI networks after injury were identified as hub genes.

### Functional enrichment analysis

Gene Ontology (GO) and Kyoto Encyclopedia of Genes and Genomes (KEGG) pathway analyses were performed on the DEOSRGs using the “org.mm.eg.db,” “clusterProfiler,” “ggplot2,” and “enrichment” packages (Gaudet and Dessimoz, 2017; Kanehisa et al., 2017). We set a significance cutoff of *P* < 0.05.

### Identification of diagnostic signature genes and immune change for spinal cord injury

Weighted correlation network analysis (WGCNA) was used to identify modules highly correlated with disease features in SCI, particularly focusing on AIS A and D grades (Jiang et al., 2021; Zhang et al., 2024). Hub genes associated with these modules were subsequently pinpointed. Using least absolute shrinkage and selection operator (LASSO) logistic regression (Jiang et al., 2021; Zhang et al., 2024), unique OSRGs were identified across different injury grades based on the pinpointed hub genes. Experimental datasets were then used to assess the diagnostic potential of these signature genes with receiver operating characteristic curve analysis. To explore immune cell status in SCI patients of varying grades, single sample gene set enrichment analysis was applied to determine the infiltration levels of immune cells in human blood (Wang et al., 2021, 2022). Spearman correlation analysis (Li et al., 2023) was performed to assess the relationship between biomarker expression and the prevalence of specific immune cells among 22 subpopulations in human blood. A significance threshold of *P* < 0.05 was used to establish correlations.

### Analysis of oxidative stress-related mechanisms at the single-cell level

The GSE162610 dataset was analyzed using a suite of R packages. The packages “Seurat,” “ggplot2,” “cowplot,” “Matrix,” “dplyr,” “ggsci,” and “SingleR” facilitated single-cell data integration, batch correction, dimensionality reduction, clustering, annotation, and visualization. For intercellular communication analysis, “Seurat,” “dplyr,” “tidyverse,” “stringr,” “CellChat,” “patchwork,” “mindr,” and “svglite” were used. The data were filtered to include only cells expressing more than 300 genes and to exclude genes present in fewer than five cells. Additionally, cells where mitochondrial genes constituted over 20% were removed. Dimensionality reduction and clustering were performed using uniform manifold approximation and projection (UMAP) and 22 principal components. Cell types were annotated using the “SingleR” package (Aran et al., 2019), CellMarker database (http://bio-bigdata.hrbmu.edu.cn/CellMarker/), and pertinent literature (Rosenberg et al., 2018; Sathyamurthy et al., 2018; Yang et al., 2022). We quantified cell subtype proportions, examined the expression of hub DEOSRGs across subtypes, and explored communication patterns among them.

### Oxidative stress–related gene set expression intensity analysis

The GSE162610 dataset was employed to assess the expression levels of OSRGs across various cell types in different SCI samples. The expression matrix was extracted from the Seurat object and a gene set specific to oxidative stress was constructed using the “GSEABase” package. The “AUCell” package was used to calculate the area under the receiver operating characteristic curve (AUC) for each cell, which quantified the OSRG intensity; higher AUC values indicated greater expression intensity. These values were incorporated into the Seurat object for further analysis. Visualization of the oxidative stress scores across the UMAP clusters was performed using the “Nebulosa” package.

### Immune infiltration analysis

A reference gene file for mouse, defining 11 subpopulations of innate immune cells, was downloaded (Li et al., 2022) and the Cell-type Identification By Estimating Relative Subsets Of RNA Transcripts (CIBERSORT) algorithm was employed to assess the infiltration of these cells in injured spinal cord tissue (Chen et al., 2018; Li et al., 2022). Spearman correlation analysis was used to explore the relationship between the expression of hub genes and the infiltration levels of specific innate immune cells among the 11 cell subpopulations in mouse spinal cord tissue. A significance threshold of *P* < 0.05 was applied to establish correlation.

### Animals

**Figure 1:**
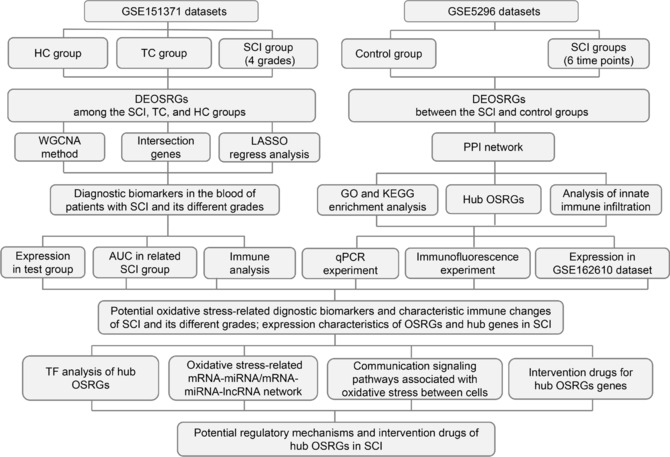
Study flowchart. AUC: Area under the curve; DEOSRGs: differentially expressed oxidative stress-related genes; GO: Gene Ontology; KEGG: Kyoto Encyclopedia of Genes and Genomes; HC: healthy uninjured control; LASSO: least absolute shrinkage and selection operator; lncRNA: long non-coding RNA; miRNA: microRNA; OSRGs: oxidative stress–related genes; PPI: protein–protein interaction; qPCR: quantitative polymerase chain reaction; SCI: spinal cord injury; TC: trauma control with noncentral nervous system injury; TF: transcription factor; WGCNA: weighted correlation network analysis.

Both mouse sequencing datasets exclusively used female mice, and therefore the experimental portion of our study also employed female mice to maintain consistency. We obtained 56 female C57BL/6J mice aged 7 weeks and weighing 21–23 g from Zhuhai Biotechnology Co., Ltd (Zhuhai, Guangdong, China, license No. SCXK (Yue) 2020-0051). The mice were housed under controlled conditions: 24 ± 2°C temperature, 55% ± 5% humidity, and a 12-hour light-dark cycle. They had access to food and water *ad libitum*, and bedding was changed regularly. All animal handling and experimental procedures complied with the guidelines approved by the Animal Care and Ethics Committee of Sun Yat-sen University (approval No. SYSU-IACUC-2021-000196, dated April 19, 2021). Mice were randomly divided into a control group (*n* = 6) and several SCI groups assessed at various post-injury time points: 0.5 hours (*n* = 3), 4 hours (*n* = 3), and 1, 3, 7, and 28 days (*n* = 6 each). The study’s workflow is shown in **Figure 1**.

### Spinal cord injury model establishment

For the SCI groups, mice were initially placed in a small animal anesthesia machine (RWD Life Science, Shenzhen, China) and anesthetized with 3% isoflurane. Maintenance anesthesia involved 1.8% inhalational isoflurane while the T8 segment was located, shaved, and disinfected. A 1.2 cm incision was made using microscissors to access the underlying tissues. The fascia and muscle layers were sequentially dissected to expose and excise the T10 vertebral lamina. A 10 g weight was then dropped from a height of 4 cm onto the spinal cord to induce SCI (Wu et al., 2022; Li et al., 2024a). During impact, the mice displayed transient spastic tail flicks and involuntary hind limb spasms, signifying successful model establishment. The site was then sutured in layers and disinfected. In the first week after injury, manual bladder expression was performed on the mice three times daily. In contrast, control mice only underwent laminectomy at the T8 segment without SCI or postoperative bladder management.

Prior to sample collection, all animals were anesthetized with an initial dose of 3% isoflurane, followed by a maintenance dose of 1.8% isoflurane. Cardiac perfusion with phosphate-buffered saline was performed on both control and SCI groups at predetermined time points. Spinal cord tissues around the T8 segment, approximately 8 mm in length, were collected for quantitative polymerase chain reaction analysis. Simultaneously, tissues approximately 1.2 mm in length centered on the T8 vertebral segment were harvested for immunofluorescence studies after perfusion with phosphate-buffered saline and 4% paraformaldehyde.

### Quantitative polymerase chain reaction

To evaluate the expression of OSRGs in spinal cord tissues from the control and various injury groups, RNA was extracted using the RNAeasy Animal RNA Extraction Kit (Beyotime, Shanghai, China). The RNA was then reverse transcribed to DNA using PrimeScript RT Master Mix (TAKARA, Dalian, China). Quantitative polymerase chain reaction on a Bio-Rad platform (Hercules, CA, USA) employing PowerUp SYBR reagent (Thermo Fisher Scientific, Waltham, MA, USA) was used to assess the expression levels of OSRGs after SCI. Primer sequences for these assays are listed in **Table 1**.

**Table 1:** Primers used for quantitative polymerase chain reaction

Gene	GenBank accession number	Sequence (5&–3&)	Product size (bp)
*Gapdh*	NM_008084	Forward: TGG AAT CCT GTG GCA TCC ATG AAA C	25
		Reverse: TAA AAC GCA GCT CAG TAA CAG TCC G	25
*Xdh*	NM_011723	Forward: ATG ACG AGG ACA ACG GTA GAT	21
		Reverse: TCA TAC TTG GAG ATC ATC ACG GT	23
*Ripk1*	NM_009068	Forward: GAA GAC AGA CCT AGA CAG CGG	21
		Reverse: CCA GTA GCT TCA CCA CTC GAC	21
*Mcl1*	NM_008562	Forward: AAA GGC GGC TGC ATA AGT C	19
		Reverse: TGG CGG TAT AGG TCG TCC TC	20
*Map2k3*	NM_008928	Forward: GCT CCC AGC GTA CCA GTT C	19
		Reverse: CGG GGT TCT TTC TTA GGC AC	20
*Hspb1*	NM_013560	Forward: ATC CCC TGA GGG CAC ACT TA	20
		Reverse: GGA ATG GTG ATC TCC GCT GAC	21
*Hbegf*	NM_001945	Forward: CGG GGA GTG CAG ATA CCT G	19
		Reverse: TTC TCC ACT GGT AGA GTC AGC	21
*Jun*	NM_010591	Forward: TTC CTC CAG TCC GAG AGC G	19
		Reverse: TGA GAA GGT CCG AGT TCT TGG	21
*Fos*	NM_010234	Forward: CGG GTT TCA ACG CCG ACT A	19
		Reverse: TTG GCA CTA GAG ACG GAC AGA	21

### Immunofluorescence staining

To analyze the protein expression of OSRGs in spinal cord tissue from the control and injury groups, tissues were dehydrated using 15% and 30% sucrose solutions (Biofroxx, Shanghai, China). Subsequently, they were embedded in optimal cutting temperature compound (SAKURA, Tokyo, Japan) and frozen at –20°C. Sections (10 μm thick) were cut using a cryostat (Thermo Fisher Scientific), fixed in pre-cooled 4% paraformaldehyde for 20 minutes, permeabilized with 0.3% Triton X-100 for 30 minutes, and blocked with 10% normal goat serum. Sections were incubated overnight at 4°C with primary antibodies: anti-Ripk1 (rabbit IgG, 1:200, Cell Signaling, Boston, MA, USA, Cat# 3493S, RRID: AB_2305314), anti-Neun (mouse IgG2b, 1:500, Thermo Fisher Scientific, Cat# MA5-33103, RRID: AB_2802653), and anti-Iba1 (mouse IgG1, 1:500, Thermo Fisher Scientific, Cat# MA5-27726, RRID: AB_2735228). The next day, sections were incubated at 37°C for 1 hour with secondary antibodies: Alexa Fluor 488 goat anti-rabbit IgG (1:200, Thermo Fisher Scientific, Cat# A-11034, RRID: AB_2576217) and Alexa Fluor 594 goat anti-mouse IgG (1:200, Thermo Fisher Scientific, Cat# A-11005, RRID: AB_2534073). Nuclei were counterstained with 4′,6-diamidino-2-phenylindole (Abcam, Cambridge, UK) for 10 minutes. Immunoreactivity was visualized using a confocal fluorescence microscope (Zeiss, Baden-Württemberg, Germany).

### MicroRNA and long non-coding RNA prediction

The microT, miRanda, and miRmap prediction tools from StarBase (https://starbase.sysu.edu.cn/index.php) were employed to identify microRNAs (miRNAs) interacting with key OSRGs in SCI. MiRNAs targeting both genes were categorized as hub miRNAs. Additionally, we used StarBase to predict interactions between these hub miRNAs and long non-coding RNAs (lncRNAs) across two datasets. We then constructed a regulatory network to map mRNA-miRNA and mRNA-miRNA-lncRNA interactions related to oxidative stress using Cytoscape software (Li et al., 2014; Kozomara et al., 2019).

### Transcription factor analysis

We investigated transcription factors linked to 25 genes by applying a fold change threshold of > 1.5 in the “Search by Target Gene” module of the KnockTF database (https://bio.liclab.net/KnockTFv2/index.php) (Feng et al., 2020). Then, the “ggraph” package was used to create regulatory networks, illustrating the interactions between these target genes and their associated transcription factors.

### Drug screening

Drugs that interact with key OSRGs were identified using the DGIdb database (https://dgidb.org/) (Yoo et al., 2015). The regulatory effects of these drugs on differentially expressed hub OSRGs were then examined at various post-SCI time points. Using Cytoscape software, an interaction map between the drugs and hub OSRGs was constructed. A murine innate immune cell gene set from the published literature was downloaded (Chen et al., 2017) and we assessed the infiltration of the immune cells in both the control and SCI groups using the CIBERSORT algorithm (Newman et al., 2015). Correlations between hub OSRGs and innate immune cells were further explored via Spearman correlation analysis (Li et al., 2022). Analyses and visual representations of the interactions among drugs, hub genes, and innate immune cells were performed using the “ggalluvial,” “readxl,” “ggplot2,” and “ggsci” packages.

### Statistical analysis

We did not use statistical methods to predetermine sample sizes; however, our samples align with a previous study (Li et al., 2024a). No animals or data points were excluded from the analysis. Investigators who were blinded to group assignments performed data collection and analysis. Statistical analyses were performed using Prism version 9.0.0 for Windows (GraphPad Software, Boston, MA, USA). Data representation varied by sample size and analysis type. Quantitative polymerase chain reaction data are presented as means ± standard error of the mean. Two-group comparisons were performed using the two-tailed unpaired Student’s *t*-test; one-way analysis of variance with Bonferroni’s *post hoc* test and Wilcoxon signed rank test were used for comparisons of multiple groups. *P* < 0.05 was considered significant.

## Results

### Identification of the expression characteristics of oxidative stress–related genes in the blood of patients with spinal cord injury

**Figure 2:**
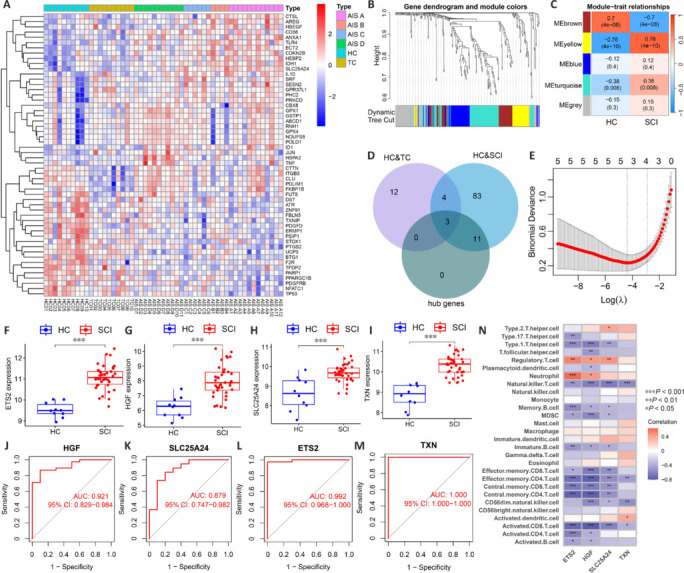
Dataset analysis reveals oxidative stress-related biomarkers in the blood of SCI patients. (A) Heatmap of DEOSRGs in the blood between different SCI groups and the HC group. (B) Dendrogram of OSRGs in SCI, where each branch corresponds to an individual gene, and each color represents a co-expression module. (C) Heatmap exhibits correlations between module signature genes and clinical features of SCI. Each row represents a module trait gene, and each column signifies a trait. (D) Identification of relatively specific DEOSRGs for SCI based on a Venn diagram. (E) LASSO logistic regression analysis. (F–I) Expression of biomarkers between the SCI and HC groups in the dataset. ***P < 0.001 (Student’s *t*-test). (J–M) AUC values of the biomarkers of SCI. (N) Heatmap of the correlation between blood biomarkers and immune cells. AIS: American Spinal Cord Injury Association Impairment Scale; AUC: area under the curve; CI: confidence interval; DEOSRGs: differentially expressed oxidative stress-related genes; HC: healthy uninjured control; LASSO: least absolute shrinkage and selection operator; SCI: spinal cord injury; TC: trauma control with non-central nervous system injury.

To investigate the expression patterns of OSRGs in SCI patients, we analyzed their expression in SCI blood samples. DEOSRGs were noted (**Figure 2A**, and **Additional Figure 1A**). Using WGCNA, a strong association was found between the MEyellow module and SCI, identifying 35 co-expressed OSRGs and 14 hub genes (**Figure 2B** and **C**). At the intersection of hub genes and DEOSRGs, 11 unique OSRGs were identified between the SCI and HC groups (**Figure 2D**). LASSO logistic regression of these 11 genes highlighted four key biomarkers that were significantly upregulated in the SCI group: *ETS2*, *HGF*, *SLC25A2*4, and *TXN* (**Figure 2E–I**). These biomarkers demonstrated high diagnostic accuracy, with AUC values of 0.992, 0.921, 0.879, and 1.000, respectively (**Figure 2J–M**). Correlation analyses showed that *ETS2* and *HGF* were positively associated with neutrophil and regulatory T cell levels; *SLC25A24* was positively associated with regulatory and type 2 helper T cells and *TXN* with activated dendritic cells (**Figure 2N**). Given the evident increase in neutrophils, regulatory T cells, and activated dendritic cells in the blood after SCI (**Additional Figure 1B**), detecting these biomarkers and immune cell changes in blood may aid in the diagnosis of SCI.

**Figure 3:**
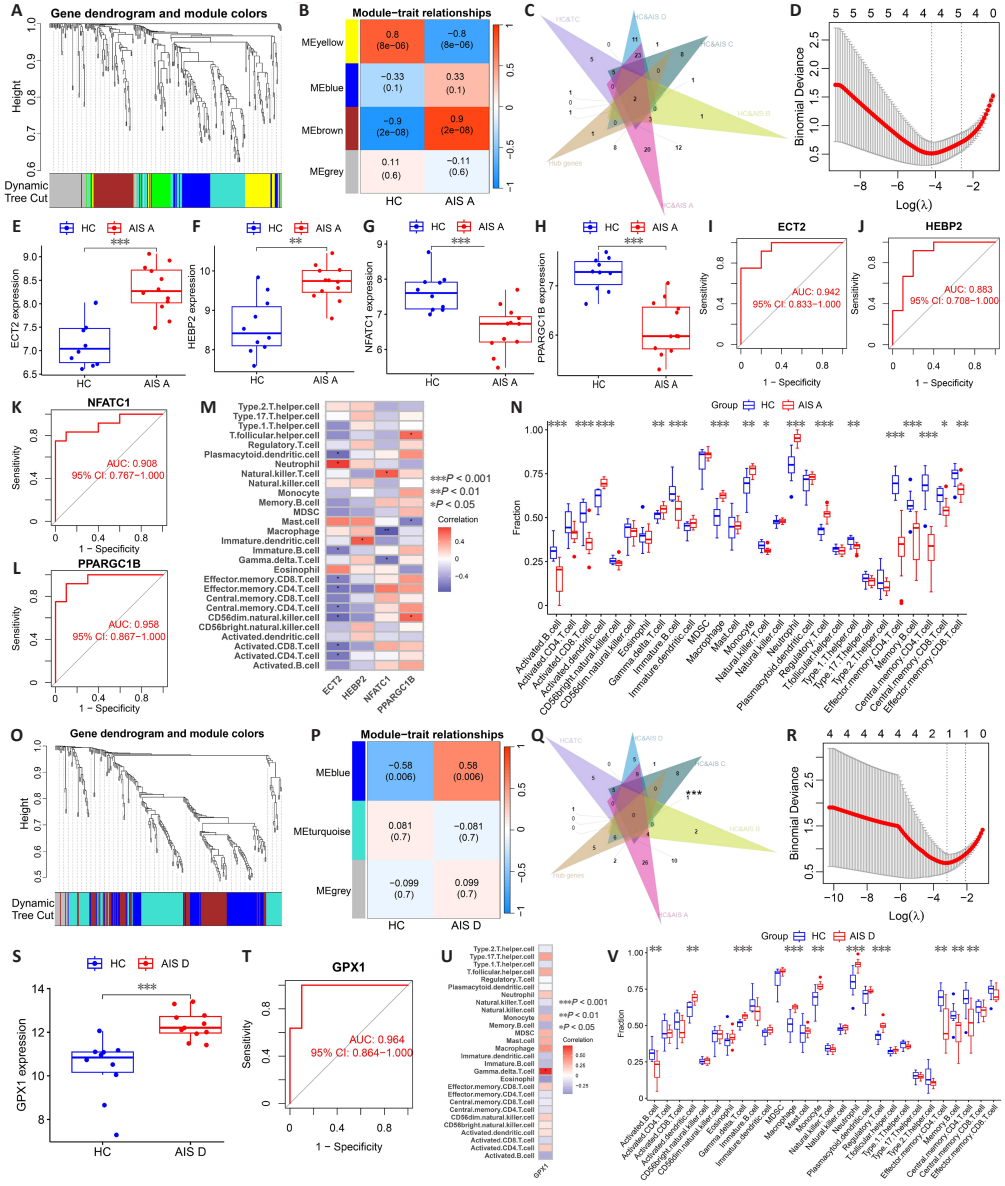
Dataset analysis reveals oxidative stress-related biomarkers in the blood of patients with AIS A and AIS D SCI. (A) Dendrogram of OSRGs in AIS A SCI. Each branch in the figure corresponds to an individual gene, and each color represents a co-expression module. (B) Heatmap displaying correlations between module signature genes and clinical features of AIS A SCI. Each row represents a module trait gene, while each column signifies a trait. (C) Identifying relatively specific DEOSRGs for AIS A SCI. (D) LASSO logistic regression analysis. (E–H) Expression of biomarkers in the dataset comprising the AIS A SCI and HC groups. (I–L) AUC values of the biomarkers of AIS A SCI. (M) Heatmap of correlation between blood biomarkers and immune cells of AIS A SCI. (N) Differences in the content of immune cells between the SCI and HC groups. (O) Dendrogram of OSRGs in AIS D SCI. Each branch in the figure corresponds to an individual gene, and each color represents a co-expression module. (P) Heatmap displaying correlations between module signature genes and clinical features of AIS D SCI. Each row represents a module trait gene, while each column signifies a trait. (Q) Identifying relatively specific DEOSRGs for AIS D SCI. (R) LASSO logistic regression analysis. (S) Expression of biomarkers in the dataset comprising the AIS D SCI and HC groups. (T) AUC values of the biomarkers of AIS D SCI. (U) Heatmap of correlation between blood biomarkers and immune cells of AIS D SCI. (V) Differences in the content of immune cells between the AIS D SCI and HC groups. Data are expressed as mean ± SEM. *P < 0.05, **P < 0.01, ***P < 0.001 (Wilcoxon signed rank test). AIS: American Spinal Cord Injury Association Impairment Scale; AUC: area under the curve; CI: confidence interval; DEOSRGs: differentially expressed oxidative stress–related genes; HC: healthy uninjured control; LASSO: least absolute shrinkage and selection operator; OSRGs: oxidative stress–related genes; SCI: spinal cord injury; TC: trauma control with noncentral nervous system injury.

In another WGCNA, the MEbrown module correlated strongly with AIS A, revealing 29 hub genes (**Figure 3A** and **B**). Eight OSRGs were uniquely identified at the intersection with DEOSRGs between the AIS A and HC groups (**Figure 3C**). Analysis via LASSO logistic regression of these eight genes identified four critical biomarkers: *ECT2*, *HEBP2*, *NFATC1*, and *PPARGC1B* (**Figure 3D**), showing significant differential expression in the AIS A group (**Figure 3E–H**). The AUC values for these biomarkers were 0.942, 0.883, 0.908, and 0.958, respectively (**Figure 3I–L**). Correlations indicated that ECT2 was positively linked with neutrophils, *HEBP2* with immature dendritic cells, *NFATC1* with natural killer T cells, and *PPARGC1B* with CD56 dim natural killer cells and T follicular helper cells (**Figure 3M**). Additionally, neutrophil content significantly increased in the blood of AIS A grade patients, while natural killer T cell content significantly decreased (**Figure 3N**). These changes in biomarkers and immune cells might assist in determining that a patient has an AIS A SCI. Lastly, the MEblue module showed a strong correlation with AIS D, identifying 47 hub genes (**Figure 3O** and **P**). Six unique OSRGs were pinpointed at the intersection with DEOSRGs between the AIS D and HC groups (**Figure 3Q**). LASSO logistic regression of these genes highlighted *GPX1* as a significant biomarker (**Figure 3R**), which was notably upregulated in the AIS D group (**Figure 3S**). The AUC for *GPX1* was 0.964 (**Figure 3T**), and its expression positively correlated with gamma delta T cell levels (**Figure 3U**). The elevated levels of these cells in AIS D patients suggest that monitoring *GPX1* and gamma delta T cells could aid in diagnosing AIS D SCI (**Figure 3V**).

### Identification and enrichment analysis of differentially expressed oxidative stress–related genes in spinal cord tissue

To characterize the tissue-level expression of OSRGs in injured spinal cord, we conducted bulk RNA sequencing on SCI tissue samples. DEOSRGs were identified across several time points after injury (0.5 and 4 hours and 1, 3, 7, and 28 days) (**Additional Figure 1C**, and **Additional Table 1**). By constructing a PPI network (**Additional Table 2**), hub genes present at four or more time points were pinpointed. These included *Xdh*, *Ripk1*, *Mcl1*, *Map2k3*, *Jun*, *Hspb1*, *Hbegf*, *Fos*, *Ucp2*, *Vcam1*, *Tnfrsf1a*, *Stat6*, *Sdc1*, *Rcan1*, *Ppp3ca*, *Map2k4*, *Il6st*, *Id1*, *Hdac1*, *Gch1*, *Fkbp1b*, *Fbxw7*, *Cbx6*, *Axl*, and *Amph* (**Additional Figure 1D**, and **Additional Table 3**). Functional enrichment analysis of the DEOSRGs revealed their involvement in various processes, including response to oxidative stress, regulation of cellular response to oxidative stress, oxidative stress-induced cell death, neuron death, apoptotic signaling pathway, and myeloid cell differentiation (**Additional Table 4**). Additionally, KEGG pathway analysis identified alterations in the TNF signaling pathway, apoptosis, peroxisome, and Toll-like receptor signaling pathway across time points (**Additional Table 5**).

**Additional Table 3:** The frequency of occurrence of hub genes in the protein-protein interaction network of differentially expressed oxidative stress-related genes between various time-point groups post-spinal cord injury and the control group

Gene	Number of time points
*Xdh*	5
*Ripk1*	5
*Mcl1*	5
*Map2k3*	5
*Jun*	5
*Hspb1*	5
*Hbegf*	5
*Fos*	5
*Ucp2*	4
*Vcam1*	4
*Tnfrsfla*	4
*Stat6*	4
*Sdc1*	4
*Rcan1*	4
*Ppp3ca*	4
*Map2k4*	4
*Il6st*	4
*Id1*	4
*Hdac1*	4
*Gch1*	4
*Fkbplb*	4
*Fbxw7*	4
*Cbx6*	4
*Ax1*	4
*Amph*	4

### Temporal expression of hub oxidative stress–related genes in spinal cord tissue

**Figure 4:**
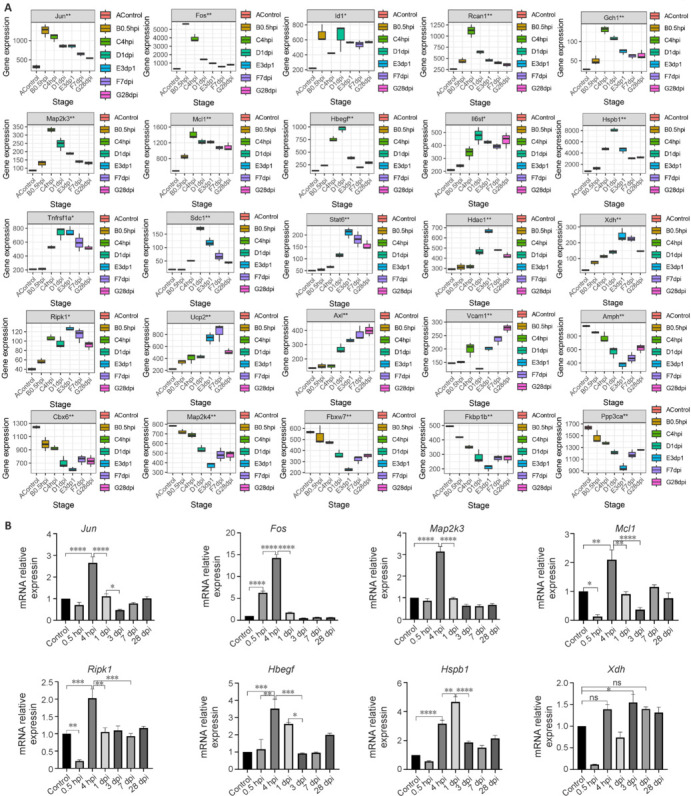
Expression trend of hub OSRGs after SCI. (A) Dataset analysis reveals the temporal dynamic expression trend of hub genes (*n* = 2 for control group; *n* = 3 for each SCI group). The y-axis indicates the relative expression levels of genes, while the x-axis represents each time point. (B) The qPCR experiments for SCI confirm the expression patterns of hub genes. Values normalized by the control group are expressed as mean ± SEM (*n* = 3 for each group). *P < 0.05, **P < 0.01, ***P < 0.001, ****P < 0.0001 (Wilcoxon signed rank test). dpi: Day post-injury; hpi: hour post-injury; OSRGs: oxidative stress–related genes; qPCR: quantitative polymerase chain reaction; SCI: spinal cord injury; SEM: standard error of the mean.

To delineate the temporal expression profiles of hub OSRGs in injured spinal cord, bulk RNA sequencing data was comprehensively analyzed. Jun and Fos exhibited peak expression 0.5 hours after SCI, whereas Id1 showed dual peaks at 0.5 hours and 1 day. The genes *Rcan1*, *Gch1*, *Map2k3*, and *Mcl1* reached their expression apex at 4 hours. *Hbegf*, *Il6st*, *Hspb1*, *Tnfrsf1a*, and *Sdc1* attained peak expression on the first day. Expression of *Stat6*, *Hdac1*, *Xdh*, and *Ripk1* peaked on the third day, while Ucp2 achieved its highest expression level on the seventh day. In contrast, Axl demonstrated a continuous increase in expression from 4 hours to 28 days. Vcam1 exhibited its lowest expression on the first day. The genes Amph, *Cbx6*, *Fbxw7*, *Fkbp1b*, *Ppp3ca*, and *Map2k4* were downregulated after injury, with their lowest expression noted on the third day. Quantitative polymerase chain reaction experiments corroborated these trends, showing consistent expression patterns for *Fos*, *Map2k3*, *Mcl1*, *Hspb1*, and Xdh, as indicated in our data analysis. Intriguingly, *Ripk1* peaked 4 hours after injury and exhibited a slight increase on the third day. This contrasts with *Jun*, which peaked at 4 hours, deviating from our initial analysis. Additionally, *Hbegf* reaches its maximum at 4 hours, unlike the peak suggested at 1 day post-injury in our data analysis. These insights enhance our understanding of the dynamic expression patterns of hub OSRGs in SCI, offering promising avenues for advancing research and treatment (**Figure 4B**).

### Expression of hub oxidative stress–related genes at the single-cell level in spinal cord tissue

**Figure 5:**
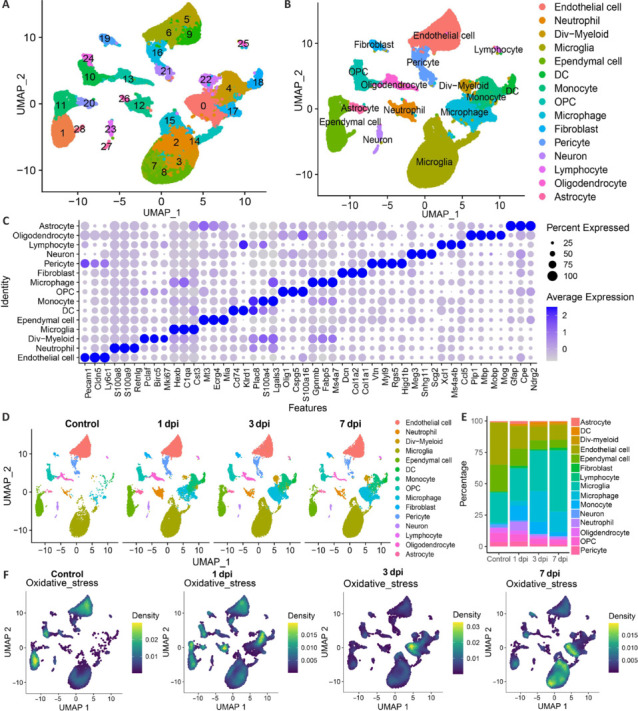
Dataset analysis demonstrates the expression characteristics of OSRGs at the single-cell level. (A) UMAP plot of single-cell clustering in SCI. (B) UMAP plot with annotations of single-cell clusters in SCI. (C) Bubble plot of cell type marker genes in SCI. The vertical axis represents the cell type, the horizontal axis represents the gene name, the size of the circle indicates the proportion of cells in the cell type that express the gene, and the color depth of the circle indicates the level of gene expression. (D) UMAP plot showing changes in single-cell clusters in SCI. (E) Proportions of different cell types in various groups. (F) Temporal expression of OSRG sets at the single-cell level in SCI. The brightness of the color represents the expression intensity of the gene set within the cell population. As the color becomes brighter, the expression intensity of the gene set within the cell population increases. OSRGs: oxidative stress–related genes. dpi: day post-injury; SCI: spinal cord injury; UMAP: uniform manifold approximation and projection.

To elucidate the expression characteristics of hub OSRGs at the single-cell level in injured spinal cord, we analyzed single-cell RNA sequencing data. This analysis facilitated the creation of a SCI-specific single-cell atlas, which delineated 29 distinct cell clusters (**Figure 5A**). After this, we annotated 15 distinct cell types, as shown in **Figures 5B** and **C**. Our longitudinal analysis of cell subtype proportions revealed that neutrophils and monocytes peaked on the first day after SCI, whereas macrophages predominated on the third day. Microglia reached their highest proportion by the seventh day (**Figures 5D** and **E**). Furthermore, the expression patterns of OSRGs were characterized across various cell types and temporal stages. Initially, in the control samples, these genes were primarily expressed in endothelial cells and pericytes. By the first day after SCI, prominent expression was observed in endothelial cells, pericytes, ependymal cells, fibroblasts, neutrophils, and differentiated myeloid cells. On the third day, expression increased significantly in macrophages and fibroblasts, while it notably declined in endothelial cells. By the seventh day, OSRGs were mainly expressed in endothelial cells, fibroblasts, and differentiated myeloid cells (**Figure 5F** and **Additional Figure 2**).

**Figure 6:**
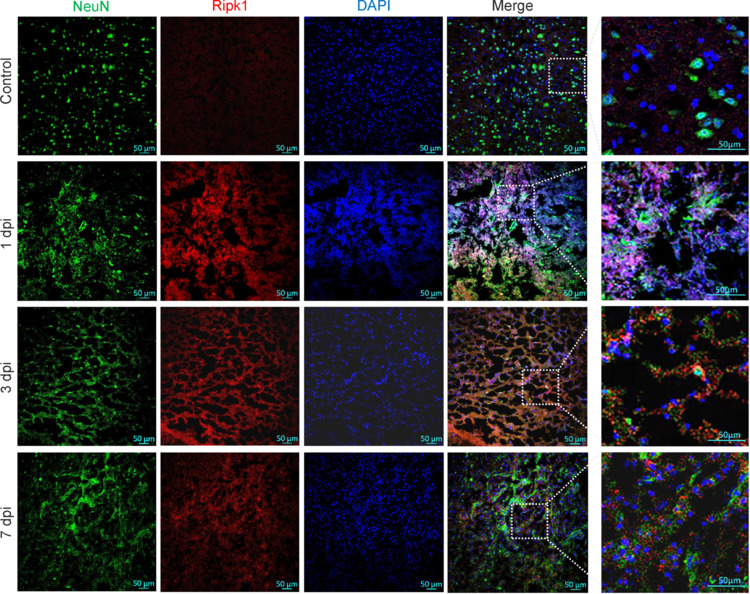
Immunofluorescence staining of spinal cord tissue for Neun (green, Alexa Fluor 488) and Ripk1 (red, Alexa Fluor 594) immunoreactivity. Ripk1 expression levels were higher and the number of Neun-expressing cells was significantly lower at various time points in the SCI groups than the control group. However, the fluorescence signals of Ripk1 and Neun overlapped after SCI. Additionally, Ripk1 expression levels were somewhat higher on day 1 after SCI than on days 3 and 7. Scale bar: 50 μm. dpi: Day post-injury; SCI: spinal cord injury.

Subsequently, we evaluated the expression of key OSRGs at the single-cell level. In both the control and 1-day post-injury groups, the genes *Xdh*, *Ripk1*, *Mcl1*, *Map2k3*, *Gch1*, and *Stat6* demonstrated elevated expression in endothelial cells, pericytes, and immune cells. The genes *Hspb1* and *Hbegf* showed increased expression in endothelial cells, ependymal cells, oligodendrocyte precursor cells (OPCs), and astrocytes, both before and after injury. Additionally, *Jun*, *Fos*, *Tnfrsf1a*, *Hdac1*, and *Ucp2* were significantly expressed across various cell types after injury. Id1 exhibited increased expression in endothelial cells and pericytes at both time points. In the 1-day post-injury group, Rcan1 was notably upregulated in ependymal cells, astrocytes, and immune cells. Il6st and Ppp3ca were consistently expressed at higher levels in various cell types in both the control and 1-day post-injury groups. *Sdc1* was upregulated in macrophages and monocytes in the 1-day post-injury group, while *Axl* showed increased expression in pericytes, fibroblasts, OPCs, and immune cells at different post-injury time points. *Vcam1*, *Amph*, *Cbx6*, *Fbxw7*, *Fkbp1b*, and *Map2k4* exhibited relatively lower expression across cell types (refer to **Additional Figures 3–6**). Furthermore, immunofluorescence experiments revealed co-expression of *Ripk1* with *Neun* and *Iba1*. Enhanced expression of *Ripk1* was observed, indicating its upregulation in neurons and microglia after SCI (**Figure 6** and **Additional Figure 7**).

### Analysis of innate immune cell infiltration in spinal cord tissue

To explore the characteristics of oxidative stress related to innate immune cell infiltration, we investigated the association between the expression of hub OSRGs and the infiltration of innate immune cells in the injured spinal cord tissue. Our findings indicated both positive and negative correlations between the expression of these hub genes and the degree of innate immune cell infiltration (**Additional Figures 8–10**). These observations suggest that specific innate immune cells play a crucial role in the oxidative stress response.

### Cell communication analysis in spinal cord tissue

**Figure 7:**
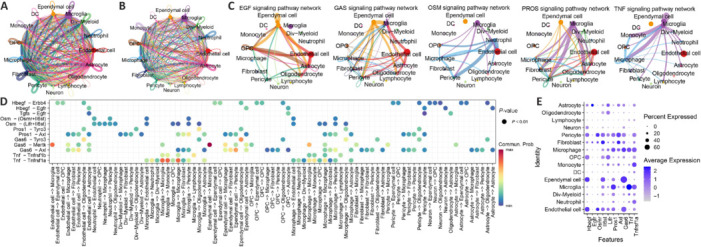
Dataset analysis shows the communication between cells in the control group. (A) Number of intercellular communications. The thickness of the lines represents the number of communications. As the lines becomes thicker, the number of communications increases. (B) Strength of intercellular communication. The thickness of the lines represents the strength of communication. As the lines become thicker, the communication intensity increases. (C) Intercommunication strength of various signaling pathways in the control group among different cell types. The thickness of the lines represents the strength of communication. As the lines become thicker, the communication intensity increases. (D) Communication characteristics of ligand-receptor pairs of OSRGs in signaling pathways among various cell types. Circle size represents the magnitude of *P*-values: as the circle becomes larger, the *P*-value decreases. Color represents the strength of communication: as the color becomes redder, the communication intensity increases; as the color becomes bluer, the communication intensity decreases. (E) Expression of ligand-receptor pairs of OSRGs in various cell types. The size of the circles represents the proportion of cells expressing a particular gene in a specific cell type, and the color intensity indicates the expression level. OSRGs: Oxidative stress–related genes; SCI: spinal cord injury.

To elucidate the potential oxidative stress-related communication regulatory mechanisms among different cells, cellular communication was analyzed across all samples. This analysis revealed variations in both the quantity and intensity of intercellular communications across various cell types, as shown in **Figure 7A** and **B**. We then conducted detailed analyses for the control group and at different post-injury time points to characterize the communication patterns between cells. The key signaling pathways in the control group that featured ligand-receptor pairs containing OSRGs included the EGF signaling pathway (*Hbegf*-*Erbb4*, *Hbegf*-*Egfr*), GAS signaling pathway (Gas6-Axl), OSM signaling pathway (*Osm*-*Osmr*+*Il6st*, *Osm*-*Lifr*+*Il6st*), PROS signaling pathway (*Pros1*-*Axl*), and TNF signaling pathway (*Tnf*-*Tnfrsf1a*), as shown in **Figure 7C** and **D**.

Expression levels of specific ligand-receptor pairs were analyzed across various cell types (**Figure 7E**), identifying distinct communication patterns. Notably, *Hbegf*-*Erbb4* interactions were primarily observed among endothelial cells, ependymal cells, OPCs, and pericytes, including between OPCs with limited processes. By contrast, *Hbegf*-*Egfr* interactions occurred between endothelial cells, ependymal cells, OPCs, pericytes, and astrocytes. *Pros1*-*Axl* pairs facilitated communication between endothelial cells, dividing myeloid cells, microglia, ependymal cells, macrophages, and pericytes, as well as between macrophages, fibroblasts, and pericytes. Additionally, *Gas6*-*Axl* pairs were involved in interactions between endothelial cells, ependymal cells, macrophages, and pericytes, and among macrophages, fibroblasts, and pericytes. Lastly, Tnf-Tnfrsf1a interactions predominantly occurred among microglia, macrophages, neutrophils, OPCs, monocytes, and pericytes, highlighting their role in communication within the spinal cord microenvironment.

**Figure 8:**
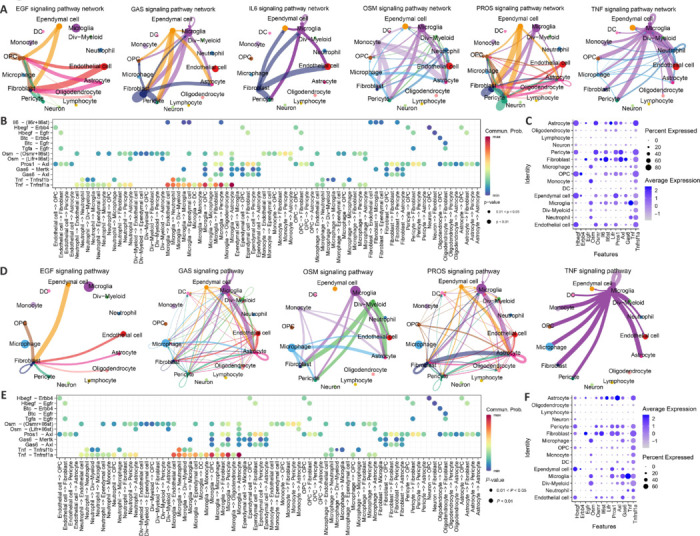
Dataset analysis demonstrates the communication between cells in the 1- and 3-day post-SCI groups. (A) Communication signal pathways among various cell types on the first day after SCI. The thickness of the lines represents the strength of communication. As the lines become thicker, the communication intensity increases. (B) Ligand-receptor pairs within communication signal pathways containing OSRGs among various cell types on the first day after SCI. Circle size represents the magnitude of *P*-values: as the circle becomes larger, the *P*-value decreases. Color represents the strength of communication: as the color becomes redder, the communication intensity increases; as the color becomes bluer, the communication intensity decreases. (C) Expression of ligand-receptor pairs within various cell types on the first day after SCI. The size of the circles represents the proportion of cells expressing a particular gene in a specific cell type, and the color intensity indicates the expression level. (D) Communication signal pathways among various cell types on the third day after SCI. The thickness of the lines represents the strength of communication. As the lines become thicker, the communication intensity increases. (E) Characteristics of ligand-receptor pairs within communication signal pathways containing OSRGs among various cell types on the third day after SCI. Circle size represents the magnitude of *P*-values: as the circle becomes larger, the *P*-value decreases. Color represents the strength of communication: as the color becomes redder, the communication intensity increases; as the color becomes bluer, the communication intensity decreases. (F) Expression of ligand-receptor pairs within various cell types on the third day after SCI. The size of the circles represents the proportion of cells expressing a particular gene in a specific cell type, and the color intensity indicates the expression level. OSRGs: Oxidative stress–related genes; SCI: spinal cord injury.

On the first day after SCI, OSRGs were identified within the ligand-receptor pairs of various signaling pathways, including EGF (*Hbegf*-*Erbb4*, *Hbegf*-*Egfr*), GAS (*Gas*-*Axl*), IL6 (*Il6*-(*Il6r*+*Il6st*)), OSM (*Osm*-(*Osmr*+*Il6st*), Osm-(*Lifr*+*Il6st*)), PROS (*Pros1*-*Axl*), and TNF (*Tnf*-*Tnfrsf1a*) (**Figure 8A** and **B**). Expression analysis across cell types (**Figure 8C**) revealed that Hbegf-Erbb4 predominantly facilitated interactions among endothelial cells, ependymal cells, OPCs, pericytes, and astrocytes. Similarly, *Hbegf*-*Egfr* was crucial for communication between the same cell groups, including fibroblasts. The *Pros1*-*Axl* pair was involved in cross-talk among endothelial cells, ependymal cells, OPCs, pericytes, fibroblasts, and astrocytes. *Gas*-*Axl* was associated with interactions between microglia and both fibroblasts and astrocytes. *Tnf*-*Tnfrsf1a* mainly supported communication across microglia, neutrophils, endothelial cells, ependymal cells, OPCs, fibroblasts, pericytes, and astrocytes, and also between macrophages and several of these cell types. *Osm*-(*Osmr*+*Il6st*) and *Osm*-(*Lifr*+*Il6st*) largely mediated communication between neutrophils, monocytes, macrophages, divergent myeloid cells, and various immune cells. *Il6*-(*Il6r*+*Il6st*) predominantly enabled communication between fibroblasts, endothelial cells, and pericytes. These results indicate that these signaling pathways and their associated ligand-receptor pairs play key roles in mediating communication and regulating oxidative stress responses among different cells on the first day after injury.

On the third day after SCI, OSRGs were identified in ligand-receptor pairs of signaling pathways, including EGF (*Hbegf*-*Erbb4*, *Hbegf*-*Egfr*), GAS (*Gas*-*Axl*), OSM (*Osm*-(*Osmr*+*Il6st*), Osm-(*Lifr*+*Il6st*)), PROS (*Pros1*-*Axl*), and TNF (*Tnf*-*Tnfrsf1a*) (**Figure 8D** and **E**). Considering the expression levels across cell types (**Figure 8F**), *Hbegf*-*Erbb4* primarily mediated communication between endothelial cells, ependymal cells, and pericytes with ependymal cells. *Hbegf*-*Egfr* played a significant role in communication between endothelial cells, ependymal cells, pericytes, and fibroblasts. *Osm*-(*Osmr*+*Il6st*) and *Osm*-(*Lifr*+*Il6st*) were primarily involved in mediating communication between microglia, macrophages, dividing myeloid cells, and other immune cells with various cell types. The *Pros1*-*Axl* ligand-receptor pair played a significant role in communication between microglia, ependymal cells, pericytes, fibroblasts, and fibroblasts, pericytes, and astrocytes. *Gas*-*Axl* ligand-receptor pairs mediated communication between microglia and fibroblasts, pericytes, and astrocytes. Additionally, *Tnf*-*Tnfrsf1a* was involved in communication between microglia and other cell types. These results suggest that these signaling pathways and ligand receptors may contribute to the communication and regulation of oxidative stress responses among different cells on the third day after injury.

**Figure 9:**
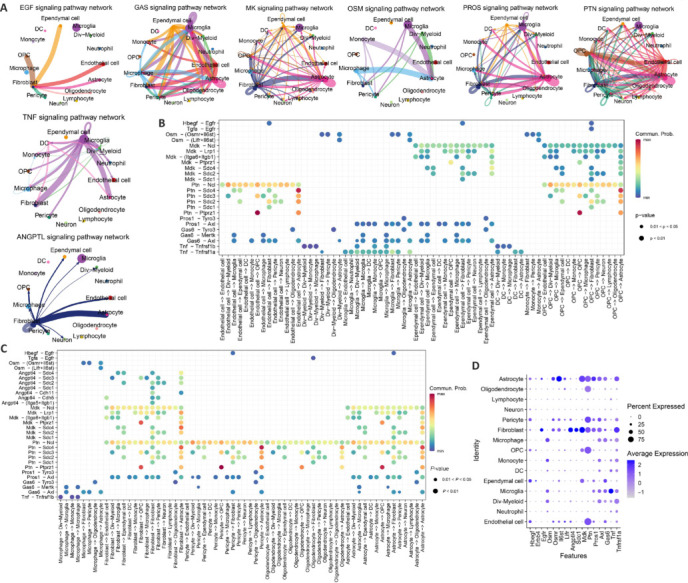
Dataset analysis demonstrates the communication between cells in SCI. (A) Communication signaling pathways between various cell types on the seventh day after SCI. The thickness of the lines represents the strength of communication. As the lines become thicker, the communication intensity increases. (B, C) Ligand-receptor pairs involved in OSRG-mediated communication signaling pathways between various cell types on the seventh day after SCI. Circle size represents the magnitude of *P*-values: as the circle increases in size, the *P*-value decreases. Color represents the strength of communication, the redder the color, the higher the communication intensity; the bluer the color, the lower the communication intensity. (D) Expression levels of ligand-receptor pairs in cells on the seventh day after SCI. The size of the circles represents the proportion of cells expressing a particular gene in a specific cell type, and the color intensity indicates the expression level. OSRG: Oxidative stress–related gene; SCI: spinal cord injury.

Seven days after SCI, OSRGs were identified within several signaling pathways, including EGF (*Hbegf*-*Erbb4*, *Hbegf*-*Egfr*), GAS (*Gas*-*Axl*), OSM (*Osm*-(*Osmr*+*Il6st*), Osm-(*Lifr*+*Il6st*)), PROS (*Pros1*-*Axl*), TNF (*Tnf*-*Tnfrsf1a*), ANGPTL (*Angptl4*-*Sdc1*), PTN (*Ptn*-*Sdc1*), and MK (*Mdk*-*Sdc1*), as shown in **Figures 9A–C**. Analysis of their expression across cell types (**Figure 9D**) revealed that Hbegf-Egfr primarily facilitates communication among endothelial cells, ependymal cells, pericytes, and fibroblasts. Osm-related pairs, *Osm*-(*Osmr*+*Il6st*) and *Osm*-(*Lifr*+*Il6st*), were mainly involved in mediating interactions between macrophages, monocytes, microglia, dividing myeloid cells, and other cell types. *Mdk*-*Sdc1* and *Angptl4*-*Sdc1* were central to communication within fibroblasts. *Ptn*-*Sdc1* played a crucial role in connecting endothelial cells, OPCs, pericytes, astrocytes, and fibroblasts. *Pros1*-*Axl* was key in communications involving microglia, ependymal cells, pericytes, astrocytes, and fibroblasts. *Gas*-*Axl* pairs contributed to communication between microglia, endothelial cells, fibroblasts, pericytes, and astrocytes. Additionally, *Tnf*-*Tnfrsf1a* facilitated communication between microglia and various cells, including fibroblasts and astrocytes. These findings show the involvement of these signaling pathways and ligand-receptor pairs in regulating oxidative stress responses among different cells on the seventh day after injury.

### Prediction of miRNA and lncRNA related to oxidative stress–related genes in spinal cord tissue

**Figure 10:**
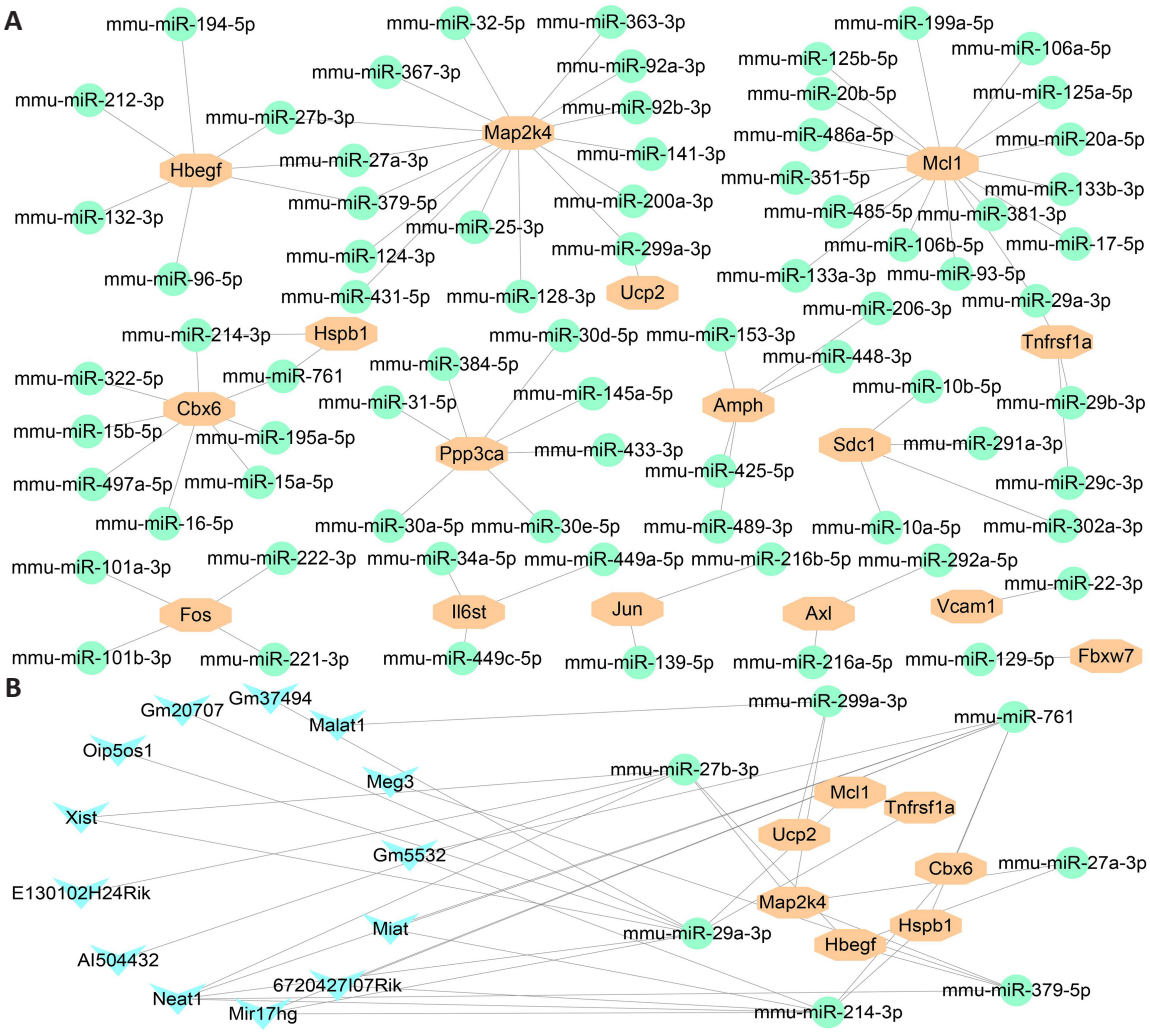
Dataset analysis demonstrates the relationship between hub OSRGs and non-coding RNAs after SCI. (A) The relationship between genes and miRNAs. Green ellipses represent miRNAs, and orange polygons represent hub OSRGs. (B) The mRNA-miRNA-lncRNA regulatory network related to oxidative stress after SCI. Green circles represent miRNAs, blue diamonds represent lncRNAs, and orange polygons represent hub OSRGs. lncRNA: LSong non-coding RNA; miRNA: microRNA; OSRGs: oxidative stress-related genes; SCI: spinal cord injury.

To identify the miRNAs and lncRNAs regulating hub OSRGs after SCI, the StarBase platform was used to predict 74 miRNAs interacting with these hub OSRGs. Among these, seven were designated as hub miRNAs owing to their interactions with at least two genes (**Figure 10A** and **Additional Table 6**). On the basis of these hub miRNAs, we further predicted 13 lncRNAs that interact with them, leading to the construction of an mRNA-miRNA-lncRNA interaction network (**Figure 10B** and **Additional Table 7**). These findings suggest that these miRNAs and lncRNAs can significantly influence the regulation of OSRG expression in SCI.

### Transcription factor prediction for oxidative stress–related genes in spinal cord tissue

**Figure 11:**
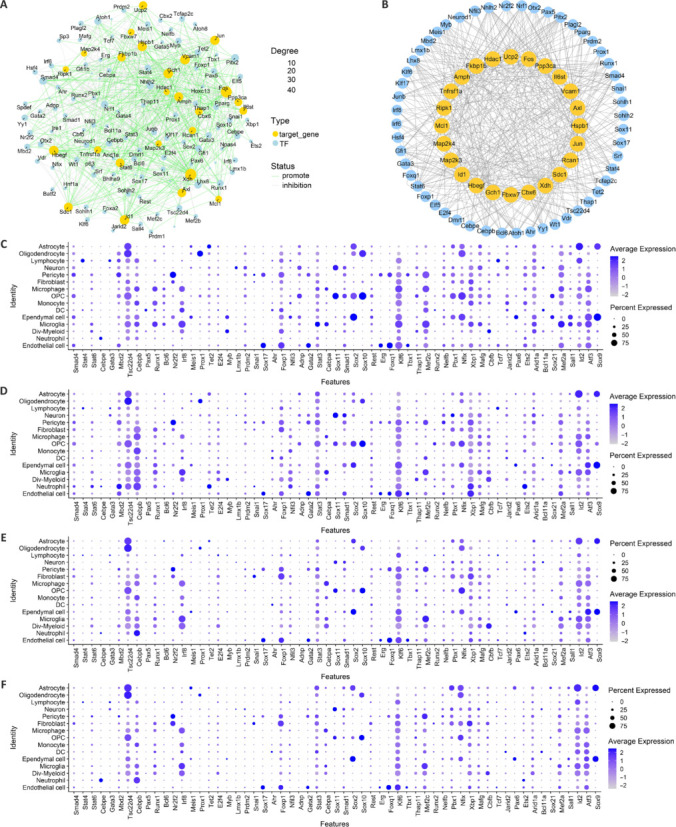
Dataset analysis predicts the TFs regulating hub OSRGs. (A) TF-target gene network diagram. Yellow circles represent target genes, and blue circles represent TFs, with larger circles indicating higher connectivity. Labeled circles in the graph denote those with a connectivity > 5. Green lines represent promotional interactions, while gray lines represent inhibitory interactions. (B) The regulatory network of TFs with high expression and a degree of at least 5 in relation to hub genes. Blue circles represent TFs, and yellow circles represent hub genes. (C) The expression of TFs with high activity in various cell types of the control group. (D) The expression of TFs with high activity in various cell types of the group one day after SCI. (E) The expression of TFs with high activity in various cell types of the group 3 days after SCI. (F) The expression of TFs with high activity in various cell types of the group 7 days after SCI. In C-F, the circle sizes depict the proportion of cells expressing a specific gene within a particular cell type, while the color intensity reflects the expression level. SCI: Spinal cord injury; TF: transcription factor.

To elucidate the transcriptional regulatory characteristics of hub OSRGs, the KnockTF database was used to identify 104 transcription factors interacting with these hub OSRGs, including those exerting inhibitory effects on their target genes (**Figure 11A** and **Additional Table 8**). We then assessed the expression levels of these transcription factors at the single-cell level, highlighting those with elevated activity in the control and various injury groups (**Figure 11C–F**), and mapped the regulatory networks involving active transcription factors with a high degree of connectivity (≥ 5) to hub genes (**Figure 11B**). By integrating these expression profiles across different cellular contexts and time points after injury (1, 3, and 7 days), we characterized the dynamic interactions between transcription factors and their target genes (**Additional Table 9**). These insights may inform targeted interventions to mitigate oxidative stress in SCI.

### Drug screening for oxidative stress–related genes in spinal cord tissue

**Figure 12:**
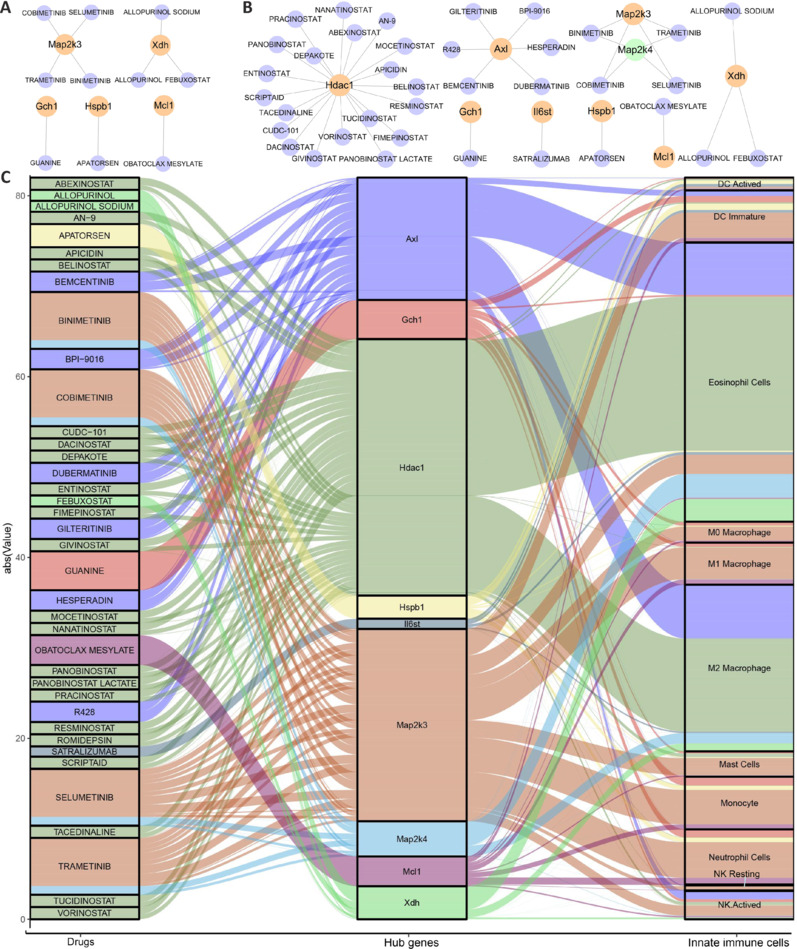
Dataset analysis predicts drugs that regulate hub OSRGs. (A) Drugs that can exert inhibitory effects on four upregulated hub genes 4 hours after SCI. Blue circles represent drugs and orange circles represent up regulated hub genes. (B) Drugs that can exert inhibitory effects on eight upregulated hub genes and one downregulated hub gene 1, 3, 7, and 28 days after SCI. Blue circles represent drugs, orange circles represent upregulated hub genes, and green circles represent downregulated hub genes. (C) The relationship among drugs, hub genes, and innate immune cells. The left column represents drugs, the middle column represents hub genes, and the right column represents innate immune cells. As the lines become wider, the degree of association increases. OSRG: oxidative stress-related gene; SCI: spinal cord injury.

To identify drugs that modulate hub OSRGs, the DGIdb database was screened for compounds targeting hub genes such as *Xdh*, *Mcl1*, *Map2K3*, *Hspb1*, *Map2K4*, *Il6St*, *Hdac1*, *Gch1*, and *Axl*, resulting in 38 potential candidates (**Additional Table 10**). Four hours after injury, 10 drugs were found to inhibit five upregulated genes (**Figure 12A**). On days 1, 3, 7, and 28, several of these drugs demonstrated inhibitory effects on specific genes among eight upregulated and one downregulated OSRGs (**Figure 12B**). Additionally, four distinct drugs influenced two differentially expressed hub genes, *Map2k3* and *Map2k4*. We further explored the interactions between these drugs, the nine hub genes, and intrinsic immune cells to provide a basis for future research into targeted modulation of oxidative stress in SCI-related innate immune responses (**Figure 12C** and **Additional Table 11**).

**Additional Table 10:** The 38 drugs that demonstrate modulatory effects on the nine hub genes

Gene	Drug	Interaction_types	Sources
*XDH*	ALLOPURINOL	Inhibitor	T dgClinicalT rial | ChemblInteractionsj TEND | PharmGKB | TTD
*XDH*	FEBUXOSTAT	Inhibitor	T dgClinicalT rial | ChemblInteractionsj TEND | PharmGKB | TTD
*XDH*	ALLOPURINOL SODIUM	Inhibitor	ChemblInteractions
*MCL1*	OBATOCLAX MESYLATE	Inhibitor	ChemblInteractions
*MAP2K3*	TRAMETINIB	Inhibitor	MyCancerGenomeClinicalT rial
*MAP2K3*	SELUMETINIB	Inhibitor	MyCancerGenomeClinicalT rial
*MAP2K3*	BINIMETINIB	Inhibitor	MyCancerGenomeClinicalTrial
*MAP2K3*	COBIMETINIB	Inhibitor	MyCancerGenomeClinicalTrial
*HSPB1*	APATORSEN	Inhibitor	TdgClinicalTrial|CancerCommons|TTD
*MAP2K4*	TRAMETINIB	Inhibitor	MyCancerGenomeClinicalTrial
*MAP2K4*	COBIMETINIB	Inhibitor	MyCancerGenomeClinicalTrial
*MAP2K4*	SELUMETINIB	Inhibitor	MyCancerGenomeClinicalTrial
*MAP2K4*	BINIMETINIB	Inhibitor	MyCancerGenomeClinicalTrial
*IL6ST*	SATRALIZUMAB	Antagonist	ChemblInteractions
*HDAC1*	ROMIDEPSIN	Inhibitorjantagonist	TALC|MyCancerGenome|TdgClinicalTrial|ChemblInteractions|TEND|TTD
*HDAC1*	PRACINOSTAT	Inhibitor	TdgClinicalTrial
*HDAC1*	SCRIPTAID	Inhibitor	DTC|TTD
*HDAC1*	PANOBINOSTAT	Inhibitor	TALC|DTC|MyCancerGenome|TdgClinicalT rial | CancerCommons| TTD
*HDAC1*	ABEXINOSTAT	Inhibitor	TALC|TdgClinicalTrial
*HDAC1*	NANATINOSTAT	Inhibitor	TALC|TTD
*HDAC1*	ENTINOSTAT	Inhibitor	TALC|DTC|TdgClinicalTrial|ChemblInteractions|TTD
*HDAC1*	VORINOSTAT	Inhibitor	TALC|DTC|MyCancerGenome|TdgClinicalTrial|ChemblInteractions|TEND|TTD
*HDAC1*	CUDC-101	Inhibitor	TALC|TdgClinicalT rial | ChemblInteractions
*HDAC1*	FIMEPINOSTAT	Inhibitor	ChemblInteractions
*HDAC1*	TACEDINALINE	Inhibitor	DTC| ChemblInteractions
*HDAC1*	DEPAKOTE	Inhibitor	TALC
*HDAC1*	RESMINOSTAT	Inhibitor	T dgClinicalT rial | TTD
*HDAC1*	BELINOSTAT	Inhibitor	TALC|DTC|MyCancerGenome|TdgClinicalT rial | ChemblInteractions
*HDAC1*	PANOBINOSTAT LACTATE	Inhibitor	ChemblInteractions
*HDAC1*	GIVINOSTAT	Inhibitor	TALC|TdgClinicalTrial|TTD
*HDAC1*	TUCIDINOSTAT	Inhibitor	TTD
*HDAC1*	MOCETINOSTAT	Inhibitor	DTC|TdgClinicalTrial| ChemblInteractions|TTD
*HDAC1*	AN-9	Inhibitor	TALC
*HDAC1*	APICIDIN	Inhibitor	DTC
*HDAC1*	DACINOSTAT	Inhibitor	TTD
*GCH1*	GUANINE	Inhibitor	TTD
*AXL*	GILTERITINIB	Inhibitor	ChemblInteractions| TTD
*AXL*	HESPERADIN	Inhibitor	DTC
*AXL*	DUBERMATINIB	Inhibitor	TTD
*AXL*	R428	Inhibitor	ChemblInteractions
*AXL*	BPI-9016	Inhibitor	ChemblInteractions
*AXL*	BEMCENTINIB	Inhibitor	TTD

## Discussion

After SCI occurs, oxidative stress responses are rapidly exhibited in spinal cord tissue and the blood (Takahashi et al., 2019; Heller et al., 2021; Zhang et al., 2021). This study identified *ETS2*, *HGF*, *SLC25A24*, and *TXN* as oxidative stress-related biomarkers of SCI. These biomarkers are upregulated after SCI and correlate with increased blood levels of neutrophils, regulatory T cells, and activated dendritic cells. *ETS2* and *HGF* are positively associated with neutrophil and regulatory T cell infiltration. *SLC25A24* correlates with regulatory T cell levels, and *TXN* with activated dendritic cells. These concurrent increases suggest that the combined elevation of these genes and cells is indicative of SCI. Monitoring these biomarkers may facilitate SCI severity diagnosis.

We also identified *ECT2*, *HEBP2*, *NFATC1*, and *PPARGC1B* as specific to AIS A SCI. After SCI, expression of both *ECT2* and *HEBP2* significantly increases, whereas expression of *NFATC1* and *PPARGC1B* decreases. In AIS A patients, there is an increase in neutrophil levels and a decrease in natural killer T cell levels. *ECT2* correlates positively with neutrophil infiltration, and *NFATC1* with natural killer T cell levels. Thus, the simultaneous elevation of *ECT2* and *HEBP2* with neutrophils and their concurrent decrease with natural killer T cells may be diagnostic for AIS A SCI. For AIS D SCI, *GPX1* was identified as a key biomarker and was significantly upregulated after injury. Patients with AIS D SCI also exhibited increased γδ T cell levels. Given the positive correlation between *GPX1* expression and γδ T cell infiltration, the concurrent rise of *GPX1* and γδ T cells could serve as a diagnostic marker for AIS D SCI. Measuring these indicators may help diagnose this specific SCI condition.

The characteristics and regulatory mechanisms of OSRG expression in spinal cord tissue may vary at different time points after SCI (David et al., 2023; Liu et al., 2023). RNA sequencing data revealed DEOSRGs as early as 0.5 hours after injury, with the highest expression peak occurring 3 days after injury, indicating an early robust oxidative stress response. Hub genes were identified by their consistent presence in PPI networks across different times and their expression patterns were analyzed over time. Single-cell analysis further delineated the expression profiles of these genes in various cell types. Elevated expression of these hub genes was observed in immune cells infiltrating from peripheral blood into spinal cord tissue, prompting investigations into correlations between OSRG expression levels and immune cell infiltration. This revealed both positive and negative associations with specific innate immune cells, highlighting their distinct roles in the oxidative stress response to SCI.

To explore the potential links between regulatory mechanisms of communication and oxidative stress, we gathered data on cell signaling pathways and ligand-receptor interactions involving hub OSRGs at various time points after SCI, as well as in control groups. Five primary signaling pathways and their associated ligand-receptor pairs were identified in the control group and different post-injury groups: the EGF signaling pathway (*Hbegf*-*Erbb4*, *Hbegf*-*Egfr*), GAS signaling pathway (*Gas*-*Axl*), OSM signaling pathway (*Osm*-(*Osmr*+*Il6st*), *Osm*-(*Lifr*+*Il6st*)), PROS signaling pathway (*Pros1*-*Axl*), and TNF signaling pathway (*Tnf*-*Tnfrsf1a*). Notably, on the first day after injury, the IL6 signaling pathway (*Il6*-(*Il6r*+*Il6st*)) showed enhanced activity compared to both the control group and the 3 days post-injury group. By the seventh day, there was a marked increase in the activity of the ANGPTL signaling pathway (*Angptl4*-*Sdc1*), PTN signaling pathway (*Ptn*-*Sdc1*), and MK signaling pathway (*Mdk*-*Sdc1*) relative to the control group. While some pathways were common across various groups, the specific cells impacted by these pathways varied. This analysis sheds light on the distinct communication patterns associated with oxidative stress at different stages after SCI.

This study indicates that the *Hbegf*-*Erbb4* and *Hbegf*-*Egfr* ligand-receptor pairs may play a pivotal role in regulating intercellular communication associated with SCI-related oxidative stress. *Hbegf*, a member of the epidermal growth factor family, has been shown to enhance antioxidant enzyme activity, reducing oxidative stress-induced damage during endometrial degradation. Conversely, inhibition of ERBB receptors, which blocks *Hbegf* signaling, diminishes these cytoprotective effects (Armant et al., 2006). Moreover, an increase in Hbegf expression is associated with prolonged *Egfr* signaling, which has been implicated in the oxidative stress response (Kalmes et al., 2001; Dreux et al., 2006). Nevertheless, the specific contributions of the *Hbegf*-*Erbb4* and *Hbegf*-*Egfr* pairs to oxidative stress management after SCI have not been investigated. Additionally, our findings reveal that activation of the *Gas6*/*Axl*-*Ampk* signaling pathway can shield cells from H_2_O_2_-induced oxidative stress, thereby enhancing cellular resilience against oxidative damage, apoptosis, and mitochondrial dysfunction (Lei et al., 2022; Liang et al., 2023). Thus, the GAS signaling pathway and its corresponding ligand-receptor pair, *Gas6*-*Axl*, likely play a regulatory role in mitigating oxidative stress after SCI. However, the precise function of *Gas6*-*Axl* in the oxidative stress response requires further exploration. Furthermore, our study highlights that *Pros1*-*Axl* is crucial in mediating intercellular communication. Prior studies have demonstrated that *Pros1*-*Axl* is involved in the proliferation and migration of papillary thyroid carcinoma cells and in modulating the immune-inflammatory response after brain injury (Wei et al., 2022; Tang et al., 2023). Nonetheless, research into the role of *Pros1*-*Axl* in oxidative stress responses and SCI is still limited.

A previous study has demonstrated that the binding of *Osm* to *Osmr* results in the dimerization of *Osmr*-*Il6st*, which in turn prolongs *Stat3* activation, significantly influencing ovarian cancer cell proliferation and migration (Geethadevi et al., 2021). Additionally, α-synuclein has been shown to impair oxidative stress repair by modulating the *IL6ST*-*AS*/*STAT3*/*HIF-1α* axis (Lin et al., 2023). However, the specific roles of *Osm*-(*Osmr*+*Il6st*) and *Osm*-(*Lifr*+*Il6st*) in oxidative stress after SCI remain unexplored. Activation of the *TNFα*-*TNFRSF1A* and *NOX4* signaling pathways augments renal inflammation and oxidative stress responses in mice (Jin et al., 2015). Moreover, *TNF*-*TNFRSF1A*-mediated AP-1 signaling transduction has been implicated in promoting nerve regeneration after SCI (Zeng, 2023), although its potential exacerbation of oxidative stress in SCI requires further investigation. Additionally, syndecan 1 (*Sdc1*), a transmembrane heparan sulfate proteoglycan belonging to the syndecan proteoglycan family (Yu et al., 2020), has been found to engage in interactions with OPG that induce oxidative stress (Le et al., 2022). Nevertheless, the effects of *Angptl4*-*Sdc1*, *Ptn*-*Sdc1*, and *Mdk*-*Sdc1* interactions on oxidative stress have not been reported and warrant further study. This research also identifies key genes, including *Xdh* (Qui et al., 2017; Liu et al., 2019), *Map2K3* (Ding et al., 2023), *Map2K4* (Yao et al., 2022), *Il6St* (Ding et al., 2023), *Hdac1* (Kong et al., 2017; Chen et al., 2022), and *Gch1* (Yin et al., 2022), which are instrumental in mediating oxidative stress responses. *Hdac1* enhances oxidative stress responses in SCI (Kong et al., 2017), while the roles of the other genes in SCI-associated oxidative stress are less well characterized.

To further elucidate the regulatory mechanisms associated with hub genes, this study analyzed transcription factors that either inhibit or activate these core genes. We compiled the expression patterns of transcription factors across various cell types and time points after SCI to identify those with elevated expression levels, detailing their respective roles in modulating core gene activity. This analysis is critical for deciphering the transcriptional regulatory mechanisms involved with OSRGs. Moreover, we assessed pharmaceutical agents that modulate the expression of upregulated or downregulated core genes at different stages after SCI. By examining the interactions between these drugs, core genes, and innate immune cells, we provided crucial insights for devising time-specific pharmacological strategies to address SCI-induced oxidative stress. Additionally, we used data from two databases to predict miRNAs that interact with core OSRGs, identifying pivotal miRNAs and their corresponding lncRNAs. This strategy enhances our understanding of the intricate regulatory networks governing oxidative stress in SCI.

This study had several limitations. First, it concentrated on evaluating the expression patterns and potential regulatory mechanisms of OSRGs after SCI through data analysis, underscoring the need for additional in-depth experimental investigations. Second, the database that was used includes only one statistically significant human blood sequencing dataset from SCI patients, highlighting the need for future studies that incorporate large-scale data analyses and validation using biological samples. These studies are essential for a more comprehensive exploration of blood biomarkers associated with SCI severity and could offer invaluable support for clinical diagnosis and treatment.

This study successfully identified diagnostic biomarkers related to oxidative stress that correlate with the severity of human SCI, enhancing both the diagnosis and assessment of SCI severity. We also delineated DEOSRGs and their hub genes in SCI, uncovering their temporal expression patterns at both the tissue and single-cell levels. These findings provide insight into the time-regulated dynamics of oxidative stress, suggesting avenues for targeted therapeutic interventions. Additionally, we characterized intercellular communication signaling pathways, including relevant ligand-receptor pairs associated with oxidative stress at various time points after SCI. Analysis of miRNAs and lncRNAs that influence these hub genes, coupled with the identification of transcription factors prominently expressed in different cells at multiple time points after injury, further elucidates their roles in modulating hub genes. These discoveries are critical for a comprehensive understanding of the regulatory mechanisms governing oxidative stress in SCI. Moreover, we analyzed and summarized drugs that inhibit these hub genes at various stages post-injury, providing a basis for time-targeted pharmacological strategies to mitigate oxidative stress in SCI. Overall, this study offers vital insights into SCI diagnostic and therapeutic research, highlighting diagnostic biomarkers linked to SCI severity, examining the expression patterns of OSRGs, and exploring potential regulatory mechanisms and drugs associated with hub OSRGs in SCI.

## Additional files:

***Additional Figure 1:*** *Identification of DEOSRGs and hub genes following SCI through dataset analysis.*

Additional Figure 1 Identification of DEOSRGs and hub genes after SCI through dataset analysis
(A) Volcano plot of the DEOSRGs in the blood between the SCI and HC groups. (B) Differences in the content of immune cells between the AIS D SCI and HC groups. (C) Heatmap displaying a selection of DEOSRGs. The vertical axis represents the gene names, and the horizontal axis represents the samples from different groups. (D) Venn diagram illustrating the differential expression of OSRGs in the PPI network at various time points after SCI. AIS: American Spinal Cord Injury Association Impairment Scale; DEOSRGs: differentially expressed oxidative stress-related genes; dpi: day post injury; HC: healthy uninjured control; hpi: hour post injury; OSRGs: oxidative stress-related genes; SCI: spinal cord injury.

***Additional Figure 2:*** *Dataset analysis shows the temporal expression bubble chart of OSRG sets at the single-cell level in SCI.*

Additional Figure 2 Dataset analysis shows the temporal expression bubble chart of OSRG sets at the single-cell level in SCI.
The vertical axis represents the cell type, the horizontal axis represents the gene set name, the size of the circle indicates the proportion of cells in the cell type that express the gene set, and the color depth of the circle indicates the density of gene set expression. dpi: day post injury; OSRGs: oxidative stress- related genes; SCI: spinal cord injury; UMAP: Uniform Manifold Approximation and Projection.

***Additional Figure 3:*** *This single-cell UMAP plot characterizes the temporal expression of the first identified group of hub OSRGs.*

Additional Figure 3 This single-cell UMAP plot characterizes the temporal expression of the first identified group of hub OSRGs.
The varying shades of color represent the relative expression levels of genes. dpi: day post injury; OSRGs: oxidative stress-related genes; SCI: spinal cord injury; UMAP: Uniform Manifold Approximation and Projection.

***Additional Figure 4:*** *This single-cell UMAP plot characterizes the temporal expression of the second identified group of hub OSRGs.*

Additional Figure 4 This single-cell UMAP plot characterizes the temporal expression of the second identified group of hub OSRGs.
The varying shades of color represent the relative expression levels of genes. dpi: day post injury; OSRGs: oxidative stress-related genes; SCI: spinal cord injury; UMAP: Uniform Manifold Approximation and Projection.

***Additional Figure 5:*** *This single-cell UMAP plot characterizes the temporal expression of the third identified group of hub OSRGs.*

Additional Figure 5 This single-cell UMAP plot characterizes the temporal expression of the third identified group of hub OSRGs.
The varying shades of color represent the relative expression levels of genes. dpi: day post injury; OSRGs: oxidative stress-related genes; SCI: spinal cord injury; UMAP: Uniform Manifold Approximation and Projection.

***Additional Figure 6:*** *This single-cell UMAP plot characterizes the temporal expression of the fourth identified group of hub OSRGs.*

Additional Figure 6 This single-cell UMAP plot characterizes the temporal expression of the fourth identified group of hub OSRGs.
The varying shades of color represent the relative expression levels of genes. dpi: day post injury; OSRGs: oxidative stress-related genes; SCI: spinal cord injury; UMAP: Uniform Manifold Approximation and Projection.

***Additional Figure 7:*** *Immunofluorescence staining of spinal cord tissue for Iba1 (green, Alexa Fluor 488) and Ripk1 (red, Alexa Fluor 594) expression.*

Additional Figure 7 Immunofluorescence staining of spinal cord tissue for *Iba1* (green, Alexa Fluor 488) and *Ripk1* (red, Alexa Fluor 594) expression.
Ibal and Ripkl expression levels were higher in the different SCI groups than the control group and the fluorescence signals from both proteins overlapped. Additionally, Ripk1 expression levels were somewhat higher on day 1 after SCI than days 3 and 7. Scale bar: 50 μm. Dapi: 4',6-diamidino-2- phenylindole; dpi: day post injury; SCI: spinal cord injury.

***Additional Figure 8:*** *Spearman correlation analysis shows the relationship between the first group of hub OSRGs and innate immune cells after SCI.*

Additional Figure 8 Spearman correlation analysis shows the relationship between the first group of hub OSRGs and innate immune cells after SCI.
The left vertical axis represents various innate immune cells, the right vertical axis represents the *P-*values of the correlations, and the bottom horizontal axis represents the correlation coefficient. The size of the circles corresponds to the correlation coefficient, and the color of the circles represents the *P*- values of the correlations; *P*-value < 0.05 was considered significant. OSRGs: oxidative stress-related genes; SCI: spinal cord injury.

***Additional Figure 9:*** *Spearman correlation analysis shows the relationship between the second group of hub OSRGs and innate immune cells after SCI.*

Additional Figure 9 Spearman correlation analysis shows the relationship between the second group of hub OSRGs and innate immune cells after SCI.
The left vertical axis represents various innate immune cells, the right vertical axis represents the *P-*values of the correlations, and the bottom horizontal axis represents the correlation coefficient. The size of the circles corresponds to the correlation coefficient, and the color of the circles represents the *P*- values of the correlations; *P*-value < 0.05 was considered significant. OSRGs: oxidative stress-related genes; SCI: spinal cord injury.

***Additional Figure 10:*** *Spearman correlation analysis shows the relationship between the third group of hub OSRGs and innate immune cells after SCI.*

Additional Figure 10 Spearman correlation analysis shows the relationship between the third group of hub OSRGs and innate immune cells after SCI.
The left vertical axis represents various innate immune cells, the right vertical axis represents the *P-*values of the correlations, and the bottom horizontal axis represents the correlation coefficient. The size of the circles corresponds to the correlation coefficient, and the color of the circles represents the *P*- values of the correlations; *P*-value < 0.05 was considered significant. OSRGs: oxidative stress-related genes; SCI: spinal cord injury.

***Additional Table 1:*** *The differentially expressed oxidative stress-related genes at various time points between the SCI and control groups.*

Additional Table 1 The differentially expressed oxidative stress-related genes between the time point groups after SCIand the control group

***Additional Table 2:*** *The protein interaction network of oxidative stress-related genes that are differentially expressed between groups at different time points after SCI and the control group.*

Additional Table 2 The protein interaction network of oxidative stress-related genes that are differentially expressed between groups at different time points after SCI andthe control group

***Additional Table 3:*** *The frequency of occurrence of hub genes in the protein-protein interaction network of differentially expressed oxidative stress-related genes between various time-point groups post-spinal cord injury and the control group.*

***Additional Table 4:*** *GO functional enrichment analysis of oxidative stress-related genes that are differentially expressed between groups at different time points after SCI and the control group.*

Additional Table 4 GO functional enrichment analysis of oxidative stress-related genes that are differentially expressed between groups at different time points after SCI and the control group

***Additional Table 5:*** *KEGG enrichment analysis of oxidative stress-related genes that are differentially expressed between groups at different time points after SCI and the control group.*

Additional Table 5 KEGG enrichment analysis of oxidative stress-related genes that are differentially expressed between groups at different time points after SCI and the control group

***Additional Table 6:*** *miRNAs that regulate hub genes.*

Additional Table 6 miRNAs that regulate hub genes

***Additional Table 7:*** *Correspondence of mRNA, miRNA, and lncRNA in the regulation of angiogenesis.*

Additional Table 7 Correspondence of mRNA, miRNA,and lncRNA in the regulation of angiogenesis

***Additional Table 8:*** *Transcription factors that have either promoting or inhibitory effects on hub genes.*

Additional Table 8 Transcription factors that have eitherpromoting or Inhibitory effects on hub genes

***Additional Table 9:*** *The regulatory effects of highly expressed transcription factors on relevant hub genes in different cell groups.*

Additional Table 9 The regulatory effects of highly expressed transcription factors on relevant hub genes in different cell groups

***Additional Table 10:*** *The 38 drugs that demonstrate modulatory effects on the nine hub genes.*

***Additional Table 11:*** *Correlation analysis among drugs, hub genes, and innate immune cells.*

Additional Table 11 Correlation analysis among drugs, hub genes, and innate immune cells

## Data Availability

*The datasets analyzed in this study are available in the GEO database, including the datasets GSE5296 (https://www.ncbi.nlm.nih.gov/geo/query/acc.cgi?acc=GSE5296), GSE162610 (https://www.ncbi.nlm.nih.gov/geo/query/acc.cgi?acc=GSE162610), GSE151371 (https://www.ncbi.nlm.nih.gov/geo/query/acc.cgi?acc=GSE151371)*.
