# Supplementary figures and images for: Integrating bulk and single-cell transcriptome profiling to uncover diagnostic biomarkers and regulatory mechanisms of oxidative stress in spinal cord injury
Source: Neural Regen Res. 2025 Jan 13;21(6):2643–57. doi: 10.4103/NRR.NRR-D-24-00693 (PMC13217428; doi:10.4103/NRR.NRR-D-24-00693)

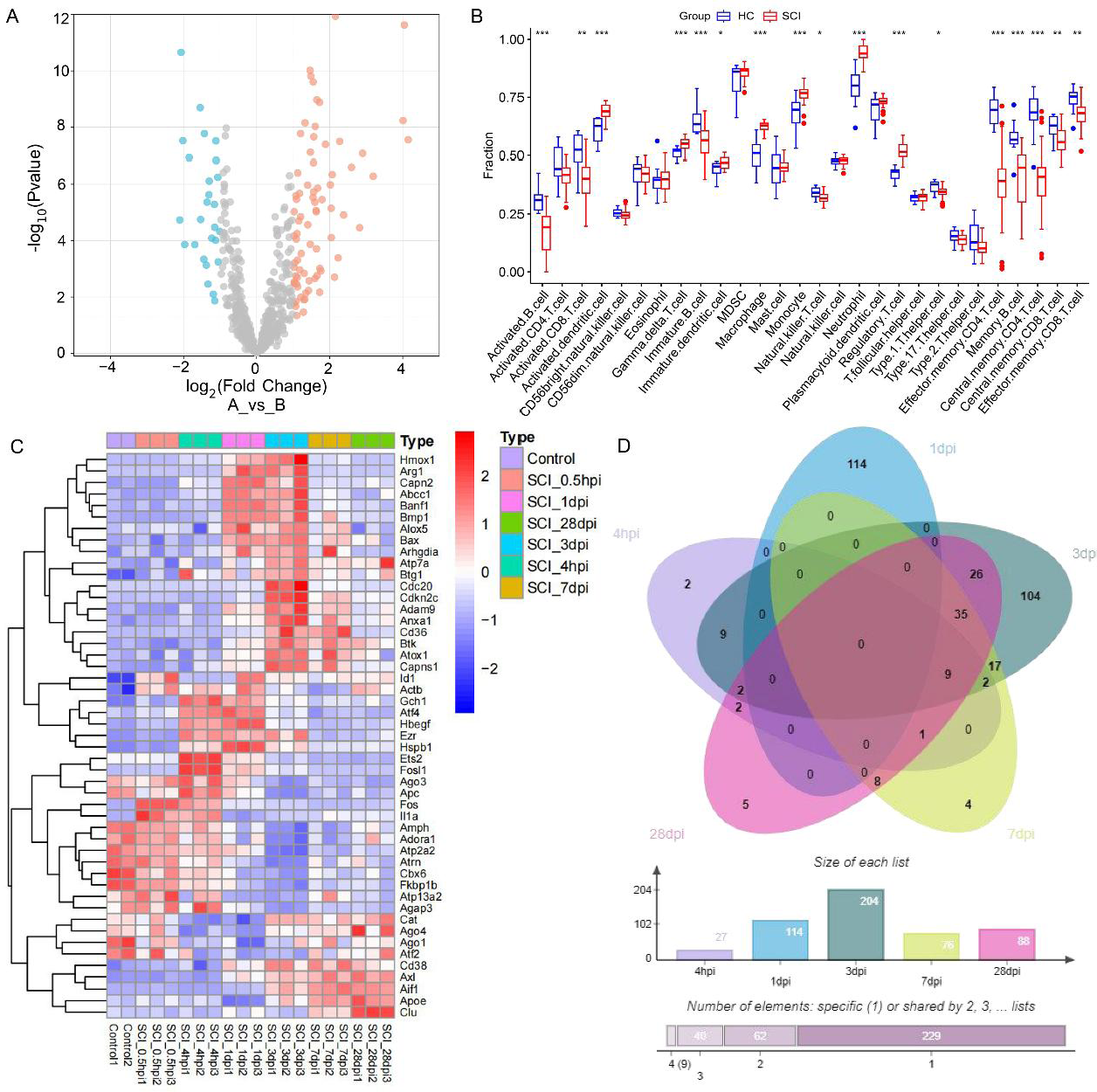

Supplement: Supplementary file 1 [file NRR-21-2643_Suppl1.tif]

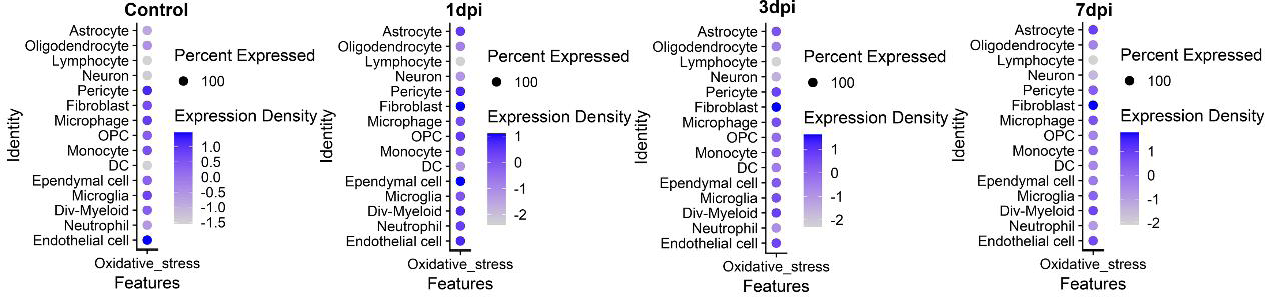

Supplement: Supplementary file 6 [file NRR-21-2643_Suppl2.tif]

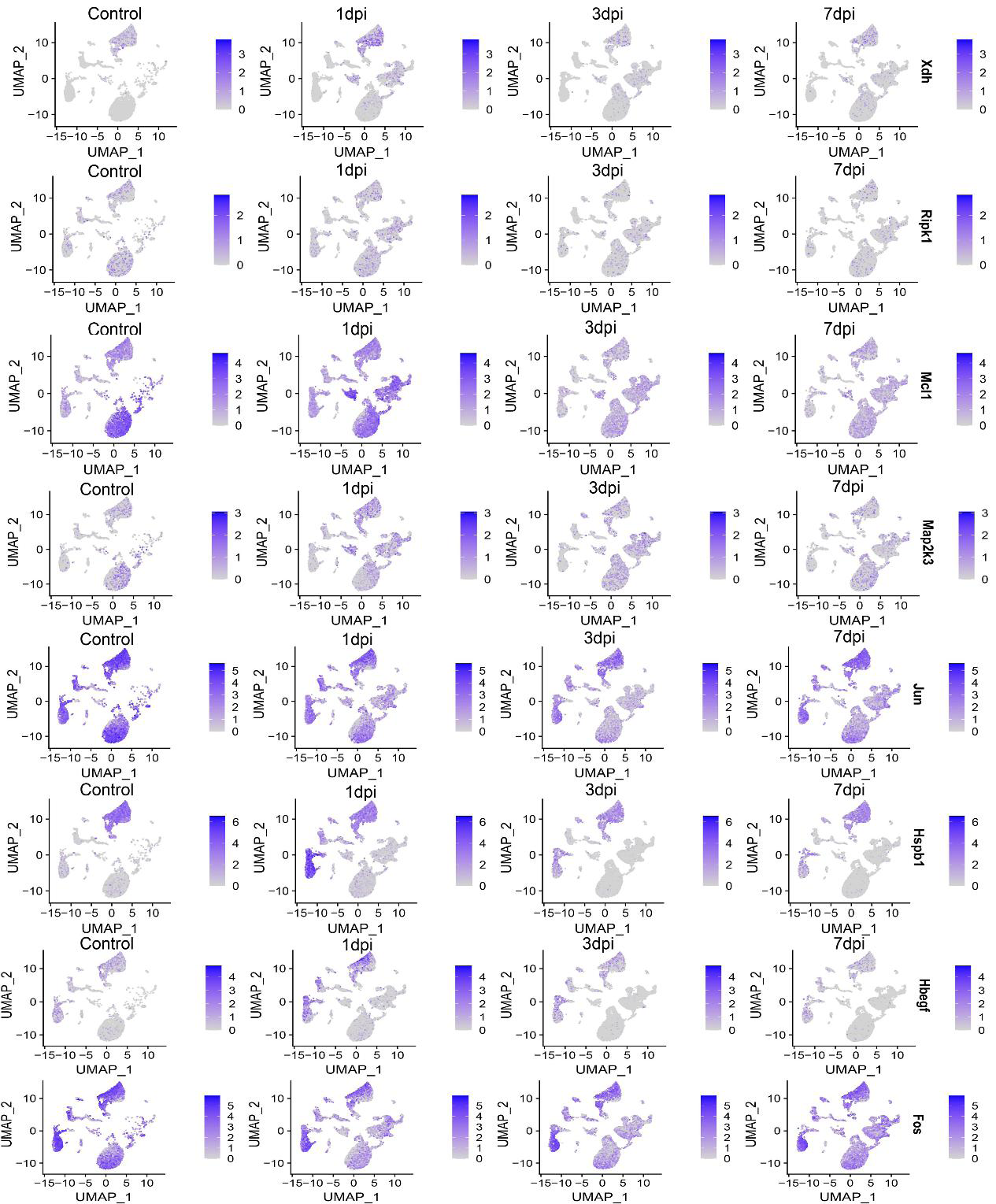

Supplement: Supplementary file 7 [file NRR-21-2643_Suppl3.tif]

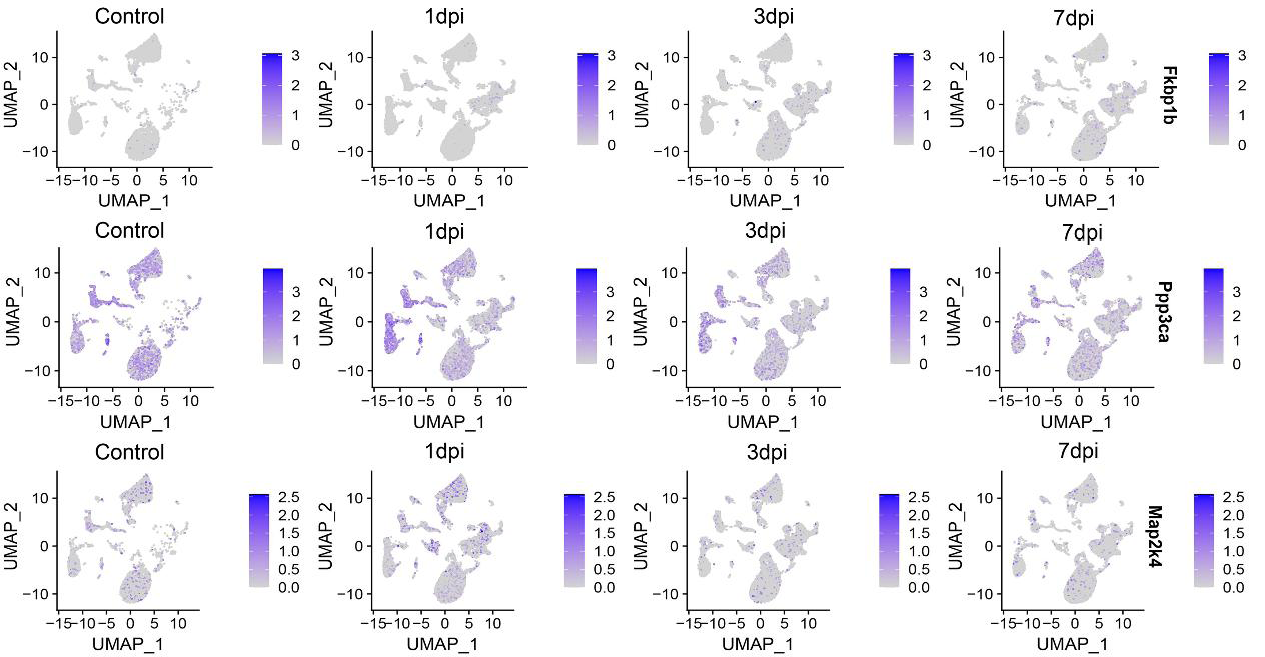

Supplement: Supplementary file 8 [file NRR-21-2643_Suppl6.tif]

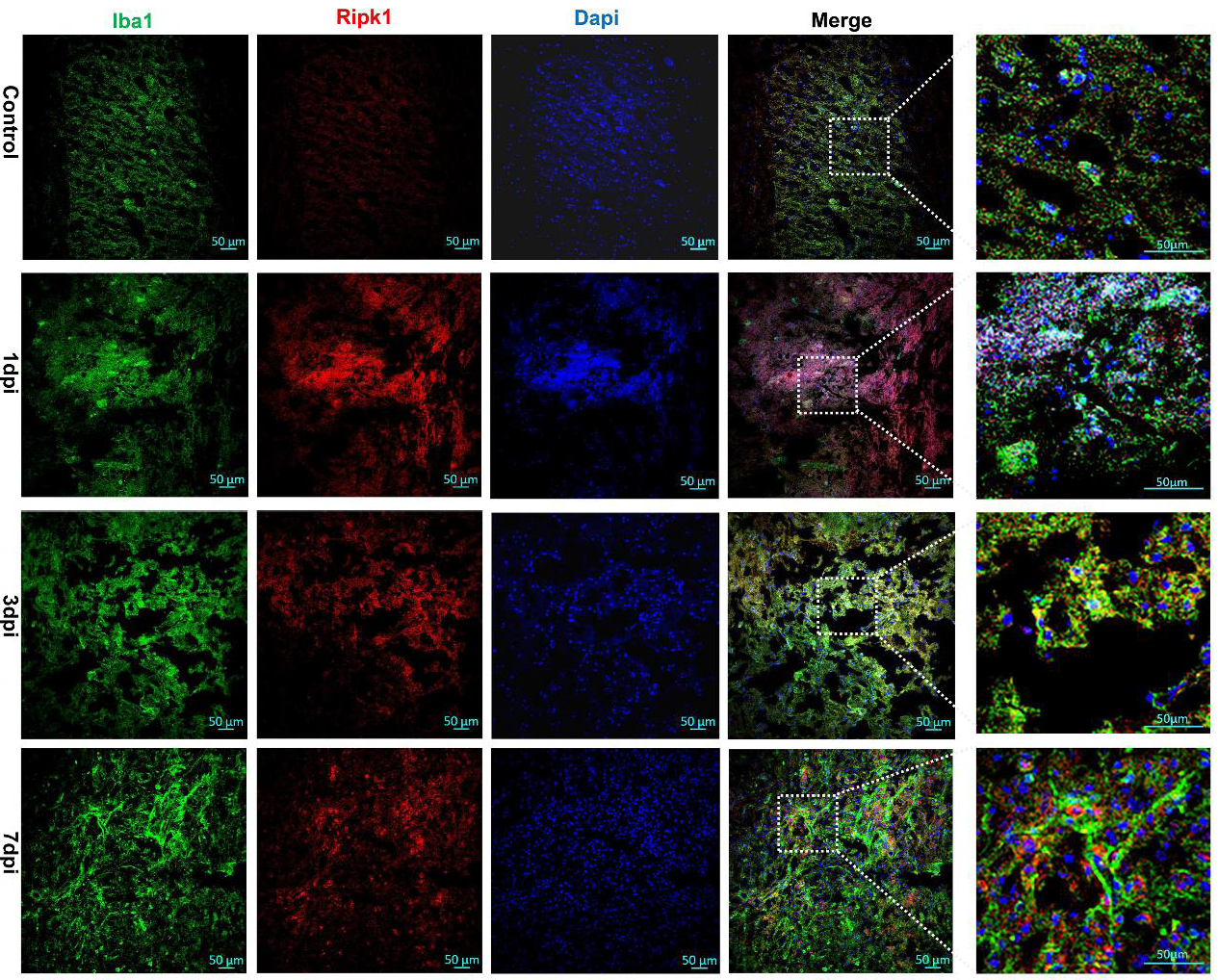

Supplement: Supplementary file 9 [file NRR-21-2643_Suppl7.tif]

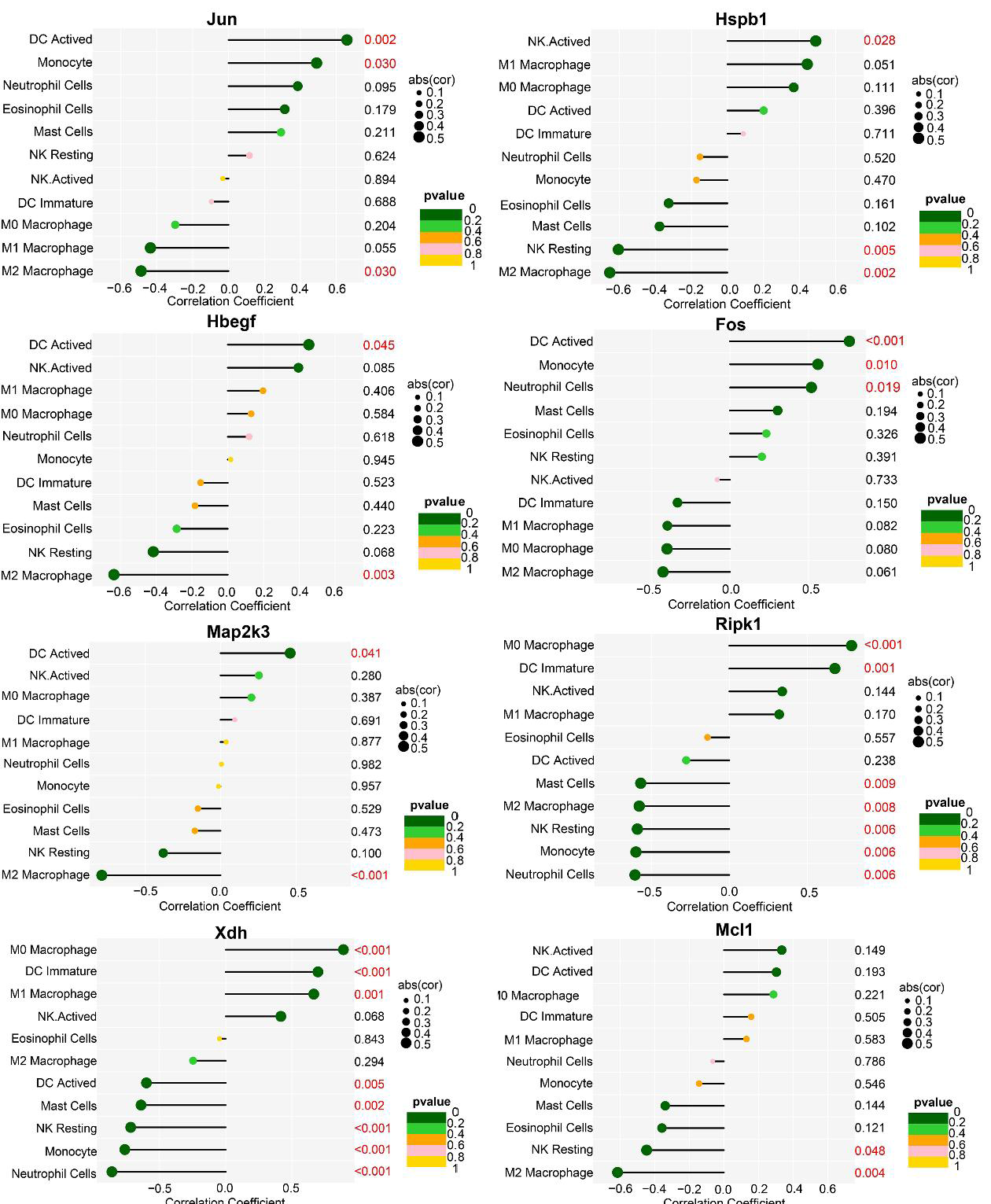

Supplement: Supplementary file 10 [file NRR-21-2643_Suppl8.tif]

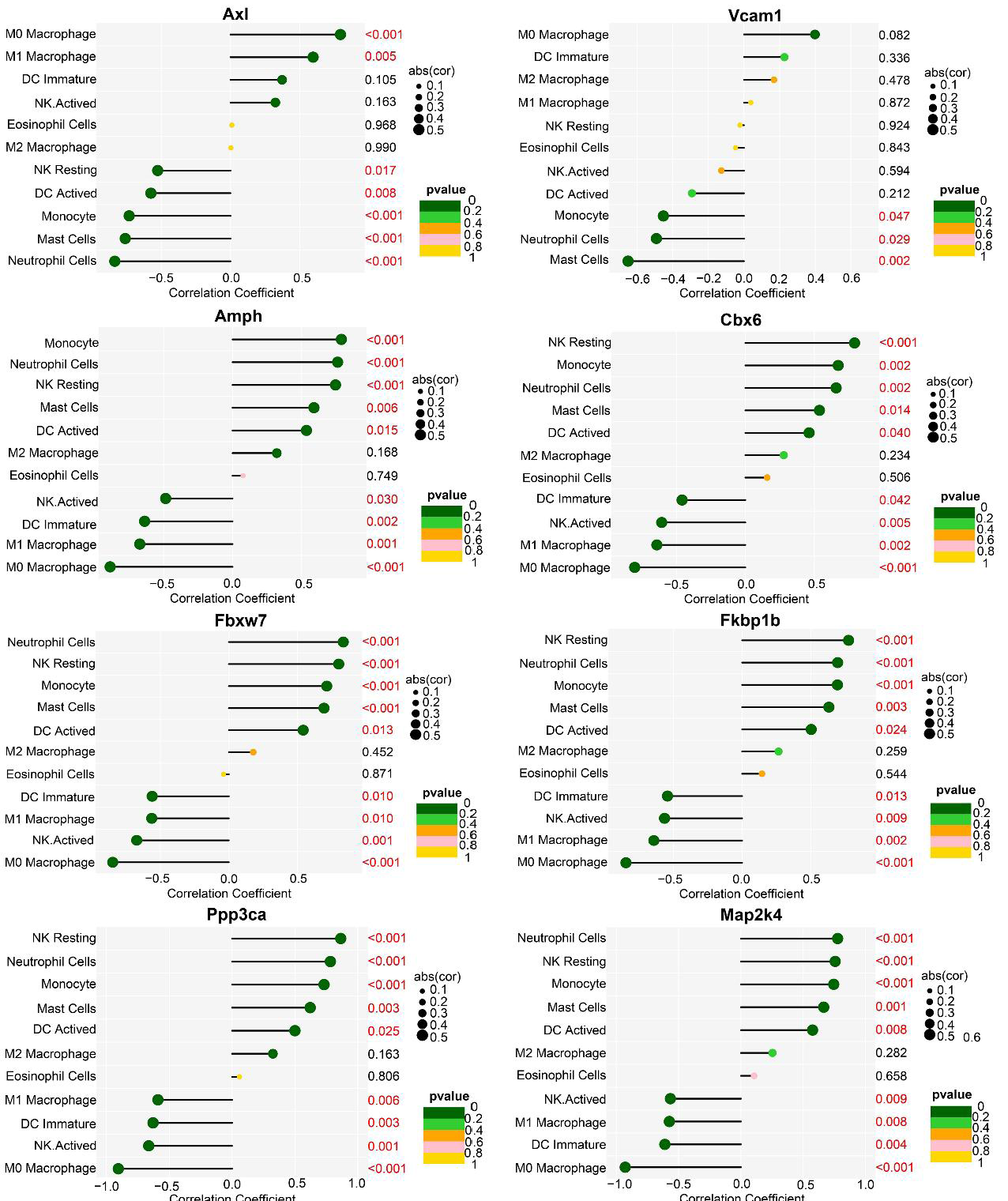

Supplement: Supplementary file 11 [file NRR-21-2643_Suppl10.tif]

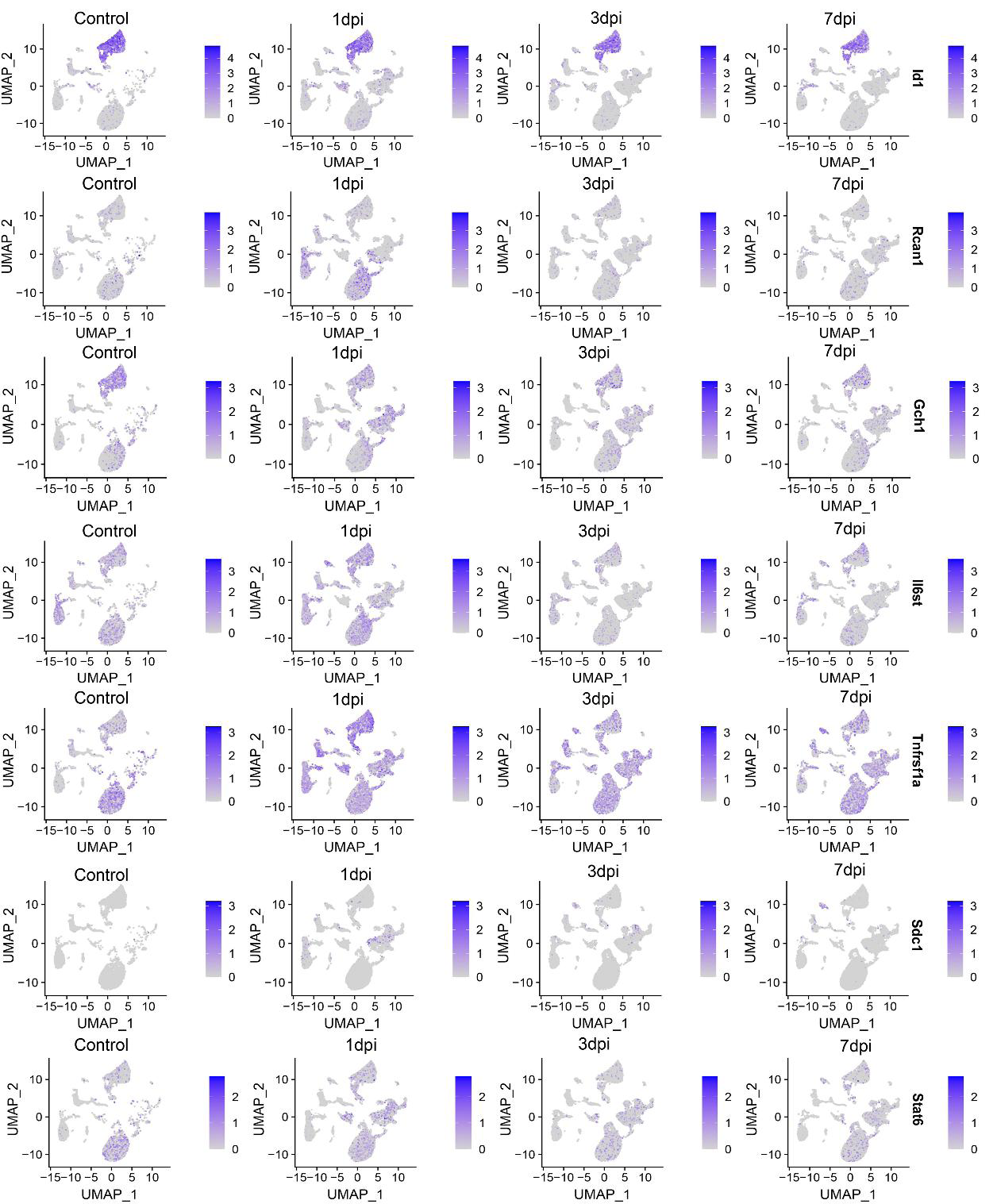

Supplement: Supplementary file 17 [file NRR-21-2643_Suppl4.tif]

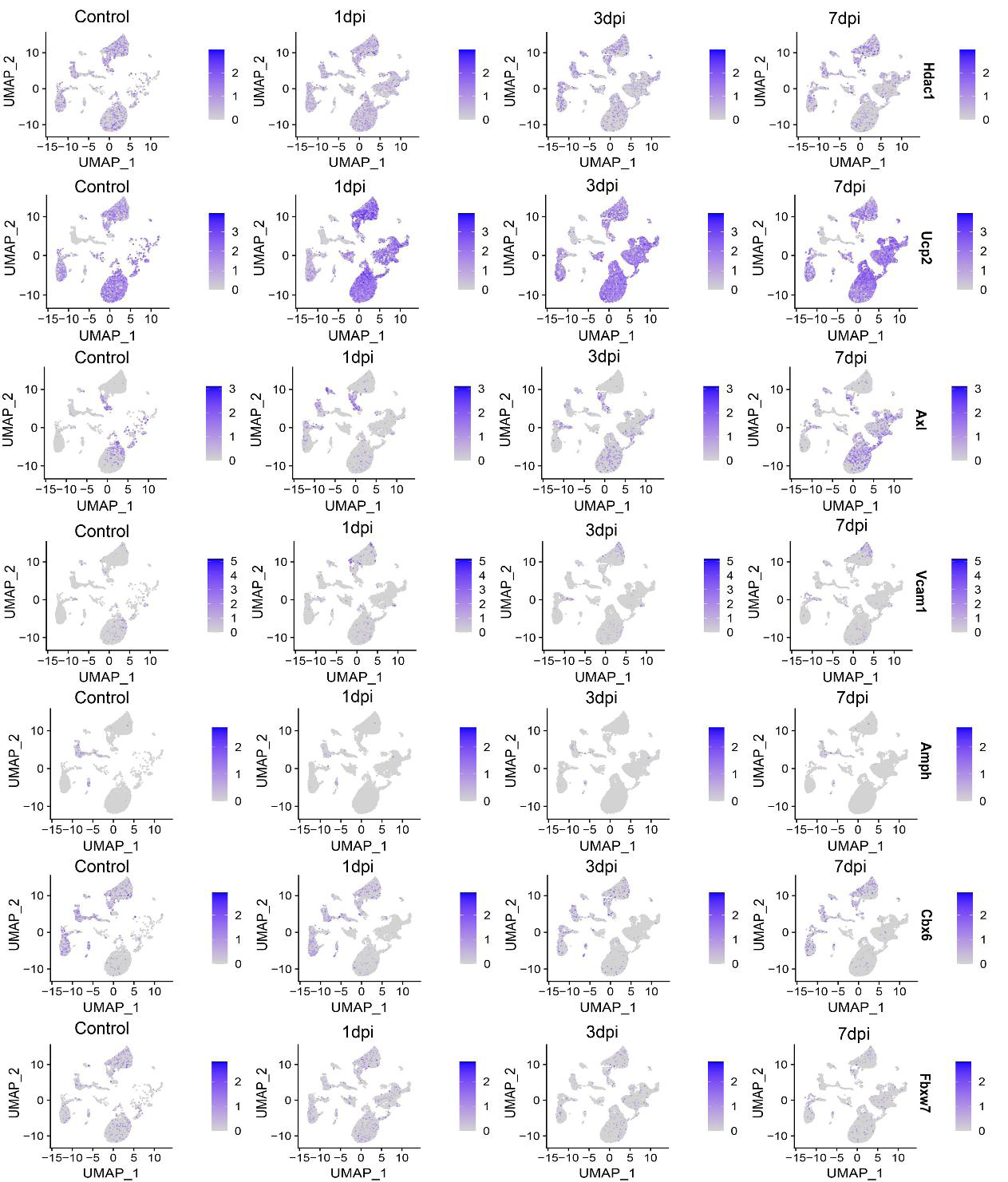

Supplement: Supplementary file 18 [file NRR-21-2643_Suppl5.tif]

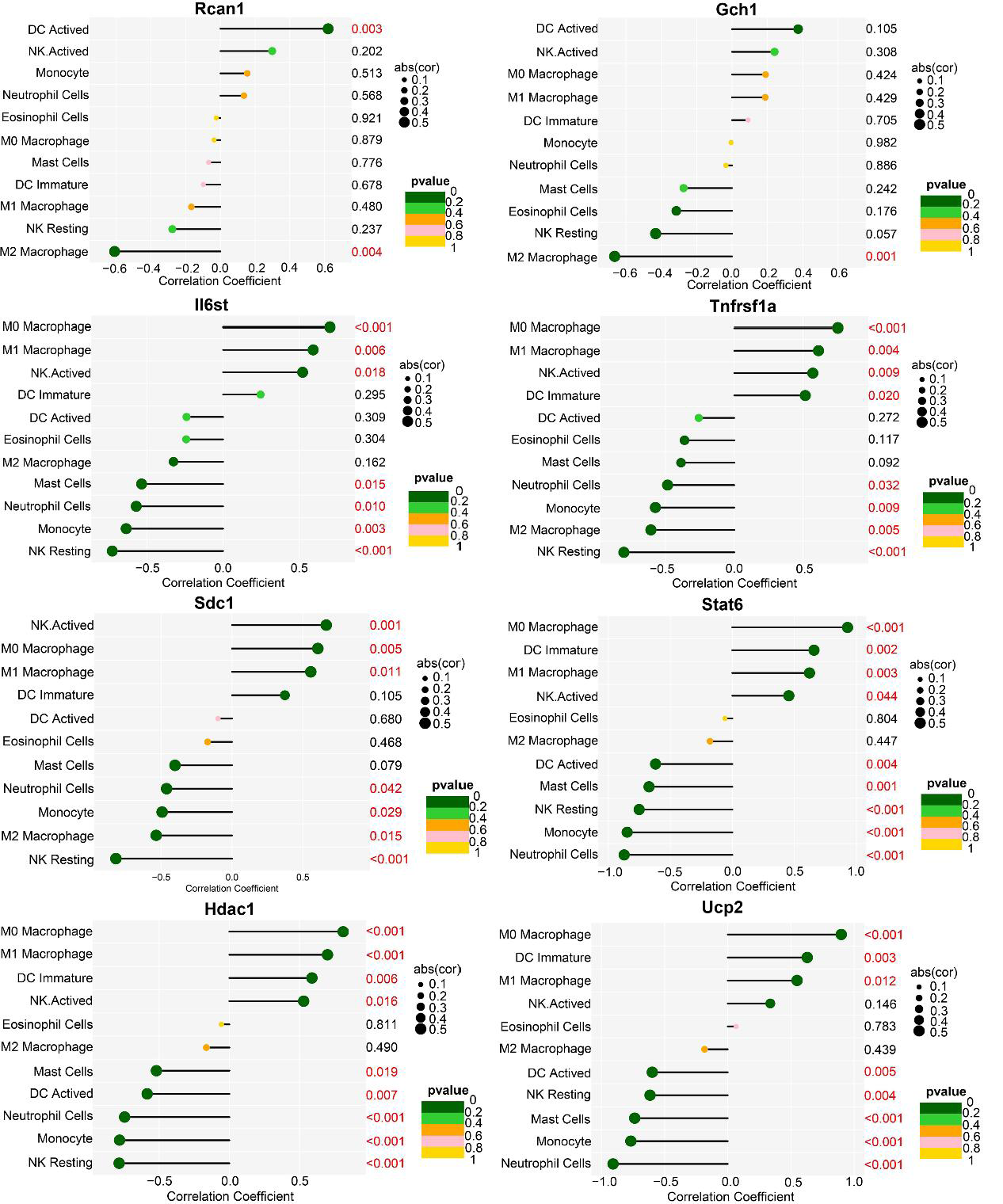

Supplement: Supplementary file 19 [file NRR-21-2643_Suppl9.tif]
