# Supplementary material for: Integrating bulk and single-cell transcriptome profiling to uncover diagnostic biomarkers and regulatory mechanisms of oxidative stress in spinal cord injury
Source: Neural Regen Res. 2025 Jan 13;21(6):2643–57. doi: 10.4103/NRR.NRR-D-24-00693 (PMC13217428; doi:10.4103/NRR.NRR-D-24-00693)
Supplement: Supplementary file 2 [file NRR-21-2643_Suppl1.pdf]

Additional Table 1 The differentially expressed oxidative stress-related genes between the time point groups after SCI and the control group

Group	Gene	logFC	AveExpr	P.Value	adj.P.Val
0.5-hour post-SCI group vs. the control group	*Fos*	5326.48985	3516.23306	2.54E-09	5.29E-05
4-hour post-SCI group vs. the control group	*Ago1*	-142.6827	386.71888	0.00010855	0.022350181
4-hour post-SCI group vs. the control group	*Atf4*	322.77662	562.35892	0.000360149	0.032328546
4-hour post-SCI group vs. the control group	*Cat*	-309.0754	612.21526	0.000758744	0.044681294
4-hour post-SCI group vs. the control group	*Cbx6*	-318.7196	1051.83424	0.000359746	0.032328546
4-hour post-SCI group vs. the control group	*Ets2*	143.71262	232.05172	2.28E-05	0.012842371
4-hour post-SCI group vs. the control group	*Ezr*	259.82646	246.39605	0.000176271	0.024036435
4-hour post-SCI group vs. the control group	*Fkbp1b*	-143.09458	407.956	0.00012843	0.022823403
4-hour post-SCI group vs. the control group	*Fos*	3615.01785	2489.34986	0.000214973	0.025863868
4-hour post-SCI group vs. the control group	*Fosl1*	312.79218	190.58759	4.97E-06	0.007900124
4-hour post-SCI group vs. the control group	*Gch1*	103.89235	89.55013	0.00016982	0.024036435
4-hour post-SCI group vs. the control group	*Hbegf*	621.35675	506.3447	6.18E-05	0.017084454
4-hour post-SCI group vs. the control group	*Hmox1*	321.46394	223.79326	5.51E-06	0.007900124
4-hour post-SCI group vs. the control group	*Hspb1*	3959.51473	3153.06144	2.54E-05	0.012911768
4-hour post-SCI group vs. the control group	*Id1*	202.51492	341.515	1.10E-05	0.011136569
4-hour post-SCI group vs. the control group	*Il1a*	57.76982	46.54505	0.000951552	0.04857434
4-hour post-SCI group vs. the control group	*Jun*	770.12277	780.51916	0.000563611	0.037989341
4-hour post-SCI group vs. the control group	*Map2k3*	246.06707	233.69404	2.74E-05	0.012946049
4-hour post-SCI group vs. the control group	*Mcl1*	934.00557	1045.54844	0.000593898	0.039273569
4-hour post-SCI group vs. the control group	*Myc*	438.05056	291.97494	0.000231642	0.026464505
4-hour post-SCI group vs. the control group	*Nfkb1*	180.68997	281.68888	0.000652336	0.041407988
4-hour post-SCI group vs. the control group	*Nr4a3*	80.17108	70.18415	0.000142154	0.022823403
4-hour post-SCI group vs. the control group	*Ppp1r15b*	116.6028	251.60168	0.000514219	0.036673123
4-hour post-SCI group vs. the control group	*Pxdn*	94.71102	214.03206	8.49E-05	0.019852584
4-hour post-SCI group vs. the control group	*Rcan1*	857.561	774.6027	0.000829114	0.046627726
4-hour post-SCI group vs. the control group	*Ripk1*	66.46169	80.00602	0.000313558	0.030075567
4-hour post-SCI group vs. the control group	*Rnh1*	366.90695	649.47702	0.000454076	0.035608535
4-hour post-SCI group vs. the control group	*Rxrb*	-107.84733	242.0565	0.000193134	0.02496823
4-hour post-SCI group vs. the control group	*Sdc1*	32.63258	38.14643	0.000952263	0.04857434
4-hour post-SCI group vs. the control group	*Slc8a1*	-42.13565	171.30216	0.000650226	0.041407988
4-hour post-SCI group vs. the control group	*Sphk1*	616.91234	410.05986	8.31E-05	0.019649039
4-hour post-SCI group vs. the control group	*Srxn1*	747.50518	668.54246	0.000812909	0.04644074
4-hour post-SCI group vs. the control group	*Tnfaip3*	113.14056	100.32858	7.36E-05	0.018451007
4-hour post-SCI group vs. the control group	*Tnfrsf1a*	317.56773	399.71094	4.75E-05	0.015913667
4-hour post-SCI group vs. the control group	*Txnrd1*	194.01323	327.30514	0.001069332	0.049583914
4-hour post-SCI group vs. the control group	*Xdh*	87.64923	79.29794	0.000652533	0.041407988
1-day post-SCI group vs. the control group	*Abcc1*	81.56012	167.36492	0.000745706	0.01635525
1-day post-SCI group vs. the control group	*Adam9*	392.63622	783.73368	0.003781236	0.03639151
1-day post-SCI group vs. the control group	*Ago4*	-29.39145	48.89588	0.003034202	0.032802795
1-day post-SCI group vs. the control group	*Amph*	-382.37328	719.76498	0.00238237	0.029062251
1-day post-SCI group vs. the control group	*Anxa1*	209.00976	201.94469	0.00080412	0.016888961
1-day post-SCI group vs. the control group	*Apoe*	-1668.75733	4184.1566	0.000830292	0.017154892
1-day post-SCI group vs. the control group	*Atf2*	-121.95537	347.94358	0.002199706	0.027697929
1-day post-SCI group vs. the control group	*Atf4*	356.86982	582.81484	0.000485617	0.013658972
1-day post-SCI group vs. the control group	*Atox1*	87.461	481.0238	0.000164432	0.009129018
1-day post-SCI group vs. the control group	*Atp13a2*	-110.24627	379.61444	0.002934615	0.032281293
1-day post-SCI group vs. the control group	*Atrn*	-95.20238	245.42162	0.000706628	0.016032307
1-day post-SCI group vs. the control group	*Axl*	132.47068	215.87176	0.003852	0.036777763
1-day post-SCI group vs. the control group	*Banf1*	441.70925	727.0848	8.51E-06	0.003934734
1-day post-SCI group vs. the control group	*Bax*	272.78887	420.55782	0.00015136	0.008873252
1-day post-SCI group vs. the control group	*Bmp1*	138.14743	134.90696	5.08E-06	0.003585212

1-day post-SCI group vs. the control group	*Btk*	43.81548	58.98163	0.004668385	0.041103112
1-day post-SCI group vs. the control group	*Capn2*	772.7698	1264.88108	4.38E-06	0.003585212
1-day post-SCI group vs. the control group	*Capns1*	332.91083	2007.569	0.00481899	0.041810109
1-day post-SCI group vs. the control group	*Cask*	-37.27393	119.94954	0.00290858	0.032116277
1-day post-SCI group vs. the control group	*Cat*	-323.70027	603.44034	0.005203444	0.043596296
1-day post-SCI group vs. the control group	*Cbx6*	-539.67913	919.25852	0.000780066	0.016631887
1-day post-SCI group vs. the control group	*Ccr1*	124.35207	86.06854	0.000104626	0.007614302
1-day post-SCI group vs. the control group	*Cdh11*	-175.23037	351.45408	0.001369688	0.021836025
1-day post-SCI group vs. the control group	*Cdk4*	228.07298	319.38624	0.000138265	0.008539621
1-day post-SCI group vs. the control group	*Cdkn1c*	-111.94191	107.66695	0.000785988	0.016727562
1-day post-SCI group vs. the control group	*Ctnna1*	290.69775	710.0798	0.002317552	0.028576733
1-day post-SCI group vs. the control group	*Ctsl*	1117.29147	1411.52108	1.28E-05	0.004315725
1-day post-SCI group vs. the control group	*Cttn*	640.67745	947.01502	1.64E-05	0.004370519
1-day post-SCI group vs. the control group	*Cyp1b1*	93.11199	81.9577	0.00248959	0.029659301
1-day post-SCI group vs. the control group	*Dynll1*	1077.89367	3763.5522	0.002250956	0.028122089
1-day post-SCI group vs. the control group	*Edn1*	81.78792	74.99811	0.001152051	0.019965687
1-day post-SCI group vs. the control group	*Eed*	54.6186	147.76356	0.000423909	0.013052128
1-day post-SCI group vs. the control group	*Eif2s1*	310.32952	468.46656	0.00042628	0.013056877
1-day post-SCI group vs. the control group	*Ercc1*	104.24033	176.1799	0.002011727	0.026602347
1-day post-SCI group vs. the control group	*Ermp1*	-99.99512	439.96728	0.003002254	0.032672642
1-day post-SCI group vs. the control group	*Ezr*	230.50783	228.80487	8.09E-05	0.007211708
1-day post-SCI group vs. the control group	*Fbxw7*	-203.1735	444.6504	0.001352694	0.02169932
1-day post-SCI group vs. the control group	*Fkbp1b*	-213.32382	365.81846	0.002158758	0.027397804
1-day post-SCI group vs. the control group	*Fos*	1110.56552	986.67846	7.04E-06	0.003585212
1-day post-SCI group vs. the control group	*Fosl1*	142.80661	88.59625	0.000552161	0.014509282
1-day post-SCI group vs. the control group	*Gch1*	81.41662	76.06469	8.61E-05	0.007211708
1-day post-SCI group vs. the control group	*Gfer*	74.58695	202.88662	0.000303632	0.011079115
1-day post-SCI group vs. the control group	*Gpr37l1*	-792.841	1685.1964	0.003721487	0.036178901
1-day post-SCI group vs. the control group	*Gpx1*	1606.8665	2158.9334	0.000968605	0.018361148
1-day post-SCI group vs. the control group	*Gpx4*	-510.40167	2611.4	0.003383589	0.034539486
1-day post-SCI group vs. the control group	*Hbegf*	825.21415	628.65914	2.98E-05	0.004737239
1-day post-SCI group vs. the control group	*Hdac1*	173.91642	396.5403	0.002758601	0.031120602
1-day post-SCI group vs. the control group	*Hebp2*	-47.49467	163.043	0.004628621	0.04099615
1-day post-SCI group vs. the control group	*Hmox1*	1350.34424	841.12144	0.004393912	0.039740349
1-day post-SCI group vs. the control group	*Hmox2*	-271.6879	955.71326	0.003975705	0.037380824
1-day post-SCI group vs. the control group	*Hspa13*	35.8548	220.04738	0.002284022	0.028361537
1-day post-SCI group vs. the control group	*Hspb1*	7357.0604	5191.58884	2.78E-06	0.003093325
1-day post-SCI group vs. the control group	*Ier3*	657.95637	582.64702	3.98E-05	0.005496505
1-day post-SCI group vs. the control group	*Il6st*	264.51005	368.95948	0.00349599	0.035118498
1-day post-SCI group vs. the control group	*Ipcef1*	-20.52484	35.72509	0.004217609	0.038638996
1-day post-SCI group vs. the control group	*Itga5*	69.21076	48.93394	0.004580258	0.040758221
1-day post-SCI group vs. the control group	*Itga7*	66.22778	152.18782	0.002163272	0.027438357
1-day post-SCI group vs. the control group	*Itsn1*	-47.54857	105.56576	0.000944229	0.018041948
1-day post-SCI group vs. the control group	*Jak2*	76.97833	253.7499	0.000601641	0.015204599
1-day post-SCI group vs. the control group	*Jun*	539.5414	642.17034	0.000285429	0.010817187
1-day post-SCI group vs. the control group	*Krt1*	-55.01967	59.04212	0.005088306	0.043095241
1-day post-SCI group vs. the control group	*Lancl1*	-285.77735	743.67554	0.003157784	0.033431387
1-day post-SCI group vs. the control group	*Lcat*	-227.8467	169.42733	0.000126925	0.008205472
1-day post-SCI group vs. the control group	*Lpo*	158.33404	188.66949	0.000694032	0.015874272
1-day post-SCI group vs. the control group	*Map2k3*	160.3853	182.28498	0.00331278	0.034304575
1-day post-SCI group vs. the control group	*Map2k4*	-243.24118	637.64984	0.000582111	0.014915047
1-day post-SCI group vs. the control group	*Map2k6*	-55.43261	73.3081	0.006260724	0.048460657
1-day post-SCI group vs. the control group	*Map4k4*	131.67347	294.14208	0.003884743	0.036921231
1-day post-SCI group vs. the control group	*Mapk14*	36.26411	118.69539	0.004076348	0.037928079
1-day post-SCI group vs. the control group	*Mapk8*	-53.38208	190.1474	0.003680734	0.036069116

1-day post-SCI group vs. the control group	*Mapkapk3*	97.47562	98.99452	0.000387675	0.012413955
1-day post-SCI group vs. the control group	*Mapt*	-198.1518	873.59752	0.005016038	0.042683492
1-day post-SCI group vs. the control group	*Mcl1*	738.7369	928.38724	9.53E-05	0.007472806
1-day post-SCI group vs. the control group	*Mdm2*	70.68817	191.8467	0.001543462	0.023212156
1-day post-SCI group vs. the control group	*Met*	61.8712	64.66526	0.004172118	0.038346118
1-day post-SCI group vs. the control group	*Mmp3*	93.11056	71.24223	0.000880723	0.017528874
1-day post-SCI group vs. the control group	*Myc*	240.46216	173.4219	0.003976225	0.037380824
1-day post-SCI group vs. the control group	*Ndufa6*	-543.06183	1710.5254	0.000301577	0.011079115
1-day post-SCI group vs. the control group	*Nme2*	2821.267	4162.0142	0.000213402	0.009892553
1-day post-SCI group vs. the control group	*Nono*	194.40102	566.58966	0.003208616	0.033678328
1-day post-SCI group vs. the control group	*Nos3*	179.58338	234.82388	0.000659698	0.015566506
1-day post-SCI group vs. the control group	*P4hb*	1085.86267	1811.4646	5.79E-05	0.006517616
1-day post-SCI group vs. the control group	*Pcna*	1256.39483	1883.0464	0.000140064	0.008570098
1-day post-SCI group vs. the control group	*Pdk2*	-306.13155	357.98052	0.000162371	0.009060585
1-day post-SCI group vs. the control group	*Pdlim1*	49.70927	102.98143	0.001831759	0.025383641
1-day post-SCI group vs. the control group	*Phc3*	-45.76987	232.85618	0.003602254	0.035703487
1-day post-SCI group vs. the control group	*Pink1*	-789.3925	1832.835	0.002299195	0.028468435
1-day post-SCI group vs. the control group	*Plekha1*	218.37862	466.55182	0.001087973	0.019371326
1-day post-SCI group vs. the control group	*Ppargc1a*	-153.56475	194.2204	0.002751528	0.031074504
1-day post-SCI group vs. the control group	*Ppargc1b*	-150.08495	260.40558	0.004453807	0.039996705
1-day post-SCI group vs. the control group	*Ppp1ca*	763.11937	1544.03592	0.00367508	0.036030674
1-day post-SCI group vs. the control group	*Ppp1r15b*	96.4004	239.48024	0.004423785	0.039894567
1-day post-SCI group vs. the control group	*Ppp3ca*	-422.0765	1383.4236	0.000412491	0.012795205
1-day post-SCI group vs. the control group	*Prodh*	-59.04578	93.55163	0.001737593	0.024691116
1-day post-SCI group vs. the control group	*Prr5l*	52.9815	277.1544	0.001615361	0.023767117
1-day post-SCI group vs. the control group	*Ptgs2*	140.12614	100.42444	0.004551248	0.040569454
1-day post-SCI group vs. the control group	*Pxdn*	48.32225	186.1988	0.002006919	0.026572523
1-day post-SCI group vs. the control group	*Pxn*	123.64603	250.45672	0.000931452	0.017952363
1-day post-SCI group vs. the control group	*Rbbp7*	355.33083	1676.253	0.00576149	0.046133649
1-day post-SCI group vs. the control group	*Rcan1*	377.80173	486.74714	8.25E-05	0.007211708
1-day post-SCI group vs. the control group	*Rela*	338.39853	606.70892	0.002579329	0.030205297
1-day post-SCI group vs. the control group	*Ripk1*	55.31126	73.31577	0.003304711	0.034255103
1-day post-SCI group vs. the control group	*Ripk3*	47.75519	34.20492	0.000993504	0.018559499
1-day post-SCI group vs. the control group	*Rnf112*	-169.30975	206.1561	0.000134891	0.008485488
1-day post-SCI group vs. the control group	*Rnh1*	817.05448	919.56554	0.000108401	0.007694518
1-day post-SCI group vs. the control group	*Rpl13a*	1140.44667	3574.903	0.000446248	0.013287839
1-day post-SCI group vs. the control group	*Sdc1*	152.46481	110.04577	1.58E-05	0.004337489
1-day post-SCI group vs. the control group	*Selenbp1*	-43.85252	75.22469	0.000316899	0.011294413
1-day post-SCI group vs. the control group	*Slc25a24*	82.17894	81.82766	0.000183144	0.009328543
1-day post-SCI group vs. the control group	*Slc4a11*	28.63979	44.86229	0.003710877	0.036130005
1-day post-SCI group vs. the control group	*Slc8a1*	-92.13801	141.30074	0.001235214	0.02074628
1-day post-SCI group vs. the control group	*Smad1*	87.13585	313.67246	0.002216878	0.027846765
1-day post-SCI group vs. the control group	*Sphk1*	300.13088	219.99098	0.003273747	0.034085893
1-day post-SCI group vs. the control group	*Spry2*	152.94797	234.25758	6.10E-05	0.006706157
1-day post-SCI group vs. the control group	*Srxn1*	489.11882	513.51064	0.000117899	0.007986818
1-day post-SCI group vs. the control group	*Stat6*	65.10242	89.40466	0.001063838	0.019105016
1-day post-SCI group vs. the control group	*Syp*	-314.62568	727.80364	0.006332232	0.048706235
1-day post-SCI group vs. the control group	*Tnfrsf1a*	524.75273	524.02194	0.000919243	0.017814832
1-day post-SCI group vs. the control group	*Trap1*	118.33977	515.70426	0.002264189	0.028245311
1-day post-SCI group vs. the control group	*Trpm2*	-53.05423	93.47741	0.000829449	0.017154892
1-day post-SCI group vs. the control group	*Tsc1*	-22.8379	86.61319	0.006054833	0.047638587
1-day post-SCI group vs. the control group	*Txnrd1*	364.4674	429.57764	0.00016987	0.009161844
1-day post-SCI group vs. the control group	*Ucp2*	205.87817	347.0516	0.000620738	0.015362721
1-day post-SCI group vs. the control group	*Vcam1*	-20.9181	134.66554	0.006111008	0.04785347
1-day post-SCI group vs. the control group	*Xdh*	114.3905	95.3427	0.000384023	0.012334964

1-day post-SCI group vs. the control group	*Ybx3*	634.19613	815.07478	0.000359467	0.01188276
3-day post-SCI group vs. the control group	*Abcc1*	87.33582	170.83034	0.005510862	0.026983891
3-day post-SCI group vs. the control group	*Actb*	1160.33367	5145.9802	0.008306206	0.035609756
3-day post-SCI group vs. the control group	*Adam9*	774.22705	1012.68818	0.002220259	0.015383645
3-day post-SCI group vs. the control group	*Adora1*	-191.08195	239.89128	0.000598294	0.007565898
3-day post-SCI group vs. the control group	*Agap3*	-87.19858	167.6002	0.008158033	0.035203948
3-day post-SCI group vs. the control group	*Ago1*	-114.5691	403.58704	0.01025195	0.040807799
3-day post-SCI group vs. the control group	*Ago3*	-20.23444	52.726214	0.004767946	0.024636874
3-day post-SCI group vs. the control group	*Aif1*	369.62494	285.482724	0.001826734	0.013584012
3-day post-SCI group vs. the control group	*Alox5*	35.46426	62.426814	0.009719883	0.039398179
3-day post-SCI group vs. the control group	*Amph*	-580.39888	600.94962	0.000104427	0.003647602
3-day post-SCI group vs. the control group	*Anxa1*	517.89963	387.278614	0.000836722	0.008970545
3-day post-SCI group vs. the control group	*Apc*	-205.75787	463.01488	9.93E-06	0.002151772
3-day post-SCI group vs. the control group	*Arg1*	2804.25968	1683.916462	0.004465201	0.023714898
3-day post-SCI group vs. the control group	*Arhgdia*	820.5035	1859.9776	0.003896972	0.021780764
3-day post-SCI group vs. the control group	*Atf2*	-124.74333	346.2708	0.000320886	0.005679882
3-day post-SCI group vs. the control group	*Atox1*	200.21853	548.67832	3.42E-05	0.002570825
3-day post-SCI group vs. the control group	*Atp13a2*	-112.1113	378.49542	0.003202229	0.019324786
3-day post-SCI group vs. the control group	*Atp2a2*	-668.33677	1088.40744	6.43E-05	0.003139632
3-day post-SCI group vs. the control group	*Atp7a*	136.6669	203.82544	0.001225457	0.010747823
3-day post-SCI group vs. the control group	*Atr*	15.55706	67.816308	0.00550642	0.026979904
3-day post-SCI group vs. the control group	*Atrn*	-132.69495	222.92608	3.78E-05	0.002670311
3-day post-SCI group vs. the control group	*Axl*	194.68435	253.19996	6.14E-05	0.003069419
3-day post-SCI group vs. the control group	*Banf1*	399.76225	701.9166	0.006850571	0.031337972
3-day post-SCI group vs. the control group	*Bax*	304.06913	439.32598	0.000253185	0.00511295
3-day post-SCI group vs. the control group	*Bmp1*	135.19879	133.137782	0.005314258	0.026349596
3-day post-SCI group vs. the control group	*Bnip3*	-502.98633	1079.9442	0.01182638	0.045009009
3-day post-SCI group vs. the control group	*Braf*	-79.09196	122.005722	0.000394483	0.006272544
3-day post-SCI group vs. the control group	*Brf2*	-17.78178	90.63558	0.005401029	0.026632794
3-day post-SCI group vs. the control group	*Btg1*	483.6878	568.51458	0.000190429	0.00451948
3-day post-SCI group vs. the control group	*Btk*	104.29449	95.269036	0.001207197	0.010651379
3-day post-SCI group vs. the control group	*Camkk2*	-44.37403	101.45673	0.009418229	0.038563389
3-day post-SCI group vs. the control group	*Capns1*	818.6605	2299.0188	2.60E-05	0.002437423
3-day post-SCI group vs. the control group	*Casp3*	55.7896	91.73926	0.009081425	0.037646355
3-day post-SCI group vs. the control group	*Cat*	102.74393	859.30686	0.001353947	0.011367908
3-day post-SCI group vs. the control group	*Cbx6*	-638.06097	860.22942	1.80E-05	0.002248755
3-day post-SCI group vs. the control group	*Ccna2*	307.29883	224.154336	0.000380157	0.006176884
3-day post-SCI group vs. the control group	*Ccr1*	101.47187	72.34042	0.000236441	0.004950986
3-day post-SCI group vs. the control group	*Ccs*	77.40893	280.34216	0.006441654	0.030065025
3-day post-SCI group vs. the control group	*Cd36*	440.53515	276.239741	0.01335843	0.048873668
3-day post-SCI group vs. the control group	*Cd38*	195.52155	387.65998	0.009612384	0.039107145
3-day post-SCI group vs. the control group	*Cdc20*	365.12938	250.300882	0.002265167	0.015591
3-day post-SCI group vs. the control group	*Cdk1*	498.64049	335.567306	0.000756336	0.008495614
3-day post-SCI group vs. the control group	*Cdk10*	-81.86012	176.68288	0.000431812	0.006485703
3-day post-SCI group vs. the control group	*Cdk4*	278.27558	349.5078	1.50E-05	0.002248755
3-day post-SCI group vs. the control group	*Cdkn2b*	47.81591	43.969205	0.007067407	0.032020248
3-day post-SCI group vs. the control group	*Cdkn2c*	288.93908	229.4808	0.000158139	0.004248676
3-day post-SCI group vs. the control group	*Chrna4*	-37.46508	45.706714	0.000591621	0.007549965
3-day post-SCI group vs. the control group	*Ctnna1*	358.53695	750.78332	0.007365549	0.032870182
3-day post-SCI group vs. the control group	*Ctsl*	1548.3818	1670.17528	0.000157742	0.004248676
3-day post-SCI group vs. the control group	*Cttn*	355.54282	775.93424	0.006626277	0.030662368
3-day post-SCI group vs. the control group	*Cygb*	-164.0204	204.29046	0.004707138	0.024432513
3-day post-SCI group vs. the control group	*Cyp1b1*	227.21859	162.421664	0.000768343	0.008574957
3-day post-SCI group vs. the control group	*Dgkk*	-48.5409	63.637548	0.0131725	0.048406163
3-day post-SCI group vs. the control group	*Dhcr24*	-197.9956	193.987088	0.002779733	0.017709632

3-day post-SCI group vs. the control group	*Dhfr*	49.29858	74.785732	0.004226284	0.022919719
3-day post-SCI group vs. the control group	*Dst*	-139.98928	394.17648	0.000670983	0.007994188
3-day post-SCI group vs. the control group	*Ect2*	155.70896	104.005547	0.001614349	0.01261398
3-day post-SCI group vs. the control group	*Eed*	67.9529	155.76414	0.00047595	0.006768284
3-day post-SCI group vs. the control group	*Eif2s1*	158.20815	377.19374	0.000278595	0.005310053
3-day post-SCI group vs. the control group	*Endog*	-110.48542	192.6676	0.000116339	0.003770647
3-day post-SCI group vs. the control group	*Etfdh*	57.97733	188.0394	0.006258056	0.029479466
3-day post-SCI group vs. the control group	*Ets1*	103.43698	136.798876	0.01368254	0.049770309
3-day post-SCI group vs. the control group	*Ezh2*	187.12776	197.126128	0.001127408	0.010313006
3-day post-SCI group vs. the control group	*Ezr*	240.22643	234.636028	0.003613947	0.0207956
3-day post-SCI group vs. the control group	*Fbxw7*	-338.15643	363.66064	1.95E-05	0.002248755
3-day post-SCI group vs. the control group	*Fkbp1b*	-285.87078	322.29028	4.31E-05	0.002779313
3-day post-SCI group vs. the control group	*Fos*	647.66855	708.94028	9.92E-05	0.003574483
3-day post-SCI group vs. the control group	*Fosl1*	44.66041	29.708534	0.009297005	0.038265349
3-day post-SCI group vs. the control group	*Gch1*	46.85022	55.324846	0.001043851	0.009943577
3-day post-SCI group vs. the control group	*Gclc*	-95.90387	337.36028	0.000575507	0.00747262
3-day post-SCI group vs. the control group	*Ggt7*	-199.55173	304.00356	0.001140553	0.010354597
3-day post-SCI group vs. the control group	*Glrx2*	-70.07258	302.7486	0.001264667	0.010961387
3-day post-SCI group vs. the control group	*Gnb2*	627.13142	1477.5961	0.005493253	0.026934411
3-day post-SCI group vs. the control group	*Gpr37*	-556.32497	1065.87452	0.01111513	0.043122138
3-day post-SCI group vs. the control group	*Gpx1*	2405.92683	2638.3696	3.12E-05	0.002554096
3-day post-SCI group vs. the control group	*Gpx3*	312.9932	405.18662	0.000436497	0.006512724
3-day post-SCI group vs. the control group	*Gpx7*	334.15896	282.231782	0.000803461	0.008789314
3-day post-SCI group vs. the control group	*Gpx8*	398.17925	351.698066	0.000185989	0.00445987
3-day post-SCI group vs. the control group	*Gsr*	127.78042	224.9762	0.003491296	0.020360839
3-day post-SCI group vs. the control group	*Gstt2*	54.269	67.040028	0.0116236	0.044489453
3-day post-SCI group vs. the control group	*Hao1*	16.33031	15.046797	0.007884375	0.034366836
3-day post-SCI group vs. the control group	*Hbegf*	252.74455	285.17738	0.000474614	0.006768284
3-day post-SCI group vs. the control group	*Hdac1*	375.76655	517.65038	3.80E-05	0.002670311
3-day post-SCI group vs. the control group	*Hebp2*	-80.9368	142.97772	0.000579437	0.007500245
3-day post-SCI group vs. the control group	*Hif1a*	337.49378	581.67902	4.27E-06	0.002030723
3-day post-SCI group vs. the control group	*Hmox1*	2604.88944	1593.848558	0.00676219	0.031070248
3-day post-SCI group vs. the control group	*Hspb1*	3786.1824	3049.06204	0.000473775	0.006768284
3-day post-SCI group vs. the control group	*Htra2*	42.92157	152.73924	0.004858823	0.024946114
3-day post-SCI group vs. the control group	*Id1*	348.29955	428.98578	3.51E-06	0.002030723
3-day post-SCI group vs. the control group	*Ier3*	443.18807	453.78604	0.001419014	0.011669444
3-day post-SCI group vs. the control group	*Igfbp2*	860.40263	1071.77728	0.00220499	0.015318646
3-day post-SCI group vs. the control group	*Il18rap*	15.27559	14.521057	0.005162138	0.025860288
3-day post-SCI group vs. the control group	*Il6st*	215.18225	339.3628	5.38E-05	0.002978006
3-day post-SCI group vs. the control group	*Impact*	-166.46747	390.59132	0.01232334	0.046303018
3-day post-SCI group vs. the control group	*Ipcef1*	-28.60314	30.878106	0.000367252	0.006055891
3-day post-SCI group vs. the control group	*Itga6*	376.07768	544.18906	0.000651115	0.007888416
3-day post-SCI group vs. the control group	*Itgb5*	613.42912	610.61662	0.000692099	0.008124841
3-day post-SCI group vs. the control group	*Jak2*	39.90713	231.50718	0.009153287	0.037845949
3-day post-SCI group vs. the control group	*Jun*	535.8263	639.94128	0.000400491	0.006300688
3-day post-SCI group vs. the control group	*Junb*	109.44258	113.956334	0.001553103	0.012314772
3-day post-SCI group vs. the control group	*Krt1*	-45.10325	64.991968	0.009015437	0.037432139
3-day post-SCI group vs. the control group	*Lancl1*	-351.48025	704.2538	0.000164381	0.004276958
3-day post-SCI group vs. the control group	*Lpo*	39.85043	117.579326	0.008775923	0.036793146
3-day post-SCI group vs. the control group	*Maoa*	42.68408	240.1429	0.01348253	0.049188298
3-day post-SCI group vs. the control group	*Map2k3*	103.8924	148.389238	8.78E-05	0.003410992
3-day post-SCI group vs. the control group	*Map2k4*	-416.54762	533.66598	0.000154568	0.004220427
3-day post-SCI group vs. the control group	*Map2k6*	-46.1591	78.87221	0.002076763	0.014740221
3-day post-SCI group vs. the control group	*Map4k4*	120.164	287.2364	0.002574928	0.016833258
3-day post-SCI group vs. the control group	*Mapk10*	-743.5281	821.52814	0.000245744	0.005044581

3-day post-SCI group vs. the control group	*Mapk14*	23.59597	111.09451	0.004770663	0.024636874
3-day post-SCI group vs. the control group	*Mapk3*	357.27283	1327.1392	0.00063411	0.007782061
3-day post-SCI group vs. the control group	*Mapk8*	-99.03708	162.7544	0.001737036	0.013142373
3-day post-SCI group vs. the control group	*Mapkapk2*	126.99605	249.15868	0.005853704	0.028086444
3-day post-SCI group vs. the control group	*Mapkapk3*	86.67108	92.5118	0.000478666	0.006782133
3-day post-SCI group vs. the control group	*Mapt*	-448.58327	723.33864	0.000216865	0.004773089
3-day post-SCI group vs. the control group	*Marcksl1*	274.52153	678.71202	0.000555756	0.007367838
3-day post-SCI group vs. the control group	*Mark3*	147.8783	422.12658	0.000384825	0.00621392
3-day post-SCI group vs. the control group	*Mcl1*	735.0429	926.17084	2.12E-05	0.002256182
3-day post-SCI group vs. the control group	*Mcm4*	176.1011	229.79596	0.001435879	0.01173396
3-day post-SCI group vs. the control group	*Melk*	107.1985	79.021598	0.002596259	0.016945294
3-day post-SCI group vs. the control group	*Met*	47.58215	56.091828	0.002954895	0.018408617
3-day post-SCI group vs. the control group	*Mgat3*	-53.06687	83.933776	0.005070325	0.025559917
3-day post-SCI group vs. the control group	*Mgmt*	17.40952	13.372234	0.002558372	0.01679596
3-day post-SCI group vs. the control group	*Mgst1*	430.73135	612.19986	0.001338207	0.011279994
3-day post-SCI group vs. the control group	*Msrb2*	-62.48097	183.02452	0.004558406	0.023987567
3-day post-SCI group vs. the control group	*Myc*	130.01396	107.152984	0.00601099	0.028647584
3-day post-SCI group vs. the control group	*Mylk*	241.13417	235.967298	0.001061655	0.010053612
3-day post-SCI group vs. the control group	*Ncf1*	342.5943	244.90548	8.67E-07	0.001942513
3-day post-SCI group vs. the control group	*Ncoa7*	-223.89417	287.9028	0.000336054	0.005819161
3-day post-SCI group vs. the control group	*Ndufa12*	-807.274	2109.9176	0.001136716	0.010354315
3-day post-SCI group vs. the control group	*Ndufa6*	-729.70883	1598.5372	0.000124417	0.003833547
3-day post-SCI group vs. the control group	*Ndufs8*	-667.84983	1835.6686	0.002568111	0.016830182
3-day post-SCI group vs. the control group	*Nfatc1*	26.87594	48.049344	0.003037997	0.018714148
3-day post-SCI group vs. the control group	*Nfe2l2*	179.34383	265.0387	0.00234367	0.015924776
3-day post-SCI group vs. the control group	*Nfkb1*	84.12043	223.74716	0.00567423	0.027539952
3-day post-SCI group vs. the control group	*Nme2*	2231.82833	3808.351	0.000661572	0.007950319
3-day post-SCI group vs. the control group	*Nono*	159.10365	545.41124	0.000225513	0.004814189
3-day post-SCI group vs. the control group	*Nox4*	20.30776	20.699571	0.006480673	0.030189956
3-day post-SCI group vs. the control group	*Nudt2*	-120.90493	357.53184	0.002741108	0.017581946
3-day post-SCI group vs. the control group	*Oxr1*	-466.8333	928.06952	0.003797236	0.021418883
3-day post-SCI group vs. the control group	*P4hb*	1959.40767	2335.5916	0.000851548	0.009024129
3-day post-SCI group vs. the control group	*Paip1*	-63.79042	94.290648	0.000712659	0.008238136
3-day post-SCI group vs. the control group	*Parp1*	100.87397	287.43438	0.002915817	0.018258664
3-day post-SCI group vs. the control group	*Pawr*	40.73152	45.52892	0.00218286	0.015190252
3-day post-SCI group vs. the control group	*Pcna*	1926.04483	2284.8364	1.80E-05	0.002248755
3-day post-SCI group vs. the control group	*Pdgfra*	134.99598	213.02144	0.001405564	0.011625559
3-day post-SCI group vs. the control group	*Pdk1*	46.26342	144.8776	0.001805095	0.013466396
3-day post-SCI group vs. the control group	*Pdk2*	-251.60355	390.69732	0.000204718	0.004636565
3-day post-SCI group vs. the control group	*Pdlim1*	52.89294	104.891626	0.007448577	0.033127069
3-day post-SCI group vs. the control group	*Phc3*	-78.4081	213.27324	0.000104623	0.003647602
3-day post-SCI group vs. the control group	*Pink1*	-1117.30017	1636.0904	3.33E-05	0.002554096
3-day post-SCI group vs. the control group	*Plekha1*	69.80098	377.40524	0.000116485	0.003770647
3-day post-SCI group vs. the control group	*Pnpla8*	-101.7818	402.37562	0.006139874	0.029057602
3-day post-SCI group vs. the control group	*Pon2*	82.15902	225.56056	0.000311504	0.005590843
3-day post-SCI group vs. the control group	*Ppargc1a*	-173.03066	182.540852	0.000222527	0.004806135
3-day post-SCI group vs. the control group	*Ppargc1b*	-219.52572	218.74112	2.12E-05	0.002256182
3-day post-SCI group vs. the control group	*Ppif*	-35.21735	121.16544	0.008129896	0.035106982
3-day post-SCI group vs. the control group	*Ppp1ca*	792.5827	1561.71392	0.001401342	0.011602046
3-day post-SCI group vs. the control group	*Ppp1r15b*	74.7324	226.47944	0.01108533	0.043046651
3-day post-SCI group vs. the control group	*Ppp3ca*	-680.5349	1228.34856	0.000225145	0.004813153
3-day post-SCI group vs. the control group	*Prdx4*	152.22322	224.76688	3.72E-06	0.002030723
3-day post-SCI group vs. the control group	*Prdx6*	1349.97188	1827.68258	1.89E-05	0.002248755
3-day post-SCI group vs. the control group	*Prkaa2*	-38.00036	83.049282	0.01157051	0.044359662
3-day post-SCI group vs. the control group	*Prodh*	-61.03757	92.356558	0.00036401	0.006034123

3-day post-SCI group vs. the control group	*Ptgs1*	137.72762	140.484786	0.000736044	0.008389937
3-day post-SCI group vs. the control group	*Ptgs2*	53.25074	48.299202	0.000804965	0.008795034
3-day post-SCI group vs. the control group	*Ptprk*	-19.5205	41.325042	0.002569391	0.016833258
3-day post-SCI group vs. the control group	*Pxdn*	153.95648	249.57934	0.001951402	0.01415702
3-day post-SCI group vs. the control group	*Pxn*	203.91477	298.61796	0.005160814	0.025860288
3-day post-SCI group vs. the control group	*Rb1*	93.44752	244.90586	0.00080378	0.008789314
3-day post-SCI group vs. the control group	*Rbbp7*	562.6355	1800.6358	0.000732942	0.008366566
3-day post-SCI group vs. the control group	*Rbpms*	32.16441	45.140462	0.003605862	0.020778631
3-day post-SCI group vs. the control group	*Rcan1*	200.5173	380.37648	0.001122439	0.010296359
3-day post-SCI group vs. the control group	*Rela*	146.07487	491.31472	0.000614968	0.007646327
3-day post-SCI group vs. the control group	*Rest*	19.61064	38.992198	0.005963704	0.028474339
3-day post-SCI group vs. the control group	*Ripk1*	87.27709	92.495264	5.15E-05	0.002945252
3-day post-SCI group vs. the control group	*Ripk3*	56.9376	39.714369	0.002693212	0.017350667
3-day post-SCI group vs. the control group	*Rnf112*	-157.14025	213.4578	0.00020439	0.004636565
3-day post-SCI group vs. the control group	*Rnf2*	87.62342	174.1527	0.004488416	0.023771476
3-day post-SCI group vs. the control group	*Rnh1*	1110.97515	1095.91794	5.58E-05	0.002978006
3-day post-SCI group vs. the control group	*Rpl13a*	705.05333	3313.667	0.000433927	0.006485703
3-day post-SCI group vs. the control group	*Rrm2b*	-37.72357	104.880158	0.009605179	0.0391061
3-day post-SCI group vs. the control group	*Rxrb*	-77.3324	260.36546	0.008792248	0.036828446
3-day post-SCI group vs. the control group	*S100a1*	359.6833	1132.14068	0.01281586	0.047489651
3-day post-SCI group vs. the control group	*Scara3*	194.70334	244.64283	0.006413928	0.029966215
3-day post-SCI group vs. the control group	*Sdc1*	100.09478	78.623754	0.000960034	0.009515727
3-day post-SCI group vs. the control group	*Sfpq*	87.41467	277.083	3.30E-05	0.002554096
3-day post-SCI group vs. the control group	*Sirpa*	306.7568	444.19278	0.01067707	0.041906937
3-day post-SCI group vs. the control group	*Sirt1*	49.73858	140.7712	0.002971803	0.018464212
3-day post-SCI group vs. the control group	*Slc23a2*	-42.35898	282.83946	0.002825102	0.01790492
3-day post-SCI group vs. the control group	*Slc25a24*	173.29894	136.499658	7.11E-05	0.00320517
3-day post-SCI group vs. the control group	*Slc4a11*	20.05936	39.714034	0.01312686	0.048323743
3-day post-SCI group vs. the control group	*Slc8a1*	-124.60312	121.821676	5.09E-05	0.002927626
3-day post-SCI group vs. the control group	*Smad1*	136.99545	343.58822	0.002010384	0.014452775
3-day post-SCI group vs. the control group	*Snca*	-198.85363	326.77172	0.0129383	0.047798689
3-day post-SCI group vs. the control group	*Sod2*	-140.88963	459.04392	0.002502077	0.016573561
3-day post-SCI group vs. the control group	*Srxn1*	257.59888	374.59868	0.000466424	0.006740501
3-day post-SCI group vs. the control group	*Stat6*	159.87416	146.267704	0.000472932	0.006768284
3-day post-SCI group vs. the control group	*Stau2*	-317.43338	312.70142	0.000912476	0.009296267
3-day post-SCI group vs. the control group	*Stk24*	-122.24258	405.4817	0.000299599	0.005479657
3-day post-SCI group vs. the control group	*Syp*	-535.65445	595.18638	0.001323317	0.011199307
3-day post-SCI group vs. the control group	*Tank*	34.6578	69.830234	0.001922552	0.014025937
3-day post-SCI group vs. the control group	*Tbc1d24*	-44.39163	160.88062	0.01211497	0.045780854
3-day post-SCI group vs. the control group	*Tfdp1*	194.51223	360.69324	0.000667629	0.007977338
3-day post-SCI group vs. the control group	*Tlr4*	29.47181	27.211953	0.001743778	0.013164667
3-day post-SCI group vs. the control group	*Tlr6*	25.03729	23.356177	0.001301262	0.011100195
3-day post-SCI group vs. the control group	*Tnfrsf1a*	540.18547	533.28158	0.000292691	0.005419993
3-day post-SCI group vs. the control group	*Tnik*	-80.75898	190.71966	0.001711432	0.013024402
3-day post-SCI group vs. the control group	*Tnrc6a*	-58.91238	326.94162	0.01290621	0.047748792
3-day post-SCI group vs. the control group	*Tnrc6c*	-71.16343	173.74574	0.001662595	0.012812015
3-day post-SCI group vs. the control group	*Tpp1*	418.56917	898.3696	0.007341695	0.032798878
3-day post-SCI group vs. the control group	*Trpm2*	-72.50394	81.807586	0.000718969	0.008286057
3-day post-SCI group vs. the control group	*Tspan31*	189.68097	602.30818	0.000984472	0.009658672
3-day post-SCI group vs. the control group	*Txnip*	305.3218	369.56928	0.001927611	0.014052992
3-day post-SCI group vs. the control group	*Txnrd1*	247.55523	359.43034	0.000430014	0.006474813
3-day post-SCI group vs. the control group	*Uba52*	724.7975	5335.674	0.002533448	0.016708235
3-day post-SCI group vs. the control group	*Ube2a*	126.50663	647.21768	0.001689367	0.012936859
3-day post-SCI group vs. the control group	*Ubqln1*	-203.80635	651.53374	0.000258586	0.005145508
3-day post-SCI group vs. the control group	*Ucp2*	516.56817	533.4656	0.001381362	0.011520967

3-day post-SCI group vs. the control group	*Vcam1*	54.39697	179.85458	0.000908252	0.00928755
3-day post-SCI group vs. the control group	*Vrk2*	33.66363	43.305966	0.003861515	0.021634878
3-day post-SCI group vs. the control group	*Xdh*	218.22403	157.64282	0.001476635	0.011943135
3-day post-SCI group vs. the control group	*Ybx3*	444.2097	701.08292	0.000666719	0.007975344
3-day post-SCI group vs. the control group	*Zc3h12a*	19.24059	15.902871	0.005371412	0.026527069
7-day post-SCI group vs. the control group	*Adam9*	421.62438	801.12658	0.001547424	0.036193674
7-day post-SCI group vs. the control group	*Aif1*	540.90047	388.24804	0.000123329	0.023069882
7-day post-SCI group vs. the control group	*Amph*	-475.24328	664.04298	0.00106252	0.032741813
7-day post-SCI group vs. the control group	*Apc*	-160.3763	490.24382	0.000926214	0.031194533
7-day post-SCI group vs. the control group	*Apoe*	3448.919	7254.7624	7.33E-05	0.022777069
7-day post-SCI group vs. the control group	*Atp2a2*	-476.74917	1203.36	0.000611822	0.029480954
7-day post-SCI group vs. the control group	*Atp7a*	82.67767	171.4319	0.002377916	0.040808265
7-day post-SCI group vs. the control group	*Axl*	239.66298	280.18714	0.001824242	0.037452109
7-day post-SCI group vs. the control group	*Btg1*	339.67387	482.10622	0.000211244	0.024447703
7-day post-SCI group vs. the control group	*Btk*	85.22746	83.82882	0.003440875	0.045443123
7-day post-SCI group vs. the control group	*Capn2*	193.519	917.3306	0.000698612	0.029875959
7-day post-SCI group vs. the control group	*Cbx6*	-489.73747	949.22352	0.000725951	0.03007147
7-day post-SCI group vs. the control group	*Cd36*	459.05098	287.34924	0.004043505	0.047142662
7-day post-SCI group vs. the control group	*Cd38*	212.40108	397.7877	0.004081657	0.047250859
7-day post-SCI group vs. the control group	*Cdc20*	54.76859	64.08441	0.004181222	0.047466133
7-day post-SCI group vs. the control group	*Cdh11*	419.04523	708.01944	0.003509841	0.045590219
7-day post-SCI group vs. the control group	*Clu*	823.8435	5455.2496	0.000574247	0.029152124
7-day post-SCI group vs. the control group	*Ctnnb1*	487.50717	1955.0058	0.004293243	0.048051197
7-day post-SCI group vs. the control group	*Ctsl*	1417.63613	1591.72788	0.000875547	0.030908799
7-day post-SCI group vs. the control group	*Cybb*	275.60892	195.64583	0.002821133	0.042986133
7-day post-SCI group vs. the control group	*Dhcr24*	-202.91278	191.03678	0.002203494	0.039668227
7-day post-SCI group vs. the control group	*Dst*	-138.35315	395.15816	0.001798446	0.03728371
7-day post-SCI group vs. the control group	*Ets2*	-45.23632	118.68236	0.000913227	0.031036628
7-day post-SCI group vs. the control group	*Ezr*	57.0589	124.73551	0.001824157	0.037452109
7-day post-SCI group vs. the control group	*Fbxw7*	-247.35893	418.13914	0.000558327	0.028980071
7-day post-SCI group vs. the control group	*Fkbp1b*	-219.87915	361.88526	0.000154299	0.023777118
7-day post-SCI group vs. the control group	*Fos*	280.98782	488.93184	0.003920326	0.046800925
7-day post-SCI group vs. the control group	*Gch1*	34.94607	48.18236	0.002214046	0.039704549
7-day post-SCI group vs. the control group	*Gclc*	-73.9547	350.52978	0.000115437	0.023069882
7-day post-SCI group vs. the control group	*Gpx1*	1448.59183	2063.9686	0.00023261	0.024522128
7-day post-SCI group vs. the control group	*Gpx3*	344.20577	423.91416	0.004254923	0.047972762
7-day post-SCI group vs. the control group	*Hbegf*	68.93875	174.8939	0.004030347	0.047142662
7-day post-SCI group vs. the control group	*Hdac1*	187.13412	404.47092	2.29E-05	0.019135385
7-day post-SCI group vs. the control group	*Hebp2*	-56.45503	157.66678	0.002458535	0.041138747
7-day post-SCI group vs. the control group	*Hif1a*	188.28068	492.15116	0.003625123	0.046036192
7-day post-SCI group vs. the control group	*Hspb1*	2260.26073	2133.50904	2.20E-05	0.019135385
7-day post-SCI group vs. the control group	*Id1*	316.36115	409.82274	0.001219336	0.034200747
7-day post-SCI group vs. the control group	*Il18bp*	101.54531	75.53672	0.001992662	0.038545794
7-day post-SCI group vs. the control group	*Il6st*	180.72798	318.69024	0.000430383	0.027310949
7-day post-SCI group vs. the control group	*Itga6*	274.35192	483.1536	0.000554996	0.028951613
7-day post-SCI group vs. the control group	*Itgb5*	816.51532	732.46834	0.00308132	0.043599311
7-day post-SCI group vs. the control group	*Jun*	332.00373	517.64774	0.002026967	0.038705767
7-day post-SCI group vs. the control group	*Lancl1*	-224.47045	780.45968	0.002462291	0.041138747
7-day post-SCI group vs. the control group	*Lcat*	150.31042	396.3216	0.004628118	0.049392235
7-day post-SCI group vs. the control group	*Map2k3*	53.85664	118.36778	0.001932588	0.037912233
7-day post-SCI group vs. the control group	*Map2k4*	-300.55952	603.25884	0.003073721	0.043599311
7-day post-SCI group vs. the control group	*Mapk10*	-599.04813	908.21612	0.00255628	0.041828944
7-day post-SCI group vs. the control group	*Mapk14*	23.12021	110.80905	0.001409211	0.035075249
7-day post-SCI group vs. the control group	*Mapk8*	-69.10992	180.7107	0.001746685	0.037201699
7-day post-SCI group vs. the control group	*Mapt*	-280.6213	824.11582	0.003214587	0.044193144

7-day post-SCI group vs. the control group	*Marcksl1*	248.67827	663.20606	0.004091592	0.047250859
7-day post-SCI group vs. the control group	*Mcl1*	590.83157	839.64404	8.62E-05	0.023069882
7-day post-SCI group vs. the control group	*Mcm4*	47.9802	152.92342	0.002044299	0.038792854
7-day post-SCI group vs. the control group	*Mgst1*	456.39582	627.59854	0.000129275	0.023069882
7-day post-SCI group vs. the control group	*Mylk*	141.2158	176.01628	0.000206188	0.024447703
7-day post-SCI group vs. the control group	*Ncf1*	274.32253	203.94242	0.001560711	0.036193674
7-day post-SCI group vs. the control group	*Ncoa7*	-155.77077	328.77684	0.001589853	0.036260524
7-day post-SCI group vs. the control group	*Ndufa12*	-746.24967	2146.5322	0.00219513	0.039604203
7-day post-SCI group vs. the control group	*Ndufa6*	-407.15017	1792.0724	0.000442482	0.027492003
7-day post-SCI group vs. the control group	*Nfe2l2*	215.54157	286.75734	4.42E-05	0.022329646
7-day post-SCI group vs. the control group	*Pdk2*	-139.89058	457.7251	0.003728318	0.046495636
7-day post-SCI group vs. the control group	*Phc1*	-56.7291	317.41984	0.003396182	0.045225934
7-day post-SCI group vs. the control group	*Pink1*	-450.63117	2036.0918	0.002963882	0.043506392
7-day post-SCI group vs. the control group	*Ppargc1a*	-125.85945	210.84358	0.002509131	0.041580449
7-day post-SCI group vs. the control group	*Ppargc1b*	-166.82298	250.36276	8.25E-05	0.023069882
7-day post-SCI group vs. the control group	*Ppp3ca*	-455.29617	1363.4918	0.001396672	0.034909875
7-day post-SCI group vs. the control group	*Prdx6*	1124.51288	1692.40718	9.19E-05	0.023069882
7-day post-SCI group vs. the control group	*Prkcd*	98.25987	159.59025	0.001537288	0.036193674
7-day post-SCI group vs. the control group	*Prr5l*	-59.71877	209.53424	9.83E-05	0.023069882
7-day post-SCI group vs. the control group	*Ptgs1*	98.45022	116.91835	6.33E-05	0.02247159
7-day post-SCI group vs. the control group	*Rbbp7*	223.75717	1597.3088	0.001688632	0.03684732
7-day post-SCI group vs. the control group	*Rcan1*	143.3928	346.10178	0.004275872	0.048051197
7-day post-SCI group vs. the control group	*Ripk1*	73.141	84.01361	0.002591091	0.042079247
7-day post-SCI group vs. the control group	*Rnh1*	678.50448	836.43554	0.001243495	0.034235259
7-day post-SCI group vs. the control group	*S100a1*	760.75363	1372.78288	0.000116329	0.023069882
7-day post-SCI group vs. the control group	*Sirpa*	464.98583	539.1302	0.000488782	0.028256555
7-day post-SCI group vs. the control group	*Slc25a24*	85.35727	83.73466	0.001548349	0.036193674
7-day post-SCI group vs. the control group	*Slc8a1*	-98.4429	137.51781	0.002323483	0.040503321
7-day post-SCI group vs. the control group	*Stat1*	124.94599	180.58054	0.000830578	0.030908799
7-day post-SCI group vs. the control group	*Stat6*	131.74846	129.39228	0.004054448	0.047142662
7-day post-SCI group vs. the control group	*Stau2*	-216.47095	373.27888	0.004625608	0.049392235
7-day post-SCI group vs. the control group	*Tlr4*	26.14574	25.21631	0.003600949	0.04588039
7-day post-SCI group vs. the control group	*Trem2*	582.00411	373.64326	0.000448229	0.027602193
7-day post-SCI group vs. the control group	*Trpm2*	-50.83481	94.80906	0.002009124	0.038563718
7-day post-SCI group vs. the control group	*Txnip*	334.85583	387.2897	0.00167681	0.036776741
7-day post-SCI group vs. the control group	*Ucp2*	627.28767	599.8973	0.003071399	0.043599311
7-day post-SCI group vs. the control group	*Vcam1*	87.669	199.8178	0.002119322	0.03896781
7-day post-SCI group vs. the control group	*Xdh*	199.0065	146.1123	0.000126198	0.023069882
28-day post-SCI group vs. the control group	*Actb*	1749.646	5499.5676	0.002525522	0.031781269
28-day post-SCI group vs. the control group	*Ago1*	-94.17783	415.8218	0.000472764	0.013862769
28-day post-SCI group vs. the control group	*Aif1*	510.83707	370.21	3.28E-07	0.00086214
28-day post-SCI group vs. the control group	*Amph*	-329.49202	751.49374	0.00172564	0.026281663
28-day post-SCI group vs. the control group	*Apc*	-147.6765	497.8637	0.001849192	0.027132261
28-day post-SCI group vs. the control group	*Apoe*	4126.71633	7661.4408	0.00227539	0.030115238
28-day post-SCI group vs. the control group	*Atp2a2*	-427.56783	1232.8688	0.00045366	0.013554999
28-day post-SCI group vs. the control group	*Axl*	264.32805	294.98618	0.000690571	0.016659841
28-day post-SCI group vs. the control group	*Bmp1*	29.39102	69.65312	0.005486394	0.048821638
28-day post-SCI group vs. the control group	*Capns1*	239.45583	1951.496	0.001237261	0.022272305
28-day post-SCI group vs. the control group	*Cbx6*	-512.1763	935.76022	0.000896238	0.019290887
28-day post-SCI group vs. the control group	*Cd36*	45.59063	39.27303	0.001739927	0.026389306
28-day post-SCI group vs. the control group	*Cd38*	142.51665	355.85704	0.003081102	0.035489793
28-day post-SCI group vs. the control group	*Cdkn2c*	57.41582	90.56684	0.000837756	0.018622047
28-day post-SCI group vs. the control group	*Clu*	2070.71883	6203.3748	1.69E-05	0.003779711
28-day post-SCI group vs. the control group	*Ctsl*	1370.8328	1563.64588	9.66E-05	0.007433528
28-day post-SCI group vs. the control group	*Cybb*	240.14968	174.37029	0.005164901	0.047046941

28-day post-SCI group vs. the control group	*Cyp1b1*	109.55499	91.8235	0.000592905	0.015424492
28-day post-SCI group vs. the control group	*Dhcr24*	-223.05395	178.95208	0.001335091	0.023086844
28-day post-SCI group vs. the control group	*Dst*	-108.44285	413.10434	0.000198323	0.009557362
28-day post-SCI group vs. the control group	*Eif2s1*	39.26425	305.8274	0.001941517	0.027792797
28-day post-SCI group vs. the control group	*Ets2*	-47.33374	117.42391	0.000694289	0.016667742
28-day post-SCI group vs. the control group	*Fbxw7*	-213.17333	438.6505	0.000202857	0.009684112
28-day post-SCI group vs. the control group	*Fkbp1b*	-222.04758	360.5842	0.000297847	0.011190238
28-day post-SCI group vs. the control group	*Fos*	488.52602	613.45476	0.002160196	0.029253295
28-day post-SCI group vs. the control group	*Gpx1*	1299.65283	1974.6052	0.000153997	0.009046085
28-day post-SCI group vs. the control group	*Gpx3*	197.6049	335.95364	0.00352218	0.038523726
28-day post-SCI group vs. the control group	*Gpx8*	224.46559	247.46987	0.000455588	0.013585387
28-day post-SCI group vs. the control group	*Gskip*	-34.99967	237.7272	0.003172854	0.036107045
28-day post-SCI group vs. the control group	*Hbegf*	158.72542	228.7659	0.001843725	0.027121707
28-day post-SCI group vs. the control group	*Hdac1*	132.67545	371.79572	0.002216316	0.02958974
28-day post-SCI group vs. the control group	*Hebp2*	-75.8629	146.02206	0.003543233	0.038672708
28-day post-SCI group vs. the control group	*Hif1a*	236.01678	520.79282	0.001254353	0.022414315
28-day post-SCI group vs. the control group	*Hmox2*	-195.2025	1001.6045	0.00022887	0.010135516
28-day post-SCI group vs. the control group	*Hspb1*	2481.37373	2266.17684	1.34E-05	0.003503026
28-day post-SCI group vs. the control group	*Id1*	352.96505	431.78508	7.07E-06	0.002724234
28-day post-SCI group vs. the control group	*Ier3*	142.0264	273.08904	0.000474441	0.013869394
28-day post-SCI group vs. the control group	*Il18bp*	55.20164	47.73052	0.002325526	0.030518663
28-day post-SCI group vs. the control group	*Il1a*	19.79459	23.75991	0.003212952	0.036483574
28-day post-SCI group vs. the control group	*Il6st*	231.13318	348.93336	0.004334236	0.042897191
28-day post-SCI group vs. the control group	*Itgb5*	697.68335	661.16916	8.35E-05	0.007124049
28-day post-SCI group vs. the control group	*Jun*	228.14773	455.33414	0.002726356	0.033030489
28-day post-SCI group vs. the control group	*Lancl1*	-289.28408	741.5715	0.000534795	0.01466564
28-day post-SCI group vs. the control group	*Lcat*	142.18615	391.44704	0.001064711	0.020514475
28-day post-SCI group vs. the control group	*Map2k3*	44.82681	112.94988	0.005425561	0.048501952
28-day post-SCI group vs. the control group	*Map2k4*	-304.21988	601.06262	0.000943359	0.019734173
28-day post-SCI group vs. the control group	*Map2k6*	-41.39796	81.7289	0.002520368	0.031754812
28-day post-SCI group vs. the control group	*Mapk1*	-140.94222	683.01792	0.005321158	0.047966473
28-day post-SCI group vs. the control group	*Mapk10*	-428.01827	1010.83404	0.001001301	0.020054014
28-day post-SCI group vs. the control group	*Mapkapk2*	73.59422	217.11758	3.97E-05	0.004975424
28-day post-SCI group vs. the control group	*Mcl1*	603.41623	847.19484	0.00086846	0.018947717
28-day post-SCI group vs. the control group	*Met*	31.72001	46.57455	0.004127982	0.041850859
28-day post-SCI group vs. the control group	*Mgst1*	491.05968	648.39686	5.94E-05	0.006287558
28-day post-SCI group vs. the control group	*Mmp14*	51.23779	106.28849	0.00567143	0.049745112
28-day post-SCI group vs. the control group	*Mpv17*	78.66752	184.65356	0.005576501	0.049265407
28-day post-SCI group vs. the control group	*Mylk*	156.69164	185.30178	0.000106243	0.007733667
28-day post-SCI group vs. the control group	*Ncf1*	154.57867	132.0961	8.88E-05	0.007337595
28-day post-SCI group vs. the control group	*Ncoa7*	-164.85007	323.32926	0.000919748	0.019494531
28-day post-SCI group vs. the control group	*Ndufa6*	-384.80717	1805.4782	0.00424654	0.042534886
28-day post-SCI group vs. the control group	*Net1*	60.49906	97.39551	0.004127684	0.041850859
28-day post-SCI group vs. the control group	*Nfe2l2*	280.58057	325.78074	2.50E-05	0.004241122
28-day post-SCI group vs. the control group	*Parp1*	30.38403	245.14042	0.003413136	0.037807887
28-day post-SCI group vs. the control group	*Pawr*	68.1886	62.00317	0.001827591	0.027093642
28-day post-SCI group vs. the control group	*Pdk1*	40.12365	141.19374	0.004948857	0.045963371
28-day post-SCI group vs. the control group	*Pdk2*	-164.48302	442.96964	0.000174835	0.009325316
28-day post-SCI group vs. the control group	*Phc1*	-58.21747	316.52682	0.00122416	0.022194835
28-day post-SCI group vs. the control group	*Plekha1*	110.06372	401.56288	2.17E-05	0.004061182
28-day post-SCI group vs. the control group	*Pon2*	98.01862	235.07632	0.000595607	0.015424492
28-day post-SCI group vs. the control group	*Ppp3ca*	-376.6375	1410.687	0.000208352	0.009851844
28-day post-SCI group vs. the control group	*Prdx4*	48.66762	162.63352	0.002568725	0.031955966
28-day post-SCI group vs. the control group	*Prdx6*	1579.90755	1965.64398	3.31E-05	0.004786417
28-day post-SCI group vs. the control group	*Prkcd*	102.30624	162.01807	5.24E-05	0.005896505

28-day post-SCI group vs. the control group	*Prodh*	59.6963	164.79688	0.003577367	0.03881172
28-day post-SCI group vs. the control group	*Ptgs1*	80.93228	106.40759	0.000857693	0.018908901
28-day post-SCI group vs. the control group	*Rb1*	90.31795	243.02812	0.000858504	0.018908901
28-day post-SCI group vs. the control group	*Rela*	111.01893	470.28116	0.002051763	0.028457967
28-day post-SCI group vs. the control group	*Ripk1*	51.31421	70.91754	0.001866919	0.027249688
28-day post-SCI group vs. the control group	*Rnf2*	51.08458	152.2294	0.003746606	0.039827305
28-day post-SCI group vs. the control group	*Rnh1*	416.20705	679.05708	0.00093486	0.019654715
28-day post-SCI group vs. the control group	*Rpl13a*	732.208	3329.9598	0.000918492	0.019487762
28-day post-SCI group vs. the control group	*S100a1*	792.98363	1392.12088	0.000111098	0.007879642
28-day post-SCI group vs. the control group	*Sdc1*	25.38336	33.7969	0.003177648	0.036141841
28-day post-SCI group vs. the control group	*Selenbp1*	58.0023	136.33758	0.00025078	0.010555886
28-day post-SCI group vs. the control group	*Sirpa*	319.42887	451.79602	0.000990276	0.020038559
28-day post-SCI group vs. the control group	*Slc7a11*	32.86242	42.98402	0.002487552	0.031508051
28-day post-SCI group vs. the control group	*Slc8a1*	-81.87948	147.45586	3.69E-05	0.004906137
28-day post-SCI group vs. the control group	*Smad1*	111.78532	328.46214	0.002891688	0.034139302
28-day post-SCI group vs. the control group	*Stat6*	106.25976	114.09906	0.000988583	0.020035415
28-day post-SCI group vs. the control group	*Stau2*	-196.88382	385.03116	0.002635765	0.032481239
28-day post-SCI group vs. the control group	*Syp*	-393.90995	680.23308	0.002346535	0.030717471
28-day post-SCI group vs. the control group	*Tlr4*	21.26145	22.28574	0.00369453	0.039515899
28-day post-SCI group vs. the control group	*Tnfaip3*	34.48963	53.13802	0.001209494	0.02208282
28-day post-SCI group vs. the control group	*Tnfrsf1a*	308.0339	393.99064	0.000164389	0.009210248
28-day post-SCI group vs. the control group	*Trem2*	479.36128	312.05756	0.000967898	0.019946359
28-day post-SCI group vs. the control group	*Trpm2*	-42.00099	100.10936	0.002037592	0.028387179
28-day post-SCI group vs. the control group	*Txnip*	337.38497	388.80718	0.001329069	0.023071926
28-day post-SCI group vs. the control group	*Ucp2*	287.44377	395.99096	0.000886985	0.019192338
28-day post-SCI group vs. the control group	*Vcam1*	130.21407	225.34484	0.000215472	0.009861104
28-day post-SCI group vs. the control group	*Xdh*	121.1574	99.40284	1.32E-05	0.003503026

SCI: spinal cord injury
