# Supplementary material for: Integrating bulk and single-cell transcriptome profiling to uncover diagnostic biomarkers and regulatory mechanisms of oxidative stress in spinal cord injury
Source: Neural Regen Res. 2025 Jan 13;21(6):2643–57. doi: 10.4103/NRR.NRR-D-24-00693 (PMC13217428; doi:10.4103/NRR.NRR-D-24-00693)
Supplement: Supplementary file 3 [file NRR-21-2643_Suppl2.pdf]

Additional Table 2 The protein interaction network of oxidative stress-related genes that are differentially expressed between groups at different time points after SCI and the control group

Group	#node1	node2	Coexpression	experimentally_ determined_interaction	database_annotated	automated_textmining	combined_score
4-hour post-SCI group vs. the control group	*Atf4*	*Hmox1*	0	0	0	0.468	0.468
4-hour post-SCI group vs. the control group	*Atf4*	*Fos*	0.061	0.769	0.217	0.401	0.885
4-hour post-SCI group vs. the control group	*Atf4*	*Fosl1*	0	0.229	0.217	0.274	0.523
4-hour post-SCI group vs. the control group	*Atf4*	*Ppp1r15b*	0	0	0	0.463	0.463
4-hour post-SCI group vs. the control group	*Atf4*	*Jun*	0.064	0.73	0	0.741	0.929
4-hour post-SCI group vs. the control group	*Cat*	*Hspb1*	0	0	0	0.462	0.462
4-hour post-SCI group vs. the control group	*Cat*	*Hmox1*	0.062	0.09	0	0.877	0.886
4-hour post-SCI group vs. the control group	*Cat*	*Txnrd1*	0.118	0.219	0	0.688	0.766
4-hour post-SCI group vs. the control group	*Cat*	*Fos*	0.062	0	0	0.488	0.499
4-hour post-SCI group vs. the control group	*Cat*	*Myc*	0.051	0.045	0	0.476	0.483
4-hour post-SCI group vs. the control group	*Cat*	*Xdh*	0.079	0	0	0.81	0.817
4-hour post-SCI group vs. the control group	*Cat*	*Jun*	0	0	0	0.682	0.682
4-hour post-SCI group vs. the control group	*Cat*	*Srxn1*	0.361	0	0	0.725	0.817
4-hour post-SCI group vs. the control group	*Ets2*	*Fos*	0.063	0.13	0.629	0.566	0.851
4-hour post-SCI group vs. the control group	*Ets2*	*Myc*	0.062	0.069	0	0.553	0.577
4-hour post-SCI group vs. the control group	*Ets2*	*Fosl1*	0.07	0.047	0.208	0.255	0.408
4-hour post-SCI group vs. the control group	*Ets2*	*Rcan1*	0.078	0	0	0.464	0.485
4-hour post-SCI group vs. the control group	*Ets2*	*Nfkb1*	0.068	0.322	0	0.293	0.514
4-hour post-SCI group vs. the control group	*Ets2*	*Id1*	0.061	0.049	0.8	0.24	0.846
4-hour post-SCI group vs. the control group	*Ets2*	*Jun*	0.061	0.456	0.629	0.522	0.897
4-hour post-SCI group vs. the control group	*Fos*	*Hmox1*	0	0	0.313	0.477	0.626
4-hour post-SCI group vs. the control group	*Fos*	*Map2k3*	0	0	0	0.435	0.435
4-hour post-SCI group vs. the control group	*Fos*	*Il1a*	0.062	0	0	0.386	0.4
4-hour post-SCI group vs. the control group	*Fos*	*Hbegf*	0.065	0	0	0.422	0.436
4-hour post-SCI group vs. the control group	*Fos*	*Mcl1*	0.089	0	0	0.411	0.441
4-hour post-SCI group vs. the control group	*Fos*	*Cat*	0.062	0	0	0.488	0.499
4-hour post-SCI group vs. the control group	*Fos*	*Nfkb1*	0.051	0.087	0.12	0.552	0.613
4-hour post-SCI group vs. the control group	*Fos*	*Nr4a3*	0.065	0.698	0	0.483	0.841
4-hour post-SCI group vs. the control group	*Fos*	*Ets2*	0.063	0.13	0.629	0.566	0.851
4-hour post-SCI group vs. the control group	*Fos*	*Myc*	0.064	0.055	0	0.85	0.856
4-hour post-SCI group vs. the control group	*Fos*	*Atf4*	0.061	0.769	0.217	0.401	0.885

4-hour post-SCI group vs. the control group	*Fos*	*Fosl1*	0.079	0	0.922	0.923	0.934
4-hour post-SCI group vs. the control group	*Fos*	*Jun*	0.662	0.982	0.932	0.995	0.999
4-hour post-SCI group vs. the control group	*Fosl1*	*Hmox1*	0.104	0	0.313	0.258	0.504
4-hour post-SCI group vs. the control group	*Fosl1*	*Tnfaip3*	0.073	0	0	0.407	0.427
4-hour post-SCI group vs. the control group	*Fosl1*	*Fos*	0.079	0	0.922	0.923	0.934
4-hour post-SCI group vs. the control group	*Fosl1*	*Myc*	0.15	0.05	0	0.563	0.616
4-hour post-SCI group vs. the control group	*Fosl1*	*Ets2*	0.07	0.047	0.208	0.255	0.408
4-hour post-SCI group vs. the control group	*Fosl1*	*Nfkb1*	0.063	0.058	0.12	0.354	0.431
4-hour post-SCI group vs. the control group	*Fosl1*	*Atf4*	0	0.229	0.217	0.274	0.523
4-hour post-SCI group vs. the control group	*Fosl1*	*Jun*	0.066	0.784	0.932	0.989	0.999
4-hour post-SCI group vs. the control group	*Gch1*	*Xdh*	0.104	0	0	0.642	0.665
4-hour post-SCI group vs. the control group	*Hbegf*	*Fos*	0.065	0	0	0.422	0.436
4-hour post-SCI group vs. the control group	*Hbegf*	*Jun*	0.063	0	0	0.409	0.423
4-hour post-SCI group vs. the control group	*Hmox1*	*Hspb1*	0.083	0	0	0.532	0.553
4-hour post-SCI group vs. the control group	*Hmox1*	*Myc*	0.088	0	0	0.42	0.449
4-hour post-SCI group vs. the control group	*Hmox1*	*Atf4*	0	0	0	0.468	0.468
4-hour post-SCI group vs. the control group	*Hmox1*	*Fosl1*	0.104	0	0.313	0.258	0.504
4-hour post-SCI group vs. the control group	*Hmox1*	*Fos*	0	0	0.313	0.477	0.626
4-hour post-SCI group vs. the control group	*Hmox1*	*Xdh*	0.068	0	0	0.619	0.629
4-hour post-SCI group vs. the control group	*Hmox1*	*Srxn1*	0.151	0	0	0.643	0.685
4-hour post-SCI group vs. the control group	*Hmox1*	*Txnrd1*	0.113	0.131	0	0.701	0.749
4-hour post-SCI group vs. the control group	*Hmox1*	*Jun*	0	0	0.39	0.713	0.817
4-hour post-SCI group vs. the control group	*Hmox1*	*Cat*	0.062	0.09	0	0.877	0.886
4-hour post-SCI group vs. the control group	*Hspb1*	*Myc*	0	0.055	0	0.446	0.454
4-hour post-SCI group vs. the control group	*Hspb1*	*Cat*	0	0	0	0.462	0.462
4-hour post-SCI group vs. the control group	*Hspb1*	*Map2k3*	0.067	0.057	0	0.528	0.548
4-hour post-SCI group vs. the control group	*Hspb1*	*Hmox1*	0.083	0	0	0.532	0.553
4-hour post-SCI group vs. the control group	*Hspb1*	*Jun*	0.062	0	0	0.588	0.597
4-hour post-SCI group vs. the control group	*Id1*	*Ets2*	0.061	0.049	0.8	0.24	0.846
4-hour post-SCI group vs. the control group	*Il1a*	*Tnfaip3*	0.408	0	0	0.438	0.653
4-hour post-SCI group vs. the control group	*Il1a*	*Fos*	0.062	0	0	0.386	0.4
4-hour post-SCI group vs. the control group	*Il1a*	*Tnfrsf1a*	0.062	0	0	0.43	0.442
4-hour post-SCI group vs. the control group	*Il1a*	*Ripk1*	0	0	0.341	0.197	0.448
4-hour post-SCI group vs. the control group	*Il1a*	*Jun*	0	0	0	0.487	0.487
4-hour post-SCI group vs. the control group	*Il1a*	*Nfkb1*	0.127	0.047	0.9	0.464	0.949
4-hour post-SCI group vs. the control group	*Jun*	*Hspb1*	0.062	0	0	0.588	0.597

4-hour post-SCI group vs. the control group	*Jun*	*Hmox1*	0	0	0.39	0.713	0.817
4-hour post-SCI group vs. the control group	*Jun*	*Map2k3*	0	0	0	0.721	0.721
4-hour post-SCI group vs. the control group	*Jun*	*Tnfaip3*	0.082	0	0	0.403	0.428
4-hour post-SCI group vs. the control group	*Jun*	*Fos*	0.662	0.982	0.932	0.995	0.999
4-hour post-SCI group vs. the control group	*Jun*	*Myc*	0.062	0.27	0.548	0.856	0.949
4-hour post-SCI group vs. the control group	*Jun*	*Ets2*	0.061	0.456	0.629	0.522	0.897
4-hour post-SCI group vs. the control group	*Jun*	*Xdh*	0	0	0	0.583	0.583
4-hour post-SCI group vs. the control group	*Jun*	*Hbegf*	0.063	0	0	0.409	0.423
4-hour post-SCI group vs. the control group	*Jun*	*Fosl1*	0.066	0.784	0.932	0.989	0.999
4-hour post-SCI group vs. the control group	*Jun*	*Cat*	0	0	0	0.682	0.682
4-hour post-SCI group vs. the control group	*Jun*	*Il1a*	0	0	0	0.487	0.487
4-hour post-SCI group vs. the control group	*Jun*	*Nfkb1*	0.063	0.047	0.204	0.725	0.778
4-hour post-SCI group vs. the control group	*Jun*	*Tnfrsf1a*	0.069	0	0	0.619	0.63
4-hour post-SCI group vs. the control group	*Jun*	*Mcl1*	0.063	0	0	0.62	0.628
4-hour post-SCI group vs. the control group	*Jun*	*Ripk1*	0	0.09	0.127	0.468	0.54
4-hour post-SCI group vs. the control group	*Jun*	*Atf4*	0.064	0.73	0	0.741	0.929
4-hour post-SCI group vs. the control group	*Map2k3*	*Hspb1*	0.067	0.057	0	0.528	0.548
4-hour post-SCI group vs. the control group	*Map2k3*	*Fos*	0	0	0	0.435	0.435
4-hour post-SCI group vs. the control group	*Map2k3*	*Myc*	0.079	0.209	0	0.306	0.45
4-hour post-SCI group vs. the control group	*Map2k3*	*Jun*	0	0	0	0.721	0.721
4-hour post-SCI group vs. the control group	*Map2k3*	*Tnfrsf1a*	0.095	0	0.676	0.245	0.759
4-hour post-SCI group vs. the control group	*Map2k3*	*Ripk1*	0.087	0.132	0.932	0.221	0.945
4-hour post-SCI group vs. the control group	*Mcl1*	*Fos*	0.089	0	0	0.411	0.441
4-hour post-SCI group vs. the control group	*Mcl1*	*Myc*	0.066	0.052	0	0.748	0.758
4-hour post-SCI group vs. the control group	*Mcl1*	*Nfkb1*	0.078	0.055	0.142	0.35	0.449
4-hour post-SCI group vs. the control group	*Mcl1*	*Tnfrsf1a*	0.068	0.077	0	0.387	0.427
4-hour post-SCI group vs. the control group	*Mcl1*	*Ripk1*	0.079	0.056	0.12	0.349	0.435
4-hour post-SCI group vs. the control group	*Mcl1*	*Jun*	0.063	0	0	0.62	0.628
4-hour post-SCI group vs. the control group	*Myc*	*Hspb1*	0	0.055	0	0.446	0.454
4-hour post-SCI group vs. the control group	*Myc*	*Hmox1*	0.088	0	0	0.42	0.449
4-hour post-SCI group vs. the control group	*Myc*	*Map2k3*	0.079	0.209	0	0.306	0.45
4-hour post-SCI group vs. the control group	*Myc*	*Sdc1*	0.072	0.045	0	0.45	0.47
4-hour post-SCI group vs. the control group	*Myc*	*Fos*	0.064	0.055	0	0.85	0.856
4-hour post-SCI group vs. the control group	*Myc*	*Cat*	0.051	0.045	0	0.476	0.483
4-hour post-SCI group vs. the control group	*Myc*	*Rxrb*	0	0.135	0.496	0.045	0.547
4-hour post-SCI group vs. the control group	*Myc*	*Ets2*	0.062	0.069	0	0.553	0.577

4-hour post-SCI group vs. the control group	*Myc*	*Fosl1*	0.15	0.05	0	0.563	0.616
4-hour post-SCI group vs. the control group	*Myc*	*Nfkb1*	0.112	0.132	0.191	0.511	0.654
4-hour post-SCI group vs. the control group	*Myc*	*Mcl1*	0.066	0.052	0	0.748	0.758
4-hour post-SCI group vs. the control group	*Myc*	*Jun*	0.062	0.27	0.548	0.856	0.949
4-hour post-SCI group vs. the control group	*Nfkb1*	*Tnfaip3*	0.212	0.14	0	0.66	0.749
4-hour post-SCI group vs. the control group	*Nfkb1*	*Fos*	0.051	0.087	0.12	0.552	0.613
4-hour post-SCI group vs. the control group	*Nfkb1*	*Myc*	0.112	0.132	0.191	0.511	0.654
4-hour post-SCI group vs. the control group	*Nfkb1*	*Ets2*	0.068	0.322	0	0.293	0.514
4-hour post-SCI group vs. the control group	*Nfkb1*	*Fosl1*	0.063	0.058	0.12	0.354	0.431
4-hour post-SCI group vs. the control group	*Nfkb1*	*Il1a*	0.127	0.047	0.9	0.464	0.949
4-hour post-SCI group vs. the control group	*Nfkb1*	*Mcl1*	0.078	0.055	0.142	0.35	0.449
4-hour post-SCI group vs. the control group	*Nfkb1*	*Jun*	0.063	0.047	0.204	0.725	0.778
4-hour post-SCI group vs. the control group	*Nfkb1*	*Ripk1*	0.089	0.132	0.676	0.45	0.84
4-hour post-SCI group vs. the control group	*Nfkb1*	*Tnfrsf1a*	0.069	0.047	0.676	0.61	0.872
4-hour post-SCI group vs. the control group	*Nr4a3*	*Fos*	0.065	0.698	0	0.483	0.841
4-hour post-SCI group vs. the control group	*Ppp1r15b*	*Atf4*	0	0	0	0.463	0.463
4-hour post-SCI group vs. the control group	*Rcan1*	*Ets2*	0.078	0	0	0.464	0.485
4-hour post-SCI group vs. the control group	*Ripk1*	*Map2k3*	0.087	0.132	0.932	0.221	0.945
4-hour post-SCI group vs. the control group	*Ripk1*	*Tnfaip3*	0.095	0.881	0.932	0.508	0.995
4-hour post-SCI group vs. the control group	*Ripk1*	*Il1a*	0	0	0.341	0.197	0.448
4-hour post-SCI group vs. the control group	*Ripk1*	*Nfkb1*	0.089	0.132	0.676	0.45	0.84
4-hour post-SCI group vs. the control group	*Ripk1*	*Tnfrsf1a*	0.113	0.999	0.932	0.988	0.999
4-hour post-SCI group vs. the control group	*Ripk1*	*Mcl1*	0.079	0.056	0.12	0.349	0.435
4-hour post-SCI group vs. the control group	*Ripk1*	*Jun*	0	0.09	0.127	0.468	0.54
4-hour post-SCI group vs. the control group	*Rxrb*	*Myc*	0	0.135	0.496	0.045	0.547
4-hour post-SCI group vs. the control group	*Sdc1*	*Myc*	0.072	0.045	0	0.45	0.47
4-hour post-SCI group vs. the control group	*Srxn1*	*Hmox1*	0.151	0	0	0.643	0.685
4-hour post-SCI group vs. the control group	*Srxn1*	*Txnrd1*	0.258	0	0	0.739	0.798
4-hour post-SCI group vs. the control group	*Srxn1*	*Cat*	0.361	0	0	0.725	0.817
4-hour post-SCI group vs. the control group	*Tnfaip3*	*Fosl1*	0.073	0	0	0.407	0.427
4-hour post-SCI group vs. the control group	*Tnfaip3*	*Jun*	0.082	0	0	0.403	0.428
4-hour post-SCI group vs. the control group	*Tnfaip3*	*Il1a*	0.408	0	0	0.438	0.653
4-hour post-SCI group vs. the control group	*Tnfaip3*	*Nfkb1*	0.212	0.14	0	0.66	0.749
4-hour post-SCI group vs. the control group	*Tnfaip3*	*Ripk1*	0.095	0.881	0.932	0.508	0.995
4-hour post-SCI group vs. the control group	*Tnfaip3*	*Tnfrsf1a*	0.083	0.981	0.864	0.532	0.998
4-hour post-SCI group vs. the control group	*Tnfrsf1a*	*Map2k3*	0.095	0	0.676	0.245	0.759

4-hour post-SCI group vs. the control group	*Tnfrsf1a*	*Tnfaip3*	0.083	0.981	0.864	0.532	0.998
4-hour post-SCI group vs. the control group	*Tnfrsf1a*	*Il1a*	0.062	0	0	0.43	0.442
4-hour post-SCI group vs. the control group	*Tnfrsf1a*	*Nfkb1*	0.069	0.047	0.676	0.61	0.872
4-hour post-SCI group vs. the control group	*Tnfrsf1a*	*Mcl1*	0.068	0.077	0	0.387	0.427
4-hour post-SCI group vs. the control group	*Tnfrsf1a*	*Jun*	0.069	0	0	0.619	0.63
4-hour post-SCI group vs. the control group	*Tnfrsf1a*	*Ripk1*	0.113	0.999	0.932	0.988	0.999
4-hour post-SCI group vs. the control group	*Txnrd1*	*Hmox1*	0.113	0.131	0	0.701	0.749
4-hour post-SCI group vs. the control group	*Txnrd1*	*Xdh*	0	0	0	0.65	0.65
4-hour post-SCI group vs. the control group	*Txnrd1*	*Cat*	0.118	0.219	0	0.688	0.766
4-hour post-SCI group vs. the control group	*Txnrd1*	*Srxn1*	0.258	0	0	0.739	0.798
4-hour post-SCI group vs. the control group	*Xdh*	*Hmox1*	0.068	0	0	0.619	0.629
4-hour post-SCI group vs. the control group	*Xdh*	*Txnrd1*	0	0	0	0.65	0.65
4-hour post-SCI group vs. the control group	*Xdh*	*Jun*	0	0	0	0.583	0.583
4-hour post-SCI group vs. the control group	*Xdh*	*Gch1*	0.104	0	0	0.642	0.665
4-hour post-SCI group vs. the control group	*Xdh*	*Cat*	0.079	0	0	0.81	0.817
1-day post-SCI group vs. the control group	*Abcc1*	*Edn1*	0	0	0.473	0.146	0.53
1-day post-SCI group vs. the control group	*Abcc1*	*Ezr*	0	0.094	0.34	0.134	0.437
1-day post-SCI group vs. the control group	*Abcc1*	*Sphk1*	0.061	0	0	0.532	0.542
1-day post-SCI group vs. the control group	*Adam9*	*Itga5*	0.089	0.091	0.212	0.192	0.402
1-day post-SCI group vs. the control group	*Adam9*	*Hbegf*	0.061	0	0	0.555	0.565
1-day post-SCI group vs. the control group	*Adam9*	*Cdh11*	0.061	0.043	0	0.392	0.406
1-day post-SCI group vs. the control group	*Ago4*	*P4hb*	0	0.142	0	0.356	0.423
1-day post-SCI group vs. the control group	*Amph*	*Mapt*	0.18	0.13	0	0.233	0.405
1-day post-SCI group vs. the control group	*Amph*	*Ppp3ca*	0.113	0.132	0.344	0.05	0.456
1-day post-SCI group vs. the control group	*Amph*	*Capn2*	0	0.406	0	0.135	0.464
1-day post-SCI group vs. the control group	*Amph*	*Myc*	0.066	0.204	0	0.376	0.496
1-day post-SCI group vs. the control group	*Amph*	*Syp*	0.187	0	0	0.436	0.522
1-day post-SCI group vs. the control group	*Amph*	*Cttn*	0.064	0	0	0.644	0.653
1-day post-SCI group vs. the control group	*Amph*	*Itsn1*	0.061	0.13	0	0.643	0.683
1-day post-SCI group vs. the control group	*Anxa1*	*Sdc1*	0.096	0	0	0.417	0.45
1-day post-SCI group vs. the control group	*Anxa1*	*Edn1*	0.082	0	0.5	0.147	0.574
1-day post-SCI group vs. the control group	*Anxa1*	*Vcam1*	0.082	0.045	0	0.385	0.414
1-day post-SCI group vs. the control group	*Anxa1*	*Ptgs2*	0.157	0	0	0.385	0.459
1-day post-SCI group vs. the control group	*Anxa1*	*Slc4a11*	0	0	0	0.474	0.474
1-day post-SCI group vs. the control group	*Anxa1*	*Tnfrsf1a*	0.17	0	0	0.453	0.526
1-day post-SCI group vs. the control group	*Apoe*	*Mapk14*	0	0	0	0.5	0.499

1-day post-SCI group vs. the control group	*Apoe*	*Hmox1*	0	0.049	0	0.552	0.556
1-day post-SCI group vs. the control group	*Apoe*	*Sdc1*	0.051	0	0.6	0.564	0.82
1-day post-SCI group vs. the control group	*Apoe*	*Edn1*	0	0	0	0.591	0.591
1-day post-SCI group vs. the control group	*Apoe*	*Xdh*	0.105	0	0	0.464	0.5
1-day post-SCI group vs. the control group	*Apoe*	*Cat*	0.081	0	0	0.551	0.569
1-day post-SCI group vs. the control group	*Apoe*	*Vcam1*	0.061	0	0	0.804	0.808
1-day post-SCI group vs. the control group	*Apoe*	*Pink1*	0.169	0	0	0.336	0.424
1-day post-SCI group vs. the control group	*Apoe*	*Nos3*	0.073	0.047	0	0.692	0.704
1-day post-SCI group vs. the control group	*Apoe*	*Mmp3*	0	0.402	0	0.442	0.652
1-day post-SCI group vs. the control group	*Apoe*	*Ptgs2*	0	0	0	0.494	0.494
1-day post-SCI group vs. the control group	*Apoe*	*Lcat*	0.116	0.13	0.72	0.692	0.924
1-day post-SCI group vs. the control group	*Apoe*	*Syp*	0.061	0	0	0.473	0.484
1-day post-SCI group vs. the control group	*Apoe*	*Gpx1*	0.078	0	0	0.464	0.484
1-day post-SCI group vs. the control group	*Apoe*	*Gch1*	0	0	0	0.419	0.418
1-day post-SCI group vs. the control group	*Apoe*	*Mapt*	0	0.708	0	0.89	0.966
1-day post-SCI group vs. the control group	*Apoe*	*Jun*	0	0	0	0.552	0.552
1-day post-SCI group vs. the control group	*Apoe*	*Ppargc1a*	0	0	0	0.438	0.438
1-day post-SCI group vs. the control group	*Atf2*	*Mapk14*	0	0.927	0.966	0.785	0.999
1-day post-SCI group vs. the control group	*Atf2*	*Hmox1*	0	0	0.313	0.213	0.436
1-day post-SCI group vs. the control group	*Atf2*	*Map2k3*	0	0	0	0.703	0.703
1-day post-SCI group vs. the control group	*Atf2*	*Map2k6*	0	0	0	0.584	0.584
1-day post-SCI group vs. the control group	*Atf2*	*Fos*	0	0.84	0	0.629	0.882
1-day post-SCI group vs. the control group	*Atf2*	*Myc*	0	0.041	0	0.409	0.409
1-day post-SCI group vs. the control group	*Atf2*	*Rela*	0	0.155	0.629	0.444	0.81
1-day post-SCI group vs. the control group	*Atf2*	*Cat*	0.062	0	0	0.479	0.491
1-day post-SCI group vs. the control group	*Atf2*	*Ppp1ca*	0.061	0.049	0.6	0	0.611
1-day post-SCI group vs. the control group	*Atf2*	*Map2k4*	0.094	0	0	0.594	0.616
1-day post-SCI group vs. the control group	*Atf2*	*Smad1*	0	0.132	0.629	0.266	0.743
1-day post-SCI group vs. the control group	*Atf2*	*Jun*	0.066	0.943	0.932	0.989	0.997
1-day post-SCI group vs. the control group	*Atf2*	*Atf4*	0	0.13	0.836	0.236	0.882
1-day post-SCI group vs. the control group	*Atf2*	*Mapk8*	0.088	0.844	0.966	0.61	0.997
1-day post-SCI group vs. the control group	*Atf2*	*Ppargc1a*	0	0	0	0.467	0.467
1-day post-SCI group vs. the control group	*Atf4*	*Hmox1*	0	0	0	0.468	0.468
1-day post-SCI group vs. the control group	*Atf4*	*Fos*	0.061	0.769	0.217	0.401	0.885
1-day post-SCI group vs. the control group	*Atf4*	*Fosl1*	0	0.229	0.217	0.274	0.523
1-day post-SCI group vs. the control group	*Atf4*	*P4hb*	0.061	0	0	0.549	0.558

1-day post-SCI group vs. the control group	*Atf4*	*Pink1*	0	0.146	0	0.375	0.444
1-day post-SCI group vs. the control group	*Atf4*	*Mapkapk3*	0.061	0	0.8	0	0.804
1-day post-SCI group vs. the control group	*Atf4*	*Ppp1ca*	0.052	0	0.6	0.112	0.633
1-day post-SCI group vs. the control group	*Atf4*	*Ppp1r15b*	0	0	0	0.463	0.463
1-day post-SCI group vs. the control group	*Atf4*	*Eif2s1*	0	0	0.9	0.75	0.974
1-day post-SCI group vs. the control group	*Atf4*	*Jun*	0.064	0.73	0	0.741	0.929
1-day post-SCI group vs. the control group	*Atf4*	*Atf2*	0	0.13	0.836	0.236	0.882
1-day post-SCI group vs. the control group	*Atox1*	*Gfer*	0.069	0	0	0.409	0.427
1-day post-SCI group vs. the control group	*Atp13a2*	*Pink1*	0.066	0	0	0.852	0.856
1-day post-SCI group vs. the control group	*Atp13a2*	*Ctnna1*	0.474	0	0	0	0.474
1-day post-SCI group vs. the control group	*Atp13a2*	*Mapt*	0.061	0.046	0	0.507	0.52
1-day post-SCI group vs. the control group	*Axl*	*Vcam1*	0.141	0	0.147	0.274	0.421
1-day post-SCI group vs. the control group	*Axl*	*Fosl1*	0.105	0.07	0.12	0.314	0.43
1-day post-SCI group vs. the control group	*Axl*	*Jun*	0.062	0.09	0.127	0.327	0.431
1-day post-SCI group vs. the control group	*Axl*	*Myc*	0.051	0.086	0.129	0.379	0.468
1-day post-SCI group vs. the control group	*Axl*	*Btk*	0	0.697	0	0.197	0.714
1-day post-SCI group vs. the control group	*Axl*	*Jak2*	0.058	0	0.8	0.305	0.826
1-day post-SCI group vs. the control group	*Banf1*	*Cdk4*	0.678	0	0	0.16	0.718
1-day post-SCI group vs. the control group	*Banf1*	*Fos*	0	0	0	0.468	0.468
1-day post-SCI group vs. the control group	*Banf1*	*Myc*	0	0	0	0.416	0.416
1-day post-SCI group vs. the control group	*Banf1*	*Stat6*	0	0	0	0.454	0.454
1-day post-SCI group vs. the control group	*Banf1*	*Hdac1*	0.084	0.099	0	0.556	0.602
1-day post-SCI group vs. the control group	*Bax*	*Mapk14*	0	0.061	0.209	0.438	0.546
1-day post-SCI group vs. the control group	*Bax*	*Hmox1*	0.084	0	0	0.493	0.516
1-day post-SCI group vs. the control group	*Bax*	*Cdk4*	0.204	0.058	0	0.328	0.452
1-day post-SCI group vs. the control group	*Bax*	*Mdm2*	0.064	0.131	0	0.464	0.525
1-day post-SCI group vs. the control group	*Bax*	*Rela*	0	0.058	0.311	0.167	0.412
1-day post-SCI group vs. the control group	*Bax*	*Cat*	0	0	0	0.493	0.493
1-day post-SCI group vs. the control group	*Bax*	*Eed*	0	0.433	0	0	0.433
1-day post-SCI group vs. the control group	*Bax*	*Ptgs2*	0	0	0	0.45	0.45
1-day post-SCI group vs. the control group	*Bax*	*Jun*	0	0	0	0.529	0.529
1-day post-SCI group vs. the control group	*Bax*	*Mapk8*	0	0.082	0.272	0.397	0.561
1-day post-SCI group vs. the control group	*Bax*	*Capn2*	0	0.047	0.8	0.132	0.82
1-day post-SCI group vs. the control group	*Bax*	*Mcl1*	0	0.422	0.72	0.61	0.865
1-day post-SCI group vs. the control group	*Bmp1*	*Ctsl*	0	0	0.5	0.331	0.651
1-day post-SCI group vs. the control group	*Bmp1*	*Cdh11*	0.069	0	0.206	0.284	0.424

1-day post-SCI group vs. the control group	*Btk*	*Axl*	0	0.697	0	0.197	0.714
1-day post-SCI group vs. the control group	*Btk*	*Mapk14*	0	0.147	0.187	0.473	0.407
1-day post-SCI group vs. the control group	*Btk*	*Mdm2*	0	0.13	0.146	0.269	0.409
1-day post-SCI group vs. the control group	*Btk*	*Sdc1*	0	0.058	0.127	0.464	0.52
1-day post-SCI group vs. the control group	*Btk*	*Fos*	0	0.07	0.676	0.266	0.759
1-day post-SCI group vs. the control group	*Btk*	*Myc*	0.051	0.086	0.129	0.394	0.481
1-day post-SCI group vs. the control group	*Btk*	*Rela*	0	0.087	0.217	0.333	0.481
1-day post-SCI group vs. the control group	*Btk*	*Vcam1*	0.065	0	0.217	0.262	0.412
1-day post-SCI group vs. the control group	*Btk*	*Met*	0.065	0.074	0.323	0.457	0.457
1-day post-SCI group vs. the control group	*Btk*	*Cttn*	0.082	0.059	0.269	0.266	0.474
1-day post-SCI group vs. the control group	*Btk*	*Stat6*	0.082	0.136	0.193	0.283	0.48
1-day post-SCI group vs. the control group	*Btk*	*Plekha1*	0	0	0	0.495	0.495
1-day post-SCI group vs. the control group	*Btk*	*Mcl1*	0	0.056	0.19	0.469	0.558
1-day post-SCI group vs. the control group	*Btk*	*Mapk8*	0.052	0.139	0.676	0.341	0.75
1-day post-SCI group vs. the control group	*Btk*	*Jak2*	0.062	0.146	0.676	0.468	0.764
1-day post-SCI group vs. the control group	*Btk*	*Jun*	0	0.136	0.676	0.442	0.83
1-day post-SCI group vs. the control group	*Capn2*	*Capns1*	0.111	0.936	0.845	0.929	0.991
1-day post-SCI group vs. the control group	*Capn2*	*Amph*	0	0.406	0	0.135	0.464
1-day post-SCI group vs. the control group	*Capn2*	*Mapk14*	0.062	0.058	0.256	0.266	0.453
1-day post-SCI group vs. the control group	*Capn2*	*Bax*	0	0.047	0.8	0.132	0.82
1-day post-SCI group vs. the control group	*Capn2*	*Ezr*	0	0.043	0.425	0.277	0.567
1-day post-SCI group vs. the control group	*Capn2*	*Eed*	0	0.433	0	0	0.433
1-day post-SCI group vs. the control group	*Capn2*	*Pxn*	0.077	0	0.629	0.433	0.789
1-day post-SCI group vs. the control group	*Capns1*	*Eed*	0	0.433	0	0	0.433
1-day post-SCI group vs. the control group	*Capns1*	*Mapk14*	0	0.134	0.34	0.158	0.476
1-day post-SCI group vs. the control group	*Capns1*	*Pxn*	0.068	0	0.629	0.161	0.685
1-day post-SCI group vs. the control group	*Capns1*	*Ezr*	0.062	0	0.629	0.203	0.698
1-day post-SCI group vs. the control group	*Capns1*	*Capn2*	0.111	0.936	0.845	0.929	0.991
1-day post-SCI group vs. the control group	*Cask*	*Sdc1*	0.051	0.144	0.676	0.511	0.854
1-day post-SCI group vs. the control group	*Cask*	*Nos3*	0.063	0.137	0.423	0.051	0.498
1-day post-SCI group vs. the control group	*Cask*	*Ppp3ca*	0.08	0.07	0.327	0.105	0.416
1-day post-SCI group vs. the control group	*Cask*	*Mapt*	0.071	0.115	0.355	0.057	0.433
1-day post-SCI group vs. the control group	*Cat*	*Prodh*	0	0	0	0.443	0.443
1-day post-SCI group vs. the control group	*Cat*	*Hmox2*	0.062	0.09	0	0.694	0.716
1-day post-SCI group vs. the control group	*Cat*	*Mapk14*	0	0.13	0	0.743	0.767
1-day post-SCI group vs. the control group	*Cat*	*Hspb1*	0	0	0	0.462	0.462

1-day post-SCI group vs. the control group	*Cat*	*Hmox1*	0.062	0.09	0	0.877	0.886
1-day post-SCI group vs. the control group	*Cat*	*Txnrd1*	0.118	0.219	0	0.688	0.766
1-day post-SCI group vs. the control group	*Cat*	*Nme2*	0	0.355	0	0.213	0.47
1-day post-SCI group vs. the control group	*Cat*	*Fos*	0.062	0	0	0.488	0.499
1-day post-SCI group vs. the control group	*Cat*	*Edn1*	0	0	0	0.536	0.536
1-day post-SCI group vs. the control group	*Cat*	*Myc*	0.051	0.045	0	0.476	0.483
1-day post-SCI group vs. the control group	*Cat*	*Xdh*	0.079	0	0	0.81	0.817
1-day post-SCI group vs. the control group	*Cat*	*P4hb*	0.089	0.134	0	0.431	0.512
1-day post-SCI group vs. the control group	*Cat*	*Jak2*	0	0.065	0.183	0.34	0.453
1-day post-SCI group vs. the control group	*Cat*	*Lpo*	0	0.203	0	0.351	0.46
1-day post-SCI group vs. the control group	*Cat*	*Atf2*	0.062	0	0	0.479	0.491
1-day post-SCI group vs. the control group	*Cat*	*Bax*	0	0	0	0.493	0.493
1-day post-SCI group vs. the control group	*Cat*	*Mapk8*	0	0	0	0.525	0.525
1-day post-SCI group vs. the control group	*Cat*	*Vcam1*	0	0	0	0.539	0.539
1-day post-SCI group vs. the control group	*Cat*	*Apoe*	0.081	0	0	0.551	0.569
1-day post-SCI group vs. the control group	*Cat*	*Ucp2*	0.076	0	0	0.565	0.582
1-day post-SCI group vs. the control group	*Cat*	*Pink1*	0	0.14	0	0.594	0.636
1-day post-SCI group vs. the control group	*Cat*	*Jun*	0	0	0	0.682	0.682
1-day post-SCI group vs. the control group	*Cat*	*Nos3*	0.049	0	0	0.696	0.699
1-day post-SCI group vs. the control group	*Cat*	*Ppargc1a*	0	0	0	0.739	0.74
1-day post-SCI group vs. the control group	*Cat*	*Ptgs2*	0.048	0.203	0	0.689	0.743
1-day post-SCI group vs. the control group	*Cat*	*Srxn1*	0.361	0	0	0.725	0.817
1-day post-SCI group vs. the control group	*Cat*	*Gpx4*	0.141	0.379	0	0.867	0.923
1-day post-SCI group vs. the control group	*Cat*	*Gpx1*	0.159	0.379	0	0.919	0.954
1-day post-SCI group vs. the control group	*Cbx6*	*Rbbp7*	0.048	0.133	0.72	0.322	0.822
1-day post-SCI group vs. the control group	*Cbx6*	*Eed*	0.057	0.261	0.72	0.449	0.878
1-day post-SCI group vs. the control group	*Cbx6*	*Phc3*	0	0.568	0.807	0.661	0.969
1-day post-SCI group vs. the control group	*Ccr1*	*Edn1*	0	0.088	0.503	0.185	0.598
1-day post-SCI group vs. the control group	*Ccr1*	*Vcam1*	0.131	0	0	0.494	0.542
1-day post-SCI group vs. the control group	*Ccr1*	*Jun*	0	0.046	0.483	0.254	0.599
1-day post-SCI group vs. the control group	*Ccr1*	*Jak2*	0.061	0.141	0.63	0.195	0.727
1-day post-SCI group vs. the control group	*Cdh11*	*Bmp1*	0.069	0	0.206	0.284	0.424
1-day post-SCI group vs. the control group	*Cdh11*	*Mmp3*	0.062	0.056	0.146	0.33	0.425
1-day post-SCI group vs. the control group	*Cdh11*	*Ctnna1*	0.062	0.162	0.686	0.269	0.795
1-day post-SCI group vs. the control group	*Cdh11*	*Adam9*	0.061	0.043	0	0.392	0.406
1-day post-SCI group vs. the control group	*Cdh11*	*Met*	0.062	0.059	0.36	0.231	0.507

1-day post-SCI group vs. the control group	*Cdk4*	*Mapk14*	0	0.235	0.185	0.581	0.469
1-day post-SCI group vs. the control group	*Cdk4*	*Ptgs2*	0	0	0	0.403	0.403
1-day post-SCI group vs. the control group	*Cdk4*	*Ppp3ca*	0.063	0.155	0.194	0.195	0.418
1-day post-SCI group vs. the control group	*Cdk4*	*Bax*	0.204	0.058	0	0.328	0.452
1-day post-SCI group vs. the control group	*Cdk4*	*Fos*	0	0	0	0.464	0.463
1-day post-SCI group vs. the control group	*Cdk4*	*Smad1*	0	0.151	0.301	0.211	0.49
1-day post-SCI group vs. the control group	*Cdk4*	*Fbxw7*	0.051	0.161	0	0.442	0.517
1-day post-SCI group vs. the control group	*Cdk4*	*Rela*	0	0.13	0	0.492	0.54
1-day post-SCI group vs. the control group	*Cdk4*	*Mcl1*	0	0.058	0	0.589	0.596
1-day post-SCI group vs. the control group	*Cdk4*	*Jun*	0.061	0.164	0	0.63	0.684
1-day post-SCI group vs. the control group	*Cdk4*	*Ppp1ca*	0.38	0.235	0.336	0.152	0.697
1-day post-SCI group vs. the control group	*Cdk4*	*Banf1*	0.678	0	0	0.16	0.718
1-day post-SCI group vs. the control group	*Cdk4*	*Hdac1*	0.089	0.104	0.585	0.524	0.817
1-day post-SCI group vs. the control group	*Cdk4*	*Mdm2*	0	0.058	0.27	0.782	0.837
1-day post-SCI group vs. the control group	*Cdk4*	*Myc*	0.104	0.352	0	0.757	0.847
1-day post-SCI group vs. the control group	*Cdk4*	*Ybx3*	0.1	0.13	0.8	0.625	0.933
1-day post-SCI group vs. the control group	*Cdk4*	*Jak2*	0.062	0.198	0.915	0.482	0.942
1-day post-SCI group vs. the control group	*Cdk4*	*Cdkn1c*	0.051	0.268	0.711	0.758	0.945
1-day post-SCI group vs. the control group	*Cdk4*	*Pcna*	0.336	0.948	0.905	0.549	0.998
1-day post-SCI group vs. the control group	*Cdkn1c*	*Cdk4*	0.051	0.268	0.711	0.758	0.945
1-day post-SCI group vs. the control group	*Cdkn1c*	*Mdm2*	0	0.206	0	0.36	0.47
1-day post-SCI group vs. the control group	*Cdkn1c*	*Myc*	0	0.041	0	0.485	0.485
1-day post-SCI group vs. the control group	*Cdkn1c*	*Jak2*	0	0.09	0.908	0.172	0.925
1-day post-SCI group vs. the control group	*Cdkn1c*	*Smad1*	0	0.137	0.296	0.155	0.442
1-day post-SCI group vs. the control group	*Cdkn1c*	*Hdac1*	0	0.164	0	0.315	0.402
1-day post-SCI group vs. the control group	*Cdkn1c*	*Jun*	0.052	0.061	0.362	0.342	0.576
1-day post-SCI group vs. the control group	*Cdkn1c*	*Mapk8*	0	0.133	0.362	0.119	0.47
1-day post-SCI group vs. the control group	*Ctnna1*	*Mapk14*	0	0.059	0.6	0.065	0.617
1-day post-SCI group vs. the control group	*Ctnna1*	*Itga5*	0.103	0.049	0.248	0.233	0.442
1-day post-SCI group vs. the control group	*Ctnna1*	*Mmp3*	0	0	0.676	0.101	0.696
1-day post-SCI group vs. the control group	*Ctnna1*	*Atp13a2*	0.474	0	0	0	0.474
1-day post-SCI group vs. the control group	*Ctnna1*	*Ezr*	0.069	0	0	0.4	0.418
1-day post-SCI group vs. the control group	*Ctnna1*	*Eed*	0	0.433	0	0	0.433
1-day post-SCI group vs. the control group	*Ctnna1*	*Pdlim1*	0.082	0.203	0.334	0.103	0.505
1-day post-SCI group vs. the control group	*Ctnna1*	*Pxn*	0.083	0.203	0.334	0.293	0.61
1-day post-SCI group vs. the control group	*Ctnna1*	*Itga7*	0.062	0.049	0.248	0.566	0.669

1-day post-SCI group vs. the control group	*Ctnna1*	*Met*	0.082	0.052	0.676	0.066	0.701
1-day post-SCI group vs. the control group	*Ctnna1*	*Cttn*	0.062	0.057	0.676	0.227	0.749
1-day post-SCI group vs. the control group	*Ctnna1*	*Cdh11*	0.062	0.162	0.686	0.269	0.795
1-day post-SCI group vs. the control group	*Ctsl*	*Mmp3*	0.05	0.047	0.5	0.26	0.62
1-day post-SCI group vs. the control group	*Ctsl*	*Bmp1*	0	0	0.5	0.331	0.651
1-day post-SCI group vs. the control group	*Cttn*	*Amph*	0.064	0	0	0.644	0.653
1-day post-SCI group vs. the control group	*Cttn*	*Sdc1*	0.064	0.059	0.284	0.639	0.742
1-day post-SCI group vs. the control group	*Cttn*	*Myc*	0	0	0	0.479	0.48
1-day post-SCI group vs. the control group	*Cttn*	*Itga5*	0	0	0.334	0.171	0.424
1-day post-SCI group vs. the control group	*Cttn*	*Btk*	0.082	0.059	0.269	0.266	0.474
1-day post-SCI group vs. the control group	*Cttn*	*Ctnna1*	0.062	0.057	0.676	0.227	0.749
1-day post-SCI group vs. the control group	*Cttn*	*Ezr*	0.052	0.062	0	0.87	0.874
1-day post-SCI group vs. the control group	*Cttn*	*Pdlim1*	0	0.137	0.419	0.115	0.517
1-day post-SCI group vs. the control group	*Cttn*	*Pxn*	0	0.348	0.629	0.984	0.996
1-day post-SCI group vs. the control group	*Cttn*	*Hdac1*	0.062	0.134	0	0.345	0.421
1-day post-SCI group vs. the control group	*Cttn*	*Eed*	0	0.433	0	0	0.433
1-day post-SCI group vs. the control group	*Cttn*	*Met*	0.062	0.087	0.146	0.342	0.454
1-day post-SCI group vs. the control group	*Cyp1b1*	*Hmox1*	0.162	0	0	0.41	0.485
1-day post-SCI group vs. the control group	*Cyp1b1*	*Gpx4*	0	0	0	0.431	0.431
1-day post-SCI group vs. the control group	*Cyp1b1*	*Ptgs2*	0.107	0.046	0	0.447	0.488
1-day post-SCI group vs. the control group	*Cyp1b1*	*Pxdn*	0.114	0.046	0	0.555	0.591
1-day post-SCI group vs. the control group	*Dynll1*	*Mapt*	0	0.438	0	0.088	0.465
1-day post-SCI group vs. the control group	*Dynll1*	*Eed*	0.08	0.433	0	0.071	0.473
1-day post-SCI group vs. the control group	*Dynll1*	*Mapk8*	0.064	0	0.9	0.063	0.904
1-day post-SCI group vs. the control group	*Edn1*	*Mapk14*	0	0	0.676	0.591	0.861
1-day post-SCI group vs. the control group	*Edn1*	*Hmox1*	0	0	0	0.617	0.617
1-day post-SCI group vs. the control group	*Edn1*	*Fos*	0	0	0	0.499	0.499
1-day post-SCI group vs. the control group	*Edn1*	*Gpr37l1*	0	0.088	0.208	0.239	0.402
1-day post-SCI group vs. the control group	*Edn1*	*Hbegf*	0.089	0	0	0.412	0.441
1-day post-SCI group vs. the control group	*Edn1*	*Mmp3*	0	0	0	0.472	0.472
1-day post-SCI group vs. the control group	*Edn1*	*Xdh*	0	0	0	0.529	0.529
1-day post-SCI group vs. the control group	*Edn1*	*Abcc1*	0	0	0.473	0.146	0.53
1-day post-SCI group vs. the control group	*Edn1*	*Cat*	0	0	0	0.536	0.536
1-day post-SCI group vs. the control group	*Edn1*	*Anxa1*	0.082	0	0.5	0.147	0.574
1-day post-SCI group vs. the control group	*Edn1*	*Apoe*	0	0	0	0.591	0.591
1-day post-SCI group vs. the control group	*Edn1*	*Ccr1*	0	0.088	0.503	0.185	0.598

1-day post-SCI group vs. the control group	*Edn1*	*Ptgs2*	0.1	0	0	0.599	0.623
1-day post-SCI group vs. the control group	*Edn1*	*Ezr*	0	0	0.629	0.197	0.689
1-day post-SCI group vs. the control group	*Edn1*	*Vcam1*	0	0	0	0.709	0.709
1-day post-SCI group vs. the control group	*Edn1*	*Jak2*	0	0	0.676	0.319	0.77
1-day post-SCI group vs. the control group	*Edn1*	*Mapk8*	0	0	0.676	0.379	0.79
1-day post-SCI group vs. the control group	*Edn1*	*Nos3*	0	0	0	0.806	0.806
1-day post-SCI group vs. the control group	*Edn1*	*Jun*	0.061	0	0.629	0.589	0.844
1-day post-SCI group vs. the control group	*Eed*	*Capns1*	0	0.433	0	0	0.433
1-day post-SCI group vs. the control group	*Eed*	*Dynll1*	0.08	0.433	0	0.071	0.473
1-day post-SCI group vs. the control group	*Eed*	*Map2k3*	0	0.433	0	0	0.433
1-day post-SCI group vs. the control group	*Eed*	*Ndufa6*	0	0.433	0	0	0.433
1-day post-SCI group vs. the control group	*Eed*	*Krt1*	0	0.433	0	0.082	0.457
1-day post-SCI group vs. the control group	*Eed*	*Pcna*	0.461	0.433	0	0	0.681
1-day post-SCI group vs. the control group	*Eed*	*Bax*	0	0.433	0	0	0.433
1-day post-SCI group vs. the control group	*Eed*	*Nono*	0.145	0.433	0	0	0.494
1-day post-SCI group vs. the control group	*Eed*	*Rbbp7*	0.162	0.862	0.961	0.905	0.999
1-day post-SCI group vs. the control group	*Eed*	*Ppp1ca*	0.061	0.439	0	0	0.451
1-day post-SCI group vs. the control group	*Eed*	*Ctnna1*	0	0.433	0	0	0.433
1-day post-SCI group vs. the control group	*Eed*	*Ezr*	0	0.433	0	0	0.433
1-day post-SCI group vs. the control group	*Eed*	*Pdlim1*	0	0.433	0	0	0.433
1-day post-SCI group vs. the control group	*Eed*	*Capn2*	0	0.433	0	0	0.433
1-day post-SCI group vs. the control group	*Eed*	*Eif2s1*	0.142	0.682	0	0	0.715
1-day post-SCI group vs. the control group	*Eed*	*Gpx4*	0	0.433	0	0	0.433
1-day post-SCI group vs. the control group	*Eed*	*Cttn*	0	0.433	0	0	0.433
1-day post-SCI group vs. the control group	*Eed*	*Hdac1*	0.062	0.86	0.548	0.432	0.962
1-day post-SCI group vs. the control group	*Eed*	*Rpl13a*	0	0.433	0	0	0.433
1-day post-SCI group vs. the control group	*Eed*	*Pxdn*	0.061	0.433	0	0	0.444
1-day post-SCI group vs. the control group	*Eed*	*Cbx6*	0.057	0.261	0.72	0.449	0.878
1-day post-SCI group vs. the control group	*Eed*	*Phc3*	0	0.147	0.9	0.374	0.942
1-day post-SCI group vs. the control group	*Eif2s1*	*P4hb*	0.062	0.358	0	0.38	0.594
1-day post-SCI group vs. the control group	*Eif2s1*	*Ppp1ca*	0.082	0.398	0.8	0.276	0.909
1-day post-SCI group vs. the control group	*Eif2s1*	*Ppp1r15b*	0	0	0	0.633	0.633
1-day post-SCI group vs. the control group	*Eif2s1*	*Rpl13a*	0.652	0	0	0.097	0.672
1-day post-SCI group vs. the control group	*Eif2s1*	*Eed*	0.142	0.682	0	0	0.715
1-day post-SCI group vs. the control group	*Eif2s1*	*Atf4*	0	0	0.9	0.75	0.974
1-day post-SCI group vs. the control group	*Ercc1*	*Hdac1*	0.052	0	0	0.42	0.427

1-day post-SCI group vs. the control group	*Ercc1*	*Pcna*	0.066	0	0.6	0	0.61
1-day post-SCI group vs. the control group	*Ezr*	*Capns1*	0.062	0	0.629	0.203	0.698
1-day post-SCI group vs. the control group	*Ezr*	*Mapk14*	0	0.199	0	0.312	0.425
1-day post-SCI group vs. the control group	*Ezr*	*Edn1*	0	0	0.629	0.197	0.689
1-day post-SCI group vs. the control group	*Ezr*	*Vcam1*	0	0.13	0.8	0.941	0.988
1-day post-SCI group vs. the control group	*Ezr*	*Ctnna1*	0.069	0	0	0.4	0.418
1-day post-SCI group vs. the control group	*Ezr*	*Eed*	0	0.433	0	0	0.433
1-day post-SCI group vs. the control group	*Ezr*	*Pdlim1*	0.061	0.049	0.338	0.156	0.434
1-day post-SCI group vs. the control group	*Ezr*	*Abcc1*	0	0.094	0.34	0.134	0.437
1-day post-SCI group vs. the control group	*Ezr*	*Jak2*	0	0.089	0.22	0.307	0.464
1-day post-SCI group vs. the control group	*Ezr*	*Met*	0.061	0.089	0.133	0.391	0.487
1-day post-SCI group vs. the control group	*Ezr*	*Map4k4*	0.048	0.137	0	0.469	0.526
1-day post-SCI group vs. the control group	*Ezr*	*Capn2*	0	0.043	0.425	0.277	0.567
1-day post-SCI group vs. the control group	*Ezr*	*Cttn*	0.052	0.062	0	0.87	0.874
1-day post-SCI group vs. the control group	*Ezr*	*Tsc1*	0.061	0.439	0	0.826	0.9
1-day post-SCI group vs. the control group	*Ezr*	*Pxn*	0.082	0.065	0.629	0.748	0.909
1-day post-SCI group vs. the control group	*Fbxw7*	*Cdk4*	0.051	0.161	0	0.442	0.517
1-day post-SCI group vs. the control group	*Fbxw7*	*Mdm2*	0.061	0.13	0	0.605	0.649
1-day post-SCI group vs. the control group	*Fbxw7*	*Txnrd1*	0	0	0	0.414	0.414
1-day post-SCI group vs. the control group	*Fbxw7*	*Myc*	0	0.852	0.585	0.94	0.996
1-day post-SCI group vs. the control group	*Fbxw7*	*Mcl1*	0.061	0.745	0	0.863	0.964
1-day post-SCI group vs. the control group	*Fbxw7*	*Jun*	0	0.762	0	0.901	0.975
1-day post-SCI group vs. the control group	*Fbxw7*	*Ppargc1a*	0	0.439	0	0.191	0.527
1-day post-SCI group vs. the control group	*Fkbp1b*	*Hspa13*	0.063	0.198	0	0.302	0.429
1-day post-SCI group vs. the control group	*Fkbp1b*	*Nme2*	0.474	0.349	0	0	0.644
1-day post-SCI group vs. the control group	*Fkbp1b*	*Ppp3ca*	0.062	0.591	0.15	0.268	0.729
1-day post-SCI group vs. the control group	*Fos*	*Ier3*	0.159	0	0	0.399	0.472
1-day post-SCI group vs. the control group	*Fos*	*Hmox2*	0	0	0.629	0.235	0.704
1-day post-SCI group vs. the control group	*Fos*	*Mapk14*	0	0.267	0.922	0.716	0.982
1-day post-SCI group vs. the control group	*Fos*	*Hmox1*	0	0	0.313	0.477	0.626
1-day post-SCI group vs. the control group	*Fos*	*Cdk4*	0	0	0	0.464	0.463
1-day post-SCI group vs. the control group	*Fos*	*Map2k3*	0	0	0	0.435	0.435
1-day post-SCI group vs. the control group	*Fos*	*Mdm2*	0.062	0.164	0	0.431	0.515
1-day post-SCI group vs. the control group	*Fos*	*Met*	0	0.071	0.12	0.364	0.434
1-day post-SCI group vs. the control group	*Fos*	*Hbegf*	0.065	0	0	0.422	0.436
1-day post-SCI group vs. the control group	*Fos*	*Mmp3*	0	0.082	0	0.416	0.441

1-day post-SCI group vs. the control group	*Fos*	*Mcl1*	0.089	0	0	0.411	0.441
1-day post-SCI group vs. the control group	*Fos*	*Stat6*	0	0.071	0.216	0.327	0.467
1-day post-SCI group vs. the control group	*Fos*	*Banf1*	0	0	0	0.468	0.468
1-day post-SCI group vs. the control group	*Fos*	*Cat*	0.062	0	0	0.488	0.499
1-day post-SCI group vs. the control group	*Fos*	*Edn1*	0	0	0	0.499	0.499
1-day post-SCI group vs. the control group	*Fos*	*Nos3*	0	0	0.241	0.372	0.503
1-day post-SCI group vs. the control group	*Fos*	*Jak2*	0	0.07	0.15	0.472	0.547
1-day post-SCI group vs. the control group	*Fos*	*Syp*	0	0	0	0.546	0.547
1-day post-SCI group vs. the control group	*Fos*	*Smad1*	0	0.142	0.388	0.227	0.558
1-day post-SCI group vs. the control group	*Fos*	*Ptgs2*	0.063	0	0	0.64	0.648
1-day post-SCI group vs. the control group	*Fos*	*Hdac1*	0.048	0.164	0	0.67	0.715
1-day post-SCI group vs. the control group	*Fos*	*Btk*	0	0.07	0.676	0.266	0.759
1-day post-SCI group vs. the control group	*Fos*	*Myc*	0.064	0.055	0	0.85	0.856
1-day post-SCI group vs. the control group	*Fos*	*Atf2*	0	0.84	0	0.629	0.882
1-day post-SCI group vs. the control group	*Fos*	*Atf4*	0.061	0.769	0.217	0.401	0.885
1-day post-SCI group vs. the control group	*Fos*	*Rela*	0	0.4	0.629	0.561	0.893
1-day post-SCI group vs. the control group	*Fos*	*Fosl1*	0.079	0	0.922	0.923	0.934
1-day post-SCI group vs. the control group	*Fos*	*Mapk8*	0	0.309	0.932	0.64	0.981
1-day post-SCI group vs. the control group	*Fos*	*Jun*	0.662	0.982	0.932	0.995	0.999
1-day post-SCI group vs. the control group	*Fosl1*	*Axl*	0.105	0.07	0.12	0.314	0.43
1-day post-SCI group vs. the control group	*Fosl1*	*Ier3*	0.187	0	0	0.351	0.45
1-day post-SCI group vs. the control group	*Fosl1*	*Mapk14*	0	0.267	0.67	0.444	0.853
1-day post-SCI group vs. the control group	*Fosl1*	*Hmox1*	0.104	0	0.313	0.258	0.504
1-day post-SCI group vs. the control group	*Fosl1*	*Fos*	0.079	0	0.922	0.923	0.934
1-day post-SCI group vs. the control group	*Fosl1*	*Myc*	0.15	0.05	0	0.563	0.616
1-day post-SCI group vs. the control group	*Fosl1*	*Met*	0.077	0.071	0.12	0.355	0.448
1-day post-SCI group vs. the control group	*Fosl1*	*Ptgs2*	0.223	0	0	0.36	0.482
1-day post-SCI group vs. the control group	*Fosl1*	*Atf4*	0	0.229	0.217	0.274	0.523
1-day post-SCI group vs. the control group	*Fosl1*	*Rela*	0.061	0.155	0.355	0.511	0.716
1-day post-SCI group vs. the control group	*Fosl1*	*Mapk8*	0	0.309	0.696	0.395	0.862
1-day post-SCI group vs. the control group	*Fosl1*	*Jun*	0.066	0.784	0.932	0.989	0.999
1-day post-SCI group vs. the control group	*Gch1*	*Nme2*	0.064	0.135	0.8	0.07	0.84
1-day post-SCI group vs. the control group	*Gch1*	*Xdh*	0.104	0	0	0.642	0.665
1-day post-SCI group vs. the control group	*Gch1*	*Nos3*	0.065	0	0	0.656	0.665
1-day post-SCI group vs. the control group	*Gch1*	*Apoe*	0	0	0	0.419	0.418
1-day post-SCI group vs. the control group	*Gfer*	*P4hb*	0.076	0	0	0.503	0.521

1-day post-SCI group vs. the control group	*Gfer*	*Atox1*	0.069	0	0	0.409	0.427
1-day post-SCI group vs. the control group	*Gpr37l1*	*Edn1*	0	0.088	0.208	0.239	0.402
1-day post-SCI group vs. the control group	*Gpx1*	*Hmox2*	0.073	0.07	0	0.443	0.478
1-day post-SCI group vs. the control group	*Gpx1*	*Mapk14*	0	0	0	0.425	0.425
1-day post-SCI group vs. the control group	*Gpx1*	*Hmox1*	0.066	0.07	0	0.746	0.76
1-day post-SCI group vs. the control group	*Gpx1*	*Txnrd1*	0.083	0.141	0	0.781	0.814
1-day post-SCI group vs. the control group	*Gpx1*	*Xdh*	0.062	0	0	0.552	0.561
1-day post-SCI group vs. the control group	*Gpx1*	*Cat*	0.159	0.379	0	0.919	0.954
1-day post-SCI group vs. the control group	*Gpx1*	*Nos3*	0	0	0	0.465	0.465
1-day post-SCI group vs. the control group	*Gpx1*	*Ptgs2*	0	0	0	0.46	0.46
1-day post-SCI group vs. the control group	*Gpx1*	*Srxn1*	0.058	0	0	0.556	0.564
1-day post-SCI group vs. the control group	*Gpx1*	*Jun*	0.051	0.045	0	0.413	0.422
1-day post-SCI group vs. the control group	*Gpx1*	*Apoe*	0.078	0	0	0.464	0.484
1-day post-SCI group vs. the control group	*Gpx1*	*Ucp2*	0.101	0	0	0.493	0.524
1-day post-SCI group vs. the control group	*Gpx1*	*Gpx4*	0.138	0	0.5	0.862	0.655
1-day post-SCI group vs. the control group	*Gpx1*	*Ppargc1a*	0	0	0.8	0.602	0.917
1-day post-SCI group vs. the control group	*Gpx1*	*Selenbp1*	0.061	0.379	0	0.872	0.919
1-day post-SCI group vs. the control group	*Gpx4*	*Hmox2*	0.089	0.07	0	0.543	0.579
1-day post-SCI group vs. the control group	*Gpx4*	*Hmox1*	0	0.07	0	0.573	0.586
1-day post-SCI group vs. the control group	*Gpx4*	*Txnrd1*	0.063	0.141	0	0.821	0.844
1-day post-SCI group vs. the control group	*Gpx4*	*Ripk3*	0.061	0.044	0	0.464	0.476
1-day post-SCI group vs. the control group	*Gpx4*	*Cyp1b1*	0	0	0	0.431	0.431
1-day post-SCI group vs. the control group	*Gpx4*	*Cat*	0.141	0.379	0	0.867	0.923
1-day post-SCI group vs. the control group	*Gpx4*	*Ptgs2*	0	0	0	0.483	0.483
1-day post-SCI group vs. the control group	*Gpx4*	*Srxn1*	0.061	0	0	0.655	0.663
1-day post-SCI group vs. the control group	*Gpx4*	*Gpx1*	0.138	0	0.5	0.862	0.655
1-day post-SCI group vs. the control group	*Gpx4*	*Ucp2*	0.064	0	0	0.4	0.415
1-day post-SCI group vs. the control group	*Gpx4*	*Eed*	0	0.433	0	0	0.433
1-day post-SCI group vs. the control group	*Hbegf*	*Fos*	0.065	0	0	0.422	0.436
1-day post-SCI group vs. the control group	*Hbegf*	*Edn1*	0.089	0	0	0.412	0.441
1-day post-SCI group vs. the control group	*Hbegf*	*Spry2*	0.062	0	0.6	0.089	0.628
1-day post-SCI group vs. the control group	*Hbegf*	*Jun*	0.063	0	0	0.409	0.423
1-day post-SCI group vs. the control group	*Hbegf*	*Stat6*	0.063	0	0.35	0.184	0.459
1-day post-SCI group vs. the control group	*Hbegf*	*Met*	0.061	0	0.147	0.401	0.478
1-day post-SCI group vs. the control group	*Hbegf*	*Ptgs2*	0.132	0	0	0.462	0.513
1-day post-SCI group vs. the control group	*Hbegf*	*P4hb*	0	0	0	0.515	0.515

1-day post-SCI group vs. the control group	*Hbegf*	*Adam9*	0.061	0	0	0.555	0.565
1-day post-SCI group vs. the control group	*Hbegf*	*Jak2*	0	0	0.676	0.203	0.731
1-day post-SCI group vs. the control group	*Hbegf*	*Pxn*	0	0	0.9	0.121	0.908
1-day post-SCI group vs. the control group	*Hbegf*	*Mmp3*	0.067	0.057	0.918	0.396	0.951
1-day post-SCI group vs. the control group	*Hdac1*	*Ercc1*	0.052	0	0	0.42	0.427
1-day post-SCI group vs. the control group	*Hdac1*	*Mapk14*	0	0.131	0	0.404	0.46
1-day post-SCI group vs. the control group	*Hdac1*	*Cdk4*	0.089	0.104	0.585	0.524	0.817
1-day post-SCI group vs. the control group	*Hdac1*	*Mdm2*	0	0.342	0	0.961	0.973
1-day post-SCI group vs. the control group	*Hdac1*	*Fos*	0.048	0.164	0	0.67	0.715
1-day post-SCI group vs. the control group	*Hdac1*	*Myc*	0.061	0.401	0.381	0.901	0.961
1-day post-SCI group vs. the control group	*Hdac1*	*Rela*	0.062	0.795	0.512	0.982	0.998
1-day post-SCI group vs. the control group	*Hdac1*	*Tnfrsf1a*	0	0	0.41	0.154	0.479
1-day post-SCI group vs. the control group	*Hdac1*	*Nono*	0.142	0.345	0	0.238	0.534
1-day post-SCI group vs. the control group	*Hdac1*	*Rbbp7*	0.224	0.813	0.956	0.981	0.999
1-day post-SCI group vs. the control group	*Hdac1*	*Ppp1ca*	0.062	0.354	0	0.197	0.472
1-day post-SCI group vs. the control group	*Hdac1*	*Smad1*	0.062	0.221	0.301	0.245	0.563
1-day post-SCI group vs. the control group	*Hdac1*	*Stat6*	0.048	0.164	0.208	0.223	0.444
1-day post-SCI group vs. the control group	*Hdac1*	*Cttn*	0.062	0.134	0	0.345	0.421
1-day post-SCI group vs. the control group	*Hdac1*	*Cdkn1c*	0	0.164	0	0.315	0.402
1-day post-SCI group vs. the control group	*Hdac1*	*Ppargc1a*	0	0	0	0.416	0.416
1-day post-SCI group vs. the control group	*Hdac1*	*Sphk1*	0.062	0	0	0.43	0.442
1-day post-SCI group vs. the control group	*Hdac1*	*Banf1*	0.084	0.099	0	0.556	0.602
1-day post-SCI group vs. the control group	*Hdac1*	*Jun*	0	0.261	0	0.787	0.836
1-day post-SCI group vs. the control group	*Hdac1*	*Eed*	0.062	0.86	0.548	0.432	0.962
1-day post-SCI group vs. the control group	*Hmox1*	*Hmox2*	0	0	0.8	0.892	0.818
1-day post-SCI group vs. the control group	*Hmox1*	*Mapk14*	0	0	0.305	0.7	0.782
1-day post-SCI group vs. the control group	*Hmox1*	*Hspb1*	0.083	0	0	0.532	0.553
1-day post-SCI group vs. the control group	*Hmox1*	*Ucp2*	0.069	0	0	0.411	0.428
1-day post-SCI group vs. the control group	*Hmox1*	*Atf2*	0	0	0.313	0.213	0.436
1-day post-SCI group vs. the control group	*Hmox1*	*Mmp3*	0.062	0	0	0.432	0.444
1-day post-SCI group vs. the control group	*Hmox1*	*Myc*	0.088	0	0	0.42	0.449
1-day post-SCI group vs. the control group	*Hmox1*	*Atf4*	0	0	0	0.468	0.468
1-day post-SCI group vs. the control group	*Hmox1*	*Rela*	0.062	0	0	0.464	0.475
1-day post-SCI group vs. the control group	*Hmox1*	*Cyp1b1*	0.162	0	0	0.41	0.485
1-day post-SCI group vs. the control group	*Hmox1*	*Fosl1*	0.104	0	0.313	0.258	0.504
1-day post-SCI group vs. the control group	*Hmox1*	*Bax*	0.084	0	0	0.493	0.516

1-day post-SCI group vs. the control group	*Hmox1*	*Mapk8*	0	0	0	0.551	0.551
1-day post-SCI group vs. the control group	*Hmox1*	*Apoe*	0	0.049	0	0.552	0.556
1-day post-SCI group vs. the control group	*Hmox1*	*Gpx4*	0	0.07	0	0.573	0.586
1-day post-SCI group vs. the control group	*Hmox1*	*Edn1*	0	0	0	0.617	0.617
1-day post-SCI group vs. the control group	*Hmox1*	*Fos*	0	0	0.313	0.477	0.626
1-day post-SCI group vs. the control group	*Hmox1*	*Xdh*	0.068	0	0	0.619	0.629
1-day post-SCI group vs. the control group	*Hmox1*	*Vcam1*	0.061	0	0	0.628	0.636
1-day post-SCI group vs. the control group	*Hmox1*	*Srxn1*	0.151	0	0	0.643	0.685
1-day post-SCI group vs. the control group	*Hmox1*	*Ppargc1a*	0	0	0	0.731	0.731
1-day post-SCI group vs. the control group	*Hmox1*	*Txnrd1*	0.113	0.131	0	0.701	0.749
1-day post-SCI group vs. the control group	*Hmox1*	*Ptgs2*	0.085	0	0	0.746	0.757
1-day post-SCI group vs. the control group	*Hmox1*	*Gpx1*	0.066	0.07	0	0.746	0.76
1-day post-SCI group vs. the control group	*Hmox1*	*Nos3*	0	0.218	0	0.757	0.802
1-day post-SCI group vs. the control group	*Hmox1*	*Jun*	0	0	0.39	0.713	0.817
1-day post-SCI group vs. the control group	*Hmox1*	*Cat*	0.062	0.09	0	0.877	0.886
1-day post-SCI group vs. the control group	*Hmox2*	*Gpx1*	0.073	0.07	0	0.443	0.478
1-day post-SCI group vs. the control group	*Hmox2*	*Mapk14*	0	0	0.34	0.342	0.547
1-day post-SCI group vs. the control group	*Hmox2*	*Gpx4*	0.089	0.07	0	0.543	0.579
1-day post-SCI group vs. the control group	*Hmox2*	*Nos3*	0	0.218	0	0.503	0.595
1-day post-SCI group vs. the control group	*Hmox2*	*Fos*	0	0	0.629	0.235	0.704
1-day post-SCI group vs. the control group	*Hmox2*	*Cat*	0.062	0.09	0	0.694	0.716
1-day post-SCI group vs. the control group	*Hmox2*	*Jun*	0	0	0.629	0.416	0.774
1-day post-SCI group vs. the control group	*Hmox2*	*Hmox1*	0	0	0.8	0.892	0.818
1-day post-SCI group vs. the control group	*Hspa13*	*Trap1*	0.171	0.393	0.411	0.175	0.722
1-day post-SCI group vs. the control group	*Hspa13*	*Fkbp1b*	0.063	0.198	0	0.302	0.429
1-day post-SCI group vs. the control group	*Hspa13*	*Xdh*	0	0.457	0	0.042	0.457
1-day post-SCI group vs. the control group	*Hspb1*	*Mapk14*	0	0.13	0.676	0.857	0.956
1-day post-SCI group vs. the control group	*Hspb1*	*Trap1*	0.058	0.134	0	0.35	0.423
1-day post-SCI group vs. the control group	*Hspb1*	*Myc*	0	0.055	0	0.446	0.454
1-day post-SCI group vs. the control group	*Hspb1*	*Cat*	0	0	0	0.462	0.462
1-day post-SCI group vs. the control group	*Hspb1*	*Pxn*	0.061	0.202	0	0.346	0.467
1-day post-SCI group vs. the control group	*Hspb1*	*Map2k6*	0	0.052	0	0.464	0.47
1-day post-SCI group vs. the control group	*Hspb1*	*Mapk8*	0	0.092	0	0.474	0.502
1-day post-SCI group vs. the control group	*Hspb1*	*Map2k3*	0.067	0.057	0	0.528	0.548
1-day post-SCI group vs. the control group	*Hspb1*	*Hmox1*	0.083	0	0	0.532	0.553
1-day post-SCI group vs. the control group	*Hspb1*	*Jun*	0.062	0	0	0.588	0.597

1-day post-SCI group vs. the control group	*Hspb1*	*Mapt*	0.061	0.13	0	0.576	0.623
1-day post-SCI group vs. the control group	*Hspb1*	*Mapkapk3*	0.07	0.671	0.966	0.421	0.993
1-day post-SCI group vs. the control group	*Ier3*	*Fosl1*	0.187	0	0	0.351	0.45
1-day post-SCI group vs. the control group	*Ier3*	*Fos*	0.159	0	0	0.399	0.472
1-day post-SCI group vs. the control group	*Ier3*	*Mcl1*	0.061	0.079	0	0.586	0.612
1-day post-SCI group vs. the control group	*Ier3*	*Rela*	0.071	0.13	0	0.836	0.856
1-day post-SCI group vs. the control group	*Il6st*	*Map2k3*	0	0	0.537	0.088	0.559
1-day post-SCI group vs. the control group	*Il6st*	*Map2k6*	0	0	0.676	0.056	0.681
1-day post-SCI group vs. the control group	*Il6st*	*Jak2*	0.062	0.877	0.932	0.522	0.995
1-day post-SCI group vs. the control group	*Il6st*	*Stat6*	0.064	0.047	0.35	0.31	0.546
1-day post-SCI group vs. the control group	*Itga5*	*Sdc1*	0.1	0	0.43	0.306	0.613
1-day post-SCI group vs. the control group	*Itga5*	*Adam9*	0.089	0.091	0.212	0.192	0.402
1-day post-SCI group vs. the control group	*Itga5*	*Cttn*	0	0	0.334	0.171	0.424
1-day post-SCI group vs. the control group	*Itga5*	*Sphk1*	0.064	0	0.376	0.107	0.433
1-day post-SCI group vs. the control group	*Itga5*	*Mmp3*	0.078	0.07	0.139	0.333	0.442
1-day post-SCI group vs. the control group	*Itga5*	*Ctnna1*	0.103	0.049	0.248	0.233	0.442
1-day post-SCI group vs. the control group	*Itga5*	*Met*	0.112	0.133	0.329	0.355	0.622
1-day post-SCI group vs. the control group	*Itga5*	*Vcam1*	0.052	0.058	0.352	0.569	0.717
1-day post-SCI group vs. the control group	*Itga5*	*Itga7*	0.061	0.297	0.72	0.668	0.84
1-day post-SCI group vs. the control group	*Itga5*	*Pxn*	0.116	0.16	0.711	0.559	0.893
1-day post-SCI group vs. the control group	*Itga7*	*Itga5*	0.061	0.297	0.72	0.668	0.84
1-day post-SCI group vs. the control group	*Itga7*	*Vcam1*	0.061	0.058	0.352	0.351	0.578
1-day post-SCI group vs. the control group	*Itga7*	*Ctnna1*	0.062	0.049	0.248	0.566	0.669
1-day post-SCI group vs. the control group	*Itga7*	*Pxn*	0.081	0.16	0.711	0.227	0.805
1-day post-SCI group vs. the control group	*Itga7*	*Met*	0.063	0.133	0.42	0.2	0.572
1-day post-SCI group vs. the control group	*Itsn1*	*Amph*	0.061	0.13	0	0.643	0.683
1-day post-SCI group vs. the control group	*Itsn1*	*Spry2*	0	0.436	0	0.798	0.881
1-day post-SCI group vs. the control group	*Itsn1*	*Rcan1*	0	0	0	0.616	0.616
1-day post-SCI group vs. the control group	*Itsn1*	*Pdlim1*	0.157	0.24	0	0.187	0.433
1-day post-SCI group vs. the control group	*Itsn1*	*Pxn*	0.157	0.24	0	0.278	0.497
1-day post-SCI group vs. the control group	*Jak2*	*Axl*	0.058	0	0.8	0.305	0.826
1-day post-SCI group vs. the control group	*Jak2*	*Mapk14*	0.062	0.147	0.42	0.633	0.617
1-day post-SCI group vs. the control group	*Jak2*	*Cdk4*	0.062	0.198	0.915	0.482	0.942
1-day post-SCI group vs. the control group	*Jak2*	*Map2k3*	0	0.084	0.376	0.226	0.45
1-day post-SCI group vs. the control group	*Jak2*	*Mdm2*	0.062	0.154	0.146	0.4	0.539
1-day post-SCI group vs. the control group	*Jak2*	*Map2k6*	0	0.084	0.376	0.181	0.438

1-day post-SCI group vs. the control group	*Jak2*	*Fos*	0	0.07	0.15	0.472	0.547
1-day post-SCI group vs. the control group	*Jak2*	*Edn1*	0	0	0.676	0.319	0.77
1-day post-SCI group vs. the control group	*Jak2*	*Myc*	0.051	0.086	0.129	0.607	0.664
1-day post-SCI group vs. the control group	*Jak2*	*Hbegf*	0	0	0.676	0.203	0.731
1-day post-SCI group vs. the control group	*Jak2*	*Rela*	0	0.064	0.676	0.399	0.801
1-day post-SCI group vs. the control group	*Jak2*	*Ccr1*	0.061	0.141	0.63	0.195	0.727
1-day post-SCI group vs. the control group	*Jak2*	*Cat*	0	0.065	0.183	0.34	0.453
1-day post-SCI group vs. the control group	*Jak2*	*Vcam1*	0.061	0	0.217	0.397	0.517
1-day post-SCI group vs. the control group	*Jak2*	*Nos3*	0	0.133	0.399	0.317	0.613
1-day post-SCI group vs. the control group	*Jak2*	*Tnfrsf1a*	0.051	0.227	0.137	0.662	0.757
1-day post-SCI group vs. the control group	*Jak2*	*Btk*	0.062	0.146	0.676	0.468	0.764
1-day post-SCI group vs. the control group	*Jak2*	*Ptgs2*	0.095	0.088	0	0.464	0.518
1-day post-SCI group vs. the control group	*Jak2*	*Mcl1*	0.061	0.056	0.19	0.61	0.682
1-day post-SCI group vs. the control group	*Jak2*	*Ezr*	0	0.089	0.22	0.307	0.464
1-day post-SCI group vs. the control group	*Jak2*	*Sphk1*	0	0	0.408	0.197	0.504
1-day post-SCI group vs. the control group	*Jak2*	*Mapk8*	0.063	0.139	0.42	0.498	0.594
1-day post-SCI group vs. the control group	*Jak2*	*Jun*	0.05	0.136	0.18	0.613	0.705
1-day post-SCI group vs. the control group	*Jak2*	*Pxn*	0.081	0.063	0.676	0.25	0.763
1-day post-SCI group vs. the control group	*Jak2*	*Met*	0.065	0.074	0.858	0.434	0.89
1-day post-SCI group vs. the control group	*Jak2*	*Cdkn1c*	0	0.09	0.908	0.172	0.925
1-day post-SCI group vs. the control group	*Jak2*	*Stat6*	0.055	0.147	0.932	0.837	0.989
1-day post-SCI group vs. the control group	*Jak2*	*Il6st*	0.062	0.877	0.932	0.522	0.995
1-day post-SCI group vs. the control group	*Jun*	*Axl*	0.062	0.09	0.127	0.327	0.431
1-day post-SCI group vs. the control group	*Jun*	*Hmox2*	0	0	0.629	0.416	0.774
1-day post-SCI group vs. the control group	*Jun*	*Mapk14*	0.049	0.4	0.932	0.903	0.995
1-day post-SCI group vs. the control group	*Jun*	*Hspb1*	0.062	0	0	0.588	0.597
1-day post-SCI group vs. the control group	*Jun*	*Hmox1*	0	0	0.39	0.713	0.817
1-day post-SCI group vs. the control group	*Jun*	*Cdk4*	0.061	0.164	0	0.63	0.684
1-day post-SCI group vs. the control group	*Jun*	*Map2k3*	0	0	0	0.721	0.721
1-day post-SCI group vs. the control group	*Jun*	*Mdm2*	0	0.045	0	0.654	0.656
1-day post-SCI group vs. the control group	*Jun*	*Map2k6*	0	0	0	0.592	0.592
1-day post-SCI group vs. the control group	*Jun*	*Fos*	0.662	0.982	0.932	0.995	0.999
1-day post-SCI group vs. the control group	*Jun*	*Edn1*	0.061	0	0.629	0.589	0.844
1-day post-SCI group vs. the control group	*Jun*	*Myc*	0.062	0.27	0.548	0.856	0.949
1-day post-SCI group vs. the control group	*Jun*	*Xdh*	0	0	0	0.583	0.583
1-day post-SCI group vs. the control group	*Jun*	*Hbegf*	0.063	0	0	0.409	0.423

1-day post-SCI group vs. the control group	*Jun*	*Fosl1*	0.066	0.784	0.932	0.989	0.999
1-day post-SCI group vs. the control group	*Jun*	*Rela*	0.061	0.46	0.629	0.859	0.97
1-day post-SCI group vs. the control group	*Jun*	*Ccr1*	0	0.046	0.483	0.254	0.599
1-day post-SCI group vs. the control group	*Jun*	*Cat*	0	0	0	0.682	0.682
1-day post-SCI group vs. the control group	*Jun*	*Vcam1*	0	0	0	0.589	0.589
1-day post-SCI group vs. the control group	*Jun*	*Nos3*	0	0	0.629	0.547	0.824
1-day post-SCI group vs. the control group	*Jun*	*Tnfrsf1a*	0.069	0	0	0.619	0.63
1-day post-SCI group vs. the control group	*Jun*	*Bax*	0	0	0	0.529	0.529
1-day post-SCI group vs. the control group	*Jun*	*Btk*	0	0.136	0.676	0.442	0.83
1-day post-SCI group vs. the control group	*Jun*	*Mmp3*	0	0.208	0	0.612	0.679
1-day post-SCI group vs. the control group	*Jun*	*Ptgs2*	0.061	0	0	0.738	0.743
1-day post-SCI group vs. the control group	*Jun*	*Map2k4*	0	0.13	0	0.827	0.843
1-day post-SCI group vs. the control group	*Jun*	*Mcl1*	0.063	0	0	0.62	0.628
1-day post-SCI group vs. the control group	*Jun*	*Jak2*	0.05	0.136	0.18	0.613	0.705
1-day post-SCI group vs. the control group	*Jun*	*Smad1*	0	0.211	0.388	0.43	0.701
1-day post-SCI group vs. the control group	*Jun*	*Gpx1*	0.051	0.045	0	0.413	0.422
1-day post-SCI group vs. the control group	*Jun*	*Pxn*	0	0	0	0.581	0.581
1-day post-SCI group vs. the control group	*Jun*	*Stat6*	0	0.134	0.216	0.552	0.669
1-day post-SCI group vs. the control group	*Jun*	*Hdac1*	0	0.261	0	0.787	0.836
1-day post-SCI group vs. the control group	*Jun*	*Ripk1*	0	0.09	0.127	0.468	0.54
1-day post-SCI group vs. the control group	*Jun*	*Ppargc1a*	0	0	0	0.542	0.542
1-day post-SCI group vs. the control group	*Jun*	*Apoe*	0	0	0	0.552	0.552
1-day post-SCI group vs. the control group	*Jun*	*Map4k4*	0	0.265	0	0.436	0.568
1-day post-SCI group vs. the control group	*Jun*	*Cdkn1c*	0.052	0.061	0.362	0.342	0.576
1-day post-SCI group vs. the control group	*Jun*	*Met*	0	0.09	0.127	0.528	0.592
1-day post-SCI group vs. the control group	*Jun*	*Atf4*	0.064	0.73	0	0.741	0.929
1-day post-SCI group vs. the control group	*Jun*	*Fbxw7*	0	0.762	0	0.901	0.975
1-day post-SCI group vs. the control group	*Jun*	*Atf2*	0.066	0.943	0.932	0.989	0.997
1-day post-SCI group vs. the control group	*Jun*	*Mapk8*	0	0.999	0.966	0.947	0.999
1-day post-SCI group vs. the control group	*Krt1*	*Eed*	0	0.433	0	0.082	0.457
1-day post-SCI group vs. the control group	*Lancl1*	*Map2k6*	0.062	0	0	0.486	0.497
1-day post-SCI group vs. the control group	*Lancl1*	*Syp*	0.067	0.698	0	0	0.706
1-day post-SCI group vs. the control group	*Lcat*	*Apoe*	0.116	0.13	0.72	0.692	0.924
1-day post-SCI group vs. the control group	*Lpo*	*Cat*	0	0.203	0	0.351	0.46
1-day post-SCI group vs. the control group	*Map2k3*	*Mapk14*	0.065	0.839	0.966	0.961	0.996
1-day post-SCI group vs. the control group	*Map2k3*	*Hspb1*	0.067	0.057	0	0.528	0.548

1-day post-SCI group vs. the control group	*Map2k3*	*Ripk3*	0.101	0.132	0.293	0.082	0.41
1-day post-SCI group vs. the control group	*Map2k3*	*Eed*	0	0.433	0	0	0.433
1-day post-SCI group vs. the control group	*Map2k3*	*Fos*	0	0	0	0.435	0.435
1-day post-SCI group vs. the control group	*Map2k3*	*Myc*	0.079	0.209	0	0.306	0.45
1-day post-SCI group vs. the control group	*Map2k3*	*Jak2*	0	0.084	0.376	0.226	0.45
1-day post-SCI group vs. the control group	*Map2k3*	*Rela*	0.079	0.241	0	0.277	0.45
1-day post-SCI group vs. the control group	*Map2k3*	*Il6st*	0	0	0.537	0.088	0.559
1-day post-SCI group vs. the control group	*Map2k3*	*Atf2*	0	0	0	0.703	0.703
1-day post-SCI group vs. the control group	*Map2k3*	*Jun*	0	0	0	0.721	0.721
1-day post-SCI group vs. the control group	*Map2k3*	*Tnfrsf1a*	0.095	0	0.676	0.245	0.759
1-day post-SCI group vs. the control group	*Map2k3*	*Map2k4*	0	0.048	0.8	0.829	0.814
1-day post-SCI group vs. the control group	*Map2k3*	*Mapkapk3*	0.109	0.081	0.9	0.452	0.935
1-day post-SCI group vs. the control group	*Map2k3*	*Ripk1*	0.087	0.132	0.932	0.221	0.945
1-day post-SCI group vs. the control group	*Map2k3*	*Mapk8*	0.062	0.135	0.922	0.75	0.958
1-day post-SCI group vs. the control group	*Map2k3*	*Map2k6*	0	0.56	0.966	0.86	0.984
1-day post-SCI group vs. the control group	*Map2k4*	*Mapk14*	0.062	0.402	0.932	0.75	0.973
1-day post-SCI group vs. the control group	*Map2k4*	*Map2k3*	0	0.048	0.8	0.829	0.814
1-day post-SCI group vs. the control group	*Map2k4*	*Map2k6*	0	0.373	0.8	0.683	0.875
1-day post-SCI group vs. the control group	*Map2k4*	*Ripk3*	0	0.106	0.341	0.163	0.419
1-day post-SCI group vs. the control group	*Map2k4*	*Tnfrsf1a*	0	0	0.629	0.301	0.729
1-day post-SCI group vs. the control group	*Map2k4*	*Mapkapk3*	0.051	0.14	0	0.574	0.406
1-day post-SCI group vs. the control group	*Map2k4*	*Atf2*	0.094	0	0	0.594	0.616
1-day post-SCI group vs. the control group	*Map2k4*	*Ripk1*	0	0.106	0.629	0.242	0.682
1-day post-SCI group vs. the control group	*Map2k4*	*Jun*	0	0.13	0	0.827	0.843
1-day post-SCI group vs. the control group	*Map2k4*	*Mapk8*	0.105	0.821	0.966	0.863	0.996
1-day post-SCI group vs. the control group	*Map2k6*	*Mapk14*	0.065	0.848	0.966	0.864	0.996
1-day post-SCI group vs. the control group	*Map2k6*	*Hspb1*	0	0.052	0	0.464	0.47
1-day post-SCI group vs. the control group	*Map2k6*	*Map2k3*	0	0.56	0.966	0.86	0.984
1-day post-SCI group vs. the control group	*Map2k6*	*Jak2*	0	0.084	0.376	0.181	0.438
1-day post-SCI group vs. the control group	*Map2k6*	*Rela*	0.05	0.311	0	0.24	0.459
1-day post-SCI group vs. the control group	*Map2k6*	*Lancl1*	0.062	0	0	0.486	0.497
1-day post-SCI group vs. the control group	*Map2k6*	*Atf2*	0	0	0	0.584	0.584
1-day post-SCI group vs. the control group	*Map2k6*	*Jun*	0	0	0	0.592	0.592
1-day post-SCI group vs. the control group	*Map2k6*	*Il6st*	0	0	0.676	0.056	0.681
1-day post-SCI group vs. the control group	*Map2k6*	*Tnfrsf1a*	0	0	0.629	0.235	0.704
1-day post-SCI group vs. the control group	*Map2k6*	*Map2k4*	0	0.373	0.8	0.683	0.875

1-day post-SCI group vs. the control group	*Map2k6*	*Mapkapk3*	0.063	0.081	0.9	0.328	0.928
1-day post-SCI group vs. the control group	*Map2k6*	*Ripk1*	0.061	0.132	0.922	0.155	0.934
1-day post-SCI group vs. the control group	*Map2k6*	*Mapk8*	0.062	0.135	0.932	0.649	0.961
1-day post-SCI group vs. the control group	*Map4k4*	*Tnfrsf1a*	0	0	0.676	0.132	0.706
1-day post-SCI group vs. the control group	*Map4k4*	*Ezr*	0.048	0.137	0	0.469	0.526
1-day post-SCI group vs. the control group	*Map4k4*	*Jun*	0	0.265	0	0.436	0.568
1-day post-SCI group vs. the control group	*Map4k4*	*Ripk1*	0	0.142	0.676	0.105	0.718
1-day post-SCI group vs. the control group	*Mapk14*	*Capns1*	0	0.134	0.34	0.158	0.476
1-day post-SCI group vs. the control group	*Mapk14*	*Hmox2*	0	0	0.34	0.342	0.547
1-day post-SCI group vs. the control group	*Mapk14*	*Btk*	0	0.147	0.187	0.473	0.407
1-day post-SCI group vs. the control group	*Mapk14*	*Ezr*	0	0.199	0	0.312	0.425
1-day post-SCI group vs. the control group	*Mapk14*	*Gpx1*	0	0	0	0.425	0.425
1-day post-SCI group vs. the control group	*Mapk14*	*Capn2*	0.062	0.058	0.256	0.266	0.453
1-day post-SCI group vs. the control group	*Mapk14*	*Hdac1*	0	0.131	0	0.404	0.46
1-day post-SCI group vs. the control group	*Mapk14*	*Ppp3ca*	0.063	0.272	0	0.284	0.468
1-day post-SCI group vs. the control group	*Mapk14*	*Cdk4*	0	0.235	0.185	0.581	0.469
1-day post-SCI group vs. the control group	*Mapk14*	*Sdc1*	0.052	0	0.354	0.237	0.491
1-day post-SCI group vs. the control group	*Mapk14*	*Trap1*	0.052	0.164	0.319	0.176	0.495
1-day post-SCI group vs. the control group	*Mapk14*	*Apoe*	0	0	0	0.5	0.499
1-day post-SCI group vs. the control group	*Mapk14*	*Mdm2*	0	0	0	0.526	0.526
1-day post-SCI group vs. the control group	*Mapk14*	*Xdh*	0	0	0	0.529	0.529
1-day post-SCI group vs. the control group	*Mapk14*	*Mmp3*	0.051	0	0	0.53	0.534
1-day post-SCI group vs. the control group	*Mapk14*	*Bax*	0	0.061	0.209	0.438	0.546
1-day post-SCI group vs. the control group	*Mapk14*	*Tnfrsf1a*	0.062	0.048	0	0.552	0.565
1-day post-SCI group vs. the control group	*Mapk14*	*Met*	0.061	0.147	0.392	0.48	0.569
1-day post-SCI group vs. the control group	*Mapk14*	*Mapt*	0.049	0.182	0.354	0.249	0.572
1-day post-SCI group vs. the control group	*Mapk14*	*Tsc1*	0	0.202	0.354	0.253	0.581
1-day post-SCI group vs. the control group	*Mapk14*	*Stat6*	0.059	0.104	0.212	0.469	0.6
1-day post-SCI group vs. the control group	*Mapk14*	*Nos3*	0.051	0	0	0.598	0.602
1-day post-SCI group vs. the control group	*Mapk14*	*Ctnna1*	0	0.059	0.6	0.065	0.617
1-day post-SCI group vs. the control group	*Mapk14*	*Jak2*	0.062	0.147	0.42	0.633	0.617
1-day post-SCI group vs. the control group	*Mapk14*	*Vcam1*	0	0	0	0.632	0.632
1-day post-SCI group vs. the control group	*Mapk14*	*Smad1*	0	0.17	0.207	0.533	0.666
1-day post-SCI group vs. the control group	*Mapk14*	*Mcl1*	0.061	0.133	0.209	0.55	0.671
1-day post-SCI group vs. the control group	*Mapk14*	*Pxn*	0	0.082	0.31	0.542	0.685
1-day post-SCI group vs. the control group	*Mapk14*	*Ptgs2*	0	0.101	0	0.737	0.753

1-day post-SCI group vs. the control group	*Mapk14*	*Ripk1*	0	0.147	0.676	0.412	0.758
1-day post-SCI group vs. the control group	*Mapk14*	*Cat*	0	0.13	0	0.743	0.767
1-day post-SCI group vs. the control group	*Mapk14*	*Hmox1*	0	0	0.305	0.7	0.782
1-day post-SCI group vs. the control group	*Mapk14*	*Mapk8*	0.066	0.209	0.702	0.872	0.783
1-day post-SCI group vs. the control group	*Mapk14*	*Myc*	0	0.162	0.548	0.619	0.843
1-day post-SCI group vs. the control group	*Mapk14*	*Fosl1*	0	0.267	0.67	0.444	0.853
1-day post-SCI group vs. the control group	*Mapk14*	*Edn1*	0	0	0.676	0.591	0.861
1-day post-SCI group vs. the control group	*Mapk14*	*Rela*	0.049	0.057	0.676	0.669	0.89
1-day post-SCI group vs. the control group	*Mapk14*	*Hspb1*	0	0.13	0.676	0.857	0.956
1-day post-SCI group vs. the control group	*Mapk14*	*Map2k4*	0.062	0.402	0.932	0.75	0.973
1-day post-SCI group vs. the control group	*Mapk14*	*Fos*	0	0.267	0.922	0.716	0.982
1-day post-SCI group vs. the control group	*Mapk14*	*Ppargc1a*	0	0.087	0.966	0.62	0.987
1-day post-SCI group vs. the control group	*Mapk14*	*Mapkapk3*	0.061	0.76	0.932	0.524	0.988
1-day post-SCI group vs. the control group	*Mapk14*	*Jun*	0.049	0.4	0.932	0.903	0.995
1-day post-SCI group vs. the control group	*Mapk14*	*Map2k3*	0.065	0.839	0.966	0.961	0.996
1-day post-SCI group vs. the control group	*Mapk14*	*Map2k6*	0.065	0.848	0.966	0.864	0.996
1-day post-SCI group vs. the control group	*Mapk14*	*Atf2*	0	0.927	0.966	0.785	0.999
1-day post-SCI group vs. the control group	*Mapk8*	*Mapk14*	0.066	0.209	0.702	0.872	0.783
1-day post-SCI group vs. the control group	*Mapk8*	*Hspb1*	0	0.092	0	0.474	0.502
1-day post-SCI group vs. the control group	*Mapk8*	*Hmox1*	0	0	0	0.551	0.551
1-day post-SCI group vs. the control group	*Mapk8*	*Dynll1*	0.064	0	0.9	0.063	0.904
1-day post-SCI group vs. the control group	*Mapk8*	*Map2k3*	0.062	0.135	0.922	0.75	0.958
1-day post-SCI group vs. the control group	*Mapk8*	*Mdm2*	0.062	0	0	0.388	0.402
1-day post-SCI group vs. the control group	*Mapk8*	*Map2k6*	0.062	0.135	0.932	0.649	0.961
1-day post-SCI group vs. the control group	*Mapk8*	*Fos*	0	0.309	0.932	0.64	0.981
1-day post-SCI group vs. the control group	*Mapk8*	*Edn1*	0	0	0.676	0.379	0.79
1-day post-SCI group vs. the control group	*Mapk8*	*Ripk3*	0	0.208	0.209	0.324	0.429
1-day post-SCI group vs. the control group	*Mapk8*	*Myc*	0	0.198	0	0.536	0.612
1-day post-SCI group vs. the control group	*Mapk8*	*Fosl1*	0	0.309	0.696	0.395	0.862
1-day post-SCI group vs. the control group	*Mapk8*	*Rela*	0	0.048	0.8	0.547	0.906
1-day post-SCI group vs. the control group	*Mapk8*	*Cat*	0	0	0	0.525	0.525
1-day post-SCI group vs. the control group	*Mapk8*	*Vcam1*	0	0	0	0.4	0.4
1-day post-SCI group vs. the control group	*Mapk8*	*Nos3*	0.052	0	0	0.409	0.416
1-day post-SCI group vs. the control group	*Mapk8*	*Tnfrsf1a*	0	0.133	0	0.49	0.539
1-day post-SCI group vs. the control group	*Mapk8*	*Bax*	0	0.082	0.272	0.397	0.561
1-day post-SCI group vs. the control group	*Mapk8*	*Btk*	0.052	0.139	0.676	0.341	0.75

1-day post-SCI group vs. the control group	*Mapk8*	*Ptgs2*	0	0	0	0.589	0.589
1-day post-SCI group vs. the control group	*Mapk8*	*Mapkapk3*	0	0.224	0.65	0.306	0.785
1-day post-SCI group vs. the control group	*Mapk8*	*Map2k4*	0.105	0.821	0.966	0.863	0.996
1-day post-SCI group vs. the control group	*Mapk8*	*Mcl1*	0	0.431	0.272	0.471	0.762
1-day post-SCI group vs. the control group	*Mapk8*	*Jak2*	0.063	0.139	0.42	0.498	0.594
1-day post-SCI group vs. the control group	*Mapk8*	*Pxn*	0	0.512	0	0.374	0.681
1-day post-SCI group vs. the control group	*Mapk8*	*Mapt*	0.062	0.228	0.629	0.258	0.774
1-day post-SCI group vs. the control group	*Mapk8*	*Jun*	0	0.999	0.966	0.947	0.999
1-day post-SCI group vs. the control group	*Mapk8*	*Ripk1*	0	0.208	0.209	0.457	0.468
1-day post-SCI group vs. the control group	*Mapk8*	*Cdkn1c*	0	0.133	0.362	0.119	0.47
1-day post-SCI group vs. the control group	*Mapk8*	*Met*	0.062	0.132	0.676	0.337	0.801
1-day post-SCI group vs. the control group	*Mapk8*	*Atf2*	0.088	0.844	0.966	0.61	0.997
1-day post-SCI group vs. the control group	*Mapkapk3*	*Mapk14*	0.061	0.76	0.932	0.524	0.988
1-day post-SCI group vs. the control group	*Mapkapk3*	*Hspb1*	0.07	0.671	0.966	0.421	0.993
1-day post-SCI group vs. the control group	*Mapkapk3*	*Map2k3*	0.109	0.081	0.9	0.452	0.935
1-day post-SCI group vs. the control group	*Mapkapk3*	*Map2k6*	0.063	0.081	0.9	0.328	0.928
1-day post-SCI group vs. the control group	*Mapkapk3*	*Map2k4*	0.051	0.14	0	0.574	0.406
1-day post-SCI group vs. the control group	*Mapkapk3*	*Mapk8*	0	0.224	0.65	0.306	0.785
1-day post-SCI group vs. the control group	*Mapkapk3*	*Atf4*	0.061	0	0.8	0	0.804
1-day post-SCI group vs. the control group	*Mapt*	*Amph*	0.18	0.13	0	0.233	0.405
1-day post-SCI group vs. the control group	*Mapt*	*Mapk14*	0.049	0.182	0.354	0.249	0.572
1-day post-SCI group vs. the control group	*Mapt*	*Hspb1*	0.061	0.13	0	0.576	0.623
1-day post-SCI group vs. the control group	*Mapt*	*Dynll1*	0	0.438	0	0.088	0.465
1-day post-SCI group vs. the control group	*Mapt*	*Ndufa6*	0	0	0.6	0	0.6
1-day post-SCI group vs. the control group	*Mapt*	*Pink1*	0.094	0	0	0.581	0.604
1-day post-SCI group vs. the control group	*Mapt*	*Cask*	0.071	0.115	0.355	0.057	0.433
1-day post-SCI group vs. the control group	*Mapt*	*Ppp1ca*	0	0.366	0	0.107	0.41
1-day post-SCI group vs. the control group	*Mapt*	*Atp13a2*	0.061	0.046	0	0.507	0.52
1-day post-SCI group vs. the control group	*Mapt*	*Ppp3ca*	0.098	0.527	0	0.152	0.606
1-day post-SCI group vs. the control group	*Mapt*	*Syp*	0.212	0	0	0.573	0.649
1-day post-SCI group vs. the control group	*Mapt*	*Mapk8*	0.062	0.228	0.629	0.258	0.774
1-day post-SCI group vs. the control group	*Mapt*	*Apoe*	0	0.708	0	0.89	0.966
1-day post-SCI group vs. the control group	*Mcl1*	*Ier3*	0.061	0.079	0	0.586	0.612
1-day post-SCI group vs. the control group	*Mcl1*	*Mapk14*	0.061	0.133	0.209	0.55	0.671
1-day post-SCI group vs. the control group	*Mcl1*	*Cdk4*	0	0.058	0	0.589	0.596
1-day post-SCI group vs. the control group	*Mcl1*	*Mdm2*	0	0.131	0	0.661	0.693

1-day post-SCI group vs. the control group	*Mcl1*	*Fos*	0.089	0	0	0.411	0.441
1-day post-SCI group vs. the control group	*Mcl1*	*Myc*	0.066	0.052	0	0.748	0.758
1-day post-SCI group vs. the control group	*Mcl1*	*Rela*	0.094	0.058	0.311	0.4	0.599
1-day post-SCI group vs. the control group	*Mcl1*	*Pink1*	0	0.208	0	0.339	0.454
1-day post-SCI group vs. the control group	*Mcl1*	*Tnfrsf1a*	0.068	0.077	0	0.387	0.427
1-day post-SCI group vs. the control group	*Mcl1*	*Bax*	0	0.422	0.72	0.61	0.865
1-day post-SCI group vs. the control group	*Mcl1*	*Btk*	0	0.056	0.19	0.469	0.558
1-day post-SCI group vs. the control group	*Mcl1*	*Ptgs2*	0.061	0	0	0.409	0.421
1-day post-SCI group vs. the control group	*Mcl1*	*Ripk1*	0.079	0.056	0.12	0.349	0.435
1-day post-SCI group vs. the control group	*Mcl1*	*Stat6*	0.069	0	0.216	0.328	0.467
1-day post-SCI group vs. the control group	*Mcl1*	*Met*	0	0.056	0.12	0.465	0.516
1-day post-SCI group vs. the control group	*Mcl1*	*Jun*	0.063	0	0	0.62	0.628
1-day post-SCI group vs. the control group	*Mcl1*	*Jak2*	0.061	0.056	0.19	0.61	0.682
1-day post-SCI group vs. the control group	*Mcl1*	*Mapk8*	0	0.431	0.272	0.471	0.762
1-day post-SCI group vs. the control group	*Mcl1*	*Fbxw7*	0.061	0.745	0	0.863	0.964
1-day post-SCI group vs. the control group	*Mdm2*	*Mapk14*	0	0	0	0.526	0.526
1-day post-SCI group vs. the control group	*Mdm2*	*Cdk4*	0	0.058	0.27	0.782	0.837
1-day post-SCI group vs. the control group	*Mdm2*	*Mapk8*	0.062	0	0	0.388	0.402
1-day post-SCI group vs. the control group	*Mdm2*	*Btk*	0	0.13	0.146	0.269	0.409
1-day post-SCI group vs. the control group	*Mdm2*	*Cdkn1c*	0	0.206	0	0.36	0.47
1-day post-SCI group vs. the control group	*Mdm2*	*Nme2*	0	0.089	0	0.445	0.473
1-day post-SCI group vs. the control group	*Mdm2*	*Ppp1ca*	0	0.227	0.198	0.247	0.492
1-day post-SCI group vs. the control group	*Mdm2*	*Ptgs2*	0.056	0.169	0	0.407	0.494
1-day post-SCI group vs. the control group	*Mdm2*	*Fos*	0.062	0.164	0	0.431	0.515
1-day post-SCI group vs. the control group	*Mdm2*	*Rela*	0	0.138	0	0.464	0.518
1-day post-SCI group vs. the control group	*Mdm2*	*Met*	0	0.082	0.146	0.438	0.52
1-day post-SCI group vs. the control group	*Mdm2*	*Bax*	0.064	0.131	0	0.464	0.525
1-day post-SCI group vs. the control group	*Mdm2*	*Jak2*	0.062	0.154	0.146	0.4	0.539
1-day post-SCI group vs. the control group	*Mdm2*	*Fbxw7*	0.061	0.13	0	0.605	0.649
1-day post-SCI group vs. the control group	*Mdm2*	*Jun*	0	0.045	0	0.654	0.656
1-day post-SCI group vs. the control group	*Mdm2*	*Mcl1*	0	0.131	0	0.661	0.693
1-day post-SCI group vs. the control group	*Mdm2*	*Myc*	0	0.055	0	0.811	0.815
1-day post-SCI group vs. the control group	*Mdm2*	*Hdac1*	0	0.342	0	0.961	0.973
1-day post-SCI group vs. the control group	*Met*	*Mapk14*	0.061	0.147	0.392	0.48	0.569
1-day post-SCI group vs. the control group	*Met*	*Mdm2*	0	0.082	0.146	0.438	0.52
1-day post-SCI group vs. the control group	*Met*	*Sdc1*	0.114	0	0.676	0.312	0.785

1-day post-SCI group vs. the control group	*Met*	*Fos*	0	0.071	0.12	0.364	0.434
1-day post-SCI group vs. the control group	*Met*	*Spry2*	0	0.141	0.128	0.281	0.414
1-day post-SCI group vs. the control group	*Met*	*Myc*	0.051	0.089	0.129	0.627	0.681
1-day post-SCI group vs. the control group	*Met*	*Itga5*	0.112	0.133	0.329	0.355	0.622
1-day post-SCI group vs. the control group	*Met*	*Hbegf*	0.061	0	0.147	0.401	0.478
1-day post-SCI group vs. the control group	*Met*	*Fosl1*	0.077	0.071	0.12	0.355	0.448
1-day post-SCI group vs. the control group	*Met*	*Vcam1*	0.062	0	0.147	0.338	0.424
1-day post-SCI group vs. the control group	*Met*	*Btk*	0.065	0.074	0.323	0.457	0.457
1-day post-SCI group vs. the control group	*Met*	*Ptgs2*	0.069	0.136	0	0.398	0.473
1-day post-SCI group vs. the control group	*Met*	*Mcl1*	0	0.056	0.12	0.465	0.516
1-day post-SCI group vs. the control group	*Met*	*Ctnna1*	0.082	0.052	0.676	0.066	0.701
1-day post-SCI group vs. the control group	*Met*	*Ezr*	0.061	0.089	0.133	0.391	0.487
1-day post-SCI group vs. the control group	*Met*	*Jak2*	0.065	0.074	0.858	0.434	0.89
1-day post-SCI group vs. the control group	*Met*	*Cdh11*	0.062	0.059	0.36	0.231	0.507
1-day post-SCI group vs. the control group	*Met*	*Pxn*	0.073	0.063	0.131	0.542	0.609
1-day post-SCI group vs. the control group	*Met*	*Itga7*	0.063	0.133	0.42	0.2	0.572
1-day post-SCI group vs. the control group	*Met*	*Cttn*	0.062	0.087	0.146	0.342	0.454
1-day post-SCI group vs. the control group	*Met*	*Jun*	0	0.09	0.127	0.528	0.592
1-day post-SCI group vs. the control group	*Met*	*Mapk8*	0.062	0.132	0.676	0.337	0.801
1-day post-SCI group vs. the control group	*Mmp3*	*Mapk14*	0.051	0	0	0.53	0.534
1-day post-SCI group vs. the control group	*Mmp3*	*Hmox1*	0.062	0	0	0.432	0.444
1-day post-SCI group vs. the control group	*Mmp3*	*Sdc1*	0.077	0.05	0.466	0.241	0.597
1-day post-SCI group vs. the control group	*Mmp3*	*Fos*	0	0.082	0	0.416	0.441
1-day post-SCI group vs. the control group	*Mmp3*	*Edn1*	0	0	0	0.472	0.472
1-day post-SCI group vs. the control group	*Mmp3*	*Ctsl*	0.05	0.047	0.5	0.26	0.62
1-day post-SCI group vs. the control group	*Mmp3*	*Myc*	0	0	0	0.442	0.442
1-day post-SCI group vs. the control group	*Mmp3*	*Itga5*	0.078	0.07	0.139	0.333	0.442
1-day post-SCI group vs. the control group	*Mmp3*	*Hbegf*	0.067	0.057	0.918	0.396	0.951
1-day post-SCI group vs. the control group	*Mmp3*	*Vcam1*	0.071	0	0	0.539	0.554
1-day post-SCI group vs. the control group	*Mmp3*	*Cdh11*	0.062	0.056	0.146	0.33	0.425
1-day post-SCI group vs. the control group	*Mmp3*	*Apoe*	0	0.402	0	0.442	0.652
1-day post-SCI group vs. the control group	*Mmp3*	*Jun*	0	0.208	0	0.612	0.679
1-day post-SCI group vs. the control group	*Mmp3*	*Ptgs2*	0.09	0	0	0.67	0.687
1-day post-SCI group vs. the control group	*Mmp3*	*Ctnna1*	0	0	0.676	0.101	0.696
1-day post-SCI group vs. the control group	*Myc*	*Axl*	0.051	0.086	0.129	0.379	0.468
1-day post-SCI group vs. the control group	*Myc*	*Amph*	0.066	0.204	0	0.376	0.496

1-day post-SCI group vs. the control group	*Myc*	*Mapk14*	0	0.162	0.548	0.619	0.843
1-day post-SCI group vs. the control group	*Myc*	*Hspb1*	0	0.055	0	0.446	0.454
1-day post-SCI group vs. the control group	*Myc*	*Hmox1*	0.088	0	0	0.42	0.449
1-day post-SCI group vs. the control group	*Myc*	*Cdk4*	0.104	0.352	0	0.757	0.847
1-day post-SCI group vs. the control group	*Myc*	*Map2k3*	0.079	0.209	0	0.306	0.45
1-day post-SCI group vs. the control group	*Myc*	*Mdm2*	0	0.055	0	0.811	0.815
1-day post-SCI group vs. the control group	*Myc*	*Sdc1*	0.072	0.045	0	0.45	0.47
1-day post-SCI group vs. the control group	*Myc*	*Nme2*	0.062	0	0	0.486	0.498
1-day post-SCI group vs. the control group	*Myc*	*Fos*	0.064	0.055	0	0.85	0.856
1-day post-SCI group vs. the control group	*Myc*	*Atf2*	0	0.041	0	0.409	0.409
1-day post-SCI group vs. the control group	*Myc*	*Banf1*	0	0	0	0.416	0.416
1-day post-SCI group vs. the control group	*Myc*	*Mmp3*	0	0	0	0.442	0.442
1-day post-SCI group vs. the control group	*Myc*	*Pxn*	0.065	0.066	0	0.42	0.45
1-day post-SCI group vs. the control group	*Myc*	*Rbbp7*	0	0.05	0.29	0.27	0.465
1-day post-SCI group vs. the control group	*Myc*	*Cttn*	0	0	0	0.479	0.48
1-day post-SCI group vs. the control group	*Myc*	*Btk*	0.051	0.086	0.129	0.394	0.481
1-day post-SCI group vs. the control group	*Myc*	*Cat*	0.051	0.045	0	0.476	0.483
1-day post-SCI group vs. the control group	*Myc*	*Cdkn1c*	0	0.041	0	0.485	0.485
1-day post-SCI group vs. the control group	*Myc*	*Stat6*	0.044	0.133	0	0.429	0.485
1-day post-SCI group vs. the control group	*Myc*	*Ppargc1a*	0.065	0	0	0.482	0.495
1-day post-SCI group vs. the control group	*Myc*	*Nono*	0.061	0.443	0	0.247	0.572
1-day post-SCI group vs. the control group	*Myc*	*Nos3*	0	0	0.475	0.263	0.596
1-day post-SCI group vs. the control group	*Myc*	*Ptgs2*	0	0	0	0.597	0.597
1-day post-SCI group vs. the control group	*Myc*	*Mapk8*	0	0.198	0	0.536	0.612
1-day post-SCI group vs. the control group	*Myc*	*Fosl1*	0.15	0.05	0	0.563	0.616
1-day post-SCI group vs. the control group	*Myc*	*Smad1*	0	0.134	0.366	0.374	0.626
1-day post-SCI group vs. the control group	*Myc*	*Jak2*	0.051	0.086	0.129	0.607	0.664
1-day post-SCI group vs. the control group	*Myc*	*Met*	0.051	0.089	0.129	0.627	0.681
1-day post-SCI group vs. the control group	*Myc*	*Mcl1*	0.066	0.052	0	0.748	0.758
1-day post-SCI group vs. the control group	*Myc*	*Rela*	0	0.269	0.548	0.617	0.862
1-day post-SCI group vs. the control group	*Myc*	*Jun*	0.062	0.27	0.548	0.856	0.949
1-day post-SCI group vs. the control group	*Myc*	*Hdac1*	0.061	0.401	0.381	0.901	0.961
1-day post-SCI group vs. the control group	*Myc*	*Fbxw7*	0	0.852	0.585	0.94	0.996
1-day post-SCI group vs. the control group	*Ndufa6*	*Eed*	0	0.433	0	0	0.433
1-day post-SCI group vs. the control group	*Ndufa6*	*Mapt*	0	0	0.6	0	0.6
1-day post-SCI group vs. the control group	*Ndufa6*	*Pink1*	0.098	0	0.6	0.212	0.691

1-day post-SCI group vs. the control group	*Nme2*	*Mdm2*	0	0.089	0	0.445	0.473
1-day post-SCI group vs. the control group	*Nme2*	*Fkbp1b*	0.474	0.349	0	0	0.644
1-day post-SCI group vs. the control group	*Nme2*	*Cat*	0	0.355	0	0.213	0.47
1-day post-SCI group vs. the control group	*Nme2*	*Myc*	0.062	0	0	0.486	0.498
1-day post-SCI group vs. the control group	*Nme2*	*Rpl13a*	0.645	0	0	0	0.645
1-day post-SCI group vs. the control group	*Nme2*	*Gch1*	0.064	0.135	0.8	0.07	0.84
1-day post-SCI group vs. the control group	*Nono*	*Myc*	0.061	0.443	0	0.247	0.572
1-day post-SCI group vs. the control group	*Nono*	*Eed*	0.145	0.433	0	0	0.494
1-day post-SCI group vs. the control group	*Nono*	*Hdac1*	0.142	0.345	0	0.238	0.534
1-day post-SCI group vs. the control group	*Nos3*	*Hmox2*	0	0.218	0	0.503	0.595
1-day post-SCI group vs. the control group	*Nos3*	*Mapk14*	0.051	0	0	0.598	0.602
1-day post-SCI group vs. the control group	*Nos3*	*Hmox1*	0	0.218	0	0.757	0.802
1-day post-SCI group vs. the control group	*Nos3*	*Trap1*	0.061	0.204	0.355	0.049	0.48
1-day post-SCI group vs. the control group	*Nos3*	*Fos*	0	0	0.241	0.372	0.503
1-day post-SCI group vs. the control group	*Nos3*	*Edn1*	0	0	0	0.806	0.806
1-day post-SCI group vs. the control group	*Nos3*	*Myc*	0	0	0.475	0.263	0.596
1-day post-SCI group vs. the control group	*Nos3*	*Xdh*	0	0	0	0.717	0.717
1-day post-SCI group vs. the control group	*Nos3*	*Rela*	0	0.051	0.482	0.278	0.614
1-day post-SCI group vs. the control group	*Nos3*	*Cat*	0.049	0	0	0.696	0.699
1-day post-SCI group vs. the control group	*Nos3*	*Vcam1*	0	0	0	0.713	0.713
1-day post-SCI group vs. the control group	*Nos3*	*Mapk8*	0.052	0	0	0.409	0.416
1-day post-SCI group vs. the control group	*Nos3*	*Gpx1*	0	0	0	0.465	0.465
1-day post-SCI group vs. the control group	*Nos3*	*Ppp3ca*	0	0	0.46	0.076	0.48
1-day post-SCI group vs. the control group	*Nos3*	*Cask*	0.063	0.137	0.423	0.051	0.498
1-day post-SCI group vs. the control group	*Nos3*	*Ppargc1a*	0	0	0	0.573	0.573
1-day post-SCI group vs. the control group	*Nos3*	*Ptgs2*	0	0.045	0	0.589	0.591
1-day post-SCI group vs. the control group	*Nos3*	*Jak2*	0	0.133	0.399	0.317	0.613
1-day post-SCI group vs. the control group	*Nos3*	*Gch1*	0.065	0	0	0.656	0.665
1-day post-SCI group vs. the control group	*Nos3*	*Apoe*	0.073	0.047	0	0.692	0.704
1-day post-SCI group vs. the control group	*Nos3*	*Jun*	0	0	0.629	0.547	0.824
1-day post-SCI group vs. the control group	*P4hb*	*Txnrd1*	0.105	0.233	0	0.379	0.541
1-day post-SCI group vs. the control group	*P4hb*	*Hbegf*	0	0	0	0.515	0.515
1-day post-SCI group vs. the control group	*P4hb*	*Ago4*	0	0.142	0	0.356	0.423
1-day post-SCI group vs. the control group	*P4hb*	*Tnfrsf1a*	0.105	0	0.217	0.293	0.461
1-day post-SCI group vs. the control group	*P4hb*	*Cat*	0.089	0.134	0	0.431	0.512
1-day post-SCI group vs. the control group	*P4hb*	*Gfer*	0.076	0	0	0.503	0.521

1-day post-SCI group vs. the control group	*P4hb*	*Atf4*	0.061	0	0	0.549	0.558
1-day post-SCI group vs. the control group	*P4hb*	*Eif2s1*	0.062	0.358	0	0.38	0.594
1-day post-SCI group vs. the control group	*Pcna*	*Ercc1*	0.066	0	0.6	0	0.61
1-day post-SCI group vs. the control group	*Pcna*	*Cdk4*	0.336	0.948	0.905	0.549	0.998
1-day post-SCI group vs. the control group	*Pcna*	*Eed*	0.461	0.433	0	0	0.681
1-day post-SCI group vs. the control group	*Pcna*	*Rbbp7*	0.583	0.215	0	0.144	0.695
1-day post-SCI group vs. the control group	*Pdlim1*	*Ctnna1*	0.082	0.203	0.334	0.103	0.505
1-day post-SCI group vs. the control group	*Pdlim1*	*Ezr*	0.061	0.049	0.338	0.156	0.434
1-day post-SCI group vs. the control group	*Pdlim1*	*Eed*	0	0.433	0	0	0.433
1-day post-SCI group vs. the control group	*Pdlim1*	*Itsn1*	0.157	0.24	0	0.187	0.433
1-day post-SCI group vs. the control group	*Pdlim1*	*Cttn*	0	0.137	0.419	0.115	0.517
1-day post-SCI group vs. the control group	*Phc3*	*Rbbp7*	0	0	0.9	0.098	0.905
1-day post-SCI group vs. the control group	*Phc3*	*Eed*	0	0.147	0.9	0.374	0.942
1-day post-SCI group vs. the control group	*Phc3*	*Cbx6*	0	0.568	0.807	0.661	0.969
1-day post-SCI group vs. the control group	*Pink1*	*Trap1*	0	0.47	0	0.693	0.83
1-day post-SCI group vs. the control group	*Pink1*	*Ndufa6*	0.098	0	0.6	0.212	0.691
1-day post-SCI group vs. the control group	*Pink1*	*Cat*	0	0.14	0	0.594	0.636
1-day post-SCI group vs. the control group	*Pink1*	*Apoe*	0.169	0	0	0.336	0.424
1-day post-SCI group vs. the control group	*Pink1*	*Atf4*	0	0.146	0	0.375	0.444
1-day post-SCI group vs. the control group	*Pink1*	*Mcl1*	0	0.208	0	0.339	0.454
1-day post-SCI group vs. the control group	*Pink1*	*Mapt*	0.094	0	0	0.581	0.604
1-day post-SCI group vs. the control group	*Pink1*	*Ppargc1a*	0.069	0	0	0.836	0.841
1-day post-SCI group vs. the control group	*Pink1*	*Atp13a2*	0.066	0	0	0.852	0.856
1-day post-SCI group vs. the control group	*Plekha1*	*Btk*	0	0	0	0.495	0.495
1-day post-SCI group vs. the control group	*Ppargc1a*	*Mapk14*	0	0.087	0.966	0.62	0.987
1-day post-SCI group vs. the control group	*Ppargc1a*	*Hmox1*	0	0	0	0.731	0.731
1-day post-SCI group vs. the control group	*Ppargc1a*	*Myc*	0.065	0	0	0.482	0.495
1-day post-SCI group vs. the control group	*Ppargc1a*	*Xdh*	0	0	0	0.431	0.431
1-day post-SCI group vs. the control group	*Ppargc1a*	*Rela*	0	0.225	0	0.901	0.92
1-day post-SCI group vs. the control group	*Ppargc1a*	*Cat*	0	0	0	0.739	0.74
1-day post-SCI group vs. the control group	*Ppargc1a*	*Pink1*	0.069	0	0	0.836	0.841
1-day post-SCI group vs. the control group	*Ppargc1a*	*Nos3*	0	0	0	0.573	0.573
1-day post-SCI group vs. the control group	*Ppargc1a*	*Gpx1*	0	0	0.8	0.602	0.917
1-day post-SCI group vs. the control group	*Ppargc1a*	*Hdac1*	0	0	0	0.416	0.416
1-day post-SCI group vs. the control group	*Ppargc1a*	*Jun*	0	0	0	0.542	0.542
1-day post-SCI group vs. the control group	*Ppargc1a*	*Fbxw7*	0	0.439	0	0.191	0.527

1-day post-SCI group vs. the control group	*Ppargc1a*	*Atf2*	0	0	0	0.467	0.467
1-day post-SCI group vs. the control group	*Ppargc1a*	*Apoe*	0	0	0	0.438	0.438
1-day post-SCI group vs. the control group	*Ppargc1a*	*Ucp2*	0	0	0	0.744	0.744
1-day post-SCI group vs. the control group	*Ppp1ca*	*Trap1*	0.069	0.257	0.177	0.101	0.419
1-day post-SCI group vs. the control group	*Ppp1ca*	*Cdk4*	0.38	0.235	0.336	0.152	0.697
1-day post-SCI group vs. the control group	*Ppp1ca*	*Mdm2*	0	0.227	0.198	0.247	0.492
1-day post-SCI group vs. the control group	*Ppp1ca*	*Mapt*	0	0.366	0	0.107	0.41
1-day post-SCI group vs. the control group	*Ppp1ca*	*Eed*	0.061	0.439	0	0	0.451
1-day post-SCI group vs. the control group	*Ppp1ca*	*Hdac1*	0.062	0.354	0	0.197	0.472
1-day post-SCI group vs. the control group	*Ppp1ca*	*Atf2*	0.061	0.049	0.6	0	0.611
1-day post-SCI group vs. the control group	*Ppp1ca*	*Atf4*	0.052	0	0.6	0.112	0.633
1-day post-SCI group vs. the control group	*Ppp1ca*	*Smad1*	0.061	0.048	0.676	0.05	0.688
1-day post-SCI group vs. the control group	*Ppp1ca*	*Ppp3ca*	0.095	0	0.72	0.381	0.747
1-day post-SCI group vs. the control group	*Ppp1ca*	*Eif2s1*	0.082	0.398	0.8	0.276	0.909
1-day post-SCI group vs. the control group	*Ppp1ca*	*Ppp1r15b*	0	0.641	0.72	0.259	0.919
1-day post-SCI group vs. the control group	*Ppp1r15b*	*Ppp1ca*	0	0.641	0.72	0.259	0.919
1-day post-SCI group vs. the control group	*Ppp1r15b*	*Ppp3ca*	0	0.215	0.72	0	0.77
1-day post-SCI group vs. the control group	*Ppp1r15b*	*Atf4*	0	0	0	0.463	0.463
1-day post-SCI group vs. the control group	*Ppp1r15b*	*Eif2s1*	0	0	0	0.633	0.633
1-day post-SCI group vs. the control group	*Ppp3ca*	*Amph*	0.113	0.132	0.344	0.05	0.456
1-day post-SCI group vs. the control group	*Ppp3ca*	*Mapk14*	0.063	0.272	0	0.284	0.468
1-day post-SCI group vs. the control group	*Ppp3ca*	*Trap1*	0	0.257	0.177	0.201	0.468
1-day post-SCI group vs. the control group	*Ppp3ca*	*Cdk4*	0.063	0.155	0.194	0.195	0.418
1-day post-SCI group vs. the control group	*Ppp3ca*	*Fkbp1b*	0.062	0.591	0.15	0.268	0.729
1-day post-SCI group vs. the control group	*Ppp3ca*	*Nos3*	0	0	0.46	0.076	0.48
1-day post-SCI group vs. the control group	*Ppp3ca*	*Cask*	0.08	0.07	0.327	0.105	0.416
1-day post-SCI group vs. the control group	*Ppp3ca*	*Ppp1ca*	0.095	0	0.72	0.381	0.747
1-day post-SCI group vs. the control group	*Ppp3ca*	*Mapt*	0.098	0.527	0	0.152	0.606
1-day post-SCI group vs. the control group	*Ppp3ca*	*Ppp1r15b*	0	0.215	0.72	0	0.77
1-day post-SCI group vs. the control group	*Ppp3ca*	*Rcan1*	0	0.839	0	0.677	0.945
1-day post-SCI group vs. the control group	*Prodh*	*Cat*	0	0	0	0.443	0.443
1-day post-SCI group vs. the control group	*Ptgs2*	*Mapk14*	0	0.101	0	0.737	0.753
1-day post-SCI group vs. the control group	*Ptgs2*	*Hmox1*	0.085	0	0	0.746	0.757
1-day post-SCI group vs. the control group	*Ptgs2*	*Cdk4*	0	0	0	0.403	0.403
1-day post-SCI group vs. the control group	*Ptgs2*	*Mdm2*	0.056	0.169	0	0.407	0.494
1-day post-SCI group vs. the control group	*Ptgs2*	*Txnrd1*	0	0.226	0	0.318	0.45

1-day post-SCI group vs. the control group	*Ptgs2*	*Fos*	0.063	0	0	0.64	0.648
1-day post-SCI group vs. the control group	*Ptgs2*	*Edn1*	0.1	0	0	0.599	0.623
1-day post-SCI group vs. the control group	*Ptgs2*	*Myc*	0	0	0	0.597	0.597
1-day post-SCI group vs. the control group	*Ptgs2*	*Xdh*	0	0	0	0.526	0.526
1-day post-SCI group vs. the control group	*Ptgs2*	*Cyp1b1*	0.107	0.046	0	0.447	0.488
1-day post-SCI group vs. the control group	*Ptgs2*	*Hbegf*	0.132	0	0	0.462	0.513
1-day post-SCI group vs. the control group	*Ptgs2*	*Anxa1*	0.157	0	0	0.385	0.459
1-day post-SCI group vs. the control group	*Ptgs2*	*Fosl1*	0.223	0	0	0.36	0.482
1-day post-SCI group vs. the control group	*Ptgs2*	*Rela*	0	0	0	0.619	0.619
1-day post-SCI group vs. the control group	*Ptgs2*	*Cat*	0.048	0.203	0	0.689	0.743
1-day post-SCI group vs. the control group	*Ptgs2*	*Vcam1*	0.076	0	0	0.671	0.683
1-day post-SCI group vs. the control group	*Ptgs2*	*Nos3*	0	0.045	0	0.589	0.591
1-day post-SCI group vs. the control group	*Ptgs2*	*Tnfrsf1a*	0.082	0	0	0.464	0.487
1-day post-SCI group vs. the control group	*Ptgs2*	*Bax*	0	0	0	0.45	0.45
1-day post-SCI group vs. the control group	*Ptgs2*	*Mmp3*	0.09	0	0	0.67	0.687
1-day post-SCI group vs. the control group	*Ptgs2*	*Mcl1*	0.061	0	0	0.409	0.421
1-day post-SCI group vs. the control group	*Ptgs2*	*Sphk1*	0.062	0	0	0.417	0.43
1-day post-SCI group vs. the control group	*Ptgs2*	*Stat6*	0.061	0	0	0.428	0.44
1-day post-SCI group vs. the control group	*Ptgs2*	*Gpx1*	0	0	0	0.46	0.46
1-day post-SCI group vs. the control group	*Ptgs2*	*Met*	0.069	0.136	0	0.398	0.473
1-day post-SCI group vs. the control group	*Ptgs2*	*Gpx4*	0	0	0	0.483	0.483
1-day post-SCI group vs. the control group	*Ptgs2*	*Apoe*	0	0	0	0.494	0.494
1-day post-SCI group vs. the control group	*Ptgs2*	*Jak2*	0.095	0.088	0	0.464	0.518
1-day post-SCI group vs. the control group	*Ptgs2*	*Mapk8*	0	0	0	0.589	0.589
1-day post-SCI group vs. the control group	*Ptgs2*	*Jun*	0.061	0	0	0.738	0.743
1-day post-SCI group vs. the control group	*Pxdn*	*Cyp1b1*	0.114	0.046	0	0.555	0.591
1-day post-SCI group vs. the control group	*Pxdn*	*Eed*	0.061	0.433	0	0	0.444
1-day post-SCI group vs. the control group	*Pxn*	*Capns1*	0.068	0	0.629	0.161	0.685
1-day post-SCI group vs. the control group	*Pxn*	*Mapk14*	0	0.082	0.31	0.542	0.685
1-day post-SCI group vs. the control group	*Pxn*	*Hspb1*	0.061	0.202	0	0.346	0.467
1-day post-SCI group vs. the control group	*Pxn*	*Myc*	0.065	0.066	0	0.42	0.45
1-day post-SCI group vs. the control group	*Pxn*	*Itga5*	0.116	0.16	0.711	0.559	0.893
1-day post-SCI group vs. the control group	*Pxn*	*Hbegf*	0	0	0.9	0.121	0.908
1-day post-SCI group vs. the control group	*Pxn*	*Vcam1*	0.062	0	0.676	0.518	0.84
1-day post-SCI group vs. the control group	*Pxn*	*Ctnna1*	0.083	0.203	0.334	0.293	0.61
1-day post-SCI group vs. the control group	*Pxn*	*Ezr*	0.082	0.065	0.629	0.748	0.909

1-day post-SCI group vs. the control group	*Pxn*	*Jak2*	0.081	0.063	0.676	0.25	0.763
1-day post-SCI group vs. the control group	*Pxn*	*Capn2*	0.077	0	0.629	0.433	0.789
1-day post-SCI group vs. the control group	*Pxn*	*Itsn1*	0.157	0.24	0	0.278	0.497
1-day post-SCI group vs. the control group	*Pxn*	*Jun*	0	0	0	0.581	0.581
1-day post-SCI group vs. the control group	*Pxn*	*Met*	0.073	0.063	0.131	0.542	0.609
1-day post-SCI group vs. the control group	*Pxn*	*Mapk8*	0	0.512	0	0.374	0.681
1-day post-SCI group vs. the control group	*Pxn*	*Itga7*	0.081	0.16	0.711	0.227	0.805
1-day post-SCI group vs. the control group	*Pxn*	*Cttn*	0	0.348	0.629	0.984	0.996
1-day post-SCI group vs. the control group	*Rbbp7*	*Myc*	0	0.05	0.29	0.27	0.465
1-day post-SCI group vs. the control group	*Rbbp7*	*Pcna*	0.583	0.215	0	0.144	0.695
1-day post-SCI group vs. the control group	*Rbbp7*	*Cbx6*	0.048	0.133	0.72	0.322	0.822
1-day post-SCI group vs. the control group	*Rbbp7*	*Phc3*	0	0	0.9	0.098	0.905
1-day post-SCI group vs. the control group	*Rbbp7*	*Eed*	0.162	0.862	0.961	0.905	0.999
1-day post-SCI group vs. the control group	*Rbbp7*	*Hdac1*	0.224	0.813	0.956	0.981	0.999
1-day post-SCI group vs. the control group	*Rcan1*	*Ppp3ca*	0	0.839	0	0.677	0.945
1-day post-SCI group vs. the control group	*Rcan1*	*Itsn1*	0	0	0	0.616	0.616
1-day post-SCI group vs. the control group	*Rela*	*Ier3*	0.071	0.13	0	0.836	0.856
1-day post-SCI group vs. the control group	*Rela*	*Mapk14*	0.049	0.057	0.676	0.669	0.89
1-day post-SCI group vs. the control group	*Rela*	*Hmox1*	0.062	0	0	0.464	0.475
1-day post-SCI group vs. the control group	*Rela*	*Cdk4*	0	0.13	0	0.492	0.54
1-day post-SCI group vs. the control group	*Rela*	*Map2k3*	0.079	0.241	0	0.277	0.45
1-day post-SCI group vs. the control group	*Rela*	*Mdm2*	0	0.138	0	0.464	0.518
1-day post-SCI group vs. the control group	*Rela*	*Map2k6*	0.05	0.311	0	0.24	0.459
1-day post-SCI group vs. the control group	*Rela*	*Fos*	0	0.4	0.629	0.561	0.893
1-day post-SCI group vs. the control group	*Rela*	*Ripk3*	0.059	0.147	0.302	0.248	0.522
1-day post-SCI group vs. the control group	*Rela*	*Myc*	0	0.269	0.548	0.617	0.862
1-day post-SCI group vs. the control group	*Rela*	*Fosl1*	0.061	0.155	0.355	0.511	0.716
1-day post-SCI group vs. the control group	*Rela*	*Bax*	0	0.058	0.311	0.167	0.412
1-day post-SCI group vs. the control group	*Rela*	*Btk*	0	0.087	0.217	0.333	0.481
1-day post-SCI group vs. the control group	*Rela*	*Mcl1*	0.094	0.058	0.311	0.4	0.599
1-day post-SCI group vs. the control group	*Rela*	*Nos3*	0	0.051	0.482	0.278	0.614
1-day post-SCI group vs. the control group	*Rela*	*Ptgs2*	0	0	0	0.619	0.619
1-day post-SCI group vs. the control group	*Rela*	*Stat6*	0.104	0.244	0.35	0.374	0.688
1-day post-SCI group vs. the control group	*Rela*	*Vcam1*	0	0.433	0	0.552	0.735
1-day post-SCI group vs. the control group	*Rela*	*Jak2*	0	0.064	0.676	0.399	0.801
1-day post-SCI group vs. the control group	*Rela*	*Atf2*	0	0.155	0.629	0.444	0.81

1-day post-SCI group vs. the control group	*Rela*	*Ripk1*	0.089	0.147	0.676	0.498	0.856
1-day post-SCI group vs. the control group	*Rela*	*Tnfrsf1a*	0.114	0	0.676	0.691	0.903
1-day post-SCI group vs. the control group	*Rela*	*Mapk8*	0	0.048	0.8	0.547	0.906
1-day post-SCI group vs. the control group	*Rela*	*Ppargc1a*	0	0.225	0	0.901	0.92
1-day post-SCI group vs. the control group	*Rela*	*Jun*	0.061	0.46	0.629	0.859	0.97
1-day post-SCI group vs. the control group	*Rela*	*Hdac1*	0.062	0.795	0.512	0.982	0.998
1-day post-SCI group vs. the control group	*Ripk1*	*Mapk14*	0	0.147	0.676	0.412	0.758
1-day post-SCI group vs. the control group	*Ripk1*	*Map2k3*	0.087	0.132	0.932	0.221	0.945
1-day post-SCI group vs. the control group	*Ripk1*	*Map2k6*	0.061	0.132	0.922	0.155	0.934
1-day post-SCI group vs. the control group	*Ripk1*	*Ripk3*	0.076	0.983	0.8	0.991	0.997
1-day post-SCI group vs. the control group	*Ripk1*	*Rela*	0.089	0.147	0.676	0.498	0.856
1-day post-SCI group vs. the control group	*Ripk1*	*Tnfrsf1a*	0.113	0.999	0.932	0.988	0.999
1-day post-SCI group vs. the control group	*Ripk1*	*Map2k4*	0	0.106	0.629	0.242	0.682
1-day post-SCI group vs. the control group	*Ripk1*	*Mcl1*	0.079	0.056	0.12	0.349	0.435
1-day post-SCI group vs. the control group	*Ripk1*	*Jun*	0	0.09	0.127	0.468	0.54
1-day post-SCI group vs. the control group	*Ripk1*	*Mapk8*	0	0.208	0.209	0.457	0.468
1-day post-SCI group vs. the control group	*Ripk1*	*Map4k4*	0	0.142	0.676	0.105	0.718
1-day post-SCI group vs. the control group	*Ripk3*	*Map2k3*	0.101	0.132	0.293	0.082	0.41
1-day post-SCI group vs. the control group	*Ripk3*	*Map2k4*	0	0.106	0.341	0.163	0.419
1-day post-SCI group vs. the control group	*Ripk3*	*Mapk8*	0	0.208	0.209	0.324	0.429
1-day post-SCI group vs. the control group	*Ripk3*	*Gpx4*	0.061	0.044	0	0.464	0.476
1-day post-SCI group vs. the control group	*Ripk3*	*Rela*	0.059	0.147	0.302	0.248	0.522
1-day post-SCI group vs. the control group	*Ripk3*	*Tnfrsf1a*	0.108	0.13	0.341	0.799	0.884
1-day post-SCI group vs. the control group	*Ripk3*	*Ripk1*	0.076	0.983	0.8	0.991	0.997
1-day post-SCI group vs. the control group	*Rpl13a*	*Nme2*	0.645	0	0	0	0.645
1-day post-SCI group vs. the control group	*Rpl13a*	*Eif2s1*	0.652	0	0	0.097	0.672
1-day post-SCI group vs. the control group	*Rpl13a*	*Eed*	0	0.433	0	0	0.433
1-day post-SCI group vs. the control group	*Sdc1*	*Mapk14*	0.052	0	0.354	0.237	0.491
1-day post-SCI group vs. the control group	*Sdc1*	*Vcam1*	0	0	0	0.402	0.402
1-day post-SCI group vs. the control group	*Sdc1*	*Slc4a11*	0	0	0	0.448	0.448
1-day post-SCI group vs. the control group	*Sdc1*	*Anxa1*	0.096	0	0	0.417	0.45
1-day post-SCI group vs. the control group	*Sdc1*	*Myc*	0.072	0.045	0	0.45	0.47
1-day post-SCI group vs. the control group	*Sdc1*	*Btk*	0	0.058	0.127	0.464	0.52
1-day post-SCI group vs. the control group	*Sdc1*	*Mmp3*	0.077	0.05	0.466	0.241	0.597
1-day post-SCI group vs. the control group	*Sdc1*	*Itga5*	0.1	0	0.43	0.306	0.613
1-day post-SCI group vs. the control group	*Sdc1*	*Cttn*	0.064	0.059	0.284	0.639	0.742

1-day post-SCI group vs. the control group	*Sdc1*	*Met*	0.114	0	0.676	0.312	0.785
1-day post-SCI group vs. the control group	*Sdc1*	*Apoe*	0.051	0	0.6	0.564	0.82
1-day post-SCI group vs. the control group	*Sdc1*	*Cask*	0.051	0.144	0.676	0.511	0.854
1-day post-SCI group vs. the control group	*Selenbp1*	*Gpx1*	0.061	0.379	0	0.872	0.919
1-day post-SCI group vs. the control group	*Slc4a11*	*Sdc1*	0	0	0	0.448	0.448
1-day post-SCI group vs. the control group	*Slc4a11*	*Anxa1*	0	0	0	0.474	0.474
1-day post-SCI group vs. the control group	*Smad1*	*Mapk14*	0	0.17	0.207	0.533	0.666
1-day post-SCI group vs. the control group	*Smad1*	*Cdk4*	0	0.151	0.301	0.211	0.49
1-day post-SCI group vs. the control group	*Smad1*	*Fos*	0	0.142	0.388	0.227	0.558
1-day post-SCI group vs. the control group	*Smad1*	*Myc*	0	0.134	0.366	0.374	0.626
1-day post-SCI group vs. the control group	*Smad1*	*Ppp1ca*	0.061	0.048	0.676	0.05	0.688
1-day post-SCI group vs. the control group	*Smad1*	*Cdkn1c*	0	0.137	0.296	0.155	0.442
1-day post-SCI group vs. the control group	*Smad1*	*Hdac1*	0.062	0.221	0.301	0.245	0.563
1-day post-SCI group vs. the control group	*Smad1*	*Jun*	0	0.211	0.388	0.43	0.701
1-day post-SCI group vs. the control group	*Smad1*	*Atf2*	0	0.132	0.629	0.266	0.743
1-day post-SCI group vs. the control group	*Sphk1*	*Itga5*	0.064	0	0.376	0.107	0.433
1-day post-SCI group vs. the control group	*Sphk1*	*Ptgs2*	0.062	0	0	0.417	0.43
1-day post-SCI group vs. the control group	*Sphk1*	*Jak2*	0	0	0.408	0.197	0.504
1-day post-SCI group vs. the control group	*Sphk1*	*Abcc1*	0.061	0	0	0.532	0.542
1-day post-SCI group vs. the control group	*Sphk1*	*Hdac1*	0.062	0	0	0.43	0.442
1-day post-SCI group vs. the control group	*Spry2*	*Met*	0	0.141	0.128	0.281	0.414
1-day post-SCI group vs. the control group	*Spry2*	*Hbegf*	0.062	0	0.6	0.089	0.628
1-day post-SCI group vs. the control group	*Spry2*	*Itsn1*	0	0.436	0	0.798	0.881
1-day post-SCI group vs. the control group	*Srxn1*	*Hmox1*	0.151	0	0	0.643	0.685
1-day post-SCI group vs. the control group	*Srxn1*	*Txnrd1*	0.258	0	0	0.739	0.798
1-day post-SCI group vs. the control group	*Srxn1*	*Cat*	0.361	0	0	0.725	0.817
1-day post-SCI group vs. the control group	*Srxn1*	*Gpx1*	0.058	0	0	0.556	0.564
1-day post-SCI group vs. the control group	*Srxn1*	*Gpx4*	0.061	0	0	0.655	0.663
1-day post-SCI group vs. the control group	*Stat6*	*Mapk14*	0.059	0.104	0.212	0.469	0.6
1-day post-SCI group vs. the control group	*Stat6*	*Fos*	0	0.071	0.216	0.327	0.467
1-day post-SCI group vs. the control group	*Stat6*	*Myc*	0.044	0.133	0	0.429	0.485
1-day post-SCI group vs. the control group	*Stat6*	*Hbegf*	0.063	0	0.35	0.184	0.459
1-day post-SCI group vs. the control group	*Stat6*	*Rela*	0.104	0.244	0.35	0.374	0.688
1-day post-SCI group vs. the control group	*Stat6*	*Tnfrsf1a*	0.113	0.066	0.35	0.272	0.555
1-day post-SCI group vs. the control group	*Stat6*	*Btk*	0.082	0.136	0.193	0.283	0.48
1-day post-SCI group vs. the control group	*Stat6*	*Ptgs2*	0.061	0	0	0.428	0.44

1-day post-SCI group vs. the control group	*Stat6*	*Mcl1*	0.069	0	0.216	0.328	0.467
1-day post-SCI group vs. the control group	*Stat6*	*Jak2*	0.055	0.147	0.932	0.837	0.989
1-day post-SCI group vs. the control group	*Stat6*	*Hdac1*	0.048	0.164	0.208	0.223	0.444
1-day post-SCI group vs. the control group	*Stat6*	*Banf1*	0	0	0	0.454	0.454
1-day post-SCI group vs. the control group	*Stat6*	*Il6st*	0.064	0.047	0.35	0.31	0.546
1-day post-SCI group vs. the control group	*Stat6*	*Jun*	0	0.134	0.216	0.552	0.669
1-day post-SCI group vs. the control group	*Syp*	*Amph*	0.187	0	0	0.436	0.522
1-day post-SCI group vs. the control group	*Syp*	*Fos*	0	0	0	0.546	0.547
1-day post-SCI group vs. the control group	*Syp*	*Apoe*	0.061	0	0	0.473	0.484
1-day post-SCI group vs. the control group	*Syp*	*Mapt*	0.212	0	0	0.573	0.649
1-day post-SCI group vs. the control group	*Syp*	*Lancl1*	0.067	0.698	0	0	0.706
1-day post-SCI group vs. the control group	*Tnfrsf1a*	*Mapk14*	0.062	0.048	0	0.552	0.565
1-day post-SCI group vs. the control group	*Tnfrsf1a*	*Map2k3*	0.095	0	0.676	0.245	0.759
1-day post-SCI group vs. the control group	*Tnfrsf1a*	*Map2k6*	0	0	0.629	0.235	0.704
1-day post-SCI group vs. the control group	*Tnfrsf1a*	*Ripk3*	0.108	0.13	0.341	0.799	0.884
1-day post-SCI group vs. the control group	*Tnfrsf1a*	*Anxa1*	0.17	0	0	0.453	0.526
1-day post-SCI group vs. the control group	*Tnfrsf1a*	*Rela*	0.114	0	0.676	0.691	0.903
1-day post-SCI group vs. the control group	*Tnfrsf1a*	*P4hb*	0.105	0	0.217	0.293	0.461
1-day post-SCI group vs. the control group	*Tnfrsf1a*	*Vcam1*	0	0	0	0.564	0.564
1-day post-SCI group vs. the control group	*Tnfrsf1a*	*Mcl1*	0.068	0.077	0	0.387	0.427
1-day post-SCI group vs. the control group	*Tnfrsf1a*	*Hdac1*	0	0	0.41	0.154	0.479
1-day post-SCI group vs. the control group	*Tnfrsf1a*	*Ptgs2*	0.082	0	0	0.464	0.487
1-day post-SCI group vs. the control group	*Tnfrsf1a*	*Mapk8*	0	0.133	0	0.49	0.539
1-day post-SCI group vs. the control group	*Tnfrsf1a*	*Stat6*	0.113	0.066	0.35	0.272	0.555
1-day post-SCI group vs. the control group	*Tnfrsf1a*	*Jun*	0.069	0	0	0.619	0.63
1-day post-SCI group vs. the control group	*Tnfrsf1a*	*Map4k4*	0	0	0.676	0.132	0.706
1-day post-SCI group vs. the control group	*Tnfrsf1a*	*Map2k4*	0	0	0.629	0.301	0.729
1-day post-SCI group vs. the control group	*Tnfrsf1a*	*Jak2*	0.051	0.227	0.137	0.662	0.757
1-day post-SCI group vs. the control group	*Tnfrsf1a*	*Ripk1*	0.113	0.999	0.932	0.988	0.999
1-day post-SCI group vs. the control group	*Trap1*	*Mapk14*	0.052	0.164	0.319	0.176	0.495
1-day post-SCI group vs. the control group	*Trap1*	*Hspb1*	0.058	0.134	0	0.35	0.423
1-day post-SCI group vs. the control group	*Trap1*	*Ppp1ca*	0.069	0.257	0.177	0.101	0.419
1-day post-SCI group vs. the control group	*Trap1*	*Ppp3ca*	0	0.257	0.177	0.201	0.468
1-day post-SCI group vs. the control group	*Trap1*	*Nos3*	0.061	0.204	0.355	0.049	0.48
1-day post-SCI group vs. the control group	*Trap1*	*Hspa13*	0.171	0.393	0.411	0.175	0.722
1-day post-SCI group vs. the control group	*Trap1*	*Pink1*	0	0.47	0	0.693	0.83

1-day post-SCI group vs. the control group	*Tsc1*	*Mapk14*	0	0.202	0.354	0.253	0.581
1-day post-SCI group vs. the control group	*Tsc1*	*Ezr*	0.061	0.439	0	0.826	0.9
1-day post-SCI group vs. the control group	*Txnrd1*	*Hmox1*	0.113	0.131	0	0.701	0.749
1-day post-SCI group vs. the control group	*Txnrd1*	*Fbxw7*	0	0	0	0.414	0.414
1-day post-SCI group vs. the control group	*Txnrd1*	*Ptgs2*	0	0.226	0	0.318	0.45
1-day post-SCI group vs. the control group	*Txnrd1*	*P4hb*	0.105	0.233	0	0.379	0.541
1-day post-SCI group vs. the control group	*Txnrd1*	*Xdh*	0	0	0	0.65	0.65
1-day post-SCI group vs. the control group	*Txnrd1*	*Cat*	0.118	0.219	0	0.688	0.766
1-day post-SCI group vs. the control group	*Txnrd1*	*Srxn1*	0.258	0	0	0.739	0.798
1-day post-SCI group vs. the control group	*Txnrd1*	*Gpx1*	0.083	0.141	0	0.781	0.814
1-day post-SCI group vs. the control group	*Txnrd1*	*Gpx4*	0.063	0.141	0	0.821	0.844
1-day post-SCI group vs. the control group	*Ucp2*	*Hmox1*	0.069	0	0	0.411	0.428
1-day post-SCI group vs. the control group	*Ucp2*	*Cat*	0.076	0	0	0.565	0.582
1-day post-SCI group vs. the control group	*Ucp2*	*Gpx1*	0.101	0	0	0.493	0.524
1-day post-SCI group vs. the control group	*Ucp2*	*Gpx4*	0.064	0	0	0.4	0.415
1-day post-SCI group vs. the control group	*Ucp2*	*Ppargc1a*	0	0	0	0.744	0.744
1-day post-SCI group vs. the control group	*Vcam1*	*Axl*	0.141	0	0.147	0.274	0.421
1-day post-SCI group vs. the control group	*Vcam1*	*Mapk14*	0	0	0	0.632	0.632
1-day post-SCI group vs. the control group	*Vcam1*	*Hmox1*	0.061	0	0	0.628	0.636
1-day post-SCI group vs. the control group	*Vcam1*	*Sdc1*	0	0	0	0.402	0.402
1-day post-SCI group vs. the control group	*Vcam1*	*Edn1*	0	0	0	0.709	0.709
1-day post-SCI group vs. the control group	*Vcam1*	*Itga5*	0.052	0.058	0.352	0.569	0.717
1-day post-SCI group vs. the control group	*Vcam1*	*Xdh*	0	0	0	0.468	0.468
1-day post-SCI group vs. the control group	*Vcam1*	*Anxa1*	0.082	0.045	0	0.385	0.414
1-day post-SCI group vs. the control group	*Vcam1*	*Rela*	0	0.433	0	0.552	0.735
1-day post-SCI group vs. the control group	*Vcam1*	*Ccr1*	0.131	0	0	0.494	0.542
1-day post-SCI group vs. the control group	*Vcam1*	*Cat*	0	0	0	0.539	0.539
1-day post-SCI group vs. the control group	*Vcam1*	*Mapk8*	0	0	0	0.4	0.4
1-day post-SCI group vs. the control group	*Vcam1*	*Btk*	0.065	0	0.217	0.262	0.412
1-day post-SCI group vs. the control group	*Vcam1*	*Met*	0.062	0	0.147	0.338	0.424
1-day post-SCI group vs. the control group	*Vcam1*	*Jak2*	0.061	0	0.217	0.397	0.517
1-day post-SCI group vs. the control group	*Vcam1*	*Mmp3*	0.071	0	0	0.539	0.554
1-day post-SCI group vs. the control group	*Vcam1*	*Tnfrsf1a*	0	0	0	0.564	0.564
1-day post-SCI group vs. the control group	*Vcam1*	*Itga7*	0.061	0.058	0.352	0.351	0.578
1-day post-SCI group vs. the control group	*Vcam1*	*Jun*	0	0	0	0.589	0.589
1-day post-SCI group vs. the control group	*Vcam1*	*Ptgs2*	0.076	0	0	0.671	0.683

1-day post-SCI group vs. the control group	*Vcam1*	*Nos3*	0	0	0	0.713	0.713
1-day post-SCI group vs. the control group	*Vcam1*	*Apoe*	0.061	0	0	0.804	0.808
1-day post-SCI group vs. the control group	*Vcam1*	*Pxn*	0.062	0	0.676	0.518	0.84
1-day post-SCI group vs. the control group	*Vcam1*	*Ezr*	0	0.13	0.8	0.941	0.988
1-day post-SCI group vs. the control group	*Xdh*	*Mapk14*	0	0	0	0.529	0.529
1-day post-SCI group vs. the control group	*Xdh*	*Hmox1*	0.068	0	0	0.619	0.629
1-day post-SCI group vs. the control group	*Xdh*	*Txnrd1*	0	0	0	0.65	0.65
1-day post-SCI group vs. the control group	*Xdh*	*Edn1*	0	0	0	0.529	0.529
1-day post-SCI group vs. the control group	*Xdh*	*Ppargc1a*	0	0	0	0.431	0.431
1-day post-SCI group vs. the control group	*Xdh*	*Hspa13*	0	0.457	0	0.042	0.457
1-day post-SCI group vs. the control group	*Xdh*	*Vcam1*	0	0	0	0.468	0.468
1-day post-SCI group vs. the control group	*Xdh*	*Apoe*	0.105	0	0	0.464	0.5
1-day post-SCI group vs. the control group	*Xdh*	*Ptgs2*	0	0	0	0.526	0.526
1-day post-SCI group vs. the control group	*Xdh*	*Gpx1*	0.062	0	0	0.552	0.561
1-day post-SCI group vs. the control group	*Xdh*	*Jun*	0	0	0	0.583	0.583
1-day post-SCI group vs. the control group	*Xdh*	*Gch1*	0.104	0	0	0.642	0.665
1-day post-SCI group vs. the control group	*Xdh*	*Nos3*	0	0	0	0.717	0.717
1-day post-SCI group vs. the control group	*Xdh*	*Cat*	0.079	0	0	0.81	0.817
1-day post-SCI group vs. the control group	*Ybx3*	*Cdk4*	0.1	0.13	0.8	0.625	0.933
3-day post-SCI group vs. the control group	*Abcc1*	*Gsr*	0.081	0.134	0	0.332	0.421
3-day post-SCI group vs. the control group	*Abcc1*	*Gclc*	0.064	0	0	0.463	0.476
3-day post-SCI group vs. the control group	*Abcc1*	*Ezr*	0	0.094	0.34	0.134	0.437
3-day post-SCI group vs. the control group	*Abcc1*	*Casp3*	0.06	0.049	0	0.405	0.422
3-day post-SCI group vs. the control group	*Abcc1*	*Actb*	0.061	0.066	0	0.479	0.503
3-day post-SCI group vs. the control group	*Actb*	*Capns1*	0.094	0.053	0.43	0.172	0.541
3-day post-SCI group vs. the control group	*Actb*	*Braf*	0.052	0.084	0.114	0.436	0.508
3-day post-SCI group vs. the control group	*Actb*	*Axl*	0.052	0.084	0.114	0.464	0.532
3-day post-SCI group vs. the control group	*Actb*	*Mapk14*	0	0.09	0	0.834	0.843
3-day post-SCI group vs. the control group	*Actb*	*Hspb1*	0.062	0.087	0.4	0.584	0.757
3-day post-SCI group vs. the control group	*Actb*	*Hmox1*	0	0.056	0	0.777	0.78
3-day post-SCI group vs. the control group	*Actb*	*Cdc20*	0.064	0.086	0	0.4	0.442
3-day post-SCI group vs. the control group	*Actb*	*Cdk4*	0.099	0.067	0	0.721	0.745
3-day post-SCI group vs. the control group	*Actb*	*Sod2*	0.051	0	0	0.674	0.677
3-day post-SCI group vs. the control group	*Actb*	*Mgst1*	0.065	0	0	0.613	0.622
3-day post-SCI group vs. the control group	*Actb*	*Ncf1*	0	0.194	0	0.603	0.666
3-day post-SCI group vs. the control group	*Actb*	*Map2k3*	0.052	0	0	0.433	0.439

3-day post-SCI group vs. the control group	*Actb*	*Cdk1*	0.083	0.067	0	0.716	0.736
3-day post-SCI group vs. the control group	*Actb*	*Arg1*	0.059	0	0	0.592	0.599
3-day post-SCI group vs. the control group	*Actb*	*Txnrd1*	0.051	0	0	0.501	0.506
3-day post-SCI group vs. the control group	*Actb*	*Sdc1*	0	0	0	0.464	0.463
3-day post-SCI group vs. the control group	*Actb*	*Hif1a*	0	0.045	0	0.747	0.748
3-day post-SCI group vs. the control group	*Actb*	*Fos*	0	0.047	0.221	0.698	0.756
3-day post-SCI group vs. the control group	*Actb*	*Ctsl*	0.062	0	0	0.404	0.417
3-day post-SCI group vs. the control group	*Actb*	*Gpx8*	0	0	0	0.527	0.527
3-day post-SCI group vs. the control group	*Actb*	*Ppif*	0.081	0.058	0	0.389	0.425
3-day post-SCI group vs. the control group	*Actb*	*Rb1*	0.061	0	0	0.436	0.447
3-day post-SCI group vs. the control group	*Actb*	*Ripk3*	0.052	0.084	0.114	0.435	0.507
3-day post-SCI group vs. the control group	*Actb*	*Myc*	0.061	0.089	0.169	0.824	0.858
3-day post-SCI group vs. the control group	*Actb*	*Mylk*	0.065	0.201	0	0.379	0.496
3-day post-SCI group vs. the control group	*Actb*	*Xdh*	0	0	0	0.468	0.468
3-day post-SCI group vs. the control group	*Actb*	*Cyp1b1*	0	0.044	0	0.466	0.467
3-day post-SCI group vs. the control group	*Actb*	*Aif1*	0.052	0.206	0	0.73	0.779
3-day post-SCI group vs. the control group	*Actb*	*Anxa1*	0.088	0.083	0	0.562	0.602
3-day post-SCI group vs. the control group	*Actb*	*Fosl1*	0	0.047	0.221	0.434	0.543
3-day post-SCI group vs. the control group	*Actb*	*Rela*	0.079	0.09	0.629	0.701	0.894
3-day post-SCI group vs. the control group	*Actb*	*P4hb*	0.066	0.066	0	0.643	0.662
3-day post-SCI group vs. the control group	*Actb*	*Prdx4*	0.062	0.082	0	0.373	0.413
3-day post-SCI group vs. the control group	*Actb*	*Alox5*	0	0.07	0	0.464	0.48
3-day post-SCI group vs. the control group	*Actb*	*Parp1*	0.061	0	0	0.81	0.814
3-day post-SCI group vs. the control group	*Actb*	*Itga6*	0.061	0.044	0.672	0.472	0.824
3-day post-SCI group vs. the control group	*Actb*	*Cat*	0.061	0.09	0	0.749	0.767
3-day post-SCI group vs. the control group	*Actb*	*Ccna2*	0.062	0.086	0	0.547	0.577
3-day post-SCI group vs. the control group	*Actb*	*Vcam1*	0	0.047	0	0.62	0.622
3-day post-SCI group vs. the control group	*Actb*	*Nfkb1*	0	0.122	0	0.675	0.703
3-day post-SCI group vs. the control group	*Actb*	*Prkaa2*	0	0.087	0	0.508	0.532
3-day post-SCI group vs. the control group	*Actb*	*Gpx7*	0	0	0	0.512	0.512
3-day post-SCI group vs. the control group	*Actb*	*Pink1*	0	0	0	0.488	0.488
3-day post-SCI group vs. the control group	*Actb*	*Cd38*	0	0	0	0.431	0.431
3-day post-SCI group vs. the control group	*Actb*	*Atp2a2*	0.066	0.066	0	0.474	0.501
3-day post-SCI group vs. the control group	*Actb*	*Tnfrsf1a*	0.063	0.047	0	0.523	0.536
3-day post-SCI group vs. the control group	*Actb*	*Nox4*	0	0.067	0	0.625	0.636
3-day post-SCI group vs. the control group	*Actb*	*Bax*	0.062	0	0	0.613	0.621

3-day post-SCI group vs. the control group	*Actb*	*Btk*	0.052	0.084	0.195	0.399	0.523
3-day post-SCI group vs. the control group	*Actb*	*Nono*	0.115	0.291	0	0.395	0.587
3-day post-SCI group vs. the control group	*Actb*	*Gsr*	0.059	0	0	0.529	0.538
3-day post-SCI group vs. the control group	*Actb*	*Ets1*	0	0.045	0	0.4	0.402
3-day post-SCI group vs. the control group	*Actb*	*Gclc*	0.06	0	0	0.591	0.599
3-day post-SCI group vs. the control group	*Actb*	*Atr*	0	0.163	0.141	0.311	0.461
3-day post-SCI group vs. the control group	*Actb*	*Ptgs2*	0	0	0	0.737	0.737
3-day post-SCI group vs. the control group	*Actb*	*Mcl1*	0	0.09	0	0.693	0.708
3-day post-SCI group vs. the control group	*Actb*	*Tlr4*	0	0.132	0	0.738	0.763
3-day post-SCI group vs. the control group	*Actb*	*Ctnna1*	0.061	0.237	0.881	0.313	0.933
3-day post-SCI group vs. the control group	*Actb*	*Mapk3*	0	0.09	0	0.859	0.867
3-day post-SCI group vs. the control group	*Actb*	*Marcksl1*	0.079	0.357	0	0.09	0.414
3-day post-SCI group vs. the control group	*Actb*	*Ptgs1*	0	0	0	0.431	0.431
3-day post-SCI group vs. the control group	*Actb*	*Tlr6*	0	0.132	0	0.471	0.521
3-day post-SCI group vs. the control group	*Actb*	*Arhgdia*	0.134	0.059	0	0.446	0.509
3-day post-SCI group vs. the control group	*Actb*	*Ezr*	0.062	0.086	0.874	0.836	0.98
3-day post-SCI group vs. the control group	*Actb*	*Jak2*	0.052	0.084	0.195	0.665	0.734
3-day post-SCI group vs. the control group	*Actb*	*Junb*	0	0.059	0.29	0.285	0.48
3-day post-SCI group vs. the control group	*Actb*	*Itgb5*	0	0.065	0.687	0.363	0.797
3-day post-SCI group vs. the control group	*Actb*	*Syp*	0	0	0	0.693	0.693
3-day post-SCI group vs. the control group	*Actb*	*Smad1*	0	0.041	0	0.537	0.537
3-day post-SCI group vs. the control group	*Actb*	*Rnf2*	0	0.433	0	0.223	0.54
3-day post-SCI group vs. the control group	*Actb*	*Mgmt*	0	0.09	0	0.464	0.491
3-day post-SCI group vs. the control group	*Actb*	*Ezh2*	0	0.056	0	0.585	0.591
3-day post-SCI group vs. the control group	*Actb*	*Gpx1*	0.062	0	0	0.614	0.623
3-day post-SCI group vs. the control group	*Actb*	*Pxn*	0.064	0	0.881	0.615	0.953
3-day post-SCI group vs. the control group	*Actb*	*Stat6*	0	0.048	0	0.524	0.528
3-day post-SCI group vs. the control group	*Actb*	*Casp3*	0.052	0.137	0.89	0.896	0.989
3-day post-SCI group vs. the control group	*Actb*	*Dst*	0.062	0.24	0.133	0.201	0.44
3-day post-SCI group vs. the control group	*Actb*	*Cdkn2b*	0	0.122	0	0.399	0.449
3-day post-SCI group vs. the control group	*Actb*	*Abcc1*	0.061	0.066	0	0.479	0.503
3-day post-SCI group vs. the control group	*Actb*	*Mapt*	0.062	0.547	0	0.48	0.76
3-day post-SCI group vs. the control group	*Actb*	*Txnip*	0.061	0.055	0	0.419	0.439
3-day post-SCI group vs. the control group	*Actb*	*Fbxw7*	0	0.073	0	0.429	0.448
3-day post-SCI group vs. the control group	*Actb*	*Bnip3*	0	0	0	0.468	0.468
3-day post-SCI group vs. the control group	*Actb*	*Ripk1*	0.052	0.084	0.114	0.4	0.476

3-day post-SCI group vs. the control group	*Actb*	*Ucp2*	0.061	0.047	0	0.496	0.51
3-day post-SCI group vs. the control group	*Actb*	*Eed*	0	0.433	0	0.205	0.53
3-day post-SCI group vs. the control group	*Actb*	*Pdgfra*	0	0.047	0.208	0.464	0.56
3-day post-SCI group vs. the control group	*Actb*	*Snca*	0	0.09	0	0.62	0.639
3-day post-SCI group vs. the control group	*Actb*	*Met*	0.052	0.084	0.114	0.59	0.643
3-day post-SCI group vs. the control group	*Actb*	*Nfe2l2*	0	0	0	0.682	0.682
3-day post-SCI group vs. the control group	*Actb*	*Ppargc1a*	0	0	0	0.698	0.698
3-day post-SCI group vs. the control group	*Actb*	*Banf1*	0.062	0.499	0	0.414	0.701
3-day post-SCI group vs. the control group	*Actb*	*Mapk8*	0	0	0	0.702	0.702
3-day post-SCI group vs. the control group	*Actb*	*Hdac1*	0.072	0.16	0	0.672	0.722
3-day post-SCI group vs. the control group	*Actb*	*Rpl13a*	0.09	0.066	0	0.704	0.726
3-day post-SCI group vs. the control group	*Actb*	*Sirt1*	0.062	0.09	0	0.719	0.739
3-day post-SCI group vs. the control group	*Actb*	*Nfatc1*	0	0	0.475	0.583	0.771
3-day post-SCI group vs. the control group	*Actb*	*Atf2*	0	0.047	0.629	0.416	0.775
3-day post-SCI group vs. the control group	*Actb*	*Jun*	0	0.059	0.629	0.815	0.929
3-day post-SCI group vs. the control group	*Actb*	*Cttn*	0.062	0.083	0.881	0.56	0.949
3-day post-SCI group vs. the control group	*Adam9*	*Hbegf*	0.061	0	0	0.555	0.565
3-day post-SCI group vs. the control group	*Adam9*	*Itgb5*	0.099	0.13	0.167	0.256	0.449
3-day post-SCI group vs. the control group	*Adora1*	*Fos*	0	0.042	0.127	0.407	0.461
3-day post-SCI group vs. the control group	*Adora1*	*Gnb2*	0	0.328	0.185	0	0.428
3-day post-SCI group vs. the control group	*Ago1*	*Ago3*	0.061	0.439	0.9	0.87	0.943
3-day post-SCI group vs. the control group	*Ago1*	*Ezh2*	0.061	0.143	0	0.433	0.504
3-day post-SCI group vs. the control group	*Ago1*	*Tnrc6a*	0.062	0.761	0.72	0.965	0.997
3-day post-SCI group vs. the control group	*Ago1*	*Stau2*	0.062	0.232	0	0.264	0.423
3-day post-SCI group vs. the control group	*Ago1*	*Hdac1*	0.05	0.067	0	0.729	0.739
3-day post-SCI group vs. the control group	*Ago1*	*Tnrc6c*	0.064	0.735	0.6	0.722	0.968
3-day post-SCI group vs. the control group	*Ago3*	*Tnrc6c*	0.062	0.587	0.6	0.566	0.923
3-day post-SCI group vs. the control group	*Ago3*	*Ago1*	0.061	0.439	0.9	0.87	0.943
3-day post-SCI group vs. the control group	*Ago3*	*Tnrc6a*	0	0.818	0.72	0.634	0.979
3-day post-SCI group vs. the control group	*Aif1*	*Axl*	0.08	0.092	0.114	0.329	0.437
3-day post-SCI group vs. the control group	*Aif1*	*Mapk14*	0	0.158	0	0.549	0.604
3-day post-SCI group vs. the control group	*Aif1*	*Hmox1*	0	0.083	0	0.497	0.519
3-day post-SCI group vs. the control group	*Aif1*	*Ncf1*	0.203	0.057	0	0.353	0.471
3-day post-SCI group vs. the control group	*Aif1*	*Arg1*	0	0	0	0.643	0.643
3-day post-SCI group vs. the control group	*Aif1*	*Fos*	0	0.132	0	0.535	0.58
3-day post-SCI group vs. the control group	*Aif1*	*Jun*	0	0	0	0.403	0.403

3-day post-SCI group vs. the control group	*Aif1*	*Nfatc1*	0.056	0.14	0.238	0.159	0.41
3-day post-SCI group vs. the control group	*Aif1*	*Pdgfra*	0	0	0	0.411	0.411
3-day post-SCI group vs. the control group	*Aif1*	*Vcam1*	0.141	0	0	0.369	0.435
3-day post-SCI group vs. the control group	*Aif1*	*Hdac1*	0.063	0.084	0	0.444	0.481
3-day post-SCI group vs. the control group	*Aif1*	*Ccr1*	0.105	0.057	0.114	0.396	0.488
3-day post-SCI group vs. the control group	*Aif1*	*Mapk3*	0	0.158	0	0.42	0.491
3-day post-SCI group vs. the control group	*Aif1*	*Ptgs2*	0.05	0	0	0.601	0.605
3-day post-SCI group vs. the control group	*Aif1*	*Mapt*	0	0.236	0	0.523	0.62
3-day post-SCI group vs. the control group	*Aif1*	*Tlr4*	0.061	0.065	0	0.675	0.69
3-day post-SCI group vs. the control group	*Aif1*	*Casp3*	0	0.052	0	0.715	0.718
3-day post-SCI group vs. the control group	*Aif1*	*Syp*	0	0	0	0.727	0.727
3-day post-SCI group vs. the control group	*Aif1*	*Snca*	0.07	0.2	0	0.701	0.758
3-day post-SCI group vs. the control group	*Aif1*	*Actb*	0.052	0.206	0	0.73	0.779
3-day post-SCI group vs. the control group	*Alox5*	*Mapk14*	0	0	0	0.463	0.463
3-day post-SCI group vs. the control group	*Alox5*	*Hmox1*	0	0	0	0.43	0.43
3-day post-SCI group vs. the control group	*Alox5*	*Mapkapk2*	0.062	0.402	0.9	0.135	0.945
3-day post-SCI group vs. the control group	*Alox5*	*Gpx8*	0	0	0.9	0.122	0.908
3-day post-SCI group vs. the control group	*Alox5*	*Vcam1*	0.061	0	0	0.393	0.405
3-day post-SCI group vs. the control group	*Alox5*	*Tlr4*	0.064	0.044	0	0.435	0.451
3-day post-SCI group vs. the control group	*Alox5*	*Actb*	0	0.07	0	0.464	0.48
3-day post-SCI group vs. the control group	*Alox5*	*Gpx7*	0	0	0.9	0.141	0.91
3-day post-SCI group vs. the control group	*Alox5*	*Gpx3*	0.062	0	0.9	0.216	0.92
3-day post-SCI group vs. the control group	*Alox5*	*Gpx1*	0	0	0.9	0.249	0.921
3-day post-SCI group vs. the control group	*Alox5*	*Ptgs1*	0.09	0	0.9	0.728	0.973
3-day post-SCI group vs. the control group	*Alox5*	*Ptgs2*	0.061	0	0.9	0.783	0.977
3-day post-SCI group vs. the control group	*Amph*	*Mapt*	0.18	0.13	0	0.233	0.405
3-day post-SCI group vs. the control group	*Amph*	*Ppp3ca*	0.113	0.132	0.344	0.05	0.456
3-day post-SCI group vs. the control group	*Amph*	*Snca*	0.184	0	0	0.363	0.458
3-day post-SCI group vs. the control group	*Amph*	*Myc*	0.066	0.204	0	0.376	0.496
3-day post-SCI group vs. the control group	*Amph*	*Syp*	0.187	0	0	0.436	0.522
3-day post-SCI group vs. the control group	*Amph*	*Cttn*	0.064	0	0	0.644	0.653
3-day post-SCI group vs. the control group	*Anxa1*	*Sdc1*	0.096	0	0	0.417	0.45
3-day post-SCI group vs. the control group	*Anxa1*	*Vcam1*	0.082	0.045	0	0.385	0.414
3-day post-SCI group vs. the control group	*Anxa1*	*Tlr4*	0.215	0.043	0	0.327	0.45
3-day post-SCI group vs. the control group	*Anxa1*	*Ptgs2*	0.157	0	0	0.385	0.459
3-day post-SCI group vs. the control group	*Anxa1*	*Slc4a11*	0	0	0	0.474	0.474

3-day post-SCI group vs. the control group	*Anxa1*	*Tnfrsf1a*	0.17	0	0	0.453	0.526
3-day post-SCI group vs. the control group	*Anxa1*	*Actb*	0.088	0.083	0	0.562	0.602
3-day post-SCI group vs. the control group	*Anxa1*	*Casp3*	0	0	0	0.612	0.612
3-day post-SCI group vs. the control group	*Apc*	*Myc*	0	0.693	0	0.513	0.844
3-day post-SCI group vs. the control group	*Apc*	*Ptgs2*	0	0.729	0	0.211	0.777
3-day post-SCI group vs. the control group	*Apc*	*Ppp1ca*	0	0.406	0	0	0.406
3-day post-SCI group vs. the control group	*Apc*	*Ctnna1*	0.065	0.798	0.72	0.157	0.949
3-day post-SCI group vs. the control group	*Apc*	*Vrk2*	0.063	0.13	0.375	0.087	0.472
3-day post-SCI group vs. the control group	*Apc*	*Tfdp1*	0	0	0	0.509	0.509
3-day post-SCI group vs. the control group	*Apc*	*Jun*	0	0.276	0	0.455	0.589
3-day post-SCI group vs. the control group	*Apc*	*Uba52*	0	0	0.6	0	0.6
3-day post-SCI group vs. the control group	*Apc*	*Hdac1*	0.061	0	0.9	0.064	0.904
3-day post-SCI group vs. the control group	*Apc*	*Casp3*	0	0	0.9	0.184	0.914
3-day post-SCI group vs. the control group	*Arg1*	*Prodh*	0.082	0	0	0.477	0.542
3-day post-SCI group vs. the control group	*Arg1*	*Mapk14*	0	0	0	0.4	0.4
3-day post-SCI group vs. the control group	*Arg1*	*Hmox1*	0	0	0	0.5	0.499
3-day post-SCI group vs. the control group	*Arg1*	*Jak2*	0.062	0.046	0	0.392	0.408
3-day post-SCI group vs. the control group	*Arg1*	*Jun*	0.062	0	0	0.403	0.416
3-day post-SCI group vs. the control group	*Arg1*	*Mapt*	0	0.433	0	0.047	0.436
3-day post-SCI group vs. the control group	*Arg1*	*Vcam1*	0	0	0	0.463	0.463
3-day post-SCI group vs. the control group	*Arg1*	*Cat*	0.076	0.058	0	0.476	0.504
3-day post-SCI group vs. the control group	*Arg1*	*Hif1a*	0	0	0	0.596	0.596
3-day post-SCI group vs. the control group	*Arg1*	*Actb*	0.059	0	0	0.592	0.599
3-day post-SCI group vs. the control group	*Arg1*	*Ptgs2*	0.048	0	0	0.598	0.601
3-day post-SCI group vs. the control group	*Arg1*	*Aif1*	0	0	0	0.643	0.643
3-day post-SCI group vs. the control group	*Arg1*	*Tlr4*	0.061	0.045	0	0.634	0.643
3-day post-SCI group vs. the control group	*Arg1*	*Stat6*	0	0	0	0.721	0.721
3-day post-SCI group vs. the control group	*Arhgdia*	*Hspb1*	0.062	0.13	0	0.376	0.446
3-day post-SCI group vs. the control group	*Arhgdia*	*Ncf1*	0.06	0.088	0	0.452	0.489
3-day post-SCI group vs. the control group	*Arhgdia*	*Gnb2*	0.423	0	0	0.09	0.452
3-day post-SCI group vs. the control group	*Arhgdia*	*Mapk3*	0.24	0.066	0	0.308	0.466
3-day post-SCI group vs. the control group	*Arhgdia*	*Junb*	0	0.047	0.411	0	0.414
3-day post-SCI group vs. the control group	*Arhgdia*	*Eed*	0	0.433	0	0	0.433
3-day post-SCI group vs. the control group	*Arhgdia*	*Pxn*	0.062	0	0	0.429	0.442
3-day post-SCI group vs. the control group	*Arhgdia*	*Actb*	0.134	0.059	0	0.446	0.509
3-day post-SCI group vs. the control group	*Arhgdia*	*Jun*	0.061	0.057	0.492	0.252	0.618

3-day post-SCI group vs. the control group	*Arhgdia*	*Casp3*	0	0.416	0.492	0.255	0.759
3-day post-SCI group vs. the control group	*Arhgdia*	*Ezr*	0.057	0.719	0	0.937	0.982
3-day post-SCI group vs. the control group	*Atf2*	*Mapk14*	0	0.927	0.966	0.785	0.999
3-day post-SCI group vs. the control group	*Atf2*	*Hmox1*	0	0	0.313	0.213	0.436
3-day post-SCI group vs. the control group	*Atf2*	*Mapkapk2*	0	0.048	0	0.552	0.556
3-day post-SCI group vs. the control group	*Atf2*	*Map2k3*	0	0	0	0.703	0.703
3-day post-SCI group vs. the control group	*Atf2*	*Map2k6*	0	0	0	0.584	0.584
3-day post-SCI group vs. the control group	*Atf2*	*Fos*	0	0.84	0	0.629	0.882
3-day post-SCI group vs. the control group	*Atf2*	*Rb1*	0.063	0.227	0.676	0.118	0.765
3-day post-SCI group vs. the control group	*Atf2*	*Myc*	0	0.041	0	0.409	0.409
3-day post-SCI group vs. the control group	*Atf2*	*Rela*	0	0.155	0.629	0.444	0.81
3-day post-SCI group vs. the control group	*Atf2*	*Cat*	0.062	0	0	0.479	0.491
3-day post-SCI group vs. the control group	*Atf2*	*Ppp1ca*	0.061	0.049	0.6	0	0.611
3-day post-SCI group vs. the control group	*Atf2*	*Map2k4*	0.094	0	0	0.594	0.616
3-day post-SCI group vs. the control group	*Atf2*	*Mapk3*	0	0.653	0.966	0.557	0.994
3-day post-SCI group vs. the control group	*Atf2*	*Junb*	0.066	0.702	0.676	0.454	0.919
3-day post-SCI group vs. the control group	*Atf2*	*Smad1*	0	0.132	0.629	0.266	0.743
3-day post-SCI group vs. the control group	*Atf2*	*Actb*	0	0.047	0.629	0.416	0.775
3-day post-SCI group vs. the control group	*Atf2*	*Nfe2l2*	0	0.086	0.293	0.262	0.482
3-day post-SCI group vs. the control group	*Atf2*	*Jun*	0.066	0.943	0.932	0.989	0.997
3-day post-SCI group vs. the control group	*Atf2*	*Mapk8*	0.088	0.844	0.966	0.61	0.997
3-day post-SCI group vs. the control group	*Atf2*	*Ppargc1a*	0	0	0	0.467	0.467
3-day post-SCI group vs. the control group	*Atf2*	*Nfatc1*	0	0.131	0.308	0.196	0.474
3-day post-SCI group vs. the control group	*Atf2*	*Mapk10*	0.051	0.456	0.961	0.492	0.988
3-day post-SCI group vs. the control group	*Atox1*	*Sod2*	0.11	0.237	0	0.25	0.446
3-day post-SCI group vs. the control group	*Atox1*	*Ppif*	0	0.55	0	0.074	0.565
3-day post-SCI group vs. the control group	*Atox1*	*Ccs*	0.089	0.345	0	0.865	0.912
3-day post-SCI group vs. the control group	*Atox1*	*Atp7a*	0	0.747	0.9	0.955	0.998
3-day post-SCI group vs. the control group	*Atp13a2*	*Pink1*	0.066	0	0	0.852	0.856
3-day post-SCI group vs. the control group	*Atp13a2*	*Ctnna1*	0.474	0	0	0	0.474
3-day post-SCI group vs. the control group	*Atp13a2*	*Mapt*	0.061	0.046	0	0.507	0.52
3-day post-SCI group vs. the control group	*Atp13a2*	*Htra2*	0.097	0	0	0.566	0.592
3-day post-SCI group vs. the control group	*Atp13a2*	*Snca*	0	0	0	0.831	0.831
3-day post-SCI group vs. the control group	*Atp2a2*	*Mapk14*	0.062	0.058	0	0.402	0.426
3-day post-SCI group vs. the control group	*Atp2a2*	*Fkbp1b*	0	0.058	0	0.669	0.674
3-day post-SCI group vs. the control group	*Atp2a2*	*P4hb*	0.082	0.132	0	0.396	0.477

3-day post-SCI group vs. the control group	*Atp2a2*	*Cat*	0	0	0	0.422	0.422
3-day post-SCI group vs. the control group	*Atp2a2*	*Rcan1*	0.057	0	0	0.402	0.412
3-day post-SCI group vs. the control group	*Atp2a2*	*Eed*	0	0.433	0	0.081	0.456
3-day post-SCI group vs. the control group	*Atp2a2*	*Casp3*	0	0.089	0	0.435	0.463
3-day post-SCI group vs. the control group	*Atp2a2*	*Mapt*	0.062	0.443	0	0.055	0.463
3-day post-SCI group vs. the control group	*Atp2a2*	*Ppp3ca*	0.09	0.054	0	0.434	0.47
3-day post-SCI group vs. the control group	*Atp2a2*	*Actb*	0.066	0.066	0	0.474	0.501
3-day post-SCI group vs. the control group	*Atp2a2*	*Ppargc1a*	0.081	0	0	0.521	0.541
3-day post-SCI group vs. the control group	*Atp2a2*	*Slc8a1*	0.081	0.169	0	0.701	0.751
3-day post-SCI group vs. the control group	*Atp2a2*	*S100a1*	0.062	0.13	0	0.825	0.845
3-day post-SCI group vs. the control group	*Atp7a*	*Mapk14*	0.062	0	0	0.463	0.474
3-day post-SCI group vs. the control group	*Atp7a*	*Mcm4*	0.427	0	0	0	0.426
3-day post-SCI group vs. the control group	*Atp7a*	*Ccs*	0	0.458	0	0.718	0.862
3-day post-SCI group vs. the control group	*Atp7a*	*Atox1*	0	0.747	0.9	0.955	0.998
3-day post-SCI group vs. the control group	*Atr*	*Cdc20*	0.097	0.399	0	0.293	0.582
3-day post-SCI group vs. the control group	*Atr*	*Cdk4*	0.062	0.174	0.358	0.448	0.689
3-day post-SCI group vs. the control group	*Atr*	*Ube2a*	0	0.086	0.131	0.447	0.522
3-day post-SCI group vs. the control group	*Atr*	*Cdk1*	0.063	0.205	0.402	0.641	0.818
3-day post-SCI group vs. the control group	*Atr*	*Fkbp1b*	0	0.215	0.201	0.122	0.401
3-day post-SCI group vs. the control group	*Atr*	*Rrm2b*	0.065	0.463	0	0.476	0.714
3-day post-SCI group vs. the control group	*Atr*	*Myc*	0	0.143	0.194	0.333	0.499
3-day post-SCI group vs. the control group	*Atr*	*Mcm4*	0.112	0.401	0.912	0.543	0.976
3-day post-SCI group vs. the control group	*Atr*	*Parp1*	0.063	0.068	0	0.504	0.529
3-day post-SCI group vs. the control group	*Atr*	*Pcna*	0.098	0.571	0	0.445	0.766
3-day post-SCI group vs. the control group	*Atr*	*Ccna2*	0.064	0.143	0.676	0.204	0.765
3-day post-SCI group vs. the control group	*Atr*	*Cdkn2b*	0.052	0.136	0.263	0.173	0.434
3-day post-SCI group vs. the control group	*Atr*	*Hdac1*	0.052	0.144	0.147	0.282	0.436
3-day post-SCI group vs. the control group	*Atr*	*Casp3*	0.061	0.083	0.14	0.358	0.46
3-day post-SCI group vs. the control group	*Atr*	*Actb*	0	0.163	0.141	0.311	0.461
3-day post-SCI group vs. the control group	*Atr*	*Sirt1*	0.113	0.154	0	0.525	0.613
3-day post-SCI group vs. the control group	*Atr*	*Uba52*	0.176	0.204	0.65	0	0.75
3-day post-SCI group vs. the control group	*Axl*	*Vcam1*	0.141	0	0.147	0.274	0.421
3-day post-SCI group vs. the control group	*Axl*	*Fosl1*	0.105	0.07	0.12	0.314	0.43
3-day post-SCI group vs. the control group	*Axl*	*Jun*	0.062	0.09	0.127	0.327	0.431
3-day post-SCI group vs. the control group	*Axl*	*Aif1*	0.08	0.092	0.114	0.329	0.437
3-day post-SCI group vs. the control group	*Axl*	*Hif1a*	0	0.097	0.12	0.359	0.446

3-day post-SCI group vs. the control group	*Axl*	*Myc*	0.051	0.086	0.129	0.379	0.468
3-day post-SCI group vs. the control group	*Axl*	*Casp3*	0	0.077	0.114	0.439	0.501
3-day post-SCI group vs. the control group	*Axl*	*Actb*	0.052	0.084	0.114	0.464	0.532
3-day post-SCI group vs. the control group	*Axl*	*Btk*	0	0.697	0	0.197	0.714
3-day post-SCI group vs. the control group	*Axl*	*Jak2*	0.058	0	0.8	0.305	0.826
3-day post-SCI group vs. the control group	*Banf1*	*Cdk4*	0.678	0	0	0.16	0.718
3-day post-SCI group vs. the control group	*Banf1*	*Fos*	0	0	0	0.468	0.468
3-day post-SCI group vs. the control group	*Banf1*	*Myc*	0	0	0	0.416	0.416
3-day post-SCI group vs. the control group	*Banf1*	*Vrk2*	0	0.307	0.9	0.415	0.955
3-day post-SCI group vs. the control group	*Banf1*	*Ezh2*	0.051	0.066	0	0.486	0.505
3-day post-SCI group vs. the control group	*Banf1*	*Stat6*	0	0	0	0.454	0.454
3-day post-SCI group vs. the control group	*Banf1*	*Actb*	0.062	0.499	0	0.414	0.701
3-day post-SCI group vs. the control group	*Banf1*	*Hdac1*	0.084	0.099	0	0.556	0.602
3-day post-SCI group vs. the control group	*Bax*	*Mapk14*	0	0.061	0.209	0.438	0.546
3-day post-SCI group vs. the control group	*Bax*	*Hmox1*	0.084	0	0	0.493	0.516
3-day post-SCI group vs. the control group	*Bax*	*Cdk4*	0.204	0.058	0	0.328	0.452
3-day post-SCI group vs. the control group	*Bax*	*Endog*	0	0	0	0.433	0.433
3-day post-SCI group vs. the control group	*Bax*	*Ppif*	0	0.077	0.72	0.312	0.806
3-day post-SCI group vs. the control group	*Bax*	*Rela*	0	0.058	0.311	0.167	0.412
3-day post-SCI group vs. the control group	*Bax*	*Parp1*	0	0.053	0	0.463	0.469
3-day post-SCI group vs. the control group	*Bax*	*Cat*	0	0	0	0.493	0.493
3-day post-SCI group vs. the control group	*Bax*	*Eed*	0	0.433	0	0	0.433
3-day post-SCI group vs. the control group	*Bax*	*Htra2*	0.103	0	0	0.395	0.434
3-day post-SCI group vs. the control group	*Bax*	*Ptgs2*	0	0	0	0.45	0.45
3-day post-SCI group vs. the control group	*Bax*	*Nfe2l2*	0	0	0	0.455	0.455
3-day post-SCI group vs. the control group	*Bax*	*Mapk3*	0	0.061	0.209	0.332	0.46
3-day post-SCI group vs. the control group	*Bax*	*Sirt1*	0	0	0.261	0.373	0.516
3-day post-SCI group vs. the control group	*Bax*	*Jun*	0	0	0	0.529	0.529
3-day post-SCI group vs. the control group	*Bax*	*Mapk8*	0	0.082	0.272	0.397	0.561
3-day post-SCI group vs. the control group	*Bax*	*Actb*	0.062	0	0	0.613	0.621
3-day post-SCI group vs. the control group	*Bax*	*Mcl1*	0	0.422	0.72	0.61	0.865
3-day post-SCI group vs. the control group	*Bax*	*Casp3*	0	0.235	0	0.839	0.871
3-day post-SCI group vs. the control group	*Bmp1*	*Ctsl*	0	0	0.5	0.331	0.651
3-day post-SCI group vs. the control group	*Bnip3*	*Pdk1*	0.223	0	0	0.444	0.55
3-day post-SCI group vs. the control group	*Bnip3*	*Sod2*	0.062	0	0	0.429	0.442
3-day post-SCI group vs. the control group	*Bnip3*	*Endog*	0.062	0	0	0.594	0.603

3-day post-SCI group vs. the control group	*Bnip3*	*Hif1a*	0.079	0.13	0	0.799	0.825
3-day post-SCI group vs. the control group	*Bnip3*	*Ctsl*	0.111	0	0	0.538	0.572
3-day post-SCI group vs. the control group	*Bnip3*	*Ppif*	0	0	0	0.467	0.467
3-day post-SCI group vs. the control group	*Bnip3*	*Cat*	0.052	0	0	0.44	0.446
3-day post-SCI group vs. the control group	*Bnip3*	*Pink1*	0.078	0.13	0	0.848	0.868
3-day post-SCI group vs. the control group	*Bnip3*	*Mcl1*	0	0.094	0	0.561	0.585
3-day post-SCI group vs. the control group	*Bnip3*	*Casp3*	0	0	0	0.604	0.604
3-day post-SCI group vs. the control group	*Bnip3*	*Actb*	0	0	0	0.468	0.468
3-day post-SCI group vs. the control group	*Bnip3*	*Hdac1*	0	0.099	0	0.69	0.709
3-day post-SCI group vs. the control group	*Bnip3*	*Sirt1*	0	0	0	0.541	0.541
3-day post-SCI group vs. the control group	*Bnip3*	*Ppargc1a*	0.062	0	0	0.602	0.611
3-day post-SCI group vs. the control group	*Braf*	*Cdk1*	0.062	0.256	0.123	0.334	0.414
3-day post-SCI group vs. the control group	*Braf*	*Ets1*	0	0.263	0.108	0.193	0.423
3-day post-SCI group vs. the control group	*Braf*	*Hif1a*	0	0.097	0.12	0.35	0.438
3-day post-SCI group vs. the control group	*Braf*	*Pxn*	0.066	0.063	0.131	0.356	0.445
3-day post-SCI group vs. the control group	*Braf*	*Mcl1*	0	0.145	0.12	0.323	0.446
3-day post-SCI group vs. the control group	*Braf*	*Eed*	0.061	0.433	0	0.058	0.454
3-day post-SCI group vs. the control group	*Braf*	*Actb*	0.052	0.084	0.114	0.436	0.508
3-day post-SCI group vs. the control group	*Braf*	*Casp3*	0	0.101	0.114	0.435	0.511
3-day post-SCI group vs. the control group	*Braf*	*Parp1*	0.061	0.056	0	0.494	0.512
3-day post-SCI group vs. the control group	*Braf*	*Jak2*	0.052	0.27	0.3	0.295	0.533
3-day post-SCI group vs. the control group	*Braf*	*Jun*	0	0.137	0.127	0.433	0.535
3-day post-SCI group vs. the control group	*Braf*	*Mark3*	0.052	0.087	0.5	0.193	0.561
3-day post-SCI group vs. the control group	*Braf*	*Mapk14*	0.064	0.272	0.305	0.495	0.58
3-day post-SCI group vs. the control group	*Braf*	*Myc*	0.051	0.144	0.129	0.5	0.599
3-day post-SCI group vs. the control group	*Braf*	*Map2k6*	0.061	0.084	0.676	0.22	0.72
3-day post-SCI group vs. the control group	*Braf*	*Map2k3*	0.061	0.084	0.676	0.26	0.724
3-day post-SCI group vs. the control group	*Braf*	*Rb1*	0.061	0.4	0.457	0.482	0.82
3-day post-SCI group vs. the control group	*Braf*	*Mapk3*	0.067	0.522	0.864	0.741	0.954
3-day post-SCI group vs. the control group	*Brf2*	*Rela*	0	0.14	0.409	0.105	0.505
3-day post-SCI group vs. the control group	*Brf2*	*Jun*	0	0.118	0.311	0.113	0.413
3-day post-SCI group vs. the control group	*Brf2*	*Junb*	0	0.118	0.311	0.113	0.413
3-day post-SCI group vs. the control group	*Brf2*	*Tfdp1*	0.064	0	0	0.449	0.462
3-day post-SCI group vs. the control group	*Btk*	*Axl*	0	0.697	0	0.197	0.714
3-day post-SCI group vs. the control group	*Btk*	*Mapk14*	0	0.147	0.187	0.473	0.407
3-day post-SCI group vs. the control group	*Btk*	*Ncf1*	0.234	0.091	0	0.213	0.405

3-day post-SCI group vs. the control group	*Btk*	*Sdc1*	0	0.058	0.127	0.464	0.52
3-day post-SCI group vs. the control group	*Btk*	*Fos*	0	0.07	0.676	0.266	0.759
3-day post-SCI group vs. the control group	*Btk*	*Myc*	0.051	0.086	0.129	0.394	0.481
3-day post-SCI group vs. the control group	*Btk*	*Rela*	0	0.087	0.217	0.333	0.481
3-day post-SCI group vs. the control group	*Btk*	*Vcam1*	0.065	0	0.217	0.262	0.412
3-day post-SCI group vs. the control group	*Btk*	*Nfkb1*	0.071	0.132	0.127	0.36	0.489
3-day post-SCI group vs. the control group	*Btk*	*Cd38*	0.064	0.042	0	0.618	0.627
3-day post-SCI group vs. the control group	*Btk*	*Gnb2*	0.061	0.058	0.681	0	0.693
3-day post-SCI group vs. the control group	*Btk*	*Mapk3*	0	0.147	0.187	0.474	0.412
3-day post-SCI group vs. the control group	*Btk*	*Nfe2l2*	0	0.087	0	0.422	0.45
3-day post-SCI group vs. the control group	*Btk*	*Met*	0.065	0.074	0.323	0.457	0.457
3-day post-SCI group vs. the control group	*Btk*	*Cttn*	0.082	0.059	0.269	0.266	0.474
3-day post-SCI group vs. the control group	*Btk*	*Stat6*	0.082	0.136	0.193	0.283	0.48
3-day post-SCI group vs. the control group	*Btk*	*Plekha1*	0	0	0	0.495	0.495
3-day post-SCI group vs. the control group	*Btk*	*Actb*	0.052	0.084	0.195	0.399	0.523
3-day post-SCI group vs. the control group	*Btk*	*Nfatc1*	0.098	0	0.183	0.43	0.543
3-day post-SCI group vs. the control group	*Btk*	*Mcl1*	0	0.056	0.19	0.469	0.558
3-day post-SCI group vs. the control group	*Btk*	*Mapk10*	0.052	0.139	0.537	0.116	0.603
3-day post-SCI group vs. the control group	*Btk*	*Mapk8*	0.052	0.139	0.676	0.341	0.75
3-day post-SCI group vs. the control group	*Btk*	*Jak2*	0.062	0.146	0.676	0.468	0.764
3-day post-SCI group vs. the control group	*Btk*	*Tlr4*	0.091	0.089	0.082	0.791	0.82
3-day post-SCI group vs. the control group	*Btk*	*Jun*	0	0.136	0.676	0.442	0.83
3-day post-SCI group vs. the control group	*Camkk2*	*Prkaa2*	0.064	0.589	0.8	0.747	0.942
3-day post-SCI group vs. the control group	*Camkk2*	*Melk*	0.064	0.284	0	0.22	0.402
3-day post-SCI group vs. the control group	*Capns1*	*Eed*	0	0.433	0	0	0.433
3-day post-SCI group vs. the control group	*Capns1*	*Mapk14*	0	0.134	0.34	0.158	0.476
3-day post-SCI group vs. the control group	*Capns1*	*Actb*	0.094	0.053	0.43	0.172	0.541
3-day post-SCI group vs. the control group	*Capns1*	*Pxn*	0.068	0	0.629	0.161	0.685
3-day post-SCI group vs. the control group	*Capns1*	*Ezr*	0.062	0	0.629	0.203	0.698
3-day post-SCI group vs. the control group	*Capns1*	*Mapk3*	0.214	0.134	0.629	0.121	0.748
3-day post-SCI group vs. the control group	*Casp3*	*Braf*	0	0.101	0.114	0.435	0.511
3-day post-SCI group vs. the control group	*Casp3*	*Axl*	0	0.077	0.114	0.439	0.501
3-day post-SCI group vs. the control group	*Casp3*	*Mapk14*	0.061	0.13	0.629	0.81	0.934
3-day post-SCI group vs. the control group	*Casp3*	*Hspb1*	0	0.13	0	0.681	0.711
3-day post-SCI group vs. the control group	*Casp3*	*Hmox1*	0	0	0	0.764	0.764
3-day post-SCI group vs. the control group	*Casp3*	*Cdk4*	0.063	0	0	0.871	0.874

3-day post-SCI group vs. the control group	*Casp3*	*Sod2*	0.052	0	0	0.621	0.625
3-day post-SCI group vs. the control group	*Casp3*	*Mgst1*	0	0	0	0.502	0.502
3-day post-SCI group vs. the control group	*Casp3*	*Endog*	0	0	0	0.72	0.72
3-day post-SCI group vs. the control group	*Casp3*	*Ncf1*	0	0	0	0.477	0.477
3-day post-SCI group vs. the control group	*Casp3*	*Map2k3*	0.052	0	0	0.399	0.405
3-day post-SCI group vs. the control group	*Casp3*	*Cdk1*	0.094	0	0	0.718	0.734
3-day post-SCI group vs. the control group	*Casp3*	*Txnrd1*	0.055	0	0	0.431	0.439
3-day post-SCI group vs. the control group	*Casp3*	*Sdc1*	0.057	0	0.177	0.473	0.555
3-day post-SCI group vs. the control group	*Casp3*	*Hif1a*	0.049	0	0	0.721	0.723
3-day post-SCI group vs. the control group	*Casp3*	*Fos*	0	0	0	0.602	0.602
3-day post-SCI group vs. the control group	*Casp3*	*Gpx8*	0	0	0	0.622	0.622
3-day post-SCI group vs. the control group	*Casp3*	*Ppif*	0.056	0.053	0	0.561	0.573
3-day post-SCI group vs. the control group	*Casp3*	*Rb1*	0	0.228	0.629	0.416	0.818
3-day post-SCI group vs. the control group	*Casp3*	*Ripk3*	0	0.077	0.194	0.665	0.729
3-day post-SCI group vs. the control group	*Casp3*	*Myc*	0	0.049	0	0.793	0.795
3-day post-SCI group vs. the control group	*Casp3*	*Xdh*	0	0	0	0.528	0.528
3-day post-SCI group vs. the control group	*Casp3*	*Aif1*	0	0.052	0	0.715	0.718
3-day post-SCI group vs. the control group	*Casp3*	*Anxa1*	0	0	0	0.612	0.612
3-day post-SCI group vs. the control group	*Casp3*	*Rela*	0	0	0.334	0.612	0.73
3-day post-SCI group vs. the control group	*Casp3*	*P4hb*	0.061	0.15	0	0.463	0.533
3-day post-SCI group vs. the control group	*Casp3*	*Parp1*	0.062	0.816	0.932	0.872	0.998
3-day post-SCI group vs. the control group	*Casp3*	*Itga6*	0	0	0.676	0.366	0.785
3-day post-SCI group vs. the control group	*Casp3*	*Cat*	0	0	0	0.766	0.766
3-day post-SCI group vs. the control group	*Casp3*	*Ccna2*	0.085	0	0	0.559	0.579
3-day post-SCI group vs. the control group	*Casp3*	*Vcam1*	0.052	0	0	0.566	0.571
3-day post-SCI group vs. the control group	*Casp3*	*Nfkb1*	0	0.133	0.138	0.552	0.635
3-day post-SCI group vs. the control group	*Casp3*	*Gpx7*	0	0	0	0.646	0.646
3-day post-SCI group vs. the control group	*Casp3*	*Pink1*	0	0	0	0.537	0.537
3-day post-SCI group vs. the control group	*Casp3*	*Atp2a2*	0	0.089	0	0.435	0.463
3-day post-SCI group vs. the control group	*Casp3*	*Tnfrsf1a*	0	0.152	0.334	0.69	0.809
3-day post-SCI group vs. the control group	*Casp3*	*Nox4*	0	0	0	0.572	0.572
3-day post-SCI group vs. the control group	*Casp3*	*Bax*	0	0.235	0	0.839	0.871
3-day post-SCI group vs. the control group	*Casp3*	*Gsr*	0	0	0	0.594	0.594
3-day post-SCI group vs. the control group	*Casp3*	*Gclc*	0.062	0	0	0.409	0.422
3-day post-SCI group vs. the control group	*Casp3*	*Atr*	0.061	0.083	0.14	0.358	0.46
3-day post-SCI group vs. the control group	*Casp3*	*Ptgs2*	0	0	0	0.717	0.717

3-day post-SCI group vs. the control group	*Casp3*	*Map2k4*	0	0.042	0.334	0.441	0.612
3-day post-SCI group vs. the control group	*Casp3*	*Mcl1*	0	0.235	0	0.872	0.898
3-day post-SCI group vs. the control group	*Casp3*	*Tlr4*	0	0.077	0.118	0.698	0.733
3-day post-SCI group vs. the control group	*Casp3*	*Mapk3*	0	0.08	0.869	0.824	0.977
3-day post-SCI group vs. the control group	*Casp3*	*Tlr6*	0	0.077	0.118	0.379	0.45
3-day post-SCI group vs. the control group	*Casp3*	*Arhgdia*	0	0.416	0.492	0.255	0.759
3-day post-SCI group vs. the control group	*Casp3*	*Ezr*	0	0.05	0.177	0.319	0.421
3-day post-SCI group vs. the control group	*Casp3*	*Jak2*	0.062	0.077	0.114	0.643	0.689
3-day post-SCI group vs. the control group	*Casp3*	*Pdlim1*	0.05	0.046	0	0.405	0.414
3-day post-SCI group vs. the control group	*Casp3*	*Syp*	0	0	0	0.591	0.591
3-day post-SCI group vs. the control group	*Casp3*	*Eif2s1*	0	0.087	0	0.475	0.501
3-day post-SCI group vs. the control group	*Casp3*	*Apc*	0	0	0.9	0.184	0.914
3-day post-SCI group vs. the control group	*Casp3*	*Stk24*	0	0.087	0.9	0.109	0.911
3-day post-SCI group vs. the control group	*Casp3*	*Mgmt*	0	0	0	0.457	0.457
3-day post-SCI group vs. the control group	*Casp3*	*Ezh2*	0.094	0.057	0	0.464	0.502
3-day post-SCI group vs. the control group	*Casp3*	*Gpx1*	0	0	0	0.618	0.618
3-day post-SCI group vs. the control group	*Casp3*	*Gpx3*	0	0	0	0.439	0.439
3-day post-SCI group vs. the control group	*Casp3*	*Pxn*	0.05	0.087	0	0.463	0.493
3-day post-SCI group vs. the control group	*Casp3*	*Htra2*	0	0	0	0.63	0.63
3-day post-SCI group vs. the control group	*Casp3*	*Pdgfra*	0	0.047	0	0.399	0.402
3-day post-SCI group vs. the control group	*Casp3*	*Ucp2*	0	0	0	0.412	0.412
3-day post-SCI group vs. the control group	*Casp3*	*Abcc1*	0.06	0.049	0	0.405	0.422
3-day post-SCI group vs. the control group	*Casp3*	*Phc3*	0	0	0	0.434	0.434
3-day post-SCI group vs. the control group	*Casp3*	*Txnip*	0	0	0	0.466	0.466
3-day post-SCI group vs. the control group	*Casp3*	*Mapk10*	0.052	0.09	0	0.453	0.487
3-day post-SCI group vs. the control group	*Casp3*	*Cttn*	0	0.354	0	0.241	0.489
3-day post-SCI group vs. the control group	*Casp3*	*Cdkn2b*	0	0.133	0.138	0.403	0.514
3-day post-SCI group vs. the control group	*Casp3*	*Ppargc1a*	0	0	0	0.552	0.552
3-day post-SCI group vs. the control group	*Casp3*	*Hdac1*	0.061	0.052	0	0.563	0.577
3-day post-SCI group vs. the control group	*Casp3*	*Bnip3*	0	0	0	0.604	0.604
3-day post-SCI group vs. the control group	*Casp3*	*Snca*	0	0	0	0.617	0.617
3-day post-SCI group vs. the control group	*Casp3*	*Nfe2l2*	0	0.067	0	0.67	0.679
3-day post-SCI group vs. the control group	*Casp3*	*Sirt1*	0	0	0	0.682	0.682
3-day post-SCI group vs. the control group	*Casp3*	*Met*	0	0.311	0.114	0.566	0.712
3-day post-SCI group vs. the control group	*Casp3*	*Mapk8*	0.052	0.13	0	0.724	0.752
3-day post-SCI group vs. the control group	*Casp3*	*Jun*	0	0	0	0.836	0.836

3-day post-SCI group vs. the control group	*Casp3*	*Ripk1*	0	0.077	0.891	0.668	0.963
3-day post-SCI group vs. the control group	*Casp3*	*Mapt*	0	0.48	0.9	0.558	0.975
3-day post-SCI group vs. the control group	*Casp3*	*Actb*	0.052	0.137	0.89	0.896	0.989
3-day post-SCI group vs. the control group	*Cat*	*Prodh*	0	0	0	0.443	0.443
3-day post-SCI group vs. the control group	*Cat*	*Mapk14*	0	0.13	0	0.743	0.767
3-day post-SCI group vs. the control group	*Cat*	*Hspb1*	0	0	0	0.462	0.462
3-day post-SCI group vs. the control group	*Cat*	*Hmox1*	0.062	0.09	0	0.877	0.886
3-day post-SCI group vs. the control group	*Cat*	*Sod2*	0.08	0.277	0.961	0.939	0.998
3-day post-SCI group vs. the control group	*Cat*	*Mgst1*	0.108	0	0	0.398	0.44
3-day post-SCI group vs. the control group	*Cat*	*Ncf1*	0.051	0	0	0.695	0.698
3-day post-SCI group vs. the control group	*Cat*	*Arg1*	0.076	0.058	0	0.476	0.504
3-day post-SCI group vs. the control group	*Cat*	*Txnrd1*	0.118	0.219	0	0.688	0.766
3-day post-SCI group vs. the control group	*Cat*	*Nme2*	0	0.355	0	0.213	0.47
3-day post-SCI group vs. the control group	*Cat*	*Hif1a*	0	0	0	0.651	0.651
3-day post-SCI group vs. the control group	*Cat*	*Fos*	0.062	0	0	0.488	0.499
3-day post-SCI group vs. the control group	*Cat*	*Gpx8*	0.141	0.379	0	0.761	0.861
3-day post-SCI group vs. the control group	*Cat*	*Ppif*	0.063	0.198	0	0.5	0.591
3-day post-SCI group vs. the control group	*Cat*	*Myc*	0.051	0.045	0	0.476	0.483
3-day post-SCI group vs. the control group	*Cat*	*Msrb2*	0.087	0	0	0.438	0.466
3-day post-SCI group vs. the control group	*Cat*	*Xdh*	0.079	0	0	0.81	0.817
3-day post-SCI group vs. the control group	*Cat*	*Maoa*	0.062	0	0	0.431	0.443
3-day post-SCI group vs. the control group	*Cat*	*P4hb*	0.089	0.134	0	0.431	0.512
3-day post-SCI group vs. the control group	*Cat*	*Prdx4*	0.284	0.362	0	0.637	0.821
3-day post-SCI group vs. the control group	*Cat*	*Parp1*	0	0	0	0.485	0.485
3-day post-SCI group vs. the control group	*Cat*	*Ptgs1*	0.061	0.203	0	0.275	0.41
3-day post-SCI group vs. the control group	*Cat*	*Prkaa2*	0.067	0.134	0	0.33	0.412
3-day post-SCI group vs. the control group	*Cat*	*Atp2a2*	0	0	0	0.422	0.422
3-day post-SCI group vs. the control group	*Cat*	*Bnip3*	0.052	0	0	0.44	0.446
3-day post-SCI group vs. the control group	*Cat*	*Oxr1*	0	0	0	0.446	0.446
3-day post-SCI group vs. the control group	*Cat*	*Jak2*	0	0.065	0.183	0.34	0.453
3-day post-SCI group vs. the control group	*Cat*	*Lpo*	0	0.203	0	0.351	0.46
3-day post-SCI group vs. the control group	*Cat*	*Txnip*	0.068	0	0	0.465	0.48
3-day post-SCI group vs. the control group	*Cat*	*Atf2*	0.062	0	0	0.479	0.491
3-day post-SCI group vs. the control group	*Cat*	*Bax*	0	0	0	0.493	0.493
3-day post-SCI group vs. the control group	*Cat*	*Mapk8*	0	0	0	0.525	0.525
3-day post-SCI group vs. the control group	*Cat*	*Vcam1*	0	0	0	0.539	0.539

3-day post-SCI group vs. the control group	*Cat*	*Snca*	0	0	0	0.551	0.551
3-day post-SCI group vs. the control group	*Cat*	*Tlr4*	0.061	0.057	0	0.559	0.575
3-day post-SCI group vs. the control group	*Cat*	*Ucp2*	0.076	0	0	0.565	0.582
3-day post-SCI group vs. the control group	*Cat*	*Pink1*	0	0.14	0	0.594	0.636
3-day post-SCI group vs. the control group	*Cat*	*Mapk3*	0	0.089	0	0.656	0.673
3-day post-SCI group vs. the control group	*Cat*	*Jun*	0	0	0	0.682	0.682
3-day post-SCI group vs. the control group	*Cat*	*Ppargc1a*	0	0	0	0.739	0.74
3-day post-SCI group vs. the control group	*Cat*	*Ptgs2*	0.048	0.203	0	0.689	0.743
3-day post-SCI group vs. the control group	*Cat*	*Glrx2*	0.139	0.226	0	0.639	0.744
3-day post-SCI group vs. the control group	*Cat*	*Casp3*	0	0	0	0.766	0.766
3-day post-SCI group vs. the control group	*Cat*	*Actb*	0.061	0.09	0	0.749	0.767
3-day post-SCI group vs. the control group	*Cat*	*Sirt1*	0.062	0	0	0.776	0.781
3-day post-SCI group vs. the control group	*Cat*	*Nox4*	0.062	0	0	0.796	0.8
3-day post-SCI group vs. the control group	*Cat*	*Srxn1*	0.361	0	0	0.725	0.817
3-day post-SCI group vs. the control group	*Cat*	*Nfe2l2*	0.079	0	0	0.834	0.84
3-day post-SCI group vs. the control group	*Cat*	*Prdx6*	0.284	0.25	0	0.751	0.856
3-day post-SCI group vs. the control group	*Cat*	*Gpx7*	0.141	0.379	0	0.788	0.877
3-day post-SCI group vs. the control group	*Cat*	*Gpx3*	0.141	0.379	0	0.8	0.884
3-day post-SCI group vs. the control group	*Cat*	*Gclc*	0.141	0.275	0	0.843	0.894
3-day post-SCI group vs. the control group	*Cat*	*Gpx1*	0.159	0.379	0	0.919	0.954
3-day post-SCI group vs. the control group	*Cat*	*Gsr*	0.233	0.275	0	0.94	0.963
3-day post-SCI group vs. the control group	*Cat*	*Hao1*	0.145	0	0.9	0.754	0.977
3-day post-SCI group vs. the control group	*Cbx6*	*Rb1*	0	0.103	0.343	0.068	0.402
3-day post-SCI group vs. the control group	*Cbx6*	*Rbbp7*	0.048	0.133	0.72	0.322	0.822
3-day post-SCI group vs. the control group	*Cbx6*	*Rnf2*	0.052	0.758	0.845	0.714	0.988
3-day post-SCI group vs. the control group	*Cbx6*	*Ezh2*	0.061	0.236	0.72	0.529	0.892
3-day post-SCI group vs. the control group	*Cbx6*	*Sirt1*	0.061	0.136	0.36	0.209	0.534
3-day post-SCI group vs. the control group	*Cbx6*	*Eed*	0.057	0.261	0.72	0.449	0.878
3-day post-SCI group vs. the control group	*Cbx6*	*Tfdp1*	0.062	0	0.407	0.065	0.434
3-day post-SCI group vs. the control group	*Cbx6*	*Phc3*	0	0.568	0.807	0.661	0.969
3-day post-SCI group vs. the control group	*Ccna2*	*Cdc20*	0.991	0.929	0.6	0.874	0.999
3-day post-SCI group vs. the control group	*Ccna2*	*Cdk4*	0.26	0.235	0.929	0.877	0.994
3-day post-SCI group vs. the control group	*Ccna2*	*Cdk1*	0.973	0.881	0.932	0.986	0.999
3-day post-SCI group vs. the control group	*Ccna2*	*Fos*	0	0.059	0	0.393	0.404
3-day post-SCI group vs. the control group	*Ccna2*	*Dhfr*	0.232	0	0	0.356	0.484
3-day post-SCI group vs. the control group	*Ccna2*	*Rb1*	0.066	0.76	0.966	0.569	0.996

3-day post-SCI group vs. the control group	*Ccna2*	*Rrm2b*	0.304	0.134	0	0.101	0.41
3-day post-SCI group vs. the control group	*Ccna2*	*Myc*	0.095	0.047	0	0.556	0.584
3-day post-SCI group vs. the control group	*Ccna2*	*Mcm4*	0.776	0.387	0.9	0.543	0.992
3-day post-SCI group vs. the control group	*Ccna2*	*Pcna*	0.569	0.56	0.789	0.226	0.965
3-day post-SCI group vs. the control group	*Ccna2*	*Jun*	0	0.044	0	0.429	0.431
3-day post-SCI group vs. the control group	*Ccna2*	*Fbxw7*	0.064	0.224	0	0.287	0.437
3-day post-SCI group vs. the control group	*Ccna2*	*Cdkn2b*	0	0.135	0.124	0.468	0.562
3-day post-SCI group vs. the control group	*Ccna2*	*Actb*	0.062	0.086	0	0.547	0.577
3-day post-SCI group vs. the control group	*Ccna2*	*Casp3*	0.085	0	0	0.559	0.579
3-day post-SCI group vs. the control group	*Ccna2*	*Cdkn2c*	0.14	0.135	0.124	0.451	0.594
3-day post-SCI group vs. the control group	*Ccna2*	*Uba52*	0	0.053	0.6	0	0.605
3-day post-SCI group vs. the control group	*Ccna2*	*Ezh2*	0.516	0.059	0	0.355	0.681
3-day post-SCI group vs. the control group	*Ccna2*	*Atr*	0.064	0.143	0.676	0.204	0.765
3-day post-SCI group vs. the control group	*Ccna2*	*Ect2*	0.927	0.071	0	0.155	0.937
3-day post-SCI group vs. the control group	*Ccna2*	*Tfdp1*	0.284	0.138	0.864	0.354	0.939
3-day post-SCI group vs. the control group	*Ccna2*	*Hdac1*	0.129	0.099	0.915	0.325	0.949
3-day post-SCI group vs. the control group	*Ccna2*	*Melk*	0.925	0.155	0	0.35	0.955
3-day post-SCI group vs. the control group	*Ccr1*	*Aif1*	0.105	0.057	0.114	0.396	0.488
3-day post-SCI group vs. the control group	*Ccr1*	*Gnb2*	0	0.198	0.429	0	0.522
3-day post-SCI group vs. the control group	*Ccr1*	*Vcam1*	0.131	0	0	0.494	0.542
3-day post-SCI group vs. the control group	*Ccr1*	*Tlr4*	0.213	0.057	0	0.495	0.593
3-day post-SCI group vs. the control group	*Ccr1*	*Jun*	0	0.046	0.483	0.254	0.599
3-day post-SCI group vs. the control group	*Ccr1*	*Jak2*	0.061	0.141	0.63	0.195	0.727
3-day post-SCI group vs. the control group	*Ccs*	*Sod2*	0	0	0	0.521	0.521
3-day post-SCI group vs. the control group	*Ccs*	*Txnrd1*	0.052	0	0	0.329	0.413
3-day post-SCI group vs. the control group	*Ccs*	*Cygb*	0	0	0	0.418	0.418
3-day post-SCI group vs. the control group	*Ccs*	*Gsr*	0.051	0	0	0.325	0.41
3-day post-SCI group vs. the control group	*Ccs*	*Atp7a*	0	0.458	0	0.718	0.862
3-day post-SCI group vs. the control group	*Ccs*	*Atox1*	0.089	0.345	0	0.865	0.912
3-day post-SCI group vs. the control group	*Cd36*	*Mapk14*	0	0	0.629	0.13	0.663
3-day post-SCI group vs. the control group	*Cd36*	*Tlr4*	0.135	0.13	0.6	0.221	0.734
3-day post-SCI group vs. the control group	*Cd36*	*Tlr6*	0.089	0.13	0.6	0.109	0.68
3-day post-SCI group vs. the control group	*Cd36*	*Itgb5*	0.062	0.057	0.5	0.074	0.535
3-day post-SCI group vs. the control group	*Cd36*	*Ppargc1a*	0	0	0	0.529	0.529
3-day post-SCI group vs. the control group	*Cd36*	*Ucp2*	0.099	0	0	0.395	0.431
3-day post-SCI group vs. the control group	*Cd38*	*Sdc1*	0	0	0	0.853	0.853

3-day post-SCI group vs. the control group	*Cd38*	*Myc*	0	0	0	0.415	0.415
3-day post-SCI group vs. the control group	*Cd38*	*Itga6*	0	0	0	0.533	0.533
3-day post-SCI group vs. the control group	*Cd38*	*Vcam1*	0.094	0	0	0.399	0.432
3-day post-SCI group vs. the control group	*Cd38*	*Tlr4*	0.062	0	0	0.403	0.416
3-day post-SCI group vs. the control group	*Cd38*	*Actb*	0	0	0	0.431	0.431
3-day post-SCI group vs. the control group	*Cd38*	*Btk*	0.064	0.042	0	0.618	0.627
3-day post-SCI group vs. the control group	*Cdc20*	*Rb1*	0.063	0.088	0	0.36	0.405
3-day post-SCI group vs. the control group	*Cdc20*	*Cdkn2c*	0.11	0.09	0.132	0.294	0.437
3-day post-SCI group vs. the control group	*Cdc20*	*Actb*	0.064	0.086	0	0.4	0.442
3-day post-SCI group vs. the control group	*Cdc20*	*Fbxw7*	0.135	0.088	0	0.501	0.447
3-day post-SCI group vs. the control group	*Cdc20*	*Hdac1*	0.159	0.163	0.133	0.212	0.455
3-day post-SCI group vs. the control group	*Cdc20*	*Rbbp7*	0.278	0.047	0.176	0.058	0.463
3-day post-SCI group vs. the control group	*Cdc20*	*Endog*	0.057	0	0	0.465	0.474
3-day post-SCI group vs. the control group	*Cdc20*	*Ezh2*	0.377	0.087	0	0.248	0.535
3-day post-SCI group vs. the control group	*Cdc20*	*Myc*	0.121	0.091	0	0.5	0.566
3-day post-SCI group vs. the control group	*Cdc20*	*Rnf2*	0.071	0	0.54	0.07	0.568
3-day post-SCI group vs. the control group	*Cdc20*	*Atr*	0.097	0.399	0	0.293	0.582
3-day post-SCI group vs. the control group	*Cdc20*	*Phc3*	0	0.066	0.54	0.116	0.587
3-day post-SCI group vs. the control group	*Cdc20*	*Tfdp1*	0.279	0.099	0	0.421	0.591
3-day post-SCI group vs. the control group	*Cdc20*	*Ppp3ca*	0	0.159	0.6	0.107	0.673
3-day post-SCI group vs. the control group	*Cdc20*	*Uba52*	0.072	0.164	0.6	0.075	0.674
3-day post-SCI group vs. the control group	*Cdc20*	*Rrm2b*	0.635	0.141	0	0.071	0.683
3-day post-SCI group vs. the control group	*Cdc20*	*Id1*	0.089	0.206	0	0.628	0.707
3-day post-SCI group vs. the control group	*Cdc20*	*Cdk4*	0.291	0.373	0	0.539	0.777
3-day post-SCI group vs. the control group	*Cdc20*	*Pcna*	0.634	0.347	0	0.339	0.828
3-day post-SCI group vs. the control group	*Cdc20*	*Mcm4*	0.903	0.049	0	0.521	0.952
3-day post-SCI group vs. the control group	*Cdc20*	*Ect2*	0.938	0.048	0	0.331	0.957
3-day post-SCI group vs. the control group	*Cdc20*	*Melk*	0.941	0.091	0	0.386	0.964
3-day post-SCI group vs. the control group	*Cdc20*	*Cdk1*	0.978	0.503	0.6	0.919	0.999
3-day post-SCI group vs. the control group	*Cdc20*	*Ccna2*	0.991	0.929	0.6	0.874	0.999
3-day post-SCI group vs. the control group	*Cdk1*	*Braf*	0.062	0.256	0.123	0.334	0.414
3-day post-SCI group vs. the control group	*Cdk1*	*Mapk14*	0.051	0.239	0.212	0.582	0.476
3-day post-SCI group vs. the control group	*Cdk1*	*Cdc20*	0.978	0.503	0.6	0.919	0.999
3-day post-SCI group vs. the control group	*Cdk1*	*Cdk4*	0.415	0.197	0.891	0.919	0.951
3-day post-SCI group vs. the control group	*Cdk1*	*Mgst1*	0	0	0	0.533	0.533
3-day post-SCI group vs. the control group	*Cdk1*	*Nfkb1*	0	0.144	0.182	0.24	0.421

3-day post-SCI group vs. the control group	*Cdk1*	*Impact*	0	0.447	0	0	0.447
3-day post-SCI group vs. the control group	*Cdk1*	*Mapk3*	0.056	0.239	0.212	0.657	0.468
3-day post-SCI group vs. the control group	*Cdk1*	*Mapt*	0	0.402	0	0.181	0.489
3-day post-SCI group vs. the control group	*Cdk1*	*Rbbp7*	0.335	0.223	0	0.112	0.501
3-day post-SCI group vs. the control group	*Cdk1*	*Rxrb*	0	0.058	0.515	0	0.523
3-day post-SCI group vs. the control group	*Cdk1*	*Dhfr*	0.303	0	0	0.369	0.542
3-day post-SCI group vs. the control group	*Cdk1*	*Hif1a*	0	0.132	0	0.505	0.552
3-day post-SCI group vs. the control group	*Cdk1*	*Eed*	0.113	0.433	0	0.196	0.561
3-day post-SCI group vs. the control group	*Cdk1*	*Uba52*	0.088	0	0.6	0.059	0.627
3-day post-SCI group vs. the control group	*Cdk1*	*Jun*	0.051	0.164	0	0.581	0.638
3-day post-SCI group vs. the control group	*Cdk1*	*Cdkn2b*	0	0.144	0.182	0.525	0.638
3-day post-SCI group vs. the control group	*Cdk1*	*Parp1*	0.121	0	0	0.653	0.682
3-day post-SCI group vs. the control group	*Cdk1*	*Cdkn2c*	0.173	0.144	0.182	0.529	0.691
3-day post-SCI group vs. the control group	*Cdk1*	*Fbxw7*	0.062	0.161	0	0.642	0.694
3-day post-SCI group vs. the control group	*Cdk1*	*Sirt1*	0.061	0.443	0	0.483	0.706
3-day post-SCI group vs. the control group	*Cdk1*	*Casp3*	0.094	0	0	0.718	0.734
3-day post-SCI group vs. the control group	*Cdk1*	*Actb*	0.083	0.067	0	0.716	0.736
3-day post-SCI group vs. the control group	*Cdk1*	*Myc*	0.114	0.145	0	0.702	0.754
3-day post-SCI group vs. the control group	*Cdk1*	*Mcl1*	0.056	0.058	0	0.765	0.773
3-day post-SCI group vs. the control group	*Cdk1*	*Ppp1ca*	0.077	0.235	0.629	0.292	0.79
3-day post-SCI group vs. the control group	*Cdk1*	*Rrm2b*	0.751	0.163	0	0.191	0.816
3-day post-SCI group vs. the control group	*Cdk1*	*Atr*	0.063	0.205	0.402	0.641	0.818
3-day post-SCI group vs. the control group	*Cdk1*	*Melk*	0.945	0.1	0	0.427	0.957
3-day post-SCI group vs. the control group	*Cdk1*	*Mcm4*	0.913	0.242	0	0.662	0.976
3-day post-SCI group vs. the control group	*Cdk1*	*Hdac1*	0.139	0.104	0.93	0.624	0.977
3-day post-SCI group vs. the control group	*Cdk1*	*Ect2*	0.953	0.13	0	0.573	0.981
3-day post-SCI group vs. the control group	*Cdk1*	*Tfdp1*	0.257	0.145	0.961	0.368	0.982
3-day post-SCI group vs. the control group	*Cdk1*	*Ezh2*	0.524	0.763	0.148	0.862	0.984
3-day post-SCI group vs. the control group	*Cdk1*	*Rb1*	0.064	0.76	0.922	0.541	0.99
3-day post-SCI group vs. the control group	*Cdk1*	*Pcna*	0.864	0.457	0.828	0.554	0.993
3-day post-SCI group vs. the control group	*Cdk1*	*Ccna2*	0.973	0.881	0.932	0.986	0.999
3-day post-SCI group vs. the control group	*Cdk10*	*Ets1*	0.061	0.267	0	0.542	0.657
3-day post-SCI group vs. the control group	*Cdk4*	*Mapk14*	0	0.235	0.185	0.581	0.469
3-day post-SCI group vs. the control group	*Cdk4*	*Cdc20*	0.291	0.373	0	0.539	0.777
3-day post-SCI group vs. the control group	*Cdk4*	*Ptgs2*	0	0	0	0.403	0.403
3-day post-SCI group vs. the control group	*Cdk4*	*Ppp3ca*	0.063	0.155	0.194	0.195	0.418

3-day post-SCI group vs. the control group	*Cdk4*	*Dhfr*	0.108	0	0	0.376	0.42
3-day post-SCI group vs. the control group	*Cdk4*	*Mgmt*	0	0.131	0	0.372	0.431
3-day post-SCI group vs. the control group	*Cdk4*	*Nfkb1*	0	0.144	0.182	0.261	0.437
3-day post-SCI group vs. the control group	*Cdk4*	*Bax*	0.204	0.058	0	0.328	0.452
3-day post-SCI group vs. the control group	*Cdk4*	*Fos*	0	0	0	0.464	0.463
3-day post-SCI group vs. the control group	*Cdk4*	*Mapk3*	0.063	0.235	0.185	0.643	0.473
3-day post-SCI group vs. the control group	*Cdk4*	*Smad1*	0	0.151	0.301	0.211	0.49
3-day post-SCI group vs. the control group	*Cdk4*	*Fbxw7*	0.051	0.161	0	0.442	0.517
3-day post-SCI group vs. the control group	*Cdk4*	*Rela*	0	0.13	0	0.492	0.54
3-day post-SCI group vs. the control group	*Cdk4*	*Hif1a*	0	0.069	0	0.549	0.562
3-day post-SCI group vs. the control group	*Cdk4*	*Mcl1*	0	0.058	0	0.589	0.596
3-day post-SCI group vs. the control group	*Cdk4*	*Sirt1*	0	0.058	0	0.597	0.605
3-day post-SCI group vs. the control group	*Cdk4*	*Mcm4*	0.281	0.224	0	0.429	0.653
3-day post-SCI group vs. the control group	*Cdk4*	*Parp1*	0.223	0	0	0.596	0.673
3-day post-SCI group vs. the control group	*Cdk4*	*Jun*	0.061	0.164	0	0.63	0.684
3-day post-SCI group vs. the control group	*Cdk4*	*Atr*	0.062	0.174	0.358	0.448	0.689
3-day post-SCI group vs. the control group	*Cdk4*	*Ppp1ca*	0.38	0.235	0.336	0.152	0.697
3-day post-SCI group vs. the control group	*Cdk4*	*Mgst1*	0	0	0	0.7	0.7
3-day post-SCI group vs. the control group	*Cdk4*	*Ezh2*	0.134	0.164	0.148	0.581	0.708
3-day post-SCI group vs. the control group	*Cdk4*	*Banf1*	0.678	0	0	0.16	0.718
3-day post-SCI group vs. the control group	*Cdk4*	*Actb*	0.099	0.067	0	0.721	0.745
3-day post-SCI group vs. the control group	*Cdk4*	*Hdac1*	0.089	0.104	0.585	0.524	0.817
3-day post-SCI group vs. the control group	*Cdk4*	*Myc*	0.104	0.352	0	0.757	0.847
3-day post-SCI group vs. the control group	*Cdk4*	*Casp3*	0.063	0	0	0.871	0.874
3-day post-SCI group vs. the control group	*Cdk4*	*Ybx3*	0.1	0.13	0.8	0.625	0.933
3-day post-SCI group vs. the control group	*Cdk4*	*Jak2*	0.062	0.198	0.915	0.482	0.942
3-day post-SCI group vs. the control group	*Cdk4*	*Cdk1*	0.415	0.197	0.891	0.919	0.951
3-day post-SCI group vs. the control group	*Cdk4*	*Tfdp1*	0.3	0.145	0.966	0.401	0.986
3-day post-SCI group vs. the control group	*Cdk4*	*Ccna2*	0.26	0.235	0.929	0.877	0.994
3-day post-SCI group vs. the control group	*Cdk4*	*Pcna*	0.336	0.948	0.905	0.549	0.998
3-day post-SCI group vs. the control group	*Cdk4*	*Cdkn2b*	0	0.825	0.922	0.985	0.999
3-day post-SCI group vs. the control group	*Cdk4*	*Cdkn2c*	0.061	0.829	0.922	0.969	0.999
3-day post-SCI group vs. the control group	*Cdk4*	*Rb1*	0.064	0.986	0.966	0.844	0.999
3-day post-SCI group vs. the control group	*Cdkn2b*	*Mapk14*	0	0.13	0.146	0.278	0.417
3-day post-SCI group vs. the control group	*Cdkn2b*	*Cdk4*	0	0.825	0.922	0.985	0.999
3-day post-SCI group vs. the control group	*Cdkn2b*	*Cdk1*	0	0.144	0.182	0.525	0.638

3-day post-SCI group vs. the control group	*Cdkn2b*	*Hif1a*	0	0.141	0.457	0.377	0.684
3-day post-SCI group vs. the control group	*Cdkn2b*	*Rb1*	0	0.079	0.581	0.542	0.807
3-day post-SCI group vs. the control group	*Cdkn2b*	*Myc*	0	0.471	0.904	0.704	0.983
3-day post-SCI group vs. the control group	*Cdkn2b*	*Rela*	0	0.091	0.263	0.335	0.515
3-day post-SCI group vs. the control group	*Cdkn2b*	*Pcna*	0	0.052	0.207	0.454	0.554
3-day post-SCI group vs. the control group	*Cdkn2b*	*Ccna2*	0	0.135	0.124	0.468	0.562
3-day post-SCI group vs. the control group	*Cdkn2b*	*Atr*	0.052	0.136	0.263	0.173	0.434
3-day post-SCI group vs. the control group	*Cdkn2b*	*Mapk3*	0	0.13	0.146	0.332	0.46
3-day post-SCI group vs. the control group	*Cdkn2b*	*Jak2*	0	0.132	0.127	0.299	0.422
3-day post-SCI group vs. the control group	*Cdkn2b*	*Smad1*	0	0.059	0.46	0.152	0.531
3-day post-SCI group vs. the control group	*Cdkn2b*	*Mgmt*	0.049	0	0	0.575	0.579
3-day post-SCI group vs. the control group	*Cdkn2b*	*Ezh2*	0.048	0.058	0	0.613	0.623
3-day post-SCI group vs. the control group	*Cdkn2b*	*Casp3*	0	0.133	0.138	0.403	0.514
3-day post-SCI group vs. the control group	*Cdkn2b*	*Actb*	0	0.122	0	0.399	0.449
3-day post-SCI group vs. the control group	*Cdkn2b*	*Pdgfra*	0	0.069	0.13	0.382	0.456
3-day post-SCI group vs. the control group	*Cdkn2b*	*Jun*	0	0.047	0.204	0.41	0.513
3-day post-SCI group vs. the control group	*Cdkn2b*	*Tfdp1*	0.062	0.081	0.544	0.291	0.684
3-day post-SCI group vs. the control group	*Cdkn2b*	*Hdac1*	0.052	0.132	0.457	0.397	0.694
3-day post-SCI group vs. the control group	*Cdkn2c*	*Cdc20*	0.11	0.09	0.132	0.294	0.437
3-day post-SCI group vs. the control group	*Cdkn2c*	*Cdk4*	0.061	0.829	0.922	0.969	0.999
3-day post-SCI group vs. the control group	*Cdkn2c*	*Cdk1*	0.173	0.144	0.182	0.529	0.691
3-day post-SCI group vs. the control group	*Cdkn2c*	*Rb1*	0	0.079	0.204	0.385	0.51
3-day post-SCI group vs. the control group	*Cdkn2c*	*Myc*	0.061	0.132	0.191	0.416	0.563
3-day post-SCI group vs. the control group	*Cdkn2c*	*Pcna*	0.08	0.052	0.207	0.263	0.422
3-day post-SCI group vs. the control group	*Cdkn2c*	*Ccna2*	0.14	0.135	0.124	0.451	0.594
3-day post-SCI group vs. the control group	*Cdkn2c*	*Ppp1ca*	0.062	0.142	0.118	0.258	0.403
3-day post-SCI group vs. the control group	*Cdkn2c*	*Hdac1*	0.061	0.132	0.166	0.245	0.418
3-day post-SCI group vs. the control group	*Cdkn2c*	*Tfdp1*	0.062	0.081	0.207	0.274	0.437
3-day post-SCI group vs. the control group	*Cdkn2c*	*Mapk10*	0	0.705	0	0.05	0.708
3-day post-SCI group vs. the control group	*Chrna4*	*Maoa*	0.061	0	0	0.4	0.412
3-day post-SCI group vs. the control group	*Chrna4*	*Jak2*	0.062	0.053	0.831	0.073	0.842
3-day post-SCI group vs. the control group	*Ctnna1*	*Mapk14*	0	0.059	0.6	0.065	0.617
3-day post-SCI group vs. the control group	*Ctnna1*	*Parp1*	0.573	0	0	0.043	0.573
3-day post-SCI group vs. the control group	*Ctnna1*	*Itga6*	0.079	0.049	0.248	0.309	0.484
3-day post-SCI group vs. the control group	*Ctnna1*	*Atp13a2*	0.474	0	0	0	0.474
3-day post-SCI group vs. the control group	*Ctnna1*	*Ezr*	0.069	0	0	0.4	0.418

3-day post-SCI group vs. the control group	*Ctnna1*	*Eed*	0	0.433	0	0	0.433
3-day post-SCI group vs. the control group	*Ctnna1*	*Pdlim1*	0.082	0.203	0.334	0.103	0.505
3-day post-SCI group vs. the control group	*Ctnna1*	*Itgb5*	0.087	0.13	0.313	0.228	0.523
3-day post-SCI group vs. the control group	*Ctnna1*	*Pxn*	0.083	0.203	0.334	0.293	0.61
3-day post-SCI group vs. the control group	*Ctnna1*	*Met*	0.082	0.052	0.676	0.066	0.701
3-day post-SCI group vs. the control group	*Ctnna1*	*Cttn*	0.062	0.057	0.676	0.227	0.749
3-day post-SCI group vs. the control group	*Ctnna1*	*Actb*	0.061	0.237	0.881	0.313	0.933
3-day post-SCI group vs. the control group	*Ctnna1*	*Apc*	0.065	0.798	0.72	0.157	0.949
3-day post-SCI group vs. the control group	*Ctsl*	*Actb*	0.062	0	0	0.404	0.417
3-day post-SCI group vs. the control group	*Ctsl*	*Tlr4*	0	0.045	0	0.435	0.437
3-day post-SCI group vs. the control group	*Ctsl*	*Bnip3*	0.111	0	0	0.538	0.572
3-day post-SCI group vs. the control group	*Ctsl*	*Bmp1*	0	0	0.5	0.331	0.651
3-day post-SCI group vs. the control group	*Cttn*	*Amph*	0.064	0	0	0.644	0.653
3-day post-SCI group vs. the control group	*Cttn*	*Ncf1*	0.063	0.13	0	0.867	0.883
3-day post-SCI group vs. the control group	*Cttn*	*Sdc1*	0.064	0.059	0.284	0.639	0.742
3-day post-SCI group vs. the control group	*Cttn*	*Myc*	0	0	0	0.479	0.48
3-day post-SCI group vs. the control group	*Cttn*	*Mylk*	0.061	0.348	0	0.95	0.967
3-day post-SCI group vs. the control group	*Cttn*	*Itga6*	0	0	0.334	0.172	0.424
3-day post-SCI group vs. the control group	*Cttn*	*Btk*	0.082	0.059	0.269	0.266	0.474
3-day post-SCI group vs. the control group	*Cttn*	*Ctnna1*	0.062	0.057	0.676	0.227	0.749
3-day post-SCI group vs. the control group	*Cttn*	*Mapk3*	0.063	0	0	0.446	0.459
3-day post-SCI group vs. the control group	*Cttn*	*Ezr*	0.052	0.062	0	0.87	0.874
3-day post-SCI group vs. the control group	*Cttn*	*Pdlim1*	0	0.137	0.419	0.115	0.517
3-day post-SCI group vs. the control group	*Cttn*	*Itgb5*	0.126	0	0.383	0.134	0.492
3-day post-SCI group vs. the control group	*Cttn*	*Pxn*	0	0.348	0.629	0.984	0.996
3-day post-SCI group vs. the control group	*Cttn*	*Casp3*	0	0.354	0	0.241	0.489
3-day post-SCI group vs. the control group	*Cttn*	*Actb*	0.062	0.083	0.881	0.56	0.949
3-day post-SCI group vs. the control group	*Cttn*	*Hdac1*	0.062	0.134	0	0.345	0.421
3-day post-SCI group vs. the control group	*Cttn*	*Eed*	0	0.433	0	0	0.433
3-day post-SCI group vs. the control group	*Cttn*	*Met*	0.062	0.087	0.146	0.342	0.454
3-day post-SCI group vs. the control group	*Cygb*	*Ccs*	0	0	0	0.418	0.418
3-day post-SCI group vs. the control group	*Cygb*	*Gpx7*	0.103	0	0	0.417	0.455
3-day post-SCI group vs. the control group	*Cyp1b1*	*Hmox1*	0.162	0	0	0.41	0.485
3-day post-SCI group vs. the control group	*Cyp1b1*	*Mgst1*	0.072	0	0.65	0.193	0.715
3-day post-SCI group vs. the control group	*Cyp1b1*	*Actb*	0	0.044	0	0.466	0.467
3-day post-SCI group vs. the control group	*Cyp1b1*	*Ptgs2*	0.107	0.046	0	0.447	0.488

3-day post-SCI group vs. the control group	*Cyp1b1*	*Pxdn*	0.114	0.046	0	0.555	0.591
3-day post-SCI group vs. the control group	*Cyp1b1*	*Gstt2*	0	0	0.65	0.189	0.704
3-day post-SCI group vs. the control group	*Dhfr*	*Cdk4*	0.108	0	0	0.376	0.42
3-day post-SCI group vs. the control group	*Dhfr*	*Cdk1*	0.303	0	0	0.369	0.542
3-day post-SCI group vs. the control group	*Dhfr*	*Rrm2b*	0.27	0	0	0.307	0.472
3-day post-SCI group vs. the control group	*Dhfr*	*Pcna*	0.456	0	0	0.088	0.483
3-day post-SCI group vs. the control group	*Dhfr*	*Ccna2*	0.232	0	0	0.356	0.484
3-day post-SCI group vs. the control group	*Dhfr*	*Myc*	0.061	0	0	0.509	0.52
3-day post-SCI group vs. the control group	*Dhfr*	*Tfdp1*	0.106	0	0.629	0.195	0.709
3-day post-SCI group vs. the control group	*Dhfr*	*Mcm4*	0.7	0	0	0.63	0.884
3-day post-SCI group vs. the control group	*Dhfr*	*Gch1*	0.063	0	0	0.88	0.901
3-day post-SCI group vs. the control group	*Dst*	*Itga6*	0.061	0.071	0.639	0.406	0.788
3-day post-SCI group vs. the control group	*Dst*	*Ezr*	0.061	0.163	0.191	0.252	0.461
3-day post-SCI group vs. the control group	*Dst*	*Itgb5*	0.062	0.255	0.167	0.096	0.403
3-day post-SCI group vs. the control group	*Dst*	*Actb*	0.062	0.24	0.133	0.201	0.44
3-day post-SCI group vs. the control group	*Ect2*	*Cdc20*	0.938	0.048	0	0.331	0.957
3-day post-SCI group vs. the control group	*Ect2*	*Cdk1*	0.953	0.13	0	0.573	0.981
3-day post-SCI group vs. the control group	*Ect2*	*Mcm4*	0.705	0	0	0.05	0.708
3-day post-SCI group vs. the control group	*Ect2*	*Pcna*	0.429	0	0	0	0.429
3-day post-SCI group vs. the control group	*Ect2*	*Ccna2*	0.927	0.071	0	0.155	0.937
3-day post-SCI group vs. the control group	*Ect2*	*Melk*	0.924	0.083	0	0.211	0.94
3-day post-SCI group vs. the control group	*Ect2*	*Ezh2*	0.458	0	0	0.129	0.508
3-day post-SCI group vs. the control group	*Eed*	*Capns1*	0	0.433	0	0	0.433
3-day post-SCI group vs. the control group	*Eed*	*Braf*	0.061	0.433	0	0.058	0.454
3-day post-SCI group vs. the control group	*Eed*	*Ube2a*	0.105	0	0	0.494	0.527
3-day post-SCI group vs. the control group	*Eed*	*Map2k3*	0	0.433	0	0	0.433
3-day post-SCI group vs. the control group	*Eed*	*Cdk1*	0.113	0.433	0	0.196	0.561
3-day post-SCI group vs. the control group	*Eed*	*Rb1*	0.079	0.328	0	0.331	0.549
3-day post-SCI group vs. the control group	*Eed*	*Ndufa6*	0	0.433	0	0	0.433
3-day post-SCI group vs. the control group	*Eed*	*Mcm4*	0.18	0.433	0	0.05	0.52
3-day post-SCI group vs. the control group	*Eed*	*Krt1*	0	0.433	0	0.082	0.457
3-day post-SCI group vs. the control group	*Eed*	*Prdx4*	0.063	0.433	0	0	0.446
3-day post-SCI group vs. the control group	*Eed*	*Parp1*	0	0.433	0	0.079	0.455
3-day post-SCI group vs. the control group	*Eed*	*Pcna*	0.461	0.433	0	0	0.681
3-day post-SCI group vs. the control group	*Eed*	*Sfpq*	0.097	0.433	0	0	0.466
3-day post-SCI group vs. the control group	*Eed*	*Atp2a2*	0	0.433	0	0.081	0.456

3-day post-SCI group vs. the control group	*Eed*	*Gnb2*	0	0.433	0	0	0.433
3-day post-SCI group vs. the control group	*Eed*	*Bax*	0	0.433	0	0	0.433
3-day post-SCI group vs. the control group	*Eed*	*Nono*	0.145	0.433	0	0	0.494
3-day post-SCI group vs. the control group	*Eed*	*Rbbp7*	0.162	0.862	0.961	0.905	0.999
3-day post-SCI group vs. the control group	*Eed*	*Ppp1ca*	0.061	0.439	0	0	0.451
3-day post-SCI group vs. the control group	*Eed*	*Ctnna1*	0	0.433	0	0	0.433
3-day post-SCI group vs. the control group	*Eed*	*Arhgdia*	0	0.433	0	0	0.433
3-day post-SCI group vs. the control group	*Eed*	*Ezr*	0	0.433	0	0	0.433
3-day post-SCI group vs. the control group	*Eed*	*Pdlim1*	0	0.433	0	0	0.433
3-day post-SCI group vs. the control group	*Eed*	*Eif2s1*	0.142	0.682	0	0	0.715
3-day post-SCI group vs. the control group	*Eed*	*Rnf2*	0.168	0.433	0.9	0.822	0.99
3-day post-SCI group vs. the control group	*Eed*	*Ezh2*	0.148	0.999	0.961	0.994	0.999
3-day post-SCI group vs. the control group	*Eed*	*Actb*	0	0.433	0	0.205	0.53
3-day post-SCI group vs. the control group	*Eed*	*Cttn*	0	0.433	0	0	0.433
3-day post-SCI group vs. the control group	*Eed*	*Hdac1*	0.062	0.86	0.548	0.432	0.962
3-day post-SCI group vs. the control group	*Eed*	*Sirt1*	0.134	0	0.72	0.543	0.879
3-day post-SCI group vs. the control group	*Eed*	*Rpl13a*	0	0.433	0	0	0.433
3-day post-SCI group vs. the control group	*Eed*	*Pxdn*	0.061	0.433	0	0	0.444
3-day post-SCI group vs. the control group	*Eed*	*Tfdp1*	0.1	0	0.6	0.043	0.625
3-day post-SCI group vs. the control group	*Eed*	*Cbx6*	0.057	0.261	0.72	0.449	0.878
3-day post-SCI group vs. the control group	*Eed*	*Phc3*	0	0.147	0.9	0.374	0.942
3-day post-SCI group vs. the control group	*Eif2s1*	*P4hb*	0.062	0.358	0	0.38	0.594
3-day post-SCI group vs. the control group	*Eif2s1*	*Paip1*	0.161	0.229	0.47	0.365	0.753
3-day post-SCI group vs. the control group	*Eif2s1*	*Nudt2*	0.143	0.4	0	0.08	0.485
3-day post-SCI group vs. the control group	*Eif2s1*	*Ppp1ca*	0.082	0.398	0.8	0.276	0.909
3-day post-SCI group vs. the control group	*Eif2s1*	*Gpr37*	0	0	0.208	0.316	0.435
3-day post-SCI group vs. the control group	*Eif2s1*	*Ppp1r15b*	0	0	0	0.633	0.633
3-day post-SCI group vs. the control group	*Eif2s1*	*Oxr1*	0	0	0	0.428	0.428
3-day post-SCI group vs. the control group	*Eif2s1*	*Sirt1*	0	0	0	0.479	0.479
3-day post-SCI group vs. the control group	*Eif2s1*	*Casp3*	0	0.087	0	0.475	0.501
3-day post-SCI group vs. the control group	*Eif2s1*	*Rpl13a*	0.652	0	0	0.097	0.672
3-day post-SCI group vs. the control group	*Eif2s1*	*Eed*	0.142	0.682	0	0	0.715
3-day post-SCI group vs. the control group	*Endog*	*Cdc20*	0.057	0	0	0.465	0.474
3-day post-SCI group vs. the control group	*Endog*	*Mapk8*	0.061	0	0	0.391	0.404
3-day post-SCI group vs. the control group	*Endog*	*Jun*	0	0	0	0.406	0.406
3-day post-SCI group vs. the control group	*Endog*	*Parp1*	0	0	0	0.426	0.426

3-day post-SCI group vs. the control group	*Endog*	*Mcl1*	0	0	0	0.427	0.427
3-day post-SCI group vs. the control group	*Endog*	*Bax*	0	0	0	0.433	0.433
3-day post-SCI group vs. the control group	*Endog*	*Pink1*	0.082	0	0	0.529	0.55
3-day post-SCI group vs. the control group	*Endog*	*Bnip3*	0.062	0	0	0.594	0.603
3-day post-SCI group vs. the control group	*Endog*	*Ppif*	0.092	0.058	0	0.611	0.638
3-day post-SCI group vs. the control group	*Endog*	*Casp3*	0	0	0	0.72	0.72
3-day post-SCI group vs. the control group	*Endog*	*Htra2*	0.065	0	0	0.884	0.887
3-day post-SCI group vs. the control group	*Etfdh*	*Ndufs8*	0.067	0	0	0.395	0.412
3-day post-SCI group vs. the control group	*Ets1*	*Braf*	0	0.263	0.108	0.193	0.423
3-day post-SCI group vs. the control group	*Ets1*	*Mapk14*	0	0.215	0.629	0.363	0.798
3-day post-SCI group vs. the control group	*Ets1*	*Hif1a*	0.061	0.074	0	0.563	0.587
3-day post-SCI group vs. the control group	*Ets1*	*Fos*	0.061	0.088	0.629	0.613	0.86
3-day post-SCI group vs. the control group	*Ets1*	*Myc*	0.075	0.069	0	0.54	0.569
3-day post-SCI group vs. the control group	*Ets1*	*Fosl1*	0.062	0.047	0.208	0.363	0.488
3-day post-SCI group vs. the control group	*Ets1*	*Parp1*	0	0.132	0	0.337	0.4
3-day post-SCI group vs. the control group	*Ets1*	*Nfkb1*	0.068	0.322	0	0.43	0.608
3-day post-SCI group vs. the control group	*Ets1*	*Actb*	0	0.045	0	0.4	0.402
3-day post-SCI group vs. the control group	*Ets1*	*Junb*	0.07	0.2	0.261	0.225	0.517
3-day post-SCI group vs. the control group	*Ets1*	*Jak2*	0.048	0.072	0.515	0.248	0.635
3-day post-SCI group vs. the control group	*Ets1*	*Cdk10*	0.061	0.267	0	0.542	0.657
3-day post-SCI group vs. the control group	*Ets1*	*Hdac1*	0.061	0.433	0.243	0.303	0.681
3-day post-SCI group vs. the control group	*Ets1*	*Id1*	0.061	0.049	0.65	0.247	0.733
3-day post-SCI group vs. the control group	*Ets1*	*Stat6*	0.087	0.087	0.676	0.443	0.829
3-day post-SCI group vs. the control group	*Ets1*	*Jun*	0.061	0.456	0.629	0.69	0.933
3-day post-SCI group vs. the control group	*Ets1*	*Mapk3*	0	0.311	0.966	0.616	0.99
3-day post-SCI group vs. the control group	*Ezh2*	*Cdc20*	0.377	0.087	0	0.248	0.535
3-day post-SCI group vs. the control group	*Ezh2*	*Cdk4*	0.134	0.164	0.148	0.581	0.708
3-day post-SCI group vs. the control group	*Ezh2*	*Cdk1*	0.524	0.763	0.148	0.862	0.984
3-day post-SCI group vs. the control group	*Ezh2*	*Hif1a*	0.088	0.058	0	0.499	0.531
3-day post-SCI group vs. the control group	*Ezh2*	*Fos*	0	0	0	0.428	0.428
3-day post-SCI group vs. the control group	*Ezh2*	*Rb1*	0.063	0.132	0.175	0.578	0.679
3-day post-SCI group vs. the control group	*Ezh2*	*Myc*	0.062	0.206	0	0.904	0.922
3-day post-SCI group vs. the control group	*Ezh2*	*Mcm4*	0.43	0.053	0	0.149	0.501
3-day post-SCI group vs. the control group	*Ezh2*	*Rela*	0	0.33	0	0.979	0.985
3-day post-SCI group vs. the control group	*Ezh2*	*Parp1*	0.109	0	0	0.368	0.413
3-day post-SCI group vs. the control group	*Ezh2*	*Pcna*	0.307	0.208	0	0.105	0.467

3-day post-SCI group vs. the control group	*Ezh2*	*Ccna2*	0.516	0.059	0	0.355	0.681
3-day post-SCI group vs. the control group	*Ezh2*	*Rbbp7*	0.174	0.899	0.961	0.894	0.999
3-day post-SCI group vs. the control group	*Ezh2*	*Melk*	0.633	0.047	0	0.733	0.899
3-day post-SCI group vs. the control group	*Ezh2*	*Mcl1*	0	0	0	0.463	0.463
3-day post-SCI group vs. the control group	*Ezh2*	*Jak2*	0	0.13	0	0.581	0.62
3-day post-SCI group vs. the control group	*Ezh2*	*Rnf2*	0.117	0.163	0.9	0.897	0.991
3-day post-SCI group vs. the control group	*Ezh2*	*Rest*	0.082	0.115	0.187	0.361	0.522
3-day post-SCI group vs. the control group	*Ezh2*	*Mgmt*	0.062	0	0	0.4	0.413
3-day post-SCI group vs. the control group	*Ezh2*	*Jun*	0	0.047	0	0.4	0.403
3-day post-SCI group vs. the control group	*Ezh2*	*Pdgfra*	0	0.044	0	0.406	0.408
3-day post-SCI group vs. the control group	*Ezh2*	*Met*	0.052	0.068	0	0.431	0.453
3-day post-SCI group vs. the control group	*Ezh2*	*Casp3*	0.094	0.057	0	0.464	0.502
3-day post-SCI group vs. the control group	*Ezh2*	*Ago1*	0.061	0.143	0	0.433	0.504
3-day post-SCI group vs. the control group	*Ezh2*	*Banf1*	0.051	0.066	0	0.486	0.505
3-day post-SCI group vs. the control group	*Ezh2*	*Ect2*	0.458	0	0	0.129	0.508
3-day post-SCI group vs. the control group	*Ezh2*	*Fbxw7*	0.062	0.13	0	0.451	0.513
3-day post-SCI group vs. the control group	*Ezh2*	*Actb*	0	0.056	0	0.585	0.591
3-day post-SCI group vs. the control group	*Ezh2*	*Cdkn2b*	0.048	0.058	0	0.613	0.623
3-day post-SCI group vs. the control group	*Ezh2*	*Tfdp1*	0.182	0	0.667	0.151	0.749
3-day post-SCI group vs. the control group	*Ezh2*	*Cbx6*	0.061	0.236	0.72	0.529	0.892
3-day post-SCI group vs. the control group	*Ezh2*	*Phc3*	0	0.087	0.9	0.162	0.916
3-day post-SCI group vs. the control group	*Ezh2*	*Sirt1*	0.052	0.266	0.72	0.959	0.991
3-day post-SCI group vs. the control group	*Ezh2*	*Hdac1*	0.088	0.845	0.548	0.979	0.998
3-day post-SCI group vs. the control group	*Ezh2*	*Eed*	0.148	0.999	0.961	0.994	0.999
3-day post-SCI group vs. the control group	*Ezr*	*Capns1*	0.062	0	0.629	0.203	0.698
3-day post-SCI group vs. the control group	*Ezr*	*Mapk14*	0	0.199	0	0.312	0.425
3-day post-SCI group vs. the control group	*Ezr*	*Vcam1*	0	0.13	0.8	0.941	0.988
3-day post-SCI group vs. the control group	*Ezr*	*Gnb2*	0.063	0	0.499	0.162	0.572
3-day post-SCI group vs. the control group	*Ezr*	*Ctnna1*	0.069	0	0	0.4	0.418
3-day post-SCI group vs. the control group	*Ezr*	*Mapk3*	0.051	0.199	0	0.415	0.516
3-day post-SCI group vs. the control group	*Ezr*	*S100a1*	0	0	0	0.707	0.707
3-day post-SCI group vs. the control group	*Ezr*	*Arhgdia*	0.057	0.719	0	0.937	0.982
3-day post-SCI group vs. the control group	*Ezr*	*Mapk10*	0.062	0.368	0	0.071	0.401
3-day post-SCI group vs. the control group	*Ezr*	*Casp3*	0	0.05	0.177	0.319	0.421
3-day post-SCI group vs. the control group	*Ezr*	*Eed*	0	0.433	0	0	0.433
3-day post-SCI group vs. the control group	*Ezr*	*Pdlim1*	0.061	0.049	0.338	0.156	0.434

3-day post-SCI group vs. the control group	*Ezr*	*Abcc1*	0	0.094	0.34	0.134	0.437
3-day post-SCI group vs. the control group	*Ezr*	*Itgb5*	0	0.048	0.327	0.195	0.439
3-day post-SCI group vs. the control group	*Ezr*	*Dst*	0.061	0.163	0.191	0.252	0.461
3-day post-SCI group vs. the control group	*Ezr*	*Jak2*	0	0.089	0.22	0.307	0.464
3-day post-SCI group vs. the control group	*Ezr*	*Stk24*	0.062	0	0.386	0.158	0.472
3-day post-SCI group vs. the control group	*Ezr*	*Met*	0.061	0.089	0.133	0.391	0.487
3-day post-SCI group vs. the control group	*Ezr*	*Map4k4*	0.048	0.137	0	0.469	0.526
3-day post-SCI group vs. the control group	*Ezr*	*Cttn*	0.052	0.062	0	0.87	0.874
3-day post-SCI group vs. the control group	*Ezr*	*Pxn*	0.082	0.065	0.629	0.748	0.909
3-day post-SCI group vs. the control group	*Ezr*	*Actb*	0.062	0.086	0.874	0.836	0.98
3-day post-SCI group vs. the control group	*Fbxw7*	*Cdc20*	0.135	0.088	0	0.501	0.447
3-day post-SCI group vs. the control group	*Fbxw7*	*Cdk4*	0.051	0.161	0	0.442	0.517
3-day post-SCI group vs. the control group	*Fbxw7*	*Ube2a*	0	0.138	0.197	0.234	0.423
3-day post-SCI group vs. the control group	*Fbxw7*	*Cdk1*	0.062	0.161	0	0.642	0.694
3-day post-SCI group vs. the control group	*Fbxw7*	*Txnrd1*	0	0	0	0.414	0.414
3-day post-SCI group vs. the control group	*Fbxw7*	*Hif1a*	0	0.231	0	0.42	0.535
3-day post-SCI group vs. the control group	*Fbxw7*	*Myc*	0	0.852	0.585	0.94	0.996
3-day post-SCI group vs. the control group	*Fbxw7*	*Ccna2*	0.064	0.224	0	0.287	0.437
3-day post-SCI group vs. the control group	*Fbxw7*	*Nfkb1*	0	0.272	0	0.493	0.616
3-day post-SCI group vs. the control group	*Fbxw7*	*Mcl1*	0.061	0.745	0	0.863	0.964
3-day post-SCI group vs. the control group	*Fbxw7*	*Mapk3*	0.064	0.163	0	0.352	0.448
3-day post-SCI group vs. the control group	*Fbxw7*	*Prdx6*	0	0.433	0	0.068	0.448
3-day post-SCI group vs. the control group	*Fbxw7*	*Ezh2*	0.062	0.13	0	0.451	0.513
3-day post-SCI group vs. the control group	*Fbxw7*	*Tnrc6a*	0	0	0	0.467	0.467
3-day post-SCI group vs. the control group	*Fbxw7*	*Actb*	0	0.073	0	0.429	0.448
3-day post-SCI group vs. the control group	*Fbxw7*	*Nfe2l2*	0	0.198	0	0.312	0.424
3-day post-SCI group vs. the control group	*Fbxw7*	*Jun*	0	0.762	0	0.901	0.975
3-day post-SCI group vs. the control group	*Fbxw7*	*Pdgfra*	0	0.064	0	0.402	0.417
3-day post-SCI group vs. the control group	*Fbxw7*	*Snca*	0.084	0.13	0	0.353	0.439
3-day post-SCI group vs. the control group	*Fbxw7*	*Ppargc1a*	0	0.439	0	0.191	0.527
3-day post-SCI group vs. the control group	*Fbxw7*	*Uba52*	0	0.749	0.6	0	0.895
3-day post-SCI group vs. the control group	*Fkbp1b*	*Atr*	0	0.215	0.201	0.122	0.401
3-day post-SCI group vs. the control group	*Fkbp1b*	*S100a1*	0	0.249	0	0.398	0.528
3-day post-SCI group vs. the control group	*Fkbp1b*	*Snca*	0.062	0	0.576	0.066	0.596
3-day post-SCI group vs. the control group	*Fkbp1b*	*Nme2*	0.474	0.349	0	0	0.644
3-day post-SCI group vs. the control group	*Fkbp1b*	*Atp2a2*	0	0.058	0	0.669	0.674

3-day post-SCI group vs. the control group	*Fkbp1b*	*Ppp3ca*	0.062	0.591	0.15	0.268	0.729
3-day post-SCI group vs. the control group	*Fkbp1b*	*Ppif*	0.159	0.657	0	0.509	0.845
3-day post-SCI group vs. the control group	*Fos*	*Ier3*	0.159	0	0	0.399	0.472
3-day post-SCI group vs. the control group	*Fos*	*Mapk14*	0	0.267	0.922	0.716	0.982
3-day post-SCI group vs. the control group	*Fos*	*Hmox1*	0	0	0.313	0.477	0.626
3-day post-SCI group vs. the control group	*Fos*	*Cdk4*	0	0	0	0.464	0.463
3-day post-SCI group vs. the control group	*Fos*	*Map2k3*	0	0	0	0.435	0.435
3-day post-SCI group vs. the control group	*Fos*	*Hif1a*	0	0.131	0	0.527	0.571
3-day post-SCI group vs. the control group	*Fos*	*Ccna2*	0	0.059	0	0.393	0.404
3-day post-SCI group vs. the control group	*Fos*	*Ezh2*	0	0	0	0.428	0.428
3-day post-SCI group vs. the control group	*Fos*	*Met*	0	0.071	0.12	0.364	0.434
3-day post-SCI group vs. the control group	*Fos*	*Hbegf*	0.065	0	0	0.422	0.436
3-day post-SCI group vs. the control group	*Fos*	*Mcl1*	0.089	0	0	0.411	0.441
3-day post-SCI group vs. the control group	*Fos*	*Adora1*	0	0.042	0.127	0.407	0.461
3-day post-SCI group vs. the control group	*Fos*	*Stat6*	0	0.071	0.216	0.327	0.467
3-day post-SCI group vs. the control group	*Fos*	*Banf1*	0	0	0	0.468	0.468
3-day post-SCI group vs. the control group	*Fos*	*Cat*	0.062	0	0	0.488	0.499
3-day post-SCI group vs. the control group	*Fos*	*Jak2*	0	0.07	0.15	0.472	0.547
3-day post-SCI group vs. the control group	*Fos*	*Syp*	0	0	0	0.546	0.547
3-day post-SCI group vs. the control group	*Fos*	*Smad1*	0	0.142	0.388	0.227	0.558
3-day post-SCI group vs. the control group	*Fos*	*Tlr4*	0.063	0.052	0	0.554	0.569
3-day post-SCI group vs. the control group	*Fos*	*Aif1*	0	0.132	0	0.535	0.58
3-day post-SCI group vs. the control group	*Fos*	*Casp3*	0	0	0	0.602	0.602
3-day post-SCI group vs. the control group	*Fos*	*Nfkb1*	0.051	0.087	0.12	0.552	0.613
3-day post-SCI group vs. the control group	*Fos*	*Ptgs2*	0.063	0	0	0.64	0.648
3-day post-SCI group vs. the control group	*Fos*	*Hdac1*	0.048	0.164	0	0.67	0.715
3-day post-SCI group vs. the control group	*Fos*	*Tfdp1*	0	0.698	0	0.108	0.719
3-day post-SCI group vs. the control group	*Fos*	*Sirt1*	0	0.439	0	0.567	0.747
3-day post-SCI group vs. the control group	*Fos*	*Nfe2l2*	0.061	0.443	0.293	0.416	0.755
3-day post-SCI group vs. the control group	*Fos*	*Actb*	0	0.047	0.221	0.698	0.756
3-day post-SCI group vs. the control group	*Fos*	*Btk*	0	0.07	0.676	0.266	0.759
3-day post-SCI group vs. the control group	*Fos*	*Rb1*	0	0.703	0.21	0.224	0.802
3-day post-SCI group vs. the control group	*Fos*	*Myc*	0.064	0.055	0	0.85	0.856
3-day post-SCI group vs. the control group	*Fos*	*Ets1*	0.061	0.088	0.629	0.613	0.86
3-day post-SCI group vs. the control group	*Fos*	*Atf2*	0	0.84	0	0.629	0.882
3-day post-SCI group vs. the control group	*Fos*	*Rela*	0	0.4	0.629	0.561	0.893

3-day post-SCI group vs. the control group	*Fos*	*Fosl1*	0.079	0	0.922	0.923	0.934
3-day post-SCI group vs. the control group	*Fos*	*Mapk10*	0	0.309	0.903	0.492	0.963
3-day post-SCI group vs. the control group	*Fos*	*Nfatc1*	0	0.142	0.676	0.916	0.974
3-day post-SCI group vs. the control group	*Fos*	*Mapk8*	0	0.309	0.932	0.64	0.981
3-day post-SCI group vs. the control group	*Fos*	*Mapk3*	0	0.267	0.966	0.746	0.993
3-day post-SCI group vs. the control group	*Fos*	*Jun*	0.662	0.982	0.932	0.995	0.999
3-day post-SCI group vs. the control group	*Fos*	*Junb*	0.822	0.804	0.932	0.886	0.999
3-day post-SCI group vs. the control group	*Fosl1*	*Axl*	0.105	0.07	0.12	0.314	0.43
3-day post-SCI group vs. the control group	*Fosl1*	*Ier3*	0.187	0	0	0.351	0.45
3-day post-SCI group vs. the control group	*Fosl1*	*Mapk14*	0	0.267	0.67	0.444	0.853
3-day post-SCI group vs. the control group	*Fosl1*	*Hmox1*	0.104	0	0.313	0.258	0.504
3-day post-SCI group vs. the control group	*Fosl1*	*Fos*	0.079	0	0.922	0.923	0.934
3-day post-SCI group vs. the control group	*Fosl1*	*Myc*	0.15	0.05	0	0.563	0.616
3-day post-SCI group vs. the control group	*Fosl1*	*Nfkb1*	0.063	0.058	0.12	0.354	0.431
3-day post-SCI group vs. the control group	*Fosl1*	*Nfe2l2*	0	0.058	0.293	0.222	0.436
3-day post-SCI group vs. the control group	*Fosl1*	*Met*	0.077	0.071	0.12	0.355	0.448
3-day post-SCI group vs. the control group	*Fosl1*	*Ptgs2*	0.223	0	0	0.36	0.482
3-day post-SCI group vs. the control group	*Fosl1*	*Ets1*	0.062	0.047	0.208	0.363	0.488
3-day post-SCI group vs. the control group	*Fosl1*	*Actb*	0	0.047	0.221	0.434	0.543
3-day post-SCI group vs. the control group	*Fosl1*	*Mapk3*	0	0.267	0.209	0.504	0.687
3-day post-SCI group vs. the control group	*Fosl1*	*Rela*	0.061	0.155	0.355	0.511	0.716
3-day post-SCI group vs. the control group	*Fosl1*	*Nfatc1*	0.062	0.074	0.676	0.294	0.774
3-day post-SCI group vs. the control group	*Fosl1*	*Mapk10*	0	0.309	0.696	0.216	0.821
3-day post-SCI group vs. the control group	*Fosl1*	*Mapk8*	0	0.309	0.696	0.395	0.862
3-day post-SCI group vs. the control group	*Fosl1*	*Junb*	0.092	0.642	0.932	0.838	0.995
3-day post-SCI group vs. the control group	*Fosl1*	*Jun*	0.066	0.784	0.932	0.989	0.999
3-day post-SCI group vs. the control group	*Gch1*	*Sod2*	0.08	0	0	0.429	0.502
3-day post-SCI group vs. the control group	*Gch1*	*Nme2*	0.064	0.135	0.8	0.07	0.84
3-day post-SCI group vs. the control group	*Gch1*	*Dhfr*	0.063	0	0	0.88	0.901
3-day post-SCI group vs. the control group	*Gch1*	*Xdh*	0.104	0	0	0.642	0.665
3-day post-SCI group vs. the control group	*Gch1*	*Nudt2*	0.062	0	0.8	0	0.807
3-day post-SCI group vs. the control group	*Gclc*	*Mapk14*	0.057	0	0	0.395	0.405
3-day post-SCI group vs. the control group	*Gclc*	*Hmox1*	0	0.097	0	0.866	0.874
3-day post-SCI group vs. the control group	*Gclc*	*Sod2*	0.067	0.698	0	0.761	0.927
3-day post-SCI group vs. the control group	*Gclc*	*Txnrd1*	0.194	0	0	0.808	0.839
3-day post-SCI group vs. the control group	*Gclc*	*Gpx8*	0.061	0	0	0.491	0.501

3-day post-SCI group vs. the control group	*Gclc*	*Prdx4*	0	0	0	0.48	0.48
3-day post-SCI group vs. the control group	*Gclc*	*Cat*	0.141	0.275	0	0.843	0.894
3-day post-SCI group vs. the control group	*Gclc*	*Ggt7*	0.062	0	0.9	0.565	0.955
3-day post-SCI group vs. the control group	*Gclc*	*Gpx7*	0.061	0	0	0.543	0.553
3-day post-SCI group vs. the control group	*Gclc*	*Nox4*	0.061	0	0	0.404	0.416
3-day post-SCI group vs. the control group	*Gclc*	*Gsr*	0.216	0	0	0.925	0.938
3-day post-SCI group vs. the control group	*Gclc*	*Casp3*	0.062	0	0	0.409	0.422
3-day post-SCI group vs. the control group	*Gclc*	*Jun*	0	0	0	0.471	0.471
3-day post-SCI group vs. the control group	*Gclc*	*Abcc1*	0.064	0	0	0.463	0.476
3-day post-SCI group vs. the control group	*Gclc*	*Gstt2*	0.079	0.142	0	0.425	0.506
3-day post-SCI group vs. the control group	*Gclc*	*Gpx3*	0.061	0	0	0.553	0.562
3-day post-SCI group vs. the control group	*Gclc*	*Actb*	0.06	0	0	0.591	0.599
3-day post-SCI group vs. the control group	*Gclc*	*Glrx2*	0.135	0	0	0.64	0.675
3-day post-SCI group vs. the control group	*Gclc*	*Gpx1*	0.061	0	0	0.775	0.779
3-day post-SCI group vs. the control group	*Gclc*	*Srxn1*	0.273	0	0	0.714	0.783
3-day post-SCI group vs. the control group	*Gclc*	*Nfe2l2*	0	0	0	0.883	0.883
3-day post-SCI group vs. the control group	*Ggt7*	*Mgst1*	0.061	0	0.65	0.308	0.752
3-day post-SCI group vs. the control group	*Ggt7*	*Gpx8*	0.065	0	0.65	0.362	0.773
3-day post-SCI group vs. the control group	*Ggt7*	*Gstt2*	0	0	0.65	0.107	0.674
3-day post-SCI group vs. the control group	*Ggt7*	*Gpx3*	0.065	0	0.65	0.229	0.725
3-day post-SCI group vs. the control group	*Ggt7*	*Gpx1*	0.065	0	0.65	0.266	0.738
3-day post-SCI group vs. the control group	*Ggt7*	*Gpx7*	0.065	0	0.65	0.383	0.78
3-day post-SCI group vs. the control group	*Ggt7*	*Prdx6*	0.062	0	0.9	0.106	0.909
3-day post-SCI group vs. the control group	*Ggt7*	*Gsr*	0.052	0	0.9	0.297	0.927
3-day post-SCI group vs. the control group	*Ggt7*	*Gclc*	0.062	0	0.9	0.565	0.955
3-day post-SCI group vs. the control group	*Glrx2*	*Sod2*	0.157	0	0	0.469	0.542
3-day post-SCI group vs. the control group	*Glrx2*	*Txnrd1*	0.081	0.476	0	0.632	0.812
3-day post-SCI group vs. the control group	*Glrx2*	*Ppif*	0.066	0.464	0	0.198	0.571
3-day post-SCI group vs. the control group	*Glrx2*	*Prdx4*	0.071	0.057	0	0.602	0.634
3-day post-SCI group vs. the control group	*Glrx2*	*Cat*	0.139	0.226	0	0.639	0.744
3-day post-SCI group vs. the control group	*Glrx2*	*Gpx7*	0.136	0.13	0	0.293	0.444
3-day post-SCI group vs. the control group	*Glrx2*	*Gsr*	0.147	0.135	0	0.818	0.857
3-day post-SCI group vs. the control group	*Glrx2*	*Gclc*	0.135	0	0	0.64	0.675
3-day post-SCI group vs. the control group	*Glrx2*	*Srxn1*	0.15	0	0	0.459	0.521
3-day post-SCI group vs. the control group	*Glrx2*	*Prdx6*	0.164	0.401	0	0.447	0.71
3-day post-SCI group vs. the control group	*Glrx2*	*Gpx1*	0.136	0.13	0	0.594	0.681

3-day post-SCI group vs. the control group	*Glrx2*	*Gpx3*	0.136	0.13	0	0.396	0.525
3-day post-SCI group vs. the control group	*Gnb2*	*Mapk14*	0.062	0.262	0.791	0.176	0.865
3-day post-SCI group vs. the control group	*Gnb2*	*Map2k3*	0	0	0.479	0.071	0.495
3-day post-SCI group vs. the control group	*Gnb2*	*Nme2*	0	0.552	0	0.057	0.559
3-day post-SCI group vs. the control group	*Gnb2*	*Ccr1*	0	0.198	0.429	0	0.522
3-day post-SCI group vs. the control group	*Gnb2*	*Prkaa2*	0.062	0	0.6	0.102	0.633
3-day post-SCI group vs. the control group	*Gnb2*	*Adora1*	0	0.328	0.185	0	0.428
3-day post-SCI group vs. the control group	*Gnb2*	*Eed*	0	0.433	0	0	0.433
3-day post-SCI group vs. the control group	*Gnb2*	*Arhgdia*	0.423	0	0	0.09	0.452
3-day post-SCI group vs. the control group	*Gnb2*	*Ubqln1*	0	0.161	0.438	0.058	0.517
3-day post-SCI group vs. the control group	*Gnb2*	*Pxn*	0.069	0.071	0.533	0	0.56
3-day post-SCI group vs. the control group	*Gnb2*	*Ezr*	0.063	0	0.499	0.162	0.572
3-day post-SCI group vs. the control group	*Gnb2*	*Mapk8*	0.062	0	0.6	0	0.608
3-day post-SCI group vs. the control group	*Gnb2*	*Mapk10*	0.062	0	0.6	0	0.608
3-day post-SCI group vs. the control group	*Gnb2*	*Ppp1ca*	0.61	0.132	0	0.061	0.655
3-day post-SCI group vs. the control group	*Gnb2*	*Btk*	0.061	0.058	0.681	0	0.693
3-day post-SCI group vs. the control group	*Gnb2*	*Mapk3*	0.223	0.262	0.673	0.176	0.825
3-day post-SCI group vs. the control group	*Gpr37*	*Eif2s1*	0	0	0.208	0.316	0.435
3-day post-SCI group vs. the control group	*Gpr37*	*Snca*	0.069	0	0	0.586	0.598
3-day post-SCI group vs. the control group	*Gpx1*	*Mapk14*	0	0	0	0.425	0.425
3-day post-SCI group vs. the control group	*Gpx1*	*Hmox1*	0.066	0.07	0	0.746	0.76
3-day post-SCI group vs. the control group	*Gpx1*	*Sod2*	0.061	0.348	0	0.859	0.906
3-day post-SCI group vs. the control group	*Gpx1*	*Mgst1*	0.141	0	0.65	0.308	0.774
3-day post-SCI group vs. the control group	*Gpx1*	*Ncf1*	0.062	0	0	0.521	0.531
3-day post-SCI group vs. the control group	*Gpx1*	*Txnrd1*	0.083	0.141	0	0.781	0.814
3-day post-SCI group vs. the control group	*Gpx1*	*Hif1a*	0	0	0	0.483	0.483
3-day post-SCI group vs. the control group	*Gpx1*	*Msrb2*	0.093	0.047	0	0.369	0.429
3-day post-SCI group vs. the control group	*Gpx1*	*Xdh*	0.062	0	0	0.552	0.561
3-day post-SCI group vs. the control group	*Gpx1*	*Prdx4*	0.098	0.224	0	0.617	0.708
3-day post-SCI group vs. the control group	*Gpx1*	*Alox5*	0	0	0.9	0.249	0.921
3-day post-SCI group vs. the control group	*Gpx1*	*Cat*	0.159	0.379	0	0.919	0.954
3-day post-SCI group vs. the control group	*Gpx1*	*Ggt7*	0.065	0	0.65	0.266	0.738
3-day post-SCI group vs. the control group	*Gpx1*	*Nox4*	0.061	0	0	0.64	0.648
3-day post-SCI group vs. the control group	*Gpx1*	*Gsr*	0.09	0.105	0.9	0.866	0.987
3-day post-SCI group vs. the control group	*Gpx1*	*Gclc*	0.061	0	0	0.775	0.779
3-day post-SCI group vs. the control group	*Gpx1*	*Ptgs2*	0	0	0	0.46	0.46

3-day post-SCI group vs. the control group	*Gpx1*	*Srxn1*	0.058	0	0	0.556	0.564
3-day post-SCI group vs. the control group	*Gpx1*	*Gstt2*	0.084	0	0.65	0.251	0.739
3-day post-SCI group vs. the control group	*Gpx1*	*Prdx6*	0.063	0.184	0	0.629	0.691
3-day post-SCI group vs. the control group	*Gpx1*	*Jun*	0.051	0.045	0	0.413	0.422
3-day post-SCI group vs. the control group	*Gpx1*	*Oxr1*	0.064	0	0	0.491	0.503
3-day post-SCI group vs. the control group	*Gpx1*	*Sirt1*	0	0.05	0	0.506	0.52
3-day post-SCI group vs. the control group	*Gpx1*	*Ucp2*	0.101	0	0	0.493	0.524
3-day post-SCI group vs. the control group	*Gpx1*	*Casp3*	0	0	0	0.618	0.618
3-day post-SCI group vs. the control group	*Gpx1*	*Actb*	0.062	0	0	0.614	0.623
3-day post-SCI group vs. the control group	*Gpx1*	*Glrx2*	0.136	0.13	0	0.594	0.681
3-day post-SCI group vs. the control group	*Gpx1*	*Nfe2l2*	0.079	0	0	0.689	0.702
3-day post-SCI group vs. the control group	*Gpx1*	*Ppargc1a*	0	0	0.8	0.602	0.917
3-day post-SCI group vs. the control group	*Gpx3*	*Hmox1*	0.062	0.07	0	0.501	0.527
3-day post-SCI group vs. the control group	*Gpx3*	*Sod2*	0.061	0.348	0	0.629	0.753
3-day post-SCI group vs. the control group	*Gpx3*	*Mgst1*	0.076	0	0.65	0.227	0.728
3-day post-SCI group vs. the control group	*Gpx3*	*Txnrd1*	0.063	0.141	0	0.733	0.768
3-day post-SCI group vs. the control group	*Gpx3*	*Prdx4*	0.061	0.224	0	0.542	0.637
3-day post-SCI group vs. the control group	*Gpx3*	*Alox5*	0.062	0	0.9	0.216	0.92
3-day post-SCI group vs. the control group	*Gpx3*	*Cat*	0.141	0.379	0	0.8	0.884
3-day post-SCI group vs. the control group	*Gpx3*	*Ggt7*	0.065	0	0.65	0.229	0.725
3-day post-SCI group vs. the control group	*Gpx3*	*Nox4*	0	0	0	0.521	0.521
3-day post-SCI group vs. the control group	*Gpx3*	*Gsr*	0.064	0.105	0.9	0.805	0.981
3-day post-SCI group vs. the control group	*Gpx3*	*Gclc*	0.061	0	0	0.553	0.562
3-day post-SCI group vs. the control group	*Gpx3*	*Srxn1*	0.058	0	0	0.498	0.507
3-day post-SCI group vs. the control group	*Gpx3*	*Gstt2*	0.065	0	0.65	0.327	0.76
3-day post-SCI group vs. the control group	*Gpx3*	*Prdx6*	0.063	0.184	0	0.516	0.597
3-day post-SCI group vs. the control group	*Gpx3*	*Nfe2l2*	0.057	0	0	0.405	0.415
3-day post-SCI group vs. the control group	*Gpx3*	*Casp3*	0	0	0	0.439	0.439
3-day post-SCI group vs. the control group	*Gpx3*	*Glrx2*	0.136	0.13	0	0.396	0.525
3-day post-SCI group vs. the control group	*Gpx3*	*Ppargc1a*	0	0	0.8	0.237	0.84
3-day post-SCI group vs. the control group	*Gpx7*	*Hmox1*	0	0.07	0	0.693	0.702
3-day post-SCI group vs. the control group	*Gpx7*	*Sod2*	0.061	0.348	0	0.489	0.659
3-day post-SCI group vs. the control group	*Gpx7*	*Mgst1*	0.049	0	0.65	0.207	0.712
3-day post-SCI group vs. the control group	*Gpx7*	*Ncf1*	0	0	0	0.403	0.403
3-day post-SCI group vs. the control group	*Gpx7*	*Txnrd1*	0.063	0.141	0	0.626	0.675
3-day post-SCI group vs. the control group	*Gpx7*	*Cygb*	0.103	0	0	0.417	0.455

3-day post-SCI group vs. the control group	*Gpx7*	*P4hb*	0.061	0.134	0.9	0.557	0.959
3-day post-SCI group vs. the control group	*Gpx7*	*Prdx4*	0.075	0.224	0	0.675	0.746
3-day post-SCI group vs. the control group	*Gpx7*	*Alox5*	0	0	0.9	0.141	0.91
3-day post-SCI group vs. the control group	*Gpx7*	*Cat*	0.141	0.379	0	0.788	0.877
3-day post-SCI group vs. the control group	*Gpx7*	*Ggt7*	0.065	0	0.65	0.383	0.78
3-day post-SCI group vs. the control group	*Gpx7*	*Srxn1*	0.058	0	0	0.398	0.409
3-day post-SCI group vs. the control group	*Gpx7*	*Sirt1*	0	0.05	0	0.4	0.416
3-day post-SCI group vs. the control group	*Gpx7*	*Glrx2*	0.136	0.13	0	0.293	0.444
3-day post-SCI group vs. the control group	*Gpx7*	*Nox4*	0	0	0	0.51	0.51
3-day post-SCI group vs. the control group	*Gpx7*	*Actb*	0	0	0	0.512	0.512
3-day post-SCI group vs. the control group	*Gpx7*	*Prdx6*	0.063	0.184	0	0.426	0.522
3-day post-SCI group vs. the control group	*Gpx7*	*Gclc*	0.061	0	0	0.543	0.553
3-day post-SCI group vs. the control group	*Gpx7*	*Ptgs2*	0	0	0	0.557	0.557
3-day post-SCI group vs. the control group	*Gpx7*	*Nfe2l2*	0	0	0	0.6	0.6
3-day post-SCI group vs. the control group	*Gpx7*	*Casp3*	0	0	0	0.646	0.646
3-day post-SCI group vs. the control group	*Gpx7*	*Gstt2*	0.065	0	0.65	0.325	0.759
3-day post-SCI group vs. the control group	*Gpx7*	*Ppargc1a*	0	0	0.8	0.243	0.842
3-day post-SCI group vs. the control group	*Gpx7*	*Gsr*	0.064	0.105	0.9	0.823	0.983
3-day post-SCI group vs. the control group	*Gpx8*	*Hmox1*	0.062	0.07	0	0.65	0.668
3-day post-SCI group vs. the control group	*Gpx8*	*Sod2*	0.061	0.348	0	0.46	0.641
3-day post-SCI group vs. the control group	*Gpx8*	*Mgst1*	0.074	0	0.65	0.239	0.732
3-day post-SCI group vs. the control group	*Gpx8*	*Txnrd1*	0.063	0.141	0	0.407	0.485
3-day post-SCI group vs. the control group	*Gpx8*	*Sirt1*	0	0.05	0	0.402	0.418
3-day post-SCI group vs. the control group	*Gpx8*	*Prdx6*	0.063	0.184	0	0.308	0.425
3-day post-SCI group vs. the control group	*Gpx8*	*Gclc*	0.061	0	0	0.491	0.501
3-day post-SCI group vs. the control group	*Gpx8*	*Nox4*	0.061	0	0	0.497	0.507
3-day post-SCI group vs. the control group	*Gpx8*	*Actb*	0	0	0	0.527	0.527
3-day post-SCI group vs. the control group	*Gpx8*	*Ptgs2*	0.067	0	0	0.544	0.556
3-day post-SCI group vs. the control group	*Gpx8*	*Casp3*	0	0	0	0.622	0.622
3-day post-SCI group vs. the control group	*Gpx8*	*Nfe2l2*	0	0	0	0.66	0.66
3-day post-SCI group vs. the control group	*Gpx8*	*Gstt2*	0.065	0	0.65	0.11	0.683
3-day post-SCI group vs. the control group	*Gpx8*	*Prdx4*	0.082	0.224	0	0.617	0.703
3-day post-SCI group vs. the control group	*Gpx8*	*Ggt7*	0.065	0	0.65	0.362	0.773
3-day post-SCI group vs. the control group	*Gpx8*	*Ppargc1a*	0	0	0.8	0.225	0.838
3-day post-SCI group vs. the control group	*Gpx8*	*Cat*	0.141	0.379	0	0.761	0.861
3-day post-SCI group vs. the control group	*Gpx8*	*Alox5*	0	0	0.9	0.122	0.908

3-day post-SCI group vs. the control group	*Gpx8*	*P4hb*	0.087	0.134	0.9	0.333	0.94
3-day post-SCI group vs. the control group	*Gpx8*	*Gsr*	0.064	0.105	0.9	0.763	0.977
3-day post-SCI group vs. the control group	*Gsr*	*Mapk14*	0.063	0	0	0.457	0.47
3-day post-SCI group vs. the control group	*Gsr*	*Hmox1*	0.105	0.088	0	0.744	0.773
3-day post-SCI group vs. the control group	*Gsr*	*Sod2*	0.121	0.047	0	0.784	0.809
3-day post-SCI group vs. the control group	*Gsr*	*Mgst1*	0.061	0	0.65	0.396	0.784
3-day post-SCI group vs. the control group	*Gsr*	*Gpx8*	0.064	0.105	0.9	0.763	0.977
3-day post-SCI group vs. the control group	*Gsr*	*Xdh*	0	0	0	0.68	0.68
3-day post-SCI group vs. the control group	*Gsr*	*P4hb*	0.094	0.233	0	0.511	0.634
3-day post-SCI group vs. the control group	*Gsr*	*Prdx4*	0.12	0.059	0	0.609	0.648
3-day post-SCI group vs. the control group	*Gsr*	*Cat*	0.233	0.275	0	0.94	0.963
3-day post-SCI group vs. the control group	*Gsr*	*Ggt7*	0.052	0	0.9	0.297	0.927
3-day post-SCI group vs. the control group	*Gsr*	*Gpx7*	0.064	0.105	0.9	0.823	0.983
3-day post-SCI group vs. the control group	*Gsr*	*Nox4*	0	0	0	0.537	0.537
3-day post-SCI group vs. the control group	*Gsr*	*Ccs*	0.051	0	0	0.325	0.41
3-day post-SCI group vs. the control group	*Gsr*	*Abcc1*	0.081	0.134	0	0.332	0.421
3-day post-SCI group vs. the control group	*Gsr*	*Sirt1*	0	0.052	0	0.417	0.424
3-day post-SCI group vs. the control group	*Gsr*	*Ppargc1a*	0	0	0	0.432	0.432
3-day post-SCI group vs. the control group	*Gsr*	*Jun*	0	0.057	0	0.464	0.472
3-day post-SCI group vs. the control group	*Gsr*	*Actb*	0.059	0	0	0.529	0.538
3-day post-SCI group vs. the control group	*Gsr*	*Ptgs2*	0	0.16	0	0.497	0.559
3-day post-SCI group vs. the control group	*Gsr*	*Casp3*	0	0	0	0.594	0.594
3-day post-SCI group vs. the control group	*Gsr*	*Prdx6*	0.08	0.098	0	0.621	0.658
3-day post-SCI group vs. the control group	*Gsr*	*Nfe2l2*	0.062	0	0	0.718	0.724
3-day post-SCI group vs. the control group	*Gsr*	*Gstt2*	0.119	0.057	0.65	0.335	0.78
3-day post-SCI group vs. the control group	*Gsr*	*Srxn1*	0.282	0	0	0.734	0.801
3-day post-SCI group vs. the control group	*Gsr*	*Glrx2*	0.147	0.135	0	0.818	0.857
3-day post-SCI group vs. the control group	*Gsr*	*Gclc*	0.216	0	0	0.925	0.938
3-day post-SCI group vs. the control group	*Gsr*	*Gpx3*	0.064	0.105	0.9	0.805	0.981
3-day post-SCI group vs. the control group	*Gsr*	*Gpx1*	0.09	0.105	0.9	0.866	0.987
3-day post-SCI group vs. the control group	*Gstt2*	*Mgst1*	0.084	0	0.65	0.38	0.784
3-day post-SCI group vs. the control group	*Gstt2*	*Gpx8*	0.065	0	0.65	0.11	0.683
3-day post-SCI group vs. the control group	*Gstt2*	*Cyp1b1*	0	0	0.65	0.189	0.704
3-day post-SCI group vs. the control group	*Gstt2*	*Ggt7*	0	0	0.65	0.107	0.674
3-day post-SCI group vs. the control group	*Gstt2*	*Gpx7*	0.065	0	0.65	0.325	0.759
3-day post-SCI group vs. the control group	*Gstt2*	*Gsr*	0.119	0.057	0.65	0.335	0.78

3-day post-SCI group vs. the control group	*Gstt2*	*Gclc*	0.079	0.142	0	0.425	0.506
3-day post-SCI group vs. the control group	*Gstt2*	*Gpx1*	0.084	0	0.65	0.251	0.739
3-day post-SCI group vs. the control group	*Gstt2*	*Prdx6*	0.105	0.094	0.65	0.192	0.74
3-day post-SCI group vs. the control group	*Gstt2*	*Gpx3*	0.065	0	0.65	0.327	0.76
3-day post-SCI group vs. the control group	*Hao1*	*Cat*	0.145	0	0.9	0.754	0.977
3-day post-SCI group vs. the control group	*Hbegf*	*Hif1a*	0	0	0.9	0.307	0.927
3-day post-SCI group vs. the control group	*Hbegf*	*Fos*	0.065	0	0	0.422	0.436
3-day post-SCI group vs. the control group	*Hbegf*	*Jun*	0.063	0	0	0.409	0.423
3-day post-SCI group vs. the control group	*Hbegf*	*Itgb5*	0.051	0	0	0.418	0.424
3-day post-SCI group vs. the control group	*Hbegf*	*Stat6*	0.063	0	0.35	0.184	0.459
3-day post-SCI group vs. the control group	*Hbegf*	*Met*	0.061	0	0.147	0.401	0.478
3-day post-SCI group vs. the control group	*Hbegf*	*Mapk3*	0	0	0	0.498	0.497
3-day post-SCI group vs. the control group	*Hbegf*	*Ptgs2*	0.132	0	0	0.462	0.513
3-day post-SCI group vs. the control group	*Hbegf*	*P4hb*	0	0	0	0.515	0.515
3-day post-SCI group vs. the control group	*Hbegf*	*Adam9*	0.061	0	0	0.555	0.565
3-day post-SCI group vs. the control group	*Hbegf*	*Uba52*	0	0	0.6	0	0.6
3-day post-SCI group vs. the control group	*Hbegf*	*Jak2*	0	0	0.676	0.203	0.731
3-day post-SCI group vs. the control group	*Hbegf*	*Ptprk*	0.057	0	0.9	0.074	0.905
3-day post-SCI group vs. the control group	*Hbegf*	*Pxn*	0	0	0.9	0.121	0.908
3-day post-SCI group vs. the control group	*Hdac1*	*Mapk14*	0	0.131	0	0.404	0.46
3-day post-SCI group vs. the control group	*Hdac1*	*Cdc20*	0.159	0.163	0.133	0.212	0.455
3-day post-SCI group vs. the control group	*Hdac1*	*Cdk4*	0.089	0.104	0.585	0.524	0.817
3-day post-SCI group vs. the control group	*Hdac1*	*Mgst1*	0	0	0	0.449	0.449
3-day post-SCI group vs. the control group	*Hdac1*	*Cdk1*	0.139	0.104	0.93	0.624	0.977
3-day post-SCI group vs. the control group	*Hdac1*	*Hif1a*	0	0.446	0.168	0.961	0.981
3-day post-SCI group vs. the control group	*Hdac1*	*Fos*	0.048	0.164	0	0.67	0.715
3-day post-SCI group vs. the control group	*Hdac1*	*Ppif*	0.052	0.284	0.327	0.258	0.615
3-day post-SCI group vs. the control group	*Hdac1*	*Rb1*	0.064	0.852	0.913	0.784	0.997
3-day post-SCI group vs. the control group	*Hdac1*	*Myc*	0.061	0.401	0.381	0.901	0.961
3-day post-SCI group vs. the control group	*Hdac1*	*Aif1*	0.063	0.084	0	0.444	0.481
3-day post-SCI group vs. the control group	*Hdac1*	*Rela*	0.062	0.795	0.512	0.982	0.998
3-day post-SCI group vs. the control group	*Hdac1*	*Parp1*	0.063	0.342	0	0.554	0.701
3-day post-SCI group vs. the control group	*Hdac1*	*Ccna2*	0.129	0.099	0.915	0.325	0.949
3-day post-SCI group vs. the control group	*Hdac1*	*Nfkb1*	0.052	0.8	0.512	0.985	0.998
3-day post-SCI group vs. the control group	*Hdac1*	*Sfpq*	0.162	0.602	0	0.216	0.715
3-day post-SCI group vs. the control group	*Hdac1*	*Tnfrsf1a*	0	0	0.41	0.154	0.479

3-day post-SCI group vs. the control group	*Hdac1*	*Nono*	0.142	0.345	0	0.238	0.534
3-day post-SCI group vs. the control group	*Hdac1*	*Rbbp7*	0.224	0.813	0.956	0.981	0.999
3-day post-SCI group vs. the control group	*Hdac1*	*Ets1*	0.061	0.433	0.243	0.303	0.681
3-day post-SCI group vs. the control group	*Hdac1*	*Atr*	0.052	0.144	0.147	0.282	0.436
3-day post-SCI group vs. the control group	*Hdac1*	*Rxrb*	0.061	0.132	0.826	0.061	0.849
3-day post-SCI group vs. the control group	*Hdac1*	*Ppp1ca*	0.062	0.354	0	0.197	0.472
3-day post-SCI group vs. the control group	*Hdac1*	*Mapk3*	0	0.131	0	0.465	0.515
3-day post-SCI group vs. the control group	*Hdac1*	*Smad1*	0.062	0.221	0.301	0.245	0.563
3-day post-SCI group vs. the control group	*Hdac1*	*Rnf2*	0.062	0.099	0	0.468	0.512
3-day post-SCI group vs. the control group	*Hdac1*	*Apc*	0.061	0	0.9	0.064	0.904
3-day post-SCI group vs. the control group	*Hdac1*	*Rest*	0.068	0.206	0.82	0.827	0.974
3-day post-SCI group vs. the control group	*Hdac1*	*Ezh2*	0.088	0.845	0.548	0.979	0.998
3-day post-SCI group vs. the control group	*Hdac1*	*Stat6*	0.048	0.164	0.208	0.223	0.444
3-day post-SCI group vs. the control group	*Hdac1*	*Casp3*	0.061	0.052	0	0.563	0.577
3-day post-SCI group vs. the control group	*Hdac1*	*Ago1*	0.05	0.067	0	0.729	0.739
3-day post-SCI group vs. the control group	*Hdac1*	*Cdkn2c*	0.061	0.132	0.166	0.245	0.418
3-day post-SCI group vs. the control group	*Hdac1*	*Cdkn2b*	0.052	0.132	0.457	0.397	0.694
3-day post-SCI group vs. the control group	*Hdac1*	*Actb*	0.072	0.16	0	0.672	0.722
3-day post-SCI group vs. the control group	*Hdac1*	*Cttn*	0.062	0.134	0	0.345	0.421
3-day post-SCI group vs. the control group	*Hdac1*	*Ppargc1a*	0	0	0	0.416	0.416
3-day post-SCI group vs. the control group	*Hdac1*	*Banf1*	0.084	0.099	0	0.556	0.602
3-day post-SCI group vs. the control group	*Hdac1*	*Txnip*	0	0.342	0	0.459	0.629
3-day post-SCI group vs. the control group	*Hdac1*	*Bnip3*	0	0.099	0	0.69	0.709
3-day post-SCI group vs. the control group	*Hdac1*	*Jun*	0	0.261	0	0.787	0.836
3-day post-SCI group vs. the control group	*Hdac1*	*Tfdp1*	0.071	0.104	0.826	0.1	0.852
3-day post-SCI group vs. the control group	*Hdac1*	*Sirt1*	0.063	0.4	0.168	0.859	0.925
3-day post-SCI group vs. the control group	*Hdac1*	*Eed*	0.062	0.86	0.548	0.432	0.962
3-day post-SCI group vs. the control group	*Hif1a*	*Braf*	0	0.097	0.12	0.35	0.438
3-day post-SCI group vs. the control group	*Hif1a*	*Axl*	0	0.097	0.12	0.359	0.446
3-day post-SCI group vs. the control group	*Hif1a*	*Mapk14*	0	0.147	0	0.66	0.698
3-day post-SCI group vs. the control group	*Hif1a*	*Hspb1*	0	0.045	0	0.408	0.41
3-day post-SCI group vs. the control group	*Hif1a*	*Hmox1*	0	0	0	0.696	0.696
3-day post-SCI group vs. the control group	*Hif1a*	*Pdk1*	0	0.131	0	0.548	0.59
3-day post-SCI group vs. the control group	*Hif1a*	*Cdk4*	0	0.069	0	0.549	0.562
3-day post-SCI group vs. the control group	*Hif1a*	*Sod2*	0	0	0	0.625	0.625
3-day post-SCI group vs. the control group	*Hif1a*	*Ube2a*	0	0.046	0.629	0.077	0.645

3-day post-SCI group vs. the control group	*Hif1a*	*Cdk1*	0	0.132	0	0.505	0.552
3-day post-SCI group vs. the control group	*Hif1a*	*Arg1*	0	0	0	0.596	0.596
3-day post-SCI group vs. the control group	*Hif1a*	*Xdh*	0	0	0	0.406	0.406
3-day post-SCI group vs. the control group	*Hif1a*	*Ucp2*	0	0	0	0.429	0.429
3-day post-SCI group vs. the control group	*Hif1a*	*Txnip*	0	0.13	0	0.4	0.455
3-day post-SCI group vs. the control group	*Hif1a*	*Stat6*	0	0.102	0	0.42	0.456
3-day post-SCI group vs. the control group	*Hif1a*	*Pink1*	0	0	0	0.477	0.477
3-day post-SCI group vs. the control group	*Hif1a*	*Gpx1*	0	0	0	0.483	0.483
3-day post-SCI group vs. the control group	*Hif1a*	*Prkaa2*	0.052	0.043	0	0.479	0.486
3-day post-SCI group vs. the control group	*Hif1a*	*Mapk8*	0.052	0	0	0.488	0.494
3-day post-SCI group vs. the control group	*Hif1a*	*Ezh2*	0.088	0.058	0	0.499	0.531
3-day post-SCI group vs. the control group	*Hif1a*	*Fbxw7*	0	0.231	0	0.42	0.535
3-day post-SCI group vs. the control group	*Hif1a*	*Smad1*	0.061	0.147	0.301	0.27	0.537
3-day post-SCI group vs. the control group	*Hif1a*	*Vcam1*	0.064	0	0	0.538	0.549
3-day post-SCI group vs. the control group	*Hif1a*	*Junb*	0.052	0.151	0.375	0.222	0.556
3-day post-SCI group vs. the control group	*Hif1a*	*Nfkb1*	0.061	0.055	0.171	0.468	0.556
3-day post-SCI group vs. the control group	*Hif1a*	*Fos*	0	0.131	0	0.527	0.571
3-day post-SCI group vs. the control group	*Hif1a*	*Ets1*	0.061	0.074	0	0.563	0.587
3-day post-SCI group vs. the control group	*Hif1a*	*Uba52*	0	0	0.6	0	0.6
3-day post-SCI group vs. the control group	*Hif1a*	*Mcl1*	0.062	0.13	0	0.552	0.602
3-day post-SCI group vs. the control group	*Hif1a*	*Tlr4*	0.062	0.054	0	0.589	0.603
3-day post-SCI group vs. the control group	*Hif1a*	*Parp1*	0	0.13	0	0.595	0.633
3-day post-SCI group vs. the control group	*Hif1a*	*Nox4*	0	0	0	0.639	0.639
3-day post-SCI group vs. the control group	*Hif1a*	*Jak2*	0.141	0.097	0.12	0.537	0.642
3-day post-SCI group vs. the control group	*Hif1a*	*Cat*	0	0	0	0.651	0.651
3-day post-SCI group vs. the control group	*Hif1a*	*Nfe2l2*	0	0	0	0.658	0.658
3-day post-SCI group vs. the control group	*Hif1a*	*Met*	0	0.097	0.12	0.611	0.663
3-day post-SCI group vs. the control group	*Hif1a*	*Ptgs2*	0.061	0	0	0.665	0.672
3-day post-SCI group vs. the control group	*Hif1a*	*Cdkn2b*	0	0.141	0.457	0.377	0.684
3-day post-SCI group vs. the control group	*Hif1a*	*Ppargc1a*	0.048	0	0	0.689	0.691
3-day post-SCI group vs. the control group	*Hif1a*	*P4hb*	0	0	0.629	0.261	0.714
3-day post-SCI group vs. the control group	*Hif1a*	*Casp3*	0.049	0	0	0.721	0.723
3-day post-SCI group vs. the control group	*Hif1a*	*Actb*	0	0.045	0	0.747	0.748
3-day post-SCI group vs. the control group	*Hif1a*	*Bnip3*	0.079	0.13	0	0.799	0.825
3-day post-SCI group vs. the control group	*Hif1a*	*Rela*	0	0.13	0	0.848	0.862
3-day post-SCI group vs. the control group	*Hif1a*	*Hbegf*	0	0	0.9	0.307	0.927

3-day post-SCI group vs. the control group	*Hif1a*	*Sirt1*	0.062	0.227	0.457	0.839	0.928
3-day post-SCI group vs. the control group	*Hif1a*	*Mapk3*	0	0.289	0.8	0.714	0.955
3-day post-SCI group vs. the control group	*Hif1a*	*Jun*	0.051	0.402	0.629	0.846	0.963
3-day post-SCI group vs. the control group	*Hif1a*	*Myc*	0	0.508	0	0.939	0.968
3-day post-SCI group vs. the control group	*Hif1a*	*Hdac1*	0	0.446	0.168	0.961	0.981
3-day post-SCI group vs. the control group	*Hmox1*	*Mapk14*	0	0	0.305	0.7	0.782
3-day post-SCI group vs. the control group	*Hmox1*	*Hspb1*	0.083	0	0	0.532	0.553
3-day post-SCI group vs. the control group	*Hmox1*	*Ucp2*	0.069	0	0	0.411	0.428
3-day post-SCI group vs. the control group	*Hmox1*	*Alox5*	0	0	0	0.43	0.43
3-day post-SCI group vs. the control group	*Hmox1*	*Atf2*	0	0	0.313	0.213	0.436
3-day post-SCI group vs. the control group	*Hmox1*	*Myc*	0.088	0	0	0.42	0.449
3-day post-SCI group vs. the control group	*Hmox1*	*Rela*	0.062	0	0	0.464	0.475
3-day post-SCI group vs. the control group	*Hmox1*	*Junb*	0.09	0	0.39	0.143	0.482
3-day post-SCI group vs. the control group	*Hmox1*	*Cyp1b1*	0.162	0	0	0.41	0.485
3-day post-SCI group vs. the control group	*Hmox1*	*Txnip*	0.096	0	0	0.463	0.494
3-day post-SCI group vs. the control group	*Hmox1*	*Arg1*	0	0	0	0.5	0.499
3-day post-SCI group vs. the control group	*Hmox1*	*Snca*	0	0	0	0.499	0.499
3-day post-SCI group vs. the control group	*Hmox1*	*Fosl1*	0.104	0	0.313	0.258	0.504
3-day post-SCI group vs. the control group	*Hmox1*	*Bax*	0.084	0	0	0.493	0.516
3-day post-SCI group vs. the control group	*Hmox1*	*Aif1*	0	0.083	0	0.497	0.519
3-day post-SCI group vs. the control group	*Hmox1*	*Gpx3*	0.062	0.07	0	0.501	0.527
3-day post-SCI group vs. the control group	*Hmox1*	*Ncf1*	0	0	0	0.545	0.545
3-day post-SCI group vs. the control group	*Hmox1*	*Mapk8*	0	0	0	0.551	0.551
3-day post-SCI group vs. the control group	*Hmox1*	*Fos*	0	0	0.313	0.477	0.626
3-day post-SCI group vs. the control group	*Hmox1*	*Xdh*	0.068	0	0	0.619	0.629
3-day post-SCI group vs. the control group	*Hmox1*	*Vcam1*	0.061	0	0	0.628	0.636
3-day post-SCI group vs. the control group	*Hmox1*	*Gpx8*	0.062	0.07	0	0.65	0.668
3-day post-SCI group vs. the control group	*Hmox1*	*Sirt1*	0	0	0	0.678	0.678
3-day post-SCI group vs. the control group	*Hmox1*	*Nox4*	0	0	0	0.685	0.685
3-day post-SCI group vs. the control group	*Hmox1*	*Srxn1*	0.151	0	0	0.643	0.685
3-day post-SCI group vs. the control group	*Hmox1*	*Tlr4*	0.056	0	0	0.682	0.687
3-day post-SCI group vs. the control group	*Hmox1*	*Hif1a*	0	0	0	0.696	0.696
3-day post-SCI group vs. the control group	*Hmox1*	*Gpx7*	0	0.07	0	0.693	0.702
3-day post-SCI group vs. the control group	*Hmox1*	*Mapk3*	0	0	0.305	0.613	0.72
3-day post-SCI group vs. the control group	*Hmox1*	*Ppargc1a*	0	0	0	0.731	0.731
3-day post-SCI group vs. the control group	*Hmox1*	*Sod2*	0.064	0	0	0.715	0.732

3-day post-SCI group vs. the control group	*Hmox1*	*Txnrd1*	0.113	0.131	0	0.701	0.749
3-day post-SCI group vs. the control group	*Hmox1*	*Ptgs2*	0.085	0	0	0.746	0.757
3-day post-SCI group vs. the control group	*Hmox1*	*Gpx1*	0.066	0.07	0	0.746	0.76
3-day post-SCI group vs. the control group	*Hmox1*	*Casp3*	0	0	0	0.764	0.764
3-day post-SCI group vs. the control group	*Hmox1*	*Gsr*	0.105	0.088	0	0.744	0.773
3-day post-SCI group vs. the control group	*Hmox1*	*Actb*	0	0.056	0	0.777	0.78
3-day post-SCI group vs. the control group	*Hmox1*	*Jun*	0	0	0.39	0.713	0.817
3-day post-SCI group vs. the control group	*Hmox1*	*Gclc*	0	0.097	0	0.866	0.874
3-day post-SCI group vs. the control group	*Hmox1*	*Cat*	0.062	0.09	0	0.877	0.886
3-day post-SCI group vs. the control group	*Hmox1*	*Nfe2l2*	0.105	0	0.419	0.898	0.942
3-day post-SCI group vs. the control group	*Hspb1*	*Mapk14*	0	0.13	0.676	0.857	0.956
3-day post-SCI group vs. the control group	*Hspb1*	*Hif1a*	0	0.045	0	0.408	0.41
3-day post-SCI group vs. the control group	*Hspb1*	*Tlr4*	0	0.057	0	0.405	0.415
3-day post-SCI group vs. the control group	*Hspb1*	*Sod2*	0	0	0	0.428	0.428
3-day post-SCI group vs. the control group	*Hspb1*	*Arhgdia*	0.062	0.13	0	0.376	0.446
3-day post-SCI group vs. the control group	*Hspb1*	*Myc*	0	0.055	0	0.446	0.454
3-day post-SCI group vs. the control group	*Hspb1*	*Cat*	0	0	0	0.462	0.462
3-day post-SCI group vs. the control group	*Hspb1*	*Pxn*	0.061	0.202	0	0.346	0.467
3-day post-SCI group vs. the control group	*Hspb1*	*Map2k6*	0	0.052	0	0.464	0.47
3-day post-SCI group vs. the control group	*Hspb1*	*Mapk8*	0	0.092	0	0.474	0.502
3-day post-SCI group vs. the control group	*Hspb1*	*Prdx6*	0.051	0.101	0	0.481	0.518
3-day post-SCI group vs. the control group	*Hspb1*	*Map2k3*	0.067	0.057	0	0.528	0.548
3-day post-SCI group vs. the control group	*Hspb1*	*Hmox1*	0.083	0	0	0.532	0.553
3-day post-SCI group vs. the control group	*Hspb1*	*Jun*	0.062	0	0	0.588	0.597
3-day post-SCI group vs. the control group	*Hspb1*	*Mapt*	0.061	0.13	0	0.576	0.623
3-day post-SCI group vs. the control group	*Hspb1*	*Snca*	0.062	0.34	0	0.494	0.659
3-day post-SCI group vs. the control group	*Hspb1*	*Casp3*	0	0.13	0	0.681	0.711
3-day post-SCI group vs. the control group	*Hspb1*	*Mapk3*	0	0.132	0.291	0.585	0.722
3-day post-SCI group vs. the control group	*Hspb1*	*Actb*	0.062	0.087	0.4	0.584	0.757
3-day post-SCI group vs. the control group	*Hspb1*	*Uba52*	0	0	0.9	0.05	0.9
3-day post-SCI group vs. the control group	*Hspb1*	*Mapkapk3*	0.07	0.671	0.966	0.421	0.993
3-day post-SCI group vs. the control group	*Hspb1*	*Mapkapk2*	0.062	0.871	0.966	0.733	0.998
3-day post-SCI group vs. the control group	*Htra2*	*Mapk14*	0.061	0.228	0	0.256	0.413
3-day post-SCI group vs. the control group	*Htra2*	*Sod2*	0.063	0.325	0	0.396	0.585
3-day post-SCI group vs. the control group	*Htra2*	*Endog*	0.065	0	0	0.884	0.887
3-day post-SCI group vs. the control group	*Htra2*	*Ppif*	0.061	0.047	0	0.531	0.544

3-day post-SCI group vs. the control group	*Htra2*	*Pink1*	0.056	0.439	0.8	0.908	0.989
3-day post-SCI group vs. the control group	*Htra2*	*Tnfrsf1a*	0	0.402	0	0.297	0.562
3-day post-SCI group vs. the control group	*Htra2*	*Bax*	0.103	0	0	0.395	0.434
3-day post-SCI group vs. the control group	*Htra2*	*Atp13a2*	0.097	0	0	0.566	0.592
3-day post-SCI group vs. the control group	*Htra2*	*Mcl1*	0	0	0	0.474	0.474
3-day post-SCI group vs. the control group	*Htra2*	*Ripk1*	0.052	0	0	0.493	0.499
3-day post-SCI group vs. the control group	*Htra2*	*Mapt*	0	0.4	0	0.25	0.531
3-day post-SCI group vs. the control group	*Htra2*	*Snca*	0	0	0	0.558	0.558
3-day post-SCI group vs. the control group	*Htra2*	*Casp3*	0	0	0	0.63	0.63
3-day post-SCI group vs. the control group	*Id1*	*Cdc20*	0.089	0.206	0	0.628	0.707
3-day post-SCI group vs. the control group	*Id1*	*Rb1*	0	0.133	0.544	0.111	0.618
3-day post-SCI group vs. the control group	*Id1*	*Ets1*	0.061	0.049	0.65	0.247	0.733
3-day post-SCI group vs. the control group	*Id1*	*Smad1*	0	0	0	0.471	0.471
3-day post-SCI group vs. the control group	*Id1*	*Txnip*	0.066	0	0	0.509	0.521
3-day post-SCI group vs. the control group	*Ier3*	*Fosl1*	0.187	0	0	0.351	0.45
3-day post-SCI group vs. the control group	*Ier3*	*Fos*	0.159	0	0	0.399	0.472
3-day post-SCI group vs. the control group	*Ier3*	*Zc3h12a*	0.125	0.086	0	0.444	0.517
3-day post-SCI group vs. the control group	*Ier3*	*Mcl1*	0.061	0.079	0	0.586	0.612
3-day post-SCI group vs. the control group	*Ier3*	*Mapk3*	0	0.193	0.6	0.247	0.736
3-day post-SCI group vs. the control group	*Ier3*	*Rela*	0.071	0.13	0	0.836	0.856
3-day post-SCI group vs. the control group	*Il18rap*	*Rela*	0	0	0.676	0.087	0.691
3-day post-SCI group vs. the control group	*Il18rap*	*Stat6*	0.061	0	0.35	0.167	0.447
3-day post-SCI group vs. the control group	*Il18rap*	*Mapk8*	0	0	0.676	0.076	0.688
3-day post-SCI group vs. the control group	*Il18rap*	*Nfkb1*	0	0	0.676	0.112	0.7
3-day post-SCI group vs. the control group	*Il6st*	*Map2k3*	0	0	0.537	0.088	0.559
3-day post-SCI group vs. the control group	*Il6st*	*Map2k6*	0	0	0.676	0.056	0.681
3-day post-SCI group vs. the control group	*Il6st*	*Mapk3*	0	0	0.9	0.26	0.922
3-day post-SCI group vs. the control group	*Il6st*	*Jak2*	0.062	0.877	0.932	0.522	0.995
3-day post-SCI group vs. the control group	*Il6st*	*Stat6*	0.064	0.047	0.35	0.31	0.546
3-day post-SCI group vs. the control group	*Il6st*	*Ptprk*	0	0.089	0.358	0.161	0.466
3-day post-SCI group vs. the control group	*Impact*	*Cdk1*	0	0.447	0	0	0.447
3-day post-SCI group vs. the control group	*Impact*	*P4hb*	0.065	0.685	0	0	0.694
3-day post-SCI group vs. the control group	*Itga6*	*Sdc1*	0.062	0	0.729	0.23	0.787
3-day post-SCI group vs. the control group	*Itga6*	*Myc*	0.061	0	0	0.431	0.442
3-day post-SCI group vs. the control group	*Itga6*	*Cttn*	0	0	0.334	0.172	0.424
3-day post-SCI group vs. the control group	*Itga6*	*Mapk3*	0.063	0.058	0.238	0.278	0.449

3-day post-SCI group vs. the control group	*Itga6*	*Ctnna1*	0.079	0.049	0.248	0.309	0.484
3-day post-SCI group vs. the control group	*Itga6*	*Cd38*	0	0	0	0.533	0.533
3-day post-SCI group vs. the control group	*Itga6*	*Vcam1*	0.051	0.058	0.352	0.384	0.595
3-day post-SCI group vs. the control group	*Itga6*	*Pdgfra*	0	0.057	0.352	0.392	0.596
3-day post-SCI group vs. the control group	*Itga6*	*Casp3*	0	0	0.676	0.366	0.785
3-day post-SCI group vs. the control group	*Itga6*	*Dst*	0.061	0.071	0.639	0.406	0.788
3-day post-SCI group vs. the control group	*Itga6*	*Met*	0.063	0.133	0.676	0.402	0.821
3-day post-SCI group vs. the control group	*Itga6*	*Actb*	0.061	0.044	0.672	0.472	0.824
3-day post-SCI group vs. the control group	*Itga6*	*Pxn*	0.081	0.16	0.711	0.416	0.852
3-day post-SCI group vs. the control group	*Itga6*	*Itgb5*	0.078	0.214	0.864	0.558	0.95
3-day post-SCI group vs. the control group	*Itgb5*	*Sdc1*	0.109	0	0.864	0.381	0.919
3-day post-SCI group vs. the control group	*Itgb5*	*Mylk*	0.061	0.089	0.756	0.125	0.793
3-day post-SCI group vs. the control group	*Itgb5*	*Hbegf*	0.051	0	0	0.418	0.424
3-day post-SCI group vs. the control group	*Itgb5*	*P4hb*	0.07	0.083	0	0.454	0.493
3-day post-SCI group vs. the control group	*Itgb5*	*Itga6*	0.078	0.214	0.864	0.558	0.95
3-day post-SCI group vs. the control group	*Itgb5*	*Vcam1*	0.073	0.056	0.317	0.222	0.472
3-day post-SCI group vs. the control group	*Itgb5*	*Ctnna1*	0.087	0.13	0.313	0.228	0.523
3-day post-SCI group vs. the control group	*Itgb5*	*Mapk3*	0.079	0	0.176	0.276	0.403
3-day post-SCI group vs. the control group	*Itgb5*	*Ezr*	0	0.048	0.327	0.195	0.439
3-day post-SCI group vs. the control group	*Itgb5*	*Pdlim1*	0	0.164	0.313	0.105	0.441
3-day post-SCI group vs. the control group	*Itgb5*	*Dst*	0.062	0.255	0.167	0.096	0.403
3-day post-SCI group vs. the control group	*Itgb5*	*Adam9*	0.099	0.13	0.167	0.256	0.449
3-day post-SCI group vs. the control group	*Itgb5*	*Cttn*	0.126	0	0.383	0.134	0.492
3-day post-SCI group vs. the control group	*Itgb5*	*Met*	0.069	0.133	0.388	0.132	0.513
3-day post-SCI group vs. the control group	*Itgb5*	*Cd36*	0.062	0.057	0.5	0.074	0.535
3-day post-SCI group vs. the control group	*Itgb5*	*Actb*	0	0.065	0.687	0.363	0.797
3-day post-SCI group vs. the control group	*Itgb5*	*Pxn*	0.079	0.164	0.856	0.467	0.933
3-day post-SCI group vs. the control group	*Jak2*	*Braf*	0.052	0.27	0.3	0.295	0.533
3-day post-SCI group vs. the control group	*Jak2*	*Axl*	0.058	0	0.8	0.305	0.826
3-day post-SCI group vs. the control group	*Jak2*	*Mapk14*	0.062	0.147	0.42	0.633	0.617
3-day post-SCI group vs. the control group	*Jak2*	*Cdk4*	0.062	0.198	0.915	0.482	0.942
3-day post-SCI group vs. the control group	*Jak2*	*Map2k3*	0	0.084	0.376	0.226	0.45
3-day post-SCI group vs. the control group	*Jak2*	*Arg1*	0.062	0.046	0	0.392	0.408
3-day post-SCI group vs. the control group	*Jak2*	*Map2k6*	0	0.084	0.376	0.181	0.438
3-day post-SCI group vs. the control group	*Jak2*	*Hif1a*	0.141	0.097	0.12	0.537	0.642
3-day post-SCI group vs. the control group	*Jak2*	*Fos*	0	0.07	0.15	0.472	0.547

3-day post-SCI group vs. the control group	*Jak2*	*Myc*	0.051	0.086	0.129	0.607	0.664
3-day post-SCI group vs. the control group	*Jak2*	*Hbegf*	0	0	0.676	0.203	0.731
3-day post-SCI group vs. the control group	*Jak2*	*Rela*	0	0.064	0.676	0.399	0.801
3-day post-SCI group vs. the control group	*Jak2*	*Ccr1*	0.061	0.141	0.63	0.195	0.727
3-day post-SCI group vs. the control group	*Jak2*	*Cat*	0	0.065	0.183	0.34	0.453
3-day post-SCI group vs. the control group	*Jak2*	*Vcam1*	0.061	0	0.217	0.397	0.517
3-day post-SCI group vs. the control group	*Jak2*	*Nfkb1*	0.147	0.132	0.676	0.454	0.851
3-day post-SCI group vs. the control group	*Jak2*	*Tnfrsf1a*	0.051	0.227	0.137	0.662	0.757
3-day post-SCI group vs. the control group	*Jak2*	*Btk*	0.062	0.146	0.676	0.468	0.764
3-day post-SCI group vs. the control group	*Jak2*	*Ets1*	0.048	0.072	0.515	0.248	0.635
3-day post-SCI group vs. the control group	*Jak2*	*Ptgs2*	0.095	0.088	0	0.464	0.518
3-day post-SCI group vs. the control group	*Jak2*	*Mcl1*	0.061	0.056	0.19	0.61	0.682
3-day post-SCI group vs. the control group	*Jak2*	*Tlr4*	0.062	0.089	0.082	0.583	0.63
3-day post-SCI group vs. the control group	*Jak2*	*Mapk3*	0.066	0.147	0.939	0.707	0.962
3-day post-SCI group vs. the control group	*Jak2*	*Ezr*	0	0.089	0.22	0.307	0.464
3-day post-SCI group vs. the control group	*Jak2*	*Sirt1*	0.062	0.085	0	0.358	0.401
3-day post-SCI group vs. the control group	*Jak2*	*Cdkn2b*	0	0.132	0.127	0.299	0.422
3-day post-SCI group vs. the control group	*Jak2*	*Mapk10*	0.063	0.139	0.42	0.24	0.536
3-day post-SCI group vs. the control group	*Jak2*	*Mapk8*	0.063	0.139	0.42	0.498	0.594
3-day post-SCI group vs. the control group	*Jak2*	*Ezh2*	0	0.13	0	0.581	0.62
3-day post-SCI group vs. the control group	*Jak2*	*Sirpa*	0.061	0.44	0	0.339	0.622
3-day post-SCI group vs. the control group	*Jak2*	*Casp3*	0.062	0.077	0.114	0.643	0.689
3-day post-SCI group vs. the control group	*Jak2*	*Jun*	0.05	0.136	0.18	0.613	0.705
3-day post-SCI group vs. the control group	*Jak2*	*Actb*	0.052	0.084	0.195	0.665	0.734
3-day post-SCI group vs. the control group	*Jak2*	*Pxn*	0.081	0.063	0.676	0.25	0.763
3-day post-SCI group vs. the control group	*Jak2*	*Chrna4*	0.062	0.053	0.831	0.073	0.842
3-day post-SCI group vs. the control group	*Jak2*	*Met*	0.065	0.074	0.858	0.434	0.89
3-day post-SCI group vs. the control group	*Jak2*	*Mark3*	0	0.087	0.9	0	0.904
3-day post-SCI group vs. the control group	*Jak2*	*Pdgfra*	0.062	0.132	0.891	0.507	0.924
3-day post-SCI group vs. the control group	*Jak2*	*Stat6*	0.055	0.147	0.932	0.837	0.989
3-day post-SCI group vs. the control group	*Jak2*	*Il6st*	0.062	0.877	0.932	0.522	0.995
3-day post-SCI group vs. the control group	*Jun*	*Braf*	0	0.137	0.127	0.433	0.535
3-day post-SCI group vs. the control group	*Jun*	*Axl*	0.062	0.09	0.127	0.327	0.431
3-day post-SCI group vs. the control group	*Jun*	*Mapk14*	0.049	0.4	0.932	0.903	0.995
3-day post-SCI group vs. the control group	*Jun*	*Hspb1*	0.062	0	0	0.588	0.597
3-day post-SCI group vs. the control group	*Jun*	*Hmox1*	0	0	0.39	0.713	0.817

3-day post-SCI group vs. the control group	*Jun*	*Cdk4*	0.061	0.164	0	0.63	0.684
3-day post-SCI group vs. the control group	*Jun*	*Sod2*	0	0	0	0.556	0.556
3-day post-SCI group vs. the control group	*Jun*	*Mgst1*	0	0	0	0.675	0.675
3-day post-SCI group vs. the control group	*Jun*	*Endog*	0	0	0	0.406	0.406
3-day post-SCI group vs. the control group	*Jun*	*Ncf1*	0	0	0	0.466	0.465
3-day post-SCI group vs. the control group	*Jun*	*Mapkapk2*	0.081	0.056	0	0.527	0.555
3-day post-SCI group vs. the control group	*Jun*	*Map2k3*	0	0	0	0.721	0.721
3-day post-SCI group vs. the control group	*Jun*	*Cdk1*	0.051	0.164	0	0.581	0.638
3-day post-SCI group vs. the control group	*Jun*	*Arg1*	0.062	0	0	0.403	0.416
3-day post-SCI group vs. the control group	*Jun*	*Map2k6*	0	0	0	0.592	0.592
3-day post-SCI group vs. the control group	*Jun*	*Hif1a*	0.051	0.402	0.629	0.846	0.963
3-day post-SCI group vs. the control group	*Jun*	*Fos*	0.662	0.982	0.932	0.995	0.999
3-day post-SCI group vs. the control group	*Jun*	*Rb1*	0	0.227	0.676	0.344	0.821
3-day post-SCI group vs. the control group	*Jun*	*Myc*	0.062	0.27	0.548	0.856	0.949
3-day post-SCI group vs. the control group	*Jun*	*Xdh*	0	0	0	0.583	0.583
3-day post-SCI group vs. the control group	*Jun*	*Aif1*	0	0	0	0.403	0.403
3-day post-SCI group vs. the control group	*Jun*	*Hbegf*	0.063	0	0	0.409	0.423
3-day post-SCI group vs. the control group	*Jun*	*Fosl1*	0.066	0.784	0.932	0.989	0.999
3-day post-SCI group vs. the control group	*Jun*	*Rela*	0.061	0.46	0.629	0.859	0.97
3-day post-SCI group vs. the control group	*Jun*	*Ccr1*	0	0.046	0.483	0.254	0.599
3-day post-SCI group vs. the control group	*Jun*	*Parp1*	0.064	0.135	0	0.57	0.622
3-day post-SCI group vs. the control group	*Jun*	*Cat*	0	0	0	0.682	0.682
3-day post-SCI group vs. the control group	*Jun*	*Ccna2*	0	0.044	0	0.429	0.431
3-day post-SCI group vs. the control group	*Jun*	*Vcam1*	0	0	0	0.589	0.589
3-day post-SCI group vs. the control group	*Jun*	*Nfkb1*	0.063	0.047	0.204	0.725	0.778
3-day post-SCI group vs. the control group	*Jun*	*Tnfrsf1a*	0.069	0	0	0.619	0.63
3-day post-SCI group vs. the control group	*Jun*	*Nox4*	0.061	0.058	0	0.542	0.56
3-day post-SCI group vs. the control group	*Jun*	*Bax*	0	0	0	0.529	0.529
3-day post-SCI group vs. the control group	*Jun*	*Btk*	0	0.136	0.676	0.442	0.83
3-day post-SCI group vs. the control group	*Jun*	*Brf2*	0	0.118	0.311	0.113	0.413
3-day post-SCI group vs. the control group	*Jun*	*Gsr*	0	0.057	0	0.464	0.472
3-day post-SCI group vs. the control group	*Jun*	*Ets1*	0.061	0.456	0.629	0.69	0.933
3-day post-SCI group vs. the control group	*Jun*	*Gclc*	0	0	0	0.471	0.471
3-day post-SCI group vs. the control group	*Jun*	*Ptgs2*	0.061	0	0	0.738	0.743
3-day post-SCI group vs. the control group	*Jun*	*Map2k4*	0	0.13	0	0.827	0.843
3-day post-SCI group vs. the control group	*Jun*	*Melk*	0	0.145	0	0.709	0.741

3-day post-SCI group vs. the control group	*Jun*	*Mcl1*	0.063	0	0	0.62	0.628
3-day post-SCI group vs. the control group	*Jun*	*Tlr4*	0	0	0	0.738	0.738
3-day post-SCI group vs. the control group	*Jun*	*Mapk3*	0	0.686	0.932	0.863	0.996
3-day post-SCI group vs. the control group	*Jun*	*Tlr6*	0	0	0	0.402	0.402
3-day post-SCI group vs. the control group	*Jun*	*Arhgdia*	0.061	0.057	0.492	0.252	0.618
3-day post-SCI group vs. the control group	*Jun*	*Jak2*	0.05	0.136	0.18	0.613	0.705
3-day post-SCI group vs. the control group	*Jun*	*Junb*	0.281	0.367	0.932	0.953	0.969
3-day post-SCI group vs. the control group	*Jun*	*Smad1*	0	0.211	0.388	0.43	0.701
3-day post-SCI group vs. the control group	*Jun*	*Apc*	0	0.276	0	0.455	0.589
3-day post-SCI group vs. the control group	*Jun*	*Ezh2*	0	0.047	0	0.4	0.403
3-day post-SCI group vs. the control group	*Jun*	*Gpx1*	0.051	0.045	0	0.413	0.422
3-day post-SCI group vs. the control group	*Jun*	*Pxn*	0	0	0	0.581	0.581
3-day post-SCI group vs. the control group	*Jun*	*Stat6*	0	0.134	0.216	0.552	0.669
3-day post-SCI group vs. the control group	*Jun*	*Casp3*	0	0	0	0.836	0.836
3-day post-SCI group vs. the control group	*Jun*	*Cdkn2b*	0	0.047	0.204	0.41	0.513
3-day post-SCI group vs. the control group	*Jun*	*Actb*	0	0.059	0.629	0.815	0.929
3-day post-SCI group vs. the control group	*Jun*	*Hdac1*	0	0.261	0	0.787	0.836
3-day post-SCI group vs. the control group	*Jun*	*Nfe2l2*	0	0.131	0.362	0.745	0.846
3-day post-SCI group vs. the control group	*Jun*	*Sirt1*	0	0.717	0	0.794	0.939
3-day post-SCI group vs. the control group	*Jun*	*Txnip*	0.083	0	0	0.43	0.455
3-day post-SCI group vs. the control group	*Jun*	*Snca*	0	0	0	0.4	0.4
3-day post-SCI group vs. the control group	*Jun*	*Tnik*	0	0.265	0	0.257	0.43
3-day post-SCI group vs. the control group	*Jun*	*Pdgfra*	0	0.073	0.199	0.311	0.444
3-day post-SCI group vs. the control group	*Jun*	*Oxr1*	0	0	0	0.446	0.447
3-day post-SCI group vs. the control group	*Jun*	*Ripk1*	0	0.09	0.127	0.468	0.54
3-day post-SCI group vs. the control group	*Jun*	*Ppargc1a*	0	0	0	0.542	0.542
3-day post-SCI group vs. the control group	*Jun*	*Map4k4*	0	0.265	0	0.436	0.568
3-day post-SCI group vs. the control group	*Jun*	*Met*	0	0.09	0.127	0.528	0.592
3-day post-SCI group vs. the control group	*Jun*	*Uba52*	0	0.757	0	0.043	0.757
3-day post-SCI group vs. the control group	*Jun*	*Nfatc1*	0.058	0.132	0.676	0.88	0.964
3-day post-SCI group vs. the control group	*Jun*	*Fbxw7*	0	0.762	0	0.901	0.975
3-day post-SCI group vs. the control group	*Jun*	*Atf2*	0.066	0.943	0.932	0.989	0.997
3-day post-SCI group vs. the control group	*Jun*	*Mapk10*	0	0.778	0.966	0.822	0.998
3-day post-SCI group vs. the control group	*Jun*	*Mapk8*	0	0.999	0.966	0.947	0.999
3-day post-SCI group vs. the control group	*Junb*	*Mapk14*	0.051	0.261	0.693	0.356	0.842
3-day post-SCI group vs. the control group	*Junb*	*Hmox1*	0.09	0	0.39	0.143	0.482

3-day post-SCI group vs. the control group	*Junb*	*Hif1a*	0.052	0.151	0.375	0.222	0.556
3-day post-SCI group vs. the control group	*Junb*	*Fos*	0.822	0.804	0.932	0.886	0.999
3-day post-SCI group vs. the control group	*Junb*	*Rb1*	0	0.133	0.316	0.264	0.525
3-day post-SCI group vs. the control group	*Junb*	*Myc*	0.08	0.135	0.318	0.387	0.623
3-day post-SCI group vs. the control group	*Junb*	*Fosl1*	0.092	0.642	0.932	0.838	0.995
3-day post-SCI group vs. the control group	*Junb*	*Rela*	0.113	0.058	0.477	0.258	0.633
3-day post-SCI group vs. the control group	*Junb*	*Nfkb1*	0.096	0.047	0.204	0.284	0.443
3-day post-SCI group vs. the control group	*Junb*	*Brf2*	0	0.118	0.311	0.113	0.413
3-day post-SCI group vs. the control group	*Junb*	*Ets1*	0.07	0.2	0.261	0.225	0.517
3-day post-SCI group vs. the control group	*Junb*	*Mapk3*	0	0.261	0.264	0.297	0.584
3-day post-SCI group vs. the control group	*Junb*	*Arhgdia*	0	0.047	0.411	0	0.414
3-day post-SCI group vs. the control group	*Junb*	*Smad1*	0	0.101	0.213	0.256	0.428
3-day post-SCI group vs. the control group	*Junb*	*Uba52*	0	0.465	0	0	0.465
3-day post-SCI group vs. the control group	*Junb*	*Actb*	0	0.059	0.29	0.285	0.48
3-day post-SCI group vs. the control group	*Junb*	*Stat6*	0.081	0.134	0.216	0.303	0.507
3-day post-SCI group vs. the control group	*Junb*	*Nfe2l2*	0.069	0.131	0.362	0.197	0.53
3-day post-SCI group vs. the control group	*Junb*	*Nfatc1*	0.078	0.087	0.676	0.213	0.756
3-day post-SCI group vs. the control group	*Junb*	*Mapk10*	0.061	0.329	0.725	0.246	0.852
3-day post-SCI group vs. the control group	*Junb*	*Mapk8*	0.061	0.329	0.725	0.33	0.868
3-day post-SCI group vs. the control group	*Junb*	*Atf2*	0.066	0.702	0.676	0.454	0.919
3-day post-SCI group vs. the control group	*Junb*	*Jun*	0.281	0.367	0.932	0.953	0.969
3-day post-SCI group vs. the control group	*Krt1*	*Eed*	0	0.433	0	0.082	0.457
3-day post-SCI group vs. the control group	*Lancl1*	*Map2k6*	0.062	0	0	0.486	0.497
3-day post-SCI group vs. the control group	*Lancl1*	*Syp*	0.067	0.698	0	0	0.706
3-day post-SCI group vs. the control group	*Lpo*	*Cat*	0	0.203	0	0.351	0.46
3-day post-SCI group vs. the control group	*Maoa*	*Chrna4*	0.061	0	0	0.4	0.412
3-day post-SCI group vs. the control group	*Maoa*	*Cat*	0.062	0	0	0.431	0.443
3-day post-SCI group vs. the control group	*Maoa*	*Snca*	0	0	0	0.586	0.587
3-day post-SCI group vs. the control group	*Maoa*	*Sirt1*	0.064	0.433	0	0.366	0.634
3-day post-SCI group vs. the control group	*Map2k3*	*Braf*	0.061	0.084	0.676	0.26	0.724
3-day post-SCI group vs. the control group	*Map2k3*	*Mapk14*	0.065	0.839	0.966	0.961	0.996
3-day post-SCI group vs. the control group	*Map2k3*	*Hspb1*	0.067	0.057	0	0.528	0.548
3-day post-SCI group vs. the control group	*Map2k3*	*Mapkapk2*	0.119	0.09	0.9	0.701	0.943
3-day post-SCI group vs. the control group	*Map2k3*	*Casp3*	0.052	0	0	0.399	0.405
3-day post-SCI group vs. the control group	*Map2k3*	*Ripk3*	0.101	0.132	0.293	0.082	0.41
3-day post-SCI group vs. the control group	*Map2k3*	*Eed*	0	0.433	0	0	0.433

3-day post-SCI group vs. the control group	*Map2k3*	*Fos*	0	0	0	0.435	0.435
3-day post-SCI group vs. the control group	*Map2k3*	*Actb*	0.052	0	0	0.433	0.439
3-day post-SCI group vs. the control group	*Map2k3*	*Myc*	0.079	0.209	0	0.306	0.45
3-day post-SCI group vs. the control group	*Map2k3*	*Jak2*	0	0.084	0.376	0.226	0.45
3-day post-SCI group vs. the control group	*Map2k3*	*Rela*	0.079	0.241	0	0.277	0.45
3-day post-SCI group vs. the control group	*Map2k3*	*Tlr4*	0	0.09	0	0.457	0.485
3-day post-SCI group vs. the control group	*Map2k3*	*Gnb2*	0	0	0.479	0.071	0.495
3-day post-SCI group vs. the control group	*Map2k3*	*Il6st*	0	0	0.537	0.088	0.559
3-day post-SCI group vs. the control group	*Map2k3*	*Atf2*	0	0	0	0.703	0.703
3-day post-SCI group vs. the control group	*Map2k3*	*Jun*	0	0	0	0.721	0.721
3-day post-SCI group vs. the control group	*Map2k3*	*Tnfrsf1a*	0.095	0	0.676	0.245	0.759
3-day post-SCI group vs. the control group	*Map2k3*	*Map2k4*	0	0.048	0.8	0.829	0.814
3-day post-SCI group vs. the control group	*Map2k3*	*Mapk10*	0.062	0.135	0.891	0.608	0.935
3-day post-SCI group vs. the control group	*Map2k3*	*Mapkapk3*	0.109	0.081	0.9	0.452	0.935
3-day post-SCI group vs. the control group	*Map2k3*	*Mapk3*	0.068	0.401	0.859	0.587	0.936
3-day post-SCI group vs. the control group	*Map2k3*	*Ripk1*	0.087	0.132	0.932	0.221	0.945
3-day post-SCI group vs. the control group	*Map2k3*	*Mapk8*	0.062	0.135	0.922	0.75	0.958
3-day post-SCI group vs. the control group	*Map2k3*	*Map2k6*	0	0.56	0.966	0.86	0.984
3-day post-SCI group vs. the control group	*Map2k4*	*Mapk14*	0.062	0.402	0.932	0.75	0.973
3-day post-SCI group vs. the control group	*Map2k4*	*Mapkapk2*	0.051	0.14	0	0.617	0.4
3-day post-SCI group vs. the control group	*Map2k4*	*Map2k3*	0	0.048	0.8	0.829	0.814
3-day post-SCI group vs. the control group	*Map2k4*	*Map2k6*	0	0.373	0.8	0.683	0.875
3-day post-SCI group vs. the control group	*Map2k4*	*Ripk3*	0	0.106	0.341	0.163	0.419
3-day post-SCI group vs. the control group	*Map2k4*	*Tnfrsf1a*	0	0	0.629	0.301	0.729
3-day post-SCI group vs. the control group	*Map2k4*	*Mapkapk3*	0.051	0.14	0	0.574	0.406
3-day post-SCI group vs. the control group	*Map2k4*	*Stk24*	0	0.077	0.486	0.284	0.573
3-day post-SCI group vs. the control group	*Map2k4*	*Mapk3*	0.062	0.197	0.372	0.504	0.585
3-day post-SCI group vs. the control group	*Map2k4*	*Casp3*	0	0.042	0.334	0.441	0.612
3-day post-SCI group vs. the control group	*Map2k4*	*Atf2*	0.094	0	0	0.594	0.616
3-day post-SCI group vs. the control group	*Map2k4*	*Ripk1*	0	0.106	0.629	0.242	0.682
3-day post-SCI group vs. the control group	*Map2k4*	*Jun*	0	0.13	0	0.827	0.843
3-day post-SCI group vs. the control group	*Map2k4*	*Mapk8*	0.105	0.821	0.966	0.863	0.996
3-day post-SCI group vs. the control group	*Map2k4*	*Mapk10*	0.105	0.867	0.966	0.912	0.997
3-day post-SCI group vs. the control group	*Map2k6*	*Braf*	0.061	0.084	0.676	0.22	0.72
3-day post-SCI group vs. the control group	*Map2k6*	*Mapk14*	0.065	0.848	0.966	0.864	0.996
3-day post-SCI group vs. the control group	*Map2k6*	*Hspb1*	0	0.052	0	0.464	0.47

3-day post-SCI group vs. the control group	*Map2k6*	*Mapkapk2*	0.063	0.13	0.9	0.603	0.94
3-day post-SCI group vs. the control group	*Map2k6*	*Map2k3*	0	0.56	0.966	0.86	0.984
3-day post-SCI group vs. the control group	*Map2k6*	*Jak2*	0	0.084	0.376	0.181	0.438
3-day post-SCI group vs. the control group	*Map2k6*	*Rela*	0.05	0.311	0	0.24	0.459
3-day post-SCI group vs. the control group	*Map2k6*	*Lancl1*	0.062	0	0	0.486	0.497
3-day post-SCI group vs. the control group	*Map2k6*	*Atf2*	0	0	0	0.584	0.584
3-day post-SCI group vs. the control group	*Map2k6*	*Jun*	0	0	0	0.592	0.592
3-day post-SCI group vs. the control group	*Map2k6*	*Il6st*	0	0	0.676	0.056	0.681
3-day post-SCI group vs. the control group	*Map2k6*	*Tnfrsf1a*	0	0	0.629	0.235	0.704
3-day post-SCI group vs. the control group	*Map2k6*	*Map2k4*	0	0.373	0.8	0.683	0.875
3-day post-SCI group vs. the control group	*Map2k6*	*Mapkapk3*	0.063	0.081	0.9	0.328	0.928
3-day post-SCI group vs. the control group	*Map2k6*	*Mapk10*	0.062	0.135	0.891	0.527	0.933
3-day post-SCI group vs. the control group	*Map2k6*	*Ripk1*	0.061	0.132	0.922	0.155	0.934
3-day post-SCI group vs. the control group	*Map2k6*	*Mapk3*	0.068	0.401	0.859	0.506	0.936
3-day post-SCI group vs. the control group	*Map2k6*	*Mapk8*	0.062	0.135	0.932	0.649	0.961
3-day post-SCI group vs. the control group	*Map4k4*	*Tnfrsf1a*	0	0	0.676	0.132	0.706
3-day post-SCI group vs. the control group	*Map4k4*	*Tlr4*	0	0.049	0.629	0.172	0.682
3-day post-SCI group vs. the control group	*Map4k4*	*Ezr*	0.048	0.137	0	0.469	0.526
3-day post-SCI group vs. the control group	*Map4k4*	*Stk24*	0	0.065	0.419	0.328	0.46
3-day post-SCI group vs. the control group	*Map4k4*	*Jun*	0	0.265	0	0.436	0.568
3-day post-SCI group vs. the control group	*Map4k4*	*Tank*	0	0	0.629	0.052	0.633
3-day post-SCI group vs. the control group	*Map4k4*	*Ripk1*	0	0.142	0.676	0.105	0.718
3-day post-SCI group vs. the control group	*Mapk10*	*Mapk14*	0.066	0.145	0.702	0.721	0.762
3-day post-SCI group vs. the control group	*Mapk10*	*Mapkapk2*	0	0.224	0.65	0.645	0.814
3-day post-SCI group vs. the control group	*Mapk10*	*Map2k3*	0.062	0.135	0.891	0.608	0.935
3-day post-SCI group vs. the control group	*Mapk10*	*Map2k6*	0.062	0.135	0.891	0.527	0.933
3-day post-SCI group vs. the control group	*Mapk10*	*Fos*	0	0.309	0.903	0.492	0.963
3-day post-SCI group vs. the control group	*Mapk10*	*Fosl1*	0	0.309	0.696	0.216	0.821
3-day post-SCI group vs. the control group	*Mapk10*	*Rela*	0	0.052	0.8	0.184	0.831
3-day post-SCI group vs. the control group	*Mapk10*	*Nfkb1*	0.051	0.058	0.8	0.278	0.853
3-day post-SCI group vs. the control group	*Mapk10*	*Gnb2*	0.062	0	0.6	0	0.608
3-day post-SCI group vs. the control group	*Mapk10*	*Btk*	0.052	0.139	0.537	0.116	0.603
3-day post-SCI group vs. the control group	*Mapk10*	*Mapkapk3*	0	0.224	0.65	0.293	0.781
3-day post-SCI group vs. the control group	*Mapk10*	*Rxrb*	0	0.047	0.42	0.049	0.428
3-day post-SCI group vs. the control group	*Mapk10*	*Map2k4*	0.105	0.867	0.966	0.912	0.997
3-day post-SCI group vs. the control group	*Mapk10*	*Mcl1*	0	0.13	0.272	0.235	0.473

3-day post-SCI group vs. the control group	*Mapk10*	*Mapk3*	0.08	0.059	0.728	0.542	0.768
3-day post-SCI group vs. the control group	*Mapk10*	*Ezr*	0.062	0.368	0	0.071	0.401
3-day post-SCI group vs. the control group	*Mapk10*	*Jak2*	0.063	0.139	0.42	0.24	0.536
3-day post-SCI group vs. the control group	*Mapk10*	*Junb*	0.061	0.329	0.725	0.246	0.852
3-day post-SCI group vs. the control group	*Mapk10*	*Pxn*	0	0.355	0	0.202	0.463
3-day post-SCI group vs. the control group	*Mapk10*	*Casp3*	0.052	0.09	0	0.453	0.487
3-day post-SCI group vs. the control group	*Mapk10*	*Cdkn2c*	0	0.705	0	0.05	0.708
3-day post-SCI group vs. the control group	*Mapk10*	*Mapt*	0.216	0.182	0.5	0.248	0.727
3-day post-SCI group vs. the control group	*Mapk10*	*Sirt1*	0.065	0.201	0	0.332	0.457
3-day post-SCI group vs. the control group	*Mapk10*	*Jun*	0	0.778	0.966	0.822	0.998
3-day post-SCI group vs. the control group	*Mapk10*	*Mapk8*	0.062	0	0.932	0.884	0.935
3-day post-SCI group vs. the control group	*Mapk10*	*Atf2*	0.051	0.456	0.961	0.492	0.988
3-day post-SCI group vs. the control group	*Mapk10*	*Met*	0.062	0.132	0.537	0.141	0.609
3-day post-SCI group vs. the control group	*Mapk10*	*Nfatc1*	0	0	0.903	0.087	0.908
3-day post-SCI group vs. the control group	*Mapk14*	*Capns1*	0	0.134	0.34	0.158	0.476
3-day post-SCI group vs. the control group	*Mapk14*	*Braf*	0.064	0.272	0.305	0.495	0.58
3-day post-SCI group vs. the control group	*Mapk14*	*Arg1*	0	0	0	0.4	0.4
3-day post-SCI group vs. the control group	*Mapk14*	*Gclc*	0.057	0	0	0.395	0.405
3-day post-SCI group vs. the control group	*Mapk14*	*Btk*	0	0.147	0.187	0.473	0.407
3-day post-SCI group vs. the control group	*Mapk14*	*Tlr6*	0	0.048	0	0.405	0.409
3-day post-SCI group vs. the control group	*Mapk14*	*Mylk*	0	0.105	0.335	0.164	0.41
3-day post-SCI group vs. the control group	*Mapk14*	*Htra2*	0.061	0.228	0	0.256	0.413
3-day post-SCI group vs. the control group	*Mapk14*	*Prkaa2*	0.062	0.201	0	0.476	0.416
3-day post-SCI group vs. the control group	*Mapk14*	*Cdkn2b*	0	0.13	0.146	0.278	0.417
3-day post-SCI group vs. the control group	*Mapk14*	*Ezr*	0	0.199	0	0.312	0.425
3-day post-SCI group vs. the control group	*Mapk14*	*Gpx1*	0	0	0	0.425	0.425
3-day post-SCI group vs. the control group	*Mapk14*	*Atp2a2*	0.062	0.058	0	0.402	0.426
3-day post-SCI group vs. the control group	*Mapk14*	*Txnip*	0.059	0.085	0	0.397	0.435
3-day post-SCI group vs. the control group	*Mapk14*	*Ppif*	0	0.052	0.25	0.273	0.437
3-day post-SCI group vs. the control group	*Mapk14*	*Snca*	0	0.136	0	0.399	0.458
3-day post-SCI group vs. the control group	*Mapk14*	*Hdac1*	0	0.131	0	0.404	0.46
3-day post-SCI group vs. the control group	*Mapk14*	*Alox5*	0	0	0	0.463	0.463
3-day post-SCI group vs. the control group	*Mapk14*	*Ppp3ca*	0.063	0.272	0	0.284	0.468
3-day post-SCI group vs. the control group	*Mapk14*	*Cdk4*	0	0.235	0.185	0.581	0.469
3-day post-SCI group vs. the control group	*Mapk14*	*Gsr*	0.063	0	0	0.457	0.47
3-day post-SCI group vs. the control group	*Mapk14*	*Atp7a*	0.062	0	0	0.463	0.474

3-day post-SCI group vs. the control group	*Mapk14*	*Cdk1*	0.051	0.239	0.212	0.582	0.476
3-day post-SCI group vs. the control group	*Mapk14*	*Sdc1*	0.052	0	0.354	0.237	0.491
3-day post-SCI group vs. the control group	*Mapk14*	*Xdh*	0	0	0	0.529	0.529
3-day post-SCI group vs. the control group	*Mapk14*	*Mgst1*	0	0	0	0.542	0.542
3-day post-SCI group vs. the control group	*Mapk14*	*Bax*	0	0.061	0.209	0.438	0.546
3-day post-SCI group vs. the control group	*Mapk14*	*Tnfrsf1a*	0.062	0.048	0	0.552	0.565
3-day post-SCI group vs. the control group	*Mapk14*	*Met*	0.061	0.147	0.392	0.48	0.569
3-day post-SCI group vs. the control group	*Mapk14*	*Mapt*	0.049	0.182	0.354	0.249	0.572
3-day post-SCI group vs. the control group	*Mapk14*	*Sod2*	0.061	0.133	0	0.523	0.577
3-day post-SCI group vs. the control group	*Mapk14*	*Stat6*	0.059	0.104	0.212	0.469	0.6
3-day post-SCI group vs. the control group	*Mapk14*	*Aif1*	0	0.158	0	0.549	0.604
3-day post-SCI group vs. the control group	*Mapk14*	*Ctnna1*	0	0.059	0.6	0.065	0.617
3-day post-SCI group vs. the control group	*Mapk14*	*Jak2*	0.062	0.147	0.42	0.633	0.617
3-day post-SCI group vs. the control group	*Mapk14*	*Sirt1*	0.051	0.045	0	0.62	0.625
3-day post-SCI group vs. the control group	*Mapk14*	*Vcam1*	0	0	0	0.632	0.632
3-day post-SCI group vs. the control group	*Mapk14*	*Nox4*	0	0.069	0	0.622	0.633
3-day post-SCI group vs. the control group	*Mapk14*	*Parp1*	0	0.058	0	0.629	0.636
3-day post-SCI group vs. the control group	*Mapk14*	*Cd36*	0	0	0.629	0.13	0.663
3-day post-SCI group vs. the control group	*Mapk14*	*Smad1*	0	0.17	0.207	0.533	0.666
3-day post-SCI group vs. the control group	*Mapk14*	*Mcl1*	0.061	0.133	0.209	0.55	0.671
3-day post-SCI group vs. the control group	*Mapk14*	*Pxn*	0	0.082	0.31	0.542	0.685
3-day post-SCI group vs. the control group	*Mapk14*	*Hif1a*	0	0.147	0	0.66	0.698
3-day post-SCI group vs. the control group	*Mapk14*	*Nfe2l2*	0	0.091	0	0.687	0.704
3-day post-SCI group vs. the control group	*Mapk14*	*Tlr4*	0	0.048	0	0.748	0.75
3-day post-SCI group vs. the control group	*Mapk14*	*Ptgs2*	0	0.101	0	0.737	0.753
3-day post-SCI group vs. the control group	*Mapk14*	*Ripk1*	0	0.147	0.676	0.412	0.758
3-day post-SCI group vs. the control group	*Mapk14*	*Mapk10*	0.066	0.145	0.702	0.721	0.762
3-day post-SCI group vs. the control group	*Mapk14*	*Cat*	0	0.13	0	0.743	0.767
3-day post-SCI group vs. the control group	*Mapk14*	*Hmox1*	0	0	0.305	0.7	0.782
3-day post-SCI group vs. the control group	*Mapk14*	*Mapk8*	0.066	0.209	0.702	0.872	0.783
3-day post-SCI group vs. the control group	*Mapk14*	*Ets1*	0	0.215	0.629	0.363	0.798
3-day post-SCI group vs. the control group	*Mapk14*	*Junb*	0.051	0.261	0.693	0.356	0.842
3-day post-SCI group vs. the control group	*Mapk14*	*Myc*	0	0.162	0.548	0.619	0.843
3-day post-SCI group vs. the control group	*Mapk14*	*Actb*	0	0.09	0	0.834	0.843
3-day post-SCI group vs. the control group	*Mapk14*	*Nfatc1*	0	0.087	0.676	0.525	0.847
3-day post-SCI group vs. the control group	*Mapk14*	*Fosl1*	0	0.267	0.67	0.444	0.853

3-day post-SCI group vs. the control group	*Mapk14*	*Gnb2*	0.062	0.262	0.791	0.176	0.865
3-day post-SCI group vs. the control group	*Mapk14*	*Nfkb1*	0.061	0.13	0.676	0.585	0.875
3-day post-SCI group vs. the control group	*Mapk14*	*Rela*	0.049	0.057	0.676	0.669	0.89
3-day post-SCI group vs. the control group	*Mapk14*	*Rb1*	0.061	0.727	0.629	0.297	0.924
3-day post-SCI group vs. the control group	*Mapk14*	*Mapk3*	0.061	0.598	0.8	0.912	0.925
3-day post-SCI group vs. the control group	*Mapk14*	*Casp3*	0.061	0.13	0.629	0.81	0.934
3-day post-SCI group vs. the control group	*Mapk14*	*Hspb1*	0	0.13	0.676	0.857	0.956
3-day post-SCI group vs. the control group	*Mapk14*	*Map2k4*	0.062	0.402	0.932	0.75	0.973
3-day post-SCI group vs. the control group	*Mapk14*	*Ncf1*	0	0.425	0.9	0.63	0.976
3-day post-SCI group vs. the control group	*Mapk14*	*Fos*	0	0.267	0.922	0.716	0.982
3-day post-SCI group vs. the control group	*Mapk14*	*Ppargc1a*	0	0.087	0.966	0.62	0.987
3-day post-SCI group vs. the control group	*Mapk14*	*Mapkapk3*	0.061	0.76	0.932	0.524	0.988
3-day post-SCI group vs. the control group	*Mapk14*	*Jun*	0.049	0.4	0.932	0.903	0.995
3-day post-SCI group vs. the control group	*Mapk14*	*Map2k3*	0.065	0.839	0.966	0.961	0.996
3-day post-SCI group vs. the control group	*Mapk14*	*Map2k6*	0.065	0.848	0.966	0.864	0.996
3-day post-SCI group vs. the control group	*Mapk14*	*Atf2*	0	0.927	0.966	0.785	0.999
3-day post-SCI group vs. the control group	*Mapk14*	*Mapkapk2*	0.083	0.978	0.932	0.969	0.999
3-day post-SCI group vs. the control group	*Mapk3*	*Capns1*	0.214	0.134	0.629	0.121	0.748
3-day post-SCI group vs. the control group	*Mapk3*	*Braf*	0.067	0.522	0.864	0.741	0.954
3-day post-SCI group vs. the control group	*Mapk3*	*Ier3*	0	0.193	0.6	0.247	0.736
3-day post-SCI group vs. the control group	*Mapk3*	*Mapk14*	0.061	0.598	0.8	0.912	0.925
3-day post-SCI group vs. the control group	*Mapk3*	*Hspb1*	0	0.132	0.291	0.585	0.722
3-day post-SCI group vs. the control group	*Mapk3*	*Hmox1*	0	0	0.305	0.613	0.72
3-day post-SCI group vs. the control group	*Mapk3*	*Cdk4*	0.063	0.235	0.185	0.643	0.473
3-day post-SCI group vs. the control group	*Mapk3*	*Sod2*	0.061	0.093	0	0.474	0.513
3-day post-SCI group vs. the control group	*Mapk3*	*Mgst1*	0	0	0	0.464	0.463
3-day post-SCI group vs. the control group	*Mapk3*	*Ncf1*	0	0.072	0.9	0.524	0.952
3-day post-SCI group vs. the control group	*Mapk3*	*Mapkapk2*	0.064	0.542	0.326	0.46	0.774
3-day post-SCI group vs. the control group	*Mapk3*	*Map2k3*	0.068	0.401	0.859	0.587	0.936
3-day post-SCI group vs. the control group	*Mapk3*	*Cdk1*	0.056	0.239	0.212	0.657	0.468
3-day post-SCI group vs. the control group	*Mapk3*	*Sdc1*	0.065	0	0.676	0.33	0.779
3-day post-SCI group vs. the control group	*Mapk3*	*Map2k6*	0.068	0.401	0.859	0.506	0.936
3-day post-SCI group vs. the control group	*Mapk3*	*Hif1a*	0	0.289	0.8	0.714	0.955
3-day post-SCI group vs. the control group	*Mapk3*	*Fos*	0	0.267	0.966	0.746	0.993
3-day post-SCI group vs. the control group	*Mapk3*	*Ppif*	0	0.052	0.25	0.277	0.441
3-day post-SCI group vs. the control group	*Mapk3*	*Rb1*	0.061	0.248	0.629	0.288	0.788

3-day post-SCI group vs. the control group	*Mapk3*	*Myc*	0	0.261	0.905	0.708	0.977
3-day post-SCI group vs. the control group	*Mapk3*	*Mylk*	0	0.131	0.629	0.324	0.705
3-day post-SCI group vs. the control group	*Mapk3*	*Xdh*	0	0	0	0.4	0.4
3-day post-SCI group vs. the control group	*Mapk3*	*Aif1*	0	0.158	0	0.42	0.491
3-day post-SCI group vs. the control group	*Mapk3*	*Hbegf*	0	0	0	0.498	0.497
3-day post-SCI group vs. the control group	*Mapk3*	*Fosl1*	0	0.267	0.209	0.504	0.687
3-day post-SCI group vs. the control group	*Mapk3*	*Rela*	0	0.05	0.676	0.566	0.855
3-day post-SCI group vs. the control group	*Mapk3*	*Parp1*	0.062	0.13	0	0.623	0.665
3-day post-SCI group vs. the control group	*Mapk3*	*Itga6*	0.063	0.058	0.238	0.278	0.449
3-day post-SCI group vs. the control group	*Mapk3*	*Cat*	0	0.089	0	0.656	0.673
3-day post-SCI group vs. the control group	*Mapk3*	*Vcam1*	0	0	0	0.561	0.561
3-day post-SCI group vs. the control group	*Mapk3*	*Nfkb1*	0	0.13	0.676	0.501	0.847
3-day post-SCI group vs. the control group	*Mapk3*	*Prkaa2*	0.062	0.201	0	0.377	0.412
3-day post-SCI group vs. the control group	*Mapk3*	*Gnb2*	0.223	0.262	0.673	0.176	0.825
3-day post-SCI group vs. the control group	*Mapk3*	*Tnfrsf1a*	0.051	0.085	0	0.44	0.471
3-day post-SCI group vs. the control group	*Mapk3*	*Nox4*	0	0.069	0	0.543	0.557
3-day post-SCI group vs. the control group	*Mapk3*	*Bax*	0	0.061	0.209	0.332	0.46
3-day post-SCI group vs. the control group	*Mapk3*	*Btk*	0	0.147	0.187	0.474	0.412
3-day post-SCI group vs. the control group	*Mapk3*	*Ets1*	0	0.311	0.966	0.616	0.99
3-day post-SCI group vs. the control group	*Mapk3*	*Ptgs2*	0	0.206	0	0.69	0.743
3-day post-SCI group vs. the control group	*Mapk3*	*Mapkapk3*	0.064	0.437	0.326	0.206	0.692
3-day post-SCI group vs. the control group	*Mapk3*	*Rxrb*	0	0.149	0.676	0	0.712
3-day post-SCI group vs. the control group	*Mapk3*	*Ppp1ca*	0.125	0.237	0	0.214	0.429
3-day post-SCI group vs. the control group	*Mapk3*	*Map2k4*	0.062	0.197	0.372	0.504	0.585
3-day post-SCI group vs. the control group	*Mapk3*	*Mcl1*	0	0.164	0.209	0.723	0.801
3-day post-SCI group vs. the control group	*Mapk3*	*Tlr4*	0	0.048	0	0.812	0.814
3-day post-SCI group vs. the control group	*Mapk3*	*Itgb5*	0.079	0	0.176	0.276	0.403
3-day post-SCI group vs. the control group	*Mapk3*	*Syp*	0	0	0	0.412	0.412
3-day post-SCI group vs. the control group	*Mapk3*	*Ppp3ca*	0.063	0.272	0	0.239	0.435
3-day post-SCI group vs. the control group	*Mapk3*	*Snca*	0	0.189	0	0.342	0.444
3-day post-SCI group vs. the control group	*Mapk3*	*Fbxw7*	0.064	0.163	0	0.352	0.448
3-day post-SCI group vs. the control group	*Mapk3*	*Cttn*	0.063	0	0	0.446	0.459
3-day post-SCI group vs. the control group	*Mapk3*	*Cdkn2b*	0	0.13	0.146	0.332	0.46
3-day post-SCI group vs. the control group	*Mapk3*	*Arhgdia*	0.24	0.066	0	0.308	0.466
3-day post-SCI group vs. the control group	*Mapk3*	*Hdac1*	0	0.131	0	0.465	0.515
3-day post-SCI group vs. the control group	*Mapk3*	*Ezr*	0.051	0.199	0	0.415	0.516

3-day post-SCI group vs. the control group	*Mapk3*	*Ripk1*	0	0.147	0.354	0.556	0.556
3-day post-SCI group vs. the control group	*Mapk3*	*Nfe2l2*	0.052	0.103	0	0.545	0.579
3-day post-SCI group vs. the control group	*Mapk3*	*Junb*	0	0.261	0.264	0.297	0.584
3-day post-SCI group vs. the control group	*Mapk3*	*Sirt1*	0.051	0.045	0	0.579	0.585
3-day post-SCI group vs. the control group	*Mapk3*	*Mark3*	0	0	0.6	0.134	0.615
3-day post-SCI group vs. the control group	*Mapk3*	*Stat6*	0.086	0.104	0.212	0.498	0.633
3-day post-SCI group vs. the control group	*Mapk3*	*Ppargc1a*	0	0.058	0.354	0.466	0.647
3-day post-SCI group vs. the control group	*Mapk3*	*Mapk10*	0.08	0.059	0.728	0.542	0.768
3-day post-SCI group vs. the control group	*Mapk3*	*Met*	0.061	0.147	0.676	0.714	0.801
3-day post-SCI group vs. the control group	*Mapk3*	*Mapk8*	0.08	0.087	0.806	0.844	0.848
3-day post-SCI group vs. the control group	*Mapk3*	*Actb*	0	0.09	0	0.859	0.867
3-day post-SCI group vs. the control group	*Mapk3*	*Pxn*	0.061	0.185	0.676	0.647	0.901
3-day post-SCI group vs. the control group	*Mapk3*	*Mapt*	0	0.228	0.865	0.286	0.919
3-day post-SCI group vs. the control group	*Mapk3*	*Il6st*	0	0	0.9	0.26	0.922
3-day post-SCI group vs. the control group	*Mapk3*	*Nfatc1*	0	0.058	0.932	0.438	0.961
3-day post-SCI group vs. the control group	*Mapk3*	*Jak2*	0.066	0.147	0.939	0.707	0.962
3-day post-SCI group vs. the control group	*Mapk3*	*Smad1*	0.061	0.359	0.932	0.511	0.977
3-day post-SCI group vs. the control group	*Mapk3*	*Casp3*	0	0.08	0.869	0.824	0.977
3-day post-SCI group vs. the control group	*Mapk3*	*Atf2*	0	0.653	0.966	0.557	0.994
3-day post-SCI group vs. the control group	*Mapk3*	*Jun*	0	0.686	0.932	0.863	0.996
3-day post-SCI group vs. the control group	*Mapk8*	*Mapk14*	0.066	0.209	0.702	0.872	0.783
3-day post-SCI group vs. the control group	*Mapk8*	*Hspb1*	0	0.092	0	0.474	0.502
3-day post-SCI group vs. the control group	*Mapk8*	*Hmox1*	0	0	0	0.551	0.551
3-day post-SCI group vs. the control group	*Mapk8*	*Sod2*	0	0	0	0.414	0.414
3-day post-SCI group vs. the control group	*Mapk8*	*Mgst1*	0	0	0	0.722	0.722
3-day post-SCI group vs. the control group	*Mapk8*	*Endog*	0.061	0	0	0.391	0.404
3-day post-SCI group vs. the control group	*Mapk8*	*Mapkapk2*	0	0.224	0.65	0.53	0.805
3-day post-SCI group vs. the control group	*Mapk8*	*Map2k3*	0.062	0.135	0.922	0.75	0.958
3-day post-SCI group vs. the control group	*Mapk8*	*Map2k6*	0.062	0.135	0.932	0.649	0.961
3-day post-SCI group vs. the control group	*Mapk8*	*Hif1a*	0.052	0	0	0.488	0.494
3-day post-SCI group vs. the control group	*Mapk8*	*Fos*	0	0.309	0.932	0.64	0.981
3-day post-SCI group vs. the control group	*Mapk8*	*Ripk3*	0	0.208	0.209	0.324	0.429
3-day post-SCI group vs. the control group	*Mapk8*	*Myc*	0	0.198	0	0.536	0.612
3-day post-SCI group vs. the control group	*Mapk8*	*Fosl1*	0	0.309	0.696	0.395	0.862
3-day post-SCI group vs. the control group	*Mapk8*	*Rela*	0	0.048	0.8	0.547	0.906
3-day post-SCI group vs. the control group	*Mapk8*	*Il18rap*	0	0	0.676	0.076	0.688

3-day post-SCI group vs. the control group	*Mapk8*	*Parp1*	0	0	0	0.472	0.472
3-day post-SCI group vs. the control group	*Mapk8*	*Cat*	0	0	0	0.525	0.525
3-day post-SCI group vs. the control group	*Mapk8*	*Vcam1*	0	0	0	0.4	0.4
3-day post-SCI group vs. the control group	*Mapk8*	*Nfkb1*	0.061	0.058	0.8	0.574	0.914
3-day post-SCI group vs. the control group	*Mapk8*	*Gnb2*	0.062	0	0.6	0	0.608
3-day post-SCI group vs. the control group	*Mapk8*	*Tnfrsf1a*	0	0.133	0	0.49	0.539
3-day post-SCI group vs. the control group	*Mapk8*	*Bax*	0	0.082	0.272	0.397	0.561
3-day post-SCI group vs. the control group	*Mapk8*	*Btk*	0.052	0.139	0.676	0.341	0.75
3-day post-SCI group vs. the control group	*Mapk8*	*Ptgs2*	0	0	0	0.589	0.589
3-day post-SCI group vs. the control group	*Mapk8*	*Mapkapk3*	0	0.224	0.65	0.306	0.785
3-day post-SCI group vs. the control group	*Mapk8*	*Rxrb*	0	0.047	0.42	0.049	0.428
3-day post-SCI group vs. the control group	*Mapk8*	*Map2k4*	0.105	0.821	0.966	0.863	0.996
3-day post-SCI group vs. the control group	*Mapk8*	*Mcl1*	0	0.431	0.272	0.471	0.762
3-day post-SCI group vs. the control group	*Mapk8*	*Tlr4*	0	0.095	0	0.707	0.724
3-day post-SCI group vs. the control group	*Mapk8*	*Mapk3*	0.08	0.087	0.806	0.844	0.848
3-day post-SCI group vs. the control group	*Mapk8*	*Jak2*	0.063	0.139	0.42	0.498	0.594
3-day post-SCI group vs. the control group	*Mapk8*	*Junb*	0.061	0.329	0.725	0.33	0.868
3-day post-SCI group vs. the control group	*Mapk8*	*Vrk2*	0.06	0.463	0	0.096	0.504
3-day post-SCI group vs. the control group	*Mapk8*	*Pxn*	0	0.512	0	0.374	0.681
3-day post-SCI group vs. the control group	*Mapk8*	*Casp3*	0.052	0.13	0	0.724	0.752
3-day post-SCI group vs. the control group	*Mapk8*	*Mapt*	0.062	0.228	0.629	0.258	0.774
3-day post-SCI group vs. the control group	*Mapk8*	*Actb*	0	0	0	0.702	0.702
3-day post-SCI group vs. the control group	*Mapk8*	*Nfe2l2*	0	0.052	0	0.483	0.488
3-day post-SCI group vs. the control group	*Mapk8*	*Sirt1*	0.065	0.201	0	0.584	0.662
3-day post-SCI group vs. the control group	*Mapk8*	*Jun*	0	0.999	0.966	0.947	0.999
3-day post-SCI group vs. the control group	*Mapk8*	*Snca*	0.071	0.13	0	0.331	0.412
3-day post-SCI group vs. the control group	*Mapk8*	*Ripk1*	0	0.208	0.209	0.457	0.468
3-day post-SCI group vs. the control group	*Mapk8*	*Met*	0.062	0.132	0.676	0.337	0.801
3-day post-SCI group vs. the control group	*Mapk8*	*Mapk10*	0.062	0	0.932	0.884	0.935
3-day post-SCI group vs. the control group	*Mapk8*	*Nfatc1*	0	0	0.932	0.571	0.969
3-day post-SCI group vs. the control group	*Mapk8*	*Atf2*	0.088	0.844	0.966	0.61	0.997
3-day post-SCI group vs. the control group	*Mapkapk2*	*Mapk14*	0.083	0.978	0.932	0.969	0.999
3-day post-SCI group vs. the control group	*Mapkapk2*	*Hspb1*	0.062	0.871	0.966	0.733	0.998
3-day post-SCI group vs. the control group	*Mapkapk2*	*Map2k4*	0.051	0.14	0	0.617	0.4
3-day post-SCI group vs. the control group	*Mapkapk2*	*Rnf2*	0	0.436	0	0	0.436
3-day post-SCI group vs. the control group	*Mapkapk2*	*Jun*	0.081	0.056	0	0.527	0.555

3-day post-SCI group vs. the control group	*Mapkapk2*	*Atf2*	0	0.048	0	0.552	0.556
3-day post-SCI group vs. the control group	*Mapkapk2*	*Mapk3*	0.064	0.542	0.326	0.46	0.774
3-day post-SCI group vs. the control group	*Mapkapk2*	*Mapk8*	0	0.224	0.65	0.53	0.805
3-day post-SCI group vs. the control group	*Mapkapk2*	*Mapk10*	0	0.224	0.65	0.645	0.814
3-day post-SCI group vs. the control group	*Mapkapk2*	*Mapkapk3*	0.116	0	0.9	0.742	0.911
3-day post-SCI group vs. the control group	*Mapkapk2*	*Map2k6*	0.063	0.13	0.9	0.603	0.94
3-day post-SCI group vs. the control group	*Mapkapk2*	*Map2k3*	0.119	0.09	0.9	0.701	0.943
3-day post-SCI group vs. the control group	*Mapkapk2*	*Alox5*	0.062	0.402	0.9	0.135	0.945
3-day post-SCI group vs. the control group	*Mapkapk3*	*Mapk14*	0.061	0.76	0.932	0.524	0.988
3-day post-SCI group vs. the control group	*Mapkapk3*	*Hspb1*	0.07	0.671	0.966	0.421	0.993
3-day post-SCI group vs. the control group	*Mapkapk3*	*Mapkapk2*	0.116	0	0.9	0.742	0.911
3-day post-SCI group vs. the control group	*Mapkapk3*	*Map2k3*	0.109	0.081	0.9	0.452	0.935
3-day post-SCI group vs. the control group	*Mapkapk3*	*Map2k6*	0.063	0.081	0.9	0.328	0.928
3-day post-SCI group vs. the control group	*Mapkapk3*	*Map2k4*	0.051	0.14	0	0.574	0.406
3-day post-SCI group vs. the control group	*Mapkapk3*	*Mapk3*	0.064	0.437	0.326	0.206	0.692
3-day post-SCI group vs. the control group	*Mapkapk3*	*Mapk10*	0	0.224	0.65	0.293	0.781
3-day post-SCI group vs. the control group	*Mapkapk3*	*Mapk8*	0	0.224	0.65	0.306	0.785
3-day post-SCI group vs. the control group	*Mapt*	*Amph*	0.18	0.13	0	0.233	0.405
3-day post-SCI group vs. the control group	*Mapt*	*Mapk14*	0.049	0.182	0.354	0.249	0.572
3-day post-SCI group vs. the control group	*Mapt*	*Hspb1*	0.061	0.13	0	0.576	0.623
3-day post-SCI group vs. the control group	*Mapt*	*Sod2*	0	0.433	0	0.199	0.526
3-day post-SCI group vs. the control group	*Mapt*	*Cdk1*	0	0.402	0	0.181	0.489
3-day post-SCI group vs. the control group	*Mapt*	*Arg1*	0	0.433	0	0.047	0.436
3-day post-SCI group vs. the control group	*Mapt*	*Ndufa6*	0	0	0.6	0	0.6
3-day post-SCI group vs. the control group	*Mapt*	*Aif1*	0	0.236	0	0.523	0.62
3-day post-SCI group vs. the control group	*Mapt*	*Pink1*	0.094	0	0	0.581	0.604
3-day post-SCI group vs. the control group	*Mapt*	*Atp2a2*	0.062	0.443	0	0.055	0.463
3-day post-SCI group vs. the control group	*Mapt*	*Ppp1ca*	0	0.366	0	0.107	0.41
3-day post-SCI group vs. the control group	*Mapt*	*Atp13a2*	0.061	0.046	0	0.507	0.52
3-day post-SCI group vs. the control group	*Mapt*	*Mapk3*	0	0.228	0.865	0.286	0.919
3-day post-SCI group vs. the control group	*Mapt*	*Ppp3ca*	0.098	0.527	0	0.152	0.606
3-day post-SCI group vs. the control group	*Mapt*	*Syp*	0.212	0	0	0.573	0.649
3-day post-SCI group vs. the control group	*Mapt*	*Prdx6*	0	0.433	0	0.163	0.506
3-day post-SCI group vs. the control group	*Mapt*	*Ndufs8*	0	0	0.6	0	0.6
3-day post-SCI group vs. the control group	*Mapt*	*Mark3*	0	0.326	0.533	0.304	0.761
3-day post-SCI group vs. the control group	*Mapt*	*Htra2*	0	0.4	0	0.25	0.531

3-day post-SCI group vs. the control group	*Mapt*	*Casp3*	0	0.48	0.9	0.558	0.975
3-day post-SCI group vs. the control group	*Mapt*	*Mapk10*	0.216	0.182	0.5	0.248	0.727
3-day post-SCI group vs. the control group	*Mapt*	*Actb*	0.062	0.547	0	0.48	0.76
3-day post-SCI group vs. the control group	*Mapt*	*Ndufa12*	0	0.433	0.6	0.071	0.77
3-day post-SCI group vs. the control group	*Mapt*	*Mapk8*	0.062	0.228	0.629	0.258	0.774
3-day post-SCI group vs. the control group	*Mapt*	*Snca*	0.158	0.561	0	0.923	0.969
3-day post-SCI group vs. the control group	*Marcksl1*	*Actb*	0.079	0.357	0	0.09	0.414
3-day post-SCI group vs. the control group	*Marcksl1*	*Pxdn*	0	0	0	0.437	0.437
3-day post-SCI group vs. the control group	*Mark3*	*Braf*	0.052	0.087	0.5	0.193	0.561
3-day post-SCI group vs. the control group	*Mark3*	*Mapk3*	0	0	0.6	0.134	0.615
3-day post-SCI group vs. the control group	*Mark3*	*Jak2*	0	0.087	0.9	0	0.904
3-day post-SCI group vs. the control group	*Mark3*	*Mapt*	0	0.326	0.533	0.304	0.761
3-day post-SCI group vs. the control group	*Mcl1*	*Braf*	0	0.145	0.12	0.323	0.446
3-day post-SCI group vs. the control group	*Mcl1*	*Ier3*	0.061	0.079	0	0.586	0.612
3-day post-SCI group vs. the control group	*Mcl1*	*Mapk14*	0.061	0.133	0.209	0.55	0.671
3-day post-SCI group vs. the control group	*Mcl1*	*Cdk4*	0	0.058	0	0.589	0.596
3-day post-SCI group vs. the control group	*Mcl1*	*Endog*	0	0	0	0.427	0.427
3-day post-SCI group vs. the control group	*Mcl1*	*Cdk1*	0.056	0.058	0	0.765	0.773
3-day post-SCI group vs. the control group	*Mcl1*	*Hif1a*	0.062	0.13	0	0.552	0.602
3-day post-SCI group vs. the control group	*Mcl1*	*Fos*	0.089	0	0	0.411	0.441
3-day post-SCI group vs. the control group	*Mcl1*	*Myc*	0.066	0.052	0	0.748	0.758
3-day post-SCI group vs. the control group	*Mcl1*	*Rela*	0.094	0.058	0.311	0.4	0.599
3-day post-SCI group vs. the control group	*Mcl1*	*Parp1*	0	0.053	0	0.661	0.665
3-day post-SCI group vs. the control group	*Mcl1*	*Nfkb1*	0.078	0.055	0.142	0.35	0.449
3-day post-SCI group vs. the control group	*Mcl1*	*Pink1*	0	0.208	0	0.339	0.454
3-day post-SCI group vs. the control group	*Mcl1*	*Tnfrsf1a*	0.068	0.077	0	0.387	0.427
3-day post-SCI group vs. the control group	*Mcl1*	*Bax*	0	0.422	0.72	0.61	0.865
3-day post-SCI group vs. the control group	*Mcl1*	*Btk*	0	0.056	0.19	0.469	0.558
3-day post-SCI group vs. the control group	*Mcl1*	*Ptgs2*	0.061	0	0	0.409	0.421
3-day post-SCI group vs. the control group	*Mcl1*	*Pawr*	0	0	0	0.414	0.414
3-day post-SCI group vs. the control group	*Mcl1*	*Ripk1*	0.079	0.056	0.12	0.349	0.435
3-day post-SCI group vs. the control group	*Mcl1*	*Ezh2*	0	0	0	0.463	0.463
3-day post-SCI group vs. the control group	*Mcl1*	*Stat6*	0.069	0	0.216	0.328	0.467
3-day post-SCI group vs. the control group	*Mcl1*	*Mapk10*	0	0.13	0.272	0.235	0.473
3-day post-SCI group vs. the control group	*Mcl1*	*Htra2*	0	0	0	0.474	0.474
3-day post-SCI group vs. the control group	*Mcl1*	*Met*	0	0.056	0.12	0.465	0.516

3-day post-SCI group vs. the control group	*Mcl1*	*Sirt1*	0.062	0	0.261	0.376	0.529
3-day post-SCI group vs. the control group	*Mcl1*	*Bnip3*	0	0.094	0	0.561	0.585
3-day post-SCI group vs. the control group	*Mcl1*	*Jun*	0.063	0	0	0.62	0.628
3-day post-SCI group vs. the control group	*Mcl1*	*Jak2*	0.061	0.056	0.19	0.61	0.682
3-day post-SCI group vs. the control group	*Mcl1*	*Actb*	0	0.09	0	0.693	0.708
3-day post-SCI group vs. the control group	*Mcl1*	*Mapk8*	0	0.431	0.272	0.471	0.762
3-day post-SCI group vs. the control group	*Mcl1*	*Mapk3*	0	0.164	0.209	0.723	0.801
3-day post-SCI group vs. the control group	*Mcl1*	*Casp3*	0	0.235	0	0.872	0.898
3-day post-SCI group vs. the control group	*Mcl1*	*Fbxw7*	0.061	0.745	0	0.863	0.964
3-day post-SCI group vs. the control group	*Mcm4*	*Cdc20*	0.903	0.049	0	0.521	0.952
3-day post-SCI group vs. the control group	*Mcm4*	*Cdk4*	0.281	0.224	0	0.429	0.653
3-day post-SCI group vs. the control group	*Mcm4*	*Cdk1*	0.913	0.242	0	0.662	0.976
3-day post-SCI group vs. the control group	*Mcm4*	*Dhfr*	0.7	0	0	0.63	0.884
3-day post-SCI group vs. the control group	*Mcm4*	*Rb1*	0.245	0.148	0	0.225	0.458
3-day post-SCI group vs. the control group	*Mcm4*	*Rrm2b*	0.751	0	0	0.096	0.765
3-day post-SCI group vs. the control group	*Mcm4*	*Myc*	0.094	0.058	0	0.356	0.402
3-day post-SCI group vs. the control group	*Mcm4*	*Atp7a*	0.427	0	0	0	0.426
3-day post-SCI group vs. the control group	*Mcm4*	*Rbbp7*	0.427	0.161	0	0	0.499
3-day post-SCI group vs. the control group	*Mcm4*	*Ezh2*	0.43	0.053	0	0.149	0.501
3-day post-SCI group vs. the control group	*Mcm4*	*Tfdp1*	0.353	0	0	0.274	0.51
3-day post-SCI group vs. the control group	*Mcm4*	*Eed*	0.18	0.433	0	0.05	0.52
3-day post-SCI group vs. the control group	*Mcm4*	*Uba52*	0	0	0.65	0.05	0.653
3-day post-SCI group vs. the control group	*Mcm4*	*Ect2*	0.705	0	0	0.05	0.708
3-day post-SCI group vs. the control group	*Mcm4*	*Melk*	0.748	0.057	0	0.163	0.784
3-day post-SCI group vs. the control group	*Mcm4*	*Pcna*	0.893	0.199	0	0.45	0.948
3-day post-SCI group vs. the control group	*Mcm4*	*Atr*	0.112	0.401	0.912	0.543	0.976
3-day post-SCI group vs. the control group	*Mcm4*	*Ccna2*	0.776	0.387	0.9	0.543	0.992
3-day post-SCI group vs. the control group	*Melk*	*Cdc20*	0.941	0.091	0	0.386	0.964
3-day post-SCI group vs. the control group	*Melk*	*Cdk1*	0.945	0.1	0	0.427	0.957
3-day post-SCI group vs. the control group	*Melk*	*Mcm4*	0.748	0.057	0	0.163	0.784
3-day post-SCI group vs. the control group	*Melk*	*Pcna*	0.486	0	0	0.062	0.497
3-day post-SCI group vs. the control group	*Melk*	*Ccna2*	0.925	0.155	0	0.35	0.955
3-day post-SCI group vs. the control group	*Melk*	*Camkk2*	0.064	0.284	0	0.22	0.402
3-day post-SCI group vs. the control group	*Melk*	*Jun*	0	0.145	0	0.709	0.741
3-day post-SCI group vs. the control group	*Melk*	*Ezh2*	0.633	0.047	0	0.733	0.899
3-day post-SCI group vs. the control group	*Melk*	*Ect2*	0.924	0.083	0	0.211	0.94

3-day post-SCI group vs. the control group	*Met*	*Mapk14*	0.061	0.147	0.392	0.48	0.569
3-day post-SCI group vs. the control group	*Met*	*Sdc1*	0.114	0	0.676	0.312	0.785
3-day post-SCI group vs. the control group	*Met*	*Hif1a*	0	0.097	0.12	0.611	0.663
3-day post-SCI group vs. the control group	*Met*	*Fos*	0	0.071	0.12	0.364	0.434
3-day post-SCI group vs. the control group	*Met*	*Myc*	0.051	0.089	0.129	0.627	0.681
3-day post-SCI group vs. the control group	*Met*	*Hbegf*	0.061	0	0.147	0.401	0.478
3-day post-SCI group vs. the control group	*Met*	*Fosl1*	0.077	0.071	0.12	0.355	0.448
3-day post-SCI group vs. the control group	*Met*	*Itga6*	0.063	0.133	0.676	0.402	0.821
3-day post-SCI group vs. the control group	*Met*	*Vcam1*	0.062	0	0.147	0.338	0.424
3-day post-SCI group vs. the control group	*Met*	*Btk*	0.065	0.074	0.323	0.457	0.457
3-day post-SCI group vs. the control group	*Met*	*Ptgs2*	0.069	0.136	0	0.398	0.473
3-day post-SCI group vs. the control group	*Met*	*Mcl1*	0	0.056	0.12	0.465	0.516
3-day post-SCI group vs. the control group	*Met*	*Ctnna1*	0.082	0.052	0.676	0.066	0.701
3-day post-SCI group vs. the control group	*Met*	*Mapk3*	0.061	0.147	0.676	0.714	0.801
3-day post-SCI group vs. the control group	*Met*	*Ezr*	0.061	0.089	0.133	0.391	0.487
3-day post-SCI group vs. the control group	*Met*	*Jak2*	0.065	0.074	0.858	0.434	0.89
3-day post-SCI group vs. the control group	*Met*	*Itgb5*	0.069	0.133	0.388	0.132	0.513
3-day post-SCI group vs. the control group	*Met*	*Ezh2*	0.052	0.068	0	0.431	0.453
3-day post-SCI group vs. the control group	*Met*	*Pxn*	0.073	0.063	0.131	0.542	0.609
3-day post-SCI group vs. the control group	*Met*	*Casp3*	0	0.311	0.114	0.566	0.712
3-day post-SCI group vs. the control group	*Met*	*Actb*	0.052	0.084	0.114	0.59	0.643
3-day post-SCI group vs. the control group	*Met*	*Cttn*	0.062	0.087	0.146	0.342	0.454
3-day post-SCI group vs. the control group	*Met*	*Jun*	0	0.09	0.127	0.528	0.592
3-day post-SCI group vs. the control group	*Met*	*Mapk8*	0.062	0.132	0.676	0.337	0.801
3-day post-SCI group vs. the control group	*Met*	*Mapk10*	0.062	0.132	0.537	0.141	0.609
3-day post-SCI group vs. the control group	*Met*	*Uba52*	0	0.046	0.9	0.05	0.901
3-day post-SCI group vs. the control group	*Mgmt*	*Cdk4*	0	0.131	0	0.372	0.431
3-day post-SCI group vs. the control group	*Mgmt*	*Myc*	0	0	0	0.414	0.414
3-day post-SCI group vs. the control group	*Mgmt*	*Parp1*	0	0	0	0.464	0.463
3-day post-SCI group vs. the control group	*Mgmt*	*Ezh2*	0.062	0	0	0.4	0.413
3-day post-SCI group vs. the control group	*Mgmt*	*Casp3*	0	0	0	0.457	0.457
3-day post-SCI group vs. the control group	*Mgmt*	*Actb*	0	0.09	0	0.464	0.491
3-day post-SCI group vs. the control group	*Mgmt*	*Pdgfra*	0	0	0	0.506	0.506
3-day post-SCI group vs. the control group	*Mgmt*	*Cdkn2b*	0.049	0	0	0.575	0.579
3-day post-SCI group vs. the control group	*Mgst1*	*Mapk14*	0	0	0	0.542	0.542
3-day post-SCI group vs. the control group	*Mgst1*	*Cdk4*	0	0	0	0.7	0.7

3-day post-SCI group vs. the control group	*Mgst1*	*Nfatc1*	0.057	0	0	0.407	0.417
3-day post-SCI group vs. the control group	*Mgst1*	*Parp1*	0	0	0	0.43	0.43
3-day post-SCI group vs. the control group	*Mgst1*	*Cat*	0.108	0	0	0.398	0.44
3-day post-SCI group vs. the control group	*Mgst1*	*Nme2*	0	0	0	0.445	0.445
3-day post-SCI group vs. the control group	*Mgst1*	*Hdac1*	0	0	0	0.449	0.449
3-day post-SCI group vs. the control group	*Mgst1*	*Nox4*	0	0	0	0.453	0.453
3-day post-SCI group vs. the control group	*Mgst1*	*Mapk3*	0	0	0	0.464	0.463
3-day post-SCI group vs. the control group	*Mgst1*	*Nfe2l2*	0.109	0	0	0.463	0.501
3-day post-SCI group vs. the control group	*Mgst1*	*Casp3*	0	0	0	0.502	0.502
3-day post-SCI group vs. the control group	*Mgst1*	*Cdk1*	0	0	0	0.533	0.533
3-day post-SCI group vs. the control group	*Mgst1*	*Myc*	0	0	0	0.587	0.587
3-day post-SCI group vs. the control group	*Mgst1*	*Actb*	0.065	0	0	0.613	0.622
3-day post-SCI group vs. the control group	*Mgst1*	*Rela*	0	0	0	0.65	0.65
3-day post-SCI group vs. the control group	*Mgst1*	*Snca*	0	0	0	0.663	0.663
3-day post-SCI group vs. the control group	*Mgst1*	*Jun*	0	0	0	0.675	0.675
3-day post-SCI group vs. the control group	*Mgst1*	*Gpx7*	0.049	0	0.65	0.207	0.712
3-day post-SCI group vs. the control group	*Mgst1*	*Cyp1b1*	0.072	0	0.65	0.193	0.715
3-day post-SCI group vs. the control group	*Mgst1*	*Ppif*	0	0	0	0.719	0.719
3-day post-SCI group vs. the control group	*Mgst1*	*Mapk8*	0	0	0	0.722	0.722
3-day post-SCI group vs. the control group	*Mgst1*	*Gpx3*	0.076	0	0.65	0.227	0.728
3-day post-SCI group vs. the control group	*Mgst1*	*Prdx6*	0.068	0	0.65	0.239	0.73
3-day post-SCI group vs. the control group	*Mgst1*	*Gpx8*	0.074	0	0.65	0.239	0.732
3-day post-SCI group vs. the control group	*Mgst1*	*Ggt7*	0.061	0	0.65	0.308	0.752
3-day post-SCI group vs. the control group	*Mgst1*	*Gpx1*	0.141	0	0.65	0.308	0.774
3-day post-SCI group vs. the control group	*Mgst1*	*Gstt2*	0.084	0	0.65	0.38	0.784
3-day post-SCI group vs. the control group	*Mgst1*	*Gsr*	0.061	0	0.65	0.396	0.784
3-day post-SCI group vs. the control group	*Msrb2*	*Txnrd1*	0.064	0.048	0	0.388	0.43
3-day post-SCI group vs. the control group	*Msrb2*	*Gpx1*	0.093	0.047	0	0.369	0.429
3-day post-SCI group vs. the control group	*Msrb2*	*Cat*	0.087	0	0	0.438	0.466
3-day post-SCI group vs. the control group	*Myc*	*Braf*	0.051	0.144	0.129	0.5	0.599
3-day post-SCI group vs. the control group	*Myc*	*Axl*	0.051	0.086	0.129	0.379	0.468
3-day post-SCI group vs. the control group	*Myc*	*Amph*	0.066	0.204	0	0.376	0.496
3-day post-SCI group vs. the control group	*Myc*	*Mapk14*	0	0.162	0.548	0.619	0.843
3-day post-SCI group vs. the control group	*Myc*	*Hspb1*	0	0.055	0	0.446	0.454
3-day post-SCI group vs. the control group	*Myc*	*Hmox1*	0.088	0	0	0.42	0.449
3-day post-SCI group vs. the control group	*Myc*	*Cdc20*	0.121	0.091	0	0.5	0.566

3-day post-SCI group vs. the control group	*Myc*	*Cdk4*	0.104	0.352	0	0.757	0.847
3-day post-SCI group vs. the control group	*Myc*	*Sod2*	0	0	0	0.511	0.511
3-day post-SCI group vs. the control group	*Myc*	*Mgst1*	0	0	0	0.587	0.587
3-day post-SCI group vs. the control group	*Myc*	*Map2k3*	0.079	0.209	0	0.306	0.45
3-day post-SCI group vs. the control group	*Myc*	*Cdk1*	0.114	0.145	0	0.702	0.754
3-day post-SCI group vs. the control group	*Myc*	*Sdc1*	0.072	0.045	0	0.45	0.47
3-day post-SCI group vs. the control group	*Myc*	*Nme2*	0.062	0	0	0.486	0.498
3-day post-SCI group vs. the control group	*Myc*	*Hif1a*	0	0.508	0	0.939	0.968
3-day post-SCI group vs. the control group	*Myc*	*Fos*	0.064	0.055	0	0.85	0.856
3-day post-SCI group vs. the control group	*Myc*	*Dhfr*	0.061	0	0	0.509	0.52
3-day post-SCI group vs. the control group	*Myc*	*Rb1*	0	0.332	0	0.504	0.655
3-day post-SCI group vs. the control group	*Myc*	*Mcm4*	0.094	0.058	0	0.356	0.402
3-day post-SCI group vs. the control group	*Myc*	*Pdgfra*	0	0.055	0	0.398	0.407
3-day post-SCI group vs. the control group	*Myc*	*Prkaa2*	0.062	0.107	0.209	0.212	0.408
3-day post-SCI group vs. the control group	*Myc*	*Atf2*	0	0.041	0	0.409	0.409
3-day post-SCI group vs. the control group	*Myc*	*Mgmt*	0	0	0	0.414	0.414
3-day post-SCI group vs. the control group	*Myc*	*Cd38*	0	0	0	0.415	0.415
3-day post-SCI group vs. the control group	*Myc*	*Banf1*	0	0	0	0.416	0.416
3-day post-SCI group vs. the control group	*Myc*	*Tlr4*	0.051	0.043	0	0.413	0.42
3-day post-SCI group vs. the control group	*Myc*	*Rest*	0.068	0.088	0.134	0.312	0.426
3-day post-SCI group vs. the control group	*Myc*	*Nfatc1*	0.062	0	0	0.429	0.441
3-day post-SCI group vs. the control group	*Myc*	*Itga6*	0.061	0	0	0.431	0.442
3-day post-SCI group vs. the control group	*Myc*	*Pxn*	0.065	0.066	0	0.42	0.45
3-day post-SCI group vs. the control group	*Myc*	*Rbbp7*	0	0.05	0.29	0.27	0.465
3-day post-SCI group vs. the control group	*Myc*	*Cttn*	0	0	0	0.479	0.48
3-day post-SCI group vs. the control group	*Myc*	*Btk*	0.051	0.086	0.129	0.394	0.481
3-day post-SCI group vs. the control group	*Myc*	*Cat*	0.051	0.045	0	0.476	0.483
3-day post-SCI group vs. the control group	*Myc*	*Stat6*	0.044	0.133	0	0.429	0.485
3-day post-SCI group vs. the control group	*Myc*	*Ppargc1a*	0.065	0	0	0.482	0.495
3-day post-SCI group vs. the control group	*Myc*	*Atr*	0	0.143	0.194	0.333	0.499
3-day post-SCI group vs. the control group	*Myc*	*Rxrb*	0	0.135	0.496	0.045	0.547
3-day post-SCI group vs. the control group	*Myc*	*Cdkn2c*	0.061	0.132	0.191	0.416	0.563
3-day post-SCI group vs. the control group	*Myc*	*Ets1*	0.075	0.069	0	0.54	0.569
3-day post-SCI group vs. the control group	*Myc*	*Nono*	0.061	0.443	0	0.247	0.572
3-day post-SCI group vs. the control group	*Myc*	*Ccna2*	0.095	0.047	0	0.556	0.584
3-day post-SCI group vs. the control group	*Myc*	*Ptgs2*	0	0	0	0.597	0.597

3-day post-SCI group vs. the control group	*Myc*	*Mapk8*	0	0.198	0	0.536	0.612
3-day post-SCI group vs. the control group	*Myc*	*Fosl1*	0.15	0.05	0	0.563	0.616
3-day post-SCI group vs. the control group	*Myc*	*Parp1*	0	0.045	0	0.621	0.623
3-day post-SCI group vs. the control group	*Myc*	*Junb*	0.08	0.135	0.318	0.387	0.623
3-day post-SCI group vs. the control group	*Myc*	*Smad1*	0	0.134	0.366	0.374	0.626
3-day post-SCI group vs. the control group	*Myc*	*Nfkb1*	0.112	0.132	0.191	0.511	0.654
3-day post-SCI group vs. the control group	*Myc*	*Jak2*	0.051	0.086	0.129	0.607	0.664
3-day post-SCI group vs. the control group	*Myc*	*Met*	0.051	0.089	0.129	0.627	0.681
3-day post-SCI group vs. the control group	*Myc*	*Nfe2l2*	0.057	0.164	0	0.686	0.731
3-day post-SCI group vs. the control group	*Myc*	*Sirt1*	0	0.206	0	0.678	0.733
3-day post-SCI group vs. the control group	*Myc*	*Mcl1*	0.066	0.052	0	0.748	0.758
3-day post-SCI group vs. the control group	*Myc*	*Casp3*	0	0.049	0	0.793	0.795
3-day post-SCI group vs. the control group	*Myc*	*Apc*	0	0.693	0	0.513	0.844
3-day post-SCI group vs. the control group	*Myc*	*Actb*	0.061	0.089	0.169	0.824	0.858
3-day post-SCI group vs. the control group	*Myc*	*Rela*	0	0.269	0.548	0.617	0.862
3-day post-SCI group vs. the control group	*Myc*	*Ezh2*	0.062	0.206	0	0.904	0.922
3-day post-SCI group vs. the control group	*Myc*	*Jun*	0.062	0.27	0.548	0.856	0.949
3-day post-SCI group vs. the control group	*Myc*	*Hdac1*	0.061	0.401	0.381	0.901	0.961
3-day post-SCI group vs. the control group	*Myc*	*Mapk3*	0	0.261	0.905	0.708	0.977
3-day post-SCI group vs. the control group	*Myc*	*Cdkn2b*	0	0.471	0.904	0.704	0.983
3-day post-SCI group vs. the control group	*Myc*	*Fbxw7*	0	0.852	0.585	0.94	0.996
3-day post-SCI group vs. the control group	*Mylk*	*Mapk14*	0	0.105	0.335	0.164	0.41
3-day post-SCI group vs. the control group	*Mylk*	*Actb*	0.065	0.201	0	0.379	0.496
3-day post-SCI group vs. the control group	*Mylk*	*Mapk3*	0	0.131	0.629	0.324	0.705
3-day post-SCI group vs. the control group	*Mylk*	*Itgb5*	0.061	0.089	0.756	0.125	0.793
3-day post-SCI group vs. the control group	*Mylk*	*Pxn*	0.062	0.049	0.864	0.414	0.919
3-day post-SCI group vs. the control group	*Mylk*	*Cttn*	0.061	0.348	0	0.95	0.967
3-day post-SCI group vs. the control group	*Ncf1*	*Mapk14*	0	0.425	0.9	0.63	0.976
3-day post-SCI group vs. the control group	*Ncf1*	*Hmox1*	0	0	0	0.545	0.545
3-day post-SCI group vs. the control group	*Ncf1*	*Sod2*	0.062	0	0	0.464	0.475
3-day post-SCI group vs. the control group	*Ncf1*	*Gpx7*	0	0	0	0.403	0.403
3-day post-SCI group vs. the control group	*Ncf1*	*Btk*	0.234	0.091	0	0.213	0.405
3-day post-SCI group vs. the control group	*Ncf1*	*Ptgs2*	0.051	0	0	0.417	0.424
3-day post-SCI group vs. the control group	*Ncf1*	*Jun*	0	0	0	0.466	0.465
3-day post-SCI group vs. the control group	*Ncf1*	*Aif1*	0.203	0.057	0	0.353	0.471
3-day post-SCI group vs. the control group	*Ncf1*	*Casp3*	0	0	0	0.477	0.477

3-day post-SCI group vs. the control group	*Ncf1*	*Arhgdia*	0.06	0.088	0	0.452	0.489
3-day post-SCI group vs. the control group	*Ncf1*	*Pxn*	0	0.163	0	0.445	0.516
3-day post-SCI group vs. the control group	*Ncf1*	*Gpx1*	0.062	0	0	0.521	0.531
3-day post-SCI group vs. the control group	*Ncf1*	*Tlr4*	0.09	0.046	0	0.528	0.554
3-day post-SCI group vs. the control group	*Ncf1*	*P4hb*	0	0.047	0	0.557	0.56
3-day post-SCI group vs. the control group	*Ncf1*	*Nfe2l2*	0	0.434	0	0.4	0.645
3-day post-SCI group vs. the control group	*Ncf1*	*Actb*	0	0.194	0	0.603	0.666
3-day post-SCI group vs. the control group	*Ncf1*	*Cat*	0.051	0	0	0.695	0.698
3-day post-SCI group vs. the control group	*Ncf1*	*Xdh*	0.089	0	0	0.718	0.732
3-day post-SCI group vs. the control group	*Ncf1*	*Cttn*	0.063	0.13	0	0.867	0.883
3-day post-SCI group vs. the control group	*Ncf1*	*Vcam1*	0.062	0	0.9	0.482	0.947
3-day post-SCI group vs. the control group	*Ncf1*	*Mapk3*	0	0.072	0.9	0.524	0.952
3-day post-SCI group vs. the control group	*Ncf1*	*Nox4*	0	0.054	0.815	0.934	0.987
3-day post-SCI group vs. the control group	*Ndufa12*	*Ndufa6*	0.818	0.99	0.8	0.688	0.999
3-day post-SCI group vs. the control group	*Ndufa12*	*Pink1*	0.062	0	0.6	0.087	0.627
3-day post-SCI group vs. the control group	*Ndufa12*	*Ndufs8*	0.852	0.978	0.8	0.696	0.999
3-day post-SCI group vs. the control group	*Ndufa12*	*Mapt*	0	0.433	0.6	0.071	0.77
3-day post-SCI group vs. the control group	*Ndufa12*	*Snca*	0	0	0.6	0	0.6
3-day post-SCI group vs. the control group	*Ndufa6*	*Eed*	0	0.433	0	0	0.433
3-day post-SCI group vs. the control group	*Ndufa6*	*Snca*	0	0	0.6	0	0.6
3-day post-SCI group vs. the control group	*Ndufa6*	*Mapt*	0	0	0.6	0	0.6
3-day post-SCI group vs. the control group	*Ndufa6*	*Pink1*	0.098	0	0.6	0.212	0.691
3-day post-SCI group vs. the control group	*Ndufa6*	*Ndufa12*	0.818	0.99	0.8	0.688	0.999
3-day post-SCI group vs. the control group	*Ndufa6*	*Ndufs8*	0.831	0.994	0.8	0.63	0.999
3-day post-SCI group vs. the control group	*Ndufs8*	*Sod2*	0.49	0	0	0.12	0.533
3-day post-SCI group vs. the control group	*Ndufs8*	*Ndufa6*	0.831	0.994	0.8	0.63	0.999
3-day post-SCI group vs. the control group	*Ndufs8*	*Etfdh*	0.067	0	0	0.395	0.412
3-day post-SCI group vs. the control group	*Ndufs8*	*Pink1*	0.062	0	0.6	0.128	0.644
3-day post-SCI group vs. the control group	*Ndufs8*	*Mapt*	0	0	0.6	0	0.6
3-day post-SCI group vs. the control group	*Ndufs8*	*Snca*	0	0	0.6	0	0.6
3-day post-SCI group vs. the control group	*Ndufs8*	*Ndufa12*	0.852	0.978	0.8	0.696	0.999
3-day post-SCI group vs. the control group	*Nfatc1*	*Mapk14*	0	0.087	0.676	0.525	0.847
3-day post-SCI group vs. the control group	*Nfatc1*	*Mgst1*	0.057	0	0	0.407	0.417
3-day post-SCI group vs. the control group	*Nfatc1*	*Fos*	0	0.142	0.676	0.916	0.974
3-day post-SCI group vs. the control group	*Nfatc1*	*Myc*	0.062	0	0	0.429	0.441
3-day post-SCI group vs. the control group	*Nfatc1*	*Aif1*	0.056	0.14	0.238	0.159	0.41

3-day post-SCI group vs. the control group	*Nfatc1*	*Fosl1*	0.062	0.074	0.676	0.294	0.774
3-day post-SCI group vs. the control group	*Nfatc1*	*Rela*	0.075	0	0	0.493	0.511
3-day post-SCI group vs. the control group	*Nfatc1*	*Nfkb1*	0.082	0	0	0.463	0.486
3-day post-SCI group vs. the control group	*Nfatc1*	*Btk*	0.098	0	0.183	0.43	0.543
3-day post-SCI group vs. the control group	*Nfatc1*	*Ppp1ca*	0	0.209	0.302	0.23	0.537
3-day post-SCI group vs. the control group	*Nfatc1*	*Mapk3*	0	0.058	0.932	0.438	0.961
3-day post-SCI group vs. the control group	*Nfatc1*	*Ppp3ca*	0	0.917	0.932	0.67	0.997
3-day post-SCI group vs. the control group	*Nfatc1*	*Rcan1*	0	0	0	0.653	0.653
3-day post-SCI group vs. the control group	*Nfatc1*	*Junb*	0.078	0.087	0.676	0.213	0.756
3-day post-SCI group vs. the control group	*Nfatc1*	*Stat6*	0.095	0	0	0.574	0.598
3-day post-SCI group vs. the control group	*Nfatc1*	*Actb*	0	0	0.475	0.583	0.771
3-day post-SCI group vs. the control group	*Nfatc1*	*Jun*	0.058	0.132	0.676	0.88	0.964
3-day post-SCI group vs. the control group	*Nfatc1*	*Mapk8*	0	0	0.932	0.571	0.969
3-day post-SCI group vs. the control group	*Nfatc1*	*Atf2*	0	0.131	0.308	0.196	0.474
3-day post-SCI group vs. the control group	*Nfatc1*	*Mapk10*	0	0	0.903	0.087	0.908
3-day post-SCI group vs. the control group	*Nfe2l2*	*Mapk14*	0	0.091	0	0.687	0.704
3-day post-SCI group vs. the control group	*Nfe2l2*	*Hmox1*	0.105	0	0.419	0.898	0.942
3-day post-SCI group vs. the control group	*Nfe2l2*	*Sod2*	0	0	0	0.707	0.707
3-day post-SCI group vs. the control group	*Nfe2l2*	*Mgst1*	0.109	0	0	0.463	0.501
3-day post-SCI group vs. the control group	*Nfe2l2*	*Ncf1*	0	0.434	0	0.4	0.645
3-day post-SCI group vs. the control group	*Nfe2l2*	*Txnrd1*	0.067	0	0	0.685	0.694
3-day post-SCI group vs. the control group	*Nfe2l2*	*Hif1a*	0	0	0	0.658	0.658
3-day post-SCI group vs. the control group	*Nfe2l2*	*Fos*	0.061	0.443	0.293	0.416	0.755
3-day post-SCI group vs. the control group	*Nfe2l2*	*Gpx8*	0	0	0	0.66	0.66
3-day post-SCI group vs. the control group	*Nfe2l2*	*Myc*	0.057	0.164	0	0.686	0.731
3-day post-SCI group vs. the control group	*Nfe2l2*	*Xdh*	0.079	0	0	0.551	0.568
3-day post-SCI group vs. the control group	*Nfe2l2*	*Fosl1*	0	0.058	0.293	0.222	0.436
3-day post-SCI group vs. the control group	*Nfe2l2*	*Rela*	0.084	0.083	0	0.403	0.455
3-day post-SCI group vs. the control group	*Nfe2l2*	*Parp1*	0	0	0	0.416	0.416
3-day post-SCI group vs. the control group	*Nfe2l2*	*Cat*	0.079	0	0	0.834	0.84
3-day post-SCI group vs. the control group	*Nfe2l2*	*Vcam1*	0	0	0	0.4	0.4
3-day post-SCI group vs. the control group	*Nfe2l2*	*Nfkb1*	0.082	0.046	0.177	0.307	0.434
3-day post-SCI group vs. the control group	*Nfe2l2*	*Gpx7*	0	0	0	0.6	0.6
3-day post-SCI group vs. the control group	*Nfe2l2*	*Pink1*	0	0	0	0.458	0.458
3-day post-SCI group vs. the control group	*Nfe2l2*	*Nox4*	0	0	0	0.632	0.632
3-day post-SCI group vs. the control group	*Nfe2l2*	*Bax*	0	0	0	0.455	0.455

3-day post-SCI group vs. the control group	*Nfe2l2*	*Btk*	0	0.087	0	0.422	0.45
3-day post-SCI group vs. the control group	*Nfe2l2*	*Gsr*	0.062	0	0	0.718	0.724
3-day post-SCI group vs. the control group	*Nfe2l2*	*Gclc*	0	0	0	0.883	0.883
3-day post-SCI group vs. the control group	*Nfe2l2*	*Ptgs2*	0	0	0	0.619	0.619
3-day post-SCI group vs. the control group	*Nfe2l2*	*Tlr4*	0.069	0	0	0.53	0.543
3-day post-SCI group vs. the control group	*Nfe2l2*	*Srxn1*	0	0	0	0.552	0.552
3-day post-SCI group vs. the control group	*Nfe2l2*	*Mapk3*	0.052	0.103	0	0.545	0.579
3-day post-SCI group vs. the control group	*Nfe2l2*	*Junb*	0.069	0.131	0.362	0.197	0.53
3-day post-SCI group vs. the control group	*Nfe2l2*	*Gpx1*	0.079	0	0	0.689	0.702
3-day post-SCI group vs. the control group	*Nfe2l2*	*Gpx3*	0.057	0	0	0.405	0.415
3-day post-SCI group vs. the control group	*Nfe2l2*	*Casp3*	0	0.067	0	0.67	0.679
3-day post-SCI group vs. the control group	*Nfe2l2*	*Actb*	0	0	0	0.682	0.682
3-day post-SCI group vs. the control group	*Nfe2l2*	*Fbxw7*	0	0.198	0	0.312	0.424
3-day post-SCI group vs. the control group	*Nfe2l2*	*Snca*	0	0	0	0.468	0.468
3-day post-SCI group vs. the control group	*Nfe2l2*	*Atf2*	0	0.086	0.293	0.262	0.482
3-day post-SCI group vs. the control group	*Nfe2l2*	*Mapk8*	0	0.052	0	0.483	0.488
3-day post-SCI group vs. the control group	*Nfe2l2*	*Txnip*	0.1	0	0	0.578	0.604
3-day post-SCI group vs. the control group	*Nfe2l2*	*Sirt1*	0	0	0	0.725	0.725
3-day post-SCI group vs. the control group	*Nfe2l2*	*Jun*	0	0.131	0.362	0.745	0.846
3-day post-SCI group vs. the control group	*Nfe2l2*	*Ppargc1a*	0	0	0	0.855	0.855
3-day post-SCI group vs. the control group	*Nfkb1*	*Mapk14*	0.061	0.13	0.676	0.585	0.875
3-day post-SCI group vs. the control group	*Nfkb1*	*Cdk4*	0	0.144	0.182	0.261	0.437
3-day post-SCI group vs. the control group	*Nfkb1*	*Sod2*	0	0.106	0	0.386	0.428
3-day post-SCI group vs. the control group	*Nfkb1*	*Cdk1*	0	0.144	0.182	0.24	0.421
3-day post-SCI group vs. the control group	*Nfkb1*	*Hif1a*	0.061	0.055	0.171	0.468	0.556
3-day post-SCI group vs. the control group	*Nfkb1*	*Fos*	0.051	0.087	0.12	0.552	0.613
3-day post-SCI group vs. the control group	*Nfkb1*	*Rb1*	0	0.079	0.204	0.277	0.424
3-day post-SCI group vs. the control group	*Nfkb1*	*Myc*	0.112	0.132	0.191	0.511	0.654
3-day post-SCI group vs. the control group	*Nfkb1*	*Fosl1*	0.063	0.058	0.12	0.354	0.431
3-day post-SCI group vs. the control group	*Nfkb1*	*Rela*	0.095	0.999	0.932	0.991	0.999
3-day post-SCI group vs. the control group	*Nfkb1*	*Il18rap*	0	0	0.676	0.112	0.7
3-day post-SCI group vs. the control group	*Nfkb1*	*Parp1*	0.063	0.429	0.138	0.32	0.644
3-day post-SCI group vs. the control group	*Nfkb1*	*Vcam1*	0.061	0.047	0	0.475	0.489
3-day post-SCI group vs. the control group	*Nfkb1*	*Snca*	0	0.151	0.211	0.196	0.414
3-day post-SCI group vs. the control group	*Nfkb1*	*Nfe2l2*	0.082	0.046	0.177	0.307	0.434
3-day post-SCI group vs. the control group	*Nfkb1*	*Junb*	0.096	0.047	0.204	0.284	0.443

3-day post-SCI group vs. the control group	*Nfkb1*	*Mcl1*	0.078	0.055	0.142	0.35	0.449
3-day post-SCI group vs. the control group	*Nfkb1*	*Nfatc1*	0.082	0	0	0.463	0.486
3-day post-SCI group vs. the control group	*Nfkb1*	*Btk*	0.071	0.132	0.127	0.36	0.489
3-day post-SCI group vs. the control group	*Nfkb1*	*Stat6*	0.098	0.13	0.151	0.399	0.546
3-day post-SCI group vs. the control group	*Nfkb1*	*Ets1*	0.068	0.322	0	0.43	0.608
3-day post-SCI group vs. the control group	*Nfkb1*	*Fbxw7*	0	0.272	0	0.493	0.616
3-day post-SCI group vs. the control group	*Nfkb1*	*Ptgs2*	0.081	0.077	0	0.586	0.618
3-day post-SCI group vs. the control group	*Nfkb1*	*Casp3*	0	0.133	0.138	0.552	0.635
3-day post-SCI group vs. the control group	*Nfkb1*	*Sirt1*	0.062	0.461	0	0.351	0.643
3-day post-SCI group vs. the control group	*Nfkb1*	*Uba52*	0	0.141	0.6	0.049	0.644
3-day post-SCI group vs. the control group	*Nfkb1*	*Actb*	0	0.122	0	0.675	0.703
3-day post-SCI group vs. the control group	*Nfkb1*	*Jun*	0.063	0.047	0.204	0.725	0.778
3-day post-SCI group vs. the control group	*Nfkb1*	*Ripk1*	0.089	0.132	0.676	0.45	0.84
3-day post-SCI group vs. the control group	*Nfkb1*	*Mapk3*	0	0.13	0.676	0.501	0.847
3-day post-SCI group vs. the control group	*Nfkb1*	*Jak2*	0.147	0.132	0.676	0.454	0.851
3-day post-SCI group vs. the control group	*Nfkb1*	*Mapk10*	0.051	0.058	0.8	0.278	0.853
3-day post-SCI group vs. the control group	*Nfkb1*	*Tnfrsf1a*	0.069	0.047	0.676	0.61	0.872
3-day post-SCI group vs. the control group	*Nfkb1*	*Mapk8*	0.061	0.058	0.8	0.574	0.914
3-day post-SCI group vs. the control group	*Nfkb1*	*Tlr4*	0.082	0.07	0.8	0.674	0.937
3-day post-SCI group vs. the control group	*Nfkb1*	*Hdac1*	0.052	0.8	0.512	0.985	0.998
3-day post-SCI group vs. the control group	*Nme2*	*Mgst1*	0	0	0	0.445	0.445
3-day post-SCI group vs. the control group	*Nme2*	*Fkbp1b*	0.474	0.349	0	0	0.644
3-day post-SCI group vs. the control group	*Nme2*	*Cat*	0	0.355	0	0.213	0.47
3-day post-SCI group vs. the control group	*Nme2*	*Myc*	0.062	0	0	0.486	0.498
3-day post-SCI group vs. the control group	*Nme2*	*Gnb2*	0	0.552	0	0.057	0.559
3-day post-SCI group vs. the control group	*Nme2*	*Ppif*	0.188	0.458	0	0.144	0.591
3-day post-SCI group vs. the control group	*Nme2*	*Rpl13a*	0.645	0	0	0	0.645
3-day post-SCI group vs. the control group	*Nme2*	*Rrm2b*	0.101	0	0.65	0.074	0.683
3-day post-SCI group vs. the control group	*Nme2*	*Gch1*	0.064	0.135	0.8	0.07	0.84
3-day post-SCI group vs. the control group	*Nme2*	*Nudt2*	0.096	0	0.9	0.061	0.907
3-day post-SCI group vs. the control group	*Nono*	*Myc*	0.061	0.443	0	0.247	0.572
3-day post-SCI group vs. the control group	*Nono*	*Sfpq*	0.709	0.841	0.629	0.986	0.982
3-day post-SCI group vs. the control group	*Nono*	*Eed*	0.145	0.433	0	0	0.494
3-day post-SCI group vs. the control group	*Nono*	*Hdac1*	0.142	0.345	0	0.238	0.534
3-day post-SCI group vs. the control group	*Nono*	*Actb*	0.115	0.291	0	0.395	0.587
3-day post-SCI group vs. the control group	*Nono*	*Rbpms*	0	0	0	0.671	0.671

3-day post-SCI group vs. the control group	*Nox4*	*Mapk14*	0	0.069	0	0.622	0.633
3-day post-SCI group vs. the control group	*Nox4*	*Hmox1*	0	0	0	0.685	0.685
3-day post-SCI group vs. the control group	*Nox4*	*Sod2*	0.067	0	0	0.645	0.655
3-day post-SCI group vs. the control group	*Nox4*	*Mgst1*	0	0	0	0.453	0.453
3-day post-SCI group vs. the control group	*Nox4*	*Ncf1*	0	0.054	0.815	0.934	0.987
3-day post-SCI group vs. the control group	*Nox4*	*Hif1a*	0	0	0	0.639	0.639
3-day post-SCI group vs. the control group	*Nox4*	*Gpx8*	0.061	0	0	0.497	0.507
3-day post-SCI group vs. the control group	*Nox4*	*Xdh*	0.062	0	0	0.737	0.742
3-day post-SCI group vs. the control group	*Nox4*	*P4hb*	0.062	0.061	0	0.699	0.712
3-day post-SCI group vs. the control group	*Nox4*	*Prdx4*	0	0	0	0.43	0.43
3-day post-SCI group vs. the control group	*Nox4*	*Cat*	0.062	0	0	0.796	0.8
3-day post-SCI group vs. the control group	*Nox4*	*Vcam1*	0.063	0	0	0.541	0.551
3-day post-SCI group vs. the control group	*Nox4*	*Gpx7*	0	0	0	0.51	0.51
3-day post-SCI group vs. the control group	*Nox4*	*Gclc*	0.061	0	0	0.404	0.416
3-day post-SCI group vs. the control group	*Nox4*	*Ppargc1a*	0	0	0	0.466	0.465
3-day post-SCI group vs. the control group	*Nox4*	*Txnip*	0	0	0	0.465	0.465
3-day post-SCI group vs. the control group	*Nox4*	*Ptgs2*	0.05	0.088	0	0.489	0.519
3-day post-SCI group vs. the control group	*Nox4*	*Gpx3*	0	0	0	0.521	0.521
3-day post-SCI group vs. the control group	*Nox4*	*Sirt1*	0	0	0	0.531	0.531
3-day post-SCI group vs. the control group	*Nox4*	*Gsr*	0	0	0	0.537	0.537
3-day post-SCI group vs. the control group	*Nox4*	*Mapk3*	0	0.069	0	0.543	0.557
3-day post-SCI group vs. the control group	*Nox4*	*Jun*	0.061	0.058	0	0.542	0.56
3-day post-SCI group vs. the control group	*Nox4*	*Casp3*	0	0	0	0.572	0.572
3-day post-SCI group vs. the control group	*Nox4*	*Nfe2l2*	0	0	0	0.632	0.632
3-day post-SCI group vs. the control group	*Nox4*	*Actb*	0	0.067	0	0.625	0.636
3-day post-SCI group vs. the control group	*Nox4*	*Gpx1*	0.061	0	0	0.64	0.648
3-day post-SCI group vs. the control group	*Nox4*	*Pxn*	0.062	0	0	0.69	0.697
3-day post-SCI group vs. the control group	*Nox4*	*Sirpa*	0	0.05	0	0.697	0.699
3-day post-SCI group vs. the control group	*Nox4*	*Tlr4*	0	0.227	0	0.988	0.991
3-day post-SCI group vs. the control group	*Nudt2*	*Nme2*	0.096	0	0.9	0.061	0.907
3-day post-SCI group vs. the control group	*Nudt2*	*Eif2s1*	0.143	0.4	0	0.08	0.485
3-day post-SCI group vs. the control group	*Nudt2*	*Gch1*	0.062	0	0.8	0	0.807
3-day post-SCI group vs. the control group	*Oxr1*	*Sod2*	0.062	0	0	0.585	0.595
3-day post-SCI group vs. the control group	*Oxr1*	*Cat*	0	0	0	0.446	0.446
3-day post-SCI group vs. the control group	*Oxr1*	*Eif2s1*	0	0	0	0.428	0.428
3-day post-SCI group vs. the control group	*Oxr1*	*Gpx1*	0.064	0	0	0.491	0.503

3-day post-SCI group vs. the control group	*Oxr1*	*Jun*	0	0	0	0.446	0.447
3-day post-SCI group vs. the control group	*Oxr1*	*Tnik*	0	0	0	0.537	0.537
3-day post-SCI group vs. the control group	*P4hb*	*Ncf1*	0	0.047	0	0.557	0.56
3-day post-SCI group vs. the control group	*P4hb*	*Txnrd1*	0.105	0.233	0	0.379	0.541
3-day post-SCI group vs. the control group	*P4hb*	*Hif1a*	0	0	0.629	0.261	0.714
3-day post-SCI group vs. the control group	*P4hb*	*Gpx8*	0.087	0.134	0.9	0.333	0.94
3-day post-SCI group vs. the control group	*P4hb*	*Impact*	0.065	0.685	0	0	0.694
3-day post-SCI group vs. the control group	*P4hb*	*Hbegf*	0	0	0	0.515	0.515
3-day post-SCI group vs. the control group	*P4hb*	*Tnfrsf1a*	0.105	0	0.217	0.293	0.461
3-day post-SCI group vs. the control group	*P4hb*	*Txnip*	0.09	0.14	0	0.378	0.471
3-day post-SCI group vs. the control group	*P4hb*	*Atp2a2*	0.082	0.132	0	0.396	0.477
3-day post-SCI group vs. the control group	*P4hb*	*Itgb5*	0.07	0.083	0	0.454	0.493
3-day post-SCI group vs. the control group	*P4hb*	*Cat*	0.089	0.134	0	0.431	0.512
3-day post-SCI group vs. the control group	*P4hb*	*Casp3*	0.061	0.15	0	0.463	0.533
3-day post-SCI group vs. the control group	*P4hb*	*Eif2s1*	0.062	0.358	0	0.38	0.594
3-day post-SCI group vs. the control group	*P4hb*	*Prdx6*	0.062	0.223	0	0.537	0.634
3-day post-SCI group vs. the control group	*P4hb*	*Gsr*	0.094	0.233	0	0.511	0.634
3-day post-SCI group vs. the control group	*P4hb*	*Actb*	0.066	0.066	0	0.643	0.662
3-day post-SCI group vs. the control group	*P4hb*	*Nox4*	0.062	0.061	0	0.699	0.712
3-day post-SCI group vs. the control group	*P4hb*	*Prdx4*	0.107	0.748	0	0.765	0.942
3-day post-SCI group vs. the control group	*P4hb*	*Gpx7*	0.061	0.134	0.9	0.557	0.959
3-day post-SCI group vs. the control group	*Paip1*	*Tnrc6a*	0	0	0	0.418	0.418
3-day post-SCI group vs. the control group	*Paip1*	*Eif2s1*	0.161	0.229	0.47	0.365	0.753
3-day post-SCI group vs. the control group	*Parp1*	*Braf*	0.061	0.056	0	0.494	0.512
3-day post-SCI group vs. the control group	*Parp1*	*Mapk14*	0	0.058	0	0.629	0.636
3-day post-SCI group vs. the control group	*Parp1*	*Cdk4*	0.223	0	0	0.596	0.673
3-day post-SCI group vs. the control group	*Parp1*	*Mgst1*	0	0	0	0.43	0.43
3-day post-SCI group vs. the control group	*Parp1*	*Endog*	0	0	0	0.426	0.426
3-day post-SCI group vs. the control group	*Parp1*	*Cdk1*	0.121	0	0	0.653	0.682
3-day post-SCI group vs. the control group	*Parp1*	*Hif1a*	0	0.13	0	0.595	0.633
3-day post-SCI group vs. the control group	*Parp1*	*Ppif*	0	0	0	0.463	0.464
3-day post-SCI group vs. the control group	*Parp1*	*Ripk3*	0.068	0.051	0	0.378	0.402
3-day post-SCI group vs. the control group	*Parp1*	*Myc*	0	0.045	0	0.621	0.623
3-day post-SCI group vs. the control group	*Parp1*	*Rela*	0	0.418	0	0.439	0.659
3-day post-SCI group vs. the control group	*Parp1*	*Ets1*	0	0.132	0	0.337	0.4
3-day post-SCI group vs. the control group	*Parp1*	*Ezh2*	0.109	0	0	0.368	0.413

3-day post-SCI group vs. the control group	*Parp1*	*Nfe2l2*	0	0	0	0.416	0.416
3-day post-SCI group vs. the control group	*Parp1*	*Ripk1*	0.068	0.051	0	0.411	0.433
3-day post-SCI group vs. the control group	*Parp1*	*Txnip*	0.063	0.201	0	0.327	0.452
3-day post-SCI group vs. the control group	*Parp1*	*Eed*	0	0.433	0	0.079	0.455
3-day post-SCI group vs. the control group	*Parp1*	*Mgmt*	0	0	0	0.464	0.463
3-day post-SCI group vs. the control group	*Parp1*	*Bax*	0	0.053	0	0.463	0.469
3-day post-SCI group vs. the control group	*Parp1*	*Mapk8*	0	0	0	0.472	0.472
3-day post-SCI group vs. the control group	*Parp1*	*Cat*	0	0	0	0.485	0.485
3-day post-SCI group vs. the control group	*Parp1*	*Atr*	0.063	0.068	0	0.504	0.529
3-day post-SCI group vs. the control group	*Parp1*	*Stat6*	0.062	0	0	0.538	0.548
3-day post-SCI group vs. the control group	*Parp1*	*Ppargc1a*	0	0	0	0.556	0.556
3-day post-SCI group vs. the control group	*Parp1*	*Ctnna1*	0.573	0	0	0.043	0.573
3-day post-SCI group vs. the control group	*Parp1*	*Uba52*	0	0	0.6	0	0.6
3-day post-SCI group vs. the control group	*Parp1*	*Jun*	0.064	0.135	0	0.57	0.622
3-day post-SCI group vs. the control group	*Parp1*	*Nfkb1*	0.063	0.429	0.138	0.32	0.644
3-day post-SCI group vs. the control group	*Parp1*	*Mapk3*	0.062	0.13	0	0.623	0.665
3-day post-SCI group vs. the control group	*Parp1*	*Mcl1*	0	0.053	0	0.661	0.665
3-day post-SCI group vs. the control group	*Parp1*	*Hdac1*	0.063	0.342	0	0.554	0.701
3-day post-SCI group vs. the control group	*Parp1*	*Sirt1*	0.061	0.13	0	0.76	0.787
3-day post-SCI group vs. the control group	*Parp1*	*Pcna*	0.142	0.446	0.6	0.067	0.799
3-day post-SCI group vs. the control group	*Parp1*	*Actb*	0.061	0	0	0.81	0.814
3-day post-SCI group vs. the control group	*Parp1*	*Casp3*	0.062	0.816	0.932	0.872	0.998
3-day post-SCI group vs. the control group	*Pawr*	*Rela*	0.051	0	0.676	0.05	0.682
3-day post-SCI group vs. the control group	*Pawr*	*Mcl1*	0	0	0	0.414	0.414
3-day post-SCI group vs. the control group	*Pcna*	*Cdc20*	0.634	0.347	0	0.339	0.828
3-day post-SCI group vs. the control group	*Pcna*	*Cdk4*	0.336	0.948	0.905	0.549	0.998
3-day post-SCI group vs. the control group	*Pcna*	*Ube2a*	0.11	0.401	0	0.391	0.647
3-day post-SCI group vs. the control group	*Pcna*	*Cdk1*	0.864	0.457	0.828	0.554	0.993
3-day post-SCI group vs. the control group	*Pcna*	*Dhfr*	0.456	0	0	0.088	0.483
3-day post-SCI group vs. the control group	*Pcna*	*Rb1*	0.113	0	0.698	0.251	0.782
3-day post-SCI group vs. the control group	*Pcna*	*Rrm2b*	0.657	0.203	0	0.185	0.757
3-day post-SCI group vs. the control group	*Pcna*	*Mcm4*	0.893	0.199	0	0.45	0.948
3-day post-SCI group vs. the control group	*Pcna*	*Parp1*	0.142	0.446	0.6	0.067	0.799
3-day post-SCI group vs. the control group	*Pcna*	*Cdkn2c*	0.08	0.052	0.207	0.263	0.422
3-day post-SCI group vs. the control group	*Pcna*	*Ect2*	0.429	0	0	0	0.429
3-day post-SCI group vs. the control group	*Pcna*	*Ezh2*	0.307	0.208	0	0.105	0.467

3-day post-SCI group vs. the control group	*Pcna*	*Melk*	0.486	0	0	0.062	0.497
3-day post-SCI group vs. the control group	*Pcna*	*Cdkn2b*	0	0.052	0.207	0.454	0.554
3-day post-SCI group vs. the control group	*Pcna*	*Eed*	0.461	0.433	0	0	0.681
3-day post-SCI group vs. the control group	*Pcna*	*Rbbp7*	0.583	0.215	0	0.144	0.695
3-day post-SCI group vs. the control group	*Pcna*	*Uba52*	0.078	0.387	0.6	0	0.754
3-day post-SCI group vs. the control group	*Pcna*	*Tfdp1*	0.302	0	0.548	0.298	0.759
3-day post-SCI group vs. the control group	*Pcna*	*Atr*	0.098	0.571	0	0.445	0.766
3-day post-SCI group vs. the control group	*Pcna*	*Ccna2*	0.569	0.56	0.789	0.226	0.965
3-day post-SCI group vs. the control group	*Pdgfra*	*Rb1*	0	0	0	0.4	0.4
3-day post-SCI group vs. the control group	*Pdgfra*	*Myc*	0	0.055	0	0.398	0.407
3-day post-SCI group vs. the control group	*Pdgfra*	*Aif1*	0	0	0	0.411	0.411
3-day post-SCI group vs. the control group	*Pdgfra*	*Itga6*	0	0.057	0.352	0.392	0.596
3-day post-SCI group vs. the control group	*Pdgfra*	*Vcam1*	0.106	0	0	0.396	0.437
3-day post-SCI group vs. the control group	*Pdgfra*	*Jak2*	0.062	0.132	0.891	0.507	0.924
3-day post-SCI group vs. the control group	*Pdgfra*	*Mgmt*	0	0	0	0.506	0.506
3-day post-SCI group vs. the control group	*Pdgfra*	*Ezh2*	0	0.044	0	0.406	0.408
3-day post-SCI group vs. the control group	*Pdgfra*	*Stat6*	0	0.143	0.653	0.093	0.707
3-day post-SCI group vs. the control group	*Pdgfra*	*Casp3*	0	0.047	0	0.399	0.402
3-day post-SCI group vs. the control group	*Pdgfra*	*Cdkn2b*	0	0.069	0.13	0.382	0.456
3-day post-SCI group vs. the control group	*Pdgfra*	*Actb*	0	0.047	0.208	0.464	0.56
3-day post-SCI group vs. the control group	*Pdgfra*	*Jun*	0	0.073	0.199	0.311	0.444
3-day post-SCI group vs. the control group	*Pdgfra*	*Fbxw7*	0	0.064	0	0.402	0.417
3-day post-SCI group vs. the control group	*Pdk1*	*Bnip3*	0.223	0	0	0.444	0.55
3-day post-SCI group vs. the control group	*Pdk1*	*Hif1a*	0	0.131	0	0.548	0.59
3-day post-SCI group vs. the control group	*Pdk1*	*Pdk2*	0	0.818	0.9	0.492	0.981
3-day post-SCI group vs. the control group	*Pdk2*	*Pdk1*	0	0.818	0.9	0.492	0.981
3-day post-SCI group vs. the control group	*Pdlim1*	*Ctnna1*	0.082	0.203	0.334	0.103	0.505
3-day post-SCI group vs. the control group	*Pdlim1*	*Ezr*	0.061	0.049	0.338	0.156	0.434
3-day post-SCI group vs. the control group	*Pdlim1*	*Casp3*	0.05	0.046	0	0.405	0.414
3-day post-SCI group vs. the control group	*Pdlim1*	*Eed*	0	0.433	0	0	0.433
3-day post-SCI group vs. the control group	*Pdlim1*	*Itgb5*	0	0.164	0.313	0.105	0.441
3-day post-SCI group vs. the control group	*Pdlim1*	*Cttn*	0	0.137	0.419	0.115	0.517
3-day post-SCI group vs. the control group	*Phc3*	*Cdc20*	0	0.066	0.54	0.116	0.587
3-day post-SCI group vs. the control group	*Phc3*	*Rbbp7*	0	0	0.9	0.098	0.905
3-day post-SCI group vs. the control group	*Phc3*	*Rnf2*	0.052	0.68	0.938	0.756	0.994
3-day post-SCI group vs. the control group	*Phc3*	*Ezh2*	0	0.087	0.9	0.162	0.916

3-day post-SCI group vs. the control group	*Phc3*	*Casp3*	0	0	0	0.434	0.434
3-day post-SCI group vs. the control group	*Phc3*	*Eed*	0	0.147	0.9	0.374	0.942
3-day post-SCI group vs. the control group	*Phc3*	*Cbx6*	0	0.568	0.807	0.661	0.969
3-day post-SCI group vs. the control group	*Phc3*	*Tfdp1*	0	0	0.6	0	0.6
3-day post-SCI group vs. the control group	*Pink1*	*Sod2*	0.061	0.146	0	0.595	0.646
3-day post-SCI group vs. the control group	*Pink1*	*Endog*	0.082	0	0	0.529	0.55
3-day post-SCI group vs. the control group	*Pink1*	*Hif1a*	0	0	0	0.477	0.477
3-day post-SCI group vs. the control group	*Pink1*	*Ppif*	0	0	0	0.556	0.556
3-day post-SCI group vs. the control group	*Pink1*	*Ndufa6*	0.098	0	0.6	0.212	0.691
3-day post-SCI group vs. the control group	*Pink1*	*Cat*	0	0.14	0	0.594	0.636
3-day post-SCI group vs. the control group	*Pink1*	*Ubqln1*	0	0.261	0	0.225	0.403
3-day post-SCI group vs. the control group	*Pink1*	*Mcl1*	0	0.208	0	0.339	0.454
3-day post-SCI group vs. the control group	*Pink1*	*Nfe2l2*	0	0	0	0.458	0.458
3-day post-SCI group vs. the control group	*Pink1*	*Actb*	0	0	0	0.488	0.488
3-day post-SCI group vs. the control group	*Pink1*	*Casp3*	0	0	0	0.537	0.537
3-day post-SCI group vs. the control group	*Pink1*	*Mapt*	0.094	0	0	0.581	0.604
3-day post-SCI group vs. the control group	*Pink1*	*Ndufa12*	0.062	0	0.6	0.087	0.627
3-day post-SCI group vs. the control group	*Pink1*	*Sirt1*	0	0.146	0	0.59	0.634
3-day post-SCI group vs. the control group	*Pink1*	*Ndufs8*	0.062	0	0.6	0.128	0.644
3-day post-SCI group vs. the control group	*Pink1*	*Ppargc1a*	0.069	0	0	0.836	0.841
3-day post-SCI group vs. the control group	*Pink1*	*Atp13a2*	0.066	0	0	0.852	0.856
3-day post-SCI group vs. the control group	*Pink1*	*Bnip3*	0.078	0.13	0	0.848	0.868
3-day post-SCI group vs. the control group	*Pink1*	*Uba52*	0	0.399	0.9	0.057	0.938
3-day post-SCI group vs. the control group	*Pink1*	*Snca*	0.062	0.265	0	0.926	0.945
3-day post-SCI group vs. the control group	*Pink1*	*Htra2*	0.056	0.439	0.8	0.908	0.989
3-day post-SCI group vs. the control group	*Plekha1*	*Btk*	0	0	0	0.495	0.495
3-day post-SCI group vs. the control group	*Pon2*	*Rb1*	0	0	0	0.439	0.439
3-day post-SCI group vs. the control group	*Ppargc1a*	*Mapk14*	0	0.087	0.966	0.62	0.987
3-day post-SCI group vs. the control group	*Ppargc1a*	*Hmox1*	0	0	0	0.731	0.731
3-day post-SCI group vs. the control group	*Ppargc1a*	*Sod2*	0	0	0.8	0.744	0.946
3-day post-SCI group vs. the control group	*Ppargc1a*	*Hif1a*	0.048	0	0	0.689	0.691
3-day post-SCI group vs. the control group	*Ppargc1a*	*Gpx8*	0	0	0.8	0.225	0.838
3-day post-SCI group vs. the control group	*Ppargc1a*	*Ppif*	0	0	0	0.533	0.533
3-day post-SCI group vs. the control group	*Ppargc1a*	*Myc*	0.065	0	0	0.482	0.495
3-day post-SCI group vs. the control group	*Ppargc1a*	*Xdh*	0	0	0	0.431	0.431
3-day post-SCI group vs. the control group	*Ppargc1a*	*Rela*	0	0.225	0	0.901	0.92

3-day post-SCI group vs. the control group	*Ppargc1a*	*Parp1*	0	0	0	0.556	0.556
3-day post-SCI group vs. the control group	*Ppargc1a*	*Cat*	0	0	0	0.739	0.74
3-day post-SCI group vs. the control group	*Ppargc1a*	*Prkaa2*	0.097	0.13	0.9	0.716	0.974
3-day post-SCI group vs. the control group	*Ppargc1a*	*Gpx7*	0	0	0.8	0.243	0.842
3-day post-SCI group vs. the control group	*Ppargc1a*	*Pink1*	0.069	0	0	0.836	0.841
3-day post-SCI group vs. the control group	*Ppargc1a*	*Sfpq*	0	0.13	0	0.42	0.474
3-day post-SCI group vs. the control group	*Ppargc1a*	*Atp2a2*	0.081	0	0	0.521	0.541
3-day post-SCI group vs. the control group	*Ppargc1a*	*Nox4*	0	0	0	0.466	0.465
3-day post-SCI group vs. the control group	*Ppargc1a*	*Gsr*	0	0	0	0.432	0.432
3-day post-SCI group vs. the control group	*Ppargc1a*	*Rxrb*	0	0.18	0.502	0.155	0.625
3-day post-SCI group vs. the control group	*Ppargc1a*	*Mapk3*	0	0.058	0.354	0.466	0.647
3-day post-SCI group vs. the control group	*Ppargc1a*	*Gpx1*	0	0	0.8	0.602	0.917
3-day post-SCI group vs. the control group	*Ppargc1a*	*Gpx3*	0	0	0.8	0.237	0.84
3-day post-SCI group vs. the control group	*Ppargc1a*	*Casp3*	0	0	0	0.552	0.552
3-day post-SCI group vs. the control group	*Ppargc1a*	*Actb*	0	0	0	0.698	0.698
3-day post-SCI group vs. the control group	*Ppargc1a*	*Hdac1*	0	0	0	0.416	0.416
3-day post-SCI group vs. the control group	*Ppargc1a*	*Nfe2l2*	0	0	0	0.855	0.855
3-day post-SCI group vs. the control group	*Ppargc1a*	*Sirt1*	0	0.716	0.676	0.987	0.998
3-day post-SCI group vs. the control group	*Ppargc1a*	*Bnip3*	0.062	0	0	0.602	0.611
3-day post-SCI group vs. the control group	*Ppargc1a*	*Jun*	0	0	0	0.542	0.542
3-day post-SCI group vs. the control group	*Ppargc1a*	*Fbxw7*	0	0.439	0	0.191	0.527
3-day post-SCI group vs. the control group	*Ppargc1a*	*Atf2*	0	0	0	0.467	0.467
3-day post-SCI group vs. the control group	*Ppargc1a*	*Snca*	0.079	0	0	0.552	0.569
3-day post-SCI group vs. the control group	*Ppargc1a*	*Cd36*	0	0	0	0.529	0.529
3-day post-SCI group vs. the control group	*Ppargc1a*	*Ucp2*	0	0	0	0.744	0.744
3-day post-SCI group vs. the control group	*Ppargc1b*	*Sirt1*	0	0	0	0.506	0.506
3-day post-SCI group vs. the control group	*Ppif*	*Mapk14*	0	0.052	0.25	0.273	0.437
3-day post-SCI group vs. the control group	*Ppif*	*Sod2*	0.089	0.402	0	0.599	0.762
3-day post-SCI group vs. the control group	*Ppif*	*Mgst1*	0	0	0	0.719	0.719
3-day post-SCI group vs. the control group	*Ppif*	*Endog*	0.092	0.058	0	0.611	0.638
3-day post-SCI group vs. the control group	*Ppif*	*Fkbp1b*	0.159	0.657	0	0.509	0.845
3-day post-SCI group vs. the control group	*Ppif*	*Nme2*	0.188	0.458	0	0.144	0.591
3-day post-SCI group vs. the control group	*Ppif*	*Actb*	0.081	0.058	0	0.389	0.425
3-day post-SCI group vs. the control group	*Ppif*	*Mapk3*	0	0.052	0.25	0.277	0.441
3-day post-SCI group vs. the control group	*Ppif*	*Parp1*	0	0	0	0.463	0.464
3-day post-SCI group vs. the control group	*Ppif*	*Bnip3*	0	0	0	0.467	0.467

3-day post-SCI group vs. the control group	*Ppif*	*Sirt1*	0	0.131	0	0.421	0.475
3-day post-SCI group vs. the control group	*Ppif*	*Snca*	0	0.085	0	0.504	0.527
3-day post-SCI group vs. the control group	*Ppif*	*Ppargc1a*	0	0	0	0.533	0.533
3-day post-SCI group vs. the control group	*Ppif*	*Ripk1*	0	0.079	0	0.526	0.544
3-day post-SCI group vs. the control group	*Ppif*	*Htra2*	0.061	0.047	0	0.531	0.544
3-day post-SCI group vs. the control group	*Ppif*	*Pink1*	0	0	0	0.556	0.556
3-day post-SCI group vs. the control group	*Ppif*	*Atox1*	0	0.55	0	0.074	0.565
3-day post-SCI group vs. the control group	*Ppif*	*Glrx2*	0.066	0.464	0	0.198	0.571
3-day post-SCI group vs. the control group	*Ppif*	*Casp3*	0.056	0.053	0	0.561	0.573
3-day post-SCI group vs. the control group	*Ppif*	*Cat*	0.063	0.198	0	0.5	0.591
3-day post-SCI group vs. the control group	*Ppif*	*Ripk3*	0	0.079	0	0.585	0.601
3-day post-SCI group vs. the control group	*Ppif*	*Hdac1*	0.052	0.284	0.327	0.258	0.615
3-day post-SCI group vs. the control group	*Ppif*	*Bax*	0	0.077	0.72	0.312	0.806
3-day post-SCI group vs. the control group	*Ppp1ca*	*Cdk4*	0.38	0.235	0.336	0.152	0.697
3-day post-SCI group vs. the control group	*Ppp1ca*	*Cdk1*	0.077	0.235	0.629	0.292	0.79
3-day post-SCI group vs. the control group	*Ppp1ca*	*Rb1*	0	0.765	0.828	0.132	0.962
3-day post-SCI group vs. the control group	*Ppp1ca*	*Prkaa2*	0.065	0.401	0	0.333	0.593
3-day post-SCI group vs. the control group	*Ppp1ca*	*Gnb2*	0.61	0.132	0	0.061	0.655
3-day post-SCI group vs. the control group	*Ppp1ca*	*Cdkn2c*	0.062	0.142	0.118	0.258	0.403
3-day post-SCI group vs. the control group	*Ppp1ca*	*Apc*	0	0.406	0	0	0.406
3-day post-SCI group vs. the control group	*Ppp1ca*	*Mapt*	0	0.366	0	0.107	0.41
3-day post-SCI group vs. the control group	*Ppp1ca*	*Mapk3*	0.125	0.237	0	0.214	0.429
3-day post-SCI group vs. the control group	*Ppp1ca*	*Eed*	0.061	0.439	0	0	0.451
3-day post-SCI group vs. the control group	*Ppp1ca*	*Hdac1*	0.062	0.354	0	0.197	0.472
3-day post-SCI group vs. the control group	*Ppp1ca*	*Nfatc1*	0	0.209	0.302	0.23	0.537
3-day post-SCI group vs. the control group	*Ppp1ca*	*Atf2*	0.061	0.049	0.6	0	0.611
3-day post-SCI group vs. the control group	*Ppp1ca*	*Smad1*	0.061	0.048	0.676	0.05	0.688
3-day post-SCI group vs. the control group	*Ppp1ca*	*Ppp3ca*	0.095	0	0.72	0.381	0.747
3-day post-SCI group vs. the control group	*Ppp1ca*	*Eif2s1*	0.082	0.398	0.8	0.276	0.909
3-day post-SCI group vs. the control group	*Ppp1ca*	*Ppp1r15b*	0	0.641	0.72	0.259	0.919
3-day post-SCI group vs. the control group	*Ppp1r15b*	*Ppp1ca*	0	0.641	0.72	0.259	0.919
3-day post-SCI group vs. the control group	*Ppp1r15b*	*Ppp3ca*	0	0.215	0.72	0	0.77
3-day post-SCI group vs. the control group	*Ppp1r15b*	*Eif2s1*	0	0	0	0.633	0.633
3-day post-SCI group vs. the control group	*Ppp3ca*	*Amph*	0.113	0.132	0.344	0.05	0.456
3-day post-SCI group vs. the control group	*Ppp3ca*	*Mapk14*	0.063	0.272	0	0.284	0.468
3-day post-SCI group vs. the control group	*Ppp3ca*	*Cdc20*	0	0.159	0.6	0.107	0.673

3-day post-SCI group vs. the control group	*Ppp3ca*	*Cdk4*	0.063	0.155	0.194	0.195	0.418
3-day post-SCI group vs. the control group	*Ppp3ca*	*Fkbp1b*	0.062	0.591	0.15	0.268	0.729
3-day post-SCI group vs. the control group	*Ppp3ca*	*Atp2a2*	0.09	0.054	0	0.434	0.47
3-day post-SCI group vs. the control group	*Ppp3ca*	*Ppp1ca*	0.095	0	0.72	0.381	0.747
3-day post-SCI group vs. the control group	*Ppp3ca*	*Mapk3*	0.063	0.272	0	0.239	0.435
3-day post-SCI group vs. the control group	*Ppp3ca*	*Mapt*	0.098	0.527	0	0.152	0.606
3-day post-SCI group vs. the control group	*Ppp3ca*	*Ppp1r15b*	0	0.215	0.72	0	0.77
3-day post-SCI group vs. the control group	*Ppp3ca*	*Rcan1*	0	0.839	0	0.677	0.945
3-day post-SCI group vs. the control group	*Ppp3ca*	*Nfatc1*	0	0.917	0.932	0.67	0.997
3-day post-SCI group vs. the control group	*Prdx4*	*Sod2*	0.12	0	0	0.458	0.547
3-day post-SCI group vs. the control group	*Prdx4*	*Txnrd1*	0.067	0.059	0	0.535	0.556
3-day post-SCI group vs. the control group	*Prdx4*	*Gpx8*	0.082	0.224	0	0.617	0.703
3-day post-SCI group vs. the control group	*Prdx4*	*P4hb*	0.107	0.748	0	0.765	0.942
3-day post-SCI group vs. the control group	*Prdx4*	*Actb*	0.062	0.082	0	0.373	0.413
3-day post-SCI group vs. the control group	*Prdx4*	*Nox4*	0	0	0	0.43	0.43
3-day post-SCI group vs. the control group	*Prdx4*	*Eed*	0.063	0.433	0	0	0.446
3-day post-SCI group vs. the control group	*Prdx4*	*Gclc*	0	0	0	0.48	0.48
3-day post-SCI group vs. the control group	*Prdx4*	*Prdx6*	0.097	0.256	0	0.742	0.508
3-day post-SCI group vs. the control group	*Prdx4*	*Glrx2*	0.071	0.057	0	0.602	0.634
3-day post-SCI group vs. the control group	*Prdx4*	*Gpx3*	0.061	0.224	0	0.542	0.637
3-day post-SCI group vs. the control group	*Prdx4*	*Gsr*	0.12	0.059	0	0.609	0.648
3-day post-SCI group vs. the control group	*Prdx4*	*Gpx1*	0.098	0.224	0	0.617	0.708
3-day post-SCI group vs. the control group	*Prdx4*	*Gpx7*	0.075	0.224	0	0.675	0.746
3-day post-SCI group vs. the control group	*Prdx4*	*Cat*	0.284	0.362	0	0.637	0.821
3-day post-SCI group vs. the control group	*Prdx4*	*Srxn1*	0.067	0.462	0	0.765	0.872
3-day post-SCI group vs. the control group	*Prdx6*	*Hspb1*	0.051	0.101	0	0.481	0.518
3-day post-SCI group vs. the control group	*Prdx6*	*Sod2*	0.214	0	0	0.591	0.695
3-day post-SCI group vs. the control group	*Prdx6*	*Mgst1*	0.068	0	0.65	0.239	0.73
3-day post-SCI group vs. the control group	*Prdx6*	*Txnrd1*	0.067	0.059	0	0.406	0.433
3-day post-SCI group vs. the control group	*Prdx6*	*Gpx8*	0.063	0.184	0	0.308	0.425
3-day post-SCI group vs. the control group	*Prdx6*	*P4hb*	0.062	0.223	0	0.537	0.634
3-day post-SCI group vs. the control group	*Prdx6*	*Prdx4*	0.097	0.256	0	0.742	0.508
3-day post-SCI group vs. the control group	*Prdx6*	*Cat*	0.284	0.25	0	0.751	0.856
3-day post-SCI group vs. the control group	*Prdx6*	*Ggt7*	0.062	0	0.9	0.106	0.909
3-day post-SCI group vs. the control group	*Prdx6*	*Gpx7*	0.063	0.184	0	0.426	0.522
3-day post-SCI group vs. the control group	*Prdx6*	*Gsr*	0.08	0.098	0	0.621	0.658

3-day post-SCI group vs. the control group	*Prdx6*	*Srxn1*	0.08	0.328	0	0.542	0.692
3-day post-SCI group vs. the control group	*Prdx6*	*Gstt2*	0.105	0.094	0.65	0.192	0.74
3-day post-SCI group vs. the control group	*Prdx6*	*Pxdn*	0.052	0	0	0.411	0.418
3-day post-SCI group vs. the control group	*Prdx6*	*Fbxw7*	0	0.433	0	0.068	0.448
3-day post-SCI group vs. the control group	*Prdx6*	*Mapt*	0	0.433	0	0.163	0.506
3-day post-SCI group vs. the control group	*Prdx6*	*Gpx3*	0.063	0.184	0	0.516	0.597
3-day post-SCI group vs. the control group	*Prdx6*	*Gpx1*	0.063	0.184	0	0.629	0.691
3-day post-SCI group vs. the control group	*Prdx6*	*Glrx2*	0.164	0.401	0	0.447	0.71
3-day post-SCI group vs. the control group	*Prkaa2*	*Mapk14*	0.062	0.201	0	0.476	0.416
3-day post-SCI group vs. the control group	*Prkaa2*	*Sod2*	0.063	0.089	0	0.383	0.427
3-day post-SCI group vs. the control group	*Prkaa2*	*Hif1a*	0.052	0.043	0	0.479	0.486
3-day post-SCI group vs. the control group	*Prkaa2*	*Myc*	0.062	0.107	0.209	0.212	0.408
3-day post-SCI group vs. the control group	*Prkaa2*	*Cat*	0.067	0.134	0	0.33	0.412
3-day post-SCI group vs. the control group	*Prkaa2*	*Mapk3*	0.062	0.201	0	0.377	0.412
3-day post-SCI group vs. the control group	*Prkaa2*	*Actb*	0	0.087	0	0.508	0.532
3-day post-SCI group vs. the control group	*Prkaa2*	*Ppp1ca*	0.065	0.401	0	0.333	0.593
3-day post-SCI group vs. the control group	*Prkaa2*	*Gnb2*	0.062	0	0.6	0.102	0.633
3-day post-SCI group vs. the control group	*Prkaa2*	*Sirt1*	0.061	0.102	0	0.82	0.836
3-day post-SCI group vs. the control group	*Prkaa2*	*Camkk2*	0.064	0.589	0.8	0.747	0.942
3-day post-SCI group vs. the control group	*Prkaa2*	*Ppargc1a*	0.097	0.13	0.9	0.716	0.974
3-day post-SCI group vs. the control group	*Prodh*	*Cat*	0	0	0	0.443	0.443
3-day post-SCI group vs. the control group	*Prodh*	*Arg1*	0.082	0	0	0.477	0.542
3-day post-SCI group vs. the control group	*Ptgs1*	*Alox5*	0.09	0	0.9	0.728	0.973
3-day post-SCI group vs. the control group	*Ptgs1*	*Cat*	0.061	0.203	0	0.275	0.41
3-day post-SCI group vs. the control group	*Ptgs1*	*Ptgs2*	0.088	0.13	0.8	0.892	0.834
3-day post-SCI group vs. the control group	*Ptgs1*	*Tlr4*	0.104	0.042	0	0.371	0.413
3-day post-SCI group vs. the control group	*Ptgs1*	*Actb*	0	0	0	0.431	0.431
3-day post-SCI group vs. the control group	*Ptgs2*	*Mapk14*	0	0.101	0	0.737	0.753
3-day post-SCI group vs. the control group	*Ptgs2*	*Hmox1*	0.085	0	0	0.746	0.757
3-day post-SCI group vs. the control group	*Ptgs2*	*Cdk4*	0	0	0	0.403	0.403
3-day post-SCI group vs. the control group	*Ptgs2*	*Sod2*	0.062	0	0	0.52	0.531
3-day post-SCI group vs. the control group	*Ptgs2*	*Ncf1*	0.051	0	0	0.417	0.424
3-day post-SCI group vs. the control group	*Ptgs2*	*Arg1*	0.048	0	0	0.598	0.601
3-day post-SCI group vs. the control group	*Ptgs2*	*Txnrd1*	0	0.226	0	0.318	0.45
3-day post-SCI group vs. the control group	*Ptgs2*	*Hif1a*	0.061	0	0	0.665	0.672
3-day post-SCI group vs. the control group	*Ptgs2*	*Fos*	0.063	0	0	0.64	0.648

3-day post-SCI group vs. the control group	*Ptgs2*	*Gpx8*	0.067	0	0	0.544	0.556
3-day post-SCI group vs. the control group	*Ptgs2*	*Myc*	0	0	0	0.597	0.597
3-day post-SCI group vs. the control group	*Ptgs2*	*Xdh*	0	0	0	0.526	0.526
3-day post-SCI group vs. the control group	*Ptgs2*	*Cyp1b1*	0.107	0.046	0	0.447	0.488
3-day post-SCI group vs. the control group	*Ptgs2*	*Aif1*	0.05	0	0	0.601	0.605
3-day post-SCI group vs. the control group	*Ptgs2*	*Hbegf*	0.132	0	0	0.462	0.513
3-day post-SCI group vs. the control group	*Ptgs2*	*Anxa1*	0.157	0	0	0.385	0.459
3-day post-SCI group vs. the control group	*Ptgs2*	*Fosl1*	0.223	0	0	0.36	0.482
3-day post-SCI group vs. the control group	*Ptgs2*	*Rela*	0	0	0	0.619	0.619
3-day post-SCI group vs. the control group	*Ptgs2*	*Alox5*	0.061	0	0.9	0.783	0.977
3-day post-SCI group vs. the control group	*Ptgs2*	*Cat*	0.048	0.203	0	0.689	0.743
3-day post-SCI group vs. the control group	*Ptgs2*	*Vcam1*	0.076	0	0	0.671	0.683
3-day post-SCI group vs. the control group	*Ptgs2*	*Nfkb1*	0.081	0.077	0	0.586	0.618
3-day post-SCI group vs. the control group	*Ptgs2*	*Gpx7*	0	0	0	0.557	0.557
3-day post-SCI group vs. the control group	*Ptgs2*	*Tnfrsf1a*	0.082	0	0	0.464	0.487
3-day post-SCI group vs. the control group	*Ptgs2*	*Nox4*	0.05	0.088	0	0.489	0.519
3-day post-SCI group vs. the control group	*Ptgs2*	*Bax*	0	0	0	0.45	0.45
3-day post-SCI group vs. the control group	*Ptgs2*	*Gsr*	0	0.16	0	0.497	0.559
3-day post-SCI group vs. the control group	*Ptgs2*	*Mcl1*	0.061	0	0	0.409	0.421
3-day post-SCI group vs. the control group	*Ptgs2*	*Snca*	0	0	0	0.43	0.43
3-day post-SCI group vs. the control group	*Ptgs2*	*Stat6*	0.061	0	0	0.428	0.44
3-day post-SCI group vs. the control group	*Ptgs2*	*Tlr6*	0.062	0.042	0	0.434	0.447
3-day post-SCI group vs. the control group	*Ptgs2*	*Gpx1*	0	0	0	0.46	0.46
3-day post-SCI group vs. the control group	*Ptgs2*	*Met*	0.069	0.136	0	0.398	0.473
3-day post-SCI group vs. the control group	*Ptgs2*	*Sirt1*	0	0	0	0.515	0.515
3-day post-SCI group vs. the control group	*Ptgs2*	*Jak2*	0.095	0.088	0	0.464	0.518
3-day post-SCI group vs. the control group	*Ptgs2*	*Mapk8*	0	0	0	0.589	0.589
3-day post-SCI group vs. the control group	*Ptgs2*	*Nfe2l2*	0	0	0	0.619	0.619
3-day post-SCI group vs. the control group	*Ptgs2*	*Casp3*	0	0	0	0.717	0.717
3-day post-SCI group vs. the control group	*Ptgs2*	*Actb*	0	0	0	0.737	0.737
3-day post-SCI group vs. the control group	*Ptgs2*	*Mapk3*	0	0.206	0	0.69	0.743
3-day post-SCI group vs. the control group	*Ptgs2*	*Jun*	0.061	0	0	0.738	0.743
3-day post-SCI group vs. the control group	*Ptgs2*	*Tlr4*	0.064	0.042	0	0.743	0.75
3-day post-SCI group vs. the control group	*Ptgs2*	*Apc*	0	0.729	0	0.211	0.777
3-day post-SCI group vs. the control group	*Ptgs2*	*Ptgs1*	0.088	0.13	0.8	0.892	0.834
3-day post-SCI group vs. the control group	*Ptprk*	*Hbegf*	0.057	0	0.9	0.074	0.905

3-day post-SCI group vs. the control group	*Ptprk*	*Uba52*	0	0	0.9	0.074	0.903
3-day post-SCI group vs. the control group	*Ptprk*	*Il6st*	0	0.089	0.358	0.161	0.466
3-day post-SCI group vs. the control group	*Pxdn*	*Cyp1b1*	0.114	0.046	0	0.555	0.591
3-day post-SCI group vs. the control group	*Pxdn*	*Marcksl1*	0	0	0	0.437	0.437
3-day post-SCI group vs. the control group	*Pxdn*	*Prdx6*	0.052	0	0	0.411	0.418
3-day post-SCI group vs. the control group	*Pxdn*	*Eed*	0.061	0.433	0	0	0.444
3-day post-SCI group vs. the control group	*Pxn*	*Capns1*	0.068	0	0.629	0.161	0.685
3-day post-SCI group vs. the control group	*Pxn*	*Braf*	0.066	0.063	0.131	0.356	0.445
3-day post-SCI group vs. the control group	*Pxn*	*Mapk14*	0	0.082	0.31	0.542	0.685
3-day post-SCI group vs. the control group	*Pxn*	*Hspb1*	0.061	0.202	0	0.346	0.467
3-day post-SCI group vs. the control group	*Pxn*	*Ncf1*	0	0.163	0	0.445	0.516
3-day post-SCI group vs. the control group	*Pxn*	*Myc*	0.065	0.066	0	0.42	0.45
3-day post-SCI group vs. the control group	*Pxn*	*Mylk*	0.062	0.049	0.864	0.414	0.919
3-day post-SCI group vs. the control group	*Pxn*	*Hbegf*	0	0	0.9	0.121	0.908
3-day post-SCI group vs. the control group	*Pxn*	*Itga6*	0.081	0.16	0.711	0.416	0.852
3-day post-SCI group vs. the control group	*Pxn*	*Vcam1*	0.062	0	0.676	0.518	0.84
3-day post-SCI group vs. the control group	*Pxn*	*Gnb2*	0.069	0.071	0.533	0	0.56
3-day post-SCI group vs. the control group	*Pxn*	*Nox4*	0.062	0	0	0.69	0.697
3-day post-SCI group vs. the control group	*Pxn*	*Ctnna1*	0.083	0.203	0.334	0.293	0.61
3-day post-SCI group vs. the control group	*Pxn*	*Mapk3*	0.061	0.185	0.676	0.647	0.901
3-day post-SCI group vs. the control group	*Pxn*	*Arhgdia*	0.062	0	0	0.429	0.442
3-day post-SCI group vs. the control group	*Pxn*	*Ezr*	0.082	0.065	0.629	0.748	0.909
3-day post-SCI group vs. the control group	*Pxn*	*Jak2*	0.081	0.063	0.676	0.25	0.763
3-day post-SCI group vs. the control group	*Pxn*	*Itgb5*	0.079	0.164	0.856	0.467	0.933
3-day post-SCI group vs. the control group	*Pxn*	*Mapk10*	0	0.355	0	0.202	0.463
3-day post-SCI group vs. the control group	*Pxn*	*Casp3*	0.05	0.087	0	0.463	0.493
3-day post-SCI group vs. the control group	*Pxn*	*Jun*	0	0	0	0.581	0.581
3-day post-SCI group vs. the control group	*Pxn*	*Met*	0.073	0.063	0.131	0.542	0.609
3-day post-SCI group vs. the control group	*Pxn*	*Mapk8*	0	0.512	0	0.374	0.681
3-day post-SCI group vs. the control group	*Pxn*	*Actb*	0.064	0	0.881	0.615	0.953
3-day post-SCI group vs. the control group	*Pxn*	*Cttn*	0	0.348	0.629	0.984	0.996
3-day post-SCI group vs. the control group	*Rb1*	*Braf*	0.061	0.4	0.457	0.482	0.82
3-day post-SCI group vs. the control group	*Rb1*	*Mapk14*	0.061	0.727	0.629	0.297	0.924
3-day post-SCI group vs. the control group	*Rb1*	*Cdc20*	0.063	0.088	0	0.36	0.405
3-day post-SCI group vs. the control group	*Rb1*	*Cdk4*	0.064	0.986	0.966	0.844	0.999
3-day post-SCI group vs. the control group	*Rb1*	*Cdk1*	0.064	0.76	0.922	0.541	0.99

3-day post-SCI group vs. the control group	*Rb1*	*Fos*	0	0.703	0.21	0.224	0.802
3-day post-SCI group vs. the control group	*Rb1*	*Pdgfra*	0	0	0	0.4	0.4
3-day post-SCI group vs. the control group	*Rb1*	*Cbx6*	0	0.103	0.343	0.068	0.402
3-day post-SCI group vs. the control group	*Rb1*	*Nfkb1*	0	0.079	0.204	0.277	0.424
3-day post-SCI group vs. the control group	*Rb1*	*Pon2*	0	0	0	0.439	0.439
3-day post-SCI group vs. the control group	*Rb1*	*Actb*	0.061	0	0	0.436	0.447
3-day post-SCI group vs. the control group	*Rb1*	*Mcm4*	0.245	0.148	0	0.225	0.458
3-day post-SCI group vs. the control group	*Rb1*	*Cdkn2c*	0	0.079	0.204	0.385	0.51
3-day post-SCI group vs. the control group	*Rb1*	*Junb*	0	0.133	0.316	0.264	0.525
3-day post-SCI group vs. the control group	*Rb1*	*Eed*	0.079	0.328	0	0.331	0.549
3-day post-SCI group vs. the control group	*Rb1*	*Id1*	0	0.133	0.544	0.111	0.618
3-day post-SCI group vs. the control group	*Rb1*	*Myc*	0	0.332	0	0.504	0.655
3-day post-SCI group vs. the control group	*Rb1*	*Ezh2*	0.063	0.132	0.175	0.578	0.679
3-day post-SCI group vs. the control group	*Rb1*	*Atf2*	0.063	0.227	0.676	0.118	0.765
3-day post-SCI group vs. the control group	*Rb1*	*Pcna*	0.113	0	0.698	0.251	0.782
3-day post-SCI group vs. the control group	*Rb1*	*Sirt1*	0.063	0.228	0.676	0.189	0.784
3-day post-SCI group vs. the control group	*Rb1*	*Mapk3*	0.061	0.248	0.629	0.288	0.788
3-day post-SCI group vs. the control group	*Rb1*	*Cdkn2b*	0	0.079	0.581	0.542	0.807
3-day post-SCI group vs. the control group	*Rb1*	*Casp3*	0	0.228	0.629	0.416	0.818
3-day post-SCI group vs. the control group	*Rb1*	*Jun*	0	0.227	0.676	0.344	0.821
3-day post-SCI group vs. the control group	*Rb1*	*Uba52*	0	0	0.9	0	0.9
3-day post-SCI group vs. the control group	*Rb1*	*Rbbp7*	0.092	0.721	0.537	0.385	0.918
3-day post-SCI group vs. the control group	*Rb1*	*Ppp1ca*	0	0.765	0.828	0.132	0.962
3-day post-SCI group vs. the control group	*Rb1*	*Tfdp1*	0.155	0.86	0.932	0.502	0.995
3-day post-SCI group vs. the control group	*Rb1*	*Ccna2*	0.066	0.76	0.966	0.569	0.996
3-day post-SCI group vs. the control group	*Rb1*	*Hdac1*	0.064	0.852	0.913	0.784	0.997
3-day post-SCI group vs. the control group	*Rbbp7*	*Cdc20*	0.278	0.047	0.176	0.058	0.463
3-day post-SCI group vs. the control group	*Rbbp7*	*Cdk1*	0.335	0.223	0	0.112	0.501
3-day post-SCI group vs. the control group	*Rbbp7*	*Rb1*	0.092	0.721	0.537	0.385	0.918
3-day post-SCI group vs. the control group	*Rbbp7*	*Myc*	0	0.05	0.29	0.27	0.465
3-day post-SCI group vs. the control group	*Rbbp7*	*Mcm4*	0.427	0.161	0	0	0.499
3-day post-SCI group vs. the control group	*Rbbp7*	*Pcna*	0.583	0.215	0	0.144	0.695
3-day post-SCI group vs. the control group	*Rbbp7*	*Sirt1*	0.064	0.086	0.36	0.292	0.56
3-day post-SCI group vs. the control group	*Rbbp7*	*Rest*	0	0.146	0.645	0.237	0.748
3-day post-SCI group vs. the control group	*Rbbp7*	*Cbx6*	0.048	0.133	0.72	0.322	0.822
3-day post-SCI group vs. the control group	*Rbbp7*	*Tfdp1*	0.269	0.139	0.806	0.267	0.899

3-day post-SCI group vs. the control group	*Rbbp7*	*Phc3*	0	0	0.9	0.098	0.905
3-day post-SCI group vs. the control group	*Rbbp7*	*Rnf2*	0.164	0.433	0.9	0.474	0.971
3-day post-SCI group vs. the control group	*Rbbp7*	*Eed*	0.162	0.862	0.961	0.905	0.999
3-day post-SCI group vs. the control group	*Rbbp7*	*Hdac1*	0.224	0.813	0.956	0.981	0.999
3-day post-SCI group vs. the control group	*Rbbp7*	*Ezh2*	0.174	0.899	0.961	0.894	0.999
3-day post-SCI group vs. the control group	*Rbpms*	*Nono*	0	0	0	0.671	0.671
3-day post-SCI group vs. the control group	*Rcan1*	*Atp2a2*	0.057	0	0	0.402	0.412
3-day post-SCI group vs. the control group	*Rcan1*	*Ppp3ca*	0	0.839	0	0.677	0.945
3-day post-SCI group vs. the control group	*Rcan1*	*Nfatc1*	0	0	0	0.653	0.653
3-day post-SCI group vs. the control group	*Rela*	*Ier3*	0.071	0.13	0	0.836	0.856
3-day post-SCI group vs. the control group	*Rela*	*Mapk14*	0.049	0.057	0.676	0.669	0.89
3-day post-SCI group vs. the control group	*Rela*	*Hmox1*	0.062	0	0	0.464	0.475
3-day post-SCI group vs. the control group	*Rela*	*Cdk4*	0	0.13	0	0.492	0.54
3-day post-SCI group vs. the control group	*Rela*	*Sod2*	0	0	0.629	0.464	0.792
3-day post-SCI group vs. the control group	*Rela*	*Mgst1*	0	0	0	0.65	0.65
3-day post-SCI group vs. the control group	*Rela*	*Map2k3*	0.079	0.241	0	0.277	0.45
3-day post-SCI group vs. the control group	*Rela*	*Map2k6*	0.05	0.311	0	0.24	0.459
3-day post-SCI group vs. the control group	*Rela*	*Hif1a*	0	0.13	0	0.848	0.862
3-day post-SCI group vs. the control group	*Rela*	*Fos*	0	0.4	0.629	0.561	0.893
3-day post-SCI group vs. the control group	*Rela*	*Ripk3*	0.059	0.147	0.302	0.248	0.522
3-day post-SCI group vs. the control group	*Rela*	*Myc*	0	0.269	0.548	0.617	0.862
3-day post-SCI group vs. the control group	*Rela*	*Fosl1*	0.061	0.155	0.355	0.511	0.716
3-day post-SCI group vs. the control group	*Rela*	*Bax*	0	0.058	0.311	0.167	0.412
3-day post-SCI group vs. the control group	*Rela*	*Nfe2l2*	0.084	0.083	0	0.403	0.455
3-day post-SCI group vs. the control group	*Rela*	*Btk*	0	0.087	0.217	0.333	0.481
3-day post-SCI group vs. the control group	*Rela*	*Brf2*	0	0.14	0.409	0.105	0.505
3-day post-SCI group vs. the control group	*Rela*	*Nfatc1*	0.075	0	0	0.493	0.511
3-day post-SCI group vs. the control group	*Rela*	*Cdkn2b*	0	0.091	0.263	0.335	0.515
3-day post-SCI group vs. the control group	*Rela*	*Rxrb*	0.061	0.325	0.27	0.094	0.524
3-day post-SCI group vs. the control group	*Rela*	*Mcl1*	0.094	0.058	0.311	0.4	0.599
3-day post-SCI group vs. the control group	*Rela*	*Uba52*	0	0	0.6	0	0.6
3-day post-SCI group vs. the control group	*Rela*	*Ptgs2*	0	0	0	0.619	0.619
3-day post-SCI group vs. the control group	*Rela*	*Junb*	0.113	0.058	0.477	0.258	0.633
3-day post-SCI group vs. the control group	*Rela*	*Parp1*	0	0.418	0	0.439	0.659
3-day post-SCI group vs. the control group	*Rela*	*Pawr*	0.051	0	0.676	0.05	0.682
3-day post-SCI group vs. the control group	*Rela*	*Stat6*	0.104	0.244	0.35	0.374	0.688

3-day post-SCI group vs. the control group	*Rela*	*Il18rap*	0	0	0.676	0.087	0.691
3-day post-SCI group vs. the control group	*Rela*	*Casp3*	0	0	0.334	0.612	0.73
3-day post-SCI group vs. the control group	*Rela*	*Vcam1*	0	0.433	0	0.552	0.735
3-day post-SCI group vs. the control group	*Rela*	*Jak2*	0	0.064	0.676	0.399	0.801
3-day post-SCI group vs. the control group	*Rela*	*Atf2*	0	0.155	0.629	0.444	0.81
3-day post-SCI group vs. the control group	*Rela*	*Mapk10*	0	0.052	0.8	0.184	0.831
3-day post-SCI group vs. the control group	*Rela*	*Mapk3*	0	0.05	0.676	0.566	0.855
3-day post-SCI group vs. the control group	*Rela*	*Ripk1*	0.089	0.147	0.676	0.498	0.856
3-day post-SCI group vs. the control group	*Rela*	*Actb*	0.079	0.09	0.629	0.701	0.894
3-day post-SCI group vs. the control group	*Rela*	*Tnfrsf1a*	0.114	0	0.676	0.691	0.903
3-day post-SCI group vs. the control group	*Rela*	*Mapk8*	0	0.048	0.8	0.547	0.906
3-day post-SCI group vs. the control group	*Rela*	*Ppargc1a*	0	0.225	0	0.901	0.92
3-day post-SCI group vs. the control group	*Rela*	*Tlr4*	0	0	0.8	0.693	0.935
3-day post-SCI group vs. the control group	*Rela*	*Jun*	0.061	0.46	0.629	0.859	0.97
3-day post-SCI group vs. the control group	*Rela*	*Ezh2*	0	0.33	0	0.979	0.985
3-day post-SCI group vs. the control group	*Rela*	*Sirt1*	0	0.554	0	0.973	0.987
3-day post-SCI group vs. the control group	*Rela*	*Hdac1*	0.062	0.795	0.512	0.982	0.998
3-day post-SCI group vs. the control group	*Rela*	*Nfkb1*	0.095	0.999	0.932	0.991	0.999
3-day post-SCI group vs. the control group	*Rest*	*Myc*	0.068	0.088	0.134	0.312	0.426
3-day post-SCI group vs. the control group	*Rest*	*Rbbp7*	0	0.146	0.645	0.237	0.748
3-day post-SCI group vs. the control group	*Rest*	*Ezh2*	0.082	0.115	0.187	0.361	0.522
3-day post-SCI group vs. the control group	*Rest*	*Hdac1*	0.068	0.206	0.82	0.827	0.974
3-day post-SCI group vs. the control group	*Ripk1*	*Mapk14*	0	0.147	0.676	0.412	0.758
3-day post-SCI group vs. the control group	*Ripk1*	*Map2k3*	0.087	0.132	0.932	0.221	0.945
3-day post-SCI group vs. the control group	*Ripk1*	*Map2k6*	0.061	0.132	0.922	0.155	0.934
3-day post-SCI group vs. the control group	*Ripk1*	*Ppif*	0	0.079	0	0.526	0.544
3-day post-SCI group vs. the control group	*Ripk1*	*Ripk3*	0.076	0.983	0.8	0.991	0.997
3-day post-SCI group vs. the control group	*Ripk1*	*Rela*	0.089	0.147	0.676	0.498	0.856
3-day post-SCI group vs. the control group	*Ripk1*	*Parp1*	0.068	0.051	0	0.411	0.433
3-day post-SCI group vs. the control group	*Ripk1*	*Nfkb1*	0.089	0.132	0.676	0.45	0.84
3-day post-SCI group vs. the control group	*Ripk1*	*Tnfrsf1a*	0.113	0.999	0.932	0.988	0.999
3-day post-SCI group vs. the control group	*Ripk1*	*Map2k4*	0	0.106	0.629	0.242	0.682
3-day post-SCI group vs. the control group	*Ripk1*	*Mcl1*	0.079	0.056	0.12	0.349	0.435
3-day post-SCI group vs. the control group	*Ripk1*	*Tlr4*	0.058	0.461	0.667	0.658	0.934
3-day post-SCI group vs. the control group	*Ripk1*	*Mapk3*	0	0.147	0.354	0.556	0.556
3-day post-SCI group vs. the control group	*Ripk1*	*Tlr6*	0.062	0.089	0.204	0.251	0.422

3-day post-SCI group vs. the control group	*Ripk1*	*Htra2*	0.052	0	0	0.493	0.499
3-day post-SCI group vs. the control group	*Ripk1*	*Casp3*	0	0.077	0.891	0.668	0.963
3-day post-SCI group vs. the control group	*Ripk1*	*Actb*	0.052	0.084	0.114	0.4	0.476
3-day post-SCI group vs. the control group	*Ripk1*	*Jun*	0	0.09	0.127	0.468	0.54
3-day post-SCI group vs. the control group	*Ripk1*	*Mapk8*	0	0.208	0.209	0.457	0.468
3-day post-SCI group vs. the control group	*Ripk1*	*Tank*	0.065	0.443	0.629	0.739	0.943
3-day post-SCI group vs. the control group	*Ripk1*	*Snca*	0	0.059	0.132	0.358	0.43
3-day post-SCI group vs. the control group	*Ripk1*	*Uba52*	0	0.055	0.9	0.05	0.902
3-day post-SCI group vs. the control group	*Ripk1*	*Tnik*	0	0.142	0.499	0.094	0.563
3-day post-SCI group vs. the control group	*Ripk1*	*Map4k4*	0	0.142	0.676	0.105	0.718
3-day post-SCI group vs. the control group	*Ripk3*	*Map2k3*	0.101	0.132	0.293	0.082	0.41
3-day post-SCI group vs. the control group	*Ripk3*	*Ppif*	0	0.079	0	0.585	0.601
3-day post-SCI group vs. the control group	*Ripk3*	*Parp1*	0.068	0.051	0	0.378	0.402
3-day post-SCI group vs. the control group	*Ripk3*	*Map2k4*	0	0.106	0.341	0.163	0.419
3-day post-SCI group vs. the control group	*Ripk3*	*Mapk8*	0	0.208	0.209	0.324	0.429
3-day post-SCI group vs. the control group	*Ripk3*	*Tank*	0	0.059	0.341	0.159	0.433
3-day post-SCI group vs. the control group	*Ripk3*	*Actb*	0.052	0.084	0.114	0.435	0.507
3-day post-SCI group vs. the control group	*Ripk3*	*Rela*	0.059	0.147	0.302	0.248	0.522
3-day post-SCI group vs. the control group	*Ripk3*	*Casp3*	0	0.077	0.194	0.665	0.729
3-day post-SCI group vs. the control group	*Ripk3*	*Tlr4*	0.064	0.089	0.667	0.599	0.871
3-day post-SCI group vs. the control group	*Ripk3*	*Tnfrsf1a*	0.108	0.13	0.341	0.799	0.884
3-day post-SCI group vs. the control group	*Ripk3*	*Uba52*	0	0.055	0.9	0.055	0.902
3-day post-SCI group vs. the control group	*Ripk3*	*Ripk1*	0.076	0.983	0.8	0.991	0.997
3-day post-SCI group vs. the control group	*Rnf2*	*Cdc20*	0.071	0	0.54	0.07	0.568
3-day post-SCI group vs. the control group	*Rnf2*	*Ube2a*	0.052	0.164	0	0.351	0.44
3-day post-SCI group vs. the control group	*Rnf2*	*Mapkapk2*	0	0.436	0	0	0.436
3-day post-SCI group vs. the control group	*Rnf2*	*Rbbp7*	0.164	0.433	0.9	0.474	0.971
3-day post-SCI group vs. the control group	*Rnf2*	*Sirt1*	0.125	0	0.36	0.149	0.481
3-day post-SCI group vs. the control group	*Rnf2*	*Hdac1*	0.062	0.099	0	0.468	0.512
3-day post-SCI group vs. the control group	*Rnf2*	*Actb*	0	0.433	0	0.223	0.54
3-day post-SCI group vs. the control group	*Rnf2*	*Tfdp1*	0.063	0.443	0.6	0.087	0.784
3-day post-SCI group vs. the control group	*Rnf2*	*Cbx6*	0.052	0.758	0.845	0.714	0.988
3-day post-SCI group vs. the control group	*Rnf2*	*Eed*	0.168	0.433	0.9	0.822	0.99
3-day post-SCI group vs. the control group	*Rnf2*	*Ezh2*	0.117	0.163	0.9	0.897	0.991
3-day post-SCI group vs. the control group	*Rnf2*	*Phc3*	0.052	0.68	0.938	0.756	0.994
3-day post-SCI group vs. the control group	*Rpl13a*	*Sod2*	0.252	0.47	0	0.18	0.66

3-day post-SCI group vs. the control group	*Rpl13a*	*Nme2*	0.645	0	0	0	0.645
3-day post-SCI group vs. the control group	*Rpl13a*	*Eif2s1*	0.652	0	0	0.097	0.672
3-day post-SCI group vs. the control group	*Rpl13a*	*Actb*	0.09	0.066	0	0.704	0.726
3-day post-SCI group vs. the control group	*Rpl13a*	*Eed*	0	0.433	0	0	0.433
3-day post-SCI group vs. the control group	*Rpl13a*	*Uba52*	0.593	0.911	0.6	0.251	0.987
3-day post-SCI group vs. the control group	*Rrm2b*	*Cdc20*	0.635	0.141	0	0.071	0.683
3-day post-SCI group vs. the control group	*Rrm2b*	*Cdk1*	0.751	0.163	0	0.191	0.816
3-day post-SCI group vs. the control group	*Rrm2b*	*Nme2*	0.101	0	0.65	0.074	0.683
3-day post-SCI group vs. the control group	*Rrm2b*	*Dhfr*	0.27	0	0	0.307	0.472
3-day post-SCI group vs. the control group	*Rrm2b*	*Ccna2*	0.304	0.134	0	0.101	0.41
3-day post-SCI group vs. the control group	*Rrm2b*	*Atr*	0.065	0.463	0	0.476	0.714
3-day post-SCI group vs. the control group	*Rrm2b*	*Pcna*	0.657	0.203	0	0.185	0.757
3-day post-SCI group vs. the control group	*Rrm2b*	*Mcm4*	0.751	0	0	0.096	0.765
3-day post-SCI group vs. the control group	*Rxrb*	*Cdk1*	0	0.058	0.515	0	0.523
3-day post-SCI group vs. the control group	*Rxrb*	*Myc*	0	0.135	0.496	0.045	0.547
3-day post-SCI group vs. the control group	*Rxrb*	*Rela*	0.061	0.325	0.27	0.094	0.524
3-day post-SCI group vs. the control group	*Rxrb*	*Mapk10*	0	0.047	0.42	0.049	0.428
3-day post-SCI group vs. the control group	*Rxrb*	*Mapk8*	0	0.047	0.42	0.049	0.428
3-day post-SCI group vs. the control group	*Rxrb*	*Ppargc1a*	0	0.18	0.502	0.155	0.625
3-day post-SCI group vs. the control group	*Rxrb*	*Mapk3*	0	0.149	0.676	0	0.712
3-day post-SCI group vs. the control group	*Rxrb*	*Hdac1*	0.061	0.132	0.826	0.061	0.849
3-day post-SCI group vs. the control group	*S100a1*	*Fkbp1b*	0	0.249	0	0.398	0.528
3-day post-SCI group vs. the control group	*S100a1*	*Atp2a2*	0.062	0.13	0	0.825	0.845
3-day post-SCI group vs. the control group	*S100a1*	*Tlr4*	0	0	0.6	0.466	0.777
3-day post-SCI group vs. the control group	*S100a1*	*Ezr*	0	0	0	0.707	0.707
3-day post-SCI group vs. the control group	*Sdc1*	*Mapk14*	0.052	0	0.354	0.237	0.491
3-day post-SCI group vs. the control group	*Sdc1*	*Vcam1*	0	0	0	0.402	0.402
3-day post-SCI group vs. the control group	*Sdc1*	*Slc4a11*	0	0	0	0.448	0.448
3-day post-SCI group vs. the control group	*Sdc1*	*Anxa1*	0.096	0	0	0.417	0.45
3-day post-SCI group vs. the control group	*Sdc1*	*Actb*	0	0	0	0.464	0.463
3-day post-SCI group vs. the control group	*Sdc1*	*Myc*	0.072	0.045	0	0.45	0.47
3-day post-SCI group vs. the control group	*Sdc1*	*Btk*	0	0.058	0.127	0.464	0.52
3-day post-SCI group vs. the control group	*Sdc1*	*Tlr4*	0	0	0	0.552	0.552
3-day post-SCI group vs. the control group	*Sdc1*	*Casp3*	0.057	0	0.177	0.473	0.555
3-day post-SCI group vs. the control group	*Sdc1*	*Cttn*	0.064	0.059	0.284	0.639	0.742
3-day post-SCI group vs. the control group	*Sdc1*	*Mapk3*	0.065	0	0.676	0.33	0.779

3-day post-SCI group vs. the control group	*Sdc1*	*Met*	0.114	0	0.676	0.312	0.785
3-day post-SCI group vs. the control group	*Sdc1*	*Itga6*	0.062	0	0.729	0.23	0.787
3-day post-SCI group vs. the control group	*Sdc1*	*Cd38*	0	0	0	0.853	0.853
3-day post-SCI group vs. the control group	*Sdc1*	*Itgb5*	0.109	0	0.864	0.381	0.919
3-day post-SCI group vs. the control group	*Sfpq*	*Eed*	0.097	0.433	0	0	0.466
3-day post-SCI group vs. the control group	*Sfpq*	*Ppargc1a*	0	0.13	0	0.42	0.474
3-day post-SCI group vs. the control group	*Sfpq*	*Hdac1*	0.162	0.602	0	0.216	0.715
3-day post-SCI group vs. the control group	*Sfpq*	*Nono*	0.709	0.841	0.629	0.986	0.982
3-day post-SCI group vs. the control group	*Sirpa*	*Nox4*	0	0.05	0	0.697	0.699
3-day post-SCI group vs. the control group	*Sirpa*	*Jak2*	0.061	0.44	0	0.339	0.622
3-day post-SCI group vs. the control group	*Sirt1*	*Mapk14*	0.051	0.045	0	0.62	0.625
3-day post-SCI group vs. the control group	*Sirt1*	*Hmox1*	0	0	0	0.678	0.678
3-day post-SCI group vs. the control group	*Sirt1*	*Cdk4*	0	0.058	0	0.597	0.605
3-day post-SCI group vs. the control group	*Sirt1*	*Sod2*	0.062	0.059	0	0.827	0.834
3-day post-SCI group vs. the control group	*Sirt1*	*Cdk1*	0.061	0.443	0	0.483	0.706
3-day post-SCI group vs. the control group	*Sirt1*	*Hif1a*	0.062	0.227	0.457	0.839	0.928
3-day post-SCI group vs. the control group	*Sirt1*	*Fos*	0	0.439	0	0.567	0.747
3-day post-SCI group vs. the control group	*Sirt1*	*Gpx8*	0	0.05	0	0.402	0.418
3-day post-SCI group vs. the control group	*Sirt1*	*Ppif*	0	0.131	0	0.421	0.475
3-day post-SCI group vs. the control group	*Sirt1*	*Rb1*	0.063	0.228	0.676	0.189	0.784
3-day post-SCI group vs. the control group	*Sirt1*	*Myc*	0	0.206	0	0.678	0.733
3-day post-SCI group vs. the control group	*Sirt1*	*Xdh*	0	0	0	0.425	0.425
3-day post-SCI group vs. the control group	*Sirt1*	*Rela*	0	0.554	0	0.973	0.987
3-day post-SCI group vs. the control group	*Sirt1*	*Maoa*	0.064	0.433	0	0.366	0.634
3-day post-SCI group vs. the control group	*Sirt1*	*Parp1*	0.061	0.13	0	0.76	0.787
3-day post-SCI group vs. the control group	*Sirt1*	*Cat*	0.062	0	0	0.776	0.781
3-day post-SCI group vs. the control group	*Sirt1*	*Vcam1*	0	0	0	0.451	0.451
3-day post-SCI group vs. the control group	*Sirt1*	*Nfkb1*	0.062	0.461	0	0.351	0.643
3-day post-SCI group vs. the control group	*Sirt1*	*Prkaa2*	0.061	0.102	0	0.82	0.836
3-day post-SCI group vs. the control group	*Sirt1*	*Gpx7*	0	0.05	0	0.4	0.416
3-day post-SCI group vs. the control group	*Sirt1*	*Pink1*	0	0.146	0	0.59	0.634
3-day post-SCI group vs. the control group	*Sirt1*	*Nox4*	0	0	0	0.531	0.531
3-day post-SCI group vs. the control group	*Sirt1*	*Bax*	0	0	0.261	0.373	0.516
3-day post-SCI group vs. the control group	*Sirt1*	*Rbbp7*	0.064	0.086	0.36	0.292	0.56
3-day post-SCI group vs. the control group	*Sirt1*	*Gsr*	0	0.052	0	0.417	0.424
3-day post-SCI group vs. the control group	*Sirt1*	*Atr*	0.113	0.154	0	0.525	0.613

3-day post-SCI group vs. the control group	*Sirt1*	*Ptgs2*	0	0	0	0.515	0.515
3-day post-SCI group vs. the control group	*Sirt1*	*Mcl1*	0.062	0	0.261	0.376	0.529
3-day post-SCI group vs. the control group	*Sirt1*	*Tlr4*	0	0.052	0	0.495	0.501
3-day post-SCI group vs. the control group	*Sirt1*	*Mapk3*	0.051	0.045	0	0.579	0.585
3-day post-SCI group vs. the control group	*Sirt1*	*Jak2*	0.062	0.085	0	0.358	0.401
3-day post-SCI group vs. the control group	*Sirt1*	*Eif2s1*	0	0	0	0.479	0.479
3-day post-SCI group vs. the control group	*Sirt1*	*Ppargc1b*	0	0	0	0.506	0.506
3-day post-SCI group vs. the control group	*Sirt1*	*Rnf2*	0.125	0	0.36	0.149	0.481
3-day post-SCI group vs. the control group	*Sirt1*	*Ezh2*	0.052	0.266	0.72	0.959	0.991
3-day post-SCI group vs. the control group	*Sirt1*	*Gpx1*	0	0.05	0	0.506	0.52
3-day post-SCI group vs. the control group	*Sirt1*	*Casp3*	0	0	0	0.682	0.682
3-day post-SCI group vs. the control group	*Sirt1*	*Actb*	0.062	0.09	0	0.719	0.739
3-day post-SCI group vs. the control group	*Sirt1*	*Hdac1*	0.063	0.4	0.168	0.859	0.925
3-day post-SCI group vs. the control group	*Sirt1*	*Nfe2l2*	0	0	0	0.725	0.725
3-day post-SCI group vs. the control group	*Sirt1*	*Mapk10*	0.065	0.201	0	0.332	0.457
3-day post-SCI group vs. the control group	*Sirt1*	*Snca*	0	0	0	0.529	0.529
3-day post-SCI group vs. the control group	*Sirt1*	*Cbx6*	0.061	0.136	0.36	0.209	0.534
3-day post-SCI group vs. the control group	*Sirt1*	*Bnip3*	0	0	0	0.541	0.541
3-day post-SCI group vs. the control group	*Sirt1*	*Mapk8*	0.065	0.201	0	0.584	0.662
3-day post-SCI group vs. the control group	*Sirt1*	*Ucp2*	0.064	0.09	0	0.642	0.668
3-day post-SCI group vs. the control group	*Sirt1*	*Eed*	0.134	0	0.72	0.543	0.879
3-day post-SCI group vs. the control group	*Sirt1*	*Jun*	0	0.717	0	0.794	0.939
3-day post-SCI group vs. the control group	*Sirt1*	*Ppargc1a*	0	0.716	0.676	0.987	0.998
3-day post-SCI group vs. the control group	*Slc4a11*	*Sdc1*	0	0	0	0.448	0.448
3-day post-SCI group vs. the control group	*Slc4a11*	*Anxa1*	0	0	0	0.474	0.474
3-day post-SCI group vs. the control group	*Slc8a1*	*Atp2a2*	0.081	0.169	0	0.701	0.751
3-day post-SCI group vs. the control group	*Smad1*	*Mapk14*	0	0.17	0.207	0.533	0.666
3-day post-SCI group vs. the control group	*Smad1*	*Cdk4*	0	0.151	0.301	0.211	0.49
3-day post-SCI group vs. the control group	*Smad1*	*Hif1a*	0.061	0.147	0.301	0.27	0.537
3-day post-SCI group vs. the control group	*Smad1*	*Fos*	0	0.142	0.388	0.227	0.558
3-day post-SCI group vs. the control group	*Smad1*	*Myc*	0	0.134	0.366	0.374	0.626
3-day post-SCI group vs. the control group	*Smad1*	*Ppp1ca*	0.061	0.048	0.676	0.05	0.688
3-day post-SCI group vs. the control group	*Smad1*	*Mapk3*	0.061	0.359	0.932	0.511	0.977
3-day post-SCI group vs. the control group	*Smad1*	*Junb*	0	0.101	0.213	0.256	0.428
3-day post-SCI group vs. the control group	*Smad1*	*Id1*	0	0	0	0.471	0.471
3-day post-SCI group vs. the control group	*Smad1*	*Cdkn2b*	0	0.059	0.46	0.152	0.531

3-day post-SCI group vs. the control group	*Smad1*	*Actb*	0	0.041	0	0.537	0.537
3-day post-SCI group vs. the control group	*Smad1*	*Hdac1*	0.062	0.221	0.301	0.245	0.563
3-day post-SCI group vs. the control group	*Smad1*	*Jun*	0	0.211	0.388	0.43	0.701
3-day post-SCI group vs. the control group	*Smad1*	*Atf2*	0	0.132	0.629	0.266	0.743
3-day post-SCI group vs. the control group	*Smad1*	*Uba52*	0	0.052	0.9	0	0.901
3-day post-SCI group vs. the control group	*Snca*	*Amph*	0.184	0	0	0.363	0.458
3-day post-SCI group vs. the control group	*Snca*	*Mapk14*	0	0.136	0	0.399	0.458
3-day post-SCI group vs. the control group	*Snca*	*Hspb1*	0.062	0.34	0	0.494	0.659
3-day post-SCI group vs. the control group	*Snca*	*Hmox1*	0	0	0	0.499	0.499
3-day post-SCI group vs. the control group	*Snca*	*Mgst1*	0	0	0	0.663	0.663
3-day post-SCI group vs. the control group	*Snca*	*Fkbp1b*	0.062	0	0.576	0.066	0.596
3-day post-SCI group vs. the control group	*Snca*	*Ppif*	0	0.085	0	0.504	0.527
3-day post-SCI group vs. the control group	*Snca*	*Ndufa6*	0	0	0.6	0	0.6
3-day post-SCI group vs. the control group	*Snca*	*Aif1*	0.07	0.2	0	0.701	0.758
3-day post-SCI group vs. the control group	*Snca*	*Maoa*	0	0	0	0.586	0.587
3-day post-SCI group vs. the control group	*Snca*	*Cat*	0	0	0	0.551	0.551
3-day post-SCI group vs. the control group	*Snca*	*Nfkb1*	0	0.151	0.211	0.196	0.414
3-day post-SCI group vs. the control group	*Snca*	*Pink1*	0.062	0.265	0	0.926	0.945
3-day post-SCI group vs. the control group	*Snca*	*Ptgs2*	0	0	0	0.43	0.43
3-day post-SCI group vs. the control group	*Snca*	*Atp13a2*	0	0	0	0.831	0.831
3-day post-SCI group vs. the control group	*Snca*	*Tlr4*	0	0.041	0	0.906	0.906
3-day post-SCI group vs. the control group	*Snca*	*Mapk3*	0	0.189	0	0.342	0.444
3-day post-SCI group vs. the control group	*Snca*	*Gpr37*	0.069	0	0	0.586	0.598
3-day post-SCI group vs. the control group	*Snca*	*Syp*	0.197	0.405	0	0.747	0.868
3-day post-SCI group vs. the control group	*Snca*	*Ndufs8*	0	0	0.6	0	0.6
3-day post-SCI group vs. the control group	*Snca*	*Htra2*	0	0	0	0.558	0.558
3-day post-SCI group vs. the control group	*Snca*	*Casp3*	0	0	0	0.617	0.617
3-day post-SCI group vs. the control group	*Snca*	*Mapt*	0.158	0.561	0	0.923	0.969
3-day post-SCI group vs. the control group	*Snca*	*Actb*	0	0.09	0	0.62	0.639
3-day post-SCI group vs. the control group	*Snca*	*Nfe2l2*	0	0	0	0.468	0.468
3-day post-SCI group vs. the control group	*Snca*	*Sirt1*	0	0	0	0.529	0.529
3-day post-SCI group vs. the control group	*Snca*	*Jun*	0	0	0	0.4	0.4
3-day post-SCI group vs. the control group	*Snca*	*Fbxw7*	0.084	0.13	0	0.353	0.439
3-day post-SCI group vs. the control group	*Snca*	*Mapk8*	0.071	0.13	0	0.331	0.412
3-day post-SCI group vs. the control group	*Snca*	*Ripk1*	0	0.059	0.132	0.358	0.43
3-day post-SCI group vs. the control group	*Snca*	*Ppargc1a*	0.079	0	0	0.552	0.569

3-day post-SCI group vs. the control group	*Snca*	*Ndufa12*	0	0	0.6	0	0.6
3-day post-SCI group vs. the control group	*Sod2*	*Mapk14*	0.061	0.133	0	0.523	0.577
3-day post-SCI group vs. the control group	*Sod2*	*Hspb1*	0	0	0	0.428	0.428
3-day post-SCI group vs. the control group	*Sod2*	*Hmox1*	0.064	0	0	0.715	0.732
3-day post-SCI group vs. the control group	*Sod2*	*Srxn1*	0.064	0	0	0.389	0.404
3-day post-SCI group vs. the control group	*Sod2*	*Mapk8*	0	0	0	0.414	0.414
3-day post-SCI group vs. the control group	*Sod2*	*Prkaa2*	0.063	0.089	0	0.383	0.427
3-day post-SCI group vs. the control group	*Sod2*	*Nfkb1*	0	0.106	0	0.386	0.428
3-day post-SCI group vs. the control group	*Sod2*	*Txnip*	0	0	0	0.436	0.436
3-day post-SCI group vs. the control group	*Sod2*	*Bnip3*	0.062	0	0	0.429	0.442
3-day post-SCI group vs. the control group	*Sod2*	*Atox1*	0.11	0.237	0	0.25	0.446
3-day post-SCI group vs. the control group	*Sod2*	*Ncf1*	0.062	0	0	0.464	0.475
3-day post-SCI group vs. the control group	*Sod2*	*Gch1*	0.08	0	0	0.429	0.502
3-day post-SCI group vs. the control group	*Sod2*	*Myc*	0	0	0	0.511	0.511
3-day post-SCI group vs. the control group	*Sod2*	*Mapk3*	0.061	0.093	0	0.474	0.513
3-day post-SCI group vs. the control group	*Sod2*	*Ccs*	0	0	0	0.521	0.521
3-day post-SCI group vs. the control group	*Sod2*	*Mapt*	0	0.433	0	0.199	0.526
3-day post-SCI group vs. the control group	*Sod2*	*Ptgs2*	0.062	0	0	0.52	0.531
3-day post-SCI group vs. the control group	*Sod2*	*Ndufs8*	0.49	0	0	0.12	0.533
3-day post-SCI group vs. the control group	*Sod2*	*Glrx2*	0.157	0	0	0.469	0.542
3-day post-SCI group vs. the control group	*Sod2*	*Prdx4*	0.12	0	0	0.458	0.547
3-day post-SCI group vs. the control group	*Sod2*	*Jun*	0	0	0	0.556	0.556
3-day post-SCI group vs. the control group	*Sod2*	*Htra2*	0.063	0.325	0	0.396	0.585
3-day post-SCI group vs. the control group	*Sod2*	*Xdh*	0	0	0	0.593	0.593
3-day post-SCI group vs. the control group	*Sod2*	*Oxr1*	0.062	0	0	0.585	0.595
3-day post-SCI group vs. the control group	*Sod2*	*Txnrd1*	0.116	0.047	0	0.557	0.607
3-day post-SCI group vs. the control group	*Sod2*	*Ucp2*	0.062	0	0	0.61	0.619
3-day post-SCI group vs. the control group	*Sod2*	*Hif1a*	0	0	0	0.625	0.625
3-day post-SCI group vs. the control group	*Sod2*	*Casp3*	0.052	0	0	0.621	0.625
3-day post-SCI group vs. the control group	*Sod2*	*Gpx8*	0.061	0.348	0	0.46	0.641
3-day post-SCI group vs. the control group	*Sod2*	*Pink1*	0.061	0.146	0	0.595	0.646
3-day post-SCI group vs. the control group	*Sod2*	*Nox4*	0.067	0	0	0.645	0.655
3-day post-SCI group vs. the control group	*Sod2*	*Gpx7*	0.061	0.348	0	0.489	0.659
3-day post-SCI group vs. the control group	*Sod2*	*Rpl13a*	0.252	0.47	0	0.18	0.66
3-day post-SCI group vs. the control group	*Sod2*	*Actb*	0.051	0	0	0.674	0.677
3-day post-SCI group vs. the control group	*Sod2*	*Prdx6*	0.214	0	0	0.591	0.695

3-day post-SCI group vs. the control group	*Sod2*	*Nfe2l2*	0	0	0	0.707	0.707
3-day post-SCI group vs. the control group	*Sod2*	*Gpx3*	0.061	0.348	0	0.629	0.753
3-day post-SCI group vs. the control group	*Sod2*	*Ppif*	0.089	0.402	0	0.599	0.762
3-day post-SCI group vs. the control group	*Sod2*	*Rela*	0	0	0.629	0.464	0.792
3-day post-SCI group vs. the control group	*Sod2*	*Gsr*	0.121	0.047	0	0.784	0.809
3-day post-SCI group vs. the control group	*Sod2*	*Sirt1*	0.062	0.059	0	0.827	0.834
3-day post-SCI group vs. the control group	*Sod2*	*Gpx1*	0.061	0.348	0	0.859	0.906
3-day post-SCI group vs. the control group	*Sod2*	*Gclc*	0.067	0.698	0	0.761	0.927
3-day post-SCI group vs. the control group	*Sod2*	*Ppargc1a*	0	0	0.8	0.744	0.946
3-day post-SCI group vs. the control group	*Sod2*	*Cat*	0.08	0.277	0.961	0.939	0.998
3-day post-SCI group vs. the control group	*Srxn1*	*Hmox1*	0.151	0	0	0.643	0.685
3-day post-SCI group vs. the control group	*Srxn1*	*Sod2*	0.064	0	0	0.389	0.404
3-day post-SCI group vs. the control group	*Srxn1*	*Txnrd1*	0.258	0	0	0.739	0.798
3-day post-SCI group vs. the control group	*Srxn1*	*Prdx4*	0.067	0.462	0	0.765	0.872
3-day post-SCI group vs. the control group	*Srxn1*	*Cat*	0.361	0	0	0.725	0.817
3-day post-SCI group vs. the control group	*Srxn1*	*Gpx7*	0.058	0	0	0.398	0.409
3-day post-SCI group vs. the control group	*Srxn1*	*Gsr*	0.282	0	0	0.734	0.801
3-day post-SCI group vs. the control group	*Srxn1*	*Gclc*	0.273	0	0	0.714	0.783
3-day post-SCI group vs. the control group	*Srxn1*	*Txnip*	0	0	0	0.403	0.403
3-day post-SCI group vs. the control group	*Srxn1*	*Gpx3*	0.058	0	0	0.498	0.507
3-day post-SCI group vs. the control group	*Srxn1*	*Glrx2*	0.15	0	0	0.459	0.521
3-day post-SCI group vs. the control group	*Srxn1*	*Nfe2l2*	0	0	0	0.552	0.552
3-day post-SCI group vs. the control group	*Srxn1*	*Gpx1*	0.058	0	0	0.556	0.564
3-day post-SCI group vs. the control group	*Srxn1*	*Prdx6*	0.08	0.328	0	0.542	0.692
3-day post-SCI group vs. the control group	*Stat6*	*Mapk14*	0.059	0.104	0.212	0.469	0.6
3-day post-SCI group vs. the control group	*Stat6*	*Arg1*	0	0	0	0.721	0.721
3-day post-SCI group vs. the control group	*Stat6*	*Hif1a*	0	0.102	0	0.42	0.456
3-day post-SCI group vs. the control group	*Stat6*	*Fos*	0	0.071	0.216	0.327	0.467
3-day post-SCI group vs. the control group	*Stat6*	*Myc*	0.044	0.133	0	0.429	0.485
3-day post-SCI group vs. the control group	*Stat6*	*Hbegf*	0.063	0	0.35	0.184	0.459
3-day post-SCI group vs. the control group	*Stat6*	*Rela*	0.104	0.244	0.35	0.374	0.688
3-day post-SCI group vs. the control group	*Stat6*	*Il18rap*	0.061	0	0.35	0.167	0.447
3-day post-SCI group vs. the control group	*Stat6*	*Parp1*	0.062	0	0	0.538	0.548
3-day post-SCI group vs. the control group	*Stat6*	*Nfkb1*	0.098	0.13	0.151	0.399	0.546
3-day post-SCI group vs. the control group	*Stat6*	*Tnfrsf1a*	0.113	0.066	0.35	0.272	0.555
3-day post-SCI group vs. the control group	*Stat6*	*Btk*	0.082	0.136	0.193	0.283	0.48

3-day post-SCI group vs. the control group	*Stat6*	*Ets1*	0.087	0.087	0.676	0.443	0.829
3-day post-SCI group vs. the control group	*Stat6*	*Ptgs2*	0.061	0	0	0.428	0.44
3-day post-SCI group vs. the control group	*Stat6*	*Mcl1*	0.069	0	0.216	0.328	0.467
3-day post-SCI group vs. the control group	*Stat6*	*Tlr4*	0.071	0.089	0	0.573	0.607
3-day post-SCI group vs. the control group	*Stat6*	*Mapk3*	0.086	0.104	0.212	0.498	0.633
3-day post-SCI group vs. the control group	*Stat6*	*Tlr6*	0.077	0.089	0	0.408	0.459
3-day post-SCI group vs. the control group	*Stat6*	*Jak2*	0.055	0.147	0.932	0.837	0.989
3-day post-SCI group vs. the control group	*Stat6*	*Junb*	0.081	0.134	0.216	0.303	0.507
3-day post-SCI group vs. the control group	*Stat6*	*Hdac1*	0.048	0.164	0.208	0.223	0.444
3-day post-SCI group vs. the control group	*Stat6*	*Banf1*	0	0	0	0.454	0.454
3-day post-SCI group vs. the control group	*Stat6*	*Actb*	0	0.048	0	0.524	0.528
3-day post-SCI group vs. the control group	*Stat6*	*Il6st*	0.064	0.047	0.35	0.31	0.546
3-day post-SCI group vs. the control group	*Stat6*	*Nfatc1*	0.095	0	0	0.574	0.598
3-day post-SCI group vs. the control group	*Stat6*	*Jun*	0	0.134	0.216	0.552	0.669
3-day post-SCI group vs. the control group	*Stat6*	*Pdgfra*	0	0.143	0.653	0.093	0.707
3-day post-SCI group vs. the control group	*Stau2*	*Ago1*	0.062	0.232	0	0.264	0.423
3-day post-SCI group vs. the control group	*Stk24*	*Map2k4*	0	0.077	0.486	0.284	0.573
3-day post-SCI group vs. the control group	*Stk24*	*Ezr*	0.062	0	0.386	0.158	0.472
3-day post-SCI group vs. the control group	*Stk24*	*Map4k4*	0	0.065	0.419	0.328	0.46
3-day post-SCI group vs. the control group	*Stk24*	*Tnik*	0	0.065	0.419	0.357	0.461
3-day post-SCI group vs. the control group	*Stk24*	*Casp3*	0	0.087	0.9	0.109	0.911
3-day post-SCI group vs. the control group	*Syp*	*Amph*	0.187	0	0	0.436	0.522
3-day post-SCI group vs. the control group	*Syp*	*Fos*	0	0	0	0.546	0.547
3-day post-SCI group vs. the control group	*Syp*	*Aif1*	0	0	0	0.727	0.727
3-day post-SCI group vs. the control group	*Syp*	*Mapk3*	0	0	0	0.412	0.412
3-day post-SCI group vs. the control group	*Syp*	*Casp3*	0	0	0	0.591	0.591
3-day post-SCI group vs. the control group	*Syp*	*Mapt*	0.212	0	0	0.573	0.649
3-day post-SCI group vs. the control group	*Syp*	*Actb*	0	0	0	0.693	0.693
3-day post-SCI group vs. the control group	*Syp*	*Lancl1*	0.067	0.698	0	0	0.706
3-day post-SCI group vs. the control group	*Syp*	*Snca*	0.197	0.405	0	0.747	0.868
3-day post-SCI group vs. the control group	*Tank*	*Ripk3*	0	0.059	0.341	0.159	0.433
3-day post-SCI group vs. the control group	*Tank*	*Tnfrsf1a*	0	0.402	0.629	0.467	0.871
3-day post-SCI group vs. the control group	*Tank*	*Zc3h12a*	0	0.387	0	0.962	0.976
3-day post-SCI group vs. the control group	*Tank*	*Tlr4*	0	0	0.6	0.5	0.791
3-day post-SCI group vs. the control group	*Tank*	*Tnik*	0	0	0.499	0.077	0.518
3-day post-SCI group vs. the control group	*Tank*	*Map4k4*	0	0	0.629	0.052	0.633

3-day post-SCI group vs. the control group	*Tank*	*Uba52*	0	0	0.9	0	0.9
3-day post-SCI group vs. the control group	*Tank*	*Ripk1*	0.065	0.443	0.629	0.739	0.943
3-day post-SCI group vs. the control group	*Tfdp1*	*Cdc20*	0.279	0.099	0	0.421	0.591
3-day post-SCI group vs. the control group	*Tfdp1*	*Cdk4*	0.3	0.145	0.966	0.401	0.986
3-day post-SCI group vs. the control group	*Tfdp1*	*Cdk1*	0.257	0.145	0.961	0.368	0.982
3-day post-SCI group vs. the control group	*Tfdp1*	*Fos*	0	0.698	0	0.108	0.719
3-day post-SCI group vs. the control group	*Tfdp1*	*Dhfr*	0.106	0	0.629	0.195	0.709
3-day post-SCI group vs. the control group	*Tfdp1*	*Rb1*	0.155	0.86	0.932	0.502	0.995
3-day post-SCI group vs. the control group	*Tfdp1*	*Mcm4*	0.353	0	0	0.274	0.51
3-day post-SCI group vs. the control group	*Tfdp1*	*Pcna*	0.302	0	0.548	0.298	0.759
3-day post-SCI group vs. the control group	*Tfdp1*	*Ccna2*	0.284	0.138	0.864	0.354	0.939
3-day post-SCI group vs. the control group	*Tfdp1*	*Rbbp7*	0.269	0.139	0.806	0.267	0.899
3-day post-SCI group vs. the control group	*Tfdp1*	*Brf2*	0.064	0	0	0.449	0.462
3-day post-SCI group vs. the control group	*Tfdp1*	*Rnf2*	0.063	0.443	0.6	0.087	0.784
3-day post-SCI group vs. the control group	*Tfdp1*	*Apc*	0	0	0	0.509	0.509
3-day post-SCI group vs. the control group	*Tfdp1*	*Ezh2*	0.182	0	0.667	0.151	0.749
3-day post-SCI group vs. the control group	*Tfdp1*	*Cdkn2c*	0.062	0.081	0.207	0.274	0.437
3-day post-SCI group vs. the control group	*Tfdp1*	*Cdkn2b*	0.062	0.081	0.544	0.291	0.684
3-day post-SCI group vs. the control group	*Tfdp1*	*Hdac1*	0.071	0.104	0.826	0.1	0.852
3-day post-SCI group vs. the control group	*Tfdp1*	*Eed*	0.1	0	0.6	0.043	0.625
3-day post-SCI group vs. the control group	*Tfdp1*	*Cbx6*	0.062	0	0.407	0.065	0.434
3-day post-SCI group vs. the control group	*Tfdp1*	*Phc3*	0	0	0.6	0	0.6
3-day post-SCI group vs. the control group	*Tlr4*	*Mapk14*	0	0.048	0	0.748	0.75
3-day post-SCI group vs. the control group	*Tlr4*	*Hspb1*	0	0.057	0	0.405	0.415
3-day post-SCI group vs. the control group	*Tlr4*	*Hmox1*	0.056	0	0	0.682	0.687
3-day post-SCI group vs. the control group	*Tlr4*	*Ncf1*	0.09	0.046	0	0.528	0.554
3-day post-SCI group vs. the control group	*Tlr4*	*Map2k3*	0	0.09	0	0.457	0.485
3-day post-SCI group vs. the control group	*Tlr4*	*Arg1*	0.061	0.045	0	0.634	0.643
3-day post-SCI group vs. the control group	*Tlr4*	*Sdc1*	0	0	0	0.552	0.552
3-day post-SCI group vs. the control group	*Tlr4*	*Hif1a*	0.062	0.054	0	0.589	0.603
3-day post-SCI group vs. the control group	*Tlr4*	*Fos*	0.063	0.052	0	0.554	0.569
3-day post-SCI group vs. the control group	*Tlr4*	*Ctsl*	0	0.045	0	0.435	0.437
3-day post-SCI group vs. the control group	*Tlr4*	*Ripk3*	0.064	0.089	0.667	0.599	0.871
3-day post-SCI group vs. the control group	*Tlr4*	*Myc*	0.051	0.043	0	0.413	0.42
3-day post-SCI group vs. the control group	*Tlr4*	*Xdh*	0.112	0	0	0.822	0.836
3-day post-SCI group vs. the control group	*Tlr4*	*Aif1*	0.061	0.065	0	0.675	0.69

3-day post-SCI group vs. the control group	*Tlr4*	*Anxa1*	0.215	0.043	0	0.327	0.45
3-day post-SCI group vs. the control group	*Tlr4*	*Rela*	0	0	0.8	0.693	0.935
3-day post-SCI group vs. the control group	*Tlr4*	*Alox5*	0.064	0.044	0	0.435	0.451
3-day post-SCI group vs. the control group	*Tlr4*	*Ccr1*	0.213	0.057	0	0.495	0.593
3-day post-SCI group vs. the control group	*Tlr4*	*Cat*	0.061	0.057	0	0.559	0.575
3-day post-SCI group vs. the control group	*Tlr4*	*Vcam1*	0.171	0	0	0.673	0.718
3-day post-SCI group vs. the control group	*Tlr4*	*Nfkb1*	0.082	0.07	0.8	0.674	0.937
3-day post-SCI group vs. the control group	*Tlr4*	*Cd38*	0.062	0	0	0.403	0.416
3-day post-SCI group vs. the control group	*Tlr4*	*Tnfrsf1a*	0.068	0	0.158	0.684	0.73
3-day post-SCI group vs. the control group	*Tlr4*	*Nox4*	0	0.227	0	0.988	0.991
3-day post-SCI group vs. the control group	*Tlr4*	*Btk*	0.091	0.089	0.082	0.791	0.82
3-day post-SCI group vs. the control group	*Tlr4*	*Ptgs2*	0.064	0.042	0	0.743	0.75
3-day post-SCI group vs. the control group	*Tlr4*	*Txnip*	0.062	0	0	0.394	0.407
3-day post-SCI group vs. the control group	*Tlr4*	*Ptgs1*	0.104	0.042	0	0.371	0.413
3-day post-SCI group vs. the control group	*Tlr4*	*Sirt1*	0	0.052	0	0.495	0.501
3-day post-SCI group vs. the control group	*Tlr4*	*Tnik*	0	0.049	0.499	0.043	0.504
3-day post-SCI group vs. the control group	*Tlr4*	*Nfe2l2*	0.069	0	0	0.53	0.543
3-day post-SCI group vs. the control group	*Tlr4*	*Stat6*	0.071	0.089	0	0.573	0.607
3-day post-SCI group vs. the control group	*Tlr4*	*Jak2*	0.062	0.089	0.082	0.583	0.63
3-day post-SCI group vs. the control group	*Tlr4*	*Map4k4*	0	0.049	0.629	0.172	0.682
3-day post-SCI group vs. the control group	*Tlr4*	*Mapk8*	0	0.095	0	0.707	0.724
3-day post-SCI group vs. the control group	*Tlr4*	*Casp3*	0	0.077	0.118	0.698	0.733
3-day post-SCI group vs. the control group	*Tlr4*	*Cd36*	0.135	0.13	0.6	0.221	0.734
3-day post-SCI group vs. the control group	*Tlr4*	*Jun*	0	0	0	0.738	0.738
3-day post-SCI group vs. the control group	*Tlr4*	*Actb*	0	0.132	0	0.738	0.763
3-day post-SCI group vs. the control group	*Tlr4*	*S100a1*	0	0	0.6	0.466	0.777
3-day post-SCI group vs. the control group	*Tlr4*	*Tank*	0	0	0.6	0.5	0.791
3-day post-SCI group vs. the control group	*Tlr4*	*Mapk3*	0	0.048	0	0.812	0.814
3-day post-SCI group vs. the control group	*Tlr4*	*Uba52*	0	0.046	0.9	0	0.9
3-day post-SCI group vs. the control group	*Tlr4*	*Tlr6*	0.116	0.13	0.8	0.989	0.903
3-day post-SCI group vs. the control group	*Tlr4*	*Snca*	0	0.041	0	0.906	0.906
3-day post-SCI group vs. the control group	*Tlr4*	*Ripk1*	0.058	0.461	0.667	0.658	0.934
3-day post-SCI group vs. the control group	*Tlr6*	*Mapk14*	0	0.048	0	0.405	0.409
3-day post-SCI group vs. the control group	*Tlr6*	*Tnfrsf1a*	0.069	0	0.158	0.326	0.425
3-day post-SCI group vs. the control group	*Tlr6*	*Ptgs2*	0.062	0.042	0	0.434	0.447
3-day post-SCI group vs. the control group	*Tlr6*	*Tlr4*	0.116	0.13	0.8	0.989	0.903

3-day post-SCI group vs. the control group	*Tlr6*	*Jun*	0	0	0	0.402	0.402
3-day post-SCI group vs. the control group	*Tlr6*	*Ripk1*	0.062	0.089	0.204	0.251	0.422
3-day post-SCI group vs. the control group	*Tlr6*	*Casp3*	0	0.077	0.118	0.379	0.45
3-day post-SCI group vs. the control group	*Tlr6*	*Stat6*	0.077	0.089	0	0.408	0.459
3-day post-SCI group vs. the control group	*Tlr6*	*Actb*	0	0.132	0	0.471	0.521
3-day post-SCI group vs. the control group	*Tlr6*	*Cd36*	0.089	0.13	0.6	0.109	0.68
3-day post-SCI group vs. the control group	*Tnfrsf1a*	*Mapk14*	0.062	0.048	0	0.552	0.565
3-day post-SCI group vs. the control group	*Tnfrsf1a*	*Map2k3*	0.095	0	0.676	0.245	0.759
3-day post-SCI group vs. the control group	*Tnfrsf1a*	*Map2k6*	0	0	0.629	0.235	0.704
3-day post-SCI group vs. the control group	*Tnfrsf1a*	*Ripk3*	0.108	0.13	0.341	0.799	0.884
3-day post-SCI group vs. the control group	*Tnfrsf1a*	*Anxa1*	0.17	0	0	0.453	0.526
3-day post-SCI group vs. the control group	*Tnfrsf1a*	*Rela*	0.114	0	0.676	0.691	0.903
3-day post-SCI group vs. the control group	*Tnfrsf1a*	*P4hb*	0.105	0	0.217	0.293	0.461
3-day post-SCI group vs. the control group	*Tnfrsf1a*	*Vcam1*	0	0	0	0.564	0.564
3-day post-SCI group vs. the control group	*Tnfrsf1a*	*Nfkb1*	0.069	0.047	0.676	0.61	0.872
3-day post-SCI group vs. the control group	*Tnfrsf1a*	*Tlr6*	0.069	0	0.158	0.326	0.425
3-day post-SCI group vs. the control group	*Tnfrsf1a*	*Mcl1*	0.068	0.077	0	0.387	0.427
3-day post-SCI group vs. the control group	*Tnfrsf1a*	*Mapk3*	0.051	0.085	0	0.44	0.471
3-day post-SCI group vs. the control group	*Tnfrsf1a*	*Hdac1*	0	0	0.41	0.154	0.479
3-day post-SCI group vs. the control group	*Tnfrsf1a*	*Ptgs2*	0.082	0	0	0.464	0.487
3-day post-SCI group vs. the control group	*Tnfrsf1a*	*Actb*	0.063	0.047	0	0.523	0.536
3-day post-SCI group vs. the control group	*Tnfrsf1a*	*Mapk8*	0	0.133	0	0.49	0.539
3-day post-SCI group vs. the control group	*Tnfrsf1a*	*Stat6*	0.113	0.066	0.35	0.272	0.555
3-day post-SCI group vs. the control group	*Tnfrsf1a*	*Htra2*	0	0.402	0	0.297	0.562
3-day post-SCI group vs. the control group	*Tnfrsf1a*	*Jun*	0.069	0	0	0.619	0.63
3-day post-SCI group vs. the control group	*Tnfrsf1a*	*Map4k4*	0	0	0.676	0.132	0.706
3-day post-SCI group vs. the control group	*Tnfrsf1a*	*Tnik*	0	0	0.676	0.142	0.71
3-day post-SCI group vs. the control group	*Tnfrsf1a*	*Map2k4*	0	0	0.629	0.301	0.729
3-day post-SCI group vs. the control group	*Tnfrsf1a*	*Tlr4*	0.068	0	0.158	0.684	0.73
3-day post-SCI group vs. the control group	*Tnfrsf1a*	*Jak2*	0.051	0.227	0.137	0.662	0.757
3-day post-SCI group vs. the control group	*Tnfrsf1a*	*Casp3*	0	0.152	0.334	0.69	0.809
3-day post-SCI group vs. the control group	*Tnfrsf1a*	*Tank*	0	0.402	0.629	0.467	0.871
3-day post-SCI group vs. the control group	*Tnfrsf1a*	*Uba52*	0	0	0.9	0	0.9
3-day post-SCI group vs. the control group	*Tnfrsf1a*	*Ripk1*	0.113	0.999	0.932	0.988	0.999
3-day post-SCI group vs. the control group	*Tnik*	*Tnfrsf1a*	0	0	0.676	0.142	0.71
3-day post-SCI group vs. the control group	*Tnik*	*Tlr4*	0	0.049	0.499	0.043	0.504

3-day post-SCI group vs. the control group	*Tnik*	*Stk24*	0	0.065	0.419	0.357	0.461
3-day post-SCI group vs. the control group	*Tnik*	*Jun*	0	0.265	0	0.257	0.43
3-day post-SCI group vs. the control group	*Tnik*	*Oxr1*	0	0	0	0.537	0.537
3-day post-SCI group vs. the control group	*Tnik*	*Tank*	0	0	0.499	0.077	0.518
3-day post-SCI group vs. the control group	*Tnik*	*Ripk1*	0	0.142	0.499	0.094	0.563
3-day post-SCI group vs. the control group	*Tnrc6a*	*Paip1*	0	0	0	0.418	0.418
3-day post-SCI group vs. the control group	*Tnrc6a*	*Zc3h12a*	0	0	0	0.521	0.521
3-day post-SCI group vs. the control group	*Tnrc6a*	*Ago3*	0	0.818	0.72	0.634	0.979
3-day post-SCI group vs. the control group	*Tnrc6a*	*Fbxw7*	0	0	0	0.467	0.467
3-day post-SCI group vs. the control group	*Tnrc6a*	*Ago1*	0.062	0.761	0.72	0.965	0.997
3-day post-SCI group vs. the control group	*Tnrc6c*	*Ago3*	0.062	0.587	0.6	0.566	0.923
3-day post-SCI group vs. the control group	*Tnrc6c*	*Ago1*	0.064	0.735	0.6	0.722	0.968
3-day post-SCI group vs. the control group	*Txnip*	*Mapk14*	0.059	0.085	0	0.397	0.435
3-day post-SCI group vs. the control group	*Txnip*	*Hmox1*	0.096	0	0	0.463	0.494
3-day post-SCI group vs. the control group	*Txnip*	*Sod2*	0	0	0	0.436	0.436
3-day post-SCI group vs. the control group	*Txnip*	*Txnrd1*	0	0.087	0	0.48	0.505
3-day post-SCI group vs. the control group	*Txnip*	*Hif1a*	0	0.13	0	0.4	0.455
3-day post-SCI group vs. the control group	*Txnip*	*P4hb*	0.09	0.14	0	0.378	0.471
3-day post-SCI group vs. the control group	*Txnip*	*Parp1*	0.063	0.201	0	0.327	0.452
3-day post-SCI group vs. the control group	*Txnip*	*Cat*	0.068	0	0	0.465	0.48
3-day post-SCI group vs. the control group	*Txnip*	*Nox4*	0	0	0	0.465	0.465
3-day post-SCI group vs. the control group	*Txnip*	*Tlr4*	0.062	0	0	0.394	0.407
3-day post-SCI group vs. the control group	*Txnip*	*Srxn1*	0	0	0	0.403	0.403
3-day post-SCI group vs. the control group	*Txnip*	*Casp3*	0	0	0	0.466	0.466
3-day post-SCI group vs. the control group	*Txnip*	*Id1*	0.066	0	0	0.509	0.521
3-day post-SCI group vs. the control group	*Txnip*	*Actb*	0.061	0.055	0	0.419	0.439
3-day post-SCI group vs. the control group	*Txnip*	*Hdac1*	0	0.342	0	0.459	0.629
3-day post-SCI group vs. the control group	*Txnip*	*Nfe2l2*	0.1	0	0	0.578	0.604
3-day post-SCI group vs. the control group	*Txnip*	*Jun*	0.083	0	0	0.43	0.455
3-day post-SCI group vs. the control group	*Txnrd1*	*Hmox1*	0.113	0.131	0	0.701	0.749
3-day post-SCI group vs. the control group	*Txnrd1*	*Sod2*	0.116	0.047	0	0.557	0.607
3-day post-SCI group vs. the control group	*Txnrd1*	*Ccs*	0.052	0	0	0.329	0.413
3-day post-SCI group vs. the control group	*Txnrd1*	*Fbxw7*	0	0	0	0.414	0.414
3-day post-SCI group vs. the control group	*Txnrd1*	*Msrb2*	0.064	0.048	0	0.388	0.43
3-day post-SCI group vs. the control group	*Txnrd1*	*Prdx6*	0.067	0.059	0	0.406	0.433
3-day post-SCI group vs. the control group	*Txnrd1*	*Casp3*	0.055	0	0	0.431	0.439

3-day post-SCI group vs. the control group	*Txnrd1*	*Ptgs2*	0	0.226	0	0.318	0.45
3-day post-SCI group vs. the control group	*Txnrd1*	*Gpx8*	0.063	0.141	0	0.407	0.485
3-day post-SCI group vs. the control group	*Txnrd1*	*Txnip*	0	0.087	0	0.48	0.505
3-day post-SCI group vs. the control group	*Txnrd1*	*Actb*	0.051	0	0	0.501	0.506
3-day post-SCI group vs. the control group	*Txnrd1*	*P4hb*	0.105	0.233	0	0.379	0.541
3-day post-SCI group vs. the control group	*Txnrd1*	*Prdx4*	0.067	0.059	0	0.535	0.556
3-day post-SCI group vs. the control group	*Txnrd1*	*Xdh*	0	0	0	0.65	0.65
3-day post-SCI group vs. the control group	*Txnrd1*	*Gpx7*	0.063	0.141	0	0.626	0.675
3-day post-SCI group vs. the control group	*Txnrd1*	*Nfe2l2*	0.067	0	0	0.685	0.694
3-day post-SCI group vs. the control group	*Txnrd1*	*Cat*	0.118	0.219	0	0.688	0.766
3-day post-SCI group vs. the control group	*Txnrd1*	*Gpx3*	0.063	0.141	0	0.733	0.768
3-day post-SCI group vs. the control group	*Txnrd1*	*Srxn1*	0.258	0	0	0.739	0.798
3-day post-SCI group vs. the control group	*Txnrd1*	*Glrx2*	0.081	0.476	0	0.632	0.812
3-day post-SCI group vs. the control group	*Txnrd1*	*Gpx1*	0.083	0.141	0	0.781	0.814
3-day post-SCI group vs. the control group	*Txnrd1*	*Gclc*	0.194	0	0	0.808	0.839
3-day post-SCI group vs. the control group	*Uba52*	*Hspb1*	0	0	0.9	0.05	0.9
3-day post-SCI group vs. the control group	*Uba52*	*Cdc20*	0.072	0.164	0.6	0.075	0.674
3-day post-SCI group vs. the control group	*Uba52*	*Ube2a*	0.048	0.402	0.6	0.105	0.768
3-day post-SCI group vs. the control group	*Uba52*	*Cdk1*	0.088	0	0.6	0.059	0.627
3-day post-SCI group vs. the control group	*Uba52*	*Hif1a*	0	0	0.6	0	0.6
3-day post-SCI group vs. the control group	*Uba52*	*Rb1*	0	0	0.9	0	0.9
3-day post-SCI group vs. the control group	*Uba52*	*Ripk3*	0	0.055	0.9	0.055	0.902
3-day post-SCI group vs. the control group	*Uba52*	*Mcm4*	0	0	0.65	0.05	0.653
3-day post-SCI group vs. the control group	*Uba52*	*Hbegf*	0	0	0.6	0	0.6
3-day post-SCI group vs. the control group	*Uba52*	*Rela*	0	0	0.6	0	0.6
3-day post-SCI group vs. the control group	*Uba52*	*Parp1*	0	0	0.6	0	0.6
3-day post-SCI group vs. the control group	*Uba52*	*Pcna*	0.078	0.387	0.6	0	0.754
3-day post-SCI group vs. the control group	*Uba52*	*Ccna2*	0	0.053	0.6	0	0.605
3-day post-SCI group vs. the control group	*Uba52*	*Nfkb1*	0	0.141	0.6	0.049	0.644
3-day post-SCI group vs. the control group	*Uba52*	*Pink1*	0	0.399	0.9	0.057	0.938
3-day post-SCI group vs. the control group	*Uba52*	*Tnfrsf1a*	0	0	0.9	0	0.9
3-day post-SCI group vs. the control group	*Uba52*	*Atr*	0.176	0.204	0.65	0	0.75
3-day post-SCI group vs. the control group	*Uba52*	*Tlr4*	0	0.046	0.9	0	0.9
3-day post-SCI group vs. the control group	*Uba52*	*Ubqln1*	0.153	0.327	0	0.114	0.45
3-day post-SCI group vs. the control group	*Uba52*	*Junb*	0	0.465	0	0	0.465
3-day post-SCI group vs. the control group	*Uba52*	*Smad1*	0	0.052	0.9	0	0.901

3-day post-SCI group vs. the control group	*Uba52*	*Apc*	0	0	0.6	0	0.6
3-day post-SCI group vs. the control group	*Uba52*	*Jun*	0	0.757	0	0.043	0.757
3-day post-SCI group vs. the control group	*Uba52*	*Fbxw7*	0	0.749	0.6	0	0.895
3-day post-SCI group vs. the control group	*Uba52*	*Tank*	0	0	0.9	0	0.9
3-day post-SCI group vs. the control group	*Uba52*	*Met*	0	0.046	0.9	0.05	0.901
3-day post-SCI group vs. the control group	*Uba52*	*Rpl13a*	0.593	0.911	0.6	0.251	0.987
3-day post-SCI group vs. the control group	*Uba52*	*Ripk1*	0	0.055	0.9	0.05	0.902
3-day post-SCI group vs. the control group	*Uba52*	*Ptprk*	0	0	0.9	0.074	0.903
3-day post-SCI group vs. the control group	*Ube2a*	*Fbxw7*	0	0.138	0.197	0.234	0.423
3-day post-SCI group vs. the control group	*Ube2a*	*Rnf2*	0.052	0.164	0	0.351	0.44
3-day post-SCI group vs. the control group	*Ube2a*	*Atr*	0	0.086	0.131	0.447	0.522
3-day post-SCI group vs. the control group	*Ube2a*	*Eed*	0.105	0	0	0.494	0.527
3-day post-SCI group vs. the control group	*Ube2a*	*Hif1a*	0	0.046	0.629	0.077	0.645
3-day post-SCI group vs. the control group	*Ube2a*	*Pcna*	0.11	0.401	0	0.391	0.647
3-day post-SCI group vs. the control group	*Ube2a*	*Uba52*	0.048	0.402	0.6	0.105	0.768
3-day post-SCI group vs. the control group	*Ubqln1*	*Pink1*	0	0.261	0	0.225	0.403
3-day post-SCI group vs. the control group	*Ubqln1*	*Gnb2*	0	0.161	0.438	0.058	0.517
3-day post-SCI group vs. the control group	*Ubqln1*	*Uba52*	0.153	0.327	0	0.114	0.45
3-day post-SCI group vs. the control group	*Ucp2*	*Hmox1*	0.069	0	0	0.411	0.428
3-day post-SCI group vs. the control group	*Ucp2*	*Sod2*	0.062	0	0	0.61	0.619
3-day post-SCI group vs. the control group	*Ucp2*	*Hif1a*	0	0	0	0.429	0.429
3-day post-SCI group vs. the control group	*Ucp2*	*Cat*	0.076	0	0	0.565	0.582
3-day post-SCI group vs. the control group	*Ucp2*	*Gpx1*	0.101	0	0	0.493	0.524
3-day post-SCI group vs. the control group	*Ucp2*	*Casp3*	0	0	0	0.412	0.412
3-day post-SCI group vs. the control group	*Ucp2*	*Actb*	0.061	0.047	0	0.496	0.51
3-day post-SCI group vs. the control group	*Ucp2*	*Sirt1*	0.064	0.09	0	0.642	0.668
3-day post-SCI group vs. the control group	*Ucp2*	*Ppargc1a*	0	0	0	0.744	0.744
3-day post-SCI group vs. the control group	*Ucp2*	*Cd36*	0.099	0	0	0.395	0.431
3-day post-SCI group vs. the control group	*Vcam1*	*Axl*	0.141	0	0.147	0.274	0.421
3-day post-SCI group vs. the control group	*Vcam1*	*Mapk14*	0	0	0	0.632	0.632
3-day post-SCI group vs. the control group	*Vcam1*	*Hmox1*	0.061	0	0	0.628	0.636
3-day post-SCI group vs. the control group	*Vcam1*	*Ncf1*	0.062	0	0.9	0.482	0.947
3-day post-SCI group vs. the control group	*Vcam1*	*Arg1*	0	0	0	0.463	0.463
3-day post-SCI group vs. the control group	*Vcam1*	*Sdc1*	0	0	0	0.402	0.402
3-day post-SCI group vs. the control group	*Vcam1*	*Hif1a*	0.064	0	0	0.538	0.549
3-day post-SCI group vs. the control group	*Vcam1*	*Xdh*	0	0	0	0.468	0.468

3-day post-SCI group vs. the control group	*Vcam1*	*Aif1*	0.141	0	0	0.369	0.435
3-day post-SCI group vs. the control group	*Vcam1*	*Anxa1*	0.082	0.045	0	0.385	0.414
3-day post-SCI group vs. the control group	*Vcam1*	*Rela*	0	0.433	0	0.552	0.735
3-day post-SCI group vs. the control group	*Vcam1*	*Alox5*	0.061	0	0	0.393	0.405
3-day post-SCI group vs. the control group	*Vcam1*	*Ccr1*	0.131	0	0	0.494	0.542
3-day post-SCI group vs. the control group	*Vcam1*	*Itga6*	0.051	0.058	0.352	0.384	0.595
3-day post-SCI group vs. the control group	*Vcam1*	*Cat*	0	0	0	0.539	0.539
3-day post-SCI group vs. the control group	*Vcam1*	*Nfe2l2*	0	0	0	0.4	0.4
3-day post-SCI group vs. the control group	*Vcam1*	*Mapk8*	0	0	0	0.4	0.4
3-day post-SCI group vs. the control group	*Vcam1*	*Btk*	0.065	0	0.217	0.262	0.412
3-day post-SCI group vs. the control group	*Vcam1*	*Met*	0.062	0	0.147	0.338	0.424
3-day post-SCI group vs. the control group	*Vcam1*	*Cd38*	0.094	0	0	0.399	0.432
3-day post-SCI group vs. the control group	*Vcam1*	*Pdgfra*	0.106	0	0	0.396	0.437
3-day post-SCI group vs. the control group	*Vcam1*	*Sirt1*	0	0	0	0.451	0.451
3-day post-SCI group vs. the control group	*Vcam1*	*Itgb5*	0.073	0.056	0.317	0.222	0.472
3-day post-SCI group vs. the control group	*Vcam1*	*Nfkb1*	0.061	0.047	0	0.475	0.489
3-day post-SCI group vs. the control group	*Vcam1*	*Jak2*	0.061	0	0.217	0.397	0.517
3-day post-SCI group vs. the control group	*Vcam1*	*Nox4*	0.063	0	0	0.541	0.551
3-day post-SCI group vs. the control group	*Vcam1*	*Mapk3*	0	0	0	0.561	0.561
3-day post-SCI group vs. the control group	*Vcam1*	*Tnfrsf1a*	0	0	0	0.564	0.564
3-day post-SCI group vs. the control group	*Vcam1*	*Casp3*	0.052	0	0	0.566	0.571
3-day post-SCI group vs. the control group	*Vcam1*	*Jun*	0	0	0	0.589	0.589
3-day post-SCI group vs. the control group	*Vcam1*	*Actb*	0	0.047	0	0.62	0.622
3-day post-SCI group vs. the control group	*Vcam1*	*Ptgs2*	0.076	0	0	0.671	0.683
3-day post-SCI group vs. the control group	*Vcam1*	*Tlr4*	0.171	0	0	0.673	0.718
3-day post-SCI group vs. the control group	*Vcam1*	*Pxn*	0.062	0	0.676	0.518	0.84
3-day post-SCI group vs. the control group	*Vcam1*	*Ezr*	0	0.13	0.8	0.941	0.988
3-day post-SCI group vs. the control group	*Vrk2*	*Apc*	0.063	0.13	0.375	0.087	0.472
3-day post-SCI group vs. the control group	*Vrk2*	*Mapk8*	0.06	0.463	0	0.096	0.504
3-day post-SCI group vs. the control group	*Vrk2*	*Banf1*	0	0.307	0.9	0.415	0.955
3-day post-SCI group vs. the control group	*Xdh*	*Mapk14*	0	0	0	0.529	0.529
3-day post-SCI group vs. the control group	*Xdh*	*Hmox1*	0.068	0	0	0.619	0.629
3-day post-SCI group vs. the control group	*Xdh*	*Sod2*	0	0	0	0.593	0.593
3-day post-SCI group vs. the control group	*Xdh*	*Ncf1*	0.089	0	0	0.718	0.732
3-day post-SCI group vs. the control group	*Xdh*	*Txnrd1*	0	0	0	0.65	0.65
3-day post-SCI group vs. the control group	*Xdh*	*Hif1a*	0	0	0	0.406	0.406

3-day post-SCI group vs. the control group	*Xdh*	*Mapk3*	0	0	0	0.4	0.4
3-day post-SCI group vs. the control group	*Xdh*	*Sirt1*	0	0	0	0.425	0.425
3-day post-SCI group vs. the control group	*Xdh*	*Ppargc1a*	0	0	0	0.431	0.431
3-day post-SCI group vs. the control group	*Xdh*	*Vcam1*	0	0	0	0.468	0.468
3-day post-SCI group vs. the control group	*Xdh*	*Actb*	0	0	0	0.468	0.468
3-day post-SCI group vs. the control group	*Xdh*	*Ptgs2*	0	0	0	0.526	0.526
3-day post-SCI group vs. the control group	*Xdh*	*Casp3*	0	0	0	0.528	0.528
3-day post-SCI group vs. the control group	*Xdh*	*Gpx1*	0.062	0	0	0.552	0.561
3-day post-SCI group vs. the control group	*Xdh*	*Nfe2l2*	0.079	0	0	0.551	0.568
3-day post-SCI group vs. the control group	*Xdh*	*Jun*	0	0	0	0.583	0.583
3-day post-SCI group vs. the control group	*Xdh*	*Gch1*	0.104	0	0	0.642	0.665
3-day post-SCI group vs. the control group	*Xdh*	*Gsr*	0	0	0	0.68	0.68
3-day post-SCI group vs. the control group	*Xdh*	*Nox4*	0.062	0	0	0.737	0.742
3-day post-SCI group vs. the control group	*Xdh*	*Cat*	0.079	0	0	0.81	0.817
3-day post-SCI group vs. the control group	*Xdh*	*Tlr4*	0.112	0	0	0.822	0.836
3-day post-SCI group vs. the control group	*Ybx3*	*Cdk4*	0.1	0.13	0.8	0.625	0.933
3-day post-SCI group vs. the control group	*Zc3h12a*	*Ier3*	0.125	0.086	0	0.444	0.517
3-day post-SCI group vs. the control group	*Zc3h12a*	*Tnrc6a*	0	0	0	0.521	0.521
3-day post-SCI group vs. the control group	*Zc3h12a*	*Tank*	0	0.387	0	0.962	0.976
7-day post-SCI group vs. the control group	*Adam9*	*Hbegf*	0.061	0	0	0.555	0.565
7-day post-SCI group vs. the control group	*Adam9*	*Itgb5*	0.099	0.13	0.167	0.256	0.449
7-day post-SCI group vs. the control group	*Adam9*	*Cdh11*	0.061	0.043	0	0.392	0.406
7-day post-SCI group vs. the control group	*Adam9*	*Prkcd*	0	0.13	0	0.388	0.445
7-day post-SCI group vs. the control group	*Aif1*	*Axl*	0.08	0.092	0.114	0.329	0.437
7-day post-SCI group vs. the control group	*Aif1*	*Mapk14*	0	0.158	0	0.549	0.604
7-day post-SCI group vs. the control group	*Aif1*	*Cybb*	0.384	0.199	0	0.565	0.767
7-day post-SCI group vs. the control group	*Aif1*	*Ncf1*	0.203	0.057	0	0.353	0.471
7-day post-SCI group vs. the control group	*Aif1*	*Fos*	0	0.132	0	0.535	0.58
7-day post-SCI group vs. the control group	*Aif1*	*Trem2*	0.197	0	0	0.728	0.772
7-day post-SCI group vs. the control group	*Aif1*	*Jun*	0	0	0	0.403	0.403
7-day post-SCI group vs. the control group	*Aif1*	*Il18bp*	0.341	0	0	0.136	0.406
7-day post-SCI group vs. the control group	*Aif1*	*Vcam1*	0.141	0	0	0.369	0.435
7-day post-SCI group vs. the control group	*Aif1*	*Hdac1*	0.063	0.084	0	0.444	0.481
7-day post-SCI group vs. the control group	*Aif1*	*Apoe*	0.079	0	0	0.464	0.485
7-day post-SCI group vs. the control group	*Aif1*	*Mapt*	0	0.236	0	0.523	0.62
7-day post-SCI group vs. the control group	*Aif1*	*Tlr4*	0.061	0.065	0	0.675	0.69

7-day post-SCI group vs. the control group	*Amph*	*Mapt*	0.18	0.13	0	0.233	0.405
7-day post-SCI group vs. the control group	*Amph*	*Ppp3ca*	0.113	0.132	0.344	0.05	0.456
7-day post-SCI group vs. the control group	*Amph*	*Capn2*	0	0.406	0	0.135	0.464
7-day post-SCI group vs. the control group	*Apc*	*Ctnnb1*	0.065	0.999	0.932	0.99	0.999
7-day post-SCI group vs. the control group	*Apc*	*Cdh11*	0	0	0.54	0	0.54
7-day post-SCI group vs. the control group	*Apc*	*Jun*	0	0.276	0	0.455	0.589
7-day post-SCI group vs. the control group	*Apc*	*Hdac1*	0.061	0	0.9	0.064	0.904
7-day post-SCI group vs. the control group	*Apoe*	*Mapk14*	0	0	0	0.5	0.499
7-day post-SCI group vs. the control group	*Apoe*	*Cybb*	0	0	0	0.552	0.552
7-day post-SCI group vs. the control group	*Apoe*	*Ncf1*	0.062	0	0	0.513	0.523
7-day post-SCI group vs. the control group	*Apoe*	*Hif1a*	0	0	0	0.4	0.4
7-day post-SCI group vs. the control group	*Apoe*	*Clu*	0.143	0	0.72	0.921	0.979
7-day post-SCI group vs. the control group	*Apoe*	*Trem2*	0.078	0.329	0	0.983	0.989
7-day post-SCI group vs. the control group	*Apoe*	*Xdh*	0.105	0	0	0.464	0.5
7-day post-SCI group vs. the control group	*Apoe*	*Aif1*	0.079	0	0	0.464	0.485
7-day post-SCI group vs. the control group	*Apoe*	*Vcam1*	0.061	0	0	0.804	0.808
7-day post-SCI group vs. the control group	*Apoe*	*Pink1*	0.169	0	0	0.336	0.424
7-day post-SCI group vs. the control group	*Apoe*	*Lcat*	0.116	0.13	0.72	0.692	0.924
7-day post-SCI group vs. the control group	*Apoe*	*Tlr4*	0	0	0	0.72	0.72
7-day post-SCI group vs. the control group	*Apoe*	*Gpx1*	0.078	0	0	0.464	0.484
7-day post-SCI group vs. the control group	*Apoe*	*Gch1*	0	0	0	0.419	0.418
7-day post-SCI group vs. the control group	*Apoe*	*Mapt*	0	0.708	0	0.89	0.966
7-day post-SCI group vs. the control group	*Apoe*	*Jun*	0	0	0	0.552	0.552
7-day post-SCI group vs. the control group	*Apoe*	*Ppargc1a*	0	0	0	0.438	0.438
7-day post-SCI group vs. the control group	*Atp2a2*	*Mapk14*	0.062	0.058	0	0.402	0.426
7-day post-SCI group vs. the control group	*Atp2a2*	*Fkbp1b*	0	0.058	0	0.669	0.674
7-day post-SCI group vs. the control group	*Atp2a2*	*Rcan1*	0.057	0	0	0.402	0.412
7-day post-SCI group vs. the control group	*Atp2a2*	*Mapt*	0.062	0.443	0	0.055	0.463
7-day post-SCI group vs. the control group	*Atp2a2*	*Ppp3ca*	0.09	0.054	0	0.434	0.47
7-day post-SCI group vs. the control group	*Atp2a2*	*Ppargc1a*	0.081	0	0	0.521	0.541
7-day post-SCI group vs. the control group	*Atp2a2*	*Slc8a1*	0.081	0.169	0	0.701	0.751
7-day post-SCI group vs. the control group	*Atp2a2*	*S100a1*	0.062	0.13	0	0.825	0.845
7-day post-SCI group vs. the control group	*Atp7a*	*Mapk14*	0.062	0	0	0.463	0.474
7-day post-SCI group vs. the control group	*Atp7a*	*Mcm4*	0.427	0	0	0	0.426
7-day post-SCI group vs. the control group	*Axl*	*Vcam1*	0.141	0	0.147	0.274	0.421
7-day post-SCI group vs. the control group	*Axl*	*Jun*	0.062	0.09	0.127	0.327	0.431

7-day post-SCI group vs. the control group	*Axl*	*Aif1*	0.08	0.092	0.114	0.329	0.437
7-day post-SCI group vs. the control group	*Axl*	*Hif1a*	0	0.097	0.12	0.359	0.446
7-day post-SCI group vs. the control group	*Axl*	*Cybb*	0.092	0.379	0	0.14	0.473
7-day post-SCI group vs. the control group	*Axl*	*Trem2*	0.078	0	0	0.452	0.473
7-day post-SCI group vs. the control group	*Axl*	*Stat1*	0.056	0.136	0.122	0.405	0.516
7-day post-SCI group vs. the control group	*Axl*	*Ctnnb1*	0	0.138	0.133	0.441	0.546
7-day post-SCI group vs. the control group	*Axl*	*Btk*	0	0.697	0	0.197	0.714
7-day post-SCI group vs. the control group	*Btk*	*Axl*	0	0.697	0	0.197	0.714
7-day post-SCI group vs. the control group	*Btk*	*Mapk14*	0	0.147	0.187	0.473	0.407
7-day post-SCI group vs. the control group	*Btk*	*Ctnnb1*	0	0.138	0.177	0.27	0.437
7-day post-SCI group vs. the control group	*Btk*	*Cybb*	0.311	0.075	0	0.233	0.469
7-day post-SCI group vs. the control group	*Btk*	*Ncf1*	0.234	0.091	0	0.213	0.405
7-day post-SCI group vs. the control group	*Btk*	*Fos*	0	0.07	0.676	0.266	0.759
7-day post-SCI group vs. the control group	*Btk*	*Trem2*	0.09	0	0.6	0.197	0.682
7-day post-SCI group vs. the control group	*Btk*	*Vcam1*	0.065	0	0.217	0.262	0.412
7-day post-SCI group vs. the control group	*Btk*	*Cd38*	0.064	0.042	0	0.618	0.627
7-day post-SCI group vs. the control group	*Btk*	*Nfe2l2*	0	0.087	0	0.422	0.45
7-day post-SCI group vs. the control group	*Btk*	*Stat6*	0.082	0.136	0.193	0.283	0.48
7-day post-SCI group vs. the control group	*Btk*	*Stat1*	0.059	0.136	0.193	0.396	0.55
7-day post-SCI group vs. the control group	*Btk*	*Mcl1*	0	0.056	0.19	0.469	0.558
7-day post-SCI group vs. the control group	*Btk*	*Mapk10*	0.052	0.139	0.537	0.116	0.603
7-day post-SCI group vs. the control group	*Btk*	*Mapk8*	0.052	0.139	0.676	0.341	0.75
7-day post-SCI group vs. the control group	*Btk*	*Tlr4*	0.091	0.089	0.082	0.791	0.82
7-day post-SCI group vs. the control group	*Btk*	*Jun*	0	0.136	0.676	0.442	0.83
7-day post-SCI group vs. the control group	*Capn2*	*Amph*	0	0.406	0	0.135	0.464
7-day post-SCI group vs. the control group	*Capn2*	*Mapk14*	0.062	0.058	0.256	0.266	0.453
7-day post-SCI group vs. the control group	*Capn2*	*Ezr*	0	0.043	0.425	0.277	0.567
7-day post-SCI group vs. the control group	*Capn2*	*Itgb5*	0.107	0.084	0.271	0.136	0.415
7-day post-SCI group vs. the control group	*Cbx6*	*Rbbp7*	0.048	0.133	0.72	0.322	0.822
7-day post-SCI group vs. the control group	*Cbx6*	*Phc1*	0.08	0.402	0.848	0.66	0.967
7-day post-SCI group vs. the control group	*Cd36*	*Mapk14*	0	0	0.629	0.13	0.663
7-day post-SCI group vs. the control group	*Cd36*	*Tlr4*	0.135	0.13	0.6	0.221	0.734
7-day post-SCI group vs. the control group	*Cd36*	*Itgb5*	0.062	0.057	0.5	0.074	0.535
7-day post-SCI group vs. the control group	*Cd36*	*Ppargc1a*	0	0	0	0.529	0.529
7-day post-SCI group vs. the control group	*Cd36*	*Ucp2*	0.099	0	0	0.395	0.431
7-day post-SCI group vs. the control group	*Cd38*	*Itga6*	0	0	0	0.533	0.533

7-day post-SCI group vs. the control group	*Cd38*	*Vcam1*	0.094	0	0	0.399	0.432
7-day post-SCI group vs. the control group	*Cd38*	*Tlr4*	0.062	0	0	0.403	0.416
7-day post-SCI group vs. the control group	*Cd38*	*Btk*	0.064	0.042	0	0.618	0.627
7-day post-SCI group vs. the control group	*Cdc20*	*Fbxw7*	0.135	0.088	0	0.501	0.447
7-day post-SCI group vs. the control group	*Cdc20*	*Hdac1*	0.159	0.163	0.133	0.212	0.455
7-day post-SCI group vs. the control group	*Cdc20*	*Rbbp7*	0.278	0.047	0.176	0.058	0.463
7-day post-SCI group vs. the control group	*Cdc20*	*Ctnnb1*	0.062	0.134	0.164	0.331	0.485
7-day post-SCI group vs. the control group	*Cdc20*	*Phc1*	0.068	0.066	0.54	0.1	0.591
7-day post-SCI group vs. the control group	*Cdc20*	*Ppp3ca*	0	0.159	0.6	0.107	0.673
7-day post-SCI group vs. the control group	*Cdc20*	*Id1*	0.089	0.206	0	0.628	0.707
7-day post-SCI group vs. the control group	*Cdc20*	*Mcm4*	0.903	0.049	0	0.521	0.952
7-day post-SCI group vs. the control group	*Cdh11*	*Ctnnb1*	0	0.199	0.705	0.976	0.994
7-day post-SCI group vs. the control group	*Cdh11*	*Adam9*	0.061	0.043	0	0.392	0.406
7-day post-SCI group vs. the control group	*Cdh11*	*Apc*	0	0	0.54	0	0.54
7-day post-SCI group vs. the control group	*Clu*	*Hspb1*	0.061	0	0	0.464	0.475
7-day post-SCI group vs. the control group	*Clu*	*Mapt*	0.086	0.13	0	0.532	0.596
7-day post-SCI group vs. the control group	*Clu*	*Lcat*	0.099	0	0.72	0.405	0.836
7-day post-SCI group vs. the control group	*Clu*	*Trem2*	0	0.13	0	0.896	0.905
7-day post-SCI group vs. the control group	*Clu*	*Apoe*	0.143	0	0.72	0.921	0.979
7-day post-SCI group vs. the control group	*Ctnnb1*	*Axl*	0	0.138	0.133	0.441	0.546
7-day post-SCI group vs. the control group	*Ctnnb1*	*Mapk14*	0.066	0.066	0.6	0.711	0.885
7-day post-SCI group vs. the control group	*Ctnnb1*	*Hspb1*	0	0.239	0	0.551	0.644
7-day post-SCI group vs. the control group	*Ctnnb1*	*Cdc20*	0.062	0.134	0.164	0.331	0.485
7-day post-SCI group vs. the control group	*Ctnnb1*	*Stat6*	0	0.042	0	0.407	0.408
7-day post-SCI group vs. the control group	*Ctnnb1*	*Ppargc1a*	0	0	0	0.431	0.431
7-day post-SCI group vs. the control group	*Ctnnb1*	*Dst*	0.062	0.145	0.219	0.2	0.432
7-day post-SCI group vs. the control group	*Ctnnb1*	*Btk*	0	0.138	0.177	0.27	0.437
7-day post-SCI group vs. the control group	*Ctnnb1*	*Mapt*	0	0.091	0	0.414	0.445
7-day post-SCI group vs. the control group	*Ctnnb1*	*Nfe2l2*	0	0	0	0.498	0.498
7-day post-SCI group vs. the control group	*Ctnnb1*	*Mgst1*	0	0	0	0.511	0.511
7-day post-SCI group vs. the control group	*Ctnnb1*	*Vcam1*	0	0	0	0.522	0.522
7-day post-SCI group vs. the control group	*Ctnnb1*	*Prkcd*	0.062	0	0	0.536	0.546
7-day post-SCI group vs. the control group	*Ctnnb1*	*Stat1*	0.062	0.052	0	0.54	0.555
7-day post-SCI group vs. the control group	*Ctnnb1*	*Tlr4*	0	0.052	0.129	0.528	0.576
7-day post-SCI group vs. the control group	*Ctnnb1*	*Mcl1*	0.078	0.056	0	0.559	0.582
7-day post-SCI group vs. the control group	*Ctnnb1*	*Itgb5*	0.062	0.117	0.21	0.451	0.593

7-day post-SCI group vs. the control group	*Ctnnb1*	*Itga6*	0.062	0	0.18	0.551	0.624
7-day post-SCI group vs. the control group	*Ctnnb1*	*Fbxw7*	0	0.13	0	0.619	0.654
7-day post-SCI group vs. the control group	*Ctnnb1*	*Mapk10*	0.098	0.262	0.43	0.354	0.722
7-day post-SCI group vs. the control group	*Ctnnb1*	*Ezr*	0.063	0.13	0	0.719	0.751
7-day post-SCI group vs. the control group	*Ctnnb1*	*Mapk8*	0.098	0.563	0.43	0.615	0.902
7-day post-SCI group vs. the control group	*Ctnnb1*	*Fos*	0	0	0.676	0.891	0.963
7-day post-SCI group vs. the control group	*Ctnnb1*	*Cdh11*	0	0.199	0.705	0.976	0.994
7-day post-SCI group vs. the control group	*Ctnnb1*	*Hif1a*	0.078	0.56	0	0.991	0.996
7-day post-SCI group vs. the control group	*Ctnnb1*	*Jun*	0	0.433	0.676	0.986	0.997
7-day post-SCI group vs. the control group	*Ctnnb1*	*Hdac1*	0.135	0.7	0.956	0.89	0.998
7-day post-SCI group vs. the control group	*Ctnnb1*	*Apc*	0.065	0.999	0.932	0.99	0.999
7-day post-SCI group vs. the control group	*Ctsl*	*Tlr4*	0	0.045	0	0.435	0.437
7-day post-SCI group vs. the control group	*Cybb*	*Axl*	0.092	0.379	0	0.14	0.473
7-day post-SCI group vs. the control group	*Cybb*	*Mapk14*	0	0.086	0.65	0.607	0.863
7-day post-SCI group vs. the control group	*Cybb*	*Ezr*	0	0	0	0.445	0.445
7-day post-SCI group vs. the control group	*Cybb*	*Btk*	0.311	0.075	0	0.233	0.469
7-day post-SCI group vs. the control group	*Cybb*	*Hif1a*	0.061	0	0	0.528	0.538
7-day post-SCI group vs. the control group	*Cybb*	*Jun*	0.061	0.058	0	0.522	0.541
7-day post-SCI group vs. the control group	*Cybb*	*Stat1*	0.157	0.07	0	0.463	0.542
7-day post-SCI group vs. the control group	*Cybb*	*Apoe*	0	0	0	0.552	0.552
7-day post-SCI group vs. the control group	*Cybb*	*Nfe2l2*	0.071	0	0	0.569	0.582
7-day post-SCI group vs. the control group	*Cybb*	*Gpx1*	0.082	0	0	0.618	0.634
7-day post-SCI group vs. the control group	*Cybb*	*Tlr4*	0.217	0.076	0	0.626	0.706
7-day post-SCI group vs. the control group	*Cybb*	*Xdh*	0.138	0	0	0.739	0.765
7-day post-SCI group vs. the control group	*Cybb*	*Aif1*	0.384	0.199	0	0.565	0.767
7-day post-SCI group vs. the control group	*Cybb*	*Prkcd*	0.112	0	0.8	0.39	0.882
7-day post-SCI group vs. the control group	*Cybb*	*Vcam1*	0.119	0	0.9	0.565	0.958
7-day post-SCI group vs. the control group	*Cybb*	*Ncf1*	0.423	0.087	0.932	0.993	0.999
7-day post-SCI group vs. the control group	*Dst*	*Ctnnb1*	0.062	0.145	0.219	0.2	0.432
7-day post-SCI group vs. the control group	*Dst*	*Itga6*	0.061	0.071	0.639	0.406	0.788
7-day post-SCI group vs. the control group	*Dst*	*Ezr*	0.061	0.163	0.191	0.252	0.461
7-day post-SCI group vs. the control group	*Dst*	*Itgb5*	0.062	0.255	0.167	0.096	0.403
7-day post-SCI group vs. the control group	*Ets2*	*Mapk14*	0.062	0.215	0.629	0.213	0.756
7-day post-SCI group vs. the control group	*Ets2*	*Fos*	0.063	0.13	0.629	0.566	0.851
7-day post-SCI group vs. the control group	*Ets2*	*Hdac1*	0.061	0	0.243	0.269	0.435
7-day post-SCI group vs. the control group	*Ets2*	*Stat1*	0	0.047	0.211	0.354	0.472

7-day post-SCI group vs. the control group	*Ets2*	*Rcan1*	0.078	0	0	0.464	0.485
7-day post-SCI group vs. the control group	*Ets2*	*Stat6*	0.078	0.067	0.544	0.151	0.622
7-day post-SCI group vs. the control group	*Ets2*	*Id1*	0.061	0.049	0.8	0.24	0.846
7-day post-SCI group vs. the control group	*Ets2*	*Jun*	0.061	0.456	0.629	0.522	0.897
7-day post-SCI group vs. the control group	*Ezr*	*Mapk14*	0	0.199	0	0.312	0.425
7-day post-SCI group vs. the control group	*Ezr*	*Ctnnb1*	0.063	0.13	0	0.719	0.751
7-day post-SCI group vs. the control group	*Ezr*	*Cybb*	0	0	0	0.445	0.445
7-day post-SCI group vs. the control group	*Ezr*	*Vcam1*	0	0.13	0.8	0.941	0.988
7-day post-SCI group vs. the control group	*Ezr*	*S100a1*	0	0	0	0.707	0.707
7-day post-SCI group vs. the control group	*Ezr*	*Mapk10*	0.062	0.368	0	0.071	0.401
7-day post-SCI group vs. the control group	*Ezr*	*Itgb5*	0	0.048	0.327	0.195	0.439
7-day post-SCI group vs. the control group	*Ezr*	*Dst*	0.061	0.163	0.191	0.252	0.461
7-day post-SCI group vs. the control group	*Ezr*	*Capn2*	0	0.043	0.425	0.277	0.567
7-day post-SCI group vs. the control group	*Fbxw7*	*Cdc20*	0.135	0.088	0	0.501	0.447
7-day post-SCI group vs. the control group	*Fbxw7*	*Ctnnb1*	0	0.13	0	0.619	0.654
7-day post-SCI group vs. the control group	*Fbxw7*	*Hif1a*	0	0.231	0	0.42	0.535
7-day post-SCI group vs. the control group	*Fbxw7*	*Mcl1*	0.061	0.745	0	0.863	0.964
7-day post-SCI group vs. the control group	*Fbxw7*	*Prdx6*	0	0.433	0	0.068	0.448
7-day post-SCI group vs. the control group	*Fbxw7*	*Nfe2l2*	0	0.198	0	0.312	0.424
7-day post-SCI group vs. the control group	*Fbxw7*	*Jun*	0	0.762	0	0.901	0.975
7-day post-SCI group vs. the control group	*Fbxw7*	*Prkcd*	0.063	0.433	0	0.096	0.477
7-day post-SCI group vs. the control group	*Fbxw7*	*Ppargc1a*	0	0.439	0	0.191	0.527
7-day post-SCI group vs. the control group	*Fkbp1b*	*S100a1*	0	0.249	0	0.398	0.528
7-day post-SCI group vs. the control group	*Fkbp1b*	*Atp2a2*	0	0.058	0	0.669	0.674
7-day post-SCI group vs. the control group	*Fkbp1b*	*Ppp3ca*	0.062	0.591	0.15	0.268	0.729
7-day post-SCI group vs. the control group	*Fos*	*Mapk14*	0	0.267	0.922	0.716	0.982
7-day post-SCI group vs. the control group	*Fos*	*Ctnnb1*	0	0	0.676	0.891	0.963
7-day post-SCI group vs. the control group	*Fos*	*Map2k3*	0	0	0	0.435	0.435
7-day post-SCI group vs. the control group	*Fos*	*Hif1a*	0	0.131	0	0.527	0.571
7-day post-SCI group vs. the control group	*Fos*	*Hbegf*	0.065	0	0	0.422	0.436
7-day post-SCI group vs. the control group	*Fos*	*Mcl1*	0.089	0	0	0.411	0.441
7-day post-SCI group vs. the control group	*Fos*	*Stat6*	0	0.071	0.216	0.327	0.467
7-day post-SCI group vs. the control group	*Fos*	*Tlr4*	0.063	0.052	0	0.554	0.569
7-day post-SCI group vs. the control group	*Fos*	*Aif1*	0	0.132	0	0.535	0.58
7-day post-SCI group vs. the control group	*Fos*	*Hdac1*	0.048	0.164	0	0.67	0.715
7-day post-SCI group vs. the control group	*Fos*	*Nfe2l2*	0.061	0.443	0.293	0.416	0.755

7-day post-SCI group vs. the control group	*Fos*	*Btk*	0	0.07	0.676	0.266	0.759
7-day post-SCI group vs. the control group	*Fos*	*Ets2*	0.063	0.13	0.629	0.566	0.851
7-day post-SCI group vs. the control group	*Fos*	*Stat1*	0	0.508	0.216	0.676	0.864
7-day post-SCI group vs. the control group	*Fos*	*Mapk10*	0	0.309	0.903	0.492	0.963
7-day post-SCI group vs. the control group	*Fos*	*Mapk8*	0	0.309	0.932	0.64	0.981
7-day post-SCI group vs. the control group	*Fos*	*Jun*	0.662	0.982	0.932	0.995	0.999
7-day post-SCI group vs. the control group	*Gch1*	*Xdh*	0.104	0	0	0.642	0.665
7-day post-SCI group vs. the control group	*Gch1*	*Apoe*	0	0	0	0.419	0.418
7-day post-SCI group vs. the control group	*Gclc*	*Mapk14*	0.057	0	0	0.395	0.405
7-day post-SCI group vs. the control group	*Gclc*	*Jun*	0	0	0	0.471	0.471
7-day post-SCI group vs. the control group	*Gclc*	*Gpx3*	0.061	0	0	0.553	0.562
7-day post-SCI group vs. the control group	*Gclc*	*Gpx1*	0.061	0	0	0.775	0.779
7-day post-SCI group vs. the control group	*Gclc*	*Nfe2l2*	0	0	0	0.883	0.883
7-day post-SCI group vs. the control group	*Gpx1*	*Mapk14*	0	0	0	0.425	0.425
7-day post-SCI group vs. the control group	*Gpx1*	*Mgst1*	0.141	0	0.65	0.308	0.774
7-day post-SCI group vs. the control group	*Gpx1*	*Cybb*	0.082	0	0	0.618	0.634
7-day post-SCI group vs. the control group	*Gpx1*	*Ncf1*	0.062	0	0	0.521	0.531
7-day post-SCI group vs. the control group	*Gpx1*	*Hif1a*	0	0	0	0.483	0.483
7-day post-SCI group vs. the control group	*Gpx1*	*Xdh*	0.062	0	0	0.552	0.561
7-day post-SCI group vs. the control group	*Gpx1*	*Gclc*	0.061	0	0	0.775	0.779
7-day post-SCI group vs. the control group	*Gpx1*	*Prdx6*	0.063	0.184	0	0.629	0.691
7-day post-SCI group vs. the control group	*Gpx1*	*Jun*	0.051	0.045	0	0.413	0.422
7-day post-SCI group vs. the control group	*Gpx1*	*Apoe*	0.078	0	0	0.464	0.484
7-day post-SCI group vs. the control group	*Gpx1*	*Ucp2*	0.101	0	0	0.493	0.524
7-day post-SCI group vs. the control group	*Gpx1*	*Nfe2l2*	0.079	0	0	0.689	0.702
7-day post-SCI group vs. the control group	*Gpx1*	*Ppargc1a*	0	0	0.8	0.602	0.917
7-day post-SCI group vs. the control group	*Gpx3*	*Mgst1*	0.076	0	0.65	0.227	0.728
7-day post-SCI group vs. the control group	*Gpx3*	*Gclc*	0.061	0	0	0.553	0.562
7-day post-SCI group vs. the control group	*Gpx3*	*Prdx6*	0.063	0.184	0	0.516	0.597
7-day post-SCI group vs. the control group	*Gpx3*	*Nfe2l2*	0.057	0	0	0.405	0.415
7-day post-SCI group vs. the control group	*Gpx3*	*Ppargc1a*	0	0	0.8	0.237	0.84
7-day post-SCI group vs. the control group	*Hbegf*	*Hif1a*	0	0	0.9	0.307	0.927
7-day post-SCI group vs. the control group	*Hbegf*	*Fos*	0.065	0	0	0.422	0.436
7-day post-SCI group vs. the control group	*Hbegf*	*Jun*	0.063	0	0	0.409	0.423
7-day post-SCI group vs. the control group	*Hbegf*	*Itgb5*	0.051	0	0	0.418	0.424
7-day post-SCI group vs. the control group	*Hbegf*	*Stat6*	0.063	0	0.35	0.184	0.459

7-day post-SCI group vs. the control group	*Hbegf*	*Stat1*	0	0	0.35	0.221	0.472
7-day post-SCI group vs. the control group	*Hbegf*	*Adam9*	0.061	0	0	0.555	0.565
7-day post-SCI group vs. the control group	*Hdac1*	*Mapk14*	0	0.131	0	0.404	0.46
7-day post-SCI group vs. the control group	*Hdac1*	*Cdc20*	0.159	0.163	0.133	0.212	0.455
7-day post-SCI group vs. the control group	*Hdac1*	*Ctnnb1*	0.135	0.7	0.956	0.89	0.998
7-day post-SCI group vs. the control group	*Hdac1*	*Mgst1*	0	0	0	0.449	0.449
7-day post-SCI group vs. the control group	*Hdac1*	*Hif1a*	0	0.446	0.168	0.961	0.981
7-day post-SCI group vs. the control group	*Hdac1*	*Fos*	0.048	0.164	0	0.67	0.715
7-day post-SCI group vs. the control group	*Hdac1*	*Ets2*	0.061	0	0.243	0.269	0.435
7-day post-SCI group vs. the control group	*Hdac1*	*Aif1*	0.063	0.084	0	0.444	0.481
7-day post-SCI group vs. the control group	*Hdac1*	*Rbbp7*	0.224	0.813	0.956	0.981	0.999
7-day post-SCI group vs. the control group	*Hdac1*	*Stat1*	0.052	0.164	0.208	0.943	0.959
7-day post-SCI group vs. the control group	*Hdac1*	*Apc*	0.061	0	0.9	0.064	0.904
7-day post-SCI group vs. the control group	*Hdac1*	*Stat6*	0.048	0.164	0.208	0.223	0.444
7-day post-SCI group vs. the control group	*Hdac1*	*Ppargc1a*	0	0	0	0.416	0.416
7-day post-SCI group vs. the control group	*Hdac1*	*Txnip*	0	0.342	0	0.459	0.629
7-day post-SCI group vs. the control group	*Hdac1*	*Jun*	0	0.261	0	0.787	0.836
7-day post-SCI group vs. the control group	*Hif1a*	*Axl*	0	0.097	0.12	0.359	0.446
7-day post-SCI group vs. the control group	*Hif1a*	*Mapk14*	0	0.147	0	0.66	0.698
7-day post-SCI group vs. the control group	*Hif1a*	*Hspb1*	0	0.045	0	0.408	0.41
7-day post-SCI group vs. the control group	*Hif1a*	*Ctnnb1*	0.078	0.56	0	0.991	0.996
7-day post-SCI group vs. the control group	*Hif1a*	*Cybb*	0.061	0	0	0.528	0.538
7-day post-SCI group vs. the control group	*Hif1a*	*Apoe*	0	0	0	0.4	0.4
7-day post-SCI group vs. the control group	*Hif1a*	*Xdh*	0	0	0	0.406	0.406
7-day post-SCI group vs. the control group	*Hif1a*	*Ucp2*	0	0	0	0.429	0.429
7-day post-SCI group vs. the control group	*Hif1a*	*Txnip*	0	0.13	0	0.4	0.455
7-day post-SCI group vs. the control group	*Hif1a*	*Stat6*	0	0.102	0	0.42	0.456
7-day post-SCI group vs. the control group	*Hif1a*	*Pink1*	0	0	0	0.477	0.477
7-day post-SCI group vs. the control group	*Hif1a*	*Gpx1*	0	0	0	0.483	0.483
7-day post-SCI group vs. the control group	*Hif1a*	*Mapk8*	0.052	0	0	0.488	0.494
7-day post-SCI group vs. the control group	*Hif1a*	*Fbxw7*	0	0.231	0	0.42	0.535
7-day post-SCI group vs. the control group	*Hif1a*	*Vcam1*	0.064	0	0	0.538	0.549
7-day post-SCI group vs. the control group	*Hif1a*	*Stat1*	0.061	0.102	0	0.532	0.57
7-day post-SCI group vs. the control group	*Hif1a*	*Fos*	0	0.131	0	0.527	0.571
7-day post-SCI group vs. the control group	*Hif1a*	*Mcl1*	0.062	0.13	0	0.552	0.602
7-day post-SCI group vs. the control group	*Hif1a*	*Tlr4*	0.062	0.054	0	0.589	0.603

7-day post-SCI group vs. the control group	*Hif1a*	*Nfe2l2*	0	0	0	0.658	0.658
7-day post-SCI group vs. the control group	*Hif1a*	*Ppargc1a*	0.048	0	0	0.689	0.691
7-day post-SCI group vs. the control group	*Hif1a*	*Hbegf*	0	0	0.9	0.307	0.927
7-day post-SCI group vs. the control group	*Hif1a*	*Jun*	0.051	0.402	0.629	0.846	0.963
7-day post-SCI group vs. the control group	*Hif1a*	*Hdac1*	0	0.446	0.168	0.961	0.981
7-day post-SCI group vs. the control group	*Hspb1*	*Mapk14*	0	0.13	0.676	0.857	0.956
7-day post-SCI group vs. the control group	*Hspb1*	*Hif1a*	0	0.045	0	0.408	0.41
7-day post-SCI group vs. the control group	*Hspb1*	*Tlr4*	0	0.057	0	0.405	0.415
7-day post-SCI group vs. the control group	*Hspb1*	*Clu*	0.061	0	0	0.464	0.475
7-day post-SCI group vs. the control group	*Hspb1*	*Mapk8*	0	0.092	0	0.474	0.502
7-day post-SCI group vs. the control group	*Hspb1*	*Prdx6*	0.051	0.101	0	0.481	0.518
7-day post-SCI group vs. the control group	*Hspb1*	*Map2k3*	0.067	0.057	0	0.528	0.548
7-day post-SCI group vs. the control group	*Hspb1*	*Jun*	0.062	0	0	0.588	0.597
7-day post-SCI group vs. the control group	*Hspb1*	*Mapt*	0.061	0.13	0	0.576	0.623
7-day post-SCI group vs. the control group	*Hspb1*	*Ctnnb1*	0	0.239	0	0.551	0.644
7-day post-SCI group vs. the control group	*Hspb1*	*Prkcd*	0	0.439	0	0.578	0.754
7-day post-SCI group vs. the control group	*Id1*	*Cdc20*	0.089	0.206	0	0.628	0.707
7-day post-SCI group vs. the control group	*Id1*	*Ets2*	0.061	0.049	0.8	0.24	0.846
7-day post-SCI group vs. the control group	*Id1*	*Txnip*	0.066	0	0	0.509	0.521
7-day post-SCI group vs. the control group	*Il18bp*	*Aif1*	0.341	0	0	0.136	0.406
7-day post-SCI group vs. the control group	*Il18bp*	*Stat1*	0.3	0.13	0	0.309	0.542
7-day post-SCI group vs. the control group	*Il6st*	*Map2k3*	0	0	0.537	0.088	0.559
7-day post-SCI group vs. the control group	*Il6st*	*Stat1*	0	0.168	0.864	0.705	0.964
7-day post-SCI group vs. the control group	*Il6st*	*Stat6*	0.064	0.047	0.35	0.31	0.546
7-day post-SCI group vs. the control group	*Itga6*	*Ctnnb1*	0.062	0	0.18	0.551	0.624
7-day post-SCI group vs. the control group	*Itga6*	*Cd38*	0	0	0	0.533	0.533
7-day post-SCI group vs. the control group	*Itga6*	*Vcam1*	0.051	0.058	0.352	0.384	0.595
7-day post-SCI group vs. the control group	*Itga6*	*Dst*	0.061	0.071	0.639	0.406	0.788
7-day post-SCI group vs. the control group	*Itga6*	*Itgb5*	0.078	0.214	0.864	0.558	0.95
7-day post-SCI group vs. the control group	*Itgb5*	*Ctnnb1*	0.062	0.117	0.21	0.451	0.593
7-day post-SCI group vs. the control group	*Itgb5*	*Mylk*	0.061	0.089	0.756	0.125	0.793
7-day post-SCI group vs. the control group	*Itgb5*	*Hbegf*	0.051	0	0	0.418	0.424
7-day post-SCI group vs. the control group	*Itgb5*	*Itga6*	0.078	0.214	0.864	0.558	0.95
7-day post-SCI group vs. the control group	*Itgb5*	*Vcam1*	0.073	0.056	0.317	0.222	0.472
7-day post-SCI group vs. the control group	*Itgb5*	*Ezr*	0	0.048	0.327	0.195	0.439
7-day post-SCI group vs. the control group	*Itgb5*	*Capn2*	0.107	0.084	0.271	0.136	0.415

7-day post-SCI group vs. the control group	*Itgb5*	*Dst*	0.062	0.255	0.167	0.096	0.403
7-day post-SCI group vs. the control group	*Itgb5*	*Adam9*	0.099	0.13	0.167	0.256	0.449
7-day post-SCI group vs. the control group	*Itgb5*	*Cd36*	0.062	0.057	0.5	0.074	0.535
7-day post-SCI group vs. the control group	*Jun*	*Axl*	0.062	0.09	0.127	0.327	0.431
7-day post-SCI group vs. the control group	*Jun*	*Mapk14*	0.049	0.4	0.932	0.903	0.995
7-day post-SCI group vs. the control group	*Jun*	*Hspb1*	0.062	0	0	0.588	0.597
7-day post-SCI group vs. the control group	*Jun*	*Ctnnb1*	0	0.433	0.676	0.986	0.997
7-day post-SCI group vs. the control group	*Jun*	*Mgst1*	0	0	0	0.675	0.675
7-day post-SCI group vs. the control group	*Jun*	*Cybb*	0.061	0.058	0	0.522	0.541
7-day post-SCI group vs. the control group	*Jun*	*Ncf1*	0	0	0	0.466	0.465
7-day post-SCI group vs. the control group	*Jun*	*Map2k3*	0	0	0	0.721	0.721
7-day post-SCI group vs. the control group	*Jun*	*Hif1a*	0.051	0.402	0.629	0.846	0.963
7-day post-SCI group vs. the control group	*Jun*	*Fos*	0.662	0.982	0.932	0.995	0.999
7-day post-SCI group vs. the control group	*Jun*	*Ets2*	0.061	0.456	0.629	0.522	0.897
7-day post-SCI group vs. the control group	*Jun*	*Xdh*	0	0	0	0.583	0.583
7-day post-SCI group vs. the control group	*Jun*	*Aif1*	0	0	0	0.403	0.403
7-day post-SCI group vs. the control group	*Jun*	*Hbegf*	0.063	0	0	0.409	0.423
7-day post-SCI group vs. the control group	*Jun*	*Vcam1*	0	0	0	0.589	0.589
7-day post-SCI group vs. the control group	*Jun*	*Btk*	0	0.136	0.676	0.442	0.83
7-day post-SCI group vs. the control group	*Jun*	*Gclc*	0	0	0	0.471	0.471
7-day post-SCI group vs. the control group	*Jun*	*Map2k4*	0	0.13	0	0.827	0.843
7-day post-SCI group vs. the control group	*Jun*	*Mcl1*	0.063	0	0	0.62	0.628
7-day post-SCI group vs. the control group	*Jun*	*Tlr4*	0	0	0	0.738	0.738
7-day post-SCI group vs. the control group	*Jun*	*Stat1*	0	0.134	0.216	0.78	0.838
7-day post-SCI group vs. the control group	*Jun*	*Apc*	0	0.276	0	0.455	0.589
7-day post-SCI group vs. the control group	*Jun*	*Gpx1*	0.051	0.045	0	0.413	0.422
7-day post-SCI group vs. the control group	*Jun*	*Stat6*	0	0.134	0.216	0.552	0.669
7-day post-SCI group vs. the control group	*Jun*	*Hdac1*	0	0.261	0	0.787	0.836
7-day post-SCI group vs. the control group	*Jun*	*Nfe2l2*	0	0.131	0.362	0.745	0.846
7-day post-SCI group vs. the control group	*Jun*	*Txnip*	0.083	0	0	0.43	0.455
7-day post-SCI group vs. the control group	*Jun*	*Prkcd*	0	0.057	0	0.486	0.495
7-day post-SCI group vs. the control group	*Jun*	*Ripk1*	0	0.09	0.127	0.468	0.54
7-day post-SCI group vs. the control group	*Jun*	*Ppargc1a*	0	0	0	0.542	0.542
7-day post-SCI group vs. the control group	*Jun*	*Apoe*	0	0	0	0.552	0.552
7-day post-SCI group vs. the control group	*Jun*	*Fbxw7*	0	0.762	0	0.901	0.975
7-day post-SCI group vs. the control group	*Jun*	*Mapk10*	0	0.778	0.966	0.822	0.998

7-day post-SCI group vs. the control group	*Jun*	*Mapk8*	0	0.999	0.966	0.947	0.999
7-day post-SCI group vs. the control group	*Lcat*	*Clu*	0.099	0	0.72	0.405	0.836
7-day post-SCI group vs. the control group	*Lcat*	*Apoe*	0.116	0.13	0.72	0.692	0.924
7-day post-SCI group vs. the control group	*Map2k3*	*Mapk14*	0.065	0.839	0.966	0.961	0.996
7-day post-SCI group vs. the control group	*Map2k3*	*Hspb1*	0.067	0.057	0	0.528	0.548
7-day post-SCI group vs. the control group	*Map2k3*	*Fos*	0	0	0	0.435	0.435
7-day post-SCI group vs. the control group	*Map2k3*	*Tlr4*	0	0.09	0	0.457	0.485
7-day post-SCI group vs. the control group	*Map2k3*	*Il6st*	0	0	0.537	0.088	0.559
7-day post-SCI group vs. the control group	*Map2k3*	*Jun*	0	0	0	0.721	0.721
7-day post-SCI group vs. the control group	*Map2k3*	*Map2k4*	0	0.048	0.8	0.829	0.814
7-day post-SCI group vs. the control group	*Map2k3*	*Mapk10*	0.062	0.135	0.891	0.608	0.935
7-day post-SCI group vs. the control group	*Map2k3*	*Ripk1*	0.087	0.132	0.932	0.221	0.945
7-day post-SCI group vs. the control group	*Map2k3*	*Mapk8*	0.062	0.135	0.922	0.75	0.958
7-day post-SCI group vs. the control group	*Map2k4*	*Mapk14*	0.062	0.402	0.932	0.75	0.973
7-day post-SCI group vs. the control group	*Map2k4*	*Map2k3*	0	0.048	0.8	0.829	0.814
7-day post-SCI group vs. the control group	*Map2k4*	*Ripk1*	0	0.106	0.629	0.242	0.682
7-day post-SCI group vs. the control group	*Map2k4*	*Prkcd*	0	0	0.676	0.132	0.689
7-day post-SCI group vs. the control group	*Map2k4*	*Jun*	0	0.13	0	0.827	0.843
7-day post-SCI group vs. the control group	*Map2k4*	*Mapk8*	0.105	0.821	0.966	0.863	0.996
7-day post-SCI group vs. the control group	*Map2k4*	*Mapk10*	0.105	0.867	0.966	0.912	0.997
7-day post-SCI group vs. the control group	*Mapk10*	*Mapk14*	0.066	0.145	0.702	0.721	0.762
7-day post-SCI group vs. the control group	*Mapk10*	*Ctnnb1*	0.098	0.262	0.43	0.354	0.722
7-day post-SCI group vs. the control group	*Mapk10*	*Map2k3*	0.062	0.135	0.891	0.608	0.935
7-day post-SCI group vs. the control group	*Mapk10*	*Fos*	0	0.309	0.903	0.492	0.963
7-day post-SCI group vs. the control group	*Mapk10*	*Btk*	0.052	0.139	0.537	0.116	0.603
7-day post-SCI group vs. the control group	*Mapk10*	*Map2k4*	0.105	0.867	0.966	0.912	0.997
7-day post-SCI group vs. the control group	*Mapk10*	*Mcl1*	0	0.13	0.272	0.235	0.473
7-day post-SCI group vs. the control group	*Mapk10*	*Ezr*	0.062	0.368	0	0.071	0.401
7-day post-SCI group vs. the control group	*Mapk10*	*Mapt*	0.216	0.182	0.5	0.248	0.727
7-day post-SCI group vs. the control group	*Mapk10*	*Jun*	0	0.778	0.966	0.822	0.998
7-day post-SCI group vs. the control group	*Mapk10*	*Mapk8*	0.062	0	0.932	0.884	0.935
7-day post-SCI group vs. the control group	*Mapk10*	*Prkcd*	0	0.162	0.879	0.157	0.899
7-day post-SCI group vs. the control group	*Mapk14*	*Gclc*	0.057	0	0	0.395	0.405
7-day post-SCI group vs. the control group	*Mapk14*	*Btk*	0	0.147	0.187	0.473	0.407
7-day post-SCI group vs. the control group	*Mapk14*	*Mylk*	0	0.105	0.335	0.164	0.41
7-day post-SCI group vs. the control group	*Mapk14*	*Ezr*	0	0.199	0	0.312	0.425

7-day post-SCI group vs. the control group	*Mapk14*	*Gpx1*	0	0	0	0.425	0.425
7-day post-SCI group vs. the control group	*Mapk14*	*Atp2a2*	0.062	0.058	0	0.402	0.426
7-day post-SCI group vs. the control group	*Mapk14*	*Txnip*	0.059	0.085	0	0.397	0.435
7-day post-SCI group vs. the control group	*Mapk14*	*Capn2*	0.062	0.058	0.256	0.266	0.453
7-day post-SCI group vs. the control group	*Mapk14*	*Hdac1*	0	0.131	0	0.404	0.46
7-day post-SCI group vs. the control group	*Mapk14*	*Ppp3ca*	0.063	0.272	0	0.284	0.468
7-day post-SCI group vs. the control group	*Mapk14*	*Atp7a*	0.062	0	0	0.463	0.474
7-day post-SCI group vs. the control group	*Mapk14*	*Apoe*	0	0	0	0.5	0.499
7-day post-SCI group vs. the control group	*Mapk14*	*Xdh*	0	0	0	0.529	0.529
7-day post-SCI group vs. the control group	*Mapk14*	*Mgst1*	0	0	0	0.542	0.542
7-day post-SCI group vs. the control group	*Mapk14*	*Prkcd*	0.062	0.248	0.234	0.602	0.55
7-day post-SCI group vs. the control group	*Mapk14*	*Mapt*	0.049	0.182	0.354	0.249	0.572
7-day post-SCI group vs. the control group	*Mapk14*	*Stat6*	0.059	0.104	0.212	0.469	0.6
7-day post-SCI group vs. the control group	*Mapk14*	*Aif1*	0	0.158	0	0.549	0.604
7-day post-SCI group vs. the control group	*Mapk14*	*Vcam1*	0	0	0	0.632	0.632
7-day post-SCI group vs. the control group	*Mapk14*	*Cd36*	0	0	0.629	0.13	0.663
7-day post-SCI group vs. the control group	*Mapk14*	*Mcl1*	0.061	0.133	0.209	0.55	0.671
7-day post-SCI group vs. the control group	*Mapk14*	*Hif1a*	0	0.147	0	0.66	0.698
7-day post-SCI group vs. the control group	*Mapk14*	*Nfe2l2*	0	0.091	0	0.687	0.704
7-day post-SCI group vs. the control group	*Mapk14*	*Tlr4*	0	0.048	0	0.748	0.75
7-day post-SCI group vs. the control group	*Mapk14*	*Ets2*	0.062	0.215	0.629	0.213	0.756
7-day post-SCI group vs. the control group	*Mapk14*	*Ripk1*	0	0.147	0.676	0.412	0.758
7-day post-SCI group vs. the control group	*Mapk14*	*Mapk10*	0.066	0.145	0.702	0.721	0.762
7-day post-SCI group vs. the control group	*Mapk14*	*Mapk8*	0.066	0.209	0.702	0.872	0.783
7-day post-SCI group vs. the control group	*Mapk14*	*Cybb*	0	0.086	0.65	0.607	0.863
7-day post-SCI group vs. the control group	*Mapk14*	*Ctnnb1*	0.066	0.066	0.6	0.711	0.885
7-day post-SCI group vs. the control group	*Mapk14*	*Stat1*	0.059	0.104	0.676	0.668	0.897
7-day post-SCI group vs. the control group	*Mapk14*	*Hspb1*	0	0.13	0.676	0.857	0.956
7-day post-SCI group vs. the control group	*Mapk14*	*Map2k4*	0.062	0.402	0.932	0.75	0.973
7-day post-SCI group vs. the control group	*Mapk14*	*Ncf1*	0	0.425	0.9	0.63	0.976
7-day post-SCI group vs. the control group	*Mapk14*	*Fos*	0	0.267	0.922	0.716	0.982
7-day post-SCI group vs. the control group	*Mapk14*	*Ppargc1a*	0	0.087	0.966	0.62	0.987
7-day post-SCI group vs. the control group	*Mapk14*	*Jun*	0.049	0.4	0.932	0.903	0.995
7-day post-SCI group vs. the control group	*Mapk14*	*Map2k3*	0.065	0.839	0.966	0.961	0.996
7-day post-SCI group vs. the control group	*Mapk8*	*Mapk14*	0.066	0.209	0.702	0.872	0.783
7-day post-SCI group vs. the control group	*Mapk8*	*Hspb1*	0	0.092	0	0.474	0.502

7-day post-SCI group vs. the control group	*Mapk8*	*Ctnnb1*	0.098	0.563	0.43	0.615	0.902
7-day post-SCI group vs. the control group	*Mapk8*	*Mgst1*	0	0	0	0.722	0.722
7-day post-SCI group vs. the control group	*Mapk8*	*Map2k3*	0.062	0.135	0.922	0.75	0.958
7-day post-SCI group vs. the control group	*Mapk8*	*Hif1a*	0.052	0	0	0.488	0.494
7-day post-SCI group vs. the control group	*Mapk8*	*Fos*	0	0.309	0.932	0.64	0.981
7-day post-SCI group vs. the control group	*Mapk8*	*Vcam1*	0	0	0	0.4	0.4
7-day post-SCI group vs. the control group	*Mapk8*	*Btk*	0.052	0.139	0.676	0.341	0.75
7-day post-SCI group vs. the control group	*Mapk8*	*Map2k4*	0.105	0.821	0.966	0.863	0.996
7-day post-SCI group vs. the control group	*Mapk8*	*Mcl1*	0	0.431	0.272	0.471	0.762
7-day post-SCI group vs. the control group	*Mapk8*	*Tlr4*	0	0.095	0	0.707	0.724
7-day post-SCI group vs. the control group	*Mapk8*	*Stat1*	0	0.144	0	0.482	0.538
7-day post-SCI group vs. the control group	*Mapk8*	*Mapt*	0.062	0.228	0.629	0.258	0.774
7-day post-SCI group vs. the control group	*Mapk8*	*Nfe2l2*	0	0.052	0	0.483	0.488
7-day post-SCI group vs. the control group	*Mapk8*	*Jun*	0	0.999	0.966	0.947	0.999
7-day post-SCI group vs. the control group	*Mapk8*	*Ripk1*	0	0.208	0.209	0.457	0.468
7-day post-SCI group vs. the control group	*Mapk8*	*Prkcd*	0	0.162	0.879	0.424	0.911
7-day post-SCI group vs. the control group	*Mapk8*	*Mapk10*	0.062	0	0.932	0.884	0.935
7-day post-SCI group vs. the control group	*Mapt*	*Amph*	0.18	0.13	0	0.233	0.405
7-day post-SCI group vs. the control group	*Mapt*	*Mapk14*	0.049	0.182	0.354	0.249	0.572
7-day post-SCI group vs. the control group	*Mapt*	*Hspb1*	0.061	0.13	0	0.576	0.623
7-day post-SCI group vs. the control group	*Mapt*	*Ctnnb1*	0	0.091	0	0.414	0.445
7-day post-SCI group vs. the control group	*Mapt*	*Clu*	0.086	0.13	0	0.532	0.596
7-day post-SCI group vs. the control group	*Mapt*	*Ndufa6*	0	0	0.6	0	0.6
7-day post-SCI group vs. the control group	*Mapt*	*Trem2*	0.062	0	0	0.595	0.604
7-day post-SCI group vs. the control group	*Mapt*	*Aif1*	0	0.236	0	0.523	0.62
7-day post-SCI group vs. the control group	*Mapt*	*Pink1*	0.094	0	0	0.581	0.604
7-day post-SCI group vs. the control group	*Mapt*	*Atp2a2*	0.062	0.443	0	0.055	0.463
7-day post-SCI group vs. the control group	*Mapt*	*Ppp3ca*	0.098	0.527	0	0.152	0.606
7-day post-SCI group vs. the control group	*Mapt*	*Prdx6*	0	0.433	0	0.163	0.506
7-day post-SCI group vs. the control group	*Mapt*	*Mapk10*	0.216	0.182	0.5	0.248	0.727
7-day post-SCI group vs. the control group	*Mapt*	*Ndufa12*	0	0.433	0.6	0.071	0.77
7-day post-SCI group vs. the control group	*Mapt*	*Mapk8*	0.062	0.228	0.629	0.258	0.774
7-day post-SCI group vs. the control group	*Mapt*	*Apoe*	0	0.708	0	0.89	0.966
7-day post-SCI group vs. the control group	*Marcksl1*	*Prkcd*	0	0.088	0.8	0.143	0.83
7-day post-SCI group vs. the control group	*Mcl1*	*Mapk14*	0.061	0.133	0.209	0.55	0.671
7-day post-SCI group vs. the control group	*Mcl1*	*Ctnnb1*	0.078	0.056	0	0.559	0.582

7-day post-SCI group vs. the control group	*Mcl1*	*Hif1a*	0.062	0.13	0	0.552	0.602
7-day post-SCI group vs. the control group	*Mcl1*	*Fos*	0.089	0	0	0.411	0.441
7-day post-SCI group vs. the control group	*Mcl1*	*Pink1*	0	0.208	0	0.339	0.454
7-day post-SCI group vs. the control group	*Mcl1*	*Btk*	0	0.056	0.19	0.469	0.558
7-day post-SCI group vs. the control group	*Mcl1*	*Ripk1*	0.079	0.056	0.12	0.349	0.435
7-day post-SCI group vs. the control group	*Mcl1*	*Stat6*	0.069	0	0.216	0.328	0.467
7-day post-SCI group vs. the control group	*Mcl1*	*Mapk10*	0	0.13	0.272	0.235	0.473
7-day post-SCI group vs. the control group	*Mcl1*	*Stat1*	0.062	0	0.216	0.484	0.587
7-day post-SCI group vs. the control group	*Mcl1*	*Jun*	0.063	0	0	0.62	0.628
7-day post-SCI group vs. the control group	*Mcl1*	*Mapk8*	0	0.431	0.272	0.471	0.762
7-day post-SCI group vs. the control group	*Mcl1*	*Fbxw7*	0.061	0.745	0	0.863	0.964
7-day post-SCI group vs. the control group	*Mcm4*	*Cdc20*	0.903	0.049	0	0.521	0.952
7-day post-SCI group vs. the control group	*Mcm4*	*Atp7a*	0.427	0	0	0	0.426
7-day post-SCI group vs. the control group	*Mcm4*	*Rbbp7*	0.427	0.161	0	0	0.499
7-day post-SCI group vs. the control group	*Mgst1*	*Mapk14*	0	0	0	0.542	0.542
7-day post-SCI group vs. the control group	*Mgst1*	*Ctnnb1*	0	0	0	0.511	0.511
7-day post-SCI group vs. the control group	*Mgst1*	*Hdac1*	0	0	0	0.449	0.449
7-day post-SCI group vs. the control group	*Mgst1*	*Nfe2l2*	0.109	0	0	0.463	0.501
7-day post-SCI group vs. the control group	*Mgst1*	*Jun*	0	0	0	0.675	0.675
7-day post-SCI group vs. the control group	*Mgst1*	*Mapk8*	0	0	0	0.722	0.722
7-day post-SCI group vs. the control group	*Mgst1*	*Gpx3*	0.076	0	0.65	0.227	0.728
7-day post-SCI group vs. the control group	*Mgst1*	*Prdx6*	0.068	0	0.65	0.239	0.73
7-day post-SCI group vs. the control group	*Mgst1*	*Gpx1*	0.141	0	0.65	0.308	0.774
7-day post-SCI group vs. the control group	*Mylk*	*Mapk14*	0	0.105	0.335	0.164	0.41
7-day post-SCI group vs. the control group	*Mylk*	*Itgb5*	0.061	0.089	0.756	0.125	0.793
7-day post-SCI group vs. the control group	*Ncf1*	*Mapk14*	0	0.425	0.9	0.63	0.976
7-day post-SCI group vs. the control group	*Ncf1*	*Cybb*	0.423	0.087	0.932	0.993	0.999
7-day post-SCI group vs. the control group	*Ncf1*	*Btk*	0.234	0.091	0	0.213	0.405
7-day post-SCI group vs. the control group	*Ncf1*	*Jun*	0	0	0	0.466	0.465
7-day post-SCI group vs. the control group	*Ncf1*	*Aif1*	0.203	0.057	0	0.353	0.471
7-day post-SCI group vs. the control group	*Ncf1*	*Apoe*	0.062	0	0	0.513	0.523
7-day post-SCI group vs. the control group	*Ncf1*	*Gpx1*	0.062	0	0	0.521	0.531
7-day post-SCI group vs. the control group	*Ncf1*	*Tlr4*	0.09	0.046	0	0.528	0.554
7-day post-SCI group vs. the control group	*Ncf1*	*Nfe2l2*	0	0.434	0	0.4	0.645
7-day post-SCI group vs. the control group	*Ncf1*	*Xdh*	0.089	0	0	0.718	0.732
7-day post-SCI group vs. the control group	*Ncf1*	*Vcam1*	0.062	0	0.9	0.482	0.947

7-day post-SCI group vs. the control group	*Ncf1*	*Prkcd*	0.088	0.402	0.9	0.523	0.97
7-day post-SCI group vs. the control group	*Ndufa12*	*Ndufa6*	0.818	0.99	0.8	0.688	0.999
7-day post-SCI group vs. the control group	*Ndufa12*	*Pink1*	0.062	0	0.6	0.087	0.627
7-day post-SCI group vs. the control group	*Ndufa12*	*Mapt*	0	0.433	0.6	0.071	0.77
7-day post-SCI group vs. the control group	*Ndufa6*	*Mapt*	0	0	0.6	0	0.6
7-day post-SCI group vs. the control group	*Ndufa6*	*Pink1*	0.098	0	0.6	0.212	0.691
7-day post-SCI group vs. the control group	*Ndufa6*	*Ndufa12*	0.818	0.99	0.8	0.688	0.999
7-day post-SCI group vs. the control group	*Nfe2l2*	*Mapk14*	0	0.091	0	0.687	0.704
7-day post-SCI group vs. the control group	*Nfe2l2*	*Ctnnb1*	0	0	0	0.498	0.498
7-day post-SCI group vs. the control group	*Nfe2l2*	*Mgst1*	0.109	0	0	0.463	0.501
7-day post-SCI group vs. the control group	*Nfe2l2*	*Cybb*	0.071	0	0	0.569	0.582
7-day post-SCI group vs. the control group	*Nfe2l2*	*Ncf1*	0	0.434	0	0.4	0.645
7-day post-SCI group vs. the control group	*Nfe2l2*	*Hif1a*	0	0	0	0.658	0.658
7-day post-SCI group vs. the control group	*Nfe2l2*	*Fos*	0.061	0.443	0.293	0.416	0.755
7-day post-SCI group vs. the control group	*Nfe2l2*	*Xdh*	0.079	0	0	0.551	0.568
7-day post-SCI group vs. the control group	*Nfe2l2*	*Vcam1*	0	0	0	0.4	0.4
7-day post-SCI group vs. the control group	*Nfe2l2*	*Pink1*	0	0	0	0.458	0.458
7-day post-SCI group vs. the control group	*Nfe2l2*	*Btk*	0	0.087	0	0.422	0.45
7-day post-SCI group vs. the control group	*Nfe2l2*	*Gclc*	0	0	0	0.883	0.883
7-day post-SCI group vs. the control group	*Nfe2l2*	*Tlr4*	0.069	0	0	0.53	0.543
7-day post-SCI group vs. the control group	*Nfe2l2*	*Stat1*	0.061	0.041	0	0.446	0.458
7-day post-SCI group vs. the control group	*Nfe2l2*	*Gpx1*	0.079	0	0	0.689	0.702
7-day post-SCI group vs. the control group	*Nfe2l2*	*Gpx3*	0.057	0	0	0.405	0.415
7-day post-SCI group vs. the control group	*Nfe2l2*	*Fbxw7*	0	0.198	0	0.312	0.424
7-day post-SCI group vs. the control group	*Nfe2l2*	*Mapk8*	0	0.052	0	0.483	0.488
7-day post-SCI group vs. the control group	*Nfe2l2*	*Txnip*	0.1	0	0	0.578	0.604
7-day post-SCI group vs. the control group	*Nfe2l2*	*Jun*	0	0.131	0.362	0.745	0.846
7-day post-SCI group vs. the control group	*Nfe2l2*	*Ppargc1a*	0	0	0	0.855	0.855
7-day post-SCI group vs. the control group	*Phc1*	*Cdc20*	0.068	0.066	0.54	0.1	0.591
7-day post-SCI group vs. the control group	*Phc1*	*Rbbp7*	0	0	0.72	0.255	0.782
7-day post-SCI group vs. the control group	*Phc1*	*Cbx6*	0.08	0.402	0.848	0.66	0.967
7-day post-SCI group vs. the control group	*Pink1*	*Hif1a*	0	0	0	0.477	0.477
7-day post-SCI group vs. the control group	*Pink1*	*Ndufa6*	0.098	0	0.6	0.212	0.691
7-day post-SCI group vs. the control group	*Pink1*	*Apoe*	0.169	0	0	0.336	0.424
7-day post-SCI group vs. the control group	*Pink1*	*Mcl1*	0	0.208	0	0.339	0.454
7-day post-SCI group vs. the control group	*Pink1*	*Nfe2l2*	0	0	0	0.458	0.458

7-day post-SCI group vs. the control group	*Pink1*	*Mapt*	0.094	0	0	0.581	0.604
7-day post-SCI group vs. the control group	*Pink1*	*Ndufa12*	0.062	0	0.6	0.087	0.627
7-day post-SCI group vs. the control group	*Pink1*	*Ppargc1a*	0.069	0	0	0.836	0.841
7-day post-SCI group vs. the control group	*Ppargc1a*	*Mapk14*	0	0.087	0.966	0.62	0.987
7-day post-SCI group vs. the control group	*Ppargc1a*	*Ctnnb1*	0	0	0	0.431	0.431
7-day post-SCI group vs. the control group	*Ppargc1a*	*Hif1a*	0.048	0	0	0.689	0.691
7-day post-SCI group vs. the control group	*Ppargc1a*	*Xdh*	0	0	0	0.431	0.431
7-day post-SCI group vs. the control group	*Ppargc1a*	*Pink1*	0.069	0	0	0.836	0.841
7-day post-SCI group vs. the control group	*Ppargc1a*	*Atp2a2*	0.081	0	0	0.521	0.541
7-day post-SCI group vs. the control group	*Ppargc1a*	*Gpx1*	0	0	0.8	0.602	0.917
7-day post-SCI group vs. the control group	*Ppargc1a*	*Gpx3*	0	0	0.8	0.237	0.84
7-day post-SCI group vs. the control group	*Ppargc1a*	*Hdac1*	0	0	0	0.416	0.416
7-day post-SCI group vs. the control group	*Ppargc1a*	*Nfe2l2*	0	0	0	0.855	0.855
7-day post-SCI group vs. the control group	*Ppargc1a*	*Jun*	0	0	0	0.542	0.542
7-day post-SCI group vs. the control group	*Ppargc1a*	*Fbxw7*	0	0.439	0	0.191	0.527
7-day post-SCI group vs. the control group	*Ppargc1a*	*Apoe*	0	0	0	0.438	0.438
7-day post-SCI group vs. the control group	*Ppargc1a*	*Cd36*	0	0	0	0.529	0.529
7-day post-SCI group vs. the control group	*Ppargc1a*	*Ucp2*	0	0	0	0.744	0.744
7-day post-SCI group vs. the control group	*Ppp3ca*	*Amph*	0.113	0.132	0.344	0.05	0.456
7-day post-SCI group vs. the control group	*Ppp3ca*	*Mapk14*	0.063	0.272	0	0.284	0.468
7-day post-SCI group vs. the control group	*Ppp3ca*	*Cdc20*	0	0.159	0.6	0.107	0.673
7-day post-SCI group vs. the control group	*Ppp3ca*	*Fkbp1b*	0.062	0.591	0.15	0.268	0.729
7-day post-SCI group vs. the control group	*Ppp3ca*	*Atp2a2*	0.09	0.054	0	0.434	0.47
7-day post-SCI group vs. the control group	*Ppp3ca*	*Prkcd*	0.064	0.211	0.195	0.204	0.463
7-day post-SCI group vs. the control group	*Ppp3ca*	*Mapt*	0.098	0.527	0	0.152	0.606
7-day post-SCI group vs. the control group	*Ppp3ca*	*Rcan1*	0	0.839	0	0.677	0.945
7-day post-SCI group vs. the control group	*Prdx6*	*Hspb1*	0.051	0.101	0	0.481	0.518
7-day post-SCI group vs. the control group	*Prdx6*	*Mgst1*	0.068	0	0.65	0.239	0.73
7-day post-SCI group vs. the control group	*Prdx6*	*Fbxw7*	0	0.433	0	0.068	0.448
7-day post-SCI group vs. the control group	*Prdx6*	*Mapt*	0	0.433	0	0.163	0.506
7-day post-SCI group vs. the control group	*Prdx6*	*Gpx3*	0.063	0.184	0	0.516	0.597
7-day post-SCI group vs. the control group	*Prdx6*	*Gpx1*	0.063	0.184	0	0.629	0.691
7-day post-SCI group vs. the control group	*Prkcd*	*Mapk14*	0.062	0.248	0.234	0.602	0.55
7-day post-SCI group vs. the control group	*Prkcd*	*Hspb1*	0	0.439	0	0.578	0.754
7-day post-SCI group vs. the control group	*Prkcd*	*Ctnnb1*	0.062	0	0	0.536	0.546
7-day post-SCI group vs. the control group	*Prkcd*	*Cybb*	0.112	0	0.8	0.39	0.882

7-day post-SCI group vs. the control group	*Prkcd*	*Ncf1*	0.088	0.402	0.9	0.523	0.97
7-day post-SCI group vs. the control group	*Prkcd*	*Map2k4*	0	0	0.676	0.132	0.689
7-day post-SCI group vs. the control group	*Prkcd*	*Ppp3ca*	0.064	0.211	0.195	0.204	0.463
7-day post-SCI group vs. the control group	*Prkcd*	*Marcksl1*	0	0.088	0.8	0.143	0.83
7-day post-SCI group vs. the control group	*Prkcd*	*Stat1*	0.076	0.764	0.966	0.604	0.996
7-day post-SCI group vs. the control group	*Prkcd*	*Adam9*	0	0.13	0	0.388	0.445
7-day post-SCI group vs. the control group	*Prkcd*	*Jun*	0	0.057	0	0.486	0.495
7-day post-SCI group vs. the control group	*Prkcd*	*Fbxw7*	0.063	0.433	0	0.096	0.477
7-day post-SCI group vs. the control group	*Prkcd*	*Mapk8*	0	0.162	0.879	0.424	0.911
7-day post-SCI group vs. the control group	*Prkcd*	*Mapk10*	0	0.162	0.879	0.157	0.899
7-day post-SCI group vs. the control group	*Ptgs1*	*Tlr4*	0.104	0.042	0	0.371	0.413
7-day post-SCI group vs. the control group	*Rbbp7*	*Cdc20*	0.278	0.047	0.176	0.058	0.463
7-day post-SCI group vs. the control group	*Rbbp7*	*Mcm4*	0.427	0.161	0	0	0.499
7-day post-SCI group vs. the control group	*Rbbp7*	*Phc1*	0	0	0.72	0.255	0.782
7-day post-SCI group vs. the control group	*Rbbp7*	*Cbx6*	0.048	0.133	0.72	0.322	0.822
7-day post-SCI group vs. the control group	*Rbbp7*	*Hdac1*	0.224	0.813	0.956	0.981	0.999
7-day post-SCI group vs. the control group	*Rcan1*	*Ets2*	0.078	0	0	0.464	0.485
7-day post-SCI group vs. the control group	*Rcan1*	*Atp2a2*	0.057	0	0	0.402	0.412
7-day post-SCI group vs. the control group	*Rcan1*	*Ppp3ca*	0	0.839	0	0.677	0.945
7-day post-SCI group vs. the control group	*Ripk1*	*Mapk14*	0	0.147	0.676	0.412	0.758
7-day post-SCI group vs. the control group	*Ripk1*	*Map2k3*	0.087	0.132	0.932	0.221	0.945
7-day post-SCI group vs. the control group	*Ripk1*	*Map2k4*	0	0.106	0.629	0.242	0.682
7-day post-SCI group vs. the control group	*Ripk1*	*Mcl1*	0.079	0.056	0.12	0.349	0.435
7-day post-SCI group vs. the control group	*Ripk1*	*Tlr4*	0.058	0.461	0.667	0.658	0.934
7-day post-SCI group vs. the control group	*Ripk1*	*Stat1*	0.062	0.136	0.676	0.426	0.829
7-day post-SCI group vs. the control group	*Ripk1*	*Jun*	0	0.09	0.127	0.468	0.54
7-day post-SCI group vs. the control group	*Ripk1*	*Mapk8*	0	0.208	0.209	0.457	0.468
7-day post-SCI group vs. the control group	*S100a1*	*Fkbp1b*	0	0.249	0	0.398	0.528
7-day post-SCI group vs. the control group	*S100a1*	*Atp2a2*	0.062	0.13	0	0.825	0.845
7-day post-SCI group vs. the control group	*S100a1*	*Tlr4*	0	0	0.6	0.466	0.777
7-day post-SCI group vs. the control group	*S100a1*	*Ezr*	0	0	0	0.707	0.707
7-day post-SCI group vs. the control group	*Sirpa*	*Trem2*	0.217	0	0	0.357	0.475
7-day post-SCI group vs. the control group	*Slc8a1*	*Atp2a2*	0.081	0.169	0	0.701	0.751
7-day post-SCI group vs. the control group	*Slc8a1*	*Stat1*	0	0.433	0	0.051	0.438
7-day post-SCI group vs. the control group	*Stat1*	*Axl*	0.056	0.136	0.122	0.405	0.516
7-day post-SCI group vs. the control group	*Stat1*	*Mapk14*	0.059	0.104	0.676	0.668	0.897

7-day post-SCI group vs. the control group	*Stat1*	*Ctnnb1*	0.062	0.052	0	0.54	0.555
7-day post-SCI group vs. the control group	*Stat1*	*Cybb*	0.157	0.07	0	0.463	0.542
7-day post-SCI group vs. the control group	*Stat1*	*Hif1a*	0.061	0.102	0	0.532	0.57
7-day post-SCI group vs. the control group	*Stat1*	*Fos*	0	0.508	0.216	0.676	0.864
7-day post-SCI group vs. the control group	*Stat1*	*Ets2*	0	0.047	0.211	0.354	0.472
7-day post-SCI group vs. the control group	*Stat1*	*Hbegf*	0	0	0.35	0.221	0.472
7-day post-SCI group vs. the control group	*Stat1*	*Vcam1*	0.097	0.044	0	0.453	0.487
7-day post-SCI group vs. the control group	*Stat1*	*Btk*	0.059	0.136	0.193	0.396	0.55
7-day post-SCI group vs. the control group	*Stat1*	*Mcl1*	0.062	0	0.216	0.484	0.587
7-day post-SCI group vs. the control group	*Stat1*	*Tlr4*	0.072	0.089	0	0.702	0.726
7-day post-SCI group vs. the control group	*Stat1*	*Slc8a1*	0	0.433	0	0.051	0.438
7-day post-SCI group vs. the control group	*Stat1*	*Nfe2l2*	0.061	0.041	0	0.446	0.458
7-day post-SCI group vs. the control group	*Stat1*	*Mapk8*	0	0.144	0	0.482	0.538
7-day post-SCI group vs. the control group	*Stat1*	*Il18bp*	0.3	0.13	0	0.309	0.542
7-day post-SCI group vs. the control group	*Stat1*	*Ripk1*	0.062	0.136	0.676	0.426	0.829
7-day post-SCI group vs. the control group	*Stat1*	*Jun*	0	0.134	0.216	0.78	0.838
7-day post-SCI group vs. the control group	*Stat1*	*Stat6*	0.07	0	0.8	0.859	0.865
7-day post-SCI group vs. the control group	*Stat1*	*Hdac1*	0.052	0.164	0.208	0.943	0.959
7-day post-SCI group vs. the control group	*Stat1*	*Il6st*	0	0.168	0.864	0.705	0.964
7-day post-SCI group vs. the control group	*Stat1*	*Prkcd*	0.076	0.764	0.966	0.604	0.996
7-day post-SCI group vs. the control group	*Stat6*	*Mapk14*	0.059	0.104	0.212	0.469	0.6
7-day post-SCI group vs. the control group	*Stat6*	*Ctnnb1*	0	0.042	0	0.407	0.408
7-day post-SCI group vs. the control group	*Stat6*	*Hif1a*	0	0.102	0	0.42	0.456
7-day post-SCI group vs. the control group	*Stat6*	*Fos*	0	0.071	0.216	0.327	0.467
7-day post-SCI group vs. the control group	*Stat6*	*Ets2*	0.078	0.067	0.544	0.151	0.622
7-day post-SCI group vs. the control group	*Stat6*	*Hbegf*	0.063	0	0.35	0.184	0.459
7-day post-SCI group vs. the control group	*Stat6*	*Btk*	0.082	0.136	0.193	0.283	0.48
7-day post-SCI group vs. the control group	*Stat6*	*Mcl1*	0.069	0	0.216	0.328	0.467
7-day post-SCI group vs. the control group	*Stat6*	*Tlr4*	0.071	0.089	0	0.573	0.607
7-day post-SCI group vs. the control group	*Stat6*	*Stat1*	0.07	0	0.8	0.859	0.865
7-day post-SCI group vs. the control group	*Stat6*	*Hdac1*	0.048	0.164	0.208	0.223	0.444
7-day post-SCI group vs. the control group	*Stat6*	*Il6st*	0.064	0.047	0.35	0.31	0.546
7-day post-SCI group vs. the control group	*Stat6*	*Jun*	0	0.134	0.216	0.552	0.669
7-day post-SCI group vs. the control group	*Tlr4*	*Mapk14*	0	0.048	0	0.748	0.75
7-day post-SCI group vs. the control group	*Tlr4*	*Hspb1*	0	0.057	0	0.405	0.415
7-day post-SCI group vs. the control group	*Tlr4*	*Ctnnb1*	0	0.052	0.129	0.528	0.576

7-day post-SCI group vs. the control group	*Tlr4*	*Cybb*	0.217	0.076	0	0.626	0.706
7-day post-SCI group vs. the control group	*Tlr4*	*Ncf1*	0.09	0.046	0	0.528	0.554
7-day post-SCI group vs. the control group	*Tlr4*	*Map2k3*	0	0.09	0	0.457	0.485
7-day post-SCI group vs. the control group	*Tlr4*	*Hif1a*	0.062	0.054	0	0.589	0.603
7-day post-SCI group vs. the control group	*Tlr4*	*Fos*	0.063	0.052	0	0.554	0.569
7-day post-SCI group vs. the control group	*Tlr4*	*Ctsl*	0	0.045	0	0.435	0.437
7-day post-SCI group vs. the control group	*Tlr4*	*Trem2*	0.182	0	0	0.607	0.665
7-day post-SCI group vs. the control group	*Tlr4*	*Xdh*	0.112	0	0	0.822	0.836
7-day post-SCI group vs. the control group	*Tlr4*	*Aif1*	0.061	0.065	0	0.675	0.69
7-day post-SCI group vs. the control group	*Tlr4*	*Vcam1*	0.171	0	0	0.673	0.718
7-day post-SCI group vs. the control group	*Tlr4*	*Cd38*	0.062	0	0	0.403	0.416
7-day post-SCI group vs. the control group	*Tlr4*	*Btk*	0.091	0.089	0.082	0.791	0.82
7-day post-SCI group vs. the control group	*Tlr4*	*Txnip*	0.062	0	0	0.394	0.407
7-day post-SCI group vs. the control group	*Tlr4*	*Ptgs1*	0.104	0.042	0	0.371	0.413
7-day post-SCI group vs. the control group	*Tlr4*	*Nfe2l2*	0.069	0	0	0.53	0.543
7-day post-SCI group vs. the control group	*Tlr4*	*Stat6*	0.071	0.089	0	0.573	0.607
7-day post-SCI group vs. the control group	*Tlr4*	*Apoe*	0	0	0	0.72	0.72
7-day post-SCI group vs. the control group	*Tlr4*	*Mapk8*	0	0.095	0	0.707	0.724
7-day post-SCI group vs. the control group	*Tlr4*	*Stat1*	0.072	0.089	0	0.702	0.726
7-day post-SCI group vs. the control group	*Tlr4*	*Cd36*	0.135	0.13	0.6	0.221	0.734
7-day post-SCI group vs. the control group	*Tlr4*	*Jun*	0	0	0	0.738	0.738
7-day post-SCI group vs. the control group	*Tlr4*	*S100a1*	0	0	0.6	0.466	0.777
7-day post-SCI group vs. the control group	*Tlr4*	*Ripk1*	0.058	0.461	0.667	0.658	0.934
7-day post-SCI group vs. the control group	*Trem2*	*Axl*	0.078	0	0	0.452	0.473
7-day post-SCI group vs. the control group	*Trem2*	*Clu*	0	0.13	0	0.896	0.905
7-day post-SCI group vs. the control group	*Trem2*	*Sirpa*	0.217	0	0	0.357	0.475
7-day post-SCI group vs. the control group	*Trem2*	*Mapt*	0.062	0	0	0.595	0.604
7-day post-SCI group vs. the control group	*Trem2*	*Tlr4*	0.182	0	0	0.607	0.665
7-day post-SCI group vs. the control group	*Trem2*	*Btk*	0.09	0	0.6	0.197	0.682
7-day post-SCI group vs. the control group	*Trem2*	*Aif1*	0.197	0	0	0.728	0.772
7-day post-SCI group vs. the control group	*Trem2*	*Apoe*	0.078	0.329	0	0.983	0.989
7-day post-SCI group vs. the control group	*Txnip*	*Mapk14*	0.059	0.085	0	0.397	0.435
7-day post-SCI group vs. the control group	*Txnip*	*Hif1a*	0	0.13	0	0.4	0.455
7-day post-SCI group vs. the control group	*Txnip*	*Tlr4*	0.062	0	0	0.394	0.407
7-day post-SCI group vs. the control group	*Txnip*	*Id1*	0.066	0	0	0.509	0.521
7-day post-SCI group vs. the control group	*Txnip*	*Hdac1*	0	0.342	0	0.459	0.629

7-day post-SCI group vs. the control group	*Txnip*	*Nfe2l2*	0.1	0	0	0.578	0.604
7-day post-SCI group vs. the control group	*Txnip*	*Jun*	0.083	0	0	0.43	0.455
7-day post-SCI group vs. the control group	*Ucp2*	*Hif1a*	0	0	0	0.429	0.429
7-day post-SCI group vs. the control group	*Ucp2*	*Gpx1*	0.101	0	0	0.493	0.524
7-day post-SCI group vs. the control group	*Ucp2*	*Ppargc1a*	0	0	0	0.744	0.744
7-day post-SCI group vs. the control group	*Ucp2*	*Cd36*	0.099	0	0	0.395	0.431
7-day post-SCI group vs. the control group	*Vcam1*	*Axl*	0.141	0	0.147	0.274	0.421
7-day post-SCI group vs. the control group	*Vcam1*	*Mapk14*	0	0	0	0.632	0.632
7-day post-SCI group vs. the control group	*Vcam1*	*Ctnnb1*	0	0	0	0.522	0.522
7-day post-SCI group vs. the control group	*Vcam1*	*Cybb*	0.119	0	0.9	0.565	0.958
7-day post-SCI group vs. the control group	*Vcam1*	*Ncf1*	0.062	0	0.9	0.482	0.947
7-day post-SCI group vs. the control group	*Vcam1*	*Hif1a*	0.064	0	0	0.538	0.549
7-day post-SCI group vs. the control group	*Vcam1*	*Xdh*	0	0	0	0.468	0.468
7-day post-SCI group vs. the control group	*Vcam1*	*Aif1*	0.141	0	0	0.369	0.435
7-day post-SCI group vs. the control group	*Vcam1*	*Itga6*	0.051	0.058	0.352	0.384	0.595
7-day post-SCI group vs. the control group	*Vcam1*	*Nfe2l2*	0	0	0	0.4	0.4
7-day post-SCI group vs. the control group	*Vcam1*	*Mapk8*	0	0	0	0.4	0.4
7-day post-SCI group vs. the control group	*Vcam1*	*Btk*	0.065	0	0.217	0.262	0.412
7-day post-SCI group vs. the control group	*Vcam1*	*Cd38*	0.094	0	0	0.399	0.432
7-day post-SCI group vs. the control group	*Vcam1*	*Itgb5*	0.073	0.056	0.317	0.222	0.472
7-day post-SCI group vs. the control group	*Vcam1*	*Stat1*	0.097	0.044	0	0.453	0.487
7-day post-SCI group vs. the control group	*Vcam1*	*Jun*	0	0	0	0.589	0.589
7-day post-SCI group vs. the control group	*Vcam1*	*Tlr4*	0.171	0	0	0.673	0.718
7-day post-SCI group vs. the control group	*Vcam1*	*Apoe*	0.061	0	0	0.804	0.808
7-day post-SCI group vs. the control group	*Vcam1*	*Ezr*	0	0.13	0.8	0.941	0.988
7-day post-SCI group vs. the control group	*Xdh*	*Mapk14*	0	0	0	0.529	0.529
7-day post-SCI group vs. the control group	*Xdh*	*Cybb*	0.138	0	0	0.739	0.765
7-day post-SCI group vs. the control group	*Xdh*	*Ncf1*	0.089	0	0	0.718	0.732
7-day post-SCI group vs. the control group	*Xdh*	*Hif1a*	0	0	0	0.406	0.406
7-day post-SCI group vs. the control group	*Xdh*	*Ppargc1a*	0	0	0	0.431	0.431
7-day post-SCI group vs. the control group	*Xdh*	*Vcam1*	0	0	0	0.468	0.468
7-day post-SCI group vs. the control group	*Xdh*	*Apoe*	0.105	0	0	0.464	0.5
7-day post-SCI group vs. the control group	*Xdh*	*Gpx1*	0.062	0	0	0.552	0.561
7-day post-SCI group vs. the control group	*Xdh*	*Nfe2l2*	0.079	0	0	0.551	0.568
7-day post-SCI group vs. the control group	*Xdh*	*Jun*	0	0	0	0.583	0.583
7-day post-SCI group vs. the control group	*Xdh*	*Gch1*	0.104	0	0	0.642	0.665

7-day post-SCI group vs. the control group	*Xdh*	*Tlr4*	0.112	0	0	0.822	0.836
28-day post-SCI group vs. the control group	*Actb*	*Capns1*	0.094	0.053	0.43	0.172	0.541
28-day post-SCI group vs. the control group	*Actb*	*Axl*	0.052	0.084	0.114	0.464	0.532
28-day post-SCI group vs. the control group	*Actb*	*Hmox2*	0	0.094	0	0.431	0.462
28-day post-SCI group vs. the control group	*Actb*	*Hspb1*	0.062	0.087	0.4	0.584	0.757
28-day post-SCI group vs. the control group	*Actb*	*Mgst1*	0.065	0	0	0.613	0.622
28-day post-SCI group vs. the control group	*Actb*	*Cybb*	0	0.09	0	0.639	0.658
28-day post-SCI group vs. the control group	*Actb*	*Ncf1*	0	0.194	0	0.603	0.666
28-day post-SCI group vs. the control group	*Actb*	*Map2k3*	0.052	0	0	0.433	0.439
28-day post-SCI group vs. the control group	*Actb*	*Sdc1*	0	0	0	0.464	0.463
28-day post-SCI group vs. the control group	*Actb*	*Hif1a*	0	0.045	0	0.747	0.748
28-day post-SCI group vs. the control group	*Actb*	*Fos*	0	0.047	0.221	0.698	0.756
28-day post-SCI group vs. the control group	*Actb*	*Ctsl*	0.062	0	0	0.404	0.417
28-day post-SCI group vs. the control group	*Actb*	*Gpx8*	0	0	0	0.527	0.527
28-day post-SCI group vs. the control group	*Actb*	*Rb1*	0.061	0	0	0.436	0.447
28-day post-SCI group vs. the control group	*Actb*	*Mylk*	0.065	0.201	0	0.379	0.496
28-day post-SCI group vs. the control group	*Actb*	*Xdh*	0	0	0	0.468	0.468
28-day post-SCI group vs. the control group	*Actb*	*Cyp1b1*	0	0.044	0	0.466	0.467
28-day post-SCI group vs. the control group	*Actb*	*Aif1*	0.052	0.206	0	0.73	0.779
28-day post-SCI group vs. the control group	*Actb*	*Rela*	0.079	0.09	0.629	0.701	0.894
28-day post-SCI group vs. the control group	*Actb*	*Prdx4*	0.062	0.082	0	0.373	0.413
28-day post-SCI group vs. the control group	*Actb*	*Parp1*	0.061	0	0	0.81	0.814
28-day post-SCI group vs. the control group	*Actb*	*Il1a*	0	0	0	0.446	0.446
28-day post-SCI group vs. the control group	*Actb*	*Slc7a11*	0	0.044	0	0.471	0.473
28-day post-SCI group vs. the control group	*Actb*	*Vcam1*	0	0.047	0	0.62	0.622
28-day post-SCI group vs. the control group	*Actb*	*Cd38*	0	0	0	0.431	0.431
28-day post-SCI group vs. the control group	*Actb*	*Atp2a2*	0.066	0.066	0	0.474	0.501
28-day post-SCI group vs. the control group	*Actb*	*Tnfrsf1a*	0.063	0.047	0	0.523	0.536
28-day post-SCI group vs. the control group	*Actb*	*Mcl1*	0	0.09	0	0.693	0.708
28-day post-SCI group vs. the control group	*Actb*	*Tlr4*	0	0.132	0	0.738	0.763
28-day post-SCI group vs. the control group	*Actb*	*Ptgs1*	0	0	0	0.431	0.431
28-day post-SCI group vs. the control group	*Actb*	*Mapk1*	0	0.09	0	0.581	0.602
28-day post-SCI group vs. the control group	*Actb*	*Itgb5*	0	0.065	0.687	0.363	0.797
28-day post-SCI group vs. the control group	*Actb*	*Syp*	0	0	0	0.693	0.693
28-day post-SCI group vs. the control group	*Actb*	*Smad1*	0	0.041	0	0.537	0.537
28-day post-SCI group vs. the control group	*Actb*	*Rnf2*	0	0.433	0	0.223	0.54

28-day post-SCI group vs. the control group	*Actb*	*Gpx1*	0.062	0	0	0.614	0.623
28-day post-SCI group vs. the control group	*Actb*	*Mmp14*	0	0.058	0	0.475	0.484
28-day post-SCI group vs. the control group	*Actb*	*Stat6*	0	0.048	0	0.524	0.528
28-day post-SCI group vs. the control group	*Actb*	*Dst*	0.062	0.24	0.133	0.201	0.44
28-day post-SCI group vs. the control group	*Actb*	*Txnip*	0.061	0.055	0	0.419	0.439
28-day post-SCI group vs. the control group	*Actb*	*Fbxw7*	0	0.073	0	0.429	0.448
28-day post-SCI group vs. the control group	*Actb*	*Ripk1*	0.052	0.084	0.114	0.4	0.476
28-day post-SCI group vs. the control group	*Actb*	*Ucp2*	0.061	0.047	0	0.496	0.51
28-day post-SCI group vs. the control group	*Actb*	*Prkcd*	0.076	0.07	0	0.522	0.554
28-day post-SCI group vs. the control group	*Actb*	*Apoe*	0.051	0.049	0	0.594	0.601
28-day post-SCI group vs. the control group	*Actb*	*Met*	0.052	0.084	0.114	0.59	0.643
28-day post-SCI group vs. the control group	*Actb*	*Nfe2l2*	0	0	0	0.682	0.682
28-day post-SCI group vs. the control group	*Actb*	*Hdac1*	0.072	0.16	0	0.672	0.722
28-day post-SCI group vs. the control group	*Actb*	*Rpl13a*	0.09	0.066	0	0.704	0.726
28-day post-SCI group vs. the control group	*Actb*	*Jun*	0	0.059	0.629	0.815	0.929
28-day post-SCI group vs. the control group	*Ago1*	*Stau2*	0.062	0.232	0	0.264	0.423
28-day post-SCI group vs. the control group	*Ago1*	*Hdac1*	0.05	0.067	0	0.729	0.739
28-day post-SCI group vs. the control group	*Aif1*	*Axl*	0.08	0.092	0.114	0.329	0.437
28-day post-SCI group vs. the control group	*Aif1*	*Cybb*	0.384	0.199	0	0.565	0.767
28-day post-SCI group vs. the control group	*Aif1*	*Ncf1*	0.203	0.057	0	0.353	0.471
28-day post-SCI group vs. the control group	*Aif1*	*Fos*	0	0.132	0	0.535	0.58
28-day post-SCI group vs. the control group	*Aif1*	*Trem2*	0.197	0	0	0.728	0.772
28-day post-SCI group vs. the control group	*Aif1*	*Jun*	0	0	0	0.403	0.403
28-day post-SCI group vs. the control group	*Aif1*	*Il18bp*	0.341	0	0	0.136	0.406
28-day post-SCI group vs. the control group	*Aif1*	*Il1a*	0.069	0	0	0.394	0.412
28-day post-SCI group vs. the control group	*Aif1*	*Vcam1*	0.141	0	0	0.369	0.435
28-day post-SCI group vs. the control group	*Aif1*	*Hdac1*	0.063	0.084	0	0.444	0.481
28-day post-SCI group vs. the control group	*Aif1*	*Apoe*	0.079	0	0	0.464	0.485
28-day post-SCI group vs. the control group	*Aif1*	*Tlr4*	0.061	0.065	0	0.675	0.69
28-day post-SCI group vs. the control group	*Aif1*	*Syp*	0	0	0	0.727	0.727
28-day post-SCI group vs. the control group	*Aif1*	*Actb*	0.052	0.206	0	0.73	0.779
28-day post-SCI group vs. the control group	*Amph*	*Ppp3ca*	0.113	0.132	0.344	0.05	0.456
28-day post-SCI group vs. the control group	*Amph*	*Syp*	0.187	0	0	0.436	0.522
28-day post-SCI group vs. the control group	*Apc*	*Jun*	0	0.276	0	0.455	0.589
28-day post-SCI group vs. the control group	*Apc*	*Hdac1*	0.061	0	0.9	0.064	0.904
28-day post-SCI group vs. the control group	*Apoe*	*Cybb*	0	0	0	0.552	0.552

28-day post-SCI group vs. the control group	*Apoe*	*Ncf1*	0.062	0	0	0.513	0.523
28-day post-SCI group vs. the control group	*Apoe*	*Sdc1*	0.051	0	0.6	0.564	0.82
28-day post-SCI group vs. the control group	*Apoe*	*Hif1a*	0	0	0	0.4	0.4
28-day post-SCI group vs. the control group	*Apoe*	*Clu*	0.143	0	0.72	0.921	0.979
28-day post-SCI group vs. the control group	*Apoe*	*Trem2*	0.078	0.329	0	0.983	0.989
28-day post-SCI group vs. the control group	*Apoe*	*Xdh*	0.105	0	0	0.464	0.5
28-day post-SCI group vs. the control group	*Apoe*	*Aif1*	0.079	0	0	0.464	0.485
28-day post-SCI group vs. the control group	*Apoe*	*Vcam1*	0.061	0	0	0.804	0.808
28-day post-SCI group vs. the control group	*Apoe*	*Lcat*	0.116	0.13	0.72	0.692	0.924
28-day post-SCI group vs. the control group	*Apoe*	*Tlr4*	0	0	0	0.72	0.72
28-day post-SCI group vs. the control group	*Apoe*	*Pon2*	0	0	0	0.448	0.448
28-day post-SCI group vs. the control group	*Apoe*	*Syp*	0.061	0	0	0.473	0.484
28-day post-SCI group vs. the control group	*Apoe*	*Gpx1*	0.078	0	0	0.464	0.484
28-day post-SCI group vs. the control group	*Apoe*	*Actb*	0.051	0.049	0	0.594	0.601
28-day post-SCI group vs. the control group	*Apoe*	*Jun*	0	0	0	0.552	0.552
28-day post-SCI group vs. the control group	*Atp2a2*	*Fkbp1b*	0	0.058	0	0.669	0.674
28-day post-SCI group vs. the control group	*Atp2a2*	*Ppp3ca*	0.09	0.054	0	0.434	0.47
28-day post-SCI group vs. the control group	*Atp2a2*	*Actb*	0.066	0.066	0	0.474	0.501
28-day post-SCI group vs. the control group	*Atp2a2*	*Slc8a1*	0.081	0.169	0	0.701	0.751
28-day post-SCI group vs. the control group	*Atp2a2*	*S100a1*	0.062	0.13	0	0.825	0.845
28-day post-SCI group vs. the control group	*Axl*	*Vcam1*	0.141	0	0.147	0.274	0.421
28-day post-SCI group vs. the control group	*Axl*	*Jun*	0.062	0.09	0.127	0.327	0.431
28-day post-SCI group vs. the control group	*Axl*	*Aif1*	0.08	0.092	0.114	0.329	0.437
28-day post-SCI group vs. the control group	*Axl*	*Hif1a*	0	0.097	0.12	0.359	0.446
28-day post-SCI group vs. the control group	*Axl*	*Cybb*	0.092	0.379	0	0.14	0.473
28-day post-SCI group vs. the control group	*Axl*	*Trem2*	0.078	0	0	0.452	0.473
28-day post-SCI group vs. the control group	*Axl*	*Actb*	0.052	0.084	0.114	0.464	0.532
28-day post-SCI group vs. the control group	*Bmp1*	*Ctsl*	0	0	0.5	0.331	0.651
28-day post-SCI group vs. the control group	*Bmp1*	*Mmp14*	0.162	0.047	0	0.375	0.458
28-day post-SCI group vs. the control group	*Capns1*	*Actb*	0.094	0.053	0.43	0.172	0.541
28-day post-SCI group vs. the control group	*Capns1*	*Mapk1*	0.065	0.134	0.629	0.06	0.679
28-day post-SCI group vs. the control group	*Cbx6*	*Rb1*	0	0.103	0.343	0.068	0.402
28-day post-SCI group vs. the control group	*Cbx6*	*Rnf2*	0.052	0.758	0.845	0.714	0.988
28-day post-SCI group vs. the control group	*Cbx6*	*Phc1*	0.08	0.402	0.848	0.66	0.967
28-day post-SCI group vs. the control group	*Cd36*	*Tlr4*	0.135	0.13	0.6	0.221	0.734
28-day post-SCI group vs. the control group	*Cd36*	*Itgb5*	0.062	0.057	0.5	0.074	0.535

28-day post-SCI group vs. the control group	*Cd36*	*Ucp2*	0.099	0	0	0.395	0.431
28-day post-SCI group vs. the control group	*Cd38*	*Sdc1*	0	0	0	0.853	0.853
28-day post-SCI group vs. the control group	*Cd38*	*Vcam1*	0.094	0	0	0.399	0.432
28-day post-SCI group vs. the control group	*Cd38*	*Tlr4*	0.062	0	0	0.403	0.416
28-day post-SCI group vs. the control group	*Cd38*	*Actb*	0	0	0	0.431	0.431
28-day post-SCI group vs. the control group	*Cdkn2c*	*Rb1*	0	0.079	0.204	0.385	0.51
28-day post-SCI group vs. the control group	*Cdkn2c*	*Hdac1*	0.061	0.132	0.166	0.245	0.418
28-day post-SCI group vs. the control group	*Cdkn2c*	*Mapk10*	0	0.705	0	0.05	0.708
28-day post-SCI group vs. the control group	*Clu*	*Hspb1*	0.061	0	0	0.464	0.475
28-day post-SCI group vs. the control group	*Clu*	*Lcat*	0.099	0	0.72	0.405	0.836
28-day post-SCI group vs. the control group	*Clu*	*Trem2*	0	0.13	0	0.896	0.905
28-day post-SCI group vs. the control group	*Clu*	*Apoe*	0.143	0	0.72	0.921	0.979
28-day post-SCI group vs. the control group	*Ctsl*	*Actb*	0.062	0	0	0.404	0.417
28-day post-SCI group vs. the control group	*Ctsl*	*Tlr4*	0	0.045	0	0.435	0.437
28-day post-SCI group vs. the control group	*Ctsl*	*Bmp1*	0	0	0.5	0.331	0.651
28-day post-SCI group vs. the control group	*Cybb*	*Axl*	0.092	0.379	0	0.14	0.473
28-day post-SCI group vs. the control group	*Cybb*	*Hmox2*	0	0	0	0.443	0.443
28-day post-SCI group vs. the control group	*Cybb*	*Tnfrsf1a*	0.093	0.13	0	0.345	0.438
28-day post-SCI group vs. the control group	*Cybb*	*Gpx8*	0	0	0	0.477	0.477
28-day post-SCI group vs. the control group	*Cybb*	*Hif1a*	0.061	0	0	0.528	0.538
28-day post-SCI group vs. the control group	*Cybb*	*Jun*	0.061	0.058	0	0.522	0.541
28-day post-SCI group vs. the control group	*Cybb*	*Apoe*	0	0	0	0.552	0.552
28-day post-SCI group vs. the control group	*Cybb*	*Nfe2l2*	0.071	0	0	0.569	0.582
28-day post-SCI group vs. the control group	*Cybb*	*Gpx1*	0.082	0	0	0.618	0.634
28-day post-SCI group vs. the control group	*Cybb*	*Actb*	0	0.09	0	0.639	0.658
28-day post-SCI group vs. the control group	*Cybb*	*Mapk1*	0	0.086	0.6	0.146	0.66
28-day post-SCI group vs. the control group	*Cybb*	*Tlr4*	0.217	0.076	0	0.626	0.706
28-day post-SCI group vs. the control group	*Cybb*	*Xdh*	0.138	0	0	0.739	0.765
28-day post-SCI group vs. the control group	*Cybb*	*Aif1*	0.384	0.199	0	0.565	0.767
28-day post-SCI group vs. the control group	*Cybb*	*Prkcd*	0.112	0	0.8	0.39	0.882
28-day post-SCI group vs. the control group	*Cybb*	*Vcam1*	0.119	0	0.9	0.565	0.958
28-day post-SCI group vs. the control group	*Cybb*	*Ncf1*	0.423	0.087	0.932	0.993	0.999
28-day post-SCI group vs. the control group	*Cyp1b1*	*Mgst1*	0.072	0	0.65	0.193	0.715
28-day post-SCI group vs. the control group	*Cyp1b1*	*Actb*	0	0.044	0	0.466	0.467
28-day post-SCI group vs. the control group	*Dst*	*Itgb5*	0.062	0.255	0.167	0.096	0.403
28-day post-SCI group vs. the control group	*Dst*	*Actb*	0.062	0.24	0.133	0.201	0.44

28-day post-SCI group vs. the control group	*Eif2s1*	*Rpl13a*	0.652	0	0	0.097	0.672
28-day post-SCI group vs. the control group	*Ets2*	*Fos*	0.063	0.13	0.629	0.566	0.851
28-day post-SCI group vs. the control group	*Ets2*	*Hdac1*	0.061	0	0.243	0.269	0.435
28-day post-SCI group vs. the control group	*Ets2*	*Stat6*	0.078	0.067	0.544	0.151	0.622
28-day post-SCI group vs. the control group	*Ets2*	*Id1*	0.061	0.049	0.8	0.24	0.846
28-day post-SCI group vs. the control group	*Ets2*	*Jun*	0.061	0.456	0.629	0.522	0.897
28-day post-SCI group vs. the control group	*Ets2*	*Mapk1*	0	0.311	0.94	0.318	0.969
28-day post-SCI group vs. the control group	*Fbxw7*	*Hif1a*	0	0.231	0	0.42	0.535
28-day post-SCI group vs. the control group	*Fbxw7*	*Mcl1*	0.061	0.745	0	0.863	0.964
28-day post-SCI group vs. the control group	*Fbxw7*	*Prdx6*	0	0.433	0	0.068	0.448
28-day post-SCI group vs. the control group	*Fbxw7*	*Actb*	0	0.073	0	0.429	0.448
28-day post-SCI group vs. the control group	*Fbxw7*	*Nfe2l2*	0	0.198	0	0.312	0.424
28-day post-SCI group vs. the control group	*Fbxw7*	*Jun*	0	0.762	0	0.901	0.975
28-day post-SCI group vs. the control group	*Fbxw7*	*Prkcd*	0.063	0.433	0	0.096	0.477
28-day post-SCI group vs. the control group	*Fkbp1b*	*S100a1*	0	0.249	0	0.398	0.528
28-day post-SCI group vs. the control group	*Fkbp1b*	*Atp2a2*	0	0.058	0	0.669	0.674
28-day post-SCI group vs. the control group	*Fkbp1b*	*Ppp3ca*	0.062	0.591	0.15	0.268	0.729
28-day post-SCI group vs. the control group	*Fos*	*Ier3*	0.159	0	0	0.399	0.472
28-day post-SCI group vs. the control group	*Fos*	*Hmox2*	0	0	0.629	0.235	0.704
28-day post-SCI group vs. the control group	*Fos*	*Map2k3*	0	0	0	0.435	0.435
28-day post-SCI group vs. the control group	*Fos*	*Hif1a*	0	0.131	0	0.527	0.571
28-day post-SCI group vs. the control group	*Fos*	*Il1a*	0.062	0	0	0.386	0.4
28-day post-SCI group vs. the control group	*Fos*	*Met*	0	0.071	0.12	0.364	0.434
28-day post-SCI group vs. the control group	*Fos*	*Hbegf*	0.065	0	0	0.422	0.436
28-day post-SCI group vs. the control group	*Fos*	*Mcl1*	0.089	0	0	0.411	0.441
28-day post-SCI group vs. the control group	*Fos*	*Stat6*	0	0.071	0.216	0.327	0.467
28-day post-SCI group vs. the control group	*Fos*	*Syp*	0	0	0	0.546	0.547
28-day post-SCI group vs. the control group	*Fos*	*Smad1*	0	0.142	0.388	0.227	0.558
28-day post-SCI group vs. the control group	*Fos*	*Tlr4*	0.063	0.052	0	0.554	0.569
28-day post-SCI group vs. the control group	*Fos*	*Aif1*	0	0.132	0	0.535	0.58
28-day post-SCI group vs. the control group	*Fos*	*Hdac1*	0.048	0.164	0	0.67	0.715
28-day post-SCI group vs. the control group	*Fos*	*Nfe2l2*	0.061	0.443	0.293	0.416	0.755
28-day post-SCI group vs. the control group	*Fos*	*Actb*	0	0.047	0.221	0.698	0.756
28-day post-SCI group vs. the control group	*Fos*	*Rb1*	0	0.703	0.21	0.224	0.802
28-day post-SCI group vs. the control group	*Fos*	*Ets2*	0.063	0.13	0.629	0.566	0.851
28-day post-SCI group vs. the control group	*Fos*	*Rela*	0	0.4	0.629	0.561	0.893

28-day post-SCI group vs. the control group	*Fos*	*Mapk10*	0	0.309	0.903	0.492	0.963
28-day post-SCI group vs. the control group	*Fos*	*Mapk1*	0	0.267	0.966	0.722	0.992
28-day post-SCI group vs. the control group	*Fos*	*Jun*	0.662	0.982	0.932	0.995	0.999
28-day post-SCI group vs. the control group	*Gpx1*	*Hmox2*	0.073	0.07	0	0.443	0.478
28-day post-SCI group vs. the control group	*Gpx1*	*Mgst1*	0.141	0	0.65	0.308	0.774
28-day post-SCI group vs. the control group	*Gpx1*	*Cybb*	0.082	0	0	0.618	0.634
28-day post-SCI group vs. the control group	*Gpx1*	*Ncf1*	0.062	0	0	0.521	0.531
28-day post-SCI group vs. the control group	*Gpx1*	*Hif1a*	0	0	0	0.483	0.483
28-day post-SCI group vs. the control group	*Gpx1*	*Xdh*	0.062	0	0	0.552	0.561
28-day post-SCI group vs. the control group	*Gpx1*	*Prdx4*	0.098	0.224	0	0.617	0.708
28-day post-SCI group vs. the control group	*Gpx1*	*Slc7a11*	0.052	0.06	0	0.463	0.479
28-day post-SCI group vs. the control group	*Gpx1*	*Prdx6*	0.063	0.184	0	0.629	0.691
28-day post-SCI group vs. the control group	*Gpx1*	*Jun*	0.051	0.045	0	0.413	0.422
28-day post-SCI group vs. the control group	*Gpx1*	*Apoe*	0.078	0	0	0.464	0.484
28-day post-SCI group vs. the control group	*Gpx1*	*Ucp2*	0.101	0	0	0.493	0.524
28-day post-SCI group vs. the control group	*Gpx1*	*Actb*	0.062	0	0	0.614	0.623
28-day post-SCI group vs. the control group	*Gpx1*	*Nfe2l2*	0.079	0	0	0.689	0.702
28-day post-SCI group vs. the control group	*Gpx1*	*Selenbp1*	0.061	0.379	0	0.872	0.919
28-day post-SCI group vs. the control group	*Gpx3*	*Mgst1*	0.076	0	0.65	0.227	0.728
28-day post-SCI group vs. the control group	*Gpx3*	*Prdx4*	0.061	0.224	0	0.542	0.637
28-day post-SCI group vs. the control group	*Gpx3*	*Prdx6*	0.063	0.184	0	0.516	0.597
28-day post-SCI group vs. the control group	*Gpx3*	*Nfe2l2*	0.057	0	0	0.405	0.415
28-day post-SCI group vs. the control group	*Gpx8*	*Hmox2*	0	0.07	0	0.397	0.415
28-day post-SCI group vs. the control group	*Gpx8*	*Mgst1*	0.074	0	0.65	0.239	0.732
28-day post-SCI group vs. the control group	*Gpx8*	*Cybb*	0	0	0	0.477	0.477
28-day post-SCI group vs. the control group	*Gpx8*	*Prdx6*	0.063	0.184	0	0.308	0.425
28-day post-SCI group vs. the control group	*Gpx8*	*Actb*	0	0	0	0.527	0.527
28-day post-SCI group vs. the control group	*Gpx8*	*Nfe2l2*	0	0	0	0.66	0.66
28-day post-SCI group vs. the control group	*Gpx8*	*Prdx4*	0.082	0.224	0	0.617	0.703
28-day post-SCI group vs. the control group	*Hbegf*	*Hif1a*	0	0	0.9	0.307	0.927
28-day post-SCI group vs. the control group	*Hbegf*	*Fos*	0.065	0	0	0.422	0.436
28-day post-SCI group vs. the control group	*Hbegf*	*Jun*	0.063	0	0	0.409	0.423
28-day post-SCI group vs. the control group	*Hbegf*	*Itgb5*	0.051	0	0	0.418	0.424
28-day post-SCI group vs. the control group	*Hbegf*	*Stat6*	0.063	0	0.35	0.184	0.459
28-day post-SCI group vs. the control group	*Hbegf*	*Met*	0.061	0	0.147	0.401	0.478
28-day post-SCI group vs. the control group	*Hbegf*	*Mmp14*	0.072	0.057	0.837	0.369	0.898

28-day post-SCI group vs. the control group	*Hdac1*	*Mgst1*	0	0	0	0.449	0.449
28-day post-SCI group vs. the control group	*Hdac1*	*Hif1a*	0	0.446	0.168	0.961	0.981
28-day post-SCI group vs. the control group	*Hdac1*	*Fos*	0.048	0.164	0	0.67	0.715
28-day post-SCI group vs. the control group	*Hdac1*	*Rb1*	0.064	0.852	0.913	0.784	0.997
28-day post-SCI group vs. the control group	*Hdac1*	*Ets2*	0.061	0	0.243	0.269	0.435
28-day post-SCI group vs. the control group	*Hdac1*	*Aif1*	0.063	0.084	0	0.444	0.481
28-day post-SCI group vs. the control group	*Hdac1*	*Rela*	0.062	0.795	0.512	0.982	0.998
28-day post-SCI group vs. the control group	*Hdac1*	*Parp1*	0.063	0.342	0	0.554	0.701
28-day post-SCI group vs. the control group	*Hdac1*	*Tnfrsf1a*	0	0	0.41	0.154	0.479
28-day post-SCI group vs. the control group	*Hdac1*	*Smad1*	0.062	0.221	0.301	0.245	0.563
28-day post-SCI group vs. the control group	*Hdac1*	*Rnf2*	0.062	0.099	0	0.468	0.512
28-day post-SCI group vs. the control group	*Hdac1*	*Apc*	0.061	0	0.9	0.064	0.904
28-day post-SCI group vs. the control group	*Hdac1*	*Stat6*	0.048	0.164	0.208	0.223	0.444
28-day post-SCI group vs. the control group	*Hdac1*	*Ago1*	0.05	0.067	0	0.729	0.739
28-day post-SCI group vs. the control group	*Hdac1*	*Cdkn2c*	0.061	0.132	0.166	0.245	0.418
28-day post-SCI group vs. the control group	*Hdac1*	*Actb*	0.072	0.16	0	0.672	0.722
28-day post-SCI group vs. the control group	*Hdac1*	*Txnip*	0	0.342	0	0.459	0.629
28-day post-SCI group vs. the control group	*Hdac1*	*Jun*	0	0.261	0	0.787	0.836
28-day post-SCI group vs. the control group	*Hif1a*	*Axl*	0	0.097	0.12	0.359	0.446
28-day post-SCI group vs. the control group	*Hif1a*	*Hmox2*	0	0	0	0.42	0.42
28-day post-SCI group vs. the control group	*Hif1a*	*Hspb1*	0	0.045	0	0.408	0.41
28-day post-SCI group vs. the control group	*Hif1a*	*Pdk1*	0	0.131	0	0.548	0.59
28-day post-SCI group vs. the control group	*Hif1a*	*Cybb*	0.061	0	0	0.528	0.538
28-day post-SCI group vs. the control group	*Hif1a*	*Apoe*	0	0	0	0.4	0.4
28-day post-SCI group vs. the control group	*Hif1a*	*Xdh*	0	0	0	0.406	0.406
28-day post-SCI group vs. the control group	*Hif1a*	*Ucp2*	0	0	0	0.429	0.429
28-day post-SCI group vs. the control group	*Hif1a*	*Txnip*	0	0.13	0	0.4	0.455
28-day post-SCI group vs. the control group	*Hif1a*	*Stat6*	0	0.102	0	0.42	0.456
28-day post-SCI group vs. the control group	*Hif1a*	*Mmp14*	0.065	0	0	0.462	0.476
28-day post-SCI group vs. the control group	*Hif1a*	*Gpx1*	0	0	0	0.483	0.483
28-day post-SCI group vs. the control group	*Hif1a*	*Fbxw7*	0	0.231	0	0.42	0.535
28-day post-SCI group vs. the control group	*Hif1a*	*Smad1*	0.061	0.147	0.301	0.27	0.537
28-day post-SCI group vs. the control group	*Hif1a*	*Vcam1*	0.064	0	0	0.538	0.549
28-day post-SCI group vs. the control group	*Hif1a*	*Fos*	0	0.131	0	0.527	0.571
28-day post-SCI group vs. the control group	*Hif1a*	*Mcl1*	0.062	0.13	0	0.552	0.602
28-day post-SCI group vs. the control group	*Hif1a*	*Tlr4*	0.062	0.054	0	0.589	0.603

28-day post-SCI group vs. the control group	*Hif1a*	*Parp1*	0	0.13	0	0.595	0.633
28-day post-SCI group vs. the control group	*Hif1a*	*Nfe2l2*	0	0	0	0.658	0.658
28-day post-SCI group vs. the control group	*Hif1a*	*Met*	0	0.097	0.12	0.611	0.663
28-day post-SCI group vs. the control group	*Hif1a*	*Actb*	0	0.045	0	0.747	0.748
28-day post-SCI group vs. the control group	*Hif1a*	*Rela*	0	0.13	0	0.848	0.862
28-day post-SCI group vs. the control group	*Hif1a*	*Mapk1*	0	0.402	0.8	0.4	0.922
28-day post-SCI group vs. the control group	*Hif1a*	*Hbegf*	0	0	0.9	0.307	0.927
28-day post-SCI group vs. the control group	*Hif1a*	*Jun*	0.051	0.402	0.629	0.846	0.963
28-day post-SCI group vs. the control group	*Hif1a*	*Hdac1*	0	0.446	0.168	0.961	0.981
28-day post-SCI group vs. the control group	*Hmox2*	*Gpx8*	0	0.07	0	0.397	0.415
28-day post-SCI group vs. the control group	*Hmox2*	*Hif1a*	0	0	0	0.42	0.42
28-day post-SCI group vs. the control group	*Hmox2*	*Cybb*	0	0	0	0.443	0.443
28-day post-SCI group vs. the control group	*Hmox2*	*Actb*	0	0.094	0	0.431	0.462
28-day post-SCI group vs. the control group	*Hmox2*	*Gpx1*	0.073	0.07	0	0.443	0.478
28-day post-SCI group vs. the control group	*Hmox2*	*Pon2*	0	0	0	0.503	0.503
28-day post-SCI group vs. the control group	*Hmox2*	*Fos*	0	0	0.629	0.235	0.704
28-day post-SCI group vs. the control group	*Hmox2*	*Nfe2l2*	0	0	0.419	0.605	0.761
28-day post-SCI group vs. the control group	*Hmox2*	*Jun*	0	0	0.629	0.416	0.774
28-day post-SCI group vs. the control group	*Hspb1*	*Hif1a*	0	0.045	0	0.408	0.41
28-day post-SCI group vs. the control group	*Hspb1*	*Tlr4*	0	0.057	0	0.405	0.415
28-day post-SCI group vs. the control group	*Hspb1*	*Mapk1*	0	0.095	0.291	0.212	0.45
28-day post-SCI group vs. the control group	*Hspb1*	*Map2k6*	0	0.052	0	0.464	0.47
28-day post-SCI group vs. the control group	*Hspb1*	*Clu*	0.061	0	0	0.464	0.475
28-day post-SCI group vs. the control group	*Hspb1*	*Prdx6*	0.051	0.101	0	0.481	0.518
28-day post-SCI group vs. the control group	*Hspb1*	*Map2k3*	0.067	0.057	0	0.528	0.548
28-day post-SCI group vs. the control group	*Hspb1*	*Jun*	0.062	0	0	0.588	0.597
28-day post-SCI group vs. the control group	*Hspb1*	*Prkcd*	0	0.439	0	0.578	0.754
28-day post-SCI group vs. the control group	*Hspb1*	*Actb*	0.062	0.087	0.4	0.584	0.757
28-day post-SCI group vs. the control group	*Hspb1*	*Mapkapk2*	0.062	0.871	0.966	0.733	0.998
28-day post-SCI group vs. the control group	*Id1*	*Rb1*	0	0.133	0.544	0.111	0.618
28-day post-SCI group vs. the control group	*Id1*	*Ets2*	0.061	0.049	0.8	0.24	0.846
28-day post-SCI group vs. the control group	*Id1*	*Smad1*	0	0	0	0.471	0.471
28-day post-SCI group vs. the control group	*Id1*	*Txnip*	0.066	0	0	0.509	0.521
28-day post-SCI group vs. the control group	*Ier3*	*Fos*	0.159	0	0	0.399	0.472
28-day post-SCI group vs. the control group	*Ier3*	*Tnfaip3*	0.191	0	0	0.492	0.572
28-day post-SCI group vs. the control group	*Ier3*	*Mcl1*	0.061	0.079	0	0.586	0.612

28-day post-SCI group vs. the control group	*Ier3*	*Mapk1*	0	0.228	0.6	0.078	0.69
28-day post-SCI group vs. the control group	*Ier3*	*Rela*	0.071	0.13	0	0.836	0.856
28-day post-SCI group vs. the control group	*Il18bp*	*Aif1*	0.341	0	0	0.136	0.406
28-day post-SCI group vs. the control group	*Il1a*	*Mapkapk2*	0.062	0	0.629	0.163	0.683
28-day post-SCI group vs. the control group	*Il1a*	*Tnfaip3*	0.408	0	0	0.438	0.653
28-day post-SCI group vs. the control group	*Il1a*	*Fos*	0.062	0	0	0.386	0.4
28-day post-SCI group vs. the control group	*Il1a*	*Aif1*	0.069	0	0	0.394	0.412
28-day post-SCI group vs. the control group	*Il1a*	*Rela*	0	0	0.9	0.405	0.938
28-day post-SCI group vs. the control group	*Il1a*	*Tnfrsf1a*	0.062	0	0	0.43	0.442
28-day post-SCI group vs. the control group	*Il1a*	*Actb*	0	0	0	0.446	0.446
28-day post-SCI group vs. the control group	*Il1a*	*Ripk1*	0	0	0.341	0.197	0.448
28-day post-SCI group vs. the control group	*Il1a*	*Jun*	0	0	0	0.487	0.487
28-day post-SCI group vs. the control group	*Il1a*	*Vcam1*	0.069	0	0	0.494	0.508
28-day post-SCI group vs. the control group	*Il1a*	*Tlr4*	0.057	0	0	0.743	0.747
28-day post-SCI group vs. the control group	*Il6st*	*Map2k3*	0	0	0.537	0.088	0.559
28-day post-SCI group vs. the control group	*Il6st*	*Map2k6*	0	0	0.676	0.056	0.681
28-day post-SCI group vs. the control group	*Il6st*	*Mapk1*	0	0	0.9	0.14	0.91
28-day post-SCI group vs. the control group	*Il6st*	*Stat6*	0.064	0.047	0.35	0.31	0.546
28-day post-SCI group vs. the control group	*Itgb5*	*Sdc1*	0.109	0	0.864	0.381	0.919
28-day post-SCI group vs. the control group	*Itgb5*	*Mylk*	0.061	0.089	0.756	0.125	0.793
28-day post-SCI group vs. the control group	*Itgb5*	*Hbegf*	0.051	0	0	0.418	0.424
28-day post-SCI group vs. the control group	*Itgb5*	*Vcam1*	0.073	0.056	0.317	0.222	0.472
28-day post-SCI group vs. the control group	*Itgb5*	*Mapk1*	0.052	0.433	0.176	0.693	0.846
28-day post-SCI group vs. the control group	*Itgb5*	*Dst*	0.062	0.255	0.167	0.096	0.403
28-day post-SCI group vs. the control group	*Itgb5*	*Met*	0.069	0.133	0.388	0.132	0.513
28-day post-SCI group vs. the control group	*Itgb5*	*Cd36*	0.062	0.057	0.5	0.074	0.535
28-day post-SCI group vs. the control group	*Itgb5*	*Actb*	0	0.065	0.687	0.363	0.797
28-day post-SCI group vs. the control group	*Jun*	*Axl*	0.062	0.09	0.127	0.327	0.431
28-day post-SCI group vs. the control group	*Jun*	*Hmox2*	0	0	0.629	0.416	0.774
28-day post-SCI group vs. the control group	*Jun*	*Hspb1*	0.062	0	0	0.588	0.597
28-day post-SCI group vs. the control group	*Jun*	*Mgst1*	0	0	0	0.675	0.675
28-day post-SCI group vs. the control group	*Jun*	*Cybb*	0.061	0.058	0	0.522	0.541
28-day post-SCI group vs. the control group	*Jun*	*Ncf1*	0	0	0	0.466	0.465
28-day post-SCI group vs. the control group	*Jun*	*Mapkapk2*	0.081	0.056	0	0.527	0.555
28-day post-SCI group vs. the control group	*Jun*	*Map2k3*	0	0	0	0.721	0.721
28-day post-SCI group vs. the control group	*Jun*	*Tnfaip3*	0.082	0	0	0.403	0.428

28-day post-SCI group vs. the control group	*Jun*	*Map2k6*	0	0	0	0.592	0.592
28-day post-SCI group vs. the control group	*Jun*	*Hif1a*	0.051	0.402	0.629	0.846	0.963
28-day post-SCI group vs. the control group	*Jun*	*Fos*	0.662	0.982	0.932	0.995	0.999
28-day post-SCI group vs. the control group	*Jun*	*Rb1*	0	0.227	0.676	0.344	0.821
28-day post-SCI group vs. the control group	*Jun*	*Ets2*	0.061	0.456	0.629	0.522	0.897
28-day post-SCI group vs. the control group	*Jun*	*Xdh*	0	0	0	0.583	0.583
28-day post-SCI group vs. the control group	*Jun*	*Aif1*	0	0	0	0.403	0.403
28-day post-SCI group vs. the control group	*Jun*	*Hbegf*	0.063	0	0	0.409	0.423
28-day post-SCI group vs. the control group	*Jun*	*Rela*	0.061	0.46	0.629	0.859	0.97
28-day post-SCI group vs. the control group	*Jun*	*Parp1*	0.064	0.135	0	0.57	0.622
28-day post-SCI group vs. the control group	*Jun*	*Il1a*	0	0	0	0.487	0.487
28-day post-SCI group vs. the control group	*Jun*	*Vcam1*	0	0	0	0.589	0.589
28-day post-SCI group vs. the control group	*Jun*	*Tnfrsf1a*	0.069	0	0	0.619	0.63
28-day post-SCI group vs. the control group	*Jun*	*Map2k4*	0	0.13	0	0.827	0.843
28-day post-SCI group vs. the control group	*Jun*	*Mcl1*	0.063	0	0	0.62	0.628
28-day post-SCI group vs. the control group	*Jun*	*Tlr4*	0	0	0	0.738	0.738
28-day post-SCI group vs. the control group	*Jun*	*Mapk1*	0	0.686	0.932	0.616	0.991
28-day post-SCI group vs. the control group	*Jun*	*Smad1*	0	0.211	0.388	0.43	0.701
28-day post-SCI group vs. the control group	*Jun*	*Apc*	0	0.276	0	0.455	0.589
28-day post-SCI group vs. the control group	*Jun*	*Gpx1*	0.051	0.045	0	0.413	0.422
28-day post-SCI group vs. the control group	*Jun*	*Mmp14*	0.071	0.091	0	0.454	0.499
28-day post-SCI group vs. the control group	*Jun*	*Stat6*	0	0.134	0.216	0.552	0.669
28-day post-SCI group vs. the control group	*Jun*	*Actb*	0	0.059	0.629	0.815	0.929
28-day post-SCI group vs. the control group	*Jun*	*Hdac1*	0	0.261	0	0.787	0.836
28-day post-SCI group vs. the control group	*Jun*	*Nfe2l2*	0	0.131	0.362	0.745	0.846
28-day post-SCI group vs. the control group	*Jun*	*Txnip*	0.083	0	0	0.43	0.455
28-day post-SCI group vs. the control group	*Jun*	*Prkcd*	0	0.057	0	0.486	0.495
28-day post-SCI group vs. the control group	*Jun*	*Ripk1*	0	0.09	0.127	0.468	0.54
28-day post-SCI group vs. the control group	*Jun*	*Apoe*	0	0	0	0.552	0.552
28-day post-SCI group vs. the control group	*Jun*	*Met*	0	0.09	0.127	0.528	0.592
28-day post-SCI group vs. the control group	*Jun*	*Fbxw7*	0	0.762	0	0.901	0.975
28-day post-SCI group vs. the control group	*Jun*	*Mapk10*	0	0.778	0.966	0.822	0.998
28-day post-SCI group vs. the control group	*Lancl1*	*Map2k6*	0.062	0	0	0.486	0.497
28-day post-SCI group vs. the control group	*Lancl1*	*Syp*	0.067	0.698	0	0	0.706
28-day post-SCI group vs. the control group	*Lcat*	*Clu*	0.099	0	0.72	0.405	0.836
28-day post-SCI group vs. the control group	*Lcat*	*Pon2*	0.061	0	0	0.5	0.511

28-day post-SCI group vs. the control group	*Lcat*	*Apoe*	0.116	0.13	0.72	0.692	0.924
28-day post-SCI group vs. the control group	*Map2k3*	*Hspb1*	0.067	0.057	0	0.528	0.548
28-day post-SCI group vs. the control group	*Map2k3*	*Mapkapk2*	0.119	0.09	0.9	0.701	0.943
28-day post-SCI group vs. the control group	*Map2k3*	*Fos*	0	0	0	0.435	0.435
28-day post-SCI group vs. the control group	*Map2k3*	*Actb*	0.052	0	0	0.433	0.439
28-day post-SCI group vs. the control group	*Map2k3*	*Rela*	0.079	0.241	0	0.277	0.45
28-day post-SCI group vs. the control group	*Map2k3*	*Tlr4*	0	0.09	0	0.457	0.485
28-day post-SCI group vs. the control group	*Map2k3*	*Il6st*	0	0	0.537	0.088	0.559
28-day post-SCI group vs. the control group	*Map2k3*	*Jun*	0	0	0	0.721	0.721
28-day post-SCI group vs. the control group	*Map2k3*	*Tnfrsf1a*	0.095	0	0.676	0.245	0.759
28-day post-SCI group vs. the control group	*Map2k3*	*Map2k4*	0	0.048	0.8	0.829	0.814
28-day post-SCI group vs. the control group	*Map2k3*	*Mapk1*	0.068	0.401	0.859	0.398	0.932
28-day post-SCI group vs. the control group	*Map2k3*	*Mapk10*	0.062	0.135	0.891	0.608	0.935
28-day post-SCI group vs. the control group	*Map2k3*	*Ripk1*	0.087	0.132	0.932	0.221	0.945
28-day post-SCI group vs. the control group	*Map2k3*	*Map2k6*	0	0.56	0.966	0.86	0.984
28-day post-SCI group vs. the control group	*Map2k4*	*Mapkapk2*	0.051	0.14	0	0.617	0.4
28-day post-SCI group vs. the control group	*Map2k4*	*Map2k3*	0	0.048	0.8	0.829	0.814
28-day post-SCI group vs. the control group	*Map2k4*	*Map2k6*	0	0.373	0.8	0.683	0.875
28-day post-SCI group vs. the control group	*Map2k4*	*Tnfrsf1a*	0	0	0.629	0.301	0.729
28-day post-SCI group vs. the control group	*Map2k4*	*Mapk1*	0.063	0.197	0.372	0.361	0.555
28-day post-SCI group vs. the control group	*Map2k4*	*Ripk1*	0	0.106	0.629	0.242	0.682
28-day post-SCI group vs. the control group	*Map2k4*	*Prkcd*	0	0	0.676	0.132	0.689
28-day post-SCI group vs. the control group	*Map2k4*	*Jun*	0	0.13	0	0.827	0.843
28-day post-SCI group vs. the control group	*Map2k4*	*Mapk10*	0.105	0.867	0.966	0.912	0.997
28-day post-SCI group vs. the control group	*Map2k6*	*Hspb1*	0	0.052	0	0.464	0.47
28-day post-SCI group vs. the control group	*Map2k6*	*Mapkapk2*	0.063	0.13	0.9	0.603	0.94
28-day post-SCI group vs. the control group	*Map2k6*	*Map2k3*	0	0.56	0.966	0.86	0.984
28-day post-SCI group vs. the control group	*Map2k6*	*Rela*	0.05	0.311	0	0.24	0.459
28-day post-SCI group vs. the control group	*Map2k6*	*Lancl1*	0.062	0	0	0.486	0.497
28-day post-SCI group vs. the control group	*Map2k6*	*Jun*	0	0	0	0.592	0.592
28-day post-SCI group vs. the control group	*Map2k6*	*Il6st*	0	0	0.676	0.056	0.681
28-day post-SCI group vs. the control group	*Map2k6*	*Tnfrsf1a*	0	0	0.629	0.235	0.704
28-day post-SCI group vs. the control group	*Map2k6*	*Map2k4*	0	0.373	0.8	0.683	0.875
28-day post-SCI group vs. the control group	*Map2k6*	*Mapk1*	0.068	0.401	0.859	0.247	0.929
28-day post-SCI group vs. the control group	*Map2k6*	*Mapk10*	0.062	0.135	0.891	0.527	0.933
28-day post-SCI group vs. the control group	*Map2k6*	*Ripk1*	0.061	0.132	0.922	0.155	0.934

28-day post-SCI group vs. the control group	*Mapk1*	*Capns1*	0.065	0.134	0.629	0.06	0.679
28-day post-SCI group vs. the control group	*Mapk1*	*Ier3*	0	0.228	0.6	0.078	0.69
28-day post-SCI group vs. the control group	*Mapk1*	*Hspb1*	0	0.095	0.291	0.212	0.45
28-day post-SCI group vs. the control group	*Mapk1*	*Cybb*	0	0.086	0.6	0.146	0.66
28-day post-SCI group vs. the control group	*Mapk1*	*Ncf1*	0	0.072	0.9	0.141	0.913
28-day post-SCI group vs. the control group	*Mapk1*	*Mapkapk2*	0.064	0.75	0.326	0.267	0.865
28-day post-SCI group vs. the control group	*Mapk1*	*Map2k3*	0.068	0.401	0.859	0.398	0.932
28-day post-SCI group vs. the control group	*Mapk1*	*Sdc1*	0.065	0	0.676	0.131	0.713
28-day post-SCI group vs. the control group	*Mapk1*	*Map2k6*	0.068	0.401	0.859	0.247	0.929
28-day post-SCI group vs. the control group	*Mapk1*	*Hif1a*	0	0.402	0.8	0.4	0.922
28-day post-SCI group vs. the control group	*Mapk1*	*Fos*	0	0.267	0.966	0.722	0.992
28-day post-SCI group vs. the control group	*Mapk1*	*Rb1*	0.061	0.248	0.629	0.248	0.776
28-day post-SCI group vs. the control group	*Mapk1*	*Mylk*	0.061	0.131	0.676	0.194	0.731
28-day post-SCI group vs. the control group	*Mapk1*	*Ets2*	0	0.311	0.94	0.318	0.969
28-day post-SCI group vs. the control group	*Mapk1*	*Rela*	0	0.054	0.676	0.31	0.77
28-day post-SCI group vs. the control group	*Mapk1*	*Parp1*	0.096	0.13	0	0.489	0.564
28-day post-SCI group vs. the control group	*Mapk1*	*Tnfrsf1a*	0.051	0.467	0	0.211	0.566
28-day post-SCI group vs. the control group	*Mapk1*	*Map2k4*	0.063	0.197	0.372	0.361	0.555
28-day post-SCI group vs. the control group	*Mapk1*	*Mcl1*	0	0.21	0.209	0.301	0.525
28-day post-SCI group vs. the control group	*Mapk1*	*Ppp3ca*	0.1	0.272	0	0.26	0.473
28-day post-SCI group vs. the control group	*Mapk1*	*Ripk1*	0	0.147	0.354	0.151	0.453
28-day post-SCI group vs. the control group	*Mapk1*	*Actb*	0	0.09	0	0.581	0.602
28-day post-SCI group vs. the control group	*Mapk1*	*Met*	0.061	0.147	0.676	0.386	0.759
28-day post-SCI group vs. the control group	*Mapk1*	*Mapk10*	0.08	0.059	0.728	0.476	0.765
28-day post-SCI group vs. the control group	*Mapk1*	*Itgb5*	0.052	0.433	0.176	0.693	0.846
28-day post-SCI group vs. the control group	*Mapk1*	*Il6st*	0	0	0.9	0.14	0.91
28-day post-SCI group vs. the control group	*Mapk1*	*Prkcd*	0.062	0.5	0.922	0.353	0.965
28-day post-SCI group vs. the control group	*Mapk1*	*Smad1*	0.061	0.48	0.932	0.219	0.97
28-day post-SCI group vs. the control group	*Mapk1*	*Jun*	0	0.686	0.932	0.616	0.991
28-day post-SCI group vs. the control group	*Mapk10*	*Mapkapk2*	0	0.224	0.65	0.645	0.814
28-day post-SCI group vs. the control group	*Mapk10*	*Map2k3*	0.062	0.135	0.891	0.608	0.935
28-day post-SCI group vs. the control group	*Mapk10*	*Map2k6*	0.062	0.135	0.891	0.527	0.933
28-day post-SCI group vs. the control group	*Mapk10*	*Fos*	0	0.309	0.903	0.492	0.963
28-day post-SCI group vs. the control group	*Mapk10*	*Rela*	0	0.052	0.8	0.184	0.831
28-day post-SCI group vs. the control group	*Mapk10*	*Map2k4*	0.105	0.867	0.966	0.912	0.997
28-day post-SCI group vs. the control group	*Mapk10*	*Mcl1*	0	0.13	0.272	0.235	0.473

28-day post-SCI group vs. the control group	*Mapk10*	*Mapk1*	0.08	0.059	0.728	0.476	0.765
28-day post-SCI group vs. the control group	*Mapk10*	*Cdkn2c*	0	0.705	0	0.05	0.708
28-day post-SCI group vs. the control group	*Mapk10*	*Jun*	0	0.778	0.966	0.822	0.998
28-day post-SCI group vs. the control group	*Mapk10*	*Prkcd*	0	0.162	0.879	0.157	0.899
28-day post-SCI group vs. the control group	*Mapk10*	*Met*	0.062	0.132	0.537	0.141	0.609
28-day post-SCI group vs. the control group	*Mapkapk2*	*Hspb1*	0.062	0.871	0.966	0.733	0.998
28-day post-SCI group vs. the control group	*Mapkapk2*	*Map2k4*	0.051	0.14	0	0.617	0.4
28-day post-SCI group vs. the control group	*Mapkapk2*	*Rnf2*	0	0.436	0	0	0.436
28-day post-SCI group vs. the control group	*Mapkapk2*	*Phc1*	0	0.443	0	0.043	0.444
28-day post-SCI group vs. the control group	*Mapkapk2*	*Jun*	0.081	0.056	0	0.527	0.555
28-day post-SCI group vs. the control group	*Mapkapk2*	*Il1a*	0.062	0	0.629	0.163	0.683
28-day post-SCI group vs. the control group	*Mapkapk2*	*Mapk10*	0	0.224	0.65	0.645	0.814
28-day post-SCI group vs. the control group	*Mapkapk2*	*Mapk1*	0.064	0.75	0.326	0.267	0.865
28-day post-SCI group vs. the control group	*Mapkapk2*	*Map2k6*	0.063	0.13	0.9	0.603	0.94
28-day post-SCI group vs. the control group	*Mapkapk2*	*Map2k3*	0.119	0.09	0.9	0.701	0.943
28-day post-SCI group vs. the control group	*Mcl1*	*Ier3*	0.061	0.079	0	0.586	0.612
28-day post-SCI group vs. the control group	*Mcl1*	*Hif1a*	0.062	0.13	0	0.552	0.602
28-day post-SCI group vs. the control group	*Mcl1*	*Fos*	0.089	0	0	0.411	0.441
28-day post-SCI group vs. the control group	*Mcl1*	*Rela*	0.094	0.058	0.311	0.4	0.599
28-day post-SCI group vs. the control group	*Mcl1*	*Parp1*	0	0.053	0	0.661	0.665
28-day post-SCI group vs. the control group	*Mcl1*	*Tnfrsf1a*	0.068	0.077	0	0.387	0.427
28-day post-SCI group vs. the control group	*Mcl1*	*Pawr*	0	0	0	0.414	0.414
28-day post-SCI group vs. the control group	*Mcl1*	*Ripk1*	0.079	0.056	0.12	0.349	0.435
28-day post-SCI group vs. the control group	*Mcl1*	*Stat6*	0.069	0	0.216	0.328	0.467
28-day post-SCI group vs. the control group	*Mcl1*	*Mapk10*	0	0.13	0.272	0.235	0.473
28-day post-SCI group vs. the control group	*Mcl1*	*Met*	0	0.056	0.12	0.465	0.516
28-day post-SCI group vs. the control group	*Mcl1*	*Mapk1*	0	0.21	0.209	0.301	0.525
28-day post-SCI group vs. the control group	*Mcl1*	*Jun*	0.063	0	0	0.62	0.628
28-day post-SCI group vs. the control group	*Mcl1*	*Actb*	0	0.09	0	0.693	0.708
28-day post-SCI group vs. the control group	*Mcl1*	*Fbxw7*	0.061	0.745	0	0.863	0.964
28-day post-SCI group vs. the control group	*Met*	*Sdc1*	0.114	0	0.676	0.312	0.785
28-day post-SCI group vs. the control group	*Met*	*Hif1a*	0	0.097	0.12	0.611	0.663
28-day post-SCI group vs. the control group	*Met*	*Fos*	0	0.071	0.12	0.364	0.434
28-day post-SCI group vs. the control group	*Met*	*Hbegf*	0.061	0	0.147	0.401	0.478
28-day post-SCI group vs. the control group	*Met*	*Vcam1*	0.062	0	0.147	0.338	0.424
28-day post-SCI group vs. the control group	*Met*	*Mcl1*	0	0.056	0.12	0.465	0.516

28-day post-SCI group vs. the control group	*Met*	*Mapk1*	0.061	0.147	0.676	0.386	0.759
28-day post-SCI group vs. the control group	*Met*	*Itgb5*	0.069	0.133	0.388	0.132	0.513
28-day post-SCI group vs. the control group	*Met*	*Mmp14*	0.1	0	0.101	0.399	0.471
28-day post-SCI group vs. the control group	*Met*	*Actb*	0.052	0.084	0.114	0.59	0.643
28-day post-SCI group vs. the control group	*Met*	*Jun*	0	0.09	0.127	0.528	0.592
28-day post-SCI group vs. the control group	*Met*	*Mapk10*	0.062	0.132	0.537	0.141	0.609
28-day post-SCI group vs. the control group	*Mgst1*	*Parp1*	0	0	0	0.43	0.43
28-day post-SCI group vs. the control group	*Mgst1*	*Hdac1*	0	0	0	0.449	0.449
28-day post-SCI group vs. the control group	*Mgst1*	*Nfe2l2*	0.109	0	0	0.463	0.501
28-day post-SCI group vs. the control group	*Mgst1*	*Actb*	0.065	0	0	0.613	0.622
28-day post-SCI group vs. the control group	*Mgst1*	*Rela*	0	0	0	0.65	0.65
28-day post-SCI group vs. the control group	*Mgst1*	*Jun*	0	0	0	0.675	0.675
28-day post-SCI group vs. the control group	*Mgst1*	*Cyp1b1*	0.072	0	0.65	0.193	0.715
28-day post-SCI group vs. the control group	*Mgst1*	*Gpx3*	0.076	0	0.65	0.227	0.728
28-day post-SCI group vs. the control group	*Mgst1*	*Prdx6*	0.068	0	0.65	0.239	0.73
28-day post-SCI group vs. the control group	*Mgst1*	*Gpx8*	0.074	0	0.65	0.239	0.732
28-day post-SCI group vs. the control group	*Mgst1*	*Gpx1*	0.141	0	0.65	0.308	0.774
28-day post-SCI group vs. the control group	*Mmp14*	*Sdc1*	0.174	0.087	0.22	0.416	0.611
28-day post-SCI group vs. the control group	*Mmp14*	*Hif1a*	0.065	0	0	0.462	0.476
28-day post-SCI group vs. the control group	*Mmp14*	*Bmp1*	0.162	0.047	0	0.375	0.458
28-day post-SCI group vs. the control group	*Mmp14*	*Hbegf*	0.072	0.057	0.837	0.369	0.898
28-day post-SCI group vs. the control group	*Mmp14*	*Met*	0.1	0	0.101	0.399	0.471
28-day post-SCI group vs. the control group	*Mmp14*	*Actb*	0	0.058	0	0.475	0.484
28-day post-SCI group vs. the control group	*Mmp14*	*Jun*	0.071	0.091	0	0.454	0.499
28-day post-SCI group vs. the control group	*Mylk*	*Actb*	0.065	0.201	0	0.379	0.496
28-day post-SCI group vs. the control group	*Mylk*	*Mapk1*	0.061	0.131	0.676	0.194	0.731
28-day post-SCI group vs. the control group	*Mylk*	*Itgb5*	0.061	0.089	0.756	0.125	0.793
28-day post-SCI group vs. the control group	*Ncf1*	*Cybb*	0.423	0.087	0.932	0.993	0.999
28-day post-SCI group vs. the control group	*Ncf1*	*Jun*	0	0	0	0.466	0.465
28-day post-SCI group vs. the control group	*Ncf1*	*Aif1*	0.203	0.057	0	0.353	0.471
28-day post-SCI group vs. the control group	*Ncf1*	*Apoe*	0.062	0	0	0.513	0.523
28-day post-SCI group vs. the control group	*Ncf1*	*Gpx1*	0.062	0	0	0.521	0.531
28-day post-SCI group vs. the control group	*Ncf1*	*Tlr4*	0.09	0.046	0	0.528	0.554
28-day post-SCI group vs. the control group	*Ncf1*	*Nfe2l2*	0	0.434	0	0.4	0.645
28-day post-SCI group vs. the control group	*Ncf1*	*Actb*	0	0.194	0	0.603	0.666
28-day post-SCI group vs. the control group	*Ncf1*	*Xdh*	0.089	0	0	0.718	0.732

28-day post-SCI group vs. the control group	*Ncf1*	*Mapk1*	0	0.072	0.9	0.141	0.913
28-day post-SCI group vs. the control group	*Ncf1*	*Vcam1*	0.062	0	0.9	0.482	0.947
28-day post-SCI group vs. the control group	*Ncf1*	*Prkcd*	0.088	0.402	0.9	0.523	0.97
28-day post-SCI group vs. the control group	*Nfe2l2*	*Hmox2*	0	0	0.419	0.605	0.761
28-day post-SCI group vs. the control group	*Nfe2l2*	*Mgst1*	0.109	0	0	0.463	0.501
28-day post-SCI group vs. the control group	*Nfe2l2*	*Cybb*	0.071	0	0	0.569	0.582
28-day post-SCI group vs. the control group	*Nfe2l2*	*Ncf1*	0	0.434	0	0.4	0.645
28-day post-SCI group vs. the control group	*Nfe2l2*	*Hif1a*	0	0	0	0.658	0.658
28-day post-SCI group vs. the control group	*Nfe2l2*	*Fos*	0.061	0.443	0.293	0.416	0.755
28-day post-SCI group vs. the control group	*Nfe2l2*	*Gpx8*	0	0	0	0.66	0.66
28-day post-SCI group vs. the control group	*Nfe2l2*	*Xdh*	0.079	0	0	0.551	0.568
28-day post-SCI group vs. the control group	*Nfe2l2*	*Rela*	0.084	0.083	0	0.403	0.455
28-day post-SCI group vs. the control group	*Nfe2l2*	*Parp1*	0	0	0	0.416	0.416
28-day post-SCI group vs. the control group	*Nfe2l2*	*Slc7a11*	0	0	0	0.616	0.616
28-day post-SCI group vs. the control group	*Nfe2l2*	*Vcam1*	0	0	0	0.4	0.4
28-day post-SCI group vs. the control group	*Nfe2l2*	*Tlr4*	0.069	0	0	0.53	0.543
28-day post-SCI group vs. the control group	*Nfe2l2*	*Gpx1*	0.079	0	0	0.689	0.702
28-day post-SCI group vs. the control group	*Nfe2l2*	*Gpx3*	0.057	0	0	0.405	0.415
28-day post-SCI group vs. the control group	*Nfe2l2*	*Actb*	0	0	0	0.682	0.682
28-day post-SCI group vs. the control group	*Nfe2l2*	*Fbxw7*	0	0.198	0	0.312	0.424
28-day post-SCI group vs. the control group	*Nfe2l2*	*Txnip*	0.1	0	0	0.578	0.604
28-day post-SCI group vs. the control group	*Nfe2l2*	*Jun*	0	0.131	0.362	0.745	0.846
28-day post-SCI group vs. the control group	*Parp1*	*Mgst1*	0	0	0	0.43	0.43
28-day post-SCI group vs. the control group	*Parp1*	*Hif1a*	0	0.13	0	0.595	0.633
28-day post-SCI group vs. the control group	*Parp1*	*Rela*	0	0.418	0	0.439	0.659
28-day post-SCI group vs. the control group	*Parp1*	*Nfe2l2*	0	0	0	0.416	0.416
28-day post-SCI group vs. the control group	*Parp1*	*Ripk1*	0.068	0.051	0	0.411	0.433
28-day post-SCI group vs. the control group	*Parp1*	*Txnip*	0.063	0.201	0	0.327	0.452
28-day post-SCI group vs. the control group	*Parp1*	*Stat6*	0.062	0	0	0.538	0.548
28-day post-SCI group vs. the control group	*Parp1*	*Mapk1*	0.096	0.13	0	0.489	0.564
28-day post-SCI group vs. the control group	*Parp1*	*Jun*	0.064	0.135	0	0.57	0.622
28-day post-SCI group vs. the control group	*Parp1*	*Mcl1*	0	0.053	0	0.661	0.665
28-day post-SCI group vs. the control group	*Parp1*	*Hdac1*	0.063	0.342	0	0.554	0.701
28-day post-SCI group vs. the control group	*Parp1*	*Actb*	0.061	0	0	0.81	0.814
28-day post-SCI group vs. the control group	*Pawr*	*Rela*	0.051	0	0.676	0.05	0.682
28-day post-SCI group vs. the control group	*Pawr*	*Mcl1*	0	0	0	0.414	0.414

28-day post-SCI group vs. the control group	*Pdk1*	*Hif1a*	0	0.131	0	0.548	0.59
28-day post-SCI group vs. the control group	*Pdk1*	*Pdk2*	0	0.818	0.9	0.492	0.981
28-day post-SCI group vs. the control group	*Pdk2*	*Pdk1*	0	0.818	0.9	0.492	0.981
28-day post-SCI group vs. the control group	*Phc1*	*Mapkapk2*	0	0.443	0	0.043	0.444
28-day post-SCI group vs. the control group	*Phc1*	*Rnf2*	0	0.999	0.896	0.958	0.999
28-day post-SCI group vs. the control group	*Phc1*	*Cbx6*	0.08	0.402	0.848	0.66	0.967
28-day post-SCI group vs. the control group	*Pon2*	*Hmox2*	0	0	0	0.503	0.503
28-day post-SCI group vs. the control group	*Pon2*	*Rb1*	0	0	0	0.439	0.439
28-day post-SCI group vs. the control group	*Pon2*	*Lcat*	0.061	0	0	0.5	0.511
28-day post-SCI group vs. the control group	*Pon2*	*Apoe*	0	0	0	0.448	0.448
28-day post-SCI group vs. the control group	*Ppp3ca*	*Amph*	0.113	0.132	0.344	0.05	0.456
28-day post-SCI group vs. the control group	*Ppp3ca*	*Fkbp1b*	0.062	0.591	0.15	0.268	0.729
28-day post-SCI group vs. the control group	*Ppp3ca*	*Atp2a2*	0.09	0.054	0	0.434	0.47
28-day post-SCI group vs. the control group	*Ppp3ca*	*Prkcd*	0.064	0.211	0.195	0.204	0.463
28-day post-SCI group vs. the control group	*Ppp3ca*	*Mapk1*	0.1	0.272	0	0.26	0.473
28-day post-SCI group vs. the control group	*Prdx4*	*Gpx8*	0.082	0.224	0	0.617	0.703
28-day post-SCI group vs. the control group	*Prdx4*	*Actb*	0.062	0.082	0	0.373	0.413
28-day post-SCI group vs. the control group	*Prdx4*	*Prdx6*	0.097	0.256	0	0.742	0.508
28-day post-SCI group vs. the control group	*Prdx4*	*Gpx3*	0.061	0.224	0	0.542	0.637
28-day post-SCI group vs. the control group	*Prdx4*	*Gpx1*	0.098	0.224	0	0.617	0.708
28-day post-SCI group vs. the control group	*Prdx6*	*Hspb1*	0.051	0.101	0	0.481	0.518
28-day post-SCI group vs. the control group	*Prdx6*	*Mgst1*	0.068	0	0.65	0.239	0.73
28-day post-SCI group vs. the control group	*Prdx6*	*Gpx8*	0.063	0.184	0	0.308	0.425
28-day post-SCI group vs. the control group	*Prdx6*	*Prdx4*	0.097	0.256	0	0.742	0.508
28-day post-SCI group vs. the control group	*Prdx6*	*Fbxw7*	0	0.433	0	0.068	0.448
28-day post-SCI group vs. the control group	*Prdx6*	*Selenbp1*	0.052	0.223	0	0.415	0.531
28-day post-SCI group vs. the control group	*Prdx6*	*Gpx3*	0.063	0.184	0	0.516	0.597
28-day post-SCI group vs. the control group	*Prdx6*	*Gpx1*	0.063	0.184	0	0.629	0.691
28-day post-SCI group vs. the control group	*Prkcd*	*Hspb1*	0	0.439	0	0.578	0.754
28-day post-SCI group vs. the control group	*Prkcd*	*Cybb*	0.112	0	0.8	0.39	0.882
28-day post-SCI group vs. the control group	*Prkcd*	*Ncf1*	0.088	0.402	0.9	0.523	0.97
28-day post-SCI group vs. the control group	*Prkcd*	*Rela*	0.072	0	0.676	0.228	0.747
28-day post-SCI group vs. the control group	*Prkcd*	*Map2k4*	0	0	0.676	0.132	0.689
28-day post-SCI group vs. the control group	*Prkcd*	*Ppp3ca*	0.064	0.211	0.195	0.204	0.463
28-day post-SCI group vs. the control group	*Prkcd*	*Mapk1*	0.062	0.5	0.922	0.353	0.965
28-day post-SCI group vs. the control group	*Prkcd*	*Actb*	0.076	0.07	0	0.522	0.554

28-day post-SCI group vs. the control group	*Prkcd*	*Jun*	0	0.057	0	0.486	0.495
28-day post-SCI group vs. the control group	*Prkcd*	*Fbxw7*	0.063	0.433	0	0.096	0.477
28-day post-SCI group vs. the control group	*Prkcd*	*Mapk10*	0	0.162	0.879	0.157	0.899
28-day post-SCI group vs. the control group	*Ptgs1*	*Tlr4*	0.104	0.042	0	0.371	0.413
28-day post-SCI group vs. the control group	*Ptgs1*	*Actb*	0	0	0	0.431	0.431
28-day post-SCI group vs. the control group	*Rb1*	*Fos*	0	0.703	0.21	0.224	0.802
28-day post-SCI group vs. the control group	*Rb1*	*Cbx6*	0	0.103	0.343	0.068	0.402
28-day post-SCI group vs. the control group	*Rb1*	*Pon2*	0	0	0	0.439	0.439
28-day post-SCI group vs. the control group	*Rb1*	*Actb*	0.061	0	0	0.436	0.447
28-day post-SCI group vs. the control group	*Rb1*	*Cdkn2c*	0	0.079	0.204	0.385	0.51
28-day post-SCI group vs. the control group	*Rb1*	*Id1*	0	0.133	0.544	0.111	0.618
28-day post-SCI group vs. the control group	*Rb1*	*Mapk1*	0.061	0.248	0.629	0.248	0.776
28-day post-SCI group vs. the control group	*Rb1*	*Jun*	0	0.227	0.676	0.344	0.821
28-day post-SCI group vs. the control group	*Rb1*	*Hdac1*	0.064	0.852	0.913	0.784	0.997
28-day post-SCI group vs. the control group	*Rela*	*Ier3*	0.071	0.13	0	0.836	0.856
28-day post-SCI group vs. the control group	*Rela*	*Mgst1*	0	0	0	0.65	0.65
28-day post-SCI group vs. the control group	*Rela*	*Map2k3*	0.079	0.241	0	0.277	0.45
28-day post-SCI group vs. the control group	*Rela*	*Tnfaip3*	0.09	0.13	0	0.552	0.614
28-day post-SCI group vs. the control group	*Rela*	*Map2k6*	0.05	0.311	0	0.24	0.459
28-day post-SCI group vs. the control group	*Rela*	*Hif1a*	0	0.13	0	0.848	0.862
28-day post-SCI group vs. the control group	*Rela*	*Fos*	0	0.4	0.629	0.561	0.893
28-day post-SCI group vs. the control group	*Rela*	*Nfe2l2*	0.084	0.083	0	0.403	0.455
28-day post-SCI group vs. the control group	*Rela*	*Mcl1*	0.094	0.058	0.311	0.4	0.599
28-day post-SCI group vs. the control group	*Rela*	*Parp1*	0	0.418	0	0.439	0.659
28-day post-SCI group vs. the control group	*Rela*	*Pawr*	0.051	0	0.676	0.05	0.682
28-day post-SCI group vs. the control group	*Rela*	*Stat6*	0.104	0.244	0.35	0.374	0.688
28-day post-SCI group vs. the control group	*Rela*	*Vcam1*	0	0.433	0	0.552	0.735
28-day post-SCI group vs. the control group	*Rela*	*Prkcd*	0.072	0	0.676	0.228	0.747
28-day post-SCI group vs. the control group	*Rela*	*Mapk1*	0	0.054	0.676	0.31	0.77
28-day post-SCI group vs. the control group	*Rela*	*Mapk10*	0	0.052	0.8	0.184	0.831
28-day post-SCI group vs. the control group	*Rela*	*Ripk1*	0.089	0.147	0.676	0.498	0.856
28-day post-SCI group vs. the control group	*Rela*	*Actb*	0.079	0.09	0.629	0.701	0.894
28-day post-SCI group vs. the control group	*Rela*	*Tnfrsf1a*	0.114	0	0.676	0.691	0.903
28-day post-SCI group vs. the control group	*Rela*	*Tlr4*	0	0	0.8	0.693	0.935
28-day post-SCI group vs. the control group	*Rela*	*Il1a*	0	0	0.9	0.405	0.938
28-day post-SCI group vs. the control group	*Rela*	*Jun*	0.061	0.46	0.629	0.859	0.97

28-day post-SCI group vs. the control group	*Rela*	*Hdac1*	0.062	0.795	0.512	0.982	0.998
28-day post-SCI group vs. the control group	*Ripk1*	*Map2k3*	0.087	0.132	0.932	0.221	0.945
28-day post-SCI group vs. the control group	*Ripk1*	*Tnfaip3*	0.095	0.881	0.932	0.508	0.995
28-day post-SCI group vs. the control group	*Ripk1*	*Map2k6*	0.061	0.132	0.922	0.155	0.934
28-day post-SCI group vs. the control group	*Ripk1*	*Rela*	0.089	0.147	0.676	0.498	0.856
28-day post-SCI group vs. the control group	*Ripk1*	*Parp1*	0.068	0.051	0	0.411	0.433
28-day post-SCI group vs. the control group	*Ripk1*	*Il1a*	0	0	0.341	0.197	0.448
28-day post-SCI group vs. the control group	*Ripk1*	*Tnfrsf1a*	0.113	0.999	0.932	0.988	0.999
28-day post-SCI group vs. the control group	*Ripk1*	*Map2k4*	0	0.106	0.629	0.242	0.682
28-day post-SCI group vs. the control group	*Ripk1*	*Mcl1*	0.079	0.056	0.12	0.349	0.435
28-day post-SCI group vs. the control group	*Ripk1*	*Tlr4*	0.058	0.461	0.667	0.658	0.934
28-day post-SCI group vs. the control group	*Ripk1*	*Mapk1*	0	0.147	0.354	0.151	0.453
28-day post-SCI group vs. the control group	*Ripk1*	*Actb*	0.052	0.084	0.114	0.4	0.476
28-day post-SCI group vs. the control group	*Ripk1*	*Jun*	0	0.09	0.127	0.468	0.54
28-day post-SCI group vs. the control group	*Rnf2*	*Mapkapk2*	0	0.436	0	0	0.436
28-day post-SCI group vs. the control group	*Rnf2*	*Hdac1*	0.062	0.099	0	0.468	0.512
28-day post-SCI group vs. the control group	*Rnf2*	*Actb*	0	0.433	0	0.223	0.54
28-day post-SCI group vs. the control group	*Rnf2*	*Cbx6*	0.052	0.758	0.845	0.714	0.988
28-day post-SCI group vs. the control group	*Rnf2*	*Phc1*	0	0.999	0.896	0.958	0.999
28-day post-SCI group vs. the control group	*Rpl13a*	*Eif2s1*	0.652	0	0	0.097	0.672
28-day post-SCI group vs. the control group	*Rpl13a*	*Actb*	0.09	0.066	0	0.704	0.726
28-day post-SCI group vs. the control group	*S100a1*	*Fkbp1b*	0	0.249	0	0.398	0.528
28-day post-SCI group vs. the control group	*S100a1*	*Atp2a2*	0.062	0.13	0	0.825	0.845
28-day post-SCI group vs. the control group	*S100a1*	*Tlr4*	0	0	0.6	0.466	0.777
28-day post-SCI group vs. the control group	*Sdc1*	*Vcam1*	0	0	0	0.402	0.402
28-day post-SCI group vs. the control group	*Sdc1*	*Actb*	0	0	0	0.464	0.463
28-day post-SCI group vs. the control group	*Sdc1*	*Tlr4*	0	0	0	0.552	0.552
28-day post-SCI group vs. the control group	*Sdc1*	*Mmp14*	0.174	0.087	0.22	0.416	0.611
28-day post-SCI group vs. the control group	*Sdc1*	*Mapk1*	0.065	0	0.676	0.131	0.713
28-day post-SCI group vs. the control group	*Sdc1*	*Met*	0.114	0	0.676	0.312	0.785
28-day post-SCI group vs. the control group	*Sdc1*	*Apoe*	0.051	0	0.6	0.564	0.82
28-day post-SCI group vs. the control group	*Sdc1*	*Cd38*	0	0	0	0.853	0.853
28-day post-SCI group vs. the control group	*Sdc1*	*Itgb5*	0.109	0	0.864	0.381	0.919
28-day post-SCI group vs. the control group	*Selenbp1*	*Prdx6*	0.052	0.223	0	0.415	0.531
28-day post-SCI group vs. the control group	*Selenbp1*	*Gpx1*	0.061	0.379	0	0.872	0.919
28-day post-SCI group vs. the control group	*Sirpa*	*Trem2*	0.217	0	0	0.357	0.475

28-day post-SCI group vs. the control group	*Slc7a11*	*Actb*	0	0.044	0	0.471	0.473
28-day post-SCI group vs. the control group	*Slc7a11*	*Gpx1*	0.052	0.06	0	0.463	0.479
28-day post-SCI group vs. the control group	*Slc7a11*	*Nfe2l2*	0	0	0	0.616	0.616
28-day post-SCI group vs. the control group	*Slc8a1*	*Atp2a2*	0.081	0.169	0	0.701	0.751
28-day post-SCI group vs. the control group	*Smad1*	*Hif1a*	0.061	0.147	0.301	0.27	0.537
28-day post-SCI group vs. the control group	*Smad1*	*Fos*	0	0.142	0.388	0.227	0.558
28-day post-SCI group vs. the control group	*Smad1*	*Mapk1*	0.061	0.48	0.932	0.219	0.97
28-day post-SCI group vs. the control group	*Smad1*	*Id1*	0	0	0	0.471	0.471
28-day post-SCI group vs. the control group	*Smad1*	*Actb*	0	0.041	0	0.537	0.537
28-day post-SCI group vs. the control group	*Smad1*	*Hdac1*	0.062	0.221	0.301	0.245	0.563
28-day post-SCI group vs. the control group	*Smad1*	*Jun*	0	0.211	0.388	0.43	0.701
28-day post-SCI group vs. the control group	*Stat6*	*Hif1a*	0	0.102	0	0.42	0.456
28-day post-SCI group vs. the control group	*Stat6*	*Fos*	0	0.071	0.216	0.327	0.467
28-day post-SCI group vs. the control group	*Stat6*	*Ets2*	0.078	0.067	0.544	0.151	0.622
28-day post-SCI group vs. the control group	*Stat6*	*Hbegf*	0.063	0	0.35	0.184	0.459
28-day post-SCI group vs. the control group	*Stat6*	*Rela*	0.104	0.244	0.35	0.374	0.688
28-day post-SCI group vs. the control group	*Stat6*	*Parp1*	0.062	0	0	0.538	0.548
28-day post-SCI group vs. the control group	*Stat6*	*Tnfrsf1a*	0.113	0.066	0.35	0.272	0.555
28-day post-SCI group vs. the control group	*Stat6*	*Mcl1*	0.069	0	0.216	0.328	0.467
28-day post-SCI group vs. the control group	*Stat6*	*Tlr4*	0.071	0.089	0	0.573	0.607
28-day post-SCI group vs. the control group	*Stat6*	*Hdac1*	0.048	0.164	0.208	0.223	0.444
28-day post-SCI group vs. the control group	*Stat6*	*Actb*	0	0.048	0	0.524	0.528
28-day post-SCI group vs. the control group	*Stat6*	*Il6st*	0.064	0.047	0.35	0.31	0.546
28-day post-SCI group vs. the control group	*Stat6*	*Jun*	0	0.134	0.216	0.552	0.669
28-day post-SCI group vs. the control group	*Stau2*	*Ago1*	0.062	0.232	0	0.264	0.423
28-day post-SCI group vs. the control group	*Syp*	*Amph*	0.187	0	0	0.436	0.522
28-day post-SCI group vs. the control group	*Syp*	*Fos*	0	0	0	0.546	0.547
28-day post-SCI group vs. the control group	*Syp*	*Aif1*	0	0	0	0.727	0.727
28-day post-SCI group vs. the control group	*Syp*	*Apoe*	0.061	0	0	0.473	0.484
28-day post-SCI group vs. the control group	*Syp*	*Actb*	0	0	0	0.693	0.693
28-day post-SCI group vs. the control group	*Syp*	*Lancl1*	0.067	0.698	0	0	0.706
28-day post-SCI group vs. the control group	*Tlr4*	*Hspb1*	0	0.057	0	0.405	0.415
28-day post-SCI group vs. the control group	*Tlr4*	*Cybb*	0.217	0.076	0	0.626	0.706
28-day post-SCI group vs. the control group	*Tlr4*	*Ncf1*	0.09	0.046	0	0.528	0.554
28-day post-SCI group vs. the control group	*Tlr4*	*Map2k3*	0	0.09	0	0.457	0.485
28-day post-SCI group vs. the control group	*Tlr4*	*Tnfaip3*	0.079	0.052	0	0.472	0.499

28-day post-SCI group vs. the control group	*Tlr4*	*Sdc1*	0	0	0	0.552	0.552
28-day post-SCI group vs. the control group	*Tlr4*	*Hif1a*	0.062	0.054	0	0.589	0.603
28-day post-SCI group vs. the control group	*Tlr4*	*Fos*	0.063	0.052	0	0.554	0.569
28-day post-SCI group vs. the control group	*Tlr4*	*Ctsl*	0	0.045	0	0.435	0.437
28-day post-SCI group vs. the control group	*Tlr4*	*Trem2*	0.182	0	0	0.607	0.665
28-day post-SCI group vs. the control group	*Tlr4*	*Xdh*	0.112	0	0	0.822	0.836
28-day post-SCI group vs. the control group	*Tlr4*	*Aif1*	0.061	0.065	0	0.675	0.69
28-day post-SCI group vs. the control group	*Tlr4*	*Rela*	0	0	0.8	0.693	0.935
28-day post-SCI group vs. the control group	*Tlr4*	*Il1a*	0.057	0	0	0.743	0.747
28-day post-SCI group vs. the control group	*Tlr4*	*Vcam1*	0.171	0	0	0.673	0.718
28-day post-SCI group vs. the control group	*Tlr4*	*Cd38*	0.062	0	0	0.403	0.416
28-day post-SCI group vs. the control group	*Tlr4*	*Tnfrsf1a*	0.068	0	0.158	0.684	0.73
28-day post-SCI group vs. the control group	*Tlr4*	*Txnip*	0.062	0	0	0.394	0.407
28-day post-SCI group vs. the control group	*Tlr4*	*Ptgs1*	0.104	0.042	0	0.371	0.413
28-day post-SCI group vs. the control group	*Tlr4*	*Nfe2l2*	0.069	0	0	0.53	0.543
28-day post-SCI group vs. the control group	*Tlr4*	*Stat6*	0.071	0.089	0	0.573	0.607
28-day post-SCI group vs. the control group	*Tlr4*	*Apoe*	0	0	0	0.72	0.72
28-day post-SCI group vs. the control group	*Tlr4*	*Cd36*	0.135	0.13	0.6	0.221	0.734
28-day post-SCI group vs. the control group	*Tlr4*	*Jun*	0	0	0	0.738	0.738
28-day post-SCI group vs. the control group	*Tlr4*	*Actb*	0	0.132	0	0.738	0.763
28-day post-SCI group vs. the control group	*Tlr4*	*S100a1*	0	0	0.6	0.466	0.777
28-day post-SCI group vs. the control group	*Tlr4*	*Ripk1*	0.058	0.461	0.667	0.658	0.934
28-day post-SCI group vs. the control group	*Tnfaip3*	*Ier3*	0.191	0	0	0.492	0.572
28-day post-SCI group vs. the control group	*Tnfaip3*	*Jun*	0.082	0	0	0.403	0.428
28-day post-SCI group vs. the control group	*Tnfaip3*	*Tlr4*	0.079	0.052	0	0.472	0.499
28-day post-SCI group vs. the control group	*Tnfaip3*	*Rela*	0.09	0.13	0	0.552	0.614
28-day post-SCI group vs. the control group	*Tnfaip3*	*Il1a*	0.408	0	0	0.438	0.653
28-day post-SCI group vs. the control group	*Tnfaip3*	*Ripk1*	0.095	0.881	0.932	0.508	0.995
28-day post-SCI group vs. the control group	*Tnfaip3*	*Tnfrsf1a*	0.083	0.981	0.864	0.532	0.998
28-day post-SCI group vs. the control group	*Tnfrsf1a*	*Cybb*	0.093	0.13	0	0.345	0.438
28-day post-SCI group vs. the control group	*Tnfrsf1a*	*Map2k3*	0.095	0	0.676	0.245	0.759
28-day post-SCI group vs. the control group	*Tnfrsf1a*	*Tnfaip3*	0.083	0.981	0.864	0.532	0.998
28-day post-SCI group vs. the control group	*Tnfrsf1a*	*Map2k6*	0	0	0.629	0.235	0.704
28-day post-SCI group vs. the control group	*Tnfrsf1a*	*Rela*	0.114	0	0.676	0.691	0.903
28-day post-SCI group vs. the control group	*Tnfrsf1a*	*Il1a*	0.062	0	0	0.43	0.442
28-day post-SCI group vs. the control group	*Tnfrsf1a*	*Vcam1*	0	0	0	0.564	0.564

28-day post-SCI group vs. the control group	*Tnfrsf1a*	*Mcl1*	0.068	0.077	0	0.387	0.427
28-day post-SCI group vs. the control group	*Tnfrsf1a*	*Hdac1*	0	0	0.41	0.154	0.479
28-day post-SCI group vs. the control group	*Tnfrsf1a*	*Actb*	0.063	0.047	0	0.523	0.536
28-day post-SCI group vs. the control group	*Tnfrsf1a*	*Stat6*	0.113	0.066	0.35	0.272	0.555
28-day post-SCI group vs. the control group	*Tnfrsf1a*	*Mapk1*	0.051	0.467	0	0.211	0.566
28-day post-SCI group vs. the control group	*Tnfrsf1a*	*Jun*	0.069	0	0	0.619	0.63
28-day post-SCI group vs. the control group	*Tnfrsf1a*	*Map2k4*	0	0	0.629	0.301	0.729
28-day post-SCI group vs. the control group	*Tnfrsf1a*	*Tlr4*	0.068	0	0.158	0.684	0.73
28-day post-SCI group vs. the control group	*Tnfrsf1a*	*Ripk1*	0.113	0.999	0.932	0.988	0.999
28-day post-SCI group vs. the control group	*Trem2*	*Axl*	0.078	0	0	0.452	0.473
28-day post-SCI group vs. the control group	*Trem2*	*Clu*	0	0.13	0	0.896	0.905
28-day post-SCI group vs. the control group	*Trem2*	*Sirpa*	0.217	0	0	0.357	0.475
28-day post-SCI group vs. the control group	*Trem2*	*Tlr4*	0.182	0	0	0.607	0.665
28-day post-SCI group vs. the control group	*Trem2*	*Aif1*	0.197	0	0	0.728	0.772
28-day post-SCI group vs. the control group	*Trem2*	*Apoe*	0.078	0.329	0	0.983	0.989
28-day post-SCI group vs. the control group	*Txnip*	*Hif1a*	0	0.13	0	0.4	0.455
28-day post-SCI group vs. the control group	*Txnip*	*Parp1*	0.063	0.201	0	0.327	0.452
28-day post-SCI group vs. the control group	*Txnip*	*Tlr4*	0.062	0	0	0.394	0.407
28-day post-SCI group vs. the control group	*Txnip*	*Id1*	0.066	0	0	0.509	0.521
28-day post-SCI group vs. the control group	*Txnip*	*Actb*	0.061	0.055	0	0.419	0.439
28-day post-SCI group vs. the control group	*Txnip*	*Hdac1*	0	0.342	0	0.459	0.629
28-day post-SCI group vs. the control group	*Txnip*	*Nfe2l2*	0.1	0	0	0.578	0.604
28-day post-SCI group vs. the control group	*Txnip*	*Jun*	0.083	0	0	0.43	0.455
28-day post-SCI group vs. the control group	*Ucp2*	*Hif1a*	0	0	0	0.429	0.429
28-day post-SCI group vs. the control group	*Ucp2*	*Gpx1*	0.101	0	0	0.493	0.524
28-day post-SCI group vs. the control group	*Ucp2*	*Actb*	0.061	0.047	0	0.496	0.51
28-day post-SCI group vs. the control group	*Ucp2*	*Cd36*	0.099	0	0	0.395	0.431
28-day post-SCI group vs. the control group	*Vcam1*	*Axl*	0.141	0	0.147	0.274	0.421
28-day post-SCI group vs. the control group	*Vcam1*	*Cybb*	0.119	0	0.9	0.565	0.958
28-day post-SCI group vs. the control group	*Vcam1*	*Ncf1*	0.062	0	0.9	0.482	0.947
28-day post-SCI group vs. the control group	*Vcam1*	*Sdc1*	0	0	0	0.402	0.402
28-day post-SCI group vs. the control group	*Vcam1*	*Hif1a*	0.064	0	0	0.538	0.549
28-day post-SCI group vs. the control group	*Vcam1*	*Xdh*	0	0	0	0.468	0.468
28-day post-SCI group vs. the control group	*Vcam1*	*Aif1*	0.141	0	0	0.369	0.435
28-day post-SCI group vs. the control group	*Vcam1*	*Rela*	0	0.433	0	0.552	0.735
28-day post-SCI group vs. the control group	*Vcam1*	*Il1a*	0.069	0	0	0.494	0.508

28-day post-SCI group vs. the control group	*Vcam1*	*Nfe2l2*	0	0	0	0.4	0.4
28-day post-SCI group vs. the control group	*Vcam1*	*Met*	0.062	0	0.147	0.338	0.424
28-day post-SCI group vs. the control group	*Vcam1*	*Cd38*	0.094	0	0	0.399	0.432
28-day post-SCI group vs. the control group	*Vcam1*	*Itgb5*	0.073	0.056	0.317	0.222	0.472
28-day post-SCI group vs. the control group	*Vcam1*	*Tnfrsf1a*	0	0	0	0.564	0.564
28-day post-SCI group vs. the control group	*Vcam1*	*Jun*	0	0	0	0.589	0.589
28-day post-SCI group vs. the control group	*Vcam1*	*Actb*	0	0.047	0	0.62	0.622
28-day post-SCI group vs. the control group	*Vcam1*	*Tlr4*	0.171	0	0	0.673	0.718
28-day post-SCI group vs. the control group	*Vcam1*	*Apoe*	0.061	0	0	0.804	0.808
28-day post-SCI group vs. the control group	*Xdh*	*Cybb*	0.138	0	0	0.739	0.765
28-day post-SCI group vs. the control group	*Xdh*	*Ncf1*	0.089	0	0	0.718	0.732
28-day post-SCI group vs. the control group	*Xdh*	*Hif1a*	0	0	0	0.406	0.406
28-day post-SCI group vs. the control group	*Xdh*	*Vcam1*	0	0	0	0.468	0.468
28-day post-SCI group vs. the control group	*Xdh*	*Actb*	0	0	0	0.468	0.468
28-day post-SCI group vs. the control group	*Xdh*	*Apoe*	0.105	0	0	0.464	0.5
28-day post-SCI group vs. the control group	*Xdh*	*Gpx1*	0.062	0	0	0.552	0.561
28-day post-SCI group vs. the control group	*Xdh*	*Nfe2l2*	0.079	0	0	0.551	0.568
28-day post-SCI group vs. the control group	*Xdh*	*Jun*	0	0	0	0.583	0.583
28-day post-SCI group vs. the control group	*Xdh*	*Tlr4*	0.112	0	0	0.822	0.836

SCI: Spinal cord injury
