# Supplementary material for: Integrating bulk and single-cell transcriptome profiling to uncover diagnostic biomarkers and regulatory mechanisms of oxidative stress in spinal cord injury
Source: Neural Regen Res. 2025 Jan 13;21(6):2643–57. doi: 10.4103/NRR.NRR-D-24-00693 (PMC13217428; doi:10.4103/NRR.NRR-D-24-00693)
Supplement: Supplementary file 4 [file NRR-21-2643_Suppl3.pdf]

Additional Table 4 GO functional enrichment analysis of oxidative stress-related genes that are differentially expressed between groups at different time points after SCI and the control group

Group	ONTOLOGY	ID	Description	GeneRatio	pvalue	p.adjust	qvalue	geneID
4-hour post-SCI group vs. the control group	BP	GO:0006979	response to oxidative stress	19/35	1.41E-26	2.54E-23	1.13E-23	Atf4/Cat/Fos/Gch1/Hmox1/Hspb1/Jun/Mcl1/Nfkb1/Nr4a3/Ppp1r15b/Pxdn/Rcan1/Ripk1/Slc8a1/Sphk1/Srxn1/Tnfaip3/Txnrd1
4-hour post-SCI group vs. the control group	BP	GO:0034599	cellular response to oxidative stress	14/35	6.13E-20	5.52E-17	2.46E-17	Atf4/Cat/Fos/Gch1/Hmox1/Hspb1/Jun/Mcl1/Nr4a3/Ripk1/Slc8a1/Sphk1/Srxn1/Tnfaip3
4-hour post-SCI group vs. the control group	BP	GO:0062197	cellular response to chemical stress	14/35	1.35E-18	8.13E-16	3.62E-16	Atf4/Cat/Fos/Gch1/Hmox1/Hspb1/Jun/Mcl1/Nr4a3/Ripk1/Slc8a1/Sphk1/Srxn1/Tnfaip3
4-hour post-SCI group vs. the control group	BP	GO:0000302	response to reactive oxygen species	11/35	2.65E-16	1.19E-13	5.31E-14	Cat/Fos/Gch1/Hmox1/Jun/Nr4a3/Ppp1r15b/Ripk1/Slc8a1/Sphk1/Tnfaip3
4-hour post-SCI group vs. the control group	BP	GO:0034614	cellular response to reactive oxygen species	9/35	8.61E-14	3.10E-11	1.38E-11	Cat/Fos/Gch1/Jun/Nr4a3/Ripk1/Slc8a1/Sphk1/Tnfaip3
4-hour post-SCI group vs. the control group	BP	GO:0042542	response to hydrogen peroxide	7/35	1.21E-10	3.64E-08	1.62E-08	Cat/Hmox1/Nr4a3/Ppp1r15b/Ripk1/Sphk1/Tnfaip3
4-hour post-SCI group vs. the control group	BP	GO:1900407	regulation of cellular response to oxidative stress	6/35	1.15E-09	2.96E-07	1.32E-07	Atf4/Gch1/Hspb1/Mcl1/Nr4a3/Ripk1
4-hour post-SCI group vs. the control group	BP	GO:1902882	regulation of response to oxidative stress	6/35	2.06E-09	4.63E-07	2.06E-07	Atf4/Gch1/Hspb1/Mcl1/Nr4a3/Ripk1
4-hour post-SCI group vs. the control group	BP	GO:0045765	regulation of angiogenesis	8/35	2.75E-09	5.49E-07	2.44E-07	Ago1/Hmox1/Hspb1/Id1/Il1a/Rnh1/Sphk1/Tnfrsf1a
4-hour post-SCI group vs. the control group	BP	GO:1901342	regulation of vasculature development	8/35	3.05E-09	5.49E-07	2.44E-07	Ago1/Hmox1/Hspb1/Id1/Il1a/Rnh1/Sphk1/Tnfrsf1a
4-hour post-SCI group vs. the control group	BP	GO:0010586	miRNA metabolic process	6/35	5.65E-09	9.26E-07	4.12E-07	Ago1/Fos/Fosl1/Jun/Myc/Nfkb1
4-hour post-SCI group vs. the control group	BP	GO:0035994	response to muscle stretch	4/35	1.06E-08	1.59E-06	7.05E-07	Fos/Jun/Nfkb1/Slc8a1
4-hour post-SCI group vs. the control group	BP	GO:0051403	stress-activated MAPK cascade	7/35	1.53E-08	2.12E-06	9.41E-07	Ezr/Il1a/Map2k3/Nfkb1/Ripk1/Sphk1/Xdh
4-hour post-SCI group vs. the control group	BP	GO:0031098	stress-activated protein kinase signaling cascade	7/35	2.01E-08	2.59E-06	1.15E-06	Ezr/Il1a/Map2k3/Nfkb1/Ripk1/Sphk1/Xdh
4-hour post-SCI group vs. the control group	BP	GO:1902895	positive regulation of miRNA transcription	5/35	2.53E-08	3.04E-06	1.35E-06	Fos/Fosl1/Jun/Myc/Nfkb1
4-hour post-SCI group vs. the control group	BP	GO:0033002	muscle cell proliferation	7/35	2.76E-08	3.11E-06	1.38E-06	Hbegf/Hmox1/Jun/Myc/Nr4a3/Rxrb/Tnfaip3
4-hour post-SCI group vs. the control group	BP	GO:2000630	positive regulation of miRNA metabolic process	5/35	3.80E-08	4.03E-06	1.79E-06	Fos/Fosl1/Jun/Myc/Nfkb1
4-hour post-SCI group vs. the control group	BP	GO:1903201	regulation of oxidative stress-induced cell death	5/35	4.06E-08	4.06E-06	1.81E-06	Atf4/Hspb1/Mcl1/Nr4a3/Ripk1
4-hour post-SCI group vs. the control group	BP	GO:1902893	regulation of miRNA transcription	5/35	7.00E-08	6.63E-06	2.95E-06	Fos/Fosl1/Jun/Myc/Nfkb1
4-hour post-SCI group vs. the control group	BP	GO:0061614	miRNA transcription	5/35	7.84E-08	7.06E-06	3.14E-06	Fos/Fosl1/Jun/Myc/Nfkb1
4-hour post-SCI group vs. the control group	BP	GO:0048660	regulation of smooth muscle cell proliferation	6/35	8.75E-08	7.50E-06	3.34E-06	Hbegf/Hmox1/Jun/Myc/Nr4a3/Tnfaip3
4-hour post-SCI group vs. the control group	BP	GO:0070301	cellular response to hydrogen peroxide	5/35	9.24E-08	7.57E-06	3.36E-06	Cat/Nr4a3/Ripk1/Sphk1/Tnfaip3
4-hour post-SCI group vs. the control group	BP	GO:0048659	smooth muscle cell proliferation	6/35	1.09E-07	8.53E-06	3.80E-06	Hbegf/Hmox1/Jun/Myc/Nr4a3/Tnfaip3
4-hour post-SCI group vs. the control group	BP	GO:0036473	cell death in response to oxidative stress	5/35	1.40E-07	1.05E-05	4.67E-06	Atf4/Hspb1/Mcl1/Nr4a3/Ripk1
4-hour post-SCI group vs. the control group	BP	GO:2000628	regulation of miRNA metabolic process	5/35	1.47E-07	1.06E-05	4.71E-06	Fos/Fosl1/Jun/Myc/Nfkb1
4-hour post-SCI group vs. the control group	BP	GO:0048661	positive regulation of smooth muscle cell proliferation	5/35	2.05E-07	1.42E-05	6.31E-06	Hbegf/Hmox1/Jun/Myc/Nr4a3
4-hour post-SCI group vs. the control group	BP	GO:0050679	positive regulation of epithelial cell proliferation	6/35	3.32E-07	2.22E-05	9.86E-06	Hmox1/Id1/Jun/Myc/Nr4a3/Tnfaip3
4-hour post-SCI group vs. the control group	BP	GO:1901214	regulation of neuron death	7/35	4.33E-07	2.78E-05	1.24E-05	Atf4/Fos/Hmox1/Jun/Mcl1/Nr4a3/Tnfrsf1a
4-hour post-SCI group vs. the control group	BP	GO:0072593	reactive oxygen species metabolic process	6/35	4.57E-07	2.84E-05	1.26E-05	Cat/Gch1/Pxdn/Ripk1/Txnrd1/Xdh
4-hour post-SCI group vs. the control group	BP	GO:0038066	p38MAPK cascade	4/35	5.43E-07	3.21E-05	1.43E-05	Ezr/Map2k3/Sphk1/Xdh
4-hour post-SCI group vs. the control group	BP	GO:1901216	positive regulation of neuron death	5/35	5.52E-07	3.21E-05	1.43E-05	Atf4/Fos/Jun/Mcl1/Tnfrsf1a
4-hour post-SCI group vs. the control group	BP	GO:0051090	regulation of DNA-binding transcription factor activity	7/35	7.84E-07	4.41E-05	1.96E-05	Cat/Fosl1/Hmox1/Id1/Ripk1/Sphk1/Tnfaip3

4-hour post-SCI group vs. the control group	BP	GO:0035924	cellular response to vascular endothelial growth factor stimulus	4/35	8.32E-07	4.54E-05	2.02E-05	Hspb1/Map2k3/Sphk1/Xdh
4-hour post-SCI group vs. the control group	BP	GO:0050678	regulation of epithelial cell proliferation	7/35	8.59E-07	4.55E-05	2.02E-05	Hmox1/Id1/Jun/Myc/Nr4a3/Tnfaip3/Xdh
4-hour post-SCI group vs. the control group	BP	GO:0070997	neuron death	7/35	9.68E-07	4.92E-05	2.19E-05	Atf4/Fos/Hmox1/Jun/Mcl1/Nr4a3/Tnfrsf1a
4-hour post-SCI group vs. the control group	BP	GO:0010563	negative regulation of phosphorus metabolic process	7/35	1.01E-06	4.92E-05	2.19E-05	Fkbp1b/Hspb1/Jun/Ppp1r15b/Slc8a1/Tnfaip3/Xdh
4-hour post-SCI group vs. the control group	BP	GO:0045936	negative regulation of phosphate metabolic process	7/35	1.01E-06	4.92E-05	2.19E-05	Fkbp1b/Hspb1/Jun/Ppp1r15b/Slc8a1/Tnfaip3/Xdh
4-hour post-SCI group vs. the control group	BP	GO:0043523	regulation of neuron apoptotic process	6/35	1.22E-06	5.79E-05	2.58E-05	Atf4/Hmox1/Jun/Mcl1/Nr4a3/Tnfrsf1a
4-hour post-SCI group vs. the control group	BP	GO:0010634	positive regulation of epithelial cell migration	5/35	1.38E-06	6.36E-05	2.83E-05	Hbegf/Hmox1/Hspb1/Jun/Map2k3
4-hour post-SCI group vs. the control group	BP	GO:0097193	intrinsic apoptotic signaling pathway	6/35	1.80E-06	8.09E-05	3.60E-05	Atf4/Hmox1/Hspb1/Mcl1/Myc/Tnfrsf1a
4-hour post-SCI group vs. the control group	BP	GO:0009612	response to mechanical stimulus	5/35	2.26E-06	9.93E-05	4.42E-05	Fos/Jun/Myc/Nfkb1/Slc8a1
4-hour post-SCI group vs. the control group	BP	GO:0051402	neuron apoptotic process	6/35	2.53E-06	0.0001061	4.72E-05	Atf4/Hmox1/Jun/Mcl1/Nr4a3/Tnfrsf1a
4-hour post-SCI group vs. the control group	BP	GO:0045766	positive regulation of angiogenesis	5/35	2.59E-06	0.0001061	4.72E-05	Hmox1/Hspb1/Il1a/Sphk1/Tnfrsf1a
4-hour post-SCI group vs. the control group	BP	GO:1904018	positive regulation of vasculature development	5/35	2.59E-06	0.0001061	4.72E-05	Hmox1/Hspb1/Il1a/Sphk1/Tnfrsf1a
4-hour post-SCI group vs. the control group	BP	GO:0032680	regulation of tumor necrosis factor production	5/35	2.88E-06	0.00011539	5.13E-05	Hspb1/Il1a/Ripk1/Tnfaip3/Tnfrsf1a
4-hour post-SCI group vs. the control group	BP	GO:0071216	cellular response to biotic stimulus	6/35	3.06E-06	0.00011948	5.31E-05	Gch1/Il1a/Map2k3/Nfkb1/Ppp1r15b/Tnfaip3
4-hour post-SCI group vs. the control group	BP	GO:1903555	regulation of tumor necrosis factor superfamily cytokine production	5/35	3.12E-06	0.00011948	5.31E-05	Hspb1/Il1a/Ripk1/Tnfaip3/Tnfrsf1a
4-hour post-SCI group vs. the control group	BP	GO:0001933	negative regulation of protein phosphorylation	6/35	3.39E-06	0.00012696	5.65E-05	Hspb1/Jun/Ppp1r15b/Slc8a1/Tnfaip3/Xdh
4-hour post-SCI group vs. the control group	BP	GO:0032640	tumor necrosis factor production	5/35	3.45E-06	0.00012696	5.65E-05	Hspb1/Il1a/Ripk1/Tnfaip3/Tnfrsf1a
4-hour post-SCI group vs. the control group	BP	GO:0071706	tumor necrosis factor superfamily cytokine production	5/35	3.72E-06	0.00013415	5.97E-05	Hspb1/Il1a/Ripk1/Tnfaip3/Tnfrsf1a
4-hour post-SCI group vs. the control group	BP	GO:0043525	positive regulation of neuron apoptotic process	4/35	3.89E-06	0.00013725	6.10E-05	Atf4/Jun/Mcl1/Tnfrsf1a
4-hour post-SCI group vs. the control group	BP	GO:0071276	cellular response to cadmium ion	3/35	4.13E-06	0.00014311	6.37E-05	Fos/Hmox1/Jun
4-hour post-SCI group vs. the control group	BP	GO:0034612	response to tumor necrosis factor	5/35	4.31E-06	0.00014662	6.52E-05	Gch1/Nfkb1/Ripk1/Sphk1/Tnfrsf1a
4-hour post-SCI group vs. the control group	BP	GO:0032872	regulation of stress-activated MAPK cascade	5/35	4.53E-06	0.00015098	6.72E-05	Ezr/Il1a/Ripk1/Sphk1/Xdh
4-hour post-SCI group vs. the control group	BP	GO:0070302	regulation of stress-activated protein kinase signaling cascade	5/35	4.86E-06	0.00015916	7.08E-05	Ezr/Il1a/Ripk1/Sphk1/Xdh
4-hour post-SCI group vs. the control group	BP	GO:2001237	negative regulation of extrinsic apoptotic signaling pathway	4/35	6.22E-06	0.00019989	8.89E-05	Hmox1/Mcl1/Ripk1/Tnfaip3
4-hour post-SCI group vs. the control group	BP	GO:0042744	hydrogen peroxide catabolic process	3/35	6.43E-06	0.0002032	9.04E-05	Cat/Pxdn/Txnrd1
4-hour post-SCI group vs. the control group	BP	GO:0042326	negative regulation of phosphorylation	6/35	7.12E-06	0.00022112	9.84E-05	Hspb1/Jun/Ppp1r15b/Slc8a1/Tnfaip3/Xdh
4-hour post-SCI group vs. the control group	BP	GO:2001233	regulation of apoptotic signaling pathway	6/35	7.87E-06	0.00024031	0.00010688	Hmox1/Hspb1/Mcl1/Myc/Ripk1/Tnfaip3
4-hour post-SCI group vs. the control group	BP	GO:0097191	extrinsic apoptotic signaling pathway	5/35	9.57E-06	0.00028663	0.00012749	Hmox1/Il1a/Mcl1/Ripk1/Tnfaip3
4-hour post-SCI group vs. the control group	BP	GO:0032760	positive regulation of tumor necrosis factor production	4/35	9.79E-06	0.00028663	0.00012749	Hspb1/Il1a/Ripk1/Tnfrsf1a
4-hour post-SCI group vs. the control group	BP	GO:2001234	negative regulation of apoptotic signaling pathway	5/35	1.02E-05	0.00028663	0.00012749	Hmox1/Hspb1/Mcl1/Ripk1/Tnfaip3
4-hour post-SCI group vs. the control group	BP	GO:1903203	regulation of oxidative stress-induced neuron death	3/35	1.03E-05	0.00028663	0.00012749	Atf4/Mcl1/Nr4a3
4-hour post-SCI group vs. the control group	BP	GO:0060047	heart contraction	5/35	1.04E-05	0.00028663	0.00012749	Fkbp1b/Gch1/Hbegf/Map2k3/Slc8a1
4-hour post-SCI group vs. the control group	BP	GO:0008630	intrinsic apoptotic signaling pathway in response to DNA damage	4/35	1.05E-05	0.00028663	0.00012749	Hmox1/Mcl1/Myc/Tnfrsf1a
4-hour post-SCI group vs. the control group	BP	GO:1903557	positive regulation of tumor necrosis factor superfamily cytokine production	4/35	1.05E-05	0.00028663	0.00012749	Hspb1/Il1a/Ripk1/Tnfrsf1a

4-hour post-SCI group vs. the control group	BP	GO:0006875	cellular metal ion homeostasis	6/35	1.10E-05	0.00029153	0.00012967	Atf4/Fkbp1b/Hmox1/Il1a/Myc/Slc8a1
4-hour post-SCI group vs. the control group	BP	GO:0002064	epithelial cell development	5/35	1.10E-05	0.00029153	0.00012967	Atf4/Ezr/Id1/Il1a/Tnfrsf1a
4-hour post-SCI group vs. the control group	BP	GO:0010632	regulation of epithelial cell migration	5/35	1.17E-05	0.0003047	0.00013552	Hbegf/Hmox1/Hspb1/Jun/Map2k3
4-hour post-SCI group vs. the control group	BP	GO:0003012	muscle system process	6/35	1.20E-05	0.00030911	0.00013749	Fkbp1b/Map2k3/Nr4a3/Slc8a1/Sphk1/Tnfrsf1a
4-hour post-SCI group vs. the control group	BP	GO:0036475	neuron death in response to oxidative stress	3/35	1.22E-05	0.00031039	0.00013806	Atf4/Mcl1/Nr4a3
4-hour post-SCI group vs. the control group	BP	GO:0003015	heart process	5/35	1.31E-05	0.00032421	0.0001442	Fkbp1b/Gch1/Hbegf/Map2k3/Slc8a1
4-hour post-SCI group vs. the control group	BP	GO:0046686	response to cadmium ion	3/35	1.33E-05	0.00032421	0.0001442	Fos/Hmox1/Jun
4-hour post-SCI group vs. the control group	BP	GO:0045446	endothelial cell differentiation	4/35	1.33E-05	0.00032421	0.0001442	Ezr/Id1/Tnfrsf1a/Xdh
4-hour post-SCI group vs. the control group	BP	GO:0090257	regulation of muscle system process	5/35	1.41E-05	0.00033918	0.00015086	Fkbp1b/Nr4a3/Slc8a1/Sphk1/Tnfrsf1a
4-hour post-SCI group vs. the control group	BP	GO:0010803	regulation of tumor necrosis factor-mediated signaling pathway	3/35	1.55E-05	0.0003678	0.00016359	Ripk1/Sphk1/Tnfrsf1a
4-hour post-SCI group vs. the control group	BP	GO:0051101	regulation of DNA binding	4/35	1.67E-05	0.00038634	0.00017184	Hmox1/Id1/Jun/Myc
4-hour post-SCI group vs. the control group	BP	GO:1901031	regulation of response to reactive oxygen species	3/35	1.67E-05	0.00038634	0.00017184	Gch1/Nr4a3/Ripk1
4-hour post-SCI group vs. the control group	BP	GO:1900744	regulation of p38MAPK cascade	3/35	1.80E-05	0.00041045	0.00018256	Ezr/Sphk1/Xdh
4-hour post-SCI group vs. the control group	BP	GO:0045601	regulation of endothelial cell differentiation	3/35	1.93E-05	0.00043509	0.00019352	Id1/Tnfrsf1a/Xdh
4-hour post-SCI group vs. the control group	BP	GO:0001667	ameboidal-type cell migration	6/35	1.96E-05	0.00043509	0.00019352	Hbegf/Hmox1/Hspb1/Jun/Map2k3/Slc8a1
4-hour post-SCI group vs. the control group	BP	GO:0003158	endothelium development	4/35	2.31E-05	0.00050113	0.0002229	Ezr/Id1/Tnfrsf1a/Xdh
4-hour post-SCI group vs. the control group	BP	GO:0032874	positive regulation of stress-activated MAPK cascade	4/35	2.31E-05	0.00050113	0.0002229	Il1a/Ripk1/Sphk1/Xdh
4-hour post-SCI group vs. the control group	BP	GO:0032768	regulation of monooxygenase activity	3/35	2.37E-05	0.00050874	0.00022628	Gch1/Il1a/Nfkb1
4-hour post-SCI group vs. the control group	BP	GO:0070304	positive regulation of stress-activated protein kinase signaling cascade	4/35	2.44E-05	0.00051777	0.0002303	Il1a/Ripk1/Sphk1/Xdh
4-hour post-SCI group vs. the control group	BP	GO:0002761	regulation of myeloid leukocyte differentiation	4/35	2.66E-05	0.00055388	0.00024636	Fos/Jun/Myc/Ripk1
4-hour post-SCI group vs. the control group	BP	GO:0030003	cellular cation homeostasis	6/35	2.68E-05	0.00055388	0.00024636	Atf4/Fkbp1b/Hmox1/Il1a/Myc/Slc8a1
4-hour post-SCI group vs. the control group	BP	GO:0010038	response to metal ion	5/35	2.73E-05	0.00055882	0.00024855	Fos/Hmox1/Il1a/Jun/Xdh
4-hour post-SCI group vs. the control group	BP	GO:0001818	negative regulation of cytokine production	5/35	2.77E-05	0.00056151	0.00024975	Ezr/Hmox1/Nfkb1/Tnfaip3/Tnfrsf1a
4-hour post-SCI group vs. the control group	BP	GO:0043618	regulation of transcription from RNA polymerase II promoter in response to stress	3/35	3.05E-05	0.00061105	0.00027179	Atf4/Hmox1/Jun
4-hour post-SCI group vs. the control group	BP	GO:0071222	cellular response to lipopolysaccharide	5/35	3.10E-05	0.00061377	0.00027299	Gch1/Il1a/Map2k3/Nfkb1/Tnfaip3
4-hour post-SCI group vs. the control group	BP	GO:0071219	cellular response to molecule of bacterial origin	5/35	3.62E-05	0.00069752	0.00031024	Gch1/Il1a/Map2k3/Nfkb1/Tnfaip3
4-hour post-SCI group vs. the control group	BP	GO:0043392	negative regulation of DNA binding	3/35	3.64E-05	0.00069752	0.00031024	Hmox1/Id1/Jun
4-hour post-SCI group vs. the control group	BP	GO:1903202	negative regulation of oxidative stress-induced cell death	3/35	3.64E-05	0.00069752	0.00031024	Atf4/Hspb1/Nr4a3
4-hour post-SCI group vs. the control group	BP	GO:0043620	regulation of DNA-templated transcription in response to stress	3/35	4.07E-05	0.00077166	0.00034322	Atf4/Hmox1/Jun
4-hour post-SCI group vs. the control group	BP	GO:0070169	positive regulation of biomineral tissue development	3/35	4.30E-05	0.00080617	0.00035857	Atf4/Rxrb/Slc8a1
4-hour post-SCI group vs. the control group	BP	GO:0010631	epithelial cell migration	5/35	4.45E-05	0.00080899	0.00035982	Hbegf/Hmox1/Hspb1/Jun/Map2k3
4-hour post-SCI group vs. the control group	BP	GO:0030857	negative regulation of epithelial cell differentiation	3/35	4.53E-05	0.00080899	0.00035982	Id1/Il1a/Xdh
4-hour post-SCI group vs. the control group	BP	GO:0043536	positive regulation of blood vessel endothelial cell migration	3/35	4.53E-05	0.00080899	0.00035982	Hmox1/Hspb1/Map2k3
4-hour post-SCI group vs. the control group	BP	GO:0110151	positive regulation of biomineralization	3/35	4.53E-05	0.00080899	0.00035982	Atf4/Rxrb/Slc8a1

4-hour post-SCI group vs. the control group	BP	GO:2001236	regulation of extrinsic apoptotic signaling pathway	4/35	4.54E-05	0.00080899	0.00035982	Hmox1/Mcl1/Ripk1/Tnfaip3
4-hour post-SCI group vs. the control group	BP	GO:0090132	epithelium migration	5/35	4.58E-05	0.00080912	0.00035988	Hbegf/Hmox1/Hspb1/Jun/Map2k3
4-hour post-SCI group vs. the control group	BP	GO:0090130	tissue migration	5/35	4.72E-05	0.00082456	0.00036675	Hbegf/Hmox1/Hspb1/Jun/Map2k3
4-hour post-SCI group vs. the control group	BP	GO:0043666	regulation of phosphoprotein phosphatase activity	3/35	4.78E-05	0.00082698	0.00036782	Fkbp1b/Ppp1r15b/Rcan1
4-hour post-SCI group vs. the control group	BP	GO:0030856	regulation of epithelial cell differentiation	4/35	4.98E-05	0.00085413	0.0003799	Id1/Il1a/Tnfrsf1a/Xdh
4-hour post-SCI group vs. the control group	BP	GO:0042743	hydrogen peroxide metabolic process	3/35	5.03E-05	0.00085413	0.0003799	Cat/Pxdn/Txnrd1
4-hour post-SCI group vs. the control group	BP	GO:0043433	negative regulation of DNA-binding transcription factor activity	4/35	5.22E-05	0.00087829	0.00039065	Cat/Hmox1/Id1/Tnfaip3
4-hour post-SCI group vs. the control group	BP	GO:1902105	regulation of leukocyte differentiation	5/35	5.97E-05	0.00099616	0.00044307	Fos/Hspb1/Jun/Myc/Ripk1
4-hour post-SCI group vs. the control group	BP	GO:0051100	negative regulation of binding	4/35	6.10E-05	0.00100856	0.00044859	Hmox1/Id1/Jun/Myc
4-hour post-SCI group vs. the control group	BP	GO:0032762	mast cell cytokine production	2/35	6.35E-05	0.00104032	0.00046272	Hmox1/Nr4a3
4-hour post-SCI group vs. the control group	BP	GO:0045787	positive regulation of cell cycle	5/35	6.74E-05	0.00109307	0.00048618	Fosl1/Il1a/Myc/Nr4a3/Sphk1
4-hour post-SCI group vs. the control group	BP	GO:0007369	gastrulation	4/35	6.95E-05	0.00110719	0.00049246	Ets2/Nr4a3/Tnfaip3/Txnrd1
4-hour post-SCI group vs. the control group	BP	GO:0071356	cellular response to tumor necrosis factor	4/35	6.95E-05	0.00110719	0.00049246	Nfkb1/Ripk1/Sphk1/Tnfrsf1a
4-hour post-SCI group vs. the control group	BP	GO:0032663	regulation of interleukin-2 production	3/35	7.36E-05	0.00116259	0.0005171	Ezr/Il1a/Tnfaip3
4-hour post-SCI group vs. the control group	BP	GO:0050727	regulation of inflammatory response	5/35	7.57E-05	0.00118606	0.00052754	Nfkb1/Ripk1/Sphk1/Tnfaip3/Tnfrsf1a
4-hour post-SCI group vs. the control group	BP	GO:0002246	wound healing involved in inflammatory response	2/35	7.76E-05	0.00119452	0.0005313	Hmox1/Il1a
4-hour post-SCI group vs. the control group	BP	GO:0045602	negative regulation of endothelial cell differentiation	2/35	7.76E-05	0.00119452	0.0005313	Id1/Xdh
4-hour post-SCI group vs. the control group	BP	GO:0043122	regulation of I-kappaB kinase/NF-kappaB signaling	4/35	8.20E-05	0.00125134	0.00055657	Hspb1/Il1a/Ripk1/Tnfaip3
4-hour post-SCI group vs. the control group	BP	GO:1904036	negative regulation of epithelial cell apoptotic process	3/35	8.39E-05	0.00126949	0.00056465	Hmox1/Id1/Tnfaip3
4-hour post-SCI group vs. the control group	BP	GO:2000144	positive regulation of DNA-templated transcription initiation	3/35	8.75E-05	0.00131336	0.00058416	Fosl1/Jun/Myc
4-hour post-SCI group vs. the control group	BP	GO:0019722	calcium-mediated signaling	4/35	9.06E-05	0.00134688	0.00059907	Fkbp1b/Rcan1/Slc8a1/Sphk1
4-hour post-SCI group vs. the control group	BP	GO:1904705	regulation of vascular associated smooth muscle cell proliferation	3/35	9.12E-05	0.00134688	0.00059907	Hmox1/Jun/Nr4a3
4-hour post-SCI group vs. the control group	BP	GO:0043619	regulation of transcription from RNA polymerase II promoter in response to oxidative stress	2/35	9.31E-05	0.00136247	0.000606	Atf4/Hmox1
4-hour post-SCI group vs. the control group	BP	GO:0002763	positive regulation of myeloid leukocyte differentiation	3/35	9.51E-05	0.00137324	0.00061079	Fos/Jun/Ripk1
4-hour post-SCI group vs. the control group	BP	GO:0032496	response to lipopolysaccharide	5/35	9.60E-05	0.00137324	0.00061079	Gch1/Il1a/Map2k3/Nfkb1/Tnfaip3
4-hour post-SCI group vs. the control group	BP	GO:0008016	regulation of heart contraction	4/35	9.61E-05	0.00137324	0.00061079	Fkbp1b/Gch1/Hbegf/Slc8a1
4-hour post-SCI group vs. the control group	BP	GO:0001885	endothelial cell development	3/35	9.90E-05	0.00139302	0.00061959	Ezr/Id1/Tnfrsf1a
4-hour post-SCI group vs. the control group	BP	GO:0032623	interleukin-2 production	3/35	9.90E-05	0.00139302	0.00061959	Ezr/Il1a/Tnfaip3
4-hour post-SCI group vs. the control group	BP	GO:0010507	negative regulation of autophagy	3/35	0.000103	0.00142756	0.00063495	Hmox1/Mcl1/Tnfaip3
4-hour post-SCI group vs. the control group	BP	GO:1990874	vascular associated smooth muscle cell proliferation	3/35	0.000103	0.00142756	0.00063495	Hmox1/Jun/Nr4a3
4-hour post-SCI group vs. the control group	BP	GO:0090520	sphingolipid mediated signaling pathway	2/35	0.0001099	0.00151071	0.00067194	Ezr/Sphk1
4-hour post-SCI group vs. the control group	BP	GO:0048146	positive regulation of fibroblast proliferation	3/35	0.0001114	0.00152053	0.0006763	Jun/Myc/Sphk1
4-hour post-SCI group vs. the control group	BP	GO:0038034	signal transduction in absence of ligand	3/35	0.0001158	0.00155646	0.00069228	Il1a/Mcl1/Ripk1
4-hour post-SCI group vs. the control group	BP	GO:0097192	extrinsic apoptotic signaling pathway in absence of ligand	3/35	0.0001158	0.00155646	0.00069228	Il1a/Mcl1/Ripk1
4-hour post-SCI group vs. the control group	BP	GO:0002237	response to molecule of bacterial origin	5/35	0.000123	0.00164153	0.00073013	Gch1/Il1a/Map2k3/Nfkb1/Tnfaip3

4-hour post-SCI group vs. the control group	BP	GO:2000142	regulation of DNA-templated transcription initiation	3/35	0.0001249	0.00165347	0.00073543	Fosl1/Jun/Myc
4-hour post-SCI group vs. the control group	BP	GO:0010882	regulation of cardiac muscle contraction by calcium ion signaling	2/35	0.0001281	0.00168402	0.00074902	Fkbp1b/Slc8a1
4-hour post-SCI group vs. the control group	BP	GO:0033209	tumor necrosis factor-mediated signaling pathway	3/35	0.0001296	0.00169078	0.00075203	Ripk1/Sphk1/Tnfrsf1a
4-hour post-SCI group vs. the control group	BP	GO:0001666	response to hypoxia	4/35	0.0001389	0.00179987	0.00080055	Cat/Hmox1/Myc/Slc8a1
4-hour post-SCI group vs. the control group	BP	GO:0010921	regulation of phosphatase activity	3/35	0.0001548	0.00199083	0.00088549	Fkbp1b/Ppp1r15b/Rcan1
4-hour post-SCI group vs. the control group	BP	GO:0045637	regulation of myeloid cell differentiation	4/35	0.0001567	0.002002	0.00089046	Fos/Jun/Myc/Ripk1
4-hour post-SCI group vs. the control group	BP	GO:0007249	I-kappaB kinase/NF-kappaB signaling	4/35	0.0001676	0.00211295	0.0009398	Hspb1/Il1a/Ripk1/Tnfaip3
4-hour post-SCI group vs. the control group	BP	GO:1903706	regulation of hemopoiesis	5/35	0.0001678	0.00211295	0.0009398	Fos/Hspb1/Jun/Myc/Ripk1
4-hour post-SCI group vs. the control group	BP	GO:0045655	regulation of monocyte differentiation	2/35	0.000191	0.00238899	0.00106258	Jun/Myc
4-hour post-SCI group vs. the control group	BP	GO:0043535	regulation of blood vessel endothelial cell migration	3/35	0.0002078	0.00256279	0.00113988	Hmox1/Hspb1/Map2k3
4-hour post-SCI group vs. the control group	BP	GO:0051341	regulation of oxidoreductase activity	3/35	0.0002078	0.00256279	0.00113988	Gch1/Il1a/Nfkb1
4-hour post-SCI group vs. the control group	BP	GO:0031668	cellular response to extracellular stimulus	4/35	0.0002134	0.0025854	0.00114994	Atf4/Fos/Fosl1/Jun
4-hour post-SCI group vs. the control group	BP	GO:0032651	regulation of interleukin-1 beta production	3/35	0.0002143	0.0025854	0.00114994	Hspb1/Sphk1/Tnfaip3
4-hour post-SCI group vs. the control group	BP	GO:0035304	regulation of protein dephosphorylation	3/35	0.0002143	0.0025854	0.00114994	Fkbp1b/Ppp1r15b/Rcan1
4-hour post-SCI group vs. the control group	BP	GO:0002573	myeloid leukocyte differentiation	4/35	0.0002168	0.0025854	0.00114994	Fos/Jun/Myc/Ripk1
4-hour post-SCI group vs. the control group	BP	GO:0036293	response to decreased oxygen levels	4/35	0.0002168	0.0025854	0.00114994	Cat/Hmox1/Myc/Slc8a1
4-hour post-SCI group vs. the control group	BP	GO:0002683	negative regulation of immune system process	5/35	0.0002285	0.00270751	0.00120425	Ezr/Hmox1/Hspb1/Myc/Tnfaip3
4-hour post-SCI group vs. the control group	BP	GO:0009408	response to heat	3/35	0.0002562	0.00299652	0.0013328	Hmox1/Hspb1/Il1a
4-hour post-SCI group vs. the control group	BP	GO:0032611	interleukin-1 beta production	3/35	0.0002562	0.00299652	0.0013328	Hspb1/Sphk1/Tnfaip3
4-hour post-SCI group vs. the control group	BP	GO:0010660	regulation of muscle cell apoptotic process	3/35	0.0002637	0.00306397	0.0013628	Atf4/Hmox1/Nr4a3
4-hour post-SCI group vs. the control group	BP	GO:0090594	inflammatory response to wounding	2/35	0.0002662	0.00307381	0.00136717	Hmox1/Il1a
4-hour post-SCI group vs. the control group	BP	GO:0048732	gland development	5/35	0.0002722	0.00312297	0.00138904	Hmox1/Jun/Nfkb1/Tnfaip3/Xdh
4-hour post-SCI group vs. the control group	BP	GO:0001704	formation of primary germ layer	3/35	0.0002869	0.00327077	0.00145478	Ets2/Nr4a3/Txnrd1
4-hour post-SCI group vs. the control group	BP	GO:1903209	positive regulation of oxidative stress-induced cell death	2/35	0.0002941	0.00331148	0.00147289	Mcl1/Ripk1
4-hour post-SCI group vs. the control group	BP	GO:0070167	regulation of biomineral tissue development	3/35	0.000295	0.00331148	0.00147289	Atf4/Rxrb/Slc8a1
4-hour post-SCI group vs. the control group	BP	GO:1901215	negative regulation of neuron death	4/35	0.000296	0.00331148	0.00147289	Atf4/Hmox1/Jun/Nr4a3
4-hour post-SCI group vs. the control group	BP	GO:1903522	regulation of blood circulation	4/35	0.0003003	0.00332913	0.00148074	Fkbp1b/Gch1/Hbegf/Slc8a1
4-hour post-SCI group vs. the control group	BP	GO:0060537	muscle tissue development	5/35	0.0003019	0.00332913	0.00148074	Fos/Myc/Rcan1/Rxrb/Slc8a1
4-hour post-SCI group vs. the control group	BP	GO:0010657	muscle cell apoptotic process	3/35	0.0003032	0.00332913	0.00148074	Atf4/Hmox1/Nr4a3
4-hour post-SCI group vs. the control group	BP	GO:0110149	regulation of biomineralization	3/35	0.0003115	0.00339806	0.0015114	Atf4/Rxrb/Slc8a1
4-hour post-SCI group vs. the control group	BP	GO:0051091	positive regulation of DNA-binding transcription factor activity	4/35	0.0003132	0.00339806	0.0015114	Cat/Fosl1/Ripk1/Sphk1
4-hour post-SCI group vs. the control group	BP	GO:0002274	myeloid leukocyte activation	4/35	0.0003176	0.00342413	0.00152299	Hmox1/Jun/Nr4a3/Sphk1
4-hour post-SCI group vs. the control group	BP	GO:0010595	positive regulation of endothelial cell migration	3/35	0.0003199	0.00342413	0.00152299	Hmox1/Hspb1/Map2k3
4-hour post-SCI group vs. the control group	BP	GO:0034312	diol biosynthetic process	2/35	0.0003232	0.00342413	0.00152299	Gch1/Sphk1
4-hour post-SCI group vs. the control group	BP	GO:1901522	positive regulation of transcription from RNA polymerase II promoter involved in cellular response to chemical stimulus	2/35	0.0003232	0.00342413	0.00152299	Atf4/Jun

4-hour post-SCI group vs. the control group	BP	GO:0002027	regulation of heart rate	3/35	0.0003462	0.0036254	0.00161251	Fkbp1b/Gch1/Slc8a1
4-hour post-SCI group vs. the control group	BP	GO:0045639	positive regulation of myeloid cell differentiation	3/35	0.0003462	0.0036254	0.00161251	Fos/Jun/Ripk1
4-hour post-SCI group vs. the control group	BP	GO:0010880	regulation of release of sequestered calcium ion into cytosol by sarcoplasmic reticulum	2/35	0.0003537	0.00368241	0.00163787	Fkbp1b/Slc8a1
4-hour post-SCI group vs. the control group	BP	GO:1904035	regulation of epithelial cell apoptotic process	3/35	0.0003739	0.00386989	0.00172126	Hmox1/Id1/Tnfaip3
4-hour post-SCI group vs. the control group	BP	GO:0006939	smooth muscle contraction	3/35	0.0003834	0.00387958	0.00172557	Fkbp1b/Slc8a1/Sphk1
4-hour post-SCI group vs. the control group	BP	GO:0032652	regulation of interleukin-1 production	3/35	0.0003834	0.00387958	0.00172557	Hspb1/Sphk1/Tnfaip3
4-hour post-SCI group vs. the control group	BP	GO:0044070	regulation of anion transport	3/35	0.0003834	0.00387958	0.00172557	Atf4/Il1a/Ripk1
4-hour post-SCI group vs. the control group	BP	GO:1903204	negative regulation of oxidative stress-induced neuron death	2/35	0.0003856	0.00387958	0.00172557	Atf4/Nr4a3
4-hour post-SCI group vs. the control group	BP	GO:2000637	positive regulation of miRNA-mediated gene silencing	2/35	0.0003856	0.00387958	0.00172557	Nfkb1/Ripk1
4-hour post-SCI group vs. the control group	BP	GO:0014808	release of sequestered calcium ion into cytosol by sarcoplasmic reticulum	2/35	0.0004188	0.00412163	0.00183323	Fkbp1b/Slc8a1
4-hour post-SCI group vs. the control group	BP	GO:0060148	positive regulation of post-transcriptional gene silencing	2/35	0.0004188	0.00412163	0.00183323	Nfkb1/Ripk1
4-hour post-SCI group vs. the control group	BP	GO:0098869	cellular oxidant detoxification	2/35	0.0004188	0.00412163	0.00183323	Cat/Gch1
4-hour post-SCI group vs. the control group	BP	GO:1900370	positive regulation of post-transcriptional gene silencing by RNA	2/35	0.0004188	0.00412163	0.00183323	Nfkb1/Ripk1
4-hour post-SCI group vs. the control group	BP	GO:0007498	mesoderm development	3/35	0.0004334	0.00421878	0.00187644	Ets2/Nr4a3/Txnrd1
4-hour post-SCI group vs. the control group	BP	GO:0043534	blood vessel endothelial cell migration	3/35	0.0004334	0.00421878	0.00187644	Hmox1/Hspb1/Map2k3
4-hour post-SCI group vs. the control group	BP	GO:0032612	interleukin-1 production	3/35	0.0004438	0.00428517	0.00190597	Hspb1/Sphk1/Tnfaip3
4-hour post-SCI group vs. the control group	BP	GO:0006883	cellular sodium ion homeostasis	2/35	0.0004534	0.00428517	0.00190597	Il1a/Slc8a1
4-hour post-SCI group vs. the control group	BP	GO:0010575	positive regulation of vascular endothelial growth factor production	2/35	0.0004534	0.00428517	0.00190597	Atf4/Il1a
4-hour post-SCI group vs. the control group	BP	GO:0032703	negative regulation of interleukin-2 production	2/35	0.0004534	0.00428517	0.00190597	Ezr/Tnfaip3
4-hour post-SCI group vs. the control group	BP	GO:1903514	release of sequestered calcium ion into cytosol by endoplasmic reticulum	2/35	0.0004534	0.00428517	0.00190597	Fkbp1b/Slc8a1
4-hour post-SCI group vs. the control group	BP	GO:0048145	regulation of fibroblast proliferation	3/35	0.0004545	0.00428517	0.00190597	Jun/Myc/Sphk1
4-hour post-SCI group vs. the control group	BP	GO:0006352	DNA-templated transcription initiation	3/35	0.0004652	0.00436406	0.00194106	Fosl1/Jun/Myc
4-hour post-SCI group vs. the control group	BP	GO:1902175	regulation of oxidative stress-induced intrinsic apoptotic signaling pathway	2/35	0.0004893	0.00454266	0.0020205	Hspb1/Mcl1
4-hour post-SCI group vs. the control group	BP	GO:0070482	response to oxygen levels	4/35	0.0004893	0.00454266	0.0020205	Cat/Hmox1/Myc/Slc8a1
4-hour post-SCI group vs. the control group	BP	GO:0071496	cellular response to external stimulus	4/35	0.0005077	0.00468868	0.00208544	Atf4/Fos/Fosl1/Jun
4-hour post-SCI group vs. the control group	BP	GO:0019932	second-messenger-mediated signaling	4/35	0.0005139	0.0047219	0.00210022	Fkbp1b/Rcan1/Slc8a1/Sphk1
4-hour post-SCI group vs. the control group	BP	GO:0030282	bone mineralization	3/35	0.0005334	0.00487685	0.00216914	Atf4/Rxrb/Slc8a1
4-hour post-SCI group vs. the control group	BP	GO:0060048	cardiac muscle contraction	3/35	0.0005454	0.00496096	0.00220655	Fkbp1b/Map2k3/Slc8a1
4-hour post-SCI group vs. the control group	BP	GO:0035195	miRNA-mediated gene silencing	3/35	0.0005575	0.00504576	0.00224427	Ago1/Nfkb1/Ripk1
4-hour post-SCI group vs. the control group	BP	GO:1900745	positive regulation of p38MAPK cascade	2/35	0.0005651	0.00506785	0.00225409	Sphk1/Xdh
4-hour post-SCI group vs. the control group	BP	GO:0006936	muscle contraction	4/35	0.0005656	0.00506785	0.00225409	Fkbp1b/Map2k3/Slc8a1/Sphk1
4-hour post-SCI group vs. the control group	BP	GO:0035303	regulation of dephosphorylation	3/35	0.0005949	0.00526232	0.00234059	Fkbp1b/Ppp1r15b/Rcan1
4-hour post-SCI group vs. the control group	BP	GO:0034311	diol metabolic process	2/35	0.000605	0.00526232	0.00234059	Gch1/Sphk1

4-hour post-SCI group vs. the control group	BP	GO:0034390	smooth muscle cell apoptotic process	2/35	0.000605	0.00526232	0.00234059	Atf4/Nr4a3
4-hour post-SCI group vs. the control group	BP	GO:0034391	regulation of smooth muscle cell apoptotic process	2/35	0.000605	0.00526232	0.00234059	Atf4/Nr4a3
4-hour post-SCI group vs. the control group	BP	GO:1902042	negative regulation of extrinsic apoptotic signaling pathway via death domain receptors	2/35	0.000605	0.00526232	0.00234059	Hmox1/Tnfaip3
4-hour post-SCI group vs. the control group	BP	GO:0001889	liver development	3/35	0.0006078	0.00526232	0.00234059	Hmox1/Jun/Tnfaip3
4-hour post-SCI group vs. the control group	BP	GO:0001959	regulation of cytokine-mediated signaling pathway	3/35	0.0006078	0.00526232	0.00234059	Ripk1/Sphk1/Tnfrsf1a
4-hour post-SCI group vs. the control group	BP	GO:0035094	response to nicotine	2/35	0.0006462	0.00552472	0.0024573	Hmox1/Nfkb1
4-hour post-SCI group vs. the control group	BP	GO:0071354	cellular response to interleukin-6	2/35	0.0006462	0.00552472	0.0024573	Nfkb1/Ripk1
4-hour post-SCI group vs. the control group	BP	GO:0061008	hepaticobiliary system development	3/35	0.0006473	0.00552472	0.0024573	Hmox1/Jun/Tnfaip3
4-hour post-SCI group vs. the control group	BP	GO:0035194	post-transcriptional gene silencing by RNA	3/35	0.0006608	0.0055872	0.00248509	Ago1/Nfkb1/Ripk1
4-hour post-SCI group vs. the control group	BP	GO:0046165	alcohol biosynthetic process	3/35	0.0006608	0.0055872	0.00248509	Gch1/Nfkb1/Sphk1
4-hour post-SCI group vs. the control group	BP	GO:0051146	striated muscle cell differentiation	4/35	0.0006876	0.00563832	0.00250782	Myc/Rcan1/Rxrb/Slc8a1
4-hour post-SCI group vs. the control group	BP	GO:0010574	regulation of vascular endothelial growth factor production	2/35	0.0006887	0.00563832	0.00250782	Atf4/Il1a
4-hour post-SCI group vs. the control group	BP	GO:0030224	monocyte differentiation	2/35	0.0006887	0.00563832	0.00250782	Jun/Myc
4-hour post-SCI group vs. the control group	BP	GO:0070296	sarcoplasmic reticulum calcium ion transport	2/35	0.0006887	0.00563832	0.00250782	Fkbp1b/Slc8a1
4-hour post-SCI group vs. the control group	BP	GO:1901099	negative regulation of signal transduction in absence of ligand	2/35	0.0006887	0.00563832	0.00250782	Mcl1/Ripk1
4-hour post-SCI group vs. the control group	BP	GO:2000352	negative regulation of endothelial cell apoptotic process	2/35	0.0006887	0.00563832	0.00250782	Id1/Tnfaip3
4-hour post-SCI group vs. the control group	BP	GO:2001240	negative regulation of extrinsic apoptotic signaling pathway in absence of ligand	2/35	0.0006887	0.00563832	0.00250782	Mcl1/Ripk1
4-hour post-SCI group vs. the control group	BP	GO:0048144	fibroblast proliferation	3/35	0.0007167	0.00584051	0.00259775	Jun/Myc/Sphk1
4-hour post-SCI group vs. the control group	BP	GO:0050999	regulation of nitric-oxide synthase activity	2/35	0.0007326	0.00591685	0.00263171	Gch1/Il1a
4-hour post-SCI group vs. the control group	BP	GO:0070741	response to interleukin-6	2/35	0.0007326	0.00591685	0.00263171	Nfkb1/Ripk1
4-hour post-SCI group vs. the control group	BP	GO:0050728	negative regulation of inflammatory response	3/35	0.0007457	0.00596918	0.00265498	Nfkb1/Tnfaip3/Tnfrsf1a
4-hour post-SCI group vs. the control group	BP	GO:0060759	regulation of response to cytokine stimulus	3/35	0.0007457	0.00596918	0.00265498	Ripk1/Sphk1/Tnfrsf1a
4-hour post-SCI group vs. the control group	BP	GO:0071887	leukocyte apoptotic process	3/35	0.0007605	0.00603401	0.00268382	Myc/Nr4a3/Ripk1
4-hour post-SCI group vs. the control group	BP	GO:1904019	epithelial cell apoptotic process	3/35	0.0007605	0.00603401	0.00268382	Hmox1/Id1/Tnfaip3
4-hour post-SCI group vs. the control group	BP	GO:0016441	post-transcriptional gene silencing	3/35	0.0007755	0.00609069	0.00270903	Ago1/Nfkb1/Ripk1
4-hour post-SCI group vs. the control group	BP	GO:1903205	regulation of hydrogen peroxide-induced cell death	2/35	0.0007778	0.00609069	0.00270903	Nr4a3/Ripk1
4-hour post-SCI group vs. the control group	BP	GO:2000108	positive regulation of leukocyte apoptotic process	2/35	0.0007778	0.00609069	0.00270903	Myc/Nr4a3
4-hour post-SCI group vs. the control group	BP	GO:0070371	ERK1 and ERK2 cascade	4/35	0.0008186	0.00638221	0.00283869	Ezr/Il1a/Jun/Myc
4-hour post-SCI group vs. the control group	BP	GO:0031099	regeneration	3/35	0.0008694	0.0067127	0.00298569	Fkbp1b/Hmox1/Jun
4-hour post-SCI group vs. the control group	BP	GO:0051092	positive regulation of NF-kappaB transcription factor activity	3/35	0.0008694	0.0067127	0.00298569	Cat/Ripk1/Sphk1
4-hour post-SCI group vs. the control group	BP	GO:0010573	vascular endothelial growth factor production	2/35	0.0008722	0.0067127	0.00298569	Atf4/Il1a
4-hour post-SCI group vs. the control group	BP	GO:0008643	carbohydrate transport	3/35	0.0008857	0.00675919	0.00300637	Ezr/Myc/Nr4a3
4-hour post-SCI group vs. the control group	BP	GO:2001235	positive regulation of apoptotic signaling pathway	3/35	0.0008857	0.00675919	0.00300637	Mcl1/Myc/Ripk1
4-hour post-SCI group vs. the control group	BP	GO:0006066	alcohol metabolic process	4/35	0.0008995	0.00683577	0.00304043	Cat/Gch1/Nfkb1/Sphk1

4-hour post-SCI group vs. the control group	BP	GO:1903532	positive regulation of secretion by cell	4/35	0.0009566	0.00723845	0.00321953	Ezr/Il1a/Sdc1/Sphk1
4-hour post-SCI group vs. the control group	BP	GO:2000377	regulation of reactive oxygen species metabolic process	3/35	0.0009702	0.00729219	0.00324343	Gch1/Ripk1/Xdh
4-hour post-SCI group vs. the control group	BP	GO:0036474	cell death in response to hydrogen peroxide	2/35	0.0009718	0.00729219	0.00324343	Nr4a3/Ripk1
4-hour post-SCI group vs. the control group	BP	GO:0071320	cellular response to cAMP	2/35	0.0010235	0.00764866	0.00340199	Ezr/Slc8a1
4-hour post-SCI group vs. the control group	BP	GO:1904707	positive regulation of vascular associated smooth muscle cell proliferation	2/35	0.0010766	0.00799073	0.00355414	Jun/Nr4a3
4-hour post-SCI group vs. the control group	BP	GO:0006937	regulation of muscle contraction	3/35	0.0010782	0.00799073	0.00355414	Fkbp1b/Slc8a1/Sphk1
4-hour post-SCI group vs. the control group	BP	GO:0042060	wound healing	4/35	0.0011102	0.00819431	0.00364468	Hbegf/Hmox1/Il1a/Sdc1
4-hour post-SCI group vs. the control group	BP	GO:0071248	cellular response to metal ion	3/35	0.0011349	0.00834233	0.00371052	Fos/Hmox1/Jun
4-hour post-SCI group vs. the control group	BP	GO:0001774	microglial cell activation	2/35	0.0011865	0.00865172	0.00384813	Jun/Sphk1
4-hour post-SCI group vs. the control group	BP	GO:0038084	vascular endothelial growth factor signaling pathway	2/35	0.0011865	0.00865172	0.00384813	Hspb1/Xdh
4-hour post-SCI group vs. the control group	BP	GO:0006941	striated muscle contraction	3/35	0.0011934	0.00866667	0.00385478	Fkbp1b/Map2k3/Slc8a1
4-hour post-SCI group vs. the control group	BP	GO:0019216	regulation of lipid metabolic process	4/35	0.0011988	0.008671	0.00385671	Il1a/Nfkb1/Nr4a3/Sphk1
4-hour post-SCI group vs. the control group	BP	GO:0001936	regulation of endothelial cell proliferation	3/35	0.0012133	0.00870612	0.00387233	Hmox1/Jun/Xdh
4-hour post-SCI group vs. the control group	BP	GO:0009266	response to temperature stimulus	3/35	0.0012133	0.00870612	0.00387233	Hmox1/Hspb1/Il1a
4-hour post-SCI group vs. the control group	BP	GO:0006984	ER-nucleus signaling pathway	2/35	0.0012435	0.00885185	0.00393714	Atf4/Ppp1r15b
4-hour post-SCI group vs. the control group	BP	GO:1990748	cellular detoxification	2/35	0.0012435	0.00885185	0.00393714	Cat/Gch1
4-hour post-SCI group vs. the control group	BP	GO:0010594	regulation of endothelial cell migration	3/35	0.0012538	0.00889045	0.00395431	Hmox1/Hspb1/Map2k3
4-hour post-SCI group vs. the control group	BP	GO:0007254	JNK cascade	3/35	0.0012744	0.00893079	0.00397226	Il1a/Nfkb1/Ripk1
4-hour post-SCI group vs. the control group	BP	GO:1903531	negative regulation of secretion by cell	3/35	0.0012744	0.00893079	0.00397226	Fkbp1b/Hmox1/Tnfrsf1a
4-hour post-SCI group vs. the control group	BP	GO:2001242	regulation of intrinsic apoptotic signaling pathway	3/35	0.0012744	0.00893079	0.00397226	Hspb1/Mcl1/Myc
4-hour post-SCI group vs. the control group	BP	GO:0045840	positive regulation of mitotic nuclear division	2/35	0.0013017	0.00905167	0.00402602	Il1a/Sphk1
4-hour post-SCI group vs. the control group	BP	GO:0060964	regulation of miRNA-mediated gene silencing	2/35	0.0013017	0.00905167	0.00402602	Nfkb1/Ripk1
4-hour post-SCI group vs. the control group	BP	GO:0031214	biomineral tissue development	3/35	0.0013162	0.00911706	0.00405511	Atf4/Rxrb/Slc8a1
4-hour post-SCI group vs. the control group	BP	GO:0001890	placenta development	3/35	0.0013374	0.00922845	0.00410465	Fosl1/Rxrb/Slc8a1
4-hour post-SCI group vs. the control group	BP	GO:0008631	intrinsic apoptotic signaling pathway in response to oxidative stress	2/35	0.0013612	0.00932154	0.00414605	Hspb1/Mcl1
4-hour post-SCI group vs. the control group	BP	GO:2001239	regulation of extrinsic apoptotic signaling pathway in absence of ligand	2/35	0.0013612	0.00932154	0.00414605	Mcl1/Ripk1
4-hour post-SCI group vs. the control group	BP	GO:0002269	leukocyte activation involved in inflammatory response	2/35	0.001422	0.00959197	0.00426634	Jun/Sphk1
4-hour post-SCI group vs. the control group	BP	GO:0030501	positive regulation of bone mineralization	2/35	0.001422	0.00959197	0.00426634	Rxrb/Slc8a1
4-hour post-SCI group vs. the control group	BP	GO:0060147	regulation of post-transcriptional gene silencing	2/35	0.001422	0.00959197	0.00426634	Nfkb1/Ripk1
4-hour post-SCI group vs. the control group	BP	GO:1900368	regulation of post-transcriptional gene silencing by RNA	2/35	0.001422	0.00959197	0.00426634	Nfkb1/Ripk1
4-hour post-SCI group vs. the control group	BP	GO:0051098	regulation of binding	4/35	0.001442	0.00969077	0.00431028	Hmox1/Id1/Jun/Myc
4-hour post-SCI group vs. the control group	BP	GO:0120163	negative regulation of cold-induced thermogenesis	2/35	0.0014841	0.00993626	0.00441947	Atf4/Id1
4-hour post-SCI group vs. the control group	BP	GO:0110148	biomineralization	3/35	0.0015148	0.01010427	0.0044942	Atf4/Rxrb/Slc8a1
4-hour post-SCI group vs. the control group	BP	GO:0001659	temperature homeostasis	3/35	0.001538	0.01022092	0.00454608	Atf4/Id1/Il1a

4-hour post-SCI group vs. the control group	BP	GO:0043124	negative regulation of I-kappaB kinase/NF-kappaB signaling	2/35	0.0015474	0.01024615	0.0045573	Ripk1/Tnfaip3
4-hour post-SCI group vs. the control group	BP	GO:0001935	endothelial cell proliferation	3/35	0.001585	0.01045604	0.00465066	Hmox1/Jun/Xdh
4-hour post-SCI group vs. the control group	BP	GO:0009991	response to extracellular stimulus	4/35	0.0016035	0.01047881	0.00466079	Atf4/Fos/Fosl1/Jun
4-hour post-SCI group vs. the control group	BP	GO:0051047	positive regulation of secretion	4/35	0.0016035	0.01047881	0.00466079	Ezr/Il1a/Sdc1/Sphk1
4-hour post-SCI group vs. the control group	BP	GO:0060966	regulation of gene silencing by RNA	2/35	0.0016121	0.01047881	0.00466079	Nfkb1/Ripk1
4-hour post-SCI group vs. the control group	BP	GO:0061900	glial cell activation	2/35	0.0016121	0.01047881	0.00466079	Jun/Sphk1
4-hour post-SCI group vs. the control group	BP	GO:0019221	cytokine-mediated signaling pathway	4/35	0.0016175	0.01047881	0.00466079	Il1a/Ripk1/Sphk1/Tnfrsf1a
4-hour post-SCI group vs. the control group	BP	GO:0031047	gene silencing by RNA	3/35	0.0016571	0.01069701	0.00475784	Ago1/Nfkb1/Ripk1
4-hour post-SCI group vs. the control group	BP	GO:0097237	cellular response to toxic substance	2/35	0.001678	0.01077793	0.00479383	Cat/Gch1
4-hour post-SCI group vs. the control group	BP	GO:0043524	negative regulation of neuron apoptotic process	3/35	0.0016816	0.01077793	0.00479383	Hmox1/Jun/Nr4a3
4-hour post-SCI group vs. the control group	BP	GO:0001503	ossification	4/35	0.0016888	0.0107854	0.00479715	Atf4/Id1/Rxrb/Slc8a1
4-hour post-SCI group vs. the control group	BP	GO:1902041	regulation of extrinsic apoptotic signaling pathway via death domain receptors	2/35	0.0017451	0.01110597	0.00493974	Hmox1/Tnfaip3
4-hour post-SCI group vs. the control group	BP	GO:0051302	regulation of cell division	3/35	0.0017565	0.0111389	0.00495438	Cat/Il1a/Myc
4-hour post-SCI group vs. the control group	BP	GO:0035196	miRNA processing	2/35	0.0018136	0.0114204	0.00507959	Ago1/Ripk1
4-hour post-SCI group vs. the control group	BP	GO:0061028	establishment of endothelial barrier	2/35	0.0018136	0.0114204	0.00507959	Ezr/Tnfrsf1a
4-hour post-SCI group vs. the control group	BP	GO:0002695	negative regulation of leukocyte activation	3/35	0.0018595	0.01162854	0.00517217	Hmox1/Hspb1/Tnfaip3
4-hour post-SCI group vs. the control group	BP	GO:0006469	negative regulation of protein kinase activity	3/35	0.0018595	0.01162854	0.00517217	Hspb1/Slc8a1/Tnfaip3
4-hour post-SCI group vs. the control group	BP	GO:0031103	axon regeneration	2/35	0.0018833	0.01169569	0.00520203	Fkbp1b/Jun
4-hour post-SCI group vs. the control group	BP	GO:0034605	cellular response to heat	2/35	0.0018833	0.01169569	0.00520203	Hmox1/Il1a
4-hour post-SCI group vs. the control group	BP	GO:0046890	regulation of lipid biosynthetic process	3/35	0.0019392	0.01193062	0.00530653	Il1a/Nfkb1/Sphk1
4-hour post-SCI group vs. the control group	BP	GO:0006692	prostanoid metabolic process	2/35	0.0019542	0.01193062	0.00530653	Sphk1/Tnfrsf1a
4-hour post-SCI group vs. the control group	BP	GO:0006693	prostaglandin metabolic process	2/35	0.0019542	0.01193062	0.00530653	Sphk1/Tnfrsf1a
4-hour post-SCI group vs. the control group	BP	GO:0032715	negative regulation of interleukin-6 production	2/35	0.0019542	0.01193062	0.00530653	Tnfaip3/Tnfrsf1a
4-hour post-SCI group vs. the control group	BP	GO:2000351	regulation of endothelial cell apoptotic process	2/35	0.0019542	0.01193062	0.00530653	Id1/Tnfaip3
4-hour post-SCI group vs. the control group	BP	GO:0051048	negative regulation of secretion	3/35	0.0019663	0.01196374	0.00532126	Fkbp1b/Hmox1/Tnfrsf1a
4-hour post-SCI group vs. the control group	BP	GO:0030099	myeloid cell differentiation	4/35	0.0019797	0.01200457	0.00533942	Fos/Jun/Myc/Ripk1
4-hour post-SCI group vs. the control group	BP	GO:0002700	regulation of production of molecular mediator of immune response	3/35	0.0019935	0.01200797	0.00534093	Hmox1/Nr4a3/Tnfaip3
4-hour post-SCI group vs. the control group	BP	GO:0007519	skeletal muscle tissue development	3/35	0.0019935	0.01200797	0.00534093	Fos/Myc/Rcan1
4-hour post-SCI group vs. the control group	BP	GO:0042692	muscle cell differentiation	4/35	0.0020451	0.01227712	0.00546064	Myc/Rcan1/Rxrb/Slc8a1
4-hour post-SCI group vs. the control group	BP	GO:1902107	positive regulation of leukocyte differentiation	3/35	0.0020768	0.01235322	0.00549449	Fos/Jun/Ripk1
4-hour post-SCI group vs. the control group	BP	GO:1903708	positive regulation of hemopoiesis	3/35	0.0020768	0.01235322	0.00549449	Fos/Jun/Ripk1
4-hour post-SCI group vs. the control group	BP	GO:0016055	Wnt signaling pathway	4/35	0.0020783	0.01235322	0.00549449	Myc/Nfkb1/Sdc1/Tnfaip3
4-hour post-SCI group vs. the control group	BP	GO:0070918	small regulatory ncRNA processing	2/35	0.0020999	0.0124404	0.00553327	Ago1/Ripk1
4-hour post-SCI group vs. the control group	BP	GO:0198738	cell-cell signaling by wnt	4/35	0.0021119	0.01247078	0.00554678	Myc/Nfkb1/Sdc1/Tnfaip3
4-hour post-SCI group vs. the control group	BP	GO:0051656	establishment of organelle localization	4/35	0.0021459	0.01263016	0.00561767	Ezr/Hmox1/Nr4a3/Sdc1
4-hour post-SCI group vs. the control group	BP	GO:0051055	negative regulation of lipid biosynthetic process	2/35	0.0021746	0.01271571	0.00565572	Nfkb1/Sphk1
4-hour post-SCI group vs. the control group	BP	GO:0072577	endothelial cell apoptotic process	2/35	0.0021746	0.01271571	0.00565572	Id1/Tnfaip3

4-hour post-SCI group vs. the control group	BP	GO:0032103	positive regulation of response to external stimulus	4/35	0.0022151	0.01291053	0.00574237	Fkbp1b/Hspb1/Ripk1/Tnfrsf1a
4-hour post-SCI group vs. the control group	BP	GO:0098754	detoxification	2/35	0.0022505	0.01307496	0.00581551	Cat/Gch1
4-hour post-SCI group vs. the control group	BP	GO:1903793	positive regulation of anion transport	2/35	0.0023278	0.01348	0.00599566	Atf4/Il1a
4-hour post-SCI group vs. the control group	BP	GO:0060538	skeletal muscle organ development	3/35	0.0023394	0.01350386	0.00600628	Fos/Myc/Rcan1
4-hour post-SCI group vs. the control group	BP	GO:0042391	regulation of membrane potential	4/35	0.0023947	0.01375733	0.00611901	Fkbp1b/Jun/Myc/Slc8a1
4-hour post-SCI group vs. the control group	BP	GO:0051591	response to cAMP	2/35	0.0024062	0.01375733	0.00611901	Ezr/Slc8a1
4-hour post-SCI group vs. the control group	BP	GO:0055078	sodium ion homeostasis	2/35	0.0024062	0.01375733	0.00611901	Il1a/Slc8a1
4-hour post-SCI group vs. the control group	BP	GO:0033003	regulation of mast cell activation	2/35	0.0024859	0.01416793	0.00630164	Hmox1/Nr4a3
4-hour post-SCI group vs. the control group	BP	GO:0050866	negative regulation of cell activation	3/35	0.0025254	0.01434764	0.00638157	Hmox1/Hspb1/Tnfaip3
4-hour post-SCI group vs. the control group	BP	GO:0033673	negative regulation of kinase activity	3/35	0.0025572	0.01440125	0.00640542	Hspb1/Slc8a1/Tnfaip3
4-hour post-SCI group vs. the control group	BP	GO:0031102	neuron projection regeneration	2/35	0.0025668	0.01440125	0.00640542	Fkbp1b/Jun
4-hour post-SCI group vs. the control group	BP	GO:0045778	positive regulation of ossification	2/35	0.0025668	0.01440125	0.00640542	Rxrb/Slc8a1
4-hour post-SCI group vs. the control group	BP	GO:0051785	positive regulation of nuclear division	2/35	0.0025668	0.01440125	0.00640542	Il1a/Sphk1
4-hour post-SCI group vs. the control group	BP	GO:0010976	positive regulation of neuron projection development	3/35	0.0025894	0.0144827	0.00644164	Fkbp1b/Hspb1/Sphk1
4-hour post-SCI group vs. the control group	BP	GO:0046173	polyol biosynthetic process	2/35	0.0026489	0.01477013	0.00656949	Gch1/Sphk1
4-hour post-SCI group vs. the control group	BP	GO:0014911	positive regulation of smooth muscle cell migration	2/35	0.0027323	0.0151413	0.00673458	Myc/Nr4a3
4-hour post-SCI group vs. the control group	BP	GO:0061077	chaperone-mediated protein folding	2/35	0.0027323	0.0151413	0.00673458	Fkbp1b/Hspb1
4-hour post-SCI group vs. the control group	BP	GO:0010656	negative regulation of muscle cell apoptotic process	2/35	0.0028169	0.01549063	0.00688996	Hmox1/Nr4a3
4-hour post-SCI group vs. the control group	BP	GO:0043542	endothelial cell migration	3/35	0.0028212	0.01549063	0.00688996	Hmox1/Hspb1/Map2k3
4-hour post-SCI group vs. the control group	BP	GO:1901617	organic hydroxy compound biosynthetic process	3/35	0.0028212	0.01549063	0.00688996	Gch1/Nfkb1/Sphk1
4-hour post-SCI group vs. the control group	BP	GO:0031330	negative regulation of cellular catabolic process	3/35	0.0028897	0.01581857	0.00703582	Hmox1/Mcl1/Tnfaip3
4-hour post-SCI group vs. the control group	BP	GO:0042098	T cell proliferation	3/35	0.0029243	0.01595967	0.00709858	Fkbp1b/Il1a/Myc
4-hour post-SCI group vs. the control group	BP	GO:0022407	regulation of cell-cell adhesion	4/35	0.0029506	0.01605462	0.00714081	Hspb1/Il1a/Nr4a3/Tnfaip3
4-hour post-SCI group vs. the control group	BP	GO:0001707	mesoderm formation	2/35	0.0029898	0.01621885	0.00721386	Nr4a3/Txnrd1
4-hour post-SCI group vs. the control group	BP	GO:0006820	anion transport	4/35	0.003015	0.0163064	0.00725279	Atf4/Il1a/Myc/Ripk1
4-hour post-SCI group vs. the control group	BP	GO:0071241	cellular response to inorganic substance	3/35	0.0030654	0.01649891	0.00733842	Fos/Hmox1/Jun
4-hour post-SCI group vs. the control group	BP	GO:0006879	cellular iron ion homeostasis	2/35	0.0030781	0.01649891	0.00733842	Hmox1/Myc
4-hour post-SCI group vs. the control group	BP	GO:0061082	myeloid leukocyte cytokine production	2/35	0.0030781	0.01649891	0.00733842	Hmox1/Nr4a3
4-hour post-SCI group vs. the control group	BP	GO:1901224	positive regulation of NIK/NF-kappaB signaling	2/35	0.0031676	0.0169122	0.00752224	Ago1/Sphk1
4-hour post-SCI group vs. the control group	BP	GO:0031348	negative regulation of defense response	3/35	0.003174	0.0169122	0.00752224	Nfkb1/Tnfaip3/Tnfrsf1a
4-hour post-SCI group vs. the control group	BP	GO:0045333	cellular respiration	3/35	0.0032107	0.01705729	0.00758678	Cat/Myc/Nr4a3
4-hour post-SCI group vs. the control group	BP	GO:0150076	neuroinflammatory response	2/35	0.0032583	0.01725923	0.0076766	Jun/Sphk1
4-hour post-SCI group vs. the control group	BP	GO:0043303	mast cell degranulation	2/35	0.0033502	0.01753971	0.00780135	Hmox1/Nr4a3
4-hour post-SCI group vs. the control group	BP	GO:0048332	mesoderm morphogenesis	2/35	0.0033502	0.01753971	0.00780135	Nr4a3/Txnrd1
4-hour post-SCI group vs. the control group	BP	GO:0055117	regulation of cardiac muscle contraction	2/35	0.0033502	0.01753971	0.00780135	Fkbp1b/Slc8a1
4-hour post-SCI group vs. the control group	BP	GO:2001244	positive regulation of intrinsic apoptotic signaling pathway	2/35	0.0033502	0.01753971	0.00780135	Mcl1/Myc
4-hour post-SCI group vs. the control group	BP	GO:0032922	circadian regulation of gene expression	2/35	0.0034433	0.01781999	0.00792601	Atf4/Id1
4-hour post-SCI group vs. the control group	BP	GO:0048678	response to axon injury	2/35	0.0034433	0.01781999	0.00792601	Fkbp1b/Jun
4-hour post-SCI group vs. the control group	BP	GO:0071868	cellular response to monoamine stimulus	2/35	0.0034433	0.01781999	0.00792601	Atf4/Nr4a3

4-hour post-SCI group vs. the control group	BP	GO:0071870	cellular response to catecholamine stimulus	2/35	0.0034433	0.01781999	0.00792601	Atf4/Nr4a3
4-hour post-SCI group vs. the control group	BP	GO:0006470	protein dephosphorylation	3/35	0.0034749	0.01793216	0.0079759	Fkbp1b/Ppp1r15b/Rcan1
4-hour post-SCI group vs. the control group	BP	GO:0002448	mast cell mediated immunity	2/35	0.0035376	0.01815162	0.00807352	Hmox1/Nr4a3
4-hour post-SCI group vs. the control group	BP	GO:0048662	negative regulation of smooth muscle cell proliferation	2/35	0.0035376	0.01815162	0.00807352	Hmox1/Tnfaip3
4-hour post-SCI group vs. the control group	BP	GO:0002279	mast cell activation involved in immune response	2/35	0.0036331	0.01858877	0.00826795	Hmox1/Nr4a3
4-hour post-SCI group vs. the control group	BP	GO:0034976	response to endoplasmic reticulum stress	3/35	0.0036716	0.01873243	0.00833185	Atf4/Jun/Ppp1r15b
4-hour post-SCI group vs. the control group	BP	GO:0010611	regulation of cardiac muscle hypertrophy	2/35	0.0037298	0.01886918	0.00839267	Nr4a3/Tnfrsf1a
4-hour post-SCI group vs. the control group	BP	GO:0071867	response to monoamine	2/35	0.0037298	0.01886918	0.00839267	Atf4/Nr4a3
4-hour post-SCI group vs. the control group	BP	GO:0071869	response to catecholamine	2/35	0.0037298	0.01886918	0.00839267	Atf4/Nr4a3
4-hour post-SCI group vs. the control group	BP	GO:0051348	negative regulation of transferase activity	3/35	0.003875	0.01954856	0.00869485	Hspb1/Slc8a1/Tnfaip3
4-hour post-SCI group vs. the control group	BP	GO:0006919	activation of cysteine-type endopeptidase activity involved in apoptotic process	2/35	0.0040271	0.02014685	0.00896096	Myc/Xdh
4-hour post-SCI group vs. the control group	BP	GO:0014743	regulation of muscle hypertrophy	2/35	0.0040271	0.02014685	0.00896096	Nr4a3/Tnfrsf1a
4-hour post-SCI group vs. the control group	BP	GO:0071277	cellular response to calcium ion	2/35	0.0040271	0.02014685	0.00896096	Fos/Jun
4-hour post-SCI group vs. the control group	BP	GO:0043627	response to estrogen	2/35	0.0041286	0.02054041	0.00913601	Hmox1/Rcan1
4-hour post-SCI group vs. the control group	BP	GO:1903036	positive regulation of response to wounding	2/35	0.0041286	0.02054041	0.00913601	Fkbp1b/Hbegf
4-hour post-SCI group vs. the control group	BP	GO:0032088	negative regulation of NF-kappaB transcription factor activity	2/35	0.0043351	0.02150836	0.00956654	Cat/Tnfaip3
4-hour post-SCI group vs. the control group	BP	GO:0043279	response to alkaloid	2/35	0.0044401	0.0219087	0.0097446	Myc/Slc8a1
4-hour post-SCI group vs. the control group	BP	GO:0051279	regulation of release of sequestered calcium ion into cytosol	2/35	0.0044401	0.0219087	0.0097446	Fkbp1b/Slc8a1
4-hour post-SCI group vs. the control group	BP	GO:0008625	extrinsic apoptotic signaling pathway via death domain receptors	2/35	0.0045463	0.02231043	0.00992328	Hmox1/Tnfaip3
4-hour post-SCI group vs. the control group	BP	GO:0050810	regulation of steroid biosynthetic process	2/35	0.0045463	0.02231043	0.00992328	Il1a/Nfkb1
4-hour post-SCI group vs. the control group	BP	GO:0045913	positive regulation of carbohydrate metabolic process	2/35	0.0046537	0.02277526	0.01013003	Myc/Nfkb1
4-hour post-SCI group vs. the control group	BP	GO:0010506	regulation of autophagy	3/35	0.0048987	0.02390952	0.01063453	Hmox1/Mcl1/Tnfaip3
4-hour post-SCI group vs. the control group	BP	GO:0006942	regulation of striated muscle contraction	2/35	0.0049828	0.02425408	0.01078778	Fkbp1b/Slc8a1
4-hour post-SCI group vs. the control group	BP	GO:0030500	regulation of bone mineralization	2/35	0.0050948	0.02473253	0.01100059	Rxrb/Slc8a1
4-hour post-SCI group vs. the control group	BP	GO:0010827	regulation of glucose transmembrane transport	2/35	0.005208	0.02521401	0.01121474	Myc/Nr4a3
4-hour post-SCI group vs. the control group	BP	GO:0006874	cellular calcium ion homeostasis	3/35	0.0052397	0.02529951	0.01125277	Atf4/Fkbp1b/Slc8a1
4-hour post-SCI group vs. the control group	BP	GO:0046683	response to organophosphorus	2/35	0.0054378	0.02618593	0.01164703	Ezr/Slc8a1
4-hour post-SCI group vs. the control group	BP	GO:0055072	iron ion homeostasis	2/35	0.0056723	0.02724199	0.01211675	Hmox1/Myc
4-hour post-SCI group vs. the control group	BP	GO:0035914	skeletal muscle cell differentiation	2/35	0.0057912	0.02773922	0.01233791	Fos/Myc
4-hour post-SCI group vs. the control group	BP	GO:0050708	regulation of protein secretion	3/35	0.0059103	0.02823446	0.01255818	Ezr/Fkbp1b/Il1a
4-hour post-SCI group vs. the control group	BP	GO:0032091	negative regulation of protein binding	2/35	0.0062783	0.02983441	0.01326981	Id1/Myc
4-hour post-SCI group vs. the control group	BP	GO:1901222	regulation of NIK/NF-kappaB signaling	2/35	0.0062783	0.02983441	0.01326981	Ago1/Sphk1
4-hour post-SCI group vs. the control group	BP	GO:0045576	mast cell activation	2/35	0.0064029	0.03034649	0.01349758	Hmox1/Nr4a3
4-hour post-SCI group vs. the control group	BP	GO:0014910	regulation of smooth muscle cell migration	2/35	0.0065287	0.03070006	0.01365484	Myc/Nr4a3
4-hour post-SCI group vs. the control group	BP	GO:0051781	positive regulation of cell division	2/35	0.0065287	0.03070006	0.01365484	Cat/Il1a

4-hour post-SCI group vs. the control group	BP	GO:2001243	negative regulation of intrinsic apoptotic signaling pathway	2/35	0.0065287	0.03070006	0.01365484	Hspb1/Mcl1
4-hour post-SCI group vs. the control group	BP	GO:0009410	response to xenobiotic stimulus	3/35	0.006573	0.03082811	0.01371179	Fos/Jun/Myc
4-hour post-SCI group vs. the control group	BP	GO:0043299	leukocyte degranulation	2/35	0.0066555	0.03105338	0.01381199	Hmox1/Nr4a3
4-hour post-SCI group vs. the control group	BP	GO:0043502	regulation of muscle adaptation	2/35	0.0066555	0.03105338	0.01381199	Nr4a3/Tnfrsf1a
4-hour post-SCI group vs. the control group	BP	GO:0072503	cellular divalent inorganic cation homeostasis	3/35	0.0067454	0.03139116	0.01396223	Atf4/Fkbp1b/Slc8a1
4-hour post-SCI group vs. the control group	BP	GO:0060395	SMAD protein signal transduction	2/35	0.0067835	0.03141773	0.01397405	Fos/Jun
4-hour post-SCI group vs. the control group	BP	GO:0051222	positive regulation of protein transport	3/35	0.0068034	0.03141773	0.01397405	Ezr/Il1a/Tnfrsf1a
4-hour post-SCI group vs. the control group	BP	GO:0090596	sensory organ morphogenesis	3/35	0.0068034	0.03141773	0.01397405	Atf4/Myc/Nr4a3
4-hour post-SCI group vs. the control group	BP	GO:0001938	positive regulation of endothelial cell proliferation	2/35	0.0069126	0.03159804	0.01405424	Hmox1/Jun
4-hour post-SCI group vs. the control group	BP	GO:0032418	lysosome localization	2/35	0.0069126	0.03159804	0.01405424	Hmox1/Nr4a3
4-hour post-SCI group vs. the control group	BP	GO:0046330	positive regulation of JNK cascade	2/35	0.0069126	0.03159804	0.01405424	Il1a/Ripk1
4-hour post-SCI group vs. the control group	BP	GO:1990849	vacuolar localization	2/35	0.0069126	0.03159804	0.01405424	Hmox1/Nr4a3
4-hour post-SCI group vs. the control group	BP	GO:0009895	negative regulation of catabolic process	3/35	0.0069793	0.03166184	0.01408262	Hmox1/Mcl1/Tnfaip3
4-hour post-SCI group vs. the control group	BP	GO:0045927	positive regulation of growth	3/35	0.0069793	0.03166184	0.01408262	Ezr/Hbegf/Sphk1
4-hour post-SCI group vs. the control group	BP	GO:0055074	calcium ion homeostasis	3/35	0.0069793	0.03166184	0.01408262	Atf4/Fkbp1b/Slc8a1
4-hour post-SCI group vs. the control group	BP	GO:0042116	macrophage activation	2/35	0.0070428	0.03186974	0.01417509	Jun/Sphk1
4-hour post-SCI group vs. the control group	BP	GO:0070372	regulation of ERK1 and ERK2 cascade	3/35	0.0070981	0.03203928	0.0142505	Ezr/Il1a/Jun
4-hour post-SCI group vs. the control group	BP	GO:0002366	leukocyte activation involved in immune response	3/35	0.0072181	0.0324994	0.01445515	Hmox1/Nr4a3/Tnfaip3
4-hour post-SCI group vs. the control group	BP	GO:0001657	ureteric bud development	2/35	0.0074402	0.03325003	0.01478902	Cat/Myc
4-hour post-SCI group vs. the control group	BP	GO:0003300	cardiac muscle hypertrophy	2/35	0.0074402	0.03325003	0.01478902	Nr4a3/Tnfrsf1a
4-hour post-SCI group vs. the control group	BP	GO:0071901	negative regulation of protein serine/threonine kinase activity	2/35	0.0074402	0.03325003	0.01478902	Slc8a1/Tnfaip3
4-hour post-SCI group vs. the control group	BP	GO:0002263	cell activation involved in immune response	3/35	0.0074617	0.03326349	0.01479501	Hmox1/Nr4a3/Tnfaip3
4-hour post-SCI group vs. the control group	BP	GO:0045833	negative regulation of lipid metabolic process	2/35	0.0075748	0.03368466	0.01498233	Nfkb1/Sphk1
4-hour post-SCI group vs. the control group	BP	GO:0070227	lymphocyte apoptotic process	2/35	0.0077106	0.03420393	0.0152133	Myc/Ripk1
4-hour post-SCI group vs. the control group	BP	GO:0015980	energy derivation by oxidation of organic compounds	3/35	0.007773	0.0343041	0.01525785	Cat/Myc/Nr4a3
4-hour post-SCI group vs. the control group	BP	GO:1904951	positive regulation of establishment of protein localization	3/35	0.007773	0.0343041	0.01525785	Ezr/Il1a/Tnfrsf1a
4-hour post-SCI group vs. the control group	BP	GO:0014074	response to purine-containing compound	2/35	0.0078475	0.0343041	0.01525785	Ezr/Slc8a1
4-hour post-SCI group vs. the control group	BP	GO:0046916	cellular transition metal ion homeostasis	2/35	0.0078475	0.0343041	0.01525785	Hmox1/Myc
4-hour post-SCI group vs. the control group	BP	GO:0072163	mesonephric epithelium development	2/35	0.0078475	0.0343041	0.01525785	Cat/Myc
4-hour post-SCI group vs. the control group	BP	GO:0072164	mesonephric tubule development	2/35	0.0078475	0.0343041	0.01525785	Cat/Myc
4-hour post-SCI group vs. the control group	BP	GO:0014897	striated muscle hypertrophy	2/35	0.0079854	0.03473855	0.01545108	Nr4a3/Tnfrsf1a
4-hour post-SCI group vs. the control group	BP	GO:0014909	smooth muscle cell migration	2/35	0.0079854	0.03473855	0.01545108	Myc/Nr4a3
4-hour post-SCI group vs. the control group	BP	GO:0046651	lymphocyte proliferation	3/35	0.0082216	0.03552412	0.01580049	Fkbp1b/Il1a/Myc
4-hour post-SCI group vs. the control group	BP	GO:0001823	mesonephros development	2/35	0.0082646	0.03552412	0.01580049	Cat/Myc
4-hour post-SCI group vs. the control group	BP	GO:0014896	muscle hypertrophy	2/35	0.0082646	0.03552412	0.01580049	Nr4a3/Tnfrsf1a
4-hour post-SCI group vs. the control group	BP	GO:0030004	cellular monovalent inorganic cation homeostasis	2/35	0.0082646	0.03552412	0.01580049	Il1a/Slc8a1
4-hour post-SCI group vs. the control group	BP	GO:1902106	negative regulation of leukocyte differentiation	2/35	0.0082646	0.03552412	0.01580049	Hspb1/Myc

4-hour post-SCI group vs. the control group	BP	GO:0007088	regulation of mitotic nuclear division	2/35	0.0085482	0.03665548	0.0163037	Il1a/Sphk1
4-hour post-SCI group vs. the control group	BP	GO:0016311	dephosphorylation	3/35	0.0086852	0.03691883	0.01642084	Fkbp1b/Ppp1r15b/Rcan1
4-hour post-SCI group vs. the control group	BP	GO:0032943	mononuclear cell proliferation	3/35	0.0086852	0.03691883	0.01642084	Fkbp1b/Il1a/Myc
4-hour post-SCI group vs. the control group	BP	GO:0006986	response to unfolded protein	2/35	0.0086916	0.03691883	0.01642084	Atf4/Hspb1
4-hour post-SCI group vs. the control group	BP	GO:1904659	glucose transmembrane transport	2/35	0.0086916	0.03691883	0.01642084	Myc/Nr4a3
4-hour post-SCI group vs. the control group	BP	GO:0008645	hexose transmembrane transport	2/35	0.0088361	0.03726888	0.01657653	Myc/Nr4a3
4-hour post-SCI group vs. the control group	BP	GO:0019218	regulation of steroid metabolic process	2/35	0.0088361	0.03726888	0.01657653	Il1a/Nfkb1
4-hour post-SCI group vs. the control group	BP	GO:1903707	negative regulation of hemopoiesis	2/35	0.0088361	0.03726888	0.01657653	Hspb1/Myc
4-hour post-SCI group vs. the control group	BP	GO:0008202	steroid metabolic process	3/35	0.0088886	0.03740257	0.01663599	Cat/Il1a/Nfkb1
4-hour post-SCI group vs. the control group	BP	GO:0019751	polyol metabolic process	2/35	0.0089817	0.03761856	0.01673206	Gch1/Sphk1
4-hour post-SCI group vs. the control group	BP	GO:0038061	NIK/NF-kappaB signaling	2/35	0.0089817	0.03761856	0.01673206	Ago1/Sphk1
4-hour post-SCI group vs. the control group	BP	GO:2000106	regulation of leukocyte apoptotic process	2/35	0.0091283	0.03814406	0.0169658	Myc/Nr4a3
4-hour post-SCI group vs. the control group	BP	GO:0072507	divalent inorganic cation homeostasis	3/35	0.0092337	0.0384049	0.01708181	Atf4/Fkbp1b/Slc8a1
4-hour post-SCI group vs. the control group	BP	GO:0002275	myeloid cell activation involved in immune response	2/35	0.009276	0.0384049	0.01708181	Hmox1/Nr4a3
4-hour post-SCI group vs. the control group	BP	GO:0015749	monosaccharide transmembrane transport	2/35	0.009276	0.0384049	0.01708181	Myc/Nr4a3
4-hour post-SCI group vs. the control group	BP	GO:0051209	release of sequestered calcium ion into cytosol	2/35	0.009276	0.0384049	0.01708181	Fkbp1b/Slc8a1
4-hour post-SCI group vs. the control group	BP	GO:0006090	pyruvate metabolic process	2/35	0.0094248	0.03884234	0.01727638	Myc/Nr4a3
4-hour post-SCI group vs. the control group	BP	GO:0051283	negative regulation of sequestering of calcium ion	2/35	0.0094248	0.03884234	0.01727638	Fkbp1b/Slc8a1
4-hour post-SCI group vs. the control group	BP	GO:0009636	response to toxic substance	2/35	0.0095747	0.03936985	0.01751101	Cat/Gch1
4-hour post-SCI group vs. the control group	BP	GO:0051282	regulation of sequestering of calcium ion	2/35	0.0097256	0.03989933	0.01774651	Fkbp1b/Slc8a1
4-hour post-SCI group vs. the control group	BP	GO:0062012	regulation of small molecule metabolic process	3/35	0.0098745	0.04033908	0.0179421	Myc/Nfkb1/Nr4a3
4-hour post-SCI group vs. the control group	BP	GO:0015718	monocarboxylic acid transport	2/35	0.0098776	0.04033908	0.0179421	Il1a/Myc
4-hour post-SCI group vs. the control group	BP	GO:0051208	sequestering of calcium ion	2/35	0.0100306	0.04087143	0.01817888	Fkbp1b/Slc8a1
4-hour post-SCI group vs. the control group	BP	GO:0007517	muscle organ development	3/35	0.0100938	0.04094358	0.01821097	Fos/Myc/Rcan1
4-hour post-SCI group vs. the control group	BP	GO:1903037	regulation of leukocyte cell-cell adhesion	3/35	0.0100938	0.04094358	0.01821097	Hspb1/Il1a/Nr4a3
4-hour post-SCI group vs. the control group	BP	GO:0002718	regulation of cytokine production involved in immune response	2/35	0.0101847	0.04112716	0.01829262	Hmox1/Nr4a3
4-hour post-SCI group vs. the control group	BP	GO:0014812	muscle cell migration	2/35	0.0101847	0.04112716	0.01829262	Myc/Nr4a3
4-hour post-SCI group vs. the control group	BP	GO:0034219	carbohydrate transmembrane transport	2/35	0.0103399	0.0415673	0.01848839	Myc/Nr4a3
4-hour post-SCI group vs. the control group	BP	GO:0043280	positive regulation of cysteine-type endopeptidase activity involved in apoptotic process	2/35	0.0103399	0.0415673	0.01848839	Myc/Xdh
4-hour post-SCI group vs. the control group	BP	GO:0043123	positive regulation of I-kappaB kinase/NF-kappaB signaling	2/35	0.0104961	0.04200775	0.0186843	Il1a/Ripk1
4-hour post-SCI group vs. the control group	BP	GO:0043500	muscle adaptation	2/35	0.0104961	0.04200775	0.0186843	Nr4a3/Tnfrsf1a
4-hour post-SCI group vs. the control group	BP	GO:0051592	response to calcium ion	2/35	0.0108117	0.04317478	0.01920337	Fos/Jun
4-hour post-SCI group vs. the control group	BP	GO:0070661	leukocyte proliferation	3/35	0.0110769	0.04413586	0.01963084	Fkbp1b/Il1a/Myc
4-hour post-SCI group vs. the control group	BP	GO:0002444	myeloid leukocyte mediated immunity	2/35	0.0112929	0.04479831	0.01992549	Hmox1/Nr4a3
4-hour post-SCI group vs. the control group	BP	GO:0033559	unsaturated fatty acid metabolic process	2/35	0.0112929	0.04479831	0.01992549	Sphk1/Tnfrsf1a
4-hour post-SCI group vs. the control group	BP	GO:0055076	transition metal ion homeostasis	2/35	0.0119489	0.04541767	0.02020097	Hmox1/Myc
4-hour post-SCI group vs. the control group	BP	GO:0002315	marginal zone B cell differentiation	1/35	0.012029	0.04541767	0.02020097	Tnfaip3

4-hour post-SCI group vs. the control group	BP	GO:0002634	regulation of germinal center formation	1/35	0.012029	0.04541767	0.02020097	Tnfaip3
4-hour post-SCI group vs. the control group	BP	GO:0003330	regulation of extracellular matrix constituent secretion	1/35	0.012029	0.04541767	0.02020097	Tnfrsf1a
4-hour post-SCI group vs. the control group	BP	GO:0006196	AMP catabolic process	1/35	0.012029	0.04541767	0.02020097	Xdh
4-hour post-SCI group vs. the control group	BP	GO:0009650	UV protection	1/35	0.012029	0.04541767	0.02020097	Cat
4-hour post-SCI group vs. the control group	BP	GO:0010501	RNA secondary structure unwinding	1/35	0.012029	0.04541767	0.02020097	Ago1
4-hour post-SCI group vs. the control group	BP	GO:0033007	negative regulation of mast cell activation involved in immune response	1/35	0.012029	0.04541767	0.02020097	Hmox1
4-hour post-SCI group vs. the control group	BP	GO:0035871	protein K11-linked deubiquitination	1/35	0.012029	0.04541767	0.02020097	Tnfaip3
4-hour post-SCI group vs. the control group	BP	GO:0036480	neuron intrinsic apoptotic signaling pathway in response to oxidative stress	1/35	0.012029	0.04541767	0.02020097	Mcl1
4-hour post-SCI group vs. the control group	BP	GO:0038130	ERBB4 signaling pathway	1/35	0.012029	0.04541767	0.02020097	Hbegf
4-hour post-SCI group vs. the control group	BP	GO:0046055	dGMP catabolic process	1/35	0.012029	0.04541767	0.02020097	Xdh
4-hour post-SCI group vs. the control group	BP	GO:0046130	purine ribonucleoside catabolic process	1/35	0.012029	0.04541767	0.02020097	Xdh
4-hour post-SCI group vs. the control group	BP	GO:0051775	response to redox state	1/35	0.012029	0.04541767	0.02020097	Fkbp1b
4-hour post-SCI group vs. the control group	BP	GO:0061687	detoxification of inorganic compound	1/35	0.012029	0.04541767	0.02020097	Cat
4-hour post-SCI group vs. the control group	BP	GO:0070922	RISC complex assembly	1/35	0.012029	0.04541767	0.02020097	Ago1
4-hour post-SCI group vs. the control group	BP	GO:0072203	cell proliferation involved in metanephros development	1/35	0.012029	0.04541767	0.02020097	Myc
4-hour post-SCI group vs. the control group	BP	GO:0086064	cell communication by electrical coupling involved in cardiac conduction	1/35	0.012029	0.04541767	0.02020097	Slc8a1
4-hour post-SCI group vs. the control group	BP	GO:0099640	axo-dendritic protein transport	1/35	0.012029	0.04541767	0.02020097	Hspb1
4-hour post-SCI group vs. the control group	BP	GO:1901724	positive regulation of cell proliferation involved in kidney development	1/35	0.012029	0.04541767	0.02020097	Myc
4-hour post-SCI group vs. the control group	BP	GO:1902884	positive regulation of response to oxidative stress	1/35	0.012029	0.04541767	0.02020097	Ripk1
4-hour post-SCI group vs. the control group	BP	GO:1903376	regulation of oxidative stress-induced neuron intrinsic apoptotic signaling pathway	1/35	0.012029	0.04541767	0.02020097	Mcl1
4-hour post-SCI group vs. the control group	BP	GO:1903589	positive regulation of blood vessel endothelial cell proliferation involved in sprouting angiogenesis	1/35	0.012029	0.04541767	0.02020097	Hmox1
4-hour post-SCI group vs. the control group	BP	GO:0035966	response to topologically incorrect protein	2/35	0.0121155	0.04564865	0.0203037	Atf4/Hspb1
4-hour post-SCI group vs. the control group	BP	GO:0002698	negative regulation of immune effector process	2/35	0.0124518	0.04662296	0.02073706	Hmox1/Tnfaip3
4-hour post-SCI group vs. the control group	BP	GO:0030177	positive regulation of Wnt signaling pathway	2/35	0.0124518	0.04662296	0.02073706	Nfkb1/Tnfaip3
4-hour post-SCI group vs. the control group	BP	GO:0030278	regulation of ossification	2/35	0.0124518	0.04662296	0.02073706	Rxrb/Slc8a1
4-hour post-SCI group vs. the control group	BP	GO:0009306	protein secretion	3/35	0.0126945	0.04706853	0.02093524	Ezr/Fkbp1b/Il1a
4-hour post-SCI group vs. the control group	BP	GO:0035592	establishment of protein localization to extracellular region	3/35	0.0127788	0.04706853	0.02093524	Ezr/Fkbp1b/Il1a
4-hour post-SCI group vs. the control group	BP	GO:0006690	icosanoid metabolic process	2/35	0.0127921	0.04706853	0.02093524	Sphk1/Tnfrsf1a
4-hour post-SCI group vs. the control group	BP	GO:0007178	transmembrane receptor protein serine/threonine kinase signaling pathway	3/35	0.0129483	0.04706853	0.02093524	Fos/Id1/Jun
4-hour post-SCI group vs. the control group	BP	GO:0032102	negative regulation of response to external stimulus	3/35	0.0129483	0.04706853	0.02093524	Nfkb1/Tnfaip3/Tnfrsf1a
4-hour post-SCI group vs. the control group	BP	GO:0014902	myotube differentiation	2/35	0.0129638	0.04706853	0.02093524	Myc/Rcan1
4-hour post-SCI group vs. the control group	BP	GO:0042471	ear morphogenesis	2/35	0.0131365	0.04706853	0.02093524	Myc/Nr4a3
4-hour post-SCI group vs. the control group	BP	GO:0002676	regulation of chronic inflammatory response	1/35	0.0132241	0.04706853	0.02093524	Tnfaip3

4-hour post-SCI group vs. the control group	BP	GO:0002887	negative regulation of myeloid leukocyte mediated immunity	1/35	0.0132241	0.04706853	0.02093524	Hmox1
4-hour post-SCI group vs. the control group	BP	GO:0009172	purine deoxyribonucleoside monophosphate catabolic process	1/35	0.0132241	0.04706853	0.02093524	Xdh
4-hour post-SCI group vs. the control group	BP	GO:0019227	neuronal action potential propagation	1/35	0.0132241	0.04706853	0.02093524	Fkbp1b
4-hour post-SCI group vs. the control group	BP	GO:0032353	negative regulation of hormone biosynthetic process	1/35	0.0132241	0.04706853	0.02093524	Nfkb1
4-hour post-SCI group vs. the control group	BP	GO:0034115	negative regulation of heterotypic cell-cell adhesion	1/35	0.0132241	0.04706853	0.02093524	Tnfaip3
4-hour post-SCI group vs. the control group	BP	GO:0050774	negative regulation of dendrite morphogenesis	1/35	0.0132241	0.04706853	0.02093524	Id1
4-hour post-SCI group vs. the control group	BP	GO:0060056	mammary gland involution	1/35	0.0132241	0.04706853	0.02093524	Nfkb1
4-hour post-SCI group vs. the control group	BP	GO:0070587	regulation of cell-cell adhesion involved in gastrulation	1/35	0.0132241	0.04706853	0.02093524	Tnfaip3
4-hour post-SCI group vs. the control group	BP	GO:0071415	cellular response to purine-containing compound	1/35	0.0132241	0.04706853	0.02093524	Slc8a1
4-hour post-SCI group vs. the control group	BP	GO:0072075	metanephric mesenchyme development	1/35	0.0132241	0.04706853	0.02093524	Myc
4-hour post-SCI group vs. the control group	BP	GO:0090009	primitive streak formation	1/35	0.0132241	0.04706853	0.02093524	Ets2
4-hour post-SCI group vs. the control group	BP	GO:0097421	liver regeneration	1/35	0.0132241	0.04706853	0.02093524	Hmox1
4-hour post-SCI group vs. the control group	BP	GO:0098870	action potential propagation	1/35	0.0132241	0.04706853	0.02093524	Fkbp1b
4-hour post-SCI group vs. the control group	BP	GO:1900747	negative regulation of vascular endothelial growth factor signaling pathway	1/35	0.0132241	0.04706853	0.02093524	Xdh
4-hour post-SCI group vs. the control group	BP	GO:1902946	protein localization to early endosome	1/35	0.0132241	0.04706853	0.02093524	Ezr
4-hour post-SCI group vs. the control group	BP	GO:1903800	positive regulation of miRNA maturation	1/35	0.0132241	0.04706853	0.02093524	Ripk1
4-hour post-SCI group vs. the control group	BP	GO:1990440	positive regulation of transcription from RNA polymerase II promoter in response to endoplasmic reticulum stress	1/35	0.0132241	0.04706853	0.02093524	Atf4
4-hour post-SCI group vs. the control group	BP	GO:0007159	leukocyte cell-cell adhesion	3/35	0.0132911	0.04712077	0.02095848	Hspb1/Il1a/Nr4a3
4-hour post-SCI group vs. the control group	BP	GO:0071692	protein localization to extracellular region	3/35	0.0132911	0.04712077	0.02095848	Ezr/Fkbp1b/Il1a
4-hour post-SCI group vs. the control group	BP	GO:0051783	regulation of nuclear division	2/35	0.0136606	0.04807562	0.02138318	Il1a/Sphk1
4-hour post-SCI group vs. the control group	BP	GO:0120161	regulation of cold-induced thermogenesis	2/35	0.0136606	0.04807562	0.02138318	Atf4/Id1
4-hour post-SCI group vs. the control group	BP	GO:0002367	cytokine production involved in immune response	2/35	0.0138373	0.04807562	0.02138318	Hmox1/Nr4a3
4-hour post-SCI group vs. the control group	BP	GO:0050729	positive regulation of inflammatory response	2/35	0.0138373	0.04807562	0.02138318	Ripk1/Tnfrsf1a
4-hour post-SCI group vs. the control group	BP	GO:0034763	negative regulation of transmembrane transport	2/35	0.014015	0.04807562	0.02138318	Fkbp1b/Myc
4-hour post-SCI group vs. the control group	BP	GO:0106106	cold-induced thermogenesis	2/35	0.014015	0.04807562	0.02138318	Atf4/Id1
4-hour post-SCI group vs. the control group	BP	GO:2001056	positive regulation of cysteine-type endopeptidase activity	2/35	0.014015	0.04807562	0.02138318	Myc/Xdh
4-hour post-SCI group vs. the control group	BP	GO:0046328	regulation of JNK cascade	2/35	0.0141937	0.04807562	0.02138318	Il1a/Ripk1
4-hour post-SCI group vs. the control group	BP	GO:0055007	cardiac muscle cell differentiation	2/35	0.0141937	0.04807562	0.02138318	Rxrb/Slc8a1
4-hour post-SCI group vs. the control group	BP	GO:0002440	production of molecular mediator of immune response	3/35	0.0142608	0.04807562	0.02138318	Hmox1/Nr4a3/Tnfaip3
4-hour post-SCI group vs. the control group	BP	GO:0006887	exocytosis	3/35	0.0142608	0.04807562	0.02138318	Hmox1/Nr4a3/Sdc1
4-hour post-SCI group vs. the control group	BP	GO:0006152	purine nucleoside catabolic process	1/35	0.0144179	0.04807562	0.02138318	Xdh
4-hour post-SCI group vs. the control group	BP	GO:0010998	regulation of translational initiation by eIF2 alpha phosphorylation	1/35	0.0144179	0.04807562	0.02138318	Ppp1r15b
4-hour post-SCI group vs. the control group	BP	GO:0032351	negative regulation of hormone metabolic process	1/35	0.0144179	0.04807562	0.02138318	Nfkb1

4-hour post-SCI group vs. the control group	BP	GO:0035331	negative regulation of hippo signaling	1/35	0.0144179	0.04807562	0.02138318	Map2k3
4-hour post-SCI group vs. the control group	BP	GO:0035726	common myeloid progenitor cell proliferation	1/35	0.0144179	0.04807562	0.02138318	Nr4a3
4-hour post-SCI group vs. the control group	BP	GO:0036490	regulation of translation in response to endoplasmic reticulum stress	1/35	0.0144179	0.04807562	0.02138318	Ppp1r15b
4-hour post-SCI group vs. the control group	BP	GO:0042362	fat-soluble vitamin biosynthetic process	1/35	0.0144179	0.04807562	0.02138318	Nfkb1
4-hour post-SCI group vs. the control group	BP	GO:0043301	negative regulation of leukocyte degranulation	1/35	0.0144179	0.04807562	0.02138318	Hmox1
4-hour post-SCI group vs. the control group	BP	GO:0045741	positive regulation of epidermal growth factor-activated receptor activity	1/35	0.0144179	0.04807562	0.02138318	Hbegf
4-hour post-SCI group vs. the control group	BP	GO:0046054	dGMP metabolic process	1/35	0.0144179	0.04807562	0.02138318	Xdh
4-hour post-SCI group vs. the control group	BP	GO:0060872	semicircular canal development	1/35	0.0144179	0.04807562	0.02138318	Nr4a3
4-hour post-SCI group vs. the control group	BP	GO:0070262	peptidyl-serine dephosphorylation	1/35	0.0144179	0.04807562	0.02138318	Ppp1r15b
4-hour post-SCI group vs. the control group	BP	GO:0070586	cell-cell adhesion involved in gastrulation	1/35	0.0144179	0.04807562	0.02138318	Tnfaip3
4-hour post-SCI group vs. the control group	BP	GO:0071281	cellular response to iron ion	1/35	0.0144179	0.04807562	0.02138318	Hmox1
4-hour post-SCI group vs. the control group	BP	GO:0072697	protein localization to cell cortex	1/35	0.0144179	0.04807562	0.02138318	Ezr
4-hour post-SCI group vs. the control group	BP	GO:0090184	positive regulation of kidney development	1/35	0.0144179	0.04807562	0.02138318	Myc
4-hour post-SCI group vs. the control group	BP	GO:0097201	negative regulation of transcription from RNA polymerase II promoter in response to stress	1/35	0.0144179	0.04807562	0.02138318	Jun
4-hour post-SCI group vs. the control group	BP	GO:1904672	regulation of somatic stem cell population maintenance	1/35	0.0144179	0.04807562	0.02138318	Myc
4-hour post-SCI group vs. the control group	BP	GO:1990253	cellular response to leucine starvation	1/35	0.0144179	0.04807562	0.02138318	Atf4
4-hour post-SCI group vs. the control group	BP	GO:2000253	positive regulation of feeding behavior	1/35	0.0144179	0.04807562	0.02138318	Nr4a3
4-hour post-SCI group vs. the control group	BP	GO:2000303	regulation of ceramide biosynthetic process	1/35	0.0144179	0.04807562	0.02138318	Sphk1
4-hour post-SCI group vs. the control group	BP	GO:0001654	eye development	3/35	0.0144414	0.04807562	0.02138318	Atf4/Jun/Pxdn
4-hour post-SCI group vs. the control group	BP	GO:0150063	visual system development	3/35	0.0147146	0.04889494	0.02174759	Atf4/Jun/Pxdn
4-hour post-SCI group vs. the control group	BP	GO:1901652	response to peptide	3/35	0.0149908	0.049721	0.02211501	Nfkb1/Nr4a3/Tnfaip3
4-hour post-SCI group vs. the control group	BP	GO:0048880	sensory system development	3/35	0.0150836	0.04990626	0.02219741	Atf4/Jun/Pxdn
4-hour post-SCI group vs. the control group	BP	GO:0072073	kidney epithelium development	2/35	0.0151021	0.04990626	0.02219741	Cat/Myc
4-hour post-SCI group vs. the control group	BP	GO:0009314	response to radiation	3/35	0.0151766	0.04993603	0.02221065	Cat/Jun/Myc
4-hour post-SCI group vs. the control group	BP	GO:0022612	gland morphogenesis	2/35	0.0154724	0.04993603	0.02221065	Nfkb1/Tnfaip3
4-hour post-SCI group vs. the control group	BP	GO:0046700	heterocycle catabolic process	3/35	0.0155521	0.04993603	0.02221065	Ago1/Hmox1/Xdh
4-hour post-SCI group vs. the control group	BP	GO:0000255	allantoin metabolic process	1/35	0.0156102	0.04993603	0.02221065	Xdh
4-hour post-SCI group vs. the control group	BP	GO:0006983	ER overload response	1/35	0.0156102	0.04993603	0.02221065	Ppp1r15b
4-hour post-SCI group vs. the control group	BP	GO:0030953	astral microtubule organization	1/35	0.0156102	0.04993603	0.02221065	Ezr
4-hour post-SCI group vs. the control group	BP	GO:0031953	negative regulation of protein autophosphorylation	1/35	0.0156102	0.04993603	0.02221065	Jun
4-hour post-SCI group vs. the control group	BP	GO:0043922	negative regulation by host of viral transcription	1/35	0.0156102	0.04993603	0.02221065	Jun
4-hour post-SCI group vs. the control group	BP	GO:0044557	relaxation of smooth muscle	1/35	0.0156102	0.04993603	0.02221065	Slc8a1
4-hour post-SCI group vs. the control group	BP	GO:0051549	positive regulation of keratinocyte migration	1/35	0.0156102	0.04993603	0.02221065	Hbegf
4-hour post-SCI group vs. the control group	BP	GO:0097048	dendritic cell apoptotic process	1/35	0.0156102	0.04993603	0.02221065	Nr4a3
4-hour post-SCI group vs. the control group	BP	GO:0097709	connective tissue replacement	1/35	0.0156102	0.04993603	0.02221065	Il1a
4-hour post-SCI group vs. the control group	BP	GO:1902548	negative regulation of cellular response to vascular endothelial growth factor stimulus	1/35	0.0156102	0.04993603	0.02221065	Xdh

4-hour post-SCI group vs. the control group	BP	GO:1902931	negative regulation of alcohol biosynthetic process	1/35	0.0156102	0.04993603	0.02221065	Nfkb1
4-hour post-SCI group vs. the control group	BP	GO:1903651	positive regulation of cytoplasmic transport	1/35	0.0156102	0.04993603	0.02221065	Ezr
4-hour post-SCI group vs. the control group	BP	GO:1905668	positive regulation of protein localization to endosome	1/35	0.0156102	0.04993603	0.02221065	Ezr
4-hour post-SCI group vs. the control group	BP	GO:2000347	positive regulation of hepatocyte proliferation	1/35	0.0156102	0.04993603	0.02221065	Tnfaip3
4-hour post-SCI group vs. the control group	BP	GO:2000668	regulation of dendritic cell apoptotic process	1/35	0.0156102	0.04993603	0.02221065	Nr4a3
4-hour post-SCI group vs. the control group	BP	GO:0031346	positive regulation of cell projection organization	3/35	0.0160289	0.05118434	0.02276588	Fkbp1b/Hspb1/Sphk1
4-hour post-SCI group vs. the control group	BP	GO:0044270	cellular nitrogen compound catabolic process	3/35	0.0162219	0.05128619	0.02281118	Ago1/Hmox1/Xdh
4-hour post-SCI group vs. the control group	BP	GO:0045667	regulation of osteoblast differentiation	2/35	0.0162245	0.05128619	0.02281118	Atf4/Id1
4-hour post-SCI group vs. the control group	BP	GO:0035637	multicellular organismal signaling	2/35	0.0166064	0.05128619	0.02281118	Fkbp1b/Slc8a1
4-hour post-SCI group vs. the control group	BP	GO:0071322	cellular response to carbohydrate stimulus	2/35	0.0167988	0.05128619	0.02281118	Fkbp1b/Nfkb1
4-hour post-SCI group vs. the control group	BP	GO:0001660	fever generation	1/35	0.0168011	0.05128619	0.02281118	Il1a
4-hour post-SCI group vs. the control group	BP	GO:0001866	NK T cell proliferation	1/35	0.0168011	0.05128619	0.02281118	Myc
4-hour post-SCI group vs. the control group	BP	GO:0009120	deoxyribonucleoside metabolic process	1/35	0.0168011	0.05128619	0.02281118	Xdh
4-hour post-SCI group vs. the control group	BP	GO:0010459	negative regulation of heart rate	1/35	0.0168011	0.05128619	0.02281118	Fkbp1b
4-hour post-SCI group vs. the control group	BP	GO:0010940	positive regulation of necrotic cell death	1/35	0.0168011	0.05128619	0.02281118	Ripk1
4-hour post-SCI group vs. the control group	BP	GO:0031054	pre-miRNA processing	1/35	0.0168011	0.05128619	0.02281118	Ago1
4-hour post-SCI group vs. the control group	BP	GO:0032536	regulation of cell projection size	1/35	0.0168011	0.05128619	0.02281118	Ezr
4-hour post-SCI group vs. the control group	BP	GO:0032769	negative regulation of monooxygenase activity	1/35	0.0168011	0.05128619	0.02281118	Nfkb1
4-hour post-SCI group vs. the control group	BP	GO:0033004	negative regulation of mast cell activation	1/35	0.0168011	0.05128619	0.02281118	Hmox1
4-hour post-SCI group vs. the control group	BP	GO:0034393	positive regulation of smooth muscle cell apoptotic process	1/35	0.0168011	0.05128619	0.02281118	Atf4
4-hour post-SCI group vs. the control group	BP	GO:0036499	PERK-mediated unfolded protein response	1/35	0.0168011	0.05128619	0.02281118	Atf4
4-hour post-SCI group vs. the control group	BP	GO:0042559	pteridine-containing compound biosynthetic process	1/35	0.0168011	0.05128619	0.02281118	Gch1
4-hour post-SCI group vs. the control group	BP	GO:0046512	sphingosine biosynthetic process	1/35	0.0168011	0.05128619	0.02281118	Sphk1
4-hour post-SCI group vs. the control group	BP	GO:0046520	sphingoid biosynthetic process	1/35	0.0168011	0.05128619	0.02281118	Sphk1
4-hour post-SCI group vs. the control group	BP	GO:0048680	positive regulation of axon regeneration	1/35	0.0168011	0.05128619	0.02281118	Fkbp1b
4-hour post-SCI group vs. the control group	BP	GO:0070278	extracellular matrix constituent secretion	1/35	0.0168011	0.05128619	0.02281118	Tnfrsf1a
4-hour post-SCI group vs. the control group	BP	GO:0070831	basement membrane assembly	1/35	0.0168011	0.05128619	0.02281118	Pxdn
4-hour post-SCI group vs. the control group	BP	GO:0090153	regulation of sphingolipid biosynthetic process	1/35	0.0168011	0.05128619	0.02281118	Sphk1
4-hour post-SCI group vs. the control group	BP	GO:1905038	regulation of membrane lipid metabolic process	1/35	0.0168011	0.05128619	0.02281118	Sphk1
4-hour post-SCI group vs. the control group	BP	GO:1905288	vascular associated smooth muscle cell apoptotic process	1/35	0.0168011	0.05128619	0.02281118	Atf4
4-hour post-SCI group vs. the control group	BP	GO:1905459	regulation of vascular associated smooth muscle cell apoptotic process	1/35	0.0168011	0.05128619	0.02281118	Atf4
4-hour post-SCI group vs. the control group	BP	GO:1905666	regulation of protein localization to endosome	1/35	0.0168011	0.05128619	0.02281118	Ezr
4-hour post-SCI group vs. the control group	BP	GO:0030879	mammary gland development	2/35	0.0169921	0.05151224	0.02291172	Nfkb1/Xdh
4-hour post-SCI group vs. the control group	BP	GO:0050714	positive regulation of protein secretion	2/35	0.0169921	0.05151224	0.02291172	Ezr/Il1a
4-hour post-SCI group vs. the control group	BP	GO:1990845	adaptive thermogenesis	2/35	0.0169921	0.05151224	0.02291172	Atf4/Id1
4-hour post-SCI group vs. the control group	BP	GO:0006631	fatty acid metabolic process	3/35	0.0172067	0.05151224	0.02291172	Nr4a3/Sphk1/Tnfrsf1a
4-hour post-SCI group vs. the control group	BP	GO:0034248	regulation of cellular amide metabolic process	3/35	0.0172067	0.05151224	0.02291172	Ago1/Ppp1r15b/Sphk1

4-hour post-SCI group vs. the control group	BP	GO:0032675	regulation of interleukin-6 production	2/35	0.0173817	0.05151224	0.02291172	Tnfaip3/Tnfrsf1a
4-hour post-SCI group vs. the control group	BP	GO:0048015	phosphatidylinositol-mediated signaling	2/35	0.0175779	0.05151224	0.02291172	Cat/Ezr
4-hour post-SCI group vs. the control group	BP	GO:0010959	regulation of metal ion transport	3/35	0.0177114	0.05151224	0.02291172	Atf4/Fkbp1b/Slc8a1
4-hour post-SCI group vs. the control group	BP	GO:0019439	aromatic compound catabolic process	3/35	0.0177114	0.05151224	0.02291172	Ago1/Hmox1/Xdh
4-hour post-SCI group vs. the control group	BP	GO:0006457	protein folding	2/35	0.017775	0.05151224	0.02291172	Fkbp1b/Hspb1
4-hour post-SCI group vs. the control group	BP	GO:0006694	steroid biosynthetic process	2/35	0.017775	0.05151224	0.02291172	Il1a/Nfkb1
4-hour post-SCI group vs. the control group	BP	GO:0010950	positive regulation of endopeptidase activity	2/35	0.0179731	0.05151224	0.02291172	Myc/Xdh
4-hour post-SCI group vs. the control group	BP	GO:0007028	cytoplasm organization	1/35	0.0179907	0.05151224	0.02291172	Fosl1
4-hour post-SCI group vs. the control group	BP	GO:0009155	purine deoxyribonucleotide catabolic process	1/35	0.0179907	0.05151224	0.02291172	Xdh
4-hour post-SCI group vs. the control group	BP	GO:0009169	purine ribonucleoside monophosphate catabolic process	1/35	0.0179907	0.05151224	0.02291172	Xdh
4-hour post-SCI group vs. the control group	BP	GO:0009170	purine deoxyribonucleoside monophosphate metabolic process	1/35	0.0179907	0.05151224	0.02291172	Xdh
4-hour post-SCI group vs. the control group	BP	GO:0010561	negative regulation of glycoprotein biosynthetic process	1/35	0.0179907	0.05151224	0.02291172	Hbegf
4-hour post-SCI group vs. the control group	BP	GO:0030213	hyaluronan biosynthetic process	1/35	0.0179907	0.05151224	0.02291172	Nfkb1
4-hour post-SCI group vs. the control group	BP	GO:0031000	response to caffeine	1/35	0.0179907	0.05151224	0.02291172	Slc8a1
4-hour post-SCI group vs. the control group	BP	GO:0032308	positive regulation of prostaglandin secretion	1/35	0.0179907	0.05151224	0.02291172	Il1a
4-hour post-SCI group vs. the control group	BP	GO:0032530	regulation of microvillus organization	1/35	0.0179907	0.05151224	0.02291172	Ezr
4-hour post-SCI group vs. the control group	BP	GO:0034392	negative regulation of smooth muscle cell apoptotic process	1/35	0.0179907	0.05151224	0.02291172	Nr4a3
4-hour post-SCI group vs. the control group	BP	GO:0036376	sodium ion export across plasma membrane	1/35	0.0179907	0.05151224	0.02291172	Slc8a1
4-hour post-SCI group vs. the control group	BP	GO:0038128	ERBB2 signaling pathway	1/35	0.0179907	0.05151224	0.02291172	Hbegf
4-hour post-SCI group vs. the control group	BP	GO:0043558	regulation of translational initiation in response to stress	1/35	0.0179907	0.05151224	0.02291172	Ppp1r15b
4-hour post-SCI group vs. the control group	BP	GO:0046085	adenosine metabolic process	1/35	0.0179907	0.05151224	0.02291172	Xdh
4-hour post-SCI group vs. the control group	BP	GO:0051280	negative regulation of release of sequestered calcium ion into cytosol	1/35	0.0179907	0.05151224	0.02291172	Fkbp1b
4-hour post-SCI group vs. the control group	BP	GO:0051547	regulation of keratinocyte migration	1/35	0.0179907	0.05151224	0.02291172	Hbegf
4-hour post-SCI group vs. the control group	BP	GO:0051782	negative regulation of cell division	1/35	0.0179907	0.05151224	0.02291172	Myc
4-hour post-SCI group vs. the control group	BP	GO:0061029	eyelid development in camera-type eye	1/35	0.0179907	0.05151224	0.02291172	Jun
4-hour post-SCI group vs. the control group	BP	GO:0070207	protein homotrimerization	1/35	0.0179907	0.05151224	0.02291172	Pxdn
4-hour post-SCI group vs. the control group	BP	GO:0070307	lens fiber cell development	1/35	0.0179907	0.05151224	0.02291172	Atf4
4-hour post-SCI group vs. the control group	BP	GO:0070572	positive regulation of neuron projection regeneration	1/35	0.0179907	0.05151224	0.02291172	Fkbp1b
4-hour post-SCI group vs. the control group	BP	GO:0072074	kidney mesenchyme development	1/35	0.0179907	0.05151224	0.02291172	Myc
4-hour post-SCI group vs. the control group	BP	GO:0098840	protein transport along microtubule	1/35	0.0179907	0.05151224	0.02291172	Hspb1
4-hour post-SCI group vs. the control group	BP	GO:0099118	microtubule-based protein transport	1/35	0.0179907	0.05151224	0.02291172	Hspb1
4-hour post-SCI group vs. the control group	BP	GO:1902337	regulation of apoptotic process involved in morphogenesis	1/35	0.0179907	0.05151224	0.02291172	Tnfrsf1a
4-hour post-SCI group vs. the control group	BP	GO:1903543	positive regulation of exosomal secretion	1/35	0.0179907	0.05151224	0.02291172	Sdc1
4-hour post-SCI group vs. the control group	BP	GO:2000811	negative regulation of anoikis	1/35	0.0179907	0.05151224	0.02291172	Mcl1

4-hour post-SCI group vs. the control group	BP	GO:0009267	cellular response to starvation	2/35	0.0181722	0.05162155	0.02296035	Atf4/Jun
4-hour post-SCI group vs. the control group	BP	GO:0048017	inositol lipid-mediated signaling	2/35	0.0181722	0.05162155	0.02296035	Cat/Ezr
4-hour post-SCI group vs. the control group	BP	GO:0051017	actin filament bundle assembly	2/35	0.0181722	0.05162155	0.02296035	Ezr/Id1
4-hour post-SCI group vs. the control group	BP	GO:0051250	negative regulation of lymphocyte activation	2/35	0.0181722	0.05162155	0.02296035	Hspb1/Tnfaip3
4-hour post-SCI group vs. the control group	BP	GO:0055067	monovalent inorganic cation homeostasis	2/35	0.0181722	0.05162155	0.02296035	Il1a/Slc8a1
4-hour post-SCI group vs. the control group	BP	GO:0002697	regulation of immune effector process	3/35	0.0183281	0.05198254	0.02312091	Hmox1/Nr4a3/Tnfaip3
4-hour post-SCI group vs. the control group	BP	GO:0062013	positive regulation of small molecule metabolic process	2/35	0.0183721	0.05202552	0.02314002	Myc/Nr4a3
4-hour post-SCI group vs. the control group	BP	GO:0006091	generation of precursor metabolites and energy	3/35	0.018432	0.05202676	0.02314057	Cat/Myc/Nr4a3
4-hour post-SCI group vs. the control group	BP	GO:0035296	regulation of tube diameter	2/35	0.0185731	0.05202676	0.02314057	Gch1/Slc8a1
4-hour post-SCI group vs. the control group	BP	GO:0097746	blood vessel diameter maintenance	2/35	0.0185731	0.05202676	0.02314057	Gch1/Slc8a1
4-hour post-SCI group vs. the control group	BP	GO:0035150	regulation of tube size	2/35	0.0187749	0.05202676	0.02314057	Gch1/Slc8a1
4-hour post-SCI group vs. the control group	BP	GO:0061572	actin filament bundle organization	2/35	0.0187749	0.05202676	0.02314057	Ezr/Id1
4-hour post-SCI group vs. the control group	BP	GO:0006914	autophagy	3/35	0.0188511	0.05202676	0.02314057	Hmox1/Mcl1/Tnfaip3
4-hour post-SCI group vs. the control group	BP	GO:0061919	process utilizing autophagic mechanism	3/35	0.0188511	0.05202676	0.02314057	Hmox1/Mcl1/Tnfaip3
4-hour post-SCI group vs. the control group	BP	GO:0051896	regulation of protein kinase B signaling	2/35	0.0189777	0.05202676	0.02314057	Hbegf/Xdh
4-hour post-SCI group vs. the control group	BP	GO:0030098	lymphocyte differentiation	3/35	0.0190626	0.05202676	0.02314057	Hspb1/Il1a/Tnfaip3
4-hour post-SCI group vs. the control group	BP	GO:0002467	germinal center formation	1/35	0.0191788	0.05202676	0.02314057	Tnfaip3
4-hour post-SCI group vs. the control group	BP	GO:0009128	purine nucleoside monophosphate catabolic process	1/35	0.0191788	0.05202676	0.02314057	Xdh
4-hour post-SCI group vs. the control group	BP	GO:0023035	CD40 signaling pathway	1/35	0.0191788	0.05202676	0.02314057	Tnfaip3
4-hour post-SCI group vs. the control group	BP	GO:0031987	locomotion involved in locomotory behavior	1/35	0.0191788	0.05202676	0.02314057	Rcan1
4-hour post-SCI group vs. the control group	BP	GO:0032239	regulation of nucleobase-containing compound transport	1/35	0.0191788	0.05202676	0.02314057	Ripk1
4-hour post-SCI group vs. the control group	BP	GO:0033084	regulation of immature T cell proliferation in thymus	1/35	0.0191788	0.05202676	0.02314057	Il1a
4-hour post-SCI group vs. the control group	BP	GO:0042416	dopamine biosynthetic process	1/35	0.0191788	0.05202676	0.02314057	Gch1
4-hour post-SCI group vs. the control group	BP	GO:0042428	serotonin metabolic process	1/35	0.0191788	0.05202676	0.02314057	Gch1
4-hour post-SCI group vs. the control group	BP	GO:0042454	ribonucleoside catabolic process	1/35	0.0191788	0.05202676	0.02314057	Xdh
4-hour post-SCI group vs. the control group	BP	GO:0045651	positive regulation of macrophage differentiation	1/35	0.0191788	0.05202676	0.02314057	Ripk1
4-hour post-SCI group vs. the control group	BP	GO:0051151	negative regulation of smooth muscle cell differentiation	1/35	0.0191788	0.05202676	0.02314057	Rcan1
4-hour post-SCI group vs. the control group	BP	GO:0070431	nucleotide-binding oligomerization domain containing 2 signaling pathway	1/35	0.0191788	0.05202676	0.02314057	Tnfaip3
4-hour post-SCI group vs. the control group	BP	GO:0070885	negative regulation of calcineurin-NFAT signaling cascade	1/35	0.0191788	0.05202676	0.02314057	Rcan1
4-hour post-SCI group vs. the control group	BP	GO:0106057	negative regulation of calcineurin-mediated signaling	1/35	0.0191788	0.05202676	0.02314057	Rcan1
4-hour post-SCI group vs. the control group	BP	GO:1901722	regulation of cell proliferation involved in kidney development	1/35	0.0191788	0.05202676	0.02314057	Myc
4-hour post-SCI group vs. the control group	BP	GO:1903978	regulation of microglial cell activation	1/35	0.0191788	0.05202676	0.02314057	Sphk1
4-hour post-SCI group vs. the control group	BP	GO:1904748	regulation of apoptotic process involved in development	1/35	0.0191788	0.05202676	0.02314057	Tnfrsf1a
4-hour post-SCI group vs. the control group	BP	GO:2001212	regulation of vasculogenesis	1/35	0.0191788	0.05202676	0.02314057	Xdh

4-hour post-SCI group vs. the control group	BP	GO:0032635	interleukin-6 production	2/35	0.0191814	0.05202676	0.02314057	Tnfaip3/Tnfrsf1a
4-hour post-SCI group vs. the control group	BP	GO:1903034	regulation of response to wounding	2/35	0.0193861	0.05250278	0.0233523	Fkbp1b/Hbegf
4-hour post-SCI group vs. the control group	BP	GO:0010952	positive regulation of peptidase activity	2/35	0.0200056	0.05362331	0.02385069	Myc/Xdh
4-hour post-SCI group vs. the control group	BP	GO:2000112	regulation of cellular macromolecule biosynthetic process	3/35	0.0202498	0.05362331	0.02385069	Ago1/Hbegf/Ppp1r15b
4-hour post-SCI group vs. the control group	BP	GO:0001886	endothelial cell morphogenesis	1/35	0.0203655	0.05362331	0.02385069	Id1
4-hour post-SCI group vs. the control group	BP	GO:0002431	Fc receptor mediated stimulatory signaling pathway	1/35	0.0203655	0.05362331	0.02385069	Nr4a3
4-hour post-SCI group vs. the control group	BP	GO:0032306	regulation of prostaglandin secretion	1/35	0.0203655	0.05362331	0.02385069	Il1a
4-hour post-SCI group vs. the control group	BP	GO:0033083	regulation of immature T cell proliferation	1/35	0.0203655	0.05362331	0.02385069	Il1a
4-hour post-SCI group vs. the control group	BP	GO:0042359	vitamin D metabolic process	1/35	0.0203655	0.05362331	0.02385069	Nfkb1
4-hour post-SCI group vs. the control group	BP	GO:0043923	positive regulation by host of viral transcription	1/35	0.0203655	0.05362331	0.02385069	Jun
4-hour post-SCI group vs. the control group	BP	GO:0044320	cellular response to leptin stimulus	1/35	0.0203655	0.05362331	0.02385069	Nr4a3
4-hour post-SCI group vs. the control group	BP	GO:0051284	positive regulation of sequestering of calcium ion	1/35	0.0203655	0.05362331	0.02385069	Fkbp1b
4-hour post-SCI group vs. the control group	BP	GO:0060546	negative regulation of necroptotic process	1/35	0.0203655	0.05362331	0.02385069	Ripk1
4-hour post-SCI group vs. the control group	BP	GO:0061179	negative regulation of insulin secretion involved in cellular response to glucose stimulus	1/35	0.0203655	0.05362331	0.02385069	Fkbp1b
4-hour post-SCI group vs. the control group	BP	GO:0071243	cellular response to arsenic-containing substance	1/35	0.0203655	0.05362331	0.02385069	Hmox1
4-hour post-SCI group vs. the control group	BP	GO:1902176	negative regulation of oxidative stress-induced intrinsic apoptotic signaling pathway	1/35	0.0203655	0.05362331	0.02385069	Hspb1
4-hour post-SCI group vs. the control group	BP	GO:1903019	negative regulation of glycoprotein metabolic process	1/35	0.0203655	0.05362331	0.02385069	Hbegf
4-hour post-SCI group vs. the control group	BP	GO:1903541	regulation of exosomal secretion	1/35	0.0203655	0.05362331	0.02385069	Sdc1
4-hour post-SCI group vs. the control group	BP	GO:1903587	regulation of blood vessel endothelial cell proliferation involved in sprouting angiogenesis	1/35	0.0203655	0.05362331	0.02385069	Hmox1
4-hour post-SCI group vs. the control group	BP	GO:2001028	positive regulation of endothelial cell chemotaxis	1/35	0.0203655	0.05362331	0.02385069	Hspb1
4-hour post-SCI group vs. the control group	BP	GO:2001171	positive regulation of ATP biosynthetic process	1/35	0.0203655	0.05362331	0.02385069	Myc
4-hour post-SCI group vs. the control group	BP	GO:1901361	organic cyclic compound catabolic process	3/35	0.02047	0.05381959	0.02393799	Ago1/Hmox1/Xdh
4-hour post-SCI group vs. the control group	BP	GO:0071897	DNA biosynthetic process	2/35	0.0210565	0.0546664	0.02431464	Myc/Sphk1
4-hour post-SCI group vs. the control group	BP	GO:0097553	calcium ion transmembrane import into cytosol	2/35	0.0210565	0.0546664	0.02431464	Fkbp1b/Slc8a1
4-hour post-SCI group vs. the control group	BP	GO:1901605	alpha-amino acid metabolic process	2/35	0.0210565	0.0546664	0.02431464	Atf4/Txnrd1
4-hour post-SCI group vs. the control group	BP	GO:0035051	cardiocyte differentiation	2/35	0.0212694	0.0546664	0.02431464	Rxrb/Slc8a1
4-hour post-SCI group vs. the control group	BP	GO:1903829	positive regulation of protein localization	3/35	0.0214772	0.0546664	0.02431464	Ezr/Il1a/Tnfrsf1a
4-hour post-SCI group vs. the control group	BP	GO:0009060	aerobic respiration	2/35	0.0214832	0.0546664	0.02431464	Cat/Myc
4-hour post-SCI group vs. the control group	BP	GO:0032388	positive regulation of intracellular transport	2/35	0.0214832	0.0546664	0.02431464	Ezr/Tnfrsf1a
4-hour post-SCI group vs. the control group	BP	GO:0043271	negative regulation of ion transport	2/35	0.0214832	0.0546664	0.02431464	Atf4/Fkbp1b
4-hour post-SCI group vs. the control group	BP	GO:0007398	ectoderm development	1/35	0.0215509	0.0546664	0.02431464	Ets2
4-hour post-SCI group vs. the control group	BP	GO:0007614	short-term memory	1/35	0.0215509	0.0546664	0.02431464	Rcan1
4-hour post-SCI group vs. the control group	BP	GO:0010644	cell communication by electrical coupling	1/35	0.0215509	0.0546664	0.02431464	Slc8a1
4-hour post-SCI group vs. the control group	BP	GO:0010918	positive regulation of mitochondrial membrane potential	1/35	0.0215509	0.0546664	0.02431464	Myc
4-hour post-SCI group vs. the control group	BP	GO:0019430	removal of superoxide radicals	1/35	0.0215509	0.0546664	0.02431464	Gch1

4-hour post-SCI group vs. the control group	BP	GO:0030656	regulation of vitamin metabolic process	1/35	0.0215509	0.0546664	0.02431464	Nfkb1
4-hour post-SCI group vs. the control group	BP	GO:0033080	immature T cell proliferation in thymus	1/35	0.0215509	0.0546664	0.02431464	Il1a
4-hour post-SCI group vs. the control group	BP	GO:0035330	regulation of hippo signaling	1/35	0.0215509	0.0546664	0.02431464	Map2k3
4-hour post-SCI group vs. the control group	BP	GO:0051000	positive regulation of nitric-oxide synthase activity	1/35	0.0215509	0.0546664	0.02431464	Gch1
4-hour post-SCI group vs. the control group	BP	GO:0055012	ventricular cardiac muscle cell differentiation	1/35	0.0215509	0.0546664	0.02431464	Rxrb
4-hour post-SCI group vs. the control group	BP	GO:0062099	negative regulation of programmed necrotic cell death	1/35	0.0215509	0.0546664	0.02431464	Ripk1
4-hour post-SCI group vs. the control group	BP	GO:0070102	interleukin-6-mediated signaling pathway	1/35	0.0215509	0.0546664	0.02431464	Ripk1
4-hour post-SCI group vs. the control group	BP	GO:0070206	protein trimerization	1/35	0.0215509	0.0546664	0.02431464	Pxdn
4-hour post-SCI group vs. the control group	BP	GO:0070423	nucleotide-binding oligomerization domain containing signaling pathway	1/35	0.0215509	0.0546664	0.02431464	Tnfaip3
4-hour post-SCI group vs. the control group	BP	GO:0070486	leukocyte aggregation	1/35	0.0215509	0.0546664	0.02431464	Nr4a3
4-hour post-SCI group vs. the control group	BP	GO:1901160	primary amino compound metabolic process	1/35	0.0215509	0.0546664	0.02431464	Gch1
4-hour post-SCI group vs. the control group	BP	GO:2000641	regulation of early endosome to late endosome transport	1/35	0.0215509	0.0546664	0.02431464	Ezr
4-hour post-SCI group vs. the control group	BP	GO:0045834	positive regulation of lipid metabolic process	2/35	0.0216979	0.05496189	0.02444607	Il1a/Nr4a3
4-hour post-SCI group vs. the control group	BP	GO:0042594	response to starvation	2/35	0.0223474	0.05644841	0.02510724	Atf4/Jun
4-hour post-SCI group vs. the control group	BP	GO:0050731	positive regulation of peptidyl-tyrosine phosphorylation	2/35	0.0223474	0.05644841	0.02510724	Hbegf/Tnfrsf1a
4-hour post-SCI group vs. the control group	BP	GO:0032695	negative regulation of interleukin-12 production	1/35	0.0227348	0.05663269	0.02518921	Nfkb1
4-hour post-SCI group vs. the control group	BP	GO:0032986	protein-DNA complex disassembly	1/35	0.0227348	0.05663269	0.02518921	Myc
4-hour post-SCI group vs. the control group	BP	GO:0033079	immature T cell proliferation	1/35	0.0227348	0.05663269	0.02518921	Il1a
4-hour post-SCI group vs. the control group	BP	GO:0035457	cellular response to interferon-alpha	1/35	0.0227348	0.05663269	0.02518921	Myc
4-hour post-SCI group vs. the control group	BP	GO:0035872	nucleotide-binding domain, leucine rich repeat containing receptor signaling pathway	1/35	0.0227348	0.05663269	0.02518921	Tnfaip3
4-hour post-SCI group vs. the control group	BP	GO:0038093	Fc receptor signaling pathway	1/35	0.0227348	0.05663269	0.02518921	Nr4a3
4-hour post-SCI group vs. the control group	BP	GO:0051132	NK T cell activation	1/35	0.0227348	0.05663269	0.02518921	Myc
4-hour post-SCI group vs. the control group	BP	GO:0140467	integrated stress response signaling	1/35	0.0227348	0.05663269	0.02518921	Atf4
4-hour post-SCI group vs. the control group	BP	GO:1901550	regulation of endothelial cell development	1/35	0.0227348	0.05663269	0.02518921	Tnfrsf1a
4-hour post-SCI group vs. the control group	BP	GO:1903140	regulation of establishment of endothelial barrier	1/35	0.0227348	0.05663269	0.02518921	Tnfrsf1a
4-hour post-SCI group vs. the control group	BP	GO:0050777	negative regulation of immune response	2/35	0.023005	0.0571476	0.02541823	Hmox1/Tnfaip3
4-hour post-SCI group vs. the control group	BP	GO:0050905	neuromuscular process	2/35	0.023005	0.0571476	0.02541823	Gch1/Nr4a3
4-hour post-SCI group vs. the control group	BP	GO:0001956	positive regulation of neurotransmitter secretion	1/35	0.0239174	0.05828852	0.02592569	Sphk1
4-hour post-SCI group vs. the control group	BP	GO:0002544	chronic inflammatory response	1/35	0.0239174	0.05828852	0.02592569	Tnfaip3
4-hour post-SCI group vs. the control group	BP	GO:0006670	sphingosine metabolic process	1/35	0.0239174	0.05828852	0.02592569	Sphk1
4-hour post-SCI group vs. the control group	BP	GO:0009110	vitamin biosynthetic process	1/35	0.0239174	0.05828852	0.02592569	Nfkb1
4-hour post-SCI group vs. the control group	BP	GO:0009159	deoxyribonucleoside monophosphate catabolic process	1/35	0.0239174	0.05828852	0.02592569	Xdh
4-hour post-SCI group vs. the control group	BP	GO:0032305	positive regulation of icosanoid secretion	1/35	0.0239174	0.05828852	0.02592569	Il1a
4-hour post-SCI group vs. the control group	BP	GO:0032495	response to muramyl dipeptide	1/35	0.0239174	0.05828852	0.02592569	Tnfaip3
4-hour post-SCI group vs. the control group	BP	GO:0035313	wound healing, spreading of epidermal cells	1/35	0.0239174	0.05828852	0.02592569	Hbegf
4-hour post-SCI group vs. the control group	BP	GO:0044321	response to leptin	1/35	0.0239174	0.05828852	0.02592569	Nr4a3
4-hour post-SCI group vs. the control group	BP	GO:0050884	neuromuscular process controlling posture	1/35	0.0239174	0.05828852	0.02592569	Gch1

4-hour post-SCI group vs. the control group	BP	GO:0060314	regulation of ryanodine-sensitive calcium-release channel activity	1/35	0.0239174	0.05828852	0.02592569	Fkbp1b
4-hour post-SCI group vs. the control group	BP	GO:0071450	cellular response to oxygen radical	1/35	0.0239174	0.05828852	0.02592569	Gch1
4-hour post-SCI group vs. the control group	BP	GO:0071451	cellular response to superoxide	1/35	0.0239174	0.05828852	0.02592569	Gch1
4-hour post-SCI group vs. the control group	BP	GO:1904754	positive regulation of vascular associated smooth muscle cell migration	1/35	0.0239174	0.05828852	0.02592569	Nr4a3
4-hour post-SCI group vs. the control group	BP	GO:0006109	regulation of carbohydrate metabolic process	2/35	0.0241187	0.0586996	0.02610853	Myc/Nfkb1
4-hour post-SCI group vs. the control group	BP	GO:0045732	positive regulation of protein catabolic process	2/35	0.024344	0.05916813	0.02631693	Ezr/Tnfaip3
4-hour post-SCI group vs. the control group	BP	GO:1903169	regulation of calcium ion transmembrane transport	2/35	0.0245703	0.05963391	0.0265241	Fkbp1b/Slc8a1
4-hour post-SCI group vs. the control group	BP	GO:0007179	transforming growth factor beta receptor signaling pathway	2/35	0.0247974	0.05963391	0.0265241	Fos/Jun
4-hour post-SCI group vs. the control group	BP	GO:0098656	anion transmembrane transport	2/35	0.0247974	0.05963391	0.0265241	Myc/Ripk1
4-hour post-SCI group vs. the control group	BP	GO:0048771	tissue remodeling	2/35	0.0250253	0.05963391	0.0265241	Il1a/Nfkb1
4-hour post-SCI group vs. the control group	BP	GO:0003177	pulmonary valve development	1/35	0.0250986	0.05963391	0.0265241	Tnfrsf1a
4-hour post-SCI group vs. the control group	BP	GO:0032310	prostaglandin secretion	1/35	0.0250986	0.05963391	0.0265241	Il1a
4-hour post-SCI group vs. the control group	BP	GO:0035234	ectopic germ cell programmed cell death	1/35	0.0250986	0.05963391	0.0265241	Il1a
4-hour post-SCI group vs. the control group	BP	GO:0043555	regulation of translation in response to stress	1/35	0.0250986	0.05963391	0.0265241	Ppp1r15b
4-hour post-SCI group vs. the control group	BP	GO:0045821	positive regulation of glycolytic process	1/35	0.0250986	0.05963391	0.0265241	Myc
4-hour post-SCI group vs. the control group	BP	GO:0046321	positive regulation of fatty acid oxidation	1/35	0.0250986	0.05963391	0.0265241	Nr4a3
4-hour post-SCI group vs. the control group	BP	GO:0046325	negative regulation of glucose import	1/35	0.0250986	0.05963391	0.0265241	Myc
4-hour post-SCI group vs. the control group	BP	GO:0046653	tetrahydrofolate metabolic process	1/35	0.0250986	0.05963391	0.0265241	Gch1
4-hour post-SCI group vs. the control group	BP	GO:0051546	keratinocyte migration	1/35	0.0250986	0.05963391	0.0265241	Hbegf
4-hour post-SCI group vs. the control group	BP	GO:0061469	regulation of type B pancreatic cell proliferation	1/35	0.0250986	0.05963391	0.0265241	Nr4a3
4-hour post-SCI group vs. the control group	BP	GO:0070230	positive regulation of lymphocyte apoptotic process	1/35	0.0250986	0.05963391	0.0265241	Myc
4-hour post-SCI group vs. the control group	BP	GO:0090050	positive regulation of cell migration involved in sprouting angiogenesis	1/35	0.0250986	0.05963391	0.0265241	Hmox1
4-hour post-SCI group vs. the control group	BP	GO:1990182	exosomal secretion	1/35	0.0250986	0.05963391	0.0265241	Sdc1
4-hour post-SCI group vs. the control group	BP	GO:0016051	carbohydrate biosynthetic process	2/35	0.0254839	0.06046967	0.02689583	Atf4/Nfkb1
4-hour post-SCI group vs. the control group	BP	GO:0043491	protein kinase B signaling	2/35	0.0259459	0.060676	0.0269876	Hbegf/Xdh
4-hour post-SCI group vs. the control group	BP	GO:0006144	purine nucleobase metabolic process	1/35	0.0262783	0.060676	0.0269876	Xdh
4-hour post-SCI group vs. the control group	BP	GO:0009158	ribonucleoside monophosphate catabolic process	1/35	0.0262783	0.060676	0.0269876	Xdh
4-hour post-SCI group vs. the control group	BP	GO:0009164	nucleoside catabolic process	1/35	0.0262783	0.060676	0.0269876	Xdh
4-hour post-SCI group vs. the control group	BP	GO:0010039	response to iron ion	1/35	0.0262783	0.060676	0.0269876	Hmox1
4-hour post-SCI group vs. the control group	BP	GO:0030810	positive regulation of nucleotide biosynthetic process	1/35	0.0262783	0.060676	0.0269876	Myc
4-hour post-SCI group vs. the control group	BP	GO:0032516	positive regulation of phosphoprotein phosphatase activity	1/35	0.0262783	0.060676	0.0269876	Ppp1r15b
4-hour post-SCI group vs. the control group	BP	GO:0035278	miRNA-mediated gene silencing by inhibition of translation	1/35	0.0262783	0.060676	0.0269876	Ago1
4-hour post-SCI group vs. the control group	BP	GO:0045649	regulation of macrophage differentiation	1/35	0.0262783	0.060676	0.0269876	Ripk1
4-hour post-SCI group vs. the control group	BP	GO:0046519	sphingoid metabolic process	1/35	0.0262783	0.060676	0.0269876	Sphk1

4-hour post-SCI group vs. the control group	BP	GO:0050849	negative regulation of calcium-mediated signaling	1/35	0.0262783	0.060676	0.0269876	Rcan1
4-hour post-SCI group vs. the control group	BP	GO:0050910	detection of mechanical stimulus involved in sensory perception of sound	1/35	0.0262783	0.060676	0.0269876	Myc
4-hour post-SCI group vs. the control group	BP	GO:0051770	positive regulation of nitric-oxide synthase biosynthetic process	1/35	0.0262783	0.060676	0.0269876	Map2k3
4-hour post-SCI group vs. the control group	BP	GO:0060009	Sertoli cell development	1/35	0.0262783	0.060676	0.0269876	Il1a
4-hour post-SCI group vs. the control group	BP	GO:0060544	regulation of necroptotic process	1/35	0.0262783	0.060676	0.0269876	Ripk1
4-hour post-SCI group vs. the control group	BP	GO:0072111	cell proliferation involved in kidney development	1/35	0.0262783	0.060676	0.0269876	Myc
4-hour post-SCI group vs. the control group	BP	GO:0097734	extracellular exosome biogenesis	1/35	0.0262783	0.060676	0.0269876	Sdc1
4-hour post-SCI group vs. the control group	BP	GO:0098719	sodium ion import across plasma membrane	1/35	0.0262783	0.060676	0.0269876	Slc8a1
4-hour post-SCI group vs. the control group	BP	GO:1900373	positive regulation of purine nucleotide biosynthetic process	1/35	0.0262783	0.060676	0.0269876	Myc
4-hour post-SCI group vs. the control group	BP	GO:1900746	regulation of vascular endothelial growth factor signaling pathway	1/35	0.0262783	0.060676	0.0269876	Xdh
4-hour post-SCI group vs. the control group	BP	GO:2000209	regulation of anoikis	1/35	0.0262783	0.060676	0.0269876	Mcl1
4-hour post-SCI group vs. the control group	BP	GO:0022408	negative regulation of cell-cell adhesion	2/35	0.0264113	0.0609049	0.02708941	Hspb1/Tnfaip3
4-hour post-SCI group vs. the control group	BP	GO:0010721	negative regulation of cell development	2/35	0.0268801	0.06174882	0.02746477	Id1/Il1a
4-hour post-SCI group vs. the control group	BP	GO:0030307	positive regulation of cell growth	2/35	0.0268801	0.06174882	0.02746477	Hbegf/Sphk1
4-hour post-SCI group vs. the control group	BP	GO:0043281	regulation of cysteine-type endopeptidase activity involved in apoptotic process	2/35	0.0268801	0.06174882	0.02746477	Myc/Xdh
4-hour post-SCI group vs. the control group	BP	GO:2000113	negative regulation of cellular macromolecule biosynthetic process	2/35	0.0271158	0.06204462	0.02759634	Ago1/Hbegf
4-hour post-SCI group vs. the control group	BP	GO:0031669	cellular response to nutrient levels	2/35	0.0273523	0.06204462	0.02759634	Atf4/Jun
4-hour post-SCI group vs. the control group	BP	GO:0034249	negative regulation of cellular amide metabolic process	2/35	0.0273523	0.06204462	0.02759634	Ago1/Sphk1
4-hour post-SCI group vs. the control group	BP	GO:1902115	regulation of organelle assembly	2/35	0.0273523	0.06204462	0.02759634	Ezr/Sdc1
4-hour post-SCI group vs. the control group	BP	GO:0000303	response to superoxide	1/35	0.0274567	0.06204462	0.02759634	Gch1
4-hour post-SCI group vs. the control group	BP	GO:0000413	protein peptidyl-prolyl isomerization	1/35	0.0274567	0.06204462	0.02759634	Fkbp1b
4-hour post-SCI group vs. the control group	BP	GO:0002043	blood vessel endothelial cell proliferation involved in sprouting angiogenesis	1/35	0.0274567	0.06204462	0.02759634	Hmox1
4-hour post-SCI group vs. the control group	BP	GO:0009151	purine deoxyribonucleotide metabolic process	1/35	0.0274567	0.06204462	0.02759634	Xdh
4-hour post-SCI group vs. the control group	BP	GO:0020027	hemoglobin metabolic process	1/35	0.0274567	0.06204462	0.02759634	Cat
4-hour post-SCI group vs. the control group	BP	GO:0031649	heat generation	1/35	0.0274567	0.06204462	0.02759634	Il1a
4-hour post-SCI group vs. the control group	BP	GO:0071359	cellular response to dsRNA	1/35	0.0274567	0.06204462	0.02759634	Nfkb1
4-hour post-SCI group vs. the control group	BP	GO:1904385	cellular response to angiotensin	1/35	0.0274567	0.06204462	0.02759634	Nfkb1
4-hour post-SCI group vs. the control group	BP	GO:2001026	regulation of endothelial cell chemotaxis	1/35	0.0274567	0.06204462	0.02759634	Hspb1
4-hour post-SCI group vs. the control group	BP	GO:0009743	response to carbohydrate	2/35	0.0275897	0.06226696	0.02769523	Fkbp1b/Nfkb1
4-hour post-SCI group vs. the control group	BP	GO:0006006	glucose metabolic process	2/35	0.0283068	0.06350906	0.02824769	Atf4/Myc
4-hour post-SCI group vs. the control group	BP	GO:0030522	intracellular receptor signaling pathway	2/35	0.0285476	0.06350906	0.02824769	Rxrb/Tnfaip3
4-hour post-SCI group vs. the control group	BP	GO:0031396	regulation of protein ubiquitination	2/35	0.0285476	0.06350906	0.02824769	Sphk1/Tnfaip3
4-hour post-SCI group vs. the control group	BP	GO:0000305	response to oxygen radical	1/35	0.0286337	0.06350906	0.02824769	Gch1

4-hour post-SCI group vs. the control group	BP	GO:0032515	negative regulation of phosphoprotein phosphatase activity	1/35	0.0286337	0.06350906	0.02824769	Fkbp1b
4-hour post-SCI group vs. the control group	BP	GO:0042026	protein refolding	1/35	0.0286337	0.06350906	0.02824769	Hspb1
4-hour post-SCI group vs. the control group	BP	GO:0042537	benzene-containing compound metabolic process	1/35	0.0286337	0.06350906	0.02824769	Txnrd1
4-hour post-SCI group vs. the control group	BP	GO:0046037	GMP metabolic process	1/35	0.0286337	0.06350906	0.02824769	Xdh
4-hour post-SCI group vs. the control group	BP	GO:0046040	IMP metabolic process	1/35	0.0286337	0.06350906	0.02824769	Xdh
4-hour post-SCI group vs. the control group	BP	GO:0051481	negative regulation of cytosolic calcium ion concentration	1/35	0.0286337	0.06350906	0.02824769	Slc8a1
4-hour post-SCI group vs. the control group	BP	GO:0140112	extracellular vesicle biogenesis	1/35	0.0286337	0.06350906	0.02824769	Sdc1
4-hour post-SCI group vs. the control group	BP	GO:1902547	regulation of cellular response to vascular endothelial growth factor stimulus	1/35	0.0286337	0.06350906	0.02824769	Xdh
4-hour post-SCI group vs. the control group	BP	GO:1990000	amyloid fibril formation	1/35	0.0286337	0.06350906	0.02824769	Ripk1
4-hour post-SCI group vs. the control group	BP	GO:2000345	regulation of hepatocyte proliferation	1/35	0.0286337	0.06350906	0.02824769	Tnfaip3
4-hour post-SCI group vs. the control group	BP	GO:0043393	regulation of protein binding	2/35	0.0292747	0.06444976	0.0286661	Id1/Myc
4-hour post-SCI group vs. the control group	BP	GO:0007623	circadian rhythm	2/35	0.0295187	0.06444976	0.0286661	Atf4/Id1
4-hour post-SCI group vs. the control group	BP	GO:0009125	nucleoside monophosphate catabolic process	1/35	0.0298094	0.06444976	0.0286661	Xdh
4-hour post-SCI group vs. the control group	BP	GO:0010763	positive regulation of fibroblast migration	1/35	0.0298094	0.06444976	0.0286661	Slc8a1
4-hour post-SCI group vs. the control group	BP	GO:0010894	negative regulation of steroid biosynthetic process	1/35	0.0298094	0.06444976	0.0286661	Nfkb1
4-hour post-SCI group vs. the control group	BP	GO:0015732	prostaglandin transport	1/35	0.0298094	0.06444976	0.0286661	Il1a
4-hour post-SCI group vs. the control group	BP	GO:0032303	regulation of icosanoid secretion	1/35	0.0298094	0.06444976	0.0286661	Il1a
4-hour post-SCI group vs. the control group	BP	GO:0034114	regulation of heterotypic cell-cell adhesion	1/35	0.0298094	0.06444976	0.0286661	Tnfaip3
4-hour post-SCI group vs. the control group	BP	GO:0034138	toll-like receptor 3 signaling pathway	1/35	0.0298094	0.06444976	0.0286661	Tnfaip3
4-hour post-SCI group vs. the control group	BP	GO:0036010	protein localization to endosome	1/35	0.0298094	0.06444976	0.0286661	Ezr
4-hour post-SCI group vs. the control group	BP	GO:0045736	negative regulation of cyclin-dependent protein serine/threonine kinase activity	1/35	0.0298094	0.06444976	0.0286661	Tnfaip3
4-hour post-SCI group vs. the control group	BP	GO:0050860	negative regulation of T cell receptor signaling pathway	1/35	0.0298094	0.06444976	0.0286661	Ezr
4-hour post-SCI group vs. the control group	BP	GO:0051590	positive regulation of neurotransmitter transport	1/35	0.0298094	0.06444976	0.0286661	Sphk1
4-hour post-SCI group vs. the control group	BP	GO:0060547	negative regulation of necrotic cell death	1/35	0.0298094	0.06444976	0.0286661	Ripk1
4-hour post-SCI group vs. the control group	BP	GO:0062098	regulation of programmed necrotic cell death	1/35	0.0298094	0.06444976	0.0286661	Ripk1
4-hour post-SCI group vs. the control group	BP	GO:1901032	negative regulation of response to reactive oxygen species	1/35	0.0298094	0.06444976	0.0286661	Nr4a3
4-hour post-SCI group vs. the control group	BP	GO:1903206	negative regulation of hydrogen peroxide-induced cell death	1/35	0.0298094	0.06444976	0.0286661	Nr4a3
4-hour post-SCI group vs. the control group	BP	GO:1903306	negative regulation of regulated secretory pathway	1/35	0.0298094	0.06444976	0.0286661	Hmox1
4-hour post-SCI group vs. the control group	BP	GO:1903798	regulation of miRNA maturation	1/35	0.0298094	0.06444976	0.0286661	Ripk1
4-hour post-SCI group vs. the control group	BP	GO:2000193	positive regulation of fatty acid transport	1/35	0.0298094	0.06444976	0.0286661	Il1a
4-hour post-SCI group vs. the control group	BP	GO:2001169	regulation of ATP biosynthetic process	1/35	0.0298094	0.06444976	0.0286661	Myc
4-hour post-SCI group vs. the control group	BP	GO:0001649	osteoblast differentiation	2/35	0.0302557	0.06533643	0.02906048	Atf4/Id1
4-hour post-SCI group vs. the control group	BP	GO:0008217	regulation of blood pressure	2/35	0.030503	0.06541788	0.02909671	Gch1/Hmox1
4-hour post-SCI group vs. the control group	BP	GO:0070374	positive regulation of ERK1 and ERK2 cascade	2/35	0.0307512	0.06541788	0.02909671	Il1a/Jun

4-hour post-SCI group vs. the control group	BP	GO:0002082	regulation of oxidative phosphorylation	1/35	0.0309836	0.06541788	0.02909671	Myc
4-hour post-SCI group vs. the control group	BP	GO:0006760	folic acid-containing compound metabolic process	1/35	0.0309836	0.06541788	0.02909671	Gch1
4-hour post-SCI group vs. the control group	BP	GO:0006817	phosphate ion transport	1/35	0.0309836	0.06541788	0.02909671	Atf4
4-hour post-SCI group vs. the control group	BP	GO:0010829	negative regulation of glucose transmembrane transport	1/35	0.0309836	0.06541788	0.02909671	Myc
4-hour post-SCI group vs. the control group	BP	GO:0016226	iron-sulfur cluster assembly	1/35	0.0309836	0.06541788	0.02909671	Xdh
4-hour post-SCI group vs. the control group	BP	GO:0031163	metallo-sulfur cluster assembly	1/35	0.0309836	0.06541788	0.02909671	Xdh
4-hour post-SCI group vs. the control group	BP	GO:0031664	regulation of lipopolysaccharide-mediated signaling pathway	1/35	0.0309836	0.06541788	0.02909671	Tnfaip3
4-hour post-SCI group vs. the control group	BP	GO:0034656	nucleobase-containing small molecule catabolic process	1/35	0.0309836	0.06541788	0.02909671	Xdh
4-hour post-SCI group vs. the control group	BP	GO:0042474	middle ear morphogenesis	1/35	0.0309836	0.06541788	0.02909671	Myc
4-hour post-SCI group vs. the control group	BP	GO:0045939	negative regulation of steroid metabolic process	1/35	0.0309836	0.06541788	0.02909671	Nfkb1
4-hour post-SCI group vs. the control group	BP	GO:0046386	deoxyribose phosphate catabolic process	1/35	0.0309836	0.06541788	0.02909671	Xdh
4-hour post-SCI group vs. the control group	BP	GO:0046685	response to arsenic-containing substance	1/35	0.0309836	0.06541788	0.02909671	Hmox1
4-hour post-SCI group vs. the control group	BP	GO:0051354	negative regulation of oxidoreductase activity	1/35	0.0309836	0.06541788	0.02909671	Nfkb1
4-hour post-SCI group vs. the control group	BP	GO:0071108	protein K48-linked deubiquitination	1/35	0.0309836	0.06541788	0.02909671	Tnfaip3
4-hour post-SCI group vs. the control group	BP	GO:0097164	ammonium ion metabolic process	1/35	0.0309836	0.06541788	0.02909671	Gch1
4-hour post-SCI group vs. the control group	BP	GO:1904030	negative regulation of cyclin-dependent protein kinase activity	1/35	0.0309836	0.06541788	0.02909671	Tnfaip3
4-hour post-SCI group vs. the control group	BP	GO:1990776	response to angiotensin	1/35	0.0309836	0.06541788	0.02909671	Nfkb1
4-hour post-SCI group vs. the control group	BP	GO:0055001	muscle cell development	2/35	0.0312498	0.06590277	0.02931238	Rcan1/Slc8a1
4-hour post-SCI group vs. the control group	BP	GO:0051651	maintenance of location in cell	2/35	0.0320039	0.06664416	0.02964213	Fkbp1b/Slc8a1
4-hour post-SCI group vs. the control group	BP	GO:0003176	aortic valve development	1/35	0.0321565	0.06664416	0.02964213	Tnfrsf1a
4-hour post-SCI group vs. the control group	BP	GO:0009264	deoxyribonucleotide catabolic process	1/35	0.0321565	0.06664416	0.02964213	Xdh
4-hour post-SCI group vs. the control group	BP	GO:0009713	catechol-containing compound biosynthetic process	1/35	0.0321565	0.06664416	0.02964213	Gch1
4-hour post-SCI group vs. the control group	BP	GO:0014829	vascular associated smooth muscle contraction	1/35	0.0321565	0.06664416	0.02964213	Slc8a1
4-hour post-SCI group vs. the control group	BP	GO:0031100	animal organ regeneration	1/35	0.0321565	0.06664416	0.02964213	Hmox1
4-hour post-SCI group vs. the control group	BP	GO:0031338	regulation of vesicle fusion	1/35	0.0321565	0.06664416	0.02964213	Sphk1
4-hour post-SCI group vs. the control group	BP	GO:0032528	microvillus organization	1/35	0.0321565	0.06664416	0.02964213	Ezr
4-hour post-SCI group vs. the control group	BP	GO:0036003	positive regulation of transcription from RNA polymerase II promoter in response to stress	1/35	0.0321565	0.06664416	0.02964213	Atf4
4-hour post-SCI group vs. the control group	BP	GO:0042423	catecholamine biosynthetic process	1/35	0.0321565	0.06664416	0.02964213	Gch1
4-hour post-SCI group vs. the control group	BP	GO:0045838	positive regulation of membrane potential	1/35	0.0321565	0.06664416	0.02964213	Myc
4-hour post-SCI group vs. the control group	BP	GO:0060004	reflex	1/35	0.0321565	0.06664416	0.02964213	Nr4a3
4-hour post-SCI group vs. the control group	BP	GO:0060252	positive regulation of glial cell proliferation	1/35	0.0321565	0.06664416	0.02964213	Myc
4-hour post-SCI group vs. the control group	BP	GO:0070920	regulation of production of small RNA involved in gene silencing by RNA	1/35	0.0321565	0.06664416	0.02964213	Ripk1
4-hour post-SCI group vs. the control group	BP	GO:1901857	positive regulation of cellular respiration	1/35	0.0321565	0.06664416	0.02964213	Myc
4-hour post-SCI group vs. the control group	BP	GO:0007204	positive regulation of cytosolic calcium ion concentration	2/35	0.0325106	0.06730072	0.02993416	Fkbp1b/Slc8a1

4-hour post-SCI group vs. the control group	BP	GO:0003018	vascular process in circulatory system	2/35	0.0327652	0.0677498	0.0301339	Gch1/Slc8a1
4-hour post-SCI group vs. the control group	BP	GO:0002089	lens morphogenesis in camera-type eye	1/35	0.0333279	0.06820865	0.03033799	Atf4
4-hour post-SCI group vs. the control group	BP	GO:0007176	regulation of epidermal growth factor-activated receptor activity	1/35	0.0333279	0.06820865	0.03033799	Hbegf
4-hour post-SCI group vs. the control group	BP	GO:0032740	positive regulation of interleukin-17 production	1/35	0.0333279	0.06820865	0.03033799	Sphk1
4-hour post-SCI group vs. the control group	BP	GO:0042430	indole-containing compound metabolic process	1/35	0.0333279	0.06820865	0.03033799	Gch1
4-hour post-SCI group vs. the control group	BP	GO:0045822	negative regulation of heart contraction	1/35	0.0333279	0.06820865	0.03033799	Fkbp1b
4-hour post-SCI group vs. the control group	BP	GO:0046128	purine ribonucleoside metabolic process	1/35	0.0333279	0.06820865	0.03033799	Xdh
4-hour post-SCI group vs. the control group	BP	GO:0046688	response to copper ion	1/35	0.0333279	0.06820865	0.03033799	Il1a
4-hour post-SCI group vs. the control group	BP	GO:0051767	nitric-oxide synthase biosynthetic process	1/35	0.0333279	0.06820865	0.03033799	Map2k3
4-hour post-SCI group vs. the control group	BP	GO:0051769	regulation of nitric-oxide synthase biosynthetic process	1/35	0.0333279	0.06820865	0.03033799	Map2k3
4-hour post-SCI group vs. the control group	BP	GO:0046777	protein autophosphorylation	2/35	0.0343091	0.07004618	0.03115529	Jun/Ripk1
4-hour post-SCI group vs. the control group	BP	GO:0010893	positive regulation of steroid biosynthetic process	1/35	0.034498	0.07004618	0.03115529	Il1a
4-hour post-SCI group vs. the control group	BP	GO:0032770	positive regulation of monooxygenase activity	1/35	0.034498	0.07004618	0.03115529	Gch1
4-hour post-SCI group vs. the control group	BP	GO:0033198	response to ATP	1/35	0.034498	0.07004618	0.03115529	Slc8a1
4-hour post-SCI group vs. the control group	BP	GO:0048384	retinoic acid receptor signaling pathway	1/35	0.034498	0.07004618	0.03115529	Rxrb
4-hour post-SCI group vs. the control group	BP	GO:0060008	Sertoli cell differentiation	1/35	0.034498	0.07004618	0.03115529	Il1a
4-hour post-SCI group vs. the control group	BP	GO:1903649	regulation of cytoplasmic transport	1/35	0.034498	0.07004618	0.03115529	Ezr
4-hour post-SCI group vs. the control group	BP	GO:0071560	cellular response to transforming growth factor beta stimulus	2/35	0.0345691	0.07011152	0.03118435	Fos/Jun
4-hour post-SCI group vs. the control group	BP	GO:2000116	regulation of cysteine-type endopeptidase activity	2/35	0.03483	0.07056109	0.03138431	Myc/Xdh
4-hour post-SCI group vs. the control group	BP	GO:0071559	response to transforming growth factor beta	2/35	0.0356172	0.0712149	0.03167512	Fos/Jun
4-hour post-SCI group vs. the control group	BP	GO:0001516	prostaglandin biosynthetic process	1/35	0.0356668	0.0712149	0.03167512	Sphk1
4-hour post-SCI group vs. the control group	BP	GO:0002507	tolerance induction	1/35	0.0356668	0.0712149	0.03167512	Tnfaip3
4-hour post-SCI group vs. the control group	BP	GO:0007214	gamma-aminobutyric acid signaling pathway	1/35	0.0356668	0.0712149	0.03167512	Atf4
4-hour post-SCI group vs. the control group	BP	GO:0010800	positive regulation of peptidyl-threonine phosphorylation	1/35	0.0356668	0.0712149	0.03167512	Sphk1
4-hour post-SCI group vs. the control group	BP	GO:0032691	negative regulation of interleukin-1 beta production	1/35	0.0356668	0.0712149	0.03167512	Tnfaip3
4-hour post-SCI group vs. the control group	BP	GO:0046033	AMP metabolic process	1/35	0.0356668	0.0712149	0.03167512	Xdh
4-hour post-SCI group vs. the control group	BP	GO:0046457	prostanoid biosynthetic process	1/35	0.0356668	0.0712149	0.03167512	Sphk1
4-hour post-SCI group vs. the control group	BP	GO:0046885	regulation of hormone biosynthetic process	1/35	0.0356668	0.0712149	0.03167512	Nfkb1
4-hour post-SCI group vs. the control group	BP	GO:0071312	cellular response to alkaloid	1/35	0.0356668	0.0712149	0.03167512	Slc8a1
4-hour post-SCI group vs. the control group	BP	GO:0150077	regulation of neuroinflammatory response	1/35	0.0356668	0.0712149	0.03167512	Sphk1
4-hour post-SCI group vs. the control group	BP	GO:1901658	glycosyl compound catabolic process	1/35	0.0356668	0.0712149	0.03167512	Xdh
4-hour post-SCI group vs. the control group	BP	GO:1903523	negative regulation of blood circulation	1/35	0.0356668	0.0712149	0.03167512	Fkbp1b
4-hour post-SCI group vs. the control group	BP	GO:0001832	blastocyst growth	1/35	0.0368341	0.07234269	0.03217674	Hbegf
4-hour post-SCI group vs. the control group	BP	GO:0002026	regulation of the force of heart contraction	1/35	0.0368341	0.07234269	0.03217674	Slc8a1
4-hour post-SCI group vs. the control group	BP	GO:0009162	deoxyribonucleoside monophosphate metabolic process	1/35	0.0368341	0.07234269	0.03217674	Xdh
4-hour post-SCI group vs. the control group	BP	GO:0010460	positive regulation of heart rate	1/35	0.0368341	0.07234269	0.03217674	Gch1

4-hour post-SCI group vs. the control group	BP	GO:0010623	programmed cell death involved in cell development	1/35	0.0368341	0.07234269	0.03217674	Il1a
4-hour post-SCI group vs. the control group	BP	GO:0035455	response to interferon-alpha	1/35	0.0368341	0.07234269	0.03217674	Myc
4-hour post-SCI group vs. the control group	BP	GO:0035767	endothelial cell chemotaxis	1/35	0.0368341	0.07234269	0.03217674	Hspb1
4-hour post-SCI group vs. the control group	BP	GO:0042558	pteridine-containing compound metabolic process	1/35	0.0368341	0.07234269	0.03217674	Gch1
4-hour post-SCI group vs. the control group	BP	GO:0043276	anoikis	1/35	0.0368341	0.07234269	0.03217674	Mcl1
4-hour post-SCI group vs. the control group	BP	GO:0045454	cell redox homeostasis	1/35	0.0368341	0.07234269	0.03217674	Txnrd1
4-hour post-SCI group vs. the control group	BP	GO:0048535	lymph node development	1/35	0.0368341	0.07234269	0.03217674	Nfkb1
4-hour post-SCI group vs. the control group	BP	GO:0050858	negative regulation of antigen receptor-mediated signaling pathway	1/35	0.0368341	0.07234269	0.03217674	Ezr
4-hour post-SCI group vs. the control group	BP	GO:0060561	apoptotic process involved in morphogenesis	1/35	0.0368341	0.07234269	0.03217674	Tnfrsf1a
4-hour post-SCI group vs. the control group	BP	GO:1901798	positive regulation of signal transduction by p53 class mediator	1/35	0.0368341	0.07234269	0.03217674	Myc
4-hour post-SCI group vs. the control group	BP	GO:1902883	negative regulation of response to oxidative stress	1/35	0.0368341	0.07234269	0.03217674	Nr4a3
4-hour post-SCI group vs. the control group	BP	GO:1903320	regulation of protein modification by small protein conjugation or removal	2/35	0.0372123	0.07300578	0.03247167	Sphk1/Tnfaip3
4-hour post-SCI group vs. the control group	BP	GO:0019318	hexose metabolic process	2/35	0.0374808	0.07335281	0.03262602	Atf4/Myc
4-hour post-SCI group vs. the control group	BP	GO:0017157	regulation of exocytosis	2/35	0.0377501	0.07335281	0.03262602	Hmox1/Sdc1
4-hour post-SCI group vs. the control group	BP	GO:0043583	ear development	2/35	0.0377501	0.07335281	0.03262602	Myc/Nr4a3
4-hour post-SCI group vs. the control group	BP	GO:0001893	maternal placenta development	1/35	0.0380001	0.07335281	0.03262602	Rxrb
4-hour post-SCI group vs. the control group	BP	GO:0002313	mature B cell differentiation involved in immune response	1/35	0.0380001	0.07335281	0.03262602	Tnfaip3
4-hour post-SCI group vs. the control group	BP	GO:0016242	negative regulation of macroautophagy	1/35	0.0380001	0.07335281	0.03262602	Hmox1
4-hour post-SCI group vs. the control group	BP	GO:0035162	embryonic hemopoiesis	1/35	0.0380001	0.07335281	0.03262602	Atf4
4-hour post-SCI group vs. the control group	BP	GO:0044030	regulation of DNA methylation	1/35	0.0380001	0.07335281	0.03262602	Myc
4-hour post-SCI group vs. the control group	BP	GO:0045742	positive regulation of epidermal growth factor receptor signaling pathway	1/35	0.0380001	0.07335281	0.03262602	Hbegf
4-hour post-SCI group vs. the control group	BP	GO:0045943	positive regulation of transcription by RNA polymerase I	1/35	0.0380001	0.07335281	0.03262602	Atf4
4-hour post-SCI group vs. the control group	BP	GO:0051150	regulation of smooth muscle cell differentiation	1/35	0.0380001	0.07335281	0.03262602	Rcan1
4-hour post-SCI group vs. the control group	BP	GO:0060795	cell fate commitment involved in formation of primary germ layer	1/35	0.0380001	0.07335281	0.03262602	Ets2
4-hour post-SCI group vs. the control group	BP	GO:0072574	hepatocyte proliferation	1/35	0.0380001	0.07335281	0.03262602	Tnfaip3
4-hour post-SCI group vs. the control group	BP	GO:0072575	epithelial cell proliferation involved in liver morphogenesis	1/35	0.0380001	0.07335281	0.03262602	Tnfaip3
4-hour post-SCI group vs. the control group	BP	GO:1904752	regulation of vascular associated smooth muscle cell migration	1/35	0.0380001	0.07335281	0.03262602	Nr4a3
4-hour post-SCI group vs. the control group	BP	GO:0051642	centrosome localization	1/35	0.0391647	0.07527816	0.03348238	Ezr
4-hour post-SCI group vs. the control group	BP	GO:1902253	regulation of intrinsic apoptotic signaling pathway by p53 class mediator	1/35	0.0391647	0.07527816	0.03348238	Myc
4-hour post-SCI group vs. the control group	BP	GO:1904706	negative regulation of vascular associated smooth muscle cell proliferation	1/35	0.0391647	0.07527816	0.03348238	Hmox1

4-hour post-SCI group vs. the control group	BP	GO:1905314	semi-lunar valve development	1/35	0.0391647	0.07527816	0.03348238	Tnfrsf1a
4-hour post-SCI group vs. the control group	BP	GO:0006520	cellular amino acid metabolic process	2/35	0.0393814	0.07561401	0.03363176	Atf4/Txnrd1
4-hour post-SCI group vs. the control group	BP	GO:0002053	positive regulation of mesenchymal cell proliferation	1/35	0.0403279	0.07629268	0.03393362	Myc
4-hour post-SCI group vs. the control group	BP	GO:0007007	inner mitochondrial membrane organization	1/35	0.0403279	0.07629268	0.03393362	Myc
4-hour post-SCI group vs. the control group	BP	GO:0009119	ribonucleoside metabolic process	1/35	0.0403279	0.07629268	0.03393362	Xdh
4-hour post-SCI group vs. the control group	BP	GO:0010614	negative regulation of cardiac muscle hypertrophy	1/35	0.0403279	0.07629268	0.03393362	Tnfrsf1a
4-hour post-SCI group vs. the control group	BP	GO:0035308	negative regulation of protein dephosphorylation	1/35	0.0403279	0.07629268	0.03393362	Fkbp1b
4-hour post-SCI group vs. the control group	BP	GO:0044319	wound healing, spreading of cells	1/35	0.0403279	0.07629268	0.03393362	Hbegf
4-hour post-SCI group vs. the control group	BP	GO:0051973	positive regulation of telomerase activity	1/35	0.0403279	0.07629268	0.03393362	Myc
4-hour post-SCI group vs. the control group	BP	GO:0060402	calcium ion transport into cytosol	1/35	0.0403279	0.07629268	0.03393362	Slc8a1
4-hour post-SCI group vs. the control group	BP	GO:0061842	microtubule organizing center localization	1/35	0.0403279	0.07629268	0.03393362	Ezr
4-hour post-SCI group vs. the control group	BP	GO:0072576	liver morphogenesis	1/35	0.0403279	0.07629268	0.03393362	Tnfaip3
4-hour post-SCI group vs. the control group	BP	GO:0090322	regulation of superoxide metabolic process	1/35	0.0403279	0.07629268	0.03393362	Gch1
4-hour post-SCI group vs. the control group	BP	GO:0090505	epiboly involved in wound healing	1/35	0.0403279	0.07629268	0.03393362	Hbegf
4-hour post-SCI group vs. the control group	BP	GO:1901186	positive regulation of ERBB signaling pathway	1/35	0.0403279	0.07629268	0.03393362	Hbegf
4-hour post-SCI group vs. the control group	BP	GO:1903959	regulation of anion transmembrane transport	1/35	0.0403279	0.07629268	0.03393362	Ripk1
4-hour post-SCI group vs. the control group	BP	GO:0002719	negative regulation of cytokine production involved in immune response	1/35	0.0414898	0.07775563	0.03458431	Hmox1
4-hour post-SCI group vs. the control group	BP	GO:0009112	nucleobase metabolic process	1/35	0.0414898	0.07775563	0.03458431	Xdh
4-hour post-SCI group vs. the control group	BP	GO:0042278	purine nucleoside metabolic process	1/35	0.0414898	0.07775563	0.03458431	Xdh
4-hour post-SCI group vs. the control group	BP	GO:0045672	positive regulation of osteoclast differentiation	1/35	0.0414898	0.07775563	0.03458431	Fos
4-hour post-SCI group vs. the control group	BP	GO:0045987	positive regulation of smooth muscle contraction	1/35	0.0414898	0.07775563	0.03458431	Sphk1
4-hour post-SCI group vs. the control group	BP	GO:0048679	regulation of axon regeneration	1/35	0.0414898	0.07775563	0.03458431	Fkbp1b
4-hour post-SCI group vs. the control group	BP	GO:0090504	epiboly	1/35	0.0414898	0.07775563	0.03458431	Hbegf
4-hour post-SCI group vs. the control group	BP	GO:1990089	response to nerve growth factor	1/35	0.0414898	0.07775563	0.03458431	Nfkb1
4-hour post-SCI group vs. the control group	BP	GO:1990090	cellular response to nerve growth factor stimulus	1/35	0.0414898	0.07775563	0.03458431	Nfkb1
4-hour post-SCI group vs. the control group	BP	GO:0005996	monosaccharide metabolic process	2/35	0.041598	0.07779644	0.03460247	Atf4/Myc
4-hour post-SCI group vs. the control group	BP	GO:0050730	regulation of peptidyl-tyrosine phosphorylation	2/35	0.041598	0.07779644	0.03460247	Hbegf/Tnfrsf1a
4-hour post-SCI group vs. the control group	BP	GO:0090068	positive regulation of cell cycle process	2/35	0.0418783	0.07823951	0.03479954	Il1a/Sphk1
4-hour post-SCI group vs. the control group	BP	GO:0048738	cardiac muscle tissue development	2/35	0.0424412	0.07878279	0.03504118	Rxrb/Slc8a1
4-hour post-SCI group vs. the control group	BP	GO:0001702	gastrulation with mouth forming second	1/35	0.0426503	0.07878279	0.03504118	Ets2
4-hour post-SCI group vs. the control group	BP	GO:0002902	regulation of B cell apoptotic process	1/35	0.0426503	0.07878279	0.03504118	Myc
4-hour post-SCI group vs. the control group	BP	GO:0009154	purine ribonucleotide catabolic process	1/35	0.0426503	0.07878279	0.03504118	Xdh
4-hour post-SCI group vs. the control group	BP	GO:0010922	positive regulation of phosphatase activity	1/35	0.0426503	0.07878279	0.03504118	Ppp1r15b
4-hour post-SCI group vs. the control group	BP	GO:0014741	negative regulation of muscle hypertrophy	1/35	0.0426503	0.07878279	0.03504118	Tnfrsf1a
4-hour post-SCI group vs. the control group	BP	GO:0034122	negative regulation of toll-like receptor signaling pathway	1/35	0.0426503	0.07878279	0.03504118	Tnfaip3
4-hour post-SCI group vs. the control group	BP	GO:0042168	heme metabolic process	1/35	0.0426503	0.07878279	0.03504118	Hmox1
4-hour post-SCI group vs. the control group	BP	GO:0070536	protein K63-linked deubiquitination	1/35	0.0426503	0.07878279	0.03504118	Tnfaip3
4-hour post-SCI group vs. the control group	BP	GO:0090183	regulation of kidney development	1/35	0.0426503	0.07878279	0.03504118	Myc

4-hour post-SCI group vs. the control group	BP	GO:1903715	regulation of aerobic respiration	1/35	0.0426503	0.07878279	0.03504118	Myc
4-hour post-SCI group vs. the control group	BP	GO:1903039	positive regulation of leukocyte cell-cell adhesion	2/35	0.0427237	0.07883752	0.03506552	Il1a/Nr4a3
4-hour post-SCI group vs. the control group	BP	GO:0006898	receptor-mediated endocytosis	2/35	0.0432909	0.07969781	0.03544816	Ezr/Sdc1
4-hour post-SCI group vs. the control group	BP	GO:0009066	aspartate family amino acid metabolic process	1/35	0.0438095	0.07969781	0.03544816	Atf4
4-hour post-SCI group vs. the control group	BP	GO:0010923	negative regulation of phosphatase activity	1/35	0.0438095	0.07969781	0.03544816	Fkbp1b
4-hour post-SCI group vs. the control group	BP	GO:0033005	positive regulation of mast cell activation	1/35	0.0438095	0.07969781	0.03544816	Nr4a3
4-hour post-SCI group vs. the control group	BP	GO:0035329	hippo signaling	1/35	0.0438095	0.07969781	0.03544816	Map2k3
4-hour post-SCI group vs. the control group	BP	GO:0044342	type B pancreatic cell proliferation	1/35	0.0438095	0.07969781	0.03544816	Nr4a3
4-hour post-SCI group vs. the control group	BP	GO:0045920	negative regulation of exocytosis	1/35	0.0438095	0.07969781	0.03544816	Hmox1
4-hour post-SCI group vs. the control group	BP	GO:0046677	response to antibiotic	1/35	0.0438095	0.07969781	0.03544816	Id1
4-hour post-SCI group vs. the control group	BP	GO:0070266	necroptotic process	1/35	0.0438095	0.07969781	0.03544816	Ripk1
4-hour post-SCI group vs. the control group	BP	GO:0070306	lens fiber cell differentiation	1/35	0.0438095	0.07969781	0.03544816	Atf4
4-hour post-SCI group vs. the control group	BP	GO:0071711	basement membrane organization	1/35	0.0438095	0.07969781	0.03544816	Pxdn
4-hour post-SCI group vs. the control group	BP	GO:0090075	relaxation of muscle	1/35	0.0438095	0.07969781	0.03544816	Slc8a1
4-hour post-SCI group vs. the control group	BP	GO:1904738	vascular associated smooth muscle cell migration	1/35	0.0438095	0.07969781	0.03544816	Nr4a3
4-hour post-SCI group vs. the control group	BP	GO:2000191	regulation of fatty acid transport	1/35	0.0438095	0.07969781	0.03544816	Il1a
4-hour post-SCI group vs. the control group	BP	GO:0071375	cellular response to peptide hormone stimulus	2/35	0.0444338	0.08075196	0.03591703	Nfkb1/Nr4a3
4-hour post-SCI group vs. the control group	BP	GO:0009069	serine family amino acid metabolic process	1/35	0.0449672	0.08114829	0.03609331	Txnrd1
4-hour post-SCI group vs. the control group	BP	GO:0010737	protein kinase A signaling	1/35	0.0449672	0.08114829	0.03609331	Ezr
4-hour post-SCI group vs. the control group	BP	GO:0030808	regulation of nucleotide biosynthetic process	1/35	0.0449672	0.08114829	0.03609331	Myc
4-hour post-SCI group vs. the control group	BP	GO:0032692	negative regulation of interleukin-1 production	1/35	0.0449672	0.08114829	0.03609331	Tnfaip3
4-hour post-SCI group vs. the control group	BP	GO:0050869	negative regulation of B cell activation	1/35	0.0449672	0.08114829	0.03609331	Tnfaip3
4-hour post-SCI group vs. the control group	BP	GO:1900371	regulation of purine nucleotide biosynthetic process	1/35	0.0449672	0.08114829	0.03609331	Myc
4-hour post-SCI group vs. the control group	BP	GO:1903580	positive regulation of ATP metabolic process	1/35	0.0449672	0.08114829	0.03609331	Myc
4-hour post-SCI group vs. the control group	BP	GO:0003007	heart morphogenesis	2/35	0.0458782	0.08216488	0.03654547	Jun/Slc8a1
4-hour post-SCI group vs. the control group	BP	GO:0006775	fat-soluble vitamin metabolic process	1/35	0.0461236	0.08216488	0.03654547	Nfkb1
4-hour post-SCI group vs. the control group	BP	GO:0009167	purine ribonucleoside monophosphate metabolic process	1/35	0.0461236	0.08216488	0.03654547	Xdh
4-hour post-SCI group vs. the control group	BP	GO:0010661	positive regulation of muscle cell apoptotic process	1/35	0.0461236	0.08216488	0.03654547	Atf4
4-hour post-SCI group vs. the control group	BP	GO:0018208	peptidyl-proline modification	1/35	0.0461236	0.08216488	0.03654547	Fkbp1b
4-hour post-SCI group vs. the control group	BP	GO:0030866	cortical actin cytoskeleton organization	1/35	0.0461236	0.08216488	0.03654547	Ezr
4-hour post-SCI group vs. the control group	BP	GO:0032885	regulation of polysaccharide biosynthetic process	1/35	0.0461236	0.08216488	0.03654547	Nfkb1
4-hour post-SCI group vs. the control group	BP	GO:0043304	regulation of mast cell degranulation	1/35	0.0461236	0.08216488	0.03654547	Hmox1
4-hour post-SCI group vs. the control group	BP	GO:0045589	regulation of regulatory T cell differentiation	1/35	0.0461236	0.08216488	0.03654547	Hspb1
4-hour post-SCI group vs. the control group	BP	GO:0048265	response to pain	1/35	0.0461236	0.08216488	0.03654547	Gch1
4-hour post-SCI group vs. the control group	BP	GO:0060674	placenta blood vessel development	1/35	0.0461236	0.08216488	0.03654547	Fosl1
4-hour post-SCI group vs. the control group	BP	GO:0070570	regulation of neuron projection regeneration	1/35	0.0461236	0.08216488	0.03654547	Fkbp1b
4-hour post-SCI group vs. the control group	BP	GO:0090049	regulation of cell migration involved in sprouting angiogenesis	1/35	0.0461236	0.08216488	0.03654547	Hmox1
4-hour post-SCI group vs. the control group	BP	GO:0002931	response to ischemia	1/35	0.0472787	0.08356128	0.03716656	Rcan1

4-hour post-SCI group vs. the control group	BP	GO:0006195	purine nucleotide catabolic process	1/35	0.0472787	0.08356128	0.03716656	Xdh
4-hour post-SCI group vs. the control group	BP	GO:0030212	hyaluronan metabolic process	1/35	0.0472787	0.08356128	0.03716656	Nfkb1
4-hour post-SCI group vs. the control group	BP	GO:0032743	positive regulation of interleukin-2 production	1/35	0.0472787	0.08356128	0.03716656	Il1a
4-hour post-SCI group vs. the control group	BP	GO:0042398	cellular modified amino acid biosynthetic process	1/35	0.0472787	0.08356128	0.03716656	Gch1
4-hour post-SCI group vs. the control group	BP	GO:0043267	negative regulation of potassium ion transport	1/35	0.0472787	0.08356128	0.03716656	Atf4
4-hour post-SCI group vs. the control group	BP	GO:0098703	calcium ion import across plasma membrane	1/35	0.0472787	0.08356128	0.03716656	Slc8a1
4-hour post-SCI group vs. the control group	BP	GO:1901020	negative regulation of calcium ion transmembrane transporter activity	1/35	0.0472787	0.08356128	0.03716656	Fkbp1b
4-hour post-SCI group vs. the control group	BP	GO:0046942	carboxylic acid transport	2/35	0.0479294	0.08462833	0.03764117	Il1a/Myc
4-hour post-SCI group vs. the control group	BP	GO:0014706	striated muscle tissue development	2/35	0.0482252	0.08468616	0.03766689	Rxrb/Slc8a1
4-hour post-SCI group vs. the control group	BP	GO:0045055	regulated exocytosis	2/35	0.0482252	0.08468616	0.03766689	Hmox1/Nr4a3
4-hour post-SCI group vs. the control group	BP	GO:0002335	mature B cell differentiation	1/35	0.0484324	0.08468616	0.03766689	Tnfaip3
4-hour post-SCI group vs. the control group	BP	GO:0006636	unsaturated fatty acid biosynthetic process	1/35	0.0484324	0.08468616	0.03766689	Sphk1
4-hour post-SCI group vs. the control group	BP	GO:0006953	acute-phase response	1/35	0.0484324	0.08468616	0.03766689	Il1a
4-hour post-SCI group vs. the control group	BP	GO:0010613	positive regulation of cardiac muscle hypertrophy	1/35	0.0484324	0.08468616	0.03766689	Nr4a3
4-hour post-SCI group vs. the control group	BP	GO:0010939	regulation of necrotic cell death	1/35	0.0484324	0.08468616	0.03766689	Ripk1
4-hour post-SCI group vs. the control group	BP	GO:0048520	positive regulation of behavior	1/35	0.0484324	0.08468616	0.03766689	Nr4a3
4-hour post-SCI group vs. the control group	BP	GO:0060259	regulation of feeding behavior	1/35	0.0484324	0.08468616	0.03766689	Nr4a3
4-hour post-SCI group vs. the control group	BP	GO:1904037	positive regulation of epithelial cell apoptotic process	1/35	0.0484324	0.08468616	0.03766689	Hmox1
4-hour post-SCI group vs. the control group	BP	GO:0032412	regulation of ion transmembrane transporter activity	2/35	0.0488188	0.08527898	0.03793057	Fkbp1b/Ripk1
4-hour post-SCI group vs. the control group	BP	GO:0006356	regulation of transcription by RNA polymerase I	1/35	0.0495847	0.08595005	0.03822905	Atf4
4-hour post-SCI group vs. the control group	BP	GO:0009126	purine nucleoside monophosphate metabolic process	1/35	0.0495847	0.08595005	0.03822905	Xdh
4-hour post-SCI group vs. the control group	BP	GO:0010464	regulation of mesenchymal cell proliferation	1/35	0.0495847	0.08595005	0.03822905	Myc
4-hour post-SCI group vs. the control group	BP	GO:0014742	positive regulation of muscle hypertrophy	1/35	0.0495847	0.08595005	0.03822905	Nr4a3
4-hour post-SCI group vs. the control group	BP	GO:0033006	regulation of mast cell activation involved in immune response	1/35	0.0495847	0.08595005	0.03822905	Hmox1
4-hour post-SCI group vs. the control group	BP	GO:0040018	positive regulation of multicellular organism growth	1/35	0.0495847	0.08595005	0.03822905	Ezr
4-hour post-SCI group vs. the control group	BP	GO:0045022	early endosome to late endosome transport	1/35	0.0495847	0.08595005	0.03822905	Ezr
4-hour post-SCI group vs. the control group	BP	GO:0061081	positive regulation of myeloid leukocyte cytokine production involved in immune response	1/35	0.0495847	0.08595005	0.03822905	Nr4a3
4-hour post-SCI group vs. the control group	BP	GO:0051924	regulation of calcium ion transport	2/35	0.0497142	0.08609161	0.03829201	Fkbp1b/Slc8a1
4-hour post-SCI group vs. the control group	CC	GO:0090575	RNA polymerase II transcription regulator complex	6/35	4.38E-07	5.60E-05	4.28E-05	Atf4/Fos/Fosl1/Jun/Myc/Rxrb
4-hour post-SCI group vs. the control group	CC	GO:0030018	Z disc	3/35	0.0005409	0.03038961	0.02324206	Fkbp1b/Hspb1/Slc8a1
4-hour post-SCI group vs. the control group	CC	GO:0031674	I band	3/35	0.0007123	0.03038961	0.02324206	Fkbp1b/Hspb1/Slc8a1
4-hour post-SCI group vs. the control group	CC	GO:0045121	membrane raft	4/35	0.0012911	0.03336119	0.02551473	Ezr/Hmox1/Ripk1/Tnfrsf1a
4-hour post-SCI group vs. the control group	CC	GO:0098857	membrane microdomain	4/35	0.0013032	0.03336119	0.02551473	Ezr/Hmox1/Ripk1/Tnfrsf1a
4-hour post-SCI group vs. the control group	CC	GO:0030017	sarcomere	3/35	0.0017807	0.03798751	0.02905295	Fkbp1b/Hspb1/Slc8a1
4-hour post-SCI group vs. the control group	CC	GO:0030016	myofibril	3/35	0.002496	0.04121312	0.0315199	Fkbp1b/Hspb1/Slc8a1
4-hour post-SCI group vs. the control group	CC	GO:0043292	contractile fiber	3/35	0.002929	0.04121312	0.0315199	Fkbp1b/Hspb1/Slc8a1
4-hour post-SCI group vs. the control group	CC	GO:0000791	euchromatin	2/35	0.0031071	0.04121312	0.0315199	Jun/Myc

4-hour post-SCI group vs. the control group	CC	GO:0030315	T-tubule	2/35	0.003289	0.04121312	0.0315199	Ezr/Slc8a1
4-hour post-SCI group vs. the control group	CC	GO:0016529	sarcoplasmic reticulum	2/35	0.0038637	0.04121312	0.0315199	Fkbp1b/Xdh
4-hour post-SCI group vs. the control group	CC	GO:0017053	transcription repressor complex	2/35	0.0038637	0.04121312	0.0315199	Jun/Myc
4-hour post-SCI group vs. the control group	CC	GO:0016528	sarcoplasm	2/35	0.0063368	0.06239346	0.04771868	Fkbp1b/Xdh
4-hour post-SCI group vs. the control group	CC	GO:0044853	plasma membrane raft	2/35	0.0118916	0.08830172	0.06753339	Ezr/Hmox1
4-hour post-SCI group vs. the control group	CC	GO:0001931	uropod	1/35	0.0132876	0.08830172	0.06753339	Ezr
4-hour post-SCI group vs. the control group	CC	GO:0031254	cell trailing edge	1/35	0.0132876	0.08830172	0.06753339	Ezr
4-hour post-SCI group vs. the control group	CC	GO:0070578	RISC-loading complex	1/35	0.0132876	0.08830172	0.06753339	Ago1
4-hour post-SCI group vs. the control group	CC	GO:0001650	fibrillar center	2/35	0.0137855	0.08830172	0.06753339	Ezr/Txnrd1
4-hour post-SCI group vs. the control group	CC	GO:0005777	peroxisome	2/35	0.0141431	0.08830172	0.06753339	Cat/Xdh
4-hour post-SCI group vs. the control group	CC	GO:0042579	microbody	2/35	0.0141431	0.08830172	0.06753339	Cat/Xdh
4-hour post-SCI group vs. the control group	CC	GO:0060187	cell pole	1/35	0.014487	0.08830172	0.06753339	Ezr
4-hour post-SCI group vs. the control group	CC	GO:0042383	sarcolemma	2/35	0.0173424	0.10090108	0.07716941	Ezr/Slc8a1
4-hour post-SCI group vs. the control group	CC	GO:0097060	synaptic membrane	3/35	0.0190902	0.10277621	0.07860351	Fosl1/Hspb1/Slc8a1
4-hour post-SCI group vs. the control group	CC	GO:0090571	RNA polymerase II transcription repressor complex	1/35	0.0192705	0.10277621	0.07860351	Myc
4-hour post-SCI group vs. the control group	CC	GO:0000164	protein phosphatase type 1 complex	1/35	0.0204629	0.10477002	0.08012839	Ppp1r15b
4-hour post-SCI group vs. the control group	CC	GO:0031430	M band	1/35	0.0228434	0.11245973	0.0860095	Hspb1
4-hour post-SCI group vs. the control group	CC	GO:0005796	Golgi lumen	1/35	0.0240315	0.11392719	0.08713182	Sdc1
4-hour post-SCI group vs. the control group	CC	GO:0097449	astrocyte projection	1/35	0.0264036	0.11770671	0.0900224	Ezr
4-hour post-SCI group vs. the control group	CC	GO:0031528	microvillus membrane	1/35	0.0275875	0.11770671	0.0900224	Ezr
4-hour post-SCI group vs. the control group	CC	GO:0033017	sarcoplasmic reticulum membrane	1/35	0.0275875	0.11770671	0.0900224	Fkbp1b
4-hour post-SCI group vs. the control group	CC	GO:0016323	basolateral plasma membrane	2/35	0.0359301	0.14835666	0.11346356	Ezr/Slc8a1
4-hour post-SCI group vs. the control group	CC	GO:1902555	endoribonuclease complex	1/35	0.0405188	0.16207513	0.12395549	Ago1
4-hour post-SCI group vs. the control group	CC	GO:0009925	basal plasma membrane	2/35	0.0439539	0.17008761	0.13008345	Ezr/Slc8a1
4-hour post-SCI group vs. the control group	CC	GO:0031672	A band	1/35	0.0451795	0.17008761	0.13008345	Hspb1
4-hour post-SCI group vs. the control group	CC	GO:1905348	endonuclease complex	1/35	0.0475016	0.17372027	0.13286172	Ago1
4-hour post-SCI group vs. the control group	MF	GO:0061629	RNA polymerase II-specific DNA-binding transcription factor binding	7/35	3.29E-07	6.29E-05	3.26E-05	Atf4/Ets2/Fos/Hspb1/Jun/Nr4a3/Rxrb
4-hour post-SCI group vs. the control group	MF	GO:0016209	antioxidant activity	4/35	3.82E-06	0.00036454	0.00018885	Cat/Pxdn/Srxn1/Txnrd1
4-hour post-SCI group vs. the control group	MF	GO:0008022	protein C-terminus binding	5/35	9.45E-06	0.00060163	0.00031167	Atf4/Ezr/Id1/Sdc1/Tnfaip3
4-hour post-SCI group vs. the control group	MF	GO:0001221	transcription coregulator binding	4/35	2.15E-05	0.00102628	0.00053166	Fos/Myc/Nfkb1/Nr4a3
4-hour post-SCI group vs. the control group	MF	GO:0001046	core promoter sequence-specific DNA binding	3/35	3.43E-05	0.0013092	0.00067823	Ago1/Fos/Myc
4-hour post-SCI group vs. the control group	MF	GO:0004601	peroxidase activity	3/35	5.05E-05	0.00160254	0.00083019	Cat/Pxdn/Txnrd1
4-hour post-SCI group vs. the control group	MF	GO:0016684	oxidoreductase activity, acting on peroxide as acceptor	3/35	5.87E-05	0.00160254	0.00083019	Cat/Pxdn/Txnrd1
4-hour post-SCI group vs. the control group	MF	GO:0070513	death domain binding	2/35	9.66E-05	0.00230636	0.00119481	Mcl1/Ripk1
4-hour post-SCI group vs. the control group	MF	GO:0035497	cAMP response element binding	2/35	0.0002229	0.00473047	0.00245061	Jun/Nr4a3
4-hour post-SCI group vs. the control group	MF	GO:0035259	nuclear glucocorticoid receptor binding	2/35	0.0002489	0.00475461	0.00246312	Ets2/Nr4a3
4-hour post-SCI group vs. the control group	MF	GO:0070412	R-SMAD binding	2/35	0.0004347	0.00754798	0.00391023	Fos/Jun
4-hour post-SCI group vs. the control group	MF	GO:0001227	DNA-binding transcription repressor activity, RNA polymerase II-specific	4/35	0.000713	0.01083392	0.0056125	Ets2/Jun/Myc/Nfkb1

4-hour post-SCI group vs. the control group	MF	GO:0001217	DNA-binding transcription repressor activity	4/35	0.0007374	0.01083392	0.0056125	Ets2/Jun/Myc/Nfkb1
4-hour post-SCI group vs. the control group	MF	GO:0016922	nuclear receptor binding	3/35	0.0009176	0.01251935	0.00648564	Ets2/Nr4a3/Rxrb
4-hour post-SCI group vs. the control group	MF	GO:0020037	heme binding	3/35	0.001024	0.01303841	0.00675454	Cat/Hmox1/Pxdn
4-hour post-SCI group vs. the control group	MF	GO:0046906	tetrapyrrole binding	3/35	0.0011976	0.01383401	0.0071667	Cat/Hmox1/Pxdn
4-hour post-SCI group vs. the control group	MF	GO:0071949	FAD binding	2/35	0.0012313	0.01383401	0.0071667	Txnrd1/Xdh
4-hour post-SCI group vs. the control group	MF	GO:0001223	transcription coactivator binding	2/35	0.0016057	0.01703795	0.0088265	Nfkb1/Nr4a3
4-hour post-SCI group vs. the control group	MF	GO:0004879	nuclear receptor activity	2/35	0.001954	0.01866049	0.00966705	Nr4a3/Rxrb
4-hour post-SCI group vs. the control group	MF	GO:0098531	ligand-activated transcription factor activity	2/35	0.001954	0.01866049	0.00966705	Nr4a3/Rxrb
4-hour post-SCI group vs. the control group	MF	GO:0140296	general transcription initiation factor binding	2/35	0.0022561	0.02027112	0.01050144	Atf4/Jun
4-hour post-SCI group vs. the control group	MF	GO:0016667	oxidoreductase activity, acting on a sulfur group of donors	2/35	0.0023349	0.02027112	0.01050144	Srxn1/Txnrd1
4-hour post-SCI group vs. the control group	MF	GO:0140678	molecular function inhibitor activity	2/35	0.0024963	0.02073016	0.01073924	Fkbp1b/Id1
4-hour post-SCI group vs. the control group	MF	GO:0043621	protein self-association	2/35	0.0043885	0.03492488	0.0180928	Id1/Tnfaip3
4-hour post-SCI group vs. the control group	MF	GO:0046332	SMAD binding	2/35	0.0049388	0.03684111	0.0190855	Fos/Jun
4-hour post-SCI group vs. the control group	MF	GO:0050660	flavin adenine dinucleotide binding	2/35	0.0051674	0.03684111	0.0190855	Txnrd1/Xdh
4-hour post-SCI group vs. the control group	MF	GO:0019888	protein phosphatase regulator activity	2/35	0.0052835	0.03684111	0.0190855	Ppp1r15b/Rcan1
4-hour post-SCI group vs. the control group	MF	GO:0043130	ubiquitin binding	2/35	0.0054008	0.03684111	0.0190855	Hspb1/Tnfaip3
4-hour post-SCI group vs. the control group	MF	GO:0003727	single-stranded RNA binding	2/35	0.0063819	0.04203279	0.02177505	Ago1/Cbx6
4-hour post-SCI group vs. the control group	MF	GO:0031625	ubiquitin protein ligase binding	3/35	0.0068012	0.04330089	0.02243199	Jun/Myc/Ripk1
4-hour post-SCI group vs. the control group	MF	GO:0019208	phosphatase regulator activity	2/35	0.0075753	0.0466734	0.02417911	Ppp1r15b/Rcan1
4-hour post-SCI group vs. the control group	MF	GO:0044389	ubiquitin-like protein ligase binding	3/35	0.0078542	0.0468796	0.02428593	Jun/Myc/Ripk1
4-hour post-SCI group vs. the control group	MF	GO:0032182	ubiquitin-like protein binding	2/35	0.0082784	0.0479146	0.02482211	Hspb1/Tnfaip3
4-hour post-SCI group vs. the control group	MF	GO:0008140	cAMP response element binding protein binding	1/35	0.012256	0.06276718	0.03251648	Atf4
4-hour post-SCI group vs. the control group	MF	GO:0015368	calcium:cation antiporter activity	1/35	0.012256	0.06276718	0.03251648	Slc8a1
4-hour post-SCI group vs. the control group	MF	GO:0016174	NAD(P)H oxidase H2O2-forming activity	1/35	0.012256	0.06276718	0.03251648	Txnrd1
4-hour post-SCI group vs. the control group	MF	GO:0016810	hydrolase activity, acting on carbon-nitrogen (but not peptide) bonds	2/35	0.0123828	0.06276718	0.03251648	Cat/Gch1
4-hour post-SCI group vs. the control group	MF	GO:0005035	death receptor activity	1/35	0.0134736	0.06276718	0.03251648	Tnfrsf1a
4-hour post-SCI group vs. the control group	MF	GO:0019855	calcium channel inhibitor activity	1/35	0.0134736	0.06276718	0.03251648	Fkbp1b
4-hour post-SCI group vs. the control group	MF	GO:0047134	protein-disulfide reductase (NAD(P)) activity	1/35	0.0134736	0.06276718	0.03251648	Txnrd1
4-hour post-SCI group vs. the control group	MF	GO:0061578	Lys63-specific deubiquitinase activity	1/35	0.0134736	0.06276718	0.03251648	Tnfaip3
4-hour post-SCI group vs. the control group	MF	GO:0044325	transmembrane transporter binding	2/35	0.0143375	0.06463239	0.03348275	Fkbp1b/Slc8a1
4-hour post-SCI group vs. the control group	MF	GO:0016668	oxidoreductase activity, acting on a sulfur group of donors, NAD(P) as acceptor	1/35	0.0146897	0.06463239	0.03348275	Txnrd1
4-hour post-SCI group vs. the control group	MF	GO:0004706	JUN kinase kinase kinase activity	1/35	0.0159043	0.06463239	0.03348275	Ripk1
4-hour post-SCI group vs. the control group	MF	GO:0016661	oxidoreductase activity, acting on other nitrogenous compounds as donors	1/35	0.0159043	0.06463239	0.03348275	Xdh
4-hour post-SCI group vs. the control group	MF	GO:0043522	leucine zipper domain binding	1/35	0.0159043	0.06463239	0.03348275	Atf4
4-hour post-SCI group vs. the control group	MF	GO:0051400	BH domain binding	1/35	0.0159043	0.06463239	0.03348275	Mcl1

4-hour post-SCI group vs. the control group	MF	GO:0030297	transmembrane receptor protein tyrosine kinase activator activity	1/35	0.0171175	0.06605432	0.03421938	Hbegf
4-hour post-SCI group vs. the control group	MF	GO:0044548	S100 protein binding	1/35	0.0171175	0.06605432	0.03421938	Ezr
4-hour post-SCI group vs. the control group	MF	GO:0003951	NAD+ kinase activity	1/35	0.0183292	0.06605432	0.03421938	Sphk1
4-hour post-SCI group vs. the control group	MF	GO:0004708	MAP kinase kinase activity	1/35	0.0183292	0.06605432	0.03421938	Map2k3
4-hour post-SCI group vs. the control group	MF	GO:0034236	protein kinase A catalytic subunit binding	1/35	0.0183292	0.06605432	0.03421938	Ezr
4-hour post-SCI group vs. the control group	MF	GO:0050664	oxidoreductase activity, acting on NAD(P)H, oxygen as acceptor	1/35	0.0183292	0.06605432	0.03421938	Txnrd1
4-hour post-SCI group vs. the control group	MF	GO:0045125	bioactive lipid receptor activity	1/35	0.0195395	0.06911188	0.03580334	Sphk1
4-hour post-SCI group vs. the control group	MF	GO:0003707	nuclear steroid receptor activity	1/35	0.0207483	0.07205322	0.0373271	Rxrb
4-hour post-SCI group vs. the control group	MF	GO:0002020	protease binding	2/35	0.0218063	0.07437496	0.03852988	Tnfaip3/Tnfrsf1a
4-hour post-SCI group vs. the control group	MF	GO:0005516	calmodulin binding	2/35	0.0231413	0.0762736	0.03951347	Slc8a1/Sphk1
4-hour post-SCI group vs. the control group	MF	GO:0140416	transcription regulator inhibitor activity	1/35	0.0231616	0.0762736	0.03951347	Id1
4-hour post-SCI group vs. the control group	MF	GO:0005123	death receptor binding	1/35	0.0243661	0.07629387	0.03952396	Ripk1
4-hour post-SCI group vs. the control group	MF	GO:0030275	LRR domain binding	1/35	0.0243661	0.07629387	0.03952396	Atf4
4-hour post-SCI group vs. the control group	MF	GO:0070628	proteasome binding	1/35	0.0243661	0.07629387	0.03952396	Id1
4-hour post-SCI group vs. the control group	MF	GO:0000979	RNA polymerase II core promoter sequence-specific DNA binding	1/35	0.0279709	0.08193236	0.04244498	Fos
4-hour post-SCI group vs. the control group	MF	GO:0030506	ankyrin binding	1/35	0.0279709	0.08193236	0.04244498	Slc8a1
4-hour post-SCI group vs. the control group	MF	GO:0071837	HMG box domain binding	1/35	0.0279709	0.08193236	0.04244498	Jun
4-hour post-SCI group vs. the control group	MF	GO:0019887	protein kinase regulator activity	2/35	0.0285605	0.08193236	0.04244498	Hbegf/Hspb1
4-hour post-SCI group vs. the control group	MF	GO:0030742	GTP-dependent protein binding	1/35	0.0291696	0.08193236	0.04244498	Gch1
4-hour post-SCI group vs. the control group	MF	GO:0070530	K63-linked polyubiquitin modification-dependent protein binding	1/35	0.0291696	0.08193236	0.04244498	Tnfaip3
4-hour post-SCI group vs. the control group	MF	GO:0140828	metal cation:monoatomic cation antiporter activity	1/35	0.0291696	0.08193236	0.04244498	Slc8a1
4-hour post-SCI group vs. the control group	MF	GO:0004709	MAP kinase kinase kinase activity	1/35	0.0303669	0.08285833	0.04292468	Ripk1
4-hour post-SCI group vs. the control group	MF	GO:0034237	protein kinase A regulatory subunit binding	1/35	0.0303669	0.08285833	0.04292468	Ezr
4-hour post-SCI group vs. the control group	MF	GO:0051537	2 iron, 2 sulfur cluster binding	1/35	0.0315628	0.08490833	0.04398668	Xdh
4-hour post-SCI group vs. the control group	MF	GO:0016725	oxidoreductase activity, acting on CH or CH2 groups	1/35	0.0327572	0.0857072	0.04440053	Xdh
4-hour post-SCI group vs. the control group	MF	GO:0035035	histone acetyltransferase binding	1/35	0.0327572	0.0857072	0.04440053	Nr4a3
4-hour post-SCI group vs. the control group	MF	GO:0016814	hydrolase activity, acting on carbon-nitrogen (but not peptide) bonds, in cyclic amidines	1/35	0.0339502	0.08645983	0.04479043	Gch1
4-hour post-SCI group vs. the control group	MF	GO:0046966	nuclear thyroid hormone receptor binding	1/35	0.0339502	0.08645983	0.04479043	Rxrb
4-hour post-SCI group vs. the control group	MF	GO:0030296	protein tyrosine kinase activator activity	1/35	0.0351418	0.08716981	0.04515824	Hbegf
4-hour post-SCI group vs. the control group	MF	GO:0043236	laminin binding	1/35	0.0351418	0.08716981	0.04515824	Pxdn
4-hour post-SCI group vs. the control group	MF	GO:0008200	ion channel inhibitor activity	1/35	0.0363319	0.08784039	0.04550563	Fkbp1b
4-hour post-SCI group vs. the control group	MF	GO:0015298	solute:cation antiporter activity	1/35	0.0363319	0.08784039	0.04550563	Slc8a1
4-hour post-SCI group vs. the control group	MF	GO:0019207	kinase regulator activity	2/35	0.036854	0.08798901	0.04558262	Hbegf/Hspb1
4-hour post-SCI group vs. the control group	MF	GO:0016248	channel inhibitor activity	1/35	0.0387079	0.09016103	0.04670784	Fkbp1b
4-hour post-SCI group vs. the control group	MF	GO:0030291	protein serine/threonine kinase inhibitor activity	1/35	0.0387079	0.09016103	0.04670784	Hspb1

4-hour post-SCI group vs. the control group	MF	GO:0042974	nuclear retinoic acid receptor binding	1/35	0.0398937	0.09071076	0.04699262	Rxrb
4-hour post-SCI group vs. the control group	MF	GO:0051019	mitogen-activated protein kinase binding	1/35	0.0398937	0.09071076	0.04699262	Gch1
4-hour post-SCI group vs. the control group	MF	GO:0003755	peptidyl-prolyl cis-trans isomerase activity	1/35	0.0422612	0.09406597	0.04873079	Fkbp1b
4-hour post-SCI group vs. the control group	MF	GO:0000993	RNA polymerase II complex binding	1/35	0.044623	0.09406597	0.04873079	Ago1
4-hour post-SCI group vs. the control group	MF	GO:0030551	cyclic nucleotide binding	1/35	0.044623	0.09406597	0.04873079	Fkbp1b
4-hour post-SCI group vs. the control group	MF	GO:0035198	miRNA binding	1/35	0.044623	0.09406597	0.04873079	Ago1
4-hour post-SCI group vs. the control group	MF	GO:0097718	disordered domain specific binding	1/35	0.044623	0.09406597	0.04873079	Ezr
4-hour post-SCI group vs. the control group	MF	GO:0005154	epidermal growth factor receptor binding	1/35	0.0458018	0.09406597	0.04873079	Hbegf
4-hour post-SCI group vs. the control group	MF	GO:0015035	protein-disulfide reductase activity	1/35	0.0458018	0.09406597	0.04873079	Txnrd1
4-hour post-SCI group vs. the control group	MF	GO:0031369	translation initiation factor binding	1/35	0.0458018	0.09406597	0.04873079	Gch1
4-hour post-SCI group vs. the control group	MF	GO:0051721	protein phosphatase 2A binding	1/35	0.0458018	0.09406597	0.04873079	Sphk1
4-hour post-SCI group vs. the control group	MF	GO:0016859	cis-trans isomerase activity	1/35	0.0469791	0.09545756	0.0494517	Fkbp1b
4-hour post-SCI group vs. the control group	MF	GO:0042805	actinin binding	1/35	0.0481551	0.09580851	0.04963351	Nfkb1
4-hour post-SCI group vs. the control group	MF	GO:0044183	protein folding chaperone	1/35	0.0481551	0.09580851	0.04963351	Hspb1
4-hour post-SCI group vs. the control group	MF	GO:0043175	RNA polymerase core enzyme binding	1/35	0.0493296	0.09713354	0.05031994	Ago1
1-day post-SCI group vs. the control group	BP	GO:0006979	response to oxidative stress	63/125	2.08E-83	7.27E-80	3.78E-80	Abcc1/Adam9/Anxa1/Apoe/Atf2/Atf4/Atox1/Atp13a2/Atrn/Axl/Cat/Cyp1b1/Edn1/Eif2s1/Ercc1/Ermp1/Fbxw7/Fos/Gch1/Gfer/Gpr37l1/Gpx1/Gpx4/Hmox1/Hmox2/Hspb1/Jak2/Jun/Lancl1/Mapk8/Mapt/Mcl1/Met/Mmp3/Ndufa6/Nono/Nos3/P4hb/Pdk2/Pink1/Plekha1/Ppargc1a/Ppargc1b/Ppp1r15b/Prr5l/Ptgs2/Pxdn/Rcan1/Rela/Ripk1/Ripk3/Rnf112/Slc25a24/Slc4a11/Slc8a1/Sphk1/Srxn1/Stat6/Trap1/Trpm2/Tsc1/Txnrd1/Ucp2
1-day post-SCI group vs. the control group	BP	GO:0034599	cellular response to oxidative stress	48/125	4.49E-65	7.84E-62	4.08E-62	Anxa1/Atf2/Atf4/Atp13a2/Axl/Cat/Cyp1b1/Edn1/Eif2s1/Ermp1/Fbxw7/Fos/Gch1/Gfer/Gpr37l1/Gpx1/Hmox1/Hspb1/Jak2/Jun/Lancl1/Mapk8/Mapt/Mcl1/Met/Mmp3/Nono/Nos3/P4hb/Pdk2/Pink1/Plekha1/Ppargc1a/Ppargc1b/Prr5l/Rela/Ripk1/Ripk3/Rnf112/Slc25a24/Slc4a11/Slc8a1/Sphk1/Srxn1/Stat6/Trap1/Trpm2/Tsc1
1-day post-SCI group vs. the control group	BP	GO:0062197	cellular response to chemical stress	50/125	8.77E-64	1.02E-60	5.31E-61	Anxa1/Atf2/Atf4/Atp13a2/Axl/Cat/Cyp1b1/Edn1/Eif2s1/Ermp1/Fbxw7/Fos/Gch1/Gfer/Gpr37l1/Gpx1/Hmox1/Hspb1/Jak2/Jun/Lancl1/Mapk8/Mapt/Mcl1/Met/Mmp3/Nono/Nos3/P4hb/Pdk2/Pink1/Plekha1/Ppargc1a/Ppargc1b/Prr5l/Ptgs2/Rela/Ripk1/Ripk3/Rnf112/Slc25a24/Slc4a11/Slc8a1/Sphk1/Srxn1/Stat6/Trap1/Trpm2/Tsc1/Ybx3
1-day post-SCI group vs. the control group	BP	GO:0000302	response to reactive oxygen species	30/125	1.22E-38	1.07E-35	5.56E-36	Adam9/Anxa1/Axl/Cat/Cyp1b1/Edn1/Fos/Gch1/Gpr37l1/Gpx1/Hmox1/Jun/Mapk8/Met/Nos3/Pdk2/Pink1/Plekha1/Ppargc1b/Ppp1r15b/Rela/Ripk1/Ripk3/Rnf112/Slc8a1/Sphk1/Stat6/Trap1/Trpm2/Ucp2
1-day post-SCI group vs. the control group	BP	GO:0034614	cellular response to reactive oxygen species	25/125	2.11E-33	1.47E-30	7.67E-31	Anxa1/Axl/Cat/Cyp1b1/Edn1/Fos/Gch1/Gpr37l1/Jun/Mapk8/Met/Nos3/Pdk2/Pink1/Plekha1/Ppargc1b/Rela/Ripk1/Ripk3/Rnf112/Slc8a1/Sphk1/Stat6/Trap1/Trpm2

1-day post-SCI group vs. the control group	BP	GO:0036473	cell death in response to oxidative stress	21/125	7.32E-30	4.26E-27	2.22E-27	Atf4/Cyp1b1/Fbxw7/Gfer/Gpr37l1/Gpx1/Hspb1/Jak2/Lancl1/Mapt/Mcl1/Met/Mmp3/Nono/P4hb/Pink1/Ripk1/Rnf112/Trap1/Trpm2/Tsc1
1-day post-SCI group vs. the control group	BP	GO:0042542	response to hydrogen peroxide	22/125	8.99E-30	4.48E-27	2.33E-27	Adam9/Anxa1/Axl/Cat/Cyp1b1/Edn1/Gpr37l1/Gpx1/Hmox1/Mapk8/Met/Pink1/Plekha1/Ppp1r15b/Rela/Ripk1/Ripk3/Rnf112/Sphk1/Stat6/Trap1/Trpm2
1-day post-SCI group vs. the control group	BP	GO:1900407	regulation of cellular response to oxidative stress	18/125	2.24E-25	9.75E-23	5.08E-23	Atf4/Fbxw7/Gch1/Gfer/Gpr37l1/Gpx1/Hspb1/Lancl1/Mcl1/Met/Mmp3/Nono/P4hb/Pink1/Ripk1/Trap1/Trpm2/Tsc1
1-day post-SCI group vs. the control group	BP	GO:0070301	cellular response to hydrogen peroxide	18/125	4.25E-25	1.65E-22	8.58E-23	Anxa1/Axl/Cat/Cyp1b1/Edn1/Gpr37l1/Mapk8/Met/Pink1/Plekha1/Rela/Ripk1/Ripk3/Rnf112/Sphk1/Stat6/Trap1/Trpm2
1-day post-SCI group vs. the control group	BP	GO:1901214	regulation of neuron death	27/125	1.29E-24	4.14E-22	2.16E-22	Abcc1/Apoe/Atf2/Atf4/Axl/Bax/Capn2/Eif2s1/Fbxw7/Fos/Gfer/Hmox1/Il6st/Itsn1/Jak2/Jun/Lancl1/Map2k4/Mapk8/Mapt/Mcl1/Nono/Pink1/Ppargc1a/Tnfrsf1a/Trpm2/Tsc1
1-day post-SCI group vs. the control group	BP	GO:1903201	regulation of oxidative stress-induced cell death	17/125	1.31E-24	4.14E-22	2.16E-22	Atf4/Fbxw7/Gfer/Gpr37l1/Gpx1/Hspb1/Lancl1/Mcl1/Met/Mmp3/Nono/P4hb/Pink1/Ripk1/Trap1/Trpm2/Tsc1
1-day post-SCI group vs. the control group	BP	GO:1902882	regulation of response to oxidative stress	18/125	1.44E-24	4.18E-22	2.18E-22	Atf4/Fbxw7/Gch1/Gfer/Gpr37l1/Gpx1/Hspb1/Lancl1/Mcl1/Met/Mmp3/Nono/P4hb/Pink1/Ripk1/Trap1/Trpm2/Tsc1
1-day post-SCI group vs. the control group	BP	GO:0070997	neuron death	28/125	1.62E-24	4.34E-22	2.26E-22	Abcc1/Apoe/Atf2/Atf4/Axl/Bax/Capn2/Eif2s1/Fbxw7/Fos/Gfer/Gpx1/Hmox1/Il6st/Itsn1/Jak2/Jun/Lancl1/Map2k4/Mapk8/Mapt/Mcl1/Nono/Pink1/Ppargc1a/Tnfrsf1a/Trpm2/Tsc1
1-day post-SCI group vs. the control group	BP	GO:0097193	intrinsic apoptotic signaling pathway	24/125	2.58E-23	6.43E-21	3.35E-21	Atf2/Atf4/Bax/Cyp1b1/Fbxw7/Gpx1/Hdac1/Hmox1/Hspb1/Ier3/Jak2/Mapt/Mcl1/Mdm2/Myc/Nono/P4hb/Pdk2/Pink1/Ptgs2/Ripk3/Tnfrsf1a/Trap1/Ybx3
1-day post-SCI group vs. the control group	BP	GO:2001233	regulation of apoptotic signaling pathway	24/125	1.13E-20	2.63E-18	1.37E-18	Bax/Ctnna1/Cttn/Fbxw7/Gpx1/Hdac1/Hmox1/Hspb1/Ier3/Jak2/Mapk8/Mcl1/Mdm2/Myc/Nono/P4hb/Pink1/Ppp1ca/Ptgs2/Rela/Ripk1/Ripk3/Trap1/Ybx3
1-day post-SCI group vs. the control group	BP	GO:0051402	neuron apoptotic process	20/125	1.77E-17	3.86E-15	2.01E-15	Apoe/Atf2/Atf4/Axl/Bax/Fbxw7/Gpx1/Hmox1/Il6st/Itsn1/Jak2/Jun/Lancl1/Map2k4/Mapk8/Mcl1/Nono/Pink1/Ppargc1a/Tnfrsf1a
1-day post-SCI group vs. the control group	BP	GO:1901216	positive regulation of neuron death	15/125	2.26E-17	4.64E-15	2.42E-15	Apoe/Atf2/Atf4/Bax/Capn2/Eif2s1/Fbxw7/Fos/Jun/Map2k4/Mapk8/Mapt/Mcl1/Tnfrsf1a/Trpm2
1-day post-SCI group vs. the control group	BP	GO:0043523	regulation of neuron apoptotic process	19/125	2.99E-17	5.80E-15	3.02E-15	Apoe/Atf2/Atf4/Axl/Bax/Fbxw7/Hmox1/Il6st/Itsn1/Jak2/Jun/Lancl1/Map2k4/Mapk8/Mcl1/Nono/Pink1/Ppargc1a/Tnfrsf1a
1-day post-SCI group vs. the control group	BP	GO:0008631	intrinsic apoptotic signaling pathway in response to ox	11/125	5.57E-17	1.02E-14	5.33E-15	Cyp1b1/Fbxw7/Gpx1/Hspb1/Jak2/Mapt/Mcl1/Nono/P4hb/Pink1/Trap1
1-day post-SCI group vs. the control group	BP	GO:2001234	negative regulation of apoptotic signaling pathway	17/125	4.53E-16	7.91E-14	4.12E-14	Bax/Ctnna1/Cttn/Gpx1/Hdac1/Hmox1/Hspb1/Ier3/Mcl1/Mdm2/Nono/Pink1/Ptgs2/Rela/Ripk1/Trap1/Ybx3
1-day post-SCI group vs. the control group	BP	GO:0072593	reactive oxygen species metabolic process	17/125	5.97E-16	9.92E-14	5.16E-14	Cat/Cyp1b1/Edn1/Gch1/Gpx1/Ier3/Mapk14/Mapt/Met/Mmp3/Nos3/Pink1/Pxdn/Ripk1/Ripk3/Txnrd1/Xdh
1-day post-SCI group vs. the control group	BP	GO:2001242	regulation of intrinsic apoptotic signaling pathway	15/125	1.73E-15	2.75E-13	1.43E-13	Bax/Fbxw7/Gpx1/Hdac1/Hspb1/Mcl1/Mdm2/Myc/Nono/P4hb/Pink1/Ptgs2/Ripk3/Trap1/Ybx3

1-day post-SCI group vs. the control group	BP	GO:0001667	ameboidal-type cell migration	20/125	1.69E-14	2.56E-12	1.33E-12	Adam9/Anxa1/Apoe/Cyp1b1/Edn1/Fbxw7/Gpx1/Hbegf/Hmox1/Hspb1/Jun/Map2k3/Map4k4/Met/Nos3/Pdlim1/Prr5l/Ptgs2/Pxn/Slc8a1
1-day post-SCI group vs. the control group	BP	GO:1903203	regulation of oxidative stress-induced neuron death	9/125	2.51E-14	3.64E-12	1.90E-12	Atf4/Fbxw7/Gfer/Lancl1/Mcl1/Nono/Pink1/Trpm2/Tsc1
1-day post-SCI group vs. the control group	BP	GO:1903202	negative regulation of oxidative stress-induced cell dea	10/125	2.61E-14	3.64E-12	1.90E-12	Atf4/Gfer/Gpr37l1/Gpx1/Hspb1/Met/Nono/Pink1/Trap1/Tsc1
1-day post-SCI group vs. the control group	BP	GO:0036475	neuron death in response to oxidative stress	9/125	4.38E-14	5.75E-12	3.00E-12	Atf4/Fbxw7/Gfer/Lancl1/Mcl1/Nono/Pink1/Trpm2/Tsc1
1-day post-SCI group vs. the control group	BP	GO:1901215	negative regulation of neuron death	16/125	4.45E-14	5.75E-12	3.00E-12	Abcc1/Apoe/Atf4/Axl/Bax/Gfer/Hmox1/Il6st/Itsn1/Jak2/Jun/Map2k4/Nono/Pink1/Ppargc1a/Tsc1
1-day post-SCI group vs. the control group	BP	GO:1902175	regulation of oxidative stress-induced intrinsic apoptot	8/125	2.00E-13	2.49E-11	1.30E-11	Fbxw7/Gpx1/Hspb1/Mcl1/Nono/P4hb/Pink1/Trap1
1-day post-SCI group vs. the control group	BP	GO:0010631	epithelial cell migration	16/125	1.05E-12	1.26E-10	6.58E-11	Adam9/Anxa1/Apoe/Cyp1b1/Fbxw7/Gpx1/Hbegf/Hmox1/Hspb1/Jun/Map2k3/Map4k4/Met/Nos3/Ptgs2/Pxn
1-day post-SCI group vs. the control group	BP	GO:0090132	epithelium migration	16/125	1.15E-12	1.34E-10	6.98E-11	Adam9/Anxa1/Apoe/Cyp1b1/Fbxw7/Gpx1/Hbegf/Hmox1/Hspb1/Jun/Map2k3/Map4k4/Met/Nos3/Ptgs2/Pxn
1-day post-SCI group vs. the control group	BP	GO:0090130	tissue migration	16/125	1.26E-12	1.42E-10	7.40E-11	Adam9/Anxa1/Apoe/Cyp1b1/Fbxw7/Gpx1/Hbegf/Hmox1/Hspb1/Jun/Map2k3/Map4k4/Met/Nos3/Ptgs2/Pxn
1-day post-SCI group vs. the control group	BP	GO:2001243	negative regulation of intrinsic apoptotic signaling patl	10/125	1.95E-11	2.13E-09	1.11E-09	Gpx1/Hdac1/Hspb1/Mcl1/Mdm2/Nono/Pink1/Ptgs2/Trap1/Ybx3
1-day post-SCI group vs. the control group	BP	GO:0009991	response to extracellular stimulus	16/125	4.29E-11	4.53E-09	2.36E-09	Apoe/Atf2/Atf4/Axl/Bax/Eif2s1/Fos/Fosl1/Jun/Mapk14/Mapk8/Mapt/Pdk2/Ppargc1a/Tsc1/Vcam1
1-day post-SCI group vs. the control group	BP	GO:0010038	response to metal ion	14/125	4.47E-11	4.58E-09	2.39E-09	Adam9/Atp13a2/Cdk4/Fos/Hmox1/Jun/Lcat/Mapk8/Mapt/Ppp1ca/Ppp3ca/Slc25a24/Trpm2/Xdh
1-day post-SCI group vs. the control group	BP	GO:0043524	negative regulation of neuron apoptotic process	12/125	5.64E-11	5.63E-09	2.93E-09	Apoe/Axl/Bax/Hmox1/Il6st/Itsn1/Jak2/Jun/Map2k4/Nono/Pink1/Ppargc1a
1-day post-SCI group vs. the control group	BP	GO:0010632	regulation of epithelial cell migration	13/125	6.41E-11	6.13E-09	3.19E-09	Adam9/Anxa1/Apoe/Fbxw7/Hbegf/Hmox1/Hspb1/Jun/Map2k3/Map4k4/Met/Nos3/Ptgs2
1-day post-SCI group vs. the control group	BP	GO:0009314	response to radiation	16/125	6.50E-11	6.13E-09	3.19E-09	Anxa1/Bax/Cat/Eif2s1/Ercc1/Fbxw7/Gpx1/Jun/Mapk14/Mapk8/Mdm2/Mmp3/Myc/Pcna/Ppp1ca/Rela
1-day post-SCI group vs. the control group	BP	GO:0010634	positive regulation of epithelial cell migration	11/125	1.12E-10	1.03E-08	5.37E-09	Adam9/Anxa1/Hbegf/Hmox1/Hspb1/Jun/Map2k3/Map4k4/Met/Nos3/Ptgs2
1-day post-SCI group vs. the control group	BP	GO:0010563	negative regulation of phosphorus metabolic process	16/125	1.31E-10	1.14E-08	5.93E-09	Apoe/Bax/Cdkn1c/Dynll1/Fkbp1b/Hspb1/Ier3/Jun/Mapt/Met/Ppargc1a/Ppp1r15b/Prr5l/Slc8a1/Spry2/Xdh
1-day post-SCI group vs. the control group	BP	GO:0045936	negative regulation of phosphate metabolic process	16/125	1.31E-10	1.14E-08	5.93E-09	Apoe/Bax/Cdkn1c/Dynll1/Fkbp1b/Hspb1/Ier3/Jun/Mapt/Met/Ppargc1a/Ppp1r15b/Prr5l/Slc8a1/Spry2/Xdh
1-day post-SCI group vs. the control group	BP	GO:0043534	blood vessel endothelial cell migration	10/125	1.44E-10	1.22E-08	6.36E-09	Anxa1/Apoe/Cyp1b1/Fbxw7/Gpx1/Hmox1/Hspb1/Map2k3/Nos3/Ptgs2
1-day post-SCI group vs. the control group	BP	GO:0033002	muscle cell proliferation	13/125	1.53E-10	1.27E-08	6.61E-09	Apoe/Edn1/Hbegf/Hmox1/Jak2/Jun/Mapk14/Mdm2/Myc/Nos3/Ppargc1a/Ptgs2/Smad1
1-day post-SCI group vs. the control group	BP	GO:0043525	positive regulation of neuron apoptotic process	9/125	1.67E-10	1.36E-08	7.07E-09	Atf2/Atf4/Bax/Fbxw7/Jun/Map2k4/Mapk8/Mcl1/Tnfrsf1a
1-day post-SCI group vs. the control group	BP	GO:0042326	negative regulation of phosphorylation	15/125	1.82E-10	1.45E-08	7.54E-09	Apoe/Bax/Cdkn1c/Dynll1/Hspb1/Ier3/Jun/Mapt/Met/Ppargc1a/Ppp1r15b/Prr5l/Slc8a1/Spry2/Xdh
1-day post-SCI group vs. the control group	BP	GO:0051091	positive regulation of DNA-binding transcription facto	13/125	1.92E-10	1.49E-08	7.76E-09	Atf2/Cat/Edn1/Fosl1/Jak2/Pink1/Ppargc1a/Ppargc1b/Ppp3ca/Rela/Ripk1/Ripk3/Sphk1

1-day post-SCI group vs. the control group	BP	GO:0035924	cellular response to vascular endothelial growth factor	8/125	2.05E-10	1.55E-08	8.08E-09	Anxa1/Hspb1/Map2k3/Mapk14/Rela/Sphk1/Spry2/Xdh
1-day post-SCI group vs. the control group	BP	GO:0043542	endothelial cell migration	12/125	4.63E-10	3.44E-08	1.79E-08	Anxa1/Apoe/Cyp1b1/Fbxw7/Gpx1/Hmox1/Hspb1/Map2k3/Met/Nos3/Ptgs2/Pxn
1-day post-SCI group vs. the control group	BP	GO:0048660	regulation of smooth muscle cell proliferation	11/125	5.32E-10	3.87E-08	2.01E-08	Apoe/Edn1/Hbegf/Hmox1/Jak2/Jun/Mdm2/Myc/Nos3/Ppargc1a/Ptgs2
1-day post-SCI group vs. the control group	BP	GO:0060047	heart contraction	12/125	6.80E-10	4.84E-08	2.52E-08	Edn1/Fkbp1b/Gch1/Gpx1/Hbegf/Jak2/Map2k3/Map2k6/Mdm2/Met/Nos3/Slc8a1
1-day post-SCI group vs. the control group	BP	GO:0051090	regulation of DNA-binding transcription factor activity	15/125	7.36E-10	5.11E-08	2.66E-08	Atf2/Cat/Cyp1b1/Edn1/Fosl1/Hmox1/Jak2/Pink1/Ppargc1a/Ppargc1b/Ppp3ca/Rela/Ripk1/Ripk3/Sphk1
1-day post-SCI group vs. the control group	BP	GO:0051403	stress-activated MAPK cascade	12/125	7.47E-10	5.11E-08	2.66E-08	Atf2/Ezr/Map2k3/Map2k4/Map2k6/Map4k4/Mapk14/Mapk8/Met/Ripk1/Sphk1/Xdh
1-day post-SCI group vs. the control group	BP	GO:0048659	smooth muscle cell proliferation	11/125	7.89E-10	5.29E-08	2.76E-08	Apoe/Edn1/Hbegf/Hmox1/Jak2/Jun/Mdm2/Myc/Nos3/Ppargc1a/Ptgs2
1-day post-SCI group vs. the control group	BP	GO:0019932	second-messenger-mediated signaling	13/125	9.56E-10	6.30E-08	3.28E-08	Apoe/Ccr1/Edn1/Fkbp1b/Mapt/Nos3/Pdk2/Ppp3ca/Rcan1/Slc8a1/Sphk1/Trpm2/Vcam1
1-day post-SCI group vs. the control group	BP	GO:0019722	calcium-mediated signaling	11/125	9.81E-10	6.34E-08	3.30E-08	Ccr1/Edn1/Fkbp1b/Mapt/Pdk2/Ppp3ca/Rcan1/Slc8a1/Sphk1/Trpm2/Vcam1
1-day post-SCI group vs. the control group	BP	GO:0048661	positive regulation of smooth muscle cell proliferation	9/125	1.16E-09	7.10E-08	3.70E-08	Edn1/Hbegf/Hmox1/Jak2/Jun/Mdm2/Myc/Ppargc1a/Ptgs2
1-day post-SCI group vs. the control group	BP	GO:0009411	response to UV	10/125	1.17E-09	7.10E-08	3.70E-08	Bax/Cat/Eif2s1/Ercc1/Fbxw7/Gpx1/Mapk8/Mmp3/Pcna/Rela
1-day post-SCI group vs. the control group	BP	GO:0003015	heart process	12/125	1.18E-09	7.10E-08	3.70E-08	Edn1/Fkbp1b/Gch1/Gpx1/Hbegf/Jak2/Map2k3/Map2k6/Mdm2/Met/Nos3/Slc8a1
1-day post-SCI group vs. the control group	BP	GO:0031098	stress-activated protein kinase signaling cascade	12/125	1.18E-09	7.10E-08	3.70E-08	Atf2/Ezr/Map2k3/Map2k4/Map2k6/Map4k4/Mapk14/Mapk8/Met/Ripk1/Sphk1/Xdh
1-day post-SCI group vs. the control group	BP	GO:0051222	positive regulation of protein transport	13/125	1.52E-09	8.97E-08	4.67E-08	Adam9/Atp13a2/Cask/Dynll1/Ezr/Fbxw7/Jak2/Mapk14/Mdm2/Prr5l/Ptgs2/Tnfrsf1a/Trpm2
1-day post-SCI group vs. the control group	BP	GO:2001235	positive regulation of apoptotic signaling pathway	10/125	1.61E-09	9.39E-08	4.89E-08	Bax/Ctnna1/Fbxw7/Jak2/Mapk8/Mcl1/Myc/Ppp1ca/Ripk1/Ripk3
1-day post-SCI group vs. the control group	BP	GO:2000377	regulation of reactive oxygen species metabolic proces	10/125	2.19E-09	1.25E-07	6.53E-08	Cyp1b1/Gch1/Ier3/Mapk14/Mapt/Mmp3/Pink1/Ripk1/Ripk3/Xdh
1-day post-SCI group vs. the control group	BP	GO:1904951	positive regulation of establishment of protein localiza	13/125	2.73E-09	1.53E-07	7.99E-08	Adam9/Atp13a2/Cask/Dynll1/Ezr/Fbxw7/Jak2/Mapk14/Mdm2/Prr5l/Ptgs2/Tnfrsf1a/Trpm2
1-day post-SCI group vs. the control group	BP	GO:0032386	regulation of intracellular transport	13/125	2.93E-09	1.61E-07	8.39E-08	Atp13a2/Ezr/Fbxw7/Ier3/Jak2/Mapk14/Mapk8/Mapt/Mdm2/Pink1/Prr5l/Ptgs2/Tnfrsf1a
1-day post-SCI group vs. the control group	BP	GO:0048732	gland development	15/125	2.96E-09	1.61E-07	8.39E-08	Anxa1/Atf2/Bax/Cdkn1c/Edn1/Gfer/Gpx1/Hmox1/Jak2/Jun/Met/Rela/Ripk3/Stat6/Xdh
1-day post-SCI group vs. the control group	BP	GO:0038066	p38MAPK cascade	7/125	3.54E-09	1.90E-07	9.89E-08	Atf2/Ezr/Map2k3/Mapk14/Met/Sphk1/Xdh
1-day post-SCI group vs. the control group	BP	GO:0051047	positive regulation of secretion	14/125	4.20E-09	2.22E-07	1.16E-07	Adam9/Atp13a2/Cask/Dynll1/Edn1/Ezr/Itsn1/Jak2/Map2k6/Pink1/Ppp3ca/Sdc1/Sphk1/Trpm2
1-day post-SCI group vs. the control group	BP	GO:1903532	positive regulation of secretion by cell	13/125	7.08E-09	3.69E-07	1.92E-07	Adam9/Atp13a2/Cask/Dynll1/Edn1/Ezr/Itsn1/Jak2/Map2k6/Pink1/Sdc1/Sphk1/Trpm2
1-day post-SCI group vs. the control group	BP	GO:0050727	regulation of inflammatory response	13/125	7.56E-09	3.88E-07	2.02E-07	Abcc1/Anxa1/Apoe/Gpx1/Gpx4/Ier3/Jak2/Krt1/Ptgs2/Rela/Ripk1/Sphk1/Tnfrsf1a

1-day post-SCI group vs. the control group	BP	GO:1902176	negative regulation of oxidative stress-induced intrinsi	5/125	8.23E-09	4.16E-07	2.17E-07	Gpx1/Hspb1/Nono/Pink1/Trap1
1-day post-SCI group vs. the control group	BP	GO:0046777	protein autophosphorylation	11/125	8.55E-09	4.17E-07	2.17E-07	Atp13a2/Eif2s1/Jak2/Jun/Mapk14/Mapkapk3/Met/Nme2/Pink1/Ripk1/Ripk3
1-day post-SCI group vs. the control group	BP	GO:0043535	regulation of blood vessel endothelial cell migration	8/125	8.57E-09	4.17E-07	2.17E-07	Anxa1/Apoe/Fbxw7/Hmox1/Hspb1/Map2k3/Nos3/Ptgs2
1-day post-SCI group vs. the control group	BP	GO:0031589	cell-substrate adhesion	13/125	8.60E-09	4.17E-07	2.17E-07	Adam9/Atrn/Axl/Cask/Cttn/Itga5/Itga7/Jak2/Map4k4/P4hb/Pxn/Tsc1/Vcam1
1-day post-SCI group vs. the control group	BP	GO:0071560	cellular response to transforming growth factor beta sti	11/125	8.92E-09	4.27E-07	2.22E-07	Adam9/Cdkn1c/Fos/Hdac1/Jun/Nos3/Ppargc1a/Pxn/Rbbp7/Smad1/Spry2
1-day post-SCI group vs. the control group	BP	GO:0050708	regulation of protein secretion	12/125	9.22E-09	4.35E-07	2.26E-07	Adam9/Anxa1/Apoe/Atp13a2/Cask/Dynll1/Ezr/Fkbp1b/Jak2/Ppp3ca/Trpm2/Ucp2
1-day post-SCI group vs. the control group	BP	GO:0031668	cellular response to extracellular stimulus	11/125	1.01E-08	4.60E-07	2.40E-07	Atf2/Atf4/Axl/Eif2s1/Fos/Fosl1/Jun/Mapk8/Pdk2/Tsc1/Vcam1
1-day post-SCI group vs. the control group	BP	GO:0045765	regulation of angiogenesis	12/125	1.03E-08	4.60E-07	2.40E-07	Atf2/Cyp1b1/Hmox1/Hspb1/Itga5/Nos3/Pxn/Rnh1/Smad1/Sphk1/Spry2/Tnfrsf1a
1-day post-SCI group vs. the control group	BP	GO:0071496	cellular response to external stimulus	12/125	1.03E-08	4.60E-07	2.40E-07	Atf2/Atf4/Axl/Eif2s1/Fos/Fosl1/Jun/Mapk8/Nos3/Pdk2/Tsc1/Vcam1
1-day post-SCI group vs. the control group	BP	GO:0001659	temperature homeostasis	10/125	1.03E-08	4.60E-07	2.40E-07	Atf4/Gpx1/Jak2/Map2k6/Ppargc1a/Ppargc1b/Ptgs2/Stat6/Trpm2/Ucp2
1-day post-SCI group vs. the control group	BP	GO:0036293	response to decreased oxygen levels	11/125	1.06E-08	4.61E-07	2.40E-07	Atf2/Cat/Edn1/Hmox1/Hmox2/Myc/P4hb/Pink1/Slc8a1/Tsc1/Ucp2
1-day post-SCI group vs. the control group	BP	GO:0071559	response to transforming growth factor beta	11/125	1.06E-08	4.61E-07	2.40E-07	Adam9/Cdkn1c/Fos/Hdac1/Jun/Nos3/Ppargc1a/Pxn/Rbbp7/Smad1/Spry2
1-day post-SCI group vs. the control group	BP	GO:0034248	regulation of cellular amide metabolic process	14/125	1.09E-08	4.71E-07	2.45E-07	Ago4/Apoe/Cdk4/Cyp1b1/Eif2s1/Pdk2/Pink1/Ppp1ca/Ppp1r15b/Rela/Rpl13a/Sphk1/Trap1/Tsc1
1-day post-SCI group vs. the control group	BP	GO:1901342	regulation of vasculature development	12/125	1.19E-08	5.04E-07	2.63E-07	Atf2/Cyp1b1/Hmox1/Hspb1/Itga5/Nos3/Pxn/Rnh1/Smad1/Sphk1/Spry2/Tnfrsf1a
1-day post-SCI group vs. the control group	BP	GO:0002761	regulation of myeloid leukocyte differentiation	9/125	1.28E-08	5.38E-07	2.80E-07	Ccr1/Fbxw7/Fos/Jun/Myc/Nme2/Ppargc1b/Ppp3ca/Ripk1
1-day post-SCI group vs. the control group	BP	GO:0032496	response to lipopolysaccharide	13/125	1.37E-08	5.71E-07	2.97E-07	Adam9/Axl/Cdk4/Gch1/Jak2/Map2k3/Mapk14/Mapk8/Mapkapk3/Nos3/Ptgs2/Rela/Rpl13a
1-day post-SCI group vs. the control group	BP	GO:0036474	cell death in response to hydrogen peroxide	6/125	1.42E-08	5.82E-07	3.03E-07	Gpr37l1/Met/Pink1/Ripk1/Rnf112/Trap1
1-day post-SCI group vs. the control group	BP	GO:0002683	negative regulation of immune system process	14/125	1.52E-08	6.15E-07	3.21E-07	Anxa1/Axl/Banf1/Btk/Ccr1/Ezr/Fbxw7/Gfer/Gpx1/Hmox1/Hspb1/Myc/Nme2/Stat6
1-day post-SCI group vs. the control group	BP	GO:0010821	regulation of mitochondrion organization	9/125	1.53E-08	6.15E-07	3.21E-07	Atp13a2/Bax/Cttn/Fbxw7/Gpx1/Ier3/Mapt/Pink1/Ppargc1a
1-day post-SCI group vs. the control group	BP	GO:0051054	positive regulation of DNA metabolic process	12/125	2.06E-08	8.17E-07	4.25E-07	Bax/Cyp1b1/Ercc1/Gfer/Jun/Map2k4/Mapk8/Met/Myc/Pcna/Ppargc1a/Stat6
1-day post-SCI group vs. the control group	BP	GO:1901031	regulation of response to reactive oxygen species	6/125	2.28E-08	8.96E-07	4.66E-07	Gch1/Gpr37l1/Met/Pink1/Ripk1/Trap1
1-day post-SCI group vs. the control group	BP	GO:0002237	response to molecule of bacterial origin	13/125	2.56E-08	9.94E-07	5.18E-07	Adam9/Axl/Cdk4/Gch1/Jak2/Map2k3/Mapk14/Mapk8/Mapkapk3/Nos3/Ptgs2/Rela/Rpl13a
1-day post-SCI group vs. the control group	BP	GO:0035994	response to muscle stretch	5/125	2.67E-08	9.97E-07	5.19E-07	Fos/Jun/Mapk14/Rela/Slc8a1
1-day post-SCI group vs. the control group	BP	GO:1903209	positive regulation of oxidative stress-induced cell dea	5/125	2.67E-08	9.97E-07	5.19E-07	Fbxw7/Mcl1/Mmp3/Ripk1/Trpm2
1-day post-SCI group vs. the control group	BP	GO:0071214	cellular response to abiotic stimulus	12/125	2.69E-08	9.97E-07	5.19E-07	Bax/Eif2s1/Ercc1/Fbxw7/Mapk14/Mdm2/Mmp3/Nos3/Pcna/Ptgs2/Slc4a11/Ybx3

1-day post-SCI group vs. the control group	BP	GO:0104004	cellular response to environmental stimulus	12/125	2.69E-08	9.97E-07	5.19E-07	Bax/Eif2s1/Ercc1/Fbxw7/Mapk14/Mdm2/Mmp3/Nos3/Pcna/Ptgs2/Slc4a11/Ybx3
1-day post-SCI group vs. the control group	BP	GO:1903829	positive regulation of protein localization	14/125	3.15E-08	1.16E-06	6.02E-07	Adam9/Atp13a2/Cask/Dynll1/Ezr/Fbxw7/Jak2/Mapk14/Mapt/Mdm2/Prr5l/Ptgs2/Tnfrsf1a/Trpm2
1-day post-SCI group vs. the control group	BP	GO:0060537	muscle tissue development	14/125	3.23E-08	1.17E-06	6.11E-07	Edn1/Fos/Gpx1/Map2k4/Mapk14/Met/Myc/Ppargc1a/Ppp3ca/Rcan1/Slc8a1/Smad1/Tsc1/Ybx3
1-day post-SCI group vs. the control group	BP	GO:1901522	positive regulation of transcription from RNA polymer	5/125	3.44E-08	1.24E-06	6.45E-07	Atf2/Atf4/Jun/Rela/Smad1
1-day post-SCI group vs. the control group	BP	GO:0001933	negative regulation of protein phosphorylation	12/125	3.70E-08	1.32E-06	6.87E-07	Apoe/Bax/Cdkn1c/Hspb1/Jun/Met/Ppargc1a/Ppp1r15b/Prr5l/Slc8a1/Spry2/Xdh
1-day post-SCI group vs. the control group	BP	GO:1902105	regulation of leukocyte differentiation	12/125	4.07E-08	1.43E-06	7.47E-07	Anxa1/Axl/Ccr1/Fbxw7/Fos/Hspb1/Jun/Myc/Nme2/Ppargc1b/Ppp3ca/Ripk1
1-day post-SCI group vs. the control group	BP	GO:0001666	response to hypoxia	10/125	4.16E-08	1.45E-06	7.56E-07	Atf2/Cat/Edn1/Hmox1/Hmox2/Myc/P4hb/Pink1/Slc8a1/Ucp2
1-day post-SCI group vs. the control group	BP	GO:1901652	response to peptide	13/125	4.87E-08	1.68E-06	8.77E-07	Abcc1/Anxa1/Cdk4/Edn1/Jak2/Mapk14/Mdm2/Pdk2/Pxn/Rela/Stat6/Tsc1/Vcam1
1-day post-SCI group vs. the control group	BP	GO:0003012	muscle system process	13/125	5.14E-08	1.76E-06	9.16E-07	Cttn/Edn1/Fkbp1b/Map2k3/Map2k4/Map2k6/Met/Nos3/Ppp3ca/Ptgs2/Slc8a1/Sphk1/Tnfrsf1a
1-day post-SCI group vs. the control group	BP	GO:1903204	negative regulation of oxidative stress-induced neuron	5/125	5.52E-08	1.84E-06	9.59E-07	Atf4/Gfer/Nono/Pink1/Tsc1
1-day post-SCI group vs. the control group	BP	GO:1990000	amyloid fibril formation	5/125	5.52E-08	1.84E-06	9.59E-07	Apoe/Mapt/Mdm2/Ripk1/Ripk3
1-day post-SCI group vs. the control group	BP	GO:1903706	regulation of hemopoiesis	13/125	5.57E-08	1.84E-06	9.59E-07	Anxa1/Axl/Ccr1/Fbxw7/Fos/Hspb1/Jun/Mapk14/Myc/Nme2/Ppargc1b/Ppp3ca/Ripk1
1-day post-SCI group vs. the control group	BP	GO:0045637	regulation of myeloid cell differentiation	10/125	5.60E-08	1.84E-06	9.59E-07	Ccr1/Fbxw7/Fos/Jun/Mapk14/Myc/Nme2/Ppargc1b/Ppp3ca/Ripk1
1-day post-SCI group vs. the control group	BP	GO:0009636	response to toxic substance	8/125	6.16E-08	2.01E-06	1.05E-06	Abcc1/Bax/Cat/Cyp1b1/Gch1/Gpx1/Nos3/Pink1
1-day post-SCI group vs. the control group	BP	GO:0036480	neuron intrinsic apoptotic signaling pathway in respon	4/125	6.82E-08	2.18E-06	1.14E-06	Fbxw7/Mcl1/Nono/Pink1
1-day post-SCI group vs. the control group	BP	GO:1903376	regulation of oxidative stress-induced neuron intrinsic	4/125	6.82E-08	2.18E-06	1.14E-06	Fbxw7/Mcl1/Nono/Pink1
1-day post-SCI group vs. the control group	BP	GO:0051896	regulation of protein kinase B signaling	9/125	7.36E-08	2.34E-06	1.22E-06	Axl/Gpx1/Hbegf/Itsn1/Met/Mmp3/Plekha1/Spry2/Xdh
1-day post-SCI group vs. the control group	BP	GO:0010594	regulation of endothelial cell migration	9/125	7.73E-08	2.43E-06	1.27E-06	Anxa1/Apoe/Fbxw7/Hmox1/Hspb1/Map2k3/Met/Nos3/Ptgs2
1-day post-SCI group vs. the control group	BP	GO:0042692	muscle cell differentiation	13/125	8.04E-08	2.51E-06	1.30E-06	Capn2/Edn1/Gpx1/Map2k4/Mapk14/Mdm2/Met/Myc/Ppp3ca/Rcan1/Slc8a1/Smad1/Tsc1
1-day post-SCI group vs. the control group	BP	GO:0097191	extrinsic apoptotic signaling pathway	10/125	9.08E-08	2.80E-06	1.46E-06	Bax/Ctnna1/Cttn/Gpx1/Hmox1/Jak2/Mcl1/Ppp1ca/Rela/Ripk1
1-day post-SCI group vs. the control group	BP	GO:0070482	response to oxygen levels	11/125	9.50E-08	2.91E-06	1.51E-06	Atf2/Cat/Edn1/Hmox1/Hmox2/Myc/P4hb/Pink1/Slc8a1/Tsc1/Ucp2
1-day post-SCI group vs. the control group	BP	GO:0010921	regulation of phosphatase activity	7/125	9.58E-08	2.91E-06	1.51E-06	Fkbp1b/Jak2/Ppargc1b/Ppp1r15b/Rcan1/Ripk3/Tsc1
1-day post-SCI group vs. the control group	BP	GO:0048511	rhythmic process	11/125	9.82E-08	2.95E-06	1.54E-06	Anxa1/Atf4/Axl/Fbxw7/Hdac1/Mapk8/Nono/Nos3/Plekha1/Ppargc1a/Ppp1ca
1-day post-SCI group vs. the control group	BP	GO:0045766	positive regulation of angiogenesis	9/125	1.03E-07	3.05E-06	1.59E-06	Cyp1b1/Hmox1/Hspb1/Itga5/Nos3/Pxn/Smad1/Sphk1/Tnfrsf1[illegible]
1-day post-SCI group vs. the control group	BP	GO:1904018	positive regulation of vasculature development	9/125	1.03E-07	3.05E-06	1.59E-06	Cyp1b1/Hmox1/Hspb1/Itga5/Nos3/Pxn/Smad1/Sphk1/Tnfrsf1[illegible]
1-day post-SCI group vs. the control group	BP	GO:0051881	regulation of mitochondrial membrane potential	7/125	1.12E-07	3.29E-06	1.72E-06	Bax/Hebp2/Mapt/Myc/Pink1/Slc4a11/Ucp2
1-day post-SCI group vs. the control group	BP	GO:0050767	regulation of neurogenesis	13/125	1.15E-07	3.33E-06	1.73E-06	Apoe/Cask/Ctnna1/Gpr37l1/Hdac1/Il6st/Mapk8/Mapt/Met/Myc/Ppp3ca/Rela/Rnf112
1-day post-SCI group vs. the control group	BP	GO:0033559	unsaturated fatty acid metabolic process	8/125	1.20E-07	3.47E-06	1.81E-06	Anxa1/Cyp1b1/Edn1/Gpx1/Gpx4/Ptgs2/Sphk1/Tnfrsf1a

1-day post-SCI group vs. the control group	BP	GO:0002573	myeloid leukocyte differentiation	10/125	1.24E-07	3.51E-06	1.83E-06	Ccr1/Fbxw7/Fos/Jun/Mapk14/Myc/Nme2/Ppargc1b/Ppp3ca/Ripk1
1-day post-SCI group vs. the control group	BP	GO:0031667	response to nutrient levels	12/125	1.25E-07	3.51E-06	1.83E-06	Apoe/Atf2/Atf4/Bax/Eif2s1/Jun/Mapk14/Mapk8/Mapt/Pdk2/Ppargc1a/Tsc1
1-day post-SCI group vs. the control group	BP	GO:0051767	nitric-oxide synthase biosynthetic process	5/125	1.26E-07	3.51E-06	1.83E-06	Edn1/Jak2/Map2k3/Map2k4/Map2k6
1-day post-SCI group vs. the control group	BP	GO:0051769	regulation of nitric-oxide synthase biosynthetic proces	5/125	1.26E-07	3.51E-06	1.83E-06	Edn1/Jak2/Map2k3/Map2k4/Map2k6
1-day post-SCI group vs. the control group	BP	GO:0042391	regulation of membrane potential	13/125	1.33E-07	3.68E-06	1.92E-06	Bax/Edn1/Fkbp1b/Hebp2/Jun/Mapt/Met/Myc/Pink1/Ppp3ca/Slc4a11/Slc8a1/Ucp2
1-day post-SCI group vs. the control group	BP	GO:0035303	regulation of dephosphorylation	8/125	1.43E-07	3.93E-06	2.05E-06	Fkbp1b/Jak2/Pink1/Ppargc1b/Ppp1r15b/Rcan1/Ripk3/Tsc1
1-day post-SCI group vs. the control group	BP	GO:0015833	peptide transport	11/125	1.54E-07	4.20E-06	2.18E-06	Abcc1/Anxa1/Cask/Dynll1/Edn1/Fkbp1b/Itsn1/Jak2/Ppp3ca/Trpm2/Ucp2
1-day post-SCI group vs. the control group	BP	GO:0009416	response to light stimulus	11/125	1.64E-07	4.43E-06	2.31E-06	Bax/Cat/Eif2s1/Ercc1/Fbxw7/Gpx1/Mapk8/Mmp3/Pcna/Ppp1ca/Rela
1-day post-SCI group vs. the control group	BP	GO:0043536	positive regulation of blood vessel endothelial cell mig	6/125	1.74E-07	4.68E-06	2.44E-06	Anxa1/Hmox1/Hspb1/Map2k3/Nos3/Ptgs2
1-day post-SCI group vs. the control group	BP	GO:0009306	protein secretion	12/125	1.98E-07	5.28E-06	2.75E-06	Adam9/Anxa1/Apoe/Atp13a2/Cask/Dynll1/Ezr/Fkbp1b/Jak2/Ppp3ca/Trpm2/Ucp2
1-day post-SCI group vs. the control group	BP	GO:0006690	icosanoid metabolic process	8/125	2.00E-07	5.28E-06	2.75E-06	Anxa1/Cyp1b1/Edn1/Gpx1/Gpx4/Ptgs2/Sphk1/Tnfrsf1a
1-day post-SCI group vs. the control group	BP	GO:0035592	establishment of protein localization to extracellular re	12/125	2.04E-07	5.34E-06	2.78E-06	Adam9/Anxa1/Apoe/Atp13a2/Cask/Dynll1/Ezr/Fkbp1b/Jak2/Ppp3ca/Trpm2/Ucp2
1-day post-SCI group vs. the control group	BP	GO:0007178	transmembrane receptor protein serine/threonine kinas	12/125	2.15E-07	5.55E-06	2.89E-06	Adam9/Atf2/Cdkn1c/Fos/Hdac1/Jak2/Jun/Mapk14/Pxn/Rbbp7/Smad1/Spry2
1-day post-SCI group vs. the control group	BP	GO:0042743	hydrogen peroxide metabolic process	6/125	2.15E-07	5.55E-06	2.89E-06	Cat/Gpx1/Mmp3/Pink1/Pxdn/Txnrd1
1-day post-SCI group vs. the control group	BP	GO:0051146	striated muscle cell differentiation	11/125	2.36E-07	6.06E-06	3.15E-06	Capn2/Edn1/Gpx1/Map2k4/Mapk14/Met/Myc/Ppp3ca/Rcan1/Slc8a1/Tsc1
1-day post-SCI group vs. the control group	BP	GO:0071692	protein localization to extracellular region	12/125	2.38E-07	6.07E-06	3.16E-06	Adam9/Anxa1/Apoe/Atp13a2/Cask/Dynll1/Ezr/Fkbp1b/Jak2/Ppp3ca/Trpm2/Ucp2
1-day post-SCI group vs. the control group	BP	GO:0007179	transforming growth factor beta receptor signaling patl	9/125	2.50E-07	6.28E-06	3.27E-06	Adam9/Cdkn1c/Fos/Hdac1/Jun/Pxn/Rbbp7/Smad1/Spry2
1-day post-SCI group vs. the control group	BP	GO:0034612	response to tumor necrosis factor	9/125	2.50E-07	6.28E-06	3.27E-06	Adam9/Gch1/Jak2/Mapk14/Rela/Ripk1/Sphk1/Tnfrsf1a/Ybx3
1-day post-SCI group vs. the control group	BP	GO:2001237	negative regulation of extrinsic apoptotic signaling pat	7/125	2.53E-07	6.31E-06	3.29E-06	Ctnna1/Cttn/Gpx1/Hmox1/Mcl1/Rela/Ripk1
1-day post-SCI group vs. the control group	BP	GO:0030003	cellular cation homeostasis	13/125	2.63E-07	6.52E-06	3.39E-06	Apoe/Atf4/Atox1/Atp13a2/Bax/Ccr1/Edn1/Fkbp1b/Hmox1/Myc/Slc4a11/Slc8a1/Trpm2
1-day post-SCI group vs. the control group	BP	GO:0071216	cellular response to biotic stimulus	11/125	2.82E-07	6.92E-06	3.60E-06	Adam9/Axl/Cdk4/Gch1/Jak2/Map2k3/Mapk14/Mapk8/Nos3/Ppp1r15b/Rela
1-day post-SCI group vs. the control group	BP	GO:0043491	protein kinase B signaling	9/125	3.07E-07	7.50E-06	3.90E-06	Axl/Gpx1/Hbegf/Itsn1/Met/Mmp3/Plekha1/Spry2/Xdh
1-day post-SCI group vs. the control group	BP	GO:0009408	response to heat	7/125	3.11E-07	7.53E-06	3.92E-06	Eif2s1/Hmox1/Hspb1/Mapk8/Mapt/Rbbp7/Trpm2
1-day post-SCI group vs. the control group	BP	GO:0010660	regulation of muscle cell apoptotic process	7/125	3.32E-07	8.00E-06	4.17E-06	Atf4/Capn2/Edn1/Hmox1/Jak2/Map2k4/Mapk8
1-day post-SCI group vs. the control group	BP	GO:0006875	cellular metal ion homeostasis	12/125	3.40E-07	8.13E-06	4.24E-06	Apoe/Atf4/Atox1/Atp13a2/Bax/Ccr1/Edn1/Fkbp1b/Hmox1/Myc/Slc8a1/Trpm2
1-day post-SCI group vs. the control group	BP	GO:1903205	regulation of hydrogen peroxide-induced cell death	5/125	3.49E-07	8.23E-06	4.28E-06	Gpr37l1/Met/Pink1/Ripk1/Trap1
1-day post-SCI group vs. the control group	BP	GO:0001503	ossification	12/125	3.49E-07	8.23E-06	4.28E-06	Atf4/Bmp1/Ccr1/Il6st/Map2k6/Mapk14/Mapk8/Ppargc1b/Ppp3ca/Ptgs2/Slc8a1/Smad1

1-day post-SCI group vs. the control group	BP	GO:0006839	mitochondrial transport	8/125	3.72E-07	8.71E-06	4.53E-06	Atf2/Bax/Fbxw7/Hebp2/Ier3/Pink1/Slc25a24/Ucp2
1-day post-SCI group vs. the control group	BP	GO:0042886	amide transport	11/125	4.09E-07	9.50E-06	4.95E-06	Abcc1/Anxa1/Cask/Dynll1/Edn1/Fkbp1b/Itsn1/Jak2/Ppp3ca/Trpm2/Ucp2
1-day post-SCI group vs. the control group	BP	GO:0070265	necrotic cell death	6/125	4.23E-07	9.77E-06	5.09E-06	Bax/Hebp2/Mapk8/Ripk1/Ripk3/Ybx3
1-day post-SCI group vs. the control group	BP	GO:0016032	viral process	11/125	4.32E-07	9.92E-06	5.16E-06	Apoe/Axl/Banf1/Bax/Ctsl/Hdac1/Jun/P4hb/Ripk1/Ripk3/Vcam1
1-day post-SCI group vs. the control group	BP	GO:0010657	muscle cell apoptotic process	7/125	4.60E-07	1.05E-05	5.46E-06	Atf4/Capn2/Edn1/Hmox1/Jak2/Map2k4/Mapk8
1-day post-SCI group vs. the control group	BP	GO:0033674	positive regulation of kinase activity	12/125	4.91E-07	1.11E-05	5.80E-06	Adam9/Axl/Edn1/Fbxw7/Hbegf/Jak2/Map2k3/Map2k4/Map2k6/Met/Pxn/Ripk3
1-day post-SCI group vs. the control group	BP	GO:0050678	regulation of epithelial cell proliferation	12/125	5.16E-07	1.16E-05	6.04E-06	Apoe/Atf2/Bax/Cask/Cdk4/Cdkn1c/Ctsl/Gpx1/Hmox1/Jun/Myc/Xdh
1-day post-SCI group vs. the control group	BP	GO:0010595	positive regulation of endothelial cell migration	7/125	5.21E-07	1.16E-05	6.07E-06	Anxa1/Hmox1/Hspb1/Map2k3/Met/Nos3/Ptgs2
1-day post-SCI group vs. the control group	BP	GO:0030099	myeloid cell differentiation	12/125	5.54E-07	1.23E-05	6.41E-06	Ccr1/Cdkn1c/Fbxw7/Fos/Jak2/Jun/Mapk14/Myc/Nme2/Ppargc1b/Ppp3ca/Ripk1
1-day post-SCI group vs. the control group	BP	GO:0030072	peptide hormone secretion	10/125	5.80E-07	1.28E-05	6.67E-06	Anxa1/Cask/Dynll1/Edn1/Fkbp1b/Itsn1/Jak2/Ppp3ca/Trpm2/Ucp2
1-day post-SCI group vs. the control group	BP	GO:2001236	regulation of extrinsic apoptotic signaling pathway	8/125	6.00E-07	1.32E-05	6.86E-06	Ctnna1/Cttn/Gpx1/Hmox1/Mcl1/Ppp1ca/Rela/Ripk1
1-day post-SCI group vs. the control group	BP	GO:0045639	positive regulation of myeloid cell differentiation	7/125	6.25E-07	1.36E-05	7.10E-06	Ccr1/Fos/Jun/Mapk14/Ppargc1b/Ppp3ca/Ripk1
1-day post-SCI group vs. the control group	BP	GO:0033157	regulation of intracellular protein transport	9/125	6.91E-07	1.50E-05	7.80E-06	Atp13a2/Fbxw7/Jak2/Mapk14/Mdm2/Pink1/Prr5l/Ptgs2/Tnfrsf1a
1-day post-SCI group vs. the control group	BP	GO:1902895	positive regulation of miRNA transcription	6/125	7.12E-07	1.52E-05	7.93E-06	Fos/Fosl1/Jun/Myc/Rela/Smad1
1-day post-SCI group vs. the control group	BP	GO:1904705	regulation of vascular associated smooth muscle cell p	6/125	7.12E-07	1.52E-05	7.93E-06	Edn1/Hmox1/Jak2/Jun/Mdm2/Ppargc1a
1-day post-SCI group vs. the control group	BP	GO:0002790	peptide secretion	10/125	7.42E-07	1.58E-05	8.22E-06	Anxa1/Cask/Dynll1/Edn1/Fkbp1b/Itsn1/Jak2/Ppp3ca/Trpm2/Ucp2
1-day post-SCI group vs. the control group	BP	GO:0033194	response to hydroperoxide	4/125	7.55E-07	1.60E-05	8.32E-06	Gpx1/Jak2/Rnf112/Trpm2
1-day post-SCI group vs. the control group	BP	GO:0032103	positive regulation of response to external stimulus	12/125	7.67E-07	1.61E-05	8.39E-06	Abcc1/Ccr1/Cttn/Edn1/Fkbp1b/Hspb1/Jak2/Met/Nono/Ptgs2/Ripk1/Tnfrsf1a
1-day post-SCI group vs. the control group	BP	GO:0002763	positive regulation of myeloid leukocyte differentiatio	6/125	7.73E-07	1.61E-05	8.41E-06	Ccr1/Fos/Jun/Ppargc1b/Ppp3ca/Ripk1
1-day post-SCI group vs. the control group	BP	GO:0010586	miRNA metabolic process	7/125	7.92E-07	1.64E-05	8.56E-06	Ago4/Fos/Fosl1/Jun/Myc/Rela/Smad1
1-day post-SCI group vs. the control group	BP	GO:1904707	positive regulation of vascular associated smooth musc	5/125	8.08E-07	1.67E-05	8.69E-06	Edn1/Jak2/Jun/Mdm2/Ppargc1a
1-day post-SCI group vs. the control group	BP	GO:0007160	cell-matrix adhesion	9/125	8.28E-07	1.70E-05	8.85E-06	Adam9/Cask/Cttn/Itga5/Itga7/Map4k4/Pxn/Tsc1/Vcam1
1-day post-SCI group vs. the control group	BP	GO:0071222	cellular response to lipopolysaccharide	10/125	8.62E-07	1.76E-05	9.16E-06	Adam9/Axl/Cdk4/Gch1/Jak2/Map2k3/Mapk14/Mapk8/Nos3/Rela
1-day post-SCI group vs. the control group	BP	GO:0050769	positive regulation of neurogenesis	10/125	8.88E-07	1.80E-05	9.38E-06	Apoe/Cask/Hdac1/Il6st/Mapk8/Mapt/Met/Myc/Rela/Rnf112
1-day post-SCI group vs. the control group	BP	GO:1990874	vascular associated smooth muscle cell proliferation	6/125	9.07E-07	1.83E-05	9.53E-06	Edn1/Hmox1/Jak2/Jun/Mdm2/Ppargc1a
1-day post-SCI group vs. the control group	BP	GO:1904037	positive regulation of epithelial cell apoptotic process	5/125	9.17E-07	1.84E-05	9.58E-06	Bax/Eif2s1/Hmox1/Jak2/Ppargc1a
1-day post-SCI group vs. the control group	BP	GO:0010720	positive regulation of cell development	11/125	9.34E-07	1.85E-05	9.64E-06	Apoe/Cask/Hdac1/Il6st/Mapk8/Mapt/Met/Myc/P4hb/Rela/Rnf112
1-day post-SCI group vs. the control group	BP	GO:0070661	leukocyte proliferation	11/125	9.34E-07	1.85E-05	9.64E-06	Anxa1/Bax/Btk/Fkbp1b/Il6st/Jak2/Myc/Ppp3ca/Ripk3/Stat6/Vcam1
1-day post-SCI group vs. the control group	BP	GO:0009266	response to temperature stimulus	8/125	9.39E-07	1.85E-05	9.64E-06	Eif2s1/Hmox1/Hspb1/Mapk8/Mapt/Rbbp7/Trpm2/Ucp2

1-day post-SCI group vs. the control group	BP	GO:0042098	T cell proliferation	9/125	9.54E-07	1.87E-05	9.73E-06	Anxa1/Bax/Fkbp1b/Il6st/Jak2/Myc/Ppp3ca/Ripk3/Vcam1
1-day post-SCI group vs. the control group	BP	GO:0046883	regulation of hormone secretion	10/125	9.71E-07	1.89E-05	9.85E-06	Anxa1/Cask/Dynll1/Edn1/Fkbp1b/Itsn1/Jak2/Ppp3ca/Trpm2/Ucp2
1-day post-SCI group vs. the control group	BP	GO:0009612	response to mechanical stimulus	8/125	1.07E-06	2.05E-05	1.07E-05	Fos/Jun/Mapk14/Mapk8/Myc/Nos3/Rela/Slc8a1
1-day post-SCI group vs. the control group	BP	GO:0051100	negative regulation of binding	8/125	1.07E-06	2.05E-05	1.07E-05	Bax/Fbxw7/Hmox1/Jak2/Jun/Mapk8/Myc/Ppp3ca
1-day post-SCI group vs. the control group	BP	GO:1903531	negative regulation of secretion by cell	8/125	1.07E-06	2.05E-05	1.07E-05	Anxa1/Apoe/Edn1/Fkbp1b/Hmox1/Ppp3ca/Tnfrsf1a/Ucp2
1-day post-SCI group vs. the control group	BP	GO:2000630	positive regulation of miRNA metabolic process	6/125	1.15E-06	2.18E-05	1.13E-05	Fos/Fosl1/Jun/Myc/Rela/Smad1
1-day post-SCI group vs. the control group	BP	GO:0060326	cell chemotaxis	10/125	1.16E-06	2.18E-05	1.13E-05	Abcc1/Anxa1/Ccr1/Edn1/Hbegf/Hspb1/Met/Rpl13a/Trpm2/Vcam1
1-day post-SCI group vs. the control group	BP	GO:0071219	cellular response to molecule of bacterial origin	10/125	1.16E-06	2.18E-05	1.13E-05	Adam9/Axl/Cdk4/Gch1/Jak2/Map2k3/Mapk14/Mapk8/Nos3/Rela
1-day post-SCI group vs. the control group	BP	GO:0071478	cellular response to radiation	8/125	1.16E-06	2.18E-05	1.14E-05	Bax/Eif2s1/Ercc1/Fbxw7/Mapk14/Mdm2/Mmp3/Pcna
1-day post-SCI group vs. the control group	BP	GO:0001890	placenta development	8/125	1.21E-06	2.26E-05	1.18E-05	Cdkn1c/Ctsl/Fosl1/Mapk14/Met/Ptgs2/Slc8a1/Vcam1
1-day post-SCI group vs. the control group	BP	GO:0014812	muscle cell migration	7/125	1.31E-06	2.42E-05	1.26E-05	Anxa1/Cyp1b1/Il6st/Mdm2/Met/Myc/Ppargc1a
1-day post-SCI group vs. the control group	BP	GO:1901653	cellular response to peptide	10/125	1.45E-06	2.67E-05	1.39E-05	Abcc1/Cdk4/Edn1/Jak2/Mdm2/Pdk2/Pxn/Rela/Stat6/Vcam1
1-day post-SCI group vs. the control group	BP	GO:0048608	reproductive structure development	10/125	1.53E-06	2.81E-05	1.46E-05	Ago4/Anxa1/Axl/Bax/Cdkn1c/Ercc1/Ermp1/Nos3/Plekha1/Ybx3
1-day post-SCI group vs. the control group	BP	GO:0032388	positive regulation of intracellular transport	8/125	1.61E-06	2.95E-05	1.54E-05	Ezr/Fbxw7/Jak2/Mapk14/Mdm2/Prr5l/Ptgs2/Tnfrsf1a
1-day post-SCI group vs. the control group	BP	GO:2000273	positive regulation of signaling receptor activity	5/125	1.65E-06	3.00E-05	1.56E-05	Edn1/Fbxw7/Hbegf/Hdac1/Jak2
1-day post-SCI group vs. the control group	BP	GO:0051098	regulation of binding	11/125	1.68E-06	3.04E-05	1.58E-05	Apoe/Bax/Eif2s1/Fbxw7/Hmox1/Jak2/Jun/Mapk8/Met/Myc/Ppp3ca
1-day post-SCI group vs. the control group	BP	GO:0061458	reproductive system development	10/125	1.71E-06	3.07E-05	1.60E-05	Ago4/Anxa1/Axl/Bax/Cdkn1c/Ercc1/Ermp1/Nos3/Plekha1/Ybx3
1-day post-SCI group vs. the control group	BP	GO:0097300	programmed necrotic cell death	5/125	1.84E-06	3.29E-05	1.71E-05	Bax/Mapk8/Ripk1/Ripk3/Ybx3
1-day post-SCI group vs. the control group	BP	GO:0018105	peptidyl-serine phosphorylation	10/125	1.90E-06	3.39E-05	1.77E-05	Bax/Mapk14/Mapk8/Mapkapk3/Met/Pdk2/Pink1/Ptgs2/Ripk1/Spry2
1-day post-SCI group vs. the control group	BP	GO:0090257	regulation of muscle system process	9/125	1.92E-06	3.40E-05	1.77E-05	Cttn/Edn1/Fkbp1b/Nos3/Ppp3ca/Ptgs2/Slc8a1/Sphk1/Tnfrsf1a
1-day post-SCI group vs. the control group	BP	GO:0010212	response to ionizing radiation	7/125	1.97E-06	3.48E-05	1.81E-05	Anxa1/Bax/Ercc1/Gpx1/Mapk14/Mdm2/Myc
1-day post-SCI group vs. the control group	BP	GO:0051770	positive regulation of nitric-oxide synthase biosyntheti	4/125	2.28E-06	3.99E-05	2.08E-05	Jak2/Map2k3/Map2k4/Map2k6
1-day post-SCI group vs. the control group	BP	GO:0001889	liver development	7/125	2.29E-06	3.99E-05	2.08E-05	Anxa1/Atf2/Gfer/Hmox1/Jun/Met/Rela
1-day post-SCI group vs. the control group	BP	GO:1902893	regulation of miRNA transcription	6/125	2.33E-06	4.05E-05	2.11E-05	Fos/Fosl1/Jun/Myc/Rela/Smad1
1-day post-SCI group vs. the control group	BP	GO:0008016	regulation of heart contraction	8/125	2.57E-06	4.43E-05	2.31E-05	Edn1/Fkbp1b/Gch1/Hbegf/Jak2/Mdm2/Nos3/Slc8a1
1-day post-SCI group vs. the control group	BP	GO:0046651	lymphocyte proliferation	10/125	2.61E-06	4.49E-05	2.34E-05	Anxa1/Bax/Btk/Fkbp1b/Il6st/Jak2/Myc/Ppp3ca/Ripk3/Vcam1
1-day post-SCI group vs. the control group	BP	GO:1903522	regulation of blood circulation	9/125	2.62E-06	4.49E-05	2.34E-05	Edn1/Fkbp1b/Gch1/Hbegf/Jak2/Mdm2/Nos3/Ptgs2/Slc8a1
1-day post-SCI group vs. the control group	BP	GO:0061008	hepaticobiliary system development	7/125	2.64E-06	4.50E-05	2.34E-05	Anxa1/Atf2/Gfer/Hmox1/Jun/Met/Rela
1-day post-SCI group vs. the control group	BP	GO:0061614	miRNA transcription	6/125	2.66E-06	4.51E-05	2.35E-05	Fos/Fosl1/Jun/Myc/Rela/Smad1
1-day post-SCI group vs. the control group	BP	GO:0045740	positive regulation of DNA replication	5/125	2.78E-06	4.68E-05	2.44E-05	Jun/Map2k4/Mapk8/Met/Pcna
1-day post-SCI group vs. the control group	BP	GO:0034644	cellular response to UV	6/125	2.84E-06	4.76E-05	2.48E-05	Bax/Eif2s1/Ercc1/Fbxw7/Mmp3/Pcna
1-day post-SCI group vs. the control group	BP	GO:0031331	positive regulation of cellular catabolic process	11/125	2.85E-06	4.76E-05	2.48E-05	Adam9/Apoe/Bax/Fbxw7/Hmox1/Mapk8/Mdm2/Myc/Pink1/Prr5l/Tsc1
1-day post-SCI group vs. the control group	BP	GO:0014902	myotube differentiation	7/125	3.04E-06	5.05E-05	2.63E-05	Capn2/Gpx1/Mapk14/Met/Myc/Ppp3ca/Rcan1

1-day post-SCI group vs. the control group	BP	GO:0016311	dephosphorylation	10/125	3.13E-06	5.14E-05	2.68E-05	Fkbp1b/Jak2/Pink1/Ppargc1b/Ppp1ca/Ppp1r15b/Ppp3ca/Rcan1/Ripk3/Tsc1
1-day post-SCI group vs. the control group	BP	GO:0032943	mononuclear cell proliferation	10/125	3.13E-06	5.14E-05	2.68E-05	Anxa1/Bax/Btk/Fkbp1b/Il6st/Jak2/Myc/Ppp3ca/Ripk3/Vcam1
1-day post-SCI group vs. the control group	BP	GO:0051048	negative regulation of secretion	8/125	3.32E-06	5.43E-05	2.83E-05	Anxa1/Apoe/Edn1/Fkbp1b/Hmox1/Ppp3ca/Tnfrsf1a/Ucp2
1-day post-SCI group vs. the control group	BP	GO:0090316	positive regulation of intracellular protein transport	7/125	3.33E-06	5.44E-05	2.83E-05	Fbxw7/Jak2/Mapk14/Mdm2/Prr5l/Ptgs2/Tnfrsf1a
1-day post-SCI group vs. the control group	BP	GO:0018209	peptidyl-serine modification	10/125	3.37E-06	5.47E-05	2.85E-05	Bax/Mapk14/Mapk8/Mapkapk3/Met/Pdk2/Pink1/Ptgs2/Ripk1/Spry2
1-day post-SCI group vs. the control group	BP	GO:0007519	skeletal muscle tissue development	8/125	3.44E-06	5.55E-05	2.89E-05	Fos/Gpx1/Mapk14/Met/Myc/Ppp3ca/Rcan1/Ybx3
1-day post-SCI group vs. the control group	BP	GO:0042176	regulation of protein catabolic process	10/125	3.46E-06	5.56E-05	2.89E-05	Adam9/Apoe/Atp13a2/Ezr/Fbxw7/Gpx1/Ier3/Mapk8/Mdm2/Rela
1-day post-SCI group vs. the control group	BP	GO:0120161	regulation of cold-induced thermogenesis	7/125	3.65E-06	5.84E-05	3.04E-05	Atf4/Jak2/Map2k6/Ppargc1a/Ppargc1b/Stat6/Ucp2
1-day post-SCI group vs. the control group	BP	GO:0006692	prostanoid metabolic process	5/125	3.70E-06	5.87E-05	3.06E-05	Anxa1/Edn1/Ptgs2/Sphk1/Tnfrsf1a
1-day post-SCI group vs. the control group	BP	GO:0006693	prostaglandin metabolic process	5/125	3.70E-06	5.87E-05	3.06E-05	Anxa1/Edn1/Ptgs2/Sphk1/Tnfrsf1a
1-day post-SCI group vs. the control group	BP	GO:1904019	epithelial cell apoptotic process	7/125	3.82E-06	5.98E-05	3.11E-05	Atf2/Bax/Eif2s1/Hmox1/Jak2/Mapk8/Ppargc1a
1-day post-SCI group vs. the control group	BP	GO:1902107	positive regulation of leukocyte differentiation	8/125	3.82E-06	5.98E-05	3.11E-05	Anxa1/Axl/Ccr1/Fos/Jun/Ppargc1b/Ppp3ca/Ripk1
1-day post-SCI group vs. the control group	BP	GO:1903708	positive regulation of hemopoiesis	8/125	3.82E-06	5.98E-05	3.11E-05	Anxa1/Axl/Ccr1/Fos/Jun/Ppargc1b/Ppp3ca/Ripk1
1-day post-SCI group vs. the control group	BP	GO:0035304	regulation of protein dephosphorylation	6/125	3.88E-06	6.03E-05	3.14E-05	Fkbp1b/Jak2/Pink1/Ppp1r15b/Rcan1/Tsc1
1-day post-SCI group vs. the control group	BP	GO:1901032	negative regulation of response to reactive oxygen spe	4/125	3.91E-06	6.03E-05	3.14E-05	Gpr37l1/Met/Pink1/Trap1
1-day post-SCI group vs. the control group	BP	GO:1903206	negative regulation of hydrogen peroxide-induced cell	4/125	3.91E-06	6.03E-05	3.14E-05	Gpr37l1/Met/Pink1/Trap1
1-day post-SCI group vs. the control group	BP	GO:0106106	cold-induced thermogenesis	7/125	3.99E-06	6.14E-05	3.20E-05	Atf4/Jak2/Map2k6/Ppargc1a/Ppargc1b/Stat6/Ucp2
1-day post-SCI group vs. the control group	BP	GO:0010823	negative regulation of mitochondrion organization	5/125	4.06E-06	6.21E-05	3.23E-05	Gpx1/Ier3/Mapt/Pink1/Ppargc1a
1-day post-SCI group vs. the control group	BP	GO:0045860	positive regulation of protein kinase activity	10/125	4.21E-06	6.41E-05	3.34E-05	Adam9/Edn1/Fbxw7/Hbegf/Jak2/Map2k3/Map2k4/Map2k6/Pxn/Ripk3
1-day post-SCI group vs. the control group	BP	GO:0051962	positive regulation of nervous system development	10/125	4.52E-06	6.86E-05	3.57E-05	Apoe/Cask/Hdac1/Il6st/Mapk8/Mapt/Met/Myc/Rela/Rnf112
1-day post-SCI group vs. the control group	BP	GO:0006631	fatty acid metabolic process	11/125	4.58E-06	6.92E-05	3.60E-05	Anxa1/Cyp1b1/Edn1/Gpx1/Gpx4/Mapk14/Pdk2/Ppargc1a/Ptgs2/Sphk1/Tnfrsf1a
1-day post-SCI group vs. the control group	BP	GO:0071276	cellular response to cadmium ion	4/125	4.60E-06	6.92E-05	3.61E-05	Fos/Hmox1/Jun/Mapk8
1-day post-SCI group vs. the control group	BP	GO:0043434	response to peptide hormone	10/125	4.86E-06	7.28E-05	3.79E-05	Anxa1/Cdk4/Edn1/Jak2/Mapk14/Pdk2/Pxn/Rela/Stat6/Tsc1
1-day post-SCI group vs. the control group	BP	GO:0006970	response to osmotic stress	6/125	4.92E-06	7.34E-05	3.82E-05	Atf2/Bax/Mapk8/Ptgs2/Slc4a11/Ybx3
1-day post-SCI group vs. the control group	BP	GO:0031099	regeneration	7/125	5.18E-06	7.69E-05	4.01E-05	Anxa1/Fkbp1b/Gfer/Gpx1/Hmox1/Jak2/Jun
1-day post-SCI group vs. the control group	BP	GO:0060538	skeletal muscle organ development	8/125	5.21E-06	7.70E-05	4.01E-05	Fos/Gpx1/Mapk14/Met/Myc/Ppp3ca/Rcan1/Ybx3
1-day post-SCI group vs. the control group	BP	GO:0006874	cellular calcium ion homeostasis	9/125	5.27E-06	7.75E-05	4.04E-05	Apoe/Atf4/Atp13a2/Bax/Ccr1/Edn1/Fkbp1b/Slc8a1/Trpm2
1-day post-SCI group vs. the control group	BP	GO:0043666	regulation of phosphoprotein phosphatase activity	5/125	5.29E-06	7.75E-05	4.04E-05	Fkbp1b/Jak2/Ppp1r15b/Rcan1/Tsc1
1-day post-SCI group vs. the control group	BP	GO:2000628	regulation of miRNA metabolic process	6/125	5.52E-06	8.06E-05	4.20E-05	Fos/Fosl1/Jun/Myc/Rela/Smad1
1-day post-SCI group vs. the control group	BP	GO:0046879	hormone secretion	10/125	5.60E-06	8.14E-05	4.24E-05	Anxa1/Cask/Dynll1/Edn1/Fkbp1b/Itsn1/Jak2/Ppp3ca/Trpm2/Ucp2
1-day post-SCI group vs. the control group	BP	GO:0071715	icosanoid transport	5/125	6.26E-06	9.06E-05	4.72E-05	Abcc1/Anxa1/Edn1/Map2k6/Ptgs2
1-day post-SCI group vs. the control group	BP	GO:0009914	hormone transport	10/125	6.74E-06	9.71E-05	5.06E-05	Anxa1/Cask/Dynll1/Edn1/Fkbp1b/Itsn1/Jak2/Ppp3ca/Trpm2/Ucp2
1-day post-SCI group vs. the control group	BP	GO:0010976	positive regulation of neuron projection development	8/125	6.77E-06	9.72E-05	5.06E-05	Apoe/Cask/Fkbp1b/Hspb1/Mapt/Met/Nme2/Sphk1
1-day post-SCI group vs. the control group	BP	GO:0007249	I-kappaB kinase/NF-kappaB signaling	8/125	7.45E-06	0.00010659	5.55E-05	Btk/Edn1/Hdac1/Hspb1/Pink1/Rela/Ripk1/Ripk3

1-day post-SCI group vs. the control group	BP	GO:0007006	mitochondrial membrane organization	6/125	7.68E-06	0.00010936	5.69E-05	Atf2/Bax/Hebp2/Ier3/Myc/Pink1
1-day post-SCI group vs. the control group	BP	GO:0050714	positive regulation of protein secretion	7/125	7.81E-06	0.00011037	5.75E-05	Adam9/Atp13a2/Cask/Dynll1/Ezr/Jak2/Trpm2
1-day post-SCI group vs. the control group	BP	GO:1990845	adaptive thermogenesis	7/125	7.81E-06	0.00011037	5.75E-05	Atf4/Jak2/Map2k6/Ppargc1a/Ppargc1b/Stat6/Ucp2
1-day post-SCI group vs. the control group	BP	GO:2000112	regulation of cellular macromolecule biosynthetic proc	11/125	8.22E-06	0.00011561	6.02E-05	Ago4/Cdk4/Cyp1b1/Eif2s1/Hbegf/Pink1/Ppp1ca/Ppp1r15b/Rpl13a/Trap1/Tsc1
1-day post-SCI group vs. the control group	BP	GO:0001516	prostaglandin biosynthetic process	4/125	8.33E-06	0.00011577	6.03E-05	Anxa1/Edn1/Ptgs2/Sphk1
1-day post-SCI group vs. the control group	BP	GO:0042744	hydrogen peroxide catabolic process	4/125	8.33E-06	0.00011577	6.03E-05	Cat/Gpx1/Pxdn/Txnrd1
1-day post-SCI group vs. the control group	BP	GO:0046457	prostanoid biosynthetic process	4/125	8.33E-06	0.00011577	6.03E-05	Anxa1/Edn1/Ptgs2/Sphk1
1-day post-SCI group vs. the control group	BP	GO:0006417	regulation of translation	10/125	8.62E-06	0.0001193	6.21E-05	Ago4/Cdk4/Cyp1b1/Eif2s1/Pink1/Ppp1ca/Ppp1r15b/Rpl13a/Trap1/Tsc1
1-day post-SCI group vs. the control group	BP	GO:0003018	vascular process in circulatory system	8/125	8.72E-06	0.0001193	6.21E-05	Abcc1/Apoe/Edn1/Gch1/Gpx1/Nos3/Ptgs2/Slc8a1
1-day post-SCI group vs. the control group	BP	GO:0030073	insulin secretion	8/125	8.72E-06	0.0001193	6.21E-05	Anxa1/Cask/Dynll1/Fkbp1b/Jak2/Ppp3ca/Trpm2/Ucp2
1-day post-SCI group vs. the control group	BP	GO:0090276	regulation of peptide hormone secretion	8/125	8.72E-06	0.0001193	6.21E-05	Cask/Dynll1/Fkbp1b/Itsn1/Jak2/Ppp3ca/Trpm2/Ucp2
1-day post-SCI group vs. the control group	BP	GO:0009650	UV protection	3/125	9.23E-06	0.0001258	6.55E-05	Cat/Ercc1/Gpx1
1-day post-SCI group vs. the control group	BP	GO:0035306	positive regulation of dephosphorylation	5/125	9.31E-06	0.00012634	6.58E-05	Jak2/Pink1/Ppargc1b/Ppp1r15b/Ripk3
1-day post-SCI group vs. the control group	BP	GO:0071248	cellular response to metal ion	7/125	9.50E-06	0.00012838	6.69E-05	Atp13a2/Fos/Hmox1/Jun/Mapk8/Slc25a24/Trpm2
1-day post-SCI group vs. the control group	BP	GO:1902883	negative regulation of response to oxidative stress	4/125	9.53E-06	0.00012838	6.69E-05	Gpr37l1/Met/Pink1/Trap1
1-day post-SCI group vs. the control group	BP	GO:0008630	intrinsic apoptotic signaling pathway in response to DN	6/125	9.96E-06	0.00013318	6.93E-05	Bax/Hmox1/Ier3/Mcl1/Myc/Tnfrsf1a
1-day post-SCI group vs. the control group	BP	GO:0071482	cellular response to light stimulus	6/125	9.96E-06	0.00013318	6.93E-05	Bax/Eif2s1/Ercc1/Fbxw7/Mmp3/Pcna
1-day post-SCI group vs. the control group	BP	GO:0002791	regulation of peptide secretion	8/125	1.02E-05	0.00013509	7.03E-05	Cask/Dynll1/Fkbp1b/Itsn1/Jak2/Ppp3ca/Trpm2/Ucp2
1-day post-SCI group vs. the control group	BP	GO:0006936	muscle contraction	9/125	1.02E-05	0.00013509	7.03E-05	Cttn/Edn1/Fkbp1b/Map2k3/Map2k6/Met/Ptgs2/Slc8a1/Sphk1
1-day post-SCI group vs. the control group	BP	GO:0035296	regulation of tube diameter	7/125	1.06E-05	0.00014011	7.30E-05	Apoe/Edn1/Gch1/Gpx1/Nos3/Ptgs2/Slc8a1
1-day post-SCI group vs. the control group	BP	GO:0097746	blood vessel diameter maintenance	7/125	1.06E-05	0.00014011	7.30E-05	Apoe/Edn1/Gch1/Gpx1/Nos3/Ptgs2/Slc8a1
1-day post-SCI group vs. the control group	BP	GO:0090087	regulation of peptide transport	8/125	1.08E-05	0.00014154	7.37E-05	Cask/Dynll1/Fkbp1b/Itsn1/Jak2/Ppp3ca/Trpm2/Ucp2
1-day post-SCI group vs. the control group	BP	GO:0034504	protein localization to nucleus	9/125	1.10E-05	0.00014291	7.44E-05	Atf2/Atp13a2/Ctnna1/Jak2/Mapk14/Mdm2/Ppp3ca/Ptgs2/Tnfrsf1a
1-day post-SCI group vs. the control group	BP	GO:0072503	cellular divalent inorganic cation homeostasis	9/125	1.10E-05	0.00014291	7.44E-05	Apoe/Atf4/Atp13a2/Bax/Ccr1/Edn1/Fkbp1b/Slc8a1/Trpm2
1-day post-SCI group vs. the control group	BP	GO:0035150	regulation of tube size	7/125	1.10E-05	0.00014331	7.46E-05	Apoe/Edn1/Gch1/Gpx1/Nos3/Ptgs2/Slc8a1
1-day post-SCI group vs. the control group	BP	GO:0031348	negative regulation of defense response	8/125	1.15E-05	0.00014802	7.71E-05	Apoe/Banf1/Ccr1/Gfer/Gpx1/Ier3/Krt1/Tnfrsf1a
1-day post-SCI group vs. the control group	BP	GO:0006986	response to unfolded protein	6/125	1.16E-05	0.00014905	7.76E-05	Atf4/Bax/Eif2s1/Ermp1/Hspa13/Hspb1
1-day post-SCI group vs. the control group	BP	GO:0055074	calcium ion homeostasis	9/125	1.21E-05	0.00015541	8.09E-05	Apoe/Atf4/Atp13a2/Bax/Ccr1/Edn1/Fkbp1b/Slc8a1/Trpm2
1-day post-SCI group vs. the control group	BP	GO:0006939	smooth muscle contraction	6/125	1.22E-05	0.00015541	8.09E-05	Cttn/Edn1/Fkbp1b/Ptgs2/Slc8a1/Sphk1
1-day post-SCI group vs. the control group	BP	GO:0051897	positive regulation of protein kinase B signaling	6/125	1.28E-05	0.00016257	8.47E-05	Axl/Gpx1/Hbegf/Itsn1/Met/Spry2
1-day post-SCI group vs. the control group	BP	GO:0008637	apoptotic mitochondrial changes	6/125	1.34E-05	0.00016998	8.85E-05	Atf2/Bax/Gpx1/Ier3/Jun/Pink1
1-day post-SCI group vs. the control group	BP	GO:1902905	positive regulation of supramolecular fiber organizatio	7/125	1.38E-05	0.00017406	9.06E-05	Cttn/Edn1/Mapk8/Mapt/Met/Pxn/Tsc1
1-day post-SCI group vs. the control group	BP	GO:2001244	positive regulation of intrinsic apoptotic signaling path	5/125	1.44E-05	0.00018099	9.42E-05	Bax/Fbxw7/Mcl1/Myc/Ripk3
1-day post-SCI group vs. the control group	BP	GO:0006470	protein dephosphorylation	8/125	1.45E-05	0.0001815	9.45E-05	Fkbp1b/Jak2/Pink1/Ppp1ca/Ppp1r15b/Ppp3ca/Rcan1/Tsc1
1-day post-SCI group vs. the control group	BP	GO:0019221	cytokine-mediated signaling pathway	10/125	1.53E-05	0.00019095	9.94E-05	Axl/Ccr1/Edn1/Il6st/Jak2/Rela/Ripk1/Sphk1/Stat6/Tnfrsf1a
1-day post-SCI group vs. the control group	BP	GO:0071356	cellular response to tumor necrosis factor	7/125	1.53E-05	0.00019095	9.94E-05	Jak2/Mapk14/Rela/Ripk1/Sphk1/Tnfrsf1a/Ybx3
1-day post-SCI group vs. the control group	BP	GO:0045672	positive regulation of osteoclast differentiation	4/125	1.56E-05	0.00019361	0.00010082	Ccr1/Fos/Ppargc1b/Ppp3ca

1-day post-SCI group vs. the control group	BP	GO:0045987	positive regulation of smooth muscle contraction	4/125	1.56E-05	0.00019361	0.00010082	Cttn/Edn1/Ptgs2/Sphk1
1-day post-SCI group vs. the control group	BP	GO:0030316	osteoclast differentiation	6/125	1.62E-05	0.00019944	0.00010386	Ccr1/Fbxw7/Fos/Mapk14/Ppargc1b/Ppp3ca
1-day post-SCI group vs. the control group	BP	GO:0015980	energy derivation by oxidation of organic compounds	9/125	1.65E-05	0.0002029	0.00010566	Bax/Cat/Il6st/Myc/Ndufa6/Pink1/Ppargc1a/Ppp1ca/Trap1
1-day post-SCI group vs. the control group	BP	GO:0043254	regulation of protein-containing complex assembly	10/125	1.66E-05	0.0002029	0.00010566	Apoe/Bax/Cttn/Lcat/Mapk8/Mapt/Met/Mmp3/Pink1/Rpl13a
1-day post-SCI group vs. the control group	BP	GO:0010922	positive regulation of phosphatase activity	4/125	1.75E-05	0.00021326	0.00011105	Jak2/Ppargc1b/Ppp1r15b/Ripk3
1-day post-SCI group vs. the control group	BP	GO:0046824	positive regulation of nucleocytoplasmic transport	5/125	1.76E-05	0.00021326	0.00011105	Jak2/Mapk14/Mdm2/Ptgs2/Tnfrsf1a
1-day post-SCI group vs. the control group	BP	GO:0072594	establishment of protein localization to organelle	10/125	1.76E-05	0.00021326	0.00011105	Atf2/Bax/Fbxw7/Jak2/Mapk14/Mapt/Pink1/Ppp3ca/Ptgs2/Tnfrsf1a
1-day post-SCI group vs. the control group	BP	GO:0002040	sprouting angiogenesis	6/125	1.77E-05	0.00021407	0.00011148	Anxa1/Fbxw7/Hmox1/Itga5/Ptgs2/Smad1
1-day post-SCI group vs. the control group	BP	GO:0051101	regulation of DNA binding	6/125	1.94E-05	0.00023346	0.00012157	Fbxw7/Hmox1/Jak2/Jun/Mapk8/Myc
1-day post-SCI group vs. the control group	BP	GO:0050796	regulation of insulin secretion	7/125	2.02E-05	0.00024212	0.00012608	Cask/Dynll1/Fkbp1b/Jak2/Ppp3ca/Trpm2/Ucp2
1-day post-SCI group vs. the control group	BP	GO:0006913	nucleocytoplasmic transport	9/125	2.08E-05	0.00024805	0.00012917	Atf2/Ier3/Jak2/Mapk14/Mdm2/Ppp3ca/Ptgs2/Tnfrsf1a/Tsc1
1-day post-SCI group vs. the control group	BP	GO:0051169	nuclear transport	9/125	2.08E-05	0.00024805	0.00012917	Atf2/Ier3/Jak2/Mapk14/Mdm2/Ppp3ca/Ptgs2/Tnfrsf1a/Tsc1
1-day post-SCI group vs. the control group	BP	GO:0010288	response to lead ion	3/125	2.18E-05	0.00025486	0.00013272	Cdk4/Mapt/Ppp1ca
1-day post-SCI group vs. the control group	BP	GO:0051549	positive regulation of keratinocyte migration	3/125	2.18E-05	0.00025486	0.00013272	Adam9/Hbegf/Map4k4
1-day post-SCI group vs. the control group	BP	GO:1901298	regulation of hydrogen peroxide-mediated programme	3/125	2.18E-05	0.00025486	0.00013272	Met/Pink1/Trap1
1-day post-SCI group vs. the control group	BP	GO:0030808	regulation of nucleotide biosynthetic process	4/125	2.18E-05	0.00025486	0.00013272	Myc/Nos3/Pdk2/Ppargc1a
1-day post-SCI group vs. the control group	BP	GO:0046686	response to cadmium ion	4/125	2.18E-05	0.00025486	0.00013272	Fos/Hmox1/Jun/Mapk8
1-day post-SCI group vs. the control group	BP	GO:1900371	regulation of purine nucleotide biosynthetic process	4/125	2.18E-05	0.00025486	0.00013272	Myc/Nos3/Pdk2/Ppargc1a
1-day post-SCI group vs. the control group	BP	GO:0051592	response to calcium ion	6/125	2.22E-05	0.00025762	0.00013415	Adam9/Fos/Jun/Ppp3ca/Slc25a24/Trpm2
1-day post-SCI group vs. the control group	BP	GO:0050777	negative regulation of immune response	7/125	2.23E-05	0.00025874	0.00013474	Anxa1/Banf1/Ccr1/Gfer/Gpx1/Hmox1/Stat6
1-day post-SCI group vs. the control group	BP	GO:0019369	arachidonic acid metabolic process	5/125	2.27E-05	0.00026017	0.00013548	Cyp1b1/Gpx1/Gpx4/Ptgs2/Sphk1
1-day post-SCI group vs. the control group	BP	GO:0038034	signal transduction in absence of ligand	5/125	2.27E-05	0.00026017	0.00013548	Bax/Ctnna1/Mcl1/Ppp1ca/Ripk1
1-day post-SCI group vs. the control group	BP	GO:0097192	extrinsic apoptotic signaling pathway in absence of lig	5/125	2.27E-05	0.00026017	0.00013548	Bax/Ctnna1/Mcl1/Ppp1ca/Ripk1
1-day post-SCI group vs. the control group	BP	GO:2000379	positive regulation of reactive oxygen species metabol	5/125	2.27E-05	0.00026017	0.00013548	Cyp1b1/Mapk14/Mapt/Ripk3/Xdh
1-day post-SCI group vs. the control group	BP	GO:0046822	regulation of nucleocytoplasmic transport	6/125	2.41E-05	0.00027477	0.00014308	Ier3/Jak2/Mapk14/Mdm2/Ptgs2/Tnfrsf1a
1-day post-SCI group vs. the control group	BP	GO:0010661	positive regulation of muscle cell apoptotic process	4/125	2.43E-05	0.00027477	0.00014308	Atf4/Capn2/Map2k4/Mapk8
1-day post-SCI group vs. the control group	BP	GO:0090049	regulation of cell migration involved in sprouting angi	4/125	2.43E-05	0.00027477	0.00014308	Anxa1/Fbxw7/Hmox1/Ptgs2
1-day post-SCI group vs. the control group	BP	GO:0044403	biological process involved in symbiotic interaction	8/125	2.50E-05	0.00028236	0.00014703	Apoe/Axl/Ctsl/Gpx1/Hdac1/Jak2/Jun/P4hb
1-day post-SCI group vs. the control group	BP	GO:0045862	positive regulation of proteolysis	9/125	2.60E-05	0.00029291	0.00015253	Adam9/Apoe/Bax/Fbxw7/Jak2/Mapk8/Mdm2/Myc/Xdh
1-day post-SCI group vs. the control group	BP	GO:0006816	calcium ion transport	10/125	2.65E-05	0.000297	0.00015466	Bax/Cask/Ccr1/Edn1/Fkbp1b/Nos3/Ppp3ca/Ptgs2/Slc8a1/Trpm2
1-day post-SCI group vs. the control group	BP	GO:0045732	positive regulation of protein catabolic process	7/125	2.71E-05	0.0003026	0.00015757	Adam9/Apoe/Ezr/Fbxw7/Ier3/Mapk8/Mdm2
1-day post-SCI group vs. the control group	BP	GO:0072507	divalent inorganic cation homeostasis	9/125	2.72E-05	0.0003026	0.00015757	Apoe/Atf4/Atp13a2/Bax/Ccr1/Edn1/Fkbp1b/Slc8a1/Trpm2
1-day post-SCI group vs. the control group	BP	GO:0007568	aging	5/125	2.73E-05	0.0003026	0.00015757	Apoe/Cdkn1c/Edn1/Eif2s1/Ercc1
1-day post-SCI group vs. the control group	BP	GO:0033209	tumor necrosis factor-mediated signaling pathway	5/125	2.73E-05	0.0003026	0.00015757	Jak2/Rela/Ripk1/Sphk1/Tnfrsf1a
1-day post-SCI group vs. the control group	BP	GO:0014013	regulation of gliogenesis	6/125	2.74E-05	0.00030272	0.00015764	Gpr37l1/Hdac1/Il6st/Myc/Rela/Rnf112
1-day post-SCI group vs. the control group	BP	GO:0010940	positive regulation of necrotic cell death	3/125	2.76E-05	0.00030429	0.00015845	Hebp2/Ripk1/Ripk3
1-day post-SCI group vs. the control group	BP	GO:0052548	regulation of endopeptidase activity	9/125	2.84E-05	0.0003115	0.00016221	Atp13a2/Bax/Gpx1/Hdac1/Jak2/Mdm2/Myc/Ptgs2/Xdh
1-day post-SCI group vs. the control group	BP	GO:0051495	positive regulation of cytoskeleton organization	7/125	2.89E-05	0.00031612	0.00016461	Cttn/Edn1/Mapk8/Mapt/Met/Pxn/Tsc1

1-day post-SCI group vs. the control group	BP	GO:0050709	negative regulation of protein secretion	5/125	2.90E-05	0.00031612	0.00016461	Anxa1/Apoe/Fkbp1b/Ppp3ca/Ucp2
1-day post-SCI group vs. the control group	BP	GO:0006636	unsaturated fatty acid biosynthetic process	4/125	2.97E-05	0.00032145	0.00016739	Anxa1/Edn1/Ptgs2/Sphk1
1-day post-SCI group vs. the control group	BP	GO:0010939	regulation of necrotic cell death	4/125	2.97E-05	0.00032145	0.00016739	Hebp2/Ripk1/Ripk3/Ybx3
1-day post-SCI group vs. the control group	BP	GO:0001959	regulation of cytokine-mediated signaling pathway	6/125	2.98E-05	0.00032175	0.00016755	Axl/Edn1/Il6st/Ripk1/Sphk1/Tnfrsf1a
1-day post-SCI group vs. the control group	BP	GO:0070663	regulation of leukocyte proliferation	8/125	3.07E-05	0.00033058	0.00017214	Anxa1/Btk/Il6st/Jak2/Ppp3ca/Ripk3/Stat6/Vcam1
1-day post-SCI group vs. the control group	BP	GO:0032872	regulation of stress-activated MAPK cascade	7/125	3.08E-05	0.00033058	0.00017214	Ezr/Map2k4/Map4k4/Met/Ripk1/Sphk1/Xdh
1-day post-SCI group vs. the control group	BP	GO:0032874	positive regulation of stress-activated MAPK cascade	6/125	3.10E-05	0.0003311	0.00017242	Map2k4/Map4k4/Met/Ripk1/Sphk1/Xdh
1-day post-SCI group vs. the control group	BP	GO:0035966	response to topologically incorrect protein	6/125	3.10E-05	0.0003311	0.00017242	Atf4/Bax/Eif2s1/Ermp1/Hspa13/Hspb1
1-day post-SCI group vs. the control group	BP	GO:0046890	regulation of lipid biosynthetic process	7/125	3.18E-05	0.00033793	0.00017597	Anxa1/Apoe/Capn2/Cdk4/Ppargc1a/Ptgs2/Sphk1
1-day post-SCI group vs. the control group	BP	GO:1900744	regulation of p38MAPK cascade	4/125	3.27E-05	0.00034657	0.00018047	Ezr/Met/Sphk1/Xdh
1-day post-SCI group vs. the control group	BP	GO:0070304	positive regulation of stress-activated protein kinase si	6/125	3.36E-05	0.00035574	0.00018525	Map2k4/Map4k4/Met/Ripk1/Sphk1/Xdh
1-day post-SCI group vs. the control group	BP	GO:0070302	regulation of stress-activated protein kinase signaling c	7/125	3.38E-05	0.00035624	0.00018551	Ezr/Map2k4/Map4k4/Met/Ripk1/Sphk1/Xdh
1-day post-SCI group vs. the control group	BP	GO:0010421	hydrogen peroxide-mediated programmed cell death	3/125	3.44E-05	0.00035821	0.00018653	Met/Pink1/Trap1
1-day post-SCI group vs. the control group	BP	GO:0010917	negative regulation of mitochondrial membrane potent	3/125	3.44E-05	0.00035821	0.00018653	Bax/Hebp2/Mapt
1-day post-SCI group vs. the control group	BP	GO:0051547	regulation of keratinocyte migration	3/125	3.44E-05	0.00035821	0.00018653	Adam9/Hbegf/Map4k4
1-day post-SCI group vs. the control group	BP	GO:0006809	nitric oxide biosynthetic process	5/125	3.45E-05	0.00035821	0.00018653	Cyp1b1/Dynll1/Jak2/Nos3/Ptgs2
1-day post-SCI group vs. the control group	BP	GO:0045670	regulation of osteoclast differentiation	5/125	3.45E-05	0.00035821	0.00018653	Ccr1/Fbxw7/Fos/Ppargc1b/Ppp3ca
1-day post-SCI group vs. the control group	BP	GO:0010506	regulation of autophagy	8/125	3.48E-05	0.00036073	0.00018784	Atp13a2/Cttn/Fbxw7/Hmox1/Mapt/Mcl1/Pink1/Tsc1
1-day post-SCI group vs. the control group	BP	GO:0006091	generation of precursor metabolites and energy	10/125	3.56E-05	0.00036734	0.00019128	Bax/Cat/Ier3/Il6st/Myc/Ndufa6/Pink1/Ppargc1a/Ppp1ca/Trap1
1-day post-SCI group vs. the control group	BP	GO:0035794	positive regulation of mitochondrial membrane permea	4/125	3.59E-05	0.0003696	0.00019247	Atf2/Bax/Hebp2/Ier3
1-day post-SCI group vs. the control group	BP	GO:0014015	positive regulation of gliogenesis	5/125	3.65E-05	0.00037456	0.00019505	Hdac1/Il6st/Myc/Rela/Rnf112
1-day post-SCI group vs. the control group	BP	GO:0043281	regulation of cysteine-type endopeptidase activity invo	7/125	3.82E-05	0.00039055	0.00020338	Bax/Gpx1/Jak2/Mdm2/Myc/Ptgs2/Xdh
1-day post-SCI group vs. the control group	BP	GO:0030098	lymphocyte differentiation	10/125	3.96E-05	0.00040435	0.00021056	Anxa1/Atf2/Axl/Bax/Btk/Ctsl/Hspb1/Ripk3/Stat6/Tsc1
1-day post-SCI group vs. the control group	BP	GO:0031669	cellular response to nutrient levels	7/125	4.05E-05	0.00041224	0.00021467	Atf2/Atf4/Eif2s1/Jun/Mapk8/Pdk2/Tsc1
1-day post-SCI group vs. the control group	BP	GO:0048144	fibroblast proliferation	6/125	4.10E-05	0.00041549	0.00021636	Bax/Cdk4/Gpx1/Jun/Myc/Sphk1
1-day post-SCI group vs. the control group	BP	GO:0097468	programmed cell death in response to reactive oxygen	3/125	4.23E-05	0.00042635	0.00022202	Met/Pink1/Trap1
1-day post-SCI group vs. the control group	BP	GO:0042060	wound healing	9/125	4.23E-05	0.00042635	0.00022202	Anxa1/Apoe/Axl/Cask/Gpx1/Hbegf/Hmox1/Jak2/Sdc1
1-day post-SCI group vs. the control group	BP	GO:1904950	negative regulation of establishment of protein localiza	6/125	4.26E-05	0.00042804	0.0002229	Anxa1/Apoe/Fkbp1b/Mapt/Ppp3ca/Ucp2
1-day post-SCI group vs. the control group	BP	GO:0034620	cellular response to unfolded protein	5/125	4.31E-05	0.00042811	0.00022293	Atf4/Bax/Eif2s1/Ermp1/Hspa13
1-day post-SCI group vs. the control group	BP	GO:1900542	regulation of purine nucleotide metabolic process	5/125	4.31E-05	0.00042811	0.00022293	Ier3/Myc/Nos3/Pdk2/Ppargc1a
1-day post-SCI group vs. the control group	BP	GO:0034198	cellular response to amino acid starvation	4/125	4.31E-05	0.00042811	0.00022293	Atf2/Atf4/Eif2s1/Mapk8
1-day post-SCI group vs. the control group	BP	GO:2001239	regulation of extrinsic apoptotic signaling pathway in a	4/125	4.31E-05	0.00042811	0.00022293	Ctnna1/Mcl1/Ppp1ca/Ripk1
1-day post-SCI group vs. the control group	BP	GO:0042593	glucose homeostasis	8/125	4.34E-05	0.00043015	0.00022399	Dynll1/Fkbp1b/Gpx1/Met/Pdk2/Ppp3ca/Ucp2/Vcam1
1-day post-SCI group vs. the control group	BP	GO:0010469	regulation of signaling receptor activity	6/125	4.42E-05	0.0004359	0.00022699	Edn1/Fbxw7/Hbegf/Hdac1/Jak2/Ppargc1a
1-day post-SCI group vs. the control group	BP	GO:0060759	regulation of response to cytokine stimulus	6/125	4.42E-05	0.0004359	0.00022699	Axl/Edn1/Il6st/Ripk1/Sphk1/Tnfrsf1a
1-day post-SCI group vs. the control group	BP	GO:0033500	carbohydrate homeostasis	8/125	4.44E-05	0.00043683	0.00022748	Dynll1/Fkbp1b/Gpx1/Met/Pdk2/Ppp3ca/Ucp2/Vcam1
1-day post-SCI group vs. the control group	BP	GO:0051924	regulation of calcium ion transport	8/125	4.55E-05	0.00044611	0.0002323	Bax/Cask/Ccr1/Fkbp1b/Nos3/Ppp3ca/Ptgs2/Slc8a1
1-day post-SCI group vs. the control group	BP	GO:0071887	leukocyte apoptotic process	6/125	4.59E-05	0.00044893	0.00023377	Anxa1/Axl/Bax/Myc/Ripk1/Ripk3
1-day post-SCI group vs. the control group	BP	GO:0001818	negative regulation of cytokine production	8/125	4.66E-05	0.00045427	0.00023655	Anxa1/Axl/Banf1/Btk/Ezr/Hmox1/Met/Tnfrsf1a
1-day post-SCI group vs. the control group	BP	GO:0035265	organ growth	7/125	4.69E-05	0.00045622	0.00023757	Atf2/Edn1/Map2k4/Mapk14/Smad1/Spry2/Ybx3

1-day post-SCI group vs. the control group	BP	GO:0043467	regulation of generation of precursor metabolites and e	6/125	4.77E-05	0.00046224	0.0002407	Ier3/Myc/Pink1/Ppargc1a/Ppp1ca/Trap1
1-day post-SCI group vs. the control group	BP	GO:0046209	nitric oxide metabolic process	5/125	4.79E-05	0.00046303	0.00024112	Cyp1b1/Dynll1/Jak2/Nos3/Ptgs2
1-day post-SCI group vs. the control group	BP	GO:1903828	negative regulation of protein localization	7/125	5.12E-05	0.00049325	0.00025685	Anxa1/Apoe/Ctnna1/Fkbp1b/Mapt/Ppp3ca/Ucp2
1-day post-SCI group vs. the control group	BP	GO:1900180	regulation of protein localization to nucleus	6/125	5.14E-05	0.0004938	0.00025714	Atp13a2/Ctnna1/Jak2/Mapk14/Ptgs2/Tnfrsf1a
1-day post-SCI group vs. the control group	BP	GO:0061448	connective tissue development	8/125	5.24E-05	0.00050235	0.00026159	Atf2/Bmp1/Cdk4/Edn1/Mapk14/Ppargc1a/Rela/Smad1
1-day post-SCI group vs. the control group	BP	GO:0007623	circadian rhythm	7/125	5.27E-05	0.00050333	0.0002621	Atf4/Fbxw7/Hdac1/Mapk8/Nono/Ppargc1a/Ppp1ca
1-day post-SCI group vs. the control group	BP	GO:2001057	reactive nitrogen species metabolic process	5/125	5.32E-05	0.00050689	0.00026396	Cyp1b1/Dynll1/Jak2/Nos3/Ptgs2
1-day post-SCI group vs. the control group	BP	GO:0071333	cellular response to glucose stimulus	6/125	5.53E-05	0.00052555	0.00027367	Dynll1/Fkbp1b/Gpx1/Ppp3ca/Ucp2/Vcam1
1-day post-SCI group vs. the control group	BP	GO:0006140	regulation of nucleotide metabolic process	5/125	5.89E-05	0.00055566	0.00028935	Ier3/Myc/Nos3/Pdk2/Ppargc1a
1-day post-SCI group vs. the control group	BP	GO:0045685	regulation of glial cell differentiation	5/125	5.89E-05	0.00055566	0.00028935	Gpr37l1/Hdac1/Il6st/Rela/Rnf112
1-day post-SCI group vs. the control group	BP	GO:0008217	regulation of blood pressure	7/125	5.89E-05	0.00055566	0.00028935	Edn1/Gch1/Gpr37l1/Hmox1/Ier3/Nos3/Ptgs2
1-day post-SCI group vs. the control group	BP	GO:0071331	cellular response to hexose stimulus	6/125	5.94E-05	0.00055881	0.00029099	Dynll1/Fkbp1b/Gpx1/Ppp3ca/Ucp2/Vcam1
1-day post-SCI group vs. the control group	BP	GO:0010332	response to gamma radiation	4/125	6.04E-05	0.00056529	0.00029436	Bax/Gpx1/Mdm2/Myc
1-day post-SCI group vs. the control group	BP	GO:1990928	response to amino acid starvation	4/125	6.04E-05	0.00056529	0.00029436	Atf2/Atf4/Eif2s1/Mapk8
1-day post-SCI group vs. the control group	BP	GO:0008643	carbohydrate transport	6/125	6.16E-05	0.00057295	0.00029836	Edn1/Ezr/Mapk14/Met/Myc/Tsc1
1-day post-SCI group vs. the control group	BP	GO:0071326	cellular response to monosaccharide stimulus	6/125	6.16E-05	0.00057295	0.00029836	Dynll1/Fkbp1b/Gpx1/Ppp3ca/Ucp2/Vcam1
1-day post-SCI group vs. the control group	BP	GO:0007044	cell-substrate junction assembly	5/125	6.19E-05	0.00057435	0.00029908	Cttn/Itga5/Map4k4/Pxn/Tsc1
1-day post-SCI group vs. the control group	BP	GO:0055001	muscle cell development	7/125	6.40E-05	0.00059248	0.00030852	Edn1/Gpx1/Map2k4/Met/Ppp3ca/Rcan1/Slc8a1
1-day post-SCI group vs. the control group	BP	GO:0048638	regulation of developmental growth	9/125	6.52E-05	0.00060056	0.00031273	Apoe/Atrn/Cdk4/Cttn/Edn1/Ezr/Mapk14/Mapt/Ybx3
1-day post-SCI group vs. the control group	BP	GO:0006820	anion transport	10/125	6.52E-05	0.00060056	0.00031273	Abcc1/Anxa1/Atf4/Edn1/Map2k6/Myc/Ptgs2/Ripk1/Slc25a24/ Slc4a11
1-day post-SCI group vs. the control group	BP	GO:0097237	cellular response to toxic substance	4/125	6.55E-05	0.00060112	0.00031302	Cat/Gch1/Nos3/Pink1
1-day post-SCI group vs. the control group	BP	GO:0015908	fatty acid transport	5/125	6.83E-05	0.00062379	0.00032483	Abcc1/Anxa1/Apoe/Edn1/Map2k6
1-day post-SCI group vs. the control group	BP	GO:0120162	positive regulation of cold-induced thermogenesis	5/125	6.83E-05	0.00062379	0.00032483	Jak2/Ppargc1a/Ppargc1b/Stat6/Ucp2
1-day post-SCI group vs. the control group	BP	GO:0032309	icosanoid secretion	4/125	7.08E-05	0.00064333	0.00033501	Anxa1/Edn1/Map2k6/Ptgs2
1-day post-SCI group vs. the control group	BP	GO:0046456	icosanoid biosynthetic process	4/125	7.08E-05	0.00064333	0.00033501	Anxa1/Edn1/Ptgs2/Sphk1
1-day post-SCI group vs. the control group	BP	GO:0032102	negative regulation of response to external stimulus	9/125	7.16E-05	0.00064898	0.00033795	Apoe/Banf1/Cask/Ccr1/Gfer/Gpx1/Ier3/Krt1/Tnfrsf1a
1-day post-SCI group vs. the control group	BP	GO:0045837	negative regulation of membrane potential	3/125	7.24E-05	0.00065143	0.00033922	Bax/Hebp2/Mapt
1-day post-SCI group vs. the control group	BP	GO:0060396	growth hormone receptor signaling pathway	3/125	7.24E-05	0.00065143	0.00033922	Jak2/Pxn/Stat6
1-day post-SCI group vs. the control group	BP	GO:0071378	cellular response to growth hormone stimulus	3/125	7.24E-05	0.00065143	0.00033922	Jak2/Pxn/Stat6
1-day post-SCI group vs. the control group	BP	GO:0007548	sex differentiation	8/125	7.35E-05	0.00065778	0.00034253	Ago4/Axl/Bax/Ercc1/Ermp1/Nos3/Plekha1/Ybx3
1-day post-SCI group vs. the control group	BP	GO:0009410	response to xenobiotic stimulus	8/125	7.35E-05	0.00065778	0.00034253	Abcc1/Cyp1b1/Fos/Gpx1/Jun/Myc/Pcna/Smad1
1-day post-SCI group vs. the control group	BP	GO:0014910	regulation of smooth muscle cell migration	5/125	7.52E-05	0.00067101	0.00034942	Cyp1b1/Il6st/Mdm2/Myc/Ppargc1a
1-day post-SCI group vs. the control group	BP	GO:0042307	positive regulation of protein import into nucleus	4/125	7.65E-05	0.00068045	0.00035433	Jak2/Mapk14/Ptgs2/Tnfrsf1a
1-day post-SCI group vs. the control group	BP	GO:0018108	peptidyl-tyrosine phosphorylation	8/125	7.85E-05	0.00069691	0.00036291	Btk/Fbxw7/Hbegf/Il6st/Itga5/Jak2/Pxn/Tnfrsf1a
1-day post-SCI group vs. the control group	BP	GO:0007611	learning or memory	8/125	8.02E-05	0.00071037	0.00036991	Amph/Apoe/Itga5/Jun/Mapt/Ptgs2/Rcan1/Tsc1
1-day post-SCI group vs. the control group	BP	GO:0071322	cellular response to carbohydrate stimulus	6/125	8.12E-05	0.00071742	0.00037359	Dynll1/Fkbp1b/Gpx1/Ppp3ca/Ucp2/Vcam1
1-day post-SCI group vs. the control group	BP	GO:0018212	peptidyl-tyrosine modification	8/125	8.20E-05	0.00072067	0.00037528	Btk/Fbxw7/Hbegf/Il6st/Itga5/Jak2/Pxn/Tnfrsf1a
1-day post-SCI group vs. the control group	BP	GO:0002042	cell migration involved in sprouting angiogenesis	4/125	8.24E-05	0.00072067	0.00037528	Anxa1/Fbxw7/Hmox1/Ptgs2
1-day post-SCI group vs. the control group	BP	GO:0034605	cellular response to heat	4/125	8.24E-05	0.00072067	0.00037528	Eif2s1/Hmox1/Mapt/Rbbp7

1-day post-SCI group vs. the control group	BP	GO:0043392	negative regulation of DNA binding	4/125	8.24E-05	0.00072067	0.00037528	Fbxw7/Hmox1/Jak2/Jun
1-day post-SCI group vs. the control group	BP	GO:0150115	cell-substrate junction organization	5/125	8.26E-05	0.00072067	0.00037528	Cttn/Itga5/Map4k4/Pxn/Tsc1
1-day post-SCI group vs. the control group	BP	GO:0043588	skin development	8/125	8.38E-05	0.00072869	0.00037946	Anxa1/Ctsl/Hdac1/Krt1/Met/Ppp3ca/Ptgs2/Rela
1-day post-SCI group vs. the control group	BP	GO:0045927	positive regulation of growth	8/125	8.56E-05	0.00074262	0.00038671	Apoe/Edn1/Ezr/Hbegf/Mapk14/Mapt/Sphk1/Ybx3
1-day post-SCI group vs. the control group	BP	GO:0120254	olefinic compound metabolic process	6/125	8.68E-05	0.00075179	0.00039149	Cyp1b1/Gpx1/Gpx4/Ppargc1a/Ptgs2/Sphk1
1-day post-SCI group vs. the control group	BP	GO:0008406	gonad development	7/125	8.81E-05	0.00075919	0.00039534	Ago4/Bax/Ercc1/Ermp1/Nos3/Plekha1/Ybx3
1-day post-SCI group vs. the control group	BP	GO:0071241	cellular response to inorganic substance	7/125	8.81E-05	0.00075919	0.00039534	Atp13a2/Fos/Hmox1/Jun/Mapk8/Slc25a24/Trpm2
1-day post-SCI group vs. the control group	BP	GO:0045687	positive regulation of glial cell differentiation	4/125	8.87E-05	0.00076237	0.00039699	Hdac1/Il6st/Rela/Rnf112
1-day post-SCI group vs. the control group	BP	GO:0006937	regulation of muscle contraction	6/125	8.98E-05	0.00076945	0.00040068	Cttn/Edn1/Fkbp1b/Ptgs2/Slc8a1/Sphk1
1-day post-SCI group vs. the control group	BP	GO:0048015	phosphatidylinositol-mediated signaling	6/125	9.28E-05	0.00079149	0.00041216	Cat/Edn1/Ezr/Jak2/Plekha1/Prr5l
1-day post-SCI group vs. the control group	BP	GO:2000116	regulation of cysteine-type endopeptidase activity	7/125	9.28E-05	0.00079149	0.00041216	Bax/Gpx1/Jak2/Mdm2/Myc/Ptgs2/Xdh
1-day post-SCI group vs. the control group	BP	GO:0001974	blood vessel remodeling	4/125	9.54E-05	0.00080749	0.00042049	Axl/Bax/Mdm2/Nos3
1-day post-SCI group vs. the control group	BP	GO:0043620	regulation of DNA-templated transcription in response	4/125	9.54E-05	0.00080749	0.00042049	Atf4/Hmox1/Jun/Rela
1-day post-SCI group vs. the control group	BP	GO:0045933	positive regulation of muscle contraction	4/125	9.54E-05	0.00080749	0.00042049	Cttn/Edn1/Ptgs2/Sphk1
1-day post-SCI group vs. the control group	BP	GO:0046887	positive regulation of hormone secretion	6/125	9.58E-05	0.0008096	0.00042159	Cask/Dynll1/Edn1/Itsn1/Jak2/Trpm2
1-day post-SCI group vs. the control group	BP	GO:0045137	development of primary sexual characteristics	7/125	9.76E-05	0.0008208	0.00042742	Ago4/Bax/Ercc1/Ermp1/Nos3/Plekha1/Ybx3
1-day post-SCI group vs. the control group	BP	GO:0045333	cellular respiration	7/125	9.76E-05	0.0008208	0.00042742	Bax/Cat/Myc/Ndufa6/Pink1/Ppargc1a/Trap1
1-day post-SCI group vs. the control group	BP	GO:0032310	prostaglandin secretion	3/125	9.88E-05	0.0008208	0.00042742	Edn1/Map2k6/Ptgs2
1-day post-SCI group vs. the control group	BP	GO:0048569	post-embryonic animal organ development	3/125	9.88E-05	0.0008208	0.00042742	Bax/Ercc1/Jak2
1-day post-SCI group vs. the control group	BP	GO:0051546	keratinocyte migration	3/125	9.88E-05	0.0008208	0.00042742	Adam9/Hbegf/Map4k4
1-day post-SCI group vs. the control group	BP	GO:0060416	response to growth hormone	3/125	9.88E-05	0.0008208	0.00042742	Jak2/Pxn/Stat6
1-day post-SCI group vs. the control group	BP	GO:0090050	positive regulation of cell migration involved in sprout	3/125	9.88E-05	0.0008208	0.00042742	Anxa1/Hmox1/Ptgs2
1-day post-SCI group vs. the control group	BP	GO:0048017	inositol lipid-mediated signaling	6/125	0.0001022	0.00084726	0.0004412	Cat/Edn1/Ezr/Jak2/Plekha1/Prr5l
1-day post-SCI group vs. the control group	BP	GO:0001678	cellular glucose homeostasis	6/125	0.000109	0.00090093	0.00046915	Dynll1/Fkbp1b/Gpx1/Ppp3ca/Ucp2/Vcam1
1-day post-SCI group vs. the control group	BP	GO:0051353	positive regulation of oxidoreductase activity	4/125	0.0001097	0.00090487	0.0004712	Apoe/Edn1/Gch1/Ripk3
1-day post-SCI group vs. the control group	BP	GO:0035967	cellular response to topologically incorrect protein	5/125	0.0001131	0.00093096	0.00048478	Atf4/Bax/Eif2s1/Ermp1/Hspa13
1-day post-SCI group vs. the control group	BP	GO:0030810	positive regulation of nucleotide biosynthetic process	3/125	0.000114	0.00093188	0.00048526	Myc/Nos3/Ppargc1a
1-day post-SCI group vs. the control group	BP	GO:0060544	regulation of necroptotic process	3/125	0.000114	0.00093188	0.00048526	Ripk1/Ripk3/Ybx3
1-day post-SCI group vs. the control group	BP	GO:1900373	positive regulation of purine nucleotide biosynthetic pr	3/125	0.000114	0.00093188	0.00048526	Myc/Nos3/Ppargc1a
1-day post-SCI group vs. the control group	BP	GO:0022604	regulation of cell morphogenesis	8/125	0.0001146	0.000934	0.00048636	Ago4/Anxa1/Cask/Ezr/Itga7/Met/P4hb/Pxn
1-day post-SCI group vs. the control group	BP	GO:0098754	detoxification	4/125	0.0001174	0.00095512	0.00049736	Abcc1/Cat/Gch1/Nos3
1-day post-SCI group vs. the control group	BP	GO:0042102	positive regulation of T cell proliferation	5/125	0.0001181	0.00095616	0.00049791	Anxa1/Il6st/Jak2/Ppp3ca/Vcam1
1-day post-SCI group vs. the control group	BP	GO:0046889	positive regulation of lipid biosynthetic process	5/125	0.0001181	0.00095616	0.00049791	Anxa1/Apoe/Capn2/Ppargc1a/Ptgs2
1-day post-SCI group vs. the control group	BP	GO:0050670	regulation of lymphocyte proliferation	7/125	0.0001192	0.00096247	0.00050119	Anxa1/Btk/Il6st/Jak2/Ppp3ca/Ripk3/Vcam1
1-day post-SCI group vs. the control group	BP	GO:0014909	smooth muscle cell migration	5/125	0.0001233	0.0009928	0.00051699	Cyp1b1/Il6st/Mdm2/Myc/Ppargc1a
1-day post-SCI group vs. the control group	BP	GO:0006606	protein import into nucleus	6/125	0.0001235	0.0009928	0.00051699	Atf2/Jak2/Mapk14/Ppp3ca/Ptgs2/Tnfrsf1a
1-day post-SCI group vs. the control group	BP	GO:0043270	positive regulation of ion transport	8/125	0.0001267	0.00101645	0.0005293	Atf4/Bax/Cask/Ccr1/Edn1/Map2k6/Pink1/Ppp3ca
1-day post-SCI group vs. the control group	BP	GO:0000303	response to superoxide	3/125	0.0001307	0.00104623	0.00054481	Gch1/Nos3/Ucp2
1-day post-SCI group vs. the control group	BP	GO:0034976	response to endoplasmic reticulum stress	7/125	0.0001313	0.00104636	0.00054488	Atf4/Bax/Eif2s1/Ermp1/Jun/P4hb/Ppp1r15b
1-day post-SCI group vs. the control group	BP	GO:0031346	positive regulation of cell projection organization	9/125	0.0001314	0.00104636	0.00054488	Apoe/Cask/Dynll1/Fkbp1b/Hspb1/Mapt/Met/Nme2/Sphk1

1-day post-SCI group vs. the control group	BP	GO:0002027	regulation of heart rate	5/125	0.0001341	0.00105841	0.00055115	Edn1/Fkbp1b/Gch1/Mdm2/Slc8a1
1-day post-SCI group vs. the control group	BP	GO:1902106	negative regulation of leukocyte differentiation	5/125	0.0001341	0.00105841	0.00055115	Anxa1/Fbxw7/Hspb1/Myc/Nme2
1-day post-SCI group vs. the control group	BP	GO:0043470	regulation of carbohydrate catabolic process	4/125	0.0001341	0.00105841	0.00055115	Ier3/Myc/Ppargc1a/Ppp1ca
1-day post-SCI group vs. the control group	BP	GO:0046902	regulation of mitochondrial membrane permeability	4/125	0.0001341	0.00105841	0.00055115	Atf2/Bax/Hebp2/Ier3
1-day post-SCI group vs. the control group	BP	GO:0032944	regulation of mononuclear cell proliferation	7/125	0.0001345	0.00105918	0.00055155	Anxa1/Btk/Il6st/Jak2/Ppp3ca/Ripk3/Vcam1
1-day post-SCI group vs. the control group	BP	GO:0071900	regulation of protein serine/threonine kinase activity	8/125	0.0001372	0.00107814	0.00056142	Adam9/Apoe/Cdkn1c/Edn1/Map2k4/Map2k6/Slc8a1/Spry2
1-day post-SCI group vs. the control group	BP	GO:0051170	import into nucleus	6/125	0.0001395	0.00109389	0.00056963	Atf2/Jak2/Mapk14/Ppp3ca/Ptgs2/Tnfrsf1a
1-day post-SCI group vs. the control group	BP	GO:0052547	regulation of peptidase activity	9/125	0.0001405	0.00109948	0.00057254	Atp13a2/Bax/Gpx1/Hdac1/Jak2/Mdm2/Myc/Ptgs2/Xdh
1-day post-SCI group vs. the control group	BP	GO:0070371	ERK1 and ERK2 cascade	8/125	0.0001455	0.00113387	0.00059044	Apoe/Ccr1/Edn1/Ezr/Fbxw7/Jun/Myc/Spry2
1-day post-SCI group vs. the control group	BP	GO:0018107	peptidyl-threonine phosphorylation	5/125	0.0001456	0.00113387	0.00059044	Atf2/Mapk8/Met/Sphk1/Spry2
1-day post-SCI group vs. the control group	BP	GO:0000305	response to oxygen radical	3/125	0.0001489	0.00115484	0.00060137	Gch1/Nos3/Ucp2
1-day post-SCI group vs. the control group	BP	GO:1903747	regulation of establishment of protein localization to m	3/125	0.0001489	0.00115484	0.00060137	Fbxw7/Mapt/Pink1
1-day post-SCI group vs. the control group	BP	GO:1904035	regulation of epithelial cell apoptotic process	5/125	0.0001516	0.00117043	0.00060948	Bax/Eif2s1/Hmox1/Jak2/Ppargc1a
1-day post-SCI group vs. the control group	BP	GO:1904659	glucose transmembrane transport	5/125	0.0001516	0.00117043	0.00060948	Edn1/Mapk14/Met/Myc/Tsc1
1-day post-SCI group vs. the control group	BP	GO:0008645	hexose transmembrane transport	5/125	0.0001579	0.00121312	0.00063171	Edn1/Mapk14/Met/Myc/Tsc1
1-day post-SCI group vs. the control group	BP	GO:1903707	negative regulation of hemopoiesis	5/125	0.0001579	0.00121312	0.00063171	Anxa1/Fbxw7/Hspb1/Myc/Nme2
1-day post-SCI group vs. the control group	BP	GO:0035051	cardiocyte differentiation	6/125	0.0001618	0.00123802	0.00064468	Edn1/Map2k4/Met/Slc8a1/Tsc1/Vcam1
1-day post-SCI group vs. the control group	BP	GO:0042698	ovulation cycle	4/125	0.0001622	0.00123802	0.00064468	Anxa1/Axl/Nos3/Plekha1
1-day post-SCI group vs. the control group	BP	GO:0043457	regulation of cellular respiration	4/125	0.0001622	0.00123802	0.00064468	Myc/Pink1/Ppargc1a/Trap1
1-day post-SCI group vs. the control group	BP	GO:0015711	organic anion transport	8/125	0.0001634	0.00124474	0.00064818	Abcc1/Anxa1/Edn1/Map2k6/Myc/Ptgs2/Slc25a24/Slc4a11
1-day post-SCI group vs. the control group	BP	GO:0015732	prostaglandin transport	3/125	0.0001687	0.00127697	0.00066496	Edn1/Map2k6/Ptgs2
1-day post-SCI group vs. the control group	BP	GO:0062098	regulation of programmed necrotic cell death	3/125	0.0001687	0.00127697	0.00066496	Ripk1/Ripk3/Ybx3
1-day post-SCI group vs. the control group	BP	GO:0098869	cellular oxidant detoxification	3/125	0.0001687	0.00127697	0.00066496	Cat/Gch1/Nos3
1-day post-SCI group vs. the control group	BP	GO:0045787	positive regulation of cell cycle	8/125	0.0001697	0.00127913	0.00066609	Anxa1/Cdk4/Edn1/Fosl1/Mdm2/Met/Myc/Sphk1
1-day post-SCI group vs. the control group	BP	GO:0050890	cognition	8/125	0.0001697	0.00127913	0.00066609	Amph/Apoe/Itga5/Jun/Mapt/Ptgs2/Rcan1/Tsc1
1-day post-SCI group vs. the control group	BP	GO:2000106	regulation of leukocyte apoptotic process	5/125	0.0001709	0.00128501	0.00066915	Anxa1/Axl/Bax/Myc/Ripk3
1-day post-SCI group vs. the control group	BP	GO:0014911	positive regulation of smooth muscle cell migration	4/125	0.0001724	0.00129353	0.00067358	Cyp1b1/Il6st/Mdm2/Myc
1-day post-SCI group vs. the control group	BP	GO:0010959	regulation of metal ion transport	9/125	0.0001742	0.00130433	0.00067921	Atf4/Bax/Cask/Ccr1/Fkbp1b/Nos3/Ppp3ca/Ptgs2/Slc8a1
1-day post-SCI group vs. the control group	BP	GO:0015749	monosaccharide transmembrane transport	5/125	0.0001777	0.00132204	0.00068843	Edn1/Mapk14/Met/Myc/Tsc1
1-day post-SCI group vs. the control group	BP	GO:0045446	endothelial cell differentiation	5/125	0.0001777	0.00132204	0.00068843	Ezr/Gpx1/Met/Tnfrsf1a/Xdh
1-day post-SCI group vs. the control group	BP	GO:0051702	biological process involved in interaction with symbio	5/125	0.0001777	0.00132204	0.00068843	Apoe/Gpx1/Hdac1/Jak2/Jun
1-day post-SCI group vs. the control group	BP	GO:0050680	negative regulation of epithelial cell proliferation	6/125	0.0001816	0.00134744	0.00070166	Apoe/Atf2/Cask/Cdkn1c/Ctsl/Xdh
1-day post-SCI group vs. the control group	BP	GO:0048738	cardiac muscle tissue development	7/125	0.0001819	0.00134744	0.00070166	Edn1/Map2k4/Mapk14/Met/Slc8a1/Smad1/Tsc1
1-day post-SCI group vs. the control group	BP	GO:0001836	release of cytochrome c from mitochondria	4/125	0.0001831	0.0013534	0.00070476	Bax/Gpx1/Jun/Pink1
1-day post-SCI group vs. the control group	BP	GO:0042063	gliogenesis	8/125	0.0001865	0.00136955	0.00071317	Anxa1/Eed/Gpr37l1/Hdac1/Il6st/Myc/Rela/Rnf112
1-day post-SCI group vs. the control group	BP	GO:0046631	alpha-beta T cell activation	6/125	0.0001868	0.00136955	0.00071317	Anxa1/Atf2/Ctsl/Jak2/Myc/Stat6
1-day post-SCI group vs. the control group	BP	GO:0048754	branching morphogenesis of an epithelial tube	6/125	0.0001868	0.00136955	0.00071317	Edn1/Met/Myc/Ppp1ca/Pxn/Spry2
1-day post-SCI group vs. the control group	BP	GO:0050731	positive regulation of peptidyl-tyrosine phosphorylatio	6/125	0.0001868	0.00136955	0.00071317	Fbxw7/Hbegf/Il6st/Itga5/Jak2/Tnfrsf1a
1-day post-SCI group vs. the control group	BP	GO:0010575	positive regulation of vascular endothelial growth fact	3/125	0.0001901	0.00138781	0.00072268	Atf4/Cyp1b1/Ptgs2
1-day post-SCI group vs. the control group	BP	GO:1903146	regulation of autophagy of mitochondrion	3/125	0.0001901	0.00138781	0.00072268	Cttn/Fbxw7/Pink1

1-day post-SCI group vs. the control group	BP	GO:0045776	negative regulation of blood pressure	4/125	0.0001943	0.00141494	0.00073681	Gch1/Gpr37l1/Ier3/Nos3
1-day post-SCI group vs. the control group	BP	GO:0051346	negative regulation of hydrolase activity	8/125	0.0001972	0.00143341	0.00074642	Anxa1/Fkbp1b/Gpx1/Mdm2/Nos3/Ptgs2/Spry2/Tsc1
1-day post-SCI group vs. the control group	BP	GO:0032640	tumor necrosis factor production	6/125	0.0001977	0.00143385	0.00074665	Axl/Hspb1/Jak2/Mapk14/Ripk1/Tnfrsf1a
1-day post-SCI group vs. the control group	BP	GO:0001676	long-chain fatty acid metabolic process	5/125	0.0001994	0.00143756	0.00074859	Cyp1b1/Gpx1/Gpx4/Ptgs2/Sphk1
1-day post-SCI group vs. the control group	BP	GO:0006476	protein deacetylation	5/125	0.0001994	0.00143756	0.00074859	Hdac1/Mapt/Pink1/Rbbp7/Ripk3
1-day post-SCI group vs. the control group	BP	GO:0018210	peptidyl-threonine modification	5/125	0.0001994	0.00143756	0.00074859	Atf2/Mapk8/Met/Sphk1/Spry2
1-day post-SCI group vs. the control group	BP	GO:0009749	response to glucose	6/125	0.0002033	0.00146228	0.00076146	Dynll1/Fkbp1b/Gpx1/Ppp3ca/Ucp2/Vcam1
1-day post-SCI group vs. the control group	BP	GO:0062012	regulation of small molecule metabolic process	8/125	0.0002046	0.00146888	0.0007649	Anxa1/Apoe/Ier3/Myc/Nos3/Pdk2/Ppargc1a/Ptgs2
1-day post-SCI group vs. the control group	BP	GO:0015718	monocarboxylic acid transport	5/125	0.0002071	0.00148059	0.000771	Anxa1/Edn1/Map2k6/Myc/Ptgs2
1-day post-SCI group vs. the control group	BP	GO:0048145	regulation of fibroblast proliferation	5/125	0.0002071	0.00148059	0.000771	Bax/Cdk4/Jun/Myc/Sphk1
1-day post-SCI group vs. the control group	BP	GO:0071375	cellular response to peptide hormone stimulus	7/125	0.0002125	0.00151209	0.0007874	Cdk4/Edn1/Jak2/Pdk2/Pxn/Rela/Stat6
1-day post-SCI group vs. the control group	BP	GO:0060143	positive regulation of syncytium formation by plasma	3/125	0.0002132	0.00151209	0.0007874	Adam9/Capn2/Mapk14
1-day post-SCI group vs. the control group	BP	GO:0097049	motor neuron apoptotic process	3/125	0.0002132	0.00151209	0.0007874	Atf2/Bax/Map2k4
1-day post-SCI group vs. the control group	BP	GO:1901857	positive regulation of cellular respiration	3/125	0.0002132	0.00151209	0.0007874	Myc/Pink1/Ppargc1a
1-day post-SCI group vs. the control group	BP	GO:0042129	regulation of T cell proliferation	6/125	0.0002148	0.00151527	0.00078905	Anxa1/Il6st/Jak2/Ppp3ca/Ripk3/Vcam1
1-day post-SCI group vs. the control group	BP	GO:0071706	tumor necrosis factor superfamily cytokine production	6/125	0.0002148	0.00151527	0.00078905	Axl/Hspb1/Jak2/Mapk14/Ripk1/Tnfrsf1a
1-day post-SCI group vs. the control group	BP	GO:0090277	positive regulation of peptide hormone secretion	5/125	0.000215	0.00151527	0.00078905	Cask/Dynll1/Itsn1/Jak2/Trpm2
1-day post-SCI group vs. the control group	BP	GO:0007517	muscle organ development	8/125	0.0002161	0.00152042	0.00079174	Fos/Gpx1/Mapk14/Met/Myc/Ppp3ca/Rcan1/Ybx3
1-day post-SCI group vs. the control group	BP	GO:0006801	superoxide metabolic process	4/125	0.000218	0.00152763	0.00079549	Edn1/Gch1/Mapt/Nos3
1-day post-SCI group vs. the control group	BP	GO:0030968	endoplasmic reticulum unfolded protein response	4/125	0.000218	0.00152763	0.00079549	Atf4/Bax/Eif2s1/Ermp1
1-day post-SCI group vs. the control group	BP	GO:0009746	response to hexose	6/125	0.0002208	0.00154391	0.00080397	Dynll1/Fkbp1b/Gpx1/Ppp3ca/Ucp2/Vcam1
1-day post-SCI group vs. the control group	BP	GO:0043405	regulation of MAP kinase activity	6/125	0.0002269	0.00158343	0.00082455	Adam9/Apoe/Edn1/Map2k4/Map2k6/Spry2
1-day post-SCI group vs. the control group	BP	GO:0006940	regulation of smooth muscle contraction	4/125	0.0002307	0.00160537	0.00083597	Cttn/Edn1/Ptgs2/Sphk1
1-day post-SCI group vs. the control group	BP	GO:0034219	carbohydrate transmembrane transport	5/125	0.0002314	0.00160537	0.00083597	Edn1/Mapk14/Met/Myc/Tsc1
1-day post-SCI group vs. the control group	BP	GO:0045807	positive regulation of endocytosis	5/125	0.0002314	0.00160537	0.00083597	Amph/Apoe/Axl/Itsn1/Ppp3ca
1-day post-SCI group vs. the control group	BP	GO:0048872	homeostasis of number of cells	8/125	0.0002324	0.0016085	0.0008376	Anxa1/Axl/Bax/Hmox1/Jak2/Mapk14/Nos3/Ripk3
1-day post-SCI group vs. the control group	BP	GO:0034284	response to monosaccharide	6/125	0.0002395	0.00165487	0.00086175	Dynll1/Fkbp1b/Gpx1/Ppp3ca/Ucp2/Vcam1
1-day post-SCI group vs. the control group	BP	GO:0150076	neuroinflammatory response	4/125	0.0002439	0.00167819	0.00087389	Jak2/Jun/Ptgs2/Sphk1
1-day post-SCI group vs. the control group	BP	GO:1905710	positive regulation of membrane permeability	4/125	0.0002439	0.00167819	0.00087389	Atf2/Bax/Hebp2/Ier3
1-day post-SCI group vs. the control group	BP	GO:0002793	positive regulation of peptide secretion	5/125	0.0002488	0.00170576	0.00088825	Cask/Dynll1/Itsn1/Jak2/Trpm2
1-day post-SCI group vs. the control group	BP	GO:0042752	regulation of circadian rhythm	5/125	0.0002488	0.00170576	0.00088825	Fbxw7/Mapk8/Nono/Ppargc1a/Ppp1ca
1-day post-SCI group vs. the control group	BP	GO:2000144	positive regulation of DNA-templated transcription ini	4/125	0.0002576	0.00176205	0.00091756	Ercc1/Fosl1/Jun/Myc
1-day post-SCI group vs. the control group	BP	GO:0050878	regulation of body fluid levels	8/125	0.0002585	0.00176468	0.00091893	Apoe/Axl/Edn1/Jak2/Krt1/Met/Ppp3ca/Xdh
1-day post-SCI group vs. the control group	BP	GO:0031334	positive regulation of protein-containing complex asse	6/125	0.0002595	0.00176468	0.00091893	Bax/Cttn/Mapk8/Mapt/Met/Mmp3
1-day post-SCI group vs. the control group	BP	GO:0048771	tissue remodeling	6/125	0.0002595	0.00176468	0.00091893	Axl/Bax/Mdm2/Nos3/Ppargc1b/Ppp3ca
1-day post-SCI group vs. the control group	BP	GO:0051450	myoblast proliferation	3/125	0.0002647	0.00179324	0.0009338	Atf2/Gpx1/Met
1-day post-SCI group vs. the control group	BP	GO:1900745	positive regulation of p38MAPK cascade	3/125	0.0002647	0.00179324	0.0009338	Met/Sphk1/Xdh
1-day post-SCI group vs. the control group	BP	GO:0006949	syncytium formation	4/125	0.0002718	0.00183079	0.00095336	Adam9/Capn2/Ercc1/Mapk14
1-day post-SCI group vs. the control group	BP	GO:0032922	circadian regulation of gene expression	4/125	0.0002718	0.00183079	0.00095336	Atf4/Hdac1/Ppargc1a/Ppp1ca
1-day post-SCI group vs. the control group	BP	GO:0048678	response to axon injury	4/125	0.0002718	0.00183079	0.00095336	Bax/Fkbp1b/Jak2/Jun

1-day post-SCI group vs. the control group	BP	GO:0060048	cardiac muscle contraction	5/125	0.0002768	0.00186045	0.0009688	Fkbp1b/Map2k3/Map2k6/Met/Slc8a1
1-day post-SCI group vs. the control group	BP	GO:0050900	leukocyte migration	8/125	0.0002773	0.00186045	0.0009688	Anxa1/Ccr1/Edn1/Itga7/Ripk3/Rpl13a/Trpm2/Vcam1
1-day post-SCI group vs. the control group	BP	GO:0014706	striated muscle tissue development	7/125	0.0002805	0.00187612	0.00097696	Edn1/Map2k4/Mapk14/Met/Slc8a1/Smad1/Tsc1
1-day post-SCI group vs. the control group	BP	GO:0002262	myeloid cell homeostasis	6/125	0.0002807	0.00187612	0.00097696	Anxa1/Axl/Bax/Hmox1/Jak2/Mapk14
1-day post-SCI group vs. the control group	BP	GO:0048662	negative regulation of smooth muscle cell proliferation	4/125	0.0002866	0.00191204	0.00099567	Apoe/Hmox1/Nos3/Ppargc1a
1-day post-SCI group vs. the control group	BP	GO:0019216	regulation of lipid metabolic process	8/125	0.0002921	0.00194469	0.00101266	Anxa1/Apoe/Capn2/Cdk4/Pdk2/Ppargc1a/Ptgs2/Sphk1
1-day post-SCI group vs. the control group	BP	GO:0034390	smooth muscle cell apoptotic process	3/125	0.0002932	0.00194469	0.00101266	Atf4/Edn1/Map2k4
1-day post-SCI group vs. the control group	BP	GO:0034391	regulation of smooth muscle cell apoptotic process	3/125	0.0002932	0.00194469	0.00101266	Atf4/Edn1/Map2k4
1-day post-SCI group vs. the control group	BP	GO:0014065	phosphatidylinositol 3-kinase signaling	5/125	0.0002967	0.00196345	0.00102244	Cat/Edn1/Jak2/Plekha1/Prr5l
1-day post-SCI group vs. the control group	BP	GO:1902903	regulation of supramolecular fiber organization	8/125	0.0002971	0.00196345	0.00102244	Apoe/Cttn/Edn1/Mapk8/Mapt/Met/Pxn/Tsc1
1-day post-SCI group vs. the control group	BP	GO:0001885	endothelial cell development	4/125	0.000302	0.00199174	0.00103717	Ezr/Gpx1/Met/Tnfrsf1a
1-day post-SCI group vs. the control group	BP	GO:0009791	post-embryonic development	5/125	0.0003071	0.00200905	0.00104618	Bax/Ercc1/Jak2/Plekha1/Slc8a1
1-day post-SCI group vs. the control group	BP	GO:0035601	protein deacylation	5/125	0.0003071	0.00200905	0.00104618	Hdac1/Mapt/Pink1/Rbbp7/Ripk3
1-day post-SCI group vs. the control group	BP	GO:0036294	cellular response to decreased oxygen levels	5/125	0.0003071	0.00200905	0.00104618	Atf2/Myc/P4hb/Pink1/Tsc1
1-day post-SCI group vs. the control group	BP	GO:0098732	macromolecule deacylation	5/125	0.0003071	0.00200905	0.00104618	Hdac1/Mapt/Pink1/Rbbp7/Ripk3
1-day post-SCI group vs. the control group	BP	GO:0001655	urogenital system development	8/125	0.0003075	0.00200905	0.00104618	Anxa1/Bax/Cat/Cdkn1c/Myc/Ppp3ca/Smad1/Tsc1
1-day post-SCI group vs. the control group	BP	GO:0010507	negative regulation of autophagy	4/125	0.0003179	0.00207345	0.00107972	Hmox1/Mcl1/Pink1/Tsc1
1-day post-SCI group vs. the control group	BP	GO:0008361	regulation of cell size	6/125	0.0003191	0.00207697	0.00108155	Apoe/Cdk4/Cttn/Edn1/Mapt/Tsc1
1-day post-SCI group vs. the control group	BP	GO:0031116	positive regulation of microtubule polymerization	3/125	0.0003236	0.00209134	0.00108903	Mapk8/Mapt/Met
1-day post-SCI group vs. the control group	BP	GO:0061036	positive regulation of cartilage development	3/125	0.0003236	0.00209134	0.00108903	Bmp1/Rela/Smad1
1-day post-SCI group vs. the control group	BP	GO:0071354	cellular response to interleukin-6	3/125	0.0003236	0.00209134	0.00108903	Il6st/Rela/Ripk1
1-day post-SCI group vs. the control group	BP	GO:0045165	cell fate commitment	7/125	0.0003237	0.00209134	0.00108903	Ctsl/Edn1/Hdac1/Rbbp7/Smad1/Spry2/Stat6
1-day post-SCI group vs. the control group	BP	GO:0055076	transition metal ion homeostasis	5/125	0.0003286	0.0021193	0.00110359	Atox1/Atp13a2/Hmox1/Hmox2/Myc
1-day post-SCI group vs. the control group	BP	GO:0051926	negative regulation of calcium ion transport	4/125	0.0003345	0.00215321	0.00112125	Fkbp1b/Nos3/Ppp3ca/Ptgs2
1-day post-SCI group vs. the control group	BP	GO:0034249	negative regulation of cellular amide metabolic process	6/125	0.0003355	0.00215595	0.00112268	Ago4/Apoe/Eif2s1/Rpl13a/Sphk1/Tsc1
1-day post-SCI group vs. the control group	BP	GO:0003158	endothelium development	5/125	0.0003398	0.00217537	0.00113279	Ezr/Gpx1/Met/Tnfrsf1a/Xdh
1-day post-SCI group vs. the control group	BP	GO:0010811	positive regulation of cell-substrate adhesion	5/125	0.0003398	0.00217537	0.00113279	Itga5/Jak2/Map4k4/P4hb/Tsc1
1-day post-SCI group vs. the control group	BP	GO:0009743	response to carbohydrate	6/125	0.000344	0.00219825	0.00114471	Dynll1/Fkbp1b/Gpx1/Ppp3ca/Ucp2/Vcam1
1-day post-SCI group vs. the control group	BP	GO:0032535	regulation of cellular component size	8/125	0.0003461	0.00220744	0.00114949	Apoe/Atp13a2/Cdk4/Cttn/Edn1/Ezr/Mapt/Tsc1
1-day post-SCI group vs. the control group	BP	GO:0048146	positive regulation of fibroblast proliferation	4/125	0.0003517	0.00223888	0.00116586	Cdk4/Jun/Myc/Sphk1
1-day post-SCI group vs. the control group	BP	GO:0010574	regulation of vascular endothelial growth factor produc	3/125	0.0003559	0.00224965	0.00117147	Atf4/Cyp1b1/Ptgs2
1-day post-SCI group vs. the control group	BP	GO:0016242	negative regulation of macroautophagy	3/125	0.0003559	0.00224965	0.00117147	Hmox1/Pink1/Tsc1
1-day post-SCI group vs. the control group	BP	GO:1901099	negative regulation of signal transduction in absence o	3/125	0.0003559	0.00224965	0.00117147	Ctnna1/Mcl1/Ripk1
1-day post-SCI group vs. the control group	BP	GO:2001240	negative regulation of extrinsic apoptotic signaling pat	3/125	0.0003559	0.00224965	0.00117147	Ctnna1/Mcl1/Ripk1
1-day post-SCI group vs. the control group	BP	GO:0051216	cartilage development	6/125	0.0003615	0.00228054	0.00118755	Atf2/Bmp1/Edn1/Mapk14/Rela/Smad1
1-day post-SCI group vs. the control group	BP	GO:0006919	activation of cysteine-type endopeptidase activity invo	4/125	0.0003694	0.00231823	0.00120718	Bax/Jak2/Myc/Xdh
1-day post-SCI group vs. the control group	BP	GO:0042306	regulation of protein import into nucleus	4/125	0.0003694	0.00231823	0.00120718	Jak2/Mapk14/Ptgs2/Tnfrsf1a
1-day post-SCI group vs. the control group	BP	GO:0071277	cellular response to calcium ion	4/125	0.0003694	0.00231823	0.00120718	Fos/Jun/Slc25a24/Trpm2
1-day post-SCI group vs. the control group	BP	GO:0006164	purine nucleotide biosynthetic process	6/125	0.0003704	0.00232042	0.00120832	Myc/Ndufa6/Nme2/Nos3/Pdk2/Ppargc1a
1-day post-SCI group vs. the control group	BP	GO:0008584	male gonad development	5/125	0.0003751	0.00234539	0.00122133	Ago4/Bax/Ercc1/Plekha1/Ybx3

1-day post-SCI group vs. the control group	BP	GO:0048639	positive regulation of developmental growth	6/125	0.0003796	0.00236926	0.00123376	Apoe/Edn1/Ezr/Mapk14/Mapt/Ybx3
1-day post-SCI group vs. the control group	BP	GO:0046546	development of primary male sexual characteristics	5/125	0.0003875	0.00240779	0.00125382	Ago4/Bax/Ercc1/Plekha1/Ybx3
1-day post-SCI group vs. the control group	BP	GO:0051224	negative regulation of protein transport	5/125	0.0003875	0.00240779	0.00125382	Anxa1/Apoe/Fkbp1b/Ppp3ca/Ucp2
1-day post-SCI group vs. the control group	BP	GO:0061180	mammary gland epithelium development	4/125	0.0003878	0.00240779	0.00125382	Bax/Gpx1/Jak2/Stat6
1-day post-SCI group vs. the control group	BP	GO:0033598	mammary gland epithelial cell proliferation	3/125	0.0003903	0.00241435	0.00125723	Bax/Gpx1/Stat6
1-day post-SCI group vs. the control group	BP	GO:0070741	response to interleukin-6	3/125	0.0003903	0.00241435	0.00125723	Il6st/Rela/Ripk1
1-day post-SCI group vs. the control group	BP	GO:0006275	regulation of DNA replication	5/125	0.0004001	0.00247087	0.00128667	Jun/Map2k4/Mapk8/Met/Pcna
1-day post-SCI group vs. the control group	BP	GO:2000142	regulation of DNA-templated transcription initiation	4/125	0.0004069	0.00250383	0.00130383	Ercc1/Fosl1/Jun/Myc
1-day post-SCI group vs. the control group	BP	GO:2000573	positive regulation of DNA biosynthetic process	4/125	0.0004069	0.00250383	0.00130383	Cyp1b1/Gfer/Myc/Pcna
1-day post-SCI group vs. the control group	BP	GO:0007007	inner mitochondrial membrane organization	3/125	0.0004267	0.00261647	0.00136249	Bax/Myc/Pink1
1-day post-SCI group vs. the control group	BP	GO:2000108	positive regulation of leukocyte apoptotic process	3/125	0.0004267	0.00261647	0.00136249	Anxa1/Bax/Myc
1-day post-SCI group vs. the control group	BP	GO:0007159	leukocyte cell-cell adhesion	8/125	0.0004282	0.00262114	0.00136492	Anxa1/Hspb1/Il6st/Itga5/Jak2/Ppp3ca/Rela/Vcam1
1-day post-SCI group vs. the control group	BP	GO:2001020	regulation of response to DNA damage stimulus	7/125	0.0004344	0.00265405	0.00138205	Ercc1/Ier3/Mapt/Mcl1/Mdm2/Myc/Pcna
1-day post-SCI group vs. the control group	BP	GO:0035264	multicellular organism growth	6/125	0.0004383	0.00267356	0.00139221	Atrn/Cdk4/Cdkn1c/Ercc1/Ezr/Plekha1
1-day post-SCI group vs. the control group	BP	GO:0001649	osteoblast differentiation	6/125	0.0004488	0.00271822	0.00141547	Atf4/Il6st/Map2k6/Mapk14/Ppp3ca/Smad1
1-day post-SCI group vs. the control group	BP	GO:0010810	regulation of cell-substrate adhesion	6/125	0.0004488	0.00271822	0.00141547	Cask/Itga5/Jak2/Map4k4/P4hb/Tsc1
1-day post-SCI group vs. the control group	BP	GO:0050866	negative regulation of cell activation	6/125	0.0004488	0.00271822	0.00141547	Anxa1/Apoe/Axl/Btk/Hmox1/Hspb1
1-day post-SCI group vs. the control group	BP	GO:0072522	purine-containing compound biosynthetic process	6/125	0.0004488	0.00271822	0.00141547	Myc/Ndufa6/Nme2/Nos3/Pdk2/Ppargc1a
1-day post-SCI group vs. the control group	BP	GO:0050728	negative regulation of inflammatory response	5/125	0.0004539	0.00274488	0.00142935	Apoe/Gpx1/Ier3/Krt1/Tnfrsf1a
1-day post-SCI group vs. the control group	BP	GO:0033673	negative regulation of kinase activity	6/125	0.0004594	0.00277299	0.00144399	Apoe/Cdkn1c/Hspb1/Mapt/Slc8a1/Spry2
1-day post-SCI group vs. the control group	BP	GO:0031112	positive regulation of microtubule polymerization or de	3/125	0.0004652	0.00279385	0.00145485	Mapk8/Mapt/Met
1-day post-SCI group vs. the control group	BP	GO:0060142	regulation of syncytium formation by plasma membrar	3/125	0.0004652	0.00279385	0.00145485	Adam9/Capn2/Mapk14
1-day post-SCI group vs. the control group	BP	GO:0090140	regulation of mitochondrial fission	3/125	0.0004652	0.00279385	0.00145485	Mapt/Pink1/Ppargc1a
1-day post-SCI group vs. the control group	BP	GO:0050729	positive regulation of inflammatory response	5/125	0.0004682	0.00280682	0.00146161	Abcc1/Jak2/Ptgs2/Ripk1/Tnfrsf1a
1-day post-SCI group vs. the control group	BP	GO:0050671	positive regulation of lymphocyte proliferation	5/125	0.0004828	0.00288938	0.0015046	Anxa1/Il6st/Jak2/Ppp3ca/Vcam1
1-day post-SCI group vs. the control group	BP	GO:0072332	intrinsic apoptotic signaling pathway by p53 class med	4/125	0.0004899	0.00292658	0.00152397	Bax/Mdm2/Myc/Pdk2
1-day post-SCI group vs. the control group	BP	GO:0061138	morphogenesis of a branching epithelium	6/125	0.0004925	0.00293709	0.00152944	Edn1/Met/Myc/Ppp1ca/Pxn/Spry2
1-day post-SCI group vs. the control group	BP	GO:0055007	cardiac muscle cell differentiation	5/125	0.0004977	0.00296355	0.00154322	Edn1/Map2k4/Met/Slc8a1/Tsc1
1-day post-SCI group vs. the control group	BP	GO:0010573	vascular endothelial growth factor production	3/125	0.0005059	0.0030072	0.00156595	Atf4/Cyp1b1/Ptgs2
1-day post-SCI group vs. the control group	BP	GO:0006887	exocytosis	8/125	0.0005095	0.0030233	0.00157434	Anxa1/Atp13a2/Cask/Ccr1/Hmox1/Itsn1/Sdc1/Syp
1-day post-SCI group vs. the control group	BP	GO:0050921	positive regulation of chemotaxis	5/125	0.000513	0.00303386	0.00157983	Ccr1/Cttn/Edn1/Hspb1/Met
1-day post-SCI group vs. the control group	BP	GO:0071453	cellular response to oxygen levels	5/125	0.000513	0.00303386	0.00157983	Atf2/Myc/P4hb/Pink1/Tsc1
1-day post-SCI group vs. the control group	BP	GO:0001654	eye development	8/125	0.0005256	0.00310006	0.00161431	Atf4/Bax/Cdkn1c/Cyp1b1/Hdac1/Jun/Pxdn/Spry2
1-day post-SCI group vs. the control group	BP	GO:0048545	response to steroid hormone	6/125	0.0005274	0.00310006	0.00161431	Adam9/Anxa1/Hdac1/Jak2/Lcat/Ppargc1b
1-day post-SCI group vs. the control group	BP	GO:0051651	maintenance of location in cell	6/125	0.0005274	0.00310006	0.00161431	Apoe/Bax/Fkbp1b/Pink1/Slc8a1/Trpm2
1-day post-SCI group vs. the control group	BP	GO:0032946	positive regulation of mononuclear cell proliferation	5/125	0.0005287	0.00310006	0.00161431	Anxa1/Il6st/Jak2/Ppp3ca/Vcam1
1-day post-SCI group vs. the control group	BP	GO:0046660	female sex differentiation	5/125	0.0005287	0.00310006	0.00161431	Axl/Bax/Ermp1/Nos3/Plekha1
1-day post-SCI group vs. the control group	BP	GO:0009895	negative regulation of catabolic process	7/125	0.0005333	0.00312181	0.00162563	Hmox1/Ier3/Mcl1/Pink1/Ppargc1a/Rela/Tsc1
1-day post-SCI group vs. the control group	BP	GO:0048041	focal adhesion assembly	4/125	0.0005356	0.0031247	0.00162714	Cttn/Map4k4/Pxn/Tsc1
1-day post-SCI group vs. the control group	BP	GO:0051384	response to glucocorticoid	4/125	0.0005356	0.0031247	0.00162714	Adam9/Anxa1/Jak2/Lcat

1-day post-SCI group vs. the control group	BP	GO:0070266	necroptotic process	3/125	0.0005489	0.00319158	0.00166197	Ripk1/Ripk3/Ybx3
1-day post-SCI group vs. the control group	BP	GO:0070306	lens fiber cell differentiation	3/125	0.0005489	0.00319158	0.00166197	Atf4/Cdkn1c/Spry2
1-day post-SCI group vs. the control group	BP	GO:0150063	visual system development	8/125	0.0005504	0.00319521	0.00166386	Atf4/Bax/Cdkn1c/Cyp1b1/Hdac1/Jun/Pxdn/Spry2
1-day post-SCI group vs. the control group	BP	GO:0007204	positive regulation of cytosolic calcium ion concentrat	6/125	0.0005517	0.00319739	0.00166499	Bax/Ccr1/Edn1/Fkbp1b/Jak2/Slc8a1
1-day post-SCI group vs. the control group	BP	GO:0030217	T cell differentiation	7/125	0.000553	0.00319998	0.00166634	Anxa1/Atf2/Ctsl/Hspb1/Ripk3/Stat6/Tsc1
1-day post-SCI group vs. the control group	BP	GO:0030100	regulation of endocytosis	6/125	0.0005769	0.00333178	0.00173497	Amph/Apoe/Axl/Itsn1/Ppp3ca/Sphk1
1-day post-SCI group vs. the control group	BP	GO:0051092	positive regulation of NF-kappaB transcription factor a	5/125	0.0005777	0.00333178	0.00173497	Cat/Rela/Ripk1/Ripk3/Sphk1
1-day post-SCI group vs. the control group	BP	GO:0048880	sensory system development	8/125	0.000585	0.00336795	0.00175381	Atf4/Bax/Cdkn1c/Cyp1b1/Hdac1/Jun/Pxdn/Spry2
1-day post-SCI group vs. the control group	BP	GO:0031281	positive regulation of cyclase activity	3/125	0.000594	0.00340321	0.00177217	Mapk14/Mapk8/Nos3
1-day post-SCI group vs. the control group	BP	GO:0071634	regulation of transforming growth factor beta producti	3/125	0.000594	0.00340321	0.00177217	Atf2/Met/Ptgs2
1-day post-SCI group vs. the control group	BP	GO:1902110	positive regulation of mitochondrial membrane permea	3/125	0.000594	0.00340321	0.00177217	Atf2/Bax/Ier3
1-day post-SCI group vs. the control group	BP	GO:0010827	regulation of glucose transmembrane transport	4/125	0.0006097	0.00348145	0.00181291	Edn1/Mapk14/Met/Myc
1-day post-SCI group vs. the control group	BP	GO:0050848	regulation of calcium-mediated signaling	4/125	0.0006097	0.00348145	0.00181291	Mapt/Pdk2/Ppp3ca/Rcan1
1-day post-SCI group vs. the control group	BP	GO:0051147	regulation of muscle cell differentiation	5/125	0.0006123	0.00349069	0.00181772	Edn1/Mapk14/Mdm2/Rcan1/Smad1
1-day post-SCI group vs. the control group	BP	GO:0033627	cell adhesion mediated by integrin	4/125	0.0006359	0.00361946	0.00188478	Adam9/Cyp1b1/Itga5/Itga7
1-day post-SCI group vs. the control group	BP	GO:0001822	kidney development	7/125	0.0006381	0.0036257	0.00188803	Bax/Cat/Cdkn1c/Myc/Ppp3ca/Smad1/Tsc1
1-day post-SCI group vs. the control group	BP	GO:0032273	positive regulation of protein polymerization	4/125	0.000663	0.00375496	0.00195534	Cttn/Mapk8/Mapt/Met
1-day post-SCI group vs. the control group	BP	GO:0046888	negative regulation of hormone secretion	4/125	0.000663	0.00375496	0.00195534	Edn1/Fkbp1b/Ppp3ca/Ucp2
1-day post-SCI group vs. the control group	BP	GO:0007613	memory	5/125	0.000667	0.00377182	0.00196411	Apoe/Itga5/Mapt/Ptgs2/Rcan1
1-day post-SCI group vs. the control group	BP	GO:0032024	positive regulation of insulin secretion	4/125	0.0006908	0.00387174	0.00201615	Cask/Dynll1/Jak2/Trpm2
1-day post-SCI group vs. the control group	BP	GO:1900182	positive regulation of protein localization to nucleus	4/125	0.0006908	0.00387174	0.00201615	Jak2/Mapk14/Ptgs2/Tnfrsf1a
1-day post-SCI group vs. the control group	BP	GO:0010803	regulation of tumor necrosis factor-mediated signaling	3/125	0.0006913	0.00387174	0.00201615	Ripk1/Sphk1/Tnfrsf1a
1-day post-SCI group vs. the control group	BP	GO:0071604	transforming growth factor beta production	3/125	0.0006913	0.00387174	0.00201615	Atf2/Met/Ptgs2
1-day post-SCI group vs. the control group	BP	GO:0098703	calcium ion import across plasma membrane	3/125	0.0006913	0.00387174	0.00201615	Ppp3ca/Slc8a1/Trpm2
1-day post-SCI group vs. the control group	BP	GO:1902686	mitochondrial outer membrane permeabilization involv	3/125	0.0006913	0.00387174	0.00201615	Atf2/Bax/Ier3
1-day post-SCI group vs. the control group	BP	GO:0002064	epithelial cell development	6/125	0.0007014	0.00392201	0.00204233	Atf4/Ezr/Gpx1/Il6st/Met/Tnfrsf1a
1-day post-SCI group vs. the control group	BP	GO:0055072	iron ion homeostasis	4/125	0.0007195	0.0040136	0.00209002	Atp13a2/Hmox1/Hmox2/Myc
1-day post-SCI group vs. the control group	BP	GO:0034329	cell junction assembly	8/125	0.0007201	0.0040136	0.00209002	Cdh11/Ctnna1/Cttn/Itga5/Map4k4/Mapt/Pxn/Tsc1
1-day post-SCI group vs. the control group	BP	GO:0033135	regulation of peptidyl-serine phosphorylation	5/125	0.0007253	0.00403607	0.00210172	Bax/Met/Pink1/Ptgs2/Spry2
1-day post-SCI group vs. the control group	BP	GO:0006163	purine nucleotide metabolic process	8/125	0.000763	0.0042327	0.00220411	Ier3/Myc/Ndufa6/Nme2/Nos3/Pdk2/Ppargc1a/Xdh
1-day post-SCI group vs. the control group	BP	GO:0001763	morphogenesis of a branching structure	6/125	0.0007631	0.0042327	0.00220411	Edn1/Met/Myc/Ppp1ca/Pxn/Spry2
1-day post-SCI group vs. the control group	BP	GO:0030879	mammary gland development	5/125	0.0007662	0.00424352	0.00220975	Bax/Gpx1/Jak2/Stat6/Xdh
1-day post-SCI group vs. the control group	BP	GO:0031960	response to corticosteroid	4/125	0.0007793	0.00429524	0.00223668	Adam9/Anxa1/Jak2/Lcat
1-day post-SCI group vs. the control group	BP	GO:0051341	regulation of oxidoreductase activity	4/125	0.0007793	0.00429524	0.00223668	Apoe/Edn1/Gch1/Ripk3
1-day post-SCI group vs. the control group	BP	GO:0072655	establishment of protein localization to mitochondrion	4/125	0.0007793	0.00429524	0.00223668	Bax/Fbxw7/Mapt/Pink1
1-day post-SCI group vs. the control group	BP	GO:0001774	microglial cell activation	3/125	0.0007983	0.00438605	0.00228397	Jak2/Jun/Sphk1
1-day post-SCI group vs. the control group	BP	GO:0038084	vascular endothelial growth factor signaling pathway	3/125	0.0007983	0.00438605	0.00228397	Hspb1/Spry2/Xdh
1-day post-SCI group vs. the control group	BP	GO:0072001	renal system development	7/125	0.0008115	0.00439637	0.00228934	Bax/Cat/Cdkn1c/Myc/Ppp3ca/Smad1/Tsc1
1-day post-SCI group vs. the control group	BP	GO:1903320	regulation of protein modification by small protein cor	6/125	0.000812	0.00439637	0.00228934	Fbxw7/Mapk8/Pink1/Rela/Sphk1/Spry2
1-day post-SCI group vs. the control group	BP	GO:0010042	response to manganese ion	2/125	0.000814	0.00439637	0.00228934	Adam9/Atp13a2

1-day post-SCI group vs. the control group	BP	GO:0032225	regulation of synaptic transmission, dopaminergic	2/125	0.000814	0.00439637	0.00228934	Pink1/Ptgs2
1-day post-SCI group vs. the control group	BP	GO:0034372	very-low-density lipoprotein particle remodeling	2/125	0.000814	0.00439637	0.00228934	Apoe/Lcat
1-day post-SCI group vs. the control group	BP	GO:0034380	high-density lipoprotein particle assembly	2/125	0.000814	0.00439637	0.00228934	Apoe/Lcat
1-day post-SCI group vs. the control group	BP	GO:0070391	response to lipoteichoic acid	2/125	0.000814	0.00439637	0.00228934	Mapk14/Rela
1-day post-SCI group vs. the control group	BP	GO:0071223	cellular response to lipoteichoic acid	2/125	0.000814	0.00439637	0.00228934	Mapk14/Rela
1-day post-SCI group vs. the control group	BP	GO:0106049	regulation of cellular response to osmotic stress	2/125	0.000814	0.00439637	0.00228934	Ptgs2/Ybx3
1-day post-SCI group vs. the control group	BP	GO:1902746	regulation of lens fiber cell differentiation	2/125	0.000814	0.00439637	0.00228934	Cdkn1c/Spry2
1-day post-SCI group vs. the control group	BP	GO:2001269	positive regulation of cysteine-type endopeptidase acti	2/125	0.000814	0.00439637	0.00228934	Bax/Jak2
1-day post-SCI group vs. the control group	BP	GO:0070665	positive regulation of leukocyte proliferation	5/125	0.0008308	0.00448036	0.00233308	Anxa1/Il6st/Jak2/Ppp3ca/Vcam1
1-day post-SCI group vs. the control group	BP	GO:0017157	regulation of exocytosis	6/125	0.0008459	0.0045546	0.00237174	Anxa1/Atp13a2/Cask/Hmox1/Sdc1/Syp
1-day post-SCI group vs. the control group	BP	GO:0006457	protein folding	5/125	0.0008532	0.00456366	0.00237645	Fkbp1b/Hspa13/Hspb1/P4hb/Trap1
1-day post-SCI group vs. the control group	BP	GO:0002021	response to dietary excess	3/125	0.0008554	0.00456366	0.00237645	Apoe/Mapk14/Ppargc1a
1-day post-SCI group vs. the control group	BP	GO:0006984	ER-nucleus signaling pathway	3/125	0.0008554	0.00456366	0.00237645	Atf4/Eif2s1/Ppp1r15b
1-day post-SCI group vs. the control group	BP	GO:0045981	positive regulation of nucleotide metabolic process	3/125	0.0008554	0.00456366	0.00237645	Myc/Nos3/Ppargc1a
1-day post-SCI group vs. the control group	BP	GO:1900544	positive regulation of purine nucleotide metabolic proc	3/125	0.0008554	0.00456366	0.00237645	Myc/Nos3/Ppargc1a
1-day post-SCI group vs. the control group	BP	GO:1990748	cellular detoxification	3/125	0.0008554	0.00456366	0.00237645	Cat/Gch1/Nos3
1-day post-SCI group vs. the control group	BP	GO:0090559	regulation of membrane permeability	4/125	0.0008755	0.00465255	0.00242274	Atf2/Bax/Hebp2/Ier3
1-day post-SCI group vs. the control group	BP	GO:0007292	female gamete generation	5/125	0.0008761	0.00465255	0.00242274	Edn1/Ercc1/Fosl1/Nos3/Ptgs2
1-day post-SCI group vs. the control group	BP	GO:0034250	positive regulation of cellular amide metabolic process	5/125	0.0008761	0.00465255	0.00242274	Apoe/Cdk4/Cyp1b1/Pink1/Rela
1-day post-SCI group vs. the control group	BP	GO:0007015	actin filament organization	8/125	0.0008918	0.00472866	0.00246237	Ctnna1/Cttn/Ezr/Met/Pdlim1/Ppargc1b/Pxn/Tsc1
1-day post-SCI group vs. the control group	BP	GO:0009267	cellular response to starvation	5/125	0.0008994	0.00475462	0.00247589	Atf2/Atf4/Eif2s1/Jun/Mapk8
1-day post-SCI group vs. the control group	BP	GO:0051017	actin filament bundle assembly	5/125	0.0008994	0.00475462	0.00247589	Ezr/Met/Pdlim1/Pxn/Tsc1
1-day post-SCI group vs. the control group	BP	GO:0045840	positive regulation of mitotic nuclear division	3/125	0.0009152	0.00482322	0.00251161	Edn1/Met/Sphk1
1-day post-SCI group vs. the control group	BP	GO:1902108	regulation of mitochondrial membrane permeability in	3/125	0.0009152	0.00482322	0.00251161	Atf2/Bax/Ier3
1-day post-SCI group vs. the control group	BP	GO:0046661	male sex differentiation	5/125	0.0009232	0.00485087	0.00252601	Ago4/Bax/Ercc1/Plekha1/Ybx3
1-day post-SCI group vs. the control group	BP	GO:0062013	positive regulation of small molecule metabolic proces	5/125	0.0009232	0.00485087	0.00252601	Anxa1/Myc/Nos3/Ppargc1a/Ptgs2
1-day post-SCI group vs. the control group	BP	GO:0006941	striated muscle contraction	5/125	0.0009474	0.00497071	0.00258842	Fkbp1b/Map2k3/Map2k6/Met/Slc8a1
1-day post-SCI group vs. the control group	BP	GO:0061572	actin filament bundle organization	5/125	0.0009721	0.0050859	0.0026484	Ezr/Met/Pdlim1/Pxn/Tsc1
1-day post-SCI group vs. the control group	BP	GO:0010001	glial cell differentiation	6/125	0.0009733	0.0050859	0.0026484	Eed/Gpr37l1/Hdac1/Il6st/Rela/Rnf112
1-day post-SCI group vs. the control group	BP	GO:0051898	negative regulation of protein kinase B signaling	3/125	0.0009774	0.0050859	0.0026484	Mmp3/Plekha1/Xdh
1-day post-SCI group vs. the control group	BP	GO:1902742	apoptotic process involved in development	3/125	0.0009774	0.0050859	0.0026484	Atf2/Bax/Tnfrsf1a
1-day post-SCI group vs. the control group	BP	GO:0043502	regulation of muscle adaptation	4/125	0.0009798	0.0050859	0.0026484	Edn1/Nos3/Ppp3ca/Tnfrsf1a
1-day post-SCI group vs. the control group	BP	GO:0070585	protein localization to mitochondrion	4/125	0.0009798	0.0050859	0.0026484	Bax/Fbxw7/Mapt/Pink1
1-day post-SCI group vs. the control group	BP	GO:0019372	lipoxygenase pathway	2/125	0.0009921	0.0050859	0.0026484	Gpx1/Gpx4
1-day post-SCI group vs. the control group	BP	GO:0034370	triglyceride-rich lipoprotein particle remodeling	2/125	0.0009921	0.0050859	0.0026484	Apoe/Lcat
1-day post-SCI group vs. the control group	BP	GO:0060068	vagina development	2/125	0.0009921	0.0050859	0.0026484	Axl/Bax
1-day post-SCI group vs. the control group	BP	GO:0071372	cellular response to follicle-stimulating hormone stimu	2/125	0.0009921	0.0050859	0.0026484	Edn1/Ppargc1a
1-day post-SCI group vs. the control group	BP	GO:0071415	cellular response to purine-containing compound	2/125	0.0009921	0.0050859	0.0026484	Slc8a1/Trpm2
1-day post-SCI group vs. the control group	BP	GO:0097421	liver regeneration	2/125	0.0009921	0.0050859	0.0026484	Gfer/Hmox1
1-day post-SCI group vs. the control group	BP	GO:1900747	negative regulation of vascular endothelial growth fact	2/125	0.0009921	0.0050859	0.0026484	Spry2/Xdh

1-day post-SCI group vs. the control group	BP	GO:1905097	regulation of guanyl-nucleotide exchange factor activit	2/125	0.0009921	0.0050859	0.0026484	Eif2s1/Met
1-day post-SCI group vs. the control group	BP	GO:2001280	positive regulation of unsaturated fatty acid biosynthet	2/125	0.0009921	0.0050859	0.0026484	Anxa1/Ptgs2
1-day post-SCI group vs. the control group	BP	GO:0051348	negative regulation of transferase activity	6/125	0.0009927	0.0050859	0.0026484	Apoe/Cdkn1c/Hspb1/Mapt/Slc8a1/Spry2
1-day post-SCI group vs. the control group	BP	GO:0006914	autophagy	8/125	0.0010096	0.00515756	0.00268572	Atp13a2/Cttn/Fbxw7/Hmox1/Mapt/Mcl1/Pink1/Tsc1
1-day post-SCI group vs. the control group	BP	GO:0061919	process utilizing autophagic mechanism	8/125	0.0010096	0.00515756	0.00268572	Atp13a2/Cttn/Fbxw7/Hmox1/Mapt/Mcl1/Pink1/Tsc1
1-day post-SCI group vs. the control group	BP	GO:0060395	SMAD protein signal transduction	4/125	0.0010164	0.00518437	0.00269968	Fos/Jak2/Jun/Smad1
1-day post-SCI group vs. the control group	BP	GO:0009165	nucleotide biosynthetic process	6/125	0.0010323	0.00525781	0.00273792	Myc/Ndufa6/Nme2/Nos3/Pdk2/Ppargc1a
1-day post-SCI group vs. the control group	BP	GO:0002269	leukocyte activation involved in inflammatory respons	3/125	0.0010424	0.00530144	0.00276064	Jak2/Jun/Sphk1
1-day post-SCI group vs. the control group	BP	GO:0007254	JNK cascade	5/125	0.0010491	0.00532022	0.00277042	Atf2/Map2k4/Map4k4/Mapk8/Ripk1
1-day post-SCI group vs. the control group	BP	GO:1903034	regulation of response to wounding	5/125	0.0010491	0.00532022	0.00277042	Anxa1/Apoe/Cask/Fkbp1b/Hbegf
1-day post-SCI group vs. the control group	BP	GO:0007229	integrin-mediated signaling pathway	4/125	0.0010925	0.0055241	0.00287659	Ctnna1/Itga5/Itga7/Pxn
1-day post-SCI group vs. the control group	BP	GO:0055013	cardiac muscle cell development	4/125	0.0010925	0.0055241	0.00287659	Edn1/Map2k4/Met/Slc8a1
1-day post-SCI group vs. the control group	BP	GO:0031214	biomineral tissue development	5/125	0.0011029	0.00556863	0.00289977	Atf4/Ccr1/Nos3/Ptgs2/Slc8a1
1-day post-SCI group vs. the control group	BP	GO:0008544	epidermis development	7/125	0.0011045	0.00556863	0.00289977	Anxa1/Ctsl/Hdac1/Krt1/Ppp3ca/Ptgs2/Rela
1-day post-SCI group vs. the control group	BP	GO:0000266	mitochondrial fission	3/125	0.0011099	0.00558812	0.00290993	Mapt/Pink1/Ppargc1a
1-day post-SCI group vs. the control group	BP	GO:0050730	regulation of peptidyl-tyrosine phosphorylation	6/125	0.0011151	0.00560586	0.00291917	Fbxw7/Hbegf/Il6st/Itga5/Jak2/Tnfrsf1a
1-day post-SCI group vs. the control group	BP	GO:0019217	regulation of fatty acid metabolic process	4/125	0.001132	0.00568271	0.00295918	Anxa1/Pdk2/Ppargc1a/Ptgs2
1-day post-SCI group vs. the control group	BP	GO:0090068	positive regulation of cell cycle process	6/125	0.0011365	0.00568922	0.00296257	Anxa1/Cdk4/Edn1/Mdm2/Met/Sphk1
1-day post-SCI group vs. the control group	BP	GO:1901293	nucleoside phosphate biosynthetic process	6/125	0.0011365	0.00568922	0.00296257	Myc/Ndufa6/Nme2/Nos3/Pdk2/Ppargc1a
1-day post-SCI group vs. the control group	BP	GO:1903037	regulation of leukocyte cell-cell adhesion	7/125	0.0011756	0.0058419	0.00304208	Anxa1/Hspb1/Il6st/Jak2/Ppp3ca/Rela/Vcam1
1-day post-SCI group vs. the control group	BP	GO:0045923	positive regulation of fatty acid metabolic process	3/125	0.0011802	0.0058419	0.00304208	Anxa1/Ppargc1a/Ptgs2
1-day post-SCI group vs. the control group	BP	GO:0048741	skeletal muscle fiber development	3/125	0.0011802	0.0058419	0.00304208	Gpx1/Ppp3ca/Rcan1
1-day post-SCI group vs. the control group	BP	GO:0031392	regulation of prostaglandin biosynthetic process	2/125	0.0011871	0.0058419	0.00304208	Anxa1/Ptgs2
1-day post-SCI group vs. the control group	BP	GO:0032354	response to follicle-stimulating hormone	2/125	0.0011871	0.0058419	0.00304208	Edn1/Ppargc1a
1-day post-SCI group vs. the control group	BP	GO:0035331	negative regulation of hippo signaling	2/125	0.0011871	0.0058419	0.00304208	Map2k3/Mapk14
1-day post-SCI group vs. the control group	BP	GO:0043619	regulation of transcription from RNA polymerase II pr	2/125	0.0011871	0.0058419	0.00304208	Atf4/Hmox1
1-day post-SCI group vs. the control group	BP	GO:0045741	positive regulation of epidermal growth factor-activate	2/125	0.0011871	0.0058419	0.00304208	Fbxw7/Hbegf
1-day post-SCI group vs. the control group	BP	GO:0070262	peptidyl-serine dephosphorylation	2/125	0.0011871	0.0058419	0.00304208	Ppp1r15b/Ppp3ca
1-day post-SCI group vs. the control group	BP	GO:0090184	positive regulation of kidney development	2/125	0.0011871	0.0058419	0.00304208	Myc/Ppp3ca
1-day post-SCI group vs. the control group	BP	GO:1990144	intrinsic apoptotic signaling pathway in response to hy	2/125	0.0011871	0.0058419	0.00304208	Atf2/Pink1
1-day post-SCI group vs. the control group	BP	GO:1990253	cellular response to leucine starvation	2/125	0.0011871	0.0058419	0.00304208	Atf2/Atf4
1-day post-SCI group vs. the control group	BP	GO:0002274	myeloid leukocyte activation	6/125	0.0012029	0.00590268	0.00307373	Adam9/Anxa1/Hmox1/Jak2/Jun/Sphk1
1-day post-SCI group vs. the control group	BP	GO:1903039	positive regulation of leukocyte cell-cell adhesion	6/125	0.0012029	0.00590268	0.00307373	Anxa1/Il6st/Jak2/Ppp3ca/Rela/Vcam1
1-day post-SCI group vs. the control group	BP	GO:0003300	cardiac muscle hypertrophy	4/125	0.001214	0.00593224	0.00308912	Edn1/Map2k4/Ppp3ca/Tnfrsf1a
1-day post-SCI group vs. the control group	BP	GO:0070167	regulation of biomineral tissue development	4/125	0.001214	0.00593224	0.00308912	Atf4/Ccr1/Nos3/Slc8a1
1-day post-SCI group vs. the control group	BP	GO:0071901	negative regulation of protein serine/threonine kinase a	4/125	0.001214	0.00593224	0.00308912	Apoe/Cdkn1c/Slc8a1/Spry2
1-day post-SCI group vs. the control group	BP	GO:0072521	purine-containing compound metabolic process	8/125	0.0012175	0.00594086	0.00309361	Ier3/Myc/Ndufa6/Nme2/Nos3/Pdk2/Ppargc1a/Xdh
1-day post-SCI group vs. the control group	BP	GO:0006898	receptor-mediated endocytosis	6/125	0.0012487	0.00607304	0.00316244	Apoe/Ctsl/Cttn/Ezr/Itsn1/Sdc1
1-day post-SCI group vs. the control group	BP	GO:0006110	regulation of glycolytic process	3/125	0.0012533	0.00607304	0.00316244	Ier3/Myc/Ppargc1a
1-day post-SCI group vs. the control group	BP	GO:0010799	regulation of peptidyl-threonine phosphorylation	3/125	0.0012533	0.00607304	0.00316244	Met/Sphk1/Spry2

1-day post-SCI group vs. the control group	BP	GO:0035307	positive regulation of protein dephosphorylation	3/125	0.0012533	0.00607304	0.00316244	Jak2/Pink1/Ppp1r15b
1-day post-SCI group vs. the control group	BP	GO:0061900	glial cell activation	3/125	0.0012533	0.00607304	0.00316244	Jak2/Jun/Sphk1
1-day post-SCI group vs. the control group	BP	GO:0043010	camera-type eye development	7/125	0.0012694	0.00614296	0.00319885	Atf4/Bax/Cdkn1c/Cyp1b1/Hdac1/Jun/Spry2
1-day post-SCI group vs. the control group	BP	GO:0071897	DNA biosynthetic process	5/125	0.0012765	0.00616009	0.00320777	Cyp1b1/Gfer/Myc/Pcna/Sphk1
1-day post-SCI group vs. the control group	BP	GO:0097553	calcium ion transmembrane import into cytosol	5/125	0.0012765	0.00616009	0.00320777	Bax/Fkbp1b/Ppp3ca/Slc8a1/Trpm2
1-day post-SCI group vs. the control group	BP	GO:0006165	nucleoside diphosphate phosphorylation	4/125	0.0013001	0.00623072	0.00324455	Ier3/Myc/Nme2/Ppargc1a
1-day post-SCI group vs. the control group	BP	GO:0033555	multicellular organismal response to stress	4/125	0.0013001	0.00623072	0.00324455	Apoe/Capn2/Gch1/Ppp3ca
1-day post-SCI group vs. the control group	BP	GO:0034446	substrate adhesion-dependent cell spreading	4/125	0.0013001	0.00623072	0.00324455	Atrn/Axl/P4hb/Pxn
1-day post-SCI group vs. the control group	BP	GO:0070227	lymphocyte apoptotic process	4/125	0.0013001	0.00623072	0.00324455	Bax/Myc/Ripk1/Ripk3
1-day post-SCI group vs. the control group	BP	GO:0110149	regulation of biomineralization	4/125	0.0013001	0.00623072	0.00324455	Atf4/Ccr1/Nos3/Slc8a1
1-day post-SCI group vs. the control group	BP	GO:0032680	regulation of tumor necrosis factor production	5/125	0.0013073	0.0062458	0.0032524	Axl/Hspb1/Jak2/Ripk1/Tnfrsf1a
1-day post-SCI group vs. the control group	BP	GO:0061136	regulation of proteasomal protein catabolic process	5/125	0.0013073	0.0062458	0.0032524	Apoe/Fbxw7/Gpx1/Mapk8/Mdm2
1-day post-SCI group vs. the control group	BP	GO:0050863	regulation of T cell activation	7/125	0.0013086	0.0062458	0.0032524	Anxa1/Hspb1/Il6st/Jak2/Ppp3ca/Ripk3/Vcam1
1-day post-SCI group vs. the control group	BP	GO:0043618	regulation of transcription from RNA polymerase II pr	3/125	0.0013291	0.00633485	0.00329877	Atf4/Hmox1/Jun
1-day post-SCI group vs. the control group	BP	GO:0043271	negative regulation of ion transport	5/125	0.0013386	0.00637178	0.00331801	Atf4/Fkbp1b/Nos3/Ppp3ca/Ptgs2
1-day post-SCI group vs. the control group	BP	GO:0046916	cellular transition metal ion homeostasis	4/125	0.0013447	0.00637444	0.00331939	Atox1/Atp13a2/Hmox1/Myc
1-day post-SCI group vs. the control group	BP	GO:0055006	cardiac cell development	4/125	0.0013447	0.00637444	0.00331939	Edn1/Map2k4/Met/Slc8a1
1-day post-SCI group vs. the control group	BP	GO:0071456	cellular response to hypoxia	4/125	0.0013447	0.00637444	0.00331939	Atf2/Myc/P4hb/Pink1
1-day post-SCI group vs. the control group	BP	GO:0010639	negative regulation of organelle organization	7/125	0.0013487	0.00638476	0.00332476	Ercc1/Gpx1/Ier3/Mapt/Met/Pink1/Ppargc1a
1-day post-SCI group vs. the control group	BP	GO:0045834	positive regulation of lipid metabolic process	5/125	0.0013705	0.00647061	0.00336947	Anxa1/Apoe/Capn2/Ppargc1a/Ptgs2
1-day post-SCI group vs. the control group	BP	GO:0110148	biomineralization	5/125	0.0013705	0.00647061	0.00336947	Atf4/Ccr1/Nos3/Ptgs2/Slc8a1
1-day post-SCI group vs. the control group	BP	GO:0014897	striated muscle hypertrophy	4/125	0.0013903	0.00653435	0.00340266	Edn1/Map2k4/Ppp3ca/Tnfrsf1a
1-day post-SCI group vs. the control group	BP	GO:0032760	positive regulation of tumor necrosis factor production	4/125	0.0013903	0.00653435	0.00340266	Hspb1/Jak2/Ripk1/Tnfrsf1a
1-day post-SCI group vs. the control group	BP	GO:0110020	regulation of actomyosin structure organization	4/125	0.0013903	0.00653435	0.00340266	Edn1/Met/Pxn/Tsc1
1-day post-SCI group vs. the control group	BP	GO:0034375	high-density lipoprotein particle remodeling	2/125	0.001399	0.00653435	0.00340266	Apoe/Lcat
1-day post-SCI group vs. the control group	BP	GO:0043922	negative regulation by host of viral transcription	2/125	0.001399	0.00653435	0.00340266	Hdac1/Jun
1-day post-SCI group vs. the control group	BP	GO:0090520	sphingolipid mediated signaling pathway	2/125	0.001399	0.00653435	0.00340266	Ezr/Sphk1
1-day post-SCI group vs. the control group	BP	GO:0150065	regulation of deacetylase activity	2/125	0.001399	0.00653435	0.00340266	Mapk8/Pink1
1-day post-SCI group vs. the control group	BP	GO:1902548	negative regulation of cellular response to vascular enc	2/125	0.001399	0.00653435	0.00340266	Spry2/Xdh
1-day post-SCI group vs. the control group	BP	GO:1903555	regulation of tumor necrosis factor superfamily cytokin	5/125	0.001403	0.0065441	0.00340774	Axl/Hspb1/Jak2/Ripk1/Tnfrsf1a
1-day post-SCI group vs. the control group	BP	GO:0006641	triglyceride metabolic process	4/125	0.0014371	0.00669418	0.00348589	Apoe/Cat/Gpx1/Il6st
1-day post-SCI group vs. the control group	BP	GO:0006260	DNA replication	6/125	0.0014456	0.0067248	0.00350183	Jun/Map2k4/Mapk8/Met/Pcna/Rbbp7
1-day post-SCI group vs. the control group	BP	GO:0022407	regulation of cell-cell adhesion	8/125	0.0014581	0.00677411	0.00352751	Anxa1/Hspb1/Il6st/Jak2/Mapk14/Ppp3ca/Rela/Vcam1
1-day post-SCI group vs. the control group	BP	GO:0042594	response to starvation	5/125	0.0014696	0.00680918	0.00354578	Atf2/Atf4/Eif2s1/Jun/Mapk8
1-day post-SCI group vs. the control group	BP	GO:0043122	regulation of I-kappaB kinase/NF-kappaB signaling	5/125	0.0014696	0.00680918	0.00354578	Hdac1/Hspb1/Pink1/Rela/Ripk1
1-day post-SCI group vs. the control group	BP	GO:0003007	heart morphogenesis	6/125	0.0014718	0.00681027	0.00354634	Atf2/Edn1/Jun/Mdm2/Nos3/Slc8a1
1-day post-SCI group vs. the control group	BP	GO:0014896	muscle hypertrophy	4/125	0.0014849	0.00682579	0.00355442	Edn1/Map2k4/Ppp3ca/Tnfrsf1a
1-day post-SCI group vs. the control group	BP	GO:0031532	actin cytoskeleton reorganization	4/125	0.0014849	0.00682579	0.00355442	Anxa1/Cttn/Ezr/Trpm2
1-day post-SCI group vs. the control group	BP	GO:0046939	nucleotide phosphorylation	4/125	0.0014849	0.00682579	0.00355442	Ier3/Myc/Nme2/Ppargc1a
1-day post-SCI group vs. the control group	BP	GO:0055017	cardiac muscle tissue growth	4/125	0.0014849	0.00682579	0.00355442	Edn1/Map2k4/Mapk14/Smad1

1-day post-SCI group vs. the control group	BP	GO:1903557	positive regulation of tumor necrosis factor superfamil	4/125	0.0014849	0.00682579	0.00355442	Hspb1/Jak2/Ripk1/Tnfrsf1a
1-day post-SCI group vs. the control group	BP	GO:0035196	miRNA processing	3/125	0.0014892	0.00683666	0.00356008	Ago4/Ppp3ca/Ripk1
1-day post-SCI group vs. the control group	BP	GO:0016485	protein processing	6/125	0.0015252	0.00699266	0.00364132	Adam9/Bmp1/Capn2/Ctsl/Mdm2/Myc
1-day post-SCI group vs. the control group	BP	GO:0000077	DNA damage checkpoint signaling	4/125	0.0015338	0.00702288	0.00365706	Atf2/Ier3/Mapk14/Mdm2
1-day post-SCI group vs. the control group	BP	GO:0031103	axon regeneration	3/125	0.0015736	0.00716829	0.00373277	Fkbp1b/Jak2/Jun
1-day post-SCI group vs. the control group	BP	GO:0046676	negative regulation of insulin secretion	3/125	0.0015736	0.00716829	0.00373277	Fkbp1b/Ppp3ca/Ucp2
1-day post-SCI group vs. the control group	BP	GO:0090199	regulation of release of cytochrome c from mitochondr	3/125	0.0015736	0.00716829	0.00373277	Bax/Gpx1/Pink1
1-day post-SCI group vs. the control group	BP	GO:0051099	positive regulation of binding	5/125	0.0015738	0.00716829	0.00373277	Apoe/Eif2s1/Jak2/Met/Myc
1-day post-SCI group vs. the control group	BP	GO:0001866	NK T cell proliferation	2/125	0.0016276	0.00732724	0.00381554	Jak2/Myc
1-day post-SCI group vs. the control group	BP	GO:0002829	negative regulation of type 2 immune response	2/125	0.0016276	0.00732724	0.00381554	Anxa1/Stat6
1-day post-SCI group vs. the control group	BP	GO:0010882	regulation of cardiac muscle contraction by calcium io	2/125	0.0016276	0.00732724	0.00381554	Fkbp1b/Slc8a1
1-day post-SCI group vs. the control group	BP	GO:0034393	positive regulation of smooth muscle cell apoptotic pr	2/125	0.0016276	0.00732724	0.00381554	Atf4/Map2k4
1-day post-SCI group vs. the control group	BP	GO:0036499	PERK-mediated unfolded protein response	2/125	0.0016276	0.00732724	0.00381554	Atf4/Eif2s1
1-day post-SCI group vs. the control group	BP	GO:0045793	positive regulation of cell size	2/125	0.0016276	0.00732724	0.00381554	Cdk4/Edn1
1-day post-SCI group vs. the control group	BP	GO:1902170	cellular response to reactive nitrogen species	2/125	0.0016276	0.00732724	0.00381554	Mapk8/Stat6
1-day post-SCI group vs. the control group	BP	GO:2001241	positive regulation of extrinsic apoptotic signaling patl	2/125	0.0016276	0.00732724	0.00381554	Ctnna1/Ppp1ca
1-day post-SCI group vs. the control group	BP	GO:2001279	regulation of unsaturated fatty acid biosynthetic proces	2/125	0.0016276	0.00732724	0.00381554	Anxa1/Ptgs2
1-day post-SCI group vs. the control group	BP	GO:0001942	hair follicle development	4/125	0.001635	0.00733218	0.00381812	Ctsl/Hdac1/Ptgs2/Rela
1-day post-SCI group vs. the control group	BP	GO:0030038	contractile actin filament bundle assembly	4/125	0.001635	0.00733218	0.00381812	Met/Pdlim1/Pxn/Tsc1
1-day post-SCI group vs. the control group	BP	GO:0043149	stress fiber assembly	4/125	0.001635	0.00733218	0.00381812	Met/Pdlim1/Pxn/Tsc1
1-day post-SCI group vs. the control group	BP	GO:0014904	myotube cell development	3/125	0.001661	0.00743917	0.00387383	Gpx1/Ppp3ca/Rcan1
1-day post-SCI group vs. the control group	BP	GO:0046942	carboxylic acid transport	6/125	0.0016653	0.00744896	0.00387893	Abcc1/Anxa1/Edn1/Map2k6/Myc/Ptgs2
1-day post-SCI group vs. the control group	BP	GO:0009206	purine ribonucleoside triphosphate biosynthetic proces	4/125	0.0016873	0.00750876	0.00391007	Myc/Ndufa6/Nme2/Ppargc1a
1-day post-SCI group vs. the control group	BP	GO:0016241	regulation of macroautophagy	4/125	0.0016873	0.00750876	0.00391007	Atp13a2/Hmox1/Pink1/Tsc1
1-day post-SCI group vs. the control group	BP	GO:0032652	regulation of interleukin-1 production	4/125	0.0016873	0.00750876	0.00391007	Anxa1/Hspb1/Jak2/Sphk1
1-day post-SCI group vs. the control group	BP	GO:0044070	regulation of anion transport	4/125	0.0016873	0.00750876	0.00391007	Atf4/Edn1/Map2k6/Ripk1
1-day post-SCI group vs. the control group	BP	GO:0007259	receptor signaling pathway via JAK-STAT	5/125	0.0017211	0.00764939	0.0039833	Cyp1b1/Il6st/Jak2/Stat6/Tnfrsf1a
1-day post-SCI group vs. the control group	BP	GO:0060562	epithelial tube morphogenesis	7/125	0.0017297	0.00767784	0.00399812	Edn1/Met/Myc/Ppp1ca/Pxn/Spry2/Tsc1
1-day post-SCI group vs. the control group	BP	GO:0009145	purine nucleoside triphosphate biosynthetic process	4/125	0.0017407	0.00771705	0.00401853	Myc/Ndufa6/Nme2/Ppargc1a
1-day post-SCI group vs. the control group	BP	GO:0001961	positive regulation of cytokine-mediated signaling patl	3/125	0.0017513	0.00772472	0.00402253	Axl/Edn1/Ripk1
1-day post-SCI group vs. the control group	BP	GO:0031113	regulation of microtubule polymerization	3/125	0.0017513	0.00772472	0.00402253	Mapk8/Mapt/Met
1-day post-SCI group vs. the control group	BP	GO:0042531	positive regulation of tyrosine phosphorylation of STA	3/125	0.0017513	0.00772472	0.00402253	Il6st/Jak2/Tnfrsf1a
1-day post-SCI group vs. the control group	BP	GO:0072132	mesenchyme morphogenesis	3/125	0.0017513	0.00772472	0.00402253	Mdm2/Myc/Nos3
1-day post-SCI group vs. the control group	BP	GO:0006109	regulation of carbohydrate metabolic process	5/125	0.0017594	0.00775072	0.00403606	Ier3/Myc/Pdk2/Ppargc1a/Ppp1ca
1-day post-SCI group vs. the control group	BP	GO:0008585	female gonad development	4/125	0.0017953	0.0078727	0.00409958	Bax/Ermp1/Nos3/Plekha1
1-day post-SCI group vs. the control group	BP	GO:0022404	molting cycle process	4/125	0.0017953	0.0078727	0.00409958	Ctsl/Hdac1/Ptgs2/Rela
1-day post-SCI group vs. the control group	BP	GO:0022405	hair cycle process	4/125	0.0017953	0.0078727	0.00409958	Ctsl/Hdac1/Ptgs2/Rela
1-day post-SCI group vs. the control group	BP	GO:0001764	neuron migration	5/125	0.0017984	0.0078727	0.00409958	Axl/Bax/Mapk8/Mapt/Met
1-day post-SCI group vs. the control group	BP	GO:0009152	purine ribonucleotide biosynthetic process	5/125	0.0017984	0.0078727	0.00409958	Myc/Ndufa6/Nme2/Pdk2/Ppargc1a
1-day post-SCI group vs. the control group	BP	GO:0048016	inositol phosphate-mediated signaling	3/125	0.0018446	0.00805495	0.00419449	Edn1/Ppp3ca/Rcan1

1-day post-SCI group vs. the control group	BP	GO:0070918	small regulatory ncRNA processing	3/125	0.0018446	0.00805495	0.00419449	Ago4/Ppp3ca/Ripk1
1-day post-SCI group vs. the control group	BP	GO:0051209	release of sequestered calcium ion into cytosol	4/125	0.0018511	0.00806296	0.00419866	Bax/Fkbp1b/Slc8a1/Trpm2
1-day post-SCI group vs. the control group	BP	GO:0098773	skin epidermis development	4/125	0.0018511	0.00806296	0.00419866	Ctsl/Hdac1/Ptgs2/Rela
1-day post-SCI group vs. the control group	BP	GO:0032308	positive regulation of prostaglandin secretion	2/125	0.0018727	0.00807633	0.00420562	Edn1/Map2k6
1-day post-SCI group vs. the control group	BP	GO:0033700	phospholipid efflux	2/125	0.0018727	0.00807633	0.00420562	Abcc1/Apoe
1-day post-SCI group vs. the control group	BP	GO:0043558	regulation of translational initiation in response to stre	2/125	0.0018727	0.00807633	0.00420562	Eif2s1/Ppp1r15b
1-day post-SCI group vs. the control group	BP	GO:0061029	eyelid development in camera-type eye	2/125	0.0018727	0.00807633	0.00420562	Hdac1/Jun
1-day post-SCI group vs. the control group	BP	GO:0097050	type B pancreatic cell apoptotic process	2/125	0.0018727	0.00807633	0.00420562	Eif2s1/Mapk8
1-day post-SCI group vs. the control group	BP	GO:1902337	regulation of apoptotic process involved in morphogen	2/125	0.0018727	0.00807633	0.00420562	Bax/Tnfrsf1a
1-day post-SCI group vs. the control group	BP	GO:1903543	positive regulation of exosomal secretion	2/125	0.0018727	0.00807633	0.00420562	Atp13a2/Sdc1
1-day post-SCI group vs. the control group	BP	GO:2000811	negative regulation of anoikis	2/125	0.0018727	0.00807633	0.00420562	Itga5/Mcl1
1-day post-SCI group vs. the control group	BP	GO:0002695	negative regulation of leukocyte activation	5/125	0.0018782	0.00808026	0.00420767	Anxa1/Axl/Btk/Hmox1/Hspb1
1-day post-SCI group vs. the control group	BP	GO:0006469	negative regulation of protein kinase activity	5/125	0.0018782	0.00808026	0.00420767	Apoe/Cdkn1c/Hspb1/Slc8a1/Spry2
1-day post-SCI group vs. the control group	BP	GO:0006090	pyruvate metabolic process	4/125	0.0019081	0.00817838	0.00425876	Ier3/Myc/Pdk2/Ppargc1a
1-day post-SCI group vs. the control group	BP	GO:0031570	DNA integrity checkpoint signaling	4/125	0.0019081	0.00817838	0.00425876	Atf2/Ier3/Mapk14/Mdm2
1-day post-SCI group vs. the control group	BP	GO:0051283	negative regulation of sequestering of calcium ion	4/125	0.0019081	0.00817838	0.00425876	Bax/Fkbp1b/Slc8a1/Trpm2
1-day post-SCI group vs. the control group	BP	GO:0002762	negative regulation of myeloid leukocyte differentiatio	3/125	0.001941	0.00829925	0.00432171	Fbxw7/Myc/Nme2
1-day post-SCI group vs. the control group	BP	GO:0034113	heterotypic cell-cell adhesion	3/125	0.001941	0.00829925	0.00432171	Itga5/Itga7/Vcam1
1-day post-SCI group vs. the control group	BP	GO:0009201	ribonucleoside triphosphate biosynthetic process	4/125	0.0019662	0.00838651	0.00436714	Myc/Ndufa6/Nme2/Ppargc1a
1-day post-SCI group vs. the control group	BP	GO:0046545	development of primary female sexual characteristics	4/125	0.0019662	0.00838651	0.00436714	Bax/Ermp1/Nos3/Plekha1
1-day post-SCI group vs. the control group	BP	GO:0097696	receptor signaling pathway via STAT	5/125	0.0020028	0.00853205	0.00444293	Cyp1b1/Il6st/Jak2/Stat6/Tnfrsf1a
1-day post-SCI group vs. the control group	BP	GO:0032612	interleukin-1 production	4/125	0.0020256	0.00859777	0.00447716	Anxa1/Hspb1/Jak2/Sphk1
1-day post-SCI group vs. the control group	BP	GO:0051282	regulation of sequestering of calcium ion	4/125	0.0020256	0.00859777	0.00447716	Bax/Fkbp1b/Slc8a1/Trpm2
1-day post-SCI group vs. the control group	BP	GO:0060419	heart growth	4/125	0.0020256	0.00859777	0.00447716	Edn1/Map2k4/Mapk14/Smad1
1-day post-SCI group vs. the control group	BP	GO:0003170	heart valve development	3/125	0.0020405	0.00861893	0.00448817	Mdm2/Nos3/Tnfrsf1a
1-day post-SCI group vs. the control group	BP	GO:0031279	regulation of cyclase activity	3/125	0.0020405	0.00861893	0.00448817	Mapk14/Mapk8/Nos3
1-day post-SCI group vs. the control group	BP	GO:0042311	vasodilation	3/125	0.0020405	0.00861893	0.00448817	Apoe/Gch1/Gpx1
1-day post-SCI group vs. the control group	BP	GO:0140353	lipid export from cell	3/125	0.0020405	0.00861893	0.00448817	Edn1/Map2k6/Ptgs2
1-day post-SCI group vs. the control group	BP	GO:0034433	steroid esterification	2/125	0.0021342	0.00890106	0.00463509	Apoe/Lcat
1-day post-SCI group vs. the control group	BP	GO:0034434	sterol esterification	2/125	0.0021342	0.00890106	0.00463509	Apoe/Lcat
1-day post-SCI group vs. the control group	BP	GO:0034435	cholesterol esterification	2/125	0.0021342	0.00890106	0.00463509	Apoe/Lcat
1-day post-SCI group vs. the control group	BP	GO:0045947	negative regulation of translational initiation	2/125	0.0021342	0.00890106	0.00463509	Eif2s1/Rpl13a
1-day post-SCI group vs. the control group	BP	GO:0047484	regulation of response to osmotic stress	2/125	0.0021342	0.00890106	0.00463509	Ptgs2/Ybx3
1-day post-SCI group vs. the control group	BP	GO:0051044	positive regulation of membrane protein ectodomain p	2/125	0.0021342	0.00890106	0.00463509	Adam9/Apoe
1-day post-SCI group vs. the control group	BP	GO:0071371	cellular response to gonadotropin stimulus	2/125	0.0021342	0.00890106	0.00463509	Edn1/Ppargc1a
1-day post-SCI group vs. the control group	BP	GO:0098885	modification of postsynaptic actin cytoskeleton	2/125	0.0021342	0.00890106	0.00463509	Cttn/Itsn1
1-day post-SCI group vs. the control group	BP	GO:0099527	postsynapse to nucleus signaling pathway	2/125	0.0021342	0.00890106	0.00463509	Jak2/Rela
1-day post-SCI group vs. the control group	BP	GO:1904748	regulation of apoptotic process involved in developme	2/125	0.0021342	0.00890106	0.00463509	Bax/Tnfrsf1a
1-day post-SCI group vs. the control group	BP	GO:0042987	amyloid precursor protein catabolic process	3/125	0.0021431	0.00890106	0.00463509	Adam9/Apoe/Rela
1-day post-SCI group vs. the control group	BP	GO:1903793	positive regulation of anion transport	3/125	0.0021431	0.00890106	0.00463509	Atf4/Edn1/Map2k6

1-day post-SCI group vs. the control group	BP	GO:2001238	positive regulation of extrinsic apoptotic signaling patl	3/125	0.0021431	0.00890106	0.00463509	Ctnna1/Ppp1ca/Ripk1
1-day post-SCI group vs. the control group	BP	GO:0031349	positive regulation of defense response	6/125	0.0021447	0.00890106	0.00463509	Abcc1/Jak2/Nono/Ptgs2/Ripk1/Tnfrsf1a
1-day post-SCI group vs. the control group	BP	GO:0006352	DNA-templated transcription initiation	4/125	0.0021481	0.00890106	0.00463509	Ercc1/Fosl1/Jun/Myc
1-day post-SCI group vs. the control group	BP	GO:0051208	sequestering of calcium ion	4/125	0.0021481	0.00890106	0.00463509	Bax/Fkbp1b/Slc8a1/Trpm2
1-day post-SCI group vs. the control group	BP	GO:0010665	regulation of cardiac muscle cell apoptotic process	3/125	0.0022488	0.00930731	0.00484664	Capn2/Jak2/Mapk8
1-day post-SCI group vs. the control group	BP	GO:0030307	positive regulation of cell growth	5/125	0.0022699	0.00938365	0.00488639	Apoe/Edn1/Hbegf/Mapt/Sphk1
1-day post-SCI group vs. the control group	BP	GO:0043280	positive regulation of cysteine-type endopeptidase acti	4/125	0.0022756	0.00939589	0.00489276	Bax/Jak2/Myc/Xdh
1-day post-SCI group vs. the control group	BP	GO:0009260	ribonucleotide biosynthetic process	5/125	0.0023169	0.00954372	0.00496974	Myc/Ndufa6/Nme2/Pdk2/Ppargc1a
1-day post-SCI group vs. the control group	BP	GO:2000113	negative regulation of cellular macromolecule biosynth	5/125	0.0023169	0.00954372	0.00496974	Ago4/Eif2s1/Hbegf/Rpl13a/Tsc1
1-day post-SCI group vs. the control group	BP	GO:0043500	muscle adaptation	4/125	0.0023413	0.00962215	0.00501058	Edn1/Nos3/Ppp3ca/Tnfrsf1a
1-day post-SCI group vs. the control group	BP	GO:0006869	lipid transport	7/125	0.0023414	0.00962215	0.00501058	Abcc1/Anxa1/Apoe/Edn1/Lcat/Map2k6/Ptgs2
1-day post-SCI group vs. the control group	BP	GO:0006446	regulation of translational initiation	3/125	0.0023577	0.00963927	0.0050195	Eif2s1/Ppp1r15b/Rpl13a
1-day post-SCI group vs. the control group	BP	GO:0060760	positive regulation of response to cytokine stimulus	3/125	0.0023577	0.00963927	0.0050195	Axl/Edn1/Ripk1
1-day post-SCI group vs. the control group	BP	GO:0071470	cellular response to osmotic stress	3/125	0.0023577	0.00963927	0.0050195	Ptgs2/Slc4a11/Ybx3
1-day post-SCI group vs. the control group	BP	GO:0031345	negative regulation of cell projection organization	5/125	0.0023645	0.00963927	0.0050195	Apoe/Mdm2/Ppp3ca/Spry2/Tsc1
1-day post-SCI group vs. the control group	BP	GO:1902115	regulation of organelle assembly	5/125	0.0023645	0.00963927	0.0050195	Dynll1/Ezr/Mapk8/Pink1/Sdc1
1-day post-SCI group vs. the control group	BP	GO:0003073	regulation of systemic arterial blood pressure	4/125	0.0024082	0.00963927	0.0050195	Edn1/Gpr37l1/Ier3/Nos3
1-day post-SCI group vs. the control group	BP	GO:0033138	positive regulation of peptidyl-serine phosphorylation	4/125	0.0024082	0.00963927	0.0050195	Met/Pink1/Ptgs2/Spry2
1-day post-SCI group vs. the control group	BP	GO:0042476	odontogenesis	4/125	0.0024082	0.00963927	0.0050195	Atf2/Bax/Edn1/Hdac1
1-day post-SCI group vs. the control group	BP	GO:0046632	alpha-beta T cell differentiation	4/125	0.0024082	0.00963927	0.0050195	Anxa1/Atf2/Ctsl/Stat6
1-day post-SCI group vs. the control group	BP	GO:2000278	regulation of DNA biosynthetic process	4/125	0.0024082	0.00963927	0.0050195	Cyp1b1/Gfer/Myc/Pcna
1-day post-SCI group vs. the control group	BP	GO:0030540	female genitalia development	2/125	0.0024119	0.00963927	0.0050195	Axl/Bax
1-day post-SCI group vs. the control group	BP	GO:0032306	regulation of prostaglandin secretion	2/125	0.0024119	0.00963927	0.0050195	Edn1/Map2k6
1-day post-SCI group vs. the control group	BP	GO:0034698	response to gonadotropin	2/125	0.0024119	0.00963927	0.0050195	Edn1/Ppargc1a
1-day post-SCI group vs. the control group	BP	GO:0045655	regulation of monocyte differentiation	2/125	0.0024119	0.00963927	0.0050195	Jun/Myc
1-day post-SCI group vs. the control group	BP	GO:0045820	negative regulation of glycolytic process	2/125	0.0024119	0.00963927	0.0050195	Ier3/Ppargc1a
1-day post-SCI group vs. the control group	BP	GO:0060546	negative regulation of necroptotic process	2/125	0.0024119	0.00963927	0.0050195	Ripk1/Ybx3
1-day post-SCI group vs. the control group	BP	GO:0061179	negative regulation of insulin secretion involved in cel	2/125	0.0024119	0.00963927	0.0050195	Fkbp1b/Ucp2
1-day post-SCI group vs. the control group	BP	GO:0090136	epithelial cell-cell adhesion	2/125	0.0024119	0.00963927	0.0050195	Ctnna1/Cyp1b1
1-day post-SCI group vs. the control group	BP	GO:0090336	positive regulation of brown fat cell differentiation	2/125	0.0024119	0.00963927	0.0050195	Mapk14/Ptgs2
1-day post-SCI group vs. the control group	BP	GO:0098760	response to interleukin-7	2/125	0.0024119	0.00963927	0.0050195	Btk/P4hb
1-day post-SCI group vs. the control group	BP	GO:0098761	cellular response to interleukin-7	2/125	0.0024119	0.00963927	0.0050195	Btk/P4hb
1-day post-SCI group vs. the control group	BP	GO:1903541	regulation of exosomal secretion	2/125	0.0024119	0.00963927	0.0050195	Atp13a2/Sdc1
1-day post-SCI group vs. the control group	BP	GO:2001028	positive regulation of endothelial cell chemotaxis	2/125	0.0024119	0.00963927	0.0050195	Hspb1/Met
1-day post-SCI group vs. the control group	BP	GO:2001171	positive regulation of ATP biosynthetic process	2/125	0.0024119	0.00963927	0.0050195	Myc/Ppargc1a
1-day post-SCI group vs. the control group	BP	GO:0031032	actomyosin structure organization	5/125	0.0024619	0.00981473	0.00511087	Edn1/Met/Pdlim1/Pxn/Tsc1
1-day post-SCI group vs. the control group	BP	GO:0031102	neuron projection regeneration	3/125	0.0024699	0.00981473	0.00511087	Fkbp1b/Jak2/Jun
1-day post-SCI group vs. the control group	BP	GO:0046622	positive regulation of organ growth	3/125	0.0024699	0.00981473	0.00511087	Edn1/Mapk14/Ybx3
1-day post-SCI group vs. the control group	BP	GO:0050873	brown fat cell differentiation	3/125	0.0024699	0.00981473	0.00511087	Mapk14/Ptgs2/Selenbp1
1-day post-SCI group vs. the control group	BP	GO:0051785	positive regulation of nuclear division	3/125	0.0024699	0.00981473	0.00511087	Edn1/Met/Sphk1

1-day post-SCI group vs. the control group	BP	GO:0043406	positive regulation of MAP kinase activity	4/125	0.0024765	0.00982982	0.00511873	Adam9/Edn1/Map2k4/Map2k6
1-day post-SCI group vs. the control group	BP	GO:0016358	dendrite development	6/125	0.0025179	0.00998272	0.00519835	Apoe/Cask/Itsn1/Mapk8/Met/Ppp3ca
1-day post-SCI group vs. the control group	BP	GO:0009150	purine ribonucleotide metabolic process	7/125	0.0025342	0.01003606	0.00522612	Ier3/Myc/Ndufa6/Nme2/Pdk2/Ppargc1a/Xdh
1-day post-SCI group vs. the control group	BP	GO:0030282	bone mineralization	4/125	0.0025461	0.01007164	0.00524465	Atf4/Ccr1/Ptgs2/Slc8a1
1-day post-SCI group vs. the control group	BP	GO:0010662	regulation of striated muscle cell apoptotic process	3/125	0.0025852	0.01021507	0.00531934	Capn2/Jak2/Mapk8
1-day post-SCI group vs. the control group	BP	GO:0031396	regulation of protein ubiquitination	5/125	0.0026134	0.01030542	0.00536639	Fbxw7/Mapk8/Pink1/Sphk1/Spry2
1-day post-SCI group vs. the control group	BP	GO:0001776	leukocyte homeostasis	4/125	0.002617	0.01030542	0.00536639	Anxa1/Axl/Bax/Ripk3
1-day post-SCI group vs. the control group	BP	GO:0009142	nucleoside triphosphate biosynthetic process	4/125	0.002617	0.01030542	0.00536639	Myc/Ndufa6/Nme2/Ppargc1a
1-day post-SCI group vs. the control group	BP	GO:0090287	regulation of cellular response to growth factor stimulu	6/125	0.0026388	0.01037959	0.00540501	Cask/Cdkn1c/Hdac1/Rbbp7/Spry2/Xdh
1-day post-SCI group vs. the control group	BP	GO:1903050	regulation of proteolysis involved in protein catabolic	5/125	0.0026654	0.01046594	0.00544998	Apoe/Fbxw7/Gpx1/Mapk8/Mdm2
1-day post-SCI group vs. the control group	BP	GO:0045931	positive regulation of mitotic cell cycle	4/125	0.0026892	0.01046594	0.00544998	Anxa1/Cdk4/Mdm2/Sphk1
1-day post-SCI group vs. the control group	BP	GO:0010659	cardiac muscle cell apoptotic process	3/125	0.0027039	0.01046594	0.00544998	Capn2/Jak2/Mapk8
1-day post-SCI group vs. the control group	BP	GO:0051851	modulation by host of symbiont process	3/125	0.0027039	0.01046594	0.00544998	Apoe/Hdac1/Jun
1-day post-SCI group vs. the control group	BP	GO:0060999	positive regulation of dendritic spine development	3/125	0.0027039	0.01046594	0.00544998	Apoe/Cask/Itsn1
1-day post-SCI group vs. the control group	BP	GO:0061077	chaperone-mediated protein folding	3/125	0.0027039	0.01046594	0.00544998	Fkbp1b/Hspa13/Hspb1
1-day post-SCI group vs. the control group	BP	GO:0090278	negative regulation of peptide hormone secretion	3/125	0.0027039	0.01046594	0.00544998	Fkbp1b/Ppp3ca/Ucp2
1-day post-SCI group vs. the control group	BP	GO:0019430	removal of superoxide radicals	2/125	0.0027057	0.01046594	0.00544998	Gch1/Nos3
1-day post-SCI group vs. the control group	BP	GO:0033631	cell-cell adhesion mediated by integrin	2/125	0.0027057	0.01046594	0.00544998	Adam9/Itga5
1-day post-SCI group vs. the control group	BP	GO:0035330	regulation of hippo signaling	2/125	0.0027057	0.01046594	0.00544998	Map2k3/Mapk14
1-day post-SCI group vs. the control group	BP	GO:0051000	positive regulation of nitric-oxide synthase activity	2/125	0.0027057	0.01046594	0.00544998	Apoe/Gch1
1-day post-SCI group vs. the control group	BP	GO:0062099	negative regulation of programmed necrotic cell death	2/125	0.0027057	0.01046594	0.00544998	Ripk1/Ybx3
1-day post-SCI group vs. the control group	BP	GO:0070102	interleukin-6-mediated signaling pathway	2/125	0.0027057	0.01046594	0.00544998	Il6st/Ripk1
1-day post-SCI group vs. the control group	BP	GO:1904424	regulation of GTP binding	2/125	0.0027057	0.01046594	0.00544998	Eif2s1/Met
1-day post-SCI group vs. the control group	BP	GO:2001267	regulation of cysteine-type endopeptidase activity invo	2/125	0.0027057	0.01046594	0.00544998	Bax/Jak2
1-day post-SCI group vs. the control group	BP	GO:0090596	sensory organ morphogenesis	6/125	0.002764	0.01067957	0.00556122	Atf4/Bax/Edn1/Hdac1/Myc/Spry2
1-day post-SCI group vs. the control group	BP	GO:0046390	ribose phosphate biosynthetic process	5/125	0.0027715	0.01069682	0.0055702	Myc/Ndufa6/Nme2/Pdk2/Ppargc1a
1-day post-SCI group vs. the control group	BP	GO:0015849	organic acid transport	6/125	0.0028067	0.01082066	0.00563469	Abcc1/Anxa1/Apoe/Edn1/Map2k6/Myc
1-day post-SCI group vs. the control group	BP	GO:0010656	negative regulation of muscle cell apoptotic process	3/125	0.0028259	0.01088245	0.00566687	Edn1/Hmox1/Jak2
1-day post-SCI group vs. the control group	BP	GO:0007264	small GTPase mediated signal transduction	7/125	0.002846	0.01093578	0.00569464	Apoe/Hmox1/Itsn1/Jun/Map4k4/Met/Spry2
1-day post-SCI group vs. the control group	BP	GO:1901987	regulation of cell cycle phase transition	7/125	0.002846	0.01093578	0.00569464	Anxa1/Atf2/Cdk4/Cdkn1c/Ier3/Mapk14/Mdm2
1-day post-SCI group vs. the control group	BP	GO:0022409	positive regulation of cell-cell adhesion	6/125	0.0028937	0.01110669	0.00578363	Anxa1/Il6st/Jak2/Ppp3ca/Rela/Vcam1
1-day post-SCI group vs. the control group	BP	GO:0009132	nucleoside diphosphate metabolic process	4/125	0.0029142	0.01117322	0.00581828	Ier3/Myc/Nme2/Ppargc1a
1-day post-SCI group vs. the control group	BP	GO:0070588	calcium ion transmembrane transport	6/125	0.0029379	0.01122871	0.00584717	Bax/Edn1/Fkbp1b/Ppp3ca/Slc8a1/Trpm2
1-day post-SCI group vs. the control group	BP	GO:2000146	negative regulation of cell motility	6/125	0.0029379	0.01122871	0.00584717	Apoe/Ctnna1/Cyp1b1/Hdac1/Ppargc1a/Rbbp7
1-day post-SCI group vs. the control group	BP	GO:0002792	negative regulation of peptide secretion	3/125	0.0029512	0.01122871	0.00584717	Fkbp1b/Ppp3ca/Ucp2
1-day post-SCI group vs. the control group	BP	GO:0015909	long-chain fatty acid transport	3/125	0.0029512	0.01122871	0.00584717	Abcc1/Anxa1/Apoe
1-day post-SCI group vs. the control group	BP	GO:0060612	adipose tissue development	3/125	0.0029512	0.01122871	0.00584717	Atf2/Cdk4/Ppargc1a
1-day post-SCI group vs. the control group	BP	GO:0070231	T cell apoptotic process	3/125	0.0029512	0.01122871	0.00584717	Bax/Ripk1/Ripk3
1-day post-SCI group vs. the control group	BP	GO:0090311	regulation of protein deacetylation	3/125	0.0029512	0.01122871	0.00584717	Mapt/Pink1/Ripk3
1-day post-SCI group vs. the control group	BP	GO:0070372	regulation of ERK1 and ERK2 cascade	6/125	0.0029826	0.01132526	0.00589745	Apoe/Ccr1/Ezr/Fbxw7/Jun/Spry2

1-day post-SCI group vs. the control group	BP	GO:0042303	molting cycle	4/125	0.002992	0.01132526	0.00589745	Ctsl/Hdac1/Ptgs2/Rela
1-day post-SCI group vs. the control group	BP	GO:0042633	hair cycle	4/125	0.002992	0.01132526	0.00589745	Ctsl/Hdac1/Ptgs2/Rela
1-day post-SCI group vs. the control group	BP	GO:0061041	regulation of wound healing	4/125	0.002992	0.01132526	0.00589745	Anxa1/Apoe/Cask/Hbegf
1-day post-SCI group vs. the control group	BP	GO:0035457	cellular response to interferon-alpha	2/125	0.0030155	0.01132526	0.00589745	Axl/Myc
1-day post-SCI group vs. the control group	BP	GO:0043691	reverse cholesterol transport	2/125	0.0030155	0.01132526	0.00589745	Apoe/Lcat
1-day post-SCI group vs. the control group	BP	GO:0051132	NK T cell activation	2/125	0.0030155	0.01132526	0.00589745	Jak2/Myc
1-day post-SCI group vs. the control group	BP	GO:0071636	positive regulation of transforming growth factor beta	2/125	0.0030155	0.01132526	0.00589745	Atf2/Ptgs2
1-day post-SCI group vs. the control group	BP	GO:0140467	integrated stress response signaling	2/125	0.0030155	0.01132526	0.00589745	Atf4/Eif2s1
1-day post-SCI group vs. the control group	BP	GO:1901741	positive regulation of myoblast fusion	2/125	0.0030155	0.01132526	0.00589745	Capn2/Mapk14
1-day post-SCI group vs. the control group	BP	GO:1902004	positive regulation of amyloid-beta formation	2/125	0.0030155	0.01132526	0.00589745	Apoe/Rela
1-day post-SCI group vs. the control group	BP	GO:1903214	regulation of protein targeting to mitochondrion	2/125	0.0030155	0.01132526	0.00589745	Fbxw7/Pink1
1-day post-SCI group vs. the control group	BP	GO:0017015	regulation of transforming growth factor beta receptor	4/125	0.0030711	0.0115093	0.00599329	Cdkn1c/Hdac1/Rbbp7/Spry2
1-day post-SCI group vs. the control group	BP	GO:0048675	axon extension	4/125	0.0030711	0.0115093	0.00599329	Apoe/Cttn/Edn1/Mapt
1-day post-SCI group vs. the control group	BP	GO:0010658	striated muscle cell apoptotic process	3/125	0.0030799	0.01152973	0.00600393	Capn2/Jak2/Mapk8
1-day post-SCI group vs. the control group	BP	GO:0070374	positive regulation of ERK1 and ERK2 cascade	5/125	0.0031085	0.01162443	0.00605324	Apoe/Ccr1/Fbxw7/Jun/Spry2
1-day post-SCI group vs. the control group	BP	GO:0030595	leukocyte chemotaxis	5/125	0.0031674	0.01183203	0.00616135	Anxa1/Ccr1/Edn1/Rpl13a/Trpm2
1-day post-SCI group vs. the control group	BP	GO:0006879	cellular iron ion homeostasis	3/125	0.003212	0.01198562	0.00624132	Atp13a2/Hmox1/Myc
1-day post-SCI group vs. the control group	BP	GO:0002698	negative regulation of immune effector process	4/125	0.0032337	0.01205395	0.00627691	Anxa1/Axl/Gfer/Hmox1
1-day post-SCI group vs. the control group	BP	GO:0009259	ribonucleotide metabolic process	7/125	0.0032662	0.012162	0.00633317	Ier3/Myc/Ndufa6/Nme2/Pdk2/Ppargc1a/Xdh
1-day post-SCI group vs. the control group	BP	GO:0000082	G1/S transition of mitotic cell cycle	5/125	0.0032876	0.01222877	0.00636794	Anxa1/Cdk4/Mdm2/Myc/Ppp3ca
1-day post-SCI group vs. the control group	BP	GO:1903844	regulation of cellular response to transforming growth	4/125	0.0033172	0.01230966	0.00641006	Cdkn1c/Hdac1/Rbbp7/Spry2
1-day post-SCI group vs. the control group	BP	GO:0010801	negative regulation of peptidyl-threonine phosphorylat	2/125	0.0033411	0.01230966	0.00641006	Met/Spry2
1-day post-SCI group vs. the control group	BP	GO:0032305	positive regulation of icosanoid secretion	2/125	0.0033411	0.01230966	0.00641006	Edn1/Map2k6
1-day post-SCI group vs. the control group	BP	GO:0032495	response to muramyl dipeptide	2/125	0.0033411	0.01230966	0.00641006	Mapk14/Rela
1-day post-SCI group vs. the control group	BP	GO:0033599	regulation of mammary gland epithelial cell proliferati	2/125	0.0033411	0.01230966	0.00641006	Bax/Gpx1
1-day post-SCI group vs. the control group	BP	GO:0043373	CD4-positive, alpha-beta T cell lineage commitment	2/125	0.0033411	0.01230966	0.00641006	Ctsl/Stat6
1-day post-SCI group vs. the control group	BP	GO:0071450	cellular response to oxygen radical	2/125	0.0033411	0.01230966	0.00641006	Gch1/Nos3
1-day post-SCI group vs. the control group	BP	GO:0071451	cellular response to superoxide	2/125	0.0033411	0.01230966	0.00641006	Gch1/Nos3
1-day post-SCI group vs. the control group	BP	GO:2000671	regulation of motor neuron apoptotic process	2/125	0.0033411	0.01230966	0.00641006	Bax/Map2k4
1-day post-SCI group vs. the control group	BP	GO:1901224	positive regulation of NIK/NF-kappaB signaling	3/125	0.0033475	0.0123125	0.00641154	Edn1/Rela/Sphk1
1-day post-SCI group vs. the control group	BP	GO:0050920	regulation of chemotaxis	5/125	0.003349	0.0123125	0.00641154	Ccr1/Cttn/Edn1/Hspb1/Met
1-day post-SCI group vs. the control group	BP	GO:0002253	activation of immune response	7/125	0.0033886	0.01244524	0.00648067	Bax/Btk/Ezr/Krt1/Nono/Plekha1/Rela
1-day post-SCI group vs. the control group	BP	GO:0000768	syncytium formation by plasma membrane fusion	3/125	0.0034864	0.01276413	0.00664672	Adam9/Capn2/Mapk14
1-day post-SCI group vs. the control group	BP	GO:0042509	regulation of tyrosine phosphorylation of STAT protei	3/125	0.0034864	0.01276413	0.00664672	Il6st/Jak2/Tnfrsf1a
1-day post-SCI group vs. the control group	BP	GO:0140253	cell-cell fusion	3/125	0.0034864	0.01276413	0.00664672	Adam9/Capn2/Mapk14
1-day post-SCI group vs. the control group	BP	GO:0044772	mitotic cell cycle phase transition	7/125	0.0035146	0.01285356	0.00669329	Anxa1/Cdk4/Cdkn1c/Ier3/Mdm2/Myc/Ppp3ca
1-day post-SCI group vs. the control group	BP	GO:0010761	fibroblast migration	3/125	0.0036289	0.01325783	0.00690381	Pdlim1/Prr5l/Slc8a1
1-day post-SCI group vs. the control group	BP	GO:0030324	lung development	5/125	0.003668	0.01331403	0.00693307	Fbxw7/Nos3/Ppp1ca/Rpl13a/Spry2
1-day post-SCI group vs. the control group	BP	GO:0003177	pulmonary valve development	2/125	0.0036824	0.01331403	0.00693307	Nos3/Tnfrsf1a
1-day post-SCI group vs. the control group	BP	GO:0010310	regulation of hydrogen peroxide metabolic process	2/125	0.0036824	0.01331403	0.00693307	Mmp3/Pink1

1-day post-SCI group vs. the control group	BP	GO:0035234	ectopic germ cell programmed cell death	2/125	0.0036824	0.01331403	0.00693307	Bax/Ybx3
1-day post-SCI group vs. the control group	BP	GO:0035584	calcium-mediated signaling using intracellular calcium	2/125	0.0036824	0.01331403	0.00693307	Trpm2/Vcam1
1-day post-SCI group vs. the control group	BP	GO:0043555	regulation of translation in response to stress	2/125	0.0036824	0.01331403	0.00693307	Eif2s1/Ppp1r15b
1-day post-SCI group vs. the control group	BP	GO:0046697	decidualization	2/125	0.0036824	0.01331403	0.00693307	Ctsl/Ptgs2
1-day post-SCI group vs. the control group	BP	GO:0070230	positive regulation of lymphocyte apoptotic process	2/125	0.0036824	0.01331403	0.00693307	Bax/Myc
1-day post-SCI group vs. the control group	BP	GO:1903902	positive regulation of viral life cycle	2/125	0.0036824	0.01331403	0.00693307	Axl/P4hb
1-day post-SCI group vs. the control group	BP	GO:1990182	exosomal secretion	2/125	0.0036824	0.01331403	0.00693307	Atp13a2/Sdc1
1-day post-SCI group vs. the control group	BP	GO:0051656	establishment of organelle localization	7/125	0.003688	0.01332019	0.00693628	Atp13a2/Ezr/Hmox1/Mapk8/Mapt/Sdc1/Spry2
1-day post-SCI group vs. the control group	BP	GO:0051235	maintenance of location	6/125	0.0037163	0.01340848	0.00698225	Apoe/Bax/Fkbp1b/Pink1/Slc8a1/Trpm2
1-day post-SCI group vs. the control group	BP	GO:0019693	ribose phosphate metabolic process	7/125	0.0037323	0.01345253	0.00700519	Ier3/Myc/Ndufa6/Nme2/Pdk2/Ppargc1a/Xdh
1-day post-SCI group vs. the control group	BP	GO:0006639	acylglycerol metabolic process	4/125	0.003849	0.01385872	0.00721671	Apoe/Cat/Gpx1/Il6st
1-day post-SCI group vs. the control group	BP	GO:0001558	regulation of cell growth	7/125	0.0038678	0.01390442	0.00724051	Apoe/Cttn/Edn1/Hbegf/Mapt/Rbbp7/Sphk1
1-day post-SCI group vs. the control group	BP	GO:0030323	respiratory tube development	5/125	0.0038696	0.01390442	0.00724051	Fbxw7/Nos3/Ppp1ca/Rpl13a/Spry2
1-day post-SCI group vs. the control group	BP	GO:0045428	regulation of nitric oxide biosynthetic process	3/125	0.0039244	0.01407202	0.00732778	Dynll1/Jak2/Ptgs2
1-day post-SCI group vs. the control group	BP	GO:0061035	regulation of cartilage development	3/125	0.0039244	0.01407202	0.00732778	Bmp1/Rela/Smad1
1-day post-SCI group vs. the control group	BP	GO:0045088	regulation of innate immune response	5/125	0.0039386	0.01410851	0.00734679	Apoe/Banf1/Ccr1/Gfer/Nono
1-day post-SCI group vs. the control group	BP	GO:0006638	neutral lipid metabolic process	4/125	0.0040383	0.01426422	0.00742787	Apoe/Cat/Gpx1/Il6st
1-day post-SCI group vs. the control group	BP	GO:2001056	positive regulation of cysteine-type endopeptidase acti	4/125	0.0040383	0.01426422	0.00742787	Bax/Jak2/Myc/Xdh
1-day post-SCI group vs. the control group	BP	GO:0002363	alpha-beta T cell lineage commitment	2/125	0.0040393	0.01426422	0.00742787	Ctsl/Stat6
1-day post-SCI group vs. the control group	BP	GO:0006656	phosphatidylcholine biosynthetic process	2/125	0.0040393	0.01426422	0.00742787	Capn2/Lcat
1-day post-SCI group vs. the control group	BP	GO:0006907	pinocytosis	2/125	0.0040393	0.01426422	0.00742787	Axl/Mapkapk3
1-day post-SCI group vs. the control group	BP	GO:0032516	positive regulation of phosphoprotein phosphatase acti	2/125	0.0040393	0.01426422	0.00742787	Jak2/Ppp1r15b
1-day post-SCI group vs. the control group	BP	GO:0034312	diol biosynthetic process	2/125	0.0040393	0.01426422	0.00742787	Gch1/Sphk1
1-day post-SCI group vs. the control group	BP	GO:0034377	plasma lipoprotein particle assembly	2/125	0.0040393	0.01426422	0.00742787	Apoe/Lcat
1-day post-SCI group vs. the control group	BP	GO:0044851	hair cycle phase	2/125	0.0040393	0.01426422	0.00742787	Ctsl/Ptgs2
1-day post-SCI group vs. the control group	BP	GO:0097734	extracellular exosome biogenesis	2/125	0.0040393	0.01426422	0.00742787	Atp13a2/Sdc1
1-day post-SCI group vs. the control group	BP	GO:0150104	transport across blood-brain barrier	2/125	0.0040393	0.01426422	0.00742787	Abcc1/Apoe
1-day post-SCI group vs. the control group	BP	GO:1900746	regulation of vascular endothelial growth factor signali	2/125	0.0040393	0.01426422	0.00742787	Spry2/Xdh
1-day post-SCI group vs. the control group	BP	GO:1903672	positive regulation of sprouting angiogenesis	2/125	0.0040393	0.01426422	0.00742787	Itga5/Smad1
1-day post-SCI group vs. the control group	BP	GO:2000209	regulation of anoikis	2/125	0.0040393	0.01426422	0.00742787	Itga5/Mcl1
1-day post-SCI group vs. the control group	BP	GO:0014068	positive regulation of phosphatidylinositol 3-kinase sig	3/125	0.0040774	0.01431778	0.00745576	Cat/Jak2/Prr5l
1-day post-SCI group vs. the control group	BP	GO:0042982	amyloid precursor protein metabolic process	3/125	0.0040774	0.01431778	0.00745576	Adam9/Apoe/Rela
1-day post-SCI group vs. the control group	BP	GO:0048488	synaptic vesicle endocytosis	3/125	0.0040774	0.01431778	0.00745576	Amph/Itsn1/Syp
1-day post-SCI group vs. the control group	BP	GO:0098586	cellular response to virus	3/125	0.0040774	0.01431778	0.00745576	Atf2/Bax/Mapk14
1-day post-SCI group vs. the control group	BP	GO:0140238	presynaptic endocytosis	3/125	0.0040774	0.01431778	0.00745576	Amph/Itsn1/Syp
1-day post-SCI group vs. the control group	BP	GO:0009913	epidermal cell differentiation	5/125	0.0040791	0.01431778	0.00745576	Anxa1/Hdac1/Krt1/Ppp3ca/Ptgs2
1-day post-SCI group vs. the control group	BP	GO:0072331	signal transduction by p53 class mediator	4/125	0.0041353	0.01450073	0.00755103	Bax/Mdm2/Myc/Pdk2
1-day post-SCI group vs. the control group	BP	GO:0051251	positive regulation of lymphocyte activation	7/125	0.00415	0.01453751	0.00757018	Anxa1/Axl/Il6st/Jak2/Ppp3ca/Stat6/Vcam1
1-day post-SCI group vs. the control group	BP	GO:0051928	positive regulation of calcium ion transport	4/125	0.0042339	0.01475787	0.00768493	Bax/Cask/Ccr1/Ppp3ca
1-day post-SCI group vs. the control group	BP	GO:0007260	tyrosine phosphorylation of STAT protein	3/125	0.0042341	0.01475787	0.00768493	Il6st/Jak2/Tnfrsf1a

1-day post-SCI group vs. the control group	BP	GO:0010611	regulation of cardiac muscle hypertrophy	3/125	0.0042341	0.01475787	0.00768493	Edn1/Ppp3ca/Tnfrsf1a
1-day post-SCI group vs. the control group	BP	GO:1903578	regulation of ATP metabolic process	3/125	0.0042341	0.01475787	0.00768493	Ier3/Myc/Ppargc1a
1-day post-SCI group vs. the control group	BP	GO:2000036	regulation of stem cell population maintenance	3/125	0.0042341	0.01475787	0.00768493	Hdac1/Myc/Rbbp7
1-day post-SCI group vs. the control group	BP	GO:0043087	regulation of GTPase activity	6/125	0.0042748	0.01488511	0.00775119	Amph/Eif2s1/Map4k4/Met/Spry2/Tsc1
1-day post-SCI group vs. the control group	BP	GO:0050870	positive regulation of T cell activation	5/125	0.0043708	0.01520395	0.00791722	Anxa1/Il6st/Jak2/Ppp3ca/Vcam1
1-day post-SCI group vs. the control group	BP	GO:0032677	regulation of interleukin-8 production	3/125	0.0043943	0.01520929	0.00792	Anxa1/Rela/Ripk1
1-day post-SCI group vs. the control group	BP	GO:0010876	lipid localization	7/125	0.0043969	0.01520929	0.00792	Abcc1/Anxa1/Apoe/Edn1/Lcat/Map2k6/Ptgs2
1-day post-SCI group vs. the control group	BP	GO:0010232	vascular transport	2/125	0.0044115	0.01520929	0.00792	Abcc1/Apoe
1-day post-SCI group vs. the control group	BP	GO:0010666	positive regulation of cardiac muscle cell apoptotic prc	2/125	0.0044115	0.01520929	0.00792	Capn2/Mapk8
1-day post-SCI group vs. the control group	BP	GO:0010880	regulation of release of sequestered calcium ion into cy	2/125	0.0044115	0.01520929	0.00792	Fkbp1b/Slc8a1
1-day post-SCI group vs. the control group	BP	GO:0030728	ovulation	2/125	0.0044115	0.01520929	0.00792	Nos3/Ptgs2
1-day post-SCI group vs. the control group	BP	GO:0051043	regulation of membrane protein ectodomain proteolysi	2/125	0.0044115	0.01520929	0.00792	Adam9/Apoe
1-day post-SCI group vs. the control group	BP	GO:1903579	negative regulation of ATP metabolic process	2/125	0.0044115	0.01520929	0.00792	Ier3/Ppargc1a
1-day post-SCI group vs. the control group	BP	GO:2001026	regulation of endothelial cell chemotaxis	2/125	0.0044115	0.01520929	0.00792	Hspb1/Met
1-day post-SCI group vs. the control group	BP	GO:0008202	steroid metabolic process	6/125	0.0044539	0.01534025	0.0079882	Apoe/Cat/Cyp1b1/Lcat/Plekha1/Ppargc1a
1-day post-SCI group vs. the control group	BP	GO:0006633	fatty acid biosynthetic process	4/125	0.0045391	0.01560291	0.00812497	Anxa1/Edn1/Ptgs2/Sphk1
1-day post-SCI group vs. the control group	BP	GO:0016052	carbohydrate catabolic process	4/125	0.0045391	0.01560291	0.00812497	Ier3/Myc/Ppargc1a/Ppp1ca
1-day post-SCI group vs. the control group	BP	GO:0010822	positive regulation of mitochondrion organization	3/125	0.0045582	0.01560704	0.00812712	Bax/Pink1/Ppargc1a
1-day post-SCI group vs. the control group	BP	GO:0016239	positive regulation of macroautophagy	3/125	0.0045582	0.01560704	0.00812712	Hmox1/Pink1/Tsc1
1-day post-SCI group vs. the control group	BP	GO:0032637	interleukin-8 production	3/125	0.0045582	0.01560704	0.00812712	Anxa1/Rela/Ripk1
1-day post-SCI group vs. the control group	BP	GO:0080164	regulation of nitric oxide metabolic process	3/125	0.0045582	0.01560704	0.00812712	Dynll1/Jak2/Ptgs2
1-day post-SCI group vs. the control group	BP	GO:0048284	organelle fusion	4/125	0.004644	0.01588538	0.00827206	Anxa1/Atp13a2/Bax/Sphk1
1-day post-SCI group vs. the control group	BP	GO:0006814	sodium ion transport	5/125	0.004677	0.01596689	0.00831451	Edn1/Nos3/Slc4a11/Slc8a1/Trpm2
1-day post-SCI group vs. the control group	BP	GO:0032868	response to insulin	5/125	0.004677	0.01596689	0.00831451	Cdk4/Mapk14/Pdk2/Rela/Tsc1
1-day post-SCI group vs. the control group	BP	GO:0002292	T cell differentiation involved in immune response	3/125	0.0047258	0.01610172	0.00838472	Anxa1/Stat6/Tsc1
1-day post-SCI group vs. the control group	BP	GO:0014743	regulation of muscle hypertrophy	3/125	0.0047258	0.01610172	0.00838472	Edn1/Ppp3ca/Tnfrsf1a
1-day post-SCI group vs. the control group	BP	GO:0010565	regulation of cellular ketone metabolic process	4/125	0.0047506	0.01615484	0.00841238	Anxa1/Pdk2/Ppargc1a/Ptgs2
1-day post-SCI group vs. the control group	BP	GO:0030216	keratinocyte differentiation	4/125	0.0047506	0.01615484	0.00841238	Anxa1/Krt1/Ppp3ca/Ptgs2
1-day post-SCI group vs. the control group	BP	GO:0009205	purine ribonucleoside triphosphate metabolic process	5/125	0.0047559	0.01615718	0.0084136	Ier3/Myc/Ndufa6/Nme2/Ppargc1a
1-day post-SCI group vs. the control group	BP	GO:0006066	alcohol metabolic process	6/125	0.0047646	0.01616198	0.0084161	Apoe/Cat/Cyp1b1/Gch1/Lcat/Sphk1
1-day post-SCI group vs. the control group	BP	GO:0009065	glutamine family amino acid catabolic process	2/125	0.004799	0.01616198	0.0084161	Nos3/Prodh
1-day post-SCI group vs. the control group	BP	GO:0010663	positive regulation of striated muscle cell apoptotic prc	2/125	0.004799	0.01616198	0.0084161	Capn2/Mapk8
1-day post-SCI group vs. the control group	BP	GO:0042026	protein refolding	2/125	0.004799	0.01616198	0.0084161	Hspa13/Hspb1
1-day post-SCI group vs. the control group	BP	GO:0042537	benzene-containing compound metabolic process	2/125	0.004799	0.01616198	0.0084161	Cyp1b1/Txnrd1
1-day post-SCI group vs. the control group	BP	GO:0043369	CD4-positive or CD8-positive, alpha-beta T cell lineag	2/125	0.004799	0.01616198	0.0084161	Ctsl/Stat6
1-day post-SCI group vs. the control group	BP	GO:0051481	negative regulation of cytosolic calcium ion concentrat	2/125	0.004799	0.01616198	0.0084161	Il6st/Slc8a1
1-day post-SCI group vs. the control group	BP	GO:0140112	extracellular vesicle biogenesis	2/125	0.004799	0.01616198	0.0084161	Atp13a2/Sdc1
1-day post-SCI group vs. the control group	BP	GO:1902547	regulation of cellular response to vascular endothelial g	2/125	0.004799	0.01616198	0.0084161	Spry2/Xdh
1-day post-SCI group vs. the control group	BP	GO:0040013	negative regulation of locomotion	6/125	0.0048286	0.0162459	0.0084598	Apoe/Ctnna1/Cyp1b1/Hdac1/Ppargc1a/Rbbp7
1-day post-SCI group vs. the control group	BP	GO:0045669	positive regulation of osteoblast differentiation	3/125	0.004897	0.01644427	0.0085631	Il6st/Ppp3ca/Smad1

1-day post-SCI group vs. the control group	BP	GO:1903036	positive regulation of response to wounding	3/125	0.004897	0.01644427	0.0085631	Anxa1/Fkbp1b/Hbegf
1-day post-SCI group vs. the control group	BP	GO:0051259	protein complex oligomerization	5/125	0.0049165	0.01649407	0.00858903	Krt1/Mapt/Pxdn/Rnf112/Trpm2
1-day post-SCI group vs. the control group	BP	GO:0044773	mitotic DNA damage checkpoint signaling	3/125	0.0050719	0.01698268	0.00884347	Atf2/Ier3/Mdm2
1-day post-SCI group vs. the control group	BP	GO:0051149	positive regulation of muscle cell differentiation	3/125	0.0050719	0.01698268	0.00884347	Edn1/Mapk14/Mdm2
1-day post-SCI group vs. the control group	BP	GO:0008360	regulation of cell shape	4/125	0.0050801	0.01699381	0.00884926	Anxa1/Ezr/Itga7/Pxn
1-day post-SCI group vs. the control group	BP	GO:1901361	organic cyclic compound catabolic process	7/125	0.0051474	0.01716992	0.00894097	Ago4/Apoe/Bax/Hmox1/Hmox2/Prr5l/Xdh
1-day post-SCI group vs. the control group	BP	GO:0007263	nitric oxide mediated signal transduction	2/125	0.0052017	0.01716992	0.00894097	Apoe/Nos3
1-day post-SCI group vs. the control group	BP	GO:0014808	release of sequestered calcium ion into cytosol by sarc	2/125	0.0052017	0.01716992	0.00894097	Fkbp1b/Slc8a1
1-day post-SCI group vs. the control group	BP	GO:0032303	regulation of icosanoid secretion	2/125	0.0052017	0.01716992	0.00894097	Edn1/Map2k6
1-day post-SCI group vs. the control group	BP	GO:0045063	T-helper 1 cell differentiation	2/125	0.0052017	0.01716992	0.00894097	Anxa1/Stat6
1-day post-SCI group vs. the control group	BP	GO:0060547	negative regulation of necrotic cell death	2/125	0.0052017	0.01716992	0.00894097	Ripk1/Ybx3
1-day post-SCI group vs. the control group	BP	GO:0097062	dendritic spine maintenance	2/125	0.0052017	0.01716992	0.00894097	Apoe/Cttn
1-day post-SCI group vs. the control group	BP	GO:1900543	negative regulation of purine nucleotide metabolic pro	2/125	0.0052017	0.01716992	0.00894097	Ier3/Ppargc1a
1-day post-SCI group vs. the control group	BP	GO:1901739	regulation of myoblast fusion	2/125	0.0052017	0.01716992	0.00894097	Capn2/Mapk14
1-day post-SCI group vs. the control group	BP	GO:1902455	negative regulation of stem cell population maintenanc	2/125	0.0052017	0.01716992	0.00894097	Hdac1/Rbbp7
1-day post-SCI group vs. the control group	BP	GO:1902993	positive regulation of amyloid precursor protein catabo	2/125	0.0052017	0.01716992	0.00894097	Apoe/Rela
1-day post-SCI group vs. the control group	BP	GO:1903798	regulation of miRNA maturation	2/125	0.0052017	0.01716992	0.00894097	Ppp3ca/Ripk1
1-day post-SCI group vs. the control group	BP	GO:2000193	positive regulation of fatty acid transport	2/125	0.0052017	0.01716992	0.00894097	Edn1/Map2k6
1-day post-SCI group vs. the control group	BP	GO:2001169	regulation of ATP biosynthetic process	2/125	0.0052017	0.01716992	0.00894097	Myc/Ppargc1a
1-day post-SCI group vs. the control group	BP	GO:0009144	purine nucleoside triphosphate metabolic process	5/125	0.0052493	0.0172781	0.0089973	Ier3/Myc/Ndufa6/Nme2/Ppargc1a
1-day post-SCI group vs. the control group	BP	GO:0009199	ribonucleoside triphosphate metabolic process	5/125	0.0052493	0.0172781	0.0089973	Ier3/Myc/Ndufa6/Nme2/Ppargc1a
1-day post-SCI group vs. the control group	BP	GO:0044843	cell cycle G1/S phase transition	5/125	0.0052493	0.0172781	0.0089973	Anxa1/Cdk4/Mdm2/Myc/Ppp3ca
1-day post-SCI group vs. the control group	BP	GO:0051604	protein maturation	6/125	0.0052945	0.01741057	0.00906628	Adam9/Bmp1/Capn2/Ctsl/Mdm2/Myc
1-day post-SCI group vs. the control group	BP	GO:0045667	regulation of osteoblast differentiation	4/125	0.0053081	0.0174388	0.00908098	Atf4/Il6st/Ppp3ca/Smad1
1-day post-SCI group vs. the control group	BP	GO:0032956	regulation of actin cytoskeleton organization	6/125	0.0053637	0.01760481	0.00916743	Cttn/Edn1/Met/Pxn/Trpm2/Tsc1
1-day post-SCI group vs. the control group	BP	GO:0060348	bone development	5/125	0.0054215	0.01777141	0.00925419	Atf2/Fbxw7/Map2k6/Mapk14/Smad1
1-day post-SCI group vs. the control group	BP	GO:0034101	erythrocyte homeostasis	4/125	0.0054246	0.01777141	0.00925419	Axl/Hmox1/Jak2/Mapk14
1-day post-SCI group vs. the control group	BP	GO:0051279	regulation of release of sequestered calcium ion into cy	3/125	0.005433	0.01778214	0.00925977	Bax/Fkbp1b/Slc8a1
1-day post-SCI group vs. the control group	BP	GO:0019058	viral life cycle	5/125	0.0055977	0.018204	0.00947945	Apoe/Axl/Banf1/Ctsl/P4hb
1-day post-SCI group vs. the control group	BP	GO:0048705	skeletal system morphogenesis	5/125	0.0055977	0.018204	0.00947945	Atf2/Mapk14/Myc/Plekha1/Ppargc1b
1-day post-SCI group vs. the control group	BP	GO:0002381	immunoglobulin production involved in immunoglobu	3/125	0.0056192	0.018204	0.00947945	Btk/Ercc1/Stat6
1-day post-SCI group vs. the control group	BP	GO:0008625	extrinsic apoptotic signaling pathway via death domair	3/125	0.0056192	0.018204	0.00947945	Bax/Gpx1/Hmox1
1-day post-SCI group vs. the control group	BP	GO:0046785	microtubule polymerization	3/125	0.0056192	0.018204	0.00947945	Mapk8/Mapt/Met
1-day post-SCI group vs. the control group	BP	GO:0002082	regulation of oxidative phosphorylation	2/125	0.0056193	0.018204	0.00947945	Myc/Pink1
1-day post-SCI group vs. the control group	BP	GO:0042474	middle ear morphogenesis	2/125	0.0056193	0.018204	0.00947945	Edn1/Myc
1-day post-SCI group vs. the control group	BP	GO:0065005	protein-lipid complex assembly	2/125	0.0056193	0.018204	0.00947945	Apoe/Lcat
1-day post-SCI group vs. the control group	BP	GO:0090335	regulation of brown fat cell differentiation	2/125	0.0056193	0.018204	0.00947945	Mapk14/Ptgs2
1-day post-SCI group vs. the control group	BP	GO:0099010	modification of postsynaptic structure	2/125	0.0056193	0.018204	0.00947945	Cttn/Itsn1
1-day post-SCI group vs. the control group	BP	GO:1903514	release of sequestered calcium ion into cytosol by endo	2/125	0.0056193	0.018204	0.00947945	Fkbp1b/Slc8a1
1-day post-SCI group vs. the control group	BP	GO:0000422	autophagy of mitochondrion	3/125	0.0058092	0.01873215	0.00975447	Cttn/Fbxw7/Pink1

1-day post-SCI group vs. the control group	BP	GO:0043154	negative regulation of cysteine-type endopeptidase acti	3/125	0.0058092	0.01873215	0.00975447	Gpx1/Mdm2/Ptgs2
1-day post-SCI group vs. the control group	BP	GO:0044774	mitotic DNA integrity checkpoint signaling	3/125	0.0058092	0.01873215	0.00975447	Atf2/Ier3/Mdm2
1-day post-SCI group vs. the control group	BP	GO:0060998	regulation of dendritic spine development	3/125	0.0058092	0.01873215	0.00975447	Apoe/Cask/Itsn1
1-day post-SCI group vs. the control group	BP	GO:0061726	mitochondrion disassembly	3/125	0.0058092	0.01873215	0.00975447	Cttn/Fbxw7/Pink1
1-day post-SCI group vs. the control group	BP	GO:0050808	synapse organization	7/125	0.0058643	0.01889249	0.00983797	Apoe/Cask/Cttn/Itsn1/Mapk14/Mapt/Tsc1
1-day post-SCI group vs. the control group	BP	GO:0045444	fat cell differentiation	5/125	0.0059621	0.01914293	0.00996838	Atf2/Gpx1/Mapk14/Ptgs2/Selenbp1
1-day post-SCI group vs. the control group	BP	GO:0014855	striated muscle cell proliferation	3/125	0.006003	0.01914293	0.00996838	Jak2/Mapk14/Smad1
1-day post-SCI group vs. the control group	BP	GO:0045824	negative regulation of innate immune response	3/125	0.006003	0.01914293	0.00996838	Banf1/Ccr1/Gfer
1-day post-SCI group vs. the control group	BP	GO:0046323	glucose import	3/125	0.006003	0.01914293	0.00996838	Mapk14/Myc/Tsc1
1-day post-SCI group vs. the control group	BP	GO:0070228	regulation of lymphocyte apoptotic process	3/125	0.006003	0.01914293	0.00996838	Bax/Myc/Ripk3
1-day post-SCI group vs. the control group	BP	GO:1902117	positive regulation of organelle assembly	3/125	0.006003	0.01914293	0.00996838	Dynll1/Mapk8/Sdc1
1-day post-SCI group vs. the control group	BP	GO:0010675	regulation of cellular carbohydrate metabolic process	4/125	0.006033	0.01914293	0.00996838	Ier3/Pdk2/Ppargc1a/Ppp1ca
1-day post-SCI group vs. the control group	BP	GO:0003176	aortic valve development	2/125	0.0060518	0.01914293	0.00996838	Nos3/Tnfrsf1a
1-day post-SCI group vs. the control group	BP	GO:0006298	mismatch repair	2/125	0.0060518	0.01914293	0.00996838	Ercc1/Pcna
1-day post-SCI group vs. the control group	BP	GO:0010165	response to X-ray	2/125	0.0060518	0.01914293	0.00996838	Anxa1/Ercc1
1-day post-SCI group vs. the control group	BP	GO:0010839	negative regulation of keratinocyte proliferation	2/125	0.0060518	0.01914293	0.00996838	Cask/Ctsl
1-day post-SCI group vs. the control group	BP	GO:0014829	vascular associated smooth muscle contraction	2/125	0.0060518	0.01914293	0.00996838	Edn1/Slc8a1
1-day post-SCI group vs. the control group	BP	GO:0031100	animal organ regeneration	2/125	0.0060518	0.01914293	0.00996838	Gfer/Hmox1
1-day post-SCI group vs. the control group	BP	GO:0031338	regulation of vesicle fusion	2/125	0.0060518	0.01914293	0.00996838	Anxa1/Sphk1
1-day post-SCI group vs. the control group	BP	GO:0034368	protein-lipid complex remodeling	2/125	0.0060518	0.01914293	0.00996838	Apoe/Lcat
1-day post-SCI group vs. the control group	BP	GO:0034369	plasma lipoprotein particle remodeling	2/125	0.0060518	0.01914293	0.00996838	Apoe/Lcat
1-day post-SCI group vs. the control group	BP	GO:0045723	positive regulation of fatty acid biosynthetic process	2/125	0.0060518	0.01914293	0.00996838	Anxa1/Ptgs2
1-day post-SCI group vs. the control group	BP	GO:0045980	negative regulation of nucleotide metabolic process	2/125	0.0060518	0.01914293	0.00996838	Ier3/Ppargc1a
1-day post-SCI group vs. the control group	BP	GO:0070168	negative regulation of biomineral tissue development	2/125	0.0060518	0.01914293	0.00996838	Ccr1/Nos3
1-day post-SCI group vs. the control group	BP	GO:0070920	regulation of production of small RNA involved in ger	2/125	0.0060518	0.01914293	0.00996838	Ppp3ca/Ripk1
1-day post-SCI group vs. the control group	BP	GO:0060541	respiratory system development	5/125	0.0061505	0.01943755	0.0101218	Fbxw7/Nos3/Ppp1ca/Rpl13a/Spry2
1-day post-SCI group vs. the control group	BP	GO:0030856	regulation of epithelial cell differentiation	4/125	0.0061598	0.01944948	0.01012801	Cdkn1c/Spry2/Tnfrsf1a/Xdh
1-day post-SCI group vs. the control group	BP	GO:0003151	outflow tract morphogenesis	3/125	0.0062006	0.01954272	0.01017657	Atf2/Edn1/Jun
1-day post-SCI group vs. the control group	BP	GO:0036465	synaptic vesicle recycling	3/125	0.0062006	0.01954272	0.01017657	Amph/Itsn1/Syp
1-day post-SCI group vs. the control group	BP	GO:0071383	cellular response to steroid hormone stimulus	4/125	0.0062885	0.01980191	0.01031153	Anxa1/Hdac1/Jak2/Ppargc1b
1-day post-SCI group vs. the control group	BP	GO:0061178	regulation of insulin secretion involved in cellular resp	3/125	0.006402	0.02012604	0.01048032	Dynll1/Fkbp1b/Ucp2
1-day post-SCI group vs. the control group	BP	GO:0046486	glycerolipid metabolic process	6/125	0.006403	0.02012604	0.01048032	Apoe/Capn2/Cat/Gpx1/Il6st/Lcat
1-day post-SCI group vs. the control group	BP	GO:0010950	positive regulation of endopeptidase activity	4/125	0.0064189	0.02015801	0.01049697	Bax/Jak2/Myc/Xdh
1-day post-SCI group vs. the control group	BP	GO:0007176	regulation of epidermal growth factor-activated recept	2/125	0.006499	0.02029988	0.01057084	Fbxw7/Hbegf
1-day post-SCI group vs. the control group	BP	GO:0032740	positive regulation of interleukin-17 production	2/125	0.006499	0.02029988	0.01057084	Jak2/Sphk1
1-day post-SCI group vs. the control group	BP	GO:0045822	negative regulation of heart contraction	2/125	0.006499	0.02029988	0.01057084	Fkbp1b/Jak2
1-day post-SCI group vs. the control group	BP	GO:0048820	hair follicle maturation	2/125	0.006499	0.02029988	0.01057084	Ctsl/Ptgs2
1-day post-SCI group vs. the control group	BP	GO:0051894	positive regulation of focal adhesion assembly	2/125	0.006499	0.02029988	0.01057084	Map4k4/Tsc1
1-day post-SCI group vs. the control group	BP	GO:0110150	negative regulation of biomineralization	2/125	0.006499	0.02029988	0.01057084	Ccr1/Nos3
1-day post-SCI group vs. the control group	BP	GO:0042770	signal transduction in response to DNA damage	4/125	0.0065511	0.02040786	0.01062707	Atf2/Ier3/Mapk14/Mdm2

1-day post-SCI group vs. the control group	BP	GO:0051250	negative regulation of lymphocyte activation	4/125	0.0065511	0.02040786	0.01062707	Anxa1/Axl/Btk/Hspb1
1-day post-SCI group vs. the control group	BP	GO:0061025	membrane fusion	4/125	0.0065511	0.02040786	0.01062707	Anxa1/Atp13a2/Ctsl/Sphk1
1-day post-SCI group vs. the control group	BP	GO:2000736	regulation of stem cell differentiation	3/125	0.0066074	0.02056478	0.01070879	Hdac1/Rbbp7/Slc4a11
1-day post-SCI group vs. the control group	BP	GO:0045786	negative regulation of cell cycle	6/125	0.0066435	0.02065869	0.01075769	Atf2/Cdkn1c/Ier3/Mapk14/Mdm2/Ptgs2
1-day post-SCI group vs. the control group	BP	GO:0034764	positive regulation of transmembrane transport	5/125	0.0067406	0.02090498	0.01088594	Bax/Edn1/Mapk14/Met/Ppp3ca
1-day post-SCI group vs. the control group	BP	GO:0048588	developmental cell growth	5/125	0.0067406	0.02090498	0.01088594	Apoe/Cttn/Edn1/Map2k4/Mapt
1-day post-SCI group vs. the control group	BP	GO:0090092	regulation of transmembrane receptor protein serine/th	5/125	0.0067406	0.02090498	0.01088594	Cdkn1c/Hdac1/Jak2/Rbbp7/Spry2
1-day post-SCI group vs. the control group	BP	GO:0040014	regulation of multicellular organism growth	3/125	0.0068166	0.02112176	0.01099882	Atrn/Cdk4/Ezr
1-day post-SCI group vs. the control group	BP	GO:0051258	protein polymerization	5/125	0.0068427	0.02118391	0.01103119	Cttn/Gpx4/Mapk8/Mapt/Met
1-day post-SCI group vs. the control group	BP	GO:0009141	nucleoside triphosphate metabolic process	5/125	0.0069459	0.02139752	0.01114242	Ier3/Myc/Ndufa6/Nme2/Ppargc1a
1-day post-SCI group vs. the control group	BP	GO:0001936	regulation of endothelial cell proliferation	4/125	0.0069585	0.02139752	0.01114242	Apoe/Hmox1/Jun/Xdh
1-day post-SCI group vs. the control group	BP	GO:0001780	neutrophil homeostasis	2/125	0.0069608	0.02139752	0.01114242	Anxa1/Axl
1-day post-SCI group vs. the control group	BP	GO:0032770	positive regulation of monooxygenase activity	2/125	0.0069608	0.02139752	0.01114242	Apoe/Gch1
1-day post-SCI group vs. the control group	BP	GO:0034367	protein-containing complex remodeling	2/125	0.0069608	0.02139752	0.01114242	Apoe/Lcat
1-day post-SCI group vs. the control group	BP	GO:0042044	fluid transport	2/125	0.0069608	0.02139752	0.01114242	Edn1/Slc4a11
1-day post-SCI group vs. the control group	BP	GO:0042659	regulation of cell fate specification	2/125	0.0069608	0.02139752	0.01114242	Hdac1/Rbbp7
1-day post-SCI group vs. the control group	BP	GO:1903649	regulation of cytoplasmic transport	2/125	0.0069608	0.02139752	0.01114242	Ezr/Mapk8
1-day post-SCI group vs. the control group	BP	GO:0002088	lens development in camera-type eye	3/125	0.0070297	0.0215904	0.01124286	Atf4/Cdkn1c/Spry2
1-day post-SCI group vs. the control group	BP	GO:0060560	developmental growth involved in morphogenesis	5/125	0.0072619	0.02228385	0.01160397	Apoe/Cttn/Edn1/Mapt/Spry2
1-day post-SCI group vs. the control group	BP	GO:0040029	epigenetic regulation of gene expression	4/125	0.0073824	0.02263365	0.01178612	Cdkn1c/Eed/Gpx1/Hdac1
1-day post-SCI group vs. the control group	BP	GO:0001963	synaptic transmission, dopaminergic	2/125	0.007437	0.0226421	0.01179052	Pink1/Ptgs2
1-day post-SCI group vs. the control group	BP	GO:0010800	positive regulation of peptidyl-threonine phosphorylati	2/125	0.007437	0.0226421	0.01179052	Met/Sphk1
1-day post-SCI group vs. the control group	BP	GO:0034311	diol metabolic process	2/125	0.007437	0.0226421	0.01179052	Gch1/Sphk1
1-day post-SCI group vs. the control group	BP	GO:0071353	cellular response to interleukin-4	2/125	0.007437	0.0226421	0.01179052	Cdk4/Stat6
1-day post-SCI group vs. the control group	BP	GO:0090200	positive regulation of release of cytochrome c from mi	2/125	0.007437	0.0226421	0.01179052	Bax/Pink1
1-day post-SCI group vs. the control group	BP	GO:0150077	regulation of neuroinflammatory response	2/125	0.007437	0.0226421	0.01179052	Ptgs2/Sphk1
1-day post-SCI group vs. the control group	BP	GO:1902042	negative regulation of extrinsic apoptotic signaling pat	2/125	0.007437	0.0226421	0.01179052	Gpx1/Hmox1
1-day post-SCI group vs. the control group	BP	GO:1903523	negative regulation of blood circulation	2/125	0.007437	0.0226421	0.01179052	Fkbp1b/Jak2
1-day post-SCI group vs. the control group	BP	GO:0006096	glycolytic process	3/125	0.0074678	0.02267625	0.0118083	Ier3/Myc/Ppargc1a
1-day post-SCI group vs. the control group	BP	GO:0031110	regulation of microtubule polymerization or depolyme	3/125	0.0074678	0.02267625	0.0118083	Mapk8/Mapt/Met
1-day post-SCI group vs. the control group	BP	GO:0045638	negative regulation of myeloid cell differentiation	3/125	0.0074678	0.02267625	0.0118083	Fbxw7/Myc/Nme2
1-day post-SCI group vs. the control group	BP	GO:0030902	hindbrain development	4/125	0.0075274	0.02283733	0.01189218	Atf2/Atrn/Gpr37l1/Smad1
1-day post-SCI group vs. the control group	BP	GO:0006757	ATP generation from ADP	3/125	0.0076927	0.02325603	0.01211021	Ier3/Myc/Ppargc1a
1-day post-SCI group vs. the control group	BP	GO:0030512	negative regulation of transforming growth factor beta	3/125	0.0076927	0.02325603	0.01211021	Hdac1/Rbbp7/Spry2
1-day post-SCI group vs. the control group	BP	GO:0043367	CD4-positive, alpha-beta T cell differentiation	3/125	0.0076927	0.02325603	0.01211021	Anxa1/Ctsl/Stat6
1-day post-SCI group vs. the control group	BP	GO:0048814	regulation of dendrite morphogenesis	3/125	0.0076927	0.02325603	0.01211021	Cask/Met/Ppp3ca
1-day post-SCI group vs. the control group	BP	GO:0048863	stem cell differentiation	5/125	0.0076987	0.02325603	0.01211021	Edn1/Hdac1/Mapk14/Rbbp7/Slc4a11
1-day post-SCI group vs. the control group	BP	GO:0010952	positive regulation of peptidase activity	4/125	0.0078229	0.02361086	0.01229499	Bax/Jak2/Myc/Xdh
1-day post-SCI group vs. the control group	BP	GO:0042475	odontogenesis of dentin-containing tooth	3/125	0.0079216	0.02366082	0.012321	Atf2/Bax/Hdac1
1-day post-SCI group vs. the control group	BP	GO:0002026	regulation of the force of heart contraction	2/125	0.0079276	0.02366082	0.012321	Nos3/Slc8a1

1-day post-SCI group vs. the control group	BP	GO:0003085	negative regulation of systemic arterial blood pressure	2/125	0.0079276	0.02366082	0.012321	Gpr37l1/Ier3
1-day post-SCI group vs. the control group	BP	GO:0009162	deoxyribonucleoside monophosphate metabolic proces	2/125	0.0079276	0.02366082	0.012321	Nme2/Xdh
1-day post-SCI group vs. the control group	BP	GO:0010460	positive regulation of heart rate	2/125	0.0079276	0.02366082	0.012321	Edn1/Gch1
1-day post-SCI group vs. the control group	BP	GO:0010623	programmed cell death involved in cell development	2/125	0.0079276	0.02366082	0.012321	Bax/Ybx3
1-day post-SCI group vs. the control group	BP	GO:0035094	response to nicotine	2/125	0.0079276	0.02366082	0.012321	Hmox1/Rela
1-day post-SCI group vs. the control group	BP	GO:0035455	response to interferon-alpha	2/125	0.0079276	0.02366082	0.012321	Axl/Myc
1-day post-SCI group vs. the control group	BP	GO:0035767	endothelial cell chemotaxis	2/125	0.0079276	0.02366082	0.012321	Hspb1/Met
1-day post-SCI group vs. the control group	BP	GO:0043276	anoikis	2/125	0.0079276	0.02366082	0.012321	Itga5/Mcl1
1-day post-SCI group vs. the control group	BP	GO:0045454	cell redox homeostasis	2/125	0.0079276	0.02366082	0.012321	Gpx1/Txnrd1
1-day post-SCI group vs. the control group	BP	GO:0060384	innervation	2/125	0.0079276	0.02366082	0.012321	Edn1/Vcam1
1-day post-SCI group vs. the control group	BP	GO:0060561	apoptotic process involved in morphogenesis	2/125	0.0079276	0.02366082	0.012321	Bax/Tnfrsf1a
1-day post-SCI group vs. the control group	BP	GO:0030168	platelet activation	3/125	0.0081545	0.02431719	0.0126628	Apoe/Axl/Jak2
1-day post-SCI group vs. the control group	BP	GO:0032651	regulation of interleukin-1 beta production	3/125	0.0083914	0.02490336	0.01296804	Hspb1/Jak2/Sphk1
1-day post-SCI group vs. the control group	BP	GO:2000117	negative regulation of cysteine-type endopeptidase acti	3/125	0.0083914	0.02490336	0.01296804	Gpx1/Mdm2/Ptgs2
1-day post-SCI group vs. the control group	BP	GO:0001893	maternal placenta development	2/125	0.0084324	0.02490336	0.01296804	Ctsl/Ptgs2
1-day post-SCI group vs. the control group	BP	GO:0002360	T cell lineage commitment	2/125	0.0084324	0.02490336	0.01296804	Ctsl/Stat6
1-day post-SCI group vs. the control group	BP	GO:0010259	multicellular organism aging	2/125	0.0084324	0.02490336	0.01296804	Edn1/Ercc1
1-day post-SCI group vs. the control group	BP	GO:0030224	monocyte differentiation	2/125	0.0084324	0.02490336	0.01296804	Jun/Myc
1-day post-SCI group vs. the control group	BP	GO:0034260	negative regulation of GTPase activity	2/125	0.0084324	0.02490336	0.01296804	Spry2/Tsc1
1-day post-SCI group vs. the control group	BP	GO:0045742	positive regulation of epidermal growth factor receptor	2/125	0.0084324	0.02490336	0.01296804	Fbxw7/Hbegf
1-day post-SCI group vs. the control group	BP	GO:0070296	sarcoplasmic reticulum calcium ion transport	2/125	0.0084324	0.02490336	0.01296804	Fkbp1b/Slc8a1
1-day post-SCI group vs. the control group	BP	GO:0070633	transepithelial transport	2/125	0.0084324	0.02490336	0.01296804	Abcc1/Edn1
1-day post-SCI group vs. the control group	BP	GO:0070670	response to interleukin-4	2/125	0.0084324	0.02490336	0.01296804	Cdk4/Stat6
1-day post-SCI group vs. the control group	BP	GO:0071674	mononuclear cell migration	4/125	0.0084367	0.02490336	0.01296804	Anxa1/Ccr1/Ripk3/Trpm2
1-day post-SCI group vs. the control group	BP	GO:1901605	alpha-amino acid metabolic process	4/125	0.008595	0.025349	0.01320009	Atf4/Nos3/Prodh/Txnrd1
1-day post-SCI group vs. the control group	BP	GO:0035773	insulin secretion involved in cellular response to gluco	3/125	0.0086323	0.02543772	0.01324629	Dynll1/Fkbp1b/Ucp2
1-day post-SCI group vs. the control group	BP	GO:0048813	dendrite morphogenesis	4/125	0.0087551	0.0257778	0.01342339	Cask/Mapk8/Met/Ppp3ca
1-day post-SCI group vs. the control group	BP	GO:0032091	negative regulation of protein binding	3/125	0.0088773	0.02606922	0.01357514	Bax/Mapk8/Myc
1-day post-SCI group vs. the control group	BP	GO:1901222	regulation of NIK/NF-kappaB signaling	3/125	0.0088773	0.02606922	0.01357514	Edn1/Rela/Sphk1
1-day post-SCI group vs. the control group	BP	GO:1901992	positive regulation of mitotic cell cycle phase transitio	3/125	0.0088773	0.02606922	0.01357514	Anxa1/Cdk4/Mdm2
1-day post-SCI group vs. the control group	BP	GO:0007612	learning	4/125	0.0089173	0.02606922	0.01357514	Amph/Jun/Ptgs2/Tsc1
1-day post-SCI group vs. the control group	BP	GO:0009060	aerobic respiration	4/125	0.0089173	0.02606922	0.01357514	Cat/Myc/Ndufa6/Pink1
1-day post-SCI group vs. the control group	BP	GO:0051260	protein homooligomerization	4/125	0.0089173	0.02606922	0.01357514	Mapt/Pxdn/Rnf112/Trpm2
1-day post-SCI group vs. the control group	BP	GO:0002828	regulation of type 2 immune response	2/125	0.0089513	0.02606922	0.01357514	Anxa1/Stat6
1-day post-SCI group vs. the control group	BP	GO:0050999	regulation of nitric-oxide synthase activity	2/125	0.0089513	0.02606922	0.01357514	Apoe/Gch1
1-day post-SCI group vs. the control group	BP	GO:0060669	embryonic placenta morphogenesis	2/125	0.0089513	0.02606922	0.01357514	Cdkn1c/Vcam1
1-day post-SCI group vs. the control group	BP	GO:0150117	positive regulation of cell-substrate junction organizati	2/125	0.0089513	0.02606922	0.01357514	Map4k4/Tsc1
1-day post-SCI group vs. the control group	BP	GO:1902253	regulation of intrinsic apoptotic signaling pathway by p	2/125	0.0089513	0.02606922	0.01357514	Mdm2/Myc
1-day post-SCI group vs. the control group	BP	GO:1904646	cellular response to amyloid-beta	2/125	0.0089513	0.02606922	0.01357514	Abcc1/Vcam1
1-day post-SCI group vs. the control group	BP	GO:1905314	semi-lunar valve development	2/125	0.0089513	0.02606922	0.01357514	Nos3/Tnfrsf1a

1-day post-SCI group vs. the control group	BP	GO:0032412	regulation of ion transmembrane transporter activity	5/125	0.0089937	0.026171	0.01362814	Edn1/Fkbp1b/Ppargc1a/Ppp3ca/Ripk1
1-day post-SCI group vs. the control group	BP	GO:0000075	cell cycle checkpoint signaling	4/125	0.0090813	0.02640391	0.01374942	Atf2/Ier3/Mapk14/Mdm2
1-day post-SCI group vs. the control group	BP	GO:0051492	regulation of stress fiber assembly	3/125	0.0091263	0.02651262	0.01380603	Met/Pxn/Tsc1
1-day post-SCI group vs. the control group	BP	GO:1904062	regulation of cation transmembrane transport	6/125	0.0093183	0.02704799	0.01408482	Bax/Edn1/Fkbp1b/Ppargc1a/Ppp3ca/Slc8a1
1-day post-SCI group vs. the control group	BP	GO:0016575	histone deacetylation	3/125	0.0093794	0.02720248	0.01416526	Hdac1/Pink1/Rbbp7
1-day post-SCI group vs. the control group	BP	GO:0060402	calcium ion transport into cytosol	2/125	0.009484	0.02741492	0.01427589	Bax/Slc8a1
1-day post-SCI group vs. the control group	BP	GO:0090322	regulation of superoxide metabolic process	2/125	0.009484	0.02741492	0.01427589	Gch1/Mapt
1-day post-SCI group vs. the control group	BP	GO:0099563	modification of synaptic structure	2/125	0.009484	0.02741492	0.01427589	Cttn/Itsn1
1-day post-SCI group vs. the control group	BP	GO:1901186	positive regulation of ERBB signaling pathway	2/125	0.009484	0.02741492	0.01427589	Fbxw7/Hbegf
1-day post-SCI group vs. the control group	BP	GO:0032970	regulation of actin filament-based process	6/125	0.0095275	0.02751761	0.01432937	Cttn/Edn1/Met/Pxn/Trpm2/Tsc1
1-day post-SCI group vs. the control group	BP	GO:0001935	endothelial cell proliferation	4/125	0.0095853	0.02763887	0.01439251	Apoe/Hmox1/Jun/Xdh
1-day post-SCI group vs. the control group	BP	GO:0017148	negative regulation of translation	4/125	0.0095853	0.02763887	0.01439251	Ago4/Eif2s1/Rpl13a/Tsc1
1-day post-SCI group vs. the control group	BP	GO:0014066	regulation of phosphatidylinositol 3-kinase signaling	3/125	0.0096365	0.02771781	0.01443362	Cat/Jak2/Prr5l
1-day post-SCI group vs. the control group	BP	GO:0032370	positive regulation of lipid transport	3/125	0.0096365	0.02771781	0.01443362	Apoe/Edn1/Map2k6
1-day post-SCI group vs. the control group	BP	GO:0046031	ADP metabolic process	3/125	0.0096365	0.02771781	0.01443362	Ier3/Myc/Ppargc1a
1-day post-SCI group vs. the control group	BP	GO:0071695	anatomical structure maturation	5/125	0.0097601	0.02805012	0.01460666	Btk/Cdkn1c/Ctsl/Edn1/Ptgs2
1-day post-SCI group vs. the control group	BP	GO:0060070	canonical Wnt signaling pathway	5/125	0.009892	0.02837559	0.01477614	Apoe/Edn1/Hdac1/Mapk14/Sdc1
1-day post-SCI group vs. the control group	BP	GO:0032611	interleukin-1 beta production	3/125	0.0098977	0.02837559	0.01477614	Hspb1/Jak2/Sphk1
1-day post-SCI group vs. the control group	BP	GO:0097061	dendritic spine organization	3/125	0.0098977	0.02837559	0.01477614	Apoe/Cask/Cttn
1-day post-SCI group vs. the control group	BP	GO:0002719	negative regulation of cytokine production involved in	2/125	0.0100306	0.02868597	0.01493777	Axl/Hmox1
1-day post-SCI group vs. the control group	BP	GO:0061436	establishment of skin barrier	2/125	0.0100306	0.02868597	0.01493777	Krt1/Met
1-day post-SCI group vs. the control group	BP	GO:0097345	mitochondrial outer membrane permeabilization	2/125	0.0100306	0.02868597	0.01493777	Bax/Ier3
1-day post-SCI group vs. the control group	BP	GO:0044262	cellular carbohydrate metabolic process	5/125	0.0101596	0.02896958	0.01508546	Ier3/Il6st/Pdk2/Ppargc1a/Ppp1ca
1-day post-SCI group vs. the control group	BP	GO:0010770	positive regulation of cell morphogenesis involved in c	3/125	0.010163	0.02896958	0.01508546	Cask/Met/P4hb
1-day post-SCI group vs. the control group	BP	GO:0046330	positive regulation of JNK cascade	3/125	0.010163	0.02896958	0.01508546	Map2k4/Map4k4/Ripk1
1-day post-SCI group vs. the control group	BP	GO:0062014	negative regulation of small molecule metabolic proces	3/125	0.010163	0.02896958	0.01508546	Apoe/Ier3/Ppargc1a
1-day post-SCI group vs. the control group	BP	GO:0022898	regulation of transmembrane transporter activity	5/125	0.0102952	0.02932254	0.01526925	Edn1/Fkbp1b/Ppargc1a/Ppp3ca/Ripk1
1-day post-SCI group vs. the control group	BP	GO:0060485	mesenchyme development	5/125	0.0104321	0.02961658	0.01542237	Edn1/Mdm2/Myc/Nos3/Spry2
1-day post-SCI group vs. the control group	BP	GO:0042116	macrophage activation	3/125	0.0104324	0.02961658	0.01542237	Jak2/Jun/Sphk1
1-day post-SCI group vs. the control group	BP	GO:0046718	viral entry into host cell	3/125	0.0104324	0.02961658	0.01542237	Axl/Ctsl/P4hb
1-day post-SCI group vs. the control group	BP	GO:0090288	negative regulation of cellular response to growth facto	3/125	0.0104324	0.02961658	0.01542237	Cask/Spry2/Xdh
1-day post-SCI group vs. the control group	BP	GO:2001257	regulation of cation channel activity	4/125	0.010465	0.02968499	0.015458	Edn1/Fkbp1b/Ppargc1a/Ppp3ca
1-day post-SCI group vs. the control group	BP	GO:0002902	regulation of B cell apoptotic process	2/125	0.0105909	0.02987202	0.01555538	Bax/Myc
1-day post-SCI group vs. the control group	BP	GO:0032816	positive regulation of natural killer cell activation	2/125	0.0105909	0.02987202	0.01555538	Axl/Jak2
1-day post-SCI group vs. the control group	BP	GO:0042168	heme metabolic process	2/125	0.0105909	0.02987202	0.01555538	Hmox1/Hmox2
1-day post-SCI group vs. the control group	BP	GO:0048710	regulation of astrocyte differentiation	2/125	0.0105909	0.02987202	0.01555538	Gpr37l1/Il6st
1-day post-SCI group vs. the control group	BP	GO:0090183	regulation of kidney development	2/125	0.0105909	0.02987202	0.01555538	Myc/Ppp3ca
1-day post-SCI group vs. the control group	BP	GO:0090312	positive regulation of protein deacetylation	2/125	0.0105909	0.02987202	0.01555538	Pink1/Ripk3
1-day post-SCI group vs. the control group	BP	GO:1903715	regulation of aerobic respiration	2/125	0.0105909	0.02987202	0.01555538	Myc/Pink1
1-day post-SCI group vs. the control group	BP	GO:0009135	purine nucleoside diphosphate metabolic process	3/125	0.010706	0.03014784	0.01569902	Ier3/Myc/Ppargc1a

1-day post-SCI group vs. the control group	BP	GO:0009179	purine ribonucleoside diphosphate metabolic process	3/125	0.010706	0.03014784	0.01569902	Ier3/Myc/Ppargc1a
1-day post-SCI group vs. the control group	BP	GO:0098739	import across plasma membrane	4/125	0.0108311	0.03047555	0.01586966	Abcc1/Ppp3ca/Slc8a1/Trpm2
1-day post-SCI group vs. the control group	BP	GO:0050851	antigen receptor-mediated signaling pathway	5/125	0.0108502	0.03050485	0.01588492	Bax/Btk/Ezr/Plekha1/Rela
1-day post-SCI group vs. the control group	BP	GO:0001704	formation of primary germ layer	3/125	0.0109837	0.03080545	0.01604146	Itga5/Smad1/Txnrd1
1-day post-SCI group vs. the control group	BP	GO:0006754	ATP biosynthetic process	3/125	0.0109837	0.03080545	0.01604146	Myc/Ndufa6/Ppargc1a
1-day post-SCI group vs. the control group	BP	GO:2000060	positive regulation of ubiquitin-dependent protein cata	3/125	0.0109837	0.03080545	0.01604146	Fbxw7/Mapk8/Mdm2
1-day post-SCI group vs. the control group	BP	GO:0140014	mitotic nuclear division	5/125	0.0111353	0.03111324	0.01620173	Banf1/Cdkn1c/Edn1/Met/Sphk1
1-day post-SCI group vs. the control group	BP	GO:0003203	endocardial cushion morphogenesis	2/125	0.0111647	0.03111324	0.01620173	Mdm2/Nos3
1-day post-SCI group vs. the control group	BP	GO:0016540	protein autoprocessing	2/125	0.0111647	0.03111324	0.01620173	Capn2/Ctsl
1-day post-SCI group vs. the control group	BP	GO:0035329	hippo signaling	2/125	0.0111647	0.03111324	0.01620173	Map2k3/Mapk14
1-day post-SCI group vs. the control group	BP	GO:0045920	negative regulation of exocytosis	2/125	0.0111647	0.03111324	0.01620173	Anxa1/Hmox1
1-day post-SCI group vs. the control group	BP	GO:0046677	response to antibiotic	2/125	0.0111647	0.03111324	0.01620173	Jak2/Mdm2
1-day post-SCI group vs. the control group	BP	GO:1902003	regulation of amyloid-beta formation	2/125	0.0111647	0.03111324	0.01620173	Apoe/Rela
1-day post-SCI group vs. the control group	BP	GO:2000191	regulation of fatty acid transport	2/125	0.0111647	0.03111324	0.01620173	Edn1/Map2k6
1-day post-SCI group vs. the control group	BP	GO:0015931	nucleobase-containing compound transport	4/125	0.0112053	0.03117651	0.01623468	Mapt/Ripk1/Slc25a24/Tsc1
1-day post-SCI group vs. the control group	BP	GO:1990138	neuron projection extension	4/125	0.0112053	0.03117651	0.01623468	Apoe/Cttn/Edn1/Mapt
1-day post-SCI group vs. the control group	BP	GO:0001657	ureteric bud development	3/125	0.0112655	0.03126909	0.01628289	Cat/Myc/Smad1
1-day post-SCI group vs. the control group	BP	GO:0009953	dorsal/ventral pattern formation	3/125	0.0112655	0.03126909	0.01628289	Bmp1/Edn1/Mapk8
1-day post-SCI group vs. the control group	BP	GO:0021761	limbic system development	3/125	0.0112655	0.03126909	0.01628289	Bax/Hdac1/Tsc1
1-day post-SCI group vs. the control group	BP	GO:1903169	regulation of calcium ion transmembrane transport	4/125	0.0113955	0.03160489	0.01645775	Bax/Fkbp1b/Ppp3ca/Slc8a1
1-day post-SCI group vs. the control group	BP	GO:0044409	entry into host	3/125	0.0115514	0.03201191	0.0166697	Axl/Ctsl/P4hb
1-day post-SCI group vs. the control group	BP	GO:0030336	negative regulation of cell migration	5/125	0.0115724	0.03204446	0.01668665	Apoe/Cyp1b1/Hdac1/Ppargc1a/Rbbp7
1-day post-SCI group vs. the control group	BP	GO:0007162	negative regulation of cell adhesion	5/125	0.0117206	0.03242926	0.01688703	Anxa1/Cask/Cyp1b1/Hspb1/Jak2
1-day post-SCI group vs. the control group	BP	GO:0010737	protein kinase A signaling	2/125	0.011752	0.03246456	0.01690541	Edn1/Ezr
1-day post-SCI group vs. the control group	BP	GO:1903580	positive regulation of ATP metabolic process	2/125	0.011752	0.03246456	0.01690541	Myc/Ppargc1a
1-day post-SCI group vs. the control group	BP	GO:0006575	cellular modified amino acid metabolic process	4/125	0.0117821	0.03249632	0.01692195	Ctsl/Gch1/Gpx1/Gpx4
1-day post-SCI group vs. the control group	BP	GO:0046578	regulation of Ras protein signal transduction	4/125	0.0117821	0.03249632	0.01692195	Apoe/Map4k4/Met/Spry2
1-day post-SCI group vs. the control group	BP	GO:0046700	heterocycle catabolic process	6/125	0.0118066	0.0325381	0.01694371	Ago4/Bax/Hmox1/Hmox2/Prr5l/Xdh
1-day post-SCI group vs. the control group	BP	GO:0030516	regulation of axon extension	3/125	0.0118416	0.03258302	0.0169671	Apoe/Cttn/Mapt
1-day post-SCI group vs. the control group	BP	GO:0042310	vasoconstriction	3/125	0.0118416	0.03258302	0.0169671	Edn1/Ptgs2/Slc8a1
1-day post-SCI group vs. the control group	BP	GO:0071902	positive regulation of protein serine/threonine kinase a	4/125	0.0119786	0.03293396	0.01714984	Adam9/Edn1/Map2k4/Map2k6
1-day post-SCI group vs. the control group	BP	GO:0014074	response to purine-containing compound	3/125	0.0121359	0.03326166	0.01732049	Ezr/Slc8a1/Trpm2
1-day post-SCI group vs. the control group	BP	GO:0072163	mesonephric epithelium development	3/125	0.0121359	0.03326166	0.01732049	Cat/Myc/Smad1
1-day post-SCI group vs. the control group	BP	GO:0072164	mesonephric tubule development	3/125	0.0121359	0.03326166	0.01732049	Cat/Myc/Smad1
1-day post-SCI group vs. the control group	BP	GO:1901800	positive regulation of proteasomal protein catabolic pr	3/125	0.0121359	0.03326166	0.01732049	Fbxw7/Mapk8/Mdm2
1-day post-SCI group vs. the control group	BP	GO:0022411	cellular component disassembly	6/125	0.0121789	0.03335348	0.01736831	Bax/Cttn/Fbxw7/Map4k4/Myc/Pink1
1-day post-SCI group vs. the control group	BP	GO:0032409	regulation of transporter activity	5/125	0.0123266	0.03367041	0.01753334	Edn1/Fkbp1b/Ppargc1a/Ppp3ca/Ripk1
1-day post-SCI group vs. the control group	BP	GO:0008210	estrogen metabolic process	2/125	0.0123526	0.03367041	0.01753334	Cyp1b1/Plekha1
1-day post-SCI group vs. the control group	BP	GO:0018208	peptidyl-proline modification	2/125	0.0123526	0.03367041	0.01753334	Fkbp1b/P4hb
1-day post-SCI group vs. the control group	BP	GO:0048265	response to pain	2/125	0.0123526	0.03367041	0.01753334	Capn2/Gch1

1-day post-SCI group vs. the control group	BP	GO:0071320	cellular response to cAMP	2/125	0.0123526	0.03367041	0.01753334	Ezr/Slc8a1
1-day post-SCI group vs. the control group	BP	GO:1904645	response to amyloid-beta	2/125	0.0123526	0.03367041	0.01753334	Abcc1/Vcam1
1-day post-SCI group vs. the control group	BP	GO:0032271	regulation of protein polymerization	4/125	0.0123777	0.03371258	0.0175553	Cttn/Mapk8/Mapt/Met
1-day post-SCI group vs. the control group	BP	GO:0030518	intracellular steroid hormone receptor signaling pathwa	3/125	0.0124344	0.03381412	0.01760817	Hdac1/Jak2/Ppargc1b
1-day post-SCI group vs. the control group	BP	GO:0106027	neuron projection organization	3/125	0.0124344	0.03381412	0.01760817	Apoe/Cask/Cttn
1-day post-SCI group vs. the control group	BP	GO:0044270	cellular nitrogen compound catabolic process	6/125	0.0126882	0.03447749	0.01795361	Ago4/Bax/Hmox1/Hmox2/Prr5l/Xdh
1-day post-SCI group vs. the control group	BP	GO:0019827	stem cell population maintenance	4/125	0.0127853	0.03471428	0.01807692	Hdac1/Mapk8/Myc/Rbbp7
1-day post-SCI group vs. the control group	BP	GO:0043267	negative regulation of potassium ion transport	2/125	0.0129663	0.03512383	0.01829018	Atf4/Nos3
1-day post-SCI group vs. the control group	BP	GO:1901020	negative regulation of calcium ion transmembrane tran	2/125	0.0129663	0.03512383	0.01829018	Fkbp1b/Ppp3ca
1-day post-SCI group vs. the control group	BP	GO:1903670	regulation of sprouting angiogenesis	2/125	0.0129663	0.03512383	0.01829018	Itga5/Smad1
1-day post-SCI group vs. the control group	BP	GO:0072330	monocarboxylic acid biosynthetic process	4/125	0.0129922	0.03516676	0.01831254	Anxa1/Edn1/Ptgs2/Sphk1
1-day post-SCI group vs. the control group	BP	GO:0001823	mesonephros development	3/125	0.0130439	0.03525194	0.0183569	Cat/Myc/Smad1
1-day post-SCI group vs. the control group	BP	GO:0043473	pigmentation	3/125	0.0130439	0.03525194	0.0183569	Atrn/Bax/Myc
1-day post-SCI group vs. the control group	BP	GO:0010721	negative regulation of cell development	4/125	0.0134126	0.0362202	0.0188611	Ctnna1/Fbxw7/Gpr37l1/Ppp3ca
1-day post-SCI group vs. the control group	BP	GO:0010613	positive regulation of cardiac muscle hypertrophy	2/125	0.0135931	0.03659446	0.01905599	Edn1/Ppp3ca
1-day post-SCI group vs. the control group	BP	GO:0033561	regulation of water loss via skin	2/125	0.0135931	0.03659446	0.01905599	Krt1/Met
1-day post-SCI group vs. the control group	BP	GO:0043368	positive T cell selection	2/125	0.0135931	0.03659446	0.01905599	Ctsl/Stat6
1-day post-SCI group vs. the control group	BP	GO:0071827	plasma lipoprotein particle organization	2/125	0.0135931	0.03659446	0.01905599	Apoe/Lcat
1-day post-SCI group vs. the control group	BP	GO:0007088	regulation of mitotic nuclear division	3/125	0.0136703	0.03674554	0.01913466	Edn1/Met/Sphk1
1-day post-SCI group vs. the control group	BP	GO:0032231	regulation of actin filament bundle assembly	3/125	0.0136703	0.03674554	0.01913466	Met/Pxn/Tsc1
1-day post-SCI group vs. the control group	BP	GO:0016055	Wnt signaling pathway	6/125	0.0137513	0.03693483	0.01923323	Apoe/Edn1/Hdac1/Mapk14/Myc/Sdc1
1-day post-SCI group vs. the control group	BP	GO:0098727	maintenance of cell number	4/125	0.0138415	0.03714844	0.01934447	Hdac1/Mapk8/Myc/Rbbp7
1-day post-SCI group vs. the control group	BP	GO:0007589	body fluid secretion	3/125	0.0139898	0.0375177	0.01953675	Edn1/Ppp3ca/Xdh
1-day post-SCI group vs. the control group	BP	GO:0198738	cell-cell signaling by wnt	6/125	0.0140265	0.03758727	0.01957298	Apoe/Edn1/Hdac1/Mapk14/Myc/Sdc1
1-day post-SCI group vs. the control group	BP	GO:0002366	leukocyte activation involved in immune response	5/125	0.014102	0.03776057	0.01966323	Anxa1/Ercc1/Hmox1/Stat6/Tsc1
1-day post-SCI group vs. the control group	BP	GO:0014742	positive regulation of muscle hypertrophy	2/125	0.0142329	0.03805248	0.01981523	Edn1/Ppp3ca
1-day post-SCI group vs. the control group	BP	GO:0018149	peptide cross-linking	2/125	0.0142329	0.03805248	0.01981523	Anxa1/Krt1
1-day post-SCI group vs. the control group	BP	GO:0009185	ribonucleoside diphosphate metabolic process	3/125	0.0143136	0.03823897	0.01991234	Ier3/Myc/Ppargc1a
1-day post-SCI group vs. the control group	BP	GO:0001892	embryonic placenta development	3/125	0.0146416	0.03899576	0.02030643	Cdkn1c/Slc8a1/Vcam1
1-day post-SCI group vs. the control group	BP	GO:0002832	negative regulation of response to biotic stimulus	3/125	0.0146416	0.03899576	0.02030643	Banf1/Ccr1/Gfer
1-day post-SCI group vs. the control group	BP	GO:0038061	NIK/NF-kappaB signaling	3/125	0.0146416	0.03899576	0.02030643	Edn1/Rela/Sphk1
1-day post-SCI group vs. the control group	BP	GO:0043401	steroid hormone mediated signaling pathway	3/125	0.0146416	0.03899576	0.02030643	Hdac1/Jak2/Ppargc1b
1-day post-SCI group vs. the control group	BP	GO:0006006	glucose metabolic process	4/125	0.0147253	0.03914549	0.0203844	Atf4/Mapk14/Myc/Pdk2
1-day post-SCI group vs. the control group	BP	GO:0098742	cell-cell adhesion via plasma-membrane adhesion mole	4/125	0.0147253	0.03914549	0.0203844	Cdh11/Itga5/Mapk14/Vcam1
1-day post-SCI group vs. the control group	BP	GO:0019439	aromatic compound catabolic process	6/125	0.0147315	0.03914549	0.0203844	Ago4/Bax/Hmox1/Hmox2/Prr5l/Xdh
1-day post-SCI group vs. the control group	BP	GO:0002263	cell activation involved in immune response	5/125	0.0147882	0.03926632	0.02044732	Anxa1/Ercc1/Hmox1/Stat6/Tsc1
1-day post-SCI group vs. the control group	BP	GO:0006778	porphyrin-containing compound metabolic process	2/125	0.0148854	0.03928537	0.02045724	Hmox1/Hmox2
1-day post-SCI group vs. the control group	BP	GO:0010453	regulation of cell fate commitment	2/125	0.0148854	0.03928537	0.02045724	Hdac1/Rbbp7
1-day post-SCI group vs. the control group	BP	GO:0032735	positive regulation of interleukin-12 production	2/125	0.0148854	0.03928537	0.02045724	Mapk14/Rela
1-day post-SCI group vs. the control group	BP	GO:0042092	type 2 immune response	2/125	0.0148854	0.03928537	0.02045724	Anxa1/Stat6

1-day post-SCI group vs. the control group	BP	GO:0045601	regulation of endothelial cell differentiation	2/125	0.0148854	0.03928537	0.02045724	Tnfrsf1a/Xdh
1-day post-SCI group vs. the control group	BP	GO:0045823	positive regulation of heart contraction	2/125	0.0148854	0.03928537	0.02045724	Edn1/Gch1
1-day post-SCI group vs. the control group	BP	GO:0048873	homeostasis of number of cells within a tissue	2/125	0.0148854	0.03928537	0.02045724	Bax/Nos3
1-day post-SCI group vs. the control group	BP	GO:0070884	regulation of calcineurin-NFAT signaling cascade	2/125	0.0148854	0.03928537	0.02045724	Ppp3ca/Rcan1
1-day post-SCI group vs. the control group	BP	GO:0030522	intracellular receptor signaling pathway	4/125	0.0149517	0.03939939	0.02051662	Hdac1/Jak2/Ppargc1b/Rela
1-day post-SCI group vs. the control group	BP	GO:0006906	vesicle fusion	3/125	0.0149738	0.03939939	0.02051662	Anxa1/Atp13a2/Sphk1
1-day post-SCI group vs. the control group	BP	GO:0045445	myoblast differentiation	3/125	0.0149738	0.03939939	0.02051662	Gpx1/Mapk14/Sdc1
1-day post-SCI group vs. the control group	BP	GO:1901989	positive regulation of cell cycle phase transition	3/125	0.0149738	0.03939939	0.02051662	Anxa1/Cdk4/Mdm2
1-day post-SCI group vs. the control group	BP	GO:0002062	chondrocyte differentiation	3/125	0.0153103	0.04022408	0.02094606	Atf2/Mapk14/Rela
1-day post-SCI group vs. the control group	BP	GO:0006413	translational initiation	3/125	0.0153103	0.04022408	0.02094606	Eif2s1/Ppp1r15b/Rpl13a
1-day post-SCI group vs. the control group	BP	GO:0048147	negative regulation of fibroblast proliferation	2/125	0.0155507	0.04073297	0.02121106	Bax/Myc
1-day post-SCI group vs. the control group	BP	GO:0060964	regulation of miRNA-mediated gene silencing	2/125	0.0155507	0.04073297	0.02121106	Ppp3ca/Ripk1
1-day post-SCI group vs. the control group	BP	GO:0106056	regulation of calcineurin-mediated signaling	2/125	0.0155507	0.04073297	0.02121106	Ppp3ca/Rcan1
1-day post-SCI group vs. the control group	BP	GO:1902991	regulation of amyloid precursor protein catabolic proc	2/125	0.0155507	0.04073297	0.02121106	Apoe/Rela
1-day post-SCI group vs. the control group	BP	GO:0002697	regulation of immune effector process	6/125	0.0156095	0.04084248	0.02126808	Anxa1/Axl/Gfer/Hmox1/Ripk3/Stat6
1-day post-SCI group vs. the control group	BP	GO:0030258	lipid modification	4/125	0.015644	0.04084248	0.02126808	Apoe/Lcat/Mapk14/Ppargc1a
1-day post-SCI group vs. the control group	BP	GO:0043393	regulation of protein binding	4/125	0.015644	0.04084248	0.02126808	Apoe/Bax/Mapk8/Myc
1-day post-SCI group vs. the control group	BP	GO:0010769	regulation of cell morphogenesis involved in differenti	3/125	0.015651	0.04084248	0.02126808	Cask/Met/P4hb
1-day post-SCI group vs. the control group	BP	GO:0090174	organelle membrane fusion	3/125	0.015651	0.04084248	0.02126808	Anxa1/Atp13a2/Sphk1
1-day post-SCI group vs. the control group	BP	GO:0002285	lymphocyte activation involved in immune response	4/125	0.0158792	0.04140698	0.02156204	Anxa1/Ercc1/Stat6/Tsc1
1-day post-SCI group vs. the control group	BP	GO:0046928	regulation of neurotransmitter secretion	3/125	0.015996	0.04161826	0.02167206	Cask/Sphk1/Syp
1-day post-SCI group vs. the control group	BP	GO:0098659	inorganic cation import across plasma membrane	3/125	0.015996	0.04161826	0.02167206	Ppp3ca/Slc8a1/Trpm2
1-day post-SCI group vs. the control group	BP	GO:0099587	inorganic ion import across plasma membrane	3/125	0.015996	0.04161826	0.02167206	Ppp3ca/Slc8a1/Trpm2
1-day post-SCI group vs. the control group	BP	GO:0002822	regulation of adaptive immune response based on soma	4/125	0.0161166	0.04187962	0.02180816	Anxa1/Jak2/Ripk3/Stat6
1-day post-SCI group vs. the control group	BP	GO:0003298	physiological muscle hypertrophy	2/125	0.0162285	0.04187962	0.02180816	Edn1/Map2k4
1-day post-SCI group vs. the control group	BP	GO:0003301	physiological cardiac muscle hypertrophy	2/125	0.0162285	0.04187962	0.02180816	Edn1/Map2k4
1-day post-SCI group vs. the control group	BP	GO:0006509	membrane protein ectodomain proteolysis	2/125	0.0162285	0.04187962	0.02180816	Adam9/Apoe
1-day post-SCI group vs. the control group	BP	GO:0032660	regulation of interleukin-17 production	2/125	0.0162285	0.04187962	0.02180816	Jak2/Sphk1
1-day post-SCI group vs. the control group	BP	GO:0042554	superoxide anion generation	2/125	0.0162285	0.04187962	0.02180816	Edn1/Mapt
1-day post-SCI group vs. the control group	BP	GO:0045940	positive regulation of steroid metabolic process	2/125	0.0162285	0.04187962	0.02180816	Apoe/Ppargc1a
1-day post-SCI group vs. the control group	BP	GO:0046006	regulation of activated T cell proliferation	2/125	0.0162285	0.04187962	0.02180816	Ppp3ca/Ripk3
1-day post-SCI group vs. the control group	BP	GO:0061049	cell growth involved in cardiac muscle cell developme	2/125	0.0162285	0.04187962	0.02180816	Edn1/Map2k4
1-day post-SCI group vs. the control group	BP	GO:0061098	positive regulation of protein tyrosine kinase activity	2/125	0.0162285	0.04187962	0.02180816	Fbxw7/Hbegf
1-day post-SCI group vs. the control group	BP	GO:0071825	protein-lipid complex subunit organization	2/125	0.0162285	0.04187962	0.02180816	Apoe/Lcat
1-day post-SCI group vs. the control group	BP	GO:1902414	protein localization to cell junction	3/125	0.0163453	0.04211585	0.02193117	Hspb1/Mapk8/Mapt
1-day post-SCI group vs. the control group	BP	GO:1903008	organelle disassembly	3/125	0.0163453	0.04211585	0.02193117	Cttn/Fbxw7/Pink1
1-day post-SCI group vs. the control group	BP	GO:0046034	ATP metabolic process	4/125	0.0163563	0.04211585	0.02193117	Ier3/Myc/Ndufa6/Ppargc1a
1-day post-SCI group vs. the control group	BP	GO:1901990	regulation of mitotic cell cycle phase transition	5/125	0.0166007	0.04271382	0.02224255	Anxa1/Cdk4/Cdkn1c/Ier3/Mdm2
1-day post-SCI group vs. the control group	BP	GO:0002286	T cell activation involved in immune response	3/125	0.0166988	0.04283979	0.02230815	Anxa1/Stat6/Tsc1
1-day post-SCI group vs. the control group	BP	GO:0046620	regulation of organ growth	3/125	0.0166988	0.04283979	0.02230815	Edn1/Mapk14/Ybx3

1-day post-SCI group vs. the control group	BP	GO:0061387	regulation of extent of cell growth	3/125	0.0166988	0.04283979	0.02230815	Apoe/Cttn/Mapt
1-day post-SCI group vs. the control group	BP	GO:1905954	positive regulation of lipid localization	3/125	0.0166988	0.04283979	0.02230815	Apoe/Edn1/Map2k6
1-day post-SCI group vs. the control group	BP	GO:0001783	B cell apoptotic process	2/125	0.0169188	0.04324515	0.02251924	Bax/Myc
1-day post-SCI group vs. the control group	BP	GO:0032768	regulation of monooxygenase activity	2/125	0.0169188	0.04324515	0.02251924	Apoe/Gch1
1-day post-SCI group vs. the control group	BP	GO:0055023	positive regulation of cardiac muscle tissue growth	2/125	0.0169188	0.04324515	0.02251924	Edn1/Mapk14
1-day post-SCI group vs. the control group	BP	GO:0060147	regulation of post-transcriptional gene silencing	2/125	0.0169188	0.04324515	0.02251924	Ppp3ca/Ripk1
1-day post-SCI group vs. the control group	BP	GO:1900368	regulation of post-transcriptional gene silencing by RN	2/125	0.0169188	0.04324515	0.02251924	Ppp3ca/Ripk1
1-day post-SCI group vs. the control group	BP	GO:0007265	Ras protein signal transduction	5/125	0.0169802	0.04337032	0.02258442	Apoe/Jun/Map4k4/Met/Spry2
1-day post-SCI group vs. the control group	BP	GO:0015748	organophosphate ester transport	3/125	0.0170566	0.043375	0.02258685	Abcc1/Apoe/Slc25a24
1-day post-SCI group vs. the control group	BP	GO:0031109	microtubule polymerization or depolymerization	3/125	0.0170566	0.043375	0.02258685	Mapk8/Mapt/Met
1-day post-SCI group vs. the control group	BP	GO:0031398	positive regulation of protein ubiquitination	3/125	0.0170566	0.043375	0.02258685	Fbxw7/Mapk8/Sphk1
1-day post-SCI group vs. the control group	BP	GO:0032649	regulation of interferon-gamma production	3/125	0.0170566	0.043375	0.02258685	Axl/Jak2/Ripk3
1-day post-SCI group vs. the control group	BP	GO:0035710	CD4-positive, alpha-beta T cell activation	3/125	0.0170566	0.043375	0.02258685	Anxa1/Ctsl/Stat6
1-day post-SCI group vs. the control group	BP	GO:2000177	regulation of neural precursor cell proliferation	3/125	0.0170566	0.043375	0.02258685	Ctnna1/Gpr37l1/Mapk8
1-day post-SCI group vs. the control group	BP	GO:1903052	positive regulation of proteolysis involved in protein ca	3/125	0.0174187	0.04426351	0.02304953	Fbxw7/Mapk8/Mdm2
1-day post-SCI group vs. the control group	BP	GO:0001990	regulation of systemic arterial blood pressure by hormo	2/125	0.0176214	0.04439065	0.02311574	Edn1/Nos3
1-day post-SCI group vs. the control group	BP	GO:0003179	heart valve morphogenesis	2/125	0.0176214	0.04439065	0.02311574	Mdm2/Nos3
1-day post-SCI group vs. the control group	BP	GO:0009394	2'-deoxyribonucleotide metabolic process	2/125	0.0176214	0.04439065	0.02311574	Nme2/Xdh
1-day post-SCI group vs. the control group	BP	GO:0019692	deoxyribose phosphate metabolic process	2/125	0.0176214	0.04439065	0.02311574	Nme2/Xdh
1-day post-SCI group vs. the control group	BP	GO:0034205	amyloid-beta formation	2/125	0.0176214	0.04439065	0.02311574	Apoe/Rela
1-day post-SCI group vs. the control group	BP	GO:0042789	mRNA transcription by RNA polymerase II	2/125	0.0176214	0.04439065	0.02311574	Atf2/Atf4
1-day post-SCI group vs. the control group	BP	GO:0043277	apoptotic cell clearance	2/125	0.0176214	0.04439065	0.02311574	Anxa1/Axl
1-day post-SCI group vs. the control group	BP	GO:0043403	skeletal muscle tissue regeneration	2/125	0.0176214	0.04439065	0.02311574	Anxa1/Gpx1
1-day post-SCI group vs. the control group	BP	GO:0060135	maternal process involved in female pregnancy	2/125	0.0176214	0.04439065	0.02311574	Ctsl/Ptgs2
1-day post-SCI group vs. the control group	BP	GO:0120163	negative regulation of cold-induced thermogenesis	2/125	0.0176214	0.04439065	0.02311574	Atf4/Map2k6
1-day post-SCI group vs. the control group	BP	GO:1900087	positive regulation of G1/S transition of mitotic cell cy	2/125	0.0176214	0.04439065	0.02311574	Anxa1/Mdm2
1-day post-SCI group vs. the control group	BP	GO:1903524	positive regulation of blood circulation	2/125	0.0176214	0.04439065	0.02311574	Edn1/Gch1
1-day post-SCI group vs. the control group	BP	GO:0044000	movement in host	3/125	0.0177851	0.0447706	0.02331359	Axl/Ctsl/P4hb
1-day post-SCI group vs. the control group	BP	GO:0000280	nuclear division	6/125	0.0177984	0.04477178	0.0233142	Ago4/Banf1/Cdkn1c/Edn1/Met/Sphk1
1-day post-SCI group vs. the control group	BP	GO:0099504	synaptic vesicle cycle	4/125	0.0178411	0.044847	0.02335337	Amph/Cask/Itsn1/Syp
1-day post-SCI group vs. the control group	BP	GO:0002429	immune response-activating cell surface receptor signa	5/125	0.017954	0.04509817	0.02348416	Bax/Btk/Ezr/Plekha1/Rela
1-day post-SCI group vs. the control group	BP	GO:0002757	immune response-activating signal transduction	5/125	0.0181531	0.04550665	0.02369688	Bax/Btk/Ezr/Plekha1/Rela
1-day post-SCI group vs. the control group	BP	GO:0001952	regulation of cell-matrix adhesion	3/125	0.0181557	0.04550665	0.02369688	Cask/Map4k4/Tsc1
1-day post-SCI group vs. the control group	BP	GO:0043123	positive regulation of I-kappaB kinase/NF-kappaB sign	3/125	0.0181557	0.04550665	0.02369688	Pink1/Rela/Ripk1
1-day post-SCI group vs. the control group	BP	GO:0002701	negative regulation of production of molecular mediato	2/125	0.0183362	0.04572917	0.02381275	Axl/Hmox1
1-day post-SCI group vs. the control group	BP	GO:0032620	interleukin-17 production	2/125	0.0183362	0.04572917	0.02381275	Jak2/Sphk1
1-day post-SCI group vs. the control group	BP	GO:0042304	regulation of fatty acid biosynthetic process	2/125	0.0183362	0.04572917	0.02381275	Anxa1/Ptgs2
1-day post-SCI group vs. the control group	BP	GO:0043124	negative regulation of I-kappaB kinase/NF-kappaB sig	2/125	0.0183362	0.04572917	0.02381275	Hdac1/Ripk1
1-day post-SCI group vs. the control group	BP	GO:0048010	vascular endothelial growth factor receptor signaling p	2/125	0.0183362	0.04572917	0.02381275	Hspb1/Mapk14
1-day post-SCI group vs. the control group	BP	GO:0050856	regulation of T cell receptor signaling pathway	2/125	0.0183362	0.04572917	0.02381275	Ezr/Rela

1-day post-SCI group vs. the control group	BP	GO:0071385	cellular response to glucocorticoid stimulus	2/125	0.0183362	0.04572917	0.02381275	Anxa1/Jak2
1-day post-SCI group vs. the control group	BP	GO:0008286	insulin receptor signaling pathway	3/125	0.0185306	0.04614805	0.02403088	Cdk4/Pdk2/Rela
1-day post-SCI group vs. the control group	BP	GO:0060996	dendritic spine development	3/125	0.0185306	0.04614805	0.02403088	Apoe/Cask/Itsn1
1-day post-SCI group vs. the control group	BP	GO:0002685	regulation of leukocyte migration	4/125	0.0188762	0.04697514	0.02446157	Anxa1/Ccr1/Edn1/Ripk3
1-day post-SCI group vs. the control group	BP	GO:0009262	deoxyribonucleotide metabolic process	2/125	0.0190632	0.04703776	0.02449418	Nme2/Xdh
1-day post-SCI group vs. the control group	BP	GO:0010543	regulation of platelet activation	2/125	0.0190632	0.04703776	0.02449418	Apoe/Jak2
1-day post-SCI group vs. the control group	BP	GO:0010677	negative regulation of cellular carbohydrate metabolic	2/125	0.0190632	0.04703776	0.02449418	Ier3/Ppargc1a
1-day post-SCI group vs. the control group	BP	GO:0010762	regulation of fibroblast migration	2/125	0.0190632	0.04703776	0.02449418	Prr5l/Slc8a1
1-day post-SCI group vs. the control group	BP	GO:0010837	regulation of keratinocyte proliferation	2/125	0.0190632	0.04703776	0.02449418	Cask/Ctsl
1-day post-SCI group vs. the control group	BP	GO:0033173	calcineurin-NFAT signaling cascade	2/125	0.0190632	0.04703776	0.02449418	Ppp3ca/Rcan1
1-day post-SCI group vs. the control group	BP	GO:0033628	regulation of cell adhesion mediated by integrin	2/125	0.0190632	0.04703776	0.02449418	Adam9/Cyp1b1
1-day post-SCI group vs. the control group	BP	GO:0044848	biological phase	2/125	0.0190632	0.04703776	0.02449418	Ctsl/Ptgs2
1-day post-SCI group vs. the control group	BP	GO:0060421	positive regulation of heart growth	2/125	0.0190632	0.04703776	0.02449418	Edn1/Mapk14
1-day post-SCI group vs. the control group	BP	GO:0060966	regulation of gene silencing by RNA	2/125	0.0190632	0.04703776	0.02449418	Ppp3ca/Ripk1
1-day post-SCI group vs. the control group	BP	GO:0071526	semaphorin-plexin signaling pathway	2/125	0.0190632	0.04703776	0.02449418	Edn1/Met
1-day post-SCI group vs. the control group	BP	GO:1902459	positive regulation of stem cell population maintenanc	2/125	0.0190632	0.04703776	0.02449418	Hdac1/Rbbp7
1-day post-SCI group vs. the control group	BP	GO:0002460	adaptive immune response based on somatic recombin	6/125	0.0191396	0.04716237	0.02455906	Anxa1/Btk/Ercc1/Jak2/Ripk3/Stat6
1-day post-SCI group vs. the control group	BP	GO:0007163	establishment or maintenance of cell polarity	4/125	0.0191407	0.04716237	0.02455906	Ctnna1/Ezr/Pdlim1/Spry2
1-day post-SCI group vs. the control group	BP	GO:0021700	developmental maturation	5/125	0.0191705	0.04720235	0.02457988	Btk/Cdkn1c/Ctsl/Edn1/Ptgs2
1-day post-SCI group vs. the control group	BP	GO:0031330	negative regulation of cellular catabolic process	4/125	0.0194075	0.04771858	0.02484871	Hmox1/Mcl1/Pink1/Tsc1
1-day post-SCI group vs. the control group	BP	GO:0051056	regulation of small GTPase mediated signal transducti	4/125	0.0194075	0.04771858	0.02484871	Apoe/Map4k4/Met/Spry2
1-day post-SCI group vs. the control group	BP	GO:0003197	endocardial cushion development	2/125	0.0198021	0.04848385	0.02524721	Mdm2/Nos3
1-day post-SCI group vs. the control group	BP	GO:0007157	heterophilic cell-cell adhesion via plasma membrane c	2/125	0.0198021	0.04848385	0.02524721	Itga5/Vcam1
1-day post-SCI group vs. the control group	BP	GO:0030574	collagen catabolic process	2/125	0.0198021	0.04848385	0.02524721	Ctsl/Mmp3
1-day post-SCI group vs. the control group	BP	GO:0042088	T-helper 1 type immune response	2/125	0.0198021	0.04848385	0.02524721	Anxa1/Stat6
1-day post-SCI group vs. the control group	BP	GO:0045429	positive regulation of nitric oxide biosynthetic process	2/125	0.0198021	0.04848385	0.02524721	Jak2/Ptgs2
1-day post-SCI group vs. the control group	BP	GO:0048806	genitalia development	2/125	0.0198021	0.04848385	0.02524721	Axl/Bax
1-day post-SCI group vs. the control group	BP	GO:0035195	miRNA-mediated gene silencing	3/125	0.0200734	0.04911355	0.02557511	Ago4/Ppp3ca/Ripk1
1-day post-SCI group vs. the control group	BP	GO:0002819	regulation of adaptive immune response	4/125	0.0202216	0.04938012	0.02571392	Anxa1/Jak2/Ripk3/Stat6
1-day post-SCI group vs. the control group	BP	GO:0042445	hormone metabolic process	4/125	0.0202216	0.04938012	0.02571392	Ctsl/Cyp1b1/Plekha1/Ppargc1a
1-day post-SCI group vs. the control group	BP	GO:0002768	immune response-regulating cell surface receptor signa	5/125	0.0202248	0.04938012	0.02571392	Bax/Btk/Ezr/Plekha1/Rela
1-day post-SCI group vs. the control group	BP	GO:0022602	ovulation cycle process	2/125	0.0205529	0.05004117	0.02605816	Nos3/Plekha1
1-day post-SCI group vs. the control group	BP	GO:0043029	T cell homeostasis	2/125	0.0205529	0.05004117	0.02605816	Bax/Ripk3
1-day post-SCI group vs. the control group	BP	GO:1902041	regulation of extrinsic apoptotic signaling pathway via	2/125	0.0205529	0.05004117	0.02605816	Gpx1/Hmox1
1-day post-SCI group vs. the control group	BP	GO:1902692	regulation of neuroblast proliferation	2/125	0.0205529	0.05004117	0.02605816	Ctnna1/Mapk8
1-day post-SCI group vs. the control group	BP	GO:0042180	cellular ketone metabolic process	4/125	0.0207759	0.05051357	0.02630415	Anxa1/Pdk2/Ppargc1a/Ptgs2
1-day post-SCI group vs. the control group	BP	GO:0045930	negative regulation of mitotic cell cycle	4/125	0.0207759	0.05051357	0.02630415	Atf2/Cdkn1c/Ier3/Mdm2
1-day post-SCI group vs. the control group	BP	GO:0090090	negative regulation of canonical Wnt signaling pathwa	3/125	0.0208705	0.05070835	0.02640558	Apoe/Hdac1/Mapk14
1-day post-SCI group vs. the control group	BP	GO:0019083	viral transcription	2/125	0.0213154	0.05153813	0.02683767	Hdac1/Jun
1-day post-SCI group vs. the control group	BP	GO:0045581	negative regulation of T cell differentiation	2/125	0.0213154	0.05153813	0.02683767	Anxa1/Hspb1

1-day post-SCI group vs. the control group	BP	GO:0045773	positive regulation of axon extension	2/125	0.0213154	0.05153813	0.02683767	Apoe/Mapt
1-day post-SCI group vs. the control group	BP	GO:0061028	establishment of endothelial barrier	2/125	0.0213154	0.05153813	0.02683767	Ezr/Tnfrsf1a
1-day post-SCI group vs. the control group	BP	GO:0071384	cellular response to corticosteroid stimulus	2/125	0.0213154	0.05153813	0.02683767	Anxa1/Jak2
1-day post-SCI group vs. the control group	BP	GO:1904407	positive regulation of nitric oxide metabolic process	2/125	0.0213154	0.05153813	0.02683767	Jak2/Ptgs2
1-day post-SCI group vs. the control group	BP	GO:2001258	negative regulation of cation channel activity	2/125	0.0213154	0.05153813	0.02683767	Fkbp1b/Ppp3ca
1-day post-SCI group vs. the control group	BP	GO:0097529	myeloid leukocyte migration	4/125	0.0213394	0.05156041	0.02684928	Anxa1/Ccr1/Edn1/Rpl13a
1-day post-SCI group vs. the control group	BP	GO:0050773	regulation of dendrite development	3/125	0.0216849	0.05235882	0.02726504	Cask/Met/Ppp3ca
1-day post-SCI group vs. the control group	BP	GO:0007520	myoblast fusion	2/125	0.0220896	0.05302729	0.02761313	Capn2/Mapk14
1-day post-SCI group vs. the control group	BP	GO:0009299	mRNA transcription	2/125	0.0220896	0.05302729	0.02761313	Atf2/Atf4
1-day post-SCI group vs. the control group	BP	GO:0010828	positive regulation of glucose transmembrane transpor	2/125	0.0220896	0.05302729	0.02761313	Mapk14/Met
1-day post-SCI group vs. the control group	BP	GO:0033013	tetrapyrrole metabolic process	2/125	0.0220896	0.05302729	0.02761313	Hmox1/Hmox2
1-day post-SCI group vs. the control group	BP	GO:0050798	activated T cell proliferation	2/125	0.0220896	0.05302729	0.02761313	Ppp3ca/Ripk3
1-day post-SCI group vs. the control group	BP	GO:0061756	leukocyte adhesion to vascular endothelial cell	2/125	0.0220896	0.05302729	0.02761313	Rela/Vcam1
1-day post-SCI group vs. the control group	BP	GO:0097720	calcineurin-mediated signaling	2/125	0.0220896	0.05302729	0.02761313	Ppp3ca/Rcan1
1-day post-SCI group vs. the control group	BP	GO:0008277	regulation of G protein-coupled receptor signaling patl	3/125	0.0220985	0.05302729	0.02761313	Edn1/Met/Syp
1-day post-SCI group vs. the control group	BP	GO:0032609	interferon-gamma production	3/125	0.0220985	0.05302729	0.02761313	Axl/Jak2/Ripk3
1-day post-SCI group vs. the control group	BP	GO:0034341	response to interferon-gamma	3/125	0.0225164	0.05391894	0.02807744	Gch1/Jak2/Rpl13a
1-day post-SCI group vs. the control group	BP	GO:0034754	cellular hormone metabolic process	3/125	0.0225164	0.05391894	0.02807744	Cyp1b1/Plekha1/Ppargc1a
1-day post-SCI group vs. the control group	BP	GO:0051588	regulation of neurotransmitter transport	3/125	0.0225164	0.05391894	0.02807744	Cask/Sphk1/Syp
1-day post-SCI group vs. the control group	BP	GO:0002831	regulation of response to biotic stimulus	5/125	0.0226764	0.05426472	0.0282575	Apoe/Banf1/Ccr1/Gfer/Nono
1-day post-SCI group vs. the control group	BP	GO:0045912	negative regulation of carbohydrate metabolic process	2/125	0.0228753	0.05470324	0.02848586	Ier3/Ppargc1a
1-day post-SCI group vs. the control group	BP	GO:0006119	oxidative phosphorylation	3/125	0.0229387	0.05477968	0.02852566	Myc/Ndufa6/Pink1
1-day post-SCI group vs. the control group	BP	GO:0030278	regulation of ossification	3/125	0.0229387	0.05477968	0.02852566	Ccr1/Mapk14/Slc8a1
1-day post-SCI group vs. the control group	BP	GO:0016236	macroautophagy	4/125	0.0230861	0.05509407	0.02868937	Atp13a2/Hmox1/Pink1/Tsc1
1-day post-SCI group vs. the control group	BP	GO:0035194	post-transcriptional gene silencing by RNA	3/125	0.0233653	0.05568399	0.02899657	Ago4/Ppp3ca/Ripk1
1-day post-SCI group vs. the control group	BP	GO:0046165	alcohol biosynthetic process	3/125	0.0233653	0.05568399	0.02899657	Apoe/Gch1/Sphk1
1-day post-SCI group vs. the control group	BP	GO:0098657	import into cell	4/125	0.0233855	0.05569414	0.02900185	Abcc1/Ppp3ca/Slc8a1/Trpm2
1-day post-SCI group vs. the control group	BP	GO:0032355	response to estradiol	2/125	0.0236725	0.05614768	0.02923802	Anxa1/Nos3
1-day post-SCI group vs. the control group	BP	GO:0032814	regulation of natural killer cell activation	2/125	0.0236725	0.05614768	0.02923802	Axl/Jak2
1-day post-SCI group vs. the control group	BP	GO:0032892	positive regulation of organic acid transport	2/125	0.0236725	0.05614768	0.02923802	Edn1/Map2k6
1-day post-SCI group vs. the control group	BP	GO:0046580	negative regulation of Ras protein signal transduction	2/125	0.0236725	0.05614768	0.02923802	Met/Spry2
1-day post-SCI group vs. the control group	BP	GO:0098926	postsynaptic signal transduction	2/125	0.0236725	0.05614768	0.02923802	Jak2/Rela
1-day post-SCI group vs. the control group	BP	GO:2000378	negative regulation of reactive oxygen species metabol	2/125	0.0236725	0.05614768	0.02923802	Mmp3/Pink1
1-day post-SCI group vs. the control group	BP	GO:0045727	positive regulation of translation	3/125	0.0237961	0.0564026	0.02937077	Cdk4/Cyp1b1/Pink1
1-day post-SCI group vs. the control group	BP	GO:0035270	endocrine system development	3/125	0.0242313	0.05739505	0.02988758	Anxa1/Cdkn1c/Edn1
1-day post-SCI group vs. the control group	BP	GO:0019318	hexose metabolic process	4/125	0.0242976	0.0575132	0.0299491	Atf4/Mapk14/Myc/Pdk2
1-day post-SCI group vs. the control group	BP	GO:0048167	regulation of synaptic plasticity	5/125	0.0243328	0.0575575	0.02997217	Apoe/Atf4/Mapt/Ptgs2/Syp
1-day post-SCI group vs. the control group	BP	GO:0003044	regulation of systemic arterial blood pressure mediatec	2/125	0.0244809	0.05771208	0.03005266	Edn1/Nos3
1-day post-SCI group vs. the control group	BP	GO:0043507	positive regulation of JUN kinase activity	2/125	0.0244809	0.05771208	0.03005266	Edn1/Map2k4
1-day post-SCI group vs. the control group	BP	GO:0046633	alpha-beta T cell proliferation	2/125	0.0244809	0.05771208	0.03005266	Jak2/Myc

1-day post-SCI group vs. the control group	BP	GO:0070169	positive regulation of biomineral tissue development	2/125	0.0244809	0.05771208	0.03005266	Atf4/Slc8a1
1-day post-SCI group vs. the control group	BP	GO:1901861	regulation of muscle tissue development	2/125	0.0244809	0.05771208	0.03005266	Ppargc1a/Ybx3
1-day post-SCI group vs. the control group	BP	GO:0000723	telomere maintenance	3/125	0.0246707	0.05808111	0.03024483	Ercc1/Myc/Pcna
1-day post-SCI group vs. the control group	BP	GO:0042471	ear morphogenesis	3/125	0.0246707	0.05808111	0.03024483	Edn1/Myc/Spry2
1-day post-SCI group vs. the control group	BP	GO:0008203	cholesterol metabolic process	3/125	0.0251145	0.05908599	0.0307681	Apoe/Cat/Lcat
1-day post-SCI group vs. the control group	BP	GO:0030857	negative regulation of epithelial cell differentiation	2/125	0.0253005	0.05928379	0.03087111	Spry2/Xdh
1-day post-SCI group vs. the control group	BP	GO:0031648	protein destabilization	2/125	0.0253005	0.05928379	0.03087111	Fbxw7/Mdm2
1-day post-SCI group vs. the control group	BP	GO:0051055	negative regulation of lipid biosynthetic process	2/125	0.0253005	0.05928379	0.03087111	Apoe/Sphk1
1-day post-SCI group vs. the control group	BP	GO:0110151	positive regulation of biomineralization	2/125	0.0253005	0.05928379	0.03087111	Atf4/Slc8a1
1-day post-SCI group vs. the control group	BP	GO:1903170	negative regulation of calcium ion transmembrane tran	2/125	0.0253005	0.05928379	0.03087111	Fkbp1b/Ppp3ca
1-day post-SCI group vs. the control group	BP	GO:1903533	regulation of protein targeting	2/125	0.0253005	0.05928379	0.03087111	Fbxw7/Pink1
1-day post-SCI group vs. the control group	BP	GO:0007093	mitotic cell cycle checkpoint signaling	3/125	0.0260149	0.06079446	0.03165776	Atf2/Ier3/Mdm2
1-day post-SCI group vs. the control group	BP	GO:0051783	regulation of nuclear division	3/125	0.0260149	0.06079446	0.03165776	Edn1/Met/Sphk1
1-day post-SCI group vs. the control group	BP	GO:0090101	negative regulation of transmembrane receptor protein	3/125	0.0260149	0.06079446	0.03165776	Hdac1/Rbbp7/Spry2
1-day post-SCI group vs. the control group	BP	GO:1903322	positive regulation of protein modification by small pr	3/125	0.0260149	0.06079446	0.03165776	Fbxw7/Mapk8/Sphk1
1-day post-SCI group vs. the control group	BP	GO:0032757	positive regulation of interleukin-8 production	2/125	0.0261313	0.06086244	0.03169316	Rela/Ripk1
1-day post-SCI group vs. the control group	BP	GO:0033619	membrane protein proteolysis	2/125	0.0261313	0.06086244	0.03169316	Adam9/Apoe
1-day post-SCI group vs. the control group	BP	GO:0045058	T cell selection	2/125	0.0261313	0.06086244	0.03169316	Ctsl/Stat6
1-day post-SCI group vs. the control group	BP	GO:0051155	positive regulation of striated muscle cell differentiatic	2/125	0.0261313	0.06086244	0.03169316	Edn1/Mapk14
1-day post-SCI group vs. the control group	BP	GO:0051496	positive regulation of stress fiber assembly	2/125	0.0261313	0.06086244	0.03169316	Pxn/Tsc1
1-day post-SCI group vs. the control group	BP	GO:0006520	cellular amino acid metabolic process	4/125	0.0265091	0.06161912	0.03208719	Atf4/Nos3/Prodh/Txnrd1
1-day post-SCI group vs. the control group	BP	GO:0099003	vesicle-mediated transport in synapse	4/125	0.0265091	0.06161912	0.03208719	Amph/Cask/Itsn1/Syp
1-day post-SCI group vs. the control group	BP	GO:1901988	negative regulation of cell cycle phase transition	4/125	0.0265091	0.06161912	0.03208719	Atf2/Ier3/Mapk14/Mdm2
1-day post-SCI group vs. the control group	BP	GO:0016441	post-transcriptional gene silencing	3/125	0.0269326	0.06224114	0.0324111	Ago4/Ppp3ca/Ripk1
1-day post-SCI group vs. the control group	BP	GO:0032200	telomere organization	3/125	0.0269326	0.06224114	0.0324111	Ercc1/Myc/Pcna
1-day post-SCI group vs. the control group	BP	GO:0034763	negative regulation of transmembrane transport	3/125	0.0269326	0.06224114	0.0324111	Fkbp1b/Myc/Ppp3ca
1-day post-SCI group vs. the control group	BP	GO:0002204	somatic recombination of immunoglobulin genes invol	2/125	0.0269729	0.06224114	0.0324111	Ercc1/Stat6
1-day post-SCI group vs. the control group	BP	GO:0002208	somatic diversification of immunoglobulins involved i	2/125	0.0269729	0.06224114	0.0324111	Ercc1/Stat6
1-day post-SCI group vs. the control group	BP	GO:0043388	positive regulation of DNA binding	2/125	0.0269729	0.06224114	0.0324111	Jak2/Myc
1-day post-SCI group vs. the control group	BP	GO:0043616	keratinocyte proliferation	2/125	0.0269729	0.06224114	0.0324111	Cask/Ctsl
1-day post-SCI group vs. the control group	BP	GO:0045190	isotype switching	2/125	0.0269729	0.06224114	0.0324111	Ercc1/Stat6
1-day post-SCI group vs. the control group	BP	GO:0050775	positive regulation of dendrite morphogenesis	2/125	0.0269729	0.06224114	0.0324111	Cask/Met
1-day post-SCI group vs. the control group	BP	GO:0070509	calcium ion import	2/125	0.0269729	0.06224114	0.0324111	Cask/Slc8a1
1-day post-SCI group vs. the control group	BP	GO:1902808	positive regulation of cell cycle G1/S phase transition	2/125	0.0269729	0.06224114	0.0324111	Anxa1/Mdm2
1-day post-SCI group vs. the control group	BP	GO:0046328	regulation of JNK cascade	3/125	0.0273978	0.06317975	0.03289987	Map2k4/Map4k4/Ripk1
1-day post-SCI group vs. the control group	BP	GO:0050807	regulation of synapse organization	4/125	0.0278254	0.06391255	0.03328146	Apoe/Cask/Itsn1/Mapk14
1-day post-SCI group vs. the control group	BP	GO:0002548	monocyte chemotaxis	2/125	0.0278255	0.06391255	0.03328146	Anxa1/Ccr1
1-day post-SCI group vs. the control group	BP	GO:0030199	collagen fibril organization	2/125	0.0278255	0.06391255	0.03328146	Cyp1b1/Pxdn
1-day post-SCI group vs. the control group	BP	GO:0051591	response to cAMP	2/125	0.0278255	0.06391255	0.03328146	Ezr/Slc8a1
1-day post-SCI group vs. the control group	BP	GO:0055078	sodium ion homeostasis	2/125	0.0278255	0.06391255	0.03328146	Edn1/Slc8a1

1-day post-SCI group vs. the control group	BP	GO:0060443	mammary gland morphogenesis	2/125	0.0278255	0.06391255	0.03328146	Bax/Stat6
1-day post-SCI group vs. the control group	BP	GO:1903900	regulation of viral life cycle	3/125	0.0278674	0.06396657	0.03330959	Axl/Banf1/P4hb
1-day post-SCI group vs. the control group	BP	GO:0010508	positive regulation of autophagy	3/125	0.0283412	0.06501141	0.03385368	Hmox1/Pink1/Tsc1
1-day post-SCI group vs. the control group	BP	GO:0007626	locomotory behavior	4/125	0.0284979	0.06532802	0.03401854	Apoe/Mapt/Rcan1/Tsc1
1-day post-SCI group vs. the control group	BP	GO:0031663	lipopolysaccharide-mediated signaling pathway	2/125	0.0286888	0.06550738	0.03411194	Mapk14/Nos3
1-day post-SCI group vs. the control group	BP	GO:0043407	negative regulation of MAP kinase activity	2/125	0.0286888	0.06550738	0.03411194	Apoe/Spry2
1-day post-SCI group vs. the control group	BP	GO:0045620	negative regulation of lymphocyte differentiation	2/125	0.0286888	0.06550738	0.03411194	Anxa1/Hspb1
1-day post-SCI group vs. the control group	BP	GO:0046470	phosphatidylcholine metabolic process	2/125	0.0286888	0.06550738	0.03411194	Capn2/Lcat
1-day post-SCI group vs. the control group	BP	GO:0048524	positive regulation of viral process	2/125	0.0286888	0.06550738	0.03411194	Axl/P4hb
1-day post-SCI group vs. the control group	BP	GO:0140115	export across plasma membrane	2/125	0.0286888	0.06550738	0.03411194	Abcc1/Slc8a1
1-day post-SCI group vs. the control group	BP	GO:0005996	monosaccharide metabolic process	4/125	0.0291801	0.0665856	0.03467341	Atf4/Mapk14/Myc/Pdk2
1-day post-SCI group vs. the control group	BP	GO:0016125	sterol metabolic process	3/125	0.0293017	0.0668193	0.0347951	Apoe/Cat/Lcat
1-day post-SCI group vs. the control group	BP	GO:0032655	regulation of interleukin-12 production	2/125	0.0295628	0.06723894	0.03501363	Mapk14/Rela
1-day post-SCI group vs. the control group	BP	GO:0032731	positive regulation of interleukin-1 beta production	2/125	0.0295628	0.06723894	0.03501363	Hspb1/Jak2
1-day post-SCI group vs. the control group	BP	GO:0051058	negative regulation of small GTPase mediated signal t	2/125	0.0295628	0.06723894	0.03501363	Met/Spry2
1-day post-SCI group vs. the control group	BP	GO:0090303	positive regulation of wound healing	2/125	0.0295628	0.06723894	0.03501363	Anxa1/Hbegf
1-day post-SCI group vs. the control group	BP	GO:0072073	kidney epithelium development	3/125	0.0297883	0.06770782	0.03525779	Cat/Myc/Smad1
1-day post-SCI group vs. the control group	BP	GO:0046173	polyol biosynthetic process	2/125	0.0304473	0.0690706	0.03596744	Gch1/Sphk1
1-day post-SCI group vs. the control group	BP	GO:0050435	amyloid-beta metabolic process	2/125	0.0304473	0.0690706	0.03596744	Apoe/Rela
1-day post-SCI group vs. the control group	BP	GO:0070098	chemokine-mediated signaling pathway	2/125	0.0304473	0.0690706	0.03596744	Ccr1/Edn1
1-day post-SCI group vs. the control group	BP	GO:0050803	regulation of synapse structure or activity	4/125	0.0305735	0.06931176	0.03609301	Apoe/Cask/Itsn1/Mapk14
1-day post-SCI group vs. the control group	BP	GO:0022612	gland morphogenesis	3/125	0.0307745	0.06967692	0.03628317	Bax/Met/Stat6
1-day post-SCI group vs. the control group	BP	GO:0032368	regulation of lipid transport	3/125	0.0307745	0.06967692	0.03628317	Apoe/Edn1/Map2k6
1-day post-SCI group vs. the control group	BP	GO:1902652	secondary alcohol metabolic process	3/125	0.0312739	0.07059591	0.03676171	Apoe/Cat/Lcat
1-day post-SCI group vs. the control group	BP	GO:0001954	positive regulation of cell-matrix adhesion	2/125	0.0313422	0.07059591	0.03676171	Map4k4/Tsc1
1-day post-SCI group vs. the control group	BP	GO:0009064	glutamine family amino acid metabolic process	2/125	0.0313422	0.07059591	0.03676171	Nos3/Prodh
1-day post-SCI group vs. the control group	BP	GO:0032615	interleukin-12 production	2/125	0.0313422	0.07059591	0.03676171	Mapk14/Rela
1-day post-SCI group vs. the control group	BP	GO:0051893	regulation of focal adhesion assembly	2/125	0.0313422	0.07059591	0.03676171	Map4k4/Tsc1
1-day post-SCI group vs. the control group	BP	GO:0060261	positive regulation of transcription initiation by RNA p	2/125	0.0313422	0.07059591	0.03676171	Ercc1/Myc
1-day post-SCI group vs. the control group	BP	GO:0070059	intrinsic apoptotic signaling pathway in response to en	2/125	0.0313422	0.07059591	0.03676171	Atf4/Bax
1-day post-SCI group vs. the control group	BP	GO:0090109	regulation of cell-substrate junction assembly	2/125	0.0313422	0.07059591	0.03676171	Map4k4/Tsc1
1-day post-SCI group vs. the control group	BP	GO:0050854	regulation of antigen receptor-mediated signaling path	2/125	0.0322475	0.07258812	0.03779913	Ezr/Rela
1-day post-SCI group vs. the control group	BP	GO:0010977	negative regulation of neuron projection development	3/125	0.0322857	0.0726271	0.03781943	Apoe/Mdm2/Tsc1
1-day post-SCI group vs. the control group	BP	GO:0070507	regulation of microtubule cytoskeleton organization	3/125	0.0327979	0.07373184	0.0383947	Mapk8/Mapt/Met
1-day post-SCI group vs. the control group	BP	GO:0110053	regulation of actin filament organization	4/125	0.0331052	0.07437478	0.0387295	Cttn/Met/Pxn/Tsc1
1-day post-SCI group vs. the control group	BP	GO:0051148	negative regulation of muscle cell differentiation	2/125	0.033163	0.07440888	0.03874726	Rcan1/Smad1
1-day post-SCI group vs. the control group	BP	GO:0060038	cardiac muscle cell proliferation	2/125	0.033163	0.07440888	0.03874726	Mapk14/Smad1
1-day post-SCI group vs. the control group	BP	GO:0009755	hormone-mediated signaling pathway	3/125	0.0338351	0.07581925	0.03948169	Hdac1/Jak2/Ppargc1b
1-day post-SCI group vs. the control group	BP	GO:0051701	biological process involved in interaction with host	3/125	0.0338351	0.07581925	0.03948169	Axl/Ctsl/P4hb
1-day post-SCI group vs. the control group	BP	GO:0001707	mesoderm formation	2/125	0.0340887	0.07614299	0.03965027	Smad1/Txnrd1

1-day post-SCI group vs. the control group	BP	GO:0009123	nucleoside monophosphate metabolic process	2/125	0.0340887	0.07614299	0.03965027	Nme2/Xdh
1-day post-SCI group vs. the control group	BP	GO:0032663	regulation of interleukin-2 production	2/125	0.0340887	0.07614299	0.03965027	Anxa1/Ezr
1-day post-SCI group vs. the control group	BP	GO:0048008	platelet-derived growth factor receptor signaling pathw	2/125	0.0340887	0.07614299	0.03965027	Jak2/Plekha1
1-day post-SCI group vs. the control group	BP	GO:0050891	multicellular organismal water homeostasis	2/125	0.0340887	0.07614299	0.03965027	Krt1/Met
1-day post-SCI group vs. the control group	BP	GO:0007584	response to nutrient	2/125	0.0350243	0.07788391	0.04055683	Mapt/Pdk2
1-day post-SCI group vs. the control group	BP	GO:0016447	somatic recombination of immunoglobulin gene segme	2/125	0.0350243	0.07788391	0.04055683	Ercc1/Stat6
1-day post-SCI group vs. the control group	BP	GO:0032233	positive regulation of actin filament bundle assembly	2/125	0.0350243	0.07788391	0.04055683	Pxn/Tsc1
1-day post-SCI group vs. the control group	BP	GO:0042440	pigment metabolic process	2/125	0.0350243	0.07788391	0.04055683	Hmox1/Hmox2
1-day post-SCI group vs. the control group	BP	GO:0046427	positive regulation of receptor signaling pathway via J.	2/125	0.0350243	0.07788391	0.04055683	Cyp1b1/Jak2
1-day post-SCI group vs. the control group	BP	GO:0061082	myeloid leukocyte cytokine production	2/125	0.0350243	0.07788391	0.04055683	Axl/Hmox1
1-day post-SCI group vs. the control group	BP	GO:0097006	regulation of plasma lipoprotein particle levels	2/125	0.0350243	0.07788391	0.04055683	Apoe/Lcat
1-day post-SCI group vs. the control group	BP	GO:0001894	tissue homeostasis	4/125	0.0357563	0.07946095	0.04137804	Bax/Nos3/Ppargc1b/Ptgs2
1-day post-SCI group vs. the control group	BP	GO:0021543	pallium development	3/125	0.0359602	0.07958083	0.04144047	Bax/Hdac1/Tsc1
1-day post-SCI group vs. the control group	BP	GO:0032675	regulation of interleukin-6 production	3/125	0.0359602	0.07958083	0.04144047	Capn2/Met/Tnfrsf1a
1-day post-SCI group vs. the control group	BP	GO:0042093	T-helper cell differentiation	2/125	0.0359699	0.07958083	0.04144047	Anxa1/Stat6
1-day post-SCI group vs. the control group	BP	GO:0042246	tissue regeneration	2/125	0.0359699	0.07958083	0.04144047	Anxa1/Gpx1
1-day post-SCI group vs. the control group	BP	GO:0046324	regulation of glucose import	2/125	0.0359699	0.07958083	0.04144047	Mapk14/Myc
1-day post-SCI group vs. the control group	BP	GO:0060425	lung morphogenesis	2/125	0.0359699	0.07958083	0.04144047	Rpl13a/Spry2
1-day post-SCI group vs. the control group	BP	GO:0150116	regulation of cell-substrate junction organization	2/125	0.0359699	0.07958083	0.04144047	Map4k4/Tsc1
1-day post-SCI group vs. the control group	BP	GO:0030178	negative regulation of Wnt signaling pathway	3/125	0.036502	0.08060483	0.0419737	Apoe/Hdac1/Mapk14
1-day post-SCI group vs. the control group	BP	GO:0050768	negative regulation of neurogenesis	3/125	0.036502	0.08060483	0.0419737	Ctnna1/Gpr37l1/Ppp3ca
1-day post-SCI group vs. the control group	BP	GO:2000058	regulation of ubiquitin-dependent protein catabolic prc	3/125	0.036502	0.08060483	0.0419737	Fbxw7/Mapk8/Mdm2
1-day post-SCI group vs. the control group	BP	GO:0002440	production of molecular mediator of immune response	5/125	0.0365288	0.08061288	0.0419779	Axl/Btk/Ercc1/Hmox1/Stat6
1-day post-SCI group vs. the control group	BP	GO:0002294	CD4-positive, alpha-beta T cell differentiation involve	2/125	0.0369253	0.08128224	0.04232645	Anxa1/Stat6
1-day post-SCI group vs. the control group	BP	GO:0042058	regulation of epidermal growth factor receptor signalir	2/125	0.0369253	0.08128224	0.04232645	Fbxw7/Hbegf
1-day post-SCI group vs. the control group	BP	GO:0045600	positive regulation of fat cell differentiation	2/125	0.0369253	0.08128224	0.04232645	Mapk14/Ptgs2
1-day post-SCI group vs. the control group	BP	GO:0060260	regulation of transcription initiation by RNA polymera	2/125	0.0369253	0.08128224	0.04232645	Ercc1/Myc
1-day post-SCI group vs. the control group	BP	GO:0043433	negative regulation of DNA-binding transcription facto	3/125	0.0375983	0.08271151	0.04307072	Cat/Cyp1b1/Hmox1
1-day post-SCI group vs. the control group	BP	GO:0001937	negative regulation of endothelial cell proliferation	2/125	0.0378904	0.08303997	0.04324176	Apoe/Xdh
1-day post-SCI group vs. the control group	BP	GO:0002293	alpha-beta T cell differentiation involved in immune re	2/125	0.0378904	0.08303997	0.04324176	Anxa1/Stat6
1-day post-SCI group vs. the control group	BP	GO:0007193	adenylate cyclase-inhibiting G protein-coupled recepto	2/125	0.0378904	0.08303997	0.04324176	Edn1/Gpr37l1
1-day post-SCI group vs. the control group	BP	GO:0042733	embryonic digit morphogenesis	2/125	0.0378904	0.08303997	0.04324176	Bax/Hdac1
1-day post-SCI group vs. the control group	BP	GO:0048332	mesoderm morphogenesis	2/125	0.0378904	0.08303997	0.04324176	Smad1/Txnrd1
1-day post-SCI group vs. the control group	BP	GO:0055117	regulation of cardiac muscle contraction	2/125	0.0378904	0.08303997	0.04324176	Fkbp1b/Slc8a1
1-day post-SCI group vs. the control group	BP	GO:0055067	monovalent inorganic cation homeostasis	3/125	0.0381527	0.08350991	0.04348648	Edn1/Pdk2/Slc8a1
1-day post-SCI group vs. the control group	BP	GO:0071466	cellular response to xenobiotic stimulus	3/125	0.0381527	0.08350991	0.04348648	Cyp1b1/Myc/Pcna
1-day post-SCI group vs. the control group	BP	GO:0051961	negative regulation of nervous system development	3/125	0.0387113	0.08467946	0.0440955	Ctnna1/Gpr37l1/Ppp3ca
1-day post-SCI group vs. the control group	BP	GO:0002287	alpha-beta T cell activation involved in immune respor	2/125	0.0388651	0.08490949	0.04421529	Anxa1/Stat6
1-day post-SCI group vs. the control group	BP	GO:0043506	regulation of JUN kinase activity	2/125	0.0388651	0.08490949	0.04421529	Edn1/Map2k4
1-day post-SCI group vs. the control group	BP	GO:0015850	organic hydroxy compound transport	4/125	0.0389328	0.08500412	0.04426456	Apoe/Lcat/Myc/Pink1

1-day post-SCI group vs. the control group	BP	GO:0001541	ovarian follicle development	2/125	0.0398494	0.08673394	0.04516534	Bax/Ermp1
1-day post-SCI group vs. the control group	BP	GO:0019080	viral gene expression	2/125	0.0398494	0.08673394	0.04516534	Hdac1/Jun
1-day post-SCI group vs. the control group	BP	GO:1904894	positive regulation of receptor signaling pathway via S	2/125	0.0398494	0.08673394	0.04516534	Cyp1b1/Jak2
1-day post-SCI group vs. the control group	BP	GO:1990868	response to chemokine	2/125	0.0398494	0.08673394	0.04516534	Ccr1/Edn1
1-day post-SCI group vs. the control group	BP	GO:1990869	cellular response to chemokine	2/125	0.0398494	0.08673394	0.04516534	Ccr1/Edn1
1-day post-SCI group vs. the control group	BP	GO:0010970	transport along microtubule	3/125	0.0404121	0.08784914	0.04574607	Dynll1/Hspb1/Mapt
1-day post-SCI group vs. the control group	BP	GO:0050792	regulation of viral process	3/125	0.0404121	0.08784914	0.04574607	Axl/Banf1/P4hb
1-day post-SCI group vs. the control group	BP	GO:0046394	carboxylic acid biosynthetic process	4/125	0.0405799	0.08814049	0.04589778	Anxa1/Edn1/Ptgs2/Sphk1
1-day post-SCI group vs. the control group	BP	GO:0032623	interleukin-2 production	2/125	0.040843	0.08814049	0.04589778	Anxa1/Ezr
1-day post-SCI group vs. the control group	BP	GO:0032732	positive regulation of interleukin-1 production	2/125	0.040843	0.08814049	0.04589778	Hspb1/Jak2
1-day post-SCI group vs. the control group	BP	GO:0032635	interleukin-6 production	3/125	0.0409874	0.08814049	0.04589778	Capn2/Met/Tnfrsf1a
1-day post-SCI group vs. the control group	BP	GO:0016053	organic acid biosynthetic process	4/125	0.0409978	0.08814049	0.04589778	Anxa1/Edn1/Ptgs2/Sphk1
1-day post-SCI group vs. the control group	BP	GO:0007596	blood coagulation	3/125	0.0415668	0.08814049	0.04589778	Apoe/Axl/Jak2
1-day post-SCI group vs. the control group	BP	GO:0055088	lipid homeostasis	3/125	0.0415668	0.08814049	0.04589778	Apoe/Atp13a2/Lcat
1-day post-SCI group vs. the control group	BP	GO:0016445	somatic diversification of immunoglobulins	2/125	0.041846	0.08814049	0.04589778	Ercc1/Stat6
1-day post-SCI group vs. the control group	BP	GO:0030104	water homeostasis	2/125	0.041846	0.08814049	0.04589778	Krt1/Met
1-day post-SCI group vs. the control group	BP	GO:0043954	cellular component maintenance	2/125	0.041846	0.08814049	0.04589778	Apoe/Cttn
1-day post-SCI group vs. the control group	BP	GO:0071479	cellular response to ionizing radiation	2/125	0.041846	0.08814049	0.04589778	Mapk14/Mdm2
1-day post-SCI group vs. the control group	BP	GO:1901184	regulation of ERBB signaling pathway	2/125	0.041846	0.08814049	0.04589778	Fbxw7/Hbegf
1-day post-SCI group vs. the control group	BP	GO:2000179	positive regulation of neural precursor cell proliferatio	2/125	0.041846	0.08814049	0.04589778	Gpr37l1/Mapk8
1-day post-SCI group vs. the control group	BP	GO:0010948	negative regulation of cell cycle process	4/125	0.0422662	0.08814049	0.04589778	Atf2/Ier3/Mapk14/Mdm2
1-day post-SCI group vs. the control group	BP	GO:0003253	cardiac neural crest cell migration involved in outflow	1/125	0.042365	0.08814049	0.04589778	Edn1
1-day post-SCI group vs. the control group	BP	GO:0003330	regulation of extracellular matrix constituent secretion	1/125	0.042365	0.08814049	0.04589778	Tnfrsf1a
1-day post-SCI group vs. the control group	BP	GO:0003357	noradrenergic neuron differentiation	1/125	0.042365	0.08814049	0.04589778	Edn1
1-day post-SCI group vs. the control group	BP	GO:0006196	AMP catabolic process	1/125	0.042365	0.08814049	0.04589778	Xdh
1-day post-SCI group vs. the control group	BP	GO:0006527	arginine catabolic process	1/125	0.042365	0.08814049	0.04589778	Nos3
1-day post-SCI group vs. the control group	BP	GO:0006560	proline metabolic process	1/125	0.042365	0.08814049	0.04589778	Prodh
1-day post-SCI group vs. the control group	BP	GO:0006707	cholesterol catabolic process	1/125	0.042365	0.08814049	0.04589778	Apoe
1-day post-SCI group vs. the control group	BP	GO:0006971	hypotonic response	1/125	0.042365	0.08814049	0.04589778	Slc4a11
1-day post-SCI group vs. the control group	BP	GO:0009157	deoxyribonucleoside monophosphate biosynthetic proc	1/125	0.042365	0.08814049	0.04589778	Nme2
1-day post-SCI group vs. the control group	BP	GO:0010501	RNA secondary structure unwinding	1/125	0.042365	0.08814049	0.04589778	Ago4
1-day post-SCI group vs. the control group	BP	GO:0010692	regulation of alkaline phosphatase activity	1/125	0.042365	0.08814049	0.04589778	Ppargc1b
1-day post-SCI group vs. the control group	BP	GO:0010873	positive regulation of cholesterol esterification	1/125	0.042365	0.08814049	0.04589778	Apoe
1-day post-SCI group vs. the control group	BP	GO:0014883	transition between fast and slow fiber	1/125	0.042365	0.08814049	0.04589778	Ppp3ca
1-day post-SCI group vs. the control group	BP	GO:0016127	sterol catabolic process	1/125	0.042365	0.08814049	0.04589778	Apoe
1-day post-SCI group vs. the control group	BP	GO:0017038	protein import	1/125	0.042365	0.08814049	0.04589778	Apoe
1-day post-SCI group vs. the control group	BP	GO:0021957	corticospinal tract morphogenesis	1/125	0.042365	0.08814049	0.04589778	Cdh11
1-day post-SCI group vs. the control group	BP	GO:0031848	protection from non-homologous end joining at telome	1/125	0.042365	0.08814049	0.04589778	Ercc1
1-day post-SCI group vs. the control group	BP	GO:0032762	mast cell cytokine production	1/125	0.042365	0.08814049	0.04589778	Hmox1
1-day post-SCI group vs. the control group	BP	GO:0033007	negative regulation of mast cell activation involved in	1/125	0.042365	0.08814049	0.04589778	Hmox1

1-day post-SCI group vs. the control group	BP	GO:0033034	positive regulation of myeloid cell apoptotic process	1/125	0.042365	0.08814049	0.04589778	Anxa1
1-day post-SCI group vs. the control group	BP	GO:0033603	positive regulation of dopamine secretion	1/125	0.042365	0.08814049	0.04589778	Pink1
1-day post-SCI group vs. the control group	BP	GO:0035562	negative regulation of chromatin binding	1/125	0.042365	0.08814049	0.04589778	Ppp3ca
1-day post-SCI group vs. the control group	BP	GO:0038130	ERBB4 signaling pathway	1/125	0.042365	0.08814049	0.04589778	Hbegf
1-day post-SCI group vs. the control group	BP	GO:0042045	epithelial fluid transport	1/125	0.042365	0.08814049	0.04589778	Edn1
1-day post-SCI group vs. the control group	BP	GO:0046055	dGMP catabolic process	1/125	0.042365	0.08814049	0.04589778	Xdh
1-day post-SCI group vs. the control group	BP	GO:0046130	purine ribonucleoside catabolic process	1/125	0.042365	0.08814049	0.04589778	Xdh
1-day post-SCI group vs. the control group	BP	GO:0046877	regulation of saliva secretion	1/125	0.042365	0.08814049	0.04589778	Ppp3ca
1-day post-SCI group vs. the control group	BP	GO:0051142	positive regulation of NK T cell proliferation	1/125	0.042365	0.08814049	0.04589778	Jak2
1-day post-SCI group vs. the control group	BP	GO:0051340	regulation of ligase activity	1/125	0.042365	0.08814049	0.04589778	Ripk3
1-day post-SCI group vs. the control group	BP	GO:0051775	response to redox state	1/125	0.042365	0.08814049	0.04589778	Fkbp1b
1-day post-SCI group vs. the control group	BP	GO:0060297	regulation of sarcomere organization	1/125	0.042365	0.08814049	0.04589778	Edn1
1-day post-SCI group vs. the control group	BP	GO:0060385	axonogenesis involved in innervation	1/125	0.042365	0.08814049	0.04589778	Edn1
1-day post-SCI group vs. the control group	BP	GO:0060638	mesenchymal-epithelial cell signaling	1/125	0.042365	0.08814049	0.04589778	Met
1-day post-SCI group vs. the control group	BP	GO:0060710	chorio-allantoic fusion	1/125	0.042365	0.08814049	0.04589778	Vcam1
1-day post-SCI group vs. the control group	BP	GO:0061687	detoxification of inorganic compound	1/125	0.042365	0.08814049	0.04589778	Cat
1-day post-SCI group vs. the control group	BP	GO:0070662	mast cell proliferation	1/125	0.042365	0.08814049	0.04589778	Stat6
1-day post-SCI group vs. the control group	BP	GO:0070666	regulation of mast cell proliferation	1/125	0.042365	0.08814049	0.04589778	Stat6
1-day post-SCI group vs. the control group	BP	GO:0070922	RISC complex assembly	1/125	0.042365	0.08814049	0.04589778	Ago4
1-day post-SCI group vs. the control group	BP	GO:0070933	histone H4 deacetylation	1/125	0.042365	0.08814049	0.04589778	Hdac1
1-day post-SCI group vs. the control group	BP	GO:0071362	cellular response to ether	1/125	0.042365	0.08814049	0.04589778	Cdk4
1-day post-SCI group vs. the control group	BP	GO:0072203	cell proliferation involved in metanephros developmen	1/125	0.042365	0.08814049	0.04589778	Myc
1-day post-SCI group vs. the control group	BP	GO:0086064	cell communication by electrical coupling involved in	1/125	0.042365	0.08814049	0.04589778	Slc8a1
1-day post-SCI group vs. the control group	BP	GO:0090269	fibroblast growth factor production	1/125	0.042365	0.08814049	0.04589778	Ptgs2
1-day post-SCI group vs. the control group	BP	GO:0090270	regulation of fibroblast growth factor production	1/125	0.042365	0.08814049	0.04589778	Ptgs2
1-day post-SCI group vs. the control group	BP	GO:0097067	cellular response to thyroid hormone stimulus	1/125	0.042365	0.08814049	0.04589778	Ppargc1a
1-day post-SCI group vs. the control group	BP	GO:0099640	axo-dendritic protein transport	1/125	0.042365	0.08814049	0.04589778	Hspb1
1-day post-SCI group vs. the control group	BP	GO:0150172	regulation of phosphatidylcholine metabolic process	1/125	0.042365	0.08814049	0.04589778	Capn2
1-day post-SCI group vs. the control group	BP	GO:1900103	positive regulation of endoplasmic reticulum unfolded	1/125	0.042365	0.08814049	0.04589778	Bax
1-day post-SCI group vs. the control group	BP	GO:1901724	positive regulation of cell proliferation involved in kid	1/125	0.042365	0.08814049	0.04589778	Myc
1-day post-SCI group vs. the control group	BP	GO:1901725	regulation of histone deacetylase activity	1/125	0.042365	0.08814049	0.04589778	Pink1
1-day post-SCI group vs. the control group	BP	GO:1901731	positive regulation of platelet aggregation	1/125	0.042365	0.08814049	0.04589778	Jak2
1-day post-SCI group vs. the control group	BP	GO:1901856	negative regulation of cellular respiration	1/125	0.042365	0.08814049	0.04589778	Trap1
1-day post-SCI group vs. the control group	BP	GO:1901858	regulation of mitochondrial DNA metabolic process	1/125	0.042365	0.08814049	0.04589778	Ppargc1a
1-day post-SCI group vs. the control group	BP	GO:1902884	positive regulation of response to oxidative stress	1/125	0.042365	0.08814049	0.04589778	Ripk1
1-day post-SCI group vs. the control group	BP	GO:1903242	regulation of cardiac muscle hypertrophy in response t	1/125	0.042365	0.08814049	0.04589778	Ppp3ca
1-day post-SCI group vs. the control group	BP	GO:1903589	positive regulation of blood vessel endothelial cell pro	1/125	0.042365	0.08814049	0.04589778	Hmox1
1-day post-SCI group vs. the control group	BP	GO:1903749	positive regulation of establishment of protein localiza	1/125	0.042365	0.08814049	0.04589778	Fbxw7
1-day post-SCI group vs. the control group	BP	GO:2000646	positive regulation of receptor catabolic process	1/125	0.042365	0.08814049	0.04589778	Apoe
1-day post-SCI group vs. the control group	BP	GO:0002703	regulation of leukocyte mediated immunity	4/125	0.0426939	0.08877172	0.04622648	Gfer/Hmox1/Ripk3/Stat6

1-day post-SCI group vs. the control group	BP	GO:0006626	protein targeting to mitochondrion	2/125	0.0428582	0.08890155	0.04629409	Fbxw7/Pink1
1-day post-SCI group vs. the control group	BP	GO:0046847	filopodium assembly	2/125	0.0428582	0.08890155	0.04629409	Ezr/Trpm2
1-day post-SCI group vs. the control group	BP	GO:0061045	negative regulation of wound healing	2/125	0.0428582	0.08890155	0.04629409	Apoe/Cask
1-day post-SCI group vs. the control group	BP	GO:2001021	negative regulation of response to DNA damage stimu	2/125	0.0428582	0.08890155	0.04629409	Ercc1/Mdm2
1-day post-SCI group vs. the control group	BP	GO:0007599	hemostasis	3/125	0.0433297	0.08977284	0.0467478	Apoe/Axl/Jak2
1-day post-SCI group vs. the control group	BP	GO:0050817	coagulation	3/125	0.0433297	0.08977284	0.0467478	Apoe/Axl/Jak2
1-day post-SCI group vs. the control group	BP	GO:0007492	endoderm development	2/125	0.0438796	0.09085807	0.04731292	Hdac1/Itga5
1-day post-SCI group vs. the control group	BP	GO:0050853	B cell receptor signaling pathway	3/125	0.0445256	0.0921411	0.04798104	Bax/Btk/Plekha1
1-day post-SCI group vs. the control group	BP	GO:0002562	somatic diversification of immune receptors via germli	2/125	0.0449099	0.09266161	0.04825208	Ercc1/Stat6
1-day post-SCI group vs. the control group	BP	GO:0002704	negative regulation of leukocyte mediated immunity	2/125	0.0449099	0.09266161	0.04825208	Gfer/Hmox1
1-day post-SCI group vs. the control group	BP	GO:0016444	somatic cell DNA recombination	2/125	0.0449099	0.09266161	0.04825208	Ercc1/Stat6
1-day post-SCI group vs. the control group	BP	GO:0046635	positive regulation of alpha-beta T cell activation	2/125	0.0449099	0.09266161	0.04825208	Anxa1/Jak2
1-day post-SCI group vs. the control group	BP	GO:0061097	regulation of protein tyrosine kinase activity	2/125	0.0449099	0.09266161	0.04825208	Fbxw7/Hbegf
1-day post-SCI group vs. the control group	BP	GO:0007369	gastrulation	3/125	0.0451297	0.09306002	0.04845955	Itga5/Smad1/Txnrd1
1-day post-SCI group vs. the control group	BP	GO:0021766	hippocampus development	2/125	0.0459492	0.09313814	0.04850023	Hdac1/Tsc1
1-day post-SCI group vs. the control group	BP	GO:0043627	response to estrogen	2/125	0.0459492	0.09313814	0.04850023	Hmox1/Rcan1
1-day post-SCI group vs. the control group	BP	GO:1901606	alpha-amino acid catabolic process	2/125	0.0459492	0.09313814	0.04850023	Nos3/Prodh
1-day post-SCI group vs. the control group	BP	GO:0002246	wound healing involved in inflammatory response	1/125	0.0465023	0.09313814	0.04850023	Hmox1
1-day post-SCI group vs. the control group	BP	GO:0002357	defense response to tumor cell	1/125	0.0465023	0.09313814	0.04850023	Rela
1-day post-SCI group vs. the control group	BP	GO:0002887	negative regulation of myeloid leukocyte mediated imr	1/125	0.0465023	0.09313814	0.04850023	Hmox1
1-day post-SCI group vs. the control group	BP	GO:0003418	growth plate cartilage chondrocyte differentiation	1/125	0.0465023	0.09313814	0.04850023	Atf2
1-day post-SCI group vs. the control group	BP	GO:0006183	GTP biosynthetic process	1/125	0.0465023	0.09313814	0.04850023	Nme2
1-day post-SCI group vs. the control group	BP	GO:0009172	purine deoxyribonucleoside monophosphate catabolic	1/125	0.0465023	0.09313814	0.04850023	Xdh
1-day post-SCI group vs. the control group	BP	GO:0009221	pyrimidine deoxyribonucleotide biosynthetic process	1/125	0.0465023	0.09313814	0.04850023	Nme2
1-day post-SCI group vs. the control group	BP	GO:0010612	regulation of cardiac muscle adaptation	1/125	0.0465023	0.09313814	0.04850023	Ppp3ca
1-day post-SCI group vs. the control group	BP	GO:0010992	ubiquitin recycling	1/125	0.0465023	0.09313814	0.04850023	Fbxw7
1-day post-SCI group vs. the control group	BP	GO:0018401	peptidyl-proline hydroxylation to 4-hydroxy-L-proline	1/125	0.0465023	0.09313814	0.04850023	P4hb
1-day post-SCI group vs. the control group	BP	GO:0019227	neuronal action potential propagation	1/125	0.0465023	0.09313814	0.04850023	Fkbp1b
1-day post-SCI group vs. the control group	BP	GO:0021940	positive regulation of cerebellar granule cell precursor	1/125	0.0465023	0.09313814	0.04850023	Gpr37l1
1-day post-SCI group vs. the control group	BP	GO:0031077	post-embryonic camera-type eye development	1/125	0.0465023	0.09313814	0.04850023	Bax
1-day post-SCI group vs. the control group	BP	GO:0031293	membrane protein intracellular domain proteolysis	1/125	0.0465023	0.09313814	0.04850023	Adam9
1-day post-SCI group vs. the control group	BP	GO:0031620	regulation of fever generation	1/125	0.0465023	0.09313814	0.04850023	Ptgs2
1-day post-SCI group vs. the control group	BP	GO:0032819	positive regulation of natural killer cell proliferation	1/125	0.0465023	0.09313814	0.04850023	Jak2
1-day post-SCI group vs. the control group	BP	GO:0033629	negative regulation of cell adhesion mediated by integ	1/125	0.0465023	0.09313814	0.04850023	Cyp1b1
1-day post-SCI group vs. the control group	BP	GO:0034384	high-density lipoprotein particle clearance	1/125	0.0465023	0.09313814	0.04850023	Apoe
1-day post-SCI group vs. the control group	BP	GO:0035672	oligopeptide transmembrane transport	1/125	0.0465023	0.09313814	0.04850023	Abcc1
1-day post-SCI group vs. the control group	BP	GO:0042117	monocyte activation	1/125	0.0465023	0.09313814	0.04850023	Adam9
1-day post-SCI group vs. the control group	BP	GO:0045602	negative regulation of endothelial cell differentiation	1/125	0.0465023	0.09313814	0.04850023	Xdh
1-day post-SCI group vs. the control group	BP	GO:0046051	UTP metabolic process	1/125	0.0465023	0.09313814	0.04850023	Nme2
1-day post-SCI group vs. the control group	BP	GO:0046618	xenobiotic export from cell	1/125	0.0465023	0.09313814	0.04850023	Abcc1

1-day post-SCI group vs. the control group	BP	GO:0048262	determination of dorsal/ventral asymmetry	1/125	0.0465023	0.09313814	0.04850023	Mapk8
1-day post-SCI group vs. the control group	BP	GO:0048263	determination of dorsal identity	1/125	0.0465023	0.09313814	0.04850023	Mapk8
1-day post-SCI group vs. the control group	BP	GO:0048563	post-embryonic animal organ morphogenesis	1/125	0.0465023	0.09313814	0.04850023	Bax
1-day post-SCI group vs. the control group	BP	GO:0050774	negative regulation of dendrite morphogenesis	1/125	0.0465023	0.09313814	0.04850023	Ppp3ca
1-day post-SCI group vs. the control group	BP	GO:0051140	regulation of NK T cell proliferation	1/125	0.0465023	0.09313814	0.04850023	Jak2
1-day post-SCI group vs. the control group	BP	GO:0060052	neurofilament cytoskeleton organization	1/125	0.0465023	0.09313814	0.04850023	Atf2
1-day post-SCI group vs. the control group	BP	GO:0060056	mammary gland involution	1/125	0.0465023	0.09313814	0.04850023	Bax
1-day post-SCI group vs. the control group	BP	GO:0060693	regulation of branching involved in salivary gland mor	1/125	0.0465023	0.09313814	0.04850023	Met
1-day post-SCI group vs. the control group	BP	GO:0061299	retina vasculature morphogenesis in camera-type eye	1/125	0.0465023	0.09313814	0.04850023	Cyp1b1
1-day post-SCI group vs. the control group	BP	GO:0070294	renal sodium ion absorption	1/125	0.0465023	0.09313814	0.04850023	Edn1
1-day post-SCI group vs. the control group	BP	GO:0070493	thrombin-activated receptor signaling pathway	1/125	0.0465023	0.09313814	0.04850023	Met
1-day post-SCI group vs. the control group	BP	GO:0071492	cellular response to UV-A	1/125	0.0465023	0.09313814	0.04850023	Mmp3
1-day post-SCI group vs. the control group	BP	GO:0072075	metanephric mesenchyme development	1/125	0.0465023	0.09313814	0.04850023	Myc
1-day post-SCI group vs. the control group	BP	GO:0072584	caveolin-mediated endocytosis	1/125	0.0465023	0.09313814	0.04850023	Itsn1
1-day post-SCI group vs. the control group	BP	GO:0086100	endothelin receptor signaling pathway	1/125	0.0465023	0.09313814	0.04850023	Edn1
1-day post-SCI group vs. the control group	BP	GO:0090713	immunological memory process	1/125	0.0465023	0.09313814	0.04850023	Tsc1
1-day post-SCI group vs. the control group	BP	GO:0098779	positive regulation of mitophagy in response to mitoch	1/125	0.0465023	0.09313814	0.04850023	Pink1
1-day post-SCI group vs. the control group	BP	GO:0098870	action potential propagation	1/125	0.0465023	0.09313814	0.04850023	Fkbp1b
1-day post-SCI group vs. the control group	BP	GO:1900454	positive regulation of long-term synaptic depression	1/125	0.0465023	0.09313814	0.04850023	Mapt
1-day post-SCI group vs. the control group	BP	GO:1901029	negative regulation of mitochondrial outer membrane p	1/125	0.0465023	0.09313814	0.04850023	Ier3
1-day post-SCI group vs. the control group	BP	GO:1902946	protein localization to early endosome	1/125	0.0465023	0.09313814	0.04850023	Ezr
1-day post-SCI group vs. the control group	BP	GO:1903800	positive regulation of miRNA maturation	1/125	0.0465023	0.09313814	0.04850023	Ripk1
1-day post-SCI group vs. the control group	BP	GO:1990440	positive regulation of transcription from RNA polymer	1/125	0.0465023	0.09313814	0.04850023	Atf4
1-day post-SCI group vs. the control group	BP	GO:2001204	regulation of osteoclast development	1/125	0.0465023	0.09313814	0.04850023	Fbxw7
1-day post-SCI group vs. the control group	BP	GO:0031647	regulation of protein stability	4/125	0.0466535	0.09338733	0.04862999	Fbxw7/Mdm2/Pink1/Tsc1
1-day post-SCI group vs. the control group	BP	GO:0007269	neurotransmitter secretion	3/125	0.0469664	0.09380655	0.04884829	Cask/Sphk1/Syp
1-day post-SCI group vs. the control group	BP	GO:0099643	signal release from synapse	3/125	0.0469664	0.09380655	0.04884829	Cask/Sphk1/Syp
1-day post-SCI group vs. the control group	BP	GO:0005977	glycogen metabolic process	2/125	0.0469974	0.09380655	0.04884829	Il6st/Ppp1ca
1-day post-SCI group vs. the control group	BP	GO:0006073	cellular glucan metabolic process	2/125	0.0469974	0.09380655	0.04884829	Il6st/Ppp1ca
1-day post-SCI group vs. the control group	BP	GO:0051865	protein autoubiquitination	2/125	0.0469974	0.09380655	0.04884829	Mdm2/Rnf112
1-day post-SCI group vs. the control group	BP	GO:0043409	negative regulation of MAPK cascade	3/125	0.0475868	0.09482031	0.04937619	Apoe/Ezr/Spry2
1-day post-SCI group vs. the control group	BP	GO:1903305	regulation of regulated secretory pathway	3/125	0.0475868	0.09482031	0.04937619	Cask/Hmox1/Syp
1-day post-SCI group vs. the control group	BP	GO:1905952	regulation of lipid localization	3/125	0.0475868	0.09482031	0.04937619	Apoe/Edn1/Map2k6
1-day post-SCI group vs. the control group	BP	GO:0006749	glutathione metabolic process	2/125	0.0480543	0.09531626	0.04963445	Gpx1/Gpx4
1-day post-SCI group vs. the control group	BP	GO:0007405	neuroblast proliferation	2/125	0.0480543	0.09531626	0.04963445	Ctnna1/Mapk8
1-day post-SCI group vs. the control group	BP	GO:0032088	negative regulation of NF-kappaB transcription factor	2/125	0.0480543	0.09531626	0.04963445	Cat/Cyp1b1
1-day post-SCI group vs. the control group	BP	GO:0044042	glucan metabolic process	2/125	0.0480543	0.09531626	0.04963445	Il6st/Ppp1ca
1-day post-SCI group vs. the control group	BP	GO:0045123	cellular extravasation	2/125	0.0480543	0.09531626	0.04963445	Ripk3/Vcam1
1-day post-SCI group vs. the control group	BP	GO:0046626	regulation of insulin receptor signaling pathway	2/125	0.0480543	0.09531626	0.04963445	Cdk4/Rela
1-day post-SCI group vs. the control group	BP	GO:0048844	artery morphogenesis	2/125	0.0480543	0.09531626	0.04963445	Apoe/Edn1

1-day post-SCI group vs. the control group	BP	GO:1901796	regulation of signal transduction by p53 class mediator	2/125	0.0480543	0.09531626	0.04963445	Mdm2/Myc
1-day post-SCI group vs. the control group	BP	GO:0006643	membrane lipid metabolic process	3/125	0.0482112	0.09551899	0.04974002	Bax/Cyp1b1/Sphk1
1-day post-SCI group vs. the control group	BP	GO:0010951	negative regulation of endopeptidase activity	3/125	0.0482112	0.09551899	0.04974002	Gpx1/Mdm2/Ptgs2
1-day post-SCI group vs. the control group	BP	GO:0030534	adult behavior	3/125	0.0488397	0.0967092	0.0503598	Mapt/Met/Tsc1
1-day post-SCI group vs. the control group	BP	GO:0043279	response to alkaloid	2/125	0.0491198	0.0972087	0.05061991	Myc/Slc8a1
1-day post-SCI group vs. the control group	CC	GO:0045121	membrane raft	14/126	2.20E-09	3.54E-07	2.35E-07	Btk/Capn2/Cask/Ctnna1/Ezr/Hmox1/Il6st/Jak2/Mapt/Nos3/Ptgs2/Ripk1/Stat6/Tnfrsf1a
1-day post-SCI group vs. the control group	CC	GO:0098857	membrane microdomain	14/126	2.27E-09	3.54E-07	2.35E-07	Btk/Capn2/Cask/Ctnna1/Ezr/Hmox1/Il6st/Jak2/Mapt/Nos3/Ptgs2/Ripk1/Stat6/Tnfrsf1a
1-day post-SCI group vs. the control group	CC	GO:0031252	cell leading edge	13/126	3.16E-08	3.29E-06	2.19E-06	Amph/Atf4/Ctnna1/Cttn/Ezr/Itga5/Itsn1/Mapt/Nme2/P4hb/Plekha1/Pxn/Tsc1
1-day post-SCI group vs. the control group	CC	GO:0090575	RNA polymerase II transcription regulator complex	9/126	1.26E-06	9.83E-05	6.54E-05	Atf2/Atf4/Fos/Fosl1/Jun/Myc/Nono/Smad1/Stat6
1-day post-SCI group vs. the control group	CC	GO:0030055	cell-substrate junction	8/126	1.64E-06	0.00010223	6.79E-05	Capn2/Cttn/Ezr/Itga5/Itga7/Jak2/Map4k4/Pxn
1-day post-SCI group vs. the control group	CC	GO:0045178	basal part of cell	9/126	7.26E-06	0.00037765	0.000251	Abcc1/Adam9/Cask/Edn1/Ezr/Lpo/Met/Slc4a11/Slc8a1
1-day post-SCI group vs. the control group	CC	GO:0005925	focal adhesion	7/126	1.11E-05	0.00048592	0.00032296	Capn2/Cttn/Ezr/Itga5/Jak2/Map4k4/Pxn
1-day post-SCI group vs. the control group	CC	GO:0030027	lamellipodium	7/126	1.25E-05	0.00048592	0.00032296	Ctnna1/Cttn/Itsn1/Nme2/P4hb/Pxn/Tsc1
1-day post-SCI group vs. the control group	CC	GO:0009925	basal plasma membrane	8/126	2.96E-05	0.00095799	0.00063672	Abcc1/Adam9/Cask/Ezr/Lpo/Met/Slc4a11/Slc8a1
1-day post-SCI group vs. the control group	CC	GO:0044853	plasma membrane raft	6/126	3.07E-05	0.00095799	0.00063672	Ctnna1/Ezr/Hmox1/Jak2/Nos3/Ptgs2
1-day post-SCI group vs. the control group	CC	GO:0097386	glial cell projection	4/126	3.77E-05	0.0010706	0.00071157	Eif2s1/Ezr/Mapt/Pink1
1-day post-SCI group vs. the control group	CC	GO:0005901	caveola	5/126	6.58E-05	0.00170955	0.00113624	Ctnna1/Hmox1/Jak2/Nos3/Ptgs2
1-day post-SCI group vs. the control group	CC	GO:0031519	PcG protein complex	4/126	7.44E-05	0.00178549	0.00118671	Cbx6/Eed/Phc3/Rbbp7
1-day post-SCI group vs. the control group	CC	GO:0042383	sarcolemma	6/126	9.32E-05	0.00207602	0.00137981	Anxa1/Ezr/Itga7/Nos3/Slc8a1/Vcam1
1-day post-SCI group vs. the control group	CC	GO:0016323	basolateral plasma membrane	7/126	0.0001084	0.0021971	0.00146029	Abcc1/Adam9/Cask/Ezr/Lpo/Slc4a11/Slc8a1
1-day post-SCI group vs. the control group	CC	GO:0097449	astrocyte projection	3/126	0.0001184	0.0021971	0.00146029	Eif2s1/Ezr/Pink1
1-day post-SCI group vs. the control group	CC	GO:0043235	receptor complex	9/126	0.0001197	0.0021971	0.00146029	Axl/Gpr37l1/Il6st/Itga5/Itga7/Jak2/Met/Ripk1/Tnfrsf1a
1-day post-SCI group vs. the control group	CC	GO:0002102	podosome	3/126	0.0001974	0.00342188	0.00227433	Cttn/Mapk8/Vcam1
1-day post-SCI group vs. the control group	CC	GO:0000791	euchromatin	4/126	0.0002289	0.0037593	0.00249859	Jak2/Jun/Myc/Ppargc1a
1-day post-SCI group vs. the control group	CC	GO:0097440	apical dendrite	3/126	0.0002748	0.00416338	0.00276716	Cask/Itsn1/Ppargc1a
1-day post-SCI group vs. the control group	CC	GO:0030018	Z disc	5/126	0.0002834	0.00416338	0.00276716	Fkbp1b/Hspb1/Pdlim1/Ppp3ca/Slc8a1
1-day post-SCI group vs. the control group	CC	GO:0034399	nuclear periphery	5/126	0.0002936	0.00416338	0.00276716	Atf4/Cask/Mapt/Nono/Pcna
1-day post-SCI group vs. the control group	CC	GO:0005884	actin filament	5/126	0.0003147	0.00426855	0.00283706	Anxa1/Cttn/Ezr/Pdlim1/Tsc1
1-day post-SCI group vs. the control group	CC	GO:0017053	transcription repressor complex	4/126	0.0003511	0.00456391	0.00303337	Hdac1/Jun/Mdm2/Myc
1-day post-SCI group vs. the control group	CC	GO:0014069	postsynaptic density	8/126	0.000377	0.00470445	0.00312678	Cask/Hspb1/Mapt/Met/Rnf112/Slc8a1/Syp/Tsc1
1-day post-SCI group vs. the control group	CC	GO:0005912	adherens junction	5/126	0.0004107	0.0047111	0.0031312	Cdh11/Ctnna1/Ezr/Pdlim1/Ppp1ca
1-day post-SCI group vs. the control group	CC	GO:0043204	perikaryon	5/126	0.0004242	0.0047111	0.0031312	Ctsl/Hspb1/Map2k4/Mapk8/Ppp1ca
1-day post-SCI group vs. the control group	CC	GO:0032279	asymmetric synapse	8/126	0.00043	0.0047111	0.0031312	Cask/Hspb1/Mapt/Met/Rnf112/Slc8a1/Syp/Tsc1
1-day post-SCI group vs. the control group	CC	GO:0031674	I band	5/126	0.0004379	0.0047111	0.0031312	Fkbp1b/Hspb1/Pdlim1/Ppp3ca/Slc8a1
1-day post-SCI group vs. the control group	CC	GO:0001726	ruffle	5/126	0.0005437	0.00565396	0.00375786	Cttn/Ezr/Itga5/Nme2/Plekha1
1-day post-SCI group vs. the control group	CC	GO:0031256	leading edge membrane	5/126	0.0006302	0.00628417	0.00417672	Amph/Atf4/Itga5/Mapt/Plekha1
1-day post-SCI group vs. the control group	CC	GO:0005635	nuclear envelope	8/126	0.0006756	0.00628417	0.00417672	Apoe/Bax/Cdk4/Gch1/Gpx4/Itsn1/Ptgs2/Smad1

1-day post-SCI group vs. the control group	CC	GO:0098984	neuron to neuron synapse	8/126	0.000696	0.00628417	0.00417672	Cask/Hspb1/Mapt/Met/Rnf112/Slc8a1/Syp/Tsc1
1-day post-SCI group vs. the control group	CC	GO:0099572	postsynaptic specialization	8/126	0.0007064	0.00628417	0.00417672	Cask/Hspb1/Mapt/Met/Rnf112/Slc8a1/Syp/Tsc1
1-day post-SCI group vs. the control group	CC	GO:0005758	mitochondrial intermembrane space	4/126	0.0007245	0.00628417	0.00417672	Capn2/Cat/Pink1/Trap1
1-day post-SCI group vs. the control group	CC	GO:0019898	extrinsic component of membrane	7/126	0.0007251	0.00628417	0.00417672	Amph/Anxa1/Apoe/Cdh11/Ctnna1/Ezr/Rnf112
1-day post-SCI group vs. the control group	CC	GO:0120111	neuron projection cytoplasm	3/126	0.0008283	0.00698459	0.00464225	Dynll1/Map2k4/Mapk8
1-day post-SCI group vs. the control group	CC	GO:0097060	synaptic membrane	8/126	0.0010961	0.00899926	0.00598129	Anxa1/Cask/Fosl1/Hspb1/Itsn1/Met/Slc8a1/Syp
1-day post-SCI group vs. the control group	CC	GO:0031970	organelle envelope lumen	4/126	0.0011866	0.00949282	0.00630933	Capn2/Cat/Pink1/Trap1
1-day post-SCI group vs. the control group	CC	GO:0150034	distal axon	7/126	0.0014297	0.0109181	0.00725663	Amph/Cttn/Itsn1/Mapt/Slc8a1/Syp/Tsc1
1-day post-SCI group vs. the control group	CC	GO:0098833	presynaptic endocytic zone	2/126	0.0014348	0.0109181	0.00725663	Amph/Itsn1
1-day post-SCI group vs. the control group	CC	GO:0098685	Schaffer collateral - CA1 synapse	4/126	0.0016595	0.0121106	0.00804922	Cask/Cdh11/Ppp3ca/Syp
1-day post-SCI group vs. the control group	CC	GO:0005652	nuclear lamina	2/126	0.0016691	0.0121106	0.00804922	Cask/Pcna
1-day post-SCI group vs. the control group	CC	GO:0030017	sarcomere	5/126	0.00182	0.01271272	0.00844941	Fkbp1b/Hspb1/Pdlim1/Ppp3ca/Slc8a1
1-day post-SCI group vs. the control group	CC	GO:0062023	collagen-containing extracellular matrix	7/126	0.0018336	0.01271272	0.00844941	Anxa1/Atrn/Bmp1/Cask/Ctsl/Krt1/Pxdn
1-day post-SCI group vs. the control group	CC	GO:0008303	caspase complex	2/126	0.0019204	0.0130251	0.00865703	Capn2/Capns1
1-day post-SCI group vs. the control group	CC	GO:1990204	oxidoreductase complex	4/126	0.0019988	0.01308332	0.00869573	Cat/Ndufa6/P4hb/Pdk2
1-day post-SCI group vs. the control group	CC	GO:0008287	protein serine/threonine phosphatase complex	3/126	0.0020128	0.01308332	0.00869573	Ppp1ca/Ppp1r15b/Ppp3ca
1-day post-SCI group vs. the control group	CC	GO:1903293	phosphatase complex	3/126	0.0021159	0.01321157	0.00878097	Ppp1ca/Ppp1r15b/Ppp3ca
1-day post-SCI group vs. the control group	CC	GO:0044309	neuron spine	5/126	0.0021172	0.01321157	0.00878097	Itsn1/Ppp1ca/Ppp3ca/Slc8a1/Syp
1-day post-SCI group vs. the control group	CC	GO:0019867	outer membrane	5/126	0.0023022	0.01377892	0.00915805	Atf2/Bax/Mcl1/Pink1/Ptgs2
1-day post-SCI group vs. the control group	CC	GO:0031968	organelle outer membrane	5/126	0.0023022	0.01377892	0.00915805	Atf2/Bax/Mcl1/Pink1/Ptgs2
1-day post-SCI group vs. the control group	CC	GO:0001533	cornified envelope	3/126	0.0024445	0.01377892	0.00915805	Anxa1/Hspb1/Krt1
1-day post-SCI group vs. the control group	CC	GO:0000164	protein phosphatase type 1 complex	2/126	0.0024731	0.01377892	0.00915805	Ppp1ca/Ppp1r15b
1-day post-SCI group vs. the control group	CC	GO:0016581	NuRD complex	2/126	0.0024731	0.01377892	0.00915805	Hdac1/Rbbp7
1-day post-SCI group vs. the control group	CC	GO:0090545	CHD-type complex	2/126	0.0024731	0.01377892	0.00915805	Hdac1/Rbbp7
1-day post-SCI group vs. the control group	CC	GO:0035098	ESC/E(Z) complex	2/126	0.0027743	0.01518584	0.01009315	Eed/Rbbp7
1-day post-SCI group vs. the control group	CC	GO:0031253	cell projection membrane	6/126	0.0028994	0.01559698	0.01036641	Atf4/Cask/Ezr/Itga5/Mapt/Plekha1
1-day post-SCI group vs. the control group	CC	GO:0030016	myofibril	5/126	0.003043	0.01609165	0.01069519	Fkbp1b/Hspb1/Pdlim1/Ppp3ca/Slc8a1
1-day post-SCI group vs. the control group	CC	GO:0005905	clathrin-coated pit	3/126	0.0036135	0.01879034	0.01248886	Cttn/Itsn1/Sphk1
1-day post-SCI group vs. the control group	CC	GO:0043292	contractile fiber	5/126	0.0038724	0.01980621	0.01316404	Fkbp1b/Hspb1/Pdlim1/Ppp3ca/Slc8a1
1-day post-SCI group vs. the control group	CC	GO:0001650	fibrillar center	4/126	0.0040275	0.02018787	0.01341771	Ezr/Nono/Selenbp1/Txnrd1
1-day post-SCI group vs. the control group	CC	GO:0016580	Sin3 complex	2/126	0.0041411	0.02018787	0.01341771	Hdac1/Rbbp7
1-day post-SCI group vs. the control group	CC	GO:0070822	Sin3-type complex	2/126	0.0041411	0.02018787	0.01341771	Hdac1/Rbbp7
1-day post-SCI group vs. the control group	CC	GO:0016234	inclusion body	3/126	0.0052547	0.02522248	0.01676393	Atf4/Gpx1/Mapt
1-day post-SCI group vs. the control group	CC	GO:0045177	apical part of cell	7/126	0.0057594	0.02722638	0.0180958	Abcc1/Anxa1/Ctsl/Ezr/Nos3/Slc4a11/Vcam1
1-day post-SCI group vs. the control group	CC	GO:0032839	dendrite cytoplasm	2/126	0.0066614	0.03102005	0.02061724	Map2k4/Mapk8
1-day post-SCI group vs. the control group	CC	GO:0016342	catenin complex	2/126	0.0081248	0.03727853	0.02477689	Cdh11/Ctnna1
1-day post-SCI group vs. the control group	CC	GO:0032838	plasma membrane bounded cell projection cytoplasm	4/126	0.0088142	0.03985571	0.0264898	Dynll1/Map2k4/Mapk8/Mapt
1-day post-SCI group vs. the control group	CC	GO:0005741	mitochondrial outer membrane	4/126	0.0094861	0.04203084	0.02793548	Atf2/Bax/Mcl1/Pink1
1-day post-SCI group vs. the control group	CC	GO:1905369	endopeptidase complex	3/126	0.0097096	0.04203084	0.02793548	Capn2/Capns1/Hspb1
1-day post-SCI group vs. the control group	CC	GO:0008305	integrin complex	2/126	0.0097189	0.04203084	0.02793548	Itga5/Itga7

1-day post-SCI group vs. the control group	CC	GO:0019897	extrinsic component of plasma membrane	4/126	0.0098341	0.04203084	0.02793548	Anxa1/Apoe/Cdh11/Ctnna1
1-day post-SCI group vs. the control group	CC	GO:0000792	heterochromatin	3/126	0.0099753	0.04205822	0.02795368	Cbx6/Eed/Hdac1
1-day post-SCI group vs. the control group	CC	GO:0043679	axon terminus	4/126	0.0105549	0.04390858	0.0291835	Amph/Itsn1/Slc8a1/Syp
1-day post-SCI group vs. the control group	CC	GO:0005902	microvillus	3/126	0.0110807	0.04548914	0.03023401	Ctsl/Ezr/Vcam1
1-day post-SCI group vs. the control group	CC	GO:0098802	plasma membrane signaling receptor complex	4/126	0.0115029	0.0466093	0.03097851	Il6st/Itga5/Itga7/Jak2
1-day post-SCI group vs. the control group	CC	GO:0043197	dendritic spine	4/126	0.0116989	0.04679553	0.03110229	Itsn1/Ppp1ca/Ppp3ca/Slc8a1
1-day post-SCI group vs. the control group	CC	GO:0042734	presynaptic membrane	4/126	0.011897	0.04696092	0.03121222	Cask/Fosl1/Itsn1/Syp
1-day post-SCI group vs. the control group	CC	GO:0034364	high-density lipoprotein particle	2/126	0.0120413	0.04696092	0.03121222	Apoe/Lcat
1-day post-SCI group vs. the control group	CC	GO:0016592	mediator complex	2/126	0.0132845	0.05116996	0.03400972	Cdk4/Ppargc1b
1-day post-SCI group vs. the control group	CC	GO:0044306	neuron projection terminus	4/126	0.0148987	0.05668774	0.03767707	Amph/Itsn1/Slc8a1/Syp
1-day post-SCI group vs. the control group	CC	GO:0000781	chromosome, telomeric region	3/126	0.0154893	0.05822484	0.0386987	Ercc1/Ppp1ca/Rbbp7
1-day post-SCI group vs. the control group	CC	GO:0005604	basement membrane	3/126	0.0158367	0.05882218	0.03909571	Atrn/Cask/Pxdn
1-day post-SCI group vs. the control group	CC	GO:0005637	nuclear inner membrane	2/126	0.0173302	0.06361215	0.04227933	Ptgs2/Smad1
1-day post-SCI group vs. the control group	CC	GO:0034358	plasma lipoprotein particle	2/126	0.0180493	0.06429531	0.04273339	Apoe/Lcat
1-day post-SCI group vs. the control group	CC	GO:1990777	lipoprotein particle	2/126	0.0180493	0.06429531	0.04273339	Apoe/Lcat
1-day post-SCI group vs. the control group	CC	GO:0030133	transport vesicle	5/126	0.0182458	0.06429531	0.04273339	Amph/Atp13a2/Rnf112/Sphk1/Syp
1-day post-SCI group vs. the control group	CC	GO:0099568	cytoplasmic region	4/126	0.0183406	0.06429531	0.04273339	Dynll1/Map2k4/Mapk8/Mapt
1-day post-SCI group vs. the control group	CC	GO:0008021	synaptic vesicle	4/126	0.0196799	0.06822377	0.04534441	Amph/Rnf112/Sphk1/Syp
1-day post-SCI group vs. the control group	CC	GO:0032994	protein-lipid complex	2/126	0.0202808	0.06953402	0.04621526	Apoe/Lcat
1-day post-SCI group vs. the control group	CC	GO:0000307	cyclin-dependent protein kinase holoenzyme complex	2/126	0.0210489	0.07138338	0.04744442	Cdk4/Pcna
1-day post-SCI group vs. the control group	CC	GO:1905368	peptidase complex	3/126	0.0219933	0.07378394	0.04903994	Capn2/Capns1/Hspb1
1-day post-SCI group vs. the control group	CC	GO:0031965	nuclear membrane	4/126	0.0231362	0.07679234	0.05103944	Cdk4/Gch1/Ptgs2/Smad1
1-day post-SCI group vs. the control group	CC	GO:0016324	apical plasma membrane	5/126	0.0235599	0.07737577	0.05142721	Abcc1/Anxa1/Ezr/Slc4a11/Vcam1
1-day post-SCI group vs. the control group	CC	GO:0098636	protein complex involved in cell adhesion	2/126	0.0250673	0.08146877	0.0541476	Itga5/Itga7
1-day post-SCI group vs. the control group	CC	GO:0070382	exocytic vesicle	4/126	0.0266097	0.08558989	0.05688667	Amph/Rnf112/Sphk1/Syp
1-day post-SCI group vs. the control group	CC	GO:0005771	multivesicular body	2/126	0.0339454	0.10807102	0.07182858	Atp13a2/Ctsl
1-day post-SCI group vs. the control group	CC	GO:0014704	intercalated disc	2/126	0.0348916	0.10886188	0.07235422	Ctnna1/Slc8a1
1-day post-SCI group vs. the control group	CC	GO:0016328	lateral plasma membrane	2/126	0.0348916	0.10886188	0.07235422	Abcc1/Anxa1
1-day post-SCI group vs. the control group	CC	GO:0030315	T-tubule	2/126	0.0377911	0.11674077	0.07759086	Ezr/Slc8a1
1-day post-SCI group vs. the control group	CC	GO:0032589	neuron projection membrane	2/126	0.0387775	0.11861341	0.0788355	Atf4/Mapt
1-day post-SCI group vs. the control group	CC	GO:0032587	ruffle membrane	2/126	0.0397736	0.12047929	0.08007564	Itga5/Plekha1
1-day post-SCI group vs. the control group	CC	GO:0016600	flotillin complex	1/126	0.0428994	0.12626986	0.0839243	Ctnna1
1-day post-SCI group vs. the control group	CC	GO:0071141	SMAD protein complex	1/126	0.0428994	0.12626986	0.0839243	Smad1
1-day post-SCI group vs. the control group	CC	GO:1905103	integral component of lysosomal membrane	1/126	0.0428994	0.12626986	0.0839243	Atp13a2
1-day post-SCI group vs. the control group	CC	GO:0016529	sarcoplasmic reticulum	2/126	0.0438539	0.12668907	0.08420293	Fkbp1b/Xdh
1-day post-SCI group vs. the control group	CC	GO:0035097	histone methyltransferase complex	2/126	0.0438539	0.12668907	0.08420293	Eed/Rbbp7
1-day post-SCI group vs. the control group	CC	GO:0005874	microtubule	5/126	0.0447689	0.1277506	0.08490847	Apoe/Dynll1/Mapt/Slc8a1/Spry2
1-day post-SCI group vs. the control group	CC	GO:0009898	cytoplasmic side of plasma membrane	3/126	0.045946	0.1277506	0.08490847	Ezr/Mapt/Ppp3ca
1-day post-SCI group vs. the control group	CC	GO:0060076	excitatory synapse	2/126	0.0470118	0.1277506	0.08490847	Met/Syp
1-day post-SCI group vs. the control group	CC	GO:0001931	uropod	1/126	0.0470876	0.1277506	0.08490847	Ezr

1-day post-SCI group vs. the control group	CC	GO:0031254	cell trailing edge	1/126	0.0470876	0.1277506	0.08490847	Ezr
1-day post-SCI group vs. the control group	CC	GO:0031932	TORC2 complex	1/126	0.0470876	0.1277506	0.08490847	Prr5l
1-day post-SCI group vs. the control group	CC	GO:0070578	RISC-loading complex	1/126	0.0470876	0.1277506	0.08490847	Ago4
1-day post-SCI group vs. the control group	MF	GO:0016209	antioxidant activity	9/126	1.72E-10	8.02E-08	5.79E-08	Apoe/Cat/Gpx1/Gpx4/Lpo/Ptgs2/Pxdn/Srxn1/Txnrd1
1-day post-SCI group vs. the control group	MF	GO:0004601	peroxidase activity	7/126	7.12E-09	1.02E-06	7.36E-07	Cat/Gpx1/Gpx4/Lpo/Ptgs2/Pxdn/Txnrd1
1-day post-SCI group vs. the control group	MF	GO:0008022	protein C-terminus binding	11/126	7.93E-09	1.02E-06	7.36E-07	Amph/Atf4/Banf1/Cask/Dynll1/Ercc1/Ezr/Jak2/Mapk14/Pcna/Sdc1
1-day post-SCI group vs. the control group	MF	GO:0016684	oxidoreductase activity, acting on peroxide as acceptor	7/126	1.02E-08	1.02E-06	7.36E-07	Cat/Gpx1/Gpx4/Lpo/Ptgs2/Pxdn/Txnrd1
1-day post-SCI group vs. the control group	MF	GO:0004674	protein serine/threonine kinase activity	14/126	1.09E-08	1.02E-06	7.36E-07	Cask/Cdk4/Map2k3/Map2k4/Map2k6/Map4k4/Mapk14/Mapk8/Mapkapk3/Nme2/Pdk2/Pink1/Ripk1/Ripk3
1-day post-SCI group vs. the control group	MF	GO:0020037	heme binding	9/126	4.51E-08	3.51E-06	2.53E-06	Cat/Cyp1b1/Hebp2/Hmox1/Hmox2/Jak2/Nos3/Ptgs2/Pxdn
1-day post-SCI group vs. the control group	MF	GO:0046906	tetrapyrrole binding	9/126	7.20E-08	4.81E-06	3.47E-06	Cat/Cyp1b1/Hebp2/Hmox1/Hmox2/Jak2/Nos3/Ptgs2/Pxdn
1-day post-SCI group vs. the control group	MF	GO:0050839	cell adhesion molecule binding	11/126	1.37E-07	8.00E-06	5.77E-06	Adam9/Cdh11/Ctnna1/Ezr/Itga5/Itga7/Nos3/P4hb/Ppp1ca/Pxn/Vcam1
1-day post-SCI group vs. the control group	MF	GO:0061629	RNA polymerase II-specific DNA-binding transcriptio	11/126	9.87E-07	5.12E-05	3.69E-05	Atf2/Atf4/Fos/Hdac1/Hspb1/Jun/Mapk14/Pcna/Ppargc1a/Ppargc1b/Rela
1-day post-SCI group vs. the control group	MF	GO:0019903	protein phosphatase binding	8/126	1.82E-06	8.16E-05	5.88E-05	Mapk14/Mapk8/Mapt/Met/Ppp1ca/Pxn/Sphk1/Stat6
1-day post-SCI group vs. the control group	MF	GO:0004713	protein tyrosine kinase activity	7/126	1.92E-06	8.16E-05	5.88E-05	Axl/Btk/Jak2/Map2k3/Map2k4/Map2k6/Met
1-day post-SCI group vs. the control group	MF	GO:0016667	oxidoreductase activity, acting on a sulfur group of do	5/126	6.02E-06	0.00023427	0.00016897	Gfer/P4hb/Selenbp1/Srxn1/Txnrd1
1-day post-SCI group vs. the control group	MF	GO:0019902	phosphatase binding	8/126	1.03E-05	0.00036848	0.00026578	Mapk14/Mapk8/Mapt/Met/Ppp1ca/Pxn/Sphk1/Stat6
1-day post-SCI group vs. the control group	MF	GO:0031625	ubiquitin protein ligase binding	9/126	1.20E-05	0.0003995	0.00028816	Fbxw7/Jun/Mdm2/Myc/Pink1/Ppargc1a/Prr5l/Rela/Ripk1
1-day post-SCI group vs. the control group	MF	GO:0016670	oxidoreductase activity, acting on a sulfur group of do	3/126	1.37E-05	0.0004266	0.0003077	Gfer/P4hb/Selenbp1
1-day post-SCI group vs. the control group	MF	GO:0044389	ubiquitin-like protein ligase binding	9/126	1.82E-05	0.00050026	0.00036083	Fbxw7/Jun/Mdm2/Myc/Pink1/Ppargc1a/Prr5l/Rela/Ripk1
1-day post-SCI group vs. the control group	MF	GO:0070513	death domain binding	3/126	1.82E-05	0.00050026	0.00036083	Bax/Mcl1/Ripk1
1-day post-SCI group vs. the control group	MF	GO:1990841	promoter-specific chromatin binding	5/126	2.13E-05	0.00055391	0.00039953	Atf2/Atf4/Ercc1/Hdac1/Ppargc1a
1-day post-SCI group vs. the control group	MF	GO:0051400	BH domain binding	3/126	2.36E-05	0.00057999	0.00041834	Bax/Mcl1/Pxn
1-day post-SCI group vs. the control group	MF	GO:0004712	protein serine/threonine/tyrosine kinase activity	4/126	3.63E-05	0.00082943	0.00059826	Map2k3/Map2k4/Map2k6/Mapk8
1-day post-SCI group vs. the control group	MF	GO:0004708	MAP kinase kinase activity	3/126	3.73E-05	0.00082943	0.00059826	Map2k3/Map2k4/Map2k6
1-day post-SCI group vs. the control group	MF	GO:0050660	flavin adenine dinucleotide binding	5/126	4.38E-05	0.00092991	0.00067073	Gfer/Nos3/Prodh/Txnrd1/Xdh
1-day post-SCI group vs. the control group	MF	GO:0005178	integrin binding	6/126	6.88E-05	0.00139782	0.00100823	Adam9/Itga5/Itga7/P4hb/Pxn/Vcam1
1-day post-SCI group vs. the control group	MF	GO:0001046	core promoter sequence-specific DNA binding	4/126	7.85E-05	0.00152747	0.00110175	Fos/Hdac1/Myc/Rela
1-day post-SCI group vs. the control group	MF	GO:0140296	general transcription initiation factor binding	4/126	0.0001216	0.00227074	0.00163786	Atf4/Ercc1/Jun/Rela
1-day post-SCI group vs. the control group	MF	GO:0000979	RNA polymerase II core promoter sequence-specific D	3/126	0.0001415	0.00254076	0.00183263	Fos/Hdac1/Rela
1-day post-SCI group vs. the control group	MF	GO:0005516	calmodulin binding	6/126	0.0002157	0.00373133	0.00269137	Cask/Mapkapk3/Nos3/Ppp3ca/Slc8a1/Sphk1
1-day post-SCI group vs. the control group	MF	GO:0045296	cadherin binding	4/126	0.0002553	0.00425883	0.00307185	Cdh11/Ctnna1/Nos3/Ppp1ca
1-day post-SCI group vs. the control group	MF	GO:0043236	laminin binding	3/126	0.0002862	0.00460944	0.00332474	Adam9/Itga7/Pxdn
1-day post-SCI group vs. the control group	MF	GO:0001221	transcription coregulator binding	5/126	0.000324	0.00504298	0.00363745	Eed/Fos/Hdac1/Myc/Rela
1-day post-SCI group vs. the control group	MF	GO:0047485	protein N-terminus binding	5/126	0.0003353	0.00505164	0.0036437	Banf1/Hdac1/Mdm2/Rela/Tsc1
1-day post-SCI group vs. the control group	MF	GO:0051019	mitogen-activated protein kinase binding	3/126	0.0004219	0.00615716	0.00444109	Gch1/Mapk14/Mapkapk3
1-day post-SCI group vs. the control group	MF	GO:0031072	heat shock protein binding	5/126	0.0004518	0.00639385	0.00461182	Bax/Hspa13/Mapt/Nos3/Tsc1

1-day post-SCI group vs. the control group	MF	GO:0016705	oxidoreductase activity, acting on paired donors, with	6/126	0.0005537	0.00760494	0.00548537	Cyp1b1/Hmox1/Hmox2/Nos3/P4hb/Ptgs2
1-day post-SCI group vs. the control group	MF	GO:0015035	protein-disulfide reductase activity	3/126	0.0006419	0.00856452	0.0061775	Gfer/P4hb/Txnrd1
1-day post-SCI group vs. the control group	MF	GO:0043548	phosphatidylinositol 3-kinase binding	3/126	0.0008033	0.01032523	0.00744748	Axl/Jak2/Met
1-day post-SCI group vs. the control group	MF	GO:0019207	kinase regulator activity	6/126	0.0008229	0.01032523	0.00744748	Cdkn1c/Hbegf/Hspb1/Itsn1/Map2k6/Spry2
1-day post-SCI group vs. the control group	MF	GO:0008140	cAMP response element binding protein binding	2/126	0.0008583	0.01032523	0.00744748	Atf2/Atf4
1-day post-SCI group vs. the control group	MF	GO:0071949	FAD binding	3/126	0.0008623	0.01032523	0.00744748	Prodh/Txnrd1/Xdh
1-day post-SCI group vs. the control group	MF	GO:0008013	beta-catenin binding	4/126	0.0009302	0.01077792	0.007774	Ctnna1/Met/Nos3/Pxn
1-day post-SCI group vs. the control group	MF	GO:0033218	amide binding	8/126	0.0009462	0.01077792	0.007774	Apoe/Cat/Ctsl/Fkbp1b/Gpr37l1/Lancl1/Ppp3ca/Rela
1-day post-SCI group vs. the control group	MF	GO:0015036	disulfide oxidoreductase activity	3/126	0.0010556	0.0117368	0.00846563	Gfer/P4hb/Txnrd1
1-day post-SCI group vs. the control group	MF	GO:0030331	nuclear estrogen receptor binding	3/126	0.0014347	0.01491972	0.01076144	Pcna/Ppargc1a/Ppargc1b
1-day post-SCI group vs. the control group	MF	GO:0051879	Hsp90 protein binding	3/126	0.0014347	0.01491972	0.01076144	Mapt/Nos3/Tsc1
1-day post-SCI group vs. the control group	MF	GO:0017166	vinculin binding	2/126	0.0014748	0.01491972	0.01076144	Ctnna1/Pxn
1-day post-SCI group vs. the control group	MF	GO:0043522	leucine zipper domain binding	2/126	0.0014748	0.01491972	0.01076144	Atf2/Atf4
1-day post-SCI group vs. the control group	MF	GO:0042277	peptide binding	7/126	0.0015016	0.01491972	0.01076144	Apoe/Cat/Ctsl/Gpr37l1/Lancl1/Ppp3ca/Rela
1-day post-SCI group vs. the control group	MF	GO:0004198	calcium-dependent cysteine-type endopeptidase activit	2/126	0.0017156	0.01669135	0.01203929	Capn2/Capns1
1-day post-SCI group vs. the control group	MF	GO:0050840	extracellular matrix binding	3/126	0.002094	0.01995701	0.01439478	Adam9/Itga7/Pxdn
1-day post-SCI group vs. the control group	MF	GO:0004707	MAP kinase activity	2/126	0.0022492	0.02100785	0.01515274	Mapk14/Mapk8
1-day post-SCI group vs. the control group	MF	GO:0019209	kinase activator activity	4/126	0.0025057	0.02272484	0.01639119	Hbegf/Itsn1/Map2k6/Spry2
1-day post-SCI group vs. the control group	MF	GO:0046982	protein heterodimerization activity	6/126	0.0025304	0.02272484	0.01639119	Atf2/Atf4/Bax/Krt1/Mcl1/P4hb
1-day post-SCI group vs. the control group	MF	GO:0019887	protein kinase regulator activity	5/126	0.0027067	0.02384955	0.01720243	Cdkn1c/Hbegf/Hspb1/Map2k6/Spry2
1-day post-SCI group vs. the control group	MF	GO:0017124	SH3 domain binding	4/126	0.0028025	0.02420916	0.01746181	Adam9/Gpx1/Lancl1/Mapt
1-day post-SCI group vs. the control group	MF	GO:0035497	cAMP response element binding	2/126	0.0028512	0.02420916	0.01746181	Atf2/Jun
1-day post-SCI group vs. the control group	MF	GO:0030275	LRR domain binding	2/126	0.0035202	0.02834387	0.02044413	Atf2/Atf4
1-day post-SCI group vs. the control group	MF	GO:0034185	apolipoprotein binding	2/126	0.0035202	0.02834387	0.02044413	Lcat/Mapt
1-day post-SCI group vs. the control group	MF	GO:0070064	proline-rich region binding	2/126	0.0035202	0.02834387	0.02044413	Cttn/Itsn1
1-day post-SCI group vs. the control group	MF	GO:0042975	peroxisome proliferator activated receptor binding	2/126	0.0038795	0.03070737	0.0221489	Mdm2/Ppargc1a
1-day post-SCI group vs. the control group	MF	GO:0016712	oxidoreductase activity, acting on paired donors, with	3/126	0.004733	0.03645432	0.02629411	Cyp1b1/Hmox1/Hmox2
1-day post-SCI group vs. the control group	MF	GO:0004497	monooxygenase activity	4/126	0.0047617	0.03645432	0.02629411	Cyp1b1/Hmox1/Hmox2/Nos3
1-day post-SCI group vs. the control group	MF	GO:0070851	growth factor receptor binding	4/126	0.004873	0.03670496	0.0264749	Hbegf/Il6st/Itga5/Jak2
1-day post-SCI group vs. the control group	MF	GO:0010857	calcium-dependent protein kinase activity	2/126	0.0050547	0.037469	0.02702599	Mapkapk3/Pink1
1-day post-SCI group vs. the control group	MF	GO:0070412	R-SMAD binding	2/126	0.0054784	0.039975	0.02883354	Fos/Jun
1-day post-SCI group vs. the control group	MF	GO:0097110	scaffold protein binding	3/126	0.0062528	0.04492362	0.03240293	Dynll1/Il6st/Nos3
1-day post-SCI group vs. the control group	MF	GO:0004602	glutathione peroxidase activity	2/126	0.0063727	0.0450327	0.03248161	Gpx1/Gpx4
1-day post-SCI group vs. the control group	MF	GO:0046332	SMAD binding	3/126	0.0064608	0.0450327	0.03248161	Fos/Jun/Smad1
1-day post-SCI group vs. the control group	MF	GO:0071813	lipoprotein particle binding	2/126	0.0068431	0.04565354	0.03292941	Apoe/Mapt
1-day post-SCI group vs. the control group	MF	GO:0071814	protein-lipid complex binding	2/126	0.0068431	0.04565354	0.03292941	Apoe/Mapt
1-day post-SCI group vs. the control group	MF	GO:0140359	ABC-type transporter activity	2/126	0.0068431	0.04565354	0.03292941	Abcc1/Atp13a2
1-day post-SCI group vs. the control group	MF	GO:0043021	ribonucleoprotein complex binding	4/126	0.0071855	0.04628369	0.03338393	Eif2s1/Mdm2/Ppp1ca/Ybx3
1-day post-SCI group vs. the control group	MF	GO:0043274	phospholipase binding	2/126	0.0073288	0.04628369	0.03338393	Btk/Met
1-day post-SCI group vs. the control group	MF	GO:0050750	low-density lipoprotein particle receptor binding	2/126	0.0073288	0.04628369	0.03338393	Apoe/Lancl1

1-day post-SCI group vs. the control group	MF	GO:0043130	ubiquitin binding	3/126	0.007334	0.04628369	0.03338393	Fbxw7/Hspb1/Mdm2
1-day post-SCI group vs. the control group	MF	GO:0051082	unfolded protein binding	3/126	0.0075627	0.04709044	0.03396583	Hspa13/Hspb1/Trap1
1-day post-SCI group vs. the control group	MF	GO:0030291	protein serine/threonine kinase inhibitor activity	2/126	0.0088763	0.05383389	0.03882981	Cdkn1c/Hspb1
1-day post-SCI group vs. the control group	MF	GO:0051059	NF-kappaB binding	2/126	0.0088763	0.05383389	0.03882981	Hdac1/Rela
1-day post-SCI group vs. the control group	MF	GO:0003727	single-stranded RNA binding	3/126	0.0092814	0.05435417	0.03920508	Ago4/Anxa1/Cbx6
1-day post-SCI group vs. the control group	MF	GO:0008237	metallopeptidase activity	4/126	0.0094157	0.05435417	0.03920508	Adam9/Bmp1/Ermp1/Mmp3
1-day post-SCI group vs. the control group	MF	GO:0070325	lipoprotein particle receptor binding	2/126	0.0094217	0.05435417	0.03920508	Apoe/Lancl1
1-day post-SCI group vs. the control group	MF	GO:0004620	phospholipase activity	3/126	0.009544	0.05435417	0.03920508	Ccr1/Hmox1/Lcat
1-day post-SCI group vs. the control group	MF	GO:0016407	acetyltransferase activity	3/126	0.009544	0.05435417	0.03920508	Atf2/Lcat/Sphk1
1-day post-SCI group vs. the control group	MF	GO:0030332	cyclin binding	2/126	0.0099817	0.05605151	0.04042935	Cdk4/Fbxw7
1-day post-SCI group vs. the control group	MF	GO:0051117	ATPase binding	3/126	0.0100821	0.05605151	0.04042935	Atox1/Ezr/Ppp3ca
1-day post-SCI group vs. the control group	MF	GO:0004197	cysteine-type endopeptidase activity	3/126	0.0115033	0.06262404	0.04517005	Capn2/Capns1/Ctsl
1-day post-SCI group vs. the control group	MF	GO:0097718	disordered domain specific binding	2/126	0.011748	0.06262404	0.04517005	Ezr/Mdm2
1-day post-SCI group vs. the control group	MF	GO:0004222	metalloendopeptidase activity	3/126	0.0118007	0.06262404	0.04517005	Adam9/Bmp1/Mmp3
1-day post-SCI group vs. the control group	MF	GO:0019208	phosphatase regulator activity	3/126	0.0118007	0.06262404	0.04517005	Map2k6/Ppp1r15b/Rcan1
1-day post-SCI group vs. the control group	MF	GO:0005154	epidermal growth factor receptor binding	2/126	0.012365	0.06416038	0.04627819	Hbegf/Itga5
1-day post-SCI group vs. the control group	MF	GO:0051721	protein phosphatase 2A binding	2/126	0.012365	0.06416038	0.04627819	Mapt/Sphk1
1-day post-SCI group vs. the control group	MF	GO:0051087	chaperone binding	3/126	0.0127192	0.06527349	0.04708107	Bax/Mapt/Tsc1
1-day post-SCI group vs. the control group	MF	GO:0032182	ubiquitin-like protein binding	3/126	0.0133538	0.06776739	0.0488799	Fbxw7/Hspb1/Mdm2
1-day post-SCI group vs. the control group	MF	GO:0042805	actinin binding	2/126	0.0136405	0.06776739	0.0488799	Pdlim1/Rela
1-day post-SCI group vs. the control group	MF	GO:0044183	protein folding chaperone	2/126	0.0136405	0.06776739	0.0488799	Hspa13/Hspb1
1-day post-SCI group vs. the control group	MF	GO:0003779	actin binding	6/126	0.0148942	0.07321659	0.05281035	Ctnna1/Cttn/Ezr/Nos3/P4hb/Pdlim1
1-day post-SCI group vs. the control group	MF	GO:0004175	endopeptidase activity	6/126	0.0158035	0.07687736	0.05545082	Adam9/Bmp1/Capn2/Capns1/Ctsl/Mmp3
1-day post-SCI group vs. the control group	MF	GO:0004715	non-membrane spanning protein tyrosine kinase activit	2/126	0.0163544	0.07750353	0.05590247	Btk/Jak2
1-day post-SCI group vs. the control group	MF	GO:0005272	sodium channel activity	2/126	0.0163544	0.07750353	0.05590247	Slc4a11/Trpm2
1-day post-SCI group vs. the control group	MF	GO:0030295	protein kinase activator activity	3/126	0.0164301	0.07750353	0.05590247	Hbegf/Map2k6/Spry2
1-day post-SCI group vs. the control group	MF	GO:0005543	phospholipid binding	6/126	0.0169122	0.07898013	0.05696753	Amph/Anxa1/Apoe/Atp13a2/Axl/Btk
1-day post-SCI group vs. the control group	MF	GO:0016298	lipase activity	3/126	0.0175364	0.07967577	0.05746928	Ccr1/Hmox1/Lcat
1-day post-SCI group vs. the control group	MF	GO:0042169	SH2 domain binding	2/126	0.0177905	0.07967577	0.05746928	Jak2/Syp
1-day post-SCI group vs. the control group	MF	GO:0050661	NADP binding	2/126	0.0177905	0.07967577	0.05746928	Cat/Nos3
1-day post-SCI group vs. the control group	MF	GO:0070888	E-box binding	2/126	0.0177905	0.07967577	0.05746928	Hdac1/Myc
1-day post-SCI group vs. the control group	MF	GO:0003697	single-stranded DNA binding	3/126	0.0179143	0.07967577	0.05746928	Anxa1/Ercc1/Ybx3
1-day post-SCI group vs. the control group	MF	GO:0001222	transcription corepressor binding	2/126	0.0216037	0.09428916	0.06800976	Eed/Hdac1
1-day post-SCI group vs. the control group	MF	GO:0030544	Hsp70 protein binding	2/126	0.0216037	0.09428916	0.06800976	Bax/Tsc1
1-day post-SCI group vs. the control group	MF	GO:0042826	histone deacetylase binding	3/126	0.0219417	0.09487741	0.06843406	Hdac1/Mapk8/Rela
1-day post-SCI group vs. the control group	MF	GO:0003712	transcription coregulator activity	6/126	0.0222734	0.09542827	0.06883139	Hdac1/Nme2/Pdlim1/Ppargc1a/Ppargc1b/Pxn
1-day post-SCI group vs. the control group	MF	GO:0030374	nuclear receptor coactivator activity	2/126	0.0232156	0.09776885	0.07051963	Ppargc1a/Ppargc1b
1-day post-SCI group vs. the control group	MF	GO:0016810	hydrolase activity, acting on carbon-nitrogen (but not p	3/126	0.0232384	0.09776885	0.07051963	Cat/Gch1/Hdac1
1-day post-SCI group vs. the control group	MF	GO:0043394	proteoglycan binding	2/126	0.0248754	0.10372143	0.07481316	Apoe/Ctsl
1-day post-SCI group vs. the control group	MF	GO:0019955	cytokine binding	3/126	0.0268962	0.11115505	0.08017495	Ccr1/Il6st/Tnfrsf1a

1-day post-SCI group vs. the control group	MF	GO:0015081	sodium ion transmembrane transporter activity	3/126	0.0273738	0.11213658	0.08088292	Slc4a11/Slc8a1/Trpm2
1-day post-SCI group vs. the control group	MF	GO:0004714	transmembrane receptor protein tyrosine kinase activit	2/126	0.0283351	0.1137478	0.08204507	Axl/Met
1-day post-SCI group vs. the control group	MF	GO:0043539	protein serine/threonine kinase activator activity	2/126	0.0283351	0.1137478	0.08204507	Map2k6/Spry2
1-day post-SCI group vs. the control group	MF	GO:0008083	growth factor activity	3/126	0.0288338	0.1137478	0.08204507	Bmp1/Gfer/Hbegf
1-day post-SCI group vs. the control group	MF	GO:0003713	transcription coactivator activity	4/126	0.0292045	0.1137478	0.08204507	Nme2/Pdlim1/Ppargc1a/Ppargc1b
1-day post-SCI group vs. the control group	MF	GO:0016887	ATP hydrolysis activity	4/126	0.0292045	0.1137478	0.08204507	Abcc1/Atp13a2/Hspa13/Trap1
1-day post-SCI group vs. the control group	MF	GO:0140678	molecular function inhibitor activity	2/126	0.0292286	0.1137478	0.08204507	Fkbp1b/Tsc1
1-day post-SCI group vs. the control group	MF	GO:0003684	damaged DNA binding	2/126	0.0301332	0.11629906	0.08388527	Ercc1/Pcna
1-day post-SCI group vs. the control group	MF	GO:0016922	nuclear receptor binding	3/126	0.0318757	0.12201582	0.08800871	Pcna/Ppargc1a/Ppargc1b
1-day post-SCI group vs. the control group	MF	GO:0004860	protein kinase inhibitor activity	2/126	0.0329128	0.12395401	0.08940671	Cdkn1c/Hspb1
1-day post-SCI group vs. the control group	MF	GO:0005080	protein kinase C binding	2/126	0.0329128	0.12395401	0.08940671	Adam9/Hspb1
1-day post-SCI group vs. the control group	MF	GO:0016597	amino acid binding	2/126	0.0377584	0.13928058	0.10046159	Nos3/Prodh
1-day post-SCI group vs. the control group	MF	GO:0019210	kinase inhibitor activity	2/126	0.0377584	0.13928058	0.10046159	Cdkn1c/Hspb1
1-day post-SCI group vs. the control group	MF	GO:0004857	enzyme inhibitor activity	5/126	0.0378772	0.13928058	0.10046159	Anxa1/Cdkn1c/Dynll1/Hspb1/Rnh1
1-day post-SCI group vs. the control group	MF	GO:0015297	antiporter activity	2/126	0.0428581	0.14898534	0.10746153	Slc4a11/Slc8a1
1-day post-SCI group vs. the control group	MF	GO:0001094	TFIID-class transcription factor complex binding	1/126	0.0434916	0.14898534	0.10746153	Ercc1
1-day post-SCI group vs. the control group	MF	GO:0015368	calcium:cation antiporter activity	1/126	0.0434916	0.14898534	0.10746153	Slc8a1
1-day post-SCI group vs. the control group	MF	GO:0016174	NAD(P)H oxidase H2O2-forming activity	1/126	0.0434916	0.14898534	0.10746153	Txnrd1
1-day post-SCI group vs. the control group	MF	GO:0031078	histone deacetylase activity (H3-K14 specific)	1/126	0.0434916	0.14898534	0.10746153	Hdac1
1-day post-SCI group vs. the control group	MF	GO:0032041	NAD-dependent histone deacetylase activity (H3-K14	1/126	0.0434916	0.14898534	0.10746153	Hdac1
1-day post-SCI group vs. the control group	MF	GO:0070878	primary miRNA binding	1/126	0.0434916	0.14898534	0.10746153	Smad1
1-day post-SCI group vs. the control group	MF	GO:0097027	ubiquitin-protein transferase activator activity	1/126	0.0434916	0.14898534	0.10746153	Fbxw7
1-day post-SCI group vs. the control group	MF	GO:1990814	DNA/DNA annealing activity	1/126	0.0434916	0.14898534	0.10746153	Anxa1
1-day post-SCI group vs. the control group	MF	GO:0008234	cysteine-type peptidase activity	3/126	0.0437852	0.14898534	0.10746153	Capn2/Capns1/Ctsl
1-day post-SCI group vs. the control group	MF	GO:0042393	histone binding	4/126	0.0440256	0.14898534	0.10746153	Ctsl/Jak2/Phc3/Rbbp7
1-day post-SCI group vs. the control group	MF	GO:0002039	p53 binding	2/126	0.0449661	0.15072213	0.10871426	Hdac1/Mdm2
1-day post-SCI group vs. the control group	MF	GO:0019199	transmembrane receptor protein kinase activity	2/126	0.0460343	0.15072213	0.10871426	Axl/Met
1-day post-SCI group vs. the control group	MF	GO:0005035	death receptor activity	1/126	0.0477361	0.15072213	0.10871426	Tnfrsf1a
1-day post-SCI group vs. the control group	MF	GO:0019855	calcium channel inhibitor activity	1/126	0.0477361	0.15072213	0.10871426	Fkbp1b
1-day post-SCI group vs. the control group	MF	GO:0030983	mismatched DNA binding	1/126	0.0477361	0.15072213	0.10871426	Pcna
1-day post-SCI group vs. the control group	MF	GO:0032404	mismatch repair complex binding	1/126	0.0477361	0.15072213	0.10871426	Pcna
1-day post-SCI group vs. the control group	MF	GO:0047134	protein-disulfide reductase (NAD(P)) activity	1/126	0.0477361	0.15072213	0.10871426	Txnrd1
1-day post-SCI group vs. the control group	MF	GO:0070410	co-SMAD binding	1/126	0.0477361	0.15072213	0.10871426	Smad1
1-day post-SCI group vs. the control group	MF	GO:0005518	collagen binding	2/126	0.0492943	0.15072213	0.10871426	Adam9/Ctsl

3-day post-SCI group vs. the control group	BP	GO:0006979	response to oxidative stress	116/222	2.31E-158	9.73E-155	4.77E-155	Abcc1/Adam9/Agap3/Aif1/Alox5/Anxa1/Atf2/Atox1/Atp13a2/Atp2a2/Atp7a/Atrn/Axl/Bnip3/Brf2/Casp3/Cat/Ccs/Cd36/Cd38/Chrna4/Cygb/Cyp1b1/Dgkk/Dhfr/Ect2/Eif2s1/Endog/Etfdh/Ezh2/Fbxw7/Fos/Gch1/Gclc/Ggt7/Glrx2/Gpr37/Gpx1/Gpx3/Gpx7/Gpx8/Gsr/Hif1a/Hmox1/Hspb1/Htra2/Impact/Jak2/Jun/Lancl1/Mapk3/Mapk8/Mapt/Mcl1/Melk/Met/Mgat3/Mgst1/Msrb2/Ncoa7/Ndufa12/Ndufa6/Ndufs8/Nfe2l2/Nfkb1/Nono/Oxr1/P4hb/Parp1/Pawr/Pdgfra/Pdk1/Pdk2/Pink1/Plekha1/Pnpla8/Ppargc1a/Ppargc1b/Ppif/Ppp1r15b/Prdx4/Prdx6/Prkaa2/Ptgs1/Ptgs2/Ptprk/Pxdn/Rbpms/Rcan1/Rela/Rest/Ripk1/Ripk3/Rnf112/Rrm2b/Sfpq/Sirpa/Sirt1/Slc25a24/Slc4a11/Slc8a1/Snca/Sod2/Srxn1/Stat6/Stk24/Tbc1d24/Tlr4/Tlr6/Trpm2/Txnip/Txnrd1/Ubqln1/Ucp2/Vrk2/Zc3h12a
3-day post-SCI group vs. the control group	BP	GO:0034599	cellular response to oxidative stress	83/222	9.12E-113	1.92E-109	9.41E-110	Agap3/Aif1/Alox5/Anxa1/Atf2/Atp13a2/Atp2a2/Atp7a/Axl/Bnip3/Brf2/Cat/Ccs/Cd36/Cyp1b1/Dhfr/Ect2/Eif2s1/Endog/Ezh2/Fbxw7/Fos/Gch1/Gpr37/Gpx1/Gsr/Hif1a/Hmox1/Hspb1/Htra2/Impact/Jak2/Jun/Lancl1/Mapk3/Mapk8/Mapt/Mcl1/Melk/Met/Mgat3/Mgst1/Ncoa7/Nfe2l2/Nono/Oxr1/P4hb/Parp1/Pawr/Pdgfra/Pdk1/Pdk2/Pink1/Plekha1/Pnpla8/Ppargc1a/Ppargc1b/Ppif/Prkaa2/Ptprk/Rela/Rest/Ripk1/Ripk3/Rnf112/Sfpq/Sirpa/Sirt1/Slc25a24/Slc4a11/Slc8a1/Snca/Sod2/Srxn1/Stat6/Stk24/Tbc1d24/Tlr4/Tlr6/Trpm2/Ubqln1/Vrk2/Zc3h12a
3-day post-SCI group vs. the control group	BP	GO:0062197	cellular response to chemical stress	87/222	4.79E-111	6.72E-108	3.29E-108	Agap3/Aif1/Alox5/Anxa1/Atf2/Atp13a2/Atp2a2/Atp7a/Axl/Bnip3/Brf2/Casp3/Cat/Ccs/Cd36/Cyp1b1/Dhfr/Ect2/Eif2s1/Endog/Ezh2/Fbxw7/Fos/Gch1/Gpr37/Gpx1/Gsr/Hif1a/Hmox1/Hspb1/Htra2/Impact/Jak2/Jun/Lancl1/Mapk3/Mapk8/Mapt/Mcl1/Melk/Met/Mgat3/Mgst1/Mylk/Ncoa7/Nfe2l2/Nono/Oxr1/P4hb/Parp1/Pawr/Pdgfra/Pdk1/Pdk2/Pink1/Plekha1/Pnpla8/Ppargc1a/Ppargc1b/Ppif/Prkaa2/Ptgs2/Ptprk/Rela/Rest/Ripk1/Ripk3/Rnf112/Sfpq/Sirpa/Sirt1/Slc25a24/Slc4a11/Slc8a1/Snca/Sod2/Srxn1/Stat6/Stk24/Tbc1d24/Tlr4/Tlr6/Trpm2/Ubqln1/Vrk2/Ybx3/Zc3h12a
3-day post-SCI group vs. the control group	BP	GO:0000302	response to reactive oxygen species	51/222	7.87E-65	8.29E-62	4.06E-62	Adam9/Agap3/Anxa1/Atp7a/Axl/Bnip3/Casp3/Cat/Ccs/Cd36/Cyp1b1/Dhfr/Ect2/Endog/Ezh2/Fos/Gch1/Glrx2/Gpr37/Gpx1/Hif1a/Hmox1/Impact/Jun/Mapk3/Mapk8/Met/Nfe2l2/Parp1/Pawr/Pdgfra/Pdk2/Pink1/Plekha1/Ppargc1b/Ppif/Ppp1r15b/Prdx6/Ptprk/Rela/Ripk1/Ripk3/Rnf112/Sirpa/Sirt1/Slc8a1/Sod2/Stat6/Stk24/Trpm2/Ucp2

3-day post-SCI group vs. the control group	BP	GO:0034614	cellular response to reactive oxygen species	41/222	2.42E-53	2.04E-50	1E-50	Agap3/Anxa1/Atp7a/Axl/Bnip3/Cat/Ccs/Cd36/Cyp1b1/Dhfr/Ect2/Endog/Ezh2/Fos/Gch1/Gpr37/Impact/Jun/Mapk3/Mapk8/Met/Nfe2l2/Parp1/Pawr/Pdgfra/Pdk2/Pink1/Plekha1/Ppargc1b/Ppif/Ptprk/Rela/Ripk1/Ripk3/Rnf112/Sirpa/Sirt1/Slc8a1/Sod2/Stat6/Trpm2
3-day post-SCI group vs. the control group	BP	GO:0036473	cell death in response to oxidative stress	34/222	4.92E-47	3.46E-44	1.69E-44	Cyp1b1/Endog/Fbxw7/Gpr37/Gpx1/Hif1a/Hspb1/Jak2/Lancl1/Mapt/Mcl1/Melk/Met/Ncoa7/Nfe2l2/Nono/Oxr1/P4hb/Parp1/Pawr/Pdk1/Pink1/Rest/Ripk1/Rnf112/Sfpq/Sirt1/Sod2/Stk24/Tbc1d24/Tlr4/Tlr6/Trpm2/Ubqln1
3-day post-SCI group vs. the control group	BP	GO:1902882	regulation of response to oxidative stress	33/222	1.71E-45	1.03E-42	5.03E-43	Alox5/Cd36/Dhfr/Endog/Fbxw7/Gch1/Ggt7/Gpr37/Gpx1/Hif1a/Hspb1/Lancl1/Mcl1/Met/Ncoa7/Nfe2l2/Nono/Oxr1/P4hb/Parp1/Pawr/Pink1/Pnpla8/Rest/Ripk1/Sfpq/Sirt1/Sod2/Tbc1d24/Tlr4/Tlr6/Trpm2/Ubqln1
3-day post-SCI group vs. the control group	BP	GO:1900407	regulation of cellular response to oxidative stress	32/222	3.32E-45	1.75E-42	8.56E-43	Alox5/Cd36/Dhfr/Endog/Fbxw7/Gch1/Gpr37/Gpx1/Hif1a/Hspb1/Lancl1/Mcl1/Met/Ncoa7/Nfe2l2/Nono/Oxr1/P4hb/Parp1/Pawr/Pink1/Pnpla8/Rest/Ripk1/Sfpq/Sirt1/Sod2/Tbc1d24/Tlr4/Tlr6/Trpm2/Ubqln1
3-day post-SCI group vs. the control group	BP	GO:0042542	response to hydrogen peroxide	33/222	5.14E-42	2.41E-39	1.18E-39	Adam9/Anxa1/Axl/Bnip3/Casp3/Cat/Cyp1b1/Ect2/Endog/Ezh2/Glrx2/Gpr37/Gpx1/Hmox1/Impact/Mapk8/Met/Nfe2l2/Pawr/Pink1/Plekha1/Ppif/Ppp1r15b/Rela/Ripk1/Ripk3/Rnf112/Sirpa/Sirt1/Sod2/Stat6/Stk24/Trpm2
3-day post-SCI group vs. the control group	BP	GO:1903201	regulation of oxidative stress-induced cell death	27/222	1.1E-37	4.62E-35	2.26E-35	Endog/Fbxw7/Gpr37/Gpx1/Hif1a/Hspb1/Lancl1/Mcl1/Met/Ncoa7/Nfe2l2/Nono/Oxr1/P4hb/Parp1/Pawr/Pink1/Rest/Ripk1/Sfpq/Sirt1/Sod2/Tbc1d24/Tlr4/Tlr6/Trpm2/Ubqln1
3-day post-SCI group vs. the control group	BP	GO:0070997	neuron death	42/222	5.2E-33	1.99E-30	9.76E-31	Abcc1/Adora1/Atf2/Atp7a/Axl/Bax/Bnip3/Braf/Casp3/Eif2s1/Fbxw7/Fos/Gclc/Gpx1/Hif1a/Hmox1/Il6st/Jak2/Jun/Lancl1/Map2k4/Mapk8/Mapt/Mcl1/Ncoa7/Nono/Oxr1/Parp1/Pawr/Pink1/Ppargc1a/Rb1/Rest/Sirt1/Slc23a2/Snca/Sod2/Tbc1d24/Tlr4/Tlr6/Tnfrsf1a/Trpm2
3-day post-SCI group vs. the control group	BP	GO:0070301	cellular response to hydrogen peroxide	25/222	6.29E-32	2.21E-29	1.08E-29	Anxa1/Axl/Bnip3/Cat/Cyp1b1/Ect2/Endog/Ezh2/Gpr37/Impact/Mapk8/Met/Nfe2l2/Pawr/Pink1/Plekha1/Ppif/Rela/Ripk1/Ripk3/Rnf112/Sirpa/Sirt1/Stat6/Trpm2
3-day post-SCI group vs. the control group	BP	GO:0097193	intrinsic apoptotic signaling pathway	36/222	1.02E-31	3.3E-29	1.62E-29	Atf2/Bax/Bnip3/Casp3/Cyp1b1/Fbxw7/Gpx1/Hdac1/Hif1a/Hmox1/Hspb1/Htra2/Ier3/Jak2/Mapt/Mcl1/Melk/Myc/Nfe2l2/Nono/P4hb/Parp1/Pdk1/Pdk2/Pink1/Ppif/Ptgs2/Ripk3/Rrm2b/Sfpq/Sirt1/Sod2/Stk24/Tnfrsf1a/Ubqln1/Ybx3

3-day post-SCI group vs. the control group	BP	GO:1901214	regulation of neuron death	39/222	1.99E-31	5.98E-29	2.93E-29	Abcc1/Adora1/Atf2/Atp7a/Axl/Bax/Braf/Casp3/Eif2s1/Fbxw7/Fos/Gclc/Hif1a/Hmox1/Il6st/Jak2/Jun/Lancl1/Map2k4/Mapk8/Mapt/Mcl1/Ncoa7/Nono/Oxr1/Parp1/Pawr/Pink1/Ppargc1a/Rest/Sirt1/Slc23a2/Snca/Sod2/Tbc1d24/Tlr4/Tlr6/Tnfrsf1a/Trpm2
3-day post-SCI group vs. the control group	BP	GO:0072593	reactive oxygen species metabolic process	33/222	2.85E-31	8E-29	3.92E-29	Alox5/Atp7a/Bnip3/Cat/Ccs/Cd36/Cyp1b1/Dhfr/Gch1/Gpx1/Gpx3/Hif1a/Ier3/Mapk14/Mapt/Met/Ncf1/Nfe2l2/Nox4/Pink1/Prdx4/Prdx6/Pxdn/Ripk1/Ripk3/Sirt1/Snca/Sod2/Tlr4/Tlr6/Txnrd1/Xdh/Zc3h12a
3-day post-SCI group vs. the control group	BP	GO:0008631	intrinsic apoptotic signaling pathway in response t	20/222	5.55E-31	1.46E-28	7.16E-29	Cyp1b1/Fbxw7/Gpx1/Hif1a/Hspb1/Jak2/Mapt/Mcl1/Melk/Nfe2l2/Nono/P4hb/Parp1/Pdk1/Pink1/Sfpq/Sirt1/Sod2/Stk24/Ubqln1
3-day post-SCI group vs. the control group	BP	GO:2001233	regulation of apoptotic signaling pathway	35/222	1.49E-26	3.68E-24	1.8E-24	Bax/Ctnna1/Cttn/Fbxw7/Gclc/Gpx1/Hdac1/Hif1a/Hmox1/Hspb1/Htra2/Ier3/Itga6/Jak2/Mapk8/Mcl1/Myc/Nfe2l2/Nono/P4hb/Parp1/Pink1/Ppif/Ppp1ca/Ptgs2/Rb1/Rela/Ripk1/Ripk3/Rrm2b/Sfpq/Sirt1/Sod2/Ubqln1/Ybx3
3-day post-SCI group vs. the control group	BP	GO:0051402	neuron apoptotic process	30/222	2.08E-23	4.88E-21	2.39E-21	Atf2/Atp7a/Axl/Bax/Bnip3/Braf/Casp3/Fbxw7/Gclc/Gpx1/Hif1a/Hmox1/Il6st/Jak2/Jun/Lancl1/Map2k4/Mapk8/Mcl1/Nono/Oxr1/Parp1/Pawr/Pink1/Ppargc1a/Rb1/Sirt1/Snca/Sod2/Tnfrsf1a
3-day post-SCI group vs. the control group	BP	GO:1902175	regulation of oxidative stress-induced intrinsic apc	14/222	2.96E-23	6.56E-21	3.22E-21	Fbxw7/Gpx1/Hif1a/Hspb1/Mcl1/Nfe2l2/Nono/P4hb/Parp1/Pink1/Sfpq/Sirt1/Sod2/Ubqln1
3-day post-SCI group vs. the control group	BP	GO:0043523	regulation of neuron apoptotic process	27/222	2.17E-21	4.58E-19	2.24E-19	Atf2/Atp7a/Axl/Bax/Braf/Casp3/Fbxw7/Gclc/Hif1a/Hmox1/Il6st/Jak2/Jun/Lancl1/Map2k4/Mapk8/Mcl1/Nono/Oxr1/Parp1/Pawr/Pink1/Ppargc1a/Sirt1/Snca/Sod2/Tnfrsf1a
3-day post-SCI group vs. the control group	BP	GO:2000377	regulation of reactive oxygen species metabolic pr	22/222	2.8E-21	5.62E-19	2.75E-19	Alox5/Bnip3/Cd36/Cyp1b1/Dhfr/Gch1/Hif1a/Ier3/Mapk14/Mapt/Nfe2l2/Nox4/Pink1/Ripk1/Ripk3/Sirt1/Snca/Sod2/Tlr4/Tlr6/Xdh/Zc3h12a
3-day post-SCI group vs. the control group	BP	GO:1903203	regulation of oxidative stress-induced neuron deatl	14/222	3.24E-21	6.21E-19	3.04E-19	Fbxw7/Hif1a/Lancl1/Mcl1/Ncoa7/Nono/Oxr1/Parp1/Pink1/Rest/Tbc1d24/Tlr4/Tlr6/Trpm2
3-day post-SCI group vs. the control group	BP	GO:0036475	neuron death in response to oxidative stress	14/222	8.42E-21	1.54E-18	7.56E-19	Fbxw7/Hif1a/Lancl1/Mcl1/Ncoa7/Nono/Oxr1/Parp1/Pink1/Rest/Tbc1d24/Tlr4/Tlr6/Trpm2
3-day post-SCI group vs. the control group	BP	GO:2001242	regulation of intrinsic apoptotic signaling pathway	22/222	2.33E-20	4.09E-18	2.01E-18	Bax/Fbxw7/Gpx1/Hdac1/Hif1a/Hspb1/Mcl1/Myc/Nfe2l2/Nono/P4hb/Parp1/Pink1/Ppif/Ptgs2/Ripk3/Rrm2b/Sfpq/Sirt1/Sod2/Ubqln1/Ybx3
3-day post-SCI group vs. the control group	BP	GO:1901216	positive regulation of neuron death	20/222	2.67E-20	4.49E-18	2.2E-18	Adora1/Atf2/Bax/Casp3/Eif2s1/Fbxw7/Fos/Jun/Map2k4/Mapk8/Mapt/Mcl1/Parp1/Pawr/Rest/Snca/Tlr4/Tlr6/Tnfrsf1a/Trpm2

3-day post-SCI group vs. the control group	BP	GO:2001234	negative regulation of apoptotic signaling pathway	24/222	8.28E-20	1.34E-17	6.57E-18	Bax/Ctnna1/Cttn/Gclc/Gpx1/Hdac1/Hif1a/Hmox1/Hspb1/Ier3/Itga6/Mcl1/Nfe2l2/Nono/Pink1/Ppif/Ptgs2/Rb1/Rela/Ripk1/Rrm2b/Sirt1/Sod2/Ybx3
3-day post-SCI group vs. the control group	BP	GO:1903202	negative regulation of oxidative stress-induced cel	14/222	2.98E-18	4.64E-16	2.28E-16	Gpr37/Gpx1/Hif1a/Hspb1/Met/Ncoa7/Nfe2l2/Nono/Oxr1/Pink1/Rest/Sirt1/Sod2/Tbc1d24
3-day post-SCI group vs. the control group	BP	GO:0010038	response to metal ion	24/222	1E-17	1.51E-15	7.38E-16	Adam9/Atp13a2/Atp7a/Bnip3/Braf/Cdk4/Ect2/Fos/Hmox1/Jun/Junb/Mapk3/Mapk8/Mapt/Mylk/Nfe2l2/Ppif/Ppp1ca/Ppp3ca/Prkaa2/Slc25a24/Snca/Trpm2/Xdh
3-day post-SCI group vs. the control group	BP	GO:1901215	negative regulation of neuron death	23/222	1.3E-17	1.88E-15	9.23E-16	Abcc1/Atp7a/Axl/Bax/Braf/Gclc/Hif1a/Hmox1/Il6st/Jak2/Jun/Map2k4/Ncoa7/Nono/Oxr1/Pink1/Ppargc1a/Rest/Sirt1/Slc23a2/Snca/Sod2/Tbc1d24
3-day post-SCI group vs. the control group	BP	GO:0046777	protein autophosphorylation	22/222	1.97E-17	2.76E-15	1.35E-15	Atp13a2/Atr/Camkk2/Eif2s1/Impact/Jak2/Jun/Mapk14/Mapk3/Mapkapk2/Mapkapk3/Mark3/Melk/Met/Nme2/Pdgfra/Pink1/Ripk1/Ripk3/Stk24/Tnik/Vrk2
3-day post-SCI group vs. the control group	BP	GO:0009314	response to radiation	27/222	6.12E-17	8.32E-15	4.08E-15	Agap3/Anxa1/Atr/Bax/Braf/Casp3/Cat/Ect2/Eif2s1/Fbxw7/Gpx1/Hif1a/Impact/Jun/Mapk10/Mapk14/Mapk8/Myc/Parp1/Pcna/Ppp1ca/Ptprk/Rela/Sirt1/Sod2/Tank/Ube2a
3-day post-SCI group vs. the control group	BP	GO:0009636	response to toxic substance	17/222	7.48E-17	9.84E-15	4.83E-15	Abcc1/Atp7a/Bax/Cat/Ccs/Cd36/Cyp1b1/Dhfr/Gch1/Gpx1/Hif1a/Mapk3/Nfe2l2/Pink1/Pon2/Prdx6/Sod2
3-day post-SCI group vs. the control group	BP	GO:1903209	positive regulation of oxidative stress-induced cell	10/222	1.88E-16	2.4E-14	1.18E-14	[illegible]Endog/Fbxw7/Mcl1/Pawr/Rest/Ripk1/[illegible]/Tlr4/Tlr6/Trp[illegible]
3-day post-SCI group vs. the control group	BP	GO:0071248	cellular response to metal ion	18/222	1.3E-15	1.61E-13	7.91E-14	Atp13a2/Atp7a/Bnip3/Braf/Ect2/Fos/Hmox1/Jun/Junb/Mapk3/Mapk8/Mylk/Nfe2l2/Ppif/Prkaa2/Slc25a24/Snca/Trpm2
3-day post-SCI group vs. the control group	BP	GO:0043524	negative regulation of neuron apoptotic process	18/222	1.47E-14	1.77E-12	8.69E-13	Atp7a/Axl/Bax/Braf/Gclc/Hif1a/Hmox1/Il6st/Jak2/Jun/Map2k4/Nono/Oxr1/Pink1/Ppargc1a/Sirt1/Snca/Sod2
3-day post-SCI group vs. the control group	BP	GO:0042743	hydrogen peroxide metabolic process	12/222	2.51E-14	2.93E-12	1.44E-12	Cat/Gpx1/Gpx3/Ncf1/Nox4/Pink1/Prdx4/Prdx6/Pxdn/Snca/Sod2/Txnrd1
3-day post-SCI group vs. the control group	BP	GO:0009991	response to extracellular stimulus	24/222	2.6E-14	2.96E-12	1.45E-12	Atf2/Axl/Bax/Cdkn2b/Eif2s1/Fos/Fosl1/Impact/Itga6/Jun/Mapk14/Mapk3/Mapk8/Mapt/Nfe2l2/Pdk2/Ppargc1a/Prkaa2/Sirt1/Sod2/Stk24/Tnrc6a/Vcam1/Zc3h12a
3-day post-SCI group vs. the control group	BP	GO:2000379	positive regulation of reactive oxygen species met	13/222	3.17E-14	3.52E-12	1.72E-12	Cd36/Cyp1b1/Mapk14/Mapt/Nfe2l2/Nox4/Ripk3/Snca/Sod2/Tlr4/Tlr6/Xdh/Zc3h12a
3-day post-SCI group vs. the control group	BP	GO:2001243	negative regulation of intrinsic apoptotic signaling	14/222	3.99E-14	4.31E-12	2.11E-12	Gpx1/Hdac1/Hif1a/Hspb1/Mcl1/Nfe2l2/Nono/Pink1/Ppif/Ptgs2/Rrm2b/Sirt1/Sod2/Ybx3
3-day post-SCI group vs. the control group	BP	GO:0009411	response to UV	16/222	5.12E-14	5.39E-12	2.64E-12	Agap3/Atr/Bax/Casp3/Cat/Eif2s1/Fbxw7/Gpx1/Impact/Mapk8/Parp1/Pcna/Ptprk/Rela/Sirt1/Ube2a
3-day post-SCI group vs. the control group	BP	GO:0000303	response to superoxide	9/222	5.81E-14	5.87E-12	2.88E-12	Atp7a/Ccs/Cd36/Dhfr/Gch1/Nfe2l2/Parp1/Sod2/Ucp2
3-day post-SCI group vs. the control group	BP	GO:0051090	regulation of DNA-binding transcription factor act	24/222	5.88E-14	5.87E-12	2.88E-12	Atf2/Cat/Cd36/Cyp1b1/Ezh2/Fosl1/Hmox1/Id1/Il18rap/Jak2/Mapk3/Pink1/Ppargc1a/Ppargc1b/Ppp3ca/Rb1/Rela/Ripk1/Ripk3/Rnf2/Sirt1/Tlr4/Tlr6/Zc3h12a

3-day post-SCI group vs. the control group	BP	GO:0051403	stress-activated MAPK cascade	19/222	6E-14	5.87E-12	2.88E-12	Atf2/Ezr/Map2k3/Map2k4/Map2k6/Map4k4/Mapk10/Mapk14/Mapk3/Mapk8/Mapkapk2/Met/Nfkb1/Ripk1/Sirpa/Tlr4/Tnik/Xdh/Zc3h12a
3-day post-SCI group vs. the control group	BP	GO:0031668	cellular response to extracellular stimulus	19/222	6.46E-14	6.18E-12	3.03E-12	Atf2/Axl/Cdkn2b/Eif2s1/Fos/Fosl1/Impact/Itga6/Jun/Map k3/Mapk8/Nfe2l2/Pdk2/Prkaa2/Sirt1/Stk24/Tnrc6a/Vcam1/Zc3h12a
3-day post-SCI group vs. the control group	BP	GO:0000305	response to oxygen radical	9/222	9.24E-14	8.65E-12	4.24E-12	Atp7a/Ccs/Cd36/Dhfr/Gch1/Nfe2l2/Parp1/Sod2/Ucp2
3-day post-SCI group vs. the control group	BP	GO:0001666	response to hypoxia	18/222	1.11E-13	1.02E-11	4.98E-12	Adora1/Atf2/Bnip3/Cat/Cd38/Chrna4/Hif1a/Hmox1/Myc/Nfe2l2/P4hb/Pdk1/Pink1/Rest/Sirt1/Slc8a1/Ubqln1/Ucp2
3-day post-SCI group vs. the control group	BP	GO:0031098	stress-activated protein kinase signaling cascade	19/222	1.24E-13	1.11E-11	5.44E-12	Atf2/Ezr/Map2k3/Map2k4/Map2k6/Map4k4/Mapk10/Mapk14/Mapk3/Mapk8/Mapkapk2/Met/Nfkb1/Ripk1/Sirpa/Tlr4/Tnik/Xdh/Zc3h12a
3-day post-SCI group vs. the control group	BP	GO:0098869	cellular oxidant detoxification	9/222	1.43E-13	1.26E-11	6.17E-12	Atp7a/Cat/Ccs/Cd36/Dhfr/Gch1/Nfe2l2/Prdx6/Sod2
3-day post-SCI group vs. the control group	BP	GO:0071214	cellular response to abiotic stimulus	21/222	2.06E-13	1.74E-11	8.52E-12	Actb/Agap3/Arhgdia/Atr/Bax/Casp3/Ect2/Eif2s1/Fbxw7/Impact/Mapk14/Mylk/Parp1/Pcna/Ptgs2/Ptprk/Rest/Sirt1/Slc4a11/Tank/Ybx3
3-day post-SCI group vs. the control group	BP	GO:0104004	cellular response to environmental stimulus	21/222	2.06E-13	1.74E-11	8.52E-12	Actb/Agap3/Arhgdia/Atr/Bax/Casp3/Ect2/Eif2s1/Fbxw7/Impact/Mapk14/Mylk/Parp1/Pcna/Ptgs2/Ptprk/Rest/Sirt1/Slc4a11/Tank/Ybx3
3-day post-SCI group vs. the control group	BP	GO:0033194	response to hydroperoxide	8/222	2.42E-13	1.96E-11	9.6E-12	Cd36/Cd38/Gpx1/Jak2/Mgst1/Oxr1/Rnf112/Trpm2
3-day post-SCI group vs. the control group	BP	GO:1902176	negative regulation of oxidative stress-induced int	8/222	2.42E-13	1.96E-11	9.6E-12	Gpx1/Hif1a/Hspb1/Nfe2l2/Nono/Pink1/Sirt1/Sod2
3-day post-SCI group vs. the control group	BP	GO:0001667	ameboidal-type cell migration	24/222	3.12E-13	2.48E-11	1.21E-11	Adam9/Anxa1/Apc/Braf/Cygb/Cyp1b1/Ets1/Fbxw7/Gpx1/Hbegf/Hif1a/Hmox1/Hspb1/Jun/Map2k3/Map4k4/Met/Nfe2l2/Pdlim1/Ptgs2/Pxn/Sirt1/Slc8a1/Zc3h12a
3-day post-SCI group vs. the control group	BP	GO:0071496	cellular response to external stimulus	20/222	3.89E-13	3.04E-11	1.49E-11	Arhgdia/Atf2/Axl/Cdkn2b/Eif2s1/Fos/Fosl1/Impact/Itga6/Jun/Mapk3/Mapk8/Nfe2l2/Pdk2/Prkaa2/Sirt1/Stk24/Tnrc6a/Vcam1/Zc3h12a
3-day post-SCI group vs. the control group	BP	GO:0003012	muscle system process	23/222	4.04E-13	3.09E-11	1.52E-11	Adora1/Aif1/Atp2a2/Cd38/Cttn/Ezh2/Fkbp1b/Map2k3/Map2k4/Map2k6/Met/Mylk/Ncf1/Nfatc1/Parp1/Pawr/Ppp3ca/Ptgs1/Ptgs2/Sirt1/Slc8a1/Tnfrsf1a/Zc3h12a
3-day post-SCI group vs. the control group	BP	GO:0010821	regulation of mitochondrion organization	15/222	5.08E-13	3.82E-11	1.87E-11	Atp13a2/Bax/Bnip3/Cttn/Endog/Fbxw7/Gclc/Gpx1/Hif1a/Htra2/Ier3/Mapt/Pink1/Ppargc1a/Ppif
3-day post-SCI group vs. the control group	BP	GO:1901031	regulation of response to reactive oxygen species	10/222	5.24E-13	3.87E-11	1.9E-11	Cd36/Dhfr/Endog/Gch1/Gpr37/Met/Nfe2l2/Pawr/Pink1/Ripk1
3-day post-SCI group vs. the control group	BP	GO:0071241	cellular response to inorganic substance	18/222	5.73E-13	4.16E-11	2.04E-11	Atp13a2/Atp7a/Bnip3/Braf/Ect2/Fos/Hmox1/Jun/Junb/Mapk3/Mapk8/Mylk/Nfe2l2/Ppif/Prkaa2/Slc25a24/Snca/Trpm2
3-day post-SCI group vs. the control group	BP	GO:0036293	response to decreased oxygen levels	18/222	8.13E-13	5.81E-11	2.85E-11	Adora1/Atf2/Bnip3/Cat/Cd38/Chrna4/Hif1a/Hmox1/Myc/Nfe2l2/P4hb/Pdk1/Pink1/Rest/Sirt1/Slc8a1/Ubqln1/Ucp2

3-day post-SCI group vs. the control group	BP	GO:0009416	response to light stimulus	20/222	8.87E-13	6.22E-11	3.05E-11	Agap3/Atr/Bax/Braf/Casp3/Cat/Eif2s1/Fbxw7/Gpx1/Hif1a/Impact/Mapk10/Mapk8/Parp1/Pcna/Ppp1ca/Ptprk/Rela/Sirt1/Ube2a
3-day post-SCI group vs. the control group	BP	GO:0002237	response to molecule of bacterial origin	22/222	9.94E-13	6.86E-11	3.36E-11	Adam9/Axl/Cd36/Cdk4/Gch1/Jak2/Map2k3/Mapk14/Mapk3/Mapk8/Mapkapk2/Mapkapk3/Mgst1/Nfkb1/Ptgs2/Rela/Rpl13a/Sirpa/Snca/Tlr4/Tlr6/Zc3h12a
3-day post-SCI group vs. the control group	BP	GO:0010563	negative regulation of phosphorus metabolic proce	23/222	1.08E-12	7.19E-11	3.53E-11	Actb/Apc/Bax/Casp3/Cdkn2b/Cdkn2c/Fkbp1b/Hspb1/Ier3/Impact/Jun/Mapt/Met/Parp1/Ppargc1a/Ppp1r15b/Rb1/Sirpa/Sirt1/Slc8a1/Snca/Xdh/Zc3h12a
3-day post-SCI group vs. the control group	BP	GO:0045936	negative regulation of phosphate metabolic proces	23/222	1.08E-12	7.19E-11	3.53E-11	Actb/Apc/Bax/Casp3/Cdkn2b/Cdkn2c/Fkbp1b/Hspb1/Ier3/Impact/Jun/Mapt/Met/Parp1/Ppargc1a/Ppp1r15b/Rb1/Sirpa/Sirt1/Slc8a1/Snca/Xdh/Zc3h12a
3-day post-SCI group vs. the control group	BP	GO:0071450	cellular response to oxygen radical	8/222	1.23E-12	7.83E-11	3.84E-11	Atp7a/Ccs/Cd36/Dhfr/Gch1/Nfe2l2/Parp1/Sod2
3-day post-SCI group vs. the control group	BP	GO:0071451	cellular response to superoxide	8/222	1.23E-12	7.83E-11	3.84E-11	Atp7a/Ccs/Cd36/Dhfr/Gch1/Nfe2l2/Parp1/Sod2
3-day post-SCI group vs. the control group	BP	GO:0010631	epithelial cell migration	20/222	1.25E-12	7.83E-11	3.84E-11	Adam9/Anxa1/Apc/Cyp1b1/Ets1/Fbxw7/Gpx1/Hbegf/Hif1a/Hmox1/Hspb1/Jun/Map2k3/Map4k4/Met/Nfe2l2/Ptgs2/Pxn/Sirt1/Zc3h12a
3-day post-SCI group vs. the control group	BP	GO:0051054	positive regulation of DNA metabolic process	20/222	1.25E-12	7.83E-11	3.84E-11	Actb/Atr/Bax/Casp3/Cdk1/Cyp1b1/Endog/Jun/Map2k4/Mapk3/Mapk8/Met/Mgmt/Myc/Nox4/Parp1/Pcna/Ppargc1a/Sirt1/Stat6
3-day post-SCI group vs. the control group	BP	GO:0090132	epithelium migration	20/222	1.39E-12	8.63E-11	4.23E-11	Adam9/Anxa1/Apc/Cyp1b1/Ets1/Fbxw7/Gpx1/Hbegf/Hif1a/Hmox1/Hspb1/Jun/Map2k3/Map4k4/Met/Nfe2l2/Ptgs2/Pxn/Sirt1/Zc3h12a
3-day post-SCI group vs. the control group	BP	GO:0090130	tissue migration	20/222	1.56E-12	9.5E-11	4.66E-11	Adam9/Anxa1/Apc/Cyp1b1/Ets1/Fbxw7/Gpx1/Hbegf/Hif1a/Hmox1/Hspb1/Jun/Map2k3/Map4k4/Met/Nfe2l2/Ptgs2/Pxn/Sirt1/Zc3h12a
3-day post-SCI group vs. the control group	BP	GO:0010634	positive regulation of epithelial cell migration	15/222	1.95E-12	1.18E-10	5.76E-11	Adam9/Anxa1/Ets1/Hbegf/Hif1a/Hmox1/Hspb1/Jun/Map2k3/Map4k4/Met/Nfe2l2/Ptgs2/Sirt1/Zc3h12a
3-day post-SCI group vs. the control group	BP	GO:0042391	regulation of membrane potential	23/222	2.19E-12	1.3E-10	6.37E-11	Adora1/Atp2a2/Bax/Bnip3/Cd36/Chrna4/Fkbp1b/Gclc/Hebp2/Jun/Mapt/Met/Myc/Parp1/Pawr/Pink1/Ppp3ca/Slc4a11/Slc8a1/Snca/Sod2/Tbc1d24/Ucp2
3-day post-SCI group vs. the control group	BP	GO:0032496	response to lipopolysaccharide	21/222	2.96E-12	1.73E-10	8.48E-11	Adam9/Axl/Cd36/Cdk4/Gch1/Jak2/Map2k3/Mapk14/Mapk3/Mapk8/Mapkapk2/Mapkapk3/Mgst1/Nfkb1/Ptgs2/Rela/Rpl13a/Sirpa/Snca/Tlr4/Zc3h12a
3-day post-SCI group vs. the control group	BP	GO:0031331	positive regulation of cellular catabolic process	22/222	3.09E-12	1.78E-10	8.73E-11	Adam9/Bax/Bnip3/Casp3/Cdc20/Endog/Fbxw7/Gclc/Hif1a/Hmox1/Mapk3/Mapk8/Myc/Paip1/Pink1/Prkaa2/Sirt1/Snca/Tnrc6a/Tnrc6c/Ubqln1/Zc3h12a
3-day post-SCI group vs. the control group	BP	GO:0070482	response to oxygen levels	19/222	3.2E-12	1.82E-10	8.92E-11	Adora1/Atf2/Bnip3/Cat/Cd36/Chrna4/Hif1a/Hmox1/Myc/Nfe2l2/P4hb/Pdk1/Pink1/Rest/Sirt1/Slc8a1/Sod2/Ubqln1/Ucp2

3-day post-SCI group vs. the control group	BP	GO:0045765	regulation of angiogenesis	19/222	3.8E-12	2.13E-10	1.05E-10	Ago1/Alox5/Atf2/Btg1/Cd36/Cyp1b1/Ets1/Hif1a/Hmox1/Hspb1/Id1/Nfe2l2/Pxn/Rnh1/S100a1/Sirt1/Smad1/Tnfrsf1a/Zc3h12a
3-day post-SCI group vs. the control group	BP	GO:0042326	negative regulation of phosphorylation	21/222	4.36E-12	2.42E-10	1.18E-10	Actb/Apc/Bax/Casp3/Cdkn2b/Cdkn2c/Hspb1/Ier3/Impact/Jun/Mapt/Met/Ppargc1a/Ppp1r15b/Rb1/Sirpa/Sirt1/Slc8a1/Snca/Xdh/Zc3h12a
3-day post-SCI group vs. the control group	BP	GO:0097237	cellular response to toxic substance	10/222	4.52E-12	2.47E-10	1.21E-10	Atp7a/Cat/Ccs/Cd36/Dhfr/Gch1/Nfe2l2/Pink1/Prdx6/Sod2
3-day post-SCI group vs. the control group	BP	GO:1901342	regulation of vasculature development	19/222	4.76E-12	2.57E-10	1.26E-10	Ago1/Alox5/Atf2/Btg1/Cd36/Cyp1b1/Ets1/Hif1a/Hmox1/Hspb1/Id1/Nfe2l2/Pxn/Rnh1/S100a1/Sirt1/Smad1/Tnfrsf1a/Zc3h12a
3-day post-SCI group vs. the control group	BP	GO:0009410	response to xenobiotic stimulus	19/222	6.28E-12	3.35E-10	1.64E-10	Abcc1/Braf/Cd38/Cyp1b1/Fos/Gclc/Gpx1/Jun/Mgst1/Myc/Mylk/Nfe2l2/Pcna/Prkaa2/Rb1/Rest/Smad1/Snca/Sod2
3-day post-SCI group vs. the control group	BP	GO:0051098	regulation of binding	21/222	8.43E-12	4.44E-10	2.17E-10	Actb/Atp2a2/Atr/Bax/Eif2s1/Fbxw7/Hmox1/Id1/Jak2/Jun/Mapk3/Mapk8/Mark3/Met/Myc/Parp1/Ppp3ca/Rb1/Rest/Sirt1/Tlr4
3-day post-SCI group vs. the control group	BP	GO:0010632	regulation of epithelial cell migration	17/222	1.02E-11	5.29E-10	2.59E-10	Adam9/Anxa1/Apc/Ets1/Fbxw7/Hbegf/Hif1a/Hmox1/Hspb1/Jun/Map2k3/Map4k4/Met/Nfe2l2/Ptgs2/Sirt1/Zc3h12a
3-day post-SCI group vs. the control group	BP	GO:0032103	positive regulation of response to external stimulu	22/222	1.06E-11	5.43E-10	2.66E-10	Abcc1/Aif1/Arg1/Braf/Ccr1/Cttn/Ets1/Fkbp1b/Hspb1/Il18rap/Jak2/Mapk3/Met/Nono/Ptgs2/Ripk1/Sfpq/Snca/Stk24/Tlr4/Tlr6/Tnfrsf1a
3-day post-SCI group vs. the control group	BP	GO:0006091	generation of precursor metabolites and energy	22/222	1.36E-11	6.9E-10	3.38E-10	Alox5/Atp7a/Bax/Bnip3/Cat/Cd36/Cdk1/Etfdh/Hif1a/Ier3/Il6st/Myc/Ndufa12/Ndufa6/Ndufs8/Pink1/Ppargc1a/Ppif/Ppp1ca/Prkaa2/Snca/Sod2
3-day post-SCI group vs. the control group	BP	GO:0001659	temperature homeostasis	15/222	2.18E-11	1.07E-09	5.25E-10	Adora1/Apc/Cd36/Gpx1/Id1/Jak2/Map2k6/Ppargc1a/Ppargc1b/Ptgs2/Rb1/Stat6/Tlr4/Trpm2/Ucp2
3-day post-SCI group vs. the control group	BP	GO:0098754	detoxification	10/222	2.18E-11	1.07E-09	5.25E-10	Abcc1/Atp7a/Cat/Ccs/Cd36/Dhfr/Gch1/Nfe2l2/Prdx6/Sod2
3-day post-SCI group vs. the control group	BP	GO:0070661	leukocyte proliferation	20/222	2.19E-11	1.07E-09	5.25E-10	Aif1/Anxa1/Arg1/Bax/Btk/Casp3/Cd38/Fkbp1b/Igfbp2/Il6st/Jak2/Junb/Mapk3/Myc/Pawr/Ppp3ca/Ripk3/Stat6/Tlr4/Vcam1
3-day post-SCI group vs. the control group	BP	GO:0033002	muscle cell proliferation	17/222	3.08E-11	1.49E-09	7.31E-10	Aif1/Apc/Cdk1/Hbegf/Hif1a/Hmox1/Jak2/Jun/Mapk14/Myc/Ppargc1a/Ptgs2/Rxrb/Sirt1/Smad1/Sod2/Tlr4
3-day post-SCI group vs. the control group	BP	GO:0048511	rhythmic process	18/222	3.14E-11	1.5E-09	7.36E-10	Adora1/Anxa1/Axl/Cdk1/Ezh2/Fbxw7/Hdac1/Id1/Mapk10/Mapk8/Nono/Pdgfra/Plekha1/Ppargc1a/Ppp1ca/Prkaa2/Sfpq/Sirt1
3-day post-SCI group vs. the control group	BP	GO:0033674	positive regulation of kinase activity	21/222	3.45E-11	1.63E-09	8E-10	Adam9/Adora1/Axl/Camkk2/Ect2/Ezh2/Fbxw7/Hbegf/Jak2/Map2k3/Map2k4/Map2k6/Met/Nox4/Pdgfra/Pxn/Ripk3/Sirt1/Snca/Tlr4/Tlr6
3-day post-SCI group vs. the control group	BP	GO:1990748	cellular detoxification	9/222	3.51E-11	1.64E-09	8.06E-10	Atp7a/Cat/Ccs/Cd36/Dhfr/Gch1/Nfe2l2/Prdx6/Sod2
3-day post-SCI group vs. the control group	BP	GO:0036480	neuron intrinsic apoptotic signaling pathway in res	6/222	3.9E-11	1.78E-09	8.74E-10	Fbxw7/Hif1a/Mcl1/Nono/Parp1/Pink1
3-day post-SCI group vs. the control group	BP	GO:1903376	regulation of oxidative stress-induced neuron intri	6/222	3.9E-11	1.78E-09	8.74E-10	Fbxw7/Hif1a/Mcl1/Nono/Parp1/Pink1

3-day post-SCI group vs. the control group	BP	GO:0019430	removal of superoxide radicals	7/222	4.21E-11	1.9E-09	9.29E-10	Atp7a/Ccs/Cd36/Dhfr/Gch1/Nfe2l2/Sod2
3-day post-SCI group vs. the control group	BP	GO:0018209	peptidyl-serine modification	19/222	4.23E-11	1.9E-09	9.29E-10	Atr/Bax/Braf/Cdk1/Mapk14/Mapk3/Mapk8/Mapkapk2/Mapkapk3/Mark3/Met/Parp1/Pdk1/Pdk2/Pink1/Ptgs2/Ripk1/Snca/Vrk2
3-day post-SCI group vs. the control group	BP	GO:0042744	hydrogen peroxide catabolic process	8/222	5.34E-11	2.37E-09	1.16E-09	Cat/Gpx1/Gpx3/Prdx4/Prdx6/Pxdn/Snca/Txnrd1
3-day post-SCI group vs. the control group	BP	GO:0034612	response to tumor necrosis factor	15/222	5.61E-11	2.46E-09	1.21E-09	Adam9/Endog/Gch1/Jak2/Mapk14/Mapk3/Nfe2l2/Nfkb1/Rela/Ripk1/Sirt1/Tank/Tnfrsf1a/Ybx3/Zc3h12a
3-day post-SCI group vs. the control group	BP	GO:0034248	regulation of cellular amide metabolic process	21/222	5.91E-11	2.57E-09	1.26E-09	Ago1/Ago3/Casp3/Cdk4/Cyp1b1/Eif2s1/Impact/Mapk3/Nfe2l2/Paip1/Pdk1/Pdk2/Pink1/Ppp1ca/Ppp1r15b/Rela/Rpl13a/Snca/Tnrc6a/Tnrc6c/Zc3h12a
3-day post-SCI group vs. the control group	BP	GO:0050727	regulation of inflammatory response	19/222	7.83E-11	3.36E-09	1.65E-09	Abcc1/Adora1/Alox5/Anxa1/Ets1/Gpx1/Ier3/Jak2/Krt1/Ncf1/Nfkb1/Ptgs2/Rb1/Rela/Ripk1/Snca/Tlr4/Tlr6/Tnfrsf1a
3-day post-SCI group vs. the control group	BP	GO:0051881	regulation of mitochondrial membrane potential	11/222	8.04E-11	3.42E-09	1.68E-09	Bax/Bnip3/Gclc/Hebp2/Mapt/Myc/Parp1/Pink1/Slc4a11/Sod2/Ucp2
3-day post-SCI group vs. the control group	BP	GO:0051100	negative regulation of binding	14/222	1.08E-10	4.54E-09	2.22E-09	Actb/Atp2a2/Bax/Fbxw7/Hmox1/Id1/Jak2/Jun/Mapk3/Mapk8/Myc/Ppp3ca/Rest/Sirt1
3-day post-SCI group vs. the control group	BP	GO:0006801	superoxide metabolic process	10/222	1.13E-10	4.73E-09	2.32E-09	Atp7a/Ccs/Cd36/Dhfr/Gch1/Mapt/Ncf1/Nfe2l2/Nox4/Sod2
3-day post-SCI group vs. the control group	BP	GO:0010586	miRNA metabolic process	12/222	1.16E-10	4.8E-09	2.35E-09	Ago1/Ets1/Fos/Fosl1/Hif1a/Jun/Myc/Nfkb1/Rela/Rest/Smad1/Zc3h12a
3-day post-SCI group vs. the control group	BP	GO:0018105	peptidyl-serine phosphorylation	18/222	1.19E-10	4.87E-09	2.39E-09	Atr/Bax/Braf/Cdk1/Mapk14/Mapk3/Mapk8/Mapkapk2/Mapkapk3/Mark3/Met/Pdk1/Pdk2/Pink1/Ptgs2/Ripk1/Snca/Vrk2
3-day post-SCI group vs. the control group	BP	GO:1904019	epithelial cell apoptotic process	13/222	1.37E-10	5.54E-09	2.72E-09	Apc/Atf2/Bax/Braf/Casp3/Eif2s1/Hmox1/Id1/Jak2/Mapk8/Nfe2l2/Ppargc1a/Rb1
3-day post-SCI group vs. the control group	BP	GO:0001503	ossification	20/222	1.38E-10	5.54E-09	2.72E-09	Alox5/Apc/Bmp1/Ccr1/Hif1a/Id1/Il6st/Junb/Map2k6/Mapk14/Mapk3/Mapk8/Nfatc1/Ppargc1b/Ppp3ca/Ptgs2/Rest/Rxrb/Slc8a1/Smad1
3-day post-SCI group vs. the control group	BP	GO:0043467	regulation of generation of precursor metabolites a	13/222	1.49E-10	5.93E-09	2.91E-09	Alox5/Atp7a/Bnip3/Cd36/Cdk1/Hif1a/Ier3/Myc/Pink1/Ppargc1a/Ppif/Ppp1ca/Prkaa2
3-day post-SCI group vs. the control group	BP	GO:0015980	energy derivation by oxidation of organic compou	18/222	1.52E-10	5.97E-09	2.93E-09	Atp7a/Bax/Bnip3/Cat/Cd36/Cdk1/Hif1a/Il6st/Myc/Ndufa12/Ndufa6/Ndufs8/Pink1/Ppargc1a/Ppif/Ppp1ca/Snca/Sod2
3-day post-SCI group vs. the control group	BP	GO:1901652	response to peptide	20/222	1.63E-10	6.35E-09	3.11E-09	Abcc1/Anxa1/Apc/Atp2a2/Cd36/Cdk4/Jak2/Mapk14/Nfe2l2/Nfkb1/Parp1/Pdk2/Pxn/Rb1/Rela/Sirt1/Stat6/Tlr4/Tlr6/Vcam1
3-day post-SCI group vs. the control group	BP	GO:0001818	negative regulation of cytokine production	17/222	1.72E-10	6.65E-09	3.26E-09	Anxa1/Arg1/Axl/Banf1/Btk/Ezh2/Ezr/Hmox1/Met/Nfkb1/Parp1/Sirpa/Sirt1/Tlr4/Tlr6/Tnfrsf1a/Zc3h12a
3-day post-SCI group vs. the control group	BP	GO:0071216	cellular response to biotic stimulus	18/222	1.75E-10	6.7E-09	3.29E-09	Adam9/Axl/Cd36/Cdk4/Gch1/Jak2/Map2k3/Mapk14/Mapk3/Mapk8/Nfkb1/Ppp1r15b/Rela/Sirpa/Tlr4/Tlr6/Txnip/Zc3h12a

3-day post-SCI group vs. the control group	BP	GO:0090257	regulation of muscle system process	16/222	1.86E-10	7.06E-09	3.46E-09	Adora1/Aif1/Atp2a2/Cttn/Fkbp1b/Ncf1/Nfatc1/Parp1/Pawr/Ppp3ca/Ptgs1/Ptgs2/Sirt1/Slc8a1/Tnfrsf1a/Zc3h12a
3-day post-SCI group vs. the control group	BP	GO:0043534	blood vessel endothelial cell migration	12/222	1.9E-10	7.16E-09	3.51E-09	Anxa1/Cyp1b1/Ets1/Fbxw7/Gpx1/Hif1a/Hmox1/Hspb1/Map2k3/Nfe2l2/Ptgs2/Sirt1
3-day post-SCI group vs. the control group	BP	GO:0031667	response to nutrient levels	19/222	1.92E-10	7.17E-09	3.51E-09	Atf2/Bax/Cdkn2b/Eif2s1/Impact/Jun/Mapk14/Mapk3/Mapk8/Mapt/Nfe2l2/Pdk2/Ppargc1a/Prkaa2/Sirt1/Sod2/Stk24/Tnrc6a/Zc3h12a
3-day post-SCI group vs. the control group	BP	GO:1903706	regulation of hemopoiesis	20/222	2E-10	7.37E-09	3.61E-09	Actb/Anxa1/Apc/Axl/Braf/Ccr1/Ets1/Fbxw7/Fos/Hif1a/Hspb1/Jun/Mapk14/Myc/Nme2/Ppargc1b/Ppp3ca/Rb1/Ripk1/Zc3h12a
3-day post-SCI group vs. the control group	BP	GO:0001933	negative regulation of protein phosphorylation	18/222	2.32E-10	8.51E-09	4.17E-09	Actb/Apc/Bax/Casp3/Cdkn2c/Hspb1/Impact/Jun/Met/Ppargc1a/Ppp1r15b/Rb1/Sirpa/Sirt1/Slc8a1/Snca/Xdh/Zc3h12a
3-day post-SCI group vs. the control group	BP	GO:2001235	positive regulation of apoptotic signaling pathway	13/222	2.65E-10	9.62E-09	4.72E-09	Bax/Ctnna1/Fbxw7/Htra2/Jak2/Mapk8/Mcl1/Myc/Ppp1ca/Ripk1/Ripk3/Sfpq/Sirt1
3-day post-SCI group vs. the control group	BP	GO:1903829	positive regulation of protein localization	21/222	2.78E-10	1E-08	4.9E-09	Adam9/Apc/Atp13a2/Cd38/Cdk1/Ect2/Ezr/Fbxw7/Hif1a/Jak2/Mapk14/Mapt/Mgat3/Parp1/Prkaa2/Ptgs2/Sirt1/Tlr4/Tnfrsf1a/Trpm2/Zc3h12a
3-day post-SCI group vs. the control group	BP	GO:0051101	regulation of DNA binding	12/222	3.05E-10	1.09E-08	5.33E-09	Fbxw7/Hmox1/Id1/Jak2/Jun/Mapk8/Myc/Parp1/Rb1/Rest/Sirt1/Tlr4
3-day post-SCI group vs. the control group	BP	GO:0051592	response to calcium ion	12/222	4.01E-10	1.42E-08	6.95E-09	Adam9/Braf/Ect2/Fos/Jun/Junb/Mylk/Ppif/Ppp3ca/Prkaa2/Slc25a24/Trpm2
3-day post-SCI group vs. the control group	BP	GO:2000628	regulation of miRNA metabolic process	11/222	4.08E-10	1.43E-08	7.01E-09	Ets1/Fos/Fosl1/Hif1a/Jun/Myc/Nfkb1/Rela/Rest/Smad1/Zc3h12a
3-day post-SCI group vs. the control group	BP	GO:0036474	cell death in response to hydrogen peroxide	8/222	4.24E-10	1.47E-08	7.23E-09	Endog/Gpr37/Met/Nfe2l2/Pawr/Pink1/Ripk1/Rnf112
3-day post-SCI group vs. the control group	BP	GO:1903204	negative regulation of oxidative stress-induced neu	7/222	4.4E-10	1.51E-08	7.43E-09	Hif1a/Ncoa7/Nono/Oxr1/Pink1/Rest/Tbc1d24
3-day post-SCI group vs. the control group	BP	GO:0043542	endothelial cell migration	15/222	4.42E-10	1.51E-08	7.43E-09	Anxa1/Cyp1b1/Ets1/Fbxw7/Gpx1/Hif1a/Hmox1/Hspb1/Map2k3/Met/Nfe2l2/Ptgs2/Pxn/Sirt1/Zc3h12a
3-day post-SCI group vs. the control group	BP	GO:0071277	cellular response to calcium ion	10/222	4.59E-10	1.55E-08	7.58E-09	Braf/Ect2/Fos/Jun/Junb/Mylk/Ppif/Prkaa2/Slc25a24/Trpm2
3-day post-SCI group vs. the control group	BP	GO:2000630	positive regulation of miRNA metabolic process	10/222	4.59E-10	1.55E-08	7.58E-09	Ets1/Fos/Fosl1/Hif1a/Jun/Myc/Nfkb1/Rela/Smad1/Zc3h12a
3-day post-SCI group vs. the control group	BP	GO:0045860	positive regulation of protein kinase activity	18/222	5.02E-10	1.68E-08	8.22E-09	Adam9/Adora1/Camkk2/Ect2/Ezh2/Fbxw7/Hbegf/Jak2/Map2k3/Map2k4/Map2k6/Nox4/Pxn/Ripk3/Sirt1/Snca/Tlr4/Tlr6
3-day post-SCI group vs. the control group	BP	GO:0043536	positive regulation of blood vessel endothelial cell	9/222	5.11E-10	1.69E-08	8.3E-09	Anxa1/Ets1/Hif1a/Hmox1/Hspb1/Map2k3/Nfe2l2/Ptgs2/Sirt1
3-day post-SCI group vs. the control group	BP	GO:0050767	regulation of neurogenesis	20/222	5.92E-10	1.95E-08	9.54E-09	Braf/Cdkn2b/Ctnna1/Ezh2/Hdac1/Hif1a/Id1/Il6st/Mapk8/Mapt/Met/Myc/Ppp3ca/Rb1/Rela/Rest/Rnf112/Stau2/Tbc1d24/Tnik
3-day post-SCI group vs. the control group	BP	GO:1901653	cellular response to peptide	17/222	5.97E-10	1.95E-08	9.55E-09	Abcc1/Apc/Cd36/Cdk4/Jak2/Nfe2l2/Nfkb1/Parp1/Pdk2/Pxn/Rb1/Rela/Sirt1/Stat6/Tlr4/Tlr6/Vcam1
3-day post-SCI group vs. the control group	BP	GO:0070663	regulation of leukocyte proliferation	16/222	6.18E-10	2E-08	9.81E-09	Aif1/Anxa1/Arg1/Btk/Casp3/Cd38/Igfbp2/Il6st/Jak2/Mapk3/Pawr/Ppp3ca/Ripk3/Stat6/Tlr4/Vcam1

3-day post-SCI group vs. the control group	BP	GO:0010506	regulation of autophagy	16/222	7.99E-10	2.57E-08	1.26E-08	Atp13a2/Bnip3/Cttn/Endog/Fbxw7/Hif1a/Hmox1/Mapk3/Mapt/Mcl1/Pink1/Prkaa2/Sirt1/Snca/Ubqln1/Zc3h12a
3-day post-SCI group vs. the control group	BP	GO:0045333	cellular respiration	15/222	8.36E-10	2.67E-08	1.31E-08	Atp7a/Bax/Bnip3/Cat/Cdk1/Hif1a/Myc/Ndufa12/Ndufa6/Ndufs8/Pink1/Ppargc1a/Ppif/Snca/Sod2
3-day post-SCI group vs. the control group	BP	GO:0002573	myeloid leukocyte differentiation	15/222	8.84E-10	2.8E-08	1.37E-08	Apc/Ccr1/Fbxw7/Fos/Jun/Junb/Mapk14/Myc/Nfatc1/Nme2/Ppargc1b/Ppp3ca/Rb1/Ripk1/Sirt1
3-day post-SCI group vs. the control group	BP	GO:0010595	positive regulation of endothelial cell migration	11/222	9.25E-10	2.91E-08	1.42E-08	Anxa1/Ets1/Hif1a/Hmox1/Hspb1/Map2k3/Met/Nfe2l2/Ptgs2/Sirt1/Zc3h12a
3-day post-SCI group vs. the control group	BP	GO:0009266	response to temperature stimulus	13/222	1.03E-09	3.2E-08	1.57E-08	Adora1/Eif2s1/Gclc/Hmox1/Hspb1/Htra2/Mapk8/Mapt/Pawr/Rbbp7/Tlr4/Trpm2/Ucp2
3-day post-SCI group vs. the control group	BP	GO:0006809	nitric oxide biosynthetic process	10/222	1.09E-09	3.37E-08	1.65E-08	Aif1/Cd36/Cyp1b1/Jak2/Ptgs2/Sirpa/Sod2/Tlr4/Tlr6/Zc3h12a
3-day post-SCI group vs. the control group	BP	GO:0048732	gland development	20/222	1.23E-09	3.77E-08	1.85E-08	Anxa1/Apc/Atf2/Bax/Braf/Ezh2/Gpx1/Hif1a/Hmox1/Jak2/Jun/Mapk3/Met/Nfkb1/Pdgfra/Rela/Ripk3/Sod2/Stat6/Xdh
3-day post-SCI group vs. the control group	BP	GO:0031669	cellular response to nutrient levels	14/222	1.24E-09	3.77E-08	1.85E-08	Atf2/Cdkn2b/Eif2s1/Impact/Jun/Mapk3/Mapk8/Nfe2l2/Pdk2/Prkaa2/Sirt1/Stk24/Tnrc6a/Zc3h12a
3-day post-SCI group vs. the control group	BP	GO:0009612	response to mechanical stimulus	13/222	1.27E-09	3.83E-08	1.88E-08	Arhgdia/Cd36/Fos/Jun/Mapk14/Mapk8/Myc/Nfkb1/Pawr/Rela/Rest/Slc8a1/Tlr4
3-day post-SCI group vs. the control group	BP	GO:0003015	heart process	15/222	1.37E-09	4.13E-08	2.02E-08	Adora1/Atp2a2/Fkbp1b/Gch1/Gpx1/Hbegf/Jak2/Map2k3/Map2k6/Met/Nox4/S100a1/Sirt1/Slc8a1/Zc3h12a
3-day post-SCI group vs. the control group	BP	GO:0071478	cellular response to radiation	13/222	1.45E-09	4.34E-08	2.13E-08	Agap3/Atr/Bax/Ect2/Eif2s1/Fbxw7/Impact/Mapk14/Parp1/Pcna/Ptprk/Sirt1/Tank
3-day post-SCI group vs. the control group	BP	GO:0043525	positive regulation of neuron apoptotic process	10/222	1.54E-09	4.54E-08	2.22E-08	Atf2/Bax/Casp3/Fbxw7/Jun/Map2k4/Mapk8/Mcl1/Pawr/Tnfrsf1a
3-day post-SCI group vs. the control group	BP	GO:1902893	regulation of miRNA transcription	10/222	1.54E-09	4.54E-08	2.22E-08	Ets1/Fos/Fosl1/Hif1a/Jun/Myc/Nfkb1/Rela/Rest/Smad1
3-day post-SCI group vs. the control group	BP	GO:0046651	lymphocyte proliferation	17/222	1.63E-09	4.76E-08	2.33E-08	Aif1/Anxa1/Arg1/Bax/Btk/Casp3/Cd38/Fkbp1b/Igfbp2/Il6st/Jak2/Myc/Pawr/Ppp3ca/Ripk3/Tlr4/Vcam1
3-day post-SCI group vs. the control group	BP	GO:0050678	regulation of epithelial cell proliferation	19/222	1.76E-09	5.1E-08	2.5E-08	Alox5/Apc/Arg1/Atf2/Atp7a/Bax/Cdk4/Cdkn2b/Ctsl/Gpx1/Hmox1/Id1/Jun/Myc/Nfatc1/Ptprk/Rb1/Sirt1/Xdh
3-day post-SCI group vs. the control group	BP	GO:0045766	positive regulation of angiogenesis	13/222	1.78E-09	5.1E-08	2.5E-08	Btg1/Cyp1b1/Ets1/Hif1a/Hmox1/Hspb1/Nfe2l2/Pxn/S100a1/Sirt1/Smad1/Tnfrsf1a/Zc3h12a
3-day post-SCI group vs. the control group	BP	GO:1904018	positive regulation of vasculature development	13/222	1.78E-09	5.1E-08	2.5E-08	Btg1/Cyp1b1/Ets1/Hif1a/Hmox1/Hspb1/Nfe2l2/Pxn/S100a1/Sirt1/Smad1/Tnfrsf1a/Zc3h12a
3-day post-SCI group vs. the control group	BP	GO:0060537	muscle tissue development	20/222	1.8E-09	5.13E-08	2.51E-08	Apc/Cdk1/Fos/Gpx1/Map2k4/Mapk14/Met/Myc/Mylk/Nox4/Pdgfra/Ppargc1a/Ppp3ca/Rb1/Rcan1/Rxrb/Sirt1/Slc8a1/Smad1/Ybx3
3-day post-SCI group vs. the control group	BP	GO:0071900	regulation of protein serine/threonine kinase activi	17/222	1.86E-09	5.24E-08	2.57E-08	Actb/Adam9/Apc/Casp3/Ccna2/Cdkn2b/Cdkn2c/Ezh2/Map2k4/Map2k6/Nox4/Rb1/Sirt1/Slc8a1/Snca/Tlr4/Tlr6
3-day post-SCI group vs. the control group	BP	GO:0061614	miRNA transcription	10/222	1.93E-09	5.41E-08	2.65E-08	Ets1/Fos/Fosl1/Hif1a/Jun/Myc/Nfkb1/Rela/Rest/Smad1

3-day post-SCI group vs. the control group	BP	GO:0030099	myeloid cell differentiation	19/222	1.96E-09	5.47E-08	2.68E-08	Apc/Casp3/Ccr1/Ets1/Fbxw7/Fos/Hif1a/Jak2/Jun/Junb/Mapk14/Myc/Nfatc1/Nme2/Ppargc1b/Ppp3ca/Rb1/Ripk1/Sirt1
3-day post-SCI group vs. the control group	BP	GO:1902105	regulation of leukocyte differentiation	17/222	2.02E-09	5.6E-08	2.75E-08	Actb/Anxa1/Apc/Axl/Braf/Ccr1/Fbxw7/Fos/Hspb1/Jun/Myc/Nme2/Ppargc1b/Ppp3ca/Rb1/Ripk1/Zc3h12a
3-day post-SCI group vs. the control group	BP	GO:0007623	circadian rhythm	14/222	2.12E-09	5.81E-08	2.85E-08	Adora1/Cdk1/Ezh2/Fbxw7/Hdac1/Id1/Mapk10/Mapk8/Nono/Ppargc1a/Ppp1ca/Prkaa2/Sfpq/Sirt1
3-day post-SCI group vs. the control group	BP	GO:0034644	cellular response to UV	10/222	2.15E-09	5.81E-08	2.85E-08	Agap3/Atr/Bax/Eif2s1/Fbxw7/Impact/Parp1/Pcna/Ptprk/Sirt1
3-day post-SCI group vs. the control group	BP	GO:0046209	nitric oxide metabolic process	10/222	2.15E-09	5.81E-08	2.85E-08	Aif1/Cd36/Cyp1b1/Jak2/Ptgs2/Sirpa/Sod2/Tlr4/Tlr6/Zc3h12a
3-day post-SCI group vs. the control group	BP	GO:0008637	apoptotic mitochondrial changes	11/222	2.15E-09	5.81E-08	2.85E-08	Atf2/Atp7a/Bax/Bnip3/Gclc/Gpx1/Ier3/Jun/Pink1/Ppif/Sod2
3-day post-SCI group vs. the control group	BP	GO:0032943	mononuclear cell proliferation	17/222	2.2E-09	5.91E-08	2.9E-08	Aif1/Anxa1/Arg1/Bax/Btk/Casp3/Cd38/Fkbp1b/Igfbp2/Il6st/Jak2/Myc/Pawr/Ppp3ca/Ripk3/Tlr4/Vcam1
3-day post-SCI group vs. the control group	BP	GO:1900180	regulation of protein localization to nucleus	12/222	2.26E-09	6.03E-08	2.95E-08	Atp13a2/Cd36/Cdk1/Ctnna1/Ect2/Jak2/Mapk14/Parp1/Ptgs2/Sirt1/Tnfrsf1a/Zc3h12a
3-day post-SCI group vs. the control group	BP	GO:0006631	fatty acid metabolic process	19/222	2.63E-09	6.96E-08	3.41E-08	Alox5/Anxa1/Cd36/Cygb/Cyp1b1/Etfdh/Gpx1/Hao1/Mapk14/Pdk1/Pdk2/Pnpla8/Ppargc1a/Prkaa2/Ptgs1/Ptgs2/Sirt1/Snca/Tnfrsf1a
3-day post-SCI group vs. the control group	BP	GO:0045637	regulation of myeloid cell differentiation	14/222	2.66E-09	6.98E-08	3.42E-08	Apc/Ccr1/Ets1/Fbxw7/Fos/Hif1a/Jun/Mapk14/Myc/Nme2/Ppargc1b/Ppp3ca/Rb1/Ripk1
3-day post-SCI group vs. the control group	BP	GO:2001057	reactive nitrogen species metabolic process	10/222	2.67E-09	6.98E-08	3.42E-08	Aif1/Cd36/Cyp1b1/Jak2/Ptgs2/Sirpa/Sod2/Tlr4/Tlr6/Zc3h12a
3-day post-SCI group vs. the control group	BP	GO:0045862	positive regulation of proteolysis	17/222	2.84E-09	7.38E-08	3.62E-08	Adam9/Bax/Cdc20/Fbxw7/Gclc/Htra2/Jak2/Mapk8/Myc/Pawr/Rest/Sirt1/Snca/Tank/Ubqln1/Xdh/Zc3h12a
3-day post-SCI group vs. the control group	BP	GO:1903522	regulation of blood circulation	15/222	2.86E-09	7.38E-08	3.62E-08	Adora1/Alox5/Atp2a2/Cd38/Fkbp1b/Gch1/Hbegf/Hif1a/Jak2/Ptgs1/Ptgs2/S100a1/Sirt1/Slc8a1/Zc3h12a
3-day post-SCI group vs. the control group	BP	GO:0007178	transmembrane receptor protein serine/threonine k	18/222	3.07E-09	7.88E-08	3.86E-08	Adam9/Atf2/Cdkn2b/Fos/Hdac1/Id1/Itgb5/Jak2/Jun/Mapk14/Mapk3/Parp1/Ptprk/Pxn/Rbbp7/Rbpms/Sirt1/Smad1
3-day post-SCI group vs. the control group	BP	GO:0045454	cell redox homeostasis	7/222	3.19E-09	8.1E-08	3.97E-08	Gclc/Gpx1/Gsr/Nfe2l2/Prdx4/Prdx6/Txnrd1
3-day post-SCI group vs. the control group	BP	GO:1902883	negative regulation of response to oxidative stress	7/222	3.19E-09	8.1E-08	3.97E-08	Ggt7/Gpr37/Met/Ncoa7/Nfe2l2/Oxr1/Pink1
3-day post-SCI group vs. the control group	BP	GO:0071219	cellular response to molecule of bacterial origin	16/222	3.22E-09	8.13E-08	3.99E-08	Adam9/Axl/Cd36/Cdk4/Gch1/Jak2/Map2k3/Mapk14/Mapk3/Mapk8/Nfkb1/Rela/Sirpa/Tlr4/Tlr6/Zc3h12a
3-day post-SCI group vs. the control group	BP	GO:0043535	regulation of blood vessel endothelial cell migratic	10/222	3.29E-09	8.25E-08	4.05E-08	Anxa1/Ets1/Fbxw7/Hif1a/Hmox1/Hspb1/Map2k3/Nfe2l2/Ptgs2/Sirt1
3-day post-SCI group vs. the control group	BP	GO:0051091	positive regulation of DNA-binding transcription f	15/222	3.32E-09	8.27E-08	4.06E-08	Atf2/Cat/Cd36/Fosl1/Il18rap/Jak2/Pink1/Ppargc1a/Ppargc1b/Ppp3ca/Rela/Ripk1/Ripk3/Tlr4/Tlr6
3-day post-SCI group vs. the control group	BP	GO:0007249	I-kappaB kinase/NF-kappaB signaling	14/222	3.34E-09	8.27E-08	4.06E-08	Btk/Cd36/Hdac1/Hspb1/Pink1/Rela/Ripk1/Ripk3/Sirpa/Sirt1/Tank/Tlr4/Tlr6/Zc3h12a
3-day post-SCI group vs. the control group	BP	GO:0032640	tumor necrosis factor production	13/222	3.64E-09	8.96E-08	4.39E-08	Axl/Cd36/Hspb1/Jak2/Mapk14/Mapkapk2/Ripk1/Sirpa/Sirt1/Tlr4/Tlr6/Tnfrsf1a/Zc3h12a

3-day post-SCI group vs. the control group	BP	GO:0014812	muscle cell migration	11/222	3.96E-09	9.69E-08	4.75E-08	Aif1/Anxa1/Atp7a/Cyp1b1/Il6st/Met/Myc/Nfe2l2/Nox4/Ppargc1a/Tlr4
3-day post-SCI group vs. the control group	BP	GO:0034504	protein localization to nucleus	16/222	4.22E-09	1.03E-07	5.04E-08	Agap3/Atf2/Atp13a2/Cd36/Cdk1/Ctnna1/Ect2/Jak2/Mapk14/Parp1/Ppp3ca/Ptgs2/Sirt1/Tnfrsf1a/Txnip/Zc3h12a
3-day post-SCI group vs. the control group	BP	GO:1902895	positive regulation of miRNA transcription	9/222	4.37E-09	1.05E-07	5.15E-08	Ets1/Fos/Fosl1/Hif1a/Jun/Myc/Nfkb1/Rela/Smad1
3-day post-SCI group vs. the control group	BP	GO:0071706	tumor necrosis factor superfamily cytokine produc	13/222	4.38E-09	1.05E-07	5.15E-08	Axl/Cd36/Hspb1/Jak2/Mapk14/Mapkapk2/Ripk1/Sirpa/Sirt1/Tlr4/Tlr6/Tnfrsf1a/Zc3h12a
3-day post-SCI group vs. the control group	BP	GO:0003018	vascular process in circulatory system	14/222	4.4E-09	1.05E-07	5.15E-08	Abcc1/Adora1/Alox5/Cd36/Cd38/Gch1/Gclc/Gpx1/Hif1a/Ptgs1/Ptgs2/Sirt1/Slc8a1/Sod2
3-day post-SCI group vs. the control group	BP	GO:0051222	positive regulation of protein transport	16/222	4.42E-09	1.05E-07	5.15E-08	Adam9/Atp13a2/Cd38/Cdk1/Ect2/Ezr/Fbxw7/Hif1a/Jak2/Mapk14/Ptgs2/Sirt1/Tlr4/Tnfrsf1a/Trpm2/Zc3h12a
3-day post-SCI group vs. the control group	BP	GO:0043588	skin development	16/222	4.82E-09	1.14E-07	5.59E-08	Anxa1/Apc/Atp7a/Casp3/Ctsl/Dhcr24/Ezh2/Hdac1/Itga6/Krt1/Met/Ppp3ca/Ptgs1/Ptgs2/Rela/Txnip
3-day post-SCI group vs. the control group	BP	GO:0042098	T cell proliferation	14/222	4.91E-09	1.15E-07	5.66E-08	Aif1/Anxa1/Arg1/Bax/Casp3/Fkbp1b/Igfbp2/Il6st/Jak2/Myc/Pawr/Ppp3ca/Ripk3/Vcam1
3-day post-SCI group vs. the control group	BP	GO:0045428	regulation of nitric oxide biosynthetic process	9/222	4.96E-09	1.16E-07	5.68E-08	Aif1/Cd36/Jak2/Ptgs2/Sirpa/Sod2/Tlr4/Tlr6/Zc3h12a
3-day post-SCI group vs. the control group	BP	GO:1903037	regulation of leukocyte cell-cell adhesion	17/222	5.05E-09	1.17E-07	5.76E-08	Actb/Aif1/Alox5/Anxa1/Arg1/Casp3/Ets1/Hspb1/Igfbp2/Il6st/Jak2/Pawr/Ppp3ca/Rela/Sirpa/Vcam1/Zc3h12a
3-day post-SCI group vs. the control group	BP	GO:0042752	regulation of circadian rhythm	11/222	5.08E-09	1.18E-07	5.76E-08	Adora1/Cdk1/Ezh2/Fbxw7/Mapk10/Mapk8/Nono/Ppargc1a/Ppp1ca/Prkaa2/Sfpq
3-day post-SCI group vs. the control group	BP	GO:1904646	cellular response to amyloid-beta	7/222	5.12E-09	1.18E-07	5.78E-08	Abcc1/Cd36/Parp1/Sirt1/Tlr4/Tlr6/Vcam1
3-day post-SCI group vs. the control group	BP	GO:0097191	extrinsic apoptotic signaling pathway	14/222	5.18E-09	1.19E-07	5.81E-08	Bax/Casp3/Ctnna1/Cttn/Gclc/Gpx1/Hmox1/Htra2/Itga6/Jak2/Mcl1/Ppp1ca/Rela/Ripk1
3-day post-SCI group vs. the control group	BP	GO:0060047	heart contraction	14/222	6.41E-09	1.45E-07	7.11E-08	Adora1/Atp2a2/Fkbp1b/Gch1/Gpx1/Hbegf/Jak2/Map2k3/Map2k6/Met/S100a1/Sirt1/Slc8a1/Zc3h12a
3-day post-SCI group vs. the control group	BP	GO:1903205	regulation of hydrogen peroxide-induced cell deatl	7/222	6.41E-09	1.45E-07	7.11E-08	Endog/Gpr37/Met/Nfe2l2/Pawr/Pink1/Ripk1
3-day post-SCI group vs. the control group	BP	GO:0010660	regulation of muscle cell apoptotic process	10/222	7.29E-09	1.64E-07	8.04E-08	Atp2a2/Bnip3/Hmox1/Jak2/Map2k4/Mapk8/Nfe2l2/Sirt1/Sod2/Zc3h12a
3-day post-SCI group vs. the control group	BP	GO:0080164	regulation of nitric oxide metabolic process	9/222	8.02E-09	1.8E-07	8.8E-08	Aif1/Cd36/Jak2/Ptgs2/Sirpa/Sod2/Tlr4/Tlr6/Zc3h12a
3-day post-SCI group vs. the control group	BP	GO:0038066	p38MAPK cascade	8/222	8.11E-09	1.81E-07	8.86E-08	Atf2/Ezr/Map2k3/Mapk14/Mapkapk2/Met/Xdh/Zc3h12a
3-day post-SCI group vs. the control group	BP	GO:0045786	negative regulation of cell cycle	17/222	8.74E-09	1.94E-07	9.5E-08	Apc/Atf2/Atr/Btg1/Casp3/Cdc20/Cdk1/Cdkn2b/Cdkn2c/Ets1/Ezh2/Ier3/Mapk14/Ptgs2/Ptprk/Rb1/Sirt1
3-day post-SCI group vs. the control group	BP	GO:1904951	positive regulation of establishment of protein loca	16/222	8.79E-09	1.94E-07	9.5E-08	Adam9/Atp13a2/Cd38/Cdk1/Ect2/Ezr/Fbxw7/Hif1a/Jak2/Mapk14/Ptgs2/Sirt1/Tlr4/Tnfrsf1a/Trpm2/Zc3h12a
3-day post-SCI group vs. the control group	BP	GO:0009267	cellular response to starvation	12/222	9.28E-09	2.04E-07	9.98E-08	Atf2/Eif2s1/Impact/Jun/Mapk3/Mapk8/Nfe2l2/Prkaa2/Sirt1/Stk24/Tnrc6a/Zc3h12a
3-day post-SCI group vs. the control group	BP	GO:0035994	response to muscle stretch	6/222	9.38E-09	2.05E-07	0.0000001	Fos/Jun/Mapk14/Nfkb1/Rela/Slc8a1
3-day post-SCI group vs. the control group	BP	GO:0010823	negative regulation of mitochondrion organization	8/222	9.43E-09	2.05E-07	0.0000001	Bnip3/Gclc/Gpx1/Ier3/Mapt/Pink1/Ppargc1a/Ppif
3-day post-SCI group vs. the control group	BP	GO:0032386	regulation of intracellular transport	16/222	9.56E-09	2.06E-07	1.01E-07	Atp13a2/Cd36/Cdk1/Ect2/Ezr/Fbxw7/Ier3/Jak2/Mapk14/Mapk3/Mapk8/Mapt/Pink1/Ptgs2/Tnfrsf1a/Zc3h12a

3-day post-SCI group vs. the control group	BP	GO:1903036	positive regulation of response to wounding	9/222	1.01E-08	2.17E-07	1.06E-07	Anxa1/Atp7a/Braf/Cd36/Fkbp1b/Hbegf/Mylk/Nfe2l2/Stk24
3-day post-SCI group vs. the control group	BP	GO:0035296	regulation of tube diameter	12/222	1.06E-08	2.25E-07	1.1E-07	Adora1/Alox5/Cd38/Gch1/Gclc/Gpx1/Hif1a/Ptgs1/Ptgs2/Sirt1/Slc8a1/Sod2
3-day post-SCI group vs. the control group	BP	GO:0097746	blood vessel diameter maintenance	12/222	1.06E-08	2.25E-07	1.1E-07	Adora1/Alox5/Cd38/Gch1/Gclc/Gpx1/Hif1a/Ptgs1/Ptgs2/Sirt1/Slc8a1/Sod2
3-day post-SCI group vs. the control group	BP	GO:0050670	regulation of lymphocyte proliferation	14/222	1.13E-08	2.38E-07	1.16E-07	Aif1/Anxa1/Arg1/Btk/Casp3/Cd38/Igfbp2/Il6st/Jak2/Pawr/Ppp3ca/Ripk3/Tlr4/Vcam1
3-day post-SCI group vs. the control group	BP	GO:0035150	regulation of tube size	12/222	1.13E-08	2.38E-07	1.16E-07	Adora1/Alox5/Cd38/Gch1/Gclc/Gpx1/Hif1a/Ptgs1/Ptgs2/Sirt1/Slc8a1/Sod2
3-day post-SCI group vs. the control group	BP	GO:0007006	mitochondrial membrane organization	10/222	1.16E-08	2.41E-07	1.18E-07	Atf2/Bax/Bnip3/Gclc/Hebp2/Ier3/Myc/Pink1/Ppif/Snca
3-day post-SCI group vs. the control group	BP	GO:0010657	muscle cell apoptotic process	10/222	1.16E-08	2.41E-07	1.18E-07	Atp2a2/Bnip3/Hmox1/Jak2/Map2k4/Mapk8/Nfe2l2/Sirt1/Sod2/Zc3h12a
3-day post-SCI group vs. the control group	BP	GO:0022407	regulation of cell-cell adhesion	19/222	1.18E-08	2.44E-07	1.2E-07	Actb/Aif1/Alox5/Anxa1/Arg1/Casp3/Ets1/Hspb1/Igfbp2/Il6st/Itga6/Jak2/Mapk14/Pawr/Ppp3ca/Rela/Sirpa/Vcam1/Zc3h12a
3-day post-SCI group vs. the control group	BP	GO:0006749	glutathione metabolic process	9/222	1.26E-08	2.59E-07	1.27E-07	Gclc/Ggt7/Gpx1/Gpx3/Gsr/Gstt2/Mgst1/Nfe2l2/Sod2
3-day post-SCI group vs. the control group	BP	GO:0051353	positive regulation of oxidoreductase activity	8/222	1.26E-08	2.59E-07	1.27E-07	Alox5/Atp7a/Ccs/Dhfr/Gch1/Ripk3/S100a1/Snca
3-day post-SCI group vs. the control group	BP	GO:0048661	positive regulation of smooth muscle cell prolifera	10/222	1.27E-08	2.59E-07	1.27E-07	Aif1/Hbegf/Hif1a/Hmox1/Jak2/Jun/Myc/Ppargc1a/Ptgs2/Tlr4
3-day post-SCI group vs. the control group	BP	GO:1901522	positive regulation of transcription from RNA poly	6/222	1.28E-08	2.6E-07	1.27E-07	Atf2/Hif1a/Jun/Nfe2l2/Rela/Smad1
3-day post-SCI group vs. the control group	BP	GO:0010594	regulation of endothelial cell migration	12/222	1.28E-08	2.6E-07	1.27E-07	Anxa1/Ets1/Fbxw7/Hif1a/Hmox1/Hspb1/Map2k3/Met/Nfe2l2/Ptgs2/Sirt1/Zc3h12a
3-day post-SCI group vs. the control group	BP	GO:0006606	protein import into nucleus	12/222	1.37E-08	2.74E-07	1.34E-07	Agap3/Atf2/Cd36/Cdk1/Ect2/Jak2/Mapk14/Ppp3ca/Ptgs2/Tnfrsf1a/Txnip/Zc3h12a
3-day post-SCI group vs. the control group	BP	GO:1903531	negative regulation of secretion by cell	12/222	1.37E-08	2.74E-07	1.34E-07	Adora1/Anxa1/Braf/Fkbp1b/Hmox1/Ppp3ca/Ptgs1/Rest/Sirt1/Snca/Tnfrsf1a/Ucp2
3-day post-SCI group vs. the control group	BP	GO:0071456	cellular response to hypoxia	10/222	1.38E-08	2.76E-07	1.35E-07	Atf2/Bnip3/Hif1a/Myc/Nfe2l2/P4hb/Pdk1/Pink1/Sirt1/Ubqln1
3-day post-SCI group vs. the control group	BP	GO:0006690	icosanoid metabolic process	11/222	1.4E-08	2.77E-07	1.36E-07	Alox5/Anxa1/Cyp1b1/Gpx1/Ncf1/Pnpla8/Ptgs1/Ptgs2/Sirt1/Tlr4/Tnfrsf1a
3-day post-SCI group vs. the control group	BP	GO:0042692	muscle cell differentiation	18/222	1.4E-08	2.78E-07	1.36E-07	Atp2a2/Ezh2/Gpx1/Map2k4/Mapk14/Met/Myc/Nfatc1/Nox4/Pdgfra/Ppp3ca/Rb1/Rcan1/Rxrb/Sirt1/Slc8a1/Smad1/Sod2
3-day post-SCI group vs. the control group	BP	GO:0032944	regulation of mononuclear cell proliferation	14/222	1.44E-08	2.84E-07	1.39E-07	Aif1/Anxa1/Arg1/Btk/Casp3/Cd38/Igfbp2/Il6st/Jak2/Pawr/Ppp3ca/Ripk3/Tlr4/Vcam1
3-day post-SCI group vs. the control group	BP	GO:0002761	regulation of myeloid leukocyte differentiation	11/222	1.5E-08	2.94E-07	1.44E-07	Apc/Ccr1/Fbxw7/Fos/Jun/Myc/Nme2/Ppargc1b/Ppp3ca/Rb1/Ripk1
3-day post-SCI group vs. the control group	BP	GO:0071222	cellular response to lipopolysaccharide	15/222	1.56E-08	3.04E-07	1.49E-07	Adam9/Axl/Cd36/Cdk4/Gch1/Jak2/Map2k3/Mapk14/Map2k3/Mapk8/Nfkb1/Rela/Sirpa/Tlr4/Zc3h12a
3-day post-SCI group vs. the control group	BP	GO:0050708	regulation of protein secretion	15/222	1.63E-08	3.16E-07	1.55E-07	Adam9/Alox5/Anxa1/Atp13a2/Cd38/Ezr/Fkbp1b/Hif1a/Jak2/Ppp3ca/Rest/Sirt1/Tlr4/Trpm2/Ucp2

3-day post-SCI group vs. the control group	BP	GO:0051170	import into nucleus	12/222	1.76E-08	3.39E-07	1.66E-07	Agap3/Atf2/Cd36/Cdk1/Ect2/Jak2/Mapk14/Ppp3ca/Ptgs2/Tnfrsf1a/Txnip/Zc3h12a
3-day post-SCI group vs. the control group	BP	GO:1904645	response to amyloid-beta	7/222	1.77E-08	3.41E-07	1.67E-07	Abcc1/Cd36/Parp1/Sirt1/Tlr4/Tlr6/Vcam1
3-day post-SCI group vs. the control group	BP	GO:0045639	positive regulation of myeloid cell differentiation	10/222	1.79E-08	3.42E-07	1.68E-07	Ccr1/Ets1/Fos/Hif1a/Jun/Mapk14/Ppargc1b/Ppp3ca/Rb1/Ripk1
3-day post-SCI group vs. the control group	BP	GO:0071482	cellular response to light stimulus	10/222	1.79E-08	3.42E-07	1.68E-07	Agap3/Aif/Bax/Eif2s1/Fbxw7/Impact/Parp1/Pcna/Ptprk/Sirt1
3-day post-SCI group vs. the control group	BP	GO:0035924	cellular response to vascular endothelial growth fa	8/222	1.92E-08	3.62E-07	1.77E-07	Anxa1/Hspb1/Map2k3/Mapk14/Mapkapk2/Pdgfra/Rela/Xdh
3-day post-SCI group vs. the control group	BP	GO:1900015	regulation of cytokine production involved in infla	8/222	1.92E-08	3.62E-07	1.77E-07	Alox5/Ezh2/Hif1a/Mapk14/Sirpa/Tlr4/Tlr6/Zc3h12a
3-day post-SCI group vs. the control group	BP	GO:0071356	cellular response to tumor necrosis factor	12/222	1.98E-08	3.73E-07	1.83E-07	Jak2/Mapk14/Mapk3/Nfe2l2/Nfkb1/Rela/Ripk1/Sirt1/Tank/Tnfrsf1a/Ybx3/Zc3h12a
3-day post-SCI group vs. the control group	BP	GO:0120161	regulation of cold-induced thermogenesis	11/222	2E-08	3.75E-07	1.84E-07	Apc/Cd36/Id1/Jak2/Map2k6/Ppargc1a/Ppargc1b/Rb1/Stat6/Tlr4/Ucp2
3-day post-SCI group vs. the control group	BP	GO:0007159	leukocyte cell-cell adhesion	17/222	2.28E-08	4.25E-07	2.08E-07	Actb/Aif1/Alox5/Anxa1/Arg1/Casp3/Ets1/Hspb1/Igfbp2/Il6st/Jak2/Pawr/Ppp3ca/Rela/Sirpa/Vcam1/Zc3h12a
3-day post-SCI group vs. the control group	BP	GO:0106106	cold-induced thermogenesis	11/222	2.3E-08	4.27E-07	2.09E-07	Apc/Cd36/Id1/Jak2/Map2k6/Ppargc1a/Ppargc1b/Rb1/Stat6/Tlr4/Ucp2
3-day post-SCI group vs. the control group	BP	GO:1904035	regulation of epithelial cell apoptotic process	10/222	2.31E-08	4.27E-07	2.09E-07	Apc/Bax/Braf/Eif2s1/Hmox1/Id1/Jak2/Nfe2l2/Ppargc1a/Rb1
3-day post-SCI group vs. the control group	BP	GO:0032680	regulation of tumor necrosis factor production	12/222	2.38E-08	4.37E-07	2.14E-07	Axl/Cd36/Hspb1/Jak2/Mapkapk2/Ripk1/Sirpa/Sirt1/Tlr4/Tlr6/Tnfrsf1a/Zc3h12a
3-day post-SCI group vs. the control group	BP	GO:0002683	negative regulation of immune system process	18/222	2.4E-08	4.39E-07	2.15E-07	Adora1/Anxa1/Arg1/Axl/Banf1/Btk/Casp3/Ccr1/Ezr/Fbxw7/Gpx1/Hmox1/Hspb1/Myc/Nme2/Pawr/Stat6/Zc3h12a
3-day post-SCI group vs. the control group	BP	GO:0006939	smooth muscle contraction	10/222	2.51E-08	4.58E-07	2.24E-07	Adora1/Cd38/Cttn/Fkbp1b/Mylk/Ncf1/Pawr/Ptgs1/Ptgs2/Slc8a1
3-day post-SCI group vs. the control group	BP	GO:0009060	aerobic respiration	12/222	2.52E-08	4.58E-07	2.25E-07	Atp7a/Bnip3/Cat/Cdk1/Hif1a/Myc/Ndufa12/Ndufa6/Ndufs8/Pink1/Ppif/Snca
3-day post-SCI group vs. the control group	BP	GO:0006936	muscle contraction	15/222	2.71E-08	4.89E-07	2.4E-07	Adora1/Atp2a2/Cd38/Cttn/Fkbp1b/Map2k3/Map2k6/Met/Mylk/Ncf1/Pawr/Ptgs1/Ptgs2/Slc8a1/Zc3h12a
3-day post-SCI group vs. the control group	BP	GO:0006914	autophagy	18/222	2.73E-08	4.89E-07	2.4E-07	Atp13a2/Atp2a2/Bnip3/Cttn/Endog/Fbxw7/Hif1a/Hmox1/Htra2/Mapk3/Mapt/Mcl1/Pink1/Prkaa2/Sirt1/Snca/Ubqln1/Zc3h12a
3-day post-SCI group vs. the control group	BP	GO:0061919	process utilizing autophagic mechanism	18/222	2.73E-08	4.89E-07	2.4E-07	Atp13a2/Atp2a2/Bnip3/Cttn/Endog/Fbxw7/Hif1a/Hmox1/Htra2/Mapk3/Mapt/Mcl1/Pink1/Prkaa2/Sirt1/Snca/Ubqln1/Zc3h12a
3-day post-SCI group vs. the control group	BP	GO:1903555	regulation of tumor necrosis factor superfamily cy	12/222	2.84E-08	5.06E-07	2.48E-07	Axl/Cd36/Hspb1/Jak2/Mapkapk2/Ripk1/Sirpa/Sirt1/Tlr4/Tlr6/Tnfrsf1a/Zc3h12a
3-day post-SCI group vs. the control group	BP	GO:1903409	reactive oxygen species biosynthetic process	8/222	2.85E-08	5.06E-07	2.48E-07	Alox5/Cd36/Ncf1/Nox4/Sirt1/Sod2/Tlr4/Tlr6
3-day post-SCI group vs. the control group	BP	GO:1900542	regulation of purine nucleotide metabolic process	9/222	2.92E-08	5.16E-07	2.53E-07	Hif1a/Ier3/Myc/Parp1/Pdk1/Pdk2/Ppargc1a/Prkaa2/Snca
3-day post-SCI group vs. the control group	BP	GO:0062012	regulation of small molecule metabolic process	16/222	3.01E-08	5.3E-07	2.6E-07	Anxa1/Apc/Cd36/Hif1a/Ier3/Myc/Nfkb1/Parp1/Pdk1/Pdk2/Ppargc1a/Prkaa2/Ptgs2/Rest/Sirt1/Snca

3-day post-SCI group vs. the control group	BP	GO:0042594	response to starvation	12/222	3.19E-08	5.6E-07	2.75E-07	Atf2/Eif2s1/Impact/Jun/Mapk3/Mapk8/Nfe2l2/Prkaa2/Sirt1/Stk24/Tnrc6a/Zc3h12a
3-day post-SCI group vs. the control group	BP	GO:0006839	mitochondrial transport	11/222	3.24E-08	5.67E-07	2.78E-07	Atf2/Bax/Bnip3/Fbxw7/Gclc/Hebp2/Ier3/Pink1/Ppif/Slc25a24/Ucp2
3-day post-SCI group vs. the control group	BP	GO:0031099	regeneration	11/222	3.47E-08	6.04E-07	2.96E-07	Anxa1/Braf/Cdk1/Dhfr/Ezh2/Fkbp1b/Gpx1/Hmox1/Jak2/Jun/Stk24
3-day post-SCI group vs. the control group	BP	GO:0035794	positive regulation of mitochondrial membrane pe	7/222	3.62E-08	6.27E-07	3.08E-07	Atf2/Bax/Bnip3/Gclc/Hebp2/Ier3/Ppif
3-day post-SCI group vs. the control group	BP	GO:0001836	release of cytochrome c from mitochondria	8/222	3.66E-08	6.32E-07	3.1E-07	Atp7a/Bax/Bnip3/Gpx1/Jun/Pink1/Ppif/Sod2
3-day post-SCI group vs. the control group	BP	GO:2000112	regulation of cellular macromolecule biosynthetic	18/222	4.13E-08	7.05E-07	3.46E-07	Ago1/Ago3/Cd36/Cdk4/Cyp1b1/Eif2s1/Hbegf/Impact/Mapk3/Paip1/Pawr/Pink1/Ppp1ca/Ppp1r15b/Rpl13a/Tnrc6a/Tnrc6c/Zc3h12a
3-day post-SCI group vs. the control group	BP	GO:0002534	cytokine production involved in inflammatory resp	8/222	4.14E-08	7.05E-07	3.46E-07	Alox5/Ezh2/Hif1a/Mapk14/Sirpa/Tlr4/Tlr6/Zc3h12a
3-day post-SCI group vs. the control group	BP	GO:0070265	necrotic cell death	8/222	4.14E-08	7.05E-07	3.46E-07	Bax/Bnip3/Hebp2/Mapk8/Ppif/Ripk1/Ripk3/Ybx3
3-day post-SCI group vs. the control group	BP	GO:0019722	calcium-mediated signaling	12/222	4.25E-08	7.21E-07	3.54E-07	Atp2a2/Camkk2/Ccr1/Fkbp1b/Mapt/Nfatc1/Pdk2/Ppp3ca/Rcan1/Slc8a1/Trpm2/Vcam1
3-day post-SCI group vs. the control group	BP	GO:0050863	regulation of T cell activation	16/222	4.36E-08	7.37E-07	3.61E-07	Actb/Aif1/Anxa1/Arg1/Braf/Casp3/Hspb1/Igfbp2/Il6st/Jak2/Pawr/Ppp3ca/Ripk3/Sirpa/Vcam1/Zc3h12a
3-day post-SCI group vs. the control group	BP	GO:1901987	regulation of cell cycle phase transition	17/222	5.02E-08	8.43E-07	4.13E-07	Actb/Aif1/Anxa1/Apc/Atf2/Atr/Cdc20/Cdk1/Cdk10/Cdk4/Cdkn2b/Cdkn2c/Ezh2/Ier3/Mapk14/Rb1/Rrm2b
3-day post-SCI group vs. the control group	BP	GO:0006109	regulation of carbohydrate metabolic process	12/222	5.02E-08	8.43E-07	4.13E-07	Cd36/Hif1a/Ier3/Myc/Nfkb1/Pdk1/Pdk2/Ppargc1a/Ppp1ca/Prkaa2/Sirt1/Snca
3-day post-SCI group vs. the control group	BP	GO:0006140	regulation of nucleotide metabolic process	9/222	5.18E-08	8.63E-07	4.23E-07	Hif1a/Ier3/Myc/Parp1/Pdk1/Pdk2/Ppargc1a/Prkaa2/Snca
3-day post-SCI group vs. the control group	BP	GO:0051341	regulation of oxidoreductase activity	9/222	5.18E-08	8.63E-07	4.23E-07	Alox5/Atp7a/Ccs/Dhfr/Gch1/Nfkb1/Ripk3/S100a1/Snca
3-day post-SCI group vs. the control group	BP	GO:0071560	cellular response to transforming growth factor bet	13/222	5.39E-08	8.94E-07	4.38E-07	Adam9/Cdkn2b/Fos/Hdac1/Itgb5/Jun/Parp1/Ppargc1a/Ptprk/Pxn/Rbbp7/Sirt1/Smad1
3-day post-SCI group vs. the control group	BP	GO:2000116	regulation of cysteine-type endopeptidase activity	13/222	5.66E-08	9.35E-07	4.58E-07	Bax/Dhcr24/Gpx1/Htra2/Jak2/Mgmt/Myc/Pawr/Ptgs2/Rest/Sirt1/Snca/Xdh
3-day post-SCI group vs. the control group	BP	GO:0070555	response to interleukin-1	9/222	5.68E-08	9.35E-07	4.58E-07	Anxa1/Mapk3/Nfkb1/Rela/Sirpa/Snca/Tank/Vrk2/Zc3h12a
3-day post-SCI group vs. the control group	BP	GO:0022411	cellular component disassembly	17/222	5.92E-08	9.66E-07	4.74E-07	Apc/Atp2a2/Bax/Bnip3/Casp3/Cdk1/Cttn/Endog/Ets1/Fbxw7/Hif1a/Htra2/Map4k4/Myc/Pink1/Prkaa2/Ubqln1
3-day post-SCI group vs. the control group	BP	GO:0007179	transforming growth factor beta receptor signaling	12/222	5.92E-08	9.66E-07	4.74E-07	Adam9/Cdkn2b/Fos/Hdac1/Itgb5/Jun/Parp1/Ptprk/Pxn/Rbbp7/Sirt1/Smad1
3-day post-SCI group vs. the control group	BP	GO:2001236	regulation of extrinsic apoptotic signaling pathway	11/222	6.2E-08	1.01E-06	4.94E-07	Ctnna1/Cttn/Gclc/Gpx1/Hmox1/Htra2/Itga6/Mcl1/Ppp1ca/Rela/Ripk1
3-day post-SCI group vs. the control group	BP	GO:0006575	cellular modified amino acid metabolic process	12/222	6.25E-08	1.01E-06	4.96E-07	Ctsl/Dhfr/Gch1/Gclc/Ggt7/Gpx1/Gpx3/Gsr/Gstt2/Mgst1/Nfe2l2/Sod2
3-day post-SCI group vs. the control group	BP	GO:0071559	response to transforming growth factor beta	13/222	6.53E-08	1.05E-06	5.17E-07	Adam9/Cdkn2b/Fos/Hdac1/Itgb5/Jun/Parp1/Ppargc1a/Ptprk/Pxn/Rbbp7/Sirt1/Smad1
3-day post-SCI group vs. the control group	BP	GO:1990845	adaptive thermogenesis	11/222	6.6E-08	1.06E-06	5.2E-07	Apc/Cd36/Id1/Jak2/Map2k6/Ppargc1a/Ppargc1b/Rb1/Stat6/Tlr4/Ucp2

3-day post-SCI group vs. the control group	BP	GO:0051048	negative regulation of secretion	12/222	7.34E-08	1.18E-06	5.76E-07	Adora1/Anxa1/Braf/Fkbp1b/Hmox1/Ppp3ca/Ptgs1/Rest/Sirt1/Snca/Tnfrsf1a/Ucp2
3-day post-SCI group vs. the control group	BP	GO:0002532	production of molecular mediator involved in infla	9/222	7.44E-08	1.18E-06	5.79E-07	Alox5/Ezh2/Hif1a/Mapk14/Ncl1/Sirpa/Tlr4/Tlr6/Zc3h12[illegible]
3-day post-SCI group vs. the control group	BP	GO:2001237	negative regulation of extrinsic apoptotic signaling	9/222	7.44E-08	1.18E-06	5.79E-07	Ctnna1/Cttn/Gclc/Gpx1/Hmox1/Itga6/Mcl1/Rela/Ripk1
3-day post-SCI group vs. the control group	BP	GO:0014910	regulation of smooth muscle cell migration	9/222	8.12E-08	1.29E-06	6.3E-07	Aif1/Atp7a/Cyp1b1/Il6st/Myc/Nfe2l2/Nox4/Ppargc1a/Tlr[illegible]
3-day post-SCI group vs. the control group	BP	GO:0048678	response to axon injury	8/222	8.29E-08	1.31E-06	6.41E-07	Bax/Braf/Dhfr/Fkbp1b/Jak2/Jun/Sod2/Stk24
3-day post-SCI group vs. the control group	BP	GO:0044772	mitotic cell cycle phase transition	17/222	8.71E-08	1.37E-06	6.71E-07	Actb/Aif1/Anxa1/Apc/Ccna2/Cdc20/Cdk1/Cdk4/Cdkn2b/Cdkn2c/Ezh2/Ier3/Myc/Nfatc1/Ppp3ca/Rb1/Rrm2b
3-day post-SCI group vs. the control group	BP	GO:0010212	response to ionizing radiation	10/222	9.16E-08	1.43E-06	7.03E-07	Anxa1/Atf/Bax/Ect2/Gpx1/Mapk14/Myc/Sirt1/Sod2/Taf[illegible]
3-day post-SCI group vs. the control group	BP	GO:0043281	regulation of cysteine-type endopeptidase activity	12/222	9.54E-08	1.49E-06	7.29E-07	Bax/Dhcr24/Gpx1/Htra2/Jak2/Mgmt/Myc/Ptgs2/Rest/Sirt1/Snca/Xdh
3-day post-SCI group vs. the control group	BP	GO:0009408	response to heat	9/222	9.65E-08	0.0000015	7.35E-07	Eif2s1/Gclc/Hmox1/Hspb1/Htra2/Mapk8/Mapt/Rbbp7/Trpm2
3-day post-SCI group vs. the control group	BP	GO:0036294	cellular response to decreased oxygen levels	10/222	9.83E-08	1.52E-06	7.46E-07	Atf2/Bnip3/Hif1a/Myc/Nfe2l2/P4hb/Pdk1/Pink1/Sirt1/Ubqln1
3-day post-SCI group vs. the control group	BP	GO:0045429	positive regulation of nitric oxide biosynthetic pro	7/222	1.07E-07	1.65E-06	8.11E-07	Aif1/Cd36/Jak2/Ptgs2/Sod2/Tlr4/Tlr6
3-day post-SCI group vs. the control group	BP	GO:0044843	cell cycle G1/S phase transition	13/222	1.09E-07	1.67E-06	8.19E-07	Actb/Aif1/Anxa1/Apc/Ccna2/Cdk4/Cdkn2b/Cdkn2c/Ezh2/Myc/Nfatc1/Ppp3ca/Rb1
3-day post-SCI group vs. the control group	BP	GO:0031349	positive regulation of defense response	14/222	1.16E-07	1.78E-06	8.7E-07	Abcc1/Arg1/Ets1/Il18rap/Jak2/Mapk3/Nono/Ptgs2/Ripk1/Sfpq/Snca/Tlr4/Tlr6/Tnfrsf1a
3-day post-SCI group vs. the control group	BP	GO:0045740	positive regulation of DNA replication	7/222	1.24E-07	1.88E-06	9.24E-07	Cdk1/Endog/Jun/Map2k4/Mapk8/Met/Pcna
3-day post-SCI group vs. the control group	BP	GO:1903034	regulation of response to wounding	11/222	1.35E-07	2.05E-06	1.01E-06	Alox5/Anxa1/Atp7a/Braf/Cd36/Fkbp1b/Hbegf/Mylk/Nfe2l2/Pdgfra/Stk24
3-day post-SCI group vs. the control group	BP	GO:0010822	positive regulation of mitochondrion organization	8/222	1.41E-07	2.12E-06	1.04E-06	Bax/Bnip3/Endog/Hif1a/Htra2/Pink1/Ppargc1a/Ppif
3-day post-SCI group vs. the control group	BP	GO:0042307	positive regulation of protein import into nucleus	7/222	1.42E-07	2.12E-06	1.04E-06	Cdk1/Ect2/Jak2/Mapk14/Ptgs2/Tnfrsf1a/Zc3h12a
3-day post-SCI group vs. the control group	BP	GO:1903426	regulation of reactive oxygen species biosynthetic	7/222	1.42E-07	2.12E-06	1.04E-06	Alox5/Cd36/Nox4/Sirt1/Sod2/Tlr4/Tlr6
3-day post-SCI group vs. the control group	BP	GO:1904407	positive regulation of nitric oxide metabolic proce	7/222	1.42E-07	2.12E-06	1.04E-06	Aif1/Cd36/Jak2/Ptgs2/Sod2/Tlr4/Tlr6
3-day post-SCI group vs. the control group	BP	GO:0052548	regulation of endopeptidase activity	15/222	1.48E-07	2.21E-06	1.08E-06	Atp13a2/Bax/Dhcr24/Gpx1/Hdac1/Htra2/Jak2/Mgmt/Myc/Pawr/Ptgs2/Rest/Sirt1/Snca/Xdh
3-day post-SCI group vs. the control group	BP	GO:0042306	regulation of protein import into nucleus	8/222	1.56E-07	2.32E-06	1.14E-06	Cd36/Cdk1/Ect2/Jak2/Mapk14/Ptgs2/Tnfrsf1a/Zc3h12a
3-day post-SCI group vs. the control group	BP	GO:0001890	placenta development	11/222	1.6E-07	2.37E-06	1.16E-06	Ctsl/Fosl1/Hif1a/Junb/Mapk14/Mapk3/Met/Ptgs2/Rxrb/Slc8a1/Vcam1
3-day post-SCI group vs. the control group	BP	GO:0043392	negative regulation of DNA binding	7/222	1.62E-07	0.0000024	1.18E-06	Fbxw7/Hmox1/Id1/Jak2/Jun/Rest/Sirt1
3-day post-SCI group vs. the control group	BP	GO:0006275	regulation of DNA replication	10/222	1.69E-07	2.49E-06	1.22E-06	Atr/Ccna2/Cdk1/Endog/Jun/Map2k4/Mapk8/Mcm4/Met/Pcna
3-day post-SCI group vs. the control group	BP	GO:0008544	epidermis development	15/222	1.83E-07	2.68E-06	1.31E-06	Anxa1/Apc/Atp7a/Casp3/Ctsl/Ezh2/Hdac1/Krt1/Ppp3ca/Ptgs1/Ptgs2/Rela/Rest/Tfdp1/Txnip
3-day post-SCI group vs. the control group	BP	GO:0050866	negative regulation of cell activation	12/222	1.92E-07	0.0000028	1.37E-06	Anxa1/Arg1/Axl/Btk/Casp3/Cygb/Gclc/Hmox1/Hspb1/Pawr/Pdgfra/Zc3h12a
3-day post-SCI group vs. the control group	BP	GO:0048144	fibroblast proliferation	10/222	1.92E-07	0.0000028	1.37E-06	Bax/Ccna2/Cdk1/Cdk4/Gpx1/Jun/Myc/Pawr/Pdgfra/Sod2

3-day post-SCI group vs. the control group	BP	GO:0043434	response to peptide hormone	15/222	1.96E-07	2.84E-06	1.39E-06	Anxa1/Apc/Atp2a2/Cdk4/Jak2/Mapk14/Nfe2l2/Nfkb1/Parp1/Pdk2/Pxn/Rb1/Rela/Sirt1/Stat6
3-day post-SCI group vs. the control group	BP	GO:0014909	smooth muscle cell migration	9/222	2.01E-07	0.0000029	1.42E-06	Aif1/Atp7a/Cyp1b1/Il6st/Myc/Nfe2l2/Nox4/Ppargc1a/Tlr4
3-day post-SCI group vs. the control group	BP	GO:0015833	peptide transport	14/222	2.08E-07	2.99E-06	1.47E-06	Abcc1/Adora1/Alox5/Anxa1/Cd38/Fkbp1b/Hif1a/Jak2/Mgst1/Ppp3ca/Rest/Sirt1/Trpm2/Ucp2
3-day post-SCI group vs. the control group	BP	GO:1903039	positive regulation of leukocyte cell-cell adhesion	13/222	2.09E-07	2.99E-06	1.47E-06	Actb/Aif1/Alox5/Anxa1/Ets1/Igfbp2/Il6st/Jak2/Pawr/Ppp3ca/Rela/Sirpa/Vcam1
3-day post-SCI group vs. the control group	BP	GO:0031589	cell-substrate adhesion	15/222	2.1E-07	2.99E-06	1.47E-06	Adam9/Atrn/Axl/Braf/Cd36/Cttn/Itga6/Itgb5/Jak2/Map4k4/P4hb/Ptprk/Pxn/Sirpa/Vcam1
3-day post-SCI group vs. the control group	BP	GO:0010976	positive regulation of neuron projection developm	12/222	2.11E-07	2.99E-06	1.47E-06	Braf/Ezh2/Fkbp1b/Hspb1/Itga6/Mapt/Met/Nfe2l2/Nme2/Sirt1/Stau2/Stk24
3-day post-SCI group vs. the control group	BP	GO:0043620	regulation of DNA-templated transcription in resp	7/222	2.11E-07	2.99E-06	1.47E-06	Cd36/Hif1a/Hmox1/Impact/Jun/Nfe2l2/Rela
3-day post-SCI group vs. the control group	BP	GO:0048660	regulation of smooth muscle cell proliferation	11/222	2.11E-07	2.99E-06	1.47E-06	Aif1/Hbegf/Hif1a/Hmox1/Jak2/Jun/Myc/Ppargc1a/Ptgs2/Sod2/Tlr4
3-day post-SCI group vs. the control group	BP	GO:0009150	purine ribonucleotide metabolic process	16/222	2.15E-07	3.03E-06	1.49E-06	Atp7a/Hif1a/Ier3/Myc/Ndufa12/Ndufa6/Ndufs8/Nme2/Nudt2/Parp1/Pdk1/Pdk2/Ppargc1a/Prkaa2/Snca/Xdh
3-day post-SCI group vs. the control group	BP	GO:0008630	intrinsic apoptotic signaling pathway in response t	9/222	2.34E-07	3.29E-06	1.61E-06	Bax/Hmox1/Htra2/Ier3/Mcl1/Myc/Sirt1/Sod2/Tnfrsf1a
3-day post-SCI group vs. the control group	BP	GO:0072594	establishment of protein localization to organelle	16/222	2.36E-07	3.31E-06	1.62E-06	Agap3/Atf2/Bax/Cd36/Cdk1/Ect2/Fbxw7/Jak2/Mapk14/Mapt/Pink1/Ppp3ca/Ptgs2/Tnfrsf1a/Txnip/Zc3h12a
3-day post-SCI group vs. the control group	BP	GO:0009141	nucleoside triphosphate metabolic process	13/222	2.37E-07	3.32E-06	1.63E-06	Atp7a/Hif1a/Ier3/Myc/Ndufa12/Ndufa6/Ndufs8/Nme2/Nudt2/Parp1/Ppargc1a/Prkaa2/Rrm2b
3-day post-SCI group vs. the control group	BP	GO:0022409	positive regulation of cell-cell adhesion	14/222	2.42E-07	3.37E-06	1.65E-06	Actb/Aif1/Alox5/Anxa1/Ets1/Igfbp2/Il6st/Itga6/Jak2/Pawr/Ppp3ca/Rela/Sirpa/Vcam1
3-day post-SCI group vs. the control group	BP	GO:0000082	G1/S transition of mitotic cell cycle	12/222	2.43E-07	3.37E-06	1.65E-06	Actb/Aif1/Anxa1/Apc/Cdk4/Cdkn2b/Cdkn2c/Ezh2/Myc/Nfatc1/Ppp3ca/Rb1
3-day post-SCI group vs. the control group	BP	GO:0071453	cellular response to oxygen levels	10/222	2.81E-07	3.89E-06	1.91E-06	Atf2/Bnip3/Hif1a/Myc/Nfe2l2/P4hb/Pdk1/Pink1/Sirt1/Ubqln1
3-day post-SCI group vs. the control group	BP	GO:0042060	wound healing	15/222	2.85E-07	3.93E-06	1.93E-06	Alox5/Anxa1/Axl/Cd36/Dst/Gpx1/Hbegf/Hif1a/Hmox1/Jak2/Mylk/Nfe2l2/Pdgfra/Sdc1/Tlr4
3-day post-SCI group vs. the control group	BP	GO:0043122	regulation of I-kappaB kinase/NF-kappaB signalin	11/222	2.91E-07	0.000004	1.96E-06	Cd36/Hdac1/Hspb1/Pink1/Rela/Ripk1/Sirt1/Tank/Tlr4/Tlr6/Zc3h12a
3-day post-SCI group vs. the control group	BP	GO:1903715	regulation of aerobic respiration	6/222	3.06E-07	4.19E-06	2.05E-06	Atp7a/Bnip3/Hif1a/Myc/Pink1/Ppif
3-day post-SCI group vs. the control group	BP	GO:0048659	smooth muscle cell proliferation	11/222	3.06E-07	4.19E-06	2.05E-06	Aif1/Hbegf/Hif1a/Hmox1/Jak2/Jun/Myc/Ppargc1a/Ptgs2/Sod2/Tlr4
3-day post-SCI group vs. the control group	BP	GO:0016241	regulation of macroautophagy	9/222	3.15E-07	4.29E-06	0.0000021	Atp13a2/Bnip3/Hif1a/Hmox1/Mapk3/Pink1/Prkaa2/Sirt1/Ubqln1
3-day post-SCI group vs. the control group	BP	GO:0051146	striated muscle cell differentiation	14/222	3.49E-07	4.75E-06	2.33E-06	Ezh2/Gpx1/Map2k4/Mapk14/Met/Myc/Nox4/Pdgfra/Ppp3ca/Rb1/Rcan1/Rxrb/Sirt1/Slc8a1
3-day post-SCI group vs. the control group	BP	GO:0031346	positive regulation of cell projection organization	16/222	3.52E-07	4.76E-06	2.33E-06	Apc/Atp7a/Braf/Ezh2/Fkbp1b/Hspb1/Itga6/Mapt/Met/Nfe2l2/Nme2/Sirt1/Stau2/Stk24/Tbc1d24/Tnik

3-day post-SCI group vs. the control group	BP	GO:0006790	sulfur compound metabolic process	14/222	3.62E-07	4.88E-06	2.39E-06	Gclc/Ggt7/Gpx1/Gpx3/Gsr/Gstt2/Mgst1/Nfe2l2/Nox4/Pdk1/Pdk2/Snca/Sod2/Xdh
3-day post-SCI group vs. the control group	BP	GO:0046677	response to antibiotic	6/222	3.63E-07	4.88E-06	2.39E-06	Endog/Ets1/Ezh2/Hif1a/Id1/Jak2
3-day post-SCI group vs. the control group	BP	GO:0042129	regulation of T cell proliferation	11/222	3.77E-07	5.05E-06	2.48E-06	Aif1/Anxa1/Arg1/Casp3/Igfbp2/Il6st/Jak2/Pawr/Ppp3ca/Ripk3/Vcam1
3-day post-SCI group vs. the control group	BP	GO:0046902	regulation of mitochondrial membrane permeabilit	7/222	3.89E-07	5.19E-06	2.55E-06	Atf2/Bax/Bnip3/Gclc/Hebp2/Ier3/Ppif
3-day post-SCI group vs. the control group	BP	GO:0009259	ribonucleotide metabolic process	16/222	3.97E-07	5.27E-06	2.58E-06	Atp7a/Hif1a/Ier3/Myc/Ndufa12/Ndufa6/Ndufs8/Nme2/Nudt2/Parp1/Pdk1/Pdk2/Ppargc1a/Prkaa2/Snca/Xdh
3-day post-SCI group vs. the control group	BP	GO:0052547	regulation of peptidase activity	16/222	3.97E-07	5.27E-06	2.58E-06	Atp13a2/Bax/Dhcr24/Gpx1/Hdac1/Htra2/Jak2/Mgmt/Myc/Pawr/Ptgs2/Rest/Sirt1/Snca/Tank/Xdh
3-day post-SCI group vs. the control group	BP	GO:0030808	regulation of nucleotide biosynthetic process	6/222	4.28E-07	5.65E-06	2.77E-06	Myc/Parp1/Pdk1/Pdk2/Ppargc1a/Snca
3-day post-SCI group vs. the control group	BP	GO:1900371	regulation of purine nucleotide biosynthetic proces	6/222	4.28E-07	5.65E-06	2.77E-06	Myc/Parp1/Pdk1/Pdk2/Ppargc1a/Snca
3-day post-SCI group vs. the control group	BP	GO:0006163	purine nucleotide metabolic process	16/222	4.47E-07	5.88E-06	2.88E-06	Atp7a/Hif1a/Ier3/Myc/Ndufa12/Ndufa6/Ndufs8/Nme2/Nudt2/Parp1/Pdk1/Pdk2/Ppargc1a/Prkaa2/Snca/Xdh
3-day post-SCI group vs. the control group	BP	GO:0006417	regulation of translation	15/222	4.51E-07	5.91E-06	0.0000029	Ago1/Ago3/Cdk4/Cyp1b1/Eif2s1/Impact/Mapk3/Paip1/Pink1/Ppp1ca/Ppp1r15b/Rpl13a/Tnrc6a/Tnrc6c/Zc3h12a
3-day post-SCI group vs. the control group	BP	GO:0031348	negative regulation of defense response	12/222	4.57E-07	5.97E-06	2.93E-06	Adora1/Alox5/Arg1/Banf1/Ccr1/Ets1/Gpx1/Ier3/Krt1/Nfkb1/Rb1/Tnfrsf1a
3-day post-SCI group vs. the control group	BP	GO:0009152	purine ribonucleotide biosynthetic process	11/222	4.61E-07	6.01E-06	2.94E-06	Myc/Ndufa12/Ndufa6/Ndufs8/Nme2/Nudt2/Parp1/Pdk1/Pdk2/Ppargc1a/Snca
3-day post-SCI group vs. the control group	BP	GO:0006476	protein deacetylation	9/222	4.82E-07	6.25E-06	3.06E-06	Hdac1/Mapt/Pink1/Prkaa2/Rbbp7/Rest/Ripk3/Sfpq/Sirt1
3-day post-SCI group vs. the control group	BP	GO:0030316	osteoclast differentiation	9/222	4.82E-07	6.25E-06	3.06E-06	Apc/Ccr1/Fbxw7/Fos/Junb/Mapk14/Nfatc1/Ppargc1b/Ppp3ca
3-day post-SCI group vs. the control group	BP	GO:0042593	glucose homeostasis	13/222	5.07E-07	6.54E-06	3.21E-06	Alox5/Cd36/Fkbp1b/Gpx1/Hif1a/Met/Nox4/Pdk2/Ppp3ca/Prkaa2/Sirt1/Ucp2/Vcam1
3-day post-SCI group vs. the control group	BP	GO:0033500	carbohydrate homeostasis	13/222	5.26E-07	6.78E-06	3.32E-06	Alox5/Cd36/Fkbp1b/Gpx1/Hif1a/Met/Nox4/Pdk2/Ppp3ca/Prkaa2/Sirt1/Ucp2/Vcam1
3-day post-SCI group vs. the control group	BP	GO:0031334	positive regulation of protein-containing complex	11/222	5.34E-07	6.86E-06	3.36E-06	Apc/Atr/Bax/Cd36/Cttn/Mapk8/Mapt/Met/Snca/Tlr4/Tlr6
3-day post-SCI group vs. the control group	BP	GO:0043457	regulation of cellular respiration	7/222	5.46E-07	6.99E-06	3.43E-06	Atp7a/Bnip3/Hif1a/Myc/Pink1/Ppargc1a/Ppif
3-day post-SCI group vs. the control group	BP	GO:0019693	ribose phosphate metabolic process	16/222	5.48E-07	6.99E-06	3.43E-06	Atp7a/Hif1a/Ier3/Myc/Ndufa12/Ndufa6/Ndufs8/Nme2/Nudt2/Parp1/Pdk1/Pdk2/Ppargc1a/Prkaa2/Snca/Xdh
3-day post-SCI group vs. the control group	BP	GO:0032872	regulation of stress-activated MAPK cascade	11/222	5.61E-07	7.13E-06	0.0000035	Ezr/Map2k4/Map4k4/Mapk3/Met/Ripk1/Sirpa/Tlr4/Tnik/Xdh/Zc3h12a
3-day post-SCI group vs. the control group	BP	GO:1900182	positive regulation of protein localization to nucle	8/222	5.65E-07	7.17E-06	3.51E-06	Cdk1/Ect2/Jak2/Mapk14/Parp1/Ptgs2/Tnfrsf1a/Zc3h12a
3-day post-SCI group vs. the control group	BP	GO:1902686	mitochondrial outer membrane permeabilization ir	6/222	5.88E-07	7.43E-06	3.64E-06	Atf2/Bax/Bnip3/Gclc/Ier3/Ppif
3-day post-SCI group vs. the control group	BP	GO:0051770	positive regulation of nitric-oxide synthase biosyn	5/222	6.01E-07	7.58E-06	3.71E-06	Jak2/Map2k3/Map2k4/Map2k6/Tlr4
3-day post-SCI group vs. the control group	BP	GO:0071322	cellular response to carbohydrate stimulus	10/222	6.03E-07	7.58E-06	3.72E-06	Fkbp1b/Gpx1/Hif1a/Nfkb1/Nox4/Ppp3ca/Prkaa2/Sirt1/Ucp2/Vcam1
3-day post-SCI group vs. the control group	BP	GO:0014911	positive regulation of smooth muscle cell migratio	7/222	6.09E-07	7.63E-06	3.74E-06	Aif1/Atp7a/Cyp1b1/Il6st/Myc/Nox4/Tlr4

3-day post-SCI group vs. the control group	BP	GO:0007015	actin filament organization	16/222	6.15E-07	7.67E-06	3.76E-06	Aif1/Braf/Ctnna1/Cttn/Ezr/Id1/Itgb5/Marcksl1/Met/Msrb2/Nox4/Pawr/Pdlim1/Ppargc1b/Pxn/Sirpa
3-day post-SCI group vs. the control group	BP	GO:0009205	purine ribonucleoside triphosphate metabolic proc	12/222	6.17E-07	7.67E-06	3.76E-06	Atp7a/Hif1a/Ier3/Myc/Ndufa12/Ndufa6/Ndufs8/Nme2/Nudt2/Parp1/Ppargc1a/Prkaa2
3-day post-SCI group vs. the control group	BP	GO:0009306	protein secretion	15/222	6.18E-07	7.67E-06	3.76E-06	Adam9/Alox5/Anxa1/Atp13a2/Cd38/Ezr/Fkbp1b/Hif1a/Jak2/Ppp3ca/Rest/Sirt1/Tlr4/Trpm2/Ucp2
3-day post-SCI group vs. the control group	BP	GO:0035592	establishment of protein localization to extracellul	15/222	6.37E-07	7.89E-06	3.87E-06	Adam9/Alox5/Anxa1/Atp13a2/Cd38/Ezr/Fkbp1b/Hif1a/Jak2/Ppp3ca/Rest/Sirt1/Tlr4/Trpm2/Ucp2
3-day post-SCI group vs. the control group	BP	GO:0070302	regulation of stress-activated protein kinase signal	11/222	6.48E-07	0.000008	3.92E-06	Ezr/Map2k4/Map4k4/Mapk3/Met/Ripk1/Sirpa/Tlr4/Tnik/Xdh/Zc3h12a
3-day post-SCI group vs. the control group	BP	GO:0042886	amide transport	14/222	6.77E-07	8.32E-06	4.08E-06	Abcc1/Adora1/Alox5/Anxa1/Cd38/Fkbp1b/Hif1a/Jak2/Mgst1/Ppp3ca/Rest/Sirt1/Trpm2/Ucp2
3-day post-SCI group vs. the control group	BP	GO:0035306	positive regulation of dephosphorylation	7/222	6.78E-07	8.32E-06	4.08E-06	Adora1/Jak2/Pawr/Pink1/Ppargc1b/Ppp1r15b/Ripk3
3-day post-SCI group vs. the control group	BP	GO:0070391	response to lipoteichoic acid	4/222	6.82E-07	8.32E-06	4.08E-06	Cd36/Mapk14/Rela/Tlr4
3-day post-SCI group vs. the control group	BP	GO:0071223	cellular response to lipoteichoic acid	4/222	6.82E-07	8.32E-06	4.08E-06	Cd36/Mapk14/Rela/Tlr4
3-day post-SCI group vs. the control group	BP	GO:0010939	regulation of necrotic cell death	6/222	6.84E-07	8.32E-06	4.08E-06	Bnip3/Hebp2/Ppif/Ripk1/Ripk3/Ybx3
3-day post-SCI group vs. the control group	BP	GO:0061448	connective tissue development	13/222	6.86E-07	8.32E-06	4.08E-06	Atf2/Atp7a/Bmp1/Cdk4/Hif1a/Mapk14/Mapk3/Parp1/Ppargc1a/Rb1/Rela/Sirt1/Smad1
3-day post-SCI group vs. the control group	BP	GO:0006937	regulation of muscle contraction	10/222	7.13E-07	8.62E-06	4.23E-06	Adora1/Atp2a2/Cttn/Fkbp1b/Ncf1/Pawr/Ptgs1/Ptgs2/Slc8a1/Zc3h12a
3-day post-SCI group vs. the control group	BP	GO:0042176	regulation of protein catabolic process	14/222	7.24E-07	8.74E-06	4.28E-06	Adam9/Apc/Atp13a2/Cdc20/Ezr/Fbxw7/Gclc/Gpx1/Ier3/Mapk8/Mgat3/Rela/Snca/Ubqln1
3-day post-SCI group vs. the control group	BP	GO:0015711	organic anion transport	14/222	7.49E-07	9.01E-06	4.42E-06	Abcc1/Adora1/Anxa1/Arg1/Cd36/Map2k6/Mgst1/Myc/Pnpla8/Ptgs2/Slc23a2/Slc25a24/Slc4a11/Snca
3-day post-SCI group vs. the control group	BP	GO:0070665	positive regulation of leukocyte proliferation	10/222	7.53E-07	9.03E-06	4.43E-06	Aif1/Anxa1/Cd38/Igfbp2/Il6st/Jak2/Mapk3/Ppp3ca/Tlr4/Vcam1
3-day post-SCI group vs. the control group	BP	GO:0071692	protein localization to extracellular region	15/222	7.66E-07	9.14E-06	4.48E-06	Adam9/Alox5/Anxa1/Atp13a2/Cd38/Ezr/Fkbp1b/Hif1a/Jak2/Ppp3ca/Rest/Sirt1/Tlr4/Trpm2/Ucp2
3-day post-SCI group vs. the control group	BP	GO:0050769	positive regulation of neurogenesis	13/222	7.66E-07	9.14E-06	4.48E-06	Braf/Hdac1/Hif1a/Il6st/Mapk8/Mapt/Met/Myc/Rela/Rnf112/Stau2/Tbc1d24/Tnik
3-day post-SCI group vs. the control group	BP	GO:0009144	purine nucleoside triphosphate metabolic process	12/222	7.92E-07	9.39E-06	0.0000046	Atp7a/Hif1a/Ier3/Myc/Ndufa12/Ndufa6/Ndufs8/Nme2/Nudt2/Parp1/Ppargc1a/Prkaa2
3-day post-SCI group vs. the control group	BP	GO:0009199	ribonucleoside triphosphate metabolic process	12/222	7.92E-07	9.39E-06	0.0000046	Atp7a/Hif1a/Ier3/Myc/Ndufa12/Ndufa6/Ndufs8/Nme2/Nudt2/Parp1/Ppargc1a/Prkaa2
3-day post-SCI group vs. the control group	BP	GO:0009260	ribonucleotide biosynthetic process	11/222	8.2E-07	0.0000097	4.76E-06	Myc/Ndufa12/Ndufa6/Ndufs8/Nme2/Nudt2/Parp1/Pdk1/Pdk2/Ppargc1a/Snca
3-day post-SCI group vs. the control group	BP	GO:0030098	lymphocyte differentiation	16/222	8.38E-07	9.89E-06	4.85E-06	Actb/Anxa1/Apc/Atf2/Atp7a/Axl/Bax/Braf/Btk/Ctsl/Ezh2/Hspb1/Nfatc1/Ripk3/Stat6/Zc3h12a
3-day post-SCI group vs. the control group	BP	GO:0046822	regulation of nucleocytoplasmic transport	9/222	8.76E-07	0.0000103	5.05E-06	Cd36/Cdk1/Ect2/Ier3/Jak2/Mapk14/Ptgs2/Tnfrsf1a/Zc3h12a

3-day post-SCI group vs. the control group	BP	GO:0051017	actin filament bundle assembly	10/222	8.85E-07	0.0000104	5.08E-06	Aif1/Braf/Ezr/Id1/Itgb5/Met/Nox4/Pawr/Pdlim1/Pxn
3-day post-SCI group vs. the control group	BP	GO:0019932	second-messenger-mediated signaling	13/222	8.86E-07	0.0000104	5.08E-06	Atp2a2/Camkk2/Ccr1/Cd36/Fkbp1b/Mapt/Nfatc1/Pdk2/Ppp3ca/Rcan1/Slc8a1/Trpm2/Vcam1
3-day post-SCI group vs. the control group	BP	GO:0009743	response to carbohydrate	11/222	0.0000009	0.0000105	5.14E-06	Casp3/Fkbp1b/Gpx1/Hif1a/Nfkb1/Nox4/Ppp3ca/Prkaa2/Sirt1/Ucp2/Vcam1
3-day post-SCI group vs. the control group	BP	GO:0033559	unsaturated fatty acid metabolic process	9/222	9.34E-07	0.0000109	5.32E-06	Alox5/Anxa1/Cyp1b1/Gpx1/Pnpla8/Ptgs1/Ptgs2/Sirt1/Tnfrsf1a
3-day post-SCI group vs. the control group	BP	GO:1903428	positive regulation of reactive oxygen species bios	5/222	9.58E-07	0.0000111	5.45E-06	Cd36/Nox4/Sod2/Tlr4/Tlr6
3-day post-SCI group vs. the control group	BP	GO:0051047	positive regulation of secretion	15/222	0.000001	0.0000116	5.69E-06	Adam9/Adora1/Atp13a2/Cd38/Ezr/Hif1a/Jak2/Map2k6/Pink1/Ppp3ca/Sdc1/Sirt1/Snca/Tlr4/Trpm2
3-day post-SCI group vs. the control group	BP	GO:0006164	purine nucleotide biosynthetic process	11/222	1.03E-06	0.0000119	5.83E-06	Myc/Ndufa12/Ndufa6/Ndufs8/Nme2/Nudt2/Parp1/Pdk1/Pdk2/Ppargc1a/Snca
3-day post-SCI group vs. the control group	BP	GO:0061572	actin filament bundle organization	10/222	1.04E-06	0.0000119	5.85E-06	Aif1/Braf/Ezr/Id1/Itgb5/Met/Nox4/Pawr/Pdlim1/Pxn
3-day post-SCI group vs. the control group	BP	GO:0009165	nucleotide biosynthetic process	12/222	1.05E-06	0.0000121	5.91E-06	Myc/Ndufa12/Ndufa6/Ndufs8/Nme2/Nudt2/Parp1/Pdk1/Pdk2/Ppargc1a/Rrm2b/Snca
3-day post-SCI group vs. the control group	BP	GO:0035601	protein deacylation	9/222	1.06E-06	0.0000121	5.92E-06	Hdac1/Mapt/Pink1/Prkaa2/Rbbp7/Rest/Ripk3/Sfpq/Sirt1
3-day post-SCI group vs. the control group	BP	GO:0098732	macromolecule deacylation	9/222	1.06E-06	0.0000121	5.92E-06	Hdac1/Mapt/Pink1/Prkaa2/Rbbp7/Rest/Ripk3/Sfpq/Sirt1
3-day post-SCI group vs. the control group	BP	GO:0060326	cell chemotaxis	13/222	1.06E-06	0.0000121	5.92E-06	Abcc1/Aif1/Alox5/Anxa1/Ccr1/Hbegf/Hspb1/Mapk3/Met/Pdgfra/Rpl13a/Trpm2/Vcam1
3-day post-SCI group vs. the control group	BP	GO:0006970	response to osmotic stress	8/222	1.07E-06	0.0000122	5.97E-06	Atf2/Bax/Casp3/Mapk8/Mylk/Ptgs2/Slc4a11/Ybx3
3-day post-SCI group vs. the control group	BP	GO:0035303	regulation of dephosphorylation	9/222	1.13E-06	0.0000127	6.25E-06	Adora1/Fkbp1b/Jak2/Pawr/Pink1/Ppargc1b/Ppp1r15b/Rcan1/Ripk3
3-day post-SCI group vs. the control group	BP	GO:1905710	positive regulation of membrane permeability	7/222	1.13E-06	0.0000128	6.26E-06	Atf2/Bax/Bnip3/Gclc/Hebp2/Ier3/Ppif
3-day post-SCI group vs. the control group	BP	GO:0032635	interleukin-6 production	10/222	1.15E-06	0.000013	6.35E-06	Aif1/Cd36/Il18rap/Mapkapk2/Met/Sirpa/Tlr4/Tlr6/Tnfrsf1a/Zc3h12a
3-day post-SCI group vs. the control group	BP	GO:0043254	regulation of protein-containing complex assembly	15/222	1.16E-06	0.000013	6.39E-06	Apc/Atr/Bax/Cd36/Cttn/Impact/Mapk8/Mapt/Met/Parp1/Pink1/Rpl13a/Snca/Tlr4/Tlr6
3-day post-SCI group vs. the control group	BP	GO:0072521	purine-containing compound metabolic process	16/222	1.16E-06	0.000013	6.39E-06	Atp7a/Hif1a/Ier3/Myc/Ndufa12/Ndufa6/Ndufs8/Nme2/Nudt2/Parp1/Pdk1/Pdk2/Ppargc1a/Prkaa2/Snca/Xdh
3-day post-SCI group vs. the control group	BP	GO:0007254	JNK cascade	10/222	1.21E-06	0.0000135	6.63E-06	Atf2/Map2k4/Map4k4/Mapk10/Mapk8/Nfkb1/Ripk1/Sirpa/Tlr4/Tnik
3-day post-SCI group vs. the control group	BP	GO:0046390	ribose phosphate biosynthetic process	11/222	1.23E-06	0.0000137	6.74E-06	Myc/Ndufa12/Ndufa6/Ndufs8/Nme2/Nudt2/Parp1/Pdk1/Pdk2/Ppargc1a/Snca
3-day post-SCI group vs. the control group	BP	GO:2001244	positive regulation of intrinsic apoptotic signaling	7/222	1.25E-06	0.0000138	6.79E-06	Bax/Fbxw7/Mcl1/Myc/Ripk3/Sfpq/Sirt1
3-day post-SCI group vs. the control group	BP	GO:1901293	nucleoside phosphate biosynthetic process	12/222	1.28E-06	0.0000142	6.94E-06	Myc/Ndufa12/Ndufa6/Ndufs8/Nme2/Nudt2/Parp1/Pdk1/Pdk2/Ppargc1a/Rrm2b/Snca
3-day post-SCI group vs. the control group	BP	GO:0018108	peptidyl-tyrosine phosphorylation	13/222	1.31E-06	0.0000145	7.09E-06	Adora1/Btk/Cd36/Fbxw7/Hbegf/Il6st/Jak2/Mapk3/Nox4/Pdgfra/Pxn/Tlr4/Tnfrsf1a
3-day post-SCI group vs. the control group	BP	GO:0032768	regulation of monooxygenase activity	6/222	1.38E-06	0.0000152	7.44E-06	Atp7a/Dhfr/Gch1/Nfkb1/S100a1/Snca
3-day post-SCI group vs. the control group	BP	GO:2000273	positive regulation of signaling receptor activity	6/222	1.38E-06	0.0000152	7.44E-06	Adora1/Fbxw7/Hbegf/Hdac1/Hif1a/Jak2

3-day post-SCI group vs. the control group	BP	GO:0018212	peptidyl-tyrosine modification	13/222	0.0000014	0.0000154	7.53E-06	Adora1/Btk/Cd36/Fbxw7/Hbegf/Il6st/Jak2/Mapk3/Nox4/Pdgfra/Pxn/Tlr4/Tnfrsf1a
3-day post-SCI group vs. the control group	BP	GO:0046034	ATP metabolic process	11/222	1.41E-06	0.0000154	7.53E-06	Atp7a/Hif1a/Ier3/Myc/Ndufa12/Ndufa6/Ndufs8/Nudt2/Parp1/Ppargc1a/Prkaa2
3-day post-SCI group vs. the control group	BP	GO:0010952	positive regulation of peptidase activity	10/222	1.41E-06	0.0000154	7.53E-06	Bax/Htra2/Jak2/Myc/Pawr/Rest/Sirt1/Snca/Tank/Xdh
3-day post-SCI group vs. the control group	BP	GO:1902905	positive regulation of supramolecular fiber organiz	10/222	1.41E-06	0.0000154	7.53E-06	Apc/Braf/Cttn/Id1/Mapk8/Mapt/Met/Nox4/Pxn/Rb1
3-day post-SCI group vs. the control group	BP	GO:0006119	oxidative phosphorylation	9/222	1.43E-06	0.0000156	7.63E-06	Atp7a/Cdk1/Myc/Ndufa12/Ndufa6/Ndufs8/Pink1/Ppif/Sn[illegible]
3-day post-SCI group vs. the control group	BP	GO:0071276	cellular response to cadmium ion	5/222	1.46E-06	0.0000158	7.75E-06	Fos/Hmox1/Jun/Mapk3/Mapk8
3-day post-SCI group vs. the control group	BP	GO:1903146	regulation of autophagy of mitochondrion	5/222	1.46E-06	0.0000158	7.75E-06	Bnip3/Cttn/Fbxw7/Hif1a/Pink1
3-day post-SCI group vs. the control group	BP	GO:0072522	purine-containing compound biosynthetic process	11/222	1.47E-06	0.0000158	7.76E-06	Myc/Ndufa12/Ndufa6/Ndufs8/Nme2/Nudt2/Parp1/Pdk1/Pdk2/Ppargc1a/Snca
3-day post-SCI group vs. the control group	BP	GO:0002763	positive regulation of myeloid leukocyte differenti	7/222	1.51E-06	0.0000162	7.93E-06	Ccr1/Fos/Jun/Ppargc1b/Ppp3ca/Rb1/Ripk1
3-day post-SCI group vs. the control group	BP	GO:2000146	negative regulation of cell motility	13/222	1.55E-06	0.0000167	8.16E-06	Adora1/Aif1/Arhgdia/Braf/Ctnna1/Cygb/Cyp1b1/Hdac1/Nfe2l2/Ppargc1a/Ptprk/Rbbp7/Stk24
3-day post-SCI group vs. the control group	BP	GO:0060135	maternal process involved in female pregnancy	6/222	1.57E-06	0.0000168	8.21E-06	Ctsl/Junb/Mapk3/Ptgs2/Rxrb/Ube2a
3-day post-SCI group vs. the control group	BP	GO:0097300	programmed necrotic cell death	6/222	1.57E-06	0.0000168	8.21E-06	Bax/Mapk8/Ppif/Ripk1/Ripk3/Ybx3
3-day post-SCI group vs. the control group	BP	GO:0042762	regulation of sulfur metabolic process	4/222	1.59E-06	0.0000169	8.26E-06	Nfe2l2/Pdk1/Pdk2/Snca
3-day post-SCI group vs. the control group	BP	GO:0043619	regulation of transcription from RNA polymerase	4/222	1.59E-06	0.0000169	8.26E-06	Cd36/Hif1a/Hmox1/Nfe2l2
3-day post-SCI group vs. the control group	BP	GO:0010639	negative regulation of organelle organization	14/222	1.62E-06	0.0000171	8.38E-06	Apc/Bnip3/Cdc20/Cdk10/Gclc/Gpx1/Ier3/Mapt/Met/Parp1/Pink1/Ppargc1a/Ppif/Snca
3-day post-SCI group vs. the control group	BP	GO:0046824	positive regulation of nucleocytoplasmic transport	7/222	1.65E-06	0.0000174	8.55E-06	Cdk1/Ect2/Jak2/Mapk14/Ptgs2/Tnfrsf1a/Zc3h12a
3-day post-SCI group vs. the control group	BP	GO:0033157	regulation of intracellular protein transport	11/222	1.67E-06	0.0000176	8.63E-06	Atp13a2/Cd36/Cdk1/Ect2/Fbxw7/Jak2/Mapk14/Pink1/Ptgs2/Tnfrsf1a/Zc3h12a
3-day post-SCI group vs. the control group	BP	GO:0030003	cellular cation homeostasis	16/222	1.73E-06	0.0000182	0.0000089	Atox1/Atp13a2/Atp2a2/Atp7a/Bax/Bnip3/Ccr1/Fkbp1b/Hif1a/Hmox1/Mapk3/Myc/Slc4a11/Slc8a1/Snca/Trpm2
3-day post-SCI group vs. the control group	BP	GO:0022900	electron transport chain	8/222	1.79E-06	0.0000188	9.21E-06	Cdk1/Etfdh/Ndufa12/Ndufs8/Pink1/Ppargc1a/Snca/Sod2
3-day post-SCI group vs. the control group	BP	GO:0071375	cellular response to peptide hormone stimulus	12/222	0.0000018	0.0000188	9.22E-06	Apc/Cdk4/Jak2/Nfe2l2/Nfkb1/Parp1/Pdk2/Pxn/Rb1/Rela/Sirt1/Stat6
3-day post-SCI group vs. the control group	BP	GO:0010720	positive regulation of cell development	14/222	1.83E-06	0.000019	9.33E-06	Braf/Hdac1/Hif1a/Il6st/Mapk8/Mapt/Met/Myc/P4hb/Rela/Rnf112/Stau2/Tbc1d24/Tnik
3-day post-SCI group vs. the control group	BP	GO:0035051	cardiocyte differentiation	10/222	0.0000019	0.0000197	9.67E-06	Map2k4/Mapk3/Met/Nox4/Pdgfra/Rest/Rxrb/Sirt1/Slc8a1/Vcam1
3-day post-SCI group vs. the control group	BP	GO:0043271	negative regulation of ion transport	10/222	1.99E-06	0.0000207	0.0000101	Adora1/Atp7a/Fkbp1b/Pawr/Ppif/Ppp3ca/Ptgs1/Ptgs2/Snca/Ubqln1
3-day post-SCI group vs. the control group	BP	GO:0019216	regulation of lipid metabolic process	14/222	2.06E-06	0.0000213	0.0000104	Adora1/Anxa1/Cd36/Cdk4/Nfkb1/Pdgfra/Pdk1/Pdk2/Ppargc1a/Prkaa2/Ptgs2/Rest/Sirt1/Snca
3-day post-SCI group vs. the control group	BP	GO:0006260	DNA replication	12/222	2.09E-06	0.0000216	0.0000106	Atr/Ccna2/Cdk1/Endog/Jun/Map2k4/Mapk8/Mcm4/Met/Pcna/Rbbp7/Rrm2b
3-day post-SCI group vs. the control group	BP	GO:0050728	negative regulation of inflammatory response	9/222	2.15E-06	0.0000221	0.0000108	Adora1/Alox5/Ets1/Gpx1/Ier3/Krt1/Nfkb1/Rb1/Tnfrsf1a
3-day post-SCI group vs. the control group	BP	GO:0045822	negative regulation of heart contraction	5/222	2.16E-06	0.0000221	0.0000108	Adora1/Atp2a2/Fkbp1b/Jak2/Zc3h12a

3-day post-SCI group vs. the control group	BP	GO:0051767	nitric-oxide synthase biosynthetic process	5/222	2.16E-06	0.0000221	0.0000108	Jak2/Map2k3/Map2k4/Map2k6/Tlr4
3-day post-SCI group vs. the control group	BP	GO:0051769	regulation of nitric-oxide synthase biosynthetic pro	5/222	2.16E-06	0.0000221	0.0000108	Jak2/Map2k3/Map2k4/Map2k6/Tlr4
3-day post-SCI group vs. the control group	BP	GO:0030073	insulin secretion	11/222	2.24E-06	0.0000229	0.0000112	Alox5/Anxa1/Cd38/Fkbp1b/Hif1a/Jak2/Ppp3ca/Rest/Sirt1/Trpm2/Ucp2
3-day post-SCI group vs. the control group	BP	GO:0050729	positive regulation of inflammatory response	9/222	2.28E-06	0.0000231	0.0000113	Abcc1/Ets1/Jak2/Ptgs2/Ripk1/Snca/Tlr4/Tlr6/Tnfrsf1a
3-day post-SCI group vs. the control group	BP	GO:1901298	regulation of hydrogen peroxide-mediated progran	4/222	2.28E-06	0.0000231	0.0000113	Endog/Met/Pawr/Pink1
3-day post-SCI group vs. the control group	BP	GO:0043618	regulation of transcription from RNA polymerase	6/222	2.28E-06	0.0000231	0.0000113	Cd36/Hif1a/Hmox1/Impact/Jun/Nfe2l2
3-day post-SCI group vs. the control group	BP	GO:0006816	calcium ion transport	15/222	2.28E-06	0.0000231	0.0000113	Atp2a2/Bax/Ccr1/Chrna4/Fkbp1b/Mylk/Nfatc1/Pawr/Ppp3ca/Ptgs2/S100a1/Slc8a1/Snca/Trpm2/Ubqln1
3-day post-SCI group vs. the control group	BP	GO:0050671	positive regulation of lymphocyte proliferation	9/222	2.41E-06	0.0000241	0.0000118	Aif1/Anxa1/Cd38/Igfbp2/Il6st/Jak2/Ppp3ca/Tlr4/Vcam1
3-day post-SCI group vs. the control group	BP	GO:2001056	positive regulation of cysteine-type endopeptidase	9/222	2.41E-06	0.0000241	0.0000118	Bax/Htra2/Jak2/Myc/Pawr/Rest/Sirt1/Snca/Xdh
3-day post-SCI group vs. the control group	BP	GO:0050731	positive regulation of peptidyl-tyrosine phosphoryl	10/222	2.41E-06	0.0000241	0.0000118	Adora1/Cd36/Fbxw7/Hbegf/Il6st/Jak2/Nox4/Pdgfra/Tlr4/Tnfrsf1a
3-day post-SCI group vs. the control group	BP	GO:0050796	regulation of insulin secretion	10/222	2.41E-06	0.0000241	0.0000118	Alox5/Cd38/Fkbp1b/Hif1a/Jak2/Ppp3ca/Rest/Sirt1/Trpm2/Ucp2
3-day post-SCI group vs. the control group	BP	GO:0046456	icosanoid biosynthetic process	6/222	2.57E-06	0.0000256	0.0000126	Alox5/Anxa1/Pnpla8/Ptgs1/Ptgs2/Sirt1
3-day post-SCI group vs. the control group	BP	GO:0071347	cellular response to interleukin-1	7/222	2.58E-06	0.0000257	0.0000126	Mapk3/Nfkb1/Rela/Sirpa/Tank/Vrk2/Zc3h12a
3-day post-SCI group vs. the control group	BP	GO:1901990	regulation of mitotic cell cycle phase transition	13/222	2.72E-06	0.0000271	0.0000133	Actb/Aif1/Anxa1/Apc/Cdc20/Cdk1/Cdk4/Cdkn2b/Cdkn2c/Ezh2/Ier3/Rb1/Rrm2b
3-day post-SCI group vs. the control group	BP	GO:0002791	regulation of peptide secretion	11/222	2.75E-06	0.0000272	0.0000133	Adora1/Alox5/Cd38/Fkbp1b/Hif1a/Jak2/Ppp3ca/Rest/Sirt1/Trpm2/Ucp2
3-day post-SCI group vs. the control group	BP	GO:0009749	response to glucose	10/222	2.77E-06	0.0000274	0.0000134	Casp3/Fkbp1b/Gpx1/Hif1a/Nox4/Ppp3ca/Prkaa2/Sirt1/Ucp2/Vcam1
3-day post-SCI group vs. the control group	BP	GO:0010508	positive regulation of autophagy	9/222	2.84E-06	0.0000279	0.0000137	Bnip3/Endog/Hif1a/Hmox1/Mapk3/Pink1/Prkaa2/Sirt1/Zc3h12a
3-day post-SCI group vs. the control group	BP	GO:0032946	positive regulation of mononuclear cell proliferatio	9/222	2.84E-06	0.0000279	0.0000137	Aif1/Anxa1/Cd38/Igfbp2/Il6st/Jak2/Ppp3ca/Tlr4/Vcam1
3-day post-SCI group vs. the control group	BP	GO:0019439	aromatic compound catabolic process	15/222	2.91E-06	0.0000286	0.000014	Ago1/Ago3/Bax/Casp3/Endog/Hmox1/Maoa/Mapkapk2/Ncf1/Paip1/Pon2/Tnrc6a/Tnrc6c/Xdh/Zc3h12a
3-day post-SCI group vs. the control group	BP	GO:0090087	regulation of peptide transport	11/222	2.98E-06	0.0000291	0.0000143	Adora1/Alox5/Cd38/Fkbp1b/Hif1a/Jak2/Ppp3ca/Rest/Sirt1/Trpm2/Ucp2
3-day post-SCI group vs. the control group	BP	GO:0071333	cellular response to glucose stimulus	9/222	2.99E-06	0.0000293	0.0000143	Fkbp1b/Gpx1/Hif1a/Nox4/Ppp3ca/Prkaa2/Sirt1/Ucp2/Vcam1
3-day post-SCI group vs. the control group	BP	GO:0060249	anatomical structure homeostasis	13/222	3.09E-06	0.0000299	0.0000147	Apc/Atp2a2/Atp7a/Bax/Cd38/Hif1a/Nox4/Ppargc1b/Ptgs1/Ptgs2/Rb1/Tlr4/Tpp1
3-day post-SCI group vs. the control group	BP	GO:0001516	prostaglandin biosynthetic process	5/222	3.09E-06	0.0000299	0.0000147	Anxa1/Pnpla8/Ptgs1/Ptgs2/Sirt1
3-day post-SCI group vs. the control group	BP	GO:0046457	prostanoid biosynthetic process	5/222	3.09E-06	0.0000299	0.0000147	Anxa1/Pnpla8/Ptgs1/Ptgs2/Sirt1
3-day post-SCI group vs. the control group	BP	GO:1903523	negative regulation of blood circulation	5/222	3.09E-06	0.0000299	0.0000147	Adora1/Atp2a2/Fkbp1b/Jak2/Zc3h12a
3-day post-SCI group vs. the control group	BP	GO:0010940	positive regulation of necrotic cell death	4/222	3.17E-06	0.0000306	0.000015	Bnip3/Hebp2/Ripk1/Ripk3
3-day post-SCI group vs. the control group	BP	GO:0009746	response to hexose	10/222	3.18E-06	0.0000306	0.000015	Casp3/Fkbp1b/Gpx1/Hif1a/Nox4/Ppp3ca/Prkaa2/Sirt1/Ucp2/Vcam1

3-day post-SCI group vs. the control group	BP	GO:0006913	nucleocytoplasmic transport	13/222	3.19E-06	0.0000306	0.000015	Agap3/Atf2/Cd36/Cdk1/Ect2/Ier3/Jak2/Mapk14/Ppp3ca/Ptgs2/Tnfrsf1a/Txnip/Zc3h12a
3-day post-SCI group vs. the control group	BP	GO:0051169	nuclear transport	13/222	3.19E-06	0.0000306	0.000015	Agap3/Atf2/Cd36/Cdk1/Ect2/Ier3/Jak2/Mapk14/Ppp3ca/Ptgs2/Tnfrsf1a/Txnip/Zc3h12a
3-day post-SCI group vs. the control group	BP	GO:0031103	axon regeneration	6/222	3.23E-06	0.0000309	0.0000152	Braf/Dhfr/Fkbp1b/Jak2/Jun/Stk24
3-day post-SCI group vs. the control group	BP	GO:0071331	cellular response to hexose stimulus	9/222	3.33E-06	0.0000318	0.0000156	Fkbp1b/Gpx1/Hif1a/Nox4/Ppp3ca/Prkaa2/Sirt1/Ucp2/Vcam1
3-day post-SCI group vs. the control group	BP	GO:0008016	regulation of heart contraction	10/222	3.48E-06	0.0000331	0.0000162	Adora1/Atp2a2/Fkbp1b/Gch1/Hbegf/Jak2/S100a1/Sirt1/Slc8a1/Zc3h12a
3-day post-SCI group vs. the control group	BP	GO:0010565	regulation of cellular ketone metabolic process	9/222	3.51E-06	0.0000332	0.0000163	Anxa1/Apc/Pdk1/Pdk2/Ppargc1a/Ptgs2/Rest/Sirt1/Snca
3-day post-SCI group vs. the control group	BP	GO:0071326	cellular response to monosaccharide stimulus	9/222	3.51E-06	0.0000332	0.0000163	Fkbp1b/Gpx1/Hif1a/Nox4/Ppp3ca/Prkaa2/Sirt1/Ucp2/Vcam1
3-day post-SCI group vs. the control group	BP	GO:0009206	purine ribonucleoside triphosphate biosynthetic pr	8/222	3.52E-06	0.0000332	0.0000163	Myc/Ndufa12/Ndufa6/Ndufs8/Nme2/Nudt2/Parp1/Ppargc1a
3-day post-SCI group vs. the control group	BP	GO:0032652	regulation of interleukin-1 production	8/222	3.52E-06	0.0000332	0.0000163	Anxa1/Cd36/Hspb1/Jak2/Sirpa/Tlr4/Tlr6/Zc3h12a
3-day post-SCI group vs. the control group	BP	GO:0032102	negative regulation of response to external stimulu	14/222	3.58E-06	0.0000337	0.0000165	Adora1/Aif1/Alox5/Arg1/Banf1/Ccr1/Ets1/Gpx1/Ier3/Krt1/Nfkb1/Pdgfra/Rb1/Tnfrsf1a
3-day post-SCI group vs. the control group	BP	GO:0006692	prostanoid metabolic process	6/222	3.61E-06	0.0000339	0.0000166	Anxa1/Pnpla8/Ptgs1/Ptgs2/Sirt1/Tnfrsf1a
3-day post-SCI group vs. the control group	BP	GO:0006693	prostaglandin metabolic process	6/222	3.61E-06	0.0000339	0.0000166	Anxa1/Pnpla8/Ptgs1/Ptgs2/Sirt1/Tnfrsf1a
3-day post-SCI group vs. the control group	BP	GO:0034284	response to monosaccharide	10/222	3.64E-06	0.000034	0.0000167	Casp3/Fkbp1b/Gpx1/Hif1a/Nox4/Ppp3ca/Prkaa2/Sirt1/Ucp2/Vcam1
3-day post-SCI group vs. the control group	BP	GO:0009145	purine nucleoside triphosphate biosynthetic proces	8/222	3.75E-06	0.000035	0.0000171	Myc/Ndufa12/Ndufa6/Ndufs8/Nme2/Nudt2/Parp1/Ppargc1a
3-day post-SCI group vs. the control group	BP	GO:0002790	peptide secretion	12/222	3.81E-06	0.0000355	0.0000174	Adora1/Alox5/Anxa1/Cd38/Fkbp1b/Hif1a/Jak2/Ppp3ca/Rest/Sirt1/Trpm2/Ucp2
3-day post-SCI group vs. the control group	BP	GO:0045913	positive regulation of carbohydrate metabolic proc	7/222	0.0000039	0.0000363	0.0000178	Cd36/Hif1a/Myc/Nfkb1/Prkaa2/Sirt1/Snca
3-day post-SCI group vs. the control group	BP	GO:0016236	macroautophagy	11/222	3.91E-06	0.0000363	0.0000178	Atp13a2/Atp2a2/Bnip3/Hif1a/Hmox1/Htra2/Mapk3/Pink1/Prkaa2/Sirt1/Ubqln1
3-day post-SCI group vs. the control group	BP	GO:0048771	tissue remodeling	10/222	4.15E-06	0.0000384	0.0000188	Atp7a/Axl/Bax/Cd38/Hif1a/Nfkb1/Nox4/Ppargc1b/Ppp3ca/Tpp1
3-day post-SCI group vs. the control group	BP	GO:0014855	striated muscle cell proliferation	7/222	4.22E-06	0.000039	0.0000191	Apc/Cdk1/Jak2/Mapk14/Rxrb/Sirt1/Smad1
3-day post-SCI group vs. the control group	BP	GO:0051702	biological process involved in interaction with syn	8/222	4.25E-06	0.0000391	0.0000192	Arg1/Gpx1/Hdac1/Jak2/Jun/Ncf1/Rest/Zc3h12a
3-day post-SCI group vs. the control group	BP	GO:0010421	hydrogen peroxide-mediated programmed cell dea	4/222	0.0000043	0.0000395	0.0000194	Endog/Met/Pawr/Pink1
3-day post-SCI group vs. the control group	BP	GO:0010917	negative regulation of mitochondrial membrane pc	4/222	0.0000043	0.0000395	0.0000194	Bax/Bnip3/Hebp2/Mapt
3-day post-SCI group vs. the control group	BP	GO:0001893	maternal placenta development	5/222	4.32E-06	0.0000395	0.0000194	Ctsl/Junb/Mapk3/Ptgs2/Rxrb
3-day post-SCI group vs. the control group	BP	GO:0022898	regulation of transmembrane transporter activity	12/222	4.37E-06	0.0000399	0.0000196	Actb/Alox5/Atp7a/Chrna4/Fkbp1b/Ppargc1a/Ppif/Ppp3ca/Ripk1/S100a1/Snca/Ubqln1
3-day post-SCI group vs. the control group	BP	GO:0010921	regulation of phosphatase activity	7/222	4.57E-06	0.0000416	0.0000204	Fkbp1b/Jak2/Pawr/Ppargc1b/Ppp1r15b/Rcan1/Ripk3
3-day post-SCI group vs. the control group	BP	GO:0002262	myeloid cell homeostasis	10/222	4.73E-06	0.000043	0.0000211	Anxa1/Axl/Bax/Casp3/Ets1/Hif1a/Hmox1/Jak2/Mapk14/Rb1
3-day post-SCI group vs. the control group	BP	GO:0040013	negative regulation of locomotion	13/222	4.76E-06	0.0000432	0.0000212	Adora1/Aif1/Arhgdia/Braf/Ctnna1/Cygb/Cyp1b1/Hdac1/Nfe2l2/Ppargc1a/Ptprk/Rbbp7/Stk24
3-day post-SCI group vs. the control group	BP	GO:0009201	ribonucleoside triphosphate biosynthetic process	8/222	0.0000048	0.0000435	0.0000213	Myc/Ndufa12/Ndufa6/Ndufs8/Nme2/Nudt2/Parp1/Ppargc1a

3-day post-SCI group vs. the control group	BP	GO:0032612	interleukin-1 production	8/222	0.0000051	0.000046	0.0000226	Anxa1/Cd36/Hspb1/Jak2/Sirpa/Tlr4/Tlr6/Zc3h12a
3-day post-SCI group vs. the control group	BP	GO:1903008	organelle disassembly	8/222	0.0000051	0.000046	0.0000226	Bnip3/Cdk1/Cttn/Fbxw7/Hif1a/Htra2/Pink1/Prkaa2
3-day post-SCI group vs. the control group	BP	GO:0046883	regulation of hormone secretion	12/222	5.16E-06	0.0000464	0.0000228	Adora1/Alox5/Anxa1/Cd38/Fkbp1b/Hif1a/Jak2/Ppp3ca/Rest/Sirt1/Trpm2/Ucp2
3-day post-SCI group vs. the control group	BP	GO:0019221	cytokine-mediated signaling pathway	14/222	5.27E-06	0.0000473	0.0000232	Arg1/Axl/Ccr1/Hif1a/Il18rap/Il6st/Jak2/Mapk3/Rela/Ripk1/Sirt1/Stat6/Tnfrsf1a/Vrk2
3-day post-SCI group vs. the control group	BP	GO:2000736	regulation of stem cell differentiation	7/222	5.33E-06	0.0000477	0.0000234	Ezh2/Hdac1/Nfe2l2/Pdgfra/Rbbp7/Rest/Slc4a11
3-day post-SCI group vs. the control group	BP	GO:0048145	regulation of fibroblast proliferation	8/222	5.42E-06	0.0000485	0.0000238	Bax/Ccna2/Cdk4/Jun/Myc/Pawr/Pdgfra/Sod2
3-day post-SCI group vs. the control group	BP	GO:1903532	positive regulation of secretion by cell	13/222	5.52E-06	0.0000493	0.0000241	Adam9/Atp13a2/Cd38/Ezr/Hif1a/Jak2/Map2k6/Pink1/Sdc1/Sirt1/Snca/Tlr4/Trpm2
3-day post-SCI group vs. the control group	BP	GO:0050714	positive regulation of protein secretion	9/222	5.56E-06	0.0000495	0.0000242	Adam9/Atp13a2/Cd38/Ezr/Hif1a/Jak2/Sirt1/Tlr4/Trpm2
3-day post-SCI group vs. the control group	BP	GO:1902107	positive regulation of leukocyte differentiation	10/222	0.0000056	0.0000497	0.0000244	Actb/Anxa1/Axl/Ccr1/Fos/Jun/Ppargc1b/Ppp3ca/Rb1/Ripk1
3-day post-SCI group vs. the control group	BP	GO:1903708	positive regulation of hemopoiesis	10/222	0.0000056	0.0000497	0.0000244	Actb/Anxa1/Axl/Ccr1/Fos/Jun/Ppargc1b/Ppp3ca/Rb1/Ripk1
3-day post-SCI group vs. the control group	BP	GO:1901361	organic cyclic compound catabolic process	15/222	5.66E-06	0.0000501	0.0000246	Ago1/Ago3/Bax/Casp3/Endog/Hmox1/Maoa/Mapkapk2/Ncf1/Nfe2l2/Paip1/Tnrc6a/Tnrc6c/Xdh/Zc3h12a
3-day post-SCI group vs. the control group	BP	GO:0051962	positive regulation of nervous system developmen	13/222	5.69E-06	0.0000502	0.0000246	Braf/Hdac1/Hif1a/Il6st/Mapk8/Mapt/Met/Myc/Rela/Rnf112/Stau2/Tbc1d24/Tnik
3-day post-SCI group vs. the control group	BP	GO:0097468	programmed cell death in response to reactive oxy	4/222	0.0000057	0.0000502	0.0000246	Endog/Met/Pawr/Pink1
3-day post-SCI group vs. the control group	BP	GO:0006875	cellular metal ion homeostasis	14/222	5.87E-06	0.0000516	0.0000253	Atox1/Atp13a2/Atp2a2/Atp7a/Bax/Bnip3/Ccr1/Fkbp1b/Hif1a/Hmox1/Myc/Slc8a1/Snca/Trpm2
3-day post-SCI group vs. the control group	BP	GO:0030336	negative regulation of cell migration	12/222	5.88E-06	0.0000516	0.0000253	Adora1/Aif1/Arhgdia/Braf/Cygb/Cyp1b1/Hdac1/Nfe2l2/Ppargc1a/Ptprk/Rbbp7/Stk24
3-day post-SCI group vs. the control group	BP	GO:0090322	regulation of superoxide metabolic process	5/222	5.89E-06	0.0000516	0.0000253	Cd36/Dhfr/Gch1/Mapt/Nfe2l2
3-day post-SCI group vs. the control group	BP	GO:0010675	regulation of cellular carbohydrate metabolic proc	9/222	6.13E-06	0.0000534	0.0000262	Cd36/Hif1a/Ier3/Pdk1/Pdk2/Ppargc1a/Ppp1ca/Sirt1/Snca
3-day post-SCI group vs. the control group	BP	GO:0032675	regulation of interleukin-6 production	9/222	6.13E-06	0.0000534	0.0000262	Aif1/Cd36/Mapkapk2/Met/Sirpa/Tlr4/Tlr6/Tnfrsf1a/Zc3h12a
3-day post-SCI group vs. the control group	BP	GO:0043280	positive regulation of cysteine-type endopeptidase	8/222	6.47E-06	0.0000563	0.0000276	Bax/Htra2/Jak2/Myc/Rest/Sirt1/Snca/Xdh
3-day post-SCI group vs. the control group	BP	GO:0010665	regulation of cardiac muscle cell apoptotic process	6/222	6.74E-06	0.0000584	0.0000286	Atp2a2/Bnip3/Jak2/Mapk8/Nfe2l2/Sirt1
3-day post-SCI group vs. the control group	BP	GO:0043470	regulation of carbohydrate catabolic process	6/222	6.74E-06	0.0000584	0.0000286	Hif1a/Ier3/Myc/Ppargc1a/Ppp1ca/Prkaa2
3-day post-SCI group vs. the control group	BP	GO:0043500	muscle adaptation	8/222	6.86E-06	0.0000593	0.0000291	Aif1/Atp2a2/Ezh2/Nfatc1/Parp1/Ppp3ca/Sirt1/Tnfrsf1a
3-day post-SCI group vs. the control group	BP	GO:0032409	regulation of transporter activity	12/222	6.91E-06	0.0000596	0.0000292	Actb/Alox5/Atp7a/Chrna4/Fkbp1b/Ppargc1a/Ppif/Ppp3ca/Ripk1/S100a1/Snca/Ubqln1
3-day post-SCI group vs. the control group	BP	GO:0045444	fat cell differentiation	11/222	7.07E-06	0.0000606	0.0000297	Alox5/Atf2/Bnip3/Gpx1/Itga6/Mapk14/Pdgfra/Ptgs2/Sirt1/Sod2/Zc3h12a
3-day post-SCI group vs. the control group	BP	GO:0010950	positive regulation of endopeptidase activity	9/222	7.08E-06	0.0000606	0.0000297	Bax/Htra2/Jak2/Myc/Pawr/Rest/Sirt1/Snca/Xdh
3-day post-SCI group vs. the control group	BP	GO:0034250	positive regulation of cellular amide metabolic prc	9/222	7.08E-06	0.0000606	0.0000297	Casp3/Cdk4/Cyp1b1/Impact/Mapk3/Nfe2l2/Paip1/Pink1/Rela
3-day post-SCI group vs. the control group	BP	GO:0043433	negative regulation of DNA-binding transcription	9/222	7.08E-06	0.0000606	0.0000297	Cat/Cyp1b1/Ezh2/Hmox1/Id1/Rb1/Rnf2/Sirt1/Zc3h12a
3-day post-SCI group vs. the control group	BP	GO:0071466	cellular response to xenobiotic stimulus	9/222	7.42E-06	0.0000634	0.0000311	Braf/Cyp1b1/Myc/Mylk/Nfe2l2/Pcna/Prkaa2/Rb1/Rest
3-day post-SCI group vs. the control group	BP	GO:0062013	positive regulation of small molecule metabolic pr	9/222	7.78E-06	0.0000662	0.0000325	Anxa1/Cd36/Hif1a/Myc/Ppargc1a/Prkaa2/Ptgs2/Sirt1/Snca

3-day post-SCI group vs. the control group	BP	GO:0035265	organ growth	10/222	7.79E-06	0.0000662	0.0000325	Apc/Atf2/Cdk1/Map2k4/Mapk14/Pdgfra/Rxrb/Sirt1/Smad1/Ybx3
3-day post-SCI group vs. the control group	BP	GO:0046700	heterocycle catabolic process	14/222	7.84E-06	0.0000665	0.0000326	Ago1/Ago3/Bax/Casp3/Endog/Hmox1/Mapkapk2/Ncf1/Nfe2l2/Paip1/Tnrc6a/Tnrc6c/Xdh/Zc3h12a
3-day post-SCI group vs. the control group	BP	GO:0010922	positive regulation of phosphatase activity	5/222	7.88E-06	0.0000668	0.0000327	Jak2/Pawr/Ppargc1b/Ppp1r15b/Ripk3
3-day post-SCI group vs. the control group	BP	GO:0001678	cellular glucose homeostasis	9/222	8.15E-06	0.0000689	0.0000338	Fkbp1b/Gpx1/Hif1a/Nox4/Ppp3ca/Prkaa2/Sirt1/Ucp2/Vcam1
3-day post-SCI group vs. the control group	BP	GO:0031102	neuron projection regeneration	6/222	8.18E-06	0.0000689	0.0000338	Braf/Dhfr/Fkbp1b/Jak2/Jun/Stk24
3-day post-SCI group vs. the control group	BP	GO:0032655	regulation of interleukin-12 production	6/222	8.18E-06	0.0000689	0.0000338	Cd36/Mapk14/Nfkb1/Rela/Tlr4/Tlr6
3-day post-SCI group vs. the control group	BP	GO:0006820	anion transport	15/222	8.34E-06	0.0000701	0.0000344	Abcc1/Adora1/Anxa1/Arg1/Cd36/Map2k6/Mgst1/Myc/Pnpla8/Ptgs2/Ripk1/Slc23a2/Slc25a24/Slc4a11/Snca
3-day post-SCI group vs. the control group	BP	GO:0009142	nucleoside triphosphate biosynthetic process	8/222	0.0000086	0.000072	0.0000353	Myc/Ndufa12/Ndufa6/Ndufs8/Nme2/Nudt2/Parp1/Ppargc1a
3-day post-SCI group vs. the control group	BP	GO:0060048	cardiac muscle contraction	8/222	0.0000086	0.000072	0.0000353	Adora1/Atp2a2/Fkbp1b/Map2k3/Map2k6/Met/Slc8a1/Zc3h12a
3-day post-SCI group vs. the control group	BP	GO:0048738	cardiac muscle tissue development	11/222	8.73E-06	0.000073	0.0000358	Apc/Cdk1/Map2k4/Mapk14/Met/Nox4/Pdgfra/Rxrb/Sirt1/Slc8a1/Smad1
3-day post-SCI group vs. the control group	BP	GO:0010662	regulation of striated muscle cell apoptotic process	6/222	8.98E-06	0.0000749	0.0000367	Atp2a2/Bnip3/Jak2/Mapk8/Nfe2l2/Sirt1
3-day post-SCI group vs. the control group	BP	GO:0044403	biological process involved in symbiotic interactio	11/222	9.04E-06	0.0000752	0.0000369	Arg1/Axl/Ctsl/Gpx1/Hdac1/Jak2/Jun/Ncf1/P4hb/Rest/Zc3h12a
3-day post-SCI group vs. the control group	BP	GO:0045920	negative regulation of exocytosis	5/222	9.06E-06	0.0000752	0.0000369	Anxa1/Braf/Hmox1/Rest/Snca
3-day post-SCI group vs. the control group	BP	GO:0035195	miRNA-mediated gene silencing	8/222	9.09E-06	0.0000754	0.000037	Ago1/Ago3/Nfkb1/Ppp3ca/Ripk1/Tnrc6a/Tnrc6c/Zc3h12a
3-day post-SCI group vs. the control group	BP	GO:0022904	respiratory electron transport chain	7/222	9.49E-06	0.0000782	0.0000384	Cdk1/Ndufa12/Ndufs8/Pink1/Ppargc1a/Snca/Sod2
3-day post-SCI group vs. the control group	BP	GO:0032651	regulation of interleukin-1 beta production	7/222	9.49E-06	0.0000782	0.0000384	Cd36/Hspb1/Jak2/Sirpa/Tlr4/Tlr6/Zc3h12a
3-day post-SCI group vs. the control group	BP	GO:0035304	regulation of protein dephosphorylation	7/222	9.49E-06	0.0000782	0.0000384	Adora1/Fkbp1b/Jak2/Pawr/Pink1/Ppp1r15b/Rcan1
3-day post-SCI group vs. the control group	BP	GO:0050900	leukocyte migration	13/222	9.77E-06	0.0000803	0.0000394	Adora1/Aif1/Alox5/Anxa1/Ccr1/Itga6/Mapk3/Pawr/Ripk3/Rpl13a/Sirpa/Trpm2/Vcam1
3-day post-SCI group vs. the control group	BP	GO:0010659	cardiac muscle cell apoptotic process	6/222	9.85E-06	0.0000807	0.0000395	Atp2a2/Bnip3/Jak2/Mapk8/Nfe2l2/Sirt1
3-day post-SCI group vs. the control group	BP	GO:0032615	interleukin-12 production	6/222	9.85E-06	0.0000807	0.0000395	Cd36/Mapk14/Nfkb1/Rela/Tlr4/Tlr6
3-day post-SCI group vs. the control group	BP	GO:0014013	regulation of gliogenesis	8/222	0.0000101	0.000083	0.0000407	Cdkn2b/Ezh2/Hdac1/Il6st/Myc/Rb1/Rela/Rnf112
3-day post-SCI group vs. the control group	BP	GO:0001649	osteoblast differentiation	10/222	0.0000103	0.0000838	0.0000411	Apc/Id1/Il6st/Junb/Map2k6/Mapk14/Nfatc1/Ppp3ca/Rest/Smad1
3-day post-SCI group vs. the control group	BP	GO:0046686	response to cadmium ion	5/222	0.0000104	0.0000843	0.0000413	Fos/Hmox1/Jun/Mapk3/Mapk8
3-day post-SCI group vs. the control group	BP	GO:1902110	positive regulation of mitochondrial membrane pe	5/222	0.0000104	0.0000843	0.0000413	Atf2/Bax/Bnip3/Gclc/Ier3
3-day post-SCI group vs. the control group	BP	GO:0032091	negative regulation of protein binding	7/222	0.0000109	0.0000879	0.0000431	Actb/Atp2a2/Bax/Id1/Mapk3/Mapk8/Myc
3-day post-SCI group vs. the control group	BP	GO:0090559	regulation of membrane permeability	7/222	0.0000109	0.0000879	0.0000431	Atf2/Bax/Bnip3/Gclc/Hebp2/Ier3/Ppif
3-day post-SCI group vs. the control group	BP	GO:0120162	positive regulation of cold-induced thermogenesis	7/222	0.0000109	0.0000879	0.0000431	Apc/Cd36/Jak2/Ppargc1a/Ppargc1b/Stat6/Ucp2
3-day post-SCI group vs. the control group	BP	GO:0001889	liver development	8/222	0.0000113	0.000091	0.0000446	Anxa1/Atf2/Ezh2/Hmox1/Jun/Met/Rela/Sod2
3-day post-SCI group vs. the control group	BP	GO:0050773	regulation of dendrite development	8/222	0.0000113	0.000091	0.0000446	Cdc20/Ezh2/Id1/Met/Ppp3ca/Stau2/Tbc1d24/Tnik
3-day post-SCI group vs. the control group	BP	GO:0010661	positive regulation of muscle cell apoptotic proces	5/222	0.0000118	0.0000949	0.0000465	Atp2a2/Bnip3/Map2k4/Mapk8/Sod2
3-day post-SCI group vs. the control group	BP	GO:0032874	positive regulation of stress-activated MAPK casc	8/222	0.0000119	0.0000955	0.0000468	Map2k4/Map4k4/Met/Ripk1/Tlr4/Tnik/Xdh/Zc3h12a

3-day post-SCI group vs. the control group	BP	GO:0045837	negative regulation of membrane potential	4/222	0.0000119	0.0000955	0.0000468	Bax/Bnip3/Hebp2/Mapt
3-day post-SCI group vs. the control group	BP	GO:0010658	striated muscle cell apoptotic process	6/222	0.0000129	0.0001027	0.0000504	Atp2a2/Bnip3/Jak2/Mapk8/Nfe2l2/Sirt1
3-day post-SCI group vs. the control group	BP	GO:0061008	hepaticobiliary system development	8/222	0.0000132	0.0001054	0.0000517	Anxa1/Atf2/Ezh2/Hmox1/Jun/Met/Rela/Sod2
3-day post-SCI group vs. the control group	BP	GO:0070304	positive regulation of stress-activated protein kina	8/222	0.0000132	0.0001054	0.0000517	Map2k4/Map4k4/Met/Ripk1/Tlr4/Tnik/Xdh/Zc3h12a
3-day post-SCI group vs. the control group	BP	GO:0043502	regulation of muscle adaptation	7/222	0.0000133	0.0001054	0.0000517	Aif1/Atp2a2/Nfatc1/Parp1/Ppp3ca/Sirt1/Tnfrsf1a
3-day post-SCI group vs. the control group	BP	GO:0042398	cellular modified amino acid biosynthetic process	5/222	0.0000134	0.0001064	0.0000522	Dhfr/Gch1/Gclc/Ggt7/Nfe2l2
3-day post-SCI group vs. the control group	BP	GO:0007160	cell-matrix adhesion	10/222	0.0000139	0.00011	0.0000539	Adam9/Cd36/Cttn/Itga6/Itgb5/Map4k4/Ptprk/Pxn/Sirpa/V<br>cam1
3-day post-SCI group vs. the control group	BP	GO:0035194	post-transcriptional gene silencing by RNA	8/222	0.0000139	0.0001101	0.000054	Ago1/Ago3/Nfkb1/Ppp3ca/Ripk1/Tnrc6a/Tnrc6c/Zc3h12<br>[illegible]
3-day post-SCI group vs. the control group	BP	GO:0071897	DNA biosynthetic process	9/222	0.000014	0.0001101	0.000054	Atr/Cyp1b1/Mapk3/Myc/Nox4/Pcna/Rrm2b/Sirt1/Tfdp1
3-day post-SCI group vs. the control group	BP	GO:0061082	myeloid leukocyte cytokine production	6/222	0.000014	0.0001103	0.0000541	Axl/Cd36/Hmox1/Mapkapk2/Sirt1/Tlr4
3-day post-SCI group vs. the control group	BP	GO:0032611	interleukin-1 beta production	7/222	0.0000142	0.0001112	0.0000545	Cd36/Hspb1/Jak2/Sirpa/Tlr4/Tlr6/Zc3h12a
3-day post-SCI group vs. the control group	BP	GO:1901617	organic hydroxy compound biosynthetic process	10/222	0.0000144	0.0001131	0.0000554	Atp7a/Dhcr24/Dhfr/Gch1/Gpr37/Nfkb1/Prkaa2/Rest/Sirt1<br>/Snca
3-day post-SCI group vs. the control group	BP	GO:0090594	inflammatory response to wounding	4/222	0.0000148	0.000116	0.0000569	Alox5/Hif1a/Hmox1/Tlr4
3-day post-SCI group vs. the control group	BP	GO:0090276	regulation of peptide hormone secretion	10/222	0.000015	0.0001169	0.0000573	Alox5/Cd38/Fkbp1b/Hif1a/Jak2/Ppp3ca/Rest/Sirt1/Trpm<br>2/Ucp2
3-day post-SCI group vs. the control group	BP	GO:0006636	unsaturated fatty acid biosynthetic process	5/222	0.0000152	0.0001182	0.0000579	Anxa1/Pnpla8/Ptgs1/Ptgs2/Sirt1
3-day post-SCI group vs. the control group	BP	GO:1904037	positive regulation of epithelial cell apoptotic proc	5/222	0.0000152	0.0001182	0.0000579	Bax/Eif2s1/Hmox1/Jak2/Ppargc1a
3-day post-SCI group vs. the control group	BP	GO:0032388	positive regulation of intracellular transport	9/222	0.0000152	0.0001182	0.000058	Cdk1/Ect2/Ezr/Fbxw7/Jak2/Mapk14/Ptgs2/Tnfrsf1a/Zc3h<br>12a
3-day post-SCI group vs. the control group	BP	GO:0006940	regulation of smooth muscle contraction	6/222	0.0000152	0.0001182	0.000058	Adora1/Cttn/Ncf1/Pawr/Ptgs1/Ptgs2
3-day post-SCI group vs. the control group	BP	GO:0046942	carboxylic acid transport	11/222	0.0000164	0.0001272	0.0000624	Abcc1/Adora1/Anxa1/Arg1/Cd36/Map2k6/Myc/Pnpla8/P<br>tgs2/Slc23a2/Snca
3-day post-SCI group vs. the control group	BP	GO:0032720	negative regulation of tumor necrosis factor produ	6/222	0.0000166	0.0001276	0.0000625	Axl/Sirpa/Sirt1/Tlr4/Tlr6/Zc3h12a
3-day post-SCI group vs. the control group	BP	GO:0150076	neuroinflammatory response	6/222	0.0000166	0.0001276	0.0000625	Aif1/Jak2/Jun/Ptgs2/Snca/Tlr4
3-day post-SCI group vs. the control group	BP	GO:1904036	negative regulation of epithelial cell apoptotic pro	6/222	0.0000166	0.0001276	0.0000625	Apc/Braf/Hmox1/Id1/Nfe2l2/Rb1
3-day post-SCI group vs. the control group	BP	GO:0014706	striated muscle tissue development	11/222	0.000017	0.0001301	0.0000638	Apc/Cdk1/Map2k4/Mapk14/Met/Nox4/Pdgfra/Rxrb/Sirt1<br>/Slc8a1/Smad1
3-day post-SCI group vs. the control group	BP	GO:0030072	peptide hormone secretion	11/222	0.000017	0.0001301	0.0000638	Alox5/Anxa1/Cd38/Fkbp1b/Hif1a/Jak2/Ppp3ca/Rest/Sirt<br>1/Trpm2/Ucp2
3-day post-SCI group vs. the control group	BP	GO:0090316	positive regulation of intracellular protein transpor	8/222	0.0000171	0.0001307	0.0000641	Cdk1/Ect2/Fbxw7/Jak2/Mapk14/Ptgs2/Tnfrsf1a/Zc3h12a
3-day post-SCI group vs. the control group	BP	GO:0019217	regulation of fatty acid metabolic process	7/222	0.0000171	0.0001307	0.0000641	Anxa1/Pdk1/Pdk2/Ppargc1a/Ptgs2/Sirt1/Snca
3-day post-SCI group vs. the control group	BP	GO:0001774	microglial cell activation	5/222	0.0000171	0.0001307	0.0000641	Aif1/Jak2/Jun/Snca/Tlr4
3-day post-SCI group vs. the control group	BP	GO:0050680	negative regulation of epithelial cell proliferation	9/222	0.0000173	0.0001315	0.0000645	Alox5/Apc/Atf2/Cdkn2b/Ctsl/Nfatc1/Ptprk/Rb1/Xdh
3-day post-SCI group vs. the control group	BP	GO:0000079	regulation of cyclin-dependent protein serine/threc	6/222	0.000018	0.0001364	0.0000669	Actb/Apc/Casp3/Ccna2/Cdkn2b/Cdkn2c
3-day post-SCI group vs. the control group	BP	GO:1903556	negative regulation of tumor necrosis factor superf	6/222	0.000018	0.0001364	0.0000669	Axl/Sirpa/Sirt1/Tlr4/Tlr6/Zc3h12a
3-day post-SCI group vs. the control group	BP	GO:0046631	alpha-beta T cell activation	9/222	0.000018	0.0001364	0.0000669	Anxa1/Atf2/Atp7a/Braf/Ctsl/Jak2/Myc/Stat6/Zc3h12a
3-day post-SCI group vs. the control group	BP	GO:0006754	ATP biosynthetic process	7/222	0.0000182	0.0001371	0.0000672	Myc/Ndufa12/Ndufa6/Ndufs8/Nudt2/Parp1/Ppargc1a
3-day post-SCI group vs. the control group	BP	GO:0010310	regulation of hydrogen peroxide metabolic process	4/222	0.0000182	0.0001371	0.0000672	Nox4/Pink1/Snca/Sod2

3-day post-SCI group vs. the control group	BP	GO:0046697	decidualization	4/222	0.0000182	0.0001371	0.0000672	Ctsl/Junb/Mapk3/Ptgs2
3-day post-SCI group vs. the control group	BP	GO:0070371	ERK1 and ERK2 cascade	12/222	0.0000185	0.0001395	0.0000684	Braf/Ccr1/Cd36/Ezr/Fbxw7/Jun/Mapk3/Myc/Nox4/Pdgfra/Sirpa/Tlr4
3-day post-SCI group vs. the control group	BP	GO:0010469	regulation of signaling receptor activity	8/222	0.0000189	0.0001414	0.0000693	Adora1/Chrna4/Fbxw7/Hbegf/Hdac1/Hif1a/Jak2/Ppargc1[illegible]
3-day post-SCI group vs. the control group	BP	GO:0060759	regulation of response to cytokine stimulus	8/222	0.0000189	0.0001414	0.0000693	Arg1/Axl/Hif1a/Il6st/Ripk1/Tlr4/Tnfrsf1a/Vrk2
3-day post-SCI group vs. the control group	BP	GO:0016311	dephosphorylation	12/222	0.0000191	0.0001427	0.0000699	Adora1/Fkbp1b/Jak2/Pawr/Pink1/Ppargc1b/Ppp1ca/Ppp1r15b/Ppp3ca/Ptprk/Rcan1/Ripk3
3-day post-SCI group vs. the control group	BP	GO:0032735	positive regulation of interleukin-12 production	5/222	0.0000193	0.0001436	0.0000704	Cd36/Mapk14/Rela/Tlr4/Tlr6
3-day post-SCI group vs. the control group	BP	GO:0009913	epidermal cell differentiation	10/222	0.0000193	0.0001436	0.0000704	Anxa1/Casp3/Ezh2/Hdac1/Krt1/Ppp3ca/Ptgs1/Ptgs2/Rest/Txnip
3-day post-SCI group vs. the control group	BP	GO:0003300	cardiac muscle hypertrophy	7/222	0.0000193	0.0001439	0.0000705	Atp2a2/Ezh2/Map2k4/Parp1/Ppp3ca/Sirt1/Tnfrsf1a
3-day post-SCI group vs. the control group	BP	GO:0002367	cytokine production involved in immune response	8/222	0.0000198	0.0001469	0.000072	Arg1/Axl/Cd36/Hmox1/Il18rap/Mapkapk2/Sirt1/Tlr4
3-day post-SCI group vs. the control group	BP	GO:0071887	leukocyte apoptotic process	8/222	0.0000198	0.0001469	0.000072	Anxa1/Axl/Bax/Hif1a/Myc/Ripk1/Ripk3/Sirt1
3-day post-SCI group vs. the control group	BP	GO:0051924	regulation of calcium ion transport	11/222	0.0000199	0.000147	0.000072	Bax/Ccr1/Fkbp1b/Mylk/Pawr/Ppp3ca/Ptgs2/S100a1/Slc8a1/Snca/Ubqln1
3-day post-SCI group vs. the control group	BP	GO:0016441	post-transcriptional gene silencing	8/222	0.0000208	0.0001537	0.0000753	Ago1/Ago3/Nfkb1/Ppp3ca/Ripk1/Tnrc6a/Tnrc6c/Zc3h12[illegible]
3-day post-SCI group vs. the control group	BP	GO:1904062	regulation of cation transmembrane transport	13/222	0.000021	0.0001547	0.0000758	Alox5/Arg1/Atp7a/Bax/Chrna4/Fkbp1b/Ppargc1a/Ppif/Ppp3ca/S100a1/Slc8a1/Snca/Ubqln1
3-day post-SCI group vs. the control group	BP	GO:0016032	viral process	12/222	0.0000213	0.000157	0.0000769	Axl/Banf1/Bax/Ctsl/Hdac1/Jun/P4hb/Rest/Ripk1/Ripk3/Vcam1/Zc3h12a
3-day post-SCI group vs. the control group	BP	GO:0060964	regulation of miRNA-mediated gene silencing	5/222	0.0000216	0.0001585	0.0000777	Nfkb1/Ppp3ca/Ripk1/Tnrc6c/Zc3h12a
3-day post-SCI group vs. the control group	BP	GO:1902108	regulation of mitochondrial membrane permeabilit	5/222	0.0000216	0.0001585	0.0000777	Atf2/Bax/Bnip3/Gclc/Ier3
3-day post-SCI group vs. the control group	BP	GO:0043255	regulation of carbohydrate biosynthetic process	7/222	0.0000218	0.0001596	0.0000782	Cd36/Hif1a/Nfkb1/Pdk2/Ppp1ca/Sirt1/Snca
3-day post-SCI group vs. the control group	BP	GO:0019233	sensory perception of pain	8/222	0.0000218	0.0001596	0.0000782	Adora1/Alox5/Chrna4/Mapk3/Pawr/Ptgs1/Ptgs2/Tlr4
3-day post-SCI group vs. the control group	BP	GO:0032970	regulation of actin filament-based process	13/222	0.0000221	0.0001609	0.0000789	Adora1/Arhgdia/Atp2a2/Braf/Cdk10/Cttn/Ect2/Id1/Met/Nox4/Pxn/Stau2/Trpm2
3-day post-SCI group vs. the control group	BP	GO:0035278	miRNA-mediated gene silencing by inhibition of t	4/222	0.0000221	0.0001611	0.000079	Ago1/Ago3/Tnrc6a/Tnrc6c
3-day post-SCI group vs. the control group	BP	GO:0098586	cellular response to virus	6/222	0.0000228	0.0001656	0.0000812	Atf2/Bax/Hif1a/Mapk14/Nfkb1/Zc3h12a
3-day post-SCI group vs. the control group	BP	GO:1904029	regulation of cyclin-dependent protein kinase activ	6/222	0.0000228	0.0001656	0.0000812	Actb/Apc/Casp3/Ccna2/Cdkn2b/Cdkn2c
3-day post-SCI group vs. the control group	BP	GO:0016570	histone modification	14/222	0.0000228	0.0001656	0.0000812	Actb/Atf2/Eed/Ezh2/Hdac1/Mapk3/Pink1/Rbbp7/Rest/Rnf2/Sfpq/Sirt1/Snca/Ube2a
3-day post-SCI group vs. the control group	BP	GO:0042102	positive regulation of T cell proliferation	7/222	0.0000232	0.000167	0.0000818	Aif1/Anxa1/Igfbp2/Il6st/Jak2/Ppp3ca/Vcam1
3-day post-SCI group vs. the control group	BP	GO:0045787	positive regulation of cell cycle	12/222	0.0000232	0.000167	0.0000818	Aif1/Anxa1/Cdk1/Cdk4/Ect2/Ezh2/Fosl1/Met/Myc/Rb1/Rrm2b/Sfpq
3-day post-SCI group vs. the control group	BP	GO:0050890	cognition	12/222	0.0000232	0.000167	0.0000818	Adora1/Amph/Braf/Casp3/Chrna4/Hif1a/Jun/Mapt/Mgat3/Ptgs1/Ptgs2/Rcan1
3-day post-SCI group vs. the control group	BP	GO:0044262	cellular carbohydrate metabolic process	11/222	0.0000239	0.0001719	0.0000843	Braf/Cd36/Hif1a/Ier3/Il6st/Pdk1/Pdk2/Ppargc1a/Ppp1ca/Sirt1/Snca
3-day post-SCI group vs. the control group	BP	GO:0010935	regulation of macrophage cytokine production	5/222	0.0000241	0.0001727	0.0000846	Axl/Cd36/Mapkapk2/Sirt1/Tlr4
3-day post-SCI group vs. the control group	BP	GO:0034198	cellular response to amino acid starvation	5/222	0.0000241	0.0001727	0.0000846	Atf2/Eif2s1/Impact/Mapk3/Mapk8

3-day post-SCI group vs. the control group	BP	GO:0046006	regulation of activated T cell proliferation	5/222	0.0000241	0.0001727	0.0000846	Arg1/Casp3/Igfbp2/Ppp3ca/Ripk3
3-day post-SCI group vs. the control group	BP	GO:2001239	regulation of extrinsic apoptotic signaling pathway	5/222	0.0000241	0.0001727	0.0000846	Ctnna1/Htra2/Mcl1/Ppp1ca/Ripk1
3-day post-SCI group vs. the control group	BP	GO:0014897	striated muscle hypertrophy	7/222	0.0000246	0.0001749	0.0000858	Atp2a2/Ezh2/Map2k4/Parp1/Ppp3ca/Sirt1/Tnfrsf1a
3-day post-SCI group vs. the control group	BP	GO:0032760	positive regulation of tumor necrosis factor produc	7/222	0.0000246	0.0001749	0.0000858	Cd36/Hspb1/Jak2/Mapkapk2/Ripk1/Tlr4/Tnfrsf1a
3-day post-SCI group vs. the control group	BP	GO:1903578	regulation of ATP metabolic process	6/222	0.0000247	0.0001753	0.000086	Hif1a/Ier3/Myc/Parp1/Ppargc1a/Prkaa2
3-day post-SCI group vs. the control group	BP	GO:0045732	positive regulation of protein catabolic process	9/222	0.0000259	0.000184	0.0000902	Adam9/Apc/Cdc20/Ezr/Fbxw7/Gclc/Ier3/Mapk8/Ubqln1
3-day post-SCI group vs. the control group	BP	GO:0042063	gliogenesis	12/222	0.0000265	0.000188	0.0000922	Anxa1/Cdkn2b/Eed/Ezh2/Hdac1/Il6st/Mapk3/Myc/Rb1/Rela/Rnf112/Tlr4
3-day post-SCI group vs. the control group	BP	GO:0002269	leukocyte activation involved in inflammatory resp	5/222	0.0000269	0.0001899	0.0000931	Aif1/Jak2/Jun/Snca/Tlr4
3-day post-SCI group vs. the control group	BP	GO:0060147	regulation of post-transcriptional gene silencing	5/222	0.0000269	0.0001899	0.0000931	Nfkb1/Ppp3ca/Ripk1/Tnrc6c/Zc3h12a
3-day post-SCI group vs. the control group	BP	GO:1900368	regulation of post-transcriptional gene silencing by	5/222	0.0000269	0.0001899	0.0000931	Nfkb1/Ppp3ca/Ripk1/Tnrc6c/Zc3h12a
3-day post-SCI group vs. the control group	BP	GO:0006470	protein dephosphorylation	10/222	0.0000272	0.0001915	0.0000939	Adora1/Fkbp1b/Jak2/Pawr/Pink1/Ppp1ca/Ppp1r15b/Ppp3ca/Ptprk/Rcan1
3-day post-SCI group vs. the control group	BP	GO:1903320	regulation of protein modification by small protein	10/222	0.0000272	0.0001915	0.0000939	Cdc20/Fbxw7/Gclc/Hif1a/Mapk8/Pink1/Rela/Tank/Ubqln1/Zc3h12a
3-day post-SCI group vs. the control group	BP	GO:0014896	muscle hypertrophy	7/222	0.0000276	0.0001928	0.0000945	Atp2a2/Ezh2/Map2k4/Parp1/Ppp3ca/Sirt1/Tnfrsf1a
3-day post-SCI group vs. the control group	BP	GO:0055017	cardiac muscle tissue growth	7/222	0.0000276	0.0001928	0.0000945	Apc/Cdk1/Map2k4/Mapk14/Rxrb/Sirt1/Smad1
3-day post-SCI group vs. the control group	BP	GO:1903557	positive regulation of tumor necrosis factor superf	7/222	0.0000276	0.0001928	0.0000945	Cd36/Hspb1/Jak2/Mapkapk2/Ripk1/Tlr4/Tnfrsf1a
3-day post-SCI group vs. the control group	BP	GO:0051092	positive regulation of NF-kappaB transcription fac	8/222	0.0000276	0.000193	0.0000946	Cat/Cd36/Il18rap/Rela/Ripk1/Ripk3/Tlr4/Tlr6
3-day post-SCI group vs. the control group	BP	GO:0002695	negative regulation of leukocyte activation	9/222	0.000028	0.000195	0.0000956	Anxa1/Arg1/Axl/Btk/Casp3/Hmox1/Hspb1/Pawr/Zc3h12[illegible]
3-day post-SCI group vs. the control group	BP	GO:0051495	positive regulation of cytoskeleton organization	9/222	0.000028	0.000195	0.0000956	Apc/Braf/Cttn/Id1/Mapk8/Mapt/Met/Nox4/Pxn
3-day post-SCI group vs. the control group	BP	GO:0016239	positive regulation of macroautophagy	6/222	0.0000287	0.0001991	0.0000976	Bnip3/Hif1a/Hmox1/Mapk3/Pink1/Sirt1
3-day post-SCI group vs. the control group	BP	GO:0030216	keratinocyte differentiation	8/222	0.0000289	0.0002008	0.0000984	Anxa1/Casp3/Ezh2/Krt1/Ppp3ca/Ptgs1/Ptgs2/Txnip
3-day post-SCI group vs. the control group	BP	GO:0017157	regulation of exocytosis	10/222	0.0000291	0.0002018	0.0000989	Anxa1/Atp13a2/Atp2a2/Braf/Chrna4/Hmox1/Rest/Sdc1/Snca/Syp
3-day post-SCI group vs. the control group	BP	GO:0042417	dopamine metabolic process	5/222	0.0000299	0.0002071	0.0001015	Atp7a/Gch1/Gpr37/Maoa/Snca
3-day post-SCI group vs. the control group	BP	GO:0071902	positive regulation of protein serine/threonine kina	9/222	0.0000303	0.0002089	0.0001024	Adam9/Ezh2/Map2k4/Map2k6/Nox4/Sirt1/Snca/Tlr4/Tlr[illegible]
3-day post-SCI group vs. the control group	BP	GO:0019369	arachidonic acid metabolic process	6/222	0.0000308	0.0002119	0.0001039	Alox5/Cyp1b1/Gpx1/Pnpla8/Ptgs1/Ptgs2
3-day post-SCI group vs. the control group	BP	GO:0038034	signal transduction in absence of ligand	6/222	0.0000308	0.0002119	0.0001039	Bax/Ctnna1/Htra2/Mcl1/Ppp1ca/Ripk1
3-day post-SCI group vs. the control group	BP	GO:0097192	extrinsic apoptotic signaling pathway in absence o	6/222	0.0000308	0.0002119	0.0001039	Bax/Ctnna1/Htra2/Mcl1/Ppp1ca/Ripk1
3-day post-SCI group vs. the control group	BP	GO:1990000	amyloid fibril formation	4/222	0.0000317	0.0002176	0.0001067	Cd36/Mapt/Ripk1/Ripk3
3-day post-SCI group vs. the control group	BP	GO:0034976	response to endoplasmic reticulum stress	10/222	0.0000322	0.0002204	0.000108	Alox5/Bax/Casp3/Eif2s1/Jun/Nfe2l2/P4hb/Ppp1r15b/Sirt1/Ubqln1
3-day post-SCI group vs. the control group	BP	GO:2001020	regulation of response to DNA damage stimulus	11/222	0.0000331	0.0002264	0.000111	Actb/Atr/Endog/Ier3/Mapt/Mcl1/Mgmt/Myc/Parp1/Pcna/Sirt1
3-day post-SCI group vs. the control group	BP	GO:0043124	negative regulation of I-kappaB kinase/NF-kappaB	5/222	0.0000332	0.0002267	0.0001111	Hdac1/Ripk1/Sirt1/Tank/Zc3h12a
3-day post-SCI group vs. the control group	BP	GO:0007519	skeletal muscle tissue development	9/222	0.0000339	0.0002312	0.0001133	Fos/Gpx1/Mapk14/Met/Myc/Ppp3ca/Rb1/Rcan1/Ybx3
3-day post-SCI group vs. the control group	BP	GO:0016358	dendrite development	11/222	0.0000351	0.0002387	0.000117	Atp7a/Cdc20/Ezh2/Gpr37/Id1/Mapk8/Met/Ppp3ca/Stau2/Tbc1d24/Tnik
3-day post-SCI group vs. the control group	BP	GO:1901988	negative regulation of cell cycle phase transition	10/222	0.0000355	0.0002412	0.0001182	Apc/Atf2/Atr/Cdc20/Cdk1/Cdkn2b/Ezh2/Ier3/Mapk14/R[illegible]

3-day post-SCI group vs. the control group	BP	GO:2000573	positive regulation of DNA biosynthetic process	6/222	0.0000356	0.0002416	0.0001185	Atr/Cyp1b1/Mapk3/Myc/Nox4/Pcna
3-day post-SCI group vs. the control group	BP	GO:0046879	hormone secretion	12/222	0.0000364	0.0002459	0.0001205	Adora1/Alox5/Anxa1/Cd38/Fkbp1b/Hif1a/Jak2/Ppp3ca/Rest/Sirt1/Trpm2/Ucp2
3-day post-SCI group vs. the control group	BP	GO:0048872	homeostasis of number of cells	12/222	0.0000364	0.0002459	0.0001205	Anxa1/Axl/Bax/Casp3/Ets1/Ezh2/Hif1a/Hmox1/Jak2/Mapk14/Rb1/Ripk3
3-day post-SCI group vs. the control group	BP	GO:0006110	regulation of glycolytic process	5/222	0.0000368	0.0002461	0.0001207	Hif1a/Ier3/Myc/Ppargc1a/Prkaa2
3-day post-SCI group vs. the control group	BP	GO:0010332	response to gamma radiation	5/222	0.0000368	0.0002461	0.0001207	Atr/Bax/Gpx1/Myc/Sod2
3-day post-SCI group vs. the control group	BP	GO:0035307	positive regulation of protein dephosphorylation	5/222	0.0000368	0.0002461	0.0001207	Adora1/Jak2/Pawr/Pink1/Ppp1r15b
3-day post-SCI group vs. the control group	BP	GO:0060966	regulation of gene silencing by RNA	5/222	0.0000368	0.0002461	0.0001207	Nfkb1/Ppp3ca/Ripk1/Tnrc6c/Zc3h12a
3-day post-SCI group vs. the control group	BP	GO:0061900	glial cell activation	5/222	0.0000368	0.0002461	0.0001207	Aif1/Jak2/Jun/Snca/Tlr4
3-day post-SCI group vs. the control group	BP	GO:1990928	response to amino acid starvation	5/222	0.0000368	0.0002461	0.0001207	Atf2/Eif2s1/Impact/Mapk3/Mapk8
3-day post-SCI group vs. the control group	BP	GO:1901032	negative regulation of response to reactive oxygen	4/222	0.0000376	0.0002506	0.0001228	Gpr37/Met/Nfe2l2/Pink1
3-day post-SCI group vs. the control group	BP	GO:1903206	negative regulation of hydrogen peroxide-induced	4/222	0.0000376	0.0002506	0.0001228	Gpr37/Met/Nfe2l2/Pink1
3-day post-SCI group vs. the control group	BP	GO:0051348	negative regulation of transferase activity	10/222	0.0000379	0.0002521	0.0001236	Actb/Apc/Casp3/Cdc20/Cdkn2c/Hspb1/Mapt/Rb1/Sirt1/Slc8a1
3-day post-SCI group vs. the control group	BP	GO:0034101	erythrocyte homeostasis	8/222	0.0000379	0.0002521	0.0001236	Axl/Casp3/Ets1/Hif1a/Hmox1/Jak2/Mapk14/Rb1
3-day post-SCI group vs. the control group	BP	GO:0045445	myoblast differentiation	7/222	0.0000385	0.0002546	0.0001248	Actb/Btg1/Gpx1/Mapk14/Rb1/Rest/Sdc1
3-day post-SCI group vs. the control group	BP	GO:1901989	positive regulation of cell cycle phase transition	7/222	0.0000385	0.0002546	0.0001248	Aif1/Anxa1/Cdk1/Cdk4/Ezh2/Rb1/Rrm2b
3-day post-SCI group vs. the control group	BP	GO:2000106	regulation of leukocyte apoptotic process	7/222	0.0000385	0.0002546	0.0001248	Anxa1/Axl/Bax/Hif1a/Myc/Ripk3/Sirt1
3-day post-SCI group vs. the control group	BP	GO:0045446	endothelial cell differentiation	7/222	0.0000406	0.0002682	0.0001315	Btg1/Ezr/Gpx1/Id1/Met/Tnfrsf1a/Xdh
3-day post-SCI group vs. the control group	BP	GO:2000113	negative regulation of cellular macromolecule bios	9/222	0.0000409	0.0002697	0.0001322	Ago1/Ago3/Eif2s1/Hbegf/Paip1/Rpl13a/Tnrc6a/Tnrc6c/Zc3h12a
3-day post-SCI group vs. the control group	BP	GO:0043279	response to alkaloid	6/222	0.000041	0.0002702	0.0001324	Casp3/Myc/Parp1/Prkaa2/Slc8a1/Snca
3-day post-SCI group vs. the control group	BP	GO:0044270	cellular nitrogen compound catabolic process	13/222	0.0000422	0.0002774	0.000136	Ago1/Ago3/Bax/Casp3/Endog/Hmox1/Mapkapk2/Ncf1/Paip1/Tnrc6a/Tnrc6c/Xdh/Zc3h12a
3-day post-SCI group vs. the control group	BP	GO:0098727	maintenance of cell number	9/222	0.0000424	0.0002781	0.0001363	Actb/Apc/Braf/Ezh2/Hdac1/Mapk8/Myc/Rbbp7/Rest
3-day post-SCI group vs. the control group	BP	GO:0050878	regulation of body fluid levels	12/222	0.0000424	0.0002781	0.0001363	Adora1/Axl/Cd36/Hif1a/Jak2/Krt1/Met/Nfe2l2/Pdgfra/Ppp3ca/Tlr4/Xdh
3-day post-SCI group vs. the control group	BP	GO:0006090	pyruvate metabolic process	7/222	0.0000428	0.0002802	0.0001374	Hif1a/Ier3/Myc/Pdk1/Pdk2/Ppargc1a/Prkaa2
3-day post-SCI group vs. the control group	BP	GO:0007626	locomotory behavior	10/222	0.000043	0.0002815	0.000138	Atp7a/Chrna4/Gpr37/Htra2/Mapk10/Mapt/Oxr1/Rcan1/Snca/Sod2
3-day post-SCI group vs. the control group	BP	GO:0002082	regulation of oxidative phosphorylation	4/222	0.0000441	0.0002871	0.0001407	Atp7a/Myc/Pink1/Ppif
3-day post-SCI group vs. the control group	BP	GO:0046685	response to arsenic-containing substance	4/222	0.0000441	0.0002871	0.0001407	Gclc/Hmox1/Ppif/Zc3h12a
3-day post-SCI group vs. the control group	BP	GO:1904030	negative regulation of cyclin-dependent protein kin	4/222	0.0000441	0.0002871	0.0001407	Actb/Apc/Casp3/Cdkn2c
3-day post-SCI group vs. the control group	BP	GO:0009914	hormone transport	12/222	0.0000446	0.0002898	0.000142	Adora1/Alox5/Anxa1/Cd38/Fkbp1b/Hif1a/Jak2/Ppp3ca/Rest/Sirt1/Trpm2/Ucp2
3-day post-SCI group vs. the control group	BP	GO:0120254	olefinic compound metabolic process	8/222	0.0000451	0.0002924	0.0001434	Alox5/Cyp1b1/Gpx1/Pnpla8/Ppargc1a/Ptgs1/Ptgs2/Rest
3-day post-SCI group vs. the control group	BP	GO:0009895	negative regulation of catabolic process	11/222	0.0000453	0.0002933	0.0001438	Adora1/Hmox1/Ier3/Mapkapk2/Mcl1/Mgat3/Paip1/Pink1/Ppargc1a/Rela/Snca

3-day post-SCI group vs. the control group	BP	GO:0050730	regulation of peptidyl-tyrosine phosphorylation	10/222	0.0000458	0.0002965	0.0001454	Adora1/Cd36/Fbxw7/Hbegf/Il6st/Jak2/Nox4/Pdgfra/Tlr4/Tnfrsf1a
3-day post-SCI group vs. the control group	BP	GO:0000422	autophagy of mitochondrion	6/222	0.000047	0.0003026	0.0001483	Bnip3/Cttn/Fbxw7/Hif1a/Htra2/Pink1
3-day post-SCI group vs. the control group	BP	GO:0008088	axo-dendritic transport	6/222	0.000047	0.0003026	0.0001483	Dst/Hif1a/Hspb1/Mapt/Sfpq/Stau2
3-day post-SCI group vs. the control group	BP	GO:0061726	mitochondrion disassembly	6/222	0.000047	0.0003026	0.0001483	Bnip3/Cttn/Fbxw7/Hif1a/Htra2/Pink1
3-day post-SCI group vs. the control group	BP	GO:0051216	cartilage development	9/222	0.0000473	0.0003031	0.0001486	Atf2/Atp7a/Bnip1/Hif1a/Mapk14/Mapk3/Rb1/Rela/Smad1
3-day post-SCI group vs. the control group	BP	GO:0002253	activation of immune response	13/222	0.0000473	0.0003031	0.0001486	Bax/Braf/Btk/Cd38/Ezr/Krt1/Nono/Pawr/Plekha1/Rela/Sfpq/Tlr4/Zc3h12a
3-day post-SCI group vs. the control group	BP	GO:0090068	positive regulation of cell cycle process	10/222	0.0000473	0.0003031	0.0001486	Aif1/Anxa1/Cdk1/Cdk4/Ect2/Ezh2/Met/Rb1/Rrm2b/Sfpq
3-day post-SCI group vs. the control group	BP	GO:0001676	long-chain fatty acid metabolic process	7/222	0.0000475	0.0003031	0.0001486	Alox5/Cd36/Cyp1b1/Gpx1/Pnpla8/Ptgs1/Ptgs2
3-day post-SCI group vs. the control group	BP	GO:0060419	heart growth	7/222	0.0000475	0.0003031	0.0001486	Apc/Cdk1/Map2k4/Mapk14/Rxrb/Sirt1/Smad1
3-day post-SCI group vs. the control group	BP	GO:1902414	protein localization to cell junction	7/222	0.0000475	0.0003031	0.0001486	Actb/Hspb1/Mapk10/Mapk8/Mapt/Stau2/Tnik
3-day post-SCI group vs. the control group	BP	GO:0030217	T cell differentiation	11/222	0.0000479	0.0003044	0.0001492	Actb/Anxa1/Apc/Atf2/Atp7a/Braf/Ctsl/Hspb1/Ripk3/Stat6/Zc3h12a
3-day post-SCI group vs. the control group	BP	GO:0070372	regulation of ERK1 and ERK2 cascade	11/222	0.0000479	0.0003044	0.0001492	Braf/Ccr1/Cd36/Ezr/Fbxw7/Jun/Mapk3/Nox4/Pdgfra/Sirpa/Tlr4
3-day post-SCI group vs. the control group	BP	GO:0010934	macrophage cytokine production	5/222	0.0000492	0.0003119	0.0001529	Axl/Cd36/Mapkapk2/Sirt1/Tlr4
3-day post-SCI group vs. the control group	BP	GO:0035196	miRNA processing	5/222	0.0000492	0.0003119	0.0001529	Ago1/Ago3/Ppp3ca/Ripk1/Zc3h12a
3-day post-SCI group vs. the control group	BP	GO:0045670	regulation of osteoclast differentiation	6/222	0.0000502	0.0003181	0.0001559	Apc/Ccr1/Fbxw7/Fos/Ppargc1b/Ppp3ca
3-day post-SCI group vs. the control group	BP	GO:0030522	intracellular receptor signaling pathway	9/222	0.0000507	0.0003209	0.0001573	Ezh2/Hdac1/Jak2/Parp1/Ppargc1b/Rela/Rxrb/Sirt1/Tlr4
3-day post-SCI group vs. the control group	BP	GO:0010042	response to manganese ion	3/222	0.0000513	0.0003216	0.0001576	Adam9/Atp13a2/Atp7a
3-day post-SCI group vs. the control group	BP	GO:0014883	transition between fast and slow fiber	3/222	0.0000513	0.0003216	0.0001576	Atp2a2/Nfatc1/Ppp3ca
3-day post-SCI group vs. the control group	BP	GO:1902884	positive regulation of response to oxidative stress	3/222	0.0000513	0.0003216	0.0001576	Endog/Pawr/Ripk1
3-day post-SCI group vs. the control group	BP	GO:2001269	positive regulation of cysteine-type endopeptidase	3/222	0.0000513	0.0003216	0.0001576	Bax/Htra2/Jak2
3-day post-SCI group vs. the control group	BP	GO:0009713	catechol-containing compound biosynthetic proces	4/222	0.0000515	0.0003216	0.0001576	Atp7a/Gch1/Gpr37/Snca
3-day post-SCI group vs. the control group	BP	GO:0032042	mitochondrial DNA metabolic process	4/222	0.0000515	0.0003216	0.0001576	Endog/Parp1/Ppargc1a/Rrm2b
3-day post-SCI group vs. the control group	BP	GO:0042423	catecholamine biosynthetic process	4/222	0.0000515	0.0003216	0.0001576	Atp7a/Gch1/Gpr37/Snca
3-day post-SCI group vs. the control group	BP	GO:1904996	positive regulation of leukocyte adhesion to vascul	4/222	0.0000515	0.0003216	0.0001576	Alox5/Ets1/Pawr/Rela
3-day post-SCI group vs. the control group	BP	GO:0002274	myeloid leukocyte activation	10/222	0.0000519	0.0003238	0.0001587	Adam9/Aif1/Anxa1/Hmox1/Il18rap/Jak2/Jun/Snca/Tlr4/Tlr6
3-day post-SCI group vs. the control group	BP	GO:0060538	skeletal muscle organ development	9/222	0.0000526	0.0003275	0.0001605	Fos/Gpx1/Mapk14/Met/Myc/Ppp3ca/Rb1/Rcan1/Ybx3
3-day post-SCI group vs. the control group	BP	GO:0002040	sprouting angiogenesis	7/222	0.0000527	0.0003275	0.0001606	Alox5/Anxa1/Fbxw7/Hmox1/Ptgs2/S100a1/Smad1
3-day post-SCI group vs. the control group	BP	GO:2000045	regulation of G1/S transition of mitotic cell cycle	8/222	0.0000533	0.0003313	0.0001624	Actb/Aif1/Anxa1/Apc/Cdkn2b/Cdkn2c/Ezh2/Rb1
3-day post-SCI group vs. the control group	BP	GO:0034764	positive regulation of transmembrane transport	10/222	0.0000535	0.0003319	0.0001627	Alox5/Atp7a/Bax/Braf/Mapk14/Met/Nfe2l2/Ppp3ca/S100a1/Snca
3-day post-SCI group vs. the control group	BP	GO:0034605	cellular response to heat	5/222	0.000054	0.0003327	0.0001631	Eif2s1/Hmox1/Htra2/Mapt/Rbbp7
3-day post-SCI group vs. the control group	BP	GO:0050798	activated T cell proliferation	5/222	0.000054	0.0003327	0.0001631	Arg1/Casp3/Igfbp2/Ppp3ca/Ripk3
3-day post-SCI group vs. the control group	BP	GO:0061756	leukocyte adhesion to vascular endothelial cell	5/222	0.000054	0.0003327	0.0001631	Alox5/Ets1/Pawr/Rela/Vcam1
3-day post-SCI group vs. the control group	BP	GO:0090199	regulation of release of cytochrome c from mitoch	5/222	0.000054	0.0003327	0.0001631	Bax/Bnip3/Gpx1/Pink1/Ppif
3-day post-SCI group vs. the control group	BP	GO:0002718	regulation of cytokine production involved in imm	7/222	0.0000554	0.0003405	0.0001669	Arg1/Axl/Cd36/Hmox1/Mapkapk2/Sirt1/Tlr4
3-day post-SCI group vs. the control group	BP	GO:0034440	lipid oxidation	7/222	0.0000554	0.0003405	0.0001669	Alox5/Cd36/Cygb/Etfdh/Hao1/Mapk14/Ppargc1a

3-day post-SCI group vs. the control group	BP	GO:0051250	negative regulation of lymphocyte activation	8/222	0.0000556	0.0003413	0.0001673	Anxa1/Arg1/Axl/Btk/Casp3/Hspb1/Pawr/Zc3h12a
3-day post-SCI group vs. the control group	BP	GO:0043393	regulation of protein binding	9/222	0.0000564	0.0003452	0.0001692	Actb/Atp2a2/Atr/Bax/Id1/Mapk3/Mapk8/Mark3/Myc
3-day post-SCI group vs. the control group	BP	GO:1903828	negative regulation of protein localization	9/222	0.0000564	0.0003452	0.0001692	Anxa1/Cd36/Ctnna1/Fkbp1b/Mapt/Ppp3ca/Rest/Sirt1/Ucp2
3-day post-SCI group vs. the control group	BP	GO:2001022	positive regulation of response to DNA damage sti	8/222	0.0000579	0.000354	0.0001735	Actb/Atr/Endog/Mgmt/Myc/Parp1/Pcna/Sirt1
3-day post-SCI group vs. the control group	BP	GO:0009308	amine metabolic process	7/222	0.0000582	0.0003555	0.0001743	Apc/Atp7a/Gch1/Gpr37/Maoa/Snca/Vcam1
3-day post-SCI group vs. the control group	BP	GO:0010959	regulation of metal ion transport	13/222	0.0000591	0.0003604	0.0001767	Adora1/Atp7a/Bax/Ccr1/Fkbp1b/Mylk/Pawr/Ppp3ca/Ptgs2/S100a1/Slc8a1/Snca/Ubqln1
3-day post-SCI group vs. the control group	BP	GO:0006941	striated muscle contraction	8/222	0.0000603	0.0003672	0.00018	Adora1/Atp2a2/Fkbp1b/Map2k3/Map2k6/Met/Slc8a1/Zc3h12a
3-day post-SCI group vs. the control group	BP	GO:0046632	alpha-beta T cell differentiation	7/222	0.0000643	0.0003903	0.0001913	Anxa1/Atf2/Atp7a/Braf/Ctsl/Stat6/Zc3h12a
3-day post-SCI group vs. the control group	BP	GO:2000278	regulation of DNA biosynthetic process	7/222	0.0000643	0.0003903	0.0001913	Atr/Cyp1b1/Mapk3/Myc/Nox4/Pcna/Tfdp1
3-day post-SCI group vs. the control group	BP	GO:0031113	regulation of microtubule polymerization	5/222	0.0000646	0.0003909	0.0001916	Apc/Mapk8/Mapt/Met/Snca
3-day post-SCI group vs. the control group	BP	GO:0042149	cellular response to glucose starvation	5/222	0.0000646	0.0003909	0.0001916	Impact/Nfe2l2/Prkaa2/Sirt1/Zc3h12a
3-day post-SCI group vs. the control group	BP	GO:0051896	regulation of protein kinase B signaling	8/222	0.0000654	0.0003952	0.0001937	Axl/Gpx1/Hbegf/Met/Nox4/Plekha1/Sirt1/Xdh
3-day post-SCI group vs. the control group	BP	GO:0022604	regulation of cell morphogenesis	11/222	0.0000662	0.0003991	0.0001957	Anxa1/Arhgdia/Braf/Ezr/Met/P4hb/Pxn/Slc23a2/Stau2/Tbc1d24/Tnik
3-day post-SCI group vs. the control group	BP	GO:0033673	negative regulation of kinase activity	9/222	0.000067	0.0004036	0.0001978	Actb/Apc/Casp3/Cdkn2c/Hspb1/Mapt/Rb1/Sirt1/Slc8a1
3-day post-SCI group vs. the control group	BP	GO:0002697	regulation of immune effector process	13/222	0.0000674	0.0004057	0.0001989	Anxa1/Arg1/Axl/Cd36/Hmox1/Il18rap/Mapkapk2/Ncf1/Ripk3/Sirt1/Stat6/Tlr4/Zc3h12a
3-day post-SCI group vs. the control group	BP	GO:0043406	positive regulation of MAP kinase activity	7/222	0.0000675	0.0004057	0.0001989	Adam9/Ezh2/Map2k4/Map2k6/Nox4/Tlr4/Tlr6
3-day post-SCI group vs. the control group	BP	GO:0032770	positive regulation of monooxygenase activity	4/222	0.0000688	0.0004128	0.0002023	Atp7a/Dhfr/Gch1/S100a1
3-day post-SCI group vs. the control group	BP	GO:0051251	positive regulation of lymphocyte activation	13/222	0.0000689	0.0004128	0.0002023	Actb/Aif1/Anxa1/Axl/Cd38/Igfbp2/Il6st/Jak2/Ppp3ca/Sirpa/Stat6/Tlr4/Vcam1
3-day post-SCI group vs. the control group	BP	GO:0050805	negative regulation of synaptic transmission	6/222	0.0000693	0.0004139	0.0002029	Adora1/Braf/Cd38/Mapt/Ptgs2/Stau2
3-day post-SCI group vs. the control group	BP	GO:0070374	positive regulation of ERK1 and ERK2 cascade	9/222	0.0000693	0.0004139	0.0002029	Braf/Ccr1/Cd36/Fbxw7/Jun/Mapk3/Nox4/Pdgfra/Tlr4
3-day post-SCI group vs. the control group	BP	GO:0048863	stem cell differentiation	10/222	0.0000701	0.0004181	0.000205	Ezh2/Hdac1/Hif1a/Mapk14/Mapk3/Nfe2l2/Pdgfra/Rbbp7/Rest/Slc4a11
3-day post-SCI group vs. the control group	BP	GO:0002246	wound healing involved in inflammatory response	3/222	0.0000702	0.0004181	0.000205	Hif1a/Hmox1/Tlr4
3-day post-SCI group vs. the control group	BP	GO:0070918	small regulatory ncRNA processing	5/222	0.0000705	0.0004194	0.0002056	Ago1/Ago3/Ppp3ca/Ripk1/Zc3h12a
3-day post-SCI group vs. the control group	BP	GO:0048638	regulation of developmental growth	12/222	0.0000708	0.0004205	0.0002061	Apc/Atrn/Cdk1/Cdk4/Cttn/Ezr/Htra2/Mapk14/Mapt/Sirt1/Slc23a2/Ybx3
3-day post-SCI group vs. the control group	BP	GO:0046888	negative regulation of hormone secretion	6/222	0.0000737	0.0004365	0.000214	Adora1/Fkbp1b/Ppp3ca/Rest/Sirt1/Ucp2
3-day post-SCI group vs. the control group	BP	GO:0060079	excitatory postsynaptic potential	6/222	0.0000737	0.0004365	0.000214	Adora1/Chrna4/Met/Ppp3ca/Snca/Tbc1d24
3-day post-SCI group vs. the control group	BP	GO:0055001	muscle cell development	9/222	0.0000741	0.0004383	0.0002149	Atp2a2/Gpx1/Map2k4/Met/Pdgfra/Ppp3ca/Rcan1/Sirt1/Slc8a1
3-day post-SCI group vs. the control group	BP	GO:0043270	positive regulation of ion transport	11/222	0.0000754	0.0004453	0.0002183	Adora1/Alox5/Atp7a/Bax/Ccr1/Map2k6/Mylk/Pink1/Ppp3ca/S100a1/Snca
3-day post-SCI group vs. the control group	BP	GO:0034655	nucleobase-containing compound catabolic proces	12/222	0.000076	0.000448	0.0002196	Ago1/Ago3/Bax/Casp3/Endog/Mapkapk2/Ncf1/Paip1/Tnrc6a/Tnrc6c/Xdh/Zc3h12a
3-day post-SCI group vs. the control group	BP	GO:0045932	negative regulation of muscle contraction	4/222	0.0000789	0.000463	0.0002269	Adora1/Ncf1/Ptgs2/Zc3h12a

3-day post-SCI group vs. the control group	BP	GO:0046885	regulation of hormone biosynthetic process	4/222	0.0000789	0.000463	0.0002269	Hif1a/Nfkb1/Ppargc1a/Rest
3-day post-SCI group vs. the control group	BP	GO:0060907	positive regulation of macrophage cytokine produc	4/222	0.0000789	0.000463	0.0002269	Cd36/Mapkapk2/Sirt1/Tlr4
3-day post-SCI group vs. the control group	BP	GO:0090200	positive regulation of release of cytochrome c fron	4/222	0.0000789	0.000463	0.0002269	Bax/Bnip3/Pink1/Ppif
3-day post-SCI group vs. the control group	BP	GO:0043367	CD4-positive, alpha-beta T cell differentiation	6/222	0.0000832	0.0004867	0.0002386	Anxa1/Atp7a/Braf/Ctsl/Stat6/Zc3h12a
3-day post-SCI group vs. the control group	BP	GO:0048814	regulation of dendrite morphogenesis	6/222	0.0000832	0.0004867	0.0002386	Id1/Met/Ppp3ca/Stau2/Tbc1d24/Tnik
3-day post-SCI group vs. the control group	BP	GO:0042311	vasodilation	5/222	0.0000835	0.0004872	0.0002388	Adora1/Gch1/Gpx1/Sirt1/Sod2
3-day post-SCI group vs. the control group	BP	GO:0043666	regulation of phosphoprotein phosphatase activity	5/222	0.0000835	0.0004872	0.0002388	Fkbp1b/Jak2/Pawr/Ppp1r15b/Rcan1
3-day post-SCI group vs. the control group	BP	GO:0009132	nucleoside diphosphate metabolic process	7/222	0.0000898	0.0005218	0.0002558	Hif1a/Ier3/Myc/Nme2/Ppargc1a/Prkaa2/Rrm2b
3-day post-SCI group vs. the control group	BP	GO:0003085	negative regulation of systemic arterial blood pres:	4/222	0.0000901	0.0005218	0.0002558	Adora1/Cd36/Ier3/Sod2
3-day post-SCI group vs. the control group	BP	GO:0031116	positive regulation of microtubule polymerization	4/222	0.0000901	0.0005218	0.0002558	Apc/Mapk8/Mapt/Met
3-day post-SCI group vs. the control group	BP	GO:0035094	response to nicotine	4/222	0.0000901	0.0005218	0.0002558	Chrna4/Hmox1/Nfkb1/Rela
3-day post-SCI group vs. the control group	BP	GO:0071354	cellular response to interleukin-6	4/222	0.0000901	0.0005218	0.0002558	Il6st/Nfkb1/Rela/Ripk1
3-day post-SCI group vs. the control group	BP	GO:0043388	positive regulation of DNA binding	5/222	0.0000907	0.0005247	0.0002572	Jak2/Myc/Parp1/Rb1/Tlr4
3-day post-SCI group vs. the control group	BP	GO:0071674	mononuclear cell migration	8/222	0.0000929	0.0005318	0.0002607	Aif1/Alox5/Anxa1/Ccr1/Mapk3/Ripk3/Sirpa/Trpm2
3-day post-SCI group vs. the control group	BP	GO:0031392	regulation of prostaglandin biosynthetic process	3/222	0.0000931	0.0005318	0.0002607	Anxa1/Ptgs2/Sirt1
3-day post-SCI group vs. the control group	BP	GO:0035331	negative regulation of hippo signaling	3/222	0.0000931	0.0005318	0.0002607	Map2k3/Mapk14/Mark3
3-day post-SCI group vs. the control group	BP	GO:0045348	positive regulation of MHC class II biosynthetic pı	3/222	0.0000931	0.0005318	0.0002607	Jak2/Sirt1/Tlr4
3-day post-SCI group vs. the control group	BP	GO:0045741	positive regulation of epidermal growth factor-acti	3/222	0.0000931	0.0005318	0.0002607	Adora1/Fbxw7/Hbegf
3-day post-SCI group vs. the control group	BP	GO:0072497	mesenchymal stem cell differentiation	3/222	0.0000931	0.0005318	0.0002607	Pdgfra/Rest/Slc4a11
3-day post-SCI group vs. the control group	BP	GO:0140052	cellular response to oxidised low-density lipoprote	3/222	0.0000931	0.0005318	0.0002607	Cd36/Tlr4/Tlr6
3-day post-SCI group vs. the control group	BP	GO:1900227	positive regulation of NLRP3 inflammasome comp	3/222	0.0000931	0.0005318	0.0002607	Cd36/Tlr4/Tlr6
3-day post-SCI group vs. the control group	BP	GO:1990144	intrinsic apoptotic signaling pathway in response t	3/222	0.0000931	0.0005318	0.0002607	Atf2/Bnip3/Pink1
3-day post-SCI group vs. the control group	BP	GO:0044272	sulfur compound biosynthetic process	6/222	0.0000937	0.0005327	0.0002611	Gclc/Ggt7/Nfe2l2/Pdk1/Pdk2/Snca
3-day post-SCI group vs. the control group	BP	GO:0001959	regulation of cytokine-mediated signaling pathway	7/222	0.000094	0.0005327	0.0002611	Arg1/Axl/Hif1a/Il6st/Ripk1/Tnfrsf1a/Vrk2
3-day post-SCI group vs. the control group	BP	GO:0042303	molting cycle	7/222	0.000094	0.0005327	0.0002611	Apc/Atp7a/Ctsl/Hdac1/Nfatc1/Ptgs2/Rela
3-day post-SCI group vs. the control group	BP	GO:0042633	hair cycle	7/222	0.000094	0.0005327	0.0002611	Apc/Atp7a/Ctsl/Hdac1/Nfatc1/Ptgs2/Rela
3-day post-SCI group vs. the control group	BP	GO:0055076	transition metal ion homeostasis	7/222	0.000094	0.0005327	0.0002611	Atox1/Atp13a2/Atp7a/Hif1a/Hmox1/Myc/Sod2
3-day post-SCI group vs. the control group	BP	GO:0061041	regulation of wound healing	7/222	0.000094	0.0005327	0.0002611	Alox5/Anxa1/Cd36/Hbegf/Mylk/Nfe2l2/Pdgfra
3-day post-SCI group vs. the control group	BP	GO:0032412	regulation of ion transmembrane transporter activi	10/222	0.0000962	0.0005443	0.0002668	Alox5/Atp7a/Chrna4/Fkbp1b/Ppargc1a/Ppif/Ppp3ca/Ripk1/S100a1/Ubqln1
3-day post-SCI group vs. the control group	BP	GO:1901605	alpha-amino acid metabolic process	8/222	0.0000964	0.0005451	0.0002672	Apc/Arg1/Atp7a/Dhfr/Gclc/Nox4/Prodh/Txnrd1
3-day post-SCI group vs. the control group	BP	GO:0071715	icosanoid transport	5/222	0.0000983	0.0005546	0.0002718	Abcc1/Anxa1/Map2k6/Pnpla8/Ptgs2
3-day post-SCI group vs. the control group	BP	GO:0003158	endothelium development	7/222	0.0000984	0.0005546	0.0002718	Btg1/Ezr/Gpx1/Id1/Met/Tnfrsf1a/Xdh
3-day post-SCI group vs. the control group	BP	GO:0048813	dendrite morphogenesis	8/222	0.0001001	0.0005637	0.0002763	Atp7a/Id1/Mapk8/Met/Ppp3ca/Stau2/Tbc1d24/Tnik
3-day post-SCI group vs. the control group	BP	GO:0051150	regulation of smooth muscle cell differentiation	4/222	0.0001023	0.0005754	0.0002821	Nfatc1/Rcan1/Sirt1/Sod2
3-day post-SCI group vs. the control group	BP	GO:0034341	response to interferon-gamma	7/222	0.0001029	0.0005779	0.0002833	Arg1/Gch1/Jak2/Rpl13a/Sirpa/Snca/Tlr4
3-day post-SCI group vs. the control group	BP	GO:0045088	regulation of innate immune response	9/222	0.0001059	0.000594	0.0002912	Arg1/Banf1/Ccr1/Il18rap/Ncf1/Nfe2l2/Nono/Sfpq/Tlr4
3-day post-SCI group vs. the control group	BP	GO:0006446	regulation of translational initiation	5/222	0.0001065	0.0005953	0.0002918	Eif2s1/Impact/Paip1/Ppp1r15b/Rpl13a
3-day post-SCI group vs. the control group	BP	GO:0071470	cellular response to osmotic stress	5/222	0.0001065	0.0005953	0.0002918	Casp3/Mylk/Ptgs2/Slc4a11/Ybx3
3-day post-SCI group vs. the control group	BP	GO:0030278	regulation of ossification	7/222	0.0001076	0.0006012	0.0002947	Alox5/Ccr1/Hif1a/Mapk14/Mapk3/Rxrb/Slc8a1

3-day post-SCI group vs. the control group	BP	GO:0033044	regulation of chromosome organization	9/222	0.0001093	0.0006082	0.0002981	Actb/Apc/Atr/Cdc20/Mapk3/Myc/Parp1/Rb1/Sfpq
3-day post-SCI group vs. the control group	BP	GO:0042180	cellular ketone metabolic process	9/222	0.0001093	0.0006082	0.0002981	Anxa1/Apc/Pdk1/Pdk2/Ppargc1a/Ptgs2/Rest/Sirt1/Snca
3-day post-SCI group vs. the control group	BP	GO:0045930	negative regulation of mitotic cell cycle	9/222	0.0001093	0.0006082	0.0002981	Apc/Atf2/Btg1/Cdc20/Cdk1/Cdkn2b/Ezh2/Ier3/Rb1
3-day post-SCI group vs. the control group	BP	GO:1901992	positive regulation of mitotic cell cycle phase trans	6/222	0.0001113	0.0006184	0.0003032	Aif1/Anxa1/Cdk1/Cdk4/Rb1/Rrm2b
3-day post-SCI group vs. the control group	BP	GO:0046165	alcohol biosynthetic process	7/222	0.0001125	0.0006244	0.0003061	Dhcr24/Dhfr/Gch1/Nfkb1/Prkaa2/Rest/Snca
3-day post-SCI group vs. the control group	BP	GO:0032731	positive regulation of interleukin-1 beta productio	5/222	0.0001151	0.0006359	0.0003117	Cd36/Hspb1/Jak2/Tlr4/Tlr6
3-day post-SCI group vs. the control group	BP	GO:0050873	brown fat cell differentiation	5/222	0.0001151	0.0006359	0.0003117	Bnip3/Itga6/Mapk14/Ptgs2/Sirt1
3-day post-SCI group vs. the control group	BP	GO:0090303	positive regulation of wound healing	5/222	0.0001151	0.0006359	0.0003117	Anxa1/Cd36/Hbegf/Mylk/Nfe2l2
3-day post-SCI group vs. the control group	BP	GO:0044346	fibroblast apoptotic process	4/222	0.0001158	0.0006381	0.0003128	Apc/Btg1/Casp3/Sirt1
3-day post-SCI group vs. the control group	BP	GO:0070741	response to interleukin-6	4/222	0.0001158	0.0006381	0.0003128	Il6st/Nfkb1/Rela/Ripk1
3-day post-SCI group vs. the control group	BP	GO:0010288	response to lead ion	3/222	0.0001203	0.0006579	0.0003225	Cdk4/Mapt/Ppp1ca
3-day post-SCI group vs. the control group	BP	GO:0043922	negative regulation by host of viral transcription	3/222	0.0001203	0.0006579	0.0003225	Hdac1/Jun/Rest
3-day post-SCI group vs. the control group	BP	GO:0044557	relaxation of smooth muscle	3/222	0.0001203	0.0006579	0.0003225	Adora1/Pawr/Slc8a1
3-day post-SCI group vs. the control group	BP	GO:0051549	positive regulation of keratinocyte migration	3/222	0.0001203	0.0006579	0.0003225	Adam9/Hbegf/Map4k4
3-day post-SCI group vs. the control group	BP	GO:0070932	histone H3 deacetylation	3/222	0.0001203	0.0006579	0.0003225	Hdac1/Sfpq/Sirt1
3-day post-SCI group vs. the control group	BP	GO:0017148	negative regulation of translation	8/222	0.0001203	0.0006579	0.0003225	Ago1/Ago3/Eif2s1/Paip1/Rpl13a/Tnrc6a/Tnrc6c/Zc3h12a
3-day post-SCI group vs. the control group	BP	GO:0010948	negative regulation of cell cycle process	10/222	0.0001232	0.0006728	0.0003298	Apc/Atf2/Atr/Cdc20/Cdk1/Cdkn2b/Ezh2/Ier3/Mapk14/R b1
3-day post-SCI group vs. the control group	BP	GO:0006584	catecholamine metabolic process	5/222	0.0001242	0.0006758	0.0003313	Atp7a/Gch1/Gpr37/Maoa/Snca
3-day post-SCI group vs. the control group	BP	GO:0009712	catechol-containing compound metabolic process	5/222	0.0001242	0.0006758	0.0003313	Atp7a/Gch1/Gpr37/Maoa/Snca
3-day post-SCI group vs. the control group	BP	GO:0042698	ovulation cycle	5/222	0.0001242	0.0006758	0.0003313	Anxa1/Axl/Pdgfra/Plekha1/Sirt1
3-day post-SCI group vs. the control group	BP	GO:0016575	histone deacetylation	6/222	0.0001245	0.0006764	0.0003315	Hdac1/Pink1/Rbbp7/Rest/Sfpq/Sirt1
3-day post-SCI group vs. the control group	BP	GO:0050870	positive regulation of T cell activation	9/222	0.0001277	0.0006928	0.0003396	Actb/Aif1/Anxa1/Igfbp2/Il6st/Jak2/Ppp3ca/Sirpa/Vcam1
3-day post-SCI group vs. the control group	BP	GO:2000108	positive regulation of leukocyte apoptotic process	4/222	0.0001304	0.0007068	0.0003465	Anxa1/Bax/Myc/Sirt1
3-day post-SCI group vs. the control group	BP	GO:0090278	negative regulation of peptide hormone secretion	5/222	0.0001339	0.0007222	0.000354	Fkbp1b/Ppp3ca/Rest/Sirt1/Ucp2
3-day post-SCI group vs. the control group	BP	GO:0031047	gene silencing by RNA	8/222	0.000134	0.0007222	0.000354	Ago1/Ago3/Nfkb1/Ppp3ca/Ripk1/Tnrc6a/Tnrc6c/Zc3h12 a
3-day post-SCI group vs. the control group	BP	GO:0050777	negative regulation of immune response	8/222	0.000134	0.0007222	0.000354	Anxa1/Arg1/Banf1/Ccr1/Gpx1/Hmox1/Stat6/Zc3h12a
3-day post-SCI group vs. the control group	BP	GO:0051099	positive regulation of binding	8/222	0.000134	0.0007222	0.000354	Eif2s1/Jak2/Mark3/Met/Myc/Parp1/Rb1/Tlr4
3-day post-SCI group vs. the control group	BP	GO:1902806	regulation of cell cycle G1/S phase transition	8/222	0.0001388	0.0007457	0.0003655	Actb/Aif1/Anxa1/Apc/Cdkn2b/Cdkn2c/Ezh2/Rb1
3-day post-SCI group vs. the control group	BP	GO:0060395	SMAD protein signal transduction	6/222	0.0001388	0.0007457	0.0003655	Fos/Jak2/Jun/Parp1/Rbpms/Smad1
3-day post-SCI group vs. the control group	BP	GO:0099565	chemical synaptic transmission, postsynaptic	6/222	0.0001388	0.0007457	0.0003655	Adora1/Chrna4/Met/Ppp3ca/Snca/Tbc1d24
3-day post-SCI group vs. the control group	BP	GO:1904950	negative regulation of establishment of protein loc	7/222	0.0001398	0.0007499	0.0003676	Anxa1/Cd36/Fkbp1b/Mapt/Ppp3ca/Rest/Ucp2
3-day post-SCI group vs. the control group	BP	GO:0032956	regulation of actin cytoskeleton organization	11/222	0.00014	0.0007499	0.0003676	Arhgdia/Braf/Cdk10/Cttn/Ect2/Id1/Met/Nox4/Pxn/Stau2/ Trpm2
3-day post-SCI group vs. the control group	BP	GO:0098657	import into cell	9/222	0.0001441	0.0007704	0.0003777	Abcc1/Actb/Arg1/Cd36/Pawr/Ppp3ca/Slc8a1/Snca/Trpm2
3-day post-SCI group vs. the control group	BP	GO:0010656	negative regulation of muscle cell apoptotic proce	5/222	0.0001442	0.0007704	0.0003777	Hmox1/Jak2/Nfe2l2/Sirt1/Zc3h12a
3-day post-SCI group vs. the control group	BP	GO:0031112	positive regulation of microtubule polymerization	4/222	0.0001464	0.000775	0.0003799	Apc/Mapk8/Mapt/Met
3-day post-SCI group vs. the control group	BP	GO:0045672	positive regulation of osteoclast differentiation	4/222	0.0001464	0.000775	0.0003799	Ccr1/Fos/Ppargc1b/Ppp3ca
3-day post-SCI group vs. the control group	BP	GO:0045987	positive regulation of smooth muscle contraction	4/222	0.0001464	0.000775	0.0003799	Cttn/Pawr/Ptgs1/Ptgs2
3-day post-SCI group vs. the control group	BP	GO:0090140	regulation of mitochondrial fission	4/222	0.0001464	0.000775	0.0003799	Bnip3/Mapt/Pink1/Ppargc1a
3-day post-SCI group vs. the control group	BP	GO:0097345	mitochondrial outer membrane permeabilization	4/222	0.0001464	0.000775	0.0003799	Bax/Bnip3/Gclc/Ier3

3-day post-SCI group vs. the control group	BP	GO:1904994	regulation of leukocyte adhesion to vascular endot	4/222	0.0001464	0.000775	0.0003799	Alox5/Ets1/Pawr/Rela
3-day post-SCI group vs. the control group	BP	GO:0010770	positive regulation of cell morphogenesis involvec	6/222	0.0001465	0.000775	0.0003799	Braf/Met/P4hb/Stau2/Tbc1d24/Tnik
3-day post-SCI group vs. the control group	BP	GO:0062014	negative regulation of small molecule metabolic pı	6/222	0.0001465	0.000775	0.0003799	Ier3/Nfkb1/Parp1/Ppargc1a/Rest/Sirt1
3-day post-SCI group vs. the control group	BP	GO:0002831	regulation of response to biotic stimulus	11/222	0.0001502	0.0007927	0.0003886	Arg1/Banf1/Ccr1/Il18rap/Mapk3/Ncf1/Nfe2l2/Nono/Sfpq/Tlr4/Zc3h12a
3-day post-SCI group vs. the control group	BP	GO:0007517	muscle organ development	11/222	0.0001502	0.0007927	0.0003886	Fos/Gpx1/Mapk14/Met/Myc/Mylk/Ppp3ca/Rb1/Rcan1/Sirt1/Ybx3
3-day post-SCI group vs. the control group	BP	GO:0002829	negative regulation of type 2 immune response	3/222	0.0001522	0.0007964	0.0003904	Anxa1/Arg1/Stat6
3-day post-SCI group vs. the control group	BP	GO:0006750	glutathione biosynthetic process	3/222	0.0001522	0.0007964	0.0003904	Gclc/Ggt7/Nfe2l2
3-day post-SCI group vs. the control group	BP	GO:0010882	regulation of cardiac muscle contraction by calciu	3/222	0.0001522	0.0007964	0.0003904	Atp2a2/Fkbp1b/Slc8a1
3-day post-SCI group vs. the control group	BP	GO:0048680	positive regulation of axon regeneration	3/222	0.0001522	0.0007964	0.0003904	Braf/Fkbp1b/Stk24
3-day post-SCI group vs. the control group	BP	GO:1902510	regulation of apoptotic DNA fragmentation	3/222	0.0001522	0.0007964	0.0003904	Bax/Casp3/Endog
3-day post-SCI group vs. the control group	BP	GO:2001241	positive regulation of extrinsic apoptotic signaling	3/222	0.0001522	0.0007964	0.0003904	Ctnna1/Htra2/Ppp1ca
3-day post-SCI group vs. the control group	BP	GO:2001279	regulation of unsaturated fatty acid biosynthetic pr	3/222	0.0001522	0.0007964	0.0003904	Anxa1/Ptgs2/Sirt1
3-day post-SCI group vs. the control group	BP	GO:0042116	macrophage activation	6/222	0.0001545	0.0008049	0.0003946	Aif1/Jak2/Jun/Snca/Tlr4/Tlr6
3-day post-SCI group vs. the control group	BP	GO:0002792	negative regulation of peptide secretion	5/222	0.000155	0.0008049	0.0003946	Fkbp1b/Ppp3ca/Rest/Sirt1/Ucp2
3-day post-SCI group vs. the control group	BP	GO:0045776	negative regulation of blood pressure	5/222	0.000155	0.0008049	0.0003946	Adora1/Cd36/Gch1/Ier3/Sod2
3-day post-SCI group vs. the control group	BP	GO:0060038	cardiac muscle cell proliferation	5/222	0.000155	0.0008049	0.0003946	Apc/Cdk1/Mapk14/Rxrb/Smad1
3-day post-SCI group vs. the control group	BP	GO:0060612	adipose tissue development	5/222	0.000155	0.0008049	0.0003946	Atf2/Cdk4/Parp1/Ppargc1a/Sirt1
3-day post-SCI group vs. the control group	BP	GO:0090311	regulation of protein deacetylation	5/222	0.000155	0.0008049	0.0003946	Mapt/Pink1/Prkaa2/Ripk3/Sirt1
3-day post-SCI group vs. the control group	BP	GO:0051259	protein complex oligomerization	9/222	0.0001576	0.0008173	0.0004006	Ect2/Krt1/Mapt/Pxdn/Rnf112/Snca/Sod2/Trpm2/Zc3h12a
3-day post-SCI group vs. the control group	BP	GO:0006638	neutral lipid metabolic process	7/222	0.0001586	0.0008214	0.0004026	Cat/Cd36/Dgkk/Gpx1/Il6st/Sirt1/Snca
3-day post-SCI group vs. the control group	BP	GO:0055007	cardiac muscle cell differentiation	7/222	0.0001653	0.000855	0.0004191	Map2k4/Met/Nox4/Pdgfra/Rxrb/Sirt1/Slc8a1
3-day post-SCI group vs. the control group	BP	GO:0048008	platelet-derived growth factor receptor signaling p	5/222	0.0001665	0.0008602	0.0004217	Jak2/Pdgfra/Plekha1/Snca/Txnip
3-day post-SCI group vs. the control group	BP	GO:0006469	negative regulation of protein kinase activity	8/222	0.0001767	0.0009119	0.000447	Actb/Apc/Casp3/Cdkn2c/Hspb1/Rb1/Sirt1/Slc8a1
3-day post-SCI group vs. the control group	BP	GO:0071901	negative regulation of protein serine/threonine kin	6/222	0.0001805	0.0009304	0.0004561	Apc/Casp3/Cdkn2c/Rb1/Sirt1/Slc8a1
3-day post-SCI group vs. the control group	BP	GO:0000002	mitochondrial genome maintenance	4/222	0.0001824	0.0009356	0.0004586	Endog/Parp1/Ppargc1a/Rrm2b
3-day post-SCI group vs. the control group	BP	GO:0070266	necroptotic process	4/222	0.0001824	0.0009356	0.0004586	Ppif/Ripk1/Ripk3/Ybx3
3-day post-SCI group vs. the control group	BP	GO:0070542	response to fatty acid	4/222	0.0001824	0.0009356	0.0004586	Cd36/Cdk4/Tlr4/Zc3h12a
3-day post-SCI group vs. the control group	BP	GO:0090075	relaxation of muscle	4/222	0.0001824	0.0009356	0.0004586	Adora1/Atp2a2/Pawr/Slc8a1
3-day post-SCI group vs. the control group	BP	GO:0014733	regulation of skeletal muscle adaptation	3/222	0.0001892	0.0009647	0.0004729	Atp2a2/Nfatc1/Ppp3ca
3-day post-SCI group vs. the control group	BP	GO:0043558	regulation of translational initiation in response to	3/222	0.0001892	0.0009647	0.0004729	Eif2s1/Impact/Ppp1r15b
3-day post-SCI group vs. the control group	BP	GO:0051547	regulation of keratinocyte migration	3/222	0.0001892	0.0009647	0.0004729	Adam9/Hbegf/Map4k4
3-day post-SCI group vs. the control group	BP	GO:0055089	fatty acid homeostasis	3/222	0.0001892	0.0009647	0.0004729	Adora1/Prkaa2/Sirt1
3-day post-SCI group vs. the control group	BP	GO:0070572	positive regulation of neuron projection regenerati	3/222	0.0001892	0.0009647	0.0004729	Braf/Fkbp1b/Stk24
3-day post-SCI group vs. the control group	BP	GO:0006520	cellular amino acid metabolic process	9/222	0.0001932	0.0009824	0.0004816	Apc/Arg1/Atp7a/Dhfr/Gclc/Hao1/Nox4/Prodh/Txnrd1
3-day post-SCI group vs. the control group	BP	GO:0099003	vesicle-mediated transport in synapse	9/222	0.0001932	0.0009824	0.0004816	Actb/Amph/Atp2a2/Braf/Chrna4/Mapk10/Snca/Syp/Tbc1d24
3-day post-SCI group vs. the control group	BP	GO:0006633	fatty acid biosynthetic process	7/222	0.0001944	0.0009874	0.000484	Alox5/Anxa1/Pnpla8/Prkaa2/Ptgs1/Ptgs2/Sirt1
3-day post-SCI group vs. the control group	BP	GO:0006165	nucleoside diphosphate phosphorylation	6/222	0.0001997	0.0010121	0.0004961	Hif1a/Ier3/Myc/Nme2/Ppargc1a/Prkaa2

3-day post-SCI group vs. the control group	BP	GO:0042310	vasoconstriction	6/222	0.0001997	0.0010121	0.0004961	Alox5/Cd38/Hif1a/Ptgs1/Ptgs2/Slc8a1
3-day post-SCI group vs. the control group	BP	GO:0071634	regulation of transforming growth factor beta prod	4/222	0.0002027	0.0010257	0.0005028	Atf2/Hif1a/Met/Ptgs2
3-day post-SCI group vs. the control group	BP	GO:0015849	organic acid transport	10/222	0.0002069	0.0010459	0.0005127	Abcc1/Adora1/Anxa1/Arg1/Cd36/Map2k6/Myc/Pnpla8/Slc23a2/Snca
3-day post-SCI group vs. the control group	BP	GO:0002700	regulation of production of molecular mediator of	8/222	0.0002087	0.0010525	0.000516	Arg1/Axl/Cd36/Hmox1/Mapkapk2/Sirt1/Stat6/Tlr4
3-day post-SCI group vs. the control group	BP	GO:0043491	protein kinase B signaling	8/222	0.0002087	0.0010525	0.000516	Axl/Gpx1/Hbegf/Met/Nox4/Plekha1/Sirt1/Xdh
3-day post-SCI group vs. the control group	BP	GO:0046916	cellular transition metal ion homeostasis	6/222	0.0002099	0.0010573	0.0005183	Atox1/Atp13a2/Atp7a/Hif1a/Hmox1/Myc
3-day post-SCI group vs. the control group	BP	GO:0008643	carbohydrate transport	7/222	0.0002104	0.0010586	0.0005189	Braf/Ezr/Mapk14/Met/Myc/Nfe2l2/Slc23a2
3-day post-SCI group vs. the control group	BP	GO:0019827	stem cell population maintenance	8/222	0.0002156	0.0010836	0.0005312	Actb/Apc/Braf/Hdac1/Mapk8/Myc/Rbbp7/Rest
3-day post-SCI group vs. the control group	BP	GO:0048608	reproductive structure development	10/222	0.0002174	0.0010912	0.0005349	Anxa1/Apc/Axl/Bax/Dhcr24/Pdgfra/Plekha1/Prdx4/Sirt1/Ybx3
3-day post-SCI group vs. the control group	BP	GO:0051147	regulation of muscle cell differentiation	7/222	0.0002188	0.0010924	0.0005355	Ezh2/Mapk14/Nfatc1/Rcan1/Sirt1/Smad1/Sod2
3-day post-SCI group vs. the control group	BP	GO:0010761	fibroblast migration	5/222	0.0002189	0.0010924	0.0005355	Apc/Braf/Cygb/Pdlim1/Slc8a1
3-day post-SCI group vs. the control group	BP	GO:0042775	mitochondrial ATP synthesis coupled electron trar	5/222	0.0002189	0.0010924	0.0005355	Cdk1/Ndufa12/Ndufs8/Pink1/Snca
3-day post-SCI group vs. the control group	BP	GO:0055117	regulation of cardiac muscle contraction	5/222	0.0002189	0.0010924	0.0005355	Adora1/Atp2a2/Fkbp1b/Slc8a1/Zc3h12a
3-day post-SCI group vs. the control group	BP	GO:0097194	execution phase of apoptosis	5/222	0.0002189	0.0010924	0.0005355	Bax/Casp3/Endog/Stk24/Zc3h12a
3-day post-SCI group vs. the control group	BP	GO:0070588	calcium ion transmembrane transport	10/222	0.0002228	0.0011104	0.0005443	Atp2a2/Bax/Fkbp1b/Pawr/Ppp3ca/S100a1/Slc8a1/Snca/Trpm2/Ubqln1
3-day post-SCI group vs. the control group	BP	GO:0014823	response to activity	4/222	0.0002245	0.001112	0.0005451	Hif1a/Ppargc1a/Prkaa2/Sod2
3-day post-SCI group vs. the control group	BP	GO:0018208	peptidyl-proline modification	4/222	0.0002245	0.001112	0.0005451	Fkbp1b/P4hb/Ppif/Prdx4
3-day post-SCI group vs. the control group	BP	GO:0033238	regulation of cellular amine metabolic process	4/222	0.0002245	0.001112	0.0005451	Apc/Atp7a/Gpr37/Snca
3-day post-SCI group vs. the control group	BP	GO:0060292	long-term synaptic depression	4/222	0.0002245	0.001112	0.0005451	Adora1/Cd38/Mapt/Stau2
3-day post-SCI group vs. the control group	BP	GO:0090049	regulation of cell migration involved in sprouting a	4/222	0.0002245	0.001112	0.0005451	Anxa1/Fbxw7/Hmox1/Ptgs2
3-day post-SCI group vs. the control group	BP	GO:1902903	regulation of supramolecular fiber organization	11/222	0.000226	0.0011184	0.0005482	Apc/Braf/Cttn/Id1/Mapk8/Mapt/Met/Nox4/Pxn/Rb1/Snca
3-day post-SCI group vs. the control group	BP	GO:0018119	peptidyl-cysteine S-nitrosylation	3/222	0.0002316	0.0011379	0.0005578	Ncoa7/Oxr1/Tbc1d24
3-day post-SCI group vs. the control group	BP	GO:0019184	nonribosomal peptide biosynthetic process	3/222	0.0002316	0.0011379	0.0005578	Gclc/Ggt7/Nfe2l2
3-day post-SCI group vs. the control group	BP	GO:0042416	dopamine biosynthetic process	3/222	0.0002316	0.0011379	0.0005578	Gch1/Gpr37/Snca
3-day post-SCI group vs. the control group	BP	GO:0042428	serotonin metabolic process	3/222	0.0002316	0.0011379	0.0005578	Atp7a/Gch1/Maoa
3-day post-SCI group vs. the control group	BP	GO:0045346	regulation of MHC class II biosynthetic process	3/222	0.0002316	0.0011379	0.0005578	Jak2/Sirt1/Tlr4
3-day post-SCI group vs. the control group	BP	GO:1903624	regulation of DNA catabolic process	3/222	0.0002316	0.0011379	0.0005578	Bax/Casp3/Endog
3-day post-SCI group vs. the control group	BP	GO:0032922	circadian regulation of gene expression	5/222	0.0002338	0.0011463	0.0005619	Hdac1/Id1/Ppargc1a/Ppp1ca/Sirt1
3-day post-SCI group vs. the control group	BP	GO:1904705	regulation of vascular associated smooth muscle c	5/222	0.0002338	0.0011463	0.0005619	Hmox1/Jak2/Jun/Ppargc1a/Sod2
3-day post-SCI group vs. the control group	BP	GO:0061458	reproductive system development	10/222	0.0002398	0.0011741	0.0005755	Anxa1/Apc/Axl/Bax/Dhcr24/Pdgfra/Plekha1/Prdx4/Sirt1/Ybx3
3-day post-SCI group vs. the control group	BP	GO:0005996	monosaccharide metabolic process	9/222	0.0002418	0.0011825	0.0005797	Cd36/Gclc/Hif1a/Mapk14/Myc/Pdk1/Pdk2/Sirt1/Slc23a2
3-day post-SCI group vs. the control group	BP	GO:0031532	actin cytoskeleton reorganization	6/222	0.0002429	0.0011841	0.0005804	Anxa1/Arhgdia/Cttn/Ezr/Tnik/Trpm2
3-day post-SCI group vs. the control group	BP	GO:0046939	nucleotide phosphorylation	6/222	0.0002429	0.0011841	0.0005804	Hif1a/Ier3/Myc/Nme2/Ppargc1a/Prkaa2
3-day post-SCI group vs. the control group	BP	GO:1902106	negative regulation of leukocyte differentiation	6/222	0.0002429	0.0011841	0.0005804	Anxa1/Fbxw7/Hspb1/Myc/Nme2/Zc3h12a
3-day post-SCI group vs. the control group	BP	GO:0071604	transforming growth factor beta production	4/222	0.0002479	0.0012055	0.0005909	Atf2/Hif1a/Met/Ptgs2
3-day post-SCI group vs. the control group	BP	GO:0098703	calcium ion import across plasma membrane	4/222	0.0002479	0.0012055	0.0005909	Pawr/Ppp3ca/Slc8a1/Trpm2

3-day post-SCI group vs. the control group	BP	GO:0030888	regulation of B cell proliferation	5/222	0.0002495	0.0012103	0.0005933	Btk/Casp3/Cd38/Pawr/Tlr4
3-day post-SCI group vs. the control group	BP	GO:0042773	ATP synthesis coupled electron transport	5/222	0.0002495	0.0012103	0.0005933	Cdk1/Ndufa12/Ndufs8/Pink1/Snca
3-day post-SCI group vs. the control group	BP	GO:0034249	negative regulation of cellular amide metabolic pro	8/222	0.0002532	0.0012272	0.0006016	Ago1/Ago3/Eif2s1/Paip1/Rpl13a/Tnrc6a/Tnrc6c/Zc3h12a
3-day post-SCI group vs. the control group	BP	GO:0045667	regulation of osteoblast differentiation	7/222	0.0002552	0.0012352	0.0006055	Apc/Id1/Il6st/Nfatc1/Ppp3ca/Rest/Smad1
3-day post-SCI group vs. the control group	BP	GO:0001885	endothelial cell development	5/222	0.0002659	0.0012797	0.0006273	Ezr/Gpx1/Id1/Met/Tnfrsf1a
3-day post-SCI group vs. the control group	BP	GO:0032732	positive regulation of interleukin-1 production	5/222	0.0002659	0.0012797	0.0006273	Cd36/Hspb1/Jak2/Tlr4/Tlr6
3-day post-SCI group vs. the control group	BP	GO:0042982	amyloid precursor protein metabolic process	5/222	0.0002659	0.0012797	0.0006273	Adam9/Casp3/Dhcr24/Pawr/Rela
3-day post-SCI group vs. the control group	BP	GO:0048488	synaptic vesicle endocytosis	5/222	0.0002659	0.0012797	0.0006273	Actb/Amph/Snca/Syp/Tbc1d24
3-day post-SCI group vs. the control group	BP	GO:0140238	presynaptic endocytosis	5/222	0.0002659	0.0012797	0.0006273	Actb/Amph/Snca/Syp/Tbc1d24
3-day post-SCI group vs. the control group	BP	GO:0031032	actomyosin structure organization	8/222	0.0002697	0.0012965	0.0006356	Braf/Ect2/Itgb5/Met/Nox4/Pdgfra/Pdlim1/Pxn
3-day post-SCI group vs. the control group	BP	GO:0017014	protein nitrosylation	3/222	0.0002796	0.0013335	0.0006537	Ncoa7/Oxr1/Tbc1d24
3-day post-SCI group vs. the control group	BP	GO:0035795	negative regulation of mitochondrial membrane pe	3/222	0.0002796	0.0013335	0.0006537	Bnip3/Gclc/Ier3
3-day post-SCI group vs. the control group	BP	GO:0045342	MHC class II biosynthetic process	3/222	0.0002796	0.0013335	0.0006537	Jak2/Sirt1/Tlr4
3-day post-SCI group vs. the control group	BP	GO:0071243	cellular response to arsenic-containing substance	3/222	0.0002796	0.0013335	0.0006537	Hmox1/Ppif/Zc3h12a
3-day post-SCI group vs. the control group	BP	GO:0090136	epithelial cell-cell adhesion	3/222	0.0002796	0.0013335	0.0006537	Ctnna1/Cyp1b1/Itgb5
3-day post-SCI group vs. the control group	BP	GO:0001942	hair follicle development	6/222	0.0002799	0.0013335	0.0006537	Apc/Atp7a/Ctsl/Hdac1/Ptgs2/Rela
3-day post-SCI group vs. the control group	BP	GO:0030038	contractile actin filament bundle assembly	6/222	0.0002799	0.0013335	0.0006537	Braf/Itgb5/Met/Nox4/Pdlim1/Pxn
3-day post-SCI group vs. the control group	BP	GO:0043149	stress fiber assembly	6/222	0.0002799	0.0013335	0.0006537	Braf/Itgb5/Met/Nox4/Pdlim1/Pxn
3-day post-SCI group vs. the control group	BP	GO:0071479	cellular response to ionizing radiation	5/222	0.0002831	0.0013441	0.0006589	Atr/Ect2/Mapk14/Sirt1/Tank
3-day post-SCI group vs. the control group	BP	GO:1990874	vascular associated smooth muscle cell proliferatic	5/222	0.0002831	0.0013441	0.0006589	Hmox1/Jak2/Jun/Ppargc1a/Sod2
3-day post-SCI group vs. the control group	BP	GO:2000036	regulation of stem cell population maintenance	5/222	0.0002831	0.0013441	0.0006589	Actb/Hdac1/Myc/Rbbp7/Rest
3-day post-SCI group vs. the control group	BP	GO:0009185	ribonucleoside diphosphate metabolic process	6/222	0.0002932	0.0013857	0.0006793	Hif1a/Ier3/Myc/Ppargc1a/Prkaa2/Rrm2b
3-day post-SCI group vs. the control group	BP	GO:0051952	regulation of amine transport	6/222	0.0002932	0.0013857	0.0006793	Adora1/Arg1/Chrna4/Pink1/Ptgs1/Snca
3-day post-SCI group vs. the control group	BP	GO:0060078	regulation of postsynaptic membrane potential	6/222	0.0002932	0.0013857	0.0006793	Adora1/Chrna4/Met/Ppp3ca/Snca/Tbc1d24
3-day post-SCI group vs. the control group	BP	GO:1903707	negative regulation of hemopoiesis	6/222	0.0002932	0.0013857	0.0006793	Anxa1/Fbxw7/Hspb1/Myc/Nme2/Zc3h12a
3-day post-SCI group vs. the control group	BP	GO:0030879	mammary gland development	7/222	0.0002963	0.0013987	0.0006856	Bax/Gpx1/Hif1a/Jak2/Nfkb1/Stat6/Xdh
3-day post-SCI group vs. the control group	BP	GO:0061081	positive regulation of myeloid leukocyte cytokine	4/222	0.0003	0.0014131	0.0006927	Cd36/Mapkapk2/Sirt1/Tlr4
3-day post-SCI group vs. the control group	BP	GO:1900744	regulation of p38MAPK cascade	4/222	0.0003	0.0014131	0.0006927	Ezr/Met/Xdh/Zc3h12a
3-day post-SCI group vs. the control group	BP	GO:0032677	regulation of interleukin-8 production	5/222	0.0003012	0.0014156	0.0006939	Anxa1/Rela/Ripk1/Tlr4/Tlr6
3-day post-SCI group vs. the control group	BP	GO:0051926	negative regulation of calcium ion transport	5/222	0.0003012	0.0014156	0.0006939	Fkbp1b/Pawr/Ppp3ca/Ptgs2/Ubqln1
3-day post-SCI group vs. the control group	BP	GO:0019395	fatty acid oxidation	6/222	0.000307	0.001441	0.0007064	Cd36/Cygb/Etfdh/Hao1/Mapk14/Ppargc1a
3-day post-SCI group vs. the control group	BP	GO:0032637	interleukin-8 production	5/222	0.0003201	0.0014995	0.000735	Anxa1/Rela/Ripk1/Tlr4/Tlr6
3-day post-SCI group vs. the control group	BP	GO:0048146	positive regulation of fibroblast proliferation	5/222	0.0003201	0.0014995	0.000735	Ccna2/Cdk4/Jun/Myc/Pdgfra
3-day post-SCI group vs. the control group	BP	GO:0022404	molting cycle process	6/222	0.0003212	0.0015013	0.0007359	Apc/Atp7a/Ctsl/Hdac1/Ptgs2/Rela
3-day post-SCI group vs. the control group	BP	GO:0022405	hair cycle process	6/222	0.0003212	0.0015013	0.0007359	Apc/Atp7a/Ctsl/Hdac1/Ptgs2/Rela
3-day post-SCI group vs. the control group	BP	GO:0051235	maintenance of location	10/222	0.0003263	0.0015234	0.0007468	Bax/Cd36/Fkbp1b/Pink1/Sirt1/Slc8a1/Snca/Tnrc6a/Trpm2/Zc3h12a
3-day post-SCI group vs. the control group	BP	GO:0006984	ER-nucleus signaling pathway	4/222	0.0003288	0.0015283	0.0007491	Atp2a2/Eif2s1/Nfe2l2/Ppp1r15b
3-day post-SCI group vs. the control group	BP	GO:0009309	amine biosynthetic process	4/222	0.0003288	0.0015283	0.0007491	Atp7a/Gch1/Gpr37/Snca
3-day post-SCI group vs. the control group	BP	GO:0042401	cellular biogenic amine biosynthetic process	4/222	0.0003288	0.0015283	0.0007491	Atp7a/Gch1/Gpr37/Snca

3-day post-SCI group vs. the control group	BP	GO:0045601	regulation of endothelial cell differentiation	4/222	0.0003288	0.0015283	0.0007491	Btg1/Id1/Tnfrsf1a/Xdh
3-day post-SCI group vs. the control group	BP	GO:0035330	regulation of hippo signaling	3/222	0.0003336	0.0015389	0.0007543	Map2k3/Mapk14/Mark3
3-day post-SCI group vs. the control group	BP	GO:0051000	positive regulation of nitric-oxide synthase activity	3/222	0.0003336	0.0015389	0.0007543	Dhfr/Gch1/S100a1
3-day post-SCI group vs. the control group	BP	GO:0060391	positive regulation of SMAD protein signal transd	3/222	0.0003336	0.0015389	0.0007543	Jak2/Parp1/Rbpms
3-day post-SCI group vs. the control group	BP	GO:0071280	cellular response to copper ion	3/222	0.0003336	0.0015389	0.0007543	Atp7a/Nfe2l2/Snca
3-day post-SCI group vs. the control group	BP	GO:1901160	primary amino compound metabolic process	3/222	0.0003336	0.0015389	0.0007543	Atp7a/Gch1/Maoa
3-day post-SCI group vs. the control group	BP	GO:1905146	lysosomal protein catabolic process	3/222	0.0003336	0.0015389	0.0007543	Atp13a2/Mgat3/Tpp1
3-day post-SCI group vs. the control group	BP	GO:2001267	regulation of cysteine-type endopeptidase activity	3/222	0.0003336	0.0015389	0.0007543	Bax/Htra2/Jak2
3-day post-SCI group vs. the control group	BP	GO:0015749	monosaccharide transmembrane transport	6/222	0.000336	0.0015446	0.0007571	Braf/Mapk14/Met/Myc/Nfe2l2/Slc23a2
3-day post-SCI group vs. the control group	BP	GO:0042100	B cell proliferation	6/222	0.000336	0.0015446	0.0007571	Bax/Btk/Casp3/Cd38/Pawr/Tlr4
3-day post-SCI group vs. the control group	BP	GO:0098773	skin epidermis development	6/222	0.000336	0.0015446	0.0007571	Apc/Atp7a/Ctsl/Hdac1/Ptgs2/Rela
3-day post-SCI group vs. the control group	BP	GO:0006919	activation of cysteine-type endopeptidase activity i	5/222	0.0003399	0.001561	0.0007652	Bax/Jak2/Myc/Snca/Xdh
3-day post-SCI group vs. the control group	BP	GO:0071383	cellular response to steroid hormone stimulus	7/222	0.0003425	0.0015712	0.0007702	Anxa1/Hdac1/Jak2/Parp1/Ppargc1b/Rest/Sirt1
3-day post-SCI group vs. the control group	BP	GO:0010769	regulation of cell morphogenesis involved in differ	6/222	0.0003513	0.0016079	0.0007882	Braf/Met/P4hb/Stau2/Tbc1d24/Tnik
3-day post-SCI group vs. the control group	BP	GO:0044106	cellular amine metabolic process	6/222	0.0003513	0.0016079	0.0007882	Apc/Atp7a/Gch1/Gpr37/Maoa/Snca
3-day post-SCI group vs. the control group	BP	GO:0048147	negative regulation of fibroblast proliferation	4/222	0.0003595	0.0016438	0.0008058	Bax/Myc/Pawr/Sod2
3-day post-SCI group vs. the control group	BP	GO:0051606	detection of stimulus	9/222	0.0003603	0.0016454	0.0008066	Adora1/Atf2/Lpo/Myc/Pawr/Rest/Sod2/Tlr4/Tlr6
3-day post-SCI group vs. the control group	BP	GO:0061180	mammary gland epithelium development	5/222	0.0003607	0.0016454	0.0008066	Bax/Gpx1/Hif1a/Jak2/Stat6
3-day post-SCI group vs. the control group	BP	GO:0015837	amine transport	6/222	0.0003671	0.0016731	0.0008201	Adora1/Arg1/Chrna4/Pink1/Ptgs1/Snca
3-day post-SCI group vs. the control group	BP	GO:0008217	regulation of blood pressure	8/222	0.0003767	0.0017149	0.0008406	Adora1/Cd36/Gch1/Hmox1/Ier3/Ptgs1/Ptgs2/Sod2
3-day post-SCI group vs. the control group	BP	GO:0007565	female pregnancy	7/222	0.0003809	0.0017319	0.000849	Atr/Ctsl/Junb/Mapk3/Ptgs2/Rxrb/Ube2a
3-day post-SCI group vs. the control group	BP	GO:0001894	tissue homeostasis	9/222	0.000389	0.001767	0.0008662	Bax/Cd38/Nox4/Ppargc1b/Ptgs1/Ptgs2/Rb1/Tlr4/Tpp1
3-day post-SCI group vs. the control group	BP	GO:0032007	negative regulation of TOR signaling	4/222	0.0003922	0.0017724	0.0008688	Endog/Hif1a/Prkaa2/Sirt1
3-day post-SCI group vs. the control group	BP	GO:0042554	superoxide anion generation	4/222	0.0003922	0.0017724	0.0008688	Mapt/Ncf1/Nox4/Sod2
3-day post-SCI group vs. the control group	BP	GO:0061098	positive regulation of protein tyrosine kinase activ	4/222	0.0003922	0.0017724	0.0008688	Adora1/Fbxw7/Hbegf/Nox4
3-day post-SCI group vs. the control group	BP	GO:0060396	growth hormone receptor signaling pathway	3/222	0.000394	0.0017724	0.0008688	Jak2/Pxn/Stat6
3-day post-SCI group vs. the control group	BP	GO:0071378	cellular response to growth hormone stimulus	3/222	0.000394	0.0017724	0.0008688	Jak2/Pxn/Stat6
3-day post-SCI group vs. the control group	BP	GO:0140467	integrated stress response signaling	3/222	0.000394	0.0017724	0.0008688	Eif2s1/Impact/Nfe2l2
3-day post-SCI group vs. the control group	BP	GO:1900225	regulation of NLRP3 inflammasome complex asse	3/222	0.000394	0.0017724	0.0008688	Cd36/Tlr4/Tlr6
3-day post-SCI group vs. the control group	BP	GO:1900452	regulation of long-term synaptic depression	3/222	0.000394	0.0017724	0.0008688	Adora1/Mapt/Stau2
3-day post-SCI group vs. the control group	BP	GO:1905709	negative regulation of membrane permeability	3/222	0.000394	0.0017724	0.0008688	Bnip3/Gclc/Ier3
3-day post-SCI group vs. the control group	BP	GO:0002757	immune response-activating signal transduction	10/222	0.0004004	0.0017959	0.0008804	Bax/Braf/Btk/Cd38/Ezr/Pawr/Plekha1/Rela/Tlr4/Zc3h12a
3-day post-SCI group vs. the control group	BP	GO:0002224	toll-like receptor signaling pathway	6/222	0.0004005	0.0017959	0.0008804	Cd36/Mapkapk2/Mapkapk3/Tlr4/Tlr6/Ubqln1
3-day post-SCI group vs. the control group	BP	GO:0015718	monocarboxylic acid transport	6/222	0.0004005	0.0017959	0.0008804	Anxa1/Cd36/Map2k6/Myc/Pnpla8/Ptgs2
3-day post-SCI group vs. the control group	BP	GO:0001936	regulation of endothelial cell proliferation	7/222	0.0004083	0.0018289	0.0008965	Alox5/Apc/Arg1/Hmox1/Jun/Sirt1/Xdh
3-day post-SCI group vs. the control group	BP	GO:0035710	CD4-positive, alpha-beta T cell activation	6/222	0.000418	0.0018706	0.000917	Anxa1/Atp7a/Braf/Ctsl/Stat6/Zc3h12a
3-day post-SCI group vs. the control group	BP	GO:0099504	synaptic vesicle cycle	8/222	0.0004233	0.0018921	0.0009275	Actb/Amph/Atp2a2/Braf/Chrna4/Snca/Syp/Tbc1d24
3-day post-SCI group vs. the control group	BP	GO:0050709	negative regulation of protein secretion	5/222	0.0004286	0.0019139	0.0009382	Anxa1/Fkbp1b/Ppp3ca/Rest/Ucp2
3-day post-SCI group vs. the control group	BP	GO:0008202	steroid metabolic process	10/222	0.0004375	0.0019517	0.0009567	Cat/Cyp1b1/Dhcr24/Nfkb1/Pdgfra/Plekha1/Ppargc1a/Prkaa2/Rest/Sirt1

3-day post-SCI group vs. the control group	BP	GO:0048545	response to steroid hormone	8/222	0.0004482	0.0019974	0.0009791	Adam9/Anxa1/Hdac1/Jak2/Parp1/Ppargc1b/Rest/Sirt1
3-day post-SCI group vs. the control group	BP	GO:0002440	production of molecular mediator of immune respo	11/222	0.0004496	0.0019994	0.0009801	Arg1/Axl/Btk/Cd36/Ezh2/Hmox1/Il18rap/Mapkapk2/Sirt1/Stat6/Tlr4
3-day post-SCI group vs. the control group	BP	GO:0006887	exocytosis	11/222	0.0004496	0.0019994	0.0009801	Anxa1/Atp13a2/Atp2a2/Braf/Ccr1/Chrna4/Hmox1/Rest/Sdc1/Snca/Syp
3-day post-SCI group vs. the control group	BP	GO:0006874	cellular calcium ion homeostasis	9/222	0.000452	0.0020078	0.0009842	Atp13a2/Atp2a2/Bax/Bnip3/Ccr1/Fkbp1b/Slc8a1/Snca/Trpm2
3-day post-SCI group vs. the control group	BP	GO:0046785	microtubule polymerization	5/222	0.0004533	0.0020113	0.0009859	Apc/Mapk8/Mapt/Met/Snca
3-day post-SCI group vs. the control group	BP	GO:0018958	phenol-containing compound metabolic process	6/222	0.0004549	0.0020142	0.0009873	Atp7a/Ctsl/Gch1/Gpr37/Maoa/Snca
3-day post-SCI group vs. the control group	BP	GO:0034219	carbohydrate transmembrane transport	6/222	0.0004549	0.0020142	0.0009873	Braf/Mapk14/Met/Myc/Nfe2l2/Slc23a2
3-day post-SCI group vs. the control group	BP	GO:0007039	protein catabolic process in the vacuole	3/222	0.0004609	0.0020322	0.0009962	Atp13a2/Mgat3/Tpp1
3-day post-SCI group vs. the control group	BP	GO:0034349	glial cell apoptotic process	3/222	0.0004609	0.0020322	0.0009962	Casp3/Mapk8/Rb1
3-day post-SCI group vs. the control group	BP	GO:0045986	negative regulation of smooth muscle contraction	3/222	0.0004609	0.0020322	0.0009962	Adora1/Ncf1/Ptgs2
3-day post-SCI group vs. the control group	BP	GO:0050965	detection of temperature stimulus involved in sens	3/222	0.0004609	0.0020322	0.0009962	Adora1/Pawr/Tlr4
3-day post-SCI group vs. the control group	BP	GO:0000266	mitochondrial fission	4/222	0.000464	0.0020395	0.0009997	Bnip3/Mapt/Pink1/Ppargc1a
3-day post-SCI group vs. the control group	BP	GO:0050974	detection of mechanical stimulus involved in senso	4/222	0.000464	0.0020395	0.0009997	Myc/Pawr/Rest/Tlr4
3-day post-SCI group vs. the control group	BP	GO:0120163	negative regulation of cold-induced thermogenesis	4/222	0.000464	0.0020395	0.0009997	Id1/Map2k6/Rb1/Tlr4
3-day post-SCI group vs. the control group	BP	GO:0043123	positive regulation of I-kappaB kinase/NF-kappaB	6/222	0.0004742	0.0020809	0.0010201	Cd36/Pink1/Rela/Ripk1/Tlr4/Tlr6
3-day post-SCI group vs. the control group	BP	GO:0007204	positive regulation of cytosolic calcium ion concer	8/222	0.0004744	0.0020809	0.0010201	Bax/Ccr1/Cd36/Cd38/Fkbp1b/Jak2/Pdgfra/Slc8a1
3-day post-SCI group vs. the control group	BP	GO:0072332	intrinsic apoptotic signaling pathway by p53 class	5/222	0.000479	0.0020988	0.0010288	Bax/Myc/Pdk2/Rrm2b/Sirt1
3-day post-SCI group vs. the control group	BP	GO:0006066	alcohol metabolic process	10/222	0.0004879	0.0021358	0.001047	Cat/Cyp1b1/Dhcr24/Dhfr/Gch1/Hao1/Nfkb1/Prkaa2/Rest/Snca
3-day post-SCI group vs. the control group	BP	GO:0002793	positive regulation of peptide secretion	6/222	0.0004942	0.0021589	0.0010583	Adora1/Cd38/Hif1a/Jak2/Sirt1/Trpm2
3-day post-SCI group vs. the control group	BP	GO:0042476	odontogenesis	6/222	0.0004942	0.0021589	0.0010583	Apc/Atf2/Bax/Hdac1/Itga6/Pdgfra
3-day post-SCI group vs. the control group	BP	GO:0048010	vascular endothelial growth factor receptor signali	4/222	0.0005031	0.0021956	0.0010763	Hif1a/Hspb1/Mapk14/Mapkapk2
3-day post-SCI group vs. the control group	BP	GO:1901991	negative regulation of mitotic cell cycle phase tran	7/222	0.0005169	0.0022532	0.0011045	Apc/Cdc20/Cdk1/Cdkn2b/Ezh2/Ier3/Rb1
3-day post-SCI group vs. the control group	BP	GO:0046394	carboxylic acid biosynthetic process	9/222	0.0005232	0.0022784	0.0011168	Alox5/Anxa1/Dhfr/Hao1/Pnpla8/Prkaa2/Ptgs1/Ptgs2/Sirt1
3-day post-SCI group vs. the control group	BP	GO:0014015	positive regulation of gliogenesis	5/222	0.0005337	0.0022997	0.0011273	Hdac1/Il6st/Myc/Rela/Rnf112
3-day post-SCI group vs. the control group	BP	GO:0019229	regulation of vasoconstriction	5/222	0.0005337	0.0022997	0.0011273	Alox5/Cd38/Hif1a/Ptgs1/Ptgs2
3-day post-SCI group vs. the control group	BP	GO:0036465	synaptic vesicle recycling	5/222	0.0005337	0.0022997	0.0011273	Actb/Amph/Snca/Syp/Tbc1d24
3-day post-SCI group vs. the control group	BP	GO:0002221	pattern recognition receptor signaling pathway	7/222	0.0005341	0.0022997	0.0011273	Cd36/Mapkapk2/Mapkapk3/Rela/Tlr4/Tlr6/Ubqln1
3-day post-SCI group vs. the control group	BP	GO:0006309	apoptotic DNA fragmentation	3/222	0.0005347	0.0022997	0.0011273	Bax/Casp3/Endog
3-day post-SCI group vs. the control group	BP	GO:0030889	negative regulation of B cell proliferation	3/222	0.0005347	0.0022997	0.0011273	Btk/Casp3/Pawr
3-day post-SCI group vs. the control group	BP	GO:0043555	regulation of translation in response to stress	3/222	0.0005347	0.0022997	0.0011273	Eif2s1/Impact/Ppp1r15b
3-day post-SCI group vs. the control group	BP	GO:0044546	NLRP3 inflammasome complex assembly	3/222	0.0005347	0.0022997	0.0011273	Cd36/Tlr4/Tlr6
3-day post-SCI group vs. the control group	BP	GO:0050665	hydrogen peroxide biosynthetic process	3/222	0.0005347	0.0022997	0.0011273	Ncf1/Nox4/Sod2
3-day post-SCI group vs. the control group	BP	GO:0051546	keratinocyte migration	3/222	0.0005347	0.0022997	0.0011273	Adam9/Hbegf/Map4k4
3-day post-SCI group vs. the control group	BP	GO:0060416	response to growth hormone	3/222	0.0005347	0.0022997	0.0011273	Jak2/Pxn/Stat6
3-day post-SCI group vs. the control group	BP	GO:0090050	positive regulation of cell migration involved in sp	3/222	0.0005347	0.0022997	0.0011273	Anxa1/Hmox1/Ptgs2
3-day post-SCI group vs. the control group	BP	GO:0016053	organic acid biosynthetic process	9/222	0.0005359	0.0023016	0.0011282	Alox5/Anxa1/Dhfr/Hao1/Pnpla8/Prkaa2/Ptgs1/Ptgs2/Sirt1

3-day post-SCI group vs. the control group	BP	GO:0030282	bone mineralization	6/222	0.0005362	0.0023016	0.0011282	Alox5/Ccr1/Hif1a/Ptgs2/Rxrb/Slc8a1
3-day post-SCI group vs. the control group	BP	GO:0010762	regulation of fibroblast migration	4/222	0.0005446	0.0023306	0.0011424	Apc/Braf/Cygb/Slc8a1
3-day post-SCI group vs. the control group	BP	GO:0044848	biological phase	4/222	0.0005446	0.0023306	0.0011424	Cdc20/Ctsl/Nfatc1/Ptgs2
3-day post-SCI group vs. the control group	BP	GO:1902459	positive regulation of stem cell population mainter	4/222	0.0005446	0.0023306	0.0011424	Actb/Hdac1/Rbbp7/Rest
3-day post-SCI group vs. the control group	BP	GO:0042445	hormone metabolic process	8/222	0.0005452	0.002331	0.0011426	Ctsl/Cyp1b1/Hif1a/Nfkb1/Pdgfra/Plekha1/Ppargc1a/Rest
3-day post-SCI group vs. the control group	BP	GO:0001776	leukocyte homeostasis	6/222	0.0005582	0.0023839	0.0011686	Anxa1/Axl/Bax/Casp3/Hif1a/Ripk3
3-day post-SCI group vs. the control group	BP	GO:0006942	regulation of striated muscle contraction	5/222	0.0005627	0.0024009	0.0011769	Adora1/Atp2a2/Fkbp1b/Slc8a1/Zc3h12a
3-day post-SCI group vs. the control group	BP	GO:0045931	positive regulation of mitotic cell cycle	6/222	0.0005809	0.0024757	0.0012136	Aif1/Anxa1/Cdk1/Cdk4/Rb1/Rrm2b
3-day post-SCI group vs. the control group	BP	GO:0048806	genitalia development	4/222	0.0005884	0.0025035	0.0012272	Axl/Bax/Dhcr24/Pdgfra
3-day post-SCI group vs. the control group	BP	GO:0097553	calcium ion transmembrane import into cytosol	7/222	0.0005886	0.0025035	0.0012272	Bax/Fkbp1b/Pawr/Ppp3ca/Slc8a1/Snca/Trpm2
3-day post-SCI group vs. the control group	BP	GO:0030500	regulation of bone mineralization	5/222	0.0005929	0.0025196	0.0012351	Alox5/Ccr1/Hif1a/Rxrb/Slc8a1
3-day post-SCI group vs. the control group	BP	GO:0050868	negative regulation of T cell activation	6/222	0.0006043	0.0025651	0.0012574	Anxa1/Arg1/Casp3/Hspb1/Pawr/Zc3h12a
3-day post-SCI group vs. the control group	BP	GO:0006907	pinocytosis	3/222	0.0006156	0.0026026	0.0012758	Axl/Mapkapk2/Mapkapk3
3-day post-SCI group vs. the control group	BP	GO:0032516	positive regulation of phosphoprotein phosphatase	3/222	0.0006156	0.0026026	0.0012758	Jak2/Pawr/Ppp1r15b
3-day post-SCI group vs. the control group	BP	GO:0050872	white fat cell differentiation	3/222	0.0006156	0.0026026	0.0012758	Atf2/Pdgfra/Sirt1
3-day post-SCI group vs. the control group	BP	GO:0060544	regulation of necroptotic process	3/222	0.0006156	0.0026026	0.0012758	Ripk1/Ripk3/Ybx3
3-day post-SCI group vs. the control group	BP	GO:0010827	regulation of glucose transmembrane transport	5/222	0.0006244	0.0026345	0.0012914	Braf/Mapk14/Met/Myc/Nfe2l2
3-day post-SCI group vs. the control group	BP	GO:0035418	protein localization to synapse	5/222	0.0006244	0.0026345	0.0012914	Hspb1/Mapk10/Mapt/Stau2/Tnik
3-day post-SCI group vs. the control group	BP	GO:0014888	striated muscle adaptation	4/222	0.0006347	0.00267	0.0013088	Atp2a2/Ezh2/Nfatc1/Ppp3ca
3-day post-SCI group vs. the control group	BP	GO:0032309	icosanoid secretion	4/222	0.0006347	0.00267	0.0013088	Anxa1/Map2k6/Pnpla8/Ptgs2
3-day post-SCI group vs. the control group	BP	GO:0032350	regulation of hormone metabolic process	4/222	0.0006347	0.00267	0.0013088	Hif1a/Nfkb1/Ppargc1a/Rest
3-day post-SCI group vs. the control group	BP	GO:0050851	antigen receptor-mediated signaling pathway	9/222	0.000647	0.0027151	0.0013309	Bax/Braf/Btk/Cd38/Ezr/Pawr/Plekha1/Rela/Zc3h12a
3-day post-SCI group vs. the control group	BP	GO:0000075	cell cycle checkpoint signaling	7/222	0.0006473	0.0027151	0.0013309	Apc/Atf2/Atr/Cdc20/Cdk1/Ier3/Mapk14
3-day post-SCI group vs. the control group	BP	GO:0110148	biomineralization	7/222	0.0006473	0.0027151	0.0013309	Adora1/Alox5/Ccr1/Hif1a/Ptgs2/Rxrb/Slc8a1
3-day post-SCI group vs. the control group	BP	GO:0030534	adult behavior	7/222	0.0006679	0.0027987	0.0013719	Chrna4/Htra2/Mapt/Met/Oxr1/Parp1/Snca
3-day post-SCI group vs. the control group	BP	GO:0044703	multi-organism reproductive process	7/222	0.000689	0.0028843	0.0014139	Atr/Ctsl/Junb/Mapk3/Ptgs2/Rxrb/Ube2a
3-day post-SCI group vs. the control group	BP	GO:0032273	positive regulation of protein polymerization	5/222	0.0006909	0.0028864	0.0014149	Apc/Cttn/Mapk8/Mapt/Met
3-day post-SCI group vs. the control group	BP	GO:0051937	catecholamine transport	5/222	0.0006909	0.0028864	0.0014149	Actb/Chrna4/Pink1/Ptgs1/Snca
3-day post-SCI group vs. the control group	BP	GO:0034329	cell junction assembly	11/222	0.0006968	0.0029082	0.0014256	Actb/Ctnna1/Cttn/Dst/Ect2/Map4k4/Mapt/Ptprk/Pxn/Snca/Stau2
3-day post-SCI group vs. the control group	BP	GO:0010666	positive regulation of cardiac muscle cell apoptoti	3/222	0.0007039	0.0029233	0.001433	Atp2a2/Bnip3/Mapk8
3-day post-SCI group vs. the control group	BP	GO:0043501	skeletal muscle adaptation	3/222	0.0007039	0.0029233	0.001433	Atp2a2/Nfatc1/Ppp3ca
3-day post-SCI group vs. the control group	BP	GO:0070498	interleukin-1-mediated signaling pathway	3/222	0.0007039	0.0029233	0.001433	Mapk3/Rela/Vrk2
3-day post-SCI group vs. the control group	BP	GO:1903579	negative regulation of ATP metabolic process	3/222	0.0007039	0.0029233	0.001433	Ier3/Parp1/Ppargc1a
3-day post-SCI group vs. the control group	BP	GO:1904385	cellular response to angiotensin	3/222	0.0007039	0.0029233	0.001433	Nfe2l2/Nfkb1/Rela
3-day post-SCI group vs. the control group	BP	GO:0032609	interferon-gamma production	6/222	0.0007052	0.0029259	0.0014342	Axl/Il18rap/Jak2/Ripk3/Tlr4/Zc3h12a
3-day post-SCI group vs. the control group	BP	GO:0001935	endothelial cell proliferation	7/222	0.0007107	0.0029456	0.0014439	Alox5/Apc/Arg1/Hmox1/Jun/Sirt1/Xdh
3-day post-SCI group vs. the control group	BP	GO:0006096	glycolytic process	5/222	0.0007261	0.0030008	0.001471	Hif1a/Ier3/Myc/Ppargc1a/Prkaa2
3-day post-SCI group vs. the control group	BP	GO:0031110	regulation of microtubule polymerization or depol	5/222	0.0007261	0.0030008	0.001471	Apc/Mapk8/Mapt/Met/Snca
3-day post-SCI group vs. the control group	BP	GO:0032024	positive regulation of insulin secretion	5/222	0.0007261	0.0030008	0.001471	Cd38/Hif1a/Jak2/Sirt1/Trpm2

3-day post-SCI group vs. the control group	BP	GO:0032868	response to insulin	8/222	0.0007315	0.0030161	0.0014785	Apc/Cdk4/Mapk14/Parp1/Pdk2/Rb1/Rela/Sirt1
3-day post-SCI group vs. the control group	BP	GO:0002042	cell migration involved in sprouting angiogenesis	4/222	0.0007349	0.0030161	0.0014785	Anxa1/Fbxw7/Hmox1/Ptgs2
3-day post-SCI group vs. the control group	BP	GO:0010828	positive regulation of glucose transmembrane tran:	4/222	0.0007349	0.0030161	0.0014785	Braf/Mapk14/Met/Nfe2l2
3-day post-SCI group vs. the control group	BP	GO:0045907	positive regulation of vasoconstriction	4/222	0.0007349	0.0030161	0.0014785	Alox5/Cd38/Ptgs1/Ptgs2
3-day post-SCI group vs. the control group	BP	GO:0046189	phenol-containing compound biosynthetic process	4/222	0.0007349	0.0030161	0.0014785	Atp7a/Gch1/Gpr37/Snca
3-day post-SCI group vs. the control group	BP	GO:0046676	negative regulation of insulin secretion	4/222	0.0007349	0.0030161	0.0014785	Fkbp1b/Ppp3ca/Rest/Ucp2
3-day post-SCI group vs. the control group	BP	GO:2000677	regulation of transcription regulatory region DNA	4/222	0.0007349	0.0030161	0.0014785	Fbxw7/Parp1/Rb1/Rest
3-day post-SCI group vs. the control group	BP	GO:0006757	ATP generation from ADP	5/222	0.0007627	0.0031241	0.0015314	Hif1a/Ier3/Myc/Ppargc1a/Prkaa2
3-day post-SCI group vs. the control group	BP	GO:0055072	iron ion homeostasis	5/222	0.0007627	0.0031241	0.0015314	Atp13a2/Hif1a/Hmox1/Myc/Sod2
3-day post-SCI group vs. the control group	BP	GO:0032715	negative regulation of interleukin-6 production	4/222	0.0007889	0.0032254	0.0015811	Sirpa/Tlr4/Tnfrsf1a/Zc3h12a
3-day post-SCI group vs. the control group	BP	GO:0045687	positive regulation of glial cell differentiation	4/222	0.0007889	0.0032254	0.0015811	Hdac1/Il6st/Rela/Rnf112
3-day post-SCI group vs. the control group	BP	GO:0006691	leukotriene metabolic process	3/222	0.0008	0.0032415	0.001589	Alox5/Ncf1/Tlr4
3-day post-SCI group vs. the control group	BP	GO:0010663	positive regulation of striated muscle cell apoptoti	3/222	0.0008	0.0032415	0.001589	Atp2a2/Bnip3/Mapk8
3-day post-SCI group vs. the control group	BP	GO:0014850	response to muscle activity	3/222	0.0008	0.0032415	0.001589	Hif1a/Ppargc1a/Prkaa2
3-day post-SCI group vs. the control group	BP	GO:0043369	CD4-positive or CD8-positive, alpha-beta T cell li	3/222	0.0008	0.0032415	0.001589	Braf/Ctsl/Stat6
3-day post-SCI group vs. the control group	BP	GO:0050961	detection of temperature stimulus involved in sens	3/222	0.0008	0.0032415	0.001589	Adora1/Pawr/Tlr4
3-day post-SCI group vs. the control group	BP	GO:0140632	inflammasome complex assembly	3/222	0.0008	0.0032415	0.001589	Cd36/Tlr4/Tlr6
3-day post-SCI group vs. the control group	BP	GO:1900016	negative regulation of cytokine production involve	3/222	0.0008	0.0032415	0.001589	Ezh2/Sirpa/Zc3h12a
3-day post-SCI group vs. the control group	BP	GO:1903747	regulation of establishment of protein localization	3/222	0.0008	0.0032415	0.001589	Fbxw7/Mapt/Pink1
3-day post-SCI group vs. the control group	BP	GO:0032410	negative regulation of transporter activity	5/222	0.0008006	0.0032415	0.001589	Fkbp1b/Ppif/Ppp3ca/Snca/Ubqln1
3-day post-SCI group vs. the control group	BP	GO:0042475	odontogenesis of dentin-containing tooth	5/222	0.0008006	0.0032415	0.001589	Atf2/Bax/Hdac1/Itga6/Pdgfra
3-day post-SCI group vs. the control group	BP	GO:0045727	positive regulation of translation	6/222	0.0008188	0.003309	0.0016221	Cdk4/Cyp1b1/Impact/Mapk3/Paip1/Pink1
3-day post-SCI group vs. the control group	BP	GO:0051224	negative regulation of protein transport	6/222	0.0008188	0.003309	0.0016221	Anxa1/Cd36/Fkbp1b/Ppp3ca/Rest/Ucp2
3-day post-SCI group vs. the control group	BP	GO:0007062	sister chromatid cohesion	4/222	0.0008457	0.0034081	0.0016706	Cdc20/Fbxw7/Rb1/Sfpq
3-day post-SCI group vs. the control group	BP	GO:0045933	positive regulation of muscle contraction	4/222	0.0008457	0.0034081	0.0016706	Cttn/Pawr/Ptgs1/Ptgs2
3-day post-SCI group vs. the control group	BP	GO:2000378	negative regulation of reactive oxygen species met	4/222	0.0008457	0.0034081	0.0016706	Bnip3/Hif1a/Pink1/Sirt1
3-day post-SCI group vs. the control group	BP	GO:0014902	myotube differentiation	6/222	0.0008493	0.003419	0.001676	Gpx1/Mapk14/Met/Myc/Ppp3ca/Rcan1
3-day post-SCI group vs. the control group	BP	GO:0002699	positive regulation of immune effector process	9/222	0.0008673	0.0034849	0.0017083	Anxa1/Arg1/Cd36/Hmox1/Il18rap/Mapkapk2/Sirt1/Stat6/Tlr4
3-day post-SCI group vs. the control group	BP	GO:0072503	cellular divalent inorganic cation homeostasis	9/222	0.0008673	0.0034849	0.0017083	Atp13a2/Atp2a2/Bax/Bnip3/Ccr1/Fkbp1b/Slc8a1/Snca/Trpm2
3-day post-SCI group vs. the control group	BP	GO:0043405	regulation of MAP kinase activity	7/222	0.0008776	0.0035198	0.0017254	Adam9/Ezh2/Map2k4/Map2k6/Nox4/Tlr4/Tlr6
3-day post-SCI group vs. the control group	BP	GO:0098739	import across plasma membrane	7/222	0.0008776	0.0035198	0.0017254	Abcc1/Arg1/Cd36/Pawr/Ppp3ca/Slc8a1/Trpm2
3-day post-SCI group vs. the control group	BP	GO:0007044	cell-substrate junction assembly	5/222	0.0008805	0.0035279	0.0017294	Cttn/Dst/Map4k4/Ptprk/Pxn
3-day post-SCI group vs. the control group	BP	GO:0007611	learning or memory	9/222	0.0008865	0.0035485	0.0017395	Amph/Braf/Casp3/Hif1a/Jun/Mapt/Ptgs1/Ptgs2/Rcan1
3-day post-SCI group vs. the control group	BP	GO:0042069	regulation of catecholamine metabolic process	3/222	0.0009039	0.0035944	0.0017619	Atp7a/Gpr37/Snca
3-day post-SCI group vs. the control group	BP	GO:0045736	negative regulation of cyclin-dependent protein se	3/222	0.0009039	0.0035944	0.0017619	Apc/Casp3/Cdkn2c
3-day post-SCI group vs. the control group	BP	GO:0062098	regulation of programmed necrotic cell death	3/222	0.0009039	0.0035944	0.0017619	Ripk1/Ripk3/Ybx3
3-day post-SCI group vs. the control group	BP	GO:1900543	negative regulation of purine nucleotide metabolic	3/222	0.0009039	0.0035944	0.0017619	Ier3/Parp1/Ppargc1a
3-day post-SCI group vs. the control group	BP	GO:1903306	negative regulation of regulated secretory pathway	3/222	0.0009039	0.0035944	0.0017619	Braf/Hmox1/Rest

3-day post-SCI group vs. the control group	BP	GO:1903798	regulation of miRNA maturation	3/222	0.0009039	0.0035944	0.0017619	Ppp3ca/Ripk1/Zc3h12a
3-day post-SCI group vs. the control group	BP	GO:2001169	regulation of ATP biosynthetic process	3/222	0.0009039	0.0035944	0.0017619	Myc/Parp1/Ppargc1a
3-day post-SCI group vs. the control group	BP	GO:0030198	extracellular matrix organization	9/222	0.000906	0.0035993	0.0017644	Atp7a/Cyp1b1/Ets1/Pdgfra/Prdx4/Pxdn/Rb1/Scara5/Tnfrsf1a
3-day post-SCI group vs. the control group	BP	GO:0043062	extracellular structure organization	9/222	0.0009259	0.0036749	0.0018014	Atp7a/Cyp1b1/Ets1/Pdgfra/Prdx4/Pxdn/Rb1/Scara5/Tnfrsf1a
3-day post-SCI group vs. the control group	BP	GO:0045229	external encapsulating structure organization	9/222	0.0009462	0.0037446	0.0018356	Atp7a/Cyp1b1/Ets1/Pdgfra/Prdx4/Pxdn/Rb1/Scara5/Tnfrsf1a
3-day post-SCI group vs. the control group	BP	GO:0045927	positive regulation of growth	9/222	0.0009462	0.0037446	0.0018356	Cd38/Cdk1/Ezr/Hbegf/Mapk14/Mapt/Sirt1/Slc23a2/Ybx3
3-day post-SCI group vs. the control group	BP	GO:0055074	calcium ion homeostasis	9/222	0.0009462	0.0037446	0.0018356	Atp13a2/Atp2a2/Bax/Bnip3/Ccr1/Fkbp1b/Slc8a1/Snca/Trpm2
3-day post-SCI group vs. the control group	BP	GO:1903169	regulation of calcium ion transmembrane transport	7/222	0.000958	0.0037881	0.0018569	Bax/Fkbp1b/Ppp3ca/S100a1/Slc8a1/Snca/Ubqln1
3-day post-SCI group vs. the control group	BP	GO:0015908	fatty acid transport	5/222	0.0009663	0.0038128	0.001869	Abcc1/Anxa1/Cd36/Map2k6/Pnpla8
3-day post-SCI group vs. the control group	BP	GO:1901222	regulation of NIK/NF-kappaB signaling	5/222	0.0009663	0.0038128	0.001869	Ago1/Ago3/Rela/Tlr4/Zc3h12a
3-day post-SCI group vs. the control group	BP	GO:0031648	protein destabilization	4/222	0.0009679	0.0038128	0.001869	Fbxw7/Id1/Sirt1/Snca
3-day post-SCI group vs. the control group	BP	GO:0048538	thymus development	4/222	0.0009679	0.0038128	0.001869	Apc/Braf/Mapk3/Ripk3
3-day post-SCI group vs. the control group	BP	GO:0006639	acylglycerol metabolic process	6/222	0.00098	0.0038533	0.0018889	Cat/Cd36/Dgkk/Gpx1/Il6st/Sirt1
3-day post-SCI group vs. the control group	BP	GO:1903322	positive regulation of protein modification by sma	6/222	0.00098	0.0038533	0.0018889	Cdc20/Fbxw7/Mapk8/Tank/Ubqln1/Zc3h12a
3-day post-SCI group vs. the control group	BP	GO:1903038	negative regulation of leukocyte cell-cell adhesion	6/222	0.001015	0.0039765	0.0019493	Anxa1/Arg1/Casp3/Hspb1/Pawr/Zc3h12a
3-day post-SCI group vs. the control group	BP	GO:0010575	positive regulation of vascular endothelial growth	3/222	0.0010161	0.0039765	0.0019493	Cyp1b1/Hif1a/Ptgs2
3-day post-SCI group vs. the control group	BP	GO:0090335	regulation of brown fat cell differentiation	3/222	0.0010161	0.0039765	0.0019493	Mapk14/Ptgs2/Sirt1
3-day post-SCI group vs. the control group	BP	GO:0097164	ammonium ion metabolic process	3/222	0.0010161	0.0039765	0.0019493	Atp7a/Gch1/Maoa
3-day post-SCI group vs. the control group	BP	GO:1990776	response to angiotensin	3/222	0.0010161	0.0039765	0.0019493	Nfe2l2/Nfkb1/Rela
3-day post-SCI group vs. the control group	BP	GO:0044706	multi-multicellular organism process	7/222	0.0010441	0.0040824	0.0020012	Atr/Ctsl/Junb/Mapk3/Ptgs2/Rxrb/Ube2a
3-day post-SCI group vs. the control group	BP	GO:0032200	telomere organization	6/222	0.001051	0.0041015	0.0020106	Atr/Ezh2/Mapk3/Myc/Parp1/Pcna
3-day post-SCI group vs. the control group	BP	GO:0034763	negative regulation of transmembrane transport	6/222	0.001051	0.0041015	0.0020106	Atp7a/Fkbp1b/Myc/Ppif/Ppp3ca/Ubqln1
3-day post-SCI group vs. the control group	BP	GO:0016051	carbohydrate biosynthetic process	7/222	0.0010741	0.0041842	0.0020511	Cd36/Hif1a/Nfkb1/Pdk2/Ppp1ca/Sirt1/Snca
3-day post-SCI group vs. the control group	BP	GO:0046890	regulation of lipid biosynthetic process	7/222	0.0010741	0.0041842	0.0020511	Anxa1/Cdk4/Nfkb1/Ppargc1a/Ptgs2/Rest/Sirt1
3-day post-SCI group vs. the control group	BP	GO:0030218	erythrocyte differentiation	6/222	0.0010879	0.0042221	0.0020696	Casp3/Ets1/Hif1a/Jak2/Mapk14/Rb1
3-day post-SCI group vs. the control group	BP	GO:0045598	regulation of fat cell differentiation	6/222	0.0010879	0.0042221	0.0020696	Alox5/Mapk14/Ptgs2/Sirt1/Sod2/Zc3h12a
3-day post-SCI group vs. the control group	BP	GO:0046328	regulation of JNK cascade	6/222	0.0010879	0.0042221	0.0020696	Map2k4/Map4k4/Ripk1/Sirpa/Tlr4/Tnik
3-day post-SCI group vs. the control group	BP	GO:0072331	signal transduction by p53 class mediator	6/222	0.0010879	0.0042221	0.0020696	Atr/Bax/Myc/Pdk2/Rrm2b/Sirt1
3-day post-SCI group vs. the control group	BP	GO:0042987	amyloid precursor protein catabolic process	4/222	0.001102	0.0042651	0.0020907	Adam9/Casp3/Dhcr24/Rela
3-day post-SCI group vs. the control group	BP	GO:0050775	positive regulation of dendrite morphogenesis	4/222	0.001102	0.0042651	0.0020907	Met/Stau2/Tbc1d24/Tnik
3-day post-SCI group vs. the control group	BP	GO:2001238	positive regulation of extrinsic apoptotic signaling	4/222	0.001102	0.0042651	0.0020907	Ctnna1/Htra2/Ppp1ca/Ripk1
3-day post-SCI group vs. the control group	BP	GO:0032147	activation of protein kinase activity	5/222	0.0011063	0.0042742	0.0020952	Camkk2/Ect2/Jak2/Ripk3/Tlr4
3-day post-SCI group vs. the control group	BP	GO:0046031	ADP metabolic process	5/222	0.0011063	0.0042742	0.0020952	Hif1a/Ier3/Myc/Ppargc1a/Prkaa2
3-day post-SCI group vs. the control group	BP	GO:0050921	positive regulation of chemotaxis	6/222	0.0011257	0.0043411	0.002128	Aif1/Ccr1/Cttn/Hspb1/Mapk3/Met
3-day post-SCI group vs. the control group	BP	GO:0051928	positive regulation of calcium ion transport	6/222	0.0011257	0.0043411	0.002128	Bax/Ccr1/Mylk/Ppp3ca/S100a1/Snca
3-day post-SCI group vs. the control group	BP	GO:0003176	aortic valve development	3/222	0.0011367	0.0043434	0.0021291	Nfatc1/Rb1/Tnfrsf1a
3-day post-SCI group vs. the control group	BP	GO:0010839	negative regulation of keratinocyte proliferation	3/222	0.0011367	0.0043434	0.0021291	Ctsl/Nfatc1/Ptprk
3-day post-SCI group vs. the control group	BP	GO:0031100	animal organ regeneration	3/222	0.0011367	0.0043434	0.0021291	Cdk1/Ezh2/Hmox1
3-day post-SCI group vs. the control group	BP	GO:0035384	thioester biosynthetic process	3/222	0.0011367	0.0043434	0.0021291	Pdk1/Pdk2/Snca

3-day post-SCI group vs. the control group	BP	GO:0045980	negative regulation of nucleotide metabolic proces	3/222	0.0011367	0.0043434	0.0021291	Ier3/Parp1/Ppargc1a
3-day post-SCI group vs. the control group	BP	GO:0070920	regulation of production of small RNA involved ir	3/222	0.0011367	0.0043434	0.0021291	Ppp3ca/Ripk1/Zc3h12a
3-day post-SCI group vs. the control group	BP	GO:0071616	acyl-CoA biosynthetic process	3/222	0.0011367	0.0043434	0.0021291	Pdk1/Pdk2/Snca
3-day post-SCI group vs. the control group	BP	GO:0097049	motor neuron apoptotic process	3/222	0.0011367	0.0043434	0.0021291	Atf2/Bax/Map2k4
3-day post-SCI group vs. the control group	BP	GO:1901857	positive regulation of cellular respiration	3/222	0.0011367	0.0043434	0.0021291	Myc/Pink1/Ppargc1a
3-day post-SCI group vs. the control group	BP	GO:2000269	regulation of fibroblast apoptotic process	3/222	0.0011367	0.0043434	0.0021291	Apc/Btg1/Sirt1
3-day post-SCI group vs. the control group	BP	GO:0150115	cell-substrate junction organization	5/222	0.0011562	0.0044142	0.0021638	Cttn/Dst/Map4k4/Ptprk/Pxn
3-day post-SCI group vs. the control group	BP	GO:0030199	collagen fibril organization	4/222	0.0011737	0.0044768	0.0021945	Atp7a/Cyp1b1/Pxdn/Rb1
3-day post-SCI group vs. the control group	BP	GO:0022408	negative regulation of cell-cell adhesion	7/222	0.001201	0.0045727	0.0022415	Anxa1/Arg1/Casp3/Hspb1/Jak2/Pawr/Zc3h12a
3-day post-SCI group vs. the control group	BP	GO:0072330	monocarboxylic acid biosynthetic process	7/222	0.001201	0.0045727	0.0022415	Alox5/Anxa1/Pnpla8/Prkaa2/Ptgs1/Ptgs2/Sirt1
3-day post-SCI group vs. the control group	BP	GO:0046330	positive regulation of JNK cascade	5/222	0.0012077	0.0045942	0.0022521	Map2k4/Map4k4/Ripk1/Tlr4/Tnik
3-day post-SCI group vs. the control group	BP	GO:0016052	carbohydrate catabolic process	6/222	0.0012455	0.0047293	0.0023183	Hif1a/Ier3/Myc/Ppargc1a/Ppp1ca/Prkaa2
3-day post-SCI group vs. the control group	BP	GO:0044839	cell cycle G2/M phase transition	6/222	0.0012455	0.0047293	0.0023183	Ccna2/Cdk1/Cdk10/Cdk4/Ier3/Rrm2b
3-day post-SCI group vs. the control group	BP	GO:0060760	positive regulation of response to cytokine stimulu	4/222	0.0012486	0.0047367	0.0023219	Axl/Hif1a/Ripk1/Tlr4
3-day post-SCI group vs. the control group	BP	GO:0055013	cardiac muscle cell development	5/222	0.0012609	0.004751	0.0023289	Map2k4/Met/Pdgfra/Sirt1/Slc8a1
3-day post-SCI group vs. the control group	BP	GO:0003299	muscle hypertrophy in response to stress	3/222	0.0012659	0.004751	0.0023289	Atp2a2/Ezh2/Ppp3ca
3-day post-SCI group vs. the control group	BP	GO:0006536	glutamate metabolic process	3/222	0.0012659	0.004751	0.0023289	Apc/Gclc/Prodh
3-day post-SCI group vs. the control group	BP	GO:0007176	regulation of epidermal growth factor-activated re	3/222	0.0012659	0.004751	0.0023289	Adora1/Fbxw7/Hbegf
3-day post-SCI group vs. the control group	BP	GO:0014898	cardiac muscle hypertrophy in response to stress	3/222	0.0012659	0.004751	0.0023289	Atp2a2/Ezh2/Ppp3ca
3-day post-SCI group vs. the control group	BP	GO:0015874	norepinephrine transport	3/222	0.0012659	0.004751	0.0023289	Actb/Ptgs1/Snca
3-day post-SCI group vs. the control group	BP	GO:0016048	detection of temperature stimulus	3/222	0.0012659	0.004751	0.0023289	Adora1/Pawr/Tlr4
3-day post-SCI group vs. the control group	BP	GO:0042430	indole-containing compound metabolic process	3/222	0.0012659	0.004751	0.0023289	Atp7a/Gch1/Maoa
3-day post-SCI group vs. the control group	BP	GO:0045648	positive regulation of erythrocyte differentiation	3/222	0.0012659	0.004751	0.0023289	Ets1/Hif1a/Mapk14
3-day post-SCI group vs. the control group	BP	GO:0046688	response to copper ion	3/222	0.0012659	0.004751	0.0023289	Atp7a/Nfe2l2/Snca
3-day post-SCI group vs. the control group	BP	GO:0061298	retina vasculature development in camera-type eye	3/222	0.0012659	0.004751	0.0023289	Cyp1b1/Hif1a/Pdgfra
3-day post-SCI group vs. the control group	BP	GO:1900017	positive regulation of cytokine production involve	3/222	0.0012659	0.004751	0.0023289	Hif1a/Tlr4/Tlr6
3-day post-SCI group vs. the control group	BP	GO:0010721	negative regulation of cell development	7/222	0.0012687	0.0047572	0.002332	Cdkn2b/Ctnna1/Fbxw7/Id1/Ppp3ca/Rb1/Rest
3-day post-SCI group vs. the control group	BP	GO:0032869	cellular response to insulin stimulus	7/222	0.0013036	0.0048839	0.0023941	Apc/Cdk4/Parp1/Pdk2/Rb1/Rela/Sirt1
3-day post-SCI group vs. the control group	BP	GO:0009135	purine nucleoside diphosphate metabolic process	5/222	0.0013158	0.004919	0.0024113	Hif1a/Ier3/Myc/Ppargc1a/Prkaa2
3-day post-SCI group vs. the control group	BP	GO:0009179	purine ribonucleoside diphosphate metabolic proc	5/222	0.0013158	0.004919	0.0024113	Hif1a/Ier3/Myc/Ppargc1a/Prkaa2
3-day post-SCI group vs. the control group	BP	GO:0051258	protein polymerization	8/222	0.0013165	0.004919	0.0024113	Aif1/Apc/Cttn/Mapk8/Mapt/Met/Msrb2/Snca
3-day post-SCI group vs. the control group	BP	GO:0010676	positive regulation of cellular carbohydrate metab	4/222	0.0013267	0.0049442	0.0024236	Cd36/Hif1a/Sirt1/Snca
3-day post-SCI group vs. the control group	BP	GO:0046622	positive regulation of organ growth	4/222	0.0013267	0.0049442	0.0024236	Cdk1/Mapk14/Sirt1/Ybx3
3-day post-SCI group vs. the control group	BP	GO:0050982	detection of mechanical stimulus	4/222	0.0013267	0.0049442	0.0024236	Myc/Pawr/Rest/Tlr4
3-day post-SCI group vs. the control group	BP	GO:0018205	peptidyl-lysine modification	10/222	0.0013372	0.0049786	0.0024405	Actb/Atf2/Atp7a/Eed/Ezh2/Mapk3/Prkaa2/Rela/Sirt1/Snca
3-day post-SCI group vs. the control group	BP	GO:2000060	positive regulation of ubiquitin-dependent protein	5/222	0.0013725	0.0051056	0.0025027	Cdc20/Fbxw7/Gclc/Mapk8/Ubqln1
3-day post-SCI group vs. the control group	BP	GO:0006282	regulation of DNA repair	7/222	0.0013758	0.0051133	0.0025065	Actb/Atr/Ier3/Mgmt/Parp1/Pcna/Sirt1
3-day post-SCI group vs. the control group	BP	GO:0000737	DNA catabolic process, endonucleolytic	3/222	0.001404	0.0051908	0.0025445	Bax/Casp3/Endog
3-day post-SCI group vs. the control group	BP	GO:0010971	positive regulation of G2/M transition of mitotic c	3/222	0.001404	0.0051908	0.0025445	Cdk1/Cdk4/Rrm2b
3-day post-SCI group vs. the control group	BP	GO:0014887	cardiac muscle adaptation	3/222	0.001404	0.0051908	0.0025445	Atp2a2/Ezh2/Ppp3ca

3-day post-SCI group vs. the control group	BP	GO:0051450	myoblast proliferation	3/222	0.001404	0.0051908	0.0025445	Atf2/Gpx1/Met
3-day post-SCI group vs. the control group	BP	GO:1900745	positive regulation of p38MAPK cascade	3/222	0.001404	0.0051908	0.0025445	Met/Xdh/Zc3h12a
3-day post-SCI group vs. the control group	BP	GO:1903649	regulation of cytoplasmic transport	3/222	0.001404	0.0051908	0.0025445	Ezr/Mapk3/Mapk8
3-day post-SCI group vs. the control group	BP	GO:0042446	hormone biosynthetic process	4/222	0.0014083	0.0051974	0.0025477	Hif1a/Nfkb1/Ppargc1a/Rest
3-day post-SCI group vs. the control group	BP	GO:0060324	face development	4/222	0.0014083	0.0051974	0.0025477	Braf/Mapk3/Pdgfra/Plekha1
3-day post-SCI group vs. the control group	BP	GO:0070167	regulation of biomineral tissue development	5/222	0.0014309	0.0052764	0.0025864	Alox5/Ccr1/Hif1a/Rxrb/Slc8a1
3-day post-SCI group vs. the control group	BP	GO:0006006	glucose metabolic process	7/222	0.0014898	0.0054773	0.002685	Cd36/Hif1a/Mapk14/Myc/Pdk1/Pdk2/Sirt1
3-day post-SCI group vs. the control group	BP	GO:0006576	cellular biogenic amine metabolic process	5/222	0.0014912	0.0054773	0.002685	Atp7a/Gch1/Gpr37/Maoa/Snca
3-day post-SCI group vs. the control group	BP	GO:0034502	protein localization to chromosome	5/222	0.0014912	0.0054773	0.002685	Atr/Cdk1/Ezh2/Rb1/Tpp1
3-day post-SCI group vs. the control group	BP	GO:1904063	negative regulation of cation transmembrane transp	5/222	0.0014912	0.0054773	0.002685	Atp7a/Fkbp1b/Ppif/Ppp3ca/Ubqln1
3-day post-SCI group vs. the control group	BP	GO:0009064	glutamine family amino acid metabolic process	4/222	0.0014932	0.0054773	0.002685	Apc/Arg1/Gclc/Prodh
3-day post-SCI group vs. the control group	BP	GO:0051851	modulation by host of symbiont process	4/222	0.0014932	0.0054773	0.002685	Hdac1/Jun/Rest/Zc3h12a
3-day post-SCI group vs. the control group	BP	GO:0048639	positive regulation of developmental growth	7/222	0.0015294	0.0056051	0.0027476	Cdk1/Ezr/Mapk14/Mapt/Sirt1/Slc23a2/Ybx3
3-day post-SCI group vs. the control group	BP	GO:0001963	synaptic transmission, dopaminergic	3/222	0.0015512	0.0056532	0.0027712	Pink1/Ptgs2/Snca
3-day post-SCI group vs. the control group	BP	GO:0034390	smooth muscle cell apoptotic process	3/222	0.0015512	0.0056532	0.0027712	Map2k4/Sirt1/Sod2
3-day post-SCI group vs. the control group	BP	GO:0034391	regulation of smooth muscle cell apoptotic process	3/222	0.0015512	0.0056532	0.0027712	Map2k4/Sirt1/Sod2
3-day post-SCI group vs. the control group	BP	GO:0006304	DNA modification	5/222	0.0015533	0.0056532	0.0027712	Cyp1b1/Ezh2/Mgmt/Myc/Parp1
3-day post-SCI group vs. the control group	BP	GO:0015844	monoamine transport	5/222	0.0015533	0.0056532	0.0027712	Actb/Chrna4/Pink1/Ptgs1/Snca
3-day post-SCI group vs. the control group	BP	GO:0034446	substrate adhesion-dependent cell spreading	5/222	0.0015533	0.0056532	0.0027712	Atrn/Axl/Braf/P4hb/Pxn
3-day post-SCI group vs. the control group	BP	GO:0070227	lymphocyte apoptotic process	5/222	0.0015533	0.0056532	0.0027712	Bax/Hif1a/Myc/Ripk1/Ripk3
3-day post-SCI group vs. the control group	BP	GO:0110149	regulation of biomineralization	5/222	0.0015533	0.0056532	0.0027712	Alox5/Ccr1/Hif1a/Rxrb/Slc8a1
3-day post-SCI group vs. the control group	BP	GO:0070507	regulation of microtubule cytoskeleton organizatio	6/222	0.0015628	0.0056829	0.0027857	Apc/Mapk8/Mapt/Met/Prkaa2/Snca
3-day post-SCI group vs. the control group	BP	GO:0002429	immune response-activating cell surface receptor s	9/222	0.0015813	0.0057455	0.0028164	Bax/Braf/Btk/Cd38/Ezr/Pawr/Plekha1/Rela/Zc3h12a
3-day post-SCI group vs. the control group	BP	GO:0014074	response to purine-containing compound	5/222	0.0016172	0.0058607	0.0028729	Adora1/Ezr/Prkaa2/Slc8a1/Trpm2
3-day post-SCI group vs. the control group	BP	GO:0055006	cardiac cell development	5/222	0.0016172	0.0058607	0.0028729	Map2k4/Met/Pdgfra/Sirt1/Slc8a1
3-day post-SCI group vs. the control group	BP	GO:1901800	positive regulation of proteasomal protein cataboli	5/222	0.0016172	0.0058607	0.0028729	Cdc20/Fbxw7/Gclc/Mapk8/Ubqln1
3-day post-SCI group vs. the control group	BP	GO:0030258	lipid modification	7/222	0.0016531	0.0059857	0.0029342	Alox5/Cd36/Cygb/Etfdh/Hao1/Mapk14/Ppargc1a
3-day post-SCI group vs. the control group	BP	GO:0033135	regulation of peptidyl-serine phosphorylation	6/222	0.0016638	0.0060191	0.0029506	Bax/Braf/Met/Pink1/Ptgs2/Snca
3-day post-SCI group vs. the control group	BP	GO:0015909	long-chain fatty acid transport	4/222	0.0016737	0.0060395	0.0029605	Abcc1/Anxa1/Cd36/Pnpla8
3-day post-SCI group vs. the control group	BP	GO:0051148	negative regulation of muscle cell differentiation	4/222	0.0016737	0.0060395	0.0029605	Ezh2/Nfatc1/Rcan1/Smad1
3-day post-SCI group vs. the control group	BP	GO:0070231	T cell apoptotic process	4/222	0.0016737	0.0060395	0.0029605	Bax/Hif1a/Ripk1/Ripk3
3-day post-SCI group vs. the control group	BP	GO:0030518	intracellular steroid hormone receptor signaling pa	5/222	0.0016831	0.0060629	0.002972	Hdac1/Jak2/Parp1/Ppargc1b/Sirt1
3-day post-SCI group vs. the control group	BP	GO:0110020	regulation of actomyosin structure organization	5/222	0.0016831	0.0060629	0.002972	Braf/Ect2/Met/Nox4/Pxn
3-day post-SCI group vs. the control group	BP	GO:0016485	protein processing	8/222	0.0016865	0.0060698	0.0029754	Adam9/Bmp1/Casp3/Ctsl/Dhcr24/Myc/Parp1/Pawr
3-day post-SCI group vs. the control group	BP	GO:0061036	positive regulation of cartilage development	3/222	0.0017078	0.0061413	0.0030105	Bmp1/Rela/Smad1
3-day post-SCI group vs. the control group	BP	GO:0006641	triglyceride metabolic process	5/222	0.0017509	0.0062858	0.0030812	Cat/Cd36/Gpx1/Il6st/Sirt1
3-day post-SCI group vs. the control group	BP	GO:0071346	cellular response to interferon-gamma	5/222	0.0017509	0.0062858	0.0030812	Arg1/Jak2/Rpl13a/Sirpa/Tlr4
3-day post-SCI group vs. the control group	BP	GO:0043473	pigmentation	5/222	0.0018207	0.0065308	0.0032014	Atp7a/Atrn/Bax/Myc/Sod2
3-day post-SCI group vs. the control group	BP	GO:0006879	cellular iron ion homeostasis	4/222	0.0018689	0.006631	0.0032505	Atp13a2/Hif1a/Hmox1/Myc
3-day post-SCI group vs. the control group	BP	GO:0032233	positive regulation of actin filament bundle asseml	4/222	0.0018689	0.006631	0.0032505	Braf/Id1/Nox4/Pxn

3-day post-SCI group vs. the control group	BP	GO:0050433	regulation of catecholamine secretion	4/222	0.0018689	0.006631	0.0032505	Chrna4/Pink1/Ptgs1/Snca
3-day post-SCI group vs. the control group	BP	GO:0002360	T cell lineage commitment	3/222	0.0018739	0.006631	0.0032505	Braf/Ctsl/Stat6
3-day post-SCI group vs. the control group	BP	GO:0010574	regulation of vascular endothelial growth factor pr	3/222	0.0018739	0.006631	0.0032505	Cyp1b1/Hif1a/Ptgs2
3-day post-SCI group vs. the control group	BP	GO:0030262	apoptotic nuclear changes	3/222	0.0018739	0.006631	0.0032505	Bax/Casp3/Endog
3-day post-SCI group vs. the control group	BP	GO:0045742	positive regulation of epidermal growth factor rece	3/222	0.0018739	0.006631	0.0032505	Adora1/Fbxw7/Hbegf
3-day post-SCI group vs. the control group	BP	GO:0050951	sensory perception of temperature stimulus	3/222	0.0018739	0.006631	0.0032505	Adora1/Pawr/Tlr4
3-day post-SCI group vs. the control group	BP	GO:0060390	regulation of SMAD protein signal transduction	3/222	0.0018739	0.006631	0.0032505	Jak2/Parp1/Rbpms
3-day post-SCI group vs. the control group	BP	GO:0070296	sarcoplasmic reticulum calcium ion transport	3/222	0.0018739	0.006631	0.0032505	Atp2a2/Fkbp1b/Slc8a1
3-day post-SCI group vs. the control group	BP	GO:1901099	negative regulation of signal transduction in absen	3/222	0.0018739	0.006631	0.0032505	Ctnna1/Mcl1/Ripk1
3-day post-SCI group vs. the control group	BP	GO:1902751	positive regulation of cell cycle G2/M phase transi	3/222	0.0018739	0.006631	0.0032505	Cdk1/Cdk4/Rrm2b
3-day post-SCI group vs. the control group	BP	GO:1905898	positive regulation of response to endoplasmic reti	3/222	0.0018739	0.006631	0.0032505	Bax/Sirt1/Ubqln1
3-day post-SCI group vs. the control group	BP	GO:2000352	negative regulation of endothelial cell apoptotic pr	3/222	0.0018739	0.006631	0.0032505	Braf/Id1/Nfe2l2
3-day post-SCI group vs. the control group	BP	GO:2000737	negative regulation of stem cell differentiation	3/222	0.0018739	0.006631	0.0032505	Ezh2/Nfe2l2/Rest
3-day post-SCI group vs. the control group	BP	GO:2001240	negative regulation of extrinsic apoptotic signaling	3/222	0.0018739	0.006631	0.0032505	Ctnna1/Mcl1/Ripk1
3-day post-SCI group vs. the control group	BP	GO:0000077	DNA damage checkpoint signaling	5/222	0.0018926	0.0066804	0.0032747	Atf2/Atr/Cdk1/Ier3/Mapk14
3-day post-SCI group vs. the control group	BP	GO:0010906	regulation of glucose metabolic process	5/222	0.0018926	0.0066804	0.0032747	Cd36/Hif1a/Pdk1/Pdk2/Sirt1
3-day post-SCI group vs. the control group	BP	GO:1902749	regulation of cell cycle G2/M phase transition	5/222	0.0018926	0.0066804	0.0032747	Cdk1/Cdk10/Cdk4/Ier3/Rrm2b
3-day post-SCI group vs. the control group	BP	GO:0072507	divalent inorganic cation homeostasis	9/222	0.0019158	0.0067567	0.0033121	Atp13a2/Atp2a2/Bax/Bnip3/Ccr1/Fkbp1b/Slc8a1/Snca/Trpm2
3-day post-SCI group vs. the control group	BP	GO:0030856	regulation of epithelial cell differentiation	6/222	0.0019381	0.0068182	0.0033422	Apc/Btg1/Ezh2/Id1/Tnfrsf1a/Xdh
3-day post-SCI group vs. the control group	BP	GO:0048015	phosphatidylinositol-mediated signaling	6/222	0.0019381	0.0068182	0.0033422	Cat/Ezr/Jak2/Pdgfra/Plekha1/Sirt1
3-day post-SCI group vs. the control group	BP	GO:0050768	negative regulation of neurogenesis	6/222	0.0019381	0.0068182	0.0033422	Cdkn2b/Ctnna1/Id1/Ppp3ca/Rb1/Rest
3-day post-SCI group vs. the control group	BP	GO:0002768	immune response-regulating cell surface receptor s	9/222	0.0019522	0.0068619	0.0033637	Bax/Braf/Btk/Cd38/Ezr/Pawr/Plekha1/Rela/Zc3h12a
3-day post-SCI group vs. the control group	BP	GO:0018107	peptidyl-threonine phosphorylation	5/222	0.0019665	0.0068978	0.0033813	Atf2/Cdk1/Cdk10/Mapk8/Met
3-day post-SCI group vs. the control group	BP	GO:0032231	regulation of actin filament bundle assembly	5/222	0.0019665	0.0068978	0.0033813	Braf/Id1/Met/Nox4/Pxn
3-day post-SCI group vs. the control group	BP	GO:0042093	T-helper cell differentiation	4/222	0.0019721	0.0068978	0.0033813	Anxa1/Atp7a/Stat6/Zc3h12a
3-day post-SCI group vs. the control group	BP	GO:0060425	lung morphogenesis	4/222	0.0019721	0.0068978	0.0033813	Id1/Mapk3/Pdgfra/Rpl13a
3-day post-SCI group vs. the control group	BP	GO:1901224	positive regulation of NIK/NF-kappaB signaling	4/222	0.0019721	0.0068978	0.0033813	Ago1/Ago3/Rela/Tlr4
3-day post-SCI group vs. the control group	BP	GO:0030595	leukocyte chemotaxis	7/222	0.0019722	0.0068978	0.0033813	Aif1/Alox5/Anxa1/Ccr1/Mapk3/Rpl13a/Trpm2
3-day post-SCI group vs. the control group	BP	GO:0006694	steroid biosynthetic process	6/222	0.0019968	0.0069781	0.0034206	Dhcr24/Nfkb1/Ppargc1a/Prkaa2/Rest/Sirt1
3-day post-SCI group vs. the control group	BP	GO:1904659	glucose transmembrane transport	5/222	0.0020424	0.0071039	0.0034823	Braf/Mapk14/Met/Myc/Nfe2l2
3-day post-SCI group vs. the control group	BP	GO:0015850	organic hydroxy compound transport	8/222	0.0020466	0.0071039	0.0034823	Actb/Cd36/Chrna4/Myc/Pink1/Ptgs1/Sirt1/Snca
3-day post-SCI group vs. the control group	BP	GO:0002828	regulation of type 2 immune response	3/222	0.0020497	0.0071039	0.0034823	Anxa1/Arg1/Stat6
3-day post-SCI group vs. the control group	BP	GO:0006921	cellular component disassembly involved in execu	3/222	0.0020497	0.0071039	0.0034823	Bax/Casp3/Endog
3-day post-SCI group vs. the control group	BP	GO:0010155	regulation of proton transport	3/222	0.0020497	0.0071039	0.0034823	Alox5/Atp7a/Ppif
3-day post-SCI group vs. the control group	BP	GO:0033598	mammary gland epithelial cell proliferation	3/222	0.0020497	0.0071039	0.0034823	Bax/Gpx1/Stat6
3-day post-SCI group vs. the control group	BP	GO:0050999	regulation of nitric-oxide synthase activity	3/222	0.0020497	0.0071039	0.0034823	Dhfr/Gch1/S100a1
3-day post-SCI group vs. the control group	BP	GO:0062149	detection of stimulus involved in sensory percepti	3/222	0.0020497	0.0071039	0.0034823	Adora1/Pawr/Tlr4
3-day post-SCI group vs. the control group	BP	GO:1902253	regulation of intrinsic apoptotic signaling pathway	3/222	0.0020497	0.0071039	0.0034823	Myc/Rrm2b/Sirt1
3-day post-SCI group vs. the control group	BP	GO:1905314	semi-lunar valve development	3/222	0.0020497	0.0071039	0.0034823	Nfatc1/Rb1/Tnfrsf1a

3-day post-SCI group vs. the control group	BP	GO:0002294	CD4-positive, alpha-beta T cell differentiation inv	4/222	0.0020792	0.0072002	0.0035295	Anxa1/Atp7a/Stat6/Zc3h12a
3-day post-SCI group vs. the control group	BP	GO:0042770	signal transduction in response to DNA damage	6/222	0.0021183	0.0073133	0.0035849	Atf2/Atr/Cdk1/Ier3/Mapk14/Sirt1
3-day post-SCI group vs. the control group	BP	GO:0048017	inositol lipid-mediated signaling	6/222	0.0021183	0.0073133	0.0035849	Cat/Ezr/Jak2/Pdgfra/Plekha1/Sirt1
3-day post-SCI group vs. the control group	BP	GO:0008645	hexose transmembrane transport	5/222	0.0021205	0.0073133	0.0035849	Braf/Mapk14/Met/Myc/Nfe2l2
3-day post-SCI group vs. the control group	BP	GO:0034766	negative regulation of ion transmembrane transpor	5/222	0.0021205	0.0073133	0.0035849	Atp7a/Fkbp1b/Ppif/Ppp3ca/Ubqln1
3-day post-SCI group vs. the control group	BP	GO:0044070	regulation of anion transport	5/222	0.0021205	0.0073133	0.0035849	Adora1/Arg1/Map2k6/Ripk1/Snca
3-day post-SCI group vs. the control group	BP	GO:0050920	regulation of chemotaxis	7/222	0.0021227	0.0073149	0.0035857	Aif1/Ccr1/Cttn/Hspb1/Mapk3/Met/Pdgfra
3-day post-SCI group vs. the control group	BP	GO:0043161	proteasome-mediated ubiquitin-dependent protein	10/222	0.0021688	0.0074676	0.0036606	Agap3/Apc/Cdc20/Fbxw7/Gclc/Mapk8/Nfe2l2/Sirt1/Ube2a/Ubqln1
3-day post-SCI group vs. the control group	BP	GO:0050679	positive regulation of epithelial cell proliferation	7/222	0.0021748	0.007476	0.0036647	Arg1/Atp7a/Hmox1/Id1/Jun/Myc/Sirt1
3-day post-SCI group vs. the control group	BP	GO:0051651	maintenance of location in cell	7/222	0.0021748	0.007476	0.0036647	Bax/Fkbp1b/Pink1/Slc8a1/Snca/Tnrc6a/Trpm2
3-day post-SCI group vs. the control group	BP	GO:0046661	male sex differentiation	6/222	0.0021811	0.0074855	0.0036694	Bax/Dhcr24/Pdgfra/Plekha1/Prdx4/Ybx3
3-day post-SCI group vs. the control group	BP	GO:0051961	negative regulation of nervous system developmer	6/222	0.0021811	0.0074855	0.0036694	Cdkn2b/Ctnna1/Id1/Ppp3ca/Rb1/Rest
3-day post-SCI group vs. the control group	BP	GO:0002293	alpha-beta T cell differentiation involved in immu	4/222	0.0021903	0.0075107	0.0036817	Anxa1/Atp7a/Stat6/Zc3h12a
3-day post-SCI group vs. the control group	BP	GO:0038061	NIK/NF-kappaB signaling	5/222	0.0022008	0.0075284	0.0036904	Ago1/Ago3/Rela/Tlr4/Zc3h12a
3-day post-SCI group vs. the control group	BP	GO:0043401	steroid hormone mediated signaling pathway	5/222	0.0022008	0.0075284	0.0036904	Hdac1/Jak2/Parp1/Ppargc1b/Sirt1
3-day post-SCI group vs. the control group	BP	GO:0051897	positive regulation of protein kinase B signaling	5/222	0.0022008	0.0075284	0.0036904	Axl/Gpx1/Hbegf/Met/Nox4
3-day post-SCI group vs. the control group	BP	GO:0051604	protein maturation	9/222	0.0022222	0.0075957	0.0037234	Adam9/Bmp1/Casp3/Ctsl/Dhcr24/Myc/Parp1/Pawr/Prdx4
3-day post-SCI group vs. the control group	BP	GO:0045165	cell fate commitment	8/222	0.0022249	0.0075987	0.0037248	Apc/Braf/Casp3/Ctsl/Hdac1/Rbbp7/Smad1/Stat6
3-day post-SCI group vs. the control group	BP	GO:0007007	inner mitochondrial membrane organization	3/222	0.0022354	0.007616	0.0037333	Bax/Myc/Pink1
3-day post-SCI group vs. the control group	BP	GO:0055094	response to lipoprotein particle	3/222	0.0022354	0.007616	0.0037333	Cd36/Tlr4/Tlr6
3-day post-SCI group vs. the control group	BP	GO:1901186	positive regulation of ERBB signaling pathway	3/222	0.0022354	0.007616	0.0037333	Adora1/Fbxw7/Hbegf
3-day post-SCI group vs. the control group	BP	GO:0042113	B cell activation	10/222	0.0022413	0.00763	0.0037402	Bax/Btk/Casp3/Cd38/Chrna4/Ezh2/Nfatc1/Pawr/Stat6/Tlr4
3-day post-SCI group vs. the control group	BP	GO:0002685	regulation of leukocyte migration	7/222	0.002282	0.007762	0.0038049	Adora1/Aif1/Anxa1/Ccr1/Mapk3/Pawr/Ripk3
3-day post-SCI group vs. the control group	BP	GO:0002287	alpha-beta T cell activation involved in immune re	4/222	0.0023053	0.0078288	0.0038377	Anxa1/Atp7a/Stat6/Zc3h12a
3-day post-SCI group vs. the control group	BP	GO:2000134	negative regulation of G1/S transition of mitotic c	4/222	0.0023053	0.0078288	0.0038377	Apc/Cdkn2b/Ezh2/Rb1
3-day post-SCI group vs. the control group	BP	GO:0002062	chondrocyte differentiation	5/222	0.0023679	0.0080218	0.0039322	Atf2/Hif1a/Mapk14/Rb1/Rela
3-day post-SCI group vs. the control group	BP	GO:0006413	translational initiation	5/222	0.0023679	0.0080218	0.0039322	Eif2s1/Impact/Paip1/Ppp1r15b/Rpl13a
3-day post-SCI group vs. the control group	BP	GO:0051209	release of sequestered calcium ion into cytosol	5/222	0.0023679	0.0080218	0.0039322	Bax/Fkbp1b/Slc8a1/Snca/Trpm2
3-day post-SCI group vs. the control group	BP	GO:0010970	transport along microtubule	6/222	0.002378	0.0080496	0.0039459	Dst/Hif1a/Hspb1/Mapt/Sfpq/Stau2
3-day post-SCI group vs. the control group	BP	GO:0030324	lung development	7/222	0.0023931	0.0080944	0.0039678	Atp7a/Fbxw7/Id1/Mapk3/Pdgfra/Ppp1ca/Rpl13a
3-day post-SCI group vs. the control group	BP	GO:0048662	negative regulation of smooth muscle cell prolifera	4/222	0.0024245	0.0081904	0.0040149	Aif1/Hmox1/Ppargc1a/Sod2
3-day post-SCI group vs. the control group	BP	GO:0002719	negative regulation of cytokine production involve	3/222	0.0024313	0.0081904	0.0040149	Arg1/Axl/Hmox1
3-day post-SCI group vs. the control group	BP	GO:0006308	DNA catabolic process	3/222	0.0024313	0.0081904	0.0040149	Bax/Casp3/Endog
3-day post-SCI group vs. the control group	BP	GO:0006767	water-soluble vitamin metabolic process	3/222	0.0024313	0.0081904	0.0040149	Dhfr/Gclc/Slc23a2
3-day post-SCI group vs. the control group	BP	GO:0048679	regulation of axon regeneration	3/222	0.0024313	0.0081904	0.0040149	Braf/Fkbp1b/Stk24
3-day post-SCI group vs. the control group	BP	GO:0022600	digestive system process	5/222	0.0024548	0.0082499	0.0040441	Cd36/Ezr/Pawr/Ppp3ca/Tlr4
3-day post-SCI group vs. the control group	BP	GO:0031570	DNA integrity checkpoint signaling	5/222	0.0024548	0.0082499	0.0040441	Atf2/Atr/Cdk1/Ier3/Mapk14
3-day post-SCI group vs. the control group	BP	GO:0051283	negative regulation of sequestering of calcium ion	5/222	0.0024548	0.0082499	0.0040441	Bax/Fkbp1b/Slc8a1/Snca/Trpm2
3-day post-SCI group vs. the control group	BP	GO:0007596	blood coagulation	6/222	0.0025164	0.0083852	0.0041104	Axl/Cd36/Jak2/Nfe2l2/Pdgfra/Tlr4

3-day post-SCI group vs. the control group	BP	GO:0040029	epigenetic regulation of gene expression	6/222	0.0025164	0.0083852	0.0041104	Eed/Ezh2/Gpx1/Hdac1/Rb1/Sirt1
3-day post-SCI group vs. the control group	BP	GO:0006971	hypotonic response	2/222	0.0025309	0.0083852	0.0041104	Mylk/Slc4a11
3-day post-SCI group vs. the control group	BP	GO:0009650	UV protection	2/222	0.0025309	0.0083852	0.0041104	Cat/Gpx1
3-day post-SCI group vs. the control group	BP	GO:0010501	RNA secondary structure unwinding	2/222	0.0025309	0.0083852	0.0041104	Ago1/Ago3
3-day post-SCI group vs. the control group	BP	GO:0010764	negative regulation of fibroblast migration	2/222	0.0025309	0.0083852	0.0041104	Braf/Cygb
3-day post-SCI group vs. the control group	BP	GO:0032225	regulation of synaptic transmission, dopaminergic	2/222	0.0025309	0.0083852	0.0041104	Pink1/Ptgs2
3-day post-SCI group vs. the control group	BP	GO:0033034	positive regulation of myeloid cell apoptotic proce	2/222	0.0025309	0.0083852	0.0041104	Anxa1/Sirt1
3-day post-SCI group vs. the control group	BP	GO:0061687	detoxification of inorganic compound	2/222	0.0025309	0.0083852	0.0041104	Atp7a/Cat
3-day post-SCI group vs. the control group	BP	GO:0070922	RISC complex assembly	2/222	0.0025309	0.0083852	0.0041104	Ago1/Ago3
3-day post-SCI group vs. the control group	BP	GO:0070933	histone H4 deacetylation	2/222	0.0025309	0.0083852	0.0041104	Hdac1/Rest
3-day post-SCI group vs. the control group	BP	GO:0071257	cellular response to electrical stimulus	2/222	0.0025309	0.0083852	0.0041104	Actb/Rest
3-day post-SCI group vs. the control group	BP	GO:0071362	cellular response to ether	2/222	0.0025309	0.0083852	0.0041104	Cdk4/Zc3h12a
3-day post-SCI group vs. the control group	BP	GO:0090269	fibroblast growth factor production	2/222	0.0025309	0.0083852	0.0041104	Aif1/Ptgs2
3-day post-SCI group vs. the control group	BP	GO:0090270	regulation of fibroblast growth factor production	2/222	0.0025309	0.0083852	0.0041104	Aif1/Ptgs2
3-day post-SCI group vs. the control group	BP	GO:0106049	regulation of cellular response to osmotic stress	2/222	0.0025309	0.0083852	0.0041104	Ptgs2/Ybx3
3-day post-SCI group vs. the control group	BP	GO:1901858	regulation of mitochondrial DNA metabolic proce	2/222	0.0025309	0.0083852	0.0041104	Endog/Ppargc1a
3-day post-SCI group vs. the control group	BP	GO:1904959	regulation of cytochrome-c oxidase activity	2/222	0.0025309	0.0083852	0.0041104	Alox5/Atp7a
3-day post-SCI group vs. the control group	BP	GO:0046928	regulation of neurotransmitter secretion	5/222	0.002544	0.008422	0.0041284	Atp2a2/Braf/Chrna4/Snca/Syp
3-day post-SCI group vs. the control group	BP	GO:0050432	catecholamine secretion	4/222	0.0025479	0.0084282	0.0041314	Chrna4/Pink1/Ptgs1/Snca
3-day post-SCI group vs. the control group	BP	GO:0002819	regulation of adaptive immune response	7/222	0.0025677	0.0084804	0.0041571	Anxa1/Arg1/Jak2/Ripk3/Sirt1/Stat6/Zc3h12a
3-day post-SCI group vs. the control group	BP	GO:0030323	respiratory tube development	7/222	0.0025677	0.0084804	0.0041571	Atp7a/Fbxw7/Id1/Mapk3/Pdgfra/Ppp1ca/Rpl13a
3-day post-SCI group vs. the control group	BP	GO:0018210	peptidyl-threonine modification	5/222	0.0026356	0.0086631	0.0042466	Atf2/Cdk1/Cdk10/Mapk8/Met
3-day post-SCI group vs. the control group	BP	GO:0051282	regulation of sequestering of calcium ion	5/222	0.0026356	0.0086631	0.0042466	Bax/Fkbp1b/Slc8a1/Snca/Trpm2
3-day post-SCI group vs. the control group	BP	GO:0000731	DNA synthesis involved in DNA repair	3/222	0.0026374	0.0086631	0.0042466	Pcna/Rrm2b/Sirt1
3-day post-SCI group vs. the control group	BP	GO:0010573	vascular endothelial growth factor production	3/222	0.0026374	0.0086631	0.0042466	Cyp1b1/Hif1a/Ptgs2
3-day post-SCI group vs. the control group	BP	GO:0014912	negative regulation of smooth muscle cell migratic	3/222	0.0026374	0.0086631	0.0042466	Aif1/Nfe2l2/Ppargc1a
3-day post-SCI group vs. the control group	BP	GO:0070316	regulation of G0 to G1 transition	3/222	0.0026374	0.0086631	0.0042466	Actb/Cdkn2b/Rrm2b
3-day post-SCI group vs. the control group	BP	GO:0090312	positive regulation of protein deacetylation	3/222	0.0026374	0.0086631	0.0042466	Pink1/Ripk3/Sirt1
3-day post-SCI group vs. the control group	BP	GO:0031214	biomineral tissue development	6/222	0.0026609	0.0087334	0.0042811	Alox5/Ccr1/Hif1a/Ptgs2/Rxrb/Slc8a1
3-day post-SCI group vs. the control group	BP	GO:0010507	negative regulation of autophagy	4/222	0.0026755	0.0087608	0.0042945	Hmox1/Mcl1/Pink1/Snca
3-day post-SCI group vs. the control group	BP	GO:0010611	regulation of cardiac muscle hypertrophy	4/222	0.0026755	0.0087608	0.0042945	Parp1/Ppp3ca/Sirt1/Tnfrsf1a
3-day post-SCI group vs. the control group	BP	GO:1905897	regulation of response to endoplasmic reticulum st	4/222	0.0026755	0.0087608	0.0042945	Alox5/Bax/Sirt1/Ubqln1
3-day post-SCI group vs. the control group	BP	GO:0048167	regulation of synaptic plasticity	9/222	0.002706	0.0088539	0.0043402	Adora1/Braf/Cd38/Cdc20/Mapt/Ptgs2/Snca/Stau2/Syp
3-day post-SCI group vs. the control group	BP	GO:0000956	nuclear-transcribed mRNA catabolic process	5/222	0.0027295	0.0089085	0.0043669	Ago1/Paip1/Tnrc6a/Tnrc6c/Zc3h12a
3-day post-SCI group vs. the control group	BP	GO:0032963	collagen metabolic process	5/222	0.0027295	0.0089085	0.0043669	Arg1/Ctsl/Cygb/Hif1a/Id1
3-day post-SCI group vs. the control group	BP	GO:0046620	regulation of organ growth	5/222	0.0027295	0.0089085	0.0043669	Apc/Cdk1/Mapk14/Sirt1/Ybx3
3-day post-SCI group vs. the control group	BP	GO:0007599	hemostasis	6/222	0.0027354	0.0089085	0.0043669	Axl/Cd36/Jak2/Nfe2l2/Pdgfra/Tlr4
3-day post-SCI group vs. the control group	BP	GO:0050817	coagulation	6/222	0.0027354	0.0089085	0.0043669	Axl/Cd36/Jak2/Nfe2l2/Pdgfra/Tlr4
3-day post-SCI group vs. the control group	BP	GO:0050906	detection of stimulus involved in sensory percepti	6/222	0.0027354	0.0089085	0.0043669	Adora1/Lpo/Myc/Pawr/Rest/Tlr4
3-day post-SCI group vs. the control group	BP	GO:0046847	filopodium assembly	4/222	0.0028074	0.0091358	0.0044783	Ezr/Itga6/Stau2/Trpm2

3-day post-SCI group vs. the control group	BP	GO:0097529	myeloid leukocyte migration	7/222	0.0028155	0.0091551	0.0044878	Aif1/Anxa1/Ccr1/Mapk3/Pawr/Rpl13a/Sirpa
3-day post-SCI group vs. the control group	BP	GO:0031109	microtubule polymerization or depolymerization	5/222	0.0028258	0.0091604	0.0044904	Apc/Mapk8/Mapt/Met/Snca
3-day post-SCI group vs. the control group	BP	GO:0032649	regulation of interferon-gamma production	5/222	0.0028258	0.0091604	0.0044904	Axl/Jak2/Ripk3/Tlr4/Zc3h12a
3-day post-SCI group vs. the control group	BP	GO:0051208	sequestering of calcium ion	5/222	0.0028258	0.0091604	0.0044904	Bax/Fkbp1b/Slc8a1/Snca/Trpm2
3-day post-SCI group vs. the control group	BP	GO:0090277	positive regulation of peptide hormone secretion	5/222	0.0028258	0.0091604	0.0044904	Cd38/Hif1a/Jak2/Sirt1/Trpm2
3-day post-SCI group vs. the control group	BP	GO:0010667	negative regulation of cardiac muscle cell apoptoti	3/222	0.002854	0.0091953	0.0045075	Jak2/Nfe2l2/Sirt1
3-day post-SCI group vs. the control group	BP	GO:0033866	nucleoside bisphosphate biosynthetic process	3/222	0.002854	0.0091953	0.0045075	Pdk1/Pdk2/Snca
3-day post-SCI group vs. the control group	BP	GO:0034030	ribonucleoside bisphosphate biosynthetic process	3/222	0.002854	0.0091953	0.0045075	Pdk1/Pdk2/Snca
3-day post-SCI group vs. the control group	BP	GO:0034033	purine nucleoside bisphosphate biosynthetic proce	3/222	0.002854	0.0091953	0.0045075	Pdk1/Pdk2/Snca
3-day post-SCI group vs. the control group	BP	GO:0035329	hippo signaling	3/222	0.002854	0.0091953	0.0045075	Map2k3/Mapk14/Mark3
3-day post-SCI group vs. the control group	BP	GO:0045023	G0 to G1 transition	3/222	0.002854	0.0091953	0.0045075	Actb/Cdkn2b/Rrm2b
3-day post-SCI group vs. the control group	BP	GO:0051953	negative regulation of amine transport	3/222	0.002854	0.0091953	0.0045075	Adora1/Ptgs1/Snca
3-day post-SCI group vs. the control group	BP	GO:0071402	cellular response to lipoprotein particle stimulus	3/222	0.002854	0.0091953	0.0045075	Cd36/Tlr4/Tlr6
3-day post-SCI group vs. the control group	BP	GO:0002244	hematopoietic progenitor cell differentiation	6/222	0.0028891	0.0093012	0.0045594	Atf2/Braf/Nfe2l2/Pdgfra/Rest/Sirpa
3-day post-SCI group vs. the control group	BP	GO:1903052	positive regulation of proteolysis involved in prote	5/222	0.0029245	0.0094081	0.0046118	Cdc20/Fbxw7/Gclc/Mapk8/Ubqln1
3-day post-SCI group vs. the control group	BP	GO:0002064	epithelial cell development	7/222	0.002946	0.00947	0.0046421	Ezr/Gpx1/Hif1a/Id1/Il6st/Met/Tnfrsf1a
3-day post-SCI group vs. the control group	BP	GO:0007162	negative regulation of cell adhesion	8/222	0.0030049	0.0096521	0.0047314	Anxa1/Arg1/Casp3/Cyp1b1/Hspb1/Jak2/Pawr/Zc3h12a
3-day post-SCI group vs. the control group	BP	GO:0006402	mRNA catabolic process	7/222	0.003013	0.0096706	0.0047405	Ago1/Ago3/Mapkapk2/Paip1/Tnrc6a/Tnrc6c/Zc3h12a
3-day post-SCI group vs. the control group	BP	GO:0045807	positive regulation of endocytosis	5/222	0.0030257	0.0096928	0.0047514	Amph/Axl/Cd36/Ppp3ca/Snca
3-day post-SCI group vs. the control group	BP	GO:0018401	peptidyl-proline hydroxylation to 4-hydroxy-L-pro	2/222	0.0030777	0.0096928	0.0047514	P4hb/Prdx4
3-day post-SCI group vs. the control group	BP	GO:0019372	lipoxygenase pathway	2/222	0.0030777	0.0096928	0.0047514	Alox5/Gpx1
3-day post-SCI group vs. the control group	BP	GO:0032353	negative regulation of hormone biosynthetic proce	2/222	0.0030777	0.0096928	0.0047514	Nfkb1/Rest
3-day post-SCI group vs. the control group	BP	GO:0045602	negative regulation of endothelial cell differentiati	2/222	0.0030777	0.0096928	0.0047514	Id1/Xdh
3-day post-SCI group vs. the control group	BP	GO:0050774	negative regulation of dendrite morphogenesis	2/222	0.0030777	0.0096928	0.0047514	Id1/Ppp3ca
3-day post-SCI group vs. the control group	BP	GO:0060056	mammary gland involution	2/222	0.0030777	0.0096928	0.0047514	Bax/Nfkb1
3-day post-SCI group vs. the control group	BP	GO:0060068	vagina development	2/222	0.0030777	0.0096928	0.0047514	Axl/Bax
3-day post-SCI group vs. the control group	BP	GO:0070493	thrombin-activated receptor signaling pathway	2/222	0.0030777	0.0096928	0.0047514	Met/Snca
3-day post-SCI group vs. the control group	BP	GO:0071415	cellular response to purine-containing compound	2/222	0.0030777	0.0096928	0.0047514	Slc8a1/Trpm2
3-day post-SCI group vs. the control group	BP	GO:0097421	liver regeneration	2/222	0.0030777	0.0096928	0.0047514	Ezh2/Hmox1
3-day post-SCI group vs. the control group	BP	GO:1900454	positive regulation of long-term synaptic depressic	2/222	0.0030777	0.0096928	0.0047514	Mapt/Stau2
3-day post-SCI group vs. the control group	BP	GO:1901029	negative regulation of mitochondrial outer membra	2/222	0.0030777	0.0096928	0.0047514	Gclc/Ier3
3-day post-SCI group vs. the control group	BP	GO:1902946	protein localization to early endosome	2/222	0.0030777	0.0096928	0.0047514	Ezr/Mgat3
3-day post-SCI group vs. the control group	BP	GO:1903599	positive regulation of autophagy of mitochondrion	2/222	0.0030777	0.0096928	0.0047514	Bnip3/Hif1a
3-day post-SCI group vs. the control group	BP	GO:1904732	regulation of electron transfer activity	2/222	0.0030777	0.0096928	0.0047514	Alox5/Atp7a
3-day post-SCI group vs. the control group	BP	GO:1905097	regulation of guanyl-nucleotide exchange factor ac	2/222	0.0030777	0.0096928	0.0047514	Eif2s1/Met
3-day post-SCI group vs. the control group	BP	GO:2001280	positive regulation of unsaturated fatty acid biosyn	2/222	0.0030777	0.0096928	0.0047514	Anxa1/Ptgs2
3-day post-SCI group vs. the control group	BP	GO:0009069	serine family amino acid metabolic process	3/222	0.0030813	0.0096928	0.0047514	Dhfr/Gclc/Txnrd1
3-day post-SCI group vs. the control group	BP	GO:0009595	detection of biotic stimulus	3/222	0.0030813	0.0096928	0.0047514	Atf2/Tlr4/Tlr6
3-day post-SCI group vs. the control group	BP	GO:0031281	positive regulation of cyclase activity	3/222	0.0030813	0.0096928	0.0047514	Mapk14/Mapk3/Mapk8
3-day post-SCI group vs. the control group	BP	GO:0050869	negative regulation of B cell activation	3/222	0.0030813	0.0096928	0.0047514	Btk/Casp3/Pawr

3-day post-SCI group vs. the control group	BP	GO:0055090	acylglycerol homeostasis	3/222	0.0030813	0.0096928	0.0047514	Adora1/Pnpla8/Sirt1
3-day post-SCI group vs. the control group	BP	GO:0070328	triglyceride homeostasis	3/222	0.0030813	0.0096928	0.0047514	Adora1/Pnpla8/Sirt1
3-day post-SCI group vs. the control group	BP	GO:1903580	positive regulation of ATP metabolic process	3/222	0.0030813	0.0096928	0.0047514	Myc/Ppargc1a/Prkaa2
3-day post-SCI group vs. the control group	BP	GO:0002292	T cell differentiation involved in immune response	4/222	0.0030844	0.0096928	0.0047514	Anxa1/Atp7a/Stat6/Zc3h12a
3-day post-SCI group vs. the control group	BP	GO:0014743	regulation of muscle hypertrophy	4/222	0.0030844	0.0096928	0.0047514	Parp1/Ppp3ca/Sirt1/Tnfrsf1a
3-day post-SCI group vs. the control group	BP	GO:0061097	regulation of protein tyrosine kinase activity	4/222	0.0030844	0.0096928	0.0047514	Adora1/Fbxw7/Hbegf/Nox4
3-day post-SCI group vs. the control group	BP	GO:0061136	regulation of proteasomal protein catabolic proces	6/222	0.0032158	0.0100982	0.0049501	Cdc20/Fbxw7/Gclc/Gpx1/Mapk8/Ubqln1
3-day post-SCI group vs. the control group	BP	GO:0045453	bone resorption	4/222	0.0032297	0.0101342	0.0049677	Cd38/Nox4/Ppargc1b/Tpp1
3-day post-SCI group vs. the control group	BP	GO:0008286	insulin receptor signaling pathway	5/222	0.0032357	0.0101381	0.0049696	Apc/Cdk4/Pdk2/Rela/Sirt1
3-day post-SCI group vs. the control group	BP	GO:0033138	positive regulation of peptidyl-serine phosphorylat	5/222	0.0032357	0.0101381	0.0049696	Braf/Met/Pink1/Ptgs2/Snca
3-day post-SCI group vs. the control group	BP	GO:0007548	sex differentiation	8/222	0.0032452	0.0101602	0.0049805	Axl/Bax/Dhcr24/Pdgfra/Plekha1/Prdx4/Sirt1/Ybx3
3-day post-SCI group vs. the control group	BP	GO:0007612	learning	6/222	0.0033016	0.0103138	0.0050558	Amph/Braf/Hif1a/Jun/Ptgs1/Ptgs2
3-day post-SCI group vs. the control group	BP	GO:0051260	protein homooligomerization	6/222	0.0033016	0.0103138	0.0050558	Ect2/Mapt/Pxdn/Rnf112/Sod2/Trpm2
3-day post-SCI group vs. the control group	BP	GO:1903305	regulation of regulated secretory pathway	6/222	0.0033016	0.0103138	0.0050558	Atp2a2/Braf/Chrna4/Hmox1/Rest/Syp
3-day post-SCI group vs. the control group	BP	GO:0008210	estrogen metabolic process	3/222	0.0033194	0.0103463	0.0050717	Cyp1b1/Pdgfra/Plekha1
3-day post-SCI group vs. the control group	BP	GO:0032885	regulation of polysaccharide biosynthetic process	3/222	0.0033194	0.0103463	0.0050717	Cd36/Nfkb1/Ppp1ca
3-day post-SCI group vs. the control group	BP	GO:0070570	regulation of neuron projection regeneration	3/222	0.0033194	0.0103463	0.0050717	Braf/Fkbp1b/Stk24
3-day post-SCI group vs. the control group	BP	GO:0001655	urogenital system development	9/222	0.0033815	0.0105321	0.0051628	Anxa1/Apc/Bax/Cat/Myc/Pdgfra/Ppp3ca/Rrm2b/Smad1
3-day post-SCI group vs. the control group	BP	GO:0045834	positive regulation of lipid metabolic process	6/222	0.0033891	0.010548	0.0051706	Adora1/Anxa1/Cd36/Pdgfra/Ppargc1a/Ptgs2
3-day post-SCI group vs. the control group	BP	GO:0019318	hexose metabolic process	7/222	0.0035152	0.0109325	0.005359	Cd36/Hif1a/Mapk14/Myc/Pdk1/Pdk2/Sirt1
3-day post-SCI group vs. the control group	BP	GO:0032088	negative regulation of NF-kappaB transcription fac	4/222	0.003534	0.0109506	0.0053679	Cat/Cyp1b1/Sirt1/Zc3h12a
3-day post-SCI group vs. the control group	BP	GO:0033209	tumor necrosis factor-mediated signaling pathway	4/222	0.003534	0.0109506	0.0053679	Jak2/Rela/Ripk1/Tnfrsf1a
3-day post-SCI group vs. the control group	BP	GO:0045123	cellular extravasation	4/222	0.003534	0.0109506	0.0053679	Pawr/Ripk3/Sirpa/Vcam1
3-day post-SCI group vs. the control group	BP	GO:0051145	smooth muscle cell differentiation	4/222	0.003534	0.0109506	0.0053679	Nfatc1/Rcan1/Sirt1/Sod2
3-day post-SCI group vs. the control group	BP	GO:1901796	regulation of signal transduction by p53 class med	4/222	0.003534	0.0109506	0.0053679	Atr/Myc/Rrm2b/Sirt1
3-day post-SCI group vs. the control group	BP	GO:0002931	response to ischemia	3/222	0.0035684	0.0110165	0.0054002	Ppif/Rcan1/Rest
3-day post-SCI group vs. the control group	BP	GO:0010664	negative regulation of striated muscle cell apoptoti	3/222	0.0035684	0.0110165	0.0054002	Jak2/Nfe2l2/Sirt1
3-day post-SCI group vs. the control group	BP	GO:1901020	negative regulation of calcium ion transmembrane	3/222	0.0035684	0.0110165	0.0054002	Fkbp1b/Ppp3ca/Ubqln1
3-day post-SCI group vs. the control group	BP	GO:1903670	regulation of sprouting angiogenesis	3/222	0.0035684	0.0110165	0.0054002	Alox5/S100a1/Smad1
3-day post-SCI group vs. the control group	BP	GO:1904707	positive regulation of vascular associated smooth r	3/222	0.0035684	0.0110165	0.0054002	Jak2/Jun/Ppargc1a
3-day post-SCI group vs. the control group	BP	GO:0048754	branching morphogenesis of an epithelial tube	6/222	0.003662	0.0112211	0.0055005	Met/Myc/Nfatc1/Pdgfra/Ppp1ca/Pxn
3-day post-SCI group vs. the control group	BP	GO:0006086	acetyl-CoA biosynthetic process from pyruvate	2/222	0.0036746	0.0112211	0.0055005	Pdk1/Pdk2
3-day post-SCI group vs. the control group	BP	GO:0007183	SMAD protein complex assembly	2/222	0.0036746	0.0112211	0.0055005	Parp1/Smad1
3-day post-SCI group vs. the control group	BP	GO:0009404	toxin metabolic process	2/222	0.0036746	0.0112211	0.0055005	Cyp1b1/Nfe2l2
3-day post-SCI group vs. the control group	BP	GO:0010572	positive regulation of platelet activation	2/222	0.0036746	0.0112211	0.0055005	Jak2/Tlr4
3-day post-SCI group vs. the control group	BP	GO:0010728	regulation of hydrogen peroxide biosynthetic proce	2/222	0.0036746	0.0112211	0.0055005	Nox4/Sod2
3-day post-SCI group vs. the control group	BP	GO:0015911	long-chain fatty acid import across plasma membra	2/222	0.0036746	0.0112211	0.0055005	Abcc1/Cd36
3-day post-SCI group vs. the control group	BP	GO:0032351	negative regulation of hormone metabolic process	2/222	0.0036746	0.0112211	0.0055005	Nfkb1/Rest
3-day post-SCI group vs. the control group	BP	GO:0032490	detection of molecule of bacterial origin	2/222	0.0036746	0.0112211	0.0055005	Tlr4/Tlr6
3-day post-SCI group vs. the control group	BP	GO:0043653	mitochondrial fragmentation involved in apoptotic	2/222	0.0036746	0.0112211	0.0055005	Bax/Bnip3

3-day post-SCI group vs. the control group	BP	GO:0061418	regulation of transcription from RNA polymerase I	2/222	0.0036746	0.0112211	0.0055005	Hif1a/Nfe2l2
3-day post-SCI group vs. the control group	BP	GO:0070262	peptidyl-serine dephosphorylation	2/222	0.0036746	0.0112211	0.0055005	Ppp1r15b/Ppp3ca
3-day post-SCI group vs. the control group	BP	GO:0090184	positive regulation of kidney development	2/222	0.0036746	0.0112211	0.0055005	Myc/Ppp3ca
3-day post-SCI group vs. the control group	BP	GO:0097201	negative regulation of transcription from RNA pol	2/222	0.0036746	0.0112211	0.0055005	Impact/Jun
3-day post-SCI group vs. the control group	BP	GO:1990253	cellular response to leucine starvation	2/222	0.0036746	0.0112211	0.0055005	Atf2/Impact
3-day post-SCI group vs. the control group	BP	GO:0045739	positive regulation of DNA repair	5/222	0.0036868	0.0112454	0.0055124	Actb/Mgmt/Parp1/Pcna/Sirt1
3-day post-SCI group vs. the control group	BP	GO:0006766	vitamin metabolic process	4/222	0.0036933	0.0112454	0.0055124	Dhfr/Gclc/Nfkb1/Slc23a2
3-day post-SCI group vs. the control group	BP	GO:0051279	regulation of release of sequestered calcium ion in	4/222	0.0036933	0.0112454	0.0055124	Bax/Fkbp1b/Slc8a1/Snca
3-day post-SCI group vs. the control group	BP	GO:1902807	negative regulation of cell cycle G1/S phase transi	4/222	0.0036933	0.0112454	0.0055124	Apc/Cdkn2b/Ezh2/Rb1
3-day post-SCI group vs. the control group	BP	GO:0008608	attachment of spindle microtubules to kinetochore	3/222	0.0038286	0.0116237	0.0056979	Apc/Ect2/Rb1
3-day post-SCI group vs. the control group	BP	GO:0010613	positive regulation of cardiac muscle hypertrophy	3/222	0.0038286	0.0116237	0.0056979	Parp1/Ppp3ca/Sirt1
3-day post-SCI group vs. the control group	BP	GO:0032689	negative regulation of interferon-gamma productio	3/222	0.0038286	0.0116237	0.0056979	Axl/Tlr4/Zc3h12a
3-day post-SCI group vs. the control group	BP	GO:0043368	positive T cell selection	3/222	0.0038286	0.0116237	0.0056979	Braf/Ctsl/Stat6
3-day post-SCI group vs. the control group	BP	GO:0050810	regulation of steroid biosynthetic process	4/222	0.0038573	0.0116774	0.0057242	Nfkb1/Ppargc1a/Rest/Sirt1
3-day post-SCI group vs. the control group	BP	GO:0055021	regulation of cardiac muscle tissue growth	4/222	0.0038573	0.0116774	0.0057242	Apc/Cdk1/Mapk14/Sirt1
3-day post-SCI group vs. the control group	BP	GO:0061912	selective autophagy	4/222	0.0038573	0.0116774	0.0057242	Htra2/Mapk3/Pink1/Ubqln1
3-day post-SCI group vs. the control group	BP	GO:2000756	regulation of peptidyl-lysine acetylation	4/222	0.0038573	0.0116774	0.0057242	Mapk3/Prkaa2/Sirt1/Snca
3-day post-SCI group vs. the control group	BP	GO:0009791	post-embryonic development	5/222	0.0039286	0.0118844	0.0058257	Bax/Jak2/Plekha1/Slc8a1/Sod2
3-day post-SCI group vs. the control group	BP	GO:0043154	negative regulation of cysteine-type endopeptidase	4/222	0.0040263	0.0121625	0.005962	Dhcr24/Gpx1/Ptgs2/Snca
3-day post-SCI group vs. the control group	BP	GO:2000300	regulation of synaptic vesicle exocytosis	4/222	0.0040263	0.0121625	0.005962	Atp2a2/Braf/Chrna4/Syp
3-day post-SCI group vs. the control group	BP	GO:0009582	detection of abiotic stimulus	5/222	0.0040536	0.0122363	0.0059981	Adora1/Myc/Pawr/Rest/Tlr4
3-day post-SCI group vs. the control group	BP	GO:0014059	regulation of dopamine secretion	3/222	0.0041	0.012341	0.0060495	Chrna4/Pink1/Snca
3-day post-SCI group vs. the control group	BP	GO:0014742	positive regulation of muscle hypertrophy	3/222	0.0041	0.012341	0.0060495	Parp1/Ppp3ca/Sirt1
3-day post-SCI group vs. the control group	BP	GO:0038084	vascular endothelial growth factor signaling pathw	3/222	0.0041	0.012341	0.0060495	Hspb1/Pdgfra/Xdh
3-day post-SCI group vs. the control group	BP	GO:1900181	negative regulation of protein localization to nucle	3/222	0.0041	0.012341	0.0060495	Cd36/Ctnna1/Sirt1
3-day post-SCI group vs. the control group	BP	GO:2001257	regulation of cation channel activity	6/222	0.0041526	0.0124904	0.0061227	Chrna4/Fkbp1b/Ppargc1a/Ppp3ca/S100a1/Ubqln1
3-day post-SCI group vs. the control group	BP	GO:0010001	glial cell differentiation	7/222	0.0041646	0.0125175	0.006136	Eed/Hdac1/Il6st/Mapk3/Rela/Rnf112/Tlr4
3-day post-SCI group vs. the control group	BP	GO:0009581	detection of external stimulus	5/222	0.0041814	0.0125412	0.0061476	Adora1/Myc/Pawr/Rest/Tlr4
3-day post-SCI group vs. the control group	BP	GO:0016079	synaptic vesicle exocytosis	5/222	0.0041814	0.0125412	0.0061476	Atp2a2/Braf/Chrna4/Snca/Syp
3-day post-SCI group vs. the control group	BP	GO:0050852	T cell receptor signaling pathway	5/222	0.0041814	0.0125412	0.0061476	Braf/Ezr/Pawr/Rela/Zc3h12a
3-day post-SCI group vs. the control group	BP	GO:0002720	positive regulation of cytokine production involve	4/222	0.0042001	0.0125705	0.006162	Cd36/Mapkapk2/Sirt1/Tlr4
3-day post-SCI group vs. the control group	BP	GO:0051262	protein tetramerization	4/222	0.0042001	0.0125705	0.006162	Krt1/Snca/Sod2/Trpm2
3-day post-SCI group vs. the control group	BP	GO:0070228	regulation of lymphocyte apoptotic process	4/222	0.0042001	0.0125705	0.006162	Bax/Hif1a/Myc/Ripk3
3-day post-SCI group vs. the control group	BP	GO:0007033	vacuole organization	6/222	0.0042563	0.0127295	0.0062399	Atf2/Atp13a2/Atp2a2/Pink1/Tpp1/Ubqln1
3-day post-SCI group vs. the control group	BP	GO:0007586	digestion	5/222	0.0043121	0.0127417	0.0062459	Cd36/Ezr/Pawr/Ppp3ca/Tlr4
3-day post-SCI group vs. the control group	BP	GO:0010811	positive regulation of cell-substrate adhesion	5/222	0.0043121	0.0127417	0.0062459	Braf/Cd36/Jak2/Map4k4/P4hb
3-day post-SCI group vs. the control group	BP	GO:0000012	single strand break repair	2/222	0.0043209	0.0127417	0.0062459	Parp1/Sirt1
3-day post-SCI group vs. the control group	BP	GO:0002756	MyD88-independent toll-like receptor signaling pa	2/222	0.0043209	0.0127417	0.0062459	Tlr4/Tlr6
3-day post-SCI group vs. the control group	BP	GO:0006271	DNA strand elongation involved in DNA replicati	2/222	0.0043209	0.0127417	0.0062459	Mcm4/Pcna
3-day post-SCI group vs. the control group	BP	GO:0031340	positive regulation of vesicle fusion	2/222	0.0043209	0.0127417	0.0062459	Anxa1/Snca

3-day post-SCI group vs. the control group	BP	GO:0031915	positive regulation of synaptic plasticity	2/222	0.0043209	0.0127417	0.0062459	Cdc20/Ptgs2
3-day post-SCI group vs. the control group	BP	GO:0031953	negative regulation of protein autophosphorylation	2/222	0.0043209	0.0127417	0.0062459	Impact/Jun
3-day post-SCI group vs. the control group	BP	GO:0032060	bleb assembly	2/222	0.0043209	0.0127417	0.0062459	Mylk/Prdx6
3-day post-SCI group vs. the control group	BP	GO:0033327	Leydig cell differentiation	2/222	0.0043209	0.0127417	0.0062459	Pdgfra/Plekha1
3-day post-SCI group vs. the control group	BP	GO:0045472	response to ether	2/222	0.0043209	0.0127417	0.0062459	Cdk4/Zc3h12a
3-day post-SCI group vs. the control group	BP	GO:0060213	positive regulation of nuclear-transcribed mRNA p	2/222	0.0043209	0.0127417	0.0062459	Tnrc6a/Tnrc6c
3-day post-SCI group vs. the control group	BP	GO:0090085	regulation of protein deubiquitination	2/222	0.0043209	0.0127417	0.0062459	Tank/Zc3h12a
3-day post-SCI group vs. the control group	BP	GO:0097709	connective tissue replacement	2/222	0.0043209	0.0127417	0.0062459	Hif1a/Ppp3ca
3-day post-SCI group vs. the control group	BP	GO:0098935	dendritic transport	2/222	0.0043209	0.0127417	0.0062459	Sfpq/Stau2
3-day post-SCI group vs. the control group	BP	GO:0150065	regulation of deacetylase activity	2/222	0.0043209	0.0127417	0.0062459	Mapk8/Pink1
3-day post-SCI group vs. the control group	BP	GO:1902931	negative regulation of alcohol biosynthetic process	2/222	0.0043209	0.0127417	0.0062459	Nfkb1/Rest
3-day post-SCI group vs. the control group	BP	GO:1905668	positive regulation of protein localization to endos	2/222	0.0043209	0.0127417	0.0062459	Ezr/Mgat3
3-day post-SCI group vs. the control group	BP	GO:2000489	regulation of hepatic stellate cell activation	2/222	0.0043209	0.0127417	0.0062459	Cygb/Gclc
3-day post-SCI group vs. the control group	BP	GO:2000774	positive regulation of cellular senescence	2/222	0.0043209	0.0127417	0.0062459	Pawr/Sirt1
3-day post-SCI group vs. the control group	BP	GO:0048041	focal adhesion assembly	4/222	0.004379	0.0128703	0.006309	Cttn/Map4k4/Ptprk/Pxn
3-day post-SCI group vs. the control group	BP	GO:0051384	response to glucocorticoid	4/222	0.004379	0.0128703	0.006309	Adam9/Anxa1/Jak2/Rest
3-day post-SCI group vs. the control group	BP	GO:0097009	energy homeostasis	4/222	0.004379	0.0128703	0.006309	Cd36/Ppargc1a/Prkaa2/Sirt1
3-day post-SCI group vs. the control group	BP	GO:0042092	type 2 immune response	3/222	0.0043828	0.0128703	0.006309	Anxa1/Arg1/Stat6
3-day post-SCI group vs. the control group	BP	GO:0045981	positive regulation of nucleotide metabolic process	3/222	0.0043828	0.0128703	0.006309	Myc/Ppargc1a/Prkaa2
3-day post-SCI group vs. the control group	BP	GO:1900544	positive regulation of purine nucleotide metabolic	3/222	0.0043828	0.0128703	0.006309	Myc/Ppargc1a/Prkaa2
3-day post-SCI group vs. the control group	BP	GO:0001822	kidney development	8/222	0.0044371	0.0130185	0.0063816	Apc/Bax/Cat/Myc/Pdgfra/Ppp3ca/Rrm2b/Smad1
3-day post-SCI group vs. the control group	BP	GO:0000086	G2/M transition of mitotic cell cycle	5/222	0.0044456	0.0130185	0.0063816	Ccna2/Cdk1/Cdk4/Ier3/Rrm2b
3-day post-SCI group vs. the control group	BP	GO:0034754	cellular hormone metabolic process	5/222	0.0044456	0.0130185	0.0063816	Cyp1b1/Pdgfra/Plekha1/Ppargc1a/Rest
3-day post-SCI group vs. the control group	BP	GO:0051588	regulation of neurotransmitter transport	5/222	0.0044456	0.0130185	0.0063816	Atp2a2/Braf/Chrna4/Snca/Syp
3-day post-SCI group vs. the control group	BP	GO:0043648	dicarboxylic acid metabolic process	4/222	0.0045629	0.0133432	0.0065408	Apc/Dhfr/Gclc/Prodh
3-day post-SCI group vs. the control group	BP	GO:0061178	regulation of insulin secretion involved in cellular	4/222	0.0045629	0.0133432	0.0065408	Fkbp1b/Hif1a/Sirt1/Ucp2
3-day post-SCI group vs. the control group	BP	GO:0001764	neuron migration	6/222	0.0045788	0.0133717	0.0065547	Axl/Bax/Mapk8/Mapt/Met/Tbc1d24
3-day post-SCI group vs. the control group	BP	GO:0002698	negative regulation of immune effector process	5/222	0.0045821	0.0133717	0.0065547	Anxa1/Arg1/Axl/Hmox1/Zc3h12a
3-day post-SCI group vs. the control group	BP	GO:1905039	carboxylic acid transmembrane transport	5/222	0.0045821	0.0133717	0.0065547	Abcc1/Arg1/Cd36/Myc/Slc23a2
3-day post-SCI group vs. the control group	BP	GO:0001782	B cell homeostasis	3/222	0.0046772	0.0136302	0.0066815	Bax/Casp3/Hif1a
3-day post-SCI group vs. the control group	BP	GO:0014046	dopamine secretion	3/222	0.0046772	0.0136302	0.0066815	Chrna4/Pink1/Snca
3-day post-SCI group vs. the control group	BP	GO:0060541	respiratory system development	7/222	0.0047084	0.0137116	0.0067214	Atp7a/Fbxw7/Id1/Mapk3/Pdgfra/Ppp1ca/Rpl13a
3-day post-SCI group vs. the control group	BP	GO:0008584	male gonad development	5/222	0.0047216	0.0137311	0.0067309	Bax/Pdgfra/Plekha1/Prdx4/Ybx3
3-day post-SCI group vs. the control group	BP	GO:1903825	organic acid transmembrane transport	5/222	0.0047216	0.0137311	0.0067309	Abcc1/Arg1/Cd36/Myc/Slc23a2
3-day post-SCI group vs. the control group	BP	GO:0030071	regulation of mitotic metaphase/anaphase transitio	4/222	0.0047519	0.0137907	0.0067601	Actb/Apc/Cdc20/Rb1
3-day post-SCI group vs. the control group	BP	GO:0032890	regulation of organic acid transport	4/222	0.0047519	0.0137907	0.0067601	Adora1/Arg1/Map2k6/Snca
3-day post-SCI group vs. the control group	BP	GO:0035019	somatic stem cell population maintenance	4/222	0.0047519	0.0137907	0.0067601	Apc/Braf/Myc/Rest
3-day post-SCI group vs. the control group	BP	GO:0032886	regulation of microtubule-based process	7/222	0.004804	0.0139322	0.0068295	Apc/Mapk8/Mapt/Met/Prkaa2/Sirt1/Snca
3-day post-SCI group vs. the control group	BP	GO:0002702	positive regulation of production of molecular me	5/222	0.004864	0.0140869	0.0069053	Cd36/Mapkapk2/Sirt1/Stat6/Tlr4
3-day post-SCI group vs. the control group	BP	GO:0046546	development of primary male sexual characteristic	5/222	0.004864	0.0140869	0.0069053	Bax/Pdgfra/Plekha1/Prdx4/Ybx3

3-day post-SCI group vs. the control group	BP	GO:0002260	lymphocyte homeostasis	4/222	0.0049462	0.0142855	0.0070027	Bax/Casp3/Hif1a/Ripk3
3-day post-SCI group vs. the control group	BP	GO:0031507	heterochromatin formation	4/222	0.0049462	0.0142855	0.0070027	Ezh2/Hdac1/Rb1/Sirt1
3-day post-SCI group vs. the control group	BP	GO:0040014	regulation of multicellular organism growth	4/222	0.0049462	0.0142855	0.0070027	Atrn/Cdk4/Ezr/Htra2
3-day post-SCI group vs. the control group	BP	GO:0050848	regulation of calcium-mediated signaling	4/222	0.0049462	0.0142855	0.0070027	Mapt/Pdk2/Ppp3ca/Rcan1
3-day post-SCI group vs. the control group	BP	GO:0032660	regulation of interleukin-17 production	3/222	0.0049833	0.0143192	0.0070192	Jak2/Parp1/Tlr4
3-day post-SCI group vs. the control group	BP	GO:0051898	negative regulation of protein kinase B signaling	3/222	0.0049833	0.0143192	0.0070192	Plekha1/Sirt1/Xdh
3-day post-SCI group vs. the control group	BP	GO:1902742	apoptotic process involved in development	3/222	0.0049833	0.0143192	0.0070192	Atf2/Bax/Tnfrsf1a
3-day post-SCI group vs. the control group	BP	GO:0001866	NK T cell proliferation	2/222	0.0050156	0.0143192	0.0070192	Jak2/Myc
3-day post-SCI group vs. the control group	BP	GO:0014820	tonic smooth muscle contraction	2/222	0.0050156	0.0143192	0.0070192	Cd38/Mylk
3-day post-SCI group vs. the control group	BP	GO:0019511	peptidyl-proline hydroxylation	2/222	0.0050156	0.0143192	0.0070192	P4hb/Prdx4
3-day post-SCI group vs. the control group	BP	GO:0031054	pre-miRNA processing	2/222	0.0050156	0.0143192	0.0070192	Ago1/Ago3
3-day post-SCI group vs. the control group	BP	GO:0032769	negative regulation of monooxygenase activity	2/222	0.0050156	0.0143192	0.0070192	Nfkb1/Snca
3-day post-SCI group vs. the control group	BP	GO:0034393	positive regulation of smooth muscle cell apoptoti	2/222	0.0050156	0.0143192	0.0070192	Map2k4/Sod2
3-day post-SCI group vs. the control group	BP	GO:0036499	PERK-mediated unfolded protein response	2/222	0.0050156	0.0143192	0.0070192	Eif2s1/Nfe2l2
3-day post-SCI group vs. the control group	BP	GO:0042559	pteridine-containing compound biosynthetic proce	2/222	0.0050156	0.0143192	0.0070192	Dhfr/Gch1
3-day post-SCI group vs. the control group	BP	GO:0045793	positive regulation of cell size	2/222	0.0050156	0.0143192	0.0070192	Atp7a/Cdk4
3-day post-SCI group vs. the control group	BP	GO:0046886	positive regulation of hormone biosynthetic proces	2/222	0.0050156	0.0143192	0.0070192	Hif1a/Ppargc1a
3-day post-SCI group vs. the control group	BP	GO:0051152	positive regulation of smooth muscle cell different	2/222	0.0050156	0.0143192	0.0070192	Sirt1/Sod2
3-day post-SCI group vs. the control group	BP	GO:1902170	cellular response to reactive nitrogen species	2/222	0.0050156	0.0143192	0.0070192	Mapk8/Stat6
3-day post-SCI group vs. the control group	BP	GO:1905063	regulation of vascular associated smooth muscle c	2/222	0.0050156	0.0143192	0.0070192	Nfatc1/Sod2
3-day post-SCI group vs. the control group	BP	GO:1905666	regulation of protein localization to endosome	2/222	0.0050156	0.0143192	0.0070192	Ezr/Mgat3
3-day post-SCI group vs. the control group	BP	GO:0018394	peptidyl-lysine acetylation	6/222	0.0050364	0.0143688	0.0070435	Actb/Atf2/Mapk3/Prkaa2/Sirt1/Snca
3-day post-SCI group vs. the control group	BP	GO:0033627	cell adhesion mediated by integrin	4/222	0.0051456	0.0146606	0.0071865	Adam9/Cyp1b1/Itga6/Itgb5
3-day post-SCI group vs. the control group	BP	GO:1902600	proton transmembrane transport	4/222	0.0051456	0.0146606	0.0071865	Alox5/Atp7a/Ppif/Slc4a11
3-day post-SCI group vs. the control group	BP	GO:0000723	telomere maintenance	5/222	0.005158	0.0146858	0.0071989	Atr/Mapk3/Myc/Parp1/Pcna
3-day post-SCI group vs. the control group	BP	GO:0032271	regulation of protein polymerization	6/222	0.0052774	0.0150157	0.0073606	Apc/Cttn/Mapk8/Mapt/Met/Snca
3-day post-SCI group vs. the control group	BP	GO:0030225	macrophage differentiation	3/222	0.0053011	0.0150408	0.0073729	Rb1/Ripk1/Sirt1
3-day post-SCI group vs. the control group	BP	GO:0030501	positive regulation of bone mineralization	3/222	0.0053011	0.0150408	0.0073729	Alox5/Rxrb/Slc8a1
3-day post-SCI group vs. the control group	BP	GO:0032881	regulation of polysaccharide metabolic process	3/222	0.0053011	0.0150408	0.0073729	Cd36/Nfkb1/Ppp1ca
3-day post-SCI group vs. the control group	BP	GO:0055023	positive regulation of cardiac muscle tissue growth	3/222	0.0053011	0.0150408	0.0073729	Cdk1/Mapk14/Sirt1
3-day post-SCI group vs. the control group	BP	GO:0090092	regulation of transmembrane receptor protein serin	7/222	0.0053041	0.0150408	0.0073729	Cdkn2b/Hdac1/Jak2/Parp1/Rbbp7/Rbpms/Sirt1
3-day post-SCI group vs. the control group	BP	GO:0060420	regulation of heart growth	4/222	0.0053505	0.0151518	0.0074274	Apc/Cdk1/Mapk14/Sirt1
3-day post-SCI group vs. the control group	BP	GO:1902099	regulation of metaphase/anaphase transition of cel	4/222	0.0053505	0.0151518	0.0074274	Actb/Apc/Cdc20/Rb1
3-day post-SCI group vs. the control group	BP	GO:0030705	cytoskeleton-dependent intracellular transport	6/222	0.005401	0.0152848	0.0074925	Dst/Hif1a/Hspb1/Mapt/Sfpq/Stau2
3-day post-SCI group vs. the control group	BP	GO:0009615	response to virus	8/222	0.0055563	0.0157048	0.0076984	Atf2/Bax/Bnip3/Hif1a/Mapk14/Nfkb1/Ripk3/Zc3h12a
3-day post-SCI group vs. the control group	BP	GO:0007091	metaphase/anaphase transition of mitotic cell cycle	4/222	0.0055607	0.0157048	0.0076984	Actb/Apc/Cdc20/Rb1
3-day post-SCI group vs. the control group	BP	GO:0032436	positive regulation of proteasomal ubiquitin-depen	4/222	0.0055607	0.0157048	0.0076984	Cdc20/Gclc/Mapk8/Ubqln1
3-day post-SCI group vs. the control group	BP	GO:0007093	mitotic cell cycle checkpoint signaling	5/222	0.0056223	0.0158185	0.0077542	Apc/Atf2/Cdc20/Cdk1/Ier3
3-day post-SCI group vs. the control group	BP	GO:0007628	adult walking behavior	3/222	0.005631	0.0158185	0.0077542	Htra2/Mapt/Oxr1
3-day post-SCI group vs. the control group	BP	GO:0009394	2'-deoxyribonucleotide metabolic process	3/222	0.005631	0.0158185	0.0077542	Nme2/Rrm2b/Xdh

3-day post-SCI group vs. the control group	BP	GO:0010907	positive regulation of glucose metabolic process	3/222	0.005631	0.0158185	0.0077542	Cd36/Hif1a/Sirt1
3-day post-SCI group vs. the control group	BP	GO:0019692	deoxyribose phosphate metabolic process	3/222	0.005631	0.0158185	0.0077542	Nme2/Rrm2b/Xdh
3-day post-SCI group vs. the control group	BP	GO:0043277	apoptotic cell clearance	3/222	0.005631	0.0158185	0.0077542	Anxa1/Axl/Cd36
3-day post-SCI group vs. the control group	BP	GO:0043403	skeletal muscle tissue regeneration	3/222	0.005631	0.0158185	0.0077542	Anxa1/Ezh2/Gpx1
3-day post-SCI group vs. the control group	BP	GO:0060323	head morphogenesis	3/222	0.005631	0.0158185	0.0077542	Braf/Pdgfra/Plekha1
3-day post-SCI group vs. the control group	BP	GO:0072001	renal system development	8/222	0.0056506	0.0158631	0.007776	Apc/Bax/Cat/Myc/Pdgfra/Ppp3ca/Rrm2b/Smad1
3-day post-SCI group vs. the control group	BP	GO:0060491	regulation of cell projection assembly	6/222	0.0056546	0.0158638	0.0077764	Apc/Atp7a/Cdk10/Cttn/Stau2/Trpm2
3-day post-SCI group vs. the control group	BP	GO:0001654	eye development	9/222	0.0056984	0.0159759	0.0078313	Apc/Bax/Cyp1b1/Hdac1/Hif1a/Jun/Pdgfra/Pxdn/Stau2
3-day post-SCI group vs. the control group	BP	GO:0001553	luteinization	2/222	0.0057582	0.0159945	0.0078404	Pdgfra/Plekha1
3-day post-SCI group vs. the control group	BP	GO:0006825	copper ion transport	2/222	0.0057582	0.0159945	0.0078404	Atox1/Atp7a
3-day post-SCI group vs. the control group	BP	GO:0031000	response to caffeine	2/222	0.0057582	0.0159945	0.0078404	Prkaa2/Slc8a1
3-day post-SCI group vs. the control group	BP	GO:0035733	hepatic stellate cell activation	2/222	0.0057582	0.0159945	0.0078404	Cygb/Gclc
3-day post-SCI group vs. the control group	BP	GO:0048308	organelle inheritance	2/222	0.0057582	0.0159945	0.0078404	Cdk1/Mapk3
3-day post-SCI group vs. the control group	BP	GO:0048313	Golgi inheritance	2/222	0.0057582	0.0159945	0.0078404	Cdk1/Mapk3
3-day post-SCI group vs. the control group	BP	GO:0051782	negative regulation of cell division	2/222	0.0057582	0.0159945	0.0078404	Myc/Txnip
3-day post-SCI group vs. the control group	BP	GO:0060211	regulation of nuclear-transcribed mRNA poly(A) t	2/222	0.0057582	0.0159945	0.0078404	Tnrc6a/Tnrc6c
3-day post-SCI group vs. the control group	BP	GO:0061029	eyelid development in camera-type eye	2/222	0.0057582	0.0159945	0.0078404	Hdac1/Jun
3-day post-SCI group vs. the control group	BP	GO:0070943	neutrophil-mediated killing of symbiont cell	2/222	0.0057582	0.0159945	0.0078404	Arg1/Ncf1
3-day post-SCI group vs. the control group	BP	GO:0097050	type B pancreatic cell apoptotic process	2/222	0.0057582	0.0159945	0.0078404	Eif2s1/Mapk8
3-day post-SCI group vs. the control group	BP	GO:1902337	regulation of apoptotic process involved in morph	2/222	0.0057582	0.0159945	0.0078404	Bax/Tnfrsf1a
3-day post-SCI group vs. the control group	BP	GO:1903543	positive regulation of exosomal secretion	2/222	0.0057582	0.0159945	0.0078404	Atp13a2/Sdc1
3-day post-SCI group vs. the control group	BP	GO:2000271	positive regulation of fibroblast apoptotic process	2/222	0.0057582	0.0159945	0.0078404	Apc/Btg1
3-day post-SCI group vs. the control group	BP	GO:0010965	regulation of mitotic sister chromatid separation	4/222	0.0057763	0.0160327	0.0078592	Actb/Apc/Cdc20/Rb1
3-day post-SCI group vs. the control group	BP	GO:0001508	action potential	5/222	0.0057833	0.0160327	0.0078592	Atp2a2/Cd36/Chrna4/Fkbp1b/Pawr
3-day post-SCI group vs. the control group	BP	GO:1903313	positive regulation of mRNA metabolic process	5/222	0.0057833	0.0160327	0.0078592	Paip1/Prdx6/Tnrc6a/Tnrc6c/Zc3h12a
3-day post-SCI group vs. the control group	BP	GO:0043087	regulation of GTPase activity	8/222	0.0059407	0.016458	0.0080676	Agap3/Amph/Ect2/Eif2s1/Ezh2/Itga6/Map4k4/Met
3-day post-SCI group vs. the control group	BP	GO:0150063	visual system development	9/222	0.0059581	0.0164604	0.0080688	Apc/Bax/Cyp1b1/Hdac1/Hif1a/Jun/Pdgfra/Pxdn/Stau2
3-day post-SCI group vs. the control group	BP	GO:0002701	negative regulation of production of molecular me	3/222	0.0059728	0.0164604	0.0080688	Arg1/Axl/Hmox1
3-day post-SCI group vs. the control group	BP	GO:0032620	interleukin-17 production	3/222	0.0059728	0.0164604	0.0080688	Jak2/Parp1/Tlr4
3-day post-SCI group vs. the control group	BP	GO:0042304	regulation of fatty acid biosynthetic process	3/222	0.0059728	0.0164604	0.0080688	Anxa1/Ptgs2/Sirt1
3-day post-SCI group vs. the control group	BP	GO:0045923	positive regulation of fatty acid metabolic process	3/222	0.0059728	0.0164604	0.0080688	Anxa1/Ppargc1a/Ptgs2
3-day post-SCI group vs. the control group	BP	GO:0048741	skeletal muscle fiber development	3/222	0.0059728	0.0164604	0.0080688	Gpx1/Ppp3ca/Rcan1
3-day post-SCI group vs. the control group	BP	GO:0050856	regulation of T cell receptor signaling pathway	3/222	0.0059728	0.0164604	0.0080688	Ezr/Pawr/Rela
3-day post-SCI group vs. the control group	BP	GO:0071385	cellular response to glucocorticoid stimulus	3/222	0.0059728	0.0164604	0.0080688	Anxa1/Jak2/Rest
3-day post-SCI group vs. the control group	BP	GO:0032204	regulation of telomere maintenance	4/222	0.0059974	0.0165067	0.0080915	Atr/Mapk3/Myc/Parp1
3-day post-SCI group vs. the control group	BP	GO:0051899	membrane depolarization	4/222	0.0059974	0.0165067	0.0080915	Chrna4/Gclc/Jun/Parp1
3-day post-SCI group vs. the control group	BP	GO:0034767	positive regulation of ion transmembrane transpor	6/222	0.0061877	0.0170082	0.0083373	Alox5/Atp7a/Bax/Ppp3ca/S100a1/Snca
3-day post-SCI group vs. the control group	BP	GO:1902115	regulation of organelle assembly	6/222	0.0061877	0.0170082	0.0083373	Cdk10/Ezr/Mapk8/Pink1/Prkaa2/Sdc1
3-day post-SCI group vs. the control group	BP	GO:0030168	platelet activation	4/222	0.0062241	0.0170305	0.0083482	Axl/Jak2/Pdgfra/Tlr4
3-day post-SCI group vs. the control group	BP	GO:0031960	response to corticosteroid	4/222	0.0062241	0.0170305	0.0083482	Adam9/Anxa1/Jak2/Rest

3-day post-SCI group vs. the control group	BP	GO:0044784	metaphase/anaphase transition of cell cycle	4/222	0.0062241	0.0170305	0.0083482	Actb/Apc/Cdc20/Rb1
3-day post-SCI group vs. the control group	BP	GO:0045685	regulation of glial cell differentiation	4/222	0.0062241	0.0170305	0.0083482	Hdac1/Il6st/Rela/Rnf112
3-day post-SCI group vs. the control group	BP	GO:0050672	negative regulation of lymphocyte proliferation	4/222	0.0062241	0.0170305	0.0083482	Arg1/Btk/Casp3/Pawr
3-day post-SCI group vs. the control group	BP	GO:0072655	establishment of protein localization to mitochond	4/222	0.0062241	0.0170305	0.0083482	Bax/Fbxw7/Mapt/Pink1
3-day post-SCI group vs. the control group	BP	GO:1901983	regulation of protein acetylation	4/222	0.0062241	0.0170305	0.0083482	Mapk3/Prkaa2/Sirt1/Snca
3-day post-SCI group vs. the control group	BP	GO:0048880	sensory system development	9/222	0.0063186	0.0172442	0.008453	Apc/Bax/Cyp1b1/Hdac1/Hif1a/Jun/Pdgfra/Pxdn/Stau2
3-day post-SCI group vs. the control group	BP	GO:0009262	deoxyribonucleotide metabolic process	3/222	0.0063268	0.0172442	0.008453	Nme2/Rrm2b/Xdh
3-day post-SCI group vs. the control group	BP	GO:0010543	regulation of platelet activation	3/222	0.0063268	0.0172442	0.008453	Jak2/Pdgfra/Tlr4
3-day post-SCI group vs. the control group	BP	GO:0010837	regulation of keratinocyte proliferation	3/222	0.0063268	0.0172442	0.008453	Ctsl/Nfatc1/Ptprk
3-day post-SCI group vs. the control group	BP	GO:0033173	calcineurin-NFAT signaling cascade	3/222	0.0063268	0.0172442	0.008453	Nfatc1/Ppp3ca/Rcan1
3-day post-SCI group vs. the control group	BP	GO:0060421	positive regulation of heart growth	3/222	0.0063268	0.0172442	0.008453	Cdk1/Mapk14/Sirt1
3-day post-SCI group vs. the control group	BP	GO:0032945	negative regulation of mononuclear cell proliferati	4/222	0.0064565	0.0175626	0.0086091	Arg1/Btk/Casp3/Pawr
3-day post-SCI group vs. the control group	BP	GO:0051306	mitotic sister chromatid separation	4/222	0.0064565	0.0175626	0.0086091	Actb/Apc/Cdc20/Rb1
3-day post-SCI group vs. the control group	BP	GO:2000117	negative regulation of cysteine-type endopeptidase	4/222	0.0064565	0.0175626	0.0086091	Dhcr24/Gpx1/Ptgs2/Snca
3-day post-SCI group vs. the control group	BP	GO:0046660	female sex differentiation	5/222	0.0064603	0.0175626	0.0086091	Axl/Bax/Pdgfra/Plekha1/Sirt1
3-day post-SCI group vs. the control group	BP	GO:0006857	oligopeptide transport	2/222	0.0065477	0.017618	0.0086363	Abcc1/Mgst1
3-day post-SCI group vs. the control group	BP	GO:0014857	regulation of skeletal muscle cell proliferation	2/222	0.0065477	0.017618	0.0086363	Jak2/Sirt1
3-day post-SCI group vs. the control group	BP	GO:0031987	locomotion involved in locomotory behavior	2/222	0.0065477	0.017618	0.0086363	Gpr37/Rcan1
3-day post-SCI group vs. the control group	BP	GO:0032239	regulation of nucleobase-containing compound tra	2/222	0.0065477	0.017618	0.0086363	Adora1/Ripk1
3-day post-SCI group vs. the control group	BP	GO:0032717	negative regulation of interleukin-8 production	2/222	0.0065477	0.017618	0.0086363	Anxa1/Tlr6
3-day post-SCI group vs. the control group	BP	GO:0045651	positive regulation of macrophage differentiation	2/222	0.0065477	0.017618	0.0086363	Rb1/Ripk1
3-day post-SCI group vs. the control group	BP	GO:0045947	negative regulation of translational initiation	2/222	0.0065477	0.017618	0.0086363	Eif2s1/Rpl13a
3-day post-SCI group vs. the control group	BP	GO:0046007	negative regulation of activated T cell proliferatio	2/222	0.0065477	0.017618	0.0086363	Arg1/Casp3
3-day post-SCI group vs. the control group	BP	GO:0047484	regulation of response to osmotic stress	2/222	0.0065477	0.017618	0.0086363	Ptgs2/Ybx3
3-day post-SCI group vs. the control group	BP	GO:0051151	negative regulation of smooth muscle cell differen	2/222	0.0065477	0.017618	0.0086363	Nfatc1/Rcan1
3-day post-SCI group vs. the control group	BP	GO:0070431	nucleotide-binding oligomerization domain contai	2/222	0.0065477	0.017618	0.0086363	Rela/Tlr4
3-day post-SCI group vs. the control group	BP	GO:0099527	postsynapse to nucleus signaling pathway	2/222	0.0065477	0.017618	0.0086363	Jak2/Rela
3-day post-SCI group vs. the control group	BP	GO:1902001	fatty acid transmembrane transport	2/222	0.0065477	0.017618	0.0086363	Abcc1/Cd36
3-day post-SCI group vs. the control group	BP	GO:1903799	negative regulation of miRNA maturation	2/222	0.0065477	0.017618	0.0086363	Ppp3ca/Zc3h12a
3-day post-SCI group vs. the control group	BP	GO:1904748	regulation of apoptotic process involved in develo	2/222	0.0065477	0.017618	0.0086363	Bax/Tnfrsf1a
3-day post-SCI group vs. the control group	BP	GO:1904923	regulation of autophagy of mitochondrion in respo	2/222	0.0065477	0.017618	0.0086363	Htra2/Pink1
3-day post-SCI group vs. the control group	BP	GO:0099111	microtubule-based transport	6/222	0.0066109	0.0177769	0.0087142	Dst/Hif1a/Hspb1/Mapt/Sfpq/Stau2
3-day post-SCI group vs. the control group	BP	GO:0006261	DNA-templated DNA replication	5/222	0.0066379	0.017838	0.0087441	Atr/Endog/Mcm4/Pcna/Rrm2b
3-day post-SCI group vs. the control group	BP	GO:0018198	peptidyl-cysteine modification	3/222	0.0066931	0.0178874	0.0087683	Ncoa7/Oxr1/Tbc1d24
3-day post-SCI group vs. the control group	BP	GO:0042088	T-helper 1 type immune response	3/222	0.0066931	0.0178874	0.0087683	Anxa1/Il18rap/Stat6
3-day post-SCI group vs. the control group	BP	GO:0045663	positive regulation of myoblast differentiation	3/222	0.0066931	0.0178874	0.0087683	Actb/Btg1/Mapk14
3-day post-SCI group vs. the control group	BP	GO:0090659	walking behavior	3/222	0.0066931	0.0178874	0.0087683	Htra2/Mapt/Oxr1
3-day post-SCI group vs. the control group	BP	GO:1902930	regulation of alcohol biosynthetic process	3/222	0.0066931	0.0178874	0.0087683	Nfkb1/Rest/Snca
3-day post-SCI group vs. the control group	BP	GO:0035773	insulin secretion involved in cellular response to g	4/222	0.0066945	0.0178874	0.0087683	Fkbp1b/Hif1a/Sirt1/Ucp2
3-day post-SCI group vs. the control group	BP	GO:0045814	negative regulation of gene expression, epigenetic	4/222	0.0066945	0.0178874	0.0087683	Ezh2/Hdac1/Rb1/Sirt1

3-day post-SCI group vs. the control group	BP	GO:0061157	mRNA destabilization	4/222	0.0066945	0.0178874	0.0087683	Paip1/Tnrc6a/Tnrc6c/Zc3h12a
3-day post-SCI group vs. the control group	BP	GO:0070828	heterochromatin organization	4/222	0.0066945	0.0178874	0.0087683	Ezh2/Hdac1/Rb1/Sirt1
3-day post-SCI group vs. the control group	BP	GO:0031396	regulation of protein ubiquitination	6/222	0.0069045	0.0184368	0.0090376	Cdc20/Fbxw7/Gclc/Mapk8/Pink1/Ubqln1
3-day post-SCI group vs. the control group	BP	GO:0048284	organelle fusion	5/222	0.0070032	0.0186885	0.009161	Anxa1/Atp13a2/Bax/Bnip3/Snca
3-day post-SCI group vs. the control group	BP	GO:0000819	sister chromatid segregation	6/222	0.0070548	0.0188	0.0092156	Actb/Apc/Cdc20/Fbxw7/Rb1/Sfpq
3-day post-SCI group vs. the control group	BP	GO:1903050	regulation of proteolysis involved in protein catab	6/222	0.0070548	0.0188	0.0092156	Cdc20/Fbxw7/Gclc/Gpx1/Mapk8/Ubqln1
3-day post-SCI group vs. the control group	BP	GO:0022602	ovulation cycle process	3/222	0.0070718	0.0188	0.0092156	Pdgfra/Plekha1/Sirt1
3-day post-SCI group vs. the control group	BP	GO:0043029	T cell homeostasis	3/222	0.0070718	0.0188	0.0092156	Bax/Casp3/Ripk3
3-day post-SCI group vs. the control group	BP	GO:0045646	regulation of erythrocyte differentiation	3/222	0.0070718	0.0188	0.0092156	Ets1/Hif1a/Mapk14
3-day post-SCI group vs. the control group	BP	GO:1902692	regulation of neuroblast proliferation	3/222	0.0070718	0.0188	0.0092156	Ctnna1/Hif1a/Mapk8
3-day post-SCI group vs. the control group	BP	GO:0051492	regulation of stress fiber assembly	4/222	0.0071878	0.0190723	0.0093492	Braf/Met/Nox4/Pxn
3-day post-SCI group vs. the control group	BP	GO:0062207	regulation of pattern recognition receptor signaling	4/222	0.0071878	0.0190723	0.0093492	Cd36/Tlr4/Tlr6/Ubqln1
3-day post-SCI group vs. the control group	BP	GO:1901019	regulation of calcium ion transmembrane transport	4/222	0.0071878	0.0190723	0.0093492	Fkbp1b/Ppp3ca/S100a1/Ubqln1
3-day post-SCI group vs. the control group	BP	GO:0045619	regulation of lymphocyte differentiation	6/222	0.0073624	0.0193235	0.0094723	Actb/Anxa1/Axl/Braf/Hspb1/Zc3h12a
3-day post-SCI group vs. the control group	BP	GO:0001886	endothelial cell morphogenesis	2/222	0.0073834	0.0193235	0.0094723	Id1/Met
3-day post-SCI group vs. the control group	BP	GO:0006264	mitochondrial DNA replication	2/222	0.0073834	0.0193235	0.0094723	Endog/Rrm2b
3-day post-SCI group vs. the control group	BP	GO:0006878	cellular copper ion homeostasis	2/222	0.0073834	0.0193235	0.0094723	Atox1/Atp7a
3-day post-SCI group vs. the control group	BP	GO:0010566	regulation of ketone biosynthetic process	2/222	0.0073834	0.0193235	0.0094723	Ppargc1a/Rest
3-day post-SCI group vs. the control group	BP	GO:0030540	female genitalia development	2/222	0.0073834	0.0193235	0.0094723	Axl/Bax
3-day post-SCI group vs. the control group	BP	GO:0042754	negative regulation of circadian rhythm	2/222	0.0073834	0.0193235	0.0094723	Adora1/Sfpq
3-day post-SCI group vs. the control group	BP	GO:0045655	regulation of monocyte differentiation	2/222	0.0073834	0.0193235	0.0094723	Jun/Myc
3-day post-SCI group vs. the control group	BP	GO:0045820	negative regulation of glycolytic process	2/222	0.0073834	0.0193235	0.0094723	Ier3/Ppargc1a
3-day post-SCI group vs. the control group	BP	GO:0051589	negative regulation of neurotransmitter transport	2/222	0.0073834	0.0193235	0.0094723	Braf/Snca
3-day post-SCI group vs. the control group	BP	GO:0060546	negative regulation of necroptotic process	2/222	0.0073834	0.0193235	0.0094723	Ripk1/Ybx3
3-day post-SCI group vs. the control group	BP	GO:0060766	negative regulation of androgen receptor signaling	2/222	0.0073834	0.0193235	0.0094723	Hdac1/Sirt1
3-day post-SCI group vs. the control group	BP	GO:0061179	negative regulation of insulin secretion involved ir	2/222	0.0073834	0.0193235	0.0094723	Fkbp1b/Ucp2
3-day post-SCI group vs. the control group	BP	GO:0070942	neutrophil mediated cytotoxicity	2/222	0.0073834	0.0193235	0.0094723	Arg1/Ncf1
3-day post-SCI group vs. the control group	BP	GO:0071498	cellular response to fluid shear stress	2/222	0.0073834	0.0193235	0.0094723	Nfe2l2/Ptgs2
3-day post-SCI group vs. the control group	BP	GO:0090336	positive regulation of brown fat cell differentiatior	2/222	0.0073834	0.0193235	0.0094723	Mapk14/Ptgs2
3-day post-SCI group vs. the control group	BP	GO:0090493	catecholamine uptake	2/222	0.0073834	0.0193235	0.0094723	Actb/Snca
3-day post-SCI group vs. the control group	BP	GO:0098760	response to interleukin-7	2/222	0.0073834	0.0193235	0.0094723	Btk/P4hb
3-day post-SCI group vs. the control group	BP	GO:0098761	cellular response to interleukin-7	2/222	0.0073834	0.0193235	0.0094723	Btk/P4hb
3-day post-SCI group vs. the control group	BP	GO:1903541	regulation of exosomal secretion	2/222	0.0073834	0.0193235	0.0094723	Atp13a2/Sdc1
3-day post-SCI group vs. the control group	BP	GO:2001028	positive regulation of endothelial cell chemotaxis	2/222	0.0073834	0.0193235	0.0094723	Hspb1/Met
3-day post-SCI group vs. the control group	BP	GO:2001171	positive regulation of ATP biosynthetic process	2/222	0.0073834	0.0193235	0.0094723	Myc/Ppargc1a
3-day post-SCI group vs. the control group	BP	GO:0006401	RNA catabolic process	7/222	0.0074327	0.0194284	0.0095237	Ago1/Ago3/Mapkapk2/Paip1/Tnrc6a/Tnrc6c/Zc3h12a
3-day post-SCI group vs. the control group	BP	GO:0045055	regulated exocytosis	7/222	0.0074327	0.0194284	0.0095237	Atp2a2/Braf/Chrna4/Hmox1/Rest/Snca/Syp
3-day post-SCI group vs. the control group	BP	GO:0019083	viral transcription	3/222	0.0074629	0.0194348	0.0095269	Hdac1/Jun/Rest
3-day post-SCI group vs. the control group	BP	GO:0045581	negative regulation of T cell differentiation	3/222	0.0074629	0.0194348	0.0095269	Anxa1/Hspb1/Zc3h12a
3-day post-SCI group vs. the control group	BP	GO:0060043	regulation of cardiac muscle cell proliferation	3/222	0.0074629	0.0194348	0.0095269	Apc/Cdk1/Mapk14

3-day post-SCI group vs. the control group	BP	GO:0060251	regulation of glial cell proliferation	3/222	0.0074629	0.0194348	0.0095269	Cdkn2b/Myc/Rb1
3-day post-SCI group vs. the control group	BP	GO:0071384	cellular response to corticosteroid stimulus	3/222	0.0074629	0.0194348	0.0095269	Anxa1/Jak2/Rest
3-day post-SCI group vs. the control group	BP	GO:2001258	negative regulation of cation channel activity	3/222	0.0074629	0.0194348	0.0095269	Fkbp1b/Ppp3ca/Ubqln1
3-day post-SCI group vs. the control group	BP	GO:0002822	regulation of adaptive immune response based on :	6/222	0.0076796	0.0199869	0.0097975	Anxa1/Arg1/Jak2/Ripk3/Stat6/Zc3h12a
3-day post-SCI group vs. the control group	BP	GO:0050779	RNA destabilization	4/222	0.0077045	0.020027	0.0098171	Paip1/Tnrc6a/Tnrc6c/Zc3h12a
3-day post-SCI group vs. the control group	BP	GO:0070585	protein localization to mitochondrion	4/222	0.0077045	0.020027	0.0098171	Bax/Fbxw7/Mapt/Pink1
3-day post-SCI group vs. the control group	BP	GO:0051955	regulation of amino acid transport	3/222	0.0078666	0.020423	0.0100112	Adora1/Arg1/Snca
3-day post-SCI group vs. the control group	BP	GO:0097720	calcineurin-mediated signaling	3/222	0.0078666	0.020423	0.0100112	Nfatc1/Ppp3ca/Rcan1
3-day post-SCI group vs. the control group	BP	GO:0007059	chromosome segregation	8/222	0.0079259	0.0205644	0.0100806	Actb/Apc/Banf1/Cdc20/Ect2/Fbxw7/Rb1/Sfpq
3-day post-SCI group vs. the control group	BP	GO:0033045	regulation of sister chromatid segregation	4/222	0.0079718	0.020658	0.0101264	Actb/Apc/Cdc20/Rb1
3-day post-SCI group vs. the control group	BP	GO:0061014	positive regulation of mRNA catabolic process	4/222	0.0079718	0.020658	0.0101264	Paip1/Tnrc6a/Tnrc6c/Zc3h12a
3-day post-SCI group vs. the control group	BP	GO:0001938	positive regulation of endothelial cell proliferation	4/222	0.0082451	0.021208	0.0103961	Arg1/Hmox1/Jun/Sirt1
3-day post-SCI group vs. the control group	BP	GO:0010389	regulation of G2/M transition of mitotic cell cycle	4/222	0.0082451	0.021208	0.0103961	Cdk1/Cdk4/Ier3/Rrm2b
3-day post-SCI group vs. the control group	BP	GO:0070664	negative regulation of leukocyte proliferation	4/222	0.0082451	0.021208	0.0103961	Arg1/Btk/Casp3/Pawr
3-day post-SCI group vs. the control group	BP	GO:0009263	deoxyribonucleotide biosynthetic process	2/222	0.0082646	0.021208	0.0103961	Nme2/Rrm2b
3-day post-SCI group vs. the control group	BP	GO:0009265	2'-deoxyribonucleotide biosynthetic process	2/222	0.0082646	0.021208	0.0103961	Nme2/Rrm2b
3-day post-SCI group vs. the control group	BP	GO:0030575	nuclear body organization	2/222	0.0082646	0.021208	0.0103961	Agap3/Ets1
3-day post-SCI group vs. the control group	BP	GO:0033574	response to testosterone	2/222	0.0082646	0.021208	0.0103961	Cdk4/Sirt1
3-day post-SCI group vs. the control group	BP	GO:0035633	maintenance of blood-brain barrier	2/222	0.0082646	0.021208	0.0103961	Ptgs1/Ptgs2
3-day post-SCI group vs. the control group	BP	GO:0046385	deoxyribose phosphate biosynthetic process	2/222	0.0082646	0.021208	0.0103961	Nme2/Rrm2b
3-day post-SCI group vs. the control group	BP	GO:0055070	copper ion homeostasis	2/222	0.0082646	0.021208	0.0103961	Atox1/Atp7a
3-day post-SCI group vs. the control group	BP	GO:0062099	negative regulation of programmed necrotic cell d	2/222	0.0082646	0.021208	0.0103961	Ripk1/Ybx3
3-day post-SCI group vs. the control group	BP	GO:0070102	interleukin-6-mediated signaling pathway	2/222	0.0082646	0.021208	0.0103961	Il6st/Ripk1
3-day post-SCI group vs. the control group	BP	GO:0070314	G1 to G0 transition	2/222	0.0082646	0.021208	0.0103961	Ezh2/Rnf112
3-day post-SCI group vs. the control group	BP	GO:0070423	nucleotide-binding oligomerization domain contai	2/222	0.0082646	0.021208	0.0103961	Rela/Tlr4
3-day post-SCI group vs. the control group	BP	GO:1904424	regulation of GTP binding	2/222	0.0082646	0.021208	0.0103961	Eif2s1/Met
3-day post-SCI group vs. the control group	BP	GO:2000641	regulation of early endosome to late endosome tra	2/222	0.0082646	0.021208	0.0103961	Ezr/Mapk3
3-day post-SCI group vs. the control group	BP	GO:0010883	regulation of lipid storage	3/222	0.0082829	0.0212161	0.0104	Cd36/Sirt1/Zc3h12a
3-day post-SCI group vs. the control group	BP	GO:0014904	myotube cell development	3/222	0.0082829	0.0212161	0.0104	Gpx1/Ppp3ca/Rcan1
3-day post-SCI group vs. the control group	BP	GO:2000351	regulation of endothelial cell apoptotic process	3/222	0.0082829	0.0212161	0.0104	Braf/Id1/Nfe2l2
3-day post-SCI group vs. the control group	BP	GO:0007173	epidermal growth factor receptor signaling pathwa	4/222	0.0085245	0.0218083	0.0106903	Adora1/Braf/Fbxw7/Hbegf
3-day post-SCI group vs. the control group	BP	GO:0007229	integrin-mediated signaling pathway	4/222	0.0085245	0.0218083	0.0106903	Ctnna1/Itga6/Itgb5/Pxn
3-day post-SCI group vs. the control group	BP	GO:0009755	hormone-mediated signaling pathway	5/222	0.0086054	0.022002	0.0107853	Hdac1/Jak2/Parp1/Ppargc1b/Sirt1
3-day post-SCI group vs. the control group	BP	GO:0061138	morphogenesis of a branching epithelium	6/222	0.0086904	0.0221936	0.0108792	Met/Myc/Nfatc1/Pdgfra/Ppp1ca/Pxn
3-day post-SCI group vs. the control group	BP	GO:0001961	positive regulation of cytokine-mediated signaling	3/222	0.0087119	0.0221936	0.0108792	Axl/Hif1a/Ripk1
3-day post-SCI group vs. the control group	BP	GO:0001974	blood vessel remodeling	3/222	0.0087119	0.0221936	0.0108792	Atp7a/Axl/Bax
3-day post-SCI group vs. the control group	BP	GO:0006111	regulation of gluconeogenesis	3/222	0.0087119	0.0221936	0.0108792	Hif1a/Pdk2/Sirt1
3-day post-SCI group vs. the control group	BP	GO:0042531	positive regulation of tyrosine phosphorylation of :	3/222	0.0087119	0.0221936	0.0108792	Il6st/Jak2/Tnfrsf1a
3-day post-SCI group vs. the control group	BP	GO:0098926	postsynaptic signal transduction	3/222	0.0087119	0.0221936	0.0108792	Chrna4/Jak2/Rela
3-day post-SCI group vs. the control group	BP	GO:0046849	bone remodeling	4/222	0.0088099	0.0224297	0.0109949	Cd38/Nox4/Ppargc1b/Tpp1

3-day post-SCI group vs. the control group	BP	GO:0043488	regulation of mRNA stability	5/222	0.008822	0.0224469	0.0110033	Mapkapk2/Paip1/Tnrc6a/Tnrc6c/Zc3h12a
3-day post-SCI group vs. the control group	BP	GO:0048016	inositol phosphate-mediated signaling	3/222	0.0091538	0.023133	0.0113397	Nfatc1/Ppp3ca/Rcan1
3-day post-SCI group vs. the control group	BP	GO:0070169	positive regulation of biomineral tissue developme	3/222	0.0091538	0.023133	0.0113397	Alox5/Rxrb/Slc8a1
3-day post-SCI group vs. the control group	BP	GO:0006085	acetyl-CoA biosynthetic process	2/222	0.0091906	0.023133	0.0113397	Pdk1/Pdk2
3-day post-SCI group vs. the control group	BP	GO:0030011	maintenance of cell polarity	2/222	0.0091906	0.023133	0.0113397	Dst/Pdlim1
3-day post-SCI group vs. the control group	BP	GO:0032700	negative regulation of interleukin-17 production	2/222	0.0091906	0.023133	0.0113397	Parp1/Tlr4
3-day post-SCI group vs. the control group	BP	GO:0035457	cellular response to interferon-alpha	2/222	0.0091906	0.023133	0.0113397	Axl/Myc
3-day post-SCI group vs. the control group	BP	GO:0035872	nucleotide-binding domain, leucine rich repeat cor	2/222	0.0091906	0.023133	0.0113397	Rela/Tlr4
3-day post-SCI group vs. the control group	BP	GO:0044539	long-chain fatty acid import into cell	2/222	0.0091906	0.023133	0.0113397	Abcc1/Cd36
3-day post-SCI group vs. the control group	BP	GO:0051132	NK T cell activation	2/222	0.0091906	0.023133	0.0113397	Jak2/Myc
3-day post-SCI group vs. the control group	BP	GO:0051988	regulation of attachment of spindle microtubules t	2/222	0.0091906	0.023133	0.0113397	Apc/Ect2
3-day post-SCI group vs. the control group	BP	GO:0060576	intestinal epithelial cell development	2/222	0.0091906	0.023133	0.0113397	Hif1a/Il6st
3-day post-SCI group vs. the control group	BP	GO:0060965	negative regulation of miRNA-mediated gene siler	2/222	0.0091906	0.023133	0.0113397	Ppp3ca/Zc3h12a
3-day post-SCI group vs. the control group	BP	GO:0071636	positive regulation of transforming growth factor b	2/222	0.0091906	0.023133	0.0113397	Atf2/Ptgs2
3-day post-SCI group vs. the control group	BP	GO:0072537	fibroblast activation	2/222	0.0091906	0.023133	0.0113397	Cygb/Gclc
3-day post-SCI group vs. the control group	BP	GO:1902004	positive regulation of amyloid-beta formation	2/222	0.0091906	0.023133	0.0113397	Casp3/Rela
3-day post-SCI group vs. the control group	BP	GO:1903214	regulation of protein targeting to mitochondrion	2/222	0.0091906	0.023133	0.0113397	Fbxw7/Pink1
3-day post-SCI group vs. the control group	BP	GO:1904177	regulation of adipose tissue development	2/222	0.0091906	0.023133	0.0113397	Parp1/Sirt1
3-day post-SCI group vs. the control group	BP	GO:2000678	negative regulation of transcription regulatory regi	2/222	0.0091906	0.023133	0.0113397	Fbxw7/Rest
3-day post-SCI group vs. the control group	BP	GO:0001558	regulation of cell growth	9/222	0.0093331	0.0234776	0.0115086	Cd38/Cdkn2c/Cttn/Hbegf/Mapt/Rb1/Rbbp7/Sirt1/Slc23a2
3-day post-SCI group vs. the control group	BP	GO:0032006	regulation of TOR signaling	4/222	0.0093995	0.0236024	0.0115698	Endog/Hif1a/Prkaa2/Sirt1
3-day post-SCI group vs. the control group	BP	GO:1904427	positive regulation of calcium ion transmembrane	4/222	0.0093995	0.0236024	0.0115698	Bax/Ppp3ca/S100a1/Snca
3-day post-SCI group vs. the control group	BP	GO:1905818	regulation of chromosome separation	4/222	0.0093995	0.0236024	0.0115698	Actb/Apc/Cdc20/Rb1
3-day post-SCI group vs. the control group	BP	GO:0006473	protein acetylation	6/222	0.0096028	0.0239845	0.0117571	Actb/Atf2/Mapk3/Prkaa2/Sirt1/Snca
3-day post-SCI group vs. the control group	BP	GO:0097305	response to alcohol	6/222	0.0096028	0.0239845	0.0117571	Cdk1/Cdk4/Ctnna1/Prkaa2/Sirt1/Tlr4
3-day post-SCI group vs. the control group	BP	GO:0002762	negative regulation of myeloid leukocyte different	3/222	0.0096086	0.0239845	0.0117571	Fbxw7/Myc/Nme2
3-day post-SCI group vs. the control group	BP	GO:0030857	negative regulation of epithelial cell differentiation	3/222	0.0096086	0.0239845	0.0117571	Ezh2/Id1/Xdh
3-day post-SCI group vs. the control group	BP	GO:0051055	negative regulation of lipid biosynthetic process	3/222	0.0096086	0.0239845	0.0117571	Nfkb1/Rest/Sirt1
3-day post-SCI group vs. the control group	BP	GO:0072577	endothelial cell apoptotic process	3/222	0.0096086	0.0239845	0.0117571	Braf/Id1/Nfe2l2
3-day post-SCI group vs. the control group	BP	GO:0090329	regulation of DNA-templated DNA replication	3/222	0.0096086	0.0239845	0.0117571	Endog/Mcm4/Pcna
3-day post-SCI group vs. the control group	BP	GO:0110151	positive regulation of biomineralization	3/222	0.0096086	0.0239845	0.0117571	Alox5/Rxrb/Slc8a1
3-day post-SCI group vs. the control group	BP	GO:1903053	regulation of extracellular matrix organization	3/222	0.0096086	0.0239845	0.0117571	Ets1/Rb1/Tnfrsf1a
3-day post-SCI group vs. the control group	BP	GO:1903170	negative regulation of calcium ion transmembrane	3/222	0.0096086	0.0239845	0.0117571	Fkbp1b/Ppp3ca/Ubqln1
3-day post-SCI group vs. the control group	BP	GO:0098813	nuclear chromosome segregation	7/222	0.0096425	0.0240437	0.0117861	Actb/Apc/Cdc20/Ect2/Fbxw7/Rb1/Sfpq
3-day post-SCI group vs. the control group	BP	GO:0046486	glycerolipid metabolic process	8/222	0.0096437	0.0240437	0.0117861	Cat/Cd36/Dgkk/Gpx1/Il6st/Pnpla8/Prdx6/Sirt1
3-day post-SCI group vs. the control group	BP	GO:0032755	positive regulation of interleukin-6 production	4/222	0.0097037	0.0241645	0.0118453	Aif1/Cd36/Tlr4/Tlr6
3-day post-SCI group vs. the control group	BP	GO:0045833	negative regulation of lipid metabolic process	4/222	0.0097037	0.0241645	0.0118453	Adora1/Nfkb1/Rest/Sirt1
3-day post-SCI group vs. the control group	BP	GO:2000058	regulation of ubiquitin-dependent protein catabolic	5/222	0.0097263	0.0242066	0.011866	Cdc20/Fbxw7/Gclc/Mapk8/Ubqln1
3-day post-SCI group vs. the control group	BP	GO:0007163	establishment or maintenance of cell polarity	6/222	0.0097931	0.0243585	0.0119404	Actb/Apc/Ctnna1/Dst/Ezr/Pdlim1
3-day post-SCI group vs. the control group	BP	GO:0006457	protein folding	5/222	0.009962	0.0247492	0.0121319	Fkbp1b/Hspb1/P4hb/Ppif/Prdx4

3-day post-SCI group vs. the control group	BP	GO:0046887	positive regulation of hormone secretion	5/222	0.009962	0.0247492	0.0121319	Cd38/Hif1a/Jak2/Sirt1/Trpm2
3-day post-SCI group vs. the control group	BP	GO:0031330	negative regulation of cellular catabolic process	6/222	0.0099862	0.0247947	0.0121542	Hmox1/Mapkapk2/Mcl1/Paip1/Pink1/Snca
3-day post-SCI group vs. the control group	BP	GO:0016525	negative regulation of angiogenesis	4/222	0.0100142	0.0248179	0.0121656	Ago1/Alox5/Atf2/Cd36
3-day post-SCI group vs. the control group	BP	GO:0003170	heart valve development	3/222	0.0100763	0.0248179	0.0121656	Nfatc1/Rb1/Tnfrsf1a
3-day post-SCI group vs. the control group	BP	GO:0010257	NADH dehydrogenase complex assembly	3/222	0.0100763	0.0248179	0.0121656	Ndufa12/Ndufa6/Ndufs8
3-day post-SCI group vs. the control group	BP	GO:0031279	regulation of cyclase activity	3/222	0.0100763	0.0248179	0.0121656	Mapk14/Mapk3/Mapk8
3-day post-SCI group vs. the control group	BP	GO:0032757	positive regulation of interleukin-8 production	3/222	0.0100763	0.0248179	0.0121656	Rela/Ripk1/Tlr4
3-day post-SCI group vs. the control group	BP	GO:0032981	mitochondrial respiratory chain complex I assembl	3/222	0.0100763	0.0248179	0.0121656	Ndufa12/Ndufa6/Ndufs8
3-day post-SCI group vs. the control group	BP	GO:0043030	regulation of macrophage activation	3/222	0.0100763	0.0248179	0.0121656	Snca/Tlr4/Tlr6
3-day post-SCI group vs. the control group	BP	GO:0045058	T cell selection	3/222	0.0100763	0.0248179	0.0121656	Braf/Ctsl/Stat6
3-day post-SCI group vs. the control group	BP	GO:0051496	positive regulation of stress fiber assembly	3/222	0.0100763	0.0248179	0.0121656	Braf/Nox4/Pxn
3-day post-SCI group vs. the control group	BP	GO:1904356	regulation of telomere maintenance via telomere le	3/222	0.0100763	0.0248179	0.0121656	Atr/Mapk3/Parp1
3-day post-SCI group vs. the control group	BP	GO:0002755	MyD88-dependent toll-like receptor signaling path	2/222	0.0101606	0.0248179	0.0121656	Tlr4/Tlr6
3-day post-SCI group vs. the control group	BP	GO:0007063	regulation of sister chromatid cohesion	2/222	0.0101606	0.0248179	0.0121656	Rb1/Sfpq
3-day post-SCI group vs. the control group	BP	GO:0014856	skeletal muscle cell proliferation	2/222	0.0101606	0.0248179	0.0121656	Jak2/Sirt1
3-day post-SCI group vs. the control group	BP	GO:0030502	negative regulation of bone mineralization	2/222	0.0101606	0.0248179	0.0121656	Ccr1/Hif1a
3-day post-SCI group vs. the control group	BP	GO:0032495	response to muramyl dipeptide	2/222	0.0101606	0.0248179	0.0121656	Mapk14/Rela
3-day post-SCI group vs. the control group	BP	GO:0033599	regulation of mammary gland epithelial cell prolife	2/222	0.0101606	0.0248179	0.0121656	Bax/Gpx1
3-day post-SCI group vs. the control group	BP	GO:0036120	cellular response to platelet-derived growth factor	2/222	0.0101606	0.0248179	0.0121656	Atp7a/Tlr4
3-day post-SCI group vs. the control group	BP	GO:0043373	CD4-positive, alpha-beta T cell lineage commitme	2/222	0.0101606	0.0248179	0.0121656	Ctsl/Stat6
3-day post-SCI group vs. the control group	BP	GO:0045019	negative regulation of nitric oxide biosynthetic prc	2/222	0.0101606	0.0248179	0.0121656	Sirpa/Zc3h12a
3-day post-SCI group vs. the control group	BP	GO:0045623	negative regulation of T-helper cell differentiation	2/222	0.0101606	0.0248179	0.0121656	Anxa1/Zc3h12a
3-day post-SCI group vs. the control group	BP	GO:0048148	behavioral response to cocaine	2/222	0.0101606	0.0248179	0.0121656	Parp1/Snca
3-day post-SCI group vs. the control group	BP	GO:0050966	detection of mechanical stimulus involved in senso	2/222	0.0101606	0.0248179	0.0121656	Pawr/Tlr4
3-day post-SCI group vs. the control group	BP	GO:0060149	negative regulation of post-transcriptional gene sil	2/222	0.0101606	0.0248179	0.0121656	Ppp3ca/Zc3h12a
3-day post-SCI group vs. the control group	BP	GO:0060967	negative regulation of gene silencing by RNA	2/222	0.0101606	0.0248179	0.0121656	Ppp3ca/Zc3h12a
3-day post-SCI group vs. the control group	BP	GO:0090141	positive regulation of mitochondrial fission	2/222	0.0101606	0.0248179	0.0121656	Bnip3/Pink1
3-day post-SCI group vs. the control group	BP	GO:1900369	negative regulation of post-transcriptional gene sil	2/222	0.0101606	0.0248179	0.0121656	Ppp3ca/Zc3h12a
3-day post-SCI group vs. the control group	BP	GO:1904406	negative regulation of nitric oxide metabolic proce	2/222	0.0101606	0.0248179	0.0121656	Sirpa/Zc3h12a
3-day post-SCI group vs. the control group	BP	GO:2000671	regulation of motor neuron apoptotic process	2/222	0.0101606	0.0248179	0.0121656	Bax/Map2k4
3-day post-SCI group vs. the control group	BP	GO:0002218	activation of innate immune response	3/222	0.0105571	0.0257268	0.0126112	Nono/Sfpq/Tlr4
3-day post-SCI group vs. the control group	BP	GO:0015695	organic cation transport	3/222	0.0105571	0.0257268	0.0126112	Actb/Ptgs1/Snca
3-day post-SCI group vs. the control group	BP	GO:0043616	keratinocyte proliferation	3/222	0.0105571	0.0257268	0.0126112	Ctsl/Nfatc1/Ptprk
3-day post-SCI group vs. the control group	BP	GO:1902808	positive regulation of cell cycle G1/S phase transit	3/222	0.0105571	0.0257268	0.0126112	Aif1/Anxa1/Ezh2
3-day post-SCI group vs. the control group	BP	GO:0008344	adult locomotory behavior	4/222	0.0106543	0.0259337	0.0127126	Htra2/Mapt/Oxr1/Snca
3-day post-SCI group vs. the control group	BP	GO:2000181	negative regulation of blood vessel morphogenesis	4/222	0.0106543	0.0259337	0.0127126	Ago1/Alox5/Atf2/Cd36
3-day post-SCI group vs. the control group	BP	GO:1901343	negative regulation of vasculature development	4/222	0.0109841	0.0267055	0.0130909	Ago1/Alox5/Atf2/Cd36
3-day post-SCI group vs. the control group	BP	GO:1905037	autophagosome organization	4/222	0.0109841	0.0267055	0.0130909	Atp13a2/Atp2a2/Pink1/Ubqln1
3-day post-SCI group vs. the control group	BP	GO:0008406	gonad development	6/222	0.0109922	0.0267098	0.013093	Bax/Pdgfra/Plekha1/Prdx4/Sirt1/Ybx3
3-day post-SCI group vs. the control group	BP	GO:0002548	monocyte chemotaxis	3/222	0.011051	0.0268062	0.0131403	Aif1/Anxa1/Ccr1

3-day post-SCI group vs. the control group	BP	GO:0015872	dopamine transport	3/222	0.011051	0.0268062	0.0131403	Chrna4/Pink1/Snca
3-day post-SCI group vs. the control group	BP	GO:0060443	mammary gland morphogenesis	3/222	0.011051	0.0268062	0.0131403	Bax/Nfkb1/Stat6
3-day post-SCI group vs. the control group	BP	GO:0003177	pulmonary valve development	2/222	0.0111738	0.0268414	0.0131575	Nfatc1/Tnfrsf1a
3-day post-SCI group vs. the control group	BP	GO:0006700	C21-steroid hormone biosynthetic process	2/222	0.0111738	0.0268414	0.0131575	Ppargc1a/Rest
3-day post-SCI group vs. the control group	BP	GO:0007252	I-kappaB phosphorylation	2/222	0.0111738	0.0268414	0.0131575	Sirpa/Tlr4
3-day post-SCI group vs. the control group	BP	GO:0032310	prostaglandin secretion	2/222	0.0111738	0.0268414	0.0131575	Map2k6/Ptgs2
3-day post-SCI group vs. the control group	BP	GO:0035067	negative regulation of histone acetylation	2/222	0.0111738	0.0268414	0.0131575	Sirt1/Snca
3-day post-SCI group vs. the control group	BP	GO:0035234	ectopic germ cell programmed cell death	2/222	0.0111738	0.0268414	0.0131575	Bax/Ybx3
3-day post-SCI group vs. the control group	BP	GO:0035584	calcium-mediated signaling using intracellular calc	2/222	0.0111738	0.0268414	0.0131575	Trpm2/Vcam1
3-day post-SCI group vs. the control group	BP	GO:0045722	positive regulation of gluconeogenesis	2/222	0.0111738	0.0268414	0.0131575	Hif1a/Sirt1
3-day post-SCI group vs. the control group	BP	GO:0045821	positive regulation of glycolytic process	2/222	0.0111738	0.0268414	0.0131575	Myc/Prkaa2
3-day post-SCI group vs. the control group	BP	GO:0046653	tetrahydrofolate metabolic process	2/222	0.0111738	0.0268414	0.0131575	Dhfr/Gch1
3-day post-SCI group vs. the control group	BP	GO:0048569	post-embryonic animal organ development	2/222	0.0111738	0.0268414	0.0131575	Bax/Jak2
3-day post-SCI group vs. the control group	BP	GO:0051900	regulation of mitochondrial depolarization	2/222	0.0111738	0.0268414	0.0131575	Gclc/Parp1
3-day post-SCI group vs. the control group	BP	GO:0060333	interferon-gamma-mediated signaling pathway	2/222	0.0111738	0.0268414	0.0131575	Arg1/Jak2
3-day post-SCI group vs. the control group	BP	GO:0070230	positive regulation of lymphocyte apoptotic proces	2/222	0.0111738	0.0268414	0.0131575	Bax/Myc
3-day post-SCI group vs. the control group	BP	GO:0090043	regulation of tubulin deacetylation	2/222	0.0111738	0.0268414	0.0131575	Mapt/Prkaa2
3-day post-SCI group vs. the control group	BP	GO:1903902	positive regulation of viral life cycle	2/222	0.0111738	0.0268414	0.0131575	Axl/P4hb
3-day post-SCI group vs. the control group	BP	GO:1990182	exosomal secretion	2/222	0.0111738	0.0268414	0.0131575	Atp13a2/Sdc1
3-day post-SCI group vs. the control group	BP	GO:0002027	regulation of heart rate	4/222	0.0113203	0.0271624	0.0133148	Fkbp1b/Gch1/Sirt1/Slc8a1
3-day post-SCI group vs. the control group	BP	GO:0005976	polysaccharide metabolic process	4/222	0.0113203	0.0271624	0.0133148	Cd36/Il6st/Nfkb1/Ppp1ca
3-day post-SCI group vs. the control group	BP	GO:1903311	regulation of mRNA metabolic process	7/222	0.011544	0.0276833	0.0135702	Mapkapk2/Paip1/Prdx6/Rest/Tnrc6a/Tnrc6c/Zc3h12a
3-day post-SCI group vs. the control group	BP	GO:0031663	lipopolysaccharide-mediated signaling pathway	3/222	0.011558	0.0276853	0.0135712	Mapk14/Mapk3/Tlr4
3-day post-SCI group vs. the control group	BP	GO:0045620	negative regulation of lymphocyte differentiation	3/222	0.011558	0.0276853	0.0135712	Anxa1/Hspb1/Zc3h12a
3-day post-SCI group vs. the control group	BP	GO:0045137	development of primary sexual characteristics	6/222	0.0118471	0.0283617	0.0139028	Bax/Pdgfra/Plekha1/Prdx4/Sirt1/Ybx3
3-day post-SCI group vs. the control group	BP	GO:0060562	epithelial tube morphogenesis	8/222	0.0119544	0.0286022	0.0140207	Casp3/Hif1a/Met/Myc/Nfatc1/Pdgfra/Ppp1ca/Pxn
3-day post-SCI group vs. the control group	BP	GO:0055088	lipid homeostasis	5/222	0.0119898	0.0286707	0.0140542	Adora1/Atp13a2/Pnpla8/Prkaa2/Sirt1
3-day post-SCI group vs. the control group	BP	GO:0007088	regulation of mitotic nuclear division	4/222	0.0120126	0.0287089	0.0140729	Apc/Cdc20/Met/Rb1
3-day post-SCI group vs. the control group	BP	GO:0031122	cytoplasmic microtubule organization	3/222	0.0120782	0.028833	0.0141338	Apc/Dst/Ezr
3-day post-SCI group vs. the control group	BP	GO:0045778	positive regulation of ossification	3/222	0.0120782	0.028833	0.0141338	Alox5/Rxrb/Slc8a1
3-day post-SCI group vs. the control group	BP	GO:0002363	alpha-beta T cell lineage commitment	2/222	0.0122296	0.0288996	0.0141664	Ctsl/Stat6
3-day post-SCI group vs. the control group	BP	GO:0003180	aortic valve morphogenesis	2/222	0.0122296	0.0288996	0.0141664	Nfatc1/Rb1
3-day post-SCI group vs. the control group	BP	GO:0010039	response to iron ion	2/222	0.0122296	0.0288996	0.0141664	Hmox1/Snca
3-day post-SCI group vs. the control group	BP	GO:0030810	positive regulation of nucleotide biosynthetic proc	2/222	0.0122296	0.0288996	0.0141664	Myc/Ppargc1a
3-day post-SCI group vs. the control group	BP	GO:0034312	diol biosynthetic process	2/222	0.0122296	0.0288996	0.0141664	Dhfr/Gch1
3-day post-SCI group vs. the control group	BP	GO:0044851	hair cycle phase	2/222	0.0122296	0.0288996	0.0141664	Ctsl/Ptgs2
3-day post-SCI group vs. the control group	BP	GO:0045649	regulation of macrophage differentiation	2/222	0.0122296	0.0288996	0.0141664	Rb1/Ripk1
3-day post-SCI group vs. the control group	BP	GO:0050910	detection of mechanical stimulus involved in senso	2/222	0.0122296	0.0288996	0.0141664	Myc/Rest
3-day post-SCI group vs. the control group	BP	GO:0051602	response to electrical stimulus	2/222	0.0122296	0.0288996	0.0141664	Actb/Rest
3-day post-SCI group vs. the control group	BP	GO:0060055	angiogenesis involved in wound healing	2/222	0.0122296	0.0288996	0.0141664	Alox5/Gpx1

3-day post-SCI group vs. the control group	BP	GO:0060253	negative regulation of glial cell proliferation	2/222	0.0122296	0.0288996	0.0141664	Cdkn2b/Rb1
3-day post-SCI group vs. the control group	BP	GO:0090026	positive regulation of monocyte chemotaxis	2/222	0.0122296	0.0288996	0.0141664	Aif1/Ccr1
3-day post-SCI group vs. the control group	BP	GO:0097734	extracellular exosome biogenesis	2/222	0.0122296	0.0288996	0.0141664	Atp13a2/Sdc1
3-day post-SCI group vs. the control group	BP	GO:0150104	transport across blood-brain barrier	2/222	0.0122296	0.0288996	0.0141664	Abcc1/Cd36
3-day post-SCI group vs. the control group	BP	GO:1900373	positive regulation of purine nucleotide biosynthet	2/222	0.0122296	0.0288996	0.0141664	Myc/Ppargc1a
3-day post-SCI group vs. the control group	BP	GO:1901387	positive regulation of voltage-gated calcium chann	2/222	0.0122296	0.0288996	0.0141664	Ppp3ca/S100a1
3-day post-SCI group vs. the control group	BP	GO:1902254	negative regulation of intrinsic apoptotic signaling	2/222	0.0122296	0.0288996	0.0141664	Rrm2b/Sirt1
3-day post-SCI group vs. the control group	BP	GO:1903672	positive regulation of sprouting angiogenesis	2/222	0.0122296	0.0288996	0.0141664	S100a1/Smad1
3-day post-SCI group vs. the control group	BP	GO:0030902	hindbrain development	5/222	0.0122615	0.0289425	0.0141874	Atf2/Atp7a/Atrn/Ezh2/Smad1
3-day post-SCI group vs. the control group	BP	GO:0043487	regulation of RNA stability	5/222	0.0122615	0.0289425	0.0141874	Mapkapk2/Paip1/Tnrc6a/Tnrc6c/Zc3h12a
3-day post-SCI group vs. the control group	BP	GO:0090596	sensory organ morphogenesis	7/222	0.0122985	0.0290134	0.0142222	Bax/Hdac1/Hif1a/Mapk3/Myc/Rest/Stau2
3-day post-SCI group vs. the control group	BP	GO:0006986	response to unfolded protein	4/222	0.0123687	0.0291463	0.0142874	Bax/Eif2s1/Hspb1/Nfe2l2
3-day post-SCI group vs. the control group	BP	GO:0007589	body fluid secretion	4/222	0.0123687	0.0291463	0.0142874	Adora1/Hif1a/Ppp3ca/Xdh
3-day post-SCI group vs. the control group	BP	GO:0000288	nuclear-transcribed mRNA catabolic process, deac	3/222	0.0126117	0.0296361	0.0145275	Paip1/Tnrc6a/Tnrc6c
3-day post-SCI group vs. the control group	BP	GO:0010171	body morphogenesis	3/222	0.0126117	0.0296361	0.0145275	Braf/Pdgfra/Plekha1
3-day post-SCI group vs. the control group	BP	GO:0010517	regulation of phospholipase activity	3/222	0.0126117	0.0296361	0.0145275	Anxa1/Pdgfra/Snca
3-day post-SCI group vs. the control group	BP	GO:0046173	polyol biosynthetic process	3/222	0.0126117	0.0296361	0.0145275	Dhfr/Gch1/Snca
3-day post-SCI group vs. the control group	BP	GO:0050435	amyloid-beta metabolic process	3/222	0.0126117	0.0296361	0.0145275	Casp3/Mgat3/Rela
3-day post-SCI group vs. the control group	BP	GO:0006310	DNA recombination	7/222	0.0126888	0.0298006	0.0146081	Actb/Ezh2/Mcm4/Nono/Parp1/Sfpq/Stat6
3-day post-SCI group vs. the control group	BP	GO:0019218	regulation of steroid metabolic process	4/222	0.0127314	0.0298675	0.0146409	Nfkb1/Ppargc1a/Rest/Sirt1
3-day post-SCI group vs. the control group	BP	GO:0038127	ERBB signaling pathway	4/222	0.0127314	0.0298675	0.0146409	Adora1/Braf/Fbxw7/Hbegf
3-day post-SCI group vs. the control group	BP	GO:0001763	morphogenesis of a branching structure	6/222	0.0127479	0.0298895	0.0146517	Met/Myc/Nfatc1/Pdgfra/Ppp1ca/Pxn
3-day post-SCI group vs. the control group	BP	GO:0001892	embryonic placenta development	4/222	0.0131009	0.0306849	0.0150416	Hif1a/Junb/Slc8a1/Vcam1
3-day post-SCI group vs. the control group	BP	GO:0016573	histone acetylation	5/222	0.0131017	0.0306849	0.0150416	Actb/Atf2/Mapk3/Sirt1/Snca
3-day post-SCI group vs. the control group	BP	GO:0019748	secondary metabolic process	3/222	0.0131585	0.0307837	0.01509	Atp7a/Cyp1b1/Nfe2l2
3-day post-SCI group vs. the control group	BP	GO:0070059	intrinsic apoptotic signaling pathway in response t	3/222	0.0131585	0.0307837	0.01509	Bax/Casp3/Sirt1
3-day post-SCI group vs. the control group	BP	GO:0000413	protein peptidyl-prolyl isomerization	2/222	0.0133273	0.0308698	0.0151322	Fkbp1b/Ppif
3-day post-SCI group vs. the control group	BP	GO:0010232	vascular transport	2/222	0.0133273	0.0308698	0.0151322	Abcc1/Cd36
3-day post-SCI group vs. the control group	BP	GO:0010880	regulation of release of sequestered calcium ion in	2/222	0.0133273	0.0308698	0.0151322	Fkbp1b/Slc8a1
3-day post-SCI group vs. the control group	BP	GO:0020027	hemoglobin metabolic process	2/222	0.0133273	0.0308698	0.0151322	Cat/Hif1a
3-day post-SCI group vs. the control group	BP	GO:0030539	male genitalia development	2/222	0.0133273	0.0308698	0.0151322	Dhcr24/Pdgfra
3-day post-SCI group vs. the control group	BP	GO:0030728	ovulation	2/222	0.0133273	0.0308698	0.0151322	Ptgs2/Sirt1
3-day post-SCI group vs. the control group	BP	GO:0036119	response to platelet-derived growth factor	2/222	0.0133273	0.0308698	0.0151322	Atp7a/Tlr4
3-day post-SCI group vs. the control group	BP	GO:0042053	regulation of dopamine metabolic process	2/222	0.0133273	0.0308698	0.0151322	Gpr37/Snca
3-day post-SCI group vs. the control group	BP	GO:0060456	positive regulation of digestive system process	2/222	0.0133273	0.0308698	0.0151322	Pawr/Ppp3ca
3-day post-SCI group vs. the control group	BP	GO:0071398	cellular response to fatty acid	2/222	0.0133273	0.0308698	0.0151322	Cdk4/Zc3h12a
3-day post-SCI group vs. the control group	BP	GO:0090201	negative regulation of release of cytochrome c fror	2/222	0.0133273	0.0308698	0.0151322	Gpx1/Ppif
3-day post-SCI group vs. the control group	BP	GO:0097284	hepatocyte apoptotic process	2/222	0.0133273	0.0308698	0.0151322	Atf2/Rb1
3-day post-SCI group vs. the control group	BP	GO:0098780	response to mitochondrial depolarisation	2/222	0.0133273	0.0308698	0.0151322	Htra2/Pink1
3-day post-SCI group vs. the control group	BP	GO:0120255	olefinic compound biosynthetic process	2/222	0.0133273	0.0308698	0.0151322	Ppargc1a/Rest

3-day post-SCI group vs. the control group	BP	GO:0140354	lipid import into cell	2/222	0.0133273	0.0308698	0.0151322	Abcc1/Cd36
3-day post-SCI group vs. the control group	BP	GO:1902894	negative regulation of miRNA transcription	2/222	0.0133273	0.0308698	0.0151322	Rela/Rest
3-day post-SCI group vs. the control group	BP	GO:2001026	regulation of endothelial cell chemotaxis	2/222	0.0133273	0.0308698	0.0151322	Hspb1/Met
3-day post-SCI group vs. the control group	BP	GO:2001224	positive regulation of neuron migration	2/222	0.0133273	0.0308698	0.0151322	Mapk8/Tbc1d24
3-day post-SCI group vs. the control group	BP	GO:0008585	female gonad development	4/222	0.0134772	0.0311827	0.0152856	Bax/Pdgfra/Plekha1/Sirt1
3-day post-SCI group vs. the control group	BP	GO:0045666	positive regulation of neuron differentiation	4/222	0.0134772	0.0311827	0.0152856	Ect2/Impact/Rest/Rnf112
3-day post-SCI group vs. the control group	BP	GO:0060041	retina development in camera-type eye	5/222	0.013683	0.0316241	0.015502	Apc/Bax/Cyp1b1/Hif1a/Pdgfra
3-day post-SCI group vs. the control group	BP	GO:0061013	regulation of mRNA catabolic process	5/222	0.013683	0.0316241	0.015502	Mapkapk2/Paip1/Tnrc6a/Tnrc6c/Zc3h12a
3-day post-SCI group vs. the control group	BP	GO:0042776	proton motive force-driven mitochondrial ATP syı	3/222	0.0137187	0.0316545	0.0155169	Ndufa12/Ndufa6/Ndufs8
3-day post-SCI group vs. the control group	BP	GO:0050854	regulation of antigen receptor-mediated signaling	3/222	0.0137187	0.0316545	0.0155169	Ezr/Pawr/Rela
3-day post-SCI group vs. the control group	BP	GO:0098930	axonal transport	3/222	0.0137187	0.0316545	0.0155169	Dst/Hif1a/Hspb1
3-day post-SCI group vs. the control group	BP	GO:0071675	regulation of mononuclear cell migration	4/222	0.0138603	0.0319637	0.0156684	Aif1/Ccr1/Mapk3/Ripk3
3-day post-SCI group vs. the control group	BP	GO:0006338	chromatin remodeling	7/222	0.0141252	0.0325568	0.0159592	Actb/Ezh2/Hdac1/Myc/Rb1/Rbbp7/Sirt1
3-day post-SCI group vs. the control group	BP	GO:0001505	regulation of neurotransmitter levels	6/222	0.0141877	0.0326829	0.016021	Atp2a2/Braf/Chrna4/Maoa/Snca/Syp
3-day post-SCI group vs. the control group	BP	GO:0090100	positive regulation of transmembrane receptor pro	4/222	0.0142502	0.032809	0.0160828	Cdkn2b/Jak2/Parp1/Rbpms
3-day post-SCI group vs. the control group	BP	GO:0009065	glutamine family amino acid catabolic process	2/222	0.0144662	0.0330532	0.0162025	Arg1/Prodh
3-day post-SCI group vs. the control group	BP	GO:0022403	cell cycle phase	2/222	0.0144662	0.0330532	0.0162025	Cdc20/Nfatc1
3-day post-SCI group vs. the control group	BP	GO:0032352	positive regulation of hormone metabolic process	2/222	0.0144662	0.0330532	0.0162025	Hif1a/Ppargc1a
3-day post-SCI group vs. the control group	BP	GO:0034405	response to fluid shear stress	2/222	0.0144662	0.0330532	0.0162025	Nfe2l2/Ptgs2
3-day post-SCI group vs. the control group	BP	GO:0034643	establishment of mitochondrion localization, micro	2/222	0.0144662	0.0330532	0.0162025	Hif1a/Mapt
3-day post-SCI group vs. the control group	BP	GO:0042537	benzene-containing compound metabolic process	2/222	0.0144662	0.0330532	0.0162025	Cyp1b1/Txnrd1
3-day post-SCI group vs. the control group	BP	GO:0043371	negative regulation of CD4-positive, alpha-beta T	2/222	0.0144662	0.0330532	0.0162025	Anxa1/Zc3h12a
3-day post-SCI group vs. the control group	BP	GO:0047497	mitochondrion transport along microtubule	2/222	0.0144662	0.0330532	0.0162025	Hif1a/Mapt
3-day post-SCI group vs. the control group	BP	GO:0051481	negative regulation of cytosolic calcium ion conce	2/222	0.0144662	0.0330532	0.0162025	Il6st/Slc8a1
3-day post-SCI group vs. the control group	BP	GO:0051882	mitochondrial depolarization	2/222	0.0144662	0.0330532	0.0162025	Gclc/Parp1
3-day post-SCI group vs. the control group	BP	GO:0090042	tubulin deacetylation	2/222	0.0144662	0.0330532	0.0162025	Mapt/Prkaa2
3-day post-SCI group vs. the control group	BP	GO:0140112	extracellular vesicle biogenesis	2/222	0.0144662	0.0330532	0.0162025	Atp13a2/Sdc1
3-day post-SCI group vs. the control group	BP	GO:2000629	negative regulation of miRNA metabolic process	2/222	0.0144662	0.0330532	0.0162025	Rela/Rest
3-day post-SCI group vs. the control group	BP	GO:2000637	positive regulation of miRNA-mediated gene silen	2/222	0.0144662	0.0330532	0.0162025	Nfkb1/Ripk1
3-day post-SCI group vs. the control group	BP	GO:0007269	neurotransmitter secretion	5/222	0.014587	0.0332932	0.0163202	Atp2a2/Braf/Chrna4/Snca/Syp
3-day post-SCI group vs. the control group	BP	GO:0099643	signal release from synapse	5/222	0.014587	0.0332932	0.0163202	Atp2a2/Braf/Chrna4/Snca/Syp
3-day post-SCI group vs. the control group	BP	GO:0046545	development of primary female sexual characterist	4/222	0.014647	0.0333759	0.0163607	Bax/Pdgfra/Plekha1/Sirt1
3-day post-SCI group vs. the control group	BP	GO:0098659	inorganic cation import across plasma membrane	4/222	0.014647	0.0333759	0.0163607	Pawr/Ppp3ca/Slc8a1/Trpm2
3-day post-SCI group vs. the control group	BP	GO:0099587	inorganic ion import across plasma membrane	4/222	0.014647	0.0333759	0.0163607	Pawr/Ppp3ca/Slc8a1/Trpm2
3-day post-SCI group vs. the control group	BP	GO:0009123	nucleoside monophosphate metabolic process	3/222	0.0148793	0.0338868	0.0166111	Nme2/Nudt2/Xdh
3-day post-SCI group vs. the control group	BP	GO:0002833	positive regulation of response to biotic stimulus	5/222	0.0148971	0.033909	0.016622	Arg1/Il18rap/Nono/Sfpq/Tlr4
3-day post-SCI group vs. the control group	BP	GO:0045580	regulation of T cell differentiation	5/222	0.0152115	0.034606	0.0169637	Actb/Anxa1/Braf/Hspb1/Zc3h12a
3-day post-SCI group vs. the control group	BP	GO:0002286	T cell activation involved in immune response	4/222	0.0154615	0.035007	0.0171603	Anxa1/Atp7a/Stat6/Zc3h12a
3-day post-SCI group vs. the control group	BP	GO:0031058	positive regulation of histone modification	4/222	0.0154615	0.035007	0.0171603	Eed/Mapk3/Pink1/Sirt1
3-day post-SCI group vs. the control group	BP	GO:1905954	positive regulation of lipid localization	4/222	0.0154615	0.035007	0.0171603	Cd36/Map2k6/Sirt1/Zc3h12a

3-day post-SCI group vs. the control group	BP	GO:0007584	response to nutrient	3/222	0.0154798	0.035007	0.0171603	Cdkn2b/Mapt/Pdk2
3-day post-SCI group vs. the control group	BP	GO:0030968	endoplasmic reticulum unfolded protein response	3/222	0.0154798	0.035007	0.0171603	Bax/Eif2s1/Nfe2l2
3-day post-SCI group vs. the control group	BP	GO:0098900	regulation of action potential	3/222	0.0154798	0.035007	0.0171603	Atp2a2/Cd36/Pawr
3-day post-SCI group vs. the control group	BP	GO:0010763	positive regulation of fibroblast migration	2/222	0.0156455	0.035007	0.0171603	Apc/Slc8a1
3-day post-SCI group vs. the control group	BP	GO:0010884	positive regulation of lipid storage	2/222	0.0156455	0.035007	0.0171603	Cd36/Zc3h12a
3-day post-SCI group vs. the control group	BP	GO:0010894	negative regulation of steroid biosynthetic process	2/222	0.0156455	0.035007	0.0171603	Nfkb1/Rest
3-day post-SCI group vs. the control group	BP	GO:0014048	regulation of glutamate secretion	2/222	0.0156455	0.035007	0.0171603	Adora1/Snca
3-day post-SCI group vs. the control group	BP	GO:0014808	release of sequestered calcium ion into cytosol by	2/222	0.0156455	0.035007	0.0171603	Fkbp1b/Slc8a1
3-day post-SCI group vs. the control group	BP	GO:0015732	prostaglandin transport	2/222	0.0156455	0.035007	0.0171603	Map2k6/Ptgs2
3-day post-SCI group vs. the control group	BP	GO:0030194	positive regulation of blood coagulation	2/222	0.0156455	0.035007	0.0171603	Cd36/Nfe2l2
3-day post-SCI group vs. the control group	BP	GO:0032780	negative regulation of ATP-dependent activity	2/222	0.0156455	0.035007	0.0171603	Ppif/Sirt1
3-day post-SCI group vs. the control group	BP	GO:0036010	protein localization to endosome	2/222	0.0156455	0.035007	0.0171603	Ezr/Mgat3
3-day post-SCI group vs. the control group	BP	GO:0043171	peptide catabolic process	2/222	0.0156455	0.035007	0.0171603	Ggt7/Tpp1
3-day post-SCI group vs. the control group	BP	GO:0045063	T-helper 1 cell differentiation	2/222	0.0156455	0.035007	0.0171603	Anxa1/Stat6
3-day post-SCI group vs. the control group	BP	GO:0050860	negative regulation of T cell receptor signaling pat	2/222	0.0156455	0.035007	0.0171603	Ezr/Pawr
3-day post-SCI group vs. the control group	BP	GO:0060148	positive regulation of post-transcriptional gene sile	2/222	0.0156455	0.035007	0.0171603	Nfkb1/Ripk1
3-day post-SCI group vs. the control group	BP	GO:0060547	negative regulation of necrotic cell death	2/222	0.0156455	0.035007	0.0171603	Ripk1/Ybx3
3-day post-SCI group vs. the control group	BP	GO:0070242	thymocyte apoptotic process	2/222	0.0156455	0.035007	0.0171603	Bax/Hif1a
3-day post-SCI group vs. the control group	BP	GO:0070734	histone H3-K27 methylation	2/222	0.0156455	0.035007	0.0171603	Eed/Ezh2
3-day post-SCI group vs. the control group	BP	GO:0097150	neuronal stem cell population maintenance	2/222	0.0156455	0.035007	0.0171603	Mapk8/Rest
3-day post-SCI group vs. the control group	BP	GO:0150105	protein localization to cell-cell junction	2/222	0.0156455	0.035007	0.0171603	Actb/Mapk8
3-day post-SCI group vs. the control group	BP	GO:1900048	positive regulation of hemostasis	2/222	0.0156455	0.035007	0.0171603	Cd36/Nfe2l2
3-day post-SCI group vs. the control group	BP	GO:1900370	positive regulation of post-transcriptional gene sile	2/222	0.0156455	0.035007	0.0171603	Nfkb1/Ripk1
3-day post-SCI group vs. the control group	BP	GO:1901028	regulation of mitochondrial outer membrane perm	2/222	0.0156455	0.035007	0.0171603	Gclc/Ier3
3-day post-SCI group vs. the control group	BP	GO:1902455	negative regulation of stem cell population mainte	2/222	0.0156455	0.035007	0.0171603	Hdac1/Rbbp7
3-day post-SCI group vs. the control group	BP	GO:1902993	positive regulation of amyloid precursor protein ca	2/222	0.0156455	0.035007	0.0171603	Casp3/Rela
3-day post-SCI group vs. the control group	BP	GO:2000679	positive regulation of transcription regulatory regi	2/222	0.0156455	0.035007	0.0171603	Parp1/Rb1
3-day post-SCI group vs. the control group	BP	GO:2000757	negative regulation of peptidyl-lysine acetylation	2/222	0.0156455	0.035007	0.0171603	Sirt1/Snca
3-day post-SCI group vs. the control group	BP	GO:0043547	positive regulation of GTPase activity	6/222	0.0157372	0.0351562	0.0172334	Agap3/Amph/Ect2/Ezh2/Itga6/Map4k4
3-day post-SCI group vs. the control group	BP	GO:0045926	negative regulation of growth	6/222	0.0157372	0.0351562	0.0172334	Apc/Cdkn2c/Hif1a/Rb1/Rbbp7/Sirt1
3-day post-SCI group vs. the control group	BP	GO:0048705	skeletal system morphogenesis	6/222	0.0157372	0.0351562	0.0172334	Atf2/Mapk14/Myc/Pdgfra/Plekha1/Ppargc1b
3-day post-SCI group vs. the control group	BP	GO:0006352	DNA-templated transcription initiation	4/222	0.0158792	0.035417	0.0173612	Brf2/Fosl1/Jun/Myc
3-day post-SCI group vs. the control group	BP	GO:0022037	metencephalon development	4/222	0.0158792	0.035417	0.0173612	Atf2/Atp7a/Atrn/Ezh2
3-day post-SCI group vs. the control group	BP	GO:0031398	positive regulation of protein ubiquitination	4/222	0.0158792	0.035417	0.0173612	Cdc20/Fbxw7/Mapk8/Ubqln1
3-day post-SCI group vs. the control group	BP	GO:0016042	lipid catabolic process	7/222	0.0159053	0.0354565	0.0173806	Adora1/Cdk4/Cyp1b1/Etfdh/Hao1/Pnpla8/Prdx6
3-day post-SCI group vs. the control group	BP	GO:0042246	tissue regeneration	3/222	0.0160938	0.0358386	0.0175679	Anxa1/Ezh2/Gpx1
3-day post-SCI group vs. the control group	BP	GO:0046324	regulation of glucose import	3/222	0.0160938	0.0358386	0.0175679	Mapk14/Myc/Nfe2l2
3-day post-SCI group vs. the control group	BP	GO:0006475	internal protein amino acid acetylation	5/222	0.0161814	0.0359956	0.0176449	Actb/Atf2/Mapk3/Sirt1/Snca
3-day post-SCI group vs. the control group	BP	GO:0018393	internal peptidyl-lysine acetylation	5/222	0.0161814	0.0359956	0.0176449	Actb/Atf2/Mapk3/Sirt1/Snca
3-day post-SCI group vs. the control group	BP	GO:0051983	regulation of chromosome segregation	4/222	0.0163039	0.0362492	0.0177692	Actb/Apc/Cdc20/Rb1

3-day post-SCI group vs. the control group	BP	GO:0015986	proton motive force-driven ATP synthesis	3/222	0.0167213	0.0370987	0.0181856	Ndufa12/Ndufa6/Ndufs8
3-day post-SCI group vs. the control group	BP	GO:0042058	regulation of epidermal growth factor receptor sig	3/222	0.0167213	0.0370987	0.0181856	Adora1/Fbxw7/Hbegf
3-day post-SCI group vs. the control group	BP	GO:0042509	regulation of tyrosine phosphorylation of STAT pr	3/222	0.0167213	0.0370987	0.0181856	Il6st/Jak2/Tnfrsf1a
3-day post-SCI group vs. the control group	BP	GO:0045600	positive regulation of fat cell differentiation	3/222	0.0167213	0.0370987	0.0181856	Mapk14/Ptgs2/Zc3h12a
3-day post-SCI group vs. the control group	BP	GO:0006760	folic acid-containing compound metabolic process	2/222	0.0168646	0.0372401	0.0182549	Dhfr/Gch1
3-day post-SCI group vs. the control group	BP	GO:0007064	mitotic sister chromatid cohesion	2/222	0.0168646	0.0372401	0.0182549	Cdc20/Rb1
3-day post-SCI group vs. the control group	BP	GO:0035743	CD4-positive, alpha-beta T cell cytokine productic	2/222	0.0168646	0.0372401	0.0182549	Arg1/Il18rap
3-day post-SCI group vs. the control group	BP	GO:0045939	negative regulation of steroid metabolic process	2/222	0.0168646	0.0372401	0.0182549	Nfkb1/Rest
3-day post-SCI group vs. the control group	BP	GO:0051354	negative regulation of oxidoreductase activity	2/222	0.0168646	0.0372401	0.0182549	Nfkb1/Snca
3-day post-SCI group vs. the control group	BP	GO:0060765	regulation of androgen receptor signaling pathway	2/222	0.0168646	0.0372401	0.0182549	Hdac1/Sirt1
3-day post-SCI group vs. the control group	BP	GO:0071459	protein localization to chromosome, centromeric r	2/222	0.0168646	0.0372401	0.0182549	Cdk1/Rb1
3-day post-SCI group vs. the control group	BP	GO:1902473	regulation of protein localization to synapse	2/222	0.0168646	0.0372401	0.0182549	Mapt/Tnik
3-day post-SCI group vs. the control group	BP	GO:1903514	release of sequestered calcium ion into cytosol by	2/222	0.0168646	0.0372401	0.0182549	Fkbp1b/Slc8a1
3-day post-SCI group vs. the control group	BP	GO:0048565	digestive tract development	4/222	0.0171747	0.0379049	0.0185808	Hif1a/Il6st/Pdgfra/Rb1
3-day post-SCI group vs. the control group	BP	GO:0031644	regulation of nervous system process	5/222	0.0171915	0.0379223	0.0185893	Adora1/Chrna4/Pawr/Tbc1d24/Tlr4
3-day post-SCI group vs. the control group	BP	GO:0001937	negative regulation of endothelial cell proliferatior	3/222	0.0173623	0.038199	0.0187249	Alox5/Apc/Xdh
3-day post-SCI group vs. the control group	BP	GO:0014009	glial cell proliferation	3/222	0.0173623	0.038199	0.0187249	Cdkn2b/Myc/Rb1
3-day post-SCI group vs. the control group	BP	GO:0030193	regulation of blood coagulation	3/222	0.0173623	0.038199	0.0187249	Cd36/Nfe2l2/Pdgfra
3-day post-SCI group vs. the control group	BP	GO:0034121	regulation of toll-like receptor signaling pathway	3/222	0.0173623	0.038199	0.0187249	Cd36/Tlr6/Ubqln1
3-day post-SCI group vs. the control group	BP	GO:2000144	positive regulation of DNA-templated transcriptior	3/222	0.0173623	0.038199	0.0187249	Fosl1/Jun/Myc
3-day post-SCI group vs. the control group	BP	GO:0003073	regulation of systemic arterial blood pressure	4/222	0.0176207	0.0387067	0.0189738	Adora1/Cd36/Ier3/Sod2
3-day post-SCI group vs. the control group	BP	GO:0006997	nucleus organization	4/222	0.0176207	0.0387067	0.0189738	Agap3/Banf1/Ets1/Sirt1
3-day post-SCI group vs. the control group	BP	GO:0009593	detection of chemical stimulus	4/222	0.0176207	0.0387067	0.0189738	Lpo/Sod2/Tlr4/Tlr6
3-day post-SCI group vs. the control group	BP	GO:0000271	polysaccharide biosynthetic process	3/222	0.018017	0.0393987	0.0193131	Cd36/Nfkb1/Ppp1ca
3-day post-SCI group vs. the control group	BP	GO:0035065	regulation of histone acetylation	3/222	0.018017	0.0393987	0.0193131	Mapk3/Sirt1/Snca
3-day post-SCI group vs. the control group	BP	GO:0043967	histone H4 acetylation	3/222	0.018017	0.0393987	0.0193131	Actb/Atf2/Sirt1
3-day post-SCI group vs. the control group	BP	GO:0000289	nuclear-transcribed mRNA poly(A) tail shortening	2/222	0.0181229	0.0393987	0.0193131	Tnrc6a/Tnrc6c
3-day post-SCI group vs. the control group	BP	GO:0014829	vascular associated smooth muscle contraction	2/222	0.0181229	0.0393987	0.0193131	Cd38/Slc8a1
3-day post-SCI group vs. the control group	BP	GO:0023019	signal transduction involved in regulation of gene	2/222	0.0181229	0.0393987	0.0193131	Parp1/Pdgfra
3-day post-SCI group vs. the control group	BP	GO:0031338	regulation of vesicle fusion	2/222	0.0181229	0.0393987	0.0193131	Anxa1/Snca
3-day post-SCI group vs. the control group	BP	GO:0032069	regulation of nuclease activity	2/222	0.0181229	0.0393987	0.0193131	Pcna/Sirt1
3-day post-SCI group vs. the control group	BP	GO:0032528	microvillus organization	2/222	0.0181229	0.0393987	0.0193131	Ezr/Tnik
3-day post-SCI group vs. the control group	BP	GO:0036003	positive regulation of transcription from RNA poly	2/222	0.0181229	0.0393987	0.0193131	Hif1a/Nfe2l2
3-day post-SCI group vs. the control group	BP	GO:0036336	dendritic cell migration	2/222	0.0181229	0.0393987	0.0193131	Alox5/Trpm2
3-day post-SCI group vs. the control group	BP	GO:0045723	positive regulation of fatty acid biosynthetic proce	2/222	0.0181229	0.0393987	0.0193131	Anxa1/Ptgs2
3-day post-SCI group vs. the control group	BP	GO:0051443	positive regulation of ubiquitin-protein transferase	2/222	0.0181229	0.0393987	0.0193131	Cdc20/Fbxw7
3-day post-SCI group vs. the control group	BP	GO:0051654	establishment of mitochondrion localization	2/222	0.0181229	0.0393987	0.0193131	Hif1a/Mapt
3-day post-SCI group vs. the control group	BP	GO:0060143	positive regulation of syncytium formation by plas	2/222	0.0181229	0.0393987	0.0193131	Adam9/Mapk14
3-day post-SCI group vs. the control group	BP	GO:0060575	intestinal epithelial cell differentiation	2/222	0.0181229	0.0393987	0.0193131	Hif1a/Il6st
3-day post-SCI group vs. the control group	BP	GO:0070168	negative regulation of biomineral tissue developm	2/222	0.0181229	0.0393987	0.0193131	Ccr1/Hif1a

3-day post-SCI group vs. the control group	BP	GO:1901223	negative regulation of NIK/NF-kappaB signaling	2/222	0.0181229	0.0393987	0.0193131	Rela/Zc3h12a
3-day post-SCI group vs. the control group	BP	GO:1901984	negative regulation of protein acetylation	2/222	0.0181229	0.0393987	0.0193131	Sirt1/Snca
3-day post-SCI group vs. the control group	BP	GO:2000647	negative regulation of stem cell proliferation	2/222	0.0181229	0.0393987	0.0193131	Cdkn2c/Nfatc1
3-day post-SCI group vs. the control group	BP	GO:0031929	TOR signaling	4/222	0.0185343	0.0402725	0.0197414	Endog/Hif1a/Prkaa2/Sirt1
3-day post-SCI group vs. the control group	BP	GO:0007259	receptor signaling pathway via JAK-STAT	5/222	0.0186023	0.0403786	0.0197934	Cyp1b1/Il6st/Jak2/Stat6/Tnfrsf1a
3-day post-SCI group vs. the control group	BP	GO:0051302	regulation of cell division	5/222	0.0186023	0.0403786	0.0197934	Apc/Cat/Ect2/Myc/Txnip
3-day post-SCI group vs. the control group	BP	GO:0019080	viral gene expression	3/222	0.0186852	0.0404543	0.0198305	Hdac1/Jun/Rest
3-day post-SCI group vs. the control group	BP	GO:0061035	regulation of cartilage development	3/222	0.0186852	0.0404543	0.0198305	Bmp1/Rela/Smad1
3-day post-SCI group vs. the control group	BP	GO:1900046	regulation of hemostasis	3/222	0.0186852	0.0404543	0.0198305	Cd36/Nfe2l2/Pdgfra
3-day post-SCI group vs. the control group	BP	GO:1990868	response to chemokine	3/222	0.0186852	0.0404543	0.0198305	Ccr1/Hif1a/Zc3h12a
3-day post-SCI group vs. the control group	BP	GO:1990869	cellular response to chemokine	3/222	0.0186852	0.0404543	0.0198305	Ccr1/Hif1a/Zc3h12a
3-day post-SCI group vs. the control group	BP	GO:0051304	chromosome separation	4/222	0.019002	0.0411189	0.0201563	Actb/Apc/Cdc20/Rb1
3-day post-SCI group vs. the control group	BP	GO:0048588	developmental cell growth	6/222	0.0191804	0.0414837	0.0203351	Cttn/Impact/Map2k4/Mapt/Sirt1/Slc23a2
3-day post-SCI group vs. the control group	BP	GO:0010833	telomere maintenance via telomere lengthening	3/222	0.0193671	0.0416161	0.0204	Atr/Mapk3/Parp1
3-day post-SCI group vs. the control group	BP	GO:0014068	positive regulation of phosphatidylinositol 3-kinas	3/222	0.0193671	0.0416161	0.0204	Cat/Jak2/Sirt1
3-day post-SCI group vs. the control group	BP	GO:0032623	interleukin-2 production	3/222	0.0193671	0.0416161	0.0204	Anxa1/Ezr/Pawr
3-day post-SCI group vs. the control group	BP	GO:0006925	inflammatory cell apoptotic process	2/222	0.0194195	0.0416161	0.0204	Anxa1/Sirt1
3-day post-SCI group vs. the control group	BP	GO:0018126	protein hydroxylation	2/222	0.0194195	0.0416161	0.0204	P4hb/Prdx4
3-day post-SCI group vs. the control group	BP	GO:0031065	positive regulation of histone deacetylation	2/222	0.0194195	0.0416161	0.0204	Pink1/Sirt1
3-day post-SCI group vs. the control group	BP	GO:0031670	cellular response to nutrient	2/222	0.0194195	0.0416161	0.0204	Cdkn2b/Pdk2
3-day post-SCI group vs. the control group	BP	GO:0032469	endoplasmic reticulum calcium ion homeostasis	2/222	0.0194195	0.0416161	0.0204	Atp2a2/Bax
3-day post-SCI group vs. the control group	BP	GO:0032480	negative regulation of type I interferon production	2/222	0.0194195	0.0416161	0.0204	Banf1/Sirpa
3-day post-SCI group vs. the control group	BP	GO:0034063	stress granule assembly	2/222	0.0194195	0.0416161	0.0204	Eif2s1/Prkaa2
3-day post-SCI group vs. the control group	BP	GO:0042104	positive regulation of activated T cell proliferation	2/222	0.0194195	0.0416161	0.0204	Igfbp2/Ppp3ca
3-day post-SCI group vs. the control group	BP	GO:0046639	negative regulation of alpha-beta T cell differentia	2/222	0.0194195	0.0416161	0.0204	Anxa1/Zc3h12a
3-day post-SCI group vs. the control group	BP	GO:0046716	muscle cell cellular homeostasis	2/222	0.0194195	0.0416161	0.0204	Apc/Hif1a
3-day post-SCI group vs. the control group	BP	GO:0048820	hair follicle maturation	2/222	0.0194195	0.0416161	0.0204	Ctsl/Ptgs2
3-day post-SCI group vs. the control group	BP	GO:0050820	positive regulation of coagulation	2/222	0.0194195	0.0416161	0.0204	Cd36/Nfe2l2
3-day post-SCI group vs. the control group	BP	GO:0098581	detection of external biotic stimulus	2/222	0.0194195	0.0416161	0.0204	Tlr4/Tlr6
3-day post-SCI group vs. the control group	BP	GO:0110150	negative regulation of biomineralization	2/222	0.0194195	0.0416161	0.0204	Ccr1/Hif1a
3-day post-SCI group vs. the control group	BP	GO:1900151	regulation of nuclear-transcribed mRNA catabolic	2/222	0.0194195	0.0416161	0.0204	Paip1/Tnrc6c
3-day post-SCI group vs. the control group	BP	GO:2000779	regulation of double-strand break repair	4/222	0.0194769	0.0417177	0.0204498	Actb/Atr/Mgmt/Parp1
3-day post-SCI group vs. the control group	BP	GO:0006898	receptor-mediated endocytosis	6/222	0.0194887	0.041722	0.0204519	Cd36/Ctsl/Cttn/Ezr/Sdc1/Snca
3-day post-SCI group vs. the control group	BP	GO:0014065	phosphatidylinositol 3-kinase signaling	4/222	0.019959	0.042707	0.0209348	Cat/Jak2/Plekha1/Sirt1
3-day post-SCI group vs. the control group	BP	GO:0007260	tyrosine phosphorylation of STAT protein	3/222	0.0200626	0.0428416	0.0210007	Il6st/Jak2/Tnfrsf1a
3-day post-SCI group vs. the control group	BP	GO:0045911	positive regulation of DNA recombination	3/222	0.0200626	0.0428416	0.0210007	Actb/Parp1/Stat6
3-day post-SCI group vs. the control group	BP	GO:0050818	regulation of coagulation	3/222	0.0200626	0.0428416	0.0210007	Cd36/Nfe2l2/Pdgfra
3-day post-SCI group vs. the control group	BP	GO:1901184	regulation of ERBB signaling pathway	3/222	0.0200626	0.0428416	0.0210007	Adora1/Fbxw7/Hbegf
3-day post-SCI group vs. the control group	BP	GO:0031056	regulation of histone modification	5/222	0.0200874	0.0428511	0.0210054	Eed/Mapk3/Pink1/Sirt1/Snca
3-day post-SCI group vs. the control group	BP	GO:0098656	anion transmembrane transport	5/222	0.0200874	0.0428511	0.0210054	Abcc1/Arg1/Myc/Ripk1/Slc23a2

3-day post-SCI group vs. the control group	BP	GO:0043543	protein acylation	6/222	0.0201155	0.0428893	0.0210241	Actb/Atf2/Mapk3/Prkaa2/Sirt1/Snca
3-day post-SCI group vs. the control group	BP	GO:0032414	positive regulation of ion transmembrane transport	4/222	0.0204485	0.0435773	0.0213613	Alox5/Atp7a/Ppp3ca/S100a1
3-day post-SCI group vs. the control group	BP	GO:0001780	neutrophil homeostasis	2/222	0.020754	0.0440653	0.0216006	Anxa1/Axl
3-day post-SCI group vs. the control group	BP	GO:0006120	mitochondrial electron transport, NADH to ubiqui	2/222	0.020754	0.0440653	0.0216006	Ndufs8/Pink1
3-day post-SCI group vs. the control group	BP	GO:0042659	regulation of cell fate specification	2/222	0.020754	0.0440653	0.0216006	Hdac1/Rbbp7
3-day post-SCI group vs. the control group	BP	GO:0048384	retinoic acid receptor signaling pathway	2/222	0.020754	0.0440653	0.0216006	Ezh2/Rxrb
3-day post-SCI group vs. the control group	BP	GO:0060045	positive regulation of cardiac muscle cell prolifera	2/222	0.020754	0.0440653	0.0216006	Cdk1/Mapk14
3-day post-SCI group vs. the control group	BP	GO:2000765	regulation of cytoplasmic translation	2/222	0.020754	0.0440653	0.0216006	Paip1/Rpl13a
3-day post-SCI group vs. the control group	BP	GO:0050864	regulation of B cell activation	6/222	0.0207558	0.0440653	0.0216006	Btk/Casp3/Cd38/Pawr/Stat6/Tlr4
3-day post-SCI group vs. the control group	BP	GO:0006305	DNA alkylation	3/222	0.0207717	0.0440653	0.0216006	Ezh2/Myc/Parp1
3-day post-SCI group vs. the control group	BP	GO:0006306	DNA methylation	3/222	0.0207717	0.0440653	0.0216006	Ezh2/Myc/Parp1
3-day post-SCI group vs. the control group	BP	GO:0110053	regulation of actin filament organization	6/222	0.0210811	0.0446992	0.0219113	Braf/Cttn/Id1/Met/Nox4/Pxn
3-day post-SCI group vs. the control group	BP	GO:0097696	receptor signaling pathway via STAT	5/222	0.0212508	0.0450363	0.0220766	Cyp1b1/Il6st/Jak2/Stat6/Tnfrsf1a
3-day post-SCI group vs. the control group	BP	GO:0090398	cellular senescence	4/222	0.0214495	0.0454345	0.0222718	Cdkn2b/Mapk14/Pawr/Sirt1
3-day post-SCI group vs. the control group	BP	GO:0120032	regulation of plasma membrane bounded cell proje	5/222	0.0216482	0.0458323	0.0224668	Apc/Atp7a/Cdk10/Stau2/Trpm2
3-day post-SCI group vs. the control group	BP	GO:0017015	regulation of transforming growth factor beta rece	4/222	0.0219611	0.046448	0.0227686	Cdkn2b/Hdac1/Rbbp7/Sirt1
3-day post-SCI group vs. the control group	BP	GO:0035966	response to topologically incorrect protein	4/222	0.0219611	0.046448	0.0227686	Bax/Eif2s1/Hspb1/Nfe2l2
3-day post-SCI group vs. the control group	BP	GO:0005979	regulation of glycogen biosynthetic process	2/222	0.0221257	0.0464926	0.0227904	Cd36/Ppp1ca
3-day post-SCI group vs. the control group	BP	GO:0008053	mitochondrial fusion	2/222	0.0221257	0.0464926	0.0227904	Bax/Bnip3
3-day post-SCI group vs. the control group	BP	GO:0010962	regulation of glucan biosynthetic process	2/222	0.0221257	0.0464926	0.0227904	Cd36/Ppp1ca
3-day post-SCI group vs. the control group	BP	GO:0030878	thyroid gland development	2/222	0.0221257	0.0464926	0.0227904	Braf/Mapk3
3-day post-SCI group vs. the control group	BP	GO:0034311	diol metabolic process	2/222	0.0221257	0.0464926	0.0227904	Dhfr/Gch1
3-day post-SCI group vs. the control group	BP	GO:0043032	positive regulation of macrophage activation	2/222	0.0221257	0.0464926	0.0227904	Tlr4/Tlr6
3-day post-SCI group vs. the control group	BP	GO:0043651	linoleic acid metabolic process	2/222	0.0221257	0.0464926	0.0227904	Alox5/Pnpla8
3-day post-SCI group vs. the control group	BP	GO:0046033	AMP metabolic process	2/222	0.0221257	0.0464926	0.0227904	Nudt2/Xdh
3-day post-SCI group vs. the control group	BP	GO:0071312	cellular response to alkaloid	2/222	0.0221257	0.0464926	0.0227904	Casp3/Slc8a1
3-day post-SCI group vs. the control group	BP	GO:0071353	cellular response to interleukin-4	2/222	0.0221257	0.0464926	0.0227904	Cdk4/Stat6
3-day post-SCI group vs. the control group	BP	GO:0090025	regulation of monocyte chemotaxis	2/222	0.0221257	0.0464926	0.0227904	Aif1/Ccr1
3-day post-SCI group vs. the control group	BP	GO:0097205	renal filtration	2/222	0.0221257	0.0464926	0.0227904	Adora1/Ppp3ca
3-day post-SCI group vs. the control group	BP	GO:1902042	negative regulation of extrinsic apoptotic signaling	2/222	0.0221257	0.0464926	0.0227904	Gpx1/Hmox1
3-day post-SCI group vs. the control group	BP	GO:0042130	negative regulation of T cell proliferation	3/222	0.0222309	0.0466904	0.0228874	Arg1/Casp3/Pawr
3-day post-SCI group vs. the control group	BP	GO:0032434	regulation of proteasomal ubiquitin-dependent pro	4/222	0.02248	0.0471902	0.0231324	Cdc20/Gclc/Mapk8/Ubqln1
3-day post-SCI group vs. the control group	BP	GO:0033143	regulation of intracellular steroid hormone recepto	3/222	0.0229809	0.0481936	0.0236242	Hdac1/Parp1/Sirt1
3-day post-SCI group vs. the control group	BP	GO:0045669	positive regulation of osteoblast differentiation	3/222	0.0229809	0.0481936	0.0236242	Il6st/Ppp3ca/Smad1
3-day post-SCI group vs. the control group	BP	GO:0002026	regulation of the force of heart contraction	2/222	0.0235338	0.0489764	0.024008	Atp2a2/Slc8a1
3-day post-SCI group vs. the control group	BP	GO:0009162	deoxyribonucleoside monophosphate metabolic pr	2/222	0.0235338	0.0489764	0.024008	Nme2/Xdh
3-day post-SCI group vs. the control group	BP	GO:0010460	positive regulation of heart rate	2/222	0.0235338	0.0489764	0.024008	Gch1/Sirt1
3-day post-SCI group vs. the control group	BP	GO:0010623	programmed cell death involved in cell developme	2/222	0.0235338	0.0489764	0.024008	Bax/Ybx3
3-day post-SCI group vs. the control group	BP	GO:0035455	response to interferon-alpha	2/222	0.0235338	0.0489764	0.024008	Axl/Myc
3-day post-SCI group vs. the control group	BP	GO:0035767	endothelial cell chemotaxis	2/222	0.0235338	0.0489764	0.024008	Hspb1/Met

3-day post-SCI group vs. the control group	BP	GO:0042558	pteridine-containing compound metabolic process	2/222	0.0235338	0.0489764	0.024008	Dhfr/Gch1
3-day post-SCI group vs. the control group	BP	GO:0043276	anoikis	2/222	0.0235338	0.0489764	0.024008	Mcl1/Tfdp1
3-day post-SCI group vs. the control group	BP	GO:0048535	lymph node development	2/222	0.0235338	0.0489764	0.024008	Nfkb1/Ripk3
3-day post-SCI group vs. the control group	BP	GO:0050858	negative regulation of antigen receptor-mediated s	2/222	0.0235338	0.0489764	0.024008	Ezr/Pawr
3-day post-SCI group vs. the control group	BP	GO:0060561	apoptotic process involved in morphogenesis	2/222	0.0235338	0.0489764	0.024008	Bax/Tnfrsf1a
3-day post-SCI group vs. the control group	BP	GO:0070198	protein localization to chromosome, telomeric regi	2/222	0.0235338	0.0489764	0.024008	Atr/Tpp1
3-day post-SCI group vs. the control group	BP	GO:1901798	positive regulation of signal transduction by p53 c	2/222	0.0235338	0.0489764	0.024008	Atr/Myc
3-day post-SCI group vs. the control group	BP	GO:2000316	regulation of T-helper 17 type immune response	2/222	0.0235338	0.0489764	0.024008	Jak2/Zc3h12a
3-day post-SCI group vs. the control group	BP	GO:0055123	digestive system development	4/222	0.0235403	0.0489764	0.024008	Hif1a/Il6st/Pdgfra/Rb1
3-day post-SCI group vs. the control group	BP	GO:1903844	regulation of cellular response to transforming gro	4/222	0.0235403	0.0489764	0.024008	Cdkn2b/Hdac1/Rbbp7/Sirt1
3-day post-SCI group vs. the control group	BP	GO:0016055	Wnt signaling pathway	8/222	0.0236902	0.049207	0.024121	Apc/Hdac1/Mapk14/Myc/Nfkb1/Prkaa2/Sdc1/Tnik
3-day post-SCI group vs. the control group	BP	GO:0030307	positive regulation of cell growth	5/222	0.0237074	0.049207	0.024121	Cd38/Hbegf/Mapt/Sirt1/Slc23a2
3-day post-SCI group vs. the control group	BP	GO:0005977	glycogen metabolic process	3/222	0.0237446	0.049207	0.024121	Cd36/Il6st/Ppp1ca
3-day post-SCI group vs. the control group	BP	GO:0006073	cellular glucan metabolic process	3/222	0.0237446	0.049207	0.024121	Cd36/Il6st/Ppp1ca
3-day post-SCI group vs. the control group	BP	GO:0044773	mitotic DNA damage checkpoint signaling	3/222	0.0237446	0.049207	0.024121	Atf2/Cdk1/Ier3
3-day post-SCI group vs. the control group	BP	GO:0051149	positive regulation of muscle cell differentiation	3/222	0.0237446	0.049207	0.024121	Mapk14/Sirt1/Sod2
3-day post-SCI group vs. the control group	BP	GO:2000142	regulation of DNA-templated transcription initiati	3/222	0.0237446	0.049207	0.024121	Fosl1/Jun/Myc
3-day post-SCI group vs. the control group	BP	GO:2000242	negative regulation of reproductive process	3/222	0.0237446	0.049207	0.024121	Apc/Prdx4/Ybx3
3-day post-SCI group vs. the control group	BP	GO:0045089	positive regulation of innate immune response	4/222	0.0240817	0.0498809	0.0244514	Il18rap/Nono/Sfpq/Tlr4
3-day post-SCI group vs. the control group	BP	GO:0198738	cell-cell signaling by wnt	8/222	0.0242401	0.0501843	0.0246001	Apc/Hdac1/Mapk14/Myc/Nfkb1/Prkaa2/Sdc1/Tnik
3-day post-SCI group vs. the control group	BP	GO:0007405	neuroblast proliferation	3/222	0.0245219	0.0506683	0.0248373	Ctnna1/Hif1a/Mapk8
3-day post-SCI group vs. the control group	BP	GO:0044042	glucan metabolic process	3/222	0.0245219	0.0506683	0.0248373	Cd36/Il6st/Ppp1ca
3-day post-SCI group vs. the control group	BP	GO:0045471	response to ethanol	3/222	0.0245219	0.0506683	0.0248373	Cdk1/Sirt1/Tlr4
3-day post-SCI group vs. the control group	BP	GO:0046626	regulation of insulin receptor signaling pathway	3/222	0.0245219	0.0506683	0.0248373	Cdk4/Rela/Sirt1
3-day post-SCI group vs. the control group	BP	GO:0032411	positive regulation of transporter activity	4/222	0.0246305	0.0508427	0.0249228	Alox5/Atp7a/Ppp3ca/S100a1
3-day post-SCI group vs. the control group	BP	GO:0035270	endocrine system development	4/222	0.0246305	0.0508427	0.0249228	Anxa1/Braf/Mapk3/Pdgfra
3-day post-SCI group vs. the control group	BP	GO:0051656	establishment of organelle localization	8/222	0.0247988	0.051165	0.0250808	Atp13a2/Ezr/Hif1a/Hmox1/Mapk8/Mapt/Sdc1/Snca
3-day post-SCI group vs. the control group	BP	GO:0000096	sulfur amino acid metabolic process	2/222	0.0249778	0.051308	0.0251509	Gclc/Nox4
3-day post-SCI group vs. the control group	BP	GO:0016242	negative regulation of macroautophagy	2/222	0.0249778	0.051308	0.0251509	Hmox1/Pink1
3-day post-SCI group vs. the control group	BP	GO:0030224	monocyte differentiation	2/222	0.0249778	0.051308	0.0251509	Jun/Myc
3-day post-SCI group vs. the control group	BP	GO:0035162	embryonic hemopoiesis	2/222	0.0249778	0.051308	0.0251509	Hif1a/Tnrc6c
3-day post-SCI group vs. the control group	BP	GO:0044030	regulation of DNA methylation	2/222	0.0249778	0.051308	0.0251509	Myc/Parp1
3-day post-SCI group vs. the control group	BP	GO:0070633	transepithelial transport	2/222	0.0249778	0.051308	0.0251509	Abcc1/Actb
3-day post-SCI group vs. the control group	BP	GO:0070670	response to interleukin-4	2/222	0.0249778	0.051308	0.0251509	Cdk4/Stat6
3-day post-SCI group vs. the control group	BP	GO:0072337	modified amino acid transport	2/222	0.0249778	0.051308	0.0251509	Abcc1/Mgst1
3-day post-SCI group vs. the control group	BP	GO:1904752	regulation of vascular associated smooth muscle c	2/222	0.0249778	0.051308	0.0251509	Atp7a/Nfe2l2
3-day post-SCI group vs. the control group	BP	GO:0006302	double-strand break repair	6/222	0.0256283	0.0526185	0.0257933	Actb/Atr/Mcm4/Mgmt/Parp1/Sfpq
3-day post-SCI group vs. the control group	BP	GO:0008625	extrinsic apoptotic signaling pathway via death do	3/222	0.0261175	0.0535708	0.0262601	Bax/Gpx1/Hmox1
3-day post-SCI group vs. the control group	BP	GO:0070373	negative regulation of ERK1 and ERK2 cascade	3/222	0.0261175	0.0535708	0.0262601	Ezr/Sirpa/Tlr4
3-day post-SCI group vs. the control group	BP	GO:0031503	protein-containing complex localization	5/222	0.0263404	0.0540017	0.0264713	Ezr/Mapk10/Sfpq/Stau2/Tnik

3-day post-SCI group vs. the control group	BP	GO:0032212	positive regulation of telomere maintenance via te	2/222	0.026457	0.054109	0.026524	Atr/Mapk3
3-day post-SCI group vs. the control group	BP	GO:0033260	nuclear DNA replication	2/222	0.026457	0.054109	0.026524	Mcm4/Pcna
3-day post-SCI group vs. the control group	BP	GO:0120178	steroid hormone biosynthetic process	2/222	0.026457	0.054109	0.026524	Ppargc1a/Rest
3-day post-SCI group vs. the control group	BP	GO:1901797	negative regulation of signal transduction by p53 c	2/222	0.026457	0.054109	0.026524	Rrm2b/Sirt1
3-day post-SCI group vs. the control group	BP	GO:1904706	negative regulation of vascular associated smooth	2/222	0.026457	0.054109	0.026524	Hmox1/Sod2
3-day post-SCI group vs. the control group	BP	GO:0043010	camera-type eye development	7/222	0.0265662	0.054306	0.0266205	Apc/Bax/Cyp1b1/Hdac1/Hif1a/Jun/Pdgfra
3-day post-SCI group vs. the control group	BP	GO:0051783	regulation of nuclear division	4/222	0.0269012	0.0549374	0.0269301	Apc/Cdc20/Met/Rb1
3-day post-SCI group vs. the control group	BP	GO:1901655	cellular response to ketone	4/222	0.0269012	0.0549374	0.0269301	Cdk4/Jak2/Prkaa2/Sirt1
3-day post-SCI group vs. the control group	BP	GO:0044774	mitotic DNA integrity checkpoint signaling	3/222	0.0269357	0.0549545	0.0269384	Atf2/Cdk1/Ier3
3-day post-SCI group vs. the control group	BP	GO:0060191	regulation of lipase activity	3/222	0.0269357	0.0549545	0.0269384	Anxa1/Pdgfra/Snca
3-day post-SCI group vs. the control group	BP	GO:0032413	negative regulation of ion transmembrane transpor	3/222	0.0277675	0.056542	0.0277166	Fkbp1b/Ppp3ca/Ubqln1
3-day post-SCI group vs. the control group	BP	GO:0034103	regulation of tissue remodeling	3/222	0.0277675	0.056542	0.0277166	Cd38/Ppargc1b/Ppp3ca
3-day post-SCI group vs. the control group	BP	GO:0045824	negative regulation of innate immune response	3/222	0.0277675	0.056542	0.0277166	Arg1/Banf1/Ccr1
3-day post-SCI group vs. the control group	BP	GO:0046323	glucose import	3/222	0.0277675	0.056542	0.0277166	Mapk14/Myc/Nfe2l2
3-day post-SCI group vs. the control group	BP	GO:0002052	positive regulation of neuroblast proliferation	2/222	0.0279709	0.0567093	0.0277986	Hif1a/Mapk8
3-day post-SCI group vs. the control group	BP	GO:0014047	glutamate secretion	2/222	0.0279709	0.0567093	0.0277986	Adora1/Snca
3-day post-SCI group vs. the control group	BP	GO:0033522	histone H2A ubiquitination	2/222	0.0279709	0.0567093	0.0277986	Rnf2/Ube2a
3-day post-SCI group vs. the control group	BP	GO:0050482	arachidonic acid secretion	2/222	0.0279709	0.0567093	0.0277986	Anxa1/Pnpla8
3-day post-SCI group vs. the control group	BP	GO:0051973	positive regulation of telomerase activity	2/222	0.0279709	0.0567093	0.0277986	Mapk3/Myc
3-day post-SCI group vs. the control group	BP	GO:0060402	calcium ion transport into cytosol	2/222	0.0279709	0.0567093	0.0277986	Bax/Slc8a1
3-day post-SCI group vs. the control group	BP	GO:0099563	modification of synaptic structure	2/222	0.0279709	0.0567093	0.0277986	Cttn/Rest
3-day post-SCI group vs. the control group	BP	GO:1903959	regulation of anion transmembrane transport	2/222	0.0279709	0.0567093	0.0277986	Arg1/Ripk1
3-day post-SCI group vs. the control group	BP	GO:1903963	arachidonate transport	2/222	0.0279709	0.0567093	0.0277986	Anxa1/Pnpla8
3-day post-SCI group vs. the control group	BP	GO:0032259	methylation	7/222	0.0286084	0.0578994	0.028382	Btg1/Eed/Ezh2/Mgmt/Myc/Parp1/Sirt1
3-day post-SCI group vs. the control group	BP	GO:0003151	outflow tract morphogenesis	3/222	0.0286128	0.0578994	0.028382	Atf2/Hif1a/Jun
3-day post-SCI group vs. the control group	BP	GO:0006094	gluconeogenesis	3/222	0.0286128	0.0578994	0.028382	Hif1a/Pdk2/Sirt1
3-day post-SCI group vs. the control group	BP	GO:0045661	regulation of myoblast differentiation	3/222	0.0286128	0.0578994	0.028382	Actb/Btg1/Mapk14
3-day post-SCI group vs. the control group	BP	GO:0002285	lymphocyte activation involved in immune respon	5/222	0.0286719	0.0579911	0.0284269	Anxa1/Atp7a/Stat6/Tlr4/Zc3h12a
3-day post-SCI group vs. the control group	BP	GO:0010876	lipid localization	8/222	0.0292796	0.0591918	0.0290155	Abcc1/Anxa1/Cd36/Map2k6/Pnpla8/Ptgs2/Sirt1/Zc3h12a
3-day post-SCI group vs. the control group	BP	GO:0048708	astrocyte differentiation	3/222	0.0294718	0.0594753	0.0291545	Il6st/Mapk3/Tlr4
3-day post-SCI group vs. the control group	BP	GO:2000781	positive regulation of double-strand break repair	3/222	0.0294718	0.0594753	0.0291545	Actb/Mgmt/Parp1
3-day post-SCI group vs. the control group	BP	GO:0006084	acetyl-CoA metabolic process	2/222	0.0295187	0.0594753	0.0291545	Pdk1/Pdk2
3-day post-SCI group vs. the control group	BP	GO:0046475	glycerophospholipid catabolic process	2/222	0.0295187	0.0594753	0.0291545	Pnpla8/Prdx6
3-day post-SCI group vs. the control group	BP	GO:0060142	regulation of syncytium formation by plasma mem	2/222	0.0295187	0.0594753	0.0291545	Adam9/Mapk14
3-day post-SCI group vs. the control group	BP	GO:0061436	establishment of skin barrier	2/222	0.0295187	0.0594753	0.0291545	Krt1/Met
3-day post-SCI group vs. the control group	BP	GO:0140718	facultative heterochromatin formation	2/222	0.0295187	0.0594753	0.0291545	Hdac1/Sirt1
3-day post-SCI group vs. the control group	BP	GO:0035264	multicellular organism growth	5/222	0.02964	0.059691	0.0292602	Atrn/Cdk4/Ezr/Htra2/Plekha1
3-day post-SCI group vs. the control group	BP	GO:0002703	regulation of leukocyte mediated immunity	6/222	0.0299521	0.0602908	0.0295543	Arg1/Hmox1/Il18rap/Ripk3/Stat6/Tlr4
3-day post-SCI group vs. the control group	BP	GO:0010810	regulation of cell-substrate adhesion	5/222	0.0301316	0.0605942	0.029703	Braf/Cd36/Jak2/Map4k4/P4hb
3-day post-SCI group vs. the control group	BP	GO:2000241	regulation of reproductive process	5/222	0.0301316	0.0605942	0.029703	Apc/Bax/Cdc20/Prdx4/Ybx3

3-day post-SCI group vs. the control group	BP	GO:0006637	acyl-CoA metabolic process	3/222	0.0303442	0.0609635	0.029884	Pdk1/Pdk2/Snca
3-day post-SCI group vs. the control group	BP	GO:0035383	thioester metabolic process	3/222	0.0303442	0.0609635	0.029884	Pdk1/Pdk2/Snca
3-day post-SCI group vs. the control group	BP	GO:0060485	mesenchyme development	6/222	0.0303675	0.0609812	0.0298927	Ezh2/Hif1a/Mapk3/Myc/Nfatc1/Pawr
3-day post-SCI group vs. the control group	BP	GO:0002902	regulation of B cell apoptotic process	2/222	0.0311	0.0621852	0.0304829	Bax/Myc
3-day post-SCI group vs. the control group	BP	GO:0022616	DNA strand elongation	2/222	0.0311	0.0621852	0.0304829	Mcm4/Pcna
3-day post-SCI group vs. the control group	BP	GO:0031069	hair follicle morphogenesis	2/222	0.0311	0.0621852	0.0304829	Atp7a/Ctsl
3-day post-SCI group vs. the control group	BP	GO:0032816	positive regulation of natural killer cell activation	2/222	0.0311	0.0621852	0.0304829	Axl/Jak2
3-day post-SCI group vs. the control group	BP	GO:0034122	negative regulation of toll-like receptor signaling p	2/222	0.0311	0.0621852	0.0304829	Tlr6/Ubqln1
3-day post-SCI group vs. the control group	BP	GO:0043516	regulation of DNA damage response, signal transd	2/222	0.0311	0.0621852	0.0304829	Atr/Sirt1
3-day post-SCI group vs. the control group	BP	GO:0048169	regulation of long-term neuronal synaptic plasticit	2/222	0.0311	0.0621852	0.0304829	Snca/Syp
3-day post-SCI group vs. the control group	BP	GO:0090183	regulation of kidney development	2/222	0.0311	0.0621852	0.0304829	Myc/Ppp3ca
3-day post-SCI group vs. the control group	BP	GO:2000515	negative regulation of CD4-positive, alpha-beta T	2/222	0.0311	0.0621852	0.0304829	Anxa1/Zc3h12a
3-day post-SCI group vs. the control group	BP	GO:0046434	organophosphate catabolic process	4/222	0.0311676	0.0622909	0.0305347	Ncf1/Pnpla8/Prdx6/Xdh
3-day post-SCI group vs. the control group	BP	GO:0034620	cellular response to unfolded protein	3/222	0.0312302	0.0623864	0.0305815	Bax/Eif2s1/Nfe2l2
3-day post-SCI group vs. the control group	BP	GO:0006909	phagocytosis	7/222	0.0314932	0.0628819	0.0308244	Aif1/Anxa1/Axl/Cd36/Met/Sirpa/Tlr4
3-day post-SCI group vs. the control group	BP	GO:0044242	cellular lipid catabolic process	5/222	0.0316374	0.0631398	0.0309508	Cyp1b1/Etfdh/Hao1/Pnpla8/Prdx6
3-day post-SCI group vs. the control group	BP	GO:0001837	epithelial to mesenchymal transition	4/222	0.0318078	0.0634498	0.0311027	Ezh2/Hif1a/Nfatc1/Pawr
3-day post-SCI group vs. the control group	BP	GO:0006112	energy reserve metabolic process	3/222	0.0321297	0.064001	0.031373	Cd36/Il6st/Ppp1ca
3-day post-SCI group vs. the control group	BP	GO:0019319	hexose biosynthetic process	3/222	0.0321297	0.064001	0.031373	Hif1a/Pdk2/Sirt1
3-day post-SCI group vs. the control group	BP	GO:0019915	lipid storage	3/222	0.0321297	0.064001	0.031373	Cd36/Sirt1/Zc3h12a
3-day post-SCI group vs. the control group	BP	GO:0140014	mitotic nuclear division	6/222	0.0325009	0.0647097	0.0317204	Actb/Apc/Banf1/Cdc20/Met/Rb1
3-day post-SCI group vs. the control group	BP	GO:0007095	mitotic G2 DNA damage checkpoint signaling	2/222	0.032714	0.0649193	0.0318231	Cdk1/Ier3
3-day post-SCI group vs. the control group	BP	GO:0016540	protein autoprocessing	2/222	0.032714	0.0649193	0.0318231	Ctsl/Parp1
3-day post-SCI group vs. the control group	BP	GO:0050901	leukocyte tethering or rolling	2/222	0.032714	0.0649193	0.0318231	Pawr/Vcam1
3-day post-SCI group vs. the control group	BP	GO:0070873	regulation of glycogen metabolic process	2/222	0.032714	0.0649193	0.0318231	Cd36/Ppp1ca
3-day post-SCI group vs. the control group	BP	GO:1902003	regulation of amyloid-beta formation	2/222	0.032714	0.0649193	0.0318231	Casp3/Rela
3-day post-SCI group vs. the control group	BP	GO:1904358	positive regulation of telomere maintenance via te	2/222	0.032714	0.0649193	0.0318231	Atr/Mapk3
3-day post-SCI group vs. the control group	BP	GO:1904738	vascular associated smooth muscle cell migration	2/222	0.032714	0.0649193	0.0318231	Atp7a/Nfe2l2
3-day post-SCI group vs. the control group	BP	GO:0050954	sensory perception of mechanical stimulus	6/222	0.0329389	0.0653348	0.0320268	Casp3/Gpx1/Myc/Pawr/Rest/Tlr4
3-day post-SCI group vs. the control group	BP	GO:0022612	gland morphogenesis	4/222	0.0331111	0.0656453	0.032179	Bax/Met/Nfkb1/Stat6
3-day post-SCI group vs. the control group	BP	GO:0031647	regulation of protein stability	6/222	0.0338265	0.0670323	0.0328589	Casp3/Fbxw7/Id1/Pink1/Sirt1/Snca
3-day post-SCI group vs. the control group	BP	GO:0045638	negative regulation of myeloid cell differentiation	3/222	0.033969	0.0672512	0.0329662	Fbxw7/Myc/Nme2
3-day post-SCI group vs. the control group	BP	GO:0097581	lamellipodium organization	3/222	0.033969	0.0672512	0.0329662	Atp7a/Cttn/Pxn
3-day post-SCI group vs. the control group	BP	GO:0000423	mitophagy	2/222	0.0343603	0.0679301	0.033299	Htra2/Pink1
3-day post-SCI group vs. the control group	BP	GO:0044786	cell cycle DNA replication	2/222	0.0343603	0.0679301	0.033299	Mcm4/Pcna
3-day post-SCI group vs. the control group	BP	GO:0086004	regulation of cardiac muscle cell contraction	2/222	0.0343603	0.0679301	0.033299	Adora1/Atp2a2
3-day post-SCI group vs. the control group	BP	GO:0008360	regulation of cell shape	4/222	0.0344452	0.0680661	0.0333656	Anxa1/Arhgdia/Ezr/Pxn
3-day post-SCI group vs. the control group	BP	GO:0002460	adaptive immune response based on somatic recon	8/222	0.0346444	0.0684277	0.0335429	Anxa1/Arg1/Btk/Il18rap/Jak2/Ripk3/Stat6/Zc3h12a
3-day post-SCI group vs. the control group	BP	GO:0030512	negative regulation of transforming growth factor	3/222	0.0349087	0.0688528	0.0337513	Hdac1/Rbbp7/Sirt1
3-day post-SCI group vs. the control group	BP	GO:0034308	primary alcohol metabolic process	3/222	0.0349087	0.0688528	0.0337513	Cyp1b1/Hao1/Rest

3-day post-SCI group vs. the control group	BP	GO:0045104	intermediate filament cytoskeleton organization	3/222	0.0349087	0.0688528	0.0337513	Atf2/Dst/Krt1
3-day post-SCI group vs. the control group	BP	GO:0000724	double-strand break repair via homologous recoml	4/222	0.0351239	0.0691799	0.0339116	Actb/Mcm4/Parp1/Sfpq
3-day post-SCI group vs. the control group	BP	GO:0002687	positive regulation of leukocyte migration	4/222	0.0351239	0.0691799	0.0339116	Aif1/Ccr1/Mapk3/Pawr
3-day post-SCI group vs. the control group	BP	GO:0007613	memory	4/222	0.0351239	0.0691799	0.0339116	Mapt/Ptgs1/Ptgs2/Rcan1
3-day post-SCI group vs. the control group	BP	GO:0006865	amino acid transport	4/222	0.0358103	0.0704686	0.0345433	Adora1/Arg1/Myc/Snca
3-day post-SCI group vs. the control group	BP	GO:0033108	mitochondrial respiratory chain complex assembly	3/222	0.0358618	0.0704686	0.0345433	Ndufa12/Ndufa6/Ndufs8
3-day post-SCI group vs. the control group	BP	GO:0035914	skeletal muscle cell differentiation	3/222	0.0358618	0.0704686	0.0345433	Fos/Myc/Rb1
3-day post-SCI group vs. the control group	BP	GO:0045103	intermediate filament-based process	3/222	0.0358618	0.0704686	0.0345433	Atf2/Dst/Krt1
3-day post-SCI group vs. the control group	BP	GO:2001251	negative regulation of chromosome organization	3/222	0.0358618	0.0704686	0.0345433	Apc/Cdc20/Parp1
3-day post-SCI group vs. the control group	BP	GO:0030100	regulation of endocytosis	5/222	0.0358804	0.0704721	0.0345451	Amph/Axl/Cd36/Ppp3ca/Snca
3-day post-SCI group vs. the control group	BP	GO:0009167	purine ribonucleoside monophosphate metabolic p	2/222	0.0360382	0.0705517	0.0345841	Nudt2/Xdh
3-day post-SCI group vs. the control group	BP	GO:0030866	cortical actin cytoskeleton organization	2/222	0.0360382	0.0705517	0.0345841	Ect2/Ezr
3-day post-SCI group vs. the control group	BP	GO:0033032	regulation of myeloid cell apoptotic process	2/222	0.0360382	0.0705517	0.0345841	Anxa1/Sirt1
3-day post-SCI group vs. the control group	BP	GO:0045622	regulation of T-helper cell differentiation	2/222	0.0360382	0.0705517	0.0345841	Anxa1/Zc3h12a
3-day post-SCI group vs. the control group	BP	GO:0060674	placenta blood vessel development	2/222	0.0360382	0.0705517	0.0345841	Fosl1/Junb
3-day post-SCI group vs. the control group	BP	GO:0071320	cellular response to cAMP	2/222	0.0360382	0.0705517	0.0345841	Ezr/Slc8a1
3-day post-SCI group vs. the control group	BP	GO:1905168	positive regulation of double-strand break repair v	2/222	0.0360382	0.0705517	0.0345841	Actb/Parp1
3-day post-SCI group vs. the control group	BP	GO:0090287	regulation of cellular response to growth factor sti	6/222	0.0365819	0.0715829	0.0350896	Cdkn2b/Hdac1/Hif1a/Rbbp7/Sirt1/Xdh
3-day post-SCI group vs. the control group	BP	GO:0032984	protein-containing complex disassembly	5/222	0.0369933	0.0723543	0.0354677	Apc/Atp2a2/Bnip3/Myc/Ubqln1
3-day post-SCI group vs. the control group	BP	GO:0035637	multicellular organismal signaling	4/222	0.0372062	0.0727032	0.0356388	Chrna4/Fkbp1b/Pawr/Slc8a1
3-day post-SCI group vs. the control group	BP	GO:0097530	granulocyte migration	4/222	0.0372062	0.0727032	0.0356388	Anxa1/Mapk3/Pawr/Sirpa
3-day post-SCI group vs. the control group	BP	GO:0006284	base-excision repair	2/222	0.0377471	0.0735894	0.0360731	Parp1/Pcna
3-day post-SCI group vs. the control group	BP	GO:0010803	regulation of tumor necrosis factor-mediated signa	2/222	0.0377471	0.0735894	0.0360731	Ripk1/Tnfrsf1a
3-day post-SCI group vs. the control group	BP	GO:0030521	androgen receptor signaling pathway	2/222	0.0377471	0.0735894	0.0360731	Hdac1/Sirt1
3-day post-SCI group vs. the control group	BP	GO:0033144	negative regulation of intracellular steroid hormon	2/222	0.0377471	0.0735894	0.0360731	Hdac1/Sirt1
3-day post-SCI group vs. the control group	BP	GO:0042119	neutrophil activation	2/222	0.0377471	0.0735894	0.0360731	Anxa1/Il18rap
3-day post-SCI group vs. the control group	BP	GO:0044728	DNA methylation or demethylation	3/222	0.037808	0.073674	0.0361146	Ezh2/Myc/Parp1
3-day post-SCI group vs. the control group	BP	GO:0000725	recombinational repair	4/222	0.0379158	0.07385	0.0362009	Actb/Mcm4/Parp1/Sfpq
3-day post-SCI group vs. the control group	BP	GO:0050808	synapse organization	8/222	0.0383166	0.0745961	0.0365666	Actb/Cdc20/Cttn/Mapk14/Mapt/Rest/Snca/Stau2
3-day post-SCI group vs. the control group	BP	GO:0001570	vasculogenesis	3/222	0.038801	0.0754693	0.0369947	Fbxw7/Junb/Xdh
3-day post-SCI group vs. the control group	BP	GO:0046364	monosaccharide biosynthetic process	3/222	0.038801	0.0754693	0.0369947	Hif1a/Pdk2/Sirt1
3-day post-SCI group vs. the control group	BP	GO:0002446	neutrophil mediated immunity	2/222	0.0394865	0.0766257	0.0375615	Arg1/Ncf1
3-day post-SCI group vs. the control group	BP	GO:0033561	regulation of water loss via skin	2/222	0.0394865	0.0766257	0.0375615	Krt1/Met
3-day post-SCI group vs. the control group	BP	GO:0051180	vitamin transport	2/222	0.0394865	0.0766257	0.0375615	Abcc1/Slc23a2
3-day post-SCI group vs. the control group	BP	GO:0060325	face morphogenesis	2/222	0.0394865	0.0766257	0.0375615	Pdgfra/Plekha1
3-day post-SCI group vs. the control group	BP	GO:2000249	regulation of actin cytoskeleton reorganization	2/222	0.0394865	0.0766257	0.0375615	Arhgdia/Trpm2
3-day post-SCI group vs. the control group	BP	GO:1903321	negative regulation of protein modification by sma	3/222	0.0398072	0.0772124	0.0378491	Cdc20/Gclc/Rela
3-day post-SCI group vs. the control group	BP	GO:0006836	neurotransmitter transport	5/222	0.0398678	0.0772944	0.0378893	Atp2a2/Braf/Chrna4/Snca/Syp
3-day post-SCI group vs. the control group	BP	GO:0045664	regulation of neuron differentiation	5/222	0.0404586	0.0784036	0.0384331	Ect2/Impact/Mapk8/Rest/Rnf112
3-day post-SCI group vs. the control group	BP	GO:0002690	positive regulation of leukocyte chemotaxis	3/222	0.0408266	0.079044	0.038747	Aif1/Ccr1/Mapk3

3-day post-SCI group vs. the control group	BP	GO:0019226	transmission of nerve impulse	3/222	0.0408266	0.079044	0.038747	Chrna4/Fkbp1b/Pawr
3-day post-SCI group vs. the control group	BP	GO:0048762	mesenchymal cell differentiation	5/222	0.0410547	0.0794491	0.0389455	Ezh2/Hif1a/Mapk3/Nfatc1/Pawr
3-day post-SCI group vs. the control group	BP	GO:0009126	purine nucleoside monophosphate metabolic proce	2/222	0.0412559	0.0796919	0.0390646	Nudt2/Xdh
3-day post-SCI group vs. the control group	BP	GO:0018149	peptide cross-linking	2/222	0.0412559	0.0796919	0.0390646	Anxa1/Krt1
3-day post-SCI group vs. the control group	BP	GO:0045022	early endosome to late endosome transport	2/222	0.0412559	0.0796919	0.0390646	Ezr/Mapk3
3-day post-SCI group vs. the control group	BP	GO:0048730	epidermis morphogenesis	2/222	0.0412559	0.0796919	0.0390646	Atp7a/Ctsl
3-day post-SCI group vs. the control group	BP	GO:0002366	leukocyte activation involved in immune response	6/222	0.0414866	0.080101	0.0392651	Anxa1/Atp7a/Hmox1/Stat6/Tlr4/Zc3h12a
3-day post-SCI group vs. the control group	BP	GO:0001909	leukocyte mediated cytotoxicity	4/222	0.0415801	0.0802447	0.0393355	Arg1/Il18rap/Ncf1/Ripk3
3-day post-SCI group vs. the control group	BP	GO:0051781	positive regulation of cell division	3/222	0.041859	0.0807459	0.0395812	Apc/Cat/Ect2
3-day post-SCI group vs. the control group	BP	GO:0014066	regulation of phosphatidylinositol 3-kinase signali	3/222	0.0429046	0.0825604	0.0404707	Cat/Jak2/Sirt1
3-day post-SCI group vs. the control group	BP	GO:0031638	zymogen activation	3/222	0.0429046	0.0825604	0.0404707	Ctsl/Dhcr24/Pawr
3-day post-SCI group vs. the control group	BP	GO:0044264	cellular polysaccharide metabolic process	3/222	0.0429046	0.0825604	0.0404707	Cd36/Il6st/Ppp1ca
3-day post-SCI group vs. the control group	BP	GO:0002021	response to dietary excess	2/222	0.0430545	0.0825604	0.0404707	Mapk14/Ppargc1a
3-day post-SCI group vs. the control group	BP	GO:0009124	nucleoside monophosphate biosynthetic process	2/222	0.0430545	0.0825604	0.0404707	Nme2/Nudt2
3-day post-SCI group vs. the control group	BP	GO:0010453	regulation of cell fate commitment	2/222	0.0430545	0.0825604	0.0404707	Hdac1/Rbbp7
3-day post-SCI group vs. the control group	BP	GO:0042181	ketone biosynthetic process	2/222	0.0430545	0.0825604	0.0404707	Ppargc1a/Rest
3-day post-SCI group vs. the control group	BP	GO:0045823	positive regulation of heart contraction	2/222	0.0430545	0.0825604	0.0404707	Gch1/Sirt1
3-day post-SCI group vs. the control group	BP	GO:0051281	positive regulation of release of sequestered calciu	2/222	0.0430545	0.0825604	0.0404707	Bax/Snca
3-day post-SCI group vs. the control group	BP	GO:0070050	neuron cellular homeostasis	2/222	0.0430545	0.0825604	0.0404707	Atp2a2/Atp7a
3-day post-SCI group vs. the control group	BP	GO:0070884	regulation of calcineurin-NFAT signaling cascade	2/222	0.0430545	0.0825604	0.0404707	Ppp3ca/Rcan1
3-day post-SCI group vs. the control group	BP	GO:1900026	positive regulation of substrate adhesion-depender	2/222	0.0430545	0.0825604	0.0404707	Braf/P4hb
3-day post-SCI group vs. the control group	BP	GO:1902116	negative regulation of organelle assembly	2/222	0.0430545	0.0825604	0.0404707	Cdk10/Pink1
3-day post-SCI group vs. the control group	BP	GO:0055067	monovalent inorganic cation homeostasis	4/222	0.0431001	0.0825726	0.0404766	Adora1/Mapk3/Pdk2/Slc8a1
3-day post-SCI group vs. the control group	BP	GO:0061025	membrane fusion	4/222	0.0431001	0.0825726	0.0404766	Anxa1/Atp13a2/Ctsl/Snca
3-day post-SCI group vs. the control group	BP	GO:0002263	cell activation involved in immune response	6/222	0.0435593	0.0833915	0.0408781	Anxa1/Atp7a/Hmox1/Stat6/Tlr4/Zc3h12a
3-day post-SCI group vs. the control group	BP	GO:0006869	lipid transport	7/222	0.0435672	0.0833915	0.0408781	Abcc1/Anxa1/Cd36/Map2k6/Pnpla8/Ptgs2/Sirt1
3-day post-SCI group vs. the control group	BP	GO:0022618	ribonucleoprotein complex assembly	4/222	0.0438717	0.0839363	0.0411451	Ago1/Ago3/Atr/Rpl13a
3-day post-SCI group vs. the control group	BP	GO:0001562	response to protozoan	2/222	0.044882	0.0852495	0.0417889	Arg1/Ier3
3-day post-SCI group vs. the control group	BP	GO:0005978	glycogen biosynthetic process	2/222	0.044882	0.0852495	0.0417889	Cd36/Ppp1ca
3-day post-SCI group vs. the control group	BP	GO:0008207	C21-steroid hormone metabolic process	2/222	0.044882	0.0852495	0.0417889	Ppargc1a/Rest
3-day post-SCI group vs. the control group	BP	GO:0009250	glucan biosynthetic process	2/222	0.044882	0.0852495	0.0417889	Cd36/Ppp1ca
3-day post-SCI group vs. the control group	BP	GO:0032508	DNA duplex unwinding	2/222	0.044882	0.0852495	0.0417889	Anxa1/Mcm4
3-day post-SCI group vs. the control group	BP	GO:0035886	vascular associated smooth muscle cell differentia	2/222	0.044882	0.0852495	0.0417889	Nfatc1/Sod2
3-day post-SCI group vs. the control group	BP	GO:0042220	response to cocaine	2/222	0.044882	0.0852495	0.0417889	Parp1/Snca
3-day post-SCI group vs. the control group	BP	GO:0045840	positive regulation of mitotic nuclear division	2/222	0.044882	0.0852495	0.0417889	Met/Rb1
3-day post-SCI group vs. the control group	BP	GO:0046326	positive regulation of glucose import	2/222	0.044882	0.0852495	0.0417889	Mapk14/Nfe2l2
3-day post-SCI group vs. the control group	BP	GO:0050892	intestinal absorption	2/222	0.044882	0.0852495	0.0417889	Cd36/Ezr
3-day post-SCI group vs. the control group	BP	GO:0071168	protein localization to chromatin	2/222	0.044882	0.0852495	0.0417889	Ezh2/Rb1
3-day post-SCI group vs. the control group	BP	GO:0085029	extracellular matrix assembly	2/222	0.044882	0.0852495	0.0417889	Atp7a/Pxdn
3-day post-SCI group vs. the control group	BP	GO:0098927	vesicle-mediated transport between endosomal cor	2/222	0.044882	0.0852495	0.0417889	Ezr/Mapk3

3-day post-SCI group vs. the control group	BP	GO:0106056	regulation of calcineurin-mediated signaling	2/222	0.044882	0.0852495	0.0417889	Ppp3ca/Rcan1
3-day post-SCI group vs. the control group	BP	GO:1902991	regulation of amyloid precursor protein catabolic p	2/222	0.044882	0.0852495	0.0417889	Casp3/Rela
3-day post-SCI group vs. the control group	BP	GO:2000772	regulation of cellular senescence	2/222	0.044882	0.0852495	0.0417889	Pawr/Sirt1
3-day post-SCI group vs. the control group	BP	GO:0000045	autophagosome assembly	3/222	0.0461192	0.087481	0.0428827	Atp2a2/Pink1/Ubqln1
3-day post-SCI group vs. the control group	BP	GO:0046718	viral entry into host cell	3/222	0.0461192	0.087481	0.0428827	Axl/Ctsl/P4hb
3-day post-SCI group vs. the control group	BP	GO:0051153	regulation of striated muscle cell differentiation	3/222	0.0461192	0.087481	0.0428827	Ezh2/Mapk14/Sirt1
3-day post-SCI group vs. the control group	BP	GO:0030183	B cell differentiation	4/222	0.046233	0.0876574	0.0429692	Bax/Btk/Ezh2/Nfatc1
3-day post-SCI group vs. the control group	BP	GO:0003298	physiological muscle hypertrophy	2/222	0.0467378	0.0882965	0.0432825	Map2k4/Sirt1
3-day post-SCI group vs. the control group	BP	GO:0003301	physiological cardiac muscle hypertrophy	2/222	0.0467378	0.0882965	0.0432825	Map2k4/Sirt1
3-day post-SCI group vs. the control group	BP	GO:0030261	chromosome condensation	2/222	0.0467378	0.0882965	0.0432825	Banf1/Cdk1
3-day post-SCI group vs. the control group	BP	GO:0031063	regulation of histone deacetylation	2/222	0.0467378	0.0882965	0.0432825	Pink1/Sirt1
3-day post-SCI group vs. the control group	BP	GO:0031297	replication fork processing	2/222	0.0467378	0.0882965	0.0432825	Atr/Pcna
3-day post-SCI group vs. the control group	BP	GO:0031952	regulation of protein autophosphorylation	2/222	0.0467378	0.0882965	0.0432825	Impact/Jun
3-day post-SCI group vs. the control group	BP	GO:0046636	negative regulation of alpha-beta T cell activation	2/222	0.0467378	0.0882965	0.0432825	Anxa1/Zc3h12a
3-day post-SCI group vs. the control group	BP	GO:0061049	cell growth involved in cardiac muscle cell develo	2/222	0.0467378	0.0882965	0.0432825	Map2k4/Sirt1
3-day post-SCI group vs. the control group	BP	GO:0043414	macromolecule methylation	6/222	0.0467884	0.0883524	0.0433099	Btg1/Eed/Ezh2/Myc/Parp1/Sirt1
3-day post-SCI group vs. the control group	BP	GO:0030509	BMP signaling pathway	4/222	0.0470356	0.0887794	0.0435192	Atf2/Id1/Mapk3/Smad1
3-day post-SCI group vs. the control group	BP	GO:0033077	T cell differentiation in thymus	3/222	0.0472165	0.0890809	0.043667	Apc/Braf/Ripk3
3-day post-SCI group vs. the control group	BP	GO:0001704	formation of primary germ layer	3/222	0.0483266	0.091053	0.0446337	Smad1/Tnrc6c/Txnrd1
3-day post-SCI group vs. the control group	BP	GO:0045921	positive regulation of exocytosis	3/222	0.0483266	0.091053	0.0446337	Atp13a2/Sdc1/Snca
3-day post-SCI group vs. the control group	BP	GO:0072091	regulation of stem cell proliferation	3/222	0.0483266	0.091053	0.0446337	Ago3/Cdkn2c/Nfatc1
3-day post-SCI group vs. the control group	BP	GO:0001783	B cell apoptotic process	2/222	0.0486213	0.0911595	0.0446859	Bax/Myc
3-day post-SCI group vs. the control group	BP	GO:0002691	regulation of cellular extravasation	2/222	0.0486213	0.0911595	0.0446859	Pawr/Ripk3
3-day post-SCI group vs. the control group	BP	GO:0007094	mitotic spindle assembly checkpoint signaling	2/222	0.0486213	0.0911595	0.0446859	Apc/Cdc20
3-day post-SCI group vs. the control group	BP	GO:0030279	negative regulation of ossification	2/222	0.0486213	0.0911595	0.0446859	Ccr1/Hif1a
3-day post-SCI group vs. the control group	BP	GO:0033028	myeloid cell apoptotic process	2/222	0.0486213	0.0911595	0.0446859	Anxa1/Sirt1
3-day post-SCI group vs. the control group	BP	GO:0036230	granulocyte activation	2/222	0.0486213	0.0911595	0.0446859	Anxa1/Il18rap
3-day post-SCI group vs. the control group	BP	GO:0048536	spleen development	2/222	0.0486213	0.0911595	0.0446859	Cdkn2b/Ripk3
3-day post-SCI group vs. the control group	BP	GO:0071173	spindle assembly checkpoint signaling	2/222	0.0486213	0.0911595	0.0446859	Apc/Cdc20
3-day post-SCI group vs. the control group	BP	GO:0071300	cellular response to retinoic acid	2/222	0.0486213	0.0911595	0.0446859	Htra2/Rxrb
3-day post-SCI group vs. the control group	BP	GO:1903115	regulation of actin filament-based movement	2/222	0.0486213	0.0911595	0.0446859	Adora1/Atp2a2
3-day post-SCI group vs. the control group	BP	GO:2000758	positive regulation of peptidyl-lysine acetylation	2/222	0.0486213	0.0911595	0.0446859	Mapk3/Prkaa2
3-day post-SCI group vs. the control group	BP	GO:0001657	ureteric bud development	3/222	0.0494495	0.0925887	0.0453865	Cat/Myc/Smad1
3-day post-SCI group vs. the control group	BP	GO:0009953	dorsal/ventral pattern formation	3/222	0.0494495	0.0925887	0.0453865	Apc/Bmp1/Mapk8
3-day post-SCI group vs. the control group	BP	GO:0032479	regulation of type I interferon production	3/222	0.0494495	0.0925887	0.0453865	Banf1/Sirpa/Tlr4
3-day post-SCI group vs. the control group	BP	GO:0060348	bone development	5/222	0.0499597	0.0935024	0.0458344	Atf2/Fbxw7/Map2k6/Mapk14/Smad1
3-day post-SCI group vs. the control group	CC	GO:0045121	membrane raft	19/223	3.04E-10	6.01E-08	3.66E-08	Actb/Atp7a/Btk/Casp3/Cd36/Ctnna1/Ezr/Hmox1/Il6st/Jak2/Mapk3/Mapt/Ptgs2/Ripk1/Stat6/Tlr4/Tlr6/Tnfrsf1a/Tpp1
3-day post-SCI group vs. the control group	CC	GO:0098857	membrane microdomain	19/223	3.17E-10	6.01E-08	3.66E-08	Actb/Atp7a/Btk/Casp3/Cd36/Ctnna1/Ezr/Hmox1/Il6st/Jak2/Mapk3/Mapt/Ptgs2/Ripk1/Stat6/Tlr4/Tlr6/Tnfrsf1a/Tpp1

3-day post-SCI group vs. the control group	CC	GO:0090575	RNA polymerase II transcription regulator comple	15/223	8.01E-10	1.01E-07	6.15E-08	Atf2/Fos/Fosl1/Hif1a/Jun/Junb/Myc/Nfe2l2/Nono/Rb1/Rxrb/Sfpq/Smad1/Stat6/Tfdp1
3-day post-SCI group vs. the control group	CC	GO:0031252	cell leading edge	18/223	3.83E-09	3.63E-07	2.21E-07	Actb/Adora1/Aif1/Amph/Apc/Atp7a/Ctnna1/Cttn/Dst/Ezr/Itgb5/Mapt/Mylk/Nme2/P4hb/Plekha1/Ptprk/Pxn
3-day post-SCI group vs. the control group	CC	GO:1990204	oxidoreductase complex	10/223	3.78E-08	2.87E-06	1.74E-06	Cat/Etfdh/Ncf1/Ndufa12/Ndufa6/Ndufs8/Nox4/P4hb/Pdk1/Pdk2
3-day post-SCI group vs. the control group	CC	GO:0031519	PcG protein complex	7/223	1.32E-07	8.31E-06	5.05E-06	Cbx6/Eed/Ezh2/Phc3/Rbbp7/Rnf2/Sirt1
3-day post-SCI group vs. the control group	CC	GO:0043235	receptor complex	16/223	2.84E-07	0.0000154	9.36E-06	Axl/Cd36/Chrna4/Gpr37/Htra2/Il18rap/Il6st/Itga6/Itgb5/Jak2/Met/Pdgfra/Ripk1/Tlr4/Tlr6/Tnfrsf1a
3-day post-SCI group vs. the control group	CC	GO:0005635	nuclear envelope	16/223	3.32E-07	0.0000157	9.56E-06	Alox5/Apc/Bax/Bnip3/Cdk4/Dst/Gch1/Mapk3/Parp1/Ptgs1/Ptgs2/Sirt1/Smad1/Snca/Stau2/Vrk2
3-day post-SCI group vs. the control group	CC	GO:0000791	euchromatin	7/223	9.85E-07	0.0000415	0.0000252	Hif1a/Jak2/Jun/Myc/Ppargc1a/Rnf2/Sirt1
3-day post-SCI group vs. the control group	CC	GO:0009925	basal plasma membrane	12/223	1.77E-06	0.000062	0.0000377	Abcc1/Adam9/Adora1/Atp7a/Cd38/Ezr/Itga6/Lpo/Met/Slc23a2/Slc4a11/Slc8a1
3-day post-SCI group vs. the control group	CC	GO:0043204	perikaryon	9/223	1.85E-06	0.000062	0.0000377	Aif1/Atp7a/Ctsl/Endog/Hspb1/Map2k4/Mapk10/Mapk8/Ppp1ca
3-day post-SCI group vs. the control group	CC	GO:0030055	cell-substrate junction	10/223	1.96E-06	0.000062	0.0000377	Cttn/Dst/Ezr/Itga6/Itgb5/Jak2/Map4k4/Mapk3/Nox4/Pxn
3-day post-SCI group vs. the control group	CC	GO:0005677	chromatin silencing complex	4/223	2.37E-06	0.0000664	0.0000404	Eed/Ezh2/Rb1/Sirt1
3-day post-SCI group vs. the control group	CC	GO:0036464	cytoplasmic ribonucleoprotein granule	11/223	2.45E-06	0.0000664	0.0000404	Actb/Ago1/Ago3/Eif2s1/Mapt/Prkaa2/Rbpms/Stau2/Tnrc6a/Tnrc6c/Zc3h12a
3-day post-SCI group vs. the control group	CC	GO:0000307	cyclin-dependent protein kinase holoenzyme comp	6/223	2.71E-06	0.0000685	0.0000417	Ccna2/Cdk1/Cdk10/Cdk4/Pcna/Rb1
3-day post-SCI group vs. the control group	CC	GO:0016323	basolateral plasma membrane	11/223	3.81E-06	0.0000901	0.0000548	Abcc1/Adam9/Adora1/Atp7a/Cd38/Ezr/Itga6/Lpo/Slc23a2/Slc4a11/Slc8a1
3-day post-SCI group vs. the control group	CC	GO:0045178	basal part of cell	12/223	4.34E-06	0.0000967	0.0000588	Abcc1/Adam9/Adora1/Atp7a/Cd38/Ezr/Itga6/Lpo/Met/Slc23a2/Slc4a11/Slc8a1
3-day post-SCI group vs. the control group	CC	GO:0035770	ribonucleoprotein granule	11/223	4.97E-06	0.0001047	0.0000637	Actb/Ago1/Ago3/Eif2s1/Mapt/Prkaa2/Rbpms/Stau2/Tnrc6a/Tnrc6c/Zc3h12a
3-day post-SCI group vs. the control group	CC	GO:0019867	outer membrane	10/223	5.83E-06	0.0001104	0.0000672	Arg1/Atf2/Bax/Bnip3/Maoa/Mcl1/Mgst1/Pink1/Ptgs2/Sn[illegible]
3-day post-SCI group vs. the control group	CC	GO:0031968	organelle outer membrane	10/223	5.83E-06	0.0001104	0.0000672	Arg1/Atf2/Bax/Bnip3/Maoa/Mcl1/Mgst1/Pink1/Ptgs2/Sn[illegible]
3-day post-SCI group vs. the control group	CC	GO:0005925	focal adhesion	9/223	8.38E-06	0.0001512	0.000092	Cttn/Dst/Ezr/Itgb5/Jak2/Map4k4/Mapk3/Nox4/Pxn
3-day post-SCI group vs. the control group	CC	GO:0030027	lamellipodium	9/223	9.63E-06	0.0001619	0.0000985	Actb/Aif1/Apc/Ctnna1/Cttn/Mylk/Nme2/P4hb/Pxn
3-day post-SCI group vs. the control group	CC	GO:0035098	ESC/E(Z) complex	4/223	9.83E-06	0.0001619	0.0000985	Eed/Ezh2/Rbbp7/Sirt1
3-day post-SCI group vs. the control group	CC	GO:0005741	mitochondrial outer membrane	9/223	0.0000171	0.0002698	0.0001641	Arg1/Atf2/Bax/Bnip3/Maoa/Mcl1/Mgst1/Pink1/Snca
3-day post-SCI group vs. the control group	CC	GO:0031970	organelle envelope lumen	7/223	0.0000182	0.0002752	0.0001674	Alox5/Cat/Ccs/Htra2/Pink1/Snca/Txnip
3-day post-SCI group vs. the control group	CC	GO:0097386	glial cell projection	5/223	0.0000201	0.0002936	0.0001786	Aif1/Eif2s1/Ezr/Mapt/Pink1
3-day post-SCI group vs. the control group	CC	GO:0045177	apical part of cell	14/223	0.0000303	0.0004254	0.0002587	Abcc1/Anxa1/Atp7a/Cd36/Ctsl/Ezr/Igfbp2/Mgst1/Nox4/Prkaa2/Slc23a2/Slc4a11/Tnik/Vcam1
3-day post-SCI group vs. the control group	CC	GO:0099572	postsynaptic specialization	13/223	0.0000413	0.000559	0.00034	Actb/Adora1/Bnip3/Chrna4/Dst/Hspb1/Mapk10/Mapt/Met/Rnf112/Slc8a1/Syp/Tnik
3-day post-SCI group vs. the control group	CC	GO:0002102	podosome	4/223	0.0000457	0.0005977	0.0003635	Actb/Cttn/Mapk8/Vcam1

3-day post-SCI group vs. the control group	CC	GO:0001725	stress fiber	6/223	0.0000643	0.0007859	0.000478	Actb/Dst/Mylk/Nox4/Pdlim1/Pxn
3-day post-SCI group vs. the control group	CC	GO:0097517	contractile actin filament bundle	6/223	0.0000643	0.0007859	0.000478	Actb/Dst/Mylk/Nox4/Pdlim1/Pxn
3-day post-SCI group vs. the control group	CC	GO:0014069	postsynaptic density	12/223	0.0000703	0.000833	0.0005067	Actb/Adora1/Bnip3/Dst/Hspb1/Mapk10/Mapt/Met/Rnf112/Slc8a1/Syp/Tnik
3-day post-SCI group vs. the control group	CC	GO:0030018	Z disc	7/223	0.0000751	0.0008628	0.0005248	Dst/Fkbp1b/Hspb1/Pdlim1/Ppp3ca/S100a1/Slc8a1
3-day post-SCI group vs. the control group	CC	GO:0005758	mitochondrial intermembrane space	6/223	0.0000824	0.0009122	0.0005548	Cat/Ccs/Htra2/Pink1/Snca/Txnip
3-day post-SCI group vs. the control group	CC	GO:0032279	asymmetric synapse	12/223	0.0000849	0.0009122	0.0005548	Actb/Adora1/Bnip3/Dst/Hspb1/Mapk10/Mapt/Met/Rnf112/Slc8a1/Syp/Tnik
3-day post-SCI group vs. the control group	CC	GO:0005884	actin filament	7/223	0.0000866	0.0009122	0.0005548	Actb/Aif1/Anxa1/Cttn/Ezr/Pawr/Pdlim1
3-day post-SCI group vs. the control group	CC	GO:0044853	plasma membrane raft	7/223	0.0000951	0.0009484	0.0005769	Cd36/Ctnna1/Ezr/Hmox1/Jak2/Mapk3/Ptgs2
3-day post-SCI group vs. the control group	CC	GO:1902911	protein kinase complex	7/223	0.0000951	0.0009484	0.0005769	Ccna2/Cdk1/Cdk10/Cdk4/Pcna/Prkaa2/Rb1
3-day post-SCI group vs. the control group	CC	GO:0005901	caveola	6/223	0.0001045	0.0010152	0.0006175	Cd36/Ctnna1/Hmox1/Jak2/Mapk3/Ptgs2
3-day post-SCI group vs. the control group	CC	GO:0032432	actin filament bundle	6/223	0.0001106	0.0010227	0.0006221	Actb/Dst/Mylk/Nox4/Pdlim1/Pxn
3-day post-SCI group vs. the control group	CC	GO:0042641	actomyosin	6/223	0.0001106	0.0010227	0.0006221	Actb/Dst/Mylk/Nox4/Pdlim1/Pxn
3-day post-SCI group vs. the control group	CC	GO:0031674	I band	7/223	0.0001359	0.0011913	0.0007246	Dst/Fkbp1b/Hspb1/Pdlim1/Ppp3ca/S100a1/Slc8a1
3-day post-SCI group vs. the control group	CC	GO:0031965	nuclear membrane	9/223	0.0001369	0.0011913	0.0007246	Alox5/Apc/Cdk4/Gch1/Ptgs2/Sirt1/Smad1/Snca/Stau2
3-day post-SCI group vs. the control group	CC	GO:0000792	heterochromatin	6/223	0.0001383	0.0011913	0.0007246	Cbx6/Eed/Ezh2/Hdac1/Rnf2/Sirt1
3-day post-SCI group vs. the control group	CC	GO:0016324	apical plasma membrane	11/223	0.0001591	0.0013396	0.0008148	Abcc1/Anxa1/Atp7a/Ezr/Igfbp2/Nox4/Prkaa2/Slc23a2/Slc4a11/Tnik/Vcam1
3-day post-SCI group vs. the control group	CC	GO:0098984	neuron to neuron synapse	12/223	0.0001686	0.0013703	0.0008335	Actb/Adora1/Bnip3/Dst/Hspb1/Mapk10/Mapt/Met/Rnf112/Slc8a1/Syp/Tnik
3-day post-SCI group vs. the control group	CC	GO:0098802	plasma membrane signaling receptor complex	8/223	0.0001699	0.0013703	0.0008335	Chrna4/Htra2/Il18rap/Il6st/Itga6/Itgb5/Jak2/Tlr6
3-day post-SCI group vs. the control group	CC	GO:0031256	leading edge membrane	7/223	0.0002226	0.0017579	0.0010692	Adora1/Aif1/Amph/Apc/Mapt/Plekha1/Ptprk
3-day post-SCI group vs. the control group	CC	GO:0000803	sex chromosome	4/223	0.0002829	0.0021879	0.0013308	Atr/Eed/Rnf2/Ube2a
3-day post-SCI group vs. the control group	CC	GO:0035097	histone methyltransferase complex	5/223	0.0003143	0.0023687	0.0014407	Eed/Ezh2/Rbbp7/Rnf2/Sirt1
3-day post-SCI group vs. the control group	CC	GO:0019898	extrinsic component of membrane	10/223	0.0003199	0.0023687	0.0014407	Amph/Anxa1/Apc/Atp2a2/Ctnna1/Ezr/Gnb2/Ncf1/Rnf112/Zc3h12a
3-day post-SCI group vs. the control group	CC	GO:0042383	sarcolemma	7/223	0.000325	0.0023687	0.0014407	Alox5/Anxa1/Cd36/Dst/Ezr/Slc8a1/Vcam1
3-day post-SCI group vs. the control group	CC	GO:1902554	serine/threonine protein kinase complex	6/223	0.0004028	0.0028806	0.0017521	Ccna2/Cdk1/Cdk10/Cdk4/Pcna/Rb1
3-day post-SCI group vs. the control group	CC	GO:0005759	mitochondrial matrix	9/223	0.0004953	0.003476	0.0021143	Cdk1/Dhfr/Etfdh/Glrx2/Pdk1/Pdk2/Ppif/Snca/Sod2
3-day post-SCI group vs. the control group	CC	GO:0009898	cytoplasmic side of plasma membrane	7/223	0.0005642	0.0038882	0.002365	Atp2a2/Dst/Ezr/Gnb2/Htra2/Mapt/Ppp3ca
3-day post-SCI group vs. the control group	CC	GO:0034399	nuclear periphery	6/223	0.000586	0.0039658	0.0024122	Actb/Alox5/Mapt/Nono/Pcna/Sfpq
3-day post-SCI group vs. the control group	CC	GO:0097449	astrocyte projection	3/223	0.0006324	0.004205	0.0025577	Eif2s1/Ezr/Pink1
3-day post-SCI group vs. the control group	CC	GO:0150034	distal axon	10/223	0.0007946	0.0051926	0.0031584	Actb/Adora1/Amph/Apc/Cttn/Mapt/Sirt1/Slc8a1/Snca/Sy[illegible]
3-day post-SCI group vs. the control group	CC	GO:0045120	pronucleus	3/223	0.0008217	0.0052787	0.0032107	Ccna2/Eed/Ezh2
3-day post-SCI group vs. the control group	CC	GO:0005912	adherens junction	6/223	0.0008591	0.0054264	0.0033006	Actb/Apc/Ctnna1/Ezr/Pdlim1/Ppp1ca
3-day post-SCI group vs. the control group	CC	GO:0030017	sarcomere	7/223	0.0009263	0.0057551	0.0035005	Dst/Fkbp1b/Hspb1/Pdlim1/Ppp3ca/S100a1/Slc8a1
3-day post-SCI group vs. the control group	CC	GO:1905369	endopeptidase complex	5/223	0.0011026	0.00674	0.0040996	Capns1/Casp3/Hspb1/Htra2/Ubqln1
3-day post-SCI group vs. the control group	CC	GO:0001726	ruffle	6/223	0.0011805	0.0070973	0.0043169	Aif1/Apc/Cttn/Ezr/Nme2/Plekha1
3-day post-SCI group vs. the control group	CC	GO:0098562	cytoplasmic side of membrane	7/223	0.0011985	0.0070973	0.0043169	Atp2a2/Dst/Ezr/Gnb2/Htra2/Mapt/Ppp3ca

3-day post-SCI group vs. the control group	CC	GO:0005902	microvillus	5/223	0.0013708	0.0079927	0.0048615	Atp7a/Ctsl/Ezr/Pdgfra/Vcam1
3-day post-SCI group vs. the control group	CC	GO:0034708	methyltransferase complex	5/223	0.0014297	0.00821	0.0049937	Eed/Ezh2/Rbbp7/Rnf2/Sirt1
3-day post-SCI group vs. the control group	CC	GO:0044306	neuron projection terminus	7/223	0.0014898	0.0084272	0.0051258	Actb/Adora1/Amph/Apc/Slc8a1/Snca/Syp
3-day post-SCI group vs. the control group	CC	GO:0014704	intercalated disc	4/223	0.0018303	0.0100713	0.0061258	Atp2a2/Ctnna1/Dst/Slc8a1
3-day post-SCI group vs. the control group	CC	GO:0030016	myofibril	7/223	0.0018336	0.0100713	0.0061258	Dst/Fkbp1b/Hspb1/Pdlim1/Ppp3ca/S100a1/Slc8a1
3-day post-SCI group vs. the control group	CC	GO:0098798	mitochondrial protein-containing complex	8/223	0.0020343	0.0110141	0.0066993	Bax/Etfdh/Ndufa12/Ndufa6/Ndufs8/Pdk1/Pdk2/Ppif
3-day post-SCI group vs. the control group	CC	GO:0043292	contractile fiber	7/223	0.0025201	0.0134522	0.0081823	Dst/Fkbp1b/Hspb1/Pdlim1/Ppp3ca/S100a1/Slc8a1
3-day post-SCI group vs. the control group	CC	GO:0016529	sarcoplasmic reticulum	4/223	0.0029024	0.0148652	0.0090418	Atp2a2/Fkbp1b/S100a1/Xdh
3-day post-SCI group vs. the control group	CC	GO:0016605	PML body	4/223	0.0029024	0.0148652	0.0090418	Agap3/Atr/Rb1/Sirt1
3-day post-SCI group vs. the control group	CC	GO:0017053	transcription repressor complex	4/223	0.0029024	0.0148652	0.0090418	Hdac1/Jun/Myc/Rest
3-day post-SCI group vs. the control group	CC	GO:0070578	RISC-loading complex	2/223	0.0031343	0.0156941	0.0095459	Ago1/Ago3
3-day post-SCI group vs. the control group	CC	GO:0031672	A band	3/223	0.0031629	0.0156941	0.0095459	Dst/Hspb1/S100a1
3-day post-SCI group vs. the control group	CC	GO:0010494	cytoplasmic stress granule	4/223	0.0031885	0.0156941	0.0095459	Eif2s1/Prkaa2/Rbpms/Stau2
3-day post-SCI group vs. the control group	CC	GO:0016234	inclusion body	4/223	0.0034932	0.0169734	0.0103241	Gpx1/Mapt/Snca/Ubqln1
3-day post-SCI group vs. the control group	CC	GO:0031253	cell projection membrane	8/223	0.0036323	0.0174259	0.0105993	Adora1/Aif1/Apc/Atp7a/Cd36/Ezr/Mapt/Plekha1
3-day post-SCI group vs. the control group	CC	GO:0043020	NADPH oxidase complex	2/223	0.003742	0.0176327	0.0107251	Ncf1/Nox4
3-day post-SCI group vs. the control group	CC	GO:0097060	synaptic membrane	10/223	0.0037685	0.0176327	0.0107251	Adora1/Anxa1/Chrna4/Fosl1/Hspb1/Itgb5/Marcksl1/Met/ Slc8a1/Syp
3-day post-SCI group vs. the control group	CC	GO:0043679	axon terminus	6/223	0.0041326	0.0191005	0.0116178	Actb/Adora1/Amph/Slc8a1/Snca/Syp
3-day post-SCI group vs. the control group	CC	GO:1905368	peptidase complex	5/223	0.0042154	0.0192484	0.0117078	Capns1/Casp3/Hspb1/Htra2/Ubqln1
3-day post-SCI group vs. the control group	CC	GO:0000932	P-body	4/223	0.0045248	0.0203385	0.0123709	Ago1/Tnrc6a/Tnrc6c/Zc3h12a
3-day post-SCI group vs. the control group	CC	GO:0032993	protein-DNA complex	6/223	0.0045614	0.0203385	0.0123709	Actb/Brf2/Fos/Mcm4/Nfe2l2/Parp1
3-day post-SCI group vs. the control group	CC	GO:0000152	nuclear ubiquitin ligase complex	3/223	0.0051133	0.0225343	0.0137065	Cdc20/Phc3/Rnf2
3-day post-SCI group vs. the control group	CC	GO:0044291	cell-cell contact zone	4/223	0.0053158	0.0229052	0.013932	Atp2a2/Ctnna1/Dst/Slc8a1
3-day post-SCI group vs. the control group	CC	GO:0005637	nuclear inner membrane	3/223	0.0054392	0.0229052	0.013932	Ptgs2/Sirt1/Smad1
3-day post-SCI group vs. the control group	CC	GO:0005778	peroxisomal membrane	3/223	0.0054392	0.0229052	0.013932	Cat/Mgst1/Pnpla8
3-day post-SCI group vs. the control group	CC	GO:0031903	microbody membrane	3/223	0.0054392	0.0229052	0.013932	Cat/Mgst1/Pnpla8
3-day post-SCI group vs. the control group	CC	GO:0030133	transport vesicle	8/223	0.0057683	0.0238927	0.0145327	Amph/Apc/Atp13a2/Atp7a/Marcksl1/Rnf112/Snca/Syp
3-day post-SCI group vs. the control group	CC	GO:0008303	caspase complex	2/223	0.0058629	0.0238927	0.0145327	Capns1/Casp3
3-day post-SCI group vs. the control group	CC	GO:0035102	PRC1 complex	2/223	0.0058629	0.0238927	0.0145327	Phc3/Rnf2
3-day post-SCI group vs. the control group	CC	GO:0005777	peroxisome	5/223	0.0061805	0.024657	0.0149976	Cat/Hao1/Mgst1/Pnpla8/Xdh
3-day post-SCI group vs. the control group	CC	GO:0042579	microbody	5/223	0.0061805	0.024657	0.0149976	Cat/Hao1/Mgst1/Pnpla8/Xdh
3-day post-SCI group vs. the control group	CC	GO:0061695	transferase complex, transferring phosphorus-cont	7/223	0.0064951	0.0256422	0.0155969	Ccna2/Cdk1/Cdk10/Cdk4/Pcna/Prkaa2/Rb1
3-day post-SCI group vs. the control group	CC	GO:0043209	myelin sheath	6/223	0.0067569	0.0260222	0.0158279	Actb/Ezr/Gnb2/Nme2/Sod2/Uba52
3-day post-SCI group vs. the control group	CC	GO:0005747	mitochondrial respiratory chain complex I	3/223	0.006866	0.0260222	0.0158279	Ndufa12/Ndufa6/Ndufs8
3-day post-SCI group vs. the control group	CC	GO:0030964	NADH dehydrogenase complex	3/223	0.006866	0.0260222	0.0158279	Ndufa12/Ndufa6/Ndufs8
3-day post-SCI group vs. the control group	CC	GO:0045271	respiratory chain complex I	3/223	0.006866	0.0260222	0.0158279	Ndufa12/Ndufa6/Ndufs8
3-day post-SCI group vs. the control group	CC	GO:0016528	sarcoplasm	4/223	0.0071646	0.026885	0.0163528	Atp2a2/Fkbp1b/S100a1/Xdh
3-day post-SCI group vs. the control group	CC	GO:0000164	protein phosphatase type 1 complex	2/223	0.007517	0.0273935	0.0166621	Ppp1ca/Ppp1r15b
3-day post-SCI group vs. the control group	CC	GO:0016581	NuRD complex	2/223	0.007517	0.0273935	0.0166621	Hdac1/Rbbp7

3-day post-SCI group vs. the control group	CC	GO:0090545	CHD-type complex	2/223	0.007517	0.0273935	0.0166621	Hdac1/Rbbp7
3-day post-SCI group vs. the control group	CC	GO:0031430	M band	2/223	0.009356	0.0335897	0.0204309	Hspb1/S100a1
3-day post-SCI group vs. the control group	CC	GO:0016363	nuclear matrix	4/223	0.0093945	0.0335897	0.0204309	Actb/Alox5/Nono/Sfpq
3-day post-SCI group vs. the control group	CC	GO:0008287	protein serine/threonine phosphatase complex	3/223	0.0098533	0.0345883	0.0210383	Ppp1ca/Ppp1r15b/Ppp3ca
3-day post-SCI group vs. the control group	CC	GO:0043296	apical junction complex	5/223	0.0098563	0.0345883	0.0210383	Actb/Apc/Cdk4/Ctnna1/Ect2
3-day post-SCI group vs. the control group	CC	GO:0008021	synaptic vesicle	6/223	0.0100214	0.034845	0.0211944	Amph/Apc/Marcksl1/Rnf112/Snca/Syp
3-day post-SCI group vs. the control group	CC	GO:1903293	phosphatase complex	3/223	0.0103324	0.0353149	0.0214803	Ppp1ca/Ppp1r15b/Ppp3ca
3-day post-SCI group vs. the control group	CC	GO:0030673	axolemma	2/223	0.0103429	0.0353149	0.0214803	Adora1/Mapt
3-day post-SCI group vs. the control group	CC	GO:0030672	synaptic vesicle membrane	4/223	0.0109942	0.0368742	0.0224287	Amph/Apc/Snca/Syp
3-day post-SCI group vs. the control group	CC	GO:0099501	exocytic vesicle membrane	4/223	0.0109942	0.0368742	0.0224287	Amph/Apc/Snca/Syp
3-day post-SCI group vs. the control group	CC	GO:0005640	nuclear outer membrane	2/223	0.0113738	0.0378129	0.0229996	Ptgs2/Snca
3-day post-SCI group vs. the control group	CC	GO:0001533	cornified envelope	3/223	0.0118498	0.039053	0.0237539	Anxa1/Hspb1/Krt1
3-day post-SCI group vs. the control group	CC	GO:0001891	phagocytic cup	2/223	0.0124479	0.0399811	0.0243184	Aif1/Tlr4
3-day post-SCI group vs. the control group	CC	GO:0016580	Sin3 complex	2/223	0.0124479	0.0399811	0.0243184	Hdac1/Rbbp7
3-day post-SCI group vs. the control group	CC	GO:0070822	Sin3-type complex	2/223	0.0124479	0.0399811	0.0243184	Hdac1/Rbbp7
3-day post-SCI group vs. the control group	CC	GO:0001741	XY body	2/223	0.0135646	0.0428415	0.0260583	Atr/Ube2a
3-day post-SCI group vs. the control group	CC	GO:0097470	ribbon synapse	2/223	0.0135646	0.0428415	0.0260583	Amph/Atp2a2
3-day post-SCI group vs. the control group	CC	GO:0031307	integral component of mitochondrial outer membr	2/223	0.014723	0.0461159	0.0280499	Bnip3/Pink1
3-day post-SCI group vs. the control group	CC	GO:0016328	lateral plasma membrane	3/223	0.0152503	0.0471819	0.0286983	Abcc1/Anxa1/Apc
3-day post-SCI group vs. the control group	CC	GO:0070382	exocytic vesicle	6/223	0.0153123	0.0471819	0.0286983	Amph/Apc/Marcksl1/Rnf112/Snca/Syp
3-day post-SCI group vs. the control group	CC	GO:0031306	intrinsic component of mitochondrial outer membr	2/223	0.0159225	0.0486665	0.0296013	Bnip3/Pink1
3-day post-SCI group vs. the control group	CC	GO:0019897	extrinsic component of plasma membrane	5/223	0.0164367	0.0498361	0.0303127	Anxa1/Apc/Atp2a2/Ctnna1/Gnb2
3-day post-SCI group vs. the control group	CC	GO:0046930	pore complex	2/223	0.0171624	0.0512171	0.0311527	Bax/Ppif
3-day post-SCI group vs. the control group	CC	GO:0097038	perinuclear endoplasmic reticulum	2/223	0.0171624	0.0512171	0.0311527	Dst/Nox4
3-day post-SCI group vs. the control group	CC	GO:0032587	ruffle membrane	3/223	0.0184616	0.0546635	0.033249	Aif1/Apc/Plekha1
3-day post-SCI group vs. the control group	CC	GO:0032839	dendrite cytoplasm	2/223	0.0197607	0.0580565	0.0353128	Map2k4/Mapk8
3-day post-SCI group vs. the control group	CC	GO:0042734	presynaptic membrane	5/223	0.0204216	0.0595369	0.0362133	Adora1/Chrna4/Fosl1/Marcksl1/Syp
3-day post-SCI group vs. the control group	CC	GO:0030426	growth cone	5/223	0.0208125	0.0602132	0.0366246	Apc/Cttn/Mapt/Sirt1/Snca
3-day post-SCI group vs. the control group	CC	GO:0044305	calyx of Held	2/223	0.0211176	0.0606331	0.03688	Actb/Adora1
3-day post-SCI group vs. the control group	CC	GO:0044309	neuron spine	5/223	0.0220143	0.0627324	0.0381569	Adora1/Ppp1ca/Ppp3ca/Slc8a1/Syp
3-day post-SCI group vs. the control group	CC	GO:0030427	site of polarized growth	5/223	0.0236855	0.0669912	0.0407473	Apc/Cttn/Mapt/Sirt1/Snca
3-day post-SCI group vs. the control group	CC	GO:0016342	catenin complex	2/223	0.0239439	0.0672202	0.0408866	Apc/Ctnna1
3-day post-SCI group vs. the control group	CC	GO:0032592	integral component of mitochondrial membrane	3/223	0.0251156	0.0699912	0.042572	Bnip3/Etfdh/Pink1
3-day post-SCI group vs. the control group	CC	GO:1902495	transmembrane transporter complex	7/223	0.0267277	0.0734574	0.0446804	Atp2a2/Chrna4/Cttn/Fkbp1b/Ndufa12/Ndufa6/Ndufs8
3-day post-SCI group vs. the control group	CC	GO:0098573	intrinsic component of mitochondrial membrane	3/223	0.026747	0.0734574	0.0446804	Bnip3/Etfdh/Pink1
3-day post-SCI group vs. the control group	CC	GO:0001650	fibrillar center	4/223	0.027706	0.0755438	0.0459494	Ezr/Nono/Sirt1/Txnrd1
3-day post-SCI group vs. the control group	CC	GO:0005746	mitochondrial respirasome	3/223	0.0284339	0.075945	0.0461934	Ndufa12/Ndufa6/Ndufs8
3-day post-SCI group vs. the control group	CC	GO:0008305	integrin complex	2/223	0.0284543	0.075945	0.0461934	Itga6/Itgb5
3-day post-SCI group vs. the control group	CC	GO:1902555	endoribonuclease complex	2/223	0.0284543	0.075945	0.0461934	Ago1/Ago3
3-day post-SCI group vs. the control group	CC	GO:0030658	transport vesicle membrane	4/223	0.0301633	0.0794218	0.0483082	Amph/Apc/Snca/Syp

3-day post-SCI group vs. the control group	CC	GO:0098803	respiratory chain complex	3/223	0.0301761	0.0794218	0.0483082	Ndufa12/Ndufa6/Ndufs8
3-day post-SCI group vs. the control group	CC	GO:0070160	tight junction	4/223	0.0307972	0.0804974	0.0489625	Actb/Apc/Cdk4/Ect2
3-day post-SCI group vs. the control group	CC	GO:0043195	terminal bouton	3/223	0.0310679	0.0806488	0.0490545	Adora1/Snca/Syp
3-day post-SCI group vs. the control group	CC	GO:0005791	rough endoplasmic reticulum	3/223	0.0319734	0.0824347	0.0501408	Ncf1/Snca/Zc3h12a
3-day post-SCI group vs. the control group	CC	GO:0005938	cell cortex	6/223	0.0324388	0.0830696	0.050527	Actb/Apc/Cttn/Ect2/Melk/Snca
3-day post-SCI group vs. the control group	CC	GO:0098858	actin-based cell projection	5/223	0.0332612	0.0846039	0.0514602	Atp7a/Ctsl/Ezr/Pdgfra/Vcam1
3-day post-SCI group vs. the control group	CC	GO:0070603	SWI/SNF superfamily-type complex	3/223	0.0347721	0.0871395	0.0530025	Actb/Hdac1/Rbbp7
3-day post-SCI group vs. the control group	CC	GO:1990351	transporter complex	7/223	0.0348425	0.0871395	0.0530025	Atp2a2/Chrna4/Cttn/Fkbp1b/Ndufa12/Ndufa6/Ndufs8
3-day post-SCI group vs. the control group	CC	GO:0032154	cleavage furrow	2/223	0.0349478	0.0871395	0.0530025	Ect2/Mylk
3-day post-SCI group vs. the control group	CC	GO:0062023	collagen-containing extracellular matrix	7/223	0.0352461	0.087309	0.0531056	Anxa1/Atrn/Bmp1/Ctsl/Itga6/Krt1/Pxdn
3-day post-SCI group vs. the control group	CC	GO:0070469	respirasome	3/223	0.0376934	0.0927648	0.0564241	Ndufa12/Ndufa6/Ndufs8
3-day post-SCI group vs. the control group	CC	GO:0016592	mediator complex	2/223	0.038389	0.0932656	0.0567287	Cdk4/Ppargc1b
3-day post-SCI group vs. the control group	CC	GO:1905348	endonuclease complex	2/223	0.038389	0.0932656	0.0567287	Ago1/Ago3
3-day post-SCI group vs. the control group	CC	GO:1904949	ATPase complex	3/223	0.0386942	0.0934082	0.0568155	Actb/Hdac1/Rbbp7
3-day post-SCI group vs. the control group	CC	GO:1902562	H4 histone acetyltransferase complex	2/223	0.0401562	0.096324	0.0585889	Actb/Atf2
3-day post-SCI group vs. the control group	CC	GO:0120111	neuron projection cytoplasm	2/223	0.0419536	0.1000026	0.0608265	Map2k4/Mapk8
3-day post-SCI group vs. the control group	CC	GO:0044295	axonal growth cone	2/223	0.0437807	0.1037056	0.0630788	Apc/Mapt
3-day post-SCI group vs. the control group	MF	GO:0016209	antioxidant activity	18/224	6.62E-21	4.05E-18	2.8E-18	Cat/Cygb/Gpx1/Gpx3/Gpx7/Gpx8/Gsr/Gstt2/Lpo/Mgst1/Prdx4/Prdx6/Ptgs1/Ptgs2/Pxdn/Sod2/Srxn1/Txnrd1
3-day post-SCI group vs. the control group	MF	GO:0004601	peroxidase activity	15/224	3.9E-19	1.19E-16	8.24E-17	Cat/Cygb/Gpx1/Gpx3/Gpx7/Gpx8/Gstt2/Lpo/Mgst1/Prdx4/Prdx6/Ptgs1/Ptgs2/Pxdn/Txnrd1
3-day post-SCI group vs. the control group	MF	GO:0016684	oxidoreductase activity, acting on peroxide as acce	15/224	9.05E-19	1.84E-16	1.28E-16	Cat/Cygb/Gpx1/Gpx3/Gpx7/Gpx8/Gstt2/Lpo/Mgst1/Prdx4/Prdx6/Ptgs1/Ptgs2/Pxdn/Txnrd1
3-day post-SCI group vs. the control group	MF	GO:0004674	protein serine/threonine kinase activity	29/224	2.12E-18	3.24E-16	2.25E-16	Atr/Braf/Camkk2/Cdk1/Cdk10/Cdk4/Map2k3/Map2k4/Map2k6/Map4k4/Mapk10/Mapk14/Mapk3/Mapk8/Mapkapk2/Mapkapk3/Mark3/Melk/Mylk/Nme2/Pdk1/Pdk2/Pink1/Prkaa2/Ripk1/Ripk3/Stk24/Tnik/Vrk2
3-day post-SCI group vs. the control group	MF	GO:0061629	RNA polymerase II-specific DNA-binding transcri	22/224	3.54E-13	4.33E-11	3E-11	Actb/Atf2/Ets1/Fos/Hdac1/Hif1a/Hspb1/Jun/Mapk14/Ncoa7/Nfatc1/Nfe2l2/Parp1/Pcna/Ppargc1a/Ppargc1b/Rb1/Rela/Rest/Rxrb/Sirt1/Tfdp1
3-day post-SCI group vs. the control group	MF	GO:0004602	glutathione peroxidase activity	7/224	1.34E-09	1.37E-07	9.46E-08	Gpx1/Gpx3/Gpx7/Gpx8/Gstt2/Mgst1/Prdx6
3-day post-SCI group vs. the control group	MF	GO:0031625	ubiquitin protein ligase binding	16/224	4.99E-09	4.36E-07	3.02E-07	Apc/Fbxw7/Gpr37/Hif1a/Jun/Myc/Pink1/Ppargc1a/Prdx6/Rb1/Rela/Ripk1/Tank/Txnip/Uba52/Ube2a
3-day post-SCI group vs. the control group	MF	GO:0051019	mitogen-activated protein kinase binding	7/224	6.2E-09	4.73E-07	3.28E-07	Gch1/Mapk14/Mapkapk2/Mapkapk3/Nfatc1/Sirt1/Stau2
3-day post-SCI group vs. the control group	MF	GO:0044389	ubiquitin-like protein ligase binding	16/224	1.05E-08	7.12E-07	4.93E-07	Apc/Fbxw7/Gpr37/Hif1a/Jun/Myc/Pink1/Ppargc1a/Prdx6/Rb1/Rela/Ripk1/Tank/Txnip/Uba52/Ube2a
3-day post-SCI group vs. the control group	MF	GO:0004712	protein serine/threonine/tyrosine kinase activity	7/224	3.69E-08	2.25E-06	1.56E-06	Braf/Map2k3/Map2k4/Map2k6/Mapk3/Mapk8/Prkaa2
3-day post-SCI group vs. the control group	MF	GO:0004708	MAP kinase kinase activity	5/224	8.21E-08	4.56E-06	3.16E-06	Braf/Map2k3/Map2k4/Map2k6/Mapk3
3-day post-SCI group vs. the control group	MF	GO:0001221	transcription coregulator binding	10/224	1.1E-07	5.61E-06	3.89E-06	Eed/Ets1/Ezh2/Fos/Hdac1/Hif1a/Myc/Nfe2l2/Nfkb1/Rela
3-day post-SCI group vs. the control group	MF	GO:0001046	core promoter sequence-specific DNA binding	7/224	1.49E-07	0.000007	4.85E-06	Ago1/Ezh2/Fos/Hdac1/Myc/Rela/Rest

3-day post-SCI group vs. the control group	MF	GO:0031072	heat shock protein binding	10/224	2.18E-07	9.51E-06	6.59E-06	Adora1/Arhgdia/Bax/Cdk1/Gpr37/Hif1a/Mapt/Nfkb1/Snca/Stau2
3-day post-SCI group vs. the control group	MF	GO:0008022	protein C-terminus binding	12/224	3.74E-07	0.0000152	0.0000106	Amph/Atp2a2/Banf1/Cdc20/Dst/Ezr/Id1/Jak2/Mapk14/Pcna/Sdc1/Sirt1
3-day post-SCI group vs. the control group	MF	GO:0000979	RNA polymerase II core promoter sequence-speci	5/224	8.74E-07	0.0000334	0.0000231	Ezh2/Fos/Hdac1/Rela/Rest
3-day post-SCI group vs. the control group	MF	GO:0004713	protein tyrosine kinase activity	9/224	9.68E-07	0.0000348	0.0000241	Axl/Btk/Jak2/Map2k3/Map2k4/Map2k6/Melk/Met/Pdgfr[illegible]
3-day post-SCI group vs. the control group	MF	GO:0033218	amide binding	15/224	3.47E-06	0.0001177	0.0000815	Cat/Cd36/Ctsl/Dhcr24/Dhfr/Fkbp1b/Gpr37/Gsr/Lancl1/Mgst1/Ppif/Ppp3ca/Rela/Tlr6/Tpp1
3-day post-SCI group vs. the control group	MF	GO:0030291	protein serine/threonine kinase inhibitor activity	5/224	4.94E-06	0.0001588	0.00011	Casp3/Cdkn2b/Cdkn2c/Hspb1/Parp1
3-day post-SCI group vs. the control group	MF	GO:0020037	heme binding	9/224	5.68E-06	0.0001736	0.0001202	Cat/Cygb/Cyp1b1/Hebp2/Hmox1/Jak2/Ptgs1/Ptgs2/Pxdn
3-day post-SCI group vs. the control group	MF	GO:0004707	MAP kinase activity	4/224	6.36E-06	0.0001796	0.0001244	Mapk10/Mapk14/Mapk3/Mapk8
3-day post-SCI group vs. the control group	MF	GO:0016667	oxidoreductase activity, acting on a sulfur group o	6/224	6.47E-06	0.0001796	0.0001244	Glrx2/Gsr/Msrb2/P4hb/Srxn1/Txnrd1
3-day post-SCI group vs. the control group	MF	GO:0046906	tetrapyrrole binding	9/224	8.83E-06	0.0002345	0.0001624	Cat/Cygb/Cyp1b1/Hebp2/Hmox1/Jak2/Ptgs1/Ptgs2/Pxdn
3-day post-SCI group vs. the control group	MF	GO:0042277	peptide binding	13/224	0.0000105	0.0002483	0.0001719	Cat/Cd36/Ctsl/Dhcr24/Gpr37/Gsr/Lancl1/Mgst1/Ppif/Ppp3ca/Rela/Tlr6/Tpp1
3-day post-SCI group vs. the control group	MF	GO:0050135	NAD(P)+ nucleosidase activity	4/224	0.0000106	0.0002483	0.0001719	Cd38/Il18rap/Tlr4/Tlr6
3-day post-SCI group vs. the control group	MF	GO:0061809	NAD+ nucleotidase, cyclic ADP-ribose generating	4/224	0.0000106	0.0002483	0.0001719	Cd38/Il18rap/Tlr4/Tlr6
3-day post-SCI group vs. the control group	MF	GO:0003953	NAD+ nucleosidase activity	4/224	0.0000133	0.000301	0.0002084	Cd38/Il18rap/Tlr4/Tlr6
3-day post-SCI group vs. the control group	MF	GO:0016799	hydrolase activity, hydrolyzing N-glycosyl compou	5/224	0.0000153	0.0003345	0.0002316	Cd38/Il18rap/Pcna/Tlr4/Tlr6
3-day post-SCI group vs. the control group	MF	GO:0043548	phosphatidylinositol 3-kinase binding	5/224	0.0000173	0.0003538	0.0002451	Axl/Jak2/Met/Pdgfra/Tlr4
3-day post-SCI group vs. the control group	MF	GO:0019903	protein phosphatase binding	9/224	0.0000174	0.0003538	0.0002451	Mapk14/Mapk8/Mapt/Met/Pawr/Ppp1ca/Pxn/Sirpa/Stat6
3-day post-SCI group vs. the control group	MF	GO:0019902	phosphatase binding	10/224	0.0000183	0.0003603	0.0002496	Mapk14/Mapk3/Mapk8/Mapt/Met/Pawr/Ppp1ca/Pxn/Sirpa/Stat6
3-day post-SCI group vs. the control group	MF	GO:0016922	nuclear receptor binding	8/224	0.0000336	0.0006419	0.0004446	Hif1a/Ncoa7/Parp1/Pcna/Ppargc1a/Ppargc1b/Rxrb/Sirt1
3-day post-SCI group vs. the control group	MF	GO:0050839	cell adhesion molecule binding	11/224	0.0000368	0.0006805	0.0004713	Adam9/Ctnna1/Dst/Ezr/Itga6/Itgb5/P4hb/Ppp1ca/Pxn/Sirpa/Vcam1
3-day post-SCI group vs. the control group	MF	GO:0030544	Hsp70 protein binding	5/224	0.000051	0.0009164	0.0006347	Bax/Cdk1/Gpr37/Snca/Stau2
3-day post-SCI group vs. the control group	MF	GO:0019887	protein kinase regulator activity	9/224	0.0000544	0.0009494	0.0006575	Apc/Casp3/Ccna2/Cdkn2b/Cdkn2c/Hbegf/Hspb1/Map2k6/Parp1
3-day post-SCI group vs. the control group	MF	GO:0003779	actin binding	13/224	0.0000612	0.0010157	0.0007034	Aif1/Ctnna1/Cttn/Dst/Ezr/Impact/Marcksl1/Msrb2/Mylk/P4hb/Pawr/Pdlim1/Snca
3-day post-SCI group vs. the control group	MF	GO:0030374	nuclear receptor coactivator activity	5/224	0.0000615	0.0010157	0.0007034	Ets1/Ncoa7/Ppargc1a/Ppargc1b/Rxrb
3-day post-SCI group vs. the control group	MF	GO:0050660	flavin adenine dinucleotide binding	6/224	0.0000667	0.0010727	0.0007429	Dhcr24/Gsr/Maoa/Prodh/Txnrd1/Xdh
3-day post-SCI group vs. the control group	MF	GO:0042826	histone deacetylase binding	7/224	0.0000971	0.0015054	0.0010426	Cdc20/Hdac1/Hif1a/Mapk8/Parp1/Rela/Sfpq
3-day post-SCI group vs. the control group	MF	GO:0004861	cyclin-dependent protein serine/threonine kinase i	3/224	0.000101	0.0015054	0.0010426	Casp3/Cdkn2b/Cdkn2c
3-day post-SCI group vs. the control group	MF	GO:0070513	death domain binding	3/224	0.000101	0.0015054	0.0010426	Bax/Mcl1/Ripk1
3-day post-SCI group vs. the control group	MF	GO:0051400	BH domain binding	3/224	0.0001306	0.0018993	0.0013154	Bax/Mcl1/Pxn
3-day post-SCI group vs. the control group	MF	GO:0004860	protein kinase inhibitor activity	5/224	0.0001523	0.0021644	0.001499	Casp3/Cdkn2b/Cdkn2c/Hspb1/Parp1
3-day post-SCI group vs. the control group	MF	GO:0019207	kinase regulator activity	9/224	0.000157	0.0021801	0.0015099	Apc/Casp3/Ccna2/Cdkn2b/Cdkn2c/Hbegf/Hspb1/Map2k6/Parp1

3-day post-SCI group vs. the control group	MF	GO:0019215	intermediate filament binding	3/224	0.0001652	0.0021943	0.0015197	Nme2/Sirt1/Ubqln1
3-day post-SCI group vs. the control group	MF	GO:0044548	S100 protein binding	3/224	0.0001652	0.0021943	0.0015197	Atp2a2/Ezr/S100a1
3-day post-SCI group vs. the control group	MF	GO:0045295	gamma-catenin binding	3/224	0.0002053	0.0026133	0.0018099	Apc/Ctnna1/Ptprk
3-day post-SCI group vs. the control group	MF	GO:0050664	oxidoreductase activity, acting on NAD(P)H, oxyg	3/224	0.0002053	0.0026133	0.0018099	Ncf1/Nox4/Txnrd1
3-day post-SCI group vs. the control group	MF	GO:0019210	kinase inhibitor activity	5/224	0.0002175	0.0026932	0.0018652	Casp3/Cdkn2b/Cdkn2c/Hspb1/Parp1
3-day post-SCI group vs. the control group	MF	GO:0072341	modified amino acid binding	6/224	0.0002204	0.0026932	0.0018652	Axl/Dhfr/Gsr/Lancl1/Mgst1/Nox4
3-day post-SCI group vs. the control group	MF	GO:0015035	protein-disulfide reductase activity	4/224	0.0002252	0.0026981	0.0018686	Glrx2/Gsr/P4hb/Txnrd1
3-day post-SCI group vs. the control group	MF	GO:0005178	integrin binding	7/224	0.0002393	0.0028101	0.0019462	Adam9/Dst/Itga6/Itgb5/P4hb/Pxn/Vcam1
3-day post-SCI group vs. the control group	MF	GO:0003713	transcription coactivator activity	9/224	0.0002438	0.0028101	0.0019462	Ets1/Ncoa7/Nme2/Pdlim1/Ppargc1a/Ppargc1b/Rbpms/Rxrb/Sirt1
3-day post-SCI group vs. the control group	MF	GO:0035173	histone kinase activity	3/224	0.0003033	0.0034314	0.0023764	Cdk1/Jak2/Prkaa2
3-day post-SCI group vs. the control group	MF	GO:1990841	promoter-specific chromatin binding	5/224	0.0003214	0.0035709	0.0024731	Atf2/Ezh2/Hdac1/Ppargc1a/Sirt1
3-day post-SCI group vs. the control group	MF	GO:0071949	FAD binding	4/224	0.0003332	0.0036352	0.0025176	Dhcr24/Prodh/Txnrd1/Xdh
3-day post-SCI group vs. the control group	MF	GO:0043295	glutathione binding	3/224	0.0003618	0.0038783	0.002686	Gsr/Lancl1/Mgst1
3-day post-SCI group vs. the control group	MF	GO:1900750	oligopeptide binding	3/224	0.0004271	0.0044337	0.0030706	Gsr/Lancl1/Mgst1
3-day post-SCI group vs. the control group	MF	GO:0043021	ribonucleoprotein complex binding	7/224	0.0004337	0.0044337	0.0030706	Eif2s1/Ezh2/Impact/Ppp1ca/Stau2/Ybx3/Zc3h12a
3-day post-SCI group vs. the control group	MF	GO:0015036	disulfide oxidoreductase activity	4/224	0.0004354	0.0044337	0.0030706	Glrx2/Gsr/P4hb/Txnrd1
3-day post-SCI group vs. the control group	MF	GO:0016538	cyclin-dependent protein serine/threonine kinase r	4/224	0.0005148	0.0051568	0.0035714	Casp3/Ccna2/Cdkn2b/Cdkn2c
3-day post-SCI group vs. the control group	MF	GO:0003712	transcription coregulator activity	12/224	0.0005572	0.0054913	0.0038031	Ets1/Ezh2/Hdac1/Ncoa7/Nme2/Pdlim1/Ppargc1a/Ppargc1b/Pxn/Rbpms/Rxrb/Sirt1
3-day post-SCI group vs. the control group	MF	GO:0017124	SH3 domain binding	6/224	0.0006197	0.0060098	0.0041622	Adam9/Gpx1/Lancl1/Mapt/Ncf1/Sirpa
3-day post-SCI group vs. the control group	MF	GO:0030331	nuclear estrogen receptor binding	4/224	0.0006526	0.0062302	0.0043148	Parp1/Pcna/Ppargc1a/Ppargc1b
3-day post-SCI group vs. the control group	MF	GO:0047485	protein N-terminus binding	6/224	0.000698	0.0064181	0.004445	Banf1/Hdac1/Id1/Parp1/Rela/Snca
3-day post-SCI group vs. the control group	MF	GO:0001222	transcription corepressor binding	4/224	0.0007038	0.0064181	0.004445	Eed/Ets1/Ezh2/Hdac1
3-day post-SCI group vs. the control group	MF	GO:0019894	kinesin binding	4/224	0.0007038	0.0064181	0.004445	Actb/Mapk8/Snca/Stau2
3-day post-SCI group vs. the control group	MF	GO:0046982	protein heterodimerization activity	9/224	0.0007868	0.0070697	0.0048962	Adora1/Atf2/Bax/Hif1a/Krt1/Mcl1/P4hb/Tlr4/Tlr6
3-day post-SCI group vs. the control group	MF	GO:0005516	calmodulin binding	7/224	0.0008353	0.0073966	0.0051226	Camkk2/Mapkapk2/Mapkapk3/Marcksl1/Mylk/Ppp3ca/Slc8a1
3-day post-SCI group vs. the control group	MF	GO:0004683	calmodulin-dependent protein kinase activity	3/224	0.0008666	0.007458	0.0051651	Camkk2/Mapkapk2/Mapkapk3
3-day post-SCI group vs. the control group	MF	GO:0010857	calcium-dependent protein kinase activity	3/224	0.0008666	0.007458	0.0051651	Mapkapk2/Mapkapk3/Pink1
3-day post-SCI group vs. the control group	MF	GO:0070412	R-SMAD binding	3/224	0.0009791	0.0083089	0.0057544	Fos/Jun/Parp1
3-day post-SCI group vs. the control group	MF	GO:0003727	single-stranded RNA binding	5/224	0.0010435	0.008616	0.0059671	Ago1/Ago3/Anxa1/Cbx6/Rbpms
3-day post-SCI group vs. the control group	MF	GO:0008013	beta-catenin binding	5/224	0.0010435	0.008616	0.0059671	Apc/Ctnna1/Met/Ptprk/Pxn
3-day post-SCI group vs. the control group	MF	GO:0005504	fatty acid binding	4/224	0.0014685	0.0116077	0.0080391	Arhgdia/Cd36/Nme2/Snca
3-day post-SCI group vs. the control group	MF	GO:0004693	cyclin-dependent protein serine/threonine kinase a	3/224	0.0015198	0.0116077	0.0080391	Cdk1/Cdk10/Cdk4
3-day post-SCI group vs. the control group	MF	GO:0016645	oxidoreductase activity, acting on the CH-NH grou	3/224	0.0015198	0.0116077	0.0080391	Dhfr/Etfdh/Prodh
3-day post-SCI group vs. the control group	MF	GO:0016702	oxidoreductase activity, acting on single donors w	3/224	0.0015198	0.0116077	0.0080391	Alox5/Ptgs1/Ptgs2
3-day post-SCI group vs. the control group	MF	GO:0043274	phospholipase binding	3/224	0.0015198	0.0116077	0.0080391	Btk/Met/Snca
3-day post-SCI group vs. the control group	MF	GO:0097472	cyclin-dependent protein kinase activity	3/224	0.0015198	0.0116077	0.0080391	Cdk1/Cdk10/Cdk4
3-day post-SCI group vs. the control group	MF	GO:0016701	oxidoreductase activity, acting on single donors w	3/224	0.0016789	0.0126646	0.0087711	Alox5/Ptgs1/Ptgs2

3-day post-SCI group vs. the control group	MF	GO:0004364	glutathione transferase activity	3/224	0.0018481	0.0134659	0.009326	Gstt2/Lancl1/Mgst1
3-day post-SCI group vs. the control group	MF	GO:0051536	iron-sulfur cluster binding	4/224	0.0018513	0.0134659	0.009326	Etfdh/Glrx2/Ndufs8/Xdh
3-day post-SCI group vs. the control group	MF	GO:0051540	metal cluster binding	4/224	0.0018513	0.0134659	0.009326	Etfdh/Glrx2/Ndufs8/Xdh
3-day post-SCI group vs. the control group	MF	GO:0051219	phosphoprotein binding	5/224	0.001899	0.0136503	0.0094538	Fbxw7/Mapk3/Rb1/Sirpa/Snca
3-day post-SCI group vs. the control group	MF	GO:0005507	copper ion binding	4/224	0.0020664	0.0146812	0.0101677	Atox1/Atp7a/Ccs/Snca
3-day post-SCI group vs. the control group	MF	GO:0030332	cyclin binding	3/224	0.0024179	0.016981	0.0117604	Cdk1/Cdk4/Fbxw7
3-day post-SCI group vs. the control group	MF	GO:0043022	ribosome binding	4/224	0.0025474	0.0176867	0.0122492	Eif2s1/Impact/Stau2/Zc3h12a
3-day post-SCI group vs. the control group	MF	GO:0016174	NAD(P)H oxidase H2O2-forming activity	2/224	0.0026725	0.0177486	0.0122921	Nox4/Txnrd1
3-day post-SCI group vs. the control group	MF	GO:0047499	calcium-independent phospholipase A2 activity	2/224	0.0026725	0.0177486	0.0122921	Pnpla8/Prdx6
3-day post-SCI group vs. the control group	MF	GO:0070878	primary miRNA binding	2/224	0.0026725	0.0177486	0.0122921	Ezh2/Smad1
3-day post-SCI group vs. the control group	MF	GO:0097027	ubiquitin-protein transferase activator activity	2/224	0.0026725	0.0177486	0.0122921	Cdc20/Fbxw7
3-day post-SCI group vs. the control group	MF	GO:0016651	oxidoreductase activity, acting on NAD(P)H	4/224	0.0029549	0.0194131	0.0134449	Ncf1/Ndufs8/Nox4/Txnrd1
3-day post-SCI group vs. the control group	MF	GO:0035198	miRNA binding	3/224	0.0030856	0.0200562	0.0138902	Ago1/Ago3/Zc3h12a
3-day post-SCI group vs. the control group	MF	GO:0032404	mismatch repair complex binding	2/224	0.0032494	0.0206811	0.014323	Atr/Pcna
3-day post-SCI group vs. the control group	MF	GO:0047134	protein-disulfide reductase (NAD(P)) activity	2/224	0.0032494	0.0206811	0.014323	Gsr/Txnrd1
3-day post-SCI group vs. the control group	MF	GO:0031406	carboxylic acid binding	6/224	0.0033993	0.021412	0.0148292	Arhgdia/Cd36/Dhfr/Gclc/Nme2/Snca
3-day post-SCI group vs. the control group	MF	GO:0042805	actinin binding	3/224	0.0038561	0.0237011	0.0164146	Nfkb1/Pdlim1/Rela
3-day post-SCI group vs. the control group	MF	GO:0008353	RNA polymerase II CTD heptapeptide repeat kina	2/224	0.0038791	0.0237011	0.0164146	Cdk1/Cdk4
3-day post-SCI group vs. the control group	MF	GO:0016668	oxidoreductase activity, acting on a sulfur group o	2/224	0.0038791	0.0237011	0.0164146	Gsr/Txnrd1
3-day post-SCI group vs. the control group	MF	GO:0016018	cyclosporin A binding	2/224	0.0045606	0.0265385	0.0183796	Ppif/Ppp3ca
3-day post-SCI group vs. the control group	MF	GO:0017166	vinculin binding	2/224	0.0045606	0.0265385	0.0183796	Ctnna1/Pxn
3-day post-SCI group vs. the control group	MF	GO:0031994	insulin-like growth factor I binding	2/224	0.0045606	0.0265385	0.0183796	Igfbp2/Itga6
3-day post-SCI group vs. the control group	MF	GO:0035325	Toll-like receptor binding	2/224	0.0045606	0.0265385	0.0183796	Cd36/Tlr6
3-day post-SCI group vs. the control group	MF	GO:0043522	leucine zipper domain binding	2/224	0.0045606	0.0265385	0.0183796	Atf2/Pawr
3-day post-SCI group vs. the control group	MF	GO:0046332	SMAD binding	4/224	0.0046311	0.0266942	0.0184875	Fos/Jun/Parp1/Smad1
3-day post-SCI group vs. the control group	MF	GO:0016810	hydrolase activity, acting on carbon-nitrogen (but r	5/224	0.0046968	0.0268202	0.0185747	Arg1/Cat/Gch1/Hdac1/Sirt1
3-day post-SCI group vs. the control group	MF	GO:0016798	hydrolase activity, acting on glycosyl bonds	5/224	0.004992	0.0282415	0.0195591	Cd38/Il18rap/Pcna/Tlr4/Tlr6
3-day post-SCI group vs. the control group	MF	GO:0004715	non-membrane spanning protein tyrosine kinase ac	3/224	0.0050511	0.0283139	0.0196093	Btk/Jak2/Melk
3-day post-SCI group vs. the control group	MF	GO:0050661	NADP binding	3/224	0.0057231	0.031503	0.0218179	Cat/Dhfr/Gsr
3-day post-SCI group vs. the control group	MF	GO:0070888	E-box binding	3/224	0.0057231	0.031503	0.0218179	Hdac1/Hif1a/Myc
3-day post-SCI group vs. the control group	MF	GO:0004857	enzyme inhibitor activity	9/224	0.0059281	0.0323401	0.0223976	Anxa1/Casp3/Cdkn2b/Cdkn2c/Hspb1/Parp1/Rnh1/Snca/Txnip
3-day post-SCI group vs. the control group	MF	GO:0017136	NAD-dependent histone deacetylase activity	2/224	0.0060759	0.0325381	0.0225348	Hdac1/Sirt1
3-day post-SCI group vs. the control group	MF	GO:0106222	lncRNA binding	2/224	0.0060759	0.0325381	0.0225348	Atp2a2/Ezh2
3-day post-SCI group vs. the control group	MF	GO:0051213	dioxygenase activity	4/224	0.0061242	0.0325381	0.0225348	Alox5/P4hb/Ptgs1/Ptgs2
3-day post-SCI group vs. the control group	MF	GO:0033293	monocarboxylic acid binding	4/224	0.0063606	0.033503	0.023203	Arhgdia/Cd36/Nme2/Snca
3-day post-SCI group vs. the control group	MF	GO:0001223	transcription coactivator binding	3/224	0.0064462	0.0336637	0.0233143	Hif1a/Nfkb1/Rela
3-day post-SCI group vs. the control group	MF	GO:0034979	NAD-dependent protein deacetylase activity	2/224	0.006908	0.0357696	0.0247728	Hdac1/Sirt1
3-day post-SCI group vs. the control group	MF	GO:0051879	Hsp90 protein binding	3/224	0.0072214	0.0365503	0.0253135	Arhgdia/Hif1a/Mapt
3-day post-SCI group vs. the control group	MF	GO:0019838	growth factor binding	5/224	0.0072383	0.0365503	0.0253135	Cd36/Igfbp2/Il6st/Itga6/Pdgfra

3-day post-SCI group vs. the control group	MF	GO:0043177	organic acid binding	5/224	0.0072383	0.0365503	0.0253135	Cd36/Dhfr/Gclc/Prodh/Snca
3-day post-SCI group vs. the control group	MF	GO:0061980	regulatory RNA binding	3/224	0.0076287	0.0376326	0.0260631	Ago1/Ago3/Zc3h12a
3-day post-SCI group vs. the control group	MF	GO:0004620	phospholipase activity	4/224	0.0076339	0.0376326	0.0260631	Ccr1/Hmox1/Pnpla8/Prdx6
3-day post-SCI group vs. the control group	MF	GO:0015399	primary active transmembrane transporter activity	5/224	0.0076374	0.0376326	0.0260631	Abcc1/Atp13a2/Atp2a2/Atp7a/Ndufs8
3-day post-SCI group vs. the control group	MF	GO:0016004	phospholipase activator activity	2/224	0.0077887	0.0377689	0.0261575	Btk/Casp3
3-day post-SCI group vs. the control group	MF	GO:0043422	protein kinase B binding	2/224	0.0077887	0.0377689	0.0261575	Pink1/Sirt1
3-day post-SCI group vs. the control group	MF	GO:0019829	ATPase-coupled cation transmembrane transporter	3/224	0.0080494	0.0387258	0.0268202	Atp13a2/Atp2a2/Atp7a
3-day post-SCI group vs. the control group	MF	GO:0051117	ATPase binding	4/224	0.0081868	0.0390791	0.0270649	Atox1/Ezr/Ppp3ca/S100a1
3-day post-SCI group vs. the control group	MF	GO:0035497	cAMP response element binding	2/224	0.008717	0.0412877	0.0285945	Atf2/Jun
3-day post-SCI group vs. the control group	MF	GO:0042626	ATPase-coupled transmembrane transporter activi	4/224	0.0096822	0.0448637	0.0310711	Abcc1/Atp13a2/Atp2a2/Atp7a
3-day post-SCI group vs. the control group	MF	GO:0005324	long-chain fatty acid transporter activity	2/224	0.0096923	0.0448637	0.0310711	Abcc1/Cd36
3-day post-SCI group vs. the control group	MF	GO:0140416	transcription regulator inhibitor activity	2/224	0.0096923	0.0448637	0.0310711	Id1/Tfdp1
3-day post-SCI group vs. the control group	MF	GO:0140296	general transcription initiation factor binding	3/224	0.0103557	0.0475741	0.0329482	Brf2/Jun/Rela
3-day post-SCI group vs. the control group	MF	GO:0005123	death receptor binding	2/224	0.0107137	0.0477817	0.033092	Casp3/Ripk1
3-day post-SCI group vs. the control group	MF	GO:0030275	LRR domain binding	2/224	0.0107137	0.0477817	0.033092	Atf2/Pawr
3-day post-SCI group vs. the control group	MF	GO:0051010	microtubule plus-end binding	2/224	0.0107137	0.0477817	0.033092	Apc/Dst
3-day post-SCI group vs. the control group	MF	GO:0060229	lipase activator activity	2/224	0.0107137	0.0477817	0.033092	Btk/Casp3
3-day post-SCI group vs. the control group	MF	GO:0004714	transmembrane receptor protein tyrosine kinase ac	3/224	0.0113745	0.0503609	0.0348782	Axl/Met/Pdgfra
3-day post-SCI group vs. the control group	MF	GO:0005506	iron ion binding	5/224	0.0116655	0.0510489	0.0353547	Alox5/Cygb/Cyp1b1/Snca/Xdh
3-day post-SCI group vs. the control group	MF	GO:0004407	histone deacetylase activity	2/224	0.0117805	0.0510489	0.0353547	Hdac1/Sirt1
3-day post-SCI group vs. the control group	MF	GO:0016641	oxidoreductase activity, acting on the CH-NH2 gro	2/224	0.0117805	0.0510489	0.0353547	Maoa/Vcam1
3-day post-SCI group vs. the control group	MF	GO:0140678	molecular function inhibitor activity	3/224	0.0119047	0.0512239	0.0354759	Fkbp1b/Id1/Tfdp1
3-day post-SCI group vs. the control group	MF	GO:0031490	chromatin DNA binding	4/224	0.012425	0.0530885	0.0367673	Ezh2/Ppargc1a/Rela/Rxrb
3-day post-SCI group vs. the control group	MF	GO:0033558	protein lysine deacetylase activity	2/224	0.0128918	0.0543236	0.0376227	Hdac1/Sirt1
3-day post-SCI group vs. the control group	MF	GO:0070840	dynein complex binding	2/224	0.0128918	0.0543236	0.0376227	Apc/Snca
3-day post-SCI group vs. the control group	MF	GO:0008234	cysteine-type peptidase activity	5/224	0.0130811	0.0547434	0.0379134	Capns1/Casp3/Ctsl/Tank/Zc3h12a
3-day post-SCI group vs. the control group	MF	GO:0016765	transferase activity, transferring alkyl or aryl (othe	3/224	0.0135798	0.0560625	0.038827	Gstt2/Lancl1/Mgst1
3-day post-SCI group vs. the control group	MF	GO:0045309	protein phosphorylated amino acid binding	3/224	0.0135798	0.0560625	0.038827	Fbxw7/Mapk3/Sirpa
3-day post-SCI group vs. the control group	MF	GO:0009931	calcium-dependent protein serine/threonine kinase	2/224	0.014047	0.0573222	0.0396994	Mapkapk2/Mapkapk3
3-day post-SCI group vs. the control group	MF	GO:0004521	endoribonuclease activity	3/224	0.0141664	0.0573222	0.0396994	Ago3/Endog/Zc3h12a
3-day post-SCI group vs. the control group	MF	GO:0005080	protein kinase C binding	3/224	0.0141664	0.0573222	0.0396994	Adam9/Hspb1/Pawr
3-day post-SCI group vs. the control group	MF	GO:0022804	active transmembrane transporter activity	8/224	0.0151038	0.0600957	0.0416202	Abcc1/Atp13a2/Atp2a2/Atp7a/Ndufs8/Slc23a2/Slc4a11/Slc8a1
3-day post-SCI group vs. the control group	MF	GO:0022853	active ion transmembrane transporter activity	6/224	0.0152261	0.0600957	0.0416202	Atp13a2/Atp2a2/Atp7a/Slc23a2/Slc4a11/Slc8a1
3-day post-SCI group vs. the control group	MF	GO:0016638	oxidoreductase activity, acting on the CH-NH2 gro	2/224	0.0152452	0.0600957	0.0416202	Maoa/Vcam1
3-day post-SCI group vs. the control group	MF	GO:0055106	ubiquitin-protein transferase regulator activity	2/224	0.0152452	0.0600957	0.0416202	Cdc20/Fbxw7
3-day post-SCI group vs. the control group	MF	GO:0004709	MAP kinase kinase kinase activity	2/224	0.0164857	0.064169	0.0444413	Braf/Ripk1
3-day post-SCI group vs. the control group	MF	GO:0016298	lipase activity	4/224	0.0164928	0.064169	0.0444413	Ccr1/Hmox1/Pnpla8/Prdx6
3-day post-SCI group vs. the control group	MF	GO:0008514	organic anion transmembrane transporter activity	5/224	0.0165936	0.064169	0.0444413	Abcc1/Cd36/Slc23a2/Slc25a24/Slc4a11
3-day post-SCI group vs. the control group	MF	GO:0009055	electron transfer activity	3/224	0.0173131	0.0665303	0.0460766	Etfdh/Ndufs8/Xdh

3-day post-SCI group vs. the control group	MF	GO:0019825	oxygen binding	2/224		0.0177679	0.0674296	0.0466994	Cygb/Sod2
3-day post-SCI group vs. the control group	MF	GO:0051537	2 iron, 2 sulfur cluster binding	2/224		0.0177679	0.0674296	0.0466994	Glrx2/Xdh
3-day post-SCI group vs. the control group	MF	GO:0016725	oxidoreductase activity, acting on CH or CH2 grou	2/224		0.0190908	0.0711249	0.0492587	Rrm2b/Xdh
3-day post-SCI group vs. the control group	MF	GO:0035035	histone acetyltransferase binding	2/224		0.0190908	0.0711249	0.0492587	Ets1/Pcna
3-day post-SCI group vs. the control group	MF	GO:0090482	vitamin transmembrane transporter activity	2/224		0.0190908	0.0711249	0.0492587	Abcc1/Slc23a2
3-day post-SCI group vs. the control group	MF	GO:0008017	microtubule binding	6/224		0.0199641	0.0739278	0.0511999	Apc/Dst/Ezr/Map4k4/Mapt/Snca
3-day post-SCI group vs. the control group	MF	GO:0140104	molecular carrier activity	3/224		0.0200887	0.0739408	0.0512089	Atox1/Atp7a/Cygb
3-day post-SCI group vs. the control group	MF	GO:0071813	lipoprotein particle binding	2/224		0.0204539	0.0739488	0.0512144	Cd36/Mapt
3-day post-SCI group vs. the control group	MF	GO:0071814	protein-lipid complex binding	2/224		0.0204539	0.0739488	0.0512144	Cd36/Mapt
3-day post-SCI group vs. the control group	MF	GO:0140359	ABC-type transporter activity	2/224		0.0204539	0.0739488	0.0512144	Abcc1/Atp13a2
3-day post-SCI group vs. the control group	MF	GO:0015085	calcium ion transmembrane transporter activity	4/224		0.0218328	0.0771924	0.0534608	Atp2a2/Marcksl1/Slc8a1/Trpm2
3-day post-SCI group vs. the control group	MF	GO:0005520	insulin-like growth factor binding	2/224		0.0218564	0.0771924	0.0534608	Igfbp2/Itga6
3-day post-SCI group vs. the control group	MF	GO:0043236	laminin binding	2/224		0.0218564	0.0771924	0.0534608	Adam9/Pxdn
3-day post-SCI group vs. the control group	MF	GO:0043325	phosphatidylinositol-3,4-bisphosphate binding	2/224		0.0218564	0.0771924	0.0534608	Ncf1/Plekha1
3-day post-SCI group vs. the control group	MF	GO:0002039	p53 binding	3/224		0.0223216	0.0783823	0.0542849	Hdac1/Hif1a/Sirt1
3-day post-SCI group vs. the control group	MF	GO:0019199	transmembrane receptor protein kinase activity	3/224		0.0230948	0.0799713	0.0553854	Axl/Met/Pdgfra
3-day post-SCI group vs. the control group	MF	GO:0015662	P-type ion transporter activity	2/224		0.0232977	0.0799713	0.0553854	Atp2a2/Atp7a
3-day post-SCI group vs. the control group	MF	GO:0038187	pattern recognition receptor activity	2/224		0.0232977	0.0799713	0.0553854	Cd36/Tlr4
3-day post-SCI group vs. the control group	MF	GO:0140358	P-type transmembrane transporter activity	2/224		0.0232977	0.0799713	0.0553854	Atp2a2/Atp7a
3-day post-SCI group vs. the control group	MF	GO:0051059	NF-kappaB binding	2/224		0.0262936	0.0897508	0.0621584	Hdac1/Rela
3-day post-SCI group vs. the control group	MF	GO:0016811	hydrolase activity, acting on carbon-nitrogen (but r	3/224		0.0271769	0.0922505	0.0638896	Cat/Hdac1/Sirt1
3-day post-SCI group vs. the control group	MF	GO:0019955	cytokine binding	4/224		0.0281189	0.0949209	0.065739	Ccr1/Cd36/Il6st/Tnfrsf1a
3-day post-SCI group vs. the control group	MF	GO:0015081	sodium ion transmembrane transporter activity	4/224		0.0287384	0.0964789	0.066818	Slc23a2/Slc4a11/Slc8a1/Trpm2
3-day post-SCI group vs. the control group	MF	GO:0097110	scaffold protein binding	3/224		0.0289104	0.0965261	0.0668507	Braf/Il6st/Mapk3
3-day post-SCI group vs. the control group	MF	GO:0004623	phospholipase A2 activity	2/224		0.0294362	0.0966964	0.0669687	Pnpla8/Prdx6
3-day post-SCI group vs. the control group	MF	GO:0008157	protein phosphatase 1 binding	2/224		0.0294362	0.0966964	0.0669687	Pawr/Ppp1ca
3-day post-SCI group vs. the control group	MF	GO:0019213	deacetylase activity	2/224		0.0294362	0.0966964	0.0669687	Hdac1/Sirt1
3-day post-SCI group vs. the control group	MF	GO:0003725	double-stranded RNA binding	3/224		0.0297987	0.0973637	0.0674308	Ago1/Ago3/Stau2
3-day post-SCI group vs. the control group	MF	GO:0005543	phospholipid binding	8/224		0.0302182	0.098209	0.0680163	Amph/Anxa1/Atp13a2/Axl/Btk/Ncf1/Snca/Tpp1
3-day post-SCI group vs. the control group	MF	GO:0003755	peptidyl-prolyl cis-trans isomerase activity	2/224		0.0310609	0.1004138	0.0695432	Fkbp1b/Ppif
3-day post-SCI group vs. the control group	MF	GO:0042393	histone binding	6/224		0.0313666	0.1008684	0.069858	Ctsl/Jak2/Phc3/Rbbp7/Sirt1/Snca
3-day post-SCI group vs. the control group	MF	GO:0051015	actin filament binding	5/224		0.0317542	0.1015803	0.0703511	Aif1/Ctnna1/Cttn/Ezr/Marcksl1
3-day post-SCI group vs. the control group	MF	GO:0015631	tubulin binding	7/224		0.0325235	0.1030519	0.0713703	Apc/Dst/Ezr/Map4k4/Mapt/Ppargc1a/Snca
3-day post-SCI group vs. the control group	MF	GO:0045182	translation regulator activity	4/224		0.032625	0.1030519	0.0713703	Ago3/Dhfr/Eif2s1/Paip1
3-day post-SCI group vs. the control group	MF	GO:0098632	cell-cell adhesion mediator activity	2/224		0.0327202	0.1030519	0.0713703	Ppp1ca/Sirpa
3-day post-SCI group vs. the control group	MF	GO:0070851	growth factor receptor binding	4/224		0.0333013	0.104344	0.0722651	Hbegf/Il6st/Jak2/Pdgfra
3-day post-SCI group vs. the control group	MF	GO:0046915	transition metal ion transmembrane transporter act	2/224		0.0344137	0.1067347	0.0739209	Atp7a/Trpm2
3-day post-SCI group vs. the control group	MF	GO:0097718	disordered domain specific binding	2/224		0.0344137	0.1067347	0.0739209	Ezr/Rb1
3-day post-SCI group vs. the control group	MF	GO:0016705	oxidoreductase activity, acting on paired donors, w	5/224		0.0350044	0.1080186	0.07481	Cyp1b1/Hmox1/P4hb/Ptgs1/Ptgs2
3-day post-SCI group vs. the control group	MF	GO:0016859	cis-trans isomerase activity	2/224		0.0379001	0.1152088	0.0797897	Fkbp1b/Ppif

3-day post-SCI group vs. the control group	MF	GO:0030159	signaling receptor complex adaptor activity	2/224	0.0379001	0.1152088	0.0797897	Gnb2/Pxn
3-day post-SCI group vs. the control group	MF	GO:0051539	4 iron, 4 sulfur cluster binding	2/224	0.0379001	0.1152088	0.0797897	Etfdh/Ndufs8
3-day post-SCI group vs. the control group	MF	GO:0005096	GTPase activator activity	5/224	0.0402531	0.1217556	0.0843238	Agap3/Arhgdia/Ect2/Jun/Tbc1d24
3-day post-SCI group vs. the control group	MF	GO:0004896	cytokine receptor activity	3/224	0.0415644	0.1251028	0.0866419	Ccr1/Il18rap/Il6st
3-day post-SCI group vs. the control group	MF	GO:0005246	calcium channel regulator activity	2/224	0.0452542	0.1355408	0.093871	Fkbp1b/Gnb2
3-day post-SCI group vs. the control group	MF	GO:0005272	sodium channel activity	2/224	0.0471687	0.1399031	0.0968922	Slc4a11/Trpm2
3-day post-SCI group vs. the control group	MF	GO:0043014	alpha-tubulin binding	2/224	0.0471687	0.1399031	0.0968922	Ppargc1a/Snca
3-day post-SCI group vs. the control group	MF	GO:0001227	DNA-binding transcription repressor activity, RN/	6/224	0.0480659	0.1418755	0.0982582	Jun/Myc/Nfatc1/Nfkb1/Rela/Rest
3-day post-SCI group vs. the control group	MF	GO:0098631	cell adhesion mediator activity	2/224	0.0491123	0.1442673	0.0999147	Ppp1ca/Sirpa
3-day post-SCI group vs. the control group	MF	GO:0001217	DNA-binding transcription repressor activity	6/224	0.0498111	0.1456201	0.1008515	Jun/Myc/Nfatc1/Nfkb1/Rela/Rest
7-day post-SCI group vs. the control group	BP	GO:0006979	response to oxidative stress	46/88	6.81E-62	2.26E-58	1.02E-58	Adam9/Aif1/Apoe/Atp2a2/Atp7a/Axl/Cd36/Cd38/Ctnnb1/Fbxw7/Fos/Gch1/Gclc/Gpx1/Gpx3/Hif1a/Hspb1/Jun/Lancl1/Mapk8/Mapt/Mcl1/Mgst1/Ncoa7/Ndufa12/Ndufa6/Nfe2l2/Pdk2/Pink1/Ppargc1a/Ppargc1b/Prdx6/Prkcd/Prr5l/Ptgs1/Rcan1/Ripk1/Sirpa/Slc25a24/Slc8a1/Stat6/Tlr4/Trem2/Trpm2/Txnip/Ucp2
7-day post-SCI group vs. the control group	BP	GO:0034599	cellular response to oxidative stress	34/88	2.88E-46	4.77E-43	2.16E-43	Aif1/Atp2a2/Atp7a/Axl/Cd36/Ctnnb1/Fbxw7/Fos/Gch1/Gpx1/Hif1a/Hspb1/Jun/Lancl1/Mapk8/Mapt/Mcl1/Mgst1/Ncoa7/Nfe2l2/Pdk2/Pink1/Ppargc1a/Ppargc1b/Prkcd/Prr5l/Ripk1/Sirpa/Slc25a24/Slc8a1/Stat6/Tlr4/Trem2/Trpm2
7-day post-SCI group vs. the control group	BP	GO:0062197	cellular response to chemical stress	35/88	1.14E-44	1.25E-41	5.68E-42	Aif1/Atp2a2/Atp7a/Axl/Cd36/Ctnnb1/Fbxw7/Fos/Gch1/Gpx1/Hif1a/Hspb1/Jun/Lancl1/Mapk8/Mapt/Mcl1/Mgst1/Mylk/Ncoa7/Nfe2l2/Pdk2/Pink1/Ppargc1a/Ppargc1b/Prkcd/Prr5l/Ripk1/Sirpa/Slc25a24/Slc8a1/Stat6/Tlr4/Trem2/Trpm2
7-day post-SCI group vs. the control group	BP	GO:0000302	response to reactive oxygen species	22/88	5.69E-29	4.71E-26	2.13E-26	Adam9/Atp7a/Axl/Cd36/Fos/Gch1/Gpx1/Hif1a/Jun/Mapk8/Nfe2l2/Pdk2/Pink1/Ppargc1b/Prdx6/Prkcd/Ripk1/Sirpa/Slc8a1/Stat6/Trpm2/Ucp2
7-day post-SCI group vs. the control group	BP	GO:1900407	regulation of cellular response to oxidative stress	16/88	2.26E-24	1.5E-21	6.79E-22	Cd36/Ctnnb1/Fbxw7/Gch1/Gpx1/Hif1a/Hspb1/Lancl1/Mcl1/Ncoa7/Nfe2l2/Pink1/Ripk1/Tlr4/Trem2/Trpm2
7-day post-SCI group vs. the control group	BP	GO:1902882	regulation of response to oxidative stress	16/88	1.17E-23	6.44E-21	2.92E-21	Cd36/Ctnnb1/Fbxw7/Gch1/Gpx1/Hif1a/Hspb1/Lancl1/Mcl1/Ncoa7/Nfe2l2/Pink1/Ripk1/Tlr4/Trem2/Trpm2
7-day post-SCI group vs. the control group	BP	GO:0070997	neuron death	24/88	2.04E-23	9.64E-21	4.37E-21	Apoe/Atp7a/Axl/Capn2/Clu/Ctnnb1/Fbxw7/Fos/Gclc/Gpx1/Hif1a/Il6st/Jun/Lancl1/Map2k4/Mapk8/Mapt/Mcl1/Ncoa7/Pink1/Ppargc1a/Tlr4/Trem2/Trpm2
7-day post-SCI group vs. the control group	BP	GO:1901214	regulation of neuron death	23/88	3.28E-23	1.36E-20	6.14E-21	Apoe/Atp7a/Axl/Capn2/Clu/Ctnnb1/Fbxw7/Fos/Gclc/Hif1a/Il6st/Jun/Lancl1/Map2k4/Mapk8/Mapt/Mcl1/Ncoa7/Pink1/Ppargc1a/Tlr4/Trem2/Trpm2
7-day post-SCI group vs. the control group	BP	GO:0034614	cellular response to reactive oxygen species	17/88	1.06E-22	3.92E-20	1.77E-20	Atp7a/Axl/Cd36/Fos/Gch1/Jun/Mapk8/Nfe2l2/Pdk2/Pink1/Ppargc1b/Prkcd/Ripk1/Sirpa/Slc8a1/Stat6/Trpm2

7-day post-SCI group vs. the control group	BP	GO:0036473	cell death in response to oxidative stress	15/88	1.21E-21	3.99E-19	1.81E-19	Ctnnb1/Fbxw7/Gpx1/Hif1a/Hspb1/Lancl1/Mapt/Mcl1/Ncoa7/Nfe2l2/Pink1/Ripk1/Tlr4/Trem2/Trpm2
7-day post-SCI group vs. the control group	BP	GO:1903201	regulation of oxidative stress-induced cell death	14/88	2.07E-21	6.23E-19	2.82E-19	Ctnnb1/Fbxw7/Gpx1/Hif1a/Hspb1/Lancl1/Mcl1/Ncoa7/Nfe2l2/Pink1/Ripk1/Tlr4/Trem2/Trpm2
7-day post-SCI group vs. the control group	BP	GO:0072593	reactive oxygen species metabolic process	17/88	1.21E-18	3.33E-16	1.51E-16	Atp7a/Cd36/Cybb/Gch1/Gpx1/Gpx3/Hif1a/Mapk14/Mapt/Ncf1/Nfe2l2/Pink1/Prdx6/Prkcd/Ripk1/Tlr4/Xdh
7-day post-SCI group vs. the control group	BP	GO:1903203	regulation of oxidative stress-induced neuron deatl	10/88	6.87E-18	1.75E-15	7.92E-16	Ctnnb1/Fbxw7/Hif1a/Lancl1/Mcl1/Ncoa7/Pink1/Tlr4/Trem2/Trpm2
7-day post-SCI group vs. the control group	BP	GO:0036475	neuron death in response to oxidative stress	10/88	1.3E-17	3.07E-15	1.39E-15	Ctnnb1/Fbxw7/Hif1a/Lancl1/Mcl1/Ncoa7/Pink1/Tlr4/Trem2/Trpm2
7-day post-SCI group vs. the control group	BP	GO:0051402	neuron apoptotic process	17/88	1.73E-16	3.81E-14	1.73E-14	Apoe/Atp7a/Axl/Ctnnb1/Fbxw7/Gclc/Gpx1/Hif1a/Il6st/Jun/Lancl1/Map2k4/Mapk8/Mcl1/Pink1/Ppargc1a/Trem2
7-day post-SCI group vs. the control group	BP	GO:1901216	positive regulation of neuron death	13/88	2E-16	4.15E-14	1.88E-14	Apoe/Capn2/Clu/Ctnnb1/Fbxw7/Fos/Jun/Map2k4/Mapk8/Mapt/Mcl1/Tlr4/Trpm2
7-day post-SCI group vs. the control group	BP	GO:0043523	regulation of neuron apoptotic process	16/88	5.17E-16	1.01E-13	4.56E-14	Apoe/Atp7a/Axl/Ctnnb1/Fbxw7/Gclc/Hif1a/Il6st/Jun/Lancl1/Map2k4/Mapk8/Mcl1/Pink1/Ppargc1a/Trem2
7-day post-SCI group vs. the control group	BP	GO:1902175	regulation of oxidative stress-induced intrinsic apc	8/88	1.12E-14	2.05E-12	9.3E-13	Fbxw7/Gpx1/Hif1a/Hspb1/Mcl1/Nfe2l2/Pink1/Trem2
7-day post-SCI group vs. the control group	BP	GO:0008631	intrinsic apoptotic signaling pathway in response t	9/88	1.18E-14	2.06E-12	9.33E-13	Fbxw7/Gpx1/Hif1a/Hspb1/Mapt/Mcl1/Nfe2l2/Pink1/Trem2
7-day post-SCI group vs. the control group	BP	GO:2001233	regulation of apoptotic signaling pathway	16/88	7.99E-14	1.32E-11	5.99E-12	Clu/Ctnnb1/Fbxw7/Gclc/Gpx1/Hdac1/Hif1a/Hspb1/Itga6/Mapk8/Mcl1/Nfe2l2/Pink1/Prkcd/Ripk1/Trem2
7-day post-SCI group vs. the control group	BP	GO:0042542	response to hydrogen peroxide	11/88	1.15E-13	1.81E-11	8.19E-12	Adam9/Axl/Gpx1/Mapk8/Nfe2l2/Pink1/Prkcd/Ripk1/Sirpa/Stat6/Trpm2
7-day post-SCI group vs. the control group	BP	GO:2000377	regulation of reactive oxygen species metabolic pr	11/88	2.47E-12	3.72E-10	1.69E-10	Cd36/Gch1/Hif1a/Mapk14/Mapt/Nfe2l2/Pink1/Prkcd/Ripk1/Tlr4/Xdh
7-day post-SCI group vs. the control group	BP	GO:0045637	regulation of myeloid cell differentiation	12/88	4.67E-12	6.72E-10	3.04E-10	Apc/Ctnnb1/Fbxw7/Fos/Hif1a/Jun/Mapk14/Ppargc1b/Ppp3ca/Ripk1/Stat1/Trem2
7-day post-SCI group vs. the control group	BP	GO:0010038	response to metal ion	13/88	6.23E-12	8.59E-10	3.89E-10	Adam9/Atp7a/Fos/Jun/Lcat/Mapk8/Mapt/Mylk/Nfe2l2/Ppp3ca/Slc25a24/Trpm2/Xdh
7-day post-SCI group vs. the control group	BP	GO:0033194	response to hydroperoxide	6/88	8E-12	1.06E-09	4.8E-10	Cd36/Cd38/Gpx1/Mgst1/Prkcd/Trpm2
7-day post-SCI group vs. the control group	BP	GO:2001234	negative regulation of apoptotic signaling pathway	12/88	9.92E-12	1.26E-09	5.72E-10	Clu/Ctnnb1/Gclc/Gpx1/Hdac1/Hif1a/Hspb1/Itga6/Mcl1/Nfe2l2/Pink1/Ripk1
7-day post-SCI group vs. the control group	BP	GO:0070301	cellular response to hydrogen peroxide	9/88	1.14E-11	1.4E-09	6.33E-10	Axl/Mapk8/Nfe2l2/Pink1/Prkcd/Ripk1/Sirpa/Stat6/Trpm2
7-day post-SCI group vs. the control group	BP	GO:1901215	negative regulation of neuron death	12/88	3.27E-11	3.84E-09	1.74E-09	Apoe/Atp7a/Axl/Ctnnb1/Gclc/Hif1a/Il6st/Jun/Map2k4/Ncoa7/Pink1/Ppargc1a
7-day post-SCI group vs. the control group	BP	GO:0006801	superoxide metabolic process	8/88	3.36E-11	3.84E-09	1.74E-09	Atp7a/Cd36/Cybb/Gch1/Mapt/Ncf1/Nfe2l2/Prkcd
7-day post-SCI group vs. the control group	BP	GO:1903706	regulation of hemopoiesis	14/88	5.7E-11	5.97E-09	2.7E-09	Apc/Axl/Ctnnb1/Fbxw7/Fos/Hif1a/Hspb1/Jun/Mapk14/Ppargc1b/Ppp3ca/Ripk1/Stat1/Trem2
7-day post-SCI group vs. the control group	BP	GO:0036480	neuron intrinsic apoptotic signaling pathway in res	5/88	5.77E-11	5.97E-09	2.7E-09	Fbxw7/Hif1a/Mcl1/Pink1/Trem2
7-day post-SCI group vs. the control group	BP	GO:1903376	regulation of oxidative stress-induced neuron intri	5/88	5.77E-11	5.97E-09	2.7E-09	Fbxw7/Hif1a/Mcl1/Pink1/Trem2

7-day post-SCI group vs. the control group	BP	GO:0045639	positive regulation of myeloid cell differentiation	9/88	6.73E-11	6.76E-09	3.06E-09	Fos/Hif1a/Jun/Mapk14/Ppargc1b/Ppp3ca/Ripk1/Stat1/Trem2
7-day post-SCI group vs. the control group	BP	GO:1990000	amyloid fibril formation	6/88	8.56E-11	8.33E-09	3.77E-09	Apoe/Cd36/Clu/Mapt/Ripk1/Trem2
7-day post-SCI group vs. the control group	BP	GO:0007249	I-kappaB kinase/NF-kappaB signaling	11/88	1.17E-10	1.11E-08	5.03E-09	Btk/Cd36/Ctnnb1/Hdac1/Hspb1/Pink1/Ripk1/Sirpa/Stat1/Tlr4/Trem2
7-day post-SCI group vs. the control group	BP	GO:0070661	leukocyte proliferation	13/88	1.58E-10	1.45E-08	6.57E-09	Aif1/Btk/Cd38/Clu/Ctnnb1/Fkbp1b/Il6st/Ppp3ca/Prkcd/Stat6/Tlr4/Trem2/Vcam1
7-day post-SCI group vs. the control group	BP	GO:2001242	regulation of intrinsic apoptotic signaling pathway	10/88	1.69E-10	1.52E-08	6.87E-09	Clu/Fbxw7/Gpx1/Hdac1/Hif1a/Hspb1/Mcl1/Nfe2l2/Pink1/Trem2
7-day post-SCI group vs. the control group	BP	GO:0019932	second-messenger-mediated signaling	12/88	1.76E-10	1.54E-08	6.97E-09	Apoe/Atp2a2/Cd36/Fkbp1b/Mapt/Pdk2/Ppp3ca/Rcan1/Slc8a1/Trem2/Trpm2/Vcam1
7-day post-SCI group vs. the control group	BP	GO:0097193	intrinsic apoptotic signaling pathway	12/88	1.83E-10	1.56E-08	7.04E-09	Clu/Fbxw7/Gpx1/Hdac1/Hif1a/Hspb1/Mapt/Mcl1/Nfe2l2/Pdk2/Pink1/Trem2
7-day post-SCI group vs. the control group	BP	GO:0051403	stress-activated MAPK cascade	11/88	2.21E-10	1.83E-08	8.27E-09	Ezr/Map2k3/Map2k4/Mapk10/Mapk14/Mapk8/Ripk1/Sirpa/Tlr4/Trem2/Xdh
7-day post-SCI group vs. the control group	BP	GO:1903202	negative regulation of oxidative stress-induced cel	7/88	2.59E-10	2.09E-08	9.48E-09	Ctnnb1/Gpx1/Hif1a/Hspb1/Ncoa7/Nfe2l2/Pink1
7-day post-SCI group vs. the control group	BP	GO:0001659	temperature homeostasis	10/88	3.22E-10	2.54E-08	1.15E-08	Apc/Cd36/Gpx1/Id1/Ppargc1a/Ppargc1b/Stat6/Tlr4/Trpm2/Ucp2
7-day post-SCI group vs. the control group	BP	GO:0031098	stress-activated protein kinase signaling cascade	11/88	3.38E-10	2.6E-08	1.18E-08	Ezr/Map2k3/Map2k4/Mapk10/Mapk14/Mapk8/Ripk1/Sirpa/Tlr4/Trem2/Xdh
7-day post-SCI group vs. the control group	BP	GO:0043524	negative regulation of neuron apoptotic process	10/88	4.37E-10	3.29E-08	1.49E-08	Apoe/Atp7a/Axl/Gclc/Hif1a/Il6st/Jun/Map2k4/Pink1/Ppargc1a
7-day post-SCI group vs. the control group	BP	GO:0019722	calcium-mediated signaling	10/88	4.6E-10	3.38E-08	1.53E-08	Atp2a2/Fkbp1b/Mapt/Pdk2/Ppp3ca/Rcan1/Slc8a1/Trem2/Trpm2/Vcam1
7-day post-SCI group vs. the control group	BP	GO:0071216	cellular response to biotic stimulus	12/88	5.26E-10	3.78E-08	1.71E-08	Adam9/Axl/Cd36/Gch1/Map2k3/Mapk14/Mapk8/Sirpa/Stat1/Tlr4/Trem2/Txnip
7-day post-SCI group vs. the control group	BP	GO:0002761	regulation of myeloid leukocyte differentiation	9/88	5.58E-10	3.86E-08	1.75E-08	Apc/Ctnnb1/Fbxw7/Fos/Jun/Ppargc1b/Ppp3ca/Ripk1/Trem2
7-day post-SCI group vs. the control group	BP	GO:0045732	positive regulation of protein catabolic process	10/88	5.6E-10	3.86E-08	1.75E-08	Adam9/Apc/Apoe/Cdc20/Clu/Ezr/Fbxw7/Gclc/Mapk8/Trem2
7-day post-SCI group vs. the control group	BP	GO:1901652	response to peptide	13/88	6.28E-10	4.24E-08	1.92E-08	Apc/Atp2a2/Cd36/Ctnnb1/Mapk14/Nfe2l2/Pdk2/Prkcd/Stat1/Stat6/Tlr4/Trem2/Vcam1
7-day post-SCI group vs. the control group	BP	GO:2001243	negative regulation of intrinsic apoptotic signaling	8/88	7.82E-10	5.18E-08	2.35E-08	Clu/Gpx1/Hdac1/Hif1a/Hspb1/Mcl1/Nfe2l2/Pink1
7-day post-SCI group vs. the control group	BP	GO:1902176	negative regulation of oxidative stress-induced int	5/88	1.39E-09	9.04E-08	4.09E-08	Gpx1/Hif1a/Hspb1/Nfe2l2/Pink1
7-day post-SCI group vs. the control group	BP	GO:0001667	ameboidal-type cell migration	13/88	1.89E-09	1.2E-07	5.44E-08	Adam9/Apc/Apoe/Fbxw7/Gpx1/Hbegf/Hif1a/Hspb1/Jun/Map2k3/Nfe2l2/Prr5l/Slc8a1
7-day post-SCI group vs. the control group	BP	GO:0032496	response to lipopolysaccharide	12/88	2.21E-09	1.37E-07	6.21E-08	Adam9/Axl/Cd36/Gch1/Map2k3/Mapk14/Mapk8/Mgst1/Sirpa/Stat1/Tlr4/Trem2
7-day post-SCI group vs. the control group	BP	GO:0071222	cellular response to lipopolysaccharide	11/88	2.24E-09	1.37E-07	6.21E-08	Adam9/Axl/Cd36/Gch1/Map2k3/Mapk14/Mapk8/Sirpa/Stat1/Tlr4/Trem2

7-day post-SCI group vs. the control group	BP	GO:0050708	regulation of protein secretion	11/88	2.32E-09	1.39E-07	6.31E-08	Adam9/Apoe/Cd38/Ezr/Fkbp1b/Hif1a/Ppp3ca/Tlr4/Trem2/Trpm2/Ucp2
7-day post-SCI group vs. the control group	BP	GO:0071219	cellular response to molecule of bacterial origin	11/88	3.14E-09	1.85E-07	8.4E-08	Adam9/Axl/Cd36/Gch1/Map2k3/Mapk14/Mapk8/Sirpa/Stat1/Tlr4/Trem2
7-day post-SCI group vs. the control group	BP	GO:1903829	positive regulation of protein localization	13/88	3.26E-09	1.9E-07	8.58E-08	Adam9/Apc/Cd38/Ezr/Fbxw7/Hif1a/Mapk14/Mapt/Prkcd/Prr5l/Tlr4/Trem2/Trpm2
7-day post-SCI group vs. the control group	BP	GO:0009636	response to toxic substance	8/88	3.83E-09	2.19E-07	9.91E-08	Atp7a/Cd36/Gch1/Gpx1/Hif1a/Nfe2l2/Pink1/Prdx6
7-day post-SCI group vs. the control group	BP	GO:0051222	positive regulation of protein transport	11/88	3.94E-09	2.2E-07	9.95E-08	Adam9/Cd38/Ezr/Fbxw7/Hif1a/Mapk14/Prkcd/Prr5l/Tlr4/Trem2/Trpm2
7-day post-SCI group vs. the control group	BP	GO:0002237	response to molecule of bacterial origin	12/88	3.99E-09	2.2E-07	9.95E-08	Adam9/Axl/Cd36/Gch1/Map2k3/Mapk14/Mapk8/Mgst1/Sirpa/Stat1/Tlr4/Trem2
7-day post-SCI group vs. the control group	BP	GO:0030316	osteoclast differentiation	8/88	4.09E-09	2.2E-07	9.95E-08	Apc/Ctnnb1/Fbxw7/Fos/Mapk14/Ppargc1b/Ppp3ca/Trem2
7-day post-SCI group vs. the control group	BP	GO:0002573	myeloid leukocyte differentiation	10/88	4.14E-09	2.2E-07	9.95E-08	Apc/Ctnnb1/Fbxw7/Fos/Jun/Mapk14/Ppargc1b/Ppp3ca/Ripk1/Trem2
7-day post-SCI group vs. the control group	BP	GO:2000379	positive regulation of reactive oxygen species meta	7/88	4.18E-09	2.2E-07	9.95E-08	Cd36/Mapk14/Mapt/Nfe2l2/Prkcd/Tlr4/Xdh
7-day post-SCI group vs. the control group	BP	GO:0010632	regulation of epithelial cell migration	10/88	4.47E-09	2.31E-07	1.05E-07	Adam9/Apc/Apoe/Fbxw7/Hbegf/Hif1a/Hspb1/Jun/Map2k3/Nfe2l2
7-day post-SCI group vs. the control group	BP	GO:1903209	positive regulation of oxidative stress-induced cell	5/88	4.53E-09	2.31E-07	1.05E-07	Fbxw7/Mcl1/Ripk1/Tlr4/Trpm2
7-day post-SCI group vs. the control group	BP	GO:0010631	epithelial cell migration	11/88	4.93E-09	2.48E-07	1.12E-07	Adam9/Apc/Apoe/Fbxw7/Gpx1/Hbegf/Hif1a/Hspb1/Jun/Map2k3/Nfe2l2
7-day post-SCI group vs. the control group	BP	GO:0090132	epithelium migration	11/88	5.25E-09	2.6E-07	1.18E-07	Adam9/Apc/Apoe/Fbxw7/Gpx1/Hbegf/Hif1a/Hspb1/Jun/Map2k3/Nfe2l2
7-day post-SCI group vs. the control group	BP	GO:0090130	tissue migration	11/88	5.59E-09	2.72E-07	1.23E-07	Adam9/Apc/Apoe/Fbxw7/Gpx1/Hbegf/Hif1a/Hspb1/Jun/Map2k3/Nfe2l2
7-day post-SCI group vs. the control group	BP	GO:0009991	response to extracellular stimulus	12/88	5.67E-09	2.72E-07	1.23E-07	Apoe/Axl/Fos/Itga6/Jun/Mapk14/Mapk8/Mapt/Nfe2l2/Pdk2/Ppargc1a/Vcam1
7-day post-SCI group vs. the control group	BP	GO:1904951	positive regulation of establishment of protein loca	11/88	6.53E-09	3.09E-07	1.4E-07	Adam9/Cd38/Ezr/Fbxw7/Hif1a/Mapk14/Prkcd/Prr5l/Tlr4/Trem2/Trpm2
7-day post-SCI group vs. the control group	BP	GO:0043122	regulation of I-kappaB kinase/NF-kappaB signalin	9/88	7.16E-09	3.34E-07	1.51E-07	Cd36/Ctnnb1/Hdac1/Hspb1/Pink1/Ripk1/Stat1/Tlr4/Trem2
7-day post-SCI group vs. the control group	BP	GO:0000303	response to superoxide	5/88	7.46E-09	3.43E-07	1.55E-07	Atp7a/Cd36/Gch1/Nfe2l2/Ucp2
7-day post-SCI group vs. the control group	BP	GO:0045670	regulation of osteoclast differentiation	7/88	7.67E-09	3.48E-07	1.58E-07	Apc/Ctnnb1/Fbxw7/Fos/Ppargc1b/Ppp3ca/Trem2
7-day post-SCI group vs. the control group	BP	GO:0032640	tumor necrosis factor production	9/88	7.85E-09	3.51E-07	1.59E-07	Axl/Cd36/Clu/Hspb1/Mapk14/Ripk1/Sirpa/Tlr4/Trem2
7-day post-SCI group vs. the control group	BP	GO:0033002	muscle cell proliferation	10/88	8.73E-09	3.86E-07	1.75E-07	Aif1/Apc/Apoe/Ctnnb1/Hbegf/Hif1a/Jun/Mapk14/Ppargc1a/Tlr4
7-day post-SCI group vs. the control group	BP	GO:0071706	tumor necrosis factor superfamily cytokine produc	9/88	8.97E-09	3.91E-07	1.77E-07	Axl/Cd36/Clu/Hspb1/Mapk14/Ripk1/Sirpa/Tlr4/Trem2
7-day post-SCI group vs. the control group	BP	GO:1902105	regulation of leukocyte differentiation	11/88	9.37E-09	3.98E-07	1.8E-07	Apc/Axl/Ctnnb1/Fbxw7/Fos/Hspb1/Jun/Ppargc1b/Ppp3ca/Ripk1/Trem2
7-day post-SCI group vs. the control group	BP	GO:0000305	response to oxygen radical	5/88	9.4E-09	3.98E-07	1.8E-07	Atp7a/Cd36/Gch1/Nfe2l2/Ucp2
7-day post-SCI group vs. the control group	BP	GO:0097237	cellular response to toxic substance	6/88	9.48E-09	3.98E-07	1.8E-07	Atp7a/Cd36/Gch1/Nfe2l2/Pink1/Prdx6

7-day post-SCI group vs. the control group	BP	GO:0030099	myeloid cell differentiation	12/88	1.08E-08	4.46E-07	2.02E-07	Apc/Ctnnb1/Fbxw7/Fos/Hif1a/Jun/Mapk14/Ppargc1b/Ppp3ca/Ripk1/Stat1/Trem2
7-day post-SCI group vs. the control group	BP	GO:0042176	regulation of protein catabolic process	11/88	1.12E-08	4.56E-07	2.07E-07	Adam9/Apc/Apoe/Cdc20/Clu/Ezr/Fbxw7/Gclc/Gpx1/Mapk8/Trem2
7-day post-SCI group vs. the control group	BP	GO:0098869	cellular oxidant detoxification	5/88	1.17E-08	4.74E-07	2.15E-07	Atp7a/Cd36/Gch1/Nfe2l2/Prdx6
7-day post-SCI group vs. the control group	BP	GO:0010563	negative regulation of phosphorus metabolic proce	12/88	1.31E-08	5.17E-07	2.34E-07	Apc/Apoe/Fkbp1b/Hspb1/Jun/Mapt/Ppargc1a/Prkcd/Prr5l/Sirpa/Slc8a1/Xdh
7-day post-SCI group vs. the control group	BP	GO:0045936	negative regulation of phosphate metabolic proces	12/88	1.31E-08	5.17E-07	2.34E-07	Apc/Apoe/Fkbp1b/Hspb1/Jun/Mapt/Ppargc1a/Prkcd/Prr5l/Sirpa/Slc8a1/Xdh
7-day post-SCI group vs. the control group	BP	GO:0010821	regulation of mitochondrion organization	8/88	1.58E-08	6.16E-07	2.79E-07	Fbxw7/Gclc/Gpx1/Hif1a/Mapt/Pink1/Ppargc1a/Trem2
7-day post-SCI group vs. the control group	BP	GO:0070391	response to lipoteichoic acid	4/88	1.65E-08	6.27E-07	2.84E-07	Cd36/Mapk14/Tlr4/Trem2
7-day post-SCI group vs. the control group	BP	GO:0071223	cellular response to lipoteichoic acid	4/88	1.65E-08	6.27E-07	2.84E-07	Cd36/Mapk14/Tlr4/Trem2
7-day post-SCI group vs. the control group	BP	GO:0120161	regulation of cold-induced thermogenesis	8/88	1.67E-08	6.27E-07	2.84E-07	Apc/Cd36/Id1/Ppargc1a/Ppargc1b/Stat6/Tlr4/Ucp2
7-day post-SCI group vs. the control group	BP	GO:0106106	cold-induced thermogenesis	8/88	1.85E-08	6.9E-07	3.12E-07	Apc/Cd36/Id1/Ppargc1a/Ppargc1b/Stat6/Tlr4/Ucp2
7-day post-SCI group vs. the control group	BP	GO:0042391	regulation of membrane potential	12/88	1.92E-08	7.07E-07	3.2E-07	Atp2a2/Cd36/Fkbp1b/Gclc/Hebp2/Jun/Mapt/Pink1/Ppp3ca/Slc8a1/Trem2/Ucp2
7-day post-SCI group vs. the control group	BP	GO:0042060	wound healing	11/88	2.24E-08	8.16E-07	3.7E-07	Apoe/Axl/Cd36/Dst/Gpx1/Hbegf/Hif1a/Mylk/Nfe2l2/Prkcd/Tlr4
7-day post-SCI group vs. the control group	BP	GO:0042743	hydrogen peroxide metabolic process	6/88	2.63E-08	9.47E-07	4.29E-07	Cybb/Gpx1/Gpx3/Ncf1/Pink1/Prdx6
7-day post-SCI group vs. the control group	BP	GO:0042326	negative regulation of phosphorylation	11/88	3.24E-08	1.16E-06	5.23E-07	Apc/Apoe/Hspb1/Jun/Mapt/Ppargc1a/Prkcd/Prr5l/Sirpa/Slc8a1/Xdh
7-day post-SCI group vs. the control group	BP	GO:0045765	regulation of angiogenesis	10/88	3.49E-08	1.23E-06	5.57E-07	Btg1/Cd36/Ctnnb1/Hif1a/Hspb1/Id1/Nfe2l2/Rnh1/S100a1/Stat1
7-day post-SCI group vs. the control group	BP	GO:1901342	regulation of vasculature development	10/88	3.94E-08	1.37E-06	6.22E-07	Btg1/Cd36/Ctnnb1/Hif1a/Hspb1/Id1/Nfe2l2/Rnh1/S100a1/Stat1
7-day post-SCI group vs. the control group	BP	GO:1990845	adaptive thermogenesis	8/88	4.11E-08	1.42E-06	6.42E-07	Apc/Cd36/Id1/Ppargc1a/Ppargc1b/Stat6/Tlr4/Ucp2
7-day post-SCI group vs. the control group	BP	GO:0009306	protein secretion	11/88	4.18E-08	1.43E-06	6.47E-07	Adam9/Apoe/Cd38/Ezr/Fkbp1b/Hif1a/Ppp3ca/Tlr4/Trem2/Trpm2/Ucp2
7-day post-SCI group vs. the control group	BP	GO:0035592	establishment of protein localization to extracellul	11/88	4.29E-08	1.44E-06	6.53E-07	Adam9/Apoe/Cd38/Ezr/Fkbp1b/Hif1a/Ppp3ca/Tlr4/Trem2/Trpm2/Ucp2
7-day post-SCI group vs. the control group	BP	GO:0003018	vascular process in circulatory system	9/88	4.31E-08	1.44E-06	6.53E-07	Apoe/Cd36/Cd38/Gch1/Gclc/Gpx1/Hif1a/Ptgs1/Slc8a1
7-day post-SCI group vs. the control group	BP	GO:0071692	protein localization to extracellular region	11/88	4.98E-08	1.65E-06	7.47E-07	Adam9/Apoe/Cd38/Ezr/Fkbp1b/Hif1a/Ppp3ca/Tlr4/Trem2/Trpm2/Ucp2
7-day post-SCI group vs. the control group	BP	GO:0071248	cellular response to metal ion	8/88	5.18E-08	0.0000017	7.69E-07	Atp7a/Fos/Jun/Mapk8/Mylk/Nfe2l2/Slc25a24/Trpm2
7-day post-SCI group vs. the control group	BP	GO:1901653	cellular response to peptide	10/88	5.3E-08	1.72E-06	7.79E-07	Apc/Cd36/Ctnnb1/Nfe2l2/Pdk2/Prkcd/Stat6/Tlr4/Trem2/Vcam1
7-day post-SCI group vs. the control group	BP	GO:0043588	skin development	10/88	5.45E-08	1.75E-06	7.94E-07	Apc/Atp7a/Ctnnb1/Ctsl/Dhcr24/Hdac1/Itga6/Ppp3ca/Ptgs1/Txnip
7-day post-SCI group vs. the control group	BP	GO:0035296	regulation of tube diameter	8/88	5.93E-08	1.87E-06	8.47E-07	Apoe/Cd38/Gch1/Gclc/Gpx1/Hif1a/Ptgs1/Slc8a1
7-day post-SCI group vs. the control group	BP	GO:0097746	blood vessel diameter maintenance	8/88	5.93E-08	1.87E-06	8.47E-07	Apoe/Cd38/Gch1/Gclc/Gpx1/Hif1a/Ptgs1/Slc8a1

7-day post-SCI group vs. the control group	BP	GO:0090322	regulation of superoxide metabolic process	5/88	6.01E-08	1.88E-06	8.51E-07	Cd36/Gch1/Mapt/Nfe2l2/Prkcd
7-day post-SCI group vs. the control group	BP	GO:0009266	response to temperature stimulus	8/88	6.2E-08	0.0000019	8.61E-07	Gclc/Hspb1/Mapk8/Mapt/Rbbp7/Tlr4/Trpm2/Ucp2
7-day post-SCI group vs. the control group	BP	GO:0035150	regulation of tube size	8/88	6.2E-08	0.0000019	8.61E-07	Apoe/Cd38/Gch1/Gclc/Gpx1/Hif1a/Ptgs1/Slc8a1
7-day post-SCI group vs. the control group	BP	GO:0043254	regulation of protein-containing complex assembly	11/88	6.99E-08	2.09E-06	9.47E-07	Apc/Apoe/Cd36/Clu/Lcat/Mapk8/Mapt/Pink1/Prkcd/Tlr4/Trem2
7-day post-SCI group vs. the control group	BP	GO:0060142	regulation of syncytium formation by plasma mem	5/88	7E-08	2.09E-06	9.47E-07	Adam9/Capn2/Mapk14/Stat1/Trem2
7-day post-SCI group vs. the control group	BP	GO:0009612	response to mechanical stimulus	8/88	7.07E-08	2.09E-06	9.47E-07	Cd36/Ctnnb1/Fos/Jun/Mapk14/Mapk8/Slc8a1/Tlr4
7-day post-SCI group vs. the control group	BP	GO:1903034	regulation of response to wounding	8/88	7.07E-08	2.09E-06	9.47E-07	Apoe/Atp7a/Cd36/Fkbp1b/Hbegf/Mylk/Nfe2l2/Prkcd
7-day post-SCI group vs. the control group	BP	GO:0031331	positive regulation of cellular catabolic process	11/88	8.05E-08	2.36E-06	1.07E-06	Adam9/Apoe/Cdc20/Clu/Fbxw7/Gclc/Hif1a/Mapk8/Pink1/Prkcd/Prr5l
7-day post-SCI group vs. the control group	BP	GO:0003012	muscle system process	11/88	8.24E-08	2.39E-06	1.08E-06	Aif1/Atp2a2/Cd38/Fkbp1b/Map2k3/Map2k4/Mylk/Ncf1/Ppp3ca/Ptgs1/Slc8a1
7-day post-SCI group vs. the control group	BP	GO:0043534	blood vessel endothelial cell migration	7/88	9.51E-08	2.74E-06	1.24E-06	Apoe/Fbxw7/Gpx1/Hif1a/Hspb1/Map2k3/Nfe2l2
7-day post-SCI group vs. the control group	BP	GO:0002763	positive regulation of myeloid leukocyte differenti	6/88	9.61E-08	2.74E-06	1.24E-06	Fos/Jun/Ppargc1b/Ppp3ca/Ripk1/Trem2
7-day post-SCI group vs. the control group	BP	GO:0046651	lymphocyte proliferation	10/88	9.81E-08	2.78E-06	1.26E-06	Aif1/Btk/Cd38/Ctnnb1/Fkbp1b/Il6st/Ppp3ca/Prkcd/Tlr4/Vcam1
7-day post-SCI group vs. the control group	BP	GO:0048660	regulation of smooth muscle cell proliferation	8/88	9.95E-08	2.79E-06	1.26E-06	Aif1/Apoe/Ctnnb1/Hbegf/Hif1a/Jun/Ppargc1a/Tlr4
7-day post-SCI group vs. the control group	BP	GO:0001933	negative regulation of protein phosphorylation	10/88	1.03E-07	2.84E-06	1.29E-06	Apc/Apoe/Hspb1/Jun/Ppargc1a/Prkcd/Prr5l/Sirpa/Slc8a1/Xdh
7-day post-SCI group vs. the control group	BP	GO:0032680	regulation of tumor necrosis factor production	8/88	1.04E-07	2.84E-06	1.29E-06	Axl/Cd36/Clu/Hspb1/Ripk1/Sirpa/Tlr4/Trem2
7-day post-SCI group vs. the control group	BP	GO:0061136	regulation of proteasomal protein catabolic proces	8/88	1.04E-07	2.84E-06	1.29E-06	Apoe/Cdc20/Clu/Fbxw7/Gclc/Gpx1/Mapk8/Trem2
7-day post-SCI group vs. the control group	BP	GO:1903555	regulation of tumor necrosis factor superfamily cy	8/88	1.17E-07	3.18E-06	1.44E-06	Axl/Cd36/Clu/Hspb1/Ripk1/Sirpa/Tlr4/Trem2
7-day post-SCI group vs. the control group	BP	GO:0032943	mononuclear cell proliferation	10/88	1.18E-07	3.18E-06	1.44E-06	Aif1/Btk/Cd38/Ctnnb1/Fkbp1b/Il6st/Ppp3ca/Prkcd/Tlr4/Vcam1
7-day post-SCI group vs. the control group	BP	GO:0045807	positive regulation of endocytosis	7/88	1.25E-07	3.35E-06	1.52E-06	Amph/Apoe/Axl/Cd36/Clu/Ppp3ca/Trem2
7-day post-SCI group vs. the control group	BP	GO:1903522	regulation of blood circulation	9/88	1.32E-07	3.48E-06	1.58E-06	Atp2a2/Cd38/Fkbp1b/Gch1/Hbegf/Hif1a/Ptgs1/S100a1/Slc8a1
7-day post-SCI group vs. the control group	BP	GO:0048659	smooth muscle cell proliferation	8/88	1.32E-07	3.48E-06	1.58E-06	Aif1/Apoe/Ctnnb1/Hbegf/Hif1a/Jun/Ppargc1a/Tlr4
7-day post-SCI group vs. the control group	BP	GO:0045862	positive regulation of proteolysis	10/88	1.38E-07	3.61E-06	1.63E-06	Adam9/Apoe/Cdc20/Clu/Fbxw7/Gclc/Mapk8/Stat1/Trem2/Xdh
7-day post-SCI group vs. the control group	BP	GO:0051091	positive regulation of DNA-binding transcription f	9/88	1.45E-07	3.76E-06	0.0000017	Cd36/Clu/Ctnnb1/Pink1/Ppargc1a/Ppargc1b/Ppp3ca/Ripk1/Tlr4
7-day post-SCI group vs. the control group	BP	GO:0051592	response to calcium ion	7/88	1.47E-07	3.78E-06	1.71E-06	Adam9/Fos/Jun/Mylk/Ppp3ca/Slc25a24/Trpm2
7-day post-SCI group vs. the control group	BP	GO:1903036	positive regulation of response to wounding	6/88	1.55E-07	3.94E-06	1.78E-06	Atp7a/Cd36/Fkbp1b/Hbegf/Mylk/Nfe2l2
7-day post-SCI group vs. the control group	BP	GO:1901031	regulation of response to reactive oxygen species	5/88	1.59E-07	4.03E-06	1.82E-06	Cd36/Gch1/Nfe2l2/Pink1/Ripk1
7-day post-SCI group vs. the control group	BP	GO:0050767	regulation of neurogenesis	11/88	1.65E-07	4.14E-06	1.88E-06	Apoe/Ctnnb1/Hdac1/Hif1a/Id1/Il6st/Mapk8/Mapt/Ppp3ca/Stau2/Trem2
7-day post-SCI group vs. the control group	BP	GO:0001774	microglial cell activation	5/88	1.8E-07	4.49E-06	2.03E-06	Aif1/Clu/Jun/Tlr4/Trem2
7-day post-SCI group vs. the control group	BP	GO:0070663	regulation of leukocyte proliferation	9/88	1.91E-07	4.73E-06	2.14E-06	Aif1/Btk/Cd38/Ctnnb1/Il6st/Ppp3ca/Stat6/Tlr4/Vcam1
7-day post-SCI group vs. the control group	BP	GO:1990748	cellular detoxification	5/88	2.04E-07	4.99E-06	2.26E-06	Atp7a/Cd36/Gch1/Nfe2l2/Prdx6

7-day post-SCI group vs. the control group	BP	GO:0019430	removal of superoxide radicals	4/88	2.36E-07	5.75E-06	2.61E-06	Atp7a/Cd36/Gch1/Nfe2l2
7-day post-SCI group vs. the control group	BP	GO:0002269	leukocyte activation involved in inflammatory res	5/88	2.88E-07	6.96E-06	3.15E-06	Aif1/Clu/Jun/Tlr4/Trem2
7-day post-SCI group vs. the control group	BP	GO:0043525	positive regulation of neuron apoptotic process	6/88	2.95E-07	7.08E-06	3.21E-06	Ctnnb1/Fbxw7/Jun/Map2k4/Mapk8/Mcl1
7-day post-SCI group vs. the control group	BP	GO:0060537	muscle tissue development	11/88	3.14E-07	7.49E-06	3.39E-06	Apc/Ctnnb1/Fos/Gpx1/Map2k4/Mapk14/Mylk/Ppargc1a/ Ppp3ca/Rcan1/Slc8a1
7-day post-SCI group vs. the control group	BP	GO:1903050	regulation of proteolysis involved in protein catab	8/88	3.61E-07	8.55E-06	3.87E-06	Apoe/Cdc20/Clu/Fbxw7/Gclc/Gpx1/Mapk8/Trem2
7-day post-SCI group vs. the control group	BP	GO:0071450	cellular response to oxygen radical	4/88	3.72E-07	8.69E-06	3.93E-06	Atp7a/Cd36/Gch1/Nfe2l2
7-day post-SCI group vs. the control group	BP	GO:0071451	cellular response to superoxide	4/88	3.72E-07	8.69E-06	3.93E-06	Atp7a/Cd36/Gch1/Nfe2l2
7-day post-SCI group vs. the control group	BP	GO:0061900	glial cell activation	5/88	3.97E-07	0.0000092	4.17E-06	Aif1/Clu/Jun/Tlr4/Trem2
7-day post-SCI group vs. the control group	BP	GO:0071496	cellular response to external stimulus	9/88	4.26E-07	0.0000098	4.44E-06	Axl/Ctnnb1/Fos/Itga6/Jun/Mapk8/Nfe2l2/Pdk2/Vcam1
7-day post-SCI group vs. the control group	BP	GO:0035994	response to muscle stretch	4/88	4.59E-07	0.0000105	4.75E-06	Fos/Jun/Mapk14/Slc8a1
7-day post-SCI group vs. the control group	BP	GO:0043535	regulation of blood vessel endothelial cell migratic	6/88	4.65E-07	0.0000105	4.78E-06	Apoe/Fbxw7/Hif1a/Hspb1/Map2k3/Nfe2l2
7-day post-SCI group vs. the control group	BP	GO:0120162	positive regulation of cold-induced thermogenesis	6/88	5.59E-07	0.0000126	0.0000057	Apc/Cd36/Ppargc1a/Ppargc1b/Stat6/Ucp2
7-day post-SCI group vs. the control group	BP	GO:0002532	production of molecular mediator involved in infla	6/88	5.94E-07	0.0000133	6.02E-06	Hif1a/Mapk14/Ncf1/Sirpa/Tlr4/Trem2
7-day post-SCI group vs. the control group	BP	GO:0010634	positive regulation of epithelial cell migration	7/88	6.03E-07	0.0000134	6.07E-06	Adam9/Hbegf/Hif1a/Hspb1/Jun/Map2k3/Nfe2l2
7-day post-SCI group vs. the control group	BP	GO:0014910	regulation of smooth muscle cell migration	6/88	6.3E-07	0.0000139	0.0000063	Aif1/Atp7a/Il6st/Nfe2l2/Ppargc1a/Tlr4
7-day post-SCI group vs. the control group	BP	GO:0038066	p38MAPK cascade	5/88	6.51E-07	0.0000143	6.47E-06	Ezr/Map2k3/Mapk14/Trem2/Xdh
7-day post-SCI group vs. the control group	BP	GO:0001503	ossification	10/88	6.97E-07	0.0000152	6.88E-06	Apc/Ctnnb1/Hif1a/Id1/Il6st/Mapk14/Mapk8/Ppargc1b/Pp p3ca/Slc8a1
7-day post-SCI group vs. the control group	BP	GO:0009408	response to heat	6/88	7.08E-07	0.0000153	6.94E-06	Gclc/Hspb1/Mapk8/Mapt/Rbbp7/Trpm2
7-day post-SCI group vs. the control group	BP	GO:0010823	negative regulation of mitochondrion organization	5/88	7.15E-07	0.0000154	6.96E-06	Gclc/Gpx1/Mapt/Pink1/Ppargc1a
7-day post-SCI group vs. the control group	BP	GO:0050714	positive regulation of protein secretion	7/88	7.42E-07	0.0000158	7.17E-06	Adam9/Cd38/Ezr/Hif1a/Tlr4/Trem2/Trpm2
7-day post-SCI group vs. the control group	BP	GO:0071241	cellular response to inorganic substance	8/88	7.45E-07	0.0000158	7.17E-06	Atp7a/Fos/Jun/Mapk8/Mylk/Nfe2l2/Slc25a24/Trpm2
7-day post-SCI group vs. the control group	BP	GO:0060047	heart contraction	8/88	7.69E-07	0.0000162	7.35E-06	Atp2a2/Fkbp1b/Gch1/Gpx1/Hbegf/Map2k3/S100a1/Slc8a 1
7-day post-SCI group vs. the control group	BP	GO:1903204	negative regulation of oxidative stress-induced neu	4/88	8.09E-07	0.000017	7.68E-06	Ctnnb1/Hif1a/Ncoa7/Pink1
7-day post-SCI group vs. the control group	BP	GO:0031668	cellular response to extracellular stimulus	8/88	8.43E-07	0.0000176	7.96E-06	Axl/Fos/Itga6/Jun/Mapk8/Nfe2l2/Pdk2/Vcam1
7-day post-SCI group vs. the control group	BP	GO:0051090	regulation of DNA-binding transcription factor act	10/88	8.6E-07	0.0000178	8.06E-06	Cd36/Clu/Ctnnb1/Id1/Pink1/Ppargc1a/Ppargc1b/Ppp3ca/ Ripk1/Tlr4
7-day post-SCI group vs. the control group	BP	GO:0033674	positive regulation of kinase activity	10/88	9.34E-07	0.0000191	8.65E-06	Adam9/Axl/Clu/Fbxw7/Hbegf/Map2k3/Map2k4/Prkcd/Tl r4/Trem2
7-day post-SCI group vs. the control group	BP	GO:0098754	detoxification	5/88	9.35E-07	0.0000191	8.65E-06	Atp7a/Cd36/Gch1/Nfe2l2/Prdx6
7-day post-SCI group vs. the control group	BP	GO:0032386	regulation of intracellular transport	9/88	9.46E-07	0.0000192	8.71E-06	Cd36/Ezr/Fbxw7/Mapk14/Mapk8/Mapt/Pink1/Prkcd/[illegible]
7-day post-SCI group vs. the control group	BP	GO:0050678	regulation of epithelial cell proliferation	10/88	9.73E-07	0.0000196	0.0000089	Apc/Apoe/Atp7a/Ctnnb1/Ctsl/Gpx1/Id1/Jun/Stat1/Xdh
7-day post-SCI group vs. the control group	BP	GO:0048661	positive regulation of smooth muscle cell prolifera	6/88	1.05E-06	0.000021	9.52E-06	Aif1/Hbegf/Hif1a/Jun/Ppargc1a/Tlr4
7-day post-SCI group vs. the control group	BP	GO:0050670	regulation of lymphocyte proliferation	8/88	1.07E-06	0.0000214	0.0000097	Aif1/Btk/Cd38/Ctnnb1/Il6st/Ppp3ca/Tlr4/Vcam1
7-day post-SCI group vs. the control group	BP	GO:1901800	positive regulation of proteasomal protein cataboli	6/88	0.0000011	0.0000217	9.84E-06	Cdc20/Clu/Fbxw7/Gclc/Mapk8/Trem2
7-day post-SCI group vs. the control group	BP	GO:0003015	heart process	8/88	1.11E-06	0.0000217	9.84E-06	Atp2a2/Fkbp1b/Gch1/Gpx1/Hbegf/Map2k3/S100a1/Slc8a 1
7-day post-SCI group vs. the control group	BP	GO:1900015	regulation of cytokine production involved in infla	5/88	1.11E-06	0.0000217	9.84E-06	Hif1a/Mapk14/Sirpa/Tlr4/Trem2
7-day post-SCI group vs. the control group	BP	GO:0060249	anatomical structure homeostasis	9/88	1.12E-06	0.0000218	9.88E-06	Apc/Atp2a2/Atp7a/Cd38/Ctnnb1/Hif1a/Ppargc1b/Ptgs1/T lr4
7-day post-SCI group vs. the control group	BP	GO:0014909	smooth muscle cell migration	6/88	1.17E-06	0.0000226	0.0000102	Aif1/Atp7a/Il6st/Nfe2l2/Ppargc1a/Tlr4

7-day post-SCI group vs. the control group	BP	GO:0060760	positive regulation of response to cytokine stimulu	5/88	0.0000012	0.0000232	0.0000105	Axl/Hif1a/Ripk1/Tlr4/Trem2
7-day post-SCI group vs. the control group	BP	GO:0032944	regulation of mononuclear cell proliferation	8/88	1.24E-06	0.0000238	0.0000108	Aif1/Btk/Cd38/Ctnnb1/Il6st/Ppp3ca/Tlr4/Vcam1
7-day post-SCI group vs. the control group	BP	GO:0060143	positive regulation of syncytium formation by plas	4/88	1.33E-06	0.0000253	0.0000114	Adam9/Capn2/Mapk14/Trem2
7-day post-SCI group vs. the control group	BP	GO:0006939	smooth muscle contraction	6/88	1.59E-06	0.00003	0.0000136	Cd38/Fkbp1b/Mylk/Ncf1/Ptgs1/Slc8a1
7-day post-SCI group vs. the control group	BP	GO:0043409	negative regulation of MAPK cascade	7/88	1.71E-06	0.0000323	0.0000146	Apc/Apoe/Ezr/Prkcd/Sirpa/Tlr4/Trem2
7-day post-SCI group vs. the control group	BP	GO:0032770	positive regulation of monooxygenase activity	4/88	1.79E-06	0.0000333	0.0000151	Apoe/Atp7a/Gch1/S100a1
7-day post-SCI group vs. the control group	BP	GO:0002534	cytokine production involved in inflammatory resp	5/88	1.79E-06	0.0000333	0.0000151	Hif1a/Mapk14/Sirpa/Tlr4/Trem2
7-day post-SCI group vs. the control group	BP	GO:0045446	endothelial cell differentiation	6/88	1.84E-06	0.000034	0.0000154	Btg1/Ctnnb1/Ezr/Gpx1/Id1/Xdh
7-day post-SCI group vs. the control group	BP	GO:0043434	response to peptide hormone	9/88	0.0000019	0.000035	0.0000158	Apc/Atp2a2/Ctnnb1/Mapk14/Nfe2l2/Pdk2/Prkcd/Stat1/Stat6
7-day post-SCI group vs. the control group	BP	GO:0045454	cell redox homeostasis	4/88	2.36E-06	0.0000431	0.0000195	Gclc/Gpx1/Nfe2l2/Prdx6
7-day post-SCI group vs. the control group	BP	GO:0000768	syncytium formation by plasma membrane fusion	5/88	0.0000024	0.0000432	0.0000196	Adam9/Capn2/Mapk14/Stat1/Trem2
7-day post-SCI group vs. the control group	BP	GO:0140253	cell-cell fusion	5/88	0.0000024	0.0000432	0.0000196	Adam9/Capn2/Mapk14/Stat1/Trem2
7-day post-SCI group vs. the control group	BP	GO:0150076	neuroinflammatory response	5/88	0.0000024	0.0000432	0.0000196	Aif1/Clu/Jun/Tlr4/Trem2
7-day post-SCI group vs. the control group	BP	GO:0014812	muscle cell migration	6/88	2.44E-06	0.0000435	0.0000197	Aif1/Atp7a/Il6st/Nfe2l2/Ppargc1a/Tlr4
7-day post-SCI group vs. the control group	BP	GO:1903052	positive regulation of proteolysis involved in prote	6/88	2.44E-06	0.0000435	0.0000197	Cdc20/Clu/Fbxw7/Gclc/Mapk8/Trem2
7-day post-SCI group vs. the control group	BP	GO:0050878	regulation of body fluid levels	9/88	2.47E-06	0.0000437	0.0000198	Apoe/Axl/Cd36/Hif1a/Nfe2l2/Ppp3ca/Prkcd/Tlr4/Xdh
7-day post-SCI group vs. the control group	BP	GO:0051101	regulation of DNA binding	6/88	2.56E-06	0.0000451	0.0000204	Ctnnb1/Fbxw7/Id1/Jun/Mapk8/Tlr4
7-day post-SCI group vs. the control group	BP	GO:0006949	syncytium formation	5/88	2.76E-06	0.0000484	0.0000219	Adam9/Capn2/Mapk14/Stat1/Trem2
7-day post-SCI group vs. the control group	BP	GO:0006575	cellular modified amino acid metabolic process	7/88	2.96E-06	0.000051	0.0000231	Ctsl/Gch1/Gclc/Gpx1/Gpx3/Mgst1/Nfe2l2
7-day post-SCI group vs. the control group	BP	GO:0048771	tissue remodeling	7/88	2.96E-06	0.000051	0.0000231	Atp7a/Axl/Cd38/Ctnnb1/Hif1a/Ppargc1b/Ppp3ca
7-day post-SCI group vs. the control group	BP	GO:0045428	regulation of nitric oxide biosynthetic process	5/88	2.96E-06	0.000051	0.0000231	Aif1/Cd36/Clu/Sirpa/Tlr4
7-day post-SCI group vs. the control group	BP	GO:1904646	cellular response to amyloid-beta	4/88	3.05E-06	0.0000521	0.0000236	Cd36/Tlr4/Trem2/Vcam1
7-day post-SCI group vs. the control group	BP	GO:0032872	regulation of stress-activated MAPK cascade	7/88	3.05E-06	0.0000521	0.0000236	Ezr/Map2k4/Ripk1/Sirpa/Tlr4/Trem2/Xdh
7-day post-SCI group vs. the control group	BP	GO:0042593	glucose homeostasis	8/88	3.28E-06	0.0000557	0.0000252	Cd36/Fkbp1b/Gpx1/Hif1a/Pdk2/Ppp3ca/Ucp2/Vcam1
7-day post-SCI group vs. the control group	BP	GO:0033500	carbohydrate homeostasis	8/88	3.36E-06	0.0000565	0.0000256	Cd36/Fkbp1b/Gpx1/Hif1a/Pdk2/Ppp3ca/Ucp2/Vcam1
7-day post-SCI group vs. the control group	BP	GO:0070302	regulation of stress-activated protein kinase signal	7/88	3.36E-06	0.0000565	0.0000256	Ezr/Map2k4/Ripk1/Sirpa/Tlr4/Trem2/Xdh
7-day post-SCI group vs. the control group	BP	GO:0055094	response to lipoprotein particle	4/88	3.45E-06	0.0000577	0.0000261	Apoe/Cd36/Tlr4/Trem2
7-day post-SCI group vs. the control group	BP	GO:0046847	filopodium assembly	5/88	3.61E-06	0.0000601	0.0000272	Ezr/Itga6/Prkcd/Stau2/Trpm2
7-day post-SCI group vs. the control group	BP	GO:1902107	positive regulation of leukocyte differentiation	7/88	0.0000037	0.0000609	0.0000276	Axl/Fos/Jun/Ppargc1b/Ppp3ca/Ripk1/Trem2
7-day post-SCI group vs. the control group	BP	GO:1903708	positive regulation of hemopoiesis	7/88	0.0000037	0.0000609	0.0000276	Axl/Fos/Jun/Ppargc1b/Ppp3ca/Ripk1/Trem2
7-day post-SCI group vs. the control group	BP	GO:0080164	regulation of nitric oxide metabolic process	5/88	3.85E-06	0.0000632	0.0000286	Aif1/Cd36/Clu/Sirpa/Tlr4
7-day post-SCI group vs. the control group	BP	GO:0045672	positive regulation of osteoclast differentiation	4/88	3.89E-06	0.0000634	0.0000287	Fos/Ppargc1b/Ppp3ca/Trem2
7-day post-SCI group vs. the control group	BP	GO:0061041	regulation of wound healing	6/88	3.97E-06	0.0000645	0.0000292	Apoe/Cd36/Hbegf/Mylk/Nfe2l2/Prkcd
7-day post-SCI group vs. the control group	BP	GO:0071277	cellular response to calcium ion	5/88	4.11E-06	0.0000663	0.00003	Fos/Jun/Mylk/Slc25a24/Trpm2
7-day post-SCI group vs. the control group	BP	GO:0003158	endothelium development	6/88	4.14E-06	0.0000666	0.0000302	Btg1/Ctnnb1/Ezr/Gpx1/Id1/Xdh
7-day post-SCI group vs. the control group	BP	GO:0050769	positive regulation of neurogenesis	8/88	0.0000043	0.0000686	0.0000311	Apoe/Ctnnb1/Hdac1/Hif1a/Il6st/Mapk8/Mapt/Stau2
7-day post-SCI group vs. the control group	BP	GO:0051098	regulation of binding	9/88	4.31E-06	0.0000686	0.0000311	Apoe/Atp2a2/Ctnnb1/Fbxw7/Id1/Jun/Mapk8/Ppp3ca/Tlr4
7-day post-SCI group vs. the control group	BP	GO:0097242	amyloid-beta clearance	4/88	4.36E-06	0.0000691	0.0000313	Apoe/Cd36/Clu/Trem2
7-day post-SCI group vs. the control group	BP	GO:0071402	cellular response to lipoprotein particle stimulus	4/88	4.88E-06	0.000077	0.0000349	Apoe/Cd36/Tlr4/Trem2

7-day post-SCI group vs. the control group	BP	GO:0006749	glutathione metabolic process	5/88	4.95E-06	0.0000777	0.0000352	Gclc/Gpx1/Gpx3/Mgst1/Nfe2l2
7-day post-SCI group vs. the control group	BP	GO:0051047	positive regulation of secretion	9/88	5.44E-06	0.000085	0.0000385	Adam9/Cd38/Ezr/Hif1a/Pink1/Ppp3ca/Tlr4/Trem2/Trpm2
7-day post-SCI group vs. the control group	BP	GO:0006936	muscle contraction	8/88	0.0000057	0.0000878	0.0000398	Atp2a2/Cd38/Fkbp1b/Map2k3/Mylk/Ncf1/Ptgs1/Slc8a1
7-day post-SCI group vs. the control group	BP	GO:0009410	response to xenobiotic stimulus	8/88	0.0000057	0.0000878	0.0000398	Cd38/Fos/Gclc/Gpx1/Jun/Mgst1/Mylk/Nfe2l2
7-day post-SCI group vs. the control group	BP	GO:0032409	regulation of transporter activity	8/88	0.0000057	0.0000878	0.0000398	Atp7a/Fkbp1b/Ppargc1a/Ppp3ca/Prkcd/Ripk1/S100a1/Trem2
7-day post-SCI group vs. the control group	BP	GO:1904950	negative regulation of establishment of protein loc	6/88	5.73E-06	0.0000879	0.0000398	Apoe/Cd36/Fkbp1b/Mapt/Ppp3ca/Ucp2
7-day post-SCI group vs. the control group	BP	GO:0050866	negative regulation of cell activation	7/88	5.82E-06	0.0000882	0.0000399	Apoe/Axl/Btk/Gclc/Hspb1/Prkcd/Trem2
7-day post-SCI group vs. the control group	BP	GO:0043619	regulation of transcription from RNA polymerase	3/88	5.86E-06	0.0000882	0.0000399	Cd36/Hif1a/Nfe2l2
7-day post-SCI group vs. the control group	BP	GO:0140052	cellular response to oxidised low-density lipoprote	3/88	5.86E-06	0.0000882	0.0000399	Cd36/Tlr4/Trem2
7-day post-SCI group vs. the control group	BP	GO:1905907	negative regulation of amyloid fibril formation	3/88	5.86E-06	0.0000882	0.0000399	Apoe/Clu/Trem2
7-day post-SCI group vs. the control group	BP	GO:0008088	axo-dendritic transport	5/88	5.92E-06	0.0000887	0.0000402	Dst/Hif1a/Hspb1/Mapt/Stau2
7-day post-SCI group vs. the control group	BP	GO:0060759	regulation of response to cytokine stimulus	6/88	5.96E-06	0.0000889	0.0000403	Axl/Hif1a/Il6st/Ripk1/Tlr4/Trem2
7-day post-SCI group vs. the control group	BP	GO:0010661	positive regulation of muscle cell apoptotic proces	4/88	6.05E-06	0.0000894	0.0000405	Atp2a2/Capn2/Map2k4/Mapk8
7-day post-SCI group vs. the control group	BP	GO:1904645	response to amyloid-beta	4/88	6.05E-06	0.0000894	0.0000405	Cd36/Tlr4/Trem2/Vcam1
7-day post-SCI group vs. the control group	BP	GO:0018108	peptidyl-tyrosine phosphorylation	8/88	0.0000061	0.0000899	0.0000407	Btk/Cd36/Fbxw7/Hbegf/Il6st/Prkcd/Tlr4/Trem2
7-day post-SCI group vs. the control group	BP	GO:0010976	positive regulation of neuron projection developm	7/88	6.16E-06	0.0000903	0.0000409	Apoe/Fkbp1b/Hspb1/Itga6/Mapt/Nfe2l2/Stau2
7-day post-SCI group vs. the control group	BP	GO:0007611	learning or memory	8/88	6.25E-06	0.0000911	0.0000413	Amph/Apoe/Hif1a/Jun/Mapt/Ptgs1/Rcan1/Trem2
7-day post-SCI group vs. the control group	BP	GO:0006809	nitric oxide biosynthetic process	5/88	6.27E-06	0.0000911	0.0000413	Aif1/Cd36/Clu/Sirpa/Tlr4
7-day post-SCI group vs. the control group	BP	GO:0072594	establishment of protein localization to organelle	9/88	6.33E-06	0.0000916	0.0000415	Cd36/Clu/Fbxw7/Mapk14/Mapt/Pink1/Ppp3ca/Prkcd/Txnip
7-day post-SCI group vs. the control group	BP	GO:0018212	peptidyl-tyrosine modification	8/88	6.39E-06	0.000092	0.0000417	Btk/Cd36/Fbxw7/Hbegf/Il6st/Prkcd/Tlr4/Trem2
7-day post-SCI group vs. the control group	BP	GO:0050671	positive regulation of lymphocyte proliferation	6/88	6.44E-06	0.0000924	0.0000418	Aif1/Cd38/Il6st/Ppp3ca/Tlr4/Vcam1
7-day post-SCI group vs. the control group	BP	GO:0070372	regulation of ERK1 and ERK2 cascade	8/88	6.99E-06	0.0000998	0.0000452	Apoe/Cd36/Ezr/Fbxw7/Jun/Sirpa/Tlr4/Trem2
7-day post-SCI group vs. the control group	BP	GO:0032946	positive regulation of mononuclear cell proliferatio	6/88	7.22E-06	0.0001027	0.0000465	Aif1/Cd38/Il6st/Ppp3ca/Tlr4/Vcam1
7-day post-SCI group vs. the control group	BP	GO:0007160	cell-matrix adhesion	7/88	7.31E-06	0.0001034	0.0000468	Adam9/Cd36/Ctnnb1/Itga6/Itgb5/Sirpa/Vcam1
7-day post-SCI group vs. the control group	BP	GO:0051881	regulation of mitochondrial membrane potential	5/88	7.44E-06	0.0001048	0.0000475	Gclc/Hebp2/Mapt/Pink1/Ucp2
7-day post-SCI group vs. the control group	BP	GO:0071333	cellular response to glucose stimulus	6/88	0.0000075	0.000105	0.0000476	Fkbp1b/Gpx1/Hif1a/Ppp3ca/Ucp2/Vcam1
7-day post-SCI group vs. the control group	BP	GO:0043542	endothelial cell migration	7/88	7.51E-06	0.000105	0.0000476	Apoe/Fbxw7/Gpx1/Hif1a/Hspb1/Map2k3/Nfe2l2
7-day post-SCI group vs. the control group	BP	GO:0006839	mitochondrial transport	6/88	7.78E-06	0.0001083	0.0000491	Fbxw7/Gclc/Hebp2/Pink1/Slc25a24/Ucp2
7-day post-SCI group vs. the control group	BP	GO:0050848	regulation of calcium-mediated signaling	5/88	7.86E-06	0.000109	0.0000494	Mapt/Pdk2/Ppp3ca/Rcan1/Trem2
7-day post-SCI group vs. the control group	BP	GO:0030100	regulation of endocytosis	7/88	7.94E-06	0.0001096	0.0000496	Amph/Apoe/Axl/Cd36/Clu/Ppp3ca/Trem2
7-day post-SCI group vs. the control group	BP	GO:0031346	positive regulation of cell projection organization	9/88	8.05E-06	0.0001105	0.0000501	Apc/Apoe/Atp7a/Fkbp1b/Hspb1/Itga6/Mapt/Nfe2l2/Stau2
7-day post-SCI group vs. the control group	BP	GO:0071331	cellular response to hexose stimulus	6/88	8.08E-06	0.0001105	0.0000501	Fkbp1b/Gpx1/Hif1a/Ppp3ca/Ucp2/Vcam1
7-day post-SCI group vs. the control group	BP	GO:0071326	cellular response to monosaccharide stimulus	6/88	8.38E-06	0.0001137	0.0000515	Fkbp1b/Gpx1/Hif1a/Ppp3ca/Ucp2/Vcam1
7-day post-SCI group vs. the control group	BP	GO:2001235	positive regulation of apoptotic signaling pathway	6/88	8.38E-06	0.0001137	0.0000515	Clu/Fbxw7/Mapk8/Mcl1/Prkcd/Ripk1
7-day post-SCI group vs. the control group	BP	GO:0046209	nitric oxide metabolic process	5/88	8.77E-06	0.0001185	0.0000537	Aif1/Cd36/Clu/Sirpa/Tlr4
7-day post-SCI group vs. the control group	BP	GO:0015980	energy derivation by oxidation of organic compou	8/88	0.0000089	0.0001198	0.0000543	Atp7a/Cd36/Hif1a/Il6st/Ndufa12/Ndufa6/Pink1/Ppargc1a
7-day post-SCI group vs. the control group	BP	GO:0045601	regulation of endothelial cell differentiation	4/88	8.99E-06	0.0001206	0.0000546	Btg1/Ctnnb1/Id1/Xdh
7-day post-SCI group vs. the control group	BP	GO:0042692	muscle cell differentiation	9/88	9.29E-06	0.0001241	0.0000562	Atp2a2/Capn2/Ctnnb1/Gpx1/Map2k4/Mapk14/Ppp3ca/Rcan1/Slc8a1

7-day post-SCI group vs. the control group	BP	GO:0071560	cellular response to transforming growth factor bet	7/88	9.35E-06	0.0001244	0.0000563	Adam9/Fos/Hdac1/Itgb5/Jun/Ppargc1a/Rbbp7
7-day post-SCI group vs. the control group	BP	GO:0010882	regulation of cardiac muscle contraction by calciu	3/88	9.65E-06	0.0001278	0.0000579	Atp2a2/Fkbp1b/Slc8a1
7-day post-SCI group vs. the control group	BP	GO:2001057	reactive nitrogen species metabolic process	5/88	9.75E-06	0.0001287	0.0000583	Aif1/Cd36/Clu/Sirpa/Tlr4
7-day post-SCI group vs. the control group	BP	GO:0071559	response to transforming growth factor beta	7/88	0.0000104	0.0001367	0.0000619	Adam9/Fos/Hdac1/Itgb5/Jun/Ppargc1a/Rbbp7
7-day post-SCI group vs. the control group	BP	GO:0042554	superoxide anion generation	4/88	0.0000108	0.0001415	0.0000641	Cybb/Mapt/Ncf1/Prkcd
7-day post-SCI group vs. the control group	BP	GO:0071322	cellular response to carbohydrate stimulus	6/88	0.0000111	0.0001454	0.0000658	Fkbp1b/Gpx1/Hif1a/Ppp3ca/Ucp2/Vcam1
7-day post-SCI group vs. the control group	BP	GO:0032651	regulation of interleukin-1 beta production	5/88	0.0000114	0.0001479	0.000067	Cd36/Hspb1/Sirpa/Tlr4/Trem2
7-day post-SCI group vs. the control group	BP	GO:0070371	ERK1 and ERK2 cascade	8/88	0.0000117	0.0001514	0.0000686	Apoe/Cd36/Ezr/Fbxw7/Jun/Sirpa/Tlr4/Trem2
7-day post-SCI group vs. the control group	BP	GO:0032768	regulation of monooxygenase activity	4/88	0.0000118	0.0001516	0.0000687	Apoe/Atp7a/Gch1/S100a1
7-day post-SCI group vs. the control group	BP	GO:2000273	positive regulation of signaling receptor activity	4/88	0.0000118	0.0001516	0.0000687	Fbxw7/Hbegf/Hdac1/Hif1a
7-day post-SCI group vs. the control group	BP	GO:0034350	regulation of glial cell apoptotic process	3/88	0.000012	0.0001533	0.0000694	Mapk8/Prkcd/Trem2
7-day post-SCI group vs. the control group	BP	GO:1905906	regulation of amyloid fibril formation	3/88	0.000012	0.0001533	0.0000694	Apoe/Clu/Trem2
7-day post-SCI group vs. the control group	BP	GO:1903320	regulation of protein modification by small protein	7/88	0.0000122	0.0001543	0.0000699	Cdc20/Ctnnb1/Fbxw7/Gclc/Hif1a/Mapk8/Pink1
7-day post-SCI group vs. the control group	BP	GO:0002683	negative regulation of immune system process	9/88	0.0000123	0.0001546	0.00007	Axl/Btk/Ctnnb1/Ezr/Fbxw7/Gpx1/Hspb1/Stat6/Trem2
7-day post-SCI group vs. the control group	BP	GO:0051251	positive regulation of lymphocyte activation	9/88	0.0000123	0.0001546	0.00007	Aif1/Axl/Cd38/Il6st/Ppp3ca/Sirpa/Stat6/Tlr4/Vcam1
7-day post-SCI group vs. the control group	BP	GO:0032675	regulation of interleukin-6 production	6/88	0.0000124	0.0001551	0.0000702	Aif1/Capn2/Cd36/Sirpa/Tlr4/Trem2
7-day post-SCI group vs. the control group	BP	GO:0070665	positive regulation of leukocyte proliferation	6/88	0.0000128	0.0001598	0.0000724	Aif1/Cd38/Il6st/Ppp3ca/Tlr4/Vcam1
7-day post-SCI group vs. the control group	BP	GO:0016032	viral process	8/88	0.000013	0.0001614	0.0000731	Apoe/Axl/Ctsl/Hdac1/Jun/Ripk1/Stat1/Vcam1
7-day post-SCI group vs. the control group	BP	GO:2001237	negative regulation of extrinsic apoptotic signaling	5/88	0.0000132	0.0001641	0.0000743	Gclc/Gpx1/Itga6/Mcl1/Ripk1
7-day post-SCI group vs. the control group	BP	GO:0050890	cognition	8/88	0.0000138	0.0001702	0.0000771	Amph/Apoe/Hif1a/Jun/Mapt/Ptgs1/Rcan1/Trem2
7-day post-SCI group vs. the control group	BP	GO:0043124	negative regulation of I-kappaB kinase/NF-kappaB	4/88	0.000014	0.0001725	0.0000781	Hdac1/Ripk1/Stat1/Trem2
7-day post-SCI group vs. the control group	BP	GO:0090257	regulation of muscle system process	7/88	0.0000142	0.0001736	0.0000786	Aif1/Atp2a2/Fkbp1b/Ncf1/Ppp3ca/Ptgs1/Slc8a1
7-day post-SCI group vs. the control group	BP	GO:0001678	cellular glucose homeostasis	6/88	0.0000151	0.0001846	0.0000836	Fkbp1b/Gpx1/Hif1a/Ppp3ca/Ucp2/Vcam1
7-day post-SCI group vs. the control group	BP	GO:0045860	positive regulation of protein kinase activity	8/88	0.0000152	0.0001853	0.0000839	Adam9/Clu/Fbxw7/Hbegf/Map2k3/Map2k4/Prkcd/Tlr4
7-day post-SCI group vs. the control group	BP	GO:0032611	interleukin-1 beta production	5/88	0.0000153	0.0001856	0.0000841	Cd36/Hspb1/Sirpa/Tlr4/Trem2
7-day post-SCI group vs. the control group	BP	GO:0051348	negative regulation of transferase activity	7/88	0.0000156	0.0001889	0.0000856	Apc/Apoe/Cdc20/Hspb1/Mapt/Prkcd/Slc8a1
7-day post-SCI group vs. the control group	BP	GO:1903532	positive regulation of secretion by cell	8/88	0.0000158	0.0001907	0.0000864	Adam9/Cd38/Ezr/Hif1a/Pink1/Tlr4/Trem2/Trpm2
7-day post-SCI group vs. the control group	BP	GO:0010660	regulation of muscle cell apoptotic process	5/88	0.000016	0.0001925	0.0000872	Atp2a2/Capn2/Map2k4/Mapk8/Nfe2l2
7-day post-SCI group vs. the control group	BP	GO:0051962	positive regulation of nervous system developmen	8/88	0.0000161	0.0001931	0.0000874	Apoe/Ctnnb1/Hdac1/Hif1a/Il6st/Mapk8/Mapt/Stau2
7-day post-SCI group vs. the control group	BP	GO:0008544	epidermis development	8/88	0.0000165	0.0001953	0.0000884	Apc/Atp7a/Ctnnb1/Ctsl/Hdac1/Ppp3ca/Ptgs1/Txnip
7-day post-SCI group vs. the control group	BP	GO:0043618	regulation of transcription from RNA polymerase I	4/88	0.0000165	0.0001953	0.0000884	Cd36/Hif1a/Jun/Nfe2l2
7-day post-SCI group vs. the control group	BP	GO:0045429	positive regulation of nitric oxide biosynthetic pro	4/88	0.0000165	0.0001953	0.0000884	Aif1/Cd36/Clu/Tlr4
7-day post-SCI group vs. the control group	BP	GO:0010594	regulation of endothelial cell migration	6/88	0.0000166	0.0001955	0.0000886	Apoe/Fbxw7/Hif1a/Hspb1/Map2k3/Nfe2l2
7-day post-SCI group vs. the control group	BP	GO:0032635	interleukin-6 production	6/88	0.0000166	0.0001955	0.0000886	Aif1/Capn2/Cd36/Sirpa/Tlr4/Trem2
7-day post-SCI group vs. the control group	BP	GO:0042116	macrophage activation	5/88	0.0000168	0.0001967	0.0000891	Aif1/Clu/Jun/Tlr4/Trem2
7-day post-SCI group vs. the control group	BP	GO:0007254	JNK cascade	6/88	0.0000172	0.0001991	0.0000902	Map2k4/Mapk10/Mapk8/Ripk1/Sirpa/Tlr4
7-day post-SCI group vs. the control group	BP	GO:0007596	blood coagulation	6/88	0.0000172	0.0001991	0.0000902	Apoe/Axl/Cd36/Nfe2l2/Prkcd/Tlr4
7-day post-SCI group vs. the control group	BP	GO:0051100	negative regulation of binding	6/88	0.0000172	0.0001991	0.0000902	Atp2a2/Fbxw7/Id1/Jun/Mapk8/Ppp3ca
7-day post-SCI group vs. the control group	BP	GO:0031589	cell-substrate adhesion	8/88	0.0000178	0.0002054	0.000093	Adam9/Axl/Cd36/Ctnnb1/Itga6/Itgb5/Sirpa/Vcam1
7-day post-SCI group vs. the control group	BP	GO:0050730	regulation of peptidyl-tyrosine phosphorylation	7/88	0.0000181	0.0002079	0.0000942	Cd36/Fbxw7/Hbegf/Il6st/Prkcd/Tlr4/Trem2

7-day post-SCI group vs. the control group	BP	GO:2000060	positive regulation of ubiquitin-dependent protein	5/88	0.0000184	0.0002113	0.0000957	Cdc20/Clu/Fbxw7/Gclc/Mapk8
7-day post-SCI group vs. the control group	BP	GO:0007599	hemostasis	6/88	0.0000189	0.0002152	0.0000974	Apoe/Axl/Cd36/Nfe2l2/Prkcd/Tlr4
7-day post-SCI group vs. the control group	BP	GO:0050817	coagulation	6/88	0.0000189	0.0002152	0.0000974	Apoe/Axl/Cd36/Nfe2l2/Prkcd/Tlr4
7-day post-SCI group vs. the control group	BP	GO:1904407	positive regulation of nitric oxide metabolic proces	4/88	0.0000193	0.0002191	0.0000992	Aif1/Cd36/Clu/Tlr4
7-day post-SCI group vs. the control group	BP	GO:0002274	myeloid leukocyte activation	7/88	0.0000199	0.000224	0.0001014	Adam9/Aif1/Clu/Jun/Prkcd/Tlr4/Trem2
7-day post-SCI group vs. the control group	BP	GO:0044403	biological process involved in symbiotic interactio	7/88	0.0000199	0.000224	0.0001014	Apoe/Axl/Ctsl/Gpx1/Hdac1/Jun/Ncf1
7-day post-SCI group vs. the control group	BP	GO:0010657	muscle cell apoptotic process	5/88	0.0000202	0.0002266	0.0001026	Atp2a2/Capn2/Map2k4/Mapk8/Nfe2l2
7-day post-SCI group vs. the control group	BP	GO:0034764	positive regulation of transmembrane transport	7/88	0.0000204	0.0002278	0.0001032	Atp7a/Mapk14/Nfe2l2/Ppp3ca/Prkcd/S100a1/Trem2
7-day post-SCI group vs. the control group	BP	GO:0022407	regulation of cell-cell adhesion	9/88	0.0000208	0.0002314	0.0001048	Aif1/Hspb1/Il6st/Itga6/Mapk14/Ppp3ca/Prkcd/Sirpa/Vcam1
7-day post-SCI group vs. the control group	BP	GO:0010639	negative regulation of organelle organization	8/88	0.0000211	0.0002348	0.0001063	Apc/Cdc20/Gclc/Gpx1/Mapt/Pink1/Ppargc1a/Prkcd
7-day post-SCI group vs. the control group	BP	GO:0051000	positive regulation of nitric-oxide synthase activity	3/88	0.0000214	0.0002367	0.0001072	Apoe/Gch1/S100a1
7-day post-SCI group vs. the control group	BP	GO:1900221	regulation of amyloid-beta clearance	3/88	0.0000214	0.0002367	0.0001072	Apoe/Clu/Trem2
7-day post-SCI group vs. the control group	BP	GO:0010720	positive regulation of cell development	8/88	0.0000228	0.0002505	0.0001134	Apoe/Ctnnb1/Hdac1/Hif1a/Il6st/Mapk8/Mapt/Stau2
7-day post-SCI group vs. the control group	BP	GO:0032760	positive regulation of tumor necrosis factor produc	5/88	0.000023	0.0002527	0.0001144	Cd36/Clu/Hspb1/Ripk1/Tlr4
7-day post-SCI group vs. the control group	BP	GO:0031667	response to nutrient levels	8/88	0.0000241	0.0002625	0.0001189	Apoe/Jun/Mapk14/Mapk8/Mapt/Nfe2l2/Pdk2/Ppargc1a
7-day post-SCI group vs. the control group	BP	GO:0001961	positive regulation of cytokine-mediated signaling	4/88	0.0000242	0.0002625	0.0001189	Axl/Hif1a/Ripk1/Trem2
7-day post-SCI group vs. the control group	BP	GO:0043620	regulation of DNA-templated transcription in resp	4/88	0.0000242	0.0002625	0.0001189	Cd36/Hif1a/Jun/Nfe2l2
7-day post-SCI group vs. the control group	BP	GO:1902903	regulation of supramolecular fiber organization	8/88	0.000025	0.0002702	0.0001224	Apc/Apoe/Clu/Id1/Mapk8/Mapt/Prkcd/Trem2
7-day post-SCI group vs. the control group	BP	GO:1903557	positive regulation of tumor necrosis factor superf	5/88	0.0000251	0.0002709	0.0001227	Cd36/Clu/Hspb1/Ripk1/Tlr4
7-day post-SCI group vs. the control group	BP	GO:1900225	regulation of NLRP3 inflammasome complex asse	3/88	0.0000254	0.0002732	0.0001237	Cd36/Tlr4/Trem2
7-day post-SCI group vs. the control group	BP	GO:0050731	positive regulation of peptidyl-tyrosine phosphoryl	6/88	0.0000264	0.0002821	0.0001278	Cd36/Fbxw7/Hbegf/Il6st/Tlr4/Trem2
7-day post-SCI group vs. the control group	BP	GO:0050796	regulation of insulin secretion	6/88	0.0000264	0.0002821	0.0001278	Cd38/Fkbp1b/Hif1a/Ppp3ca/Trpm2/Ucp2
7-day post-SCI group vs. the control group	BP	GO:0043536	positive regulation of blood vessel endothelial cell	4/88	0.0000279	0.0002958	0.000134	Hif1a/Hspb1/Map2k3/Nfe2l2
7-day post-SCI group vs. the control group	BP	GO:0051353	positive regulation of oxidoreductase activity	4/88	0.0000279	0.0002958	0.000134	Apoe/Atp7a/Gch1/S100a1
7-day post-SCI group vs. the control group	BP	GO:0001942	hair follicle development	5/88	0.0000285	0.0003015	0.0001365	Apc/Atp7a/Ctnnb1/Ctsl/Hdac1
7-day post-SCI group vs. the control group	BP	GO:0009749	response to glucose	6/88	0.0000288	0.000304	0.0001377	Fkbp1b/Gpx1/Hif1a/Ppp3ca/Ucp2/Vcam1
7-day post-SCI group vs. the control group	BP	GO:0032652	regulation of interleukin-1 production	5/88	0.0000297	0.0003122	0.0001414	Cd36/Hspb1/Sirpa/Tlr4/Trem2
7-day post-SCI group vs. the control group	BP	GO:0034349	glial cell apoptotic process	3/88	0.0000298	0.0003126	0.0001416	Mapk8/Prkcd/Trem2
7-day post-SCI group vs. the control group	BP	GO:0009746	response to hexose	6/88	0.0000314	0.0003281	0.0001486	Fkbp1b/Gpx1/Hif1a/Ppp3ca/Ucp2/Vcam1
7-day post-SCI group vs. the control group	BP	GO:0042987	amyloid precursor protein catabolic process	4/88	0.000032	0.0003313	0.0001501	Adam9/Apoe/Clu/Dhcr24
7-day post-SCI group vs. the control group	BP	GO:0032412	regulation of ion transmembrane transporter activi	7/88	0.000032	0.0003313	0.0001501	Atp7a/Fkbp1b/Ppargc1a/Ppp3ca/Ripk1/S100a1/Trem2
7-day post-SCI group vs. the control group	BP	GO:0008637	apoptotic mitochondrial changes	5/88	0.0000322	0.0003313	0.0001501	Atp7a/Gclc/Gpx1/Jun/Pink1
7-day post-SCI group vs. the control group	BP	GO:0022404	molting cycle process	5/88	0.0000322	0.0003313	0.0001501	Apc/Atp7a/Ctnnb1/Ctsl/Hdac1
7-day post-SCI group vs. the control group	BP	GO:0022405	hair cycle process	5/88	0.0000322	0.0003313	0.0001501	Apc/Atp7a/Ctnnb1/Ctsl/Hdac1
7-day post-SCI group vs. the control group	BP	GO:0008016	regulation of heart contraction	6/88	0.0000332	0.0003408	0.0001543	Atp2a2/Fkbp1b/Gch1/Hbegf/S100a1/Slc8a1
7-day post-SCI group vs. the control group	BP	GO:0051702	biological process involved in interaction with syn	5/88	0.0000335	0.0003417	0.0001548	Apoe/Gpx1/Hdac1/Jun/Ncf1
7-day post-SCI group vs. the control group	BP	GO:0098773	skin epidermis development	5/88	0.0000335	0.0003417	0.0001548	Apc/Atp7a/Ctnnb1/Ctsl/Hdac1
7-day post-SCI group vs. the control group	BP	GO:0010665	regulation of cardiac muscle cell apoptotic process	4/88	0.0000342	0.0003451	0.0001563	Atp2a2/Capn2/Mapk8/Nfe2l2
7-day post-SCI group vs. the control group	BP	GO:0035924	cellular response to vascular endothelial growth fa	4/88	0.0000342	0.0003451	0.0001563	Hspb1/Map2k3/Mapk14/Xdh
7-day post-SCI group vs. the control group	BP	GO:0034284	response to monosaccharide	6/88	0.0000342	0.0003451	0.0001563	Fkbp1b/Gpx1/Hif1a/Ppp3ca/Ucp2/Vcam1

7-day post-SCI group vs. the control group	BP	GO:0007178	transmembrane receptor protein serine/threonine k	8/88	0.0000344	0.0003463	0.0001569	Adam9/Fos/Hdac1/Id1/Itgb5/Jun/Mapk14/Rbbp7
7-day post-SCI group vs. the control group	BP	GO:0044546	NLRP3 inflammasome complex assembly	3/88	0.0000347	0.0003484	0.0001578	Cd36/Tlr4/Trem2
7-day post-SCI group vs. the control group	BP	GO:0045165	cell fate commitment	7/88	0.0000356	0.0003562	0.0001613	Apc/Ctnnb1/Ctsl/Ets2/Hdac1/Rbbp7/Stat6
7-day post-SCI group vs. the control group	BP	GO:0007179	transforming growth factor beta receptor signaling	6/88	0.0000361	0.0003594	0.0001628	Adam9/Fos/Hdac1/Itgb5/Jun/Rbbp7
7-day post-SCI group vs. the control group	BP	GO:0034612	response to tumor necrosis factor	6/88	0.0000361	0.0003594	0.0001628	Adam9/Gch1/Mapk14/Nfe2l2/Ripk1/Stat1
7-day post-SCI group vs. the control group	BP	GO:0031663	lipopolysaccharide-mediated signaling pathway	4/88	0.0000365	0.0003617	0.0001638	Mapk14/Stat1/Tlr4/Trem2
7-day post-SCI group vs. the control group	BP	GO:0031334	positive regulation of protein-containing complex	6/88	0.0000371	0.0003672	0.0001663	Apc/Cd36/Clu/Mapk8/Mapt/Tlr4
7-day post-SCI group vs. the control group	BP	GO:0032612	interleukin-1 production	5/88	0.0000377	0.000371	0.000168	Cd36/Hspb1/Sirpa/Tlr4/Trem2
7-day post-SCI group vs. the control group	BP	GO:1902414	protein localization to cell junction	5/88	0.0000377	0.000371	0.000168	Hspb1/Mapk10/Mapk8/Mapt/Stau2
7-day post-SCI group vs. the control group	BP	GO:0090303	positive regulation of wound healing	4/88	0.0000389	0.0003811	0.0001726	Cd36/Hbegf/Mylk/Nfe2l2
7-day post-SCI group vs. the control group	BP	GO:0022898	regulation of transmembrane transporter activity	7/88	0.0000396	0.0003864	0.000175	Atp7a/Fkbp1b/Ppargc1a/Ppp3ca/Ripk1/S100a1/Trem2
7-day post-SCI group vs. the control group	BP	GO:0051770	positive regulation of nitric-oxide synthase biosyn	3/88	0.0000401	0.0003896	0.0001764	Map2k3/Map2k4/Tlr4
7-day post-SCI group vs. the control group	BP	GO:1901522	positive regulation of transcription from RNA poly	3/88	0.0000401	0.0003896	0.0001764	Hif1a/Jun/Nfe2l2
7-day post-SCI group vs. the control group	BP	GO:0007519	skeletal muscle tissue development	6/88	0.0000414	0.0003978	0.0001802	Ctnnb1/Fos/Gpx1/Mapk14/Ppp3ca/Rcan1
7-day post-SCI group vs. the control group	BP	GO:0010662	regulation of striated muscle cell apoptotic process	4/88	0.0000414	0.0003978	0.0001802	Atp2a2/Capn2/Mapk8/Nfe2l2
7-day post-SCI group vs. the control group	BP	GO:0043457	regulation of cellular respiration	4/88	0.0000414	0.0003978	0.0001802	Atp7a/Hif1a/Pink1/Ppargc1a
7-day post-SCI group vs. the control group	BP	GO:1903409	reactive oxygen species biosynthetic process	4/88	0.0000414	0.0003978	0.0001802	Cd36/Cybb/Ncf1/Tlr4
7-day post-SCI group vs. the control group	BP	GO:0048511	rhythmic process	7/88	0.0000421	0.000403	0.0001825	Axl/Fbxw7/Hdac1/Id1/Mapk10/Mapk8/Ppargc1a
7-day post-SCI group vs. the control group	BP	GO:0010659	cardiac muscle cell apoptotic process	4/88	0.0000441	0.0004185	0.0001895	Atp2a2/Capn2/Mapk8/Nfe2l2
7-day post-SCI group vs. the control group	BP	GO:0014911	positive regulation of smooth muscle cell migratio	4/88	0.0000441	0.0004185	0.0001895	Aif1/Atp7a/Il6st/Tlr4
7-day post-SCI group vs. the control group	BP	GO:0032615	interleukin-12 production	4/88	0.0000441	0.0004185	0.0001895	Cd36/Mapk14/Prkcd/Tlr4
7-day post-SCI group vs. the control group	BP	GO:0043123	positive regulation of I-kappaB kinase/NF-kappaB	5/88	0.0000457	0.0004322	0.0001957	Cd36/Ctnnb1/Pink1/Ripk1/Tlr4
7-day post-SCI group vs. the control group	BP	GO:0010666	positive regulation of cardiac muscle cell apoptoti	3/88	0.000046	0.0004343	0.0001967	Atp2a2/Capn2/Mapk8
7-day post-SCI group vs. the control group	BP	GO:0009150	purine ribonucleotide metabolic process	8/88	0.0000467	0.0004391	0.0001989	Atp7a/Hif1a/Ndufa12/Ndufa6/Pdk2/Ppargc1a/Trem2/Xdh
7-day post-SCI group vs. the control group	BP	GO:0001836	release of cytochrome c from mitochondria	4/88	0.0000469	0.0004399	0.0001992	Atp7a/Gpx1/Jun/Pink1
7-day post-SCI group vs. the control group	BP	GO:0009743	response to carbohydrate	6/88	0.0000498	0.0004662	0.0002112	Fkbp1b/Gpx1/Hif1a/Ppp3ca/Ucp2/Vcam1
7-day post-SCI group vs. the control group	BP	GO:0016358	dendrite development	7/88	0.0000505	0.0004716	0.0002136	Apoe/Atp7a/Cdc20/Id1/Mapk8/Ppp3ca/Stau2
7-day post-SCI group vs. the control group	BP	GO:0009314	response to radiation	8/88	0.0000523	0.0004842	0.0002193	Fbxw7/Gpx1/Hif1a/Jun/Mapk10/Mapk14/Mapk8/Prkcd
7-day post-SCI group vs. the control group	BP	GO:0010663	positive regulation of striated muscle cell apoptoti	3/88	0.0000525	0.0004842	0.0002193	Atp2a2/Capn2/Mapk8
7-day post-SCI group vs. the control group	BP	GO:0140632	inflammasome complex assembly	3/88	0.0000525	0.0004842	0.0002193	Cd36/Tlr4/Trem2
7-day post-SCI group vs. the control group	BP	GO:1903747	regulation of establishment of protein localization	3/88	0.0000525	0.0004842	0.0002193	Fbxw7/Mapt/Pink1
7-day post-SCI group vs. the control group	BP	GO:0010658	striated muscle cell apoptotic process	4/88	0.0000528	0.000486	0.0002201	Atp2a2/Capn2/Mapk8/Nfe2l2
7-day post-SCI group vs. the control group	BP	GO:0015833	peptide transport	7/88	0.0000558	0.000512	0.0002319	Cd38/Fkbp1b/Hif1a/Mgst1/Ppp3ca/Trpm2/Ucp2
7-day post-SCI group vs. the control group	BP	GO:0097006	regulation of plasma lipoprotein particle levels	4/88	0.000056	0.0005124	0.000232	Apoe/Cd36/Lcat/Trem2
7-day post-SCI group vs. the control group	BP	GO:0001666	response to hypoxia	6/88	0.0000566	0.0005154	0.0002334	Cd38/Hif1a/Nfe2l2/Pink1/Slc8a1/Ucp2
7-day post-SCI group vs. the control group	BP	GO:0060538	skeletal muscle organ development	6/88	0.0000566	0.0005154	0.0002334	Ctnnb1/Fos/Gpx1/Mapk14/Ppp3ca/Rcan1
7-day post-SCI group vs. the control group	BP	GO:0043161	proteasome-mediated ubiquitin-dependent protein	8/88	0.0000576	0.000523	0.0002369	Apc/Cdc20/Clu/Ctnnb1/Fbxw7/Gclc/Mapk8/Nfe2l2
7-day post-SCI group vs. the control group	BP	GO:0090090	negative regulation of canonical Wnt signaling pat	5/88	0.0000589	0.0005327	0.0002412	Apc/Apoe/Ctnnb1/Hdac1/Mapk14
7-day post-SCI group vs. the control group	BP	GO:1903828	negative regulation of protein localization	6/88	0.0000596	0.0005376	0.0002435	Apoe/Cd36/Fkbp1b/Mapt/Ppp3ca/Ucp2
7-day post-SCI group vs. the control group	BP	GO:0035303	regulation of dephosphorylation	5/88	0.000061	0.0005482	0.0002483	Fkbp1b/Pink1/Ppargc1b/Prkcd/Rcan1

7-day post-SCI group vs. the control group	BP	GO:0007623	circadian rhythm	6/88	0.0000611	0.0005482	0.0002483	Fbxw7/Hdac1/Id1/Mapk10/Mapk8/Ppargc1a
7-day post-SCI group vs. the control group	BP	GO:0032720	negative regulation of tumor necrosis factor produ	4/88	0.0000628	0.0005605	0.0002538	Axl/Sirpa/Tlr4/Trem2
7-day post-SCI group vs. the control group	BP	GO:0001959	regulation of cytokine-mediated signaling pathway	5/88	0.0000631	0.0005605	0.0002538	Axl/Hif1a/Il6st/Ripk1/Trem2
7-day post-SCI group vs. the control group	BP	GO:0042303	molting cycle	5/88	0.0000631	0.0005605	0.0002538	Apc/Atp7a/Ctnnb1/Ctsl/Hdac1
7-day post-SCI group vs. the control group	BP	GO:0042633	hair cycle	5/88	0.0000631	0.0005605	0.0002538	Apc/Atp7a/Ctnnb1/Ctsl/Hdac1
7-day post-SCI group vs. the control group	BP	GO:0046034	ATP metabolic process	6/88	0.0000642	0.0005684	0.0002574	Atp7a/Hif1a/Ndufa12/Ndufa6/Ppargc1a/Trem2
7-day post-SCI group vs. the control group	BP	GO:0009259	ribonucleotide metabolic process	8/88	0.0000644	0.0005686	0.0002575	Atp7a/Hif1a/Ndufa12/Ndufa6/Pdk2/Ppargc1a/Trem2/Xdh
7-day post-SCI group vs. the control group	BP	GO:0051054	positive regulation of DNA metabolic process	7/88	0.0000652	0.000574	0.00026	Ctnnb1/Jun/Map2k4/Mapk8/Ppargc1a/Prkcd/Stat6
7-day post-SCI group vs. the control group	BP	GO:0001649	osteoblast differentiation	6/88	0.0000658	0.0005779	0.0002617	Apc/Ctnnb1/Id1/Il6st/Mapk14/Ppp3ca
7-day post-SCI group vs. the control group	BP	GO:0001937	negative regulation of endothelial cell proliferatior	4/88	0.0000664	0.0005783	0.0002619	Apc/Apoe/Stat1/Xdh
7-day post-SCI group vs. the control group	BP	GO:0030193	regulation of blood coagulation	4/88	0.0000664	0.0005783	0.0002619	Apoe/Cd36/Nfe2l2/Prkcd
7-day post-SCI group vs. the control group	BP	GO:1903556	negative regulation of tumor necrosis factor superf	4/88	0.0000664	0.0005783	0.0002619	Axl/Sirpa/Tlr4/Trem2
7-day post-SCI group vs. the control group	BP	GO:0045730	respiratory burst	3/88	0.0000671	0.0005805	0.0002629	Cybb/Ncf1/Trem2
7-day post-SCI group vs. the control group	BP	GO:0071276	cellular response to cadmium ion	3/88	0.0000671	0.0005805	0.0002629	Fos/Jun/Mapk8
7-day post-SCI group vs. the control group	BP	GO:1903146	regulation of autophagy of mitochondrion	3/88	0.0000671	0.0005805	0.0002629	Fbxw7/Hif1a/Pink1
7-day post-SCI group vs. the control group	BP	GO:0033673	negative regulation of kinase activity	6/88	0.0000674	0.0005814	0.0002633	Apc/Apoe/Hspb1/Mapt/Prkcd/Slc8a1
7-day post-SCI group vs. the control group	BP	GO:0006163	purine nucleotide metabolic process	8/88	0.0000685	0.0005879	0.0002663	Atp7a/Hif1a/Ndufa12/Ndufa6/Pdk2/Ppargc1a/Trem2/Xdh
7-day post-SCI group vs. the control group	BP	GO:0006816	calcium ion transport	8/88	0.0000685	0.0005879	0.0002663	Atp2a2/Ctnnb1/Fkbp1b/Mylk/Ppp3ca/S100a1/Slc8a1/Trpm2
7-day post-SCI group vs. the control group	BP	GO:0070374	positive regulation of ERK1 and ERK2 cascade	6/88	0.0000691	0.0005911	0.0002677	Apoe/Cd36/Fbxw7/Jun/Tlr4/Trem2
7-day post-SCI group vs. the control group	BP	GO:0033157	regulation of intracellular protein transport	6/88	0.0000708	0.0006041	0.0002736	Cd36/Fbxw7/Mapk14/Pink1/Prkcd/Prr5l
7-day post-SCI group vs. the control group	BP	GO:0055001	muscle cell development	6/88	0.0000725	0.0006172	0.0002795	Atp2a2/Gpx1/Map2k4/Ppp3ca/Rcan1/Slc8a1
7-day post-SCI group vs. the control group	BP	GO:0051146	striated muscle cell differentiation	7/88	0.000073	0.0006198	0.0002807	Capn2/Gpx1/Map2k4/Mapk14/Ppp3ca/Rcan1/Slc8a1
7-day post-SCI group vs. the control group	BP	GO:1900046	regulation of hemostasis	4/88	0.000074	0.0006267	0.0002838	Apoe/Cd36/Nfe2l2/Prkcd
7-day post-SCI group vs. the control group	BP	GO:0006790	sulfur compound metabolic process	7/88	0.0000744	0.0006283	0.0002846	Gclc/Gpx1/Gpx3/Mgst1/Nfe2l2/Pdk2/Xdh
7-day post-SCI group vs. the control group	BP	GO:0051224	negative regulation of protein transport	5/88	0.0000748	0.0006305	0.0002856	Apoe/Cd36/Fkbp1b/Ppp3ca/Ucp2
7-day post-SCI group vs. the control group	BP	GO:0019693	ribose phosphate metabolic process	8/88	0.0000763	0.0006414	0.0002905	Atp7a/Hif1a/Ndufa12/Ndufa6/Pdk2/Ppargc1a/Trem2/Xdh
7-day post-SCI group vs. the control group	BP	GO:0014902	myotube differentiation	5/88	0.0000773	0.0006469	0.000293	Capn2/Gpx1/Mapk14/Ppp3ca/Rcan1
7-day post-SCI group vs. the control group	BP	GO:0032411	positive regulation of transporter activity	5/88	0.0000773	0.0006469	0.000293	Atp7a/Ppp3ca/Prkcd/S100a1/Trem2
7-day post-SCI group vs. the control group	BP	GO:0042982	amyloid precursor protein metabolic process	4/88	0.000078	0.000651	0.0002948	Adam9/Apoe/Clu/Dhcr24
7-day post-SCI group vs. the control group	BP	GO:0010959	regulation of metal ion transport	8/88	0.0000786	0.0006545	0.0002964	Atp7a/Ctnnb1/Fkbp1b/Mylk/Ppp3ca/S100a1/Slc8a1/Trem2
7-day post-SCI group vs. the control group	BP	GO:0007015	actin filament organization	8/88	0.0000811	0.0006729	0.0003047	Aif1/Ezr/Id1/Itgb5/Marcksl1/Ppargc1b/Prkcd/Sirpa
7-day post-SCI group vs. the control group	BP	GO:0050818	regulation of coagulation	4/88	0.0000822	0.0006809	0.0003084	Apoe/Cd36/Nfe2l2/Prkcd
7-day post-SCI group vs. the control group	BP	GO:0051235	maintenance of location	7/88	0.0000831	0.0006852	0.0003103	Apoe/Cd36/Fkbp1b/Pink1/Slc8a1/Trem2/Trpm2
7-day post-SCI group vs. the control group	BP	GO:0030073	insulin secretion	6/88	0.0000836	0.0006852	0.0003103	Cd38/Fkbp1b/Hif1a/Ppp3ca/Trpm2/Ucp2
7-day post-SCI group vs. the control group	BP	GO:0090276	regulation of peptide hormone secretion	6/88	0.0000836	0.0006852	0.0003103	Cd38/Fkbp1b/Hif1a/Ppp3ca/Trpm2/Ucp2
7-day post-SCI group vs. the control group	BP	GO:0045648	positive regulation of erythrocyte differentiation	3/88	0.0000842	0.0006852	0.0003103	Hif1a/Mapk14/Stat1
7-day post-SCI group vs. the control group	BP	GO:0046688	response to copper ion	3/88	0.0000842	0.0006852	0.0003103	Atp7a/Lcat/Nfe2l2
7-day post-SCI group vs. the control group	BP	GO:0051767	nitric-oxide synthase biosynthetic process	3/88	0.0000842	0.0006852	0.0003103	Map2k3/Map2k4/Tlr4
7-day post-SCI group vs. the control group	BP	GO:0051769	regulation of nitric-oxide synthase biosynthetic pro	3/88	0.0000842	0.0006852	0.0003103	Map2k3/Map2k4/Tlr4

7-day post-SCI group vs. the control group	BP	GO:0006091	generation of precursor metabolites and energy	8/88	0.0000874	0.0007091	0.0003212	Atp7a/Cd36/Hif1a/Il6st/Ndufa12/Ndufa6/Pink1/Ppargc1a
7-day post-SCI group vs. the control group	BP	GO:0042098	T cell proliferation	6/88	0.0000876	0.0007091	0.0003212	Aif1/Ctnnb1/Fkbp1b/Il6st/Ppp3ca/Vcam1
7-day post-SCI group vs. the control group	BP	GO:0010469	regulation of signaling receptor activity	5/88	0.0000881	0.0007118	0.0003224	Fbxw7/Hbegf/Hdac1/Hif1a/Ppargc1a
7-day post-SCI group vs. the control group	BP	GO:1904019	epithelial cell apoptotic process	5/88	0.000091	0.0007326	0.0003318	Apc/Id1/Mapk8/Nfe2l2/Ppargc1a
7-day post-SCI group vs. the control group	BP	GO:0010822	positive regulation of mitochondrion organization	4/88	0.0000911	0.0007326	0.0003318	Hif1a/Pink1/Ppargc1a/Trem2
7-day post-SCI group vs. the control group	BP	GO:0071900	regulation of protein serine/threonine kinase activi	7/88	0.0000926	0.0007398	0.000335	Adam9/Apc/Apoe/Map2k4/Prkcd/Slc8a1/Tlr4
7-day post-SCI group vs. the control group	BP	GO:0006914	autophagy	8/88	0.0000927	0.0007398	0.000335	Atp2a2/Clu/Fbxw7/Hif1a/Mapt/Mcl1/Pink1/Trem2
7-day post-SCI group vs. the control group	BP	GO:0061919	process utilizing autophagic mechanism	8/88	0.0000927	0.0007398	0.000335	Atp2a2/Clu/Fbxw7/Hif1a/Mapt/Mcl1/Pink1/Trem2
7-day post-SCI group vs. the control group	BP	GO:0002791	regulation of peptide secretion	6/88	0.0000938	0.0007459	0.0003378	Cd38/Fkbp1b/Hif1a/Ppp3ca/Trpm2/Ucp2
7-day post-SCI group vs. the control group	BP	GO:0043467	regulation of generation of precursor metabolites a	5/88	0.0000939	0.0007459	0.0003378	Atp7a/Cd36/Hif1a/Pink1/Ppargc1a
7-day post-SCI group vs. the control group	BP	GO:0030098	lymphocyte differentiation	8/88	0.0000955	0.0007564	0.0003426	Apc/Atp7a/Axl/Btk/Ctnnb1/Ctsl/Hspb1/Stat6
7-day post-SCI group vs. the control group	BP	GO:0090087	regulation of peptide transport	6/88	0.0000982	0.0007762	0.0003515	Cd38/Fkbp1b/Hif1a/Ppp3ca/Trpm2/Ucp2
7-day post-SCI group vs. the control group	BP	GO:0042886	amide transport	7/88	0.000103	0.0008121	0.0003678	Cd38/Fkbp1b/Hif1a/Mgst1/Ppp3ca/Trpm2/Ucp2
7-day post-SCI group vs. the control group	BP	GO:0042744	hydrogen peroxide catabolic process	3/88	0.0001039	0.0008174	0.0003702	Gpx1/Gpx3/Prdx6
7-day post-SCI group vs. the control group	BP	GO:0045333	cellular respiration	6/88	0.000105	0.0008244	0.0003734	Atp7a/Hif1a/Ndufa12/Ndufa6/Pink1/Ppargc1a
7-day post-SCI group vs. the control group	BP	GO:0036293	response to decreased oxygen levels	6/88	0.0001074	0.000841	0.0003809	Cd38/Hif1a/Nfe2l2/Pink1/Slc8a1/Ucp2
7-day post-SCI group vs. the control group	BP	GO:0072521	purine-containing compound metabolic process	8/88	0.0001135	0.0008867	0.0004016	Atp7a/Hif1a/Ndufa12/Ndufa6/Pdk2/Ppargc1a/Trem2/Xdh
7-day post-SCI group vs. the control group	BP	GO:0031116	positive regulation of microtubule polymerization	3/88	0.0001148	0.0008924	0.0004042	Apc/Mapk8/Mapt
7-day post-SCI group vs. the control group	BP	GO:1902883	negative regulation of response to oxidative stress	3/88	0.0001148	0.0008924	0.0004042	Ncoa7/Nfe2l2/Pink1
7-day post-SCI group vs. the control group	BP	GO:0050709	negative regulation of protein secretion	4/88	0.0001164	0.0009032	0.000409	Apoe/Fkbp1b/Ppp3ca/Ucp2
7-day post-SCI group vs. the control group	BP	GO:0098657	import into cell	6/88	0.0001173	0.0009077	0.0004111	Cd36/Ppp3ca/Prkcd/Slc8a1/Trem2/Trpm2
7-day post-SCI group vs. the control group	BP	GO:0009205	purine ribonucleoside triphosphate metabolic proc	6/88	0.0001199	0.0009256	0.0004192	Atp7a/Hif1a/Ndufa12/Ndufa6/Ppargc1a/Trem2
7-day post-SCI group vs. the control group	BP	GO:0070296	sarcoplasmic reticulum calcium ion transport	3/88	0.0001264	0.0009734	0.0004409	Atp2a2/Fkbp1b/Slc8a1
7-day post-SCI group vs. the control group	BP	GO:0007613	memory	5/88	0.0001312	0.0010085	0.0004568	Apoe/Mapt/Ptgs1/Rcan1/Trem2
7-day post-SCI group vs. the control group	BP	GO:0062012	regulation of small molecule metabolic process	7/88	0.0001332	0.0010211	0.0004624	Apc/Apoe/Cd36/Hif1a/Pdk2/Ppargc1a/Trem2
7-day post-SCI group vs. the control group	BP	GO:0045667	regulation of osteoblast differentiation	5/88	0.0001351	0.0010336	0.0004681	Apc/Ctnnb1/Id1/Il6st/Ppp3ca
7-day post-SCI group vs. the control group	BP	GO:0009144	purine nucleoside triphosphate metabolic process	6/88	0.0001363	0.0010381	0.0004702	Atp7a/Hif1a/Ndufa12/Ndufa6/Ppargc1a/Trem2
7-day post-SCI group vs. the control group	BP	GO:0009199	ribonucleoside triphosphate metabolic process	6/88	0.0001363	0.0010381	0.0004702	Atp7a/Hif1a/Ndufa12/Ndufa6/Ppargc1a/Trem2
7-day post-SCI group vs. the control group	BP	GO:0050999	regulation of nitric-oxide synthase activity	3/88	0.0001387	0.0010537	0.0004772	Apoe/Gch1/S100a1
7-day post-SCI group vs. the control group	BP	GO:0007517	muscle organ development	7/88	0.00014	0.0010611	0.0004806	Ctnnb1/Fos/Gpx1/Mapk14/Mylk/Ppp3ca/Rcan1
7-day post-SCI group vs. the control group	BP	GO:2001236	regulation of extrinsic apoptotic signaling pathway	5/88	0.0001473	0.0011139	0.0005045	Gclc/Gpx1/Itga6/Mcl1/Ripk1
7-day post-SCI group vs. the control group	BP	GO:0010001	glial cell differentiation	6/88	0.0001483	0.0011187	0.0005067	Clu/Ctnnb1/Hdac1/Il6st/Tlr4/Trem2
7-day post-SCI group vs. the control group	BP	GO:0002052	positive regulation of neuroblast proliferation	3/88	0.0001518	0.00114	0.0005163	Ctnnb1/Hif1a/Mapk8
7-day post-SCI group vs. the control group	BP	GO:1903205	regulation of hydrogen peroxide-induced cell deatl	3/88	0.0001518	0.00114	0.0005163	Nfe2l2/Pink1/Ripk1
7-day post-SCI group vs. the control group	BP	GO:0050863	regulation of T cell activation	7/88	0.000157	0.0011768	0.0005329	Aif1/Ctnnb1/Hspb1/Il6st/Ppp3ca/Sirpa/Vcam1
7-day post-SCI group vs. the control group	BP	GO:0035418	protein localization to synapse	4/88	0.0001599	0.001193	0.0005403	Hspb1/Mapk10/Mapt/Stau2
7-day post-SCI group vs. the control group	BP	GO:1900542	regulation of purine nucleotide metabolic process	4/88	0.0001599	0.001193	0.0005403	Hif1a/Pdk2/Ppargc1a/Trem2
7-day post-SCI group vs. the control group	BP	GO:0006937	regulation of muscle contraction	5/88	0.0001603	0.0011932	0.0005404	Atp2a2/Fkbp1b/Ncf1/Ptgs1/Slc8a1
7-day post-SCI group vs. the control group	BP	GO:0030178	negative regulation of Wnt signaling pathway	5/88	0.0001648	0.0012187	0.0005519	Apc/Apoe/Ctnnb1/Hdac1/Mapk14
7-day post-SCI group vs. the control group	BP	GO:0030856	regulation of epithelial cell differentiation	5/88	0.0001648	0.0012187	0.0005519	Apc/Btg1/Ctnnb1/Id1/Xdh

7-day post-SCI group vs. the control group	BP	GO:2000058	regulation of ubiquitin-dependent protein catabolic	5/88	0.0001648	0.0012187	0.0005519	Cdc20/Clu/Fbxw7/Gclc/Mapk8
7-day post-SCI group vs. the control group	BP	GO:0031112	positive regulation of microtubule polymerization	3/88	0.0001657	0.0012193	0.0005522	Apc/Mapk8/Mapt
7-day post-SCI group vs. the control group	BP	GO:0090140	regulation of mitochondrial fission	3/88	0.0001657	0.0012193	0.0005522	Mapt/Pink1/Ppargc1a
7-day post-SCI group vs. the control group	BP	GO:0046486	glycerolipid metabolic process	7/88	0.0001675	0.0012301	0.0005571	Apoe/Capn2/Cd36/Gpx1/Il6st/Lcat/Prdx6
7-day post-SCI group vs. the control group	BP	GO:0034250	positive regulation of cellular amide metabolic prc	5/88	0.0001742	0.0012763	0.0005781	Apoe/Clu/Nfe2l2/Pink1/Prkcd
7-day post-SCI group vs. the control group	BP	GO:0014912	negative regulation of smooth muscle cell migratic	3/88	0.0001803	0.0013126	0.0005945	Aif1/Nfe2l2/Ppargc1a
7-day post-SCI group vs. the control group	BP	GO:0031069	hair follicle morphogenesis	3/88	0.0001803	0.0013126	0.0005945	Atp7a/Ctnnb1/Ctsl
7-day post-SCI group vs. the control group	BP	GO:1903715	regulation of aerobic respiration	3/88	0.0001803	0.0013126	0.0005945	Atp7a/Hif1a/Pink1
7-day post-SCI group vs. the control group	BP	GO:0032436	positive regulation of proteasomal ubiquitin-depen	4/88	0.0001818	0.0013202	0.0005979	Cdc20/Clu/Gclc/Mapk8
7-day post-SCI group vs. the control group	BP	GO:0019216	regulation of lipid metabolic process	7/88	0.0001843	0.0013354	0.0006048	Apoe/Capn2/Cd36/Pdk2/Ppargc1a/Prkcd/Trem2
7-day post-SCI group vs. the control group	BP	GO:0001936	regulation of endothelial cell proliferation	5/88	0.0001941	0.0014035	0.0006356	Apc/Apoe/Jun/Stat1/Xdh
7-day post-SCI group vs. the control group	BP	GO:0009141	nucleoside triphosphate metabolic process	6/88	0.0001967	0.0014194	0.0006429	Atp7a/Hif1a/Ndufa12/Ndufa6/Ppargc1a/Trem2
7-day post-SCI group vs. the control group	BP	GO:0010970	transport along microtubule	5/88	0.0001993	0.0014351	0.00065	Dst/Hif1a/Hspb1/Mapt/Stau2
7-day post-SCI group vs. the control group	BP	GO:0006140	regulation of nucleotide metabolic process	4/88	0.0002057	0.001468	0.0006649	Hif1a/Pdk2/Ppargc1a/Trem2
7-day post-SCI group vs. the control group	BP	GO:0030168	platelet activation	4/88	0.0002057	0.001468	0.0006649	Apoe/Axl/Prkcd/Tlr4
7-day post-SCI group vs. the control group	BP	GO:0045685	regulation of glial cell differentiation	4/88	0.0002057	0.001468	0.0006649	Ctnnb1/Hdac1/Il6st/Trem2
7-day post-SCI group vs. the control group	BP	GO:0051341	regulation of oxidoreductase activity	4/88	0.0002057	0.001468	0.0006649	Apoe/Atp7a/Gch1/S100a1
7-day post-SCI group vs. the control group	BP	GO:0071375	cellular response to peptide hormone stimulus	6/88	0.0002086	0.0014854	0.0006728	Apc/Ctnnb1/Nfe2l2/Pdk2/Prkcd/Stat6
7-day post-SCI group vs. the control group	BP	GO:0006606	protein import into nucleus	5/88	0.0002101	0.0014885	0.0006741	Cd36/Mapk14/Ppp3ca/Prkcd/Txnip
7-day post-SCI group vs. the control group	BP	GO:1903531	negative regulation of secretion by cell	5/88	0.0002101	0.0014885	0.0006741	Apoe/Fkbp1b/Ppp3ca/Ptgs1/Ucp2
7-day post-SCI group vs. the control group	BP	GO:0030808	regulation of nucleotide biosynthetic process	3/88	0.0002121	0.0014885	0.0006741	Pdk2/Ppargc1a/Trem2
7-day post-SCI group vs. the control group	BP	GO:0034381	plasma lipoprotein particle clearance	3/88	0.0002121	0.0014885	0.0006741	Apoe/Cd36/Trem2
7-day post-SCI group vs. the control group	BP	GO:0036474	cell death in response to hydrogen peroxide	3/88	0.0002121	0.0014885	0.0006741	Nfe2l2/Pink1/Ripk1
7-day post-SCI group vs. the control group	BP	GO:0046686	response to cadmium ion	3/88	0.0002121	0.0014885	0.0006741	Fos/Jun/Mapk8
7-day post-SCI group vs. the control group	BP	GO:1900371	regulation of purine nucleotide biosynthetic proces	3/88	0.0002121	0.0014885	0.0006741	Pdk2/Ppargc1a/Trem2
7-day post-SCI group vs. the control group	BP	GO:0035304	regulation of protein dephosphorylation	4/88	0.0002141	0.0014992	0.000679	Fkbp1b/Pink1/Prkcd/Rcan1
7-day post-SCI group vs. the control group	BP	GO:1902904	negative regulation of supramolecular fiber organi	5/88	0.0002213	0.0015466	0.0007004	Apc/Apoe/Clu/Prkcd/Trem2
7-day post-SCI group vs. the control group	BP	GO:0048863	stem cell differentiation	6/88	0.0002252	0.0015703	0.0007112	Ctnnb1/Hdac1/Hif1a/Mapk14/Nfe2l2/Rbbp7
7-day post-SCI group vs. the control group	BP	GO:0001890	placenta development	5/88	0.0002271	0.0015803	0.0007157	Ctsl/Hif1a/Mapk14/Slc8a1/Vcam1
7-day post-SCI group vs. the control group	BP	GO:0060292	long-term synaptic depression	3/88	0.0002293	0.0015921	0.0007211	Cd38/Mapt/Stau2
7-day post-SCI group vs. the control group	BP	GO:0051170	import into nucleus	5/88	0.000233	0.0016146	0.0007312	Cd36/Mapk14/Ppp3ca/Prkcd/Txnip
7-day post-SCI group vs. the control group	BP	GO:0010506	regulation of autophagy	6/88	0.0002384	0.0016459	0.0007454	Fbxw7/Hif1a/Mapt/Mcl1/Pink1/Trem2
7-day post-SCI group vs. the control group	BP	GO:0045766	positive regulation of angiogenesis	5/88	0.000239	0.0016459	0.0007454	Btg1/Hif1a/Hspb1/Nfe2l2/S100a1
7-day post-SCI group vs. the control group	BP	GO:1904018	positive regulation of vasculature development	5/88	0.000239	0.0016459	0.0007454	Btg1/Hif1a/Hspb1/Nfe2l2/S100a1
7-day post-SCI group vs. the control group	BP	GO:0042398	cellular modified amino acid biosynthetic process	3/88	0.0002474	0.0016961	0.0007682	Gch1/Gclc/Nfe2l2
7-day post-SCI group vs. the control group	BP	GO:0098703	calcium ion import across plasma membrane	3/88	0.0002474	0.0016961	0.0007682	Ppp3ca/Slc8a1/Trpm2
7-day post-SCI group vs. the control group	BP	GO:0048813	dendrite morphogenesis	5/88	0.0002643	0.0018085	0.0008191	Atp7a/Id1/Mapk8/Ppp3ca/Stau2
7-day post-SCI group vs. the control group	BP	GO:0030072	peptide hormone secretion	6/88	0.0002665	0.00182	0.0008243	Cd38/Fkbp1b/Hif1a/Ppp3ca/Trpm2/Ucp2
7-day post-SCI group vs. the control group	BP	GO:0009060	aerobic respiration	5/88	0.0002709	0.0018423	0.0008344	Atp7a/Hif1a/Ndufa12/Ndufa6/Pink1
7-day post-SCI group vs. the control group	BP	GO:0032388	positive regulation of intracellular transport	5/88	0.0002709	0.0018423	0.0008344	Ezr/Fbxw7/Mapk14/Prkcd/Prr5l

7-day post-SCI group vs. the control group	BP	GO:1904062	regulation of cation transmembrane transport	7/88	0.0002732	0.0018544	0.0008399	Atp7a/Fkbp1b/Ppargc1a/Ppp3ca/S100a1/Slc8a1/Trem2
7-day post-SCI group vs. the control group	BP	GO:0045834	positive regulation of lipid metabolic process	5/88	0.0002776	0.0018805	0.0008517	Apoe/Capn2/Cd36/Ppargc1a/Prkcd
7-day post-SCI group vs. the control group	BP	GO:0015850	organic hydroxy compound transport	6/88	0.0002815	0.001903	0.0008619	Apoe/Cd36/Lcat/Pink1/Ptgs1/Trem2
7-day post-SCI group vs. the control group	BP	GO:0048730	epidermis morphogenesis	3/88	0.0002861	0.0019263	0.0008724	Atp7a/Ctnnb1/Ctsl
7-day post-SCI group vs. the control group	BP	GO:1900744	regulation of p38MAPK cascade	3/88	0.0002861	0.0019263	0.0008724	Ezr/Trem2/Xdh
7-day post-SCI group vs. the control group	BP	GO:0050680	negative regulation of epithelial cell proliferation	5/88	0.0002915	0.0019572	0.0008864	Apc/Apoe/Ctsl/Stat1/Xdh
7-day post-SCI group vs. the control group	BP	GO:0051924	regulation of calcium ion transport	6/88	0.0002919	0.0019572	0.0008864	Ctnnb1/Fkbp1b/Mylk/Ppp3ca/S100a1/Slc8a1
7-day post-SCI group vs. the control group	BP	GO:0001818	negative regulation of cytokine production	6/88	0.0002972	0.0019887	0.0009007	Axl/Btk/Ezr/Sirpa/Tlr4/Trem2
7-day post-SCI group vs. the control group	BP	GO:0001935	endothelial cell proliferation	5/88	0.0002986	0.0019941	0.0009031	Apc/Apoe/Jun/Stat1/Xdh
7-day post-SCI group vs. the control group	BP	GO:0019221	cytokine-mediated signaling pathway	7/88	0.0003023	0.0020147	0.0009124	Axl/Hif1a/Il6st/Ripk1/Stat1/Stat6/Trem2
7-day post-SCI group vs. the control group	BP	GO:0002021	response to dietary excess	3/88	0.0003069	0.0020372	0.0009227	Apoe/Mapk14/Ppargc1a
7-day post-SCI group vs. the control group	BP	GO:0032735	positive regulation of interleukin-12 production	3/88	0.0003069	0.0020372	0.0009227	Cd36/Mapk14/Tlr4
7-day post-SCI group vs. the control group	BP	GO:0002790	peptide secretion	6/88	0.0003081	0.0020405	0.0009242	Cd38/Fkbp1b/Hif1a/Ppp3ca/Trpm2/Ucp2
7-day post-SCI group vs. the control group	BP	GO:0006754	ATP biosynthetic process	4/88	0.0003129	0.0020683	0.0009368	Ndufa12/Ndufa6/Ppargc1a/Trem2
7-day post-SCI group vs. the control group	BP	GO:0006875	cellular metal ion homeostasis	7/88	0.00032	0.0021114	0.0009563	Apoe/Atp2a2/Atp7a/Fkbp1b/Hif1a/Slc8a1/Trpm2
7-day post-SCI group vs. the control group	BP	GO:0071901	negative regulation of protein serine/threonine kin	4/88	0.0003243	0.0021352	0.000967	Apc/Apoe/Prkcd/Slc8a1
7-day post-SCI group vs. the control group	BP	GO:0046326	positive regulation of glucose import	3/88	0.0003287	0.0021598	0.0009782	Mapk14/Nfe2l2/Prkcd
7-day post-SCI group vs. the control group	BP	GO:0042129	regulation of T cell proliferation	5/88	0.0003363	0.002201	0.0009968	Aif1/Ctnnb1/Il6st/Ppp3ca/Vcam1
7-day post-SCI group vs. the control group	BP	GO:2001257	regulation of cation channel activity	5/88	0.0003363	0.002201	0.0009968	Fkbp1b/Ppargc1a/Ppp3ca/S100a1/Trem2
7-day post-SCI group vs. the control group	BP	GO:0070482	response to oxygen levels	6/88	0.0003424	0.0022368	0.001013	Cd38/Hif1a/Nfe2l2/Pink1/Slc8a1/Ucp2
7-day post-SCI group vs. the control group	BP	GO:0033555	multicellular organismal response to stress	4/88	0.000348	0.0022642	0.0010254	Apoe/Capn2/Gch1/Ppp3ca
7-day post-SCI group vs. the control group	BP	GO:0042310	vasoconstriction	4/88	0.000348	0.0022642	0.0010254	Cd38/Hif1a/Ptgs1/Slc8a1
7-day post-SCI group vs. the control group	BP	GO:0010935	regulation of macrophage cytokine production	3/88	0.0003514	0.0022788	0.0010321	Axl/Cd36/Tlr4
7-day post-SCI group vs. the control group	BP	GO:0043405	regulation of MAP kinase activity	5/88	0.0003523	0.0022788	0.0010321	Adam9/Apoe/Map2k4/Prkcd/Tlr4
7-day post-SCI group vs. the control group	BP	GO:0098739	import across plasma membrane	5/88	0.0003523	0.0022788	0.0010321	Cd36/Ppp3ca/Prkcd/Slc8a1/Trpm2
7-day post-SCI group vs. the control group	BP	GO:0010595	positive regulation of endothelial cell migration	4/88	0.0003603	0.0023105	0.0010464	Hif1a/Hspb1/Map2k3/Nfe2l2
7-day post-SCI group vs. the control group	BP	GO:0032642	regulation of chemokine production	4/88	0.0003603	0.0023105	0.0010464	Aif1/Sirpa/Tlr4/Trem2
7-day post-SCI group vs. the control group	BP	GO:0042102	positive regulation of T cell proliferation	4/88	0.0003603	0.0023105	0.0010464	Aif1/Il6st/Ppp3ca/Vcam1
7-day post-SCI group vs. the control group	BP	GO:0046889	positive regulation of lipid biosynthetic process	4/88	0.0003603	0.0023105	0.0010464	Apoe/Capn2/Ppargc1a/Prkcd
7-day post-SCI group vs. the control group	BP	GO:0046883	regulation of hormone secretion	6/88	0.0003607	0.0023105	0.0010464	Cd38/Fkbp1b/Hif1a/Ppp3ca/Trpm2/Ucp2
7-day post-SCI group vs. the control group	BP	GO:0009152	purine ribonucleotide biosynthetic process	5/88	0.0003689	0.0023585	0.0010682	Ndufa12/Ndufa6/Pdk2/Ppargc1a/Trem2
7-day post-SCI group vs. the control group	BP	GO:0006641	triglyceride metabolic process	4/88	0.0003858	0.0024504	0.0011098	Apoe/Cd36/Gpx1/Il6st
7-day post-SCI group vs. the control group	BP	GO:0030301	cholesterol transport	4/88	0.0003858	0.0024504	0.0011098	Apoe/Cd36/Lcat/Trem2
7-day post-SCI group vs. the control group	BP	GO:0006469	negative regulation of protein kinase activity	5/88	0.0003861	0.0024504	0.0011098	Apc/Apoe/Hspb1/Prkcd/Slc8a1
7-day post-SCI group vs. the control group	BP	GO:0030336	negative regulation of cell migration	6/88	0.0003862	0.0024504	0.0011098	Aif1/Apoe/Hdac1/Nfe2l2/Ppargc1a/Rbbp7
7-day post-SCI group vs. the control group	BP	GO:0055017	cardiac muscle tissue growth	4/88	0.000399	0.0025171	0.00114	Apc/Ctnnb1/Map2k4/Mapk14
7-day post-SCI group vs. the control group	BP	GO:0060326	cell chemotaxis	6/88	0.0003995	0.0025171	0.00114	Aif1/Hbegf/Hspb1/Prkcd/Trpm2/Vcam1
7-day post-SCI group vs. the control group	BP	GO:0000266	mitochondrial fission	3/88	0.0003998	0.0025171	0.00114	Mapt/Pink1/Ppargc1a
7-day post-SCI group vs. the control group	BP	GO:0043277	apoptotic cell clearance	3/88	0.0003998	0.0025171	0.00114	Axl/Cd36/Trem2
7-day post-SCI group vs. the control group	BP	GO:0010042	response to manganese ion	2/88	0.0004048	0.002525	0.0011435	Adam9/Atp7a

7-day post-SCI group vs. the control group	BP	GO:0014883	transition between fast and slow fiber	2/88	0.0004048	0.002525	0.0011435	Atp2a2/Ppp3ca
7-day post-SCI group vs. the control group	BP	GO:0017038	protein import	2/88	0.0004048	0.002525	0.0011435	Apoe/Clu
7-day post-SCI group vs. the control group	BP	GO:0034372	very-low-density lipoprotein particle remodeling	2/88	0.0004048	0.002525	0.0011435	Apoe/Lcat
7-day post-SCI group vs. the control group	BP	GO:0034380	high-density lipoprotein particle assembly	2/88	0.0004048	0.002525	0.0011435	Apoe/Lcat
7-day post-SCI group vs. the control group	BP	GO:0001708	cell fate specification	4/88	0.0004126	0.0025686	0.0011633	Apc/Ctnnb1/Hdac1/Rbbp7
7-day post-SCI group vs. the control group	BP	GO:0051048	negative regulation of secretion	5/88	0.0004222	0.0026186	0.001186	Apoe/Fkbp1b/Ppp3ca/Ptgs1/Ucp2
7-day post-SCI group vs. the control group	BP	GO:0120032	regulation of plasma membrane bounded cell proje	5/88	0.0004222	0.0026186	0.001186	Apc/Atp7a/Prkcd/Stau2/Trpm2
7-day post-SCI group vs. the control group	BP	GO:0032873	negative regulation of stress-activated MAPK casc	3/88	0.0004255	0.0026193	0.0011863	Ezr/Sirpa/Trem2
7-day post-SCI group vs. the control group	BP	GO:0048010	vascular endothelial growth factor receptor signali	3/88	0.0004255	0.0026193	0.0011863	Hif1a/Hspb1/Mapk14
7-day post-SCI group vs. the control group	BP	GO:0048741	skeletal muscle fiber development	3/88	0.0004255	0.0026193	0.0011863	Gpx1/Ppp3ca/Rcan1
7-day post-SCI group vs. the control group	BP	GO:0070303	negative regulation of stress-activated protein kina	3/88	0.0004255	0.0026193	0.0011863	Ezr/Sirpa/Trem2
7-day post-SCI group vs. the control group	BP	GO:0030705	cytoskeleton-dependent intracellular transport	5/88	0.0004316	0.0026523	0.0012012	Dst/Hif1a/Hspb1/Mapt/Stau2
7-day post-SCI group vs. the control group	BP	GO:0015918	sterol transport	4/88	0.0004407	0.0026978	0.0012219	Apoe/Cd36/Lcat/Trem2
7-day post-SCI group vs. the control group	BP	GO:1904035	regulation of epithelial cell apoptotic process	4/88	0.0004407	0.0026978	0.0012219	Apc/Id1/Nfe2l2/Ppargc1a
7-day post-SCI group vs. the control group	BP	GO:0060491	regulation of cell projection assembly	5/88	0.000451	0.0027483	0.0012447	Apc/Atp7a/Prkcd/Stau2/Trpm2
7-day post-SCI group vs. the control group	BP	GO:0010543	regulation of platelet activation	3/88	0.0004522	0.0027483	0.0012447	Apoe/Prkcd/Tlr4
7-day post-SCI group vs. the control group	BP	GO:0010762	regulation of fibroblast migration	3/88	0.0004522	0.0027483	0.0012447	Apc/Prr5l/Slc8a1
7-day post-SCI group vs. the control group	BP	GO:0044848	biological phase	3/88	0.0004522	0.0027483	0.0012447	Cdc20/Ctnnb1/Ctsl
7-day post-SCI group vs. the control group	BP	GO:0009206	purine ribonucleoside triphosphate biosynthetic pr	4/88	0.0004552	0.0027563	0.0012483	Ndufa12/Ndufa6/Ppargc1a/Trem2
7-day post-SCI group vs. the control group	BP	GO:0032602	chemokine production	4/88	0.0004552	0.0027563	0.0012483	Aif1/Sirpa/Tlr4/Trem2
7-day post-SCI group vs. the control group	BP	GO:0009416	response to light stimulus	6/88	0.0004562	0.0027575	0.0012489	Fbxw7/Gpx1/Hif1a/Mapk10/Mapk8/Prkcd
7-day post-SCI group vs. the control group	BP	GO:0009145	purine nucleoside triphosphate biosynthetic proces	4/88	0.0004701	0.0028282	0.0012809	Ndufa12/Ndufa6/Ppargc1a/Trem2
7-day post-SCI group vs. the control group	BP	GO:0010721	negative regulation of cell development	5/88	0.0004709	0.0028282	0.0012809	Ctnnb1/Fbxw7/Id1/Ppp3ca/Trem2
7-day post-SCI group vs. the control group	BP	GO:0022409	positive regulation of cell-cell adhesion	6/88	0.0004714	0.0028282	0.0012809	Aif1/Il6st/Itga6/Ppp3ca/Sirpa/Vcam1
7-day post-SCI group vs. the control group	BP	GO:0045927	positive regulation of growth	6/88	0.0004714	0.0028282	0.0012809	Apoe/Cd38/Ezr/Hbegf/Mapk14/Mapt
7-day post-SCI group vs. the control group	BP	GO:0070588	calcium ion transmembrane transport	6/88	0.0004791	0.0028641	0.0012972	Atp2a2/Fkbp1b/Ppp3ca/S100a1/Slc8a1/Trpm2
7-day post-SCI group vs. the control group	BP	GO:2000146	negative regulation of cell motility	6/88	0.0004791	0.0028641	0.0012972	Aif1/Apoe/Hdac1/Nfe2l2/Ppargc1a/Rbbp7
7-day post-SCI group vs. the control group	BP	GO:0048806	genitalia development	3/88	0.00048	0.0028642	0.0012972	Axl/Ctnnb1/Dhcr24
7-day post-SCI group vs. the control group	BP	GO:0009260	ribonucleotide biosynthetic process	5/88	0.0004812	0.0028642	0.0012972	Ndufa12/Ndufa6/Pdk2/Ppargc1a/Trem2
7-day post-SCI group vs. the control group	BP	GO:0030217	T cell differentiation	6/88	0.0004869	0.0028642	0.0012972	Apc/Atp7a/Ctnnb1/Ctsl/Hspb1/Stat6
7-day post-SCI group vs. the control group	BP	GO:0031345	negative regulation of cell projection organization	5/88	0.0004916	0.0028642	0.0012972	Apoe/Cd38/Id1/Ppp3ca/Prkcd
7-day post-SCI group vs. the control group	BP	GO:0002246	wound healing involved in inflammatory response	2/88	0.0004938	0.0028642	0.0012972	Hif1a/Tlr4
7-day post-SCI group vs. the control group	BP	GO:0002679	respiratory burst involved in defense response	2/88	0.0004938	0.0028642	0.0012972	Ncf1/Trem2
7-day post-SCI group vs. the control group	BP	GO:0034351	negative regulation of glial cell apoptotic process	2/88	0.0004938	0.0028642	0.0012972	Prkcd/Trem2
7-day post-SCI group vs. the control group	BP	GO:0034370	triglyceride-rich lipoprotein particle remodeling	2/88	0.0004938	0.0028642	0.0012972	Apoe/Lcat
7-day post-SCI group vs. the control group	BP	GO:0034384	high-density lipoprotein particle clearance	2/88	0.0004938	0.0028642	0.0012972	Apoe/Trem2
7-day post-SCI group vs. the control group	BP	GO:0045602	negative regulation of endothelial cell differentiati	2/88	0.0004938	0.0028642	0.0012972	Id1/Xdh
7-day post-SCI group vs. the control group	BP	GO:0048262	determination of dorsal/ventral asymmetry	2/88	0.0004938	0.0028642	0.0012972	Ctnnb1/Mapk8
7-day post-SCI group vs. the control group	BP	GO:0048263	determination of dorsal identity	2/88	0.0004938	0.0028642	0.0012972	Ctnnb1/Mapk8
7-day post-SCI group vs. the control group	BP	GO:0050774	negative regulation of dendrite morphogenesis	2/88	0.0004938	0.0028642	0.0012972	Id1/Ppp3ca

7-day post-SCI group vs. the control group	BP	GO:0060768	regulation of epithelial cell proliferation involved i	2/88	0.0004938	0.0028642	0.0012972	Apc/Ctnnb1
7-day post-SCI group vs. the control group	BP	GO:0061517	macrophage proliferation	2/88	0.0004938	0.0028642	0.0012972	Clu/Trem2
7-day post-SCI group vs. the control group	BP	GO:0071415	cellular response to purine-containing compound	2/88	0.0004938	0.0028642	0.0012972	Slc8a1/Trpm2
7-day post-SCI group vs. the control group	BP	GO:1900454	positive regulation of long-term synaptic depressic	2/88	0.0004938	0.0028642	0.0012972	Mapt/Stau2
7-day post-SCI group vs. the control group	BP	GO:0042100	B cell proliferation	4/88	0.0005009	0.0029005	0.0013136	Btk/Cd38/Prkcd/Tlr4
7-day post-SCI group vs. the control group	BP	GO:0045646	regulation of erythrocyte differentiation	3/88	0.0005089	0.0029314	0.0013276	Hif1a/Mapk14/Stat1
7-day post-SCI group vs. the control group	BP	GO:0045740	positive regulation of DNA replication	3/88	0.0005089	0.0029314	0.0013276	Jun/Map2k4/Mapk8
7-day post-SCI group vs. the control group	BP	GO:1902692	regulation of neuroblast proliferation	3/88	0.0005089	0.0029314	0.0013276	Ctnnb1/Hif1a/Mapk8
7-day post-SCI group vs. the control group	BP	GO:0022600	digestive system process	4/88	0.0005169	0.0029719	0.001346	Cd36/Ezr/Ppp3ca/Tlr4
7-day post-SCI group vs. the control group	BP	GO:0099111	microtubule-based transport	5/88	0.0005238	0.0030065	0.0013616	Dst/Hif1a/Hspb1/Mapt/Stau2
7-day post-SCI group vs. the control group	BP	GO:0009201	ribonucleoside triphosphate biosynthetic process	4/88	0.0005332	0.003055	0.0013836	Ndufa12/Ndufa6/Ppargc1a/Trem2
7-day post-SCI group vs. the control group	BP	GO:0006164	purine nucleotide biosynthetic process	5/88	0.0005349	0.0030595	0.0013857	Ndufa12/Ndufa6/Pdk2/Ppargc1a/Trem2
7-day post-SCI group vs. the control group	BP	GO:0018105	peptidyl-serine phosphorylation	6/88	0.0005358	0.0030598	0.0013858	Mapk14/Mapk8/Pdk2/Pink1/Prkcd/Ripk1
7-day post-SCI group vs. the control group	BP	GO:0010934	macrophage cytokine production	3/88	0.0005389	0.0030721	0.0013913	Axl/Cd36/Tlr4
7-day post-SCI group vs. the control group	BP	GO:0031396	regulation of protein ubiquitination	5/88	0.0005461	0.0031078	0.0014075	Cdc20/Fbxw7/Gclc/Mapk8/Pink1
7-day post-SCI group vs. the control group	BP	GO:0006476	protein deacetylation	4/88	0.0005498	0.0031181	0.0014122	Hdac1/Mapt/Pink1/Rbbp7
7-day post-SCI group vs. the control group	BP	GO:0060419	heart growth	4/88	0.0005498	0.0031181	0.0014122	Apc/Ctnnb1/Map2k4/Mapk14
7-day post-SCI group vs. the control group	BP	GO:0032963	collagen metabolic process	4/88	0.0005668	0.0032036	0.0014509	Ctsl/Hif1a/Id1/Prkcd
7-day post-SCI group vs. the control group	BP	GO:1905954	positive regulation of lipid localization	4/88	0.0005668	0.0032036	0.0014509	Apoe/Cd36/Prkcd/Trem2
7-day post-SCI group vs. the control group	BP	GO:0010828	positive regulation of glucose transmembrane trans	3/88	0.00057	0.0032051	0.0014516	Mapk14/Nfe2l2/Prkcd
7-day post-SCI group vs. the control group	BP	GO:0043392	negative regulation of DNA binding	3/88	0.00057	0.0032051	0.0014516	Fbxw7/Id1/Jun
7-day post-SCI group vs. the control group	BP	GO:0046676	negative regulation of insulin secretion	3/88	0.00057	0.0032051	0.0014516	Fkbp1b/Ppp3ca/Ucp2
7-day post-SCI group vs. the control group	BP	GO:0030258	lipid modification	5/88	0.000581	0.0032536	0.0014736	Apoe/Cd36/Lcat/Mapk14/Ppargc1a
7-day post-SCI group vs. the control group	BP	GO:0046390	ribose phosphate biosynthetic process	5/88	0.000581	0.0032536	0.0014736	Ndufa12/Ndufa6/Pdk2/Ppargc1a/Trem2
7-day post-SCI group vs. the control group	BP	GO:0032490	detection of molecule of bacterial origin	2/88	0.0005914	0.0032536	0.0014736	Tlr4/Trem2
7-day post-SCI group vs. the control group	BP	GO:0035331	negative regulation of hippo signaling	2/88	0.0005914	0.0032536	0.0014736	Map2k3/Mapk14
7-day post-SCI group vs. the control group	BP	GO:0036005	response to macrophage colony-stimulating factor	2/88	0.0005914	0.0032536	0.0014736	Tlr4/Trem2
7-day post-SCI group vs. the control group	BP	GO:0036006	cellular response to macrophage colony-stimulatin	2/88	0.0005914	0.0032536	0.0014736	Tlr4/Trem2
7-day post-SCI group vs. the control group	BP	GO:0042762	regulation of sulfur metabolic process	2/88	0.0005914	0.0032536	0.0014736	Nfe2l2/Pdk2
7-day post-SCI group vs. the control group	BP	GO:0043587	tongue morphogenesis	2/88	0.0005914	0.0032536	0.0014736	Ctnnb1/Hdac1
7-day post-SCI group vs. the control group	BP	GO:0045741	positive regulation of epidermal growth factor-acti	2/88	0.0005914	0.0032536	0.0014736	Fbxw7/Hbegf
7-day post-SCI group vs. the control group	BP	GO:0060767	epithelial cell proliferation involved in prostate gla	2/88	0.0005914	0.0032536	0.0014736	Apc/Ctnnb1
7-day post-SCI group vs. the control group	BP	GO:0061418	regulation of transcription from RNA polymerase	2/88	0.0005914	0.0032536	0.0014736	Hif1a/Nfe2l2
7-day post-SCI group vs. the control group	BP	GO:1900227	positive regulation of NLRP3 inflammasome com	2/88	0.0005914	0.0032536	0.0014736	Cd36/Tlr4
7-day post-SCI group vs. the control group	BP	GO:1902947	regulation of tau-protein kinase activity	2/88	0.0005914	0.0032536	0.0014736	Apoe/Clu
7-day post-SCI group vs. the control group	BP	GO:0014904	myotube cell development	3/88	0.0006022	0.0033076	0.001498	Gpx1/Ppp3ca/Rcan1
7-day post-SCI group vs. the control group	BP	GO:0009308	amine metabolic process	4/88	0.0006201	0.0034004	0.00154	Apc/Atp7a/Gch1/Vcam1
7-day post-SCI group vs. the control group	BP	GO:0072522	purine-containing compound biosynthetic process	5/88	0.00063	0.0034489	0.001562	Ndufa12/Ndufa6/Pdk2/Ppargc1a/Trem2
7-day post-SCI group vs. the control group	BP	GO:0043270	positive regulation of ion transport	6/88	0.0006355	0.0034621	0.001568	Atp7a/Mylk/Pink1/Ppp3ca/S100a1/Trem2
7-day post-SCI group vs. the control group	BP	GO:0007062	sister chromatid cohesion	3/88	0.0006355	0.0034621	0.001568	Cdc20/Ctnnb1/Fbxw7

7-day post-SCI group vs. the control group	BP	GO:0031113	regulation of microtubule polymerization	3/88	0.0006355	0.0034621	0.001568	Apc/Mapk8/Mapt
7-day post-SCI group vs. the control group	BP	GO:0042476	odontogenesis	4/88	0.0006576	0.0035703	0.001617	Apc/Ctnnb1/Hdac1/Itga6
7-day post-SCI group vs. the control group	BP	GO:0042752	regulation of circadian rhythm	4/88	0.0006576	0.0035703	0.001617	Fbxw7/Mapk10/Mapk8/Ppargc1a
7-day post-SCI group vs. the control group	BP	GO:0043406	positive regulation of MAP kinase activity	4/88	0.0006769	0.0036692	0.0016618	Adam9/Map2k4/Prkcd/Tlr4
7-day post-SCI group vs. the control group	BP	GO:0048732	gland development	7/88	0.0006844	0.0037037	0.0016774	Apc/Ctnnb1/Gpx1/Hif1a/Jun/Stat6/Xdh
7-day post-SCI group vs. the control group	BP	GO:0032060	bleb assembly	2/88	0.0006975	0.0037322	0.0016903	Mylk/Prdx6
7-day post-SCI group vs. the control group	BP	GO:0034375	high-density lipoprotein particle remodeling	2/88	0.0006975	0.0037322	0.0016903	Apoe/Lcat
7-day post-SCI group vs. the control group	BP	GO:0043922	negative regulation by host of viral transcription	2/88	0.0006975	0.0037322	0.0016903	Hdac1/Jun
7-day post-SCI group vs. the control group	BP	GO:0045603	positive regulation of endothelial cell differentiatic	2/88	0.0006975	0.0037322	0.0016903	Btg1/Ctnnb1
7-day post-SCI group vs. the control group	BP	GO:0051549	positive regulation of keratinocyte migration	2/88	0.0006975	0.0037322	0.0016903	Adam9/Hbegf
7-day post-SCI group vs. the control group	BP	GO:0097709	connective tissue replacement	2/88	0.0006975	0.0037322	0.0016903	Hif1a/Ppp3ca
7-day post-SCI group vs. the control group	BP	GO:0150065	regulation of deacetylase activity	2/88	0.0006975	0.0037322	0.0016903	Mapk8/Pink1
7-day post-SCI group vs. the control group	BP	GO:0030857	negative regulation of epithelial cell differentiatior	3/88	0.0007057	0.0037639	0.0017047	Ctnnb1/Id1/Xdh
7-day post-SCI group vs. the control group	BP	GO:0051489	regulation of filopodium assembly	3/88	0.0007057	0.0037639	0.0017047	Prkcd/Stau2/Trpm2
7-day post-SCI group vs. the control group	BP	GO:0016311	dephosphorylation	6/88	0.0007165	0.0038041	0.0017229	Fkbp1b/Pink1/Ppargc1b/Ppp3ca/Prkcd/Rcan1
7-day post-SCI group vs. the control group	BP	GO:0009142	nucleoside triphosphate biosynthetic process	4/88	0.0007167	0.0038041	0.0017229	Ndufa12/Ndufa6/Ppargc1a/Trem2
7-day post-SCI group vs. the control group	BP	GO:0060048	cardiac muscle contraction	4/88	0.0007167	0.0038041	0.0017229	Atp2a2/Fkbp1b/Map2k3/Slc8a1
7-day post-SCI group vs. the control group	BP	GO:0051651	maintenance of location in cell	5/88	0.0007232	0.0038323	0.0017357	Apoe/Fkbp1b/Pink1/Slc8a1/Trpm2
7-day post-SCI group vs. the control group	BP	GO:0042311	vasodilation	3/88	0.0007426	0.0039289	0.0017794	Apoe/Gch1/Gpx1
7-day post-SCI group vs. the control group	BP	GO:0018209	peptidyl-serine modification	6/88	0.0007489	0.0039559	0.0017916	Mapk14/Mapk8/Pdk2/Pink1/Prkcd/Ripk1
7-day post-SCI group vs. the control group	BP	GO:0014013	regulation of gliogenesis	4/88	0.0007795	0.0040916	0.0018531	Ctnnb1/Hdac1/Il6st/Trem2
7-day post-SCI group vs. the control group	BP	GO:0032414	positive regulation of ion transmembrane transport	4/88	0.0007795	0.0040916	0.0018531	Atp7a/Ppp3ca/S100a1/Trem2
7-day post-SCI group vs. the control group	BP	GO:0035601	protein deacylation	4/88	0.0007795	0.0040916	0.0018531	Hdac1/Mapt/Pink1/Rbbp7
7-day post-SCI group vs. the control group	BP	GO:0098732	macromolecule deacylation	4/88	0.0007795	0.0040916	0.0018531	Hdac1/Mapt/Pink1/Rbbp7
7-day post-SCI group vs. the control group	BP	GO:0050808	synapse organization	7/88	0.0007994	0.0041894	0.0018974	Apoe/Cdc20/Ctnnb1/Mapk14/Mapt/Stau2/Trem2
7-day post-SCI group vs. the control group	BP	GO:0097191	extrinsic apoptotic signaling pathway	5/88	0.000811	0.0042162	0.0019095	Gclc/Gpx1/Itga6/Mcl1/Ripk1
7-day post-SCI group vs. the control group	BP	GO:0006750	glutathione biosynthetic process	2/88	0.0008122	0.0042162	0.0019095	Gclc/Nfe2l2
7-day post-SCI group vs. the control group	BP	GO:0010940	positive regulation of necrotic cell death	2/88	0.0008122	0.0042162	0.0019095	Hebp2/Ripk1
7-day post-SCI group vs. the control group	BP	GO:0014820	tonic smooth muscle contraction	2/88	0.0008122	0.0042162	0.0019095	Cd38/Mylk
7-day post-SCI group vs. the control group	BP	GO:0046886	positive regulation of hormone biosynthetic proces	2/88	0.0008122	0.0042162	0.0019095	Hif1a/Ppargc1a
7-day post-SCI group vs. the control group	BP	GO:1902170	cellular response to reactive nitrogen species	2/88	0.0008122	0.0042162	0.0019095	Mapk8/Stat6
7-day post-SCI group vs. the control group	BP	GO:0040013	negative regulation of locomotion	6/88	0.0008171	0.0042349	0.001918	Aif1/Apoe/Hdac1/Nfe2l2/Ppargc1a/Rbbp7
7-day post-SCI group vs. the control group	BP	GO:0030003	cellular cation homeostasis	7/88	0.0008184	0.0042353	0.0019182	Apoe/Atp2a2/Atp7a/Fkbp1b/Hif1a/Slc8a1/Trpm2
7-day post-SCI group vs. the control group	BP	GO:0050773	regulation of dendrite development	4/88	0.0008235	0.004255	0.0019271	Cdc20/Id1/Ppp3ca/Stau2
7-day post-SCI group vs. the control group	BP	GO:0045088	regulation of innate immune response	5/88	0.0008419	0.0043434	0.0019671	Apoe/Ncf1/Nfe2l2/Tlr4/Trem2
7-day post-SCI group vs. the control group	BP	GO:0007586	digestion	4/88	0.0008461	0.0043516	0.0019708	Cd36/Ezr/Ppp3ca/Tlr4
7-day post-SCI group vs. the control group	BP	GO:0032874	positive regulation of stress-activated MAPK casc	4/88	0.0008461	0.0043516	0.0019708	Map2k4/Ripk1/Tlr4/Xdh
7-day post-SCI group vs. the control group	BP	GO:0042063	gliogenesis	6/88	0.0008529	0.0043794	0.0019834	Clu/Ctnnb1/Hdac1/Il6st/Tlr4/Trem2
7-day post-SCI group vs. the control group	BP	GO:0046777	protein autophosphorylation	5/88	0.0008577	0.0043975	0.0019916	Jun/Mapk14/Pink1/Prkcd/Ripk1
7-day post-SCI group vs. the control group	BP	GO:0032434	regulation of proteasomal ubiquitin-dependent pro	4/88	0.0008692	0.0044426	0.002012	Cdc20/Clu/Gclc/Mapk8

7-day post-SCI group vs. the control group	BP	GO:0034341	response to interferon-gamma	4/88	0.0008692	0.0044426	0.002012	Gch1/Sirpa/Stat1/Tlr4
7-day post-SCI group vs. the control group	BP	GO:0006119	oxidative phosphorylation	4/88	0.0008927	0.0045487	0.0020601	Atp7a/Ndufa12/Ndufa6/Pink1
7-day post-SCI group vs. the control group	BP	GO:0070304	positive regulation of stress-activated protein kina:	4/88	0.0008927	0.0045487	0.0020601	Map2k4/Ripk1/Tlr4/Xdh
7-day post-SCI group vs. the control group	BP	GO:0031122	cytoplasmic microtubule organization	3/88	0.0009023	0.0045706	0.00207	Apc/Dst/Ezr
7-day post-SCI group vs. the control group	BP	GO:0032655	regulation of interleukin-12 production	3/88	0.0009023	0.0045706	0.00207	Cd36/Mapk14/Tlr4
7-day post-SCI group vs. the control group	BP	GO:0032731	positive regulation of interleukin-1 beta productio	3/88	0.0009023	0.0045706	0.00207	Cd36/Hspb1/Tlr4
7-day post-SCI group vs. the control group	BP	GO:0050727	regulation of inflammatory response	6/88	0.0009025	0.0045706	0.00207	Apoe/Gpx1/Ncf1/Ripk1/Tlr4/Trem2
7-day post-SCI group vs. the control group	BP	GO:0002064	epithelial cell development	5/88	0.0009232	0.004668	0.0021141	Ezr/Gpx1/Hif1a/Id1/Il6st
7-day post-SCI group vs. the control group	BP	GO:0010917	negative regulation of mitochondrial membrane po	2/88	0.0009353	0.0046863	0.0021224	Hebp2/Mapt
7-day post-SCI group vs. the control group	BP	GO:0014733	regulation of skeletal muscle adaptation	2/88	0.0009353	0.0046863	0.0021224	Atp2a2/Ppp3ca
7-day post-SCI group vs. the control group	BP	GO:0051547	regulation of keratinocyte migration	2/88	0.0009353	0.0046863	0.0021224	Adam9/Hbegf
7-day post-SCI group vs. the control group	BP	GO:0061029	eyelid development in camera-type eye	2/88	0.0009353	0.0046863	0.0021224	Hdac1/Jun
7-day post-SCI group vs. the control group	BP	GO:2000271	positive regulation of fibroblast apoptotic process	2/88	0.0009353	0.0046863	0.0021224	Apc/Btg1
7-day post-SCI group vs. the control group	BP	GO:2001138	regulation of phospholipid transport	2/88	0.0009353	0.0046863	0.0021224	Apoe/Prkcd
7-day post-SCI group vs. the control group	BP	GO:0050870	positive regulation of T cell activation	5/88	0.0009401	0.0047011	0.0021291	Aif1/Il6st/Ppp3ca/Sirpa/Vcam1
7-day post-SCI group vs. the control group	BP	GO:0006690	icosanoid metabolic process	4/88	0.0009411	0.0047011	0.0021291	Gpx1/Ncf1/Ptgs1/Tlr4
7-day post-SCI group vs. the control group	BP	GO:0002831	regulation of response to biotic stimulus	6/88	0.0009544	0.0047531	0.0021527	Apoe/Ncf1/Nfe2l2/Stat1/Tlr4/Trem2
7-day post-SCI group vs. the control group	BP	GO:1903037	regulation of leukocyte cell-cell adhesion	6/88	0.0009544	0.0047531	0.0021527	Aif1/Hspb1/Il6st/Ppp3ca/Sirpa/Vcam1
7-day post-SCI group vs. the control group	BP	GO:0006275	regulation of DNA replication	4/88	0.0009659	0.0048035	0.0021755	Jun/Map2k4/Mapk8/Mcm4
7-day post-SCI group vs. the control group	BP	GO:0032613	interleukin-10 production	3/88	0.0009896	0.004892	0.0022156	Prkcd/Tlr4/Trem2
7-day post-SCI group vs. the control group	BP	GO:0051851	modulation by host of symbiont process	3/88	0.0009896	0.004892	0.0022156	Apoe/Hdac1/Jun
7-day post-SCI group vs. the control group	BP	GO:0061077	chaperone-mediated protein folding	3/88	0.0009896	0.004892	0.0022156	Clu/Fkbp1b/Hspb1
7-day post-SCI group vs. the control group	BP	GO:0090278	negative regulation of peptide hormone secretion	3/88	0.0009896	0.004892	0.0022156	Fkbp1b/Ppp3ca/Ucp2
7-day post-SCI group vs. the control group	BP	GO:0060828	regulation of canonical Wnt signaling pathway	5/88	0.0009922	0.0048977	0.0022181	Apc/Apoe/Ctnnb1/Hdac1/Mapk14
7-day post-SCI group vs. the control group	BP	GO:0046879	hormone secretion	6/88	0.0010085	0.0049703	0.002251	Cd38/Fkbp1b/Hif1a/Ppp3ca/Trpm2/Ucp2
7-day post-SCI group vs. the control group	BP	GO:0090316	positive regulation of intracellular protein transpor	4/88	0.001017	0.005005	0.0022667	Fbxw7/Mapk14/Prkcd/Prr5l
7-day post-SCI group vs. the control group	BP	GO:0035306	positive regulation of dephosphorylation	3/88	0.0010352	0.0050795	0.0023005	Pink1/Ppargc1b/Prkcd
7-day post-SCI group vs. the control group	BP	GO:0098930	axonal transport	3/88	0.0010352	0.0050795	0.0023005	Dst/Hif1a/Hspb1
7-day post-SCI group vs. the control group	BP	GO:0006470	protein dephosphorylation	5/88	0.0010465	0.0051274	0.0023222	Fkbp1b/Pink1/Ppp3ca/Prkcd/Rcan1
7-day post-SCI group vs. the control group	BP	GO:0019184	nonribosomal peptide biosynthetic process	2/88	0.0010668	0.0051654	0.0023394	Gclc/Nfe2l2
7-day post-SCI group vs. the control group	BP	GO:0034433	steroid esterification	2/88	0.0010668	0.0051654	0.0023394	Apoe/Lcat
7-day post-SCI group vs. the control group	BP	GO:0034434	sterol esterification	2/88	0.0010668	0.0051654	0.0023394	Apoe/Lcat
7-day post-SCI group vs. the control group	BP	GO:0034435	cholesterol esterification	2/88	0.0010668	0.0051654	0.0023394	Apoe/Lcat
7-day post-SCI group vs. the control group	BP	GO:0042428	serotonin metabolic process	2/88	0.0010668	0.0051654	0.0023394	Atp7a/Gch1
7-day post-SCI group vs. the control group	BP	GO:0051044	positive regulation of membrane protein ectodoma	2/88	0.0010668	0.0051654	0.0023394	Adam9/Apoe
7-day post-SCI group vs. the control group	BP	GO:0060788	ectodermal placode formation	2/88	0.0010668	0.0051654	0.0023394	Ctnnb1/Hdac1
7-day post-SCI group vs. the control group	BP	GO:0071697	ectodermal placode morphogenesis	2/88	0.0010668	0.0051654	0.0023394	Ctnnb1/Hdac1
7-day post-SCI group vs. the control group	BP	GO:0006639	acylglycerol metabolic process	4/88	0.00107	0.0051734	0.002343	Apoe/Cd36/Gpx1/Il6st
7-day post-SCI group vs. the control group	BP	GO:0002792	negative regulation of peptide secretion	3/88	0.0010822	0.0052095	0.0023594	Fkbp1b/Ppp3ca/Ucp2
7-day post-SCI group vs. the control group	BP	GO:0060038	cardiac muscle cell proliferation	3/88	0.0010822	0.0052095	0.0023594	Apc/Ctnnb1/Mapk14

7-day post-SCI group vs. the control group	BP	GO:0070265	necrotic cell death	3/88	0.0010822	0.0052095	0.0023594	Hebp2/Mapk8/Ripk1
7-day post-SCI group vs. the control group	BP	GO:0009914	hormone transport	6/88	0.0011237	0.0053993	0.0024453	Cd38/Fkbp1b/Hif1a/Ppp3ca/Trpm2/Ucp2
7-day post-SCI group vs. the control group	BP	GO:0006638	neutral lipid metabolic process	4/88	0.0011248	0.0053993	0.0024453	Apoe/Cd36/Gpx1/Il6st
7-day post-SCI group vs. the control group	BP	GO:0046328	regulation of JNK cascade	4/88	0.001153	0.0055236	0.0025016	Map2k4/Ripk1/Sirpa/Tlr4
7-day post-SCI group vs. the control group	BP	GO:0050900	leukocyte migration	6/88	0.0011541	0.0055236	0.0025016	Aif1/Itga6/Sirpa/Trem2/Trpm2/Vcam1
7-day post-SCI group vs. the control group	BP	GO:0061082	myeloid leukocyte cytokine production	3/88	0.00118	0.0056393	0.002554	Axl/Cd36/Tlr4
7-day post-SCI group vs. the control group	BP	GO:0009411	response to UV	4/88	0.0011817	0.0056393	0.002554	Fbxw7/Gpx1/Mapk8/Prkcd
7-day post-SCI group vs. the control group	BP	GO:0060100	positive regulation of phagocytosis, engulfment	2/88	0.0012066	0.005709	0.0025856	Cd36/Trem2
7-day post-SCI group vs. the control group	BP	GO:0061179	negative regulation of insulin secretion involved in	2/88	0.0012066	0.005709	0.0025856	Fkbp1b/Ucp2
7-day post-SCI group vs. the control group	BP	GO:0071696	ectodermal placode development	2/88	0.0012066	0.005709	0.0025856	Ctnnb1/Hdac1
7-day post-SCI group vs. the control group	BP	GO:0090083	regulation of inclusion body assembly	2/88	0.0012066	0.005709	0.0025856	Apoe/Clu
7-day post-SCI group vs. the control group	BP	GO:1905155	positive regulation of membrane invagination	2/88	0.0012066	0.005709	0.0025856	Cd36/Trem2
7-day post-SCI group vs. the control group	BP	GO:2001171	positive regulation of ATP biosynthetic process	2/88	0.0012066	0.005709	0.0025856	Ppargc1a/Trem2
7-day post-SCI group vs. the control group	BP	GO:0046324	regulation of glucose import	3/88	0.0012309	0.0058157	0.0026339	Mapk14/Nfe2l2/Prkcd
7-day post-SCI group vs. the control group	BP	GO:0006909	phagocytosis	6/88	0.0012327	0.0058157	0.0026339	Aif1/Axl/Cd36/Sirpa/Tlr4/Trem2
7-day post-SCI group vs. the control group	BP	GO:0046434	organophosphate catabolic process	4/88	0.0012706	0.0059862	0.0027111	Ncf1/Prdx6/Prkcd/Xdh
7-day post-SCI group vs. the control group	BP	GO:1904036	negative regulation of epithelial cell apoptotic proc	3/88	0.0012832	0.0060328	0.0027322	Apc/Id1/Nfe2l2
7-day post-SCI group vs. the control group	BP	GO:0009165	nucleotide biosynthetic process	5/88	0.001286	0.0060328	0.0027322	Ndufa12/Ndufa6/Pdk2/Ppargc1a/Trem2
7-day post-SCI group vs. the control group	BP	GO:0050807	regulation of synapse organization	5/88	0.001286	0.0060328	0.0027322	Apoe/Cdc20/Mapk14/Stau2/Trem2
7-day post-SCI group vs. the control group	BP	GO:0051092	positive regulation of NF-kappaB transcription fac	4/88	0.0013013	0.0060959	0.0027608	Cd36/Clu/Ripk1/Tlr4
7-day post-SCI group vs. the control group	BP	GO:0007626	locomotory behavior	5/88	0.0013295	0.0062103	0.0028126	Apoe/Atp7a/Mapk10/Mapt/Rcan1
7-day post-SCI group vs. the control group	BP	GO:0008643	carbohydrate transport	4/88	0.0013325	0.0062103	0.0028126	Ezr/Mapk14/Nfe2l2/Prkcd
7-day post-SCI group vs. the control group	BP	GO:0010761	fibroblast migration	3/88	0.0013369	0.0062103	0.0028126	Apc/Prr5l/Slc8a1
7-day post-SCI group vs. the control group	BP	GO:0014009	glial cell proliferation	3/88	0.0013369	0.0062103	0.0028126	Clu/Ctnnb1/Trem2
7-day post-SCI group vs. the control group	BP	GO:0055117	regulation of cardiac muscle contraction	3/88	0.0013369	0.0062103	0.0028126	Atp2a2/Fkbp1b/Slc8a1
7-day post-SCI group vs. the control group	BP	GO:2001244	positive regulation of intrinsic apoptotic signaling	3/88	0.0013369	0.0062103	0.0028126	Clu/Fbxw7/Mcl1
7-day post-SCI group vs. the control group	BP	GO:0002862	negative regulation of inflammatory response to an	2/88	0.0013548	0.0062319	0.0028224	Gpx1/Trem2
7-day post-SCI group vs. the control group	BP	GO:0007398	ectoderm development	2/88	0.0013548	0.0062319	0.0028224	Ctnnb1/Ets2
7-day post-SCI group vs. the control group	BP	GO:0035330	regulation of hippo signaling	2/88	0.0013548	0.0062319	0.0028224	Map2k3/Mapk14
7-day post-SCI group vs. the control group	BP	GO:0070102	interleukin-6-mediated signaling pathway	2/88	0.0013548	0.0062319	0.0028224	Il6st/Ripk1
7-day post-SCI group vs. the control group	BP	GO:0071280	cellular response to copper ion	2/88	0.0013548	0.0062319	0.0028224	Atp7a/Nfe2l2
7-day post-SCI group vs. the control group	BP	GO:1901160	primary amino compound metabolic process	2/88	0.0013548	0.0062319	0.0028224	Atp7a/Gch1
7-day post-SCI group vs. the control group	BP	GO:1903727	positive regulation of phospholipid metabolic proc	2/88	0.0013548	0.0062319	0.0028224	Capn2/Prkcd
7-day post-SCI group vs. the control group	BP	GO:0005996	monosaccharide metabolic process	5/88	0.0013741	0.0063122	0.0028588	Cd36/Gclc/Hif1a/Mapk14/Pdk2
7-day post-SCI group vs. the control group	BP	GO:0032922	circadian regulation of gene expression	3/88	0.001392	0.0063679	0.002884	Hdac1/Id1/Ppargc1a
7-day post-SCI group vs. the control group	BP	GO:0048678	response to axon injury	3/88	0.001392	0.0063679	0.002884	Fkbp1b/Jun/Trem2
7-day post-SCI group vs. the control group	BP	GO:1902895	positive regulation of miRNA transcription	3/88	0.001392	0.0063679	0.002884	Fos/Hif1a/Jun
7-day post-SCI group vs. the control group	BP	GO:1901293	nucleoside phosphate biosynthetic process	5/88	0.0013968	0.0063811	0.00289	Ndufa12/Ndufa6/Pdk2/Ppargc1a/Trem2
7-day post-SCI group vs. the control group	BP	GO:0048738	cardiac muscle tissue development	5/88	0.0014431	0.0065834	0.0029816	Apc/Ctnnb1/Map2k4/Mapk14/Slc8a1
7-day post-SCI group vs. the control group	BP	GO:0030888	regulation of B cell proliferation	3/88	0.0014485	0.0065899	0.0029845	Btk/Cd38/Tlr4

7-day post-SCI group vs. the control group	BP	GO:0048662	negative regulation of smooth muscle cell prolifera	3/88	0.0014485	0.0065899	0.0029845	Aif1/Apoe/Ppargc1a
7-day post-SCI group vs. the control group	BP	GO:0050803	regulation of synapse structure or activity	5/88	0.0014667	0.0066542	0.0030137	Apoe/Cdc20/Mapk14/Stau2/Trem2
7-day post-SCI group vs. the control group	BP	GO:1903039	positive regulation of leukocyte cell-cell adhesion	5/88	0.0014667	0.0066542	0.0030137	Aif1/Il6st/Ppp3ca/Sirpa/Vcam1
7-day post-SCI group vs. the control group	BP	GO:0001885	endothelial cell development	3/88	0.0015064	0.0067515	0.0030578	Ezr/Gpx1/Id1
7-day post-SCI group vs. the control group	BP	GO:0032732	positive regulation of interleukin-1 production	3/88	0.0015064	0.0067515	0.0030578	Cd36/Hspb1/Tlr4
7-day post-SCI group vs. the control group	BP	GO:0010544	negative regulation of platelet activation	2/88	0.0015112	0.0067515	0.0030578	Apoe/Prkcd
7-day post-SCI group vs. the control group	BP	GO:0043691	reverse cholesterol transport	2/88	0.0015112	0.0067515	0.0030578	Apoe/Lcat
7-day post-SCI group vs. the control group	BP	GO:0045837	negative regulation of membrane potential	2/88	0.0015112	0.0067515	0.0030578	Hebp2/Mapt
7-day post-SCI group vs. the control group	BP	GO:0060576	intestinal epithelial cell development	2/88	0.0015112	0.0067515	0.0030578	Hif1a/Il6st
7-day post-SCI group vs. the control group	BP	GO:1900452	regulation of long-term synaptic depression	2/88	0.0015112	0.0067515	0.0030578	Mapt/Stau2
7-day post-SCI group vs. the control group	BP	GO:1901741	positive regulation of myoblast fusion	2/88	0.0015112	0.0067515	0.0030578	Capn2/Mapk14
7-day post-SCI group vs. the control group	BP	GO:1902004	positive regulation of amyloid-beta formation	2/88	0.0015112	0.0067515	0.0030578	Apoe/Clu
7-day post-SCI group vs. the control group	BP	GO:1902430	negative regulation of amyloid-beta formation	2/88	0.0015112	0.0067515	0.0030578	Apoe/Clu
7-day post-SCI group vs. the control group	BP	GO:1903214	regulation of protein targeting to mitochondrion	2/88	0.0015112	0.0067515	0.0030578	Fbxw7/Pink1
7-day post-SCI group vs. the control group	BP	GO:0006898	receptor-mediated endocytosis	5/88	0.0015146	0.0067515	0.0030578	Apoe/Cd36/Clu/Ctsl/Ezr
7-day post-SCI group vs. the control group	BP	GO:0051258	protein polymerization	5/88	0.0015146	0.0067515	0.0030578	Aif1/Apc/Mapk8/Mapt/Prkcd
7-day post-SCI group vs. the control group	BP	GO:0034101	erythrocyte homeostasis	4/88	0.0015306	0.0068135	0.0030858	Axl/Hif1a/Mapk14/Stat1
7-day post-SCI group vs. the control group	BP	GO:0010507	negative regulation of autophagy	3/88	0.0015657	0.0069421	0.0031441	Mcl1/Pink1/Trem2
7-day post-SCI group vs. the control group	BP	GO:1903578	regulation of ATP metabolic process	3/88	0.0015657	0.0069421	0.0031441	Hif1a/Ppargc1a/Trem2
7-day post-SCI group vs. the control group	BP	GO:2000179	positive regulation of neural precursor cell prolifer	3/88	0.0015657	0.0069421	0.0031441	Ctnnb1/Hif1a/Mapk8
7-day post-SCI group vs. the control group	BP	GO:0007159	leukocyte cell-cell adhesion	6/88	0.0016085	0.0071223	0.0032257	Aif1/Hspb1/Il6st/Ppp3ca/Sirpa/Vcam1
7-day post-SCI group vs. the control group	BP	GO:0030879	mammary gland development	4/88	0.0016369	0.0072383	0.0032782	Gpx1/Hif1a/Stat6/Xdh
7-day post-SCI group vs. the control group	BP	GO:0036120	cellular response to platelet-derived growth factor	2/88	0.0016758	0.0073805	0.0033426	Atp7a/Tlr4
7-day post-SCI group vs. the control group	BP	GO:0043373	CD4-positive, alpha-beta T cell lineage commitme	2/88	0.0016758	0.0073805	0.0033426	Ctsl/Stat6
7-day post-SCI group vs. the control group	BP	GO:0090594	inflammatory response to wounding	2/88	0.0016758	0.0073805	0.0033426	Hif1a/Tlr4
7-day post-SCI group vs. the control group	BP	GO:0010675	regulation of cellular carbohydrate metabolic proc	4/88	0.0017106	0.0075241	0.0034076	Cd36/Hif1a/Pdk2/Ppargc1a
7-day post-SCI group vs. the control group	BP	GO:0006260	DNA replication	5/88	0.001718	0.0075466	0.0034179	Jun/Map2k4/Mapk8/Mcm4/Rbbp7
7-day post-SCI group vs. the control group	BP	GO:0050768	negative regulation of neurogenesis	4/88	0.0017483	0.0076674	0.0034726	Ctnnb1/Id1/Ppp3ca/Trem2
7-day post-SCI group vs. the control group	BP	GO:0042306	regulation of protein import into nucleus	3/88	0.0017525	0.0076674	0.0034726	Cd36/Mapk14/Prkcd
7-day post-SCI group vs. the control group	BP	GO:2000630	positive regulation of miRNA metabolic process	3/88	0.0017525	0.0076674	0.0034726	Fos/Hif1a/Jun
7-day post-SCI group vs. the control group	BP	GO:0045453	bone resorption	3/88	0.0018177	0.0079213	0.0035876	Cd38/Ctnnb1/Ppargc1b
7-day post-SCI group vs. the control group	BP	GO:0045669	positive regulation of osteoblast differentiation	3/88	0.0018177	0.0079213	0.0035876	Ctnnb1/Il6st/Ppp3ca
7-day post-SCI group vs. the control group	BP	GO:0061180	mammary gland epithelium development	3/88	0.0018177	0.0079213	0.0035876	Gpx1/Hif1a/Stat6
7-day post-SCI group vs. the control group	BP	GO:0001894	tissue homeostasis	5/88	0.001827	0.0079513	0.0036011	Cd38/Ctnnb1/Ppargc1b/Ptgs1/Tlr4
7-day post-SCI group vs. the control group	BP	GO:0007252	I-kappaB phosphorylation	2/88	0.0018485	0.0079925	0.0036198	Sirpa/Tlr4
7-day post-SCI group vs. the control group	BP	GO:0035584	calcium-mediated signaling using intracellular calc	2/88	0.0018485	0.0079925	0.0036198	Trpm2/Vcam1
7-day post-SCI group vs. the control group	BP	GO:0050665	hydrogen peroxide biosynthetic process	2/88	0.0018485	0.0079925	0.0036198	Cybb/Ncf1
7-day post-SCI group vs. the control group	BP	GO:0051546	keratinocyte migration	2/88	0.0018485	0.0079925	0.0036198	Adam9/Hbegf
7-day post-SCI group vs. the control group	BP	GO:0060099	regulation of phagocytosis, engulfment	2/88	0.0018485	0.0079925	0.0036198	Cd36/Trem2
7-day post-SCI group vs. the control group	BP	GO:0051017	actin filament bundle assembly	4/88	0.0018649	0.0080529	0.0036471	Aif1/Ezr/Id1/Itgb5

7-day post-SCI group vs. the control group	BP	GO:0001654	eye development	6/88	0.0018814	0.0081137	0.0036747	Apc/Ctnnb1/Hdac1/Hif1a/Jun/Stau2
7-day post-SCI group vs. the control group	BP	GO:0051961	negative regulation of nervous system developmer	4/88	0.0019049	0.0081936	0.0037109	Ctnnb1/Id1/Ppp3ca/Trem2
7-day post-SCI group vs. the control group	BP	GO:0062013	positive regulation of small molecule metabolic pr	4/88	0.0019049	0.0081936	0.0037109	Cd36/Hif1a/Ppargc1a/Trem2
7-day post-SCI group vs. the control group	BP	GO:0006941	striated muscle contraction	4/88	0.0019455	0.0083553	0.0037841	Atp2a2/Fkbp1b/Map2k3/Slc8a1
7-day post-SCI group vs. the control group	BP	GO:0150063	visual system development	6/88	0.0019491	0.0083553	0.0037841	Apc/Ctnnb1/Hdac1/Hif1a/Jun/Stau2
7-day post-SCI group vs. the control group	BP	GO:0007405	neuroblast proliferation	3/88	0.0019526	0.0083553	0.0037841	Ctnnb1/Hif1a/Mapk8
7-day post-SCI group vs. the control group	BP	GO:0048844	artery morphogenesis	3/88	0.0019526	0.0083553	0.0037841	Apoe/Ctnnb1/Mylk
7-day post-SCI group vs. the control group	BP	GO:0014706	striated muscle tissue development	5/88	0.0019702	0.0084196	0.0038132	Apc/Ctnnb1/Map2k4/Mapk14/Slc8a1
7-day post-SCI group vs. the control group	BP	GO:0061572	actin filament bundle organization	4/88	0.0019867	0.0084795	0.0038404	Aif1/Ezr/Id1/Itgb5
7-day post-SCI group vs. the control group	BP	GO:0006874	cellular calcium ion homeostasis	5/88	0.0019997	0.0085186	0.0038581	Apoe/Atp2a2/Fkbp1b/Slc8a1/Trpm2
7-day post-SCI group vs. the control group	BP	GO:0051896	regulation of protein kinase B signaling	4/88	0.0020286	0.0085186	0.0038581	Axl/Gpx1/Hbegf/Xdh
7-day post-SCI group vs. the control group	BP	GO:0002363	alpha-beta T cell lineage commitment	2/88	0.0020293	0.0085186	0.0038581	Ctsl/Stat6
7-day post-SCI group vs. the control group	BP	GO:0006656	phosphatidylcholine biosynthetic process	2/88	0.0020293	0.0085186	0.0038581	Capn2/Lcat
7-day post-SCI group vs. the control group	BP	GO:0010885	regulation of cholesterol storage	2/88	0.0020293	0.0085186	0.0038581	Cd36/Trem2
7-day post-SCI group vs. the control group	BP	GO:0030810	positive regulation of nucleotide biosynthetic proc	2/88	0.0020293	0.0085186	0.0038581	Ppargc1a/Trem2
7-day post-SCI group vs. the control group	BP	GO:0034377	plasma lipoprotein particle assembly	2/88	0.0020293	0.0085186	0.0038581	Apoe/Lcat
7-day post-SCI group vs. the control group	BP	GO:0044851	hair cycle phase	2/88	0.0020293	0.0085186	0.0038581	Ctnnb1/Ctsl
7-day post-SCI group vs. the control group	BP	GO:0150104	transport across blood-brain barrier	2/88	0.0020293	0.0085186	0.0038581	Apoe/Cd36
7-day post-SCI group vs. the control group	BP	GO:1900373	positive regulation of purine nucleotide biosynthet	2/88	0.0020293	0.0085186	0.0038581	Ppargc1a/Trem2
7-day post-SCI group vs. the control group	BP	GO:1901387	positive regulation of voltage-gated calcium chann	2/88	0.0020293	0.0085186	0.0038581	Ppp3ca/S100a1
7-day post-SCI group vs. the control group	BP	GO:1902992	negative regulation of amyloid precursor protein c	2/88	0.0020293	0.0085186	0.0038581	Apoe/Clu
7-day post-SCI group vs. the control group	BP	GO:1905153	regulation of membrane invagination	2/88	0.0020293	0.0085186	0.0038581	Cd36/Trem2
7-day post-SCI group vs. the control group	BP	GO:0048880	sensory system development	6/88	0.0020422	0.0085618	0.0038776	Apc/Ctnnb1/Hdac1/Hif1a/Jun/Stau2
7-day post-SCI group vs. the control group	BP	GO:0046785	microtubule polymerization	3/88	0.0020936	0.008755	0.0039651	Apc/Mapk8/Mapt
7-day post-SCI group vs. the control group	BP	GO:0070373	negative regulation of ERK1 and ERK2 cascade	3/88	0.0020936	0.008755	0.0039651	Ezr/Sirpa/Tlr4
7-day post-SCI group vs. the control group	BP	GO:0000422	autophagy of mitochondrion	3/88	0.0021664	0.0090366	0.0040927	Fbxw7/Hif1a/Pink1
7-day post-SCI group vs. the control group	BP	GO:0061726	mitochondrion disassembly	3/88	0.0021664	0.0090366	0.0040927	Fbxw7/Hif1a/Pink1
7-day post-SCI group vs. the control group	BP	GO:0010232	vascular transport	2/88	0.0022182	0.009172	0.004154	Apoe/Cd36
7-day post-SCI group vs. the control group	BP	GO:0010880	regulation of release of sequestered calcium ion in	2/88	0.0022182	0.009172	0.004154	Fkbp1b/Slc8a1
7-day post-SCI group vs. the control group	BP	GO:0030539	male genitalia development	2/88	0.0022182	0.009172	0.004154	Ctnnb1/Dhcr24
7-day post-SCI group vs. the control group	BP	GO:0036119	response to platelet-derived growth factor	2/88	0.0022182	0.009172	0.004154	Atp7a/Tlr4
7-day post-SCI group vs. the control group	BP	GO:0043501	skeletal muscle adaptation	2/88	0.0022182	0.009172	0.004154	Atp2a2/Ppp3ca
7-day post-SCI group vs. the control group	BP	GO:0051043	regulation of membrane protein ectodomain protec	2/88	0.0022182	0.009172	0.004154	Adam9/Apoe
7-day post-SCI group vs. the control group	BP	GO:1904385	cellular response to angiotensin	2/88	0.0022182	0.009172	0.004154	Nfe2l2/Prkcd
7-day post-SCI group vs. the control group	BP	GO:0022411	cellular component disassembly	6/88	0.0022385	0.0092191	0.0041753	Apc/Atp2a2/Fbxw7/Hif1a/Pink1/Trem2
7-day post-SCI group vs. the control group	BP	GO:0014855	striated muscle cell proliferation	3/88	0.0022408	0.0092191	0.0041753	Apc/Ctnnb1/Mapk14
7-day post-SCI group vs. the control group	BP	GO:0034103	regulation of tissue remodeling	3/88	0.0022408	0.0092191	0.0041753	Cd38/Ppargc1b/Ppp3ca
7-day post-SCI group vs. the control group	BP	GO:0046323	glucose import	3/88	0.0022408	0.0092191	0.0041753	Mapk14/Nfe2l2/Prkcd
7-day post-SCI group vs. the control group	BP	GO:1902905	positive regulation of supramolecular fiber organiz	4/88	0.0022468	0.0092274	0.0041791	Apc/Id1/Mapk8/Mapt
7-day post-SCI group vs. the control group	BP	GO:0060070	canonical Wnt signaling pathway	5/88	0.0022483	0.0092274	0.0041791	Apc/Apoe/Ctnnb1/Hdac1/Mapk14

7-day post-SCI group vs. the control group	BP	GO:0044262	cellular carbohydrate metabolic process	5/88	0.0023139	0.0094493	0.0042796	Cd36/Hif1a/Il6st/Pdk2/Ppargc1a
7-day post-SCI group vs. the control group	BP	GO:0061448	connective tissue development	5/88	0.0023139	0.0094493	0.0042796	Atp7a/Ctnnb1/Hif1a/Mapk14/Ppargc1a
7-day post-SCI group vs. the control group	BP	GO:0003151	outflow tract morphogenesis	3/88	0.0023167	0.0094493	0.0042796	Ctnnb1/Hif1a/Jun
7-day post-SCI group vs. the control group	BP	GO:0010921	regulation of phosphatase activity	3/88	0.0023167	0.0094493	0.0042796	Fkbp1b/Ppargc1b/Rcan1
7-day post-SCI group vs. the control group	BP	GO:0019229	regulation of vasoconstriction	3/88	0.0023167	0.0094493	0.0042796	Cd38/Hif1a/Ptgs1
7-day post-SCI group vs. the control group	BP	GO:0071356	cellular response to tumor necrosis factor	4/88	0.0023852	0.0096488	0.0043699	Mapk14/Nfe2l2/Ripk1/Stat1
7-day post-SCI group vs. the control group	BP	GO:0006942	regulation of striated muscle contraction	3/88	0.0023942	0.0096488	0.0043699	Atp2a2/Fkbp1b/Slc8a1
7-day post-SCI group vs. the control group	BP	GO:0048708	astrocyte differentiation	3/88	0.0023942	0.0096488	0.0043699	Il6st/Tlr4/Trem2
7-day post-SCI group vs. the control group	BP	GO:0061178	regulation of insulin secretion involved in cellular	3/88	0.0023942	0.0096488	0.0043699	Fkbp1b/Hif1a/Ucp2
7-day post-SCI group vs. the control group	BP	GO:0006691	leukotriene metabolic process	2/88	0.0024151	0.0096488	0.0043699	Ncf1/Tlr4
7-day post-SCI group vs. the control group	BP	GO:0010878	cholesterol storage	2/88	0.0024151	0.0096488	0.0043699	Cd36/Trem2
7-day post-SCI group vs. the control group	BP	GO:0014850	response to muscle activity	2/88	0.0024151	0.0096488	0.0043699	Hif1a/Ppargc1a
7-day post-SCI group vs. the control group	BP	GO:0032352	positive regulation of hormone metabolic process	2/88	0.0024151	0.0096488	0.0043699	Hif1a/Ppargc1a
7-day post-SCI group vs. the control group	BP	GO:0032930	positive regulation of superoxide anion generation	2/88	0.0024151	0.0096488	0.0043699	Mapt/Prkcd
7-day post-SCI group vs. the control group	BP	GO:0034383	low-density lipoprotein particle clearance	2/88	0.0024151	0.0096488	0.0043699	Cd36/Trem2
7-day post-SCI group vs. the control group	BP	GO:0034643	establishment of mitochondrion localization, micro	2/88	0.0024151	0.0096488	0.0043699	Hif1a/Mapt
7-day post-SCI group vs. the control group	BP	GO:0043369	CD4-positive or CD8-positive, alpha-beta T cell li	2/88	0.0024151	0.0096488	0.0043699	Ctsl/Stat6
7-day post-SCI group vs. the control group	BP	GO:0043586	tongue development	2/88	0.0024151	0.0096488	0.0043699	Ctnnb1/Hdac1
7-day post-SCI group vs. the control group	BP	GO:0047497	mitochondrion transport along microtubule	2/88	0.0024151	0.0096488	0.0043699	Hif1a/Mapt
7-day post-SCI group vs. the control group	BP	GO:0051481	negative regulation of cytosolic calcium ion conce	2/88	0.0024151	0.0096488	0.0043699	Il6st/Slc8a1
7-day post-SCI group vs. the control group	BP	GO:1900016	negative regulation of cytokine production involve	2/88	0.0024151	0.0096488	0.0043699	Sirpa/Trem2
7-day post-SCI group vs. the control group	BP	GO:1903428	positive regulation of reactive oxygen species bios	2/88	0.0024151	0.0096488	0.0043699	Cd36/Tlr4
7-day post-SCI group vs. the control group	BP	GO:1902893	regulation of miRNA transcription	3/88	0.0024733	0.0098574	0.0044644	Fos/Hif1a/Jun
7-day post-SCI group vs. the control group	BP	GO:2000736	regulation of stem cell differentiation	3/88	0.0024733	0.0098574	0.0044644	Hdac1/Nfe2l2/Rbbp7
7-day post-SCI group vs. the control group	BP	GO:0097553	calcium ion transmembrane import into cytosol	4/88	0.0024807	0.009875	0.0044724	Fkbp1b/Ppp3ca/Slc8a1/Trpm2
7-day post-SCI group vs. the control group	BP	GO:0035051	cardiocyte differentiation	4/88	0.0025294	0.0100569	0.0045547	Ctnnb1/Map2k4/Slc8a1/Vcam1
7-day post-SCI group vs. the control group	BP	GO:0010827	regulation of glucose transmembrane transport	3/88	0.002554	0.0101424	0.0045935	Mapk14/Nfe2l2/Prkcd
7-day post-SCI group vs. the control group	BP	GO:0007612	learning	4/88	0.0025788	0.0101726	0.0046072	Amph/Hif1a/Jun/Ptgs1
7-day post-SCI group vs. the control group	BP	GO:0043271	negative regulation of ion transport	4/88	0.0025788	0.0101726	0.0046072	Atp7a/Fkbp1b/Ppp3ca/Ptgs1
7-day post-SCI group vs. the control group	BP	GO:1905952	regulation of lipid localization	4/88	0.0025788	0.0101726	0.0046072	Apoe/Cd36/Prkcd/Trem2
7-day post-SCI group vs. the control group	BP	GO:0006631	fatty acid metabolic process	6/88	0.0026158	0.0101726	0.0046072	Cd36/Gpx1/Mapk14/Pdk2/Ppargc1a/Ptgs1
7-day post-SCI group vs. the control group	BP	GO:0034248	regulation of cellular amide metabolic process	6/88	0.0026158	0.0101726	0.0046072	Apoe/Clu/Nfe2l2/Pdk2/Pink1/Prkcd
7-day post-SCI group vs. the control group	BP	GO:0007263	nitric oxide mediated signal transduction	2/88	0.0026199	0.0101726	0.0046072	Apoe/Cd36
7-day post-SCI group vs. the control group	BP	GO:0010763	positive regulation of fibroblast migration	2/88	0.0026199	0.0101726	0.0046072	Apc/Slc8a1
7-day post-SCI group vs. the control group	BP	GO:0014808	release of sequestered calcium ion into cytosol by	2/88	0.0026199	0.0101726	0.0046072	Fkbp1b/Slc8a1
7-day post-SCI group vs. the control group	BP	GO:0030194	positive regulation of blood coagulation	2/88	0.0026199	0.0101726	0.0046072	Cd36/Nfe2l2
7-day post-SCI group vs. the control group	BP	GO:0070841	inclusion body assembly	2/88	0.0026199	0.0101726	0.0046072	Apoe/Clu
7-day post-SCI group vs. the control group	BP	GO:0097062	dendritic spine maintenance	2/88	0.0026199	0.0101726	0.0046072	Apoe/Trem2
7-day post-SCI group vs. the control group	BP	GO:1900048	positive regulation of hemostasis	2/88	0.0026199	0.0101726	0.0046072	Cd36/Nfe2l2
7-day post-SCI group vs. the control group	BP	GO:1901032	negative regulation of response to reactive oxygen	2/88	0.0026199	0.0101726	0.0046072	Nfe2l2/Pink1

7-day post-SCI group vs. the control group	BP	GO:1901739	regulation of myoblast fusion	2/88	0.0026199	0.0101726	0.0046072	Capn2/Mapk14
7-day post-SCI group vs. the control group	BP	GO:1902455	negative regulation of stem cell population mainte	2/88	0.0026199	0.0101726	0.0046072	Hdac1/Rbbp7
7-day post-SCI group vs. the control group	BP	GO:1902993	positive regulation of amyloid precursor protein ca	2/88	0.0026199	0.0101726	0.0046072	Apoe/Clu
7-day post-SCI group vs. the control group	BP	GO:1903206	negative regulation of hydrogen peroxide-induced	2/88	0.0026199	0.0101726	0.0046072	Nfe2l2/Pink1
7-day post-SCI group vs. the control group	BP	GO:1903798	regulation of miRNA maturation	2/88	0.0026199	0.0101726	0.0046072	Ppp3ca/Ripk1
7-day post-SCI group vs. the control group	BP	GO:2001169	regulation of ATP biosynthetic process	2/88	0.0026199	0.0101726	0.0046072	Ppargc1a/Trem2
7-day post-SCI group vs. the control group	BP	GO:0019915	lipid storage	3/88	0.0026363	0.0101883	0.0046143	Apoe/Cd36/Trem2
7-day post-SCI group vs. the control group	BP	GO:0033627	cell adhesion mediated by integrin	3/88	0.0026363	0.0101883	0.0046143	Adam9/Itga6/Itgb5
7-day post-SCI group vs. the control group	BP	GO:0050805	negative regulation of synaptic transmission	3/88	0.0026363	0.0101883	0.0046143	Cd38/Mapt/Stau2
7-day post-SCI group vs. the control group	BP	GO:0061614	miRNA transcription	3/88	0.0026363	0.0101883	0.0046143	Fos/Hif1a/Jun
7-day post-SCI group vs. the control group	BP	GO:0032273	positive regulation of protein polymerization	3/88	0.0027202	0.010476	0.0047446	Apc/Mapk8/Mapt
7-day post-SCI group vs. the control group	BP	GO:0046888	negative regulation of hormone secretion	3/88	0.0027202	0.010476	0.0047446	Fkbp1b/Ppp3ca/Ucp2
7-day post-SCI group vs. the control group	BP	GO:0050766	positive regulation of phagocytosis	3/88	0.0027202	0.010476	0.0047446	Cd36/Sirpa/Trem2
7-day post-SCI group vs. the control group	BP	GO:0032103	positive regulation of response to external stimulu	6/88	0.0027914	0.0107378	0.0048631	Aif1/Fkbp1b/Hspb1/Ripk1/Tlr4/Trem2
7-day post-SCI group vs. the control group	BP	GO:0031110	regulation of microtubule polymerization or depol	3/88	0.0028058	0.0107431	0.0048655	Apc/Mapk8/Mapt
7-day post-SCI group vs. the control group	BP	GO:0032024	positive regulation of insulin secretion	3/88	0.0028058	0.0107431	0.0048655	Cd38/Hif1a/Trpm2
7-day post-SCI group vs. the control group	BP	GO:0035050	embryonic heart tube development	3/88	0.0028058	0.0107431	0.0048655	Ctnnb1/Hif1a/Slc8a1
7-day post-SCI group vs. the control group	BP	GO:2001259	positive regulation of cation channel activity	3/88	0.0028058	0.0107431	0.0048655	Ppp3ca/S100a1/Trem2
7-day post-SCI group vs. the control group	BP	GO:0002082	regulation of oxidative phosphorylation	2/88	0.0028327	0.010759	0.0048727	Atp7a/Pink1
7-day post-SCI group vs. the control group	BP	GO:0010742	macrophage derived foam cell differentiation	2/88	0.0028327	0.010759	0.0048727	Cd36/Stat1
7-day post-SCI group vs. the control group	BP	GO:0032727	positive regulation of interferon-alpha production	2/88	0.0028327	0.010759	0.0048727	Stat1/Tlr4
7-day post-SCI group vs. the control group	BP	GO:0065005	protein-lipid complex assembly	2/88	0.0028327	0.010759	0.0048727	Apoe/Lcat
7-day post-SCI group vs. the control group	BP	GO:0097164	ammonium ion metabolic process	2/88	0.0028327	0.010759	0.0048727	Atp7a/Gch1
7-day post-SCI group vs. the control group	BP	GO:1903514	release of sequestered calcium ion into cytosol by	2/88	0.0028327	0.010759	0.0048727	Fkbp1b/Slc8a1
7-day post-SCI group vs. the control group	BP	GO:1990776	response to angiotensin	2/88	0.0028327	0.010759	0.0048727	Nfe2l2/Prkcd
7-day post-SCI group vs. the control group	BP	GO:0090287	regulation of cellular response to growth factor sti	5/88	0.0028892	0.0109504	0.0049594	Ctnnb1/Hdac1/Hif1a/Rbbp7/Xdh
7-day post-SCI group vs. the control group	BP	GO:0043367	CD4-positive, alpha-beta T cell differentiation	3/88	0.002893	0.0109504	0.0049594	Atp7a/Ctsl/Stat6
7-day post-SCI group vs. the control group	BP	GO:0048814	regulation of dendrite morphogenesis	3/88	0.002893	0.0109504	0.0049594	Id1/Ppp3ca/Stau2
7-day post-SCI group vs. the control group	BP	GO:0030111	regulation of Wnt signaling pathway	5/88	0.0029283	0.0110712	0.0050141	Apc/Apoe/Ctnnb1/Hdac1/Mapk14
7-day post-SCI group vs. the control group	BP	GO:0002697	regulation of immune effector process	6/88	0.0029444	0.0111196	0.005036	Axl/Cd36/Ncf1/Stat6/Tlr4/Trem2
7-day post-SCI group vs. the control group	BP	GO:0034504	protein localization to nucleus	5/88	0.0029677	0.0111821	0.0050644	Cd36/Mapk14/Ppp3ca/Prkcd/Txnip
7-day post-SCI group vs. the control group	BP	GO:0072503	cellular divalent inorganic cation homeostasis	5/88	0.0029677	0.0111821	0.0050644	Apoe/Atp2a2/Fkbp1b/Slc8a1/Trpm2
7-day post-SCI group vs. the control group	BP	GO:0042475	odontogenesis of dentin-containing tooth	3/88	0.0029819	0.0112227	0.0050827	Ctnnb1/Hdac1/Itga6
7-day post-SCI group vs. the control group	BP	GO:0009713	catechol-containing compound biosynthetic proces	2/88	0.0030533	0.0113368	0.0051344	Atp7a/Gch1
7-day post-SCI group vs. the control group	BP	GO:0014829	vascular associated smooth muscle contraction	2/88	0.0030533	0.0113368	0.0051344	Cd38/Slc8a1
7-day post-SCI group vs. the control group	BP	GO:0034368	protein-lipid complex remodeling	2/88	0.0030533	0.0113368	0.0051344	Apoe/Lcat
7-day post-SCI group vs. the control group	BP	GO:0034369	plasma lipoprotein particle remodeling	2/88	0.0030533	0.0113368	0.0051344	Apoe/Lcat
7-day post-SCI group vs. the control group	BP	GO:0036003	positive regulation of transcription from RNA poly	2/88	0.0030533	0.0113368	0.0051344	Hif1a/Nfe2l2
7-day post-SCI group vs. the control group	BP	GO:0042423	catecholamine biosynthetic process	2/88	0.0030533	0.0113368	0.0051344	Atp7a/Gch1
7-day post-SCI group vs. the control group	BP	GO:0051443	positive regulation of ubiquitin-protein transferase	2/88	0.0030533	0.0113368	0.0051344	Cdc20/Fbxw7

7-day post-SCI group vs. the control group	BP	GO:0051654	establishment of mitochondrion localization	2/88	0.0030533	0.0113368	0.0051344	Hif1a/Mapt
7-day post-SCI group vs. the control group	BP	GO:0060575	intestinal epithelial cell differentiation	2/88	0.0030533	0.0113368	0.0051344	Hif1a/Il6st
7-day post-SCI group vs. the control group	BP	GO:0070920	regulation of production of small RNA involved ir	2/88	0.0030533	0.0113368	0.0051344	Ppp3ca/Ripk1
7-day post-SCI group vs. the control group	BP	GO:1901857	positive regulation of cellular respiration	2/88	0.0030533	0.0113368	0.0051344	Pink1/Ppargc1a
7-day post-SCI group vs. the control group	BP	GO:2000269	regulation of fibroblast apoptotic process	2/88	0.0030533	0.0113368	0.0051344	Apc/Btg1
7-day post-SCI group vs. the control group	BP	GO:0044272	sulfur compound biosynthetic process	3/88	0.0030724	0.0113823	0.005155	Gclc/Nfe2l2/Pdk2
7-day post-SCI group vs. the control group	BP	GO:0072655	establishment of protein localization to mitochond	3/88	0.0030724	0.0113823	0.005155	Fbxw7/Mapt/Pink1
7-day post-SCI group vs. the control group	BP	GO:0055074	calcium ion homeostasis	5/88	0.0031295	0.0115808	0.0052449	Apoe/Atp2a2/Fkbp1b/Slc8a1/Trpm2
7-day post-SCI group vs. the control group	BP	GO:0022904	respiratory electron transport chain	3/88	0.0031646	0.0116977	0.0052979	Ndufa12/Pink1/Ppargc1a
7-day post-SCI group vs. the control group	BP	GO:0006109	regulation of carbohydrate metabolic process	4/88	0.0032238	0.0119031	0.0053909	Cd36/Hif1a/Pdk2/Ppargc1a
7-day post-SCI group vs. the control group	BP	GO:0001570	vasculogenesis	3/88	0.0032585	0.0119058	0.0053921	Ctnnb1/Fbxw7/Xdh
7-day post-SCI group vs. the control group	BP	GO:0035773	insulin secretion involved in cellular response to g	3/88	0.0032585	0.0119058	0.0053921	Fkbp1b/Hif1a/Ucp2
7-day post-SCI group vs. the control group	BP	GO:0003299	muscle hypertrophy in response to stress	2/88	0.0032817	0.0119058	0.0053921	Atp2a2/Ppp3ca
7-day post-SCI group vs. the control group	BP	GO:0006536	glutamate metabolic process	2/88	0.0032817	0.0119058	0.0053921	Apc/Gclc
7-day post-SCI group vs. the control group	BP	GO:0007176	regulation of epidermal growth factor-activated re	2/88	0.0032817	0.0119058	0.0053921	Fbxw7/Hbegf
7-day post-SCI group vs. the control group	BP	GO:0014898	cardiac muscle hypertrophy in response to stress	2/88	0.0032817	0.0119058	0.0053921	Atp2a2/Ppp3ca
7-day post-SCI group vs. the control group	BP	GO:0032928	regulation of superoxide anion generation	2/88	0.0032817	0.0119058	0.0053921	Mapt/Prkcd
7-day post-SCI group vs. the control group	BP	GO:0042430	indole-containing compound metabolic process	2/88	0.0032817	0.0119058	0.0053921	Atp7a/Gch1
7-day post-SCI group vs. the control group	BP	GO:0045822	negative regulation of heart contraction	2/88	0.0032817	0.0119058	0.0053921	Atp2a2/Fkbp1b
7-day post-SCI group vs. the control group	BP	GO:0046716	muscle cell cellular homeostasis	2/88	0.0032817	0.0119058	0.0053921	Apc/Hif1a
7-day post-SCI group vs. the control group	BP	GO:0048820	hair follicle maturation	2/88	0.0032817	0.0119058	0.0053921	Ctnnb1/Ctsl
7-day post-SCI group vs. the control group	BP	GO:0050820	positive regulation of coagulation	2/88	0.0032817	0.0119058	0.0053921	Cd36/Nfe2l2
7-day post-SCI group vs. the control group	BP	GO:0090077	foam cell differentiation	2/88	0.0032817	0.0119058	0.0053921	Cd36/Stat1
7-day post-SCI group vs. the control group	BP	GO:0098581	detection of external biotic stimulus	2/88	0.0032817	0.0119058	0.0053921	Tlr4/Trem2
7-day post-SCI group vs. the control group	BP	GO:1900017	positive regulation of cytokine production involve	2/88	0.0032817	0.0119058	0.0053921	Hif1a/Tlr4
7-day post-SCI group vs. the control group	BP	GO:0001764	neuron migration	4/88	0.003282	0.0119058	0.0053921	Axl/Ctnnb1/Mapk8/Mapt
7-day post-SCI group vs. the control group	BP	GO:0006911	phagocytosis, engulfment	4/88	0.003341	0.0120933	0.005477	Aif1/Cd36/Sirpa/Trem2
7-day post-SCI group vs. the control group	BP	GO:1903169	regulation of calcium ion transmembrane transport	4/88	0.003341	0.0120933	0.005477	Fkbp1b/Ppp3ca/S100a1/Slc8a1
7-day post-SCI group vs. the control group	BP	GO:0032091	negative regulation of protein binding	3/88	0.0033541	0.0121141	0.0054865	Atp2a2/Id1/Mapk8
7-day post-SCI group vs. the control group	BP	GO:1903321	negative regulation of protein modification by sma	3/88	0.0033541	0.0121141	0.0054865	Cdc20/Ctnnb1/Gclc
7-day post-SCI group vs. the control group	BP	GO:0051495	positive regulation of cytoskeleton organization	4/88	0.0034007	0.0122691	0.0055567	Apc/Id1/Mapk8/Mapt
7-day post-SCI group vs. the control group	BP	GO:0062207	regulation of pattern recognition receptor signaling	3/88	0.0034514	0.0124249	0.0056272	Cd36/Tlr4/Trem2
7-day post-SCI group vs. the control group	BP	GO:1901019	regulation of calcium ion transmembrane transport	3/88	0.0034514	0.0124249	0.0056272	Fkbp1b/Ppp3ca/S100a1
7-day post-SCI group vs. the control group	BP	GO:0014887	cardiac muscle adaptation	2/88	0.0035178	0.0125684	0.0056922	Atp2a2/Ppp3ca
7-day post-SCI group vs. the control group	BP	GO:0034367	protein-containing complex remodeling	2/88	0.0035178	0.0125684	0.0056922	Apoe/Lcat
7-day post-SCI group vs. the control group	BP	GO:0042659	regulation of cell fate specification	2/88	0.0035178	0.0125684	0.0056922	Hdac1/Rbbp7
7-day post-SCI group vs. the control group	BP	GO:0051450	myoblast proliferation	2/88	0.0035178	0.0125684	0.0056922	Ctnnb1/Gpx1
7-day post-SCI group vs. the control group	BP	GO:0060571	morphogenesis of an epithelial fold	2/88	0.0035178	0.0125684	0.0056922	Ctnnb1/Hif1a
7-day post-SCI group vs. the control group	BP	GO:1901863	positive regulation of muscle tissue development	2/88	0.0035178	0.0125684	0.0056922	Ctnnb1/Ppargc1a
7-day post-SCI group vs. the control group	BP	GO:1903649	regulation of cytoplasmic transport	2/88	0.0035178	0.0125684	0.0056922	Ezr/Mapk8

7-day post-SCI group vs. the control group	BP	GO:0071902	positive regulation of protein serine/threonine kina	4/88	0.0035223	0.0125708	0.0056933	Adam9/Map2k4/Prkcd/Tlr4
7-day post-SCI group vs. the control group	BP	GO:0016575	histone deacetylation	3/88	0.0035504	0.0126574	0.0057325	Hdac1/Pink1/Rbbp7
7-day post-SCI group vs. the control group	BP	GO:0046890	regulation of lipid biosynthetic process	4/88	0.0035841	0.0127367	0.0057684	Apoe/Capn2/Ppargc1a/Prkcd
7-day post-SCI group vs. the control group	BP	GO:0048736	appendage development	4/88	0.0035841	0.0127367	0.0057684	Atp7a/Ctnnb1/Hdac1/Itga6
7-day post-SCI group vs. the control group	BP	GO:0060173	limb development	4/88	0.0035841	0.0127367	0.0057684	Atp7a/Ctnnb1/Hdac1/Itga6
7-day post-SCI group vs. the control group	BP	GO:0002262	myeloid cell homeostasis	4/88	0.0036467	0.0128707	0.0058291	Axl/Hif1a/Mapk14/Stat1
7-day post-SCI group vs. the control group	BP	GO:0032271	regulation of protein polymerization	4/88	0.0036467	0.0128707	0.0058291	Apc/Mapk8/Mapt/Prkcd
7-day post-SCI group vs. the control group	BP	GO:0009798	axis specification	3/88	0.0036511	0.0128707	0.0058291	Apc/Ctnnb1/Ets2
7-day post-SCI group vs. the control group	BP	GO:0032370	positive regulation of lipid transport	3/88	0.0036511	0.0128707	0.0058291	Apoe/Prkcd/Trem2
7-day post-SCI group vs. the control group	BP	GO:0043502	regulation of muscle adaptation	3/88	0.0036511	0.0128707	0.0058291	Aif1/Atp2a2/Ppp3ca
7-day post-SCI group vs. the control group	BP	GO:0070585	protein localization to mitochondrion	3/88	0.0036511	0.0128707	0.0058291	Fbxw7/Mapt/Pink1
7-day post-SCI group vs. the control group	BP	GO:0071214	cellular response to abiotic stimulus	5/88	0.0036529	0.0128707	0.0058291	Ctnnb1/Fbxw7/Mapk14/Mylk/Prkcd
7-day post-SCI group vs. the control group	BP	GO:0104004	cellular response to environmental stimulus	5/88	0.0036529	0.0128707	0.0058291	Ctnnb1/Fbxw7/Mapk14/Mylk/Prkcd
7-day post-SCI group vs. the control group	BP	GO:0002700	regulation of production of molecular mediator of	4/88	0.0037101	0.0130444	0.0059078	Axl/Cd36/Stat6/Tlr4
7-day post-SCI group vs. the control group	BP	GO:0043491	protein kinase B signaling	4/88	0.0037101	0.0130444	0.0059078	Axl/Gpx1/Hbegf/Xdh
7-day post-SCI group vs. the control group	BP	GO:0097061	dendritic spine organization	3/88	0.0037535	0.013142	0.005952	Apoe/Stau2/Trem2
7-day post-SCI group vs. the control group	BP	GO:2000628	regulation of miRNA metabolic process	3/88	0.0037535	0.013142	0.005952	Fos/Hif1a/Jun
7-day post-SCI group vs. the control group	BP	GO:0043032	positive regulation of macrophage activation	2/88	0.0037616	0.013142	0.005952	Tlr4/Trem2
7-day post-SCI group vs. the control group	BP	GO:0046885	regulation of hormone biosynthetic process	2/88	0.0037616	0.013142	0.005952	Hif1a/Ppargc1a
7-day post-SCI group vs. the control group	BP	GO:0060907	positive regulation of macrophage cytokine produc	2/88	0.0037616	0.013142	0.005952	Cd36/Tlr4
7-day post-SCI group vs. the control group	BP	GO:1903523	negative regulation of blood circulation	2/88	0.0037616	0.013142	0.005952	Atp2a2/Fkbp1b
7-day post-SCI group vs. the control group	BP	GO:0019827	stem cell population maintenance	4/88	0.0037742	0.0131719	0.0059655	Apc/Hdac1/Mapk8/Rbbp7
7-day post-SCI group vs. the control group	BP	GO:0046330	positive regulation of JNK cascade	3/88	0.0038577	0.0134492	0.0060911	Map2k4/Ripk1/Tlr4
7-day post-SCI group vs. the control group	BP	GO:0099024	plasma membrane invagination	4/88	0.0039046	0.0135985	0.0061587	Aif1/Cd36/Sirpa/Trem2
7-day post-SCI group vs. the control group	BP	GO:0030307	positive regulation of cell growth	4/88	0.003971	0.0138005	0.0062502	Apoe/Cd38/Hbegf/Mapt
7-day post-SCI group vs. the control group	BP	GO:0043281	regulation of cysteine-type endopeptidase activity	4/88	0.003971	0.0138005	0.0062502	Dhcr24/Gpx1/Stat1/Xdh
7-day post-SCI group vs. the control group	BP	GO:0002026	regulation of the force of heart contraction	2/88	0.0040132	0.0139033	0.0062968	Atp2a2/Slc8a1
7-day post-SCI group vs. the control group	BP	GO:0032647	regulation of interferon-alpha production	2/88	0.0040132	0.0139033	0.0062968	Stat1/Tlr4
7-day post-SCI group vs. the control group	BP	GO:0071354	cellular response to interleukin-6	2/88	0.0040132	0.0139033	0.0062968	Il6st/Ripk1
7-day post-SCI group vs. the control group	BP	GO:0046849	bone remodeling	3/88	0.0040714	0.0140902	0.0063815	Cd38/Ctnnb1/Ppargc1b
7-day post-SCI group vs. the control group	BP	GO:0031669	cellular response to nutrient levels	4/88	0.004106	0.0141656	0.0064156	Jun/Mapk8/Nfe2l2/Pdk2
7-day post-SCI group vs. the control group	BP	GO:0034767	positive regulation of ion transmembrane transport	4/88	0.004106	0.0141656	0.0064156	Atp7a/Ppp3ca/S100a1/Trem2
7-day post-SCI group vs. the control group	BP	GO:0098727	maintenance of cell number	4/88	0.004106	0.0141656	0.0064156	Apc/Hdac1/Mapk8/Rbbp7
7-day post-SCI group vs. the control group	BP	GO:0006913	nucleocytoplasmic transport	5/88	0.0041855	0.0144101	0.0065263	Cd36/Mapk14/Ppp3ca/Prkcd/Txnip
7-day post-SCI group vs. the control group	BP	GO:0051169	nuclear transport	5/88	0.0041855	0.0144101	0.0065263	Cd36/Mapk14/Ppp3ca/Prkcd/Txnip
7-day post-SCI group vs. the control group	BP	GO:0002360	T cell lineage commitment	2/88	0.0042723	0.0145874	0.0066066	Ctsl/Stat6
7-day post-SCI group vs. the control group	BP	GO:0010875	positive regulation of cholesterol efflux	2/88	0.0042723	0.0145874	0.0066066	Apoe/Trem2
7-day post-SCI group vs. the control group	BP	GO:0045742	positive regulation of epidermal growth factor rece	2/88	0.0042723	0.0145874	0.0066066	Fbxw7/Hbegf
7-day post-SCI group vs. the control group	BP	GO:0060795	cell fate commitment involved in formation of prii	2/88	0.0042723	0.0145874	0.0066066	Ctnnb1/Ets2
7-day post-SCI group vs. the control group	BP	GO:1901099	negative regulation of signal transduction in absen	2/88	0.0042723	0.0145874	0.0066066	Mcl1/Ripk1

7-day post-SCI group vs. the control group	BP	GO:1904752	regulation of vascular associated smooth muscle c	2/88	0.0042723	0.0145874	0.0066066	Atp7a/Nfe2l2
7-day post-SCI group vs. the control group	BP	GO:2000352	negative regulation of endothelial cell apoptotic pr	2/88	0.0042723	0.0145874	0.0066066	Id1/Nfe2l2
7-day post-SCI group vs. the control group	BP	GO:2001240	negative regulation of extrinsic apoptotic signaling	2/88	0.0042723	0.0145874	0.0066066	Mcl1/Ripk1
7-day post-SCI group vs. the control group	BP	GO:0003300	cardiac muscle hypertrophy	3/88	0.0042921	0.0145948	0.00661	Atp2a2/Map2k4/Ppp3ca
7-day post-SCI group vs. the control group	BP	GO:0009953	dorsal/ventral pattern formation	3/88	0.0042921	0.0145948	0.00661	Apc/Ctnnb1/Mapk8
7-day post-SCI group vs. the control group	BP	GO:0022900	electron transport chain	3/88	0.0042921	0.0145948	0.00661	Ndufa12/Pink1/Ppargc1a
7-day post-SCI group vs. the control group	BP	GO:0032479	regulation of type I interferon production	3/88	0.0042921	0.0145948	0.00661	Sirpa/Stat1/Tlr4
7-day post-SCI group vs. the control group	BP	GO:0051216	cartilage development	4/88	0.0043142	0.0146551	0.0066373	Atp7a/Ctnnb1/Hif1a/Mapk14
7-day post-SCI group vs. the control group	BP	GO:0006006	glucose metabolic process	4/88	0.0043852	0.0148809	0.0067395	Cd36/Hif1a/Mapk14/Pdk2
7-day post-SCI group vs. the control group	BP	GO:0007006	mitochondrial membrane organization	3/88	0.0044051	0.0149027	0.0067494	Gclc/Hebp2/Pink1
7-day post-SCI group vs. the control group	BP	GO:0032755	positive regulation of interleukin-6 production	3/88	0.0044051	0.0149027	0.0067494	Aif1/Cd36/Tlr4
7-day post-SCI group vs. the control group	BP	GO:1904063	negative regulation of cation transmembrane trans	3/88	0.0044051	0.0149027	0.0067494	Atp7a/Fkbp1b/Ppp3ca
7-day post-SCI group vs. the control group	BP	GO:0010324	membrane invagination	4/88	0.004457	0.015032	0.006808	Aif1/Cd36/Sirpa/Trem2
7-day post-SCI group vs. the control group	BP	GO:0035265	organ growth	4/88	0.004457	0.015032	0.006808	Apc/Ctnnb1/Map2k4/Mapk14
7-day post-SCI group vs. the control group	BP	GO:0048639	positive regulation of developmental growth	4/88	0.004457	0.015032	0.006808	Apoe/Ezr/Mapk14/Mapt
7-day post-SCI group vs. the control group	BP	GO:0016525	negative regulation of angiogenesis	3/88	0.0045199	0.0152135	0.0068902	Cd36/Ctnnb1/Stat1
7-day post-SCI group vs. the control group	BP	GO:0043255	regulation of carbohydrate biosynthetic process	3/88	0.0045199	0.0152135	0.0068902	Cd36/Hif1a/Pdk2
7-day post-SCI group vs. the control group	BP	GO:0000819	sister chromatid segregation	4/88	0.0045295	0.0152156	0.0068911	Apc/Cdc20/Ctnnb1/Fbxw7
7-day post-SCI group vs. the control group	BP	GO:0033598	mammary gland epithelial cell proliferation	2/88	0.004539	0.0152156	0.0068911	Gpx1/Stat6
7-day post-SCI group vs. the control group	BP	GO:0044346	fibroblast apoptotic process	2/88	0.004539	0.0152156	0.0068911	Apc/Btg1
7-day post-SCI group vs. the control group	BP	GO:0070741	response to interleukin-6	2/88	0.004539	0.0152156	0.0068911	Il6st/Ripk1
7-day post-SCI group vs. the control group	BP	GO:0009100	glycoprotein metabolic process	5/88	0.0046063	0.0154101	0.0069792	Atp7a/Ctnnb1/Ctsl/Hbegf/Hif1a
7-day post-SCI group vs. the control group	BP	GO:0021700	developmental maturation	5/88	0.0046063	0.0154101	0.0069792	Btk/Cdc20/Ctnnb1/Ctsl/Hif1a
7-day post-SCI group vs. the control group	BP	GO:0014074	response to purine-containing compound	3/88	0.0046366	0.0154801	0.0070109	Ezr/Slc8a1/Trpm2
7-day post-SCI group vs. the control group	BP	GO:0071456	cellular response to hypoxia	3/88	0.0046366	0.0154801	0.0070109	Hif1a/Nfe2l2/Pink1
7-day post-SCI group vs. the control group	BP	GO:0043393	regulation of protein binding	4/88	0.004677	0.0155993	0.0070649	Apoe/Atp2a2/Id1/Mapk8
7-day post-SCI group vs. the control group	BP	GO:0014897	striated muscle hypertrophy	3/88	0.004755	0.0158118	0.0071611	Atp2a2/Map2k4/Ppp3ca
7-day post-SCI group vs. the control group	BP	GO:0106027	neuron projection organization	3/88	0.004755	0.0158118	0.0071611	Apoe/Stau2/Trem2
7-day post-SCI group vs. the control group	BP	GO:2000181	negative regulation of blood vessel morphogenesis	3/88	0.004755	0.0158118	0.0071611	Cd36/Ctnnb1/Stat1
7-day post-SCI group vs. the control group	BP	GO:0032607	interferon-alpha production	2/88	0.0048132	0.0159731	0.0072342	Stat1/Tlr4
7-day post-SCI group vs. the control group	BP	GO:1901186	positive regulation of ERBB signaling pathway	2/88	0.0048132	0.0159731	0.0072342	Fbxw7/Hbegf
7-day post-SCI group vs. the control group	BP	GO:0072507	divalent inorganic cation homeostasis	5/88	0.0048276	0.0160049	0.0072486	Apoe/Atp2a2/Fkbp1b/Slc8a1/Trpm2
7-day post-SCI group vs. the control group	BP	GO:0032606	type I interferon production	3/88	0.0048753	0.0161146	0.0072983	Sirpa/Stat1/Tlr4
7-day post-SCI group vs. the control group	BP	GO:0071346	cellular response to interferon-gamma	3/88	0.0048753	0.0161146	0.0072983	Sirpa/Stat1/Tlr4
7-day post-SCI group vs. the control group	BP	GO:1901343	negative regulation of vasculature development	3/88	0.0048753	0.0161146	0.0072983	Cd36/Ctnnb1/Stat1
7-day post-SCI group vs. the control group	BP	GO:0052548	regulation of endopeptidase activity	5/88	0.004941	0.0163157	0.0073893	Dhcr24/Gpx1/Hdac1/Stat1/Xdh
7-day post-SCI group vs. the control group	BP	GO:0002027	regulation of heart rate	3/88	0.0049973	0.0164525	0.0074513	Fkbp1b/Gch1/Slc8a1
7-day post-SCI group vs. the control group	BP	GO:0014896	muscle hypertrophy	3/88	0.0049973	0.0164525	0.0074513	Atp2a2/Map2k4/Ppp3ca
7-day post-SCI group vs. the control group	BP	GO:1902106	negative regulation of leukocyte differentiation	3/88	0.0049973	0.0164525	0.0074513	Ctnnb1/Fbxw7/Hspb1
7-day post-SCI group vs. the control group	BP	GO:0014002	astrocyte development	2/88	0.0050948	0.0167567	0.0075891	Tlr4/Trem2

7-day post-SCI group vs. the control group	BP	GO:0010906	regulation of glucose metabolic process	3/88	0.0051212	0.016827	0.0076209	Cd36/Hif1a/Pdk2
7-day post-SCI group vs. the control group	BP	GO:0007589	body fluid secretion	3/88	0.0053746	0.0176027	0.0079722	Hif1a/Ppp3ca/Xdh
7-day post-SCI group vs. the control group	BP	GO:0060840	artery development	3/88	0.0053746	0.0176027	0.0079722	Apoe/Ctnnb1/Mylk
7-day post-SCI group vs. the control group	BP	GO:1904659	glucose transmembrane transport	3/88	0.0053746	0.0176027	0.0079722	Mapk14/Nfe2l2/Prkcd
7-day post-SCI group vs. the control group	BP	GO:0001702	gastrulation with mouth forming second	2/88	0.0053839	0.0176027	0.0079722	Ctnnb1/Ets2
7-day post-SCI group vs. the control group	BP	GO:0048710	regulation of astrocyte differentiation	2/88	0.0053839	0.0176027	0.0079722	Il6st/Trem2
7-day post-SCI group vs. the control group	BP	GO:0008645	hexose transmembrane transport	3/88	0.0055041	0.0179247	0.0081181	Mapk14/Nfe2l2/Prkcd
7-day post-SCI group vs. the control group	BP	GO:0010586	miRNA metabolic process	3/88	0.0055041	0.0179247	0.0081181	Fos/Hif1a/Jun
7-day post-SCI group vs. the control group	BP	GO:0034766	negative regulation of ion transmembrane transpor	3/88	0.0055041	0.0179247	0.0081181	Atp7a/Fkbp1b/Ppp3ca
7-day post-SCI group vs. the control group	BP	GO:1903707	negative regulation of hemopoiesis	3/88	0.0055041	0.0179247	0.0081181	Ctnnb1/Fbxw7/Hspb1
7-day post-SCI group vs. the control group	BP	GO:0048545	response to steroid hormone	4/88	0.0055462	0.0180264	0.0081641	Adam9/Hdac1/Lcat/Ppargc1b
7-day post-SCI group vs. the control group	BP	GO:0050679	positive regulation of epithelial cell proliferation	4/88	0.0055462	0.0180264	0.0081641	Atp7a/Ctnnb1/Id1/Jun
7-day post-SCI group vs. the control group	BP	GO:0001892	embryonic placenta development	3/88	0.0056354	0.0182124	0.0082484	Hif1a/Slc8a1/Vcam1
7-day post-SCI group vs. the control group	BP	GO:0019395	fatty acid oxidation	3/88	0.0056354	0.0182124	0.0082484	Cd36/Mapk14/Ppargc1a
7-day post-SCI group vs. the control group	BP	GO:0051897	positive regulation of protein kinase B signaling	3/88	0.0056354	0.0182124	0.0082484	Axl/Gpx1/Hbegf
7-day post-SCI group vs. the control group	BP	GO:0009954	proximal/distal pattern formation	2/88	0.0056804	0.0182124	0.0082484	Apc/Ctnnb1
7-day post-SCI group vs. the control group	BP	GO:0016540	protein autoprocessing	2/88	0.0056804	0.0182124	0.0082484	Capn2/Ctsl
7-day post-SCI group vs. the control group	BP	GO:0019934	cGMP-mediated signaling	2/88	0.0056804	0.0182124	0.0082484	Apoe/Cd36
7-day post-SCI group vs. the control group	BP	GO:0035329	hippo signaling	2/88	0.0056804	0.0182124	0.0082484	Map2k3/Mapk14
7-day post-SCI group vs. the control group	BP	GO:0045671	negative regulation of osteoclast differentiation	2/88	0.0056804	0.0182124	0.0082484	Ctnnb1/Fbxw7
7-day post-SCI group vs. the control group	BP	GO:0045686	negative regulation of glial cell differentiation	2/88	0.0056804	0.0182124	0.0082484	Ctnnb1/Trem2
7-day post-SCI group vs. the control group	BP	GO:0046677	response to antibiotic	2/88	0.0056804	0.0182124	0.0082484	Hif1a/Id1
7-day post-SCI group vs. the control group	BP	GO:0070542	response to fatty acid	2/88	0.0056804	0.0182124	0.0082484	Cd36/Tlr4
7-day post-SCI group vs. the control group	BP	GO:0090075	relaxation of muscle	2/88	0.0056804	0.0182124	0.0082484	Atp2a2/Slc8a1
7-day post-SCI group vs. the control group	BP	GO:1902003	regulation of amyloid-beta formation	2/88	0.0056804	0.0182124	0.0082484	Apoe/Clu
7-day post-SCI group vs. the control group	BP	GO:1904738	vascular associated smooth muscle cell migration	2/88	0.0056804	0.0182124	0.0082484	Atp7a/Nfe2l2
7-day post-SCI group vs. the control group	BP	GO:0007204	positive regulation of cytosolic calcium ion concer	4/88	0.005715	0.018288	0.0082826	Cd36/Cd38/Fkbp1b/Slc8a1
7-day post-SCI group vs. the control group	BP	GO:1901617	organic hydroxy compound biosynthetic process	4/88	0.005715	0.018288	0.0082826	Apoe/Atp7a/Dhcr24/Gch1
7-day post-SCI group vs. the control group	BP	GO:0045445	myoblast differentiation	3/88	0.0057686	0.0184238	0.0083441	Btg1/Gpx1/Mapk14
7-day post-SCI group vs. the control group	BP	GO:0048709	oligodendrocyte differentiation	3/88	0.0057686	0.0184238	0.0083441	Clu/Ctnnb1/Hdac1
7-day post-SCI group vs. the control group	BP	GO:0043010	camera-type eye development	5/88	0.005853	0.0186755	0.0084581	Apc/Ctnnb1/Hdac1/Hif1a/Jun
7-day post-SCI group vs. the control group	BP	GO:0030324	lung development	4/88	0.0058872	0.0187647	0.0084985	Atp7a/Ctnnb1/Fbxw7/Id1
7-day post-SCI group vs. the control group	BP	GO:0002062	chondrocyte differentiation	3/88	0.0059036	0.0187647	0.0084985	Ctnnb1/Hif1a/Mapk14
7-day post-SCI group vs. the control group	BP	GO:0015749	monosaccharide transmembrane transport	3/88	0.0059036	0.0187647	0.0084985	Mapk14/Nfe2l2/Prkcd
7-day post-SCI group vs. the control group	BP	GO:0051209	release of sequestered calcium ion into cytosol	3/88	0.0059036	0.0187647	0.0084985	Fkbp1b/Slc8a1/Trpm2
7-day post-SCI group vs. the control group	BP	GO:0048167	regulation of synaptic plasticity	5/88	0.005983	0.0189479	0.0085815	Apoe/Cd38/Cdc20/Mapt/Stau2
7-day post-SCI group vs. the control group	BP	GO:0009595	detection of biotic stimulus	2/88	0.0059842	0.0189479	0.0085815	Tlr4/Trem2
7-day post-SCI group vs. the control group	BP	GO:0031281	positive regulation of cyclase activity	2/88	0.0059842	0.0189479	0.0085815	Mapk14/Mapk8
7-day post-SCI group vs. the control group	BP	GO:1903580	positive regulation of ATP metabolic process	2/88	0.0059842	0.0189479	0.0085815	Ppargc1a/Trem2
7-day post-SCI group vs. the control group	BP	GO:0006090	pyruvate metabolic process	3/88	0.0060406	0.0190719	0.0086376	Hif1a/Pdk2/Ppargc1a

7-day post-SCI group vs. the control group	BP	GO:0044106	cellular amine metabolic process	3/88	0.0060406	0.0190719	0.0086376	Apc/Atp7a/Gch1
7-day post-SCI group vs. the control group	BP	GO:0051283	negative regulation of sequestering of calcium ion	3/88	0.0060406	0.0190719	0.0086376	Fkbp1b/Slc8a1/Trpm2
7-day post-SCI group vs. the control group	BP	GO:0030323	respiratory tube development	4/88	0.0061521	0.0194053	0.0087886	Atp7a/Ctnnb1/Fbxw7/Id1
7-day post-SCI group vs. the control group	BP	GO:0021782	glial cell development	3/88	0.0061794	0.0194362	0.0088026	Clu/Tlr4/Trem2
7-day post-SCI group vs. the control group	BP	GO:0098659	inorganic cation import across plasma membrane	3/88	0.0061794	0.0194362	0.0088026	Ppp3ca/Slc8a1/Trpm2
7-day post-SCI group vs. the control group	BP	GO:0099587	inorganic ion import across plasma membrane	3/88	0.0061794	0.0194362	0.0088026	Ppp3ca/Slc8a1/Trpm2
7-day post-SCI group vs. the control group	BP	GO:0014823	response to activity	2/88	0.0062952	0.0197255	0.0089336	Hif1a/Ppargc1a
7-day post-SCI group vs. the control group	BP	GO:0033238	regulation of cellular amine metabolic process	2/88	0.0062952	0.0197255	0.0089336	Apc/Atp7a
7-day post-SCI group vs. the control group	BP	GO:0048265	response to pain	2/88	0.0062952	0.0197255	0.0089336	Capn2/Gch1
7-day post-SCI group vs. the control group	BP	GO:0071320	cellular response to cAMP	2/88	0.0062952	0.0197255	0.0089336	Ezr/Slc8a1
7-day post-SCI group vs. the control group	BP	GO:0001676	long-chain fatty acid metabolic process	3/88	0.0063202	0.0197289	0.0089352	Cd36/Gpx1/Ptgs1
7-day post-SCI group vs. the control group	BP	GO:0050764	regulation of phagocytosis	3/88	0.0063202	0.0197289	0.0089352	Cd36/Sirpa/Trem2
7-day post-SCI group vs. the control group	BP	GO:0051282	regulation of sequestering of calcium ion	3/88	0.0063202	0.0197289	0.0089352	Fkbp1b/Slc8a1/Trpm2
7-day post-SCI group vs. the control group	BP	GO:1903008	organelle disassembly	3/88	0.0063202	0.0197289	0.0089352	Fbxw7/Hif1a/Pink1
7-day post-SCI group vs. the control group	BP	GO:0045930	negative regulation of mitotic cell cycle	4/88	0.0063329	0.0197501	0.0089448	Apc/Btg1/Cdc20/Ctnnb1
7-day post-SCI group vs. the control group	BP	GO:0009913	epidermal cell differentiation	4/88	0.0064247	0.0200174	0.0090659	Hdac1/Ppp3ca/Ptgs1/Txnip
7-day post-SCI group vs. the control group	BP	GO:0045786	negative regulation of cell cycle	5/88	0.006454	0.0200898	0.0090987	Apc/Btg1/Cdc20/Ctnnb1/Mapk14
7-day post-SCI group vs. the control group	BP	GO:0002224	toll-like receptor signaling pathway	3/88	0.0064628	0.0200984	0.0091026	Cd36/Tlr4/Trem2
7-day post-SCI group vs. the control group	BP	GO:2000116	regulation of cysteine-type endopeptidase activity	4/88	0.0065173	0.0202489	0.0091707	Dhcr24/Gpx1/Stat1/Xdh
7-day post-SCI group vs. the control group	BP	GO:0015748	organophosphate ester transport	3/88	0.0066074	0.020338	0.0092111	Apoe/Prkcd/Slc25a24
7-day post-SCI group vs. the control group	BP	GO:0031109	microtubule polymerization or depolymerization	3/88	0.0066074	0.020338	0.0092111	Apc/Mapk8/Mapt
7-day post-SCI group vs. the control group	BP	GO:0031398	positive regulation of protein ubiquitination	3/88	0.0066074	0.020338	0.0092111	Cdc20/Fbxw7/Mapk8
7-day post-SCI group vs. the control group	BP	GO:0035710	CD4-positive, alpha-beta T cell activation	3/88	0.0066074	0.020338	0.0092111	Atp7a/Ctsl/Stat6
7-day post-SCI group vs. the control group	BP	GO:0051208	sequestering of calcium ion	3/88	0.0066074	0.020338	0.0092111	Fkbp1b/Slc8a1/Trpm2
7-day post-SCI group vs. the control group	BP	GO:0090277	positive regulation of peptide hormone secretion	3/88	0.0066074	0.020338	0.0092111	Cd38/Hif1a/Trpm2
7-day post-SCI group vs. the control group	BP	GO:2000177	regulation of neural precursor cell proliferation	3/88	0.0066074	0.020338	0.0092111	Ctnnb1/Hif1a/Mapk8
7-day post-SCI group vs. the control group	BP	GO:0002861	regulation of inflammatory response to antigenic s	2/88	0.0066135	0.020338	0.0092111	Gpx1/Trem2
7-day post-SCI group vs. the control group	BP	GO:0002931	response to ischemia	2/88	0.0066135	0.020338	0.0092111	Rcan1/Trem2
7-day post-SCI group vs. the control group	BP	GO:1901020	negative regulation of calcium ion transmembrane	2/88	0.0066135	0.020338	0.0092111	Fkbp1b/Ppp3ca
7-day post-SCI group vs. the control group	BP	GO:1904707	positive regulation of vascular associated smooth m	2/88	0.0066135	0.020338	0.0092111	Jun/Ppargc1a
7-day post-SCI group vs. the control group	BP	GO:0002718	regulation of cytokine production involved in imm	3/88	0.0067538	0.020731	0.009389	Axl/Cd36/Tlr4
7-day post-SCI group vs. the control group	BP	GO:0034440	lipid oxidation	3/88	0.0067538	0.020731	0.009389	Cd36/Mapk14/Ppargc1a
7-day post-SCI group vs. the control group	BP	GO:0018958	phenol-containing compound metabolic process	3/88	0.0069022	0.0211473	0.0095776	Atp7a/Ctsl/Gch1
7-day post-SCI group vs. the control group	BP	GO:0034219	carbohydrate transmembrane transport	3/88	0.0069022	0.0211473	0.0095776	Mapk14/Nfe2l2/Prkcd
7-day post-SCI group vs. the control group	BP	GO:0010939	regulation of necrotic cell death	2/88	0.006939	0.0211622	0.0095843	Hebp2/Ripk1
7-day post-SCI group vs. the control group	BP	GO:0032689	negative regulation of interferon-gamma productic	2/88	0.006939	0.0211622	0.0095843	Axl/Tlr4
7-day post-SCI group vs. the control group	BP	GO:0032733	positive regulation of interleukin-10 production	2/88	0.006939	0.0211622	0.0095843	Tlr4/Trem2
7-day post-SCI group vs. the control group	BP	GO:0043368	positive T cell selection	2/88	0.006939	0.0211622	0.0095843	Ctsl/Stat6
7-day post-SCI group vs. the control group	BP	GO:0071827	plasma lipoprotein particle organization	2/88	0.006939	0.0211622	0.0095843	Apoe/Lcat
7-day post-SCI group vs. the control group	BP	GO:0043500	muscle adaptation	3/88	0.0070526	0.0214688	0.0097232	Aif1/Atp2a2/Ppp3ca

7-day post-SCI group vs. the control group	BP	GO:0048565	digestive tract development	3/88	0.0070526	0.0214688	0.0097232	Ctnnb1/Hif1a/Il6st
7-day post-SCI group vs. the control group	BP	GO:0032868	response to insulin	4/88	0.0071907	0.021852	0.0098967	Apc/Mapk14/Pdk2/Prkcd
7-day post-SCI group vs. the control group	BP	GO:0002793	positive regulation of peptide secretion	3/88	0.0072048	0.021852	0.0098967	Cd38/Hif1a/Trpm2
7-day post-SCI group vs. the control group	BP	GO:0008286	insulin receptor signaling pathway	3/88	0.0072048	0.021852	0.0098967	Apc/Pdk2/Prkcd
7-day post-SCI group vs. the control group	BP	GO:0046632	alpha-beta T cell differentiation	3/88	0.0072048	0.021852	0.0098967	Atp7a/Ctsl/Stat6
7-day post-SCI group vs. the control group	BP	GO:0038084	vascular endothelial growth factor signaling pathw	2/88	0.0072717	0.0219944	0.0099612	Hspb1/Xdh
7-day post-SCI group vs. the control group	BP	GO:0061081	positive regulation of myeloid leukocyte cytokine	2/88	0.0072717	0.0219944	0.0099612	Cd36/Tlr4
7-day post-SCI group vs. the control group	BP	GO:1903725	regulation of phospholipid metabolic process	2/88	0.0072717	0.0219944	0.0099612	Capn2/Prkcd
7-day post-SCI group vs. the control group	BP	GO:0032535	regulation of cellular component size	5/88	0.0073969	0.0223526	0.0101235	Apoe/Atp7a/Ezr/Mapt/Prkcd
7-day post-SCI group vs. the control group	BP	GO:0019318	hexose metabolic process	4/88	0.0074928	0.0226218	0.0102454	Cd36/Hif1a/Mapk14/Pdk2
7-day post-SCI group vs. the control group	BP	GO:0031929	TOR signaling	3/88	0.0075152	0.0226688	0.0102666	Hif1a/Prr5l/Trem2
7-day post-SCI group vs. the control group	BP	GO:0006984	ER-nucleus signaling pathway	2/88	0.0076114	0.0227519	0.0103043	Atp2a2/Nfe2l2
7-day post-SCI group vs. the control group	BP	GO:0009309	amine biosynthetic process	2/88	0.0076114	0.0227519	0.0103043	Atp7a/Gch1
7-day post-SCI group vs. the control group	BP	GO:0010453	regulation of cell fate commitment	2/88	0.0076114	0.0227519	0.0103043	Hdac1/Rbbp7
7-day post-SCI group vs. the control group	BP	GO:0010874	regulation of cholesterol efflux	2/88	0.0076114	0.0227519	0.0103043	Apoe/Trem2
7-day post-SCI group vs. the control group	BP	GO:0035794	positive regulation of mitochondrial membrane pe	2/88	0.0076114	0.0227519	0.0103043	Gclc/Hebp2
7-day post-SCI group vs. the control group	BP	GO:0042401	cellular biogenic amine biosynthetic process	2/88	0.0076114	0.0227519	0.0103043	Atp7a/Gch1
7-day post-SCI group vs. the control group	BP	GO:0045981	positive regulation of nucleotide metabolic proces	2/88	0.0076114	0.0227519	0.0103043	Ppargc1a/Trem2
7-day post-SCI group vs. the control group	BP	GO:0070050	neuron cellular homeostasis	2/88	0.0076114	0.0227519	0.0103043	Atp2a2/Atp7a
7-day post-SCI group vs. the control group	BP	GO:0070884	regulation of calcineurin-NFAT signaling cascade	2/88	0.0076114	0.0227519	0.0103043	Ppp3ca/Rcan1
7-day post-SCI group vs. the control group	BP	GO:1900544	positive regulation of purine nucleotide metabolic	2/88	0.0076114	0.0227519	0.0103043	Ppargc1a/Trem2
7-day post-SCI group vs. the control group	BP	GO:0046822	regulation of nucleocytoplasmic transport	3/88	0.0076733	0.0229161	0.0103787	Cd36/Mapk14/Prkcd
7-day post-SCI group vs. the control group	BP	GO:0048638	regulation of developmental growth	5/88	0.0077054	0.0229912	0.0104127	Apc/Apoe/Ezr/Mapk14/Mapt
7-day post-SCI group vs. the control group	BP	GO:0050892	intestinal absorption	2/88	0.0079582	0.0236392	0.0107061	Cd36/Ezr
7-day post-SCI group vs. the control group	BP	GO:0060964	regulation of miRNA-mediated gene silencing	2/88	0.0079582	0.0236392	0.0107061	Ppp3ca/Ripk1
7-day post-SCI group vs. the control group	BP	GO:0097028	dendritic cell differentiation	2/88	0.0079582	0.0236392	0.0107061	Trem2/Trpm2
7-day post-SCI group vs. the control group	BP	GO:0106056	regulation of calcineurin-mediated signaling	2/88	0.0079582	0.0236392	0.0107061	Ppp3ca/Rcan1
7-day post-SCI group vs. the control group	BP	GO:1902991	regulation of amyloid precursor protein catabolic p	2/88	0.0079582	0.0236392	0.0107061	Apoe/Clu
7-day post-SCI group vs. the control group	BP	GO:0030900	forebrain development	5/88	0.0081035	0.0240275	0.010882	Atp7a/Axl/Ctnnb1/Hdac1/Hif1a
7-day post-SCI group vs. the control group	BP	GO:0032102	negative regulation of response to external stimulu	5/88	0.0081035	0.0240275	0.010882	Aif1/Apoe/Gpx1/Prkcd/Trem2
7-day post-SCI group vs. the control group	BP	GO:0036294	cellular response to decreased oxygen levels	3/88	0.0081594	0.0241716	0.0109473	Hif1a/Nfe2l2/Pink1
7-day post-SCI group vs. the control group	BP	GO:0099003	vesicle-mediated transport in synapse	4/88	0.0082301	0.0243592	0.0110322	Amph/Atp2a2/Ctnnb1/Mapk10
7-day post-SCI group vs. the control group	BP	GO:0006509	membrane protein ectodomain proteolysis	2/88	0.008312	0.0244706	0.0110827	Adam9/Apoe
7-day post-SCI group vs. the control group	BP	GO:0045940	positive regulation of steroid metabolic process	2/88	0.008312	0.0244706	0.0110827	Apoe/Ppargc1a
7-day post-SCI group vs. the control group	BP	GO:0050850	positive regulation of calcium-mediated signaling	2/88	0.008312	0.0244706	0.0110827	Ppp3ca/Trem2
7-day post-SCI group vs. the control group	BP	GO:0061098	positive regulation of protein tyrosine kinase activ	2/88	0.008312	0.0244706	0.0110827	Fbxw7/Hbegf
7-day post-SCI group vs. the control group	BP	GO:0071825	protein-lipid complex subunit organization	2/88	0.008312	0.0244706	0.0110827	Apoe/Lcat
7-day post-SCI group vs. the control group	BP	GO:2001239	regulation of extrinsic apoptotic signaling pathway	2/88	0.008312	0.0244706	0.0110827	Mcl1/Ripk1
7-day post-SCI group vs. the control group	BP	GO:0035966	response to topologically incorrect protein	3/88	0.0086632	0.0253301	0.011472	Clu/Hspb1/Nfe2l2
7-day post-SCI group vs. the control group	BP	GO:0007094	mitotic spindle assembly checkpoint signaling	2/88	0.0086728	0.0253301	0.011472	Apc/Cdc20

7-day post-SCI group vs. the control group	BP	GO:0032373	positive regulation of sterol transport	2/88	0.0086728	0.0253301	0.011472	Apoe/Trem2
7-day post-SCI group vs. the control group	BP	GO:0032376	positive regulation of cholesterol transport	2/88	0.0086728	0.0253301	0.011472	Apoe/Trem2
7-day post-SCI group vs. the control group	BP	GO:0034142	toll-like receptor 4 signaling pathway	2/88	0.0086728	0.0253301	0.011472	Tlr4/Trem2
7-day post-SCI group vs. the control group	BP	GO:0060147	regulation of post-transcriptional gene silencing	2/88	0.0086728	0.0253301	0.011472	Ppp3ca/Ripk1
7-day post-SCI group vs. the control group	BP	GO:0071173	spindle assembly checkpoint signaling	2/88	0.0086728	0.0253301	0.011472	Apc/Cdc20
7-day post-SCI group vs. the control group	BP	GO:0071542	dopaminergic neuron differentiation	2/88	0.0086728	0.0253301	0.011472	Ctnnb1/Hif1a
7-day post-SCI group vs. the control group	BP	GO:1900368	regulation of post-transcriptional gene silencing by	2/88	0.0086728	0.0253301	0.011472	Ppp3ca/Ripk1
7-day post-SCI group vs. the control group	BP	GO:0032970	regulation of actin filament-based process	5/88	0.0088558	0.0258418	0.0117037	Atp2a2/Id1/Prkcd/Stau2/Trpm2
7-day post-SCI group vs. the control group	BP	GO:0030278	regulation of ossification	3/88	0.0090091	0.0261732	0.0118538	Hif1a/Mapk14/Slc8a1
7-day post-SCI group vs. the control group	BP	GO:0060541	respiratory system development	4/88	0.0090135	0.0261732	0.0118538	Atp7a/Ctnnb1/Fbxw7/Id1
7-day post-SCI group vs. the control group	BP	GO:0010559	regulation of glycoprotein biosynthetic process	2/88	0.0090405	0.0261732	0.0118538	Ctnnb1/Hbegf
7-day post-SCI group vs. the control group	BP	GO:0010907	positive regulation of glucose metabolic process	2/88	0.0090405	0.0261732	0.0118538	Cd36/Hif1a
7-day post-SCI group vs. the control group	BP	GO:0030890	positive regulation of B cell proliferation	2/88	0.0090405	0.0261732	0.0118538	Cd38/Tlr4
7-day post-SCI group vs. the control group	BP	GO:0034205	amyloid-beta formation	2/88	0.0090405	0.0261732	0.0118538	Apoe/Clu
7-day post-SCI group vs. the control group	BP	GO:0042417	dopamine metabolic process	2/88	0.0090405	0.0261732	0.0118538	Atp7a/Gch1
7-day post-SCI group vs. the control group	BP	GO:0097300	programmed necrotic cell death	2/88	0.0090405	0.0261732	0.0118538	Mapk8/Ripk1
7-day post-SCI group vs. the control group	BP	GO:0120163	negative regulation of cold-induced thermogenesis	2/88	0.0090405	0.0261732	0.0118538	Id1/Tlr4
7-day post-SCI group vs. the control group	BP	GO:0006869	lipid transport	5/88	0.009117	0.0263715	0.0119436	Apoe/Cd36/Lcat/Prkcd/Trem2
7-day post-SCI group vs. the control group	BP	GO:0009855	determination of bilateral symmetry	3/88	0.009185	0.0264989	0.0120013	Ctnnb1/Hif1a/Mapk8
7-day post-SCI group vs. the control group	BP	GO:0046165	alcohol biosynthetic process	3/88	0.009185	0.0264989	0.0120013	Apoe/Dhcr24/Gch1
7-day post-SCI group vs. the control group	BP	GO:0055123	digestive system development	3/88	0.009185	0.0264989	0.0120013	Ctnnb1/Hif1a/Il6st
7-day post-SCI group vs. the control group	BP	GO:0002702	positive regulation of production of molecular med	3/88	0.0093629	0.0269652	0.0122125	Cd36/Stat6/Tlr4
7-day post-SCI group vs. the control group	BP	GO:0009799	specification of symmetry	3/88	0.0093629	0.0269652	0.0122125	Ctnnb1/Hif1a/Mapk8
7-day post-SCI group vs. the control group	BP	GO:0002440	production of molecular mediator of immune respo	5/88	0.0093834	0.026998	0.0122274	Axl/Btk/Cd36/Stat6/Tlr4
7-day post-SCI group vs. the control group	BP	GO:0035137	hindlimb morphogenesis	2/88	0.0094151	0.026998	0.0122274	Atp7a/Ctnnb1
7-day post-SCI group vs. the control group	BP	GO:0045841	negative regulation of mitotic metaphase/anaphase	2/88	0.0094151	0.026998	0.0122274	Apc/Cdc20
7-day post-SCI group vs. the control group	BP	GO:0071174	mitotic spindle checkpoint signaling	2/88	0.0094151	0.026998	0.0122274	Apc/Cdc20
7-day post-SCI group vs. the control group	BP	GO:1901385	regulation of voltage-gated calcium channel activi	2/88	0.0094151	0.026998	0.0122274	Ppp3ca/S100a1
7-day post-SCI group vs. the control group	BP	GO:0048588	developmental cell growth	4/88	0.0097225	0.0278555	0.0126157	Apoe/Ctnnb1/Map2k4/Mapt
7-day post-SCI group vs. the control group	BP	GO:0006110	regulation of glycolytic process	2/88	0.0097965	0.0278745	0.0126243	Hif1a/Ppargc1a
7-day post-SCI group vs. the control group	BP	GO:0033046	negative regulation of sister chromatid segregation	2/88	0.0097965	0.0278745	0.0126243	Apc/Cdc20
7-day post-SCI group vs. the control group	BP	GO:0033048	negative regulation of mitotic sister chromatid seg	2/88	0.0097965	0.0278745	0.0126243	Apc/Cdc20
7-day post-SCI group vs. the control group	BP	GO:0033173	calcineurin-NFAT signaling cascade	2/88	0.0097965	0.0278745	0.0126243	Ppp3ca/Rcan1
7-day post-SCI group vs. the control group	BP	GO:0035307	positive regulation of protein dephosphorylation	2/88	0.0097965	0.0278745	0.0126243	Pink1/Prkcd
7-day post-SCI group vs. the control group	BP	GO:0060966	regulation of gene silencing by RNA	2/88	0.0097965	0.0278745	0.0126243	Ppp3ca/Ripk1
7-day post-SCI group vs. the control group	BP	GO:1902459	positive regulation of stem cell population mainter	2/88	0.0097965	0.0278745	0.0126243	Hdac1/Rbbp7
7-day post-SCI group vs. the control group	BP	GO:2000816	negative regulation of mitotic sister chromatid sep	2/88	0.0097965	0.0278745	0.0126243	Apc/Cdc20
7-day post-SCI group vs. the control group	BP	GO:0008203	cholesterol metabolic process	3/88	0.0099088	0.0281699	0.0127581	Apoe/Dhcr24/Lcat
7-day post-SCI group vs. the control group	BP	GO:0002065	columnar/cuboidal epithelial cell differentiation	3/88	0.0100948	0.028674	0.0129864	Ctnnb1/Hif1a/Il6st
7-day post-SCI group vs. the control group	BP	GO:0034332	adherens junction organization	2/88	0.0101846	0.0287812	0.013035	Cdh11/Ctnnb1

7-day post-SCI group vs. the control group	BP	GO:0042088	T-helper 1 type immune response	2/88	0.0101846	0.0287812	0.013035	Il18bp/Stat6
7-day post-SCI group vs. the control group	BP	GO:0045663	positive regulation of myoblast differentiation	2/88	0.0101846	0.0287812	0.013035	Btg1/Mapk14
7-day post-SCI group vs. the control group	BP	GO:1901021	positive regulation of calcium ion transmembrane	2/88	0.0101846	0.0287812	0.013035	Ppp3ca/S100a1
7-day post-SCI group vs. the control group	BP	GO:1902100	negative regulation of metaphase/anaphase transiti	2/88	0.0101846	0.0287812	0.013035	Apc/Cdc20
7-day post-SCI group vs. the control group	BP	GO:1903018	regulation of glycoprotein metabolic process	2/88	0.0101846	0.0287812	0.013035	Ctnnb1/Hbegf
7-day post-SCI group vs. the control group	BP	GO:0050728	negative regulation of inflammatory response	3/88	0.0102828	0.029009	0.0131382	Apoe/Gpx1/Trem2
7-day post-SCI group vs. the control group	BP	GO:1903322	positive regulation of protein modification by sma	3/88	0.0102828	0.029009	0.0131382	Cdc20/Fbxw7/Mapk8
7-day post-SCI group vs. the control group	BP	GO:0050864	regulation of B cell activation	4/88	0.0103404	0.0291467	0.0132005	Btk/Cd38/Stat6/Tlr4
7-day post-SCI group vs. the control group	BP	GO:0001508	action potential	3/88	0.0104728	0.0294199	0.0133242	Atp2a2/Cd36/Fkbp1b
7-day post-SCI group vs. the control group	BP	GO:0002367	cytokine production involved in immune response	3/88	0.0104728	0.0294199	0.0133242	Axl/Cd36/Tlr4
7-day post-SCI group vs. the control group	BP	GO:0050729	positive regulation of inflammatory response	3/88	0.0104728	0.0294199	0.0133242	Ripk1/Tlr4/Trem2
7-day post-SCI group vs. the control group	BP	GO:0071887	leukocyte apoptotic process	3/88	0.0104728	0.0294199	0.0133242	Axl/Hif1a/Ripk1
7-day post-SCI group vs. the control group	BP	GO:0007018	microtubule-based movement	5/88	0.0105031	0.0294799	0.0133514	Dst/Hif1a/Hspb1/Mapt/Stau2
7-day post-SCI group vs. the control group	BP	GO:0014888	striated muscle adaptation	2/88	0.0105796	0.0295443	0.0133806	Atp2a2/Ppp3ca
7-day post-SCI group vs. the control group	BP	GO:0030195	negative regulation of blood coagulation	2/88	0.0105796	0.0295443	0.0133806	Apoe/Prkcd
7-day post-SCI group vs. the control group	BP	GO:0031577	spindle checkpoint signaling	2/88	0.0105796	0.0295443	0.0133806	Apc/Cdc20
7-day post-SCI group vs. the control group	BP	GO:0032350	regulation of hormone metabolic process	2/88	0.0105796	0.0295443	0.0133806	Hif1a/Ppargc1a
7-day post-SCI group vs. the control group	BP	GO:0051646	mitochondrion localization	2/88	0.0105796	0.0295443	0.0133806	Hif1a/Mapt
7-day post-SCI group vs. the control group	BP	GO:1905819	negative regulation of chromosome separation	2/88	0.0105796	0.0295443	0.0133806	Apc/Cdc20
7-day post-SCI group vs. the control group	BP	GO:0034763	negative regulation of transmembrane transport	3/88	0.0106649	0.0297576	0.0134772	Atp7a/Fkbp1b/Ppp3ca
7-day post-SCI group vs. the control group	BP	GO:0030218	erythrocyte differentiation	3/88	0.010859	0.0302737	0.0137109	Hif1a/Mapk14/Stat1
7-day post-SCI group vs. the control group	BP	GO:0019083	viral transcription	2/88	0.0109812	0.030339	0.0137405	Hdac1/Jun
7-day post-SCI group vs. the control group	BP	GO:0035196	miRNA processing	2/88	0.0109812	0.030339	0.0137405	Ppp3ca/Ripk1
7-day post-SCI group vs. the control group	BP	GO:0042307	positive regulation of protein import into nucleus	2/88	0.0109812	0.030339	0.0137405	Mapk14/Prkcd
7-day post-SCI group vs. the control group	BP	GO:0045773	positive regulation of axon extension	2/88	0.0109812	0.030339	0.0137405	Apoe/Mapt
7-day post-SCI group vs. the control group	BP	GO:0051985	negative regulation of chromosome segregation	2/88	0.0109812	0.030339	0.0137405	Apc/Cdc20
7-day post-SCI group vs. the control group	BP	GO:0060043	regulation of cardiac muscle cell proliferation	2/88	0.0109812	0.030339	0.0137405	Apc/Mapk14
7-day post-SCI group vs. the control group	BP	GO:0090207	regulation of triglyceride metabolic process	2/88	0.0109812	0.030339	0.0137405	Apoe/Cd36
7-day post-SCI group vs. the control group	BP	GO:1900047	negative regulation of hemostasis	2/88	0.0109812	0.030339	0.0137405	Apoe/Prkcd
7-day post-SCI group vs. the control group	BP	GO:1903426	regulation of reactive oxygen species biosynthetic	2/88	0.0109812	0.030339	0.0137405	Cd36/Tlr4
7-day post-SCI group vs. the control group	BP	GO:2001258	negative regulation of cation channel activity	2/88	0.0109812	0.030339	0.0137405	Fkbp1b/Ppp3ca
7-day post-SCI group vs. the control group	BP	GO:0003007	heart morphogenesis	4/88	0.0109832	0.030339	0.0137405	Ctnnb1/Hif1a/Jun/Slc8a1
7-day post-SCI group vs. the control group	BP	GO:0031333	negative regulation of protein-containing complex	3/88	0.0110552	0.030411	0.0137731	Clu/Prkcd/Trem2
7-day post-SCI group vs. the control group	BP	GO:0050921	positive regulation of chemotaxis	3/88	0.0110552	0.030411	0.0137731	Aif1/Hspb1/Trem2
7-day post-SCI group vs. the control group	BP	GO:0051928	positive regulation of calcium ion transport	3/88	0.0110552	0.030411	0.0137731	Mylk/Ppp3ca/S100a1
7-day post-SCI group vs. the control group	BP	GO:0071453	cellular response to oxygen levels	3/88	0.0110552	0.030411	0.0137731	Hif1a/Nfe2l2/Pink1
7-day post-SCI group vs. the control group	BP	GO:1900180	regulation of protein localization to nucleus	3/88	0.0110552	0.030411	0.0137731	Cd36/Mapk14/Prkcd
7-day post-SCI group vs. the control group	BP	GO:0042113	B cell activation	5/88	0.011096	0.0304978	0.0138124	Btk/Cd38/Prkcd/Stat6/Tlr4
7-day post-SCI group vs. the control group	BP	GO:0016485	protein processing	4/88	0.0112474	0.0308885	0.0139893	Adam9/Capn2/Ctsl/Dhcr24
7-day post-SCI group vs. the control group	BP	GO:0016064	immunoglobulin mediated immune response	4/88	0.0113811	0.030945	0.0140149	Btk/Prkcd/Stat6/Trem2

7-day post-SCI group vs. the control group	BP	GO:0007520	myoblast fusion	2/88	0.0113895	0.030945	0.0140149	Capn2/Mapk14
7-day post-SCI group vs. the control group	BP	GO:0030850	prostate gland development	2/88	0.0113895	0.030945	0.0140149	Apc/Ctnnb1
7-day post-SCI group vs. the control group	BP	GO:0031103	axon regeneration	2/88	0.0113895	0.030945	0.0140149	Fkbp1b/Jun
7-day post-SCI group vs. the control group	BP	GO:0034605	cellular response to heat	2/88	0.0113895	0.030945	0.0140149	Mapt/Rbbp7
7-day post-SCI group vs. the control group	BP	GO:0045124	regulation of bone resorption	2/88	0.0113895	0.030945	0.0140149	Cd38/Ppargc1b
7-day post-SCI group vs. the control group	BP	GO:0045907	positive regulation of vasoconstriction	2/88	0.0113895	0.030945	0.0140149	Cd38/Ptgs1
7-day post-SCI group vs. the control group	BP	GO:0046189	phenol-containing compound biosynthetic process	2/88	0.0113895	0.030945	0.0140149	Atp7a/Gch1
7-day post-SCI group vs. the control group	BP	GO:0050819	negative regulation of coagulation	2/88	0.0113895	0.030945	0.0140149	Apoe/Prkcd
7-day post-SCI group vs. the control group	BP	GO:0051438	regulation of ubiquitin-protein transferase activity	2/88	0.0113895	0.030945	0.0140149	Cdc20/Fbxw7
7-day post-SCI group vs. the control group	BP	GO:0090199	regulation of release of cytochrome c from mitoch	2/88	0.0113895	0.030945	0.0140149	Gpx1/Pink1
7-day post-SCI group vs. the control group	BP	GO:0097720	calcineurin-mediated signaling	2/88	0.0113895	0.030945	0.0140149	Ppp3ca/Rcan1
7-day post-SCI group vs. the control group	BP	GO:2000677	regulation of transcription regulatory region DNA	2/88	0.0113895	0.030945	0.0140149	Ctnnb1/Fbxw7
7-day post-SCI group vs. the control group	BP	GO:0034329	cell junction assembly	5/88	0.0116072	0.0314849	0.0142595	Cdh11/Ctnnb1/Dst/Mapt/Stau2
7-day post-SCI group vs. the control group	BP	GO:0052547	regulation of peptidase activity	5/88	0.0116072	0.0314849	0.0142595	Dhcr24/Gpx1/Hdac1/Stat1/Xdh
7-day post-SCI group vs. the control group	BP	GO:0016125	sterol metabolic process	3/88	0.011656	0.0315913	0.0143077	Apoe/Dhcr24/Lcat
7-day post-SCI group vs. the control group	BP	GO:0019724	B cell mediated immunity	4/88	0.0117881	0.0317596	0.0143839	Btk/Prkcd/Stat6/Trem2
7-day post-SCI group vs. the control group	BP	GO:0002714	positive regulation of B cell mediated immunity	2/88	0.0118044	0.0317596	0.0143839	Stat6/Trem2
7-day post-SCI group vs. the control group	BP	GO:0002891	positive regulation of immunoglobulin mediated ir	2/88	0.0118044	0.0317596	0.0143839	Stat6/Trem2
7-day post-SCI group vs. the control group	BP	GO:0010883	regulation of lipid storage	2/88	0.0118044	0.0317596	0.0143839	Cd36/Trem2
7-day post-SCI group vs. the control group	BP	GO:0032715	negative regulation of interleukin-6 production	2/88	0.0118044	0.0317596	0.0143839	Sirpa/Tlr4
7-day post-SCI group vs. the control group	BP	GO:0033047	regulation of mitotic sister chromatid segregation	2/88	0.0118044	0.0317596	0.0143839	Apc/Cdc20
7-day post-SCI group vs. the control group	BP	GO:0045687	positive regulation of glial cell differentiation	2/88	0.0118044	0.0317596	0.0143839	Hdac1/Il6st
7-day post-SCI group vs. the control group	BP	GO:0048483	autonomic nervous system development	2/88	0.0118044	0.0317596	0.0143839	Ctnnb1/Vcam1
7-day post-SCI group vs. the control group	BP	GO:2000351	regulation of endothelial cell apoptotic process	2/88	0.0118044	0.0317596	0.0143839	Id1/Nfe2l2
7-day post-SCI group vs. the control group	BP	GO:0031099	regeneration	3/88	0.0118604	0.0318843	0.0144404	Fkbp1b/Gpx1/Jun
7-day post-SCI group vs. the control group	BP	GO:0002253	activation of immune response	5/88	0.0119216	0.0320229	0.0145031	Btk/Cd38/Ezr/Tlr4/Trem2
7-day post-SCI group vs. the control group	BP	GO:0010565	regulation of cellular ketone metabolic process	3/88	0.0120668	0.0323343	0.0146442	Apc/Pdk2/Ppargc1a
7-day post-SCI group vs. the control group	BP	GO:0030216	keratinocyte differentiation	3/88	0.0120668	0.0323343	0.0146442	Ppp3ca/Ptgs1/Txnip
7-day post-SCI group vs. the control group	BP	GO:1901136	carbohydrate derivative catabolic process	3/88	0.0120668	0.0323343	0.0146442	Ctsl/Prkcd/Xdh
7-day post-SCI group vs. the control group	BP	GO:0001974	blood vessel remodeling	2/88	0.0122258	0.0326548	0.0147893	Atp7a/Axl
7-day post-SCI group vs. the control group	BP	GO:0006111	regulation of gluconeogenesis	2/88	0.0122258	0.0326548	0.0147893	Hif1a/Pdk2
7-day post-SCI group vs. the control group	BP	GO:0048546	digestive tract morphogenesis	2/88	0.0122258	0.0326548	0.0147893	Ctnnb1/Hif1a
7-day post-SCI group vs. the control group	BP	GO:2000378	negative regulation of reactive oxygen species met	2/88	0.0122258	0.0326548	0.0147893	Hif1a/Pink1
7-day post-SCI group vs. the control group	BP	GO:0016055	Wnt signaling pathway	5/88	0.0122417	0.0326707	0.0147965	Apc/Apoe/Ctnnb1/Hdac1/Mapk14
7-day post-SCI group vs. the control group	BP	GO:0032368	regulation of lipid transport	3/88	0.0122753	0.0327341	0.0148252	Apoe/Prkcd/Trem2
7-day post-SCI group vs. the control group	BP	GO:0198738	cell-cell signaling by wnt	5/88	0.0124583	0.0331954	0.0150341	Apc/Apoe/Ctnnb1/Hdac1/Mapk14
7-day post-SCI group vs. the control group	BP	GO:1902652	secondary alcohol metabolic process	3/88	0.0124858	0.033242	0.0150553	Apoe/Dhcr24/Lcat
7-day post-SCI group vs. the control group	BP	GO:0048016	inositol phosphate-mediated signaling	2/88	0.0126538	0.0335813	0.0152089	Ppp3ca/Rcan1
7-day post-SCI group vs. the control group	BP	GO:0048713	regulation of oligodendrocyte differentiation	2/88	0.0126538	0.0335813	0.0152089	Ctnnb1/Hdac1
7-day post-SCI group vs. the control group	BP	GO:0070918	small regulatory ncRNA processing	2/88	0.0126538	0.0335813	0.0152089	Ppp3ca/Ripk1

7-day post-SCI group vs. the control group	BP	GO:1901861	regulation of muscle tissue development	2/88	0.0126538	0.0335813	0.0152089	Ctnnb1/Ppargc1a
7-day post-SCI group vs. the control group	BP	GO:0051656	establishment of organelle localization	5/88	0.0126775	0.0336172	0.0152252	Ctnnb1/Ezr/Hif1a/Mapk8/Mapt
7-day post-SCI group vs. the control group	BP	GO:0002762	negative regulation of myeloid leukocyte different	2/88	0.0130883	0.0344857	0.0156185	Ctnnb1/Fbxw7
7-day post-SCI group vs. the control group	BP	GO:0006695	cholesterol biosynthetic process	2/88	0.0130883	0.0344857	0.0156185	Apoe/Dhcr24
7-day post-SCI group vs. the control group	BP	GO:0031648	protein destabilization	2/88	0.0130883	0.0344857	0.0156185	Fbxw7/Id1
7-day post-SCI group vs. the control group	BP	GO:0048538	thymus development	2/88	0.0130883	0.0344857	0.0156185	Apc/Ctnnb1
7-day post-SCI group vs. the control group	BP	GO:0072577	endothelial cell apoptotic process	2/88	0.0130883	0.0344857	0.0156185	Id1/Nfe2l2
7-day post-SCI group vs. the control group	BP	GO:1902653	secondary alcohol biosynthetic process	2/88	0.0130883	0.0344857	0.0156185	Apoe/Dhcr24
7-day post-SCI group vs. the control group	BP	GO:1903170	negative regulation of calcium ion transmembrane	2/88	0.0130883	0.0344857	0.0156185	Fkbp1b/Ppp3ca
7-day post-SCI group vs. the control group	BP	GO:1903533	regulation of protein targeting	2/88	0.0130883	0.0344857	0.0156185	Fbxw7/Pink1
7-day post-SCI group vs. the control group	BP	GO:0001558	regulation of cell growth	5/88	0.0131238	0.034513	0.0156309	Apoe/Cd38/Hbegf/Mapt/Rbbp7
7-day post-SCI group vs. the control group	BP	GO:0007009	plasma membrane organization	3/88	0.0131299	0.034513	0.0156309	Atp2a2/Clu/Prkcd
7-day post-SCI group vs. the control group	BP	GO:0070507	regulation of microtubule cytoskeleton organizatio	3/88	0.0131299	0.034513	0.0156309	Apc/Mapk8/Mapt
7-day post-SCI group vs. the control group	BP	GO:0071695	anatomical structure maturation	4/88	0.013212	0.0347012	0.0157161	Btk/Ctnnb1/Ctsl/Hif1a
7-day post-SCI group vs. the control group	BP	GO:0009395	phospholipid catabolic process	2/88	0.0135292	0.0351994	0.0159417	Prdx6/Prkcd
7-day post-SCI group vs. the control group	BP	GO:0010257	NADH dehydrogenase complex assembly	2/88	0.0135292	0.0351994	0.0159417	Ndufa12/Ndufa6
7-day post-SCI group vs. the control group	BP	GO:0014014	negative regulation of gliogenesis	2/88	0.0135292	0.0351994	0.0159417	Ctnnb1/Trem2
7-day post-SCI group vs. the control group	BP	GO:0031279	regulation of cyclase activity	2/88	0.0135292	0.0351994	0.0159417	Mapk14/Mapk8
7-day post-SCI group vs. the control group	BP	GO:0032757	positive regulation of interleukin-8 production	2/88	0.0135292	0.0351994	0.0159417	Ripk1/Tlr4
7-day post-SCI group vs. the control group	BP	GO:0032981	mitochondrial respiratory chain complex I assemb	2/88	0.0135292	0.0351994	0.0159417	Ndufa12/Ndufa6
7-day post-SCI group vs. the control group	BP	GO:0033619	membrane protein proteolysis	2/88	0.0135292	0.0351994	0.0159417	Adam9/Apoe
7-day post-SCI group vs. the control group	BP	GO:0043030	regulation of macrophage activation	2/88	0.0135292	0.0351994	0.0159417	Tlr4/Trem2
7-day post-SCI group vs. the control group	BP	GO:0043666	regulation of phosphoprotein phosphatase activity	2/88	0.0135292	0.0351994	0.0159417	Fkbp1b/Rcan1
7-day post-SCI group vs. the control group	BP	GO:0045058	T cell selection	2/88	0.0135292	0.0351994	0.0159417	Ctsl/Stat6
7-day post-SCI group vs. the control group	BP	GO:0045839	negative regulation of mitotic nuclear division	2/88	0.0135292	0.0351994	0.0159417	Apc/Cdc20
7-day post-SCI group vs. the control group	BP	GO:1905953	negative regulation of lipid localization	2/88	0.0135292	0.0351994	0.0159417	Apoe/Trem2
7-day post-SCI group vs. the control group	BP	GO:0043388	positive regulation of DNA binding	2/88	0.0139765	0.0363061	0.016443	Ctnnb1/Tlr4
7-day post-SCI group vs. the control group	BP	GO:0070509	calcium ion import	2/88	0.0139765	0.0363061	0.016443	Ctnnb1/Slc8a1
7-day post-SCI group vs. the control group	BP	GO:0120254	olefinic compound metabolic process	3/88	0.0142451	0.0369747	0.0167458	Gpx1/Ppargc1a/Ptgs1
7-day post-SCI group vs. the control group	BP	GO:0098813	nuclear chromosome segregation	4/88	0.0142714	0.0370141	0.0167636	Apc/Cdc20/Ctnnb1/Fbxw7
7-day post-SCI group vs. the control group	BP	GO:0010876	lipid localization	5/88	0.0144057	0.0372219	0.0168577	Apoe/Cd36/Lcat/Prkcd/Trem2
7-day post-SCI group vs. the control group	BP	GO:0002066	columnar/cuboidal epithelial cell development	2/88	0.0144302	0.0372219	0.0168577	Hif1a/Il6st
7-day post-SCI group vs. the control group	BP	GO:0007595	lactation	2/88	0.0144302	0.0372219	0.0168577	Hif1a/Xdh
7-day post-SCI group vs. the control group	BP	GO:0009948	anterior/posterior axis specification	2/88	0.0144302	0.0372219	0.0168577	Ctnnb1/Ets2
7-day post-SCI group vs. the control group	BP	GO:0043470	regulation of carbohydrate catabolic process	2/88	0.0144302	0.0372219	0.0168577	Hif1a/Ppargc1a
7-day post-SCI group vs. the control group	BP	GO:0046902	regulation of mitochondrial membrane permeabili	2/88	0.0144302	0.0372219	0.0168577	Gclc/Hebp2
7-day post-SCI group vs. the control group	BP	GO:0051591	response to cAMP	2/88	0.0144302	0.0372219	0.0168577	Ezr/Slc8a1
7-day post-SCI group vs. the control group	BP	GO:0021543	pallium development	3/88	0.0144744	0.0373067	0.0168961	Ctnnb1/Hdac1/Hif1a
7-day post-SCI group vs. the control group	BP	GO:0048015	phosphatidylinositol-mediated signaling	3/88	0.0147057	0.0378736	0.0171529	Ezr/Prr5l/Trem2
7-day post-SCI group vs. the control group	BP	GO:0043407	negative regulation of MAP kinase activity	2/88	0.0148902	0.0382891	0.0173411	Apoe/Prkcd

7-day post-SCI group vs. the control group	BP	GO:0046470	phosphatidylcholine metabolic process	2/88	0.0148902	0.0382891	0.0173411	Capn2/Lcat
7-day post-SCI group vs. the control group	BP	GO:0006457	protein folding	3/88	0.0149392	0.0383259	0.0173577	Clu/Fkbp1b/Hspb1
7-day post-SCI group vs. the control group	BP	GO:0006694	steroid biosynthetic process	3/88	0.0149392	0.0383259	0.0173577	Apoe/Dhcr24/Ppargc1a
7-day post-SCI group vs. the control group	BP	GO:0046887	positive regulation of hormone secretion	3/88	0.0149392	0.0383259	0.0173577	Cd38/Hif1a/Trpm2
7-day post-SCI group vs. the control group	BP	GO:0006910	phagocytosis, recognition	3/88	0.0151748	0.0389001	0.0176178	Cd36/Sirpa/Trem2
7-day post-SCI group vs. the control group	BP	GO:0031647	regulation of protein stability	4/88	0.0152213	0.0389891	0.0176581	Clu/Fbxw7/Id1/Pink1
7-day post-SCI group vs. the control group	BP	GO:0010676	positive regulation of cellular carbohydrate metabo	2/88	0.0153565	0.0391537	0.0177327	Cd36/Hif1a
7-day post-SCI group vs. the control group	BP	GO:0030858	positive regulation of epithelial cell differentiation	2/88	0.0153565	0.0391537	0.0177327	Btg1/Ctnnb1
7-day post-SCI group vs. the control group	BP	GO:0031102	neuron projection regeneration	2/88	0.0153565	0.0391537	0.0177327	Fkbp1b/Jun
7-day post-SCI group vs. the control group	BP	GO:0032653	regulation of interleukin-10 production	2/88	0.0153565	0.0391537	0.0177327	Tlr4/Trem2
7-day post-SCI group vs. the control group	BP	GO:0050873	brown fat cell differentiation	2/88	0.0153565	0.0391537	0.0177327	Itga6/Mapk14
7-day post-SCI group vs. the control group	BP	GO:1903078	positive regulation of protein localization to plasm	2/88	0.0153565	0.0391537	0.0177327	Ezr/Trem2
7-day post-SCI group vs. the control group	BP	GO:2001020	regulation of response to DNA damage stimulus	4/88	0.0153834	0.0391758	0.0177427	Clu/Mapt/Mcl1/Prkcd
7-day post-SCI group vs. the control group	BP	GO:0009267	cellular response to starvation	3/88	0.0154125	0.0391758	0.0177427	Jun/Mapk8/Nfe2l2
7-day post-SCI group vs. the control group	BP	GO:0048017	inositol lipid-mediated signaling	3/88	0.0154125	0.0391758	0.0177427	Ezr/Prr5l/Trem2
7-day post-SCI group vs. the control group	BP	GO:0051250	negative regulation of lymphocyte activation	3/88	0.0154125	0.0391758	0.0177427	Axl/Btk/Hspb1
7-day post-SCI group vs. the control group	BP	GO:0016570	histone modification	5/88	0.0156421	0.0397289	0.0179932	Ctnnb1/Hdac1/Phc1/Pink1/Rbbp7
7-day post-SCI group vs. the control group	BP	GO:0006584	catecholamine metabolic process	2/88	0.015829	0.039989	0.018111	Atp7a/Gch1
7-day post-SCI group vs. the control group	BP	GO:0009712	catechol-containing compound metabolic process	2/88	0.015829	0.039989	0.018111	Atp7a/Gch1
7-day post-SCI group vs. the control group	BP	GO:0016126	sterol biosynthetic process	2/88	0.015829	0.039989	0.018111	Apoe/Dhcr24
7-day post-SCI group vs. the control group	BP	GO:0042446	hormone biosynthetic process	2/88	0.015829	0.039989	0.018111	Hif1a/Ppargc1a
7-day post-SCI group vs. the control group	BP	GO:0046850	regulation of bone remodeling	2/88	0.015829	0.039989	0.018111	Cd38/Ppargc1b
7-day post-SCI group vs. the control group	BP	GO:0050435	amyloid-beta metabolic process	2/88	0.015829	0.039989	0.018111	Apoe/Clu
7-day post-SCI group vs. the control group	BP	GO:0070098	chemokine-mediated signaling pathway	2/88	0.015829	0.039989	0.018111	Hif1a/Trem2
7-day post-SCI group vs. the control group	BP	GO:1901361	organic cyclic compound catabolic process	5/88	0.0161559	0.0407838	0.0184709	Apoe/Ncf1/Nfe2l2/Prr5l/Xdh
7-day post-SCI group vs. the control group	BP	GO:0009064	glutamine family amino acid metabolic process	2/88	0.0163077	0.0410418	0.0185878	Apc/Gclc
7-day post-SCI group vs. the control group	BP	GO:0019748	secondary metabolic process	2/88	0.0163077	0.0410418	0.0185878	Atp7a/Nfe2l2
7-day post-SCI group vs. the control group	BP	GO:0033344	cholesterol efflux	2/88	0.0163077	0.0410418	0.0185878	Apoe/Trem2
7-day post-SCI group vs. the control group	BP	GO:0060999	positive regulation of dendritic spine development	2/88	0.0163077	0.0410418	0.0185878	Apoe/Stau2
7-day post-SCI group vs. the control group	BP	GO:0035107	appendage morphogenesis	3/88	0.0163842	0.0411719	0.0186467	Atp7a/Ctnnb1/Hdac1
7-day post-SCI group vs. the control group	BP	GO:0035108	limb morphogenesis	3/88	0.0163842	0.0411719	0.0186467	Atp7a/Ctnnb1/Hdac1
7-day post-SCI group vs. the control group	BP	GO:0002460	adaptive immune response based on somatic recon	5/88	0.016417	0.041223	0.0186698	Btk/Il18bp/Prkcd/Stat6/Trem2
7-day post-SCI group vs. the control group	BP	GO:0002699	positive regulation of immune effector process	4/88	0.0165486	0.0415221	0.0188053	Cd36/Stat6/Tlr4/Trem2
7-day post-SCI group vs. the control group	BP	GO:0090596	sensory organ morphogenesis	4/88	0.0167195	0.041919	0.0189851	Ctnnb1/Hdac1/Hif1a/Stau2
7-day post-SCI group vs. the control group	BP	GO:0042776	proton motive force-driven mitochondrial ATP syn	2/88	0.0167925	0.0420702	0.0190535	Ndufa12/Ndufa6
7-day post-SCI group vs. the control group	BP	GO:0055088	lipid homeostasis	3/88	0.0168827	0.0422643	0.0191414	Apoe/Lcat/Trem2
7-day post-SCI group vs. the control group	BP	GO:0009895	negative regulation of catabolic process	4/88	0.0172388	0.0430397	0.0194926	Mcl1/Pink1/Ppargc1a/Trem2
7-day post-SCI group vs. the control group	BP	GO:0048608	reproductive structure development	4/88	0.0172388	0.0430397	0.0194926	Apc/Axl/Ctnnb1/Dhcr24
7-day post-SCI group vs. the control group	BP	GO:0009166	nucleotide catabolic process	2/88	0.0172835	0.0430397	0.0194926	Ncf1/Xdh
7-day post-SCI group vs. the control group	BP	GO:0015909	long-chain fatty acid transport	2/88	0.0172835	0.0430397	0.0194926	Apoe/Cd36

7-day post-SCI group vs. the control group	BP	GO:0045776	negative regulation of blood pressure	2/88	0.0172835	0.0430397	0.0194926	Cd36/Gch1
7-day post-SCI group vs. the control group	BP	GO:0070231	T cell apoptotic process	2/88	0.0172835	0.0430397	0.0194926	Hif1a/Ripk1
7-day post-SCI group vs. the control group	BP	GO:0090311	regulation of protein deacetylation	2/88	0.0172835	0.0430397	0.0194926	Mapt/Pink1
7-day post-SCI group vs. the control group	BP	GO:0007389	pattern specification process	5/88	0.017353	0.0431481	0.0195417	Apc/Ctnnb1/Ets2/Hif1a/Mapk8
7-day post-SCI group vs. the control group	BP	GO:0007409	axonogenesis	5/88	0.017353	0.0431481	0.0195417	Apc/Apoe/Cdh11/Dst/Mapt
7-day post-SCI group vs. the control group	BP	GO:0071478	cellular response to radiation	3/88	0.0173897	0.0432069	0.0195683	Fbxw7/Mapk14/Prkcd
7-day post-SCI group vs. the control group	BP	GO:0032387	negative regulation of intracellular transport	2/88	0.0177805	0.0440785	0.0199631	Cd36/Mapt
7-day post-SCI group vs. the control group	BP	GO:0032608	interferon-beta production	2/88	0.0177805	0.0440785	0.0199631	Sirpa/Tlr4
7-day post-SCI group vs. the control group	BP	GO:0032648	regulation of interferon-beta production	2/88	0.0177805	0.0440785	0.0199631	Sirpa/Tlr4
7-day post-SCI group vs. the control group	BP	GO:0002366	leukocyte activation involved in immune response	4/88	0.0179466	0.044424	0.0201196	Atp7a/Stat6/Tlr4/Trem2
7-day post-SCI group vs. the control group	BP	GO:0061458	reproductive system development	4/88	0.0179466	0.044424	0.0201196	Apc/Axl/Ctnnb1/Dhcr24
7-day post-SCI group vs. the control group	BP	GO:0002221	pattern recognition receptor signaling pathway	3/88	0.018166	0.0449335	0.0203503	Cd36/Tlr4/Trem2
7-day post-SCI group vs. the control group	BP	GO:0007584	response to nutrient	2/88	0.0182835	0.0450894	0.0204209	Mapt/Pdk2
7-day post-SCI group vs. the control group	BP	GO:0050433	regulation of catecholamine secretion	2/88	0.0182835	0.0450894	0.0204209	Pink1/Ptgs1
7-day post-SCI group vs. the control group	BP	GO:0051784	negative regulation of nuclear division	2/88	0.0182835	0.0450894	0.0204209	Apc/Cdc20
7-day post-SCI group vs. the control group	BP	GO:0098900	regulation of action potential	2/88	0.0182835	0.0450894	0.0204209	Atp2a2/Cd36
7-day post-SCI group vs. the control group	BP	GO:0006820	anion transport	5/88	0.0183239	0.0451554	0.0204508	Cd36/Mgst1/Prkcd/Ripk1/Slc25a24
7-day post-SCI group vs. the control group	BP	GO:0002263	cell activation involved in immune response	4/88	0.0186725	0.04598	0.0208243	Atp7a/Stat6/Tlr4/Trem2
7-day post-SCI group vs. the control group	BP	GO:0071674	mononuclear cell migration	3/88	0.0186942	0.0459994	0.0208331	Aif1/Sirpa/Trpm2
7-day post-SCI group vs. the control group	BP	GO:0006940	regulation of smooth muscle contraction	2/88	0.0187925	0.0460703	0.0208652	Ncf1/Ptgs1
7-day post-SCI group vs. the control group	BP	GO:0042093	T-helper cell differentiation	2/88	0.0187925	0.0460703	0.0208652	Atp7a/Stat6
7-day post-SCI group vs. the control group	BP	GO:0060425	lung morphogenesis	2/88	0.0187925	0.0460703	0.0208652	Ctnnb1/Id1
7-day post-SCI group vs. the control group	BP	GO:1901224	positive regulation of NIK/NF-kappaB signaling	2/88	0.0187925	0.0460703	0.0208652	Tlr4/Trem2
7-day post-SCI group vs. the control group	BP	GO:1904377	positive regulation of protein localization to cell pe	2/88	0.0187925	0.0460703	0.0208652	Ezr/Trem2
7-day post-SCI group vs. the control group	BP	GO:1901605	alpha-amino acid metabolic process	3/88	0.0189615	0.0464499	0.0210371	Apc/Atp7a/Gclc
7-day post-SCI group vs. the control group	BP	GO:0001960	negative regulation of cytokine-mediated signaling	2/88	0.0193075	0.0470888	0.0213264	Il6st/Trem2
7-day post-SCI group vs. the control group	BP	GO:0002294	CD4-positive, alpha-beta T cell differentiation inv	2/88	0.0193075	0.0470888	0.0213264	Atp7a/Stat6
7-day post-SCI group vs. the control group	BP	GO:0008347	glial cell migration	2/88	0.0193075	0.0470888	0.0213264	Ctnnb1/Trem2
7-day post-SCI group vs. the control group	BP	GO:0015986	proton motive force-driven ATP synthesis	2/88	0.0193075	0.0470888	0.0213264	Ndufa12/Ndufa6
7-day post-SCI group vs. the control group	BP	GO:0042058	regulation of epidermal growth factor receptor sign	2/88	0.0193075	0.0470888	0.0213264	Fbxw7/Hbegf
7-day post-SCI group vs. the control group	BP	GO:1905710	positive regulation of membrane permeability	2/88	0.0193075	0.0470888	0.0213264	Gclc/Hebp2
7-day post-SCI group vs. the control group	BP	GO:0048592	eye morphogenesis	3/88	0.0195023	0.0475289	0.0215258	Ctnnb1/Hif1a/Stau2
7-day post-SCI group vs. the control group	BP	GO:0000075	cell cycle checkpoint signaling	3/88	0.019776	0.0481464	0.0218054	Apc/Cdc20/Mapk14
7-day post-SCI group vs. the control group	BP	GO:0002293	alpha-beta T cell differentiation involved in immun	2/88	0.0198284	0.0481464	0.0218054	Atp7a/Stat6
7-day post-SCI group vs. the control group	BP	GO:0034121	regulation of toll-like receptor signaling pathway	2/88	0.0198284	0.0481464	0.0218054	Cd36/Trem2
7-day post-SCI group vs. the control group	BP	GO:0042733	embryonic digit morphogenesis	2/88	0.0198284	0.0481464	0.0218054	Ctnnb1/Hdac1
7-day post-SCI group vs. the control group	BP	GO:0042775	mitochondrial ATP synthesis coupled electron tran	2/88	0.0198284	0.0481464	0.0218054	Ndufa12/Pink1
7-day post-SCI group vs. the control group	BP	GO:0002287	alpha-beta T cell activation involved in immune re	2/88	0.0203552	0.049209	0.0222867	Atp7a/Stat6
7-day post-SCI group vs. the control group	BP	GO:0021885	forebrain cell migration	2/88	0.0203552	0.049209	0.0222867	Axl/Ctnnb1
7-day post-SCI group vs. the control group	BP	GO:0032371	regulation of sterol transport	2/88	0.0203552	0.049209	0.0222867	Apoe/Trem2

7-day post-SCI group vs. the control group	BP	GO:0032374	regulation of cholesterol transport	2/88	0.0203552	0.049209	0.0222867	Apoe/Trem2
7-day post-SCI group vs. the control group	BP	GO:0032481	positive regulation of type I interferon production	2/88	0.0203552	0.049209	0.0222867	Stat1/Tlr4
7-day post-SCI group vs. the control group	BP	GO:1904705	regulation of vascular associated smooth muscle c	2/88	0.0203552	0.049209	0.0222867	Jun/Ppargc1a
7-day post-SCI group vs. the control group	BP	GO:0042594	response to starvation	3/88	0.0206096	0.0497152	0.0225159	Jun/Mapk8/Nfe2l2
7-day post-SCI group vs. the control group	BP	GO:0046631	alpha-beta T cell activation	3/88	0.0206096	0.0497152	0.0225159	Atp7a/Ctsl/Stat6
7-day post-SCI group vs. the control group	BP	GO:1904064	positive regulation of cation transmembrane transp	3/88	0.0206096	0.0497152	0.0225159	Ppp3ca/S100a1/Trem2
7-day post-SCI group vs. the control group	BP	GO:0019080	viral gene expression	2/88	0.0208878	0.0502398	0.0227535	Hdac1/Jun
7-day post-SCI group vs. the control group	BP	GO:0042773	ATP synthesis coupled electron transport	2/88	0.0208878	0.0502398	0.0227535	Ndufa12/Pink1
7-day post-SCI group vs. the control group	BP	GO:1990868	response to chemokine	2/88	0.0208878	0.0502398	0.0227535	Hif1a/Trem2
7-day post-SCI group vs. the control group	BP	GO:1990869	cellular response to chemokine	2/88	0.0208878	0.0502398	0.0227535	Hif1a/Trem2
7-day post-SCI group vs. the control group	BP	GO:0007265	Ras protein signal transduction	4/88	0.0209586	0.0503736	0.0228141	Aif1/Apoe/Dhcr24/Jun
7-day post-SCI group vs. the control group	BP	GO:0050821	protein stabilization	3/88	0.021176	0.0508592	0.023034	Clu/Fbxw7/Pink1
7-day post-SCI group vs. the control group	BP	GO:0014068	positive regulation of phosphatidylinositol 3-kinas	2/88	0.0214261	0.0512741	0.023222	Prr5l/Trem2
7-day post-SCI group vs. the control group	BP	GO:0046824	positive regulation of nucleocytoplasmic transport	2/88	0.0214261	0.0512741	0.023222	Mapk14/Prkcd
7-day post-SCI group vs. the control group	BP	GO:0050432	catecholamine secretion	2/88	0.0214261	0.0512741	0.023222	Pink1/Ptgs1
7-day post-SCI group vs. the control group	BP	GO:0060761	negative regulation of response to cytokine stimulu	2/88	0.0214261	0.0512741	0.023222	Il6st/Trem2
7-day post-SCI group vs. the control group	BP	GO:0098586	cellular response to virus	2/88	0.0214261	0.0512741	0.023222	Hif1a/Mapk14
7-day post-SCI group vs. the control group	BP	GO:0050777	negative regulation of immune response	3/88	0.0214623	0.0512866	0.0232276	Gpx1/Stat6/Trem2
7-day post-SCI group vs. the control group	BP	GO:0051099	positive regulation of binding	3/88	0.0214623	0.0512866	0.0232276	Apoe/Ctnnb1/Tlr4
7-day post-SCI group vs. the control group	BP	GO:0072001	renal system development	4/88	0.0217572	0.0519538	0.0235298	Apc/Ctnnb1/Ppp3ca/Stat1
7-day post-SCI group vs. the control group	BP	GO:0043954	cellular component maintenance	2/88	0.0219702	0.052274	0.0236748	Apoe/Trem2
7-day post-SCI group vs. the control group	BP	GO:1901184	regulation of ERBB signaling pathway	2/88	0.0219702	0.052274	0.0236748	Fbxw7/Hbegf
7-day post-SCI group vs. the control group	BP	GO:1901292	nucleoside phosphate catabolic process	2/88	0.0219702	0.052274	0.0236748	Ncf1/Xdh
7-day post-SCI group vs. the control group	BP	GO:1990874	vascular associated smooth muscle cell proliferatic	2/88	0.0219702	0.052274	0.0236748	Jun/Ppargc1a
7-day post-SCI group vs. the control group	BP	GO:2000036	regulation of stem cell population maintenance	2/88	0.0219702	0.052274	0.0236748	Hdac1/Rbbp7
7-day post-SCI group vs. the control group	BP	GO:0002757	immune response-activating signal transduction	4/88	0.0221634	0.0526958	0.0238659	Btk/Cd38/Ezr/Tlr4
7-day post-SCI group vs. the control group	BP	GO:0006626	protein targeting to mitochondrion	2/88	0.0225201	0.0533905	0.0241805	Fbxw7/Pink1
7-day post-SCI group vs. the control group	BP	GO:0032677	regulation of interleukin-8 production	2/88	0.0225201	0.0533905	0.0241805	Ripk1/Tlr4
7-day post-SCI group vs. the control group	BP	GO:0051926	negative regulation of calcium ion transport	2/88	0.0225201	0.0533905	0.0241805	Fkbp1b/Ppp3ca
7-day post-SCI group vs. the control group	BP	GO:0061045	negative regulation of wound healing	2/88	0.0225201	0.0533905	0.0241805	Apoe/Prkcd
7-day post-SCI group vs. the control group	BP	GO:0007259	receptor signaling pathway via JAK-STAT	3/88	0.0226292	0.0536107	0.0242802	Il6st/Stat1/Stat6
7-day post-SCI group vs. the control group	BP	GO:0008202	steroid metabolic process	4/88	0.0229898	0.0543959	0.0246358	Apoe/Dhcr24/Lcat/Ppargc1a
7-day post-SCI group vs. the control group	BP	GO:0002437	inflammatory response to antigenic stimulus	2/88	0.0230756	0.0543959	0.0246358	Gpx1/Trem2
7-day post-SCI group vs. the control group	BP	GO:0002712	regulation of B cell mediated immunity	2/88	0.0230756	0.0543959	0.0246358	Stat6/Trem2
7-day post-SCI group vs. the control group	BP	GO:0002889	regulation of immunoglobulin mediated immune r	2/88	0.0230756	0.0543959	0.0246358	Stat6/Trem2
7-day post-SCI group vs. the control group	BP	GO:0007492	endoderm development	2/88	0.0230756	0.0543959	0.0246358	Ctnnb1/Hdac1
7-day post-SCI group vs. the control group	BP	GO:0016239	positive regulation of macroautophagy	2/88	0.0230756	0.0543959	0.0246358	Hif1a/Pink1
7-day post-SCI group vs. the control group	BP	GO:0032637	interleukin-8 production	2/88	0.0230756	0.0543959	0.0246358	Ripk1/Tlr4
7-day post-SCI group vs. the control group	BP	GO:0015931	nucleobase-containing compound transport	3/88	0.0232253	0.0546711	0.0247605	Mapt/Ripk1/Slc25a24
7-day post-SCI group vs. the control group	BP	GO:1990138	neuron projection extension	3/88	0.0232253	0.0546711	0.0247605	Apoe/Ctnnb1/Mapt

7-day post-SCI group vs. the control group	BP	GO:0015711	organic anion transport	4/88	0.02341	0.0550667	0.0249396	Cd36/Mgst1/Prkcd/Slc25a24
7-day post-SCI group vs. the control group	BP	GO:0002292	T cell differentiation involved in immune response	2/88	0.0236367	0.0553641	0.0250743	Atp7a/Stat6
7-day post-SCI group vs. the control group	BP	GO:0006919	activation of cysteine-type endopeptidase activity i	2/88	0.0236367	0.0553641	0.0250743	Stat1/Xdh
7-day post-SCI group vs. the control group	BP	GO:0019369	arachidonic acid metabolic process	2/88	0.0236367	0.0553641	0.0250743	Gpx1/Ptgs1
7-day post-SCI group vs. the control group	BP	GO:0038034	signal transduction in absence of ligand	2/88	0.0236367	0.0553641	0.0250743	Mcl1/Ripk1
7-day post-SCI group vs. the control group	BP	GO:0061097	regulation of protein tyrosine kinase activity	2/88	0.0236367	0.0553641	0.0250743	Fbxw7/Hbegf
7-day post-SCI group vs. the control group	BP	GO:0097192	extrinsic apoptotic signaling pathway in absence o	2/88	0.0236367	0.0553641	0.0250743	Mcl1/Ripk1
7-day post-SCI group vs. the control group	BP	GO:0002695	negative regulation of leukocyte activation	3/88	0.02383	0.0557774	0.0252615	Axl/Btk/Hspb1
7-day post-SCI group vs. the control group	BP	GO:0006066	alcohol metabolic process	4/88	0.024049	0.0562501	0.0254756	Apoe/Dhcr24/Gch1/Lcat
7-day post-SCI group vs. the control group	BP	GO:0043627	response to estrogen	2/88	0.0242034	0.0565316	0.0256031	Ctnnb1/Rcan1
7-day post-SCI group vs. the control group	BP	GO:0048002	antigen processing and presentation of peptide anti	2/88	0.0242034	0.0565316	0.0256031	Ctsl/Trem2
7-day post-SCI group vs. the control group	BP	GO:0016051	carbohydrate biosynthetic process	3/88	0.024753	0.0576649	0.0261164	Cd36/Hif1a/Pdk2
7-day post-SCI group vs. the control group	BP	GO:0097696	receptor signaling pathway via STAT	3/88	0.024753	0.0576649	0.0261164	Il6st/Stat1/Stat6
7-day post-SCI group vs. the control group	BP	GO:0005977	glycogen metabolic process	2/88	0.0247757	0.0576649	0.0261164	Cd36/Il6st
7-day post-SCI group vs. the control group	BP	GO:0006073	cellular glucan metabolic process	2/88	0.0247757	0.0576649	0.0261164	Cd36/Il6st
7-day post-SCI group vs. the control group	BP	GO:2000107	negative regulation of leukocyte apoptotic process	2/88	0.0247757	0.0576649	0.0261164	Axl/Hif1a
7-day post-SCI group vs. the control group	BP	GO:0061351	neural precursor cell proliferation	3/88	0.0250649	0.058297	0.0264026	Ctnnb1/Hif1a/Mapk8
7-day post-SCI group vs. the control group	BP	GO:0033209	tumor necrosis factor-mediated signaling pathway	2/88	0.0253535	0.0588031	0.0266318	Ripk1/Stat1
7-day post-SCI group vs. the control group	BP	GO:0044042	glucan metabolic process	2/88	0.0253535	0.0588031	0.0266318	Cd36/Il6st
7-day post-SCI group vs. the control group	BP	GO:0045123	cellular extravasation	2/88	0.0253535	0.0588031	0.0266318	Sirpa/Vcam1
7-day post-SCI group vs. the control group	BP	GO:0051145	smooth muscle cell differentiation	2/88	0.0253535	0.0588031	0.0266318	Ctnnb1/Rcan1
7-day post-SCI group vs. the control group	BP	GO:0099173	postsynapse organization	3/88	0.0256951	0.0595537	0.0269718	Apoe/Stau2/Trem2
7-day post-SCI group vs. the control group	BP	GO:0006644	phospholipid metabolic process	4/88	0.0258045	0.0597237	0.0270488	Capn2/Lcat/Prdx6/Prkcd
7-day post-SCI group vs. the control group	BP	GO:0051604	protein maturation	4/88	0.0258045	0.0597237	0.0270488	Adam9/Capn2/Ctsl/Dhcr24
7-day post-SCI group vs. the control group	BP	GO:0003208	cardiac ventricle morphogenesis	2/88	0.0259368	0.059946	0.0271495	Ctnnb1/Hif1a
7-day post-SCI group vs. the control group	BP	GO:0051279	regulation of release of sequestered calcium ion in	2/88	0.0259368	0.059946	0.0271495	Fkbp1b/Slc8a1
7-day post-SCI group vs. the control group	BP	GO:0032956	regulation of actin cytoskeleton organization	4/88	0.0260293	0.0601178	0.0272273	Id1/Prkcd/Stau2/Trpm2
7-day post-SCI group vs. the control group	BP	GO:0007059	chromosome segregation	4/88	0.0262552	0.0605974	0.0274445	Apc/Cdc20/Ctnnb1/Fbxw7
7-day post-SCI group vs. the control group	BP	GO:0002381	immunoglobulin production involved in immunog	2/88	0.0265255	0.0610511	0.02765	Btk/Stat6
7-day post-SCI group vs. the control group	BP	GO:0032722	positive regulation of chemokine production	2/88	0.0265255	0.0610511	0.02765	Aif1/Tlr4
7-day post-SCI group vs. the control group	BP	GO:0050810	regulation of steroid biosynthetic process	2/88	0.0265255	0.0610511	0.02765	Apoe/Ppargc1a
7-day post-SCI group vs. the control group	BP	GO:0055021	regulation of cardiac muscle tissue growth	2/88	0.0265255	0.0610511	0.02765	Apc/Mapk14
7-day post-SCI group vs. the control group	BP	GO:0008361	regulation of cell size	3/88	0.0266564	0.0613096	0.027767	Apoe/Atp7a/Mapt
7-day post-SCI group vs. the control group	BP	GO:0032869	cellular response to insulin stimulus	3/88	0.026981	0.0620133	0.0280857	Apc/Pdk2/Prkcd
7-day post-SCI group vs. the control group	BP	GO:0043154	negative regulation of cysteine-type endopeptidase	2/88	0.0271196	0.0622024	0.0281714	Dhcr24/Gpx1
7-day post-SCI group vs. the control group	BP	GO:0045913	positive regulation of carbohydrate metabolic proc	2/88	0.0271196	0.0622024	0.0281714	Cd36/Hif1a
7-day post-SCI group vs. the control group	BP	GO:0060998	regulation of dendritic spine development	2/88	0.0271196	0.0622024	0.0281714	Apoe/Stau2
7-day post-SCI group vs. the control group	BP	GO:1902115	regulation of organelle assembly	3/88	0.0273078	0.0625906	0.0283472	Ezr/Mapk8/Pink1
7-day post-SCI group vs. the control group	BP	GO:0048872	homeostasis of number of cells	4/88	0.0276356	0.0632981	0.0286676	Axl/Hif1a/Mapk14/Stat1
7-day post-SCI group vs. the control group	BP	GO:0002720	positive regulation of cytokine production involve	2/88	0.0277191	0.0634018	0.0287146	Cd36/Tlr4

7-day post-SCI group vs. the control group	BP	GO:0032413	negative regulation of ion transmembrane transpor	2/88	0.0277191	0.0634018	0.0287146	Fkbp1b/Ppp3ca
7-day post-SCI group vs. the control group	BP	GO:0002067	glandular epithelial cell differentiation	2/88	0.028324	0.0644735	0.0292	Ctnnb1/Hif1a
7-day post-SCI group vs. the control group	BP	GO:0006094	gluconeogenesis	2/88	0.028324	0.0644735	0.0292	Hif1a/Pdk2
7-day post-SCI group vs. the control group	BP	GO:0014015	positive regulation of gliogenesis	2/88	0.028324	0.0644735	0.0292	Hdac1/Il6st
7-day post-SCI group vs. the control group	BP	GO:0045661	regulation of myoblast differentiation	2/88	0.028324	0.0644735	0.0292	Btg1/Mapk14
7-day post-SCI group vs. the control group	BP	GO:0051384	response to glucocorticoid	2/88	0.028324	0.0644735	0.0292	Adam9/Lcat
7-day post-SCI group vs. the control group	BP	GO:0060415	muscle tissue morphogenesis	2/88	0.028324	0.0644735	0.0292	Ctnnb1/Mylk
7-day post-SCI group vs. the control group	BP	GO:0097009	energy homeostasis	2/88	0.028324	0.0644735	0.0292	Cd36/Ppargc1a
7-day post-SCI group vs. the control group	BP	GO:0031503	protein-containing complex localization	3/88	0.0286362	0.0650947	0.0294813	Ezr/Mapk10/Stau2
7-day post-SCI group vs. the control group	BP	GO:0098742	cell-cell adhesion via plasma-membrane adhesion	3/88	0.0286362	0.0650947	0.0294813	Cdh11/Mapk14/Vcam1
7-day post-SCI group vs. the control group	BP	GO:0031397	negative regulation of protein ubiquitination	2/88	0.0289341	0.0656817	0.0297472	Cdc20/Gclc
7-day post-SCI group vs. the control group	BP	GO:0043648	dicarboxylic acid metabolic process	2/88	0.0289341	0.0656817	0.0297472	Apc/Gclc
7-day post-SCI group vs. the control group	BP	GO:0030522	intracellular receptor signaling pathway	3/88	0.0289735	0.0657108	0.0297603	Hdac1/Ppargc1b/Tlr4
7-day post-SCI group vs. the control group	BP	GO:0021675	nerve development	2/88	0.0295494	0.0657108	0.0297603	Ctnnb1/Vcam1
7-day post-SCI group vs. the control group	BP	GO:0030071	regulation of mitotic metaphase/anaphase transitio	2/88	0.0295494	0.0657108	0.0297603	Apc/Cdc20
7-day post-SCI group vs. the control group	BP	GO:0030500	regulation of bone mineralization	2/88	0.0295494	0.0657108	0.0297603	Hif1a/Slc8a1
7-day post-SCI group vs. the control group	BP	GO:0072384	organelle transport along microtubule	2/88	0.0295494	0.0657108	0.0297603	Hif1a/Mapt
7-day post-SCI group vs. the control group	BP	GO:0006196	AMP catabolic process	1/88	0.0299965	0.0657108	0.0297603	Xdh
7-day post-SCI group vs. the control group	BP	GO:0006707	cholesterol catabolic process	1/88	0.0299965	0.0657108	0.0297603	Apoe
7-day post-SCI group vs. the control group	BP	GO:0006971	hypotonic response	1/88	0.0299965	0.0657108	0.0297603	Mylk
7-day post-SCI group vs. the control group	BP	GO:0008298	intracellular mRNA localization	1/88	0.0299965	0.0657108	0.0297603	Stau2
7-day post-SCI group vs. the control group	BP	GO:0009650	UV protection	1/88	0.0299965	0.0657108	0.0297603	Gpx1
7-day post-SCI group vs. the control group	BP	GO:0010692	regulation of alkaline phosphatase activity	1/88	0.0299965	0.0657108	0.0297603	Ppargc1b
7-day post-SCI group vs. the control group	BP	GO:0010873	positive regulation of cholesterol esterification	1/88	0.0299965	0.0657108	0.0297603	Apoe
7-day post-SCI group vs. the control group	BP	GO:0016127	sterol catabolic process	1/88	0.0299965	0.0657108	0.0297603	Apoe
7-day post-SCI group vs. the control group	BP	GO:0021957	corticospinal tract morphogenesis	1/88	0.0299965	0.0657108	0.0297603	Cdh11
7-day post-SCI group vs. the control group	BP	GO:0030450	regulation of complement activation, classical path	1/88	0.0299965	0.0657108	0.0297603	Trem2
7-day post-SCI group vs. the control group	BP	GO:0032225	regulation of synaptic transmission, dopaminergic	1/88	0.0299965	0.0657108	0.0297603	Pink1
7-day post-SCI group vs. the control group	BP	GO:0033603	positive regulation of dopamine secretion	1/88	0.0299965	0.0657108	0.0297603	Pink1
7-day post-SCI group vs. the control group	BP	GO:0035562	negative regulation of chromatin binding	1/88	0.0299965	0.0657108	0.0297603	Ppp3ca
7-day post-SCI group vs. the control group	BP	GO:0038130	ERBB4 signaling pathway	1/88	0.0299965	0.0657108	0.0297603	Hbegf
7-day post-SCI group vs. the control group	BP	GO:0042421	norepinephrine biosynthetic process	1/88	0.0299965	0.0657108	0.0297603	Atp7a
7-day post-SCI group vs. the control group	BP	GO:0046055	dGMP catabolic process	1/88	0.0299965	0.0657108	0.0297603	Xdh
7-day post-SCI group vs. the control group	BP	GO:0046130	purine ribonucleoside catabolic process	1/88	0.0299965	0.0657108	0.0297603	Xdh
7-day post-SCI group vs. the control group	BP	GO:0046877	regulation of saliva secretion	1/88	0.0299965	0.0657108	0.0297603	Ppp3ca
7-day post-SCI group vs. the control group	BP	GO:0051775	response to redox state	1/88	0.0299965	0.0657108	0.0297603	Fkbp1b
7-day post-SCI group vs. the control group	BP	GO:0060484	lung-associated mesenchyme development	1/88	0.0299965	0.0657108	0.0297603	Ctnnb1
7-day post-SCI group vs. the control group	BP	GO:0060710	chorio-allantoic fusion	1/88	0.0299965	0.0657108	0.0297603	Vcam1
7-day post-SCI group vs. the control group	BP	GO:0060982	coronary artery morphogenesis	1/88	0.0299965	0.0657108	0.0297603	Ctnnb1
7-day post-SCI group vs. the control group	BP	GO:0061687	detoxification of inorganic compound	1/88	0.0299965	0.0657108	0.0297603	Atp7a

7-day post-SCI group vs. the control group	BP	GO:0061762	CAMKK-AMPK signaling cascade	1/88	0.0299965	0.0657108	0.0297603	Trem2
7-day post-SCI group vs. the control group	BP	GO:0070601	centromeric sister chromatid cohesion	1/88	0.0299965	0.0657108	0.0297603	Ctnnb1
7-day post-SCI group vs. the control group	BP	GO:0070662	mast cell proliferation	1/88	0.0299965	0.0657108	0.0297603	Stat6
7-day post-SCI group vs. the control group	BP	GO:0070666	regulation of mast cell proliferation	1/88	0.0299965	0.0657108	0.0297603	Stat6
7-day post-SCI group vs. the control group	BP	GO:0070933	histone H4 deacetylation	1/88	0.0299965	0.0657108	0.0297603	Hdac1
7-day post-SCI group vs. the control group	BP	GO:0071649	regulation of chemokine (C-C motif) ligand 5 proc	1/88	0.0299965	0.0657108	0.0297603	Sirpa
7-day post-SCI group vs. the control group	BP	GO:0071670	smooth muscle cell chemotaxis	1/88	0.0299965	0.0657108	0.0297603	Aif1
7-day post-SCI group vs. the control group	BP	GO:0086064	cell communication by electrical coupling involve	1/88	0.0299965	0.0657108	0.0297603	Slc8a1
7-day post-SCI group vs. the control group	BP	GO:0090129	positive regulation of synapse maturation	1/88	0.0299965	0.0657108	0.0297603	Cdc20
7-day post-SCI group vs. the control group	BP	GO:0090269	fibroblast growth factor production	1/88	0.0299965	0.0657108	0.0297603	Aif1
7-day post-SCI group vs. the control group	BP	GO:0090270	regulation of fibroblast growth factor production	1/88	0.0299965	0.0657108	0.0297603	Aif1
7-day post-SCI group vs. the control group	BP	GO:0097067	cellular response to thyroid hormone stimulus	1/88	0.0299965	0.0657108	0.0297603	Ppargc1a
7-day post-SCI group vs. the control group	BP	GO:0097501	stress response to metal ion	1/88	0.0299965	0.0657108	0.0297603	Atp7a
7-day post-SCI group vs. the control group	BP	GO:0098883	synapse pruning	1/88	0.0299965	0.0657108	0.0297603	Trem2
7-day post-SCI group vs. the control group	BP	GO:0099640	axo-dendritic protein transport	1/88	0.0299965	0.0657108	0.0297603	Hspb1
7-day post-SCI group vs. the control group	BP	GO:0150172	regulation of phosphatidylcholine metabolic proce	1/88	0.0299965	0.0657108	0.0297603	Capn2
7-day post-SCI group vs. the control group	BP	GO:1901725	regulation of histone deacetylase activity	1/88	0.0299965	0.0657108	0.0297603	Pink1
7-day post-SCI group vs. the control group	BP	GO:1901858	regulation of mitochondrial DNA metabolic proce	1/88	0.0299965	0.0657108	0.0297603	Ppargc1a
7-day post-SCI group vs. the control group	BP	GO:1902884	positive regulation of response to oxidative stress	1/88	0.0299965	0.0657108	0.0297603	Ripk1
7-day post-SCI group vs. the control group	BP	GO:1903242	regulation of cardiac muscle hypertrophy in respor	1/88	0.0299965	0.0657108	0.0297603	Ppp3ca
7-day post-SCI group vs. the control group	BP	GO:1903749	positive regulation of establishment of protein loca	1/88	0.0299965	0.0657108	0.0297603	Fbxw7
7-day post-SCI group vs. the control group	BP	GO:1904779	regulation of protein localization to centrosome	1/88	0.0299965	0.0657108	0.0297603	Apc
7-day post-SCI group vs. the control group	BP	GO:1904959	regulation of cytochrome-c oxidase activity	1/88	0.0299965	0.0657108	0.0297603	Atp7a
7-day post-SCI group vs. the control group	BP	GO:2000646	positive regulation of receptor catabolic process	1/88	0.0299965	0.0657108	0.0297603	Apoe
7-day post-SCI group vs. the control group	BP	GO:0048469	cell maturation	3/88	0.0299984	0.0657108	0.0297603	Btk/Ctnnb1/Hif1a
7-day post-SCI group vs. the control group	BP	GO:0002285	lymphocyte activation involved in immune respon	3/88	0.0303442	0.0663805	0.0300636	Atp7a/Stat6/Tlr4
7-day post-SCI group vs. the control group	BP	GO:0050871	positive regulation of B cell activation	3/88	0.0303442	0.0663805	0.0300636	Cd38/Stat6/Tlr4
7-day post-SCI group vs. the control group	BP	GO:0006112	energy reserve metabolic process	2/88	0.0307958	0.0672351	0.0304507	Cd36/Il6st
7-day post-SCI group vs. the control group	BP	GO:0019319	hexose biosynthetic process	2/88	0.0307958	0.0672351	0.0304507	Hif1a/Pdk2
7-day post-SCI group vs. the control group	BP	GO:0045995	regulation of embryonic development	2/88	0.0307958	0.0672351	0.0304507	Ctnnb1/Nfe2l2
7-day post-SCI group vs. the control group	BP	GO:0003002	regionalization	4/88	0.0310233	0.0676872	0.0306554	Apc/Ctnnb1/Ets2/Mapk8
7-day post-SCI group vs. the control group	BP	GO:2000241	regulation of reproductive process	3/88	0.0313944	0.0682974	0.0309318	Apc/Cdc20/Ctnnb1
7-day post-SCI group vs. the control group	BP	GO:0034644	cellular response to UV	2/88	0.0314267	0.0682974	0.0309318	Fbxw7/Prkcd
7-day post-SCI group vs. the control group	BP	GO:0046683	response to organophosphorus	2/88	0.0314267	0.0682974	0.0309318	Ezr/Slc8a1
7-day post-SCI group vs. the control group	BP	GO:0051937	catecholamine transport	2/88	0.0314267	0.0682974	0.0309318	Pink1/Ptgs1
7-day post-SCI group vs. the control group	BP	GO:0060420	regulation of heart growth	2/88	0.0314267	0.0682974	0.0309318	Apc/Mapk14
7-day post-SCI group vs. the control group	BP	GO:1902099	regulation of metaphase/anaphase transition of cel	2/88	0.0314267	0.0682974	0.0309318	Apc/Cdc20
7-day post-SCI group vs. the control group	BP	GO:0001655	urogenital system development	4/88	0.0315264	0.0684691	0.0310096	Apc/Ctnnb1/Ppp3ca/Stat1
7-day post-SCI group vs. the control group	BP	GO:0008217	regulation of blood pressure	3/88	0.0317487	0.0689068	0.0312078	Cd36/Gch1/Ptgs1
7-day post-SCI group vs. the control group	BP	GO:0006096	glycolytic process	2/88	0.0320627	0.069361	0.0314135	Hif1a/Ppargc1a

7-day post-SCI group vs. the control group	BP	GO:0007091	metaphase/anaphase transition of mitotic cell cycle	2/88	0.0320627	0.069361	0.0314135	Apc/Cdc20
7-day post-SCI group vs. the control group	BP	GO:0015914	phospholipid transport	2/88	0.0320627	0.069361	0.0314135	Apoe/Prkcd
7-day post-SCI group vs. the control group	BP	GO:0045638	negative regulation of myeloid cell differentiation	2/88	0.0320627	0.069361	0.0314135	Ctnnb1/Fbxw7
7-day post-SCI group vs. the control group	BP	GO:1900182	positive regulation of protein localization to nucleu	2/88	0.0320627	0.069361	0.0314135	Mapk14/Prkcd
7-day post-SCI group vs. the control group	BP	GO:0006757	ATP generation from ADP	2/88	0.0327038	0.0699038	0.0316593	Hif1a/Ppargc1a
7-day post-SCI group vs. the control group	BP	GO:0010965	regulation of mitotic sister chromatid separation	2/88	0.0327038	0.0699038	0.0316593	Apc/Cdc20
7-day post-SCI group vs. the control group	BP	GO:0030512	negative regulation of transforming growth factor	2/88	0.0327038	0.0699038	0.0316593	Hdac1/Rbbp7
7-day post-SCI group vs. the control group	BP	GO:0031016	pancreas development	2/88	0.0327038	0.0699038	0.0316593	Clu/Ctnnb1
7-day post-SCI group vs. the control group	BP	GO:0003266	regulation of secondary heart field cardioblast prol	1/88	0.0329468	0.0699038	0.0316593	Ctnnb1
7-day post-SCI group vs. the control group	BP	GO:0006521	regulation of cellular amino acid metabolic proces	1/88	0.0329468	0.0699038	0.0316593	Apc
7-day post-SCI group vs. the control group	BP	GO:0009172	purine deoxyribonucleoside monophosphate catab	1/88	0.0329468	0.0699038	0.0316593	Xdh
7-day post-SCI group vs. the control group	BP	GO:0010612	regulation of cardiac muscle adaptation	1/88	0.0329468	0.0699038	0.0316593	Ppp3ca
7-day post-SCI group vs. the control group	BP	GO:0010992	ubiquitin recycling	1/88	0.0329468	0.0699038	0.0316593	Fbxw7
7-day post-SCI group vs. the control group	BP	GO:0019227	neuronal action potential propagation	1/88	0.0329468	0.0699038	0.0316593	Fkbp1b
7-day post-SCI group vs. the control group	BP	GO:0019372	lipoxygenase pathway	1/88	0.0329468	0.0699038	0.0316593	Gpx1
7-day post-SCI group vs. the control group	BP	GO:0031293	membrane protein intracellular domain proteolysis	1/88	0.0329468	0.0699038	0.0316593	Adam9
7-day post-SCI group vs. the control group	BP	GO:0042117	monocyte activation	1/88	0.0329468	0.0699038	0.0316593	Adam9
7-day post-SCI group vs. the control group	BP	GO:0048617	embryonic foregut morphogenesis	1/88	0.0329468	0.0699038	0.0316593	Ctnnb1
7-day post-SCI group vs. the control group	BP	GO:0048664	neuron fate determination	1/88	0.0329468	0.0699038	0.0316593	Ctnnb1
7-day post-SCI group vs. the control group	BP	GO:0060068	vagina development	1/88	0.0329468	0.0699038	0.0316593	Axl
7-day post-SCI group vs. the control group	BP	GO:0071372	cellular response to follicle-stimulating hormone s	1/88	0.0329468	0.0699038	0.0316593	Ppargc1a
7-day post-SCI group vs. the control group	BP	GO:0071609	chemokine (C-C motif) ligand 5 production	1/88	0.0329468	0.0699038	0.0316593	Sirpa
7-day post-SCI group vs. the control group	BP	GO:0072526	pyridine-containing compound catabolic process	1/88	0.0329468	0.0699038	0.0316593	Ncf1
7-day post-SCI group vs. the control group	BP	GO:0090009	primitive streak formation	1/88	0.0329468	0.0699038	0.0316593	Ets2
7-day post-SCI group vs. the control group	BP	GO:0098779	positive regulation of mitophagy in response to mi	1/88	0.0329468	0.0699038	0.0316593	Pink1
7-day post-SCI group vs. the control group	BP	GO:0098870	action potential propagation	1/88	0.0329468	0.0699038	0.0316593	Fkbp1b
7-day post-SCI group vs. the control group	BP	GO:1900747	negative regulation of vascular endothelial growth	1/88	0.0329468	0.0699038	0.0316593	Xdh
7-day post-SCI group vs. the control group	BP	GO:1901029	negative regulation of mitochondrial outer membra	1/88	0.0329468	0.0699038	0.0316593	Gclc
7-day post-SCI group vs. the control group	BP	GO:1902946	protein localization to early endosome	1/88	0.0329468	0.0699038	0.0316593	Ezr
7-day post-SCI group vs. the control group	BP	GO:1903599	positive regulation of autophagy of mitochondrion	1/88	0.0329468	0.0699038	0.0316593	Hif1a
7-day post-SCI group vs. the control group	BP	GO:1903800	positive regulation of miRNA maturation	1/88	0.0329468	0.0699038	0.0316593	Ripk1
7-day post-SCI group vs. the control group	BP	GO:1904732	regulation of electron transfer activity	1/88	0.0329468	0.0699038	0.0316593	Atp7a
7-day post-SCI group vs. the control group	BP	GO:2000434	regulation of protein neddylation	1/88	0.0329468	0.0699038	0.0316593	Hif1a
7-day post-SCI group vs. the control group	BP	GO:2001204	regulation of osteoclast development	1/88	0.0329468	0.0699038	0.0316593	Fbxw7
7-day post-SCI group vs. the control group	BP	GO:0000082	G1/S transition of mitotic cell cycle	3/88	0.0331869	0.070323	0.0318492	Aif1/Apc/Ppp3ca
7-day post-SCI group vs. the control group	BP	GO:0099504	synaptic vesicle cycle	3/88	0.0331869	0.070323	0.0318492	Amph/Atp2a2/Ctnnb1
7-day post-SCI group vs. the control group	BP	GO:0032410	negative regulation of transporter activity	2/88	0.0333499	0.0703983	0.0318833	Fkbp1b/Ppp3ca
7-day post-SCI group vs. the control group	BP	GO:0033108	mitochondrial respiratory chain complex assembly	2/88	0.0333499	0.0703983	0.0318833	Ndufa12/Ndufa6
7-day post-SCI group vs. the control group	BP	GO:0048644	muscle organ morphogenesis	2/88	0.0333499	0.0703983	0.0318833	Ctnnb1/Mylk
7-day post-SCI group vs. the control group	BP	GO:0051899	membrane depolarization	2/88	0.0333499	0.0703983	0.0318833	Gclc/Jun

7-day post-SCI group vs. the control group	BP	GO:1903035	negative regulation of response to wounding	2/88	0.0333499	0.0703983	0.0318833	Apoe/Prkcd
7-day post-SCI group vs. the control group	BP	GO:2001251	negative regulation of chromosome organization	2/88	0.0333499	0.0703983	0.0318833	Apc/Cdc20
7-day post-SCI group vs. the control group	BP	GO:0050920	regulation of chemotaxis	3/88	0.0335516	0.070779	0.0320557	Aif1/Hspb1/Trem2
7-day post-SCI group vs. the control group	BP	GO:0031960	response to corticosteroid	2/88	0.0340011	0.0716358	0.0324438	Adam9/Lcat
7-day post-SCI group vs. the control group	BP	GO:0044784	metaphase/anaphase transition of cell cycle	2/88	0.0340011	0.0716358	0.0324438	Apc/Cdc20
7-day post-SCI group vs. the control group	BP	GO:0002312	B cell activation involved in immune response	2/88	0.0346572	0.0728791	0.0330068	Stat6/Tlr4
7-day post-SCI group vs. the control group	BP	GO:0051306	mitotic sister chromatid separation	2/88	0.0346572	0.0728791	0.0330068	Apc/Cdc20
7-day post-SCI group vs. the control group	BP	GO:2000117	negative regulation of cysteine-type endopeptidase	2/88	0.0346572	0.0728791	0.0330068	Dhcr24/Gpx1
7-day post-SCI group vs. the control group	BP	GO:0007163	establishment or maintenance of cell polarity	3/88	0.0350317	0.0729372	0.0330331	Apc/Dst/Ezr
7-day post-SCI group vs. the control group	BP	GO:0021954	central nervous system neuron development	2/88	0.0353182	0.0729372	0.0330331	Atp7a/Cdh11
7-day post-SCI group vs. the control group	BP	GO:0046364	monosaccharide biosynthetic process	2/88	0.0353182	0.0729372	0.0330331	Hif1a/Pdk2
7-day post-SCI group vs. the control group	BP	GO:0009952	anterior/posterior pattern specification	3/88	0.035407	0.0729372	0.0330331	Apc/Ctnnb1/Ets2
7-day post-SCI group vs. the control group	BP	GO:0031330	negative regulation of cellular catabolic process	3/88	0.035407	0.0729372	0.0330331	Mcl1/Pink1/Trem2
7-day post-SCI group vs. the control group	BP	GO:0000727	double-strand break repair via break-induced repli	1/88	0.0358882	0.0729372	0.0330331	Mcm4
7-day post-SCI group vs. the control group	BP	GO:0002579	positive regulation of antigen processing and prese	1/88	0.0358882	0.0729372	0.0330331	Trem2
7-day post-SCI group vs. the control group	BP	GO:0003263	cardioblast proliferation	1/88	0.0358882	0.0729372	0.0330331	Ctnnb1
7-day post-SCI group vs. the control group	BP	GO:0003264	regulation of cardioblast proliferation	1/88	0.0358882	0.0729372	0.0330331	Ctnnb1
7-day post-SCI group vs. the control group	BP	GO:0003306	Wnt signaling pathway involved in heart developn	1/88	0.0358882	0.0729372	0.0330331	Ctnnb1
7-day post-SCI group vs. the control group	BP	GO:0003337	mesenchymal to epithelial transition involved in m	1/88	0.0358882	0.0729372	0.0330331	Ctnnb1
7-day post-SCI group vs. the control group	BP	GO:0006086	acetyl-CoA biosynthetic process from pyruvate	1/88	0.0358882	0.0729372	0.0330331	Pdk2
7-day post-SCI group vs. the control group	BP	GO:0006152	purine nucleoside catabolic process	1/88	0.0358882	0.0729372	0.0330331	Xdh
7-day post-SCI group vs. the control group	BP	GO:0006534	cysteine metabolic process	1/88	0.0358882	0.0729372	0.0330331	Gclc
7-day post-SCI group vs. the control group	BP	GO:0007440	foregut morphogenesis	1/88	0.0358882	0.0729372	0.0330331	Ctnnb1
7-day post-SCI group vs. the control group	BP	GO:0009404	toxin metabolic process	1/88	0.0358882	0.0729372	0.0330331	Nfe2l2
7-day post-SCI group vs. the control group	BP	GO:0010572	positive regulation of platelet activation	1/88	0.0358882	0.0729372	0.0330331	Tlr4
7-day post-SCI group vs. the control group	BP	GO:0010744	positive regulation of macrophage derived foam ce	1/88	0.0358882	0.0729372	0.0330331	Cd36
7-day post-SCI group vs. the control group	BP	GO:0014824	artery smooth muscle contraction	1/88	0.0358882	0.0729372	0.0330331	Cd38
7-day post-SCI group vs. the control group	BP	GO:0015911	long-chain fatty acid import across plasma membra	1/88	0.0358882	0.0729372	0.0330331	Cd36
7-day post-SCI group vs. the control group	BP	GO:0031272	regulation of pseudopodium assembly	1/88	0.0358882	0.0729372	0.0330331	Apc
7-day post-SCI group vs. the control group	BP	GO:0031274	positive regulation of pseudopodium assembly	1/88	0.0358882	0.0729372	0.0330331	Apc
7-day post-SCI group vs. the control group	BP	GO:0032354	response to follicle-stimulating hormone	1/88	0.0358882	0.0729372	0.0330331	Ppargc1a
7-day post-SCI group vs. the control group	BP	GO:0032825	positive regulation of natural killer cell differentiat	1/88	0.0358882	0.0729372	0.0330331	Axl
7-day post-SCI group vs. the control group	BP	GO:0034135	regulation of toll-like receptor 2 signaling pathway	1/88	0.0358882	0.0729372	0.0330331	Trem2
7-day post-SCI group vs. the control group	BP	GO:0034333	adherens junction assembly	1/88	0.0358882	0.0729372	0.0330331	Ctnnb1
7-day post-SCI group vs. the control group	BP	GO:0035864	response to potassium ion	1/88	0.0358882	0.0729372	0.0330331	Mylk
7-day post-SCI group vs. the control group	BP	GO:0035865	cellular response to potassium ion	1/88	0.0358882	0.0729372	0.0330331	Mylk
7-day post-SCI group vs. the control group	BP	GO:0039654	fusion of virus membrane with host endosome mei	1/88	0.0358882	0.0729372	0.0330331	Ctsl
7-day post-SCI group vs. the control group	BP	GO:0045348	positive regulation of MHC class II biosynthetic pi	1/88	0.0358882	0.0729372	0.0330331	Tlr4
7-day post-SCI group vs. the control group	BP	GO:0046054	dGMP metabolic process	1/88	0.0358882	0.0729372	0.0330331	Xdh
7-day post-SCI group vs. the control group	BP	GO:0048241	epinephrine transport	1/88	0.0358882	0.0729372	0.0330331	Ptgs1

7-day post-SCI group vs. the control group	BP	GO:0048251	elastic fiber assembly	1/88	0.0358882	0.0729372	0.0330331	Atp7a
7-day post-SCI group vs. the control group	BP	GO:0048312	intracellular distribution of mitochondria	1/88	0.0358882	0.0729372	0.0330331	Mapt
7-day post-SCI group vs. the control group	BP	GO:0060439	trachea morphogenesis	1/88	0.0358882	0.0729372	0.0330331	Ctnnb1
7-day post-SCI group vs. the control group	BP	GO:0061684	chaperone-mediated autophagy	1/88	0.0358882	0.0729372	0.0330331	Clu
7-day post-SCI group vs. the control group	BP	GO:0070244	negative regulation of thymocyte apoptotic process	1/88	0.0358882	0.0729372	0.0330331	Hif1a
7-day post-SCI group vs. the control group	BP	GO:0070262	peptidyl-serine dephosphorylation	1/88	0.0358882	0.0729372	0.0330331	Ppp3ca
7-day post-SCI group vs. the control group	BP	GO:0070944	neutrophil-mediated killing of bacterium	1/88	0.0358882	0.0729372	0.0330331	Ncf1
7-day post-SCI group vs. the control group	BP	GO:0071073	positive regulation of phospholipid biosynthetic pr	1/88	0.0358882	0.0729372	0.0330331	Capn2
7-day post-SCI group vs. the control group	BP	GO:0071732	cellular response to nitric oxide	1/88	0.0358882	0.0729372	0.0330331	Mapk8
7-day post-SCI group vs. the control group	BP	GO:0072182	regulation of nephron tubule epithelial cell differe	1/88	0.0358882	0.0729372	0.0330331	Ctnnb1
7-day post-SCI group vs. the control group	BP	GO:0072497	mesenchymal stem cell differentiation	1/88	0.0358882	0.0729372	0.0330331	Ctnnb1
7-day post-SCI group vs. the control group	BP	GO:0072697	protein localization to cell cortex	1/88	0.0358882	0.0729372	0.0330331	Ezr
7-day post-SCI group vs. the control group	BP	GO:0090184	positive regulation of kidney development	1/88	0.0358882	0.0729372	0.0330331	Ppp3ca
7-day post-SCI group vs. the control group	BP	GO:0090209	negative regulation of triglyceride metabolic proce	1/88	0.0358882	0.0729372	0.0330331	Apoe
7-day post-SCI group vs. the control group	BP	GO:0097090	presynaptic membrane organization	1/88	0.0358882	0.0729372	0.0330331	Apoe
7-day post-SCI group vs. the control group	BP	GO:0097201	negative regulation of transcription from RNA pol	1/88	0.0358882	0.0729372	0.0330331	Jun
7-day post-SCI group vs. the control group	BP	GO:0099004	calmodulin dependent kinase signaling pathway	1/88	0.0358882	0.0729372	0.0330331	Trem2
7-day post-SCI group vs. the control group	BP	GO:1900272	negative regulation of long-term synaptic potentiat	1/88	0.0358882	0.0729372	0.0330331	Apoe
7-day post-SCI group vs. the control group	BP	GO:1903025	regulation of RNA polymerase II regulatory region	1/88	0.0358882	0.0729372	0.0330331	Fbxw7
7-day post-SCI group vs. the control group	BP	GO:1990144	intrinsic apoptotic signaling pathway in response t	1/88	0.0358882	0.0729372	0.0330331	Pink1
7-day post-SCI group vs. the control group	BP	GO:2000303	regulation of ceramide biosynthetic process	1/88	0.0358882	0.0729372	0.0330331	Prkcd
7-day post-SCI group vs. the control group	BP	GO:2001054	negative regulation of mesenchymal cell apoptotic	1/88	0.0358882	0.0729372	0.0330331	Hif1a
7-day post-SCI group vs. the control group	BP	GO:0015908	fatty acid transport	2/88	0.0359841	0.0729372	0.0330331	Apoe/Cd36
7-day post-SCI group vs. the control group	BP	GO:0042632	cholesterol homeostasis	2/88	0.0359841	0.0729372	0.0330331	Apoe/Lcat
7-day post-SCI group vs. the control group	BP	GO:0050772	positive regulation of axonogenesis	2/88	0.0359841	0.0729372	0.0330331	Apoe/Mapt
7-day post-SCI group vs. the control group	BP	GO:0090559	regulation of membrane permeability	2/88	0.0359841	0.0729372	0.0330331	Gclc/Hebp2
7-day post-SCI group vs. the control group	BP	GO:1901222	regulation of NIK/NF-kappaB signaling	2/88	0.0359841	0.0729372	0.0330331	Tlr4/Trem2
7-day post-SCI group vs. the control group	BP	GO:0042445	hormone metabolic process	3/88	0.0365452	0.0740293	0.0335278	Ctsl/Hif1a/Ppargc1a
7-day post-SCI group vs. the control group	BP	GO:0019935	cyclic-nucleotide-mediated signaling	2/88	0.0366549	0.0741607	0.0335873	Apoe/Cd36
7-day post-SCI group vs. the control group	BP	GO:0055092	sterol homeostasis	2/88	0.0366549	0.0741607	0.0335873	Apoe/Lcat
7-day post-SCI group vs. the control group	BP	GO:0033044	regulation of chromosome organization	3/88	0.0373145	0.0753894	0.0341437	Apc/Cdc20/Ctnnb1
7-day post-SCI group vs. the control group	BP	GO:0042180	cellular ketone metabolic process	3/88	0.0373145	0.0753894	0.0341437	Apc/Pdk2/Ppargc1a
7-day post-SCI group vs. the control group	BP	GO:0006970	response to osmotic stress	2/88	0.0373305	0.0753894	0.0341437	Mapk8/Mylk
7-day post-SCI group vs. the control group	BP	GO:0014066	regulation of phosphatidylinositol 3-kinase signali	2/88	0.0380109	0.0758999	0.034375	Prr5l/Trem2
7-day post-SCI group vs. the control group	BP	GO:0031638	zymogen activation	2/88	0.0380109	0.0758999	0.034375	Ctsl/Dhcr24
7-day post-SCI group vs. the control group	BP	GO:0032147	activation of protein kinase activity	2/88	0.0380109	0.0758999	0.034375	Prkcd/Tlr4
7-day post-SCI group vs. the control group	BP	GO:0044264	cellular polysaccharide metabolic process	2/88	0.0380109	0.0758999	0.034375	Cd36/Il6st
7-day post-SCI group vs. the control group	BP	GO:0046031	ADP metabolic process	2/88	0.0380109	0.0758999	0.034375	Hif1a/Ppargc1a
7-day post-SCI group vs. the control group	BP	GO:0097529	myeloid leukocyte migration	3/88	0.038092	0.0758999	0.034375	Aif1/Sirpa/Trem2
7-day post-SCI group vs. the control group	BP	GO:0031348	negative regulation of defense response	3/88	0.0384839	0.0758999	0.034375	Apoe/Gpx1/Trem2

7-day post-SCI group vs. the control group	BP	GO:0033045	regulation of sister chromatid segregation	2/88	0.038696	0.0758999	0.034375	Apc/Cdc20
7-day post-SCI group vs. the control group	BP	GO:0042158	lipoprotein biosynthetic process	2/88	0.038696	0.0758999	0.034375	Apoe/Lcat
7-day post-SCI group vs. the control group	BP	GO:0060395	SMAD protein signal transduction	2/88	0.038696	0.0758999	0.034375	Fos/Jun
7-day post-SCI group vs. the control group	BP	GO:0000255	allantoin metabolic process	1/88	0.0388208	0.0758999	0.034375	Xdh
7-day post-SCI group vs. the control group	BP	GO:0000279	M phase	1/88	0.0388208	0.0758999	0.034375	Cdc20
7-day post-SCI group vs. the control group	BP	GO:0002069	columnar/cuboidal epithelial cell maturation	1/88	0.0388208	0.0758999	0.034375	Hif1a
7-day post-SCI group vs. the control group	BP	GO:0002756	MyD88-independent toll-like receptor signaling pa	1/88	0.0388208	0.0758999	0.034375	Tlr4
7-day post-SCI group vs. the control group	BP	GO:0006271	DNA strand elongation involved in DNA replicati	1/88	0.0388208	0.0758999	0.034375	Mcm4
7-day post-SCI group vs. the control group	BP	GO:0006568	tryptophan metabolic process	1/88	0.0388208	0.0758999	0.034375	Atp7a
7-day post-SCI group vs. the control group	BP	GO:0006586	indolalkylamine metabolic process	1/88	0.0388208	0.0758999	0.034375	Atp7a
7-day post-SCI group vs. the control group	BP	GO:0009950	dorsal/ventral axis specification	1/88	0.0388208	0.0758999	0.034375	Ctnnb1
7-day post-SCI group vs. the control group	BP	GO:0010288	response to lead ion	1/88	0.0388208	0.0758999	0.034375	Mapt
7-day post-SCI group vs. the control group	BP	GO:0010872	regulation of cholesterol esterification	1/88	0.0388208	0.0758999	0.034375	Apoe
7-day post-SCI group vs. the control group	BP	GO:0014010	Schwann cell proliferation	1/88	0.0388208	0.0758999	0.034375	Ctnnb1
7-day post-SCI group vs. the control group	BP	GO:0018158	protein oxidation	1/88	0.0388208	0.0758999	0.034375	Gpx1
7-day post-SCI group vs. the control group	BP	GO:0021860	pyramidal neuron development	1/88	0.0388208	0.0758999	0.034375	Atp7a
7-day post-SCI group vs. the control group	BP	GO:0030174	regulation of DNA-templated DNA replication ini	1/88	0.0388208	0.0758999	0.034375	Mcm4
7-day post-SCI group vs. the control group	BP	GO:0030949	positive regulation of vascular endothelial growth	1/88	0.0388208	0.0758999	0.034375	Hif1a
7-day post-SCI group vs. the control group	BP	GO:0030953	astral microtubule organization	1/88	0.0388208	0.0758999	0.034375	Ezr
7-day post-SCI group vs. the control group	BP	GO:0031915	positive regulation of synaptic plasticity	1/88	0.0388208	0.0758999	0.034375	Cdc20
7-day post-SCI group vs. the control group	BP	GO:0031953	negative regulation of protein autophosphorylation	1/88	0.0388208	0.0758999	0.034375	Jun
7-day post-SCI group vs. the control group	BP	GO:0034374	low-density lipoprotein particle remodeling	1/88	0.0388208	0.0758999	0.034375	Apoe
7-day post-SCI group vs. the control group	BP	GO:0034756	regulation of iron ion transport	1/88	0.0388208	0.0758999	0.034375	Atp7a
7-day post-SCI group vs. the control group	BP	GO:0043589	skin morphogenesis	1/88	0.0388208	0.0758999	0.034375	Itga6
7-day post-SCI group vs. the control group	BP	GO:0044557	relaxation of smooth muscle	1/88	0.0388208	0.0758999	0.034375	Slc8a1
7-day post-SCI group vs. the control group	BP	GO:0048548	regulation of pinocytosis	1/88	0.0388208	0.0758999	0.034375	Axl
7-day post-SCI group vs. the control group	BP	GO:0060272	embryonic skeletal joint morphogenesis	1/88	0.0388208	0.0758999	0.034375	Ctnnb1
7-day post-SCI group vs. the control group	BP	GO:0070508	cholesterol import	1/88	0.0388208	0.0758999	0.034375	Cd36
7-day post-SCI group vs. the control group	BP	GO:0070932	histone H3 deacetylation	1/88	0.0388208	0.0758999	0.034375	Hdac1
7-day post-SCI group vs. the control group	BP	GO:0071803	positive regulation of podosome assembly	1/88	0.0388208	0.0758999	0.034375	Mapk8
7-day post-SCI group vs. the control group	BP	GO:0090520	sphingolipid mediated signaling pathway	1/88	0.0388208	0.0758999	0.034375	Ezr
7-day post-SCI group vs. the control group	BP	GO:0097048	dendritic cell apoptotic process	1/88	0.0388208	0.0758999	0.034375	Axl
7-day post-SCI group vs. the control group	BP	GO:0097066	response to thyroid hormone	1/88	0.0388208	0.0758999	0.034375	Ppargc1a
7-day post-SCI group vs. the control group	BP	GO:0098935	dendritic transport	1/88	0.0388208	0.0758999	0.034375	Stau2
7-day post-SCI group vs. the control group	BP	GO:1900121	negative regulation of receptor binding	1/88	0.0388208	0.0758999	0.034375	Atp2a2
7-day post-SCI group vs. the control group	BP	GO:1901298	regulation of hydrogen peroxide-mediated progran	1/88	0.0388208	0.0758999	0.034375	Pink1
7-day post-SCI group vs. the control group	BP	GO:1901533	negative regulation of hematopoietic progenitor ce	1/88	0.0388208	0.0758999	0.034375	Nfe2l2
7-day post-SCI group vs. the control group	BP	GO:1902548	negative regulation of cellular response to vascula	1/88	0.0388208	0.0758999	0.034375	Xdh
7-day post-SCI group vs. the control group	BP	GO:1902931	negative regulation of alcohol biosynthetic process	1/88	0.0388208	0.0758999	0.034375	Apoe
7-day post-SCI group vs. the control group	BP	GO:1902950	regulation of dendritic spine maintenance	1/88	0.0388208	0.0758999	0.034375	Apoe

7-day post-SCI group vs. the control group	BP	GO:1903651	positive regulation of cytoplasmic transport	1/88	0.0388208	0.0758999	0.034375	Ezr
7-day post-SCI group vs. the control group	BP	GO:1903977	positive regulation of glial cell migration	1/88	0.0388208	0.0758999	0.034375	Trem2
7-day post-SCI group vs. the control group	BP	GO:1904753	negative regulation of vascular associated smooth	1/88	0.0388208	0.0758999	0.034375	Nfe2l2
7-day post-SCI group vs. the control group	BP	GO:1905668	positive regulation of protein localization to endos	1/88	0.0388208	0.0758999	0.034375	Ezr
7-day post-SCI group vs. the control group	BP	GO:2000489	regulation of hepatic stellate cell activation	1/88	0.0388208	0.0758999	0.034375	Gclc
7-day post-SCI group vs. the control group	BP	GO:2000668	regulation of dendritic cell apoptotic process	1/88	0.0388208	0.0758999	0.034375	Axl
7-day post-SCI group vs. the control group	BP	GO:2000672	negative regulation of motor neuron apoptotic proc	1/88	0.0388208	0.0758999	0.034375	Map2k4
7-day post-SCI group vs. the control group	BP	GO:0062014	negative regulation of small molecule metabolic pr	2/88	0.0393859	0.0769593	0.0348548	Apoe/Ppargc1a
7-day post-SCI group vs. the control group	BP	GO:0000045	autophagosome assembly	2/88	0.0400805	0.0780861	0.0353651	Atp2a2/Pink1
7-day post-SCI group vs. the control group	BP	GO:0007173	epidermal growth factor receptor signaling pathwa	2/88	0.0400805	0.0780861	0.0353651	Fbxw7/Hbegf
7-day post-SCI group vs. the control group	BP	GO:0007229	integrin-mediated signaling pathway	2/88	0.0400805	0.0780861	0.0353651	Itga6/Itgb5
7-day post-SCI group vs. the control group	BP	GO:0046718	viral entry into host cell	2/88	0.0400805	0.0780861	0.0353651	Axl/Ctsl
7-day post-SCI group vs. the control group	BP	GO:0055013	cardiac muscle cell development	2/88	0.0400805	0.0780861	0.0353651	Map2k4/Slc8a1
7-day post-SCI group vs. the control group	BP	GO:0016236	macroautophagy	3/88	0.0404743	0.0788071	0.0356916	Atp2a2/Hif1a/Pink1
7-day post-SCI group vs. the control group	BP	GO:0009135	purine nucleoside diphosphate metabolic process	2/88	0.0407797	0.0791855	0.035863	Hif1a/Ppargc1a
7-day post-SCI group vs. the control group	BP	GO:0009179	purine ribonucleoside diphosphate metabolic proc	2/88	0.0407797	0.0791855	0.035863	Hif1a/Ppargc1a
7-day post-SCI group vs. the control group	BP	GO:0019217	regulation of fatty acid metabolic process	2/88	0.0407797	0.0791855	0.035863	Pdk2/Ppargc1a
7-day post-SCI group vs. the control group	BP	GO:0033077	T cell differentiation in thymus	2/88	0.0407797	0.0791855	0.035863	Apc/Ctnnb1
7-day post-SCI group vs. the control group	BP	GO:0001704	formation of primary germ layer	2/88	0.0414835	0.0791855	0.035863	Ctnnb1/Ets2
7-day post-SCI group vs. the control group	BP	GO:0001840	neural plate development	1/88	0.0417445	0.0791855	0.035863	Ctnnb1
7-day post-SCI group vs. the control group	BP	GO:0002829	negative regulation of type 2 immune response	1/88	0.0417445	0.0791855	0.035863	Stat6
7-day post-SCI group vs. the control group	BP	GO:0009120	deoxyribonucleoside metabolic process	1/88	0.0417445	0.0791855	0.035863	Xdh
7-day post-SCI group vs. the control group	BP	GO:0010457	centriole-centriole cohesion	1/88	0.0417445	0.0791855	0.035863	Ctnnb1
7-day post-SCI group vs. the control group	BP	GO:0010459	negative regulation of heart rate	1/88	0.0417445	0.0791855	0.035863	Fkbp1b
7-day post-SCI group vs. the control group	BP	GO:0010936	negative regulation of macrophage cytokine produ	1/88	0.0417445	0.0791855	0.035863	Axl
7-day post-SCI group vs. the control group	BP	GO:0015867	ATP transport	1/88	0.0417445	0.0791855	0.035863	Slc25a24
7-day post-SCI group vs. the control group	BP	GO:0032253	dense core granule localization	1/88	0.0417445	0.0791855	0.035863	Mapk8
7-day post-SCI group vs. the control group	BP	GO:0032494	response to peptidoglycan	1/88	0.0417445	0.0791855	0.035863	Trem2
7-day post-SCI group vs. the control group	BP	GO:0032536	regulation of cell projection size	1/88	0.0417445	0.0791855	0.035863	Ezr
7-day post-SCI group vs. the control group	BP	GO:0034393	positive regulation of smooth muscle cell apoptoti	1/88	0.0417445	0.0791855	0.035863	Map2k4
7-day post-SCI group vs. the control group	BP	GO:0036499	PERK-mediated unfolded protein response	1/88	0.0417445	0.0791855	0.035863	Nfe2l2
7-day post-SCI group vs. the control group	BP	GO:0042559	pteridine-containing compound biosynthetic proce	1/88	0.0417445	0.0791855	0.035863	Gch1
7-day post-SCI group vs. the control group	BP	GO:0045793	positive regulation of cell size	1/88	0.0417445	0.0791855	0.035863	Atp7a
7-day post-SCI group vs. the control group	BP	GO:0045906	negative regulation of vasoconstriction	1/88	0.0417445	0.0791855	0.035863	Hif1a
7-day post-SCI group vs. the control group	BP	GO:0048680	positive regulation of axon regeneration	1/88	0.0417445	0.0791855	0.035863	Fkbp1b
7-day post-SCI group vs. the control group	BP	GO:0048711	positive regulation of astrocyte differentiation	1/88	0.0417445	0.0791855	0.035863	Il6st
7-day post-SCI group vs. the control group	BP	GO:0051764	actin crosslink formation	1/88	0.0417445	0.0791855	0.035863	Aif1
7-day post-SCI group vs. the control group	BP	GO:0060263	regulation of respiratory burst	1/88	0.0417445	0.0791855	0.035863	Ncf1
7-day post-SCI group vs. the control group	BP	GO:0060346	bone trabecula formation	1/88	0.0417445	0.0791855	0.035863	Ppargc1b
7-day post-SCI group vs. the control group	BP	GO:0070344	regulation of fat cell proliferation	1/88	0.0417445	0.0791855	0.035863	Trem2

7-day post-SCI group vs. the control group	BP	GO:0072160	nephron tubule epithelial cell differentiation	1/88	0.0417445	0.0791855	0.035863	Ctnnb1
7-day post-SCI group vs. the control group	BP	GO:0090153	regulation of sphingolipid biosynthetic process	1/88	0.0417445	0.0791855	0.035863	Prkcd
7-day post-SCI group vs. the control group	BP	GO:0090331	negative regulation of platelet aggregation	1/88	0.0417445	0.0791855	0.035863	Prkcd
7-day post-SCI group vs. the control group	BP	GO:0097113	AMPA glutamate receptor clustering	1/88	0.0417445	0.0791855	0.035863	Apoe
7-day post-SCI group vs. the control group	BP	GO:0097688	glutamate receptor clustering	1/88	0.0417445	0.0791855	0.035863	Apoe
7-day post-SCI group vs. the control group	BP	GO:1900038	negative regulation of cellular response to hypoxia	1/88	0.0417445	0.0791855	0.035863	Pink1
7-day post-SCI group vs. the control group	BP	GO:1901524	regulation of mitophagy	1/88	0.0417445	0.0791855	0.035863	Pink1
7-day post-SCI group vs. the control group	BP	GO:1901950	dense core granule transport	1/88	0.0417445	0.0791855	0.035863	Mapk8
7-day post-SCI group vs. the control group	BP	GO:1902036	regulation of hematopoietic stem cell differentiatic	1/88	0.0417445	0.0791855	0.035863	Nfe2l2
7-day post-SCI group vs. the control group	BP	GO:1902902	negative regulation of autophagosome assembly	1/88	0.0417445	0.0791855	0.035863	Pink1
7-day post-SCI group vs. the control group	BP	GO:1902969	mitotic DNA replication	1/88	0.0417445	0.0791855	0.035863	Mcm4
7-day post-SCI group vs. the control group	BP	GO:1904948	midbrain dopaminergic neuron differentiation	1/88	0.0417445	0.0791855	0.035863	Ctnnb1
7-day post-SCI group vs. the control group	BP	GO:1905038	regulation of membrane lipid metabolic process	1/88	0.0417445	0.0791855	0.035863	Prkcd
7-day post-SCI group vs. the control group	BP	GO:1905666	regulation of protein localization to endosome	1/88	0.0417445	0.0791855	0.035863	Ezr
7-day post-SCI group vs. the control group	BP	GO:1905809	negative regulation of synapse organization	1/88	0.0417445	0.0791855	0.035863	Apoe
7-day post-SCI group vs. the control group	BP	GO:2000343	positive regulation of chemokine (C-X-C motif) li	1/88	0.0417445	0.0791855	0.035863	Tlr4
7-day post-SCI group vs. the control group	BP	GO:2000644	regulation of receptor catabolic process	1/88	0.0417445	0.0791855	0.035863	Apoe
7-day post-SCI group vs. the control group	BP	GO:2000822	regulation of behavioral fear response	1/88	0.0417445	0.0791855	0.035863	Apoe
7-day post-SCI group vs. the control group	BP	GO:2001140	positive regulation of phospholipid transport	1/88	0.0417445	0.0791855	0.035863	Apoe
7-day post-SCI group vs. the control group	BP	GO:0021761	limbic system development	2/88	0.0421919	0.07976	0.0361232	Ctnnb1/Hdac1
7-day post-SCI group vs. the control group	BP	GO:0032006	regulation of TOR signaling	2/88	0.0421919	0.07976	0.0361232	Hif1a/Trem2
7-day post-SCI group vs. the control group	BP	GO:0060993	kidney morphogenesis	2/88	0.0421919	0.07976	0.0361232	Ctnnb1/Ppp3ca
7-day post-SCI group vs. the control group	BP	GO:0070167	regulation of biomineral tissue development	2/88	0.0421919	0.07976	0.0361232	Hif1a/Slc8a1
7-day post-SCI group vs. the control group	BP	GO:1904427	positive regulation of calcium ion transmembrane	2/88	0.0421919	0.07976	0.0361232	Ppp3ca/S100a1
7-day post-SCI group vs. the control group	BP	GO:1905818	regulation of chromosome separation	2/88	0.0421919	0.07976	0.0361232	Apc/Cdc20
7-day post-SCI group vs. the control group	BP	GO:0006576	cellular biogenic amine metabolic process	2/88	0.0429048	0.0810153	0.0366917	Atp7a/Gch1
7-day post-SCI group vs. the control group	BP	GO:0044409	entry into host	2/88	0.0429048	0.0810153	0.0366917	Axl/Ctsl
7-day post-SCI group vs. the control group	BP	GO:0007264	small GTPase mediated signal transduction	4/88	0.0435129	0.0813598	0.0368477	Aif1/Apoe/Dhcr24/Jun
7-day post-SCI group vs. the control group	BP	GO:1901987	regulation of cell cycle phase transition	4/88	0.0435129	0.0813598	0.0368477	Aif1/Apc/Cdc20/Mapk14
7-day post-SCI group vs. the control group	BP	GO:0000041	transition metal ion transport	2/88	0.0436223	0.0813598	0.0368477	Atp7a/Trpm2
7-day post-SCI group vs. the control group	BP	GO:0006165	nucleoside diphosphate phosphorylation	2/88	0.0436223	0.0813598	0.0368477	Hif1a/Ppargc1a
7-day post-SCI group vs. the control group	BP	GO:0015844	monoamine transport	2/88	0.0436223	0.0813598	0.0368477	Pink1/Ptgs1
7-day post-SCI group vs. the control group	BP	GO:0030516	regulation of axon extension	2/88	0.0436223	0.0813598	0.0368477	Apoe/Mapt
7-day post-SCI group vs. the control group	BP	GO:0070227	lymphocyte apoptotic process	2/88	0.0436223	0.0813598	0.0368477	Hif1a/Ripk1
7-day post-SCI group vs. the control group	BP	GO:0110149	regulation of biomineralization	2/88	0.0436223	0.0813598	0.0368477	Hif1a/Slc8a1
7-day post-SCI group vs. the control group	BP	GO:0034976	response to endoplasmic reticulum stress	3/88	0.0437657	0.0813598	0.0368477	Clu/Jun/Nfe2l2
7-day post-SCI group vs. the control group	BP	GO:0044843	cell cycle G1/S phase transition	3/88	0.0437657	0.0813598	0.0368477	Aif1/Apc/Ppp3ca
7-day post-SCI group vs. the control group	BP	GO:0046700	heterocycle catabolic process	4/88	0.044125	0.0813598	0.0368477	Ncf1/Nfe2l2/Prr5l/Xdh
7-day post-SCI group vs. the control group	BP	GO:0046916	cellular transition metal ion homeostasis	2/88	0.0443443	0.0813598	0.0368477	Atp7a/Hif1a
7-day post-SCI group vs. the control group	BP	GO:0055006	cardiac cell development	2/88	0.0443443	0.0813598	0.0368477	Map2k4/Slc8a1

7-day post-SCI group vs. the control group	BP	GO:0001711	endodermal cell fate commitment	1/88	0.0446595	0.0813598	0.0368477	Ctnnb1
7-day post-SCI group vs. the control group	BP	GO:0002730	regulation of dendritic cell cytokine production	1/88	0.0446595	0.0813598	0.0368477	Tlr4
7-day post-SCI group vs. the control group	BP	GO:0006570	tyrosine metabolic process	1/88	0.0446595	0.0813598	0.0368477	Atp7a
7-day post-SCI group vs. the control group	BP	GO:0006825	copper ion transport	1/88	0.0446595	0.0813598	0.0368477	Atp7a
7-day post-SCI group vs. the control group	BP	GO:0009155	purine deoxyribonucleotide catabolic process	1/88	0.0446595	0.0813598	0.0368477	Xdh
7-day post-SCI group vs. the control group	BP	GO:0009169	purine ribonucleoside monophosphate catabolic pr	1/88	0.0446595	0.0813598	0.0368477	Xdh
7-day post-SCI group vs. the control group	BP	GO:0009170	purine deoxyribonucleoside monophosphate metal	1/88	0.0446595	0.0813598	0.0368477	Xdh
7-day post-SCI group vs. the control group	BP	GO:0010421	hydrogen peroxide-mediated programmed cell dea	1/88	0.0446595	0.0813598	0.0368477	Pink1
7-day post-SCI group vs. the control group	BP	GO:0010561	negative regulation of glycoprotein biosynthetic pr	1/88	0.0446595	0.0813598	0.0368477	Hbegf
7-day post-SCI group vs. the control group	BP	GO:0010831	positive regulation of myotube differentiation	1/88	0.0446595	0.0813598	0.0368477	Mapk14
7-day post-SCI group vs. the control group	BP	GO:0010887	negative regulation of cholesterol storage	1/88	0.0446595	0.0813598	0.0368477	Trem2
7-day post-SCI group vs. the control group	BP	GO:0010889	regulation of sequestering of triglyceride	1/88	0.0446595	0.0813598	0.0368477	Trem2
7-day post-SCI group vs. the control group	BP	GO:0010988	regulation of low-density lipoprotein particle clear	1/88	0.0446595	0.0813598	0.0368477	Trem2
7-day post-SCI group vs. the control group	BP	GO:0021781	glial cell fate commitment	1/88	0.0446595	0.0813598	0.0368477	Ctnnb1
7-day post-SCI group vs. the control group	BP	GO:0031000	response to caffeine	1/88	0.0446595	0.0813598	0.0368477	Slc8a1
7-day post-SCI group vs. the control group	BP	GO:0031269	pseudopodium assembly	1/88	0.0446595	0.0813598	0.0368477	Apc
7-day post-SCI group vs. the control group	BP	GO:0032372	negative regulation of sterol transport	1/88	0.0446595	0.0813598	0.0368477	Apoe
7-day post-SCI group vs. the control group	BP	GO:0032375	negative regulation of cholesterol transport	1/88	0.0446595	0.0813598	0.0368477	Apoe
7-day post-SCI group vs. the control group	BP	GO:0032488	Cdc42 protein signal transduction	1/88	0.0446595	0.0813598	0.0368477	Apoe
7-day post-SCI group vs. the control group	BP	GO:0032530	regulation of microvillus organization	1/88	0.0446595	0.0813598	0.0368477	Ezr
7-day post-SCI group vs. the control group	BP	GO:0032688	negative regulation of interferon-beta production	1/88	0.0446595	0.0813598	0.0368477	Sirpa
7-day post-SCI group vs. the control group	BP	GO:0032736	positive regulation of interleukin-13 production	1/88	0.0446595	0.0813598	0.0368477	Tlr4
7-day post-SCI group vs. the control group	BP	GO:0033700	phospholipid efflux	1/88	0.0446595	0.0813598	0.0368477	Apoe
7-day post-SCI group vs. the control group	BP	GO:0035733	hepatic stellate cell activation	1/88	0.0446595	0.0813598	0.0368477	Gclc
7-day post-SCI group vs. the control group	BP	GO:0036376	sodium ion export across plasma membrane	1/88	0.0446595	0.0813598	0.0368477	Slc8a1
7-day post-SCI group vs. the control group	BP	GO:0038128	ERBB2 signaling pathway	1/88	0.0446595	0.0813598	0.0368477	Hbegf
7-day post-SCI group vs. the control group	BP	GO:0038203	TORC2 signaling	1/88	0.0446595	0.0813598	0.0368477	Prr5l
7-day post-SCI group vs. the control group	BP	GO:0043455	regulation of secondary metabolic process	1/88	0.0446595	0.0813598	0.0368477	Atp7a
7-day post-SCI group vs. the control group	BP	GO:0044794	positive regulation by host of viral process	1/88	0.0446595	0.0813598	0.0368477	Apoe
7-day post-SCI group vs. the control group	BP	GO:0046085	adenosine metabolic process	1/88	0.0446595	0.0813598	0.0368477	Xdh
7-day post-SCI group vs. the control group	BP	GO:0046479	glycosphingolipid catabolic process	1/88	0.0446595	0.0813598	0.0368477	Prkcd
7-day post-SCI group vs. the control group	BP	GO:0048021	regulation of melanin biosynthetic process	1/88	0.0446595	0.0813598	0.0368477	Atp7a
7-day post-SCI group vs. the control group	BP	GO:0048102	autophagic cell death	1/88	0.0446595	0.0813598	0.0368477	Trem2
7-day post-SCI group vs. the control group	BP	GO:0051280	negative regulation of release of sequestered calciu	1/88	0.0446595	0.0813598	0.0368477	Fkbp1b
7-day post-SCI group vs. the control group	BP	GO:0051782	negative regulation of cell division	1/88	0.0446595	0.0813598	0.0368477	Txnip
7-day post-SCI group vs. the control group	BP	GO:0055089	fatty acid homeostasis	1/88	0.0446595	0.0813598	0.0368477	Apoe
7-day post-SCI group vs. the control group	BP	GO:0060742	epithelial cell differentiation involved in prostate g	1/88	0.0446595	0.0813598	0.0368477	Ctnnb1
7-day post-SCI group vs. the control group	BP	GO:0070341	fat cell proliferation	1/88	0.0446595	0.0813598	0.0368477	Trem2
7-day post-SCI group vs. the control group	BP	GO:0070572	positive regulation of neuron projection regenerati	1/88	0.0446595	0.0813598	0.0368477	Fkbp1b
7-day post-SCI group vs. the control group	BP	GO:0070943	neutrophil-mediated killing of symbiont cell	1/88	0.0446595	0.0813598	0.0368477	Ncf1

7-day post-SCI group vs. the control group	BP	GO:0072283	metanephric renal vesicle morphogenesis	1/88	0.0446595	0.0813598	0.0368477	Ctnnb1
7-day post-SCI group vs. the control group	BP	GO:0097050	type B pancreatic cell apoptotic process	1/88	0.0446595	0.0813598	0.0368477	Mapk8
7-day post-SCI group vs. the control group	BP	GO:0098840	protein transport along microtubule	1/88	0.0446595	0.0813598	0.0368477	Hspb1
7-day post-SCI group vs. the control group	BP	GO:0099118	microtubule-based protein transport	1/88	0.0446595	0.0813598	0.0368477	Hspb1
7-day post-SCI group vs. the control group	BP	GO:0150079	negative regulation of neuroinflammatory respons	1/88	0.0446595	0.0813598	0.0368477	Trem2
7-day post-SCI group vs. the control group	BP	GO:1900376	regulation of secondary metabolite biosynthetic pr	1/88	0.0446595	0.0813598	0.0368477	Atp7a
7-day post-SCI group vs. the control group	BP	GO:1903365	regulation of fear response	1/88	0.0446595	0.0813598	0.0368477	Apoe
7-day post-SCI group vs. the control group	BP	GO:1904925	positive regulation of autophagy of mitochondrion	1/88	0.0446595	0.0813598	0.0368477	Pink1
7-day post-SCI group vs. the control group	BP	GO:1905665	positive regulation of calcium ion import across pl	1/88	0.0446595	0.0813598	0.0368477	Ppp3ca
7-day post-SCI group vs. the control group	BP	GO:2000811	negative regulation of anoikis	1/88	0.0446595	0.0813598	0.0368477	Mcl1
7-day post-SCI group vs. the control group	BP	GO:2001053	regulation of mesenchymal cell apoptotic process	1/88	0.0446595	0.0813598	0.0368477	Hif1a
7-day post-SCI group vs. the control group	BP	GO:0006520	cellular amino acid metabolic process	3/88	0.0450336	0.0818838	0.037085	Apc/Atp7a/Gclc
7-day post-SCI group vs. the control group	BP	GO:1901988	negative regulation of cell cycle phase transition	3/88	0.0450336	0.0818838	0.037085	Apc/Cdc20/Mapk14
7-day post-SCI group vs. the control group	BP	GO:0030518	intracellular steroid hormone receptor signaling pa	2/88	0.0450707	0.0818838	0.037085	Hdac1/Ppargc1b
7-day post-SCI group vs. the control group	BP	GO:0061326	renal tubule development	2/88	0.0450707	0.0818838	0.037085	Ctnnb1/Stat1
7-day post-SCI group vs. the control group	BP	GO:0099175	regulation of postsynapse organization	2/88	0.0450707	0.0818838	0.037085	Apoe/Stau2
7-day post-SCI group vs. the control group	BP	GO:0019058	viral life cycle	3/88	0.0454602	0.0824558	0.0373441	Apoe/Axl/Ctsl
7-day post-SCI group vs. the control group	BP	GO:0045926	negative regulation of growth	3/88	0.0454602	0.0824558	0.0373441	Apc/Hif1a/Rbbp7
7-day post-SCI group vs. the control group	BP	GO:0048705	skeletal system morphogenesis	3/88	0.0454602	0.0824558	0.0373441	Ctnnb1/Mapk14/Ppargc1b
7-day post-SCI group vs. the control group	BP	GO:1905037	autophagosome organization	2/88	0.0458015	0.0829839	0.0375833	Atp2a2/Pink1
7-day post-SCI group vs. the control group	BP	GO:1905477	positive regulation of protein localization to memb	2/88	0.0458015	0.0829839	0.0375833	Ezr/Trem2
7-day post-SCI group vs. the control group	BP	GO:0005976	polysaccharide metabolic process	2/88	0.0465367	0.08402	0.0380525	Cd36/Il6st
7-day post-SCI group vs. the control group	BP	GO:0008630	intrinsic apoptotic signaling pathway in response t	2/88	0.0465367	0.08402	0.0380525	Clu/Mcl1
7-day post-SCI group vs. the control group	BP	GO:0021987	cerebral cortex development	2/88	0.0465367	0.08402	0.0380525	Ctnnb1/Hif1a
7-day post-SCI group vs. the control group	BP	GO:0031532	actin cytoskeleton reorganization	2/88	0.0465367	0.08402	0.0380525	Ezr/Trpm2
7-day post-SCI group vs. the control group	BP	GO:0046939	nucleotide phosphorylation	2/88	0.0465367	0.08402	0.0380525	Hif1a/Ppargc1a
7-day post-SCI group vs. the control group	BP	GO:0071482	cellular response to light stimulus	2/88	0.0465367	0.08402	0.0380525	Fbxw7/Prkcd
7-day post-SCI group vs. the control group	BP	GO:0045444	fat cell differentiation	3/88	0.0471871	0.08402	0.0380525	Gpx1/Itga6/Mapk14
7-day post-SCI group vs. the control group	BP	GO:1903076	regulation of protein localization to plasma memb	2/88	0.0472763	0.08402	0.0380525	Ezr/Trem2
7-day post-SCI group vs. the control group	BP	GO:0002371	dendritic cell cytokine production	1/88	0.0475656	0.08402	0.0380525	Tlr4
7-day post-SCI group vs. the control group	BP	GO:0006857	oligopeptide transport	1/88	0.0475656	0.08402	0.0380525	Mgst1
7-day post-SCI group vs. the control group	BP	GO:0006968	cellular defense response	1/88	0.0475656	0.08402	0.0380525	Ncf1
7-day post-SCI group vs. the control group	BP	GO:0009128	purine nucleoside monophosphate catabolic proces	1/88	0.0475656	0.08402	0.0380525	Xdh
7-day post-SCI group vs. the control group	BP	GO:0018119	peptidyl-cysteine S-nitrosylation	1/88	0.0475656	0.08402	0.0380525	Ncoa7
7-day post-SCI group vs. the control group	BP	GO:0019064	fusion of virus membrane with host plasma memb	1/88	0.0475656	0.08402	0.0380525	Ctsl
7-day post-SCI group vs. the control group	BP	GO:0019377	glycolipid catabolic process	1/88	0.0475656	0.08402	0.0380525	Prkcd
7-day post-SCI group vs. the control group	BP	GO:0021819	layer formation in cerebral cortex	1/88	0.0475656	0.08402	0.0380525	Ctnnb1
7-day post-SCI group vs. the control group	BP	GO:0021859	pyramidal neuron differentiation	1/88	0.0475656	0.08402	0.0380525	Atp7a
7-day post-SCI group vs. the control group	BP	GO:0023035	CD40 signaling pathway	1/88	0.0475656	0.08402	0.0380525	Trem2
7-day post-SCI group vs. the control group	BP	GO:0031987	locomotion involved in locomotory behavior	1/88	0.0475656	0.08402	0.0380525	Rcan1

7-day post-SCI group vs. the control group	BP	GO:0032239	regulation of nucleobase-containing compound tra	1/88	0.0475656	0.08402	0.0380525	Ripk1
7-day post-SCI group vs. the control group	BP	GO:0035112	genitalia morphogenesis	1/88	0.0475656	0.08402	0.0380525	Ctnnb1
7-day post-SCI group vs. the control group	BP	GO:0039663	membrane fusion involved in viral entry into host	1/88	0.0475656	0.08402	0.0380525	Ctsl
7-day post-SCI group vs. the control group	BP	GO:0042159	lipoprotein catabolic process	1/88	0.0475656	0.08402	0.0380525	Apoe
7-day post-SCI group vs. the control group	BP	GO:0042416	dopamine biosynthetic process	1/88	0.0475656	0.08402	0.0380525	Gch1
7-day post-SCI group vs. the control group	BP	GO:0042454	ribonucleoside catabolic process	1/88	0.0475656	0.08402	0.0380525	Xdh
7-day post-SCI group vs. the control group	BP	GO:0042541	hemoglobin biosynthetic process	1/88	0.0475656	0.08402	0.0380525	Hif1a
7-day post-SCI group vs. the control group	BP	GO:0042640	anagen	1/88	0.0475656	0.08402	0.0380525	Ctnnb1
7-day post-SCI group vs. the control group	BP	GO:0045346	regulation of MHC class II biosynthetic process	1/88	0.0475656	0.08402	0.0380525	Tlr4
7-day post-SCI group vs. the control group	BP	GO:0045651	positive regulation of macrophage differentiation	1/88	0.0475656	0.08402	0.0380525	Ripk1
7-day post-SCI group vs. the control group	BP	GO:0046541	saliva secretion	1/88	0.0475656	0.08402	0.0380525	Ppp3ca
7-day post-SCI group vs. the control group	BP	GO:0051151	negative regulation of smooth muscle cell differen	1/88	0.0475656	0.08402	0.0380525	Rcan1
7-day post-SCI group vs. the control group	BP	GO:0055119	relaxation of cardiac muscle	1/88	0.0475656	0.08402	0.0380525	Atp2a2
7-day post-SCI group vs. the control group	BP	GO:0061548	ganglion development	1/88	0.0475656	0.08402	0.0380525	Ctnnb1
7-day post-SCI group vs. the control group	BP	GO:0070431	nucleotide-binding oligomerization domain contai	1/88	0.0475656	0.08402	0.0380525	Tlr4
7-day post-SCI group vs. the control group	BP	GO:0070885	negative regulation of calcineurin-NFAT signaling	1/88	0.0475656	0.08402	0.0380525	Rcan1
7-day post-SCI group vs. the control group	BP	GO:0071371	cellular response to gonadotropin stimulus	1/88	0.0475656	0.08402	0.0380525	Ppargc1a
7-day post-SCI group vs. the control group	BP	GO:0072498	embryonic skeletal joint development	1/88	0.0475656	0.08402	0.0380525	Ctnnb1
7-day post-SCI group vs. the control group	BP	GO:0090128	regulation of synapse maturation	1/88	0.0475656	0.08402	0.0380525	Cdc20
7-day post-SCI group vs. the control group	BP	GO:0097468	programmed cell death in response to reactive oxy	1/88	0.0475656	0.08402	0.0380525	Pink1
7-day post-SCI group vs. the control group	BP	GO:0106057	negative regulation of calcineurin-mediated signal	1/88	0.0475656	0.08402	0.0380525	Rcan1
7-day post-SCI group vs. the control group	BP	GO:1902001	fatty acid transmembrane transport	1/88	0.0475656	0.08402	0.0380525	Cd36
7-day post-SCI group vs. the control group	BP	GO:1903799	negative regulation of miRNA maturation	1/88	0.0475656	0.08402	0.0380525	Ppp3ca
7-day post-SCI group vs. the control group	BP	GO:1903818	positive regulation of voltage-gated potassium cha	1/88	0.0475656	0.08402	0.0380525	Trem2
7-day post-SCI group vs. the control group	BP	GO:1903978	regulation of microglial cell activation	1/88	0.0475656	0.08402	0.0380525	Trem2
7-day post-SCI group vs. the control group	BP	GO:1904923	regulation of autophagy of mitochondrion in respo	1/88	0.0475656	0.08402	0.0380525	Pink1
7-day post-SCI group vs. the control group	BP	GO:2000288	positive regulation of myoblast proliferation	1/88	0.0475656	0.08402	0.0380525	Ctnnb1
7-day post-SCI group vs. the control group	BP	GO:2001212	regulation of vasculogenesis	1/88	0.0475656	0.08402	0.0380525	Xdh
7-day post-SCI group vs. the control group	BP	GO:0007088	regulation of mitotic nuclear division	2/88	0.0480202	0.0846873	0.0383548	Apc/Cdc20
7-day post-SCI group vs. the control group	BP	GO:0018107	peptidyl-threonine phosphorylation	2/88	0.0480202	0.0846873	0.0383548	Mapk8/Prkcd
7-day post-SCI group vs. the control group	BP	GO:0120034	positive regulation of plasma membrane bounded	2/88	0.0480202	0.0846873	0.0383548	Apc/Atp7a
7-day post-SCI group vs. the control group	BP	GO:0021537	telencephalon development	3/88	0.0485034	0.0854485	0.0386995	Ctnnb1/Hdac1/Hif1a
7-day post-SCI group vs. the control group	BP	GO:0032886	regulation of microtubule-based process	3/88	0.0485034	0.0854485	0.0386995	Apc/Mapk8/Mapt
7-day post-SCI group vs. the control group	BP	GO:0006986	response to unfolded protein	2/88	0.0487683	0.0858696	0.0388902	Hspb1/Nfe2l2
7-day post-SCI group vs. the control group	BP	GO:0044772	mitotic cell cycle phase transition	4/88	0.0488707	0.0860042	0.0389512	Aif1/Apc/Cdc20/Ppp3ca
7-day post-SCI group vs. the control group	BP	GO:0009185	ribonucleoside diphosphate metabolic process	2/88	0.0495208	0.0867326	0.0392811	Hif1a/Ppargc1a
7-day post-SCI group vs. the control group	BP	GO:0016241	regulation of macroautophagy	2/88	0.0495208	0.0867326	0.0392811	Hif1a/Pink1
7-day post-SCI group vs. the control group	BP	GO:0019218	regulation of steroid metabolic process	2/88	0.0495208	0.0867326	0.0392811	Apoe/Ppargc1a
7-day post-SCI group vs. the control group	BP	GO:0038127	ERBB signaling pathway	2/88	0.0495208	0.0867326	0.0392811	Fbxw7/Hbegf
7-day post-SCI group vs. the control group	BP	GO:0051952	regulation of amine transport	2/88	0.0495208	0.0867326	0.0392811	Pink1/Ptgs1

7-day post-SCI group vs. the control group	CC	GO:0045121	membrane raft	12/88	2.93E-09	4.23E-07	2.06E-07	Atp7a/Btk/Capn2/Cd36/Ctnnb1/Ezr/Il6st/Mapt/Ripk1/Stat6/Tlr4/Trem2
7-day post-SCI group vs. the control group	CC	GO:0098857	membrane microdomain	12/88	3.01E-09	4.23E-07	2.06E-07	Atp7a/Btk/Capn2/Cd36/Ctnnb1/Ezr/Il6st/Mapt/Ripk1/Stat6/Tlr4/Trem2
7-day post-SCI group vs. the control group	CC	GO:0030018	Z disc	7/88	1.6E-07	0.000015	0.0000073	Ctnnb1/Dst/Fkbp1b/Hspb1/Ppp3ca/S100a1/Slc8a1
7-day post-SCI group vs. the control group	CC	GO:0043204	perikaryon	7/88	2.91E-07	0.0000171	8.35E-06	Aif1/Atp7a/Ctsl/Hspb1/Map2k4/Mapk10/Mapk8
7-day post-SCI group vs. the control group	CC	GO:0031674	I band	7/88	3.05E-07	0.0000171	8.35E-06	Ctnnb1/Dst/Fkbp1b/Hspb1/Ppp3ca/S100a1/Slc8a1
7-day post-SCI group vs. the control group	CC	GO:0031252	cell leading edge	10/88	5.03E-07	0.0000236	0.0000115	Aif1/Amph/Apc/Atp7a/Ctnnb1/Dst/Ezr/Itgb5/Mapt/Mylk
7-day post-SCI group vs. the control group	CC	GO:0030017	sarcomere	7/88	2.59E-06	0.0001038	0.0000506	Ctnnb1/Dst/Fkbp1b/Hspb1/Ppp3ca/S100a1/Slc8a1
7-day post-SCI group vs. the control group	CC	GO:0030016	myofibril	7/88	5.66E-06	0.0001988	0.0000968	Ctnnb1/Dst/Fkbp1b/Hspb1/Ppp3ca/S100a1/Slc8a1
7-day post-SCI group vs. the control group	CC	GO:0043292	contractile fiber	7/88	8.19E-06	0.0002258	0.00011	Ctnnb1/Dst/Fkbp1b/Hspb1/Ppp3ca/S100a1/Slc8a1
7-day post-SCI group vs. the control group	CC	GO:0097386	glial cell projection	4/88	9.16E-06	0.0002258	0.00011	Aif1/Ezr/Mapt/Pink1
7-day post-SCI group vs. the control group	CC	GO:0019898	extrinsic component of membrane	8/88	9.21E-06	0.0002258	0.00011	Amph/Apc/Apoe/Atp2a2/Cdh11/Ctnnb1/Ezr/Ncf1
7-day post-SCI group vs. the control group	CC	GO:0090575	RNA polymerase II transcription regulator comple	7/88	9.64E-06	0.0002258	0.00011	Ctnnb1/Fos/Hif1a/Jun/Nfe2l2/Stat1/Stat6
7-day post-SCI group vs. the control group	CC	GO:0016323	basolateral plasma membrane	7/88	0.0000107	0.0002319	0.0001129	Adam9/Atp7a/Cd38/Ctnnb1/Ezr/Itga6/Slc8a1
7-day post-SCI group vs. the control group	CC	GO:0005902	microvillus	5/88	0.000018	0.0003616	0.0001761	Atp7a/Ctnnb1/Ctsl/Ezr/Vcam1
7-day post-SCI group vs. the control group	CC	GO:0009925	basal plasma membrane	7/88	0.000022	0.000412	0.0002006	Adam9/Atp7a/Cd38/Ctnnb1/Ezr/Itga6/Slc8a1
7-day post-SCI group vs. the control group	CC	GO:1990204	oxidoreductase complex	5/88	0.0000357	0.0006269	0.0003053	Cybb/Ncf1/Ndufa12/Ndufa6/Pdk2
7-day post-SCI group vs. the control group	CC	GO:0045178	basal part of cell	7/88	0.0000383	0.0006327	0.0003081	Adam9/Atp7a/Cd38/Ctnnb1/Ezr/Itga6/Slc8a1
7-day post-SCI group vs. the control group	CC	GO:0014704	intercalated disc	4/88	0.0000538	0.000808	0.0003935	Atp2a2/Ctnnb1/Dst/Slc8a1
7-day post-SCI group vs. the control group	CC	GO:0000791	euchromatin	4/88	0.000057	0.000808	0.0003935	Ctnnb1/Hif1a/Jun/Ppargc1a
7-day post-SCI group vs. the control group	CC	GO:0031253	cell projection membrane	7/88	0.0000575	0.000808	0.0003935	Aif1/Apc/Atp7a/Cd36/Ctnnb1/Ezr/Mapt
7-day post-SCI group vs. the control group	CC	GO:0097038	perinuclear endoplasmic reticulum	3/88	0.0000681	0.0009111	0.0004437	Capn2/Clu/Dst
7-day post-SCI group vs. the control group	CC	GO:0016529	sarcoplasmic reticulum	4/88	0.0000882	0.0011268	0.0005487	Atp2a2/Fkbp1b/S100a1/Xdh
7-day post-SCI group vs. the control group	CC	GO:0016342	catenin complex	3/88	0.0001164	0.0014223	0.0006926	Apc/Cdh11/Ctnnb1
7-day post-SCI group vs. the control group	CC	GO:0042383	sarcolemma	5/88	0.0001594	0.0018662	0.0009088	Cd36/Dst/Ezr/Slc8a1/Vcam1
7-day post-SCI group vs. the control group	CC	GO:0044291	cell-cell contact zone	4/88	0.0001701	0.0019116	0.0009309	Atp2a2/Ctnnb1/Dst/Slc8a1
7-day post-SCI group vs. the control group	CC	GO:0031672	A band	3/88	0.0002151	0.002239	0.0010904	Dst/Hspb1/S100a1
7-day post-SCI group vs. the control group	CC	GO:0034364	high-density lipoprotein particle	3/88	0.0002151	0.002239	0.0010904	Apoe/Clu/Lcat
7-day post-SCI group vs. the control group	CC	GO:0016528	sarcoplasm	4/88	0.000236	0.0023678	0.0011531	Atp2a2/Fkbp1b/S100a1/Xdh
7-day post-SCI group vs. the control group	CC	GO:0009898	cytoplasmic side of plasma membrane	5/88	0.0002444	0.0023678	0.0011531	Atp2a2/Dst/Ezr/Mapt/Ppp3ca
7-day post-SCI group vs. the control group	CC	GO:0030055	cell-substrate junction	5/88	0.0002635	0.0024683	0.001202	Capn2/Dst/Ezr/Itga6/Itgb5
7-day post-SCI group vs. the control group	CC	GO:0019897	extrinsic component of plasma membrane	5/88	0.000298	0.002701	0.0013153	Apc/Apoe/Atp2a2/Cdh11/Ctnnb1
7-day post-SCI group vs. the control group	CC	GO:0043235	receptor complex	7/88	0.0003533	0.0031026	0.0015109	Axl/Cd36/Il6st/Itga6/Itgb5/Ripk1/Tlr4
7-day post-SCI group vs. the control group	CC	GO:0005635	nuclear envelope	7/88	0.0003787	0.0032243	0.0015702	Apc/Apoe/Ctnnb1/Dst/Gch1/Ptgs1/Stau2
7-day post-SCI group vs. the control group	CC	GO:0034358	plasma lipoprotein particle	3/88	0.0004054	0.0032547	0.001585	Apoe/Clu/Lcat
7-day post-SCI group vs. the control group	CC	GO:1990777	lipoprotein particle	3/88	0.0004054	0.0032547	0.001585	Apoe/Clu/Lcat
7-day post-SCI group vs. the control group	CC	GO:0098562	cytoplasmic side of membrane	5/88	0.0004411	0.0034432	0.0016768	Atp2a2/Dst/Ezr/Mapt/Ppp3ca
7-day post-SCI group vs. the control group	CC	GO:0032994	protein-lipid complex	3/88	0.0004868	0.0036969	0.0018004	Apoe/Clu/Lcat
7-day post-SCI group vs. the control group	CC	GO:0031519	PcG protein complex	3/88	0.0005161	0.0038162	0.0018584	Cbx6/Phc1/Rbbp7

7-day post-SCI group vs. the control group	CC	GO:0043020	NADPH oxidase complex	2/88	0.0005971	0.0043018	0.0020949	Cybb/Ncf1
7-day post-SCI group vs. the control group	CC	GO:0098858	actin-based cell projection	5/88	0.0006969	0.0048264	0.0023504	Atp7a/Ctnnb1/Ctsl/Ezr/Vcam1
7-day post-SCI group vs. the control group	CC	GO:0030877	beta-catenin destruction complex	2/88	0.0007042	0.0048264	0.0023504	Apc/Ctnnb1
7-day post-SCI group vs. the control group	CC	GO:0045177	apical part of cell	7/88	0.0007476	0.005002	0.0024359	Atp7a/Cd36/Ctnnb1/Ctsl/Ezr/Mgst1/Vcam1
7-day post-SCI group vs. the control group	CC	GO:0044853	plasma membrane raft	4/88	0.0008157	0.0053304	0.0025958	Cd36/Ctnnb1/Ezr/Trem2
7-day post-SCI group vs. the control group	CC	GO:0005912	adherens junction	4/88	0.0009579	0.0061174	0.0029791	Apc/Cdh11/Ctnnb1/Ezr
7-day post-SCI group vs. the control group	CC	GO:0043198	dendritic shaft	3/88	0.0011461	0.0071566	0.0034851	Ctnnb1/Slc8a1/Stau2
7-day post-SCI group vs. the control group	CC	GO:0016581	NuRD complex	2/88	0.0012181	0.0072828	0.0035466	Hdac1/Rbbp7
7-day post-SCI group vs. the control group	CC	GO:0090545	CHD-type complex	2/88	0.0012181	0.0072828	0.0035466	Hdac1/Rbbp7
7-day post-SCI group vs. the control group	CC	GO:0031256	leading edge membrane	4/88	0.0013561	0.0079386	0.003866	Aif1/Amph/Apc/Mapt
7-day post-SCI group vs. the control group	CC	GO:0014069	postsynaptic density	6/88	0.0014006	0.0080318	0.0039114	Ctnnb1/Dst/Hspb1/Mapk10/Mapt/Slc8a1
7-day post-SCI group vs. the control group	CC	GO:0031430	M band	2/88	0.0015255	0.0085354	0.0041566	Hspb1/S100a1
7-day post-SCI group vs. the control group	CC	GO:0032279	asymmetric synapse	6/88	0.0015491	0.0085354	0.0041566	Ctnnb1/Dst/Hspb1/Mapk10/Mapt/Slc8a1
7-day post-SCI group vs. the control group	CC	GO:0017053	transcription repressor complex	3/88	0.0016489	0.0089105	0.0043393	Ctnnb1/Hdac1/Jun
7-day post-SCI group vs. the control group	CC	GO:0016234	inclusion body	3/88	0.0019103	0.0098306	0.0047873	Clu/Gpx1/Mapt
7-day post-SCI group vs. the control group	CC	GO:0005925	focal adhesion	4/88	0.0019383	0.0098306	0.0047873	Capn2/Dst/Ezr/Itgb5
7-day post-SCI group vs. the control group	CC	GO:0001891	phagocytic cup	2/88	0.0020486	0.0098306	0.0047873	Aif1/Tlr4
7-day post-SCI group vs. the control group	CC	GO:0016580	Sin3 complex	2/88	0.0020486	0.0098306	0.0047873	Hdac1/Rbbp7
7-day post-SCI group vs. the control group	CC	GO:0070822	Sin3-type complex	2/88	0.0020486	0.0098306	0.0047873	Hdac1/Rbbp7
7-day post-SCI group vs. the control group	CC	GO:0097449	astrocyte projection	2/88	0.0020486	0.0098306	0.0047873	Ezr/Pink1
7-day post-SCI group vs. the control group	CC	GO:0030027	lamellipodium	4/88	0.0020641	0.0098306	0.0047873	Aif1/Apc/Ctnnb1/Mylk
7-day post-SCI group vs. the control group	CC	GO:0031528	microvillus membrane	2/88	0.0022392	0.01011	0.0049234	Ctnnb1/Ezr
7-day post-SCI group vs. the control group	CC	GO:0097470	ribbon synapse	2/88	0.0022392	0.01011	0.0049234	Amph/Atp2a2
7-day post-SCI group vs. the control group	CC	GO:0098984	neuron to neuron synapse	6/88	0.002241	0.01011	0.0049234	Ctnnb1/Dst/Hspb1/Mapk10/Mapt/Slc8a1
7-day post-SCI group vs. the control group	CC	GO:0099572	postsynaptic specialization	6/88	0.0022666	0.01011	0.0049234	Ctnnb1/Dst/Hspb1/Mapk10/Mapt/Slc8a1
7-day post-SCI group vs. the control group	CC	GO:0005874	microtubule	6/88	0.0023449	0.0102957	0.0050138	Apc/Apoe/Dst/Mapt/Slc8a1/Stau2
7-day post-SCI group vs. the control group	CC	GO:0005758	mitochondrial intermembrane space	3/88	0.0028439	0.0121742	0.0059286	Capn2/Pink1/Txnip
7-day post-SCI group vs. the control group	CC	GO:0002102	podosome	2/88	0.0028594	0.0121742	0.0059286	Mapk8/Vcam1
7-day post-SCI group vs. the control group	CC	GO:0032993	protein-DNA complex	4/88	0.0032208	0.0135083	0.0065783	Ctnnb1/Fos/Mcm4/Nfe2l2
7-day post-SCI group vs. the control group	CC	GO:0032839	dendrite cytoplasm	2/88	0.0033126	0.0136888	0.0066662	Map2k4/Mapk8
7-day post-SCI group vs. the control group	CC	GO:0097440	apical dendrite	2/88	0.0035509	0.014461	0.0070422	Clu/Ppargc1a
7-day post-SCI group vs. the control group	CC	GO:0000792	heterochromatin	3/88	0.0037004	0.0148543	0.0072338	Cbx6/Hdac1/Phc1
7-day post-SCI group vs. the control group	CC	GO:0031970	organelle envelope lumen	3/88	0.0041262	0.0163303	0.0079526	Capn2/Pink1/Txnip
7-day post-SCI group vs. the control group	CC	GO:0008305	integrin complex	2/88	0.0048582	0.0189606	0.0092335	Itga6/Itgb5
7-day post-SCI group vs. the control group	CC	GO:0098685	Schaffer collateral - CA1 synapse	3/88	0.0053171	0.020463	0.0099651	Cdh11/Ctnnb1/Ppp3ca
7-day post-SCI group vs. the control group	CC	GO:0099568	cytoplasmic region	4/88	0.0053888	0.020463	0.0099651	Ctnnb1/Map2k4/Mapk8/Mapt
7-day post-SCI group vs. the control group	CC	GO:0150034	distal axon	5/88	0.0061698	0.0231164	0.0112573	Amph/Apc/Clu/Mapt/Slc8a1
7-day post-SCI group vs. the control group	CC	GO:0031965	nuclear membrane	4/88	0.0069143	0.0255646	0.0124495	Apc/Ctnnb1/Gch1/Stau2
7-day post-SCI group vs. the control group	CC	GO:0120111	neuron projection cytoplasm	2/88	0.0073393	0.0267835	0.0130431	Map2k4/Mapk8
7-day post-SCI group vs. the control group	CC	GO:0044295	axonal growth cone	2/88	0.0076821	0.0273249	0.0133067	Apc/Mapt

7-day post-SCI group vs. the control group	CC	GO:0099522	cytosolic region	2/88	0.0076821	0.0273249	0.0133067	Marcksl1/Prkcd
7-day post-SCI group vs. the control group	CC	GO:0000152	nuclear ubiquitin ligase complex	2/88	0.008389	0.0294665	0.0143497	Cdc20/Phc1
7-day post-SCI group vs. the control group	CC	GO:0005903	brush border	3/88	0.0098517	0.034177	0.0166436	Atp7a/Cd36/Ezr
7-day post-SCI group vs. the control group	CC	GO:0005747	mitochondrial respiratory chain complex I	2/88	0.0102785	0.034384	0.0167444	Ndufa12/Ndufa6
7-day post-SCI group vs. the control group	CC	GO:0030964	NADH dehydrogenase complex	2/88	0.0102785	0.034384	0.0167444	Ndufa12/Ndufa6
7-day post-SCI group vs. the control group	CC	GO:0045271	respiratory chain complex I	2/88	0.0102785	0.034384	0.0167444	Ndufa12/Ndufa6
7-day post-SCI group vs. the control group	CC	GO:0001726	ruffle	3/88	0.0111987	0.0370215	0.0180289	Aif1/Apc/Ezr
7-day post-SCI group vs. the control group	CC	GO:0098636	protein complex involved in cell adhesion	2/88	0.0127697	0.0417244	0.020319	Itga6/Itgb5
7-day post-SCI group vs. the control group	CC	GO:0097060	synaptic membrane	5/88	0.0145574	0.0470186	0.0228972	Ctnnb1/Hspb1/Itgb5/Marcksl1/Slc8a1
7-day post-SCI group vs. the control group	CC	GO:0016328	lateral plasma membrane	2/88	0.0179415	0.0572904	0.0278994	Apc/Ctnnb1
7-day post-SCI group vs. the control group	CC	GO:0032838	plasma membrane bounded cell projection cytopla	3/88	0.0189305	0.0597692	0.0291066	Map2k4/Mapk8/Mapt
7-day post-SCI group vs. the control group	CC	GO:0030315	T-tubule	2/88	0.0194818	0.0608266	0.0296215	Ezr/Slc8a1
7-day post-SCI group vs. the control group	CC	GO:0005741	mitochondrial outer membrane	3/88	0.0200251	0.0618357	0.0301129	Mcl1/Mgst1/Pink1
7-day post-SCI group vs. the control group	CC	GO:0032587	ruffle membrane	2/88	0.0205385	0.0627319	0.0305493	Aif1/Apc
7-day post-SCI group vs. the control group	CC	GO:0030133	transport vesicle	4/88	0.0216896	0.0655352	0.0319145	Amph/Apc/Atp7a/Marcksl1
7-day post-SCI group vs. the control group	CC	GO:0098802	plasma membrane signaling receptor complex	3/88	0.0232124	0.0693902	0.0337918	Il6st/Itga6/Itgb5
7-day post-SCI group vs. the control group	CC	GO:0030426	growth cone	3/88	0.0241268	0.0713645	0.0347533	Apc/Clu/Mapt
7-day post-SCI group vs. the control group	CC	GO:0031526	brush border membrane	2/88	0.0244199	0.0714791	0.0348091	Atp7a/Cd36
7-day post-SCI group vs. the control group	CC	GO:0098862	cluster of actin-based cell projections	3/88	0.0250605	0.0725979	0.0353539	Atp7a/Cd36/Ezr
7-day post-SCI group vs. the control group	CC	GO:0000118	histone deacetylase complex	2/88	0.026168	0.0732701	0.0356813	Hdac1/Rbbp7
7-day post-SCI group vs. the control group	CC	GO:0019867	outer membrane	3/88	0.0263355	0.0732701	0.0356813	Mcl1/Mgst1/Pink1
7-day post-SCI group vs. the control group	CC	GO:0030427	site of polarized growth	3/88	0.0263355	0.0732701	0.0356813	Apc/Clu/Mapt
7-day post-SCI group vs. the control group	CC	GO:0031968	organelle outer membrane	3/88	0.0263355	0.0732701	0.0356813	Mcl1/Mgst1/Pink1
7-day post-SCI group vs. the control group	CC	GO:0016324	apical plasma membrane	4/88	0.0268897	0.0740785	0.0360749	Atp7a/Ctnnb1/Ezr/Vcam1
7-day post-SCI group vs. the control group	CC	GO:1902495	transmembrane transporter complex	4/88	0.0275878	0.0752639	0.0366522	Atp2a2/Fkbp1b/Ndufa12/Ndufa6
7-day post-SCI group vs. the control group	CC	GO:0005746	mitochondrial respirasome	2/88	0.0279655	0.0755606	0.0367967	Ndufa12/Ndufa6
7-day post-SCI group vs. the control group	CC	GO:0044306	neuron projection terminus	3/88	0.0283123	0.0757692	0.0368983	Amph/Apc/Slc8a1
7-day post-SCI group vs. the control group	CC	GO:0098803	respiratory chain complex	2/88	0.0291907	0.0769923	0.0374939	Ndufa12/Ndufa6
7-day post-SCI group vs. the control group	CC	GO:0001725	stress fiber	2/88	0.0298112	0.0769923	0.0374939	Dst/Mylk
7-day post-SCI group vs. the control group	CC	GO:0097517	contractile actin filament bundle	2/88	0.0298112	0.0769923	0.0374939	Dst/Mylk
7-day post-SCI group vs. the control group	CC	GO:0016600	flotillin complex	1/88	0.0301393	0.0769923	0.0374939	Ctnnb1
7-day post-SCI group vs. the control group	CC	GO:0070852	cell body fiber	1/88	0.0301393	0.0769923	0.0374939	Apc
7-day post-SCI group vs. the control group	CC	GO:0070603	SWI/SNF superfamily-type complex	2/88	0.0323456	0.0779419	0.0379564	Hdac1/Rbbp7
7-day post-SCI group vs. the control group	CC	GO:1990351	transporter complex	4/88	0.0330391	0.0779419	0.0379564	Atp2a2/Fkbp1b/Ndufa12/Ndufa6
7-day post-SCI group vs. the control group	CC	GO:0001931	uropod	1/88	0.0331034	0.0779419	0.0379564	Ezr
7-day post-SCI group vs. the control group	CC	GO:0031254	cell trailing edge	1/88	0.0331034	0.0779419	0.0379564	Ezr
7-day post-SCI group vs. the control group	CC	GO:0031932	TORC2 complex	1/88	0.0331034	0.0779419	0.0379564	Prr5l
7-day post-SCI group vs. the control group	CC	GO:0036019	endolysosome	1/88	0.0331034	0.0779419	0.0379564	Prkcd
7-day post-SCI group vs. the control group	CC	GO:0042555	MCM complex	1/88	0.0331034	0.0779419	0.0379564	Mcm4
7-day post-SCI group vs. the control group	CC	GO:0071162	CMG complex	1/88	0.0331034	0.0779419	0.0379564	Mcm4

7-day post-SCI group vs. the control group	CC	GO:1990909	Wnt signalosome	1/88	0.0331034	0.0779419	0.0379564	Ctnnb1
7-day post-SCI group vs. the control group	CC	GO:0070469	respirasome	2/88	0.0343001	0.0779419	0.0379564	Ndufa12/Ndufa6
7-day post-SCI group vs. the control group	CC	GO:0005901	caveola	2/88	0.0349616	0.0779419	0.0379564	Cd36/Ctnnb1
7-day post-SCI group vs. the control group	CC	GO:1904949	ATPase complex	2/88	0.0349616	0.0779419	0.0379564	Hdac1/Rbbp7
7-day post-SCI group vs. the control group	CC	GO:0008021	synaptic vesicle	3/88	0.0350789	0.0779419	0.0379564	Amph/Apc/Marcksl1
7-day post-SCI group vs. the control group	CC	GO:0032432	actin filament bundle	2/88	0.0356281	0.0779419	0.0379564	Dst/Mylk
7-day post-SCI group vs. the control group	CC	GO:0042641	actomyosin	2/88	0.0356281	0.0779419	0.0379564	Dst/Mylk
7-day post-SCI group vs. the control group	CC	GO:0031143	pseudopodium	1/88	0.0360585	0.0779419	0.0379564	Capn2
7-day post-SCI group vs. the control group	CC	GO:0031261	DNA replication preinitiation complex	1/88	0.0360585	0.0779419	0.0379564	Mcm4
7-day post-SCI group vs. the control group	CC	GO:0060187	cell pole	1/88	0.0360585	0.0779419	0.0379564	Ezr
7-day post-SCI group vs. the control group	CC	GO:0097441	basal dendrite	1/88	0.0360585	0.0779419	0.0379564	Mapk8
7-day post-SCI group vs. the control group	CC	GO:0098684	photoreceptor ribbon synapse	1/88	0.0360585	0.0779419	0.0379564	Amph
7-day post-SCI group vs. the control group	CC	GO:0030175	filopodium	2/88	0.0369759	0.0793147	0.0386249	Ezr/Vcam1
7-day post-SCI group vs. the control group	CC	GO:1905369	endopeptidase complex	2/88	0.0376571	0.0800024	0.0389598	Capn2/Hspb1
7-day post-SCI group vs. the control group	CC	GO:0005916	fascia adherens	1/88	0.0390047	0.0800024	0.0389598	Ctnnb1
7-day post-SCI group vs. the control group	CC	GO:0042627	chylomicron	1/88	0.0390047	0.0800024	0.0389598	Apoe
7-day post-SCI group vs. the control group	CC	GO:0098833	presynaptic endocytic zone	1/88	0.0390047	0.0800024	0.0389598	Amph
7-day post-SCI group vs. the control group	CC	GO:0099026	anchored component of presynaptic membrane	1/88	0.0390047	0.0800024	0.0389598	Marcksl1
7-day post-SCI group vs. the control group	CC	GO:1990907	beta-catenin-TCF complex	1/88	0.0390047	0.0800024	0.0389598	Ctnnb1
7-day post-SCI group vs. the control group	CC	GO:0005770	late endosome	3/88	0.0401479	0.0811624	0.0395247	Apoe/Atp7a/Ctsl
7-day post-SCI group vs. the control group	CC	GO:0099240	intrinsic component of synaptic membrane	3/88	0.0401479	0.0811624	0.0395247	Itgb5/Marcksl1/Slc8a1
7-day post-SCI group vs. the control group	CC	GO:0031010	ISWI-type complex	1/88	0.041942	0.0824176	0.0401359	Rbbp7
7-day post-SCI group vs. the control group	CC	GO:0034362	low-density lipoprotein particle	1/88	0.041942	0.0824176	0.0401359	Apoe
7-day post-SCI group vs. the control group	CC	GO:0038201	TOR complex	1/88	0.041942	0.0824176	0.0401359	Prr5l
7-day post-SCI group vs. the control group	CC	GO:0090533	cation-transporting ATPase complex	1/88	0.041942	0.0824176	0.0401359	Atp2a2
7-day post-SCI group vs. the control group	CC	GO:0070382	exocytic vesicle	3/88	0.0442864	0.0857341	0.041751	Amph/Apc/Marcksl1
7-day post-SCI group vs. the control group	CC	GO:0008303	caspase complex	1/88	0.0448704	0.0857341	0.041751	Capn2
7-day post-SCI group vs. the control group	CC	GO:0035102	PRC1 complex	1/88	0.0448704	0.0857341	0.041751	Phc1
7-day post-SCI group vs. the control group	CC	GO:1990124	messenger ribonucleoprotein complex	1/88	0.0448704	0.0857341	0.041751	Stau2
7-day post-SCI group vs. the control group	CC	GO:0030672	synaptic vesicle membrane	2/88	0.0454604	0.0857341	0.041751	Amph/Apc
7-day post-SCI group vs. the control group	CC	GO:0099501	exocytic vesicle membrane	2/88	0.0454604	0.0857341	0.041751	Amph/Apc
7-day post-SCI group vs. the control group	CC	GO:0090571	RNA polymerase II transcription repressor comple	1/88	0.04779	0.0889337	0.0433092	Ctnnb1
7-day post-SCI group vs. the control group	CC	GO:0098533	ATPase dependent transmembrane transport comp	1/88	0.04779	0.0889337	0.0433092	Atp2a2
7-day post-SCI group vs. the control group	MF	GO:0061629	RNA polymerase II-specific DNA-binding transcri	13/88	1.53E-10	5.55E-08	3.33E-08	Ctnnb1/Ets2/Fos/Hdac1/Hif1a/Hspb1/Jun/Mapk14/Ncoa7/Nfe2l2/Ppargc1a/Ppargc1b/Stat1
7-day post-SCI group vs. the control group	MF	GO:0044389	ubiquitin-like protein ligase binding	12/88	4.95E-10	8.98E-08	5.39E-08	Apc/Clu/Fbxw7/Hif1a/Jun/Pink1/Ppargc1a/Prdx6/Prr5l/Ripk1/Stat1/Txnip
7-day post-SCI group vs. the control group	MF	GO:0031625	ubiquitin protein ligase binding	11/88	3.93E-09	4.75E-07	2.85E-07	Apc/Clu/Fbxw7/Hif1a/Jun/Pink1/Ppargc1a/Prdx6/Prr5l/Ripk1/Txnip
7-day post-SCI group vs. the control group	MF	GO:0016209	antioxidant activity	6/88	2.87E-07	0.000026	0.0000156	Apoe/Gpx1/Gpx3/Mgst1/Prdx6/Ptgs1

7-day post-SCI group vs. the control group	MF	GO:0050839	cell adhesion molecule binding	9/88	4.98E-07	0.0000334	0.00002	Adam9/Cdh11/Ctnnb1/Dst/Ezr/Itga6/Itgb5/Sirpa/Vcam1
7-day post-SCI group vs. the control group	MF	GO:0016922	nuclear receptor binding	7/88	5.51E-07	0.0000334	0.00002	Ctnnb1/Ets2/Hif1a/Ncoa7/Ppargc1a/Ppargc1b/Stat1
7-day post-SCI group vs. the control group	MF	GO:0008022	protein C-terminus binding	8/88	6.65E-07	0.0000345	0.0000207	Amph/Atp2a2/Cdc20/Ctnnb1/Dst/Ezr/Id1/Mapk14
7-day post-SCI group vs. the control group	MF	GO:0004601	peroxidase activity	5/88	1.02E-06	0.0000431	0.0000259	Gpx1/Gpx3/Mgst1/Prdx6/Ptgs1
7-day post-SCI group vs. the control group	MF	GO:0004674	protein serine/threonine kinase activity	10/88	1.13E-06	0.0000431	0.0000259	Map2k3/Map2k4/Mapk10/Mapk14/Mapk8/Mylk/Pdk2/Pink1/Prkcd/Ripk1
7-day post-SCI group vs. the control group	MF	GO:0016684	oxidoreductase activity, acting on peroxide as acce	5/88	1.32E-06	0.0000431	0.0000259	Gpx1/Gpx3/Mgst1/Prdx6/Ptgs1
7-day post-SCI group vs. the control group	MF	GO:0033218	amide binding	10/88	1.41E-06	0.0000431	0.0000259	Apoe/Cd36/Clu/Ctsl/Dhcr24/Fkbp1b/Lancl1/Mgst1/Ppp3ca/Trem2
7-day post-SCI group vs. the control group	MF	GO:0004602	glutathione peroxidase activity	4/88	1.43E-06	0.0000431	0.0000259	Gpx1/Gpx3/Mgst1/Prdx6
7-day post-SCI group vs. the control group	MF	GO:0071813	lipoprotein particle binding	4/88	1.66E-06	0.0000431	0.0000259	Apoe/Cd36/Mapt/Trem2
7-day post-SCI group vs. the control group	MF	GO:0071814	protein-lipid complex binding	4/88	1.66E-06	0.0000431	0.0000259	Apoe/Cd36/Mapt/Trem2
7-day post-SCI group vs. the control group	MF	GO:0042277	peptide binding	9/88	2.64E-06	0.0000638	0.0000383	Apoe/Cd36/Clu/Ctsl/Dhcr24/Lancl1/Mgst1/Ppp3ca/Trem2
7-day post-SCI group vs. the control group	MF	GO:0017124	SH3 domain binding	6/88	3.41E-06	0.0000774	0.0000464	Adam9/Gpx1/Lancl1/Mapt/Ncf1/Sirpa
7-day post-SCI group vs. the control group	MF	GO:0001221	transcription coregulator binding	6/88	3.72E-06	0.0000795	0.0000477	Ctnnb1/Fos/Hdac1/Hif1a/Nfe2l2/Stat1
7-day post-SCI group vs. the control group	MF	GO:0044548	S100 protein binding	3/88	0.0000102	0.0002058	0.0001235	Atp2a2/Ezr/S100a1
7-day post-SCI group vs. the control group	MF	GO:0004707	MAP kinase activity	3/88	0.0000156	0.0002986	0.0001792	Mapk10/Mapk14/Mapk8
7-day post-SCI group vs. the control group	MF	GO:0019903	protein phosphatase binding	6/88	0.0000245	0.0004446	0.0002669	Ctnnb1/Mapk14/Mapk8/Mapt/Sirpa/Stat6
7-day post-SCI group vs. the control group	MF	GO:0034185	apolipoprotein binding	3/88	0.0000315	0.000545	0.0003272	Lcat/Mapt/Trem2
7-day post-SCI group vs. the control group	MF	GO:0000979	RNA polymerase II core promoter sequence-speci	3/88	0.0000487	0.0008028	0.0004819	Fos/Hdac1/Stat1
7-day post-SCI group vs. the control group	MF	GO:0001540	amyloid-beta binding	4/88	0.0000535	0.0008125	0.0004877	Apoe/Cd36/Clu/Trem2
7-day post-SCI group vs. the control group	MF	GO:0004713	protein tyrosine kinase activity	5/88	0.0000537	0.0008125	0.0004877	Axl/Btk/Map2k3/Map2k4/Prkcd
7-day post-SCI group vs. the control group	MF	GO:0019902	phosphatase binding	6/88	0.0000904	0.0013133	0.0007883	Ctnnb1/Mapk14/Mapk8/Mapt/Sirpa/Stat6
7-day post-SCI group vs. the control group	MF	GO:0050750	low-density lipoprotein particle receptor binding	3/88	0.000099	0.0013828	0.0008301	Apoe/Clu/Lancl1
7-day post-SCI group vs. the control group	MF	GO:0005178	integrin binding	5/88	0.0001234	0.0016589	0.0009958	Adam9/Dst/Itga6/Itgb5/Vcam1
7-day post-SCI group vs. the control group	MF	GO:0051019	mitogen-activated protein kinase binding	3/88	0.0001466	0.0018347	0.0011013	Gch1/Mapk14/Stau2
7-day post-SCI group vs. the control group	MF	GO:0070325	lipoprotein particle receptor binding	3/88	0.0001466	0.0018347	0.0011013	Apoe/Clu/Lancl1
7-day post-SCI group vs. the control group	MF	GO:0004712	protein serine/threonine/tyrosine kinase activity	3/88	0.0003023	0.0036573	0.0021953	Map2k3/Map2k4/Mapk8
7-day post-SCI group vs. the control group	MF	GO:0072341	modified amino acid binding	4/88	0.0003606	0.0042221	0.0025343	Axl/Lancl1/Mgst1/Trem2
7-day post-SCI group vs. the control group	MF	GO:0008035	high-density lipoprotein particle binding	2/88	0.0004202	0.0046222	0.0027745	Cd36/Trem2
7-day post-SCI group vs. the control group	MF	GO:0097027	ubiquitin-protein transferase activator activity	2/88	0.0004202	0.0046222	0.0027745	Cdc20/Fbxw7
7-day post-SCI group vs. the control group	MF	GO:0030331	nuclear estrogen receptor binding	3/88	0.0005069	0.0054119	0.0032486	Ctnnb1/Ppargc1a/Ppargc1b
7-day post-SCI group vs. the control group	MF	GO:0001046	core promoter sequence-specific DNA binding	3/88	0.0005374	0.0055734	0.0033455	Fos/Hdac1/Stat1
7-day post-SCI group vs. the control group	MF	GO:0030374	nuclear receptor coactivator activity	3/88	0.0006018	0.006022	0.0036148	Ncoa7/Ppargc1a/Ppargc1b
7-day post-SCI group vs. the control group	MF	GO:0070513	death domain binding	2/88	0.0006138	0.006022	0.0036148	Mcl1/Ripk1
7-day post-SCI group vs. the control group	MF	GO:0015085	calcium ion transmembrane transporter activity	4/88	0.0008128	0.0075648	0.0045409	Atp2a2/Marcksl1/Slc8a1/Trpm2
7-day post-SCI group vs. the control group	MF	GO:0042826	histone deacetylase binding	4/88	0.0008128	0.0075648	0.0045409	Cdc20/Hdac1/Hif1a/Mapk8
7-day post-SCI group vs. the control group	MF	GO:0004708	MAP kinase kinase activity	2/88	0.0009706	0.0085936	0.0051584	Map2k3/Map2k4
7-day post-SCI group vs. the control group	MF	GO:0050664	oxidoreductase activity, acting on NAD(P)H, oxyg	2/88	0.0009706	0.0085936	0.0051584	Cybb/Ncf1
7-day post-SCI group vs. the control group	MF	GO:0044325	transmembrane transporter binding	4/88	0.0011754	0.0101591	0.0060981	Atp2a2/Ctnnb1/Fkbp1b/Slc8a1

7-day post-SCI group vs. the control group	MF	GO:0030169	low-density lipoprotein particle binding	2/88	0.0014058	0.0110938	0.0066592	Cd36/Trem2
7-day post-SCI group vs. the control group	MF	GO:0043295	glutathione binding	2/88	0.0014058	0.0110938	0.0066592	Lancl1/Mgst1
7-day post-SCI group vs. the control group	MF	GO:0050135	NAD(P)+ nucleosidase activity	2/88	0.0014058	0.0110938	0.0066592	Cd38/Tlr4
7-day post-SCI group vs. the control group	MF	GO:0061809	NAD+ nucleotidase, cyclic ADP-ribose generating	2/88	0.0014058	0.0110938	0.0066592	Cd38/Tlr4
7-day post-SCI group vs. the control group	MF	GO:0003953	NAD+ nucleosidase activity	2/88	0.0015681	0.0118585	0.0071182	Cd38/Tlr4
7-day post-SCI group vs. the control group	MF	GO:1900750	oligopeptide binding	2/88	0.0015681	0.0118585	0.0071182	Lancl1/Mgst1
7-day post-SCI group vs. the control group	MF	GO:1990841	promoter-specific chromatin binding	3/88	0.0016516	0.0122356	0.0073446	Hdac1/Ppargc1a/Stat1
7-day post-SCI group vs. the control group	MF	GO:0051010	microtubule plus-end binding	2/88	0.0017388	0.0126236	0.0075774	Apc/Dst
7-day post-SCI group vs. the control group	MF	GO:0048156	tau protein binding	2/88	0.0021055	0.0149863	0.0089957	Apoe/Clu
7-day post-SCI group vs. the control group	MF	GO:0046332	SMAD binding	3/88	0.0023627	0.0164935	0.0099004	Ctnnb1/Fos/Jun
7-day post-SCI group vs. the control group	MF	GO:0010857	calcium-dependent protein kinase activity	2/88	0.0025056	0.0166603	0.0100005	Pink1/Prkcd
7-day post-SCI group vs. the control group	MF	GO:0055106	ubiquitin-protein transferase regulator activity	2/88	0.0025056	0.0166603	0.0100005	Cdc20/Fbxw7
7-day post-SCI group vs. the control group	MF	GO:0050660	flavin adenine dinucleotide binding	3/88	0.0025243	0.0166603	0.0100005	Cybb/Dhcr24/Xdh
7-day post-SCI group vs. the control group	MF	GO:0070412	R-SMAD binding	2/88	0.002718	0.0176183	0.0105756	Fos/Jun
7-day post-SCI group vs. the control group	MF	GO:0005516	calmodulin binding	4/88	0.0029759	0.0189515	0.0113758	Marcksl1/Mylk/Ppp3ca/Slc8a1
7-day post-SCI group vs. the control group	MF	GO:0005543	phospholipid binding	6/88	0.0030937	0.0193623	0.0116224	Amph/Apoe/Axl/Btk/Ncf1/Trem2
7-day post-SCI group vs. the control group	MF	GO:0051117	ATPase binding	3/88	0.0037413	0.0228437	0.0137122	Ezr/Ppp3ca/S100a1
7-day post-SCI group vs. the control group	MF	GO:0015662	P-type ion transporter activity	2/88	0.0039017	0.0228437	0.0137122	Atp2a2/Atp7a
7-day post-SCI group vs. the control group	MF	GO:0038187	pattern recognition receptor activity	2/88	0.0039017	0.0228437	0.0137122	Cd36/Tlr4
7-day post-SCI group vs. the control group	MF	GO:0140358	P-type transmembrane transporter activity	2/88	0.0039017	0.0228437	0.0137122	Atp2a2/Atp7a
7-day post-SCI group vs. the control group	MF	GO:0004364	glutathione transferase activity	2/88	0.0041624	0.0239833	0.0143962	Lancl1/Mgst1
7-day post-SCI group vs. the control group	MF	GO:0004623	phospholipase A2 activity	2/88	0.0049916	0.0283116	0.0169944	Lcat/Prdx6
7-day post-SCI group vs. the control group	MF	GO:0001530	lipopolysaccharide binding	2/88	0.0052835	0.0295063	0.0177115	Tlr4/Trem2
7-day post-SCI group vs. the control group	MF	GO:0046915	transition metal ion transmembrane transporter act	2/88	0.0058903	0.0319131	0.0191562	Atp7a/Trpm2
7-day post-SCI group vs. the control group	MF	GO:0097718	disordered domain specific binding	2/88	0.0058903	0.0319131	0.0191562	Ctnnb1/Ezr
7-day post-SCI group vs. the control group	MF	GO:0015631	tubulin binding	5/88	0.0067503	0.0360349	0.0216303	Apc/Dst/Ezr/Mapt/Ppargc1a
7-day post-SCI group vs. the control group	MF	GO:0016799	hydrolase activity, hydrolyzing N-glycosyl compou	2/88	0.0068572	0.0360748	0.0216543	Cd38/Tlr4
7-day post-SCI group vs. the control group	MF	GO:0043548	phosphatidylinositol 3-kinase binding	2/88	0.0071944	0.0370929	0.0222654	Axl/Tlr4
7-day post-SCI group vs. the control group	MF	GO:0019207	kinase regulator activity	4/88	0.0072551	0.0370929	0.0222654	Apc/Hbegf/Hspb1/Trem2
7-day post-SCI group vs. the control group	MF	GO:0071949	FAD binding	2/88	0.007539	0.0380092	0.0228155	Dhcr24/Xdh
7-day post-SCI group vs. the control group	MF	GO:0004715	non-membrane spanning protein tyrosine kinase ac	2/88	0.0082502	0.0410249	0.0246257	Btk/Prkcd
7-day post-SCI group vs. the control group	MF	GO:0047485	protein N-terminus binding	3/88	0.0084137	0.0412727	0.0247744	Clu/Hdac1/Id1
7-day post-SCI group vs. the control group	MF	GO:0003713	transcription coactivator activity	4/88	0.0088909	0.0429406	0.0257756	Ctnnb1/Ncoa7/Ppargc1a/Ppargc1b
7-day post-SCI group vs. the control group	MF	GO:0070888	E-box binding	2/88	0.0089903	0.0429406	0.0257756	Hdac1/Hif1a
7-day post-SCI group vs. the control group	MF	GO:0008017	microtubule binding	4/88	0.0097304	0.0448421	0.026917	Apc/Dst/Ezr/Mapt
7-day post-SCI group vs. the control group	MF	GO:0001223	transcription coactivator binding	2/88	0.009759	0.0448421	0.026917	Hif1a/Stat1
7-day post-SCI group vs. the control group	MF	GO:0032813	tumor necrosis factor receptor superfamily binding	2/88	0.009759	0.0448421	0.026917	Ripk1/Stat1
7-day post-SCI group vs. the control group	MF	GO:0031072	heat shock protein binding	3/88	0.0100385	0.0453459	0.0272194	Hif1a/Mapt/Stau2
7-day post-SCI group vs. the control group	MF	GO:0046873	metal ion transmembrane transporter activity	5/88	0.0101185	0.0453459	0.0272194	Atp2a2/Atp7a/Marcksl1/Slc8a1/Trpm2
7-day post-SCI group vs. the control group	MF	GO:0019955	cytokine binding	3/88	0.0104226	0.0461391	0.0276955	Cd36/Il18bp/Il6st

7-day post-SCI group vs. the control group	MF	GO:0051879	Hsp90 protein binding	2/88	0.010556	0.0461664	0.0277119	Hif1a/Mapt
7-day post-SCI group vs. the control group	MF	GO:0001222	transcription corepressor binding	2/88	0.0109649	0.046282	0.0277813	Hdac1/Stat1
7-day post-SCI group vs. the control group	MF	GO:0008374	O-acyltransferase activity	2/88	0.0109649	0.046282	0.0277813	Lcat/Prdx6
7-day post-SCI group vs. the control group	MF	GO:0019894	kinesin binding	2/88	0.0109649	0.046282	0.0277813	Mapk8/Stau2
7-day post-SCI group vs. the control group	MF	GO:0019829	ATPase-coupled cation transmembrane transporte	2/88	0.0113807	0.047485	0.0285034	Atp2a2/Atp7a
7-day post-SCI group vs. the control group	MF	GO:0019838	growth factor binding	3/88	0.0118336	0.0482861	0.0289843	Cd36/Il6st/Itga6
7-day post-SCI group vs. the control group	MF	GO:1901681	sulfur compound binding	4/88	0.0118387	0.0482861	0.0289843	Apoe/Hbegf/Lancl1/Mgst1
7-day post-SCI group vs. the control group	MF	GO:0043394	proteoglycan binding	2/88	0.0126692	0.0507721	0.0304765	Apoe/Ctsl
7-day post-SCI group vs. the control group	MF	GO:0003779	actin binding	5/88	0.012728	0.0507721	0.0304765	Aif1/Dst/Ezr/Marcksl1/Mylk
7-day post-SCI group vs. the control group	MF	GO:0020037	heme binding	3/88	0.0138019	0.0542848	0.032585	Cybb/Hebp2/Ptgs1
7-day post-SCI group vs. the control group	MF	GO:0042393	histone binding	4/88	0.0139077	0.0542848	0.032585	Ctsl/Phc1/Rbbp7/Stat1
7-day post-SCI group vs. the control group	MF	GO:0140678	molecular function inhibitor activity	2/88	0.0149508	0.0577356	0.0346564	Fkbp1b/Id1
7-day post-SCI group vs. the control group	MF	GO:0046982	protein heterodimerization activity	4/88	0.0153518	0.0586602	0.0352114	Cybb/Hif1a/Mcl1/Tlr4
7-day post-SCI group vs. the control group	MF	GO:0046906	tetrapyrrole binding	3/88	0.0159456	0.0602944	0.0361924	Cybb/Hebp2/Ptgs1
7-day post-SCI group vs. the control group	MF	GO:0016765	transferase activity, transferring alkyl or aryl (othe	2/88	0.0163982	0.0607404	0.0364601	Lancl1/Mgst1
7-day post-SCI group vs. the control group	MF	GO:0045309	protein phosphorylated amino acid binding	2/88	0.0163982	0.0607404	0.0364601	Fbxw7/Sirpa
7-day post-SCI group vs. the control group	MF	GO:0005080	protein kinase C binding	2/88	0.0168935	0.0619429	0.0371819	Adam9/Hspb1
7-day post-SCI group vs. the control group	MF	GO:0003712	transcription coregulator activity	5/88	0.0182217	0.0661447	0.0397041	Ctnnb1/Hdac1/Ncoa7/Ppargc1a/Ppargc1b
7-day post-SCI group vs. the control group	MF	GO:0001786	phosphatidylserine binding	2/88	0.0184172	0.0661925	0.0397328	Axl/Trem2
7-day post-SCI group vs. the control group	MF	GO:0009055	electron transfer activity	2/88	0.0194641	0.0685968	0.041176	Cybb/Xdh
7-day post-SCI group vs. the control group	MF	GO:0045296	cadherin binding	2/88	0.0194641	0.0685968	0.041176	Cdh11/Ctnnb1
7-day post-SCI group vs. the control group	MF	GO:0140104	molecular carrier activity	2/88	0.021631	0.0755006	0.0453201	Atp7a/Clu
7-day post-SCI group vs. the control group	MF	GO:0016651	oxidoreductase activity, acting on NAD(P)H	2/88	0.0227504	0.0786513	0.0472113	Cybb/Ncf1
7-day post-SCI group vs. the control group	MF	GO:0002039	p53 binding	2/88	0.0233189	0.0798562	0.0479345	Hdac1/Hif1a
7-day post-SCI group vs. the control group	MF	GO:0005518	collagen binding	2/88	0.0256508	0.087021	0.0522353	Adam9/Ctsl
7-day post-SCI group vs. the control group	MF	GO:0097110	scaffold protein binding	2/88	0.0280735	0.094358	0.0566394	Il6st/Trem2
7-day post-SCI group vs. the control group	MF	GO:0019887	protein kinase regulator activity	3/88	0.0289898	0.0964568	0.0578993	Apc/Hbegf/Hspb1
7-day post-SCI group vs. the control group	MF	GO:0005041	low-density lipoprotein particle receptor activity	1/88	0.0305579	0.0964568	0.0578993	Cd36
7-day post-SCI group vs. the control group	MF	GO:0008429	phosphatidylethanolamine binding	1/88	0.0305579	0.0964568	0.0578993	Trem2
7-day post-SCI group vs. the control group	MF	GO:0015368	calcium:cation antiporter activity	1/88	0.0305579	0.0964568	0.0578993	Slc8a1
7-day post-SCI group vs. the control group	MF	GO:0031078	histone deacetylase activity (H3-K14 specific)	1/88	0.0305579	0.0964568	0.0578993	Hdac1
7-day post-SCI group vs. the control group	MF	GO:0032041	NAD-dependent histone deacetylase activity (H3-I	1/88	0.0305579	0.0964568	0.0578993	Hdac1
7-day post-SCI group vs. the control group	MF	GO:0047499	calcium-independent phospholipase A2 activity	1/88	0.0305579	0.0964568	0.0578993	Prdx6
7-day post-SCI group vs. the control group	MF	GO:0043130	ubiquitin binding	2/88	0.0312255	0.0977141	0.058654	Fbxw7/Hspb1
7-day post-SCI group vs. the control group	MF	GO:0051015	actin filament binding	3/88	0.0318199	0.0980468	0.0588537	Aif1/Ezr/Marcksl1
7-day post-SCI group vs. the control group	MF	GO:0051082	unfolded protein binding	2/88	0.031872	0.0980468	0.0588537	Clu/Hspb1
7-day post-SCI group vs. the control group	MF	GO:0005388	P-type calcium transporter activity	1/88	0.0335625	0.1015265	0.0609424	Atp2a2
7-day post-SCI group vs. the control group	MF	GO:0019855	calcium channel inhibitor activity	1/88	0.0335625	0.1015265	0.0609424	Fkbp1b
7-day post-SCI group vs. the control group	MF	GO:0005539	glycosaminoglycan binding	3/88	0.0359393	0.104492	0.0627225	Apoe/Hbegf/Trem2
7-day post-SCI group vs. the control group	MF	GO:0008013	beta-catenin binding	2/88	0.0365432	0.104492	0.0627225	Apc/Ctnnb1

7-day post-SCI group vs. the control group	MF	GO:0005347	ATP transmembrane transporter activity	1/88	0.0365578	0.104492	0.0627225	Slc25a24
7-day post-SCI group vs. the control group	MF	GO:0032036	myosin heavy chain binding	1/88	0.0365578	0.104492	0.0627225	Axl
7-day post-SCI group vs. the control group	MF	GO:0045294	alpha-catenin binding	1/88	0.0365578	0.104492	0.0627225	Ctnnb1
7-day post-SCI group vs. the control group	MF	GO:0070097	delta-catenin binding	1/88	0.0365578	0.104492	0.0627225	Ctnnb1
7-day post-SCI group vs. the control group	MF	GO:0097655	serpin family protein binding	1/88	0.0365578	0.104492	0.0627225	Ctsl
7-day post-SCI group vs. the control group	MF	GO:0004620	phospholipase activity	2/88	0.0372309	0.1047771	0.0628936	Lcat/Prdx6
7-day post-SCI group vs. the control group	MF	GO:0004706	JUN kinase kinase kinase activity	1/88	0.039544	0.1047771	0.0628936	Ripk1
7-day post-SCI group vs. the control group	MF	GO:0015245	fatty acid transmembrane transporter activity	1/88	0.039544	0.1047771	0.0628936	Cd36
7-day post-SCI group vs. the control group	MF	GO:0016018	cyclosporin A binding	1/88	0.039544	0.1047771	0.0628936	Ppp3ca
7-day post-SCI group vs. the control group	MF	GO:0016661	oxidoreductase activity, acting on other nitrogenou	1/88	0.039544	0.1047771	0.0628936	Xdh
7-day post-SCI group vs. the control group	MF	GO:0031994	insulin-like growth factor I binding	1/88	0.039544	0.1047771	0.0628936	Itga6
7-day post-SCI group vs. the control group	MF	GO:0035325	Toll-like receptor binding	1/88	0.039544	0.1047771	0.0628936	Cd36
7-day post-SCI group vs. the control group	MF	GO:0051400	BH domain binding	1/88	0.039544	0.1047771	0.0628936	Mcl1
7-day post-SCI group vs. the control group	MF	GO:1990226	histone methyltransferase binding	1/88	0.039544	0.1047771	0.0628936	Ctnnb1
7-day post-SCI group vs. the control group	MF	GO:1990405	protein antigen binding	1/88	0.039544	0.1047771	0.0628936	Sirpa
7-day post-SCI group vs. the control group	MF	GO:0004197	cysteine-type endopeptidase activity	2/88	0.0421819	0.1071883	0.0643409	Capn2/Ctsl
7-day post-SCI group vs. the control group	MF	GO:0042626	ATPase-coupled transmembrane transporter activi	2/88	0.0421819	0.1071883	0.0643409	Atp2a2/Atp7a
7-day post-SCI group vs. the control group	MF	GO:0004198	calcium-dependent cysteine-type endopeptidase ac	1/88	0.042521	0.1071883	0.0643409	Capn2
7-day post-SCI group vs. the control group	MF	GO:0016595	glutamate binding	1/88	0.042521	0.1071883	0.0643409	Gclc
7-day post-SCI group vs. the control group	MF	GO:0030297	transmembrane receptor protein tyrosine kinase ac	1/88	0.042521	0.1071883	0.0643409	Hbegf
7-day post-SCI group vs. the control group	MF	GO:0043560	insulin receptor substrate binding	1/88	0.042521	0.1071883	0.0643409	Prkcd
7-day post-SCI group vs. the control group	MF	GO:0070411	I-SMAD binding	1/88	0.042521	0.1071883	0.0643409	Ctnnb1
7-day post-SCI group vs. the control group	MF	GO:0022853	active ion transmembrane transporter activity	3/88	0.0436694	0.109324	0.0656229	Atp2a2/Atp7a/Slc8a1
7-day post-SCI group vs. the control group	MF	GO:0051087	chaperone binding	2/88	0.0451159	0.109354	0.0656409	Atp7a/Mapt
7-day post-SCI group vs. the control group	MF	GO:0015278	calcium-release channel activity	1/88	0.0454889	0.109354	0.0656409	Trpm2
7-day post-SCI group vs. the control group	MF	GO:0017136	NAD-dependent histone deacetylase activity	1/88	0.0454889	0.109354	0.0656409	Hdac1
7-day post-SCI group vs. the control group	MF	GO:0034236	protein kinase A catalytic subunit binding	1/88	0.0454889	0.109354	0.0656409	Ezr
7-day post-SCI group vs. the control group	MF	GO:0045295	gamma-catenin binding	1/88	0.0454889	0.109354	0.0656409	Apc
7-day post-SCI group vs. the control group	MF	GO:0106222	lncRNA binding	1/88	0.0454889	0.109354	0.0656409	Atp2a2
7-day post-SCI group vs. the control group	MF	GO:0032182	ubiquitin-like protein binding	2/88	0.0466105	0.1105857	0.0663803	Fbxw7/Hspb1
7-day post-SCI group vs. the control group	MF	GO:0051219	phosphoprotein binding	2/88	0.0466105	0.1105857	0.0663803	Fbxw7/Sirpa
7-day post-SCI group vs. the control group	MF	GO:0004698	calcium-dependent protein kinase C activity	1/88	0.0484476	0.1127339	0.0676698	Prkcd
7-day post-SCI group vs. the control group	MF	GO:0030228	lipoprotein particle receptor activity	1/88	0.0484476	0.1127339	0.0676698	Cd36
7-day post-SCI group vs. the control group	MF	GO:0034979	NAD-dependent protein deacetylase activity	1/88	0.0484476	0.1127339	0.0676698	Hdac1
28-day post-SCI group vs. the control group	BP	GO:0006979	response to oxidative stress	49/99	2.52E-64	8.25E-61	4.06E-61	Aif1/Apoe/Atp2a2/Axl/Cd36/Cd38/Cyp1b1/Eif2s1/Fbxw7/Fos/Gpx1/Gpx3/Gpx8/Gskip/Hif1a/Hmox2/Hspb1/Jun/Lancl1/Mapk1/Mcl1/Met/Mgst1/Mpv17/Ncoa7/Ndufa6/Net1/Nfe2l2/Parp1/Pawr/Pdk1/Pdk2/Plekha1/Prdx4/Prdx6/Prkcd/Ptgs1/Rela/Ripk1/Sirpa/Slc7a11/Slc8a1/Stat6/Tlr4/Tnfaip3/Trem2/Trpm2/Txnip/Ucp2

28-day post-SCI group vs. the control group	BP	GO:0034599	cellular response to oxidative stress	38/99	1.61E-51	2.64E-48	1.30E-48	Aif1/Atp2a2/Axl/Cd36/Cyp1b1/Eif2s1/Fbxw7/Fos/Gpx1/Gskip/Hif1a/Hspb1/Jun/Lancl1/Mapk1/Mcl1/Met/Mgst1/Mpv17/Ncoa7/Net1/Nfe2l2/Parp1/Pawr/Pdk1/Pdk2/Plekha1/Prkcd/Rela/Ripk1/Sirpa/Slc7a11/Slc8a1/Stat6/Tlr4/Tnfaip3/Trem2/Trpm2
28-day post-SCI group vs. the control group	BP	GO:0062197	cellular response to chemical stress	39/99	1.69E-49	1.84E-46	9.08E-47	Aif1/Atp2a2/Axl/Cd36/Cyp1b1/Eif2s1/Fbxw7/Fos/Gpx1/Gskip/Hif1a/Hspb1/Jun/Lancl1/Mapk1/Mcl1/Met/Mgst1/Mpv17/Mylk/Ncoa7/Net1/Nfe2l2/Parp1/Pawr/Pdk1/Pdk2/Plekha1/Prkcd/Rela/Ripk1/Sirpa/Slc7a11/Slc8a1/Stat6/Tlr4/Tnfaip3/Trem2/Trpm2
28-day post-SCI group vs. the control group	BP	GO:0000302	response to reactive oxygen species	26/99	1.03E-34	8.45E-32	4.16E-32	Axl/Cd36/Cyp1b1/Fos/Gpx1/Hif1a/Jun/Mapk1/Met/Mpv17/Net1/Nfe2l2/Parp1/Pawr/Pdk2/Plekha1/Prdx6/Prkcd/Rela/Ripk1/Sirpa/Slc8a1/Stat6/Tnfaip3/Trpm2/Ucp2
28-day post-SCI group vs. the control group	BP	GO:0034614	cellular response to reactive oxygen species	22/99	1.41E-30	9.25E-28	4.55E-28	Axl/Cd36/Cyp1b1/Fos/Jun/Mapk1/Met/Mpv17/Net1/Nfe2l2/Parp1/Pawr/Pdk2/Plekha1/Prkcd/Rela/Ripk1/Sirpa/Slc8a1/Stat6/Tnfaip3/Trpm2
28-day post-SCI group vs. the control group	BP	GO:0036473	cell death in response to oxidative stress	19/99	2.93E-28	1.60E-25	7.87E-26	Cyp1b1/Fbxw7/Gpx1/Gskip/Hif1a/Hspb1/Lancl1/Mcl1/Met/Ncoa7/Nfe2l2/Parp1/Pawr/Pdk1/Ripk1/Slc7a11/Tlr4/Trem2/Trpm2
28-day post-SCI group vs. the control group	BP	GO:1900407	regulation of cellular response to oxidative stress	17/99	2.16E-25	1.01E-22	4.97E-23	Cd36/Fbxw7/Gpx1/Hif1a/Hspb1/Lancl1/Mcl1/Met/Ncoa7/Nfe2l2/Parp1/Pawr/Ripk1/Slc7a11/Tlr4/Trem2/Trpm2
28-day post-SCI group vs. the control group	BP	GO:1902882	regulation of response to oxidative stress	17/99	1.24E-24	5.09E-22	2.51E-22	Cd36/Fbxw7/Gpx1/Hif1a/Hspb1/Lancl1/Mcl1/Met/Ncoa7/Nfe2l2/Parp1/Pawr/Ripk1/Slc7a11/Tlr4/Trem2/Trpm2
28-day post-SCI group vs. the control group	BP	GO:1903201	regulation of oxidative stress-induced cell death	16/99	1.78E-24	6.47E-22	3.19E-22	Fbxw7/Gpx1/Hif1a/Hspb1/Lancl1/Mcl1/Met/Ncoa7/Nfe2l2/Parp1/Pawr/Ripk1/Slc7a11/Tlr4/Trem2/Trpm2
28-day post-SCI group vs. the control group	BP	GO:0070301	cellular response to hydrogen peroxide	14/99	1.37E-19	4.48E-17	2.21E-17	Axl/Cyp1b1/Met/Net1/Nfe2l2/Pawr/Plekha1/Prkcd/Rela/Ripk1/Sirpa/Stat6/Tnfaip3/Trpm2
28-day post-SCI group vs. the control group	BP	GO:0042542	response to hydrogen peroxide	15/99	1.55E-19	4.61E-17	2.27E-17	Axl/Cyp1b1/Gpx1/Met/Net1/Nfe2l2/Pawr/Plekha1/Prkcd/Rela/Ripk1/Sirpa/Stat6/Tnfaip3/Trpm2
28-day post-SCI group vs. the control group	BP	GO:0070997	neuron death	22/99	1.88E-19	5.12E-17	2.52E-17	Apoe/Axl/Clu/Eif2s1/Fbxw7/Fos/Gpx1/Hif1a/Il6st/Jun/Lancl1/Map2k4/Mcl1/Ncoa7/Parp1/Pawr/Rb1/Slc7a11/Tlr4/Tnfrsf1a/Trem2/Trpm2
28-day post-SCI group vs. the control group	BP	GO:0008631	intrinsic apoptotic signaling pathway in response to ox	11/99	3.90E-18	9.81E-16	4.83E-16	Cyp1b1/Fbxw7/Gpx1/Gskip/Hif1a/Hspb1/Mcl1/Nfe2l2/Parp1/Pdk1/Trem2
28-day post-SCI group vs. the control group	BP	GO:1901214	regulation of neuron death	20/99	6.06E-18	1.42E-15	6.97E-16	Apoe/Axl/Clu/Eif2s1/Fbxw7/Fos/Hif1a/Il6st/Jun/Lancl1/Map2k4/Mcl1/Ncoa7/Parp1/Pawr/Slc7a11/Tlr4/Tnfrsf1a/Trem2/Trpm2
28-day post-SCI group vs. the control group	BP	GO:0072593	reactive oxygen species metabolic process	17/99	1.00E-17	2.18E-15	1.08E-15	Cd36/Cybb/Cyp1b1/Gpx1/Gpx3/Hif1a/Ier3/Met/Mpv17/Ncf1/Nfe2l2/Prdx4/Prdx6/Prkcd/Ripk1/Tlr4/Xdh
28-day post-SCI group vs. the control group	BP	GO:1903203	regulation of oxidative stress-induced neuron death	10/99	2.35E-17	4.80E-15	2.36E-15	Fbxw7/Hif1a/Lancl1/Mcl1/Ncoa7/Parp1/Slc7a11/Tlr4/Trem2/Trpm2
28-day post-SCI group vs. the control group	BP	GO:0036475	neuron death in response to oxidative stress	10/99	4.43E-17	8.53E-15	4.20E-15	Fbxw7/Hif1a/Lancl1/Mcl1/Ncoa7/Parp1/Slc7a11/Tlr4/Trem2/Trpm2

28-day post-SCI group vs. the control group	BP	GO:1901216	positive regulation of neuron death	13/99	9.89E-16	1.80E-13	8.85E-14	Apoe/Clu/Eif2s1/Fbxw7/Fos/Jun/Map2k4/Mcl1/Parp1/Pawr/Tlr4/Tnfrsf1a/Trpm2
28-day post-SCI group vs. the control group	BP	GO:0097193	intrinsic apoptotic signaling pathway	16/99	1.03E-14	1.78E-12	8.77E-13	Clu/Cyp1b1/Fbxw7/Gpx1/Gskip/Hdac1/Hif1a/Hspb1/Ier3/Mcl1/Nfe2l2/Parp1/Pdk1/Pdk2/Tnfrsf1a/Trem2
28-day post-SCI group vs. the control group	BP	GO:1902175	regulation of oxidative stress-induced intrinsic apoptot	8/99	2.95E-14	4.83E-12	2.38E-12	Fbxw7/Gpx1/Hif1a/Hspb1/Mcl1/Nfe2l2/Parp1/Trem2
28-day post-SCI group vs. the control group	BP	GO:0010563	negative regulation of phosphorus metabolic process	17/99	2.56E-13	3.81E-11	1.88E-11	Actb/Apc/Apoe/Cdkn2c/Fkbp1b/Gskip/Hspb1/Ier3/Jun/Met/Parp1/Prkcd/Rb1/Sirpa/Slc8a1/Tnfaip3/Xdh
28-day post-SCI group vs. the control group	BP	GO:0045936	negative regulation of phosphate metabolic process	17/99	2.56E-13	3.81E-11	1.88E-11	Actb/Apc/Apoe/Cdkn2c/Fkbp1b/Gskip/Hspb1/Ier3/Jun/Met/Parp1/Prkcd/Rb1/Sirpa/Slc8a1/Tnfaip3/Xdh
28-day post-SCI group vs. the control group	BP	GO:0051402	neuron apoptotic process	15/99	4.53E-13	6.44E-11	3.17E-11	Apoe/Axl/Fbxw7/Gpx1/Hif1a/Il6st/Jun/Lancl1/Map2k4/Mcl1/Parp1/Pawr/Rb1/Tnfrsf1a/Trem2
28-day post-SCI group vs. the control group	BP	GO:2001233	regulation of apoptotic signaling pathway	16/99	5.43E-13	7.40E-11	3.64E-11	Clu/Fbxw7/Gpx1/Hdac1/Hif1a/Hspb1/Ier3/Mcl1/Nfe2l2/Parp1/Prkcd/Rb1/Rela/Ripk1/Tnfaip3/Trem2
28-day post-SCI group vs. the control group	BP	GO:0003012	muscle system process	16/99	1.68E-12	2.20E-10	1.08E-10	Aif1/Atp2a2/Cd38/Fkbp1b/Map2k3/Map2k4/Map2k6/Met/Mylk/Ncf1/Parp1/Pawr/Ppp3ca/Ptgs1/Slc8a1/Tnfrsf1a
28-day post-SCI group vs. the control group	BP	GO:0032680	regulation of tumor necrosis factor production	12/99	1.97E-12	2.48E-10	1.22E-10	Axl/Cd36/Clu/Hspb1/Il1a/Mapkapk2/Ripk1/Sirpa/Tlr4/Tnfaip3/Tnfrsf1a/Trem2
28-day post-SCI group vs. the control group	BP	GO:1903555	regulation of tumor necrosis factor superfamily cytoki	12/99	2.38E-12	2.89E-10	1.42E-10	Axl/Cd36/Clu/Hspb1/Il1a/Mapkapk2/Ripk1/Sirpa/Tlr4/Tnfaip3/Tnfrsf1a/Trem2
28-day post-SCI group vs. the control group	BP	GO:0051403	stress-activated MAPK cascade	13/99	2.57E-12	3.00E-10	1.48E-10	Il1a/Map2k3/Map2k4/Map2k6/Mapk1/Mapk10/Mapkapk2/Met/Ripk1/Sirpa/Tlr4/Trem2/Xdh
28-day post-SCI group vs. the control group	BP	GO:0032640	tumor necrosis factor production	12/99	3.05E-12	3.44E-10	1.70E-10	Axl/Cd36/Clu/Hspb1/Il1a/Mapkapk2/Ripk1/Sirpa/Tlr4/Tnfaip3/Tnfrsf1a/Trem2
28-day post-SCI group vs. the control group	BP	GO:0071706	tumor necrosis factor superfamily cytokine production	12/99	3.66E-12	3.99E-10	1.97E-10	Axl/Cd36/Clu/Hspb1/Il1a/Mapkapk2/Ripk1/Sirpa/Tlr4/Tnfaip3/Tnfrsf1a/Trem2
28-day post-SCI group vs. the control group	BP	GO:0031098	stress-activated protein kinase signaling cascade	13/99	4.26E-12	4.50E-10	2.22E-10	Il1a/Map2k3/Map2k4/Map2k6/Mapk1/Mapk10/Mapkapk2/Met/Ripk1/Sirpa/Tlr4/Trem2/Xdh
28-day post-SCI group vs. the control group	BP	GO:0042326	negative regulation of phosphorylation	15/99	5.97E-12	6.11E-10	3.01E-10	Actb/Apc/Apoe/Cdkn2c/Gskip/Hspb1/Ier3/Jun/Met/Prkcd/Rb1/Sirpa/Slc8a1/Tnfaip3/Xdh
28-day post-SCI group vs. the control group	BP	GO:0001933	negative regulation of protein phosphorylation	14/99	1.42E-11	1.41E-09	6.95E-10	Actb/Apc/Apoe/Cdkn2c/Gskip/Hspb1/Jun/Met/Prkcd/Rb1/Sirpa/Slc8a1/Tnfaip3/Xdh
28-day post-SCI group vs. the control group	BP	GO:0033194	response to hydroperoxide	6/99	1.65E-11	1.59E-09	7.81E-10	Cd36/Cd38/Gpx1/Mgst1/Prkcd/Trpm2
28-day post-SCI group vs. the control group	BP	GO:0043523	regulation of neuron apoptotic process	13/99	2.25E-11	2.10E-09	1.03E-09	Apoe/Axl/Fbxw7/Hif1a/Il6st/Jun/Lancl1/Map2k4/Mcl1/Parp1/Pawr/Tnfrsf1a/Trem2
28-day post-SCI group vs. the control group	BP	GO:2001234	negative regulation of apoptotic signaling pathway	12/99	4.12E-11	3.74E-09	1.84E-09	Clu/Gpx1/Hdac1/Hif1a/Hspb1/Ier3/Mcl1/Nfe2l2/Rb1/Rela/Ripk1/Tnfaip3
28-day post-SCI group vs. the control group	BP	GO:0042060	wound healing	14/99	4.93E-11	4.36E-09	2.15E-09	Apoe/Axl/Cd36/Dst/Gpx1/Hbegf/Hif1a/Il1a/Mylk/Nfe2l2/Prkcd/Sdc1/Slc7a11/Tlr4
28-day post-SCI group vs. the control group	BP	GO:0001659	temperature homeostasis	11/99	5.26E-11	4.52E-09	2.23E-09	Apc/Cd36/Gpx1/Id1/Il1a/Map2k6/Rb1/Stat6/Tlr4/Trpm2/Ucp2
28-day post-SCI group vs. the control group	BP	GO:0070661	leukocyte proliferation	14/99	5.66E-11	4.75E-09	2.34E-09	Aif1/Cd38/Clu/Fkbp1b/Il1a/Il6st/Mapk1/Pawr/Ppp3ca/Prkcd/Stat6/Tlr4/Trem2/Vcam1
28-day post-SCI group vs. the control group	BP	GO:1903209	positive regulation of oxidative stress-induced cell dea	6/99	7.15E-11	5.85E-09	2.88E-09	Fbxw7/Mcl1/Pawr/Ripk1/Tlr4/Trpm2

28-day post-SCI group vs. the control group	BP	GO:0036480	neuron intrinsic apoptotic signaling pathway in respon	5/99	1.05E-10	8.18E-09	4.03E-09	Fbxw7/Hif1a/Mcl1/Parp1/Trem2
28-day post-SCI group vs. the control group	BP	GO:1903376	regulation of oxidative stress-induced neuron intrinsic	5/99	1.05E-10	8.18E-09	4.03E-09	Fbxw7/Hif1a/Mcl1/Parp1/Trem2
28-day post-SCI group vs. the control group	BP	GO:2000377	regulation of reactive oxygen species metabolic proces	10/99	2.18E-10	1.66E-08	8.16E-09	Cd36/Cyp1b1/Hif1a/Ier3/Mpv17/Nfe2l2/Prkcd/Ripk1/Tlr4/Xdb
28-day post-SCI group vs. the control group	BP	GO:1901652	response to peptide	14/99	2.50E-10	1.85E-08	9.13E-09	Apc/Atp2a2/Cd36/Nfe2l2/Parp1/Pdk2/Prkcd/Rb1/Rela/Stat6/Tlr4/Tnfaip3/Trem2/Vcam1
28-day post-SCI group vs. the control group	BP	GO:0009636	response to toxic substance	9/99	3.92E-10	2.85E-08	1.40E-08	Cd36/Cyp1b1/Gpx1/Hif1a/Mapk1/Nfe2l2/Pon2/Prdx6/Slc7a11
28-day post-SCI group vs. the control group	BP	GO:2001242	regulation of intrinsic apoptotic signaling pathway	10/99	5.52E-10	3.92E-08	1.93E-08	Clu/Fbxw7/Gpx1/Hdac1/Hif1a/Hspb1/Mcl1/Nfe2l2/Parp1/Trem2
28-day post-SCI group vs. the control group	BP	GO:1903202	negative regulation of oxidative stress-induced cell dea	7/99	5.98E-10	4.17E-08	2.05E-08	Gpx1/Hif1a/Hspb1/Met/Ncoa7/Nfe2l2/Slc7a11
28-day post-SCI group vs. the control group	BP	GO:0045765	regulation of angiogenesis	12/99	6.90E-10	4.70E-08	2.31E-08	Ago1/Cd36/Cyp1b1/Hif1a/Hspb1/Id1/Il1a/Nfe2l2/Rnh1/S100a1/Smad1/Tnfrsf1a
28-day post-SCI group vs. the control group	BP	GO:0032496	response to lipopolysaccharide	13/99	7.59E-10	5.07E-08	2.49E-08	Axl/Cd36/Il1a/Map2k3/Mapk1/Mapkapk2/Mgst1/Rela/Rpl13a/Sirpa/Tlr4/Tnfaip3/Trem2
28-day post-SCI group vs. the control group	BP	GO:1901342	regulation of vasculature development	12/99	7.98E-10	5.22E-08	2.57E-08	Ago1/Cd36/Cyp1b1/Hif1a/Hspb1/Id1/Il1a/Nfe2l2/Rnh1/S100a1/Smad1/Tnfrsf1a
28-day post-SCI group vs. the control group	BP	GO:1901653	cellular response to peptide	12/99	1.14E-09	7.32E-08	3.60E-08	Apc/Cd36/Nfe2l2/Parp1/Pdk2/Prkcd/Rb1/Rela/Stat6/Tlr4/Trem2/Vcam1
28-day post-SCI group vs. the control group	BP	GO:0010631	epithelial cell migration	12/99	1.41E-09	8.84E-08	4.35E-08	Apc/Apoe/Cyp1b1/Fbxw7/Gpx1/Hbegf/Hif1a/Hspb1/Jun/Map2k3/Met/Nfe2l2
28-day post-SCI group vs. the control group	BP	GO:0002237	response to molecule of bacterial origin	13/99	1.44E-09	8.89E-08	4.38E-08	Axl/Cd36/Il1a/Map2k3/Mapk1/Mapkapk2/Mgst1/Rela/Rpl13a/Sirpa/Tlr4/Tnfaip3/Trem2
28-day post-SCI group vs. the control group	BP	GO:0090132	epithelium migration	12/99	1.51E-09	9.12E-08	4.49E-08	Apc/Apoe/Cyp1b1/Fbxw7/Gpx1/Hbegf/Hif1a/Hspb1/Jun/Map2k3/Met/Nfe2l2
28-day post-SCI group vs. the control group	BP	GO:0090130	tissue migration	12/99	1.61E-09	9.59E-08	4.72E-08	Apc/Apoe/Cyp1b1/Fbxw7/Gpx1/Hbegf/Hif1a/Hspb1/Jun/Map2k3/Met/Nfe2l2
28-day post-SCI group vs. the control group	BP	GO:0006469	negative regulation of protein kinase activity	10/99	1.99E-09	1.16E-07	5.73E-08	Actb/Apc/Apoe/Cdkn2c/Gskip/Hspb1/Prkcd/Rb1/Slc8a1/Tnfaip3
28-day post-SCI group vs. the control group	BP	GO:1902105	regulation of leukocyte differentiation	12/99	2.83E-09	1.62E-07	7.99E-08	Actb/Apc/Axl/Fbxw7/Fos/Hspb1/Jun/Mmp14/Ppp3ca/Rb1/Ripk1/Trem2
28-day post-SCI group vs. the control group	BP	GO:1903706	regulation of hemopoiesis	13/99	3.20E-09	1.81E-07	8.90E-08	Actb/Apc/Axl/Fbxw7/Fos/Hif1a/Hspb1/Jun/Mmp14/Ppp3ca/Rb1/Ripk1/Trem2
28-day post-SCI group vs. the control group	BP	GO:0032760	positive regulation of tumor necrosis factor production	8/99	4.64E-09	2.57E-07	1.27E-07	Cd36/Clu/Hspb1/Il1a/Mapkapk2/Ripk1/Tlr4/Tnfrsf1a
28-day post-SCI group vs. the control group	BP	GO:0034248	regulation of cellular amide metabolic process	13/99	5.20E-09	2.84E-07	1.40E-07	Ago1/Apoe/Clu/Cyp1b1/Eif2s1/Mapk1/Nfe2l2/Pdk1/Pdk2/Prkcd/Rela/Rpl13a/Slc7a11
28-day post-SCI group vs. the control group	BP	GO:1903557	positive regulation of tumor necrosis factor superfamil	8/99	5.35E-09	2.87E-07	1.41E-07	Cd36/Clu/Hspb1/Il1a/Mapkapk2/Ripk1/Tlr4/Tnfrsf1a
28-day post-SCI group vs. the control group	BP	GO:0033673	negative regulation of kinase activity	10/99	5.86E-09	3.09E-07	1.52E-07	Actb/Apc/Apoe/Cdkn2c/Gskip/Hspb1/Prkcd/Rb1/Slc8a1/Tnfaip3
28-day post-SCI group vs. the control group	BP	GO:0032675	regulation of interleukin-6 production	9/99	6.34E-09	3.29E-07	1.62E-07	Aif1/Cd36/Mapkapk2/Met/Sirpa/Tlr4/Tnfaip3/Tnfrsf1a/Trem2
28-day post-SCI group vs. the control group	BP	GO:0007249	I-kappaB kinase/NF-kappaB signaling	10/99	6.94E-09	3.55E-07	1.75E-07	Cd36/Hdac1/Hspb1/Il1a/Rela/Ripk1/Sirpa/Tlr4/Tnfaip3/Trem2
28-day post-SCI group vs. the control group	BP	GO:0001667	ameboidal-type cell migration	13/99	8.27E-09	4.16E-07	2.05E-07	Apc/Apoe/Cyp1b1/Fbxw7/Gpx1/Hbegf/Hif1a/Hspb1/Jun/Map2k3/Met/Nfe2l2/Slc8a1

28-day post-SCI group vs. the control group	BP	GO:0043534	blood vessel endothelial cell migration	8/99	9.82E-09	4.87E-07	2.40E-07	Apoe/Cyp1b1/Fbxw7/Gpx1/Hif1a/Hspb1/Map2k3/Nfe2l2
28-day post-SCI group vs. the control group	BP	GO:0032635	interleukin-6 production	9/99	1.00E-08	4.89E-07	2.41E-07	Aif1/Cd36/Mapkapk2/Met/Sirpa/Tlr4/Tnfaip3/Tnfrsf1a/Trem2
28-day post-SCI group vs. the control group	BP	GO:1901522	positive regulation of transcription from RNA polymer	5/99	1.06E-08	5.11E-07	2.52E-07	Hif1a/Jun/Nfe2l2/Rela/Smad1
28-day post-SCI group vs. the control group	BP	GO:0006936	muscle contraction	11/99	1.23E-08	5.81E-07	2.86E-07	Atp2a2/Cd38/Fkbp1b/Map2k3/Map2k6/Met/Mylk/Ncf1/Pawr/Ptgs1/Slc8a1
28-day post-SCI group vs. the control group	BP	GO:0010632	regulation of epithelial cell migration	10/99	1.42E-08	6.64E-07	3.27E-07	Apc/Apoe/Fbxw7/Hbegf/Hif1a/Hspb1/Jun/Map2k3/Met/Nfe2l2
28-day post-SCI group vs. the control group	BP	GO:0051098	regulation of binding	12/99	1.61E-08	7.40E-07	3.64E-07	Actb/Apoe/Atp2a2/Eif2s1/Fbxw7/Id1/Jun/Met/Parp1/Ppp3ca/Rb1/Tlr4
28-day post-SCI group vs. the control group	BP	GO:1990000	amyloid fibril formation	5/99	1.71E-08	7.75E-07	3.82E-07	Apoe/Cd36/Clu/Ripk1/Trem2
28-day post-SCI group vs. the control group	BP	GO:0043122	regulation of I-kappaB kinase/NF-kappaB signaling	9/99	2.04E-08	9.14E-07	4.50E-07	Cd36/Hdac1/Hspb1/Il1a/Rela/Ripk1/Tlr4/Tnfaip3/Trem2
28-day post-SCI group vs. the control group	BP	GO:0090257	regulation of muscle system process	10/99	2.07E-08	9.14E-07	4.50E-07	Aif1/Atp2a2/Fkbp1b/Ncf1/Parp1/Pawr/Ppp3ca/Ptgs1/Slc8a1/Tnfrsf1a
28-day post-SCI group vs. the control group	BP	GO:0051348	negative regulation of transferase activity	10/99	2.39E-08	1.04E-06	5.13E-07	Actb/Apc/Apoe/Cdkn2c/Gskip/Hspb1/Prkcd/Rb1/Slc8a1/Tnfaip3
28-day post-SCI group vs. the control group	BP	GO:0071216	cellular response to biotic stimulus	11/99	2.51E-08	1.08E-06	5.32E-07	Axl/Cd36/Il1a/Map2k3/Mapk1/Rela/Sirpa/Tlr4/Tnfaip3/Trem2/Txnip
28-day post-SCI group vs. the control group	BP	GO:0070391	response to lipoteichoic acid	4/99	2.66E-08	1.12E-06	5.50E-07	Cd36/Rela/Tlr4/Trem2
28-day post-SCI group vs. the control group	BP	GO:0071223	cellular response to lipoteichoic acid	4/99	2.66E-08	1.12E-06	5.50E-07	Cd36/Rela/Tlr4/Trem2
28-day post-SCI group vs. the control group	BP	GO:0033002	muscle cell proliferation	10/99	2.76E-08	1.14E-06	5.62E-07	Aif1/Apc/Apoe/Hbegf/Hif1a/Jun/Mapk1/Smad1/Tlr4/Tnfaip3
28-day post-SCI group vs. the control group	BP	GO:0071900	regulation of protein serine/threonine kinase activity	11/99	3.09E-08	1.26E-06	6.23E-07	Actb/Apc/Apoe/Cdkn2c/Map2k4/Map2k6/Prkcd/Rb1/Slc8a1/Tlr4/Tnfaip3
28-day post-SCI group vs. the control group	BP	GO:0051090	regulation of DNA-binding transcription factor activity	12/99	3.33E-08	1.34E-06	6.62E-07	Cd36/Clu/Cyp1b1/Id1/Mapk1/Ppp3ca/Rb1/Rela/Ripk1/Rnf2/Tlr4/Tnfaip3
28-day post-SCI group vs. the control group	BP	GO:0002761	regulation of myeloid leukocyte differentiation	8/99	3.42E-08	1.36E-06	6.71E-07	Apc/Fbxw7/Fos/Jun/Ppp3ca/Rb1/Ripk1/Trem2
28-day post-SCI group vs. the control group	BP	GO:0032872	regulation of stress-activated MAPK cascade	9/99	3.60E-08	1.42E-06	6.99E-07	Il1a/Map2k4/Mapk1/Met/Ripk1/Sirpa/Tlr4/Trem2/Xdh
28-day post-SCI group vs. the control group	BP	GO:0070302	regulation of stress-activated protein kinase signaling c	9/99	4.08E-08	1.59E-06	7.83E-07	Il1a/Map2k4/Mapk1/Met/Ripk1/Sirpa/Tlr4/Trem2/Xdh
28-day post-SCI group vs. the control group	BP	GO:0120161	regulation of cold-induced thermogenesis	8/99	4.24E-08	1.63E-06	8.03E-07	Apc/Cd36/Id1/Map2k6/Rb1/Stat6/Tlr4/Ucp2
28-day post-SCI group vs. the control group	BP	GO:0070663	regulation of leukocyte proliferation	10/99	4.47E-08	1.70E-06	8.37E-07	Aif1/Cd38/Il1a/Il6st/Mapk1/Pawr/Ppp3ca/Stat6/Tlr4/Vcam1
28-day post-SCI group vs. the control group	BP	GO:1902107	positive regulation of leukocyte differentiation	9/99	4.62E-08	1.72E-06	8.46E-07	Actb/Axl/Fos/Jun/Mmp14/Ppp3ca/Rb1/Ripk1/Trem2
28-day post-SCI group vs. the control group	BP	GO:1903708	positive regulation of hemopoiesis	9/99	4.62E-08	1.72E-06	8.46E-07	Actb/Axl/Fos/Jun/Mmp14/Ppp3ca/Rb1/Ripk1/Trem2
28-day post-SCI group vs. the control group	BP	GO:0106106	cold-induced thermogenesis	8/99	4.71E-08	1.73E-06	8.52E-07	Apc/Cd36/Id1/Map2k6/Rb1/Stat6/Tlr4/Ucp2
28-day post-SCI group vs. the control group	BP	GO:0042743	hydrogen peroxide metabolic process	6/99	5.35E-08	1.94E-06	9.56E-07	Cybb/Gpx1/Gpx3/Ncf1/Prdx4/Prdx6
28-day post-SCI group vs. the control group	BP	GO:2001243	negative regulation of intrinsic apoptotic signaling pat	7/99	5.45E-08	1.94E-06	9.56E-07	Clu/Gpx1/Hdac1/Hif1a/Hspb1/Mcl1/Nfe2l2
28-day post-SCI group vs. the control group	BP	GO:0050727	regulation of inflammatory response	11/99	5.46E-08	1.94E-06	9.56E-07	Apoe/Gpx1/Ier3/Ncf1/Rb1/Rela/Ripk1/Tlr4/Tnfaip3/Tnfrsf1a/Trem2
28-day post-SCI group vs. the control group	BP	GO:0042762	regulation of sulfur metabolic process	4/99	6.24E-08	2.20E-06	1.08E-06	Nfe2l2/Pdk1/Pdk2/Slc7a11
28-day post-SCI group vs. the control group	BP	GO:0051251	positive regulation of lymphocyte activation	12/99	6.71E-08	2.34E-06	1.15E-06	Actb/Aif1/Axl/Cd38/Il1a/Il6st/Mmp14/Ppp3ca/Sirpa/Stat6/Tlr4/Vcam1
28-day post-SCI group vs. the control group	BP	GO:0042391	regulation of membrane potential	12/99	7.37E-08	2.54E-06	1.25E-06	Atp2a2/Cd36/Fkbp1b/Hebp2/Jun/Met/Parp1/Pawr/Ppp3ca/Slc8a1/Trem2/Ucp2
28-day post-SCI group vs. the control group	BP	GO:0010038	response to metal ion	10/99	7.51E-08	2.56E-06	1.26E-06	Fos/Il1a/Jun/Lcat/Mapk1/Mylk/Nfe2l2/Ppp3ca/Trpm2/Xdh

28-day post-SCI group vs. the control group	BP	GO:0050878	regulation of body fluid levels	11/99	7.97E-08	2.69E-06	1.32E-06	Apoe/Axl/Cd36/Hif1a/Met/Nfe2l2/Ppp3ca/Prkcd/Slc7a11/Tlr4/Xdh
28-day post-SCI group vs. the control group	BP	GO:0045637	regulation of myeloid cell differentiation	9/99	8.66E-08	2.88E-06	1.42E-06	Apc/Fbxw7/Fos/Hif1a/Jun/Ppp3ca/Rb1/Ripk1/Trem2
28-day post-SCI group vs. the control group	BP	GO:0071901	negative regulation of protein serine/threonine kinase a	7/99	8.71E-08	2.88E-06	1.42E-06	Apc/Apoe/Cdkn2c/Prkcd/Rb1/Slc8a1/Tnfaip3
28-day post-SCI group vs. the control group	BP	GO:1904646	cellular response to amyloid-beta	5/99	9.30E-08	3.04E-06	1.50E-06	Cd36/Parp1/Tlr4/Trem2/Vcam1
28-day post-SCI group vs. the control group	BP	GO:0071222	cellular response to lipopolysaccharide	10/99	9.63E-08	3.12E-06	1.54E-06	Axl/Cd36/Il1a/Map2k3/Mapk1/Rela/Sirpa/Tlr4/Tnfaip3/Trem2
28-day post-SCI group vs. the control group	BP	GO:0050708	regulation of protein secretion	10/99	9.93E-08	3.18E-06	1.57E-06	Apoe/Cd38/Fkbp1b/Hif1a/Il1a/Ppp3ca/Tlr4/Trem2/Trpm2/Ucp2
28-day post-SCI group vs. the control group	BP	GO:1990845	adaptive thermogenesis	8/99	1.04E-07	3.29E-06	1.62E-06	Apc/Cd36/Id1/Map2k6/Rb1/Stat6/Tlr4/Ucp2
28-day post-SCI group vs. the control group	BP	GO:0019932	second-messenger-mediated signaling	10/99	1.12E-07	3.52E-06	1.74E-06	Apoe/Atp2a2/Cd36/Fkbp1b/Pdk2/Ppp3ca/Slc8a1/Trem2/Trpm2/Vcam1
28-day post-SCI group vs. the control group	BP	GO:0043542	endothelial cell migration	9/99	1.16E-07	3.63E-06	1.79E-06	Apoe/Cyp1b1/Fbxw7/Gpx1/Hif1a/Hspb1/Map2k3/Met/Nfe2l2
28-day post-SCI group vs. the control group	BP	GO:0070665	positive regulation of leukocyte proliferation	8/99	1.19E-07	3.68E-06	1.81E-06	Aif1/Cd38/Il1a/Il6st/Mapk1/Ppp3ca/Tlr4/Vcam1
28-day post-SCI group vs. the control group	BP	GO:1903829	positive regulation of protein localization	12/99	1.26E-07	3.84E-06	1.89E-06	Apc/Cd38/Fbxw7/Hif1a/Il1a/Mapk1/Parp1/Prkcd/Tlr4/Tnfrsf1a/Trem2/Trpm2
28-day post-SCI group vs. the control group	BP	GO:0045639	positive regulation of myeloid cell differentiation	7/99	1.27E-07	3.84E-06	1.89E-06	Fos/Hif1a/Jun/Ppp3ca/Rb1/Ripk1/Trem2
28-day post-SCI group vs. the control group	BP	GO:0071219	cellular response to molecule of bacterial origin	10/99	1.30E-07	3.90E-06	1.92E-06	Axl/Cd36/Il1a/Map2k3/Mapk1/Rela/Sirpa/Tlr4/Tnfaip3/Trem2
28-day post-SCI group vs. the control group	BP	GO:0022407	regulation of cell-cell adhesion	12/99	1.38E-07	4.09E-06	2.02E-06	Actb/Aif1/Hspb1/Il1a/Il6st/Pawr/Ppp3ca/Prkcd/Rela/Sirpa/Tnfaip3/Vcam1
28-day post-SCI group vs. the control group	BP	GO:0009410	response to xenobiotic stimulus	10/99	1.42E-07	4.18E-06	2.06E-06	Cd38/Cyp1b1/Fos/Gpx1/Jun/Mgst1/Mylk/Nfe2l2/Rb1/Smad1
28-day post-SCI group vs. the control group	BP	GO:0060047	heart contraction	9/99	1.55E-07	4.53E-06	2.23E-06	Atp2a2/Fkbp1b/Gpx1/Hbegf/Map2k3/Map2k6/Met/S100a1/Slc8a1
28-day post-SCI group vs. the control group	BP	GO:0051222	positive regulation of protein transport	10/99	1.59E-07	4.62E-06	2.27E-06	Cd38/Fbxw7/Hif1a/Il1a/Mapk1/Prkcd/Tlr4/Tnfrsf1a/Trem2/Trpm2
28-day post-SCI group vs. the control group	BP	GO:0006939	smooth muscle contraction	7/99	1.61E-07	4.62E-06	2.28E-06	Cd38/Fkbp1b/Mylk/Ncf1/Pawr/Ptgs1/Slc8a1
28-day post-SCI group vs. the control group	BP	GO:0043588	skin development	10/99	1.69E-07	4.80E-06	2.37E-06	Apc/Ctsl/Dhcr24/Hdac1/Il1a/Met/Ppp3ca/Ptgs1/Rela/Txnip
28-day post-SCI group vs. the control group	BP	GO:0002763	positive regulation of myeloid leukocyte differentiatio	6/99	1.94E-07	5.48E-06	2.70E-06	Fos/Jun/Ppp3ca/Rb1/Ripk1/Trem2
28-day post-SCI group vs. the control group	BP	GO:0098657	import into cell	9/99	2.04E-07	5.70E-06	2.81E-06	Actb/Cd36/Pawr/Ppp3ca/Prkcd/Slc7a11/Slc8a1/Trem2/Trpm2
28-day post-SCI group vs. the control group	BP	GO:0045766	positive regulation of angiogenesis	8/99	2.20E-07	6.05E-06	2.98E-06	Cyp1b1/Hif1a/Hspb1/Il1a/Nfe2l2/S100a1/Smad1/Tnfrsf1a
28-day post-SCI group vs. the control group	BP	GO:1904018	positive regulation of vasculature development	8/99	2.20E-07	6.05E-06	2.98E-06	Cyp1b1/Hif1a/Hspb1/Il1a/Nfe2l2/S100a1/Smad1/Tnfrsf1a
28-day post-SCI group vs. the control group	BP	GO:1904645	response to amyloid-beta	5/99	2.22E-07	6.05E-06	2.98E-06	Cd36/Parp1/Tlr4/Trem2/Vcam1
28-day post-SCI group vs. the control group	BP	GO:0003015	heart process	9/99	2.33E-07	6.31E-06	3.10E-06	Atp2a2/Fkbp1b/Gpx1/Hbegf/Map2k3/Map2k6/Met/S100a1/Slc8a1
28-day post-SCI group vs. the control group	BP	GO:0043254	regulation of protein-containing complex assembly	11/99	2.38E-07	6.39E-06	3.14E-06	Apc/Apoe/Cd36/Clu/Lcat/Met/Parp1/Prkcd/Rpl13a/Tlr4/Trem2
28-day post-SCI group vs. the control group	BP	GO:0001503	ossification	11/99	2.44E-07	6.48E-06	3.19E-06	Apc/Bmp1/Hif1a/Id1/Il6st/Map2k6/Mapk1/Mmp14/Ppp3ca/Slc8a1/Smad1
28-day post-SCI group vs. the control group	BP	GO:1904951	positive regulation of establishment of protein localiza	10/99	2.50E-07	6.59E-06	3.24E-06	Cd38/Fbxw7/Hif1a/Il1a/Mapk1/Prkcd/Tlr4/Tnfrsf1a/Trem2/Trpm2
28-day post-SCI group vs. the control group	BP	GO:0014812	muscle cell migration	7/99	2.68E-07	7.00E-06	3.45E-06	Aif1/Cyp1b1/Il6st/Met/Net1/Nfe2l2/Tlr4
28-day post-SCI group vs. the control group	BP	GO:0009314	response to radiation	11/99	2.80E-07	7.27E-06	3.58E-06	Eif2s1/Fbxw7/Gpx1/Hif1a/Jun/Mapk10/Net1/Parp1/Prkcd/Rela/Slc7a11

28-day post-SCI group vs. the control group	BP	GO:0045807	positive regulation of endocytosis	7/99	2.82E-07	7.27E-06	3.58E-06	Amph/Apoe/Axl/Cd36/Clu/Ppp3ca/Trem2
28-day post-SCI group vs. the control group	BP	GO:1901031	regulation of response to reactive oxygen species	5/99	2.87E-07	7.33E-06	3.61E-06	Cd36/Met/Nfe2l2/Pawr/Ripk1
28-day post-SCI group vs. the control group	BP	GO:2000379	positive regulation of reactive oxygen species metabol	6/99	2.89E-07	7.33E-06	3.61E-06	Cd36/Cyp1b1/Nfe2l2/Prkcd/Tlr4/Xdh
28-day post-SCI group vs. the control group	BP	GO:1902176	negative regulation of oxidative stress-induced intrinsi	4/99	2.96E-07	7.45E-06	3.67E-06	Gpx1/Hif1a/Hspb1/Nfe2l2
28-day post-SCI group vs. the control group	BP	GO:0046651	lymphocyte proliferation	10/99	3.02E-07	7.53E-06	3.71E-06	Aif1/Cd38/Fkbp1b/Il1a/Il6st/Pawr/Ppp3ca/Prkcd/Tlr4/Vcam1
28-day post-SCI group vs. the control group	BP	GO:0001774	microglial cell activation	5/99	3.25E-07	8.06E-06	3.97E-06	Aif1/Clu/Jun/Tlr4/Trem2
28-day post-SCI group vs. the control group	BP	GO:0033674	positive regulation of kinase activity	11/99	3.36E-07	8.26E-06	4.07E-06	Axl/Clu/Fbxw7/Hbegf/Map2k3/Map2k4/Map2k6/Met/Prkcd/Tlr4/Trem2
28-day post-SCI group vs. the control group	BP	GO:0032943	mononuclear cell proliferation	10/99	3.63E-07	8.85E-06	4.36E-06	Aif1/Cd38/Fkbp1b/Il1a/Il6st/Pawr/Ppp3ca/Prkcd/Tlr4/Vcam1
28-day post-SCI group vs. the control group	BP	GO:0019722	calcium-mediated signaling	8/99	3.88E-07	9.39E-06	4.62E-06	Atp2a2/Fkbp1b/Pdk2/Ppp3ca/Slc8a1/Trem2/Trpm2/Vcam1
28-day post-SCI group vs. the control group	BP	GO:1903039	positive regulation of leukocyte cell-cell adhesion	9/99	4.15E-07	9.97E-06	4.91E-06	Actb/Aif1/Il1a/Il6st/Pawr/Ppp3ca/Rela/Sirpa/Vcam1
28-day post-SCI group vs. the control group	BP	GO:0006809	nitric oxide biosynthetic process	6/99	4.83E-07	1.15E-05	5.68E-06	Aif1/Cd36/Clu/Cyp1b1/Sirpa/Tlr4
28-day post-SCI group vs. the control group	BP	GO:0002269	leukocyte activation involved in inflammatory respons	5/99	5.19E-07	1.23E-05	6.05E-06	Aif1/Clu/Jun/Tlr4/Trem2
28-day post-SCI group vs. the control group	BP	GO:0050767	regulation of neurogenesis	11/99	5.55E-07	1.30E-05	6.42E-06	Apoe/Hdac1/Hif1a/Id1/Il6st/Met/Ppp3ca/Rb1/Rela/Stau2/Trem2
28-day post-SCI group vs. the control group	BP	GO:0062012	regulation of small molecule metabolic process	10/99	5.58E-07	1.30E-05	6.42E-06	Apc/Apoe/Cd36/Hif1a/Ier3/Parp1/Pdk1/Pdk2/Slc7a11/Trem2
28-day post-SCI group vs. the control group	BP	GO:0043525	positive regulation of neuron apoptotic process	6/99	5.94E-07	1.38E-05	6.78E-06	Fbxw7/Jun/Map2k4/Mcl1/Pawr/Tnfrsf1a
28-day post-SCI group vs. the control group	BP	GO:1903037	regulation of leukocyte cell-cell adhesion	10/99	6.01E-07	1.38E-05	6.82E-06	Actb/Aif1/Hspb1/Il1a/Il6st/Pawr/Ppp3ca/Rela/Sirpa/Vcam1
28-day post-SCI group vs. the control group	BP	GO:1900542	regulation of purine nucleotide metabolic process	6/99	6.35E-07	1.45E-05	7.15E-06	Hif1a/Ier3/Parp1/Pdk1/Pdk2/Trem2
28-day post-SCI group vs. the control group	BP	GO:0061900	glial cell activation	5/99	7.16E-07	1.63E-05	8.00E-06	Aif1/Clu/Jun/Tlr4/Trem2
28-day post-SCI group vs. the control group	BP	GO:0046209	nitric oxide metabolic process	6/99	7.24E-07	1.63E-05	8.04E-06	Aif1/Cd36/Clu/Cyp1b1/Sirpa/Tlr4
28-day post-SCI group vs. the control group	BP	GO:0035994	response to muscle stretch	4/99	7.37E-07	1.65E-05	8.13E-06	Fos/Jun/Rela/Slc8a1
28-day post-SCI group vs. the control group	BP	GO:0050728	negative regulation of inflammatory response	7/99	7.61E-07	1.68E-05	8.28E-06	Apoe/Gpx1/Ier3/Rb1/Tnfaip3/Tnfrsf1a/Trem2
28-day post-SCI group vs. the control group	BP	GO:0060759	regulation of response to cytokine stimulus	7/99	7.61E-07	1.68E-05	8.28E-06	Axl/Hif1a/Il6st/Ripk1/Tlr4/Tnfrsf1a/Trem2
28-day post-SCI group vs. the control group	BP	GO:0042593	glucose homeostasis	9/99	7.93E-07	1.74E-05	8.57E-06	Cd36/Fkbp1b/Gpx1/Hif1a/Met/Pdk2/Ppp3ca/Ucp2/Vcam1
28-day post-SCI group vs. the control group	BP	GO:0033500	carbohydrate homeostasis	9/99	8.16E-07	1.78E-05	8.76E-06	Cd36/Fkbp1b/Gpx1/Hif1a/Met/Pdk2/Ppp3ca/Ucp2/Vcam1
28-day post-SCI group vs. the control group	BP	GO:2001057	reactive nitrogen species metabolic process	6/99	8.24E-07	1.78E-05	8.78E-06	Aif1/Cd36/Clu/Cyp1b1/Sirpa/Tlr4
28-day post-SCI group vs. the control group	BP	GO:0050671	positive regulation of lymphocyte proliferation	7/99	8.33E-07	1.79E-05	8.83E-06	Aif1/Cd38/Il1a/Il6st/Ppp3ca/Tlr4/Vcam1
28-day post-SCI group vs. the control group	BP	GO:0050900	leukocyte migration	10/99	8.41E-07	1.80E-05	8.85E-06	Aif1/Il1a/Mapk1/Mmp14/Pawr/Rpl13a/Sirpa/Trem2/Trpm2/Vcam1
28-day post-SCI group vs. the control group	BP	GO:0051770	positive regulation of nitric-oxide synthase biosyntheti	4/99	8.98E-07	1.91E-05	9.40E-06	Map2k3/Map2k4/Map2k6/Tlr4
28-day post-SCI group vs. the control group	BP	GO:0006140	regulation of nucleotide metabolic process	6/99	9.34E-07	1.96E-05	9.64E-06	Hif1a/Ier3/Parp1/Pdk1/Pdk2/Trem2
28-day post-SCI group vs. the control group	BP	GO:0043535	regulation of blood vessel endothelial cell migration	6/99	9.34E-07	1.96E-05	9.64E-06	Apoe/Fbxw7/Hif1a/Hspb1/Map2k3/Nfe2l2
28-day post-SCI group vs. the control group	BP	GO:0032946	positive regulation of mononuclear cell proliferation	7/99	9.53E-07	1.99E-05	9.78E-06	Aif1/Cd38/Il1a/Il6st/Ppp3ca/Tlr4/Vcam1
28-day post-SCI group vs. the control group	BP	GO:0032651	regulation of interleukin-1 beta production	6/99	9.93E-07	2.06E-05	1.01E-05	Cd36/Hspb1/Sirpa/Tlr4/Tnfaip3/Trem2
28-day post-SCI group vs. the control group	BP	GO:0060537	muscle tissue development	11/99	1.05E-06	2.15E-05	1.06E-05	Apc/Fos/Gpx1/Map2k4/Mapk1/Met/Mylk/Ppp3ca/Rb1/Slc8a1/Smad1
28-day post-SCI group vs. the control group	BP	GO:0000303	response to superoxide	4/99	1.08E-06	2.22E-05	1.09E-05	Cd36/Nfe2l2/Parp1/Ucp2
28-day post-SCI group vs. the control group	BP	GO:0038066	p38MAPK cascade	5/99	1.17E-06	2.38E-05	1.17E-05	Map2k3/Mapkapk2/Met/Trem2/Xdh
28-day post-SCI group vs. the control group	BP	GO:0043620	regulation of DNA-templated transcription in response	5/99	1.28E-06	2.59E-05	1.27E-05	Cd36/Hif1a/Jun/Nfe2l2/Rela

28-day post-SCI group vs. the control group	BP	GO:0009306	protein secretion	10/99	1.30E-06	2.59E-05	1.27E-05	Apoe/Cd38/Fkbp1b/Hif1a/Il1a/Ppp3ca/Tlr4/Trem2/Trpm2/Ucp2
28-day post-SCI group vs. the control group	BP	GO:0000305	response to oxygen radical	4/99	1.30E-06	2.59E-05	1.27E-05	Cd36/Nfe2l2/Parp1/Ucp2
28-day post-SCI group vs. the control group	BP	GO:0035592	establishment of protein localization to extracellular re	10/99	1.33E-06	2.63E-05	1.29E-05	Apoe/Cd38/Fkbp1b/Hif1a/Il1a/Ppp3ca/Tlr4/Trem2/Trpm2/Ucp2
28-day post-SCI group vs. the control group	BP	GO:0010634	positive regulation of epithelial cell migration	7/99	1.34E-06	2.65E-05	1.30E-05	Hbegf/Hif1a/Hspb1/Jun/Map2k3/Met/Nfe2l2
28-day post-SCI group vs. the control group	BP	GO:0060326	cell chemotaxis	9/99	1.37E-06	2.68E-05	1.32E-05	Aif1/Hbegf/Hspb1/Mapk1/Met/Prkcd/Rpl13a/Trpm2/Vcam1
28-day post-SCI group vs. the control group	BP	GO:0032611	interleukin-1 beta production	6/99	1.42E-06	2.76E-05	1.36E-05	Cd36/Hspb1/Sirpa/Tlr4/Tnfaip3/Trem2
28-day post-SCI group vs. the control group	BP	GO:0007159	leukocyte cell-cell adhesion	10/99	1.51E-06	2.91E-05	1.43E-05	Actb/Aif1/Hspb1/Il1a/Il6st/Pawr/Ppp3ca/Rela/Sirpa/Vcam1
28-day post-SCI group vs. the control group	BP	GO:0071692	protein localization to extracellular region	10/99	1.51E-06	2.91E-05	1.43E-05	Apoe/Cd38/Fkbp1b/Hif1a/Il1a/Ppp3ca/Tlr4/Trem2/Trpm2/Ucp2
28-day post-SCI group vs. the control group	BP	GO:0009416	response to light stimulus	9/99	1.68E-06	3.22E-05	1.58E-05	Eif2s1/Fbxw7/Gpx1/Hif1a/Mapk10/Parp1/Prkcd/Rela/Slc7a11
28-day post-SCI group vs. the control group	BP	GO:0022409	positive regulation of cell-cell adhesion	9/99	1.77E-06	3.36E-05	1.66E-05	Actb/Aif1/Il1a/Il6st/Pawr/Ppp3ca/Rela/Sirpa/Vcam1
28-day post-SCI group vs. the control group	BP	GO:1904030	negative regulation of cyclin-dependent protein kinase	4/99	1.82E-06	3.44E-05	1.69E-05	Actb/Apc/Cdkn2c/Tnfaip3
28-day post-SCI group vs. the control group	BP	GO:0097529	myeloid leukocyte migration	8/99	1.95E-06	3.66E-05	1.80E-05	Aif1/Il1a/Mapk1/Mmp14/Pawr/Rpl13a/Sirpa/Trem2
28-day post-SCI group vs. the control group	BP	GO:0051054	positive regulation of DNA metabolic process	9/99	1.96E-06	3.66E-05	1.80E-05	Actb/Cyp1b1/Jun/Map2k4/Mapk1/Met/Parp1/Prkcd/Stat6
28-day post-SCI group vs. the control group	BP	GO:0035924	cellular response to vascular endothelial growth factor	5/99	1.99E-06	3.70E-05	1.82E-05	Hspb1/Map2k3/Mapkapk2/Rela/Xdh
28-day post-SCI group vs. the control group	BP	GO:0034250	positive regulation of cellular amide metabolic process	7/99	2.01E-06	3.72E-05	1.83E-05	Apoe/Clu/Cyp1b1/Mapk1/Nfe2l2/Prkcd/Rela
28-day post-SCI group vs. the control group	BP	GO:0031668	cellular response to extracellular stimulus	8/99	2.07E-06	3.81E-05	1.88E-05	Axl/Eif2s1/Fos/Jun/Mapk1/Nfe2l2/Pdk2/Vcam1
28-day post-SCI group vs. the control group	BP	GO:0002573	myeloid leukocyte differentiation	8/99	2.14E-06	3.91E-05	1.92E-05	Apc/Fbxw7/Fos/Jun/Ppp3ca/Rb1/Ripk1/Trem2
28-day post-SCI group vs. the control group	BP	GO:0060760	positive regulation of response to cytokine stimulus	5/99	2.16E-06	3.93E-05	1.93E-05	Axl/Hif1a/Ripk1/Tlr4/Trem2
28-day post-SCI group vs. the control group	BP	GO:0009266	response to temperature stimulus	7/99	2.35E-06	4.25E-05	2.09E-05	Eif2s1/Hspb1/Il1a/Pawr/Tlr4/Trpm2/Ucp2
28-day post-SCI group vs. the control group	BP	GO:0051767	nitric-oxide synthase biosynthetic process	4/99	2.48E-06	4.42E-05	2.18E-05	Map2k3/Map2k4/Map2k6/Tlr4
28-day post-SCI group vs. the control group	BP	GO:0051769	regulation of nitric-oxide synthase biosynthetic proces	4/99	2.48E-06	4.42E-05	2.18E-05	Map2k3/Map2k4/Map2k6/Tlr4
28-day post-SCI group vs. the control group	BP	GO:0010594	regulation of endothelial cell migration	7/99	2.54E-06	4.51E-05	2.22E-05	Apoe/Fbxw7/Hif1a/Hspb1/Map2k3/Met/Nfe2l2
28-day post-SCI group vs. the control group	BP	GO:0050670	regulation of lymphocyte proliferation	8/99	2.63E-06	4.58E-05	2.26E-05	Aif1/Cd38/Il1a/Il6st/Pawr/Ppp3ca/Tlr4/Vcam1
28-day post-SCI group vs. the control group	BP	GO:0007596	blood coagulation	7/99	2.63E-06	4.58E-05	2.26E-05	Apoe/Axl/Cd36/Nfe2l2/Prkcd/Slc7a11/Tlr4
28-day post-SCI group vs. the control group	BP	GO:0009612	response to mechanical stimulus	7/99	2.63E-06	4.58E-05	2.26E-05	Cd36/Fos/Jun/Pawr/Rela/Slc8a1/Tlr4
28-day post-SCI group vs. the control group	BP	GO:1903034	regulation of response to wounding	7/99	2.63E-06	4.58E-05	2.26E-05	Apoe/Cd36/Fkbp1b/Hbegf/Mylk/Nfe2l2/Prkcd
28-day post-SCI group vs. the control group	BP	GO:0007599	hemostasis	7/99	2.94E-06	5.07E-05	2.50E-05	Apoe/Axl/Cd36/Nfe2l2/Prkcd/Slc7a11/Tlr4
28-day post-SCI group vs. the control group	BP	GO:0050817	coagulation	7/99	2.94E-06	5.07E-05	2.50E-05	Apoe/Axl/Cd36/Nfe2l2/Prkcd/Slc7a11/Tlr4
28-day post-SCI group vs. the control group	BP	GO:1904035	regulation of epithelial cell apoptotic process	6/99	3.01E-06	5.15E-05	2.54E-05	Apc/Eif2s1/Id1/Nfe2l2/Rb1/Tnfaip3
28-day post-SCI group vs. the control group	BP	GO:0032944	regulation of mononuclear cell proliferation	8/99	3.04E-06	5.18E-05	2.55E-05	Aif1/Cd38/Il1a/Il6st/Pawr/Ppp3ca/Tlr4/Vcam1
28-day post-SCI group vs. the control group	BP	GO:0010586	miRNA metabolic process	6/99	3.16E-06	5.33E-05	2.63E-05	Ago1/Fos/Hif1a/Jun/Rela/Smad1
28-day post-SCI group vs. the control group	BP	GO:0032652	regulation of interleukin-1 production	6/99	3.16E-06	5.33E-05	2.63E-05	Cd36/Hspb1/Sirpa/Tlr4/Tnfaip3/Trem2
28-day post-SCI group vs. the control group	BP	GO:0042744	hydrogen peroxide catabolic process	4/99	3.30E-06	5.53E-05	2.72E-05	Gpx1/Gpx3/Prdx4/Prdx6
28-day post-SCI group vs. the control group	BP	GO:0070371	ERK1 and ERK2 cascade	9/99	3.32E-06	5.54E-05	2.73E-05	Apoe/Cd36/Fbxw7/Il1a/Jun/Mapk1/Sirpa/Tlr4/Trem2
28-day post-SCI group vs. the control group	BP	GO:0048660	regulation of smooth muscle cell proliferation	7/99	3.53E-06	5.86E-05	2.88E-05	Aif1/Apoe/Hbegf/Hif1a/Jun/Tlr4/Tnfaip3
28-day post-SCI group vs. the control group	BP	GO:0006801	superoxide metabolic process	5/99	3.72E-06	6.12E-05	3.02E-05	Cd36/Cybb/Ncf1/Nfe2l2/Prkcd
28-day post-SCI group vs. the control group	BP	GO:0042176	regulation of protein catabolic process	9/99	3.73E-06	6.12E-05	3.02E-05	Apc/Apoe/Clu/Fbxw7/Gpx1/Ier3/Rela/Tnfaip3/Trem2

28-day post-SCI group vs. the control group	BP	GO:0045454	cell redox homeostasis	4/99	3.77E-06	6.17E-05	3.04E-05	Gpx1/Nfe2l2/Prdx4/Prdx6
28-day post-SCI group vs. the control group	BP	GO:1901215	negative regulation of neuron death	8/99	3.92E-06	6.38E-05	3.14E-05	Apoe/Axl/Hif1a/Il6st/Jun/Map2k4/Ncoa7/Slc7a11
28-day post-SCI group vs. the control group	BP	GO:0050730	regulation of peptidyl-tyrosine phosphorylation	8/99	4.03E-06	6.49E-05	3.19E-05	Cd36/Fbxw7/Hbegf/Il6st/Prkcd/Tlr4/Tnfrsf1a/Trem2
28-day post-SCI group vs. the control group	BP	GO:1903522	regulation of blood circulation	8/99	4.03E-06	6.49E-05	3.19E-05	Atp2a2/Cd38/Fkbp1b/Hbegf/Hif1a/Ptgs1/S100a1/Slc8a1
28-day post-SCI group vs. the control group	BP	GO:0032612	interleukin-1 production	6/99	4.23E-06	6.78E-05	3.34E-05	Cd36/Hspb1/Sirpa/Tlr4/Tnfaip3/Trem2
28-day post-SCI group vs. the control group	BP	GO:0032720	negative regulation of tumor necrosis factor productior	5/99	4.29E-06	6.78E-05	3.34E-05	Axl/Sirpa/Tlr4/Tnfaip3/Trem2
28-day post-SCI group vs. the control group	BP	GO:0150076	neuroinflammatory response	5/99	4.29E-06	6.78E-05	3.34E-05	Aif1/Clu/Jun/Tlr4/Trem2
28-day post-SCI group vs. the control group	BP	GO:1904036	negative regulation of epithelial cell apoptotic process	5/99	4.29E-06	6.78E-05	3.34E-05	Apc/Id1/Nfe2l2/Rb1/Tnfaip3
28-day post-SCI group vs. the control group	BP	GO:0050731	positive regulation of peptidyl-tyrosine phosphorylatio	7/99	4.35E-06	6.85E-05	3.37E-05	Cd36/Fbxw7/Hbegf/Il6st/Tlr4/Tnfrsf1a/Trem2
28-day post-SCI group vs. the control group	BP	GO:1905954	positive regulation of lipid localization	6/99	4.43E-06	6.93E-05	3.41E-05	Apoe/Cd36/Il1a/Map2k6/Prkcd/Trem2
28-day post-SCI group vs. the control group	BP	GO:0048659	smooth muscle cell proliferation	7/99	4.51E-06	7.02E-05	3.46E-05	Aif1/Apoe/Hbegf/Hif1a/Jun/Tlr4/Tnfaip3
28-day post-SCI group vs. the control group	BP	GO:1903556	negative regulation of tumor necrosis factor superfami	5/99	4.60E-06	7.14E-05	3.51E-05	Axl/Sirpa/Tlr4/Tnfaip3/Trem2
28-day post-SCI group vs. the control group	BP	GO:1902895	positive regulation of miRNA transcription	5/99	4.93E-06	7.61E-05	3.75E-05	Fos/Hif1a/Jun/Rela/Smad1
28-day post-SCI group vs. the control group	BP	GO:0051101	regulation of DNA binding	6/99	5.09E-06	7.79E-05	3.83E-05	Fbxw7/Id1/Jun/Parp1/Rb1/Tlr4
28-day post-SCI group vs. the control group	BP	GO:0043434	response to peptide hormone	9/99	5.09E-06	7.79E-05	3.83E-05	Apc/Atp2a2/Nfe2l2/Parp1/Pdk2/Prkcd/Rb1/Rela/Stat6
28-day post-SCI group vs. the control group	BP	GO:0071375	cellular response to peptide hormone stimulus	8/99	5.27E-06	8.00E-05	3.94E-05	Apc/Nfe2l2/Parp1/Pdk2/Prkcd/Rb1/Rela/Stat6
28-day post-SCI group vs. the control group	BP	GO:0045428	regulation of nitric oxide biosynthetic process	5/99	5.28E-06	8.00E-05	3.94E-05	Aif1/Cd36/Clu/Sirpa/Tlr4
28-day post-SCI group vs. the control group	BP	GO:0098739	import across plasma membrane	7/99	5.52E-06	8.24E-05	4.06E-05	Cd36/Pawr/Ppp3ca/Prkcd/Slc7a11/Slc8a1/Trpm2
28-day post-SCI group vs. the control group	BP	GO:0055094	response to lipoprotein particle	4/99	5.52E-06	8.24E-05	4.06E-05	Apoe/Cd36/Tlr4/Trem2
28-day post-SCI group vs. the control group	BP	GO:1903205	regulation of hydrogen peroxide-induced cell death	4/99	5.52E-06	8.24E-05	4.06E-05	Met/Nfe2l2/Pawr/Ripk1
28-day post-SCI group vs. the control group	BP	GO:0042982	amyloid precursor protein metabolic process	5/99	5.65E-06	8.40E-05	4.14E-05	Apoe/Clu/Dhcr24/Pawr/Rela
28-day post-SCI group vs. the control group	BP	GO:0045732	positive regulation of protein catabolic process	7/99	5.89E-06	8.72E-05	4.29E-05	Apc/Apoe/Clu/Fbxw7/Ier3/Tnfaip3/Trem2
28-day post-SCI group vs. the control group	BP	GO:0050863	regulation of T cell activation	9/99	6.19E-06	9.11E-05	4.49E-05	Actb/Aif1/Hspb1/Il1a/Il6st/Pawr/Ppp3ca/Sirpa/Vcam1
28-day post-SCI group vs. the control group	BP	GO:0002246	wound healing involved in inflammatory response	3/99	6.28E-06	9.17E-05	4.51E-05	Hif1a/Il1a/Tlr4
28-day post-SCI group vs. the control group	BP	GO:0061517	macrophage proliferation	3/99	6.28E-06	9.17E-05	4.51E-05	Clu/Mapk1/Trem2
28-day post-SCI group vs. the control group	BP	GO:0048732	gland development	10/99	6.32E-06	9.18E-05	4.52E-05	Apc/Gpx1/Hif1a/Jun/Mapk1/Met/Rela/Stat6/Tnfaip3/Xdh
28-day post-SCI group vs. the control group	BP	GO:0060048	cardiac muscle contraction	6/99	6.35E-06	9.20E-05	4.53E-05	Atp2a2/Fkbp1b/Map2k3/Map2k6/Met/Slc8a1
28-day post-SCI group vs. the control group	BP	GO:0080164	regulation of nitric oxide metabolic process	5/99	6.87E-06	9.90E-05	4.88E-05	Aif1/Cd36/Clu/Sirpa/Tlr4
28-day post-SCI group vs. the control group	BP	GO:0097242	amyloid-beta clearance	4/99	6.97E-06	0.00010005	4.93E-05	Apoe/Cd36/Clu/Trem2
28-day post-SCI group vs. the control group	BP	GO:2000630	positive regulation of miRNA metabolic process	5/99	7.32E-06	0.00010462	5.15E-05	Fos/Hif1a/Jun/Rela/Smad1
28-day post-SCI group vs. the control group	BP	GO:1903036	positive regulation of response to wounding	5/99	7.80E-06	0.00011043	5.44E-05	Cd36/Fkbp1b/Hbegf/Mylk/Nfe2l2
28-day post-SCI group vs. the control group	BP	GO:0071402	cellular response to lipoprotein particle stimulus	4/99	7.80E-06	0.00011043	5.44E-05	Apoe/Cd36/Tlr4/Trem2
28-day post-SCI group vs. the control group	BP	GO:0001959	regulation of cytokine-mediated signaling pathway	6/99	7.87E-06	0.00011046	5.44E-05	Axl/Hif1a/Il6st/Ripk1/Tnfrsf1a/Trem2
28-day post-SCI group vs. the control group	BP	GO:0061041	regulation of wound healing	6/99	7.87E-06	0.00011046	5.44E-05	Apoe/Cd36/Hbegf/Mylk/Nfe2l2/Prkcd
28-day post-SCI group vs. the control group	BP	GO:0032874	positive regulation of stress-activated MAPK cascade	6/99	8.20E-06	0.00011467	5.65E-05	Il1a/Map2k4/Met/Ripk1/Tlr4/Xdh
28-day post-SCI group vs. the control group	BP	GO:0043619	regulation of transcription from RNA polymerase II pr	3/99	8.35E-06	0.00011477	5.65E-05	Cd36/Hif1a/Nfe2l2
28-day post-SCI group vs. the control group	BP	GO:0140052	cellular response to oxidised low-density lipoprotein p	3/99	8.35E-06	0.00011477	5.65E-05	Cd36/Tlr4/Trem2
28-day post-SCI group vs. the control group	BP	GO:1902947	regulation of tau-protein kinase activity	3/99	8.35E-06	0.00011477	5.65E-05	Apoe/Clu/Rb1
28-day post-SCI group vs. the control group	BP	GO:1905907	negative regulation of amyloid fibril formation	3/99	8.35E-06	0.00011477	5.65E-05	Apoe/Clu/Trem2
28-day post-SCI group vs. the control group	BP	GO:0001818	negative regulation of cytokine production	8/99	8.54E-06	0.00011683	5.75E-05	Axl/Met/Parp1/Sirpa/Tlr4/Tnfaip3/Tnfrsf1a/Trem2

28-day post-SCI group vs. the control group	BP	GO:0030808	regulation of nucleotide biosynthetic process	4/99	8.69E-06	0.0001175	5.79E-05	Parp1/Pdk1/Pdk2/Trem2
28-day post-SCI group vs. the control group	BP	GO:0036474	cell death in response to hydrogen peroxide	4/99	8.69E-06	0.0001175	5.79E-05	Met/Nfe2l2/Pawr/Ripk1
28-day post-SCI group vs. the control group	BP	GO:1900371	regulation of purine nucleotide biosynthetic process	4/99	8.69E-06	0.0001175	5.79E-05	Parp1/Pdk1/Pdk2/Trem2
28-day post-SCI group vs. the control group	BP	GO:0006749	glutathione metabolic process	5/99	8.82E-06	0.0001187	5.84E-05	Gpx1/Gpx3/Mgst1/Nfe2l2/Slc7a11
28-day post-SCI group vs. the control group	BP	GO:0070304	positive regulation of stress-activated protein kinase si	6/99	8.91E-06	0.00011942	5.88E-05	Il1a/Map2k4/Met/Ripk1/Tlr4/Xdh
28-day post-SCI group vs. the control group	BP	GO:0006690	icosanoid metabolic process	6/99	9.66E-06	0.00012899	6.35E-05	Cyp1b1/Gpx1/Ncf1/Ptgs1/Tlr4/Tnfrsf1a
28-day post-SCI group vs. the control group	BP	GO:0001666	response to hypoxia	7/99	1.06E-05	0.00014159	6.97E-05	Cd38/Hif1a/Hmox2/Nfe2l2/Pdk1/Slc8a1/Ucp2
28-day post-SCI group vs. the control group	BP	GO:0098703	calcium ion import across plasma membrane	4/99	1.07E-05	0.00014179	6.98E-05	Pawr/Ppp3ca/Slc8a1/Trpm2
28-day post-SCI group vs. the control group	BP	GO:0097709	connective tissue replacement	3/99	1.08E-05	0.00014283	7.03E-05	Hif1a/Il1a/Ppp3ca
28-day post-SCI group vs. the control group	BP	GO:0071496	cellular response to external stimulus	8/99	1.11E-05	0.00014554	7.17E-05	Axl/Eif2s1/Fos/Jun/Mapk1/Nfe2l2/Pdk2/Vcam1
28-day post-SCI group vs. the control group	BP	GO:0032102	negative regulation of response to external stimulus	9/99	1.12E-05	0.00014554	7.17E-05	Aif1/Apoe/Gpx1/Ier3/Prkcd/Rb1/Tnfaip3/Tnfrsf1a/Trem2
28-day post-SCI group vs. the control group	BP	GO:0045670	regulation of osteoclast differentiation	5/99	1.12E-05	0.00014554	7.17E-05	Apc/Fbxw7/Fos/Ppp3ca/Trem2
28-day post-SCI group vs. the control group	BP	GO:1904019	epithelial cell apoptotic process	6/99	1.22E-05	0.00015882	7.82E-05	Apc/Eif2s1/Id1/Nfe2l2/Rb1/Tnfaip3
28-day post-SCI group vs. the control group	BP	GO:0050866	negative regulation of cell activation	7/99	1.27E-05	0.00016403	8.08E-05	Apoe/Axl/Hspb1/Pawr/Prkcd/Tnfaip3/Trem2
28-day post-SCI group vs. the control group	BP	GO:1902893	regulation of miRNA transcription	5/99	1.32E-05	0.00017038	8.39E-05	Fos/Hif1a/Jun/Rela/Smad1
28-day post-SCI group vs. the control group	BP	GO:0070374	positive regulation of ERK1 and ERK2 cascade	7/99	1.34E-05	0.00017233	8.48E-05	Apoe/Cd36/Fbxw7/Il1a/Jun/Tlr4/Trem2
28-day post-SCI group vs. the control group	BP	GO:0009411	response to UV	6/99	1.37E-05	0.00017499	8.62E-05	Eif2s1/Fbxw7/Gpx1/Parp1/Prkcd/Rela
28-day post-SCI group vs. the control group	BP	GO:0010882	regulation of cardiac muscle contraction by calcium io	3/99	1.37E-05	0.00017499	8.62E-05	Atp2a2/Fkbp1b/Slc8a1
28-day post-SCI group vs. the control group	BP	GO:0009991	response to extracellular stimulus	9/99	1.43E-05	0.00018097	8.91E-05	Apoe/Axl/Eif2s1/Fos/Jun/Mapk1/Nfe2l2/Pdk2/Vcam1
28-day post-SCI group vs. the control group	BP	GO:0051047	positive regulation of secretion	9/99	1.43E-05	0.00018097	8.91E-05	Cd38/Hif1a/Il1a/Map2k6/Ppp3ca/Sdc1/Tlr4/Trem2/Trpm2
28-day post-SCI group vs. the control group	BP	GO:0019221	cytokine-mediated signaling pathway	9/99	1.46E-05	0.00018276	9.00E-05	Axl/Hif1a/Il1a/Il6st/Rela/Ripk1/Stat6/Tnfrsf1a/Trem2
28-day post-SCI group vs. the control group	BP	GO:0015833	peptide transport	8/99	1.46E-05	0.00018276	9.00E-05	Cd38/Fkbp1b/Hif1a/Mgst1/Ppp3ca/Slc7a11/Trpm2/Ucp2
28-day post-SCI group vs. the control group	BP	GO:0018108	peptidyl-tyrosine phosphorylation	8/99	1.46E-05	0.00018276	9.00E-05	Cd36/Fbxw7/Hbegf/Il6st/Prkcd/Tlr4/Tnfrsf1a/Trem2
28-day post-SCI group vs. the control group	BP	GO:0061614	miRNA transcription	5/99	1.48E-05	0.00018314	9.02E-05	Fos/Hif1a/Jun/Rela/Smad1
28-day post-SCI group vs. the control group	BP	GO:0071333	cellular response to glucose stimulus	6/99	1.48E-05	0.00018314	9.02E-05	Fkbp1b/Gpx1/Hif1a/Ppp3ca/Ucp2/Vcam1
28-day post-SCI group vs. the control group	BP	GO:0018212	peptidyl-tyrosine modification	8/99	1.53E-05	0.00018898	9.30E-05	Cd36/Fbxw7/Hbegf/Il6st/Prkcd/Tlr4/Tnfrsf1a/Trem2
28-day post-SCI group vs. the control group	BP	GO:0071331	cellular response to hexose stimulus	6/99	1.59E-05	0.00019567	9.63E-05	Fkbp1b/Gpx1/Hif1a/Ppp3ca/Ucp2/Vcam1
28-day post-SCI group vs. the control group	BP	GO:0071326	cellular response to monosaccharide stimulus	6/99	1.65E-05	0.00020218	9.95E-05	Fkbp1b/Gpx1/Hif1a/Ppp3ca/Ucp2/Vcam1
28-day post-SCI group vs. the control group	BP	GO:0072594	establishment of protein localization to organelle	9/99	1.66E-05	0.000203	9.99E-05	Cd36/Clu/Fbxw7/Mapk1/Ppp3ca/Prkcd/Tnfaip3/Tnfrsf1a/Txnip
28-day post-SCI group vs. the control group	BP	GO:0070372	regulation of ERK1 and ERK2 cascade	8/99	1.67E-05	0.00020343	0.00010016	Apoe/Cd36/Fbxw7/Il1a/Jun/Sirpa/Tlr4/Trem2
28-day post-SCI group vs. the control group	BP	GO:0003018	vascular process in circulatory system	7/99	1.68E-05	0.0002035	0.0001002	Apoe/Cd36/Cd38/Gpx1/Hif1a/Ptgs1/Slc8a1
28-day post-SCI group vs. the control group	BP	GO:1905906	regulation of amyloid fibril formation	3/99	1.71E-05	0.00020685	0.00010184	Apoe/Clu/Trem2
28-day post-SCI group vs. the control group	BP	GO:0010935	regulation of macrophage cytokine production	4/99	1.72E-05	0.00020685	0.00010184	Axl/Cd36/Mapkapk2/Tlr4
28-day post-SCI group vs. the control group	BP	GO:0030100	regulation of endocytosis	7/99	1.73E-05	0.00020685	0.00010184	Amph/Apoe/Axl/Cd36/Clu/Ppp3ca/Trem2
28-day post-SCI group vs. the control group	BP	GO:0042098	T cell proliferation	7/99	1.77E-05	0.00021177	0.00010427	Aif1/Fkbp1b/Il1a/Il6st/Pawr/Ppp3ca/Vcam1
28-day post-SCI group vs. the control group	BP	GO:2000273	positive regulation of signaling receptor activity	4/99	1.88E-05	0.00022385	0.00011022	Fbxw7/Hbegf/Hdac1/Hif1a
28-day post-SCI group vs. the control group	BP	GO:0002687	positive regulation of leukocyte migration	6/99	1.90E-05	0.00022574	0.00011115	Aif1/Il1a/Mapk1/Mmp14/Pawr/Trem2
28-day post-SCI group vs. the control group	BP	GO:0030168	platelet activation	5/99	1.92E-05	0.00022674	0.00011164	Apoe/Axl/Prkcd/Slc7a11/Tlr4
28-day post-SCI group vs. the control group	BP	GO:0006790	sulfur compound metabolic process	8/99	2.03E-05	0.00023924	0.00011779	Gpx1/Gpx3/Mgst1/Nfe2l2/Pdk1/Pdk2/Slc7a11/Xdh
28-day post-SCI group vs. the control group	BP	GO:0120163	negative regulation of cold-induced thermogenesis	4/99	2.05E-05	0.00024054	0.00011843	Id1/Map2k6/Rb1/Tlr4

28-day post-SCI group vs. the control group	BP	GO:0070431	nucleotide-binding oligomerization domain containing	3/99	2.10E-05	0.00024587	0.00012106	Rela/Tlr4/Tnfaip3
28-day post-SCI group vs. the control group	BP	GO:0031348	negative regulation of defense response	7/99	2.14E-05	0.00024849	0.00012235	Apoe/Gpx1/Ier3/Rb1/Tnfaip3/Tnfrsf1a/Trem2
28-day post-SCI group vs. the control group	BP	GO:0050678	regulation of epithelial cell proliferation	9/99	2.14E-05	0.00024849	0.00012235	Apc/Apoe/Ctsl/Gpx1/Id1/Jun/Rb1/Tnfaip3/Xdh
28-day post-SCI group vs. the control group	BP	GO:0071322	cellular response to carbohydrate stimulus	6/99	2.19E-05	0.00025285	0.0001245	Fkbp1b/Gpx1/Hif1a/Ppp3ca/Ucp2/Vcam1
28-day post-SCI group vs. the control group	BP	GO:0002064	epithelial cell development	7/99	2.20E-05	0.00025285	0.0001245	Gpx1/Hif1a/Id1/Il1a/Il6st/Met/Tnfrsf1a
28-day post-SCI group vs. the control group	BP	GO:0043124	negative regulation of I-kappaB kinase/NF-kappaB sig	4/99	2.23E-05	0.00025621	0.00012615	Hdac1/Ripk1/Tnfaip3/Trem2
28-day post-SCI group vs. the control group	BP	GO:0036293	response to decreased oxygen levels	7/99	2.25E-05	0.00025651	0.0001263	Cd38/Hif1a/Hmox2/Nfe2l2/Pdk1/Slc8a1/Ucp2
28-day post-SCI group vs. the control group	BP	GO:0050870	positive regulation of T cell activation	7/99	2.25E-05	0.00025651	0.0001263	Actb/Aif1/Il1a/Il6st/Ppp3ca/Sirpa/Vcam1
28-day post-SCI group vs. the control group	BP	GO:0030099	myeloid cell differentiation	9/99	2.26E-05	0.00025651	0.0001263	Apc/Fbxw7/Fos/Hif1a/Jun/Ppp3ca/Rb1/Ripk1/Trem2
28-day post-SCI group vs. the control group	BP	GO:0050714	positive regulation of protein secretion	6/99	2.27E-05	0.00025651	0.0001263	Cd38/Hif1a/Il1a/Tlr4/Trem2/Trpm2
28-day post-SCI group vs. the control group	BP	GO:0002532	production of molecular mediator involved in inflamm	5/99	2.34E-05	0.00026357	0.00012977	Hif1a/Ncf1/Sirpa/Tlr4/Trem2
28-day post-SCI group vs. the control group	BP	GO:2001237	negative regulation of extrinsic apoptotic signaling pat	5/99	2.34E-05	0.00026357	0.00012977	Gpx1/Mcl1/Rela/Ripk1/Tnfaip3
28-day post-SCI group vs. the control group	BP	GO:0042692	muscle cell differentiation	9/99	2.42E-05	0.00027079	0.00013333	Atp2a2/Gpx1/Map2k4/Met/Mmp14/Ppp3ca/Rb1/Slc8a1/Smad1
28-day post-SCI group vs. the control group	BP	GO:0006937	regulation of muscle contraction	6/99	2.43E-05	0.00027079	0.00013333	Atp2a2/Fkbp1b/Ncf1/Pawr/Ptgs1/Slc8a1
28-day post-SCI group vs. the control group	BP	GO:0014910	regulation of smooth muscle cell migration	5/99	2.46E-05	0.00027387	0.00013484	Aif1/Cyp1b1/Il6st/Nfe2l2/Tlr4
28-day post-SCI group vs. the control group	BP	GO:0032370	positive regulation of lipid transport	5/99	2.58E-05	0.00028542	0.00014053	Apoe/Il1a/Map2k6/Prkcd/Trem2
28-day post-SCI group vs. the control group	BP	GO:0043502	regulation of muscle adaptation	5/99	2.58E-05	0.00028542	0.00014053	Aif1/Atp2a2/Parp1/Ppp3ca/Tnfrsf1a
28-day post-SCI group vs. the control group	BP	GO:0043618	regulation of transcription from RNA polymerase II pr	4/99	2.63E-05	0.0002885	0.00014205	Cd36/Hif1a/Jun/Nfe2l2
28-day post-SCI group vs. the control group	BP	GO:0045429	positive regulation of nitric oxide biosynthetic process	4/99	2.63E-05	0.0002885	0.00014205	Aif1/Cd36/Clu/Tlr4
28-day post-SCI group vs. the control group	BP	GO:0071248	cellular response to metal ion	6/99	2.68E-05	0.00029332	0.00014442	Fos/Jun/Mapk1/Mylk/Nfe2l2/Trpm2
28-day post-SCI group vs. the control group	BP	GO:2000628	regulation of miRNA metabolic process	5/99	2.71E-05	0.00029533	0.00014541	Fos/Hif1a/Jun/Rela/Smad1
28-day post-SCI group vs. the control group	BP	GO:0032103	positive regulation of response to external stimulus	9/99	2.88E-05	0.00031271	0.00015397	Aif1/Fkbp1b/Hspb1/Mapk1/Met/Ripk1/Tlr4/Tnfrsf1a/Trem2
28-day post-SCI group vs. the control group	BP	GO:0042886	amide transport	8/99	2.95E-05	0.00031621	0.00015569	Cd38/Fkbp1b/Hif1a/Mgst1/Ppp3ca/Slc7a11/Trpm2/Ucp2
28-day post-SCI group vs. the control group	BP	GO:0001678	cellular glucose homeostasis	6/99	2.96E-05	0.00031621	0.00015569	Fkbp1b/Gpx1/Hif1a/Ppp3ca/Ucp2/Vcam1
28-day post-SCI group vs. the control group	BP	GO:0006941	striated muscle contraction	6/99	2.96E-05	0.00031621	0.00015569	Atp2a2/Fkbp1b/Map2k3/Map2k6/Met/Slc8a1
28-day post-SCI group vs. the control group	BP	GO:0035296	regulation of tube diameter	6/99	2.96E-05	0.00031621	0.00015569	Apoe/Cd38/Gpx1/Hif1a/Ptgs1/Slc8a1
28-day post-SCI group vs. the control group	BP	GO:0097746	blood vessel diameter maintenance	6/99	2.96E-05	0.00031621	0.00015569	Apoe/Cd38/Gpx1/Hif1a/Ptgs1/Slc8a1
28-day post-SCI group vs. the control group	BP	GO:0042116	macrophage activation	5/99	2.97E-05	0.00031692	0.00015604	Aif1/Clu/Jun/Tlr4/Trem2
28-day post-SCI group vs. the control group	BP	GO:0018209	peptidyl-serine modification	8/99	3.01E-05	0.00031984	0.00015748	Mapk1/Mapkapk2/Met/Parp1/Pdk1/Pdk2/Prkcd/Ripk1
28-day post-SCI group vs. the control group	BP	GO:0070423	nucleotide-binding oligomerization domain containing	3/99	3.05E-05	0.0003212	0.00015815	Rela/Tlr4/Tnfaip3
28-day post-SCI group vs. the control group	BP	GO:1900221	regulation of amyloid-beta clearance	3/99	3.05E-05	0.0003212	0.00015815	Apoe/Clu/Trem2
28-day post-SCI group vs. the control group	BP	GO:0035150	regulation of tube size	6/99	3.06E-05	0.0003212	0.00015815	Apoe/Cd38/Gpx1/Hif1a/Ptgs1/Slc8a1
28-day post-SCI group vs. the control group	BP	GO:0010934	macrophage cytokine production	4/99	3.07E-05	0.0003212	0.00015815	Axl/Cd36/Mapkapk2/Tlr4
28-day post-SCI group vs. the control group	BP	GO:1904407	positive regulation of nitric oxide metabolic process	4/99	3.07E-05	0.0003212	0.00015815	Aif1/Cd36/Clu/Tlr4
28-day post-SCI group vs. the control group	BP	GO:0051896	regulation of protein kinase B signaling	6/99	3.16E-05	0.00032868	0.00016183	Axl/Gpx1/Hbegf/Met/Plekha1/Xdh
28-day post-SCI group vs. the control group	BP	GO:0010001	glial cell differentiation	7/99	3.29E-05	0.00034138	0.00016809	Clu/Hdac1/Il6st/Mapk1/Rela/Tlr4/Trem2
28-day post-SCI group vs. the control group	BP	GO:0006606	protein import into nucleus	6/99	3.36E-05	0.00034481	0.00016977	Cd36/Mapk1/Ppp3ca/Prkcd/Tnfrsf1a/Txnip
28-day post-SCI group vs. the control group	BP	GO:0007254	JNK cascade	6/99	3.36E-05	0.00034481	0.00016977	Il1a/Map2k4/Mapk10/Ripk1/Sirpa/Tlr4
28-day post-SCI group vs. the control group	BP	GO:0051100	negative regulation of binding	6/99	3.36E-05	0.00034481	0.00016977	Actb/Atp2a2/Fbxw7/Id1/Jun/Ppp3ca
28-day post-SCI group vs. the control group	BP	GO:1903531	negative regulation of secretion by cell	6/99	3.36E-05	0.00034481	0.00016977	Apoe/Fkbp1b/Ppp3ca/Ptgs1/Tnfrsf1a/Ucp2

28-day post-SCI group vs. the control group	BP	GO:0003300	cardiac muscle hypertrophy	5/99	3.41E-05	0.00034865	0.00017166	Atp2a2/Map2k4/Parp1/Ppp3ca/Tnfrsf1a
28-day post-SCI group vs. the control group	BP	GO:0030098	lymphocyte differentiation	9/99	3.52E-05	0.00035839	0.00017646	Actb/Apc/Axl/Ctsl/Hspb1/Il1a/Mmp14/Stat6/Tnfaip3
28-day post-SCI group vs. the control group	BP	GO:0032715	negative regulation of interleukin-6 production	4/99	3.57E-05	0.00036048	0.00017749	Sirpa/Tlr4/Tnfaip3/Tnfrsf1a
28-day post-SCI group vs. the control group	BP	GO:1902904	negative regulation of supramolecular fiber organizatio	6/99	3.58E-05	0.00036048	0.00017749	Apc/Apoe/Clu/Met/Prkcd/Trem2
28-day post-SCI group vs. the control group	BP	GO:0042063	gliogenesis	8/99	3.60E-05	0.00036048	0.00017749	Clu/Hdac1/Il6st/Mapk1/Rb1/Rela/Tlr4/Trem2
28-day post-SCI group vs. the control group	BP	GO:0045860	positive regulation of protein kinase activity	8/99	3.60E-05	0.00036048	0.00017749	Clu/Fbxw7/Hbegf/Map2k3/Map2k4/Map2k6/Prkcd/Tlr4
28-day post-SCI group vs. the control group	BP	GO:0035872	nucleotide-binding domain, leucine rich repeat contain	3/99	3.61E-05	0.00036048	0.00017749	Rela/Tlr4/Tnfaip3
28-day post-SCI group vs. the control group	BP	GO:1900225	regulation of NLRP3 inflammasome complex assembl	3/99	3.61E-05	0.00036048	0.00017749	Cd36/Tlr4/Trem2
28-day post-SCI group vs. the control group	BP	GO:1902004	positive regulation of amyloid-beta formation	3/99	3.61E-05	0.00036048	0.00017749	Apoe/Clu/Rela
28-day post-SCI group vs. the control group	BP	GO:0001890	placenta development	6/99	3.69E-05	0.0003673	0.00018084	Ctsl/Hif1a/Mapk1/Met/Slc8a1/Vcam1
28-day post-SCI group vs. the control group	BP	GO:0048661	positive regulation of smooth muscle cell proliferation	5/99	3.73E-05	0.00036954	0.00018195	Aif1/Hbegf/Hif1a/Jun/Tlr4
28-day post-SCI group vs. the control group	BP	GO:1903532	positive regulation of secretion by cell	8/99	3.74E-05	0.00036956	0.00018196	Cd38/Hif1a/Il1a/Map2k6/Sdc1/Tlr4/Trem2/Trpm2
28-day post-SCI group vs. the control group	BP	GO:0051170	import into nucleus	6/99	3.81E-05	0.00037543	0.00018485	Cd36/Mapk1/Ppp3ca/Prkcd/Tnfrsf1a/Txnip
28-day post-SCI group vs. the control group	BP	GO:0001961	positive regulation of cytokine-mediated signaling path	4/99	3.84E-05	0.00037736	0.0001858	Axl/Hif1a/Ripk1/Trem2
28-day post-SCI group vs. the control group	BP	GO:0008544	epidermis development	8/99	3.89E-05	0.0003792	0.0001867	Apc/Ctsl/Hdac1/Il1a/Ppp3ca/Ptgs1/Rela/Txnip
28-day post-SCI group vs. the control group	BP	GO:0010595	positive regulation of endothelial cell migration	5/99	3.90E-05	0.0003792	0.0001867	Hif1a/Hspb1/Map2k3/Met/Nfe2l2
28-day post-SCI group vs. the control group	BP	GO:0042102	positive regulation of T cell proliferation	5/99	3.90E-05	0.0003792	0.0001867	Aif1/Il1a/Il6st/Ppp3ca/Vcam1
28-day post-SCI group vs. the control group	BP	GO:0002221	pattern recognition receptor signaling pathway	6/99	3.93E-05	0.00038142	0.0001878	Cd36/Mapkapk2/Rela/Tlr4/Tnfaip3/Trem2
28-day post-SCI group vs. the control group	BP	GO:0014897	striated muscle hypertrophy	5/99	4.07E-05	0.00039251	0.00019326	Atp2a2/Map2k4/Parp1/Ppp3ca/Tnfrsf1a
28-day post-SCI group vs. the control group	BP	GO:0014909	smooth muscle cell migration	5/99	4.07E-05	0.00039251	0.00019326	Aif1/Cyp1b1/Il6st/Nfe2l2/Tlr4
28-day post-SCI group vs. the control group	BP	GO:0034349	glial cell apoptotic process	3/99	4.24E-05	0.00040455	0.00019918	Prkcd/Rb1/Trem2
28-day post-SCI group vs. the control group	BP	GO:0071450	cellular response to oxygen radical	3/99	4.24E-05	0.00040455	0.00019918	Cd36/Nfe2l2/Parp1
28-day post-SCI group vs. the control group	BP	GO:0071451	cellular response to superoxide	3/99	4.24E-05	0.00040455	0.00019918	Cd36/Nfe2l2/Parp1
28-day post-SCI group vs. the control group	BP	GO:0090594	inflammatory response to wounding	3/99	4.24E-05	0.00040455	0.00019918	Hif1a/Il1a/Tlr4
28-day post-SCI group vs. the control group	BP	GO:0044403	biological process involved in symbiotic interaction	7/99	4.27E-05	0.00040614	0.00019997	Apoe/Axl/Ctsl/Gpx1/Hdac1/Jun/Ncf1
28-day post-SCI group vs. the control group	BP	GO:0043536	positive regulation of blood vessel endothelial cell mig	4/99	4.43E-05	0.00041891	0.00020626	Hif1a/Hspb1/Map2k3/Nfe2l2
28-day post-SCI group vs. the control group	BP	GO:0014896	muscle hypertrophy	5/99	4.43E-05	0.00041891	0.00020626	Atp2a2/Map2k4/Parp1/Ppp3ca/Tnfrsf1a
28-day post-SCI group vs. the control group	BP	GO:0043409	negative regulation of MAPK cascade	6/99	4.57E-05	0.00042963	0.00021153	Apc/Apoe/Prkcd/Sirpa/Tlr4/Trem2
28-day post-SCI group vs. the control group	BP	GO:1905952	regulation of lipid localization	6/99	4.57E-05	0.00042963	0.00021153	Apoe/Cd36/Il1a/Map2k6/Prkcd/Trem2
28-day post-SCI group vs. the control group	BP	GO:0044546	NLRP3 inflammasome complex assembly	3/99	4.94E-05	0.00046271	0.00022782	Cd36/Tlr4/Trem2
28-day post-SCI group vs. the control group	BP	GO:0042987	amyloid precursor protein catabolic process	4/99	5.07E-05	0.00047412	0.00023344	Apoe/Clu/Dhcr24/Rela
28-day post-SCI group vs. the control group	BP	GO:0050796	regulation of insulin secretion	6/99	5.14E-05	0.00047917	0.00023593	Cd38/Fkbp1b/Hif1a/Ppp3ca/Trpm2/Ucp2
28-day post-SCI group vs. the control group	BP	GO:1900015	regulation of cytokine production involved in inflamm	4/99	5.42E-05	0.00050378	0.00024804	Hif1a/Sirpa/Tlr4/Trem2
28-day post-SCI group vs. the control group	BP	GO:0009749	response to glucose	6/99	5.61E-05	0.00051805	0.00025507	Fkbp1b/Gpx1/Hif1a/Ppp3ca/Ucp2/Vcam1
28-day post-SCI group vs. the control group	BP	GO:0051099	positive regulation of binding	6/99	5.61E-05	0.00051805	0.00025507	Apoe/Eif2s1/Met/Parp1/Rb1/Tlr4
28-day post-SCI group vs. the control group	BP	GO:0043524	negative regulation of neuron apoptotic process	6/99	5.77E-05	0.00053153	0.00026171	Apoe/Axl/Hif1a/Il6st/Jun/Map2k4
28-day post-SCI group vs. the control group	BP	GO:0031663	lipopolysaccharide-mediated signaling pathway	4/99	5.79E-05	0.00053168	0.00026178	Mapk1/Tlr4/Tnfaip3/Trem2
28-day post-SCI group vs. the control group	BP	GO:1902903	regulation of supramolecular fiber organization	8/99	5.85E-05	0.00053591	0.00026386	Apc/Apoe/Clu/Id1/Met/Prkcd/Rb1/Trem2
28-day post-SCI group vs. the control group	BP	GO:0045446	endothelial cell differentiation	5/99	5.90E-05	0.00053793	0.00026486	Gpx1/Id1/Met/Tnfrsf1a/Xdh
28-day post-SCI group vs. the control group	BP	GO:0051702	biological process involved in interaction with symbio	5/99	5.90E-05	0.00053793	0.00026486	Apoe/Gpx1/Hdac1/Jun/Ncf1

28-day post-SCI group vs. the control group	BP	GO:0042129	regulation of T cell proliferation	6/99	5.93E-05	0.00053922	0.00026549	Aif1/Il1a/Il6st/Pawr/Ppp3ca/Vcam1
28-day post-SCI group vs. the control group	BP	GO:0009746	response to hexose	6/99	6.10E-05	0.0005531	0.00027233	Fkbp1b/Gpx1/Hif1a/Ppp3ca/Ucp2/Vcam1
28-day post-SCI group vs. the control group	BP	GO:0090303	positive regulation of wound healing	4/99	6.17E-05	0.00055747	0.00027448	Cd36/Hbegf/Mylk/Nfe2l2
28-day post-SCI group vs. the control group	BP	GO:1904385	cellular response to angiotensin	3/99	6.54E-05	0.00058943	0.00029021	Nfe2l2/Prkcd/Rela
28-day post-SCI group vs. the control group	BP	GO:1903409	reactive oxygen species biosynthetic process	4/99	6.57E-05	0.00059045	0.00029072	Cd36/Cybb/Ncf1/Tlr4
28-day post-SCI group vs. the control group	BP	GO:0034284	response to monosaccharide	6/99	6.64E-05	0.00059341	0.00029217	Fkbp1b/Gpx1/Hif1a/Ppp3ca/Ucp2/Vcam1
28-day post-SCI group vs. the control group	BP	GO:0030316	osteoclast differentiation	5/99	6.64E-05	0.00059341	0.00029217	Apc/Fbxw7/Fos/Ppp3ca/Trem2
28-day post-SCI group vs. the control group	BP	GO:0002224	toll-like receptor signaling pathway	5/99	6.90E-05	0.00061333	0.00030198	Cd36/Mapkapk2/Tlr4/Tnfaip3/Trem2
28-day post-SCI group vs. the control group	BP	GO:0032963	collagen metabolic process	5/99	6.90E-05	0.00061333	0.00030198	Ctsl/Hif1a/Id1/Mmp14/Prkcd
28-day post-SCI group vs. the control group	BP	GO:0014911	positive regulation of smooth muscle cell migration	4/99	6.99E-05	0.00061798	0.00030427	Aif1/Cyp1b1/Il6st/Tlr4
28-day post-SCI group vs. the control group	BP	GO:0032615	interleukin-12 production	4/99	6.99E-05	0.00061798	0.00030427	Cd36/Prkcd/Rela/Tlr4
28-day post-SCI group vs. the control group	BP	GO:0007179	transforming growth factor beta receptor signaling pat	6/99	7.01E-05	0.0006182	0.00030438	Fos/Hdac1/Itgb5/Jun/Parp1/Smad1
28-day post-SCI group vs. the control group	BP	GO:0006575	cellular modified amino acid metabolic process	6/99	7.21E-05	0.00063187	0.00031111	Ctsl/Gpx1/Gpx3/Mgst1/Nfe2l2/Slc7a11
28-day post-SCI group vs. the control group	BP	GO:0048771	tissue remodeling	6/99	7.21E-05	0.00063187	0.00031111	Axl/Cd38/Hif1a/Il1a/Mmp14/Ppp3ca
28-day post-SCI group vs. the control group	BP	GO:0140632	inflammasome complex assembly	3/99	7.46E-05	0.00065046	0.00032026	Cd36/Tlr4/Trem2
28-day post-SCI group vs. the control group	BP	GO:1903204	negative regulation of oxidative stress-induced neuron	3/99	7.46E-05	0.00065046	0.00032026	Hif1a/Ncoa7/Slc7a11
28-day post-SCI group vs. the control group	BP	GO:0051048	negative regulation of secretion	6/99	7.81E-05	0.00067964	0.00033463	Apoe/Fkbp1b/Ppp3ca/Ptgs1/Tnfrsf1a/Ucp2
28-day post-SCI group vs. the control group	BP	GO:0002534	cytokine production involved in inflammatory respons	4/99	7.89E-05	0.00068438	0.00033696	Hif1a/Sirpa/Tlr4/Trem2
28-day post-SCI group vs. the control group	BP	GO:0043123	positive regulation of I-kappaB kinase/NF-kappaB sig	5/99	8.02E-05	0.00068886	0.00033917	Cd36/Il1a/Rela/Ripk1/Tlr4
28-day post-SCI group vs. the control group	BP	GO:0043500	muscle adaptation	5/99	8.02E-05	0.00068886	0.00033917	Aif1/Atp2a2/Parp1/Ppp3ca/Tnfrsf1a
28-day post-SCI group vs. the control group	BP	GO:0002700	regulation of production of molecular mediator of imm	6/99	8.02E-05	0.00068886	0.00033917	Axl/Cd36/Mapkapk2/Stat6/Tlr4/Tnfaip3
28-day post-SCI group vs. the control group	BP	GO:0043491	protein kinase B signaling	6/99	8.02E-05	0.00068886	0.00033917	Axl/Gpx1/Hbegf/Met/Plekha1/Xdh
28-day post-SCI group vs. the control group	BP	GO:1905517	macrophage migration	4/99	8.37E-05	0.00071643	0.00035274	Mapk1/Mmp14/Rpl13a/Trem2
28-day post-SCI group vs. the control group	BP	GO:0045736	negative regulation of cyclin-dependent protein serine/	3/99	8.45E-05	0.00071817	0.0003536	Apc/Cdkn2c/Tnfaip3
28-day post-SCI group vs. the control group	BP	GO:0098869	cellular oxidant detoxification	3/99	8.45E-05	0.00071817	0.0003536	Cd36/Nfe2l2/Prdx6
28-day post-SCI group vs. the control group	BP	GO:1902993	positive regulation of amyloid precursor protein catab	3/99	8.45E-05	0.00071817	0.0003536	Apoe/Clu/Rela
28-day post-SCI group vs. the control group	BP	GO:0051592	response to calcium ion	5/99	8.63E-05	0.00073147	0.00036015	Fos/Jun/Mylk/Ppp3ca/Trpm2
28-day post-SCI group vs. the control group	BP	GO:0050769	positive regulation of neurogenesis	7/99	8.76E-05	0.00073872	0.00036372	Apoe/Hdac1/Hif1a/Il6st/Met/Rela/Stau2
28-day post-SCI group vs. the control group	BP	GO:0070482	response to oxygen levels	7/99	8.76E-05	0.00073872	0.00036372	Cd38/Hif1a/Hmox2/Nfe2l2/Pdk1/Slc8a1/Ucp2
28-day post-SCI group vs. the control group	BP	GO:0061082	myeloid leukocyte cytokine production	4/99	8.87E-05	0.00074364	0.00036614	Axl/Cd36/Mapkapk2/Tlr4
28-day post-SCI group vs. the control group	BP	GO:0097006	regulation of plasma lipoprotein particle levels	4/99	8.87E-05	0.00074364	0.00036614	Apoe/Cd36/Lcat/Trem2
28-day post-SCI group vs. the control group	BP	GO:0010721	negative regulation of cell development	6/99	8.91E-05	0.00074575	0.00036718	Fbxw7/Id1/Il1a/Ppp3ca/Rb1/Trem2
28-day post-SCI group vs. the control group	BP	GO:0032869	cellular response to insulin stimulus	6/99	9.15E-05	0.00076342	0.00037588	Apc/Parp1/Pdk2/Prkcd/Rb1/Rela
28-day post-SCI group vs. the control group	BP	GO:0046822	regulation of nucleocytoplasmic transport	5/99	9.28E-05	0.00077219	0.0003802	Cd36/Ier3/Mapk1/Prkcd/Tnfrsf1a
28-day post-SCI group vs. the control group	BP	GO:1901224	positive regulation of NIK/NF-kappaB signaling	4/99	9.39E-05	0.00077935	0.00038373	Ago1/Rela/Tlr4/Trem2
28-day post-SCI group vs. the control group	BP	GO:0010575	positive regulation of vascular endothelial growth fact	3/99	9.53E-05	0.00078338	0.00038571	Cyp1b1/Hif1a/Il1a
28-day post-SCI group vs. the control group	BP	GO:0045730	respiratory burst	3/99	9.53E-05	0.00078338	0.00038571	Cybb/Ncf1/Trem2
28-day post-SCI group vs. the control group	BP	GO:0071276	cellular response to cadmium ion	3/99	9.53E-05	0.00078338	0.00038571	Fos/Jun/Mapk1
28-day post-SCI group vs. the control group	BP	GO:1990776	response to angiotensin	3/99	9.53E-05	0.00078338	0.00038571	Nfe2l2/Prkcd/Rela
28-day post-SCI group vs. the control group	BP	GO:0009743	response to carbohydrate	6/99	9.63E-05	0.00078969	0.00038882	Fkbp1b/Gpx1/Hif1a/Ppp3ca/Ucp2/Vcam1

28-day post-SCI group vs. the control group	BP	GO:0014013	regulation of gliogenesis	5/99	0.0001032	0.00084352	0.00041532	Hdac1/Il6st/Rb1/Rela/Trem2
28-day post-SCI group vs. the control group	BP	GO:0000079	regulation of cyclin-dependent protein serine/threonine	4/99	0.000105	0.00085402	0.00042049	Actb/Apc/Cdkn2c/Tnfaip3
28-day post-SCI group vs. the control group	BP	GO:0030193	regulation of blood coagulation	4/99	0.000105	0.00085402	0.00042049	Apoe/Cd36/Nfe2l2/Prkcd
28-day post-SCI group vs. the control group	BP	GO:0009150	purine ribonucleotide metabolic process	8/99	0.0001081	0.00087705	0.00043183	Hif1a/Ier3/Ndufa6/Parp1/Pdk1/Pdk2/Trem2/Xdh
28-day post-SCI group vs. the control group	BP	GO:0032409	regulation of transporter activity	7/99	0.0001113	0.00090147	0.00044385	Actb/Fkbp1b/Ppp3ca/Prkcd/Ripk1/S100a1/Trem2
28-day post-SCI group vs. the control group	BP	GO:0003158	endothelium development	5/99	0.0001144	0.00092392	0.00045491	Gpx1/Id1/Met/Tnfrsf1a/Xdh
28-day post-SCI group vs. the control group	BP	GO:1900046	regulation of hemostasis	4/99	0.000117	0.00094231	0.00046396	Apoe/Cd36/Nfe2l2/Prkcd
28-day post-SCI group vs. the control group	BP	GO:0034504	protein localization to nucleus	7/99	0.000118	0.0009485	0.00046701	Cd36/Mapk1/Parp1/Ppp3ca/Prkcd/Tnfrsf1a/Txnip
28-day post-SCI group vs. the control group	BP	GO:0006536	glutamate metabolic process	3/99	0.0001195	0.00095576	0.00047058	Apc/Prodh/Slc7a11
28-day post-SCI group vs. the control group	BP	GO:0046688	response to copper ion	3/99	0.0001195	0.00095576	0.00047058	Il1a/Lcat/Nfe2l2
28-day post-SCI group vs. the control group	BP	GO:0007611	learning or memory	7/99	0.0001203	0.00095989	0.00047262	Amph/Apoe/Hif1a/Jun/Ptgs1/Slc7a11/Trem2
28-day post-SCI group vs. the control group	BP	GO:0001885	endothelial cell development	4/99	0.0001233	0.00097909	0.00048207	Gpx1/Id1/Met/Tnfrsf1a
28-day post-SCI group vs. the control group	BP	GO:1904029	regulation of cyclin-dependent protein kinase activity	4/99	0.0001233	0.00097909	0.00048207	Actb/Apc/Cdkn2c/Tnfaip3
28-day post-SCI group vs. the control group	BP	GO:0001649	osteoblast differentiation	6/99	0.0001267	0.00100349	0.00049409	Apc/Id1/Il6st/Map2k6/Ppp3ca/Smad1
28-day post-SCI group vs. the control group	BP	GO:0070588	calcium ion transmembrane transport	7/99	0.0001299	0.00102163	0.00050302	Atp2a2/Fkbp1b/Pawr/Ppp3ca/S100a1/Slc8a1/Trpm2
28-day post-SCI group vs. the control group	BP	GO:0050818	regulation of coagulation	4/99	0.0001299	0.00102163	0.00050302	Apoe/Cd36/Nfe2l2/Prkcd
28-day post-SCI group vs. the control group	BP	GO:1903578	regulation of ATP metabolic process	4/99	0.0001299	0.00102163	0.00050302	Hif1a/Ier3/Parp1/Trem2
28-day post-SCI group vs. the control group	BP	GO:0051224	negative regulation of protein transport	5/99	0.0001308	0.00102619	0.00050526	Apoe/Cd36/Fkbp1b/Ppp3ca/Ucp2
28-day post-SCI group vs. the control group	BP	GO:0010976	positive regulation of neuron projection development	6/99	0.000133	0.00104047	0.00051229	Apoe/Fkbp1b/Hspb1/Met/Nfe2l2/Stau2
28-day post-SCI group vs. the control group	BP	GO:0055001	muscle cell development	6/99	0.0001395	0.00108874	0.00053606	Atp2a2/Gpx1/Map2k4/Met/Ppp3ca/Slc8a1
28-day post-SCI group vs. the control group	BP	GO:0000082	G1/S transition of mitotic cell cycle	6/99	0.0001428	0.00111219	0.0005476	Actb/Aif1/Apc/Cdkn2c/Ppp3ca/Rb1
28-day post-SCI group vs. the control group	BP	GO:0060907	positive regulation of macrophage cytokine production	3/99	0.0001474	0.00114503	0.00056377	Cd36/Mapkapk2/Tlr4
28-day post-SCI group vs. the control group	BP	GO:0018105	peptidyl-serine phosphorylation	7/99	0.0001481	0.00114507	0.00056379	Mapk1/Mapkapk2/Met/Pdk1/Pdk2/Prkcd/Ripk1
28-day post-SCI group vs. the control group	BP	GO:0009259	ribonucleotide metabolic process	8/99	0.0001481	0.00114507	0.00056379	Hif1a/Ier3/Ndufa6/Parp1/Pdk1/Pdk2/Trem2/Xdh
28-day post-SCI group vs. the control group	BP	GO:0010821	regulation of mitochondrion organization	5/99	0.000149	0.00114678	0.00056463	Fbxw7/Gpx1/Hif1a/Ier3/Trem2
28-day post-SCI group vs. the control group	BP	GO:1904950	negative regulation of establishment of protein localiza	5/99	0.000149	0.00114678	0.00056463	Apoe/Cd36/Fkbp1b/Ppp3ca/Ucp2
28-day post-SCI group vs. the control group	BP	GO:0042306	regulation of protein import into nucleus	4/99	0.0001513	0.00115895	0.00057063	Cd36/Mapk1/Prkcd/Tnfrsf1a
28-day post-SCI group vs. the control group	BP	GO:0071277	cellular response to calcium ion	4/99	0.0001513	0.00115895	0.00057063	Fos/Jun/Mylk/Trpm2
28-day post-SCI group vs. the control group	BP	GO:0051146	striated muscle cell differentiation	7/99	0.0001536	0.00117418	0.00057813	Gpx1/Map2k4/Met/Mmp14/Ppp3ca/Rb1/Slc8a1
28-day post-SCI group vs. the control group	BP	GO:0002685	regulation of leukocyte migration	6/99	0.0001568	0.0011945	0.00058813	Aif1/Il1a/Mapk1/Mmp14/Pawr/Trem2
28-day post-SCI group vs. the control group	BP	GO:0006163	purine nucleotide metabolic process	8/99	0.0001574	0.0011945	0.00058813	Hif1a/Ier3/Ndufa6/Parp1/Pdk1/Pdk2/Trem2/Xdh
28-day post-SCI group vs. the control group	BP	GO:0006816	calcium ion transport	8/99	0.0001574	0.0011945	0.00058813	Atp2a2/Fkbp1b/Mylk/Pawr/Ppp3ca/S100a1/Slc8a1/Trpm2
28-day post-SCI group vs. the control group	BP	GO:0061180	mammary gland epithelium development	4/99	0.0001589	0.00120115	0.0005914	Gpx1/Hif1a/Mapk1/Stat6
28-day post-SCI group vs. the control group	BP	GO:0071214	cellular response to abiotic stimulus	7/99	0.0001594	0.00120115	0.0005914	Actb/Eif2s1/Fbxw7/Mylk/Net1/Parp1/Prkcd
28-day post-SCI group vs. the control group	BP	GO:0104004	cellular response to environmental stimulus	7/99	0.0001594	0.00120115	0.0005914	Actb/Eif2s1/Fbxw7/Mylk/Net1/Parp1/Prkcd
28-day post-SCI group vs. the control group	BP	GO:0030073	insulin secretion	6/99	0.0001605	0.00120406	0.00059284	Cd38/Fkbp1b/Hif1a/Ppp3ca/Trpm2/Ucp2
28-day post-SCI group vs. the control group	BP	GO:0090276	regulation of peptide hormone secretion	6/99	0.0001605	0.00120406	0.00059284	Cd38/Fkbp1b/Hif1a/Ppp3ca/Trpm2/Ucp2
28-day post-SCI group vs. the control group	BP	GO:0061036	positive regulation of cartilage development	3/99	0.0001628	0.00120996	0.00059574	Bmp1/Rela/Smad1
28-day post-SCI group vs. the control group	BP	GO:0071354	cellular response to interleukin-6	3/99	0.0001628	0.00120996	0.00059574	Il6st/Rela/Ripk1
28-day post-SCI group vs. the control group	BP	GO:1902883	negative regulation of response to oxidative stress	3/99	0.0001628	0.00120996	0.00059574	Met/Ncoa7/Nfe2l2

28-day post-SCI group vs. the control group	BP	GO:1905523	positive regulation of macrophage migration	3/99	0.0001628	0.00120996	0.00059574	Mapk1/Mmp14/Trem2
28-day post-SCI group vs. the control group	BP	GO:0030324	lung development	6/99	0.0001642	0.00121812	0.00059976	Fbxw7/Id1/Mapk1/Mmp14/Rpl13a/Slc7a11
28-day post-SCI group vs. the control group	BP	GO:0019233	sensory perception of pain	5/99	0.0001691	0.00124822	0.00061458	Il1a/Mapk1/Pawr/Ptgs1/Tlr4
28-day post-SCI group vs. the control group	BP	GO:0046328	regulation of JNK cascade	5/99	0.0001691	0.00124822	0.00061458	Il1a/Map2k4/Ripk1/Sirpa/Tlr4
28-day post-SCI group vs. the control group	BP	GO:0097191	extrinsic apoptotic signaling pathway	6/99	0.0001719	0.00126646	0.00062356	Gpx1/Il1a/Mcl1/Rela/Ripk1/Tnfaip3
28-day post-SCI group vs. the control group	BP	GO:0050921	positive regulation of chemotaxis	5/99	0.0001744	0.00127884	0.00062966	Aif1/Hspb1/Mapk1/Met/Trem2
28-day post-SCI group vs. the control group	BP	GO:1900180	regulation of protein localization to nucleus	5/99	0.0001744	0.00127884	0.00062966	Cd36/Mapk1/Parp1/Prkcd/Tnfrsf1a
28-day post-SCI group vs. the control group	BP	GO:0019693	ribose phosphate metabolic process	8/99	0.0001749	0.00127952	0.00062999	Hif1a/Ier3/Ndufa6/Parp1/Pdk1/Pdk2/Trem2/Xdh
28-day post-SCI group vs. the control group	BP	GO:0030323	respiratory tube development	6/99	0.0001759	0.00128396	0.00063218	Fbxw7/Id1/Mapk1/Mmp14/Rpl13a/Slc7a11
28-day post-SCI group vs. the control group	BP	GO:0010574	regulation of vascular endothelial growth factor produc	3/99	0.0001792	0.00129878	0.00063948	Cyp1b1/Hif1a/Il1a
28-day post-SCI group vs. the control group	BP	GO:0070296	sarcoplasmic reticulum calcium ion transport	3/99	0.0001792	0.00129878	0.00063948	Atp2a2/Fkbp1b/Slc8a1
28-day post-SCI group vs. the control group	BP	GO:2000352	negative regulation of endothelial cell apoptotic proces	3/99	0.0001792	0.00129878	0.00063948	Id1/Nfe2l2/Tnfaip3
28-day post-SCI group vs. the control group	BP	GO:0002791	regulation of peptide secretion	6/99	0.0001799	0.00129878	0.00063948	Cd38/Fkbp1b/Hif1a/Ppp3ca/Trpm2/Ucp2
28-day post-SCI group vs. the control group	BP	GO:0045088	regulation of innate immune response	6/99	0.0001799	0.00129878	0.00063948	Apoe/Ncf1/Nfe2l2/Tlr4/Tnfaip3/Trem2
28-day post-SCI group vs. the control group	BP	GO:0050709	negative regulation of protein secretion	4/99	0.0001836	0.00131956	0.00064971	Apoe/Fkbp1b/Ppp3ca/Ucp2
28-day post-SCI group vs. the control group	BP	GO:0046777	protein autophosphorylation	6/99	0.000184	0.00131956	0.00064971	Eif2s1/Jun/Mapkapk2/Met/Prkcd/Ripk1
28-day post-SCI group vs. the control group	BP	GO:0071241	cellular response to inorganic substance	6/99	0.000184	0.00131956	0.00064971	Fos/Jun/Mapk1/Mylk/Nfe2l2/Trpm2
28-day post-SCI group vs. the control group	BP	GO:0071560	cellular response to transforming growth factor beta sti	6/99	0.0001881	0.00134352	0.0006615	Fos/Hdac1/Itgb5/Jun/Parp1/Smad1
28-day post-SCI group vs. the control group	BP	GO:0090087	regulation of peptide transport	6/99	0.0001881	0.00134352	0.0006615	Cd38/Fkbp1b/Hif1a/Ppp3ca/Trpm2/Ucp2
28-day post-SCI group vs. the control group	BP	GO:0060249	anatomical structure homeostasis	7/99	0.0001907	0.00135895	0.0006691	Apc/Atp2a2/Cd38/Hif1a/Ptgs1/Rb1/Tlr4
28-day post-SCI group vs. the control group	BP	GO:0006913	nucleocytoplasmic transport	7/99	0.0001941	0.00137709	0.00067803	Cd36/Ier3/Mapk1/Ppp3ca/Prkcd/Tnfrsf1a/Txnip
28-day post-SCI group vs. the control group	BP	GO:0051169	nuclear transport	7/99	0.0001941	0.00137709	0.00067803	Cd36/Ier3/Mapk1/Ppp3ca/Prkcd/Tnfrsf1a/Txnip
28-day post-SCI group vs. the control group	BP	GO:0033598	mammary gland epithelial cell proliferation	3/99	0.0001966	0.00138237	0.00068063	Gpx1/Mapk1/Stat6
28-day post-SCI group vs. the control group	BP	GO:0050999	regulation of nitric-oxide synthase activity	3/99	0.0001966	0.00138237	0.00068063	Apoe/Il1a/S100a1
28-day post-SCI group vs. the control group	BP	GO:0070741	response to interleukin-6	3/99	0.0001966	0.00138237	0.00068063	Il6st/Rela/Ripk1
28-day post-SCI group vs. the control group	BP	GO:0002697	regulation of immune effector process	8/99	0.0001967	0.00138237	0.00068063	Axl/Cd36/Mapkapk2/Ncf1/Stat6/Tlr4/Tnfaip3/Trem2
28-day post-SCI group vs. the control group	BP	GO:0051092	positive regulation of NF-kappaB transcription factor a	5/99	0.0001969	0.00138237	0.00068063	Cd36/Clu/Rela/Ripk1/Tlr4
28-day post-SCI group vs. the control group	BP	GO:0002683	negative regulation of immune system process	8/99	0.0001996	0.00139819	0.00068842	Axl/Fbxw7/Gpx1/Hspb1/Pawr/Stat6/Tnfaip3/Trem2
28-day post-SCI group vs. the control group	BP	GO:0008088	axo-dendritic transport	4/99	0.0002015	0.00140811	0.0006933	Dst/Hif1a/Hspb1/Stau2
28-day post-SCI group vs. the control group	BP	GO:2001235	positive regulation of apoptotic signaling pathway	5/99	0.0002029	0.0014152	0.00069679	Clu/Fbxw7/Mcl1/Prkcd/Ripk1
28-day post-SCI group vs. the control group	BP	GO:0071559	response to transforming growth factor beta	6/99	0.0002055	0.00143027	0.00070422	Fos/Hdac1/Itgb5/Jun/Parp1/Smad1
28-day post-SCI group vs. the control group	BP	GO:0032368	regulation of lipid transport	5/99	0.000209	0.00145163	0.00071473	Apoe/Il1a/Map2k6/Prkcd/Trem2
28-day post-SCI group vs. the control group	BP	GO:0090322	regulation of superoxide metabolic process	3/99	0.0002151	0.00149043	0.00073384	Cd36/Nfe2l2/Prkcd
28-day post-SCI group vs. the control group	BP	GO:0016032	viral process	7/99	0.000223	0.00154197	0.00075921	Apoe/Axl/Ctsl/Hdac1/Jun/Ripk1/Vcam1
28-day post-SCI group vs. the control group	BP	GO:0032868	response to insulin	6/99	0.0002242	0.00154683	0.0007616	Apc/Parp1/Pdk2/Prkcd/Rb1/Rela
28-day post-SCI group vs. the control group	BP	GO:0048708	astrocyte differentiation	4/99	0.0002306	0.00158544	0.00078062	Il6st/Mapk1/Tlr4/Trem2
28-day post-SCI group vs. the control group	BP	GO:0045862	positive regulation of proteolysis	7/99	0.0002307	0.00158544	0.00078062	Apoe/Clu/Fbxw7/Mmp14/Pawr/Trem2/Xdh
28-day post-SCI group vs. the control group	BP	GO:0045672	positive regulation of osteoclast differentiation	3/99	0.0002346	0.00160399	0.00078975	Fos/Ppp3ca/Trem2
28-day post-SCI group vs. the control group	BP	GO:0050890	cognition	7/99	0.0002347	0.00160399	0.00078975	Amph/Apoe/Hif1a/Jun/Ptgs1/Slc7a11/Trem2
28-day post-SCI group vs. the control group	BP	GO:0045667	regulation of osteoblast differentiation	5/99	0.0002349	0.00160399	0.00078975	Apc/Id1/Il6st/Ppp3ca/Smad1

28-day post-SCI group vs. the control group	BP	GO:2000112	regulation of cellular macromolecule biosynthetic proc	8/99	0.0002543	0.00173279	0.00085317	Ago1/Cd36/Cyp1b1/Eif2s1/Hbegf/Mapk1/Pawr/Rpl13a
28-day post-SCI group vs. the control group	BP	GO:0010573	vascular endothelial growth factor production	3/99	0.0002553	0.00173613	0.00085481	Cyp1b1/Hif1a/Il1a
28-day post-SCI group vs. the control group	BP	GO:2001236	regulation of extrinsic apoptotic signaling pathway	5/99	0.0002558	0.00173613	0.00085481	Gpx1/Mcl1/Rela/Ripk1/Tnfaip3
28-day post-SCI group vs. the control group	BP	GO:0072521	purine-containing compound metabolic process	8/99	0.0002578	0.00174616	0.00085975	Hif1a/Ier3/Ndufa6/Parp1/Pdk1/Pdk2/Trem2/Xdh
28-day post-SCI group vs. the control group	BP	GO:0044843	cell cycle G1/S phase transition	6/99	0.00026	0.00175685	0.00086501	Actb/Aif1/Apc/Cdkn2c/Ppp3ca/Rb1
28-day post-SCI group vs. the control group	BP	GO:0030879	mammary gland development	5/99	0.0002631	0.00177453	0.00087372	Gpx1/Hif1a/Mapk1/Stat6/Xdh
28-day post-SCI group vs. the control group	BP	GO:0051962	positive regulation of nervous system development	7/99	0.0002683	0.00180564	0.00088903	Apoe/Hdac1/Hif1a/Il6st/Met/Rela/Stau2
28-day post-SCI group vs. the control group	BP	GO:0051604	protein maturation	7/99	0.0002727	0.00183189	0.00090196	Bmp1/Ctsl/Dhcr24/Mmp14/Parp1/Pawr/Prdx4
28-day post-SCI group vs. the control group	BP	GO:0034644	cellular response to UV	4/99	0.000274	0.00183664	0.0009043	Eif2s1/Fbxw7/Parp1/Prkcd
28-day post-SCI group vs. the control group	BP	GO:0090075	relaxation of muscle	3/99	0.0002772	0.00185036	0.00091105	Atp2a2/Pawr/Slc8a1
28-day post-SCI group vs. the control group	BP	GO:1902003	regulation of amyloid-beta formation	3/99	0.0002772	0.00185036	0.00091105	Apoe/Clu/Rela
28-day post-SCI group vs. the control group	BP	GO:0010675	regulation of cellular carbohydrate metabolic process	5/99	0.0002782	0.00185307	0.00091239	Cd36/Hif1a/Ier3/Pdk1/Pdk2
28-day post-SCI group vs. the control group	BP	GO:1900182	positive regulation of protein localization to nucleus	4/99	0.0002857	0.00189706	0.00093405	Mapk1/Parp1/Prkcd/Tnfrsf1a
28-day post-SCI group vs. the control group	BP	GO:0030856	regulation of epithelial cell differentiation	5/99	0.0002859	0.00189706	0.00093405	Apc/Id1/Il1a/Tnfrsf1a/Xdh
28-day post-SCI group vs. the control group	BP	GO:0048814	regulation of dendrite morphogenesis	4/99	0.0002978	0.00197173	0.00097081	Id1/Met/Ppp3ca/Stau2
28-day post-SCI group vs. the control group	BP	GO:0009165	nucleotide biosynthetic process	6/99	0.0003001	0.00197576	0.0009728	Mapk1/Ndufa6/Parp1/Pdk1/Pdk2/Trem2
28-day post-SCI group vs. the control group	BP	GO:0034381	plasma lipoprotein particle clearance	3/99	0.0003002	0.00197576	0.0009728	Apoe/Cd36/Trem2
28-day post-SCI group vs. the control group	BP	GO:0046686	response to cadmium ion	3/99	0.0003002	0.00197576	0.0009728	Fos/Jun/Mapk1
28-day post-SCI group vs. the control group	BP	GO:0043433	negative regulation of DNA-binding transcription facto	5/99	0.0003019	0.00197925	0.00097452	Cyp1b1/Id1/Rb1/Rnf2/Tnfaip3
28-day post-SCI group vs. the control group	BP	GO:2000045	regulation of G1/S transition of mitotic cell cycle	5/99	0.0003019	0.00197925	0.00097452	Actb/Aif1/Apc/Cdkn2c/Rb1
28-day post-SCI group vs. the control group	BP	GO:0051017	actin filament bundle assembly	5/99	0.0003102	0.00202933	0.00099917	Aif1/Id1/Itgb5/Met/Pawr
28-day post-SCI group vs. the control group	BP	GO:0030003	cellular cation homeostasis	8/99	0.0003164	0.00206567	0.00101706	Apoe/Atp2a2/Fkbp1b/Hif1a/Il1a/Mapk1/Slc8a1/Trpm2
28-day post-SCI group vs. the control group	BP	GO:0060541	respiratory system development	6/99	0.0003187	0.00207695	0.00102262	Fbxw7/Id1/Mapk1/Mmp14/Rpl13a/Slc7a11
28-day post-SCI group vs. the control group	BP	GO:0044272	sulfur compound biosynthetic process	4/99	0.000323	0.00209626	0.00103212	Nfe2l2/Pdk1/Pdk2/Slc7a11
28-day post-SCI group vs. the control group	BP	GO:0045685	regulation of glial cell differentiation	4/99	0.000323	0.00209626	0.00103212	Hdac1/Il6st/Rela/Trem2
28-day post-SCI group vs. the control group	BP	GO:1901293	nucleoside phosphate biosynthetic process	6/99	0.0003317	0.00214829	0.00105774	Mapk1/Ndufa6/Parp1/Pdk1/Pdk2/Trem2
28-day post-SCI group vs. the control group	BP	GO:0061572	actin filament bundle organization	5/99	0.000336	0.00217212	0.00106947	Aif1/Id1/Itgb5/Met/Pawr
28-day post-SCI group vs. the control group	BP	GO:0048738	cardiac muscle tissue development	6/99	0.000345	0.00222141	0.00109374	Apc/Map2k4/Mapk1/Met/Slc8a1/Smad1
28-day post-SCI group vs. the control group	BP	GO:0051091	positive regulation of DNA-binding transcription facto	6/99	0.000345	0.00222141	0.00109374	Cd36/Clu/Ppp3ca/Rela/Ripk1/Tlr4
28-day post-SCI group vs. the control group	BP	GO:0002274	myeloid leukocyte activation	6/99	0.0003518	0.00226086	0.00111317	Aif1/Clu/Jun/Prkcd/Tlr4/Trem2
28-day post-SCI group vs. the control group	BP	GO:0010720	positive regulation of cell development	7/99	0.0003582	0.00229633	0.00113063	Apoe/Hdac1/Hif1a/Il6st/Met/Rela/Stau2
28-day post-SCI group vs. the control group	BP	GO:0034764	positive regulation of transmembrane transport	6/99	0.0003587	0.00229633	0.00113063	Met/Nfe2l2/Ppp3ca/Prkcd/S100a1/Trem2
28-day post-SCI group vs. the control group	BP	GO:0015908	fatty acid transport	4/99	0.0003636	0.0023141	0.00113938	Apoe/Cd36/Il1a/Map2k6
28-day post-SCI group vs. the control group	BP	GO:0120162	positive regulation of cold-induced thermogenesis	4/99	0.0003636	0.0023141	0.00113938	Apc/Cd36/Stat6/Ucp2
28-day post-SCI group vs. the control group	BP	GO:1901222	regulation of NIK/NF-kappaB signaling	4/99	0.0003636	0.0023141	0.00113938	Ago1/Rela/Tlr4/Trem2
28-day post-SCI group vs. the control group	BP	GO:0062207	regulation of pattern recognition receptor signaling pat	4/99	0.000378	0.00240059	0.00118196	Cd36/Tlr4/Tnfaip3/Trem2
28-day post-SCI group vs. the control group	BP	GO:0019216	regulation of lipid metabolic process	7/99	0.0003811	0.00241585	0.00118948	Apoe/Cd36/Il1a/Pdk1/Pdk2/Prkcd/Trem2
28-day post-SCI group vs. the control group	BP	GO:0071478	cellular response to radiation	5/99	0.0003826	0.00242068	0.00119186	Eif2s1/Fbxw7/Net1/Parp1/Prkcd
28-day post-SCI group vs. the control group	BP	GO:0006909	phagocytosis	7/99	0.000393	0.00248165	0.00122188	Aif1/Axl/Cd36/Met/Sirpa/Tlr4/Trem2
28-day post-SCI group vs. the control group	BP	GO:0050864	regulation of B cell activation	6/99	0.000395	0.00248931	0.00122565	Cd38/Mmp14/Pawr/Stat6/Tlr4/Tnfaip3

28-day post-SCI group vs. the control group	BP	GO:0061081	positive regulation of myeloid leukocyte cytokine prod	3/99	0.0004045	0.00253953	0.00125038	Cd36/Mapkapk2/Tlr4
28-day post-SCI group vs. the control group	BP	GO:1900744	regulation of p38MAPK cascade	3/99	0.0004045	0.00253953	0.00125038	Met/Trem2/Xdh
28-day post-SCI group vs. the control group	BP	GO:0031638	zymogen activation	4/99	0.0004078	0.00255545	0.00125821	Ctsl/Dhcr24/Mmp14/Pawr
28-day post-SCI group vs. the control group	BP	GO:0071356	cellular response to tumor necrosis factor	5/99	0.0004233	0.00263766	0.00129869	Mapk1/Nfe2l2/Rela/Ripk1/Tnfrsf1a
28-day post-SCI group vs. the control group	BP	GO:0009408	response to heat	4/99	0.0004233	0.00263766	0.00129869	Eif2s1/Hspb1/Il1a/Trpm2
28-day post-SCI group vs. the control group	BP	GO:0060395	SMAD protein signal transduction	4/99	0.0004233	0.00263766	0.00129869	Fos/Jun/Parp1/Smad1
28-day post-SCI group vs. the control group	BP	GO:0006984	ER-nucleus signaling pathway	3/99	0.0004338	0.00268206	0.00132055	Atp2a2/Eif2s1/Nfe2l2
28-day post-SCI group vs. the control group	BP	GO:0032735	positive regulation of interleukin-12 production	3/99	0.0004338	0.00268206	0.00132055	Cd36/Rela/Tlr4
28-day post-SCI group vs. the control group	BP	GO:0045601	regulation of endothelial cell differentiation	3/99	0.0004338	0.00268206	0.00132055	Id1/Tnfrsf1a/Xdh
28-day post-SCI group vs. the control group	BP	GO:1990748	cellular detoxification	3/99	0.0004338	0.00268206	0.00132055	Cd36/Nfe2l2/Prdx6
28-day post-SCI group vs. the control group	BP	GO:0046330	positive regulation of JNK cascade	4/99	0.0004393	0.00271127	0.00133493	Il1a/Map2k4/Ripk1/Tlr4
28-day post-SCI group vs. the control group	BP	GO:0097553	calcium ion transmembrane import into cytosol	5/99	0.0004448	0.00273987	0.00134902	Fkbp1b/Pawr/Ppp3ca/Slc8a1/Trpm2
28-day post-SCI group vs. the control group	BP	GO:0016485	protein processing	6/99	0.0004504	0.00276954	0.00136362	Bmp1/Ctsl/Dhcr24/Mmp14/Parp1/Pawr
28-day post-SCI group vs. the control group	BP	GO:0035051	cardiocyte differentiation	5/99	0.0004558	0.00279224	0.0013748	Map2k4/Mapk1/Met/Slc8a1/Vcam1
28-day post-SCI group vs. the control group	BP	GO:0061136	regulation of proteasomal protein catabolic process	5/99	0.0004558	0.00279224	0.0013748	Apoe/Clu/Fbxw7/Gpx1/Trem2
28-day post-SCI group vs. the control group	BP	GO:0045840	positive regulation of mitotic nuclear division	3/99	0.0004643	0.00283369	0.00139521	Il1a/Met/Rb1
28-day post-SCI group vs. the control group	BP	GO:1902991	regulation of amyloid precursor protein catabolic proc	3/99	0.0004643	0.00283369	0.00139521	Apoe/Clu/Rela
28-day post-SCI group vs. the control group	BP	GO:0007612	learning	5/99	0.0004671	0.00284527	0.00140091	Amph/Hif1a/Jun/Ptgs1/Slc7a11
28-day post-SCI group vs. the control group	BP	GO:0042554	superoxide anion generation	3/99	0.0004963	0.00301732	0.00148562	Cybb/Ncf1/Prkcd
28-day post-SCI group vs. the control group	BP	GO:0050680	negative regulation of epithelial cell proliferation	5/99	0.0005022	0.00303925	0.00149642	Apc/Apoe/Ctsl/Rb1/Xdh
28-day post-SCI group vs. the control group	BP	GO:0014706	striated muscle tissue development	6/99	0.0005027	0.00303925	0.00149642	Apc/Map2k4/Mapk1/Met/Slc8a1/Smad1
28-day post-SCI group vs. the control group	BP	GO:0030072	peptide hormone secretion	6/99	0.0005027	0.00303925	0.00149642	Cd38/Fkbp1b/Hif1a/Ppp3ca/Trpm2/Ucp2
28-day post-SCI group vs. the control group	BP	GO:0007178	transmembrane receptor protein serine/threonine kinas	7/99	0.0005066	0.00305588	0.00150461	Fos/Hdac1/Id1/Itgb5/Jun/Parp1/Smad1
28-day post-SCI group vs. the control group	BP	GO:0014883	transition between fast and slow fiber	2/99	0.000512	0.00305588	0.00150461	Atp2a2/Ppp3ca
28-day post-SCI group vs. the control group	BP	GO:0017038	protein import	2/99	0.000512	0.00305588	0.00150461	Apoe/Clu
28-day post-SCI group vs. the control group	BP	GO:0034372	very-low-density lipoprotein particle remodeling	2/99	0.000512	0.00305588	0.00150461	Apoe/Lcat
28-day post-SCI group vs. the control group	BP	GO:0034380	high-density lipoprotein particle assembly	2/99	0.000512	0.00305588	0.00150461	Apoe/Lcat
28-day post-SCI group vs. the control group	BP	GO:0051775	response to redox state	2/99	0.000512	0.00305588	0.00150461	Fkbp1b/Slc7a11
28-day post-SCI group vs. the control group	BP	GO:1902884	positive regulation of response to oxidative stress	2/99	0.000512	0.00305588	0.00150461	Pawr/Ripk1
28-day post-SCI group vs. the control group	BP	GO:0032768	regulation of monooxygenase activity	3/99	0.0005296	0.00315499	0.00155341	Apoe/Il1a/S100a1
28-day post-SCI group vs. the control group	BP	GO:0015850	organic hydroxy compound transport	6/99	0.0005305	0.00315499	0.00155341	Actb/Apoe/Cd36/Lcat/Ptgs1/Trem2
28-day post-SCI group vs. the control group	BP	GO:0042310	vasoconstriction	4/99	0.0005442	0.00323061	0.00159064	Cd38/Hif1a/Ptgs1/Slc8a1
28-day post-SCI group vs. the control group	BP	GO:0051924	regulation of calcium ion transport	6/99	0.0005497	0.00325736	0.00160381	Fkbp1b/Mylk/Pawr/Ppp3ca/S100a1/Slc8a1
28-day post-SCI group vs. the control group	BP	GO:0032642	regulation of chemokine production	4/99	0.0005633	0.00332377	0.00163651	Aif1/Sirpa/Tlr4/Trem2
28-day post-SCI group vs. the control group	BP	GO:0034205	amyloid-beta formation	3/99	0.0005643	0.00332377	0.00163651	Apoe/Clu/Rela
28-day post-SCI group vs. the control group	BP	GO:0043277	apoptotic cell clearance	3/99	0.0005643	0.00332377	0.00163651	Axl/Cd36/Trem2
28-day post-SCI group vs. the control group	BP	GO:1902806	regulation of cell cycle G1/S phase transition	5/99	0.000565	0.00332377	0.00163651	Actb/Aif1/Apc/Cdkn2c/Rb1
28-day post-SCI group vs. the control group	BP	GO:0045165	cell fate commitment	6/99	0.0005694	0.00334406	0.0016465	Apc/Ctsl/Ets2/Hdac1/Smad1/Stat6
28-day post-SCI group vs. the control group	BP	GO:0032970	regulation of actin filament-based process	7/99	0.0005766	0.00337985	0.00166412	Atp2a2/Id1/Il1a/Met/Prkcd/Stau2/Trpm2
28-day post-SCI group vs. the control group	BP	GO:0002790	peptide secretion	6/99	0.0005795	0.00339105	0.00166963	Cd38/Fkbp1b/Hif1a/Ppp3ca/Trpm2/Ucp2

28-day post-SCI group vs. the control group	BP	GO:0048010	vascular endothelial growth factor receptor signaling p	3/99	0.0006004	0.00350229	0.00172441	Hif1a/Hspb1/Mapkapk2
28-day post-SCI group vs. the control group	BP	GO:0006869	lipid transport	7/99	0.0006015	0.00350229	0.00172441	Apoe/Cd36/Il1a/Lcat/Map2k6/Prkcd/Trem2
28-day post-SCI group vs. the control group	BP	GO:0006641	triglyceride metabolic process	4/99	0.0006028	0.00350229	0.00172441	Apoe/Cd36/Gpx1/Il6st
28-day post-SCI group vs. the control group	BP	GO:0030301	cholesterol transport	4/99	0.0006028	0.00350229	0.00172441	Apoe/Cd36/Lcat/Trem2
28-day post-SCI group vs. the control group	BP	GO:0043405	regulation of MAP kinase activity	5/99	0.0006054	0.00351096	0.00172868	Apoe/Map2k4/Map2k6/Prkcd/Tlr4
28-day post-SCI group vs. the control group	BP	GO:0044262	cellular carbohydrate metabolic process	6/99	0.0006106	0.00351497	0.00173065	Cd36/Hif1a/Ier3/Il6st/Pdk1/Pdk2
28-day post-SCI group vs. the control group	BP	GO:0061448	connective tissue development	6/99	0.0006106	0.00351497	0.00173065	Bmp1/Hif1a/Parp1/Rb1/Rela/Smad1
28-day post-SCI group vs. the control group	BP	GO:0006109	regulation of carbohydrate metabolic process	5/99	0.0006193	0.00351497	0.00173065	Cd36/Hif1a/Ier3/Pdk1/Pdk2
28-day post-SCI group vs. the control group	BP	GO:0008016	regulation of heart contraction	5/99	0.0006193	0.00351497	0.00173065	Atp2a2/Fkbp1b/Hbegf/S100a1/Slc8a1
28-day post-SCI group vs. the control group	BP	GO:0022898	regulation of transmembrane transporter activity	6/99	0.0006212	0.00351497	0.00173065	Actb/Fkbp1b/Ppp3ca/Ripk1/S100a1/Trem2
28-day post-SCI group vs. the control group	BP	GO:0008630	intrinsic apoptotic signaling pathway in response to DI	4/99	0.0006233	0.00351497	0.00173065	Clu/Ier3/Mcl1/Tnfrsf1a
28-day post-SCI group vs. the control group	BP	GO:0055017	cardiac muscle tissue growth	4/99	0.0006233	0.00351497	0.00173065	Apc/Map2k4/Mapk1/Smad1
28-day post-SCI group vs. the control group	BP	GO:0071482	cellular response to light stimulus	4/99	0.0006233	0.00351497	0.00173065	Eif2s1/Fbxw7/Parp1/Prkcd
28-day post-SCI group vs. the control group	BP	GO:0002679	respiratory burst involved in defense response	2/99	0.0006243	0.00351497	0.00173065	Ncf1/Trem2
28-day post-SCI group vs. the control group	BP	GO:0006521	regulation of cellular amino acid metabolic process	2/99	0.0006243	0.00351497	0.00173065	Apc/Slc7a11
28-day post-SCI group vs. the control group	BP	GO:0034351	negative regulation of glial cell apoptotic process	2/99	0.0006243	0.00351497	0.00173065	Prkcd/Trem2
28-day post-SCI group vs. the control group	BP	GO:0034370	triglyceride-rich lipoprotein particle remodeling	2/99	0.0006243	0.00351497	0.00173065	Apoe/Lcat
28-day post-SCI group vs. the control group	BP	GO:0034384	high-density lipoprotein particle clearance	2/99	0.0006243	0.00351497	0.00173065	Apoe/Trem2
28-day post-SCI group vs. the control group	BP	GO:0045602	negative regulation of endothelial cell differentiation	2/99	0.0006243	0.00351497	0.00173065	Id1/Xdh
28-day post-SCI group vs. the control group	BP	GO:0050774	negative regulation of dendrite morphogenesis	2/99	0.0006243	0.00351497	0.00173065	Id1/Ppp3ca
28-day post-SCI group vs. the control group	BP	GO:0071415	cellular response to purine-containing compound	2/99	0.0006243	0.00351497	0.00173065	Slc8a1/Trpm2
28-day post-SCI group vs. the control group	BP	GO:1905097	regulation of guanyl-nucleotide exchange factor activit	2/99	0.0006243	0.00351497	0.00173065	Eif2s1/Met
28-day post-SCI group vs. the control group	BP	GO:0009152	purine ribonucleotide biosynthetic process	5/99	0.0006335	0.0035604	0.00175302	Ndufa6/Parp1/Pdk1/Pdk2/Trem2
28-day post-SCI group vs. the control group	BP	GO:0010543	regulation of platelet activation	3/99	0.000638	0.00357349	0.00175946	Apoe/Prkcd/Tlr4
28-day post-SCI group vs. the control group	BP	GO:1905521	regulation of macrophage migration	3/99	0.000638	0.00357349	0.00175946	Mapk1/Mmp14/Trem2
28-day post-SCI group vs. the control group	BP	GO:0010906	regulation of glucose metabolic process	4/99	0.0006443	0.00360252	0.00177375	Cd36/Hif1a/Pdk1/Pdk2
28-day post-SCI group vs. the control group	BP	GO:0048511	rhythmic process	6/99	0.000654	0.00364444	0.0017944	Axl/Fbxw7/Hdac1/Id1/Mapk10/Plekha1
28-day post-SCI group vs. the control group	BP	GO:0006875	cellular metal ion homeostasis	7/99	0.000654	0.00364444	0.0017944	Apoe/Atp2a2/Fkbp1b/Hif1a/Il1a/Slc8a1/Trpm2
28-day post-SCI group vs. the control group	BP	GO:0034612	response to tumor necrosis factor	5/99	0.0006626	0.00368587	0.00181479	Mapk1/Nfe2l2/Rela/Ripk1/Tnfrsf1a
28-day post-SCI group vs. the control group	BP	GO:0007088	regulation of mitotic nuclear division	4/99	0.0006658	0.00369735	0.00182045	Apc/Il1a/Met/Rb1
28-day post-SCI group vs. the control group	BP	GO:0046883	regulation of hormone secretion	6/99	0.0006766	0.00373705	0.00183999	Cd38/Fkbp1b/Hif1a/Ppp3ca/Trpm2/Ucp2
28-day post-SCI group vs. the control group	BP	GO:0002753	cytoplasmic pattern recognition receptor signaling patl	3/99	0.0006771	0.00373705	0.00183999	Rela/Tlr4/Tnfaip3
28-day post-SCI group vs. the control group	BP	GO:0097237	cellular response to toxic substance	3/99	0.0006771	0.00373705	0.00183999	Cd36/Nfe2l2/Prdx6
28-day post-SCI group vs. the control group	BP	GO:0031334	positive regulation of protein-containing complex asse	5/99	0.0006775	0.00373705	0.00183999	Apc/Cd36/Clu/Met/Tlr4
28-day post-SCI group vs. the control group	BP	GO:0001942	hair follicle development	4/99	0.0006878	0.00378092	0.00186159	Apc/Ctsl/Hdac1/Rela
28-day post-SCI group vs. the control group	BP	GO:0015918	sterol transport	4/99	0.0006878	0.00378092	0.00186159	Apoe/Cd36/Lcat/Trem2
28-day post-SCI group vs. the control group	BP	GO:0032602	chemokine production	4/99	0.0007102	0.00389802	0.00191925	Aif1/Sirpa/Tlr4/Trem2
28-day post-SCI group vs. the control group	BP	GO:0045740	positive regulation of DNA replication	3/99	0.0007176	0.00393172	0.00193584	Jun/Map2k4/Met
28-day post-SCI group vs. the control group	BP	GO:0001892	embryonic placenta development	4/99	0.0007333	0.00399538	0.00196719	Hif1a/Mapk1/Slc8a1/Vcam1
28-day post-SCI group vs. the control group	BP	GO:0038061	NIK/NF-kappaB signaling	4/99	0.0007333	0.00399538	0.00196719	Ago1/Rela/Tlr4/Trem2

28-day post-SCI group vs. the control group	BP	GO:0051897	positive regulation of protein kinase B signaling	4/99	0.0007333	0.00399538	0.00196719	Axl/Gpx1/Hbegf/Met
28-day post-SCI group vs. the control group	BP	GO:0007162	negative regulation of cell adhesion	6/99	0.0007357	0.00399538	0.00196719	Cyp1b1/Hspb1/Mmp14/Pawr/Prkcd/Tnfaip3
28-day post-SCI group vs. the control group	BP	GO:2001020	regulation of response to DNA damage stimulus	6/99	0.0007357	0.00399538	0.00196719	Actb/Clu/Ier3/Mcl1/Parp1/Prkcd
28-day post-SCI group vs. the control group	BP	GO:0007519	skeletal muscle tissue development	5/99	0.0007397	0.00399538	0.00196719	Fos/Gpx1/Met/Ppp3ca/Rb1
28-day post-SCI group vs. the control group	BP	GO:0006086	acetyl-CoA biosynthetic process from pyruvate	2/99	0.0007475	0.00399538	0.00196719	Pdk1/Pdk2
28-day post-SCI group vs. the control group	BP	GO:0007183	SMAD protein complex assembly	2/99	0.0007475	0.00399538	0.00196719	Parp1/Smad1
28-day post-SCI group vs. the control group	BP	GO:0009404	toxin metabolic process	2/99	0.0007475	0.00399538	0.00196719	Cyp1b1/Nfe2l2
28-day post-SCI group vs. the control group	BP	GO:0032490	detection of molecule of bacterial origin	2/99	0.0007475	0.00399538	0.00196719	Tlr4/Trem2
28-day post-SCI group vs. the control group	BP	GO:0036005	response to macrophage colony-stimulating factor	2/99	0.0007475	0.00399538	0.00196719	Tlr4/Trem2
28-day post-SCI group vs. the control group	BP	GO:0036006	cellular response to macrophage colony-stimulating fa	2/99	0.0007475	0.00399538	0.00196719	Tlr4/Trem2
28-day post-SCI group vs. the control group	BP	GO:0045741	positive regulation of epidermal growth factor-activate	2/99	0.0007475	0.00399538	0.00196719	Fbxw7/Hbegf
28-day post-SCI group vs. the control group	BP	GO:0061418	regulation of transcription from RNA polymerase II pr	2/99	0.0007475	0.00399538	0.00196719	Hif1a/Nfe2l2
28-day post-SCI group vs. the control group	BP	GO:1900227	positive regulation of NLRP3 inflammasome complex	2/99	0.0007475	0.00399538	0.00196719	Cd36/Tlr4
28-day post-SCI group vs. the control group	BP	GO:0099173	postsynapse organization	5/99	0.0007559	0.0040186	0.00197862	Actb/Apoe/Slc7a11/Stau2/Trem2
28-day post-SCI group vs. the control group	BP	GO:0022404	molting cycle process	4/99	0.0007568	0.0040186	0.00197862	Apc/Ctsl/Hdac1/Rela
28-day post-SCI group vs. the control group	BP	GO:0022405	hair cycle process	4/99	0.0007568	0.0040186	0.00197862	Apc/Ctsl/Hdac1/Rela
28-day post-SCI group vs. the control group	BP	GO:0045445	myoblast differentiation	4/99	0.0007568	0.0040186	0.00197862	Actb/Gpx1/Rb1/Sdc1
28-day post-SCI group vs. the control group	BP	GO:0035196	miRNA processing	3/99	0.0007596	0.00402069	0.00197965	Ago1/Ppp3ca/Ripk1
28-day post-SCI group vs. the control group	BP	GO:0042307	positive regulation of protein import into nucleus	3/99	0.0007596	0.00402069	0.00197965	Mapk1/Prkcd/Tnfrsf1a
28-day post-SCI group vs. the control group	BP	GO:0042100	B cell proliferation	4/99	0.0007808	0.00411961	0.00202835	Cd38/Pawr/Prkcd/Tlr4
28-day post-SCI group vs. the control group	BP	GO:0098773	skin epidermis development	4/99	0.0007808	0.00411961	0.00202835	Apc/Ctsl/Hdac1/Rela
28-day post-SCI group vs. the control group	BP	GO:0042113	B cell activation	7/99	0.0008013	0.00419524	0.00206559	Cd38/Mmp14/Pawr/Prkcd/Stat6/Tlr4/Tnfaip3
28-day post-SCI group vs. the control group	BP	GO:0010828	positive regulation of glucose transmembrane transpor	3/99	0.0008032	0.00419524	0.00206559	Met/Nfe2l2/Prkcd
28-day post-SCI group vs. the control group	BP	GO:0043392	negative regulation of DNA binding	3/99	0.0008032	0.00419524	0.00206559	Fbxw7/Id1/Jun
28-day post-SCI group vs. the control group	BP	GO:0046676	negative regulation of insulin secretion	3/99	0.0008032	0.00419524	0.00206559	Fkbp1b/Ppp3ca/Ucp2
28-day post-SCI group vs. the control group	BP	GO:0061756	leukocyte adhesion to vascular endothelial cell	3/99	0.0008032	0.00419524	0.00206559	Pawr/Rela/Vcam1
28-day post-SCI group vs. the control group	BP	GO:2000677	regulation of transcription regulatory region DNA binc	3/99	0.0008032	0.00419524	0.00206559	Fbxw7/Parp1/Rb1
28-day post-SCI group vs. the control group	BP	GO:0006090	pyruvate metabolic process	4/99	0.0008054	0.00419524	0.00206559	Hif1a/Ier3/Pdk1/Pdk2
28-day post-SCI group vs. the control group	BP	GO:0022600	digestive system process	4/99	0.0008054	0.00419524	0.00206559	Cd36/Pawr/Ppp3ca/Tlr4
28-day post-SCI group vs. the control group	BP	GO:0031346	positive regulation of cell projection organization	7/99	0.0008119	0.00422238	0.00207895	Apc/Apoe/Fkbp1b/Hspb1/Met/Nfe2l2/Stau2
28-day post-SCI group vs. the control group	BP	GO:0009260	ribonucleotide biosynthetic process	5/99	0.0008233	0.00427448	0.0021046	Ndufa6/Parp1/Pdk1/Pdk2/Trem2
28-day post-SCI group vs. the control group	BP	GO:0098659	inorganic cation import across plasma membrane	4/99	0.0008306	0.00429879	0.00211657	Pawr/Ppp3ca/Slc8a1/Trpm2
28-day post-SCI group vs. the control group	BP	GO:0099587	inorganic ion import across plasma membrane	4/99	0.0008306	0.00429879	0.00211657	Pawr/Ppp3ca/Slc8a1/Trpm2
28-day post-SCI group vs. the control group	BP	GO:0031345	negative regulation of cell projection organization	5/99	0.0008408	0.00433114	0.0021325	Apoe/Cd38/Id1/Ppp3ca/Prkcd
28-day post-SCI group vs. the control group	BP	GO:0031669	cellular response to nutrient levels	5/99	0.0008408	0.00433114	0.0021325	Eif2s1/Jun/Mapk1/Nfe2l2/Pdk2
28-day post-SCI group vs. the control group	BP	GO:0034249	negative regulation of cellular amide metabolic proces	5/99	0.0008408	0.00433114	0.0021325	Ago1/Apoe/Clu/Eif2s1/Rpl13a
28-day post-SCI group vs. the control group	BP	GO:0045687	positive regulation of glial cell differentiation	3/99	0.0008484	0.00435643	0.00214496	Hdac1/Il6st/Rela
28-day post-SCI group vs. the control group	BP	GO:2000351	regulation of endothelial cell apoptotic process	3/99	0.0008484	0.00435643	0.00214496	Id1/Nfe2l2/Tnfaip3
28-day post-SCI group vs. the control group	BP	GO:0015849	organic acid transport	6/99	0.0008521	0.00436167	0.00214753	Apoe/Cd36/Il1a/Map2k6/Prkcd/Slc7a11
28-day post-SCI group vs. the control group	BP	GO:0030198	extracellular matrix organization	6/99	0.0008521	0.00436167	0.00214753	Cyp1b1/Mmp14/Mpv17/Prdx4/Rb1/Tnfrsf1a

28-day post-SCI group vs. the control group	BP	GO:0001676	long-chain fatty acid metabolic process	4/99	0.0008563	0.00436271	0.00214804	Cd36/Cyp1b1/Gpx1/Ptgs1
28-day post-SCI group vs. the control group	BP	GO:0060419	heart growth	4/99	0.0008563	0.00436271	0.00214804	Apc/Map2k4/Mapk1/Smad1
28-day post-SCI group vs. the control group	BP	GO:1902414	protein localization to cell junction	4/99	0.0008563	0.00436271	0.00214804	Actb/Hspb1/Mapk10/Stau2
28-day post-SCI group vs. the control group	BP	GO:0043062	extracellular structure organization	6/99	0.0008658	0.00440454	0.00216864	Cyp1b1/Mmp14/Mpv17/Prdx4/Rb1/Tnfrsf1a
28-day post-SCI group vs. the control group	BP	GO:0009895	negative regulation of catabolic process	6/99	0.0008798	0.00442905	0.00218071	Ier3/Mapkapk2/Mcl1/Rela/Tnfaip3/Trem2
28-day post-SCI group vs. the control group	BP	GO:0045229	external encapsulating structure organization	6/99	0.0008798	0.00442905	0.00218071	Cyp1b1/Mmp14/Mpv17/Prdx4/Rb1/Tnfrsf1a
28-day post-SCI group vs. the control group	BP	GO:0048608	reproductive structure development	6/99	0.0008798	0.00442905	0.00218071	Apc/Axl/Dhcr24/Il1a/Plekha1/Prdx4
28-day post-SCI group vs. the control group	BP	GO:0032060	bleb assembly	2/99	0.0008815	0.00442905	0.00218071	Mylk/Prdx6
28-day post-SCI group vs. the control group	BP	GO:0034375	high-density lipoprotein particle remodeling	2/99	0.0008815	0.00442905	0.00218071	Apoe/Lcat
28-day post-SCI group vs. the control group	BP	GO:0043922	negative regulation by host of viral transcription	2/99	0.0008815	0.00442905	0.00218071	Hdac1/Jun
28-day post-SCI group vs. the control group	BP	GO:0044557	relaxation of smooth muscle	2/99	0.0008815	0.00442905	0.00218071	Pawr/Slc8a1
28-day post-SCI group vs. the control group	BP	GO:1901298	regulation of hydrogen peroxide-mediated programme	2/99	0.0008815	0.00442905	0.00218071	Met/Pawr
28-day post-SCI group vs. the control group	BP	GO:0002253	activation of immune response	7/99	0.0008899	0.00446445	0.00219814	Cd38/Mapk1/Pawr/Plekha1/Rela/Tlr4/Trem2
28-day post-SCI group vs. the control group	BP	GO:0051216	cartilage development	5/99	0.0008951	0.00448362	0.00220758	Bmp1/Hif1a/Rb1/Rela/Smad1
28-day post-SCI group vs. the control group	BP	GO:0030217	T cell differentiation	6/99	0.0009081	0.0045421	0.00223637	Actb/Apc/Ctsl/Hspb1/Il1a/Stat6
28-day post-SCI group vs. the control group	BP	GO:0006164	purine nucleotide biosynthetic process	5/99	0.0009137	0.00456311	0.00224671	Ndufa6/Parp1/Pdk1/Pdk2/Trem2
28-day post-SCI group vs. the control group	BP	GO:0044772	mitotic cell cycle phase transition	7/99	0.000925	0.00461254	0.00227106	Actb/Aif1/Apc/Cdkn2c/Ier3/Ppp3ca/Rb1
28-day post-SCI group vs. the control group	BP	GO:0030522	intracellular receptor signaling pathway	5/99	0.0009327	0.00464354	0.00228632	Hdac1/Parp1/Rela/Tlr4/Tnfaip3
28-day post-SCI group vs. the control group	BP	GO:0002718	regulation of cytokine production involved in immune	4/99	0.0009367	0.004652	0.00229048	Axl/Cd36/Mapkapk2/Tlr4
28-day post-SCI group vs. the control group	BP	GO:0061458	reproductive system development	6/99	0.0009372	0.004652	0.00229048	Apc/Axl/Dhcr24/Il1a/Plekha1/Prdx4
28-day post-SCI group vs. the control group	BP	GO:0070918	small regulatory ncRNA processing	3/99	0.0009434	0.00467574	0.00230217	Ago1/Ppp3ca/Ripk1
28-day post-SCI group vs. the control group	BP	GO:0006631	fatty acid metabolic process	7/99	0.0009491	0.00469643	0.00231236	Cd36/Cyp1b1/Gpx1/Pdk1/Pdk2/Ptgs1/Tnfrsf1a
28-day post-SCI group vs. the control group	BP	GO:0060538	skeletal muscle organ development	5/99	0.0009519	0.00469643	0.00231236	Fos/Gpx1/Met/Ppp3ca/Rb1
28-day post-SCI group vs. the control group	BP	GO:1903050	regulation of proteolysis involved in protein catabolic	5/99	0.0009519	0.00469643	0.00231236	Apoe/Clu/Fbxw7/Gpx1/Trem2
28-day post-SCI group vs. the control group	BP	GO:0046390	ribose phosphate biosynthetic process	5/99	0.0009913	0.00486435	0.00239504	Ndufa6/Parp1/Pdk1/Pdk2/Trem2
28-day post-SCI group vs. the control group	BP	GO:1903828	negative regulation of protein localization	5/99	0.0009913	0.00486435	0.00239504	Apoe/Cd36/Fkbp1b/Ppp3ca/Ucp2
28-day post-SCI group vs. the control group	BP	GO:0030857	negative regulation of epithelial cell differentiation	3/99	0.0009934	0.00486435	0.00239504	Id1/Il1a/Xdh
28-day post-SCI group vs. the control group	BP	GO:0051489	regulation of filopodium assembly	3/99	0.0009934	0.00486435	0.00239504	Prkcd/Stau2/Trpm2
28-day post-SCI group vs. the control group	BP	GO:0072577	endothelial cell apoptotic process	3/99	0.0009934	0.00486435	0.00239504	Id1/Nfe2l2/Tnfaip3
28-day post-SCI group vs. the control group	BP	GO:0010959	regulation of metal ion transport	7/99	0.0010114	0.00494493	0.00243471	Fkbp1b/Mylk/Pawr/Ppp3ca/S100a1/Slc8a1/Trem2
28-day post-SCI group vs. the control group	BP	GO:0008286	insulin receptor signaling pathway	4/99	0.0010223	0.00495771	0.002441	Apc/Pdk2/Prkcd/Rela
28-day post-SCI group vs. the control group	BP	GO:0006750	glutathione biosynthetic process	2/99	0.0010261	0.00495771	0.002441	Nfe2l2/Slc7a11
28-day post-SCI group vs. the control group	BP	GO:0010940	positive regulation of necrotic cell death	2/99	0.0010261	0.00495771	0.002441	Hebp2/Ripk1
28-day post-SCI group vs. the control group	BP	GO:0014820	tonic smooth muscle contraction	2/99	0.0010261	0.00495771	0.002441	Cd38/Mylk
28-day post-SCI group vs. the control group	BP	GO:0032494	response to peptidoglycan	2/99	0.0010261	0.00495771	0.002441	Rela/Trem2
28-day post-SCI group vs. the control group	BP	GO:0036499	PERK-mediated unfolded protein response	2/99	0.0010261	0.00495771	0.002441	Eif2s1/Nfe2l2
28-day post-SCI group vs. the control group	BP	GO:0097113	AMPA glutamate receptor clustering	2/99	0.0010261	0.00495771	0.002441	Apoe/Slc7a11
28-day post-SCI group vs. the control group	BP	GO:0097688	glutamate receptor clustering	2/99	0.0010261	0.00495771	0.002441	Apoe/Slc7a11
28-day post-SCI group vs. the control group	BP	GO:0007015	actin filament organization	7/99	0.0010372	0.00500371	0.00246365	Aif1/Id1/Itgb5/Met/Pawr/Prkcd/Sirpa
28-day post-SCI group vs. the control group	BP	GO:0032757	positive regulation of interleukin-8 production	3/99	0.001045	0.00502671	0.00247498	Rela/Ripk1/Tlr4

28-day post-SCI group vs. the control group	BP	GO:0098754	detoxification	3/99	0.001045	0.00502671	0.00247498	Cd36/Nfe2l2/Prdx6
28-day post-SCI group vs. the control group	BP	GO:0043406	positive regulation of MAP kinase activity	4/99	0.001052	0.00504833	0.00248562	Map2k4/Map2k6/Prkcd/Tlr4
28-day post-SCI group vs. the control group	BP	GO:0046034	ATP metabolic process	5/99	0.0010526	0.00504833	0.00248562	Hif1a/Ier3/Ndufa6/Parp1/Trem2
28-day post-SCI group vs. the control group	BP	GO:0072522	purine-containing compound biosynthetic process	5/99	0.0010736	0.00514172	0.0025316	Ndufa6/Parp1/Pdk1/Pdk2/Trem2
28-day post-SCI group vs. the control group	BP	GO:0043388	positive regulation of DNA binding	3/99	0.0010983	0.00525204	0.00258592	Parp1/Rb1/Tlr4
28-day post-SCI group vs. the control group	BP	GO:0032386	regulation of intracellular transport	6/99	0.0011102	0.00530162	0.00261033	Cd36/Fbxw7/Ier3/Mapk1/Prkcd/Tnfrsf1a
28-day post-SCI group vs. the control group	BP	GO:0001776	leukocyte homeostasis	4/99	0.0011132	0.00530816	0.00261355	Axl/Hif1a/Slc7a11/Tnfaip3
28-day post-SCI group vs. the control group	BP	GO:0033157	regulation of intracellular protein transport	5/99	0.0011386	0.00542128	0.00266925	Cd36/Fbxw7/Mapk1/Prkcd/Tnfrsf1a
28-day post-SCI group vs. the control group	BP	GO:0051235	maintenance of location	6/99	0.0011442	0.00543477	0.00267589	Apoe/Cd36/Fkbp1b/Slc8a1/Trem2/Trpm2
28-day post-SCI group vs. the control group	BP	GO:0033559	unsaturated fatty acid metabolic process	4/99	0.0011448	0.00543477	0.00267589	Cyp1b1/Gpx1/Ptgs1/Tnfrsf1a
28-day post-SCI group vs. the control group	BP	GO:0006914	autophagy	7/99	0.0011597	0.00548966	0.00270292	Atp2a2/Clu/Fbxw7/Hif1a/Mcl1/Tnfaip3/Trem2
28-day post-SCI group vs. the control group	BP	GO:0061919	process utilizing autophagic mechanism	7/99	0.0011597	0.00548966	0.00270292	Atp2a2/Clu/Fbxw7/Hif1a/Mcl1/Tnfaip3/Trem2
28-day post-SCI group vs. the control group	BP	GO:1901990	regulation of mitotic cell cycle phase transition	6/99	0.0011614	0.00548995	0.00270306	Actb/Aif1/Apc/Cdkn2c/Ier3/Rb1
28-day post-SCI group vs. the control group	BP	GO:0010876	lipid localization	7/99	0.001174	0.00552015	0.00271793	Apoe/Cd36/Il1a/Lcat/Map2k6/Prkcd/Trem2
28-day post-SCI group vs. the control group	BP	GO:0043270	positive regulation of ion transport	6/99	0.0011789	0.00552015	0.00271793	Il1a/Map2k6/Mylk/Ppp3ca/S100a1/Trem2
28-day post-SCI group vs. the control group	BP	GO:0010421	hydrogen peroxide-mediated programmed cell death	2/99	0.0011813	0.00552015	0.00271793	Met/Pawr
28-day post-SCI group vs. the control group	BP	GO:0014733	regulation of skeletal muscle adaptation	2/99	0.0011813	0.00552015	0.00271793	Atp2a2/Ppp3ca
28-day post-SCI group vs. the control group	BP	GO:0032308	positive regulation of prostaglandin secretion	2/99	0.0011813	0.00552015	0.00271793	Il1a/Map2k6
28-day post-SCI group vs. the control group	BP	GO:0034350	regulation of glial cell apoptotic process	2/99	0.0011813	0.00552015	0.00271793	Prkcd/Trem2
28-day post-SCI group vs. the control group	BP	GO:0061029	eyelid development in camera-type eye	2/99	0.0011813	0.00552015	0.00271793	Hdac1/Jun
28-day post-SCI group vs. the control group	BP	GO:2001138	regulation of phospholipid transport	2/99	0.0011813	0.00552015	0.00271793	Apoe/Prkcd
28-day post-SCI group vs. the control group	BP	GO:0007265	Ras protein signal transduction	6/99	0.0011966	0.00558334	0.00274904	Aif1/Apoe/Dhcr24/Jun/Met/Net1
28-day post-SCI group vs. the control group	BP	GO:0050920	regulation of chemotaxis	5/99	0.0012065	0.00562172	0.00276794	Aif1/Hspb1/Mapk1/Met/Trem2
28-day post-SCI group vs. the control group	BP	GO:0007160	cell-matrix adhesion	5/99	0.0012534	0.00583188	0.00287141	Cd36/Itgb5/Mmp14/Sirpa/Vcam1
28-day post-SCI group vs. the control group	BP	GO:0032655	regulation of interleukin-12 production	3/99	0.0012682	0.00587595	0.00289311	Cd36/Rela/Tlr4
28-day post-SCI group vs. the control group	BP	GO:0032731	positive regulation of interleukin-1 beta production	3/99	0.0012682	0.00587595	0.00289311	Cd36/Hspb1/Tlr4
28-day post-SCI group vs. the control group	BP	GO:0051785	positive regulation of nuclear division	3/99	0.0012682	0.00587595	0.00289311	Il1a/Met/Rb1
28-day post-SCI group vs. the control group	BP	GO:0001889	liver development	4/99	0.0012772	0.00588414	0.00289714	Jun/Met/Rela/Tnfaip3
28-day post-SCI group vs. the control group	BP	GO:0042303	molting cycle	4/99	0.0012772	0.00588414	0.00289714	Apc/Ctsl/Hdac1/Rela
28-day post-SCI group vs. the control group	BP	GO:0042633	hair cycle	4/99	0.0012772	0.00588414	0.00289714	Apc/Ctsl/Hdac1/Rela
28-day post-SCI group vs. the control group	BP	GO:0050773	regulation of dendrite development	4/99	0.0012772	0.00588414	0.00289714	Id1/Met/Ppp3ca/Stau2
28-day post-SCI group vs. the control group	BP	GO:0002757	immune response-activating signal transduction	6/99	0.0013068	0.00601206	0.00296013	Cd38/Mapk1/Pawr/Plekha1/Rela/Tlr4
28-day post-SCI group vs. the control group	BP	GO:0007586	digestion	4/99	0.0013119	0.00601862	0.00296336	Cd36/Pawr/Ppp3ca/Tlr4
28-day post-SCI group vs. the control group	BP	GO:0035966	response to topologically incorrect protein	4/99	0.0013119	0.00601862	0.00296336	Clu/Eif2s1/Hspb1/Nfe2l2
28-day post-SCI group vs. the control group	BP	GO:0050435	amyloid-beta metabolic process	3/99	0.0013284	0.00608556	0.00299632	Apoe/Clu/Rela
28-day post-SCI group vs. the control group	BP	GO:0006857	oligopeptide transport	2/99	0.0013471	0.00609445	0.00300069	Mgst1/Slc7a11
28-day post-SCI group vs. the control group	BP	GO:0019184	nonribosomal peptide biosynthetic process	2/99	0.0013471	0.00609445	0.00300069	Nfe2l2/Slc7a11
28-day post-SCI group vs. the control group	BP	GO:0023035	CD40 signaling pathway	2/99	0.0013471	0.00609445	0.00300069	Tnfaip3/Trem2
28-day post-SCI group vs. the control group	BP	GO:0034433	steroid esterification	2/99	0.0013471	0.00609445	0.00300069	Apoe/Lcat
28-day post-SCI group vs. the control group	BP	GO:0034434	sterol esterification	2/99	0.0013471	0.00609445	0.00300069	Apoe/Lcat

28-day post-SCI group vs. the control group	BP	GO:0034435	cholesterol esterification	2/99	0.0013471	0.00609445	0.00300069	Apoe/Lcat
28-day post-SCI group vs. the control group	BP	GO:0045651	positive regulation of macrophage differentiation	2/99	0.0013471	0.00609445	0.00300069	Rb1/Ripk1
28-day post-SCI group vs. the control group	BP	GO:0045947	negative regulation of translational initiation	2/99	0.0013471	0.00609445	0.00300069	Eif2s1/Rpl13a
28-day post-SCI group vs. the control group	BP	GO:0097468	programmed cell death in response to reactive oxygen	2/99	0.0013471	0.00609445	0.00300069	Met/Pawr
28-day post-SCI group vs. the control group	BP	GO:0061008	hepaticobiliary system development	4/99	0.0013833	0.00621249	0.00305881	Jun/Met/Rela/Tnfaip3
28-day post-SCI group vs. the control group	BP	GO:0008202	steroid metabolic process	6/99	0.0013845	0.00621249	0.00305881	Apoe/Cyp1b1/Dhcr24/Il1a/Lcat/Plekha1
28-day post-SCI group vs. the control group	BP	GO:1901361	organic cyclic compound catabolic process	7/99	0.0013889	0.00621249	0.00305881	Ago1/Apoe/Hmox2/Mapkapk2/Ncf1/Nfe2l2/Xdh
28-day post-SCI group vs. the control group	BP	GO:0009064	glutamine family amino acid metabolic process	3/99	0.0013903	0.00621249	0.00305881	Apc/Prodh/Slc7a11
28-day post-SCI group vs. the control group	BP	GO:0019748	secondary metabolic process	3/99	0.0013903	0.00621249	0.00305881	Cyp1b1/Nfe2l2/Slc7a11
28-day post-SCI group vs. the control group	BP	GO:0032613	interleukin-10 production	3/99	0.0013903	0.00621249	0.00305881	Prkcd/Tlr4/Trem2
28-day post-SCI group vs. the control group	BP	GO:0051851	modulation by host of symbiont process	3/99	0.0013903	0.00621249	0.00305881	Apoe/Hdac1/Jun
28-day post-SCI group vs. the control group	BP	GO:0061077	chaperone-mediated protein folding	3/99	0.0013903	0.00621249	0.00305881	Clu/Fkbp1b/Hspb1
28-day post-SCI group vs. the control group	BP	GO:0090278	negative regulation of peptide hormone secretion	3/99	0.0013903	0.00621249	0.00305881	Fkbp1b/Ppp3ca/Ucp2
28-day post-SCI group vs. the control group	BP	GO:0015711	organic anion transport	6/99	0.0014247	0.00635762	0.00313027	Cd36/Il1a/Map2k6/Mgst1/Prkcd/Slc7a11
28-day post-SCI group vs. the control group	BP	GO:0098930	axonal transport	3/99	0.0014539	0.00647306	0.00318711	Dst/Hif1a/Hspb1
28-day post-SCI group vs. the control group	BP	GO:0033044	regulation of chromosome organization	5/99	0.0014545	0.00647306	0.00318711	Actb/Apc/Mapk1/Parp1/Rb1
28-day post-SCI group vs. the control group	BP	GO:0002702	positive regulation of production of molecular mediato	4/99	0.0014574	0.00647697	0.00318903	Cd36/Mapkapk2/Stat6/Tlr4
28-day post-SCI group vs. the control group	BP	GO:0009913	epidermal cell differentiation	5/99	0.0014812	0.00657407	0.00323684	Hdac1/Il1a/Ppp3ca/Ptgs1/Txnip
28-day post-SCI group vs. the control group	BP	GO:0014902	myotube differentiation	4/99	0.0014954	0.00661909	0.00325901	Gpx1/Met/Mmp14/Ppp3ca
28-day post-SCI group vs. the control group	BP	GO:0032411	positive regulation of transporter activity	4/99	0.0014954	0.00661909	0.00325901	Ppp3ca/Prkcd/S100a1/Trem2
28-day post-SCI group vs. the control group	BP	GO:0002792	negative regulation of peptide secretion	3/99	0.0015194	0.00665243	0.00327543	Fkbp1b/Ppp3ca/Ucp2
28-day post-SCI group vs. the control group	BP	GO:0060038	cardiac muscle cell proliferation	3/99	0.0015194	0.00665243	0.00327543	Apc/Mapk1/Smad1
28-day post-SCI group vs. the control group	BP	GO:0001886	endothelial cell morphogenesis	2/99	0.0015233	0.00665243	0.00327543	Id1/Met
28-day post-SCI group vs. the control group	BP	GO:0032306	regulation of prostaglandin secretion	2/99	0.0015233	0.00665243	0.00327543	Il1a/Map2k6
28-day post-SCI group vs. the control group	BP	GO:0060100	positive regulation of phagocytosis, engulfment	2/99	0.0015233	0.00665243	0.00327543	Cd36/Trem2
28-day post-SCI group vs. the control group	BP	GO:0061179	negative regulation of insulin secretion involved in cel	2/99	0.0015233	0.00665243	0.00327543	Fkbp1b/Ucp2
28-day post-SCI group vs. the control group	BP	GO:0090083	regulation of inclusion body assembly	2/99	0.0015233	0.00665243	0.00327543	Apoe/Clu
28-day post-SCI group vs. the control group	BP	GO:0090136	epithelial cell-cell adhesion	2/99	0.0015233	0.00665243	0.00327543	Cyp1b1/Itgb5
28-day post-SCI group vs. the control group	BP	GO:1905155	positive regulation of membrane invagination	2/99	0.0015233	0.00665243	0.00327543	Cd36/Trem2
28-day post-SCI group vs. the control group	BP	GO:2001028	positive regulation of endothelial cell chemotaxis	2/99	0.0015233	0.00665243	0.00327543	Hspb1/Met
28-day post-SCI group vs. the control group	BP	GO:0090316	positive regulation of intracellular protein transport	4/99	0.0015736	0.00686291	0.00337906	Fbxw7/Mapk1/Prkcd/Tnfrsf1a
28-day post-SCI group vs. the control group	BP	GO:0006639	acylglycerol metabolic process	4/99	0.0016545	0.00718343	0.00353687	Apoe/Cd36/Gpx1/Il6st
28-day post-SCI group vs. the control group	BP	GO:0010469	regulation of signaling receptor activity	4/99	0.0016545	0.00718343	0.00353687	Fbxw7/Hbegf/Hdac1/Hif1a
28-day post-SCI group vs. the control group	BP	GO:0051783	regulation of nuclear division	4/99	0.0016545	0.00718343	0.00353687	Apc/Il1a/Met/Rb1
28-day post-SCI group vs. the control group	BP	GO:0098900	regulation of action potential	3/99	0.0016559	0.00718343	0.00353687	Atp2a2/Cd36/Pawr
28-day post-SCI group vs. the control group	BP	GO:0006820	anion transport	7/99	0.0016711	0.00723996	0.0035647	Cd36/Il1a/Map2k6/Mgst1/Prkcd/Ripk1/Slc7a11
28-day post-SCI group vs. the control group	BP	GO:0032956	regulation of actin cytoskeleton organization	6/99	0.0016846	0.00728897	0.00358884	Id1/Il1a/Met/Prkcd/Stau2/Trpm2
28-day post-SCI group vs. the control group	BP	GO:0001508	action potential	4/99	0.0016961	0.0072999	0.00359421	Atp2a2/Cd36/Fkbp1b/Pawr
28-day post-SCI group vs. the control group	BP	GO:0002367	cytokine production involved in immune response	4/99	0.0016961	0.0072999	0.00359421	Axl/Cd36/Mapkapk2/Tlr4
28-day post-SCI group vs. the control group	BP	GO:0050729	positive regulation of inflammatory response	4/99	0.0016961	0.0072999	0.00359421	Ripk1/Tlr4/Tnfrsf1a/Trem2

28-day post-SCI group vs. the control group	BP	GO:0071887	leukocyte apoptotic process	4/99	0.0016961	0.0072999	0.00359421	Axl/Hif1a/Ripk1/Slc7a11
28-day post-SCI group vs. the control group	BP	GO:0002862	negative regulation of inflammatory response to antige	2/99	0.0017099	0.00731115	0.00359976	Gpx1/Trem2
28-day post-SCI group vs. the control group	BP	GO:0019430	removal of superoxide radicals	2/99	0.0017099	0.00731115	0.00359976	Cd36/Nfe2l2
28-day post-SCI group vs. the control group	BP	GO:0051000	positive regulation of nitric-oxide synthase activity	2/99	0.0017099	0.00731115	0.00359976	Apoe/S100a1
28-day post-SCI group vs. the control group	BP	GO:0070102	interleukin-6-mediated signaling pathway	2/99	0.0017099	0.00731115	0.00359976	Il6st/Ripk1
28-day post-SCI group vs. the control group	BP	GO:1904424	regulation of GTP binding	2/99	0.0017099	0.00731115	0.00359976	Eif2s1/Met
28-day post-SCI group vs. the control group	BP	GO:0006940	regulation of smooth muscle contraction	3/99	0.0017269	0.00736446	0.00362601	Ncf1/Pawr/Ptgs1
28-day post-SCI group vs. the control group	BP	GO:0060425	lung morphogenesis	3/99	0.0017269	0.00736446	0.00362601	Id1/Mapk1/Rpl13a
28-day post-SCI group vs. the control group	BP	GO:0009205	purine ribonucleoside triphosphate metabolic process	5/99	0.0017382	0.00739426	0.00364067	Hif1a/Ier3/Ndufa6/Parp1/Trem2
28-day post-SCI group vs. the control group	BP	GO:0006638	neutral lipid metabolic process	4/99	0.0017384	0.00739426	0.00364067	Apoe/Cd36/Gpx1/Il6st
28-day post-SCI group vs. the control group	BP	GO:0002831	regulation of response to biotic stimulus	6/99	0.0017549	0.00743572	0.00366109	Apoe/Ncf1/Nfe2l2/Tlr4/Tnfaip3/Trem2
28-day post-SCI group vs. the control group	BP	GO:0007517	muscle organ development	6/99	0.0017549	0.00743572	0.00366109	Fos/Gpx1/Met/Mylk/Ppp3ca/Rb1
28-day post-SCI group vs. the control group	BP	GO:0031589	cell-substrate adhesion	6/99	0.0017549	0.00743572	0.00366109	Axl/Cd36/Itgb5/Mmp14/Sirpa/Vcam1
28-day post-SCI group vs. the control group	BP	GO:0031333	negative regulation of protein-containing complex asse	4/99	0.0018251	0.00772296	0.00380252	Clu/Prkcd/Rpl13a/Trem2
28-day post-SCI group vs. the control group	BP	GO:0046879	hormone secretion	6/99	0.0018521	0.00782711	0.0038538	Cd38/Fkbp1b/Hif1a/Ppp3ca/Trpm2/Ucp2
28-day post-SCI group vs. the control group	BP	GO:0001937	negative regulation of endothelial cell proliferation	3/99	0.0018745	0.00787098	0.00387539	Apc/Apoe/Xdh
28-day post-SCI group vs. the control group	BP	GO:0014009	glial cell proliferation	3/99	0.0018745	0.00787098	0.00387539	Clu/Rb1/Trem2
28-day post-SCI group vs. the control group	BP	GO:0034121	regulation of toll-like receptor signaling pathway	3/99	0.0018745	0.00787098	0.00387539	Cd36/Tnfaip3/Trem2
28-day post-SCI group vs. the control group	BP	GO:0055117	regulation of cardiac muscle contraction	3/99	0.0018745	0.00787098	0.00387539	Atp2a2/Fkbp1b/Slc8a1
28-day post-SCI group vs. the control group	BP	GO:2001244	positive regulation of intrinsic apoptotic signaling path	3/99	0.0018745	0.00787098	0.00387539	Clu/Fbxw7/Mcl1
28-day post-SCI group vs. the control group	BP	GO:0006085	acetyl-CoA biosynthetic process	2/99	0.0019068	0.00793527	0.00390705	Pdk1/Pdk2
28-day post-SCI group vs. the control group	BP	GO:0010544	negative regulation of platelet activation	2/99	0.0019068	0.00793527	0.00390705	Apoe/Prkcd
28-day post-SCI group vs. the control group	BP	GO:0032700	negative regulation of interleukin-17 production	2/99	0.0019068	0.00793527	0.00390705	Parp1/Tlr4
28-day post-SCI group vs. the control group	BP	GO:0043691	reverse cholesterol transport	2/99	0.0019068	0.00793527	0.00390705	Apoe/Lcat
28-day post-SCI group vs. the control group	BP	GO:0060576	intestinal epithelial cell development	2/99	0.0019068	0.00793527	0.00390705	Hif1a/Il6st
28-day post-SCI group vs. the control group	BP	GO:0140467	integrated stress response signaling	2/99	0.0019068	0.00793527	0.00390705	Eif2s1/Nfe2l2
28-day post-SCI group vs. the control group	BP	GO:1902430	negative regulation of amyloid-beta formation	2/99	0.0019068	0.00793527	0.00390705	Apoe/Clu
28-day post-SCI group vs. the control group	BP	GO:0009144	purine nucleoside triphosphate metabolic process	5/99	0.0019269	0.00799839	0.00393813	Hif1a/Ier3/Ndufa6/Parp1/Trem2
28-day post-SCI group vs. the control group	BP	GO:0009199	ribonucleoside triphosphate metabolic process	5/99	0.0019269	0.00799839	0.00393813	Hif1a/Ier3/Ndufa6/Parp1/Trem2
28-day post-SCI group vs. the control group	BP	GO:0048678	response to axon injury	3/99	0.0019512	0.00808912	0.0039828	Fkbp1b/Jun/Trem2
28-day post-SCI group vs. the control group	BP	GO:0006839	mitochondrial transport	4/99	0.0019607	0.00810814	0.00399217	Fbxw7/Hebp2/Ier3/Ucp2
28-day post-SCI group vs. the control group	BP	GO:0046434	organophosphate catabolic process	4/99	0.0019607	0.00810814	0.00399217	Ncf1/Prdx6/Prkcd/Xdh
28-day post-SCI group vs. the control group	BP	GO:0010639	negative regulation of organelle organization	6/99	0.0019792	0.00817428	0.00402473	Apc/Gpx1/Ier3/Met/Parp1/Prkcd
28-day post-SCI group vs. the control group	BP	GO:0099003	vesicle-mediated transport in synapse	5/99	0.0020266	0.00833913	0.0041059	Actb/Amph/Atp2a2/Mapk10/Syp
28-day post-SCI group vs. the control group	BP	GO:0030888	regulation of B cell proliferation	3/99	0.0020298	0.00833913	0.0041059	Cd38/Pawr/Tlr4
28-day post-SCI group vs. the control group	BP	GO:0048662	negative regulation of smooth muscle cell proliferation	3/99	0.0020298	0.00833913	0.0041059	Aif1/Apoe/Tnfaip3
28-day post-SCI group vs. the control group	BP	GO:0061035	regulation of cartilage development	3/99	0.0020298	0.00833913	0.0041059	Bmp1/Rela/Smad1
28-day post-SCI group vs. the control group	BP	GO:0046486	glycerolipid metabolic process	6/99	0.0020319	0.00833913	0.0041059	Apoe/Cd36/Gpx1/Il6st/Lcat/Prdx6
28-day post-SCI group vs. the control group	BP	GO:0010565	regulation of cellular ketone metabolic process	4/99	0.0020549	0.00841265	0.00414209	Apc/Pdk1/Pdk2/Slc7a11
28-day post-SCI group vs. the control group	BP	GO:0030216	keratinocyte differentiation	4/99	0.0020549	0.00841265	0.00414209	Il1a/Ppp3ca/Ptgs1/Txnip

28-day post-SCI group vs. the control group	BP	GO:0009914	hormone transport	6/99	0.0020586	0.00841713	0.0041443	Cd38/Fkbp1b/Hif1a/Ppp3ca/Trpm2/Ucp2
28-day post-SCI group vs. the control group	BP	GO:0032623	interleukin-2 production	3/99	0.0021103	0.00853669	0.00420317	Il1a/Pawr/Tnfaip3
28-day post-SCI group vs. the control group	BP	GO:0032732	positive regulation of interleukin-1 production	3/99	0.0021103	0.00853669	0.00420317	Cd36/Hspb1/Tlr4
28-day post-SCI group vs. the control group	BP	GO:0046824	positive regulation of nucleocytoplasmic transport	3/99	0.0021103	0.00853669	0.00420317	Mapk1/Prkcd/Tnfrsf1a
28-day post-SCI group vs. the control group	BP	GO:0048488	synaptic vesicle endocytosis	3/99	0.0021103	0.00853669	0.00420317	Actb/Amph/Syp
28-day post-SCI group vs. the control group	BP	GO:0140238	presynaptic endocytosis	3/99	0.0021103	0.00853669	0.00420317	Actb/Amph/Syp
28-day post-SCI group vs. the control group	BP	GO:0032305	positive regulation of icosanoid secretion	2/99	0.0021139	0.00853669	0.00420317	Il1a/Map2k6
28-day post-SCI group vs. the control group	BP	GO:0032495	response to muramyl dipeptide	2/99	0.0021139	0.00853669	0.00420317	Rela/Tnfaip3
28-day post-SCI group vs. the control group	BP	GO:0043373	CD4-positive, alpha-beta T cell lineage commitment	2/99	0.0021139	0.00853669	0.00420317	Ctsl/Stat6
28-day post-SCI group vs. the control group	BP	GO:0050965	detection of temperature stimulus involved in sensory p	2/99	0.0021139	0.00853669	0.00420317	Pawr/Tlr4
28-day post-SCI group vs. the control group	BP	GO:0050966	detection of mechanical stimulus involved in sensory p	2/99	0.0021139	0.00853669	0.00420317	Pawr/Tlr4
28-day post-SCI group vs. the control group	BP	GO:0031667	response to nutrient levels	6/99	0.0021682	0.0087449	0.00430569	Apoe/Eif2s1/Jun/Mapk1/Nfe2l2/Pdk2
28-day post-SCI group vs. the control group	BP	GO:0010507	negative regulation of autophagy	3/99	0.0021929	0.00881186	0.00433865	Mcl1/Tnfaip3/Trem2
28-day post-SCI group vs. the control group	BP	GO:0010611	regulation of cardiac muscle hypertrophy	3/99	0.0021929	0.00881186	0.00433865	Parp1/Ppp3ca/Tnfrsf1a
28-day post-SCI group vs. the control group	BP	GO:0045911	positive regulation of DNA recombination	3/99	0.0021929	0.00881186	0.00433865	Actb/Parp1/Stat6
28-day post-SCI group vs. the control group	BP	GO:0032677	regulation of interleukin-8 production	3/99	0.0022774	0.0091178	0.00448929	Rela/Ripk1/Tlr4
28-day post-SCI group vs. the control group	BP	GO:0046847	filopodium assembly	3/99	0.0022774	0.0091178	0.00448929	Prkcd/Stau2/Trpm2
28-day post-SCI group vs. the control group	BP	GO:0051926	negative regulation of calcium ion transport	3/99	0.0022774	0.0091178	0.00448929	Fkbp1b/Pawr/Ppp3ca
28-day post-SCI group vs. the control group	BP	GO:0007252	I-kappaB phosphorylation	2/99	0.0023313	0.0092656	0.00456206	Sirpa/Tlr4
28-day post-SCI group vs. the control group	BP	GO:0032310	prostaglandin secretion	2/99	0.0023313	0.0092656	0.00456206	Il1a/Map2k6
28-day post-SCI group vs. the control group	BP	GO:0035584	calcium-mediated signaling using intracellular calcium	2/99	0.0023313	0.0092656	0.00456206	Trpm2/Vcam1
28-day post-SCI group vs. the control group	BP	GO:0046697	decidualization	2/99	0.0023313	0.0092656	0.00456206	Ctsl/Mapk1
28-day post-SCI group vs. the control group	BP	GO:0050665	hydrogen peroxide biosynthetic process	2/99	0.0023313	0.0092656	0.00456206	Cybb/Ncf1
28-day post-SCI group vs. the control group	BP	GO:0060099	regulation of phagocytosis, engulfment	2/99	0.0023313	0.0092656	0.00456206	Cd36/Trem2
28-day post-SCI group vs. the control group	BP	GO:0032637	interleukin-8 production	3/99	0.0023639	0.00938368	0.0046202	Rela/Ripk1/Tlr4
28-day post-SCI group vs. the control group	BP	GO:0097530	granulocyte migration	4/99	0.0024093	0.00955254	0.00470334	Il1a/Mapk1/Pawr/Sirpa
28-day post-SCI group vs. the control group	BP	GO:0014743	regulation of muscle hypertrophy	3/99	0.0024524	0.00967629	0.00476427	Parp1/Ppp3ca/Tnfrsf1a
28-day post-SCI group vs. the control group	BP	GO:0019369	arachidonic acid metabolic process	3/99	0.0024524	0.00967629	0.00476427	Cyp1b1/Gpx1/Ptgs1
28-day post-SCI group vs. the control group	BP	GO:0038034	signal transduction in absence of ligand	3/99	0.0024524	0.00967629	0.00476427	Il1a/Mcl1/Ripk1
28-day post-SCI group vs. the control group	BP	GO:0097192	extrinsic apoptotic signaling pathway in absence of lig	3/99	0.0024524	0.00967629	0.00476427	Il1a/Mcl1/Ripk1
28-day post-SCI group vs. the control group	BP	GO:0006898	receptor-mediated endocytosis	5/99	0.0025426	0.00994001	0.00489412	Apoe/Cd36/Clu/Ctsl/Sdc1
28-day post-SCI group vs. the control group	BP	GO:0045669	positive regulation of osteoblast differentiation	3/99	0.0025429	0.00994001	0.00489412	Il6st/Ppp3ca/Smad1
28-day post-SCI group vs. the control group	BP	GO:0002363	alpha-beta T cell lineage commitment	2/99	0.0025587	0.00994001	0.00489412	Ctsl/Stat6
28-day post-SCI group vs. the control group	BP	GO:0006907	pinocytosis	2/99	0.0025587	0.00994001	0.00489412	Axl/Mapkapk2
28-day post-SCI group vs. the control group	BP	GO:0010885	regulation of cholesterol storage	2/99	0.0025587	0.00994001	0.00489412	Cd36/Trem2
28-day post-SCI group vs. the control group	BP	GO:0034377	plasma lipoprotein particle assembly	2/99	0.0025587	0.00994001	0.00489412	Apoe/Lcat
28-day post-SCI group vs. the control group	BP	GO:0045649	regulation of macrophage differentiation	2/99	0.0025587	0.00994001	0.00489412	Rb1/Ripk1
28-day post-SCI group vs. the control group	BP	GO:0072578	neurotransmitter-gated ion channel clustering	2/99	0.0025587	0.00994001	0.00489412	Apoe/Slc7a11
28-day post-SCI group vs. the control group	BP	GO:0150104	transport across blood-brain barrier	2/99	0.0025587	0.00994001	0.00489412	Apoe/Cd36
28-day post-SCI group vs. the control group	BP	GO:1901387	positive regulation of voltage-gated calcium channel ac	2/99	0.0025587	0.00994001	0.00489412	Ppp3ca/S100a1

28-day post-SCI group vs. the control group	BP	GO:1902992	negative regulation of amyloid precursor protein catab	2/99	0.0025587	0.00994001	0.00489412	Apoe/Clu
28-day post-SCI group vs. the control group	BP	GO:1903672	positive regulation of sprouting angiogenesis	2/99	0.0025587	0.00994001	0.00489412	S100a1/Smad1
28-day post-SCI group vs. the control group	BP	GO:1905153	regulation of membrane invagination	2/99	0.0025587	0.00994001	0.00489412	Cd36/Trem2
28-day post-SCI group vs. the control group	BP	GO:0009141	nucleoside triphosphate metabolic process	5/99	0.0025827	0.0100215	0.00493424	Hif1a/Ier3/Ndufa6/Parp1/Trem2
28-day post-SCI group vs. the control group	BP	GO:2000242	negative regulation of reproductive process	3/99	0.0026355	0.01021394	0.00502899	Apc/Il1a/Prdx4
28-day post-SCI group vs. the control group	BP	GO:0050768	negative regulation of neurogenesis	4/99	0.0026868	0.01040053	0.00512086	Id1/Ppp3ca/Rb1/Trem2
28-day post-SCI group vs. the control group	BP	GO:0007568	aging	3/99	0.0027301	0.01053078	0.00518499	Apoe/Eif2s1/Mapk1
28-day post-SCI group vs. the control group	BP	GO:0033209	tumor necrosis factor-mediated signaling pathway	3/99	0.0027301	0.01053078	0.00518499	Rela/Ripk1/Tnfrsf1a
28-day post-SCI group vs. the control group	BP	GO:0045123	cellular extravasation	3/99	0.0027301	0.01053078	0.00518499	Pawr/Sirpa/Vcam1
28-day post-SCI group vs. the control group	BP	GO:0006457	protein folding	4/99	0.0027448	0.01057503	0.00520678	Clu/Fkbp1b/Hspb1/Prdx4
28-day post-SCI group vs. the control group	BP	GO:0010232	vascular transport	2/99	0.0027962	0.01069732	0.00526699	Apoe/Cd36
28-day post-SCI group vs. the control group	BP	GO:0010880	regulation of release of sequestered calcium ion into cy	2/99	0.0027962	0.01069732	0.00526699	Fkbp1b/Slc8a1
28-day post-SCI group vs. the control group	BP	GO:0043501	skeletal muscle adaptation	2/99	0.0027962	0.01069732	0.00526699	Atp2a2/Ppp3ca
28-day post-SCI group vs. the control group	BP	GO:0060456	positive regulation of digestive system process	2/99	0.0027962	0.01069732	0.00526699	Pawr/Ppp3ca
28-day post-SCI group vs. the control group	BP	GO:1903579	negative regulation of ATP metabolic process	2/99	0.0027962	0.01069732	0.00526699	Ier3/Parp1
28-day post-SCI group vs. the control group	BP	GO:2001026	regulation of endothelial cell chemotaxis	2/99	0.0027962	0.01069732	0.00526699	Hspb1/Met
28-day post-SCI group vs. the control group	BP	GO:0009267	cellular response to starvation	4/99	0.0028634	0.01090349	0.0053685	Eif2s1/Jun/Mapk1/Nfe2l2
28-day post-SCI group vs. the control group	BP	GO:0051250	negative regulation of lymphocyte activation	4/99	0.0028634	0.01090349	0.0053685	Axl/Hspb1/Pawr/Tnfaip3
28-day post-SCI group vs. the control group	BP	GO:0055067	monovalent inorganic cation homeostasis	4/99	0.0028634	0.01090349	0.0053685	Il1a/Mapk1/Pdk2/Slc8a1
28-day post-SCI group vs. the control group	BP	GO:0071466	cellular response to xenobiotic stimulus	4/99	0.0028634	0.01090349	0.0053685	Cyp1b1/Mylk/Nfe2l2/Rb1
28-day post-SCI group vs. the control group	BP	GO:0046661	male sex differentiation	4/99	0.002924	0.01110835	0.00546936	Dhcr24/Il1a/Plekha1/Prdx4
28-day post-SCI group vs. the control group	BP	GO:0051961	negative regulation of nervous system development	4/99	0.002924	0.01110835	0.00546936	Id1/Ppp3ca/Rb1/Trem2
28-day post-SCI group vs. the control group	BP	GO:0010506	regulation of autophagy	5/99	0.0030103	0.01142303	0.0056243	Fbxw7/Hif1a/Mcl1/Tnfaip3/Trem2
28-day post-SCI group vs. the control group	BP	GO:0006691	leukotriene metabolic process	2/99	0.0030436	0.01144311	0.00563419	Ncf1/Tlr4
28-day post-SCI group vs. the control group	BP	GO:0010878	cholesterol storage	2/99	0.0030436	0.01144311	0.00563419	Cd36/Trem2
28-day post-SCI group vs. the control group	BP	GO:0034383	low-density lipoprotein particle clearance	2/99	0.0030436	0.01144311	0.00563419	Cd36/Trem2
28-day post-SCI group vs. the control group	BP	GO:0043369	CD4-positive or CD8-positive, alpha-beta T cell lineag	2/99	0.0030436	0.01144311	0.00563419	Ctsl/Stat6
28-day post-SCI group vs. the control group	BP	GO:0050961	detection of temperature stimulus involved in sensory	2/99	0.0030436	0.01144311	0.00563419	Pawr/Tlr4
28-day post-SCI group vs. the control group	BP	GO:0051481	negative regulation of cytosolic calcium ion concentra	2/99	0.0030436	0.01144311	0.00563419	Il6st/Slc8a1
28-day post-SCI group vs. the control group	BP	GO:1900016	negative regulation of cytokine production involved in	2/99	0.0030436	0.01144311	0.00563419	Sirpa/Trem2
28-day post-SCI group vs. the control group	BP	GO:1903428	positive regulation of reactive oxygen species biosynth	2/99	0.0030436	0.01144311	0.00563419	Cd36/Tlr4
28-day post-SCI group vs. the control group	BP	GO:0001936	regulation of endothelial cell proliferation	4/99	0.0030478	0.01144574	0.00563548	Apc/Apoe/Jun/Xdh
28-day post-SCI group vs. the control group	BP	GO:0010970	transport along microtubule	4/99	0.003111	0.01166977	0.00574579	Dst/Hif1a/Hspb1/Stau2
28-day post-SCI group vs. the control group	BP	GO:0002720	positive regulation of cytokine production involved in	3/99	0.0031295	0.01171227	0.00576671	Cd36/Mapkapk2/Tlr4
28-day post-SCI group vs. the control group	BP	GO:0014855	striated muscle cell proliferation	3/99	0.0031295	0.01171227	0.00576671	Apc/Mapk1/Smad1
28-day post-SCI group vs. the control group	BP	GO:0014015	positive regulation of gliogenesis	3/99	0.0032346	0.01206436	0.00594007	Hdac1/Il6st/Rela
28-day post-SCI group vs. the control group	BP	GO:0019229	regulation of vasoconstriction	3/99	0.0032346	0.01206436	0.00594007	Cd38/Hif1a/Ptgs1
28-day post-SCI group vs. the control group	BP	GO:0036465	synaptic vesicle recycling	3/99	0.0032346	0.01206436	0.00594007	Actb/Amph/Syp
28-day post-SCI group vs. the control group	BP	GO:0046942	carboxylic acid transport	5/99	0.0032422	0.01207736	0.00594647	Cd36/Il1a/Map2k6/Prkcd/Slc7a11
28-day post-SCI group vs. the control group	BP	GO:0007263	nitric oxide mediated signal transduction	2/99	0.0033009	0.01207736	0.00594647	Apoe/Cd36

28-day post-SCI group vs. the control group	BP	GO:0010763	positive regulation of fibroblast migration	2/99	0.0033009	0.01207736	0.00594647	Apc/Slc8a1
28-day post-SCI group vs. the control group	BP	GO:0014808	release of sequestered calcium ion into cytosol by sarc	2/99	0.0033009	0.01207736	0.00594647	Fkbp1b/Slc8a1
28-day post-SCI group vs. the control group	BP	GO:0015732	prostaglandin transport	2/99	0.0033009	0.01207736	0.00594647	Il1a/Map2k6
28-day post-SCI group vs. the control group	BP	GO:0030194	positive regulation of blood coagulation	2/99	0.0033009	0.01207736	0.00594647	Cd36/Nfe2l2
28-day post-SCI group vs. the control group	BP	GO:0032303	regulation of icosanoid secretion	2/99	0.0033009	0.01207736	0.00594647	Il1a/Map2k6
28-day post-SCI group vs. the control group	BP	GO:0070841	inclusion body assembly	2/99	0.0033009	0.01207736	0.00594647	Apoe/Clu
28-day post-SCI group vs. the control group	BP	GO:0097062	dendritic spine maintenance	2/99	0.0033009	0.01207736	0.00594647	Apoe/Trem2
28-day post-SCI group vs. the control group	BP	GO:1900048	positive regulation of hemostasis	2/99	0.0033009	0.01207736	0.00594647	Cd36/Nfe2l2
28-day post-SCI group vs. the control group	BP	GO:1900543	negative regulation of purine nucleotide metabolic pro	2/99	0.0033009	0.01207736	0.00594647	Ier3/Parp1
28-day post-SCI group vs. the control group	BP	GO:1901032	negative regulation of response to reactive oxygen spe	2/99	0.0033009	0.01207736	0.00594647	Met/Nfe2l2
28-day post-SCI group vs. the control group	BP	GO:1903206	negative regulation of hydrogen peroxide-induced cell	2/99	0.0033009	0.01207736	0.00594647	Met/Nfe2l2
28-day post-SCI group vs. the control group	BP	GO:1903798	regulation of miRNA maturation	2/99	0.0033009	0.01207736	0.00594647	Ppp3ca/Ripk1
28-day post-SCI group vs. the control group	BP	GO:2000193	positive regulation of fatty acid transport	2/99	0.0033009	0.01207736	0.00594647	Il1a/Map2k6
28-day post-SCI group vs. the control group	BP	GO:2000679	positive regulation of transcription regulatory region D	2/99	0.0033009	0.01207736	0.00594647	Parp1/Rb1
28-day post-SCI group vs. the control group	BP	GO:2001169	regulation of ATP biosynthetic process	2/99	0.0033009	0.01207736	0.00594647	Parp1/Trem2
28-day post-SCI group vs. the control group	BP	GO:0002440	production of molecular mediator of immune response	6/99	0.0033249	0.0121515	0.00598298	Axl/Cd36/Mapkapk2/Stat6/Tlr4/Tnfaip3
28-day post-SCI group vs. the control group	BP	GO:0006874	cellular calcium ion homeostasis	5/99	0.0033385	0.01215949	0.00598691	Apoe/Atp2a2/Fkbp1b/Slc8a1/Trpm2
28-day post-SCI group vs. the control group	BP	GO:0006942	regulation of striated muscle contraction	3/99	0.0033419	0.01215949	0.00598691	Atp2a2/Fkbp1b/Slc8a1
28-day post-SCI group vs. the control group	BP	GO:0043648	dicarboxylic acid metabolic process	3/99	0.0033419	0.01215949	0.00598691	Apc/Prodh/Slc7a11
28-day post-SCI group vs. the control group	BP	GO:0061178	regulation of insulin secretion involved in cellular resp	3/99	0.0033419	0.01215949	0.00598691	Fkbp1b/Hif1a/Ucp2
28-day post-SCI group vs. the control group	BP	GO:0032412	regulation of ion transmembrane transporter activity	5/99	0.0033875	0.01231153	0.00606177	Fkbp1b/Ppp3ca/Ripk1/S100a1/Trem2
28-day post-SCI group vs. the control group	BP	GO:0001654	eye development	6/99	0.0034026	0.01235269	0.00608204	Apc/Cyp1b1/Hdac1/Hif1a/Jun/Stau2
28-day post-SCI group vs. the control group	BP	GO:1902905	positive regulation of supramolecular fiber organizatio	4/99	0.0034406	0.01247688	0.00614318	Apc/Id1/Met/Rb1
28-day post-SCI group vs. the control group	BP	GO:0030071	regulation of mitotic metaphase/anaphase transition	3/99	0.0034514	0.01248825	0.00614878	Actb/Apc/Rb1
28-day post-SCI group vs. the control group	BP	GO:0051881	regulation of mitochondrial membrane potential	3/99	0.0034514	0.01248825	0.00614878	Hebp2/Parp1/Ucp2
28-day post-SCI group vs. the control group	BP	GO:0150063	visual system development	6/99	0.0035217	0.01272855	0.00626709	Apc/Cyp1b1/Hdac1/Hif1a/Jun/Stau2
28-day post-SCI group vs. the control group	BP	GO:0010827	regulation of glucose transmembrane transport	3/99	0.003563	0.01279709	0.00630084	Met/Nfe2l2/Prkcd
28-day post-SCI group vs. the control group	BP	GO:0035418	protein localization to synapse	3/99	0.003563	0.01279709	0.00630084	Hspb1/Mapk10/Stau2
28-day post-SCI group vs. the control group	BP	GO:0050848	regulation of calcium-mediated signaling	3/99	0.003563	0.01279709	0.00630084	Pdk2/Ppp3ca/Trem2
28-day post-SCI group vs. the control group	BP	GO:0006883	cellular sodium ion homeostasis	2/99	0.003568	0.01279709	0.00630084	Il1a/Slc8a1
28-day post-SCI group vs. the control group	BP	GO:0065005	protein-lipid complex assembly	2/99	0.003568	0.01279709	0.00630084	Apoe/Lcat
28-day post-SCI group vs. the control group	BP	GO:1903146	regulation of autophagy of mitochondrion	2/99	0.003568	0.01279709	0.00630084	Fbxw7/Hif1a
28-day post-SCI group vs. the control group	BP	GO:1903514	release of sequestered calcium ion into cytosol by endo	2/99	0.003568	0.01279709	0.00630084	Fkbp1b/Slc8a1
28-day post-SCI group vs. the control group	BP	GO:0007369	gastrulation	4/99	0.0036493	0.01307445	0.00643741	Ets2/Rnf2/Smad1/Tnfaip3
28-day post-SCI group vs. the control group	BP	GO:0019915	lipid storage	3/99	0.0036768	0.01315835	0.00647871	Apoe/Cd36/Trem2
28-day post-SCI group vs. the control group	BP	GO:0048880	sensory system development	6/99	0.0036852	0.01317425	0.00648654	Apc/Cyp1b1/Hdac1/Hif1a/Jun/Stau2
28-day post-SCI group vs. the control group	BP	GO:0071674	mononuclear cell migration	4/99	0.0037208	0.01328679	0.00654195	Aif1/Mapk1/Sirpa/Trpm2
28-day post-SCI group vs. the control group	BP	GO:0060070	canonical Wnt signaling pathway	5/99	0.0037444	0.01335662	0.00657634	Apc/Apoe/Gskip/Hdac1/Sdc1
28-day post-SCI group vs. the control group	BP	GO:0035023	regulation of Rho protein signal transduction	3/99	0.0037928	0.01345094	0.00662277	Apoe/Met/Net1
28-day post-SCI group vs. the control group	BP	GO:0046888	negative regulation of hormone secretion	3/99	0.0037928	0.01345094	0.00662277	Fkbp1b/Ppp3ca/Ucp2

28-day post-SCI group vs. the control group	BP	GO:0050766	positive regulation of phagocytosis	3/99	0.0037928	0.01345094	0.00662277	Cd36/Sirpa/Trem2
28-day post-SCI group vs. the control group	BP	GO:1902099	regulation of metaphase/anaphase transition of cell cyc	3/99	0.0037928	0.01345094	0.00662277	Actb/Apc/Rb1
28-day post-SCI group vs. the control group	BP	GO:0007264	small GTPase mediated signal transduction	6/99	0.0038116	0.01345094	0.00662277	Aif1/Apoe/Dhcr24/Jun/Met/Net1
28-day post-SCI group vs. the control group	BP	GO:1901987	regulation of cell cycle phase transition	6/99	0.0038116	0.01345094	0.00662277	Actb/Aif1/Apc/Cdkn2c/Ier3/Rb1
28-day post-SCI group vs. the control group	BP	GO:0003176	aortic valve development	2/99	0.0038449	0.01345094	0.00662277	Rb1/Tnfrsf1a
28-day post-SCI group vs. the control group	BP	GO:0014829	vascular associated smooth muscle contraction	2/99	0.0038449	0.01345094	0.00662277	Cd38/Slc8a1
28-day post-SCI group vs. the control group	BP	GO:0032042	mitochondrial DNA metabolic process	2/99	0.0038449	0.01345094	0.00662277	Mpv17/Parp1
28-day post-SCI group vs. the control group	BP	GO:0034368	protein-lipid complex remodeling	2/99	0.0038449	0.01345094	0.00662277	Apoe/Lcat
28-day post-SCI group vs. the control group	BP	GO:0034369	plasma lipoprotein particle remodeling	2/99	0.0038449	0.01345094	0.00662277	Apoe/Lcat
28-day post-SCI group vs. the control group	BP	GO:0035384	thioester biosynthetic process	2/99	0.0038449	0.01345094	0.00662277	Pdk1/Pdk2
28-day post-SCI group vs. the control group	BP	GO:0036003	positive regulation of transcription from RNA polymer	2/99	0.0038449	0.01345094	0.00662277	Hif1a/Nfe2l2
28-day post-SCI group vs. the control group	BP	GO:0045980	negative regulation of nucleotide metabolic process	2/99	0.0038449	0.01345094	0.00662277	Ier3/Parp1
28-day post-SCI group vs. the control group	BP	GO:0060575	intestinal epithelial cell differentiation	2/99	0.0038449	0.01345094	0.00662277	Hif1a/Il6st
28-day post-SCI group vs. the control group	BP	GO:0070920	regulation of production of small RNA involved in ger	2/99	0.0038449	0.01345094	0.00662277	Ppp3ca/Ripk1
28-day post-SCI group vs. the control group	BP	GO:0071616	acyl-CoA biosynthetic process	2/99	0.0038449	0.01345094	0.00662277	Pdk1/Pdk2
28-day post-SCI group vs. the control group	BP	GO:1904996	positive regulation of leukocyte adhesion to vascular e	2/99	0.0038449	0.01345094	0.00662277	Pawr/Rela
28-day post-SCI group vs. the control group	BP	GO:0048813	dendrite morphogenesis	4/99	0.0038665	0.01351222	0.00665295	Id1/Met/Ppp3ca/Stau2
28-day post-SCI group vs. the control group	BP	GO:0046700	heterocycle catabolic process	6/99	0.0038976	0.01360629	0.00669926	Ago1/Hmox2/Mapkapk2/Ncf1/Nfe2l2/Xdh
28-day post-SCI group vs. the control group	BP	GO:0007091	metaphase/anaphase transition of mitotic cell cycle	3/99	0.003911	0.01360943	0.00670081	Actb/Apc/Rb1
28-day post-SCI group vs. the control group	BP	GO:0032024	positive regulation of insulin secretion	3/99	0.003911	0.01360943	0.00670081	Cd38/Hif1a/Trpm2
28-day post-SCI group vs. the control group	BP	GO:2001259	positive regulation of cation channel activity	3/99	0.003911	0.01360943	0.00670081	Ppp3ca/S100a1/Trem2
28-day post-SCI group vs. the control group	BP	GO:0032388	positive regulation of intracellular transport	4/99	0.0039408	0.01368417	0.00673761	Fbxw7/Mapk1/Prkcd/Tnfrsf1a
28-day post-SCI group vs. the control group	BP	GO:0043271	negative regulation of ion transport	4/99	0.0039408	0.01368417	0.00673761	Fkbp1b/Pawr/Ppp3ca/Ptgs1
28-day post-SCI group vs. the control group	BP	GO:0045834	positive regulation of lipid metabolic process	4/99	0.0040161	0.01393074	0.00685901	Apoe/Cd36/Il1a/Prkcd
28-day post-SCI group vs. the control group	BP	GO:0010965	regulation of mitotic sister chromatid separation	3/99	0.0040314	0.01396909	0.00687789	Actb/Apc/Rb1
28-day post-SCI group vs. the control group	BP	GO:0050851	antigen receptor-mediated signaling pathway	5/99	0.0041276	0.01413597	0.00696006	Cd38/Mapk1/Pawr/Plekha1/Rela
28-day post-SCI group vs. the control group	BP	GO:0003299	muscle hypertrophy in response to stress	2/99	0.0041315	0.01413597	0.00696006	Atp2a2/Ppp3ca
28-day post-SCI group vs. the control group	BP	GO:0007176	regulation of epidermal growth factor-activated recepto	2/99	0.0041315	0.01413597	0.00696006	Fbxw7/Hbegf
28-day post-SCI group vs. the control group	BP	GO:0014898	cardiac muscle hypertrophy in response to stress	2/99	0.0041315	0.01413597	0.00696006	Atp2a2/Ppp3ca
28-day post-SCI group vs. the control group	BP	GO:0015874	norepinephrine transport	2/99	0.0041315	0.01413597	0.00696006	Actb/Ptgs1
28-day post-SCI group vs. the control group	BP	GO:0016048	detection of temperature stimulus	2/99	0.0041315	0.01413597	0.00696006	Pawr/Tlr4
28-day post-SCI group vs. the control group	BP	GO:0045822	negative regulation of heart contraction	2/99	0.0041315	0.01413597	0.00696006	Atp2a2/Fkbp1b
28-day post-SCI group vs. the control group	BP	GO:0046716	muscle cell cellular homeostasis	2/99	0.0041315	0.01413597	0.00696006	Apc/Hif1a
28-day post-SCI group vs. the control group	BP	GO:0050820	positive regulation of coagulation	2/99	0.0041315	0.01413597	0.00696006	Cd36/Nfe2l2
28-day post-SCI group vs. the control group	BP	GO:0061298	retina vasculature development in camera-type eye	2/99	0.0041315	0.01413597	0.00696006	Cyp1b1/Hif1a
28-day post-SCI group vs. the control group	BP	GO:0098581	detection of external biotic stimulus	2/99	0.0041315	0.01413597	0.00696006	Tlr4/Trem2
28-day post-SCI group vs. the control group	BP	GO:1900017	positive regulation of cytokine production involved in	2/99	0.0041315	0.01413597	0.00696006	Hif1a/Tlr4
28-day post-SCI group vs. the control group	BP	GO:0140014	mitotic nuclear division	5/99	0.0042421	0.01448837	0.00713357	Actb/Apc/Il1a/Met/Rb1
28-day post-SCI group vs. the control group	BP	GO:0001935	endothelial cell proliferation	4/99	0.0042477	0.01448837	0.00713357	Apc/Apoe/Jun/Xdh
28-day post-SCI group vs. the control group	BP	GO:0042594	response to starvation	4/99	0.0042477	0.01448837	0.00713357	Eif2s1/Jun/Mapk1/Nfe2l2

28-day post-SCI group vs. the control group	BP	GO:0044784	metaphase/anaphase transition of cell cycle	3/99	0.004279	0.01456477	0.00717118	Actb/Apc/Rb1
28-day post-SCI group vs. the control group	BP	GO:0051341	regulation of oxidoreductase activity	3/99	0.004279	0.01456477	0.00717118	Apoe/Il1a/S100a1
28-day post-SCI group vs. the control group	BP	GO:0002312	B cell activation involved in immune response	3/99	0.0044062	0.01491535	0.0073438	Stat6/Tlr4/Tnfaip3
28-day post-SCI group vs. the control group	BP	GO:0035304	regulation of protein dephosphorylation	3/99	0.0044062	0.01491535	0.0073438	Fkbp1b/Pawr/Prkcd
28-day post-SCI group vs. the control group	BP	GO:0051306	mitotic sister chromatid separation	3/99	0.0044062	0.01491535	0.0073438	Actb/Apc/Rb1
28-day post-SCI group vs. the control group	BP	GO:0030336	negative regulation of cell migration	5/99	0.004418	0.01491535	0.0073438	Aif1/Apoe/Cyp1b1/Hdac1/Nfe2l2
28-day post-SCI group vs. the control group	BP	GO:0001780	neutrophil homeostasis	2/99	0.0044276	0.01491535	0.0073438	Axl/Slc7a11
28-day post-SCI group vs. the control group	BP	GO:0014887	cardiac muscle adaptation	2/99	0.0044276	0.01491535	0.0073438	Atp2a2/Ppp3ca
28-day post-SCI group vs. the control group	BP	GO:0032770	positive regulation of monooxygenase activity	2/99	0.0044276	0.01491535	0.0073438	Apoe/S100a1
28-day post-SCI group vs. the control group	BP	GO:0034367	protein-containing complex remodeling	2/99	0.0044276	0.01491535	0.0073438	Apoe/Lcat
28-day post-SCI group vs. the control group	BP	GO:0051450	myoblast proliferation	2/99	0.0044276	0.01491535	0.0073438	Gpx1/Met
28-day post-SCI group vs. the control group	BP	GO:1900745	positive regulation of p38MAPK cascade	2/99	0.0044276	0.01491535	0.0073438	Met/Xdh
28-day post-SCI group vs. the control group	BP	GO:0050777	negative regulation of immune response	4/99	0.0044882	0.01510392	0.00743664	Gpx1/Stat6/Tnfaip3/Trem2
28-day post-SCI group vs. the control group	BP	GO:0035773	insulin secretion involved in cellular response to gluco	3/99	0.0045357	0.01524795	0.00750756	Fkbp1b/Hif1a/Ucp2
28-day post-SCI group vs. the control group	BP	GO:0016055	Wnt signaling pathway	6/99	0.0045894	0.01541259	0.00758862	Apc/Apoe/Gskip/Hdac1/Sdc1/Tnfaip3
28-day post-SCI group vs. the control group	BP	GO:0016358	dendrite development	5/99	0.0045991	0.01542932	0.00759686	Apoe/Id1/Met/Ppp3ca/Stau2
28-day post-SCI group vs. the control group	BP	GO:2001257	regulation of cation channel activity	4/99	0.0046536	0.01559612	0.00767899	Fkbp1b/Ppp3ca/S100a1/Trem2
28-day post-SCI group vs. the control group	BP	GO:0032091	negative regulation of protein binding	3/99	0.0046674	0.01562662	0.007694	Actb/Atp2a2/Id1
28-day post-SCI group vs. the control group	BP	GO:0198738	cell-cell signaling by wnt	6/99	0.004688	0.01567952	0.00772005	Apc/Apoe/Gskip/Hdac1/Sdc1/Tnfaip3
28-day post-SCI group vs. the control group	BP	GO:0007548	sex differentiation	5/99	0.0047227	0.01573462	0.00774718	Axl/Dhcr24/Il1a/Plekha1/Prdx4
28-day post-SCI group vs. the control group	BP	GO:0010800	positive regulation of peptidyl-threonine phosphorylati	2/99	0.0047334	0.01573462	0.00774718	Mapk1/Met
28-day post-SCI group vs. the control group	BP	GO:0032691	negative regulation of interleukin-1 beta production	2/99	0.0047334	0.01573462	0.00774718	Tnfaip3/Trem2
28-day post-SCI group vs. the control group	BP	GO:0043032	positive regulation of macrophage activation	2/99	0.0047334	0.01573462	0.00774718	Tlr4/Trem2
28-day post-SCI group vs. the control group	BP	GO:1902042	negative regulation of extrinsic apoptotic signaling pat	2/99	0.0047334	0.01573462	0.00774718	Gpx1/Tnfaip3
28-day post-SCI group vs. the control group	BP	GO:1903523	negative regulation of blood circulation	2/99	0.0047334	0.01573462	0.00774718	Atp2a2/Fkbp1b
28-day post-SCI group vs. the control group	BP	GO:1901019	regulation of calcium ion transmembrane transporter a	3/99	0.0048015	0.01594484	0.00785068	Fkbp1b/Ppp3ca/S100a1
28-day post-SCI group vs. the control group	BP	GO:0007259	receptor signaling pathway via JAK-STAT	4/99	0.004823	0.01599988	0.00787778	Cyp1b1/Il6st/Stat6/Tnfrsf1a
28-day post-SCI group vs. the control group	BP	GO:0030111	regulation of Wnt signaling pathway	5/99	0.0048486	0.01606881	0.00791172	Apc/Apoe/Gskip/Hdac1/Tnfaip3
28-day post-SCI group vs. the control group	BP	GO:0002699	positive regulation of immune effector process	5/99	0.0049125	0.01624755	0.00799973	Cd36/Mapkapk2/Stat6/Tlr4/Trem2
28-day post-SCI group vs. the control group	BP	GO:0072503	cellular divalent inorganic cation homeostasis	5/99	0.0049125	0.01624755	0.00799973	Apoe/Atp2a2/Fkbp1b/Slc8a1/Trpm2
28-day post-SCI group vs. the control group	BP	GO:0019439	aromatic compound catabolic process	6/99	0.0049415	0.016327	0.00803884	Ago1/Hmox2/Mapkapk2/Ncf1/Pon2/Xdh
28-day post-SCI group vs. the control group	BP	GO:0001558	regulation of cell growth	6/99	0.0049934	0.01648178	0.00811505	Apoe/Cd38/Cdkn2c/Hbegf/Mmp14/Rb1
28-day post-SCI group vs. the control group	BP	GO:0002026	regulation of the force of heart contraction	2/99	0.0050486	0.01658025	0.00816354	Atp2a2/Slc8a1
28-day post-SCI group vs. the control group	BP	GO:0003085	negative regulation of systemic arterial blood pressure	2/99	0.0050486	0.01658025	0.00816354	Cd36/Ier3
28-day post-SCI group vs. the control group	BP	GO:0031116	positive regulation of microtubule polymerization	2/99	0.0050486	0.01658025	0.00816354	Apc/Met
28-day post-SCI group vs. the control group	BP	GO:0035094	response to nicotine	2/99	0.0050486	0.01658025	0.00816354	Mapk1/Rela
28-day post-SCI group vs. the control group	BP	GO:0035767	endothelial cell chemotaxis	2/99	0.0050486	0.01658025	0.00816354	Hspb1/Met
28-day post-SCI group vs. the control group	BP	GO:0009798	axis specification	3/99	0.0050765	0.01663217	0.0081891	Apc/Ets2/Rnf2
28-day post-SCI group vs. the control group	BP	GO:0006911	phagocytosis, engulfment	4/99	0.0050847	0.01663217	0.0081891	Aif1/Cd36/Sirpa/Trem2
28-day post-SCI group vs. the control group	BP	GO:1903169	regulation of calcium ion transmembrane transport	4/99	0.0050847	0.01663217	0.0081891	Fkbp1b/Ppp3ca/S100a1/Slc8a1

28-day post-SCI group vs. the control group	BP	GO:1905475	regulation of protein localization to membrane	4/99	0.0050847	0.01663217	0.0081891	Actb/Mmp14/Slc7a11/Trem2
28-day post-SCI group vs. the control group	BP	GO:0045927	positive regulation of growth	5/99	0.005174	0.01687377	0.00830806	Apoe/Cd38/Hbegf/Mapk1/Mmp14
28-day post-SCI group vs. the control group	BP	GO:0055074	calcium ion homeostasis	5/99	0.005174	0.01687377	0.00830806	Apoe/Atp2a2/Fkbp1b/Slc8a1/Trpm2
28-day post-SCI group vs. the control group	BP	GO:0002695	negative regulation of leukocyte activation	4/99	0.0051741	0.01687377	0.00830806	Axl/Hspb1/Pawr/Tnfaip3
28-day post-SCI group vs. the control group	BP	GO:0033045	regulation of sister chromatid segregation	3/99	0.0052175	0.01698161	0.00836115	Actb/Apc/Rb1
28-day post-SCI group vs. the control group	BP	GO:0097061	dendritic spine organization	3/99	0.0052175	0.01698161	0.00836115	Apoe/Stau2/Trem2
28-day post-SCI group vs. the control group	BP	GO:2000146	negative regulation of cell motility	5/99	0.0052408	0.01704056	0.00839018	Aif1/Apoe/Cyp1b1/Hdac1/Nfe2l2
28-day post-SCI group vs. the control group	BP	GO:0071902	positive regulation of protein serine/threonine kinase a	4/99	0.0053559	0.01726502	0.00850069	Map2k4/Map2k6/Prkcd/Tlr4
28-day post-SCI group vs. the control group	BP	GO:0010660	regulation of muscle cell apoptotic process	3/99	0.0053609	0.01726502	0.00850069	Atp2a2/Map2k4/Nfe2l2
28-day post-SCI group vs. the control group	BP	GO:0062014	negative regulation of small molecule metabolic proces	3/99	0.0053609	0.01726502	0.00850069	Apoe/Ier3/Parp1
28-day post-SCI group vs. the control group	BP	GO:0001893	maternal placenta development	2/99	0.0053732	0.01726502	0.00850069	Ctsl/Mapk1
28-day post-SCI group vs. the control group	BP	GO:0002360	T cell lineage commitment	2/99	0.0053732	0.01726502	0.00850069	Ctsl/Stat6
28-day post-SCI group vs. the control group	BP	GO:0010875	positive regulation of cholesterol efflux	2/99	0.0053732	0.01726502	0.00850069	Apoe/Trem2
28-day post-SCI group vs. the control group	BP	GO:0045742	positive regulation of epidermal growth factor receptor	2/99	0.0053732	0.01726502	0.00850069	Fbxw7/Hbegf
28-day post-SCI group vs. the control group	BP	GO:0050951	sensory perception of temperature stimulus	2/99	0.0053732	0.01726502	0.00850069	Pawr/Tlr4
28-day post-SCI group vs. the control group	BP	GO:0060795	cell fate commitment involved in formation of primary	2/99	0.0053732	0.01726502	0.00850069	Ets2/Smad1
28-day post-SCI group vs. the control group	BP	GO:0072337	modified amino acid transport	2/99	0.0053732	0.01726502	0.00850069	Mgst1/Slc7a11
28-day post-SCI group vs. the control group	BP	GO:1901099	negative regulation of signal transduction in absence o	2/99	0.0053732	0.01726502	0.00850069	Mcl1/Ripk1
28-day post-SCI group vs. the control group	BP	GO:2001240	negative regulation of extrinsic apoptotic signaling pat	2/99	0.0053732	0.01726502	0.00850069	Mcl1/Ripk1
28-day post-SCI group vs. the control group	BP	GO:0097696	receptor signaling pathway via STAT	4/99	0.0054484	0.01748937	0.00861116	Cyp1b1/Il6st/Stat6/Tnfrsf1a
28-day post-SCI group vs. the control group	BP	GO:0055013	cardiac muscle cell development	3/99	0.0055066	0.01765881	0.00869458	Map2k4/Met/Slc8a1
28-day post-SCI group vs. the control group	BP	GO:0002262	myeloid cell homeostasis	4/99	0.0055419	0.01773747	0.00873331	Axl/Hif1a/Rb1/Slc7a11
28-day post-SCI group vs. the control group	BP	GO:0120032	regulation of plasma membrane bounded cell projectio	4/99	0.0055419	0.01773747	0.00873331	Apc/Prkcd/Stau2/Trpm2
28-day post-SCI group vs. the control group	BP	GO:0030705	cytoskeleton-dependent intracellular transport	4/99	0.0056366	0.0180227	0.00887375	Dst/Hif1a/Hspb1/Stau2
28-day post-SCI group vs. the control group	BP	GO:0062149	detection of stimulus involved in sensory perception of	2/99	0.0057072	0.01821291	0.0089674	Pawr/Tlr4
28-day post-SCI group vs. the control group	BP	GO:1905314	semi-lunar valve development	2/99	0.0057072	0.01821291	0.0089674	Rb1/Tnfrsf1a
28-day post-SCI group vs. the control group	BP	GO:0006754	ATP biosynthetic process	3/99	0.0058051	0.01850717	0.00911229	Ndufa6/Parp1/Trem2
28-day post-SCI group vs. the control group	BP	GO:0022408	negative regulation of cell-cell adhesion	4/99	0.005829	0.01854749	0.00913214	Hspb1/Pawr/Prkcd/Tnfaip3
28-day post-SCI group vs. the control group	BP	GO:0060491	regulation of cell projection assembly	4/99	0.005829	0.01854749	0.00913214	Apc/Prkcd/Stau2/Trpm2
28-day post-SCI group vs. the control group	BP	GO:0099024	plasma membrane invagination	4/99	0.0059269	0.01884056	0.00927643	Aif1/Cd36/Sirpa/Trem2
28-day post-SCI group vs. the control group	BP	GO:1905818	regulation of chromosome separation	3/99	0.0059579	0.0189206	0.00931584	Actb/Apc/Rb1
28-day post-SCI group vs. the control group	BP	GO:0030307	positive regulation of cell growth	4/99	0.0060259	0.01911796	0.00941302	Apoe/Cd38/Hbegf/Mmp14
28-day post-SCI group vs. the control group	BP	GO:1901186	positive regulation of ERBB signaling pathway	2/99	0.0060505	0.01917735	0.00944226	Fbxw7/Hbegf
28-day post-SCI group vs. the control group	BP	GO:0010657	muscle cell apoptotic process	3/99	0.0061131	0.0193384	0.00952155	Atp2a2/Map2k4/Nfe2l2
28-day post-SCI group vs. the control group	BP	GO:0032755	positive regulation of interleukin-6 production	3/99	0.0061131	0.0193384	0.00952155	Aif1/Cd36/Tlr4
28-day post-SCI group vs. the control group	BP	GO:2000113	negative regulation of cellular macromolecule biosynth	4/99	0.0061259	0.01936027	0.00953232	Ago1/Eif2s1/Hbegf/Rpl13a
28-day post-SCI group vs. the control group	BP	GO:0043255	regulation of carbohydrate biosynthetic process	3/99	0.0062707	0.0197987	0.00974819	Cd36/Hif1a/Pdk2
28-day post-SCI group vs. the control group	BP	GO:0006084	acetyl-CoA metabolic process	2/99	0.0064029	0.0200805	0.00988694	Pdk1/Pdk2
28-day post-SCI group vs. the control group	BP	GO:0009112	nucleobase metabolic process	2/99	0.0064029	0.0200805	0.00988694	Mapk1/Xdh
28-day post-SCI group vs. the control group	BP	GO:0014002	astrocyte development	2/99	0.0064029	0.0200805	0.00988694	Tlr4/Trem2

28-day post-SCI group vs. the control group	BP	GO:0031112	positive regulation of microtubule polymerization or de	2/99	0.0064029	0.0200805	0.00988694	Apc/Met
28-day post-SCI group vs. the control group	BP	GO:0045987	positive regulation of smooth muscle contraction	2/99	0.0064029	0.0200805	0.00988694	Pawr/Ptgs1
28-day post-SCI group vs. the control group	BP	GO:0046466	membrane lipid catabolic process	2/99	0.0064029	0.0200805	0.00988694	Cyp1b1/Prkcd
28-day post-SCI group vs. the control group	BP	GO:1904994	regulation of leukocyte adhesion to vascular endothelia	2/99	0.0064029	0.0200805	0.00988694	Pawr/Rela
28-day post-SCI group vs. the control group	BP	GO:0046889	positive regulation of lipid biosynthetic process	3/99	0.0064307	0.02009062	0.00989192	Apoe/Il1a/Prkcd
28-day post-SCI group vs. the control group	BP	GO:0055006	cardiac cell development	3/99	0.0064307	0.02009062	0.00989192	Map2k4/Met/Slc8a1
28-day post-SCI group vs. the control group	BP	GO:0071456	cellular response to hypoxia	3/99	0.0064307	0.02009062	0.00989192	Hif1a/Nfe2l2/Pdk1
28-day post-SCI group vs. the control group	BP	GO:1901800	positive regulation of proteasomal protein catabolic pro	3/99	0.0064307	0.02009062	0.00989192	Clu/Fbxw7/Trem2
28-day post-SCI group vs. the control group	BP	GO:0099111	microtubule-based transport	4/99	0.0065372	0.02040372	0.01004608	Dst/Hif1a/Hspb1/Stau2
28-day post-SCI group vs. the control group	BP	GO:0106027	neuron projection organization	3/99	0.0065932	0.02055884	0.01012246	Apoe/Stau2/Trem2
28-day post-SCI group vs. the control group	BP	GO:0006006	glucose metabolic process	4/99	0.0066428	0.02069384	0.01018893	Cd36/Hif1a/Pdk1/Pdk2
28-day post-SCI group vs. the control group	BP	GO:0010324	membrane invagination	4/99	0.0067495	0.02093362	0.01030698	Aif1/Cd36/Sirpa/Trem2
28-day post-SCI group vs. the control group	BP	GO:0035265	organ growth	4/99	0.0067495	0.02093362	0.01030698	Apc/Map2k4/Mapk1/Smad1
28-day post-SCI group vs. the control group	BP	GO:0071346	cellular response to interferon-gamma	3/99	0.006758	0.02093362	0.01030698	Rpl13a/Sirpa/Tlr4
28-day post-SCI group vs. the control group	BP	GO:0001702	gastrulation with mouth forming second	2/99	0.0067645	0.02093362	0.01030698	Ets2/Rnf2
28-day post-SCI group vs. the control group	BP	GO:0014912	negative regulation of smooth muscle cell migration	2/99	0.0067645	0.02093362	0.01030698	Aif1/Nfe2l2
28-day post-SCI group vs. the control group	BP	GO:0034122	negative regulation of toll-like receptor signaling pathw	2/99	0.0067645	0.02093362	0.01030698	Tnfaip3/Trem2
28-day post-SCI group vs. the control group	BP	GO:0048710	regulation of astrocyte differentiation	2/99	0.0067645	0.02093362	0.01030698	Il6st/Trem2
28-day post-SCI group vs. the control group	BP	GO:0000819	sister chromatid segregation	4/99	0.0068574	0.02120082	0.01043854	Actb/Apc/Fbxw7/Rb1
28-day post-SCI group vs. the control group	BP	GO:0030004	cellular monovalent inorganic cation homeostasis	3/99	0.0069253	0.02139074	0.01053206	Il1a/Mapk1/Slc8a1
28-day post-SCI group vs. the control group	BP	GO:0002429	immune response-activating cell surface receptor signa	5/99	0.0070359	0.02171186	0.01069016	Cd38/Mapk1/Pawr/Plekha1/Rela
28-day post-SCI group vs. the control group	BP	GO:0043393	regulation of protein binding	4/99	0.0070765	0.02175155	0.0107097	Actb/Apoe/Atp2a2/Id1
28-day post-SCI group vs. the control group	BP	GO:0045619	regulation of lymphocyte differentiation	4/99	0.0070765	0.02175155	0.0107097	Actb/Axl/Hspb1/Mmp14
28-day post-SCI group vs. the control group	BP	GO:1903076	regulation of protein localization to plasma membrane	3/99	0.0070951	0.02175155	0.0107097	Actb/Mmp14/Trem2
28-day post-SCI group vs. the control group	BP	GO:0000002	mitochondrial genome maintenance	2/99	0.0071353	0.02175155	0.0107097	Mpv17/Parp1
28-day post-SCI group vs. the control group	BP	GO:0016540	protein autoprocessing	2/99	0.0071353	0.02175155	0.0107097	Ctsl/Parp1
28-day post-SCI group vs. the control group	BP	GO:0019934	cGMP-mediated signaling	2/99	0.0071353	0.02175155	0.0107097	Apoe/Cd36
28-day post-SCI group vs. the control group	BP	GO:0033866	nucleoside bisphosphate biosynthetic process	2/99	0.0071353	0.02175155	0.0107097	Pdk1/Pdk2
28-day post-SCI group vs. the control group	BP	GO:0034030	ribonucleoside bisphosphate biosynthetic process	2/99	0.0071353	0.02175155	0.0107097	Pdk1/Pdk2
28-day post-SCI group vs. the control group	BP	GO:0034033	purine nucleoside bisphosphate biosynthetic process	2/99	0.0071353	0.02175155	0.0107097	Pdk1/Pdk2
28-day post-SCI group vs. the control group	BP	GO:0046677	response to antibiotic	2/99	0.0071353	0.02175155	0.0107097	Hif1a/Id1
28-day post-SCI group vs. the control group	BP	GO:0050901	leukocyte tethering or rolling	2/99	0.0071353	0.02175155	0.0107097	Pawr/Vcam1
28-day post-SCI group vs. the control group	BP	GO:0070542	response to fatty acid	2/99	0.0071353	0.02175155	0.0107097	Cd36/Tlr4
28-day post-SCI group vs. the control group	BP	GO:2000191	regulation of fatty acid transport	2/99	0.0071353	0.02175155	0.0107097	Il1a/Map2k6
28-day post-SCI group vs. the control group	BP	GO:0007623	circadian rhythm	4/99	0.0071878	0.02187096	0.0107685	Fbxw7/Hdac1/Id1/Mapk10
28-day post-SCI group vs. the control group	BP	GO:0050871	positive regulation of B cell activation	4/99	0.0071878	0.02187096	0.0107685	Cd38/Mmp14/Stat6/Tlr4
28-day post-SCI group vs. the control group	BP	GO:0018107	peptidyl-threonine phosphorylation	3/99	0.0072673	0.02209237	0.01087751	Mapk1/Met/Prkcd
28-day post-SCI group vs. the control group	BP	GO:0006986	response to unfolded protein	3/99	0.007442	0.02256048	0.011108	Eif2s1/Hspb1/Nfe2l2
28-day post-SCI group vs. the control group	BP	GO:0007589	body fluid secretion	3/99	0.007442	0.02256048	0.011108	Hif1a/Ppp3ca/Xdh
28-day post-SCI group vs. the control group	BP	GO:1904659	glucose transmembrane transport	3/99	0.007442	0.02256048	0.011108	Met/Nfe2l2/Prkcd

28-day post-SCI group vs. the control group	BP	GO:0009595	detection of biotic stimulus	2/99	0.007515	0.02269759	0.0111755	Tlr4/Trem2
28-day post-SCI group vs. the control group	BP	GO:0032692	negative regulation of interleukin-1 production	2/99	0.007515	0.02269759	0.0111755	Tnfaip3/Trem2
28-day post-SCI group vs. the control group	BP	GO:0050869	negative regulation of B cell activation	2/99	0.007515	0.02269759	0.0111755	Pawr/Tnfaip3
28-day post-SCI group vs. the control group	BP	GO:0071634	regulation of transforming growth factor beta producti	2/99	0.007515	0.02269759	0.0111755	Hif1a/Met
28-day post-SCI group vs. the control group	BP	GO:0008645	hexose transmembrane transport	3/99	0.0076191	0.02292753	0.01128872	Met/Nfe2l2/Prkcd
28-day post-SCI group vs. the control group	BP	GO:0009206	purine ribonucleoside triphosphate biosynthetic proces	3/99	0.0076191	0.02292753	0.01128872	Ndufa6/Parp1/Trem2
28-day post-SCI group vs. the control group	BP	GO:0038127	ERBB signaling pathway	3/99	0.0076191	0.02292753	0.01128872	Fbxw7/Hbegf/Mapk1
28-day post-SCI group vs. the control group	BP	GO:0044070	regulation of anion transport	3/99	0.0076191	0.02292753	0.01128872	Il1a/Map2k6/Ripk1
28-day post-SCI group vs. the control group	BP	GO:0009145	purine nucleoside triphosphate biosynthetic process	3/99	0.0077988	0.02344651	0.01154424	Ndufa6/Parp1/Trem2
28-day post-SCI group vs. the control group	BP	GO:0030595	leukocyte chemotaxis	4/99	0.0078799	0.02358838	0.0116141	Aif1/Mapk1/Rpl13a/Trpm2
28-day post-SCI group vs. the control group	BP	GO:0072507	divalent inorganic cation homeostasis	5/99	0.0078974	0.02358838	0.0116141	Apoe/Atp2a2/Fkbp1b/Slc8a1/Trpm2
28-day post-SCI group vs. the control group	BP	GO:0008210	estrogen metabolic process	2/99	0.0079037	0.02358838	0.0116141	Cyp1b1/Plekha1
28-day post-SCI group vs. the control group	BP	GO:0010661	positive regulation of muscle cell apoptotic process	2/99	0.0079037	0.02358838	0.0116141	Atp2a2/Map2k4
28-day post-SCI group vs. the control group	BP	GO:0018208	peptidyl-proline modification	2/99	0.0079037	0.02358838	0.0116141	Fkbp1b/Prdx4
28-day post-SCI group vs. the control group	BP	GO:0033238	regulation of cellular amine metabolic process	2/99	0.0079037	0.02358838	0.0116141	Apc/Slc7a11
28-day post-SCI group vs. the control group	BP	GO:0060292	long-term synaptic depression	2/99	0.0079037	0.02358838	0.0116141	Cd38/Stau2
28-day post-SCI group vs. the control group	BP	GO:1905168	positive regulation of double-strand break repair via hc	2/99	0.0079037	0.02358838	0.0116141	Actb/Parp1
28-day post-SCI group vs. the control group	BP	GO:0008637	apoptotic mitochondrial changes	3/99	0.0079809	0.0237515	0.01169441	Gpx1/Ier3/Jun
28-day post-SCI group vs. the control group	BP	GO:2000106	regulation of leukocyte apoptotic process	3/99	0.0079809	0.0237515	0.01169441	Axl/Hif1a/Slc7a11
28-day post-SCI group vs. the control group	BP	GO:0002768	immune response-regulating cell surface receptor signa	5/99	0.0079874	0.0237515	0.01169441	Cd38/Mapk1/Pawr/Plekha1/Rela
28-day post-SCI group vs. the control group	BP	GO:0040013	negative regulation of locomotion	5/99	0.0079874	0.0237515	0.01169441	Aif1/Apoe/Cyp1b1/Hdac1/Nfe2l2
28-day post-SCI group vs. the control group	BP	GO:0052548	regulation of endopeptidase activity	5/99	0.0080781	0.02399941	0.01181647	Dhcr24/Gpx1/Hdac1/Pawr/Xdh
28-day post-SCI group vs. the control group	BP	GO:0099504	synaptic vesicle cycle	4/99	0.0081199	0.02410194	0.01186696	Actb/Amph/Atp2a2/Syp
28-day post-SCI group vs. the control group	BP	GO:0002062	chondrocyte differentiation	3/99	0.0081655	0.0241714	0.01190115	Hif1a/Rb1/Rela
28-day post-SCI group vs. the control group	BP	GO:0015749	monosaccharide transmembrane transport	3/99	0.0081655	0.0241714	0.01190115	Met/Nfe2l2/Prkcd
28-day post-SCI group vs. the control group	BP	GO:0051209	release of sequestered calcium ion into cytosol	3/99	0.0081655	0.0241714	0.01190115	Fkbp1b/Slc8a1/Trpm2
28-day post-SCI group vs. the control group	BP	GO:0002861	regulation of inflammatory response to antigenic stimu	2/99	0.0083012	0.02444043	0.01203361	Gpx1/Trem2
28-day post-SCI group vs. the control group	BP	GO:0010803	regulation of tumor necrosis factor-mediated signaling	2/99	0.0083012	0.02444043	0.01203361	Ripk1/Tnfrsf1a
28-day post-SCI group vs. the control group	BP	GO:0042398	cellular modified amino acid biosynthetic process	2/99	0.0083012	0.02444043	0.01203361	Nfe2l2/Slc7a11
28-day post-SCI group vs. the control group	BP	GO:0071604	transforming growth factor beta production	2/99	0.0083012	0.02444043	0.01203361	Hif1a/Met
28-day post-SCI group vs. the control group	BP	GO:1901020	negative regulation of calcium ion transmembrane tran	2/99	0.0083012	0.02444043	0.01203361	Fkbp1b/Ppp3ca
28-day post-SCI group vs. the control group	BP	GO:1903670	regulation of sprouting angiogenesis	2/99	0.0083012	0.02444043	0.01203361	S100a1/Smad1
28-day post-SCI group vs. the control group	BP	GO:0051283	negative regulation of sequestering of calcium ion	3/99	0.0083526	0.02456967	0.01209725	Fkbp1b/Slc8a1/Trpm2
28-day post-SCI group vs. the control group	BP	GO:0051651	maintenance of location in cell	4/99	0.0083648	0.0245833	0.01210396	Apoe/Fkbp1b/Slc8a1/Trpm2
28-day post-SCI group vs. the control group	BP	GO:0009201	ribonucleoside triphosphate biosynthetic process	3/99	0.0085423	0.02505987	0.01233861	Ndufa6/Parp1/Trem2
28-day post-SCI group vs. the control group	BP	GO:0021782	glial cell development	3/99	0.0085423	0.02505987	0.01233861	Clu/Tlr4/Trem2
28-day post-SCI group vs. the control group	BP	GO:0007204	positive regulation of cytosolic calcium ion concentrat	4/99	0.0086144	0.02524891	0.01243168	Cd36/Cd38/Fkbp1b/Slc8a1
28-day post-SCI group vs. the control group	BP	GO:0008608	attachment of spindle microtubules to kinetochore	2/99	0.0087076	0.02536298	0.01248785	Apc/Rb1
28-day post-SCI group vs. the control group	BP	GO:0010613	positive regulation of cardiac muscle hypertrophy	2/99	0.0087076	0.02536298	0.01248785	Parp1/Ppp3ca
28-day post-SCI group vs. the control group	BP	GO:0010939	regulation of necrotic cell death	2/99	0.0087076	0.02536298	0.01248785	Hebp2/Ripk1

28-day post-SCI group vs. the control group	BP	GO:0032689	negative regulation of interferon-gamma production	2/99	0.0087076	0.02536298	0.01248785	Axl/Tlr4
28-day post-SCI group vs. the control group	BP	GO:0032733	positive regulation of interleukin-10 production	2/99	0.0087076	0.02536298	0.01248785	Tlr4/Trem2
28-day post-SCI group vs. the control group	BP	GO:0043368	positive T cell selection	2/99	0.0087076	0.02536298	0.01248785	Ctsl/Stat6
28-day post-SCI group vs. the control group	BP	GO:0071827	plasma lipoprotein particle organization	2/99	0.0087076	0.02536298	0.01248785	Apoe/Lcat
28-day post-SCI group vs. the control group	BP	GO:0018210	peptidyl-threonine modification	3/99	0.0087344	0.02537325	0.0124929	Mapk1/Met/Prkcd
28-day post-SCI group vs. the control group	BP	GO:0050764	regulation of phagocytosis	3/99	0.0087344	0.02537325	0.0124929	Cd36/Sirpa/Trem2
28-day post-SCI group vs. the control group	BP	GO:0051282	regulation of sequestering of calcium ion	3/99	0.0087344	0.02537325	0.0124929	Fkbp1b/Slc8a1/Trpm2
28-day post-SCI group vs. the control group	BP	GO:0031330	negative regulation of cellular catabolic process	4/99	0.0088689	0.02574104	0.01267399	Mapkapk2/Mcl1/Tnfaip3/Trem2
28-day post-SCI group vs. the control group	BP	GO:0015718	monocarboxylic acid transport	3/99	0.0089291	0.02589278	0.0127487	Cd36/Il1a/Map2k6
28-day post-SCI group vs. the control group	BP	GO:0014742	positive regulation of muscle hypertrophy	2/99	0.0091228	0.02634786	0.01297277	Parp1/Ppp3ca
28-day post-SCI group vs. the control group	BP	GO:0038084	vascular endothelial growth factor signaling pathway	2/99	0.0091228	0.02634786	0.01297277	Hspb1/Xdh
28-day post-SCI group vs. the control group	BP	GO:0002040	sprouting angiogenesis	3/99	0.0091263	0.02634786	0.01297277	Fbxw7/S100a1/Smad1
28-day post-SCI group vs. the control group	BP	GO:0051208	sequestering of calcium ion	3/99	0.0091263	0.02634786	0.01297277	Fkbp1b/Slc8a1/Trpm2
28-day post-SCI group vs. the control group	BP	GO:0090277	positive regulation of peptide hormone secretion	3/99	0.0091263	0.02634786	0.01297277	Cd38/Hif1a/Trpm2
28-day post-SCI group vs. the control group	BP	GO:0042445	hormone metabolic process	4/99	0.0092597	0.02670954	0.01315085	Ctsl/Cyp1b1/Hif1a/Plekha1
28-day post-SCI group vs. the control group	BP	GO:0051983	regulation of chromosome segregation	3/99	0.009326	0.02685343	0.01322169	Actb/Apc/Rb1
28-day post-SCI group vs. the control group	BP	GO:1903052	positive regulation of proteolysis involved in protein c	3/99	0.009326	0.02685343	0.01322169	Clu/Fbxw7/Trem2
28-day post-SCI group vs. the control group	BP	GO:0048872	homeostasis of number of cells	5/99	0.0094243	0.02711237	0.01334919	Axl/Hif1a/Rb1/Slc7a11/Tnfaip3
28-day post-SCI group vs. the control group	BP	GO:0043010	camera-type eye development	5/99	0.009526	0.02727252	0.01342804	Apc/Cyp1b1/Hdac1/Hif1a/Jun
28-day post-SCI group vs. the control group	BP	GO:0042180	cellular ketone metabolic process	4/99	0.0095265	0.02727252	0.01342804	Apc/Pdk1/Pdk2/Slc7a11
28-day post-SCI group vs. the control group	BP	GO:0009308	amine metabolic process	3/99	0.0095283	0.02727252	0.01342804	Apc/Slc7a11/Vcam1
28-day post-SCI group vs. the control group	BP	GO:0034219	carbohydrate transmembrane transport	3/99	0.0095283	0.02727252	0.01342804	Met/Nfe2l2/Prkcd
28-day post-SCI group vs. the control group	BP	GO:0010874	regulation of cholesterol efflux	2/99	0.0095466	0.02727252	0.01342804	Apoe/Trem2
28-day post-SCI group vs. the control group	BP	GO:0035794	positive regulation of mitochondrial membrane permea	2/99	0.0095466	0.02727252	0.01342804	Hebp2/Ier3
28-day post-SCI group vs. the control group	BP	GO:0046627	negative regulation of insulin receptor signaling pathw	2/99	0.0095466	0.02727252	0.01342804	Prkcd/Rela
28-day post-SCI group vs. the control group	BP	GO:1902624	positive regulation of neutrophil migration	2/99	0.0095466	0.02727252	0.01342804	Il1a/Pawr
28-day post-SCI group vs. the control group	BP	GO:1990778	protein localization to cell periphery	5/99	0.0096284	0.02748225	0.0135313	Actb/Mapk10/Mmp14/Tnfrsf1a/Trem2
28-day post-SCI group vs. the control group	BP	GO:0048167	regulation of synaptic plasticity	5/99	0.0097317	0.02773279	0.01365466	Apoe/Cd38/Mapk1/Stau2/Syp
28-day post-SCI group vs. the control group	BP	GO:0048565	digestive tract development	3/99	0.0097332	0.02773279	0.01365466	Hif1a/Il6st/Rb1
28-day post-SCI group vs. the control group	BP	GO:2000116	regulation of cysteine-type endopeptidase activity	4/99	0.0097981	0.02789354	0.01373381	Dhcr24/Gpx1/Pawr/Xdh
28-day post-SCI group vs. the control group	BP	GO:0002793	positive regulation of peptide secretion	3/99	0.0099406	0.02827448	0.01392137	Cd38/Hif1a/Trpm2
28-day post-SCI group vs. the control group	BP	GO:0001782	B cell homeostasis	2/99	0.0099791	0.02828571	0.0139269	Hif1a/Tnfaip3
28-day post-SCI group vs. the control group	BP	GO:0046326	positive regulation of glucose import	2/99	0.0099791	0.02828571	0.0139269	Nfe2l2/Prkcd
28-day post-SCI group vs. the control group	BP	GO:0060964	regulation of miRNA-mediated gene silencing	2/99	0.0099791	0.02828571	0.0139269	Ppp3ca/Ripk1
28-day post-SCI group vs. the control group	BP	GO:0097028	dendritic cell differentiation	2/99	0.0099791	0.02828571	0.0139269	Trem2/Trpm2
28-day post-SCI group vs. the control group	BP	GO:0032660	regulation of interleukin-17 production	2/99	0.0104202	0.02933255	0.01444233	Parp1/Tlr4
28-day post-SCI group vs. the control group	BP	GO:0034198	cellular response to amino acid starvation	2/99	0.0104202	0.02933255	0.01444233	Eif2s1/Mapk1
28-day post-SCI group vs. the control group	BP	GO:0045940	positive regulation of steroid metabolic process	2/99	0.0104202	0.02933255	0.01444233	Apoe/Il1a
28-day post-SCI group vs. the control group	BP	GO:0050850	positive regulation of calcium-mediated signaling	2/99	0.0104202	0.02933255	0.01444233	Ppp3ca/Trem2
28-day post-SCI group vs. the control group	BP	GO:0051898	negative regulation of protein kinase B signaling	2/99	0.0104202	0.02933255	0.01444233	Plekha1/Xdh

28-day post-SCI group vs. the control group	BP	GO:0061098	positive regulation of protein tyrosine kinase activity	2/99	0.0104202	0.02933255	0.01444233	Fbxw7/Hbegf
28-day post-SCI group vs. the control group	BP	GO:0071825	protein-lipid complex subunit organization	2/99	0.0104202	0.02933255	0.01444233	Apoe/Lcat
28-day post-SCI group vs. the control group	BP	GO:2001239	regulation of extrinsic apoptotic signaling pathway in a	2/99	0.0104202	0.02933255	0.01444233	Mcl1/Ripk1
28-day post-SCI group vs. the control group	BP	GO:0045786	negative regulation of cell cycle	5/99	0.0104757	0.02946354	0.01450682	Apc/Cdkn2c/Ier3/Rb1/Tnfaip3
28-day post-SCI group vs. the control group	BP	GO:0009142	nucleoside triphosphate biosynthetic process	3/99	0.0105782	0.02970068	0.01462358	Ndufa6/Parp1/Trem2
28-day post-SCI group vs. the control group	BP	GO:0051304	chromosome separation	3/99	0.0105782	0.02970068	0.01462358	Actb/Apc/Rb1
28-day post-SCI group vs. the control group	BP	GO:0060828	regulation of canonical Wnt signaling pathway	4/99	0.0106432	0.02985767	0.01470088	Apc/Apoe/Gskip/Hdac1
28-day post-SCI group vs. the control group	BP	GO:0035195	miRNA-mediated gene silencing	3/99	0.0107959	0.03025996	0.01489895	Ago1/Ppp3ca/Ripk1
28-day post-SCI group vs. the control group	BP	GO:0030225	macrophage differentiation	2/99	0.0108698	0.03031115	0.01492415	Rb1/Ripk1
28-day post-SCI group vs. the control group	BP	GO:0032373	positive regulation of sterol transport	2/99	0.0108698	0.03031115	0.01492415	Apoe/Trem2
28-day post-SCI group vs. the control group	BP	GO:0032376	positive regulation of cholesterol transport	2/99	0.0108698	0.03031115	0.01492415	Apoe/Trem2
28-day post-SCI group vs. the control group	BP	GO:0034142	toll-like receptor 4 signaling pathway	2/99	0.0108698	0.03031115	0.01492415	Tlr4/Trem2
28-day post-SCI group vs. the control group	BP	GO:0060147	regulation of post-transcriptional gene silencing	2/99	0.0108698	0.03031115	0.01492415	Ppp3ca/Ripk1
28-day post-SCI group vs. the control group	BP	GO:1900368	regulation of post-transcriptional gene silencing by RN	2/99	0.0108698	0.03031115	0.01492415	Ppp3ca/Ripk1
28-day post-SCI group vs. the control group	BP	GO:0006470	protein dephosphorylation	4/99	0.0110829	0.03085301	0.01519094	Fkbp1b/Pawr/Ppp3ca/Prkcd
28-day post-SCI group vs. the control group	BP	GO:1903320	regulation of protein modification by small protein con	4/99	0.0110829	0.03085301	0.01519094	Fbxw7/Hif1a/Rela/Tnfaip3
28-day post-SCI group vs. the control group	BP	GO:0019318	hexose metabolic process	4/99	0.0112321	0.03115503	0.01533965	Cd36/Hif1a/Pdk1/Pdk2
28-day post-SCI group vs. the control group	BP	GO:0000018	regulation of DNA recombination	3/99	0.0112391	0.03115503	0.01533965	Actb/Parp1/Stat6
28-day post-SCI group vs. the control group	BP	GO:0032414	positive regulation of ion transmembrane transporter a	3/99	0.0112391	0.03115503	0.01533965	Ppp3ca/S100a1/Trem2
28-day post-SCI group vs. the control group	BP	GO:0036294	cellular response to decreased oxygen levels	3/99	0.0112391	0.03115503	0.01533965	Hif1a/Nfe2l2/Pdk1
28-day post-SCI group vs. the control group	BP	GO:0090090	negative regulation of canonical Wnt signaling pathwa	3/99	0.0112391	0.03115503	0.01533965	Apc/Apoe/Hdac1
28-day post-SCI group vs. the control group	BP	GO:0010559	regulation of glycoprotein biosynthetic process	2/99	0.0113278	0.03124213	0.01538253	Hbegf/Pawr
28-day post-SCI group vs. the control group	BP	GO:0010907	positive regulation of glucose metabolic process	2/99	0.0113278	0.03124213	0.01538253	Cd36/Hif1a
28-day post-SCI group vs. the control group	BP	GO:0030890	positive regulation of B cell proliferation	2/99	0.0113278	0.03124213	0.01538253	Cd38/Tlr4
28-day post-SCI group vs. the control group	BP	GO:0048246	macrophage chemotaxis	2/99	0.0113278	0.03124213	0.01538253	Mapk1/Rpl13a
28-day post-SCI group vs. the control group	BP	GO:0050974	detection of mechanical stimulus involved in sensory p	2/99	0.0113278	0.03124213	0.01538253	Pawr/Tlr4
28-day post-SCI group vs. the control group	BP	GO:0060135	maternal process involved in female pregnancy	2/99	0.0113278	0.03124213	0.01538253	Ctsl/Mapk1
28-day post-SCI group vs. the control group	BP	GO:0035303	regulation of dephosphorylation	3/99	0.0114645	0.03159263	0.01555511	Fkbp1b/Pawr/Prkcd
28-day post-SCI group vs. the control group	BP	GO:0006417	regulation of translation	5/99	0.0114886	0.03163224	0.01557461	Ago1/Cyp1b1/Eif2s1/Mapk1/Rpl13a
28-day post-SCI group vs. the control group	BP	GO:0050852	T cell receptor signaling pathway	3/99	0.0116926	0.03214898	0.01582904	Mapk1/Pawr/Rela
28-day post-SCI group vs. the control group	BP	GO:0001941	postsynaptic membrane organization	2/99	0.0117942	0.03214898	0.01582904	Apoe/Slc7a11
28-day post-SCI group vs. the control group	BP	GO:0002347	response to tumor cell	2/99	0.0117942	0.03214898	0.01582904	Rela/Txnip
28-day post-SCI group vs. the control group	BP	GO:0003382	epithelial cell morphogenesis	2/99	0.0117942	0.03214898	0.01582904	Id1/Met
28-day post-SCI group vs. the control group	BP	GO:0032620	interleukin-17 production	2/99	0.0117942	0.03214898	0.01582904	Parp1/Tlr4
28-day post-SCI group vs. the control group	BP	GO:0032873	negative regulation of stress-activated MAPK cascade	2/99	0.0117942	0.03214898	0.01582904	Sirpa/Trem2
28-day post-SCI group vs. the control group	BP	GO:0048741	skeletal muscle fiber development	2/99	0.0117942	0.03214898	0.01582904	Gpx1/Ppp3ca
28-day post-SCI group vs. the control group	BP	GO:0050856	regulation of T cell receptor signaling pathway	2/99	0.0117942	0.03214898	0.01582904	Pawr/Rela
28-day post-SCI group vs. the control group	BP	GO:0070303	negative regulation of stress-activated protein kinase si	2/99	0.0117942	0.03214898	0.01582904	Sirpa/Trem2
28-day post-SCI group vs. the control group	BP	GO:0072348	sulfur compound transport	2/99	0.0117942	0.03214898	0.01582904	Mgst1/Slc7a11
28-day post-SCI group vs. the control group	BP	GO:1900077	negative regulation of cellular response to insulin stim	2/99	0.0117942	0.03214898	0.01582904	Prkcd/Rela

28-day post-SCI group vs. the control group	BP	GO:1901385	regulation of voltage-gated calcium channel activity	2/99	0.0117942	0.03214898	0.01582904	Ppp3ca/S100a1
28-day post-SCI group vs. the control group	BP	GO:0034976	response to endoplasmic reticulum stress	4/99	0.0118415	0.03225116	0.01587935	Clu/Eif2s1/Jun/Nfe2l2
28-day post-SCI group vs. the control group	BP	GO:0060348	bone development	4/99	0.0121541	0.0330267	0.0162612	Fbxw7/Map2k6/Mmp14/Smad1
28-day post-SCI group vs. the control group	BP	GO:0007266	Rho protein signal transduction	3/99	0.0121566	0.0330267	0.0162612	Apoe/Met/Net1
28-day post-SCI group vs. the control group	BP	GO:0034341	response to interferon-gamma	3/99	0.0121566	0.0330267	0.0162612	Rpl13a/Sirpa/Tlr4
28-day post-SCI group vs. the control group	BP	GO:0006110	regulation of glycolytic process	2/99	0.0122689	0.0331119	0.01630315	Hif1a/Ier3
28-day post-SCI group vs. the control group	BP	GO:0010762	regulation of fibroblast migration	2/99	0.0122689	0.0331119	0.01630315	Apc/Slc8a1
28-day post-SCI group vs. the control group	BP	GO:0010799	regulation of peptidyl-threonine phosphorylation	2/99	0.0122689	0.0331119	0.01630315	Mapk1/Met
28-day post-SCI group vs. the control group	BP	GO:0035307	positive regulation of protein dephosphorylation	2/99	0.0122689	0.0331119	0.01630315	Pawr/Prkcd
28-day post-SCI group vs. the control group	BP	GO:0060966	regulation of gene silencing by RNA	2/99	0.0122689	0.0331119	0.01630315	Ppp3ca/Ripk1
28-day post-SCI group vs. the control group	BP	GO:0089718	amino acid import across plasma membrane	2/99	0.0122689	0.0331119	0.01630315	Prkcd/Slc7a11
28-day post-SCI group vs. the control group	BP	GO:1902459	positive regulation of stem cell population maintenanc	2/99	0.0122689	0.0331119	0.01630315	Actb/Hdac1
28-day post-SCI group vs. the control group	BP	GO:1990928	response to amino acid starvation	2/99	0.0122689	0.0331119	0.01630315	Eif2s1/Mapk1
28-day post-SCI group vs. the control group	BP	GO:0030278	regulation of ossification	3/99	0.0123925	0.03336281	0.01642668	Hif1a/Mapk1/Slc8a1
28-day post-SCI group vs. the control group	BP	GO:0045621	positive regulation of lymphocyte differentiation	3/99	0.0123925	0.03336281	0.01642668	Actb/Axl/Mmp14
28-day post-SCI group vs. the control group	BP	GO:1905039	carboxylic acid transmembrane transport	3/99	0.0123925	0.03336281	0.01642668	Cd36/Prkcd/Slc7a11
28-day post-SCI group vs. the control group	BP	GO:0045926	negative regulation of growth	4/99	0.0124719	0.03354894	0.01651833	Apc/Cdkn2c/Hif1a/Rb1
28-day post-SCI group vs. the control group	BP	GO:0008584	male gonad development	3/99	0.012631	0.03383788	0.01666059	Il1a/Plekha1/Prdx4
28-day post-SCI group vs. the control group	BP	GO:0035194	post-transcriptional gene silencing by RNA	3/99	0.012631	0.03383788	0.01666059	Ago1/Ppp3ca/Ripk1
28-day post-SCI group vs. the control group	BP	GO:0055123	digestive system development	3/99	0.012631	0.03383788	0.01666059	Hif1a/Il6st/Rb1
28-day post-SCI group vs. the control group	BP	GO:1903825	organic acid transmembrane transport	3/99	0.012631	0.03383788	0.01666059	Cd36/Prkcd/Slc7a11
28-day post-SCI group vs. the control group	BP	GO:1904375	regulation of protein localization to cell periphery	3/99	0.012631	0.03383788	0.01666059	Actb/Mmp14/Trem2
28-day post-SCI group vs. the control group	BP	GO:0030574	collagen catabolic process	2/99	0.0127519	0.03402243	0.01675146	Ctsl/Mmp14
28-day post-SCI group vs. the control group	BP	GO:0042088	T-helper 1 type immune response	2/99	0.0127519	0.03402243	0.01675146	Il18bp/Stat6
28-day post-SCI group vs. the control group	BP	GO:0048806	genitalia development	2/99	0.0127519	0.03402243	0.01675146	Axl/Dhcr24
28-day post-SCI group vs. the control group	BP	GO:1901021	positive regulation of calcium ion transmembrane trans	2/99	0.0127519	0.03402243	0.01675146	Ppp3ca/S100a1
28-day post-SCI group vs. the control group	BP	GO:1903018	regulation of glycoprotein metabolic process	2/99	0.0127519	0.03402243	0.01675146	Hbegf/Pawr
28-day post-SCI group vs. the control group	BP	GO:0046546	development of primary male sexual characteristics	3/99	0.0128722	0.0343153	0.01689566	Il1a/Plekha1/Prdx4
28-day post-SCI group vs. the control group	BP	GO:0050807	regulation of synapse organization	4/99	0.0129585	0.03451729	0.01699511	Apoe/Slc7a11/Stau2/Trem2
28-day post-SCI group vs. the control group	BP	GO:0006275	regulation of DNA replication	3/99	0.013116	0.03490831	0.01718763	Jun/Map2k4/Met
28-day post-SCI group vs. the control group	BP	GO:0014888	striated muscle adaptation	2/99	0.0132431	0.03513236	0.01729795	Atp2a2/Ppp3ca
28-day post-SCI group vs. the control group	BP	GO:0030195	negative regulation of blood coagulation	2/99	0.0132431	0.03513236	0.01729795	Apoe/Prkcd
28-day post-SCI group vs. the control group	BP	GO:0032309	icosanoid secretion	2/99	0.0132431	0.03513236	0.01729795	Il1a/Map2k6
28-day post-SCI group vs. the control group	BP	GO:1902041	regulation of extrinsic apoptotic signaling pathway via	2/99	0.0132431	0.03513236	0.01729795	Gpx1/Tnfaip3
28-day post-SCI group vs. the control group	BP	GO:0008203	cholesterol metabolic process	3/99	0.0136114	0.03605103	0.01775027	Apoe/Dhcr24/Lcat
28-day post-SCI group vs. the control group	BP	GO:0048144	fibroblast proliferation	3/99	0.0136114	0.03605103	0.01775027	Gpx1/Jun/Pawr
28-day post-SCI group vs. the control group	BP	GO:0005996	monosaccharide metabolic process	4/99	0.013626	0.03606033	0.01775485	Cd36/Hif1a/Pdk1/Pdk2
28-day post-SCI group vs. the control group	BP	GO:0019083	viral transcription	2/99	0.0137424	0.03616375	0.01780577	Hdac1/Jun
28-day post-SCI group vs. the control group	BP	GO:0060043	regulation of cardiac muscle cell proliferation	2/99	0.0137424	0.03616375	0.01780577	Apc/Mapk1
28-day post-SCI group vs. the control group	BP	GO:0072666	establishment of protein localization to vacuole	2/99	0.0137424	0.03616375	0.01780577	Clu/Tnfaip3

28-day post-SCI group vs. the control group	BP	GO:0090207	regulation of triglyceride metabolic process	2/99	0.0137424	0.03616375	0.01780577	Apoe/Cd36
28-day post-SCI group vs. the control group	BP	GO:1900047	negative regulation of hemostasis	2/99	0.0137424	0.03616375	0.01780577	Apoe/Prkcd
28-day post-SCI group vs. the control group	BP	GO:1903426	regulation of reactive oxygen species biosynthetic proc	2/99	0.0137424	0.03616375	0.01780577	Cd36/Tlr4
28-day post-SCI group vs. the control group	BP	GO:2001258	negative regulation of cation channel activity	2/99	0.0137424	0.03616375	0.01780577	Fkbp1b/Ppp3ca
28-day post-SCI group vs. the control group	BP	GO:0090068	positive regulation of cell cycle process	4/99	0.0137962	0.03627603	0.01786105	Aif1/Il1a/Met/Rb1
28-day post-SCI group vs. the control group	BP	GO:1904062	regulation of cation transmembrane transport	5/99	0.0139745	0.03671524	0.0180773	Fkbp1b/Ppp3ca/S100a1/Slc8a1/Trem2
28-day post-SCI group vs. the control group	BP	GO:0031103	axon regeneration	2/99	0.0142499	0.0373189	0.01837453	Fkbp1b/Jun
28-day post-SCI group vs. the control group	BP	GO:0034605	cellular response to heat	2/99	0.0142499	0.0373189	0.01837453	Eif2s1/Il1a
28-day post-SCI group vs. the control group	BP	GO:0045907	positive regulation of vasoconstriction	2/99	0.0142499	0.0373189	0.01837453	Cd38/Ptgs1
28-day post-SCI group vs. the control group	BP	GO:0050819	negative regulation of coagulation	2/99	0.0142499	0.0373189	0.01837453	Apoe/Prkcd
28-day post-SCI group vs. the control group	BP	GO:0050803	regulation of synapse structure or activity	4/99	0.014315	0.03745948	0.01844374	Apoe/Slc7a11/Stau2/Trem2
28-day post-SCI group vs. the control group	BP	GO:0016441	post-transcriptional gene silencing	3/99	0.0146341	0.03823341	0.0188248	Ago1/Ppp3ca/Ripk1
28-day post-SCI group vs. the control group	BP	GO:0043467	regulation of generation of precursor metabolites and e	3/99	0.0146341	0.03823341	0.0188248	Cd36/Hif1a/Ier3
28-day post-SCI group vs. the control group	BP	GO:0051258	protein polymerization	4/99	0.0146677	0.03829041	0.01885286	Aif1/Apc/Met/Prkcd
28-day post-SCI group vs. the control group	BP	GO:0002714	positive regulation of B cell mediated immunity	2/99	0.0147653	0.03836165	0.01888794	Stat6/Trem2
28-day post-SCI group vs. the control group	BP	GO:0002891	positive regulation of immunoglobulin mediated immu	2/99	0.0147653	0.03836165	0.01888794	Stat6/Trem2
28-day post-SCI group vs. the control group	BP	GO:0006692	prostanoid metabolic process	2/99	0.0147653	0.03836165	0.01888794	Ptgs1/Tnfrsf1a
28-day post-SCI group vs. the control group	BP	GO:0006693	prostaglandin metabolic process	2/99	0.0147653	0.03836165	0.01888794	Ptgs1/Tnfrsf1a
28-day post-SCI group vs. the control group	BP	GO:0010883	regulation of lipid storage	2/99	0.0147653	0.03836165	0.01888794	Cd36/Trem2
28-day post-SCI group vs. the control group	BP	GO:0014904	myotube cell development	2/99	0.0147653	0.03836165	0.01888794	Gpx1/Ppp3ca
28-day post-SCI group vs. the control group	BP	GO:0055007	cardiac muscle cell differentiation	3/99	0.0148964	0.03867161	0.01904055	Map2k4/Met/Slc8a1
28-day post-SCI group vs. the control group	BP	GO:0051928	positive regulation of calcium ion transport	3/99	0.0151614	0.03929707	0.01934851	Mylk/Ppp3ca/S100a1
28-day post-SCI group vs. the control group	BP	GO:0071453	cellular response to oxygen levels	3/99	0.0151614	0.03929707	0.01934851	Hif1a/Nfe2l2/Pdk1
28-day post-SCI group vs. the control group	BP	GO:0006111	regulation of gluconeogenesis	2/99	0.0152887	0.03931555	0.0193576	Hif1a/Pdk2
28-day post-SCI group vs. the control group	BP	GO:0007062	sister chromatid cohesion	2/99	0.0152887	0.03931555	0.0193576	Fbxw7/Rb1
28-day post-SCI group vs. the control group	BP	GO:0010823	negative regulation of mitochondrion organization	2/99	0.0152887	0.03931555	0.0193576	Gpx1/Ier3
28-day post-SCI group vs. the control group	BP	GO:0016574	histone ubiquitination	2/99	0.0152887	0.03931555	0.0193576	Phc1/Rnf2
28-day post-SCI group vs. the control group	BP	GO:0031113	regulation of microtubule polymerization	2/99	0.0152887	0.03931555	0.0193576	Apc/Met
28-day post-SCI group vs. the control group	BP	GO:0032892	positive regulation of organic acid transport	2/99	0.0152887	0.03931555	0.0193576	Il1a/Map2k6
28-day post-SCI group vs. the control group	BP	GO:0042531	positive regulation of tyrosine phosphorylation of STA	2/99	0.0152887	0.03931555	0.0193576	Il6st/Tnfrsf1a
28-day post-SCI group vs. the control group	BP	GO:0045933	positive regulation of muscle contraction	2/99	0.0152887	0.03931555	0.0193576	Pawr/Ptgs1
28-day post-SCI group vs. the control group	BP	GO:0070527	platelet aggregation	2/99	0.0152887	0.03931555	0.0193576	Prkcd/Slc7a11
28-day post-SCI group vs. the control group	BP	GO:1902622	regulation of neutrophil migration	2/99	0.0152887	0.03931555	0.0193576	Il1a/Pawr
28-day post-SCI group vs. the control group	BP	GO:0016125	sterol metabolic process	3/99	0.0159722	0.04104102	0.02020717	Apoe/Dhcr24/Lcat
28-day post-SCI group vs. the control group	BP	GO:0048863	stem cell differentiation	4/99	0.0161332	0.04142202	0.02039476	Hdac1/Hif1a/Mapk1/Nfe2l2
28-day post-SCI group vs. the control group	BP	GO:0031099	regeneration	3/99	0.0162479	0.04164268	0.0205034	Fkbp1b/Gpx1/Jun
28-day post-SCI group vs. the control group	BP	GO:0031331	positive regulation of cellular catabolic process	5/99	0.016354	0.04164268	0.0205034	Apoe/Clu/Fbxw7/Hif1a/Prkcd
28-day post-SCI group vs. the control group	BP	GO:0006695	cholesterol biosynthetic process	2/99	0.0163592	0.04164268	0.0205034	Apoe/Dhcr24
28-day post-SCI group vs. the control group	BP	GO:0031529	ruffle organization	2/99	0.0163592	0.04164268	0.0205034	Aif1/Plekha1
28-day post-SCI group vs. the control group	BP	GO:0031648	protein destabilization	2/99	0.0163592	0.04164268	0.0205034	Fbxw7/Id1

28-day post-SCI group vs. the control group	BP	GO:0034113	heterotypic cell-cell adhesion	2/99	0.0163592	0.04164268	0.0205034	Tnfaip3/Vcam1
28-day post-SCI group vs. the control group	BP	GO:0048538	thymus development	2/99	0.0163592	0.04164268	0.0205034	Apc/Mapk1
28-day post-SCI group vs. the control group	BP	GO:0051353	positive regulation of oxidoreductase activity	2/99	0.0163592	0.04164268	0.0205034	Apoe/S100a1
28-day post-SCI group vs. the control group	BP	GO:1902653	secondary alcohol biosynthetic process	2/99	0.0163592	0.04164268	0.0205034	Apoe/Dhcr24
28-day post-SCI group vs. the control group	BP	GO:1903053	regulation of extracellular matrix organization	2/99	0.0163592	0.04164268	0.0205034	Rb1/Tnfrsf1a
28-day post-SCI group vs. the control group	BP	GO:1903170	negative regulation of calcium ion transmembrane tran	2/99	0.0163592	0.04164268	0.0205034	Fkbp1b/Ppp3ca
28-day post-SCI group vs. the control group	BP	GO:0008643	carbohydrate transport	3/99	0.0165262	0.04200239	0.02068051	Met/Nfe2l2/Prkcd
28-day post-SCI group vs. the control group	BP	GO:1901136	carbohydrate derivative catabolic process	3/99	0.0165262	0.04200239	0.02068051	Ctsl/Prkcd/Xdh
28-day post-SCI group vs. the control group	BP	GO:0007018	microtubule-based movement	5/99	0.0168002	0.04250566	0.0209283	Dst/Hif1a/Hspb1/Met/Stau2
28-day post-SCI group vs. the control group	BP	GO:0022612	gland morphogenesis	3/99	0.0168071	0.04250566	0.0209283	Met/Stat6/Tnfaip3
28-day post-SCI group vs. the control group	BP	GO:0001894	tissue homeostasis	4/99	0.0168993	0.04250566	0.0209283	Cd38/Ptgs1/Rb1/Tlr4
28-day post-SCI group vs. the control group	BP	GO:0003170	heart valve development	2/99	0.0169061	0.04250566	0.0209283	Rb1/Tnfrsf1a
28-day post-SCI group vs. the control group	BP	GO:0009395	phospholipid catabolic process	2/99	0.0169061	0.04250566	0.0209283	Prdx6/Prkcd
28-day post-SCI group vs. the control group	BP	GO:0014014	negative regulation of gliogenesis	2/99	0.0169061	0.04250566	0.0209283	Rb1/Trem2
28-day post-SCI group vs. the control group	BP	GO:0042311	vasodilation	2/99	0.0169061	0.04250566	0.0209283	Apoe/Gpx1
28-day post-SCI group vs. the control group	BP	GO:0043030	regulation of macrophage activation	2/99	0.0169061	0.04250566	0.0209283	Tlr4/Trem2
28-day post-SCI group vs. the control group	BP	GO:0043666	regulation of phosphoprotein phosphatase activity	2/99	0.0169061	0.04250566	0.0209283	Fkbp1b/Pawr
28-day post-SCI group vs. the control group	BP	GO:0044058	regulation of digestive system process	2/99	0.0169061	0.04250566	0.0209283	Pawr/Ppp3ca
28-day post-SCI group vs. the control group	BP	GO:0045058	T cell selection	2/99	0.0169061	0.04250566	0.0209283	Ctsl/Stat6
28-day post-SCI group vs. the control group	BP	GO:0140353	lipid export from cell	2/99	0.0169061	0.04250566	0.0209283	Il1a/Map2k6
28-day post-SCI group vs. the control group	BP	GO:1904356	regulation of telomere maintenance via telomere lengtl	2/99	0.0169061	0.04250566	0.0209283	Mapk1/Parp1
28-day post-SCI group vs. the control group	BP	GO:1905953	negative regulation of lipid localization	2/99	0.0169061	0.04250566	0.0209283	Apoe/Trem2
28-day post-SCI group vs. the control group	BP	GO:1902652	secondary alcohol metabolic process	3/99	0.0170908	0.04293695	0.02114066	Apoe/Dhcr24/Lcat
28-day post-SCI group vs. the control group	BP	GO:0015695	organic cation transport	2/99	0.0174608	0.04373213	0.02153217	Actb/Ptgs1
28-day post-SCI group vs. the control group	BP	GO:0050775	positive regulation of dendrite morphogenesis	2/99	0.0174608	0.04373213	0.02153217	Met/Stau2
28-day post-SCI group vs. the control group	BP	GO:0070509	calcium ion import	2/99	0.0174608	0.04373213	0.02153217	Pawr/Slc8a1
28-day post-SCI group vs. the control group	BP	GO:1903793	positive regulation of anion transport	2/99	0.0174608	0.04373213	0.02153217	Il1a/Map2k6
28-day post-SCI group vs. the control group	BP	GO:0022411	cellular component disassembly	5/99	0.0175619	0.0439517	0.02164029	Apc/Atp2a2/Fbxw7/Hif1a/Trem2
28-day post-SCI group vs. the control group	BP	GO:0007613	memory	3/99	0.0176661	0.04417874	0.02175207	Apoe/Ptgs1/Trem2
28-day post-SCI group vs. the control group	BP	GO:0007009	plasma membrane organization	3/99	0.0179578	0.04469563	0.02200657	Atp2a2/Clu/Prkcd
28-day post-SCI group vs. the control group	BP	GO:0002066	columnar/cuboidal epithelial cell development	2/99	0.0180231	0.04469563	0.02200657	Hif1a/Il6st
28-day post-SCI group vs. the control group	BP	GO:0007595	lactation	2/99	0.0180231	0.04469563	0.02200657	Hif1a/Xdh
28-day post-SCI group vs. the control group	BP	GO:0009948	anterior/posterior axis specification	2/99	0.0180231	0.04469563	0.02200657	Ets2/Rnf2
28-day post-SCI group vs. the control group	BP	GO:0010665	regulation of cardiac muscle cell apoptotic process	2/99	0.0180231	0.04469563	0.02200657	Atp2a2/Nfe2l2
28-day post-SCI group vs. the control group	BP	GO:0030199	collagen fibril organization	2/99	0.0180231	0.04469563	0.02200657	Cyp1b1/Rb1
28-day post-SCI group vs. the control group	BP	GO:0043470	regulation of carbohydrate catabolic process	2/99	0.0180231	0.04469563	0.02200657	Hif1a/Ier3
28-day post-SCI group vs. the control group	BP	GO:0046902	regulation of mitochondrial membrane permeability	2/99	0.0180231	0.04469563	0.02200657	Hebp2/Ier3
28-day post-SCI group vs. the control group	BP	GO:0051930	regulation of sensory perception of pain	2/99	0.0180231	0.04469563	0.02200657	Il1a/Tlr4
28-day post-SCI group vs. the control group	BP	GO:0055078	sodium ion homeostasis	2/99	0.0180231	0.04469563	0.02200657	Il1a/Slc8a1
28-day post-SCI group vs. the control group	BP	GO:0071715	icosanoid transport	2/99	0.0180231	0.04469563	0.02200657	Il1a/Map2k6

28-day post-SCI group vs. the control group	BP	GO:0044270	cellular nitrogen compound catabolic process	5/99	0.0181875	0.04506905	0.02219043	Ago1/Hmox2/Mapkapk2/Ncf1/Xdh
28-day post-SCI group vs. the control group	BP	GO:0034101	erythrocyte homeostasis	3/99	0.0182521	0.04519513	0.02225251	Axl/Hif1a/Rb1
28-day post-SCI group vs. the control group	BP	GO:0052547	regulation of peptidase activity	5/99	0.0185057	0.04578833	0.02254458	Dhcr24/Gpx1/Hdac1/Pawr/Xdh
28-day post-SCI group vs. the control group	BP	GO:0035637	multicellular organismal signaling	3/99	0.0185492	0.04586125	0.02258048	Fkbp1b/Pawr/Slc8a1
28-day post-SCI group vs. the control group	BP	GO:0006446	regulation of translational initiation	2/99	0.018593	0.04586562	0.02258263	Eif2s1/Rpl13a
28-day post-SCI group vs. the control group	BP	GO:0043407	negative regulation of MAP kinase activity	2/99	0.018593	0.04586562	0.02258263	Apoe/Prkcd
28-day post-SCI group vs. the control group	BP	GO:0051931	regulation of sensory perception	2/99	0.018593	0.04586562	0.02258263	Il1a/Tlr4
28-day post-SCI group vs. the control group	BP	GO:0010676	positive regulation of cellular carbohydrate metabolic	2/99	0.0191705	0.04707717	0.02317916	Cd36/Hif1a
28-day post-SCI group vs. the control group	BP	GO:0031102	neuron projection regeneration	2/99	0.0191705	0.04707717	0.02317916	Fkbp1b/Jun
28-day post-SCI group vs. the control group	BP	GO:0031122	cytoplasmic microtubule organization	2/99	0.0191705	0.04707717	0.02317916	Apc/Dst
28-day post-SCI group vs. the control group	BP	GO:0032653	regulation of interleukin-10 production	2/99	0.0191705	0.04707717	0.02317916	Tlr4/Trem2
28-day post-SCI group vs. the control group	BP	GO:0050982	detection of mechanical stimulus	2/99	0.0191705	0.04707717	0.02317916	Pawr/Tlr4
28-day post-SCI group vs. the control group	BP	GO:0060711	labyrinthine layer development	2/99	0.0191705	0.04707717	0.02317916	Mapk1/Vcam1
28-day post-SCI group vs. the control group	BP	GO:0120254	olefinic compound metabolic process	3/99	0.0194565	0.04774356	0.02350726	Cyp1b1/Gpx1/Ptgs1
28-day post-SCI group vs. the control group	BP	GO:0010662	regulation of striated muscle cell apoptotic process	2/99	0.0197555	0.04829618	0.02377936	Atp2a2/Nfe2l2
28-day post-SCI group vs. the control group	BP	GO:0016126	sterol biosynthetic process	2/99	0.0197555	0.04829618	0.02377936	Apoe/Dhcr24
28-day post-SCI group vs. the control group	BP	GO:0042698	ovulation cycle	2/99	0.0197555	0.04829618	0.02377936	Axl/Plekha1
28-day post-SCI group vs. the control group	BP	GO:0060324	face development	2/99	0.0197555	0.04829618	0.02377936	Mapk1/Plekha1
28-day post-SCI group vs. the control group	BP	GO:0070098	chemokine-mediated signaling pathway	2/99	0.0197555	0.04829618	0.02377936	Hif1a/Trem2
28-day post-SCI group vs. the control group	BP	GO:0030178	negative regulation of Wnt signaling pathway	3/99	0.0200748	0.04904007	0.02414562	Apc/Apoe/Hdac1
28-day post-SCI group vs. the control group	BP	GO:0010659	cardiac muscle cell apoptotic process	2/99	0.0203479	0.04959622	0.02441945	Atp2a2/Nfe2l2
28-day post-SCI group vs. the control group	BP	GO:0033344	cholesterol efflux	2/99	0.0203479	0.04959622	0.02441945	Apoe/Trem2
28-day post-SCI group vs. the control group	BP	GO:0060999	positive regulation of dendritic spine development	2/99	0.0203479	0.04959622	0.02441945	Apoe/Stau2
28-day post-SCI group vs. the control group	BP	GO:0006694	steroid biosynthetic process	3/99	0.020388	0.04961987	0.02443109	Apoe/Dhcr24/Il1a
28-day post-SCI group vs. the control group	BP	GO:0046887	positive regulation of hormone secretion	3/99	0.020388	0.04961987	0.02443109	Cd38/Hif1a/Trpm2
28-day post-SCI group vs. the control group	BP	GO:0006910	phagocytosis, recognition	3/99	0.0207039	0.05035121	0.02479118	Cd36/Sirpa/Trem2
28-day post-SCI group vs. the control group	BP	GO:0031349	positive regulation of defense response	4/99	0.0208478	0.05066366	0.02494502	Ripk1/Tlr4/Tnfrsf1a/Trem2
28-day post-SCI group vs. the control group	BP	GO:0001836	release of cytochrome c from mitochondria	2/99	0.0209478	0.05079326	0.02500883	Gpx1/Jun
28-day post-SCI group vs. the control group	BP	GO:0035306	positive regulation of dephosphorylation	2/99	0.0209478	0.05079326	0.02500883	Pawr/Prkcd
28-day post-SCI group vs. the control group	BP	GO:0050854	regulation of antigen receptor-mediated signaling path	2/99	0.0209478	0.05079326	0.02500883	Pawr/Rela
28-day post-SCI group vs. the control group	BP	GO:0098813	nuclear chromosome segregation	4/99	0.02107	0.05105194	0.0251362	Actb/Apc/Fbxw7/Rb1
28-day post-SCI group vs. the control group	BP	GO:0062013	positive regulation of small molecule metabolic proces	3/99	0.0213437	0.05163856	0.02542503	Cd36/Hif1a/Trem2
28-day post-SCI group vs. the control group	BP	GO:2001022	positive regulation of response to DNA damage stimul	3/99	0.0213437	0.05163856	0.02542503	Actb/Parp1/Prkcd
28-day post-SCI group vs. the control group	BP	GO:0072659	protein localization to plasma membrane	4/99	0.0215188	0.05188083	0.02554431	Actb/Mmp14/Tnfrsf1a/Trem2
28-day post-SCI group vs. the control group	BP	GO:0009166	nucleotide catabolic process	2/99	0.0215549	0.05188083	0.02554431	Ncf1/Xdh
28-day post-SCI group vs. the control group	BP	GO:0015909	long-chain fatty acid transport	2/99	0.0215549	0.05188083	0.02554431	Apoe/Cd36
28-day post-SCI group vs. the control group	BP	GO:0032835	glomerulus development	2/99	0.0215549	0.05188083	0.02554431	Mpv17/Ppp3ca
28-day post-SCI group vs. the control group	BP	GO:0045776	negative regulation of blood pressure	2/99	0.0215549	0.05188083	0.02554431	Cd36/Ier3
28-day post-SCI group vs. the control group	BP	GO:0070231	T cell apoptotic process	2/99	0.0215549	0.05188083	0.02554431	Hif1a/Ripk1
28-day post-SCI group vs. the control group	BP	GO:0070265	necrotic cell death	2/99	0.0215549	0.05188083	0.02554431	Hebp2/Ripk1

28-day post-SCI group vs. the control group	BP	GO:0006091	generation of precursor metabolites and energy	5/99	0.0218924	0.05265439	0.02592519	Cd36/Hif1a/Ier3/Il6st/Ndufa6
28-day post-SCI group vs. the control group	BP	GO:0050954	sensory perception of mechanical stimulus	4/99	0.0219735	0.05281055	0.02600207	Gpx1/Mpv17/Pawr/Tlr4
28-day post-SCI group vs. the control group	BP	GO:0010658	striated muscle cell apoptotic process	2/99	0.0221693	0.05308621	0.0261378	Atp2a2/Nfe2l2
28-day post-SCI group vs. the control group	BP	GO:0032608	interferon-beta production	2/99	0.0221693	0.05308621	0.0261378	Sirpa/Tlr4
28-day post-SCI group vs. the control group	BP	GO:0032648	regulation of interferon-beta production	2/99	0.0221693	0.05308621	0.0261378	Sirpa/Tlr4
28-day post-SCI group vs. the control group	BP	GO:0032663	regulation of interleukin-2 production	2/99	0.0221693	0.05308621	0.0261378	Il1a/Tnfaip3
28-day post-SCI group vs. the control group	BP	GO:0048008	platelet-derived growth factor receptor signaling pathw	2/99	0.0221693	0.05308621	0.0261378	Plekha1/Txnip
28-day post-SCI group vs. the control group	BP	GO:0030968	endoplasmic reticulum unfolded protein response	2/99	0.0227909	0.05449494	0.02683141	Eif2s1/Nfe2l2
28-day post-SCI group vs. the control group	BP	GO:0042440	pigment metabolic process	2/99	0.0227909	0.05449494	0.02683141	Hmox2/Slc7a11
28-day post-SCI group vs. the control group	BP	GO:0040029	epigenetic regulation of gene expression	3/99	0.0229905	0.05485196	0.02700719	Gpx1/Hdac1/Rb1
28-day post-SCI group vs. the control group	BP	GO:0051494	negative regulation of cytoskeleton organization	3/99	0.0229905	0.05485196	0.02700719	Apc/Met/Prkcd
28-day post-SCI group vs. the control group	BP	GO:0055088	lipid homeostasis	3/99	0.0229905	0.05485196	0.02700719	Apoe/Lcat/Trem2
28-day post-SCI group vs. the control group	BP	GO:0046324	regulation of glucose import	2/99	0.0234197	0.05579447	0.02747125	Nfe2l2/Prkcd
28-day post-SCI group vs. the control group	BP	GO:0072665	protein localization to vacuole	2/99	0.0234197	0.05579447	0.02747125	Clu/Tnfaip3
28-day post-SCI group vs. the control group	BP	GO:0001960	negative regulation of cytokine-mediated signaling pat	2/99	0.0240556	0.0571429	0.02813517	Il6st/Trem2
28-day post-SCI group vs. the control group	BP	GO:0042058	regulation of epidermal growth factor receptor signalir	2/99	0.0240556	0.0571429	0.02813517	Fbxw7/Hbegf
28-day post-SCI group vs. the control group	BP	GO:0042509	regulation of tyrosine phosphorylation of STAT protei	2/99	0.0240556	0.0571429	0.02813517	Il6st/Tnfrsf1a
28-day post-SCI group vs. the control group	BP	GO:1905710	positive regulation of membrane permeability	2/99	0.0240556	0.0571429	0.02813517	Hebp2/Ier3
28-day post-SCI group vs. the control group	BP	GO:0000280	nuclear division	5/99	0.0242973	0.05767515	0.02839723	Actb/Apc/Il1a/Met/Rb1
28-day post-SCI group vs. the control group	BP	GO:1901991	negative regulation of mitotic cell cycle phase transitic	3/99	0.0243565	0.05777384	0.02844582	Apc/Ier3/Rb1
28-day post-SCI group vs. the control group	BP	GO:0090596	sensory organ morphogenesis	4/99	0.024579	0.0582594	0.0286849	Hdac1/Hif1a/Mapk1/Stau2
28-day post-SCI group vs. the control group	BP	GO:0010569	regulation of double-strand break repair via homologoı	2/99	0.0246985	0.05843036	0.02876907	Actb/Parp1
28-day post-SCI group vs. the control group	BP	GO:0010761	fibroblast migration	2/99	0.0246985	0.05843036	0.02876907	Apc/Slc8a1
28-day post-SCI group vs. the control group	BP	GO:0050853	B cell receptor signaling pathway	3/99	0.0247047	0.05843036	0.02876907	Cd38/Mapk1/Plekha1
28-day post-SCI group vs. the control group	BP	GO:0060041	retina development in camera-type eye	3/99	0.0250557	0.05921752	0.02915664	Apc/Cyp1b1/Hif1a
28-day post-SCI group vs. the control group	BP	GO:0032371	regulation of sterol transport	2/99	0.0253484	0.05973682	0.02941232	Apoe/Trem2
28-day post-SCI group vs. the control group	BP	GO:0032374	regulation of cholesterol transport	2/99	0.0253484	0.05973682	0.02941232	Apoe/Trem2
28-day post-SCI group vs. the control group	BP	GO:0032922	circadian regulation of gene expression	2/99	0.0253484	0.05973682	0.02941232	Hdac1/Id1
28-day post-SCI group vs. the control group	BP	GO:2000134	negative regulation of G1/S transition of mitotic cell cy	2/99	0.0253484	0.05973682	0.02941232	Apc/Rb1
28-day post-SCI group vs. the control group	BP	GO:0007219	Notch signaling pathway	3/99	0.0257656	0.06063253	0.02985334	Fbxw7/Il6st/Mmp14
28-day post-SCI group vs. the control group	BP	GO:1901605	alpha-amino acid metabolic process	3/99	0.0257656	0.06063253	0.02985334	Apc/Prodh/Slc7a11
28-day post-SCI group vs. the control group	BP	GO:0002460	adaptive immune response based on somatic recombin	5/99	0.0258588	0.06080824	0.02993985	Il18bp/Prkcd/Stat6/Tnfaip3/Trem2
28-day post-SCI group vs. the control group	BP	GO:0008542	visual learning	2/99	0.0260053	0.06097728	0.03002308	Hif1a/Slc7a11
28-day post-SCI group vs. the control group	BP	GO:0019080	viral gene expression	2/99	0.0260053	0.06097728	0.03002308	Hdac1/Jun
28-day post-SCI group vs. the control group	BP	GO:1990868	response to chemokine	2/99	0.0260053	0.06097728	0.03002308	Hif1a/Trem2
28-day post-SCI group vs. the control group	BP	GO:1990869	cellular response to chemokine	2/99	0.0260053	0.06097728	0.03002308	Hif1a/Trem2
28-day post-SCI group vs. the control group	BP	GO:0002366	leukocyte activation involved in immune response	4/99	0.0263306	0.06169574	0.03037683	Stat6/Tlr4/Tnfaip3/Trem2
28-day post-SCI group vs. the control group	BP	GO:0010833	telomere maintenance via telomere lengthening	2/99	0.026669	0.06239944	0.03072331	Mapk1/Parp1
28-day post-SCI group vs. the control group	BP	GO:0060761	negative regulation of response to cytokine stimulus	2/99	0.026669	0.06239944	0.03072331	Il6st/Trem2
28-day post-SCI group vs. the control group	BP	GO:0030534	adult behavior	3/99	0.0272177	0.06363773	0.031333	Met/Parp1/Slc7a11

28-day post-SCI group vs. the control group	BP	GO:0007389	pattern specification process	5/99	0.027277	0.06364974	0.03133891	Apc/Bmp1/Ets2/Hif1a/Rnf2
28-day post-SCI group vs. the control group	BP	GO:0007260	tyrosine phosphorylation of STAT protein	2/99	0.0273396	0.06364974	0.03133891	Il6st/Tnfrsf1a
28-day post-SCI group vs. the control group	BP	GO:0043954	cellular component maintenance	2/99	0.0273396	0.06364974	0.03133891	Apoe/Trem2
28-day post-SCI group vs. the control group	BP	GO:1901184	regulation of ERBB signaling pathway	2/99	0.0273396	0.06364974	0.03133891	Fbxw7/Hbegf
28-day post-SCI group vs. the control group	BP	GO:1901292	nucleoside phosphate catabolic process	2/99	0.0273396	0.06364974	0.03133891	Ncf1/Xdh
28-day post-SCI group vs. the control group	BP	GO:2000036	regulation of stem cell population maintenance	2/99	0.0273396	0.06364974	0.03133891	Actb/Hdac1
28-day post-SCI group vs. the control group	BP	GO:0002263	cell activation involved in immune response	4/99	0.0273644	0.06366201	0.03134496	Stat6/Tlr4/Tnfaip3/Trem2
28-day post-SCI group vs. the control group	BP	GO:0000070	mitotic sister chromatid segregation	3/99	0.0275875	0.0641355	0.03157808	Actb/Apc/Rb1
28-day post-SCI group vs. the control group	BP	GO:0001822	kidney development	4/99	0.0278903	0.06476928	0.03189013	Apc/Mpv17/Ppp3ca/Smad1
28-day post-SCI group vs. the control group	BP	GO:0050808	synapse organization	5/99	0.0278997	0.06476928	0.03189013	Actb/Apoe/Slc7a11/Stau2/Trem2
28-day post-SCI group vs. the control group	BP	GO:0017148	negative regulation of translation	3/99	0.0279599	0.06481709	0.03191367	Ago1/Eif2s1/Rpl13a
28-day post-SCI group vs. the control group	BP	GO:1904064	positive regulation of cation transmembrane transport	3/99	0.0279599	0.06481709	0.03191367	Ppp3ca/S100a1/Trem2
28-day post-SCI group vs. the control group	BP	GO:0061045	negative regulation of wound healing	2/99	0.028017	0.06490335	0.03195615	Apoe/Prkcd
28-day post-SCI group vs. the control group	BP	GO:0015980	energy derivation by oxidation of organic compounds	4/99	0.0286904	0.06616009	0.03257492	Cd36/Hif1a/Il6st/Ndufa6
28-day post-SCI group vs. the control group	BP	GO:0002437	inflammatory response to antigenic stimulus	2/99	0.0287011	0.06616009	0.03257492	Gpx1/Trem2
28-day post-SCI group vs. the control group	BP	GO:0002712	regulation of B cell mediated immunity	2/99	0.0287011	0.06616009	0.03257492	Stat6/Trem2
28-day post-SCI group vs. the control group	BP	GO:0002889	regulation of immunoglobulin mediated immune respo	2/99	0.0287011	0.06616009	0.03257492	Stat6/Trem2
28-day post-SCI group vs. the control group	BP	GO:0007632	visual behavior	2/99	0.0287011	0.06616009	0.03257492	Hif1a/Slc7a11
28-day post-SCI group vs. the control group	BP	GO:0010822	positive regulation of mitochondrion organization	2/99	0.0287011	0.06616009	0.03257492	Hif1a/Trem2
28-day post-SCI group vs. the control group	BP	GO:0043113	receptor clustering	2/99	0.0287011	0.06616009	0.03257492	Apoe/Slc7a11
28-day post-SCI group vs. the control group	BP	GO:0016042	lipid catabolic process	4/99	0.0289602	0.06671038	0.03284586	Apoe/Cyp1b1/Prdx6/Prkcd
28-day post-SCI group vs. the control group	BP	GO:0031047	gene silencing by RNA	3/99	0.0290934	0.0669229	0.0329505	Ago1/Ppp3ca/Ripk1
28-day post-SCI group vs. the control group	BP	GO:0031644	regulation of nervous system process	3/99	0.0290934	0.0669229	0.0329505	Il1a/Pawr/Tlr4
28-day post-SCI group vs. the control group	BP	GO:0061097	regulation of protein tyrosine kinase activity	2/99	0.0293918	0.06756193	0.03326514	Fbxw7/Hbegf
28-day post-SCI group vs. the control group	BP	GO:0033143	regulation of intracellular steroid hormone receptor sig	2/99	0.0300892	0.06901945	0.03398277	Hdac1/Parp1
28-day post-SCI group vs. the control group	BP	GO:0048002	antigen processing and presentation of peptide antigen	2/99	0.0300892	0.06901945	0.03398277	Ctsl/Trem2
28-day post-SCI group vs. the control group	BP	GO:0071347	cellular response to interleukin-1	2/99	0.0300892	0.06901945	0.03398277	Rela/Sirpa
28-day post-SCI group vs. the control group	BP	GO:0051302	regulation of cell division	3/99	0.0306421	0.07023847	0.03458298	Apc/Il1a/Txnip
28-day post-SCI group vs. the control group	BP	GO:0005977	glycogen metabolic process	2/99	0.0307931	0.07038737	0.03465629	Cd36/Il6st
28-day post-SCI group vs. the control group	BP	GO:0006073	cellular glucan metabolic process	2/99	0.0307931	0.07038737	0.03465629	Cd36/Il6st
28-day post-SCI group vs. the control group	BP	GO:2000107	negative regulation of leukocyte apoptotic process	2/99	0.0307931	0.07038737	0.03465629	Axl/Hif1a
28-day post-SCI group vs. the control group	BP	GO:2000573	positive regulation of DNA biosynthetic process	2/99	0.0307931	0.07038737	0.03465629	Cyp1b1/Mapk1
28-day post-SCI group vs. the control group	BP	GO:0032088	negative regulation of NF-kappaB transcription factor	2/99	0.0315036	0.07181062	0.03535704	Cyp1b1/Tnfaip3
28-day post-SCI group vs. the control group	BP	GO:0044042	glucan metabolic process	2/99	0.0315036	0.07181062	0.03535704	Cd36/Il6st
28-day post-SCI group vs. the control group	BP	GO:0046626	regulation of insulin receptor signaling pathway	2/99	0.0315036	0.07181062	0.03535704	Prkcd/Rela
28-day post-SCI group vs. the control group	BP	GO:0048844	artery morphogenesis	2/99	0.0315036	0.07181062	0.03535704	Apoe/Mylk
28-day post-SCI group vs. the control group	BP	GO:0072001	renal system development	4/99	0.0317409	0.07230127	0.03559862	Apc/Mpv17/Ppp3ca/Smad1
28-day post-SCI group vs. the control group	BP	GO:0043279	response to alkaloid	2/99	0.0322205	0.07316869	0.03602571	Parp1/Slc8a1
28-day post-SCI group vs. the control group	BP	GO:0051279	regulation of release of sequestered calcium ion into cy	2/99	0.0322205	0.07316869	0.03602571	Fkbp1b/Slc8a1
28-day post-SCI group vs. the control group	BP	GO:1902807	negative regulation of cell cycle G1/S phase transition	2/99	0.0322205	0.07316869	0.03602571	Apc/Rb1

28-day post-SCI group vs. the control group	BP	GO:0051495	positive regulation of cytoskeleton organization	3/99	0.0322336	0.07316869	0.03602571	Apc/Id1/Met
28-day post-SCI group vs. the control group	BP	GO:0098656	anion transmembrane transport	3/99	0.0322336	0.07316869	0.03602571	Prkcd/Ripk1/Slc7a11
28-day post-SCI group vs. the control group	BP	GO:0016311	dephosphorylation	4/99	0.0326048	0.07331697	0.03609872	Fkbp1b/Pawr/Ppp3ca/Prkcd
28-day post-SCI group vs. the control group	BP	GO:0043087	regulation of GTPase activity	4/99	0.0326048	0.07331697	0.03609872	Amph/Eif2s1/Met/Net1
28-day post-SCI group vs. the control group	BP	GO:0046578	regulation of Ras protein signal transduction	3/99	0.0326381	0.07331697	0.03609872	Apoe/Met/Net1
28-day post-SCI group vs. the control group	BP	GO:0008625	extrinsic apoptotic signaling pathway via death domair	2/99	0.0329438	0.07331697	0.03609872	Gpx1/Tnfaip3
28-day post-SCI group vs. the control group	BP	GO:0032722	positive regulation of chemokine production	2/99	0.0329438	0.07331697	0.03609872	Aif1/Tlr4
28-day post-SCI group vs. the control group	BP	GO:0046785	microtubule polymerization	2/99	0.0329438	0.07331697	0.03609872	Apc/Met
28-day post-SCI group vs. the control group	BP	GO:0050810	regulation of steroid biosynthetic process	2/99	0.0329438	0.07331697	0.03609872	Apoe/Il1a
28-day post-SCI group vs. the control group	BP	GO:0055021	regulation of cardiac muscle tissue growth	2/99	0.0329438	0.07331697	0.03609872	Apc/Mapk1
28-day post-SCI group vs. the control group	BP	GO:0070373	negative regulation of ERK1 and ERK2 cascade	2/99	0.0329438	0.07331697	0.03609872	Sirpa/Tlr4
28-day post-SCI group vs. the control group	BP	GO:0016051	carbohydrate biosynthetic process	3/99	0.0334552	0.07331697	0.03609872	Cd36/Hif1a/Pdk2
28-day post-SCI group vs. the control group	BP	GO:0046890	regulation of lipid biosynthetic process	3/99	0.0334552	0.07331697	0.03609872	Apoe/Il1a/Prkcd
28-day post-SCI group vs. the control group	BP	GO:0000422	autophagy of mitochondrion	2/99	0.0336735	0.07331697	0.03609872	Fbxw7/Hif1a
28-day post-SCI group vs. the control group	BP	GO:0034109	homotypic cell-cell adhesion	2/99	0.0336735	0.07331697	0.03609872	Prkcd/Slc7a11
28-day post-SCI group vs. the control group	BP	GO:0043154	negative regulation of cysteine-type endopeptidase acti	2/99	0.0336735	0.07331697	0.03609872	Dhcr24/Gpx1
28-day post-SCI group vs. the control group	BP	GO:0045913	positive regulation of carbohydrate metabolic process	2/99	0.0336735	0.07331697	0.03609872	Cd36/Hif1a
28-day post-SCI group vs. the control group	BP	GO:0060998	regulation of dendritic spine development	2/99	0.0336735	0.07331697	0.03609872	Apoe/Stau2
28-day post-SCI group vs. the control group	BP	GO:0061726	mitochondrion disassembly	2/99	0.0336735	0.07331697	0.03609872	Fbxw7/Hif1a
28-day post-SCI group vs. the control group	BP	GO:1900076	regulation of cellular response to insulin stimulus	2/99	0.0336735	0.07331697	0.03609872	Prkcd/Rela
28-day post-SCI group vs. the control group	BP	GO:2000300	regulation of synaptic vesicle exocytosis	2/99	0.0336735	0.07331697	0.03609872	Atp2a2/Syp
28-day post-SCI group vs. the control group	BP	GO:0002315	marginal zone B cell differentiation	1/99	0.0336886	0.07331697	0.03609872	Tnfaip3
28-day post-SCI group vs. the control group	BP	GO:0002634	regulation of germinal center formation	1/99	0.0336886	0.07331697	0.03609872	Tnfaip3
28-day post-SCI group vs. the control group	BP	GO:0003330	regulation of extracellular matrix constituent secretion	1/99	0.0336886	0.07331697	0.03609872	Tnfrsf1a
28-day post-SCI group vs. the control group	BP	GO:0006196	AMP catabolic process	1/99	0.0336886	0.07331697	0.03609872	Xdh
28-day post-SCI group vs. the control group	BP	GO:0006560	proline metabolic process	1/99	0.0336886	0.07331697	0.03609872	Prodh
28-day post-SCI group vs. the control group	BP	GO:0006707	cholesterol catabolic process	1/99	0.0336886	0.07331697	0.03609872	Apoe
28-day post-SCI group vs. the control group	BP	GO:0006971	hypotonic response	1/99	0.0336886	0.07331697	0.03609872	Mylk
28-day post-SCI group vs. the control group	BP	GO:0008298	intracellular mRNA localization	1/99	0.0336886	0.07331697	0.03609872	Stau2
28-day post-SCI group vs. the control group	BP	GO:0009650	UV protection	1/99	0.0336886	0.07331697	0.03609872	Gpx1
28-day post-SCI group vs. the control group	BP	GO:0010501	RNA secondary structure unwinding	1/99	0.0336886	0.07331697	0.03609872	Ago1
28-day post-SCI group vs. the control group	BP	GO:0010873	positive regulation of cholesterol esterification	1/99	0.0336886	0.07331697	0.03609872	Apoe
28-day post-SCI group vs. the control group	BP	GO:0016127	sterol catabolic process	1/99	0.0336886	0.07331697	0.03609872	Apoe
28-day post-SCI group vs. the control group	BP	GO:0030450	regulation of complement activation, classical pathway	1/99	0.0336886	0.07331697	0.03609872	Trem2
28-day post-SCI group vs. the control group	BP	GO:0032836	glomerular basement membrane development	1/99	0.0336886	0.07331697	0.03609872	Mpv17
28-day post-SCI group vs. the control group	BP	GO:0035562	negative regulation of chromatin binding	1/99	0.0336886	0.07331697	0.03609872	Ppp3ca
28-day post-SCI group vs. the control group	BP	GO:0035871	protein K11-linked deubiquitination	1/99	0.0336886	0.07331697	0.03609872	Tnfaip3
28-day post-SCI group vs. the control group	BP	GO:0038130	ERBB4 signaling pathway	1/99	0.0336886	0.07331697	0.03609872	Hbegf
28-day post-SCI group vs. the control group	BP	GO:0046055	dGMP catabolic process	1/99	0.0336886	0.07331697	0.03609872	Xdh
28-day post-SCI group vs. the control group	BP	GO:0046130	purine ribonucleoside catabolic process	1/99	0.0336886	0.07331697	0.03609872	Xdh

28-day post-SCI group vs. the control group	BP	GO:0046877	regulation of saliva secretion	1/99	0.0336886	0.07331697	0.03609872	Ppp3ca
28-day post-SCI group vs. the control group	BP	GO:0060638	mesenchymal-epithelial cell signaling	1/99	0.0336886	0.07331697	0.03609872	Met
28-day post-SCI group vs. the control group	BP	GO:0060710	chorio-allantoic fusion	1/99	0.0336886	0.07331697	0.03609872	Vcam1
28-day post-SCI group vs. the control group	BP	GO:0061762	CAMKK-AMPK signaling cascade	1/99	0.0336886	0.07331697	0.03609872	Trem2
28-day post-SCI group vs. the control group	BP	GO:0070662	mast cell proliferation	1/99	0.0336886	0.07331697	0.03609872	Stat6
28-day post-SCI group vs. the control group	BP	GO:0070666	regulation of mast cell proliferation	1/99	0.0336886	0.07331697	0.03609872	Stat6
28-day post-SCI group vs. the control group	BP	GO:0070922	RISC complex assembly	1/99	0.0336886	0.07331697	0.03609872	Ago1
28-day post-SCI group vs. the control group	BP	GO:0070933	histone H4 deacetylation	1/99	0.0336886	0.07331697	0.03609872	Hdac1
28-day post-SCI group vs. the control group	BP	GO:0071257	cellular response to electrical stimulus	1/99	0.0336886	0.07331697	0.03609872	Actb
28-day post-SCI group vs. the control group	BP	GO:0071649	regulation of chemokine (C-C motif) ligand 5 producti	1/99	0.0336886	0.07331697	0.03609872	Sirpa
28-day post-SCI group vs. the control group	BP	GO:0071670	smooth muscle cell chemotaxis	1/99	0.0336886	0.07331697	0.03609872	Aif1
28-day post-SCI group vs. the control group	BP	GO:0086064	cell communication by electrical coupling involved in	1/99	0.0336886	0.07331697	0.03609872	Slc8a1
28-day post-SCI group vs. the control group	BP	GO:0090269	fibroblast growth factor production	1/99	0.0336886	0.07331697	0.03609872	Aif1
28-day post-SCI group vs. the control group	BP	GO:0090270	regulation of fibroblast growth factor production	1/99	0.0336886	0.07331697	0.03609872	Aif1
28-day post-SCI group vs. the control group	BP	GO:0098883	synapse pruning	1/99	0.0336886	0.07331697	0.03609872	Trem2
28-day post-SCI group vs. the control group	BP	GO:0099640	axo-dendritic protein transport	1/99	0.0336886	0.07331697	0.03609872	Hspb1
28-day post-SCI group vs. the control group	BP	GO:0106104	regulation of glutamate receptor clustering	1/99	0.0336886	0.07331697	0.03609872	Slc7a11
28-day post-SCI group vs. the control group	BP	GO:1901858	regulation of mitochondrial DNA metabolic process	1/99	0.0336886	0.07331697	0.03609872	Mpv17
28-day post-SCI group vs. the control group	BP	GO:1903242	regulation of cardiac muscle hypertrophy in response t	1/99	0.0336886	0.07331697	0.03609872	Ppp3ca
28-day post-SCI group vs. the control group	BP	GO:1903749	positive regulation of establishment of protein localiza	1/99	0.0336886	0.07331697	0.03609872	Fbxw7
28-day post-SCI group vs. the control group	BP	GO:1904717	regulation of AMPA glutamate receptor clustering	1/99	0.0336886	0.07331697	0.03609872	Slc7a11
28-day post-SCI group vs. the control group	BP	GO:1904779	regulation of protein localization to centrosome	1/99	0.0336886	0.07331697	0.03609872	Apc
28-day post-SCI group vs. the control group	BP	GO:2000389	regulation of neutrophil extravasation	1/99	0.0336886	0.07331697	0.03609872	Pawr
28-day post-SCI group vs. the control group	BP	GO:2000646	positive regulation of receptor catabolic process	1/99	0.0336886	0.07331697	0.03609872	Apoe
28-day post-SCI group vs. the control group	BP	GO:0009100	glycoprotein metabolic process	4/99	0.033778	0.07346269	0.03617047	Ctsl/Hbegf/Hif1a/Pawr
28-day post-SCI group vs. the control group	BP	GO:0032271	regulation of protein polymerization	3/99	0.0338677	0.07360879	0.0362424	Apc/Met/Prkcd
28-day post-SCI group vs. the control group	BP	GO:0032413	negative regulation of ion transmembrane transporter a	2/99	0.0344095	0.07463765	0.03674898	Fkbp1b/Ppp3ca
28-day post-SCI group vs. the control group	BP	GO:0034103	regulation of tissue remodeling	2/99	0.0344095	0.07463765	0.03674898	Cd38/Ppp3ca
28-day post-SCI group vs. the control group	BP	GO:0046323	glucose import	2/99	0.0344095	0.07463765	0.03674898	Nfe2l2/Prkcd
28-day post-SCI group vs. the control group	BP	GO:0045787	positive regulation of cell cycle	4/99	0.034674	0.07516137	0.03700684	Aif1/Il1a/Met/Rb1
28-day post-SCI group vs. the control group	BP	GO:0019827	stem cell population maintenance	3/99	0.0347007	0.07516947	0.03701082	Actb/Apc/Hdac1
28-day post-SCI group vs. the control group	BP	GO:0006066	alcohol metabolic process	4/99	0.0349757	0.07571502	0.03727944	Apoe/Cyp1b1/Dhcr24/Lcat
28-day post-SCI group vs. the control group	BP	GO:0003151	outflow tract morphogenesis	2/99	0.0351518	0.07594552	0.03739292	Hif1a/Jun
28-day post-SCI group vs. the control group	BP	GO:0006094	gluconeogenesis	2/99	0.0351518	0.07594552	0.03739292	Hif1a/Pdk2
28-day post-SCI group vs. the control group	BP	GO:0010921	regulation of phosphatase activity	2/99	0.0351518	0.07594552	0.03739292	Fkbp1b/Pawr
28-day post-SCI group vs. the control group	BP	GO:0002637	regulation of immunoglobulin production	2/99	0.0359003	0.07746026	0.03813873	Stat6/Tnfaip3
28-day post-SCI group vs. the control group	BP	GO:2000781	positive regulation of double-strand break repair	2/99	0.0359003	0.07746026	0.03813873	Actb/Parp1
28-day post-SCI group vs. the control group	BP	GO:0043281	regulation of cysteine-type endopeptidase activity invo	3/99	0.03597	0.07755953	0.03818761	Dhcr24/Gpx1/Xdh
28-day post-SCI group vs. the control group	BP	GO:0006637	acyl-CoA metabolic process	2/99	0.0366549	0.07777039	0.03829143	Pdk1/Pdk2
28-day post-SCI group vs. the control group	BP	GO:0030500	regulation of bone mineralization	2/99	0.0366549	0.07777039	0.03829143	Hif1a/Slc8a1

28-day post-SCI group vs. the control group	BP	GO:0032890	regulation of organic acid transport	2/99	0.0366549	0.07777039	0.03829143	Il1a/Map2k6
28-day post-SCI group vs. the control group	BP	GO:0035383	thioester metabolic process	2/99	0.0366549	0.07777039	0.03829143	Pdk1/Pdk2
28-day post-SCI group vs. the control group	BP	GO:2000736	regulation of stem cell differentiation	2/99	0.0366549	0.07777039	0.03829143	Hdac1/Nfe2l2
28-day post-SCI group vs. the control group	BP	GO:0034767	positive regulation of ion transmembrane transport	3/99	0.0368294	0.07777039	0.03829143	Ppp3ca/S100a1/Trem2
28-day post-SCI group vs. the control group	BP	GO:0098727	maintenance of cell number	3/99	0.0368294	0.07777039	0.03829143	Actb/Apc/Hdac1
28-day post-SCI group vs. the control group	BP	GO:0002357	defense response to tumor cell	1/99	0.036995	0.07777039	0.03829143	Rela
28-day post-SCI group vs. the control group	BP	GO:0002676	regulation of chronic inflammatory response	1/99	0.036995	0.07777039	0.03829143	Tnfaip3
28-day post-SCI group vs. the control group	BP	GO:0009172	purine deoxyribonucleoside monophosphate catabolic	1/99	0.036995	0.07777039	0.03829143	Xdh
28-day post-SCI group vs. the control group	BP	GO:0010612	regulation of cardiac muscle adaptation	1/99	0.036995	0.07777039	0.03829143	Ppp3ca
28-day post-SCI group vs. the control group	BP	GO:0010992	ubiquitin recycling	1/99	0.036995	0.07777039	0.03829143	Fbxw7
28-day post-SCI group vs. the control group	BP	GO:0018401	peptidyl-proline hydroxylation to 4-hydroxy-L-proline	1/99	0.036995	0.07777039	0.03829143	Prdx4
28-day post-SCI group vs. the control group	BP	GO:0019227	neuronal action potential propagation	1/99	0.036995	0.07777039	0.03829143	Fkbp1b
28-day post-SCI group vs. the control group	BP	GO:0019372	lipoxygenase pathway	1/99	0.036995	0.07777039	0.03829143	Gpx1
28-day post-SCI group vs. the control group	BP	GO:0033629	negative regulation of cell adhesion mediated by integ	1/99	0.036995	0.07777039	0.03829143	Cyp1b1
28-day post-SCI group vs. the control group	BP	GO:0034115	negative regulation of heterotypic cell-cell adhesion	1/99	0.036995	0.07777039	0.03829143	Tnfaip3
28-day post-SCI group vs. the control group	BP	GO:0035672	oligopeptide transmembrane transport	1/99	0.036995	0.07777039	0.03829143	Slc7a11
28-day post-SCI group vs. the control group	BP	GO:0043353	enucleate erythrocyte differentiation	1/99	0.036995	0.07777039	0.03829143	Rb1
28-day post-SCI group vs. the control group	BP	GO:0051901	positive regulation of mitochondrial depolarization	1/99	0.036995	0.07777039	0.03829143	Parp1
28-day post-SCI group vs. the control group	BP	GO:0060068	vagina development	1/99	0.036995	0.07777039	0.03829143	Axl
28-day post-SCI group vs. the control group	BP	GO:0060693	regulation of branching involved in salivary gland mor	1/99	0.036995	0.07777039	0.03829143	Met
28-day post-SCI group vs. the control group	BP	GO:0060768	regulation of epithelial cell proliferation involved in pr	1/99	0.036995	0.07777039	0.03829143	Apc
28-day post-SCI group vs. the control group	BP	GO:0061299	retina vasculature morphogenesis in camera-type eye	1/99	0.036995	0.07777039	0.03829143	Cyp1b1
28-day post-SCI group vs. the control group	BP	GO:0070493	thrombin-activated receptor signaling pathway	1/99	0.036995	0.07777039	0.03829143	Met
28-day post-SCI group vs. the control group	BP	GO:0070587	regulation of cell-cell adhesion involved in gastrulatio	1/99	0.036995	0.07777039	0.03829143	Tnfaip3
28-day post-SCI group vs. the control group	BP	GO:0071609	chemokine (C-C motif) ligand 5 production	1/99	0.036995	0.07777039	0.03829143	Sirpa
28-day post-SCI group vs. the control group	BP	GO:0072526	pyridine-containing compound catabolic process	1/99	0.036995	0.07777039	0.03829143	Ncf1
28-day post-SCI group vs. the control group	BP	GO:0072584	caveolin-mediated endocytosis	1/99	0.036995	0.07777039	0.03829143	Mapk1
28-day post-SCI group vs. the control group	BP	GO:0090009	primitive streak formation	1/99	0.036995	0.07777039	0.03829143	Ets2
28-day post-SCI group vs. the control group	BP	GO:0098870	action potential propagation	1/99	0.036995	0.07777039	0.03829143	Fkbp1b
28-day post-SCI group vs. the control group	BP	GO:1900454	positive regulation of long-term synaptic depression	1/99	0.036995	0.07777039	0.03829143	Stau2
28-day post-SCI group vs. the control group	BP	GO:1900747	negative regulation of vascular endothelial growth fact	1/99	0.036995	0.07777039	0.03829143	Xdh
28-day post-SCI group vs. the control group	BP	GO:1901029	negative regulation of mitochondrial outer membrane p	1/99	0.036995	0.07777039	0.03829143	Ier3
28-day post-SCI group vs. the control group	BP	GO:1903599	positive regulation of autophagy of mitochondrion	1/99	0.036995	0.07777039	0.03829143	Hif1a
28-day post-SCI group vs. the control group	BP	GO:1903800	positive regulation of miRNA maturation	1/99	0.036995	0.07777039	0.03829143	Ripk1
28-day post-SCI group vs. the control group	BP	GO:1905634	regulation of protein localization to chromatin	1/99	0.036995	0.07777039	0.03829143	Rb1
28-day post-SCI group vs. the control group	BP	GO:2000434	regulation of protein neddylation	1/99	0.036995	0.07777039	0.03829143	Hif1a
28-day post-SCI group vs. the control group	BP	GO:2001204	regulation of osteoclast development	1/99	0.036995	0.07777039	0.03829143	Fbxw7
28-day post-SCI group vs. the control group	BP	GO:0006282	regulation of DNA repair	3/99	0.037263	0.07828348	0.03854406	Actb/Ier3/Parp1
28-day post-SCI group vs. the control group	BP	GO:0002260	lymphocyte homeostasis	2/99	0.0374156	0.07840262	0.03860272	Hif1a/Tnfaip3
28-day post-SCI group vs. the control group	BP	GO:0014032	neural crest cell development	2/99	0.0374156	0.07840262	0.03860272	Hif1a/Mapk1

28-day post-SCI group vs. the control group	BP	GO:0031507	heterochromatin formation	2/99	0.0374156	0.07840262	0.03860272	Hdac1/Rb1
28-day post-SCI group vs. the control group	BP	GO:0034620	cellular response to unfolded protein	2/99	0.0374156	0.07840262	0.03860272	Eif2s1/Nfe2l2
28-day post-SCI group vs. the control group	BP	GO:0007059	chromosome segregation	4/99	0.0380771	0.07965221	0.03921797	Actb/Apc/Fbxw7/Rb1
28-day post-SCI group vs. the control group	BP	GO:0006112	energy reserve metabolic process	2/99	0.0381824	0.07965221	0.03921797	Cd36/Il6st
28-day post-SCI group vs. the control group	BP	GO:0006835	dicarboxylic acid transport	2/99	0.0381824	0.07965221	0.03921797	Prkcd/Slc7a11
28-day post-SCI group vs. the control group	BP	GO:0008593	regulation of Notch signaling pathway	2/99	0.0381824	0.07965221	0.03921797	Il6st/Mmp14
28-day post-SCI group vs. the control group	BP	GO:0019319	hexose biosynthetic process	2/99	0.0381824	0.07965221	0.03921797	Hif1a/Pdk2
28-day post-SCI group vs. the control group	BP	GO:0033627	cell adhesion mediated by integrin	2/99	0.0381824	0.07965221	0.03921797	Cyp1b1/Itgb5
28-day post-SCI group vs. the control group	BP	GO:0050805	negative regulation of synaptic transmission	2/99	0.0381824	0.07965221	0.03921797	Cd38/Stau2
28-day post-SCI group vs. the control group	BP	GO:0032273	positive regulation of protein polymerization	2/99	0.0389552	0.08105757	0.03990992	Apc/Met
28-day post-SCI group vs. the control group	BP	GO:0051937	catecholamine transport	2/99	0.0389552	0.08105757	0.03990992	Actb/Ptgs1
28-day post-SCI group vs. the control group	BP	GO:0060079	excitatory postsynaptic potential	2/99	0.0389552	0.08105757	0.03990992	Met/Ppp3ca
28-day post-SCI group vs. the control group	BP	GO:0060420	regulation of heart growth	2/99	0.0389552	0.08105757	0.03990992	Apc/Mapk1
28-day post-SCI group vs. the control group	BP	GO:0006096	glycolytic process	2/99	0.039734	0.08140173	0.04007937	Hif1a/Ier3
28-day post-SCI group vs. the control group	BP	GO:0015914	phospholipid transport	2/99	0.039734	0.08140173	0.04007937	Apoe/Prkcd
28-day post-SCI group vs. the control group	BP	GO:0031110	regulation of microtubule polymerization or depolyme	2/99	0.039734	0.08140173	0.04007937	Apc/Met
28-day post-SCI group vs. the control group	BP	GO:0035050	embryonic heart tube development	2/99	0.039734	0.08140173	0.04007937	Hif1a/Slc8a1
28-day post-SCI group vs. the control group	BP	GO:0001781	neutrophil apoptotic process	1/99	0.0402902	0.08140173	0.04007937	Slc7a11
28-day post-SCI group vs. the control group	BP	GO:0002176	male germ cell proliferation	1/99	0.0402902	0.08140173	0.04007937	Prdx4
28-day post-SCI group vs. the control group	BP	GO:0002579	positive regulation of antigen processing and presentat	1/99	0.0402902	0.08140173	0.04007937	Trem2
28-day post-SCI group vs. the control group	BP	GO:0006152	purine nucleoside catabolic process	1/99	0.0402902	0.08140173	0.04007937	Xdh
28-day post-SCI group vs. the control group	BP	GO:0006534	cysteine metabolic process	1/99	0.0402902	0.08140173	0.04007937	Slc7a11
28-day post-SCI group vs. the control group	BP	GO:0010572	positive regulation of platelet activation	1/99	0.0402902	0.08140173	0.04007937	Tlr4
28-day post-SCI group vs. the control group	BP	GO:0010744	positive regulation of macrophage derived foam cell di	1/99	0.0402902	0.08140173	0.04007937	Cd36
28-day post-SCI group vs. the control group	BP	GO:0014824	artery smooth muscle contraction	1/99	0.0402902	0.08140173	0.04007937	Cd38
28-day post-SCI group vs. the control group	BP	GO:0015911	long-chain fatty acid import across plasma membrane	1/99	0.0402902	0.08140173	0.04007937	Cd36
28-day post-SCI group vs. the control group	BP	GO:0031272	regulation of pseudopodium assembly	1/99	0.0402902	0.08140173	0.04007937	Apc
28-day post-SCI group vs. the control group	BP	GO:0031274	positive regulation of pseudopodium assembly	1/99	0.0402902	0.08140173	0.04007937	Apc
28-day post-SCI group vs. the control group	BP	GO:0032825	positive regulation of natural killer cell differentiation	1/99	0.0402902	0.08140173	0.04007937	Axl
28-day post-SCI group vs. the control group	BP	GO:0034088	maintenance of mitotic sister chromatid cohesion	1/99	0.0402902	0.08140173	0.04007937	Rb1
28-day post-SCI group vs. the control group	BP	GO:0034135	regulation of toll-like receptor 2 signaling pathway	1/99	0.0402902	0.08140173	0.04007937	Trem2
28-day post-SCI group vs. the control group	BP	GO:0034333	adherens junction assembly	1/99	0.0402902	0.08140173	0.04007937	Actb
28-day post-SCI group vs. the control group	BP	GO:0035331	negative regulation of hippo signaling	1/99	0.0402902	0.08140173	0.04007937	Map2k3
28-day post-SCI group vs. the control group	BP	GO:0035864	response to potassium ion	1/99	0.0402902	0.08140173	0.04007937	Mylk
28-day post-SCI group vs. the control group	BP	GO:0035865	cellular response to potassium ion	1/99	0.0402902	0.08140173	0.04007937	Mylk
28-day post-SCI group vs. the control group	BP	GO:0039654	fusion of virus membrane with host endosome membra	1/99	0.0402902	0.08140173	0.04007937	Ctsl
28-day post-SCI group vs. the control group	BP	GO:0043587	tongue morphogenesis	1/99	0.0402902	0.08140173	0.04007937	Hdac1
28-day post-SCI group vs. the control group	BP	GO:0045348	positive regulation of MHC class II biosynthetic proce	1/99	0.0402902	0.08140173	0.04007937	Tlr4
28-day post-SCI group vs. the control group	BP	GO:0046054	dGMP metabolic process	1/99	0.0402902	0.08140173	0.04007937	Xdh
28-day post-SCI group vs. the control group	BP	GO:0048241	epinephrine transport	1/99	0.0402902	0.08140173	0.04007937	Ptgs1

28-day post-SCI group vs. the control group	BP	GO:0051971	positive regulation of transmission of nerve impulse	1/99	0.0402902	0.08140173	0.04007937	Pawr
28-day post-SCI group vs. the control group	BP	GO:0060020	Bergmann glial cell differentiation	1/99	0.0402902	0.08140173	0.04007937	Mapk1
28-day post-SCI group vs. the control group	BP	GO:0060439	trachea morphogenesis	1/99	0.0402902	0.08140173	0.04007937	Mapk1
28-day post-SCI group vs. the control group	BP	GO:0060767	epithelial cell proliferation involved in prostate gland c	1/99	0.0402902	0.08140173	0.04007937	Apc
28-day post-SCI group vs. the control group	BP	GO:0061684	chaperone-mediated autophagy	1/99	0.0402902	0.08140173	0.04007937	Clu
28-day post-SCI group vs. the control group	BP	GO:0070213	protein auto-ADP-ribosylation	1/99	0.0402902	0.08140173	0.04007937	Parp1
28-day post-SCI group vs. the control group	BP	GO:0070244	negative regulation of thymocyte apoptotic process	1/99	0.0402902	0.08140173	0.04007937	Hif1a
28-day post-SCI group vs. the control group	BP	GO:0070262	peptidyl-serine dephosphorylation	1/99	0.0402902	0.08140173	0.04007937	Ppp3ca
28-day post-SCI group vs. the control group	BP	GO:0070586	cell-cell adhesion involved in gastrulation	1/99	0.0402902	0.08140173	0.04007937	Tnfaip3
28-day post-SCI group vs. the control group	BP	GO:0070944	neutrophil-mediated killing of bacterium	1/99	0.0402902	0.08140173	0.04007937	Ncf1
28-day post-SCI group vs. the control group	BP	GO:0090184	positive regulation of kidney development	1/99	0.0402902	0.08140173	0.04007937	Ppp3ca
28-day post-SCI group vs. the control group	BP	GO:0090209	negative regulation of triglyceride metabolic process	1/99	0.0402902	0.08140173	0.04007937	Apoe
28-day post-SCI group vs. the control group	BP	GO:0097090	presynaptic membrane organization	1/99	0.0402902	0.08140173	0.04007937	Apoe
28-day post-SCI group vs. the control group	BP	GO:0097201	negative regulation of transcription from RNA polyme	1/99	0.0402902	0.08140173	0.04007937	Jun
28-day post-SCI group vs. the control group	BP	GO:0099004	calmodulin dependent kinase signaling pathway	1/99	0.0402902	0.08140173	0.04007937	Trem2
28-day post-SCI group vs. the control group	BP	GO:1900272	negative regulation of long-term synaptic potentiation	1/99	0.0402902	0.08140173	0.04007937	Apoe
28-day post-SCI group vs. the control group	BP	GO:1903025	regulation of RNA polymerase II regulatory region seq	1/99	0.0402902	0.08140173	0.04007937	Fbxw7
28-day post-SCI group vs. the control group	BP	GO:2000303	regulation of ceramide biosynthetic process	1/99	0.0402902	0.08140173	0.04007937	Prkcd
28-day post-SCI group vs. the control group	BP	GO:2000674	regulation of type B pancreatic cell apoptotic process	1/99	0.0402902	0.08140173	0.04007937	Eif2s1
28-day post-SCI group vs. the control group	BP	GO:2001054	negative regulation of mesenchymal cell apoptotic pro	1/99	0.0402902	0.08140173	0.04007937	Hif1a
28-day post-SCI group vs. the control group	BP	GO:0030258	lipid modification	3/99	0.040372	0.08151652	0.04013589	Apoe/Cd36/Lcat
28-day post-SCI group vs. the control group	BP	GO:0006757	ATP generation from ADP	2/99	0.0405186	0.08161114	0.04018248	Hif1a/Ier3
28-day post-SCI group vs. the control group	BP	GO:0031016	pancreas development	2/99	0.0405186	0.08161114	0.04018248	Clu/Met
28-day post-SCI group vs. the control group	BP	GO:0043367	CD4-positive, alpha-beta T cell differentiation	2/99	0.0405186	0.08161114	0.04018248	Ctsl/Stat6
28-day post-SCI group vs. the control group	BP	GO:0055072	iron ion homeostasis	2/99	0.0405186	0.08161114	0.04018248	Hif1a/Hmox2
28-day post-SCI group vs. the control group	BP	GO:0002285	lymphocyte activation involved in immune response	3/99	0.0408266	0.08218073	0.04046292	Stat6/Tlr4/Tnfaip3
28-day post-SCI group vs. the control group	BP	GO:0002822	regulation of adaptive immune response based on soma	3/99	0.0412837	0.08279554	0.04076564	Stat6/Tnfaip3/Trem2
28-day post-SCI group vs. the control group	BP	GO:0032204	regulation of telomere maintenance	2/99	0.0413092	0.08279554	0.04076564	Mapk1/Parp1
28-day post-SCI group vs. the control group	BP	GO:0032410	negative regulation of transporter activity	2/99	0.0413092	0.08279554	0.04076564	Fkbp1b/Ppp3ca
28-day post-SCI group vs. the control group	BP	GO:0035914	skeletal muscle cell differentiation	2/99	0.0413092	0.08279554	0.04076564	Fos/Rb1
28-day post-SCI group vs. the control group	BP	GO:0051899	membrane depolarization	2/99	0.0413092	0.08279554	0.04076564	Jun/Parp1
28-day post-SCI group vs. the control group	BP	GO:1903035	negative regulation of response to wounding	2/99	0.0413092	0.08279554	0.04076564	Apoe/Prkcd
28-day post-SCI group vs. the control group	BP	GO:2001251	negative regulation of chromosome organization	2/99	0.0413092	0.08279554	0.04076564	Apc/Parp1
28-day post-SCI group vs. the control group	BP	GO:2000241	regulation of reproductive process	3/99	0.0422059	0.08454098	0.04162503	Apc/Il1a/Prdx4
28-day post-SCI group vs. the control group	BP	GO:0008217	regulation of blood pressure	3/99	0.0426708	0.08534825	0.0420225	Cd36/Ier3/Ptgs1
28-day post-SCI group vs. the control group	BP	GO:0007044	cell-substrate junction assembly	2/99	0.0429076	0.08534825	0.0420225	Dst/Mmp14
28-day post-SCI group vs. the control group	BP	GO:0070555	response to interleukin-1	2/99	0.0429076	0.08534825	0.0420225	Rela/Sirpa
28-day post-SCI group vs. the control group	BP	GO:2000117	negative regulation of cysteine-type endopeptidase acti	2/99	0.0429076	0.08534825	0.0420225	Dhcr24/Gpx1
28-day post-SCI group vs. the control group	BP	GO:0000012	single strand break repair	1/99	0.0435743	0.08534825	0.0420225	Parp1
28-day post-SCI group vs. the control group	BP	GO:0000255	allantoin metabolic process	1/99	0.0435743	0.08534825	0.0420225	Xdh

28-day post-SCI group vs. the control group	BP	GO:0001731	formation of translation preinitiation complex	1/99	0.0435743	0.08534825	0.0420225	Rpl13a
28-day post-SCI group vs. the control group	BP	GO:0002069	columnar/cuboidal epithelial cell maturation	1/99	0.0435743	0.08534825	0.0420225	Hif1a
28-day post-SCI group vs. the control group	BP	GO:0002756	MyD88-independent toll-like receptor signaling pathw	1/99	0.0435743	0.08534825	0.0420225	Tlr4
28-day post-SCI group vs. the control group	BP	GO:0010872	regulation of cholesterol esterification	1/99	0.0435743	0.08534825	0.0420225	Apoe
28-day post-SCI group vs. the control group	BP	GO:0018158	protein oxidation	1/99	0.0435743	0.08534825	0.0420225	Gpx1
28-day post-SCI group vs. the control group	BP	GO:0030949	positive regulation of vascular endothelial growth fact	1/99	0.0435743	0.08534825	0.0420225	Hif1a
28-day post-SCI group vs. the control group	BP	GO:0031953	negative regulation of protein autophosphorylation	1/99	0.0435743	0.08534825	0.0420225	Jun
28-day post-SCI group vs. the control group	BP	GO:0033148	positive regulation of intracellular estrogen receptor si	1/99	0.0435743	0.08534825	0.0420225	Parp1
28-day post-SCI group vs. the control group	BP	GO:0033327	Leydig cell differentiation	1/99	0.0435743	0.08534825	0.0420225	Plekha1
28-day post-SCI group vs. the control group	BP	GO:0034086	maintenance of sister chromatid cohesion	1/99	0.0435743	0.08534825	0.0420225	Rb1
28-day post-SCI group vs. the control group	BP	GO:0034374	low-density lipoprotein particle remodeling	1/99	0.0435743	0.08534825	0.0420225	Apoe
28-day post-SCI group vs. the control group	BP	GO:0048012	hepatocyte growth factor receptor signaling pathway	1/99	0.0435743	0.08534825	0.0420225	Met
28-day post-SCI group vs. the control group	BP	GO:0048548	regulation of pinocytosis	1/99	0.0435743	0.08534825	0.0420225	Axl
28-day post-SCI group vs. the control group	BP	GO:0051549	positive regulation of keratinocyte migration	1/99	0.0435743	0.08534825	0.0420225	Hbegf
28-day post-SCI group vs. the control group	BP	GO:0070508	cholesterol import	1/99	0.0435743	0.08534825	0.0420225	Cd36
28-day post-SCI group vs. the control group	BP	GO:0070932	histone H3 deacetylation	1/99	0.0435743	0.08534825	0.0420225	Hdac1
28-day post-SCI group vs. the control group	BP	GO:0071635	negative regulation of transforming growth factor beta	1/99	0.0435743	0.08534825	0.0420225	Met
28-day post-SCI group vs. the control group	BP	GO:0097048	dendritic cell apoptotic process	1/99	0.0435743	0.08534825	0.0420225	Axl
28-day post-SCI group vs. the control group	BP	GO:0098935	dendritic transport	1/99	0.0435743	0.08534825	0.0420225	Stau2
28-day post-SCI group vs. the control group	BP	GO:1900121	negative regulation of receptor binding	1/99	0.0435743	0.08534825	0.0420225	Atp2a2
28-day post-SCI group vs. the control group	BP	GO:1901533	negative regulation of hematopoietic progenitor cell di	1/99	0.0435743	0.08534825	0.0420225	Nfe2l2
28-day post-SCI group vs. the control group	BP	GO:1902548	negative regulation of cellular response to vascular enc	1/99	0.0435743	0.08534825	0.0420225	Xdh
28-day post-SCI group vs. the control group	BP	GO:1902931	negative regulation of alcohol biosynthetic process	1/99	0.0435743	0.08534825	0.0420225	Apoe
28-day post-SCI group vs. the control group	BP	GO:1902950	regulation of dendritic spine maintenance	1/99	0.0435743	0.08534825	0.0420225	Apoe
28-day post-SCI group vs. the control group	BP	GO:1903977	positive regulation of glial cell migration	1/99	0.0435743	0.08534825	0.0420225	Trem2
28-day post-SCI group vs. the control group	BP	GO:1904181	positive regulation of membrane depolarization	1/99	0.0435743	0.08534825	0.0420225	Parp1
28-day post-SCI group vs. the control group	BP	GO:1904753	negative regulation of vascular associated smooth mus	1/99	0.0435743	0.08534825	0.0420225	Nfe2l2
28-day post-SCI group vs. the control group	BP	GO:2000347	positive regulation of hepatocyte proliferation	1/99	0.0435743	0.08534825	0.0420225	Tnfaip3
28-day post-SCI group vs. the control group	BP	GO:2000668	regulation of dendritic cell apoptotic process	1/99	0.0435743	0.08534825	0.0420225	Axl
28-day post-SCI group vs. the control group	BP	GO:2000672	negative regulation of motor neuron apoptotic process	1/99	0.0435743	0.08534825	0.0420225	Map2k4
28-day post-SCI group vs. the control group	BP	GO:2000774	positive regulation of cellular senescence	1/99	0.0435743	0.08534825	0.0420225	Pawr
28-day post-SCI group vs. the control group	BP	GO:0044242	cellular lipid catabolic process	3/99	0.0436085	0.08536404	0.04203028	Cyp1b1/Prdx6/Prkcd
28-day post-SCI group vs. the control group	BP	GO:0001570	vasculogenesis	2/99	0.0437155	0.08536913	0.04203278	Fbxw7/Xdh
28-day post-SCI group vs. the control group	BP	GO:0045814	negative regulation of gene expression, epigenetic	2/99	0.0437155	0.08536913	0.04203278	Hdac1/Rb1
28-day post-SCI group vs. the control group	BP	GO:0046364	monosaccharide biosynthetic process	2/99	0.0437155	0.08536913	0.04203278	Hif1a/Pdk2
28-day post-SCI group vs. the control group	BP	GO:0070828	heterochromatin organization	2/99	0.0437155	0.08536913	0.04203278	Hdac1/Rb1
28-day post-SCI group vs. the control group	BP	GO:0003333	amino acid transmembrane transport	2/99	0.044529	0.08669896	0.04268755	Prkcd/Slc7a11
28-day post-SCI group vs. the control group	BP	GO:0042632	cholesterol homeostasis	2/99	0.044529	0.08669896	0.04268755	Apoe/Lcat
28-day post-SCI group vs. the control group	BP	GO:0090559	regulation of membrane permeability	2/99	0.044529	0.08669896	0.04268755	Hebp2/Ier3
28-day post-SCI group vs. the control group	BP	GO:1901992	positive regulation of mitotic cell cycle phase transitio	2/99	0.044529	0.08669896	0.04268755	Aif1/Rb1

28-day post-SCI group vs. the control group	BP	GO:1903321	negative regulation of protein modification by small pr	2/99	0.044529	0.08669896	0.04268755	Rela/Tnfaip3
28-day post-SCI group vs. the control group	BP	GO:0003002	regionalization	4/99	0.0447408	0.0870595	0.04286506	Apc/Bmp1/Ets2/Rnf2
28-day post-SCI group vs. the control group	BP	GO:0002690	positive regulation of leukocyte chemotaxis	2/99	0.0453481	0.0879796	0.04331809	Aif1/Mapk1
28-day post-SCI group vs. the control group	BP	GO:0019226	transmission of nerve impulse	2/99	0.0453481	0.0879796	0.04331809	Fkbp1b/Pawr
28-day post-SCI group vs. the control group	BP	GO:0019935	cyclic-nucleotide-mediated signaling	2/99	0.0453481	0.0879796	0.04331809	Apoe/Cd36
28-day post-SCI group vs. the control group	BP	GO:0048864	stem cell development	2/99	0.0453481	0.0879796	0.04331809	Hif1a/Mapk1
28-day post-SCI group vs. the control group	BP	GO:0055092	sterol homeostasis	2/99	0.0453481	0.0879796	0.04331809	Apoe/Lcat
28-day post-SCI group vs. the control group	BP	GO:0001655	urogenital system development	4/99	0.045441	0.0881075	0.04338106	Apc/Mpv17/Ppp3ca/Smad1
28-day post-SCI group vs. the control group	BP	GO:0048545	response to steroid hormone	3/99	0.0455146	0.08814586	0.04339995	Hdac1/Lcat/Parp1
28-day post-SCI group vs. the control group	BP	GO:0050679	positive regulation of epithelial cell proliferation	3/99	0.0455146	0.08814586	0.04339995	Id1/Jun/Tnfaip3
28-day post-SCI group vs. the control group	BP	GO:0051781	positive regulation of cell division	2/99	0.0461728	0.08888478	0.04376377	Apc/Il1a
28-day post-SCI group vs. the control group	BP	GO:1901617	organic hydroxy compound biosynthetic process	3/99	0.0464831	0.08888478	0.04376377	Apoe/Dhcr24/Slc7a11
28-day post-SCI group vs. the control group	BP	GO:0001660	fever generation	1/99	0.0468473	0.08888478	0.04376377	Il1a
28-day post-SCI group vs. the control group	BP	GO:0002829	negative regulation of type 2 immune response	1/99	0.0468473	0.08888478	0.04376377	Stat6
28-day post-SCI group vs. the control group	BP	GO:0006206	pyrimidine nucleobase metabolic process	1/99	0.0468473	0.08888478	0.04376377	Mapk1
28-day post-SCI group vs. the control group	BP	GO:0009120	deoxyribonucleoside metabolic process	1/99	0.0468473	0.08888478	0.04376377	Xdh
28-day post-SCI group vs. the control group	BP	GO:0010459	negative regulation of heart rate	1/99	0.0468473	0.08888478	0.04376377	Fkbp1b
28-day post-SCI group vs. the control group	BP	GO:0010936	negative regulation of macrophage cytokine productio	1/99	0.0468473	0.08888478	0.04376377	Axl
28-day post-SCI group vs. the control group	BP	GO:0019511	peptidyl-proline hydroxylation	1/99	0.0468473	0.08888478	0.04376377	Prdx4
28-day post-SCI group vs. the control group	BP	GO:0031054	pre-miRNA processing	1/99	0.0468473	0.08888478	0.04376377	Ago1
28-day post-SCI group vs. the control group	BP	GO:0034393	positive regulation of smooth muscle cell apoptotic pro	1/99	0.0468473	0.08888478	0.04376377	Map2k4
28-day post-SCI group vs. the control group	BP	GO:0036093	germ cell proliferation	1/99	0.0468473	0.08888478	0.04376377	Prdx4
28-day post-SCI group vs. the control group	BP	GO:0042983	amyloid precursor protein biosynthetic process	1/99	0.0468473	0.08888478	0.04376377	Pawr
28-day post-SCI group vs. the control group	BP	GO:0042984	regulation of amyloid precursor protein biosynthetic pr	1/99	0.0468473	0.08888478	0.04376377	Pawr
28-day post-SCI group vs. the control group	BP	GO:0045176	apical protein localization	1/99	0.0468473	0.08888478	0.04376377	Actb
28-day post-SCI group vs. the control group	BP	GO:0045842	positive regulation of mitotic metaphase/anaphase tran	1/99	0.0468473	0.08888478	0.04376377	Rb1
28-day post-SCI group vs. the control group	BP	GO:0045906	negative regulation of vasoconstriction	1/99	0.0468473	0.08888478	0.04376377	Hif1a
28-day post-SCI group vs. the control group	BP	GO:0046886	positive regulation of hormone biosynthetic process	1/99	0.0468473	0.08888478	0.04376377	Hif1a
28-day post-SCI group vs. the control group	BP	GO:0048680	positive regulation of axon regeneration	1/99	0.0468473	0.08888478	0.04376377	Fkbp1b
28-day post-SCI group vs. the control group	BP	GO:0048711	positive regulation of astrocyte differentiation	1/99	0.0468473	0.08888478	0.04376377	Il6st
28-day post-SCI group vs. the control group	BP	GO:0051764	actin crosslink formation	1/99	0.0468473	0.08888478	0.04376377	Aif1
28-day post-SCI group vs. the control group	BP	GO:0060263	regulation of respiratory burst	1/99	0.0468473	0.08888478	0.04376377	Ncf1
28-day post-SCI group vs. the control group	BP	GO:0070278	extracellular matrix constituent secretion	1/99	0.0468473	0.08888478	0.04376377	Tnfrsf1a
28-day post-SCI group vs. the control group	BP	GO:0070344	regulation of fat cell proliferation	1/99	0.0468473	0.08888478	0.04376377	Trem2
28-day post-SCI group vs. the control group	BP	GO:0090153	regulation of sphingolipid biosynthetic process	1/99	0.0468473	0.08888478	0.04376377	Prkcd
28-day post-SCI group vs. the control group	BP	GO:0090331	negative regulation of platelet aggregation	1/99	0.0468473	0.08888478	0.04376377	Prkcd
28-day post-SCI group vs. the control group	BP	GO:0098908	regulation of neuronal action potential	1/99	0.0468473	0.08888478	0.04376377	Pawr
28-day post-SCI group vs. the control group	BP	GO:1902036	regulation of hematopoietic stem cell differentiation	1/99	0.0468473	0.08888478	0.04376377	Nfe2l2
28-day post-SCI group vs. the control group	BP	GO:1902170	cellular response to reactive nitrogen species	1/99	0.0468473	0.08888478	0.04376377	Stat6
28-day post-SCI group vs. the control group	BP	GO:1905038	regulation of membrane lipid metabolic process	1/99	0.0468473	0.08888478	0.04376377	Prkcd

28-day post-SCI group vs. the control group	BP	GO:1905809	negative regulation of synapse organization	1/99	0.0468473	0.08888478	0.04376377	Apoe
28-day post-SCI group vs. the control group	BP	GO:2000343	positive regulation of chemokine (C-X-C motif) ligand	1/99	0.0468473	0.08888478	0.04376377	Tlr4
28-day post-SCI group vs. the control group	BP	GO:2000644	regulation of receptor catabolic process	1/99	0.0468473	0.08888478	0.04376377	Apoe
28-day post-SCI group vs. the control group	BP	GO:2000822	regulation of behavioral fear response	1/99	0.0468473	0.08888478	0.04376377	Apoe
28-day post-SCI group vs. the control group	BP	GO:2001140	positive regulation of phospholipid transport	1/99	0.0468473	0.08888478	0.04376377	Apoe
28-day post-SCI group vs. the control group	BP	GO:0007163	establishment or maintenance of cell polarity	3/99	0.0469711	0.08892243	0.0437823	Actb/Apc/Dst
28-day post-SCI group vs. the control group	BP	GO:0006885	regulation of pH	2/99	0.047003	0.08892243	0.0437823	Mapk1/Pdk2
28-day post-SCI group vs. the control group	BP	GO:0032147	activation of protein kinase activity	2/99	0.047003	0.08892243	0.0437823	Prkcd/Tlr4
28-day post-SCI group vs. the control group	BP	GO:0044264	cellular polysaccharide metabolic process	2/99	0.047003	0.08892243	0.0437823	Cd36/Il6st
28-day post-SCI group vs. the control group	BP	GO:0046031	ADP metabolic process	2/99	0.047003	0.08892243	0.0437823	Hif1a/Ier3
28-day post-SCI group vs. the control group	BP	GO:0009952	anterior/posterior pattern specification	3/99	0.0474617	0.08968643	0.04415847	Apc/Ets2/Rnf2
28-day post-SCI group vs. the control group	BP	GO:0051056	regulation of small GTPase mediated signal transducti	3/99	0.0474617	0.08968643	0.04415847	Apoe/Met/Net1
28-day post-SCI group vs. the control group	BP	GO:0014033	neural crest cell differentiation	2/99	0.0478387	0.09019048	0.04440665	Hif1a/Mapk1
28-day post-SCI group vs. the control group	BP	GO:0042158	lipoprotein biosynthetic process	2/99	0.0478387	0.09019048	0.04440665	Apoe/Lcat
28-day post-SCI group vs. the control group	BP	GO:0099565	chemical synaptic transmission, postsynaptic	2/99	0.0478387	0.09019048	0.04440665	Met/Ppp3ca
28-day post-SCI group vs. the control group	BP	GO:0150115	cell-substrate junction organization	2/99	0.0478387	0.09019048	0.04440665	Dst/Mmp14
28-day post-SCI group vs. the control group	BP	GO:0010770	positive regulation of cell morphogenesis involved in c	2/99	0.0486799	0.09126218	0.04493432	Met/Stau2
28-day post-SCI group vs. the control group	BP	GO:0002819	regulation of adaptive immune response	3/99	0.0489487	0.09126218	0.04493432	Stat6/Tnfaip3/Trem2
28-day post-SCI group vs. the control group	BP	GO:0007173	epidermal growth factor receptor signaling pathway	2/99	0.0495264	0.09126218	0.04493432	Fbxw7/Hbegf
28-day post-SCI group vs. the control group	BP	GO:0046718	viral entry into host cell	2/99	0.0495264	0.09126218	0.04493432	Axl/Ctsl
28-day post-SCI group vs. the control group	BP	GO:0008406	gonad development	3/99	0.0499526	0.09126218	0.04493432	Il1a/Plekha1/Prdx4
28-day post-SCI group vs. the control group	BP	GO:0045930	negative regulation of mitotic cell cycle	3/99	0.0499526	0.09126218	0.04493432	Apc/Ier3/Rb1
28-day post-SCI group vs. the control group	CC	GO:0030018	Z disc	6/99	6.25E-06	0.00061071	0.00040065	Dst/Fkbp1b/Hspb1/Ppp3ca/S100a1/Slc8a1
28-day post-SCI group vs. the control group	CC	GO:0045121	membrane raft	9/99	8.96E-06	0.00061071	0.00040065	Actb/Cd36/Il6st/Mapk1/Ripk1/Stat6/Tlr4/Tnfrsf1a/Trem2
28-day post-SCI group vs. the control group	CC	GO:0098857	membrane microdomain	9/99	9.14E-06	0.00061071	0.00040065	Actb/Cd36/Il6st/Mapk1/Ripk1/Stat6/Tlr4/Tnfrsf1a/Trem2
28-day post-SCI group vs. the control group	CC	GO:0043204	perikaryon	6/99	1.03E-05	0.00061071	0.00040065	Aif1/Ctsl/Hspb1/Map2k4/Mapk1/Mapk10
28-day post-SCI group vs. the control group	CC	GO:0031674	I band	6/99	1.08E-05	0.00061071	0.00040065	Dst/Fkbp1b/Hspb1/Ppp3ca/S100a1/Slc8a1
28-day post-SCI group vs. the control group	CC	GO:0090575	RNA polymerase II transcription regulator complex	7/99	2.09E-05	0.00098999	0.00064948	Fos/Hif1a/Jun/Nfe2l2/Rb1/Smad1/Stat6
28-day post-SCI group vs. the control group	CC	GO:1990204	oxidoreductase complex	5/99	6.28E-05	0.00221249	0.00145149	Cybb/Ncf1/Ndufa6/Pdk1/Pdk2
28-day post-SCI group vs. the control group	CC	GO:0030017	sarcomere	6/99	6.45E-05	0.00221249	0.00145149	Dst/Fkbp1b/Hspb1/Ppp3ca/S100a1/Slc8a1
28-day post-SCI group vs. the control group	CC	GO:0014069	postsynaptic density	8/99	7.09E-05	0.00221249	0.00145149	Actb/Dst/Hspb1/Mapk1/Mapk10/Met/Slc8a1/Syp
28-day post-SCI group vs. the control group	CC	GO:0032279	asymmetric synapse	8/99	8.14E-05	0.00221249	0.00145149	Actb/Dst/Hspb1/Mapk1/Mapk10/Met/Slc8a1/Syp
28-day post-SCI group vs. the control group	CC	GO:0031252	cell leading edge	8/99	8.57E-05	0.00221249	0.00145149	Actb/Aif1/Amph/Apc/Dst/Itgb5/Mylk/Plekha1
28-day post-SCI group vs. the control group	CC	GO:0043235	receptor complex	8/99	0.000121	0.00247297	0.00162237	Axl/Cd36/Il6st/Itgb5/Met/Ripk1/Tlr4/Tnfrsf1a
28-day post-SCI group vs. the control group	CC	GO:0030016	myofibril	6/99	0.0001239	0.00247297	0.00162237	Dst/Fkbp1b/Hspb1/Ppp3ca/S100a1/Slc8a1
28-day post-SCI group vs. the control group	CC	GO:0098984	neuron to neuron synapse	8/99	0.0001352	0.00247297	0.00162237	Actb/Dst/Hspb1/Mapk1/Mapk10/Met/Slc8a1/Syp
28-day post-SCI group vs. the control group	CC	GO:0099572	postsynaptic specialization	8/99	0.0001373	0.00247297	0.00162237	Actb/Dst/Hspb1/Mapk1/Mapk10/Met/Slc8a1/Syp
28-day post-SCI group vs. the control group	CC	GO:0016529	sarcoplasmic reticulum	4/99	0.0001393	0.00247297	0.00162237	Atp2a2/Fkbp1b/S100a1/Xdh
28-day post-SCI group vs. the control group	CC	GO:0043292	contractile fiber	6/99	0.0001685	0.00281541	0.00184702	Dst/Fkbp1b/Hspb1/Ppp3ca/S100a1/Slc8a1
28-day post-SCI group vs. the control group	CC	GO:0031672	A band	3/99	0.0003044	0.00455056	0.00298536	Dst/Hspb1/S100a1

28-day post-SCI group vs. the control group	CC	GO:0034364	high-density lipoprotein particle	3/99	0.0003044	0.00455056	0.00298536	Apoe/Clu/Lcat
28-day post-SCI group vs. the control group	CC	GO:0016528	sarcoplasm	4/99	0.0003703	0.00525778	0.00344932	Atp2a2/Fkbp1b/S100a1/Xdh
28-day post-SCI group vs. the control group	CC	GO:0000792	heterochromatin	4/99	0.0004152	0.00561549	0.003684	Cbx6/Hdac1/Phc1/Rnf2
28-day post-SCI group vs. the control group	CC	GO:0097386	glial cell projection	3/99	0.0004399	0.0056781	0.00372507	Aif1/Eif2s1/Slc7a11
28-day post-SCI group vs. the control group	CC	GO:0034358	plasma lipoprotein particle	3/99	0.0005722	0.00677109	0.00444211	Apoe/Clu/Lcat
28-day post-SCI group vs. the control group	CC	GO:1990777	lipoprotein particle	3/99	0.0005722	0.00677109	0.00444211	Apoe/Clu/Lcat
28-day post-SCI group vs. the control group	CC	GO:0032994	protein-lipid complex	3/99	0.0006865	0.00779881	0.00511634	Apoe/Clu/Lcat
28-day post-SCI group vs. the control group	CC	GO:0031519	PcG protein complex	3/99	0.0007276	0.0078182	0.00512906	Cbx6/Phc1/Rnf2
28-day post-SCI group vs. the control group	CC	GO:0043020	NADPH oxidase complex	2/99	0.0007547	0.0078182	0.00512906	Cybb/Ncf1
28-day post-SCI group vs. the control group	CC	GO:0005635	nuclear envelope	7/99	0.0007708	0.0078182	0.00512906	Apc/Apoe/Dst/Parp1/Ptgs1/Smad1/Stau2
28-day post-SCI group vs. the control group	CC	GO:0044306	neuron projection terminus	5/99	0.0008956	0.00877097	0.00575412	Actb/Amph/Apc/Slc8a1/Syp
28-day post-SCI group vs. the control group	CC	GO:0035102	PRC1 complex	2/99	0.0011926	0.0112898	0.00740658	Phc1/Rnf2
28-day post-SCI group vs. the control group	CC	GO:0014704	intercalated disc	3/99	0.0016086	0.01473657	0.0096678	Atp2a2/Dst/Slc8a1
28-day post-SCI group vs. the control group	CC	GO:0000791	euchromatin	3/99	0.0016786	0.01489796	0.00977368	Hif1a/Jun/Rnf2
28-day post-SCI group vs. the control group	CC	GO:0031430	M band	2/99	0.0019249	0.01623411	0.01065025	Hspb1/S100a1
28-day post-SCI group vs. the control group	CC	GO:0032587	ruffle membrane	3/99	0.0019779	0.01623411	0.01065025	Aif1/Apc/Plekha1
28-day post-SCI group vs. the control group	CC	GO:0150034	distal axon	6/99	0.0020007	0.01623411	0.01065025	Actb/Amph/Apc/Clu/Slc8a1/Syp
28-day post-SCI group vs. the control group	CC	GO:0031256	leading edge membrane	4/99	0.0020909	0.01649492	0.01082135	Aif1/Amph/Apc/Plekha1
28-day post-SCI group vs. the control group	CC	GO:0001891	phagocytic cup	2/99	0.0025829	0.01906447	0.01250709	Aif1/Tlr4
28-day post-SCI group vs. the control group	CC	GO:0097449	astrocyte projection	2/99	0.0025829	0.01906447	0.01250709	Eif2s1/Slc7a11
28-day post-SCI group vs. the control group	CC	GO:0042383	sarcolemma	4/99	0.002618	0.01906447	0.01250709	Cd36/Dst/Slc8a1/Vcam1
28-day post-SCI group vs. the control group	CC	GO:0097470	ribbon synapse	2/99	0.0028226	0.02004022	0.01314721	Amph/Atp2a2
28-day post-SCI group vs. the control group	CC	GO:0030027	lamellipodium	4/99	0.0031647	0.02192132	0.01438129	Actb/Aif1/Apc/Mylk
28-day post-SCI group vs. the control group	CC	GO:0001725	stress fiber	3/99	0.003498	0.02273007	0.01491187	Actb/Dst/Mylk
28-day post-SCI group vs. the control group	CC	GO:0097517	contractile actin filament bundle	3/99	0.003498	0.02273007	0.01491187	Actb/Dst/Mylk
28-day post-SCI group vs. the control group	CC	GO:0002102	podosome	2/99	0.0036016	0.02273007	0.01491187	Actb/Vcam1
28-day post-SCI group vs. the control group	CC	GO:0097038	perinuclear endoplasmic reticulum	2/99	0.0036016	0.02273007	0.01491187	Clu/Dst
28-day post-SCI group vs. the control group	CC	GO:0044291	cell-cell contact zone	3/99	0.0037264	0.02300651	0.01509323	Atp2a2/Dst/Slc8a1
28-day post-SCI group vs. the control group	CC	GO:0032839	dendrite cytoplasm	2/99	0.0041703	0.02519903	0.01653161	Map2k4/Mapk1
28-day post-SCI group vs. the control group	CC	GO:0043679	axon terminus	4/99	0.0045647	0.02610837	0.01712817	Actb/Amph/Slc8a1/Syp
28-day post-SCI group vs. the control group	CC	GO:0032432	actin filament bundle	3/99	0.0045965	0.02610837	0.01712817	Actb/Dst/Mylk
28-day post-SCI group vs. the control group	CC	GO:0042641	actomyosin	3/99	0.0045965	0.02610837	0.01712817	Actb/Dst/Mylk
28-day post-SCI group vs. the control group	CC	GO:0032993	protein-DNA complex	4/99	0.0049048	0.02731318	0.01791858	Actb/Fos/Nfe2l2/Parp1
28-day post-SCI group vs. the control group	CC	GO:0031253	cell projection membrane	5/99	0.0050118	0.0273724	0.01795743	Aif1/Apc/Cd36/Plekha1/Slc7a11
28-day post-SCI group vs. the control group	CC	GO:0019898	extrinsic component of membrane	5/99	0.0062137	0.03329616	0.02184366	Amph/Apc/Apoe/Atp2a2/Ncf1
28-day post-SCI group vs. the control group	CC	GO:0030672	synaptic vesicle membrane	3/99	0.0066805	0.03449578	0.02263066	Amph/Apc/Syp
28-day post-SCI group vs. the control group	CC	GO:0099501	exocytic vesicle membrane	3/99	0.0066805	0.03449578	0.02263066	Amph/Apc/Syp
28-day post-SCI group vs. the control group	CC	GO:0098685	Schaffer collateral - CA1 synapse	3/99	0.0073633	0.03734227	0.02449808	Actb/Ppp3ca/Syp
28-day post-SCI group vs. the control group	CC	GO:0000803	sex chromosome	2/99	0.0087882	0.04350799	0.02854305	Phc1/Rnf2
28-day post-SCI group vs. the control group	CC	GO:0036464	cytoplasmic ribonucleoprotein granule	4/99	0.0088854	0.04350799	0.02854305	Actb/Ago1/Eif2s1/Stau2

28-day post-SCI group vs. the control group	CC	GO:0120111	neuron projection cytoplasm	2/99	0.0092071	0.04431892	0.02907505	Map2k4/Mapk1
28-day post-SCI group vs. the control group	CC	GO:0000152	nuclear ubiquitin ligase complex	2/99	0.0105162	0.04977651	0.03265546	Phc1/Rnf2
28-day post-SCI group vs. the control group	CC	GO:0005884	actin filament	3/99	0.0111592	0.05195413	0.03408407	Actb/Aif1/Pawr
28-day post-SCI group vs. the control group	CC	GO:0035770	ribonucleoprotein granule	4/99	0.0114151	0.05228854	0.03430345	Actb/Ago1/Eif2s1/Stau2
28-day post-SCI group vs. the control group	CC	GO:0044853	plasma membrane raft	3/99	0.0116131	0.05235093	0.03434438	Cd36/Mapk1/Trem2
28-day post-SCI group vs. the control group	CC	GO:0005777	peroxisome	3/99	0.0148215	0.06475859	0.04248433	Mgst1/Mpv17/Xdh
28-day post-SCI group vs. the control group	CC	GO:0042579	microbody	3/99	0.0148215	0.06475859	0.04248433	Mgst1/Mpv17/Xdh
28-day post-SCI group vs. the control group	CC	GO:0001726	ruffle	3/99	0.0153552	0.06508758	0.04270015	Aif1/Apc/Plekha1
28-day post-SCI group vs. the control group	CC	GO:0030658	transport vesicle membrane	3/99	0.0153552	0.06508758	0.04270015	Amph/Apc/Syp
28-day post-SCI group vs. the control group	CC	GO:0005874	microtubule	5/99	0.0182068	0.07604005	0.04988543	Apc/Apoe/Dst/Slc8a1/Stau2
28-day post-SCI group vs. the control group	CC	GO:0005925	focal adhesion	3/99	0.0216113	0.08895096	0.05835552	Dst/Itgb5/Mapk1
28-day post-SCI group vs. the control group	CC	GO:0043198	dendritic shaft	2/99	0.0223683	0.09075148	0.05953674	Slc8a1/Stau2
28-day post-SCI group vs. the control group	CC	GO:0097060	synaptic membrane	5/99	0.0230296	0.09211844	0.06043352	Hspb1/Itgb5/Met/Slc8a1/Syp
28-day post-SCI group vs. the control group	CC	GO:0009898	cytoplasmic side of plasma membrane	3/99	0.0250116	0.09865693	0.06472304	Atp2a2/Dst/Ppp3ca
28-day post-SCI group vs. the control group	CC	GO:0030055	cell-substrate junction	3/99	0.0260848	0.10148041	0.06657536	Dst/Itgb5/Mapk1
28-day post-SCI group vs. the control group	CC	GO:0045177	apical part of cell	5/99	0.0267343	0.10260173	0.06731099	Cd36/Ctsl/Mgst1/Slc7a11/Vcam1
28-day post-SCI group vs. the control group	CC	GO:0019897	extrinsic component of plasma membrane	3/99	0.0279276	0.10562503	0.0692944	Apc/Apoe/Atp2a2
28-day post-SCI group vs. the control group	CC	GO:0017053	transcription repressor complex	2/99	0.0282659	0.10562503	0.0692944	Hdac1/Jun
28-day post-SCI group vs. the control group	CC	GO:0010494	cytoplasmic stress granule	2/99	0.0296523	0.1091262	0.07159132	Eif2s1/Stau2
28-day post-SCI group vs. the control group	CC	GO:0031526	brush border membrane	2/99	0.0303555	0.1091262	0.07159132	Cd36/Slc7a11
28-day post-SCI group vs. the control group	CC	GO:0060076	excitatory synapse	2/99	0.0303555	0.1091262	0.07159132	Met/Syp
28-day post-SCI group vs. the control group	CC	GO:0016234	inclusion body	2/99	0.0310654	0.1102821	0.07234963	Clu/Gpx1
28-day post-SCI group vs. the control group	CC	GO:0070852	cell body fiber	1/99	0.0338487	0.11448562	0.07510732	Apc
28-day post-SCI group vs. the control group	CC	GO:0071141	SMAD protein complex	1/99	0.0338487	0.11448562	0.07510732	Smad1
28-day post-SCI group vs. the control group	CC	GO:0140092	bBAF complex	1/99	0.0338487	0.11448562	0.07510732	Actb
28-day post-SCI group vs. the control group	CC	GO:0044309	neuron spine	3/99	0.0338619	0.11448562	0.07510732	Ppp3ca/Slc8a1/Syp
28-day post-SCI group vs. the control group	CC	GO:0098562	cytoplasmic side of membrane	3/99	0.0346989	0.11593515	0.07605827	Atp2a2/Dst/Ppp3ca
28-day post-SCI group vs. the control group	CC	GO:0036019	endolysosome	1/99	0.0371705	0.12133826	0.07960293	Prkcd
28-day post-SCI group vs. the control group	CC	GO:0070578	RISC-loading complex	1/99	0.0371705	0.12133826	0.07960293	Ago1
28-day post-SCI group vs. the control group	CC	GO:0005791	rough endoplasmic reticulum	2/99	0.0377429	0.12180666	0.07991022	Ncf1/Slc7a11
28-day post-SCI group vs. the control group	CC	GO:0070603	SWI/SNF superfamily-type complex	2/99	0.0400803	0.12492908	0.08195866	Actb/Hdac1
28-day post-SCI group vs. the control group	CC	GO:0031143	pseudopodium	1/99	0.0404811	0.12492908	0.08195866	Mapk1
28-day post-SCI group vs. the control group	CC	GO:0098684	photoreceptor ribbon synapse	1/99	0.0404811	0.12492908	0.08195866	Amph
28-day post-SCI group vs. the control group	CC	GO:0005901	caveola	2/99	0.0432798	0.12492908	0.08195866	Cd36/Mapk1
28-day post-SCI group vs. the control group	CC	GO:1904949	ATPase complex	2/99	0.0432798	0.12492908	0.08195866	Actb/Hdac1
28-day post-SCI group vs. the control group	CC	GO:0005677	chromatin silencing complex	1/99	0.0437803	0.12492908	0.08195866	Rb1
28-day post-SCI group vs. the control group	CC	GO:0019908	nuclear cyclin-dependent protein kinase holoenzyme c	1/99	0.0437803	0.12492908	0.08195866	Rb1
28-day post-SCI group vs. the control group	CC	GO:0030877	beta-catenin destruction complex	1/99	0.0437803	0.12492908	0.08195866	Apc
28-day post-SCI group vs. the control group	CC	GO:0042627	chylomicron	1/99	0.0437803	0.12492908	0.08195866	Apoe
28-day post-SCI group vs. the control group	CC	GO:0098833	presynaptic endocytic zone	1/99	0.0437803	0.12492908	0.08195866	Amph

28-day post-SCI group vs. the control group	CC	GO:0098871	postsynaptic actin cytoskeleton	1/99	0.0437803	0.12492908	0.08195866	Actb
28-day post-SCI group vs. the control group	CC	GO:1905369	endopeptidase complex	2/99	0.0465714	0.12492908	0.08195866	Capns1/Hspb1
28-day post-SCI group vs. the control group	CC	GO:0008021	synaptic vesicle	3/99	0.0470329	0.12492908	0.08195866	Amph/Apc/Syp
28-day post-SCI group vs. the control group	CC	GO:0016586	RSC-type complex	1/99	0.0470683	0.12492908	0.08195866	Actb
28-day post-SCI group vs. the control group	CC	GO:0034362	low-density lipoprotein particle	1/99	0.0470683	0.12492908	0.08195866	Apoe
28-day post-SCI group vs. the control group	CC	GO:0035060	brahma complex	1/99	0.0470683	0.12492908	0.08195866	Actb
28-day post-SCI group vs. the control group	CC	GO:0071564	npBAF complex	1/99	0.0470683	0.12492908	0.08195866	Actb
28-day post-SCI group vs. the control group	CC	GO:0090533	cation-transporting ATPase complex	1/99	0.0470683	0.12492908	0.08195866	Atp2a2
28-day post-SCI group vs. the control group	CC	GO:0140288	GBAF complex	1/99	0.0470683	0.12492908	0.08195866	Actb
28-day post-SCI group vs. the control group	MF	GO:0016209	antioxidant activity	8/99	6.93E-10	2.37E-07	1.53E-07	Apoe/Gpx1/Gpx3/Gpx8/Mgst1/Prdx4/Prdx6/Ptgs1
28-day post-SCI group vs. the control group	MF	GO:0004601	peroxidase activity	7/99	1.31E-09	2.37E-07	1.53E-07	Gpx1/Gpx3/Gpx8/Mgst1/Prdx4/Prdx6/Ptgs1
28-day post-SCI group vs. the control group	MF	GO:0016684	oxidoreductase activity, acting on peroxide as acceptor	7/99	1.88E-09	2.37E-07	1.53E-07	Gpx1/Gpx3/Gpx8/Mgst1/Prdx4/Prdx6/Ptgs1
28-day post-SCI group vs. the control group	MF	GO:0061629	RNA polymerase II-specific DNA-binding transcriptio	12/99	7.99E-09	7.55E-07	4.88E-07	Actb/Ets2/Fos/Hdac1/Hif1a/Hspb1/Jun/Ncoa7/Nfe2l2/Parp1/Rb1/Rela
28-day post-SCI group vs. the control group	MF	GO:0004602	glutathione peroxidase activity	5/99	3.53E-08	2.67E-06	1.72E-06	Gpx1/Gpx3/Gpx8/Mgst1/Prdx6
28-day post-SCI group vs. the control group	MF	GO:0031625	ubiquitin protein ligase binding	10/99	1.59E-07	9.94E-06	6.42E-06	Apc/Clu/Fbxw7/Hif1a/Jun/Prdx6/Rb1/Rela/Ripk1/Txnip
28-day post-SCI group vs. the control group	MF	GO:0004708	MAP kinase kinase activity	4/99	1.84E-07	9.94E-06	6.42E-06	Map2k3/Map2k4/Map2k6/Mapk1
28-day post-SCI group vs. the control group	MF	GO:0044389	ubiquitin-like protein ligase binding	10/99	2.58E-07	1.22E-05	7.88E-06	Apc/Clu/Fbxw7/Hif1a/Jun/Prdx6/Rb1/Rela/Ripk1/Txnip
28-day post-SCI group vs. the control group	MF	GO:0004674	protein serine/threonine kinase activity	11/99	4.13E-07	1.73E-05	1.12E-05	Map2k3/Map2k4/Map2k6/Mapk1/Mapk10/Mapkapk2/Mylk/Pdk1/Pdk2/Prkcd/Ripk1
28-day post-SCI group vs. the control group	MF	GO:0033218	amide binding	11/99	5.26E-07	1.99E-05	1.29E-05	Apoe/Cd36/Clu/Ctsl/Dhcr24/Fkbp1b/Lancl1/Mgst1/Ppp3ca/Rela/Trem2
28-day post-SCI group vs. the control group	MF	GO:0042277	peptide binding	10/99	8.23E-07	2.83E-05	1.83E-05	Apoe/Cd36/Clu/Ctsl/Dhcr24/Lancl1/Mgst1/Ppp3ca/Rela/Trem2
28-day post-SCI group vs. the control group	MF	GO:0019207	kinase regulator activity	8/99	2.45E-06	7.73E-05	4.99E-05	Apc/Cdkn2c/Gskip/Hbegf/Hspb1/Map2k6/Parp1/Trem2
28-day post-SCI group vs. the control group	MF	GO:0004713	protein tyrosine kinase activity	6/99	6.47E-06	0.00018821	0.0001216	Axl/Map2k3/Map2k4/Map2k6/Met/Prkcd
28-day post-SCI group vs. the control group	MF	GO:0019887	protein kinase regulator activity	7/99	1.03E-05	0.00027918	0.00018037	Apc/Cdkn2c/Gskip/Hbegf/Hspb1/Map2k6/Parp1
28-day post-SCI group vs. the control group	MF	GO:0004712	protein serine/threonine/tyrosine kinase activity	4/99	1.40E-05	0.00035373	0.00022853	Map2k3/Map2k4/Map2k6/Mapk1
28-day post-SCI group vs. the control group	MF	GO:0001046	core promoter sequence-specific DNA binding	4/99	3.06E-05	0.00072281	0.00046698	Ago1/Fos/Hdac1/Rela
28-day post-SCI group vs. the control group	MF	GO:0000979	RNA polymerase II core promoter sequence-specific D	3/99	6.91E-05	0.00153739	0.00099325	Fos/Hdac1/Rela
28-day post-SCI group vs. the control group	MF	GO:0004860	protein kinase inhibitor activity	4/99	7.51E-05	0.00157789	0.00101941	Cdkn2c/Gskip/Hspb1/Parp1
28-day post-SCI group vs. the control group	MF	GO:0001540	amyloid-beta binding	4/99	8.48E-05	0.00168661	0.00108965	Apoe/Cd36/Clu/Trem2
28-day post-SCI group vs. the control group	MF	GO:0070412	R-SMAD binding	3/99	8.93E-05	0.00168855	0.0010909	Fos/Jun/Parp1
28-day post-SCI group vs. the control group	MF	GO:0019210	kinase inhibitor activity	4/99	0.0001009	0.00178638	0.00115411	Cdkn2c/Gskip/Hspb1/Parp1
28-day post-SCI group vs. the control group	MF	GO:0001221	transcription coregulator binding	5/99	0.0001049	0.00178638	0.00115411	Fos/Hdac1/Hif1a/Nfe2l2/Rela
28-day post-SCI group vs. the control group	MF	GO:0047485	protein N-terminus binding	5/99	0.0001087	0.00178638	0.00115411	Clu/Hdac1/Id1/Parp1/Rela
28-day post-SCI group vs. the control group	MF	GO:0071813	lipoprotein particle binding	3/99	0.0001263	0.00190952	0.00123366	Apoe/Cd36/Trem2
28-day post-SCI group vs. the control group	MF	GO:0071814	protein-lipid complex binding	3/99	0.0001263	0.00190952	0.00123366	Apoe/Cd36/Trem2
28-day post-SCI group vs. the control group	MF	GO:0050750	low-density lipoprotein particle receptor binding	3/99	0.0001405	0.00204276	0.00131974	Apoe/Clu/Lancl1
28-day post-SCI group vs. the control group	MF	GO:0008022	protein C-terminus binding	6/99	0.0001695	0.00237303	0.00153312	Amph/Atp2a2/Dst/Id1/Sdc1/Tnfaip3
28-day post-SCI group vs. the control group	MF	GO:0030291	protein serine/threonine kinase inhibitor activity	3/99	0.0001893	0.00255535	0.00165091	Cdkn2c/Hspb1/Parp1

28-day post-SCI group vs. the control group	MF	GO:0070325	lipoprotein particle receptor binding	3/99	0.0002077	0.00270711	0.00174895	Apoe/Clu/Lancl1
28-day post-SCI group vs. the control group	MF	GO:0046332	SMAD binding	4/99	0.0002264	0.00285287	0.00184313	Fos/Jun/Parp1/Smad1
28-day post-SCI group vs. the control group	MF	GO:0050660	flavin adenine dinucleotide binding	4/99	0.0002476	0.00301896	0.00195043	Cybb/Dhcr24/Prodh/Xdh
28-day post-SCI group vs. the control group	MF	GO:0020037	heme binding	5/99	0.000256	0.00302385	0.00195359	Cybb/Cyp1b1/Hebp2/Hmox2/Ptgs1
28-day post-SCI group vs. the control group	MF	GO:0046906	tetrapyrrole binding	5/99	0.0003289	0.00376758	0.00243408	Cybb/Cyp1b1/Hebp2/Hmox2/Ptgs1
28-day post-SCI group vs. the control group	MF	GO:0043548	phosphatidylinositol 3-kinase binding	3/99	0.0003977	0.0044211	0.00285629	Axl/Met/Tlr4
28-day post-SCI group vs. the control group	MF	GO:0071949	FAD binding	3/99	0.0004272	0.00461349	0.00298059	Dhcr24/Prodh/Xdh
28-day post-SCI group vs. the control group	MF	GO:0008035	high-density lipoprotein particle binding	2/99	0.0005314	0.00557965	0.00360479	Cd36/Trem2
28-day post-SCI group vs. the control group	MF	GO:0072341	modified amino acid binding	4/99	0.0005637	0.00575918	0.00372078	Axl/Lancl1/Mgst1/Trem2
28-day post-SCI group vs. the control group	MF	GO:0051219	phosphoprotein binding	4/99	0.000625	0.00621703	0.00401657	Fbxw7/Mapk1/Rb1/Sirpa
28-day post-SCI group vs. the control group	MF	GO:0070513	death domain binding	2/99	0.0007758	0.0075197	0.00485818	Mcl1/Ripk1
28-day post-SCI group vs. the control group	MF	GO:0044548	S100 protein binding	2/99	0.0010649	0.01006302	0.00650131	Atp2a2/S100a1
28-day post-SCI group vs. the control group	MF	GO:0017124	SH3 domain binding	4/99	0.0011595	0.01069036	0.00690661	Gpx1/Lancl1/Ncf1/Sirpa
28-day post-SCI group vs. the control group	MF	GO:0050664	oxidoreductase activity, acting on NAD(P)H, oxygen a	2/99	0.0012259	0.01103317	0.00712808	Cybb/Ncf1
28-day post-SCI group vs. the control group	MF	GO:0042826	histone deacetylase binding	4/99	0.0012607	0.01108264	0.00716005	Hdac1/Hif1a/Parp1/Rela
28-day post-SCI group vs. the control group	MF	GO:0019902	phosphatase binding	5/99	0.001387	0.01151655	0.00744038	Mapk1/Met/Pawr/Sirpa/Stat6
28-day post-SCI group vs. the control group	MF	GO:0004707	MAP kinase activity	2/99	0.0013979	0.01151655	0.00744038	Mapk1/Mapk10
28-day post-SCI group vs. the control group	MF	GO:0045309	protein phosphorylated amino acid binding	3/99	0.0014015	0.01151655	0.00744038	Fbxw7/Mapk1/Sirpa
28-day post-SCI group vs. the control group	MF	GO:0019955	cytokine binding	4/99	0.0016847	0.0131499	0.00849562	Cd36/Il18bp/Il6st/Tnfrsf1a
28-day post-SCI group vs. the control group	MF	GO:0030169	low-density lipoprotein particle binding	2/99	0.0017742	0.0131499	0.00849562	Cd36/Trem2
28-day post-SCI group vs. the control group	MF	GO:0043295	glutathione binding	2/99	0.0017742	0.0131499	0.00849562	Lancl1/Mgst1
28-day post-SCI group vs. the control group	MF	GO:0050135	NAD(P)+ nucleosidase activity	2/99	0.0017742	0.0131499	0.00849562	Cd38/Tlr4
28-day post-SCI group vs. the control group	MF	GO:0061809	NAD+ nucleotidase, cyclic ADP-ribose generating	2/99	0.0017742	0.0131499	0.00849562	Cd38/Tlr4
28-day post-SCI group vs. the control group	MF	GO:0003953	NAD+ nucleosidase activity	2/99	0.0019784	0.01411028	0.00911608	Cd38/Tlr4
28-day post-SCI group vs. the control group	MF	GO:1900750	oligopeptide binding	2/99	0.0019784	0.01411028	0.00911608	Lancl1/Mgst1
28-day post-SCI group vs. the control group	MF	GO:0005178	integrin binding	4/99	0.0021482	0.01454482	0.00939682	Dst/Itgb5/Mmp14/Vcam1
28-day post-SCI group vs. the control group	MF	GO:0016922	nuclear receptor binding	4/99	0.0021482	0.01454482	0.00939682	Ets2/Hif1a/Ncoa7/Parp1
28-day post-SCI group vs. the control group	MF	GO:0034185	apolipoprotein binding	2/99	0.0021933	0.01454482	0.00939682	Lcat/Trem2
28-day post-SCI group vs. the control group	MF	GO:0051010	microtubule plus-end binding	2/99	0.0021933	0.01454482	0.00939682	Apc/Dst
28-day post-SCI group vs. the control group	MF	GO:0048156	tau protein binding	2/99	0.0026545	0.01729989	0.01117676	Apoe/Clu
28-day post-SCI group vs. the control group	MF	GO:0009931	calcium-dependent protein serine/threonine kinase acti	2/99	0.0029007	0.01858417	0.01200648	Mapkapk2/Prkcd
28-day post-SCI group vs. the control group	MF	GO:0010857	calcium-dependent protein kinase activity	2/99	0.0031572	0.0198906	0.01285051	Mapkapk2/Prkcd
28-day post-SCI group vs. the control group	MF	GO:0004857	enzyme inhibitor activity	6/99	0.0033574	0.02080468	0.01344106	Cdkn2c/Gskip/Hspb1/Parp1/Rnh1/Txnip
28-day post-SCI group vs. the control group	MF	GO:0043130	ubiquitin binding	3/99	0.0037546	0.02289071	0.01478876	Fbxw7/Hspb1/Tnfaip3
28-day post-SCI group vs. the control group	MF	GO:0019903	protein phosphatase binding	4/99	0.0040532	0.02431913	0.01571161	Met/Pawr/Sirpa/Stat6
28-day post-SCI group vs. the control group	MF	GO:0050839	cell adhesion molecule binding	5/99	0.004527	0.02629986	0.01699128	Dst/Itgb5/Mmp14/Sirpa/Vcam1
28-day post-SCI group vs. the control group	MF	GO:0005516	calmodulin binding	4/99	0.0045376	0.02629986	0.01699128	Mapkapk2/Mylk/Ppp3ca/Slc8a1
28-day post-SCI group vs. the control group	MF	GO:0043325	phosphatidylinositol-3,4-bisphosphate binding	2/99	0.004592	0.02629986	0.01699128	Ncf1/Plekha1
28-day post-SCI group vs. the control group	MF	GO:0038187	pattern recognition receptor activity	2/99	0.0049089	0.0276951	0.01789268	Cd36/Tlr4
28-day post-SCI group vs. the control group	MF	GO:0004364	glutathione transferase activity	2/99	0.0052356	0.0291038	0.01880279	Lancl1/Mgst1

28-day post-SCI group vs. the control group	MF	GO:0051059	NF-kappaB binding	2/99	0.005572	0.03052501	0.01972098	Hdac1/Rela
28-day post-SCI group vs. the control group	MF	GO:0051019	mitogen-activated protein kinase binding	2/99	0.0059181	0.03195781	0.02064665	Mapkapk2/Stau2
28-day post-SCI group vs. the control group	MF	GO:0004623	phospholipase A2 activity	2/99	0.0062738	0.03340133	0.02157925	Lcat/Prdx6
28-day post-SCI group vs. the control group	MF	GO:0001530	lipopolysaccharide binding	2/99	0.006639	0.03485475	0.02251825	Tlr4/Trem2
28-day post-SCI group vs. the control group	MF	GO:0032182	ubiquitin-like protein binding	3/99	0.0069407	0.03593935	0.02321896	Fbxw7/Hspb1/Tnfaip3
28-day post-SCI group vs. the control group	MF	GO:0004177	aminopeptidase activity	2/99	0.0073977	0.03778832	0.0244135	Ctsl/Mmp14
28-day post-SCI group vs. the control group	MF	GO:0016799	hydrolase activity, hydrolyzing N-glycosyl compounds	2/99	0.0086055	0.04337171	0.02802071	Cd38/Tlr4
28-day post-SCI group vs. the control group	MF	GO:0019209	kinase activator activity	3/99	0.0100232	0.04985233	0.03220758	Hbegf/Map2k6/Trem2
28-day post-SCI group vs. the control group	MF	GO:0070888	E-box binding	2/99	0.0112654	0.05530269	0.03572884	Hdac1/Hif1a
28-day post-SCI group vs. the control group	MF	GO:0015085	calcium ion transmembrane transporter activity	3/99	0.0115846	0.05614096	0.03627041	Atp2a2/Slc8a1/Trpm2
28-day post-SCI group vs. the control group	MF	GO:0001223	transcription coactivator binding	2/99	0.0122224	0.05848196	0.03778283	Hif1a/Rela
28-day post-SCI group vs. the control group	MF	GO:0008374	O-acyltransferase activity	2/99	0.0137222	0.06403714	0.04137181	Lcat/Prdx6
28-day post-SCI group vs. the control group	MF	GO:0019894	kinesin binding	2/99	0.0137222	0.06403714	0.04137181	Actb/Stau2
28-day post-SCI group vs. the control group	MF	GO:0044325	transmembrane transporter binding	3/99	0.0151067	0.06963798	0.04499029	Atp2a2/Fkbp1b/Slc8a1
28-day post-SCI group vs. the control group	MF	GO:0043394	proteoglycan binding	2/99	0.0158392	0.07213533	0.04660372	Apoe/Ctsl
28-day post-SCI group vs. the control group	MF	GO:0140296	general transcription initiation factor binding	2/99	0.0169468	0.07626078	0.04926901	Jun/Rela
28-day post-SCI group vs. the control group	MF	GO:1901681	sulfur compound binding	4/99	0.0175621	0.07809964	0.05045702	Apoe/Hbegf/Lancl1/Mgst1
28-day post-SCI group vs. the control group	MF	GO:0004714	transmembrane receptor protein tyrosine kinase activit	2/99	0.0180865	0.07949655	0.05135951	Axl/Met
28-day post-SCI group vs. the control group	MF	GO:0140678	molecular function inhibitor activity	2/99	0.0186682	0.08111024	0.05240205	Fkbp1b/Id1
28-day post-SCI group vs. the control group	MF	GO:0016765	transferase activity, transferring alkyl or aryl (other tha	2/99	0.0204601	0.08788559	0.05677933	Lancl1/Mgst1
28-day post-SCI group vs. the control group	MF	GO:0005080	protein kinase C binding	2/99	0.0210728	0.08950032	0.05782254	Hspb1/Pawr
28-day post-SCI group vs. the control group	MF	GO:0052689	carboxylic ester hydrolase activity	3/99	0.0214062	0.08990598	0.05808462	Lcat/Pon2/Prdx6
28-day post-SCI group vs. the control group	MF	GO:0005543	phospholipid binding	5/99	0.0225614	0.09294459	0.06004775	Amph/Apoe/Axl/Ncf1/Trem2
28-day post-SCI group vs. the control group	MF	GO:0046982	protein heterodimerization activity	4/99	0.0226214	0.09294459	0.06004775	Cybb/Hif1a/Mcl1/Tlr4
28-day post-SCI group vs. the control group	MF	GO:0001786	phosphatidylserine binding	2/99	0.0229562	0.09330598	0.06028122	Axl/Trem2
28-day post-SCI group vs. the control group	MF	GO:0008234	cysteine-type peptidase activity	3/99	0.0237798	0.09562503	0.06177947	Capns1/Ctsl/Tnfaip3
28-day post-SCI group vs. the control group	MF	GO:0009055	electron transfer activity	2/99	0.024249	0.0964856	0.06233545	Cybb/Xdh
28-day post-SCI group vs. the control group	MF	GO:0043022	ribosome binding	2/99	0.0262428	0.10333116	0.06675809	Eif2s1/Stau2
28-day post-SCI group vs. the control group	MF	GO:0002020	protease binding	3/99	0.027033	0.10534496	0.06805912	Apc/Tnfaip3/Tnfrsf1a
28-day post-SCI group vs. the control group	MF	GO:0016651	oxidoreductase activity, acting on NAD(P)H	2/99	0.0283008	0.10916027	0.07052404	Cybb/Ncf1
28-day post-SCI group vs. the control group	MF	GO:0002039	p53 binding	2/99	0.0290008	0.10962302	0.07082301	Hdac1/Hif1a
28-day post-SCI group vs. the control group	MF	GO:0016712	oxidoreductase activity, acting on paired donors, with i	2/99	0.0290008	0.10962302	0.07082301	Cyp1b1/Hmox2
28-day post-SCI group vs. the control group	MF	GO:0019199	transmembrane receptor protein kinase activity	2/99	0.0297077	0.11118324	0.071831	Axl/Met
28-day post-SCI group vs. the control group	MF	GO:0043621	protein self-association	2/99	0.0318692	0.11686663	0.07550281	Id1/Tnfaip3
28-day post-SCI group vs. the control group	MF	GO:0038024	cargo receptor activity	2/99	0.0340909	0.11686663	0.07550281	Cd36/Sdc1
28-day post-SCI group vs. the control group	MF	GO:0005041	low-density lipoprotein particle receptor activity	1/99	0.034318	0.11686663	0.07550281	Cd36
28-day post-SCI group vs. the control group	MF	GO:0008429	phosphatidylethanolamine binding	1/99	0.034318	0.11686663	0.07550281	Trem2
28-day post-SCI group vs. the control group	MF	GO:0015368	calcium:cation antiporter activity	1/99	0.034318	0.11686663	0.07550281	Slc8a1
28-day post-SCI group vs. the control group	MF	GO:0031078	histone deacetylase activity (H3-K14 specific)	1/99	0.034318	0.11686663	0.07550281	Hdac1
28-day post-SCI group vs. the control group	MF	GO:0032041	NAD-dependent histone deacetylase activity (H3-K14	1/99	0.034318	0.11686663	0.07550281	Hdac1

28-day post-SCI group vs. the control group	MF	GO:0047499	calcium-independent phospholipase A2 activity	1/99	0.034318	0.11686663	0.07550281	Prdx6
28-day post-SCI group vs. the control group	MF	GO:0070878	primary miRNA binding	1/99	0.034318	0.11686663	0.07550281	Smad1
28-day post-SCI group vs. the control group	MF	GO:0097027	ubiquitin-protein transferase activator activity	1/99	0.034318	0.11686663	0.07550281	Fbxw7
28-day post-SCI group vs. the control group	MF	GO:0097110	scaffold protein binding	2/99	0.0348446	0.11760062	0.07597701	Il6st/Trem2
28-day post-SCI group vs. the control group	MF	GO:0003725	double-stranded RNA binding	2/99	0.0356048	0.11910271	0.07694745	Ago1/Stau2
28-day post-SCI group vs. the control group	MF	GO:0005035	death receptor activity	1/99	0.037685	0.11970522	0.07733671	Tnfrsf1a
28-day post-SCI group vs. the control group	MF	GO:0005388	P-type calcium transporter activity	1/99	0.037685	0.11970522	0.07733671	Atp2a2
28-day post-SCI group vs. the control group	MF	GO:0016670	oxidoreductase activity, acting on a sulfur group of doı	1/99	0.037685	0.11970522	0.07733671	Selenbp1
28-day post-SCI group vs. the control group	MF	GO:0019855	calcium channel inhibitor activity	1/99	0.037685	0.11970522	0.07733671	Fkbp1b
28-day post-SCI group vs. the control group	MF	GO:0061578	Lys63-specific deubiquitinase activity	1/99	0.037685	0.11970522	0.07733671	Tnfaip3
28-day post-SCI group vs. the control group	MF	GO:0070410	co-SMAD binding	1/99	0.037685	0.11970522	0.07733671	Smad1
28-day post-SCI group vs. the control group	MF	GO:0051082	unfolded protein binding	2/99	0.0395007	0.12410603	0.08017989	Clu/Hspb1
28-day post-SCI group vs. the control group	MF	GO:0004861	cyclin-dependent protein serine/threonine kinase inhib	1/99	0.0410404	0.12410603	0.08017989	Cdkn2c
28-day post-SCI group vs. the control group	MF	GO:0008353	RNA polymerase II CTD heptapeptide repeat kinase ac	1/99	0.0410404	0.12410603	0.08017989	Mapk1
28-day post-SCI group vs. the control group	MF	GO:0017154	semaphorin receptor activity	1/99	0.0410404	0.12410603	0.08017989	Met
28-day post-SCI group vs. the control group	MF	GO:0032036	myosin heavy chain binding	1/99	0.0410404	0.12410603	0.08017989	Axl
28-day post-SCI group vs. the control group	MF	GO:0097655	serpin family protein binding	1/99	0.0410404	0.12410603	0.08017989	Ctsl
28-day post-SCI group vs. the control group	MF	GO:0008238	exopeptidase activity	2/99	0.0443783	0.12427561	0.08028945	Ctsl/Mmp14
28-day post-SCI group vs. the control group	MF	GO:0004706	JUN kinase kinase kinase activity	1/99	0.0443841	0.12427561	0.08028945	Ripk1
28-day post-SCI group vs. the control group	MF	GO:0015245	fatty acid transmembrane transporter activity	1/99	0.0443841	0.12427561	0.08028945	Cd36
28-day post-SCI group vs. the control group	MF	GO:0016018	cyclosporin A binding	1/99	0.0443841	0.12427561	0.08028945	Ppp3ca
28-day post-SCI group vs. the control group	MF	GO:0016661	oxidoreductase activity, acting on other nitrogenous cc	1/99	0.0443841	0.12427561	0.08028945	Xdh
28-day post-SCI group vs. the control group	MF	GO:0035325	Toll-like receptor binding	1/99	0.0443841	0.12427561	0.08028945	Cd36
28-day post-SCI group vs. the control group	MF	GO:0043522	leucine zipper domain binding	1/99	0.0443841	0.12427561	0.08028945	Pawr
28-day post-SCI group vs. the control group	MF	GO:0051400	BH domain binding	1/99	0.0443841	0.12427561	0.08028945	Mcl1
28-day post-SCI group vs. the control group	MF	GO:0101020	estrogen 16-alpha-hydroxylase activity	1/99	0.0443841	0.12427561	0.08028945	Cyp1b1
28-day post-SCI group vs. the control group	MF	GO:1990405	protein antigen binding	1/99	0.0443841	0.12427561	0.08028945	Sirpa
28-day post-SCI group vs. the control group	MF	GO:0003727	single-stranded RNA binding	2/99	0.045212	0.12447321	0.08041711	Ago1/Cbx6
28-day post-SCI group vs. the control group	MF	GO:0008013	beta-catenin binding	2/99	0.045212	0.12447321	0.08041711	Apc/Met
28-day post-SCI group vs. the control group	MF	GO:0016705	oxidoreductase activity, acting on paired donors, with :	3/99	0.0456665	0.12447321	0.08041711	Cyp1b1/Hmox2/Ptgs1
28-day post-SCI group vs. the control group	MF	GO:0004620	phospholipase activity	2/99	0.0460515	0.12447321	0.08041711	Lcat/Prdx6
28-day post-SCI group vs. the control group	MF	GO:0004198	calcium-dependent cysteine-type endopeptidase activit	1/99	0.0477164	0.12447321	0.08041711	Capns1
28-day post-SCI group vs. the control group	MF	GO:0030297	transmembrane receptor protein tyrosine kinase activat	1/99	0.0477164	0.12447321	0.08041711	Hbegf
28-day post-SCI group vs. the control group	MF	GO:0042301	phosphate ion binding	1/99	0.0477164	0.12447321	0.08041711	Rela
28-day post-SCI group vs. the control group	MF	GO:0043560	insulin receptor substrate binding	1/99	0.0477164	0.12447321	0.08041711	Prkcd
28-day post-SCI group vs. the control group	MF	GO:0070411	I-SMAD binding	1/99	0.0477164	0.12447321	0.08041711	Smad1
28-day post-SCI group vs. the control group	MF	GO:0051117	ATPase binding	2/99	0.0477477	0.12447321	0.08041711	Ppp3ca/S100a1
28-day post-SCI group vs. the control group	MF	GO:0005539	glycosaminoglycan binding	3/99	0.0481578	0.1246824	0.08055226	Apoe/Hbegf/Trem2

SCI: spinal cord injury; GO: Gene Ontology; BP: biological process; MF: :molecular function; CC: cellular component
