# Supplementary material for: Integrating bulk and single-cell transcriptome profiling to uncover diagnostic biomarkers and regulatory mechanisms of oxidative stress in spinal cord injury
Source: Neural Regen Res. 2025 Jan 13;21(6):2643–57. doi: 10.4103/NRR.NRR-D-24-00693 (PMC13217428; doi:10.4103/NRR.NRR-D-24-00693)
Supplement: Supplementary file 5 [file NRR-21-2643_Suppl4.pdf]

Additional Table 5 KEGG enrichment analysis of oxidative stress-related genes that are differentially expressed between groups at different time points after SCI and the control group

Group	ID	Description	pvalue	p.adjust	qvalue	geneID
4-hour post-SCI group and the control group	mmu04668	TNF signaling pathway - Mus musculus (house mouse)	1.45E-09	2.41E-07	1.19E-07	Atf4/Fos/Jun/Map2k3/Nfkb1/Ripk1/Tnfaip3/Tnfrsf1a
4-hour post-SCI group and the control group	mmu04210	Apoptosis - Mus musculus (house mouse)	1.43E-07	1.02E-05	5.02E-06	Atf4/Fos/Jun/Mcl1/Nfkb1/Ripk1/Tnfrsf1a
4-hour post-SCI group and the control group	mmu04010	MAPK signaling pathway - Mus musculus (house mouse)	1.91E-07	1.02E-05	5.02E-06	Atf4/Fos/Hspb1/Il1a/Jun/Map2k3/Myc/Nfkb1/Tnfrsf1a
4-hour post-SCI group and the control group	mmu05418	Fluid shear stress and atherosclerosis - Mus musculus (house mouse)	2.44E-07	1.02E-05	5.02E-06	Fos/Hmox1/Il1a/Jun/Nfkb1/Sdc1/Tnfrsf1a
4-hour post-SCI group and the control group	mmu05166	Human T-cell leukemia virus 1 infection - Mus musculus (house mouse)	6.28E-07	2.10E-05	1.03E-05	Atf4/Ets2/Fos/Fosl1/Jun/Myc/Nfkb1/Tnfrsf1a
4-hour post-SCI group and the control group	mmu04380	Osteoclast differentiation - Mus musculus (house mouse)	2.18E-06	6.06E-05	2.98E-05	Fos/Fosl1/Il1a/Jun/Nfkb1/Tnfrsf1a
4-hour post-SCI group and the control group	mmu05417	Lipid and atherosclerosis - Mus musculus (house mouse)	3.28E-06	7.82E-05	3.85E-05	Atf4/Fos/Jun/Map2k3/Nfkb1/Rxrb/Tnfrsf1a
4-hour post-SCI group and the control group	mmu04932	Non-alcoholic fatty liver disease - Mus musculus (house mouse)	6.87E-06	0.000141374	6.95E-05	Atf4/Fos/Il1a/Jun/Nfkb1/Tnfrsf1a
4-hour post-SCI group and the control group	mmu04657	IL-17 signaling pathway - Mus musculus (house mouse)	8.69E-06	0.000141374	6.95E-05	Fos/Fosl1/Jun/Nfkb1/Tnfaip3
4-hour post-SCI group and the control group	mmu05161	Hepatitis B - Mus musculus (house mouse)	8.85E-06	0.000141374	6.95E-05	Atf4/Fos/Jun/Map2k3/Myc/Nfkb1
4-hour post-SCI group and the control group	mmu05132	Salmonella infection - Mus musculus (house mouse)	9.31E-06	0.000141374	6.95E-05	Fos/Jun/Map2k3/Myc/Nfkb1/Ripk1/Tnfrsf1a
4-hour post-SCI group and the control group	mmu04620	Toll-like receptor signaling pathway - Mus musculus (house mouse)	1.24E-05	0.000172629	8.49E-05	Fos/Jun/Map2k3/Nfkb1/Ripk1
4-hour post-SCI group and the control group	mmu05167	Kaposi sarcoma-associated herpesvirus infection - Mus musculus (house mouse)	5.37E-05	0.000656175	0.000322607	Fos/Jun/Myc/Nfkb1/Rcan1/Tnfrsf1a
4-hour post-SCI group and the control group	mmu05207	Chemical carcinogenesis - receptor activation - Mus musculus (house mouse)	5.50E-05	0.000656175	0.000322607	Atf4/Fos/Jun/Myc/Nfkb1/Rxrb
4-hour post-SCI group and the control group	mmu05140	Leishmaniasis - Mus musculus (house mouse)	6.00E-05	0.000664958	0.000326925	Fos/Il1a/Jun/Nfkb1
4-hour post-SCI group and the control group	mmu05169	Epstein-Barr virus infection - Mus musculus (house mouse)	6.37E-05	0.000664958	0.000326925	Jun/Map2k3/Myc/Nfkb1/Ripk1/Tnfaip3
4-hour post-SCI group and the control group	mmu05162	Measles - Mus musculus (house mouse)	7.45E-05	0.000731049	0.000359419	Fos/Il1a/Jun/Nfkb1/Tnfaip3
4-hour post-SCI group and the control group	mmu05170	Human immunodeficiency virus 1 infection - Mus musculus (house mouse)	7.88E-05	0.000731049	0.000359419	Fos/Jun/Map2k3/Nfkb1/Ripk1/Tnfrsf1a
4-hour post-SCI group and the control group	mmu05133	Pertussis - Mus musculus (house mouse)	8.72E-05	0.000766432	0.000376815	Fos/Il1a/Jun/Nfkb1
4-hour post-SCI group and the control group	mmu05235	PD-L1 expression and PD-1 checkpoint pathway in cancer - Mus musculus (house mouse)	0.000146749	0.001225356	0.000602444	Fos/Jun/Map2k3/Nfkb1
4-hour post-SCI group and the control group	mmu04912	GnRH signaling pathway - Mus musculus (house mouse)	0.000160113	0.001273278	0.000626005	Atf4/Hbegf/Jun/Map2k3
4-hour post-SCI group and the control group	mmu05142	Chagas disease - Mus musculus (house mouse)	0.00026942	0.002018233	0.000992261	Fos/Jun/Nfkb1/Tnfrsf1a
4-hour post-SCI group and the control group	mmu04064	NF-kappa B signaling pathway - Mus musculus (house mouse)	0.000290045	0.002018233	0.000992261	Nfkb1/Ripk1/Tnfaip3/Tnfrsf1a
4-hour post-SCI group and the control group	mmu04659	Th17 cell differentiation - Mus musculus (house mouse)	0.000290045	0.002018233	0.000992261	Fos/Jun/Nfkb1/Rxrb
4-hour post-SCI group and the control group	mmu04928	Parathyroid hormone synthesis, secretion and action - Mus musculus (house mouse)	0.000323081	0.002158183	0.001061067	Atf4/Fos/Hbegf/Rxrb
4-hour post-SCI group and the control group	mmu05030	Cocaine addiction - Mus musculus (house mouse)	0.000425514	0.002733111	0.001343729	Atf4/Jun/Nfkb1
4-hour post-SCI group and the control group	mmu05208	Chemical carcinogenesis - reactive oxygen species - Mus musculus (house mouse)	0.000543831	0.003363695	0.001653755	Cat/Fos/Hmox1/Jun/Nfkb1
4-hour post-SCI group and the control group	mmu04926	Relaxin signaling pathway - Mus musculus (house mouse)	0.000634463	0.003784118	0.001860455	Atf4/Fos/Jun/Nfkb1
4-hour post-SCI group and the control group	mmu04915	Estrogen signaling pathway - Mus musculus (house mouse)	0.000711777	0.004098854	0.002015195	Atf4/Fos/Hbegf/Jun
4-hour post-SCI group and the control group	mmu05135	Yersinia infection - Mus musculus (house mouse)	0.000752842	0.004190819	0.002060409	Fos/Jun/Map2k3/Nfkb1
4-hour post-SCI group and the control group	mmu04936	Alcoholic liver disease - Mus musculus (house mouse)	0.000886111	0.004631415	0.002277027	Map2k3/Nfkb1/Ripk1/Tnfrsf1a
4-hour post-SCI group and the control group	mmu05321	Inflammatory bowel disease - Mus musculus (house mouse)	0.000903646	0.004631415	0.002277027	Il1a/Jun/Nfkb1
4-hour post-SCI group and the control group	mmu05171	Coronavirus disease - COVID-19 - Mus musculus (house mouse)	0.00091519	0.004631415	0.002277027	Fos/Hbegf/Jun/Nfkb1/Tnfrsf1a
4-hour post-SCI group and the control group	mmu05163	Human cytomegalovirus infection - Mus musculus (house mouse)	0.001036637	0.005091718	0.002503334	Atf4/Myc/Nfkb1/Ripk1/Tnfrsf1a
4-hour post-SCI group and the control group	mmu05031	Amphetamine addiction - Mus musculus (house mouse)	0.001233719	0.005886602	0.002894138	Atf4/Fos/Jun
4-hour post-SCI group and the control group	mmu04920	Adipocytokine signaling pathway - Mus musculus (house mouse)	0.001340224	0.006217151	0.003056652	Nfkb1/Rxrb/Tnfrsf1a
4-hour post-SCI group and the control group	mmu05160	Hepatitis C - Mus musculus (house mouse)	0.00162395	0.00732972	0.003603644	Myc/Nfkb1/Ripk1/Tnfrsf1a
4-hour post-SCI group and the control group	mmu04217	Necroptosis - Mus musculus (house mouse)	0.001971176	0.008662799	0.00425905	Il1a/Ripk1/Tnfaip3/Tnfrsf1a
4-hour post-SCI group and the control group	mmu04662	B cell receptor signaling pathway - Mus musculus (house mouse)	0.002101284	0.008702248	0.004278445	Fos/Jun/Nfkb1
4-hour post-SCI group and the control group	mmu04012	ErbB signaling pathway - Mus musculus (house mouse)	0.002174574	0.008702248	0.004278445	Hbegf/Jun/Myc
4-hour post-SCI group and the control group	mmu05152	Tuberculosis - Mus musculus (house mouse)	0.002184862	0.008702248	0.004278445	Il1a/Nfkb1/Sphk1/Tnfrsf1a
4-hour post-SCI group and the control group	mmu05206	MicroRNAs in cancer - Mus musculus (house mouse)	0.002188589	0.008702248	0.004278445	Ezr/Hmox1/Mcl1/Myc/Nfkb1
4-hour post-SCI group and the control group	mmu04218	Cellular senescence - Mus musculus (house mouse)	0.002366997	0.009016595	0.004432994	Il1a/Map2k3/Myc/Nfkb1
4-hour post-SCI group and the control group	mmu05323	Rheumatoid arthritis - Mus musculus (house mouse)	0.002403947	0.009016595	0.004432994	Fos/Il1a/Jun
4-hour post-SCI group and the control group	mmu04658	Th1 and Th2 cell differentiation - Mus musculus (house mouse)	0.002483613	0.009016595	0.004432994	Fos/Jun/Nfkb1
4-hour post-SCI group and the control group	mmu05210	Colorectal cancer - Mus musculus (house mouse)	0.002483613	0.009016595	0.004432994	Fos/Jun/Myc
4-hour post-SCI group and the control group	mmu04211	Longevity regulating pathway - Mus musculus (house mouse)	0.002647826	0.009408233	0.004625541	Atf4/Cat/Nfkb1
4-hour post-SCI group and the control group	mmu05022	Pathways of neurodegeneration - multiple diseases - Mus musculus (house mouse)	0.002825338	0.009707767	0.004772807	Atf4/Cat/Il1a/Map2k3/Nfkb1/Tnfrsf1a
4-hour post-SCI group and the control group	mmu01522	Endocrine resistance - Mus musculus (house mouse)	0.002906517	0.009707767	0.004772807	Fos/Hbegf/Jun
4-hour post-SCI group and the control group	mmu05222	Small cell lung cancer - Mus musculus (house mouse)	0.002906517	0.009707767	0.004772807	Myc/Nfkb1/Rxrb
4-hour post-SCI group and the control group	mmu05205	Proteoglycans in cancer - Mus musculus (house mouse)	0.003498241	0.011455024	0.005631843	Ezr/Hbegf/Myc/Sdc1
4-hour post-SCI group and the control group	mmu04933	AGE-RAGE signaling pathway in diabetic complications - Mus musculus (house mouse)	0.003670995	0.01178954	0.005796307	Il1a/Jun/Nfkb1
4-hour post-SCI group and the control group	mmu04621	NOD-like receptor signaling pathway - Mus musculus (house mouse)	0.00421755	0.013289262	0.006533643	Jun/Nfkb1/Ripk1/Tnfaip3
4-hour post-SCI group and the control group	mmu05145	Toxoplasmosis - Mus musculus (house mouse)	0.004548115	0.014065468	0.006915263	Map2k3/Nfkb1/Tnfrsf1a
4-hour post-SCI group and the control group	mmu05202	Transcriptional misregulation in cancer - Mus musculus (house mouse)	0.00479981	0.014573967	0.007165266	Myc/Nfkb1/Nr4a3/Rxrb
4-hour post-SCI group and the control group	mmu04935	Growth hormone synthesis, secretion and action - Mus musculus (house mouse)	0.005542596	0.016528812	0.008126362	Atf4/Fos/Map2k3
4-hour post-SCI group and the control group	mmu05216	Thyroid cancer - Mus musculus (house mouse)	0.005811725	0.017027334	0.00837146	Myc/Rxrb
4-hour post-SCI group and the control group	mmu04919	Thyroid hormone signaling pathway - Mus musculus (house mouse)	0.005946659	0.017122276	0.008418137	Myc/Rcan1/Rxrb
4-hour post-SCI group and the control group	mmu04722	Neurotrophin signaling pathway - Mus musculus (house mouse)	0.006085182	0.01722416	0.008468229	Atf4/Jun/Nfkb1
4-hour post-SCI group and the control group	mmu04660	T cell receptor signaling pathway - Mus musculus (house mouse)	0.006225636	0.017328021	0.008519292	Fos/Jun/Nfkb1
4-hour post-SCI group and the control group	mmu04071	Sphingolipid signaling pathway - Mus musculus (house mouse)	0.006806898	0.018635279	0.009162003	Nfkb1/Sphk1/Tnfrsf1a
4-hour post-SCI group and the control group	mmu05219	Bladder cancer - Mus musculus (house mouse)	0.007100817	0.019126395	0.009403459	Hbegf/Myc

4-hour post-SCI group and the control group	mmu05224	Breast cancer - Mus musculus (house mouse)	0.010383592	0.02752476	0.013532501	Fos/Jun/Myc
4-hour post-SCI group and the control group	mmu04921	Oxytocin signaling pathway - Mus musculus (house mouse)	0.011572197	0.030196201	0.01484591	Fos/Jun/Rcan1
4-hour post-SCI group and the control group	mmu04978	Mineral absorption - Mus musculus (house mouse)	0.01208667	0.031053444	0.015267372	Hmox1/Slc8a1
4-hour post-SCI group and the control group	mmu04370	VEGF signaling pathway - Mus musculus (house mouse)	0.013855213	0.035057887	0.01723615	Hspb1/Sphk1
4-hour post-SCI group and the control group	mmu04310	Wnt signaling pathway - Mus musculus (house mouse)	0.015840726	0.0394836	0.019412044	Fosl1/Jun/Myc
4-hour post-SCI group and the control group	mmu05164	Influenza A - Mus musculus (house mouse)	0.016086916	0.039507573	0.019423831	Il1a/Nfkb1/Tnfrsf1a
4-hour post-SCI group and the control group	mmu05225	Hepatocellular carcinoma - Mus musculus (house mouse)	0.016335279	0.039536109	0.01943786	Hmox1/Myc/Txnrd1
4-hour post-SCI group and the control group	mmu04137	Mitophagy - animal - Mus musculus (house mouse)	0.018732142	0.044689538	0.021971535	Atf4/Jun
4-hour post-SCI group and the control group	mmu05221	Acute myeloid leukemia - Mus musculus (house mouse)	0.019782959	0.04653175	0.022877255	Myc/Nfkb1
4-hour post-SCI group and the control group	mmu04622	RIG-I-like receptor signaling pathway - Mus musculus (house mouse)	0.020317531	0.047125385	0.023169115	Nfkb1/Ripk1
4-hour post-SCI group and the control group	mmu04917	Prolactin signaling pathway - Mus musculus (house mouse)	0.021957418	0.050231354	0.024696159	Fos/Nfkb1
4-hour post-SCI group and the control group	mmu04623	Cytosolic DNA-sensing pathway - Mus musculus (house mouse)	0.022515971	0.05081307	0.024982158	Nfkb1/Ripk1
4-hour post-SCI group and the control group	mmu05220	Chronic myeloid leukemia - Mus musculus (house mouse)	0.02308042	0.051392402	0.025266986	Myc/Nfkb1
4-hour post-SCI group and the control group	mmu04151	PI3K-Akt signaling pathway - Mus musculus (house mouse)	0.025060301	0.055066714	0.027073455	Atf4/Mcl1/Myc/Nfkb1
4-hour post-SCI group and the control group	mmu05014	Amyotrophic lateral sclerosis - Mus musculus (house mouse)	0.026190468	0.056802703	0.027926951	Atf4/Cat/Map2k3/Tnfrsf1a
4-hour post-SCI group and the control group	mmu05010	Alzheimer disease - Mus musculus (house mouse)	0.029519062	0.062714545	0.030833498	Atf4/Il1a/Nfkb1/Tnfrsf1a
4-hour post-SCI group and the control group	mmu04146	Peroxisome - Mus musculus (house mouse)	0.02966736	0.062714545	0.030833498	Cat/Xdh
4-hour post-SCI group and the control group	mmu04024	cAMP signaling pathway - Mus musculus (house mouse)	0.031551991	0.065864782	0.032382307	Fos/Jun/Nfkb1
4-hour post-SCI group and the control group	mmu05203	Viral carcinogenesis - Mus musculus (house mouse)	0.033376336	0.06881294	0.033831764	Atf4/Jun/Nfkb1
4-hour post-SCI group and the control group	mmu05231	Choline metabolism in cancer - Mus musculus (house mouse)	0.03691214	0.075174725	0.036959524	Fos/Jun
4-hour post-SCI group and the control group	mmu05215	Prostate cancer - Mus musculus (house mouse)	0.037601679	0.07565639	0.037196334	Atf4/Nfkb1
4-hour post-SCI group and the control group	mmu05146	Amoebiasis - Mus musculus (house mouse)	0.043294966	0.086074516	0.042318388	Hspb1/Nfkb1
4-hour post-SCI group and the control group	mmu04350	TGF-beta signaling pathway - Mus musculus (house mouse)	0.04550877	0.088371682	0.043447786	Id1/Myc
4-hour post-SCI group and the control group	mmu04931	Insulin resistance - Mus musculus (house mouse)	0.04550877	0.088371682	0.043447786	Nfkb1/Tnfrsf1a
4-hour post-SCI group and the control group	mmu04625	C-type lectin receptor signaling pathway - Mus musculus (house mouse)	0.047007784	0.089207953	0.043858937	Jun/Nfkb1
4-hour post-SCI group and the control group	mmu04725	Cholinergic synapse - Mus musculus (house mouse)	0.047007784	0.089207953	0.043858937	Atf4/Fos
4-hour post-SCI group and the control group	mmu04066	HIF-1 signaling pathway - Mus musculus (house mouse)	0.048524999	0.091052526	0.044765818	Hmox1/Nfkb1
1-day post-SCI group and the control group	mmu04668	TNF signaling pathway - Mus musculus (house mouse)	1E-15	2.26E-13	1.1E-13	Atf2/Atf4/Edn1/Fos/Jun/Map2k3/Map2k4/Map2k6/Mapk14/Mapk8/Mmp3/Ptgs2/Rela/Ripk1/Ripk3/Tnfrsf1a/Vcam1
1-day post-SCI group and the control group	mmu05417	Lipid and atherosclerosis - Mus musculus (house mouse)	3.68E-11	3.71E-09	1.8E-09	Atf4/Bax/Eif2s1/Fos/Jak2/Jun/Map2k3/Map2k4/Map2k6/Mapk14/Mapk8/Mmp3/Nos3/Ppp3ca/Rela/Tnfrsf1a/Vcam1
1-day post-SCI group and the control group	mmu05161	Hepatitis B - Mus musculus (house mouse)	6.39E-11	3.71E-09	1.8E-09	Atf2/Atf4/Bax/Fos/Jak2/Jun/Map2k3/Map2k4/Map2k6/Mapk14/Mapk8/Myc/Pcna/Rela/Stat6
1-day post-SCI group and the control group	mmu05167	Kaposi sarcoma-associated herpesvirus infection - Mus musculus (house mou	6.57E-11	3.71E-09	1.8E-09	Bax/Ccr1/Cdk4/Fos/Il6st/Jak2/Jun/Map2k4/Map2k6/Mapk14/Mapk8/Myc/Ppp3ca/Ptgs2/Rcan1/Rela/Tnfrsf1a
1-day post-SCI group and the control group	mmu05418	Fluid shear stress and atherosclerosis - Mus musculus (house mouse)	1.85E-10	8.36E-09	4.05E-09	Ctsl/Edn1/Fos/Hmox1/Jun/Map2k4/Map2k6/Mapk14/Mapk8/Nos3/Rela/Sdc1/Tnfrsf1a/Vcam1
1-day post-SCI group and the control group	mmu04010	MAPK signaling pathway - Mus musculus (house mouse)	8.52E-10	3.21E-08	1.55E-08	Atf2/Atf4/Fos/Hspb1/Jun/Map2k3/Map2k4/Map2k6/Map4k4/Mapk14/Mapk8/Mapkapk3/Mapt/Met/Myc/Ppp3ca/Rela/Tnfrsf1a
1-day post-SCI group and the control group	mmu05163	Human cytomegalovirus infection - Mus musculus (house mouse)	4.4E-09	0.000000142	6.88E-08	Atf2/Atf4/Bax/Ccr1/Cdk4/Map2k6/Mapk14/Mdm2/Myc/Ppp3ca/Ptgs2/Pxn/Rela/Ripk1/Tnfrsf1a/Tsc1
1-day post-SCI group and the control group	mmu04210	Apoptosis - Mus musculus (house mouse)	9.77E-09	0.000000276	0.000000134	Atf4/Bax/Capn2/Ctsl/Eif2s1/Fos/Jun/Mapk8/Mcl1/Rela/Ripk1/Tnfrsf1a
1-day post-SCI group and the control group	mmu04933	AGE-RAGE signaling pathway in diabetic complications - Mus musculus (ho	6.01E-08	0.0000015	0.000000726	Bax/Cdk4/Edn1/Jak2/Jun/Mapk14/Mapk8/Nos3/Rela/Vcam1
1-day post-SCI group and the control group	mmu05169	Epstein-Barr virus infection - Mus musculus (house mouse)	6.63E-08	0.0000015	0.000000726	Bax/Btk/Cdk4/Hdac1/Jun/Map2k3/Map2k4/Map2k6/Mapk14/Mapk8/Mdm2/Myc/Rela/Ripk1
1-day post-SCI group and the control group	mmu04370	VEGF signaling pathway - Mus musculus (house mouse)	9.98E-08	0.00000205	0.000000993	Hspb1/Mapk14/Mapkapk3/Nos3/Ppp3ca/Ptgs2/Pxn/Sphk1
1-day post-SCI group and the control group	mmu05132	Salmonella infection - Mus musculus (house mouse)	0.000000205	0.00000386	0.00000187	Bax/Dynll1/Fos/Jun/Map2k3/Map2k4/Map2k6/Mapk14/Mapk8/Myc/Rela/Ripk1/Ripk3/Tnfrsf1a
1-day post-SCI group and the control group	mmu04380	Osteoclast differentiation - Mus musculus (house mouse)	0.000000568	0.00000982	0.00000475	Btk/Fos/Fosl1/Jun/Map2k6/Mapk14/Mapk8/Ppp3ca/Rela/Tnfrsf1a
1-day post-SCI group and the control group	mmu04926	Relaxin signaling pathway - Mus musculus (house mouse)	0.00000061	0.00000982	0.00000475	Atf2/Atf4/Edn1/Fos/Jun/Map2k4/Mapk14/Mapk8/Nos3/Rela
1-day post-SCI group and the control group	mmu04620	Toll-like receptor signaling pathway - Mus musculus (house mouse)	0.000000651	0.00000982	0.00000475	Fos/Jun/Map2k3/Map2k4/Map2k6/Mapk14/Mapk8/Rela/Ripk1
1-day post-SCI group and the control group	mmu05022	Pathways of neurodegeneration - multiple diseases - Mus musculus (house mo	0.000000838	0.0000117	0.00000566	Atf4/Bax/Capn2/Cat/Eif2s1/Gpx1/Map2k3/Map2k6/Mapk14/Mapk8/Mapt/Ndufa6/Pink1/Ppp3ca/Ptgs2/Rela/Tnfrsf1a/Trap1
1-day post-SCI group and the control group	mmu05205	Proteoglycans in cancer - Mus musculus (house mouse)	0.000000905	0.0000117	0.00000566	Ctsl/Cttn/Ezr/Hbegf/Itga5/Mapk14/Mdm2/Met/Myc/Ppp1ca/Pxn/Sdc1
1-day post-SCI group and the control group	mmu05135	Yersinia infection - Mus musculus (house mouse)	0.00000093	0.0000117	0.00000566	Fos/Itga5/Jun/Map2k3/Map2k4/Map2k6/Mapk14/Mapk8/Pxn/Rela

1-day post-SCI group and the control group	mmu04659	Th17 cell differentiation - Mus musculus (house mouse)	0.000000988	0.0000117	0.00000569	Fos/Il6st/Jak2/Jun/Mapk14/Mapk8/Ppp3ca/Rela/Stat6
1-day post-SCI group and the control group	mmu05166	Human T-cell leukemia virus 1 infection - Mus musculus (house mouse)	0.00000118	0.0000133	0.00000645	Atf2/Atf4/Bax/Cdk4/Fos/Fosl1/Jun/Map2k4/Mapk8/Myc/Ppp3ca/Rela/Tnfrsf1a
1-day post-SCI group and the control group	mmu05208	Chemical carcinogenesis - reactive oxygen species - Mus musculus (house mo	0.0000021	0.0000223	0.0000108	Cat/Cyp1b1/Fos/Hmox1/Jun/Lpo/Map2k4/Mapk14/Mapk8/Met/Ndufa6/Rela
1-day post-SCI group and the control group	mmu04218	Cellular senescence - Mus musculus (house mouse)	0.00000217	0.0000223	0.0000108	Capn2/Cdk4/Map2k3/Map2k6/Mapk14/Mdm2/Myc/Ppp1ca/Ppp3ca/Rela/Tsc1
1-day post-SCI group and the control group	mmu04935	Growth hormone synthesis, secretion and action - Mus musculus (house mous	0.00000246	0.0000236	0.0000115	Atf2/Atf4/Fos/Jak2/Map2k3/Map2k4/Map2k6/Mapk14/Mapk8
1-day post-SCI group and the control group	mmu04658	Th1 and Th2 cell differentiation - Mus musculus (house mouse)	0.00000261	0.0000236	0.0000115	Fos/Jak2/Jun/Mapk14/Mapk8/Ppp3ca/Rela/Stat6
1-day post-SCI group and the control group	mmu05235	PD-L1 expression and PD-1 checkpoint pathway in cancer - Mus musculus (h	0.00000261	0.0000236	0.0000115	Fos/Jak2/Jun/Map2k3/Map2k6/Mapk14/Ppp3ca/Rela
1-day post-SCI group and the control group	mmu04912	GnRH signaling pathway - Mus musculus (house mouse)	0.0000031	0.000027	0.0000131	Atf4/Hbegf/Jun/Map2k3/Map2k4/Map2k6/Mapk14/Mapk8
1-day post-SCI group and the control group	mmu04932	Non-alcoholic fatty liver disease - Mus musculus (house mouse)	0.00000348	0.0000291	0.0000141	Atf4/Bax/Eif2s1/Fos/Jun/Mapk14/Mapk8/Ndufa6/Rela/Tnfrsf1a
1-day post-SCI group and the control group	mmu01522	Endocrine resistance - Mus musculus (house mouse)	0.00000398	0.000031	0.000015	Bax/Cdk4/Fos/Hbegf/Jun/Mapk14/Mapk8/Mdm2
1-day post-SCI group and the control group	mmu04657	IL-17 signaling pathway - Mus musculus (house mouse)	0.00000398	0.000031	0.000015	Fos/Fosl1/Jun/Mapk14/Mapk8/Mmp3/Ptgs2/Rela
1-day post-SCI group and the control group	mmu05170	Human immunodeficiency virus 1 infection - Mus musculus (house mouse)	0.00000472	0.0000356	0.0000172	Bax/Fos/Jun/Map2k3/Map2k6/Mapk14/Mapk8/Ppp3ca/Pxn/Rela/Ripk1/Tnfrsf1a
1-day post-SCI group and the control group	mmu05031	Amphetamine addiction - Mus musculus (house mouse)	0.00000547	0.0000399	0.0000193	Atf2/Atf4/Fos/Hdac1/Jun/Ppp1ca/Ppp3ca
1-day post-SCI group and the control group	mmu04936	Alcoholic liver disease - Mus musculus (house mouse)	0.0000114	0.0000807	0.0000391	Map2k3/Map2k4/Map2k6/Mapk14/Mapk8/Ppargc1a/Rela/Ripk1/Tnfrsf1a
1-day post-SCI group and the control group	mmu04211	Longevity regulating pathway - Mus musculus (house mouse)	0.0000319	0.00021838	0.000105782	Atf2/Atf4/Bax/Cat/Ppargc1a/Rela/Tsc1
1-day post-SCI group and the control group	mmu04071	Sphingolipid signaling pathway - Mus musculus (house mouse)	0.0000374	0.000248464	0.000120355	Abcc1/Bax/Mapk14/Mapk8/Nos3/Rela/Sphk1/Tnfrsf1a
1-day post-SCI group and the control group	mmu05206	MicroRNAs in cancer - Mus musculus (house mouse)	0.0000488	0.000312813	0.000151526	Abcc1/Cyp1b1/Ezr/Hdac1/Hmox1/Itga5/Mcl1/Mdm2/Met/Myc/Ptgs2/Spry2
1-day post-SCI group and the control group	mmu04664	Fc epsilon RI signaling pathway - Mus musculus (house mouse)	0.0000498	0.000312813	0.000151526	Btk/Map2k3/Map2k4/Map2k6/Mapk14/Mapk8
1-day post-SCI group and the control group	mmu04151	PI3K-Akt signaling pathway - Mus musculus (house mouse)	0.0000674	0.000411712	0.000199432	Atf2/Atf4/Cdk4/Itga5/Itga7/Jak2/Mcl1/Mdm2/Met/Myc/Nos3/Rela/Tsc1
1-day post-SCI group and the control group	mmu05140	Leishmaniasis - Mus musculus (house mouse)	0.0000696	0.000414051	0.000200565	Fos/Jak2/Jun/Mapk14/Ptgs2/Rela
1-day post-SCI group and the control group	mmu05142	Chagas disease - Mus musculus (house mouse)	0.000076	0.000440667	0.000213458	Fos/Jun/Map2k4/Mapk14/Mapk8/Rela/Tnfrsf1a
1-day post-SCI group and the control group	mmu05203	Viral carcinogenesis - Mus musculus (house mouse)	0.0000973	0.000549743	0.000266294	Atf2/Atf4/Bax/Cdk4/Hdac1/Il6st/Jun/Mdm2/Pxn/Rela
1-day post-SCI group and the control group	mmu05145	Toxoplasmosis - Mus musculus (house mouse)	0.000108894	0.000595074	0.000288252	Jak2/Map2k3/Map2k6/Mapk14/Mapk8/Rela/Tnfrsf1a
1-day post-SCI group and the control group	mmu05220	Chronic myeloid leukemia - Mus musculus (house mouse)	0.000110589	0.000595074	0.000288252	Bax/Cdk4/Hdac1/Mdm2/Myc/Rela
1-day post-SCI group and the control group	mmu04931	Insulin resistance - Mus musculus (house mouse)	0.000115349	0.000606255	0.000293668	Mapk8/Nos3/Ppargc1a/Ppargc1b/Ppp1ca/Rela/Tnfrsf1a
1-day post-SCI group and the control group	mmu05133	Pertussis - Mus musculus (house mouse)	0.00011897	0.000611073	0.000296002	Fos/Itga5/Jun/Mapk14/Mapk8/Rela
1-day post-SCI group and the control group	mmu04625	C-type lectin receptor signaling pathway - Mus musculus (house mouse)	0.000129195	0.000648846	0.000314299	Jun/Mapk14/Mapk8/Mdm2/Ppp3ca/Ptgs2/Rela
1-day post-SCI group and the control group	mmu04921	Oxytocin signaling pathway - Mus musculus (house mouse)	0.000147412	0.000724242	0.00035082	Fos/Jun/Nos3/Ppp1ca/Ppp3ca/Ptgs2/Rcan1/Trpm2
1-day post-SCI group and the control group	mmu05171	Coronavirus disease - COVID-19 - Mus musculus (house mouse)	0.000193659	0.000931212	0.000451076	Fos/Hbegf/Il6st/Jun/Mapk14/Mapk8/Mmp3/Rela/Rpl13a/Tnfrsf1a
1-day post-SCI group and the control group	mmu04660	T cell receptor signaling pathway - Mus musculus (house mouse)	0.000220163	0.001036602	0.000502127	Cdk4/Fos/Jun/Mapk14/Mapk8/Ppp3ca/Rela
1-day post-SCI group and the control group	mmu05014	Amyotrophic lateral sclerosis - Mus musculus (house mouse)	0.000313568	0.001446252	0.00070056	Atf4/Bax/Cat/Eif2s1/Gpx1/Map2k3/Map2k6/Mapk14/Ndufa6/Pink1/Ppp3ca/Tnfrsf1a
1-day post-SCI group and the control group	mmu04217	Necroptosis - Mus musculus (house mouse)	0.000369132	0.001668477	0.000808205	Bax/Capn2/Jak2/Mapk8/Ripk1/Ripk3/Stat6/Tnfrsf1a
1-day post-SCI group and the control group	mmu04728	Dopaminergic synapse - Mus musculus (house mouse)	0.000409256	0.001813567	0.000878486	Atf2/Atf4/Fos/Mapk14/Mapk8/Ppp1ca/Ppp3ca
1-day post-SCI group and the control group	mmu05152	Tuberculosis - Mus musculus (house mouse)	0.00044589	0.001937907	0.000938716	Bax/Jak2/Mapk14/Mapk8/Ppp3ca/Rela/Sphk1/Tnfrsf1a
1-day post-SCI group and the control group	mmu04137	Mitophagy - animal - Mus musculus (house mouse)	0.000590098	0.002516267	0.001218872	Atf4/Jun/Mapk8/Pink1/Rela
1-day post-SCI group and the control group	mmu05162	Measles - Mus musculus (house mouse)	0.000629032	0.002632615	0.00127523	Bax/Cdk4/Eif2s1/Fos/Jun/Mapk8/Rela
1-day post-SCI group and the control group	mmu04064	NF-kappa B signaling pathway - Mus musculus (house mouse)	0.000644348	0.002647685	0.00128253	Btk/Ptgs2/Rela/Ripk1/Tnfrsf1a/Vcam1
1-day post-SCI group and the control group	mmu04920	Adipocytokine signaling pathway - Mus musculus (house mouse)	0.000719759	0.002904741	0.001407047	Jak2/Mapk8/Ppargc1a/Rela/Tnfrsf1a
1-day post-SCI group and the control group	mmu05219	Bladder cancer - Mus musculus (house mouse)	0.000736547	0.002920343	0.001414605	Cdk4/Hbegf/Mdm2/Myc
1-day post-SCI group and the control group	mmu04917	Prolactin signaling pathway - Mus musculus (house mouse)	0.000869643	0.00338861	0.001641432	Fos/Jak2/Mapk14/Mapk8/Rela
1-day post-SCI group and the control group	mmu05100	Bacterial invasion of epithelial cells - Mus musculus (house mouse)	0.000981738	0.003677703	0.001781468	Ctnna1/Cttn/Itga5/Met/Pxn
1-day post-SCI group and the control group	mmu04510	Focal adhesion - Mus musculus (house mouse)	0.000983951	0.003677703	0.001781468	Capn2/Itga5/Itga7/Jun/Mapk8/Met/Ppp1ca/Pxn
1-day post-SCI group and the control group	mmu05165	Human papillomavirus infection - Mus musculus (house mouse)	0.000992654	0.003677703	0.001781468	Bax/Cdk4/Hdac1/Itga5/Itga7/Mdm2/Ptgs2/Pxn/Rela/Tnfrsf1a/Tsc1
1-day post-SCI group and the control group	mmu05030	Cocaine addiction - Mus musculus (house mouse)	0.00134117	0.004853526	0.002351033	Atf2/Atf4/Jun/Rela
1-day post-SCI group and the control group	mmu04722	Neurotrophin signaling pathway - Mus musculus (house mouse)	0.001352974	0.004853526	0.002351033	Atf4/Bax/Jun/Mapk14/Mapk8/Rela
1-day post-SCI group and the control group	mmu05160	Hepatitis C - Mus musculus (house mouse)	0.001392665	0.004917848	0.00238219	Bax/Cdk4/Eif2s1/Myc/Rela/Ripk1/Tnfrsf1a
1-day post-SCI group and the control group	mmu04662	B cell receptor signaling pathway - Mus musculus (house mouse)	0.001459902	0.005075966	0.002458782	Btk/Fos/Jun/Ppp3ca/Rela
1-day post-SCI group and the control group	mmu04530	Tight junction - Mus musculus (house mouse)	0.001492337	0.005110122	0.002475327	Cdk4/Cttn/Ezr/Jun/Mapk8/Pcna/Ybx3
1-day post-SCI group and the control group	mmu03083	Polycomb repressive complex - Mus musculus (house mouse)	0.001540101	0.00511857	0.002479419	Cbx6/Eed/Hdac1/Phc3/Rbbp7
1-day post-SCI group and the control group	mmu04012	ErbB signaling pathway - Mus musculus (house mouse)	0.001540101	0.00511857	0.002479419	Hbegf/Jun/Map2k4/Mapk8/Myc
1-day post-SCI group and the control group	mmu05010	Alzheimer disease - Mus musculus (house mouse)	0.001564707	0.005124983	0.002482526	Apoe/Atf4/Capn2/Eif2s1/Mapk8/Mapt/Ndufa6/Ppp3ca/Ptgs2/Rela/Tnfrsf1a
1-day post-SCI group and the control group	mmu05202	Transcriptional misregulation in cancer - Mus musculus (house mouse)	0.001845176	0.005956364	0.002885244	Bax/Hdac1/Mdm2/Met/Mmp3/Myc/Rela/Smad1
1-day post-SCI group and the control group	mmu05210	Colorectal cancer - Mus musculus (house mouse)	0.001893654	0.005956364	0.002885244	Bax/Fos/Jun/Mapk8/Myc

1-day post-SCI group and the control group	mmu05207	Chemical carcinogenesis - receptor activation - Mus musculus (house mouse)	0.001897603	0.005956364	0.002885244	Atf2/Atf4/Cyp1b1/Fos/Jak2/Jun/Myc/Rela
1-day post-SCI group and the control group	mmu04978	Mineral absorption - Mus musculus (house mouse)	0.002081862	0.006445216	0.003122042	Atox1/Hmox1/Hmox2/Slc8a1
1-day post-SCI group and the control group	mmu04915	Estrogen signaling pathway - Mus musculus (house mouse)	0.002192011	0.00669452	0.003242804	Atf2/Atf4/Fos/Hbegf/Jun/Nos3
1-day post-SCI group and the control group	mmu05222	Small cell lung cancer - Mus musculus (house mouse)	0.002415097	0.007277492	0.003525194	Bax/Cdk4/Myc/Ptgs2/Rela
1-day post-SCI group and the control group	mmu04215	Apoptosis - multiple species - Mus musculus (house mouse)	0.003979283	0.011833131	0.005731931	Bax/Mapk8/Tnfrsf1a
1-day post-SCI group and the control group	mmu04110	Cell cycle - Mus musculus (house mouse)	0.004680609	0.013737892	0.006654592	Cdk4/Cdkn1c/Hdac1/Mdm2/Myc/Pcna
1-day post-SCI group and the control group	mmu05012	Parkinson disease - Mus musculus (house mouse)	0.005049321	0.014630083	0.007086766	Atf4/Bax/Eif2s1/Mapk8/Mapt/Ndufa6/Pink1/Trap1
1-day post-SCI group and the control group	mmu05415	Diabetic cardiomyopathy - Mus musculus (house mouse)	0.005327346	0.015240256	0.007382332	Mapk14/Mapk8/Ndufa6/Nos3/Pdk2/Ppp1ca/Rela
1-day post-SCI group and the control group	mmu05020	Prion disease - Mus musculus (house mouse)	0.005399821	0.015254493	0.007389228	Atf2/Atf4/Bax/Eif2s1/Mapk14/Mapk8/Ndufa6/Ppp3c[illegible]
1-day post-SCI group and the control group	mmu04622	RIG-I-like receptor signaling pathway - Mus musculus (house mouse)	0.005617378	0.015673179	0.007592038	Mapk14/Mapk8/Rela/Ripk1
1-day post-SCI group and the control group	mmu05218	Melanoma - Mus musculus (house mouse)	0.005902672	0.01626834	0.007880333	Bax/Cdk4/Mdm2/Met
1-day post-SCI group and the control group	mmu04621	NOD-like receptor signaling pathway - Mus musculus (house mouse)	0.006041628	0.016450699	0.007968667	Jun/Mapk14/Mapk8/Rela/Ripk1/Ripk3/Trpm2
1-day post-SCI group and the control group	mmu04670	Leukocyte transendothelial migration - Mus musculus (house mouse)	0.006918888	0.018615103	0.009017097	Ctnna1/Ezr/Mapk14/Pxn/Vcam1
1-day post-SCI group and the control group	mmu05212	Pancreatic cancer - Mus musculus (house mouse)	0.007140282	0.018984749	0.009196152	Bax/Cdk4/Mapk8/Rela
1-day post-SCI group and the control group	mmu05412	Arrhythmogenic right ventricular cardiomyopathy - Mus musculus (house mo	0.007474453	0.019642168	0.009514604	Ctnna1/Itga5/Itga7/Slc8a1
1-day post-SCI group and the control group	mmu04022	cGMP-PKG signaling pathway - Mus musculus (house mouse)	0.007917582	0.020298807	0.009832678	Atf2/Atf4/Nos3/Ppp1ca/Ppp3ca/Slc8a1
1-day post-SCI group and the control group	mmu05164	Influenza A - Mus musculus (house mouse)	0.007917582	0.020298807	0.009832678	Bax/Cdk4/Eif2s1/Jak2/Rela/Tnfrsf1a
1-day post-SCI group and the control group	mmu04141	Protein processing in endoplasmic reticulum - Mus musculus (house mouse)	0.00813517	0.020298807	0.009832678	Atf4/Bax/Capn2/Eif2s1/Mapk8/P4hb
1-day post-SCI group and the control group	mmu05225	Hepatocellular carcinoma - Mus musculus (house mouse)	0.00813517	0.020298807	0.009832678	Bax/Cdk4/Hmox1/Met/Myc/Txnrd1
1-day post-SCI group and the control group	mmu01521	EGFR tyrosine kinase inhibitor resistance - Mus musculus (house mouse)	0.008173413	0.020298807	0.009832678	Axl/Bax/Jak2/Met
1-day post-SCI group and the control group	mmu04750	Inflammatory mediator regulation of TRP channels - Mus musculus (house m	0.00904623	0.022222261	0.010764393	Map2k3/Map2k6/Mapk14/Mapk8/Ppp1ca
1-day post-SCI group and the control group	mmu05323	Rheumatoid arthritis - Mus musculus (house mouse)	0.011394985	0.027691038	0.013413451	Ctsl/Fos/Jun/Mmp3
1-day post-SCI group and the control group	mmu05410	Hypertrophic cardiomyopathy - Mus musculus (house mouse)	0.013273588	0.031886233	0.01544559	Edn1/Itga5/Itga7/Slc8a1
1-day post-SCI group and the control group	mmu04550	Signaling pathways regulating pluripotency of stem cells - Mus musculus (ho	0.013403505	0.031886233	0.01544559	Il6st/Jak2/Mapk14/Myc/Smad1
1-day post-SCI group and the control group	mmu05224	Breast cancer - Mus musculus (house mouse)	0.016254257	0.03826523	0.018535556	Bax/Cdk4/Fos/Jun/Myc
1-day post-SCI group and the control group	mmu05231	Choline metabolism in cancer - Mus musculus (house mouse)	0.017014626	0.039642324	0.019202616	Fos/Jun/Mapk8/Tsc1
1-day post-SCI group and the control group	mmu05215	Prostate cancer - Mus musculus (house mouse)	0.017597544	0.040582091	0.019657836	Atf4/Mdm2/Mmp3/Rela
1-day post-SCI group and the control group	mmu05144	Malaria - Mus musculus (house mouse)	0.019598915	0.044740958	0.021672378	Met/Sdc1/Vcam1
1-day post-SCI group and the control group	mmu04261	Adrenergic signaling in cardiomyocytes - Mus musculus (house mouse)	0.020479317	0.045862094	0.022215453	Atf2/Atf4/Mapk14/Ppp1ca/Slc8a1
1-day post-SCI group and the control group	mmu05213	Endometrial cancer - Mus musculus (house mouse)	0.020520197	0.045862094	0.022215453	Bax/Ctnna1/Myc
1-day post-SCI group and the control group	mmu04922	Glucagon signaling pathway - Mus musculus (house mouse)	0.020698821	0.045862094	0.022215453	Atf2/Atf4/Ppargc1a/Ppp3ca
1-day post-SCI group and the control group	mmu04928	Parathyroid hormone synthesis, secretion and action - Mus musculus (house n	0.023407734	0.051360658	0.02487894	Atf2/Atf4/Fos/Hbegf
1-day post-SCI group and the control group	mmu05321	Inflammatory bowel disease - Mus musculus (house mouse)	0.024440772	0.053111677	0.025727128	Jun/Rela/Stat6
1-day post-SCI group and the control group	mmu04024	cAMP signaling pathway - Mus musculus (house mouse)	0.025377181	0.05462136	0.026458414	Edn1/Fos/Jun/Mapk8/Ppp1ca/Rela
1-day post-SCI group and the control group	mmu04066	HIF-1 signaling pathway - Mus musculus (house mouse)	0.027859103	0.059397711	0.028772063	Edn1/Hmox1/Nos3/Rela
1-day post-SCI group and the control group	mmu04714	Thermogenesis - Mus musculus (house mouse)	0.028937732	0.06071138	0.0294084	Atf2/Map2k3/Mapk14/Ndufa6/Ppargc1a/Tsc1
1-day post-SCI group and the control group	mmu04630	JAK-STAT signaling pathway - Mus musculus (house mouse)	0.029012518	0.06071138	0.0294084	Il6st/Jak2/Mcl1/Myc/Stat6
1-day post-SCI group and the control group	mmu04310	Wnt signaling pathway - Mus musculus (house mouse)	0.02965049	0.061370954	0.029727896	Fosl1/Jun/Mapk8/Myc/Ppp3ca
1-day post-SCI group and the control group	mmu04720	Long-term potentiation - Mus musculus (house mouse)	0.029870818	0.061370954	0.029727896	Atf4/Ppp1ca/Ppp3ca
1-day post-SCI group and the control group	mmu04919	Thyroid hormone signaling pathway - Mus musculus (house mouse)	0.032784101	0.066749611	0.0323333	Hdac1/Mdm2/Myc/Rcan1
1-day post-SCI group and the control group	mmu01523	Antifolate resistance - Mus musculus (house mouse)	0.034204678	0.069020155	0.033433144	Abcc1/Rela
1-day post-SCI group and the control group	mmu00480	Glutathione metabolism - Mus musculus (house mouse)	0.035885121	0.071140679	0.034460319	Gpx1/Gpx4/Lancl1
1-day post-SCI group and the control group	mmu05223	Non-small cell lung cancer - Mus musculus (house mouse)	0.035885121	0.071140679	0.034460319	Bax/Cdk4/Met
1-day post-SCI group and the control group	mmu04115	p53 signaling pathway - Mus musculus (house mouse)	0.037157449	0.07258232	0.035158646	Bax/Cdk4/Mdm2
1-day post-SCI group and the control group	mmu04611	Platelet activation - Mus musculus (house mouse)	0.037254642	0.07258232	0.035158646	Btk/Mapk14/Nos3/Ppp1ca
1-day post-SCI group and the control group	mmu04918	Thyroid hormone synthesis - Mus musculus (house mouse)	0.038452753	0.073646797	0.035674275	Atf2/Atf4/Gpx1
1-day post-SCI group and the control group	mmu05214	Glioma - Mus musculus (house mouse)	0.038452753	0.073646797	0.035674275	Bax/Cdk4/Mdm2
1-day post-SCI group and the control group	mmu04623	Cytosolic DNA-sensing pathway - Mus musculus (house mouse)	0.039770944	0.075531374	0.036587158	Rela/Ripk1/Ripk3
1-day post-SCI group and the control group	mmu04068	FoxO signaling pathway - Mus musculus (house mouse)	0.043061975	0.081100052	0.039284608	Cat/Mapk14/Mapk8/Mdm2
1-day post-SCI group and the control group	mmu01524	Platinum drug resistance - Mus musculus (house mouse)	0.046701765	0.08722809	0.04225301	Bax/Ercc1/Mdm2
1-day post-SCI group and the control group	mmu04371	Apelin signaling pathway - Mus musculus (house mouse)	0.049352972	0.091424358	0.044285669	Nos3/Ppargc1a/Slc8a1/Sphk1
3-day post-SCI group and the control group	mmu05417	Lipid and atherosclerosis - Mus musculus (house mouse)	4.5E-16	1.13E-13	4.88E-14	Bax/Casp3/Cd36/Eif2s1/Fos/Jak2/Jun/Map2k3/Map2k4/Map2k6/Mapk10/Mapk14/Mapk3/Mapk8/Ncf1/Nfatc1/Nfe2l2/Nfkb1/Ppp3ca/Rela/Rxrb/Sod2/Tank/Tlr4/Tlr6/Tnfrsf1a/Vcam1
3-day post-SCI group and the control group	mmu05167	Kaposi sarcoma-associated herpesvirus infection - Mus musculus (house mou	1.14E-15	1.43E-13	6.19E-14	Bax/Casp3/Ccr1/Cdk4/Fos/Gnb2/Hif1a/Il6st/Jak2/Jun/Map2k4/Map2k6/Mapk10/Mapk14/Mapk3/Mapk8/Mapkapk2/Myc/Nfatc1/Nfkb1/Ppp3ca/Ptgs2/Rb1/Rcan1/Rela/Tnfrsf1a/Uba52
3-day post-SCI group and the control group	mmu05161	Hepatitis B - Mus musculus (house mouse)	5.66E-15	4.74E-13	2.05E-13	Atf2/Bax/Braf/Casp3/Ccna2/Fos/Jak2/Jun/Map2k3/Map2k4/Map2k6/Mapk10/Mapk14/Mapk3/Mapk8/Myc/Nfatc1/Nfkb1/Pcna/Rb1/Rela/Stat6/Tlr4
3-day post-SCI group and the control group	mmu05208	Chemical carcinogenesis - reactive oxygen species - Mus musculus (house mo	8.24E-15	5.17E-13	2.23E-13	Braf/Cat/Cyp1b1/Fos/Gstt2/Hif1a/Hmox1/Jun/Lpo/Map2k4/Mapk10/Mapk14/Mapk3/Mapk8/Met/Mgst1/Ncf1/Ndufa12/Ndufa6/Ndufs8/Nfe2l2/Nfkb1/Nox4/Ppif/Rela/Sod2

3-day post-SCI group and the control group	mmu04668	TNF signaling pathway - Mus musculus (house mouse)	8.05E-14	4.04E-12	1.75E-12	Atf2/Casp3/Fos/Jun/Junb/Map2k3/Map2k4/Map2k6/Mapk10/Mapk14/Mapk3/Mapk8/Nfkb1/Ptgs2/Rela/Ripk1/Ripk3/Tnfrsf1a/Vcam1
3-day post-SCI group and the control group	mmu05022	Pathways of neurodegeneration - multiple diseases - Mus musculus (house mo	1.5E-13	6.26E-12	2.71E-12	Apc/Atp2a2/Bax/Braf/Casp3/Cat/Ccs/Eif2s1/Gpr37/Gpx1/Gpx3/Gpx7/Gpx8/Htra2/Map2k3/Map2k6/Mapk10/Mapk14/Mapk3/Mapk8/Mapt/Ndufa12/Ndufa6/Ndufs8/Nfkb1/Nox4/Pink1/Ppif/Ppp3ca/Ptgs2/Rela/Snca/Tank/Tnfrsf1a/Uba52
3-day post-SCI group and the control group	mmu05418	Fluid shear stress and atherosclerosis - Mus musculus (house mouse)	7.69E-13	2.76E-11	1.19E-11	Actb/Ctsl/Fos/Gstt2/Hmox1/Jun/Map2k4/Map2k6/Mapk10/Mapk14/Mapk8/Mgst1/Ncf1/Nfe2l2/Nfkb1/Prkaa2/Rela/Sdc1/Tnfrsf1a/Vcam1
3-day post-SCI group and the control group	mmu04210	Apoptosis - Mus musculus (house mouse)	1.86E-11	5.84E-10	2.52E-10	Actb/Bax/Casp3/Ctsl/Eif2s1/Endog/Fos/Htra2/Jun/Mapk10/Mapk3/Mapk8/Mcl1/Nfkb1/Parp1/Rela/Ripk1/Tnfrsf1a
3-day post-SCI group and the control group	mmu05166	Human T-cell leukemia virus 1 infection - Mus musculus (house mouse)	5.29E-11	1.47E-09	6.37E-10	Atf2/Atr/Bax/Ccna2/Cdc20/Cdk4/Cdkn2b/Cdkn2c/Ets1/Fos/Fosl1/Jun/Map2k4/Mapk10/Mapk3/Mapk8/Myc/Nfatc1/Nfkb1/Ppp3ca/Rb1/Rela/Tnfrsf1a
3-day post-SCI group and the control group	mmu04010	MAPK signaling pathway - Mus musculus (house mouse)	6.6E-11	1.51E-09	6.51E-10	Atf2/Braf/Casp3/Fos/Hspb1/Jun/Map2k3/Map2k4/Map2k6/Map4k4/Mapk10/Mapk14/Mapk3/Mapk8/Mapkapk2/Mapkapk3/Mapt/Met/Myc/Nfatc1/Nfkb1/Pdgfra/Ppp3ca/Rela/Tnfrsf1a
3-day post-SCI group and the control group	mmu04380	Osteoclast differentiation - Mus musculus (house mouse)	6.61E-11	1.51E-09	6.51E-10	Btk/Fos/Fosl1/Jun/Junb/Map2k6/Mapk10/Mapk14/Mapk3/Mapk8/Ncf1/Nfatc1/Nfkb1/Ppp3ca/Rela/Sirpa/Tnfrsf1a
3-day post-SCI group and the control group	mmu04936	Alcoholic liver disease - Mus musculus (house mouse)	3.13E-10	6.26E-09	2.7E-09	Camkk2/Casp3/Map2k3/Map2k4/Map2k6/Mapk10/Mapk14/Mapk8/Nfkb1/Nox4/Ppargc1a/Prkaa2/Rela/Ripk1/Sirt1/Tlr4/Tnfrsf1a
3-day post-SCI group and the control group	mmu04659	Th17 cell differentiation - Mus musculus (house mouse)	3.24E-10	6.26E-09	2.7E-09	Fos/Hif1a/Il6st/Jak2/Jun/Mapk10/Mapk14/Mapk3/Mapk8/Nfatc1/Nfkb1/Ppp3ca/Rela/Rxrb/Stat6
3-day post-SCI group and the control group	mmu04218	Cellular senescence - Mus musculus (house mouse)	4.01E-10	7.18E-09	3.1E-09	Atr/Ccna2/Cdk1/Cdk4/Cdkn2b/Ets1/Map2k3/Map2k6/Mapk14/Mapk3/Mapkapk2/Myc/Nfatc1/Nfkb1/Ppp1ca/Ppp3ca/Rb1/Rela/Sirt1
3-day post-SCI group and the control group	mmu05145	Toxoplasmosis - Mus musculus (house mouse)	5.57E-10	9.32E-09	4.03E-09	Alox5/Casp3/Itga6/Jak2/Map2k3/Map2k6/Mapk10/Mapk14/Mapk3/Mapk8/Nfkb1/Ppif/Rela/Tlr4/Tnfrsf1a
3-day post-SCI group and the control group	mmu05170	Human immunodeficiency virus 1 infection - Mus musculus (house mouse)	9.89E-10	1.55E-08	6.7E-09	Atr/Bax/Casp3/Cdk1/Fos/Gnb2/Jun/Map2k3/Map2k6/Mapk10/Mapk14/Mapk3/Mapk8/Nfatc1/Nfkb1/Ppp3ca/Pxn/Rela/Ripk1/Tlr4/Tnfrsf1a
3-day post-SCI group and the control group	mmu04620	Toll-like receptor signaling pathway - Mus musculus (house mouse)	1.7E-09	2.52E-08	1.09E-08	Fos/Jun/Map2k3/Map2k4/Map2k6/Mapk10/Mapk14/Mapk3/Mapk8/Nfkb1/Rela/Ripk1/Tlr4/Tlr6
3-day post-SCI group and the control group	mmu04933	AGE-RAGE signaling pathway in diabetic complications - Mus musculus (ho	1.95E-09	2.72E-08	1.17E-08	Bax/Casp3/Cdk4/Jak2/Jun/Mapk10/Mapk14/Mapk3/Mapk8/Nfatc1/Nfkb1/Nox4/Rela/Vcam1
3-day post-SCI group and the control group	mmu00480	Glutathione metabolism - Mus musculus (house mouse)	3.36E-09	4.26E-08	1.84E-08	Gclc/Ggt7/Gpx1/Gpx3/Gpx7/Gpx8/Gsr/Gstt2/Lancl1/Mgst1/Prdx6/Rrm2b
3-day post-SCI group and the control group	mmu05235	PD-L1 expression and PD-1 checkpoint pathway in cancer - Mus musculus (h	3.39E-09	4.26E-08	1.84E-08	Fos/Hif1a/Jak2/Jun/Map2k3/Map2k6/Mapk14/Mapk3/Nfatc1/Nfkb1/Ppp3ca/Rela/Tlr4
3-day post-SCI group and the control group	mmu05135	Yersinia infection - Mus musculus (house mouse)	1.14E-08	0.000000136	5.89E-08	Actb/Fos/Jun/Map2k3/Map2k4/Map2k6/Mapk10/Mapk14/Mapk3/Mapk8/Nfatc1/Nfkb1/Pxn/Rela/Tlr4
3-day post-SCI group and the control group	mmu05132	Salmonella infection - Mus musculus (house mouse)	1.47E-08	0.000000168	7.26E-08	Actb/Bax/Casp3/Fos/Jun/Map2k3/Map2k4/Map2k6/Mapk10/Mapk14/Mapk3/Mapk8/Myc/Nfkb1/Rela/Ripk1/Ripk3/Tlr4/Tlr6/Tnfrsf1a
3-day post-SCI group and the control group	mmu05163	Human cytomegalovirus infection - Mus musculus (house mouse)	0.000000018	0.000000196	8.49E-08	Atf2/Bax/Casp3/Ccr1/Cdk4/Gnb2/Map2k6/Mapk14/Mapk3/Myc/Nfatc1/Nfkb1/Pdgfra/Ppp3ca/Ptgs2/Pxn/Rb1/Rela/Ripk1/Tnfrsf1a
3-day post-SCI group and the control group	mmu05140	Leishmaniasis - Mus musculus (house mouse)	2.94E-08	0.000000308	0.000000133	Fos/Jak2/Jun/Mapk14/Mapk3/Marcksl1/Ncf1/Nfkb1/Ptgs2/Rela/Tlr4
3-day post-SCI group and the control group	mmu04920	Adipocytokine signaling pathway - Mus musculus (house mouse)	3.43E-08	0.00000034	0.000000147	Camkk2/Cd36/Jak2/Mapk10/Mapk8/Nfkb1/Ppargc1a/Prkaa2/Rela/Rxrb/Tnfrsf1a
3-day post-SCI group and the control group	mmu04658	Th1 and Th2 cell differentiation - Mus musculus (house mouse)	3.52E-08	0.00000034	0.000000147	Fos/Jak2/Jun/Mapk10/Mapk14/Mapk3/Mapk8/Nfatc1/Nfkb1/Ppp3ca/Rela/Stat6
3-day post-SCI group and the control group	mmu01522	Endocrine resistance - Mus musculus (house mouse)	6.63E-08	0.000000616	0.000000266	Bax/Braf/Cdk4/Cdkn2c/Fos/Hbegf/Jun/Mapk10/Mapk14/Mapk3/Mapk8/Rb1
3-day post-SCI group and the control group	mmu04932	Non-alcoholic fatty liver disease - Mus musculus (house mouse)	8.13E-08	0.000000729	0.000000315	Bax/Casp3/Eif2s1/Fos/Jun/Mapk10/Mapk14/Mapk8/Ndufa12/Ndufa6/Ndufs8/Nfkb1/Prkaa2/Rela/Tnfrsf1a
3-day post-SCI group and the control group	mmu05205	Proteoglycans in cancer - Mus musculus (house mouse)	9.58E-08	0.000000829	0.000000358	Actb/Braf/Casp3/Ctsl/Cttn/Ezr/Hbegf/Hif1a/Itgb5/Mapk14/Mapk3/Met/Myc/Ppp1ca/Pxn/Sdc1/Tlr4

3-day post-SCI group and the control group	mmu05169	Epstein-Barr virus infection - Mus musculus (house mouse)	0.000000102	0.000000853	0.000000368	Bax/Btk/Casp3/Ccna2/Cdk4/Hdac1/Jun/Map2k3/Map2k4/Map2k6/Mapk10/Mapk14/Mapk8/Myc/Nfkb1/Rb1/Rela/Ripk1
3-day post-SCI group and the control group	mmu05142	Chagas disease - Mus musculus (house mouse)	0.000000209	0.00000169	0.000000732	Fos/Jun/Map2k4/Mapk10/Mapk14/Mapk3/Mapk8/Nfkb1/Rela/Tlr4/Tlr6/Tnfrsf1a
3-day post-SCI group and the control group	mmu04921	Oxytocin signaling pathway - Mus musculus (house mouse)	0.000000415	0.00000326	0.00000141	Actb/Camkk2/Cd38/Fos/Jun/Mapk3/Mylk/Nfatc1/Ppp1ca/Ppp3ca/Prkaa2/Ptgs2/Rcan1/Trpm2
3-day post-SCI group and the control group	mmu05203	Viral carcinogenesis - Mus musculus (house mouse)	0.000000471	0.00000359	0.00000155	Atf2/Bax/Casp3/Ccna2/Cdc20/Cdk1/Cdk4/Cdkn2b/Hdac1/Il6st/Jun/Mapk3/Mapkapk2/Nfkb1/Pxn/Rb1/Rela
3-day post-SCI group and the control group	mmu04657	IL-17 signaling pathway - Mus musculus (house mouse)	0.000000587	0.00000421	0.00000182	Casp3/Fos/Fosl1/Jun/Mapk10/Mapk14/Mapk3/Mapk8/Nfkb1/Ptgs2/Rela
3-day post-SCI group and the control group	mmu05222	Small cell lung cancer - Mus musculus (house mouse)	0.000000587	0.00000421	0.00000182	Bax/Casp3/Cdk4/Cdkn2b/Itga6/Myc/Nfkb1/Ptgs2/Rb1/Rela/Rxrb
3-day post-SCI group and the control group	mmu05415	Diabetic cardiomyopathy - Mus musculus (house mouse)	0.000000787	0.00000548	0.00000237	Atp2a2/Cd36/Gsr/Mapk10/Mapk14/Mapk8/Ncf1/Ndufa12/Ndufa6/Ndufs8/Nfkb1/Parp1/Pdk2/Ppif/Ppp1ca/Rela
3-day post-SCI group and the control group	mmu05133	Pertussis - Mus musculus (house mouse)	0.000000808	0.00000548	0.00000237	Casp3/Fos/Jun/Mapk10/Mapk14/Mapk3/Mapk8/Nfkb1/Rela/Tlr4
3-day post-SCI group and the control group	mmu05014	Amyotrophic lateral sclerosis - Mus musculus (house mouse)	0.00000166	0.000011	0.00000474	Actb/Bax/Casp3/Cat/Ccs/Eif2s1/Gpx1/Gpx3/Gpx7/Gpx8/Map2k3/Map2k6/Mapk14/Ndufa12/Ndufa6/Ndufs8/Pink1/Ppp3ca/Tank/Tnfrsf1a/Ubqln1
3-day post-SCI group and the control group	mmu04664	Fc epsilon RI signaling pathway - Mus musculus (house mouse)	0.00000192	0.0000124	0.00000535	Alox5/Btk/Map2k3/Map2k4/Map2k6/Mapk10/Mapk14/Mapk3/Mapk8
3-day post-SCI group and the control group	mmu05225	Hepatocellular carcinoma - Mus musculus (house mouse)	0.00000197	0.0000124	0.00000535	Actb/Apc/Bax/Braf/Cdk4/Gstt2/Hmox1/Mapk3/Met/Mgst1/Myc/Nfe2l2/Rb1/Txnrd1
3-day post-SCI group and the control group	mmu05210	Colorectal cancer - Mus musculus (house mouse)	0.00000282	0.0000173	0.00000745	Apc/Bax/Braf/Casp3/Fos/Jun/Mapk10/Mapk3/Mapk8/Myc
3-day post-SCI group and the control group	mmu04110	Cell cycle - Mus musculus (house mouse)	0.00000293	0.0000175	0.00000757	Atr/Ccna2/Cdc20/Cdk1/Cdk4/Cdkn2b/Cdkn2c/Hdac1/Mcm4/Myc/Pcna/Rb1/Tfdp1
3-day post-SCI group and the control group	mmu05012	Parkinson disease - Mus musculus (house mouse)	0.00000339	0.0000198	0.00000854	Bax/Casp3/Eif2s1/Gpr37/Htra2/Maoa/Mapk10/Mapk8/Mapt/Ndufa12/Ndufa6/Ndufs8/Nfe2l2/Pink1/Ppif/Snca/Uba52
3-day post-SCI group and the control group	mmu04211	Longevity regulating pathway - Mus musculus (house mouse)	0.00000347	0.0000198	0.00000854	Atf2/Bax/Camkk2/Cat/Nfkb1/Ppargc1a/Prkaa2/Rela/Sirt1/Sod2
3-day post-SCI group and the control group	mmu04625	C-type lectin receptor signaling pathway - Mus musculus (house mouse)	0.00000379	0.0000212	0.00000914	Jun/Mapk10/Mapk14/Mapk3/Mapk8/Mapkapk2/Nfatc1/Nfkb1/Ppp3ca/Ptgs2/Rela
3-day post-SCI group and the control group	mmu04935	Growth hormone synthesis, secretion and action - Mus musculus (house mous	0.00000582	0.0000318	0.0000137	Atf2/Fos/Jak2/Junb/Map2k3/Map2k4/Map2k6/Mapk10/Mapk14/Mapk3/Mapk8
3-day post-SCI group and the control group	mmu05212	Pancreatic cancer - Mus musculus (house mouse)	0.00000638	0.0000333	0.0000144	Bax/Braf/Cdk4/Mapk10/Mapk3/Mapk8/Nfkb1/Rb1/Rela
3-day post-SCI group and the control group	mmu05220	Chronic myeloid leukemia - Mus musculus (house mouse)	0.00000638	0.0000333	0.0000144	Bax/Braf/Cdk4/Hdac1/Mapk3/Myc/Nfkb1/Rb1/Rela
3-day post-SCI group and the control group	mmu04370	VEGF signaling pathway - Mus musculus (house mouse)	0.00000668	0.0000342	0.0000148	Hspb1/Mapk14/Mapk3/Mapkapk2/Mapkapk3/Ppp3ca/Ptgs2/Pxn
3-day post-SCI group and the control group	mmu04722	Neurotrophin signaling pathway - Mus musculus (house mouse)	0.00000806	0.0000405	0.0000175	Arhgdia/Bax/Braf/Jun/Mapk10/Mapk14/Mapk3/Mapk8/Mapkapk2/Nfkb1/Rela
3-day post-SCI group and the control group	mmu04660	T cell receptor signaling pathway - Mus musculus (house mouse)	0.00000873	0.000043	0.0000186	Cdk4/Fos/Jun/Mapk10/Mapk14/Mapk3/Mapk8/Nfatc1/Nfkb1/Ppp3ca/Rela
3-day post-SCI group and the control group	mmu05010	Alzheimer disease - Mus musculus (house mouse)	0.0000109	0.0000527	0.0000228	Apc/Atp2a2/Braf/Casp3/Eif2s1/Mapk10/Mapk3/Mapk8/Mapt/Ndufa12/Ndufa6/Ndufs8/Nfkb1/Nox4/Ppif/Ppp3ca/Ptgs2/Rela/Snca/Tnfrsf1a
3-day post-SCI group and the control group	mmu04926	Relaxin signaling pathway - Mus musculus (house mouse)	0.0000149	0.0000703	0.0000304	Atf2/Fos/Gnb2/Jun/Map2k4/Mapk10/Mapk14/Mapk3/Mapk8/Nfkb1/Rela
3-day post-SCI group and the control group	mmu05152	Tuberculosis - Mus musculus (house mouse)	0.0000151	0.0000703	0.0000304	Bax/Casp3/Jak2/Mapk10/Mapk14/Mapk3/Mapk8/Nfkb1/Ppp3ca/Rela/Tlr4/Tlr6/Tnfrsf1a
3-day post-SCI group and the control group	mmu04215	Apoptosis - multiple species - Mus musculus (house mouse)	0.0000162	0.0000737	0.0000319	Bax/Casp3/Htra2/Mapk10/Mapk8/Tnfrsf1a
3-day post-SCI group and the control group	mmu04068	FoxO signaling pathway - Mus musculus (house mouse)	0.0000172	0.0000773	0.0000334	Bnip3/Braf/Cat/Cdkn2b/Mapk10/Mapk14/Mapk3/Mapk8/Prkaa2/Sirt1/Sod2
3-day post-SCI group and the control group	mmu04931	Insulin resistance - Mus musculus (house mouse)	0.0000211	0.0000927	0.0000401	Cd36/Mapk10/Mapk8/Nfkb1/Ppargc1a/Ppargc1b/Ppp1ca/Prkaa2/Rela/Tnfrsf1a
3-day post-SCI group and the control group	mmu04137	Mitophagy - animal - Mus musculus (house mouse)	0.0000222	0.0000962	0.0000416	Bnip3/Hif1a/Jun/Mapk10/Mapk8/Pink1/Rela/Uba52
3-day post-SCI group and the control group	mmu05031	Amphetamine addiction - Mus musculus (house mouse)	0.0000248	0.000105395	0.0000455	Atf2/Fos/Hdac1/Jun/Maoa/Ppp1ca/Ppp3ca/Sirt1
3-day post-SCI group and the control group	mmu04912	GnRH signaling pathway - Mus musculus (house mouse)	0.0000257	0.000107342	0.0000464	Hbegf/Jun/Map2k3/Map2k4/Map2k6/Mapk10/Mapk14/Mapk3/Mapk8
3-day post-SCI group and the control group	mmu05160	Hepatitis C - Mus musculus (house mouse)	0.0000322	0.000132622	0.0000573	Bax/Braf/Casp3/Cdk4/Eif2s1/Mapk3/Myc/Nfkb1/Rb1/Rela/Ripk1/Tnfrsf1a
3-day post-SCI group and the control group	mmu05207	Chemical carcinogenesis - receptor activation - Mus musculus (house mouse)	0.0000378	0.000153203	0.0000662	Atf2/Chrna4/Cyp1b1/Fos/Gstt2/Jak2/Jun/Mapk3/Mgst1/Myc/Nfkb1/Rb1/Rela/Rxrb

3-day post-SCI group and the control group	mmu04917	Prolactin signaling pathway - Mus musculus (house mouse)	0.0000415	0.000165228	0.0000714	Fos/Jak2/Mapk10/Mapk14/Mapk3/Mapk8/Nfkb1/Rela
3-day post-SCI group and the control group	mmu05162	Measles - Mus musculus (house mouse)	0.0000446	0.00017473	0.0000755	Bax/Casp3/Cdk4/Eif2s1/Fos/Jun/Mapk10/Mapk8/Nfkb1/Rela/Tlr4
3-day post-SCI group and the control group	mmu04510	Focal adhesion - Mus musculus (house mouse)	0.0000537	0.00020751	0.0000896	Actb/Braf/Itga6/Itgb5/Jun/Mapk10/Mapk3/Mapk8/Met/Mylk/Pdgfra/Ppp1ca/Pxn
3-day post-SCI group and the control group	mmu04071	Sphingolipid signaling pathway - Mus musculus (house mouse)	0.0000681	0.000258955	0.000111857	Abcc1/Adora1/Bax/Mapk10/Mapk14/Mapk3/Mapk8/Nfkb1/Rela/Tnfrsf1a
3-day post-SCI group and the control group	mmu05219	Bladder cancer - Mus musculus (house mouse)	0.0000704	0.000263755	0.000113931	Braf/Cdk4/Hbegf/Mapk3/Myc/Rb1
3-day post-SCI group and the control group	mmu05016	Huntington disease - Mus musculus (house mouse)	0.0000739	0.000272651	0.000117773	Bax/Casp3/Gpx1/Gpx3/Gpx7/Gpx8/Hdac1/Mapk10/Mapk8/Ndufa12/Ndufa6/Ndufs8/Ppargc1a/Ppif/Rest/Sod2
3-day post-SCI group and the control group	mmu05206	MicroRNAs in cancer - Mus musculus (house mouse)	0.0000768	0.000279425	0.0001207	Abcc1/Apc/Casp3/Cyp1b1/Ezh2/Ezr/Hdac1/Hmox1/Mapk3/Mcl1/Met/Myc/Nfkb1/Pdgfra/Ptgs2/Sirt1
3-day post-SCI group and the control group	mmu04064	NF-kappa B signaling pathway - Mus musculus (house mouse)	0.0000873	0.000313136	0.000135261	Btk/Nfkb1/Parp1/Ptgs2/Rela/Ripk1/Tlr4/Tnfrsf1a/Vcam1
3-day post-SCI group and the control group	mmu04662	B cell receptor signaling pathway - Mus musculus (house mouse)	0.000095	0.000334629	0.000144545	Btk/Fos/Jun/Mapk3/Nfatc1/Nfkb1/Ppp3ca/Rela
3-day post-SCI group and the control group	mmu05321	Inflammatory bowel disease - Mus musculus (house mouse)	0.000096	0.000334629	0.000144545	Il18rap/Jun/Nfatc1/Nfkb1/Rela/Stat6/Tlr4
3-day post-SCI group and the control group	mmu04621	NOD-like receptor signaling pathway - Mus musculus (house mouse)	0.000101407	0.000346311	0.000149591	Jun/Mapk10/Mapk14/Mapk3/Mapk8/Nfkb1/Rela/Ripk1/Ripk3/Tank/Tlr4/Trpm2/Txnip
3-day post-SCI group and the control group	mmu03083	Polycomb repressive complex - Mus musculus (house mouse)	0.000103479	0.000346311	0.000149591	Cbx6/Eed/Ezh2/Hdac1/Phc3/Rbbp7/Rnf2/Tfdp1
3-day post-SCI group and the control group	mmu04012	ErbB signaling pathway - Mus musculus (house mouse)	0.000103479	0.000346311	0.000149591	Braf/Hbegf/Jun/Map2k4/Mapk10/Mapk3/Mapk8/Myc
3-day post-SCI group and the control group	mmu05171	Coronavirus disease - COVID-19 - Mus musculus (house mouse)	0.000113367	0.000374409	0.000161729	Fos/Hbegf/Il6st/Jun/Mapk10/Mapk14/Mapk3/Mapk8/Nfkb1/Rela/Rpl13a/Tlr4/Tnfrsf1a/Uba52
3-day post-SCI group and the control group	mmu04140	Autophagy - animal - Mus musculus (house mouse)	0.000184823	0.000602476	0.000260243	Bnip3/Camkk2/Ctsl/Eif2s1/Hif1a/Mapk10/Mapk3/Mapk8/Prkaa2/Tank
3-day post-SCI group and the control group	mmu05164	Influenza A - Mus musculus (house mouse)	0.000217655	0.000700403	0.000302544	Actb/Bax/Casp3/Cdk4/Eif2s1/Jak2/Mapk3/Nfkb1/Rela/Tlr4/Tnfrsf1a
3-day post-SCI group and the control group	mmu04622	RIG-I-like receptor signaling pathway - Mus musculus (house mouse)	0.000227584	0.000723082	0.00031234	Mapk10/Mapk14/Mapk8/Nfkb1/Rela/Ripk1/Tank
3-day post-SCI group and the control group	mmu05020	Prion disease - Mus musculus (house mouse)	0.000235486	0.000738837	0.000319145	Atf2/Bax/Casp3/Eif2s1/Mapk10/Mapk14/Mapk3/Mapk8/Ncf1/Ndufa12/Ndufa6/Ndufs8/Ppif/Ppp3ca
3-day post-SCI group and the control group	mmu05218	Melanoma - Mus musculus (house mouse)	0.000248439	0.000760467	0.000328489	Bax/Braf/Cdk4/Mapk3/Met/Pdgfra/Rb1
3-day post-SCI group and the control group	mmu05223	Non-small cell lung cancer - Mus musculus (house mouse)	0.000248439	0.000760467	0.000328489	Bax/Braf/Cdk4/Mapk3/Met/Rb1/Rxrb
3-day post-SCI group and the control group	mmu05226	Gastric cancer - Mus musculus (house mouse)	0.000288786	0.000873317	0.000377235	Apc/Bax/Braf/Cdkn2b/Ctnna1/Mapk3/Met/Myc/Rb1/Rxrb
3-day post-SCI group and the control group	mmu05231	Choline metabolism in cancer - Mus musculus (house mouse)	0.000303828	0.000907866	0.000392158	Dgkk/Fos/Hif1a/Jun/Mapk10/Mapk3/Mapk8/Pdgfra
3-day post-SCI group and the control group	mmu05216	Thyroid cancer - Mus musculus (house mouse)	0.000425126	0.001255371	0.000542266	Bax/Braf/Mapk3/Myc/Rxrb
3-day post-SCI group and the control group	mmu01521	EGFR tyrosine kinase inhibitor resistance - Mus musculus (house mouse)	0.000441421	0.001288334	0.000556504	Axl/Bax/Braf/Jak2/Mapk3/Met/Pdgfra
3-day post-SCI group and the control group	mmu05213	Endometrial cancer - Mus musculus (house mouse)	0.000496184	0.00143152	0.000618354	Apc/Bax/Braf/Ctnna1/Mapk3/Myc
3-day post-SCI group and the control group	mmu04024	cAMP signaling pathway - Mus musculus (house mouse)	0.000546605	0.001559068	0.000673449	Adora1/Atp2a2/Braf/Fos/Jun/Mapk10/Mapk3/Mapk8/Nfatc1/Nfkb1/Ppp1ca/Rela
3-day post-SCI group and the control group	mmu05165	Human papillomavirus infection - Mus musculus (house mouse)	0.000579955	0.001622351	0.000700785	Apc/Atr/Bax/Casp3/Ccna2/Cdk4/Hdac1/Itga6/Itgb5/Mapk3/Nfkb1/Ptgs2/Pxn/Rb1/Rela/Tnfrsf1a
3-day post-SCI group and the control group	mmu04728	Dopaminergic synapse - Mus musculus (house mouse)	0.000581719	0.001622351	0.000700785	Atf2/Fos/Gnb2/Maoa/Mapk10/Mapk14/Mapk8/Ppp1ca/Ppp3ca
3-day post-SCI group and the control group	mmu04530	Tight junction - Mus musculus (house mouse)	0.000709069	0.001955782	0.000844813	Actb/Cdk4/Cttn/Ezr/Jun/Mapk10/Mapk8/Pcna/Prkaa2/Ybx3
3-day post-SCI group and the control group	mmu04550	Signaling pathways regulating pluripotency of stem cells - Mus musculus (hou	0.000756814	0.002064786	0.000891898	Apc/Id1/Il6st/Jak2/Mapk14/Mapk3/Myc/Rest/Smad1
3-day post-SCI group and the control group	mmu04022	cGMP-PKG signaling pathway - Mus musculus (house mouse)	0.000889591	0.002400939	0.001037101	Adora1/Atf2/Atp2a2/Mapk3/Mylk/Nfatc1/Ppif/Ppp1ca/Ppp3ca/Slc8a1
3-day post-SCI group and the control group	mmu04217	Necroptosis - Mus musculus (house mouse)	0.000971652	0.002594518	0.001120719	Bax/Jak2/Mapk10/Mapk8/Parp1/Ripk1/Ripk3/Stat6/Tlr4/Tnfrsf1a
3-day post-SCI group and the control group	mmu05224	Breast cancer - Mus musculus (house mouse)	0.001072015	0.002832377	0.001223463	Apc/Bax/Braf/Cdk4/Fos/Jun/Mapk3/Myc/Rb1
3-day post-SCI group and the control group	mmu04914	Progesterone-mediated oocyte maturation - Mus musculus (house mouse)	0.001104047	0.002886622	0.001246895	Braf/Ccna2/Cdk1/Mapk10/Mapk14/Mapk3/Mapk8
3-day post-SCI group and the control group	mmu04723	Retrograde endocannabinoid signaling - Mus musculus (house mouse)	0.001124652	0.002910182	0.001257072	Gnb2/Mapk10/Mapk14/Mapk3/Mapk8/Ndufa12/Ndufa6/Ndufs8/Ptgs2
3-day post-SCI group and the control group	mmu05211	Renal cell carcinoma - Mus musculus (house mouse)	0.001162479	0.002975707	0.001285376	Braf/Ets1/Hif1a/Jun/Mapk3/Met
3-day post-SCI group and the control group	mmu04919	Thyroid hormone signaling pathway - Mus musculus (house mouse)	0.001173685	0.002975707	0.001285376	Actb/Atp2a2/Hdac1/Hif1a/Mapk3/Myc/Rcan1/Rxrb
3-day post-SCI group and the control group	mmu05230	Central carbon metabolism in cancer - Mus musculus (house mouse)	0.001255087	0.003150267	0.001360778	Hif1a/Mapk3/Met/Myc/Pdgfra/Pdk1
3-day post-SCI group and the control group	mmu05221	Acute myeloid leukemia - Mus musculus (house mouse)	0.001353236	0.003362992	0.001452666	Braf/Ccna2/Mapk3/Myc/Nfkb1/Rela
3-day post-SCI group and the control group	mmu05030	Cocaine addiction - Mus musculus (house mouse)	0.001430957	0.003521276	0.001521038	Atf2/Jun/Maoa/Nfkb1/Rela
3-day post-SCI group and the control group	mmu01523	Antifolate resistance - Mus musculus (house mouse)	0.001518424	0.003700237	0.001598341	Abcc1/Dhfr/Nfkb1/Rela
3-day post-SCI group and the control group	mmu04115	p53 signaling pathway - Mus musculus (house mouse)	0.001683103	0.004062105	0.001754652	Atr/Bax/Casp3/Cdk1/Cdk4/Rrm2b
3-day post-SCI group and the control group	mmu04151	PI3K-Akt signaling pathway - Mus musculus (house mouse)	0.001740174	0.004159845	0.001796872	Atf2/Cdk4/Gnb2/Itga6/Itgb5/Jak2/Mapk3/Mcl1/Met/Myc/Nfkb1/Pdgfra/Prkaa2/Rela/Tlr4
3-day post-SCI group and the control group	mmu04918	Thyroid hormone synthesis - Mus musculus (house mouse)	0.001805603	0.004235574	0.001829583	Atf2/Gpx1/Gpx3/Gpx7/Gpx8/Gsr
3-day post-SCI group and the control group	mmu05214	Glioma - Mus musculus (house mouse)	0.001805603	0.004235574	0.001829583	Bax/Braf/Cdk4/Mapk3/Pdgfra/Rb1
3-day post-SCI group and the control group	mmu04726	Serotonergic synapse - Mus musculus (house mouse)	0.002056056	0.004778427	0.002064072	Alox5/Braf/Casp3/Gnb2/Maoa/Mapk3/Ptgs1/Ptgs2
3-day post-SCI group and the control group	mmu05412	Arrhythmogenic right ventricular cardiomyopathy - Mus musculus (house mo	0.002213908	0.005098083	0.002202149	Actb/Atp2a2/Ctnna1/Itga6/Itgb5/Slc8a1

3-day post-SCI group and the control group	mmu04810	Regulation of actin cytoskeleton - Mus musculus (house mouse)	0.002387785	0.005448492	0.002353511	Actb/Apc/Braf/Ezr/Itga6/Itgb5/Mapk3/Mylk/Pdgfra/Ppp1ca/Pxn
3-day post-SCI group and the control group	mmu01524	Platinum drug resistance - Mus musculus (house mouse)	0.002688301	0.006078951	0.002625842	Atp7a/Bax/Casp3/Gstt2/Mapk3/Mgst1
3-day post-SCI group and the control group	mmu05144	Malaria - Mus musculus (house mouse)	0.00308518	0.006860525	0.002963448	Cd36/Met/Sdc1/Tlr4/Vcam1
3-day post-SCI group and the control group	mmu04350	TGF-beta signaling pathway - Mus musculus (house mouse)	0.003088603	0.006860525	0.002963448	Cdkn2b/Hdac1/Id1/Mapk3/Myc/Smad1/Tfdp1
3-day post-SCI group and the control group	mmu04066	HIF-1 signaling pathway - Mus musculus (house mouse)	0.003769056	0.008298535	0.003584605	Hif1a/Hmox1/Mapk3/Nfkb1/Pdk1/Rela/Tlr4
3-day post-SCI group and the control group	mmu05134	Legionellosis - Mus musculus (house mouse)	0.003859194	0.008423109	0.003638416	Bnip3/Casp3/Nfkb1/Rela/Tlr4
3-day post-SCI group and the control group	mmu04213	Longevity regulating pathway - multiple species - Mus musculus (house mous	0.004145552	0.008970116	0.003874699	Cat/Hdac1/Prkaa2/Sirt1/Sod2
3-day post-SCI group and the control group	mmu04670	Leukocyte transendothelial migration - Mus musculus (house mouse)	0.004773376	0.01024032	0.004423372	Actb/Ctnna1/Ezr/Mapk14/Ncf1/Pxn/Vcam1
3-day post-SCI group and the control group	mmu05410	Hypertrophic cardiomyopathy - Mus musculus (house mouse)	0.005097942	0.010843928	0.004684104	Actb/Atp2a2/Itga6/Itgb5/Prkaa2/Slc8a1
3-day post-SCI group and the control group	mmu05202	Transcriptional misregulation in cancer - Mus musculus (house mouse)	0.005870793	0.012382933	0.005348887	Bax/Ccna2/Cdkn2c/Hdac1/Met/Myc/Nfkb1/Rela/Rxrb/Smad1
3-day post-SCI group and the control group	mmu04611	Platelet activation - Mus musculus (house mouse)	0.006231023	0.013033224	0.005629784	Actb/Btk/Mapk14/Mapk3/Mylk/Ppp1ca/Ptgs1
3-day post-SCI group and the control group	mmu04062	Chemokine signaling pathway - Mus musculus (house mouse)	0.006477383	0.013436556	0.005804006	Braf/Ccr1/Gnb2/Jak2/Mapk3/Ncf1/Nfkb1/Pxn/Rela
3-day post-SCI group and the control group	mmu04934	Cushing syndrome - Mus musculus (house mouse)	0.007457032	0.015341926	0.006627043	Apc/Atf2/Braf/Cdk4/Cdkn2b/Cdkn2c/Mapk3/Rb1
3-day post-SCI group and the control group	mmu05215	Prostate cancer - Mus musculus (house mouse)	0.007649085	0.015609109	0.006742454	Braf/Mapk3/Nfkb1/Pdgfra/Rb1/Rela
3-day post-SCI group and the control group	mmu04623	Cytosolic DNA-sensing pathway - Mus musculus (house mouse)	0.009899938	0.020039391	0.008656143	Casp3/Nfkb1/Rela/Ripk1/Ripk3
3-day post-SCI group and the control group	mmu05100	Bacterial invasion of epithelial cells - Mus musculus (house mouse)	0.010451288	0.020952565	0.009050594	Actb/Ctnna1/Cttn/Met/Pxn
3-day post-SCI group and the control group	mmu04310	Wnt signaling pathway - Mus musculus (house mouse)	0.010518021	0.020952565	0.009050594	Apc/Fosl1/Jun/Mapk10/Mapk8/Myc/Nfatc1/Ppp3ca
3-day post-SCI group and the control group	mmu04613	Neutrophil extracellular trap formation - Mus musculus (house mouse)	0.010680938	0.021109571	0.009118414	Actb/Hdac1/Mapk14/Mapk3/Ncf1/Nfkb1/Ppif/Rela/Tlr4
3-day post-SCI group and the control group	mmu04910	Insulin signaling pathway - Mus musculus (house mouse)	0.010897464	0.021369247	0.009230582	Braf/Mapk10/Mapk3/Mapk8/Ppargc1a/Ppp1ca/Prkaa2
3-day post-SCI group and the control group	mmu05146	Amoebiasis - Mus musculus (house mouse)	0.011014481	0.021431276	0.009257377	Arg1/Casp3/Hspb1/Nfkb1/Rela/Tlr4
3-day post-SCI group and the control group	mmu04928	Parathyroid hormone synthesis, secretion and action - Mus musculus (house n	0.011498517	0.022200983	0.009589856	Atf2/Braf/Fos/Hbegf/Mapk3/Rxrb
3-day post-SCI group and the control group	mmu04015	Rap1 signaling pathway - Mus musculus (house mouse)	0.012697349	0.024328508	0.010508854	Actb/Braf/Id1/Map2k3/Map2k6/Mapk14/Mapk3/Met/Pdgfra
3-day post-SCI group and the control group	mmu04978	Mineral absorption - Mus musculus (house mouse)	0.014437911	0.027453906	0.01185889	Atox1/Atp7a/Hmox1/Slc8a1
3-day post-SCI group and the control group	mmu04114	Oocyte meiosis - Mus musculus (house mouse)	0.019222217	0.036276515	0.015669872	Cdc20/Cdk1/Mapk14/Mapk3/Ppp1ca/Ppp3ca
3-day post-SCI group and the control group	mmu04714	Thermogenesis - Mus musculus (house mouse)	0.019950579	0.037367827	0.016141272	Actb/Atf2/Map2k3/Mapk14/Ndufa12/Ndufa6/Ndufs8/Ppargc1a/Prkaa2
3-day post-SCI group and the control group	mmu04390	Hippo signaling pathway - Mus musculus (house mouse)	0.020098234	0.037367827	0.016141272	Actb/Apc/Ctnna1/Id1/Myc/Ppp1ca/Smad1
3-day post-SCI group and the control group	mmu04014	Ras signaling pathway - Mus musculus (house mouse)	0.0220223	0.040644098	0.017556478	Ets1/Gnb2/Mapk10/Mapk3/Mapk8/Met/Nfkb1/Pdgfra/Rela
3-day post-SCI group and the control group	mmu00983	Drug metabolism - other enzymes - Mus musculus (house mouse)	0.022306362	0.04086786	0.017653133	Gstt2/Mgst1/Nme2/Rrm2b/Xdh
3-day post-SCI group and the control group	mmu04152	AMPK signaling pathway - Mus musculus (house mouse)	0.02376133	0.042907149	0.018534017	Camkk2/Ccna2/Cd36/Ppargc1a/Prkaa2/Sirt1
3-day post-SCI group and the control group	mmu04750	Inflammatory mediator regulation of TRP channels - Mus musculus (house m	0.02376133	0.042907149	0.018534017	Map2k3/Map2k6/Mapk10/Mapk14/Mapk8/Ppp1ca
3-day post-SCI group and the control group	mmu05414	Dilated cardiomyopathy - Mus musculus (house mouse)	0.024222873	0.043428152	0.018759067	Actb/Atp2a2/Itga6/Itgb5/Slc8a1
3-day post-SCI group and the control group	mmu05034	Alcoholism - Mus musculus (house mouse)	0.027271521	0.048547175	0.020970262	Atf2/Braf/Camkk2/Gnb2/Hdac1/Maoa/Mapk3/Ppp1ca
3-day post-SCI group and the control group	mmu04720	Long-term potentiation - Mus musculus (house mouse)	0.029429866	0.052020396	0.022470542	Braf/Mapk3/Ppp1ca/Ppp3ca
3-day post-SCI group and the control group	mmu04141	Protein processing in endoplasmic reticulum - Mus musculus (house mouse)	0.032822558	0.05722767	0.024719858	Bax/Eif2s1/Mapk10/Mapk8/Nfe2l2/P4hb/Ubqln1
3-day post-SCI group and the control group	mmu04371	Apelin signaling pathway - Mus musculus (house mouse)	0.032831811	0.05722767	0.024719858	Gnb2/Mapk3/Mylk/Ppargc1a/Prkaa2/Slc8a1
3-day post-SCI group and the control group	mmu04020	Calcium signaling pathway - Mus musculus (house mouse)	0.033283785	0.057615379	0.024887331	Atp2a2/Cd38/Met/Mylk/Nfatc1/Pdgfra/Ppif/Ppp3ca/Slc8a1
3-day post-SCI group and the control group	mmu04922	Glucagon signaling pathway - Mus musculus (house mouse)	0.035383363	0.060830301	0.026276037	Atf2/Ppargc1a/Ppp3ca/Prkaa2/Sirt1
3-day post-SCI group and the control group	mmu04145	Phagosome - Mus musculus (house mouse)	0.040345285	0.068888888	0.029756995	Actb/Cd36/Ctsl/Itgb5/Ncf1/Tlr4/Tlr6
3-day post-SCI group and the control group	mmu04725	Cholinergic synapse - Mus musculus (house mouse)	0.046277611	0.078484327	0.033901806	Chrna4/Fos/Gnb2/Jak2/Mapk3
7-day post-SCI group and the control group	mmu05417	Lipid and atherosclerosis - Mus musculus (house mouse)	1.62E-09	0.000000196	0.000000109	Cd36/Cybb/Fos/Jun/Map2k3/Map2k4/Mapk10/Mapk14/Mapk8/Ncf1/Nfe2l2/Ppp3ca/Tlr4/Vcam1
7-day post-SCI group and the control group	mmu05418	Fluid shear stress and atherosclerosis - Mus musculus (house mouse)	1.95E-09	0.000000196	0.000000109	Ctnnb1/Ctsl/Fos/Jun/Map2k4/Mapk10/Mapk14/Mapk8/Mgst1/Ncf1/Nfe2l2/Vcam1
7-day post-SCI group and the control group	mmu04380	Osteoclast differentiation - Mus musculus (house mouse)	5.67E-09	0.000000339	0.000000188	Btk/Fos/Jun/Mapk10/Mapk14/Mapk8/Ncf1/Ppp3ca/Sirpa/Stat1/Trem2
7-day post-SCI group and the control group	mmu04620	Toll-like receptor signaling pathway - Mus musculus (house mouse)	6.75E-09	0.000000339	0.000000188	Fos/Jun/Map2k3/Map2k4/Mapk10/Mapk14/Mapk8/Ripk1/Stat1/Tlr4
7-day post-SCI group and the control group	mmu04659	Th17 cell differentiation - Mus musculus (house mouse)	1.09E-08	0.000000438	0.000000243	Fos/Hif1a/Il6st/Jun/Mapk10/Mapk14/Mapk8/Ppp3ca/Stat1/Stat6
7-day post-SCI group and the control group	mmu05208	Chemical carcinogenesis - reactive oxygen species - Mus musculus (house mo	2.25E-08	0.000000754	0.000000418	Fos/Hif1a/Jun/Map2k4/Mapk10/Mapk14/Mapk8/Mgst1/Ncf1/Ndufa12/Ndufa6/Nfe2l2/Prkcd
7-day post-SCI group and the control group	mmu05140	Leishmaniasis - Mus musculus (house mouse)	0.000000084	0.00000241	0.00000134	Cybb/Fos/Jun/Mapk14/Marcksl1/Ncf1/Stat1/Tlr4
7-day post-SCI group and the control group	mmu05167	Kaposi sarcoma-associated herpesvirus infection - Mus musculus (house mou	0.000000219	0.00000551	0.00000306	Ctnnb1/Fos/Hif1a/Il6st/Jun/Map2k4/Mapk10/Mapk14/Mapk8/Ppp3ca/Rcan1/Stat1
7-day post-SCI group and the control group	mmu04668	TNF signaling pathway - Mus musculus (house mouse)	0.000000343	0.00000766	0.00000425	Fos/Jun/Map2k3/Map2k4/Mapk10/Mapk14/Mapk8/Ripk1/Vcam1
7-day post-SCI group and the control group	mmu04658	Th1 and Th2 cell differentiation - Mus musculus (house mouse)	0.000000508	0.00000929	0.00000516	Fos/Jun/Mapk10/Mapk14/Mapk8/Ppp3ca/Stat1/Stat6
7-day post-SCI group and the control group	mmu05235	PD-L1 expression and PD-1 checkpoint pathway in cancer - Mus musculus (h	0.000000508	0.00000929	0.00000516	Fos/Hif1a/Jun/Map2k3/Mapk14/Ppp3ca/Stat1/Tlr4
7-day post-SCI group and the control group	mmu04912	GnRH signaling pathway - Mus musculus (house mouse)	0.000000605	0.0000101	0.00000563	Hbegf/Jun/Map2k3/Map2k4/Mapk10/Mapk14/Mapk8/Prkcd

7-day post-SCI group and the control group	mmu05161	Hepatitis B - Mus musculus (house mouse)	0.000000714	0.000011	0.00000612	Fos/Jun/Map2k3/Map2k4/Mapk10/Mapk14/Mapk8/Stat1/Stat6/Tlr4
7-day post-SCI group and the control group	mmu05022	Pathways of neurodegeneration - multiple diseases - Mus musculus (house mo	0.000000906	0.000013	0.00000719	Apc/Atp2a2/Capn2/Ctnnb1/Cybb/Gpx1/Gpx3/Map2k3/Mapk10/Mapk14/Mapk8/Mapt/Ndufa12/Ndufa6/Pink1/Ppp3ca
7-day post-SCI group and the control group	mmu05415	Diabetic cardiomyopathy - Mus musculus (house mouse)	0.000000966	0.000013	0.00000719	Atp2a2/Cd36/Cybb/Mapk10/Mapk14/Mapk8/Ncf1/Ndufa12/Ndufa6/Pdk2/Prkcd
7-day post-SCI group and the control group	mmu04621	NOD-like receptor signaling pathway - Mus musculus (house mouse)	0.00000122	0.0000153	0.00000849	Cybb/Jun/Mapk10/Mapk14/Mapk8/Prkcd/Ripk1/Stat1/Tlr4/Trpm2/Txnip
7-day post-SCI group and the control group	mmu04933	AGE-RAGE signaling pathway in diabetic complications - Mus musculus (ho	0.00000147	0.0000174	0.00000964	Cybb/Jun/Mapk10/Mapk14/Mapk8/Prkcd/Stat1/Vcam1
7-day post-SCI group and the control group	mmu04936	Alcoholic liver disease - Mus musculus (house mouse)	0.00000193	0.0000215	0.0000119	Ctnnb1/Map2k3/Map2k4/Mapk10/Mapk14/Mapk8/Ppargc1a/Ripk1/Tlr4
7-day post-SCI group and the control group	mmu05135	Yersinia infection - Mus musculus (house mouse)	0.0000129	0.000136457	0.0000757	Fos/Jun/Map2k3/Map2k4/Mapk10/Mapk14/Mapk8/Tlr4
7-day post-SCI group and the control group	mmu04210	Apoptosis - Mus musculus (house mouse)	0.0000136	0.000136842	0.000076	Capn2/Ctsl/Fos/Jun/Mapk10/Mapk8/Mcl1/Ripk1
7-day post-SCI group and the control group	mmu04664	Fc epsilon RI signaling pathway - Mus musculus (house mouse)	0.0000148	0.000141436	0.0000785	Btk/Map2k3/Map2k4/Mapk10/Mapk14/Mapk8
7-day post-SCI group and the control group	mmu05169	Epstein-Barr virus infection - Mus musculus (house mouse)	0.0000163	0.000148859	0.0000826	Btk/Hdac1/Jun/Map2k3/Map2k4/Mapk10/Mapk14/Mapk8/Ripk1/Stat1
7-day post-SCI group and the control group	mmu05142	Chagas disease - Mus musculus (house mouse)	0.0000191	0.000166932	0.0000927	Fos/Jun/Map2k4/Mapk10/Mapk14/Mapk8/Tlr4
7-day post-SCI group and the control group	mmu00480	Glutathione metabolism - Mus musculus (house mouse)	0.0000244	0.000204745	0.000113658	Gclc/Gpx1/Gpx3/Lancl1/Mgst1/Prdx6
7-day post-SCI group and the control group	mmu05145	Toxoplasmosis - Mus musculus (house mouse)	0.0000276	0.00022214	0.000123314	Itga6/Map2k3/Mapk10/Mapk14/Mapk8/Stat1/Tlr4
7-day post-SCI group and the control group	mmu05171	Coronavirus disease - COVID-19 - Mus musculus (house mouse)	0.0000311	0.000240685	0.000133609	Cybb/Fos/Hbegf/Il6st/Jun/Mapk10/Mapk14/Mapk8/Stat1/Tlr4
7-day post-SCI group and the control group	mmu04625	C-type lectin receptor signaling pathway - Mus musculus (house mouse)	0.0000329	0.000245259	0.000136148	Jun/Mapk10/Mapk14/Mapk8/Ppp3ca/Prkcd/Stat1
7-day post-SCI group and the control group	mmu05132	Salmonella infection - Mus musculus (house mouse)	0.0000357	0.000248989	0.000138219	Ctnnb1/Fos/Jun/Map2k3/Map2k4/Mapk10/Mapk14/Mapk8/Ripk1/Tlr4
7-day post-SCI group and the control group	mmu05133	Pertussis - Mus musculus (house mouse)	0.0000359	0.000248989	0.000138219	Fos/Jun/Mapk10/Mapk14/Mapk8/Tlr4
7-day post-SCI group and the control group	mmu04935	Growth hormone synthesis, secretion and action - Mus musculus (house mous	0.0000437	0.000292531	0.00016239	Fos/Map2k3/Map2k4/Mapk10/Mapk14/Mapk8/Stat1
7-day post-SCI group and the control group	mmu05010	Alzheimer disease - Mus musculus (house mouse)	0.0000555	0.000360163	0.000199933	Apc/Apoe/Atp2a2/Capn2/Ctnnb1/Cybb/Mapk10/Mapk8/Mapt/Ndufa12/Ndufa6/Ppp3ca
7-day post-SCI group and the control group	mmu05210	Colorectal cancer - Mus musculus (house mouse)	0.0000765	0.000480527	0.00026675	Apc/Ctnnb1/Fos/Jun/Mapk10/Mapk8
7-day post-SCI group and the control group	mmu04217	Necroptosis - Mus musculus (house mouse)	0.0000832	0.000507007	0.000281449	Capn2/Cybb/Mapk10/Mapk8/Ripk1/Stat1/Stat6/Tlr4
7-day post-SCI group and the control group	mmu01522	Endocrine resistance - Mus musculus (house mouse)	0.000104198	0.000615993	0.000341949	Fos/Hbegf/Jun/Mapk10/Mapk14/Mapk8
7-day post-SCI group and the control group	mmu05170	Human immunodeficiency virus 1 infection - Mus musculus (house mouse)	0.000135047	0.000775555	0.000430525	Fos/Jun/Map2k3/Mapk10/Mapk14/Mapk8/Ppp3ca/Ripk1/Tlr4
7-day post-SCI group and the control group	mmu04010	MAPK signaling pathway - Mus musculus (house mouse)	0.000152587	0.000851943	0.00047293	Fos/Hspb1/Jun/Map2k3/Map2k4/Mapk10/Mapk14/Mapk8/Mapt/Ppp3ca
7-day post-SCI group and the control group	mmu04137	Mitophagy - animal - Mus musculus (house mouse)	0.000219721	0.001193621	0.000662602	Hif1a/Jun/Mapk10/Mapk8/Pink1
7-day post-SCI group and the control group	mmu04510	Focal adhesion - Mus musculus (house mouse)	0.000232625	0.0012172	0.000675691	Capn2/Ctnnb1/Itga6/Itgb5/Jun/Mapk10/Mapk8/Mylk
7-day post-SCI group and the control group	mmu04921	Oxytocin signaling pathway - Mus musculus (house mouse)	0.000236173	0.0012172	0.000675691	Cd38/Fos/Jun/Mylk/Ppp3ca/Rcan1/Trpm2
7-day post-SCI group and the control group	mmu05205	Proteoglycans in cancer - Mus musculus (house mouse)	0.000248692	0.001249679	0.000693721	Ctnnb1/Ctsl/Ezr/Hbegf/Hif1a/Itgb5/Mapk14/Tlr4
7-day post-SCI group and the control group	mmu04931	Insulin resistance - Mus musculus (house mouse)	0.000262488	0.001273346	0.000706859	Cd36/Mapk10/Mapk8/Ppargc1a/Ppargc1b/Prkcd
7-day post-SCI group and the control group	mmu04932	Non-alcoholic fatty liver disease - Mus musculus (house mouse)	0.000266072	0.001273346	0.000706859	Fos/Jun/Mapk10/Mapk14/Mapk8/Ndufa12/Ndufa6
7-day post-SCI group and the control group	mmu05020	Prion disease - Mus musculus (house mouse)	0.000300202	0.001403271	0.000778983	Cybb/Mapk10/Mapk14/Mapk8/Ncf1/Ndufa12/Ndufa6/Ppp3ca/Prkcd
7-day post-SCI group and the control group	mmu04917	Prolactin signaling pathway - Mus musculus (house mouse)	0.000326899	0.001493333	0.000828978	Fos/Mapk10/Mapk14/Mapk8/Stat1
7-day post-SCI group and the control group	mmu05412	Arrhythmogenic right ventricular cardiomyopathy - Mus musculus (house mo	0.000393417	0.001753902	0.000973625	Atp2a2/Ctnnb1/Itga6/Itgb5/Slc8a1
7-day post-SCI group and the control group	mmu04670	Leukocyte transendothelial migration - Mus musculus (house mouse)	0.000401391	0.001753902	0.000973625	Ctnnb1/Cybb/Ezr/Mapk14/Ncf1/Vcam1
7-day post-SCI group and the control group	mmu04919	Thyroid hormone signaling pathway - Mus musculus (house mouse)	0.000419813	0.001795371	0.000996645	Atp2a2/Ctnnb1/Hdac1/Hif1a/Rcan1/Stat1
7-day post-SCI group and the control group	mmu04660	T cell receptor signaling pathway - Mus musculus (house mouse)	0.000458639	0.001920551	0.001066134	Fos/Jun/Mapk10/Mapk14/Mapk8/Ppp3ca
7-day post-SCI group and the control group	mmu04012	ErbB signaling pathway - Mus musculus (house mouse)	0.000588128	0.002412524	0.001339238	Hbegf/Jun/Map2k4/Mapk10/Mapk8
7-day post-SCI group and the control group	mmu04926	Relaxin signaling pathway - Mus musculus (house mouse)	0.000617065	0.0024806	0.001377029	Fos/Jun/Map2k4/Mapk10/Mapk14/Mapk8
7-day post-SCI group and the control group	mmu05166	Human T-cell leukemia virus 1 infection - Mus musculus (house mouse)	0.000930815	0.003615684	0.002007135	Cdc20/Ets2/Fos/Jun/Map2k4/Mapk10/Mapk8/Ppp3ca
7-day post-SCI group and the control group	mmu04657	IL-17 signaling pathway - Mus musculus (house mouse)	0.000935401	0.003615684	0.002007135	Fos/Jun/Mapk10/Mapk14/Mapk8
7-day post-SCI group and the control group	mmu05162	Measles - Mus musculus (house mouse)	0.001138104	0.004316205	0.002396008	Fos/Jun/Mapk10/Mapk8/Stat1/Tlr4
7-day post-SCI group and the control group	mmu05231	Choline metabolism in cancer - Mus musculus (house mouse)	0.001184345	0.004408394	0.002447184	Fos/Hif1a/Jun/Mapk10/Mapk8
7-day post-SCI group and the control group	mmu05321	Inflammatory bowel disease - Mus musculus (house mouse)	0.001588336	0.005804647	0.003222271	Jun/Stat1/Stat6/Tlr4
7-day post-SCI group and the control group	mmu05031	Amphetamine addiction - Mus musculus (house mouse)	0.002358117	0.008463957	0.004698505	Fos/Hdac1/Jun/Ppp3ca
7-day post-SCI group and the control group	mmu04622	RIG-I-like receptor signaling pathway - Mus musculus (house mouse)	0.002618095	0.009073052	0.005036625	Mapk10/Mapk14/Mapk8/Ripk1
7-day post-SCI group and the control group	mmu04920	Adipocytokine signaling pathway - Mus musculus (house mouse)	0.002618095	0.009073052	0.005036625	Cd36/Mapk10/Mapk8/Ppargc1a
7-day post-SCI group and the control group	mmu04310	Wnt signaling pathway - Mus musculus (house mouse)	0.002712178	0.009239794	0.005129186	Apc/Ctnnb1/Jun/Mapk10/Mapk8/Ppp3ca
7-day post-SCI group and the control group	mmu04722	Neurotrophin signaling pathway - Mus musculus (house mouse)	0.002999508	0.010048351	0.005578032	Jun/Mapk10/Mapk14/Mapk8/Prkcd
7-day post-SCI group and the control group	mmu05016	Huntington disease - Mus musculus (house mouse)	0.003069475	0.010114171	0.00561457	Gpx1/Gpx3/Hdac1/Mapk10/Mapk8/Ndufa12/Ndufa6/Ppargc1a
7-day post-SCI group and the control group	mmu05152	Tuberculosis - Mus musculus (house mouse)	0.003398575	0.01101796	0.006116281	Mapk10/Mapk14/Mapk8/Ppp3ca/Stat1/Tlr4
7-day post-SCI group and the control group	mmu04145	Phagosome - Mus musculus (house mouse)	0.003588783	0.011449926	0.006356073	Cd36/Ctsl/Cybb/Itgb5/Ncf1/Tlr4
7-day post-SCI group and the control group	mmu04750	Inflammatory mediator regulation of TRP channels - Mus musculus (house m	0.003694063	0.011601668	0.006440308	Map2k3/Mapk10/Mapk14/Mapk8/Prkcd

7-day post-SCI group and the control group	mmu04662	B cell receptor signaling pathway - Mus musculus (house mouse)	0.004601266	0.014228531	0.00789853	Btk/Fos/Jun/Ppp3ca
7-day post-SCI group and the control group	mmu04728	Dopaminergic synapse - Mus musculus (house mouse)	0.004790882	0.014405769	0.007996918	Fos/Mapk10/Mapk14/Mapk8/Ppp3ca
7-day post-SCI group and the control group	mmu03083	Polycomb repressive complex - Mus musculus (house mouse)	0.004801923	0.014405769	0.007996918	Cbx6/Hdac1/Phc1/Rbbp7
7-day post-SCI group and the control group	mmu05323	Rheumatoid arthritis - Mus musculus (house mouse)	0.00543906	0.016033862	0.008900704	Ctsl/Fos/Jun/Tlr4
7-day post-SCI group and the control group	mmu05012	Parkinson disease - Mus musculus (house mouse)	0.005566482	0.016033862	0.008900704	Mapk10/Mapk8/Mapt/Ndufa12/Ndufa6/Nfe2l2/Pink1
7-day post-SCI group and the control group	mmu04550	Signaling pathways regulating pluripotency of stem cells - Mus musculus (hou	0.005583932	0.016033862	0.008900704	Apc/Ctnnb1/Id1/Il6st/Mapk14
7-day post-SCI group and the control group	mmu04140	Autophagy - animal - Mus musculus (house mouse)	0.005925735	0.016775673	0.009312497	Ctsl/Hif1a/Mapk10/Mapk8/Prkcd
7-day post-SCI group and the control group	mmu05410	Hypertrophic cardiomyopathy - Mus musculus (house mouse)	0.006373398	0.017792403	0.009876904	Atp2a2/Itga6/Itgb5/Slc8a1
7-day post-SCI group and the control group	mmu04666	Fc gamma R-mediated phagocytosis - Mus musculus (house mouse)	0.006878216	0.018777695	0.010423858	Amph/Marcksl1/Ncf1/Prkcd
7-day post-SCI group and the control group	mmu04930	Type II diabetes mellitus - Mus musculus (house mouse)	0.006913181	0.018777695	0.010423858	Mapk10/Mapk8/Prkcd
7-day post-SCI group and the control group	mmu04723	Retrograde endocannabinoid signaling - Mus musculus (house mouse)	0.00703958	0.018866075	0.010472919	Mapk10/Mapk14/Mapk8/Ndufa12/Ndufa6
7-day post-SCI group and the control group	mmu05414	Dilated cardiomyopathy - Mus musculus (house mouse)	0.007140282	0.018884166	0.010482962	Atp2a2/Itga6/Itgb5/Slc8a1
7-day post-SCI group and the control group	mmu04979	Cholesterol metabolism - Mus musculus (house mouse)	0.007743699	0.02021407	0.011221217	Apoe/Cd36/Lcat
7-day post-SCI group and the control group	mmu05014	Amyotrophic lateral sclerosis - Mus musculus (house mouse)	0.010039799	0.025871789	0.014361926	Gpx1/Gpx3/Map2k3/Mapk14/Ndufa12/Ndufa6/Pink1/Ppp3ca
7-day post-SCI group and the control group	mmu04064	NF-kappa B signaling pathway - Mus musculus (house mouse)	0.010468139	0.026634127	0.014785114	Btk/Ripk1/Tlr4/Vcam1
7-day post-SCI group and the control group	mmu05144	Malaria - Mus musculus (house mouse)	0.011100807	0.027890777	0.015482704	Cd36/Tlr4/Vcam1
7-day post-SCI group and the control group	mmu04370	VEGF signaling pathway - Mus musculus (house mouse)	0.011638777	0.028881409	0.016032623	Hspb1/Mapk14/Ppp3ca
7-day post-SCI group and the control group	mmu04611	Platelet activation - Mus musculus (house mouse)	0.018799324	0.04608127	0.025580595	Btk/Mapk14/Mylk/Ptgs1
7-day post-SCI group and the control group	mmu04915	Estrogen signaling pathway - Mus musculus (house mouse)	0.023024406	0.055757899	0.030952277	Fos/Hbegf/Jun/Prkcd
7-day post-SCI group and the control group	mmu05212	Pancreatic cancer - Mus musculus (house mouse)	0.02390292	0.057196273	0.031750746	Mapk10/Mapk8/Stat1
7-day post-SCI group and the control group	mmu04613	Neutrophil extracellular trap formation - Mus musculus (house mouse)	0.027082196	0.064041428	0.035550623	Cybb/Hdac1/Mapk14/Ncf1/Tlr4
7-day post-SCI group and the control group	mmu04215	Apoptosis - multiple species - Mus musculus (house mouse)	0.027759018	0.064878636	0.036015373	Mapk10/Mapk8
7-day post-SCI group and the control group	mmu05224	Breast cancer - Mus musculus (house mouse)	0.031717591	0.073278573	0.040678339	Apc/Ctnnb1/Fos/Jun
7-day post-SCI group and the control group	mmu04024	cAMP signaling pathway - Mus musculus (house mouse)	0.035649527	0.080942084	0.0449325	Atp2a2/Fos/Jun/Mapk10/Mapk8
7-day post-SCI group and the control group	mmu04512	ECM-receptor interaction - Mus musculus (house mouse)	0.035840027	0.080942084	0.0449325	Cd36/Itga6/Itgb5
7-day post-SCI group and the control group	mmu04914	Progesterone-mediated oocyte maturation - Mus musculus (house mouse)	0.03895839	0.087007071	0.04829929	Mapk10/Mapk14/Mapk8
7-day post-SCI group and the control group	mmu04714	Thermogenesis - Mus musculus (house mouse)	0.039871718	0.087111036	0.048357003	Map2k3/Mapk14/Ndufa12/Ndufa6/Ppargc1a
7-day post-SCI group and the control group	mmu04810	Regulation of actin cytoskeleton - Mus musculus (house mouse)	0.039871718	0.087111036	0.048357003	Apc/Ezr/Itga6/Itgb5/Mylk
7-day post-SCI group and the control group	mmu04640	Hematopoietic cell lineage - Mus musculus (house mouse)	0.041111931	0.088854818	0.04932501	Cd36/Cd38/Itga6
7-day post-SCI group and the control group	mmu04216	Ferroptosis - Mus musculus (house mouse)	0.041875372	0.08954202	0.049706489	Cybb/Gclc
28-day post-SCI group and the control group	mmu05418	Fluid shear stress and atherosclerosis - Mus musculus (house mouse)	1.02E-12	2.06E-10	9.9E-11	Actb/Ctsl/Fos/Il1a/Jun/Map2k4/Map2k6/Mapk10/Mgst1/Ncf1/Nfe2l2/Rela/Sdc1/Tnfrsf1a/Vcam1
28-day post-SCI group and the control group	mmu05417	Lipid and atherosclerosis - Mus musculus (house mouse)	1.9E-12	2.06E-10	9.9E-11	Cd36/Cybb/Eif2s1/Fos/Jun/Map2k3/Map2k4/Map2k6/Mapk1/Mapk10/Ncf1/Nfe2l2/Ppp3ca/Rela/Tlr4/Tnfrsf1a/Vcam1
28-day post-SCI group and the control group	mmu04668	TNF signaling pathway - Mus musculus (house mouse)	1.02E-11	7.37E-10	3.54E-10	Fos/Jun/Map2k3/Map2k4/Map2k6/Mapk1/Mapk10/Mmp14/Rela/Ripk1/Tnfaip3/Tnfrsf1a/Vcam1
28-day post-SCI group and the control group	mmu04380	Osteoclast differentiation - Mus musculus (house mouse)	6.23E-10	3.38E-08	1.62E-08	Fos/Il1a/Jun/Map2k6/Mapk1/Mapk10/Ncf1/Ppp3ca/Rela/Sirpa/Tnfrsf1a/Trem2
28-day post-SCI group and the control group	mmu04210	Apoptosis - Mus musculus (house mouse)	1.26E-09	5.48E-08	2.63E-08	Actb/Ctsl/Eif2s1/Fos/Jun/Mapk1/Mapk10/Mcl1/Parp1/Rela/Ripk1/Tnfrsf1a
28-day post-SCI group and the control group	mmu05208	Chemical carcinogenesis - reactive oxygen species - Mus musculus (house mc	3.94E-09	0.000000142	6.84E-08	Cyp1b1/Fos/Hif1a/Jun/Map2k4/Mapk1/Mapk10/Met/Mgst1/Ncf1/Ndufa6/Nfe2l2/Prkcd/Rela
28-day post-SCI group and the control group	mmu04620	Toll-like receptor signaling pathway - Mus musculus (house mouse)	9.93E-09	0.000000308	0.000000148	Fos/Jun/Map2k3/Map2k4/Map2k6/Mapk1/Mapk10/Rela/Ripk1/Tlr4
28-day post-SCI group and the control group	mmu05167	Kaposi sarcoma-associated herpesvirus infection - Mus musculus (house mou	4.06E-08	0.0000011	0.000000529	Fos/Hif1a/Il6st/Jun/Map2k4/Map2k6/Mapk1/Mapk10/Mapkapk2/Ppp3ca/Rb1/Rela/Tnfrsf1a
28-day post-SCI group and the control group	mmu05235	PD-L1 expression and PD-1 checkpoint pathway in cancer - Mus musculus (h	4.69E-08	0.00000113	0.000000543	Fos/Hif1a/Jun/Map2k3/Map2k6/Mapk1/Ppp3ca/Rela/Tlr4
28-day post-SCI group and the control group	mmu04912	GnRH signaling pathway - Mus musculus (house mouse)	5.72E-08	0.00000124	0.000000596	Hbegf/Jun/Map2k3/Map2k4/Map2k6/Mapk1/Mapk10/Mmp14/Prkcd
28-day post-SCI group and the control group	mmu05161	Hepatitis B - Mus musculus (house mouse)	0.000000107	0.00000206	0.000000991	Fos/Jun/Map2k3/Map2k4/Map2k6/Mapk1/Mapk10/Rb1/Rela/Stat6/Tlr4
28-day post-SCI group and the control group	mmu05140	Leishmaniasis - Mus musculus (house mouse)	0.000000114	0.00000206	0.000000991	Cybb/Fos/Il1a/Jun/Mapk1/Ncf1/Rela/Tlr4
28-day post-SCI group and the control group	mmu05135	Yersinia infection - Mus musculus (house mouse)	0.000000178	0.00000286	0.00000138	Actb/Fos/Jun/Map2k3/Map2k4/Map2k6/Mapk1/Mapk10/Rela/Tlr4
28-day post-SCI group and the control group	mmu04010	MAPK signaling pathway - Mus musculus (house mouse)	0.000000185	0.00000286	0.00000138	Fos/Hspb1/Il1a/Jun/Map2k3/Map2k4/Map2k6/Mapk1/Mapk10/Mapkapk2/Met/Ppp3ca/Rela/Tnfrsf1a
28-day post-SCI group and the control group	mmu04659	Th17 cell differentiation - Mus musculus (house mouse)	0.00000022	0.00000318	0.00000153	Fos/Hif1a/Il6st/Jun/Mapk1/Mapk10/Ppp3ca/Rela/Stat6
28-day post-SCI group and the control group	mmu05132	Salmonella infection - Mus musculus (house mouse)	0.00000125	0.0000169	0.00000812	Actb/Fos/Jun/Map2k3/Map2k4/Map2k6/Mapk1/Mapk10/Rela/Ripk1/Tlr4/Tnfrsf1a
28-day post-SCI group and the control group	mmu05022	Pathways of neurodegeneration - multiple diseases - Mus musculus (house mc	0.00000157	0.00002	0.00000961	Apc/Atp2a2/Cybb/Eif2s1/Gpx1/Gpx3/Gpx8/Il1a/Map2k3/Map2k6/Mapk1/Mapk10/Ndufa6/Ppp3ca/Rela/Tnfrsf1a
28-day post-SCI group and the control group	mmu04621	NOD-like receptor signaling pathway - Mus musculus (house mouse)	0.00000181	0.0000218	0.0000105	Cybb/Jun/Mapk1/Mapk10/Prkcd/Rela/Ripk1/Tlr4/Tnfaip3/Trpm2/Txnip

28-day post-SCI group and the control group	mmu04933	AGE-RAGE signaling pathway in diabetic complications - Mus musculus (ho	0.00000198	0.0000226	0.0000109	Cybb/Il1a/Jun/Mapk1/Mapk10/Prkcd/Rela/Vcam1
28-day post-SCI group and the control group	mmu05142	Chagas disease - Mus musculus (house mouse)	0.0000023	0.0000249	0.000012	Fos/Jun/Map2k4/Mapk1/Mapk10/Rela/Tlr4/Tnfrsf1a
28-day post-SCI group and the control group	mmu05133	Pertussis - Mus musculus (house mouse)	0.00000357	0.0000369	0.0000177	Fos/Il1a/Jun/Mapk1/Mapk10/Rela/Tlr4
28-day post-SCI group and the control group	mmu05170	Human immunodeficiency virus 1 infection - Mus musculus (house mouse)	0.00000503	0.0000496	0.0000238	Fos/Jun/Map2k3/Map2k6/Mapk1/Mapk10/Ppp3ca/Rela/Ripk1/Tlr4/Tnfrsf1a
28-day post-SCI group and the control group	mmu05171	Coronavirus disease - COVID-19 - Mus musculus (house mouse)	0.00000715	0.0000672	0.0000323	Cybb/Fos/Hbegf/Il6st/Jun/Mapk1/Mapk10/Rela/Rpl13a/Tlr4/Tnfrsf1a
28-day post-SCI group and the control group	mmu05166	Human T-cell leukemia virus 1 infection - Mus musculus (house mouse)	0.00000743	0.0000672	0.0000323	Cdkn2c/Ets2/Fos/Jun/Map2k4/Mapk1/Mapk10/Ppp3ca/Rb1/Rela/Tnfrsf1a
28-day post-SCI group and the control group	mmu04658	Th1 and Th2 cell differentiation - Mus musculus (house mouse)	0.00000874	0.0000759	0.0000364	Fos/Jun/Mapk1/Mapk10/Ppp3ca/Rela/Stat6
28-day post-SCI group and the control group	mmu05415	Diabetic cardiomyopathy - Mus musculus (house mouse)	0.0000105	0.0000874	0.000042	Atp2a2/Cd36/Cybb/Mapk10/Ncf1/Ndufa6/Parp1/Pdk2/Prkcd/Rela
28-day post-SCI group and the control group	mmu01522	Endocrine resistance - Mus musculus (house mouse)	0.0000126	0.000101368	0.0000487	Cdkn2c/Fos/Hbegf/Jun/Mapk1/Mapk10/Rb1
28-day post-SCI group and the control group	mmu04217	Necroptosis - Mus musculus (house mouse)	0.0000156	0.00012113	0.0000582	Cybb/Il1a/Mapk10/Parp1/Ripk1/Stat6/Tlr4/Tnfaip3/Tnfrsf1a
28-day post-SCI group and the control group	mmu05169	Epstein-Barr virus infection - Mus musculus (house mouse)	0.000023	0.000170782	0.000082	Hdac1/Jun/Map2k3/Map2k4/Map2k6/Mapk10/Rb1/Rela/Ripk1/Tnfaip3
28-day post-SCI group and the control group	mmu04936	Alcoholic liver disease - Mus musculus (house mouse)	0.0000236	0.000170782	0.000082	Map2k3/Map2k4/Map2k6/Mapk10/Rela/Ripk1/Tlr4/Tnfrsf1a
28-day post-SCI group and the control group	mmu04064	NF-kappa B signaling pathway - Mus musculus (house mouse)	0.0000279	0.000195565	0.0000939	Parp1/Rela/Ripk1/Tlr4/Tnfaip3/Tnfrsf1a/Vcam1
28-day post-SCI group and the control group	mmu05162	Measles - Mus musculus (house mouse)	0.0000289	0.000196003	0.0000941	Eif2s1/Fos/Il1a/Jun/Mapk10/Rela/Tlr4/Tnfaip3
28-day post-SCI group and the control group	mmu00480	Glutathione metabolism - Mus musculus (house mouse)	0.0000305	0.000200707	0.0000964	Gpx1/Gpx3/Gpx8/Lancl1/Mgst1/Prdx6
28-day post-SCI group and the control group	mmu05145	Toxoplasmosis - Mus musculus (house mouse)	0.0000356	0.000227196	0.000109107	Map2k3/Map2k6/Mapk1/Mapk10/Rela/Tlr4/Tnfrsf1a
28-day post-SCI group and the control group	mmu04625	C-type lectin receptor signaling pathway - Mus musculus (house mouse)	0.0000424	0.000256223	0.000123047	Jun/Mapk1/Mapk10/Mapkapk2/Ppp3ca/Prkcd/Rela
28-day post-SCI group and the control group	mmu04921	Oxytocin signaling pathway - Mus musculus (house mouse)	0.0000425	0.000256223	0.000123047	Actb/Cd38/Fos/Jun/Mapk1/Mylk/Ppp3ca/Trpm2
28-day post-SCI group and the control group	mmu04932	Non-alcoholic fatty liver disease - Mus musculus (house mouse)	0.0000488	0.000286357	0.000137518	Eif2s1/Fos/Il1a/Jun/Mapk10/Ndufa6/Rela/Tnfrsf1a
28-day post-SCI group and the control group	mmu05205	Proteoglycans in cancer - Mus musculus (house mouse)	0.0000546	0.000311775	0.000149724	Actb/Ctsl/Hbegf/Hif1a/Itgb5/Mapk1/Met/Sdc1/Tlr4
28-day post-SCI group and the control group	mmu05010	Alzheimer disease - Mus musculus (house mouse)	0.0000821	0.000456551	0.000219251	Apc/Apoe/Atp2a2/Cybb/Eif2s1/Il1a/Mapk1/Mapk10/Ndufa6/Ppp3ca/Rela/Tnfrsf1a
28-day post-SCI group and the control group	mmu05144	Malaria - Mus musculus (house mouse)	0.000113833	0.000617542	0.000296564	Cd36/Met/Sdc1/Tlr4/Vcam1
28-day post-SCI group and the control group	mmu04657	IL-17 signaling pathway - Mus musculus (house mouse)	0.000129347	0.000684592	0.000328764	Fos/Jun/Mapk1/Mapk10/Rela/Tnfaip3
28-day post-SCI group and the control group	mmu04218	Cellular senescence - Mus musculus (house mouse)	0.000155517	0.000803506	0.00038587	Il1a/Map2k3/Map2k6/Mapk1/Mapkapk2/Ppp3ca/Rb1/Rela
28-day post-SCI group and the control group	mmu05321	Inflammatory bowel disease - Mus musculus (house mouse)	0.000170214	0.000858988	0.000412514	Il1a/Jun/Rela/Stat6/Tlr4
28-day post-SCI group and the control group	mmu04664	Fc epsilon RI signaling pathway - Mus musculus (house mouse)	0.000228958	0.00112918	0.000542269	Map2k3/Map2k4/Map2k6/Mapk1/Mapk10
28-day post-SCI group and the control group	mmu04066	HIF-1 signaling pathway - Mus musculus (house mouse)	0.000393182	0.001896009	0.000910526	Cybb/Hif1a/Mapk1/Pdk1/Rela/Tlr4
28-day post-SCI group and the control group	mmu05020	Prion disease - Mus musculus (house mouse)	0.000402484	0.001898674	0.000911806	Cybb/Eif2s1/Il1a/Mapk1/Mapk10/Ncf1/Ndufa6/Ppp3ca/Prkcd
28-day post-SCI group and the control group	mmu04935	Growth hormone synthesis, secretion and action - Mus musculus (house mous	0.000451934	0.002086588	0.001002048	Fos/Map2k3/Map2k4/Map2k6/Mapk1/Mapk10
28-day post-SCI group and the control group	mmu04722	Neurotrophin signaling pathway - Mus musculus (house mouse)	0.000540692	0.002444381	0.001173872	Jun/Mapk1/Mapk10/Mapkapk2/Prkcd/Rela
28-day post-SCI group and the control group	mmu04660	T cell receptor signaling pathway - Mus musculus (house mouse)	0.000564869	0.002501561	0.001201332	Fos/Jun/Mapk1/Mapk10/Ppp3ca/Rela
28-day post-SCI group and the control group	mmu05164	Influenza A - Mus musculus (house mouse)	0.000630746	0.00273744	0.001314608	Actb/Eif2s1/Il1a/Mapk1/Rela/Tlr4/Tnfrsf1a
28-day post-SCI group and the control group	mmu05225	Hepatocellular carcinoma - Mus musculus (house mouse)	0.000652763	0.002775288	0.001332785	Actb/Apc/Mapk1/Met/Mgst1/Nfe2l2/Rb1
28-day post-SCI group and the control group	mmu04662	B cell receptor signaling pathway - Mus musculus (house mouse)	0.000665046	0.002775288	0.001332785	Fos/Jun/Mapk1/Ppp3ca/Rela
28-day post-SCI group and the control group	mmu04012	ErbB signaling pathway - Mus musculus (house mouse)	0.0007025	0.002876276	0.001381282	Hbegf/Jun/Map2k4/Mapk1/Mapk10
28-day post-SCI group and the control group	mmu04926	Relaxin signaling pathway - Mus musculus (house mouse)	0.000758552	0.003048257	0.001463873	Fos/Jun/Map2k4/Mapk1/Mapk10/Rela
28-day post-SCI group and the control group	mmu05152	Tuberculosis - Mus musculus (house mouse)	0.000798031	0.003148594	0.001512058	Il1a/Mapk1/Mapk10/Ppp3ca/Rela/Tlr4/Tnfrsf1a
28-day post-SCI group and the control group	mmu05323	Rheumatoid arthritis - Mus musculus (house mouse)	0.000824375	0.003194452	0.001534081	Ctsl/Fos/Il1a/Jun/Tlr4
28-day post-SCI group and the control group	mmu04145	Phagosome - Mus musculus (house mouse)	0.000851766	0.003242687	0.001557245	Actb/Cd36/Ctsl/Cybb/Itgb5/Ncf1/Tlr4
28-day post-SCI group and the control group	mmu05210	Colorectal cancer - Mus musculus (house mouse)	0.000868307	0.003248666	0.001560116	Apc/Fos/Jun/Mapk1/Mapk10
28-day post-SCI group and the control group	mmu05014	Amyotrophic lateral sclerosis - Mus musculus (house mouse)	0.001048734	0.00385721	0.001852359	Actb/Eif2s1/Gpx1/Gpx3/Gpx8/Map2k3/Map2k6/Ndufa6/Ppp3ca/Tnfrsf1a
28-day post-SCI group and the control group	mmu04140	Autophagy - animal - Mus musculus (house mouse)	0.001250867	0.004523969	0.002172558	Ctsl/Eif2s1/Hif1a/Mapk1/Mapk10/Prkcd
28-day post-SCI group and the control group	mmu05231	Choline metabolism in cancer - Mus musculus (house mouse)	0.001409474	0.005004863	0.002403499	Fos/Hif1a/Jun/Mapk1/Mapk10
28-day post-SCI group and the control group	mmu04370	VEGF signaling pathway - Mus musculus (house mouse)	0.001429961	0.005004863	0.002403499	Hspb1/Mapk1/Mapkapk2/Ppp3ca
28-day post-SCI group and the control group	mmu04510	Focal adhesion - Mus musculus (house mouse)	0.001607228	0.005536007	0.002658572	Actb/Itgb5/Jun/Mapk1/Mapk10/Met/Mylk
28-day post-SCI group and the control group	mmu04613	Neutrophil extracellular trap formation - Mus musculus (house mouse)	0.0018472	0.006263162	0.003007776	Actb/Cybb/Hdac1/Mapk1/Ncf1/Rela/Tlr4
28-day post-SCI group and the control group	mmu04931	Insulin resistance - Mus musculus (house mouse)	0.002347868	0.007838266	0.003764193	Cd36/Mapk10/Prkcd/Rela/Tnfrsf1a
28-day post-SCI group and the control group	mmu04137	Mitophagy - animal - Mus musculus (house mouse)	0.002575088	0.00834021	0.004005243	Hif1a/Jun/Mapk10/Rela
28-day post-SCI group and the control group	mmu05211	Renal cell carcinoma - Mus musculus (house mouse)	0.002575088	0.00834021	0.004005243	Hif1a/Jun/Mapk1/Met
28-day post-SCI group and the control group	mmu05031	Amphetamine addiction - Mus musculus (house mouse)	0.002716183	0.0085422	0.004102245	Fos/Hdac1/Jun/Ppp3ca
28-day post-SCI group and the control group	mmu05230	Central carbon metabolism in cancer - Mus musculus (house mouse)	0.002716183	0.0085422	0.004102245	Hif1a/Mapk1/Met/Pdk1
28-day post-SCI group and the control group	mmu05160	Hepatitis C - Mus musculus (house mouse)	0.002763113	0.008565651	0.004113507	Eif2s1/Mapk1/Rb1/Rela/Ripk1/Tnfrsf1a
28-day post-SCI group and the control group	mmu05207	Chemical carcinogenesis - receptor activation - Mus musculus (house mouse)	0.002877327	0.008794084	0.004223208	Cyp1b1/Fos/Jun/Mapk1/Mgst1/Rb1/Rela
28-day post-SCI group and the control group	mmu04920	Adipocytokine signaling pathway - Mus musculus (house mouse)	0.003014124	0.009084236	0.004362548	Cd36/Mapk10/Rela/Tnfrsf1a
28-day post-SCI group and the control group	mmu05203	Viral carcinogenesis - Mus musculus (house mouse)	0.003173841	0.009434568	0.004530789	Hdac1/Il6st/Jun/Mapk1/Mapkapk2/Rb1/Rela
28-day post-SCI group and the control group	mmu04919	Thyroid hormone signaling pathway - Mus musculus (house mouse)	0.003424063	0.010040833	0.004821938	Actb/Atp2a2/Hdac1/Hif1a/Mapk1

28-day post-SCI group and the control group	mmu04917	Prolactin signaling pathway - Mus musculus (house mouse)	0.003501816	0.010131921	0.004865681	Fos/Mapk1/Mapk10/Rela
28-day post-SCI group and the control group	mmu05212	Pancreatic cancer - Mus musculus (house mouse)	0.003855208	0.010864677	0.005217575	Mapk1/Mapk10/Rb1/Rela
28-day post-SCI group and the control group	mmu05220	Chronic myeloid leukemia - Mus musculus (house mouse)	0.003855208	0.010864677	0.005217575	Hdac1/Mapk1/Rb1/Rela
28-day post-SCI group and the control group	mmu05412	Arrhythmogenic right ventricular cardiomyopathy - Mus musculus (house mo	0.004040655	0.01124131	0.005398446	Actb/Atp2a2/Itgb5/Slc8a1
28-day post-SCI group and the control group	mmu05219	Bladder cancer - Mus musculus (house mouse)	0.004946649	0.01358763	0.006525226	Hbegf/Mapk1/Rb1
28-day post-SCI group and the control group	mmu04915	Estrogen signaling pathway - Mus musculus (house mouse)	0.005303818	0.014386606	0.006908921	Fos/Hbegf/Jun/Mapk1/Prkcd
28-day post-SCI group and the control group	mmu03083	Polycomb repressive complex - Mus musculus (house mouse)	0.005510372	0.014762354	0.007089367	Cbx6/Hdac1/Phc1/Rnf2
28-day post-SCI group and the control group	mmu05163	Human cytomegalovirus infection - Mus musculus (house mouse)	0.00583195	0.01543333	0.007411592	Map2k6/Mapk1/Ppp3ca/Rb1/Rela/Ripk1/Tnfrsf1a
28-day post-SCI group and the control group	mmu04550	Signaling pathways regulating pluripotency of stem cells - Mus musculus (hou	0.006573313	0.017185649	0.008253113	Apc/Id1/Il6st/Mapk1/Smad1
28-day post-SCI group and the control group	mmu05410	Hypertrophic cardiomyopathy - Mus musculus (house mouse)	0.00730099	0.018860892	0.00905762	Actb/Atp2a2/Itgb5/Slc8a1
28-day post-SCI group and the control group	mmu04930	Type II diabetes mellitus - Mus musculus (house mouse)	0.007693389	0.01964077	0.009432143	Mapk1/Mapk10/Prkcd
28-day post-SCI group and the control group	mmu04666	Fc gamma R-mediated phagocytosis - Mus musculus (house mouse)	0.007875378	0.019871593	0.009542992	Amph/Mapk1/Ncf1/Prkcd
28-day post-SCI group and the control group	mmu05224	Breast cancer - Mus musculus (house mouse)	0.008042753	0.020060659	0.009633787	Apc/Fos/Jun/Mapk1/Rb1
28-day post-SCI group and the control group	mmu05414	Dilated cardiomyopathy - Mus musculus (house mouse)	0.008173413	0.020154895	0.009679042	Actb/Atp2a2/Itgb5/Slc8a1
28-day post-SCI group and the control group	mmu04979	Cholesterol metabolism - Mus musculus (house mouse)	0.008613599	0.021001698	0.010085705	Apoe/Cd36/Lcat
28-day post-SCI group and the control group	mmu04015	Rap1 signaling pathway - Mus musculus (house mouse)	0.009410665	0.022690159	0.01089656	Actb/Id1/Map2k3/Map2k6/Mapk1/Met
28-day post-SCI group and the control group	mmu04024	cAMP signaling pathway - Mus musculus (house mouse)	0.011620359	0.027710086	0.013307293	Atp2a2/Fos/Jun/Mapk1/Mapk10/Rela
28-day post-SCI group and the control group	mmu04928	Parathyroid hormone synthesis, secretion and action - Mus musculus (house n	0.013145818	0.031006984	0.014890572	Fos/Hbegf/Mapk1/Mmp14
28-day post-SCI group and the control group	mmu04350	TGF-beta signaling pathway - Mus musculus (house mouse)	0.013983797	0.03262886	0.01566945	Hdac1/Id1/Mapk1/Smad1
28-day post-SCI group and the control group	mmu04022	cGMP-PKG signaling pathway - Mus musculus (house mouse)	0.015491415	0.035762096	0.017174133	Atp2a2/Mapk1/Mylk/Ppp3ca/Slc8a1
28-day post-SCI group and the control group	mmu04670	Leukocyte transendothelial migration - Mus musculus (house mouse)	0.018172502	0.041509821	0.019934379	Actb/Cybb/Ncf1/Vcam1
28-day post-SCI group and the control group	mmu04020	Calcium signaling pathway - Mus musculus (house mouse)	0.020069408	0.045365223	0.02178587	Atp2a2/Cd38/Met/Mylk/Ppp3ca/Slc8a1
28-day post-SCI group and the control group	mmu04611	Platelet activation - Mus musculus (house mouse)	0.021356553	0.04777703	0.022944099	Actb/Mapk1/Mylk/Ptgs1
28-day post-SCI group and the control group	mmu04071	Sphingolipid signaling pathway - Mus musculus (house mouse)	0.021918442	0.048527228	0.023304368	Mapk1/Mapk10/Rela/Tnfrsf1a
28-day post-SCI group and the control group	mmu04622	RIG-I-like receptor signaling pathway - Mus musculus (house mouse)	0.02213915	0.048527228	0.023304368	Mapk10/Rela/Ripk1
28-day post-SCI group and the control group	mmu04750	Inflammatory mediator regulation of TRP channels - Mus musculus (house m	0.022489344	0.048801877	0.023436264	Map2k3/Map2k6/Mapk10/Prkcd
28-day post-SCI group and the control group	mmu05218	Melanoma - Mus musculus (house mouse)	0.022963845	0.048854455	0.023461514	Mapk1/Met/Rb1
28-day post-SCI group and the control group	mmu05223	Non-small cell lung cancer - Mus musculus (house mouse)	0.022963845	0.048854455	0.023461514	Mapk1/Met/Rb1
28-day post-SCI group and the control group	mmu04918	Thyroid hormone synthesis - Mus musculus (house mouse)	0.024663024	0.051959964	0.024952881	Gpx1/Gpx3/Gpx8
28-day post-SCI group and the control group	mmu01521	EGFR tyrosine kinase inhibitor resistance - Mus musculus (house mouse)	0.029201025	0.060929062	0.029260137	Axl/Mapk1/Met
28-day post-SCI group and the control group	mmu04215	Apoptosis - multiple species - Mus musculus (house mouse)	0.029819112	0.061626164	0.029594908	Mapk10/Tnfrsf1a
28-day post-SCI group and the control group	mmu05165	Human papillomavirus infection - Mus musculus (house mouse)	0.033224973	0.068017162	0.032664075	Apc/Hdac1/Itgb5/Mapk1/Rb1/Rela/Tnfrsf1a
28-day post-SCI group and the control group	mmu05226	Gastric cancer - Mus musculus (house mouse)	0.038167656	0.077405433	0.037172631	Apc/Mapk1/Met/Rb1
28-day post-SCI group and the control group	mmu04512	ECM-receptor interaction - Mus musculus (house mouse)	0.039509974	0.079385781	0.038123659	Cd36/Itgb5/Sdc1
28-day post-SCI group and the control group	mmu05202	Transcriptional misregulation in cancer - Mus musculus (house mouse)	0.041086414	0.081795888	0.039281072	Cdkn2c/Hdac1/Met/Rela/Smad1
28-day post-SCI group and the control group	mmu05016	Huntington disease - Mus musculus (house mouse)	0.042461428	0.083382098	0.040042822	Gpx1/Gpx3/Gpx8/Hdac1/Mapk10/Ndufa6
28-day post-SCI group and the control group	mmu04520	Adherens junction - Mus musculus (house mouse)	0.04291858	0.083382098	0.040042822	Actb/Mapk1/Met
28-day post-SCI group and the control group	mmu05206	MicroRNAs in cancer - Mus musculus (house mouse)	0.043035922	0.083382098	0.040042822	Apc/Cyp1b1/Hdac1/Mapk1/Mcl1/Met
28-day post-SCI group and the control group	mmu04390	Hippo signaling pathway - Mus musculus (house mouse)	0.043926709	0.08435483	0.04050996	Actb/Apc/Id1/Smad1
28-day post-SCI group and the control group	mmu04216	Ferroptosis - Mus musculus (house mouse)	0.044908998	0.08542373	0.041023281	Cybb/Slc7a11
28-day post-SCI group and the control group	mmu04640	Hematopoietic cell lineage - Mus musculus (house mouse)	0.045270641	0.08542373	0.041023281	Cd36/Cd38/Il1a
28-day post-SCI group and the control group	mmu04810	Regulation of actin cytoskeleton - Mus musculus (house mouse)	0.045873405	0.085814905	0.041211135	Actb/Apc/Itgb5/Mapk1/Mylk
28-day post-SCI group and the control group	mmu04934	Cushing syndrome - Mus musculus (house mouse)	0.04832478	0.089628011	0.043042314	Apc/Cdkn2c/Mapk1/Rb1
28-day post-SCI group and the control group	mmu04014	Ras signaling pathway - Mus musculus (house mouse)	0.048753247	0.089656395	0.043055945	Ets2/Mapk1/Mapk10/Met/Rela

KEGG: Kyoto Encyclopedia of Genes and Genomes; SCI: spinal cord injury.
