# Supplementary material for: Integrating bulk and single-cell transcriptome profiling to uncover diagnostic biomarkers and regulatory mechanisms of oxidative stress in spinal cord injury
Source: Neural Regen Res. 2025 Jan 13;21(6):2643–57. doi: 10.4103/NRR.NRR-D-24-00693 (PMC13217428; doi:10.4103/NRR.NRR-D-24-00693)
Supplement: Supplementary file 12 [file NRR-21-2643_Suppl5.pdf]

Additional Table 6 miRNAs that regulate hub genes

**miRNA**	***Gene***
mmu-miR-101a-3p	*Fos*
mmu-miR-101a-3p	*Fos*
mmu-miR-101a-3p	*Ripk1*
mmu-miR-101b-3p	*Fos*
mmu-miR-103-3p	*Axl*
mmu-miR-106a-5p	*Mcl1*
mmu-miR-106b-5p	*Mcl1*
mmu-miR-107-3p	*Axl*
mmu-miR-10a-5p	*Sdc1*
mmu-miR-10b-5p	*Sdc1*
mmu-miR-124-3p	*Map2k4*
mmu-miR-124-3p	*Map2k4*
mmu-miR-124-3p	*Ppp3ca*
mmu-miR-125a-5p	*Mcl1*
mmu-miR-125b-5p	*Mcl1*
mmu-miR-126a-3p	*Vcam1*
mmu-miR-128-3p	*Map2k4*
mmu-miR-129-5p	*Fbxw7*
mmu-miR-132-3p	*Hbegf*
mmu-miR-132-3p	*Vcam1*
mmu-miR-133a-3p	*Mcl1*
mmu-miR-133b-3p	*Mcl1*
mmu-miR-134-5p	*Stat6*
mmu-miR-135a-5p	*Il6st*
mmu-miR-135b-5p	*Il6st*
mmu-miR-135b-5p	*Vcam1*
mmu-miR-138-5p	*Hbegf*
mmu-miR-139-5p	*Fos*
mmu-miR-139-5p	*Jun*
mmu-miR-140-5p	*Axl*
mmu-miR-141-3p	*Cbx6*
mmu-miR-141-3p	*Map2k4*
mmu-miR-141-3p	*Map2k4*
mmu-miR-141-3p	*Map2k4*
mmu-miR-144-3p	*Vcam1*
mmu-miR-145a-5p	*Ppp3ca*
mmu-miR-149-5p	*Fkbp1b*
mmu-miR-149-5p	*Tnfrsf1a*
mmu-miR-153-3p	*Amph*
mmu-miR-153-3p	*Amph*
mmu-miR-153-3p	*Amph*
mmu-miR-153-3p	*Mcl1*

mmu-miR-15a-5p	*Axl*
mmu-miR-15a-5p	*Cbx6*
mmu-miR-15b-5p	*Axl*
mmu-miR-15b-5p	*Cbx6*
mmu-miR-16-5p	*Axl*
mmu-miR-16-5p	*Cbx6*
mmu-miR-17-5p	*Mcl1*
mmu-miR-187-3p	*Ppp3ca*
mmu-miR-194-5p	*Hbegf*
mmu-miR-194-5p	*Hbegf*
mmu-miR-194-5p	*Ppp3ca*
mmu-miR-195a-5p	*Axl*
mmu-miR-195a-5p	*Cbx6*
mmu-miR-199a-5p	*Mcl1*
mmu-miR-200a-3p	*Cbx6*
mmu-miR-200a-3p	*Map2k4*
mmu-miR-200a-3p	*Map2k4*
mmu-miR-200a-3p	*Map2k4*
mmu-miR-205-5p	*Mcl1*
mmu-miR-206-3p	*Amph*
mmu-miR-20a-5p	*Mcl1*
mmu-miR-20b-5p	*Mcl1*
mmu-miR-212-3p	*Hbegf*
mmu-miR-212-3p	*Vcam1*
mmu-miR-214-3p	*Cbx6*
mmu-miR-214-3p	*Hspb1*
mmu-miR-214-3p	*Hspb1*
mmu-miR-214-3p	*Map2k4*
mmu-miR-214-3p	*Map2k4*
mmu-miR-216a-5p	*Axl*
mmu-miR-216a-5p	*Axl*
mmu-miR-216b-5p	*Jun*
mmu-miR-217-5p	*Il6st*
mmu-miR-217-5p	*Mcl1*
mmu-miR-221-3p	*Fos*
mmu-miR-222-3p	*Fos*
mmu-miR-222-3p	*Map2k4*
mmu-miR-22-3p	*Vcam1*
mmu-miR-224-5p	*Mcl1*
mmu-miR-24-3p	*Tnfrsf1a*
mmu-miR-25-3p	*Map2k4*
mmu-miR-25-3p	*Map2k4*
mmu-miR-27a-3p	*Hbegf*
mmu-miR-27a-3p	*Map2k4*
mmu-miR-27a-3p	*Map2k4*

mmu-miR-27b-3p	*Hbegf*
mmu-miR-27b-3p	*Map2k4*
mmu-miR-27b-3p	*Map2k4*
mmu-miR-291a-3p	*Sdc1*
mmu-miR-292a-5p	*Axl*
mmu-miR-299a-3p	*Map2k4*
mmu-miR-299a-3p	*Ucp2*
mmu-miR-29a-3p	*Fos*
mmu-miR-29a-3p	*Mcl1*
mmu-miR-29a-3p	*Tnfrsf1a*
mmu-miR-29b-3p	*Fos*
mmu-miR-29b-3p	*Tnfrsf1a*
mmu-miR-29c-3p	*Fos*
mmu-miR-29c-3p	*Tnfrsf1a*
mmu-miR-302a-3p	*Mcl1*
mmu-miR-302a-3p	*Sdc1*
mmu-miR-30a-5p	*Ppp3ca*
mmu-miR-30a-5p	*Ppp3ca*
mmu-miR-30d-5p	*Ppp3ca*
mmu-miR-30d-5p	*Ppp3ca*
mmu-miR-30e-5p	*Ppp3ca*
mmu-miR-30e-5p	*Ppp3ca*
mmu-miR-31-5p	*Ppp3ca*
mmu-miR-320-3p	*Mcl1*
mmu-miR-322-5p	*Axl*
mmu-miR-322-5p	*Cbx6*
mmu-miR-32-5p	*Map2k4*
mmu-miR-32-5p	*Map2k4*
mmu-miR-326-3p	*Il6st*
mmu-miR-328-3p	*Mcl1*
mmu-miR-330-5p	*Il6st*
mmu-miR-330-5p	*Sdc1*
mmu-miR-335-5p	*Map2k3*
mmu-miR-335-5p	*Map2k3*
mmu-miR-338-3p	*Axl*
mmu-miR-34a-5p	*Il6st*
mmu-miR-34b-5p	*Il6st*
mmu-miR-34c-5p	*Il6st*
mmu-miR-351-5p	*Mcl1*
mmu-miR-363-3p	*Map2k4*
mmu-miR-363-3p	*Map2k4*
mmu-miR-365-3p	*Map2k3*
mmu-miR-367-3p	*Map2k4*
mmu-miR-367-3p	*Map2k4*
mmu-miR-370-3p	*Il6st*

mmu-miR-377-3p	*Fkbp1b*
mmu-miR-378a-3p	*Mcl1*
mmu-miR-379-5p	*Hbegf*
mmu-miR-379-5p	*Map2k4*
mmu-miR-381-3p	*Mcl1*
mmu-miR-384-5p	*Ppp3ca*
mmu-miR-384-5p	*Ppp3ca*
mmu-miR-411-5p	*Mcl1*
mmu-miR-411-5p	*Mcl1*
mmu-miR-425-5p	*Amph*
mmu-miR-425-5p	*Amph*
mmu-miR-425-5p	*Cbx6*
mmu-miR-431-5p	*Map2k4*
mmu-miR-433-3p	*Ppp3ca*
mmu-miR-448-3p	*Amph*
mmu-miR-448-3p	*Amph*
mmu-miR-449a-5p	*Il6st*
mmu-miR-449b	*Il6st*
mmu-miR-449c-5p	*Il6st*
mmu-miR-485-5p	*Cbx6*
mmu-miR-485-5p	*Mcl1*
mmu-miR-485-5p	*Mcl1*
mmu-miR-486a-5p	*Mcl1*
mmu-miR-489-3p	*Amph*
mmu-miR-489-3p	*Amph*
mmu-miR-497a-5p	*Axl*
mmu-miR-497a-5p	*Cbx6*
mmu-miR-503-5p	*Amph*
mmu-miR-504-5p	*Cbx6*
mmu-miR-505-3p	*Vcam1*
mmu-miR-539-5p	*Sdc1*
mmu-miR-542-3p	*Ripk1*
mmu-miR-542-3p	*Ripk1*
mmu-miR-653-5p	*Vcam1*
mmu-miR-761	*Cbx6*
mmu-miR-761	*Hbegf*
mmu-miR-761	*Hspb1*
mmu-miR-761	*Hspb1*
mmu-miR-761	*Map2k4*
mmu-miR-873a-5p	*Map2k3*
mmu-miR-873a-5p	*Map2k3*
mmu-miR-874-3p	*Map2k4*
mmu-miR-874-3p	*Map2k4*
mmu-miR-874-3p	*Ppp3ca*
mmu-miR-876-3p	*Axl*

mmu-miR-92a-3p	*Map2k4*
mmu-miR-92a-3p	*Map2k4*
mmu-miR-92b-3p	*Map2k4*
mmu-miR-92b-3p	*Map2k4*
mmu-miR-93-5p	*Mcl1*
mmu-miR-96-5p	*Cbx6*
mmu-miR-96-5p	*Hbegf*

miRNA: microRNA
