# Supplementary material for: Integrating bulk and single-cell transcriptome profiling to uncover diagnostic biomarkers and regulatory mechanisms of oxidative stress in spinal cord injury
Source: Neural Regen Res. 2025 Jan 13;21(6):2643–57. doi: 10.4103/NRR.NRR-D-24-00693 (PMC13217428; doi:10.4103/NRR.NRR-D-24-00693)
Supplement: Supplementary file 13 [file NRR-21-2643_Suppl6.pdf]

Additional Table 7 Correspondence of mRNA, miRNA, and lncRNA in the regulation of angiogenesis

miRNA	ID
mmu-miR-101a-3p	*Fos*
mmu-miR-101a-3p	*Ripk1*
mmu-miR-124-3p	*Map2k4*
mmu-miR-124-3p	*Ppp3ca*
mmu-miR-132-3p	*Hbegf*
mmu-miR-132-3p	*Vcam1*
mmu-miR-135b-5p	*Il6st*
mmu-miR-135b-5p	*Vcam1*
mmu-miR-139-5p	*Fos*
mmu-miR-139-5p	*Jun*
mmu-miR-141-3p	*Cbx6*
mmu-miR-141-3p	*Map2k4*
mmu-miR-149-5p	*Fkbp1b*
mmu-miR-149-5p	*Tnfrsf1a*
mmu-miR-153-3p	*Amph*
mmu-miR-153-3p	*Mcl1*
mmu-miR-15a-5p	*Axl*
mmu-miR-15a-5p	*Cbx6*
mmu-miR-15b-5p	*Axl*
mmu-miR-15b-5p	*Cbx6*
mmu-miR-16-5p	*Axl*
mmu-miR-16-5p	*Cbx6*
mmu-miR-194-5p	*Hbegf*
mmu-miR-194-5p	*Ppp3ca*
mmu-miR-200a-3p	*Cbx6*
mmu-miR-200a-3p	*Map2k4*
mmu-miR-212-3p	*Hbegf*
mmu-miR-212-3p	*Vcam1*
mmu-miR-214-3p	*Cbx6*
mmu-miR-214-3p	*Hspb1*
mmu-miR-214-3p	*Map2k4*
mmu-miR-217-5p	*Il6st*
mmu-miR-217-5p	*Mcl1*
mmu-miR-222-3p	*Fos*
mmu-miR-222-3p	*Map2k4*
mmu-miR-27a-3p	*Hbegf*
mmu-miR-27a-3p	*Map2k4*
mmu-miR-27b-3p	*Hbegf*
mmu-miR-27b-3p	*Map2k4*
mmu-miR-299a-3p	*Map2k4*
mmu-miR-299a-3p	*Ucp2*
mmu-miR-29a-3p	*Fos*

mmu-miR-29a-3p	*Mcl1*
mmu-miR-29a-3p	*Tnfrsf1a*
mmu-miR-29b-3p	*Fos*
mmu-miR-29b-3p	*Tnfrsf1a*
mmu-miR-29c-3p	*Fos*
mmu-miR-29c-3p	*Tnfrsf1a*
mmu-miR-302a-3p	*Mcl1*
mmu-miR-302a-3p	*Sdc1*
mmu-miR-322-5p	*Axl*
mmu-miR-322-5p	*Cbx6*
mmu-miR-330-5p	*Il6st*
mmu-miR-330-5p	*Sdc1*
mmu-miR-379-5p	*Hbegf*
mmu-miR-379-5p	*Map2k4*
mmu-miR-425-5p	*Amph*
mmu-miR-425-5p	*Cbx6*
mmu-miR-485-5p	*Cbx6*
mmu-miR-485-5p	*Mcl1*
mmu-miR-497a-5p	*Axl*
mmu-miR-497a-5p	*Cbx6*
mmu-miR-761	*Cbx6*
mmu-miR-761	*Hbegf*
mmu-miR-761	*Hspb1*
mmu-miR-761	*Map2k4*
mmu-miR-874-3p	*Map2k4*
mmu-miR-874-3p	*Ppp3ca*
mmu-miR-96-5p	*Cbx6*
mmu-miR-96-5p	*Hbegf*
mmu-miR-15a-5p	*Gm5532*
mmu-miR-15a-5p	*Miat*
mmu-miR-15a-5p	*Gm44053*
mmu-miR-15a-5p	*Gm10619*
mmu-miR-15a-5p	*H19*
mmu-miR-15a-5p	*Meg3*
mmu-miR-15a-5p	*Snhg18*
mmu-miR-15a-5p	*Gm20732*
mmu-miR-15a-5p	*Malat1*
mmu-miR-15a-5p	*Neat1*
mmu-miR-15a-5p	*Xist*
mmu-miR-15b-5p	*Gm5532*
mmu-miR-15b-5p	*Miat*
mmu-miR-15b-5p	*Gm44053*
mmu-miR-15b-5p	*H19*
mmu-miR-15b-5p	*Meg3*
mmu-miR-15b-5p	*Snhg18*
mmu-miR-15b-5p	*Gm20732*

mmu-miR-15b-5p	*Malat1*
mmu-miR-15b-5p	*Neat1*
mmu-miR-15b-5p	*Xist*
mmu-miR-16-5p	*Gm5532*
mmu-miR-16-5p	*Miat*
mmu-miR-16-5p	*Gm44053*
mmu-miR-16-5p	*H19*
mmu-miR-16-5p	*Meg3*
mmu-miR-16-5p	*Snhg18*
mmu-miR-16-5p	*Gm20732*
mmu-miR-16-5p	*Malat1*
mmu-miR-16-5p	*Neat1*
mmu-miR-16-5p	*Xist*
mmu-miR-27a-3p	*Gm28653*
mmu-miR-27a-3p	*Mir124-2hg*
mmu-miR-27a-3p	*E130102H24Rik*
mmu-miR-27a-3p	*Meg3*
mmu-miR-27a-3p	*Neat1*
mmu-miR-27a-3p	*Xist*
mmu-miR-27b-3p	*Gm28653*
mmu-miR-27b-3p	*Mir124-2hg*
mmu-miR-27b-3p	*AI504432*
mmu-miR-27b-3p	*E130102H24Rik*
mmu-miR-27b-3p	*Meg3*
mmu-miR-27b-3p	*Neat1*
mmu-miR-27b-3p	*Xist*
mmu-miR-29a-3p	*Oip5os1*
mmu-miR-29a-3p	*Gm23925*
mmu-miR-29a-3p	*Gm20707*
mmu-miR-29a-3p	*Gm37494*
mmu-miR-29a-3p	*Mir17hg*
mmu-miR-29a-3p	*Neat1*
mmu-miR-29a-3p	*Xist*
mmu-miR-29a-3p	*Xist*
mmu-miR-29b-3p	*Oip5os1*
mmu-miR-29b-3p	*Gm20707*
mmu-miR-29b-3p	*Gm37494*
mmu-miR-29b-3p	*Mir17hg*
mmu-miR-29b-3p	*Neat1*
mmu-miR-29b-3p	*Xist*
mmu-miR-29c-3p	*Oip5os1*
mmu-miR-29c-3p	*Gm23925*
mmu-miR-29c-3p	*Gm20707*
mmu-miR-29c-3p	*Gm37494*
mmu-miR-29c-3p	*Mir17hg*
mmu-miR-29c-3p	*Neat1*

mmu-miR-29c-3p	*Xist*
mmu-miR-96-5p	*2610037D02Rik*
mmu-miR-96-5p	*Malat1*
mmu-miR-96-5p	*Xist*
mmu-miR-101a-3p	*Malat1*
mmu-miR-101a-3p	*Neat1*
mmu-miR-101a-3p	*Xist*
mmu-miR-101a-3p	*Jpx*
mmu-miR-124-3p	*Gm37584*
mmu-miR-124-3p	*AI504432*
mmu-miR-124-3p	*Gm9866*
mmu-miR-124-3p	*Meg3*
mmu-miR-124-3p	*Carmn*
mmu-miR-124-3p	*Malat1*
mmu-miR-124-3p	*Neat1*
mmu-miR-124-3p	*Neat1*
mmu-miR-124-3p	*Xist*
mmu-miR-132-3p	*lincRNA*
mmu-miR-135b-5p	*Gm28653*
mmu-miR-135b-5p	*4833445I07Rik*
mmu-miR-135b-5p	*Meg3*
mmu-miR-135b-5p	*Dubr*
mmu-miR-135b-5p	*Malat1*
mmu-miR-135b-5p	*Neat1*
mmu-miR-139-5p	*Oip5os1*
mmu-miR-139-5p	*C130071C03Rik*
mmu-miR-139-5p	*Mir124a-1hg*
mmu-miR-139-5p	*Malat1*
mmu-miR-139-5p	*Neat1*
mmu-miR-139-5p	*Jpx*
mmu-miR-141-3p	*Oip5os1*
mmu-miR-141-3p	*AI504432*
mmu-miR-141-3p	*Gm5878*
mmu-miR-141-3p	*Gm37494*
mmu-miR-141-3p	*Neat1*
mmu-miR-141-3p	*Xist*
mmu-miR-149-5p	*2900097C17Rik*
mmu-miR-149-5p	*H19*
mmu-miR-149-5p	*Malat1*
mmu-miR-149-5p	*Xist*
mmu-miR-153-3p	*Oip5os1*
mmu-miR-153-3p	*Miat*
mmu-miR-153-3p	*Mir17hg*
mmu-miR-153-3p	*BC002059*
mmu-miR-153-3p	*Malat1*
mmu-miR-194-5p	*Oip5os1*

mmu-miR-194-5p	*Gm37494*
mmu-miR-194-5p	*Malat1*
mmu-miR-200a-3p	*Oip5os1*
mmu-miR-200a-3p	*AI504432*
mmu-miR-200a-3p	*Gm5878*
mmu-miR-200a-3p	*Gm37494*
mmu-miR-200a-3p	*Neat1*
mmu-miR-200a-3p	*Xist*
mmu-miR-212-3p	*Neat1*
mmu-miR-212-3p	*Xist*
mmu-miR-214-3p	*Gm5532*
mmu-miR-214-3p	*Miat*
mmu-miR-214-3p	*Gm37494*
mmu-miR-214-3p	*Meg3*
mmu-miR-214-3p	*6720427I07Rik*
mmu-miR-214-3p	*Mir17hg*
mmu-miR-214-3p	*Neat1*
mmu-miR-214-3p	*Cdr1os*
mmu-miR-217-5p	*AI504432*
mmu-miR-217-5p	*Gm8953*
mmu-miR-217-5p	*Meg3*
mmu-miR-217-5p	*Malat1*
mmu-miR-217-5p	*Neat1*
mmu-miR-217-5p	*Xist*
mmu-miR-222-3p	*Oip5os1*
mmu-miR-222-3p	*Miat*
mmu-miR-222-3p	*Meg3*
mmu-miR-299a-3p	*Gm23925*
mmu-miR-299a-3p	*Malat1*
mmu-miR-299a-3p	*Neat1*
mmu-miR-302a-3p	*F730311O21Rik*
mmu-miR-302a-3p	*Meg3*
mmu-miR-302a-3p	*Mir17hg*
mmu-miR-302a-3p	*Malat1*
mmu-miR-302a-3p	*Neat1*
mmu-miR-302a-3p	*Xist*
mmu-miR-322-5p	*Gm5532*
mmu-miR-322-5p	*Miat*
mmu-miR-322-5p	*Gm44053*
mmu-miR-322-5p	*Gm37494*
mmu-miR-322-5p	*H19*
mmu-miR-322-5p	*Meg3*
mmu-miR-322-5p	*Snhg18*
mmu-miR-322-5p	*Gm20732*
mmu-miR-322-5p	*Malat1*
mmu-miR-322-5p	*Neat1*

mmu-miR-322-5p	*Xist*
mmu-miR-330-5p	*Oip5os1*
mmu-miR-330-5p	*Gm21269*
mmu-miR-330-5p	*Meg3*
mmu-miR-330-5p	*Mirg*
mmu-miR-330-5p	*Neat1*
mmu-miR-379-5p	*Oip5os1*
mmu-miR-379-5p	*Meg3*
mmu-miR-379-5p	*3222401L13Rik*
mmu-miR-379-5p	*Neat1*
mmu-miR-425-5p	*Xist*
mmu-miR-485-5p	*Gm26532*
mmu-miR-485-5p	*Meg3*
mmu-miR-485-5p	*Malat1*
mmu-miR-485-5p	*Neat1*
mmu-miR-497a-5p	*Gm5532*
mmu-miR-497a-5p	*Miat*
mmu-miR-497a-5p	*Gm44053*
mmu-miR-497a-5p	*Gm10619*
mmu-miR-497a-5p	*H19*
mmu-miR-497a-5p	*Meg3*
mmu-miR-497a-5p	*Snhg18*
mmu-miR-497a-5p	*Gm20732*
mmu-miR-497a-5p	*Malat1*
mmu-miR-497a-5p	*Neat1*
mmu-miR-497a-5p	*Xist*
mmu-miR-761	*Gm5532*
mmu-miR-761	*Miat*
mmu-miR-761	*Gm37494*
mmu-miR-761	*Meg3*
mmu-miR-761	*6720427I07Rik*
mmu-miR-761	*Mir17hg*
mmu-miR-761	*Neat1*
mmu-miR-761	*Cdr1os*
mmu-miR-874-3p	*Oip5os1*
mmu-miR-874-3p	*Meg3*
mmu-miR-874-3p	*1700056N10Rik*
mmu-miR-874-3p	*Neat1*

lncRNA: Long non-coding RNA; miRNA: microRNA
