# Supplementary material for: Integrating bulk and single-cell transcriptome profiling to uncover diagnostic biomarkers and regulatory mechanisms of oxidative stress in spinal cord injury
Source: Neural Regen Res. 2025 Jan 13;21(6):2643–57. doi: 10.4103/NRR.NRR-D-24-00693 (PMC13217428; doi:10.4103/NRR.NRR-D-24-00693)
Supplement: Supplementary file 14 [file NRR-21-2643_Suppl7.pdf]

Additional Table 8 Transcription factors that have either promoting or Inhibitory effects on hub genes

Target gene	Transcription factor	Status
Amph	Adnp	Inhibit
Amph	Bcl6	Promote
Amph	Bhlha9	Inhibit
Amph	Cebpb	Promote
Amph	Dmrt1	Inhibit
Amph	Elf5	Inhibit
Amph	Foxa2	Promote
Amph	Gata2	Promote
Amph	Gata4	Inhibit
Amph	Gfi1	Promote
Amph	Hnf1a	Promote
Amph	Mkx	Promote
Amph	Nr2f2	Promote
Amph	Nrf1	Inhibit
Amph	Pitx2	Promote
Amph	Prdm2	Inhibit
Amph	Prox1	Promote
Amph	Runx1	Inhibit
Amph	Smad4	Inhibit
Amph	Snai1	Inhibit
Amph	Spdef	Inhibit
Amph	Srf	Inhibit
Amph	Stat3	Inhibit
Amph	Stat4	Promote
Amph	Stat6	Inhibit
Amph	Vdr	Inhibit
Amph	Wt1	Promote
Amph	Yy1	Promote
Axl	Cbfb	Promote
Axl	Cebpa	Promote
Axl	Gata2	Promote
Axl	Gfi1	Inhibit
Axl	Hsf4	Promote
Axl	Irf8	Promote
Axl	Mbd2	Inhibit
Axl	Meis1	Promote
Axl	Neurod1	Inhibit
Axl	Nhlh2	Promote
Axl	Nrf1	Inhibit
Axl	Otx2	Promote
Axl	Pitx2	Inhibit
Axl	Pparg	Inhibit
Axl	Smad4	Inhibit

Axl	Sohlh1	Inhibit
Axl	Sohlh2	Inhibit
Axl	Sox10	Inhibit
Axl	Sox11	Inhibit
Axl	Stat3	Inhibit
Axl	Stat6	Inhibit
Axl	Tcf7	Promote
Axl	Tet2	Inhibit
Axl	Tsc22d4	Promote
Cbx6	Atoh1	Promote
Cbx6	Atoh8	Inhibit
Cbx6	Bcl6	Promote
Cbx6	Cebpe	Promote
Cbx6	E2f4	Promote
Cbx6	Gata3	Promote
Cbx6	Gata5	Promote
Cbx6	Gfi1	Promote
Cbx6	Gfi1b	Promote
Cbx6	Lhx8	Inhibit
Cbx6	Myb	Promote
Cbx6	Nhlh2	Promote
Cbx6	Nrf1	Inhibit
Cbx6	Smad4	Promote
Cbx6	Stat4	Promote
Cbx6	Tcfap2c	Inhibit
Cbx6	Wt1	Inhibit
Cbx6	Yy1	Promote
Fbxw7	Atoh1	Promote
Fbxw7	Lhx8	Promote
Fbxw7	Meis1	Promote
Fbxw7	Nhlh2	Promote
Fbxw7	Nrf1	Inhibit
Fbxw7	p63	Promote
Fbxw7	Smad1	Promote
Fbxw7	Sox17	Inhibit
Fbxw7	Stat4	Inhibit
Fbxw7	Stat6	Promote
Fbxw7	Tet2	Promote
Fbxw7	Thap1	Promote
Fbxw7	Tsc22d4	Inhibit
Fkbp1b	Adnp	Inhibit
Fkbp1b	Cbx2	Promote
Fkbp1b	Cebpb	Promote
Fkbp1b	E2f4	Promote
Fkbp1b	Elf5	Inhibit
Fkbp1b	Gata3	Inhibit

Fkbp1b	Gfi1	Inhibit
Fkbp1b	Gfi1b	Inhibit
Fkbp1b	Junb	Promote
Fkbp1b	Meis1	Inhibit
Fkbp1b	Myb	Promote
Fkbp1b	Nrf1	Inhibit
Fkbp1b	Pitx2	Promote
Fkbp1b	Plagl2	Promote
Fkbp1b	Pparg	Inhibit
Fkbp1b	Prdm2	Promote
Fkbp1b	Rest	Inhibit
Fkbp1b	Runx1	Inhibit
Fkbp1b	Smad4	Inhibit
Fkbp1b	Srf	Inhibit
Fkbp1b	Thap1	Promote
Fkbp1b	Wt1	Inhibit
Fkbp1b	Yy1	Promote
Fos	Adnp	Inhibit
Fos	Atoh8	Promote
Fos	Batf2	Promote
Fos	Cebpe	Inhibit
Fos	E2f4	Inhibit
Fos	Erg	Promote
Fos	Foxp1	Inhibit
Fos	Gata1	Inhibit
Fos	Gata3	Inhibit
Fos	Gfi1b	Promote
Fos	Hoxc13	Promote
Fos	Hsf4	Promote
Fos	Irf6	Inhibit
Fos	Jarid2	Promote
Fos	Junb	Promote
Fos	Klf17	Promote
Fos	Klf6	Inhibit
Fos	Lmx1b	Inhibit
Fos	Mbd2	Promote
Fos	Meis1	Inhibit
Fos	Nhlh2	Promote
Fos	Nr2f2	Inhibit
Fos	Nrf1	Inhibit
Fos	Otx2	Inhibit
Fos	Pax5	Promote
Fos	Pitx2	Inhibit
Fos	Plagl2	Inhibit
Fos	Pparg	Inhibit
Fos	Prdm2	Inhibit

Fos	Sall4	Promote
Fos	Smad4	Inhibit
Fos	Sohlh1	Inhibit
Fos	Sohlh2	Inhibit
Fos	Sox17	Inhibit
Fos	Sox2	Inhibit
Fos	Sox21	Inhibit
Fos	Sp3	Inhibit
Fos	Srf	Promote
Fos	Stat3	Promote
Fos	Stat4	Promote
Fos	Tcfap2c	Inhibit
Fos	Thap1	Promote
Fos	Thap11	Promote
Fos	Tsc22d4	Promote
Fos	Vdr	Inhibit
Fos	Yy1	Promote
Gch1	Atoh1	Inhibit
Gch1	Atoh8	Promote
Gch1	Bcl6	Inhibit
Gch1	Cbx2	Promote
Gch1	Cebpa	Promote
Gch1	Cebpb	Inhibit
Gch1	Foxa2	Inhibit
Gch1	Gata3	Inhibit
Gch1	Gfi1	Inhibit
Gch1	Hoxc13	Inhibit
Gch1	Il4r	Promote
Gch1	Irf8	Promote
Gch1	Klf17	Promote
Gch1	Lhx8	Promote
Gch1	Lmx1b	Inhibit
Gch1	Mbd2	Inhibit
Gch1	Meis1	Inhibit
Gch1	Myb	Inhibit
Gch1	Neurod1	Inhibit
Gch1	Npas4	Promote
Gch1	p63	Inhibit
Gch1	Pax5	Promote
Gch1	Pitx2	Inhibit
Gch1	Plagl2	Promote
Gch1	Runx1	Inhibit
Gch1	Runx2	Inhibit
Gch1	Sall4	Promote
Gch1	Sohlh2	Promote
Gch1	Stat3	Inhibit

Gch1	Stat4	Inhibit
Gch1	Tcfap2c	Promote
Gch1	Tet2	Inhibit
Gch1	Yy1	Promote
Hbegf	Atoh1	Promote
Hbegf	Bcl6	Inhibit
Hbegf	Bhlha9	Promote
Hbegf	Cebpb	Inhibit
Hbegf	Cebpe	Inhibit
Hbegf	Gata3	Inhibit
Hbegf	Gfi1	Inhibit
Hbegf	Hsf4	Promote
Hbegf	Irf6	Inhibit
Hbegf	Junb	Inhibit
Hbegf	Klf6	Inhibit
Hbegf	Lhx8	Inhibit
Hbegf	Mbd2	Inhibit
Hbegf	Mef2a	Promote
Hbegf	Mef2b	Inhibit
Hbegf	Mef2c	Inhibit
Hbegf	Myb	Inhibit
Hbegf	Neurod1	Inhibit
Hbegf	Nhlh2	Promote
Hbegf	Pax6	Promote
Hbegf	Pitx2	Promote
Hbegf	Prox1	Inhibit
Hbegf	Runx1	Inhibit
Hbegf	Runx2	Promote
Hbegf	Smad4	Promote
Hbegf	Snai1	Inhibit
Hbegf	Sox11	Inhibit
Hbegf	Sox17	Inhibit
Hbegf	Spdef	Promote
Hbegf	Stat6	Promote
Hbegf	Tsc22d4	Promote
Hbegf	Vdr	Promote
Hdac1	Cebpb	Promote
Hdac1	Cebpe	Promote
Hdac1	Junb	Promote
Hdac1	Nfil3	Inhibit
Hdac1	Nhlh2	Promote
Hdac1	Nrf1	Inhibit
Hdac1	Rest	Inhibit
Hdac1	Smad4	Inhibit
Hdac1	Spdef	Inhibit
Hdac1	Yy1	Promote

Hspb1	Ahr	Inhibit
Hspb1	Bcl6	Promote
Hspb1	Cbx2	Promote
Hspb1	Cebpb	Inhibit
Hspb1	Cebpe	Inhibit
Hspb1	Dmrt1	Inhibit
Hspb1	Elf5	Inhibit
Hspb1	Ets2	Inhibit
Hspb1	Hnf1a	Promote
Hspb1	Hsf4	Promote
Hspb1	Lhx8	Inhibit
Hspb1	Lmx1b	Inhibit
Hspb1	Meis1	Inhibit
Hspb1	Myb	Inhibit
Hspb1	Nelfb	Inhibit
Hspb1	Nr2f2	Inhibit
Hspb1	Pbx1	Inhibit
Hspb1	Pitx2	Promote
Hspb1	Pparg	Inhibit
Hspb1	Prox1	Inhibit
Hspb1	Runx1	Promote
Hspb1	Runx2	Promote
Hspb1	Smad1	Promote
Hspb1	Snai1	Inhibit
Hspb1	Sox17	Inhibit
Hspb1	Sp3	Promote
Hspb1	Tcfap2c	Inhibit
Hspb1	Tet2	Inhibit
Hspb1	Wt1	Promote
Hspb1	Yy1	Inhibit
Id1	Arid1a	Inhibit
Id1	Atoh1	Inhibit
Id1	Bcl6	Promote
Id1	Cebpe	Inhibit
Id1	Dmrt1	Inhibit
Id1	En2	Inhibit
Id1	Gata2	Inhibit
Id1	Gata3	Inhibit
Id1	Gata5	Promote
Id1	Gfi1	Inhibit
Id1	Ire1	Inhibit
Id1	Irf8	Promote
Id1	Klf6	Promote
Id1	Meis1	Promote
Id1	Myb	Inhibit
Id1	Nfix	Promote

Id1	Nr2f2	Inhibit
Id1	Nrf1	Inhibit
Id1	Pitx2	Inhibit
Id1	Runx1	Inhibit
Id1	Sall4	Inhibit
Id1	Snai1	Inhibit
Id1	Sohlh1	Inhibit
Id1	Sohlh2	Inhibit
Id1	Sox17	Inhibit
Id1	Stat6	Inhibit
Id1	Tcfap2c	Inhibit
Id1	Thap11	Inhibit
Id1	Tsc22d4	Promote
Id1	Vdr	Promote
Id1	Xbp1	Inhibit
Id1	Yy1	Inhibit
Il6st	E2f4	Promote
Il6st	Elf5	Inhibit
Il6st	Gfi1	Promote
Il6st	Irf6	Inhibit
Il6st	Klf17	Promote
Il6st	Lhx8	Inhibit
Il6st	Lmx1b	Inhibit
Il6st	Nelfb	Promote
Il6st	Neurod1	Inhibit
Il6st	Nhlh2	Promote
Il6st	Nrf1	Inhibit
Il6st	Pax5	Inhibit
Il6st	Pitx2	Promote
Il6st	Pparg	Inhibit
Il6st	Runx1	Inhibit
Il6st	Smad4	Inhibit
Il6st	Stat4	Promote
Il6st	Stat6	Promote
Il6st	Tcf7	Promote
Jun	Ahr	Inhibit
Jun	Atoh1	Promote
Jun	Bcl11a	Promote
Jun	Cbx2	Inhibit
Jun	Cebpb	Promote
Jun	Erg	Promote
Jun	Ets2	Inhibit
Jun	Gfi1	Inhibit
Jun	Irf8	Inhibit
Jun	Lhx8	Inhibit
Jun	Lmx1b	Inhibit

Jun	Mafg	Promote
Jun	Mbd2	Inhibit
Jun	Myb	Inhibit
Jun	Nfix	Inhibit
Jun	Nhlh2	Promote
Jun	Nrf1	Inhibit
Jun	Otx2	Promote
Jun	Pbx1	Promote
Jun	Pitx2	Inhibit
Jun	Prox1	Inhibit
Jun	Sall1	Inhibit
Jun	Smad1	Promote
Jun	Smad4	Promote
Jun	Sohlh1	Inhibit
Jun	Sohlh2	Inhibit
Jun	Sox2	Inhibit
Jun	Stat4	Inhibit
Jun	Stat6	Promote
Jun	Tsc22d4	Promote
Map2k3	Atoh1	Inhibit
Map2k3	Bcl6	Inhibit
Map2k3	Cebpb	Promote
Map2k3	Gata3	Inhibit
Map2k3	Gfi1	Inhibit
Map2k3	Irf8	Promote
Map2k3	Nhlh2	Promote
Map2k3	Pitx2	Promote
Map2k3	Runx1	Promote
Map2k3	Smad4	Promote
Map2k3	Tet2	Inhibit
Map2k4	Atoh1	Inhibit
Map2k4	Cebpb	Promote
Map2k4	Cebpe	Promote
Map2k4	Lhx8	Promote
Map2k4	Nhlh2	Promote
Map2k4	Nrf1	Inhibit
Map2k4	Otx2	Promote
Map2k4	Pitx2	Promote
Map2k4	Stat4	Promote
Map2k4	Thap1	Promote
Mcl1	Atoh1	Inhibit
Mcl1	Irf8	Promote
Mcl1	Lhx8	Promote
Mcl1	Myb	Inhibit
Mcl1	Nfil3	Inhibit
Mcl1	Pax5	Promote

Mcl1	Pitx2	Inhibit
Mcl1	Prdm1	Inhibit
Mcl1	Runx2	Inhibit
Mcl1	Smad4	Promote
Mcl1	Sox10	Promote
Mcl1	Tcfap2c	Inhibit
Mcl1	Tet2	Inhibit
Ppp3ca	Dmrt1	Inhibit
Ppp3ca	Gata3	Promote
Ppp3ca	Hsf4	Promote
Ppp3ca	Lhx8	Promote
Ppp3ca	Mbd2	Inhibit
Ppp3ca	Myb	Inhibit
Ppp3ca	Nhlh2	Promote
Ppp3ca	Otx2	Promote
Ppp3ca	Pax5	Promote
Ppp3ca	Pitx2	Promote
Ppp3ca	Prdm1	Inhibit
Ppp3ca	Runx1	Inhibit
Ppp3ca	Smad4	Inhibit
Ppp3ca	Snai1	Promote
Ppp3ca	Sox10	Promote
Ppp3ca	Stat4	Promote
Ppp3ca	Tet2	Promote
Ppp3ca	Thap1	Promote
Ppp3ca	Wt1	Promote
Rcan1	Ahr	Inhibit
Rcan1	Arid1a	Promote
Rcan1	Atoh1	Inhibit
Rcan1	Gata3	Inhibit
Rcan1	Ire1	Promote
Rcan1	Mbd2	Promote
Rcan1	Nhlh2	Promote
Rcan1	Plagl2	Promote
Rcan1	Smad1	Promote
Rcan1	Smad4	Promote
Rcan1	Sox2	Inhibit
Rcan1	Srf	Promote
Rcan1	Tcfap2c	Promote
Rcan1	Thap11	Inhibit
Rcan1	Tsc22d4	Promote
Rcan1	Xbp1	Inhibit
Ripk1	Atoh1	Inhibit
Ripk1	E2f4	Promote
Ripk1	Junb	Promote
Ripk1	Lhx8	Promote

Ripk1	Nhlh2	Promote
Ripk1	Pax5	Inhibit
Ripk1	Pax6	Inhibit
Ripk1	Pitx2	Inhibit
Ripk1	Prdm1	Inhibit
Ripk1	Smad4	Promote
Ripk1	Sp3	Promote
Sdc1	Ahr	Inhibit
Sdc1	Atoh1	Promote
Sdc1	Bcl11a	Promote
Sdc1	Cebpe	Promote
Sdc1	Elf5	Inhibit
Sdc1	Erg	Inhibit
Sdc1	Foxq1	Inhibit
Sdc1	Gata4	Inhibit
Sdc1	Gfi1	Inhibit
Sdc1	Id2	Inhibit
Sdc1	Irf6	Inhibit
Sdc1	Lhx8	Promote
Sdc1	Mbd2	Promote
Sdc1	Meis1	Promote
Sdc1	Nfil3	Inhibit
Sdc1	Nfix	Inhibit
Sdc1	Nhlh2	Promote
Sdc1	Pax5	Promote
Sdc1	Pparg	Inhibit
Sdc1	Smad4	Inhibit
Sdc1	Stat6	Promote
Sdc1	Tcfap2c	Promote
Sdc1	Tet2	Inhibit
Sdc1	Vdr	Promote
Sdc1	Wt1	Inhibit
Sdc1	Xbp1	Inhibit
Sdc1	Yy1	Promote
Stat6	Foxp1	Promote
Stat6	Hsf4	Promote
Stat6	Irf6	Inhibit
Stat6	Lhx8	Promote
Stat6	Neurod1	Inhibit
Stat6	Nfil3	Inhibit
Stat6	Nhlh2	Promote
Stat6	Nrf1	Inhibit
Stat6	Pitx2	Inhibit
Stat6	Rest	Inhibit
Stat6	Smad4	Promote
Stat6	Sox11	Inhibit

Stat6	Stat6	Promote
Stat6	Yy1	Inhibit
Tnfrsf1a	Atoh1	Inhibit
Tnfrsf1a	Bhlha9	Promote
Tnfrsf1a	Gfi1	Inhibit
Tnfrsf1a	Junb	Promote
Tnfrsf1a	Lhx8	Inhibit
Tnfrsf1a	Neurod1	Inhibit
Tnfrsf1a	Nhlh2	Promote
Tnfrsf1a	Nr2f2	Inhibit
Tnfrsf1a	Nrf1	Inhibit
Tnfrsf1a	Otx2	Promote
Tnfrsf1a	Pax5	Inhibit
Tnfrsf1a	Pitx2	Inhibit
Tnfrsf1a	Runx1	Inhibit
Tnfrsf1a	Sox2	Inhibit
Tnfrsf1a	Spdef	Promote
Tnfrsf1a	Stat4	Inhibit
Ucp2	Batf2	Promote
Ucp2	Cebpb	Inhibit
Ucp2	Cebpe	Inhibit
Ucp2	Junb	Promote
Ucp2	Klf17	Promote
Ucp2	Lhx8	Inhibit
Ucp2	Mafg	Promote
Ucp2	Neurod1	Inhibit
Ucp2	Nhlh2	Promote
Ucp2	Nr2f2	Inhibit
Ucp2	Nrf1	Inhibit
Ucp2	Otx2	Promote
Ucp2	Pax5	Promote
Ucp2	Pitx2	Inhibit
Ucp2	Plagl2	Inhibit
Ucp2	Runx1	Inhibit
Ucp2	Sall4	Promote
Ucp2	Snai1	Inhibit
Ucp2	Srf	Inhibit
Ucp2	Stat4	Inhibit
Ucp2	Thap11	Promote
Ucp2	Wt1	Promote
Ucp2	Yy1	Inhibit
Vcam1	Adnp	Promote
Vcam1	Batf2	Inhibit
Vcam1	Cebpa	Inhibit
Vcam1	E2f4	Inhibit
Vcam1	Elf5	Inhibit

Vcam1	Foxp1	Promote
Vcam1	Gata2	Promote
Vcam1	Gfi1	Inhibit
Vcam1	Irf8	Promote
Vcam1	Junb	Promote
Vcam1	Klf6	Inhibit
Vcam1	Mafg	Promote
Vcam1	Mef2b	Promote
Vcam1	Mef2c	Promote
Vcam1	Nelfb	Promote
Vcam1	Neurod1	Inhibit
Vcam1	Nhlh2	Promote
Vcam1	Nr2f2	Inhibit
Vcam1	Nrf1	Inhibit
Vcam1	Otx2	Promote
Vcam1	Pitx2	Inhibit
Vcam1	Pparg	Inhibit
Vcam1	Prdm2	Inhibit
Vcam1	Prox1	Promote
Vcam1	Smad4	Inhibit
Vcam1	Snai1	Promote
Vcam1	Sox11	Inhibit
Vcam1	Stat4	Inhibit
Vcam1	Stat6	Inhibit
Vcam1	Tbx1	Inhibit
Vcam1	Tet2	Inhibit
Vcam1	Thap1	Promote
Vcam1	Tsc22d4	Promote
Xdh	Ahr	Promote
Xdh	Atf3	Inhibit
Xdh	Atoh1	Inhibit
Xdh	Bcl6	Promote
Xdh	Cbfb	Inhibit
Xdh	Cebpa	Inhibit
Xdh	Cebpe	Inhibit
Xdh	Dmrt1	Inhibit
Xdh	E2f4	Inhibit
Xdh	Elf5	Promote
Xdh	Foxa2	Inhibit
Xdh	Foxp1	Promote
Xdh	Gata3	Inhibit
Xdh	Gfi1b	Promote
Xdh	Irf6	Inhibit
Xdh	Jarid2	Inhibit
Xdh	Junb	Promote
Xdh	Klf17	Inhibit

Xdh	Klf6	Inhibit
Xdh	Lhx8	Promote
Xdh	Lmx1b	Inhibit
Xdh	Mbd2	Inhibit
Xdh	Mef2b	Promote
Xdh	Mef2c	Promote
Xdh	Myb	Inhibit
Xdh	Neurod1	Inhibit
Xdh	Nfil3	Inhibit
Xdh	Npas4	Promote
Xdh	Nr2f2	Inhibit
Xdh	Nrf1	Inhibit
Xdh	Otx2	Inhibit
Xdh	p63	Promote
Xdh	Pbx1	Inhibit
Xdh	Plagl2	Inhibit
Xdh	Pparg	Inhibit
Xdh	Prdm2	Inhibit
Xdh	Prox1	Inhibit
Xdh	Runx1	Inhibit
Xdh	Smad4	Inhibit
Xdh	Sohlh1	Promote
Xdh	Sohlh2	Promote
Xdh	Sox11	Inhibit
Xdh	Sox9	Inhibit
Xdh	Sp3	Promote
Xdh	Srf	Inhibit
Xdh	Stat6	Promote
Xdh	Tbx1	Inhibit
Xdh	Tet2	Inhibit
Xdh	Tsc22d4	Promote
