# Supplementary material for: Integrating bulk and single-cell transcriptome profiling to uncover diagnostic biomarkers and regulatory mechanisms of oxidative stress in spinal cord injury
Source: Neural Regen Res. 2025 Jan 13;21(6):2643–57. doi: 10.4103/NRR.NRR-D-24-00693 (PMC13217428; doi:10.4103/NRR.NRR-D-24-00693)
Supplement: Supplementary file 15 [file NRR-21-2643_Suppl8.pdf]

Additional Table 9 The regulatory effects of highly expressed transcription factors on relevant hub genes in different cell groups

Group	Target gene	TF	Interaction type	Cell types with elevated transcription factor activity
Control group	*Amph*	Smad4_DataSet_11_127	Inhibit	Pericyte, OPC, Ependymal cell, Endothelial cell
Control group	*Axl*	Smad4_DataSet_11_127	Inhibit	Pericyte, OPC, Ependymal cell, Endothelial cell
Control group	*Fkbp1b*	Smad4_DataSet_11_127	Inhibit	Pericyte, OPC, Ependymal cell, Endothelial cell
Control group	*Fos*	Smad4_DataSet_11_127	Inhibit	Pericyte, OPC, Ependymal cell, Endothelial cell
Control group	*Gch1*	Smad4_DataSet_11_127	Promote	Pericyte, OPC, Ependymal cell, Endothelial cell
Control group	*Hbegf*	Smad4_DataSet_11_127	Promote	Pericyte, OPC, Ependymal cell, Endothelial cell
Control group	*Hdac1*	Smad4_DataSet_11_127	Inhibit	Pericyte, OPC, Ependymal cell, Endothelial cell
Control group	*Hspb1*	Smad4_DataSet_11_127	Inhibit	Pericyte, OPC, Ependymal cell, Endothelial cell
Control group	*Id1*	Smad4_DataSet_11_127	Inhibit	Pericyte, OPC, Ependymal cell, Endothelial cell
Control group	*Il6st*	Smad4_DataSet_11_127	Inhibit	Pericyte, OPC, Ependymal cell, Endothelial cell
Control group	*Jun*	Smad4_DataSet_11_127	Promote	Pericyte, OPC, Ependymal cell, Endothelial cell
Control group	*Map2k3*	Smad4_DataSet_11_127	Promote	Pericyte, OPC, Ependymal cell, Endothelial cell
Control group	*Mcl1*	Smad4_DataSet_11_127	Promote	Pericyte, OPC, Ependymal cell, Endothelial cell
Control group	*Rcan1*	Smad4_DataSet_11_127	Promote	Pericyte, OPC, Ependymal cell, Endothelial cell
Control group	*Ripk1*	Smad4_DataSet_11_127	Promote	Pericyte, OPC, Ependymal cell, Endothelial cell
Control group	*Sdc1*	Smad4_DataSet_11_127	Inhibit	Pericyte, OPC, Ependymal cell, Endothelial cell
Control group	*Stat6*	Smad4_DataSet_11_127	Promote	Pericyte, OPC, Ependymal cell, Endothelial cell
Control group	*Ucp2*	Smad4_DataSet_11_127	Promote	Pericyte, OPC, Ependymal cell, Endothelial cell
Control group	*Vcam1*	Smad4_DataSet_11_127	Inhibit	Pericyte, OPC, Ependymal cell, Endothelial cell
Control group	*Xdh*	Smad4_DataSet_11_127	Inhibit	Pericyte, OPC, Ependymal cell, Endothelial cell
Control group	*Amph*	Smad4_DataSet_11_128	Inhibit	Pericyte, OPC, Ependymal cell, Endothelial cell
Control group	*Cbx6*	Smad4_DataSet_11_128	Promote	Pericyte, OPC, Ependymal cell, Endothelial cell
Control group	*Fkbp1b*	Smad4_DataSet_11_128	Inhibit	Pericyte, OPC, Ependymal cell, Endothelial cell
Control group	*Gch1*	Smad4_DataSet_11_128	Inhibit	Pericyte, OPC, Ependymal cell, Endothelial cell
Control group	*Hbegf*	Smad4_DataSet_11_128	Promote	Pericyte, OPC, Ependymal cell, Endothelial cell
Control group	*Hspb1*	Smad4_DataSet_11_128	Promote	Pericyte, OPC, Ependymal cell, Endothelial cell
Control group	*Id1*	Smad4_DataSet_11_128	Promote	Pericyte, OPC, Ependymal cell, Endothelial cell
Control group	*Jun*	Smad4_DataSet_11_128	Promote	Pericyte, OPC, Ependymal cell, Endothelial cell
Control group	*Ppp3ca*	Smad4_DataSet_11_128	Inhibit	Pericyte, OPC, Ependymal cell, Endothelial cell
Control group	*Sdc1*	Smad4_DataSet_11_128	Inhibit	Pericyte, OPC, Ependymal cell, Endothelial cell
Control group	*Ucp2*	Smad4_DataSet_11_128	Inhibit	Pericyte, OPC, Ependymal cell, Endothelial cell
Control group	*Vcam1*	Smad4_DataSet_11_128	Inhibit	Pericyte, OPC, Ependymal cell, Endothelial cell
Control group	*Xdh*	Smad4_DataSet_11_128	Inhibit	Pericyte, OPC, Ependymal cell, Endothelial cell
Control group	*Amph*	Stat4_DataSet_11_152	Promote	Lymphocyte
Control group	*Cbx6*	Stat4_DataSet_11_152	Promote	Lymphocyte
Control group	*Fbxw7*	Stat4_DataSet_11_152	Inhibit	Lymphocyte

Control group	*Fos*	Stat4_DataSet_11_152	Promote	Lymphocyte
Control group	*Gch1*	Stat4_DataSet_11_152	Inhibit	Lymphocyte
Control group	*Il6st*	Stat4_DataSet_11_152	Promote	Lymphocyte
Control group	*Jun*	Stat4_DataSet_11_152	Inhibit	Lymphocyte
Control group	*Map2k4*	Stat4_DataSet_11_152	Promote	Lymphocyte
Control group	*Ppp3ca*	Stat4_DataSet_11_152	Promote	Lymphocyte
Control group	*Tnfrsf1a*	Stat4_DataSet_11_152	Inhibit	Lymphocyte
Control group	*Ucp2*	Stat4_DataSet_11_152	Inhibit	Lymphocyte
Control group	*Vcam1*	Stat4_DataSet_11_152	Inhibit	Lymphocyte
Control group	*Amph*	Stat6_DataSet_11_153	Inhibit	Microglia, Div-Myeloid
Control group	*Axl*	Stat6_DataSet_11_153	Inhibit	Microglia, Div-Myeloid
Control group	*Fbxw7*	Stat6_DataSet_11_153	Promote	Microglia, Div-Myeloid
Control group	*Hbegf*	Stat6_DataSet_11_153	Promote	Microglia, Div-Myeloid
Control group	*Id1*	Stat6_DataSet_11_153	Inhibit	Microglia, Div-Myeloid
Control group	*Il6st*	Stat6_DataSet_11_153	Promote	Microglia, Div-Myeloid
Control group	*Jun*	Stat6_DataSet_11_153	Promote	Microglia, Div-Myeloid
Control group	*Sdc1*	Stat6_DataSet_11_153	Promote	Microglia, Div-Myeloid
Control group	*Stat6*	Stat6_DataSet_11_153	Promote	Microglia, Div-Myeloid
Control group	*Vcam1*	Stat6_DataSet_11_153	Inhibit	Microglia, Div-Myeloid
Control group	*Xdh*	Stat6_DataSet_11_153	Promote	Microglia, Div-Myeloid
Control group	*Cbx6*	Cebpe_DataSet_11_023	Promote	Neutrophil
Control group	*Fos*	Cebpe_DataSet_11_023	Inhibit	Neutrophil
Control group	*Hbegf*	Cebpe_DataSet_11_023	Inhibit	Neutrophil
Control group	*Hdac1*	Cebpe_DataSet_11_023	Promote	Neutrophil
Control group	*Hspb1*	Cebpe_DataSet_11_023	Inhibit	Neutrophil
Control group	*Id1*	Cebpe_DataSet_11_023	Inhibit	Neutrophil
Control group	*Map2k4*	Cebpe_DataSet_11_023	Promote	Neutrophil
Control group	*Sdc1*	Cebpe_DataSet_11_023	Promote	Neutrophil
Control group	*Ucp2*	Cebpe_DataSet_11_023	Inhibit	Neutrophil
Control group	*Xdh*	Cebpe_DataSet_11_023	Inhibit	Neutrophil
Control group	*Axl*	Mbd2_DataSet_11_076	Inhibit	Endothelial cell, OPC, Pericyte, Lymphocyte
Control group	*Fos*	Mbd2_DataSet_11_076	Promote	Endothelial cell, OPC, Pericyte, Lymphocyte
Control group	*Gch1*	Mbd2_DataSet_11_076	Inhibit	Endothelial cell, OPC, Pericyte, Lymphocyte
Control group	*Hbegf*	Mbd2_DataSet_11_076	Inhibit	Endothelial cell, OPC, Pericyte, Lymphocyte
Control group	*Jun*	Mbd2_DataSet_11_076	Inhibit	Endothelial cell, OPC, Pericyte, Lymphocyte
Control group	*Ppp3ca*	Mbd2_DataSet_11_076	Inhibit	Endothelial cell, OPC, Pericyte, Lymphocyte
Control group	*Rcan1*	Mbd2_DataSet_11_076	Promote	Endothelial cell, OPC, Pericyte, Lymphocyte
Control group	*Sdc1*	Mbd2_DataSet_11_076	Promote	Endothelial cell, OPC, Pericyte, Lymphocyte
Control group	*Xdh*	Mbd2_DataSet_11_076	Inhibit	Endothelial cell, OPC, Pericyte, Lymphocyte

Control group	*Axl*	Tsc22d4_DataSet_11_169	Promote	Endothelial cell, OPC, Pericyte, Lymphocyte
Control group	*Fbxw7*	Tsc22d4_DataSet_11_169	Inhibit	Endothelial cell, OPC, Pericyte, Lymphocyte
Control group	*Fos*	Tsc22d4_DataSet_11_169	Promote	Endothelial cell, OPC, Pericyte, Lymphocyte
Control group	*Hbegf*	Tsc22d4_DataSet_11_169	Promote	Endothelial cell, OPC, Pericyte, Lymphocyte
Control group	*Id1*	Tsc22d4_DataSet_11_169	Promote	Endothelial cell, OPC, Pericyte, Lymphocyte
Control group	*Jun*	Tsc22d4_DataSet_11_169	Promote	Endothelial cell, OPC, Pericyte, Lymphocyte
Control group	*Rcan1*	Tsc22d4_DataSet_11_169	Promote	Endothelial cell, OPC, Pericyte, Lymphocyte
Control group	*Vcam1*	Tsc22d4_DataSet_11_169	Promote	Endothelial cell, OPC, Pericyte, Lymphocyte
Control group	*Xdh*	Tsc22d4_DataSet_11_169	Promote	Endothelial cell, OPC, Pericyte, Lymphocyte
Control group	*Amph*	Cebpb_DataSet_11_020	Promote	Microglia, Monocyte, Microphage
Control group	*Fkbp1b*	Cebpb_DataSet_11_020	Promote	Microglia, Monocyte, Microphage
Control group	*Id1*	Cebpb_DataSet_11_020	Inhibit	Microglia, Monocyte, Microphage
Control group	*Vcam1*	Cebpb_DataSet_11_020	Promote	Microglia, Monocyte, Microphage
Control group	*Xdh*	Cebpb_DataSet_11_020	Promote	Microglia, Monocyte, Microphage
Control group	*Fos*	Cebpb_DataSet_11_021	Inhibit	Microglia, Monocyte, Microphage
Control group	*Hbegf*	Cebpb_DataSet_11_021	Inhibit	Microglia, Monocyte, Microphage
Control group	*Hdac1*	Cebpb_DataSet_11_021	Promote	Microglia, Monocyte, Microphage
Control group	*Hspb1*	Cebpb_DataSet_11_021	Inhibit	Microglia, Monocyte, Microphage
Control group	*Id1*	Cebpb_DataSet_11_021	Inhibit	Microglia, Monocyte, Microphage
Control group	*Map2k4*	Cebpb_DataSet_11_021	Promote	Microglia, Monocyte, Microphage
Control group	*Ucp2*	Cebpb_DataSet_11_021	Inhibit	Microglia, Monocyte, Microphage
Control group	*Vcam1*	Cebpb_DataSet_11_021	Inhibit	Microglia, Monocyte, Microphage
Control group	*Xdh*	Cebpb_DataSet_11_021	Inhibit	Microglia, Monocyte, Microphage
Control group	*Fos*	Cebpb_DataSet_11_022	Promote	Microglia, Monocyte, Microphage
Control group	*Gch1*	Cebpb_DataSet_11_022	Inhibit	Microglia, Monocyte, Microphage
Control group	*Id1*	Cebpb_DataSet_11_022	Promote	Microglia, Monocyte, Microphage
Control group	*Jun*	Cebpb_DataSet_11_022	Promote	Microglia, Monocyte, Microphage
Control group	*Map2k3*	Cebpb_DataSet_11_022	Promote	Microglia, Monocyte, Microphage
Control group	*Fos*	Pax5_DataSet_11_100	Promote	DC
Control group	*Gch1*	Pax5_DataSet_11_100	Promote	DC
Control group	*Il6st*	Pax5_DataSet_11_100	Inhibit	DC
Control group	*Mcl1*	Pax5_DataSet_11_100	Promote	DC
Control group	*Ppp3ca*	Pax5_DataSet_11_100	Promote	DC
Control group	*Ripk1*	Pax5_DataSet_11_100	Inhibit	DC
Control group	*Sdc1*	Pax5_DataSet_11_100	Promote	DC
Control group	*Tnfrsf1a*	Pax5_DataSet_11_100	Inhibit	DC
Control group	*Ucp2*	Pax5_DataSet_11_100	Promote	DC
Control group	*Amph*	Runx1_DataSet_11_114	Inhibit	Microglia, Monocyte, Microphage
Control group	*Fos*	Runx1_DataSet_11_114	Promote	Microglia, Monocyte, Microphage

Control group	*Hspb1*	Runx1_DataSet_11_114	Promote	Microglia, Monocyte, Microphage
Control group	*Map2k3*	Runx1_DataSet_11_114	Promote	Microglia, Monocyte, Microphage
Control group	*Sdc1*	Runx1_DataSet_11_114	Promote	Microglia, Monocyte, Microphage
Control group	*Fkbp1b*	Runx1_DataSet_11_115	Inhibit	Microglia, Monocyte, Microphage
Control group	*Fos*	Runx1_DataSet_11_115	Inhibit	Microglia, Monocyte, Microphage
Control group	*Gch1*	Runx1_DataSet_11_115	Inhibit	Microglia, Monocyte, Microphage
Control group	*Jun*	Runx1_DataSet_11_115	Inhibit	Microglia, Monocyte, Microphage
Control group	*Sdc1*	Runx1_DataSet_11_115	Inhibit	Microglia, Monocyte, Microphage
Control group	*Ucp2*	Runx1_DataSet_11_115	Inhibit	Microglia, Monocyte, Microphage
Control group	*Fos*	Runx1_DataSet_11_116	Promote	Microglia, Monocyte, Microphage
Control group	*Gch1*	Runx1_DataSet_11_116	Inhibit	Microglia, Monocyte, Microphage
Control group	*Hbegf*	Runx1_DataSet_11_116	Inhibit	Microglia, Monocyte, Microphage
Control group	*Il6st*	Runx1_DataSet_11_116	Inhibit	Microglia, Monocyte, Microphage
Control group	*Jun*	Runx1_DataSet_11_116	Promote	Microglia, Monocyte, Microphage
Control group	*Ppp3ca*	Runx1_DataSet_11_116	Inhibit	Microglia, Monocyte, Microphage
Control group	*Sdc1*	Runx1_DataSet_11_116	Promote	Microglia, Monocyte, Microphage
Control group	*Tnfrsf1a*	Runx1_DataSet_11_116	Inhibit	Microglia, Monocyte, Microphage
Control group	*Xdh*	Runx1_DataSet_11_116	Inhibit	Microglia, Monocyte, Microphage
Control group	*Amph*	Runx1_DataSet_11_117	Inhibit	Microglia, Monocyte, Microphage
Control group	*Id1*	Runx1_DataSet_11_117	Inhibit	Microglia, Monocyte, Microphage
Control group	*Amph*	Bcl6_DataSet_11_014	Promote	Microglia, Microphage
Control group	*Cbx6*	Bcl6_DataSet_11_014	Promote	Microglia, Microphage
Control group	*Fos*	Bcl6_DataSet_11_014	Inhibit	Microglia, Microphage
Control group	*Gch1*	Bcl6_DataSet_11_014	Inhibit	Microglia, Microphage
Control group	*Hbegf*	Bcl6_DataSet_11_014	Inhibit	Microglia, Microphage
Control group	*Il6st*	Bcl6_DataSet_11_014	Inhibit	Microglia, Microphage
Control group	*Map2k3*	Bcl6_DataSet_11_014	Inhibit	Microglia, Microphage
Control group	*Xdh*	Bcl6_DataSet_11_014	Promote	Microglia, Microphage
Control group	*Amph*	Bcl6_DataSet_11_015	Promote	Microglia, Microphage
Control group	*Fos*	Bcl6_DataSet_11_015	Promote	Microglia, Microphage
Control group	*Hspb1*	Bcl6_DataSet_11_015	Promote	Microglia, Microphage
Control group	*Id1*	Bcl6_DataSet_11_015	Promote	Microglia, Microphage
Control group	*Il6st*	Bcl6_DataSet_11_015	Promote	Microglia, Microphage
Control group	*Amph*	Nr2f2_DataSet_11_094	Promote	Fibroblast, Pericyte
Control group	*Hspb1*	Nr2f2_DataSet_11_094	Inhibit	Fibroblast, Pericyte
Control group	*Vcam1*	Nr2f2_DataSet_11_094	Inhibit	Fibroblast, Pericyte
Control group	*Amph*	Nr2f2_DataSet_11_095	Promote	Fibroblast, Pericyte
Control group	*Fos*	Nr2f2_DataSet_11_095	Inhibit	Fibroblast, Pericyte
Control group	*Hspb1*	Nr2f2_DataSet_11_095	Inhibit	Fibroblast, Pericyte

Control group	*Id1*	Nr2f2_DataSet_11_095	Inhibit	Fibroblast, Pericyte
Control group	*Tnfrsf1a*	Nr2f2_DataSet_11_095	Inhibit	Fibroblast, Pericyte
Control group	*Ucp2*	Nr2f2_DataSet_11_095	Inhibit	Fibroblast, Pericyte
Control group	*Vcam1*	Nr2f2_DataSet_11_095	Inhibit	Fibroblast, Pericyte
Control group	*Xdh*	Nr2f2_DataSet_11_095	Inhibit	Fibroblast, Pericyte
Control group	*Axl*	Irf8_DataSet_11_063	Promote	Microglia, Microphage, DC
Control group	*Gch1*	Irf8_DataSet_11_063	Promote	Microglia, Microphage, DC
Control group	*Id1*	Irf8_DataSet_11_063	Promote	Microglia, Microphage, DC
Control group	*Jun*	Irf8_DataSet_11_063	Inhibit	Microglia, Microphage, DC
Control group	*Map2k3*	Irf8_DataSet_11_063	Promote	Microglia, Microphage, DC
Control group	*Mcl1*	Irf8_DataSet_11_063	Promote	Microglia, Microphage, DC
Control group	*Vcam1*	Irf8_DataSet_11_063	Promote	Microglia, Microphage, DC
Control group	*Axl*	Meis1_DataSet_11_081	Promote	Endothelial cell, Astrocyte
Control group	*Fbxw7*	Meis1_DataSet_11_081	Promote	Endothelial cell, Astrocyte
Control group	*Fkbp1b*	Meis1_DataSet_11_081	Inhibit	Endothelial cell, Astrocyte
Control group	*Hspb1*	Meis1_DataSet_11_081	Inhibit	Endothelial cell, Astrocyte
Control group	*Il6st*	Meis1_DataSet_11_081	Promote	Endothelial cell, Astrocyte
Control group	*Sdc1*	Meis1_DataSet_11_081	Promote	Endothelial cell, Astrocyte
Control group	*Vcam1*	Meis1_DataSet_11_081	Promote	Endothelial cell, Astrocyte
Control group	*Fos*	Meis1_DataSet_11_082	Inhibit	Endothelial cell, Astrocyte
Control group	*Gch1*	Meis1_DataSet_11_082	Inhibit	Endothelial cell, Astrocyte
Control group	*Id1*	Meis1_DataSet_11_082	Promote	Endothelial cell, Astrocyte
Control group	*Il6st*	Meis1_DataSet_11_082	Inhibit	Endothelial cell, Astrocyte
Control group	*Sdc1*	Meis1_DataSet_11_082	Promote	Endothelial cell, Astrocyte
Control group	*Vcam1*	Meis1_DataSet_11_082	Inhibit	Endothelial cell, Astrocyte
Control group	*Amph*	Prox1_DataSet_11_109	Promote	Oligodendrocyte
Control group	*Hbegf*	Prox1_DataSet_11_109	Inhibit	Oligodendrocyte
Control group	*Hspb1*	Prox1_DataSet_11_109	Inhibit	Oligodendrocyte
Control group	*Jun*	Prox1_DataSet_11_109	Inhibit	Oligodendrocyte
Control group	*Vcam1*	Prox1_DataSet_11_109	Promote	Oligodendrocyte
Control group	*Xdh*	Prox1_DataSet_11_109	Inhibit	Oligodendrocyte
Control group	*Fos*	Tet2_DataSet_11_159	Promote	Astrocyte
Control group	*Gch1*	Tet2_DataSet_11_159	Inhibit	Astrocyte
Control group	*Vcam1*	Tet2_DataSet_11_159	Inhibit	Astrocyte
Control group	*Xdh*	Tet2_DataSet_11_159	Inhibit	Astrocyte
Control group	*Fos*	Tet2_DataSet_11_161	Inhibit	Astrocyte
Control group	*Gch1*	Tet2_DataSet_11_161	Inhibit	Astrocyte
Control group	*Hspb1*	Tet2_DataSet_11_161	Inhibit	Astrocyte
Control group	*Map2k3*	Tet2_DataSet_11_161	Inhibit	Astrocyte

Control group	*Fos*	Tet2_DataSet_11_162	Inhibit	Astrocyte
Control group	*Axl*	Tet2_DataSet_11_163	Inhibit	Astrocyte
Control group	*Fbxw7*	Tet2_DataSet_11_163	Promote	Astrocyte
Control group	*Fos*	Tet2_DataSet_11_163	Inhibit	Astrocyte
Control group	*Mcl1*	Tet2_DataSet_11_163	Inhibit	Astrocyte
Control group	*Ppp3ca*	Tet2_DataSet_11_163	Promote	Astrocyte
Control group	*Sdc1*	Tet2_DataSet_11_163	Inhibit	Astrocyte
Control group	*Cbx6*	E2f4_DataSet_11_033	Promote	Div-Myeloid, Ependymal cell
Control group	*Fkbp1b*	E2f4_DataSet_11_033	Promote	Div-Myeloid, Ependymal cell
Control group	*Fos*	E2f4_DataSet_11_033	Inhibit	Div-Myeloid, Ependymal cell
Control group	*Ripk1*	E2f4_DataSet_11_033	Promote	Div-Myeloid, Ependymal cell
Control group	*Vcam1*	E2f4_DataSet_11_033	Inhibit	Div-Myeloid, Ependymal cell
Control group	*Xdh*	E2f4_DataSet_11_033	Inhibit	Div-Myeloid, Ependymal cell
Control group	*Il6st*	E2f4_DataSet_11_034	Promote	Div-Myeloid, Ependymal cell
Control group	*Fos*	Lmx1b_DataSet_11_072	Inhibit	Neuron
Control group	*Gch1*	Lmx1b_DataSet_11_072	Inhibit	Neuron
Control group	*Hspb1*	Lmx1b_DataSet_11_072	Inhibit	Neuron
Control group	*Il6st*	Lmx1b_DataSet_11_072	Inhibit	Neuron
Control group	*Jun*	Lmx1b_DataSet_11_072	Inhibit	Neuron
Control group	*Xdh*	Lmx1b_DataSet_11_072	Inhibit	Neuron
Control group	*Amph*	Prdm2_DataSet_11_108	Inhibit	Neuron
Control group	*Fkbp1b*	Prdm2_DataSet_11_108	Promote	Neuron
Control group	*Fos*	Prdm2_DataSet_11_108	Inhibit	Neuron
Control group	*Vcam1*	Prdm2_DataSet_11_108	Inhibit	Neuron
Control group	*Xdh*	Prdm2_DataSet_11_108	Inhibit	Neuron
Control group	*Amph*	Snai1_DataSet_11_129	Inhibit	Pericyte
Control group	*Hbegf*	Snai1_DataSet_11_129	Inhibit	Pericyte
Control group	*Vcam1*	Snai1_DataSet_11_129	Promote	Pericyte
Control group	*Amph*	Snai1_DataSet_11_130	Inhibit	Pericyte
Control group	*Fos*	Snai1_DataSet_11_130	Inhibit	Pericyte
Control group	*Hbegf*	Snai1_DataSet_11_130	Inhibit	Pericyte
Control group	*Hspb1*	Snai1_DataSet_11_130	Inhibit	Pericyte
Control group	*Ucp2*	Snai1_DataSet_11_130	Inhibit	Pericyte
Control group	*Id1*	Snai1_DataSet_11_131	Inhibit	Pericyte
Control group	*Ppp3ca*	Snai1_DataSet_11_131	Promote	Pericyte
Control group	*Ucp2*	Snai1_DataSet_11_131	Inhibit	Pericyte
Control group	*Fos*	Snai1_DataSet_11_132	Promote	Pericyte
Control group	*Hspb1*	Snai1_DataSet_11_132	Inhibit	Pericyte
Control group	*Fbxw7*	Sox17_DataSet_11_139	Inhibit	Endothelial cell, Pericyte

Control group	*Fos*	Sox17_DataSet_11_139	Inhibit	Endothelial cell, Pericyte
Control group	*Hbegf*	Sox17_DataSet_11_139	Inhibit	Endothelial cell, Pericyte
Control group	*Hspb1*	Sox17_DataSet_11_139	Inhibit	Endothelial cell, Pericyte
Control group	*Id1*	Sox17_DataSet_11_139	Inhibit	Endothelial cell, Pericyte
Control group	*Fos*	Ahr_DataSet_11_002	Inhibit	DC
Control group	*Jun*	Ahr_DataSet_11_002	Inhibit	DC
Control group	*Rcan1*	Ahr_DataSet_11_002	Inhibit	DC
Control group	*Sdc1*	Ahr_DataSet_11_002	Inhibit	DC
Control group	*Xdh*	Ahr_DataSet_11_002	Promote	DC
Control group	*Fos*	Ahr_DataSet_11_003	Promote	DC
Control group	*Hspb1*	Ahr_DataSet_11_003	Inhibit	DC
Control group	*Fos*	Foxp1_DataSet_11_044	Inhibit	Endothelial cell
Control group	*Gch1*	Foxp1_DataSet_11_044	Promote	Endothelial cell
Control group	*Stat6*	Foxp1_DataSet_11_044	Promote	Endothelial cell
Control group	*Vcam1*	Foxp1_DataSet_11_044	Promote	Endothelial cell
Control group	*Xdh*	Foxp1_DataSet_11_044	Promote	Endothelial cell
Control group	*Hdac1*	Nfil3_DataSet_11_090	Inhibit	Ependymal cell, Monocyte
Control group	*Mcl1*	Nfil3_DataSet_11_090	Inhibit	Ependymal cell, Monocyte
Control group	*Sdc1*	Nfil3_DataSet_11_090	Inhibit	Ependymal cell, Monocyte
Control group	*Stat6*	Nfil3_DataSet_11_090	Inhibit	Ependymal cell, Monocyte
Control group	*Xdh*	Nfil3_DataSet_11_090	Inhibit	Ependymal cell, Monocyte
Control group	*Amph*	Adnp_DataSet_11_001	Inhibit	OPC,Neuron
Control group	*Fkbp1b*	Adnp_DataSet_11_001	Inhibit	OPC,Neuron
Control group	*Fos*	Adnp_DataSet_11_001	Inhibit	OPC,Neuron
Control group	*Vcam1*	Adnp_DataSet_11_001	Promote	OPC,Neuron
Control group	*Amph*	Gata2_DataSet_11_047	Promote	Endothelial cell, Pericyte
Control group	*Axl*	Gata2_DataSet_11_047	Promote	Endothelial cell, Pericyte
Control group	*Id1*	Gata2_DataSet_11_047	Inhibit	Endothelial cell, Pericyte
Control group	*Vcam1*	Gata2_DataSet_11_047	Promote	Endothelial cell, Pericyte
Control group	*Amph*	Stat3_DataSet_11_151	Inhibit	Microglia
Control group	*Axl*	Stat3_DataSet_11_151	Inhibit	Microglia
Control group	*Fos*	Stat3_DataSet_11_151	Promote	Microglia
Control group	*Gch1*	Stat3_DataSet_11_151	Inhibit	Microglia
Control group	*Axl*	Cebpa_DataSet_11_019	Promote	Microglia, Microphage
Control group	*Gch1*	Cebpa_DataSet_11_019	Promote	Microglia, Microphage
Control group	*Vcam1*	Cebpa_DataSet_11_019	Inhibit	Microglia, Microphage
Control group	*Xdh*	Cebpa_DataSet_11_019	Inhibit	Microglia, Microphage
Control group	*Axl*	Sox11_DataSet_11_137	Inhibit	OPC, Neuron
Control group	*Stat6*	Sox11_DataSet_11_137	Inhibit	OPC, Neuron

Control group	*Vcam1*	Sox11_DataSet_11_137	Inhibit	OPC, Neuron
Control group	*Xdh*	Sox11_DataSet_11_137	Inhibit	OPC, Neuron
Control group	*Hbegf*	Sox11_DataSet_11_138	Inhibit	OPC, Neuron
Control group	*Fbxw7*	Smad1_DataSet_11_126	Promote	OPC, Neuron
Control group	*Hspb1*	Smad1_DataSet_11_126	Promote	OPC, Neuron
Control group	*Jun*	Smad1_DataSet_11_126	Promote	OPC, Neuron
Control group	*Rcan1*	Smad1_DataSet_11_126	Promote	OPC, Neuron
Control group	*Tnfrsf1a*	Sox2_DataSet_11_140	Inhibit	Ependymal cell, OPC, Astrocyte
Control group	*Fos*	Sox2_DataSet_11_141	Inhibit	Ependymal cell, OPC, Astrocyte
Control group	*Jun*	Sox2_DataSet_11_141	Inhibit	Ependymal cell, OPC, Astrocyte
Control group	*Rcan1*	Sox2_DataSet_11_141	Inhibit	Ependymal cell, OPC, Astrocyte
Control group	*Tnfrsf1a*	Sox2_DataSet_11_141	Inhibit	Ependymal cell, OPC, Astrocyte
Control group	*Axl*	Sox10_DataSet_11_136	Inhibit	OPC, Oligodendrocyte
Control group	*Mcl1*	Sox10_DataSet_11_136	Promote	OPC, Oligodendrocyte
Control group	*Ppp3ca*	Sox10_DataSet_11_136	Promote	OPC, Oligodendrocyte
Control group	*Hdac1*	Rest_DataSet_11_110	Inhibit	Ependymal cell
Control group	*Xdh*	Rest_DataSet_11_112	Promote	Ependymal cell
Control group	*Fkbp1b*	Rest_DataSet_11_113	Inhibit	Ependymal cell
Control group	*Stat6*	Rest_DataSet_11_113	Inhibit	Ependymal cell
Control group	*Xdh*	Rest_DataSet_11_113	Inhibit	Ependymal cell
Control group	*Fos*	Erg_DataSet_11_038	Promote	Endothelial cell, Pericyte
Control group	*Jun*	Erg_DataSet_11_038	Promote	Endothelial cell, Pericyte
Control group	*Sdc1*	Erg_DataSet_11_038	Inhibit	Endothelial cell, Pericyte
Control group	*Fos*	Foxq1_DataSet_11_045	Inhibit	Endothelial cell, Pericyte
Control group	*Gch1*	Foxq1_DataSet_11_045	Inhibit	Endothelial cell, Pericyte
Control group	*Sdc1*	Foxq1_DataSet_11_045	Inhibit	Endothelial cell, Pericyte
Control group	*Id1*	Klf6_DataSet_11_067	Promote	Microglia, Microphage
Control group	*Xdh*	Klf6_DataSet_11_067	Inhibit	Microglia, Microphage
Control group	*Fos*	Klf6_DataSet_11_068	Inhibit	Microglia, Microphage
Control group	*Hbegf*	Klf6_DataSet_11_068	Inhibit	Microglia, Microphage
Control group	*Vcam1*	Klf6_DataSet_11_068	Inhibit	Microglia, Microphage
Control group	*Fos*	Tbx1_DataSet_11_154	Inhibit	Endothelial cell, Pericyte
Control group	*Vcam1*	Tbx1_DataSet_11_154	Inhibit	Endothelial cell, Pericyte
Control group	*Xdh*	Tbx1_DataSet_11_154	Inhibit	Endothelial cell, Pericyte
Control group	*Fos*	Tbx1_DataSet_11_155	Promote	Endothelial cell, Pericyte
Control group	*Rcan1*	Thap11_DataSet_11_167	Inhibit	OPC
Control group	*Fos*	Thap11_DataSet_11_168	Promote	OPC
Control group	*Id1*	Thap11_DataSet_11_168	Inhibit	OPC
Control group	*Ucp2*	Thap11_DataSet_11_168	Promote	OPC

Control group	*Hbegf*	Mef2c_DataSet_11_079	Inhibit	Microglia, Microphage, Pericyte, DC
Control group	*Vcam1*	Mef2c_DataSet_11_079	Promote	Microglia, Microphage, Pericyte, DC
Control group	*Xdh*	Mef2c_DataSet_11_079	Promote	Microglia, Microphage, Pericyte, DC
Control group	*Gch1*	Runx2_DataSet_11_119	Inhibit	DC
Control group	*Hbegf*	Runx2_DataSet_11_119	Promote	DC
Control group	*Hbegf*	Runx2_DataSet_11_120	Promote	DC
Control group	*Hspb1*	Runx2_DataSet_11_120	Promote	DC
Control group	*Mcl1*	Runx2_DataSet_11_120	Inhibit	DC
Control group	*Hspb1*	Nelfb_DataSet_11_086	Inhibit	Endothelial cell
Control group	*Il6st*	Nelfb_DataSet_11_086	Promote	Endothelial cell
Control group	*Vcam1*	Nelfb_DataSet_11_086	Promote	Endothelial cell
Control group	*Hspb1*	Pbx1_DataSet_11_102	Inhibit	Ependymal cell, OPC, Astrocyte
Control group	*Jun*	Pbx1_DataSet_11_102	Promote	Ependymal cell, OPC, Astrocyte
Control group	*Xdh*	Pbx1_DataSet_11_102	Inhibit	Ependymal cell, OPC, Astrocyte
Control group	*Id1*	Nfix_DataSet_11_091	Promote	Ependymal cell, OPC, Oligodendrocyte
Control group	*Jun*	Nfix_DataSet_11_091	Inhibit	Ependymal cell, OPC, Oligodendrocyte
Control group	*Sdc1*	Nfix_DataSet_11_091	Inhibit	Ependymal cell, OPC, Oligodendrocyte
Control group	*Id1*	Xbp1_DataSet_11_181	Inhibit	Microglia, Monocyte, Microphage, Fibroblast
Control group	*Rcan1*	Xbp1_DataSet_11_181	Inhibit	Microglia, Monocyte, Microphage, Fibroblast
Control group	*Sdc1*	Xbp1_DataSet_11_181	Inhibit	Microglia, Monocyte, Microphage, Fibroblast
Control group	*Jun*	Mafg_DataSet_11_073	Promote	Microglia, Neuron, Astrocyte
Control group	*Ucp2*	Mafg_DataSet_11_073	Promote	Microglia, Neuron, Astrocyte
Control group	*Vcam1*	Mafg_DataSet_11_073	Promote	Microglia, Neuron, Astrocyte
Control group	*Axl*	Cbfb_DataSet_11_017	Promote	Div-Myeloid
Control group	*Xdh*	Cbfb_DataSet_11_017	Inhibit	Div-Myeloid
Control group	*Axl*	Tcf7_DataSet_11_156	Promote	Endothelial cell
Control group	*Il6st*	Tcf7_DataSet_11_156	Promote	Endothelial cell
Control group	*Fos*	Jarid2_DataSet_11_064	Promote	DC,Monocyte
Control group	*Xdh*	Jarid2_DataSet_11_064	Inhibit	DC,Monocyte
Control group	*Hbegf*	Pax6_DataSet_11_101	Promote	Ependymal cell
Control group	*Ripk1*	Pax6_DataSet_11_101	Inhibit	Ependymal cell
Control group	*Hspb1*	Ets2_DataSet_11_040	Inhibit	Endothelial cell, Monocyte
Control group	*Jun*	Ets2_DataSet_11_040	Inhibit	Endothelial cell, Monocyte
Control group	*Id1*	Arid1a_DataSet_11_004	Inhibit	Div-Myeloid, Microglia
Control group	*Rcan1*	Arid1a_DataSet_11_004	Promote	Div-Myeloid, Microglia
Control group	*Jun*	Bcl11a_DataSet_11_013	Promote	DC, Neuron
Control group	*Sdc1*	Bcl11a_DataSet_11_013	Promote	DC, Neuron
Control group	*Fos*	Sox21_DataSet_11_142	Inhibit	Ependymal cell, OPC
Control group	*Hbegf*	Mef2a_DataSet_11_077	Promote	Microglia, Microphage

Control group	*Jun*	Sall1_DataSet_11_122	Inhibit	Microglia, Ependymal cell
Control group	*Sdc1*	Id2_DataSet_11_059	Inhibit	Astrocyte, Lymphocyte, Microglia
Control group	*Xdh*	Atf3_DataSet_11_006	Inhibit	Microglia, Ependymal cell, Microphage
Control group	*Xdh*	Sox9_DataSet_11_144	Inhibit	Ependymal cell, Astrocyte
1-day post-SCI group	*Amph*	Smad4_DataSet_11_127	Inhibit	Pericyte, Neutrophil
1-day post-SCI group	*Axl*	Smad4_DataSet_11_127	Inhibit	Pericyte, Neutrophil
1-day post-SCI group	*Fkbp1b*	Smad4_DataSet_11_127	Inhibit	Pericyte, Neutrophil
1-day post-SCI group	*Fos*	Smad4_DataSet_11_127	Inhibit	Pericyte, Neutrophil
1-day post-SCI group	*Gch1*	Smad4_DataSet_11_127	Promote	Pericyte, Neutrophil
1-day post-SCI group	*Hbegf*	Smad4_DataSet_11_127	Promote	Pericyte, Neutrophil
1-day post-SCI group	*Hdac1*	Smad4_DataSet_11_127	Inhibit	Pericyte, Neutrophil
1-day post-SCI group	*Hspb1*	Smad4_DataSet_11_127	Inhibit	Pericyte, Neutrophil
1-day post-SCI group	*Id1*	Smad4_DataSet_11_127	Inhibit	Pericyte, Neutrophil
1-day post-SCI group	*Il6st*	Smad4_DataSet_11_127	Inhibit	Pericyte, Neutrophil
1-day post-SCI group	*Jun*	Smad4_DataSet_11_127	Promote	Pericyte, Neutrophil
1-day post-SCI group	*Map2k3*	Smad4_DataSet_11_127	Promote	Pericyte, Neutrophil
1-day post-SCI group	*Mcl1*	Smad4_DataSet_11_127	Promote	Pericyte, Neutrophil
1-day post-SCI group	*Rcan1*	Smad4_DataSet_11_127	Promote	Pericyte, Neutrophil
1-day post-SCI group	*Ripk1*	Smad4_DataSet_11_127	Promote	Pericyte, Neutrophil
1-day post-SCI group	*Sdc1*	Smad4_DataSet_11_127	Inhibit	Pericyte, Neutrophil
1-day post-SCI group	*Stat6*	Smad4_DataSet_11_127	Promote	Pericyte, Neutrophil
1-day post-SCI group	*Ucp2*	Smad4_DataSet_11_127	Promote	Pericyte, Neutrophil
1-day post-SCI group	*Vcam1*	Smad4_DataSet_11_127	Inhibit	Pericyte, Neutrophil
1-day post-SCI group	*Xdh*	Smad4_DataSet_11_127	Inhibit	Pericyte, Neutrophil
1-day post-SCI group	*Amph*	Smad4_DataSet_11_128	Inhibit	Pericyte, Neutrophil
1-day post-SCI group	*Cbx6*	Smad4_DataSet_11_128	Promote	Pericyte, Neutrophil
1-day post-SCI group	*Fkbp1b*	Smad4_DataSet_11_128	Inhibit	Pericyte, Neutrophil
1-day post-SCI group	*Gch1*	Smad4_DataSet_11_128	Inhibit	Pericyte, Neutrophil
1-day post-SCI group	*Hbegf*	Smad4_DataSet_11_128	Promote	Pericyte, Neutrophil
1-day post-SCI group	*Hspb1*	Smad4_DataSet_11_128	Promote	Pericyte, Neutrophil
1-day post-SCI group	*Id1*	Smad4_DataSet_11_128	Promote	Pericyte, Neutrophil
1-day post-SCI group	*Jun*	Smad4_DataSet_11_128	Promote	Pericyte, Neutrophil
1-day post-SCI group	*Ppp3ca*	Smad4_DataSet_11_128	Inhibit	Pericyte, Neutrophil
1-day post-SCI group	*Sdc1*	Smad4_DataSet_11_128	Inhibit	Pericyte, Neutrophil
1-day post-SCI group	*Ucp2*	Smad4_DataSet_11_128	Inhibit	Pericyte, Neutrophil
1-day post-SCI group	*Vcam1*	Smad4_DataSet_11_128	Inhibit	Pericyte, Neutrophil
1-day post-SCI group	*Xdh*	Smad4_DataSet_11_128	Inhibit	Pericyte, Neutrophil
1-day post-SCI group	*Amph*	Stat4_DataSet_11_152	Promote	Lymphocyte
1-day post-SCI group	*Cbx6*	Stat4_DataSet_11_152	Promote	Lymphocyte

1-day post-SCI group	*Fbxw7*	Stat4_DataSet_11_152	Inhibit	Lymphocyte
1-day post-SCI group	*Fos*	Stat4_DataSet_11_152	Promote	Lymphocyte
1-day post-SCI group	*Gch1*	Stat4_DataSet_11_152	Inhibit	Lymphocyte
1-day post-SCI group	*Il6st*	Stat4_DataSet_11_152	Promote	Lymphocyte
1-day post-SCI group	*Jun*	Stat4_DataSet_11_152	Inhibit	Lymphocyte
1-day post-SCI group	*Map2k4*	Stat4_DataSet_11_152	Promote	Lymphocyte
1-day post-SCI group	*Ppp3ca*	Stat4_DataSet_11_152	Promote	Lymphocyte
1-day post-SCI group	*Tnfrsf1a*	Stat4_DataSet_11_152	Inhibit	Lymphocyte
1-day post-SCI group	*Ucp2*	Stat4_DataSet_11_152	Inhibit	Lymphocyte
1-day post-SCI group	*Vcam1*	Stat4_DataSet_11_152	Inhibit	Lymphocyte
1-day post-SCI group	*Amph*	Stat6_DataSet_11_153	Inhibit	Neutrophil
1-day post-SCI group	*Axl*	Stat6_DataSet_11_153	Inhibit	Neutrophil
1-day post-SCI group	*Fbxw7*	Stat6_DataSet_11_153	Promote	Neutrophil
1-day post-SCI group	*Hbegf*	Stat6_DataSet_11_153	Promote	Neutrophil
1-day post-SCI group	*Id1*	Stat6_DataSet_11_153	Inhibit	Neutrophil
1-day post-SCI group	*Il6st*	Stat6_DataSet_11_153	Promote	Neutrophil
1-day post-SCI group	*Jun*	Stat6_DataSet_11_153	Promote	Neutrophil
1-day post-SCI group	*Sdc1*	Stat6_DataSet_11_153	Promote	Neutrophil
1-day post-SCI group	*Stat6*	Stat6_DataSet_11_153	Promote	Neutrophil
1-day post-SCI group	*Vcam1*	Stat6_DataSet_11_153	Inhibit	Neutrophil
1-day post-SCI group	*Xdh*	Stat6_DataSet_11_153	Promote	Neutrophil
1-day post-SCI group	*Cbx6*	Cebpe_DataSet_11_023	Promote	Neutrophil
1-day post-SCI group	*Fos*	Cebpe_DataSet_11_023	Inhibit	Neutrophil
1-day post-SCI group	*Hbegf*	Cebpe_DataSet_11_023	Inhibit	Neutrophil
1-day post-SCI group	*Hdac1*	Cebpe_DataSet_11_023	Promote	Neutrophil
1-day post-SCI group	*Hspb1*	Cebpe_DataSet_11_023	Inhibit	Neutrophil
1-day post-SCI group	*Id1*	Cebpe_DataSet_11_023	Inhibit	Neutrophil
1-day post-SCI group	*Map2k4*	Cebpe_DataSet_11_023	Promote	Neutrophil
1-day post-SCI group	*Sdc1*	Cebpe_DataSet_11_023	Promote	Neutrophil
1-day post-SCI group	*Ucp2*	Cebpe_DataSet_11_023	Inhibit	Neutrophil
1-day post-SCI group	*Xdh*	Cebpe_DataSet_11_023	Inhibit	Neutrophil
1-day post-SCI group	*Cbx6*	Gata3_DataSet_11_048	Promote	Lymphocyte, Neuron
1-day post-SCI group	*Fkbp1b*	Gata3_DataSet_11_048	Inhibit	Lymphocyte, Neuron
1-day post-SCI group	*Fos*	Gata3_DataSet_11_048	Inhibit	Lymphocyte, Neuron
1-day post-SCI group	*Gch1*	Gata3_DataSet_11_048	Inhibit	Lymphocyte, Neuron
1-day post-SCI group	*Hbegf*	Gata3_DataSet_11_048	Inhibit	Lymphocyte, Neuron
1-day post-SCI group	*Id1*	Gata3_DataSet_11_048	Inhibit	Lymphocyte, Neuron
1-day post-SCI group	*Map2k3*	Gata3_DataSet_11_048	Inhibit	Lymphocyte, Neuron
1-day post-SCI group	*Ppp3ca*	Gata3_DataSet_11_048	Promote	Lymphocyte, Neuron

1-day post-SCI group	*Rcan1*	Gata3_DataSet_11_048	Inhibit	Lymphocyte, Neuron
1-day post-SCI group	*Xdh*	Gata3_DataSet_11_048	Inhibit	Lymphocyte, Neuron
1-day post-SCI group	*Axl*	Mbd2_DataSet_11_076	Inhibit	Endothelial cell, Neutrophil, Pericyte
1-day post-SCI group	*Fos*	Mbd2_DataSet_11_076	Promote	Endothelial cell, Neutrophil, Pericyte
1-day post-SCI group	*Gch1*	Mbd2_DataSet_11_076	Inhibit	Endothelial cell, Neutrophil, Pericyte
1-day post-SCI group	*Hbegf*	Mbd2_DataSet_11_076	Inhibit	Endothelial cell, Neutrophil, Pericyte
1-day post-SCI group	*Jun*	Mbd2_DataSet_11_076	Inhibit	Endothelial cell, Neutrophil, Pericyte
1-day post-SCI group	*Ppp3ca*	Mbd2_DataSet_11_076	Inhibit	Endothelial cell, Neutrophil, Pericyte
1-day post-SCI group	*Rcan1*	Mbd2_DataSet_11_076	Promote	Endothelial cell, Neutrophil, Pericyte
1-day post-SCI group	*Sdc1*	Mbd2_DataSet_11_076	Promote	Endothelial cell, Neutrophil, Pericyte
1-day post-SCI group	*Xdh*	Mbd2_DataSet_11_076	Inhibit	Endothelial cell, Neutrophil, Pericyte
1-day post-SCI group	*Axl*	Tsc22d4_DataSet_11_169	Promote	Ependymal cell, OPC, Oligodendrocyte, Astrocyte, Neutrophil
1-day post-SCI group	*Fbxw7*	Tsc22d4_DataSet_11_169	Inhibit	Ependymal cell, OPC, Oligodendrocyte, Astrocyte, Neutrophil
1-day post-SCI group	*Fos*	Tsc22d4_DataSet_11_169	Promote	Ependymal cell, OPC, Oligodendrocyte, Astrocyte, Neutrophil
1-day post-SCI group	*Hbegf*	Tsc22d4_DataSet_11_169	Promote	Ependymal cell, OPC, Oligodendrocyte, Astrocyte, Neutrophil
1-day post-SCI group	*Id1*	Tsc22d4_DataSet_11_169	Promote	Ependymal cell, OPC, Oligodendrocyte, Astrocyte, Neutrophil
1-day post-SCI group	*Jun*	Tsc22d4_DataSet_11_169	Promote	Ependymal cell, OPC, Oligodendrocyte, Astrocyte, Neutrophil
1-day post-SCI group	*Rcan1*	Tsc22d4_DataSet_11_169	Promote	Ependymal cell, OPC, Oligodendrocyte, Astrocyte, Neutrophil
1-day post-SCI group	*Vcam1*	Tsc22d4_DataSet_11_169	Promote	Ependymal cell, OPC, Oligodendrocyte, Astrocyte, Neutrophil
1-day post-SCI group	*Xdh*	Tsc22d4_DataSet_11_169	Promote	Ependymal cell, OPC, Oligodendrocyte, Astrocyte, Neutrophil
1-day post-SCI group	*Amph*	Cebpb_DataSet_11_020	Promote	Neutrophil, Monocyte, Microphage, Microglia, Div-Myeloid
1-day post-SCI group	*Fkbp1b*	Cebpb_DataSet_11_020	Promote	Neutrophil, Monocyte, Microphage, Microglia, Div-Myeloid
1-day post-SCI group	*Id1*	Cebpb_DataSet_11_020	Inhibit	Neutrophil, Monocyte, Microphage, Microglia, Div-Myeloid
1-day post-SCI group	*Vcam1*	Cebpb_DataSet_11_020	Promote	Neutrophil, Monocyte, Microphage, Microglia, Div-Myeloid
1-day post-SCI group	*Xdh*	Cebpb_DataSet_11_020	Promote	Neutrophil, Monocyte, Microphage, Microglia, Div-Myeloid
1-day post-SCI group	*Fos*	Cebpb_DataSet_11_021	Inhibit	Neutrophil, Monocyte, Microphage, Microglia, Div-Myeloid
1-day post-SCI group	*Hbegf*	Cebpb_DataSet_11_021	Inhibit	Neutrophil, Monocyte, Microphage, Microglia, Div-Myeloid
1-day post-SCI group	*Hdac1*	Cebpb_DataSet_11_021	Promote	Neutrophil, Monocyte, Microphage, Microglia, Div-Myeloid
1-day post-SCI group	*Hspb1*	Cebpb_DataSet_11_021	Inhibit	Neutrophil, Monocyte, Microphage, Microglia, Div-Myeloid
1-day post-SCI group	*Id1*	Cebpb_DataSet_11_021	Inhibit	Neutrophil, Monocyte, Microphage, Microglia, Div-Myeloid
1-day post-SCI group	*Map2k4*	Cebpb_DataSet_11_021	Promote	Neutrophil, Monocyte, Microphage, Microglia, Div-Myeloid
1-day post-SCI group	*Ucp2*	Cebpb_DataSet_11_021	Inhibit	Neutrophil, Monocyte, Microphage, Microglia, Div-Myeloid
1-day post-SCI group	*Vcam1*	Cebpb_DataSet_11_021	Inhibit	Neutrophil, Monocyte, Microphage, Microglia, Div-Myeloid
1-day post-SCI group	*Xdh*	Cebpb_DataSet_11_021	Inhibit	Neutrophil, Monocyte, Microphage, Microglia, Div-Myeloid
1-day post-SCI group	*Fos*	Cebpb_DataSet_11_022	Promote	Neutrophil, Monocyte, Microphage, Microglia, Div-Myeloid
1-day post-SCI group	*Gch1*	Cebpb_DataSet_11_022	Inhibit	Neutrophil, Monocyte, Microphage, Microglia, Div-Myeloid
1-day post-SCI group	*Id1*	Cebpb_DataSet_11_022	Promote	Neutrophil, Monocyte, Microphage, Microglia, Div-Myeloid
1-day post-SCI group	*Jun*	Cebpb_DataSet_11_022	Promote	Neutrophil, Monocyte, Microphage, Microglia, Div-Myeloid
1-day post-SCI group	*Map2k3*	Cebpb_DataSet_11_022	Promote	Neutrophil, Monocyte, Microphage, Microglia, Div-Myeloid

1-day post-SCI group	*Fos*	Pax5_DataSet_11_100	Promote	DC
1-day post-SCI group	*Gch1*	Pax5_DataSet_11_100	Promote	DC
1-day post-SCI group	*Il6st*	Pax5_DataSet_11_100	Inhibit	DC
1-day post-SCI group	*Mcl1*	Pax5_DataSet_11_100	Promote	DC
1-day post-SCI group	*Ppp3ca*	Pax5_DataSet_11_100	Promote	DC
1-day post-SCI group	*Ripk1*	Pax5_DataSet_11_100	Inhibit	DC
1-day post-SCI group	*Sdc1*	Pax5_DataSet_11_100	Promote	DC
1-day post-SCI group	*Tnfrsf1a*	Pax5_DataSet_11_100	Inhibit	DC
1-day post-SCI group	*Ucp2*	Pax5_DataSet_11_100	Promote	DC
1-day post-SCI group	*Amph*	Runx1_DataSet_11_114	Inhibit	Ependymal cell, Microglia, Neutrophil, Fibroblast
1-day post-SCI group	*Fos*	Runx1_DataSet_11_114	Promote	Ependymal cell, Microglia, Neutrophil, Fibroblast
1-day post-SCI group	*Hspb1*	Runx1_DataSet_11_114	Promote	Ependymal cell, Microglia, Neutrophil, Fibroblast
1-day post-SCI group	*Map2k3*	Runx1_DataSet_11_114	Promote	Ependymal cell, Microglia, Neutrophil, Fibroblast
1-day post-SCI group	*Sdc1*	Runx1_DataSet_11_114	Promote	Ependymal cell, Microglia, Neutrophil, Fibroblast
1-day post-SCI group	*Fkbp1b*	Runx1_DataSet_11_115	Inhibit	Ependymal cell, Microglia, Neutrophil, Fibroblast
1-day post-SCI group	*Fos*	Runx1_DataSet_11_115	Inhibit	Ependymal cell, Microglia, Neutrophil, Fibroblast
1-day post-SCI group	*Gch1*	Runx1_DataSet_11_115	Inhibit	Ependymal cell, Microglia, Neutrophil, Fibroblast
1-day post-SCI group	*Jun*	Runx1_DataSet_11_115	Inhibit	Ependymal cell, Microglia, Neutrophil, Fibroblast
1-day post-SCI group	*Sdc1*	Runx1_DataSet_11_115	Inhibit	Ependymal cell, Microglia, Neutrophil, Fibroblast
1-day post-SCI group	*Ucp2*	Runx1_DataSet_11_115	Inhibit	Ependymal cell, Microglia, Neutrophil, Fibroblast
1-day post-SCI group	*Fos*	Runx1_DataSet_11_116	Promote	Ependymal cell, Microglia, Neutrophil, Fibroblast
1-day post-SCI group	*Gch1*	Runx1_DataSet_11_116	Inhibit	Ependymal cell, Microglia, Neutrophil, Fibroblast
1-day post-SCI group	*Hbegf*	Runx1_DataSet_11_116	Inhibit	Ependymal cell, Microglia, Neutrophil, Fibroblast
1-day post-SCI group	*Il6st*	Runx1_DataSet_11_116	Inhibit	Ependymal cell, Microglia, Neutrophil, Fibroblast
1-day post-SCI group	*Jun*	Runx1_DataSet_11_116	Promote	Ependymal cell, Microglia, Neutrophil, Fibroblast
1-day post-SCI group	*Ppp3ca*	Runx1_DataSet_11_116	Inhibit	Ependymal cell, Microglia, Neutrophil, Fibroblast
1-day post-SCI group	*Sdc1*	Runx1_DataSet_11_116	Promote	Ependymal cell, Microglia, Neutrophil, Fibroblast
1-day post-SCI group	*Tnfrsf1a*	Runx1_DataSet_11_116	Inhibit	Ependymal cell, Microglia, Neutrophil, Fibroblast
1-day post-SCI group	*Xdh*	Runx1_DataSet_11_116	Inhibit	Ependymal cell, Microglia, Neutrophil, Fibroblast
1-day post-SCI group	*Amph*	Runx1_DataSet_11_117	Inhibit	Ependymal cell, Microglia, Neutrophil, Fibroblast
1-day post-SCI group	*Id1*	Runx1_DataSet_11_117	Inhibit	Ependymal cell, Microglia, Neutrophil, Fibroblast
1-day post-SCI group	*Amph*	Nr2f2_DataSet_11_094	Promote	Pericyte
1-day post-SCI group	*Hspb1*	Nr2f2_DataSet_11_094	Inhibit	Pericyte
1-day post-SCI group	*Vcam1*	Nr2f2_DataSet_11_094	Inhibit	Pericyte
1-day post-SCI group	*Amph*	Nr2f2_DataSet_11_095	Promote	Pericyte
1-day post-SCI group	*Fos*	Nr2f2_DataSet_11_095	Inhibit	Pericyte
1-day post-SCI group	*Hspb1*	Nr2f2_DataSet_11_095	Inhibit	Pericyte
1-day post-SCI group	*Id1*	Nr2f2_DataSet_11_095	Inhibit	Pericyte
1-day post-SCI group	*Tnfrsf1a*	Nr2f2_DataSet_11_095	Inhibit	Pericyte

1-day post-SCI group	*Ucp2*	Nr2f2_DataSet_11_095	Inhibit	Pericyte
1-day post-SCI group	*Vcam1*	Nr2f2_DataSet_11_095	Inhibit	Pericyte
1-day post-SCI group	*Xdh*	Nr2f2_DataSet_11_095	Inhibit	Pericyte
1-day post-SCI group	*Axl*	Irf8_DataSet_11_063	Promote	Microglia, Div-Myeloid
1-day post-SCI group	*Gch1*	Irf8_DataSet_11_063	Promote	Microglia, Div-Myeloid
1-day post-SCI group	*Id1*	Irf8_DataSet_11_063	Promote	Microglia, Div-Myeloid
1-day post-SCI group	*Jun*	Irf8_DataSet_11_063	Inhibit	Microglia, Div-Myeloid
1-day post-SCI group	*Map2k3*	Irf8_DataSet_11_063	Promote	Microglia, Div-Myeloid
1-day post-SCI group	*Mcl1*	Irf8_DataSet_11_063	Promote	Microglia, Div-Myeloid
1-day post-SCI group	*Vcam1*	Irf8_DataSet_11_063	Promote	Microglia, Div-Myeloid
1-day post-SCI group	*Axl*	Meis1_DataSet_11_081	Promote	Ependymal cell
1-day post-SCI group	*Fbxw7*	Meis1_DataSet_11_081	Promote	Ependymal cell
1-day post-SCI group	*Fkbp1b*	Meis1_DataSet_11_081	Inhibit	Ependymal cell
1-day post-SCI group	*Hspb1*	Meis1_DataSet_11_081	Inhibit	Ependymal cell
1-day post-SCI group	*Il6st*	Meis1_DataSet_11_081	Promote	Ependymal cell
1-day post-SCI group	*Sdc1*	Meis1_DataSet_11_081	Promote	Ependymal cell
1-day post-SCI group	*Vcam1*	Meis1_DataSet_11_081	Promote	Ependymal cell
1-day post-SCI group	*Fos*	Meis1_DataSet_11_082	Inhibit	Ependymal cell
1-day post-SCI group	*Gch1*	Meis1_DataSet_11_082	Inhibit	Ependymal cell
1-day post-SCI group	*Id1*	Meis1_DataSet_11_082	Promote	Ependymal cell
1-day post-SCI group	*Il6st*	Meis1_DataSet_11_082	Inhibit	Ependymal cell
1-day post-SCI group	*Sdc1*	Meis1_DataSet_11_082	Promote	Ependymal cell
1-day post-SCI group	*Vcam1*	Meis1_DataSet_11_082	Inhibit	Ependymal cell
1-day post-SCI group	*Amph*	Prox1_DataSet_11_109	Promote	Oligodendrocyte
1-day post-SCI group	*Hbegf*	Prox1_DataSet_11_109	Inhibit	Oligodendrocyte
1-day post-SCI group	*Hspb1*	Prox1_DataSet_11_109	Inhibit	Oligodendrocyte
1-day post-SCI group	*Jun*	Prox1_DataSet_11_109	Inhibit	Oligodendrocyte
1-day post-SCI group	*Vcam1*	Prox1_DataSet_11_109	Promote	Oligodendrocyte
1-day post-SCI group	*Xdh*	Prox1_DataSet_11_109	Inhibit	Oligodendrocyte
1-day post-SCI group	*Fos*	Tet2_DataSet_11_159	Promote	Astrocyte, Neutrophil
1-day post-SCI group	*Gch1*	Tet2_DataSet_11_159	Inhibit	Astrocyte, Neutrophil
1-day post-SCI group	*Vcam1*	Tet2_DataSet_11_159	Inhibit	Astrocyte, Neutrophil
1-day post-SCI group	*Xdh*	Tet2_DataSet_11_159	Inhibit	Astrocyte, Neutrophil
1-day post-SCI group	*Fos*	Tet2_DataSet_11_161	Inhibit	Astrocyte, Neutrophil
1-day post-SCI group	*Gch1*	Tet2_DataSet_11_161	Inhibit	Astrocyte, Neutrophil
1-day post-SCI group	*Hspb1*	Tet2_DataSet_11_161	Inhibit	Astrocyte, Neutrophil
1-day post-SCI group	*Map2k3*	Tet2_DataSet_11_161	Inhibit	Astrocyte, Neutrophil
1-day post-SCI group	*Fos*	Tet2_DataSet_11_162	Inhibit	Astrocyte, Neutrophil
1-day post-SCI group	*Axl*	Tet2_DataSet_11_163	Inhibit	Astrocyte, Neutrophil

1-day post-SCI group	*Fbxw7*	Tet2_DataSet_11_163	Promote	Astrocyte, Neutrophil
1-day post-SCI group	*Fos*	Tet2_DataSet_11_163	Inhibit	Astrocyte, Neutrophil
1-day post-SCI group	*Mcl1*	Tet2_DataSet_11_163	Inhibit	Astrocyte, Neutrophil
1-day post-SCI group	*Ppp3ca*	Tet2_DataSet_11_163	Promote	Astrocyte, Neutrophil
1-day post-SCI group	*Sdc1*	Tet2_DataSet_11_163	Inhibit	Astrocyte, Neutrophil
1-day post-SCI group	*Cbx6*	E2f4_DataSet_11_033	Promote	Microglia, Div-Myeloid
1-day post-SCI group	*Fkbp1b*	E2f4_DataSet_11_033	Promote	Microglia, Div-Myeloid
1-day post-SCI group	*Fos*	E2f4_DataSet_11_033	Inhibit	Microglia, Div-Myeloid
1-day post-SCI group	*Ripk1*	E2f4_DataSet_11_033	Promote	Microglia, Div-Myeloid
1-day post-SCI group	*Vcam1*	E2f4_DataSet_11_033	Inhibit	Microglia, Div-Myeloid
1-day post-SCI group	*Xdh*	E2f4_DataSet_11_033	Inhibit	Microglia, Div-Myeloid
1-day post-SCI group	*Il6st*	E2f4_DataSet_11_034	Promote	Microglia, Div-Myeloid
1-day post-SCI group	*Cbx6*	Myb_DataSet_11_084	Promote	Div-Myeloid
1-day post-SCI group	*Fkbp1b*	Myb_DataSet_11_084	Promote	Div-Myeloid
1-day post-SCI group	*Gch1*	Myb_DataSet_11_084	Inhibit	Div-Myeloid
1-day post-SCI group	*Mcl1*	Myb_DataSet_11_084	Inhibit	Div-Myeloid
1-day post-SCI group	*Ppp3ca*	Myb_DataSet_11_084	Inhibit	Div-Myeloid
1-day post-SCI group	*Xdh*	Myb_DataSet_11_084	Inhibit	Div-Myeloid
1-day post-SCI group	*Hbegf*	Myb_DataSet_11_085	Inhibit	Div-Myeloid
1-day post-SCI group	*Hspb1*	Myb_DataSet_11_085	Inhibit	Div-Myeloid
1-day post-SCI group	*Id1*	Myb_DataSet_11_085	Inhibit	Div-Myeloid
1-day post-SCI group	*Jun*	Myb_DataSet_11_085	Inhibit	Div-Myeloid
1-day post-SCI group	*Fos*	Lmx1b_DataSet_11_072	Inhibit	Neuron
1-day post-SCI group	*Gch1*	Lmx1b_DataSet_11_072	Inhibit	Neuron
1-day post-SCI group	*Hspb1*	Lmx1b_DataSet_11_072	Inhibit	Neuron
1-day post-SCI group	*Il6st*	Lmx1b_DataSet_11_072	Inhibit	Neuron
1-day post-SCI group	*Jun*	Lmx1b_DataSet_11_072	Inhibit	Neuron
1-day post-SCI group	*Xdh*	Lmx1b_DataSet_11_072	Inhibit	Neuron
1-day post-SCI group	*Amph*	Prdm2_DataSet_11_108	Inhibit	Pericyte
1-day post-SCI group	*Fkbp1b*	Prdm2_DataSet_11_108	Promote	Pericyte
1-day post-SCI group	*Fos*	Prdm2_DataSet_11_108	Inhibit	Pericyte
1-day post-SCI group	*Vcam1*	Prdm2_DataSet_11_108	Inhibit	Pericyte
1-day post-SCI group	*Xdh*	Prdm2_DataSet_11_108	Inhibit	Pericyte
1-day post-SCI group	*Amph*	Snai1_DataSet_11_129	Inhibit	Neutrophil
1-day post-SCI group	*Hbegf*	Snai1_DataSet_11_129	Inhibit	Neutrophil
1-day post-SCI group	*Vcam1*	Snai1_DataSet_11_129	Promote	Neutrophil
1-day post-SCI group	*Amph*	Snai1_DataSet_11_130	Inhibit	Neutrophil
1-day post-SCI group	*Fos*	Snai1_DataSet_11_130	Inhibit	Neutrophil
1-day post-SCI group	*Hbegf*	Snai1_DataSet_11_130	Inhibit	Neutrophil

1-day post-SCI group	*Hspb1*	Snai1_DataSet_11_130	Inhibit	Neutrophil
1-day post-SCI group	*Ucp2*	Snai1_DataSet_11_130	Inhibit	Neutrophil
1-day post-SCI group	*Id1*	Snai1_DataSet_11_131	Inhibit	Neutrophil
1-day post-SCI group	*Ppp3ca*	Snai1_DataSet_11_131	Promote	Neutrophil
1-day post-SCI group	*Ucp2*	Snai1_DataSet_11_131	Inhibit	Neutrophil
1-day post-SCI group	*Fos*	Snai1_DataSet_11_132	Promote	Neutrophil
1-day post-SCI group	*Hspb1*	Snai1_DataSet_11_132	Inhibit	Neutrophil
1-day post-SCI group	*Fbxw7*	Sox17_DataSet_11_139	Inhibit	Endothelial cell, Pericyte
1-day post-SCI group	*Fos*	Sox17_DataSet_11_139	Inhibit	Endothelial cell, Pericyte
1-day post-SCI group	*Hbegf*	Sox17_DataSet_11_139	Inhibit	Endothelial cell, Pericyte
1-day post-SCI group	*Hspb1*	Sox17_DataSet_11_139	Inhibit	Endothelial cell, Pericyte
1-day post-SCI group	*Id1*	Sox17_DataSet_11_139	Inhibit	Endothelial cell, Pericyte
1-day post-SCI group	*Fos*	Ahr_DataSet_11_002	Inhibit	DC,Monocyte
1-day post-SCI group	*Jun*	Ahr_DataSet_11_002	Inhibit	DC,Monocyte
1-day post-SCI group	*Rcan1*	Ahr_DataSet_11_002	Inhibit	DC,Monocyte
1-day post-SCI group	*Sdc1*	Ahr_DataSet_11_002	Inhibit	DC,Monocyte
1-day post-SCI group	*Xdh*	Ahr_DataSet_11_002	Promote	DC,Monocyte
1-day post-SCI group	*Fos*	Ahr_DataSet_11_003	Promote	DC,Monocyte
1-day post-SCI group	*Hspb1*	Ahr_DataSet_11_003	Inhibit	DC,Monocyte
1-day post-SCI group	*Fos*	Foxp1_DataSet_11_044	Inhibit	Endothelial cell, Pericyte, Fibroblast, Lymphocyte
1-day post-SCI group	*Gch1*	Foxp1_DataSet_11_044	Promote	Endothelial cell, Pericyte, Fibroblast, Lymphocyte
1-day post-SCI group	*Stat6*	Foxp1_DataSet_11_044	Promote	Endothelial cell, Pericyte, Fibroblast, Lymphocyte
1-day post-SCI group	*Vcam1*	Foxp1_DataSet_11_044	Promote	Endothelial cell, Pericyte, Fibroblast, Lymphocyte
1-day post-SCI group	*Xdh*	Foxp1_DataSet_11_044	Promote	Endothelial cell, Pericyte, Fibroblast, Lymphocyte
1-day post-SCI group	*Hdac1*	Nfil3_DataSet_11_090	Inhibit	Microglia, Div-Myeloid, Neutrophil
1-day post-SCI group	*Mcl1*	Nfil3_DataSet_11_090	Inhibit	Microglia, Div-Myeloid, Neutrophil
1-day post-SCI group	*Sdc1*	Nfil3_DataSet_11_090	Inhibit	Microglia, Div-Myeloid, Neutrophil
1-day post-SCI group	*Stat6*	Nfil3_DataSet_11_090	Inhibit	Microglia, Div-Myeloid, Neutrophil
1-day post-SCI group	*Xdh*	Nfil3_DataSet_11_090	Inhibit	Microglia, Div-Myeloid, Neutrophil
1-day post-SCI group	*Amph*	Adnp_DataSet_11_001	Inhibit	OPC, Neuron, Astrocyte
1-day post-SCI group	*Fkbp1b*	Adnp_DataSet_11_001	Inhibit	OPC, Neuron, Astrocyte
1-day post-SCI group	*Fos*	Adnp_DataSet_11_001	Inhibit	OPC, Neuron, Astrocyte
1-day post-SCI group	*Vcam1*	Adnp_DataSet_11_001	Promote	OPC, Neuron, Astrocyte
1-day post-SCI group	*Amph*	Gata2_DataSet_11_047	Promote	Endothelial cell, Pericyte, Neuron
1-day post-SCI group	*Axl*	Gata2_DataSet_11_047	Promote	Endothelial cell, Pericyte, Neuron
1-day post-SCI group	*Id1*	Gata2_DataSet_11_047	Inhibit	Endothelial cell, Pericyte, Neuron
1-day post-SCI group	*Vcam1*	Gata2_DataSet_11_047	Promote	Endothelial cell, Pericyte, Neuron
1-day post-SCI group	*Amph*	Stat3_DataSet_11_151	Inhibit	Endothelial cell, Neutrophil, OPC, Neuron, Pericyte, Astrocyte
1-day post-SCI group	*Axl*	Stat3_DataSet_11_151	Inhibit	Endothelial cell, Neutrophil, OPC, Neuron, Pericyte, Astrocyte

1-day post-SCI group	*Fos*	Stat3_DataSet_11_151	Promote	Endothelial cell, Neutrophil, OPC, Neuron, Pericyte, Astrocyte
1-day post-SCI group	*Gch1*	Stat3_DataSet_11_151	Inhibit	Endothelial cell, Neutrophil, OPC, Neuron, Pericyte, Astrocyte
1-day post-SCI group	*Axl*	Cebpa_DataSet_11_019	Promote	Microglia, Microphage
1-day post-SCI group	*Gch1*	Cebpa_DataSet_11_019	Promote	Microglia, Microphage
1-day post-SCI group	*Vcam1*	Cebpa_DataSet_11_019	Inhibit	Microglia, Microphage
1-day post-SCI group	*Xdh*	Cebpa_DataSet_11_019	Inhibit	Microglia, Microphage
1-day post-SCI group	*Axl*	Sox11_DataSet_11_137	Inhibit	OPC, Neuron
1-day post-SCI group	*Stat6*	Sox11_DataSet_11_137	Inhibit	OPC, Neuron
1-day post-SCI group	*Vcam1*	Sox11_DataSet_11_137	Inhibit	OPC, Neuron
1-day post-SCI group	*Xdh*	Sox11_DataSet_11_137	Inhibit	OPC, Neuron
1-day post-SCI group	*Hbegf*	Sox11_DataSet_11_138	Inhibit	OPC, Neuron
1-day post-SCI group	*Fbxw7*	Smad1_DataSet_11_126	Promote	Endothelial cell, Ependymal cell, Neuron, Pericyte
1-day post-SCI group	*Hspb1*	Smad1_DataSet_11_126	Promote	Endothelial cell, Ependymal cell, Neuron, Pericyte
1-day post-SCI group	*Jun*	Smad1_DataSet_11_126	Promote	Endothelial cell, Ependymal cell, Neuron, Pericyte
1-day post-SCI group	*Rcan1*	Smad1_DataSet_11_126	Promote	Endothelial cell, Ependymal cell, Neuron, Pericyte
1-day post-SCI group	*Tnfrsf1a*	Sox2_DataSet_11_140	Inhibit	Ependymal cell, OPC
1-day post-SCI group	*Fos*	Sox2_DataSet_11_141	Inhibit	Ependymal cell, OPC
1-day post-SCI group	*Jun*	Sox2_DataSet_11_141	Inhibit	Ependymal cell, OPC
1-day post-SCI group	*Rcan1*	Sox2_DataSet_11_141	Inhibit	Ependymal cell, OPC
1-day post-SCI group	*Tnfrsf1a*	Sox2_DataSet_11_141	Inhibit	Ependymal cell, OPC
1-day post-SCI group	*Axl*	Sox10_DataSet_11_136	Inhibit	OPC, Oligodendrocyte
1-day post-SCI group	*Mcl1*	Sox10_DataSet_11_136	Promote	OPC, Oligodendrocyte
1-day post-SCI group	*Ppp3ca*	Sox10_DataSet_11_136	Promote	OPC, Oligodendrocyte
1-day post-SCI group	*Hdac1*	Rest_DataSet_11_110	Inhibit	DC
1-day post-SCI group	*Xdh*	Rest_DataSet_11_112	Promote	DC
1-day post-SCI group	*Fkbp1b*	Rest_DataSet_11_113	Inhibit	DC
1-day post-SCI group	*Stat6*	Rest_DataSet_11_113	Inhibit	DC
1-day post-SCI group	*Xdh*	Rest_DataSet_11_113	Inhibit	DC
1-day post-SCI group	*Fos*	Erg_DataSet_11_038	Promote	Endothelial cell, Pericyte
1-day post-SCI group	*Jun*	Erg_DataSet_11_038	Promote	Endothelial cell, Pericyte
1-day post-SCI group	*Sdc1*	Erg_DataSet_11_038	Inhibit	Endothelial cell, Pericyte
1-day post-SCI group	*Fos*	Foxq1_DataSet_11_045	Inhibit	Endothelial cell, Pericyte
1-day post-SCI group	*Gch1*	Foxq1_DataSet_11_045	Inhibit	Endothelial cell, Pericyte
1-day post-SCI group	*Sdc1*	Foxq1_DataSet_11_045	Inhibit	Endothelial cell, Pericyte
1-day post-SCI group	*Id1*	Klf6_DataSet_11_067	Promote	Endothelial cell, Ependymal cell, Pericyte
1-day post-SCI group	*Xdh*	Klf6_DataSet_11_067	Inhibit	Endothelial cell, Ependymal cell, Pericyte
1-day post-SCI group	*Fos*	Klf6_DataSet_11_068	Inhibit	Endothelial cell, Ependymal cell, Pericyte
1-day post-SCI group	*Hbegf*	Klf6_DataSet_11_068	Inhibit	Endothelial cell, Ependymal cell, Pericyte
1-day post-SCI group	*Vcam1*	Klf6_DataSet_11_068	Inhibit	Endothelial cell, Ependymal cell, Pericyte

1-day post-SCI group	*Fos*	Tbx1_DataSet_11_154	Inhibit	Endothelial cell
1-day post-SCI group	*Vcam1*	Tbx1_DataSet_11_154	Inhibit	Endothelial cell
1-day post-SCI group	*Xdh*	Tbx1_DataSet_11_154	Inhibit	Endothelial cell
1-day post-SCI group	*Fos*	Tbx1_DataSet_11_155	Promote	Endothelial cell
1-day post-SCI group	*Rcan1*	Thap11_DataSet_11_167	Inhibit	Div-Myeloid
1-day post-SCI group	*Fos*	Thap11_DataSet_11_168	Promote	Div-Myeloid
1-day post-SCI group	*Id1*	Thap11_DataSet_11_168	Inhibit	Div-Myeloid
1-day post-SCI group	*Ucp2*	Thap11_DataSet_11_168	Promote	Div-Myeloid
1-day post-SCI group	*Hbegf*	Mef2c_DataSet_11_079	Inhibit	Microglia, DC, Pericyte
1-day post-SCI group	*Vcam1*	Mef2c_DataSet_11_079	Promote	Microglia, DC, Pericyte
1-day post-SCI group	*Xdh*	Mef2c_DataSet_11_079	Promote	Microglia, DC, Pericyte
1-day post-SCI group	*Gch1*	Runx2_DataSet_11_119	Inhibit	OPC
1-day post-SCI group	*Hbegf*	Runx2_DataSet_11_119	Promote	OPC
1-day post-SCI group	*Hbegf*	Runx2_DataSet_11_120	Promote	OPC
1-day post-SCI group	*Hspb1*	Runx2_DataSet_11_120	Promote	OPC
1-day post-SCI group	*Mcl1*	Runx2_DataSet_11_120	Inhibit	OPC
1-day post-SCI group	*Hspb1*	Nelfb_DataSet_11_086	Inhibit	Pericyte
1-day post-SCI group	*Il6st*	Nelfb_DataSet_11_086	Promote	Pericyte
1-day post-SCI group	*Vcam1*	Nelfb_DataSet_11_086	Promote	Pericyte
1-day post-SCI group	*Hspb1*	Pbx1_DataSet_11_102	Inhibit	Neuron, OPC, Fibroblast
1-day post-SCI group	*Jun*	Pbx1_DataSet_11_102	Promote	Neuron, OPC, Fibroblast
1-day post-SCI group	*Xdh*	Pbx1_DataSet_11_102	Inhibit	Neuron, OPC, Fibroblast
1-day post-SCI group	*Id1*	Nfix_DataSet_11_091	Promote	Ependymal cell, OPC, Neuron
1-day post-SCI group	*Jun*	Nfix_DataSet_11_091	Inhibit	Ependymal cell, OPC, Neuron
1-day post-SCI group	*Sdc1*	Nfix_DataSet_11_091	Inhibit	Ependymal cell, OPC, Neuron
1-day post-SCI group	*Id1*	Xbp1_DataSet_11_181	Inhibit	Endothelial cell, Neutrophil, Pericyte
1-day post-SCI group	*Rcan1*	Xbp1_DataSet_11_181	Inhibit	Endothelial cell, Neutrophil, Pericyte
1-day post-SCI group	*Sdc1*	Xbp1_DataSet_11_181	Inhibit	Endothelial cell, Neutrophil, Pericyte
1-day post-SCI group	*Jun*	Mafg_DataSet_11_073	Promote	Microglia, Neutrophil, OPC, Oligodendrocyte
1-day post-SCI group	*Ucp2*	Mafg_DataSet_11_073	Promote	Microglia, Neutrophil, OPC, Oligodendrocyte
1-day post-SCI group	*Vcam1*	Mafg_DataSet_11_073	Promote	Microglia, Neutrophil, OPC, Oligodendrocyte
1-day post-SCI group	*Axl*	Cbfb_DataSet_11_017	Promote	Div-Myeloid, Microglia
1-day post-SCI group	*Xdh*	Cbfb_DataSet_11_017	Inhibit	Div-Myeloid, Microglia
1-day post-SCI group	*Axl*	Tcf7_DataSet_11_156	Promote	Lymphocyte
1-day post-SCI group	*Il6st*	Tcf7_DataSet_11_156	Promote	Lymphocyte
1-day post-SCI group	*Fos*	Jarid2_DataSet_11_064	Promote	DC,Monocyte
1-day post-SCI group	*Xdh*	Jarid2_DataSet_11_064	Inhibit	DC,Monocyte
1-day post-SCI group	*Hbegf*	Pax6_DataSet_11_101	Promote	Ependymal cell
1-day post-SCI group	*Ripk1*	Pax6_DataSet_11_101	Inhibit	Ependymal cell

1-day post-SCI group	*Hspb1*	Ets2_DataSet_11_040	Inhibit	Neutrophil
1-day post-SCI group	*Jun*	Ets2_DataSet_11_040	Inhibit	Neutrophil
1-day post-SCI group	*Id1*	Arid1a_DataSet_11_004	Inhibit	Neuron, Pericyte, Pericyte
1-day post-SCI group	*Rcan1*	Arid1a_DataSet_11_004	Promote	Neuron, Pericyte, Pericyte
1-day post-SCI group	*Jun*	Bcl11a_DataSet_11_013	Promote	DC
1-day post-SCI group	*Sdc1*	Bcl11a_DataSet_11_013	Promote	DC
1-day post-SCI group	*Fos*	Sox21_DataSet_11_142	Inhibit	Ependymal cell, OPC
1-day post-SCI group	*Hbegf*	Mef2a_DataSet_11_077	Promote	Microglia, Microphage, Neutrophil, Endothelial cell, Monocyte, Pericyte
1-day post-SCI group	*Jun*	Sall1_DataSet_11_122	Inhibit	Microglia, Ependymal cell
1-day post-SCI group	*Sdc1*	Id2_DataSet_11_059	Inhibit	Microphage, OPC, Microglia, Monocyte, Astrocyte
1-day post-SCI group	*Xdh*	Atf3_DataSet_11_006	Inhibit	Microglia, Ependymal cell, Microphage
1-day post-SCI group	*Xdh*	Sox9_DataSet_11_144	Inhibit	Ependymal cell, Astrocyte
3-day post-SCI group	*Amph*	Smad4_DataSet_11_127	Inhibit	Pericyte
3-day post-SCI group	*Axl*	Smad4_DataSet_11_127	Inhibit	Pericyte
3-day post-SCI group	*Fkbp1b*	Smad4_DataSet_11_127	Inhibit	Pericyte
3-day post-SCI group	*Fos*	Smad4_DataSet_11_127	Inhibit	Pericyte
3-day post-SCI group	*Gch1*	Smad4_DataSet_11_127	Promote	Pericyte
3-day post-SCI group	*Hbegf*	Smad4_DataSet_11_127	Promote	Pericyte
3-day post-SCI group	*Hdac1*	Smad4_DataSet_11_127	Inhibit	Pericyte
3-day post-SCI group	*Hspb1*	Smad4_DataSet_11_127	Inhibit	Pericyte
3-day post-SCI group	*Id1*	Smad4_DataSet_11_127	Inhibit	Pericyte
3-day post-SCI group	*Il6st*	Smad4_DataSet_11_127	Inhibit	Pericyte
3-day post-SCI group	*Jun*	Smad4_DataSet_11_127	Promote	Pericyte
3-day post-SCI group	*Map2k3*	Smad4_DataSet_11_127	Promote	Pericyte
3-day post-SCI group	*Mcl1*	Smad4_DataSet_11_127	Promote	Pericyte
3-day post-SCI group	*Rcan1*	Smad4_DataSet_11_127	Promote	Pericyte
3-day post-SCI group	*Ripk1*	Smad4_DataSet_11_127	Promote	Pericyte
3-day post-SCI group	*Sdc1*	Smad4_DataSet_11_127	Inhibit	Pericyte
3-day post-SCI group	*Stat6*	Smad4_DataSet_11_127	Promote	Pericyte
3-day post-SCI group	*Ucp2*	Smad4_DataSet_11_127	Promote	Pericyte
3-day post-SCI group	*Vcam1*	Smad4_DataSet_11_127	Inhibit	Pericyte
3-day post-SCI group	*Xdh*	Smad4_DataSet_11_127	Inhibit	Pericyte
3-day post-SCI group	*Amph*	Smad4_DataSet_11_128	Inhibit	Pericyte
3-day post-SCI group	*Cbx6*	Smad4_DataSet_11_128	Promote	Pericyte
3-day post-SCI group	*Fkbp1b*	Smad4_DataSet_11_128	Inhibit	Pericyte
3-day post-SCI group	*Gch1*	Smad4_DataSet_11_128	Inhibit	Pericyte
3-day post-SCI group	*Hbegf*	Smad4_DataSet_11_128	Promote	Pericyte
3-day post-SCI group	*Hspb1*	Smad4_DataSet_11_128	Promote	Pericyte
3-day post-SCI group	*Id1*	Smad4_DataSet_11_128	Promote	Pericyte

3-day post-SCI group	*Jun*	Smad4_DataSet_11_128	Promote	Pericyte
3-day post-SCI group	*Ppp3ca*	Smad4_DataSet_11_128	Inhibit	Pericyte
3-day post-SCI group	*Sdc1*	Smad4_DataSet_11_128	Inhibit	Pericyte
3-day post-SCI group	*Ucp2*	Smad4_DataSet_11_128	Inhibit	Pericyte
3-day post-SCI group	*Vcam1*	Smad4_DataSet_11_128	Inhibit	Pericyte
3-day post-SCI group	*Xdh*	Smad4_DataSet_11_128	Inhibit	Pericyte
3-day post-SCI group	*Amph*	Stat6_DataSet_11_153	Inhibit	Div-Myeloid
3-day post-SCI group	*Axl*	Stat6_DataSet_11_153	Inhibit	Div-Myeloid
3-day post-SCI group	*Fbxw7*	Stat6_DataSet_11_153	Promote	Div-Myeloid
3-day post-SCI group	*Hbegf*	Stat6_DataSet_11_153	Promote	Div-Myeloid
3-day post-SCI group	*Id1*	Stat6_DataSet_11_153	Inhibit	Div-Myeloid
3-day post-SCI group	*Il6st*	Stat6_DataSet_11_153	Promote	Div-Myeloid
3-day post-SCI group	*Jun*	Stat6_DataSet_11_153	Promote	Div-Myeloid
3-day post-SCI group	*Sdc1*	Stat6_DataSet_11_153	Promote	Div-Myeloid
3-day post-SCI group	*Stat6*	Stat6_DataSet_11_153	Promote	Div-Myeloid
3-day post-SCI group	*Vcam1*	Stat6_DataSet_11_153	Inhibit	Div-Myeloid
3-day post-SCI group	*Xdh*	Stat6_DataSet_11_153	Promote	Div-Myeloid
3-day post-SCI group	*Cbx6*	Cebpe_DataSet_11_023	Promote	Neutrophil
3-day post-SCI group	*Fos*	Cebpe_DataSet_11_023	Inhibit	Neutrophil
3-day post-SCI group	*Hbegf*	Cebpe_DataSet_11_023	Inhibit	Neutrophil
3-day post-SCI group	*Hdac1*	Cebpe_DataSet_11_023	Promote	Neutrophil
3-day post-SCI group	*Hspb1*	Cebpe_DataSet_11_023	Inhibit	Neutrophil
3-day post-SCI group	*Id1*	Cebpe_DataSet_11_023	Inhibit	Neutrophil
3-day post-SCI group	*Map2k4*	Cebpe_DataSet_11_023	Promote	Neutrophil
3-day post-SCI group	*Sdc1*	Cebpe_DataSet_11_023	Promote	Neutrophil
3-day post-SCI group	*Ucp2*	Cebpe_DataSet_11_023	Inhibit	Neutrophil
3-day post-SCI group	*Xdh*	Cebpe_DataSet_11_023	Inhibit	Neutrophil
3-day post-SCI group	*Axl*	Mbd2_DataSet_11_076	Inhibit	Div-Myeloid, Pericyte
3-day post-SCI group	*Fos*	Mbd2_DataSet_11_076	Promote	Div-Myeloid, Pericyte
3-day post-SCI group	*Gch1*	Mbd2_DataSet_11_076	Inhibit	Div-Myeloid, Pericyte
3-day post-SCI group	*Hbegf*	Mbd2_DataSet_11_076	Inhibit	Div-Myeloid, Pericyte
3-day post-SCI group	*Jun*	Mbd2_DataSet_11_076	Inhibit	Div-Myeloid, Pericyte
3-day post-SCI group	*Ppp3ca*	Mbd2_DataSet_11_076	Inhibit	Div-Myeloid, Pericyte
3-day post-SCI group	*Rcan1*	Mbd2_DataSet_11_076	Promote	Div-Myeloid, Pericyte
3-day post-SCI group	*Sdc1*	Mbd2_DataSet_11_076	Promote	Div-Myeloid, Pericyte
3-day post-SCI group	*Xdh*	Mbd2_DataSet_11_076	Inhibit	Div-Myeloid, Pericyte
3-day post-SCI group	*Axl*	Tsc22d4_DataSet_11_169	Promote	Ependymal cell, OPC, Oligodendrocyte,Astrocyte
3-day post-SCI group	*Fbxw7*	Tsc22d4_DataSet_11_169	Inhibit	Ependymal cell, OPC, Oligodendrocyte,Astrocyte
3-day post-SCI group	*Fos*	Tsc22d4_DataSet_11_169	Promote	Ependymal cell, OPC, Oligodendrocyte,Astrocyte

3-day post-SCI group	*Hbegf*	Tsc22d4_DataSet_11_169	Promote	Ependymal cell, OPC, Oligodendrocyte,Astrocyte
3-day post-SCI group	*Id1*	Tsc22d4_DataSet_11_169	Promote	Ependymal cell, OPC, Oligodendrocyte,Astrocyte
3-day post-SCI group	*Jun*	Tsc22d4_DataSet_11_169	Promote	Ependymal cell, OPC, Oligodendrocyte,Astrocyte
3-day post-SCI group	*Rcan1*	Tsc22d4_DataSet_11_169	Promote	Ependymal cell, OPC, Oligodendrocyte,Astrocyte
3-day post-SCI group	*Vcam1*	Tsc22d4_DataSet_11_169	Promote	Ependymal cell, OPC, Oligodendrocyte,Astrocyte
3-day post-SCI group	*Xdh*	Tsc22d4_DataSet_11_169	Promote	Ependymal cell, OPC, Oligodendrocyte,Astrocyte
3-day post-SCI group	*Amph*	Cebpb_DataSet_11_020	Promote	Neutrophil, Monocyte, Microphage, Microglia, Div-Myeloid
3-day post-SCI group	*Fkbp1b*	Cebpb_DataSet_11_020	Promote	Neutrophil, Monocyte, Microphage, Microglia, Div-Myeloid
3-day post-SCI group	*Id1*	Cebpb_DataSet_11_020	Inhibit	Neutrophil, Monocyte, Microphage, Microglia, Div-Myeloid
3-day post-SCI group	*Vcam1*	Cebpb_DataSet_11_020	Promote	Neutrophil, Monocyte, Microphage, Microglia, Div-Myeloid
3-day post-SCI group	*Xdh*	Cebpb_DataSet_11_020	Promote	Neutrophil, Monocyte, Microphage, Microglia, Div-Myeloid
3-day post-SCI group	*Fos*	Cebpb_DataSet_11_021	Inhibit	Neutrophil, Monocyte, Microphage, Microglia, Div-Myeloid
3-day post-SCI group	*Hbegf*	Cebpb_DataSet_11_021	Inhibit	Neutrophil, Monocyte, Microphage, Microglia, Div-Myeloid
3-day post-SCI group	*Hdac1*	Cebpb_DataSet_11_021	Promote	Neutrophil, Monocyte, Microphage, Microglia, Div-Myeloid
3-day post-SCI group	*Hspb1*	Cebpb_DataSet_11_021	Inhibit	Neutrophil, Monocyte, Microphage, Microglia, Div-Myeloid
3-day post-SCI group	*Id1*	Cebpb_DataSet_11_021	Inhibit	Neutrophil, Monocyte, Microphage, Microglia, Div-Myeloid
3-day post-SCI group	*Map2k4*	Cebpb_DataSet_11_021	Promote	Neutrophil, Monocyte, Microphage, Microglia, Div-Myeloid
3-day post-SCI group	*Ucp2*	Cebpb_DataSet_11_021	Inhibit	Neutrophil, Monocyte, Microphage, Microglia, Div-Myeloid
3-day post-SCI group	*Vcam1*	Cebpb_DataSet_11_021	Inhibit	Neutrophil, Monocyte, Microphage, Microglia, Div-Myeloid
3-day post-SCI group	*Xdh*	Cebpb_DataSet_11_021	Inhibit	Neutrophil, Monocyte, Microphage, Microglia, Div-Myeloid
3-day post-SCI group	*Fos*	Cebpb_DataSet_11_022	Promote	Neutrophil, Monocyte, Microphage, Microglia, Div-Myeloid
3-day post-SCI group	*Gch1*	Cebpb_DataSet_11_022	Inhibit	Neutrophil, Monocyte, Microphage, Microglia, Div-Myeloid
3-day post-SCI group	*Id1*	Cebpb_DataSet_11_022	Promote	Neutrophil, Monocyte, Microphage, Microglia, Div-Myeloid
3-day post-SCI group	*Jun*	Cebpb_DataSet_11_022	Promote	Neutrophil, Monocyte, Microphage, Microglia, Div-Myeloid
3-day post-SCI group	*Map2k3*	Cebpb_DataSet_11_022	Promote	Neutrophil, Monocyte, Microphage, Microglia, Div-Myeloid
3-day post-SCI group	*Amph*	Runx1_DataSet_11_114	Inhibit	DC,Microglia, Neutrophil, Div-Myeloid
3-day post-SCI group	*Fos*	Runx1_DataSet_11_114	Promote	DC,Microglia, Neutrophil, Div-Myeloid
3-day post-SCI group	*Hspb1*	Runx1_DataSet_11_114	Promote	DC,Microglia, Neutrophil, Div-Myeloid
3-day post-SCI group	*Map2k3*	Runx1_DataSet_11_114	Promote	DC,Microglia, Neutrophil, Div-Myeloid
3-day post-SCI group	*Sdc1*	Runx1_DataSet_11_114	Promote	DC,Microglia, Neutrophil, Div-Myeloid
3-day post-SCI group	*Fkbp1b*	Runx1_DataSet_11_115	Inhibit	DC,Microglia, Neutrophil, Div-Myeloid
3-day post-SCI group	*Fos*	Runx1_DataSet_11_115	Inhibit	DC,Microglia, Neutrophil, Div-Myeloid
3-day post-SCI group	*Gch1*	Runx1_DataSet_11_115	Inhibit	DC,Microglia, Neutrophil, Div-Myeloid
3-day post-SCI group	*Jun*	Runx1_DataSet_11_115	Inhibit	DC,Microglia, Neutrophil, Div-Myeloid
3-day post-SCI group	*Sdc1*	Runx1_DataSet_11_115	Inhibit	DC,Microglia, Neutrophil, Div-Myeloid
3-day post-SCI group	*Ucp2*	Runx1_DataSet_11_115	Inhibit	DC,Microglia, Neutrophil, Div-Myeloid
3-day post-SCI group	*Fos*	Runx1_DataSet_11_116	Promote	DC,Microglia, Neutrophil, Div-Myeloid
3-day post-SCI group	*Gch1*	Runx1_DataSet_11_116	Inhibit	DC,Microglia, Neutrophil, Div-Myeloid
3-day post-SCI group	*Hbegf*	Runx1_DataSet_11_116	Inhibit	DC,Microglia, Neutrophil, Div-Myeloid

3-day post-SCI group	*Il6st*	Runx1_DataSet_11_116	Inhibit	DC,Microglia, Neutrophil, Div-Myeloid
3-day post-SCI group	*Jun*	Runx1_DataSet_11_116	Promote	DC,Microglia, Neutrophil, Div-Myeloid
3-day post-SCI group	*Ppp3ca*	Runx1_DataSet_11_116	Inhibit	DC,Microglia, Neutrophil, Div-Myeloid
3-day post-SCI group	*Sdc1*	Runx1_DataSet_11_116	Promote	DC,Microglia, Neutrophil, Div-Myeloid
3-day post-SCI group	*Tnfrsf1a*	Runx1_DataSet_11_116	Inhibit	DC,Microglia, Neutrophil, Div-Myeloid
3-day post-SCI group	*Xdh*	Runx1_DataSet_11_116	Inhibit	DC,Microglia, Neutrophil, Div-Myeloid
3-day post-SCI group	*Amph*	Runx1_DataSet_11_117	Inhibit	DC,Microglia, Neutrophil, Div-Myeloid
3-day post-SCI group	*Id1*	Runx1_DataSet_11_117	Inhibit	DC,Microglia, Neutrophil, Div-Myeloid
3-day post-SCI group	*Amph*	Nr2f2_DataSet_11_094	Promote	Pericyte, Fibroblast
3-day post-SCI group	*Hspb1*	Nr2f2_DataSet_11_094	Inhibit	Pericyte, Fibroblast
3-day post-SCI group	*Vcam1*	Nr2f2_DataSet_11_094	Inhibit	Pericyte, Fibroblast
3-day post-SCI group	*Amph*	Nr2f2_DataSet_11_095	Promote	Pericyte, Fibroblast
3-day post-SCI group	*Fos*	Nr2f2_DataSet_11_095	Inhibit	Pericyte, Fibroblast
3-day post-SCI group	*Hspb1*	Nr2f2_DataSet_11_095	Inhibit	Pericyte, Fibroblast
3-day post-SCI group	*Id1*	Nr2f2_DataSet_11_095	Inhibit	Pericyte, Fibroblast
3-day post-SCI group	*Tnfrsf1a*	Nr2f2_DataSet_11_095	Inhibit	Pericyte, Fibroblast
3-day post-SCI group	*Ucp2*	Nr2f2_DataSet_11_095	Inhibit	Pericyte, Fibroblast
3-day post-SCI group	*Vcam1*	Nr2f2_DataSet_11_095	Inhibit	Pericyte, Fibroblast
3-day post-SCI group	*Xdh*	Nr2f2_DataSet_11_095	Inhibit	Pericyte, Fibroblast
3-day post-SCI group	*Axl*	Irf8_DataSet_11_063	Promote	Microglia, Div-Myeloid, DC, Monocyte, Lymphocyte
3-day post-SCI group	*Gch1*	Irf8_DataSet_11_063	Promote	Microglia, Div-Myeloid, DC, Monocyte, Lymphocyte
3-day post-SCI group	*Id1*	Irf8_DataSet_11_063	Promote	Microglia, Div-Myeloid, DC, Monocyte, Lymphocyte
3-day post-SCI group	*Jun*	Irf8_DataSet_11_063	Inhibit	Microglia, Div-Myeloid, DC, Monocyte, Lymphocyte
3-day post-SCI group	*Map2k3*	Irf8_DataSet_11_063	Promote	Microglia, Div-Myeloid, DC, Monocyte, Lymphocyte
3-day post-SCI group	*Mcl1*	Irf8_DataSet_11_063	Promote	Microglia, Div-Myeloid, DC, Monocyte, Lymphocyte
3-day post-SCI group	*Vcam1*	Irf8_DataSet_11_063	Promote	Microglia, Div-Myeloid, DC, Monocyte, Lymphocyte
3-day post-SCI group	*Axl*	Meis1_DataSet_11_081	Promote	Ependymal cell
3-day post-SCI group	*Fbxw7*	Meis1_DataSet_11_081	Promote	Ependymal cell
3-day post-SCI group	*Fkbp1b*	Meis1_DataSet_11_081	Inhibit	Ependymal cell
3-day post-SCI group	*Hspb1*	Meis1_DataSet_11_081	Inhibit	Ependymal cell
3-day post-SCI group	*Il6st*	Meis1_DataSet_11_081	Promote	Ependymal cell
3-day post-SCI group	*Sdc1*	Meis1_DataSet_11_081	Promote	Ependymal cell
3-day post-SCI group	*Vcam1*	Meis1_DataSet_11_081	Promote	Ependymal cell
3-day post-SCI group	*Fos*	Meis1_DataSet_11_082	Inhibit	Ependymal cell
3-day post-SCI group	*Gch1*	Meis1_DataSet_11_082	Inhibit	Ependymal cell
3-day post-SCI group	*Id1*	Meis1_DataSet_11_082	Promote	Ependymal cell
3-day post-SCI group	*Il6st*	Meis1_DataSet_11_082	Inhibit	Ependymal cell
3-day post-SCI group	*Sdc1*	Meis1_DataSet_11_082	Promote	Ependymal cell
3-day post-SCI group	*Vcam1*	Meis1_DataSet_11_082	Inhibit	Ependymal cell

3-day post-SCI group	*Amph*	Prox1_DataSet_11_109	Promote	Oligodendrocyte
3-day post-SCI group	*Hbegf*	Prox1_DataSet_11_109	Inhibit	Oligodendrocyte
3-day post-SCI group	*Hspb1*	Prox1_DataSet_11_109	Inhibit	Oligodendrocyte
3-day post-SCI group	*Jun*	Prox1_DataSet_11_109	Inhibit	Oligodendrocyte
3-day post-SCI group	*Vcam1*	Prox1_DataSet_11_109	Promote	Oligodendrocyte
3-day post-SCI group	*Xdh*	Prox1_DataSet_11_109	Inhibit	Oligodendrocyte
3-day post-SCI group	*Fos*	Tet2_DataSet_11_159	Promote	Astrocyte
3-day post-SCI group	*Gch1*	Tet2_DataSet_11_159	Inhibit	Astrocyte
3-day post-SCI group	*Vcam1*	Tet2_DataSet_11_159	Inhibit	Astrocyte
3-day post-SCI group	*Xdh*	Tet2_DataSet_11_159	Inhibit	Astrocyte
3-day post-SCI group	*Fos*	Tet2_DataSet_11_161	Inhibit	Astrocyte
3-day post-SCI group	*Gch1*	Tet2_DataSet_11_161	Inhibit	Astrocyte
3-day post-SCI group	*Hspb1*	Tet2_DataSet_11_161	Inhibit	Astrocyte
3-day post-SCI group	*Map2k3*	Tet2_DataSet_11_161	Inhibit	Astrocyte
3-day post-SCI group	*Fos*	Tet2_DataSet_11_162	Inhibit	Astrocyte
3-day post-SCI group	*Axl*	Tet2_DataSet_11_163	Inhibit	Astrocyte
3-day post-SCI group	*Fbxw7*	Tet2_DataSet_11_163	Promote	Astrocyte
3-day post-SCI group	*Fos*	Tet2_DataSet_11_163	Inhibit	Astrocyte
3-day post-SCI group	*Mcl1*	Tet2_DataSet_11_163	Inhibit	Astrocyte
3-day post-SCI group	*Ppp3ca*	Tet2_DataSet_11_163	Promote	Astrocyte
3-day post-SCI group	*Sdc1*	Tet2_DataSet_11_163	Inhibit	Astrocyte
3-day post-SCI group	*Cbx6*	E2f4_DataSet_11_033	Promote	Lymphocyte, Div-Myeloid
3-day post-SCI group	*Fkbp1b*	E2f4_DataSet_11_033	Promote	Lymphocyte, Div-Myeloid
3-day post-SCI group	*Fos*	E2f4_DataSet_11_033	Inhibit	Lymphocyte, Div-Myeloid
3-day post-SCI group	*Ripk1*	E2f4_DataSet_11_033	Promote	Lymphocyte, Div-Myeloid
3-day post-SCI group	*Vcam1*	E2f4_DataSet_11_033	Inhibit	Lymphocyte, Div-Myeloid
3-day post-SCI group	*Xdh*	E2f4_DataSet_11_033	Inhibit	Lymphocyte, Div-Myeloid
3-day post-SCI group	*Il6st*	E2f4_DataSet_11_034	Promote	Lymphocyte, Div-Myeloid
3-day post-SCI group	*Amph*	Prdm2_DataSet_11_108	Inhibit	Pericyte
3-day post-SCI group	*Fkbp1b*	Prdm2_DataSet_11_108	Promote	Pericyte
3-day post-SCI group	*Fos*	Prdm2_DataSet_11_108	Inhibit	Pericyte
3-day post-SCI group	*Vcam1*	Prdm2_DataSet_11_108	Inhibit	Pericyte
3-day post-SCI group	*Xdh*	Prdm2_DataSet_11_108	Inhibit	Pericyte
3-day post-SCI group	*Amph*	Snai1_DataSet_11_129	Inhibit	Fibroblast
3-day post-SCI group	*Hbegf*	Snai1_DataSet_11_129	Inhibit	Fibroblast
3-day post-SCI group	*Vcam1*	Snai1_DataSet_11_129	Promote	Fibroblast
3-day post-SCI group	*Amph*	Snai1_DataSet_11_130	Inhibit	Fibroblast
3-day post-SCI group	*Fos*	Snai1_DataSet_11_130	Inhibit	Fibroblast
3-day post-SCI group	*Hbegf*	Snai1_DataSet_11_130	Inhibit	Fibroblast

3-day post-SCI group	*Hspb1*	Snai1_DataSet_11_130	Inhibit	Fibroblast
3-day post-SCI group	*Ucp2*	Snai1_DataSet_11_130	Inhibit	Fibroblast
3-day post-SCI group	*Id1*	Snai1_DataSet_11_131	Inhibit	Fibroblast
3-day post-SCI group	*Ppp3ca*	Snai1_DataSet_11_131	Promote	Fibroblast
3-day post-SCI group	*Ucp2*	Snai1_DataSet_11_131	Inhibit	Fibroblast
3-day post-SCI group	*Fos*	Snai1_DataSet_11_132	Promote	Fibroblast
3-day post-SCI group	*Hspb1*	Snai1_DataSet_11_132	Inhibit	Fibroblast
3-day post-SCI group	*Fbxw7*	Sox17_DataSet_11_139	Inhibit	Endothelial cell, Pericyte
3-day post-SCI group	*Fos*	Sox17_DataSet_11_139	Inhibit	Endothelial cell, Pericyte
3-day post-SCI group	*Hbegf*	Sox17_DataSet_11_139	Inhibit	Endothelial cell, Pericyte
3-day post-SCI group	*Hspb1*	Sox17_DataSet_11_139	Inhibit	Endothelial cell, Pericyte
3-day post-SCI group	*Id1*	Sox17_DataSet_11_139	Inhibit	Endothelial cell, Pericyte
3-day post-SCI group	*Fos*	Foxp1_DataSet_11_044	Inhibit	Endothelial cell, Pericyte, Fibroblast
3-day post-SCI group	*Gch1*	Foxp1_DataSet_11_044	Promote	Endothelial cell, Pericyte, Fibroblast
3-day post-SCI group	*Stat6*	Foxp1_DataSet_11_044	Promote	Endothelial cell, Pericyte, Fibroblast
3-day post-SCI group	*Vcam1*	Foxp1_DataSet_11_044	Promote	Endothelial cell, Pericyte, Fibroblast
3-day post-SCI group	*Xdh*	Foxp1_DataSet_11_044	Promote	Endothelial cell, Pericyte, Fibroblast
3-day post-SCI group	*Hdac1*	Nfil3_DataSet_11_090	Inhibit	Lymphocyte
3-day post-SCI group	*Mcl1*	Nfil3_DataSet_11_090	Inhibit	Lymphocyte
3-day post-SCI group	*Sdc1*	Nfil3_DataSet_11_090	Inhibit	Lymphocyte
3-day post-SCI group	*Stat6*	Nfil3_DataSet_11_090	Inhibit	Lymphocyte
3-day post-SCI group	*Xdh*	Nfil3_DataSet_11_090	Inhibit	Lymphocyte
3-day post-SCI group	*Amph*	Gata2_DataSet_11_047	Promote	Endothelial cell, Pericyte
3-day post-SCI group	*Axl*	Gata2_DataSet_11_047	Promote	Endothelial cell, Pericyte
3-day post-SCI group	*Id1*	Gata2_DataSet_11_047	Inhibit	Endothelial cell, Pericyte
3-day post-SCI group	*Vcam1*	Gata2_DataSet_11_047	Promote	Endothelial cell, Pericyte
3-day post-SCI group	*Amph*	Stat3_DataSet_11_151	Inhibit	Ependymal cell, DC, Pericyte
3-day post-SCI group	*Axl*	Stat3_DataSet_11_151	Inhibit	Ependymal cell, DC, Pericyte
3-day post-SCI group	*Fos*	Stat3_DataSet_11_151	Promote	Ependymal cell, DC, Pericyte
3-day post-SCI group	*Gch1*	Stat3_DataSet_11_151	Inhibit	Ependymal cell, DC, Pericyte
3-day post-SCI group	*Axl*	Cebpa_DataSet_11_019	Promote	Microglia, Div-Myeloid, Monocyte, Microphage
3-day post-SCI group	*Gch1*	Cebpa_DataSet_11_019	Promote	Microglia, Div-Myeloid, Monocyte, Microphage
3-day post-SCI group	*Vcam1*	Cebpa_DataSet_11_019	Inhibit	Microglia, Div-Myeloid, Monocyte, Microphage
3-day post-SCI group	*Xdh*	Cebpa_DataSet_11_019	Inhibit	Microglia, Div-Myeloid, Monocyte, Microphage
3-day post-SCI group	*Axl*	Sox11_DataSet_11_137	Inhibit	OPC, Neuron
3-day post-SCI group	*Stat6*	Sox11_DataSet_11_137	Inhibit	OPC, Neuron
3-day post-SCI group	*Vcam1*	Sox11_DataSet_11_137	Inhibit	OPC, Neuron
3-day post-SCI group	*Xdh*	Sox11_DataSet_11_137	Inhibit	OPC, Neuron
3-day post-SCI group	*Hbegf*	Sox11_DataSet_11_138	Inhibit	OPC, Neuron

3-day post-SCI group	*Fbxw7*	Smad1_DataSet_11_126	Promote	Endothelial cell, Ependymal cell, Neuron, Pericyte
3-day post-SCI group	*Hspb1*	Smad1_DataSet_11_126	Promote	Endothelial cell, Ependymal cell, Neuron, Pericyte
3-day post-SCI group	*Jun*	Smad1_DataSet_11_126	Promote	Endothelial cell, Ependymal cell, Neuron, Pericyte
3-day post-SCI group	*Rcan1*	Smad1_DataSet_11_126	Promote	Endothelial cell, Ependymal cell, Neuron, Pericyte
3-day post-SCI group	*Tnfrsf1a*	Sox2_DataSet_11_140	Inhibit	Ependymal cell, Astrocyte
3-day post-SCI group	*Fos*	Sox2_DataSet_11_141	Inhibit	Ependymal cell, Astrocyte
3-day post-SCI group	*Jun*	Sox2_DataSet_11_141	Inhibit	Ependymal cell, Astrocyte
3-day post-SCI group	*Rcan1*	Sox2_DataSet_11_141	Inhibit	Ependymal cell, Astrocyte
3-day post-SCI group	*Tnfrsf1a*	Sox2_DataSet_11_141	Inhibit	Ependymal cell, Astrocyte
3-day post-SCI group	*Axl*	Sox10_DataSet_11_136	Inhibit	OPC, Oligodendrocyte
3-day post-SCI group	*Mcl1*	Sox10_DataSet_11_136	Promote	OPC, Oligodendrocyte
3-day post-SCI group	*Ppp3ca*	Sox10_DataSet_11_136	Promote	OPC, Oligodendrocyte
3-day post-SCI group	*Hdac1*	Rest_DataSet_11_110	Inhibit	DC
3-day post-SCI group	*Xdh*	Rest_DataSet_11_112	Promote	DC
3-day post-SCI group	*Fkbp1b*	Rest_DataSet_11_113	Inhibit	DC
3-day post-SCI group	*Stat6*	Rest_DataSet_11_113	Inhibit	DC
3-day post-SCI group	*Xdh*	Rest_DataSet_11_113	Inhibit	DC
3-day post-SCI group	*Fos*	Erg_DataSet_11_038	Promote	Endothelial cell
3-day post-SCI group	*Jun*	Erg_DataSet_11_038	Promote	Endothelial cell
3-day post-SCI group	*Sdc1*	Erg_DataSet_11_038	Inhibit	Endothelial cell
3-day post-SCI group	*Fos*	Foxq1_DataSet_11_045	Inhibit	Endothelial cell, Pericyte
3-day post-SCI group	*Gch1*	Foxq1_DataSet_11_045	Inhibit	Endothelial cell, Pericyte
3-day post-SCI group	*Sdc1*	Foxq1_DataSet_11_045	Inhibit	Endothelial cell, Pericyte
3-day post-SCI group	*Id1*	Klf6_DataSet_11_067	Promote	Endothelial cell, Ependymal cell, Microglia, Pericyte, Fibroblast, Lymphocyte
3-day post-SCI group	*Xdh*	Klf6_DataSet_11_067	Inhibit	Endothelial cell, Ependymal cell, Microglia, Pericyte, Fibroblast, Lymphocyte
3-day post-SCI group	*Fos*	Klf6_DataSet_11_068	Inhibit	Endothelial cell, Ependymal cell, Microglia, Pericyte, Fibroblast, Lymphocyte
3-day post-SCI group	*Hbegf*	Klf6_DataSet_11_068	Inhibit	Endothelial cell, Ependymal cell, Microglia, Pericyte, Fibroblast, Lymphocyte
3-day post-SCI group	*Vcam1*	Klf6_DataSet_11_068	Inhibit	Endothelial cell, Ependymal cell, Microglia, Pericyte, Fibroblast, Lymphocyte
3-day post-SCI group	*Fos*	Tbx1_DataSet_11_154	Inhibit	Endothelial cell, Pericyte
3-day post-SCI group	*Vcam1*	Tbx1_DataSet_11_154	Inhibit	Endothelial cell, Pericyte
3-day post-SCI group	*Xdh*	Tbx1_DataSet_11_154	Inhibit	Endothelial cell, Pericyte
3-day post-SCI group	*Fos*	Tbx1_DataSet_11_155	Promote	Endothelial cell, Pericyte
3-day post-SCI group	*Rcan1*	Thap11_DataSet_11_167	Inhibit	Neuron, OPC, Pericyte
3-day post-SCI group	*Fos*	Thap11_DataSet_11_168	Promote	Neuron, OPC, Pericyte

3-day post-SCI group	*Id1*	Thap11_DataSet_11_168	Inhibit	Neuron, OPC, Pericyte
3-day post-SCI group	*Ucp2*	Thap11_DataSet_11_168	Promote	Neuron, OPC, Pericyte
3-day post-SCI group	*Hbegf*	Mef2c_DataSet_11_079	Inhibit	Endothelial cell, Microglia, Pericyte
3-day post-SCI group	*Vcam1*	Mef2c_DataSet_11_079	Promote	Endothelial cell, Microglia, Pericyte
3-day post-SCI group	*Xdh*	Mef2c_DataSet_11_079	Promote	Endothelial cell, Microglia, Pericyte
3-day post-SCI group	*Hspb1*	Nelfb_DataSet_11_086	Inhibit	Endothelial cell
3-day post-SCI group	*Il6st*	Nelfb_DataSet_11_086	Promote	Endothelial cell
3-day post-SCI group	*Vcam1*	Nelfb_DataSet_11_086	Promote	Endothelial cell
3-day post-SCI group	*Hspb1*	Pbx1_DataSet_11_102	Inhibit	Ependymal cell, Fibroblast, Neuron, Astrocyte
3-day post-SCI group	*Jun*	Pbx1_DataSet_11_102	Promote	Ependymal cell, Fibroblast, Neuron, Astrocyte
3-day post-SCI group	*Xdh*	Pbx1_DataSet_11_102	Inhibit	Ependymal cell, Fibroblast, Neuron, Astrocyte
3-day post-SCI group	*Id1*	Nfix_DataSet_11_091	Promote	Ependymal cell, OPC, Oligodendrocyte, Astrocyte
3-day post-SCI group	*Jun*	Nfix_DataSet_11_091	Inhibit	Ependymal cell, OPC, Oligodendrocyte, Astrocyte
3-day post-SCI group	*Sdc1*	Nfix_DataSet_11_091	Inhibit	Ependymal cell, OPC, Oligodendrocyte, Astrocyte
3-day post-SCI group	*Id1*	Xbp1_DataSet_11_181	Inhibit	Endothelial cell, Neutrophil, Pericyte, Fibroblast
3-day post-SCI group	*Rcan1*	Xbp1_DataSet_11_181	Inhibit	Endothelial cell, Neutrophil, Pericyte, Fibroblast
3-day post-SCI group	*Sdc1*	Xbp1_DataSet_11_181	Inhibit	Endothelial cell, Neutrophil, Pericyte, Fibroblast
3-day post-SCI group	*Jun*	Mafg_DataSet_11_073	Promote	Microglia, Endothelial cell, Monocyte, Microphage, Pericyte
3-day post-SCI group	*Ucp2*	Mafg_DataSet_11_073	Promote	Microglia, Endothelial cell, Monocyte, Microphage, Pericyte
3-day post-SCI group	*Vcam1*	Mafg_DataSet_11_073	Promote	Microglia, Endothelial cell, Monocyte, Microphage, Pericyte
3-day post-SCI group	*Axl*	Cbfb_DataSet_11_017	Promote	Div-Myeloid, Lymphocyte
3-day post-SCI group	*Xdh*	Cbfb_DataSet_11_017	Inhibit	Div-Myeloid, Lymphocyte
3-day post-SCI group	*Axl*	Tcf7_DataSet_11_156	Promote	Lymphocyte, Endothelial cell
3-day post-SCI group	*Il6st*	Tcf7_DataSet_11_156	Promote	Lymphocyte, Endothelial cell
3-day post-SCI group	*Fos*	Jarid2_DataSet_11_064	Promote	DC
3-day post-SCI group	*Xdh*	Jarid2_DataSet_11_064	Inhibit	DC
3-day post-SCI group	*Hbegf*	Pax6_DataSet_11_101	Promote	Ependymal cell
3-day post-SCI group	*Ripk1*	Pax6_DataSet_11_101	Inhibit	Ependymal cell
3-day post-SCI group	*Hspb1*	Ets2_DataSet_11_040	Inhibit	Neutrophil
3-day post-SCI group	*Jun*	Ets2_DataSet_11_040	Inhibit	Neutrophil
3-day post-SCI group	*Id1*	Arid1a_DataSet_11_004	Inhibit	Neuron, Pericyte, Astrocyte, Lymphocyte
3-day post-SCI group	*Rcan1*	Arid1a_DataSet_11_004	Promote	Neuron, Pericyte, Astrocyte, Lymphocyte
3-day post-SCI group	*Jun*	Bcl11a_DataSet_11_013	Promote	DC, Neuron
3-day post-SCI group	*Sdc1*	Bcl11a_DataSet_11_013	Promote	DC, Neuron
3-day post-SCI group	*Fos*	Sox21_DataSet_11_142	Inhibit	Ependymal cell, OPC
3-day post-SCI group	*Hbegf*	Mef2a_DataSet_11_077	Promote	Microglia, Microphage, Neutrophil, Endothelial cell, Monocyte, Pericyte, Div-Myeloid
3-day post-SCI group	*Jun*	Sall1_DataSet_11_122	Inhibit	Microglia, Ependymal cell
3-day post-SCI group	*Sdc1*	Id2_DataSet_11_059	Inhibit	Microphage, Div-Myeloid, Microglia, Astrocyte

3-day post-SCI group	*Xdh*	Atf3_DataSet_11_006	Inhibit	Microglia, Ependymal cell, Endothelial cell
3-day post-SCI group	*Xdh*	Sox9_DataSet_11_144	Inhibit	Ependymal cell
7-day post-SCI group	*Amph*	Inhibit	Smad4_DataSet_11_127	Pericyte, Fibroblast
7-day post-SCI group	*Axl*	Inhibit	Smad4_DataSet_11_127	Pericyte, Fibroblast
7-day post-SCI group	*Fkbp1b*	Inhibit	Smad4_DataSet_11_127	Pericyte, Fibroblast
7-day post-SCI group	*Fos*	Inhibit	Smad4_DataSet_11_127	Pericyte, Fibroblast
7-day post-SCI group	*Gch1*	Promote	Smad4_DataSet_11_127	Pericyte, Fibroblast
7-day post-SCI group	*Hbegf*	Promote	Smad4_DataSet_11_127	Pericyte, Fibroblast
7-day post-SCI group	*Hdac1*	Inhibit	Smad4_DataSet_11_127	Pericyte, Fibroblast
7-day post-SCI group	*Hspb1*	Inhibit	Smad4_DataSet_11_127	Pericyte, Fibroblast
7-day post-SCI group	*Id1*	Inhibit	Smad4_DataSet_11_127	Pericyte, Fibroblast
7-day post-SCI group	*Il6st*	Inhibit	Smad4_DataSet_11_127	Pericyte, Fibroblast
7-day post-SCI group	*Jun*	Promote	Smad4_DataSet_11_127	Pericyte, Fibroblast
7-day post-SCI group	*Map2k3*	Promote	Smad4_DataSet_11_127	Pericyte, Fibroblast
7-day post-SCI group	*Mcl1*	Promote	Smad4_DataSet_11_127	Pericyte, Fibroblast
7-day post-SCI group	*Rcan1*	Promote	Smad4_DataSet_11_127	Pericyte, Fibroblast
7-day post-SCI group	*Ripk1*	Promote	Smad4_DataSet_11_127	Pericyte, Fibroblast
7-day post-SCI group	*Sdc1*	Inhibit	Smad4_DataSet_11_127	Pericyte, Fibroblast
7-day post-SCI group	*Stat6*	Promote	Smad4_DataSet_11_127	Pericyte, Fibroblast
7-day post-SCI group	*Ucp2*	Promote	Smad4_DataSet_11_127	Pericyte, Fibroblast
7-day post-SCI group	*Vcam1*	Inhibit	Smad4_DataSet_11_127	Pericyte, Fibroblast
7-day post-SCI group	*Xdh*	Inhibit	Smad4_DataSet_11_127	Pericyte, Fibroblast
7-day post-SCI group	*Amph*	Inhibit	Smad4_DataSet_11_128	Pericyte, Fibroblast
7-day post-SCI group	*Cbx6*	Promote	Smad4_DataSet_11_128	Pericyte, Fibroblast
7-day post-SCI group	*Fkbp1b*	Inhibit	Smad4_DataSet_11_128	Pericyte, Fibroblast
7-day post-SCI group	*Gch1*	Inhibit	Smad4_DataSet_11_128	Pericyte, Fibroblast
7-day post-SCI group	*Hbegf*	Promote	Smad4_DataSet_11_128	Pericyte, Fibroblast
7-day post-SCI group	*Hspb1*	Promote	Smad4_DataSet_11_128	Pericyte, Fibroblast
7-day post-SCI group	*Id1*	Promote	Smad4_DataSet_11_128	Pericyte, Fibroblast
7-day post-SCI group	*Jun*	Promote	Smad4_DataSet_11_128	Pericyte, Fibroblast
7-day post-SCI group	*Ppp3ca*	Inhibit	Smad4_DataSet_11_128	Pericyte, Fibroblast
7-day post-SCI group	*Sdc1*	Inhibit	Smad4_DataSet_11_128	Pericyte, Fibroblast
7-day post-SCI group	*Ucp2*	Inhibit	Smad4_DataSet_11_128	Pericyte, Fibroblast
7-day post-SCI group	*Vcam1*	Inhibit	Smad4_DataSet_11_128	Pericyte, Fibroblast
7-day post-SCI group	*Xdh*	Inhibit	Smad4_DataSet_11_128	Pericyte, Fibroblast
7-day post-SCI group	*Amph*	Inhibit	Stat6_DataSet_11_153	Pericyte
7-day post-SCI group	*Axl*	Inhibit	Stat6_DataSet_11_153	Pericyte
7-day post-SCI group	*Fbxw7*	Promote	Stat6_DataSet_11_153	Pericyte
7-day post-SCI group	*Hbegf*	Promote	Stat6_DataSet_11_153	Pericyte

7-day post-SCI group	*Id1*	Inhibit	Stat6_DataSet_11_153	Pericyte
7-day post-SCI group	*Il6st*	Promote	Stat6_DataSet_11_153	Pericyte
7-day post-SCI group	*Jun*	Promote	Stat6_DataSet_11_153	Pericyte
7-day post-SCI group	*Sdc1*	Promote	Stat6_DataSet_11_153	Pericyte
7-day post-SCI group	*Stat6*	Promote	Stat6_DataSet_11_153	Pericyte
7-day post-SCI group	*Vcam1*	Inhibit	Stat6_DataSet_11_153	Pericyte
7-day post-SCI group	*Xdh*	Promote	Stat6_DataSet_11_153	Pericyte
7-day post-SCI group	*Cbx6*	Promote	Cebpe_DataSet_11_023	Neutrophil
7-day post-SCI group	*Fos*	Inhibit	Cebpe_DataSet_11_023	Neutrophil
7-day post-SCI group	*Hbegf*	Inhibit	Cebpe_DataSet_11_023	Neutrophil
7-day post-SCI group	*Hdac1*	Promote	Cebpe_DataSet_11_023	Neutrophil
7-day post-SCI group	*Hspb1*	Inhibit	Cebpe_DataSet_11_023	Neutrophil
7-day post-SCI group	*Id1*	Inhibit	Cebpe_DataSet_11_023	Neutrophil
7-day post-SCI group	*Map2k4*	Promote	Cebpe_DataSet_11_023	Neutrophil
7-day post-SCI group	*Sdc1*	Promote	Cebpe_DataSet_11_023	Neutrophil
7-day post-SCI group	*Ucp2*	Inhibit	Cebpe_DataSet_11_023	Neutrophil
7-day post-SCI group	*Xdh*	Inhibit	Cebpe_DataSet_11_023	Neutrophil
7-day post-SCI group	*Cbx6*	Promote	Gata3_DataSet_11_048	Lymphocyte, Neuron
7-day post-SCI group	*Fkbp1b*	Inhibit	Gata3_DataSet_11_048	Lymphocyte, Neuron
7-day post-SCI group	*Fos*	Inhibit	Gata3_DataSet_11_048	Lymphocyte, Neuron
7-day post-SCI group	*Gch1*	Inhibit	Gata3_DataSet_11_048	Lymphocyte, Neuron
7-day post-SCI group	*Hbegf*	Inhibit	Gata3_DataSet_11_048	Lymphocyte, Neuron
7-day post-SCI group	*Id1*	Inhibit	Gata3_DataSet_11_048	Lymphocyte, Neuron
7-day post-SCI group	*Map2k3*	Inhibit	Gata3_DataSet_11_048	Lymphocyte, Neuron
7-day post-SCI group	*Ppp3ca*	Promote	Gata3_DataSet_11_048	Lymphocyte, Neuron
7-day post-SCI group	*Rcan1*	Inhibit	Gata3_DataSet_11_048	Lymphocyte, Neuron
7-day post-SCI group	*Xdh*	Inhibit	Gata3_DataSet_11_048	Lymphocyte, Neuron
7-day post-SCI group	*Axl*	Inhibit	Mbd2_DataSet_11_076	Pericyte
7-day post-SCI group	*Fos*	Promote	Mbd2_DataSet_11_076	Pericyte
7-day post-SCI group	*Gch1*	Inhibit	Mbd2_DataSet_11_076	Pericyte
7-day post-SCI group	*Hbegf*	Inhibit	Mbd2_DataSet_11_076	Pericyte
7-day post-SCI group	*Jun*	Inhibit	Mbd2_DataSet_11_076	Pericyte
7-day post-SCI group	*Ppp3ca*	Inhibit	Mbd2_DataSet_11_076	Pericyte
7-day post-SCI group	*Rcan1*	Promote	Mbd2_DataSet_11_076	Pericyte
7-day post-SCI group	*Sdc1*	Promote	Mbd2_DataSet_11_076	Pericyte
7-day post-SCI group	*Xdh*	Inhibit	Mbd2_DataSet_11_076	Pericyte
7-day post-SCI group	*Axl*	Promote	Tsc22d4_DataSet_11_169	Ependymal cell, OPC, Oligodendrocyte, Astrocyte
7-day post-SCI group	*Fbxw7*	Inhibit	Tsc22d4_DataSet_11_169	Ependymal cell, OPC, Oligodendrocyte, Astrocyte
7-day post-SCI group	*Fos*	Promote	Tsc22d4_DataSet_11_169	Ependymal cell, OPC, Oligodendrocyte, Astrocyte

7-day post-SCI group	*Hbegf*	Promote	Tsc22d4_DataSet_11_169	Ependymal cell, OPC, Oligodendrocyte, Astrocyte
7-day post-SCI group	*Id1*	Promote	Tsc22d4_DataSet_11_169	Ependymal cell, OPC, Oligodendrocyte, Astrocyte
7-day post-SCI group	*Jun*	Promote	Tsc22d4_DataSet_11_169	Ependymal cell, OPC, Oligodendrocyte, Astrocyte
7-day post-SCI group	*Rcan1*	Promote	Tsc22d4_DataSet_11_169	Ependymal cell, OPC, Oligodendrocyte, Astrocyte
7-day post-SCI group	*Vcam1*	Promote	Tsc22d4_DataSet_11_169	Ependymal cell, OPC, Oligodendrocyte, Astrocyte
7-day post-SCI group	*Xdh*	Promote	Tsc22d4_DataSet_11_169	Ependymal cell, OPC, Oligodendrocyte, Astrocyte
7-day post-SCI group	*Amph*	Promote	Cebpb_DataSet_11_020	Neutrophil, Monocyte, Microphage, Microglia, Div-Myeloid
7-day post-SCI group	*Fkbp1b*	Promote	Cebpb_DataSet_11_020	Neutrophil, Monocyte, Microphage, Microglia, Div-Myeloid
7-day post-SCI group	*Id1*	Inhibit	Cebpb_DataSet_11_020	Neutrophil, Monocyte, Microphage, Microglia, Div-Myeloid
7-day post-SCI group	*Vcam1*	Promote	Cebpb_DataSet_11_020	Neutrophil, Monocyte, Microphage, Microglia, Div-Myeloid
7-day post-SCI group	*Xdh*	Promote	Cebpb_DataSet_11_020	Neutrophil, Monocyte, Microphage, Microglia, Div-Myeloid
7-day post-SCI group	*Fos*	Inhibit	Cebpb_DataSet_11_021	Neutrophil, Monocyte, Microphage, Microglia, Div-Myeloid
7-day post-SCI group	*Hbegf*	Inhibit	Cebpb_DataSet_11_021	Neutrophil, Monocyte, Microphage, Microglia, Div-Myeloid
7-day post-SCI group	*Hdac1*	Promote	Cebpb_DataSet_11_021	Neutrophil, Monocyte, Microphage, Microglia, Div-Myeloid
7-day post-SCI group	*Hspb1*	Inhibit	Cebpb_DataSet_11_021	Neutrophil, Monocyte, Microphage, Microglia, Div-Myeloid
7-day post-SCI group	*Id1*	Inhibit	Cebpb_DataSet_11_021	Neutrophil, Monocyte, Microphage, Microglia, Div-Myeloid
7-day post-SCI group	*Map2k4*	Promote	Cebpb_DataSet_11_021	Neutrophil, Monocyte, Microphage, Microglia, Div-Myeloid
7-day post-SCI group	*Ucp2*	Inhibit	Cebpb_DataSet_11_021	Neutrophil, Monocyte, Microphage, Microglia, Div-Myeloid
7-day post-SCI group	*Vcam1*	Inhibit	Cebpb_DataSet_11_021	Neutrophil, Monocyte, Microphage, Microglia, Div-Myeloid
7-day post-SCI group	*Xdh*	Inhibit	Cebpb_DataSet_11_021	Neutrophil, Monocyte, Microphage, Microglia, Div-Myeloid
7-day post-SCI group	*Fos*	Promote	Cebpb_DataSet_11_022	Neutrophil, Monocyte, Microphage, Microglia, Div-Myeloid
7-day post-SCI group	*Gch1*	Inhibit	Cebpb_DataSet_11_022	Neutrophil, Monocyte, Microphage, Microglia, Div-Myeloid
7-day post-SCI group	*Id1*	Promote	Cebpb_DataSet_11_022	Neutrophil, Monocyte, Microphage, Microglia, Div-Myeloid
7-day post-SCI group	*Jun*	Promote	Cebpb_DataSet_11_022	Neutrophil, Monocyte, Microphage, Microglia, Div-Myeloid
7-day post-SCI group	*Map2k3*	Promote	Cebpb_DataSet_11_022	Neutrophil, Monocyte, Microphage, Microglia, Div-Myeloid
7-day post-SCI group	*Amph*	Inhibit	Runx1_DataSet_11_114	Microglia, Microphage, Fibroblast, Div-Myeloid, DC, Monocyte
7-day post-SCI group	*Fos*	Promote	Runx1_DataSet_11_114	Microglia, Microphage, Fibroblast, Div-Myeloid, DC, Monocyte
7-day post-SCI group	*Hspb1*	Promote	Runx1_DataSet_11_114	Microglia, Microphage, Fibroblast, Div-Myeloid, DC, Monocyte
7-day post-SCI group	*Map2k3*	Promote	Runx1_DataSet_11_114	Microglia, Microphage, Fibroblast, Div-Myeloid, DC, Monocyte
7-day post-SCI group	*Sdc1*	Promote	Runx1_DataSet_11_114	Microglia, Microphage, Fibroblast, Div-Myeloid, DC, Monocyte
7-day post-SCI group	*Fkbp1b*	Inhibit	Runx1_DataSet_11_115	Microglia, Microphage, Fibroblast, Div-Myeloid, DC, Monocyte
7-day post-SCI group	*Fos*	Inhibit	Runx1_DataSet_11_115	Microglia, Microphage, Fibroblast, Div-Myeloid, DC, Monocyte
7-day post-SCI group	*Gch1*	Inhibit	Runx1_DataSet_11_115	Microglia, Microphage, Fibroblast, Div-Myeloid, DC, Monocyte
7-day post-SCI group	*Jun*	Inhibit	Runx1_DataSet_11_115	Microglia, Microphage, Fibroblast, Div-Myeloid, DC, Monocyte
7-day post-SCI group	*Sdc1*	Inhibit	Runx1_DataSet_11_115	Microglia, Microphage, Fibroblast, Div-Myeloid, DC, Monocyte
7-day post-SCI group	*Ucp2*	Inhibit	Runx1_DataSet_11_115	Microglia, Microphage, Fibroblast, Div-Myeloid, DC, Monocyte
7-day post-SCI group	*Fos*	Promote	Runx1_DataSet_11_116	Microglia, Microphage, Fibroblast, Div-Myeloid, DC, Monocyte
7-day post-SCI group	*Gch1*	Inhibit	Runx1_DataSet_11_116	Microglia, Microphage, Fibroblast, Div-Myeloid, DC, Monocyte
7-day post-SCI group	*Hbegf*	Inhibit	Runx1_DataSet_11_116	Microglia, Microphage, Fibroblast, Div-Myeloid, DC, Monocyte

7-day post-SCI group	*Il6st*	Inhibit	Runx1_DataSet_11_116	Microglia, Microphage, Fibroblast, Div-Myeloid, DC, Monocyte
7-day post-SCI group	*Jun*	Promote	Runx1_DataSet_11_116	Microglia, Microphage, Fibroblast, Div-Myeloid, DC, Monocyte
7-day post-SCI group	*Ppp3ca*	Inhibit	Runx1_DataSet_11_116	Microglia, Microphage, Fibroblast, Div-Myeloid, DC, Monocyte
7-day post-SCI group	*Sdc1*	Promote	Runx1_DataSet_11_116	Microglia, Microphage, Fibroblast, Div-Myeloid, DC, Monocyte
7-day post-SCI group	*Tnfrsf1a*	Inhibit	Runx1_DataSet_11_116	Microglia, Microphage, Fibroblast, Div-Myeloid, DC, Monocyte
7-day post-SCI group	*Xdh*	Inhibit	Runx1_DataSet_11_116	Microglia, Microphage, Fibroblast, Div-Myeloid, DC, Monocyte
7-day post-SCI group	*Amph*	Inhibit	Runx1_DataSet_11_117	Microglia, Microphage, Fibroblast, Div-Myeloid, DC, Monocyte
7-day post-SCI group	*Id1*	Inhibit	Runx1_DataSet_11_117	Microglia, Microphage, Fibroblast, Div-Myeloid, DC, Monocyte
7-day post-SCI group	*Amph*	Promote	Nr2f2_DataSet_11_094	Pericyte, Fibroblast
7-day post-SCI group	*Hspb1*	Inhibit	Nr2f2_DataSet_11_094	Pericyte, Fibroblast
7-day post-SCI group	*Vcam1*	Inhibit	Nr2f2_DataSet_11_094	Pericyte, Fibroblast
7-day post-SCI group	*Amph*	Promote	Nr2f2_DataSet_11_095	Pericyte, Fibroblast
7-day post-SCI group	*Fos*	Inhibit	Nr2f2_DataSet_11_095	Pericyte, Fibroblast
7-day post-SCI group	*Hspb1*	Inhibit	Nr2f2_DataSet_11_095	Pericyte, Fibroblast
7-day post-SCI group	*Id1*	Inhibit	Nr2f2_DataSet_11_095	Pericyte, Fibroblast
7-day post-SCI group	*Tnfrsf1a*	Inhibit	Nr2f2_DataSet_11_095	Pericyte, Fibroblast
7-day post-SCI group	*Ucp2*	Inhibit	Nr2f2_DataSet_11_095	Pericyte, Fibroblast
7-day post-SCI group	*Vcam1*	Inhibit	Nr2f2_DataSet_11_095	Pericyte, Fibroblast
7-day post-SCI group	*Xdh*	Inhibit	Nr2f2_DataSet_11_095	Pericyte, Fibroblast
7-day post-SCI group	*Axl*	Promote	Irf8_DataSet_11_063	Microglia, Div-Myeloid, DC, Monocyte, Microphage
7-day post-SCI group	*Gch1*	Promote	Irf8_DataSet_11_063	Microglia, Div-Myeloid, DC, Monocyte, Microphage
7-day post-SCI group	*Id1*	Promote	Irf8_DataSet_11_063	Microglia, Div-Myeloid, DC, Monocyte, Microphage
7-day post-SCI group	*Jun*	Inhibit	Irf8_DataSet_11_063	Microglia, Div-Myeloid, DC, Monocyte, Microphage
7-day post-SCI group	*Map2k3*	Promote	Irf8_DataSet_11_063	Microglia, Div-Myeloid, DC, Monocyte, Microphage
7-day post-SCI group	*Mcl1*	Promote	Irf8_DataSet_11_063	Microglia, Div-Myeloid, DC, Monocyte, Microphage
7-day post-SCI group	*Vcam1*	Promote	Irf8_DataSet_11_063	Microglia, Div-Myeloid, DC, Monocyte, Microphage
7-day post-SCI group	*Axl*	Promote	Meis1_DataSet_11_081	Ependymal cell
7-day post-SCI group	*Fbxw7*	Promote	Meis1_DataSet_11_081	Ependymal cell
7-day post-SCI group	*Fkbp1b*	Inhibit	Meis1_DataSet_11_081	Ependymal cell
7-day post-SCI group	*Hspb1*	Inhibit	Meis1_DataSet_11_081	Ependymal cell
7-day post-SCI group	*Il6st*	Promote	Meis1_DataSet_11_081	Ependymal cell
7-day post-SCI group	*Sdc1*	Promote	Meis1_DataSet_11_081	Ependymal cell
7-day post-SCI group	*Vcam1*	Promote	Meis1_DataSet_11_081	Ependymal cell
7-day post-SCI group	*Fos*	Inhibit	Meis1_DataSet_11_082	Ependymal cell
7-day post-SCI group	*Gch1*	Inhibit	Meis1_DataSet_11_082	Ependymal cell
7-day post-SCI group	*Id1*	Promote	Meis1_DataSet_11_082	Ependymal cell
7-day post-SCI group	*Il6st*	Inhibit	Meis1_DataSet_11_082	Ependymal cell
7-day post-SCI group	*Sdc1*	Promote	Meis1_DataSet_11_082	Ependymal cell
7-day post-SCI group	*Vcam1*	Inhibit	Meis1_DataSet_11_082	Ependymal cell

7-day post-SCI group	*Amph*	Promote	Prox1_DataSet_11_109	Oligodendrocyte
7-day post-SCI group	*Hbegf*	Inhibit	Prox1_DataSet_11_109	Oligodendrocyte
7-day post-SCI group	*Hspb1*	Inhibit	Prox1_DataSet_11_109	Oligodendrocyte
7-day post-SCI group	*Jun*	Inhibit	Prox1_DataSet_11_109	Oligodendrocyte
7-day post-SCI group	*Vcam1*	Promote	Prox1_DataSet_11_109	Oligodendrocyte
7-day post-SCI group	*Xdh*	Inhibit	Prox1_DataSet_11_109	Oligodendrocyte
7-day post-SCI group	*Fos*	Promote	Tet2_DataSet_11_159	Astrocyte
7-day post-SCI group	*Gch1*	Inhibit	Tet2_DataSet_11_159	Astrocyte
7-day post-SCI group	*Vcam1*	Inhibit	Tet2_DataSet_11_159	Astrocyte
7-day post-SCI group	*Xdh*	Inhibit	Tet2_DataSet_11_159	Astrocyte
7-day post-SCI group	*Fos*	Inhibit	Tet2_DataSet_11_161	Astrocyte
7-day post-SCI group	*Gch1*	Inhibit	Tet2_DataSet_11_161	Astrocyte
7-day post-SCI group	*Hspb1*	Inhibit	Tet2_DataSet_11_161	Astrocyte
7-day post-SCI group	*Map2k3*	Inhibit	Tet2_DataSet_11_161	Astrocyte
7-day post-SCI group	*Fos*	Inhibit	Tet2_DataSet_11_162	Astrocyte
7-day post-SCI group	*Axl*	Inhibit	Tet2_DataSet_11_163	Astrocyte
7-day post-SCI group	*Fbxw7*	Promote	Tet2_DataSet_11_163	Astrocyte
7-day post-SCI group	*Fos*	Inhibit	Tet2_DataSet_11_163	Astrocyte
7-day post-SCI group	*Mcl1*	Inhibit	Tet2_DataSet_11_163	Astrocyte
7-day post-SCI group	*Ppp3ca*	Promote	Tet2_DataSet_11_163	Astrocyte
7-day post-SCI group	*Sdc1*	Inhibit	Tet2_DataSet_11_163	Astrocyte
7-day post-SCI group	*Cbx6*	Promote	E2f4_DataSet_11_033	Div-Myeloid
7-day post-SCI group	*Fkbp1b*	Promote	E2f4_DataSet_11_033	Div-Myeloid
7-day post-SCI group	*Fos*	Inhibit	E2f4_DataSet_11_033	Div-Myeloid
7-day post-SCI group	*Ripk1*	Promote	E2f4_DataSet_11_033	Div-Myeloid
7-day post-SCI group	*Vcam1*	Inhibit	E2f4_DataSet_11_033	Div-Myeloid
7-day post-SCI group	*Xdh*	Inhibit	E2f4_DataSet_11_033	Div-Myeloid
7-day post-SCI group	*Il6st*	Promote	E2f4_DataSet_11_034	Div-Myeloid
7-day post-SCI group	*Amph*	Inhibit	Prdm2_DataSet_11_108	Neuron
7-day post-SCI group	*Fkbp1b*	Promote	Prdm2_DataSet_11_108	Neuron
7-day post-SCI group	*Fos*	Inhibit	Prdm2_DataSet_11_108	Neuron
7-day post-SCI group	*Vcam1*	Inhibit	Prdm2_DataSet_11_108	Neuron
7-day post-SCI group	*Xdh*	Inhibit	Prdm2_DataSet_11_108	Neuron
7-day post-SCI group	*Amph*	Inhibit	Snai1_DataSet_11_129	Pericyte, Fibroblast
7-day post-SCI group	*Hbegf*	Inhibit	Snai1_DataSet_11_129	Pericyte, Fibroblast
7-day post-SCI group	*Vcam1*	Promote	Snai1_DataSet_11_129	Pericyte, Fibroblast
7-day post-SCI group	*Amph*	Inhibit	Snai1_DataSet_11_130	Pericyte, Fibroblast
7-day post-SCI group	*Fos*	Inhibit	Snai1_DataSet_11_130	Pericyte, Fibroblast
7-day post-SCI group	*Hbegf*	Inhibit	Snai1_DataSet_11_130	Pericyte, Fibroblast

7-day post-SCI group	*Hspb1*	Inhibit	Snai1_DataSet_11_130	Pericyte, Fibroblast
7-day post-SCI group	*Ucp2*	Inhibit	Snai1_DataSet_11_130	Pericyte, Fibroblast
7-day post-SCI group	*Id1*	Inhibit	Snai1_DataSet_11_131	Pericyte, Fibroblast
7-day post-SCI group	*Ppp3ca*	Promote	Snai1_DataSet_11_131	Pericyte, Fibroblast
7-day post-SCI group	*Ucp2*	Inhibit	Snai1_DataSet_11_131	Pericyte, Fibroblast
7-day post-SCI group	*Fos*	Promote	Snai1_DataSet_11_132	Pericyte, Fibroblast
7-day post-SCI group	*Hspb1*	Inhibit	Snai1_DataSet_11_132	Pericyte, Fibroblast
7-day post-SCI group	*Fbxw7*	Inhibit	Sox17_DataSet_11_139	Endothelial cell, Pericyte
7-day post-SCI group	*Fos*	Inhibit	Sox17_DataSet_11_139	Endothelial cell, Pericyte
7-day post-SCI group	*Hbegf*	Inhibit	Sox17_DataSet_11_139	Endothelial cell, Pericyte
7-day post-SCI group	*Hspb1*	Inhibit	Sox17_DataSet_11_139	Endothelial cell, Pericyte
7-day post-SCI group	*Id1*	Inhibit	Sox17_DataSet_11_139	Endothelial cell, Pericyte
7-day post-SCI group	*Fos*	Inhibit	Ahr_DataSet_11_002	DC
7-day post-SCI group	*Jun*	Inhibit	Ahr_DataSet_11_002	DC
7-day post-SCI group	*Rcan1*	Inhibit	Ahr_DataSet_11_002	DC
7-day post-SCI group	*Sdc1*	Inhibit	Ahr_DataSet_11_002	DC
7-day post-SCI group	*Xdh*	Promote	Ahr_DataSet_11_002	DC
7-day post-SCI group	*Fos*	Promote	Ahr_DataSet_11_003	DC
7-day post-SCI group	*Hspb1*	Inhibit	Ahr_DataSet_11_003	DC
7-day post-SCI group	*Fos*	Inhibit	Foxp1_DataSet_11_044	Endothelial cell, Pericyte, Fibroblast
7-day post-SCI group	*Gch1*	Promote	Foxp1_DataSet_11_044	Endothelial cell, Pericyte, Fibroblast
7-day post-SCI group	*Stat6*	Promote	Foxp1_DataSet_11_044	Endothelial cell, Pericyte, Fibroblast
7-day post-SCI group	*Vcam1*	Promote	Foxp1_DataSet_11_044	Endothelial cell, Pericyte, Fibroblast
7-day post-SCI group	*Xdh*	Promote	Foxp1_DataSet_11_044	Endothelial cell, Pericyte, Fibroblast
7-day post-SCI group	*Amph*	Promote	Gata2_DataSet_11_047	Endothelial cell, Pericyte
7-day post-SCI group	*Axl*	Promote	Gata2_DataSet_11_047	Endothelial cell, Pericyte
7-day post-SCI group	*Id1*	Inhibit	Gata2_DataSet_11_047	Endothelial cell, Pericyte
7-day post-SCI group	*Vcam1*	Promote	Gata2_DataSet_11_047	Endothelial cell, Pericyte
7-day post-SCI group	*Amph*	Inhibit	Stat3_DataSet_11_151	DC,Fibroblast, Pericyte, Astrocyte
7-day post-SCI group	*Axl*	Inhibit	Stat3_DataSet_11_151	DC,Fibroblast, Pericyte, Astrocyte
7-day post-SCI group	*Fos*	Promote	Stat3_DataSet_11_151	DC,Fibroblast, Pericyte, Astrocyte
7-day post-SCI group	*Gch1*	Inhibit	Stat3_DataSet_11_151	DC,Fibroblast, Pericyte, Astrocyte
7-day post-SCI group	*Axl*	Promote	Cebpa_DataSet_11_019	Microglia, Div-Myeloid, Monocyte, Microphage
7-day post-SCI group	*Gch1*	Promote	Cebpa_DataSet_11_019	Microglia, Div-Myeloid, Monocyte, Microphage
7-day post-SCI group	*Vcam1*	Inhibit	Cebpa_DataSet_11_019	Microglia, Div-Myeloid, Monocyte, Microphage
7-day post-SCI group	*Xdh*	Inhibit	Cebpa_DataSet_11_019	Microglia, Div-Myeloid, Monocyte, Microphage
7-day post-SCI group	*Axl*	Inhibit	Sox11_DataSet_11_137	OPC, Neuron
7-day post-SCI group	*Stat6*	Inhibit	Sox11_DataSet_11_137	OPC, Neuron
7-day post-SCI group	*Vcam1*	Inhibit	Sox11_DataSet_11_137	OPC, Neuron

7-day post-SCI group	*Xdh*	Inhibit	Sox11_DataSet_11_137	OPC, Neuron
7-day post-SCI group	*Hbegf*	Inhibit	Sox11_DataSet_11_138	OPC, Neuron
7-day post-SCI group	*Fbxw7*	Promote	Smad1_DataSet_11_126	Endothelial cell, Astrocyte, Neuron, Pericyte
7-day post-SCI group	*Hspb1*	Promote	Smad1_DataSet_11_126	Endothelial cell, Astrocyte, Neuron, Pericyte
7-day post-SCI group	*Jun*	Promote	Smad1_DataSet_11_126	Endothelial cell, Astrocyte, Neuron, Pericyte
7-day post-SCI group	*Rcan1*	Promote	Smad1_DataSet_11_126	Endothelial cell, Astrocyte, Neuron, Pericyte
7-day post-SCI group	*Tnfrsf1a*	Inhibit	Sox2_DataSet_11_140	Ependymal cell, OPC, Astrocyte
7-day post-SCI group	*Fos*	Inhibit	Sox2_DataSet_11_141	Ependymal cell, OPC, Astrocyte
7-day post-SCI group	*Jun*	Inhibit	Sox2_DataSet_11_141	Ependymal cell, OPC, Astrocyte
7-day post-SCI group	*Rcan1*	Inhibit	Sox2_DataSet_11_141	Ependymal cell, OPC, Astrocyte
7-day post-SCI group	*Tnfrsf1a*	Inhibit	Sox2_DataSet_11_141	Ependymal cell, OPC, Astrocyte
7-day post-SCI group	*Axl*	Inhibit	Sox10_DataSet_11_136	OPC, Oligodendrocyte
7-day post-SCI group	*Mcl1*	Promote	Sox10_DataSet_11_136	OPC, Oligodendrocyte
7-day post-SCI group	*Ppp3ca*	Promote	Sox10_DataSet_11_136	OPC, Oligodendrocyte
7-day post-SCI group	*Hdac1*	Inhibit	Rest_DataSet_11_110	Fibroblast
7-day post-SCI group	*Xdh*	Promote	Rest_DataSet_11_112	Fibroblast
7-day post-SCI group	*Fkbp1b*	Inhibit	Rest_DataSet_11_113	Fibroblast
7-day post-SCI group	*Stat6*	Inhibit	Rest_DataSet_11_113	Fibroblast
7-day post-SCI group	*Xdh*	Inhibit	Rest_DataSet_11_113	Fibroblast
7-day post-SCI group	*Fos*	Promote	Erg_DataSet_11_038	Endothelial cell
7-day post-SCI group	*Jun*	Promote	Erg_DataSet_11_038	Endothelial cell
7-day post-SCI group	*Sdc1*	Inhibit	Erg_DataSet_11_038	Endothelial cell
7-day post-SCI group	*Fos*	Inhibit	Foxq1_DataSet_11_045	Endothelial cell
7-day post-SCI group	*Gch1*	Inhibit	Foxq1_DataSet_11_045	Endothelial cell
7-day post-SCI group	*Sdc1*	Inhibit	Foxq1_DataSet_11_045	Endothelial cell
7-day post-SCI group	*Id1*	Promote	Klf6_DataSet_11_067	Endothelial cell, Microglia, Pericyte, Fibroblast, Lymphocyte
7-day post-SCI group	*Xdh*	Inhibit	Klf6_DataSet_11_067	Endothelial cell, Microglia, Pericyte, Fibroblast, Lymphocyte
7-day post-SCI group	*Fos*	Inhibit	Klf6_DataSet_11_068	Endothelial cell, Microglia, Pericyte, Fibroblast, Lymphocyte
7-day post-SCI group	*Hbegf*	Inhibit	Klf6_DataSet_11_068	Endothelial cell, Microglia, Pericyte, Fibroblast, Lymphocyte
7-day post-SCI group	*Vcam1*	Inhibit	Klf6_DataSet_11_068	Endothelial cell, Microglia, Pericyte, Fibroblast, Lymphocyte
7-day post-SCI group	*Fos*	Inhibit	Tbx1_DataSet_11_154	Endothelial cell
7-day post-SCI group	*Vcam1*	Inhibit	Tbx1_DataSet_11_154	Endothelial cell
7-day post-SCI group	*Xdh*	Inhibit	Tbx1_DataSet_11_154	Endothelial cell
7-day post-SCI group	*Fos*	Promote	Tbx1_DataSet_11_155	Endothelial cell
7-day post-SCI group	*Rcan1*	Inhibit	Thap11_DataSet_11_167	OPC, Neuron
7-day post-SCI group	*Fos*	Promote	Thap11_DataSet_11_168	OPC, Neuron
7-day post-SCI group	*Id1*	Inhibit	Thap11_DataSet_11_168	OPC, Neuron
7-day post-SCI group	*Ucp2*	Promote	Thap11_DataSet_11_168	OPC, Neuron
7-day post-SCI group	*Hbegf*	Inhibit	Mef2c_DataSet_11_079	Microglia, Pericyte

7-day post-SCI group	*Vcam1*	Promote	Mef2c_DataSet_11_079	Microglia, Pericyte
7-day post-SCI group	*Xdh*	Promote	Mef2c_DataSet_11_079	Microglia, Pericyte
7-day post-SCI group	*Hspb1*	Inhibit	Nelfb_DataSet_11_086	Pericyte
7-day post-SCI group	*Il6st*	Promote	Nelfb_DataSet_11_086	Pericyte
7-day post-SCI group	*Vcam1*	Promote	Nelfb_DataSet_11_086	Pericyte
7-day post-SCI group	*Hspb1*	Inhibit	Pbx1_DataSet_11_102	Neuron, Pericyte, Fibroblast, Astrocyte
7-day post-SCI group	*Jun*	Promote	Pbx1_DataSet_11_102	Neuron, Pericyte, Fibroblast, Astrocyte
7-day post-SCI group	*Xdh*	Inhibit	Pbx1_DataSet_11_102	Neuron, Pericyte, Fibroblast, Astrocyte
7-day post-SCI group	*Id1*	Promote	Nfix_DataSet_11_091	Ependymal cell, OPC, Astrocyte, Oligodendrocyte
7-day post-SCI group	*Jun*	Inhibit	Nfix_DataSet_11_091	Ependymal cell, OPC, Astrocyte, Oligodendrocyte
7-day post-SCI group	*Sdc1*	Inhibit	Nfix_DataSet_11_091	Ependymal cell, OPC, Astrocyte, Oligodendrocyte
7-day post-SCI group	*Id1*	Inhibit	Xbp1_DataSet_11_181	Endothelial cell, Fibroblast
7-day post-SCI group	*Rcan1*	Inhibit	Xbp1_DataSet_11_181	Endothelial cell, Fibroblast
7-day post-SCI group	*Sdc1*	Inhibit	Xbp1_DataSet_11_181	Endothelial cell, Fibroblast
7-day post-SCI group	*Jun*	Promote	Mafg_DataSet_11_073	Endothelial cell, Ependymal cell, Neuron
7-day post-SCI group	*Ucp2*	Promote	Mafg_DataSet_11_073	Endothelial cell, Ependymal cell, Neuron
7-day post-SCI group	*Vcam1*	Promote	Mafg_DataSet_11_073	Endothelial cell, Ependymal cell, Neuron
7-day post-SCI group	*Axl*	Promote	Cbfb_DataSet_11_017	Div-Myeloid, OPC, Fibroblast, Lymphocyte
7-day post-SCI group	*Xdh*	Inhibit	Cbfb_DataSet_11_017	Div-Myeloid, OPC, Fibroblast, Lymphocyte
7-day post-SCI group	*Fos*	Promote	Jarid2_DataSet_11_064	DC
7-day post-SCI group	*Xdh*	Inhibit	Jarid2_DataSet_11_064	DC
7-day post-SCI group	*Hbegf*	Promote	Pax6_DataSet_11_101	Ependymal cell, Astrocyte
7-day post-SCI group	*Ripk1*	Inhibit	Pax6_DataSet_11_101	Ependymal cell, Astrocyte
7-day post-SCI group	*Hspb1*	Inhibit	Ets2_DataSet_11_040	Neutrophil, Ependymal cell
7-day post-SCI group	*Jun*	Inhibit	Ets2_DataSet_11_040	Neutrophil, Ependymal cell
7-day post-SCI group	*Id1*	Inhibit	Arid1a_DataSet_11_004	Ependymal cell, Div-Myeloid, Pericyte, Lymphocyte
7-day post-SCI group	*Rcan1*	Promote	Arid1a_DataSet_11_004	Ependymal cell, Div-Myeloid, Pericyte, Lymphocyte
7-day post-SCI group	*Jun*	Promote	Bcl11a_DataSet_11_013	DC, Neuron
7-day post-SCI group	*Sdc1*	Promote	Bcl11a_DataSet_11_013	DC, Neuron
7-day post-SCI group	*Fos*	Inhibit	Sox21_DataSet_11_142	Ependymal cell, OPC, Astrocyte
7-day post-SCI group	*Hbegf*	Promote	Mef2a_DataSet_11_077	Microglia, Microphage, Endothelial cell, Monocyte, Pericyte
7-day post-SCI group	*Jun*	Inhibit	Sall1_DataSet_11_122	Astrocyte, Ependymal cell
7-day post-SCI group	*Sdc1*	Inhibit	Id2_DataSet_11_059	Microphage, DC, Microglia, Lymphocyte, Astrocyte
7-day post-SCI group	*Xdh*	Inhibit	Atf3_DataSet_11_006	Endothelial cell, Microglia, Ependymal cell, DC, Monocyte, Microphage
7-day post-SCI group	*Xdh*	Inhibit	Sox9_DataSet_11_144	Ependymal cell, Astrocyte

DC: dendritic cell; OPC: Oligodendrocyte precursor cell; SCI: spinal cord injury.
