# Supplementary material for: Integrating bulk and single-cell transcriptome profiling to uncover diagnostic biomarkers and regulatory mechanisms of oxidative stress in spinal cord injury
Source: Neural Regen Res. 2025 Jan 13;21(6):2643–57. doi: 10.4103/NRR.NRR-D-24-00693 (PMC13217428; doi:10.4103/NRR.NRR-D-24-00693)
Supplement: Supplementary file 16 [file NRR-21-2643_Suppl9.pdf]

Additional Table 11 Correlation analysis among drugs, hub genes, and innate immune cells

Drug	interaction_types between drugs and genes	Gene	Immune cell	Correlation between genesand immune cells	pvalue of the correlation between genes and immune cells
ALLOPURINOL	Inhibitor	*Xdh*	Mast Cells	-0.640060967	0.002368759
ALLOPURINOL	Inhibitor	*Xdh*	Neutrophil Cells	-0.860150376	0
ALLOPURINOL	Inhibitor	*Xdh*	Eosinophil Cells	-0.047322772	0.842953367
ALLOPURINOL	Inhibitor	*Xdh*	M0 Macrophage	0.889419463	1.56E-07
ALLOPURINOL	Inhibitor	*Xdh*	M1 Macrophage	0.665284427	0.001369306
ALLOPURINOL	Inhibitor	*Xdh*	M2 Macrophage	-0.24679421	0.294193082
ALLOPURINOL	Inhibitor	*Xdh*	Monocyte	-0.762406015	0.000141345
ALLOPURINOL	Inhibitor	*Xdh*	NK Resting	-0.718202428	0.000361873
ALLOPURINOL	Inhibitor	*Xdh*	NK.Actived	0.416416198	0.067799022
ALLOPURINOL	Inhibitor	*Xdh*	DC Actived	-0.59921692	0.005236945
ALLOPURINOL	Inhibitor	*Xdh*	DC Immature	0.696935372	0.000638489
FEBUXOSTAT	Inhibitor	*Xdh*	Mast Cells	-0.640060967	0.002368759
FEBUXOSTAT	Inhibitor	*Xdh*	Neutrophil Cells	-0.860150376	0
FEBUXOSTAT	Inhibitor	*Xdh*	Eosinophil Cells	-0.047322772	0.842953367
FEBUXOSTAT	Inhibitor	*Xdh*	M0 Macrophage	0.889419463	1.56E-07
FEBUXOSTAT	Inhibitor	*Xdh*	M1 Macrophage	0.665284427	0.001369306
FEBUXOSTAT	Inhibitor	*Xdh*	M2 Macrophage	-0.24679421	0.294193082
FEBUXOSTAT	Inhibitor	*Xdh*	Monocyte	-0.762406015	0.000141345
FEBUXOSTAT	Inhibitor	*Xdh*	NK Resting	-0.718202428	0.000361873
FEBUXOSTAT	Inhibitor	*Xdh*	NK.Actived	0.416416198	0.067799022
FEBUXOSTAT	Inhibitor	*Xdh*	DC Actived	-0.59921692	0.005236945
FEBUXOSTAT	Inhibitor	*Xdh*	DC Immature	0.696935372	0.000638489
ALLOPURINOL SODIUM	Inhibitor	*Xdh*	Mast Cells	-0.640060967	0.002368759
ALLOPURINOL SODIUM	Inhibitor	*Xdh*	Neutrophil Cells	-0.860150376	0

ALLOPURINOL SODIUM	Inhibitor	*Xdh*	Eosinophil Cells	-0.047322772	0.842953367
ALLOPURINOL SODIUM	Inhibitor	*Xdh*	M0 Macrophage	0.889419463	1.56E-07
ALLOPURINOL SODIUM	Inhibitor	*Xdh*	M1 Macrophage	0.665284427	0.001369306
ALLOPURINOL SODIUM	Inhibitor	*Xdh*	M2 Macrophage	-0.24679421	0.294193082
ALLOPURINOL SODIUM	Inhibitor	*Xdh*	Monocyte	-0.762406015	0.000141345
ALLOPURINOL SODIUM	Inhibitor	*Xdh*	NK Resting	-0.718202428	0.000361873
ALLOPURINOL SODIUM	Inhibitor	*Xdh*	NK.Actived	0.416416198	0.067799022
ALLOPURINOL SODIUM	Inhibitor	*Xdh*	DC Actived	-0.59921692	0.005236945
ALLOPURINOL SODIUM	Inhibitor	*Xdh*	DC Immature	0.696935372	0.000638489
OBATOCLAX MESYLATE	Inhibitor	*Mcl1*	Mast Cells	-0.338983267	0.14372311
OBATOCLAX MESYLATE	Inhibitor	*Mcl1*	Neutrophil Cells	-0.064685977	0.78643634
OBATOCLAX MESYLATE	Inhibitor	*Mcl1*	Eosinophil Cells	-0.357744101	0.12146919
OBATOCLAX MESYLATE	Inhibitor	*Mcl1*	M0 Macrophage	0.286479574	0.220744693
OBATOCLAX MESYLATE	Inhibitor	*Mcl1*	M1 Macrophage	0.130647684	0.582994803
OBATOCLAX MESYLATE	Inhibitor	*Mcl1*	M2 Macrophage	-0.614575083	0.003934911
OBATOCLAX MESYLATE	Inhibitor	*Mcl1*	Monocyte	-0.143663041	0.545670963
OBATOCLAX MESYLATE	Inhibitor	*Mcl1*	NK Resting	-0.446392775	0.048498962
OBATOCLAX MESYLATE	Inhibitor	*Mcl1*	NK.Actived	0.334493865	0.14944568
OBATOCLAX MESYLATE	Inhibitor	*Mcl1*	DC Actived	0.303563737	0.193215423
OBATOCLAX MESYLATE	Inhibitor	*Mcl1*	DC Immature	0.15816055	0.505421718
TRAMETINIB	Inhibitor	*Map2k3*	Mast Cells	-0.170180916	0.473172965
TRAMETINIB	Inhibitor	*Map2k3*	Neutrophil Cells	0.006015038	0.982248374
TRAMETINIB	Inhibitor	*Map2k3*	Eosinophil Cells	-0.149496939	0.52930141
TRAMETINIB	Inhibitor	*Map2k3*	M0 Macrophage	0.204551324	0.386995256
TRAMETINIB	Inhibitor	*Map2k3*	M1 Macrophage	0.036874887	0.877339389
TRAMETINIB	Inhibitor	*Map2k3*	M2 Macrophage	-0.785665971	4.04041E-05
TRAMETINIB	Inhibitor	*Map2k3*	Monocyte	-0.013533835	0.956905273
TRAMETINIB	Inhibitor	*Map2k3*	NK Resting	-0.378798749	0.099556518

TRAMETINIB	Inhibitor	*Map2k3*	NK.Actived	0.25408446	0.279697214
TRAMETINIB	Inhibitor	*Map2k3*	DC Actived	0.460936093	0.04081115
TRAMETINIB	Inhibitor	*Map2k3*	DC Immature	0.094645544	0.691436202
SELUMETINIB	Inhibitor	*Map2k3*	Mast Cells	-0.170180916	0.473172965
SELUMETINIB	Inhibitor	*Map2k3*	Neutrophil Cells	0.006015038	0.982248374
SELUMETINIB	Inhibitor	*Map2k3*	Eosinophil Cells	-0.149496939	0.52930141
SELUMETINIB	Inhibitor	*Map2k3*	M0 Macrophage	0.204551324	0.386995256
SELUMETINIB	Inhibitor	*Map2k3*	M1 Macrophage	0.036874887	0.877339389
SELUMETINIB	Inhibitor	*Map2k3*	M2 Macrophage	-0.785665971	4.04041E-05
SELUMETINIB	Inhibitor	*Map2k3*	Monocyte	-0.013533835	0.956905273
SELUMETINIB	Inhibitor	*Map2k3*	NK Resting	-0.378798749	0.099556518
SELUMETINIB	Inhibitor	*Map2k3*	NK.Actived	0.25408446	0.279697214
SELUMETINIB	Inhibitor	*Map2k3*	DC Actived	0.460936093	0.04081115
SELUMETINIB	Inhibitor	*Map2k3*	DC Immature	0.094645544	0.691436202
BINIMETINIB	Inhibitor	*Map2k3*	Mast Cells	-0.170180916	0.473172965
BINIMETINIB	Inhibitor	*Map2k3*	Neutrophil Cells	0.006015038	0.982248374
BINIMETINIB	Inhibitor	*Map2k3*	Eosinophil Cells	-0.149496939	0.52930141
BINIMETINIB	Inhibitor	*Map2k3*	M0 Macrophage	0.204551324	0.386995256
BINIMETINIB	Inhibitor	*Map2k3*	M1 Macrophage	0.036874887	0.877339389
BINIMETINIB	Inhibitor	*Map2k3*	M2 Macrophage	-0.785665971	4.04041E-05
BINIMETINIB	Inhibitor	*Map2k3*	Monocyte	-0.013533835	0.956905273
BINIMETINIB	Inhibitor	*Map2k3*	NK Resting	-0.378798749	0.099556518
BINIMETINIB	Inhibitor	*Map2k3*	NK.Actived	0.25408446	0.279697214
BINIMETINIB	Inhibitor	*Map2k3*	DC Actived	0.460936093	0.04081115
BINIMETINIB	Inhibitor	*Map2k3*	DC Immature	0.094645544	0.691436202
COBIMETINIB	Inhibitor	*Map2k3*	Mast Cells	-0.170180916	0.473172965
COBIMETINIB	Inhibitor	*Map2k3*	Neutrophil Cells	0.006015038	0.982248374
COBIMETINIB	Inhibitor	*Map2k3*	Eosinophil Cells	-0.149496939	0.52930141

COBIMETINIB	Inhibitor	*Map2k3*	M0 Macrophage	0.204551324	0.386995256
COBIMETINIB	Inhibitor	*Map2k3*	M1 Macrophage	0.036874887	0.877339389
COBIMETINIB	Inhibitor	*Map2k3*	M2 Macrophage	-0.785665971	4.04041E-05
COBIMETINIB	Inhibitor	*Map2k3*	Monocyte	-0.013533835	0.956905273
COBIMETINIB	Inhibitor	*Map2k3*	NK Resting	-0.378798749	0.099556518
COBIMETINIB	Inhibitor	*Map2k3*	NK.Actived	0.25408446	0.279697214
COBIMETINIB	Inhibitor	*Map2k3*	DC Actived	0.460936093	0.04081115
COBIMETINIB	Inhibitor	*Map2k3*	DC Immature	0.094645544	0.691436202
APATORSEN	Inhibitor	*Hspb1*	Mast Cells	-0.376648075	0.101652021
APATORSEN	Inhibitor	*Hspb1*	Neutrophil Cells	-0.152688992	0.520442511
APATORSEN	Inhibitor	*Hspb1*	Eosinophil Cells	-0.326004399	0.160697293
APATORSEN	Inhibitor	*Hspb1*	M0 Macrophage	0.367572998	0.110848272
APATORSEN	Inhibitor	*Hspb1*	M1 Macrophage	0.442665096	0.050637415
APATORSEN	Inhibitor	*Hspb1*	M2 Macrophage	-0.652702899	0.001810803
APATORSEN	Inhibitor	*Hspb1*	Monocyte	-0.171493055	0.469716614
APATORSEN	Inhibitor	*Hspb1*	NK Resting	-0.604790211	0.00472867
APATORSEN	Inhibitor	*Hspb1*	NK.Actived	0.489826108	0.028359041
APATORSEN	Inhibitor	*Hspb1*	DC Actived	0.200582621	0.396471193
APATORSEN	Inhibitor	*Hspb1*	DC Immature	0.088225613	0.711476214
TRAMETINIB	Inhibitor	*Map2k4*	Mast Cells	0.66415738	0.001404745
TRAMETINIB	Inhibitor	*Map2k4*	Neutrophil Cells	0.77593985	8.58E-05
TRAMETINIB	Inhibitor	*Map2k4*	Eosinophil Cells	0.10540072	0.658307737
TRAMETINIB	Inhibitor	*Map2k4*	M0 Macrophage	-0.928814532	3.44E-09
TRAMETINIB	Inhibitor	*Map2k4*	M1 Macrophage	-0.574633662	0.008046006
TRAMETINIB	Inhibitor	*Map2k4*	M2 Macrophage	0.252831989	0.28215547
TRAMETINIB	Inhibitor	*Map2k4*	Monocyte	0.744360902	0.000253438
TRAMETINIB	Inhibitor	*Map2k4*	NK Resting	0.756082303	0.000115005
TRAMETINIB	Inhibitor	*Map2k4*	NK.Actived	-0.566396609	0.009224716

TRAMETINIB	Inhibitor	*Map2k4*	DC Actived	0.574633662	0.008046006
TRAMETINIB	Inhibitor	*Map2k4*	DC Immature	-0.610893968	0.004219453
COBIMETINIB	Inhibitor	*Map2k4*	Mast Cells	0.66415738	0.001404745
COBIMETINIB	Inhibitor	*Map2k4*	Neutrophil Cells	0.77593985	8.58336E-05
COBIMETINIB	Inhibitor	*Map2k4*	Eosinophil Cells	0.10540072	0.658307737
COBIMETINIB	Inhibitor	*Map2k4*	M0 Macrophage	-0.928814532	3.43626E-09
COBIMETINIB	Inhibitor	*Map2k4*	M1 Macrophage	-0.574633662	0.008046006
COBIMETINIB	Inhibitor	*Map2k4*	M2 Macrophage	0.252831989	0.28215547
COBIMETINIB	Inhibitor	*Map2k4*	Monocyte	0.744360902	0.000253438
COBIMETINIB	Inhibitor	*Map2k4*	NK Resting	0.756082303	0.000115005
COBIMETINIB	Inhibitor	*Map2k4*	NK.Actived	-0.566396609	0.009224716
COBIMETINIB	Inhibitor	*Map2k4*	DC Actived	0.574633662	0.008046006
COBIMETINIB	Inhibitor	*Map2k4*	DC Immature	-0.610893968	0.004219453
SELUMETINIB	Inhibitor	*Map2k4*	Mast Cells	0.66415738	0.001404745
SELUMETINIB	Inhibitor	*Map2k4*	Neutrophil Cells	0.77593985	8.58E-05
SELUMETINIB	Inhibitor	*Map2k4*	Eosinophil Cells	0.10540072	0.658307737
SELUMETINIB	Inhibitor	*Map2k4*	M0 Macrophage	-0.928814532	3.44E-09
SELUMETINIB	Inhibitor	*Map2k4*	M1 Macrophage	-0.574633662	0.008046006
SELUMETINIB	Inhibitor	*Map2k4*	M2 Macrophage	0.252831989	0.28215547
SELUMETINIB	Inhibitor	*Map2k4*	Monocyte	0.744360902	0.000253438
SELUMETINIB	Inhibitor	*Map2k4*	NK Resting	0.756082303	0.000115005
SELUMETINIB	Inhibitor	*Map2k4*	NK.Actived	-0.566396609	0.009224716
SELUMETINIB	Inhibitor	*Map2k4*	DC Actived	0.574633662	0.008046006
SELUMETINIB	Inhibitor	*Map2k4*	DC Immature	-0.610893968	0.004219453
BINIMETINIB	Inhibitor	*Map2k4*	Mast Cells	0.66415738	0.001404745
BINIMETINIB	Inhibitor	*Map2k4*	Neutrophil Cells	0.77593985	8.58336E-05
BINIMETINIB	Inhibitor	*Map2k4*	Eosinophil Cells	0.10540072	0.658307737
BINIMETINIB	Inhibitor	*Map2k4*	M0 Macrophage	-0.928814532	3.43626E-09

BINIMETINIB	Inhibitor	*Map2k4*	M1 Macrophage	-0.574633662	0.008046006
BINIMETINIB	Inhibitor	*Map2k4*	M2 Macrophage	0.252831989	0.28215547
BINIMETINIB	Inhibitor	*Map2k4*	Monocyte	0.744360902	0.000253438
BINIMETINIB	Inhibitor	*Map2k4*	NK Resting	0.756082303	0.000115005
BINIMETINIB	Inhibitor	*Map2k4*	NK.Actived	-0.566396609	0.009224716
BINIMETINIB	Inhibitor	*Map2k4*	DC Actived	0.574633662	0.008046006
BINIMETINIB	Inhibitor	*Map2k4*	DC Immature	-0.610893968	0.004219453
SATRALIZUMAB	Antagonist	*Il6st*	Mast Cells	-0.534639161	0.015154416
SATRALIZUMAB	Antagonist	*Il6st*	Neutrophil Cells	-0.569924812	0.009858661
SATRALIZUMAB	Antagonist	*Il6st*	Eosinophil Cells	-0.241991449	0.303989478
SATRALIZUMAB	Antagonist	*Il6st*	M0 Macrophage	0.701535283	0.000566992
SATRALIZUMAB	Antagonist	*Il6st*	M1 Macrophage	0.591534652	0.006010574
SATRALIZUMAB	Antagonist	*Il6st*	M2 Macrophage	-0.325285335	0.161676426
SATRALIZUMAB	Antagonist	*Il6st*	Monocyte	-0.636090226	0.003205118
SATRALIZUMAB	Antagonist	*Il6st*	NK Resting	-0.727293598	0.000279525
SATRALIZUMAB	Antagonist	*Il6st*	NK.Actived	0.522284723	0.018159196
SATRALIZUMAB	Antagonist	*Il6st*	DC Actived	-0.239686768	0.308759852
SATRALIZUMAB	Antagonist	*Il6st*	DC Immature	0.246293519	0.295205225
ROMIDEPSIN	Inhibitor|Antagonist	*Hdac1*	Mast Cells	-0.519578902	0.018876851
ROMIDEPSIN	Inhibitor|Antagonist	*Hdac1*	Neutrophil Cells	-0.745864662	0.000242084
ROMIDEPSIN	Inhibitor|Antagonist	*Hdac1*	Eosinophil Cells	-0.05700243	0.81133678
ROMIDEPSIN	Inhibitor|Antagonist	*Hdac1*	M0 Macrophage	0.810629323	1.46E-05
ROMIDEPSIN	Inhibitor|Antagonist	*Hdac1*	M1 Macrophage	0.699086407	0.000604158
ROMIDEPSIN	Inhibitor|Antagonist	*Hdac1*	M2 Macrophage	-0.163774751	0.490229533
ROMIDEPSIN	Inhibitor|Antagonist	*Hdac1*	Monocyte	-0.781954887	6.72E-05
ROMIDEPSIN	Inhibitor|Antagonist	*Hdac1*	NK Resting	-0.784871008	4.16E-05
ROMIDEPSIN	Inhibitor|Antagonist	*Hdac1*	NK.Actived	0.529342625	0.016389437
ROMIDEPSIN	Inhibitor|Antagonist	*Hdac1*	DC Actived	-0.585388838	0.006695006

ROMIDEPSIN	Inhibitor|Antagonist	*Hdac1*	DC Immature	0.588308099	0.006362344
PRACINOSTAT	Inhibitor	*Hdac1*	Mast Cells	-0.519578902	0.018876851
PRACINOSTAT	Inhibitor	*Hdac1*	Neutrophil Cells	-0.745864662	0.000242084
PRACINOSTAT	Inhibitor	*Hdac1*	Eosinophil Cells	-0.05700243	0.81133678
PRACINOSTAT	Inhibitor	*Hdac1*	M0 Macrophage	0.810629323	1.46218E-05
PRACINOSTAT	Inhibitor	*Hdac1*	M1 Macrophage	0.699086407	0.000604158
PRACINOSTAT	Inhibitor	*Hdac1*	M2 Macrophage	-0.163774751	0.490229533
PRACINOSTAT	Inhibitor	*Hdac1*	Monocyte	-0.781954887	6.72096E-05
PRACINOSTAT	Inhibitor	*Hdac1*	NK Resting	-0.784871008	4.1642E-05
PRACINOSTAT	Inhibitor	*Hdac1*	NK.Actived	0.529342625	0.016389437
PRACINOSTAT	Inhibitor	*Hdac1*	DC Actived	-0.585388838	0.006695006
PRACINOSTAT	Inhibitor	*Hdac1*	DC Immature	0.588308099	0.006362344
SCRIPTAID	Inhibitor	*Hdac1*	Mast Cells	-0.519578902	0.018876851
SCRIPTAID	Inhibitor	*Hdac1*	Neutrophil Cells	-0.745864662	0.000242084
SCRIPTAID	Inhibitor	*Hdac1*	Eosinophil Cells	-0.05700243	0.81133678
SCRIPTAID	Inhibitor	*Hdac1*	M0 Macrophage	0.810629323	1.46E-05
SCRIPTAID	Inhibitor	*Hdac1*	M1 Macrophage	0.699086407	0.000604158
SCRIPTAID	Inhibitor	*Hdac1*	M2 Macrophage	-0.163774751	0.490229533
SCRIPTAID	Inhibitor	*Hdac1*	Monocyte	-0.781954887	6.72E-05
SCRIPTAID	Inhibitor	*Hdac1*	NK Resting	-0.784871008	4.16E-05
SCRIPTAID	Inhibitor	*Hdac1*	NK.Actived	0.529342625	0.016389437
SCRIPTAID	Inhibitor	*Hdac1*	DC Actived	-0.585388838	0.006695006
SCRIPTAID	Inhibitor	*Hdac1*	DC Immature	0.588308099	0.006362344
PANOBINOSTAT	Inhibitor	*Hdac1*	Mast Cells	-0.519578902	0.018876851
PANOBINOSTAT	Inhibitor	*Hdac1*	Neutrophil Cells	-0.745864662	0.000242084
PANOBINOSTAT	Inhibitor	*Hdac1*	Eosinophil Cells	-0.05700243	0.81133678
PANOBINOSTAT	Inhibitor	*Hdac1*	M0 Macrophage	0.810629323	1.46218E-05
PANOBINOSTAT	Inhibitor	*Hdac1*	M1 Macrophage	0.699086407	0.000604158

PANOBINOSTAT	Inhibitor	*Hdac1*	M2 Macrophage	-0.163774751	0.490229533
PANOBINOSTAT	Inhibitor	*Hdac1*	Monocyte	-0.781954887	6.72096E-05
PANOBINOSTAT	Inhibitor	*Hdac1*	NK Resting	-0.784871008	4.1642E-05
PANOBINOSTAT	Inhibitor	*Hdac1*	NK.Actived	0.529342625	0.016389437
PANOBINOSTAT	Inhibitor	*Hdac1*	DC Actived	-0.585388838	0.006695006
PANOBINOSTAT	Inhibitor	*Hdac1*	DC Immature	0.588308099	0.006362344
ABEXINOSTAT	Inhibitor	*Hdac1*	Mast Cells	-0.519578902	0.018876851
ABEXINOSTAT	Inhibitor	*Hdac1*	Neutrophil Cells	-0.745864662	0.000242084
ABEXINOSTAT	Inhibitor	*Hdac1*	Eosinophil Cells	-0.05700243	0.81133678
ABEXINOSTAT	Inhibitor	*Hdac1*	M0 Macrophage	0.810629323	1.46E-05
ABEXINOSTAT	Inhibitor	*Hdac1*	M1 Macrophage	0.699086407	0.000604158
ABEXINOSTAT	Inhibitor	*Hdac1*	M2 Macrophage	-0.163774751	0.490229533
ABEXINOSTAT	Inhibitor	*Hdac1*	Monocyte	-0.781954887	6.72E-05
ABEXINOSTAT	Inhibitor	*Hdac1*	NK Resting	-0.784871008	4.16E-05
ABEXINOSTAT	Inhibitor	*Hdac1*	NK.Actived	0.529342625	0.016389437
ABEXINOSTAT	Inhibitor	*Hdac1*	DC Actived	-0.585388838	0.006695006
ABEXINOSTAT	Inhibitor	*Hdac1*	DC Immature	0.588308099	0.006362344
NANATINOSTAT	Inhibitor	*Hdac1*	Mast Cells	-0.519578902	0.018876851
NANATINOSTAT	Inhibitor	*Hdac1*	Neutrophil Cells	-0.745864662	0.000242084
NANATINOSTAT	Inhibitor	*Hdac1*	Eosinophil Cells	-0.05700243	0.81133678
NANATINOSTAT	Inhibitor	*Hdac1*	M0 Macrophage	0.810629323	1.46218E-05
NANATINOSTAT	Inhibitor	*Hdac1*	M1 Macrophage	0.699086407	0.000604158
NANATINOSTAT	Inhibitor	*Hdac1*	M2 Macrophage	-0.163774751	0.490229533
NANATINOSTAT	Inhibitor	*Hdac1*	Monocyte	-0.781954887	6.72096E-05
NANATINOSTAT	Inhibitor	*Hdac1*	NK Resting	-0.784871008	4.1642E-05
NANATINOSTAT	Inhibitor	*Hdac1*	NK.Actived	0.529342625	0.016389437
NANATINOSTAT	Inhibitor	*Hdac1*	DC Actived	-0.585388838	0.006695006
NANATINOSTAT	Inhibitor	*Hdac1*	DC Immature	0.588308099	0.006362344

ENTINOSTAT	Inhibitor	*Hdac1*	Mast Cells	-0.519578902	0.018876851
ENTINOSTAT	Inhibitor	*Hdac1*	Neutrophil Cells	-0.745864662	0.000242084
ENTINOSTAT	Inhibitor	*Hdac1*	Eosinophil Cells	-0.05700243	0.81133678
ENTINOSTAT	Inhibitor	*Hdac1*	M0 Macrophage	0.810629323	1.46E-05
ENTINOSTAT	Inhibitor	*Hdac1*	M1 Macrophage	0.699086407	0.000604158
ENTINOSTAT	Inhibitor	*Hdac1*	M2 Macrophage	-0.163774751	0.490229533
ENTINOSTAT	Inhibitor	*Hdac1*	Monocyte	-0.781954887	6.72E-05
ENTINOSTAT	Inhibitor	*Hdac1*	NK Resting	-0.784871008	4.16E-05
ENTINOSTAT	Inhibitor	*Hdac1*	NK.Actived	0.529342625	0.016389437
ENTINOSTAT	Inhibitor	*Hdac1*	DC Actived	-0.585388838	0.006695006
ENTINOSTAT	Inhibitor	*Hdac1*	DC Immature	0.588308099	0.006362344
VORINOSTAT	Inhibitor	*Hdac1*	Mast Cells	-0.519578902	0.018876851
VORINOSTAT	Inhibitor	*Hdac1*	Neutrophil Cells	-0.745864662	0.000242084
VORINOSTAT	Inhibitor	*Hdac1*	Eosinophil Cells	-0.05700243	0.81133678
VORINOSTAT	Inhibitor	*Hdac1*	M0 Macrophage	0.810629323	1.46218E-05
VORINOSTAT	Inhibitor	*Hdac1*	M1 Macrophage	0.699086407	0.000604158
VORINOSTAT	Inhibitor	*Hdac1*	M2 Macrophage	-0.163774751	0.490229533
VORINOSTAT	Inhibitor	*Hdac1*	Monocyte	-0.781954887	6.72096E-05
VORINOSTAT	Inhibitor	*Hdac1*	NK Resting	-0.784871008	4.1642E-05
VORINOSTAT	Inhibitor	*Hdac1*	NK.Actived	0.529342625	0.016389437
VORINOSTAT	Inhibitor	*Hdac1*	DC Actived	-0.585388838	0.006695006
VORINOSTAT	Inhibitor	*Hdac1*	DC Immature	0.588308099	0.006362344
CUDC-101	Inhibitor	*Hdac1*	Mast Cells	-0.519578902	0.018876851
CUDC-102	Inhibitor	*Hdac1*	Neutrophil Cells	-0.745864662	0.000242084
CUDC-103	Inhibitor	*Hdac1*	Eosinophil Cells	-0.05700243	0.81133678
CUDC-104	Inhibitor	*Hdac1*	M0 Macrophage	0.810629323	1.46E-05
CUDC-105	Inhibitor	*Hdac1*	M1 Macrophage	0.699086407	0.000604158
CUDC-106	Inhibitor	*Hdac1*	M2 Macrophage	-0.163774751	0.490229533

CUDC-107	Inhibitor	*Hdac1*	Monocyte	-0.781954887	6.72E-05
CUDC-108	Inhibitor	*Hdac1*	NK Resting	-0.784871008	4.16E-05
CUDC-109	Inhibitor	*Hdac1*	NK.Actived	0.529342625	0.016389437
CUDC-110	Inhibitor	*Hdac1*	DC Actived	-0.585388838	0.006695006
CUDC-111	Inhibitor	*Hdac1*	DC Immature	0.588308099	0.006362344
FIMEPINOSTAT	Inhibitor	*Hdac1*	Mast Cells	-0.519578902	0.018876851
FIMEPINOSTAT	Inhibitor	*Hdac1*	Neutrophil Cells	-0.745864662	0.000242084
FIMEPINOSTAT	Inhibitor	*Hdac1*	Eosinophil Cells	-0.05700243	0.81133678
FIMEPINOSTAT	Inhibitor	*Hdac1*	M0 Macrophage	0.810629323	1.46218E-05
FIMEPINOSTAT	Inhibitor	*Hdac1*	M1 Macrophage	0.699086407	0.000604158
FIMEPINOSTAT	Inhibitor	*Hdac1*	M2 Macrophage	-0.163774751	0.490229533
FIMEPINOSTAT	Inhibitor	*Hdac1*	Monocyte	-0.781954887	6.72096E-05
FIMEPINOSTAT	Inhibitor	*Hdac1*	NK Resting	-0.784871008	4.1642E-05
FIMEPINOSTAT	Inhibitor	*Hdac1*	NK.Actived	0.529342625	0.016389437
FIMEPINOSTAT	Inhibitor	*Hdac1*	DC Actived	-0.585388838	0.006695006
FIMEPINOSTAT	Inhibitor	*Hdac1*	DC Immature	0.588308099	0.006362344
TACEDINALINE	Inhibitor	*Hdac1*	Mast Cells	-0.519578902	0.018876851
TACEDINALINE	Inhibitor	*Hdac1*	Neutrophil Cells	-0.745864662	0.000242084
TACEDINALINE	Inhibitor	*Hdac1*	Eosinophil Cells	-0.05700243	0.81133678
TACEDINALINE	Inhibitor	*Hdac1*	M0 Macrophage	0.810629323	1.46E-05
TACEDINALINE	Inhibitor	*Hdac1*	M1 Macrophage	0.699086407	0.000604158
TACEDINALINE	Inhibitor	*Hdac1*	M2 Macrophage	-0.163774751	0.490229533
TACEDINALINE	Inhibitor	*Hdac1*	Monocyte	-0.781954887	6.72E-05
TACEDINALINE	Inhibitor	*Hdac1*	NK Resting	-0.784871008	4.16E-05
TACEDINALINE	Inhibitor	*Hdac1*	NK.Actived	0.529342625	0.016389437
TACEDINALINE	Inhibitor	*Hdac1*	DC Actived	-0.585388838	0.006695006
TACEDINALINE	Inhibitor	*Hdac1*	DC Immature	0.588308099	0.006362344
DEPAKOTE	Inhibitor	*Hdac1*	Mast Cells	-0.519578902	0.018876851

DEPAKOTE	Inhibitor	*Hdac1*	Neutrophil Cells	-0.745864662	0.000242084
DEPAKOTE	Inhibitor	*Hdac1*	Eosinophil Cells	-0.05700243	0.81133678
DEPAKOTE	Inhibitor	*Hdac1*	M0 Macrophage	0.810629323	1.46218E-05
DEPAKOTE	Inhibitor	*Hdac1*	M1 Macrophage	0.699086407	0.000604158
DEPAKOTE	Inhibitor	*Hdac1*	M2 Macrophage	-0.163774751	0.490229533
DEPAKOTE	Inhibitor	*Hdac1*	Monocyte	-0.781954887	6.72096E-05
DEPAKOTE	Inhibitor	*Hdac1*	NK Resting	-0.784871008	4.1642E-05
DEPAKOTE	Inhibitor	*Hdac1*	NK.Actived	0.529342625	0.016389437
DEPAKOTE	Inhibitor	*Hdac1*	DC Actived	-0.585388838	0.006695006
DEPAKOTE	Inhibitor	*Hdac1*	DC Immature	0.588308099	0.006362344
RESMINOSTAT	Inhibitor	*Hdac1*	Mast Cells	-0.519578902	0.018876851
RESMINOSTAT	Inhibitor	*Hdac1*	Neutrophil Cells	-0.745864662	0.000242084
RESMINOSTAT	Inhibitor	*Hdac1*	Eosinophil Cells	-0.05700243	0.81133678
RESMINOSTAT	Inhibitor	*Hdac1*	M0 Macrophage	0.810629323	1.46E-05
RESMINOSTAT	Inhibitor	*Hdac1*	M1 Macrophage	0.699086407	0.000604158
RESMINOSTAT	Inhibitor	*Hdac1*	M2 Macrophage	-0.163774751	0.490229533
RESMINOSTAT	Inhibitor	*Hdac1*	Monocyte	-0.781954887	6.72E-05
RESMINOSTAT	Inhibitor	*Hdac1*	NK Resting	-0.784871008	4.16E-05
RESMINOSTAT	Inhibitor	*Hdac1*	NK.Actived	0.529342625	0.016389437
RESMINOSTAT	Inhibitor	*Hdac1*	DC Actived	-0.585388838	0.006695006
RESMINOSTAT	Inhibitor	*Hdac1*	DC Immature	0.588308099	0.006362344
BELINOSTAT	Inhibitor	*Hdac1*	Mast Cells	-0.519578902	0.018876851
BELINOSTAT	Inhibitor	*Hdac1*	Neutrophil Cells	-0.745864662	0.000242084
BELINOSTAT	Inhibitor	*Hdac1*	Eosinophil Cells	-0.05700243	0.81133678
BELINOSTAT	Inhibitor	*Hdac1*	M0 Macrophage	0.810629323	1.46218E-05
BELINOSTAT	Inhibitor	*Hdac1*	M1 Macrophage	0.699086407	0.000604158
BELINOSTAT	Inhibitor	*Hdac1*	M2 Macrophage	-0.163774751	0.490229533
BELINOSTAT	Inhibitor	*Hdac1*	Monocyte	-0.781954887	6.72096E-05

BELINOSTAT	Inhibitor	*Hdac1*	NK Resting	-0.784871008	4.1642E-05
BELINOSTAT	Inhibitor	*Hdac1*	NK.Actived	0.529342625	0.016389437
BELINOSTAT	Inhibitor	*Hdac1*	DC Actived	-0.585388838	0.006695006
BELINOSTAT	Inhibitor	*Hdac1*	DC Immature	0.588308099	0.006362344
PANOBINOSTAT LACTAT	Inhibitor	*Hdac1*	Mast Cells	-0.519578902	0.018876851
PANOBINOSTAT LACTAT	Inhibitor	*Hdac1*	Neutrophil Cells	-0.745864662	0.000242084
PANOBINOSTAT LACTAT	Inhibitor	*Hdac1*	Eosinophil Cells	-0.05700243	0.81133678
PANOBINOSTAT LACTAT	Inhibitor	*Hdac1*	M0 Macrophage	0.810629323	1.46E-05
PANOBINOSTAT LACTAT	Inhibitor	*Hdac1*	M1 Macrophage	0.699086407	0.000604158
PANOBINOSTAT LACTAT	Inhibitor	*Hdac1*	M2 Macrophage	-0.163774751	0.490229533
PANOBINOSTAT LACTAT	Inhibitor	*Hdac1*	Monocyte	-0.781954887	6.72E-05
PANOBINOSTAT LACTAT	Inhibitor	*Hdac1*	NK Resting	-0.784871008	4.16E-05
PANOBINOSTAT LACTAT	Inhibitor	*Hdac1*	NK.Actived	0.529342625	0.016389437
PANOBINOSTAT LACTAT	Inhibitor	*Hdac1*	DC Actived	-0.585388838	0.006695006
PANOBINOSTAT LACTAT	Inhibitor	*Hdac1*	DC Immature	0.588308099	0.006362344
GIVINOSTAT	Inhibitor	*Hdac1*	Mast Cells	-0.519578902	0.018876851
GIVINOSTAT	Inhibitor	*Hdac1*	Neutrophil Cells	-0.745864662	0.000242084
GIVINOSTAT	Inhibitor	*Hdac1*	Eosinophil Cells	-0.05700243	0.81133678
GIVINOSTAT	Inhibitor	*Hdac1*	M0 Macrophage	0.810629323	1.46218E-05
GIVINOSTAT	Inhibitor	*Hdac1*	M1 Macrophage	0.699086407	0.000604158
GIVINOSTAT	Inhibitor	*Hdac1*	M2 Macrophage	-0.163774751	0.490229533
GIVINOSTAT	Inhibitor	*Hdac1*	Monocyte	-0.781954887	6.72096E-05
GIVINOSTAT	Inhibitor	*Hdac1*	NK Resting	-0.784871008	4.1642E-05
GIVINOSTAT	Inhibitor	*Hdac1*	NK.Actived	0.529342625	0.016389437
GIVINOSTAT	Inhibitor	*Hdac1*	DC Actived	-0.585388838	0.006695006
GIVINOSTAT	Inhibitor	*Hdac1*	DC Immature	0.588308099	0.006362344
TUCIDINOSTAT	Inhibitor	*Hdac1*	Mast Cells	-0.519578902	0.018876851
TUCIDINOSTAT	Inhibitor	*Hdac1*	Neutrophil Cells	-0.745864662	0.000242084

TUCIDINOSTAT	Inhibitor	*Hdac1*	Eosinophil Cells	-0.05700243	0.81133678
TUCIDINOSTAT	Inhibitor	*Hdac1*	M0 Macrophage	0.810629323	1.46E-05
TUCIDINOSTAT	Inhibitor	*Hdac1*	M1 Macrophage	0.699086407	0.000604158
TUCIDINOSTAT	Inhibitor	*Hdac1*	M2 Macrophage	-0.163774751	0.490229533
TUCIDINOSTAT	Inhibitor	*Hdac1*	Monocyte	-0.781954887	6.72E-05
TUCIDINOSTAT	Inhibitor	*Hdac1*	NK Resting	-0.784871008	4.16E-05
TUCIDINOSTAT	Inhibitor	*Hdac1*	NK.Actived	0.529342625	0.016389437
TUCIDINOSTAT	Inhibitor	*Hdac1*	DC Actived	-0.585388838	0.006695006
TUCIDINOSTAT	Inhibitor	*Hdac1*	DC Immature	0.588308099	0.006362344
MOCETINOSTAT	Inhibitor	*Hdac1*	Mast Cells	-0.519578902	0.018876851
MOCETINOSTAT	Inhibitor	*Hdac1*	Neutrophil Cells	-0.745864662	0.000242084
MOCETINOSTAT	Inhibitor	*Hdac1*	Eosinophil Cells	-0.05700243	0.81133678
MOCETINOSTAT	Inhibitor	*Hdac1*	M0 Macrophage	0.810629323	1.46218E-05
MOCETINOSTAT	Inhibitor	*Hdac1*	M1 Macrophage	0.699086407	0.000604158
MOCETINOSTAT	Inhibitor	*Hdac1*	M2 Macrophage	-0.163774751	0.490229533
MOCETINOSTAT	Inhibitor	*Hdac1*	Monocyte	-0.781954887	6.72096E-05
MOCETINOSTAT	Inhibitor	*Hdac1*	NK Resting	-0.784871008	4.1642E-05
MOCETINOSTAT	Inhibitor	*Hdac1*	NK.Actived	0.529342625	0.016389437
MOCETINOSTAT	Inhibitor	*Hdac1*	DC Actived	-0.585388838	0.006695006
MOCETINOSTAT	Inhibitor	*Hdac1*	DC Immature	0.588308099	0.006362344
AN-9	Inhibitor	*Hdac1*	Mast Cells	-0.519578902	0.018876851
AN-10	Inhibitor	*Hdac1*	Neutrophil Cells	-0.745864662	0.000242084
AN-11	Inhibitor	*Hdac1*	Eosinophil Cells	-0.05700243	0.81133678
AN-12	Inhibitor	*Hdac1*	M0 Macrophage	0.810629323	1.46218E-05
AN-13	Inhibitor	*Hdac1*	M1 Macrophage	0.699086407	0.000604158
AN-14	Inhibitor	*Hdac1*	M2 Macrophage	-0.163774751	0.490229533
AN-15	Inhibitor	*Hdac1*	Monocyte	-0.781954887	6.72096E-05
AN-16	Inhibitor	*Hdac1*	NK Resting	-0.784871008	4.1642E-05

AN-17	Inhibitor	*Hdac1*	NK.Actived	0.529342625	0.016389437
AN-18	Inhibitor	*Hdac1*	DC Actived	-0.585388838	0.006695006
AN-19	Inhibitor	*Hdac1*	DC Immature	0.588308099	0.006362344
APICIDIN	Inhibitor	*Hdac1*	Mast Cells	-0.519578902	0.018876851
APICIDIN	Inhibitor	*Hdac1*	Neutrophil Cells	-0.745864662	0.000242084
APICIDIN	Inhibitor	*Hdac1*	Eosinophil Cells	-0.05700243	0.81133678
APICIDIN	Inhibitor	*Hdac1*	M0 Macrophage	0.810629323	1.46E-05
APICIDIN	Inhibitor	*Hdac1*	M1 Macrophage	0.699086407	0.000604158
APICIDIN	Inhibitor	*Hdac1*	M2 Macrophage	-0.163774751	0.490229533
APICIDIN	Inhibitor	*Hdac1*	Monocyte	-0.781954887	6.72E-05
APICIDIN	Inhibitor	*Hdac1*	NK Resting	-0.784871008	4.16E-05
APICIDIN	Inhibitor	*Hdac1*	NK.Actived	0.529342625	0.016389437
APICIDIN	Inhibitor	*Hdac1*	DC Actived	-0.585388838	0.006695006
APICIDIN	Inhibitor	*Hdac1*	DC Immature	0.588308099	0.006362344
DACINOSTAT	Inhibitor	*Hdac1*	Mast Cells	-0.519578902	0.018876851
DACINOSTAT	Inhibitor	*Hdac1*	Neutrophil Cells	-0.745864662	0.000242084
DACINOSTAT	Inhibitor	*Hdac1*	Eosinophil Cells	-0.05700243	0.81133678
DACINOSTAT	Inhibitor	*Hdac1*	M0 Macrophage	0.810629323	1.46E-05
DACINOSTAT	Inhibitor	*Hdac1*	M1 Macrophage	0.699086407	0.000604158
DACINOSTAT	Inhibitor	*Hdac1*	M2 Macrophage	-0.163774751	0.490229533
DACINOSTAT	Inhibitor	*Hdac1*	Monocyte	-0.781954887	6.72E-05
DACINOSTAT	Inhibitor	*Hdac1*	NK Resting	-0.784871008	4.16E-05
DACINOSTAT	Inhibitor	*Hdac1*	NK.Actived	0.529342625	0.016389437
DACINOSTAT	Inhibitor	*Hdac1*	DC Actived	-0.585388838	0.006695006
DACINOSTAT	Inhibitor	*Hdac1*	DC Immature	0.588308099	0.006362344
GUANINE	Inhibitor	*Gch1*	Mast Cells	-0.274096696	0.242228413
GUANINE	Inhibitor	*Gch1*	Neutrophil Cells	-0.034586466	0.886238253
GUANINE	Inhibitor	*Gch1*	Eosinophil Cells	-0.315126642	0.175950817

GUANINE	Inhibitor	*Gch1*	M0 Macrophage	0.189399374	0.423850447
GUANINE	Inhibitor	*Gch1*	M1 Macrophage	0.187447344	0.428730665
GUANINE	Inhibitor	*Gch1*	M2 Macrophage	-0.66340095	0.00142896
GUANINE	Inhibitor	*Gch1*	Monocyte	-0.006015038	0.982248374
GUANINE	Inhibitor	*Gch1*	NK Resting	-0.431830574	0.057263317
GUANINE	Inhibitor	*Gch1*	NK.Actived	0.239968657	0.308173968
GUANINE	Inhibitor	*Gch1*	DC Actived	0.373358235	0.104919247
GUANINE	Inhibitor	*Gch1*	DC Immature	0.090343474	0.70484434
GILTERITINIB	Inhibitor	*Axl*	Mast Cells	-0.762049057	9.42E-05
GILTERITINIB	Inhibitor	*Axl*	Neutrophil Cells	-0.837593985	0
GILTERITINIB	Inhibitor	*Axl*	Eosinophil Cells	0.009679658	0.96769282
GILTERITINIB	Inhibitor	*Axl*	M0 Macrophage	0.795477373	2.75E-05
GILTERITINIB	Inhibitor	*Axl*	M1 Macrophage	0.597680467	0.005384717
GILTERITINIB	Inhibitor	*Axl*	M2 Macrophage	0.003018889	0.989921772
GILTERITINIB	Inhibitor	*Axl*	Monocyte	-0.733834586	0.000345147
GILTERITINIB	Inhibitor	*Axl*	NK Resting	-0.527287859	0.016889743
GILTERITINIB	Inhibitor	*Axl*	NK.Actived	0.324663477	0.162526508
GILTERITINIB	Inhibitor	*Axl*	DC Actived	-0.576170116	0.007840403
GILTERITINIB	Inhibitor	*Axl*	DC Immature	0.37320459	0.105073674
HESPERADIN	Inhibitor	*Axl*	Mast Cells	-0.762049057	9.42474E-05
HESPERADIN	Inhibitor	*Axl*	Neutrophil Cells	-0.837593985	0
HESPERADIN	Inhibitor	*Axl*	Eosinophil Cells	0.009679658	0.96769282
HESPERADIN	Inhibitor	*Axl*	M0 Macrophage	0.795477373	2.75467E-05
HESPERADIN	Inhibitor	*Axl*	M1 Macrophage	0.597680467	0.005384717
HESPERADIN	Inhibitor	*Axl*	M2 Macrophage	0.003018889	0.989921772
HESPERADIN	Inhibitor	*Axl*	Monocyte	-0.733834586	0.000345147
HESPERADIN	Inhibitor	*Axl*	NK Resting	-0.527287859	0.016889743
HESPERADIN	Inhibitor	*Axl*	NK.Actived	0.324663477	0.162526508

HESPERADIN	Inhibitor	*Axl*	DC Actived	-0.576170116	0.007840403
HESPERADIN	Inhibitor	*Axl*	DC Immature	0.37320459	0.105073674
DUBERMATINIB	Inhibitor	*Axl*	Mast Cells	-0.762049057	9.42E-05
DUBERMATINIB	Inhibitor	*Axl*	Neutrophil Cells	-0.837593985	0
DUBERMATINIB	Inhibitor	*Axl*	Eosinophil Cells	0.009679658	0.96769282
DUBERMATINIB	Inhibitor	*Axl*	M0 Macrophage	0.795477373	2.75E-05
DUBERMATINIB	Inhibitor	*Axl*	M1 Macrophage	0.597680467	0.005384717
DUBERMATINIB	Inhibitor	*Axl*	M2 Macrophage	0.003018889	0.989921772
DUBERMATINIB	Inhibitor	*Axl*	Monocyte	-0.733834586	0.000345147
DUBERMATINIB	Inhibitor	*Axl*	NK Resting	-0.527287859	0.016889743
DUBERMATINIB	Inhibitor	*Axl*	NK.Actived	0.324663477	0.162526508
DUBERMATINIB	Inhibitor	*Axl*	DC Actived	-0.576170116	0.007840403
DUBERMATINIB	Inhibitor	*Axl*	DC Immature	0.37320459	0.105073674
R428	Inhibitor	*Axl*	Mast Cells	-0.762049057	9.42474E-05
R429	Inhibitor	*Axl*	Neutrophil Cells	-0.837593985	0
R430	Inhibitor	*Axl*	Eosinophil Cells	0.009679658	0.96769282
R431	Inhibitor	*Axl*	M0 Macrophage	0.795477373	2.75467E-05
R432	Inhibitor	*Axl*	M1 Macrophage	0.597680467	0.005384717
R433	Inhibitor	*Axl*	M2 Macrophage	0.003018889	0.989921772
R434	Inhibitor	*Axl*	Monocyte	-0.733834586	0.000345147
R435	Inhibitor	*Axl*	NK Resting	-0.527287859	0.016889743
R436	Inhibitor	*Axl*	NK.Actived	0.324663477	0.162526508
R437	Inhibitor	*Axl*	DC Actived	-0.576170116	0.007840403
R438	Inhibitor	*Axl*	DC Immature	0.37320459	0.105073674
BPI-9016	Inhibitor	*Axl*	Mast Cells	-0.762049057	9.42E-05
BPI-9017	Inhibitor	*Axl*	Neutrophil Cells	-0.837593985	0
BPI-9018	Inhibitor	*Axl*	Eosinophil Cells	0.009679658	0.96769282
BPI-9019	Inhibitor	*Axl*	M0 Macrophage	0.795477373	2.75E-05

BPI-9020	Inhibitor	*Axl*	M1 Macrophage	0.597680467	0.005384717
BPI-9021	Inhibitor	*Axl*	M2 Macrophage	0.003018889	0.989921772
BPI-9022	Inhibitor	*Axl*	Monocyte	-0.733834586	0.000345147
BPI-9023	Inhibitor	*Axl*	NK Resting	-0.527287859	0.016889743
BPI-9024	Inhibitor	*Axl*	NK.Actived	0.324663477	0.162526508
BPI-9025	Inhibitor	*Axl*	DC Actived	-0.576170116	0.007840403
BPI-9026	Inhibitor	*Axl*	DC Immature	0.37320459	0.105073674
BEMCENTINIB	Inhibitor	*Axl*	Mast Cells	-0.762049057	9.42474E-05
BEMCENTINIB	Inhibitor	*Axl*	Neutrophil Cells	-0.837593985	0
BEMCENTINIB	Inhibitor	*Axl*	Eosinophil Cells	0.009679658	0.96769282
BEMCENTINIB	Inhibitor	*Axl*	M0 Macrophage	0.795477373	2.75467E-05
BEMCENTINIB	Inhibitor	*Axl*	M1 Macrophage	0.597680467	0.005384717
BEMCENTINIB	Inhibitor	*Axl*	M2 Macrophage	0.003018889	0.989921772
BEMCENTINIB	Inhibitor	*Axl*	Monocyte	-0.733834586	0.000345147
BEMCENTINIB	Inhibitor	*Axl*	NK Resting	-0.527287859	0.016889743
BEMCENTINIB	Inhibitor	*Axl*	NK.Actived	0.324663477	0.162526508
BEMCENTINIB	Inhibitor	*Axl*	DC Actived	-0.576170116	0.007840403
BEMCENTINIB	Inhibitor	*Axl*	DC Immature	0.37320459	0.105073674
